# Exploring the key genomic variation in monkeypox virus during the 2022 outbreak

**DOI:** 10.1186/s12863-023-01171-0

**Published:** 2023-11-16

**Authors:** Jie Zhu, Jian Yu, Hao Qin, Xinlei Chen, Chuanchang Wu, Xiaodan Hong, Yafei Zhang, Zhenhua Zhang

**Affiliations:** 1 Institute of Clinical Virology, Department of Infectious Diseases, The Second Affiliated Hospital of Anhui Medical University, Furong Road 678, Hefei, 230601 Anhui China; 2 Department of Infectious Diseases, The Third People’s Hospital of Hefei, Hefei, China

**Keywords:** Monkeypox virus, Genomic variation, Phylogenetic tree, Reference sequence, Protein structure prediction

## Abstract

**Background:**

In 2022, a global outbreak of monkeypox occurred with a significant shift in its epidemiological characteristics. The monkeypox virus (MPXV) belongs to the B.1 lineage, and its genomic variations that were linked to the outbreak were investigated in this study. Previous studies have suggested that viral genomic variation plays a crucial role in the pathogenicity and transmissibility of viruses. Therefore, understanding the genomic variation of MPXV is crucial for controlling future outbreaks.

**Methods:**

This study employed bioinformatics and phylogenetic approaches to evaluate the key genomic variation in the B.1 lineage of MPXV. A total of 979 MPXV strains were screened, and 212 representative strains were analyzed to identify specific substitutions in the viral genome. Reference sequences were constructed for each of the 10 lineages based on the most common nucleotide at each site. A total of 49 substitutions were identified, with 23 non-synonymous substitutions. Class I variants, which had significant effects on protein conformation likely to affect viral characteristics, were classified among the non-synonymous substitutions.

**Results:**

The phylogenetic analysis revealed 10 relatively monophyletic branches. The study identified 49 substitutions specific to the B.1 lineage, with 23 non-synonymous substitutions that were classified into Class I, II, and III variants. The Class I variants were likely responsible for the observed changes in the characteristics of circulating MPXV in 2022. These key mutations, particularly Class I variants, played a crucial role in the pathogenicity and transmissibility of MPXV.

**Conclusion:**

This study provides an understanding of the genomic variation of MPXV in the B.1 lineage linked to the recent outbreak of monkeypox. The identification of key mutations, particularly Class I variants, sheds light on the molecular mechanisms underlying the observed changes in the characteristics of circulating MPXV. Further studies can focus on functional domains affected by these mutations, enabling the development of effective control strategies against future monkeypox outbreaks.

**Supplementary Information:**

The online version contains supplementary material available at 10.1186/s12863-023-01171-0.

## Introduction

Monkeypox (MPX) is a zoonotic disease caused by Monkeypox virus (MPXV) [1]. Although previously concentrated in Africa [2], a global outbreak began in May 2022, with over 16 000 confirmed cases reported in more than 75 countries and territories that were not previously endemic just 2 months later. The World Health Organization declared the outbreak a public health emergency of international concern [3].

MPXV is a type of enveloped *Orthopoxvirus*. It has a double-stranded genome of 197 kb encoding more than 200 proteins [4]. The previously endemic Congo Basin and West African strains are now known as clade I and clade IIa, respectively, while the recently evolved West African branch is referred to as clade IIb [5]. Epidemiological statistics have shown that the incidence of the Congo Basin strain has increased continuously from 0.64/100 000 in 2001 to 2.82/100 000 in 2013, with the rate of suspected and confirmed cases reaching 500/100 000 in 2016. Few of the Congo Basin isolates have spread to other areas. The incidence of the West African strain was low but has spread repeatedly to countries outside Africa [2]. Virulence differs between Congo Basin and West African strains, with fatality rates of nearly 10% for clade I and less than 3% for clade IIa [6–8].

Clade IIb encompasses most of the circulating strains from 2017 to 2019, the B.1 lineage that caused the 2022 MPX outbreak, and the A.2 lineage, which caused a minor endemic in 2022 [9]. The 2022 global MPX epidemic was primarily transmitted from person to person, with a significant proportion of transmission occurring among men who have sex with men [10]. The median R0 for MPX transmission in Europe was 2.44 in 2022, with the highest estimates in Portugal and Germany [11]. Typical clinical features of the disease are fever, rash, and swollen lymph nodes [3], and these were consistent up to 2022. The rashes of newly infected patients occurred mostly in the genital region, rather than on the hands and face [12]. Furthermore, the first death outside of Africa was reported in 2022; however, the overall case fatality rate was lower than that of the Congo Basin and early West African strains at 1.18% [13]. It is reasonable to conclude that the 2022 MPXV strains exhibited significant changes in biological characteristics [14], prompting research on the molecular basis of these changes. Whole genome sequencing and phylogenetic analyses have shown that MPXV in 2022 was closely related to strains circulating from Nigeria to the United Kingdom in 2018–2019. However, the mean number of single nucleotide polymorphisms (SNPs) differed by as much as 50, which was far more (about 6–12 times) than expected based on previous estimates of the replacement rate of poxviruses [15, 16]. This likely reflected the continued and accelerated evolution of MPXV.

Different types of mutations can lead to changes in the virus’s biological characteristics. Previous classic studies have identified virulence-related genes in MPXV, such as D10L, D14L, and B10R [17]. The deletion of the D14L gene is more common in West African strains than in Congo Basin strains [18]. Additionally, research suggests that the B18R region and the virus’s ability to bind to the envelope may be closely related to changes in the virus’s infectivity and transmissibility [19]. Moreover, there are sites related to immune evasion, such as those with interferon inhibitory effects, such as B9R, B16R, C1L, D11L, D9L, and those achieving immune evasion through interfering with TNF-α and IL-1beta receptors, such as J2L and B14R [20]. Of course, these functions do not exist in isolation; they work in concert to respond to the virus’s selective pressures. Recent studies have focused on viral phylogeny and functional domains of 2022 pandemic isolates [21, 22]. However, the focus on a small sample of classical strains may introduce bias in the identification of critical genetic variants. It is challenging to fully unveil the virus’s evolutionary history and the contributions of observed mutations.To identify key mutations in MPXV evolution, we selected 212 of 979 complete genome sequences from public databases. Through phylogenetic analysis, we identified 10 monophyletic branches and established corresponding reference sequences (RSs) to eliminate uninformative mutations and to obtain the maximum commonality for each branch. Non-synonymous substitutions based on the alignment of these RSs were considered universal mutations in circulating strains, and those leading to structural changes in the protein were likely to affect the biological and clinical features of MPXV. Our team was the first to apply this method to select over 3000 HBV sequences reported from different countries. We constructed HBV subtypes by selecting the most frequent nucleotides at each position using infectious nature plasmids constructed based on four subtype-specific reference sequences, and in vitro and in vivo studies confirmed that these reference sequences possessed complete biological functionality [23]. This demonstrates the reliability of using this method for virus subtyping.

## Materials and methods

### Sequence acquisition and selection of MPXV strains

In the “nucleotide” module of the NCBI website (http://www.ncbi.nlm.nih.gov/genbank/), we employed the search terms “monkeypox virus” and “complete” with the date set to before September 25, 2022. This search yielded a total of 954 full-length sequences of MPXV were obtained, all of which originated from human infections (Additional file 1). To prevent potential analytical bias, we retained sequences from no more than four strains uploaded by the same author. Sequences containing more than 10% N bases were excluded from our analysis. It’s worth noting that the A.2 lineage, which was only locally endemic in 2022, was analyzed separately. Given that only three strains of the A.2 lineage were obtained from the NCBI database, we obtained an additional 25 strainsfrom the GSAID database (https://www.epicov.org/) using the same retrieval criteria.

### Establishment of a phylogenetic tree and RSs

A phylogenetic analysis of the selected isolates was performed using IQ-TREE, adopting maximum-likelihood estimation and best substitution model. The rapid bootstrap method was used for evaluating branch support. The selection of branches to construct RSs was guided by the following principles: (1) Classical isolate (Zaire-96-I-16) with well-defined functional regions served as original RS; (2) To maintain consistency with classification rules, corresponding RSs were established for clade I and clade IIa; (3) To better understand the recent mutations, RSs for the 2022 circulating strains were established independently, without being based solely on the overall of separation of all isolated strains. Given the abundance of strains in the B.1 lineage, two reference sequences were constructed based on the branching clustering situation. RSs were also constructed for the branches adjacent to the 2022 RSs within clade IIb; (4) When a selected branch contained only one isolate, it was automatically used as the RS for that branch. In cases with multiple isolates, RSs were constructed as followed method; (5) The most frequently observed nucleotide at each site among all isolates on the corresponding branch was selected; (6) All RSs were included in a new phylogenetic analysis to evaluate clades and distances.

Moreover, phylogenetic trees were embellished using the iTOL website (https://itol.embl.de/itol.cgi). The RSs were constructed using MEGA_X_10.0.2 software.

### Homology analysis of the RSs

To verify the reliability and specificity of all artificially constructed RSs, intragroup homology of RSs was evaluated. Identifying sites within each RS where base substitutions occurred and tallying the number of isolates exhibiting these substitutions; Calculating the heterogeneity rate (HR) for each site, which was defined as the ratio of the number of isolates with a substitution at that site to the total number of isolates within the RS branch; Using a statistical cutoff point of 20%; Sites with a HR of less than 5% were deemed to have negligible impact on the stability of the RS and were excluded from consideration; Comparing the number of substitution sites and the HR of each RS to assess the reliability of the constructed RSs.

### Alignment of each RS

We utilized an early isolated strain with clearly defined open reading frames (ORF) as the original reference strain. Each RS was compared with this reference strain to ascertain the start-end sites as well as the length. This process allowed us to identify ORFs within each RS. Most importantly, it facilitated the identification of all mutations in each RS relative to the original reference strain, encompassing both coding and non-coding regions (NCRs).

### Identification and classification of key mutation sites

This study primarily focused on identifying specific mutations in the RSs corresponding to the B.1 lineage of MPXV, which was responsible for the 2022 pandemic. We selected unique mutations present in the RSs of the B.1 lineage and also specific site mutations that were shared by the B.1 lineage and neighboring RSs for further analyses. To identify non-synonymous mutations in ORFs, we translated nucleotide sequences into amino acid (AA) sequences. Protein conformation models of ORFs containing non-synonymous mutations in each RS were constructed using ColabFold. Differences in protein conformation were assessed by comparing the root mean square deviation (RMSD) and predicted local distance difference test (pLDDT). Mutations were categorized into Classes I, II, and III based on their significance. Non-synonymous mutations within the B.1 lineage that were predicted to result in significant changes in protein conformation were considered the most likely to affect virus characteristics (Class I).

The SWISS-MODEL website (https://swissmodel.expasy.org/) was used to visually display the differences in protein conformation models.

### Statistical analysis

The analysis encompassed determining the total counts of the three substitution types in the constructed RSs. The total counts the three substitution types in the constructed RSs were determined, and the proportions of polymorphic sites with HR > 20% and 5% ≤ HR ≤ 20% were distinguished. Pairwise comparisons of evaluation indexes between RSs were performed by chi-squared (χ2) or Fisher’s exact tests. Values of *P* < 0.05 were considered statistically significant. Statistical analyses were performed using the “rcompanion” package in R 4.2.2. GraphPad Prism 8 was used to generate plots. The two parameters, i.e., total polymorphic sites and proportion of polymorphic sites with HR > 20%, were evaluated for three mutation types, SNPs, insertions, and deletions.

All of the alignments for nucleotide or amino acid sequences were performed by SnapGene Viewer 5.3 software.

## Results

### Details of MPXV strains included in the analysis

**Fig. 1:**
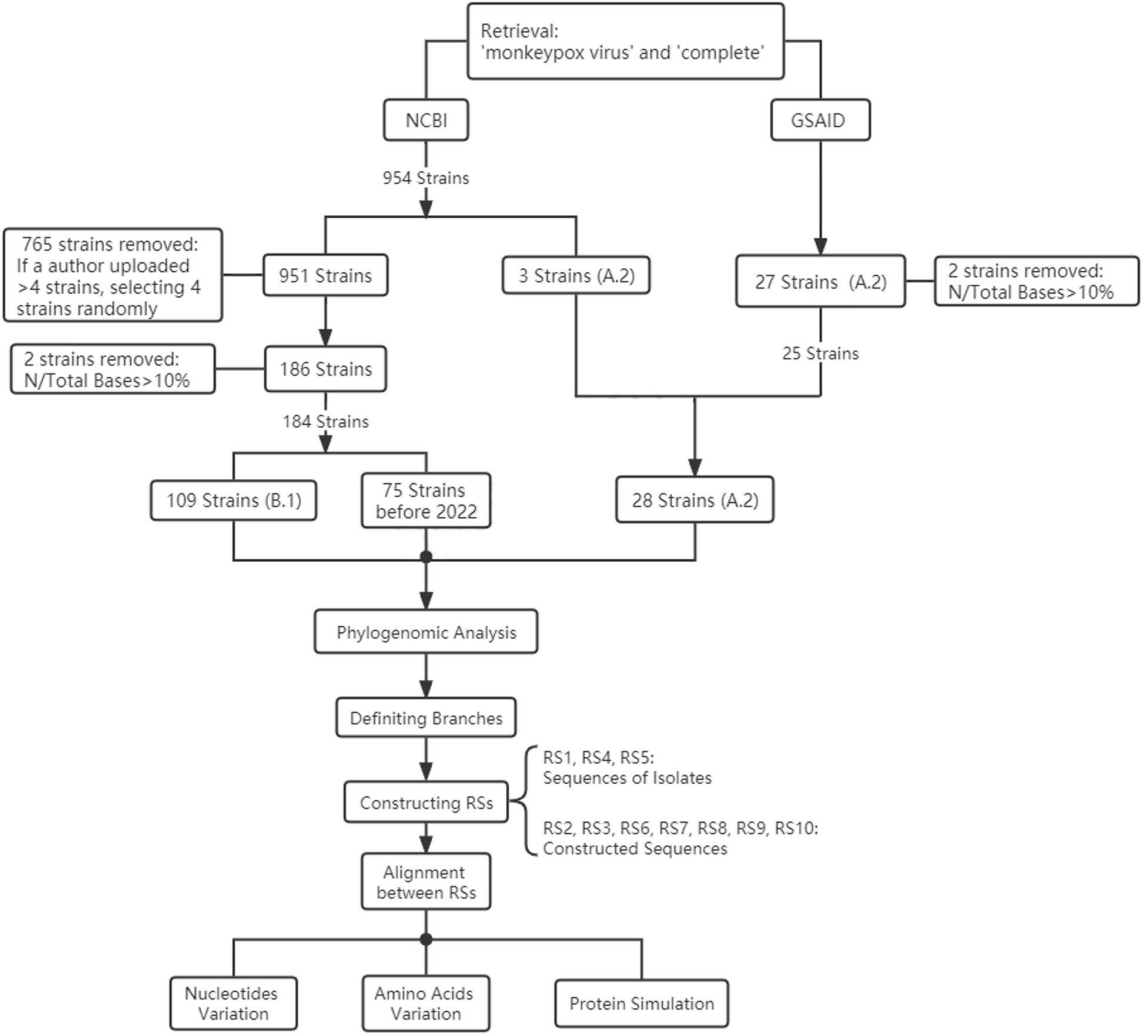
Flow chart of data acquisition, screening, and analysis

Following screening based on several criteria, a total of 212 strains were included in subsequent analyses. Among these, 187 strains from NCBI (Additional file 2), including 75 isolates obtained prior to 2022, 109 strains of the B.1 lineage and 3 strains of the A.2 lineage prevalent in 2022, and 25 strains of the A.2 lineage (Additional file 3), were downloaded from GASAID (Fig. 1).

### Phylogenetic analysis and RSs

**Fig. 2:**
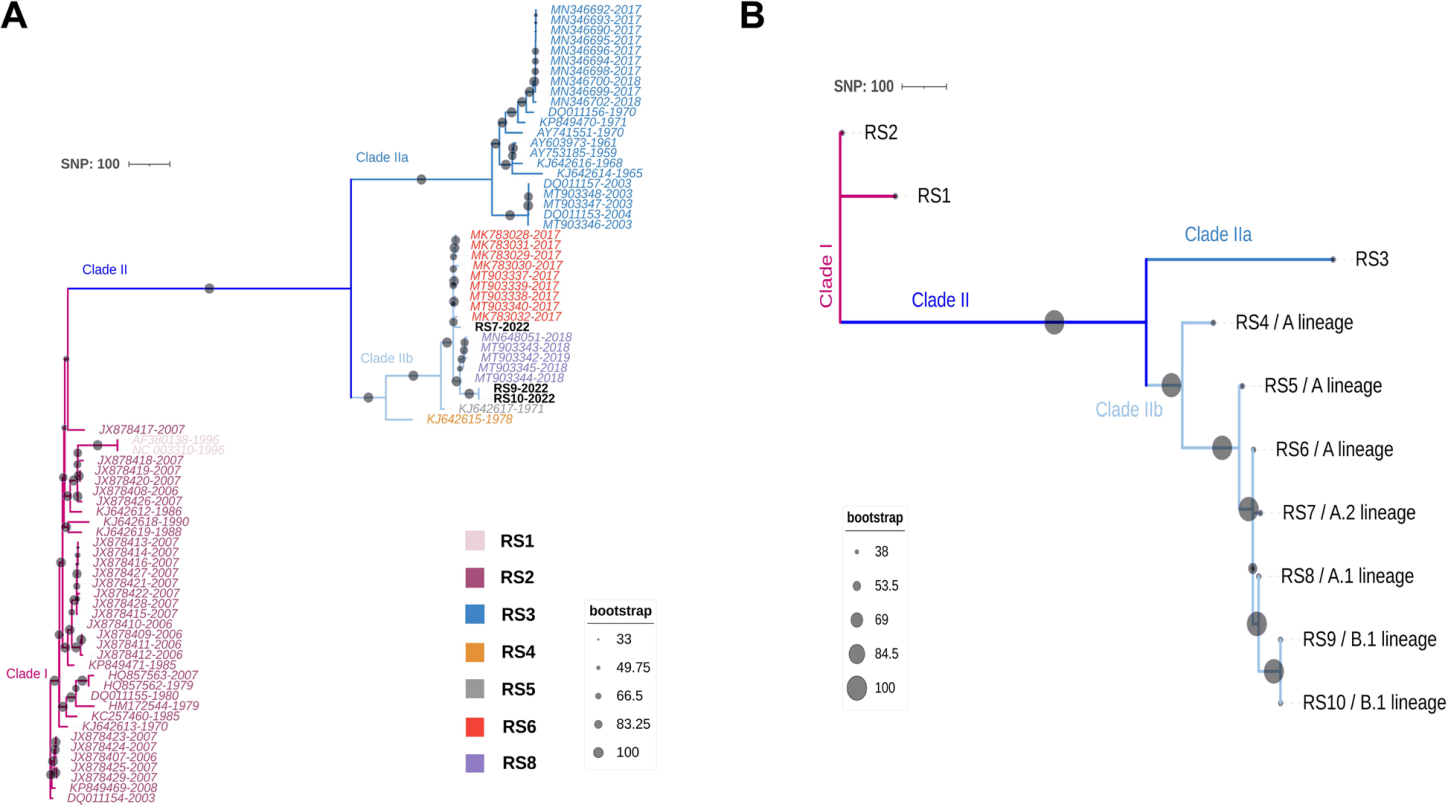
Phylogenetic tree of isolates and RSs. SNP are used as the length scale. **A**, Phylogenetic tree of all 212 isolates (isolates of A.2 (RS7) and B.1 (RS9, RS10) lineages were folded; colors represent the RSs to which the isolates belong); **B**, Phylogenetic tree based on 10 RSs (colors represent three clade classifications); lineages corresponding to the RSs in clade IIb are marked

The phylogenetic tree revealed the presence of 37 isolates of the Congo Basin strain (clade I) and 175 isolates of the West Africa strain (clades IIa and IIb). Despite some temporal overlap, it was evident that the Congo Basin and West African strains had undergone substantial divergence over time. Within the West African strain, there were two main branches: one including 22 strains belonging to clade IIa and the other encompassed 153 strains in clade IIb. Clade IIb showed a clear time gradient, with a close genetic relationship between the 2022 epidemic strains and those from to 2017–2019. The A.2 lineage was located between the 2017 and 2018 isolates, while the B.1 lineage displayed significant variation and differed by approximately 50 SNPs from the 2018–2019 epidemic strains. To gain a more detailed resolution, branches belonging to the B.1 and A.2 lineages were used to construct separate phylogenetic trees for greater resolution (Fig. 2A).

**Fig. 3:**
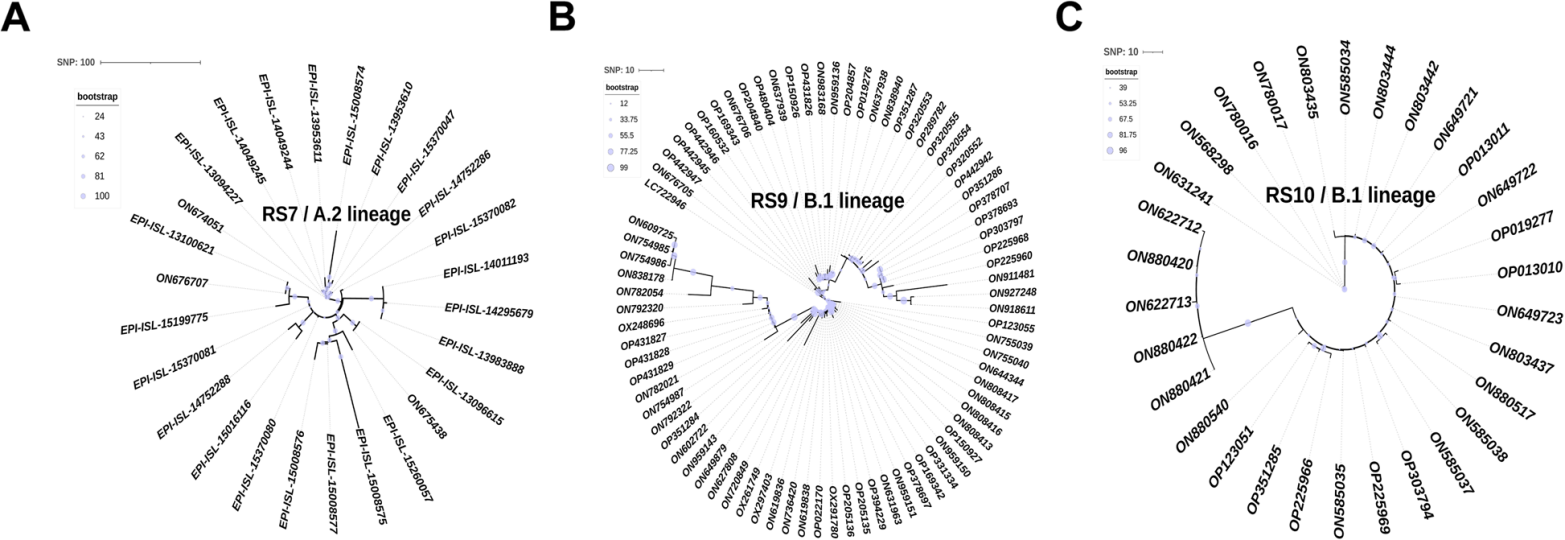
Phylogenetic trees of RS7 (**A**), RS9 (**B**), and RS10 (**C**); sequences of EPI ISL_15022589 and EPI ISL_14952916 in RS7 had too many ‘N’ bases and are not shown

In the Congo Basin branch, AF380138-1996 and NC003310-1996 were separate from other isolates and had identical genomic sequences. AF380138, representing the classic isolate Zaire-96-I-16 with well-established ORF borders and starting-ending sites [4], was designated as RS1. The remaining Congo Basin isolates formed RS2 (35 isolates), while RS3 (22 isolates) corresponded to the relatively independent clade IIa. Within clade IIb, early isolates KJ642615 and KJ642617 each occupying a separate branch, corresponding to RS4 and RS5, respectively. RS7 (28 isolates) was established independently based on A.2 lineage isolates. The B.1 lineage, which comprised over 50% of the included isolates, clustered together and constituted the two final branches. To minimize heterogeneity, RS9 (79 isolates) and RS10 (30 isolates) were evaluated separately. The neighboring branches of 2022 isolates led to the establishment of RS6 (nine isolates) and RS8 (five isolates) (Additional files 4, 5, 6, 7, 8, 9, 10, 11, 12 and 13). Among all RSs, RS7 and RS3 showed the greatest internal variation, with approximately 100 SNPs, whereas RS8 showed the least variation among internal isolates (Figs. 2A-B and 3A).

The internal relationships within RS7, 9, and 10 were evaluated separately. The genomic sequences of EPI ISL_15022589 and EPI ISL_14952916 from RS7 contained numerous ‘N’ bases and are not shown in the figure (Fig. 3A). The isolates of RS9 and 10 showed high homology, with approximately 60 SNPs separating the most distantly related ON91148 and ON9725 isolates in RS9 (Fig. 3B) and 50 SNPs separating the most distantly related isolates in RS10 (Fig. 3C).

### Homology of each RS with internal isolates

**Fig. 4:**
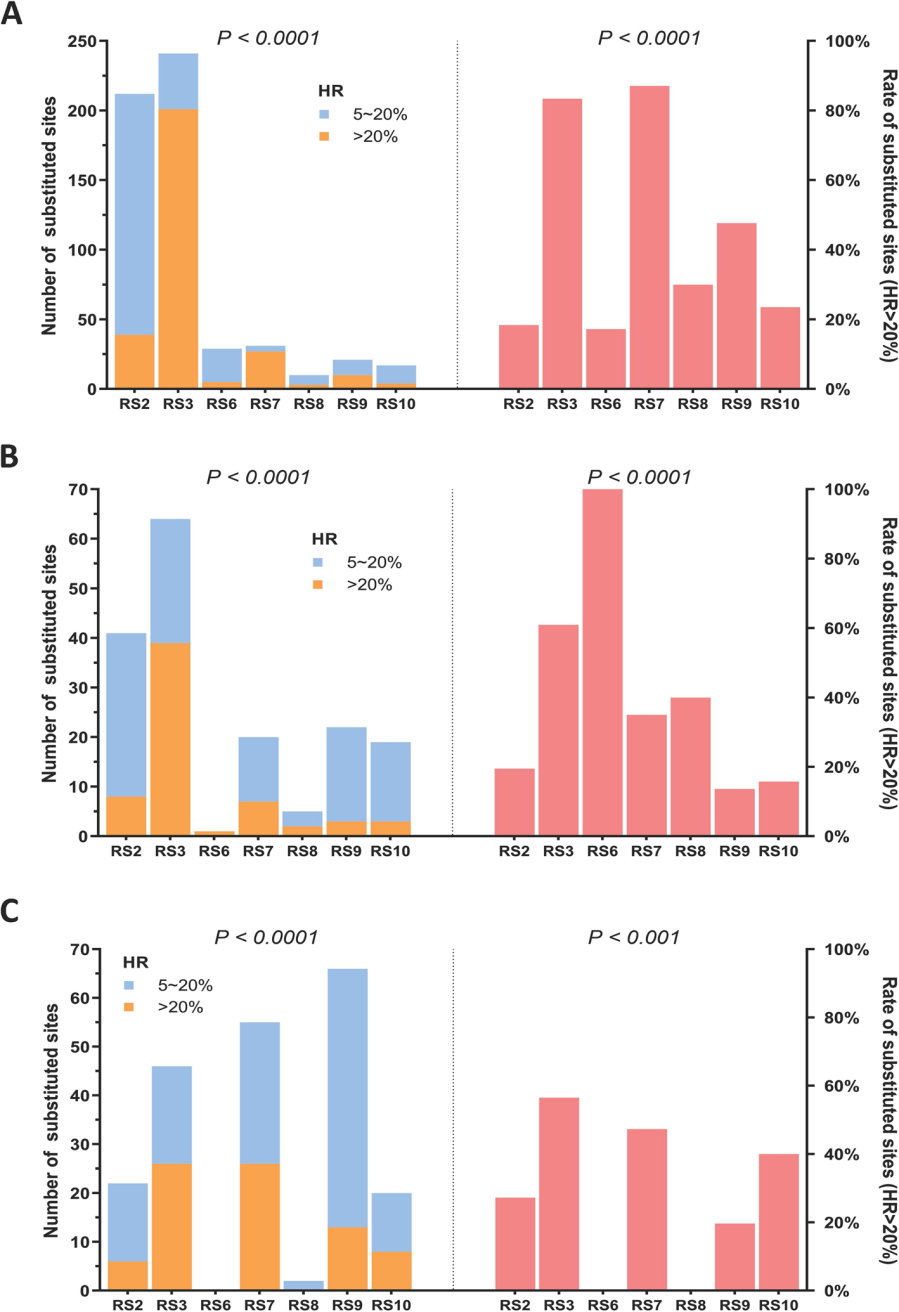
Homology based on the constructed RSs. The total number of sites and the proportion of mutant sites with HR > 20% for three substitution types: SNPs (both overall *P < 0.0001*) (**A**), insertions (both overall *P < 0.0001*) (**B**), and deletions (overall *P < 0.0001* and *P < 0.001*) (**C**)

A comparison of each RS with corresponding isolates showed few mutations and low HR values. The numbers of differential sites were 222 (RS2), 85 (RS3), 24 (RS6), 46 (RS7), 12 (RS8), 83 (RS9), and 41 (RS10) for 5% ≤ HR ≤ 20% and were 53 (RS2), 266 (RS3), 6 (RS6), 60 (RS7), 5 (RS8), 26 (RS9), and 15 (RS10) for HR > 20%. Few sites were in ORFs (Additional file 14). Furthermore, the RSs showed significant differences in the number of polymorphic sites and rate of polymorphic sites with HR > 20% for the three substitution types (SNP, insertion, and deletion) (rate of polymorphic sites with HR > 20%: *P* < 0.001 for deletions and *P* < 0.0001 for others). The numbers of SNP sites with HR > 20% were relatively low for RS7 (27 sites), RS8 (3 sites), RS9 (10 sites), and RS10 (4 sites). The ratios of SNP sites with an HR > 20% were significantly higher in RS3 (83.40%) and RS7 (87.10%) than in other lineages (Fig. 4A). A relatively lower number and proportion of sites with HR > 20% were observed in RS7 (7, 35.0%), RS8 (2, 40.0%), RS9 (3, 13.64%), and RS10 (3, 15.79%) (Fig. 4B). The total numbers of deletion sites in RS7 (55) and RS9 (56) were significantly higher than those in other lineages; however, the proportion with HR > 20% was only 19.7% in RS9. There were very few deletions in RS6 (0) and RS8 (2) (Fig. 4C). The overall intragroup heterogeneities in RS8, RS9, and RS10 were relatively low.

### Alignment of ORF and NCRs of the RSs

The start-stop sites and ORF lengths were compared for each RS (Additional file 15). D14L, D15L, D16L, and D17L in RS3-RS10 showed whole-segment deletions when compared against RS1. ORFs with length differences in RS3-RS10 (West African clade) are listed in Table 1, including four newly emerged regions that may have independent coding functions.

**Table 1:** The ORFs with length differences and the newly added regions between the RSs belonging to West African strains and RS1

ORF	Sequence Length								
	RS1	RS3	RS4	RS5	RS6	RS7	RS8	RS9	RS10
J1L	741	759							
D4L	252	253	255	254	254	254	254	254	254
D11L	462	471	477	474	468	468	468	468	468
D13L	948	951	951	951	946	946	946	951	951
O1L	1329	1328							
C2L	1128	1125					1126		
C9L	1464	1484	1459	1454	1449	1449	1449	1449	1449
C11L	1032			1033	1036	1036	1036	1036	1036
C12L	222			224	224	224	224	224	224
C14L	195					185	185		
H5R	642	633	633	633			633	633	633
H7R	441	435							
A10L	303	321							
A26L	228	227							
A28L	1563	1527	1533	1539	1530	1530	1530	1530	1530
A32L	234	237	237	237				237	237
A33R	429							438	438
A48R	255	112	155	146	146	146	146	129	129
A50R	1665	1667	1667	1667				1667	1667
B1R	213	2457	2462	2475	2476	2476	2476	2476	2476
B5R	1686		1677						
B7R	531							533	533
B10R	666	664	664						
B14R	981	978	1017	1049	1049	1013	1049	1017	1013
B16R	1059			1065				1071	1071
B17R	2382	2364	2364	2364				2364	2364
B18R	213	214	214	214				214	214
B21R	5640	5643	5643	5643			5643	5643	5643
N3R	531		530						
J1R	1764	1763	1763	1763			1763	1763	1763
J3R	741	759							
New 1					109	109	109		
New 2			402	402					
New 3				162					
New 4				297					

Unfilled blanks are the same length as RS1; Start-stop site of newly added regions: New1: RS6 (13,791–13,683), RS7 (13,412–13,304), RS8 (13,410–13,302); New2: RS4 (47,852–48,253), RS5 (47,716–48,117); New3: RS5 (121,932–122,093); New4: RS5 (169,825–170,121)

*Abbreviations*: *ORF *Open reading frame, *RS *Reference sequence

Relative to RS1, RS2-RS10 showed 1785 variant sites in 181 ORFs and 675 NCRs, including 1495 SNPs, 141 insertions, and 149 deletions. As both RS2 and RS1 belonged to the Congo Basin strain, only 172 sites differentiated the two. Most of the remaining variants showed general differences between the West African and Congo Basin strains. There were only six differences between RS9 and RS10, four of which were differences in the length of the insertion and two of which were deletions of short sequences only occurring in the NCR of RS10. RS9 and RS10 relative to RS1 differed at 1776 sites; among these, 1711 had appeared in RS3-RS7, 16 appeared in RS8, and 49 were unique to RS9 and 10 (Additional file 16).

### Screening of key mutation sites

To identify key mutations in the virus, only variants shared by RS9 (representing the B.1 lineage) and RS10 were examined. Unique variants in RS9 were selected as well as differences shared by RS5 or RS8 with RS9 and other RSs. A total of 44 nucleotide substitutions, including 28 in ORFs and 5 in NCRs, were unique to RS9. Additionally, 23 were non-synonymous substitutions, distributed in J1L (S105L), J2L (S54F), D9L (A423D), C3L (S36F), C9L (R48C), C15L (P78S), C18L (E125K), C19L (E353K), F8L (L108F), F9R (D56N), G9R (S30L, D88N), G10R (M142R), M4R (E162K), A19R (E62K, R243Q), A24R (S307L), A47R (H221Y), B21R (D209N, P722S, M1740I), J2R (S54F), and J3R (S105L) regions. Moreover, 13 nucleotide substitutions in 11 ORFs and three in NCRs were shared and specific to RS8 and RS9, among which nine were non-synonymous substitutions, distributed in G8L (D196N), L6R (S734L), H4L (H740Y), A11L (D98N), A14L (A17T), A19R (E435K), A24R (D100N), and B9R (R108I and L263F) (Table 2).

**Table 2:** Important variable regions and classification of recent monkeypox viruses

Type	Region	Nucleotide Site	RS1	RS5	RS8	RS9	AA Site	RS1	RS5	RS8	RS9	Class
**ORF**	**J2L**	2626	G			**A**	54	S			**F**	**I**
	**C9L**	32398	G			**A**	276	L				**I**
		33084	G			**A**	48	R			**C**	
	**C15L**	36486	G			**A**	78	P			**S**	**I**
	**A47R**	152326	C			**T**	221	H			**Y**	**I**
	**J1L**	1298	G			**A**	105	S			**L**	**II**
	**D9L**	14058	G			**T**	423	A			**D**	**II**
	**C3L**	27697	G			**A**	36	S			**F**	**II**
	**C18L**	39229	G			**A**	611	F				**II**
		40387	G			**A**	225	I				
		40689	C			**T**	125	E			**K**	
	**C19L**	41146	C			**T**	359	E				**II**
		41166	C			**T**	353	E			**K**	
	**F8L**	55158	G			**A**	518	V				**II**
		56390	G			**A**	108	L			**F**	
	**F9R**	56908	G			**A**	56	D			**N**	**II**
	**G9R**	75339	C			**T**	30	S			**L**	**II**
		75512	G			**A**	88	D			**N**	
	**G10R**	76478	G			**A**	142	M			**I**	**II**
	**M4R**	79656	G			**A**	162	E			**K**	**II**
	**A19R**	126413	G			**A**	62	E			**K**	**II**
		126957	G			**A**	243	R			**Q**	
		127532	G		**A**	**A**	435	E		**K**	**K**	
	**A24R**	130359	G		**A**	**A**	100	D		**N**	**N**	**II**
		130981	C			**T**	307	S			**L**	
	**B21R**	181670	G			**A**	209	D			**N**	**II**
		183209	C			**T**	722	P			**S**	
		186265	G			**A**	1740	M			**I**	
	**J2R**	194233	C			**T**	54	S			**F**	**II**
	**J3R**	195561	C			**T**	105	S			**L**	**II**
	**J3L**	3147	G			**A**	498	I	^a^	^a^	^a^	**III**
		3558	G			**A**	361	I				
		3854	C			**T**	263	D				
	**D3R**	7747	C			**T**	64	I				**III**
	**O1L**	23728	G			**A**	237	F				**III**
	**C1L** ^**b**^	25599	C		**T**	**T**	185	S				**III**
	**I7L**	66571	G			**A**	140	I				**III**
	**G8L** ^**b**^	74635	C		**T**	**T**	196	D		**N**	**N**	**III**
	**L6R** ^**b**^	83548	G			**A**	50	K				**III**
		84646	C			**T**	356	N				
		84724	G			**A**	442	T				
		85599	C		**T**	**T**	734	S		**L**	**L**	
		86860	C			**T**	1154	F				
	**H4L** ^**b**^	89503	G		**A**	**A**	740	H		**Y**	**Y**	**III**
		89570	G		**A**	**A**	717	F				
	**E3R**	97316	G			**A**	125	V				**III**
	**A11L** ^**b**^	121579	C		**T**	**T**	98	D		**N**	**N**	**III**
	**A14L** ^**b**^	123603	C		**T**	**T**	17	A		**T**	**T**	**III**
	**A45L** ^**b**^	150269	G		**A**	**A**	328	I				**III**
	**B5R** ^**b**^	164557	C		**T**	**T**	500	L				**III**
	**B9R** ^**b**^	167383	G		**T**	**T**	108	R		**I**	**I**	**III**
		167847	C		**T**	**T**	263	L		**F**	**F**	
	**B11R**	169968	G			**A**	75	R				**III**
	**J1R**	193005	G			**A**	263	D	^a^	^a^	^a^	**III**
		193301	C			**T**	361	I				
		193712	C			**T**	498	I				
	**B14R**	172992-172993	Add	(AT)_34_	(AT)_34_	**(AT)**_**18**_			^a^	^a^	^a^	**III**
**NCR**		15486	G			**A**						**II**
		153420	A			**C**						**II**
		157756	G			**A**						**II**
		177827	G			**A**						**II**
		135368-135369	Add	(T)_19_	(T)_18_	**(T)**_**17**_						**II**
		94010^**b**^	G		**A**	**A**						**III**
		187115^**b**^	C		**T**	**T**						**III**
		178879-178896^**b**^ (TATATACAT)_2_	Del		**✓**	**✓**						**III**

^a^Terminator appeared before the mutation; ^b^Mutation common and unique to RS8 and RS9; (AT)34: 34 cycles of AT, and similar formats have the same interpretation

*Abbreviation*: *RS *Reference sequence, *AA *Amino acid, *ORF *Open reading frame, *NCR *Non-coding region, *Del *Deletion

### Protein conformational diversity

ORFs with non-synonymous substitutions were used to simulate protein conformation, and the pLDDT and RMSD were evaluated. The models satisfying pLDDT > 70 were reliable and RMSD > 1 indicated a significant difference in conformations.

**Fig. 5:**
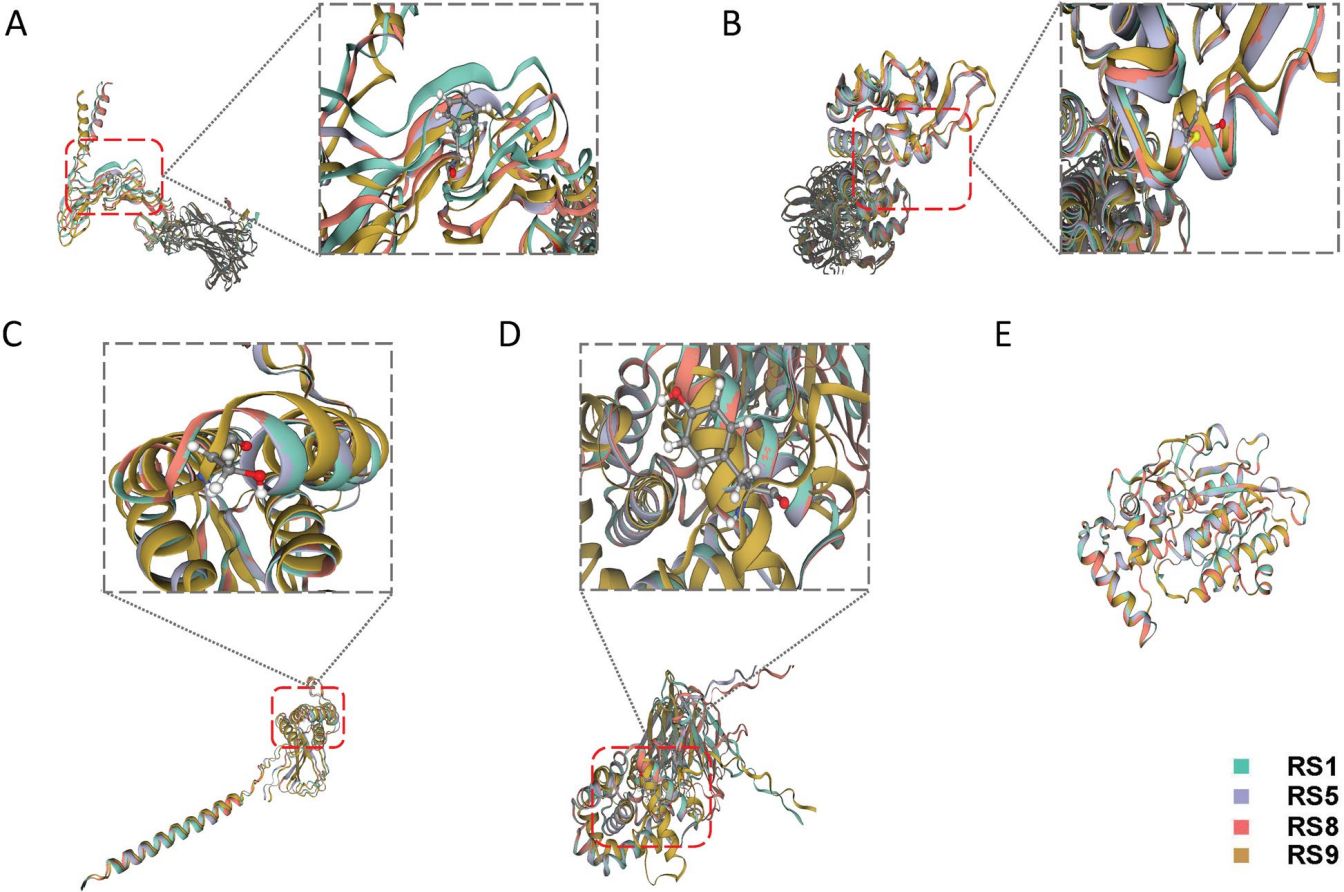
Protein conformation differences in the key ORFs of RS9. RS1 (green), RS5 (purple), RS8 (red), and RS9 (yellow) were compared. **A**, Compared with the conformation of J2L, the local structure of S54F RS9 mutant differed substantially. **B**, Compared with the conformation of C9L, the downstream structure of the R48C mutant differed significantly. **C**, The predicted protein conformation of C15L showed that RS9 was locally separated near P78S. **D**, Compared with the protein conformation of A47R, the difference of RS9 near H221Y was significant. **E**, The AA sequence of A45L was the identical for all RSs and served as a control

Four ORFs met the criteria, and all corresponding substitution sites were specific to RS9 (Table 3). The J2L protein of RS9 harbored a unique AA mutation, S54F (Fig. 5A). The protein structure of C9L in RS9 was an outlier downstream of that of R48C (Fig. 5B). Similarly, local regions with the most significant differences in C15L (Fig. 5C) and A47R (Fig. 5D) were specific AA mutations P78S and H221Y. To better show the magnitude of the differences, A45L with the same AA sequence and protein conformation of the RSs was selected as a control (Fig. 5E). Furthermore, no significant protein conformational differences were found in the shared and specific ORFs of RS9 and RS8 (Additional file 17). The complete pictures of the conformation model of the four key sites were shown in Additional file 18.

**Table 3:** Relevant parameters of protein conformation models for the key variable regions

ORF	pLDDT (RS9)	RMSD (RS9 vs. RS8)	Relevant References
J2L	86.1	1.093	[4]
C9L	90.6	2.375	[24]
C15L	78.7	1.572	[25]
A47R	70.2	3.475	[26]^a^
J1L	78.1	0.036	[27]
D9L	84.9	0.532	[28]
C3L	80.8	0.313	[29]
C18L	88.3	0.819	[30]^a^
C19L	92	0.088	[31]^a^
F8L	90.1	0.863	[32]
F9R	95	0.142	[33]^a^
G9R	89.4	0.601	[32]
G10R	84.5	0.1	[34]^a^
M4R	80.2	0.075	[35]
A19R	87.9	0.212	[36]^a^
A24R	89.3	0.122	[26]^a^
B21R	62.9	4.615	[37]
J2R	86.1	0.602	[38]^a^
J3R	78.1	0.035	None

^a^ORF has not been studied in monkeypox virus, and the literature is related to isoforms of other orthopoxviruses

*Abbreviations*: *ORF *Open reading frame, *RS *Reference sequence, pLDDT predicted local distance difference test, *RMSD *Root mean square deviation

### Classification of the key mutation sites

To preliminarily evaluate the core mutations influenced the biological characteristics of MPXV in 2022 and facilitate further research, the key mutations were divided into three grades (Class I–III), with a primary focus on class I mutations. Class I included specific non-synonymous mutations in RS9 with substantial differences in the corresponding protein conformation (RS9 vs. RS8). Class II included non-synonymous mutations unique to RS9, shared and unique non-synonymous mutations in RS8 and RS9 predicted to alter protein conformation, and unique mutations in the NCR of RS9. Class III included synonymous mutations unique to RS9 and shared and unique mutations in ORFs and NCRs of RS8 and RS9, without conformational differences. Finally, 4 ORFs were assigned to class I, 15 ORFs and 5 non-coding mutations were assigned to Class II, and 17 ORFs and 3 non-coding mutations were assigned to Class III (Table 2).

## Discussion

MPXV caused an unexpected global epidemic in 2022, leading to significant changes in its mode of transmission and clinical presentation [39].Understanding mutations in key gene functional sites of the strains responsible for this outbreak is crucial for various aspects, including origin tracing, molecular analyses of replication, transmission, pathogenicity, prediction of epidemic trends, and the identification of therapeutic targets.Understanding mutations in key gene functional sites of the strains responsible for this outbreak is crucial for various aspects, including origin tracing, molecular analyses of replication, transmission, pathogenicity, prediction of epidemic trends, and the identification of therapeutic targets.

Previous comparative genomic studies of MPXV have focused on the difference between West Africa and the Congo Basin strain [4, 40, 41], as well as the genetic variation in the 2022 pandemic strains [14, 21, 22], however, these studies all focused on strains isolated in a certain region. In this study, RSs were constructed based on a large sample size to more clearly reflect the evolution of MPXV genotypes. This method helped filter out less informative variants and minimized biases resulting from location, researcher, and sequencing errors, making it possible to identify loci associated with the virus’s biological characteristics. This approach has been successfully applied to construct RSs for different genotypes of Hepatitis B virus and SARS-CoV-2 [23, 42, 43].

The phylogenetic analysis showed clear differentiation between the West African and Congo Basin strains, with a particular focus on the West African lineage, especially clade IIb, which exhibited a high degree of relatedness. These finding results indicated that MPXV gained a few critical mutations in recent years. Constructed RSs showed high homology with their respective isolate sequences to the sequences of their isolates, with RS8 (5 isolates), RS9 (79 isolates), and RS10 (30 isolates) showing the lowest total numbers of substitutions and heterogeneity rates. Substitutions with HR values greater than 20% were primarily located outside of coding regions, confirming the reliability of the RSs for further comparative analyses. The focus of this analysis was on the B.1 lineage corresponding to RS9 and RS10 due to the sudden outbreak and rapid changes in MPX characteristics in 2022. [12]. Ten specific polymorphic sites common to RS9 were first screened. The B.1 lineage is now believed to have evolved from the A.1 lineage circulating in 2017–2019 [15, 44]. Ten mutations shared and specific to RS8 and RS9 were considered the secondary focus. Mutations that appeared in the early RSs were eliminated because they did not cause a pandemic. Through this process, 65 relatively important mutation sites were obtained by screening the RSs of recently circulating strains, most of which were SNPs (62 sites). Among the SNPs, there were 38 G-to-A and 23 C-to-T mutations, consistent with results published in 2022 [15, 22, 44], supporting the accuracy of the RSs. Moreover, the A.2 lineage with a low prevalence in 2022 [45] clustered between the 2017–2019 isolates in the phylogenetic tree and was considered a local recurrence of early strains. To further narrow the important sites, missense mutations were screened and proteins conformational models were obtained for the relevant ORFs, providing a basis for predicting mutations likely to affect protein structure and, consequently, the virus’s characteristics. The importance of variant sites was ranked by assignment to Classes I–III. Studies of variation in the NCRs of MPXV are limited; however, these regions may play regulatory roles. This study has played a filtering role in the accumulation of numerous mutations in the virus over different periods and across long evolutionary timescales. It helps provide strong clues for a deeper understanding of the virus’s characteristics and its association with the genome. Additionally, it sheds light on the sudden outbreak of monkeypox in recent years.

Meanwhile, most of the G-to-A and C-to-T mutations in MPXV were believed to be caused by the action of host Apolipoprotein B mRNA Editing Catalytic Polypeptide-like 3 (APOBEC3). Mutations mediated by APOBEC33 often do not completely destroy the virus but are more likely to generate hyper-mutated that alter viral features [15, 22].

Four variants were assigned to Class I: J2L (S54F), C9L (R48C), C15L (P78S), and A47R (H221Y). Due to the apparent increase in transmission capacity in the viral epidemiological characteristics, further research is needed to determine whether these mutation sites are involved in changes in the virus’s abilities such as cell entry, immune evasion, and replication. This will provide assistance for further epidemic prediction and drug development. The functions of ORFs in MPXV are not well-studied, suggesting that new variants may be key determinants of the epidemic. In limited studies, J2L was identified as an inverted terminal ORF, encoding a TNF binding protein [4]. It is speculated that new mutations in this region may enhance virus replication and spread through natural immune evasion mechanisms. C9L may reduce the stability of G-quadruplex (RG4), a non-canonical secondary structure of RNA [24]. Although the role of RG4 in MPXV remains unclear, RG4 can inhibit the expression and life cycle of proteins in other viruses [46]. Further studies are needed to determine whether the C9L mutation increases the self-replication ability by inhibiting RG4 function in MPXV. There is limited research on C15L and A47R in MPXV; however, some studies suggest that C15L may be a good epitope antigen for vaccine design [47]. Additionally, the C15 protein in Ectromelia virus, a member of the immunomodulatory protein B22 family, inhibits CD4 + T cell activation, and it may have a similar function in other orthopoxviruses [25]. Furthermore, A52R may block the activation of nuclear factor-B via Toll-like receptors (TLRs). It can also disrupt the formation of protein signaling complexes, such as interleukin-1 receptor associated kinase-2 and tumor necrosis factor receptor associated factor-6, thereby weakening the innate immune response [26]. If A47R has a similar function, its mutation might explain the reduced virulence, which is in line with the selection pressure under viral mutation. The protein conformation of B21R exhibited significant differences compared to RS9; however, it was not included in Class I variation owing to low confidence. Nevertheless, previous studies have found that B21R has high immunogenicity and multiple alternative targets to improve vaccine efficacy. In the development of a vaccine targeting this region, it’s important to take into account sites that are prone to mutation [37]. Among other secondary mutations, both F8L and G9R underwent mutations in the 2022 pandemic strains and are involved in the formation of the DNA replication complex (RC) [32]. Therefore, although the protein conformation models of F8L and G9R showed no obvious changes, slight alterations may have influenced RC formation, making it a continued focus for further research. It should be noted that RS7 corresponding to the A.2 lineage was intermediate between the 2017 strains and the 2018–2019 strains in a phylogenetic analysis, consistent with previous results [15]. This indirectly supports the presence of key mutations originating from the B.1 lineage.

This study provides targets for future research. However, it had limitations. Candidate sites were identified by a bioinformatics approach and were not verified experimentally. The classification of key sites only partially represents their importance. Additionally, the protein conformation models were specific to a single ORF, and mutations without significant structural changes may still influence the biological characteristics of the virus.

## Conclusion

Characterizing mutation profiles of the 2022 MPX epidemic strains is crucial for a deeper understanding the changes in virus characteristics. However, studies focusing on representative mutations that are expected to affect the function of corresponding proteins are limited. In this study, we categorized MPXV isolates into clusters by a phylogenetic analysis and established RSs to to highlight distinct mutations within each group. The characteristic mutation sites and types in the 2022 pandemic strains were screened and classified based on changes in amino acid sequences and protein conformation. Our findings provide insight into the molecular biological basis of the 2022 MPX epidemic.

### Supplementary Information

**Additional file 1.** Details of 954 MPXV isolates from the NCBI database.

**Additional file 2.** Details of 187 MPXV isolates from the NCBI database included in the phylogenetic tree analysis.

**Additional file 3.** The detailed information of 25 isolates downloaded from the GASAID database.

**Additional file 4. **Full length sequence of RS1.

**Additional file 5. **Full length sequence of RS2.

**Additional file 6. **Full length sequence of RS3.

**Additional file 7. **Full length sequence of RS4.

**Additional file 8. **Full length sequence of RS5.

**Additional file 9. **Full length sequence of RS6.

**Additional file 10. **Full length sequence of RS7.

**Additional file 11. **Full length sequence of RS8.

**Additional file 12. **Full length sequence of RS9.

**Additional file 13. **Full length sequence of RS10.

**Additional file 14.** Each sheet showed the heterogeneity within the artificially constructed RS.

**Additional file 15. **All aligned ORFs of RSs and their starting-ending sites and lengths.

**Additional file 16.** All of the nucleotide substitutions of RSs with RS1 as reference, including SNP, insertion (Add), deletion (Del) types.

**Additional file 17. **The parameters of protein conformation model in the key ORFs.

**Additional file 18.**

## Data Availability

All data relevant for interpretation of this study are presented in the article and Supplementary material. Any further information is available from the corresponding author on reasonable request.

## Abbreviations

MPX: Monkeypox
MPXV: Monkeypox virus
SNPs: Single nucleotide polymorphisms
RSs: Reference sequences
HR: Heterogeneity rate
ORF: Open reading frames
NCRs: Non-coding regions
AA: Amino acid
RMSD: Root mean square deviation
pLDDT: Predicted local distance difference test

## References

[1] Ullah A, Shahid FA, Haq MU, Tahir Ul Qamar M, Irfan M, Shaker B, Ahmad S, Alrumaihi F, Allemailem KS, Almatroudi A. An integrative reverse vaccinology, immunoinformatic, docking and simulation approaches towards designing of multi-epitopes based vaccine against monkeypox virus. J Biomol Struct Dyn. 2023;41:821-7834. 10.1080/07391102.2022.2125441 PMC9527787 36129135

[2] Bunge EM, Hoet B, Chen L, Lienert F, Weidenthaler H, Baer LR, Steffen R (2022). The changing epidemiology of human monkeypox-A potential threat? A systematic review. PLoS Negl Trop Dis.

[3] Singhal T, Kabra SK, Lodha R (2022). Monkeypox: a review. Indian J Pediatr.

[4] Shchelkunov SN, Totmenin AV, Safronov PF, Mikheev MV, Gutorov VV, Ryazankina OI, Petrov NA, Babkin IV, Uvarova EA, Sandakhchiev LS (2002). Analysis of the monkeypox virus genome. Virology.

[5] Ulaeto D, Agafonov A, Burchfield J, Carter L, Happi C, Jakob R, Krpelanova E, Kuppalli K, Lefkowitz EJ, Mauldin MR (2023). New nomenclature for mpox (monkeypox) and monkeypox virus clades. Lancet Infect Dis.

[6] Beer EM, Rao VB (2019). A systematic review of the epidemiology of human monkeypox outbreaks and implications for outbreak strategy. PLoS Negl Trop Dis.

[7] Likos AM, Sammons SA, Olson VA, Frace AM, Li Y, Olsen-Rasmussen M, Davidson W, Galloway R, Khristova ML, Reynolds MG, et al. A tale of two clades: monkeypox viruses. J Gen Virol. 2005;86:2661–72. 10.1099/vir.0.81215-0 16186219

[8] Yinka-Ogunleye A, Aruna O, Dalhat M, Ogoina D, McCollum A, Disu Y, Mamadu I, Akinpelu A, Ahmad A, Burga J (2019). Outbreak of human monkeypox in Nigeria in 2017-18: a clinical and epidemiological report. Lancet Infect Dis.

[9] Luna N, Ramírez AL, Muñoz M, Ballesteros N, Patiño LH, Castañeda SA, Bonilla-Aldana DK, Paniz-Mondolfi A, Ramírez JD (2022). Phylogenomic analysis of the monkeypox virus (MPXV) 2022 outbreak: emergence of a novel viral lineage?. Travel Med Infect Dis.

[10] Haider N, Guitian J, Simons D, Asogun D, Ansumana R, Honeyborne I, Velavan TP, Ntoumi F, Valdoleiros SR, Petersen E (2022). Increased outbreaks of monkeypox highlight gaps in actual disease burden in Sub-saharan Africa and in animal reservoirs. Int J Infect Dis.

[11] Branda F, Pierini M, Mazzoli S. Monkeypox: early estimation of basic reproduction number R0 in Europe. J Med Virol. 2023;95:e28270. 10.1002/jmv.28270 36319946

[12] Venkatesan P (2022). Global monkeypox outbreak. Lancet Infect Dis.

[13] Sah R, Mohanty A, Abdelaal A, Reda A, Rodriguez-Morales AJ, Henao-Martinez AF (2022). First monkeypox deaths outside Africa: no room for complacency. Ther Adv Infect Dis.

[14] Happi C, Adetifa I, Mbala P, Njouom R, Nakoune E, Happi A, Ndodo N, Ayansola O, Mboowa G, Bedford T (2022). Urgent need for a non-discriminatory and non-stigmatizing nomenclature for monkeypox virus. PLoS Biol.

[15] Isidro J, Borges V, Pinto M, Sobral D, Santos JD, Nunes A, Mixão V, Ferreira R, Santos D, Duarte S (2022). Phylogenomic characterization and signs of microevolution in the 2022 multi-country outbreak of monkeypox virus. Nat Med.

[16] Mukherjee AG, Wanjari UR, Kannampuzha S, Das S, Murali R, Namachivayam A, Renu K, Ramanathan G, Doss C. GP, Vellingiri B, et al. The pathophysiological and immunological background of the monkeypox virus infection: an update. J Med Virol. 2023;95:e28206. 10.1002/jmv.28206 36217803

[17] Chen N, Li G, Liszewski MK, Atkinson JP, Jahrling PB, Feng Z, Schriewer J, Buck C, Wang C, Lefkowitz EJ (2005). Virulence differences between monkeypox virus isolates from West Africa and the Congo basin. Virology.

[18] Estep RD, Messaoudi I, O’Connor MA, Li H, Sprague J, Barron A, Engelmann F, Yen B, Powers MF, Jones JM (2011). Deletion of the monkeypox virus inhibitor of complement enzymes locus impacts the adaptive immune response to monkeypox virus in a nonhuman primate model of Infection. J Virol.

[19] Guan H, Gul I, Xiao C, Ma S, Liang Y, Yu D, Liu Y, Liu H, Zhang CY, Li J, Qin P (2023). Emergence, phylogeography, and adaptive evolution of mpox virus. New Microbes New Infect.

[20] Rabaan AA, Alasiri NA, Aljeldah M, Alshukairiis AN, AlMusa Z, Alfouzan WA, Abuzaid AA, Alamri AA, Al-Afghani HM, Al-Baghli N (2023). An updated review on monkeypox viral disease: emphasis on genomic diversity. Biomedicines.

[21] Wang L, Shang J, Weng S, Aliyari SR, Ji C, Cheng G, Wu A. Genomic annotation and molecular evolution of monkeypox virus outbreak in 2022. J Med Virol. 2023;95:e28036. 10.1002/jmv.28036 PMC10087776 35906185

[22] Gigante CM, Korber B, Seabolt MH, Wilkins K, Davidson W, Rao AK, Zhao H, Smith TG, Hughes CM, Minhaj F, et al. Multiple lineages of monkeypox virus detected in the United States, 2021–2022. Science. 2022;378:560–5. 10.1126/science.add4153 PMC10258808 36264825

[23] Wang C, Liu Z, Chen Z, Huang X, Xu M, He T, Zhang Z (2020). The establishment of reference sequence for SARS-CoV-2 and variation analysis. J Med Virol.

[24] Dai Y, Teng X, Hu D, Zhang Q, Li J. A peculiar evolutionary feature of monkeypox virus. bioRxiv. 2022:2022.2006.2018.496696.

[25] Forsyth KS, Roy NH, Peauroi E, DeHaven BC, Wold ED, Hersperger AR, Burkhardt JK, Eisenlohr LC (2020). Ectromelia-encoded virulence factor C15 specifically inhibits antigen presentation to CD4 + T cells post peptide loading. PLoS Pathog.

[26] Shchelkunov SN, Sergeev AA, Titova KA, Pyankov SA, Starostina EV, Borgoyakova MB, Kisakova LA, Kisakov DN, Karpenko LI, Yakubitskiy SN (2022). Comparison of the effectiveness of transepidemal and intradermal immunization of mice with the vacinia virus. Acta Naturae.

[27] Jones JM, Messauodi I, Estep RD, Orzechowska B, Wong SW (2008). Monkeypox virus viral chemokine inhibitor (MPV vCCI), a potent inhibitor of rhesus macrophage inflammatory protein-1. Cytokine.

[28] Lum FM, Torres-Ruesta A, Tay MZ, Lin RTP, Lye DC, Rénia L, Ng LFP (2022). Monkeypox: disease epidemiology, host immunity and clinical interventions. Nat Rev Immunol.

[29] Subbaram K, Shaik Syed Ali P, Ali S (2022). Monkeypox: epidemiology, mode of transmission, clinical features, genetic clades and molecular properties. Eur Rev Med Pharmacol Sci.

[30] Zhang WH, Wilcock D, Smith GL (2000). Vaccinia virus F12L protein is required for actin tail formation, normal plaque size, and virulence. J Virol.

[31] Monticelli SR, Bryk P, Ward BM (2020). The molluscum contagiosum gene MC021L partially compensates for the loss of its vaccinia virus homolog, F13L. J Virol.

[32] Kannan SR, Sachdev S, Reddy AS, Kandasamy SL, Byrareddy SN, Lorson CL, Singh K (2022). Mutations in the monkeypox virus replication complex: potential contributing factors to the 2022 outbreak. J Autoimmun.

[33] Senkevich TG, Weisberg AS, Moss B (2000). Vaccinia virus E10R protein is associated with the membranes of intracellular mature virions and has a role in morphogenesis. Virology.

[34] Shchelkunov SN, Blinov VM, Totmenin AV, Marennikova SS, Kolykhalov AA, Frolov IV, Chizhikov VE, Gytorov VV, Gashikov PV, Belanov EF (1993). Nucleotide sequence analysis of variola virus HindIII M, L, I genome fragments. Virus Res.

[35] Brown JN, Estep RD, Lopez-Ferrer D, Brewer HM, Clauss TR, Manes NP, O’Connor M, Li H, Adkins JN, Wong SW, Smith RD (2010). Characterization of macaque pulmonary fluid proteome during monkeypox infection: dynamics of host response. Mol Cell Proteomics.

[36] Simpson DA, Condit RC (1994). The vaccinia virus A18R protein plays a role in viral transcription during both the early and the late phases of infection. J Virol.

[37] Hammarlund E, Lewis MW, Carter SV, Amanna I, Hansen SG, Strelow LI, Wong SW, Yoshihara P, Hanifin JM, Slifka MK (2005). Multiple diagnostic techniques identify previously vaccinated individuals with protective immunity against monkeypox. Nat Med.

[38] Hu F-Q, Smith CA, Pickup DJ (1994). Cowpox Virus contains two copies of an early gene encoding a Soluble secreted form of the type II TNF receptor. Virology.

[39] Reed KD, Melski JW, Graham MB, Regnery RL, Sotir MJ, Wegner MV, Kazmierczak JJ, Stratman EJ, Li Y, Fairley JA, et al. The detection of monkeypox in humans in the Western Hemisphere. N Engl J Med. 2004;350:342–50. 10.1056/NEJMoa032299 14736926

[40] Weaver JR, Isaacs SN (2008). Monkeypox virus and insights into its immunomodulatory proteins. Immunol Rev.

[41] Berthet N, Descorps-Declère S, Besombes C, Curaudeau M, Nkili Meyong AA, Selekon B, Labouba I, Gonofio EC, Ouilibona RS, Simo Tchetgna HD (2021). Genomic history of human monkey pox infections in the Central African Republic between 2001 and 2018. Sci Rep.

[42] Yu J, Sun S, Tang Q, Wang C, Yu L, Ren L, Li J, Zhang Z (2022). Establishing reference sequences for each clade of SARS-CoV-2 to provide a basis for virus variation and function research. J Med Virol.

[43] Cai Q, Zhu H, Zhang Y, Li X, Zhang Z (2016). Hepatitis B virus genotype A: design of reference sequences for sub-genotypes. Virus Genes.

[44] Khosravi E, Keikha M (2022). B.1 as a new human monkeypox sublineage that linked with the monkeypox virus (MPXV) 2022 outbreak – correspondence. Int J Surg.

[45] Gong Q, Wang C, Chuai X, Chiu S (2022). Monkeypox virus: a re-emergent threat to humans. Virol Sin.

[46] Métifiot M, Amrane S, Litvak S, Andreola ML (2014). G-quadruplexes in viruses: function and potential therapeutic applications. Nucleic Acids Res.

[47] Swetha RG, Basu S, Ramaiah S, Anbarasu A (2022). Multi-epitope vaccine for monkeypox using pan-genome and reverse vaccinology approaches. Viruses.

