# Supplementary material for: Exploring the key genomic variation in monkeypox virus during the 2022 outbreak
Source: BMC Genom Data. 2023 Nov 16;24:67. doi: 10.1186/s12863-023-01171-0 (PMC10652487; doi:10.1186/s12863-023-01171-0)
Supplement: Supplementary file 4 — Additional file 4. Full length sequence of RS1. [file 12863_2023_1171_MOESM4_ESM.docx]

**Additional file4. Full length sequence of RS1.**

GTTAGTAAATTATATACATAATTTTATAATTAATTTAATTTTACTATTTTATTTAGTGTCTAGAAAAAAA

TGTGTGACCCACGACCGTAGGAAACTCTAGAGGGTAAGAAAAATCAATCGTTTATAGAGACCATCAGAAA

GAGGTTTAATATTTTTGTGAGACCCATCGAAGAGAGAAAGGATAAAAACTTTTTACGACTCCATCAGAAA

GAGGTTTAATATTTTTGTGAGACCCATCGACGAGAGAAAGAGATGGTTAGTCAAGATATTTTTCTTAGTA

CAAAAGTCAATGTTTTAAAATATATGGACGAGAATTAATTTGTCTGTATAAAAACTTGTGTGAAATTATG

TACTAGAGAAAAAACGTGAGCAGTGTCCCCTACATGGATTTTACAGATCATTTATATTCCAAAAATATTA

ACTATATACGTTTATTATATGATGTTAACGTGTAAATTATAAACATTATTTTATGATGCAATTGTCTGAC

AACCTAGATTGGCATAAGGATATTGATAAGCTCTACGAGAATATATTGTTGGACGTTATCGTTTACGAAA

TAGTTGAGACATCAGAAAGAGGTTTAATATTTTTGTGAGACCATCGAAGAGAGAAAGAGAATAAAAATAT

TTTTTTGTAAAACTTTTTATGAGACAAGAGAGAAAGAGAATACGAATAGTGATCATATCGTATCACATAT

TGAAACAGAAAGAAGAAGTAACGAGAGGTAACTTTTTGTGAATGTAGTTAAACATTTTTGTTTTGCAAAC

CGGAATATAGTACCCGGTACACTTTTTTAATTCGTGGTGCGGTGTCTGAATCGTTCGATTAACCCAACTC

ATCCATTTTCAGATGAATAGAGTTATCGATTCAGACACATGCTTTGAGTTTTGTTGAATCGATGAGTGAA

GTATCATCGGTTGCACCTTCAGATGCCGATCCGTCGACATACTTGAATCCATCCTTGACTTCAAGTTCAG

ATGATTCCTCACACATGTCTCCGATACGTACGCTAAACTCTAGGTTCTTAACACATTTTGTATCAACGAT

CGTTGAACCGATGATATCTTTGTAACTCACTTTCTTATGTGAGATGTTAGACCCAAGTACTGGATGGGTC

TTGATGTCGCTGTCTTTCTCTTCTTCGCTACATCTGATGTCGATAGACATCTCACAGTCTTTGATCATAG

CCAGAGCTTCTTCACGCGTGATCGCGGGAGAGTCCTTACCTTGTCCTGGTGACACGCTGGACAATCTAGT

ATTCACAGTGTTTCCATCAGAGGATTCGGAGATGGATGAAATCTTTGGGCATTTGGTGAATCCAAAGTTC

ATGTTAAGACCCGCGCCGACGATAGTGTAATAAGTGGTGGGATCTCCTTTTACAACTTCTTCGGATACCT

CATCATCTTCGGTCTCTGTAACTTCCGTTACGGATTGACAAATCTTATCATTGGTCGGTGTTTGGTCTTG

CTTTGTGACTTTGATAATAACATCGATTCCCATATGATGTTTGTTTTCTTCTTCAGTACACGAGGATGAG

GATTGTTGAAGACTAGTAGGCATAGCAGCTGCCACTAGGCACATGCATGCCAGGACAATATATTGTTTCA

TGATTGCTATTGATTGATTACTGTTCTAGATGATTCTACTTTCTTACCATATAATAAATTAGAATATATT

TTCTACTTTTACGAGAAATTAATTATTGTATTTATTATTTATGGGTAAAAAAAAACTTACTATAAGTGGG

TGGGATTCTGGGAATTAGTGATCAGTTTATGTATATCGCAACTAGCGGGCATATGGCTATTGACATCGAG

AACATTACCCATATGATAAGAGATTGTATCCGTTTCGTAGTCTTGAGTATTGGTATTACTATATAGTATG

TAGATGTCGACGCTAGATAGACAGTCGCCCACTAGAGTTACCGTCTCTGAATGCGGCATGATAGTATCAT

TCTTTGTTTTCGTTAACTGTTTGGAAGATGAATCTTTGTTGTTACATTTAATCTCGAAATTCAGAGTACA

TATCTTTGAAGTATTCTGATATCTATTTTCTCCTGTAAAGAATCCTGAAGTTGCTACATTATTAAGGACA

GAGAAGTATTCTGCACGAAAGACGGGATCACAATCTTTATGATTCATGGTAATAGTTAGTTCCGACGTTG

AGATGGATTCGCTGAGACCGGTAGTGGTCGTCCGAGTACACGATGTGTCGTTGACTGGATACAGGTTAAT

TTCCACATCGATATAGTTAAATGTATTGCTGGTTACGGGTTCGCATTTATCTGTGGAAGAGACGGTGTGA

GAATATGTTCCGGGACCACACGGAGAACAGATGACGTCTCCGGTAGACGTGTATCCGGATACTCCGTATC

CTATTCCACACTTTGTTTTAGAAATACATGTTCTACACCCTGATGATCCTTTGAGAAGACAATAATATCC

TGGAGAGCATTCACAGATTCTATTGTGAGTCGTGTTACACGATCGCGTCTCTACCTGATTACTATCACAT

CTTCCGTTACAACTTAGACAAGCCTGTAAATGATTATTGTGAGATGTAAAGGTATCCGAACCACACGGCG

TACATTGTGTATTAGTCTTGCTATCACATAATCTGGAAGCGTAAGTTCCCGGAGGACACGATAGACAACA

TAGATTACGGCTTCTGTATTCGTTGTCTTTACATTTTCCATTGGATGGTGCATGTGGTGCTAAATCTCTT

CCGTTTATTATTATACATGAGAGAAACAATATATACGAGTATAATACGGACCTCATGATTTAATAATAGT

AGTAATCGTCGTCTTGTTACTGTTTGTTTCCTACTTCTCCAATCATATAGATTATTTTTTAAATATTTTC

TTTCTATCATGGATAATATTTGTAATGGTTCTTTCCGTACAACATACTGTTTAGATGGTAGTCGCTTAGC

TTGGTTATGATATTGCGCATAATTTCCGGAGGCAAATACGATAGTCTAGATTGACTATCGATGGTAGACT

CTAATTTATTGAGTGCTTTGTCGACGAGTTTACTTTTATGCTCCATCGATAGATGACACTGTTCTATGAG

ATCGTCGTACATGGGAAATGAAATGCGTTTGTCTGAATGTATGGCTTCGAGATAGGTGTGATACCGGATG

TCTTCTGTTCTCAATACCGTATACAAGTTGGTGTCTGAGATTCGAATCTCTTTGAGGAGACTTATGTCAC

GACTACATTTTTCGATGATGGAATCTATCTTATCGAATGATATATTTTTCATAAATACACTTTTATAGTC

CTCGTTTAAACAGAATTTAGTATGTAGTTCCGCAAATGACTCGTCCCTTAATAGGCAGTAGGCTATTATC

TTCTTTACGTAGTGATCGTCGTAGGGAGAGAACTCCGACATCTTGTAGAACAACGATTTAATCATAGGTA

GAGATACTTTCAGTCTGTGGTGGATGATGTCATTAACGACATCCGCCTTGTATATGATGTTTCTGTTTTC

AAACACCAAGTCGAATACTGTCTTATCGTCTTTAGTCGGAAGGTTGATGTCGTATCCGATGTATACGAGG

TATGAGGCAACATTGTTATTGCAATTCTGGAAGGCGGTATGAAGAGGAGTCATTGTATTATAGTATTCGT

CTTTCTGAATGTCGAATCTATCTAGTAGATACCGTAGTATATTGAGAGAGCGACTTCCATATCCTTGATT

ATGTTTTATGAATAGATAAAGTAGATGTTGTCCTTCTTCCTTTTGTAATTTCCCGTATTTTTGTTCGTGC

CAATTGAGTAACATTATGAGAATATGACCTGTTGCACAATCGTTCTTTATGTATTCCATGATGGGTGTAC

AATCAAGATTATTACGTATCCTCGTATCGGCTCCTCGAGATAAAAGAGCATACACCACACGAGGACTATG

TTTGGTATACTGTTGAAGGTAAGTGTGTAACGGCGTATTTCCGATTTTCGTAACCGCGTTAATGTTTGCT

CCATGATCTATTATCGCGTAGATGAATCGCTTCTCAGCTCGCATCTTAGTGTGACTCTTTGACTTGTAAT

AATTGCTTTCGTGGAACACGGATATGTGTTTACAGTAGTAATGAAGAGAAGTGAGTCCATCCTCATCGAC

GCAATTAGGGTCAGATCCTTTAGTCAATAATTTGTACAGAACGTAATAGTTTAAGCTCCCATTGAATTTA

TATCTAAGATAACACAGCAATAGATCGGATGATTTACTAAAGTCATCAATGGGGTCCGTTAGTATATCAA

AGATCTTGTTATCGATTGATAGTGAATGAATCAGATAGTGGTGTAGAGGAATATGTCCTTTTTTATCCTT

GCTATCAAAGTTACGCATGCCGTGGCGTAACAATATCTTTAATACAGATGGATTAAATCGTGTATTCATC

GTATAGCAATGTAATGGAGAGTTACCACATTTTAGTCGTTTATTCAGATCGCAGTGTTTAATAACTAATT

TAAACAGATGAGATGATATATCCACATCAAAGAATGCGAGATACATATGACAGACATTATTGACAGAAAT

GTGACCTTCATTATCACCGTCGTCCATAAATGCGTTAGGTACGTACCACATACTATCGTTAACGATGCGC

ACAATCTCGTCCATTTTCATCCATCTTCATAATGATTTACTTTTTCATAATTAGAGAAAAAGATCAAGGT

ATAAAAATTAGAAGTGTTAGACTATAAACTAACTTATAAAACTAACTTATGACTTAACTAACTTATGACT

ATTAACTCATTTCAAGAAAGGTGGGTGGAGAGAACTCTATATGACAGCTTGTGAAACAATTAGATCCCTA

ATTTCTAATGGAAGTTTTGATAGGAGATTGTCATCAGTTGATACATTGTTTATTATCTCATCTATTAGAG

CACGTCTGTTTAGAGCTTTAGTGACCTGCTCGGTTACTTCTGTGTAAATCTTGAATCCTTTAGTGATACA

CTGTGTCAAAACTGGATGTTTAGAATACCTATGTAGAATATGGGAAGCATGCTTGTTTTTGTCTCTATTA

TAGATTAACTCATACATGGTTGTATTATGAATTTTCATCTGCCTAATGTACTCCAATTCTTGTTTACAAT

CAATTATATAATCAAAGAGTGATGATGCATACACATTACAAAGTGAATAATCTACCATCATAAAATACTT

GATACAGAGCTTTATCACATCATGGTTTTCAATTGTATTATTAAGTATAGCTAATTTTATACAGTCAATA

GACAATGGTTCTCTAAGCAATATTTCTAATATTTTAAGATGTGTTTCCCTACGGGCGATGACAGATCCCC

TATCCACGGCCACGTCAAGACATGTATATCCATTACTCATTACTGCGTTGACATTTGCTCCATTTTCTAA

TAGCCATGATACTAAATCTATATAACCTGCATAGATAGCGCGATAAAGCAAGGTCCTTCCACCAGCATCT

AGTTGATTGATATCTTCAATATATGGGATACAAAGCTTATAAATTTCTAATACTGTGGGTTCATCTACAA

GGAATCCCCTAGTATACTGAATTATTTTATATAGATCTAATTTAACATCATTTTCATCTGGGATACCACA

ATTCAAAATAAACTCAACAACACTACTTTCCTTTTTACATATTCCCCTAAAATAGGCATTCAAGCATTCT

ATTTTATATATTACAGCCCCATGATCTACCATAAAATCAACAATGTCTATTTCTACATATGCATTAGATA

GATAGTAAAGTAAGAGATCTTGTACAGAATTACAATTCTTAATAATTATAGAGAAAATATCTTCCATATA

ATTCTTTGACACTAATGCAGATATAATATCTTTATATGTAATATATGCAAACAGTCTATCTACTATATAC

TGATCAATATTATCTCTATGAATCCTAAAATAATCATACAGAACATCTACAGGATCACAAATTGGTTCAA

GGAGAAATCTATCAAATATTTTCCTGTCAACAACTGGTTCTAGAACATAACAGTCAACACCTAATCCATG

TTTTTTATAGTCATCTACCAAAGATAATGACCAAAGATCGAGGTCGTCGTGAAACTGCTCATCGACAGCC

ATGAAATCTGCCGACTCCATGGCGCGAATCGCACTGTCTTATTCGCCATTGATTTTCATTTTTTATAATT

ATGTACATGTTTTCCTTCTATTCTCAAGAGTCTACAAAAATATATTTTTTTTCGATATCTAAGTACTAAG

TTTTTTTACTGTTTTTGTTACTGTCTTCCATTCTTCTAACTAAAGATCTGAGATAAATTATACAATCTTC

GCTATCGAACCATTTTTGTAGTCTAAAGCCTGAAGTAATTAACCAACTGTTTTTATTAGTGGCTTTTTTC

GATCTATCCTCGTCCTCATCATCCTTATATTATTATCATTATTATCATAGTCTATTAAACACAAATCATC

TACGTTTATAACAACATTCTCATTATTAATTAGTTCTGTAGTAATATCTTTAATAATTTGGCTATACATC

TGTTCAATACTATCTATTGATGATTTCTTTTTTAAGACTTAAACTAGTTATGGTAATGACGATGAAATCG

AGTAGTAACTTCTAATAAAGACTTGATATCATTATCATATGTTTGATCGTCATAGTTAATAGTGTGGCTA

AATGGTACTGTTAATAAGTTTATAGACAATATCATAGTATTTTCTTTCCAGAATTAGATTATTTTTTTAA

ATACTGATCCTCACAATTCCGTGATGTAGCAGTAGTTGGTGCATGGTCTATATCGTTAAAATGTATCATA

TATAATAGTTTTCTGACGTGGAGTACAGAATTTTCGATTAATGAGTTCATGGTAAGGAAGGGCAAATGTC

TGTATATAATATACATAAGTTAATAGTTTTTTATCATATTTTCTAATACCATAATAAAAATTATCATTAT

GTATAATCATCACTGTCGCTATCATTATTGCGTTTGTGTAGTTCTGCCCTATCATCTACATCACTGTCAC

TCTCACTATATCTTCTAAAATTACAAACAACTGGATATTCGATAACAGCATTTGTGTAGTTTTTGTCTTT

TACAGTATATACGTTATTGTCAAAATCTAAACAAATATTAGCATAATACATCTATAAGATCAGGATCCAT

GTTCGAGCATACTAGCCATGTATATTTGTAACTTCGTCATACAGCGTTAGATCAATAGAATAAACAATCG

TGTGACGCAACTTTTTTACGATCTAGTTGTATGAGTTTATCGTTTACATAAGCAATTAACGGCTTTAACA

GATGATCTGAGTAATAATATACCTCTGTTATACGTTTAATGTTCACGGTCTTAGTATTTTTAGATATCAA

TTGTGATTTACACCATATTCGACTCCCTGTGTGCAACGTTAGAAATTCTAAATCTATAGTATTATCTATT

ACAGCGTAAAACACATTCAATATTGTATTGTTATTTTTATATTATTTACACAATTAACAATGTATTATTA

GTTTATATTACTGAATTAATAATATAAAATTCCCAATCTTGTCATAAACACAAAATCCATTAAAAATGTC

GATAAAATATCTGATGTTGTTGTTCGCTGCTATGATAATCAGATCATTAGCCGATAGTGGTAACGCTATC

GAAACAACATCGCCAGAAATTACAAACGCTACAACAGATATTCCAGCTATCAGATTATGTGGTCCAGAGG

GAGATGGATATTGTTTACACGGTGACTGTATCCACGCTAGAGATATCGACGGTATGTATTGTAGATGCTC

TCATGGTTATACAGGCATTAGATGTCAGCATGTAGTATTAGTAGACTATCAACGTTCAGGAAAACCAGAC

ACTACAACATCATATATCCCATCTCTAGGTATTGTGCTTGTATTAGTAGGCATTATTATTACGTGTTGTC

TATTATCTGTTTATAGGTTCACTCGAAGAACTAAACTACCTATACAAGATATGGTTGTGTTATATTTTTT

ATAAAATTTTTTTATGAGTATTTTTACAAAAATGTATATGTATAAAAAAATACTAAGTATGCGATGTATC

CTGTATTATTTGTATTTATCTAAACAATACTTCTGCCTCTAGATGGGATACAAAAATTTTTTATTTCAGC

ATATTAAAGTAAAATCTAGTTACCTTGAAAATGAATACAGTGGGTGGTTCCGTATCACCAGTAAGAACAT

AATAGTCGAATACAGTATCCGATTGAGATTTTGCATACAATACTAGTCTAGAAAGAAATTTATAATCATC

TTCTGTGACAGGAGTCCATATATCTGTATCATCGTCTAGTTTATCAGTGTCCTATGATATATTCCTGTTA

TCATCATTAGTTAATGAAAATAACTCTCGTGCTTCAGAAAAGTCAAATATTGTATCCATACATCTCCAAA

ACTATCACTTATACGTTTATCTTTAACGAACATATACCTGATGGTTATTTACTAACAGACATTTTTCAAG

ATCTATTGACAATAACTCCTATAGTTTCCACATCAACCAAGTAATGATCATCTATTGTTATATAACAATA

ACATAACTCTTTTCCATTTTTATCAGTATATATATATATATATATATATCAACGTCGTTGTAGTGAATAG

TAGTCATTGATCTATTATATGAAACGGATATGTCTAGTTAATATTTTCTTTGATTTAAAGTCTAGAGTCT

TTACAAACATAATATCCTTATCCGACTTTATATTTCCTGTAGGGTGGCATAATTTTATTCTGCCTCCACA

ATCAGTGTTTCCAAATATATTACTAGACAATATTCCATATGGTTATTAGTTAAGGGACCCGATTAGAACA

CGTACGCGCTTATTCATCATTTGGATCGTATTTCATAAAAGTTATTATGTTATCGATGTCAACACATTCT

ACATTTTTTAATCGTCTATATAGTATTTTTCTGATATTTTCTATAATATCAGAATTGTCTTCCATAGGAA

GTTGTATACTATCGGAATCAGTTACATGTTTAAATAATTCTCTGATGTCATTCCTTATACAATCAAATTC

ATTATTAAACAGTTTAATAGTCTGTAGACCTTTATCGTCGTACATATCCATTGTCTTATTAGTTACGCTT

ATTTTTATGGGTTTTACATTGCTTTATTATATTTTATAAGAATGATTGTTTGACAATGTCGTAGTATAGA

TATATTATTAGAGGAGGTATAATTATAAAAAGTTTCTGAGTACGATGTTATAAGAGGAGAGGACACATTA

ACAATCATACATCAATTAACTCATTCTTATAACATTGTAATCAAAAGAATTGCAATTTTGATGTATAACA

ACTGTCAATGGAATTGTATATTACAAATTACGGTATGTTGTAACGACAAATACCGATCGGTAATTGTCTC

TGTCGCTGTAATAGAATTAATTATATATCTATTACACCGACCTTGTATCATAATAAAGTTGTGGTAGTAT

GATCTCCATATTTATAATTTAGTACTTTGTATTTAGTATTTTTGGAATCATAAAAAAGTTTTACTAATTT

AAAATTTAAAAAGTATTTACATTTTTTTCACTGTTTAGTCGCGGATATGGAATTCGATCCTGTCAAAATC

AATACATCATCTATAGATCATGTAACAATATTACAATACATAGATGAACCAAATGATATAAGACTAACAG

TATGCATTATCCGAAATATTAATAACATTACATATTATATCAATATCACAAAAATAAATCCACATTTGGC

TAATCGATTTCGGGCTTGGAAAAAACGTATCGCCGGAAGGGACTATATGACTAACTTATCTAGAGATACA

GGAATACAACAATCAAAACTTACTGAAACTATACGTAACTGTCAAAAAAATAAAAACATATATGGTCTAT

ATATACACTACAATTTAGTTATTAATGTGGTTATTGATTGGATAACCGATGTGATTGTTCAATCAATATT

AAGAGGGTTGGTAAATTGGTACATAGCTAATAATACATATACTCCAAATACACCCAATAATACTACAACC

ATTTCTGAGTTGGATATCATCAAAATACTGGATAAATACGAGGACATGTATAGAGTAAGTAAAGAAAAAG

AATGTGGAATTTGCTATGAAGTTGTTTACTCAAAACGATTAGAAAACGATAGATACTTTGGTTTATTGGA

TTCGTGTAATCATATATTTTGCATAACATGTATCAATATATGGCATAGAACACGAAGAGAAACCGGTGCG

TTAGATAATTGTCCTATATGCCGTACCCGTTTTAGAAAAATAACAATGAGCAAGTTCTATAAGCTAGTTA

ACTAATAAATAAAAAGTTTAATTATCGACGATATATGTCGTTATTTTTCTCTCATATGAAAGATTAATTT

GATTCTAATATAATCTTCAGTATTGGATGAATCTCAATTCAAATTAATTCCATTAGATTAGATTAGATTA

GATTAGATTAGATTAGATTAGATTAGATTAGATTAGATTAGATTAGATTAGATTAGATTAGATTAGATCA

TAAATAAAAATAGTAGCACGCACTACTTCAGCCAAATATTCTTTTTTGAAACGCCATCTAGCGTAATGAG

AACACAAGTGAACCTATAATGAGCAAATTTATTAGTATCGGTTACATGAAGGACTTTACGTAGAGTGGTG

ATTCCTCCATCTGTGGTACGAACGGTTTCATCTTCTTTGATGCCATCACCCAGATGTTCTATAAACTTGG

TATCCTCGTCCGATTTCATATCATTTGCCAACCAATACATATAGCTAAACCCAGGCATACGTTCCACACA

TCCGGAACAATGAAATTCTCCAGAAGATGTTACAATGGCTAGATTTGGACATTTGGTTTCAACCGCGTTA

ACATATGAGTGAACACACCCATACATGAAAGCGATGAGAAATAGGATTCTCATCTTGCCAAAATATCACT

ATAAAAAATTTATTTATCAATTTTAAAGGTATAAAAAAATACTTATTGTTGCTCGAATATTTTGTATTTG

ATGGTATACGGAAGATTAGAAATGTAGGTATTATCATCAACTGATTCTATGATGGTTTTATGAATTTTAT

TATGCTTCACTATTGCATCGGAAATAATATCATATGCTTCCACGTATATTTTATTTTGTTTTGACTCATA

ATACGCACGTATTTCTGGATTATTGGCATATCGATGAATAATTTTAGCTCCATGCTCAGTAAATATTAAT

GAGAACATAGTGTTGCCTCCTACCATTATTTTTTTCATCTCATTCAATTCTTGATTGCAGAGATCTATAT

AATCATTATAGCGTTGACTTATGGACTCTGGAATCTTAGACGATGTACAGTCATCTATAATCATGGTATA

TTTAATACATTGTTTTATAGCATAGGCATTATCTACGATATTAGATACTTCACTCAATGAATCAATCACA

CAATCTAATGTAGGTTTATGACATAATAGCATTTTCAGCAGTTCAATGTTTCTAGATTCGTTGATGGCAA

TGGCTATACATGTATATCCGTTATTTGATCTAATGTTGACATCTGAACCGGATTCTAGCAGTAAAGATAC

TAGAGATTGTTTATTATATCTAACAGCCTTGTGAAGAAGTGTTTCTCCTCGTTTGTCAATCATGTTAATG

TCTTTAAGATAAGGTAGGCAAATGTTTATAGTACTAAGAATTGGGCAAGTATAAGACATGTCACAAAGAC

CCTTTTTGTATGTATAAGTGTAGAAATTATAACATCCATAGTTGGATTCACATAGGTGTCCAATCGGGAT

CTCTCCATCATCGAGATGATTGACGGCATCTCCCCCTTCCTTTTTTAGTAGATATTTCATCGTGTAAGAA

TCAATATTAATATTTCTAAAGTATCTGTGTATAGCCTCTTTATTTACCACAGCTCCATATTCCAACATGC

ATTCCACTAGAGGGATATCGATATCGCCGAATGTCATATACTCAATTAGTATATGTTGGAGGACATCCGA

GTTCATTGTTTTCAATATCAAAGAGATGGTTTCCTTATCATTTCTCCATAGTGGTACAATACTACGCATT

ATTCCGTGCGGCTTTCCATTCTCCAAAAACAATTTTACCAAATCTAAATCTACATCTTTATTGTATCTAT

AATCACTATTTAGATAATCAGCCATAATTCCTCGAGTGCAACATGTTAGATCGTCTATATATAAATAAGC

CGTGTTATCTATTCCTTTCATTAACAATTTAACGATGTCTATATCTATATGAGATGACTTAATATAATAT

TGAAGAGCTGTACAATAGTTTTTATCTATAGAAGACGGCTTGATTCCGTGATTAATTAGACATTTAACAA

CTTCCGGACGCACATATGCTCTCGTATCCGACTCTGAATACAGATGAGAGATGATATACAGATGCAATAC

GGTACCGCAATTTCGTGGTTGATAATCATCATACGCGTATCCGTACTCGTCATCCTCATAAAGAACACTG

CAGCCATTTTCTATGAACAAATCAATAATTTCAGGAACAGGATCATCTGTCATTACATAATTTTCTATAA

CTGAACGATGGTTTTCACATTTAACACTCAAGTCAAATCCATGTTCTACCAACACCTTTATCAAGTCAAC

GTCTACATTTTTTGATTTAATATAGCTGAATATATTAAAGTCATTTATGTTGCTATATCCAGTAGCTTCT

AGTAGAGCCATCGCTATATCCTTATTGACTTTAACATGTCTACTATTTGTGTATTCTTCTATTGGGGTAA

ACTGTCTCCAATTTTTGTGTAATGGATTAGTGCCACTGTCTAGTAGTAGTTTGACGACCTCAACATTATT

ACAATGCTCATTGAAAAGGTATGCGTGTAAAGCATTATTCTTGAATTGGTTCCTGGTATCATTAGGATCT

CTGTCTCTCAACATCTGTTTAAGTTCATCGAGAACCACCTCCTCATTTTCCAGATAGTCAAACATTTTGA

CTGAATAGAAGTGAATGAGCTACTGTGAACTCTATACACCCGCACAACTAATGTCATTAAATATCATTTT

TGAATGTATTTATACCATGTCAAAAACTTGTACAATTATTAATAAAAATAATTAGTGTTTAAATTTTACC

AGTTCCAGATTTTACACCTCCGTTAACACCTCCATTAACCCCACTTTTTACACCACTGGACGATCCTCCT

CCCCACATTCCACTGCCACCAGATGTATAAGTTTTAGATCCTTTATTACTACCATCATGTCCATGGATAA

AGACACTCCACATGCCGCCACTACTACCCCCTTTAGAAGACATATTAATAAGACAAGTTTAACAATAAAA

TTAATCACGAGTACCCTACTCCAACCACTATTATATGATTATAGTTTCTATTTTTACAGTACCTTGACTA

AAGTCTCTAGTCACAAGATCAATACTACCAACCTACACTATATATGATTATAGTTTCTATTTTTATAGGA

ACGCGTACGAGAAAATCAAATGTCTAAGTTCTAACGGTAGTGTTGATAAACGATTGTTATCCGCGGATAC

CTCATCTATCATGTTGTCTATTTTCTTACTTTGTTCTATTAACCTATTAGCATTATATATTATTTGATTA

TAAAACTTATATTGCTTATTAGCCCAATCTGTAAATATCGGATTATTAACATATCGTTTCTTTGTAGGTT

TATTTAACTTGTACATCACTGTAAGCATGTCCGTACCATTTATTTTAATTTGACACATATCAGCAATTTC

TTTTTCGCAGTCGGTTATATATTCTATATAAGATGGATACGTATCACATATGTACTTATAGTCTACTAAT

ATGAAGTACTTAATACATATTTTCAGTAACGATTTAGCCTTATTACCTATTAATAAGTGCCTGTCGTTGG

ATAGGTAATCAACTGTTTTCTTAATACATTCGATGGTTGGTAATTTACTCAAAATAATTTCCAATATCTT

AATATATATTTCTGCTATTTCTGGTATACATGCATGTGCCATTATAACACAAATACCAATACATGTAGAC

CCATATGTTGTTGTTATATTAATATCTGCGCCATTATCTATTAACCATTCTACTAGTGCAACACTATGCG

ACTCGATACAATAATAAAGTATACTACGTCCATGTTTATCTATTTTGTTTATATCATCGATATACGGCTT

ACAAATTTTTAGTATCGATAACACTTCTGACTCGTGAATAAATAAGGTAGGGAATAACGGCATAATATTT

ATTATGTTATCATCATTAACAACTACGTTTCCATTTTTTAAAATATACTCTACAACTTTAGGATCCCTAT

TGTCAAATCTTTTAAAATATTTATTTATATGCTTAAATCTATATAATATAGCTCCTTCCCTAATCATACA

TTTGATAACATTGATGTACACTGTATGATAAGATACATATTCTGACAATAGATCTTGTATAGAATCTGTA

TATCTTTTAAGAATTGAGGATATTATGACATTATTACGTAAACTATTACACAATTCTAAAATATAAAACG

TATCATGGGCAGATAATAGTTTATCCACTATATAATTATCTATTTTATGATTTTTCTTCCTATATTGTTT

ACGTAAATAGATAGATAGAATATGCATTAGTTCATTACCGCTATAGTTACTATCGAATAACACGTCAAAT

ATTTCCCGTTTAATATCGCATTTGTCAACATAATAATAGAGTATGGTACGTTCACGATAAGTATAATGAC

ACATTTCGTTTTCGTGCGAAATTAAATAGTTTATCACGTCCAAAGATGTCACATAACCATCTTGTGACCT

AGTAATAATATAATAATAGAGAACTGTTTTACCCATTCTATTATCATAATCAGTGGTGTAGTCATAATCT

AAATAATCAAACTCGTCATCCCAATTAAAATAAATATAATCAGTACATTGAATGGGTATGATATTGTACC

CATACTGTATGTTGCTACATGTAGGTATTCCTTTATCCAATAATAGTTTAAATACATCTATATTAGGATT

TGATGTTGTCGCGTATTTCTCTACAATATTAATACCATTTTTGATACTATTTATTTCTATACCTTTCGAA

ATTAGTAATTTCAATAAGTCTATATCGATGTTATCAGAACATAGATATTCAAATATATCAAAATCATTGA

TATTTTTATAGTCGACTGACGACAATAACAAAATCACAACATCGTTTTTGATATTATTATTTTTTTTGGT

AACGTATGCCTTTAATGGAGTTTCACCATCATACTCATATAATGGATTTGCACCACTTTCTATTAATGAT

TGTGCAATACTGGCATCGATGTTAAATGTTTTACAACTATCATAGAGTATCTTATCGTTAACCATGATTG

GTTGTTGACGTTATCACATTTTTTGGTTTCTTTCATTTCAGTTATGTATGGATTTAGCACGTTTGGGAAG

CATGAGCTCATATGATTTCAGTACTGTAGTGTCAGTACTATTAGTTTCGATCAGATCAATGTCTAGATCT

ATAGAATCAAAACACGATAGGTCAGAAGATAATGAATATCTGTACGCTTCTTCTTGTACTGTAACTTCTG

GTTTTGTTAGATGGTTGCATCGTGCTTTAACGTCAATGGTACAAATTTTATCCTCGCTTTGTGTATCATA

TTCGTCTCTAGTATAAAATTCTATATTCAAATTATCATGCGATGTGTATACGCTAACGGTATCAATAAAC

GGAGCACAGCATTTAGTCAACAGTAATCCAAATTTTTTTAAAGTATATCTTAACGAAAGAAGTTGTCATC

GTTAGAGTGTGGTAAATCATTGTCTACGGTACTAGATCCTCATAAGTGTATATATCTAGAGTAATATTTA

ATTTATCAAATGGTTGATAATATGGATGTCGTGGCAATTTCCTAATACGGAAATAAGACATAAACACGCA

ATAAATCTAATTGCGGACATGTTACACTCCTTAAAAATACGAATAAAAACTTTGGCTTTTAGTAAGTGTC

ATTTAACACTATACTCATATTAATCCATGGACTCATAATCTCTATACGGGATTAACGGATGTTCTATATA

CGGGGATGAGTAGTTCTCTTCTTTAATTTTATACTTTTTACTAATCATATTTAGACTGATGTATGGGTAA

TAGTGTTTGAAGAGCTCGTTCTCATCATCAGAATAAATCAATATCTCTGTTTTTTTATTATACAGATGTA

TTACAGCCTCATATATTACGTAATATAACGTGTAATCTACCTTATTAACTTTCACCGCATAGTTGTTTGC

AAATACGGTTAATCCTTTGACCTCGTCGATTTCCGACCAATCTGGGCGTATAACGAATCTTAACTTTAAT

TTCTTGTAATCATTCGAAATAATTTTTAGTTTGCATCCGTAGTTATCTCCTCTATGTAACTGTAAATTTC

TCAACGCGATATCTCCATTAATAATGATGTCGAATTCGTGTTGTATACCCATACTGAATTGATGAACGAA

TACCGACGGTGTGTGTTAATAGTAATTTACTTTTCATCTTTACATACTTGGTAATAGTTTTACTATCATA

AGTTTATAAATTCCACAAGCTACTATGGAATATACCAACCATCTTAGTATAGAACACATGTCTTAAAGTT

ATTAATTAATTACATGTTGTTTTATATATCGCTACGAATTTAAACAGAGAAATCAGTTAGGAAGAAAAAA

TTATCTATCATCATCTATTGGATAACGTCTCTGTATTCTACGATAGAGTGCTATTTTAAGATGTGACAGA

TCCGTGTCATCAAATATATACTCCATTAAAATGATTATTCCGGCAGCGAACTTGATATTGGATACATCAC

GACCTTTGTTAATATCCACGACAATAGACAGCAATCCCATGGTTCCATAAACAGTGAGTTTATCTTTCTT

TGAAGTGATATTTTGTAGAGATCTTATAAAACTGTCGAATGACATCGTATTTATATCTTTAGCTAAATCA

TATATGTTACCATCATAATATCTAACAGCATCTATCTTAAACGTTTCCATCGCTGTAAAGACGTTTCCGA

TAGATGGTCTCGTTTCATCAGTCATACTGAGCCAACAAATGTAATCGTGTATAACATCTTTGATAGAATC

AGACTCTAAAGAAAAGGAATCGGCTTTATTATACACATTCATGATAAACTTAATGAAAAATGTTTTTCGT

TGTTTAAGTTGGATGAATAGTATGTCTTAATAATTGTTATTATTTCACTAATTAATATTTAGTAACGAGT

ACACTCTATAAAAACGAGAATGACATAACTAATCATAACTAGTTATCAAAGAATGTCTAGGACGCGTAAT

TTTTTATGGTATAGATCCTGTAAGCGTTGTCTGTATTCTGGAGCTATTTTCTCTATCGCATTAGTGAGTT

CAGAATATGTTATAAATTTAAATCGAATAACGAACATAACTTTAGTAAAGTCGTCTATATTAACTCTTTT

ATTTTCTAGCCATCGTAATACCATGTTTAAGATAGTATATTCTCTAGTTACTACGATCTCATCGTTGTCT

AGAATATCGCATACTGAATCTACATCCAATTTTAGAAATTGGTCTGTGTTACATATCTCTTCTATATTAT

TGTTGATATATTGTCGTAGAAAACTATTACGTAGACCATTTTCTTTATAAAACGAATATATAGTACTCCA

ATTATCTTTACCGATATATTTGCATACATAATCCATTCTCTCAATCACTACATCTTTAAGAGTTTGGTTG

TTAAGATATTTGGCTAAACTATATAATTCTATTAGATCATCAACAGAATCAGTATATATTTTTCTAGATC

CAAAGATGAACTCTTTGGCATCCTCTATAATATTATCAGAAAAGATATTTTCGTGTTTTAGTTTATCAAG

ATCTAACCTGTTCATATCCATGATTAACGACGTCATATAACCACATAAAATAAAAATCCATTTTCATTTT

TAGCACAATACTATTCATAATTGATATTGATGTAATATTTTGTTACTTTGAACGTAAAGACAGTACACGG

GTCCGTATCTCCAACAAGCACGTAGTAATCAAATTTGGTGTTGTTAAACTTCGCAATATTCATCAATTTA

GATAGAAACTTATACTCATCATCTGTTTTAGGAATCCATGTATTATTACTTTCCAACTTATCATTATCCC

AGGCTATGTTTCGCCCATCATCGTTGTACAGAGTGAATAATTCTTTTGTATTCGGTAGTTCAAATATATG

ATCCATGCATATATCGACAAAGCTATTGTAGATGTGATTTTTCCTAAATCTAATATAAAACTCGTTTACT

AGCAAACATTTTCCTGATTTATCGACCAAGACACACATGGTTTCTAAATCTATCAAGTGGTGGGGATCCA

TAGTTATAACGCAGTAACATAGATTATTACCTTCTTGACTGTCGCTAATATCTATATACTTATTGTTATC

GTATTGGATTCTACATATAGATGGCTTGTATATCAAAGATATAGAACACATAACCAATTTATATTCTCGC

TTTGTATTTTCGAATCTAAAGTTAAGAGATTTAGAAAACATTATATCCTCGGATGATGATATCACTGTTT

CCAGAGTAGGATATATTAAAGTCTTTAAAGATTTTGTCCGATTCAAATAAATCACTAAATAATATCCCAT

ATTATCATCTGTTATAGTCATGTCATTAAATCTATTATATTTTATGAAAGATATATCACTGCTCACCTCT

ATATTTCGTACATTTTTAAACTGTTTGTATAATATCTCTCTAATACAATCAGATATATCTATTGTGTCGG

TAGACGATACCGTTACATTTGAATTAATGGTGTTCCATTTTACAACTTTTAACAAGTTGACCAATTCATT

TCTAATAGTATCAAACTCTCCATGATTAAATATTTTAATAGTATCCATTTTATATCACTACGGACATAAA

CCATTGTATAATTTTTATGTTTATTAGTGTACACATTTTGGAAGTAAGTTCCGGCTGCCATGTATTTCCT

GGAGAGCAAGTAGATGATGAGGAACCAGATAGTTTATATCCATACTTGCACTTAAAGTCTACATTGTAGT

TGTATGAGAGTATGATCTTTTAAGCCGCTAGAAGTTTTCCGTTTGATATAGGATGTGGACATTTAACAAT

CTGACACGTGGGTGGATTGGACCATTCTCCTCCTGAACACATGACACCAGAGTTACCAATCAACGAATAT

CCACTATTGCAACTATAAGTTACAATGCTCCCATCGATATAAAAATCCTCGTATCCGTTATGTCTTCCGT

TGGATATAGATGGAGGTGATTGGCATTTAACAGATTCGCAAATAGGTGCCTCAGGATTCCATACCATAGA

TCCAGTAGATCCTAATTCACAATACGATTTAGATTCACCGATCAAATGATATCCGCTATTACAAGAGTAC

GTTATACTAGAGCCAAAGTCTACTCCGCCAATATCAAGTTGGCCATTATCGATATCTCGAGGCGATGGGC

ATCTCCGTTTAATACATTGATTAAAGAGTGTCCATCCGGTACCGGTACATTTAGCATATATGGGTCCCAT

TTTTTGCTTTCTGTATCCAGGTAGACATAGATATTCTATAGTGTCTCCTATGTTGTAATTAGCATCAGTC

TCTACACTATTCTTAAATTTCATATTAATGGGGCGTGACGGAATAGTACAGTATGATAGAACACATCCTA

TTCCCAACAATGTCAGGAACGTCACGCTCTCCACCTTCATATTTATTTATCCGTAAAATGTTATCCTGGA

CATCGTACAAATAATAAAAAGCCCATATATATGTTCGCTATTGTAGAAATTGTTTTTCACAGTTGCTCAA

AAACAATGGCAGTGACTTATGAGTTAGTTACACTTTGGAGTCTCATCTTTAGTAAACATATCATAATATT

CGATATTACGAGTTGACATATCGAACAAATTCCAAGTATTTGATTTTGGATAATATTCGTATTTTGCATC

TGCTATAATTAAGATATAATCACCACAAGAACACACGAACGTCTTTCCTACATGGTTAAAGTACATGTAC

AATTCTATCCATTTGTCTTCCTTAACTATATATTTGTATAGATAATTACGAGTCTCATGAGTAATTCCAG

TAATTGCATAGATGTCACCATCGTATTCTACAGCATAAACTATACTATGACGTCTAGGCATGGGAGACTT

TTTTATCCAACGATTTTTAGTGAAACATTCCACATCGTTTAATACTACATATTTCTCATAGTGGTATAAA

CTCCACCCATTACATATATATCATCGTTTACGAATACTGATGCGCCTGAATATCTAGGAGTGATTAAGTT

TGGAAGTCTTTTCCATTTCGAAGTGCCGTGTTTCAAATATTCTGCTATACCCGTTGAAATAGAAAATTCT

AATCCTCCTATTACATATAACTTTCCATCGTTAACACAAGTACTAACTTCTGATTTTAACGACGACATAT

TAGTAACCGTTTTCCATTTTTTTTGTTTTAAGATCTACCCGCGATACGGAATAAACATGTCTATTGTTAA

TCATGCCGCCAATAATGTATAGACAATTATGTAAAACATTTGCATCATAGAATTGTCTATCTGTATTACC

GACTATCGTCCAATATTCTGTTCTAGGAGAGTAATGGGTTATTGTGGATATATAATCAGAGTTTTTAATG

ACTACTATATTATGTTTTATACCATTTCGTGTCACAGCTTTGTAGATTTGGATATAGTTAATCCCAACAA

TGCTATAGCATTGCATATAGCATTAGTCATAAACTTGGGATGTAAAATGTTGATGATATCTACATCGTTT

GGATTTTTATGTATCCACTTTAATAATATTATAGCGTAACATCCTCATGATTTACGTTAACGTTTTCGTG

TGATAAGATAGTGGTCAGTTCATCCTTTGATAATTTTCCAAATTCTGGATCGGATGTCACCGCAGTAATA

TTGTTGATTATTTCTGACATCGACGCATTATATAGTTTTTTAATTCCATATCTTTTAGAAAAGTTAAACA

TCCTTATACAATTTGTGGAATTAATATTATGAATCATGGTTTTTACACATAGATCTACTACAGGCGGAAC

ATCAATTATTATGGCAGCGACTAGTATCATTTCTACATTGTTTATGGTGATGTTTATCTTCTTCCAGCGC

ATATAGTCTAATATCGATTCAAACGCGTGATAGTTTATACCATTCAATATAATCGCTTCATCCTTTAGAT

GGTGATCCTGAATGTGTTTAAAAAATTATACGGAGACGCCGTAATAATTTCCCCATTGATAGAAAATATC

ACGCGTTCCATTCTCTTGAAGTACTATAAGTAATTATAATATAATGTAAAGGTTTATATATTCAATATTT

TTTTTATAAAAAAAATCATTTCGACATTAATTCCTTTTTAAATTTCCGTCTATCATCTATAGAAACATAT

TCTATGAATTTATAAAATGCTTTTACGTATCCTATCGTAGGCGATAGAACCGCTAAAAAGCCTATCGAAT

TTCTACAAAAGAATCTGTTATATGGTATAGGGAGAGTATAAAACATTAAATGTCCGTACTTATTAAAGTA

TTCAGTAGCCAATCCTAACTCTTTCGAATAATTATTAATGGCTCTTATTCTGTACGAATCTATTTTTTTG

AACAATGGACCTAGTGGTATATCTTGTTCTATGTATCTAAAATAATGTCTGACTAGATCCGTTAGTTTAA

TATCCGCAGTCATCTTGTCTAGAATGGCAAATCTAACTGCGGGTTTAGGTGTAGGCGTTAGTTTAGTTTC

TATATCTACATCTATGTCTTTATCTAACACCAAAAATATAATAGCTAATATTTTATTACAATCATCCGGA

TATTCTTCTACGATCTCACTAACTAATGTTTCTTTGGTTATACTAGTATAGTCACGATCAGACAAATAAA

GAAAATCAGATGATCGATGAATAATACATTTAAATTCATCATCTGTAAGATTTTTGAGATGTCTCATTAA

AATATTATTAGTGTCAGTTCTCATTATCATATATTGACAGCAGCTATTACACTTATTTTATTTTTCTGTA

TTTTATTACTTTTCACCATATAGATCAGTCATTAGATCATCAAAATACTTTTCAATCATCCTAAAGAGTA

TGGTGAACGAATCTTCCCATCTAATTTCTGAACGTCTACCAATGTCTCTAGCCACTTTGGCACTAATAGC

GATCATTCGCTTAACATCTTCTACATTATTAACTGGTTGATTCAATCTATCTAGCAATGGACCGTCGGAT

AGCGTCATTCTCATGTTCTTAATCAATGTACATACATCGTCATCATCTACCAATTCATCAAACAATATAA

GCTTTTTAAAATCATCATTATAATAGGATGGATCGCCGTCATTTCTCCAAAGAATATATCTAATAAGTAG

AGTCCTCATGCTTAGTAATTTAACTATTTTAGTTAACAACTATTTTTTATGTTAAATCAATTAGTAACAC

CGCTATGTTTAATACTTATTCATATTTTAGTTTTAGGATCGAGAATCAATACAAAAATTAATACATCAAT

TTTGGAAATACTTAGTTTCCACGTAGTCAATGAAACATTTGAGCTCATCGTAAAGGACGTTCTCGTACAG

GACGTAACTATAAATTGGTTTATATTTGTTCAAGATAGATACAAATCCGATAACTTTTTTGACGAATTCT

ACGGGATTCACTTTAAAAGTGTCATACCGGGTTCTTTTTATTCTTTTAAACAGATCGATTGTGTGATGTT

GATTAGGTCTTTTACGAATTTGATACAGAATAGCGTTTACATATCCACCATAGTAATCAATAGCCATTTG

TTCGTATGTCATAAATTCTTTAATTATATGACACTGTGTATTATTTAGTTCGTCCTTGTTCATCATTAGG

AATCTATCCAATATGGCAATTATATTAGAACTATAACTGCGTTGTATGCGCATGTTGATGTGTCTGTTTA

TACAATCAATTATACTAGGATCCATACCACTACATTCGGGTAAAATTGTAGCATCATATACCATTTCTAG

TACTTTAGGTTCATTGTTATCCATTGCAGAGGACGTCATGATCGAATCCAAAAAAATATATTATTTTTAT

GTTATTTTGTTAAAAATAATCATCAAATACTTCGTAAGATACTCCTTCATGAACATAATCAGTTACAAAA

CGTTTATATGAAGTAAAGTATCTACGATTTTTACAAAAGTCAGGATGCATAAGTACAAAGTACGCGATAA

ACGGAATAATAATAGATTTATCTAGTTTATCTTTTTCTATCTCTTTCATAGTTATATACATGGTCTCAGA

AGTCGGATTATGTAACATCAGCTTCGATAAAATGACTGGGTTATTTAGTCTTACACATTCGCTAATACAT

GTATGACCGTTAACTATAGAGTCTACACTAAAATGATTGAATAATAGATAGTCTACCATTGTTTCGTATT

CAGATAGTACAGCGTAGTACATGGCATCTTCACAAATTATATCATTATCTAATAGATATTTGACGCATCT

TATGGATCCCACTTCAACAGCCATCTTAAAATCGGTAGAATCATATTGCTTTCCTTTATCGTTAATAATT

TCTAGAACATCATCTCTATCATAAAAGATACAAATATTAACTGTTTGATCAGTAATAACATTGCTAGTCG

ATATCAATTTGTTAATAAGATGCGCTGGGCTCAATGTCTTAATAAGAAGTGTAAGAGGACTATCTCCGAA

TTTGTTTTGTTTATTAACATCCGTTGATGGAAGTAAAAGATTTATAATGTCTACATACTTGACTGTTTTA

GAGCATACAATATGGAGAGGCGTATTTCCATCATGATCTGGTTTTGAGGGACTAATTCCTAGTTTCATCA

TCCATGAGATTGTAGAAGCTTTTGGATTGTCTGACATAAGATGTCTATGAATATGATTTTTGCCAAATTT

ATCCACTATCCTGGCTTCGAATCCGATAGACATTATTTTTTTAAACACTCTTTCTGAAGGATCTGTATAC

GCCAACAACGGACCACATCCTTCTTCATCAACCGAGTTGTTAATCTTGGCTCCATACTGTACCAATAAAT

TTATTCTCTCTATGACTTCATCATCTGTTCCCGAGAGATAATATAGAGGTGTTTTATTATGTTTATCACA

TGCGTTTGGATCTGCGCCGTGCACCAGCAGCATCGCGACTATTCTATTATTATTAATTTTAGAAGCTATA

TGCAATGGATAATTTCCATCATCATCCGTCTCATTTGGAGAGTATCCTCTATGAAGAAGTTCTTCTATAA

ATCGTTCATCTAGTCCTTTAATGCCACAATACGCATGTAGAATGTGATAATTTCCAGAGGGTTCGATAAC

TTGTAGCATATTCCTAAATACATCTAAATTTTTACTATTATATTTGGCATAAAGAGATAGATAATACTCG

ACCGACATAATGTTGTGTTGTCCATTATAGTATAAAAATTAATATTTCTATTTCTATTTCTATATATTTG

CAACAATTTACTCTCTATAACAAATATCATAACTTAGTTCTTTTATGTCAAGAAGGCACTGGTTTAATTC

ATCTATAAATGTCACGCCATAACTACCACGCATACTATACTCAGAATTATGATAAAGATATTTATTCTTG

GGGTGTAAGTAATGGGGATTAATCTTTGTTGGATCAGTCTCTAAGTTAACACATGTCACACATGATCCAT

TTATAGTTATATCACACGATGATGATTTATGAATTGATTCCGGAAGATCGCTATTGTATTTTGTAGTTCC

ACAATTCATTTCCATACATGTTATTGTCACACTAATATTATGATGAACTTTATCTAGCCGCTGAGTGGTA

AACAACAGAACAGATAGTTTATTATCTTTACCAACACCCTCAGCCGCTGCCACAAATCTCTGATCCGTAT

CCATGATGGTCATGTTTATTTTTAGTCCGTATCCAGTCAACACTATGTTAGCATTTCTGTCGATATAGCT

TTCACTCATATGACACTCACCAATAATTGTAGAATTAATGTCGTAATTTACACCAATAGTGAGTTCGGCG

ACAAAGTACCAGTACCGGTAATCTTGTCGAGGAGGACATATAGTATTCTTGTATTCTACCGAATACCCGA

GAGATGCGATACAAAAGAGTAAGACTAATTTGTAAACCATCTTACTCAAAATATGCGACAATAGTACGAT

GCAATGAGTAAGACAATAGGAAATCTATCTTATACACATAATTATTCTATCAATTTTACCAATTAGTTAG

TGTAATGTTAACAAAAATGTGGGATAATTTAATAGTTTTTCCTTACATAATTGACATACATGAGTCTGAG

TTCCTCGTTTTTGCTAATTATTTCGTCCAATTTATTATTCTTGACATCGTCAAGATCTTTTGTATAGGAG

TCAGACTTGTATTCAACATGTTTTTCTATAATCATCTTAGCTATTTCGGCATCATCCAATAGTACATTTT

CCAGATTAACAGAATAGATATTAATGTCGTATTTGAACAGAGCCTGTAACATCTCAATGTCTTTATTATC

TATAGCCAATTTGATGTCCGGAATGAAGAGAAGGGAATTGGTGTTTGTCGACGTCATATAGTCGAGCAAG

AGAATCATCATATCCACGTGTCCATTTTTTATAGTGGTGTGAATACAACTAAGGAGAATAGCTAGATCAA

AAGGAGATGGTATCTCTGAAAGAAAGTAGGAAACAATACTTACATCATTAAGCATGACAGCATGATAAAA

TGAAGTTTTCCATCCAGTTTTCCCATAGAACATCAGTCTCCAATTTTTCTTAACAAACAGTTTTACCGTT

TGCATGTTACCACTATCAACCGCATAATACAATGCGGTGTTTCCTTTGTCATCAAATTGTGAATCATCCA

TTCCACTGAATAGCAAAATCTTTACTATTTTGGTATCTTCTAATGTGGCTGCCTGATGTAATGGAAATTC

ATTATCTAGAAGATTTTTCAATGCTCCAGCGTTCAACAACGTACATACTAGACGCACGTTATTATCAGCT

ATTGCATAATACAAGACACTATGACCGTTGATATCCGCCTTAAATGCATCTTTGCTAGAGAGAAAGCTTT

TCAGTTGCTTAGACTTCCAAGTATTAATTCGTGACAGATCCATGTCTGAAACGAGACGCTAATTAGTGTA

TATTTTTTCATTTTTTATAATTTTGTCATATTGCACCAGAATTAATAATATCTCTAATAGATCTGATTAG

TAGATACATGGCTATCGCAAAACAACATATACACATTTAATAAAAATAATATTCATTAAGAAGATTCAGA

TTCCACTGTACCCATCAATATAAATAAAATAATTATTCCTTACATCGTACCATAAACAATATATTAAGTA

GATTCCACCTTACCCATAAACAATATAAATCCAGTTATATCATGTCTAATGATGAACACAAATGGTGTAT

TAAATTCCAGTTCTTCAGGAGATGATCTCGCCGTAGCTACCATGATAGTAGATGCCTCCGCTACAGTTCC

TTGTTCGTCTACATCTATCTTTACATTCTGAAACATTTTATAAATATATAATGGGTCCCTAGTCATATGT

TTAAACGACGCCTTATCTGGATTAAACATACTAGGAGCCATCATTTCGGCTATCGACTTAATATCCCTCT

TGTTTTCGATAGAAAATCTAGGGAGTTTAAGATTGTACATTTTATTCCCTAATTGAGATGACCAATATTC

TAATTTTGCAGCCGTGATAGAATCTGTGAAATGGGTCATATTATCACCTATTGCCAGGTACATACTAATA

TTAGCATCCTTATACAGAAGGCGCACCATATCATATTCTTCGTCATCGATTGTGATTGTATTTCCTTGCA

ATTTAGTAACTACGTTCATCATGGGAACCGTTTTCGTACCGTACTTATTAGTAAAACTAGCATTGTGTGT

TTTAGTGATATCAAACGGATATTGCCATGTACCTTTAAAATATATAGTATTAATGATTGCCCATAGAGTA

TTATCGTCGAGCATAGTAGAATCAACTACATTAGACATACCAGATCTACGTTCTACTATAGAATTAATTT

TATTAACCGCATCTCGTCTAAAGTTTAATCTATATAGGCCGAATCTATGATATTGTTGATAATACGACGG

TTTAATACACACAGTACTATCGACGAAACTTTGATACGTTAGATCGGTGTACGTATATTTAGATGTTTTC

ATCTTAGCTAATCCTGATATTAATTCTGTAAATGCTGGACCCAGATCTCTTTTTCTCAAATTCATAGTAT

TCAATAATTCTACTCTAGTATTACCTGATGCAGACAATAGCGACATAAACATAGAAAACGAATACCCAAA

CGGTGAGAAGACAATATTATCATTATCATCCTCATCCCCATTTTGAATATTTTTATACGCTAATATACCG

GCATTGATAAATCCCTGCAGACGATATGCGGATACTGAACACGCTAATGATAGTATCAATAACGCAATCA

TGATTTTTATGGTATTAATAATTAACCTTATTTTTATGTTTGGTATAAAATTTATTGATGTCTACACATC

CTTTTGTATAATCAACTCTAATCACTTTAACTTTTACAGTTTTCCCTACAAGTTTATCCCTATATTCAAC

ATATCTATCCATATGCATCTCTTAACACTCTGCCAAGATAGCTTCAGAGTGAGGATAGTCAAAAAGATAA

ATATATAGAGTATAATCATTCTCGTATACTCTGCCCTTTATTACATCGCCCGCATTGGGCAACGAATAAC

AAAATGCAAGCATCTTGTTAACAGGCTCGTAAATTGGGATAAAATTATGTTTTTATTGTTTATCTATTTT

ATTCAAGAGAATATTCAGGAAGTTCCTTTTCCGGTTGTATCTCGTCGCAGTATATATCATTTGTACATTG

TTTCATATTTTTTAATAGTCTACACCTTTTAGTAGGACTAGTATCGTACAATTCATAGCTGTATTTTGAA

TTCCAATCACGTATAAAAATATCTTCCAATTGTTGACGAAGACCTAATCCATCATCCGGTGTAATATTAA

TAGATGCTCCACATATATCCGTAAAGTAATTTCCTGTCCAATTTGATGTACCTATATACGCCGTTTTATC

GGTTACCATATATTTTGCATGGTTTACCCTAGAATACGGAATGGGAGGATCAGCATCTGGTACAATAAAT

AGCTTTACTTCTATATCTATGTTTTTAGATTTTAGCATAGCTATAGATCTTAAAAAGTTTCTCATGATAA

ACGAAGATCGTTGCCAGCAACTAATCAATAGCTTAACGGATACTTGTCTGTCTATAGCGGATCTTCTTAA

TTCATCTTCTATATAAGGCCAAAACAAAATTTTACCCGCCTTTGAATAAATAATAGGAATAAAGTTCATA

ACAGATACATAAACGAATTTACTCGCATTTCTGATACATGACAATAAAGCGGTTAAATCATTGGTTCTTT

CCATAGTACATAATTGTTGTGGTGCAGAAGCAATAAATACAGAGTGTGGAACACCGCTTACGTTAATACT

AAGAGGATGATCTGTATTATAATACGACGGATAAAAGTTTTTCCAATTATATGGTAGATTGTTAACTCCA

AGATACCAGTATACCTCAAAAATTTGAGTGAGATCCGCTGCCAAGTTCCTATTATTGAAGATCGCAATAC

CCAATTCCTTGACCTGAGTTAGTGATCTCCAATCCATGTTAGCGCTTCCTAAATAAATATGTGTATTATC

AGATATCCAAAATTTTGTATGAAGAACTCCTCCTAGGATATTTGTAATATCTATGTATCGTACTTCAACT

CCGGCCATTTGTAGTCTTTCAACATCCTTTAATGGTTTGTTGGATTTATTGACGGCTACTCTAACTCTTA

CTCCTCTTTTGGGTAATTGTACAATCTCGTTTAATATTACCGTGCCGAAATTCGTACCCACTTCATCCGA

TAAACTCCAATAAAAAGATGATATATCTAGTGTTTTTATGGTATTGGATAGAATTTCCCTCCACATGTTA

AATGTAGTCAAATATACTTTATCAAATTGCATACCTATAGGAATAGTCTCTGTAATCACTGCGATTGTAT

TATCCGGATTCATTTTATTTGTTAAAAAATAATCCTATATCACTTCACTCTATTAAAAATCCAAGTTTCT

ATTTCTTTCATGACTGATTTTTTAACTTCATCCGTTTCCTTATGAAGATGATGTTTGGCACCTTCATAAA

TTTTTATTTCCCTATTACAATTTGCATGTTGCATGAAATAATATGCACCTGAAACATCGCTAATCTCATT

GTTTGTTCCCTGGAGTATGAGAGTCGGGGTGTCAATCTTGGGAATTATTTTTCTAACCTTGTTGGTAGCC

TTCAAGACCTGACTAGCAAATCCAGCCTTAATTTTTTCATGATTGACTAATGGATCGTATTGGTATTTAT

AAACTTCATCCATATCTCTAGATACTGATTCTGGACATAGCTTTCCGACTGACGCATTTGGTGTAATGGT

TCCCATAAGTTTTGCAGCTAGCAGATTCAGTCTTGGAACAGCGTCTGCATTAACTAGAGGAGACATTAGA

ATCATTGCTGTAAACAAGTTTGGATTATCGCAAGCAGCCAGTATAGAAATTGTTGCTCCCATGGAATGAC

CCAATAAGAAGACTGGAACTCCTGGATAAGTAGATTTAATAGTCACTACGTGCTGTACCACATCTCTAAC

ATACTTACCAAAGTCATCAATCATCATTTTTTCACCATTACTTCTTCCATGGCCAATATGATCATGTGAG

AATACTAAAATTCCTAACGATGATATGTTTTCAGCTAGTTCGTCATAACGTCCAGAATGTTCACCAGCTC

CATGACTTATGAATACTAATGCCTTAGGATATGTAATAGGTTTCCAATATTTACAATATATGTAATCATT

GTCCAGATTGAACATACAGTTTGCACTCATGATTCACTATATAACTATCAATATTAACAGTTCGTTTAAT

GATCATATTATTTTTATGTTTTATTGATAATTGTAAAAATATACAATTAAATCAATATAGAGGAAGGAGA

CGGTACTGTATTTTGTGAGATAGTCATGGAGACTAAATCAGATTATGAGGATGCTGTTTTTTACTTTGTG

GATGATGATGAAATATGTAGTCGCGACTCCATCATTGATCTAATAGATGAATATATCACGTGGAGAAATC

ATGTTATAGTGTTTAATAAAGATATTACCAGTTGTGGAAGACTGTACAAGGAATTGATAAAGTTCGATGA

TGCCGCTATACGGTACTATGGTATTGATAAAATTAATGAGATTGTCGAGGCTATGAGTGAAGGAGACCAC

TACATCAATCTTACAGAAGTCCATGATCAGGAAAGTCTATTCGCTACCATAGGAATATGTGCTAAAATCG

CTGAACATTGGGGATACAAAAAGATTTCAGAATCTAAATTCCAATCATTGGGAAACATTACAGATCTGAT

GACCGACGATAATATAAACATCTTGATACTTTTTCTAGAAAAAAAATTGAATTGATGATATAGGTGTCTT

CATAACGCATTATTACGTTAGCATTCTATTATCCTATCATGTATTTGAGAGTCTTATATGTAGCAAACAT

GATAACTGCAATACCCATAATCTTTAGATATTCACGCGTGCTATGGATGGCATTATCCCGCGGTGTGGAA

ATGTACGTTATATAATCTACAAAATAATCATCGCATATAGTATGAGATAGTAGAGTAAACATTTTTATCG

TTTCTACTGGGTTCATACATCGTCTACCCAATTCGGTAATGAATGAAATTGTCGCCAATCTTACACCCAA

ACCCTTGTTGTTCATTAGTATAGTATTAACTTCATTATTTATGTCATAAACTGTAAATGATTCTGTAGAT

GCCATATCACACATGATATTCATGTCACTATTATAATCATTATTAACTTTATCACAATACGTGTTGATAA

TATCTACATATGATCTAGTTTTTGTGGGTAATTGTCCATACAAGTCGTCTAAACGTTGTTTACTCATATA

GTATTGAACAGCCATCATTACATGGTCCCGTTCCGTTGATAGATAATCGAGTATGTTAGTAGACTTGTCA

AATCTATATACCATATTTTCTGGAAGCGGATATACATAGTCGCGATCATCATTATCACTAGCCTCATCCT

CTATATCATGTACATGTACATAATCTATGATATTATTATACATAAACATCGACAACATACTATTGTCTAT

TATCTAAGTCCTGTTGATCCAAACCCTTGATCTCCTCTATCTGTACTATCTAGAGATTGTACTTCTTCAA

GTTCTGGATAATATATACGTTGATAGATTAGCTGAGCTATTCTATCTCCAGTATTTACATTAAACGTACA

TTTTCCATTATTAATAAGAATGACTCCTATGTTTCCCCTATAATCTTCGTCTATTACACCGCCTCCTATA

TCAATGCCTTTTAGGGACAGACCAGACCTAGGAGCTATTCTACCATAGCAGAACTTAGGCATGGACATAC

TAATATCTGTCTTAATTAACTGTCGTTCTCCAGGAGGGATAGTATAATCGTAAGCGCTATACAAATCATA

TCCGGCAGCACCCGGCGATTGCCTAGTAGGCGATTTAGCTCTGTTAGTTTCCTTAACAAATCTAACTGGT

GAGTTAATATTCATGTTGAACATAAAAAATATCATTTTATTTCAAAATTATTTACCATCCCATCCCATCC

CATCCCATCCCATTCCATATATTCCATGAATAAGTGCGATTATTGTACACTTCTATAGTATCTATATACG

ATCCACGATAAAATCCTCCTATCAATAGCAGTTTATTATCCACTATGATCAATTCTGGATTATCCCTCGG

ATAAATAGGATCATCTATCAGAGTCCATGTATTACTGGATTCACAATAAAATTCCGCATTTCTACCAACC

AAGAATAACCTTCTACCAAACACTAACGCACATGATTTATAATGAGGATAATAAGTGGATGGTCCAAACT

GCCACTGATCATGATTGGGTAGCAAATATTCTGTAGTTGTATCAGTTTCAGAATGTCCTCCCATTACGTA

TATAACATTGTTTATGGATGCCACTGCTGGATTACATCTAGGTTTCAGAAGACTCGGCATATTAACCCAA

GCAGCATCCCCGTGGAACCAACGCTCAACAGATGTGGGATTTGGTAGACCTCCTACTACGTATAATTTAT

TGTTAGCGGGTATCCCGCTAGCATACAGTCTGGGGCTATTCATCGGAGGAATTGGAATCCAATTGTTTGA

TATATAATTTACCGCTATAGCATTGTTATGTATTTCATTGTTCATCCATCCACCGATGAGATATACTACT

TCTCCAACATGAGTACTTGTACACATATGGAATATATCTATAATTTGATCCATGTTCATAGGATACTCTA

TGAATGGATACTTGTATGATTTGCGTGGTTGTTTATCACAATGAAATATTTTGTTACAGTCTAGTATCCA

TTTTACATTATGTATACCTCTGGGAGAAAGATAATTTGACCTGATTACATTTTTGATAAGAAGTAGCAGA

TTTCCTAATCTATTTCTTCGCCTCATATACCACTTAATGACAAAATCAACTACATAATCCTCATCTGGAA

CATTTAGTTCGTCGCTTTCTAGAATAAGTTTCATAGATAGATAATCAAAATTGTCTATGATGTCATCTTC

TAGTTCCAAAAAGAGTTTGGTAATAAAGTCTTTAGTATGACATAAGAGATTGGATAGTCCGTATTCTATA

CCCATCATGTAACACACGATACAATATTCCTTTCTAAAATCTCGTAGGATAAAGTATATACAAGTGTAGA

TGATAAATTCTACAGATGTTAATATAGAAGCACGTAATAAATTGACGACGTTATGACTATCTATATATAC

CTTTCCAGTATATGAGTAAATAACTATAGAAGTTAGACTGTGAATGTCAAGGTCTAGACAAACCCTCGTA

ACTGGATCTTTATTTTTTGTGTATTTTTGACGTAAATGTGTGCGAAAGTATGGAGATAACTTTTTCAATA

TTGTAGAATTGACTATTATATTGCCTCCTATAGCTTCAATAATTGTTTTGAATTTCTTAGTCGTATACAA

TGCTAATATATTCTTACAGTACACAGTATTGACAAATATCGGCATTTATGTTTCTTTAAAAGTCAACATC

TAAAGAAAAATGATTGTCTTCTTGAGACATAACTCCCATTTTTTGGTATTCACCCACACGTTTTTCGAAA

AAATTAGTTTTTCCTTCCAATGATATATTTTCCATGAAATCAAACGGATTGGTAACATTGTAAATTTTTT

TAAATCCCAATTCAGAAATCAATCTATCTGCGACGAATTCTATATATGTTTTCATCATTTCACAATTCAT

TCCTATGAGTTTAACTGGAAGAGCCACAGTAAGAAATTCTTGTTCAATGGATACCGCATTTGTTATAATA

AATCTAACGGTTTCTTCACTCGGTGGATGTAATAAATGTTTAAACATCAAACATGCGAAATCGCAGTGCA

GACCCTCGTCTCTACTAATTAATTCGTTAGAAAACGTGAGTCCGGGCATTAGGCCACGCTTTTTAAGCCA

AAATATGGAAGCGAATGATCCGGAAAAGAAGATTCCTTCTACTGCAGCAAAGGCAATAAGTCTCTCTCCA

TAACCGGCGCTGTCATGTATCCACTTTTGAGCCCAATCGGCCTTCTTTTTTACACAAGGCATCGTTTCTA

TGGCATTAAAGAGATAGTTTTTTTCATTACTATCTTTAACATAAGTATCGATCAAAAGACTATACATTTC

CGAATGAATGTTTTCAATGGCCATCTGAAATCCGTAGAAACATCTAGCCTCGGTAATCTGCACTTCTGTA

CAAAATCGTTCTGCTAAATTTTCATTCACTATTCCATCACTGGCTGCAAAAAACGCCAATACATGTTTTA

TAAAATATTTTTCGTCTGGTGTTAGTTTATTCCAATCATTGATATCTTTAGATATATCCACTTCTTCCAC

TGTCCAAAATGATGCCTCTGCCTTTTTATACATATTCCAGATGTCATGATATTGGATTGGGAAAATAACA

AATCTATTTGGATTTGGTGCAAGGATAGGTTCCATAACTAAATTAACAATAGTAGTAATTTTTTTTTCAG

TTATCTGTATGACTGTACTTGGATCTTTTGTATATCGCTATCGCCGCAATCACTACAATAATTACAAGTA

TTATTGATAGCATTGTTATTACTACTATCATAATTAAATTATCGACATTCGTGGGTGCTGAATAATCGTT

ATCATCATTTTGTAATTGTGACATCATACTAGATAAATCATTTGTGAGATTGTTGTGGGAAGCGGGCACG

GAAGATGCATTATCATTATTATTTAACGCCTCCCATTCGGATTCACAAATGTTACGCACGTTCAACGTTT

TATGGAAACTATAATTTTGTGAAAACAGATAACAAGAAAACTCGTCATCGTTCAAATTTTTAACGATAGT

AAACCGATTAAACGTCGAGCTAATTTCTAACGCTAGCGACTCTGTTGGATATGGGTTTCCAGATATATAT

CTTTTCAGTTCCCCTACGTATCTATAATCATCTGTAGGAAATGGAAGATATTTCCATTTATCTACTGTTC

CTAATATCATATGCGGTGGTGTAGAACCATTAAGCGCGAAAGATGTTATTTCGCATCGTATTTTAACTTC

GCAATAATTTCTGGTTAGATAACGCACTCTACCAGTCAAGTCAATGATATTAGCCTTTACAGATATATTC

ATAGTAGTCGTAACGATGACTCCATCTTTTAGATGCGATACTCCTTTGTATGTACCAGAATCTTCGTACC

TCAAACTCGATATATTTAAACAAGTTAATGATATATTAACGCGTTTTATGAATGATGATATATAACCAGA

AGTTTTATCCTCTGTGGCTAGCGCTATAACCTTATCATTATAATACCAACTAGTGTAATTAATATGTGAC

ACGACAGTGTGGGTACAAATATGTACATTATCGTCTACGTCGTATTTGATACATCCGCATACAGCCAACA

AATATAAAATTACAAAAACTCTAACGACGTTCGTACACATCTTGATGCGGTTTAATAAATGTTTTGATTT

CAATTTATTGTAAAAAAGATTCGGTTTTATACTGTTCGATATTCTCATTGCTTATATTCTCATCTATCAT

CTCCACACAGTCAAATCCATGGTTAACATGTACCTCATCAACCGGTAAAAGACTATCGGATTCTTCTATC

ATCATAACTCGAGAATATTTAATTTGGTGGTCATTATTAATCAAGTCAATTATCTTATTTTTAACAAACG

TAAGTATTTTACTCATTTTTTATAAAAACTTTTAGAAATATACAGACTCTATCGTGTGTCTATATCTTCT

TTTTATATCCAATGTATTTATGTCTGATTTTTCTTCATTTATCATATATAATGGTCCAAATTCTACACGT

GCTTCGGATTCATCCAGATCATTAAGGTTCTTATAATCGCAACATCCTTCTCTTCCATCTTCTACATCTT

CCTTCTTATTCTTAGCGTCACAGAATCTACCACAGCAGGATCCCATGACGAGCGTCACATTAAACTAATT

CATTTTCAATTATAATATACTGATTAGTAATGACCATTAAAATAAAAATATTCTTCATAACCGGTAAGAA

AGTAAAAAGTTCACATTGAAACTATGTCAGTAGTTATACATCATGAGATGATATACTCTATTTTGGTGGA

GGATTATATGATATAATTCGTGGATAATCATTCTTAAGACACATTTCTTCATTCGTAAATCTTTTCACAT

TAAATGAGTGTCCATATTTTGCAATTTCTTCATATGATGGCGGTGTACGTGGACGAGGCTGCTCCTGTTC

TTGTAGTCGCCGACTGTCGTGTTTGCGTTTAGATCCCTCCATTATCGCGATTGCGTAGTGAGTACTATTT

ATACCTTGTAATTAAATTTTTTTATTAATTAAACGTATAAAAACGTTCCGTATCTGTATTTAAGAGCCAG

ATTTCGTCTAATAGAACAAATAGCTACAGTAAAAATAACTAGAATAATCGCTACACCCACTAGAAACCAC

GGATCGTAATACGGCAATCGGTTTTCGATAATAGGTGGAACGTATATTTTATTTAAGGACTTAACAATTG

TCTGTAAACCACAATTTGCTTCCGCCGATCCTGTATTAACTATCTGTAAAAGCATATGTTGGCCGGGCGG

AGCCGAACATTCTCCGATATTCAATTTTTGTATATCTATAATGTTATTAACCTCCGCATACGCATTACAG

TTCTTTTCTAGCTTGGATACTACACTAGGTACATCATCTAAATCTATTCCTATTTCCTCAGCGATAGCTC

TTCTATCCTTTTCCGGAAGTAATGAAATCACTTCAATAAATGATTCAACCATGAGTGTGAAACTAAGTCG

AGAATTACTCATGCATTTGTTAGTTATTCGGAGCGCGCAATTTTTAAACTGTCCTATAACCTCTCCTATA

TGAATAGCACAAGTGACATTAGTAGGGATAGAATGTTGAGCTAATTTTTGTAAATAACTATCTATAAAAA

GATTATACAAAGTTTTAAACTCTTTAGTTTCCGCCATTTATCCAGTCTGAGAAAATGTCTCTCATAATAA

ATTTTTCCAAGAAACTAATTGGGTGAAGAATGGAAACCTTTAATCTATATTTATCACAGTCTGTTTTGGT

ACACATAATGAATTCTTCTAATGCTGTACTAAATTCGATATCTTTTTCGATTTCTGGATATGTTTTTAAT

AAAGTATGAACAAAGAAATGGAAATCGTAATACCAGTTATGTTCAACTTTGAAATTGTTTTTTATTTTCT

TGTTAATGATACCAGCCACTTGGGAAAAGTCAAAGTCGTTTAATGCCGATTTAATACGTTCATTAAAAAC

AAACTTTTTATTCTTTAGATGAATTATTATTGGTTCATTGGAATCAAAAAGTAAGATATTATCGGGTTTA

AGATCTGCATGTAAAAAGTTGTCACAACAGGGTAGTTCGTAGATTTTAATGTATAACAGAGACATCTGTA

AAAAGATAAACTTTATGTATTGTACCAAAGATTTAAATCCTAATTTGATAGCTAACTCGGTATCTACTTT

ATCTGCCGAATACAGTACTAGGGGAAAAATTATAATATTTCCTCTTTCGTATTCGTAATTAGTTCTCTTT

TCATGTTCGAAAAAGTGAAACATGCGGTTAAAATAGTTTATAACATTAATATTACTGTTAATAACTGCAG

GATAAAAGTGGGATAGTAATTTCACGAATTTGATACTGTCCTTTCTCTCGTTAAACGCCTTTAGAAAAAC

TTTAGAAGAATATCTCAATGAGAGTTCCTGACCATCCATAGTTTGTATCAATAATAGCAACATATGAAGA

ACCCGCTTATACAGAGTATGTAAAAATGTTAATTTATAGTTTAATCCCATGGCCCACGCACACACGATTA

ATTTTTTTTCATCTCCCTTTAGATTGTTGTATAGAAATTTGGGTACTGTGAACTCCGCCGTAGTTTCCAT

GGGACTATATAATTTTGTGGCCTCGAATACAAATTTTACTACATAGTTATCTATCTTAAAGACTATACCA

TATCCTCCTGTAGATATGTGATAAAAATCGTCGTTTATAGGATAAAATCGTTTATCTTTTTGTTGGAAAA

AAGATGAATTAATGTAATCATTCTCTTCTATCTTTAGTAGTGTTTCCTTATTAAAATTCTTAAAATAATT

TAACAATCTAACTGACGGAGCCCAATTTTGGTGTAAATCTAATTGGGACATTATGTTGTTAAAATATAAA

CAGTCTCCTAATATAACAGTATCTGATAATCTATGGGGAGACATCCATTGATATTCAGGGGATGAATCAT

TGGCAACACCCATTTATTGTACAAAAAGCCCCAATTTACAAACGAAAGTCCAGGTTTGATAGAGACAAAC

TATTAACTATTTTGTCTCTGTTTTTAACACCTCCACAGTTTTTAATTTCTTTGGTAATGAAATTATTCAC

AATATCAGTATCTTCTTTATCTACCAGAGATTTTACTAACTTGATAACCTTGGCTGTCTCATTCAATAGG

GTAGTGATATTTGTATGTATGATATTGATATCTTTTTGAATTGTTTCTTTTAGAAGTGATTCTTTGATGG

TATCAGCATACGAATTACAATAATGCAGAAACTCAGTTAACATGCAGGAATTATAGTAAGCCAATTCCAA

TTGTTGCCTGTATTGTATTAGAGTATTAATATGCGCAATGATGTCCTTGCGTTTCTCTGATAGAATGCGA

GCAGCGATTTTGGCGTTATCATTTGACGATATTTCTGGAATAACGAATCCTGTTTCTACTAACTTCTTGG

TAGGACAAAGTGAAACAATCAAGAAAATAGCTTCTCCTCCTATTTGTGGAAGAAATTGAACTCCTCTAGA

TGATCTACTGACGATAGTATCTCCTTGACAGATATTGGACCGAACTACGGAAGTACCTGGAATGTAAAGC

CCTGAAACCCCCTCATTTTTTAAGCAGATTGTTGCCGTAAATCCTGCACTATGCCCAAGATAGAGAGCTC

CTTTGGTGAATCCATCACTATGTTTCAGTTTAACCAAGAAACAGTCAGCTGGTCTAAAATTTCCATCTCT

ATCTAATACAGAATCCAACTTGATGTCAGGGACTATGACCGGTTTAATGTTATATGTAACATTGAGTAAA

TCCTTAAGTTCATAATCATCGTTGTCATCAGTTATGTACGATCCAAACAATGTTTCTACCGGCATGGTGG

ATACGAAGATGCTATCCATCAGAATGTTTCCCTGATTAGTATTTTCTATATAGCTATTCTTCTTTAAACG

ATTTTCCGAATCAGTAACTATGTTCATTTTTTTAGGAGTAGGACGTCTAGCCAGTATGGAAGAGGATTTT

CTAGATACTCTCTTCAACATCTTTGATCTCAATGGAATGCAAAACCCCATGGTGTAACAACCAACGATAA

AAATAATATTGTTTTTTCACTTTTTATAATTTTACCATCTGACTCATGGATTCATTAATATCTTTATAAG

AGCTACTAACGTATAATTCTTTATAACTGAACTGAGATATATACACCGGATCTATGGTTTCCATAATTGA

GTAAATGAATGCTCGGCAATAACTAATGGCAAATGTATAGAACAACGAAATTATACTAGAGTTGTTAAAG

TTAATATTTTCTATGAGTTGTTCCAATAAATTATTTGTTGTGACTGCGTTCAAGTCATAAATTATCTTGA

TACTATCCAGTAAACAGTCTTTAAGTTCTGGAATATTATCATCCCATTGTAAAGCCCCTAGTTCGACTAT

CGAATATCCTGCTCTGATAGCAGTTTCAATATCGACGGACGTCAATACTGTAATAAAGGTGGTAGTATTG

TCATCATCGTGATAAACTACGGGAATATGGTCGTTAGTAGGTACCGTGACTTTACACAACGCGATATATA

ACTTTCCTTTTGTACCATTTTTAACGTAGTTGGGACGTCCTGCAGGGTATTGTTTTGAAGAAATGATATC

GAGAACAGATTTGATACGATATTTGTTGGATTCCTGATTATTCACTATAATATAATCTAGACAGATAGAT

GATTCGATAAATAGAGAAGGTATATCGTTGGTAGGATAATACATCCCCATTCCAGTATTCTCGGATACTC

TATTGATGACACTAGTTAAGAACATGTCTTCTATTCTAGAAAACGAAAACATCCTACATGGACTCATTAA

AACTTCTAACGCTCCTGATTGTGTTTCGAATGCCTCGTACAAGGATTTCAAGGATGCCATAGATTCTTTG

ACCAACGATTTAGAATTGCGTTTAGCATCTGATTTTTTTATTAAATCAAATGGTCGGCTCTCTGGTTTAC

TACCCCAATGATAACAATAGTCTTGTAAAGATAAACCGCAAGAAAATTTATACGCATCCATCCAAATAAC

CCTAGCACCGTCGGATGATATTAATGTATTATTATAGATTTTCCATCCACAGTTATTGGGCCAGTATACT

GTTAGCAACGGTATATCGAATAGATTACTCATGTAACCTACTAGAATGATAGTTCGTGTACTAGTCATAA

TATCTTTAATCCAATCTAAGAAATCTAAAATTAGATCTTTTACACTATTAAAGTTAACAAAGGTATTACC

CGGGTACGTGGATATCATATATGGTATTGGTCCATTATCAGTAATGGCTCCATAAACTGATACGGCGATG

GTTTTTATATGTGTTTGATCTAATGAGGACGAAATTCGCGCCCACAATTCATCTCTAGATATGTATTTAA

TATCGAACGGTAACACATCAATCTCGGGACGCGTATATGTTTCTAAATTCTTAATCCAAATATAATGATG

ACCTATATGCCCTATTATCATACTGTCAACTATAGTATACCTAGAGAACTTTCGATACATCTGCTGTTTC

CTGTAATCGTTAAATTTTACAAATCTATAACATGCTAAACCTTTTGACGACAGCCATTCATTAATTTCTG

ATATGGAATCTGTAATCTCAATACCGTATCGTTCTAAAGCCAGTGCTATATCTCCCTGTTCGTGGGAACG

CTTTCGTATAATATCGATCAATGGATAATATGAAGTTTTTGGAGAATAATATGATTCATGATCTATTTCG

TCCATAAACAATCTAGACATAGGAATTGGAGGCGATGATCTTAATTTTGTGCAATGGGTCAATCCTATAA

CTTCTAATATTGTAATATTCATCATCGACATAACACTATCTATGTTATCATCGTATATTAGTATACCACG

ACCTTCTTCATTTCGTGCCAAAATGATATACAGTCTTAAATAATTACGCAATATCTCAATAGTTTCATAA

TTGTTAGCTGTTTTCATCAAGGTTTGTATCCTGTTTAACATGATGGCGTTCTATAACGTCTCTATTTTCT

ATTTTTTAATTTTTTAAATTTTTAACGATTTACTGTGGCTAGATACCCAATCTCTCTCAAATATTTTTTT

AGCCTCGCTTACAAGCTGTTTATCTATACTATTAAAACTGACGAATCCGTGATTTTGGTAATGGGTTCCG

TCGAAATTTGCCGAAGTGATATGAACATATTCGTCGTCGACTATCAACAATTTTGTATTATTCTGAATAG

TGAAAACCTTCACAGATAGATCATTTTGAACACACAACGCATCTAGACTTCTGGCGGTTGCCATAGAATA

TACGTCGTTCTTATCCCAATTACCAACTAGAAGTCTGATCTTAACTCCTCTATTAATGGCTGCTTCTATA

ATGGAGTTGTAAATGTCAGGCCAATAGTAGCTACTACCGTCGACACGTGTAGTGGGAACTATGGCCAAAT

GTTCAATATCTATACTAGTCTTAGCCGACTTGAGTTTATCAATAACTACATCAGTGTCTAGATCTCTAGA

ATATCCCAATAGGTGTTCTGGAGAATCAGTAAAGAACACTCCACCTATAGGATTCTTAATATGATACGCA

GTGCTAACTGGCAGACAACAAGCCGCAGAGCATAAATTCAACCATGAATTTTTTGCGCTATTAAAGGCTT

TAAAAGTATCAAATCTTCTACGAAGATCTGTGGCCAGCGGAGGATAATCAGAATATACGCCTAACGTTTT

AATCGTATGTATAGATCCTCCAGTAAATGACGCGTTTCCTACATAACATCTTTCATCATCAGACACCCAA

AAACAACCGAGTAGTAGTCCCACATTATTTTTTTTATCTATATTAACGGTTATAAAATTTATATCCGGGG

AGTGACTTTGTAGCTCTCCCAGATTTCTTTTCCCTCGTTCATCTAGCAAAACTATTATTTTAATCCCTTT

TTCAGATACCTCTTTTAGTTTATCAAAAATAAGCGCTCCCCTAGTAGTACTCAGAGGATTACAACAAAAA

GATGCTATGTATATATATTTCTTAGCTAGAGTGATAATTTCGTTAAAACATTCAAATGTTGTCAAATGAT

CGGATCTAAAATCCATATTTTCTGGTAGTGTTTCTACCAGCCTACATTTTGCTCCCGCAGGTACCGATAC

AAATGGCCACATTTAGTTAACATAAAAACTTATATATCCTGTTCTATCAACGATTCTAGAATATCATCGG

CTATATCGCTAAAATTTTCATCAAAGTCGACATCACAACCTAACTCAGTCAATATATTAAGAAGTTCCAT

GATGTCATCTTCGTCTATTTCTATATCCGTATCCATTGTAGATTGTTGACCGATTATCGAGTTTAAATCA

TTACTAATACTCAATCCTTCAGAATACAATCTGTGTTTCATTGTAAATTTATAGGCGGTGTATTTAAGTT

GGTAGATTTTCAATTATGTATCATTATAGCAACAGTAATTCTTGCTCCTCCTTGATTTTAGCATCCTCTT

CATTATTTTCTTCTACGTACATAAGCATGTCCAATACGTTAGACAACACACCGACGATGGTGGCCGCCAC

AGACACGAATATGACTAGACCGATGACCATTTAAAAAATACTCTCTAGCTTTAACTTAAACTGTATCGAT

CATTCTTTTAGCACATGTATAATATAAAAACATTATTCTATTTCGAATTTAGGCTTCCAAAAATTTTTCA

TCCGTAAACCGATAATAATATATATAGACTTGTTAATAGTCGGAATAAATATATTAATGCTTAAACTATC

ATCATCTCCACGATTAGAGATACAATATTTACATTCTTTTTGCTGTTTCGAAACTTTATCAATACACGTT

AATACAAACCCAGGAAGGAGATATTGAAACTGAGGCTGTTGAAAATGAAACGGTGAATACAATAATTCAG

ATAATGTAAAATCATGATTCCGTATTCTGATGATATTAGAACTGCTAATGGATGTCGATGGTATGTATCT

AGGAGTATCTATTTTAACAAAGCATCGATTTGCTAATATACAATTATCCTTTTGATTAATTGTTATTTTA

TTCATATTCTTAAAAGGTTTCATATTTATCAATTCTTCTACATTAAAAATTTCCATTTTTAATTTATCTA

GCCCCGCAATACTCCTCATTACGTTTCATTTTTTGTCTAGAATGCCCATTTTGTTCATCTTGGTACATAG

ATTATCCAATTGAGAAGCGCATTTAGTAGTTTTGTACATTTTAAGTTTATTAACGAATCGTCGAAAACTA

GTTATAGTTAACATTTTATTATTTGATACCCTGATATTAATACCCCTGCCGTTACTATTATTTATAACTG

ATGTAACCCACGTAACATTGGAATTAATTATCGATAGTAATGCATCGACACTTCCAAAATTGTCTATTAT

AAACTCACCGATAATTTTTTTATTGCATGTTTTCATATTCATTAGGATTATCAAATCTTTAATCTTATTA

CGATTGTATGCGTTGATATTACAAGACGTCATTCTAAAAGACGGAGGATTTCCATCAAATGCCAGACAAT

CACGTACAAAGTACATGGAAATAGGTTTTGTTCTATTACGCATCATAGATTCATATAAAACACCCGTAGA

AATACTAATTTGTTTTACTCTATAAAATACTATTGCATCTATTTCATCGTTTTGTATAACGTCTTTCCAA

GTGTCAAATTCTAATTTTTTTTCATTGATAGTACCAAATTCTTCTATCTCTTTAACTACTTGCATAGATA

GGTAATTACAGTGATGCCTACATGCCGTTTTTTGAAACTGAATAGATGCATCTAGAAGCGATGCTACGCT

AGTCACGATCACCACTTTCATATTTAGAATATATGTATGTAAAAATATAGTAGAATTTCATTTTGTTTTT

TCTATGCTATAAATGAATTCTCATTTTGTATCTGCACATACTCCGTTTTATATCAATACCAAAGAAGGAA

GATATCTGGTTCTAAAAGCCGTTAAAGTATGCGATGTTAGAACTGTAGAATGCGAAGGAAGTAAAGCTTC

CTGCGTACTCAAAGTAGATAAACCCTCATCACCCACGTGTGAGAGAAGACCTTCGTCCCCGTCCAGATGC

GAGAGAATGAATAACCCTGGAAAACAAGTCCCGTTTATGAGAACGGACATGTTACAAAATATGTTTGCTG

CTAATCGCGACAACGTAACGTCAAGACTTTTGAACTAAAATACAATTATATCTTTTTCGATATTAATAAA

TCCGTGTCTCCCGGGTTTTTTATCTCTTTCAGTATGTGAATAGATAGGTATTTTATCTCTATTCATCATC

GAATTTAAGAGATCCGATAAACATTGTTTGTATTCTCCAGATGTCAGCATCTGATACAACAATATATGTG

CACATAAACCTCTGGCACTTATTTCATGTACCTTCCCCTTATCACTAAGGAGAATAGTATTTGAGAAATA

TGTATACATGATATTATCATGAATTAGATATACAGAATTTGTAACACTCTCGAAATCACACGATGTGTCG

GCGTTAAGATCTAATATATCACTCGATAACACATTTTCATCTAGATACACTAGACATTTTTTAAAGCTAA

AATAGTCTTTAGTAGTAACAGTAACTATGCGATTATTTTCATCGATGATACATTTCATCGGCATATTATT

ACGCGTACCATCAAAGACTATACCATGTGTATATCTAACGTATTCTAGCATAGTTGCCATACGTGCATTA

AACTTTTCAGGATCTTTGGATAGATCTTCCAATCTATCTATTTGAGAAAACATTTTTATCATGTTCAATA

GTTGAAACGTCGGATCCACTATATAGATATTATCTATAAAGATTTTAGGAACTATGTTCATGGTATCCTG

GCGAATATTAAAACTATCAATGATATGATTATCGTTTTCATCTTTTATCACCATATAGTTTCTAAGATAT

GGGATTTTACTTAATATAATATTATTTCCCGTAATAAATTTTATTAGAAATGCCAAATCTATAAGAAAAG

TCCTAGAATTAGTCTGAAGAATATCTATATCACCGTACCGTATATTTGGATTAATTAGATATAGAGAATA

TGATCCGTAACATATACAACTTTTATTATGACGTCTAAGATATTCTTCCATCAACTTATTAACATTTTTG

ACTAGGGAAGATACATTATGACGTCCCATTACTTTTGCCTTGTCTATTACAGCGACGTTCATAGAATTTA

GCATATCTCTTGCCAATTCTTCCATTGATGTTACATTATAAGAAATTTTAGATGAAATTACATTTGGAGC

TTTAATAGTAAGAACTCCTAATATATCCGTGTATGTGGTCACTAATACAGATTGTAGTTCTATAATCGTA

AATAATTTACCTATATTATATGTTTGAGTTTGTTTAGAAAAGTAGCTAAGTATACGATCTTTTATTTCTG

ATGCCGATGTATCAACATCGAAAAAAAATCTTTTTTTATTCTTTTTTACTAACGATACGAATATGTCTTT

GTTAAAAACAGTTATTTTCTGAATATTTCTAGCTTGTAATTTTAACATATGATATTCGTTCACACTAGGT

ACTCTGCCTAAATAGGTTTCTATAATCTTTAATGTAATATTAGGAAGAGTATTCTGATCAGGATTCCTAT

TCATTTTGAGGATTTAAAACTCTGATTATTGTCTAATATGGTCTCAACACAAACTTTTTCACAGAGCGAT

AGAGTTTTTGATAACTCGTTTTTCTTAAGAAATATAAAACTACTGTCTCCAGAGCTCGCTCTATCTTTTA

TTTTATCTAATTCGATACAAACTCCTGATACTGGTTCAGAAAGTAATTCATTAATTTTCAGTCCTTTATA

GAAGATATTTAATATAGATAATACAAAATCTTCAGTTCTTGATATCGATCTGATTGATCCTAGAACTAGA

TATATTAATAACGTGCTCATTAGGCAGTTTATGGCAGCTTGATAATTAGATATAGTATATTCCAGTTCAT

ATTTATTAGATACCGCATTGCCCAGATTTTGATATTCTATGAATTCCTCTGAAAATAAATCCAAAATAAC

TAGACATTCTATTTTTTGTGGATTAGTGTACTCTCTTCCCTCTATCATGTTCACTACTGGTGTCCACAAT

GATAAATATCTAGAGGGAATATAATATAGTCCATATGATGCCAATCTAGCAATGTCGAATAACTGTAATT

TTATTCTTCGCTCTTCATTATGAATTGAATCTTGAGGTATAAACCTAACACAAATTATATCATTAGACTT

TTCGTATGTAATGTCTTTCATGTTATAAGTTTTTAATCCTGGAATAGAATCTATTTTAATGAGGCTTTTA

AATGCAGCGTTCTCCAACGAGTCAAAGCATAATACTCTGTTGGTTTTCTTATATTCAATATTACGATTTT

CTTCTTTGAATGGAATAGGTTTTTGAATTAGTTTATAATTACAACATAATAGATAAGGAAGTGTGTAAAT

AGTACGCGGAAAAAACATAATAGCTCCCCTGTTTTCATCCATGGTTTTAAGTAAATGATCACTGGCTTCT

TTAGTCAATGGATATTCGAACATTAACCGTTTCATCATCATTGGACAGAATCCATATTTCTTAATGTAAA

GAGTGATCAAATCATTGTGTTTATTGTACCATCTTGTTGTAAATGTGTATTCGGTTATCGGATCTGCTCC

TTTTTCTATTAAAGTATCGATATCGATCTCGTCTAAGAATTCAACTATATCGACATATTTCATTTGTATA

CACATAACCATTACTAACGTAGAATGTATAGGAAGAGATGTAACGGGAACAGGGTTTGTTGATTCGCAAA

CTATTCTAATACATAATTCTTCTGTTAATACGTCTTGCACGTAATCTATTATAGATGCCAAGATATCTAT

ATAATTATTTTGTAAGATGATATTAACTATGTGATCTATATAAGTAGTGTAATAATTCATGTATTTCGAT

ATATGTTCCAACTCTGTCTTTGTGATGTCTAGTTTCGTAATATCTATAGCGTCCTCAAAAAATATATTCG

CATATATTCCCAAGTCTTCAGTTCTATCTTCTAAAAAATCTTCAACGTATGGAATATAATAATCTATTTT

ACCTCTTCTGATGTCATTAATGATATAGTTTTTGACACTATTTTCCGTCAATTGATTCTTATTCACTATG

TCTAAAAACCGGATAGCGTCCCTAGGACGAACTACTGCCATTAATATCTCTATTATAGCTTCTGGACATA

AATCATCTATTATACCAGAATTAATGGGAACTATTCCGTATCTATCTAACATAGTTTTAAGAAAGTCAGA

ATCTAAGACCTGATGTTCATATATTGGTTCATACATGAAATGATCTCTATTGATGATAGTGACTATTTCA

TTCTCTGAAAATTGGTAACTCATTCTATACACGCTTTCCTTGTTGATAAAGGATAGTATATACTCGATGG

AATTTGTACCAACAAACTGTTCTCTTATGAATCGTATATCATCATCTGAAATGATCATGTAAGGCATACA

TTTAACAATGAGAGACTTGTCTCCTGTTATCAATATACTATTCTTGTGATAATTTATGTGTGAGGCAAAT

TTGTCCACGTTCTTTAATTTTGTTATAGTAGATATCAAATCCAATGGAGATACAGTTCTTGGCTTAAACA

GATATAGTTTTTCTGGAACGAATTCTACAACATTATTATAAAGGACTTTGGGTATATAAGTGGGATGAAA

TCCTATTTTAATTAATGCGATAGCCTTGTCCTCGTGCAGATATCCAAACGCTTTTGTGATAGTATGGCAT

TCATTGTCTAGAAACGCTCTACGAATATCTGTAACAGATATCATCTTTAGAGAATACTAGTCGCGTTAAT

AGTACTAAAATTTGTATTTTTTAATCTATCTCAATAAAAAATTAATATGTATGATTCAATGTATAACTAA

ACTACTAACTGTTATTGATAACTAGAATCAGAATCTAATGATGACATAACTAAGAAGTTTATCTACAGCC

AATTTAGCTGCATTATTTTTAGCATCTCGTTTAGATTTTCCATCTGCCTTATCGAATACTCTTCCGTCAA

TGTCTACACAGGCATAAAATGTAGGAGAGTTACTAGGCCCCACTGATTCAATACGAAAAGACCAATCTCT

CCTAGTTATTTGGCAGTACTCATTAATAACGGTGACAGGGTTAACACCTTTCCAATAAATAATTTTTTTA

ACCGGAATAACATCATCAAAAGACTTATTATCCTCTCTCATTGATTTTTCGCGGGATACATCATCTATTA

TAGCATCAGCATCAGAATCTGTAGGCCGTGTATCAGCATCCATTGTCGTAGACCAACGAGGAGGAGTATC

GTCGGAACTGTACACCATAGTACTACGTTGAAGATCATACAGAGCTTTATTAACTTCTCGCTTCTCCATA

TTAAGTTGTTTAGTTAGTTGTGCAGTAGCTCCTTAGTCCAATGTTTTTAATAACCGCACACAATCTCTGT

GTCAGAACGCTCGTCAATATAGATCGTAGAAATTTTTTAGAGAGAACTAACACAACTAGCAATAAAACTG

ATCTTATTTTATCATTTTTTTATTCATCATCCTCTGGTGGTTCGTCGTTCCTATCGAATGTAGCTCTGAT

TAACCCGTCATCTATAGGTGATGCTGGTTCTGGAGATTCTGGAGGAGATGGATTATTATCCGGAAGAATC

TCTGTTATTTCCTTGTTTTCATGTATCGATTGCGTTGTAACATTAAGATTGCGAAATGCTCTAAATTTGG

GAGGCTTAAAGTGTTGTTTACAATCTCTACACGCGTGTCTAACTAATGGAGGTTCGTCAGCGGCTCTAGT

TTGAATCATCATCGGTGTAGTATTCCTACTTTTACAGTTAGGACACGGTGTATTGTATTTCTCGTCGAGA

ACGTTAAAATAATCGTTGTAACTCACATCCTTTATTTTATCTATATTGTATTCTACTCCTTTCTTAATGC

ATTTTATACCGAACAAGAGATAGCGAAGGAATTCTTTTTCGGTACCGCTAGTACCCTTAATCATATCACA

TAGTGTTTTATATTCTAAATTTGTGGCAATGGACGGTTTATTTCTATACGATAGTTTGTTTCTGGAATCC

TTTGAGTATTCTATACCAATATTATTCTTTGATTCGAATTTAGTTTCTTCGATATTAGATTTTGTATTAC

CTATATTCTTGATGTAGTACTTTGATGATTTTTCCATGGCCCATTCTATTAAGTTTTCCAAGTTGGCATC

ATCCACATATTGTGATAGTAATTCTCGGATATCAGTAGTGACTACCGCCATTGATATTTGTTCATTTGAT

GAGTAACTACTAATGTATACATTTTCCATTTATAACACTTATGTATTAACTTTGTTTATTTATATTTTTT

CATTATTATGTTGATATTAATAATCGTATTGTGGTTATATGGCTACAATTTCATAATGAGTTGAAGTCAG

TGTCCTATGATCAATGACGATAGCTTTACTCTGAAAGAAAGTATCAAATCGATAGTGCAGAGTCAACAAT

GAAAATGGATAAGACGATGACAAAGTTTCAGAATAGAGTCAAAATGGTAAAAGAAATAAATCAGACGATA

AGAGCAGCACAAACTCATTGAGACATTGAAACTAGGATATATAAAATTTAAGTGAATGATTAGGACAGGA

CTACTACTCTAGAAGATATAGCACCATCTATTATTCCAAATAATCAGAAAACTTATAAACTATTCTCGGA

CATTTCAGTCATTGGCAAAGCATCACAGAATCCGAGTAAGATGATATATGCTCGCTGCTTTACATGTTTC

CCAATTTGTTTGGAGATGACCATAGATTCATTTGTTATAGAATGCATCCAATGAGTAAAATCAAACACAA

AATCTTCGTTCAAACTTAATCTTATTAGAATATTAGTGGAAGAAAGATTCTATAATAATGAATGCAGAGA

TTATAAATGGAGAATAATTGGAACACAAGTTGATAAAATATTGATAGCTAAATATACAATAGATGCAATG

TATCGCATAAGACCGATATATATAATACAAAGCAGTACAGATACAATGATGATGTAGAAAATGGATTCAT

TGGATTGGATAAACTAAAATTAAACATTGTTCATGATATAGTTGAATCATGTATACCTGTTCGTATGCCT

GTGGCTAAGATACTGTGTAAAGAAATGGTAAATAAATACTTTGAGAATCTTTAAGAGTGTATTGACTTTG

TTAGTGAATATGCATTCCATCTTTCTCCAATACTAATTCAAATTGTTAAATTAATAATGGAATAGTATAA

ATAGTTATTAGTGATAAGATAGTAAAAATAATTATTAGAATAGTGTAGTATCATAGATAACTCTCTTCTA

TAAAAAATGGATTTTATTCGTAGAAAGTATCTTATATACACAGTAGAAAATAATATAGATTTTTTAAAGG

ATGATACATTAAGTAAAGTAAACAATTTTACCCTCAATCATGTACTAGCTCTCAAGTATCTAGTTAGCAA

TTTTCCTCAACATGTTATTACTAAGGATGTATTAGCTAATACCAATTTTTTTGTTTTTATACATATGGTA

CGATGCTGTAAAGTATACGAAGCGGTTTTACGACACGCATTTGATGCACCCACGTTGTACGTTAAAGCAT

TGACTAAGAATTATTTATCGTTTAGTAACACAATACAGTCGTACAAGGAAACCGTGCATAAACTAACACA

AGATGAAAAATTTTTAGAGGTTGCCGAATACATGGACGAATTAGGAGAACTTATAGGCGTAAATTATGAC

TTAGTTCTTAATCCATTATTTCACGGAGGGGAACCCATCAAAGATATGGAAATCATTTTTTTAAAACTGT

TTAAGAAAACAGACTTCAAAGTTGTTAAAAAATTAAGTGTTATAAGATTACTTATTTGGGCATACCTAAG

CAAGAAAGATACAGGCATAGAGTTTGCGGATAATGATAGACAAGATATATATACTCTATTTCAACAAACT

GGTAGAATAGTCCATAGCAATCTAACAGAAACGTTTAGGGATTATATCTTTCCCGGAGATAAGACTAGCT

ATTGGGTGTGGTTAAACGAAAGTATAGCTAATGATGCGGATATCGTTATTAATAGATCCGCCATTACCAT

GTATGATAAAATTCTTAGTTATATATACTCTGAGATAAAACAGGGACGCGTTAATAAAAACATGCTTAAG

TTAGTTTATATCTTTGAGCCTGAAAAAGATATCAGAGAACTTCTGCTAGAAATCATATATGATATTCCTG

GAGATATCCTATCTATTATTGATGCAAAAAACGATGATTGGAAAAAATATTTTATTAGTTTTTACAAAGC

TAATTTTATTAACGGTAATACATTTATTAGTGATAGAACGTTTAACGAGGACTTATTCAGAGTTGTTGTT

CAAATAGATCCCGAATATTTCGATAATGAACGAATTATGTCTTTATTCTATACGAGTGCTGCGGACATTA

AACGATTTGATGAGTTAGATATTAATAACAGTTATATATCTAATATAATTTATGAGGTGAACGATATCAC

ATTAGATACAATGGATGATATGAAGAAGTGTCAAATCTTTAACGAGGATACGTTGTATTATGTTAAGGAA

TACAATACATACCTGTTTTTGCACGAGTCGGATCCCATGGTCATAGAGAACGGAATACTAAAGAAACTGT

CATCTATAAAATCCAAGAGTAGACGGCTGAACTTGTTTAGCAAAAACATTTTAAAATATTATTTAGACGG

ACAATTGGCTCGTCTAGGTCTTGTGTTAGATGATTATAAAGGAGACTTATTAGTTAAAATGATAAACCAT

CTCAAATCTGTGGAGGATGTATCCGCATTCGTTAGATTTTCTACAGATAAAAACCCTAGTATTCTTCCAT

CGCTAATCAAAACTATTTTAGCTAGTTATAATATTTCCATCATCGTCTTATTTCAAAGGTTTTTAAGAGA

TAATCTATATCATGTAGAAGAATTCTTGGATAAAAGCATCCATCTAACCAAGACTGATAAGAAATATATA

CTTCAATTGATAAGGCACGGTAGATCATAGAACAAACCAAATATATTATTAATAATTTGTATATACATAG

ATATAATTATCATATATTAAAAAATAACACATTTTTGATAAATGGAAACTGTTGCAACAATTCAGACTCC

CACCAAATTAATGAATAAAGAAAATGCAGAAATGATTTTGGAAAAAATTGTTAATCATATAGCTATGTAT

ATTAGTGACGAATCAATATATTCAGAAAATAATCCTGAATATATTGATTTTCGTAACAGATACGGAGACT

ATAGATCTCTCATTATAAAAAGTGATCACGAGTTTGTAAAGCTATGTAAAGATCATGCAGAGAAAAGTTC

TCCAGAAACGCAACAAATGATTATCAAACACATATACGAACAATATCTTATTCCAGTATCTGAAGTACTG

TTAAAACCTATAATGTCCATGGGTGACATATTTACATATAACGGATGTAAAGACAATGAATGGATGCTAG

AACAACTCTCTACCCTAAACTTTAACAATCTCTACACATGGAACTCATGTAGCATAGGCAATGTAACGCG

TCTGTTTTATACATTTTTTAGTTATCTGATGAAAGATAAACTAAATATATAAGTATAATCCCATTAATAC

TTTAACCTGATGTATTATTACCTGCATCTTATTAGAATATTAACCTAACTAAAAGACATAAAAAGCGGGG

ATATAAATATTATGGCAGCAACCGTTCCGCGTTTTGACGATGTGTACAAAAATGCACAAAGAAGAATTCT

AGATCAAGAAACATTTTTTAGTAGAGGTCTAAGTAGACCGTTAATGAAAAACACATATCTATTTGATAAT

TACGCGTATGGATGGATACCAGAAACTGCAATTTGGAGTAGTAGATACGCAAACCTAGATGCTAGTGACT

ATTATCCCATTTCGTTGGGATTACTTAAAAAGTTTGAATTTCTCATGTCTCTATATAAAGGTCCTATTCC

CGTATATGAAGAAAAAGTAAATACTGAATTCATTGCTAATGGATCTTTCTCCGGTAGATACGTATCATAT

CTTAGAAAGTTTTCTGCCCTTCCAACAAACGAGTTTATTAGTTTTTTATTATTGACCTCCATCCCTATCT

ATAATATCTTATTCTGGTTTAAAAACACACAGTTTGATATTACTAAACACACATTATTCAGATACGTCTA

TACAGATAATACCAAACACCTTGCGTTGGCTAGGTATATACATCAAACAGGAGACTATAAGCCTTTGTTT

AGTCGTCTCAAAGAGAATTATATATTTACCGGTCCCGTTCCAATAGGTATCAAAGATATAGATTACCCTA

ATCTTAGTAGAGCAAGAAGTCCATCCGATTATGAGACATTAGCTAATATTAGTACTATATTGTACTTTAC

CAAGTATGATCCAGTATTAATGTTTTTATTGTTTTACGTACCTGGGTATTCAATTACTACAAAAATTACT

CCAGCCGTAGAATATCTAATGGATAAACTGAATCTAACAAAGAGCGACGTACAACTGTTGTAAATTATTT

TTATGCTTCGTAAAATGTAGGTCTTGAACCAAACATTCTTTGAAAAAATGAGATGCATAAAACTTTATTA

TCCAATAGATTAACTATTTCAGACGTCAATCGTTTAAAGTAAACTTCGTAAAATATTCTTTGATTGCTGC

CGAGTTTAAAACTTCTATCGATAATTGTTTCATATGTTTTAATATTTACAAGTTTTTTGGTCCATGGTAC

ATTAGCTGGACAGATATATGCAAAATAATATCGTTCTCCAAGTTCTATAGTCTCTGGATTGTTTTTATTA

TATTCAGTAACCAAATACATATTAGGGTTATCTGCGGATTTATAATTTGAGTGATGCATTCGACTCAACA

TAAATAATTCTAGAGGAGACGATCTACTATCAAATTCGGATCGTAAATCTGTTTCTAAAGAACGGAGAAT

ATCTATACATACCTGATTAGAATTCATCCGTCCTTCAGACAACATCTCAGACAGTCTGGTCTTGTATGTC

TTAATCATATTCTTATGAAACTTGGAAACATCTCTTCTAGTTTCACTAGTACCTTTATTAATTCTCTCAG

GTACAGATTTTGAATTCGACGATGCCGAGTATTTCATCGTTGTATATTTCTTCTTCGATTGCATAATCAA

ATTCTTATATACCGCCTCAAACTCTATTTTAAAATTATCAAACAATACTCTACTATTAATCAGTCGTTCT

AACTCCTTTGCTATTTCTATGGACTTATCTACATCTTGACTGTCTATCTCTGTAAACACGGAGTCGGTAT

CTCCATACACGCTACGAAAACGAAATCTATAATCTATAGGCAACGATGTTTTCACAATCGGATTAATATC

TCTATCGTCCATATAAAATGGATTACTTAATGTATTGGCAAACCGTAACATACCGTTGGATAACTCTGCT

CCATTTAGTACCGATTCTAGATACAAGATCATTCTACGTCCTATGGATGTGCAACTCTTAGCCGAAGCGT

ATGAGTATAGAGCACTATTTCTAAATCCCATCAGACCATATACTGAGTTGGCTACTATCTTGTACGTATA

TTGCATGGAATCATAGATGGCCTTTTCAGTTGAACTGGTAGCCTGTTTTAACATCTTTTTATATCTGGCT

CTCTCTGCCAAAAATGTTCTTAATAGTCTAGGAATGGTTCCTTCTATTGATCTATCGAAAATTGCTATTT

CAGAGATGAGGTTCGGTAGTCTAGGTTCACAATGAACCGTAATATATCTAGGAGGTGGATATTTCTGAAG

CAAGAGCTGATTATTTATTTCTTCTTCCAATCTATTGGTACTAACAACGACACCGACTAATGTTTCCGGA

GATAGATTTCCAAAGATACACACATTAGGATACAGACTGTTATAATCAAAGATTAATACATTATTACTAA

ACATTTTTTGTTTTGGAGCAAATACCTTACCGCCTTCATAAGGAAACTTTTGTTTTGTTTCTGATCTGAC

TAAGATAGTTTTAGTTTCCAACAATAGCTTTAACAGTGGACCCTTGATGACTGTACTCGCTCTATATTCG

AATACCATGGATTGAGGAAGCACATATGTTGACGCACCAGCGTCTGTTTTTGTTTCTACTCCATAATACT

CCCACAAATACTGACACAAACAAGCATCATGAATACAGTATCTAGCCATATCTAAAGCTATGTTTAGATT

ATAATCCTTATACATCTGAGCTAAATCAATGTCATCCTTTCCGAAAGATAATTTATATGTATCATTAGGT

AAAGTAGGACATGATAGTACGACTTTAAATCCATTTTCCCAAATATCTTTACGAATTACTTTACATATAA

TATCCTCATCAACAGTCACATAATTACCTGTTGTTAAAACCTTTGCAAATGTATCGGCTTTGCCTTTCGC

GTCCGTAGTATCGTCACCGATGAACGTCATTTCTCTAACTCCTCTATTTAATACTTTACCCATGCAACTG

AACGCGTTCTTGGATATAGAATCCAATTTGTACGAATCCAATTTTTCAGATTTTTGAATGAATGAATATA

GATCGAAAAATATAGTTCCATTATTGTTATTAACGTGAAACGTAGTATTGGCCATGCCGCATACTCCCTT

ATGACTAGACTGATTTCTCTCATAAATACAGAGATGTACAGCTTCCTTTTTGTCTGGAGATCTAAAGATA

ATCTTCTCTCCTGTTAATAACTCTAGACGATTAGTAATATATCTCAGATCAAAGTTATGTCCGTTAAAGG

TAACGACGTAGTCGAACGTTAGTTCCAACAATTGTTTAGCTATTCGTAACAAAACTATTTCAGAACATAG

AACTAGTTCTCGTTCGTAATCCATTTCCATTAGCGACTGTATCCTCAAACATCCTCTATCGACGGCTTCT

TGTATTTCCTGTTCCGTTAACATCTCTTCATTAATGAGCGTAAACAGTAATCGTTTACCACTTAAATCGA

TATAACAGTAACTTGTATGCGAGATTGGGTTAATAAATACAGAAGGAAACTTCTTATCGAAGTGACACTC

TATATCTAGAAATAAGTACGATCTTGGGATATCGAATCTAGGTATTTCTTTAGCGAAACAGTTACGTGGA

TCGTCACAATGATAACATCCATTGTTAATCTTTGTCAAATATTGCTCGTCCAACGAGTAACATCCGTCTG

GAGATATCCCGTTAGAAATATAAAACCAACTAATATTGAGAAATTCATCCATGGTGGCATTTTGTATGCT

GCGTTTCTTTGGCTCTTCTATCAACCACATATCTGCGACGGAGCATTTTCTATCTTTAATATCTAGATTA

TAACTTATTGTCTCGTCAATGTCTATAGTTCTCATCTTTCCCATCGGCCTCGCATTAAATGGAGGAGGAG

ATAATGACTGATATATTTCGTCCGTCACTACGTAATAAAAGTAATGAGGAAATCGTATAAATACTGTCTC

GCCATTTCGACATCTGGATTTCAGATATAAAAATCTGTTTTCACCGTGACTTTCAAACCAATTAATACAC

CTAACATCCATTTCTAGAATTTAGAAATATATTTTCATTTAAATGAATCCCAAACATTGGGGAAGAGCCA

TATGGACCATTATTTTTATAGTACTTTCGCAAGCGGGTTTAGACGGCAACATAGAAGCGTGTAAACGAAA

AATATATACTATAGTCAGCACTCTTCCATGTCCTGCATGTAGACGACACGCGACTATCGCTATAGAGGAC

AATAATGTCATGTCTAGCGATGATCTGAATTATATTTATTATTTTTTCATCAGATTATTTAACAATTTGG

CATTTGATCCCAAATACGCAATCGATGTGTCAAAGGTTAAACCTTTATAAACTTAACCCATTATAAAACT

TATGATTAGTCACGACTGAAATAACCGCGTGATTATTTTTTGGTATAATTCTACACGGCATGGTTTCTGT

GACTATGAATTCAACACCTGTTATCTTAGTGAAATCTTTAACAAACAGCAAGGGTTCGTCAAAGACATAA

AACTCATTGTTTACGATCGAAATAGACCCCCTATCACACTTAAAATAAAAAATATCCTTATCCTTTACCA

CCAAATAAAATTCTGATTGGTCAATGTGAATGTATTCACTTAACAGTTCCACAAATTTATTTATTAACTC

CGAGGCACATACATCGTCGGTATTTTTTATGACAAACTTTACTCTTCCAGCATCCGTTTCTAAAAAAATA

TTAACGAGTTCCATTTATATCATCCAATATTATTGAAATGACGTTGATGGACAGATGATACAAATAAGAA

GGTACGGTACCTTTGTCCACCATCTCCTCCAATTCATACTCTATTTTGTCATTAACTTTAATGTGTGAAA

ACAGTACGCCACATGCTTCCATGACAGTGTGTAACACTTTGGATACAAAATGTTTGACATTAGTATAATT

GTCCAAGACTGTCAATCTATAATAGATAGTAGCTATAATATATTCTATGATGGTATTGAAGAAGATGACA

ACCTTGGCATATTGATCATTTAACACAGACATGGTATCAACAAATAGCTTAAATGAAAGAGAATCAGTAA

TTGGAATAAGCGTCTTCTCGATGTAGTGTCCGTATACCAACATGTCTGATATTTTGATGTATTCCATTAA

ATTATTTAGTTTTTTCTTTTTATTCTCGTTAAACAGAATTTCTGTCAATGGACCCCAACATCGTTGACCT

ATTAAGTTTTGATTGATTTTTCCGTGTAAGGCGTATCTAGTCAGATCGTATAGCCTATCCAATAATCCAT

CGTCTGTGCGTAGATCACATCGTACACTTTTTAATTTTCTATAGAAGAGTGACAGACATCTGGAGCAATT

ACAGACAGCAATTTCTTTATTCTCTACAGATGTAAGATACTTGAAGACATTCCTATGATGATGCAGAATT

TTGGATAACACGGTATTGATGGTATCTGTTACCATAATTCCTTTGACTGATAGTGTCAAAGTACAAGATT

TCCAATCTTTTGCAATTTTCAGTACCATTATCTTTGTTTTGATATCTATATCAGACAGCATGGTACGTCT

GACAACACAGGGATTAAGACGGAAAGATGAAATGATTCTCTCAACATCTTCAATAGATACCTTGCTATTT

TTTTTGGCATTATCTATATGTGAGAGAATATCCTCTAGAGAATCAGTATCCTTTTTGATGATAGTGGATC

TCAATGACATGGGACGTCTAAACCTTCTTATTCTATCACCAGATTGCATGGTGATTTGTCTTCTTTCTTT

TATCATGATGTAATCTCTAAATTCATCGGCAAATTGTCTATATCTAAAATCATAATATGAGATGTTTACC

TCTACAAATATCTGTTCGTCCAATGTTAGAGTATCTATATCAGTTTTGTATTCCAAATTAAACATGGCAA

CGGATTTAATTTTATATTCCTCTATTAAGTCCTCGTCGATAATAACAGAATGTAGATAATCATTTAATCC

ATCGTACATTGTTGGAAGATGCTCGTTGACAAAATCTTTAATTGTCTTGATGAAGGTGGGACTATATCTA

ACATCTTGATTAATAAAATTTATAACATTGTCCATAGGATACTTTGTAACTAGTTTTATACACATCTCTT

CATCGGTAAGTTTAGACAGAATATCGTGAACAGGTGGTATATTATATTCATCAGATATACGAAGAATAAT

GTCCAAATCTATATTGTTTAATATATTATATAGATGTAGTGTAGCTCCTACAGGAATATCTTTAACTAAG

TCAATGATTTCATCAACAGTTAGATCTATTTTAAAGTTAATCATATAGGCATTGATTTTTAAAAGGTATG

TAGCCTTGACTACATTCTCATTAATTAACCATTCCAAGTCACTGTGTGTAAGAAGATTATATTCTATCAT

AAGCTTGACTACATTTGGTCCCGATACCATTAAAGAATTCTTATGATATAAGGAAACAGCTTTTAGGTAC

TCATCTACTCTACAAGAATTTTGGAGAGCCTTAACGATATCAGTGACGTTTATTATTTCAGGAGGAAAGA

ACCTAACATTGAGAATATCTGAATTAATAGCTTCCAGATACAGTGATTTTGGCAATAGTCCGTGTAATCC

ATAATCCAGTAACACGAGCTGGTGCTTGCTAGACACCTTTTCAATGTTTAATTTTTTTGAAATAAGCTTT

GATAAAGCCTTCCTCGCAAATTCCGGATACATGAACATGTCGCCAACATGATTAAGTATTGTTTTTCATT

ATTTTTATATTTTCTCAACAAGTTCTCAATACCCCAATAGATAATAGAATATCACCCAATGCGTCCATGT

TGTCTATTTCCAACAGGTCGCTATATCCACCAATAGAAGTTTTCCCAAAAAAGATTCTAGGAACAGTTCT

ACCACCAGTAATTTGTTCAAAATAGTCACGCAATTCATTTTCGGGTTTAAATTCTTTAATATCTACAATT

TCATACGCTCCTCTTTTGAAACTAAACTTATTTAGAATATCCAGTGCGTTTCTACAAAAAGGACACGTAA

ACTTGACAAAAATTGTCACTTTGTTATTGGCCAACCTTTGTTGTACAAATTCCTCGGCCATTTTTAATAT

TTAAGTGATACAAAACTATCTCGACTTATTTAACTCTTTAGTCGAGATATATGGACACAGATAGTTATAT

GATAACCAACTACAGAAGACAAACGCTATAAAAAACATAATTACAACGAGCATATTTATAAATATTTTTA

TTCAGTATTACTTGATATAGTAATATTAGGTACAGTCAAACATTCAACCACTCTAGATACATTAACTCTC

TCATTTTCTTTAACAAATTCTGCAATATCTTCGTAAAAAGATTCTTGAAACTTTTTAGAATATCTATCGA

CTCTAGATGAAATAGCGTTCGTCAACATACTATGTTTTGTATACATAAAGGCGCCCATTTTAACAGTTTC

TAGTGACAAAATGCTAGCGATCCTAGGATCCTTTAGAATCACATAGATTGACGATTCGTCTCTCTTAGTA

ACTCTAGTAAAATAATCATACAATCTAGTACGCGAAATAATATTATCCTTGACTTGAGGAGATCTAAACA

ATCTAGTTTTGAGAACATCGATAAGTTCATCGGGAATGACATACATACTATCTTTAATAGAACTCTTTTC

ATCCAGTTGAATGGATTCGTCCTTAACCAACTGATTAATGAGATCTTCTATTTTATCATTTTCCAGATGA

TATGTATGTCCATTAAAGTTAAATTGTGTAGCGCTTCTTTTTAGCCTAGCAGCCAATACTTTAACATCAC

TAATATCGATATACAAAGGAGATGATTTATCGATGGTATTAAGAATTCGTTTTTCGACATCCGTCAAAAC

CAATTCCTTTTTGCCTGTATCATCCAGTTTGCCATTCTTTGTAAAGAAATTATTTTCTACTAGACTATTA

ATAAGACTGATAAGGATTCCTCCATAATTGCACAATCCAAACTTTTTCACAAAACTAGACTTTACGAGAT

CTACAGGAATGCGTACTTCAGGTTTCTTAGCTTGTGATTTTTTCTTTTGCGGACATTTTCTAGTGACCAA

CTCATCTACCATTTCATTGATTTTAGCAGTGAAATAAGCTTTCAATGCACGGGCACTGATACTATTGAAA

ACGAGTTGATCTTCAAATTCCGCCATTTAAGTTCACCAAACAACTTTTAAATACAAATATATCAATAGTA

GTAGAATAAGAACTATAAAAAAAATAATAATTAACCAATACCAACCCCAACAACCTGTATTATTAGTTGA

TGTGACAGTTTTCTCATCACTTAGAACAGATTTAACAATTTCTATAAAGTCTGTCAAATCATCTTCCTGA

GAACCCATAAATACACCAAATATAGCAGCGTACAACTTATCCATTTATACATTGAATATTGGCTTTTCTT

TATCGCTATCTTCATCATATTCATCATCAATATCGACAAGTCCCAGATTACGAACCAGATCTTCTTCTAC

ATTTTCAGTCATTGATACGCGTTCACTATCTCCAGAGAGTCCGATAACGTTAGCCACCACTTCTCTATCA

ATGATTAGTTTCTTGAGCGCGAATGTAATTTTTGTTTCCGTTCCGGATCTATAGAAAACTACAGGTGTGA

TAATTGCCTTGGCTAATTGTCTTTCTCTTTTACTGAGTGATTCTAGTTCACCTTCTATAGATCTGAGAAT

GGATGATTCTCCAGTCGAAACATATTCTACCATGGCTCCGTTTAATTTGTTGATGAAGATGGATTCATCC

TTAAATGTTTTCTCTGTAATAGTTTCCGCCGAAAGACTATGCAAAGAATTTGGAATGCGTTCCTTGTGCG

TAATGTTTCCATAGACAGCTTCTAGAAGTTGATACAACATAGGACTAGCCGCGGTAACTTTTATTTTTAG

AAAGTATCCATCGCTTCTATCTTGTTTAGATTTATTTTTATAAAGTTTAGTCTCTCCTTCCAACATAATA

AAAGTGGAAGTCATCTGACTAGATAAACTATCAGTAAGTTTTATAGAGATAGATGAACAATTAGCGTATT

GAGAAGCATTTAGTGTAACGTATTCGATACATTTTGCATTAGATTTACTAATCGATTTTGCATACTCTAT

AACACCCGCACAAGTCTGTAGAGAATCGCTAGATGCTGTAGGTCTTGGTGAAGTTTCAACTCTCTTCTTG

ATTACCTTACTCATGATTAAACCTAAATAATTGTACTTTGCAATATAATGATATATATTTTCACTTTATC

TCATTTGAGAATAAAAATGTTTTTGTTAACCGCTGCATGATGTACAGATTTCGGAATCGCAAACCACTTG

TGGTTTTATTTTATCCTTGTCCAATGTGAATTGAATGGGAGCGGATGCGGGTTTCGTACGTAGATAGTAC

ATTCCCGTTTTTAGACCGAGACTCCATCCGTAAAAATGCATACTCGTTAGTTTGGAATAACTCGGATCTG

CTATATGGATATTCATAGATTGACTTTGATCGATGAAGGCTCCCCTGTCTGCAGCCATTTTTATGATCGT

CTTTTGTGCAATTTCCCAAATAGTTTTATAAACTCGCTTAATATCTTCTGGAAGGTTTGTATTCTGAATG

GATCCACCATCTACCATAATCCTATTCTTGATCTCATCATTCCATAATTTTCTCTCGGTTAAAACTCTAA

GGAGATGCGGGTTAACTACTTGGAATTCTCCAGACAATACTCTCCGAGTGTAAATATTACTGGTATACGG

TTCCACCGACTCATTATTTCCCAAAATTTGAGCAGTTGATGCAGTCGGCATAGGTGCCACCAATAAACTA

TTTCTAAGACCGTATGTTCTGATTTTATCTTTTAGATGTTCCCAATTCCAAAGATCCGACGGTACAACAT

TCCAAAGATCATATTGTAGAATACCGTTACTGGCGTACGATCCTACATATGTATCATATGGTCCTTCCTT

CTCAGCTAGTTTACAACTCGCCTCTAATGCACCGTAATAAATGGTTTCAAAGATCTTCTTATTTAGATCT

TGTGCTTCCGGGCTATCAAATGGATAATTTAAGAGAATAAACGCGTCCGCTAATCCTTGAACACCAATAC

CGATAGGTCTATGTCTCTTATTAGAGATTTCAGCTTCTGGAATAGGATAATAATTAATATCTATAATTTT

ATTGAGATTTCTGACAATTACTTTGACCACATCCTTCAGTTTGAGAAAATCAAATCGCCCATCTATTACA

AACATGTTCAATGCAACAGATGCCAGATTACACACGGCTACCTCATTAGCATCCGCATATTGTATTATCT

CAGTGCAAAGATTACTACACTTGATAGTTCCTAAATTTTGTTGATTACTCTTTTTGTTACACGCATCCTT

ATAAAGAATGAATGGAGTACCAGTTTCAATCTGAGATTCTATAATCGCTTTCCAGACGACTCGAGCCTTT

ATTATAGATTTGTATCTCCTTTCTCTTTCGTATAGAGTATACAATCGTTCGAACTCGTCTCCCCAAACAT

TGTCCAATCTAGGACATTCATCCGGACACATCAACGACCACTCTCCGTCATCCTTCACTCGTTTCATAAA

GAGATCAGGAATCCAAAGAGCTATAAATAGATCTCTTGTTCTATGTTCCTCGTTTCCTGTATTCTTTTTA

AGATCGAGGAACGCCATAATATCAGAATGCCACGGTTCCAAGTATATGGCCATAACTCCAGGCCGTTTGT

TTCCTCCCTGATCTATGTATCTAGCGGTGTTATTATAAACTCTCAACATTGGAATAATACCGTTTGATAT

ACCATTGGTACCGGAGATATAGCCTCCACTGGCACGAATATTACTAATTGATAGACCTATTCCCCCTGCC

ATTTTAGAGATTAATGCGCATCGTTTTAACGTGTCATAGATGCCTTCTATGCTATCATCGATCATGTTAA

GTAGAAAACAGCTAGACATTTGGTGACGAGTAGTTCCCGCATTAAATAAGGTAGGAGAAGCGTGCGTAAA

CCATTTTTCAGAAAGTAGATTGTACGTCTCAATAGCTGAGTCTATATCCCATTGATGAATTCCTACTGCG

ACACGCATTAACATGTGCTGAGGTCTTTCAACAATTTTGTTGTTTATTTTCAACAAGTAGGATTTTTCCA

AAGTTTTAAAACCAAAATAGTTGTATGAAAAGTCTCGTTCGTAAATAATAACCGAATTGAGCTTATCCTT

ATATTTGTTAACTATATCCATGGTAATACTTGAAATAATCGGAGAATGTTTCCCATTTTTAGGATTAACA

TAGTTGAATAAATCCTCCATCACTTCACTAAATAGTTTTTTTGTTTCCTTGTGTAGATTTGATATGGCTA

TTCTGGCGGCTAGAATGGCATAATCCGGATGTTGTGTAGTACAAGTGGCTGCTATTTCGGCTGCCAGAGT

GTCCAATTCTACCGTTGTTACTCCATTATATATTCCTTGAATAACCTTCATAGCTATTTTAATAGGATCT

ATATGATCCGTGTTTAAGCCATAGCACAATTTTCTAATACGAGACGTGATTTTATCAAACATGACATTTT

CCTTGTATCCATTTCGTTTAATGACAAACATTTTTGTTGGTGTAATAAAAAAAAATTATTTAATTTTTCA

TTAATAGGGATTTGACGTATGTAGCGTACAAAATTATCGTTCCTGGTATATAGATAAAGAGTCCTATATA

TTTGAAAATCGTTACGGTTCGATTAAACTTTAATGATTGCATAGTGAATATATCATTAGGATTTAACTCC

TTGACTATCATGGCGGTGCCAGAAATTACCATCAAAAGCATTAATACAGTTATACAGATCGCAGTTAGAA

CGGTTATAGCATCCACCATTTATATCTAAAAATTAGATCAAAGAATATGTGACAACGTCCTAGTTGTATA

CTGAGAATTGACGAAACAATGTTTCTTACATATTTTTTTCTTATTAGTAACCGACTTAATAGTAGGAACT

GGAAAACTAGACTTGATTATTCTATAAGTATAGATACCCTTCCAAATAATGTTCTCTTTGATAAAAGTTC

CAGAAAATGTAGAATTTTTTAAAAAGTTATCTTTTGCTATTACTAATATCGTGGTTAGACGCTTATTATT

AATATGAGTGATGAAATCCACACCGCTTCTAGATATCGCTTTTATTTCCACATTAGATGGTAAATCCAAT

AGTGAAACTATCTTTTTAGGAATGTATGGACTTGCGTTTAGAGGAGTGAACGTCTTCGGAGTAGTAAAGG

ATGATTCGTCAAATGAATAAACAATTTCACAAATGGATGTTAATGTATTAGTAGGAAATTTTTTGACGCT

AGTGGAATTGAAGATTCTAATGGATGATGTTCTACCTATTTCATCCGATAACATGTTAATTTCCAACACC

AACGGTTTTAATATTTCGATGATATACGGTAGTCTCTCTTTCGGACTTATATAGCTTATTCCACAATACG

AGTCATTATATACTCCAAAAAACAAAATAACTAGTATAAAATCTTTATCGAATGGGAAAAACGAAATTAT

CGATATAGGTATAGAATCCGGAACATTGAACGTATTAATACTTAATTCTTTTTCAGTGGTAAGAACCGAT

AGGTTATTGACATTGTATGGTTTTAAATATTCTATAACTTGAGACTTGATAGATATTAATGACGAATTGA

AAATTATTTTTATCACCACGTGTGTTTCAGGATCATCGTCGACGCCAGTCAACCAACCGAATGGAGTAAA

ATAAATATCATTAATATATGCTCTAGATATTAGTATTTTTATTAATCCTTTGATTATCATCTTCTCGTAC

GCGAATGATTCCATGATCAAGAGTGATTTGAGAACATCCTCCGGAGTATTAATGGGCTTAGTAAACAGTC

CATCGTTGCAATAATAAAAGTTGTCCAAGTTAAAGGATATTATGCATTCGTTTAAAGATATCACCTCATC

TAACGGAGACAATTTTTTGGTAGGTTTTAGAGACTTTGAAGCTACTTGTTTAACAAAGTTATTCATCGTC

GTCTACTATTCTATTTAATTTTGTAGTTAATTTATCACATATCACATTAATTGACTTTTTGGTCCACTTT

TCCATACGTTTATATTCTTTTAATCCTGCGTTATCCGTTTCCGTTATATACAGGGATAGATCTTGCAAGT

TAAATAGAATGCTCTTAAATAATGTCATTTTTTTATCCGCTAAAAATTTAAAGAATGTATAAACTTTTTT

CAAAGATTTAAAACTTTTAGGTGGAGTTCTGGTACACAATATCATAAACAAACTAATAAACATCCCACAT

TCAGATTCCAACAATTGATTAACTTCCACATTAATACAGCCTATTTTCGCTCCAAATGTACATTCGAAAA

ATCTGAATAAAACATCAATATCGCAATTTGTATTATCCAATACAGAATGTCTGTGATTCGTGTTAAAACC

ATCGGAAAAAGAATAGAAATAAAAATTATTATAATGGTGGAATTCAGTTGGAATATTGCCTCCGGAGTCA

TAAAAGGATACTAAACATTGTTTTTTATCATAAATTACACATTTCCAATGAGACAAATAACAAAATCCAA

ACATTACAAATCTAGAGGTAGAACTTTTAATTTTGTCTTTAAGTATATACGATAAGATATGTTTATTCAT

AAACGCGTCAAATTTTTCATGAATAGCTAAGGAGTTTAAGAATCTCATGTCAAATTGTCCTATATAATCC

ACTTCGGATCCATAAGCAAACTGAGAGACTAAGTTCTTAATACTTCGATTGCTCATCCAGGCTCCTCTCT

CAGGCTCTATTTTCATCTTGACGACCTTTGGATTTTCACCAGTATGTATTCCTTTACGTGATAAATCATC

GATTTTCAAATCCATTTGTGAGAAGTCTATCGCCTTAGATACTTTTTCCCGTAGTTGAGGTTTAAAGAAA

TACGCTAACGGTATACTAGTAGGTAACTCAAAGACATCATATATAGAATGGTAACGCGTCGTTAACTCGT

CGGTTAACTCTTTCTTTTGATCGAGTTCGTCGCTACTATTGGGTCTGCTCAGGTGCCCCGACTCTACTAG

TTCCAACATCATACCGATAGGAATACAAGACACTTTGCCAGCGGTTGTAGATTTATCATATTTCTCCACC

ACATATCCGTTACAATTTGTTAAGAATTTAGATACATCTATATTGCTACATAATCCAGCTAGTGAATATA

TATGACATAATAAATTGGTAAATCCTAGTTCTGGTATTTTACTAATTACTAAATCTGTATATCTTTCCAT

TTATCATGGAAAAGAATTTACCAGATATCTTCTTTTTTCCAAACTGCGTTAATGTATTCTCTTACAAATA

TTCACAAGATGAATTCAGTAATATGAGTAAAACGGAACGTGATAATTTCTCATTGGCTGTGTTTCCAGTG

ATAAAACATAGATGGCATAACGCACACGTTGTAAAACATAAAGGAATATACAAAGTTAGTACAGAAGCAC

GTGGAAAAAAAGTATCTCCTCCATCACTAGGAAAACCCGCACATATAAACCTAATGTCGAAGCAATATAT

ATATAGTGAGTATGCAATAAGCTTTGAATGTTATAGTTTTCTAAAATGTATAACAAATACAGAAATCAAT

TCGTTCGATGAGTATATATTAAGAGGACTATTAGAAGCTGGTAATAGTTTACAGATATTTTCCAATTCCG

TAGGCAAACGAATAGATACTATAGGTGTACTAGGGAATAAGTATCCATTTAGCAAAATTCCATTGGCCTC

ATTAACTCCTAAAGCACAACGAGAGATATTTTTAGCGTGGATTTCTCATAGACCTGTAGTTTTAACTGGA

GGAACCGGAGTGGGTAAGACGTCACAGGTACCCAAGTTATTGCTTTGGTTTAATTATTTATTTGGTGGAT

TCTCTACTCTAGATAAAATCACTGACTTTCACGAAAGACCAGTCATTCTATCTCTTCCTAGGATAGCTTT

AGTTAGATTGCATAGCAATACCATTTTAAAATCATTGGGATTTAAGGTACTAGATGGATCTCCTATCTCT

TTACGGTACGGATCTATACCGGAAGAATTAATAAACAAACAACCAAAAAAATATGGAATTGTATTTTCTA

CCCATAAGTTATCTCTAACAAAACTATTTAGTTATGGCACTATTATTATAGACGAAGTTCATGAGCATGA

TCAAATAGGAGATATTATTATAGCAGTAGCGAGAAAGCATCATACGAAAATAGATTCTATGTTTTTAATG

ACTGCCACGTTAGAGGATGACAGGGAACGTCTAAAAATATTTTTACCTAATCCCGCATTTATACATATTC

CTGGAGATACACTGTTTAAAATTAGCGAGGTATTTATTCATAATAAGATAAATCCATCTTCCAGAATGGC

ATATATAGAAGAAGAAAAGAGAAATTTAGTTACCGCTATACAGATGTATACTCCTCCTGATGGATCATCC

GGTATAGTCTTTGTGGCATCCGTTGCACAGTGTCACGAATATAAATCATATTTAGAAAAAAGATTACCGT

ATGATATGTATATTATTCATGGTAAGGTCTTAGATATAGACGAAATATTAGAAAAAGTGTATTCATCACC

TAATGTATCGATAATTATTTCTACTCCTTATTTGGAATCCAGCGTTACTATACGCAATGTTACACACATT

TATGATATGGGTAGAGTTTTTGTCCCCGCTCCTTTTGGAGGATCACAACAATTTATTTCTAAATCTATGA

GAGATCAACGAAAAGGAAGAGTAGGAAGAGTTAATCCTGGAACATACGTATATTTCTATGATCTGTCTTA

TATGAAGTCTATACAGCGAATAGATTCAGAATTTCTACATAATTATATATTGTACGCTAATAAGTTTAAT

CTAACACTCCCCGAAGATTTGTTTATAATCCCTACAAATTTGGATATTCTATGGCGTACAAAGGAATATA

TAGACTCGTTCGATATTAGTACAGAAACATGGAATAAATTATTATCCAATTATTATATGAAGATGATAGA

GTATGCTAAACTTTATGTACTAAGTCCTATTCTCGCTGAGGAGTTGGATAACTTTGAGAGGACGGGAGAA

TTAACTAGTATTGTACAAGAAGCCATTTTATCTCTAAATTTACGAATTAAGATTTTAAAATTTAAACATA

AAGATGATGATACGTATATACACTTTTGTAGAATATTATTCGGCGTCTATAACGGAACAAACGCTACTAT

ATATTATCATAGACCTCTAACGGGATATATGAATATGATTTCAGATACTATATTTGTTCCTGTAGATAAT

AACTAAAAATCAAACTCTAATGACCACATCTTTTTTTAGAGATGAAAAATTTTCCACATCTCCTTTTGTA

GACACGACTAAACATTTTGCAGAAAAAAGTTTATTATTATTTAGATAATCGTATACTTCATCAGTGTAGA

TAGTAAATGTGAACAGATAAAAGGTATTCTTGCTCAATAGATTGGTAAATTCCATAGAATATATTAATCC

TTTCTTCTTGAGATCCCACATCATTTCAACCAAAGACGTTTTATCCAATGATTTACCTCGTACTATACCA

CATACAAAACTAGATTTTGCAGTGATGTCGTACCTGGTATTCCTACCAAACAAAATTTTACTTTTAGTTC

TTTTAGAAAATTCTAAGGTAGAATCTCTATTTGTCAATATGTCATCTATGGAATTACCACTAGCAAAAAA

TGATAGAAATATATATTGATACATCGCAGCTGGTTTTGATCTACTATACTTTAAAAACGAATCAGATTCC

ATAATTGCCTGTATATCATCAGCTGAAAAACTATGTTTTACACGTATTCCTTCGGCATTTCTTTTTAATG

ATATATCTTGTTTAGACAATGATAAAGTTATCATGTCCATGGGAGATGCGTCTCCGTATCGTATAAATAT

TTCATTAGATGTTAGACGCTTCATTAGGGGTATACTTCTATAAGGTTTCTTAATTAGTCCATCATTGGTT

GCGTCAAGAACTACTATCTGATGTTGTTGGGTATCTCTAGTGTTACACATGGCCTTACTAAAGTTTGGGT

AAATAACTATGATATCTCTATTAATTATAGATGCATATATTTCATTCGTCAAGGATATTAATATCGACTT

ACTATCGTCATTAATACGTGTAATATAATCATATAAATCATGCGATAGCCAAGGAAAATTCAAATAGATG

TTCATCATATAATCGTCGCTATAATTCATATTAATACTTTGACATTGACTAATTTGTAATATAGCCTCGC

CACGAAGAAAGCTCTCGTATTCAGTTTCATCGATAAAGGATACCGTTAAATATAACTGGTTGCCGATAGT

CTCATAGTCTATTAAGTGGTAAGTTTCGTACAAATACAGAATCCCTAAAATATTATCTAATGTGGGATTA

ATCCTTACCATAACTGTATAAAATGGAGCCGGAGTCATAACTATTTTACCGTTTGTACTTACTGGAATAG

ATGAAGGAATAATCTCCGGACATGATGGTAAAGACCCAAATGTCTGTTTGAAGAAATCCAATGTTCCAGG

TCCTAATCTCTTGACAAAAATTACGATATTCGATCCCGATATCCTTTGCATTCTATTTACCAGCATATCA

CGAACTATATTAAGATTATCTATCATGTCTATTCTCCCACCGTTATATAAATCGCCTCCGCTAAGAAACG

TTAGTATATCCATACAATGGAATACTTCATTTCTAAAATAGTATTCGTTTTCTAATTCTTTAATGTGAAA

TCGTATACTAGAAAGGGAAAAATTATCTTTGAGTTTTCCATTAGAAAAGAACCACGAAACTAATGTTCTG

ATTGCGTCTGTCTCCGTCGCTGAATTAATAGATTTACACCAAAAACTCATATAACTTCTAGATGTAGAAG

CATTCGCTAAAAAATTAGTAGAATCAAAGGATATAAGTAGATGTTCCAACAAGTGAGCAATTCCCAAGAT

TTCATCTATATCATTCTCGAATCCGAAATTAGAAATTCCCAAGTAGATATCCTTTTTCATCCGATCATTG

ATGAAAATACGAACTTTATTCGGTAAGACGATCATTTACTAAGGAGTAAAATAGGAAGTAACGTTCGTAT

ATCGTTATCGTCGTATAAATTAAAGGTGTGTTTTTTGCCATTAAGAGACATTATAATTTTACCAATATTG

GAATTATAATATAGGTGTATTTGAGCACTAGAAACGGTCGATGCATCGGTAAATATAGCTGTATCTAATG

TTCTAGTCGGTATTTCTTCATTTCGCTGTCTAATGATAGCGTTTTCTCTATCTGTTTCCATTACAGCTGC

CTGAAGTTTATTGGTCGGGTAATATGTAAAATAATAAGAAATACATACGAATAACAAAAATAAAATAAGA

TATAATAAAGATGCCATTTAGAGATCTAATTTTGTTCAACTTGTCCAAATTCCTACTTACAGAAGATGAG

GAATCGTTGGAGATAGTATCTTCCTTATGTAGAGGATTTGAAATATCTTACGATGACTTAATATCGTACT

TTCCAGATAGGAAATACCATAAATATATTTCTAAGGTATTTGAACATGTAGATTTATCGGAGGAATTAAG

TATGGAATTCCATGATACAACTCTGAGAGATTTAGTATATCTTAGATTGTACAAGTATTCCAAGTATATA

CGGCCGTGTTATAAATTAGGAGATAATCTAAAAGGTATAGTTGTTATAAAGGACAGAAATATATATATTA

GAGAAGCAAATGATGACTTGATAGAATATCTCCTCAAGGAATACACTCCTCAGATTTATACATATTCTAA

TGAGCGAGTTCCCATAGCTGGTTCAAAATTAATTCTTTGTGGATTTTCTCAAGTTACATTTATGGCGTAT

ACAACGTCGCATATAACAACAAATAAAAAGGTAGATGTTCTCGTTTCCAAAAAATGTATAGATGAACTAG

TCGATCCAATAAATTATCAAATACTTCAAAATTTATTTGATAAAGGAAGCGGAACAATAAACAAAATACT

CAGGAAGATATTTTATTCGGTAACAGGTGGCCAAACTCCATAGGTAGCTTTTTCTATTTCGGATTTTAGA

ATTTCCAAATTCACCAGCGATTTATCGGTTTTGGTGAAATCCAAGGATTTATTAATGTCCACAAATGCCA

TTTGTTTTGTCTGTGGATTGTATTTGAAAATGGAAACGATGTAGTTAGATAGATGCGCTGCGAAGTTTCC

TATTAGGGTTCCGCGCTTCACGTCACCCAACATACTTGAATCACCATCCTTTAAAAAAAATGATAAGATA

TCAACATGGAGTATATCATACTCGGATTTTAATTCTTCTACTGCCTCACTGACATTTTCACAAATACTAC

AATACGGTTTACCGAAAATAATCAGTACGTTCTTCATTTATGGGTATCAAAAACTTAAAATCGTTACTGT

TGGAAAATAAATCACTGACGATATTAGATGATAATTTATACAAAGTATACAATGGAATATTTGTGGATAC

AATGAGTATTTATATAGCCGTCGCCAATTGTGTCAGAAACTTAGAAGAGTTAACTACGGTATTCATAAAA

TACGTAAACGGATGGGTAAAAAAGGGAGGACATGTAACCCTTTTTATCGATAGAGGAAGTATAAAAATTA

AACAAGACGTTAGAGACAAGAGACGTAAATATTCTAAATTAACCAAGGACAGAAAAATGTTAGAATTAGA

AAAGTGTACATCCGAAATACAAAATGTTACCGGATTTATGGAAGAAGAAATAAAGGCAGAAATGCAATTA

AAAATCGATAAACTCACATTTCAAATATATTTATCTGATTATGATAACATAAAAATATCATTGAATGAGA

TACTAACACATTTCAACAATAATGAGAATGTTACATTATTTTATTGTGATGAACGAGACGCAGAATTCGT

TATGTGTCTAGAGGCTAAAACACAGTTCTCTACCACAGGAGAATGGCCGTTAATAATAAGTACCGATCAG

GATACTATGCTATTCGCGTCTGCTGATAATCATCCTAAGATGATAATAAACTTAACTCAACTGTTTAAAT

TTGTTCCCTCGGCAGAGGATAACTATTTAGCAAAATTAACTGCATTAGTGAATGGATGTGATTTCTTTCC

TGGACTCTATGGGGCATCTATAACACCCAACAACTTAAACAAAATACAATTGTTTAGTGATTTTACAATC

GATAATATAGTCACTAGTTTGGCAATTAAAAATTATTATAGAAAGACTAACTCTACCGTAGACGTGCGTA

ATATTGTTACGTTTATAAACGATTACGCTAATTTAGAAGATGTCTACTCGTATATTCCTCCTTGTCAATG

CACTGTTCAAGAATTTATATTCTCCGCATTAGATGAAAAATGGAATGAATTTAAATCATCTTATTTAGAG

AGCGTGCCGTTACCCTGCCAATTAATGTACGCATTAGAACCACGTAAGGAGATTGATGTTTCAGAAGTTA

AAACTTTATCATCTTATATAGATTTCGAAAATACTAAATCAGATATCGATGTTATAAAATCTATATCCTC

GATTTTTGGATATTCTAATGAAAACTGTAACACCATAGTGTTCGGCATCTATAAGGATAATTTACTACTG

AGTATAAATAATTCATTTTACTTTAACGATAGTCTGTTAATAACCAATACTAAAAGTGATAATATAATAA

ATATAGGTTACTAGATTAAAAATGGTGTTCCAGCTCGTGTGTTCTACGTGCGGCAAAGATATTTCTCACG

AACGATATAAATTGATTATACGAAAAAAATCATTAAAGGATGTACTAGTCAGTGTAAAGAACGAATGTTG

TAGGTTAAAATTATCTACACAAATAGAACCTCAACGTAACTTAACAGTGCAACCTCTATTGGATATAAAC

TAATGGATCCGGTTAATTTTATCAAGACATATGCGCCTAGAGGTTCTATTATTTCTATTAATTATGCCAT

GTCATTAACTAGTCATTTGAATCCATCGATAGAAAAACATGTGGGTATTTATTATGGTACGTTATTATCG

GAACACTTGGTAGTTGAATCTACCTATAGAAAAGGAGTTAGAATAATCCCATTGGATAGATTTTTTGAAG

GATATCTTAATGCAAAAGTATACATGTTAGAGAATATTCAAGTTATGAAAATAGCAGCTGATATGTCGTT

AACTTTACTAGGTATTCCATATGGATTTGGTCATGATAGAATGTATTGTTTTAAATTGGTAGCTGAATGT

TATAAAAATGCCGGTATTGATACATCGTCTAAACGAATATTAGGTAAAGATATTTTTCTGAGCCAAAACT

TTACAGATGATAATAGATGGATAAAGATATATGATTCTAATAATTTAACATTTTGGCAAATTGATTACCT

TAAAGGGTGAGTTAATATGCATAACTACTCCTCCGTTGTTTTTTCCCTCGTTCTTTTTCTTAACGTTGTT

TGCCATCACTCTCATAATGTAAAGATATTCTAAAATGGTAAACTTTTGCATATCGGATGCAGAAATTGGT

ATAAATGTTGTAATTGTATTATTTCCCGTCAATGGACTAGTCACAGCTCCATCAGTTTTATATCCTTTAG

AGTATTTCTCACTCGTGTCTAGCATTCTAGAGCATTCCATGATTTGTTTATCGTTGATATTGGCCGGAAA

GATAGATTTTTTATTTTTTATTATATTACTATTGGCAATTGTAGATATAACTTCTGGTAAATATTTTTCT

ACCTTTTCATTCTCTTCTATTTTTAAGCCGGCTATATATTCTGCTATATTGTTGCTAGTATCAATACCTT

TTCTGGCTAAGAAGTCATATGTGGTATTCACTATATCAGTTTTAACTGGTAGTTCCATTAGCCTTTCCAC

TTCTGCAGAATAATCAGAAATTGGTTCTTTACCAGAAAATCCAGCTACTATAATAGGCTCACCGATGATC

ATTGGCAAAATCCTATATTGTACCAGATTAATGAGAGCATATTTCATTTCCAATAATTCTGCTAGTTCTT

GAGACATTGATTTATTTGATGAATCTATTTGGTTCTCTAGATACTCTACCATTTCTGCCGCATACAATAA

CTTGTTAGATAAAATCAGGGTTATCAAAGTGTTTAGTGTGGCTAGAATAGTGGGCTTGCACGTATTAAAG

AATGCTGTAGTATGAGTAAACCGTTTTAACGAATTATATAGTCTCCAGAAATCTGTGGCGTTGCATACAT

GAACTGAATGACATCGAAGATTGTCCAATATTTTTAATAGCTGCTCTTTGTCCATTATTTCTATATTTGA

CTCGCAACAATTGTAGATACCATTAATCACTGATTCCTTTTTCGATGCCGGACAATAGCACAATTGTTTA

GCTTTGGACTCTATGTATTCAGAATTAATAGATATATCTCTCAATACAGATTGCACTATACATTTTGAAA

CTATGTCAAAAATTGTAGAACGACGCTGTTCTGTAGCCATTTAACTTTAAATAATTTACAAAAATTTAAA

ATGAGCATCCGTATAAAAATCGATAAATTGCGCCAAATTGTGGCATATTTTTCAGAGTTCAGCGAAGAAG

TGTCTATAAATGTAGACTCGACGGATGAGTTAATGTATATTTTTGCCGCCTTGGGCGGATCTGTAAACAT

TTGGGCCATTATACCTCTCAGTGCATCAGTGTTCTACCGCGGAGCCGAAAATATTGTGTTTAACCTTCCA

GTGTCCAAGGTAAAATCGTGTTTGTGTAGTTTTCACAATGATGCTATCATAGATATAGAACCTGATCTGG

AAAATAATCTAGTAAAACTTTCTAGTTATCATGTAGTAAGTGTCGATTGTAACAAGGAACTGATGCCTAT

TAGGACAGATACTACTATTTGTTTAAGTATAGATCAAAAGAAATCTTACGTATTTAATTTTCACAAGTAT

GAAGAAAAATGTTGTGGTAGAACCGTCATTCATCTAGAATGGTTGTTGGGCTTTATCAAGTGTATTAGTC

AGCATCAGCATTTGGCTATTATGTTTAAAGATGACAATATTATTATGAAGACTCCTGGTAATACTGATGC

GTTTTCCAGGGAATATTCTATGACTGAATGTTCTCAAGAACTACAAAAGTTTTCTTTCAAAATAGCTATC

TCGTCTCTCAACAAACTACGAGGATTCAAAAAGAGAGTCAATGTTTTTGAAACTAGAATCGTAATGGATA

ATGACGATAACATTCTAGGAATGTTGTTTTCGGATAGAGTTCAATCCTTTAAGATTAACATCTTTATGGC

GTTTTTAGACTAATACTTTCAATGAGATATATATGGGTGGCGGAGTAAGTGTTGAGCTCCCTAAACGGGA

TCCGCCTCCGGGAGTACCCACTGATGAGATGTTATTAAACGTGGATAAAATGCATGACGTGATAGCTCCC

GCTAAGCTTTTAGAATATGTGCATATAGGACCACTAACAAAAGATAAAGAGGATAAAGTAAAGAAAAGAT

ATCCAGAGTTTAGATTAGTCAACACAGGACCCGGTGGTCTTTCGGCATTATTAAGACAATCATATAATGG

AACCGCACCCAATTGCTGTCGCACTTTTAATCGTACTCATTATTGGAAGAAGGATGGAAAGATATCAGAT

AAGTATGAAGAGGGCGCAGTATTAGAATCGTGTTGGCCCGACGTCCACGACACTGGAAAATGCGATGTTG

ATTTATTCGACTGGTGTCAGGGGGATACGTTCGATATGAACATATGCCATCAGTGGATCGGTTCAGCCTT

TAATAGGAGTGATAGAACTGTAGAGGGTCGACAATCGTTAATAAATCTGTATAATAAGATGCAAACATTA

TGTAGTAAAGATGCTAGTGTACCAATATGTGAATTATTTTTGCATCATTTACGCGCACACAATACAGAAG

ATAGCAAAGAGATGATCGATTATATTCTAAGACAACAGTCGGCGGACTTTAAACAGAAATATATGAGATG

TAGTTATCCCACTAGAGATAAGTTAGAAGAGTCATTAAAATATGCGGAACCTCGAGAATGTTGGGATCCA

GAGTGTTCGAATGCCAATGTTAATTTCTTACTAACACGTAATTATAATAATTTAGGACTTTGCAATATTG

TACGATGTAATACGAGCGTGAATAACTTACAGATGGATAAAACTTCCTCATTAAGATTATCATGTGGATT

AAGCAATAGTGATAGATTTTCTACTGTTCCCGTCAATAGAGCAAAAGTAGTTCAACATAATATTAAACAT

TCGTTCGACCTAAAATTGCATTTGATCAGTTTATTATCTCTCTTGGTAATATGGATACTAATTGTAGCTA

TTTAAATGGGTGCCGCAGCAAGCATACAGACGACTGTGAATACACTCAGTGAACGTATCTCGTCTAAATT

AGAACAAGAAGCGAACGCTAGTGCTCAAACAAAATGTGATATAGAAATCGGAAATTTTTATATCCGACAA

AACCATGGATGTAACATCACTGTTAAAAATATGTGCTCTGCGGACGCGGATGCTCAGTTGGATGCTGTGT

TATCAGCCGCTACAGAAACATATAGTGGATTAACACCGGAACAAAAAGCATACGTACCAGCTATGTTTAC

TGCTGCGTTAAACATTCAGACGAGTGTAAACACTGTTGTTAGAGATTTTGAAAATTATGTGAAACAGACT

TGTAATTCTAGCGCTGTTGTCGATAACAAATTAAAGATACAAAACGTAATTATAGATGAATGTTACGGAG

CCCCAGGATCTCCAACAAATTTGGAATTTATTAATACAGGATCTAGCAAAGGAAATTGTGCCATTAAGGC

GTTGATGCAATTGACTACTAAGGCCACTACTCAAATAGCACCTAGACAAGTTGCTGGTACAGGTGTTCAG

TTTTATATGATTGTTATCGGTGTTATAATATTGGCAGCGTTGTTTATGTACTATGCCAAGCGTATGCTGT

TCACATCCACCAATGATAAAATCAAACTTATTTTAGCCAATAAGGAAAACGTCCATTGGACTACTTACAT

GGACACATTCTTTAGAACTTCTCCGATGATTATTGCTACCACGGATATACAAAACTGAAAATATATTGAT

AATATTTTAATAGATTAACATGGAAGTTATCGCTGATCGTCTAGACGATATAGTGAAACAAAATATAGCG

GATGAAAAATTTGTAGATTTTGTTATACACGGTCTAGAGCATCAATGTCCTGCTATACTTCGACCATTAA

TTAGGTTGTTTATTGATATACTATTATTTGTTATAGTAATTTATATTTTTACGGTACGTCTAGTAAGTAG

AAATTATCAAATGTTGTTGGTGTTGGTGGCGCTAGTCATCACATTAACTATTTTTTTATTACTTTATACT

ATAATAGTACTAGACTGACTTCTAACAAACATCTCACCTGCCATAAATAAATGCTTGATATTAAAGTCTT

CTATTTCTAACACTATTCCATCTGTGGAAAATAATACTCTGACATTATCGCTAATTGATACATCGGTAAG

TGATATGCCTATAAAGTAATAATCTTCTTTGGGTACATATACCAGTGTACCAGGTTCTAACAACCTATTT

ACTGGTGCTCCTGTAGCATACTTTTTTTTTACCTTGAGAATATCCATTGTTTGCTTGGTCAATAGCGATA

TGTGATTTTTTATCAACCACTCAAAAAAGTAATTGGAGTGTTCATATCCTCTACGGGCTATTGTCTCATG

GCCGTGTATGAAATTTAAGTAACACGACTGTGGTAGATTTGTTCTATAGAGCCGGTTGCCGCAAATAGAT

AGAACTACCAATATGTTTGTACAAATGTTAAACATTAATTGATTAACAGAAAAAACAATGTTCGTTCTGG

GAATAGAAACCAGATTAAAACAAAATTCATTAGAATATATGCCACGTTTATACATGGAATATAAAATAAC

TACAGTTTGAAAAATAACAGTATCATTTAAACATTTAACTTGCGGGGTTAATCTCACAACTTTACTGTTT

TTGAACTGTTCAAAATATAGCATAGATCCATGAGAAATACGTTTAGCCGCCTTTAATAGAGGAAATCCAA

CCGCCTTTCTGGATCTCACCAACGACGATAGTTCTGACCAGCAACTCATTTCTTCATCATCCACCTGTTT

TAACATATAATAGGCAGGAGATAGATATCCGTCATTGCAATATTCCTTCTCGTAGGCACACAATCTAATA

TTGATAAAATCTCCATTCTCTTCTCTGTATTTATTATCTTGTCTCGGTGGCTGATTAGGCTGTGGTCTAT

CGTTGTTGAATCTATTTTGGTCATTAAATCTTTCATTTCTTCCTGGTATATTTCTATCACCTCGTTTGGT

TGGATTTTTGTCTATATTATCGTTTGTAACATCGGTACGGGTATTCATTTATCACAAAAAAAACTTCTCT

AAATGAGTCTACTGCTAGAAAACCTCATCGAAGAAGATACCATATTTTTTGCAGGAAGTATATCTGAGTA

TGATGATTTACAAATGGTTATTGCTGGTGCAAAATCCAAATTTCCAAGATCTATGCTTTCTATTTTTAAT

ATAGTACCTAGAACGATGTCAAAATATGAGTTGGAGTTGATTCATAACGAGAATATCACAGGGGCAATGT

TTACCACAATGTATAATATAAGAAACAATTTGGGTCTAGGCGATGATAAACTAACTATTGAAGCCATTGA

AAACTATTTCTTGGATCCTAACAATGAGGTTATGCCTCTTATCATTAATAATACGGATATGACTACCGTC

ATTCCTAAAAAAAGTGGTAGGAGAAAGAATAAGAACATGGTTATCTTCCGTCAAGGATCATCACCTATCT

TGTGTATTTTCGAAACTCGTAAAAAGATTAATATTTATAAAGAAAATATGGAATCCGTATCGACTGAGTA

TACACCTATCGGAGACAACAAGGCTTTGATATCTAAATATGCGGGAATTAATATCCTGAATGTGTATTCT

CCTTCCACGTCCATGAGATTGAATGCCATTTACGGATTCACCAATAAAAATAAACTAGAGAAACTTAGTA

CTAATAAGGAACTAGAATCGTATAGTTCTAGCCCTCTTCAAGAACCCATTAGGTTAAATGATTTTCTGGG

ACTATTGGAATGTGTTAAAAAGAATATTCCTCTAACAGATATTCCGACAAAGGATTGATTACTATAAATG

GAGAATGTTCCTAATGTATACTTTAATCCTGTGTTTATAGAGCCCACGTTTAAACATTCTTTATTAAGTG

TTTATAAACACAGATTAATAGTTTTATTTGAAGTATTCGTTGTATTCATTCTAATATATGTATTTTTTAG

ATCTGAATTAAATATGTTCTTCATGCCTAAACGAAAAATACCCGATCCTATTGATAGATTACGACGTGCT

AATCTAGCGTGTGAAGACGATAAATTAATGATCTATGGATTACCATGGATAACAACTCAAACATCTGCGT

TATCAATAAATAGTAAACCGATAGTGTATAAAGATTGTGCAAAGCTTTTGCGATCAATAAATGGATCACA

ACCAGTATCTCTTAACGATGTTCTTCGCAGATGATGATTCATTTTTTAAGTATTTTGCTAGTCAAGATGA

TGAATCTTCATTATCTGATATATTGCAAATCACTCAATATCTAGACTTTCTGTTATTATTATTGATCCAA

TCAAAAAATAAATTAGAAGCTGTGGGTCATTGTTATGAATCTCTTTCAGAGGAATACAGACAATTGACAA

AATTCACAGACTCTCAAGATTTTAAAAAACTGTTTAACAAGGTCCCTATTGTTACAGATGGAAGGGTCAA

ACTTAATAAAGGATATTTGTTCGACTTTGTGATTAGTTTGATGCGATTCAAAAAAGAATCAGCTCTAGCT

ACCACCGCAATAGATCCTGTTAGATACATAGATCCTCGTCGTGATATCGCATTTTCTAACGTGATGGATA

TATTAAAGTCGAATAAAGTTGAACAATAATTAATTCTTTATTGTTATCATGAACGGCGGACATATTCAGT

TGATAATCGGCCCCATGCTTTCAGGTAAAAGTACAGAATTAATTAGACGAGTTAGACGTTATCAAATAGC

TCAATATAAATGTGTGACTATAAAATATTCTAACGATAATAGATACGGAACGGGACTATGGACGCATGAT

AAGAATAATTTTGCAGCATTGGAAGTAACTAAACTATGTGATGTCTTGGAAGCAATTACAGATTTCTCCG

TGATAGGTATCGATGAAGGACAGTTCTTTCCAGACATTGTTGAATTCTGTGAGCGTATGGCAAACGAAGG

AAAAATAGTTATAGTAGCCGCGCTCGATGGGACATTTCAACGTAGACCGTTTAATAATATTTTGAATCTT

ATTCCATTATCTGAAATGGTGGTAAAACTAACTGCAGTGTGTATGAAATGCTTTAAGGAGGCTTCCTTTT

CTAAACGATTAGGTGCAGAAACCGAGATAGAAATAATAGGAGGTAATGATATGTATCAATCTGTGTGTAG

AAAGTGTTACATCGACTCATAATATTATATTTTTTATCTAAAAAACTAAAAATAAACATTGATTAAATTT

TAATATAATACTTAAAAATGGATGTTGTGTCGTTAGATAAACCGTTTATGTATTTTGAGGAAATTGATAA

TGAGTTAGATTACGAACCAGAAAGTGCAAATGAGGTCGCAAAAAAACTGCCGTATCAAGGACAGTTAAAA

CTATTACTAGGAGAATTATTTTTTCTTAGTAAGTTACAGCGACACGGTATATTAGATGGTGCCACCGTAG

TGTATATAGGATCTGCTCCAGGTACACATATACGTTATTTGAGAGATCATTTCTATAATTTAGGAGTGAT

CATCAAATGGATGCTAATTGACGGCCGCCATCATGATCCTATTCTAAATGGATTGCGTGATGTGACTCTA

GTGACTCGGTTTGTTGATGAGGAATATCTACGATCCATCAAAAAACAACTACATCCTTCTAAGATTATTT

TAATTTCTGATGTGAGATCCAAACGAGGAGGAAATGAACCTAGTACTGCGGATTTACTAAGTAATTATGC

TCTACAAAATGTCATGATTAGTATTTTAAACCCCGTGGCGTCTAGTCTTAAATGGAGATGCCCGTTTCCA

GATCAATGGATCAAGGACTTTTATATCCCACACGGTAATAAAATGTTACAACCTTTTGCTCCTTCATATT

CAGCTGAAATGAGATTATTAAGTATTTATACCGGTGAGAATATGAGACTGACTCGAGTTACCAAATCAGA

CGCTGTAAATTATGAAAAAAAGATGTATTACCTTAATAAGATAGTCCGCAACAAAGTAGTTGTTAACTTT

GATTATCCTAATCAGGAATATGACTATTTTCACATGTACTTTATGTTGAGGACCGTATACTGCAATAAAA

CATTTCCTACTACTAAAGCAAAGATACTATTTCTACAACAATCTATATTTCGTTTCTTAAATATTCCAAC

GACATCAACTGAAAAAGTTAGTCATGAACCAATACAACGTAAAATATCTAGCAAAGATTCTATGTCTAAA

AACAGAAATAGCAAGAGATCCGTACGCGGTAATAAATAGAAACGTACTACTGAGATATACTACCGATATA

GAGTATAATGATTTAGTTACTTTAATAACCGTTAGACATAAAATTGATTCTATGAAAACTGTGTTTCAGG

TATTTAACGAATCATCCATAAATTATACTCCGGTTGATGATGATTATGGAGAACCAATCATTATAACATC

GTATCTTCAAAAAGGTCATAACAAGTTTCCTGTAAATTTTCTATACATAGATGTGGTAATATCTGACTTA

TTTCCTAGCTTTGTTAGACTAGATACTACAGAAACTAATATAGTTAATAGTGTACTACAAACAGGCGATG

GTAAAAAGACTCTTCGTCTTCCTAAAATGTTAGAGACGGAAATAGTTGTCAAGATTCTCTATCGTCCTAA

TATACCATTAAAAATTGTTAGATTTTTCCGCAATAACATGGTAACTGGAGTAGAGATAGCCGATAGATCT

GTTATTTCAGTCGCTGATTAATCAATTAGTAGAGATGAGATAAGAACATTATAATAATCAATAATATATC

TTATATCTGTTTAGAAAAATGCTAATATTAAAATAGCTAACGCTAGTAATCCAATCGGAAGCCATTTGAT

ATCTATAATAGGGTATCTAATTTCCTGATTCAGATAGCGTACGGCTATATTCTCGGTAGCTACTCGTTTG

GAATCACAAACATTATTTACATCTAATTTACTATCTGTAATGGAAACGTTTCCCAATGAAATGGTACAAT

CAGATACATTACATCTTGATATATTTTTTTTTAAAGAGGCTGGTAACAACGCATCGCTTCGTTTACATGG

CTCGTACCAACAATAATAGGGTAATCTTGTATCTATTCCTATCCGTACTATACTTTTATCAGGATAAATA

CATTTACATCGTATATCGTCTTTGTTAGCATCACAGAATGCATAAATTTGTTCGTCCGTCATGATAAAAA

TTTAAAGTGTAAATATAACTATTATTTTTATAGTTGTAATAAAAAGGGAAATTTGATTGTATACCTTCGG

TTCTTTAAAAGAAACTGACTTGATAAAAATGGCTGTAATCTCTAAGGTTACGTATAGTCTATACGATCAA

AAAGAGATTAATGCTACAGATATTATCATTAGTCATATTAAAAATGACGACGATATCGGTACCGTTAAAG

ATGGTAGACTAGGTGCTATGGATGGGGCATTATGTAAGACTTGTGGGAAAACGGAATTGGAATGTTTCGG

TCACTGGGGTAAAGTAAGTATTTATAAAACTCATATAGTTAAGCCTGAATTTATTTCAGAAATTATTCGT

TTACTGAATCATATATGTATTCATTGCGGATTATTGCGTTCACGAGAACCGTATTCCGACGATATTAACC

TAAAAGAGTTATCGGGACACGCTCTTAGGAGATTAAAGGATAAAATATTATCCAAGAAAAAGTCATGTTG

GAACAGCGAATGTATGCAACCGTATCAAAAAATTACTTTTTCAAAGAAAAAGGTTTGTTTCGTCAACAAG

TTGGATGATATTAACGTTCCTAATTCTCTCATCTATCAAAAGTTAATTTCTATTCATGAAAAGTTTTGGC

CATTATTAGAAATTCATCAATATCCAGCTAACTTATTTTATACAGACTACTTTCCCATCCCTCCGTTGAT

TATTAGACCGGCTATTAGTTTTTGGATAGATAGTATACCCAAAGAGACAAATGAATTAACTTACTTATTA

GGTATGATCGTTAAGAATTGTAACTTGAATGCTGATGAACAGGTTATCCAGAAGGCGGTAATAGAATACG

ATGATATTAAAATTATTTCTAATAACACTACCAGTATCAATTTATCATATATCACATCCGGCAAAAATAA

TATGATTAGAAGTTATATCGTCGCTCGGCGAAAGATCAGACCAGCTAGATCTGTAATTGGTCCCAGTACA

TCTATCACCGTTAATGAGGTAGGAATGCCCACATATATTAGAAATACACTTACAGAAAAGATATTTGTTA

ATGCCTTTACAGTGGATAAAGTTAAACAACTATTAGCATCAAACCAAGTTAAATTTTACTTTAATAAACG

ATTAAACCAATTAACAAGAATACGTCAAGGAAAGTTTATCAAAAATAAAATACATTTATTGCCTGGTGAT

TGGGTAGAAGTAGCTGTTCAAGAATATACAAGTATTATTTTTGGAAGACAACCGTCTCTACATAGATACA

ACGTCATCGCTTCATCTATCAGAGCCACCGAAGGAGATACTATCAAAATATCTCCCGGAATTGCCAACTC

TCAAAATGCTGATTTCGACGGAGATGAAGAATGGATGATATTGGAGCAAAATCCTAAAGTCGTAGTTGAA

CAAAGTATTCTTATGTATCCGACGACGTTACTCAAACACGATATTCATGGAGCCCCCGTTTATGGATCTA

TTCAAGATGAAATCGTAGCAGCGTATTCATTGTTTAGGATACAAGATCTTCGTTTAGATGAAGTATTGAA

CATCTTGGGGAAATATGGAAGAGAGTTCGATCCTAAAGGTAAATGTAAATTCAGCGGTAAAGATATCTAT

ACTGACTTGATAGGTGAAAAGATTAATTATCCGGGTCTCTTAAAGGATGGCGAAATTATTGCAAACGACG

TAGATAGTAATTTTGTTGTAGCTATGAGGCATCTGTCATTGGCTGGACTCTTATCCGATCATAAATCGAA

CGTGGAAGGTATCAACTTTATTATCAAGTCATCTTATGTTTTTAAGAGATATCTATCTATTTACGGTTTT

GGGGTGACATTCAAAGATCTGAGACCAAATTCGACGTTCACTAATAAATTGGAGGCTATCAACGTAGAAA

AAATAGAACTTATCAAAGAAGCATACGCCAAATATCTCAAAGATGTAAGAGACGGGAAAATAGTTCCATT

ATCTAAAGCTTTAGAGGCGGACTACTTGGAATCCATGTTATCCAACTTGACAAATCTTAATATCAGAGAG

ATAGAAGAACATATGAGACAAACGCTGATAGATGATCCAGATAATAACCTCCTGAAAATGGCCAAAGCGG

GTTATAAAGTAAATCCCACAGAACTAATGTATATTCTAGGTACTTATGGACAACAGAGGATAGATGGCGA

ACCAGCAGAGACTCGAGTATTGGGTAGAGTCTTACCTTACTATCTTCCAGACTCTAAGGATCCAGAAGGA

AGAGGTTATATTCTTAATTCTTTAACAAAAGGATTAACGGGTTCTCAATATTACTTTTCGATGCTGGTTG

CAAGATCTCAATCTACTGATATCGTCTGTGAAACATCACGTACCGGAACACTGGCTAGAAAAATCATTAA

AAAGATGGAGGATATGGTGGTCGACGGATACGGACAAGTAGTTATAGGTAATACGCTCATCAAGTACGCA

GCCAATTATACCAAAATTCTAGGCTCAGTATGTAAACCTGTAGATCTTATCTATCCAGATGAGTCCATGA

CTTGGTATTTGGAAATTAGTGCTTTGTGGAATAAAATAAAACAGGGATTCGTTTACTCTCAGAAACAGAA

ACTTGCAAAGAAGACATTGGCGCCGTTTAATTTCCTAGTATTCGTCAAACCCACCACTGAGGATAATGCT

ATTAAGGTTAAGGATCTGTACGATATGATTCATAACGTCATTGATGATGTGAGAGAGAAATACTTCTTTA

CGGTATCTAATATAGATTTTATGGAGTATATATTCTTGACGCATCTTAATCCTTCTAGAATTAGAATTAC

AAAAGAAACGGCTATCACTATCTTTGAAAAGTTCTATGAAAAACTCAATTATACTCTAGGTGGTGGAACT

CCTATTGGAATTATTTCTGCACAGGTATTGTCTGAGAAGTTTACACAACAAGCCCTGTTCAGTTTTCACA

CTACTGAAAAGAGTGGTGCTGTAAAACAAAAACTTGGTTTCAACGAGTTTAATAACTTGACTAATTTGAG

TAAGAATAAGACCGAAATTATCACTCTGGTATCCGATGATATCTCTAAACTTCAATCTGTTAAGATTAAT

TTCGAATTTGTATGTTTGGGAGAATTAAATCCAGACATCACTCTTCGAAAAGAAACAGATAGATATGTAG

TAGACATAATAGTCAATAGATTATACATCAAGAGAGCAGAAATAACCGAATTAGTCGTCGAATATATGAT

TGAACGATTTATCTCCTTTAGCGTCATTGTAAAGGAATGGGGCATGGAGACATTCATTGAGGACGAGGAT

AATATTAGATTTACTGTCTACCTAAATTTCGTTGAACCGGAGGAATTGAATCTTAGTAAGTTTATGATGG

TTCTTCCGGGTGCCGCCAACAAGGGCAAGATTAGTAAATTCAAGATTCCTATCTCTGACTATACGGGATA

TAACGACTTCAATCAAACAAAAAAGCTCAATAAGATGACTGTAGAACTCATGAATCTAAAAGAATTGGGT

TCTTTCGATTTGGAGAACGTCAACGTGTATCCTGGAGTATGGAATACATACGATATCTTCGGTATTGAGG

CCGCTCGTGGATACTTGTGCGAAGCCATGTTAAACACCTATGGAGAAGGTTTCGATTATCTGTACCAGCC

TTGTGATCTTCTCGCTAGTTTACTATGTGCTAGTTACGAACCAGAATCAGTTAATAAATTCAAGTTCGGT

GCAGCTAGTACTCTTAAGAGAGCTACGTTCGGAGATAATAAAGCATTGTTAAACGCGGCTCTTCATAAAA

AGTCAGAACCTATTAACGATAATAGTAGCTGCCACTTTTTTAGCAAGGTCCCTAATATAGGAACTGGATA

TTACAAATACTTTATCGACTTGGGTCTTCTCATGAGAATGGAAAGGAAATTATCTGATAAGATATCTTCT

CAAAAGATCAAGGAGATAGAAGAAACAGAAGACTTTTAATTCTTATCAATAACATATTTTTCTATGATCT

GTCTTTTAAACGATGGATTTTCCACAAATGCGCCTCTCAAGTCCCTCATAGAATGATACACGTATAAAAA

ATATAGCATAGGTGATGACTCCTTATTTTTAGACATTAGATATGCCAAAATCATAGCCCCGCTTCTATTT

ACTCCCGCAACACAATGAACCAACACGGGCTCGTTTCGTTGATCACATTTAGATAAGAAGGCGGTCACGT

CGTCAAAATATTTACTAATATCAGTAGTTGTATCATCTACCAACGGTATATGAATAATATTAATATTAGA

GTTAGGTAATGTATATTTATCCATCGTCAAATTTAAAACATATTTGAACTTAACTTCAGATGATGGTGCA

TCCATAGCATTTTTATAATTTCCCAAATACACATTATTTGTTACTCTTGTCATTATAGTGGGAGATTTGG

CTCTGTGCATATCTCCAGTTGAACGTAGTAGTAAGTATTTATACAAACTTTTCTTATCCATTTATAACGT

ACAAATGGATAAAACTACTTTATCAGTAAACGCATGCAATTTAGAATACGTTAGAGAAAAGGCTATAGTA

GGCGTACAAGCAGCCAAGACATCAACACTTATATTTTTTGTTATTATATTGGCAATTAGTGCGCTATTAC

TCTGGTTTCAGACGTCTGATAATCCAGTCTTTAATGAATTAACGAGATATATGCGAATTAAAAATACGGT

TAACGATTGGAAATCATTAACGGATAGCAAAACAAAATTAGAAAGTGATAGAGGTAGACTTCTAGCCGCT

GGTAAGGATGATATATTCGAATTCAAATGTGTGGATTTCGGCGCCTATTTTATAGCTATGCGATTGGATA

AGAAAACATATCTGCCGCAAGCTATTAGGCGAGGTACTGGAGACGCGTGGATGGTTAAAAAGGCGGCAAA

AGTCGATCCATCTGCTCAACAATTTTGTCAGTATTTGATAAAACACAAGTCTAATAATGTTATTACTTGT

GGTAATGAGATGTTAAATGAATTAGGTTATAGCGGTTATTTTATGTCACCGCATTGGTGTTCCGATCTTA

GTAATATGGAATAAGTGTTAGATAAATGCGGTAACAAATGTTCCTGTAAGGAACCATAACAGTTTAGATT

TAACATTAAAGATGAGCATAAACATAATAAACAAAATTACAATCAAACCTATAACATTAATATCAAACAA

TCCAAAAAATGAAATCAGTGGAGTAGTAAACGTGTACATGACTCCTGGATAACGTTTAGCAGCTACCGTT

CCTATTCTAGACCAAAAATTTGGTTTCATGGTTTCGAAGCGGTGTTCTGCAACAAGACGAGGATCGTGTT

CTACATATTTGGCAGAGTTATCCATTATTTGCCTGTTAATCTTCATTTCGTTTTCGATTCTGGCTATTTC

AAAATAAAATCCCGATGATAGACCTCCAGACTTTATAATTTCATCTACGATGTTCAGCGCCGTAGTAACT

CTAATAATATAGGCTGATAAGCTAACATCATACCCTCCTGTATATGTGAATATGGCATGATCTTTGTCTA

TTACAAGCTCGGTTTTAACTTTATTTCCTGTAATAATTTCTCTCATCTGTAGGATATCTATTTTCTTGTC

ATGTATTGCCTTCAAGACGGGACGAAGAAACGTAATATCCTCAATAACGTTATCGTTTTCTATAATAACT

ACATATTCTACATTTTTATTTTCTAGCTCGATAAAAAATTTAGAATCCCATAGGGCTAAATGTCTAGCGA

TATTTCTTTTCGTTTCCTCTGTACACATAGTGTTACAAAACCCTGAAAAGAAGTGAGTATACTTGTCATC

ATCTCTAATATTTCCTCCAGTCCATTGTATAAACACATAATCCTTGTAATGATCTGGATCATCATTGACT

ATCACAACATCTCTTTTTTCTTGCATAACTTCATTGTCCTTCACATCATCGAACTTCTGATCATTAATAT

GCTCATGAACATTAGGAAATGTTTCTGATGGAGGTCTATCAATAACTGGCACAACAATAACAGGAGTTTT

CGCCGCCGCCATTTAGTTATTGAAATTAATCATATACAACTCTCTAATACGAGTTATATTTTCGTCTATC

CATTGTTTCACATTGACATATTTCGACAAAAAGATATAAAATGCGTATTCCAATGCTTCTCTGTTTAATG

AATTACTAAAATATACAAACACGTCACTGTCTGGTAATAAATGATATCTTAGAATATTGTAACAATTTAT

TTTGTATTGTACATGTTCGTGATCTATGAGTTCTTCTTCGAATGGCATAGGATCTCCGAATCTGAAAACG

TATAAATAGGAGTTAGAATAATAATATTTGAGAGTATTGGTAATGTATAAACTCTTTAGCGGTATAATTA

GTTTTTTTCTCTCGATTTCTATTTTTAGATGTGATGGAAAAATGACTAATTTTGTAGCATTAGTATCATG

AACTCTAATCAAAATCTTAATATCTTCGTCACACGTTAGCTCTTTGAAGTTTTTAAGAGATGCATCAGTT

GGTTTTACAGATGGAGTAGGTGTAACAATTTTTTGTTTAATGCATGTATGTATTGGAGCCATTGTCTTAA

CTATAATGGTGCTTGTATCGAAAAACTTTAATGCGGATAACGGAAGCTCTTCGCCGCGACTTTCTACGTC

GTAATTGGGTTCTAATGCCGATCTCTGAATGGATACTAGTTTTCTAAGTTCTAATGTGATTCTCTGAAAA

TGTAAATCCAATTCCTCCGGCATTATAGATGTGTATACATCGGTAAATAAAACTATAGTATCCAACGATC

CCTTCTCGCAAATTCTAGTCTTAACCAAGAAATCGTATATAACTACGGAGATGGCGTATTTAAGAGTGGA

TTCTTCTACCGTTTTGTTCTTGGATTTCATATAAGAAACTATAAAGTCCGCACTACTGTTAAGAATGATC

ACTAACGCAACTATATAGTTCAAATTAAGCATCTTGGAAACATAAAATAACTCTGTAGATGATACTTGAC

TTTCGAATAAGTTTGCAGACAAACGAAGAAAGAACAGACCTCTCTTAATTTCAGAAGAAAACTTTTTTTC

GTATTCCTGACGTCTAGAGTTTATATCAATAAGAAAGTTAAGAATTAGTCGGTTAATGTTGTATTTCATT

ACCCAAGTTTGAGATTTCATAATATTGTCAAAAGACATGATAATATTAAAGATAAAGCGCTGACTATGAA

CGAAATAGCTATATGGTTCGCTCAAGAATATAGTCTTGTTAAACGTGGAAACGATAACTGTATTTTTAAT

CACGTCAGCGACATCTAAATTAAATATAGGTATATTTATTCCACACACACTACAATATGCCACACCATCT

TCATAATAAATAAATTCGTTAGCAAAATTATTAATTTTAGTGAAATAGTTAGCGTCAACTTTCATAGCTT

CCTTCAATCTAATTTGATGCTCACATGGCGCGAATTCTACTCTAACATCCCTTTTCCATGCCTCAGGTTC

ATCGATCTCTATAATATCTAGTTTCTTGCGTTTCACAAACACAGGCTCGTCTCTCGCGATGAGATCTGTA

TAGTAACTATGTAAATGATAACTAGATAGAAAGATGTAGCTATATAGATGACGATCCTTTAAGAGAGGTA

TAATAACTTTACCCCAATCAGATAGACTGTTGTTATGGTCTTCGGAAAAAGAATTTTTATAAATTTTTCC

AGTATTTTCTAAATATACATACTTGATATCTAAGAAATCCTTAATGATAATAGGAATGGATAATCCGTCT

ATTTTATAAAGAAATACATATCGCATATTATACTTTTTTTTGGAAATTGGAATACCGATGTGTCTACATA

AATACGCAAAGTCTAAATATTTTTTAGAGAATCTTAGTTGGTCCAAATTCTTTTCCAAGTACGGTAATAG

ATTTTTCATATTGAACGGTATCTTCTTGATCTCTGGTTCTAATTCCGCATTAAATGATGAAACTAAGTCA

CTATTTTTATAACTAACGATTACATCACCTCTAACATCATCATTTACCAGGATACTGATCTTCTTTTGTC

GTAAATACATGTCTAATGTGTTAAAAAAAAGATCATACAAGTTATACGTCATTTCATCTGTAGTATTCTT

GTCATTGAAGGATAAACTCGTACTAATCTCTTCTTTAACAGTCTGTTCAAATTTATATCCTATATACGAA

AAAATAGCAACCAGTGTTTGATCATCCGCGTCAATATTCTGTTCTATCGTAGTGTATAGCAATCTTATAT

CTTCTTCTGTGATAGTCGATACGTTATAAAGGTTGATAACGAAAATATTTTTATTTCGTGAAATAAAGTC

ATTGTAGGATTTTGGACTTATATTCGTGTCTAGTAGATATGATTTTATTTTTGGAATGATCTCAATTAAA

ATAGTCTCTTTAGAGTCCATTTAAAGTTACAAACAACTAGGAAATTGGTTTATGATGTATAATTTTTTTA

GTTTTTATAGATTCTTTATTCTATACTTAAAAAATGAAAATAAATACAAAGGTTCTTGAGGGTTGTGTTA

ATTGAAAGCGAGAAATAATCATAAATTATTTCATTATCGCGATATCCGTTAAGTTTGTATCGTAATGGCG

TGGTCAATTACGAATAAAGCGGATACTAGTAGTTTCACAAAGATGGCTGAAATCAGAGCTCATCTAAGAA

ATAGCGCTGAAAATAAAGATAAAAACGAGGATATTTTCCCGGAAGATGTAATAATTCCATCTACTAAGCC

CAAAACCAAACGAACCACTACTCCTCGTAAACCAGCGGCTACTAAAAGATCAACCAAAAAGGATAAAGAA

AAGGAGGAAGTGGAAGAAGTAGAAGAAGTAGTTATAGAGGAATATCATCAAACAACTGAAGAAAATTCTC

CACCTCCGTCATCATCTCCTGGAGTCGGCGACATTGTAGAAAGCGTGGCCGCTGTAGAGCTCGATGATAG

CGACGGGGATGATGAACCTATGGTACAAGTTGAAGCTGGTAAAGTAAATCATAGTGCTAGAAGCGATCTC

TCTGACCTAAAGGTGGCTACCGACAATATCGTTAAAGATCTTAAGAAAATTATTACTAGAATCTCTGCAG

TATCGACTGTTCTAGAGGATGTTCAAGCAGCTGGTATCTCTAGACAATTTACTTCTATGACTAAAGCTAT

TACAACACTATCTGATCTAGTCACCGAGGGAAAATCTAAAGTTGTTCGTAAAAAAGTTAAAACTTGTAAG

AAGTAAATGCGTGCACTTTTTTATAAAGATGGTAAACTGTTTACCGATAATAATTTTTTAAATCCTGTAT

CAGACGATAATCCAGCGTATGAGGTTTTGCAACATGTTAAAATTCCTACTCATTTAACAGATGTAGTAGT

ATATGAACAAACGTGGGAAGAGGCATTAACTAGATTAATTTTTGTGGGAAGTGATTCAAAAGGACGTAGA

CAATACTTTTACGGAAAAATGCATGTACAGAATCGCAATGCTAAAAGAGATCGTATTTTTGTTAGAGTAT

ATAACGTTATGAAACGAATTAATTGTTTTATAAACAAAAATATAAAGAAATCGTCCACAGATTCCAATTA

TCAGTTGGCGGTTTTTATGTTAATGGAAACTATGTTTTTTATTAGATTTGGTAAAATGAAATATCTTAAG

GAGAATGAAACAGTAGGGTTATTAACACTAAAAAATAAACACATAGAAATAAGTCCCGATGAAATAGTTA

TCAAGTTTGTAGGAAAGGACAAAGTTTCACATGAATTTGTTGTTCATAAGTCTAATAGACTATATAAACC

GTTATTGAAACTGACTGATGATTCTAGTCCCGAAGAATTTCTGTTCAACAAACTAAGTGAACGAAAGGTA

TACGAATGTATCAAACAGTTTGGTATTAGAATCAAGGATCTCCGAACGTATGGAGTCAATTATACGTTTT

TATATAATTTTTGGACAAATGTAAAGTCCGTATCTCCTCTTCCATCACCAAAAAAGTTGATAGCGTTAAC

TATCAAACAAACTGCTGAAGTGGTAGGTCATACTCCATCAATTTCAAAAAGAGCTTATATGGCAACGACT

ATTTTAGAAATGGTAAAGGATAAAAATTTTTTAGACGTAGTATCTAAAACTACGTTCGATGAATTCCTAT

CTATAGTCGTAGATCACGTTAAATCATCTACGGATGGATGATAATAGATCTTTACACAAATAATTACAAG

ACCGATAAATGGAAATGGATAAACGGATGAAATCTCTCGCTATGACAGCTTTCTTCGGAGAGCTAAACAC

GTTAGATATTATGGCATTGATAATGTCTATATTTAAACGCCATCCAAACAATACCATTTTTTCAGTGGAT

AAGGATGGTCAATTTATGATTGATTTCGAATACGATAATTATAAGGCTTCTCAATATTTGGATCTGACCC

TCACTCCGATATCTGGAAATGAATGCAAGACTCACGCATCGAGTATAGCCGAACAATTGGCGTGTGTGGA

TATTATTAAAGAGGATATTAGCGAATATATCAAAACTACTCCCCGTCTTAAACGATTTATAAAAAAATAC

CGCAATAGATCATATACTCGTATCAGTCGAGATACAGAAAAGCTTAAAATAGCTCTAGCTAAAGGCATAG

ATTACGAATATATAAAAGACGCTTGTTAATAAGTAAATGAAAAAAAACTAGTCGTTTATAATAAAACACG

ATATGGATGCCAACATAGTATCATCTTCTACTATTGCGACGTATATAGACGCTTTAGCGAAGAATGCTTC

AGAATTAGAACAGAGGTCTACCGCATACGAAATAAATAATGAATTGGAACTAGTATTTATTAAACCGCCA

TTGATTACGTTGACAAATGTAGTAAATATCTCCACGATTCAGGAATCGTTTATTCGATTTACCGTTACTA

ATAAGGAAGGTATCAAAATTAGAACTAAGATTCCATTATCTAAGGTACATGGTCTAGATGTAAAAAATGT

ACAGTTGGTAGATGCTATAGATAACATAGTTTGGGAAAAGAAATCATTAGTGACGGAAAATCGTCTTCAC

AAAGAATGCTTGTTGAGACTATCGACAGAGGAACGTCATATATTTTTGGATTACAAGAAATATGGATCCT

CTATCCGACTAGAATTAGTCAATCTTATTCAAGCAAAAACAAAAAACTTTACGATAGACTTTAAGCTAAA

ATATTTTCTAGGATCTGGTGCTCAATCTAAAAGTTCTTTATTGCACGCTATTAATCATCCAAAGTCAAGG

CCTAATACATCTCTGGAAATAGAATTTACACCTAGAGACAATGAAACAGTTCCATATGATGAACTAATAA

AGGAATTGACGACTCTCTCGCGTCATATATTTATGGCTTCTCCAGAGAATGTAATTCTTTCTCCACCTAT

TAACGCACCTATAAAGACTTTTATGTTGCCTAAACAAGATATAGTAGGTCTGGATCTGGAAAATCTATAT

GCCGTAACTAAGACTGATGGCATTCCTATAACTATCAGAGTTACATCAAAAGGGTTGTATTGTTATTTTA

CACATCTTGGTTATATTATTAGATATCCAGTTAAGAGAACAATAGATTCCGAAGTAGTAGTCTTTGGTGA

GGCAGTTAAGGATAAGAACTGGACCGTATATCTCATTAAGCTAATAGAGCCCGTAAATGCAATCAGTGAT

AGACTAGAAGAAAGTAAGTATGTTGAATCTAAACTAGTGGATATTTGTGATCGGATAGTATTCAAGTCAA

AGAAATACGAAGGTCCGTTTACTACAACTAGTGAAGTCGTCGATATGTTATCTACATATTTACCAAAGCA

ACCAGAAGGTGTTATTCTGTTCTATTCAAAGGGACCTAAATCTAACATTGATTTTAAAATCAAAAAGGAG

AATACTATAGACCAAACTGCAAATGTAGTATTTAGGTACATGTCCAGTGAACCAATTATCTTTGGAGAGT

CGTCTATCTTTATAGAGTATAAGAAATTTACCAACGATAAAGGCTTTCCTAAAGAATATGGTTCTGGTAA

GATTGTGTTATATAACGGCGTTAATTATCTAAATAATATCTATTGTTTGGAATATATTAATACACATAAT

GAAGTGGGTATTAAGTCCGTTGTTGTACCTATTAAGTTTATAGCAGAATTCTTAGTCAATGGAGAAATAC

TTAAACCTAGAATCGATAAAACCATGAAATATATTAACTCAGAAGACTATTATGGAAATCAACATAATAT

CATAGTCGAACATTTAAGAGATCAAAGCATCAAAATAGGAGATGTCTTTAACGAGGATAAACTATCGGAT

ATTGGACATCAATACGCCGCCAACAACGATAAATTTAGATTAAATCCAGAAGTTAGTTATTTTACTAATA

AACGAACTAGAGGGCCGTTGGGAATTTTATCAAACTACGTCAAGACTCTTCTTATTTCTATGTATTGTTC

CAAAACATTTTTAGACGATTCCAACAAACGAAAGGTATTAGCGATTGATTTTGGAAACGGTGCTGACCTG

GAAAAATACTTTTATGGAGAGATTGCGTTATTGGTAGCGACGGATCCGGATGCTGATGCTATAGCTAGAG

GAAATGAAAGATACAACAAATTAAATTCTGGAATTAAAACCAAGTACTACAAATTTGACTACATTCAGGA

AACTATTCGATCCGATACATTTGTCTCTAGTGTCAGAGAAGTATTCTATTTTGGAAAGTTTAATATCATT

GACTGGCAGTTCGCTATTCATTATTCTTTTCATCCAAGACATTATGCTACAGTCATGAATAACTTATCCG

AACTAACTGCTTCTGGAGGCAAGGTATTAATTACTACCATGGATGGAGACAAATTATCAAAATTAACCGA

TAAAAAGACTTTTATAATTCATAAGAATCTACCTAGTAGCGAAAACTATATGTCTGTAGAAAAAATAGCT

GATGATAGAATAGTGGTATATAATCCATCAACAATGTCTACTCCAATGACTGAATACATTATCAAAAAGA

ACGATATAGTCAGAGTGTTTAATGAATACGGATTTGTTCTTGTAGATAATGTTGATTTCGCTACAATTAT

AGAACGAAGTAAAAAGTTTATTAATGGCGCATCTACAATGGAAGATAGACCGTCTACAAGAAACTTTTTC

GAACTAAATAGAGGAGCCATTAAATGTGAAGGTTTAGATGTCGAAGACTTACTTAGTTACTATGTTGTTT

ATGTCTTTTCTAAGCGGTAAATAATAATATGGTATGGGTTCTGATATCCCCGTTCTAAATGCATTAAATA

ATTCCAATAGAGCGATTTTTGTTCCTATAGGACCTTCCAACTGTGGATACTCTGTATTATTAATAGATAT

ATTAATACTTTTGTAGGGTAACAGAGGTTCTACGTCTTCTAAAAATAAAAGTTTTATAACATCTGGCCTG

TTCATAAATAAAAACTTGGCGATTCTATATATACTCTTATTATCAAATCTAGCCATTGTCTTATAGATGT

GAGCTACTGTAGGTGTACCATTTGATTTTCTTTCTAATACTATATATTTCTCTCGAAGAAGTTCTTGCAG

ATCATCTGGGAATAAAATACTACTATTGAGTAAATCAGTTATTTTTTTTATATCGATATTGATGGACATT

TTTATAGTTAAGGATAATAAGTATCCCAAAGTAGATAACGACGATAACGAAGTATTTATACTTTTAGGAA

ATCACAATGACTTTATCAGATCAAAATTAACAAAATTAAAGGAGCATGTATTTTTTTCTGAATATATTGT

GACTCCAGATACATATGGATCTTTATGCGTCGAATTAAATGGGTCTAGTTTTCAGCACGGTGGTAGATAT

ATAGAGGTGGAGGAATTTATAGATGCTGGAAGACAAGTTAGATGGTGTTCTACATCCAATCATATATCTG

AAGATATACACACTGATAAATTTGTCATTTATGATATTTATACGTTTGATTCGTTCAAGAATAAACGATT

GGTATTTGTACAGGTGCCTCCATCATTAGGAGATGATAGCTATTTAACTAATCCGTTATTGTCTCCGTAT

TATCGTAATTCAGTAGCCAGACAAATGGTCAATGATATGATTTTTAATCAAGATTCATTTTTAAAATATT

TATTAGAACATCTGATTAGAAGCCACTATAGAGTTTCTAAACATATAACAATAGTTAGATACAAGGATAC

CGAAGAATTAAATCTAACAAGAATATGTTATAATAGAGATAAGTTTAAGGCATTTGTATTCGCTTGGTTT

AACGGCGTTTCGGAAAATGAAAAGGTACTAGATACGTATAAAAAGGTATCTGATTTGATATAATGAATTC

AGTGACTATATCACACGCACCATATACTATTACTTATCACGATGATTGGGAACCAGTAATGAGTCAATTG

GTAGAGTTTTATAACGAAGTAGCCAGTTGGCTGCTACGAGACGAGACGTCGCCTATTCCTGATAAGTTCT

TTATACAATTGAAACAGCCGCTTAGAAATAAACGAGTATGTGTGTGTGGTATAGATCCGTATCCAAAAGA

TGGAACTGGTGTACCGTTCGAATCACCAAATTTTACAAAAAAATCAATTAAGGAGATAGCTTCATCTATA

TCTAGATTAACCGGAGTAATTGATTATAAAGGTTATAACCTTAATATAATAGACGGGGTTATACCCTGGA

ATTATTACTTAAGTTGTAAATTAGGAGAAACAAAAAGTCACGCGATTTACTGGGATAAGATTTCCAAGTT

ACTGCTGCAGCATATAACTAAACACGTTAGTGTTCTTTATTGTTTGGGTAAAACAGATTTCTCGAATATA

CGGGCAAAGTTAGAATCCCCGGTAACTACCATAGTGGGATATCATCCAGCGGCCAGAGACCACCAATTCG

AGAAAGATCGATCATTTGAAATTATCAACGTTTTACTGGAATTAGACAACAAGACACCTATAAATTGGGC

TCAAGGGTTTATTTATTAATGCTTTAGTGAAATTTTAACTTGTGTTCTAAATGGATGCGGCTATTAGAGG

TAATGATGTTATCTTTGTTCTTAAGACTATAGGTGTCCCGTCAGCATGCAGACAAAATGAAGATCCAAGA

TTCGTAGAAGCATTTAAATGCGACGAGTTAGAAAGATATATTGATAATAATCCAGAATGTACACTATTCG

AAAGTCTTAGGGATGAGGAAGCATACTCTATAGTCAGAATTTTCATGGATGTAGATTTAGACGCGTGTCT

AGACGAAATAGATTATTTAACGGCTATTCAAGATTTTATTATCGAGGTGTCAAACTGTGTAGCTAGATTC

GCATTTACAGAATGCGGTGCCATTCATGAAAATGTAATAAAATCCATGAGATCTAATTTTTCATTGACTA

AGTCTACAAATAGAGATAAAACAAGTTTTCATATTATCTTTTTAGACACGTATACCACTATGGATACATT

GATAGCTATGAAACGAACACTATTAGAATTAAGTAGATCATCTGAAAATCCACTAACAAGATCGATAGAC

ACTGCCGTATATAGGAGAAAAACAACTCTTCGGGTTGTAGGTACTAGGAAAAATCCAAATTGCGACACTA

TTCATGTAATGCAACCACCTCATGATAATATAGAAGATTACCTATTCACTTACGTGGATATGAACAACAA

TAGTTATTACTTTTCTCTACAACGACGATTGGAGGATTTAGTTCCTGATAAGTTATGGGAACCAGGGTTT

ATTTCGTTCGAAGACGCTATAAAAAGAGTTTCAAAAATATTCATTAATTCTATAATAAACTTTAATGATC

TCGATGAAAATAATTTTACAACGGTACCACTGGTCATAGATTATGTAACACCTTGTGCATTATGTAAAAA

ACGATCGCATAAACATCCGCATCAACTATCGTTGGAAAATGGTGCTATTAGAATTTACAAAACTGGTAAT

CCACATAGTTGTAAAGTTAAAATTGTTCCGTTGGATGGTAATAAACTGTTTAATATTGCACAAAGAATTT

TAGACACTAACTCTGTTTTATTAACCGAACGAGGAGACCATATAGTTTGGATTAATAATTCATGGAAATT

TAACAGCGAAGAACCATTGATAACAAAACTAATTCTATCAATAAGACATCAACTACCTAAGGAATATTCA

AGCGAATTACTCTGTCCGAGGAAACGAAAGACTGTAGAAGCTAACATACGAGACATGTTAGTAGATTCAG

TAGAGACCGATACCTATCCGGATAAACTTCCGTTTAAAAATGGTGTATTGGACCTGGTAGACGGAATGTT

TTACTCTGGAGATGATGCTAAAAAATATACGTGTACTGTATCGACCGGATTTAAATTTGACGATACAAAA

TTCGTCGAAAACAGTCCAGAAATGGAAGAGTTAATGAATATCATTAACGATATCCAACCATTAACGGATG

AAAATAAGAAAAATAGAGAGCTGTATGAAAAAACATTATCTAGTTGTTTATGTGGTGCTACCAAAGGATG

TTTAACATTCTTTTTTGGAGAAACCGCAACTGGAAAGTCGACAACCAAACGTTTGTTAAAGTCTGCTATC

GGTGACCTGTTTGTCGAGACGGGTCAAACAATTTTAACAGATGTATTGGATAAAGGACCTAATCCATTTA

TCGCTAATATGCATTTAAAAAGATCTGTATTCTGTAGCGAACTACCTGATTTTGCATGTAGTGGATCAAA

GAAAATTAGATCTGATAATATTAAAAAGTTGACAGAACCTTGTGTCATTGGAAGACCGTGTTTCTCCAAT

AAAATTAATAATAGAAACCATGCGACAATCATTATCGATACTAATTACAAACCTGTCTTTGATAGGATAG

ATAACGCATTAATGAGAAGAATTGCCGTCGTGCGATTCAGAACACACTTTTCTCAACCTTCTGGTAGAGA

GGCTGCTGAAAATAATGACGCGTACGATAAAGTCAAACTATTAGACGAGGGATTAGATGGTAAAATACAG

AATAATAGATATAGATTCGCATTTCTATACTTGTTGGTTAAATGGTACAAAAAATATCATATTCCTATTA

TGAAACTATATCCTACACCGGAAGAGATTCCGGACTTTGCATTCTATCTCAAAATAGGTACTCTGTTGGT

ATCTAGCTCTGTAAAGCATATTCCATTAATGACAGACCTCTCCAAAAAGGGATATATATTGTACGATAAT

GTGGTTACTCTTCCGTTGACTACTTTCCAACAGAAAATATCCAAGTATTTTAATTCTAGACTATTTGGAC

ACGATATAGAGAGCTTCATCAATAGACATAAGAAATTTGCCAATGTTAGTGATGAATATCTGCAATATAT

ATTCATAGAGGATATTTCATCTCCGTAAATATATGCCATATATTTATAGAAGATATCACATATCTAAATG

AATACCGGAATCATAGATTTATTTGATAATCATGTTGATAGTATACCAACTATATTACCTCATCAGTTAG

CTACTTTAGATTATCTAGTTAGAACTATCATAGATGAGAACAGAAGCGTGTTATTGTTCCATATTATGGG

ATCGGGTAAAACAATAATCGCTTTGTTGTTCGCCTTGGTAGCTTCCAGATTTAAAAAGGTTTACATTTTA

GTACCGAACATCAACATCTTAAAAATTTTCAATTATAATATGGGTGTAGCTATGAACTTGTTTAATGACG

AATTCATAGCTGAGAATATCTTTATTCATTCCACAACAAGTTTTTATTCTCTTAATTATAACGATAACGT

CATTAATTATAACGGATTAAGTCGCTACAATAACTCTATTTTTATCGTTGATGAGGCACATAATATTTTT

GGGAATAATACTGGAGAACTTATGACCGTGATAAAAAATAAAAACAAGATTCCTTTTCTACTATTGTCTG

GATCTCCCATTACTAACACACCTAATACGCTGGGTCATATTATAGATTTAATGTCCGAAGAGACGATAGA

TTTTGGTGAGATTATTAGTCGTGGTAAGAAAGTAATTCAGACACTTCTTAACGAACGCGGAGTGAATGTA

CTCAAGGATTTGCTTAAAGGAAGAATATCATATTACGAAATGCCGGACAAAGATCTACCAACAATAAGAT

ATCACGGACGTAAATTTCTAGATACTCGAGTAGTATATTGTCACATGTCTAAACTTCAAGAGAGAGATTA

TATGATTACTAGACGACAGCTATGTTATCATGAAATGTTTGATAAAAATATGTATAACGTGTCAATGGCA

GTATTGGGACAACTTAATCTGATGAATAATTTAGATACGTTATTTCAGGAACAGGATAAGGAATTGTACC

CAAATCTGAAAATAAATAATGGAGTGTTATACGGTGAAGAATTGGTAACGTTAAACATTAGTTCCAAATT

TAAGTACTTTATCAATCGGATACAGACACTCAAGGGAAAACACTTTATATACTTCTCTAATTCTACATAT

GGTGGATTGGTAATTAAATATATCATGCTCAGTAATGGATATTCTGAATATAATGGTTCTCAGGGAACTA

ATCCACATATGATAAACGGCAAACCAAAAACATTTGCTATCGTTACTAGTAAAATGAAATCGTCTTTAGA

GGATCTATTAGATGTGTATAATTCTCCTGAAAACGATGATGGCAGTCAATTGATGTTTTTGTTTTCGTCA

AACATTATGTCTGAATCCTATACTCTGAAAGAGGTAAGGCATATTTGGTTTATGACTATCCCGGATACTT

TTTCTCAATACAACCAAATTCTTGGACGATCTATTAGAAAATTCTCTTACGCCGATATTTCTGAACCCGT

TAATGTATATCTTTTAGCAGCCGTATATTCAGATTTCAATGACGAAGTGACGTCATTAAACGATTATACA

CAGGATGAATTGATTAATGTTTTACCCTTTGACATCAAAAAGCTGTTGTATCTAAAATTTAAGACTAAAG

AAACGAATAGAATATACTCTATTCTTCAAGAGATGTCTGAAACGTATTCTCTTCCACCACATCCATCAAT

TGTAAAAGTTTTATTGGGAGAATTGGTCAGACAATTTTTTTATAATAATTCTCGTATTAAGTATAACGAC

TCCAAGTTACTTAAAATGGTTACATCAGTTATAAAAAATAAAGAAGACGCTAGGAATTACATAGATGATA

TTGTAAACGGTCACTTCTTTGTATCGAATAAAGTATTTGATAAATCTCTTTTATACAAATACGAAAACGA

TATTATTACAGTACCGTTTAGACTTTCCTACGAACCATTTGTTTGGGGAGTTAACTTTCGTAAAGAATAT

AACGTGGTATCTTCTCCATAAAACTGATGAGATATATAAAGAAATAAATGTCGAGCTTTGTTACCAATGG

ATATCTTCCAGTTACATTGGAACCACATGAGTTGACATTAGACATAAAAACTAATATTAGGAATGCCGTA

TATAAGGCGTATCTCCATAGAGAAATTAGTGGTAAAATGGCCAAGAAAATAGAAATTCGTGAAGACGTGG

AATTACCTCTCGGCGAAATAGTTAATAATTCTGTAGTTATAAACGTTCCGTGTGTAATAACCTACGCATA

TTATCACGTTGGGGATATAGTCAGAGGAACATTAAACATCGAAGATGAATCAAATGTAACTATTCAATGT

GGAGATTTAATCTGTAAACTAAGTAGAGATTCGGGTACTGTATCATTTAGCGATTCAAAGTACTGCTTTT

TTCGAAATGGTAATGCGTATGATAACGGCATCGAAGTCTCCGCCGTTCTAATGGAGGCTCAACAAGGTAC

CGAATCTAGTTTTGTTTTTCTCGCGAATATCGTTGACTCATAAGAAAGAGAATAGCGGTGAGTATAAATA

CGAATACTATGGCAATAATTGCGAATGTTTTATTCCCTTCGATATATTTTTGATAATATGAAAAACATGC

CTCTCTCAAATCAGACAACCATTTCATAAAATAGTTCTCTCGCACTGGTGAGGTGGTTGCAGCTCGTATA

ATCTCCCCAGAATAATATACTTGCGTGTCGTCGTTCAATTTATACGGATTTCTATAATTCTCTGTTATAT

AATGAGGTTTACCCTCATGATTAGACGACGACAATAGTGTTCTGAATTTAGATAGTTGATCAGAATGAAT

GTTTATTGGTGTTGGAAAAATTATCCATGCTGCGTCTGCAGAGTGGTTGATAGTTGTTCCTAGATATGTA

AAATAATCCAACGTACTAGGTAGCAAATTGTCTAGATAAAATACTGAATCAAATGGCGCAGACATATTAG

CGGATCTAATGGAATCCAATTGATTGACTATCTTTTGAAAATATACATTTTTATGATCTGATACTTGTAA

GAATATAGCAATAATGATAATTCCATCATCGTGTTTTTTTGCCTCTTCATAAGAACTATATTTTTTCTTA

TTCCAATGAACCAGATTAATCTCTCCAGAGTATTTGTATACATCTATCAAGTGATTGGATCCATAATCGT

CTTCCTTCCCCCAATATATATGTATTGTTGATAACACATATTCATTGGGGAGAAACCCTCCACTTATATA

TCCTCCTTTAAAATTAATCCTTACTAGTTTTCCAGTATTCTGGATAGTGGTTGGTTTCGACTCATTATAA

TGTATGTCTAACGTCTTCAATCGCGTGTCAGAAATTGCTTTTTTAGTTTCTATATTAATAGGAGATAGTT

GTTGAGGCATAGTAAAAATGAAATGATAACTGTCTAGAAATAGCTCTTAGTATGGGATTTACAATGGATG

AGGAAGTGATATTTGAAACTCCTAGAGAATTAATATCTATTAAACGAATAAAAGATATTCCAAGATCAAA

AGACACGCACGTGTTTGCTGCGTGTATAACAAGTGACGGATATCCGTTAATAGGAGCTAGAAGAACTTCA

TTCGCATTCCAGGCGATATTATCTCAACAAAATTCAGATTCTATCTTTAGAGTATCCACTAAACTATTAC

GGTTTATGTACTACAATGAACTAAGAGAAATCTTTAGACGGTTGAGAAAAGGTTCTATCAACAATATCGA

TCCTCACTTCGAAGAGTTAATATTATTGGGTGGTAAACTAGATAAAAAGGAATCTATTAAAGATTGTTTA

AGAAGAGAATTAAAAGAGGAAAGTGATGAACATATAACAGTAAAAGAATTCGGAAATGTAATTCTAAAAC

TTACAACGAGTGATAAATTATTTAATAAAGTATATATAGGTTATTGCATGGCATGTTTTATTAATCAATC

GTTGGAGGATTTATCACATACTAGTATTTACAATGTAGAAATTAGAAAGATTAAATCGTTAAATGATTGT

ATTAACGACGATAAATACGAATATCTGTCTTATATTTATAATATACTAATTAATAGTAAATGAGCTTTTA

CAGATCTAGTATAATTAGTCAGATTATTAAGTATAATAGACGACTAGCTAAGTCTATTATTTGCGAGGAT

GACTCTCAAATTATTACACTCACGGCATTCGTTAACCAATGCCTATGGTGTCATAAACGAGTATCCGTGT

CCGCTATTTTATTAACTACTGATAACAAAATATTAGTATGTAACAGACGAGATAGTTTTCTCTATTCTGA

AATAATTAGAACTAGAAACATGTATAGAAAGAAACGATTATTTCTGAATTATTCCAATTATTTGAACAAA

CAGGAAAGAAGTATACTATCGTCATTTTTTTCTCTAGATCCAGCTACTGCTGATAATGATAGAATAAACG

CTATTTATCCGGGTGGTATACCCAAAAGGGGTGAGAACGTTCCAGAGTGTTTATCCAGGGAAATCAAAGA

AGAAGTTAATATAGACAATTCTTTTGTATTCATAGACACTCGTTTTTTTATTCATGGTATCATAGAAGAT

ACCATTATTAACAAATTTTTTGAGGTAATTTTCTTTGTTGGAAGAATATCTCTAACGAGTGATCAAATTA

TTGATACATTTAAAAGTAATCATGAAATAAAGGATCTAATATTTTTAGATCCGAATTCAGGTAATGGACT

CCAATACGAAATTGCAAAATATGCTCTAGATACTGCAAAACTTAAATGTTACGGTCATAGAGGATGTTAT

TATGAATCATTAAAAAAATTAACTGAGGATGATTGATTAGAAAATATAAATTAATTTACCATCGTGTATT

TTTATAACGGGATTGTCTGGCATATCATGTAGATAGTTACCGTCTACATCGTATACTCTACCATCTACGC

CTTTAAATCCTCTATTTATTGATATTAATCTATTAGAATTGGAATACCAAATATTAGTACCCTCAATTAG

TTTATTGGTAATATTTTTTTTAGACGATAGATCGATGGCTCTTGAAACCAAGGTTTTCCAACCGGACTCA

TTGTCGATCGGTGAGAAGTCTTTTTCATTAGCATGAATCCATTCTAATGATGTATGTTTAAACACTCTAA

ACAATTGTACAAATTCTTTTGATTTGTTTTGAATGATTTCAAATAGGTCTTCGTCTACAGTAGGCATACC

ATTAGATAATCTAGCCATTATAAAGTGCACGTTTACATATCTACGTTCTGGAGGAGTAAGAACGTGACTA

TTGAGACGAATGGCTCTTCCTACTATCTGACGAAGAGACGCCTCGTTCCATGTCATATCTAAAATGAAGA

TATCATTGATTGAGAAGAAACTAATACCCTCGCCTCCGCTAGAAGAGAATACGCATGTTTTAATGTATTC

TCCGTTAGTGTTTGATTCTTGGTTAAACTCAGCCACAGCCTTGATTCTAGTATCTTTTGTTCTAGATGAG

AACTCTATATTAGAGATACCAAAGACTTTGAAATATAGTAATAAGATTTCTATTCCTGACTGATTAACAA

ATGGTTCAAAGACTAGACATTTACCATGGGATGCTAATATTCCCAAACATACATCTATAAATTTGACGCT

TTTCTCTTTTAATTCAGTAAATAGAGAGATATCAGCCGCAATAGCATCCCCTCCCAATAGTTCTCCCTTT

TTAAAGGTGTCTAATGCGGATTTAGAAAATTCTCTATCTCTTAATGAATTTTTAAAATCATTATATAGGG

TTGCTATCTCTTGTGTGTATTCTCCCGGATCACGATTTTGTCTTTCAGGAAAGCTATCGAATGTAAACGT

AGTAGCCATACGTCTCAGAATTCTAAATGATGATATACCAGTTTTTATTTCTGCGAGTTTAGCCTTTTGA

TAAATCTCTTCTTGCTTTTTTGACATATTAACGTATCGCATTAATACTGTTTTCTTAGCGAATGATGCAG

ACCCTTCCACATCATCAAAAATAGAAAACTCGTTATTAACTATGTACGAACATAGGCCTCCTAGTTTGGA

GACTAATTCTTTTTCATCGACTAGACGTTTATTCTCAAATAGCGATTGGTGTTGTAAGGATCCTGGTCGC

AGTAAGTTAACCAACATGGTGAATTCTTGCACACTATTAACGATAGGTGTAGCCGATAAACAAATCATCT

TATGGTTTTTTAACGCAGTGGTCTTAGATAAAAAATTATATACTGACCGAGTAGGACGGATCTTACCATC

TTCTTTGATTAATGATTTAGAAATGAAGTTATGACATTCATCAATGATGACGCATATTCTACTCTTGGAA

TTAATAGTTTTGATATTAGTAAAAAATTTATTTCTAAAATTTTGATCATCGTAATTAATAAAAATACAAT

CCTTCGTTATCTCTGGAGCGTATCTGAGTATAGTGTTTATCCAAGGATCTTCTATCAAAGCCTTTTTTAC

CAATAAGATAATTGCCCAATTTGTATAAATATCCTTAAGATGTTTGAGAATATATACAGTAGTCATTGTT

TTACCGACACCTGTTTCATGGAACAATAAAAGAGAATGCATACTGTCTAATCCTAAGAAAACTCTTGCTA

CAAAATGTTGATAATCCTTGAGGCGTACTACGTCTGACCCCATCATTTCAACGGGCATATTAGTAGTTCT

GCGTAAGGCATAATCGATATAGGCCGCGTGTGATTTACTCATTTATGAGTGATAAGTAATAACTATGTTT

TAAAAATCACAGCAGTAGTTTAACTAGTCTTCTCTGATGTTTGTTTTCGATACTTTTTGAATCAGAAGTC

ATACTAGAATAAAGCAGCGAGTGAACGTAATAGAGAGCTTCGTATACTCTATTCGAAAACTCTAAGAACT

TATTAATGAATTCCGTATCCACTGGATCGTTTAAAATACTAAATTGAACAGTGTTCACATCCTTCCAAGA

CGAAGACTTAGTGACGGACTTAACATGAGACATAAATAAATCCAAATTTTTTTTATAAACATCACTAGCC

ACCATAATGGCGCTATCTTTCAACCAACTATCGCTTACGCATTTTAACAGTCTAACATTTTTAAAGAGAC

TACAATATATTCTCATAGTATCGATTACACCTCTACCGAATAGAGTGGGAAGTTTAATAATACAATATTT

TTCGTTTACAAAATCAAATAATGGTCGAAACACGTCGAAGGTTAACATCTTATAATCGCTAATGTATAGA

TTGTTTTCAGTGAGATGATTATTAGATTTAATAGCATCTCGTTCACGTTTGAACAGTTTATTGCGTGCGC

TGAGGTCGGCAACTACGGCATCCGCTCTAGTACTCCTCCCATAATACTTTACGCTATTAATCTTTAAAAT

TTCATAGACTTTATCTAGATCGCTTTCTGGTAACATGATATCATGTGTAAAAAGTTTTAACATGTCGGTC

GGCATTCTATTTAGATCATTAACTCTAGAAATCTGAAGAAAGTAATTAGCTCCATATTCCAGACTAGGTA

ATGGGCTTTTACCTAAAGACAAGTTAAGTTCTGGCAATGTTTCATAAAATGGAAGAAGGACATGTGTCCC

CTCCCGGATATTTTTTACAATTTCATCCATTTACAACTCTATAGTTTGTTTTCATTATTATTAGTTATTA

TCTCCCATAATCTTGGTAATACTTACACCTTGATCATAAGATACCTTATACAGGTCATTACATACAACTA

CCAATTGTTTTTGTACATAATAGATTGGATGATTGACATCCATGGTGGAATAAACTACTCGAACAGATAG

TTTATCTTTCCCCCTAGATACATTGGCCGTAATAGTTGTCGGCCTAAAGAATATCTTTGGTGTAAAGTTA

AAAGTTAGGGTTCTTGTTCCATTATTGCTTTTTGTCAGTAGTTCGTTATAAATTCTCGAGATGGGCCCGT

TCTCTGAATATAGAACATCATTTCCAAATCTAACTTCTAGTCTAGAAATAATATCGGTCTTATTTTTAAA

ATCTATTCCCTTGATGAATGGATCGTTAATAAACAAATCCTTGGCCTTTGATTCGGCTGATCTATTATCT

CCGTTATAGACGTTACGTTGACTAGTCCAAAGACTTACAGGAATAGATGTATCGATGATGTTGATAGTAT

GTGATATGTGAGCAAAGACTGTTCTCTTGGTGGCGTCGCTATATGTTCCAGTAATAGCGGAAAACTTTTT

AGAAATGTTATATATAAAAGAATTTTTTCGGGTTCCAAACATTAACAGATTAGTATGAAGATAAACACTC

ATATTATCAGGAACATTATCAATTTTTACATAAACATCGGCATCTTGAATAGAAACAACACCATCTTCTG

GAACCTCTACGATCTCGGCAGATTCCGGATAACCAGTCGGTGGACCATCACTAACAATAACTAGATCATC

CAACAATCTACTCACATATGCGTCTATATAATCTTTTTCATCTTGTGAGTACCCTGGATACGAAATAAAT

TTGTTATCCGTATTTCCATAATAAGGTTTAGTATAAACAGAGAGAGATGTTGCTGCATGAACTTCGGTTA

CTGTCGCCGTTGGTTGGTTTATTTGACCTATTACTCTCCTAGGTTTCTCTATAAATGATGGTTTAATTTG

TACATTCTTAACCATATATCCAATAAAGCTCAATTCAGGAACATAAACAAATTCTTTGTTGAACGTTTCA

AAGTCGAACGAAGAGTCACGAATAACGATATCGGATACTGGATTGAAGGTCACCGTTACCGTAATTTTTG

AATCGGATAGTTTAAGACTACTGAATGTATCTTCCACATCAAACGGAGTTTTAATATAAACGTATACTGT

AGATGGTTCTTTAATAGTGTCATTAGGAGTTAGACCAATAGAAATATCATTAAGTTCACTAGAATATCCA

GAATGTTTCAAAGCAATTGTATTATTGATACAATTATTATATAATTCTTCGCCATCAATTTCCCAAATAA

CACCGTTACACGAAGAGACAGATACATGATTAATACATTTATATCCAACATATGGTACGTAACCGAATCT

TCCCATACCTTTAACTTCTGGAAGTTCCAAACTCAGAACCAAATGATTAAGCGCAGTAATATACTGATCC

CTAATTTCGAAGCTAGCGATAGCCTGATTGTCGGGCCCATCGTTTGTCATAACTCCGGATAGAGAAATAT

ATTGCGGCATATATAAAGTTGGAATTTGACTATCAACTGCGAAGACATTAGACCGTTTAATAAAGTCATC

CCCACCGATCAAAGAATTAATGATAGTATTATTCATTTTCTATTTAAAATGGAAAAAAGCTTACAATAAA

CTCCGTAGAGAAATATCTATAATTTGTGAGTTTTCCTTAAAGTAACAGCTTCCGTAAACACCGTCTTTAT

CTCTTAATAAGTTTATTGTATTTATGACCTTTTCCTTATCTTCATAGAATACTAAAGGCAATAAAGAAAT

TTTTGGTTCTTCTCTAAGAGCTACGTGAGACTTAACCATAGACGCCAACGAATCCCTACATATTTTAGAA

CAGAAATACCCAACTTCACCACCCTTGAATGTCTCAATACTAATAGGTCTAAAAACCAAATCTTGATTAC

AAAACCAACACTTATCAATTACACTATTTGTCTTAATAGACATATCTGCCATAGATTTATAATACTTTGG

TAGTATACAAGCGAGTGCTTCTTCTTTAGCGGGCTTAAAGACTGCTTTAGGTGCTGAAATAACCACATCT

GGAAGACTTACTCGCTTAGCCATTTAATTACGGAACTATTTTTTTATACTTCTAATGAACAAGTAGAAAA

CCTCTCATCTACAAAAACATACTCGTGTCCATAATCCTCTACCATAGTAACACGTTTTTTAGATCTCATA

TGTGCTAAAAAGTTTTCCCATACTAATTGGTTACTATTATTTTTCGTATAATTTCTAACAGTTTGAGGTT

TTAGATTTTTAGTTACAGAAGTGATATCGAATATTTTATCCAAAAAGAATGAGTAATTAATTGTCTTAGA

AGGAGTGTTTTCTTGGCAAAAGAATACCAAGTGCTTAAATATTTCTACTACTTCATTAATCTTTTCTGTA

CTCAGATTCAGTTTCTCATCTTTTACTTGATTGATTATTTCAAAGACTAACTTATAATCCTTTTTATTTA

TTCTCTCGTTAGCCTTAAGAAAACTAGATACAAAATTTGCATCTACATCATCCGTGGATATTTGATTTTT

TTCCATGATATCCAATAGTTCCGAGATAATTTCTCCAGAACATTGATGAGACAATAATCTCCGCAATACA

TTTCTCAAATGAATAAGTTTATTAGACACGTGGAAGTTTGACTTTTTTTGTACCTTTGTACATTTTTGAA

ATACAGACTCGCAAAAAATACAATATTCATATCCTTGTTCAGATACTATACCGTTGTGTCTACAACAGCT

ACATAATCGTAGATTCATGTTAACACTCTACGTATCTCGTCGTCCAATATTTTATATAAAAACATTTTAT

TTCTAGACGTTGTCAGAAAATCCTGTAATATTTTTAGTTTTTTTGGTTGTGAATAAAGTATCGCCCTAAT

AATATTGGTACCGTCTTCCGACAATATAGTAGTTAAATTATCCGAGCATGTAGAAGAACACCGCTTAGGC

GGATTCAGTACAATGTTATATTTTTCGTACCAACTCATTTAAATATCATAATCTAAAATAGTTCTGTAAT

ATGTCTAGCGCTAATATATTGATCATAATCCTGTGCATAAATTAAGATACAACAATGTCTTGAAATCATC

GACATGGCTTCTTCCATAGTTAGAAGATCATCGTCAAAGTTAGCAACGTGATTCATCAACATTTGCTGTT

TTGAGGCAGCAAATACTGAACCATCACCATTCAACCATTCATAAAAACCATCGTCTGAATCCATTGATAA

TTTCTTGTACTGGTTTTTGAGAGCTCGCATCAATCTAGCATTTCTAGCTCCCGGATTGAAAACAGAAAGA

GGATCGTACATCCAGGGTCCATTTTCTGTAAATAGAATCGTATAATGTCCCTTCAAGAAGATATCAGACG

ATCCACAATCAAAGAATTGGTCTCCGAGTTTGTAACAGACTGCGGACTTTAACCTATACATGATACCGTT

TAGCATGATTTCTGGTGATACGTCAATAGGAGTATCATCTATTAGAGATCTAAAGCCGGTGTAACATTCT

CCACCAAACATATTCTTATTCTGACGTCGTTCTACATAAAACATCATTGCTCCATTAACGATAACAGGTG

AATGAACAGCACTACCCATCACATTAGTTCCCAATGGATCAATGTGTGTAACTCCAGAACATCTTCCATA

GCCTATGTTAGGAGGAGCGAACACCACTCTTCCACTATTGCCATCGAATGCCATAGAATAAATATCCTTG

GAATTGATAGAAATCGGACTGTCGGATGTTGTTATCATCTTCATAGGATTAACAACGATGTATGGTGCAG

CCTGAAGTTTCATATCATAACTGATGCCGTTCATAGGTCTAGCCACAGAAACCAACGTAGGTCTAAATCC

AACTATAGACAAAATAGAAGCTAATATCTGTTCCTCATCTGTCATAACTTGAGAGCATCCAGTATGAATA

ATCTTCATTAGATGGGGATCTACCGCATCATCATCGTTACAATAAAAAATTCCCATTCTAATGTTCATAA

TTGCTTTTCTAATCATGGTATGAATGTTTGCTCTCTGAATCTCTGTGGAAATTAGATCTGATACACCTGT

AATCACTATCGGATTATCCTCCGTAAGACGATTAACCAACAACATATAATTATAAGACTTTACTCTTCTA

AATTCATAAAGTTGCTGGATTAGACTATATGTGTCTCCATGTACATACGCGTTCTCGAGCGCAGGAAGTT

TAATACCGAATAGTGCCATCAGAATAGGATGAATGTAGTAATTAGTTTCTGGTTTTCTATAAATAAAAGA

CAAATCTTGTGAACTAGACATATCGGTAAAATGCATGGATTGGAATCGTGTAGTCGACAGAAGAATATGA

TGATTAGATGGAGAGTATATTTTATCTAACTCTTTGAGTTGGTCACCGATTCTAGGACTAGCTCGAGAAT

GAATAAGTACTAAGGGATGAGTACATTTCACAGAAACACTGGCGTTGTTCAACGTACTCTTTACATGGGA

AAGGAGTTGAAATAGCTCGTTTCTATTTGTCCTGACAATATTTAGTTTATTCATAATATTAAGCATATCC

TGAATAGTAAAGTTAGATGTGTCATACTTGTTAGTAGTTAGATATTTAGCAATTGCATTCCCATCATTTC

TCAATCTCGTACTCCAATCATGTGTGGATGCTACTTCGTCGATGGAAACCATACAATCCTTTTTGATAGG

CTGTTGAGATTGATCATTTCCTGTACGTTTAGGTTTGGTACGTTGATTTCTAGCCCCTGCGGATATAAAG

TCATCGTCTACAATTTGGGATAATGAATTACATACACTACAAGACAAAGATTTATCAGAAGTGTGAATAT

GATCTTCATCTACCAAAGAAAGAGTTTGATTAGTATAACTAGATTTTAGTCCCGCGTTAGATGTTAAAAA

AACATCGCTATTGACCACGGCTTCCATTATTTATATTCGTAGTTTTTACTCGAAAGCGTGATTTTAATAT

CCAATCTTATTACTTTTGGAATCGTTCAAAACCTTTGACTAGTTGTATAATTTGATCTATTGCCCTACGC

GTATACTCCCTTGCATCATATACGTTCGTCACCAGATCGTTTGTTTCGGCCTGAAGTTGACGCATATCTT

TTTCAACACTCGACATGAGATCCTTAAGGGTCATATCGTCTAGATTTTGTTGAGATGCTGCTCCTGGATT

TGGATTTTGTTGTGCTGTTGTACATACTGTACCACCAGTAGGTGTGGGAGTACATACAGTGGCCACAATA

GGAGGTTGAAGAGGTGTAACCGTTGGAGTAGTACAAGAAATACTTCCATCCGATTGTTGTGTACATGTGG

TTGTTGGTAACGTCTGAGAAGGTTGGGTAGATGGCGGTGTCGTCATCTTTTGATCTTTATTAAATTTAGA

GATAATATCCTGAACAGTATTGCTCGGCGTCAACGCTGGAAGGAGTGTACTCGCCGGCGCATCAGTATCT

GCAGACAGCCAATCAAAAAGATTAGACATATCAGATGATGTATTAGTTTGTTGTCGTGGTTTTAGTACAG

GAGCAGTACTACTAGGTAGAAGAATAGGAGCCGGTGTAGGTGTCGGAACCGGCTGTGGAGTTATATGAAT

AGTTGGTTGTAGCGGTTGGGTAGGCTGTCTGCTGGCGACCATCATATTATCTCTAGCTAGTTGTTCTCGC

AACTGTCTTTGATAATACGACTCTTGAGACTTTAGTCCTATTTCAATCGCTTCATCCTTTTTCGTATCCG

GATCCTTTTCTTCAGAATAATAGATTGACGACTTTGGTGTAGAGGATTCTGCCAGCCCCTGTGAGAACTT

GTTAAAGAAGTCCATTTAAGGCTTTAAAATTGAATTGCGATTATAAGATTAAATGGCAGACACGGACGAT

ATTATCGACTATGAATCCGATGATCTCACCGAATACGAGGATGATGAAGAAGATGGAGAGTCACTAGAAA

CTAGTGATATAGATCCCAAATCTTCTTATAAGATTGTAGAATCAACATCCACTCATATAGAAGATGCGCA

TTCCAATCTTAAACATATAGGGAATCATATATCTGCTCTTAAACGACGCTATACTAGACGTATAAGTCTA

TTTGAAATAGCGGGTATAATAGCAGAAAGCTATAACTTACTTCAACGAGGAAGGTTACCTCTAGTTTCAG

AATTTTCTGACGAAACGATGAAGCAAAATATGCTACATGTAATTATACAAGAGATAGAGGAGGGTTCTTG

TCCTATAGTCATCGAAAAGAACGGAGAATTGTTGTCGGTAAACGATTTTGATAAAGATGGTCTAAAATTC

CATCTAGACTATATTATCAAAATTTGGAAACTTCAAAAACGATATTAGAATTTATACGAATATCGTTCTC

TAAATGTTACAATCAAGTCTCTCATATTCAGCAGTTTATTGTCGTACTTTATATCGTGTTCATTAACGAT

ATTTTGCAAAATAGTAATGATTCTATCTTCCTTCGATAGATATTCTTCAGAGATTATTGTCTTATATTCT

TTCTTGTTATCCGATATGAATTTGATAAGACTTTGAACATTATTAATACCCGTCTGTTTAATTTTTTCTA

TAGATATTTTAGTTTTGGTAGATTCTATGGTGTCTGTTAATAGGCATCCAACATCGACATTCGACGTCAA

TTGTCTATAAATCAGAGTATAAATTTTAGAAATAACATTAGCAAATTGTTGTGCGTTGATGTCGTTATTC

TGAAACAGTATGATTTTAGGTAGCATTTTCTTAACAAAGAGAACGTATTTATTGTTACTCAGTTGAACAG

ATGATATATCCAGATTACTAACGCATCTGATTCCATATACCAAACTTTCAGAAGAAATGGTGTACAATTG

TTTGTATTCATTCAATGTCTCCTTTTCAGAAATTAGTTTAGAGTCGAATACTGCAATAATTTTCAAGAGA

TAGTTTTCATCAGATAAGATTTTATTTAGTGTAGATATGATAAAACTATTGTTTTGTTGGAGAACTTGAT

ACGCCGCATTCTCTGTAGTCGACGCTCTCAAATGGGAAACAATCTCTATTATTTTTTTGGAATCGGATAC

TATATCTTCGGTATCTTGACGCAGTCTAGTATACATAGAGTTAAGAGAAATTAGAGTTTGTACATTAAGC

AACATGTCTCTAAATGTGGCTACAAACTTTTCTTTTTCCACATCATCTAGTTTATTATATACCGATTTCA

CAACGGCACCAGATTTAAGGAACCAGAATGAAAAACTCTGATAACTACAATATTTCATCATAGTTACGAT

TTTATCATCTTCTATAGTTGGTGTGATAACACATACCTTTTTCTCCAAGACTGGAACCAACGTCATAAAA

ATGTTTAAATCAAAATCCATATCAACATCTGATGCGCTAAGACCAGTCTCGCGTTCAAGATTATCTTTAC

TAATGGTGACGAACTCATCGTATAGAACTCTAAGTTTGTCCATTATTTATTTACAGATTTAGTTGTTTAA

TTTATTTGTGCTCTTCCAGAGTTGGGATAGTATTTTTCTAACGTCGGTATTATATTATTAGGATCTACGT

TCATATGTATCATAATATTAATCATCCACGTTTTGATAAATCTATCTTTAGCTTCTGAAATAACGTATTT

AAACAAAGGAGAAAAATATTTAGTTACGGCATCAGACGCAATAACATTTTTTGTAAATGTAACGTATTTA

GACGACAGATCTTCGTTAAAAAGTTTTCCATCTATGTAGAATCCATCGGTTGTTAACACCATTCCCGCGT

CAGAGTGAATAGGAGTTTGAATAGTTTGTTTTGGAAATAGATCCTTCAATAACTTATAGTTGGGTGGGGA

AAAATCAATTTTATCACTAGACTCTTTCTTTTTTACTATCATTACCTCATGAACTATTTCTTGAATGAGT

ATATGTATTTTCTTTCCTATATCGGTCGCGTTCATTGGAAAATATATCATGTCGTTAACTATAAGAATAT

TTTTATCCTCGTTTACAAACTGAATAATATCAGATATAGTTCGTAAACGAACTATATCATCACCAGCACA

ACATCTAACTATATGATATCCACTAGTTTCCTTTAGCCGTTTATTATCTTGTTCCATATTAGCAGTCATT

CCATCATTTAAGAAGGCGTCAAAGATAATAGGGAGAAATGACATTTTGGATTCTGTTACGACTTTACCAA

AATTAAGGATATACGGACTTACTATCTTTTTCTCAACGTCGATTTGATGAACACACGATGAAAATGTACT

TCGATGAGATTGATCATGTAGAAAACAACAAGGGATACAATATTTCCGCATATCATGAAATATATTAAGA

AATCCCACTTTATTATATTTCCCCAAAGGATCAATGCATGTAAACATTATGCCGTTATCATTAATAAAGA

CTTCTTTCTCATCGGATCTGTAAAAGTTGTTACTGATTTTTTTCATTCCAGGATCTAGATAATTAATAAT

AATGGGTTTTCTATTCTTATTCTTTGTATTTTGACATATCCTAGACCAGTAAACAGTTTCCACTTTGGTA

AAATCAGAAGACTTTTGAACGCTATTAAACATGGCATTAATGGCAATAACTAAAAATGTAAAATATTTTT

CTATGTTAGGAATATGGTTTTTCACTTTAATAGATATATGGTTTTTTGCCAAAATGATAGATATTTTTTT

ATCCGATGATAGCAAAATATTATTAGTCGCCGTCTCTATAAAAATGAAGCTAGTCTCGATATCCAATTTT

ATTCTAGAATTGATAGGAGTCGCCAAATGTACCTTATACGTTATATCTCCCTTGATGCGTTCCATTTGTG

TATCTATATCGGACACAAGATCTGTAAATAGTTTTACGTTATTAATCATCACGGTATCGCCATCGCTAGA

TAATGCTAATGTACTATCCAAGTCCCAAATGGAGAGATTTAACTGTTCATCGTTTAGAATAAAATGATTA

CCTGTCATATTAATAAAGTGTTCATCGTATCTAGATAACAACGACTTATAATTAATGTCCAAGTCTTGAA

CTCGCTGAATGATCTTTTTTAACCCAGTTAGTTTTAGATTGGTACGAAATATATTGTTAAACTTTGATTC

TACAGTAATGTCCAAATCTAGTTGTGGAAATACTTCCATCAACATTGTTTCAAACTTGATAATATTATTA

TCTACATCTTCGTACGATCCAAATTCCGGAATAGATGTATCGCACGCTCTGGCCACCCAGATAACCAAAA

AGTCACACGCTCCAGAATATACATTGTATAAAAAGCTATCGTTTTTTAGTAGTGTTTTTTTCTGAGTATA

TACGAAAGGATTAAAAATAGTATTATCAACGTAACTATATTCCAAATTATTCTTATGAGAATAGATAATA

ATATCGTCCTTAATATCTAACAAATTTCCTAAATATCCCTTTAATTGAGTCATTCGAAGCGTTAATAAAA

TATGTCTCTTAACTATTTCCGGCCGTTGTATATTTAAATGACTTCGTAAGAAATAATATATAGGCGACTT

CTCATCTATGTAATCATATGGAGTGAGATATAGGGCTCGTTCTACCTCCTGCCCCTTACCCACCTGTAAT

ACCAATTGTGGACTCACTATATATCGCATATTTATATCGTGGGGTAAAGTGAAAATCTACTACCGATGAT

GTAAGTCTTACAATGTTCGAACCAGTACCAGATCTTAATTTGGAGGCCTCCGTAGAGCTAGGGGAGGTAA

ATATAGATCAAACAACACCTATGATAAAGGAGAATAGCGGTTTTATATCCCGTAGTAGACGTCTATTCGC

CCATAGATCTAAGGATGATGAGAGAAAACTAGCACTACGATTCTTTTTACAAAGACTTTATTTTTTAGAT

CATAGAGAGATTCATTATTTGTTCAGATGCGTCGACGCTGTAAAAGACGTCACTATTACCAAAAAAAATA

ACATTATCGTGGCGCCTTATATAGCACTTTTAACTATCGCATCAAAAGGATGCAAACTTACAGAAACAAT

GATTGAAGCATTCTTTCCAGAACTATATAATGAACATAGTAAGAAATTCAAATTCAACTCTCAAGTATCC

ATCATCCAAGAAAAACTCGGATACCAGTCTGGAAACTATCACGTTTATGATTTTGAACCGTATTACTCTA

CAGTAGCTCTGGCTATTCGAGATGAACATTCATCTGGCATTTTTAATATCCGTCAAGAGAGTTATCTTGT

AAGTTCATTATCTGAAATAACATATAGATTTTATCTAATTAATCTAAAATCTGATCTTGTTCAATGGAGT

GCTAGTACGGGCGCTGTAATTAATCAAATGGTAAATACTGTATTGATTACAGTGTATGACAAATTACAAC

TGGCCATAGAAAATGATTCACAATTTACATGTTCATTGGCTGTGGAATCAGAACTTCCAATAAAATTACT

TAAAGATAGAAATGAATTATTTACAAAATTCATTAACGAGTTAAAAAAGACCAGTTCATTCAAGATAAGC

AAACGCGATAAGGATACGCTATTAAAACATTTTACTTATGACTGGAGTTAGAATTTATAGACGACTCATT

TCGTTTATCATTATTACTATTACTATCATTATTAGTATTCTTCTTGTCATCTTGTTCAGAAATATACAGC

AATGCTATACCTAATACCAAATACATTATCATGCTTGCAATGGCTCTAACAACAACGAACCAAAATGAAT

TTGGTCGTAGCTTTTGTTCACAAAAATACATAAAGAAATGTCTACATAAATCTATGGCGCCATTGGCTAC

TTGAAATAGCGCCAGTCCTCCTACAGATTTTAATATAGCTGTATAACATGACATTTATTCATCATCAAAA

GAGACAGAGTCACCATCTGTCATATTTAGATTTTTTTTCATGTGTTCAAAGTATCCTCTACTCATTTCAT

TATAATAGTTTATCATACTTAGAATTTTAGGACGGATCAATGAGTAAGATTTGACTAGATCGTCAGTAGT

AATTTGTGCATCATCTATTCTGCATCCGCTTCGTCGAATAATGTATAGCATCGCTTTGAGATTCTCCATA

GCTATCAAGTCTTTATATAATGACATGGAAATATCTGTGAATGCTTTATACTTCTCCAACATCGATGCCT

TAACATCATCACATACTTTAGCATTGAAAATACGTTCTATTGTGTAGATGGATGTAGCAAGATTTTTAAA

CAACAATGCCATCTTACATGATGATTGTCTCAAGTCTCCAATCGTTTGTTTAGAACGATTAGCTACAGAG

TCCAATGCTTGGCTAACTAGCATATTATTATCTTTAGAAATTGTATTCTTCAATGAGGCGTTTATCATAT

CTGTGATTTCGTTAGTCATATTACAGTCTGACTGGGTTGTAATGTTATCCAACATATCACCTATGGATAC

GGTACACGTACCAGCATTTGTAATAATCCTATCTAAGATGTTGTATGGCATTGCGCAGAAAATATCTTCT

CCTGTAATATCTCCACTCTCGATAAATCTACTCAGATTATTCTTAAATGCCTTATTCTCTGGAGAAAAGA

TATCAGTGTCCATCATTTCATTAATAGTATACGCAGAAAAGATACCACGAGTATCAATTCTATCCAAGAT

ACTTATCGGTTCCGAGTCACAGATAATGGTTTCCTCTCCTTCGGGAGATCCTGCATAGAAATATCTAGGA

CAATAGTTTCTATACTGTCTGTAACTCTGATAATCTCTAAAGTCACTAACTGATACCATGAAATTGAGAA

GATCAAACGCTGAAGTAATCAATTTTTCTGCCTCGTTTTTACTACAACTAGTTTTCATCAATGTAGTGAC

GATGTATTGTTTAGTTACTCTTGGTCTAATACTGATGATAGAGATATTATTGCTTCCCATAATGGATCTT

CTAGTAGTCACCTTAAAGCCCATTGATGCGAATAGCAGATAGATAAAGTCTTGGTATGACTCCTTTCTAA

TATAGTACGGACTACCTTTGTCACCCAACTTTATACCCACATAAGCCATAACAACCTCTTTAATAGCCGT

TTCATGAGGTTTATCAGCCATGAGCCTGAGTAGTTGAAAGAATCGCATGAATCCCGTCTCAGAAAGTCCT

ATATGCATGATAGATTTATCTTTCCTGGGAAACTCTCGTATAGTTATAGATGAAATACTCTTCAAAGTTT

CTGAAATAAGATTAGTAACAGTCTTACCTCCGACTACTCTGGGTAACAAACATACTCTAATAGGTGTTTT

CTCTGCGGAGATAATATCAGAAAGGATAGAGCAATAAGTAGTATTATTGTGATTATAAAGACCGAATACA

TAACAGGTAGAATTTATAAACATCATGTCCTGAAGGGTTTTAGACTTGTATTCCTCGTAATCTATACCGT

CCCAAAACATGGATTTGGTAACTTTGATAGCCGTAGATCTTTGTTCCTTCGCCAACAGGTTAAAGAAATT

AATAAAGAATTTGTTGTTTCTATTTATGTCCACAAATTGCACGTTTGGAAGCGCCACGGTTACATTCACT

GCAGCATTTTGAGGATCGCGAGTATGAAGTACAATGTTATTGTTTACTGGTATATCTGGAAAGAAATCTA

CCAGTCTAGGAATAAGAGATTGATATCGCATAGAAATAGTAAAGTTTATAATCTCATCATTGTAGATTAC

TCTGTTACCATTGTAATAAATTGGTACTCTATCATAATCATTGACAAAGTACTGTTCATACATGATGAGA

TGTTTATATGTTGGCATAGTAGTGAGATCGACGTTTGGTAATGGCAATGTATTAAGATTAACTCCATAAT

GTCTAGCAGCATCTGTGATGTTATAAGTGATGTCAAAGCGGGGTTGATCTTGTGCTGTTATATATTGTCT

AACACCTATAAGATTATCAAAATCTTGTCTGCTTAATACACCGTTAACAATTTTTGCCTTGAATTCTTTT

ATTGGTGCATTAATAACATCCTTATAGAGGATGTTAAACAAATAAGTATTATCAAAGTTAAGATCTGGGT

ATTTCTTTTCTGCTAGAACATCCATTGAGTCGGAGCCATCTGGTTTAATATAACCACCGATAAATCTAGC

TCTGTATTCTGTATCCGTCAATCTAATATTAAGAAGGTGTTGAGTGAAAGGTGGAAGATCGTAAAAGCTG

TGAGTATTAATAATAGGGTTAGTTTCCGAACTAATGTTAATTGGATGATTAATAATATCTATATTTCCAG

CGTTAAGTGTAACATTAAACAGTTTTAATTCACGTGACGTGGTATCAATTAAATAATTAATGCCCAATTT

GGATATAGTAGCCTGAAGCTCATCTTGTTTAGTTACGGATCCTAATGAGTTATTAAGAAATACATCGAAC

GGATGAACGAAGGTTGTTTTAAGTTGATCACATACTTTGTAATCTAGACATAGATGTGGAAGAACGGTAG

AAACTATACGAAATAGATATTCAGAGTCCTCTAATTGATCAAGAGTAACTATTGACTTAATAGGCATCAT

TTATTTAGTATTAAATGACGACCGTACCAGTGACAGATATACAAAACGACTTAATTACAGAGTTTTCAGA

AGATAATTATCCATCTAACAAAAATTATGAAATAACTCTTCGCCAAATGTCTATTCTAACTCACGTTAAC

AACGTGGTAGATAGAGAACATAATGCCGCCGTAGTGTCATCTCCAGAGGAAATATCATCACAACTTAATG

AAGATCTATTTCCAGATGATGATTCACCGGCCACTATTATCGAACGAGTACAACCTCATACTACTATTAT

TGACGATACGCCACCTCCTACTTTTCGTAGAGAGTTATTGATATCGGAACAACGTCAACAACGAGAAAAA

AGATTTAATATTACAGTATCAAAAAATTCTGAAGCAATAATGGAATCTAGATCTATGATAACTTCTATGC

CAACACAAACACCATCCTTGGGAGTAGTTTATGATAAAGATAAAAGAATTCAGATGCTAGAGGATGAAGT

GGTTAATCTTAGAAATCAACGATCTAATACAAAATCATCTGATAATTTAGATAATTTTACCAGAATACTA

TTTGGTAAGACTCCGTATAAATCAACAGAAGTTAATAAGCGTATAGCCATCGTTAATTATGCAAATTTGA

ACGGGTCCCCCTTATCAGTCGAGGACTTGGATGTTTGTTCGGAGGATGAAATAGATAGAATCTATAAAAC

GATTAAACAATATCACGAAAGTAGAAAACGAAAAATTATCGTCACTAACGTGATTATTATTGTCATAAAC

ATTATCGAGCAGGCATTGCTAAAACTCGGATTTGAAGAAATCAAAGGACTGAGTACCGATATCACTTCAG

AAATTATCGATGTGGAGATCGGAGATGACTGCGATGCTGTAGCATCAAAACTAGGAATCGGTAACAGTCC

GGTTCTTAATATTGTATTGTTTATACTCAAGATATTCGTTAAACGAATTAAAATTATTTAATTTAATACA

TTCCCATATCCAGACAACAATCGTCTGGATTAATCTGTTCCTGTCGTCTCATACCGGACGACATATTAAT

CTTTTTATTAGTGGGCATCTTTTTAGATGGTTTCTTTTTCCCAGCATTAACTGATTCGATACCTAGAAGA

TCGTGATTGATTTCTCCGACCATTCCACGAACTTCTAATTGGCCGTCTCTAACGGTACCATAAACTATTT

TACCAGCATTAGTAACAGCTTGGACAATCTGACCATCCATTGCGTTGAATGATGTAGTTGCTGTTGTTCT

ACGTCTAGGAGCACCAGAGGTATTTTTAGAGCTCTTGGATGTTGATGTAGAAGACGAGGATTTTGATTTT

GGTTTACATGTAATACATTTTGAACTCTTTGATTTTGTATCACATGCGCCGGCAGTCACATCTGTTTGAG

AATTAAGATTATTGTTGCCTCCTTTGACGGCTGCATCTCCACCGATCTGCGCTAGTAGATTTTTAAGCTG

TGGTGTAATCTTATTAACTGTTTCGATATAATCATCGTAACTACTTCTAACGGCTAAATTTTTTTTATCC

GCCATTTAGAAGCTAAAAATATTTTTATTTATGCAGAAGATTTAACTAGATTATACAATGAACTAATATG

ATCCTTTTCTAGATTATTTACGAACTTGGTATTTCTTGTTTCTGGAGGAGGAGAATTTAAATTCGGACTT

GGATTCGGATTTTGTGGGTTCTTGATCTTATTATACAGCGTGTATAGGATGGCGACGGTAACTGCTACAC

AAATACCGATCAACAGAAGAATACCAATCATTTATTGACAATAACTTCACTATGATCAAGTATGTAATAA

TCATCTTTTCACTAAGTAAGTAGTAATAATGATTCAACAATGACAAGATATATGGACGATAATAATTTAG

TTCATGGAAATATCGCTATGATTGGTGTGAATGACTCCGCTAACTCTGTGGGGTGCACAGTGCTTTCCCC

ACATAGAATAAATTAGCATTCCGACTGTGATAATAATACCAAGTATAAACGCCATAATACTCAATACTTT

CCATGTACGAGTGGGACTGGTAGACTTACTAAAGTCAATAAAGGCGAAGATACACGAAAGAATCAAAAGA

ATGATTCCAGCGATTAGCACGCCGGAAAAATAATTTCCAATCATAAGCATCATGTCCATTTAACTAATAA

AAATTTTAAATCGCCGAATAAACAAAGTGGAATATAAACCATATAAAAACAATAGTTTGTACTGCAAAAA

TAATATCTATTTTTGTTTTCGAAGATATGGTAAAATTAAATAGTAGTACACAGCATGTTATAACTAACAG

CAGCAACGGCTCGTAATTACTTATCATTTACTAGACGAAAAGGTGGTGGGATATTTTCTTGCTCAAATAA

TACGAATATATCACCCATCCATTTTATACGATGTTTATATACTCTAATCTTTAATAGATCTATAGATGAC

GGGTTTACCAACAATATAGATTTTATCGATTCATCTAATTTAAACCCTTCCTTAAACGTGAATGATCTAT

TATCTGGCATAATGATGACCCTACCTGATGAATCTGACAATGTACTGGGCCATGTAGAATAAATTATCAA

CGAATTATCGTCTACGAACATTTATATCATTTGTTTTAATTTTAGGACGTGAATAAATAGATATAAAATA

GAAAATAACAGATATTACAACCAGTGTTATGGACGCACCCAACCATGTAGGCAGTTTTATTTTATCGTTT

ACTACAGGTTCTCCTGGATGTACGTCACCAACTACAGACGTAGTTCTAGTACAATTAGACGTAAGTTCCG

CTTGGGAATTTTTTAACACTAAAGAGTTAACGTTAATCGTACACCCAACGTATTTACATCTAGCTCTTTG

AACATCTTGATTATAATATAACCATTTTCTATCTCTAGATTCGTCAGTGCACTCATGTAACCAACATACC

CTAGGTCCTAAATATTTATCTCCGGAATTAGATTTTGGATAATTCGCGCACCAACAATTTCTATTTCCTT

TATGGTCGTTACAAAAGACGTATAATGCCGTATCCCCAAAAGTAAAATAATCAGGACGAATAATTCTAAT

AAACTCAGAACAATATCTCGCATCCATATGTTTGGAGCAAATATCGGAATAAGTAGACATAGCCGGTTTC

CGTTTTACACGTAACCATTCTAAACAATTGGGGTTTCCAGGATCGTTTCTACAAAAACCAGTCATGAAAT

CGTCACAATGTTCTGTCTTGTAATTATTATTAAATATTTTTGGACAGTGTTTGGTATTTGTCTTAGAACA

ACATTTTGCCACGCTATCACTATCACCCAGGAGATAATCCTTTTTTATAAAATGACACCGTTGCCCGGAT

GCTATATAATCAGTAGCATATTTTAAATCCTTAATATACTCAGGAGTTACCTCGTTCTGATAATAGATTA

ATGATCCAGGACGAAATTTGAAAGAACTACATGGTTCTCCATGAATTAATACATATTGTTTAGCAAATTC

AGGAACTATAAAACTACTACAATGATCTATCGACATACCATCTATCAAACAAAATTTGGGTTTAATTTCT

CCTGGAGACGTTTCATAATAATACATATAACTTTCTTCGGCAAACCTAACAGCTCTATTATATTCAGGAT

AATTAAAATCTAATACCATATATTTGTCTCGTATATCTGCTATTCCTGTCTCTATTTTGATTCTATTAAG

AGTAACAGCTGCCCCCATTCTTAATAATCATCAGTATTTAAACTGTTAAATGTTGGTATATCAACATCTA

TCTTATTTCCCGCAATATAAGGTTTGTTGCAGGTATACTGTTCAGGAATGGGTACATTTATACTTCTTTT

ATAGTCCTGTCTTTCGATGTTCATCACAAATGCAAAGAACAGAATAAACAAAATAATGTAAGAAATAATA

TTAAATATCTGTGAATTCGTAAATACATTGATTGCCATAATAATTACAGCAGCTACAATACACACAATAG

ACATTCCCACAGTGTTGCCATTACCTCCACGATACATTTGAGTTACTAAGCAATAGGTAATAACTAAGCT

AGTAAGAGGCAATAGAAAAGATGAGATAAATATCATCAATATAGAGATTAGAGGAGGGCTATATAGAGCC

AAGACGAACAAAATCAAACCGAGTAACGTTCTAACATCATTATTTTTGAAGATTCCCAAATAATCATTCA

TTATTCCTCCATAATCGTTTTGCATCATACCCCCATCTTTAGGCATAAACGATTGCTGCTGTTCCTCTGT

AAATAAATCTTTATCAAGCACTCCAGCACCCGCAGAGAAGTCATCAAGCATATTGTAATATCTTAAATAA

CTCATTTATATATTAAAAAATGTCACTATTAAAGATGGAGTATAATCTTTATGCCGAACTAAAAAAAATG

ACTTGTGGTCAGACCATAAGTCTTTTTAATGAAGACGGCGATTTCGTAGAAGTTGAACCAGGATCATCCT

TTAAGTTTCTAATACCTAAGGGATTTTACTCCTCTCCTTGTGTAAAGACGAGTCTAGTATTCGAGACATT

AACAACGACCGATAATAAAATTACTAGTATCAATCCAACAAATGCGCCAAAGTTATATCCTCTTCAACGC

AAAGTCGTATCTGAAGTAGTTTCTAATATGAGGAAAATGATCGAATTAAAACGTCCTCTATACATCACTC

TTCACTTGGCATGTGGATTTGGTAAGACTATTACCACGTGTTATCTTATGACCACACACGGCAGAAAAAC

CATCATTTGCGTACCCAATAAAATGTTAATACATCAATGGAAGACACAGGTAGAGGCAGTCGGATTGGAA

CATAAGATATCTATAGATGGAGTTAGTAGTCTATTAAAGGAACTAAAGACTCAAAGTCCGGATGTATTAA

TCGTAGTCAGTAGACATCTGACAAACGATGCATTTTGTAAATATATCAATAAGCATTATGATTTGTTTAT

CTTGGATGAATCACATACGTATAATCTGATGAACAATACAGCAGTTACAAGATTTTTAGCGTATTATCCT

CCGATGATGTGTTATTTTTTAACTGCTACACCTAGACCAGCTAACCGAATTTATTGTAACAGTGTTATTA

ATATTGCCAAGTTATCAGATCTAAAAAAAACTATCTATACAGTAGATAGTTTTTTTGAGCCATATTCCAC

AGACAATATTAGAAATATGGTAAAACGACTAGATGGACCATCTAATAAATATCATATATATACCGAGAAG

TTATTATCTGTAGACGAGCCTAGAAACCAACTTATTCTTGATACCCTGGTAGAAGAATTCAAGTCAGGAA

CTATTAATAGAATTTTAGTTATTACTAAACTACGTGAACATATGGTATTCTTCTACAAACGATTATTAGA

TCCTTTCGGACCAGAGGTTGTATTTATAGGAGACGCCCAAAATAGACGTACTCCAGATATGGTCAAATCA

ATTAAGGAACTAAATAGATTTATATTCGTATCCACCTTATTTTATTCCGGCACTGGTTTAGATATTCCGA

GTTTGGATTCTTTGTTCATTTGCTCGGCAGTAATCAACAATATGCAAATAGAGCAATTACTAGGGAGGGT

ATGTCGAGAAACAGAACTATTAGATAGGACGGTATATGTATTTCCTAACACATCCATCAAAGAAATAAAG

TACATGATAGGAAATTTCGTGCAACGAATTATTAGTCTGTCTGTAGATAAACTCGGATTTAAACAAGAAA

GTTATCGGAAACATCAGGAATCTGAACCCGCTTCCGTGCCAACATCCTCCAGAGAAGAACGTGTATTAAA

TAGAATATTTAACTCGCAAAATCGTTAAGAAGTTTAAGAGACGATCCACATGCTGAGCAGGCCAGTGTAT

TACCCCTCATAGTATTAATATAATCCAATGATACTTTTGTGATGTCGGAAATCTTAACCAATTTAGACTG

ACAGGCAGAACACGTCATGCAATCATCACCGTCATCGATAACTGTAGTCTTGGGCTTCTTTTTGCGACTC

TTCATTCCGGAACGCATATTGGTGCTATCCATTTAGGTAGTAAAAAATAAGTCAGAATATGCCCTATAAC

ACGATCGTGCAAAACCTGGTATATCGTCTCTATCTTTATCACAATATAGTGTATCAACATCTTTATTATT

GACCTCGTTTATCTTGGAACATGGAATGGGAACATTTTTGTTAACGGCCACCTTTGCCTTAATTCCAGAT

GTTGTAAAATTATAACCAAACAGTCTATCATCGACACAAATGAAATTCTTGTTTAGACGTTTGTAGTTTA

CGTATGCGGCTCGTTCTCGTCTCATTTTTTCAGATATTGCAGGTACTATAATATTAAAAATAAGAATGAA

ATAACATAGGATTAAAAATAAAGTTATCATGACTTCTAGTGCTGATTTAACTAACTTAAAAGAATTACTT

AGTCTGTACAAAAGTTTGAGATTTTCAGATTCTGTGGCTATAGAGAAGTATAATTCTTTGGTAGAATGGG

GAACATCTACTTACTGGAAAATAGGCGTACAAAAGGTAACTAATGTCGAGACGTCCATATCTGATTATTA

TGATGAGGTAAAAAATAAACCGTTCAATATTGATCCGGGGTATTATATTTTCTTACCAGTATACTTTGGA

AGCGTCTTTATTTATTCGAAGGGTAAAAATATGGTAGAACTTGGATCTGGAAACTCTTTTCAAATACCGG

ATGAGATTCGAAGTGCGTGTAACAAAGTATTAGATAGTGATAACGGAATAGACTTTCTGAGATTTGTTTT

GTTAAACAATAGATGGATAATGGAAGACGCTATATCAAAATACCAGTCTCCAGTTAATATATTTAAACTA

GCTAGTGAGTACGGATTAAACATACCCAACTATTTAGAAATTGAAATAGAGGAAGACACATTATTTGACG

ATGAGTTATACTCTATTATGGAACGCTCTTTCGATGATACATTTCCAAAAATATCTATATCGTATATTAA

GTTGGGAGAACTTAAACGGCAAGTTGTAGACTTTTTCAAATTCTCATTCATGTATATTGAGTCAATCAAG

GTAGATCGTATAGGAGATAATATTTTTATTCCTAGCGTTATAACAAAATCAGGAAAAAAGATATTAGTAA

AAGATGTAGACCATTTAATACGATCCAAGGTTAGAGAACATACATTTGTAAAAGTAAAAAAGAAAAACAC

ATTTTCCATTTTATACGACTATGATGGGAACGGAACAGAAACTAGAGGAGAAGTAATAAAACGAATTATA

GACACTATAGGACGAGACTATTATGTTAATGGAAAGTATTTCTCTAAGGTTGGTAGTGCAGGCTTAAAGC

AATTGACTAATAAATTAGATATTAACGAGTGCGCAACTGTCGATGAGTTAGTTGATGAGATTAATAAATC

CGGAACTGTAAAACGAAAAATAAAAAACCAATCAGTATTTGATTTAAGCAGAGAATGTTTGGGATATCCA

GAAGCGGATTTTATAACGTTAGTTAATAACATGCGGTTCAAAATAGAAAATTGTAAGGTCGTAAATTTCA

ATATTGAAAATACTAATTGTTTAAATAACCCGAGTATTGAAACTATATATGGAAACTTCAACCAGTTCGT

CTCAATCTTTAATACCGTTACCGATGTCAAAAAAAGATTATTCGAGTGAAATAATATGCGCCTTTGATAT

AGGTGCAAAAAATCCTGCCAGAACTGTTTTAGAAGTCAAGGATAACTCCGTTAGGGTATTGGATATATCA

AAATTAGACTGGAGTTCTGATTGGGAAAGGCGCATAGCTCAAGATTTGTCACAATATGAATACACTACAG

TTCTTCTAGAACGTCAGCCTAGAAGGTCACCGTACGTCAAATTTATCTATTTTATTAAAGGCTTTTTATA

TCATACATCTGCTGCCAAAGTTATTTGCGTCTCACCTGTCATGTCTGGTAATTCATATAGAGATCGAAAA

AAGAGATCTGTTGAAGCATTTCTTGATTGGATGGACACATTCGGATTGCGAGACTCCGTTCCGGATAGAC

GCAAATTAGACGATGTAGCGGATAGTTTCAATTTGGCTATGAGATACGTATTAGATAAATGGAATACTAA

TTATACACCTTATAATAGGTGTAAATATAGAAATTACATAAAAAAAATGTAATAACGTTAGTAACGCCAT

TATGGATAATCTATTTACCTTTCTACATGAAATAGAAGATAGATATGCCAGAACTATTTTTAACTTTCAT

CTAATAAGTTGCGATGAAATAGGAGATATATATGGTCTTATGAAAGAACGCATTTCCTCAGAGGATATGT

TTGACAATATAGTATATAATAAAGATATACATCCTGCCATTAAGAAACTAGTTTATTGCGACATCCAACT

TACTAAACATATTATTAATCAGAATACGTATCCGGTATTTAACGATTCTTCACAAGTGAAATGTTGTCAT

TATTTCGATATAAACTCAGATAATAGCAATATTAGCTCTCGTACAGTAGAGATATTTGAGAGTGAAAAGT

CATCTCTTGTATCATATATTAAAACTACCAATAAGAAGAGAAAGGTCAATTACGGCGAAATAAAGAAAAC

TGTACATGGAGGCACTAATGCAAATTACTTTTCCGGTAAAAAGTCTGATGAGTATCTGAGCACTACAGTC

AGGTCCAACATTAATCAACCTTGGATCAAAACCATTTCTAAGAGAATGAGAGTAGATATCATTAATCACT

CTATAGTAACGCGTGGAAAAAGCTCTATATTACAAACTATAGAAATTATTTTTACTAATAGAACATGTGT

GAAAATATTCAAGGATTCTACTATGCACATTATTCTATCCAAGGACAAGGATGAAAAGGGATGTATAAAC

ATGATTGATAAATTATTCTATGTATATTATAATTTATTTCTGTTGTTCGAGGATATCATCCAAAACGAGT

ACTTTAAAGAAGTAGCTAATGTTGTAAACCATGTACTCATGGCTACGGCATTAGATGAGAAATTATTCCT

AATTAAGAAAATGGCTGAACACGATGTTTATGGAGTTAGCAATTTCAAAATAGGGATGTTTAACCTGACA

TTTATTAAGTCGTTGGATCATACCGTTTTCCCCTCTCTGTTAGATGAGGATAGCAAAATAAAGTTTTTTA

AGGGGAAAAAGCTCAATATTGTAGCATTACGATCTCTGGAGGATTGTACAAATTACGTGACTAAATCCGA

GAATATGATAGAAATGATGAAGGAAAGATCGACTATTTTAAATAGCATAGATATAGAAACGGAATCGGTA

GATCGTCTAAAAGAATTGCTTCTAAAATGAAAAAAAACACTGTTTCAGAAATGGATCAACGACTCGGGTA

TAAGTTTTTGGTGCCTGATCCTAAAGCCGGAGTTTTTTATAGACCGTTACATTTCCAATATGTATCGTAT

TCTAATTTTATATTGCATCGATTGCATGAAATCTTGACCGTCAAGCGGCCACTCTTATCGTTTAAGAATA

ATACAGAACGAATTATGATAGAAATTAGCAATGTTAAAGTGACTCCTCCAGATTACTCACCTATAATTGC

GAGTATTAAAGGTAAGAGTTATGACGCATTAGCCACGTTCACTGTAAATATCTTTAAAGAGGTAATGACC

AAAGAGGGTATATCCATCACTAAAATAAGTAGTTATGAGGGAAAAGATTCTCATTTGATAAAAATTCCGC

TACTAATAGGATATGGGAATAAAAATCCACTTGATACAGCCAAGTATCTTGTTCCTAATGTCATAGGTGG

AGTCTTTATCAATAAACAATCTGTCGAAAAAGTAGGAATTAATCTAGTAGAAAAGATTACAACATGGCCA

AAATTTAGGGTTGTTAAGCCAAACTCATTCACTTTCTCGTTTTCCTCCGTATCCCCTCCTAATGTATTAC

CGACAAGATATCGCCATTACAAGATATCTCTGGATATATCACAATTGGAAGCGTCTAATATATCATCGAC

AAAGACATTTATAACGGTCAATATTGTTTTGCTGTCTCAATATTTATCTAGAGTGAGTCTAGAATTCATT

AGACGTAGTTTATCATACGATATGCCTCCAGAAGTTGTCTATCTAGTAAACGCGATAATAGATAGTGCTA

AACGACTTACCGAATCTATTACTGACTTTGATATTGATACATACATTAATGACCTGGTGGAAGCTGAACA

CATTAAACAAAAATCTCAGTTAACGATTAACGAGTTTAAATATGAAATGCTGCATAACTTTTTACCTCAT

ATGAACTATACACCCGATCAACTAAAGGGATTTTATATGATATCTTTACTAAGAAAGTTTCTCTACTGTA

TCTACCACACTTCTAGATATCCAGATAGAGATTCGATGGTTTGTCATCGCATCCTAACGTACGGCAAATA

TTTTGAGACGTTGGCACATGATGAATTAGAGAATTACATAGGTAACATCCGAAACGATATCATGAACAAT

CACAAGAACAGAGGCACTTACGCAGTAAACATTCATGTACTAACAACTCCTGGACTTAATCATGCATTTT

CTAGTCTATTGAGTGGAAAGTTCAAAAAGTCAGACGGTAGTTATCGAACACATCCTCACTATTCATGGAT

GCAGAATATTTCTATTCCTAGAAGTGTTGGATTTTATCCGGATCAAGTAAAGATTTCAAAGATGTTTTCT

GTCAGAAAATACCATCCAAGCCAATATCTTTACTTTTGTTCATCAGACGTTCCGGAAAGAGGTCCTCAGG

TAGGTTTAGTATCTCAATTGTCTGTCTTGAGTTCCATTACAAATATACTAACGTCTGAGTATTTGGATTT

GGAAAAGAAAATTTGTGAGTATATCAGATCATATTATAAAGATGATATAAGTTACTTTGAAACAGGATTT

CCAATCACTATAGAAAATGCTCTAGTCGCATCTCTTAATCCAAATATGATATGTGATTTTGTAACTGACT

TTAGACGTAGAAAACGGATGGGATTCTTCGGTAACTTGGAGGTAGGTATTACTTTAGTTAGGGATCACAT

GAATGAAATTCGCATTAATATTGGAGCAGGAAGATTAGTCAGACCATTCTTGGTTGTGGATAACGGAGAG

CTCATGACGGATGTGTGTCCGGAGTTAGAAAGCAGATTAGACGACATGACATTCTCTGACATTCAGAAAG

AGTTTCCACATGTCATCGAAATGGTAGATATAGACCAATTTACTTTTAGTAACGTATGTGAATCGGTTCA

AAAATTTAGAATGATGTCAAAGGATGAAAGAAAGCAATACGATTTATGTGACTTTCCTGCCGAATTTAGA

GATGGATATGTAGCATCTTCACTAGTGGGAATCAATCACAATTCTGGACCCAGAGCTATTCTTGGATGTG

CTCAAGCTAAACAAGCTATCTCTTGTCTGAGTTCGGATATACGAAATAAAATAGACAATGGAATTCATTT

GATGTATCCAGAGAGGCCAATTGTGATTAGTAAGGCTTTAGAAACTTCAAAGATTGCGGCTAATTGCTTC

GGACAACATGTTACTATAGCATTAATGTCGTACAAAGGTATCAATCAAGAGGATGGAATTATCATCAAAA

AACAATTTATTCAGAGAGGCGGTCTCGATATTGTTACAGCCAAGAAACATCAAGTAGAAATTCCATTGGA

AAACTTTAATAACAAAGAAAGAGATAGGTCTAACGCCTATTCGAAATTAGAAAGTAATGGATTAGTTAGA

CTGAATGCTTTCTTGGAATCCGGAGACGCTATGGCAAGAAATATCTCATCAAGAACTCTTGAAGATGATT

TTGCTAGAGATAATCAGATTAGCTTTGATGTTTCCGAGAAATATACAGATATGTACAAATCTCGCGTTGA

ACGAGTACAAGTAGAACTTACTGACAAAGTTAAGGTGCGAGTATTAACCATGAAAGAAAGAAGACCCATT

CTAGGAGACAAATTTACTACTAGAACGAGTCAAAAGGGAACAGTCGCGTATATCGCAGATGAAACGGAAC

TTCCGTACGACGAAAATGGTATCACACCAGATGTCATTATTAATTCTACATCCATCTTCTCTAGAAAAAC

TATATCTATGTTGATAGAAGTTATTTTAACAGCCGCATATTCTACTAAGCCGTACAACAATAAGGGAGAA

AACCGACCTGTCTGTTTTCCTAGTAGTAACGAAACATCCATTGATGCATATATGCAATTCGCTAAACAAT

GTTATGAGTATTCAAATCCGAAATTGTCCGAGGAAGAATTATCGGATAAAATCTTTTGTGAAAAGATTCT

CTATGATCCTGAAACGGATAAGCCTTATGAATCCAAAGTATTTTTTGGACCAATTTATTACTTGCGTCTG

AGACATTTAACTCAGGACAAGGCAACCGTTAGATGTAGAGGTAAAAAGACGAAGCTCATTAGACAAGCGA

ATGAGGGACGAAAACGTGGAGGAGGTATCAAGTTTGGAGAAATGGAGAGAGACTGTTTAATAGCACATGG

CGCAGCCAATACTATTACAGAAGTTTTAAAAGACTCAGAAGAGGATTATCAAGATGTGTATATTTGTGAA

AATTGTGGAGACATAGCAGCACAAATCAAAAGTATTAATACATGTCTTAGATGTTCAAAACTTAATCTCT

CTCCTCTCTTAACAAAAATTGATACCACACACGTATCTAAAGTATTTCTTACTCAAATGAACGCCAGAGG

CGTAAAAGTTAAATTAGATTTCGAACGAAGGCCTCCTTCGTTTTATAAACCATTAGATAAAGTTGATCTT

AAACCGTCTTTTCTGGTATAATATTGTTTAGTAGATACTCATCAAGATTATCAAGATAAGCTAATTCACT

AAACATATTATCGGATTCGGTATTGTTACTCGAGAATAGAGTTCGTTATGCTCCTGATATTCGGAAATCT

GTGGAGTTTCAGGTTTTGGTGGAAGTGTAACTGCTACTTGGTGGGATACTGAAGGATATTTCAGAGAGTT

GTGGATGTTCGGGTTCGACATCCACCGATGGTGTCACGCCACTAATCGGTTCGGTAACGTCTGTGGATGG

AGGTGCTACTTCTACAGAACCTGTAGCCTCAGTTGTCAACGGAGATACATCTTCAATGCGCGGAAATGTA

TAATTTGGTAATGGTTTCTCATGTGGATCTTAAGAAGAAGAGGTAAGATATCTACGAAAGATACCGATCA

CGTTCTAGTTCTCTTTTGTAGAACTTTAACTTTTTCTTTCTCAGCATCTAGTTGATATTCCGACCTCTTC

ACGTTTCACATGGGTTACCTCCGCAGTTTTTACAAGCGATTTCACGTTCCAGATCACGTTCAGCCTTCAT

ACGTCTCTCCCTCTCTCTATCGAGTTTATCAGAGCAGTCTTTCTGAAGGCGATCGAACTCCATAAATTTC

TCCAACGCTTTGATTGTTTCCATAGATTTCCGAAGTTTAGCTTCTAGGACGGCGATTCTTTTTTCTTTCG

AATTCACGGGGTACAACCGTTTCCATTACCACCATCTCTACGTTTCTTTTCTAGATCGGCAATCTTTCTC

AACATTTCATCCCCATGCCTTTTCATTCCTCGAGTCTATCGTCGTCGAAATATCGTTCCAGCTCCTTTTC

GACCTCAATAACTTTAGCACGTTGTCTCATCAAGCTCTCTCTTGTAGTACTATCATTTTTATCTGATTCC

CTGGCACGTTTAAGATCTTCATGTAATTGAGTCAGCTCTTGACACAATCTCTTAACTAACTTCCTCTCTT

GCTTCTTCGTCATAGTACTTACAATCACTATGGGATCCATTGTTACCACGTCTGTACTCGGCGAGCTCAC

GTTTAAGAGATTCAATTTCCAGTTTGTACATTGATTTCATTATTACGTCCGCAGTCGTTCAACTGTATTT

CAAGATCTGAGATTCTAGATTGTAATCTCTGTAGCATTTCCACGGCATTCACTCAGTTGTCTTTCAAGAT

CTGAGATTCTAGATTGGAGTCTGCTAATCTCTGTAAGATTTCCTCCTCCGCTCTCGATGCAGTCGGTCAA

CTTATTCTCTAGTTCTCTAATACGTGAACGCAGTGCATCAACTTCTTGTGTGTCTTCTTGATTGCGTGTG

CATTCATCGAGTCTAGATTCGAGATCTCTAACGTGTCGTCGTTCTTCCTCAAGTTCTCTGTGTACTACAG

AAAGCGTGTCCCTATCTTGTTGATATTTAGCAATTTCTGATTCTAGAGTACTGATTCTGCTCACGTATGT

ACTAATAGTTGTCTTATCCTTATCAAGATCCTCCTTGTATTTGTCACATTCCTTGATATCCATACGAAGT

CTGGACAGTTCCCATTCGACATTACGACGTTTATCGATTTCAGCTCGGAGATCGTCGTCGCGTTGTTTTA

GCCACATACGACTGAGTTCAAGTTCTCGTTGACAAGATCCATCTACTTTTCCATCCCTAATAGTATCCAG

TTCCTTTTCTAGTTCTGACCGCATTTCTCGTTCCATATCAAGAGATTCTCTCAATTCTCGTATAGTCTTC

TTATCAATTTCTGATGAATCTGAACCATCATCTGTCCCATTTTGTTGCATATCCCTGAGTTCTTTGATCT

CTGTTGTAAGTCTGTCGATTCTTTCGGTTTTATAAACAGAATCCCTTTCCAAAGTCCTAATCTTACTGAG

TTTATCACTAAGTTCTTCATTCAATTCAGTGAGTTTTCTCTTGGCTTCTTCCAAGTCTGTTTTAAACTCT

CCATCATTTCCGCATTCTTCCTCGCATTTATCTAACCATTCAATTAGTTTATTAATGACTAGTTGGTAAT

CAGCGATTCCTATAACCGTTCTTGTATTTGTGGGAACATAATTAGGATCTTCTAATGGATTGTATGGCTT

GATAGCATCATCTTTATCATTATTAGGTGGGGGATGGACAACCTTAATTGGTTGGTCCTCCTTATCTCCT

CCAGTAGCATGTGGTTCTTCAATACCAGTATTAGTAATAGGCTTAGACAAATGCTTGTCGTACGCGGGCA

CTTCCTCATCCATCAAGTATTTATAATCGGGTTCTGTTTCAGAATATTCTTTTCTAAGAGACGCGACTTC

AGGAGTTAGTAGAAGAACTCTGTTTCTGTATCTATCAACGCTGGAATCGATACTCAAGTTAAGGATAGCG

AATACCTCATCGTCATCATCCGTATCTTCTGAAACGCCATCATATGACATTTCATGAAGTCTAACGTATT

GATAAACAGAATCAGATTTAGTATTAAACAGATCCTTGACCTTTTTAGTAAATGCATATGTATATTTTAG

ATCTCCAGATTTCATAATATGATCGCATGCCTTAAATGTCAATGCTTCCATGATATAGTCTGGAACACTA

ATGGGTGACGAAAAAGATACAGCACCATATGCTACGTTGATAAATAGATCTGAACCACTAAGTAGATAAT

GATTAATGTTAAGGAAGAGGAAATATTCAGTATATAGATATGCCTTAGCATCATATCTTGTACTAAACAC

GCTAAACAGTTTATTGATGTGATCAATTTCCAACAGAACAATTAGAGCGGCAGGAATACCAACAAACATA

TTACCACATCCGTATTTTCTATGAATATCACATATCATGTTAAAAAATCTTGATAGAAGAGCGAATATCT

CGTCTGACTTAATGAGATGTAGTTCAGCAGCATAAGTCATAACTGTAAATAGAACATACTTTCCTGTAGT

GTTGATTCTAGACTCCACATCAACACCATTATTAAAAATAGTTTTATATACATCTTTAATCTGCTCTCCG

TTAATCGTCGAACGTTCTAGTATACGGAAACACTTTGATTTCTTATCTGTAGTTAATGACTTAGTGATAT

CACGAAGAATATTACGAATTACATTTCTTGTTTTTCTTGTGAGACCTGATTCAGAACTCAACTCATCATT

CCATAGTTTTTCTACCTCAGTGGCGAAATCTTTGGAGTGTTTGGTACATTTTTTAATAAGGTTCGTGACC

TCCATTTATTATAAAAAATTTTTATTCAAAACTTAACTACAATCGGGTAATTATAAGATCGTAGATCTCC

CATGTGGTGGAATACTACCATCTATCGCATGTGGATGGACAGTAGGTAATGGCCATGGGAACAGTAATGT

TTGCATATTTATCTTTCTTGCTAGTATTACTGTATATTGTCCCAATGTTTCAATGTGATGTTCTAACCTA

TCAACTGCCACTGTATCACAACAATAATGTCCGATGGAATTAAGATTATGATCCAATGTGTTTAATATAT

GATTATCAAGTCTTATACGATCAGCGTCTTTTTTGACAGGATCAGGCTCTTCTACAGGAAGAAGTTTCGG

CCTCTTATGATAGTCATGTCTGGGAAATGGTGGTCTAGGATGAGGATCCGGTATCGGAGTGGGTTTTGGA

TTATAATCATCATCATCATCATCATCATCATCATCATCATCATCATCATCATCATCATCATCATCATCAT

CATCTATGATATCATCATCATCATCTTCGATATTTATTTTGCTATCTTGATGATGTCCTGTATCAGTTGC

ATTTTCAGCACTCGACTGAATACTAGTACATTCATTGTCTATTATTAACGTATTTCTAAACCCAAAATGT

ATGTGTTGAACATCACTACTATAGTTGATGAGTCTTATAGCATGAATTCGCTTATCGTTATCGGGTTTAT

CTTCTGTCACCTTAACAATTCCTTTTTTATTAAACTCTGCATAATCATAACCATTTCTATTGTTTGTTCT

AATATAAACGAGTATAGCATCATTGCTAAATTTTTCAATCGTATCGAAAACAGAATATCCTAAACCATAT

AATATATATTCAGGAACACTCAAACTAAATGTCCAGGATTCTCCTAAATACGTAAACTTTAATAGTGCTA

AATCATTCAAAAATCTACCGCTTATAGATAGATAGTACATGAATGCGTATAGTAGTCTACCGATCTCTTT

ATTATGAAAACCGACATTACGATCATATATTTCGTGATATACATGTGACCCGTTTACGTTAAACCATAAA

TACATGGGTGATCCTATAAACATGAATTTATTTCTAATTCTCAGAGCCATAGTTAATTGACCGTGTAATA

TTTGTTTACATGCATACTTGATACGATCATTAATAAGATTTTTATCATTGCTCGTTATTTCAGAATCGTA

TATATAAGGAGTACCATCATGATTCTTACCAGATATTATACAAAATACTATATATAAAATATATTGACCC

ACGTTAGTAATCATGTAAATGTTTAATGTTTTAAATTTTGTATTTAATGATCCATCATCATACGCTAGCA

TGGTCTTGTGATATTCATTCTTTAAAATATAATATTGTGTTAGCCATTGCATTGGAGCTCCTAATGGAGA

TTTTCTATTCTCGTCCATTTTAGGATATGCTTTCATAAAGTCCCTAATAACTTCGTGAATAATGTTTCTA

TGTTTTCTACTGATGCATGTATTTGCTTCGATTTTTTTATCCCATGTTTCATCTATCATAGATTTAAACG

CAGTAATGCTCGCAACATTAACATCTTGAACCGTTGGTACAATTCCGTTCCATAAATTTATAATGTTCGC

CATTTATATAACTCATTTTTTGAATATACTTTTAATTGAACAAAAGAGTTAAGTTACTCATATGGACGCC

GTCCAGTCTGTACATCAATCTTTTTAGCCAGAGATATCATAGCCGCTCTTAGAGTTTCAGCGTGATTTTC

CAACCTAAATAGAACTTCATCGTTGTGTTTACAACACTTTTCTATTTGTTCAAACTTTGTTGTTATATTA

GTAATCTTTTTTTCCAAATTAGTTAGCCGTTGTTTGAGAGTTTCCTCATTGTCGTCTCCATAGGCTTTAA

CAATTGCTTCGCGTTTAGTCTCTGGATTTTTAGCAGCCTTTGTAGAGAAAAATTCAGTTGCTGGAATTGC

AAGATCGTCATCTCCGGGGAAAAGAGTTCCGTCCATTTAAAGTACAGATTTTAGAAACTGACACTCTGTG

TTATTTATATTTGGCGCAATACATGGATTATAAATATCGATGTTAATAACATCAGAAAATGTAAAGTCTA

TACATTGTCGCATCGTGTTAAATTTTCTAATGGATCTAGTATTATTGGGTCCAACTTCTGCCTGAAATCC

AAATATGGAAGCGGATACAAAACCGTTTCCTGGATAAACCACACATCTCCACTTTTGCTTTACATCAGAA

ATTGTGTCATTGACATCTTGAACTCTCCTATCTAATGCCGGTGTTCCACCTATAGATTTTGAATACTCGA

ATGCTGCATGAGTAGCATTGAATTCCTTAATATTGCCATAATTTTCATATATTGAGTAACTCTGGATAAA

AAGTAAACACACCGCAGCCGTCGCTACTACAATAAAAAAAATTGATAGAGAGTTCATTTATAATCTATTA

GAAGCTGACAAAATTTTTTTACACGCGTCAGACAATGCTTTAATAAATAGTTCAACATCTACTTTTGTCA

TATCGAACCGATGGTATGATTCTAACCTAGAATTACATCCGAAAAAGTTGACCATGTTCATAGTCATTAA

GTCATTAACAAACAACATTCCAGACTCTGGATTATAAGACGATACTGTTTCGTCACAATCACCCACCTTA

ATCATGTGATTATGAATATTGGCTATTAGAGCACCTTCTAAGAAATCTATAATATCTTTGAAACACGATT

TAAAATCAAACCACGAATATACTTCTACGAAGAAAGTTAGTTTACCCATAGGAGATATAACTATAAATGG

AGATCTAGATACAAAATCCGGATCTATGATAGTTTTAACATTATTATATTCTCTATTAAATACCTCCACA

TCTAAAAATGTTAATTTTGAAACCATGTCTTCGTTTATTACCGTACCTGAACTAAACGCTATAAGCTCTA

TTGTTTGAGAACTCTTTAAACGATATTCTTGAAATACATGTAACAAAGTTTCCTTTAACTCGGTCGGTTT

ATCTACCATAGTTACAGAATTTGTATCCTTATCTATAATATAATAATCAAAATCGTATAAAGTTATATAA

TTATCGTGTTCAGATTGTGATCTTTTCAAATAGACTAAAAACCCCATTTCTCTAGTAAGTATCTTATGTA

TATGTTTGTAAAATATCTTCATGGTGGGAATATGCTCTACAGCAGTTAGCCATTCCTCATTGACAGCTGT

AGATGTATTAGACAAAACTACTCCAATGTTTAACAAGGGCCATTTTACGAGATTATTAAATCCTTGTTTG

ATAAATGTAGCCAATGCGGGTTCGAGTTCAACGACGATTGAATTCTCTTCCCGTGGATGCTGCATGATGA

ACGACGGGATGTTGTTCTATTGATTTGGAATTCTTTTTCGACTTTTTGTTTATATTAAATATTTTAAAAT

TTATGGCTGATAGTAATTCATGTACTACGGATAATGTAGACGTGTATTGCACATCGATATCTTTATTATT

AGATAAATTTATCAATAAATGTGAGAAGTTTGCCTCGTTAAGGTCTTCCATTTAAATATTATATAAATAT

TTGTGTTTGTATTTTATTCGTCTTTTATGGGATAGTTTTTAACTAGTAAAGCTGTAATTACATACTTTGT

CCGTAAAACATAAATATAAATACCCGCTTTTATCAAACGTTCCAAAAAGTCGGCAGCCGACATTTTTAAC

ATGGCATCTATTTTAAATACACTTAGGTTTTTAGAAAAAACATCATTTTATAATTGTAACGATTCAATAA

CTAAAGAAAAGATTAAGATTAAACATAAGGGAATGTCATTTGTATTTTATAAGCCAAAGCATTCTACCGT

TGTTAAATACTTGTCTGGAGGAGGTATATATCATGATGATTTGGTTGTATTGGGGAAGGTAACAATTAAT

GATCTAAAGATGATGCTATTTTACATGGATTTATCATATCATGGAGTGACAAGTAGTGGAGCAATTTACA

AATTGGGATCGTCTATCGATAGACTTTCTCTAAATAGGACTATTGTTACAAAAGTTAATAACAATTATAA

CAATTATAACAATTATAATTGTTATAATAATTATAATTGTTATAATTATGATGATACATTTTTTGACGAT

GATGATTGATCACTATTACACAATTTTGTTTTTGTACTTTCTAATATAGTGTTTAGGTTCTTTTTCATAT

GAGAATATTGACTTACTAAAATATCTATGTTTAACTTTTGTTCTATAACGTCCTTATCGGCGGTATCGGT

ACATATACGTAATTCACCTTCACAAAATACGGAGTCTTCGATAATAATAGCCAATCGATTATTGGATCTA

GCTGTCTGTATCATATTCAACATGTTTAATATATCCTTTCGTTTCCCCTTTACAGGCATCGATCGTAGCA

TATTTTCCGCGTCTGAGATGGAAATGTTAAAACTGCAAAAATGCGTAATGTTAGCCCGTCCTAATATTGG

TACGTGTCTATAAGTTTGGCATAGTAGAATAATAGACGTGTTTAAATGCCTTCCAAAGTTTAAGAATTCT

ATTAGAGTATTACATTTTGATAGTTTATCACCTACATCATCAAAAATAAGTAAAAAGTGTGCTGATTTTT

TATGATTTTGTGCGACAGCAATACATTTTTCTATGTTACTTTTAGTTCGTATCAGATTATATTCTAGAGC

TTCCTGACTACTAACGAAATTAATATGATTTGGCCAAATGTATCCATCATAATCTGGGTTATAAACGGGT

GTAAACAAGAATATATGTTTATATTTTTTAACTAGTGTAGAAAACAGAGATAGTAAATAGATAGTTCTTC

CAGATCCAGATCCTCCTGTTAAAACCATTCTAAACGGCATTTTTAATAAATTTTCTCTTGAAAATTGTTT

TTCTTGAAAACAATTCATAATTATATTTACAGTTACTAAATTAATTTGATAATAAATCAAAATATGGAAA

ACTAAGGTCGTTAGTAGGGAGGAGAACAACGAAGACATATCGTGATATAAATAACATTTATTATCATGAT

GACACCAGAAAACGACGAAGAGCAGACATCTGTGTTCTCCGCTACTGTTTACGGAGACAAAATTCAGGGA

AAGAATAAACGCAAACGCGTGATTGGTCTATGTATTAGAATATCTATGGTTATTTCACTACTATCTATGA

TTACCATGTCCGCGTTTCTCATAGTGCGCCAAAATCAATGCATGTCTGCTAACGAGGCTGCTATTACTGA

CTCCGCTGTTGCCGTTGCTGCGGCATCATCTACTCATAGAAAGGTTGCGTCTAGCACTACACAATATGAT

CACAAAGAAAGCTGTAATGGTTTATATTACCAGGGTTCTTGTTATATATTACATTCAGACTATAAGTCAT

TCGAGGATGCTAAAGCAAACTGCGCTGCGGAATCATCAACGCTACCCAATAAATCCGATGTCTTGACTAC

CTGGCTCATTGATTATGTTGAGGATACATGGGGATCTGATGGTAATCCAATTACAAAAACTACATCCGAT

TATCAAGATTCTGATGTATCACAAGAAGTTAGAAAGTATTTTTGTACATAAATAAATGAAATCGCTTAAT

AGACAAACTGTAAGTAGGTTTAGGAAGTTGTCGGTGCCGGCCGCTATAATGATGTTACTCTCAACCATTA

TTAGCGGCATAGGAACATTTCTACATTACAGAGAAGAACTGATGCCTAGTGCTTGCGCCAATGGATGGAT

ACAATACGATAAACATTGTTATCTGGATACCAACATTAAAATGTCTACGGATAATGCAGTTTATCAGTGT

CGCAAATTACGAGCTAGATTGCCTGGACCTGATACTAGACATCTGAGAGTATTGTTTAGTATTTTTTATA

AAGATTATTGGGTAAGTTTAAAAAAGACCAATGATAAATGGTTAGATATTAATAATGATAAAGATATAGA

TATTAGTAAATTAACAAATTTTAAGCAACTAAACAGCACAACGGATTCTGAAGCGTGTTATATATACAAG

TCTGGAAAACTGGTTAAAACAGTATGTAAAAGTACTCAATCTGTACTATGCGTTAAAAGATTCTACAAGT

GATAACAAAAAATGAATTAATAGTAAGTCGTTAACGTACGCCGCCATGGACGCCGCGTTTGTTATTACTC

CAATGGGTGTGTTGACTATAACAGATACATTGTATGATGATCTCGATATCTCAATCATGGACTTTATAGG

ACCATACATTATAGGTAACATAAAAATTGTCCAAATAGATGCACGGGATATAAAATATTCCGACATGCAA

AAATGCTACTTTAGCTATAAGGGTAAAATAGTTCCTCAGGATTCTAATGATTTGGCTAGATTCAACATTT

ATAGTATTTGTACCGCATACAGATCAAAAAATACCATCATCATAGCATGCGACTATGATATCATGTTAGA

TATAGAAGGTAAACATCAACCATTTTATCTATTCCCATCTATTGATGTTTTTAACGCTACAATCATAGAA

GCGTATAATCTGTATACAGCTGGAGATTATCATCTGATCATCAATCCTTCAGATAATCTGAAAATGAAAT

TGTCGTTTAATTCTTCATTTTGTATATCAGACGGCAATGGATGGATTATAATTGATGGGAAATGTAATAG

TAATTTTTTATCATAAAAGTTGTAAAGTAAATAATAAACAATAAATATTGAACTAGTAGTATGTTGTATA

TTGAGCAATCAGAGATGATGCTGGTACCTCTTATCACGGTGACCGTAGTTGCGGGAACAATATTAGTATG

TTATATATTATATATTTGTAGGAAAAAGATACGTACTGTCTATAATGACAATAAAATTATCATGACAAAA

TTAAAAAAGATAAAGAGTCCTAATTCCAGCAAATCTAGTAAATCAACTGATAGCGAATCAGACTGGGAGG

ATCACTGTAGTGCTATGGAACAAAACTATGACGTAGATAATATTTCTAGAAATGAGATATTGAACGATGA

TAGCTTCGCTGGTAGTTTAATATGGGATAACGAATCCAATGTCATGGCGCCTAGCACAAAACACATTTAC

GATAGTGTTGCTGGAAGCACGCTGCTAATAAATAATGATCGTAATGAACTGACTATTTATCAGAATACTA

CAGTAGTAATTAATGATACAGAGACTGTTGAAATACTTAATGAAGATACCAAACAGATTCCTAGCTATTC

TTCCAATCCTTTCGTAAATTATAATAAAACCAGTATTTGTAGCAAGTCAAATCCGTTCATTGCAGAACTC

AACAATAAATTTAGTGATAATAATCCGTTTAGGAGAGCACATAGCGACGATTATCTTAATAAGCAATAAC

AATAACAATAACAAGATCATGAACACGATGATATAGAATCATCGGTTGTATCATTGTCTGATTAGTTTCC

TTTTTATAAAATTGAAGTAATATTTAGTATTAATTACCGCCGATGCATTATACAAATATGGAGATATTCC

CTGTATTCGGCATTTCTAAAATTAGCAATTTTATTGCTAATAATGACTGTAGATATTATATAGATGTAGA

GCATCAAAAAATTATATCTGATGAGATCAATAGACAGATGGATGAAACGGTACTTCTTACCAACATCTTA

AGCGTAGAAGTTGTAAATGGCAATGAGATGTACCATCTTATTCCCCATAGACTATCGACGATTATACTCT

GTATTAGTTCTGTTGGAGGATGTGTTATCTCTATAGATAATGACGTCAATGATAAAAATATTCTAACATT

TCCCATTAATCATGCTGTAATCATATCCCCACTGAGTAAATGTGTCGTAGTTAGCAAGGGCCCTACAACC

ACACTGGTTGTTAAAGCGGATATACCCAGCAAACGATTGGTAACATCGTTTACAAACGACATACTGTATG

TAAACAATCTATCACTGATTAATTATTTACCGTTGTCTGTATTCATTATTAGACGAGTCACTGACTATTT

GGATAGACACATATGCGATCAGATATTTGCTAATAATAAGTGGTATTCCATTATAACTATCGACGATAAG

CAATATCCTATTCCATCAAATTGTATAGGTATGTCTTCTGCCAAGTACATAAATTCGAGCATCGAGCAAG

ATATTTTGATCCATGTTTGTAACCTCGAGCATCCATTCGACTCAGTCTACAAAAAAATGCAGTCGTACAA

TTCTCTACCTATCAAGGAACAAATATTGTATGGTAGAATTGATAATATAAATATGAGCATTAGTATTTCG

GTGGATTAATAGATTTCTCTAGTATGGGATCATTAATCATCTCTAAATACATCATAAAAAAGCTATTATC

AAATACTGTACTGAATGGATTCATTCTTTTCTCTTTTTATGAAACTCTGTTGTATATCTACGGATAAAAC

TAGAAGCAAAAAATCTGATAGGAAGAATAATGATTATATGGAGGAACACGATTATTATAAAATAACAATA

GTTCCTGGTTCCTCTTCCACGTCTACTAGCTCGTGGTATTATACACATGCCTAGTAATAGTCTCTTTGCG

TTGACGGAAAGCAGACTAGAAATAACAGGCCAAAATGTTCAGACACCATAATAGTTCCCAACCCAGATAA

TAACAGAGTTCCATCAACACATTCCTTTAAACTCAATCCCAAACCCAAAACCGTTAAAATGTATCCAGCC

AATTGATAATAGATAATGAGGTGTACAGCACATGATAATTTACACAGTAACCAAAATGAAAACACTTTAG

TAATTATAAGAAATATAGACGGTAATGTCATCATCAACAATCCAATAATATGCCTGAGAGTAAACATTGA

CGGATAAAACAAAAATGCCCCGCATAACTCTATCATGGCAATAACGCAACCAAACACTTGTAAAATTCCT

AAATTAGTAGAAAATACAACTGATATCGATGTATAAGCGATTTCGAGGAATAATAAGAACAAAGTAATTC

CCGTAAAGATAAACATCAACATTGTTTGGTAATCATTAAACCAATTAGTATGACGTTGAACTAATTTCAC

AGTAGATTTTATTCCAGTATTATCCCCGCATGTATACGTACCTGGTAAGATATCTTTATATTCCATAATC

AATGAGACATCACTATCCGATAACGAATGAAGTCTAGCACTAGTATGCCATTTACTTAATATGGTCGTCT

TGGAAGTTTTATTATAAGTTAAAATATCATGATTGTCCAATTTCCATCTAATATACTTTGTCGGATTATC

TATAATACATGGAATAATGATGGTATCATTACATGCTGTATACTCTATAGTCTTTGTAGATGTTATAACC

ACAAAAGTACAGAGGTATATCAACAATATTCTAACTCTTAACATTTTTATTTATTTAAAATGATACCTTT

GTTATTTATTTTATTCTTATTTTGCTAACGGTATCGAATGGCATAAGTTTGAAACGAGTGAAGAAATAAT

TTCTACTTACTTAATAGATGATGTGGTAACGGGTGTTATTAATGGGGCTGTATATACATTTTCAAATAAT

GAACTAAACAAAACTGGGTTAACTAATAACAATAATTATATCACAACATCTATAAAAGTAGAGGATAATG

ATACATTAGTAGTATGCGGAACCAATAACGGAAATCCCAAATGTTGGAAAATAGACGGTTCATACTACCC

AAAACATATAGGTAGAGGATACGATCATCAAAATAGCAAAGTAACGATAATCAGTCACAATGAATGTGTA

CTATCCGACATAAACATATCAAAAGAAGGAATTAAACGATGGAGAAGATTTGACGGACCATGTGGTTATG

ATTAAACGAGTTAAGTTTTTTAAGAAGCCTTAGAAGAGGGCTATTGGGTATGAAAATCCGAAATATTAAA

CCAGACAACCCCATATAATTTTATAGCTAAGAATACCGCGAAGAATGGAACTAAAAACGGAAATATTTGT

AGCACAACGAATAACTCCCAAACTGCATTCATGTTACACTATATAACAATTACAATACATTTTTATCATA

ACACTACTTCGGTTAGATGTTTTAGAAAAAAATAAATATCACTACCGTTCTTGTTTTATAAAAATAACAA

TTAACAATTATCAATTTTTTTCTTTAATATTTTACGTGGTTGACCATTCTTGGTGGTAAAATAATCTCTT

AGTGTTGGAATGGAATGCTGTTTAATGTTTCCGCACTCATCGTATATTTTGACGTATGCAGTCACATCGT

TTACGCAATAGTCAGACTGTAGTTCTATCATGCTTCCTACGTTAGAAGGAGGAACAGTTTTAAAGTCTCT

TGGTTTTAATCTATTGTCATTAGTTTTCATGAAATCCTTTGTTTTATCCACTTCACATTTTAAATAAATG

TCAACTATACATTCTTCTGTTAATTTTACTAGATCATCATAGGTCATAGAATTTATAGGTTCCGTAGTCC

ATGGATCCAAACTAGCAAACTTCGCGTATACAGTATCGCGATTAGTGTATACACCAACTGTATGAAAATT

AAGAAAACAGTTTAATAAATCTACAGAAATATTTAATCCTCCGTTTGATACAGATGCGCCATATTTATGG

ATTTCGGATTCACACGTTGTTTGTCTAAGTGGTTCGTCTAGTGTTGCTTCTACATAGACTTCGATTCCCA

TATATTCTTTATTGTCAGAATCACATACCGATTTATCATACGCTGGTTCACTTGTTTGAAAACTAAATGG

TAGTAGATACATCAAAATAATAAATAATAAGTACATTCTGCAATATTGTTATCGTAATTGGAAAATTGGT

ATTCAAGTGAGCTGGATTATGTGAGTATTGGATTGTATATTTTATTTTATTTTATATTTTATATTTTATA

TTTTATTTTATATTTTGTAGTAAGAATAGAATGCTAAATGTCAAGTTTATTCGAATAGATGTCTTATTAA

AAACATATATAATAAATAACAATGGCTGAATGGCATAAAATTATCGAGGATATCTCAAAAAATAATAAGT

TCGAGGATGCCGCCATCGTTGATTACAAGACTACAAAGAATGTTCTAGCGGCTATTCCTAACAGAACATT

TGCAAAGATTAATCCGGGTGAAGTTATTCCCCTCATCACTAATCATAATATTCTAAAACCTCTTATTGGT

CAGAAATTTTGTATTGTATATACTAACTCTCTAATGGATGAGAACACGTATGCTATGGAGTTGCTTACTG

GGTACGCCCCTGTATCTCCGATCGTTATAGCGAGAACTCATACCGCACTTATATTTTTGATGGGTAAGCC

AACAACATCCAGACGTGATGTGTATAGAACATGTAGAGATCACGCTACCCGTGTACGTGCAACTGGTAAT

TAAAATAAAAAGTAATATTCATATGTAGTGTCAATTTTAAATGATGATGATGAAATGGATAATATCCATA

TTGACGATGTCAATAATGCCGGTATTGACATACAGTTCATCGATTTTTAGATTTCATTCAGAGGATATTG

AATTATGTTATGGGAATTTGTATTTTGATAGGATCTATAATAATGTAGTAAATATAAAATATATTCCTGA

GCATATTCCATATAGATATAATTTTATTAATCGTACGTTCTCCGTAGATGAACTAGATGATAATGTCTTT

TTTACACATGGTTATTTTTTAAAACACAAATATGGTTGTTCACTTAATCCTAGTTTGATTGTCTCATTAT

CAGGAAACTTAAAATATAATGATATACAATGCTCAGTAAATGTATCATGTCTCATTAAAAATTTGGTAAC

GAGTACATCTACTATATTAACATCTAAACATAAGACTTATTCTCTATATCGGTCCACGTGTATTGCTATA

ATAGGATACGATTCTATTATATGGTATAAAGATATAAATGACAGGTATAATGACATCTATGATTTTACTG

CAATATGTATGCTAATAGCGTCTACATTGATAGTGATCATATACGTGTTTAAAAAAATAAAAATGAACTC

TTAATTATGTTATACTATTAGAAATGGATAAAATCAAAATTACGATTGATTCAAAAATTGGTAATGTTGT

TACCATATCGTATAACTTGGAAAAGATAACTATTGATGTCACACCAAAAAAGAAAAAAGAAAAGGATGTA

TTATTAGCGCAATCAGTTGCTGTCGAAGAGGCAAAAGATGTCAAGGTGGAAGAAAAAAATATTATCGATA

TTGAAGATGACGATGATATGGATATAGAAAACACGTAATACGATCTATAAAAATAAGGTATTAAATACTT

TTTATTTACGGTACTCTTGTAGTGGTGATACCACTAATCGATTTTTTTTTAAAAAAAATACTTATTCTGA

TTCTTCTAGCCATTTCCGTGTTCGTTCGAATGCCACATCGACGTCAAAGATAGGGGAGTAGTTGAAATCT

AGTTCTGCATTGTTGGTACGCACCTCAAATGTAGTGTTGGATATCTTCAACGTATAGTTGTTGAGTATTG

ATGGTTTTCTAAATAGAATTCTCTTCATATCATTCTTGCACGCGTACATTTTTAGCATCCATCTTGGAAT

CCTAGATCCTTGTTCTATTCCCAATGGTTTCATCAATAGAAGATTAAACATATCGTAAGAACACGATGGA

GAGTAATCGTAGCAAAAGTAAGCATTTCCTTTAATCGCAGATCCCGGATACTGGATATATTTTGCAGCCA

ACACGTGCATCCATGCAACATTTCCTACATATACCCGGCTATGCACAGCGTCATCATCGACTGTACGATA

CATAATGTTACCGTGTTGCTTACATTGCTCGTAAAAGACTTTCGTCAATTTGTCTCCTTCTCCGTAAATT

CCAGTGGGTCTTAGGCAACAAGTATACAATTTTGCGCCATTCATGATTACGGAATTATTGGCTTTCATAA

CCAGTTGCTCGGCCATACGTTTACTTTTTGCGTATACATGTCCTGGTGATATATCATAAAGGGTATGCTC

ATGACCGATGAATGGATTACCGTGTTTATTTGGTCCTATTGCTTCCATGCTACTAGTATAGATCAAATAC

TTGATTCCTAGGTCCACACAAGCTGCCAATATAGTCTGTGTTCCATAATAGTTTACTTTCATGATTTCAT

TATCAGTGTATTTTCCAAATACATCCACTAGAGCAGCCGTATGAATAATCAGATTTACCCCATCTAGCGC

TTCTCTCACCTTATCAAAGTCGTTTATATCACATTGTATATAGTTTATAACCTTAACTTTCGAGGTTATT

GGTTGTGGATCTTCTACAATATCTATGACTCTTATTTCTTGAACATCATCTGCGCTAATTAAAAGTTTTA

CTATATACCTGCCTAGAAATCCGGCACCGCCAGTAACCGCGTACACGGCCATTGCTGCCACTCATAATAT

CAGACTACTTATTCTATTTTACTAAATAATGGCTGTTTGTATAATAGACCACGATAATATCAGAGGAGTT

ATTTACGTTGAACAAGTCCATGGAAAAGATAAAGTTTTAGGATCAGTTATTGGATTAAAATCCGGAACGT

ATAGTTTGATAATTCATCGTTACGGAGATATTAGTCGAGGATGTGATTCCATAGGCAGTCCAGAAATATT

TATCGGTAACATCTTTGTAAACAGATATGGTGTAGCATATGTTTATTTAGATACAGATGTAAATATATCT

ACAATTATTGGAAAGGCGTTATCTATTTCAAAAAATGATCAGAGATTAGCGTGTGGAGTTATTGGTATTT

CTTACATAAATGAAAAGATAATACATTTTCTTACAATTAACGAGAATGGCGTTTGATATATCAGTTAATG

CGTCTAAAACAATAAATGCATTAGTTTACTTTTCTACTCAGCAAAATAAATTAGTCATACGTAATGAAGT

TAATGATATACACTACACTGTCGAATTTGATAGGGACAAAGTAGTTGATACGTTTATTTCATATAATAGA

CATAATGACTCCATAGAGATAAGAGGGGTGCTTCCAGAGGAAACTAATATTGGTCGCGTGGTTAATACGC

CGGTTAGTATGACTTACTTGTATAATAAGTATAGTTTTAAACCGATTTTAGCAGAATATATAAGACACAG

AAATACTATATCCGGCAATATTTATTCGGCATTGATGACACTGGATGATTTGGTTATTAAACAGTATGGA

GACATTGATCTATTATTTAATGAGAAACTTAAAGTAGACTCCGATTCGGGACTATTTGACTTTGTCAACT

TTGTAAAGGATATGATATGTTGTGATTCTAGAATAGTAGTAGCTCTATCTAGTCTAGTATCTAAACATTG

GGAATTGACAAATAAAAAGTATAGGTGTATGGCATTAGCCGAACATATAGCTGATAGTATTCCAATATCT

GAGCTATCTAGACTACGATACAATCTATGTAAGTATCTACGCGGACACACCGATAGCATAGAGGATGAAT

TTGATCATTTTGAAGACGATGATTCGTCTACATGTTCTGCCGTAACCGACAGGGAAACGGATGTATAATT

TTTTTATAGTGTGATGGATATGATGGATATGATATATGATGGATATTATGATGGATATGATGGATATTAT

GATGGATATGATGGATATGATGGATATGATGGATATGATAAATATGATAAAAATATAATTGTTGTATATG

ATGGATATGATGGATATGATGGATATGATGGATATGATGGATATGATGGATATGATAAATATGATAAAAA

TATAATTGTTGTATCCATTCCCATTCAAATCACCTTATATGATTCTGTAACACAATGAAGGAGTCTCATA

GATATATAGAGGTCAGATACTGGTTTGATAAACTTTTTATTCCACATGAGTATGTTTGACTTATGGTTAG

ACACGCATACTTTAACAAATCACTGAAAATTGGAGTTAGGTATTCCTCTCAGAATCAGTTGCCGTTCTGG

AACATTAAATGTATTTTTTATGATATACTCCAACGCATTTATGTGGGTATACAACAAGTCATTAATAATG

AGTATTTCCAAGAGTTTTAGCTGTCTAGTATTTAACAAGAGAAGAGATTTCATCAGACTGTTTATGAACT

CGAATACCGCCTCATTGTCGCTTATATTGATGACATCGAATTCCCAATATCATCTCATCAGTGATGAGTA

GCTCAATCTTGTTATCGGGATCCAATTTCTAAAGATGTCATTAAACCCTCGATCGTGAATGGATTTATCA

TCATCGTTTTTATGTTGGACATGAGCTTAGTCCGTTTGTCCACATCTATATACGATGATTTCTGAATTAT

TTCATATATCTCTCGTTAACTCCAGGAACTTGTCAGGGATCTAACTTTAATATGTTCTCGTCTAAGAGAT

GAAAATCTTTGGATGGTGGCATGTGACTTTTCTCTAAAGGATGATGTTGCCCGATCCTCTCTTAAATGAA

TCCATCTTATCCTTGGACAAGATGGACAGTCTATTTTCCTTAGATGGTTTAATATTTTTTACCCATGATC

TATAAAGGTAGACAGACCTAATCGTCTCGGATGACCATATATTATTTTCAGTTTTATTATACGCATAAAT

TGTAAAAAATATGTTAGGTTTACGAAAATGTCTCGTGGGGCATTAATCGTTTTTGAAGGATTGGACAAAT

CTGGAAAAACAACACAATGTATGAACATCATGGAATCTATACCGGCAAACACGATAAAATATCTTAACTT

TCCTCAGCGATCCACAGTCACTGGAAAGATGATAGATGACTATCTAACTCGTAAAAAAACCTATAATGAT

CATATAGTTAATCTATTATTTTGTGCAAATAGATGGGAGTTTGCATCTTTTATACAAGAACAACTAGAAC

AGGGAATTACTTTAATAGTTGACAGATACGCGTTCTCTGGAGTAGCGTATGCCACCGCTAAAGGCGCGTC

AATGACTCTCAGTAAGAGTTATGAATCTGGATTGCCTAAACCCGACTTAGTTATATTCTTGGAATCTGGT

AGCAAAGAAATTAATAGAAACGTCGGCGAGGAAATTTATGAAGATGTTGAATTCCAACAAAAGGTATTAC

AAGAATATAAAAAAATGATTGAAGAAGGAGATATTCATTGGCAAATTATTTCTTCTGAATTCGAGGAAGA

TGTAAAGAAGGAGTTGATTAAGAATATAGTTATAGAGGCTATACACACGGTTACTGGACCAGTGGGGCAA

CTGTGGATGTAATAAAATGAAATTACATTTTTATAAATAGATGTTAGTACAGTGTTATAAATGGATGAAG

CATATTACTCTGGCAACTTGGAATCAGTACTCGGGGATACGTGTCCGATATGCATACCGAACTCGCATCA

ATATCTCAATTAGTTGCCAAGATAGAAACTATAGATAATGATTATTAAACAAGGACATTGTAAATTTTAT

CATATGTAGATCAAACTTGGATAATCCATTTATCTCTTTCCTAGATACTGCATATACTATCATAGATCAA

GAGATCTATCAGAACGAGTTGATTAATTCATTAGACGATAATGAAATTATCGATTGTATAGTTAACAAGT

TTATGAGCTTTTATAAGGATAACCTAGAAAATATGGTAGATGCTATCATTACTCTAAAATATTATAATTA

ATAATCCAGATTTTAAAACTACGTATGTGGAAGTACTCGGTTCCAGAATAGCTGATATAGATATTAAACA

AGTGATACGTAAGAATATAATACAATTGTCTAATGATCCGCGAACGATATTTGTGAAAATATTAAAAAAA

AATACTTTTTTTATTAAATGACGTCTCTTCGCGAATTTAGAAAATTATGCTGTGATATATATCACGCATC

AGGATATAAAGAAAAATCTAAATTAATTAGAGACTTTATAACAGATAGAGATGATACCGATACATATTTG

ATCATTAAGCTATTGCTTCCCGGATTAGACGATAGAATGTATAACATGAACGATAAACAAATTATAAAAT

TATATAGTATAATATTTAAACAATCTCAGGAAGATATGCTACAAGATTTAGGATACGGATATATAGGAGA

CACTATTAGGACATTCTTCAAAGAGAACACGGAAATCCGTCCACGAGATAAAAGCATTTTAACTTTAGAA

GAAGTGGATAGTTTTTTAACTACGTTATCATCAGTAACTAAAGAATCACATCAAATAAAATTATTGACTG

ATATAGCATCTGTTTGTACATGTAATGATTTAAAATGTGTAGTCATGCTTATTGATAAAGATCTAAAAAT

TAAAGCGGGTCCTCGGTACGTGCTTAACGCTATTAGTCCTCATGCCTATGATGTTTTTAGAAAATCTAAT

AACTTGAAAGAGATAATAGAAAATGCAGCTAAACAAAATCTAGACTCTATATCTATTTCTGTTATGACTC

CAATTAATCCCATGTTAGCGGAATCATGTGATTCTGTCAATAAGGCGTTTAAAAAATTTCCATCAGGAAT

GTTTGCGGAAGTCAAATACGATGGTGAAAGAGTACAAGTTCATAAAAAAAATAACGAGTTTGCATTCTTT

AGTAGAAACATGAAACCAGTACTCTCTCATAAAGTGGATTATCTCAAAGAATACATACCGAAAGCATTTA

AAAAAGCTACGTCTATCGTATTGGATTCTGAAATTGTTCTTGTAGACGAACATAATGTACCGCTACCGTT

TGGAAGTTTAGGTATACACAAAAAGAAAGAATATAAAAACTCTAACATGTGTTTGTTCGTGTTTGACTGT

TTATACTTTGATGGATTCGATATGACAGACATTCCATTGTATGAACGAAGATCTTTTCTCAAAGATGTTA

TGGTCGAAATACCCAATAGAATAGTATTCTCAGAGTTGACGAATATTAGTAACGAGTCTCAGTTAACTGA

TGTATTAGATGATGCACTAACGAGAAAATTAGAAGGATTGGTCTTAAAAGATATTAATGGCGTATACGAA

CCGGGAAAGAGAAGATGGTTAAAAATAAAGCGAGACTATTTGAACGAGGGTTCCATGGCAGATTCTGCCG

ATTTAGTAGTACTAGGTGCCTACTATGGTAAAGGAGGAAAGGGTGGTATCATGGCAGTCTTTCTAATGGG

TTGTTACGACGATGAATCCGGTAAATGGAAGACGGTAACTAAATGTTCCGGTCACGATGATAATACGTTA

AGGGTTTTGCAAGACCAATTAACGATGGTTAAAATTAACAAGGATCCCAAAAAAATTCCAGAGTGGTTGG

TAGTTAATAAAATCTATATTCCCGATTTTGTAGTAGATGATCCGAAACAATCTCAGATATGGGAAATTTC

AGGAGCAGAGTTTACATCTTCCAAGTCACATACAGCGAATGGAATATCAATTAGATTTCCTAGATTTACT

AGGATTAGAGAAGATAAAACGTGGAAAGAATCTACTCATCTAAACGATTTAGTAAACTTGACTAAATCTT

AATAGTTACATATAAACTGAAAAATAAAATAATACTATTTTAGTTGGTGGTCGCCATGGATGGTGTTATC

GTATACTGTCTAAATGCGTTAGTAAAACATGGCGAGGAAATAAATCATATAAAAAATGATTTCATGATTA

AACCATGTTGTGAAAGAGTTTGTGAAAAAGTCAAGAACGTTCACATCGGCGGACAATCTAAAAACAATAC

AGTGATTGCAGATTTGCCATATCTGGATAATGCTGTATCAGATGTATGCAAATCAATATATAAAAAGAAT

GTATCAAGAATATCCAGATTTGCTAATATGATAAAAATAGATGACGATGACAAGACTCCTACCGGTGTAT

ATAATTATTTTAAACCTAAAGATACTATTCCTGTTATTATATCCATAGGAAAGGATAAAGATGTCTGTGA

ACTATTAATCTCATATGATAAAGCGTGTGCGTGTATAAAGTTAAATTTATATAAAGTAGCCATTCTTCCC

ATGGATGTTTCCTTTTTTACCAAAGGAAATGCATCATTGATTATTCTCCTGTTTGATTTCTCTATCGATG

CGGCACCTCTCTTAAGAAGTGTAACCGATAATAATGTTATTATATCTAGACACCAGCGTCTACATGACGA

GCTTCCGAGTTCCAATTGGTTCAAGTTTTACATAAGTATAAAGTCCGACTATTGTTCTATATTATATATG

GTTGTTGATGGATCTATGATGTATGCGATAGCTGATAATAGAACTCACGCAATTATTAGCAAAAATATAT

TAGACAATACTACGATTAACGATGAGTGTAGATGCTGTTATTCTGAACCACAGATTAGGATTCTTGATAG

AGATGAGATGCTCAATGGATCATCGTGTTATATGAACAGACATTGTATTATGATGAATTTACCTGATGTA

GGCGAATTTGGATCTAGTATGTTGGGGAAATATGAACCTGACATGATTAAGATTGCTCTTTCGGTGGCTG

GTAATTTAATAAGAAATCGAGACTACATTCCCGGGAGACGAGGCTATAGCTACTACGTTTACGGTATAGC

CTCTAGATAATTTTTTTAAGCACGAAATAAAAACATAATTTTAAACAATCTATTTCATACTATTTTGTGT

GATCAACATGAACATAAAGATAGATGATATGATATATTAGTATTTCTGGTGATAAATTTACGGCGACTGC

TAGGAGGGAAAATGAAGAAAGAAAAAATATCTACCTCTCCAAAAAGAAAAACTACTGATGTTATCAAACC

TGATTATCTTGAGTACAATGACTTGTTAGATAGAGATGAGATGTCTACTATTCTAGAGGAATATTAGGCC

TTAGAATAAAATATGGACGACTCTTAACGAAATTAGAAAATTCGATAATGATGTTGAAGAACAATTCGGT

ACTATAGAAGAACTCAAGCAGAAGCTTAGATTAAATTCTGAAGAGGGAGCAGATAATTTTTATAGATTAT

ATAAAGGTACAAAAACAGGATATCGTCAAACTTACTGTATACGATTGCATATATCTATGATAGGATTGTA

TGCGTGCGTGGTAGATGTTTGGAGAAATGAGAAACTGTTTTCTAGATGGAAATATTGGTTACAAGCGATT

AAACTGTTTATTGATGATCACATGCTTGATAAGATAAAATCTATACTGTAGAATAGACTAGTGTATGTGG

AAATGTCATAGAAAGTTAAAAGTTAATGAGAGCAAAAATATATAAGGTTGTATTCCATATTTGTTATTTT

TTTCTGTAATAGTTAGAAAATACATTCGATGGTCTATCTACCAGATTATTATGTGTTATAAGGTACTTTT

CTCATAATAAACTAGAGTATGAGTAAGATAGTGTTTTTCAAAACATATAAATCTAAAATTGATGGATGAG

ATATACAGCTATTAATTTCGAAAATATATTTTAATCTGATAACTTTAAACATGGATTTTTGATGGTGGTT

TAAGTTTAAAAAAGATTTTGTTATTGTAGTATGATAATATCAAAAAGATGGATATAAAGAATTGGTCAGT

GTATAATAAATTATATGTAGGAGGAGGAATATCTGATGATGTTCAAACTAATACATCTGAAACATACGAT

AAAGAAAAAGATTGTTGGACATTGGATAATGGTCACTTGGTACCACATAATTATATAATGTATAAATGCG

AACCGGTTAAACATAGATATCCATTGGAAAAAAACACAGTACACGAATGATTTTCTAAAGTATTTGGAAA

GTTTTATAGGTAGTTGATAGAACAAAATACATAATTTTGTAAAAATAAATCACTTTTTATACTAATATGA

CACAATTACCAATACTTTTGTTACTAATATCATTAGTATACGTTACACCTTCTCCTCAGACATCTAAAAA

AATAGGTGATGATGCAACTATATCATGTAGTCGAAATAATACAAATTACTACGTTGTTATGAGTGCTTGG

TATAAGGAGCCCAATTCCATTATTCTCTTAGCTGCCAAAAGCGACGTCTTGTATTTTGATAATTATACCA

AGGATAAAATATCTTACGACTCTCCATACGATGATCTAGTTACAACTATCACAATTAAATCATTGACTGC

TGGAGATGCCGGTACTTATATATGTGCATTCTTTATGACATCGACTACAAATGATACTGATAAAGTAGAT

TATGAAGAATACTCCATAGAGTTGATTGTAAATACAGATAGTGAATCGACTATAGACATAATACTATCTG

GATCTACACCGGAAACTATTTCTGAGAAACCAGAGGATATAGATAATTCTAATTGCTCGTCTGTATTCGA

AATCGCGACTCCGGAACCAATTACTGATAATGTAGAAGACCATACAGACACCGTCACATACACTAGTGAT

AGCATTAATACAGTAAATGCATCATCTGGAGAATCCACAACAGACGAGACTCCGGAACCAATTACTGATA

AAGAAGAAGATCATACAGTAACAGACACTGTCTCATACACTACAGTAAGTACATCATCTGGAATTGTCAC

TACTAAATCAACCACCGATGATGCGGATCTTTATGATACATACAATGATAATGATACAGTACCGCCAACT

ACTGTAGGTGGTAGTACAACCTCTATTAGCAATTATAAAACCAAGGACTTTGTAGAAATATTTGGTATTA

CCACATTAATTATATTGTCGGCAGTGGCGATTTTCTGTATTACGTATTATATATGTAATAAACACCCACG

TAAATACAAAACAGAGAACAAAGTCTAGATTTTTGACTTACATAAATATCTGGGATAATAAAATCTATCA

TATTGAGAGGACCATCTGGTTCAGGAAAGACAGCCATAACCAAAAGACTGTTAAAAGACTATGGGAATAT

ATTTGGATTTGTGGTGTCCCATACCACTAGATTTCCTCGTCCTATGGAACGACGAGAAGGTGTTGATTAC

CATTACTTAACAGAGAGGCAATCTGGAAGGGAATAGCCGCCGGAAACTTTCTAGAACATACTGAGTTTTT

AGGAAATATTTACGGAACTTCTAAAACAGCTGTAAATACAGCGGCTATTAATAATCGTATTTGCGCGATG

GATTTAAACATCAACGGTGTTAGAAGTCTTAAAAATACTTACCTAATGCATTACTTGGGTATATAAGACC

TACCTCTCTTAAAATGGTTGAGGCCAATCTTCGTCGTAGAAACACTGAAGCGGACGACGAATCTCATCGT

CGCGTGATGTTGGCAAAAAACGGATATGGATGAGGTCAACGAAGCAGGTCTATTCGACACTATTATTATT

GAAGATGATGTGAATTTAGCATATAGTAAGTGTTAATTCAGATACTACAGGACCGTATTAGAATGTATTT

TAACACTAATTAGAGACTTAAGATTTGACTTAAAACTTGATAATTAATAATATAACTCGTTTTTATATGT

GGCTATTTCAACGTCTAATGTATTAGTTAAATATTAAAACTTACCACGTAAAACTTAAAATTTAAAATGG

TATTTCATTGACAGATCATACATTATGAAGTTTCAAGGACTTGTGTTAATTGACAATTGCAAAAATCAAT

GGGTCGTTGGACCATTAATAGGAAAAGGTGGATTCGGTAGTATTTATACTACTAATGACAATAATTATGT

AGTAAAAATAGAGCCCAAAGCTAACGGATCATTATTTACCGAACAGGCATTTTATACTAGAGTACTTAAA

CCATCCGTTATCGAAGAATGGAAAAAATCTCACAATATAAAGCACGTAGGTCTTATCACGTGCAAGGCAT

TTGGTTTATACAAATCCATTAATGTGGAATATCGATTCTTGGTAATTAATAGATTAGGTGCAGATCTAGA

TGCGGTGATCAGAGCCAATAATAATAGACTACCAGAAAGGTCGGTGATGTTGATCGGAATCGAAATCTTA

AATACCATACAATTTATGCACGAGCAAGGATATTCTCACGGAGATATTAAAGCGAGTAATATAGTCTTGG

ATCAAATAGATAAGAATAAATTATATCTAGTGGATTACGGATTGGTTTCTAAATTCATGTCTAACGGCGA

ACATGTTCCATTTATAAGAAATCCAAATAAAATGGATAACGGTACTCTAGAATTTACACCTATAGATTCG

CATAAAGGATACGTTGTATCTAGACGTGGTGATCTAGAAACACTTGGATATTGTATGATTAGATGGTTGG

GAGGTATCTTGCCATGGACTAAGATATCTGAAACAAAGAATTCTGCATTAGTAAGTGCTGCAAAACAGAA

ATATGTTAACAATACTGCGACTTTGTTAATGACCAGTTTGCAATATGCACCTAGAGAATTGCTGCAATAT

ATTACCATGGTAAACTCTTTGACATATTTTGAGGAACCCAATTACGACGAGTTTCGTCGAGTATTAATGA

ATGGAGTTATGTAAAATTTTTGTTGATAAAAAAATTAAAAAAATAACTTAGTTATTATCACTCTCGCGAG

TGCAATAGAAACACGGCGATGTTTTACGCACACGCTTTCGGTGGGTACGACGAGAACCTTCATGCATTTC

CTAGAATATCATCGACGGTTGCCAATGATGTCAGGAAATATTCTGTTGTGTCAGTTTATAATAAAAAGTA

TAACATTGTAAAAAACAAATATATGTGGTGTAACAGTCAAGTGAACAAGAGATATATTGGAGCACTACTG

CCTATGTTTGAGTGCAATGAATATCTACAAATTGGAGATCCAATCCATGATCTAGAAGGAAATCAAATCT

CTATTGTCACATATCGCCACAAAAACTACTATGCTCTAAGTGGAATTGGGTACGAGAGTCTAGACTTGTG

TTTGGAAGGAGTAGGGATTCATCATCACGTACTTGAAACAGGAAACGCGGTATATGGAAAAGTTCAACAT

GAGTATTCTACTATCAAAGAGAAGGCCAAAGAAATGAATGCACTCAAACCAGGACCTATCATCGATTACC

ACGTCTGGATAGGAGATTGTGTCTGCCAAGTTACTACTGTAGACGTGCATGGAAAGGAAATTATGAGAAT

GAGATTCAAAAGGGGTGCGGTGCTTCCGATTCCAAATCTGGTAAAAGTTAAAGTTGGGGAGGAAAATGAT

ACAATAAATCTTTCCACTTCCATATCAGCTCTCCTGAATTCCGGTGGCGGCACCATCGAGGTAACATCTA

AGGAAGAACGTGTGGATTATGTACTCATGAAACGTTTGGAATCTATACATCATCTGTGGTCTGTAGTGTA

TGATCATCTTAATGTTGTGAATGGCGAAGAACGATGCTATATACATATGCATTCATCTCATCAAAGTCCT

ATGCTGAGTACTGTAAAAACAAATTTGTATATGAAGACTATGGGAGCATGTCTTCAAATGGACTCCATGG

AAGCTCTAGAGTATCTTAGTGAACTGAAGGAATCAGGTGGGCGGAGTCCCAGACCAGAATTGCAGAAATT

TGAATATCCAGATGGAGTGAAAGACACTGAATCAATTGAGAGATTGGCAGAGGAGTTCTTCAATAGATCA

GAACTTCAGGCTGGTGAATCAGTCAAATTTGGTAATTCTATTAATGTTAAACATACATCTGTTTCAGCTA

AGCAACTAAGAACACGTATACGGCAGCAGCTTCCTTCTATACTCTCATCTTTTGCCAACACAAAGGGTGG

ATATTTGTTCATTGGAGTTGATAATAATACACACAAAGTAATTGGATTCACGGTGGGTCACGACTACCTC

AAACTGGTAGAGAGAGATATAGAAAAGTATATCCAAAAACTTCCTGTTGTGCATTTCTGCAAGAAAAAAG

AGGACATCAAGTACGCATGTAGATTCATCAAGGTGTATAAACCTGGTGATGAGACTACCTCGACATATGT

GTGCGCAATCAAAGTGGAAAGATGCTGCTGTGCTGTGTTTGCGGATTGGCCAGAATCATGGTACATGGAT

ACTAGTGGTAGTATGAAGAAGTATTCTCCAGATGAATGGGTGTCACATATAAAATTTTAATTAGGGTAAG

GTAAAACTATATATAATAACTAACAATTTGTGTATCATATAGACAATTAATTAGGTAACTGTTATCTCTT

TTTAACTAACTAACTAACTCTTATATACTATTAATAATACATCTATTAATAATACATCTATTAATAATAC

ATCTATTAATCATTGATTAGTTTATTGCTTTAATTGTTTTTGTAAACTAACACTGTTCATTGAAAAGGGA

TAACATGTTACAGAATATAAATTATATATGGATTTTTTTAAAAAGGAAATACTTGACTGGAGTATATATT

TATTTCTTCATTACATAACACGTCTGTGTTCTAATTCTTCCAATTCTTCCACATCTCATATAATACAGGA

ATATAATCTTGTTCGAAAATACGAGAAAGTGGATAAAACAATAGTTGATTTTTTATCTAGGTGGCCAAAT

TTATTCCATATTTTAGAATATGGGGAAAATATTCTACATATTTATTTTATAGATGCTGCTAATACGAATA

TTATGATTTTTTTTCTAGATAGAGTATTAAATATTAATAAGAACCGTGGGTCATTTATACATAATCTCGG

GTTATCATCCATTAATATAAAAGAATATGTATATCAATTAGTTAATAATGATCATCTAGATAATAGTATA

AGACTAATGCTTGAAAATGGACGTAGAACAAGACATTTTTTGTCTTATATATTGGATACAGTTAATATCT

ATATAAGTATTTTAATAAATCATAGATTTTATATAGATGCCGAAGACAGTTACGGTTGTACATTATTACA

TAGATGTATATATAACTATAGGAAATCAGAATCAGAATCATATAATGAATTAATTAAGATATTGTTAAAT

AATGGATCAGATGTAGATAAAAAAGATACGTACGGAAACACACCGTTTATCCTATTATGTAAACACGATA

TCGACAACGCGGAATTGTTTGAGATATGTTTAGAGAATGCTAATATAGACTCTGTAGACTTTAATGGATA

TACACCTCTTCATTATGTCTCATGTCGTAATAAATATGATTTTGTAAAGTTATTAATTTCTAAAGGAGCA

AATGTTAATGCACGTAATAGATTCGGAACTACTCCATTTTATTGTGGAATTATACACGGTATCTCGCTTA

TAAAACTATATTTGGAATCAGACACAGAGTTAGAAATAGATAATGAACATATAGTTCGTCATTTAATAAT

TTTTGATGCTGTTGAATCTTTAGATTATCTATTGTCCAGAGGAGTTATTGATATTAACTATCGTACTATA

TACAACGAAACATCTATTTACGACGCTGTCAGTTATAATGCGTATAATACGTTAGTCTATCTATTAAACA

GAAATGGTGATTTTGAGACGATTACTACTAGTGGATGTACATGTATTTCGGAAGCAGTCGCGAACAACAA

CAAAATAATAATGGATATACTATTGTCTAAACGACCATCTTTGAAAATTATGATACCATCTATGATAGCA

ATTACTAAACATAAACAACATAATGCAGATTTATTGAAAATGTGTATAAAATATACTGCGTGTATGACCG

ATTATGATACTCTTATAGATGTACAATCGCTACATCAATATAAATGGTATATTTTAAAATGTTTTGATGA

AATAGATATCATGAAGAGATGTTATATAAAAAATAAAACTGTATTCCAATTAGTTTTTTGTATCAAAGAC

ATTAATACTTTAATGAGATACGGTAGACATCCTTCTTTCGTGAAATGTAATATTCTCGACGTATACGGAA

GTCATGTACGTAATATCATAGCATCTATTAGATATCGTCAGAGATTAATTAGTCTATTATCCAAGAAGCT

GGATGCTGGAGATAAATGGTCGTGTTTTCCTAACGAAATAAAATATAAAATATTGGAAAACTTTAACGAT

AACGAACTGACCACATATCTAAAAATCTTATAAACACTATTAAAATATAAAATCTAAGTAGGATAAAAAC

ACTACATCATTGTTTCCTTTTAGTGCTCGACAGTGTATGCTATTTTTAACACTCATAAATAAAAATGAAA

ACGATTTCCGTTGTTACGTTGTTATGCGTACTACCTGCTGTTGTTTATTCAACATGTACTGTACCCACTA

TGAATAACGCTAAATTAACGTCTACCGAAACATCGTTTAATGATAAACAGAAAGTTACGTTTACATGTGA

TTCAGGATATCATTCTTTGGATCCAAATGCTGTCTGCGAAACAGATAAATGGAAATACGAAAATCCATGC

AAGAAAATGTGCACAGTTTCTGATTATGTCTCTGAACTATATGATAAGCCATTATACGAAGTGAATTCCA

CCATGACACTAAGTTGCAACGGTGAAACAAAATATTTTCGTTGTGAAGAAAAAAATGGAAATACTTCTTG

GAATGATACTGTCACGTGTCCTAATGCGGAATGTCAACCTCTTCAATTAGAACACGGATCGTGTCAACCA

GTTAAAGAAAAATACTCATTTGGGGAATATATGACTATCAACTGTGATGTTGGATATGAGGTTATTGGTG

TTTCGTATATAAGTTGTACGGCTAATTCTTGGAATGTTATTCCATCATGTCAACAAAAATGTGATATACC

GTCTCTATCTAATGGATTAATTTCCGGATCTACATTTTCTATCGGTGGCGTTATACATCTTAGTTGTAAA

AGTGGTTTTACACTAACGGGGTCTCCATCATCCACATGTATCGACGGTAAATGGAATCCCATACTCCCAA

CATGTGTACGATCTAACGAAGAATTTGATCCAGTGGATGATGGTCCCGACGATGAGACAGATCTGAGCAA

ACTCTCGAAAGACGTTGTACAATATGAACAAGAAATAGAATCGTTAGAAGCAACTTATCATATAATCATA

ATGGCGTTGACAATTATGGGTGTCATATTTCTAATCTCTATTATAGTATTAGTTTGTTCCTGTGACAAAA

ATAATGACCAATATAAGTTCCATAAATTGCTACCGTGAATATAAATCCGTTAAAATAATTAATAATTAAT

AATTAATAATTAATAACGAACAAGTATCAAAAGATTAAAGAATTAGCTAGAATCAATTAGATGTCTTCTT

CAGTGGATGTTGATATCTACGATGCTGTTAGAGCATTTTTACTCAGGCACTATTATGACAAGAGATTTAT

TGTGTATGGAAGAAGTAACACCATATTACATAATATATACAGGCTATTTACAAGATGCACCGTTATACAG

TTCGATGATATAGTACGTACTATGCCAAATGAATCACGTGTTAAACAATGGGTGATGGATACACTTAATG

GTATAATGATGAATGAATGCGATACTGTATGTGTGGGTACCGGACTACGATTCATGGAAATGTTTTTCGA

TTACAATAAAAATAATCCCAAAAATAGCATCAACAATCAAATAATGTATGATATAATTAATAGCGTAACC

ATAATTCTAGCTAATGAGAGATATAGAAGCGCGTTTAACGACGATAGAATATACATCCGTAGAACTATGA

TGGACAAATTGTACGAATACGCATCTCTAACTACTATTGGTACGATCACTGGAGGTGTTTGTTATTATCT

GTTGATGCATCTAGTTAGTTTGTATAAATAATTATTTCGATATACTAGTAAAATTTTAAGATTTTAAATG

TATAAAAAACTAATAACGTTTTTATTTGTAATAGGTGCAGTTGCATCCTATTCGAATAATGAGTACACTC

CGTTTAATAAACTGAGTGTAAAACTCTATATAGATGGAGTAGATAATATAGAAAATTCATATACTGATGA

TAATAATGAATTGGTGTTAAATTTTAAAGAGTACACAATTTCTATTATTACAGAGTCATGCGACGTCGGA

TTTGATTCCATAGATATAGATGTTATAAACGACTATAAAATTATTGATATGTATACCATTGACTCGTCTA

CTATTCAACGCAGAGAACATACGTGTAGAATATCTACCAAATTATCATGCTATTATGATAAGTACCCTTA

TATCCACAAATATGAGGGTGATGAACGACAATATTCTATTACTGCAGAGGGAAAATGCTATAAAGGAATA

AAATATGAAATAAGTATGATGAACGATGATACTCTATTGAGAAAACATACTCTTAAAATTGGATTTACTT

ATATATTCGATCGTCATGGGCATAGTAATACATATTATTCAAAATATGATTTTTAAAAATTTAAAATATA

TTATCACTTCAGTGACAGTAGTCAAATAACAAACAACACCATGAGATATATTATAATTCTCGCAGTTTTG

TTCATTAATAGTATACATGCTAAAATAACTAGTTATAAGTTTGAATCCGTCAATTTTGATTCCAAAATTG

AATGGACTGGGGATGGTCTATACAATATATCCCTTAAAAATTATGGCATCAAGACGTGGCAAACAATGTA

TACAAATGTACCAGAAGGAACATACGACATATCCGGATTTCCAAAGAATGATTTCGTATCTTTCTGGGTT

AAATTTGAACAAGGCGACTATAAAGTGGAAGAGTATTGTACGGGACTATGTGTCGAAGTAAAAATTGGAC

CACCAACTGTAAGATTGACTGAATATGACGATCATATCAATTTGTTCATCGAGCATCCGTATGCTACTAG

AGGTAGCAAGAAGATTCCTATTTACAAACGCGGTGACATGTGTGATATCTACTTGTTGTATACGGCTAAC

TTCACATTCGGAGATTCTGAAGAACCAGTAACATATGATATCGATGACTACGATTGCACGTCTACAGGTT

GCAGCATAGACTTTGCCACAACAGAAAAAGTGTGTGTGACAGCACAGGGAGCCACAGAAGGGTTTCTCGA

AAAAATTACTCCATGGAGTTCGGAAGTATGTCTGACACCTAAAAAGAATGTATATACGTGCGCAATTAGA

TCTAAAGAAGATGTTCCCAATTTCAAGGACAAAATAGCCAGAGTTATCACGAGAAAATTTAATAAACAGT

CTCAATCTTATTTGACTAAATTTCTCGGTAGCACATCGAATGATGTTACAACTTTTCTTAGCATTCTTGA

CTAAATATTCATAACTAATTTTTATTAATGATACAAAAATGAAATAAAACTGCATATTATACACTGGTTA

ACGCCCTTGGCCTAACCATTTTTAAGATGAGGTCCCTGATTATAGTCCTTCTGTTCCCCTCTATCATCTA

CTCCATGTCTATTAGACGATGCGAGAAGACTGAAGAGGAAACATGGGGATTAAAAATAGGGTTGTGTATA

ATTGCCAAAGATTTCTATCCCGAAAGAACTGATTGCAGTGTTCATCGCCCAACTGCAAGTGGAGGATTGA

TAACTGAAGGCAATGGATTCAGAGTAGTTATATATGATCAATGTACAGAACCACATGACTTTATTATCAC

CGATACTCAACAAACACGTCTTGGATCATCTCATACATATATTAAATTCAGTAACATGAATACAGGTGTC

CCATCTAGTATTCCAAAATGTTCCAGAACTCTCTCTATTTCTGTATATTGTGATCAAGAGGCGGGAGACA

TAAAATTTGAGGAGTATACTCAAGAATCAAGTGATATCAGTATTAGAGTTAAGTATGATTCATCATGTAT

TGATTATCTGGGTATTAATCAAAGTTTCATGAATGAATGTATTCGAAGAATTACAACATGGGATAGAGAA

TCATGTGTCAGAATTGATACACAGACTATAAATAAATATCTTAAGTCTTGCACCAACACAAAATTCGACC

GTAATGTCTACAAAAGGTACATACTGAAGAGTAAAGCACTCCATGCTAAAACAGAGTTGTAATAGATATA

AAATACTTTTTATAATAATTAGGCTAGAAAAATCTCACTCACATGTAATCTTAAAAAATGATATGATAGT

TCTTACAAGTAGAGATTGAGTTTTAAATGGATTCTATTAATTACCGGGGACTTAACAATTCGTTCTGATC

TACAGACATTGGTTAATAAATCATCTTATTTTGCCGATATATTAAAATGTGGAAACTCCACTAATAATAT

TACATTGTGTGACTTTCAAGATGATGCGATATATAGGGTTATACAGTTTAACAATTATATAATAGAGATA

GAAAGTACAAAAGATGTAGAATCAATGATATGGCACGCTAAACAGTTGGGTGTGGAATCATTGCTAAAAG

AATGTCAAAATTATTTGCTTAGAATATTACGTATATAATTGTTTAGAAATTTATAGAATAACTAATATTA

ATACATTATCGTATATCTACAACGATGTAAGAAACTTCATATTGGATAATATTACTATTAATATATAAGG

ATCCAGATTTTATATATTTGCCTAAATACATTATTATAGATTTACTAGGACAATCACCTAAATGTTTTTA

ACGAAGATAATGTGGTAAAGATTATATACACTTATATATCTTCCGATATCTACAAGGATATTTCCATATC

ATCATTGTGTAAACTAAATAACGTTTTCTATGGCATTTAATAAGGCATTGGATATGTGGAAAAGATGATG

GTGTATGGAAGTTAGTACATCATTATCAACTTCTCCTTATTGATTGAAAATGAAAATATAAATAGTTTTT

ATATATAGCGGTATCTACCCTATAGTTTTATTGCTTACTACTAACATGGATACAGATACAGATACAGATA

CAGATACAGATATAGATACAGATACAGATGTTAGATAGAGAGAAAGAAGAAATACTAAAAAATGTAGAAA

TTGAAAATAATAAAAACATTAACAAGAATCATCCCAGTGAATATATTAGAGAAGCACTCGTTATTAATAC

CAGTAGTAATAGTGATTCCATTGATAAAGAAGTTATAGAATATATCAGTCACGATGTAGGAATATAGATC

ATATCTACTAATTTTTATAATCGATACAAAACATAAAAAACAACTCGTTATTACATAGCAGGCATGGAAT

CCTTCAAGTATTGTTTTGATAACGATGGTAAGGAATGGATTATCGGAAATACTTTATATTCTGGTAATTC

AATACTCTATAAGGTCAGAAAAAATTTCACTAGTTCGTTCTACAATTACGTAATGAAGATAGATCATAAA

TCACACAAGCCATTGTTGTCCGAAATACGATTCTATATATCTGTATTGGATCCTTTGACTATCGACAACT

GGACACGGGAACGTGGTATAAAGTATTTGGCTATTCCAGATCTGTATGGAATTGGAGAAACCGATGATTA

TATGTTCTTCGTTATAAAGAATTTGGGAAGAGTATTCGCCCCAAAGGATAGTGAATCAGTTTTCGAAGCA

TGTGTCACTATGATAAACACGTTAGAGTTTATACACTCTCGAGGATTTACTCATGGAAAAATAGAACCGA

TGAATATACTGATTAGAAATAAACGTATTTCACTAATTGACTATTCTAGAACTAACAAACTATACAAAAG

TGGAACACATATAGATTACAACGAGGACATGATAACTTCAGGAAATATCAATTATATGTGTGTAGACAAT

CATCTTGGAGCAACAGTTTCAAGACGAGGAGATTTAGAAATGTTGGGATATTGCATGATAGAATGGTTCG

GTGGTAAACTTCCATGGAAAAACGAAAGTAGTATAAAAGTAATAAAACAAAAAAAAGAATATAAACAATT

TATAGCTACTTTTTTTGAGGACTGTTTTCCTGAAGGAAATGAACCTCTGGAATTAGTTAGATATATAGAA

TTAGTATACATGTTAGATTATTCTCAAACTCCTAATTATGACAGACTACGTAGACTGTTTATACAAGATT

GAAATTATATTCTTTTTTTTATAGAGTGTGGGGTAGTGTTACGGATATCTGATATCTAATATCTAATATT

AATATTAGACTATCTCTATCGCGCTACACGACCAATATCGATTACTATGGATATCTTTAGGGAAATCGCA

TCTTCTATGAAAGGAAAGAATGTATTCATTTCTCCAGCGTCAATCTCGTCAGTATTGACAATACTGTATT

ATGGAGCTAATGGATCCACTGCTGAACAGCTATCAAAATATGTAGAAAAGGAGGAGAACATGGATAAGGT

TAGCGCTCAGAATATCTCATTCAAATCCATGAATAAAGTATATGGGCGATATTCTGCCGTGTTTAAAGAT

TCCTTTTTGGGAAAAATTGGCGATAAGTTTCAAACTGTTGACTTCACTGATTGTCGCACTATAGATGCAA

TCAATAAGTGTGTAGATATCTTTACTGAGGGAAAAATCAATCCACTATTGGATGAACCATTGTCTCCTGA

TACCTGTCTCCTAGCAATTAGTGCCGTATACTTTAAAGCAAAATGGTTGATGCCATTCGAAAAGGAATTT

ACCAGTGATTATCCCTTTTACGTATCTCCAACGGAAATGGTAGATGTAAGTATGATGTCTATTTACGGCG

AGCCATTTAATCACGCATCTGTAAAAGAATCATTCGGTAACTTTTCAATCATAGAACTGTCATATGTTGG

AGATACTAGTATGATGGTCATTCTTCCAAACAAGATTGATGGATTAGAATCCATAGAACAAAATCTAACA

GATACAAATTTTAAGAAATGGTGTAACTCTCTGGAAGCTACGTTTATCGATGTGCACATTCCTAAGTTTA

AGGTAATAGGTTCGTATAATCTTGTGGATACGCTAATAAAGTTGGGACTGACAGATGTGTTCTATTCAAC

TGGTGATTATATCAATATGTGTAATTCAGATGTGAGTGTTGACGCTATGATTCACAAAACGTATATAGAT

GTCAATGAAGAGTATACAGAAGCAGCTGCAGCAACTTCTGTACTAGTGGCAGACTGTGCATCAACAGTTA

CAAATGAGTTCTGTGCAGATCATCCGTTCATCTATGTGATTAGACATGTCGATGGTAAAATTCTTTTCGT

TGGTAGATATTGCTCTCCAACAACTAATTAAGCACATTCTTAATATTAGAATATTATATAGTTAAGATTT

TACTAACAGGTTAACATTTTTTTTAAAAAATAGAAAAAACATGTGGTATTAGTGCAGGTCGTTATTCTTC

CAATTGCAATTGGTAAGATGACGGCCAACTTTAGTACCCACGTCTTTTCACCACAGCACTGTGGATGTGA

CAGACTGACCAGTATTGATGACGTCAGACAATGTTTGACTGAATATATTTATTGGTCGTCGTATGCATAC

CGCAACAGGCAATGCGCTGGACAACTGTATGGCACACTCCTCTCTTTTAAAGATGATGCGGAATCAGTGT

TCATCGACGTTCGTGAGCTGGTAAAAAATATGCCGTGGGATAATGTTAAGGATTGTACAGAGATCATCCG

TTGTTATATACCGGATGAGCAAAAAACCATCAGAGAGATTTCGGCCATCATTGGACTTTGTGCATATGCT

GCTACTTACTGGGGAGGTGAAGACCATCCCACTAGTAACAGTCTGAACGCATTGTTTGTGATGCTTGGGA

TGCTCAATTACATGGATTATACCATCATATTCTGGCGTATGAATTGATGAGTTACAGCTTGACATTTCTT

CTTTCCTCCCTCTTCTTCTACTTTTCCCAGAAACAAACTTTTTTTACCTACTATAAAATAAAATGAGTAT

ACTACCTGTTATATTTCTTCCTATATTTTTTTATTCTCCATTCGTTCAGACTTTTAACGTGCCTGAATGT

ATCGACAAAGGGCAATATTTTGCATCATTCATGGAGTTAGAAAACGAGCCAGTAATCTTACCATGTCCTC

AAATAAATACGCTATCATCCGGATATAATATATTAGATATTTTATGGGAAAAACGAGGAGCGGATAATGA

TAGAATTATACAGATAGATAATGGTAGCAATATGCTAATTCTGAACCCGACACAATCAGACTCTGGTATT

TATATATGCATTACCACGAACGAAACCTACTGTGACATGATGTCGTTAAATTTGACAATCGTGTCTGTCT

CAGAATCAAATATAGATCTTATCTCGTATCCACAAATAGTAAATGAGAGATCTACTGGTGAAATGGTATG

TCCCAATATTAATGCATTTATTTCTAGTAACGTAAACGCAGATATTATATGGAGCGGACATCGACGCCTT

AGAAATAAGAGACTTAAACAACGGACACCTGGAATTATTACCATAGAAGATGTTAGAAAAAATGATGCTG

GTTATTATACATGTGTTTTAGAATATATATATATGGGCAAAACATATAACGTAACCAGAATTATAAAATT

AGAGGTACGTGATAGAATAATACCTCCTACTATGAAATTACCAGAAGGAGTAGTAACTTCAATAGGTAGT

AATTTGACTATTACATGCAGAGTATCGTTGAGACTTCCCACAACGGACGCTGACGTCTTTTGGATAAGTA

ATGGTATGTATTACGAAGAAGAAGACGAGGACGGAGACGGTAGAATAAGTGTAGCAAATAAAATCTATAT

GACTGATAAGAGACGTGTTATTACATCCTGGTTAAACATTAATCCTGTCAAGGAAGAAGATGCTACAACG

TTTACGTGTATGGCGTTTACTATTCCTAGCATCAGCAAAACAGTTACTGTTAGTATAACGTGAATGTATG

TTGTTACATTTCCATATCAATTGAGTTTATAAGAATTTTTATACATTATCTTCCAACAAACAATTGACGA

ACGTATTGCTATGATTAACTCCCACAATACTATGCATATTATTAATCATTAACTTGCAGACTATACCTAG

TAGTGCTATTTTGACATACTCATGTTCTTGTGTAATTGCAGTATCTATATTATTAAAGTACGTAAATCTA

GCTATAGTTTTATTATTTAATTTTAGATAATATACTGTCTCCGTATTTTTAAAAAATTGCCACATCCTTT

ATTAAATCATGAATGGGAATTTCTGTGTCATCGTTAGTATATTGTGAACAACAAGAGCTGATATCTATAG

GAAAGGGTGGAATGCGATACATTGATCTATGTAGTTTTAAAACATAAGCGAACTTTGAAGAATTTATATA

AATCATCTCACGAGATATTGTTCTCTGTCATATTCATACACCTGCATAAACTTTCTATACATCTTACAAT

GTGTTATTTTATGATCATATTTACATATTTACTGGTATATCAAAGATGTTAGATTAGTTAATGGGAATCG

TCTATAATAATGAATATTAAACAATTATAGGAGGAATTTATACCTACAAAAACATCATAAAAATGAGTCA

TCGTCCGATTTATGTTTTAAATATACTAACATTACTACCTTCAGAAATTATATACGAAATATTATACATG

CTGACAATTAACGATCTTTATAATATATAGTATCCACCTACCAAAGTATAATTGTATTTTTTCTCATGTG

ATGTGTGTAAAAAACTGATATTATATAATTATCTTAGTACCTATGATGAAGATGAAGATGATGGTCCGTA

TATATTTTGTATCATTATCGTTATTGCTATTCCATAGTTACGCCATAGACATCGAAAATGAAATCACCGA

ATTCTTCAATAAAATGAGAGATACTCTACCAGCTAAAGACTCTAAATGGTTGAATCCAGTATGTATGTTT

GGAGGCACAATGAATGATATGGCCGCTCTAGGAGAGCCATTCAGTGCAAAGTGTCCTCCTATTGAAGACA

GTCTTTTATCGCATAGATATAAAGACTATGTGGTTAAATGGGAAAGGCTAGAAAAGAATAGACGGCGACA

GGTTTCTAATAAACGTGTCAAACATGGTGATTTATGGATAGCCAACTATACATCTAAATTCAGTAACCGT

AGGTATTTATGTACCGTAACCACAAAGAATGGTGACTGTGTTCAGGGTGTAGTTAGATCTCATGTGTGGA

AACCTTCTTCATGCATTCCAAAAACATATGAACTAGGTACTTATGATAAGTATGGCATAGACTTATACTG

TGGAATTCTTTATGCGAAACATTATAATAATATAACTTGGTATAAAGATAATAAGGAAATTAATATCGAC

GATTTTAAGTATTCACAAGCGGGAAAGGAATTAATTATTCATAATCCAGAGTTAGAAGATAGTGGAAGAT

ACGACTGTTACGTTCATTACGACGACGTTAGAATCAAGAATGATATCGTAGTATCAAGATGTAAAATACT

TACGGTTATACCGTCACAAGACCACAGGTTTAAACTAATACTAGATCCGAAAATCAACGTAACGATAGGA

GAACCTGCCAATATAACATGCAGTGCTGTGTCAACGTCATTATTTGTCGACGATGTACTGATTGAATGGG

AAAATCCATCCGGATGGATTATAGGATTAGATTTTGGTGTATACTCTATTTTAACTAGTAGAGGCGGTAT

CACCGAGGCGACTTTGTATTTTGAAAATGTTACTGAAGAATATATAGGCAATACATATACATGTCGTGGA

CACAACTATTATTTTGATAAAACTCTTACAACTACAGTAGTATTGGAGTAAATACACAATGCATTTTTAT

ATACATTACTGAATTATTATTATTAATTATATCGTATTTGTGCTATAGAATGGATGAAGATACGCGACTA

TCTAGGTATTTGTATCTCACCGATAGAGAACATATAAATGTAGACTCTATTAAACAGTTGTGTAAAATAT

CAGATCCTAATGCATGTTATAGATGTGGATGTACGGCTTTACATGAGTACTTTTATAATTATAGATCAGT

CAACGGAAAATACAAGTATAGATACAACGGTTACTATCAATATTATTCATCTAGCGATTATGAAAATTAT

AATGAATATTATTATGATGATTATGATAGAACTGGTATGAACAGTGAGAGTGATAATATATCAATCAAAA

CAGAATATGAGAATGAATATGAATTCTATGATGAAACACAAGATCAAAGTACACAACTAGTAGATTACGA

CATTAAACTCAAAACCAATGAGGATGATTTTGTTGATGAATTCTATGGTTATGATAGATCAGTGGGTGTC

CATGATTATATAGATGTATCAATTAATAAAGTAGTATATGGAAGAGAGTCTCACGTAAGATGGCGGGATA

TATGGCAAGAACATAATGATGGCGTATACAGTATAGGAAAGGAGTGCATAGATAATATATACGAAGACAG

ACATACCGTAGACGAATTCTACAAGATAGACAGCGTATCAGATGTAGATGACGCAGAACATATATCTCAG

ATAACTAATGATGTATCTACACAAACATGGGAAAAGAAATCAGAGTTAGATAGATACATGGAAATGTATC

CTCGTCATAGATATGGTAAGCATTCTGTCTTTAAGGGATTTTCTGACAAAGTTAGAAAAAATGATTTAGA

CATGAATGTGGTAAAAGAATTACTTTCTAACGGTGCATCTCTAACAATCAAGGATAGCAGTAATAAGGAT

CCAATTGCTGTTTATTTTAGAAGAACAATAATGAATTTAGAAATGATTGATATCATTAACAAACATACAA

CTATCTATGAACGCAGGTATATAGTACACTCCTATCTAAAAAATTATAGAAATTTCGATTATCCATTTTT

CAGAAAGTTAGTTTTGACTAATAAACATTGTCTCAACAATTATTATAATATAAGCGACAGCAAATATGGA

ACACCACTACATATATTAGCATCTAATAAAAAAATAATAACTCCTAATTACATGAAGTTATTAGTGTATA

ACGGAAATGATATAAACGCACGAGGTGAAGATACACAAATGCGAACTCCATTACACAAATATTTGTGTAA

ATTTGTATATCATAATATTGAATATGGTATCCGATACTATAATGAAAAGATTATAGACGCATTTATAGAG

TTAGGAGCCGATCTAACTATTCCAAATGACGATGGAATGATACCAGTAGTTTACTGTATACACTCAAATG

CCGAATATGGTTATAACAATATTACTAACATAAAGATAATACGTAAACTACTTAATCTTAGTAGACATGC

GTCACATAATCTATTTAGAGATCGAGTCATGCACGATTATATAAGTAATACATATATTGATCTTGAGTGT

TTAGATATCATTAGATCACTGGATGGGTACGATATTAATTGTTACTTTGAAGGACGTACACCACTTCATT

GCGCTATACAATATAACTTCACTCAGATTGCTGAGTACTTATTAGATCGAGGAGCTGATATATCATTAAA

GACAGACGATGGTAAAACTGTATTTGATTTATCGTTATGTAGTTACATTCCTCTTAAATGGACTAGCTTT

TTGATTAGTCGTCTACCGCCTAAAAGTGTCATATGCTCACTGACTAACCATATAATAGATTATGTTCTTA

CGAACAATAGACGTATTATTTGGCAGAGTCAAATGATTAATAAGTACGTACTGTTACTGGACCCATCCTT

TTATTATAGATTCAGAAATGCTATCGAAAACAAATTAGACCAATACAATAATCGTTATAATATGTTCGAA

CACGATAGGGACGTTAATGAAAAGTATGGCAAAGTCTTACATGACCTCGATACATATATCAAGGATGTAC

AAGTATTAAAATCTACTTCCATCACTAATAATATAACACTATACGACACTATTATAAATAATAAGTCAGA

GTTTCCTATACGTCGTGCAAACGACAAACAATTAATTAATCTCATAAAATCCAATACATATCATAATCTT

ATCGAAAAAGTTATTAAAAATACATTAGAGAAATATACTTTAACTAATATAGTCCTCGAGTATATGATCT

CATCTCAATCTCAATCTCAATCTCAATCTCAATCATCTTATTTGAGTCGTATTCCTAATGAGATATTACT

CGAAATATTATATAAACTCGACATGTACGATTTACGTAATCTATATACAAGATATATGAGAGAGAATGAT

ATCACAGAGTATCATATAGAGAATACGAGGTCTGTTTCTACACAGACATGAATAATGAATACACATACAA

CGTTTTTTTTTTAATCTTAGATATAACACTAATTACATCAAGATTATATATTGAAATCGTAATTTGAGTT

GTCTGATCATCATGGATATCGAAAATGATATACGTAACATTAGCAATCTTTTTTAGATGATTGATATATT

ATTATGCGATGTAATCATAACTATCGGAGATGTAGAAATTAAAGCGCATAAAACTATTTTGGCTGCCGGA

TCTACGTATTTTAAAACAATGTTCACAACACCTATGATAGCGAGAGATCTAGCAACTAGAGTAAATCTAC

AGATGTTCGATAAGATGCCGTCAAAAATATTGTACAGTACTTATACAATAGGTATATAAGTTCTATGAAT

GTGATAGACGTATTAAAATGCACCGACTAAGAACGTAGAACGAACTATAGAATGTTATACAATGGGTGAT

GATAAGTAGAAGATGTTACCCGATATACCCATAGCATTATCTAGTTATGGCATGTGTGTATTAGATCAAT

ACATATACATTATAGGCGGTCGTACCCAACACTGATTATACATCGGTACATACAGTAAATAGCATAGATA

TGGAGGAGGATACAAATATTTCAAATAAAGTTATGAGATACGCACTGTCAATAATATATGGAAGACATTA

CCTAACTTCTGAACTGGAACTATAAATCCAGGCTCTTGCATAAAGATGAATATATATGTTGTATGCGACA

TCAAAGATGAAAAAATGTTAAGACTTATATATTTAGATATAACACGAATATGTATAACGGATGGGAATTG

GTAACGATGACAGAAAGCAGATTGTCAGCTCTGCATACTATTCTTCATGACAATACCATAATGATGTTAC

ATTGTTATGAAACGTATATGTTACAAGATACATTTAATGTGCTTACGGAACATATATTTAGAAACATCTA

CTAACGATTTTTTATGCTTGTATTATTAATGGTATGTAATATGATTTAATTGATTGTGTACACGATACCA

ATTTGTCAAGTATGAATACGGAGTACAAACATAAACTGAAGTTTAACATTATTTATTTATGATATACATT

ATATACATTATATACATTATATACATTATATACATTATATACATTATATACATTATATACATTATATACA

TTATATACATTATATACATTATATACATTATATACATTATATACATTATATACATTATATACATTATATA

TCGTTATATATCGTTATATATCGTTATTGTTTGGTCTATGCCATGGATATCTTTAAAGAACTAATCTTAA

AACATACGGATGAAAATGTTTTGATTTCTCCAGTTTCCATTTTATCTACTTTATCTATTCTGAATCATGG

AGTAGCTGGTTCTACAGCTGAACAACTATCAAAATATATAGAGAATATGAATGAGAATACACCCGATGAT

AAGAAGGATGACAATAATGACATGGACGTAGATATTCCGTATTGTGCGACACTAGCTACCGCAAATAAAA

TATACGGTAGTGATAGTATCGAGTTCCATGCCTCCTTCCTACAAAAAATAAAAGACGATTTTCAAACTGT

AAACTTTAATAATGCTAACCAAACAAAGGAACTAATCAACGAATGGGTTAAGACAATGACAAATGGTAAA

ATTAATTCCTTATTGACTAGTCCGCTATCCATTAATACTCGTATGACAGTTATTAGCGCCGTCCATTTTA

AAGCAATGTGGAAATATCCATTTTCTAAACATCTTACATATACAGACAAGTTTTATATTTCTAAGAATAT

AGTTACCAGTGTTGATATGATGGTGGGTACCGAGAATGACTTGCAATATGTACATATTAATGAATTATTC

GGAGGATTCTCTATTATCGATATTCCATACGAGGGAAACTCTAGTATGGTGATTATACTGCCGGACGACA

TAGAAGGTATATATAACATAGAAAAAAATATAACAGATGAAAAATTTAAAAAATGGTGTGGTATGTTATC

TACTAAAAGTATAGACTTGTATATGCCAAAGTTTAAAGTGGAAATGACGGAACCGTATAATCTGGTACCG

ATTCTAGAAAATTTAGGACTTACTAATATATTTGGATATTATGCAGATTTTAGTAAGATGTGTAATGAAA

CTATCACTGTAGAAAAATTTCTACATACGGCGTTTATAGATGTTAATGAGGAGTATACAGAAGCATCGGC

CGTTACAGGAGTATTCATGACTAACTTTTCGATGGTATATCGTATGAAGGTCTACATAAACCATCCATTC

ATATACATGATTAAAGATAACACCGGACATACACTTTTTATAGGGAAATACTGCTATCCGCAATAAATAT

AAACAATAGACTTTTATCACGTTATCTCATGTATAAAATATTACAAATAGTATAGCATAAACTAAAGTCG

ATACATACATTAAAACTTAAAAAATAATGTAATTTACAATTAATAGTATAAACTAAAAAAATTAAAAAAT

TAAAAACAATATCATTATTATAAGTAATATCAAAATGACGATATACGGATTAATAGCGTATCTTGTATTC

GTGACTTCATCCATCGCTAGTCCATTTTACATTCCCGTTATTCCGCCCATTTCGGAAGATAAATCGTTCA

ATAGTGTAGAGGTATTAGTTTCTTTGTTTCCCGATGACCAAAAAGACTATACAGTAACTTCTCAGTTCAA

TAACTACACTATCGGTACCAAAGACTGGACTATCAACGTACTATCCACACCTGATGGTCTGGACATACCA

TTGACTAATATAACTTATTGGTCACGGTTTACTATAGGTCGTGCATTGTTCAAATCAGAGTCTGAGGATA

TTTTCCAAAAGAAAATGAGTATTCTAGGTGTTTCTATAGAATGTAAGAAGCCGTCGACATTACTTACTTT

TTTAACCGTGCGTAAAATGACTCGAGTATTTAATAGATTTCCAGATATGGCTTATTATCGAGGAGACTGT

CTAGAAGCCGTTTATGTAACAATGACTTATAAAAATACTAAAACTGGAGAGACTGATTACACGTACCTCT

CTAATGGGGGGTTGCCTGCATACTATCGTAATGGGGTCGATGGTTGATTATTGATTAGTATATTCCTTAT

TCTTTTTATTCACACAAAAAGAACATTTTTATAAACATGAAACCACTGTCTAAATGTAATTATGATCTTG

ATTTATAGATGATGATCAGCCTTCAGAGGATTTTGACCAGCATGTTTAATATGAAAAAAAACATAACTAT

TAAGCGCTATTGCGCTATTGTGCTTAATTATTTTGCTCTATAAACTGAATATATATAGCCACAATTATTG

ACGGGCTTGTTTGTGACCGGTAATCATGAATTTACAGAAATTATCTCTGGCTATATATCTTACGGTGACA

TGTTCGTGGTGTTATGAAACATGTATGAGAAAAACTGCGTTGTATCATGACATTCAATTGGAGCATGTAG

AAGACAATAAAGATAGTGTAGCATCGCTACCGTACAAGTATCTACAAGTAGTCAAACAAAGAGAACGTAG

TAGATTGTTGGCTACATTTAATTGGACGGATATAGCTGAGGGTGTTAGAAATGAGTTCATTAAAATATGT

GATATCAACGGAACATATCTATATAATTATACTATTGATGTTAGTATAATTATTGATTCCACGGAAGAAC

TACCAACAGTTACTCCAATTACAACATATGAACCTTCTATATATAATTATACTATCGATTATAGCACTGT

TATTACTACTGAAGAACTACAAGTGACTCCAACATATGCGCCTGTAACAACTCCTCTTCCAACATCAGCA

GTTCCTTATGATCAACGATCGAATAACAATGTAAGTACTATATCTATTCAGATACTGAGTAAAATATTGG

GAGTCAATGAAACAGAATTAACTAATTATCTTATTATGCATAAAAATGACACTGTTGACAATAACACCAT

GGTTGATGATGAGACATCTGACAATAACACATTGCATGGTAATATAGGATTTTTGGAAATAAATAATTGT

TACAATGTTTCTGTGTCAGATGCTAGTTTTAGAATAACATTAGTAAACGATACTTCTGAAGAAATTTTGC

TAATGCTAACAGGAACTAGTTCATCCGACACCTTCATATCTTCCACCAATATCACTGAATGTTTGAAAAC

ATTAATCAATAATGTGTCGATTAATGATGTACTTATAACACAAAATATGAATGTAACATCTAATTGTGAT

AAATGCTCAATGAATTTGATGGCATCCGTTATTCCTGCAGTTAATGAATTTAACAATACGTTGATGAAAA

TTGGTGTAAAAGATGATGAAAACAATACGGTATATAACTATTATATTTGTAAACTAACTACAAATTCTAC

ATGTGATGAGTTAATCAATTTAGATGAAGTCATTAACAACATAACTCTGACAAATATTATACGCAATAGT

GTTTCGACAACTAACAGCAGAAAAAGACGAGATCTGAATGGTGAGTTTGAATTTTCCACTTCCAAGGAAT

TAGATTGTCTTTACGAATCATATGGTGTAAACGATGATATAAGTCATTGTTTTGCATCACCTAGACGTAG

ACGATCTGACGACAAAAAGGAGTACATGGACATGAAATTATTCGACCACGCGAAAAAAGATTTAGGAATA

GACAGTGTTATTCCTAGAGGTACAACCCATTTCCAAGTAGGTGCATCTGGTGCAAGTGGTGGTGTTGTAG

GAGATAGTTTCCCATTTCAAAATGTTAAATCGCGTGCCAGTCTATTGGCGGAAAAAATAATGCCTAGAGT

ACCTATTACTGCTACCGAAGCTGATCTATATGCAACTGTAAATAGACAACCCAAGTTACCAGCAGGTGTT

AAAAGTACTCCGTTTACAGAGGCGCTTGCGTCTACGATAAACCAAAAGCTTTCTAATGTTAGAGAGGTAA

CTTATGCTTCGTCCAATCTGCCAGGATCAAGTGGCTATGTTCATAGACCATCTGATTCTGTTATTTACAG

CAGTATAAGACGGTCACGTTTACCTAGTGATAGCGATAGTGATTATGAGGATATACAAACTGTTGTTAAG

GAATATAATGAAAGATATGGTAGATCAGTCAGTAGAACACAGTCATCAAGTAGTGAAAGCGATTTTGAAG

ATATAGATACTGTTGTTAGGGAATATAGACAAAAATATGGCAATGCAATGGCAAAAGGACGTAGTAGTTC

CCCTAAACCTGATCCATTATATAGTACTGTTAAGAAAACAACTAAAAGTCTATCTACTGGTGTAGACATA

GTTACAAAACAATCAGACTATTCTCTATTACCTGACGTTAATACTGGCAGTTCTATTGTGTCACCTCTCA

CCAGAAAAGGAGCTACTAGACGACGACCTAGACGCCCTACAAATGATGGTCTACAGAGTCCAAATCCTCC

TCTCCGTAATCCACTTCCTCAACATGATGATTATTCTCCTCCACAAGTACACAGACCTCCGACACTTCCT

CCTAAACCAGTCCAAAATCCGACACAACTTCCCCCTAGACCAGTAGGTCAATTACCTCCTCCTATAGATC

AACCAGATAAAGGATTTAGTAAGTTTGTATCACCTAGACGGTGTAGAAGAGCAAGCTCTGGAGTCATATG

TGGTATGATACAATCAAAACCAAACGATGATACCTATTCACTTCTTCAACGGCCAAAAATTGAACCAGAA

TATGCGGAGGTTGGTAATGGTATACCCAAGAACAATGTTCCTGTAATAGGTAATAAACATAGTAAAAAAT

ATACATCGACGATGTCAAAAATATCAACAAAATTTGATAAATCTACGGCATTTGGAGCAGCAATGTTACT

AACTGGTCAGCAGGCCATTAGCCAACAGACTAGATCAACTACGTTGAGTAGAAAAGATCAGATGAGCAAG

GAAGAAAAGATATTCGAAGCAGTTACAATGAGTCTATCAACTATAGGTTCAACGTTGACGTCTGCAGGTA

TGACGGGTGGTCCAAAACTAATGATTGCAGGAATGGCTATAACGGCTATAACTGGTATAATAGATACGAT

AAAAGATATATATTACATGTTTTCAGGACAGGAGAGGCCAGTAGATCCTGTTATTAAATTATTTAATAAG

TACGCTGGCTTAATGTCCGATAATAATAAAATGGGTGTAAGAAAATGTTTGACACCCGGTGACGACACAC

TTATTTATATCGCATACAGAAACGATACCAGTTTTAAACAGAATACGGATGCGATGGCTTTGTATTTCTT

AGATGTTATCGACTCAGAGATCCTATATCTAAACACATCAAATTTAGTTCTAGAGTATCAACTAAAGGTG

GCTTGCCCCATAGGAACATTAAGATCTGTAGATGTGGACATAACTGCGTATACAATATTATATGATACAG

CGGATAATATTAAGAAATACAAGTTTATCAGAATGGCAACGCTACTATCCAAACATCCAGTTATTAGATT

GACATGTGGTTTAGCAGCAACATTGGTGATTAAACCGTACGAGGTACCCATCAGTGATATGCAACTACTA

AAAATGGCGACGCCTGGTGAACCAGAATCCACTAAATCTATACCATCCGATGTCTGTGATAGGTATCCTC

TAAAGAAATTCTATCTTTTAGCTGGTGGTTGTCCCTATGATACATCTCAAACTTTTATTGTACATACTAC

TTGCAGTATTCTACTAAGAACAGCTACACGGGATCAGTTTAGAAACAGATGGGTGTTACAAAATCCATTT

AGACAAGAAGGGACATATAAGCAACTGTTTACCTTTAGCAAATACGATTTTAACGACACCATAATCGATC

CTAATGGTGTGGTGGGTCATGCTAGCTTTTGTACCAATAGAAGCAGCAACCAATGTTTCTGGTCCGAACC

TATGATATTGGAAGATGTATCATCGTGTAGTTCTAGAACTAGAAAAATATACGTAAAACTGGGAATATTT

AATGCTGAAGGTTTTAATAGTTTTGTACTAAATTGTCCAACTGGGTCTACACCTACATACATCAAACATA

AAAATGCGGACAGTAACAATGTTATCATAGAGCTACCTGTAGGTGATTACGGCACAGCCAAATTGTATTC

AGCAACAAAACCATCGAGGATAGCTGTGTTCTGCACACATAACTATGATAAACGATTCAAATCAGATATT

ATAGTTCTAATGTTTAATAAAAACAGCGGTATTCCATTTTGGAGCATGTACACAGGAAGTGTAACTAGTA

AAAATAGAATGTTTACCACATTGGCTAGAGGAATGCCGTTTAGATCAACGTATTGCGATAACAGACGACG

ATCAGGTTGTTATTATGCAGGAATACCATTTCATGAAGATAGTGTAGAAGCAGATATACATTATGGACCA

GAAATAATGTTAAAGGAAACATATGACATAAACAGTATTGACCCACGAGTTATAACAAAGTCAAAGACCC

ATTTTCCTACTCCATTGAGTGTAAAATTCATGGTTGACAATTTAGGAAATGGATATGACAACCCTAATTC

ATTTTGGGAAGATGCTAAAACTAAGAAACGGACATATAGTGCAATGACGATAAAAGTCCTACCATGTACA

GTGAGAAATAAAAATATAGACTTTGGATATAACTATGGAGATATTATTTCTAATATGGTTTATCTACAAT

CTACTAGTCAGGATTATGGAGATGGTACCAAATATACATTTAAATCCGTAACTAGATCAGATCATGAGTG

TGAATCTAGCTTAGATCTGACGTCTAAGGAAGTAACTGTGACATGTCCTGCGTTTAGTATACCAAGAAAT

ATATCAACATATGAAGGTCTATGCTTTAGTGTTACTACATCTAAAGATCATTGTGCTACAGGTATTGGTT

GGTTAAAATCTAGTGGCTATGGGAAGGAAGATGCTGATAAACCACGTGCTTGTTTTCATCATTGGAATTA

TTACACACTGTCGTTGGATTATTACTGTTCATACGAAGATATTTGGAGAAGCACCTGGCCTGACTATGAT

CCATGTAAGTCATATATCCATATAGAGTATAGAGATACATGGATAGAATCTAACGTGTTACAGCAACCTC

CTTACACATTCGAATTCATTCATGACAATTCTAACGAATATGTGGATAAAGAAATTAGTAACAAATTAAA

TGATCTGTACAATGAATACAAGAAGATTATGGAATATAGCGACGGATCATTGCCGGCGTCTATAAACAGA

TTAGCAAAGGCATTGACTTCAGAGGGTAGAGAAATAGCAAGTGTTAATATAGATGGTAATCTGTTAGATA

TCGCATATCAAGCAGATAAGGAAAAGATGGCCGACATACAGACAAGAATAAATGATATTATTAGAGATTT

GTTTATACACACTCTATCAGACAAAGATATAAAAGACATTATAGAATCCGAAGAAGGTAAGAGATGTTGT

ATAATAGATGTTAAGAACAATCGTGTTAAAAAGTACTATTCTATTGATAATTATCTATGCGGTACTTTAG

ATGATTATATATACACCGTTGTAGAATATAACAAATCCTATGTGTTAGTAAACGATACTTATATGAGCTA

TGACTATCTTGAATCATCAGGTGTAGTTGTTCTATCATGTTATGAAATGACTATAATCTCCTTGGATACA

AAAGACGCCAAAGATGCTATAGAAGATGTGATAGTAGCAAGTGCGGTAGCCGAAGCATTGAATGACATGT

TTAAGGAATTTGATAAAAACGTAAGTGCTATTATAATAAAAGAAGAAGATAATTATCTAAACAGTTCGCC

CGATATCTACCATATAATATATATCATAGGTGGCACTATTCTGCTATTGTTAGTCATTATTTTAATATTG

GCAATTTATATAGCGCGCAATAAATACAGAACCAGGAAATATGAAATAATGAAATATGACAATATGAGCA

TTAAATCTGATCATCATGATAGTCTTGAAACAGTGTCTATGGAAATTATTGATAATCGGTACTAATAAAA

TAGTTTAACTCTTTTAGAACCAGTTTGGTACTGTAATTTCAGTTCATTACTCGTTGAGAATATTGATGAT

TTTTTTTAAAATGAGTATCGGTAGTTACATATTACCATATCATCCATTATATAATCGATGATGCATGTAT

TAAAATACTTTCCGAATAAGTCTTCTAAATATTGTATTAATTATGAAAAACTATGCTATGTGAGATGATT

CAAAGATGTTTAATGATACGATACTAGATTTTATCTCTAGCGAGATTGTTTAGAATCATTTATCATAACT

ATGTTTAATAAATTCATCAACGAATATCGATAAAGACCTCTTGTAATTCGAGTATAGGAAGCAGTATTAC

CATATCAACTTCCGAGTTAACAATTACTCTAAAACATGAGGATTGTACTCCTGTCTTTATTGGAGATTAC

TATTCAGTCGTTGATAAACTAGTAACCTCAGGTTTCTTTACAAACGATAAAGTACAACATCAAGACCTCA

CAACGCAGTGCAAGATTAATCTAGAAATCAAATGTAATTATGGAGGAGAATCTAGACAACTAACACCCAC

GGCGAAGTATACTTTATGCCTCATTCAGAAACGGTAACTGTAGTAGGAGACTGTCTCTCTAATCTCGATG

TATATATAATATATACCAATACGGACGCGATATATTCCGACATGAATGGCGTCGCTTATCATATGTTATA

TCCTAAATGTTGATCATATTCCACAAATGATTGTGAACGAGATTAAATCATCTAACAAATAATTAGTTTT

TATGACATTAACATATAATAAATAAATTAATCATTATTGACTTAACGATGACGAAAGTTATCATCATCTT

AGGATTCTTGATTATTAATACAAATTCGTTGTGTCTATGAAATGTGAACAATGTGTCTCATATTATAATA

CACAAGAATTAAAGTGTTGTAAACTATCTAAGCCAGGAACATATTCAGATCATCGATGTGATAAATACAG

CGATACCATCTGTGGACATTGTCCAAGTGACACATTCACGTCAATATATAATCGTTCTCCTCGGTGTCAT

AGTTGTAGAGGTCACACCTTGTACACCTACCACAAATAGAATCTGTCATTGTGACTCGAATAGTTATCGT

CTCCTTAAAGCTTCTGATGGTAACTGTGTTACATGTGCTCCTAAAACAAAATATGGTCGTGTGTACGGAA

AGAAAGGAGAAAATGATATGGAATACCATTTGTAAGAAATGTCGGAAGGGTACTTATTCAGATATTGTAT

CTGACTCTGATCAATGTAAACCTATGACAAGATAAGACTTACTCGCATCTACTGGATAGACATAAATATC

CTCCTCGTAATAATGAAATATAATATAATATACACTAATTATTAATATCAATCGAGTATTAACATATAAG

TTATTTTTAAACCCCTTTTGGGTTCCGTCCTAAACGGCGTTTCGGTCTGTGTCGCCACCATGGTCACACC

GAGCCTCTGCGTGCTCCTCCATCGAGGACGACTTCAACTATGACAGCTCGGTGGCGTCTGCCAGCGTGCA

CATACGAATGGCATTTCTAAGAAAAGTCTACGGTATCCTTGTCTACAATTTCCTTTAACAACGGCAACAG

CTGCAGTATTTTTATACTTTGAATGCATTCGGACATTTATACAAGGGAGTCCTGTTCTAATATTGGCATC

AATGTTCGGATCTATAGGCTTGATTTTCGCATTGACTTTACACAGACATAAACATCCCCTGAATCTGTAC

CTACTTTGTGGATTTACACTGTTAGAATCTCTAACGCTGGCCTCTGTTGTTACTTTCTATGATGCGCGTA

TCGTTATACAAGCTTTCATGTTGACTACTGCAGTGTTTCTTGCTCTGACTACATGTACTCTACAATCAAA

GAGAGATTTCAGTAAACTTGTAACAGGATTGTTTGCTGCTTTCTGGATTTTAATTTTGTCAGGAGTCTTG

AGGATAAAGTTTAAAATAGAATTAATAAAGAACATATAGGTCATTTTTTAAACATGGATAGAAACCAAGG

TTGTTAGTTAATAATATACAAGATATTTTTTCTCACTCTGATCCATGTAAACCAAGGACGAGAGACACTC

TCATTCCTCATTCACGACACCATTAAAAATGGAAATTAAAGCCCTCTATTAAGCACAGACGGCTACAGGT

CTACCATCAGGTTAACCTTCGTCTACCTTCACAATGGCCTTTCCTTGTGCCCAGTTCAGTCCCTGTCATT

GCCACGCTACTAAGGACTCCCTGAATACCGTGACTGACGTCAGACATTGTCTGACTGAATACATCCTGTG

GGTTTCTCATAGATGGACCCATAGAGAAAGCGCAGGGCCTCTCTACAGGCTTCTCATCTCTTTCAGAATT

GATGCAATGGAGCTATTTGGTAGCGAGTTGAAGGAGTTCTCGGATTCACTTCCGTGGGACAATATCGACA

ATTGCGTGGAGATCATTAAATGTTTCATCAGAAATGACTCCATGAAAACCGCCAAAGAACTTTGTGCAAT

AATTGGACTTTGTACTCAATCAGCTATTGTCACTGGAAGAGTCTTCAATGATAAGTATATCGACATACTA

CTTATGCTGCGAAAGATTCTGAATGAGAACGACTATCTCACCCTCTTGGATCATATCCTCACTGCTAAAT

ACTAAATCTCCTTCATGCTCTCTCACTAATACTCTTACTCACTACACTTTTTATCATCTTATGATGAATG

ATTGCCTTCATCATTTTTCGTGGAATATAATATAGGAATAATTAGCACCAGAATAGCTATGGATATCTCG

TTAAGAATATTCTCGATAAGAGACATAATGTAGACATAGTTATTATATCCTTCTTAGATAAGTGTTACGC

TACTGGAAAGTTTCCATCGTTATTATTACGTGAAGATGATATCATTAAACCAACATTGAGATTGGCTCTT

ATGTTAGCTGGATTGAATTACTGTAATAAATGCATCGAGTATAGAGGGATATAGCAATTCTCGATAATAG

TCATGCAATATTTGAATGAGACTGATAATTTAGGTAATACAGTACTACACACACATATCTTTCTAGATTA

TATATCGTTAAAAATCTGTAAGAGGTATATTTCTCATAAGTATCCACTGTGTAATATTATTAATGGATAT

ATAGATAACACAATAGGGACTAATAGTATTGTAAAAGATATAATCGACTATTTGTACATATCCAGATATC

TATATTCCTACTAGTTTGCTGCGTAGTTGCATCATTGATATGCATGATTTATCAGGATTCAGAGATGAAT

TACTAAGTAAACTACAATCCCACAATAAGTAAGAATCAAATATCAAAAACTCACTTTTGATTTTTCTAGT

CTTAAGTAATACATATATTTATTAATAGACCTATGAAATAAAAAAGTTAACAATGGATTCGCGTATAGCT

ATTTACGTATTAGTATCGGCATCTCTTTTGTATCTTGTTAATTGTCACAAACTAGTACATTACTTCAATC

TGAAAATAAATGGAAGTGATATAACTAATACAGCAGATATATTGCTGGACAATTATCCAATTATGACCTT

TGATGGAAAGGATATTTATCCATCTATCGCGTTCATGGTCGGTAATAAACTTTTCCTAGATCTTTATAAA

AATATCTTTGTAGAATTTTTCAGACTATTTCGAGTATCTGTAAGTAGTCAATACGAGGAATTAGAATATT

ATTATTCATGTGATTATACTAACAACCGTCCTACAATTAAACAACATTACTTTTATAACGGCGAAGAATA

TACTGAAATTGATAGATCGAAAAAAGCCACTAATAAAAACAGTTGGTTAATTACTTCAGGCTTTAGACTA

CAAAAATGGTTCGATAGCGAAGATTGTATAATTTATCTCAGATCTTTAGTTAGAAGAATGGAAGACAGTA

ACAAAAACAGTAAAAAAACTTAGTACTTAGATATCGAAAAAAAATATATTTTTGTAGACTCTTGAGAATA

GAAGGAAAACATGTACATAATTATAAAAAATGAAAATCAATGGCGAATAAGACAGTGCGATTCGCGCCAT

GGAGTCGGCAGATTTCATGGCTGTCGATGAGCAGTTTCACGACGACCTCGATCTTTGGTCATTATCTTTG

GTAGATGACTATAAAAAACATGGATTAGGTGTTGACTGTTATGTTCTAGAACCAGTTGTTGACAGGAAAA

TATTTGATAGATTTCTCCTTGAACCAATTTGTGATCCTGTAGATGTTCTGTATGATTATTTTAGGATTCA

TAGAGATAATATTGATCAGTATATAGTAGATAGACTGTTTGCATATATTACATATAAAGATATTATATCT

GCATTAGTGTCAAAGAATTATATGGAAGATATTTTCTCTATAATTATTAAGAATTGTAATTCTGTACAAG

ATCTCTTACTTTACTATCTATCTAATGCATATGTAGAAATAGACATTGTTGATTTTATGGTAGATCATGG

GGCTGTAATATATAAAATAGAATGCTTGAATGCCTATTTTAGGGGAATATGTAAAAAGGAAAGTAGTGTT

GTTGAGTTTATTTTGAATTGTGGTATCCCAGATGAAAATGATGTTAAATTAGATCTATATAAAATAATTC

AGTATACTAGGGGATTCCTTGTAGATGAACCCACAGTATTAGAAATTTATAAGCTTTGTATCCCATATAT

TGAAGATATCAATCAACTAGATGCTGGTGGAAGGACCTTGCTTTATCGCGCTATCTATGCAGGTTATATA

GATTTAGTATCATGGCTATTAGAAAATGGAGCAAATGTCAACGCAGTAATGAGTAATGGATATACATGTC

TTGACGTGGCCGTGGATAGGGGATCTGTCATCGCCCGTAGGGAAACACATCTTAAAATATTAGAAATATT

GCTTAGAGAACCATTGTCTATTGACTGTATAAAATTAGCTATACTTAATAATACAATTGAAAACCATGAT

GTGATAAAGCTCTGTATCAAGTATTTTATGATGGTAGATTATTCACTTTGTAATGTGTATGCATCATCAC

TCTTTGATTATATAATTGATTGTAAACAAGAATTGGAGTACATTAGGCAGATGAAAATTCATAATACAAC

CATGTATGAGTTAATCTATAATAGAGACAAAAACAAGCATGCTTCCCATATTCTACATAGGTATTCTAAA

CATCCAGTTTTGACACAGTGTATCACTAAAGGATTCAAGATTTACACAGAAGTAACCGAGCAGGTCACTA

AAGCTCTAAACAGACGTGCTCTAATAGATGAGATAATAAACAATGTATCAACTGATGACAATCTCCTATC

AAAACTTCCATTAGAAATTAGGGATCTAATTGTTTCACAAGCTGTCATATAGAGTTCTCTCCACCCACCT

TTCTTGAAATGAGTTAATAGTCATAAGTTAGTTAAGTCATAAGTTAGTTTTATAAGTTAGTTTATAGTCT

AACACTTCTAATTTTTATACCTTGATCTTTTTCTCTAATTATGAAAAAGTAAATCATTATGAAGATGGAT

GAAAATGGACGAGATTGTGCGCATCGTTAACGATAGTATGTGGTACGTACCTAACGCATTTATGGACGAC

GGTGATAATGAAGGTCACATTTCTGTCAATAATGTCTGTCATATGTATCTCGCATTCTTTGATGTGGATA

TATCATCTCATCTGTTTAAATTAGTTATTAAACACTGCGATCTGAATAAACGACTAAAATGTGGTAACTC

TCCATTACATTGCTATACGATGAATACACGATTTAATCCATCTGTATTAAAGATATTGTTACGCCACGGC

ATGCGTAACTTTGATAGCAAGGATAAAAAAGGACATATTCCTCTACACCACTATCTGATTCATTCACTAT

CAATCGATAACAAGATCTTTGATATACTAACGGACCCCATTGATGACTTTAGTAAATCATCCGATCTATT

GCTGTGTTATCTTAGATATAAATTCAATGGGAGCTTAAACTATTACGTTCTGTACAAATTATTGACTAAA

GGATCTGACCCTAATTGCGTCGATGAGGATGGACTCACTTCTCTTCATTACTACTGTAAACACATATCCG

TGTTCCACGAAAGCAATTATTACAAGTCAAAGAGTCACACTAAGATGCGAGCTGAGAAGCGATTCATCTA

CGCGATAATAGATCATGGAGCAAACATTAACGCGGTTACGAAAATCGGAAATACGCCGTTACACACTTAC

CTTCAACAGTATACCAAACATAGTCCTCGTGTGGTGTATGCTCTTTTATCTCGAGGAGCCGATACGAGGA

TACGTAATAATCTTGATTGTACACCCATCATGGAATACATAAAGAACGATTGTGCAACAGGTCATATTCT

CATAATGTTACTCAATTGGCACGAACAAAAATACGGGAAATTACAAAAGGAAGAAGGACAACATCTACTT

TATCTATTCATAAAACATAATCAAGGATATGGAAGTCGCTCTCTCAATATACTACGGTATCTACTAGATA

GATTCGACATTCAGAAAGACGAATACTATAATACAATGACTCCTCTTCATACCGCCTTCCAGAATTGCAA

TAACAATGTTGCCTCATACCTCGTATACATCGGATACGACATCAACCTTCCGACTAAAGACGATAAGACA

GTATTCGACTTGGTGTTTGAAAACAGAAACATCATATACAAGGCGGATGTCGTTAATGACATCATCCACC

ACAGACTGAAAGTATCTCTACCTATGATTAAATCGTTGTTCTACAAGATGTCGGAGTTCTCTCCCTACGA

CGATCACTACGTAAAGAAGATAATAGCCTACTGCCTATTAAGGGACGAGTCATTTGCGGAACTACATACT

AAATTCTGTTTAAACGAGGACTATAAAAGTGTATTTATGAAAAATATATCATTCGATAAGATAGATTCCA

TCATCGAAAAATGTAGTCGTGACATAAGTCTCCTCAAAGAGATTCGAATCTCAGACACCAACTTGTATAC

GGTATTGAGAACAGAAGACATCCGGTATCACACCTATCTCGAAGCCATACATTCAGACAAACGCATTTCA

TTTCCCATGTACGACGATCTCATAGAACAGTGTCATCTATCGATGGAGCATAAAAGTAAACTCGTCGACA

AAGCACTCAATAAATTAGAGTCTACCATCGATAGTCAATCTAGACTATCGTATTTGCCTCCGGAAATTAT

GCGCAATATCATAACCAAGCTAAGCGACTACCATCTAAACAGTATGTTGTACGGAAAGAACCATTACAAA

TATTATCCATGATAGAAAGAAAATATTTAAAAAATAATCTATATGATTGGAGAAGTAGGAAACAAACAGT

AACAAGACGACGATTACTACTATTATTAAATCATGAGGTCCGTATTATACTCGTATATATTGTTTCTCTC

ATGTATAATAATAAACGGAAGAGATTTAGCACCACATGCACCATCCAATGGAAAATGTAAAGACAACGAA

TACAGAAGCCGTAATCTATGTTGTCTATCGTGTCCTCCGGGAACTTACGCTTCCAGATTATGTGATAGCA

AGACTAATACACAATGTACGCCGTGTGGTTCGGATACCTTTACATCTCACAATAATCATTTACAGGCTTG

TCTAAGTTGTAACGGAAGATGTGATAGTAATCAGGTAGAGACGCGATCGTGTAACACGACTCACAATAGA

ATCTGTGAATGCTCTCCAGGATATTATTGTCTTCTCAAAGGATCATCAGGGTGTAGAACATGTATTTCTA

AAACAAAGTGTGGAATAGGATACGGAGTATCCGGATACACGTCTACCGGAGACGTCATCTGTTCTCCGTG

TGGTCCCGGAACATATTCTCACACCGTCTCTTCCACAGATAAATGCGAACCCGTAACCAGCAATACATTT

AACTATATCGATGTGGAAATTAACCTGTATCCAGTCAACGACACATCGTGTACTCGGACGACCACTACCG

GTCTCAGCGAATCCATCTCAACGTCGGAACTAACTATTACCATGAATCATAAAGATTGTGATCCCGTCTT

TCGTGCAGAATACTTCTCTGTCCTTAATAATGTAGCAACTTCAGGATTCTTTACAGGAGAAAATAGATAT

CAGAATACTTCAAAGATATGTACTCTGAATTTCGAGATTAAATGTAACAACAAAGATTCATCTTCCAAAC

AGTTAACGAAAACAAAGAATGATACTATCATGCCGCATTCAGAGACGGTAACTCTAGTGGGCGACTGTCT

ATCTAGCGTCGACATCTACATACTATATAGTAATACCAATACTCAAGACTACGAAACGGATACAATCTCT

TATCATATGGGTAATGTTCTCGATGTCAATAGCCATATGCCCGCTAGTTGCGATATACATAAACTGATCA

CTAATTCCCAGAATCCCACCCACTTATAGTAAGTTTTTTTTTACCCATAAATAATAAATACAATAATTAA

TTTCTCGTAAAAGTAGAAAATATATTCTAATTTATTATATGGTAAGAAAGTAGAATCATCTAGAACAGTA

ATCAATCAATAGCAATCATGAAACAATATATTGTCCTGGCATGCATGTGCCTAGTGGCAGCTGCTATGCC

TACTAGTCTTCAACAATCCTCATCCTCGTGTACTGAAGAAGAAAACAAACATCATATGGGAATCGATGTT

ATTATCAAAGTCACAAAGCAAGACCAAACACCGACCAATGATAAGATTTGTCAATCCGTAACGGAAGTTA

CAGAGACCGAAGATGATGAGGTATCCGAAGAAGTTGTAAAAGGAGATCCCACCACTTATTACACTATCGT

CGGCGCGGGTCTTAACATGAACTTTGGATTCACCAAATGCCCAAAGATTTCATCCATCTCCGAATCCTCT

GATGGAAACACTGTGAATACTAGATTGTCCAGCGTGTCACCAGGACAAGGTAAGGACTCTCCCGCGATCA

CGCGTGAAGAAGCTCTGGCTATGATCAAAGACTGTGAGATGTCTATCGACATCAGATGTAGCGAAGAAGA

GAAAGACAGCGACATCAAGACCCATCCAGTACTTGGGTCTAACATCTCACATAAGAAAGTGAGTTACAAA

GATATCATCGGTTCAACGATCGTTGATACAAAATGTGTTAAGAACCTAGAGTTTAGCGTACGTATCGGAG

ACATGTGTGAGGAATCATCTGAACTTGAAGTCAAGGATGGATTCAAGTATGTCGACGGATCGGCATCTGA

AGGTGCAACCGATGATACTTCACTCATCGATTCAACAAAACTCAAAGCATGTGTCTGAATCGATAACTCT

ATTCATCTGAAAATGGATGAGTTGGGTTAATCGAACGATTCAGACACCGCACCACGAATTAAAAAAGTGT

ACCGGGTACTATATTCCGGTTTGCAAAACAAAAATGTTTAACTACATTCACAAAAAGTTACCTCTCGTTA

CTTCTTCTTTCTGTTTCAATATGTGATACGATATGATCACTATTCGTATTCTCTTTCTCTCTTGTCTCAT

AAAAAGTTTTACAAAAAAATATTTTTATTCTCTTTCTCTCTTCGATGGTCTCACAAAAATATTAAACCTC

TTTCTGATGTCTCAACTATTTCGTAAACGATAACGTCCAACAATATATTCTCGTAGAGCTTATCAATATC

CTTATGCCAATCTAGGTTGTCAGACAATTGCATCATAAAATAATGTTTATAATTTACACGTTAACATCAT

ATAATAAACGTATATAGTTAATATTTTTGGAATATAAATGATCTGTAAAATCCATGTAGGGGACACTGCT

CACGTTTTTTCTCTAGTACATAATTTCACACAAGTTTTTATACAGACAAATTAATTCTCGTCCATATATT

TTAAAACATTGACTTTTGTACTAAGAAAAATATCTTGACTAACCATCTCTTTCTCTCGTCGATGGGTCTC

ACAAAAATATTAAACCTCTTTCTGATGGAGTCGTAAAAAGTTTTTATCCTTTCTCTCTTCGATGGGTCTC

ACAAAAATATTAAACCTCTTTCTGATGGTCTCTATAAACGATTGATTTTTCTTACCCTCTAGAGTTTCCT

ACGGTCGTGGGTCACACATTTTTTTCTAGACACTAAATAAAATAGTAAAATTAAATTAATTATAAAATTA

TGTATATAATTTACTAAC
