# Supplementary material for: Exploring the key genomic variation in monkeypox virus during the 2022 outbreak
Source: BMC Genom Data. 2023 Nov 16;24:67. doi: 10.1186/s12863-023-01171-0 (PMC10652487; doi:10.1186/s12863-023-01171-0)
Supplement: Supplementary file 5 — Additional file 5. Full length sequence of RS2. [file 12863_2023_1171_MOESM5_ESM.docx]

**Additional file5. Full length sequence of RS2.**

GTTAGTAAATTATATACATAATTTTATAATTAATTTAATTTTACTATTTTATTTAGTGTCTAGAAAAAAATGTGTGACCCACGACCGTAGGAAACTCTAGAGGGTAAGAAAAATCAATCGTTTATAGAGACCATCAGAAAGAGGTTTAATATTTTTGTGAGACCCATCGAAGAGAGAAAGGATAAAAACTTTTTAcgactccatcagaaagaggtttaatatttttgtgagattgtgagacccatcgacgagagaaaggacccatcgacgagagaaaggataaaaactttttacgactccatcagaaagaggtttaatatttgataaaaactttttaCGACTCCATCAGAAAGAGGTTTAATATTTTTGTGAGACCCATCGACGAGAGAAAGAGATGGTTAGTCAAGATATTTTTCTTAGTACAAAAGTCAATGTTTTAAAATATATGGACGAGAATTAATTTGTCTGTATAAAAACTTGTGTGAAATTATGTACTAGAGAAAAAACGTGAGCAGTGTCCCCTACATGGATTTTACAGATCATTTATATTCCAAAAATATTAACTATATACGTTTATTATATGATGTTAACGTGTAAATTATAAACATTATTTTATGATGCAATTGTCTGACAACCATTGGCATAAGGATATTGATAAGCTCTACGAGAATATATTGTTGGACGTTATCGTTTACGAAATAGTTGAGACATCAGAAAGAGGTTTAATATTTTTGTGAGACCATCGAAGAGAGAAAGAGAATAAAAATATTTTTTTGTAAAACTTTTTTATGAGACAAGAGAGAAAGAGAATACGAATAGTGATCATATCGTATCACATATTGAAACAGAAAGAAGAAGTAACGAGAGGTAACTTTTTGTGAATGTAGTTAAACATTTTTGTTTTGCAAACCGGAATATAGTACCCGGTACACTTTTTTAATTCGTGGTGCGGTGTCTGAATCGTTCGATTAACCCAACTCATCCATTTTCAGATGAATAGAGTTATCGATTCAGACACATGCTTTGAGTTTTGTTGAATCGATGAGTGAAGTATCATCGGTTGCACCTTCAGATGCCGATCCGTCGACATACTTGAATCCATCCTTGACTTCAAGTTCAGATGATTCCTCACACATGTCTCCGATACGTACGCTAAACTCTAGGTTCTTAACACATTTTGTATCAACGATCGTTGAACCGATGATATCTTTGTAACTCACTTTCTTATGTGAGATGTTAGACCCAAGTACTGGATGGGTCTTGATGTCGCTGTCTTTCTCTTCTTCGCTACATCTGATGTCGATAGACATCTCACAGTCTTTGATCATAGCCAGAGCTTCTTCACGCGTGATCGCGGGAGAGTCCTTACCTTGTCCTGGTGACACGCTGGACAATCTAGTATTCACAGTGTTTCCATCAGAGGATTCGGAGATGGATGAAATCTTTGGGCATTTGGTGAATCCAAAGTTCATGTTAAGACCCGCGCCGACGATAGTGTAATAAGTGGTGGGATCTCCTTTTACAACTtcttcggatacctcatcaTCTTCGGATACCTCATCATCTTCGGTCTCTGTAACTTCCGTTACGGATTGACAAATCTTATCATTGGTCGGTGTTTGGTCTTGCTTTGTGACTTTGATAATAACATCGATTCCCATATGATGTTTGTTTTCTTCTTCAGTACACGAGGATGAGGATTGTTGAAGACTAGTAGGCATAGCAGCTGCCACTAGGCACATGCATGCCAGGACAATATATTGTTTCATGATTGCTATTGATTGATTACTGTTCTAGATGATTCTACTTTCTTACCATATAATAAATTAGAATATATTTTCTACTTTTACGAGAAATTAATTATTGTATTTATTATTTATGGGTAAAAAAAACTTACTATAAGTGGGTGGGATTCTGGGAATTAGTGATCAGTTTATGTATATCGCAACTAGCGGGCATATGGCTATTGACATCGAGAACATTACCCATATGATAAGAGATTGTATCCGTTTCGTAGTCTTGAGTATTGGTATTACTATATAGTATGTAGATGTCGACGCTAGATAGACAGTCGCCCACTAGAGTTACCGTCTCTGAATGCGGCATGATAGTATCATTCTTTGTTTTCGTTAACTGTTTGGAAGATGAATCTTTGTTGTTACATTTAATCTCGAAATTCAGAGTACATATCTTTGAAGTATTCTGATATCTATTTTCTCCTGTAAAGAATCCTGAAGTTGCTACATTATTAAGGACAGAGAAGTATTCTGCACGAAAGACGGGATCACAATCTTTATGATTCATGGTAATAGTTAGTTCCGACGTTGAGATGGATTCGCTGAGACCGGTAGTGGTCGTCCGAGTACACGATGTGTCGTTGACTGGATACAGGTTAATTTCCACATCGATATAGTTAAATGTATTGCTGGTTACGGGTTCGCATTTATCTGTGGAAGAGACGGTGTGAGAATATGTTCCGGGACCACACGGAGAACAGATGACGTCTCCGGTAGACGTGTATCCGGATACTCCGTATCCTATTCCACACTTTGTTTTAGAAATACATGTTCTACACCCTGATGATCCTTTGAGAAGACAATAATATCCTGGAGAGCATTCACAGATTCTATTGTGAGTCGTGTTACACGATCGCGTCTCTACCTGATTACTATCACATCTTCCGTTACAACTTAGACAAGCCTGTAAATGATTATTGTGAGATGTAAAGGTATCCGAACCACACGGCGTACATTGTGTATTAGTCTTGCTATCACATAATCTGGAAGCGTAAGTTCCCGGAGGACACGATAGACAACATAGATTACGGCTTCTGTATTCGTTGTCTTTACATTTTCCATTGGATGGTGCATGTGGTGCTATATCTCTTCCGTTTATTATTATACATGAGAGAAACAATATATACGAGTATAATACGGACCTCATGATTTAATAATGTAGTAATCGTCGTCTTGTTACTGTTTGTTTCCTACTTCTCCAATCATATAGATTATTTTTTAAATATTTTCTTTCTATCATGGATAATATTTGTAATGGTTCTTTCCGTACAACATACTGTTTAGATGGTAGTCGCTTAGCTTGGTTATGATATTGCGCATAATTTCCGGAGGCAAATACGATAGTCTAGATTGACTATCGATGGTAGACTCTAATTTATTGAGTGCTTTGTCGACGAGTTTACTTTTATGCTCCATCGATAGATGACACTGTTCTATGAGATCGTCGTACATGGGAAATGAAATGCGTTTGTCTGAATGTATGGCTTCGAGATAGGTGTGATACCGGATGTCTTCTGTTCTCAATACCGTATACAAGTTGGTGTCTGAGATTCGAATCTCTTTGAGGAGACTTATGTCACGACTACATTTTTCGATGATGGAATCTATCTTATCGAATGATATATTTTTCATAAATACACTTTTATAGTCCTCGTTTAAACAGAATTTAGTATGTAGTTCCGCAAATGACTCGTCCCTTAATAGGCAGTAGGCTATTATCTTCTTTACGTAGTGATCGTCGTAGGGAGAGAACTCCGACATCTTGTAGAACAACGATTTAATCATAGGTAGAGATACTTTCAGTCTGTGGTGGATGATGTCATTAACGACATCCGCCTTGTATATGATGTTTCTGTTTTCAAACACCAAGTCGAATACTGTCTTATCGTCTTTAGTCGGAAGGTTGATGTCGTATCCGATGTATACGAGGTATGAGGCAACATTGTTATTGCAATTCTGGAAGGCGGTATGAAGAGGAGTCATTGTATTATAGTATTCGTCTTTCTGAATGTCGAATCTATCTAGTAGATACCGTAGTATATTGAGAGAGCGACTTC

CATATCCTTGATTATGTTTTATGAATAGATAAAGTAGATGTTGTCCTTCTTCCTTTTGTAATTTCCCGTATTTTTGTTCGTGCCAATTGAGTAACATTATGAGAATATGACCTGTTGCACAATCGTTCTTTATGTATTCCATGATGGGTGTACAATCAAGATTATTACGTATCCTCGTATCGGCTCCTCGAGATAAAAGAGCATACACCACACGAGGACTATGTTTGGTATACTGTTGAAGGTAAGTGTGTAACGGCGTATTTCCGATTTTCGTAACCGCGTTAATGTTTGCTCCATGATCTATTATCGCGTAGATGAATCGCTTCTCAGCTCGCATCTTAGTGTGACTCTTTGACTTGTAATAATTGCTTTCGTGGAACACGGATATGTGTTTACAGTAGTAATGAAGAGAAGTGAGTCCATCCTCATCGACGCAATTAGGGTCAGATCCTTTAGTCAATAATTTGTACAGAACGTAATAGTTTAAGCTCCCATTGAATTTATATCTAAGATAACACAGCAATAGATCGGATGATTTACTAAAGTCATCAATGGGGTCCGTTAGTATATCAAAGATCTTGTTATCGATTGATAGTGAATGAATCAGATAGTGGTGTAGAGGAATATGTCCTTTTTTATCCTTGCTATCAAAGTTACGCATGCCGTGGCGTAACAATATCTTTAATACAGATGGATTAAATCGTGTATTCATCGTATAGCAATGTAATGGAGAGTTACCACATTTTAGTCGTTTATTCAGATCGCAGTGTTTAATAACTAATTTAAACAGATGAGATGATATATCCACATCAAAGAATGCGAGATACATATGACAGACATTATTGACAGAAATGTGACCTTCATTATCACCGTCGTCCATAAATGCGTTAGGTACGTACCACATACTATCGTTAACGATGCGCACAATCTCGTCCATTTTCATCCATCTTCATAATGATTTACTTTTTCATAATTAGAGAAAAAGATCAAGGTATAAAAATTAGAAGTGTTAGACTATAAACTAACTTATAAAACTAACTTATGACTTAACTAACTTATGACTATTAACTCATTTCAAGAAAGGTGGGTGGAGAGAACTCTATATGACAGCTTGTGAAACAATTAGATCCCTAATTTCTAATGGAAGTTTTGATAGGAGATTGTCATCAGTTGATACATTGTTTATTATCTCATCTATTAGAGCACGTCTGTTTAGAGCTTTAGTGACCTGCTCGGTTACTTCTGTGTAAATCTTGAATCCTTTAGTGATACACTGTGTCAAAACTGGATGTTTAGAATACCTATGTAGAATATGGGAAGCATGCTTGTTTTTGTCTCTATTATAGATTAACTCATACATGGTTGTATTATGAATTTTCATCTGCCTAATGTACTCCAATTCTTGTTTACAATCAATTATATAATCAAAGAGTGATGATGCATACACATTACAAAGTGAATAATCTACCATCATAAAATACTTGATACAGAGCTTTATCACATCATGGTTTTCAATTGTATTATTAAGTATAGCTAATTTTATACAGTCAATAGACAATGGTTCTCTAAGCAATATTTCTAATATTTTAAGATGTGcTTCCCTACGGGCGATGACAGATCCCCTATCCACGGCCACGTCAAGACATGTATATCCATTACTCATTACTGCGTTGACATTTGCTCCATTTTCTAATAGCCATGATACTAAATCTATATAACCTGCATAGATAGCGCGATAAAGCAAGGTCCTTCCACCAGCATCTAGTTGATTGATATCTTCAATATATGGGATACAAAGCTTATAAATTTCTAATACTGTGGGTTCATCTACAAGGAATCCCCTAGTATACTGAATTATTTTATATAGATCTAATTTAACATCATTTTCATCTGGGATACCACAATTCAAAATAAACTCAACAACACTACTTTCCTTTTTACATATTCCCCTAAAATAGGCATTCAAGCATTCTATTTTATATATTACAGCCCCATGATCTACCATAAAATCAACAATGTCTATTTCTACATATGCATTAGATAGATAGTAAAGTAAGAGATCTTGTACAGAATTACAATTCTTAATAATTATAGAGAAAATATCTTCCATATAATTCTTTGACACTAATGCAGATATAATATCTTTATATGTAATATATGCAAACAGTCTATCTACTATATACTGATCAATATTATCTCTATGAATCCTAAAATAATCATACAGAACATCTACAGGATCACAAATTGGTTCAAGGAGAAATCTATCAAATATTTTCCTGTCAACAACTGGTTCTAGAACATAACAGTCAACACCTAATCCATGTTTTTTATAGTCATCTACCAAAGATAATGACCAAAGATCGAGGTCGTCGTGAAACTGCTCATCGACAGCCATGAAATCTGCCGACTCCATGGCGCGAATCGCACTGTCTTATTCGCCATTGATTTTCATTTTTTATAATTATGTACATGTTTTCCTTCTATTCTCAAGAGTCTACAAAAATATATTTTTTTTCGATATCTAAGTACTAAGTTTTTTTACTGTTTTTGTTACTGTCTTCCATTCTTCTAACTAAAGATCTGAGATAAATTATACAATCTTCGCTATCGAACCATTTTTGTAGTCTAAAGCCTGAAGTAATTAACCAACTGTTTTTATTAGTGGCTTTTTTCGATCTATCCTCGTCCTCATCATCCTTATATTATTATCATTATTATCATAGTCTATTAAACACAAATCATCTACGTTTATAACAACATTCTCATTATTAATTAGTTCTGTAGTAATATCTTTAATAATTTGGCTATACATCTGTTCAATACTATCTATTGATGATTTCTTTTTTAAGACTTAAACTAGTTATGGTAAT

GACGATGAAATCGAGTAGTAACTTCTAATAAAGACTTGATATCATTATCATATGTTTGATCGTCATAGTTAATAGTGTGGCTAAATGGTACTGTTAATAAGTTTATAGACAATATCATAGTATTTTCTTTCCAGAATTAGATTATTTTTTTAAATACTGATCCTCACAATTCCGTGATGTAGCAGTAGTTGGTGCATGGTCTATATCGTTAAAATGTATCATATATAATAGTTTTCTGACGTGGAGTACAGAATTTTCGATTAATGAGTTCATGGTAAGGAAGGGCAAATGTCTGTATATAATATACATAAGTTAATAGTTTTTTATCATATTTTCTAATACCATAATAAAAATTATCATTATGTATAATCATCACTGTCGCTATCATTATTGCGTTTGTGTAGTTCTGCCCTATCATCTACATCACTGTCACTCTCACTATATCTTCTAAAATTACAAACAACTGGATATTCGATAACAGCATTTGTGTAGTTTTTGTCTTTTACAGTATATACGTTATTGTCAAAATCTAAACAAATATTAGCATAATACATCTATAAGATCAGGATCCATGTTCGAGCATACTAGCCATGTATATTTGTAACTTCGTCATACAGCGTTAGATCAATAGAATAAACAATCGTGTGACGCAACTTTTTTACGATCTAGTTGTATGAGTTTATCGTTTACATAAGCAATTAACGGCTTTAACAGATGATCTGAGTAATAATATACCTCTGTTATACGTTTAATGTTCACGGTCTTAGTATTTTTAGATATCAATTGTGATTTACACCATATTCGACTCCCTGTGTGCAACGTTAGAAATTCTAAATCTATAGTATTATCTATTACAGCGTAAAACACATTCAATATTGTATTGTTATTTTTATATTATTTACacaattAACAATGTATTATTAGTTTATATTACTGAATTAATAATATAAAATTCCCAATCTTGTCATAAACACAAAATCCATTAAAAATGTCGATAAAATATCTGATGTTGTTGTTCGCTGCTATGATAATCAGATCATTAGCCGATAGTGGTAACGCTATCGAAACAACATCGCCAGAAATTACAAACGCTACAACAGATATTCCAGCTATCAGATTATGTGGTCCAGAGGGAGATGGATATTGTTTACACGGTGACTGTATaCACGCTAGAGATATCGACGGTATGTATTGTAGATGCTCTCATGGTTATACAGGCATTAGATGTCAGCATGTAGTATTAGTAGACTATCAACGTTCAGGAAAACCAGACACTACAACATCATATATCCCATCTCTAGGTATTGTGCTTGTATTAGTAGGCATTATTATTACGTGTTGTCTATTATCTGTTTATAGGTTCACTCGAAGAACTAAACTACCTATACAAGATATGGTTGTGTTATATTTTTTATAAAATTTTTTTATGAGTATTTTTACAAAAATGTATATGTATAAAAAAATACTAAGTATGCG

ATGTATCCTGTATTATTTGTATTTATCTAAACAATACTTCTGCCTCTAGATGGGATACAAAAATTTTTTATTTCAGCATATTAAAGTAAAATCTAGTTACCTTGAAAATGAATACAGTGGGTGGTTCCGTATCACCAGTAAGAACATAATAGTCGAATACAGTATCCGATTGAGATTTTGCATACAATACTAGTCTAGAAAGAAATTTATAATCATCTTCTGTGACAGGAGTCCATATATCTGTATCATCGTCTAGTTTATCAGTGTCCTATGcTATATTCCTGTTATCATCATTAGTTAATGAAAATAACTCTCGTGCTTCAGAAAAGTCAAATATTGTATCCATACATCTCCAAAACTATCACTTATACGTTTATCTTTAACGAACATATACCTGATGGTTATTTACTAACAGACATTTTTCAAGATCTATTGACAATAACTCCTATAGTTTCCACATCAACCAAGTAATGATCATCTATTGTTATAT

AACAATAACATAACTCTTTTCCATTTTTATCAGTATATATATATATATATATATATCAACGTCGTTGTAGTGAATAGTAGTCATTGATCTATTATATGAAACGGATATGTCTAGTTAATATTTTCTTTGATTTAAAGTCTAGAGTCTTTACAAACATAATATCCTTATCCGACTTTATATTTCCTGTAGGGTGGCATAATTTTATTCTGCCTCCACAATCAGTGTTTCCAAATATATTACTAGACAATATTCCATATGGTTATTAGTTAAGGGACCCGATTAGAACACGTACGCGCTTATTCATCATTTGGATCGTATTTCATAAAAGTTATTATGTTATCGATGTCAACACATTCTACATTTTTTAATCGTCTATATAGTATTTTTCTGATATTTTCTATAATATCAGAATTGTCTTCCATAGGAAGTTGTATACTATCGGAATCAGTTACATGTTTAAATAATTCTCTGATGTCATTCCTTATACAATCAAATTCATTATTAAACAGTTTAATAGTCTGTAGACCTTTATCGTCGTACATATCCATTGTCTTATTAGTTACGCTTATTTTTATGGGTTTTACATTGCTTTATTATATTTTATAAGAATGATTGTTTGACAATGTCGTAGTATAGATATATTATTAGAGGAGGTATAATTATAAAAAGTTTCTGAGTACGATGTTATAAGAGGAGAGGACACATTAACAATCATACATCAATTAACTCATTCTTATAACATTGTAATCAAAAGAATTGCAATTTTGATGTATAACAACTGTCAATGGAATTGTATATTACAAATTACGGTATGTTGTAACGACAAATACCGATCGGTAATTGTCTCTGTCGCTGTAATAGAATTAATTATATATCTATTACACCGGCCTTGTATCATAATAAAGTTGTGGTAGTATGATCTCCATATTTATAATTTAGTACTTTGTATTTAGTATTTTTGGAATCATAAAAAAGTTTTACTAATTTAAAATTTAAAAAGTATTTACATTTTTTTCACTGTTTAGTCGCGGATATGGAATTCGATCCTGTCAAAATCAATACATCATCTATAGATCATGTAACAATATTACAATACATAGATGAACCAAATGATATAAGACTAACAGTATGCATTATCCGAAATATTAATAACATTACATATTATATCAATATCACAAAAATAAATCCACATTTGGCTAATCGATTTCGGGCTTGGAAAAAACGTATCGCCGGAAGGGACTATATGACTAACTTATCTAGAGATACAGGAATACAACAATCAAAACTTACTGAAACTATACGTAACTGTCAAAAAAATAAAAACATATATGGTCTATATATACACTACAATTTAGTTATTAATGTGGTTATTGATTGGATAACCGATGTGATTGTTCAATCAATATTAAGAGGGTTGGTAAATTGGTACATAGCTAATAATACATATACTCCAAATACACCCAATAATACTACAACCATTTCTGAGTTGGATATCATCAAAATACTGGATAAATACGAGGACATGTATAGAGTAAGTAAAGAAAAAGAATGTGGAATTTGCTATGAAGTTGTTTACTCAAAACGATTAGAAAACGATAGATACTTTGGTTTATTGGATTCGTGTAATCATATATTTTGCATAACATGTATCAATATATGGCATAGAACACGAAGAGAAACCGGTGCGTTAGATAATTGTCCTATATGCCGTACCCGTTTTAGAAAAATAACAATGAGCAAGTTCTATAAGCTAGTTAACTAATAAATAAAAAGTTTAATTATCGACGATATATGTCGTTATTTTTCTCTCATATGAAAGATTAATTTGATTCTAATATAATCTTCAGTATTGGATGAATCTCAATTCAAATTAATTCCATTAGATTAGATTAGATTAGATTAGATTAGATTAGATTAGATTAGATTAGATTagattagattagattagattagattagattagattagattagattagattAGATTAGATTAGATTAGATTAGATTAGATTAGATCATAAATAAAAATAGTAGCACGCACTACTTCAGCCAAATATTCTTTTTTGAAACGCCATCTAGCGTAATGAGAACACAAGTGAACCTATAATGAGCAAATTTATTAGTATCGGTTACATGAAGGACTTTACGTAGAGTGGTGATTCCTCCATCTGTGGTACGAACGGTTTCATCTTCTTTGATGCCATCACCCAGATGTTCTATAAACTTGGTATCCTCGTCCGATTTCATATCATTTGCCAACCAATACATATAGCTAAACCCAGGCATACG

TTCCACACATCCGGAACAATGAAATTCTCCAGAAGATGTTACAATGGCTAGATTTGGACATTTGGTTTCA

ACCGCGTTAACATATGAGTGAACACACCCATACATGAAAGCGATGAGAAATAGGATTCTCATCTTGCCAA

AATATCACTATAAAAAATTTATTTATCAATTTTAAAGGTATAAAAAAATACTTATTGTTGCTCGAATATT

TTGTATTTGATGGTATACGGAAGATTAGAAATGTAGGTATTATCATCAACTGATTCTATGATGGTTTTAT

GAATTTTATTATGCTTCACTATTGCATCGGAAATAATATCATATGCTTCCACGTATATTTTATTTTGTTT

TGACTCATAATACGCACGTATTTCTGGATTATTGGCATATCGATGAATAATTTTAGCTCCATGCTCAGTA

AATATTAATGAGAACATAGTGTTGCCTCCTACCATTATTTTTTTCATCTCATTCAATTCTTGATTGCAGA

GATCTATATAATCATTATAGCGTTGACTTATGGACTCTGGAATCTTAGACGATGTACAGTCATCTATAAT

CATGGTATATTTAATACATTGTTTTATAGCATAGGCATTATCTACGATATTAGATACTTCACTCAATGAA

TCAATCACACAATCTAATGTAGGTTTATGACATAATAGCATTTTCAGCAGTTCAATGTTTCTAGATTCGT

TGATGGCAATGGCTATACATGTATATCCGTTATTTGATCTAATGTTGACATCTGAACCGGATTCTAGCAG

TAAAGATACTAGAGATTGTTTATTATATCTAACAGCCTTGTGAAGAAGTGTTTCTCCTCGTTTGTCAATC

ATGTTAATGTCTTTAAGATAAGGTAGGCAAATGTTTATAGTACTAAGAATTGGGCAAGTATAAGACATGT

CACAAAGACCCTTTTTGTATGTATAAGTGTAGAAATTATAACATCCATAGTTGGATTCACATAGGTGTCC

AATCGGGATCTCTCCATCATCGAGATGATTGACGGCATCTCCCCCTTCCTTTTTTAGTAGATATTTCATC

GTGTAAGAATCAATATTAATATTTCTAAAGTATCTGTGTATAGCCTCTTTATTTACCACAGCTCCATATT

CCAACATGCATTCCACTAGAGGGATATCGATATCGCCGAATGTCATATACTCAATTAGTATATGTTGGAG

GACATCCGAGTTCATTGTTTTCAATATCAAAGAGATGGTTTCCTTATCATTTCTCCATAGTGGTACAATA

CTACGCATTATTCCGTGCGGCTTTCCATTCTCCAAAAACAATTTTACCAAATCTAAATCTACATCTTTAT

TGTATCTATAATCACTATTTAGATAATCAGCCATAATTCCTCGAGTGCAACATGTTAGATCGTCTATATA

TAAATAAGCCGTGTTATCTATTCCTTTCATTAACAATTTAACGATGTCTATATCTATATGAGATGACTTA

ATATAATATTGAAGAGCTGTACAATAGTTTTTATCTATAGAAGACGGCTTGATTCCGTGATTAATTAGAC

ATTTAACAACTTCCGGACGCACATATGCTCTCGTATCCGACTCTGAATACAGATGAGAGATGATATACAG

ATGCAATACGGTACCGCAATTTCGTGGTTGATAATCATCATACGCGTATCCGTACTCGTCATCCTCATAA

AGAACACTGCAGCCATTTTCTATGAACAAATCAATAATTTCAGGAACAGGATCATCTGTCATTACATAAT

TTTCTATAACTGAACGATGGTTTTCACATTTAACACTCAAGTCAAATCCATGTTCTACCAACACCTTTAT

CAAGTCAACGTCTACATTTTTTGATTTAATATAGCTGAATATATTAAAGTCATTTATGTTGCTATATCCA

GTAGCTTCTAGTAGAGCCATCGCTATATCCTTATTGACTTTAACATGTCTACTATTTGTGTATTCTTCTA

TTGGGGTAAACTGTCTCCAATTTTTGTGTAATGGATTAGTGCCACTGTCTAGTAGTAGTTTGACGACCTC

AACATTATTACAATGCTCATTGAAAAGGTATGCGTGTAAAGCATTATTCTTGAATTGGTTCCTGGTATCA

TTAGGATCTCTGTCTCTCAACATCTGTTTAAGTTCATCGAGAACCACCTCCTCATTTTCCAGATAGTCAA

ACATTTTGACTGAATAGAAGTGAATGAGCTACTGTGAACTCTATACACCCGCACAACTAATGTCATTAAA

TATCATTTTTGAATGTATTTATACCATGTCAAAAACTTGTACAATTATTAATAAAAATAATTAGTGTTTA

AATTTTACCAGTTCCAGATTTTACACCTCCGTTAACACCTCCATTAACCCCACTTTTTACACCACTGGAC

GATCCTCCTCCCCACATTCCACTGCCACCAGATGTATAAGTTTTAGATCCTTTATTACTACCATCATGTC

CATGGATAAAGACACTCCACATGCCGCCACTACTACCCCCTTTAGAAGACATATTAATAAGACAAGTTTA

ACAATAAAATTAATCACGAGTACCCTACTCCAACCACTATTATATGATTATAGTTTCTATTTTTACAGTA

CCTTGACTAAAGTCTCTAGTCACAAGATCAATACTACCAACCTACACTATATATGATTATAGTTTCTATT

TTTATAGGAACGCGTACGAGAAAATCAAATGTCTAAGTTCTAACGGTAGTGTTGATAAACGATTGTTATC

CGCGGATACCTCATCTATCATGTTGTCTATTTTCTTACTTTGTTCTATTAACCTATTAGCATTATATATT

ATTTGATTATAAAACTTATATTGCTTATTAGCCCAATCTGTAAATATCGGATTATTAACATATCGTTTCT

TTGTAGGTTTATTTAACTTGTACATCACTGTAAGCATGTCCGTACCATTTATTTTAATTTGACACATATC

AGCAATTTCTTTTTCGCAGTCGGTTATATATTCTATATAAGATGGATACGTATCACATATGTACTTATAG

TCTACTAATATGAAGTACTTAATACATATTTTCAGTAACGATTTAGCCTTATTACCTATTAATAAGTGCC

TGTCGTTGGATAGGTAATCAACTGTTTTCTTAATACATTCGATGGTTGGTAATTTACTCAAAATAATTTC

CAATATCTTAATATATATTTCTGCTATTTCTGGTATACATGCATGTGCCATTATAACACAAATACCAATA

CATGTAGACCCATATGTTGTTGTTATATTAATATCTGCGCCATTATCTATTAACCATTCTACTAGTGCAA

CACTATGCGACTCGATACAATAATAAAGTATACTACGTCCATGTTTATCTATTTTGTTTATATCATCGAT

ATACGGCTTACAAATTTTTAGTATCGATAACACTTCTGACTCGTGAATAAATAAGGTAGGGAATAACGGC

ATAATATTTATTATGTTATCATCATTAACAACTACGTTTCCATTTTTTAAAATATACTCTACAACTTTAG

GATCCCTATTGTCAAATCTTTTAAAATATTTATTTATATGCTTAAATCTATATAATATAGCTCCTTCCCT

AATCATACATTTGATAACATTGATGTACACTGTATGATAAGATACATATTCTGACAATAGATCTTGTATA

GAATCTGTATATCTTTTAAGAATTGAGGATATTATGACATTATTACGTAAACTATTACACAATTCTAAAA

TATAAAACGTATCATGGGCAGATAATAGTTTATCCACTATATAATTATCTATTTTATGATTTTTCTTCCT

ATATTGTTTACGTAAATAGATAGATAGAATATGCATTAGTTCATTACCGCTATAGTTACTATCGAATAAC

ACGTCAAATATTTCCCGTTTAATATCGCATTTGTCAACATAATAATAGAGTATGGTACGTTCACGATAAG

TATAATGACACATTTCGTTTTCGTGCGAAATTAAATAGTTTATCACGTCCAAAGATGTCACATAACCATC

TTGTGACCTAGTAATAATATAATAATAGAGAACTGTTTTACCCATTCTATTATCATAATCAGTGGTGTAG

TCATAATCTAAATAATCAAACTCGTCATCCCAATTAAAATAAATATAATCAGTACATTGAATGGGTATGA

TATTGTACCCATACTGTATGTTGCTACATGTAGGTATTCCTTTATCCAATAATAGTTTAAATACATCTAT

ATTAGGATTTGATGTTGTCGCGTATTTCTCTACAATATTAATACCATTTTTGATACTATTTATTTCTATA

CCTTTCGAAATTAGTAATTTCAATAAGTCTATATCGATGTTATCAGAACATAGATATTCAAATATATCAA

AATCATTGATATTTTTATAGTCGACTGACGACAATAACAAAATCACAACATCGTTTTTGATATTATTATT

TTTTTTGGTAACGTATGCCTTTAATGGAGTTTCACCATCATACTCATATAATGGATTTGCACCACTTTCT

ATTAATGATTGTGCAcTACTGGCATCGATGTTAAATGTTTTACAACTATCATAGAGTATCTTATCGTTAA

CCATGATTGGTTGTTGACGTTATCACATTTTTTGGTTTCTTTCATTTCAGTTATGTATGGATTTAGCACG

TTTGGGAAGCATGAGCTCATATGATTTCAGTACTGTAGTGTCAGTACTATTAGTTTCGATCAGATCAATG

TCTAGATCTATAGAATCAAAACACGATAGGTCAGAAGATAATGAATATCTGTACGCTTCTTCTTGTACTG

TAACTTCTGGTTTTGTTAGATGGTTGCATCGTGCTTTAACGTCAATGGTACAAATTTTATCCTCGCTTTG

TGTATCATATTCGTCTCTAGTATAAAATTCTATATTCAAATTATCATGCGATGTGTATACGCTAACGGTA

TCAATAAACGGAGCACAGCATTTAGTCAACAGTAATCCAAATTTTTTTAAAGTATATCTTAACGAAAGAA

GTTGTCATCGTTAGAGTGTGGTAAATCATTGTCTACGGTACTAGATCCTCATAAGTGTATATATCTAGAG

TAATATTTAATTTATCAAATGGTTGATAATATGGATGTCGTGGCAATTTCCTAATACGGAAATAAGACAT

AAACACGCAATAAATCTAATTGCGGACATGTTACACTCCTTAAAAATACGAATAAAAACTTTGGCTTTTA

GTAAGTGTCATTTAACACTATACTCATATTAATCCATGGACTCATAATCTCTATACGGGATTAACGGATG

TTCTATATACGGGGATGAGTAGTTCTCTTCTTTAATTTTATACTTTTTACTAATCATATTTAGACTGATG

TATGGGTAATAGTGTTTGAAGAGCTCGTTCTCATCATCAGAATAAATCAATATCTCTGTTTTTTTATTAT

ACAGATGTATTACAGCCTCATATATTACGTAATATAACGTGTAATCTACCTTATTAACTTTCACCGCATA

GTTGTTTGCAAATACGGTTAATCCTTTGACCTCGTCGATTTCCGACCAATCTGGGCGTATAACGAATCTT

AACTTTAATTTCTTGTAATCATTCGAAATAATTTTTAGTTTGCATCCGTAGTTATCTCCTCTATGTAACT

GTAAATTTCTCAACGCGATATCTCCATTAATAATGATGTCGAATTCGTGTTGTATACCCATACTGAATTG

ATGAACGAATACCGACGGTGTGTGTTAATAGTAATTTACTTTTCATCTTTACATACTTGGTAATAGTTTT

ACTATCATAAGTTTATAAATTCCACAAGCTACTATGGAATATACCAACCATCTTAGTATAGAACACATGT

CTTAAAGTTATTAATTAATTACATGTTGTTTTATATATCGCTACGAATTTAAACAGAGAAATCAGTTAGG

AAGAAAAAATTATCTATCATCATCTATTGGATAACGTCTCTGTATTCTACGATAGAGTGCTATTTTAAGA

TGTGACAGATCCGTGTCATCAAATATATACTCCATTAAAATGATTATTCCGGCAGCGAACTTGATATTGG

ATACATCACGACCTTTGTTAATATCCACGACAATAGACAGCAATCCCATGGTTCCATAAACAGTGAGTTT

ATCTTTCTTTGAAGTGATATTTTGTAGAGATCTTATAAAACTGTCGAATGACATCGTATTTATATCTTTA

GCTAAATCATATATGTTACCATCATAATATCTAACAGCATCTATCTTAAACGTTTCCATCGCTGTAAAGA

CGTTTCCGATAGATGGTCTCGTTTCATCAGTCATACTGAGCCAACAAATGTAATCGTGTATAACATCTTT

GATAGAATCAGACTCTAAAGAAAAGGAATCGGCTTTATTATACACATTCATGATAAACTTAATGAAAAAT

GTTTTTCGTTGTTTAAGTTGGATGAATAGTATGTCTTAATAATTGTTATTATTTCACTAATTAATATTTA

GTAACGAGTACACTCTATAAAAACGAGAATGACATAACTAATCATAACTAGTTATCAAAGAATGTCTAGG

ACGCGTAATTTTTTATGGTATAGATCCTGTAAGCGTTGTCTGTATTCTGGAGCTATTTTCTCTATCGCAT

TAGTGAGTTCAGAATATGTTATAAATTTAAATCGAATAACGAACATAACTTTAGTAAAGTCGTCTATATT

AACTCTTTTATTTTCTAGCCATCGTAATACCATGTTTAAGATAGTATATTCTCTAGTTACTACGATCTCA

TCGTTGTCTAGAATATCGCATACTGAATCTACATCCAATTTTAGAAATTGGTCTGTGTTACATATCTCTT

CTATATTATTGTTGATATATTGTCGTAGAAAACTATTACGTAGACCATTTTCTTTATAAAACGAATATAT

AGTACTCCAATTATCTTTACCGATATATTTGCATACATAATCCATTCTCTCAATCACTACATCTTTAAGA

GTTTGGTTGTTAAGATATTTGGCTAAACTATATAATTCTATTAGATCATCAACAGAATCAGTATATATTT

TTCTAGATCCAAAGATGAACTCTTTGGCATCCTCTATAATATTATCAGAAAAGATATTTTCGTGTTTTAG

TTTATCAAGATCTAACCTGTTCATATCCATGATTAACGACGTCATATAACCACATAAAATAAAAATCCAT

TTTCATTTTTAGCACAATACTATTCATAATTGATATTGATGTAATATTTTGTTACTTTGAACGTAAAGAC

AGTACACGGGTCCGTATCTCCAACAAGCACGTAGTAATCAAATTTGGTGTTGTTAAACTTCGCAATATTC

ATCAATTTAGATAGAAACTTATACTCATCATCTGTTTTAGGAATCCATGTATTATTACTTTCCAACTTAT

CATTATCCCAGGCTATGTTTCGCCCATCATCGTTGTACAGAGTGAATAATTCTTTTGTATTCGGTAGTTC

AAATATATGATCCATGCATATATCGACAAAGCTATTGTAGATGTGATTTTTCCTAAATCTAATATAAAAC

TCGTTTACTAGCAAACATTTTCCTGATTTATCGACCAAGACACACATGGTTTCTAAATCTATCAAGTGGT

GGGGATCCATAGTTATAACGCAGTAACATAGATTATTACCTTCTTGACTGTCGCTAATATCTATATACTT

ATTGTTATCGTATTGGATTCTACATATAGATGGCTTGTATATCAAAGATATAGAACACATAACCAATTTA

TATTCTCGCTTTGTATTTTCGAATCTAAAGTTAAGAGATTTAGAAAACATTATATCCTCGGATGATGATA

TCACTGTTTCCAGAGTAGGATATATTAAAGTCTTTAAAGATTTTGTCCGATTCAAATAAATCACTAAATA

ATATCCCATATTATCATCTGTTATAGTCATGTCATTAAATCTATTATATTTTATGAAAGATATATCACTG

CTCACCTCTATATTTCGTACATTTTTAAACTGTTTGTATAATATCTCTCTAATACAATCAGATATATCTA

TTGTGTCGGTAGACGATACCGTTACATTTGAATTAATGGTGTTCCATTTTACAACTTTTAACAAGTTGAC

CAATTCATTTCTAATAGTATCAAACTCTCCATGATTAAATATTTTAATAGTATCCATTTTATATCACTAC

GGACATAAACCATTGTATAATTTTTATGTTTATTAGTGTACACATTTTGGAAGTAAGTTCCGGCTGCCAT

GTATTTCCTGGAGAGCAAGTAGATGATGAGGAACCAGATAGTTTATATCCATACTTGCACTTAAAGTCTA

CATTGTAGTTGTATGAGtGTATGATCTTTTAAGCCGCTAGAAGTTTTCCGTTTGATATAGGATGTGGACA

TTTAACAATCTGACACGTGGGTGGATTGGACCATTCTCCTCCTGAACACATGACACCAGAGTTACCAATC

AACGAATATCCACTATTGCAACTATAAGTTACAATGCTCCCATCGATATAAAAATCCTCGTATCCGTTAT

GTCTTCCGTTGGATATAGATGGAGGTGATTGGCATTTAACAGATTCGCAAATAGGTGCCTCAGGATTCCA

TACCATAGATCCAGTAGATCCTAATTCACAATACGATTTAGATTCACCGATCAAATGATATCCGCTATTA

CAAGAGTACGTTATACTAGAGCCAAAGTCTACTCCGCCAATATCAAGTTGGCCATTATCGATATCTCGAG

GCGATGGGCATCTCCGTTTAATACATTGATTAAAGAGTGTCCATCCGGTACCGGTACATTTAGCATATAT

GGGTCCCATTTTTTGCTTTCTGTATCCAGGTAGACATAGATATTCTATAGTGTCTCCTATGTTGTAATTA

GCATCAGTCTCTACACTATTCTTAAATTTCATATTAATGGGGCGTGACGGAATAGTACAGTATGATAGAA

CACATCCTATTCCCAACAATGTCAGGAACGTCACGCTCTCCACCTTCATATTTATTTATCCGTAAAATGT

TATCCTGGACATCGTACAAATAATAAAAAGCCCATATATATGTTCGCTATTGTAGAAATTGTTTTTCACA

GTTGCTCAAAAACAATGGCAGTGACTTATGAGTTAGTTACACTTTGGAGTCTCATCTTTAGTAAACATAT

CATAATATTCGATATTACGAGTTGACATATCGAACAAATTCCAAGTATTTGATTTTGGATAATATTCGTA

TTTTGCATCTGCTATAATTAAGATATAATCACCACAAGAACACACGAACGTCTTTCCTACATGGTTAAAG

TACATGTACAATTCTATCCATTTGTCTTCCTTAACTATATATTTGTATAGATAATTACGAGTCTCATGAG

TAATTCCAGTAATTGCATAGATGTCACCATCGTATTCTACAGCATAAACTATACTATGACGTCTAGGCAT

GGGAGACTTTTTTATCCAACGATTTTTAGTGAAACATTCCACATCGTTTAATACTACATATTTCTCATAG

TGGTATAAACTCCACCCATTACATATATATCATCGTTTACGAATACTGATGCGCCTGAATATCTAGGAGT

GATTAAGTTTGGAAGTCTTTTCCATTTCGAAGTGCCGTGTTTCAAATATTCTGCTATACCCGTTGAAATA

GAAAATTCTAATCCTCCTATTACATATAACTTTCCATCGTTAACACAAGTACTAACTTCTGATTTTAACG

ACGACATATTAGTAACCGTTTTCCATTTTTTTTGTTTTAAGATCTACCCGCGATACGGAATAAACATGTC

TATTGTTAATCATGCCGCCAATAATGTATAGACAATTATGTAAAACATTTGCATCATAGAATTGTCTATC

TGTATTACCGACTATCGTCCAATATTCTGTTCTAGGAGAGTAATGGGTTATTGTGGATATATAATCAGAG

TTTTTAATGACTACTATATTATGTTTTATACCATTTCGTGTCACAGCTTTGTAGATTTGGATATAGTTAA

TCCCAACAATGCTATAGCATTGCATATAGCATTAGTCATAAACTTGGGATGTAAAATGTTGATGATATCT

ACATCGTTTGGATTTTTATGTATCCACTTTAATAATATTATAGCGTAACATCCTCATGATTTACGTTAAC

GTTTTCGTGTGATAAGATAGTGGTCAGTTCATCCTTTGATAATTTTCCAAATTCTGGATCGGATGTCACC

GCAGTAATATTGTTGATTATTTCTGACATCGACGCATTATATAGTTTTTTAATTCCATATCTTTTAGAAA

AGTTAAACATCCTTATACAATTTGTGGAATTAATATTATGAATCATGGTTTTTACACATAGATCTACTAC

AGGCGGAACATCAATTATTATGGCAGCGACTAGTATCATTTCTACATTGTTTATGGTGATGTTTATCTTC

TTCCAGCGCATATAGTCTAATATCGATTCAAACGCGTGATAGTTTATACCATTCAATATAATCGCTTCAT

CCTTTAGATGGTGATCCTGAATGTGTTTAAAAAATTATACGGAGACGCCGTAATAATTTCCCCATTGATA

GAAAATATCACGCGTTCCATTCTCTTGAAGTACTATAAGTAATTATAATATAATGTAAAGGTTTATATAT

TCAATATTTTTTTATAAAAAAAATCATTTCGACATTAATTCCTTTTTAAATTTCCGTCTATCATCTATAG

AAACATATTCTATGAATTTATAAAATGCTTTTACGTATCCTATCGTAGGCGATAGAACCGCTAAAAAGCC

TATCGAATTTCTACAAAAGAATCTGTTATATGGTATAGGGAGAGTATAAAACATTAAATGTCCGTACTTA

TTAAAGTATTCAGTAGCCAATCCTAACTCTTTCGAATAATTATTAATGGCTCTTATTCTGTACGAATCTA

TTTTTTTGAACAATGGACCTAGTGGTATATCTTGTTCTATGTATCTAAAATAATGTCTGACTAGATCCGT

TAGTTTAATATCCGCAGTCATCTTGTCTAGAATGGCAAATCTAACTGCGGGTTTAGGCGTAGGCGTTAGT

TTAGTTTCTATATCTACATCTATGTCTTTATCTAACACCAAAAATATAATAGCTAATATTTTATTACAAT

CATCCGGATATTCTTCTACGATCTCACTAACTAATGTTTCTTTGGTTATACTAGTATAGTCACGATCAGA

CAAATAAAGAAAATCAGATGATCGATGAATAATACATTTAAATTCATCATCTGTAAGATTTTTGAGATGT

CTCATTAAAATATTATTAGTGTCAGTTCTCATTATCATATATTGACAGCAGCTATTACACTTATTTTATT

TTTCTGTATTTTATTACTTTTCACCATATAGATCAGTCATTAGATCATCAAAATACTTTTCAATCATCCT

AAAGAGTATGGTGAACGAATCTTCCCATCTAATTTCTGAACGTCTACCAATGTCTCTAGCCACTTTGGCA

CTAATAGCGATCATTCGCTTAACATCTTCTACATTATTAACTGGTTGATTCAATCTATCTAGCAATGGAC

CGTCGGATAGCGTCATTCTCATGTTCTTAATCAATGTACATACATCGTCATCATCTACCAATTCATCAAA

CAATATAAGCTTTTTAAAATCATCATTATAATAGGATGGATCGCCGTCATTTCTCCAAAGAATATATCTA

ATAAGTAGAGTCCTCATGCTTAGTAATTTAACTATTTTAGTTAACAACTATTTTTTATGTTAAATCAATT

AGTAACACCGCTATGTTTAATACTTATTCATATTTTAGTTTTAGGATCGAGAATCAATACAAAAATTAAT

ACATCAATTTTGGAAATACTTAGTTTCCACGTAGTCAATGAAACATTTGAGCTCATCGTAAAGGACGTTC

TCGTACAGGACGTAACTATAAATTGGTTTATATTTGTTCAAGATAGATACAAATCCGATAACTTTTTTGA

CGAATTCTACGGGATTCACTTTAAAAGTGTCATACCGGGTTCTTTTTATTCTTTTAAACAGATCGATTGT

GTGATGTTGATTAGGTCTTTTACGAATTTGATACAGAATAGCGTTTACATATCCACCATAGTAATCAATA

GCCATTTGTTCGTATGTCATAAATTCTTTAATTATATGACACTGTGTATTATTTAGTTCGTCCTTGTTCA

TCATTAGGAATCTATCCAATATGGCAATTATATTAGAACTATAACTGCGTTGTATGCGCATGTTGATGTG

TCTGTTTATACAATCAATTATACTAGGATCCATACCACTACATTCGGGTAAAATTGTAGCATCATATACC

ATTTCTAGTACTTTAGGTTCATTGTTATCCATTGCAGAGGACGTCATGATCGAATCCaAAAAAAATATATT

ATTTTTATGTTATTTTGTTAAAAATAATCATCAAATACTTCGTAAGATACTCCTTCATGAACATAATCAG

TTACAAAACGTTTATATGAAGTAAAGTATCTACGATTTTTACAAAAGTCAGGATGCATAAGTACAAAGTA

CGCGATAAACGGAATAATAATAGATTTATCTAGTTTATCTTTTTCTATCTCTTTCATAGTTATATACATG

GTCTCAGAAGTCGGATTATGTAACATCAGCTTCGATAAAATGACTGGGTTATTTAGTCTTACACATTCGC

TcATACATGTATGACCGTTAACTATAGAGTCTACACTAAAATGATTGAATAATAGATAGTCTACCATTGT

TTCGTATTCAGATAGTACAGCGTAGTACATGGCATCTTCACAAATTATATCATTATCTAATAGATATTTG

ACGCATCTTATGGATCCCACTTCAACAGCCATCTTAAAATCGGTAGAATCATATTGCTTTCCTTTATCGT

TAATAATTTCTAGAACATCATCTCTATCATAAAAGATACAAATATTAACTGTTTGATCAGTAATAACATT

GCTAGTCGATATCAATTTGTTAATAAGATGCGCTGGGCTCAATGTCTTAATAAGAAGTGTAAGAGGACTA

TCTCCGAATTTGTTTTGTTTATTAACATCCGTTGATGGAAGTAAAAGATTTATAATGTCTACATACTTGA

CTGTTTTAGAGCATACAATATGGAGAGGCGTATTTCCATCATGATCTGGTTTTGAGGGACTAATTCCTAG

TTTCATCATCCATGAGATTGTAGAAGCTTTTGGATTGTCTGACATAAGATGTCTATGAATATGATTTTTG

CCAAATTTATCCACTATCCTGGCTTCGAATCCGATAGACATTATTTTTTTAAACACTCTTTCTGAAGGAT

CTGTATACGCCAACAACGGACCACATCCTTCTTCATCAACCGAGTTGTTAATCTTGGCTCCATACTGTAC

CAATAAATTTATTCTCTCTATGACTTCATCATCTGTTCCCGAGAGATAATATAGAGGTGTTTTATTATGT

TTATCACATGCGTTTGGATCTGCGCCGTGCACCAGCAGCATCGCGACTATTCTATTATTATTAATTTTAG

AAGCTATATGCAATGGATAATTTCCATCATCATCCGTCTCATTTGGAGAGTATCCTCTATGAAGAAGTTC

TTCTATAAATCGTTCATCTAGTCCTTTAATGCCACAATACGCATGTAGAATGTGATAATTTCCAGAGGGT

TCGATAACTTGTAGCATATTCCTAAATACATCTAAATTTTTACTATTATATTTGGCATAAAGAGATAGAT

AATACTCGACCGACATAATGTTGTGTTGTCCATTATAGTATAAAAATTAATATTTCTATTTCTATTTCTA

TATATTTGCAACAATTTACTCTCTATAACAAATATCATAACTTAGTTCTTTTATGTCAAGAAGGCACTGG

TTTAATTCATCTATAAATGTCACGCCATAACTACCACGCATACTATACTCAGAATTATGATAAAGATATT

TATTCTTGGGGTGTAAGTAATGGGGATTAATCTTTGTTGGATCAGTCTCTAAGTTAACACATGTCACACA

TGATCCATTTATAGTTATATCACACGATGATGATTTATGAATTGATTCCGGAAGATCGCTATTGTATTTT

GTAGTTCCACAATTCATTTCCATACATGTTATTGTCACACTAATATTATGATGAACTTTATCTAGCCGCT

GAGTGGTAAACAACAGAACAGATAGTTTATTATCTTTACCAACACCCTCAGCCGCTGCCACAAATCTCTG

ATCCGTATCCATGATGGTCATGTTTATTTTTAGTCCGTATCCAGTCAACACTATGTTAGCATTTCTGTCG

ATATAGCTTTCACTCATATGACACTCACCAATAATTGTAGAATTAATGTCGTAATTTACACCAATAGTGA

GTTCGGCGACAAAGTACCAGTACCGGTAATCTTGTCGAGGAGGACATATAGTATTCTTGTATTCTACCGA

ATACCCGAGAGATGCGATACAAAAGAGTAAGACTAATTTGTAAACCATCTTACTCAAAATATGCGACAAT

AGTACGATGCAATGAGTAAGACAATAGGAAATCTATCTTATACACATAATTATTCTATCAATTTTACCAA

TTAGTTAGTGTAATGTTAACAAAAATGTGGGATAATTTAATAGTTTTTCCTTACATAATTGACATACATG

AGTCTGAGTTCCTCGTTTTTGCTAATTATTTCGTCCAATTTATTATTCTTGACATCGTCAAGATCTTTTG

TATAGGAGTCAGACTTGTATTCAACATGTTTTTCTATAATCATCTTAGCTATTTCGGCATCATCCAATAG

TACATTTTCCAGATTAACAGAATAGATATTAATGTCGTATTTGAACAGAGCCTGTAACATCTCAATGTCT

TTATTATCTATAGCCAATTTGATGTCCGGAATGAAGAGAAGGGAATTGGTGTTTGTCGACGTCATATAGT

CGAGCAAGAGAATCATCATATCCACGTGTCCATTTTTTATAGTGGTGTGAATACAACTAAGGAGAATAGC

TAGATCAAAAGGAGATGGTATCTCTGAAAGAAAGTAGGAAACAATACTTACATCATTAAGCATGACAGCA

TGATAAAATGAAGTTTTCCATCCAGTTTTCCCATAGAACATCAGTCTCCAATTTTTCTTAACAAACAGTT

TTACCGTTTGCATGTTACCACTATCAACCGCATAATACAATGCGGTGTTTCCTTTGTCATCAAATTGTGA

ATCATCCATTCCACTGAATAGCAAAATCTTTACTATTTTGGTATCTTCTAATGTGGCTGCCTGATGTAAT

GGAAATTCATTATCTAGAAGATTTTTCAATGCTCCAGCGTTCAACAACGTACATACTAGACGCACGTTAT

TATCAGCTATTGCATAATACAAGACACTATGACCgTTGATATCCGCCTTAAATGCATCTTTGCTAGAGAG

AAAGCTTTTCAGTTGCTTAGACTTCCAAGTATTAATTCGTGACAGATCCATGTCTGAAACGAGACGCTAA

TTAGTGTATATTTTTTCATTTTTTATAATTTTGTCATATTGCACCAGAATTAATAATATCTCTAATAGAT

CTGATTAGTAGATACATGGCTATCGCAAAACAACATATACACATTTAATAAAAATAATATTCATTAAGAA

GATTCAGATTCCACTGTACCCATCAATATAAATAAAATAATTATTCCTTACATCGTACCATAAACAATAT

ATTAAGTAGATTCCACCTTACCCATAAACAATATAAATCCAGTAATATCATGTCTAATGATGAACACAAA

TGGTGTATTAAATTCCAGTTCTTCAGGAGATGATCTCGCCGTAGCTACCATGATAGTAGATGCCTCCGCT

ACAGTTCCTTGTTCGTCTACATCTATCTTTACATTCTGAAACATTTTATAAATATATAATGGGTCCCTAG

TCATATGTTTAAACGACGCCTTATCTGGATTAAACATACTAGGAGCCATCATTTCGGCTATCGACTTAAT

ATCCCTCTTGTTTTCGATAGAAAATCTAGGGAGTTTAAGATTGTACATTTTATTCCCTAATTGAGATGAC

CAATATTCTAATTTTGCAGCCGTGATAGAATCTGTGAAATGGGTCATATTATCACCTATTGCCAGGTACA

TACTAATATTAGCATCCTTATACAGAAGGCGCACCATATCATATTCTTCGTCATCGATTGTGATTGTATT

TCCTTGCAATTTAGTAACTACGTTCATCATGGGAACCGTTTTCGTACCGTACTTATTAGTAAAACTAGCA

TTGTGTGTTTTAGTGATATCAAACGGATATTGCCACGTACCTTTAAAATATATAGTATTAATGATTGCCC

ATAGAGTATTATCGTCGAGCATAGTAGAATCAACTACATTAGACATACCAGATCTACGTTCTACTATAGA

ATTAATTTTATTAACCGCATCTCGTCTAAAGTTTAATCTATATAGGCCGAATCTATGATATTGTTGATAA

TACGACGGTTTAATACACACAGTACTATCGACGAAACTTTGATACGTTAGATCGGTGTACGTATATTTAG

ATGTTTTCATCTTAGCTAATCCTGATATTAATTCTGTAAATGCTGGACCCAGATCTCTTTTTCTCAAATT

CATAGTATTCAATAATTCTACTCTAGTATTACCTGATGCAGACAATAGCGACATAAACATAGAAAACGAA

TACCCAAACGGTGAGAAGACAATATTATCATTATCATCCTCATCCCCATTTTGAATATTTTTATACGCTA

ATATACCGGCATTGATAAATCCCTGCAGACGATATGCGGATACTGAACACGCTAATGATAGTATCAATAA

CGCAATCATGATTTTTATGGTATTAATAATTAACCTTATTTTTATGTTTGGTATAAAATTTATTGATGTC

TACACATCCTTTTGTATAATCAACTCTAATCACTTTAACTTTTACAGTTTTCCCTACAAGTTTATCCCTA

TATTCAACATATCTATCCATATGCATCTCTTAACACTCTGCCAAGATAGCTTCAGAGTGAGGATAGTCAA

AAAGATAAATATATAGAGTATAATCATTCTCGTATACTCTGCCCTTTATTACATCGCCCGCATTGGGCAA

CGAATAACAAAATGCAAGCATCTTGTTAACAGGCTCGTAAATTGGGATAAAATTATGTTTTTATTGTTTA

TCTATTTTATTCAAGAGAATATTCAGGAAGTTCCTTTTCCGGTTGTATCTCGTCGCAGTATATATCATTT

GTACATTGTTTCATATTTTTTAATAGTCTACACCTTTTAGTAGGACTAGTATCGTACAATTCATAGCTGT

ATTTTGAATTCCAATCACGTATAAAAATATCTTCCAATTGTTGACGAAGACCTAATCCATCATCCGGTGT

AATATTAATAGATGCTCCACATATATCCGTAAAGTAATTTCCTGTCCAATTTGATGTACCTATATACGCC

GTTTTATCGGTTACCATATATTTTGCATGGTTTACCCTAGAATACGGAATGGGAGGATCAGCATCTGGTA

CAATAAATAGCTTTACTTCTATATCTATGTTTTTAGATTTTAGCATAGCTATAGATCTTAAAAAGTTTCT

CATGATAAACGAAGATCGTTGCCAGCAACTAATCAATAGCTTAACGGATACTTGTCTGTCTATAGCGGAT

CTTCTTAATTCATCTTCTATATAAGGCCAAAACAAAATTTTACCCGCCTTTGAATAAATAATAGGAATAA

AGTTCATAACAGATACATAAACGAATTTACTCGCATTTCCGATACATGACAATAAAGCGGTTAAATCATT

GGTTCTTTCCATAGTACATAATTGTTGTGGTGCAGAAGCAATAAATACAGAGTGTGGAACACCGCTTACG

TTAATACTAAGAGGATGATCTGTATTATAATACGACGGATAAAAGTTTTTCCAATTATATGGTAGATTGT

TAACTCCAAGATACCAGTATACCTCAAAAATTTGAGTGAGATCCGCTGCCAAGTTCCTATTATTGAAGAT

CGCAATACCCAATTCCTTGACCTGAGTTAGTGATCTCCAATCCATGTTAGCGCTTCCTAAATAAATATGT

GTATTATCAGATATCCAAAATTTTGTATGAAGAACTCCTCCTAGGATATTTGTAATATCTATGTATCGTA

CTTCAACTCCGGCCATTTGTAGTCTTTCAACATCCTTTAATGGTTTGTTGGATTTATTGACGGCTACTCT

AACTCTTACTCCTCTTTTGGGTAATTGTACAATCTCGTTTAATATTACCGTGCCGAAATTCGTACCCACT

TCATCCGATAAACTCCAATAAAAAGATGATATATCTAGTGTTTTTATGGTATTGGATAGAATTTCCCTCC

ACATGTTAAATGTAGTCAAATATACTTTATCAAATTGCATACCTATAGGAATAGTCTCTGTAATCACTGC

GATTGTATTATCCGGATTCATTTTATTTGTTAAAAAAATAATCCTATATCACTTCACTCTATTAAAAATC

CAAGTTTCTATTTCTTTCATGACTGATTTTTTAACTTCATCCGTTTCCTTATGAAGATGATGTTTGGCAC

CTTCATAAATTTTTATTTCCCTATTACAATTTGCATGTTGCATGAAATAATATGCACCTGAAACATCGCT

AATCTCATTGTTTGTTCCCTGGAGTATGAGAGTCGGGGTGTTAATCTTGGGAATTATTTTTCTAACCTTG

TTGGTAGCCTTCAAGACCTGACTAGCAAATCCAGCCTTAATTTTTTCATGATTGACTAATGGATCGTATT

GGTATTTATAAACTTCATCCATATCTCTAGATACTGATTCTGGACATAGCTTTCCGACTGACGCATTTGG

TGTAATGGTTCCCATAAGTTTTGCAGCTAGCAGATTCAGTCTTGGAACAGCGTCTGCATTAACTAGAGGA

GACATTAGAATCATTGCTGTAAACAAGTTTGGATTATCGCAAGCAGCCAGTATAGAAATTGTTGCTCCCA

TGGAATGACCCAATAAGAAGACTGGAACTCCTGGATAAGTAGATTTAATAGTCACTACGTGCTGTACCAC

ATCTCTAACATACTTACCAAAGTCATCAATCATCATTTTTTCACCATTACTTCTTCCATGGCCAATATGA

TCATGTGAGAATACTAAAATTCCTAACGATGATATGTTTTCAGCTAGTTCGTCATAACGTCCAGAATGTT

CACCAGCTCCATGACTTATGAATACTAATGCCTTAGGATATGTAATAGGTTTCCAATATTTACAATATAT

GTAATCATTGTCCAGATTGAACATACAGTTTGCACTCATGATTCACTATATAACTATCAATATTAACAGT

TCGTTTAATGATCATATTATTTTTATGTTTTATTGATAATTGTAAAAATATACAATTAAATCAATATAGA

GGAAGGAGACGGTACTGTATTTTGTGAGATAGTCATGGAGACTAAATCAGATTATGAGGATGCTGTTTTT

TACTTTGTGGATGATGATGAAATATGTAGTCGCGACTCCATCATTGATCTAATAGATGAATATATCACGT

GGAGAAATCATGTTATAGTGTTTAATAAAGATATTACCAGTTGTGGAAGACTGTACAAGGAATTGATAAA

GTTCGATGATGCCGCTATACGGTACTATGGTATTGATAAAATTAATGAGATTGTCGAGGCTATGAGTGAA

GGAGACCACTACATCAATCTTACAGAAGTCCATGATCAGGAAAGTCTATTCGCTACCATAGGAATATGTG

CTAAAATCGCTGAACATTGGGGATACAAAAAGATTTCAGAATCTAAATTCCAATCATTGGGAAACATTAC

AGATCTGATGACCGACGATAATATAAACATCTTGATACTTTTTCTAGAAAAAAAATTGAATTGATGATAT

AGGTGTCTTCATAACGCATTATTACGTTAGCATTCTATTATCCTATCATGTATTTGAGAGTCTTATATGT

AGCAAACATGATAACTGCAATACCCATAATCTTTAGATATTCACGCGTGCTATGGATGGCATTATCCCGC

GGTGTGGAAATGTACGTTATATAATCTACAAAATAATCATCGCATATAGTATGAGATAGTAGAGTAAACA

TTTTTATCGTTTCTACTGGGTTCATACATCGTCTACCCAATTCGGTAATGAATGAAATTGTCGCCAATCT

TACACCCAAACCCTTGTTGTTCATTAGTATAGTATTAACTTCATTATTTATGTCATAAACTGTAAATGAT

TCTGTAGATGCCATATCACACATGATATTCATGTCACTATTATAATCATTATTAACTTTATCACAATACG

TGTTGATAATATCTACATATGATCTAGTTTTTGTGGGTAATTGCCTATACAAGTCGTCTAAACGTTGTTT

ACTCATATAGTATTGAACAGCCATCATTACATGGTCCCGTTCCGTTGATAGATAATCGAGTATGTTAGTA

GACTTGTCAAATCTATATACCATATTTTCTGGAAGCGGATATACATAGTCGCGATCATCATTATCACTAG

CCTCATCCTCTATATCATGTACATGTACATAATCTATGATATTATTATACATAAACATCGACAACATACT

ATTGTCTATTATCTAAGTCCTGTTGATCCAAACCCTTGATCTCCTCTATCTGTACTATCTAGAGATTGTA

CTTCTTCAAGTTCTGGATAATATATACGTTGATAGATTAGCTGAGCTATTCTATCTCCAGTATTTACATT

AAACGTACATTTTCCATTATTAATAAGAATGACTCCTATGTTTCCCCTATAATCTTCGTCTATTACACCG

CCTCCTATATCAATGCCTTTTAGGGACAGACCAGACCTAGGAGCTATTCTACCATAGCAGAACTTAGGCA

TGGACATACTAATATCTGTCTTAATTAACTGTCGTTCTCCAGGAGGGATAGTATAATCGTAAGCGCTATA

CAAATCATATCCGGCAGCACCCGGCGATTGCCTAGTAGGCGATTTAGCTCTGTTAGTTTCCTTAACAAAT

CTAACTGGTGAGTTAATATTCATGTTGAACATAAAAAATATCATTTTATTTCAAAATTATTTACCATTCC

ATTCCATCCCATCCCATTCCATATATTCCATGAATAAGTGCGATTATTGTACACTTCTATAGTAT

CTATATACGATCCACGATAAAATCCTCCTATCAATAGCAGTTTATTATCCACTATGATCAATTCTGGATT

ATCCCTCGGATAAATAGGATCATCTATCAGAGTCCATGTATTACTGGATTCACAATAAAATTCCGCATTT

CTACCAACCAAGAATAACCTTCTACCAAACACTAACGCACATGATTTATAATGAGGATAATAAGTGGATG

GTCCAAACTGCCACTGATCATGATTGGGTAGCAAATATTCTGTAGTTGTATCAGTTTCAGAATGTCCTCC

CATTACGTATATAACATTGTTTATGGATGCCACTGCTGGATTACATCTAGGTTTCAGAAGACTCGGCATA

TTAACCCAAGCAGCATCCCCGTGGAACCAACGCTCAACAGATGTGGGATTTGGTAGACCTCCTACTACGT

ATAATTTATTGTTAGCGGGTATCCCGCTAGCATACAGTCTGGGGCTATTCATCGGAGGAATTGGAATCCA

ATTGTTTGATATATAATTTACCGCTATAGCATTGTTATGTATTTCATTGTTCATCCATCCACCGATGAGA

TATACTACTTCTCCAACATGAGTACTTGTACACATATGGAATATATCTATAATTTGATCCATGTTCATAG

GATACTCTATGAATGGATACTTGTATGATTTGCGTGGTTGTTTATCACAATGAAATATTTTGTTACAGTC

TAGTATCCATTTTACATTATGTATACCTCTGGGAGAAAGATAATTTGACCTGATTACATTTTTGATAAGA

AGTAGCAGATTTCCTAATCTATTTCTTCGCCTCATATACCACTTAATGACAAAATCAACTACATAATCCT

CATCTGGAACATTTAGTTCGTCGCTTTCTAGAATAAGTTTCATAGATAGATAATCAAAATTGTCTATGAT

GTCATCTTCTAGTTCCAAAAAGTGTTTGGTAATAAAGTCTTTAGTATGACATAAGAGATTGGATAGTCCG

TATTCTATACCCATCATGTAACACTCGATACAATATTCCTTTCTAAAATCTCGTAGGATAAAGTTTATAC

AAGTGTAGATGATAAATTCTACAGATGTTAATATAGAAGCACGTAATAAATTGACGACGTTATGACTATC

TATATATACCTTTCCAGTATATGAGTAAATAACTATAGAAGTTAGACTGTGAATGTCAAGGTCTAGACAA

ACCCTCGTAACTGGATCTTTATTTTTTGTGTATTTTTGACGTAAATGTGTGCGAAAGTATGGAGATAACT

TTTTCAATATTGTAGAATTGACTATTATATTGCCTCCTATAGCTTCAATAATTGTTTTGAATTTCTTAGT

CGTATACAATGCTAATATATTCTTACAGTACACAGTATTGACAAATATCGGCATTTATGTTTCTTTAAAA

GTCAACATCTAAAGAAAAATGATTGTCTTCTTGAGACATAACTCCCATTTTTTGGTATTCACCCACACGT

TTTTCGAAAAAATTAGTTTTTCCTTCCAATGATATATTTTCCATGAAATCAAACGGATTGGTAACATTGT

AAATTTTTTTAAATCCCAATTCAGAAATCAATCTATCTGCGACGAATTCTATATATGTTTTCATCATTTC

ACAATTCATTCCTATGAGTTTAACTGGAAGAGCCACAGTAAGAAATTCTTGTTCAATGGATACCGCATTT

GTTATAATAAATCTAACGGTTTCTTCACTCGGTGGATGTAATAAATGTTTAAACATCAAACATGCGAAAT

CGCAGTGCAGACCCTCGTCTCTACTAATTAATTCGTTAGAAAACGTGAGTCCGGGCATTAGGCCACGCTT

TTTAAGCCAAAATATGGAAGCGAATGATCCGGAAAAGAAGATTCCTTCTACTGCAGCAAAGGCAATAAGT

CTCTCTCCATAACCGGCGCTGTCATGTATCCACTTTTGAGCCCAATCGGCCTTCTTTTTTACACAAGGCA

TCGTTTCTATGGCATTAAAGAGATAGTTTTTTTCATTACTATCTTTAACATAAGTATCGATCAAAAGACT

ATACATTTCCGAATGAATGTTTTCAATGGCCATCTGAAATCCGTAGAAACATCTAGCCTCGGTAATCTGC

ACTTCTGTACAAAATCGTTCTGCTAAATTTTCATTCACTATTCCATCACTGGCTGCAAAAAACGCCAATA

CATGTTTTATAAAATATTTTTCGTCTGGTGTTAGTTTATTCCAGTCATTGATATCTTTAGATATATCCAC

TTCTTCCACTGTCCAAAATGATGCCTCTGCCTTTTTATACATATTCCAGATGTCATGATATTGGATTGGG

AAAATAACAAATCTATTTGGATTTGGTGCAAGGATAGGTTCCATAACTAAATTAACAATAGTAGTAATTT

TTTTTCAGTTATCTGTATGACTGTACTTGGATCTTTTGTATATCGCTATCGCCGCAATCACTACAATAAT

TACAAGTATTATTGATAGCATTGTTATTACTACTATCATAATTAAATTATCGACATTCATGGGTGTTGAA

TAATCGTTATCATCATTTTGTAATTGTGACATCATACTAGATAAATCATTTGTGAGATTGTTGTGGGAAG

CGGGCACGGAAGATGCATTATCATTATTATTTAACGCCTCCCATTCGGATTCACAAATGTTACGCACGTT

CAACGTTTTATGGAAACTATAATTTTGTGAAAACAGATAACAAGAAAACTCGTCATCGTTCAAATTTTTA

ACGATAGTAAACCGATTAAACGTCGAGCTAATTTCTAACGCTAGCGACTCTGTTGGATATGGGTTTCCAG

ATATATATCTTTTCAGTTCCCCTACGTATCTATAATCATCTGTAGGAAATGGAAGATATTTCCATTTATC

TACTGTTCCTAATATCATATGCGGTGGTGTAGAACCATTAAGCGCGAAAGATGTTATTTCGCATCGTATT

TTAACTTCGCAATAATTTCTGGTTAGATAACGCACTCTACCAGTCAAGTCAATGATATTAGCCTTTACAG

ATATATTCATAGTAGTCGTAACGATGACTCCATCTTTTAGATGCGATACTCCTTTGTATGTACCAGAATC

TTCGTACCTCAAACTCGATATATTTAAACAAGTTAATGATATATTAACGCGTTTTATGAATGATGATATA

TAACCAGAAGTTTTATCCTCTGTGGCTAGCGCTATAACCTTATCATTATAATACCAACTAGTGTAATTAA

TATGTGACACGACAGTGTGGGTACAAATATGTACATTATCGTCTACGTCGTATTTGATACATCCGCATAC

AGCCAACAAATATAAAATTACAAAAACTCTAACGACGTTCGTACACATCTTGATGTGGTTTAATAAATGT

TTTGATTTCAATTTATTGTAAAAAAGATTCGGTTTTATACTGTTCGATATTCTCATTGCTTATATTCTCA

TCTATCATCTCCACACAGTCAAATCCATGGTTAACATGTACCTCATCAACCGGTAAAAGACTATCGGATT

CTTCTATCATCATAACTCGAGAATATTTAATTTGGTGGTCATTATTAATCAAGTCAATTATCTTATTTTT

AACAAACGTAAGTATTTTACTCATTTTTTATAAAAACTTTTAGAAATATACAGACTCTATCGTGTGTCTA

TATCTTCTTTTTATATCCAATGTATTTATGTCTGATTTTTCTTCATTTATCATATATAATGGTCCAAATT

CTACACGTGCTTCGGATTCATCCAGATCATTAAGGTTCTTATAATCGCAACATCCTTCTCTTCCATCTTC

TACATCTTCCTTCTTATTCTTAGCGTCACAGAATCTACCACAGCAGGATCCCATGACGAGCGTCACATTA

AACTAATTCATTTTCAATTATAATATACTGATTAGTAATGACCATTAAAATAAAAATATTCTTCATAACC

GGTAAGAAAGTAAAAAGTTCACATTGAAACTATGTCAGTAGTTATACATCATGAGATGATATACTCTATT

TTGGTGGAGGATTATATGATATAATTCGTGGATAATCATTCTTAAGACACATTTCTTCATTCGTAAATCT

TTTCACATTAAATGAGTGTCCATATTTTGCAATTTCTTCATATGATGGCGGTGTACGTGGACGAGGCTGC

TCCTGTTCTTGTAGTCGCCGACTGTCGTGTTTGCGTTTAGATCCCTCCATTATCGCGATTGCGTAGTGAG

TACTATTTATACCTTGTAATTAAATTTTTTTATTAATTAAACGTATAAAAACGTTCCGTATCTGTATTTA

AGAGCCAGATTTCGTCTAATAGAACAAATAGCTACAGTAAAAATAACTAGAATAATCGCTACACCCACTA

GAAACCACGGATCGTAATACGGCAATCGGTTTTCGATAATAGGTGGAACGTATATTTTATTTAAGGACTT

AACAATTGTCTGTAAACCACAATTTGCTTCCGCCGATCCTGTATTAACTATCTGTAAAAGCATATGTTGG

CCGGGCGGAGCCGAACATTCTCCGATATTCAATTTTTGTATATCTATAATGTTATTAACCTCCGCATACG

CATTACAGTTCTTTTCTAGCTTGGATACTACACTAGGTACATCATCTAAATCTATTCCTATTTCCTCAGC

GATAGCTCTTCTATCCTTTTCCGGAAGTAATGAAATCACTTCAATAAATGATTCAACCATGAGTGTGAAA

CTAAGTCGAGAATTACTCATGCATTTGTTAGTTATTCGGAGCGCGCAATTTTTAAACTGTCCTATAACCT

CTCCTATATGAATAGCACAAGTGACATTAGTAGGGATAGAATGTTGAGCTAATTTTTGTAAATAACTATC

TATAAAAAGATTATACAAAGTTTTAAACTCTTTAGTTTCCGCCATTTATCCAGTCTGAGAAAATGTCTCT

CATAATAAATTTTTCCAAGAAACTAATTGGGTGAAGAATGGAAACCTTTAATCTATATTTATCACAGTCT

GTTTTGGTACACATGATGAATTCTTCTAATGCTGTACTAAATTCGATATCTTTTTCGATTTCTGGATATG

TTTTTAATAAAGTATGAACAAAGAAATGGAAATCGTAATACCAGTTATGTTCAACTTTGAAATTGTTTTT

TATTTTCTTGTTAATGATTCCAGCCACTTGGGAAAAGTCAAAGTCGTTTAATGCCGATTTAATACGTTCA

TTAAAAACAAACTTTTTATTCTTTAGATGAATTATTATTGGTTCATTGGAATCAAAAAGTAAGATATTAT

CGGGTTTAAGATCTGCATGTAAAAAGTTGTCACAACAGGGTAGTTCGTAGATTTTAATGTATAACAGAGA

CATCTGTAAAAAGATAAACTTTATGTATTGTACCAAAGATTTAAATCCTAATTTGATAGCTAACTCGGTA

TCTACTTTATCTGCCGAATACAGTGCTAGGGGAAAAATTATAATATTTCCTCTTTCGTATTCGTAATTAG

TTCTCTTTTCATGTTCGAAAAAGTGAAACATGCGGTTAAAATAGTTTATAACATTAATATTACTGTTAAT

AACTGCAGGATAAAAGTGGGATAGTAATTTCACGAATTTGATACTGTCCTTTCTCTCGTTAAACGCCTTT

AGAAAAACTTTAGAAGAATATCTCAATGAGAGTTCCTGACCATCCATAGTTTGTATCAATAATAGCAACA

TATGAAGAACCCGCTTATACAGAGTATGTAAAAATGTTAATTTATAGTTTAATCCCATGGCCCACGCACA

CACGATTAATTTTTTTTCATCTCCCTTTAGATTGTTGTATAGAAATTTGGGTACTGTGAACTCCGCCGTA

GTTTCCATGGGACTATATAATTTTGTGGCCTCGAATACAAATTTTACTACATAGTTATCTATCTTAAAGA

CTATACCATATCCTCCTGTAGATATGTGATAAAAATCGTCGTTTATAGGATAAAATCGTTTATCTTTTTG

TTGGAAAAAGGATGAATTAATGTAATCATTCTCTTCTATCTTTAGTAGTGTTTCCTTATTAAAATTCTTA

AAATAATTTAACAATCTAACTGACGGAGCCCAATTTTGGTGTAAATCTAATTGGGACATTATGTTGTTAA

AATATAAACAGTCTCCTAATATAACAGTATCTGATAATCTATGGGGAGACATCCATTGATATTCAGGGGA

TGAATCATTGGCAACACCCATTTATTGTACAAAAAGCCCCAATTTACAAACGAAAGTCCAGGTTTGATAG

AGACAAACTATTAACTATTTTGTCTCTGTTTTTAACACCTCCACAGTTTTTAATTTCTTTGGTAATGAAA

TTATTCACAATATCAGTATCTTCTTTATCTACCAGAGATTTTACTAACTTGATAACCTTGGCTGTCTCAT

TCAATAGGGTAGTGATATTTGTATGTATGATATTGATATCTTTTTGAATTGTTTCTTTTAGAAGTGATTC

TTTGATGGTATCAGCATACGAATTACAATAATGCAGAAACTCAGTTAACATGCAGGAATTATAGTAAGCC

AATTCCAATTGTTGCCTGTATTGTATTAGAGTATTAATATGCGCAATGATGTCCTTGCGTTTCTCTGATA

GAATGCGAGCAGCGATTTTGGCGTTATCATTTGACGATATTTCTGGAATAACGAATCCTGTTTCTACTAA

CTTCTTGGTAGGACAAAGTGAAACAATCAAGAAAATAGCTTCTCCTCCTATTTGTGGAAGAAATTGAACT

CCTCTAGATGATCTACTGACGATAGTATCTCCTTGACAGATATTGGACCGAACTACGGAAGTACCTGGAA

TGTAAAGCCCTGAAACCCCCTCATTTTTTAAGCAGATTGTTGCCGTAAATCCTGCACTATGCCCAAGATA

GAGAGCTCCTTTGGTGAATCCATCACTATGTTTCAGTTTAACCAAGAAACAGTCAGCTGGTCTAAAATTT

CCATCTCTATCTAATACAGAATCCAACTTGATGTCAGGGACTATGACCGGTTTAATGTTATATGTAACAT

TGAGTAAATCCTTAAGTTCATAATCATCGTTGTCATCAGTTATGTACGATCCAAACAATGTTTCTACCGG

CATGGTGGATACGAAGATGCTATCCATCAGAATGTTTCCCTGATTAGTATTTTCTATATAGCTATTCTTC

TTTAAACGATTTTCCGAATCAGTAACTATGTTCATTTTTTTAGGAGTAGGACGTCTAGCCAGTATGGAAG

AGGATTTTCTAGATACTCTCTTCAACATCTTTGATCTCAATGGAATGCAAAACCCCATGGTGTAACAACC

AACGATAAAAATAATATTGTTTTTTCACTTTTTATAATTTTACCATCTGACTCATGGATTCATTAATATC

TTTATAAGAGCTACTAACGTATAATTCTTTATAACTGAACTGAGATATATACACCGGATCTATGGTTTCC

ATAATTGAGTAAATGAATGCTCGGCAATAACTAATGGCAAATGTATAGAACAACGAAATTATACTAGAGT

TGTTAAAGTTAATATTTTCTATGAGTTGTTCCAATAAATTATTTGTTGTGACTGCGTTCAAGTCATAAAT

TATCTTGATACTATCCAGTAAACAGTCTTTAAGTTCTGGAATATTATCATCCCATTGTAAAGCCCCTAGT

TCGACTATCGAATATCCTGCTCTGATAGCAGTTTCAATATCGACGGACGTCAATACTGTAATAAAGGTGG

TAGTATTGTCATCATCGTGATAAACTACGGGAATATGGTCGTTAGTAGGTACCGTGACTTTACACAACGC

GATATATAACTTTCCTTTTGTACCATTTTTAACGTAGTTGGGACGTCCTGCAGGGTATTGTTTTGAAGAA

ATGATATCGAGAACAGATTTGATACGATATTTGTTGGATTCCTGATTATTCACTATAATATAATCTAGAC

AGATAGATGATTCGATAAATAGAGAAGGTATATCGTTGGTAGGATAATACATCCCCATTCCAGTATTCTC

GGATACTCTATTGATGACACTAGTTAAGAACATGTCTTCTATTCTAGAAAACGAAAACATCCTACATGGA

CTCATTAAAACTTCTAACGCTCCTGATTGTGTTTCGAATGCCTCGTACAAGGATTTCAAGGATGCCATAG

ATTCTTTGACCAACGATTTAGTATTGCGTTTAGCATCTGATTTTTTTATTAAATCAAATGGTCGGCTCTC

TGGTTTACTACCCCAATGATAACAATAGTCTTGTAAAGATAAACCGCAAGAAAATTTATACGCATCCATC

CAAATAACCCTAGCACCGTCGGATGATATTAATGTATTATTATAGATTTTCCATCCACAGTTATTGGGCC

AGTATACTGTTAGCAACGGTATATCGAATAGATTACTCATGTAACCTACTAGAATGATAGTTCGTGTACT

AGTCATAATATCTTTAATCCAATCTAAGAAATCTAAAATTAGATCTTTTACACTATTAAAGTTAACAAAG

GTATTACCCGGGTACGTGGATATCATATATGGTATTGGTCCATTATCAGTAATGGCTCCATAAACTGATA

CGGCGATGGTTTTTATATGTGTTTGATCTAATGAGGACGAAATTCGCGCCCACAATTCATCTCTAGATAT

GCATTTAATATCGAACGGTAACACATCAATCTCGGGACGCGTATATGTTTCTAAATTCTTAATCCAAATA

TAATGATGACCTATATGCCCTATTATCATACTGTCAACTATAGTATACCTAGAGAACTTTCGATACATCT

GCTGTTTCCTGTAATCGTTAAATTTTACAAATCTATAACATGCTAAACCTTTTGACGACAGCCATTCATT

AATTTCTGATATGGAATCTGTATTCTCAATACCGTATCGTTCTAAAGCCAGTGCTATATCTCCCTGTTCG

TGGGAACGCTTTCGTATAATATCGATCAATGGATAATATGAAGTTTTTGGAGAATAATATGATTCATGAT

CTATTTCGTCCATAAACAATCTAGACATAGGAATTGGAGGCGATGATCTTAATTTTGTGCAATGGGTCAA

TCCTATAACTTCTAATATTGTAATATTCATCATCGACATAACACTATCTATGTTATCATCGTATATTAGT

ATACCACGACCTTCTTCATTTCGTGCCAAAATGATATACAGTCTTAAATAATTACGCAATATCTCAATAG

TTTCATAATTGTTAGCTGTTTTCATCAAGGTTTGTATCCTGTTTAACATGATGGCGTTCTATAACGTCTC

TATTTTCTATTTTTAATTTTTTAAATTTTTAACGATTTACTGTGGCTAGATACCCAATCTCTCTCAAATA

TTTTTTTAGCCTCGCTTACAAGCTGTTTATCTATACTATTAAAACTGACGAATCCGTGATTTTGGTAATG

GGTTCCGTCGAAATTTGCCGAAGTGATATGAACATATTCGTCGTCGACTATCAACAATTTTGTATTATTC

TGAATAGTGAAAACCTTCACAGATAGATCATTTTGAACACACAACGCATCTAGACTTCTGGCGGTTGCCA

TAGAATATACGTCGTTCTTATCCCAATTACCAACTAGAAGTCTGATCTTAACTCCTCTATTAATGGCTGC

TTCTATAATGGAGTTGTAAATGTCAGGCCAATAGTAGCTATTACCGTCGACACGTGTAGTGGGAACTATG

GCCAAATGTTCAATATCTATACTAGTCTTAGCCGACTTGAGTTTATCAATAACTACATCAGTGTCTAGAT

CTCTAGAATATCCCAATAGGTGTTCTGGAGAATCAGTAAAGAACACTCCACCTATAGGATTCTTAATATG

ATACGCAGTGCTAACTGGCAGACAACAAGCCGCAGAGCATAAATTCAACCATGAATTTTTTGCGCTATTA

AAGGCTTTAAAAGTATCAAATCTTCTACGAAGATCTGTGGCCAGCGGAGGATAATCAGAATATACGCCTA

ACGTTTTAATCGTATGTATAGATCCTCCAGTAAATGACGCGTTTCCTACATAACATCTTTCATCATCAGA

CACCCAAAAACAACCGAGTAGTAGTCCCACATTATTTTTTTTATCTATATTAACGGTTATAAAATTTATA

TCCGGGGAGTGACTTTGTAGCTCTCCCAGATTTCTTTTCCCTCGTTCATCTAGCAAAACTATTATTTTAA

TCCCTTTTTCAGATACCTCTTTTAGTTTATCAAAAATAAGCGCTCCCCTAGTAGTACTCAGAGGATTACA

ACAAAAAGATGCTATGTATATATATTTCTTAGCTAGAGTGATAATTTCGTTAAAACATTCAAATGTTGTC

AAATGATCGGATCTAAAATCCATATTTTCTGGTAGTGTTTCTACCAGCCTACATTTTGCTCCCGCAGGTA

CCGATGCAAATGGCCACATTTAGTTAACATAAAAACTTATATATCCTGTTCTATCAACGATTCTAGAATA

TCATCGGCTATATCGCTAAAATTTTCATCAAAGTCGACATCACAACCTAACTCAGTCAATATATTAAGAA

GTTCCATGATGTCATCTTCGTCTATTTCTATATCCGTATCCATTGTAGATTGTTGACCGATTATCGAGTT

TAAATCATTACTAATACTCAATCCTTCAGAATACAATCTGTGTTTCATTGTAAATTTATAGGCGGTGTAT

TTAAGTTGGTAGATTTTCAATTATGTATCAATATAGCAACAGTAATTCTTGCTCCTCCTTGATTTTAGCA

TCCTCTTCATTATTTTCTTCTACGTACATAAGCATGTCCAATACGTTAGACAACACACCGACGATGGTGG

CCGCCACAGACACGAATATGACTAGACCGATGACCATTTAAAAAATACTCTCTAGCTTTAACTTAAACTG

TATCGATCATTCTTTTAGCACATGTATAATATAAAAACATTATTCTATTTCGAATTTAGGCTTCCAAAAA

TTTTTCATCCGTAAACCGATAATAATATATATAGACTTGTTAATAGTCGGAATAAATATATTAATGCTTA

AACTATCATCATCTCCACGATTAGAGATACAATATTTACATTCTTTTTGCTGTTTCGAAACTTTATCAAT

ACACGTTAATACAAACCCAGGAAGGAGATATTGAAACTGAGGCTGTTGAAAATGAAACGGTGAATACAAT

AATTCAGATAATGTAAAATCATGATTCCGTATTCTGATGATATTAGAACTGCTAATGGATGTCGATGGTA

TGTATCTAGGAGTATCTATTTTAACAAAGCATCGATTTGCTAATATACAATTATCCTTTTGATTAATTGT

TATTTTATTCATATTCTTAAAAGGTTTCATATTTATCAATTCTTCTACATTAAAAATTTCCATTTTTAAT

TTATCTAGCCCCGCAATACTCCTCATTACGTTTCATTTTTTGTCTAGAATGCCCATTTTGTTCATCTTGG

TACATAGATTATCCAATTGAGAAGCGCATTTAGTAGTTTTGTACATTTTAAGTTTATTAACGAATCGTCG

AAAACTAGTTATAGTTAACATTTTATTATTTGATACCCTGATATTAATACCCCTGCCGTTACTATTATTT

ATAACTGATGTAACCCACGTAACATTGGAATTAATTATCGATAGTAATGCATCGACACTTCCAAAATTGT

CTATTATAAACTCACCGATAATTTTTTTATTGCATGTTTTCATATTCATTAGGATTATCAAATCTTTAAT

CTTATTACGATTGTATGCGTTGATATTACAAGACGTCATTCTAAAAGACGGAGGATTTCCATCAAATGCC

AGACAATCACGTACAAAGTACATGGAAATAGGTTTTGTTCTATTACGCATCATAGATTCATATAAAACAC

CCGTAGAAATACTAATTTGTTTTACTCTATAAAATACTATTGCATCTATTTCATCGTTTTGTATAACGTC

TTTCCAAGTGTCAAATTCTAATTTTTTTTCATTGATAGTACCAAATTCTTCTATCTCTTTAACTACTTGC

ATAGATAGGTAATTACAGTGATGCCTACATGCCGTTTTTTGAAACTGAATAGATGCATCTAGAAGCGATG

CTACGCTAGTCACGATCACCACTTTCATATTTAGAATATATGTATGTAAAAATATAGTAGAATTTCATTT

TGTTTTTTCTATGCTATAAATGAATTCTCATTTTGTATCTGCACATACTCCGTTTTATATCAATACCAAA

GAAGGAAGATATCTGGTTCTAAAAGCCGTTAAAGTATGCGATGTTAGAACTGTAGAATGCGAAGGAAGTA

AAGCTTCCTGCGTACTCAAAGTAGATAAACCCTCATCACCCACGTGTGAGAGAAGACCTTCGTCCCCGTC

CAGATGCGAGAGAATGAATAACCCTGGAAAACAAGTCCCGTTTATGAGAACGGACATGTTACAAAATATG

TTTGCTGCTAATCGCGACAACGTAACGTCAAGACTTTTGAACTAAAATACAATTATATCTTTTTCGATAT

TAATAAATCCGTGTCTCCCGGGTTTTTTATCTCTTTCAGTATGTGAATAGATAGGTATTTTATCTCTATT

CATCATCGAATTTAAGAGATCCGATAAACATTGTTTGTATTCTCCAGATGTCAGCATCTGATACAACAAT

ATATGTGCACATAAACCTCTGGCACTTATTTCATGTACCTTCCCCTTATCACTAAGGAGAATAGTATTTG

AGAAATATGTATACATGATATTATCATGAATTAGATATACAGAATTTGTAACACTCTCGAAATCACACGA

TGTGTCGGCGTTAAGATCTAATATATCACTCGATAACACATTTTCATCTAGATACACTAGACATTTTTTA

AAGCTAAAATAGTCTTTAGTAGTAACAGTAACTATGCGATTATTTTCATCGATGATACATTTCATCGGCA

TATTATTACGCGTACCATCAAAGACTATACCATGTGTATATCTAACGTATTCTAGCATAGTTGCCATACG

TGCATTAAACTTTTCAGGATCTTTGGATAGATCTTCCAATCTATCTATTTGAGAAAACATTTTTATCATG

TTCAATAGTTGAAACGTCGGATCCACTATATAGATATTATCTATAAAGATTTTAGGAACTATGTTCATGG

TATCCTGGCGAATATTAAAACTATCAATGATATGATTATCGTTTTCATCTTTTATCACCATATAGTTTCT

AAGATATGGGATTTTACTTAATATAATATTATTTCCCGTAATAAATTTTATTAGAAATGCCAAATCTATA

AGAAAAGTCCTAGAATTAGTCTGAAGAATATCTATATCACCGTACCGTATATTTGGATTAATTAGATATA

GAGAATATGATCCGTAACATATACAACTTTTATTATGACGTCTAAGATATTCTTCCATCAACTTATTAAC

ATTTTTGACTAGGGAAGATACATTATGACGTCCCATTACTTTTGCCTTGTCTATTACAGCGACGTTCATA

GAATTTAGCATATCTCTTGCCAATTCTTCCATTGATGTTACATTATAAGAAATTTTAGATGAAATTACAT

TTGGAGCTTTAATAGTAAGAACTCCTAATATATCCGTGTATGTGGTCACTAATACAGATTGTAGTTCTAT

AATCGTAAATAATTTACCTATATTATATGTTTGAGTTTGTTTAGAAAAGTAGCTAAGTATACGATCTTTT

ATTTCTGATGCCGATGTATCAACATCGAAAAAAAATCTTTTTTTATTCTTTTTTACTAACGATACGAATA

TGTCTTTGTTAAAAACAGTTATTTTCTGAATATTTCTAGCTTGTAATTTTAACATATGATATTCGTTCAC

ACTAGGTACTCTGCCTAAATAGGTTTCTATAATCTTTAATGTAATATTAGGAAGAGTATTCTGATCAGGA

TTCCTATTCATTTTGAGGATTTAAAACTCTGATTATTGTCTAATATGGTCTCAACACAAACTTTTTCACA

GAGCGATAGAGTTTTTGATAACTCGTTTTTCTTAAGAAATATAAAACTACTGTCTCCAGAGCTCGCTCTA

TCTTTTATTTTATCTAATTCGATACAAACTCCTGATACTGGTTCAGAAAGTAATTCATTAATTTTCAGTC

CTTTATAGAAGATATTTAATATAGATAATACAAAATCTTCAGTTCTTGATATCGATCTGATTGATCCTAG

AACTAGATATATTAATAACGTGCTCATTAGGCAGTTTATGGCAGCTTGATAATTAGATATAGTATATTCC

AGTTCATATTTATTAGATACCGCATTGCCCAGATTTTGATATTCTATGAATTCCTCTGAAAATAAATCCA

AAATAACTAGACATTCTATTTTTTGTGGATTAGTGTACTCTCTTCCCTCTATCATGTTCACTACTGGTGT

CCACAATGATAAATATCTAGAGGGAATATAATATAGTCCATATGATGCCAATCTAGCAATGTCGAATAAC

TGTAATTTTATTCTTCGCTCTTCATTATGAATTGAATCTTGAGGTATAAACCTAACACAAATTATATCAT

TAGACTTTTCGTATGTAATGTCTTTCATGTTATAAGTTTTTAATCCTGGAATAGAATCTATTTTAATGAG

GCTTTTAAATGCAGCGTTCTCCAACGAGTCAAAGCATAATACTCTGTTGGTTTTCTTATATTCAATATTA

CGATTTTCTTCTTTGAATGGAATAGGTTTTTGAATTAGTTTATAATTACAACATAATAGATAAGGAAGTG

TGTAAATAGTACGCGGAAAAAACATAATAGCTCCCCTGTTTTCATCCATGGTTTTAAGTAAATGATCACT

GGCTTCTTTAGTCAATGGATATTCGAACATTAACCGTTTCATCATCATTGGACAGAATCCATATTTCTTA

ATGTAAAGAGTGATCAAATCATTGTGTTTATTGTACCATCTTGTTGTAAATGTGTATTCGGTTATCGGAT

CTGCTCCTTTTTCTATTAAAGTATCGATATCGATCTCGTCTAAGAATTCAACTATATCGACATATTTCAT

TTGTATACACATAACCATTACTAACGTAGAATGTATAGGAAGAGATGTAACGGGAACAGGGTTTGTTGAT

TCGCAAACTATTCTAATACATAATTCTTCTGTTAATACGTCTTGCACGTAATCTATTATAGATGCCAAGA

TATCTATATAATTATTTTGTAAGATGATATTAACTATGTGATCTATATAAGTAGTGTAATAATTCATGTA

TTTCGATATATGTTCCAACTCTGTCTTTGTGATGTCTAGTTTCGTAATATCTATAGCGTCCTCAAAAAAT

ATATTCGCATATATTCCCAAGTCTTCAGTTCTATCTTCTAAAAAATCTTCAACGTATGGAATATAATAAT

CTATTTTACCTCTTCTGATGTCATTAATGATATAGTTTTTGACACTATTTTCCGTCAATTGATTCTTATT

CACTATGTCTAAAAACCGGATAGCGTCCCTAGGACGAACTACTGCCATTAATATCTCTATTATAGCTTCT

GGACATAAATCATCTATTATACCAGAATTAATGGGAACTATTCCGTATCTATCTAACATAGTTTTAAGAA

AGTCAGAATCTAAGACCTGATGTTCATATATTGGTTCATACATGAAATGATCTCTATTGATGATAGTGAC

TATTTCATTCTCTGAAAATTGGTAACTCATTCTATACACGCTTTCCTTGTTGATAAAGGATAGTATATAC

TCGATGGAATTTGTACCAACAAACTGTTCTCTTATGAATCGTATATCATCATCTGAAATGATCATGTAAG

GCATACATTTAACAATGAGAGACTTGTCTCCTGTTATCAATATACTATTCTTGTGATAATTTATGTGTGA

GGCAAATTTGTCCACGTTCTTTAATTTTGTTATAGTAGATATCAAATCCAATGGAGATACAGTTCTTGGC

TTAAACAGATATAGTTTTTCTGGAACGAATTCTACAACATTATTATAAAGGACTTTGGGTATATAAGTGG

GATGAAATCCTATTTTAATTAATGCGATAGCCTTGTCCTCGTGCAGATATCCAAACGCTTTTGTGATAGT

ATGGCATTCATTGTCTAGAAACGCTCTACGAATATCTGTAACAGATATCATCTTTAGAGAATACTAGTCG

CGTTAATAGTACTAAAATTTGTATTTTTTAATCTATCTCAATAAAAAATTAATATGTATGATTCAATGTA

TAACTAAACTACTAACTGTTATTGATAACTAGAATCAGAATCTAATGATGACATAACTAAGAAGTTTATC

TACAGCCAATTTAGCTGCATTATTTTTAGCATCTCGTTTAGATTTTCCATCTGCCTTATCGAATACTCTT

CCGTCAATGTCTACACAGGCATAAAATGTAGGAGAGTTACTAGGCCCCACTGATTCAATACGAAAAGACC

AATCTCTCCTAGTTATTTGGCAGTACTCATTAATAACGGTGACAGGGTTAACACCTTTCCAATAAATAAT

TTTTTTAACCGGAATAACATCATCAAAAGACTTATTATCCTCTCTCATTGATTTTTCGCGGGATACATCA

TCTATTATAGCATCAGCATCAGAATCTGTAGGCCGTGTATCAGCATCCATTGTCGTAGACCAACGAGGAG

GAGTATCGTCGGAACTGTACACCATAGTACTACGTTGAAGATCATACAGAGCTTTATTAACTTCTCGCTT

CTCCATATTAAGTTGTTTAGTTAGTTGTGCAGTAGCTCCTTAGTCCAATGTTTTTAATAACCGCACACAA

TCTCTGTGTCAGAACGCTCGTCAATATAGATCGTAGAAATTTTTTAGAGAGAACTAACACAACTAGCAAT

AAAACTGATCTTATTTTATCATTTTTTTATTCATCATCCTCTGGTGGTTCGTCGTTCCTATCGAATGTAG

CTCTGATTAACCCGTCATCTATAGGTGATGCTGGTTCTGGAGATTCTGGAGGAGATGGATTATTATCCGG

AAGAATCTCTGTTATTTCCTTGTTTTCATGTATCGATTGCGTTGTAACATTAAGATTGCGAAATGCTCTA

AATTTGGGAGGCTTAAAGTGTTGTTTACAATCTCTACACGCGTGTCTAACTAATGGAGGTTCGTCAGCGG

CTCTAGTTTGAATCATCATCGGTGTAGTATTCCTACTTTTACAGTTAGGACACGGTGTATTGTATTTCTC

GTCGAGAACGTTAAAATAATCGTTGTAACTCACATCCTTTATTTTATCTATATTGTATTCTACTCCTTTC

TTAATGCATTTTATACCGAACAAGAGATAGCGAAGGAATTCTTTTTCGGTACCGCTAGTACCCTTAATCA

TATCACATAGTGTTTTATATTCTAAATTTGTGGCAATGGACGGTTTATTTCTATACGATAGTTTGTTTCT

GGAATCCTTTGAGTATTCTATACCAATATTATTCTTTGATTCGAATTTAGTTTCTTCGATATTAGATTTT

GTATTACCTATATTCTTGATGTAGTACTTTGATGATTTTTCCATGGCCCATTCTATTAAGTTTTCCAAGT

TGGCATCATCCACATATTGTGATAGTAATTCTCGGATATCAGTAGTGACTACCGCCATTGATATTTGTTC

ATTTGATGAGTAACTACTAATGTATACATTTTCCATTTATAACACTTATGTATTAACTTTGTTTATTTAT

ATTTTTTCATTATTATGTTGATATTAATAATCGTATTGTGGTTATATGGCTACAATTTCATAATGAGTTG

AAGTCAGTGTCCTATGATCAATGACGATAGCTTTACTCTGAAAGAAAGTATCAAATCGATAGTGCAGAGT

CAACAATGAAAATGGATAAGACGATGACAAAGTTTCAGAATAGAGTCAAAATGGTAAAAGAAATAAATCA

GACGATAAGAGCAGCACAAACTCATTGAGACATTGAAACTAGGATATATAAAATTTAAGTGAATGATTAG

GACTACTACTCTAGAAGATATAGCACCATCTATTATTCCAAATAATCAGAAAACTTATAAACTAT

TCTCGGACATTTCAGTCATTGGCAAAGCATCACAGAATCCGAGTAAGATGATATATGCTCGCTGCTTTAC

ATGTTTCCCAATTTGTTTGGAGATGACCATAGATTCATTTGTTATAGAATGCATCCAATGAGTAAAATCA

AACACAAAATCTTCGTTCAAACTTAATCTTATTAGAATATTAGTGGAAGAAAGATTCTATAATAATGAAT

GCAGAGATTATAAATGGAGAATAATTGGAACACAAGTTGATAAAATATTGATAGCTAAATATACAATAGA

TGCAATGTATCGCATAAGACCGATATATATAATACAAAGCAGTACAGATACAATGATGATGTAGAAAATG

GATTCATTGGATTGGATAAACTAAAATTAAACATTGTTCATGATATAGTTGAATCATGTATACCTGTTCG

TATGCCTGTGGCTAAGATACTGTGTAAAGAAATGGTAAATAAATACTTTGAGAATCTTTAAGAGTGTATT

GACTTTGTTAGTGAATATGCATTCCATCTTTCTCCAATACTAATTCAAATTGTTAAATTAATAATGGAAT

AGTATAAATAGTTATTAGTGATAAGATAGTAAAAATAATTATTAGAATAGTGTAGTATCATAGATAACTC

TCTTCTATAAAAAATGGATTTTATTCGTAGAAAGTATCTTATATACACAGTAGAAAATAATATAGATTTT

TTAAAGGATGATACATTAAGTAAAGTAAACAATTTTACCCTCAATCATGTACTAGCTCTCAAGTATCTAG

TTAGCAATTTTCCTCAACATGTTATTACTAAGGATGTATTAGCTAATACCAATTTTTTTGTTTTTATACA

TATGGTACGATGCTGTAAAGTATACGAAGCGGTTTTACGACACGCATTTGATGCACCCACGTTGTACGTT

AAAGCATTGACTAAGAATTATTTATCGTTTAGTAACACAATACAGTCGTACAAGGAAACCGTGCATAAAC

TAACACAAGATGAAAAATTTTTAGAGGTTGCCGAATACATGGACGAATTAGGAGAACTTATAGGCGTAAA

TTATGACTTAGTTCTTAATCCATTATTTCACGGAGGGGAACCCATCAAAGATATGGAAATCATTTTTTTA

AAACTGTTTAAGAAAACAGACTTCAAAGTTGTTAAAAAATTAAGTGTTATAAGATTACTTATTTGGGCAT

ACCTAAGCAAGAAAGATACAGGCATAGAGTTTGCGGATAATGATAGACAAGATATATATACTCTATTTCA

ACAAACTGGTAGAATAGTCCATAGCAATCTAACAGAAACGTTTAGGGATTATATCTTTCCCGGAGATAAG

ACTAGCTATTGGGTGTGGTTAAACGAAAGTATAGCTAATGATGCGGATATCGTTATTAATAGATCCGCCA

TTACCATGTATGATAAAATTCTTAGTTATATATACTCTGAGATAAAACAGGGACGCGTTAATAAAAACAT

GCTTAAGTTAGTTTATATCTTTGAGCCTGAAAAAGATATCAGAGAACTTCTGCTAGAAATCATATATGAT

ATTCCTGGAGATATCCTATCTATTATTGATGCAAAAAACGATGATTGGAAAAAATATTTTATTAGTTTTT

ACAAAGCTAATTTTATTAACGGTAATACATTTATTAGTGATAGAACGTTTAACGAGGACTTATTCAGAGT

TGTTGTTCAAATAGATCCCGAATATTTCGATAATGAACGAATTATGTCTTTATTCTATACGAGTGCTGCG

GACATTAAACGATTTGATGAGTTAGATATTAATAACAGTTATATATCTAATATAATTTATGAGGTGAACG

ATATCACATTAGATACAATGGATGATATGAAGAAGTGTCAAATCTTTAACGAGGATACGTTGTATTATGT

TAAGGAATACAATACATACCTGTTTTTGCACGAGTCGGATCCCATGGTCATAGAGAACGGAATACTAAAG

AAACTGTCATCTATAAAATCCAAGAGTAGACGGCTGAACTTGTTTAGCAAAAACATTTTAAAATATTATT

TAGACGGACAATTGGCTCGTCTAGGTCTTGTGTTAGATGATTATAAAGGAGACTTATTAGTTAAAATGAT

AAACCATCTCAAATCTGTGGAGGATGTATCCGCATTCGTTAGATTTTCTACAGATAAAAACCCTAGTATT

CTTCCATCGCTAATCAAAACTATTTTAGCTAGTTATAATATTTCCATCATCGTCTTATTTCAAAGGTTTT

TAAGAGATAATCTATATCATGTAGAAGAATTCTTGGATAAAAGCATCCATCTAACCAAGACTGATAAGAA

ATATATACTTCAATTGATAAGGCACGGTAGATCATAGAACAAACCAAATATATTATTAATAATTTGTATA

TACATAGATATAATTATCATATATTAAAAAATAACACATTTTTGATAAATGGAAACTGTTGCAACAATTC

AGACTCCCACCAAATTAATGAATAAAGAAAATGCAGAAATGATTTTGGAAAAAATTGTTAATCATATAGC

TATGTATATTAGTGACGAATCAATATATTCAGAAAATAATCCTGAATATATTGATTTTCGTAACAGATAC

GGAGACTATAGATCTCTCATTATAAAAAGTGATCACGAGTTTGTAAAGCTATGTAAAGATCATGCAGAGA

AAAGTTCTCCAGAAACGCAACAAATGATTATCAAACACATATACGAACAATATCTTATTCCAGTATCTGA

AGTACTATTAAAACCTATAATGTCCATGGGTGACATATTTACATATAACGGATGTAAAGACAATGAATGG

ATGCTAGAACAACTCTCTACCCTAAACTTTAACAATCTCTACACATGGAACTCATGTAGCATAGGCAATG

TAACGCGTCTGTTTTATACATTTTTTAGTTATCTGATGAAAGATAAACTAAATATATAAGTATAATCCCA

TTAATACTTTAACCTGATGTATTATTACCTGCATCTTATTAGAATATTAACCTAACTAAAAGACATAAAA

AGCGGGGATATAAATATTATGGCAGCAACCGTTCCGCGTTTTGACGATGTGTACAAAAATGCACAAAGAA

GAATTCTAGATCAAGAAACATTTTTTAGTAGAGGTCTAAGTAGACCGTTAATGAAAAACACATATCTATT

TGATAATTACGCGTATGGATGGATACCAGAAACTGCAATTTGGAGTAGTAGATACGCAAACCTAGATGCT

AGTGACTATTATCCCATTTCGTTGGGATTACTTAAAAAGTTTGAATTTCTCATGTCTCTATATAAAGGTC

CTATTCCCGTATATGAAGAAAAAGTAAATACTGAATTCATTGCTAATGGATCTTTCTCCGGTAGATACGT

ATCATATCTTAGAAAGTTTTCTGCCCTTCCAACAAACGAGTTTATTAGTTTTTTATTATTGACCTCCATC

CCTATCTATAATATCTTATTCTGGTTTAAAAACACACAGTTTGATATTACTAAACACACATTATTCAGAT

ACGTCTATACAGATAATACCAAACACCTTGCGTTGGCTAGGTATATACATCAAACAGGAGACTATAAGCC

TTTGTTTAGTCGTCTCAAAGAGAATTATATATTTACCGGTCCCGTTCCAATAGGTATCAAAGATATAGAT

TACCCTAATCTTAGTAGAGCAAGAAGTCCATCCGATTATGAGACATTAGCTAATATTAGTACTATATTGT

ACTTTACCAAGTATGATCCAGTATTAATGTTTTTATTGTTTTACGTACCTGGGTATTCAATTACTACAAA

AATTACTCCAGCCGTAGAATATCTAATGGATAAACTGAATCTAACAAAGAGCGACGTACAACTGTTGTAA

ATTATTTTTATGCTTCGTAAAATGTAGGTCTTGAACCAAACATTCTTTGAAAAAATGAGATGCATAAAAC

TTTATTATCCAATAGATTAACTATTTCAGACGTCAATCGTTTAAAGTAAACTTCGTAAAATATTCTTTGA

TTGCTGCCGAGTTTAAAACTTCTATCGATAATTGTTTCATATGTTTTAATATTTACAAGTTTTTTGGTCC

ATGGTACATTAGCTGGACAGATATATGCAAAATAATATCGTTCTCCAAGTTCTATAGTCTCTGGATTGTT

TTTATTATATTCAGTAACCAAATACATATTAGGGTTATCTGCGGATTTATAATTTGAGTGATGCATTCGA

CTCAACATAAATAATTCTAGAGGAGACGATCTACTATCAAATTCGGATCGTAAATCTGTTTCTAAAGAAC

GGAGAATATCTATACATACCTGATTAGAATTCATCCGTCCTTCAGACAACATCTCAGACAGTCTGGTCTT

GTATGTCTTAATCATATTCTTATGAAACTTGGAAACATCTCTTCTAGTTTCACTAGTACCTTTATTAATT

CTCTCAGGTACAGATTTTGAATTCGACGATGCCGAGTATTTCATCGTTGTATATTTCTTCTTCGATTGCA

TAATCAAATTCTTATATACCGCCTCAAACTCTATTTTAAAATTATTAAACAATACTCTACTATTAATCAG

TCGTTCTAACTCCTTTGCTATTTCTATGGACTTATCTACATCTTGACTGTCTATCTCTGTAAACACGGAG

TCGGTATCTCCATACACGCTACGAAAACGAAATCTATAATCTATAGGCAACGATGTTTTCACAATCGGAT

TAATATCTCTATCGTCCATATAAAATGGATTACTTAATGTATTGGCAAACCGTAACATACCGTTGGATAA

CTCTGCTCCATTTAGTACCGATTCTAGATACAAGATCATTCTACGTCCTATGGATGTGCAACTCTTAGCC

GAAGCGTATGAGTATAGAGCACTATTTCTAAATCCCATCAGACCATATACTGAGTTGGCTACTATCTTGT

ACGTATATTGCATGGAATCATAGATGGCCTTTTCAGTTGAACTGGTAGCCTGTTTTAACATCTTTTTATA

TCTGGCTCTCTCTGCCAAAAATGTTCTTAATAGTCTAGGAATGGTTCCTTCTATTGATCTATCGAAAATT

GCTATTTCAGAGATGAGGTTCGGTAGTCTAGGTTCACAATGAACCGTAATATATCTAGGAGGTGGATATT

TCTGAAGCAAGAGCTGATTATTTATTTCTTCTTCCAATCTATTGGTACTAACAACGACACCGACTAATGT

TTCCGGAGATAGATTTCCAAAGATACACACATTAGGATACAGACTGTTATAATCAAAGATTAATACATTA

TTACTAAACATTTTTTGTTTTGGAGCAAATACCTTACCGCCTTCATAAGGAAACTTTTGTTTTGTTTCTG

ATCTGACTAAGATAGTTTTAGTTTCCAACAATAGCTTTAACAGTGGACCCTTGATGACTGTACTCGCTCT

ATATTCGAATACCATGGATTGAGGAAGCACATATGTTGACGCACCAGCGTCTGTTTTTGTTTCTACTCCA

TAATACTCCCACAAATACTGACACAAACAAGCATCATGAATACAGTATCTAGCCATATCTAAAGCTATGT

TTAGATTATAATCCTTATACATCTGAGCTAAATCAATGTCATCCTTTCCGAAAGATAATTTATATGTATC

ATTAGGTAAAGTAGGACATGATAGTACGACTTTAAATCCATTTTCCCAAATATCTTTACGAATTACTTTA

CATATAATATCCTCATCAACAGTCACATAATTACCTGTTGTTAAAACCTTTGCAAATGTATCGGCTTTGC

CTTTCGCGTCCGTAGTATCGTCACCGATGAACGTCATTTCTCTAACTCCTCTATTTAATACTTTACCCAT

GCAACTGAACGCGTTCTTGGATATAGAATCCAATTTGTACGAATCCAATTTTTCAGATTTTTGAATGAAT

GAATATAGATCGAAAAATATAGTTCCATTATTGTTATTAACGTGAAACGTAGTATTGGCCATGCCGCATA

CTCCCTTATGACTAGACTGATTTCTCTCATAAATACAGAGATGTACAGCTTCCTTTTTGTCTGGAGATCT

AAAGATAATCTTCTCTCCTGTTAATAACTCTAGACGATTAGTAATATATCTCAGATCAAAGTTATGTCCG

TTAAAGGTAACGACGTAGTCGAACGTTAGTTCCAACAATTGTTTAGCTATTCGTAACAAAACTATTTCAG

AACATAGAACTAGTTCTCGTTCGTAATCCATTTCCATTAGCGACTGTATCCTCAAACATCCTCTATCGAC

GGCTTCTTGTATTTCCTGTTCCGTTAACATCTCTTCATTAATGAGCGTAAACAGTAATCGTTTACCACTT

AAATCGATATAACAGTAACTTGTATGCGAGATTGGGTTAATAAATACAGAAGGAAACTTCTTATCGAAGT

GACACTCTATATCTAGAAATAAGTACGATCTTGGGATATCGAATCTAGGTATTTCTTTAGCGAAACAGTT

ACGTGGATCGTCACAATGATAACATCCATTGTTAATCTTTGTCAAATATTGCTCGTCCAACGAGTAACAT

CCGTCTGGAGATATCCCGTTAGAAATATAAAACCAACTAATATTGAGAAATTCATCCATGGTGGCATTTT

GTATGCTGCGTTTCTTTGGCTCTTCTATCAACCACATATCTGCGACGGAGCATTTTCTATCTTTAATATC

TAGATTATAACTTATTGTCTCGTCAATGTCTATAGTTCTCATCTTTCCCATCGGCCTCGCATTAAATGGA

GGAGGAGATAATGACTGATATATTTCGTCCGTCACTACGTAATAAAAGTAATGAGGAAATCGTATAAATA

CTGTCTCGCCATTTCGACATCTGGATTTCAGATATAAAAATCTGTTTTCACCGTGACTTTCAAACCAATT

AATACACCTAACATCCATTTCTAGAATTTAGAAATATATTTTCATTTAAATGAATCCCAAACATTGGGGA

AGAGCCATATGGACCATTATTTTTATAGTACTTTCGCAAGCGGGTTTAGACGGCAACATAGAAGCGTGTA

AACGAAAACTATATACTATAGTCAGCACTCTTCCATGTCCTGCATGTAGACGACACGCGACTATCGCTAT

AGAGGACAATAATGTCATGTCTAGCGATGATCTGAATTATATTTATTATTTTTTCATCAGATTATTTAAC

AATTTGGCATTTGATCCCAAATACGCAATCGATGTGTCAAAGGTTAAACCTTTATAAACTTAACCCATTA

TAAAACTTATGATTAGTCACGACTGAAATAACCGCGTGATTATTTTTTGGTATAATTCTACACGGCATGG

TTTCTGTGACTATGAATTCAACACCTGTTATCTTAGTGAAATCTTTAACAAACAGCAAGGGTTCGTCAAA

GACATAAAACTCATTGTTTACGATCGAAATAGACCCCCTATCACACTTAAAATAAAAAATATCCTTATCC

TTTACCACCAAATAAAATTCTGATTGGTCAATGTGAATGTATTCACTTAACAGTTCCACAAATTTATTTA

TTAACTCCGAGGCACATACATCGTCGGTATTTTTTATGACAAACTTTACTCTTCCAGCATCCGTTTCTAA

AAAAATATTAACGAGTTCCATTTATATCATCCAATATTATTGAAATGACGTTGATGGACAGATGATACAA

ATAAGAAGGTACGGTACCTTTGTCCACCATCTCCTCCAATTCATACTCTATTTTGTCATTAACTTTAATG

TGTGAAAACAGTACGCCACATGCTTCCATGACAGTGTGTAACACTTTGGATACAAAATGTTTGACATTAG

TATAATTGTCCAAGACTGTCAATCTATAATAGATAGTAGCTATAATATATTCTATGATGGTATTGAAGAA

GATGACAACCTTGGCATATTGATCATTTAACACAGACATGGTATCAACAAATAGCTTAAATGAAAGAGAA

TCAGTAATTGGAATAAGCGTCTTCTCGATGTAGTGTCCGTATACCAACATGTCTGATATTTTGATGTATT

CCATTAAATTATTTAGTTTTTTCTTTTTATTCTCGTTAAACAGAATTTCTGTCAATGGACCCCAACATCG

TTGACCTATTAAGTTTTGATTGATTTTTCCGTGTAAGGCGTATCTAGTCAGATCGTATAGCCTATCCAAT

AATCCATCGTCTGTGCGTAGATCACATCGTACACTTTTTAATTTTCTATAGAAGAGTGACAGACATCTGG

AGCAATTACAGACAGCAATTTCTTTATTCTCTACAGATGTAAGATACTTGAAGACATTCCTATGATGATG

CAGAATTTTGGATAACACGGTATTGATGGTATCTGTTACCATAATTCCTTTGACTGATAGTGTCAAAGTA

CAAGATTTCCAATCTTTTGCAATTTTCAGTACCATTATCTTTGTTTTGATATCTATATCAGACAGCATGG

TACGTCTGACAACACAGGGATTAAGACGGAAAGATGAAATGATTCTCTCAACATCTTCAATAGATACCTT

GCTATTTTTTTTGGCATTATCTATATGTGAGAGAATATCCTCTAGAGAATCAGTATCCTTTTTGATGATA

GTGGATCTCAATGACATGGGACGTCTAAACCTTCTTATTCTATCACCAGATTGCATGGTGATTTGTCTTC

TTTCTTTTATCATGATGTAATCTCTAAATTCATCGGCAAATTGTCTATATCTAAAATCATAATATGAGAT

GTTTACCTCTACAAATATCTGTTCGTCCAATGTTAGAGTATCTATATCAGTTTTGTATTCCAAATTAAAC

ATGGCAACGGATTTAATTTTATATTCCTCTATTAAGTCCTCGTCGATAATAACAGAATGTAGATAATCAT

TTAATCCATCGTACATGGTTGGAAGATGCTCGTTGACAAAATCTTTAATTGTCTTGATGAAGGTGGGACT

ATATCTAACATCTTGATTAATAAAATTTATAACATTGTCCATAGGATACTTTGTAACTAGTTTTATACAC

ATCTCTTCATCGGTAAGTTTAGACAGAATATCGTGAACAGGTGGTATATTATATTCATCAGATATACGAA

GAATAATGTCCAAATCTATATTGTTTAATATATTATATAGATGTAGTGTAGCTCCTACAGGAATATCTTT

AACTAAGTCAATGATTTCATCAACAGTTAGATCTATTTTAAAGTTAATCATATAGGCATTGATTTTTAAA

AGGTATGTAGCCTTGACTACATTCTCATTAATTAACCATTCCAAGTCACTGTGTGTAAGAAGATTATATT

CTATCATAAGCTTGACTACATTTGGTCCCGATACCATTAAAGAATTCTTATGATATAAGGAAACAGCTTT

TAGGTACTCATCTACTCTACAAGAATTTTGGAGAGCCTTAACGATATCAGTGACGTTTATTATTTCAGGA

GGAAAGAACCTAACATTGAGAATATCTGAATTAATAGCTTCCAGATACAGTGATTTTGGCAATAGTCCGT

GTAATCCATAATCCAGTAACACGAGCTGGTGCTTGCTAGACACCTTTTCAATGTTTAATTTTTTTGAAAT

AAGCTTTGATAAAGCCTTCCTCGCAAATTCCGGATACATGAACATGTCGCCAACATGATTAAGTATTGTT

TTTCATTATTTTTATATTTTCTCAACAAGTTCTCAATACCCCAATAGATAATAGAATATCACCCAATGCG

TCCATGTTGTCTATTTCCAACAGGTCGCTATATCCACCAATAGAAGTTTTCCCAAAAAAGATTCTAGGAA

CAGTTCTACCACCAGTAATTTGTTCAAAATAGTCACGCAATTCATTTTCGGGTTTAAATTCTTTAATATC

TACAATTTCATACGCTCCTCTTTTGAAACTAAACTTATTTAGAATATCCAGTGCGTTTCTACAAAAAGGA

CACGTAAACTTGACAAAAATTGTCACTTTGTTATTGGCCAACCTTTGTTGTACAAATTCCTCGGCCATTT

TTAATATTTAAGTGATACAAAACTATCTCGACTTATTTAACTCTTTAGTCGAGATATATGGACACAGATA

GTTATATGATAACCAACTACAGAAGACAAACGCTATAAAAAACATAATTACAACGAGCATATTTATAAAT

ATTTTTATTCAGTATTACTTGATATAGTAATATTAGGCACAGTCAAACATTCAACCACTCTAGATACATT

AACTCTCTCATTTTCTTTAACAAATTCTGCAATATCTTCGTAAAAAGATTCTTGAAACTTTTTAGAATAT

CTATCGACTCTAGATGAAATAGCGTTCGTCAACATACTATGTTTTGTATACATAAAGGCGCCCATTTTAA

CAGTTTCTAGTGACAAAATGCTAGCGATCCTAGGATCCTTTAGAATCACATAGATTGACGATTCGTCTCT

CTTAGTAACTCTAGTAAAATAATCATACAATCTAGTACGCGAAATAATATTATCCTTGACTTGAGGAGAT

CTAAACAATCTAGTTTTGAGAACATCGATAAGTTCATCGGGAATGACATACATACTATCTTTAATAGAAC

TCTTTTCATCCAGTTGAATGGATTCGTCCTTAACCAACTGATTAATGAGATCTTCTATTTTATCATTTTC

CAGATGATATGTATGTCCATTAAAGTTAAATTGTGTAGCGCTTCTTTTTAGCCTAGCAGCCAATACTTTA

ACATCACTAATATCGATATACAAAGGAGATGATTTATCGATGGTATTAAGAATTCGTTTTTCGACATCCG

TCAAAACCAATTCCTTTTTGCCTGTATCATCCAGTTTGCCATTCTTTGTAAAGAAATTATTTTCTACTAG

ACTATTAATAAGACTGATAAGGATTCCTCCATAATTGCACAATCCAAACTTTTTCACAAAACTAGACTTT

ACGAGATCTACAGGAATGCGTACTTCAGGTTTCTTAGCTTGTGATTTTTTCTTTTGCGGACATTTTCTAG

TGACCAACTCATCTACCATTTCATTGATTTTAGCAGTGAAATAAGCTTTCAATGCACGGGCACTGATACT

ATTGAAAACGAGTTGATCTTCAAATTCCGCCATTTAAGTTCACCAAACAACTTTTAAATACAAATATATC

AATAGTAGTAGAATAAGAACTATAAAAAAAATAATAATTAACCAATACCAACCCCAACAACCTGTATTAT

TAGTTGATGTGACAGTTTTCTCATCACTTAGAACAGATTTAACAATTTCTATAAAGTCTGTCAAATCATC

TTCCTGAGAACCCATAAATACACCAAATATAGCAGCGTACAACTTATCCATTTATACATTGAATATTGGC

TTTTCTTTATCGCTATCTTCATCATATTCATCATCAATATCGACAAGTCCCAGATTACGAACCAGATCTT

CTTCTACATTTTCAGTCATTGATACGCGTTCACTATCTCCAGAGAGTCCGATAACGTTAGCCACCACTTC

TCTATCAATGATTAGTTTCTTGAGCGCGAATGTAATTTTTGTTTCCGTTCCGGATCTATAGAAAACTACA

GGTGTGATAATTGCCTTGGCTAATTGTCTTTCTCTTTTACTGAGTGATTCTAGTTCACCTTCTATAGATC

TGAGAATGGATGATTCTCCAGTCGAAACATATTCTACCATGGCTCCGTTTAATTTGTTGATGAAGATGGA

TTCATCCTTAAATGTTTTCTCTGTAATAGTTTCCGCCGAAAGACTATGCAAAGAATTTGGAATGCGTTCC

TTGTGCGTAATGTTTCCATAGACAGCTTCTAGAAGTTGATACAACATAGGACTAGCCGCGGTAACTTTTA

TTTTTAGAAAGTATCCATCGCTTCTATCTTGTTTAGATTTATTTTTATAAAGTTTAGTCTCTCCTTCCAA

CATAATAAAAGTGGAAGTCATCTGACTAGATAAACTATCAGTAAGTTTTATAGAGATAGATGAACAATTA

GCGTATTGAGAAGCATTTAGTGTAACGTATTCGATACATTTTGCATTAGATTTACTAATCGATTTTGCAT

ACTCTATAACACCCGCACAAGTCTGTAGAGAATCGCTAGATGCTGTAGGTCTTGGTGAAGTTTCAACTCT

CTTCTTGATTACCTTACTCATGATTAAACCTAAATAATTGTACTTTGCAATATAATGATATATATTTTCA

CTTTATCTCATTTGAGAATAAAAATGTTTTTGTTAACCGCTGCATGATGTACAGATTTCGGAATCGCAAA

CCACTTGTGGTTTTATTTTATCCTTGTCCAATGTGAATTGAATGGGAGCGGATGCGGGTTTCGTACGTAG

ATAGTACATTCCCGTTTTTAGACCGAGACTCCATCCGTAAAAATGCATACTCGTTAGTTTGGAATAACTC

GGATCTGCTATATGGATATTCATAGATTGACTTTGATCGATGAAGGCTCCCCTGTCTGCAGCCATTTTTA

TGATCGTCTTTTGTGGAATTTCCCAAATAGTTTTATAAACTCGCTTAATATCTTCTGGAAGGTTTGTATT

CTGAATGGATCCACCATCTACCATAATCCTATTCTTGATCTCATCATTCCATAATTTTCTCTCGGTTAAA

ACTCTAAGGAGATGCGGGTTAACTACTTGGAATTCTCCAGACAATACTCTCCGAGTGTAAATATTACTGG

TATACGGTTCCACCGACTCATTATTTCCCAAAATTTGAGCAGTTGATGCAGTCGGCATAGGTGCCACCAA

TAAACTATTTCTAAGACCGTATGTTCTGATTTTATCTTTTAGATGTTCCCAATTCCAAAGATCCGACGGT

ACAACATTCCAAAGATCATATTGTAGAATACCGTTACTGGCGTACGATCCTACATATGTATCATATGGTC

CTTCCTTCTCAGCTAGTTTACAACTCGCCTCTAATGCACCGTAATAAATGGTTTCAAAGATCTTCTTATT

TAGATCTTGTGCTTCCGGGCTATCAAATGGATAATTTAAGAGAATAAACGCGTCCGCTAATCCTTGAACA

CCAATACCGATAGGTCTATGTCTCTTATTAGAGATTTCAGCTTCTGGAATAGGATAATAATTAATATCTA

TAATTTTATTGAGATTTCTGACAATTACTTTGACCACATCCTTCAGTTTGAGAAAATCAAATCGCCCATC

TATTACAAACATGTTCAATGCAACAGATGCCAGATTACACACGGCTACCTCATTAGCATCCGCATATTGT

ATTATCTCAGTGCAAAGATTACTACACTTGATAGTTCCTAAATTTTGTTGATTACTCTTTTTGTTACACG

CATCCTTATAAAGAATGAATGGAGTACCAGTTTCAATCTGAGATTCTATAATCGCTTTCCAGACGACTCG

AGCCTTTATTATAGATTTGTATCTCCTTTCTCTTTCGTATAGTGTATACAATCGTTCGAACTCGTCTCCC

CAAACATTGTCCAATCCAGGACATTCATCCGGACACATCAACGACCACTCTCCGTCATCCTTCACTCGTT

TCATAAAGAGATCAGGAATCCAAAGAGCTATAAATAGATCTCTTGTTCTATGTTCCTCGTTTCCTGTATT

CTTTTTAAGATCGAGGAACGCCATAATATCAGAATGCCACGGTTCCAAGTATATGGCCATAACTCCAGGC

CGTTTGTTTCCTCCCTGATCTATGTATCTAGCGGTGTTATTATAAACTCTCAACATTGGAATAATACCGT

TTGATATACCATTGGTACCGGAGATATAGCTTCCACTGGCACGAATATTACTAATTGATAGACCTATTCC

CCCTGCCATTTTAGAGATTAATGCGCATCGTTTTAACGTGTCATAGATGCCTTCTATGCTATCATCGATC

ATGTTAAGTAGAAAACAGCTAGACATTTGGTGACGAGTAGTTCCCGCATTAAATAAGGTAGGAGAAGCGT

GCGTAAACCATTTTTCAGAAAGTAGATTGTACGTCTCAATAGCTGAGTCTATATCCCATTGATGAATTCC

TACTGCGACACGCATTAACATGTGCTGAGGTCTTTCAACAATTTTGTTGTTTATTTTCAACAAGTAGGAT

TTTTCCAAAGTTTTAAAACCAAAATAGTTGTATGAAAAGTCTCGTTCGTAAATAATAACCGAATTGAGCT

TATCCTTATATTTGTTAACTATATCCATGGTAATACTTGAAATAATCGGAGAATGTTTCCCATTTTTAGG

ATTAACATAGTTGAATAAATCCTCCATCACTTCACTAAATAGTTTTTTTGTTTCCTTGTGTAGATTTGAT

ATGGCTATTCTGGCGGCTAGAATGGCATAATCCGGATGTTGTGTAGTACAAGTGGCTGCTATTTCGGCTG

CCAGAGTGTCCAATTCTACCGTTGTTACTCCATTATATATTCCTTGAATAACCTTCATAGCTATTTTAAT

AGGATCTATATGATCAGTGTTTAAGCCATAGCACAATTTTCTAATACGAGACGTGATTTTATCAAACATG

ACATTTTCCTTGTATCCATTTCGTTTAATGACAAACATTTTTGTTGGTGTAATAAAAAAAAATTATTTAA

TTTTTCATTAATAGGGATTTGACGTATGTAGCGTACAAAATTATCGTTCCTGGTATATAGATAAAGAGTC

CTATATATTTGAAAATCGTTACGGTTCGATTAAACTTTAATGATTGCATTGTGAATATATCATTAGGATT

TAACTCCTTGACTATCATGGCGGTGCCAGAAATTACCATCAAAAGCATTAATACAGTTATACAGATCGCA

GTTAGAACGGTTATAGCATCCACCATTTATATCTAAAAATTAGATCAAAGAATATGTGACAACGTCCTAG

TTGTATACTGAGAATTGACGAAACAATGTTTCTTACATATTTTTTTCTTATTAGTAACCGACTTAATAGT

AGGAACTGGAAAACTAGACTTGATTATTCTATAAGTATAGATACCCTTCCAAATAATGTTCTCTTTGATA

AAAGTTCCAGAAAATGTAGAATTTTTTAAAAAGTTATCTTTTGCTATTACTAATATCGTGGTTAGACGCT

TATTATTAATATGAGTGATGAAATCCACACCGCTTCTAGATATCGCTTTTATTTCCACATTAGATGGTAA

ATCCAATAGTGAAACTATCTTTTTAGGAATGTATGGACTTGCGTTTAGAGGAGTGAACGTCTTCGGAGTA

GTAAAGGATGATTCGTCAAATGAATAAACAATTTCACAAATGGATGTTAATGTATTAGTAGGAAATTTTT

TGACGCTAGTGGAATTGAAGATTCTAATGGATGATGTTCTACCTATTTCATCCGATAACATGTTAATTTC

CAACACCAACGGTTTTAATATTTCGATGATATACGGTAGTCTCTCTTTCGGACTTATATAGCTTATTCCA

CAATACGAGTCATTATATACTCCAAAAAACAAAATAACTAGTATAAAATCTGTATCGAATGGGAAAAACG

AAATTATCGATATAGGTATAGAATCCGGAACATTGAACGTATTAATACTTAATTCTTTTTCAGTGGTAAG

AACCGATAGGTTATTGACATTGTATGGTTTTAAATATTCTATAACTTGAGACTTGATAGATATTAATGAC

GAATTGAAAATTATTTTTATCACCACGTGTGTTTCAGGATCATCGTCGACGCCAGTCAACCAACCGAATG

GAGTAAAATAAATATCATTAATATATGCTCTAGATATTAGTATTTTTATTAATCCTTTGATTATCATCTT

CTCGTACGCGAATGATTCCATGATCAAGAGTGATTTGAGAACATCCTCCGGAGTATTAATGGGCTTAGTA

AACAGTCCATCGTTGCAATAATAAAAGTTGTCCAAGTTAAAGGATATTATGCATTCGTTTAAAGATATCA

CCTCATCTAACGGAGACAATTTTTTGGTAGGTTTTAGAGACTTTGAAGCTACTTGTTTAACAAAGTTATT

CATCGTCGTCTACTATTCTATTTAATTTTGTAGTTAATTTATCACATATCACATTAATTGACTTTTTGGT

CCACTTTTCCATACGTTTATATTCTTTTAATCCTGCGTTATCCGTTTCCGTTATATACAGGGATAGATCT

TGCAAGTTAAATAGAATGCTCTTAAATAATGTCATTTTTTTATCCGCTAAAAATTTAAAGAATGTATAAA

CTTTTTTCAAAGATTTAAAACTTTTAGGTGGAGTTCTGGTACACAATATCATAAACAAACTAATAAACAT

CCCACATTCAGATTCCAACAATTGATTAACTTCCACATTAATACAGCCTATTTTCGCTCCAAATGTACAT

TCGAAAAATCTGAATAAAACATCAATATCGCAATTTGTATTATCCAATACAGAATGTCTGTGATTCGTGT

TAAAACCATCGGAAAAAGAATAGAAATAAAAATTATTATAATGGTGGAATTCAGTTGGAATATTGCCTCC

GGAGTCATAAAAGGATACTAAACATTGTTTTTTATCATAAATTACACATTTCCAATGAGACAAATAACAA

AATCCAAACATTACAAATCTAGAGGTAGAACTTTTAATTTTGTCTTTAAGTATATACGATAAGATATGTT

TATTCATAAACGCGTCAAATTTTTCATGAATAGCTAAGGAGTTTAAGAATCTCATGTCAAATTGTCCTAT

ATAATCCACTTCGGATCCATAAGCAAACTGAGAGACTAAGTTCTTAATACTTCGATTGCTCATCCAGGCT

CCTCTCTCAGGCTCTATTTTCATCTTGACGACCTTTGGATTTTCACCAGTATGTATTCCTTTACGTGATA

AATCATCGATTTTCAAATCCATTTGTGAGAAGTCTATCGCCTTAGATACTTTTTCCCGTAGTTGAGGTTT

AAAGAAATACGCTAACGGTATACTAGTAGGTAACTCAAAGACATCATATATAGAATGGTAACGCGTCGTT

AACTCGTCGGTTAACTCTTTCTTTTGATCGAGTTCGTCGCTACTATTGGGTCTGCTCAGGTGCCCCGACT

CTACTAGTTCCAACATCATACCGATAGGAATACAAGACACTTTGCCAGCGGTTGTAGATTTATCATATTT

CTCCACCACATATCCGTTACAATTTGTTAAGAATTTAGATACATCTATATTGCTACATAATCCAGCTAGT

GAATATATATGACATAATAAATTGGTAAATCCTAGTTCTGGTATTTTACTAATTACTAAATCTGTATATC

TTTCCATTTATCATGGAAAAGAATTTACCAGATATCTTCTTTTTTCCAAACTGCGTTAATGTATTCTCTT

ACAAATATTCACAAGATGAATTCAGTAATATGAGTAAAACGGAACGTGATAATTTCTCATTGGCTGTGTT

TCCAGTGATAAAACATAGATGGCATAACGCACACGTTGTAAAACATAAAGGAATATACAAAGTTAGTACA

GAAGCACGTGGAAAAAAAGTATCTCCTCCATCACTAGGAAAACCCGCACATATAAACCTAATGTCGAAGC

AATATATATATAGTGAGTATGCAATAAGCTTTGAATGTTATAGTTTTCTAAAATGTATAACAAATACAGA

AATCAATTCGTTCGATGAGTATATATTAAGAGGACTATTAGAAGCTGGTAATAGTTTACAGATATTTTCC

AATTCCGTAGGTAAACGAATAGATACTATAGGTGTACTAGGGAATAAGTATCCATTTAGCAAAATTCCAT

TGGCCTCATTAACTCCTAAAGCACAACGAGAGATATTTTTAGCGTGGATTTCTCATAGACCTGTAGTTTT

AACTGGAGGAACCGGAGTGGGTAAGACGTCACAGGTACCCAAGTTATTGCTTTGGTTTAATTATTTATTT

GGTGGATTCTCTACTCTAGATAAAATCACTGACTTTCACGAAAGACCAGTCATTCTATCTCTTCCTAGGA

TAGCTTTAGTTAGATTGCATAGCAATACCATTTTAAAATCATTGGGATTTAAGGTACTAGATGGATCTCC

TATCTCTTTACGGTACGGATCTATACCGGAAGAATTAATAAACAAACAACCAAAAAAATATGGAATTGTA

TTTTCTACCCATAAGTTATCTCTAACAAAACTATTTAGTTATGGCACTATTATTATAGACGAAGTTCATG

AGCATGATCAAATAGGAGATATTATTATAGCAGTAGCGAGAAAGCATCATACGAAAATAGATTCTATGTT

TTTAATGACTGCCACGTTAGAGGATGACAGGGAACGTCTAAAAATATTTTTACCTAATCCCGCATTTATA

CATATTCCTGGAGATACACTGTTTAAAATTAGCGAGGTATTTATTCATAATAAGATAAATCCATCTTCCA

GAATGGCATATATAGAAGAAGAAAAGAGAAATTTAGTTACTGCTATACAGATGTATACTCCTCCTGATGG

ATCATCCGGTATAGTCTTTGTGGCATCCGTTGCACAGTGTCACGAATATAAATCATATTTAGAAAAAAGA

TTACCGTATGATATGTATATTATTCATGGTAAGGTCTTAGATATAGACGAAATATTAGAAAAAGTGTATT

CATCACCTAATGTATCGATAATTATTTCTACTCCTTATTTGGAATCCAGCGTTACTATACGCAATGTTAC

ACACATTTATGATATGGGTAGAGTTTTTGTCCCCGCTCCTTTTGGAGGATCACAACAATTTATTTCTAAA

TCTATGAGAGATCAACGAAAAGGAAGAGTAGGAAGAGTTAATCCTGGAACATACGTATATTTCTATGATC

TGTCTTATATGAAGTCTATACAGCGAATAGATTCAGAATTTCTACATAATTATATATTGTACGCTAATAA

GTTTAATCTAACACTCCCCGAAGATTTGTTTATAATCCCTACAAATTTGGATATTCTATGGCGTACAAAG

GAATATATAGACTCGTTCGATATTAGTACAGAAACATGGAATAAATTATTATCCAATTATTATATGAAGA

TGATAGAGTATGCTAAACTTTATGTACTAAGTCCTATTCTCGCTGAGGAGTTGGATAACTTTGAGAGGAC

GGGAGAATTAACTAGTATTGTACAAGAAGCCATTTTATCTCTAAATTTACGAATTAAGATTTTAAAATTT

AAACATAAAGATGATGATACGTATATACACTTTTGTAGAATATTATTCGGCGTCTATAACGGAACAAACG

CTACTATATATTATCATAGACCTCTAACGGGATATATGAATATGATTTCAGATACTATATTTGTTCCTGT

AGATAATAACTAAAAATCAAACTCTAATGACCACATCTTTTTTTAGAGATGAAAAATTTTCCACATCTCC

TTTTGTAGACACGACTAAACATTTTGCAGAAAAAAGTTTATTATTATTTAGATAATCGTATACTTCATCA

GTGTAGATAGTAAATGTGAACAGATAAAAGGTATTCTTGCTCAATAGATTGGTAAATTCCATAGAATATA

TTAATCCTTTCTTCTTGAGATCCCACATCATTTCAACCAAAGACGTTTTATCCAATGATTTACCTCGTAC

TATACCACATACAAAACTAGATTTTGCAGTGATGTCGTACCTGGTATTCCTACCAAACAAAATTTTACTT

TTAGTTCTTTTAGAAAATTCTAAGGTAGAATCTCTATTTGTCAATATGTCATCTATGGAATTACCACTAG

CAAAAAATGATAGAAATATATATTGATACATCGCAGCTGGTTTTGATCTACTATACTTTAAAAACGAATC

AGATTCCATAATTGCCTGTATATCATCAGCTGAAAAACTATGTTTTACACGTATTCCTTCGGCATTTCTT

TTTAATGATATATCTTGTTTAGACAATGATAAAGTTATCATGTCCATGAGAGACGCGTCTCCGTATCGTA

TAAATATTTCATTAGATGTTAGACGCTTCATTAGGGGTATACTTCTATAAGGTTTCTTAATTAGTCCATC

ATTGGTTGCGTCAAGAACTACTATCTGATGTTGTTGGGTATCTCTAGTGTTACACATGGCCTTACTAAAG

TTTGGGTAAATAACTATGATATCTCTATTAATTATAGATGCATATATTTCATTCGTCAAGGATATTAATA

TCGACTTACTATCGTCATTAATACGTGTAATATAATCATATAAATCATGCGATAGCCAAGGAAAATTCAA

ATAGATGTTCATCATATAATCGTCGCTATAATTCATATTAATACTTTGACATTGACTAATTTGTAATATA

GCCTCGCCACGAAGAAAGCTCTCGTATTCAGTTTCATCGATAAAGGATACCGTTAAATATAACTGGTTGC

CGATAGTCTCATAGTCTATTAAGTGGTAAGTTTCGTACAAATACAGAATCCCTAAAATATTATCTAATGT

GGGATTAATCCTTACCATAACTGTATAAAATGGAGCCGGAGTCATAACTATTTTACCGTTTGTACTTACT

GGAATAGATGAAGGAATAATCTCCGGACATGATGGTAAAGACCCAAATGTCTGTTTGAAGAAATCCAATG

TTCCAGGTCCTAATCTCTTGACAAAAATTACGATATTCGATCCCGATATCCTTTGCATTCTATTTACCAG

CATATCACGAACTATATTAAGATTATCTATCATGTCTATTCTCCCACCGTTATATAAATCGCCTCCGCTA

AGAAACGTTAGTATATCCATACAATGGAATACTTCATTTCTAAAATAGTATTCGTTTTCTAATTCTTTAA

TGTGAAATCGTATACTAGAAAGGGAAAAATTATCTTTGAGTTTTCCATTAGAAAAGAACCACGAAACTAA

TGTTCTGATTGCGTCTGTCTCCGTCGCTGAATTAATAGATTTACACCAAAAACTCATATAACTTCTAGAT

GTAGAAGCATTCGCTAAAAAATTAGTAGAATCAAAGGATATAAGTAGATGTTCCAACAAGTGAGCAATTC

CCAAGATTTCATCTATATCATTCTCGAATCCGAAATTAGAAATTCCCAAGTAGATATCCTTTTTCATCCG

ATCATTGATGAAAATACGAACTTTATTCGGTAAGACGATCATTTACTAAGGAGTAAAATAGGAAGTAACG

TTCGTATATCGTTATCGTCGTATAAATTAAAGGTGTGTTTTTTGCCATTAAGAGACATTATAATTTTACC

AATATTGGAATTATAATATAGGTGTATTTGAGCACTAGAAACGGTCGATGCATCGGTAAATATAGCTGTA

TCTAATGTTCTAGTCGGTATTTCTTCATTTCGCTGTCTAATGATAGCGTTTTCTCTATCTGTTTCCATTA

CAGCTGCCTGAAGTTTATTGGTCGGGTAATATGTAAAATAATAAGAAATACATACGAATAACAAAAATAA

AATAAGATATAATAAAGATGCCATTTAGAGATCTAATTTTGTTCAACTTGTCCAAATTCCTACTTACAGA

AGATGAGGAATCGTTGGAGATAGTATCTTCCTTATGTAGAGGATTTGAAATATCTTACGATGACTTAATA

TCGTACTTTCCAGATAGGAAATACCATAAATATATTTCTAAGGTATTTGAACATGTAGATTTATCGGAGG

AATTAAGTATGGAATTCCATGATACAACTCTGAGAGATTTAGTATATCTTAGATTGTACAAGTATTCCAA

GTATATACGGCCGTGTTATAAATTAGGAGATAATCTAAAAGGTATAGTTGTTATAAAGGACAGAAATATA

TATATTAGAGAAGCAAATGATGACTTGATAGAATATCTCCTCAAGGAATACACTCCTCAGATTTATACAT

ATTCTAATGAGCGAGTTCCCATAGCTGGTTCAAAATTAATTCTTTGTGGATTTTCTCAAGTTACATTTAT

GGCGTATACAACGTCGCATATAACAACAAATAAAAAGGTAGATGTTCTCGTTTCCAAAAAATGTATAGAT

GAACTAGTCGATCCAATAAATTATCAAATACTTCAAAATTTATTTGATAAAGGAAGCGGAACAATAAACA

AAATACTCAGGAAGATATTTTATTCGGTAACAGGTGGCCAAACTCCATAGGTAGCTTTTTCTATTTCGGA

TTTTAGAATTTCCAAATTCACCAGCGATTTATCGGTTTTGGTGAAATCCAAGGATTTATTAATGTCCACA

AATGCCATTTGTTTTGTCTGTGGATTGTATTTGAAAATGGAAACGATGTAGTTAGATAGATGCGCTGCGA

AGTTTCCTATTAGGGTTCCGCGCTTCACGTCACCCAACATACTTGAATCACCATCCTTTAAAAAAAATGA

TAAGATATCAACATGGAGTATATCATACTCGGATTTTAATTCTTCTACTGCCTCACTGACATTTTCACAA

ATACTACAATACGGTTTACCGAAAATAATCAGTACGTTCTTCATTTATGGGTATCAAAAACTTAAAATCG

TTACTGTTGGAAAATAAATCACTGACGATATTAGATGATAATTTATACAAAGTATACAATGGAATATTTG

TGGATACAATGAGTATTTATATAGCCGTCGCCAATTGTGTCAGAAACTTAGAAGAGTTAACTACGGTATT

CATAAAATACGTAAACGGATGGGTAAAAAAGGGAGGACATGTAACCCTTTTTATCGATAGAGGAAGTATA

AAAATTAAACAAGACGTTAGAGACAAGAGACGTAAATATTCTAAATTAACCAAGGACAGAAAAATGTTAG

AATTAGAAAAGTGTACATCCGAAATACAAAATGTTACCGGATTTATGGAAGAAGAAATAAAGGCAGAAAT

GCAATTAAAAATCGATAAACTCACATTTCAAATATATTTATCTGATTATGATAACATAAAAATATCATTG

AATGAGATACTAACACATTTCAACAATAATGAGAATGTTACATTATTTTATTGTGATGAACGAGACGCAG

AATTCGTTATGTGTCTAGAGGCTAAAACACAGTTCTCTACCACAGGAGAATGGCCGTTAATAATAAGTAC

CGATCAGGATACTATGCTATTCGCGTCTGCTGATAATCATCCTAAGATGATAAAAAACTTAACTCAACTG

TTTAAATTTGTTCCCTCGGCAGAGGATAACTATTTAGCAAAATTAACTGCATTAGTGAATGGATGTGATT

TCTTTCCTGGACTCTATGGGGCATCTATAACACCCAACAACTTAAACAAAATACAATTGTTTAGTGATTT

TACAATCGATAATATAGTCACTAGTTTGGCAATTAAAAATTATTATAGAAAGACTAACTCTACCGTAGAC

GTGCGTAATATTGTTACGTTTATAAACGATTACGCTAATTTAGAAGATGTCTACTCGTATATTCCTCCTT

GTCAATGCACTGTTCAAGAATTTATATTCTCCGCATTAGATGAAAAATGGAATGAATTTAAATCATCTTA

TTTAGAGAGCGTGCCGTTACCCTGCCAATTAATGTACGCATTAGAACCACGTAAGGAGATTGATGTTTCA

GAAGTTAAAACTTTATCATCTTATATAGATTTCGAAAATACTAAATCAGATATCGATGTTATAAAATCTA

TATCCTCGATTTTTGGATATTCTAATGAAAACTGTAACACCATAGTGTTCGGCATCTATAAGGATAATTT

ACTACTGAGTATAAATAATTCATTTTACTTTAACGATAGTCTGTTAATAACCAATACTAAAAGTGATAAT

ATAATAAATATAGGTTACTAGATTAAAAAATGGTGTTCCAGCTCGTGTGTTCTACGTGCGGCAAAGATATT

TCTCACGAACGATATAAATTGATTATACGAAAAAAATCATTAAAGGATGTACTAGTCAGTGTAAAGAACG

AATGTTGTAGGTTAAAATTATCTACACAAATAGAACCTCAACGTAACTTAACAGTGCAACCTCTATTGGA

TATAAACTAATGGATCCGGTTAATTTTATCAAGACATATGCGCCTAGAGGTTCTATTATTTTTATTAATT

ATGCCATGTCATTAACTAGTCATTTGAATCCATCGATAGAAAAACATGTGGGTATTTATTATGGTACGTT

ATTATCGGAACACTTGGTAGTTGAATCTACCTATAGAAAAGGAGTTAGAATAATCCCATTGGATAGATTT

TTTGAAGGATATCTTAATGCAAAAGTATACATGTTAGAGAATATTCAAGTTATGAAAATAGCAGCTGATA

TGTCGTTAACTTTACTAGGTATTCCATATGGATTTGGTCATGATAGAATGTATTGTTTTAAATTGGTAGC

TGAATGTTATAAAAATGCCGGTATTGATACATCGTCTAAACGAATATTAGGTAAAGATATTTTTCTGAGC

CAAAACTTTACAGATGATAATAGATGGATAAAGATATATGATTCTAATAATTTAACATTTTGGCAAATTG

ATTACCTTAAAGGGTGAGTTAATATGCATAACTACTCCTCCGTTGTTTTTTCCCTCGTTCTTTTTCTTAA

CGTTGTTTGCCATCACTCTCATAATGTAAAGATATTCTAAAATGGTAAACTTTTGCATATCGGATGCAGA

AATTGGTATAAATGTTGTAATTGTATTATTTCCCGTCAATGGACTAGTCACAGCTCCATCAGTTTTATAT

CCTTTAGAGTATTTCTCACTCGTGTCTAGCATTCTAGAGCATTCCATGATTTGTTTATCGTTGATATTGG

CCGGAAAGATAGATTTTTTATTTTTTATTATATTACTATTGGCAATTGTAGATATAACTTCTGGTAAATA

TTTTTCTACCTTTTCATTCTCTTCTATTTTTAAGCCGGCTATATATTCTGCTATATTGTTGCTAGTATCA

ATACCTTTTCTGGCTAAGAAGTCATATGTGGTATTCACTATATCAGTTTTAACTGGTAGTTCCATTAGCC

TTTCCACTTCTGCAGAATAATCAGAAATTGGTTCTTTACCAGAAAATCCAGCTACTATAATAGGCTCACC

GATGATCATTGGCAAAATCCTATATTGTACCAGATTAATGAGAGCATATTTCATTTCCAATAATTCTGCT

AGTTCTTGAGACATTGATTTATTTGATGAATCTATTTGGTTCTCTAGATACTCTACCATTTCTGCCGCAT

ACAATAACTTGTTAGATAAAATCAGGGTTATCAAAGTGTTTAGTGTGGCTAGAATAGTGGGCTTGCACGT

ATTAAAGAATGCTGTAGTATGAGTAAACCGTTTTAACGAATTATATAGTCTCCAGAAATCTGTGGCGTTG

CATACATGAACTGAATGACATCGAAGATTGTCCAATATTTTTAATAGCTGCTCTTTGTCCATTATTTCTA

TATTTGACTCGCAACAATTGTAGATACCATTAATCACTGATTCCTTTTTCGATGCCGGACAATAGCACAA

TTGTTTAGCTTTGGACTCTATGTATTCAGAATTAATAGATATATCTCTCAATACAGATTGCACTATACAT

TTTGAAACTATGTCAAAAATTGTAGAACGACGCTGTTCTGTAGCCATTTAACTTTAAATAATTTACAAAA

ATTTAAAATGAGCATCCGTATAAAAATCGATAAATTGCGCCAAATTGTGGCATATTTTTCAGAGTTCAGC

GAAGAAGTGTCTATAAATGTAGACTCGACGGATGAGTTAATGTATATTTTTGCCGCCTTGGGCGGATCTG

TAAACATTTGGGCCATTATACCTCTCAGTGCATCAGTGTTCTACCGCGGAGCCGAAAATATTGTGTTTAA

CCTTCCAGTGTCCAAGGTAAAATCGTGTTTGTGTAGTTTTCACAATGATGCTATCATAGATATAGAACCT

GATCTGGAAAATAATCTAGTAAAACTTTCTAGTTATCATGTAGTAAGTGTCGATTGTAACAAGGAACTGA

TGCCTATTAGGACAGATACTACTATTTGTTTAAGTATAGATCAAAAGAAATCTTACGTATTTAATTTTCA

CAAGTATGAAGAAAAATGTTGTGGTAGAACCGTCATTCATCTAGAATGGTTGTTGGGCTTTATCAAGTGT

ATTAGTCAGCATCAGCATTTGGCTATTATGTTTAAAGATGACAATATTATTATGAAGACTCCTGGTAATA

CTGATGCGTTTTCCAGGGAATATTCTATGACTGAATGTTCTCAAGAACTACAAAAGTTTTCTTTCAAAAT

AGCTATCTCGTCTCTCAACAAACTACGAGGATTCAAAAAGAGAGTCAATGTTTTTGAAACTAGAATCGTA

ATGGATAATGACGATAACATTCTAGGAATGTTGTTTTCGGATAGAGTTCAATCCTTTAAGATTAACATCT

TTATGGCGTTTTTAGACTAATACTTTCAATGAGATAAATATGGGTGGCGGAGTAAGTGTTGAGCTCCCTA

AACGGGATCCGCCTCCGGGAGTACCCACTGATGAGATGTTATTAAACGTGGATAAAATGCATGACGTGAT

AGCTCCCGCTAAGCTTTTAGAATATGTGCATATAGGACCACTAACAAAAGATAAAGAGGATAAAGTAAAG

AAAAGATATCCAGAGTTTAGATTAGTCAACACAGGACCCGGTGGTCTTTCGGCATTATTAAGACAATCAT

ATAATGGAACCGCACCCAATTGCTGTCGCACTTTTAATCGTACTCATTATTGGAAGAAGGATGGAAAGAT

ATCAGATAAGTATGAAGAGGGTGCAGTATTAGAATCGTGTTGGCCCGACGTCCACGACACTGGAAAATGC

GATGTTGATTTATTCGACTGGTGTCAGGGGGATACGTTCGATATGAACATATGCCATCAGTGGATCGGTT

CAGCCTTTAATAGGAGTGATAGAACTGTAGAGGGTCGACAATCGTTAATAAATCTGTATAATAAGATGCA

AACATTATGTAGTAAAGATGCTAGTGTACCAATATGTGAATTATTTTTGCATCATTTACGCGCACACAAT

ACAGAAGATAGCAAAGAGATGATCGATTATATTCTAAGACAACAGTCGGCGGACTTTAAACAGAAATATA

TGAGATGTAGTTATCCCACTAGAGATAAGTTAGAAGAGTCATTAAAATATGCGGAACCTCGAGAATGTTG

GGATCCAGAGTGTTCGAATGCCAATGTTAATTTCTTACTAACACGTAATTATAATAATTTAGGACTTTGC

AATATTGTACGATGTAATACGAGCGTGAATAACTTACAGATGGATAAAACTTCCTCATTAAGATTATCAT

GTGGATTAAGCAATAGTGATAGATTTTCTACTGTTCCCGTCAATAGAGCAAAAGTAGTTCAACATAATAT

TAAACATTCGTTCGACCTAAAATTGCATTTGATCAGTTTATTATCTCTCTTGGTAATATGGATACTAATT

GTAGCTATTTAAATGGGTGCCGCAGCAAGCATACAGACGACTGTGAATACACTCAGTGAACGTATCTCGT

CTAAATTAGAACAAGAAGCGAACGCTAGTGCTCAAACAAAATGTGATATAGAAATCGGAAATTTTTATAT

CCGACAAAACCATGGATGTAACATCACTGTTAAAAATATGTGCTCTGCGGACGCGGATGCTCAGTTGGAT

GCTGTGTTATCAGCCGCTACAGAAACATATAGTGGATTAACACCGGAACAAAAAGCATACGTACCAGCTA

TGTTTACTGCTGCGTTAAACATTCAGACGAGTGTAAACACTGTTGTTAGAGATTTTGAAAATTATGTGAA

ACAGACTTGTAATTCTAGCGCTGTTGTCGATAACAAATTAAAGATACAAAACGTAATTATAGATGAATGT

TACGGAGCCCCAGGATCTCCAACAAATTTGGAATTTATTAATACAGGATCTAGCAAAGGAAATTGTGCCA

TTAAGGCGTTGATGCAATTGACTACTAAGGCCACTACTCAAATAGCACCTAGACAAGTTGCTGGTACAGG

AGTTCAGTTTTATATGATTGTTATCGGTGTTATAATATTGGCAGCGTTGTTTATGTACTATGCCAAGCGT

ATGCTGTTCACATCCACCAATGATAAAATCAAACTTATTTTAGCCAATAAGGAAAACGTCCATTGGACTA

CTTACATGGACACATTCTTTAGAACTTCTCCGATGATTATTGCTACCACGGATATACAAAACTGAAAATA

TATTGATAATATTTTAATAGATTAACATGGAAGTTATCGCTGATCGTCTAGACGATATAGTGAAACAAAA

TATAGCGGATGAAAAATTTGTAGATTTTGTTATACACGGTCTAGAGCATCAATGTCCTGCTATACTTCGA

CCATTAATTAGGTTGTTTATTGATATACTATTATTTGTTATAGTAATTTATATTTTTACGGTACGTCTAG

TAAGTAGAAATTATCAAATGTTGTTGGTGTTGGTGGCGCTAGTCATCACATTAACTATTTTTTTATTACT

TTATACTATAATAGTACTAGACTGACTTCTAACAAACATCTCACCTGCCATAAATAAATGCTTGATATTA

AAGTCTTCTATTTCTAACACTATTCCATCTGTGGAAAATAATACTCTGACATTATCGCTAATTGATACAT

CGGTAAGTGATATGCCTATAAAGTAATAATCTTCTTTGGGCACATATACCAGTGTACCAGGTTCTAACAA

CCTATTTACTGGTGCTCCTGTAGCATACTTTTTTTTTACCTTGAGAATATCCATTGTTTGCTTGGTCAAT

AGCGATATGTGATTTTTTATCAACCACTCAAAAAAGTAATTGGAGTGTTCATATCCTCTACGGGCTATTG

TCTCATGGCCGTGTATGAAATTTAAGTAACACGACTGTGGTAGATTTGTTCTATAGAGCCGGTTGCCGCA

AATAGATAGAACTACCAATATGTCTGTACAAATGTTAAACATTAATTGATTAACAGAAAAAACAATGTTC

GTTCTGGGAATAGAAACCAGATTAAAACAAAATTCATTAGAATATATGCCACGTTTATACATGGAATATA

AAATAACTACAGTTTGAAAAATAACAGTATCATTTAAACATTTAACTTGCGGGGTTAATCTCACAACTTT

ACTGTTTTTGAACTGTTCAAAATATAGCATAGATCCATGAGAAATACGTTTAGCCGCCTTTAATAGAGGA

AATCCAACCGCCTTTCTGGATCTCACCAACGACGATAGTTCTGACCAGCAACTCATTTCTTCATCATCCA

CCTGTTTTAACATATAATAGGCAGGAGATAGATATCCGTCATTGCAATATTCCTTCTCGTAGGCACACAA

TCTAATATTGATAAAATCTCCATTCTCTTCTCTGTATTTATTATCTTGTCTCGGTGGCTGATTAGGCTGT

GGTCTATCGTTGTTGAATCTATTTTGGTCATTAAATCTTTCATTTCTTCCTGGTATATTTCTATCACCTC

GTTTGGTTGGATTTTTGTCTATATTATCGTTTGTAACATCGGTACGGGTATTCATTTATCACAAAAAAAA

CTTCTCTAAATGAGTCTACTGCTAGAAAACCTCATCGAAGAAGATACCATATTTTTTGCAGGAAGTATAT

CTGAGTATGATGATTTACAAATGGTTATTGCTGGTGCAAAATCCAAATTTCCAAGATCTATGCTTTCTAT

TTTTAATATAGTACCTAGAACGATGTCAAAATATGAGTTGGAGTTGATTCATAACGAGAATATCACAGGG

GCAATGTTTACCACAATGTATAATATAAGAAACAATTTGGGTCTAGGCGATGATAAACTAACTATTGAAG

CCATTGAAAACTATTTCTTGGATCCTAACAATGAGGTTATGCCTCTTATCATTAATAATACGGATATGAC

TACCGTCATTCCTAAAAAAAGTGGTAGGAGAAAGAATAAGAACATGGTTATCTTCCGTCAAGGATCATCA

CCTATCTTGTGTATTTTCGAAACTCGTAAAAAGATTAATATTTATAAAGAAAATATGGAATCCGTATCGA

CTGAGTATACACCTATCGGAGACAACAAGGCTTTGATATCTAAATATGCGGGAATTAATATCCTGAATGT

GTATTCTCCTTCCACGTCCATGAGATTGAATGCCATTTACGGATTCACCAATAAAAATAAACTAGAGAAA

CTTAGTACTAATAAGGAACTAGAATCGTATAGTTCTAGCCCTCTTCAAGAACCCATTAGGTTAAATGATT

TTCTGGGACTATTGGAATGTGTTAAAAAGAATATTCCTCTAACAGATATTCCGACAAAGGATTGATTACT

ATAAATGGAGAATGTTCCTAATGTATACTTTAATCCTGTGTTTATAGAGCCCACGTTTAAACATTCTTTA

TTAAGTGTTTATAAACACAGATTAATAGTTTTATTTGAAGTATTCGTTGTATTCATTCTAATATATGTAT

TTTTTAGATCTGAATTAAATATGTTCTTCATGCCTAAACGAAAAATACCCGATCCTATTGATAGATTACG

ACGTGCTAATCTAGCGTGTGAAGACGATAAATTAATGATCTATGGATTACCATGGATAACAACTCAAACA

TCTGCGTTATCAATAAATAGTAAACCGATAGTGTATAAAGATTGTGCAAAGCTTTTGCGATCAATAAATG

GATCACAACCAGTATCTCTTAACGATGTTCTTCGCAGATGATGATTCATTTTTTAAGTATTTTGCTAGTC

AAGATGATGAATCTTCATTATCTGATATATTGCAAATCACTCAATATCTAGACTTTCTGTTATTATTATT

GATCCAATCAAAAAATAAATTAGAAGCTGTGGGTCATTGTTATGAATCTCTTTCAGAGGAATACAGACAA

TTGACAAAATTCACAGACTCTCAAGATTTTAAAAAACTGTTTAACAAGGTCCCTATTGTTACAGATGGAA

GGGTCAAACTTAATAAAGGATATTTGTTCGACTTTGTGATTAGTTTGATGCGATTCAAAAAAGAATCAGC

TCTAGCTACCACCGCAATAGATCCTGTTAGATACATAGATCCTCGTCGTGATATCGCATTTTCTAACGTG

ATGGATATATTAAAGTCGAATAAAGTTGAACAATAATTAATTCTTTATTGTTATCATGAACGGCGGACAT

ATTCAGTTGATAATCGGCCCCATGTTTTCAGGTAAAAGTACAGAATTAATTAGACGAGTTAGACGTTATC

AAATAGCTCAATATAAATGTGTGACTATAAAATATTCTAACGATAATAGATACGGAACGGGACTATGGAC

GCATGATAAGAATAATTTTGCAGCATTGGAAGTAACTAAACTATGTGATGTCTTGGAAGCAATTACAGAT

TTCTCCGTGATAGGTATCGATGAAGGACAGTTCTTTCCAGACATTGTTGAATTCTGTGAGCGTATGGCAA

ACGAAGGAAAAATAGTTATAGTAGCCGCGCTCGATGGGACATTTCAACGTAGACCGTTTAATAATATTTT

GAATCTTATTCCATTATCTGAAATGGTGGTAAAACTAACTGCAGTGTGTATGAAATGCTTTAAGGAGGCT

TCCTTTTCTAAACGATTAGGTGCAGAAACCGAGATAGAAATAATAGGAGGTAATGATATGTATCAATCTG

TGTGTAGAAAGTGTTACATCGACTCATAATATTATATTTTTTATCTAAAAAACTAAAAATAAACATTGAT

TAAATTTTAATATAATACTTAAAAATGGATGTTGTGTCGTTAGATAAACCGTTTATGTATTTTGAGGAAA

TTGATAATGAGTTAGATTACGAACCAGAAAGTGCAAATGAGGTCGCAAAAAAACTGCCGTATCAAGGACA

GTTAAAACTATTACTAGGAGAATTATTTTTTCTTAGTAAGTTACAGCGACACGGTATATTAGATGGTGCC

ACCGTAGTGTATATAGGATCTGCTCCAGGTACACATATACGTTATTTGAGAGATCATTTCTATAATTTAG

GAGTGATCATCAAATGGATGCTAATTGACGGCCGCCATCATGATCCTATTCTAAATGGATTGCGTGATGT

GACTCTAGTGACTCGGTTTGTTGATGAGGAATATCTACGATCCATCAAAAAACAACTACATCCTTCTAAG

ATTATTTTAATTTCTGATGTGAGATCCAAACGAGGAGGAAATGAACCTAGTACTGCGGATTTACTAAGTA

ATTATGCTCTACAAAATGTCATGATTAGTATTTTAAACCCCGTGGCGTCTAGTCTTAAATGGAGATGCCC

GTTTCCAGATCAATGGATCAAGGACTTTTATATCCCACACGGTAATAAAATGTTACAACCTTTTGCTCCT

TCATATTCAGCTGAAATGAGATTATTAAGTATTTATACCGGTGAGAATATGAGACTGACTCGAGTTACCA

AATCAGACGCTGTAAATTATGAAAAAAAGATGTATTACCTTAATAAGATAGTCCGCAACAAAGTAGTTGT

TAACTTTGATTATCCTAATCAGGAATATGACTATTTTCACATGTACTTTATGTTGAGGACCGTATACTGC

AATAAAACATTTCCTACTACTAAAGCAAAGATACTATTTCTACAACAATCTATATTTCGTTTCTTAAATA

TTCCAACGACATCAACTGAAAAAGTTAGTCATGAACCAATACAACGTAAAATATCTAGCAAAGATTCTAT

GTCTAAAAACAGAAATAGCAAGAGATCCGTACGCGGTAATAAATAGAAACGTACTACTGAGATATACTAC

CGATATAGAGTATAATGATTTAGTTACTTTAATAACCGTTAGACATAAAATTGATTCTATGAAAACTGTG

TTTCAGGTATTTAACGAATCATCCATAAATTATACTCCGGTTGATGATGATTATGGAGAACCAATCATTA

TAACATCGTATCTTCAAAAAGGTCATAACAAGTTTCCTGTAAATTTTCTATACATAGATGTGGTAATATC

TGACTTATTTCCTAGCTTTGTTAGACTAGATACTACAGAAACTAATATAGTTAATAGTGTACTACAAACA

GGCGATGGTAAAAAGACTCTTCGTCTTCCTAAAATGTTAGAGACGGAAATAGTTGTCAAGATTCTCTATC

GTCCTAATATACCATTAAAAATTGTTAGATTTTTCCGCAATAACATGGTAACTGGAGTAGAGATAGCCGA

TAGATCTGTTATTTCAGTCGCTGATTAATCAATTAGTAGAGATGAGATAAGAACATTATAATAATCAATA

ATATATCTTATATCTGTTTAGAAAAATGCTAATATTAAAATAGCTAACGCTAGTAATCCAATCGGAAGCC

ATTTGATATCTATAATAGGGTATCTAATTTCCTGATTCAGATAGCGTACGGCTATATTCTCGGTAGCTAC

TCGTTTGGAATCACAAACATTATTTACATCTAATTTACTATCTGTAATGGAAACGTTTCCCAATGAAATG

GTACAATCAGATACATTACATCTTGATATATTTTTTTTTAAAGAGGCTGGTAACAACGCATCGCTTCGTT

TACATGGCTCGTACCAACAATAATAGGGTAATCTTGTATCTATTCCTATCCGTACTATACTTTTATCAGG

ATAAATACATTTACATCGTATATCGTCTTTGTTAGCATCACAGAATGCATAAATTTGTTCGTCCGTCATG

ATAAAAATTTAAAGTGTAAATATAACTATTATTTTTATAGTTGTAATAAAAAGGGAAATTTGATTGTATA

CCTTCGGTTCTTTAAAAGAAACTGACTTGATAAAAATGGCTGTAATCTCTAAGGTTACGTATAGTCTATA

CGATCAAAAAGAGATTAATGCTACAGATATTATCATTAGTCATATTAAAAATGACGACGATATCGGTACC

GTTAAAGATGGTAGACTAGGTGCTATGGATGGGGCATTATGTAAGACTTGTGGGAAAACGGAATTGGAAT

GTTTCGGTCACTGGGGTAAAGTAAGTATTTATAAAACTCATATAGTTAAGCCTGAATTTATTTCAGAAAT

TATTCGTTTACTGAATCATATATGTATTCATTGCGGATTATTGCGTTCACGAGAACCGTATTCCGACGAT

ATTAACCTAAAAGAGTTATCGGGACACGCTCTTAGGAGATTAAAGGATAAAATATTATCCAAGAAAAAGT

CATGTTGGAACAGCGAATGTATGCAACCGTATCAAAAAATTACTTTTTCAAAGAAAAAGGTTTGTTTCGT

CAACAAGTTGGATGATATTAACGTTCCTAATTCTCTCATCTATCAAAAGTTAATTTCTATTCATGAAAAG

TTTTGGCCATTATTAGAAATTCATCAATATCCAGCTAACTTATTTTATACAGACTACTTTCCCATCCCTC

CGTTGATTATTAGACCGGCTATTAGTTTTTGGATAGATAGTATACCCAAAGAGACAAATGAATTAACTTA

CTTATTAGGTATGATCGTTAAGAATTGTAACTTGAATGCTGATGAACAGGTTATCCAGAAGGCGGTAATA

GAATACGATGATATTAAAATTATTTCTAATAACACTACCAGTATCAATTTATCATATATCACATCCGGCA

AAAATAATATGATTAGAAGTTATATCGTCGCTCGGCGAAAAGATCAGACCGCTAGATCTGTAATTGGTCC

CAGTACATCTATCACCGTTAATGAGGTAGGAATGCCCACATATATTAGAAATACACTTACAGAAAAGATA

TTTGTTAATGCCTTTACAGTGGATAAAGTTAAACAACTATTAGCATCAAACCAAGTTAAATTTTACTTTA

ATAAACGATTAAACCAATTAACAAGAATACGTCAAGGAAAGTTTATCAAAAATAAAATACATTTATTGCC

TGGTGATTGGGTAGAAGTAGCTGTTCAAGAATATACAAGTATTATTTTTGGAAGACAACCGTCTCTACAT

AGATACAACGTCATCGCTTCATCTATCAGAGCTACCGAAGGAGATACTATCAAAATATCTCCCGGAATTG

CCAACTCTCAAAATGCTGATTTCGACGGAGATGAAGAATGGATGATATTGGAGCAAAATCCTAAAGCCGT

AGTTGAACAAAGTATTCTTATGTATCCGACGACGTTACTCAAACACGATATTCATGGAGCCCCCGTTTAT

GGATCTATTCAAGATGAAATCGTAGCAGCGTATTCATTGTTTAGGATACAAGATCTTTGTTTAGATGAAG

TATTGAACATCTTGGGGAAATATGGAAGAGAGTTCGATCCTAAAGGTAAATGTAAATTCAGCGGTAAAGA

TATCTATACTTACTTGATAGGTGAAAAGATTAATTATCCGGGTCTCTTAAAGGATGGCGAAATTATTGCA

AACGACGTAGATAGTAATTTTGTTGTAGCTATGAGGCATCTGTCATTGGCTGGACTCTTATCCGATCATA

AATCGAACGTGGAAGGTATCAACTTTATTATCAAGTCATCTTATGTTTTTAAGAGATATCTATCTATTTA

CGGTTTTGGGGTGACATTCAAAGATCTGAGACCAAATTCGACGTTCACTAATAAATTGGAGGCTATCAAC

GTAGAAAAAATAGAACTTATCAAAGAAGCATACGCCAAATATCTCAAAGATGTAAGAGACGGGAAAATAG

TTCCATTATCTAAAGCTTTAGAGGCGGACTACTTGGAATCCATGTTATCCAACTTGACAAATCTTAATAT

CAGAGAGATAGAAGAACATATGAGACAAACGCTGATAGATGATCCAGATAATAACCTCCTGAAAATGGCC

AAAGCGGGTTATAAAGTAAATCCCACAGAACTAATGTATATTCTAGGTACTTATGGACAACAGAGGATAG

ATGGCGAACCAGCAGAGACTCGAGTATTGGGTAGAGTCTTACCTTACTATCTTCCAGACTCTAAGGATCC

AGAAGGAAGAGGTTATATTCTTAATTCTTTAACAAAAGGATTAACGGGTTCTCAATATTACTTTTCGATG

CTGGTTGCAAGATCTCAATCTACTGATATCGTCTGTGAAACATCACGTACCGGAACACTGGCTAGAAAAA

TCATTAAAAAGATGGAGGATATGGTGGTCGACGGATACGGACAAGTAGTTATAGGTAATACGCTCATCAA

GTACGCAGCCAATTATACCAAAATTCTAGGCTCAGTATGTAAACCTGTAGATCTTATCTATCCAGATGAG

TCCATGACTTGGTATTTGGAAATTAGTGCTTTGTGGAATAAAATAAAACAGGGATTCGTTTACTCTCAGA

AACAGAAACTTGCAAAGAAGACATTGGCGCCGTTTAATTTCCTAGTATTCGTCAAACCCACCACTGAGGA

TAATGCTATTAAGGTTAAGGATCTGTACGATATGATTCATAACGTCATTGATGATGTGAGAGAGAAATAC

TTCTTTACGGTATCTAATATAGATTTTATGGAGTATATATTCTTGACGCATCTTAATCCTTCTAGAATTA

GAATTACAAAAGAAACGGCTATCACTATCTTTGAAAAGTTCTATGAAAAACTCAATTATACTCTAGGTGG

TGGAACTCCTATTGGAATTATTTCTGCACAGGTATTGTCTGAGAAGTTTACACAACAAGCCCTGTCCAGT

TTTCACACTACTGAAAAGAGTGGTGCTGTAAAACAAAAACTTGGTTTCAACGAGTTTAATAACTTGACTA

ATTTGAGTAAGAATAAGACCGAAATTATCACTCTGGTATCCGATGATATCTCTAAACTTCAATCTGTTAA

GATTAATTTCGAATTTGTATGTTTGGGAGAATTAAATCCAGACATCACTCTTCGAAAAGAAACAGATAGA

TATGTAGTAGACATAATAGTCAATAGATTATACATCAAGAGAGCAGAAATAACCGAATTAGTCGTCGAAT

ATATGATTGAACGATTTATCTCCTTTAGCGTCATTGTAAAGGAATGGGGCATGGAGACATTCATTGAGGA

CGAGGATAATATTAGATTTACTGTCTACCTAAATTTCGTTGAACCGGAGGAATTGAATCTTAGTAAGTTT

ATGATGGTTCTTCCGGGTGCCGCCAACAAGGGCAAGATTAGTAAATTCAAGATTCCTATCTCTGACTATA

CGGGATATAACGACTTCAATCAAACAAAAAAGCTCAATAAGATGACTGTAGAACTCATGAATCTAAAAGA

ATTGGGTTCTTTCGATTTGGAGAACGTCAACGTGTATCCTGGAGTATGGAATACATACGATATCTTCGGT

ATTGAGGCCGCTCGTGGATACTTGTGCGAAGCCATGTTAAACACCTATGGAGAAGGTTTCGATTATCTGT

ACCAGCCTTGTGATCTTCTCGCTAGTTTACTATGTGCTAGTTACGAACCAGAATCAGTTAATAAATTCAA

GTTCGGTGCAGCTAGTACTCTTAAGAGAGCTACGTTCGGAGATAATAAAGCATTGTTAAACGCGGCTCTT

CATAAAAAGTCAGAACCTATTAACGATAATAGTAGCTGCCACTTTTTTAGCAAGGTCCCTAATATAGGAA

CTGGATATTACAAATACTTTATCGACTTGGGTCTTCTCATGAGAATGGAAAGGAAATTATCTGATAAGAT

ATCTTCTCAAAAGATCAAGGAGATAGAAGAAACAGAAGACTTTTAATTCTTATCAATAACATATTTTTCT

ATGATCTGTCTTTTAAACGATGGATTTTCCACAAATGCGCCTCTCAAGTCCCTCATAGAATGATACACGT

ATAAAAAATATAGCATAGGTGATGACTCCTTATTTTTAGACATTAGATATGCCAAAATCATAGCCCCGCT

TCTATTTACTCCCGCAACACAATGAACCAACACGGGCTCGTTTCGTTGATCACATTTAGATAAGAAGGCG

GTCACGTCGTCAAAATATTTACTAATATCAGTAGTTGTATCATCTACCAACGGTATATGAATAATATTAA

TATTAGAGTTAGGTAATGTATATTTATCCATCGTCAAATTTAAAACATATTTGAACTTAACTTCAGATGA

TGGTGCATCCATAGCATTTTTATAATTTCCCAAATACACATTATTTGTTACTCTTGTCATTATAGTGGGA

GATTTGGCTCTGTGCATATCTCCAGTTGAACGTAGTAGTAAGTATTTATACAAACTTTTCTTATCCATTT

ATAACGTACAAATGGATAAAACTACTTTATCAGTAAACGCATGCAATTTAGAATACGTTAGAGAAAAGGC

TATAGTAGGCGTACAAGCAGCCAAGACATCAACACTTATATTTTTTGTTATTATATTGGCAATTAGTGCG

CTATTACTCTGGTTTCAGACGTCTGATAATCCAGTCTTTAATGAATTAACGAGATATATGCGAATTAAAA

ATACGGTTAACGATTGGAAATCATTAACGGATAGCAAAACAAAATTAGAAAGTGATAGAGGTAGACTTCT

AGCCGCTGGTAAGGATGATATATTCGAATTCAAATGTGTGGATTTCGGCGCCTATTTTATAGCTATGCGA

TTGGATAAGAAAACATATCTGCCGCAAGCTATTAGGCGAGGTACTGGAGACGCGTGGATGGTTAAAAAGG

CGGCAAAAGTCGATCCATCTGCTCAACAATTTTGTCAGTATTTGATAAAACACAAGTCTAATAATGTTAT

TACTTGTGGTAATGAGATGTTAAATGAATTAGGTTATAGCGGTTATTTTATGTCACCGCATTGGTGTTCC

GATCTTAGTAATATGGAATAAGTGTTAGATAAATGCGGTAACAAATGTTCCTGTAAGGAACCATAACAGT

TTAGATTTAACATTAAAGATGAGCATAAACATAATAAACAAAATTACAATCAAACCTATAACATTAATAT

CAAACAATCCAAAAAATGAAATCAGTGGAGTAGTAAACGTGTACATGACTCCTGGATAACGTTTAGCAGC

TACCGTTCCTATTCTAGACCAAAAATTTGGTTTCATGGTTTCGAAGCGGTGTTCTGCAACAAGACGAGGA

TCGTGTTCTACATATTTGGCAGAGTTATCCATTATTTGCCTGTTAATCTTCATTTCGTTTTCGATTCTGG

CTATTTCAAAATAAAATCCCGATGATAGACCTCCAGACTTTATAATTTCATCTACGATGTTCAGCGCCGT

AGTAACTCTAATAATATAGGCTGATAAGCTAACATCATACCCTCCTGTATATGTGAATATGGCATGATCT

TTGTCTATTACAAGCTCGGTTTTAACTTTATTTCCTGTAATAATTTCTCTCATCTGTAGGATATCTATTT

TCTTGTCATGTATTGCCTTCAAGACGGGACGAAGAAACGTAATATCCTCAATAACGTTATCGTTTTCTAT

AATAACTACATATTCTACATTTTTATTTTCTAGCTCGATAAAAAATTTAGAATCCCATAGGGCTAAATGT

CTAGCGATATTTCTTTTCGTTTCCTCTGTACACATAGTGTTACAAAACCCTGAAAAGAAGTGAGTATACT

TGTCATCATCTCTAATATTTCCTCCAGTCCATTGTATAAACACATAATCCTTGTAATGATCTGGATCATC

ATTGACTATCACAACATCTCTTTTTTCTTGCATAACTTCATTGTCCTTCACATCATCGAACTTCTGATCA

TTAATATGCTCATGAACATTAGGAAATGTTTCTGATGGAGGTCTATCAATAACTGGCACAACAATAACAG

GAGTTTTCGCCGCCGCCATTTAGTTATTGAAATTAATCATATACAACTCTCTAATACGAGTTATATTTTC

GTCTATCCATTGTTTCACATTGACATATTTCGACAAAAAGATATAAAATGCGTATTCCAATGCTTCTCTG

TTTAATGAATTACTAAAATATACAAACACGTCACTGTCTGGTAATAAATGATATCTTAGAATATTGTAAC

AATTTATTTTGTATTGCACATGTTCGTGATCTATGAGTTCTTCTTCGAATGGCATAGGATCTCCGAATCT

GAAAACGTATAAATAGGAGTTAGAATAATAATATTTGAGAGTATTGGTAATGTATAAACTCTTTAGCGGT

ATAATTAGTTTTTTTCTCTCGATTTCTATTTTTAGATGTGATGGAAAAATGACTAATTTTGTAGCATTAG

TATCATGAACTCTAATCAAAATCTTAATATCTTCGTCACACGTTAGCTCTTTGAAGTTTTTAAGAGATGC

ATCAGTTGGTTTTACAGATGGAGTAGGTGTAACAATTTTTTGTTTAATGCATGTATGTATTGGAGCCATT

GTCTTAACTATAATGGTGCTTGTATCGAAAAACTTTAATGCGGATAACGGAAGCTCTTCGCCGCGACTTT

CTACGTCGTAATTGGGTTCTAATGCCGATCTCTGAATGGATACTAGTTTTCTAAGTTCTAATGTGATTCT

CTGAAAATGTAAATCCAATTCCTCCGGCATTATAGATGTGTATACATCGGTAAATAAAACTATAGTATCC

AACGATCCCTTCTCGCAAATTCTAGTCTTAACCAAGAAATCGTATATAACTACGGAGATGGCGTATTTAA

GAGTGGATTCTTCTACCGTTTTGTTCTTGGATTTCATATAAGAAACTATAAAGTCCGCACTACTGTTAAG

AATGATCACTAACGCAACTATATAGTTCAAATTAAGCATCTTGGAAACATAAAATAACTCTGTAGATGAT

ACTTGACTTTCGAATAAGTTTGCAGACAAACGAAGAAAGAACAGACCTCTCTTAATTTCAGAAGAAAACT

TTTTTTCGTATTCCTGACGTCTAGAGTTTATATCAATAAGAAAGTTAAGAATTAGTCGGTTAATGTTGTA

TTTCATTACCCAAGTTTGAGATTTCATAATATTGTCAAAAGACATGATAATATTAAAGATAAAGCGCTGA

CTATGAACGAAATAGCTATATGGTTCGCTCAAGAATATAGTCTTGTTAAACGTGGAAACGATAACTGTAT

TTTTAATCACGTCAGCGGCATCTAAATTAAATATAGGTATATTTATTCCACACACACTACAATATGCCAC

ACCATCTTCATAATAAATAAATTCGTTAGCAAAATTATTAATTTTAGTGAAATAGTTAGCGTCAACTTTC

ATAGCTTCCTTCAATCTAATTTGATGCTCACATGGCGCGAATTCTACTCTAACATCCCTTTTCCATGCCT

CAGGTTCATCGATCTCTATAATATCTAGTTTCTTGCGTTTCACAAACACAGGCTCGTCTCTCGCGATGAG

ATCTGTATAGTAACTATGTAAATGATAACTAGATAGAAAGATGTAGCTATATAGATGACGATCCTTTAAG

AGAGGTATAATAACTTTACCCCAATCAGATAGACTGTTGTTATGGTCTTCGGAAAAAGAATTTTTATAAA

TTTTTCCAGTATTTTCTAAATATACATACTTGATATCTAAGAAATCCTTAATGATAATAGGAATGGATAA

TCCGTCTATTTTATAAAGAAATACATATCGCATATTATACTTTTTTTTGGAAATTGGAATACCGATGTGT

CTACATAAATACGCAAAGTCTAAATATTTTTTAGAGAATCTTAGTTGGTCCAAATTCTTTTCCAAGTACG

GTAATAGATTTTTCATATTGAACGGTATCTTCTTGATCTCTGGTTCTAATTCCGCATTAAATGATGAAAC

TAAGTCACTATTTTTATAACTAACGATTACATCACCTCTAACATCATCATTTACCAGGATACTGATCTTC

TTTTGTCGTAAATACATGTCTAATGTGTTAAAAAAAAGATCATACAAGTTATACGTCATTTCATCTGTAG

TATTCTTGTCATTGAAGGATAAACTCGTACTAATCTCTTCTTTAACAGTCTGTTCAAATTTATATCCTAT

ATACGAAAAAATAGCAACCAGTGTTTGATCATCCGCGTCAATATTCTGTTCTATCGTAGTGTATAGCAAT

CTTATATCTTCTTCTGTGATAGTCGATACGTTATAAAGGTTGATAACGAAAATATTTTTATTTCGTGAAA

TAAAGTCATTGTAGGATTTTGGACTTATATTCGTGTCTAGTAGATATGATTTTATTTTTGGAATGATCTC

AATTAAAATAGTCTCTTTAGAGTCCATTTAAAGTTACAAACAACTAGGAAATTGGTTTATGATGTATAAT

TTTTTTAGTTTTTATAGATTCTTTATTCTATACTTAAAAAATGAAAATAAATACAAAGGTTCTTGAGGGT

TGTGTTAATTGAAAGCGAGAAATAATCATAAATTATTTCATTATCGCGATATCCGTTAAGTTTGTATCGT

AATGGCGTGGTCAATTACGAATAAAGCGGATACTAGTAGTTTCACAAAGATGGCTGAAATCAGAGCTCAT

CTAAGAAATAGCGCTGAAAATAAAGATAAAAACGAGGATATTTTCCCGGAAGATGTAATAATTCCATCTA

CTAAGCCCAAAACCAAACGAACCACTACTCCTCGTAAACCAGCGGCTACTAAAAGATCAACCAAAAAGGA

TAAAGAAAAGGAGGAAGTGGAAGAAGTAGAAGAAGTAGTTATAGAGGAATATCATCAAACAACTGAAGAA

AATTCTCCACCTCCGTCATCATCTCCTGGAGTCGGCGACATTGTAGAAAGCGTGGCCGCTGTAGAGCTCG

ATGATAGCGACGGGGATGATGAACCTATGGTACAAGTTGAAGCTGGTAAAGTAAATCATAGTGCTAGAAG

CGATCTCTCTGACCTAAAGGTGGCTACCGACAATATCGTTAAAGATCTTAAGAAAATTATTACTAGAATC

TCTGCAGTATCGACTGTTCTAGAGGATGTTCAAGCAGCTGGTATCTCTAGACAATTTACTTCTATGACTA

AAGCTATTACAACACTATCTGATCTAGTCACCGAGGGAAAATCTAAAGTTGTTCGTAAAAAAGTTAAAAC

TTGTAAGAAGTAAATGCGTGCACTTTTTTATAAAGATGGTAAACTGTTTACCGATAATAATTTTTTAAAT

CCTGTATCAGACGATAATCCAGCGTATGAGGTTTTGCAACATGTTAAAATTCCTACTCATTTAACAGATG

TAGTAGTATATGAACAAACGTGGGAAGAGGCATTAACTAGATTAATTTTTGTGGGAAGTGATTCAAAAGG

ACGTAGACAATACTTTTACGGAAAAATGCATGTACAGAATCGCAATGCTAAAAGAGATCGTATTTTTGTT

AGAGTATATAACGTTATGAAACGAATTAATTGTTTTATAAACAAAAATATAAAGAAATCGTCCACAGATT

CCAATTATCAGTTGGCGGTTTTTATGTTAATGGAAACTATGTTTTTTATTAGATTTGGTAAAATGAAATA

TCTTAAGGAGAATGAAACAGTAGGGTTATTAACACTAAAAAATAAACACATAGAAATAAGTCCCGATGAA

ATAGTTATCAAGTTTGTAGGAAAGGACAAAGTTTCACATGAATTTGTTGTTCATAAGTCTAATAGACTAT

ATAAACCGTTATTGAAACTGACTGATGATTCTAGTCCCGAAGAATTTCTGTTCAACAAACTAAGTGAACG

AAAGGTATACGAATGTATCAAACAGTTTGGTATTAGAATCAAGGATCTCCGAACGTATGGAGTCAATTAT

ACGTTTTTATATAATTTTTGGACAAATGTAAAGTCCGTATCTCCTCTTCCATCACCAAAAAAGTTGATAG

CGTTAACTATCAAACAAACTGCTGAAGTGGTAGGTCATACTCCATCAATTTCAAAAAGAGCTTATATGGC

AACGACTATTTTAGAAATGGTAAAGGATAAAAATTTTTTAGACGTAGTATCTAAAACTACGTTCGATGAA

TTCCTATCTATAGTCGTAGATCACGTTAAATCATCTACGGATGGATGATAATAGATCTTTACACAAATAA

TTACAAGACCGATAAATGGAAATGGATAAACGGATGAAATCTCTCGCTATGACAGCTTTCTTCGGAGAGC

TAAACACGTTAGATATTATGGCATTGATAATGTCTATATTTAAACGCCATCCAAACAATACCATTTTTTC

AGTGGATAAGGATGGTCAATTTATGATTGATTTCGAATACGATAATTATAAGGCTTCTCAATATTTGGAT

CTGACCCTCACTCCGATATCTGGAAATGAATGCAAGACTCACGCATCGAGTATAGCCGAACAATTGGCGT

GTGTGGATATTATTAAAGAGGATATTAGCGAATATATCAAAACTACTCCCCGTCTTAAACGATTTATAAA

AAAATACCGCAATAGATCATATACTCGTATCAGTCGAGATACAGAAAAGCTTAAAATAGCTCTAGCTAAA

GGCATAGATTACGAATATATAAAAGACGCTTGTTAATAAGTAAATGAAAAAAAACTAGTCGTTTATAATA

AAACACGATATGGATGCCAACATAGTATCATCTTCTACTATTGCGACGTATATAGACGCTTTAGCGAAGA

ATGCTTCAGAATTAGAACAGAGGTCTACCGCATACGAAATAAATAATGAATTGGAACTAGTATTTATTAA

ACCGCCATTGATTACGTTGACAAATGTAGTAAATATCTCCACGATTCAGGAATCGTTTATTCGATTTACC

GTTACTAATAAGGAAGGTATCAAAATTAGAACTAAGATTCCATTATCTAAGGTACATGGTCTAGATGTAA

AAAATGTACAGTTGGTAGATGCTATAGATAACATAGTTTGGGAAAAGAAATCATTAGTGACGGAAAATCG

TCTTCACAAAGAATGCTTGTTGAGACTATCGACAGAGGAACGTCATATATTTTTGGATTACAAGAAATAT

GGATCCTCTATCCGACTAGAATTAGTCAATCTTATTCAAGCAAAAACAAAAAACTTTACGATAGACTTTA

AGCTAAAATATTTTCTAGGATCTGGTGCTCAATCTAAAAGTTCTTTATTGCACGCTATTAATCATCCAAA

GTCAAGGCCTAATACATCTCTGGAAATAGAATTTACACCTAGAGACAATGAAACAGTTCCATATGATGAA

CTAATAAAGGAATTGACGACTCTCTCGCGTCATATATTTATGGCTTCTCCAGAGAATGTAATTCTTTCTC

CACCTATTAACGCACCTATAAAGACTTTTATGTTGCCTAAACAAGATATAGTAGGTCTGGATCTGGAAAA

TCTATATGCCGTAACTAAGACTGATGGCATTCCTATAACTATCAGAGTTACATCAAAAGGGTTGTATTGT

TATTTTACACATCTTGGTTATATTATTAGATATCCAGTTAAGAGAACAATAGATTCCGAAGTAGTAGTCT

TTGGTGAGGCAGTTAAGGATAAGAACTGGACCGTATATCTCATTAAGCTAATAGAGCCCGTAAATGCAAT

CAGTGATAGACTAGAAGAAAGTAAGTATGTTGAATCTAAACTAGTGGATATTTGTGATCGGATAGTATTC

AAGTCAAAGAAATACGAAGGTCCGTTTACTACAACTAGTGAAGTCGTCGATATGTTATCTACATATTTAC

CAAAGCAACCAGAAGGTGTTATTCTGTTCTATTCAAAGGGACCTAAATCTAACATTGATTTTAAAATCAA

AAAGGAGAATACTATAGACCAAACTGCAAATGTAGTATTTAGGTACATGTCCAGTGAACCAATTATCTTT

GGAGAGTCGTCTATCTTTATAGAGTATAAGAAATTTACCAACGATAAAGGCTTTCCTAAAGAATATGGTT

CTGGTAAGATTGTGTTATATAACGGCGTTAATTATCTAAATAATATCTATTGTTTGGAATATATTAATAC

ACATAATGAAGTGGGTATTAAGTCCGTTGTTGTACCTATTAAGTTTATAGCAGAATTCTTAGTCAATGGA

GAAATACTTAAACCTAGAATCGATAAAACCATGAAATATATTAACTCAGAAGACTATTATGGAAATCAAC

ATAATATCATAGTCGAACATTTAAGAGATCAAAGCATCAAAATAGGAGATGTCTTTAACGAGGATAAACT

ATCGGATATTGGACATCAATACGCCGCCAACAACGATAAATTTAGATTAAATCCAGAAGTTAGTTATTTT

ACTAATAAACGAACTAGAGGGCCGTTGGGAATTTTATCAAACTACGTCAAGACTCTTCTTATTTCTATGT

ATTGTTCCAAAACATTTTTAGACGATTCCAACAAACGAAAGGTATTAGCGATTGATTTTGGAAACGGTGC

TGACCTGGAAAAATACTTTTATGGAGAGATTGCGTTATTGGTAGCGACGGATCCGGATGCTGATGCTATA

GCTAGAGGAAATGAAAGATACAACAAATTAAATTCTGGAATTAAAACCAAGTACTACAAATTTGACTACA

TTCAGGAAACTATTCGATCCGATACATTTGTCTCTAGTGTCAGAGAAGTATTCTATTTTGGAAAGTTTAA

TATCATTGACTGGCAGTTCGCTATTCATTATTCTTTTCATCCAAGACATTATGCTACAGTCATGAATAAC

TTATCCGAACTAACTGCTTCTGGAGGCAAGGTATTAATTACTACCATGGATGGAGACAAATTATCAAAAT

TAACCGATAAAAAGACTTTTATAATTCATAAGAATCTACCTAGTAGCGAAAACTATATGTCTGTAGAAAA

AATAGCTGATGATAGAATAGTGGTATATAATCCATCAACAATGTCTACTCCAATGACTGAATACATTATC

AAAAAGAACGATATAGTCAGAGTGTTTAATGAATACGGATTTGTTCTTGTAGATAATGTTGATTTCGCTA

CAATTATAGAACGAAGTAAAAAGTTTATTAATGGCGCATCTACAATGGAAGATAGACCGTCTACAAGAAA

CTTTTTCGAACTAAATAGAGGAGCCATTAAATGTGAAGGTTTAGATGTCGAAGACTTACTTAGTTACTAT

GTTGTTTATGTCTTTTCTAAGCGGTAAATAATAATATGGTATGGGTTCTGATATCCCCGTTCTAAATGCA

TTAAATAATTCCAATAGAGCGATTTTTGTTCCTATAGGACCTTCCAACTGTGGATACTCTGTATTATTAA

TAGATATATTAATACTTTTGTAGGGTAACAGAGGTTCTACGTCTTCTAAAAATAAAAGTTTTATAACATC

TGGCCTGTTCATAAATAAAAACTTGGCGATTCTATATATACTCTTATTATCAAATCTAGCCATTGTCTTA

TAGATGTGAGCTACTGTAGGTGTACCATTTGATTTTCTTTCTAATACTATATATTTCTCTCGAAGAAGTT

CTTGCAGATCATCTGGGAATAAAATACTACTATTGAGTAAATCAGTTATTTTTTTTATATCGATATTGAT

GGACATTTTTATAGTTAAGGATAATAAGTATCCCAAAGTAGATAACGACGATAACGAAGTATTTATACTT

TTAGGAAATCACAATGACTTTATCAGATCAAAATTAACAAAATTAAAGGAGCATGTATTTTTTTCTGAAT

ATATTGTGACTCCAGATACATATGGATCTTTATGCGTCGAATTAAATGGGTCTAGTTTTCAGCACGGTGG

TAGATATATAGAGGTGGAGGAATTTATAGATGCTGGAAGACAAGTTAGATGGTGTTCTACATCCAATCAT

ATATCTGAAGATATACACACTGATAAATTTGTCATTTATGATATTTATACGTTTGATTCGTTCAAGAATA

AACGATTGGTATTTGTACAGGTGCCTCCATCATTAGGAGATGATAGCTATTTAACTAATCCGTTATTGTC

TCCGTATTATCGTAATTCAGTAGCCAGACAAATGGTCAATGATATGATTTTTAATCAAGATTCATTTTTA

AAATATTTATTAGAACATCTGATTAGAAGCCACTATAGAGTTTCTAAACATATAACAATAGTTAGATACA

AGGATACCGAAGAATTAAATCTAACAAGAATATGTTATAATAGAGATAAGTTTAAGGCATTTGTATTCGC

TTGGTTTAACGGCGTTTCGGAAAATGAAAAGGTACTAGATACGTATAAAAAGGTATCTGATTTGATATAA

TGAATTCAGTGACTATATCACACGCACCATATACTATTACTTATCACGATGATTGGGAACCAGTAATGAG

TCAATTGGTAGAGTTTTATAACGAAGTAGCCAGTTGGCTGCTACGAGACGAGACGTCGCCTATTCCTGAT

AAGTTCTTTATACAATTGAAACAGCCGCTTAGAAATAAACGAGTATGTGTGTGTGGTATAGATCCGTATC

CAAAAGATGGAACTGGTGTACCGTTCGAATCACCAAATTTTACAAAAAAATCAATTAAGGAGATAGCTTC

ATCTATATCTAGATTAACCGGAGTAATTGATTATAAAGGTTATAACCTTAATATAATAGACGGGGTTATA

CCCTGGAATTATTACTTAAGTTGTAAATTAGGAGAAACAAAAAGTCACGCGATTTACTGGGATAAGATTT

CCAAGTTACTGCTGCAGCATATAACTAAACACGTTAGTGTTCTTTATTGTTTGGGTAAAACAGATTTCTC

GAATATACGGGCAAAGTTAGAATCCCCGGTAACTACCATAGTGGGATATCATCCAGCGGCCAGAGACCAC

CAATTCGAGAAAGATCGATCATTTGAAATTATCAACGTTTTACTGGAATTAGACAACAAGACACCTATAA

ATTGGGCTCAAGGGTTTATTTATTAATGCTTTAGTGAAATTTTAACTTGTGTTCTAAATGGATGCGGCTA

TTAGAGGTAATGATGTTATCTTTGTTCTTAAGACTATAGGTGTCCCGTCAGCATGCAGACAAAATGAAGA

TCCAAGATTCGTAGAAGCATTTAAATGCGACGAGTTAGAAAGATATATTGATAATAATCCAGAATGTACA

CTATTCGAAAGTCTTAGGGATGAGGAAGCATACTCTATAGTCAGAATTTTCATGGATGTAGATTTAGACG

CGTGTCTAGACGAAATAGATTATTTAACGGCTATTCAAGATTTTATTATCGAGGTGTCAAACTGTGTAGC

TAGATTCGCATTTACAGAATGCGGTGCCATTCATGAAAATGTAATAAAATCCATGAGATCTAATTTTTCA

TTGACTAAGTCTACAAATAGAGATAAAACAAGTTTTCATATTATCTTTTTAGACACGTATACCACTATGG

ATACATTGATAGCTATGAAACGAACACTATTAGAATTAAGTAGATCATCTGAAAATCCACTAACAAGATC

GATAGACACTGCCGTATATAGGAGAAAAACAACTCTTCGGGTTGTAGGTACTAGGAAAAATCCAAATTGC

GACACTATTCATGTAATGCAACCACCTCATGATAATATAGAAGATTACCTATTCACTTACGTGGATATGA

ACAACAATAGTTATTACTTTTCTCTACAACGACGATTGGAGGATTTAGTTCCTGATAAGTTATGGGAACC

AGGGTTTATTTCGTTCGAAGACGCTATAAAAAGAGTTTCAAAAATATTCATTAATTCTATAATAAACTTT

AATGATCTCGATGAAAATAATTTTACAACGGTACCACTGGTCATAGATTATGTAACACCTTGTGCATTAT

GTAAAAAACGATCGCATAAACATCCGCATCAACTATCGTTGGAAAATGGTGCTATTAGAATTTACAAAAC

TGGTAATCCACATAGTTGTAAAGTTAAAATTGTTCCGTTGGATGGTAATAAACTGTTTAATATTGCACAA

AGAATTTTAGACACTAACTCTGTTTTATTAACCGAACGAGGAGACCATATAGTTTGGATTAATAATTCAT

GGAAATTTAACAGCGAAGAACCATTGATAACAAAACTAATTCTATCAATAAGACATCAACTACCTAAGGA

ATATTCAAGCGAATTACTCTGTCCGAGGAAACGAAAGACTGTAGAAGCTAACATACGAGACATGTTAGTA

GATTCAGTAGAGACCGATACCTATCCGGATAAACTTCCGTTTAAAAATGGTGTATTGGACCTGGTAGACG

GAATGTTTTACTCTGGAGATGATGCTAAAAAATATACGTGTACTGTATCGACCGGATTTAAATTTGACGA

TACAAAATTCGTCGAAAACAGTCCAGAAATGGAAGAGTTAATGAATATCATTAACGATATCCAACCATTA

ACGGATGAAAATAAGAAAAATAGAGAGCTGTATGAAAAAACATTATCTAGTTGTTTATGTGGTGCTACCA

AAGGATGTTTAACATTCTTTTTTGGAGAAACCGCAACTGGAAAGTCGACAACCAAACGTTTGTTAAAGTC

TGCTATCGGTGACCTGTTTGTCGAGACGGGTCAAACAATTTTAACAGATGTATTGGATAAAGGACCTAAT

CCATTTATCGCTAATATGCATTTAAAAAGATCTGTATTCTGTAGCGAACTACCTGATTTTGCATGTAGTG

GATCAAAGAAAATTAGATCTGATAATATTAAAAAGTTGACAGAACCTTGTGTCATTGGAAGACCGTGTTT

CTCCAATAAAATTAATAATAGAAACCATGCGACAATCATTATCGATACTAATTACAAACCTGTCTTTGAT

AGGATAGATAACGCATTAATGAGAAGAATTGCCGTCGTGCGATTCAGAACACACTTTTCTCAACCTTCTG

GTAGAGAGGCTGCTGAAAATAATGACGCGTACGATAAAGTCAAACTATTAGACGAGGGATTAGATGGTAA

AATACAGAATAATAGATATAGATTCGCATTTCTATACTTGTTGGTTAAATGGTACAAAAAATATCATATT

CCTATTATGAAACTATATCCTACACCGGAAGAGATTCCGGACTTTGCATTCTATCTCAAAATAGGTACTC

TGTTGGTATCTAGCTCTGTAAAGCATATTCCATTAATGACAGACCTCTCCAAAAAGGGATATATATTGTA

CGATAATGTGGTTACTCTTCCGTTGACTACTTTCCAACAGAAAATATCCAAGTATTTTAATTCTAGACTA

TTTGGACACGATATAGAGAGCTTCATCAATAGACATAAGAAATTTGCCAATGTTAGTGATGAATATCTGC

AATATATATTCATAGAGGATATTTCATCTCCGTAAATATATGCCATATATTTATAGAAGATATCACATAT

CTAAATGAATACCGGAATCATAGATTTATTTGATAATCATGTTGATAGTATACCAACTATATTACCTCAT

CAGTTAGCTACTTTAGATTATCTAGTTAGAACTATCATAGATGAGAACAGAAGCGTGTTATTGTTCCATA

TTATGGGATCGGGTAAAACAATAATCGCTTTGTTGTTCGCCTTGGTAGCTTCCAGATTTAAAAAGGTTTA

CATTTTAGTACCGAACATCAACATCTTAAAAATTTTCAATTATAATATGGGTGTAGCTATGAACTTGTTT

AATGACGAATTCATAGCTGAGAATATCTTTATTCATTCCACAACAAGTTTTTATTCTCTTAATTATAACG

ATAACGTCATTAATTATAACGGATTAAGTCGCTACAATAACTCTATTTTTATCGTTGATGAGGCGCATAA

TATTTTTGGGAATAATACTGGAGAACTTATGACCGTGATAAAAAATAAAAACAAGATTCCTTTTCTACTA

TTGTCTGGATCTCCCATTACTAACACACCTAATACGCTGGGTCATATTATAGATTTAATGTCCGAAGAGA

CGATAGATTTTGGTGAGATTATTAGTCGTGGTAAGAAAGTAATTCAGACACTTCTTAACGAACGCGGAGT

GAATGTACTCAAGGATTTGCTTAAAGGAAGAATATCATATTACGAAATGCCGGACAAAGATCTACCAACA

ATAAGATATCACGGACGTAAATTTCTAGATACTCGAGTAGTATATTGTCACATGTCTAAACTTCAAGAGA

GAGATTATATGATTACTAGACGACAGCTATGTTATCATGAAATGTTTGATAAAAATATGTATAACGTGTC

AATGGCAGTATTGGGACAACTTAATCTGATGAATAATTTAGATACGTTATTTCAGGAACAGGATAAGGAA

TTGTACCCAAATCTGAAAATAAATAATGGAGTGTTATACGGTGAAGAATTGGTAACGTTAAACATTAGTT

CCAAATTTAAGTACTTTATCAATCGGATACAGACACTCAAGGGAAAACACTTTATATACTTCTCTAATTC

TACATATGGTGGATTGGTAATTAAATATATCATGCTCAGTAATGGATATTCTGAATATAATGGTTCTCAG

GGAACTAATCCACATATGATAAACGGCAAACCAAAAACATTTGCTATCGTTACTAGTAAAATGAAATCGT

CTTTAGAGGATCTATTAGATGTGTATAATTCTCCTGAAAACGATGATGGCAGTCAATTGATGTTTTTGTT

TTCGTCAAACATTATGTCTGAATCCTATACTCTGAAAGAGGTAAGGCATATTTGGTTTATGACTATCCCG

GATACTTTTTCTCAATACAACCAAATTCTTGGACGATCTATTAGAAAATTCTCTTACGCCGATATTTCTG

AACCCGTTAATGTATATCTTTTAGCAGCCGTATATTCAGATTTCAATGACGAAGTGACGTCATTAAACGA

TTATACACAGGATGAATTGATTAATGTTTTACCCTTTGACATCAAAAAGCTGTTGTATCTAAAATTTAAG

ACTAAAGAAACGAATAGAATATACTCTATTCTTCAAGAGATGTCTGAAACGTATTCTCTTCCACCACATC

CATCAATTGTAAAAGTTTTATTGGGAGAATTGGTCAGACAATTTTTTTATAATAATTCTCGTATTAAGTA

TAACGACTCCAAGTTACTTAAAATGGTTACATCAGTTATAAAAAATAAAGAAGACGCTAGGAATTACATA

GATGATATTGTAAACGGTCACTTCTTTGTATCGAATAAAGTATTTGATAAATCTCTTTTATACAAATACG

AAAACGATATTATTACAGTACCGTTTAGACTTTCCTACGAACCATTTGTTTGGGGAGTTAACTTTCGTAA

AGAATATAACGTGGTATCTTCTCCATAAAACTGATGAGATATATAAAGAAATAAATGTCGAGCTTTGTTA

CCAATGGATATCTTCCAGTTACATTGGAACCACATGAGTTGACATTAGACATAAAAACTAATATTAGGAA

TGCCGTATATAAGGCGTATCTCCATAGAGAAATTAGTGGTAAAATGGCCAAGAAAATAGAAATTCGTGAA

GACGTGGAATTACCTCTCGGCGAAATAGTTAATAATTCTGTAGTTATAAACGTTCCGTGTGTAATAACCT

ACGCATATTATCACGTTGGGGATATAGTCAGAGGAACATTAAACATCGAAGATGAATCAAATGTAACTAT

TCAATGTGGAGATTTAATCTGTAAACTAAGTAGAGATTCGGGTACTGTATCATTTAGCGATTCAAAGTAC

TGCTTTTTTCGAAATGGTAATGCGTATGATAACGGCATCGAAGTCTCCGCCGTTCTAATGGAGGCTCAAC

AAGGTACCGAATCTAGTTTTGTTTTTCTCGCGAATATCGTTGACTCATAAGAAAGAGAATAGCGGTGAGT

ATAAATACGAATACTATGGCAATAATTGCGAATGTTTTATTCCCTTCGATATATTTTTGATAATATGAAA

AACATGCCTCTCTCAAATCAGACAACCATTTCATAAAATAGTTCTCTCGCACTGGTGAGGTGGTTGCAGC

TCGTATAATCTCCCCAGAATAATATACTTGCGTGTCGTCGTTCAATTTATACGGATTTCTATAATTCTCT

GTTATATAATGAGGTTTACCCTCATGATTAGACGACGACAATAGTGTTCTGAATTTAGATAGTTGATCAG

AATGAATGTTTATTGGTGTTGGAAAAATTATCCATGCTGCGTCTGCAGAGTGGTTGATAGTTGTTCCTAG

ATATGTAAAATAATCCAACGTACTAGGTAGCAAATTGTCTAGATAAAATACTGAATCAAATGGCGCAGAC

ATATTAGCGGATCTAATGGAATCCAATTGATTGACTATCTTTTGAAAATATACATTTTTATGATCTGATA

CTTGTAAGAATATAGCAATAATGATAATTCCATCATCGTGTTTTTTTGCCTCTTCATAAGAACTATATTT

TTTCTTATTCCAATGAACCAGATTAATCTCTCCAGAGTATTTGTATACATCTATCAAGTGATTGGATCCA

TAATCGTCTTCCTTCCCCCAATATATATGTATTGTTGATAACACATATTCATTGGGGAGAAACCCTCCAC

TTATATATCCTCCTTTAAAATTAATCCTTACTAGTTTTCCAGTATTCTGGATAGTGGTTGGTTTCGACTC

ATTATAATGTATGTCTAACGTCTTCAATCGCGTGTCAGAAATTGCTTTTTTAGTTTCTATATTAATAGGA

GATAGTTGTTGAGGCATAGTAAAAATGAAATGATAACTGTCTAGAAATAGCTCTTAGTATGGGATTTACA

ATGGATGAGGAAGTGATATTTGAAACTCCTAGAGAATTAATATCTATTAAACGAATAAAAGATATTCCAA

GATCAAAAGACACGCACGTGTTTGCTGCGTGTATAACAAGTGACGGATATCCGTTAATAGGAGCTAGAAG

AACTTCATTCGCATTCCAGGCGATATTATCTCAACAAAATTCAGATTCTATCTTTAGAGTATCCACTAAA

CTATTACGGTTTATGTACTACAATGAACTAAGAGAAATCTTTAGACGGTTGAGAAAAGGTTCTATCAACA

ATATCGATCCTCACTTCGAAGAGTTAATATTATTGGGTGGTAAACTAGATAAAAAGGAATCTATTAAAGA

TTGTTTAAGAAGAGAATTAAAAGAGGAAAGTGATGAACATATAACAGTAAAAGAATTCGGAAATGTAATT

CTAAAACTTACAACGAGTGATAAATTATTTAATAAAGTATATATAGGTTATTGCATGGCATGTTTTATTA

ATCAATCGTTGGAGGATTTATCACATACTAGTATTTACAATGTAGAAATTAGAAAGATTAAATCGTTAAA

TGATTGTATTAACGACGATAAATACGAATATCTGTCTTATATTTATAATATACTAATTAATAGTAAATGA

GCTTTTACAGATCTAGTATAATTAGTCAGATTATTAAGTATAATAGACGACTAGCTAAGTCTATTATTTG

CGAGGATGACTCTCAAATTATTACACTCACGGCATTCGTTAACCAATGCCTATGGTGTCATAAACGAGTA

TCCGTGTCCGCTATTTTATTAACTACTGATAACAAAATATTAGTATGTAACAGACGAGATAGTTTTCTCT

ATTCTGAAATAATTAGAACTAGAAACATGTATAGAAAGAAACGATTATTTCTGAATTATTCCAATTATTT

GAACAAACAGGAAAGAAGTATACTATCGTCATTTTTTTCTCTAGATCCAGCTACTGCTGATAATGATAGA

ATAAACGCTATTTATCCGGGTGGTATACCCAAAAGGGGTGAGAACGTTCCAGAGTGTTTATCCAGGGAAA

TCAAAGAAGAAGTTAATATAGACAATTCTTTTGTATTCATAGACACTCGTTTTTTTATTCATGGTATCAT

AGAAGATACCATTATTAACAAATTTTTTGAGGTAATTTTCTTTGTTGGAAGAATATCTCTAACGAGTGAT

CAAATTATTGATACATTTAAAAGTAATCATGAAATAAAGGATCTAATATTTTTAGATCCGAATTCAGGTA

ATGGACTCCAATACGAAATTGCAAAATATGCTCTAGATACTGCAAAACTTAAATGTTACGGTCATAGAGG

ATGTTATTATGAATCATTAAAAAAATTAACTGAGGATGATTGATTAGAAAATATAAATTAATTTACCATC

GTGTATTTTTATAACGGGATTGTCTGGCATATCATGTAGATAGTTACCGTCTACATCGTATACTCTACCA

TCTACGCCTTTAAATCCTCTATTTATTGATATTAATCTATTAGAATTGGAATACCAAATATTAGTACCCT

CAATTAGTTTATTGGTAATATTTTTTTTAGACGATAGATCGATGGCTCTTGAAACCAAGGTTTTCCAACC

GGACTCATTGTCGATCGGTGAGAAGTCTTTTTCATTAGCATGAATCCATTCTAATGATGTATGTTTAAAC

ACTCTAAACAATTGTACAAATTCTTTTGATTTGTTTTGAATGATTTCAAATAGGTCTTCGTCTACAGTAG

GCATACCATTAGATAATCTAGCCATTATAAAGTGCACGTTTACATATCTACGTTCTGGAGGAGTAAGAAC

GTGACTATTGAGACGAATGGCTCTTCCTACTATCTGACGAAGAGACGCCTCGTTCCATGTCATATCTAAA

ATGAAGATATCATTGATTGAGAAGAAACTAATACCCTCGCCTCCGCTAGAAGAGAATACGCATGTTTTAA

TGTATTCTCCGTTAGTGTTTGATTCTTGGTTAAACTCAGCCACAGCCTTGATTCTAGTATCTTTTGTTCT

AGATGAGAACTCTATATTAGAGATACCAAAGACTTTGAAATATAGTAATAAGATTTCTATTCCTGACTGA

TTAACAAATGGTTCAAAGACTAGACATTTACCATGGGATGCTAATATTCCCAAACATACATCTATAAATT

TGACGCTTTTCTCTTTTAATTCAGTAAATAGAGAGATATCAGCCGCAATAGCATCCCCTCCCAATAGTTC

TCCCTTTTTAAAGGTGTCTAATGCGGATTTAGAAAATTCTCTATCTCTTAATGAATTTTTAAAATCATTA

TATAGGGTTGCTATCTCTTGTGTGTATTCTCCCGGATCACGATTTTGTCTTTCAGGAAAGCTATCGAATG

TAAACGTAGTAGCCATACGTCTCAGAATTCTAAATGATGATATACCAGTTTTTATTTCTGCGAGTTTAGC

CTTTTGATAAATCTCTTCTTGCTTTTTTGACATATTAACGTATCGCATTAATACTGTTTTCTTAGCGAAT

GATGCAGACCCTTCCACATCATCAAAAATAGAAAACTCGTTATTAACTATGTACGAACATAGGCCTCCTA

GTTTGGAGACTAATTCTTTTTCATCGACTAGACGTTTATTCTCAAATAGCGATTGGTGTTGTAAGGATCC

TGGTCGCAGTAAGTTAACCAACATGGTGAATTCTTGCACACTATTAACGATAGGTGTAGCCGATAAACAA

ATCATCTTATGGTTTTTTAACGCAGTGGTCTTAGATAAAAAATTATATACTGACCGAGTAGGACGGATCT

TACCATCTTCTTTGATTAATGATTTAGAAATGAAGTTATGACATTCATCAATGATGACGCATATTCTACT

CTTGGAATTAATAGTTTTGATATTAGTAAAAAATTTATTTCTAAAATTTTGATCATCGTAATTAATAAAA

ATACAATCCTTCGTTATCTCTGGAGCGTATCTGAGTATAGTGTTTATCCAAGGATCTTCTATCAAAGCCT

TTTTTACCAATAAGATAATTGCCCAATTTGTATAAATATCCTTAAGATGTTTGAGAATATATACAGTAGT

CATTGTTTTACCGACACCTGTTTCATGGAACAATAAAAGAGAATGCATACTGTCTAATCCTAAGAAAACT

CTTGCTACAAAATGTTGATAATCCTTGAGGCGTACTACGTCTGACCCCATCATTTCAACGGGCATATTAG

TAGTTCTGCGTAAGGCATAATCGATATAGGCCGCGTGTGATTTACTCATTTATGAGTGATAAGTAATAAC

TATGTTTTAAAAATCACAGCAGTAGTTTAACTAGTCTTCTCTGATGTTTGTTTTCGATACTTTTTGAATC

AGAAGTCATACTAGAATAAAGCAGCGAGTGAACGTAATAGAGAGCTTCGTATACTCTATTCGAAAACTCT

AAGAACTTATTAATGAATTCCGTATCCACTGGATCGTTTAAAATACTAAATTGAACAGTGTTCACATCCT

TCCAAGACGAAGACTTAGTGACGGACTTAACATGAGACATAAATAAATCCAAATTTTTTTTATAAACATC

ACTAGCCACCATAATGGCGCTATCTTTCAACCAACTATCGCTTACGCATTTTAACAGTCTAACATTTTTA

AAGAGACTACAATATATTCTCATAGTATCGATTACACCTCTACCGAATAGAGTGGGAAGTTTAATAATAC

AATATTTTTCGTTTACAAAATCAAATAATGGTCGAAACACGTCGAAGGTTAACATCTTATAATCGCTAAT

GTATAGATTGTTTTCAGTGAGATGATTATTAGATTTAATAGCATCTCGTTCACGTTTGAACAGTTTATTG

CGTGCGCTGAGGTCGGCAACTACGGCATCCGCTCTAGTACTCCTCCCATAATACTTTACGCTATTAATCT

TTAAAATTTCATAGACTTTATCTAGATCGCTTTCTGGTAACATGATATCATGTGTAAAAAGTTTTAACAT

GTCGGTCGGCATTCTATTTAGATCATTAACTCTAGAAATCTGAAGAAAGTAATTAGCTCCATATTCCAGA

CTAGGTAATGGGCTTTTACCTAAAGACAAGTTAAGTTCTGGCAATGTTTCATAAAATGGAAGAAGGACAT

GTGTCCCCTCCCGGATATTTTTTACAATTTCATCCATTTACAACTCTATAGTTTGTTTTCATTATTATTA

GTTATTATCTCCCATAATCTTGGTAATACTTACACCTTGATCATAAGATACCTTATACAGGTCATTACAT

ACAACTACCAATTGTTTTTGTACATAATAGATTGGATGATTGACATCCATGGTGGAATAAACTACTCGAA

CAGATAGTTTATCTTTCCCCCTAGATACATTGGCCGTAATAGTTGTCGGCCTAAAGAATATCTTTGGTGT

AAAGTTAAAAGTTAGGGTTCTTGTTCCATTATTGCTTTTTGTCAGTAGTTCGTTATAAATTCTCGAGATG

GGCCCGTTCTCTGAATATAGAACATCATTTCCAAATCTAACTTCTAGTCTAGAAATAATATCGGTCTTAT

TTTTAAAATCTATTCCCTTGATGAATGGATCGTTAATAAACAAATCCTTGGCCTTTGATTCGGCTGATCT

ATTATCTCCGTTATAGACGTTACGTTGACTAGTCCAAAGACTTACAGGAATAGATGTATCGATGATGTTG

ATAGTATGTGATATGTGAGCAAAGACTGTTCTCTTGGTGGCGTCGCTATATGTTCCAGTAATAGCGGAAA

ACTTTTTAGAAATGTTATATATAAAAGAATTTTTTCGGGTTCCAAACATTAACAGATTAGTATGAAGATA

AACACTCATATTATCAGGAACATTATCAATTTTTACATAAACATCGGCATCTTGAATAGAAACAACACCA

TCTTCTGGAACCTCTACGATCTCGGCAGATTCCGGATAACCAGTCGGTGGACCATCACTAACAATAACTA

GATCATCCAACAATCTACTCACATATGCGTCTATATAATCTTTTTCATCTTGTGAGTACCCTGGATACGA

AATAAATTTGTTATCCGTATTTCCATAATAAGGTTTAGTATAAACAGAGAGAGATGTTGCTGCATGAACT

TCGGTTACTGTCGCCGTTGGTTGGTTTATTTGACCTATTACTCTCCTAGGTTTCTCTATAAATGATGGTT

TAATTTGTACATTCTTAACCATATATCCAATAAAGCTCAATTCAGGAACATAAACAAATTCTTTGTTGAA

CGTTTCAAAGTCGAACGAAGAGTCACGAATAACGATATCGGATACTGGATTGAAGGTCACCGTTACCGTA

ATTTTTGAATCGGATAGTTTAAGACTACTGAATGTATCTTCCACATCAAACGGAGTTTTAATATAAACGT

ATACTGTAGATGGTTCTTTAATAGTGTCATTAGGAGTTAGACCAATAGAAATATCATTAAGTTCACTAGA

ATATCCAGAATGTTTCAAAGCAATTGTATTATTGATACAATTATTATATAATTCTTCGCCATCAATTTCC

CAAATAACACCGTTACACGAAGAGACAGATACATGATTAATACATTTATATCCAACATATGGTACGTAAC

CGAATCTTCCCATACCTTTAACTTCTGGAAGTTCCAAACTCAGAACCAAATGATTAAGCGCAGTAATATA

CTGATCCCTAATTTCGAAGCTAGCGATAGCCTGATTGTCGGGCCCATCGTTTGTCATAACTCCGGATAGA

GAAATATATTGCGGCATATATAAAGTTGGAATTTGACTATCAACTGCGAAGACATTAGACCGTTTAATAA

AGTCATCCCCACCGATCAAAGAATTAATGATAGTATTATTCATTTTCTATTTAAAATGGAAAAAAGCTTA

CAATAAACTCCGTAGAGAAATATCTATAATTTGTGAGTTTTCCTTAAAGTAACAGCTTCCGTAAACACCG

TCTTTATCTCTTAATAAGTTTATTGTATTTATGACCTTTTCCTTATCTTCATAGAATACTAAAGGCAATA

AAGAAATTTTTGGTTCTTCTCTAAGAGCTACGTGAGACTTAACCATAGACGCCAACGAATCCCTACATAT

TTTAGAACAGAAATACCCAACTTCACCACCCTTGAATGTCTCAATACTAATAGGTCTAAAAACCAAATCT

TGATTACAAAACCAACACTTATCAATTACACTATTTGTCTTAATAGACATATCTGCCATAGATTTATAAT

ACTTTGGTAGTATACAAGCGAGTGCTTCTTCTTTAGCGGGCTTAAAGACTGCTTTAGGTGCTGAAATAAC

CACATCTGGAAGACTTACTCGCTTAGCCATTTAATTACGGAACTATTTTTTTATACTTCTAATGAACAAG

TAGAAAACCTCTCATCTACAAAAACATACTCGTGTCCATAATCCTCTACCATAGTAACACGTTTTTTAGA

TCTCATATGTGCTAAAAAGTTTTCCCATACTAATTGGTTACTATTATTTTTCGTATAATTTTTAACAGTT

TGAGGTTTTAGATTTTTAGTTACAGAAGTGATATCGAATATTTTATCCAAAAAGAATGAGTAATTAATTG

TCTTAGAAGGAGTGTTTTCTTGGCAAAAGAATACCAAGTGCTTAAATATTTCTACTACTTCATTAATCTT

TTCTGTACTCAGATTCAGTTTCTCATCTTTTACTTGATTGATTATTTCAAAGACTAACTTATAATCCTTT

TTATTTATTCTCTCGTTAGCCTTAAGAAAACTAGATACAAAATTTGCATCTACATCATCCGTGGATATTT

GATTTTTTTCCATGATATCCAATAGTTCCGAGATAATTTCTCCAGAACATTGATGAGACAATAATCTCCG

CAATACATTTCTCAAATGAATAAGTTTATTAGACACGTGGAAGTTTGACTTTTTTTGTACCTTTGTACAT

TTTTGAAATACAGACTCGCAAAAAATACAATATTCATATCCTTGTTCAGATACTATACCGTTGTGTCTAC

AACAGCTACATAATCGTAGATTCATGTTAACACTCTACGTATCTCGTCGTCCAATATTTTATATAAAAAC

ATTTTATTTCTAGACGTTGTCAGAAAATCCTGTAATATTTTTAGTTTTTTTGGTTGTGAATAAAGTATCG

CCCTAATAATATTGGTACCGTCTTCCGACAATATAGTAGTTAAATTATCCGAGCATGTAGAAGAACACCG

CTTAGGCGGATTCAGTACAATGTTATATTTTTCGTACCAACTCATTTAAATATCATAATCTAAAATAGTT

CTGTAATATGTCTAGCGCTAATATATTGATCATAATCCTGTGCATAAATTAAGATACAACAATGTCTTGA

AATCATCGACATGGCTTCTTCCATAGTTAGAAGATCATCGTCAAAGTTAGCAACGTGATTCATCAACATT

TGCTGTTTTGAGGCAGCAAATACTGAACCATCACCATTCAACCATTCATAAAAACCATCGTCTGAATCCA

TTGATAATTTCTTGTACTGGTTTTTGAGAGCTCGCATCAATCTAGCATTTCTAGCTCCCGGATTGAAAAC

AGAAAGAGGATCGTACATCCAGGGTCCATTTTCTGTAAATAGAATCGTATAATGTCCCTTCAAGAAGATA

TCAGACGATCCACAATCAAAGAATTGGTCTCCGAGTTTGTAACAGACTGCGGACTTTAACCTATACATGA

TACCGTTTAGCATGATTTCTGGTGATACGTCAATCGGAGTATCATCTATTAGAGATCTAAAGCCGGTGTA

ACATTCTCCACCAAACATATTCTTATTCTGACGTCGTTCTACATAAAACATCATTGCTCCATTAACGATA

ACAGGTGAATGAACAGCACTACCCATCACATTAGTTCCCAATGGATCAATGTGTGTAACTCCAGAACATC

TTCCATAGCCTATGTTAGGAGGAGCGAACACCACTCTTCCACTATTGCCATCGAATGCCATAGAATAAAT

ATCCTTGGAATTGATAGAAATCGGACTGTCGGATGTTGTTATCATCTTCATAGGATTAACAACGATGTAT

GGTGCAGCCTGAAGTTTCATATCATAACTGATGCCGTTCATAGGTCTAGCCACAGAAACCAACGTAGGTC

TAAATCCAACTATAGACAAAATAGAAGCTAATATCTGTTCCTCATCTGTCATAACTTGAGAGCATCCAGT

ATGAATAATCTTCATTAGATGGGGATCTACCGCATCATCATCGTTACAATAAAAAATTCCCATTCTAATG

TTCATAATTGCTTTTCTAATCATGGTATGAATGTTTGCTCTCTGAATCTCTGTGGAAATTAGATCTGATA

CACCTGTAATCACTATCGGATTATCCTCCGTAAGACGATTAACCAACAACATATAATTATAAGACTTTAC

TCTTCTAAATTCATAAAGTTGCTGGATTAGACTATATGTGTCTCCATGTACATACGCGTTCTCGAGCGCA

GGAAGTTTAATACCGAATAGTGCCATCAGAATAGGATGAATGTAGTAATTAGTTTCTGGTTTTCTATAAA

TAAAAGACAAATCTTGTGAACTAGACATATCGGTAAAATGCATGGATTGGAATCGTGTAGTCGACAGAAG

AATATGATGATTAGATGGAGAGTATATTTTATCTAACTCTTTGAGTTGGTCACCGATTCTAGGACTAGCT

CGAGAATGAATAAGTACTAAGGGATGAGTACATTTCACAGAAACACTGGCGTTGTTCAACGTACTCTTTA

CATGGGAAAGGAGTTGAAATAGCTCGTTTCTATTTGTCCTGACAATATTTAGTTTATTCATAATATTAAG

CATATCCTGAATAGTAAAGTTAGATGTGTCATACTTGTTAGTAGTTAGATATTTAGCAATTGCATTCCCA

TCATTTCTCAATCTCGTACTCCAATCATGTGTGGATGCTACTTCGTCGATGGAAACCATACAATCCTTTT

TGATAGGCTGTTGAGATTGATCATTTCCTGTACGTTTAGGTTTGGTACGTTGATTTCTAGCCCCTGCGGA

TATAAAGTCATCGTCTACAATTTGGGATAATGAATTACATACACTACAAGACAAAGATTTATCAGAAGTG

TGAATATGATCTTCATCTACCAAAGAAAGAGTTTGATTAGTATAACTAGATTTTAGTCCCGCGTTAGATG

TTAAAAAAACATCGCTATTGACCACGGCTTCCATTATTTATATTCGTAGTTTTTACTCGAAAGCGTGATT

TTAATATCCAATCTTATTACTTTTGGAATCGTTCAAAACCTTTGACTAGTTGTATAATTTGATCTATTGC

CCTACGCGTATACTCCCTTGCATCATATACGTTCGTCACCAGATCGTTTGTTTCGGCCTGAAGTTGACGC

ATATCTTTTTCAACACTCGACATGAGATCCTTAAGGGTCATATCGTCTAGATTTTGTTGAGATGCTGCTC

CTGGATTTGGATTTTGTTGTGCTGTTGTACATACTGTACCACCAGTAGGTGTGGGAGTACATACAGTGGC

CACAATAGGAGGTTGAAGAGGTGTAACCGTTGGAGTAGTACAAGAAATACTTCCATCCGATTGTTGTGTA

CATGTGGTTGTTGGTAACGTCTGAGAAGGTTGGGTAGATGGCGGTGTCGTCATCTTTTGATCTTTATTAA

ATTTAGAGATAATATCCTGAACAGTATTGCTCGGCGTCAACGCTGGAAGGAGTGTACTCGCCGGCGCATC

AGTATCTGCAGACAGCCAATCAAAAAGATTAGACATATCAGATGATGTATTAGTTTGTTGTCGTGGTTTT

AGTACAGGAGCAGTACTACTAGGTAGAAGAATAGGAGCCGGTGTAGGTGTCGGAACCGGCTGTGGAGTTA

TATGAATAGTTGGTTGTAGCGGTTGGGTAGGCTGTCTGCTGGCGACCATCATATTATCTCTAGCTAGTTG

TTCTCGCAACTGTCTTTGATAATACGACTCTTGAGACTTTAGTCCTATTTCAATCGCTTCATCCTTTTTC

GTATCCGGATCCTTTTCTTCAGAATAATAGATTGACGACTTTGGTGTAGAGGATTCTGCCAGCCCCTGTG

AGAACTTGTTAAAGAAGTCCATTTAAGGCTTTAAAATTGAATTGCGATTATAAGATTAAATGGCAGACAC

GGACGATATTATCGACTATGAATCCGATGATCTCACCGAATACGAGGATGATGAAGAAGATGGAGAGTCA

CTAGAAACTAGTGATATAGATCCCAAATCTTCTTATAAGATTGTAGAATCAACATCCACTCATATAGAAG

ATGCGCATTCCAATCTTAAACATATAGGGAATCATATATCTGCTCTTAAACGACGCTATACTAGACGTAT

AAGTCTATTTGAAATAGCGGGTATAATAGCAGAAAGCTATAACTTACTTCAACGAGGAAGGTTACCTCTA

GTTTCAGAATTTTCTGACGAAACGATGAAGCAAAATATGCTACATGTAATTATACAAGAGATAGAGGAGG

GTTCTTGTCCTATAGTCATCGAAAAGAACGGAGAATTGTTGTCGGTAAACGATTTTGATAAAGATGGTCT

AAAATTCCATCTAGACTATATTATCAAAATTTGGAAACTTCAAAAACGATATTAGAATTTATACGAATAT

CGTTCTCTAAATGTTACAATCAAGTCTCTCATATTCAGCAGTTTATTGTCGTACTTTATATCGTGTTCAT

TAACGATATTTTGCAAAATAGTAATGATTCTATCTTCCTTCGATAGATATTCTTCAGAGATTATTGTCTT

ATATTCTTTCTTGTTATCCGATATGAATTTGATAAGACTTTGAACATTATTAATACCCGTCTGTTTAATT

TTTTCTATAGATATTTTAGTTTTGGTAGATTCTATGGTGTCTGTTAATAGGCATCCAACATCGACATTCG

ACGTCAATTGTCTATAAATCAGAGTATAAATTTTAGAAATAACATTAGCAAATTGTTGTGCGTTGATGTC

GTTATTCTGAAACAGTATGATTTTAGGTAGCATTTTCTTAACAAAGAGAACGTATTTATTGTTACTCAGT

TGAACAGATGATATATCCAGATTACTAACGCATCTGATTCCATATACCAAACTTTCAGAAGAAATGGTGT

ACAATTGTTTGTATTCATTCAATGTCTCCTTTTCAGAAATTAGTTTAGAGTCGAATACTGCAATAATTTT

CAAGAGATAGTTTTCATCAGATAAGATTTTATTTAGTGTAGATATGATAAAACTATTGTTTTGTTGGAGA

ACTTGATACGCCGCATTCTCTGTAGTCGACGCTCTCAAATGGGAAACAATCTCTATTATTTTTTTGGAAT

CGGATACTATATCTTCGGTATCTTGACGCAGTCTAGTATACATAGAGTTAAGAGAAATTAGAGTTTGTAC

ATTAAGCAACATGTCTCTAAATGTGGCTACAAACTTTTCTTTTTCCACATCATCTAGTTTATTATATACC

GATTTCACAACGGCACCAGATTTAAGGAACCAGAATGAAAAACTCTGATAACTACAATATTTCATCATAG

TTACGATTTTATCATCTTCTATAGTTGGTGTGATAACACATACCTTTTTCTCCAAGACTGGAACCAACGT

CATAAAAATGTTTAAATCAAAATCCATATCAACATCTGATGCGCTAAGACCAGTCTCGCGTTCAAGATTA

TCTTTACTAATGGTGACGAACTCATCGTATAGAACTCTAAGTTTGTCCATTATTTATTTACAGATTTAGT

TGTTTAATTTATTTGTGCTCTTCCAGAGTTGGGATAGTATTTTTCTAACGTCGGTATTATATTATTAGGA

TCTACGTTCATATGTATCATAATATTAATCATCCACGTTTTGATAAATCTATCTTTAGCTTCTGAAATAA

CGTATTTAAACAAAGGAGAAAAATATTTAGTTACGGCATCAGACGCAATAACATTTTTTGTAAATGTAAC

GTATTTAGACGACAGATCTTCGTTAAAAAGTTTTCCATCTATGTAGAATCCATCGGTTGTTAACACCATT

CCCGCGTCAGAGTGAATAGGAGTTTGAATAGTTTGTTTTGGAAATAGATCCTTCAATAACTTATAGTTGG

GTGGGGAAAAATCAATTTTATCACTAGACTCTTTCTTTTTTACTATCATTACCTCATGAACTATTTCTTG

AATGAGTATATGTATTTTCTTTCCTATATCGGTCGCGTTCATTGGAAAATATATCATGTCGTTAACTATA

AGAATATTTTTATCCTCGTTTACAAACTGAATAATATCAGATATAGTTCGTAAACGAACTATATCATCAC

CAGCACAACATCTAACTATATGATATCCACTAGTTTCCTTTAGCCGTTTATTATCTTGTTCCATATTAGC

AGTCATTCCATCATTTAAGAAGGCGTCAAAGATAATAGGGAGAAATGACATTTTGGATTCTGTTACGACT

TTACCAAAATTAAGGATATACGGACTTACTATCTTTTTCTCAACGTCGATTTGATGAACACACGATGAAA

ATGTACTTCGATGAGATTGATCATGTAGAAAACAACAAGGGATACAATATTTCCGCATATCATGAAATAT

ATTAAGAAATCCCACTTTATTATATTTCCCCAAAGGATCAATGCATGTAAACATTATGCCGTTATCATTA

ATAAAGACTTCTTTCTCATCGGATCTGTAAAAGTTGTTACTGATTTTTTTCATTCCAGGATCTAGATAAT

TAATAATAATGGGTTTTCTATTCTTATTCTTTGTATTTTGACATATCCTAGACCAGTAAACAGTTTCCAC

TTTGGTAAAATCAGAAGACTTTTGAACGCTATTAAACATGGCATTAATGGCAATAACTAAAAATGTAAAA

TATTTTTCTATGTTAGGAATATGGTTTTTCACTTTAATAGATATATGGTTTTTTGCCAAAATGATAGATA

TTTTTTTATCCGATGATAGCAAAATATTATTAGTCGCCGTCTCTATAAAAATGAAGCTAGTCTCGATATC

CAATTTTATTCTAGAATTGATAGGAGTCGCCAAATGTACCTTATACGTTATATCTCCCTTGATGCGTTCC

ATTTGTGTATCTATATCGGACACAAGATCTGTAAATAGTTTTACGTTATTAATCATCACGGTATCGCCAT

CGCTAGATAATGCTAATGTACTATCCAAGTCCCAAATGGAGAGATTTAACTGTTCATCGTTTAGAATAAA

ATGATTACCTGTCATATTAATAAAGTGTTCATCGTATCTAGATAACAACGACTTATAATTAATGTCCAAG

TCTTGAACTCGCTGAATGATCTTTTTTAACCCAGTTAGTTTTAGATTGGTACGAAATATATTGTTAAACT

TTGATTCTACAGTAATGTCCAAATCTAGTTGTGGAAATACTTCCATCAACATTGTTTCAAACTTGATAAT

ATTATTATCTACATCTTCGTACGATCCAAATTCCGGAATAGATGTATCGCACGCTCTGGCCACCCAGATA

ACCAAAAAGTCACACGCTCCAGAATATACATTGTATAAAAAGCTATCGTTTTTTAGTAGTGTTTTTTTCT

GAGTATATACGAAAGGATTAAAAATAGTATTATCAACGTAACTATATTCCAAATTATTCTTATGAGAATA

GATAATAATATCGTCCTTAATATCTAACAAATTTCCTAAATATCCCTTTAATTGAGTCATTCGAAGCGTT

AATAAAATATGTCTCTTAACTATTTCCGGCCGTTGTATATTTAAATGACTTCGTAAGAAATAATATATAG

GCGACTTCTCATCTATGTAATCATATGGAGTGAGATATAGGGCTCGTTCTACCTCCTGCCCCTTACCCAC

CTGTAATACCAATTGTGGACTCACTATATATCGCATATTTATATCGTGGGGTAAAGTGAAAATCTACTAC

CGATGATGTAAGTCTTACAATGTTCGAACCAGTACCAGATCTTAATTTGGAGGCCTCCGTAGAACTAGGG

GAGGTAAATATAGATCAAACAACACCTATGATAAAGGAGAATAGCGGTTTTATATCCCGTAGTAGACGTC

TATTCGCCCATAGATCTAAGGATGATGAGAGAAAACTAGCACTACGATTCTTTTTACAAAGACTTTATTT

TTTAGATCATAGAGAGATTCATTATTTGTTCAGATGCGTCGACGCTGTAAAAGACGTCACTATTACCAAA

AAAAATAACATTATCGTGGCGCCTTATATAGCACTTTTAACTATCGCATCAAAAGGATGCAAACTTACAG

AAACAATGATTGAAGCATTCTTTCCAGAACTATATAATGAACATAGTAAGAAATTCAAATTCAACTCTCA

AGTATCCATCATCCAAGAAAAACTCGGATACCAGTCTGGAAACTATCACGTTTATGATTTTGAACCGTAT

TACTCTACAGTAGCTCTGGCTATTCGAGATGAACATTCATCTGGCATTTTTAATATCCGTCAAGAGAGTT

ATCTTGTAAGTTCATTATCTGAAATAACATATAGATTTTATCTAATTAATCTAAAATCTGATCTTGTTCA

ATGGAGTGCTAGTACGGGCGCTGTAATTAATCAAATGGTAAATACTGTATTGATTACAGTGTATGACAAA

TTACAACTGGCCATAGAAAATGATTCACAATTTACATGTTCATTGGCTGTGGAATCAGAACTTCCAATAA

AATTACTTAAAGATAGAAATGAATTATTTACAAAATTCATTAACGAGTTAAAAAAGACCAGTTCATTCAA

GATAAGCAAACGCGATAAGGATACGCTATTAAAACATTTTACTTATGACTGGAGTTAGAATTTATAGACG

ACTCATTTCGTTTATCATTATTACTATTACTATCATTATTAGTATTCTTCTTGTCATCTTGTTCAGAAAT

ATACAGCAATGCTATACCTAATACCAAATACATTATCATGCTTGCAATGGCTCTAACAACAACGAACCAA

AATGAATTTGGTCGTAGCTTTTGTTCACAAAAATACATAAAGAAATGTCTACATAAATCTATGGCGCCAT

TGGCTACTTGAAATAGCGCCAGTCCTCCTACAGATTTTAATATAGCTGTATAACATGACATTTATTCATC

ATCAAAAGAGACAGAGTCACCATCTGTCATATTTAGATTTTTTTTCATGTGTTCAAAGTATCCTCTACTC

ATTTCATTATAATAGTTTATCATACTTAGAATTTTAGGACGGATCAATGAGTAAGATTTGACTAGATCGT

CAGTAGTAATTTGTGCATCATCTATTCTGCATCCGCTTCGTCGAATAATGTATAGCATCGCTTTGAGATT

CTCCATAGCTATCAAGTCTTTATATAATGACATGGAAATATCTGTGAATGCTTTATACTTCTCCAACATC

GATGCCTTAACATCATCACATACTTTAGCATTGAAAATACGTTCTATTGTGTAGATGGATGTAGCAAGAT

TTTTAAACAACAATGCCATCTTACATGATGATTGTCTCAAGTCTCCAATCGTTTGTTTAGAACGATTAGC

TACAGAGTCCAATGCTTGGCTAACTAGCATATTATTATCTTTAGAAATTGTATTCTTCAATGAGGCGTTT

ATCATATCTGTGATTTCGTTAGTCATATTACAGTCTGACTGGGTTGTAATGTTATCCAACATATCACCTA

TGGATACGGTACACGTACCAGCATTTGTAATAATCCTATCTAAGATGTTGTATGGCATTGCGCAGAAAAT

ATCTTCTCCTGTAATATCTCCACTCTCGATAAATCTACTCAGATTATTCTTAAATGCCTTATTCTCTGGA

GAAAAGATATCAGTGTCCATCATTTCATTAATAGTATACGCAGAAAAGATACCACGAGTATCAATTCTAT

CCAAGATACTTATCGGTTCCGAGTCACAGATAATGGTTTCCTCTCCTTCGGGAGATCCTGCATAGAAATA

TCTAGGACAATAGTTTCTATACTGTCTGTAACTCTGATAATCTCTAAAGTCACTAACTGATACCATGAAA

TTGAGAAGATCAAACGCTGAAGTAATCAATTTTTCTGCCTCGTTTTTACTACAACTAGTTTTCATCAATG

TAGTGACGATGTATTGTTTAGTTACTCTTGGTCTAATACTGATGATAGAGATATTATTGCTTCCCATAAT

GGATCTTCTAGTAGTCACCTTAAAGCCCATTGATGCGAATAGCAGATAGATAAAGTCTTGGTATGACTCC

TTTCTAATATAGTACGGACTACCTTTGTCACCCAACTTTATACCCACATAAGCCATAACAACCTCTTTAA

TAGCCGTTTCATGAGGTTTATCAGCCATGAGCCTGAGTAGTTGAAAGAATCGCATGAATCCCGTCTCAGA

AAGTCCTATATGCATGATAGATTTATCTTTCCTGGGAAACTCTCGTATAGTTATAGATGAAATACTCTTC

AAAGTTTCTGAAATAAGATTAGTAACAGTCTTACCTCCGACTACTCTGGGTAACAAACATACTCTAATAG

GTGTTTTCTCTGCGGAGATAATATCAGAAAGGATAGAGCAATAAGTAGTATTATTGTGATTATAAAGACC

GAATACATAACAGGTAGAATTTATAAACATCATGTCCTGAAGGGTTTTAGACTTGTATTCCTCGTAATCT

ATACCGTCCCAAAACATGGATTTGGTAACTTTGATAGCCGTAGATCTTTGTTCCTTCGCCAACAGGTTAA

AGAAATTAATAAAGAATTTGTTGTTTCTATTTATGTCCACAAATTGCACGTTTGGAAGCGCCACGGTTAC

ATTCACTGCAGCATTTTGAGGATCGCGAGTATGAAGTACAATGTTATTGTTTACTGGTATATCTGGAAAG

AAATCTACCAGTCTAGGAATAAGAGATTGATATCGCATAGAAATAGTAAAGTTTATAATCTCATCATTGT

AGATTACTCTGTTACCATTGTAATAAATTGGTACTCTATCATAATCATTGACAAAGTACTGTTCATACAT

GATGAGATGTTTATATGTTGGCATAGTAGTGAGATCGACGTTTGGTAATGGCAATGTATTAAGATTAACT

CCATAATGTCTAGCAGCATCTGTGATGTTATAAGTGATGTCAAAGCGGGGTTGATCTTGTGCTGTTATAT

ATTGTCTAACACCTATAAGATTATCAAAATCTTGTCTGCTTAATACACCGTTAACAATTTTTGCCTTGAA

TTCTTTTATTGGTGCATTAATAACATCCTTATAGAGGATGTTAAACAAATAAGTATTATCAAAGTTAAGA

TCTGGGTATTTCTTTTCTGCTAGAACATCCATTGAGTCGGAGCCATCTGGTTTAATATAACCACCGATAA

ATCTAGCTCTGTATTCTGTATCCGTCAATCTAATATTAAGAAGGTGTTGAGTGAAAGGTGGAAGATCGTA

AAAGCTGTGAGTATTAATAATAGGGTTAGTTTCCGAACTAATGTTAATTGGATGATTAATAATATCTATA

TTTCCAGCGTTAAGTGTAACATTAAACAGTTTTAATTCACGTGACGTGGTATCAATTAAATAATTAATGC

CCAATTTGGATATAGTAGCCTGAAGCTCATCTTGTTTAGTTACGGATCCTAATGAGTTATTAAGAAATAC

ATCGAACGGATGAACGAAGGTTGTTTTAAGTTGATCACATACTTTGTAATCTAGACATAGATGTGGAAGA

ACGGTAGAAACTATACGAAATAGATATTCAGAGTCCTCTAATTGATCAAGAGTAACTATTGACTTAATAG

GCATCATTTATTTAGTATTAAATGACGACCGTACCAGTGACAGATATACAAAACGACTTAATTACAGAGT

TTTCAGAAGATAATTATCCATCTAACAAAAATTATGAAATAACTCTTCGCCAAATGTCTATTCTAACTCA

CGTTAACAACGTGGTAGATAGAGAACATAATGCCGCCGTAGTGTCATCTCCAGAGGAAATATCATCACAA

CTTAATGAAGATCTATTTCCAGATGATGATTCACCGGCCACTATTATCGAACGAGTACAACCTCATACTA

CTATTATTGACGATACGCCACCTCCTACTTTTCGTAGAGAGTTATTGATATCGGAACAACGTCAACAACG

AGAAAAAAGATTTAATATTACAGTATCAAAAAATTCTGAAGCAATAATGGAATCTAGATCTATGATAACT

TCTATGCCAACACAAACACCATCCTTGGGAGTAGTTTATGATAAAGATAAAAGAATTCAGATGCTAGAGG

ATGAAGTGGTTAATCTTAGAAATCAACGATCTAATACAAAATCATCTGATAATTTAGATAATTTTACCAG

AATACTATTTGGTAAGACTCCGTATAAATCAACAGAAGTTAATAAGCGTATAGCCATCGTTAATTATGCA

AATTTGAACGGGTCCCCCTTATCAGTCGAGGACTTGGATGTTTGTTCGGAGGATGAAATAGATAGAATCT

ATAAAACGATTAAACAATATCACGAAAGTAGAAAACGAAAAATTATCGTCACTAACGTGATTATTATTGT

CATAAACATTATCGAGCAGGCATTGCTAAAACTCGGATTTGAAGAAATCAAAGGACTGAGTACCGATATC

ACTTCAGAAATTATCGATGTGGAGATCGGAGATGACTGCGATGCTGTAGCATCAAAACTAGGAATCGGTA

ACAGTCCGGTTCTTAATATTGTATTGTTTATACTCAAGATATTCGTTAAACGAATTAAAATTATTTAATT

TAATACATTCCCATATCCAGACAACAATCGTCTGGATTAATCTGTTCCTGTCGTCTCATACCGGACGACA

TATTAATCTTTTTATTAGTGGGCATCTTTTTAGATGGTTTCTTTTTCCCAGCATTAACTGATTCGATACC

TAGAAGATCGTGATTGATTTCTCCGACCATTCCACGAACTTCTAATTGGCCGTCTCTAACGGTACCATAA

ACTATTTTACCAGCATTAGTAACAGCTTGGACAATCTGACCATCCATTGCGTTGAATGATGTAGTTGCTG

TTGTTCTACGTCTAGGAGCACCAGAGGTATTTTTAGAGCTCTTGGATGTTGATGTAGAAGACGAGGATTT

TGATTTTGGTTTACATGTAATACATTTTGAACTCTTTGATTTTGTATCACATGCGCCGGCAGTCACATCT

GTTTGAGAATTAAGATTATTGTTGCCTCCTTTGACGGCTGCATCTCCACCGATCTGCGCTAGTAGATTTT

TAAGCTGTGGTGTAATCTTATTAACTGTTTCGATATAATCATCGTAACTACTTCTAACGGCTAAATTTTT

TTTATCCGCCATTTAGAAGCTAAAAATATTTTTATTTATGCAGAAGATTTAACTAGATTATACAATGAAC

TAATATGATCCTTTTCTAGATTATTTACGAACTTGGTATTTCTTGTTTCTGGAGGAGGAGAATTTAAATT

CGGACTTGGATTCGGATTTTGTGGGTTCTTGATCTTATTATACAGCGTGTATAGGATGGCGACGGTAACT

GCTACACAAATACCGATCAACAGAAGAATACCAATCATTTATTGACAATAACTTCACTATGATCAAGTAT

GTAATAATCATCTTTTCACTAAGTAAGTAGTAATAATGATTCAACAATGACAAGATATATGGACGATAAT

AATTTAGTTCATGGAAATATCGCTATGATTGGTGTGAATGACTCCGCTAACTCTGTGGGGTGCACAGTGC

TTTCCCCACATAGAATAAATTAGCATTCCGACTGTGATAATAATACCAAGTATAAACGCCATAATACTCA

ATACTTTCCATGTACGAGTGGGACTGGTAGACTTACTAAAGTCAATAAAGGCGAAGATACACGAAAGAAT

CAAAAGAATGATTCCAGCGATTAGCACGCCGGAAAAATAATTTCCAATCATAAGCATCATGTCCATTTAA

CTAATAAAAATTTTAAATCGCCGAATAAACAAAGTGGAATATAAACCATATAAAAACAATAGTTTGTACT

GCAAAAATAATATCTATTTTTGTTTTCGAAGATATGGTAAAATTAAATAGTAGTACACAGCATGTTATAA

CTAACAGCAGCAACGGCTCGTAATTACTTATCATTTACTAGACGAAAAGGTGGTGGGATATTTTCTTGCT

CAAATAATACGAATATATCACCCATCCATTTTATACGATGTTTATATACTCTAATCTTTAATAGATCTAT

AGATGACGGGTTTACCAACAATATAGATTTTATCGATTCATCTAATTTAAACCCTTCCTTAAACGTGAAT

GATCTATTATCTGGCATAATGATGACCCTACCTGATGAATCTGACAATGTACTGGGCCATGTAGAATAAA

TTATCAACGAATTATCGTCTACGAACATTTATATCATTTGTTTTAATTTTAGGACGTGAATAAATAGATA

TAAAATAGAAAATAACAGATATTACAACCAGTGTTATGGACGCACCCAACCATGTAGGCAGTTTTATTTT

ATCGTTTACTACAGGTTCTCCTGGATGTACGTCACCAACTGCAGACGTAGTTCTAGTACAATTAGACGTA

AGTTCCGCTTGGGAATTTTTTAACGCTAAAGAGTTAACGTTAATCGTACACCCAACGTATTTACATCTAG

TTCTTTGAACATCTTGATTATAATATAACCATTTTCTATCTCTAGATTCGTCAGTGCACTCATGTAACCA

ACATACCCTAGGTCCTAAATATTTATCTCCGGAATTAGATTTTGGATAATTCGCGCACCAACAATTTCTA

TTTCCTTTATGGTCGTTACAAAAGACGTATAATGCCGTATCCCCAAAAGTAAAATAATCAGGACGAATAA

TTCTAATAAACTCAGAACAATATCTCGCATCCATATGTTTGGAGCAAATATCGGAATAAGTAGACATAGC

CGGTTTCCGTTTTACACGTAACCATTCTAAACAATTGGGGTTTCCAGGATCGTTTCTACAAAAACCAGTC

ATGAAATCGTCACAATGTTCTGTCTTGTAATTATTATTAAATATTTTTGGACAGTGTTTGGTATTTGTCT

TAGAACAACATTTTGCCACGCTATCACTATCACCCAGGAGATAATCCTTTTTTATAAAATGACATCGTTG

CCCGGATGCTATATAATCAGTAGCATATTTTAAATCCTTAATATATTCAGGAGTTACCTCGTTCTGATAA

TAGATTAATGATCCAGGACGAAATTTGAAAGAACTACATGGTTCTCCATGAATTAATACATATTGTTTAG

CAAATTCAGGAACTATAAAACTACTACAATGATCTATCGACATACCATCTATCAAACAAAATTTGGGTTT

AATTTCTCCTGGAGACGTTTCATAATAATACATATAACTTTCTTCGGCAAACCTAACAGCTCTATTATAT

TCAGGATAATTAAAATCTAATACCATATATTTGTCTCGTATATCTGCTATTCCTGTCTCTATTTTGATTC

TATTAAGAGTAACAGCTGCCCCCATTCTTAATAATCATCAGTATTTAAACTGTTAAATGTTGGTATATCA

ACATCTATCTTATTTCCCGCAATATAAGGTTTGTTGCAGGTATACTGTTCAGGAATGGGTACATTTATAC

TTCTTTTATAGTCCTGTCTTTCGATGTTCATCACAAATGCAAAGAACAGAATAAACAAAATAATGTAAGA

AATAATATTAAATATCTGTGAATTCGTAAATACATTGATTGCCATAATAATTACAGCAGCTACAATACAC

ACAATAGACATTCCCACAGTGTTGCCATTACCTCCACGATACATTTGAGTTACTAAGCAATAGGTAATAA

CTAAGCTAGTAAGAGGCAATAGAAAAGATGAGATAAATATCATCAATATAGAGATTAGAGGAGGGCTATA

TAGAGCCAAGACGAACAAAATCAAACCGAGTAACGTTCTAACATCATTATTTTTGAAGATTCCCAAATAA

TCATTCATTATTCCTCCATAATCGTTTTGCATCATACCCCCATCTTTAGGCATAAACGATTGCTGCTGTT

CCTCTGTAAATAAATCTTTATCAAGCACTCCAGCACCCGCAGAGAAGTCATCAAGCATATTGTAATATCT

TAAATAACTCATTTATATATTAAAAAATGTCACTATTAAAGATGGAGTATAATCTTTATGCCGAACTAAA

AAAAATGACTTGTGGTCAGACCATAAGTCTTTTTAATGAAGACGGCGATTTCGTAGAAGTTGAACCAGGA

TCATCCTTTAAGTTTCTAATACCTAAGGGATTTTACTCCTCTCCTTGTGTAAAGACGAGTCTAGTATTCG

AGACATTAACAACGACCGATAATAAAATTACTAGTATCAATCCAACAAATGCGCCAAAGTTATATCCTCT

TCAACGCAAAGTCGTATCTGAAGTAGTTTCTAATATGAGGAAAATGATCGAATTAAAACGTCCTCTATAC

ATCACTCTTCACTTGGCATGTGGATTTGGTAAGACTATTACCACGTGTTATCTTATGACCACACACGGCA

GAAAAACCATCATTTGCGTACCCAATAAAATGTTAATACATCAATGGAAGACACAGGTAGAGGCAGTCGG

ATTGGAACATAAGATATCTATAGATGGAGTTAGTAGTCTATTAAAGGAACTAAAGACTCAAAGTCCGGAT

GTATTAATCGTAGTCAGTAGACATCTGACAAACGATGCATTTTGTAAATATATCAATAAGCATTATGATT

TGTTTATCTTGGATGAATCACATACGTATAATCTGATGAACAATACAGCAGTTACAAGATTTTTAGCGTA

TTATCCTCCGATGATGTGTTATTTTTTAACTGCTACACCTAGACCAGCTAACCGAATTTATTGTAACAGT

GTTATTAATATTGCCAAGTTATCAGATCTAAAAAAAACTATCTATACAGTAGATAGTTTTTTTGAGCCAT

ATTCCACAGACAATATTAGAAATATGGTAAAACGACTAGATGGACCATCTAATAAATATCATATATATAC

CGAGAAGTTATTATCTGTAGACGAGCCTAGAAACCAACTTATTCTTGATACCCTGGTAGAAGAATTCAAG

TCAGGAACTATTAATAGAATTTTAGTTATTACTAAACTACGTGAACATATGGTATTCTTCTACAAACGAT

TATTAGATCTTTTCGGACCAGAGGTTGTATTTATAGGAGACGCCCAAAATAGACGTACTCCAGATATGGT

CAAATCAATTAAGGAACTAAATAGATTTATATTCGTATCCACCTTATTTTATTCCGGCACTGGTTTAGAT

ATTCCGAGTTTGGATTCTTTGTTCATTTGCTCGGCAGTAATCAACAATATGCAAATAGAGCAATTACTAG

GGAGGGTATGTCGAGAAACAGAACTATTAGATAGGACGGTATATGTATTTCCTAACACATCCATCAAAGA

AATAAAGTACATGATAGGAAATTTCGTGCAACGAATTATTAGTCTGTCTGTAGATAAACTCGGATTTAAA

CAAGAAAGTTATCGGAAACATCAGGAATCTGAACCCGCTTCCGTGCCAACATCCTCCAGAGAAGAACGTG

TATTAAATAGAATATTTAACTCGCAAAATCGTTAAGAAGTTTAAGAGACGATCCACATGCTGAGCAGGCC

AGTGTATTACCCCTCATAGTATTAATATAATCCAATGATACTTTTGTGATGTCGGAAATCTTAACCAATT

TAGACTGACAGGCAGAACACGTCATGCAATCATCATCGTCATCGATAACTGTAGTCTTGGGCTTCTTTTT

GCGACTCTTCATTCCGGAACGCATATTGGTGCTATCCATTTAGGTAGTAAAAAATAAGTCAGAATATGCC

CTATAACACGATCGTGCAAAACCTGGTATATCGTCTCTATCTTTATCACAATATAGTGTATCAACATCTT

TATTATTGACCTCGTTTATCTTGGAACATGGAATGGGAACATTTTTGTTAACGGCCACCTTTGCCTTAAT

TCCAGATGTTGTAAAATTATAACTAAACAGTCTATCATCGACACAAATGAAATTCTTGTTTAGACGTTTG

TAGTTTACGTATGCGGCTCGTTCTCGTCTCATTTTTTCAGATATTGCAGGTACTATAATATTAAAAATAA

GAATGAAATAACATAGGATTAAAAATAAAGTTATCATGACTTCTAGTGCTGATTTAACTAACTTAAAAGA

ATTACTTAGTCTGTACAAAAGTTTGAGATTTTCAGATTCTGTGGCTATAGAGAAGTATAATTCTTTGGTA

GAATGGGGAACATCTACTTACTGGAAAATAGGCGTACAAAAGGTAACTAATGTCGAGACGTCCATATCTG

ATTATTATGATGAGGTAAAAAATAAACCGTTTAATATTGATCCGGGGTATTATATTTTCTTACCAGTATA

TTTTGGAAGCGTCTTTATTTATTCGAAGGGTAAAAATATGGTAGAACTTGGATCTGGAAACTCTTTTCAA

ATACCGGATGAGATTCGAAGTGCGTGTAACAAAGTATTAGATAGTGATAACGGAATAGACTTTCTGAGAT

TTGTTTTGTTAAACAATAGATGGATAATGGAAGACGCTATATCAAAATACCAGTCTCCAGTTAATATATT

TAAACTAGCTAGTGAGTACGGATTAAACATACCCAACTATTTAGAAATTGAAATAGAGGAAGACACATTA

TTTGACGATGAGTTATACTCTATTATGGAACGCTCTTTCGATGATACATTTCCAAAAATATCTATATCGT

ATATTAAGTTGGGAGAACTTAAACGGCAAGTTGTAGACTTTTTCAAATTCTCATTCATGTATATTGAGTC

AATCAAGGTAGATCGTATAGGAGATAATATTTTTATTCCTAGCGTTATAACAAAATCAGGAAAAAAGATA

TTAGTAAAAGATGTAGACCATTTAATACGATCCAAGGTTAGAGAACATACATTTGTAAAAGTAAAAAAGA

AAAACACATTTTCCATTTTATACGACTATGATGGGAACGGAACAGAAACTAGAGGAGAAGTAATAAAACG

AATTATAGACACTATAGGACGAGACTATTATGTTAATGGAAAGTATTTCTCTAAGGTTGGTAGTGCAGGC

TTAAAGCAATTGACTAATAAATTAGATATTAATGAGTGCGCAACTGTCGATGAGTTAGTTGATGAGATTA

ATAAATCCGGAACTGTAAAACGAAAAATAAAAAACCAATCAGTATTTGATTTAAGCAGAGAATGTTTGGG

ATATCCAGAAGCGGATTTTATAACGTTAGTTAATAACATGCGGTTCAAAATAGAAAATTGTAAGGTCGTA

AATTTCAATATTGAAAATACTAATTGTTTAAATAACCCGAGTATTGAAACTATATATGGAAACTTCAACC

AGTTCGTCTCAATCTTTAATACCGTTACCGATGTCAAAAAAAGATTATTCGAGTGAAATAATATGCGCCT

TTGATATAGGTGCAAAAAATCCTGCCAGAACTGTTTTAGAAGTCAAGGATAACTCCGTTAGGGTATTGGA

TATATCAAAATTAGACTGGAGTTCTGATTGGGAAAGGCGCATAGCTCAAGATTTGTCACAATATGAATAC

ACTACAGTTCTTCTAGAACGTCAGCCTAGAAGGTCACCGTACGTCAAATTTATCTATTTTATTAAAGGCT

TTTTATATCATACATCTGCTGCCAAAGTTATTTGCGTCTCACCTGTCATGTCTGGTAATTCATATAGAGA

TCGAAAAAAGAGATCTGTTGAAGCATTTCTTGATTGGATGGACACATTCGGATTGCGAGACTCCGTTCCG

GATAGACGCAAATTAGACGATGTAGCGGATAGTTTCAATTTGGCTATGAGATACGTATTAGATAAATGGA

ATACTAATTATACACCTTATAATAGGTGTAAATATAGAAATTACATAAAAAAAATGTAATAACGTTAGTA

ACGCCATTATGGATAATCTATTTACCTTTCTACATGAAATAGAAGATAGATATGCCAGAACTATTTTTAA

CTTTCATCTAATAAGTTGCGATGAAATAGGAGATATATATGGTCTTATGAAAGAACGCATTTCCTCAGAG

GATATGTTTGACAATATAGTATATAATAAAGATATACATCCTGCCATTAAGAAACTAGTTTATTGCGACA

TCCAACTTACTAAACATATTATTAATCAGAATACGTATCCGGTATTTAACGATTCTTCACAAGTGAAATG

TTGTCATTATTTCGATATAAACTCAGATAATAGCAATATTAGCTCTCGTACAGTAGAGATATTTGAGAGT

GAAAAGTCATCTCTTGTATCATATATTAAAACTACCAATAAGAAGAGAAAGGTCAATTACGGCGAAATAA

AGAAAACTGTACATGGAGGCACTAATGCAAATTACTTTTCCGGTAAAAAGTCTGATGAGTATCTGAGCAC

TACAGTCAGGTCCAACATTAATCAACCTTGGATCAAAACCATTTCTAAGAGAATGAGAGTAGATATCATT

AATCACTCTATAGTAACGCGTGGAAAAAGCTCTATATTACAAACTATAGAAATTATTTTTACTAATAGAA

CATGTGTGAAAATATTCAAGGATTCTACTATGCACATTATTCTATCCAAGGACAAGGATGAAAAGGGATG

TATAAACATGATTGATAAATTATTCTATGTATATTATAATTTATTTCTGTTGTTCGAGGATATCATCCAA

AACGAGTACTTTAAAGAAGTAGCTAATGTTGTAAACCATGTACTCATGGCTACGGCATTAGATGAGAAAT

TATTCCTAATTAAGAAAATGGCTGAACACGATGTTTATGGAGTTAGCAATTTCAAAATAGGGATGTTTAA

CCTGACATTTATTAAGTCGTTGGATCATACCGTTTTCCCCTCTCTGTTAGATGAGGATAGCAAAATAAAG

TTTTTTAAGGGGAAAAAGCTCAATATTGTAGCATTACGATCTCTGGAGGATTGTACAAATTACGTGACTA

AATCCGAGAATATGATAGAAATGATGAAGGAAAGATCGACTATTTTAAATAGCATAGATATAGAAACGGA

ATCGGTAGATCGTCTAAAAGAATTGCTTCTAAAATGAAAAAAAACACTGTTTCAGAAATGGATCAACGAC

TCGGGTATAAGTTTTTGGTGCCTGATCCTAAAGCCGGAGTTTTTTATAGACCGTTACATTTCCAATATGT

ATCGTATTCTAATTTTATATTGCATCGATTGCATGAAATCTTGACCGTCAAGCGGCCACTCTTATCGTTT

AAGAATAATACAGAACGAATTATGATAGAAATTAGCAATGTTAAAGTGACTCCTCCAGATTACTCACCTA

TAATTGCGAGTATTAAAGGTAAGAGTTATGACGCATTAGCCACGTTCACTGTAAATATCTTTAAAGAGGT

AATGACCAAAGAGGGTATATCCATCACTAAAATAAGTAGTTATGAGGGAAAAGATTCTCATTTGATAAAA

ATTCCGCTACTAATAGGATATGGGAATAAAAATCCACTTGATACAGCCAAGTATCTTGTTCCTAATGTCA

TAGGTGGAGTCTTTATCAATAAACAATCTGTCGAAAAAGTAGGAATTAATCTAGTAGAAAAGATTACAAC

ATGGCCAAAATTTAGGGTTGTTAAGCCAAACTCATTCACTTTCTCGTTTTCCTCCGTATCCCCTCCTAAT

GTATTACCGACAAGATATCGCCATTACAAGATATCTCTGGATATATCACAATTGGAAGCGTCGAATATAT

CATCGACAAAGACATTTATAACGGTCAATATTGTTTTGCTGTCTCAATATTTATCTAGAGTGAGTCTAGA

ATTCATTAGACGTAGTTTATCATACGATATGCCTCCAGAAGTTGTCTATCTAGTAAACGCGATAATAGAT

AGTGCTAAACGACTTACCGAATCTATTACTGACTTTGATATTGATACATACATTAATGACCTGGTGGAAG

CTGAACACATTAAACAAAAATCTCAGTTAACGATTAACGAGTTTAAATATGAAATGCTGCATAACTTTTT

ACCTCATATGAACTATACACCCGATCAACTAAAGGGATTTTATATGATATCTTTACTAAGAAAGTTTCTC

TACTGTATCTACCACACTTCTAGATATCCAGATAGAGATTCGATGGTTTGTCATCGCATCCTAACGTACG

GCAAATATTTTGAGACGTTGGCACATGATGAATTAGAGAATTACATAGGTAACATCCGAAACGATATCAT

GAACAATCACAAGAACAGAGGCACTTACGCAGTAAACATTCATGTACTAACAACTCCTGGACTTAATCAT

GCATTTTCTAGTCTATTGAGTGGAAAGTTCAAAAAGTCAGACGGTAGTTATCGAACACATCCTCACTATT

CATGGATGCAGAATATTTCTATTCCTAGAAGTGTTGGATTTTATCCGGATCAAGTAAAGATTTCAAAGAT

GTTTTCTGTCAGAAAATACCATCCAAGCCAATATCTTTACTTTTGTTCATCAGACGTTCCGGAAAGAGGT

CCTCAGGTAGGTTTAGTATCTCAATTGTCTGTCTTGAGTTCCATTACAAATATACTAACGTCTGAGTATT

TGGATTTGGAAAAGAAAATTTGTGAGTATATCAGATCATATTATAAAGATGATATAAGTTACTTTGAAAC

AGGATTTCCAATCACTATAGAAAATGCTCTAGTCGCATCTCTTAATCCAAATATGATATGTGATTTTGTA

ACTGACTTTAGACGTAGAAAACGGATGGGATTCTTCGGTAACTTGGAGGTAGGTATTACTTTAGTTAGGG

ATCACATGAATGAAATTCGCATTAATATTGGAGCAGGAAGATTAGTCAGACCATTCTTGGTTGTGGATAA

CGGAGAGCTCATGATGGATGTGTGTCCGGAGTTAGAAAGCAGATTAGACGACATGACATTCTCTGACATT

CAGAAAGAGTTTCCACATGTCATCGAAATGGTAGATATAGAACAATTTACTTTTAGTAACGTATGTGAAT

CGGTTCAAAAATTTAGAATGATGTCAAAGGATGAAAGAAAGCAATACGATTTATGTGACTTTCCTGCCGA

ATTTAGAGATGGATATGTAGCATCTTCACTAGTGGGAATCAATCACAATTCTGGACCCAGAGCTATTCTT

GGATGTGCTCAAGCTAAACAAGCTATCTCTTGTCTGAGTTCGGATATACGAAATAAAATAGACAATGGAA

TTCATTTGATGTATCCAGAGAGGCCAATTGTGATTAGTAAGGCTTTAGAAACTTCAAAGATTGCGGCTAA

TTGCTTCGGACAACATGTTACTATAGCATTAATGTCGTACAAAGGTATCAATCAAGAGGATGGAATTATC

ATCAAAAAACAATTTATTCAGAGAGGCGGTCTCGATATTGTTACAGCCAAGAAACATCAAGTAGAAATTC

CATTGGAAAACTTTAATAACAAAGAAAGAGATAGGTCTAACGCCTATTCGAAATTAGAAAGTAATGGATT

AGTTAGACTGAATGCTTTCTTGGAATCCGGAGACGCTATGGCAAGAAATATCTCATCAAGAACTCTTGAA

GATGATTTTGCTAGAGATAATCAGATTAGCTTTGATGTTTCCGAGAAATATACAGATATGTACAAATCTC

GCGTTGAACGAGTACAAGTAGAACTTACTGACAAAGTTAAGGTGCGAGTATTAACCATGAAAGAAAGAAG

ACCCATTCTAGGAGACAAATTTACTACTAGAACGAGTCAAAAGGGAACAGTCGCGTATATCGCAGATGAA

ACGGAACTTCCGTACGACGAAAATGGTATCACACCAGATGTCATTATTAATTCTACATCCATCTTCTCTA

GAAAAACTATATCTATGTTGATAGAAGTTATTTTAACAGCCGCATATTCTACTAAGCCGTACAACAATAA

GGGAGAAAACCGACCTGTCTGTTTTCCTAGTAGTAACGAAACATCCATTGATGCATATATGCAATTCGCT

AAACAATGTTATGAGTATTCAAATCCGAAATTGTCCGAGGAAGAATTATCGGATAAAATCTTTTGTGAAA

AGATTCTCTATGATCCTGAAACGGATAAGCCTTATGAATCCAAAGTATTTTTTGGACCAATTTATTACTT

GCGTCTGAGACATTTAACTCAGGACAAGGCAACCGTTAGATGTAGAGGTAAAAAGACGAAGCTCATTAGA

CAAGCGAATGAGGGACGAAAACGTGGAGGAGGTATCAAGTTTGGAGAAATGGAGAGAGACTGTTTAATAG

CACATGGCGCAGCCAATACTATTACAGAAGTTTTAAAAGACTCAGAAGAGGATTATCAAGATGTGTATAT

TTGTGAAAATTGTGGAGACATAGCAGCACAAATCAAAAGTATTAATACATGTCTTAGATGTTCAAAACTT

AATCTCTCTCCTCTCTTAACAAAAATTGATACCACACACGTATCTAAAGTATTTCTTACTCAAATGAACG

CCAGAGGCGTAAAAGTTAAATTAGATTTCGAACGAAGGCCTCCTTCGTTTTATAAACCATTAGATAAAGT

TGATCTTAAACCGTCTTTTCTGGTATAATATTGTTTAGTAGATACTCATCAAGATTATCAAGATAAGCTA

ATTCACTAAACATATTATCGGATTCGGTATTGTTACTCGAGAATAGAGTTCGTTATGCTCCTGATATTCG

GAAATCTGTGGAGTTTCAGGTTTTGGTGGAAGTGTAACTGCTACTTGGTGGGATACTGAAGGATATTTCA

GAGAGTTGTGGATGTTCGGGTTCGACATCCACCGATGGTGTCACGCCACTAATCGGTTCGGTAACGTCTG

TGGATGGAGGTGCTACTTCTACAGAACCTGTAGCCTCAGTTGTCAACGGAGATACATCTTCAATGCGCGG

AAATGTATAATTTGGTAATGGTTTCTCATGTGGATCTTAAGAAGAAGAGGTAAGATATCTACGAAAGATA

CCGATCACGTTCTAGTTCTCTTTTGTAGAACTTTAACTTTTTCTTTCTCAGCATCTAGTTGATATTCCGA

CCTCTTCACGTTTCACATGGGTTACCTCCGCAGTTTTTACAAGCGATTTCACGTTCCAGATCACGTTCAG

CCTTCATACGTCTCTCCCTCTCTCTATCGAGTTTATCAGAGCAGTCTTTCTGAAGGCGATCGAACTCCAT

AAATTTCTCCAACGCTTTGATTGTTTCCATAGATTTCCGAAGTTTAGCTTCTAGGACGGCGATTCTTTTT

TCTTTCGAATTCACGGGGTACAACCGTTTCCATTACCACCATCTCTACGTTTCTTTTCTAGATCGGCAAT

CTTTCTCAACATTTCATCCCCATGCCTTTTCATTCCTCGAGTCTATCGTCGTCGAAATATCGTTCCAGCT

CCTTTTCGACCTCAATAACTTTAGCACGTTGTCTCATCAAGCTCTCTCTTGTAGTACTATCATTTTTATC

TGATTCCCTGGCACGTTTAAGATCTTCATGTAATTGAGTCAGCTCTTGACACAATCTCTTAACTAACTTC

CTCTCTTGCTTCTTCGTCATAGTACTTACAATCACTATGGGATCCATTGTTACCACGTCTGTACTCGGCG

AGCTCACGTTTAAGAGATTCAATTTCCAGTTTGTACATTGATTTCATTATTACGTCCGCAGTCGTTCAAC

TGTATTTCAAGATCTGAGATTCTAGATTGTAATCTCTGTAGCATTTCCACGGCATTCACTCAGTTGTCTT

TCAAGATCTGAGATTCTAGATTGGAGTCTGCTAATCTCTGTAAGATTTCCTCCTCCGCTCTCGATGCAGT

CGGTCAACTTATTCTCTAGTTCTCTAATACGTGAACGCAGTGCATCAACTTCTTGTGTGTCTTCTTGATT

GCGTGTGCATTCATCGAGTCTAGATTCGAGATCTCTAACGTGTCGTCGTTCTTCCTCAAGTTCTCTGTGT

ACTACAGAAAGCGTGTCCCTATCTTGTTGATATTTAGCAATTTCTGATTCTAGAGTACTGATTCTACTCA

CGTATGTACTAATAGTTGTCTTATCCTTATCAAGATCCTCCTTGTATTTGTCACATTCCTTGATATCCAT

ACGAAGTCTGGACAGTTCCCATTCGACATTACGACGTTTATCGATTTCAGCTCGGAGATCGTCGTCGCGT

TGTTTTAGCCACATACGACTGAGTTCAAGTTCTCGTTGACAAGATCCATCTACTTTTCCATCCCTAATAG

TATCCAGTTCCTTTTCTAGTTCTGACCGCATTTCTCGTTCCATATCAAGAGATTCTCTCAATTCTCGTAT

AGTCTTCTTATCAATTTCTGATGAATCTGAACCATCATCTGTCCCATTTTGTTGCATATCCCTGAGTTCT

TTGATCTCTGTTGTAAGTCTGTCGATTCTTTCGGTTTTATAAACAGAATCCCTTTCCAAAGTCCTAATCT

TACTGAGTTTATCACTAAGTTCTTCATTCAATTCAGTGAGTTTTCTCTTGGCTTCTTCCAAGTCTGTTTT

AAACTCTCCATCATTTCCGCATTCTTCCTCGCATTTATCTAACCATTCAATTAGTTTATTAATGACTAGT

TGGTAATCAGCGATTCCTATAGCCGTTCTTGTATTTGTGGGAACATAATTAGGATCTTCTAATGGATTGT

ATGGCTTGATAGCATCATCTTTATCATTATTAGGTGGGGGATGGACAACCTTAATTGGTTGGTCCTCCTT

ATCTCCTCCAGTAGCATGTGGTTCTTCAATACCAGTATTAGTAATAGGCTTAGACAAATGCTTGTCGTAC

GCGGGCACTTCCTCATCCATCAAGTATTTATAATCGGGTTCTGTTTCAGAATATTCTTTTCTAAGAGACG

CGACTTCAGGAGTTAGTAGAAGAACTCTGTTTCTGTATCTATCAACGCTGGAATCGATACTCAAGTTAAG

GATAGCGAATACCTCATCGTCATCATCCGTATCTTCTGAAACGCCATCATATGACATTTCATGAAGTCTA

ACGTATTGATAAACAGAATCAGATTTAGTATTAAACAGATCCTTGACCTTTTTAGTAAATGCATATGTAT

ATTTTAGATCTCCAGATTTCATAATATGATCGCATGCCTTAAATGTCAATGCTTCCATGATATAGTCTGG

AACACTAATGGGTGACGAAAAAGATACAGCACCATATGCTACGTTGATAAATAGATCTGAACCACTAAGT

AGATAATGATTAATGTTAAGGAAGAGGAAATATTCAGTATATAGATATGCCTTAGCATCATATCTTGTAC

TAAACACGCTAAACAGTTTATTGATGTGATCAATTTCCAACAGAACAATTAGAGCGGCAGGAATACCAAC

AAACATATTACCACATCCGTATTTTCTATGAATATCACATATCATGTTAAAAAATCTTGATAGAAGAGCG

AATATCTCGTCTGACTTAATGAGATGTAGTTCAGCAGCATAAGTCATAACTGTAAATAGAACATACTTTC

CTGTAGTGTTGATTCTAGACTCCACATCAACACCATTATTAAAAATAGTTTTATATACATCTTTAATCTG

CTCTCCGTTAATCGTCGAACGTTCTAGTATACGGAAACACTTTGATTTCTTATCTGTAGTTAATGACTTA

GTGATATCACGAAGAATATTACGAATTACATTTCTTGTTTTTCTTGTGAGACCTGATTCAGAACTCAACT

CATCATTCCATAGTTTTTCTACCTCAGTGGCGAAATCTTTGGAGTGTTTGGTACATTTTTTAATAAGGTT

CGTGACCTCCATTTATTATAAAAAATTTTTATTCAAAACTTAACTACAATCGGGTAATTATAAGATCGTA

GATCTCCCATGTGGTGGAATACTACCATCTATCGCATGTGGATGGACAGTAGGTAATGGCCATGGGAACA

GTAATGTTTGCATATTTATCTTTCTTGCTAGTATTACTGTATATTGTCCCAATGTTTCAATGTGATGTTC

TAACCTATCAACTGCCACTGTATCACAACAATAATGTCCGATGGAATTAAGATTATGATCCAATGTGTTT

AATATATGATTATCAAGTCTTATACGATCAGCGTCTTTTTTGACAGGATCAGGCTCTTCTACAGGAAGAA

GTTTCGGCCTCTTATGATAGTCATGTCTGGGAAATGGTGGTCTAGGATGAGGATCCGGTATCGGAGTGGG

TTTTGGATTATAATCATCATCATCATCATCATCATCATCATCATCATCATCATCATCATCATCATCATCA

TCATCTATGATATCATCATCATCATCATCTTCGATATTTATTTTGCTATCTTGATGATGTCCTGTAT

CAGTTGCATTTTCAGCACTCGACTGAATATTAGTACATTCATTGTCTATTATTAACGTATTTCTAAACCC

AAAATGTATGTGTTGAACATCACTACTATAGTTGATGAGTCTTATAGCATGAATTCGCTTATCGTTATCG

GGTTTATCTTCTGTCACCTTAACAATTCCTTTTTTATTAAACTCTGCATAATCATAACCATTTCTATTGT

TTGTTCTAATATAAACGAGTATAGCATCATTGCTAAATTTTTCAATCGTATCGAAAACAGAATATCCTAA

ACCATATAATATATATTCAGGAACACTCAAACTAAATGTCCAGGATTCTCCTAAATACGTAAACTTTAAT

AGTGCTAAATCATTCAAAAATCTACCGCTTATAGATAGATAGTACATGAATGCGTATAGTAGTCTACCGA

TCTCTTTATTATGAAAACCGACATTACGATCATATATTTCGTGATATACATGTGACCCGTTTACGTTAAA

CCATAAATACATGGGTGATCCTATAAACATGAATTTATTTCTAATTCTCAGAGCCATAGTTAATTGACCG

TGTAATATTTGTTTACATGCATACTTGATACGATCATTAATAAGATTTTTATCATTGCTCGTTATTTCAG

AATCGTATATATAAGGAGTACCATCATGATTCTTACCAGATATTATACAAAATACTATATATAAAATATA

TTGACCCACGTTAGTAATCATGTAAATGTTTAATGTTTTAAATTTTGTATTTAATGATCCATCATCATAC

GCTAGCATGGTCTTGTGATATTCATTCTTTAAAATATAATATTGTGTTAGCCATTGCATTGGAGCTCCTA

ATGGAGATTTTCTATTCTCGTCCATTTTAGGATATGCTTTCATAAAGTCCCTAATAACTTCGTGAATAAT

GTTTCTATGTTTTCTACTGATGCATGTATTTGCTTCGATTTTTTTATCCCATGTTTCATCTATCATAGAT

TTAAACGCAGTAATGCTCGCAACATTAACATCTTGAACCGTTGGTACAATTCCGTTCCATAAATTTATAA

TGTTCGCCATTTATATAACTCATTTTTTGAATATACTTTTAATTGAACAAAAGAGTTAAGTTACTCATAT

GGACGCCGTCCAGTCTGTACATCAATCTTTTTAGCCAGAGATATCATAGCCGCTCTTAGAGTTTCAGCGT

GATTTTCCAACCTAAATAGAACTTCATCGTTGTGTTTACAACACTTTTCTATTTGTTCAAACTTTGTTGT

TATATTAGTAATCTTTTTTTCCAAATTAGTTAGCCGTTGTTTGAGAGTTTCCTCATTGTCGTCTCCATAG

GCTTTAACAATTGCTTCGCGTTTAGTCTCTGGATTTTTAGCAGCCTTTGTAGAGAAAAATTCAGTTGCTG

GAATTGCAAGATCGTCATCTCCGGGGAAAAGAGTTCCGTCCATTTAAAGTACAGATTTTAGAAACTGACA

CTCTGTGTTATTTATATTTGGCGCAATACATGGATTATAAATATCGATGTTAATAACATCAGAAAATGTA

AAGTCTATACATTGTCGCATCGTGTTAAATTTTCTAATGGATCTAGTATTATTGGGTCCAACTTCTGCCT

GAAATCCAAATATGGAAGCGGATACAAAACCGTTTCCTGGATAAACCACACATCTCCACTTTTGCTTTAC

ATCAGAAATTGTGTCATTGACATCTTGAACTCTCCTATCTAATGCCGGTGTTCCACCTATAGATTTTGAA

TACTCGAATGCTGCATGAGTAGCATTGAATTCCTTAATATTGCCATAATTTTCATATATTGAGTAACTCT

GGATAAAAAGTAAACACACCGCAGCCGTCGCTACTACAATAAAAAAAATTGATAGAGAGTTCATTTATAA

TCTATTAGAAGCTGACAAAATTTTTTTACACGCGTCAGACAATGCTTTAATAAATAGTTCAACATCTACT

TTTGTCATATCGAACCGATGGTATGATTCTAACCTAGAATTACATCCGAAAAAGTTGACCATGTTCATAG

TCATTAAGTCATTAACAAACAACATTCCAGACTCTGGATTATAAGACGATACTGTTTCGTCACAATCACC

CACCTTAATCATGTGATTATGAATATTGGCTATTAGAGCACCTTCTAAGAAATCTATAATATCTTTGAAA

CACGATTTAAAATCAAACCACGAATATACTTCTACGAAGAAAGTTAGTTTACCCATAGGAGATATAACTA

TAAATGGAGATCTAGATACAAAATCCGGATCTATGATAGTTTTAACATTATTATATTCTCTATTAAATAC

CTCCACATCTAAAAATGTTAATTTTGAAACTATGTCTTCGTTTATTACCGTACCTGAACTAAACGCTATA

AGCTCTATTGTTTGAGAACTCTTTAAACGATATTCTTGAAATACATGTAACAAAGTTTCCTTTAACTCGG

TCGGTTTATCTACCATAGTTACAGAATTTGTATCCTTATCTATAATATAATAATCAAAATCGTATAAAGT

TATATAATTATCGTGTTCAGATTGTGATCTTTTCAAATAGACTAAAAACCCCATTTCTCTAGTAAGTATC

TTATGTATATGTTTGTAAAATATCTTCATGGTGGGAATATGCTCTACAGCAGTTAGCCATTCCTCATTGA

CAGCTGTAGATGTATTAGACAAAACTACTCCAATGTTTAACAAGGGCCATTTTACGAGATTATTAAATCC

TTGTTTGATAAATGTAGCCAATGCGGGTTCGAGTTCAACGACGATTGAATTCTCTTCCCGTGGATGCTGC

ATGATGAACGACGGGATGTTGTTCTATTGATTTGGAATTCTTTTTCGACTTTTTGTTTATATTAAATATT

TTAAAATTTATGGCTGATAGTAATTCATGTACTACGGATAATGTAGACGTGTATTGCACATCGATATCTT

TATTATTAGATAAATTTATCAATAAATGTGAGAAGTTTGCCTCGTTAAGGTCTTCCATTTAAATATTATA

TAAATATTTGTGTTTGTATTTTATTCGTCTTTTATGGGATAGTTTTTAACTAGTAAAGCTGTAATTACAT

ACTTTGTCCGTAAAACATAAATATAAATACCCGCTTTTATCAAACGTTCCAAAAAGTCGGCAGCTGACAT

TTTTAACATGGCATCTATTTTAAATACACTTAGGTTTTTAGAAAAAACATCATTTTATAATTGTAACGAT

TCAATAACTAAAGAAAAGATTAAGATTAAACATAAGGGAATGTCATTTGTATTTTATAAGCCAAAGCATT

CTACCGTTGTTAAATACTTGTCTGGAGGAGGTATATATCATGATGATTTGGTTGTATTGGGGAAGGTAAC

AATTAATGATCTAAAGATGATGCTATTTTACATGGATTTATCATATCATGGAGTGACAAGTAGTGGAGCA

ATTTACAAATTGGGATCGTCTATCGATAGACTTTCTCTAAATAGGACTATTGTTACAAAAGTTAATAACA

ATTATAACAATTATAACAattataacaATTATAATTGTTATAATAATTATAATTGTTATAATTATGATGATACATTTTT

TGACGATGATGATTGATCACTATTACACAATTTTGTTTTTGTACTTTCTAATATAGTGTTTAGGTTCTTT

TTCATATGAGAATATTGACTTACTAAAATATCTATGTTTAACTTTTGTTCTATAACGTCCTTATCGGCGG

TATCGGTACATATACGTAATTCACCTTCACAAAATACGGAGTCTTCGATAATAATAGCCAATCGATTATT

GGATCTAGCTGTCTGTATCATATTCAACATGTTTAATATATCCTTTCGTTTCCCCTTTACAGGCATCGAT

CGTAGCATATTTTCCGCGTCTGAGATGGAAATGTTAAAACTGCAAAAATGCGTAATGTTAGCCCGTCCTA

ATATTGGTACGTGTCTATAAGTTTGGCATAGTAGAATAATAGACGTGTTTAAATGCCTTCCAAAGTTTAA

GAATTCTATTAGAGTATTACATTTTGATAGTTTATCACCTACATCATCAAAAATAAGTAAAAAGTGTGCT

GATTTTTTATGATTTTGTGCGACAGCAATACATTTTTCTATGTTACTTTTAGTTCGTATCAGATTATATT

CTAGAGCTTCCTGACTACTAACGAAATTAATATGATTTGGCCAAATGTATCCATCATAATCTGGGTTATA

AACGGGTGTAAACAAGAATATATGTTTATATTTTTTAACTAGTGTAGAAAACAGAGATAGTAAATAGATA

GTTTTTCCAGATCCAGATCCTCCTGTTAAAACCATTCTAAACGGCATTTTTAATAAATTTTCTCTTGAAA

ATTGTTTTTCTTGAAAACAATTCATAATTATATTTACAGTTACTAAATTAATTTGATAATAAATCAAAAT

ATGGAAAACTAAGGTCGTTAGTAGGGAGGAGAACAACGAAGACATATCGTGATATAAATAACATTTATTA

TCATGATGACACCAGAAAACGACGAAGAGCAGACATCTGTGTTCTCCGCTACTGTTTACGGAGACAAAAT

TCAGGGAAAGAATAAACGCAAACGCGTGATTGGTCTATGTATTAGAATATCTATGGTTATTTCACTACTA

TCTATGATTACCATGTCCGCGTTTCTCATAGTGCGCCTAAATCAATGCATGTCTGCTAACGAGGCTGCTA

TTACTGACTCCGCTGTTGCCGTTGCTGCGGCATCATCTACTCATAGAAAGGTTGCGTCTAGCACTACACA

ATATGATCACAAAGAAAGCTGTAATGGTTTATATTACCAGGGTTCTTGTTATATATTACATTCAGACTAT

AAGTCATTCGAGGATGCTAAAGCAAACTGCGCTGCGGAATCATCAACGCTACCCAATAAATCCGATGTCT

TGACTACCTGGCTCATTGATTATGTTGAGGATACATGGGGATCTGATGGTAATCCAATTACAAAAACTAC

ATCCGATTATCAAGATTCTGATGTATCACAAGAAGTTAGAAAGTATTTTTGTACATAAATAAATGAAATC

GCTTAATAGACAAACTGTAAGTAGGTTTAGGAAGTTGTCGGTGCCGGCCGCTATAATGATGTTACTCTCA

ACCATTATTAGCGGCATAGGAACATTTCTACATTACAGAGAccgaagatgatgaggtatAGAACTGATGCCTAGTGCTTGCGCCAATG

GATGGATACAATACGATAAACATTGTTATCTGGATACCAACATTAAAATGTCTACGGATAATGCAGTTTA

TCAGTGTCGCAAATTACGAGCTAGATTGCCTAGACCTGATACTAGACATCTGAGAGTATTGTTTAGTATT

TTTTATAAAGATTATTGGGTAAGTTTAAAAAAGACCAATGATAAATGGTTAGATATTAATAATGATAAAG

ATATAGATATTAGTAAATTAACAAATTTTAAGCAACTAAACAGCACAACGGATTCTGAGGCGTGTTATAT

ATACAAGTCTGGAAAACTGGTTAAAACAGTATGTAAAAGTACTCAATCTGTACTATGCGTTAAAAGATTC

TACAAGTGATAACAAAAAATGAATTAATAGTAAGTCGTTAACGTACGCCGCCATGGACGCCGCGTTTGTT

ATTACTCCAATGGGTGTGTTGACTATAACAGATACATTGTATGATGATCTCGATATCTCAATCATGGACT

TTATAGGACCATACATTATAGGTAACATAAAAATTGTCCAAATAGATGCACGGGATATAAAATATTCCGA

CATGCAAAAATGCTACTTTAGCTATAAGGGTAAAATAGTTCCTCAGGATTCTAATGATTTGGCTAGATTC

AACATTTATAGTATTTGTACCGCATACAGATCAAAAAATACCATCATCATAGCATGCGACTATGATATCA

TGTTAGATATAGAAGGTAAACATCAACCATTTTATCTATTCCCATCTATTGATGTTTTTAACGCTACAAT

CATAGAAGCGTATAATCTGTATACAGCTGGAGATTATCATCTGATCATCAATCCTTCAGATAATCTGAAA

ATGAAATTGTCGTTTAATTCTTCATTTTGTATATCAGACGGCAATGGATGGATTATAATTGATGGGAAAT

GTAATAGTAATTTTTTATCATAAAAGTTGTAAAGTAAATAATAAACAATAAATATTGAACTAGTAGTATG

TTGTATATTGAGCAATCAGAGATGATGCTGGTACCTCTTATCACGGTGACCGTAGTTGCGGGAACAATAT

TAGTATGTTATATATTATATATTTGTAGGAAAAAGATACGTACTGTCTATAATGACAATAAAATTATCAT

GACAAAATTAAAAAAGATAAAGAGTCCTAATTCCAGCAAATCTAGTAAATCAACTGATAGCGAATCAGAC

TGGGAGGATCACTGTAGTGCTATGGAACAAAACAATGACGTAGATAATATTTCTAGAAATGAGATATTGA

ACGATGATAGCTTCGCTGGTAGTTTAATATGGGATAACGAATCCAATGTCATGGCGCCTAGCACAGAACA

CATTTACGATAGTGTTGCTGGAAGCACGCTGCTAATAAATAATGATCGTAATGAACAGACTATTTATCAG

AATACTACAGTAGTAATTAATGATACAGAGACTGTTGAAATACTTAATGAAGATACCAAACAGATTCCTA

GCTATTCTTCCAATCCTTTCGTAAATTATAATAAAACCAGTATTTGTAGCAAGTCAAATCCGTTCATTGC

AGAACTCAACAATAAATTTAGTGATAATAATCCGTTTAGGAGAGCACATAGCGACGATTATCTTAATAAG

CAATAACAAGATCATGAACACGATGATATAGAATCATCGGTTGTATCATTGTCTGATT

AGTTTCCTTTTTATAAAATTGAAGTAATATTTAGTATTAATTACCGCCGATGCATTATACAAATATGGAG

ATATTCCCTGTATTCGGCATTTCTAAAATTAGCAATTTTATTGCTAATAATGACTGTAGATATTATATAG

ATGTAGAGCATCAAAAAATTATATCTGATGAGATCAATAGACAGATGGATGAAACGGTACTTCTTACCAA

CATCTTAAGCGTAGAAGTTGTAAATGACAATGAGATGTACCATCTTATTCCCCATAGACTATCGACGATT

ATACTCTGTATTAGTTCTGTTGGAGGATGTGTTATCTCTATAGATAATGACGTCAATGACAAAAATATTC

TAACATTTCCCATTAATCATGCTGTAATCATATCCCCACTGAGTAAATGTGTCGTAGTTAGCAAGGGCCC

TACAACCATACTGGTTGTTAAAGCGGATATACCCAGCAAACGATTGGTAACATCGTTTACAAACGACATA

CTGTATGTAAACAATCTATCACTGATTAATTATTTACCGTTGTCTGTATTCATTATTAGACGAGTCACTG

ACTATTTGGATAGACACATATGCGATCAGATATTTGCTAATAATAAGTGGTATTCCATTATAACTATCGA

CGATAAGCAATATCCTATTCCATCAAATTGTATAGGTATGTCTTCTGCCAAGTACATAAATTCGAGCATC

GAGCAAGATATTTTGATCCATGTTTGTAACCTCGAGCATCCATTCGACTCAGTCTACAAAAAAATGCAGT

CGTACAATTCTCTACCTATCAAGGAACAAATATTGTATGGTAGAATTGATAATATAAATATGAGCATTAG

TATTTCGGTGGATTAATAGATTTCTCTAGTATGGGATCATTAATCATCTCTAAATACATCATAAAAAAGC

TATTATCAAATACTGTACTGAATGGATTCATTCTTTTCTCTTTTTATGAAACTCTGTTGTATATCTACGG

ATAAAACTAGAAGCAAAAAATCTGATAGGAAGAATAATGATTATATGGAGGAACACGATTATTATAAAAT

AACAATAGTTCCTGGTTCCTCTTCCACGTCTACTAGCTCGTGGTATTATACACATGCCTAGTAATAGTCT

CTTTGCGTTGACGGAAAGCAGACTAGAAATAACAGGCCAAAATGTTCAGACACCATAATAGTTCCCAACC

CAGATAATAACAGAGTTCCATCAACACATTCCTTTAAACTCAATCCCAAACCCAAAACCGTTAAAATGTA

TCCAGCCAATTGATAATAGATAATGAGGTGTACAGCACATGATAATTTACACAGTAACCAAAATGAAAAC

ACTTTAGTAATTATAAGAAATATAGACGGTAATGTCATCATCAACAATCCAATAATATGCCTGAGAGTAA

ACATTGACGGATAAAACAAAAATGCCCCGCATAACTCTATCATGGCAATAACGCAACCAAACACTTGTAA

AATTCCTAAATTAGTAGAAAATACAACTGATATCGATGTATAAGCGATTTCGAGGAATAATAAGAACAAA

GTAATTCCCGTAAAGATAAACATCAACATTGTTTGGTAATCATTAAACCAATTAGTATGACGTTGAACTA

ATTTCACAGTAGATTTTATTCCAGTATTATCCCCGCATGTATACGTACCTGGTAAGATATCTTTATATTC

CATAATCAATGAGACATCACTATCCGATAACGAATGAAGTCTAGCACTAGTATGCCATTTACTTAATATG

GTCGTCTTGGAAGTTTTATTATAAGTTAAAATATCATGATTGTCCAATTTCCATCTAATATACTTTGTCG

GATTATCTATAATACATGGAATAATGATGGTATCATTACATGCTGTATACTCTATAGTCTTTGTAGATGT

TATAACCACAAAAGTACAGAGGTATATCAACAATATTCTAACTCTTAACATTTTTATTTATTTAAAATGA

TACCTTTGTTATTTATTTTATTCTTATTTTGCTAACGGTATCGAATGGCATAAGTTTGAAACGAGTGAAG

AAATAATTTCTACTTACTTAATAGATGATGTGGTAACGGGTGTTATTAATGGGGCTGTATATACATTTTC

AAATAATGAACTAAACAAAACTGGGTTAACTAATAACAATAATTATATCACAACATCTATAAAAGTAGAG

GATAATGATACATTAGTAGTATGCGGAACCAATAACGGAAATCCCAAATGTTGGAAAATAGACGGTTCAT

ACTACCCAAAACATATAGGTAGAGGATACGATCATCAAAATAGCAAAGTAACGATAATCAGTCACAATGA

ATGTGTACTATCCGACATAAACATATCAAAAGAAGGAATTAAACGATGGAGAAGATTTGACGGACCATGT

GGTTATGATTAAACGAGTTAAGTTTTTTAAGAAGCCTTAGAAGAGGGCTATTGGGTATGAAAATCCGAAA

TATTAAACCAGACAACCCCATATAATTTTATAGCTAAGAATGCCGCGAAGAATGGAACTAAAAACGGAAA

TATTTGTAGCACAACGAATAACTCCCAAACTGCATTCATGTTACACTATATAACAATTACAATACATTTT

TATCATAACACTACTTCGGTTAGATGTTTTAGAAAAAAATAAATATCACTACCGTTCTTGTTTTATAAAA

ATAACAATTAACAATTATCAATTTTTTTCTTTAATATTTTACGTGGTTGACCATTCTTGGTGGTAAAATA

ATCTCTTAGTGTTGGAATGGAATGCTGTTTAATGTTTCCGCACTCATCGTATATTTTGACGTATGCAGTC

ACATCGTTTACGCAATAGTCAGACTGTAGTTCTATCATGCTTCCTACGTTAGAAGGAGGAACAGTTTTAA

AGTCTCTTGGTTTTAATCTATTGTCATTAGTTTTCATGAAATCCTTTGTTTTATCCACTTCACATTTTAA

ATAAATGTCAACTATACATTCTTCTGTTAATTTTACTAGATCATCATGGGTCATAGAATTTATAGGTTCC

GTAGTCCATGGATCCAAACTAGCAAACTTCGCGTATACAGTATCGCGATTAGTGTATACACCAACTGTAT

GAAAATTAAGAAAACAGTTTAATAAATCTACAGAAATATTTAATCCTCCGTTTGATACAGATGCGCCATA

TTTATGGATTTCGGATTCACACGTTGTTTGTCTAAGTGGTTCGTCTAGTGTTGCTTCTACATAGACTTCG

ATTCCCATATATTCTTTATTGTCAGAATCACATACCGATTTATCATACGCTGGTTCACTTGTTTGAAAAC

TAAATGGTAGTAGATACATCAAAATAATAAATAATAAGTACATTCTGCAATATTGTTATCGTAATTGGAA

AATTGGTATTCAAGTGAGCTGGATTATGTGAGTATTGGATTGTATATTTTATTTTAttgtatattttattttatttTATTTTATATTTTATA

TTTTATATTTTATTTTATATTTTGTAGTAAGAATAGAATGCTAAATGTCAAGTTTATTCGAATAGATGTC

TTATTAAAAACATATATAATAAATAACAATGGCTGAATGGCATAAAATTATCGAGGATATCTCAAAAAAT

AATAAGTTCGAGGATGCCGCCATCGTTGATTACAAGACTACAAAGAATGTTCTAGCGGCTATTCCTAACA

GAACATTTGCAAAGATTAATCCGGGTGAAGTTATTCCCCTCATCACTAATCATAATATTCTAAAACCTCT

TATTGGTCAGAAATTTTGTATTGTATATACTAACTCTCTAATGGATGAGAACACGTATGCTATGGAGTTG

CTTACTGGGTACGCCCCTGTATCTCCGATCGTTATAGCGAGAACTCATACCGCACTTATATTTTTGATGG

GTAAGCCAACAACATCCAGACGTGATGTGTATAGAACATGTAGAGATCACGCTACCCGTGTACGTGCAAC

TGGTAATTAAAATAAAAAGTAATATTCATATGTAGTGTCAATTTTAAATGATGATGATGAAATGGATAAT

ATCCATATTGACGATGTCAATAATGCCGGTATTGACATACAGTTCATCGATTTTTAGATTTCATTCAGAG

GATATTGAATTATGTTATGGGAATTTGTATTTTGATAGGATCTATAATAATGTAGTAAATATAAAATATA

TTCCTGAGCATATTCCATATAGATATAATTTTATTAATCGTACGTTCTCCGTAGATGAACTAGATGATAA

TGTCTTTTTTACACATGGTTATTTTTTAAAACACAAATATGGTTGTTCACTTAATCCTAGTTTGATTGTC

TCATTATCAGGAAACTTAAAATATAATGATATACAATGCTCAGTAAATGTATCATGTCTCATTAAAAATT

TGGTAACGAGTACATCTACTATATTAACATCTAAACATAAGACTTATTCTCTATATCGGTCCACGTGTAT

TGCTATAATAGGATACGATTCTATTATATGGTATAAAGATATAAATGACAGGTATAATGACATCTATGAT

TTTACTGCAATATGTATGCTAATAGCGTCTACATTGATAGTGATCATATACGTGTTTAAAAAAATAAAAA

TGAACTCTTAATTATGTTATACTATTAGAAATGGATAAAATCAAAATTACGATTGATTCAAAAATTGGTA

ATGTTGTTACCATATCGTATAACTTGGAAAAGATAACTATTGATATCACACCAAAAAAGAAAAAAGAAAA

GGATGTATTATTAGCGCAATCAGTTGCTGTCGAAGAGGCAAAAGATGTCAAGGTGGAAGAAAAAAATATT

ATCGATATTGAAGATGACGATGATATGGATATAGAAAACACGTAATACGATCTATAAAAATAAGGTATTA

AATACTTTTTATTTACGGTACTCTTGTAGTGGTGATACCACTAATCGATTTTTTTTTTTAAAAAAAATACTT

ATTCTGATTCTTCTAGCCATTTCCGTGTTCGTTCGAATGCCACATCGACGTCAAAGATAGGGGAGTAGTT

GAAATCTAGTTCTGCATTGTTGGTACGCACCTCAAATGTAGTGTTGGATATCTTCAACGTATAGTTGTTG

AGTATTGATGGTTTTCTAAATAGAATTCTCTTCATATCATTCTTGCACGCGTACATTTTTAGCATCCATC

TTGGAATCCTAGATCCTTGTTCTATTCCCAATGGTTTCATCAATAGAAGATTAAACATATCGTAAGAACA

CGATGGAGAGTAATCGTAGCAAAAGTAAGCATTTCCTTTAATCGCAGATCCCGGATACTGGATATATTTT

GCAGCCAACACGTGCATCCATGCAACATTTCCTACATATACCCGGCTATGCACAGCGTCATCATCGACTG

TACGATACATAATGTTACCGTGTTGCTTACATTGCTCGTAAAAGACTTTCGTCAATTTGTCTCCTTCTCC

GTAAATTCCAGTGGGTCTTAGGCAACAAGTATACAATTTTGCGCCATTCATGATTACGGAATTATTGGCT

TTCATAACCAGTTGCTCGGCCATACGTTTACTTTTTGCGTATACATGTCCTGGTGATATATCATAAAGGG

TATGCTCATGACCGATGAATGGATTACCGTGTTTATTTGGTCCTATTGCTTCCATGCTACTAGTATAGAT

CAAATACTTGATTCCTAGGTCCACACAAGCTGCCAATATAGTCTGTGTTCCATAATAGTTTACTTTCATG

ATTTCATTATCAGTGTATTTTCCAAATACATCCACTAGAGCAGCCGTATGAATAATCAGATTTACCCCAT

CTAGCGCTTCTCTCACCTTATCAAAGTCGTTTATATCACATTGTATATAGTTTATAACCTTAACTTTCGA

GGTTATTGGTTGTGGATCTTCTACAATATCTATGACTCTTATTTCTTGAACATCATCTGCGCTAATTAAA

AGTTTTACTATATACCTGCCTAGAAATCCGGCACCGCCAGTAACCGCGTACACGGCCATTGCTGCCACTC

ATAATATCAGACTACTTATTCTATTTTACTAAATAATGGCTGTTTGTATAATAGACCACGATAATATCAG

AGGAGTTATTTACGTTGAACAAGTCCATGGAAAAGATAAAGTTTTAGGATCAGTTATTGGATTAAAATCC

GGAACGTATAGTTTGATAATTCATCGTTACGGAGATATTAGTCGAGGATGTGATTCCATAGGCAGTCCAG

AAATATTTATCGGTAACATCTTTGTAAACAGATATGGTGTAGCATATGTTTATTTAGATACAGATGTAAA

TATATCTACAATTATTGGAAAGGCGTTATCTATTTCAAAAAATGATCAGAGATTAGCGTGTGGAGTTATT

GGTATTTCTTACATAAATGAAAAGATAATACATTTTCTTACAATTAACGAGAATGGCGTTTGATATATCA

GTTAATGCGTCTAAAACAATAAATGCATTAGTTTACTTTTCTACTCAGCAAAATAAATTAGTCATACGTA

ATGAAGTTAATGATATACACTACACTGTCGAATTTGATAGGGACAAAGTAGTTGATACGTTTATTTCATA

TAATAGACATAATGACTCCATAGAGATAAGAGGGGTGCTTCCAGAGGAAACTAATATTGGTCGCGTGGTT

AATACGCCGGTTAGTATGACTTACTTGTATAATAAGTATAGTTTTAAACCGATTTTAGCAGAATATATAA

GACACAGAAATACTATATCCGGCAATATTTATTCGGCATTGATGACACTGGATGATTTGGTTATTAAACA

GTATGGAGACATTGATCTATTATTTAATGAGAAACTTAAAGTAGACTCCGATTCGGGACTATTTGACTTT

GTCAACTTTGTAAAGGATATGATATGTTGTGATTCTAGAATAGTAGTAGCTCTATCTAGTCTAGTATCTA

AACATTGGGAATTGACAAATAAAAAGTATAGGTGTATGGCATTAGCCGAACATATAGCTGATAGTATTCC

AATATCTGAGCTATCTAGACTACGATACAATCTATGTAAGTATCTACGCGGACACACCGATAGCATAGAG

GATGAATTTGATCATTTTGAAGACGATGATTCGTCTACATGTTCTGCCGTAACCGACAGGGAAACGGATG

TATAATTTTTTTATAGTGTGATGGATATGATGGATATGATATATGATGGATATTAT

ATATGATGGATATGATGGATATGATGGATATGATGGATATGATGGATATGATGGATATGATAAATATG

ATAAAAATATAATTGTTGTATCCATTCCCATTCAAATCACCTTATATGATTCTGTAACACAATGAAGGAG

TCTCATAGATATATAGAGGTCAGATACTGGTTTGATAAACTTTTTATTCCACATGAGTATGTTTGACTTA

TGGTTAGACACGCATACTTTAACAAATCACTGAAAATTGGAGTTAGGTATTCCTCTCAGAATCAGTTGCC

GTTCTGGAACATTAAATGTATTTTTTATGATATACTCCAACGCATTTATGTGGGTATACAACAAGTCATT

AATAATGAGTATTTCCAAGAGTTTTAGTTGTCTAGTATTTAACAAGAGAAGAGATTTCATCAGACTGTTT

ATGAACTCGAATACCGCCTCATTGTCGCTTATATTGATGACATCGAATTCCCAATATCATCTCATCAGTG

ATGAGTAGCTCAATCTTGTTATCGGGATCCAATTTCTAAAGATGTCATTAAACCCTCGATCGTGAATGGA

TTTATCATCATCGTTTTTATGTTGGACATGAGCTTAGTCCGTTTGTCCACATCTATATACGATGATTTCT

GAATTATTTCATATATCTCTCGTTAACTCCAGGAACTTGTCAGGGATCTAACTTTAATATGTTCTCGTCT

AAGAGATGAAAATCTTTGGATGGTGGCATGTGACTTTTCTCTAAAGGATGATGTTGCCCGATCCTCTCTT

AAATGAATCCATCTTATCCTTGGACAAGATGGACAGTCTATTTTCCTTAGATGGTTTAATATTTTTTACC

CATGATCTATAAAGGTAGACAGACCTAATCGTCTCGGATGACCATATATTATTTTCAGTTTTATTATACG

CATAAATTGTAAAAAATATGTTAGGTTTACGAAAATGTCTCGTGGGGCATTAATCGTTTTTGAAGGATTG

GACAAATCTGGAAAAACAACACAATGTATGAACATCATGGAATCTATACCGGCAAACACGATAAAATATC

TTAACTTTCCTCAGCGATCCACAGTCACTGGAAAGATGATAGATGACTATCTAACTCGTAAAAAAACCTA

TAATGATCATATAGTTAATCTATTATTTTGTGCAAATAGATGGGAGTTTGCATCTTTTATACAAGAACAA

CTAGAACAGGGAATTACTTTAATAGTTGACAGATACGCGTTCTCTGGAGTAGCGTATGCCACCGCTAAAG

GCGCGTCAATGACTCTCAGTAAGAGTTATGAATCTGGATTGCCTAAACCCGACTTAGTTATATTCTTGGA

ATCTGGTAGCAAAGAAATTAATAGAAACGTCGGCGAGGAAATTTATGAAGATGTTGAATTCCAACAAAAG

GTATTACAAGAATATAAAAAAATGATTGAAGAAGGAGATATTCATTGGCAAATTATTTCTTCTGAATTCG

AGGAAGATGTAAAGAAGGAGTTGATTAAGAATATAGTTATAGAGGCTATACACACGGTTACTGGACCAGT

GGGGCAACTGTGGATGTAATAAAATGAAATTACATTTTTATAAATAGATGTTAGTACAGTGTTATAAATG

GATGAAGCATATTACTCTGGCAACTTGGAATCAGTACTCGGGGATACGTGTCCGATATGCATACCGAACT

CGCATCAATATCTCAATTAGTTGCCAAGATAGAAACTATAGATAATGATTATTAAACAAGGACATTGTAA

ATTTTATCATATGTAGATCAAACTTGGATAATCCATTTATCTCTTTCCTAGATACTGCATATACTATCAT

AGATCAAGAGATCTATCAGAACGAGTTGATTAATTCATTAGACGATAATGAAATTATCGATTGTATAGTT

AACAAGTTTATGAGCTTTTATAAGGATAACCTAGAAAATATGGTAGATGCTATCATTACTCTAAAATATT

ATAATTAATAATCCAGATTTTAAAACTACGTATGTGGAAGTACTCGGTTCCAGAATAGCTGATATAGATA

TTAAACAAGTGATACGTAAGAATATAATACAATTGTCTAATGATCCGCGAACGATATTTGTGAAAATATT

AAAAAAAAATACTTTTTTTATTAAATGACGTCTCTTCGCGAATTTAGAAAATTATGCTGTGATATATATC

ACGCATCAGGATATAAAGAAAAATCTAAATTAATTAGAGACTTTATAACAGATAGAGATGATACCGATAC

ATATTTGATCATTAAGCTATTGCTTCCCGGATTAGACGATAGAATGTATAACATGAACGATAAACAAATT

ATAAAATTATATAGTATAATATTTAAACAATCTCAGGAAGATATGCTACAAGATTTAGGATACGGATATA

TAGGAGACACTATTAGGACATTCTTCAAAGAGAACACGGAAATCCGTCCACGAGATAAAAGCATTTTAAC

TTTAGAAGAAGTGGATAGTTTTTTAACTACGTTATCATCAGTAACTAAAGAATCACATCAAATAAAATTA

TTGACTGATATAGCATCTGTTTGTACATGTAATGATTTAAAATGTGTAGTCATGCTTATTGATAAAGATC

TAAAAATTAAAGCGGGTCCTCGGTACGTGCTTAACGCTATTAGTCCTCATGCCTATGATGTTTTTAGAAA

ATCTAATAACTTGAAAGAGATAATAGAAAATGCAGCTAAACAAAATCTAGACTCTATATCTATTTCTGTT

ATGACTCCAATTAATCCCATGTTAGCGGAATCATGTGATTCTGTCAATAAGGCGTTTAAAAAATTTCCAT

CAGGAATGTTTGCGGAAGTCAAATACGATGGTGAAAGAGTACAAGTTCATAAAAAAAATAACGAGTTTGC

ATTCTTTAGTAGAAACATGAAACCAGTACTCTCTCATAAAGTGGATTATCTCAAAGAATACATACCGAAA

GCATTTAAAAAAGCTACGTCTATCGTATTGGATTCTGAAATTGTTCTTGTAGACGAACATAATGTACCGC

TACCGTTTGGAAGTTTAGGTATACACAAAAAGAAAGAATATAAAAACTCTAACATGTGTTTGTTCGTGTT

TGACTGTTTATACTTTGATGGATTCGATATGACAGACATTCCATTGTATGAACGAAGATCTTTTCTCAAA

GATGTTATGGTCGAAATACCCAATAGAATAGTATTCTCAGAGTTGACGAATATTAGTAACGAGTCTCAGT

TAACTGATGTATTAGATGATGCACTAACGAGAAAATTAGAAGGATTGGTCTTAAAAGATATTAATGGCGT

ATACGAACCGGGAAAGAGAAGATGGTTAAAAATAAAGCGAGACTATTTGAACGAGGGTTCCATGGCAGAT

TCTGCCGATTTAGTAGTACTAGGTGCCTACTATGGTAAAGGAGGAAAGGGTGGTATCATGGCAGTCTTTC

TAATGGGTTGTTACGACGATGAATCCGGTAAATGGAAGACGGTAACTAAATGTTCCGGTCACGATGATAA

TACGTTAAGGGTTTTGCAAGACCAATTAACGATGGTTAAAATTAACAAGGATCCCAAAAAAATTCCAGAG

TGGTTGGTAGTTAATAAAATCTATATTCCCGATTTTGTAGTAGATGATCCGAAACAATCTCAGATATGGG

AAATTTCAGGAGCAGAGTTTACATCTTCCAAGTCACATACAGCGAATGGAATATCAATTAGATTTCCTAG

ATTTACTAGGATTAGAGAAGATAAAACGTGGAAAGAATCTACTCATCTAAACGATTTAGTAAACTTGACT

AAATCTTAATAGTTACATATAAACTGAAAAATAAAATAATACTATTTTAGTTGGTGGTCGCCATGGATGG

TGTTATCGTATACTGTCTAAATGCGTTAGTAAAACATGGCGAGGAAATAAATCATATAAAAAATGATTTC

ATGATTAAACCATGTTGTGAAAGAGTTTGTGAAAAAGTCAAGAACGTTCACATCGGCGGACAATCTAAAA

ACAATACAGTGATTGCAGATTTGCCATATCTGGATAATGCTGTATCAGATGTATGCAAATCAATATATAA

AAAGAATGTATCAAGAATATCCAGATTTGCTAATATGATAAAAATAGATGACGATGACAAGACTCCTACC

GGTGTATATAATTATTTTAAACCTAAAGATGCTATTCCTGTTATTATATCCATAGGAAAGGATAAAGATG

TCTGTGAACTATTAATCTCATATGATAAAGCGTGTGCGTGTATAAAGTTAAATTTATATAAAGTAGCCAT

TCTTCCCATGGATGTTTCCTTTTTTACCAAAGGAAATGCATCATTGATTATTCTCCTGTTTGATTTCTCT

ATCGATGCGGCACCTCTCTTAAGAAGTGTAACCGATAATAATGTTATTATATCTAGACACCAGCGTCTAC

ATGACGAGCTTCCGAGTTCCAATTGGTTCAAGTTTTACATAAGTATAAAGTCCGACTATTGTTCTATATT

ATATATGGTTGTTGATGGATCTATGATGTATGCGATAGCTGATAATAGAACTCACGCAATTATTAGCAAA

AATATATTAGACAATACTACGATTAACGATGAGTGTAGATGCTGTTATTCTGAACCACAGATTAGGATTC

TTGATAGAGATGAGATGCTCAATGGATCATCGTGTTATATGAACAGACATTGTATTATGATGAATTTACC

TGATGTAGGCGAATTTGGATCTAGTATGTTGGGGAAATATGAACCTGACATGATTAAGATTGCTCTTTCG

GTGGCTGGTAATTTAATAAGAAATCGAGACTACATTCCCGGGAGACGAGGCTATAGCTACTACGTTTACG

GTATAGCCTCTAGATAATTTTTTTAAGCACGAAATAAAAACATAATTTTAAACAATCTATTTCATACTAT

TTTGTGTGATCAACATGAACATAAAGATAGATGATATGATATATTAGTATTTCTGGTGATAAATTTACGG

CGACTGCTAGGAGGGAAAATGAAGAAAGAAAAAATATCTACCTCTCCAAAAAGAAAAACTACTGATGTTA

TCAAACCTGATTATCTTGAGTACAATGACTTGTTAGATAGAGATGAGATGTCTACTATTCTAGAGGAATA

TTAGGCCTTAGAATAAAATATGGACGACTCTTAACGAAATTAGAAAATTCGATAATGATGTTGAAGAACA

ATTCGGTACTATAGAAGAACTCAAGCAGAAGCTTAGATTAAATTCTGAAGAGGGAGCAGATAATTTTTAT

AGATTATATAAAGGTACAAAAACAGGATATCGTCAAACTTACTGTATACGATTGCATATATCTATGATAG

GATTGTATGCGTGCGTGGTAGATGTTTGGAGAAATGAGAAACTGTTTTCTAGATGGAAATATTGGTTACA

AGCGATTAAACTGTTTATTGATGATCACATGCTTGATAAGATAAAATCTATACTGTAGAATAGACTAGTG

TATGTGGAAATGTCATAGAAAGTTAAAAGTTAATGAGAGCAAAAATATATAAGGTTGTATTCCATATTTG

TTATTTTTTTCTGTAATAGTTAGAAAATACATTCGATGGTCTATCTACCAGATTATTATGTGTTATAAGG

TACTTTTCTCATAATAAACTAGAGTATGAGTAAGATAGTGTTTTTCAAAACATATAAATCTAAAATTGAT

GGATGAGATATACAGCTATTAATTTCGAAAATATATTTTAATCTGATAACTTTAAACATGGATTTTTGAT

GGTGGTTTAAGTTTAAAAAAGATTTTGTTATTGTAGTATGATAATATCAAAAAGATGGATATAAAGAATT

GGTCAGTGTATAATAAATTATATGTAGGAGGAGGAATATCTGATGATGTTCAAACTAATACATCTGAAAC

ATACGATAAAGAAAAAGATTGTTGGACATTGGATAATGGTCACTTGGTACCACATAATTATATAATGTAT

AAATGCGAACCGGTTAAACATAGATATCCATTGGAAAAAACACAGTACACGAATGATTTTCTAAAGTAT

TTGGAAAGTTTTATAGGTAGTTGATAGAACAAAATACATAATTTTGTAAAAATAAATCACTTTTTATACT

AATATGACACAATTACCAATACTTTTGTTACTAATATCATTAGTATACGCTACACCTTCTCCTCAGACAT

CTAAAAAAATAGGTGATGATGCAACTATATCATGTAGTCGAAATAATACAAATTACTACGTTGTTATGAG

TGCTTGGTATAAGGAGCCCAATTCCATTATTCTCTTAGCTGCCAAAAGCGACGTCTTGTATTTTGATAAT

TATACCAAGGATAAAATATCTTACGACTCTCCATACGATGATCTAGTTACAACTATCACAATTAAATCAT

TGACTGCTGGAGATGCCGGTACTTATATATGTGCATTCTTTATGACATCGACTACAAATGATACTGATAA

AGTAGATTATGAAGAATACTCCATAGAGTTGATTGTAAATACAGATAGTGAATCGACTATAGACATAATA

CTATCTGGATCTACACCGGAAACTATTTCTGAGAAACCAGAGGATATAGATAATTCTAATTGCTCGTCTG

TATTCGAAATCGCGACTCCGGAACCAATTACTGATAATGTAGAAGACCATACAGACACCGTCACATACAC

TAGTGATAGCATTAATACAGTAAATGCATCATCTGGAGAATCCACAACAGACGAGACTCCGGAACCAATT

ACTGATAAAGAAGAAGATCATACAGTAACAGACACTGTCTCATACACTACAGTAAGTACATCATCTGGAA

TTGTCACTACTAAATCAACCACCGATGATGCGGATCTTTATGATACATACAATGATAATGATACAGTACC

GCCAACTACTGTAGGTGGTAGTACAACCTCTATTAGCAATTATAAAACCAAGGACTTTGTAGAAATATTT

GGTATTACCACATTAATTATATTGTCGGCAGTGGCGATTTTCTGTATTACGTATTATATATGTAATAAAC

ACCCACGTAAATACAAAACAGAGAACAAAGTCTAGATTTTTGACTTACATAAATATCTGGGATAATAAAA

TCTATCATATTGAGAGGACCATCTGGTTCAGGAAAGACAGCCATAACCAAAAGACTGTTAAAAGACTATG

GGAATATATTTGGATTTGTGGTGTCCCATACCACTAGATTTCCTCGTCCTATGGAACGACGAGAAGGTGT

TGATTACCATTACTTAACAGAGAGGCAATCTGGAAGGGAATAGCCGCCGGAAACTTTCTAGAACATACTG

AGTTTTTAGGAAATATTTACGGAACTTCTAAAACAGCTGTAAATACAGCGGCTATTAATAATCGTATTTG

CGCGATGGATTTAAACATCAACGGTGTTAGAAGTCTTAAAAATACTTACCTAATGCATTACTTGGGTATA

TAAGACCTACCTCTCTTAAAATGGTTGAGGCCAATCTTCGTCGTAGAAACACTGAAGCGGACGACGAATC

TCATCGTCGCGTGATGTTGGCAAAAAACGGATATGGATGAGGTCAACGAAGCAGGTCTATTCGACACTAT

TATTATTGAAGATGATGTGAATTTAGCATATAGTAAGTGTTAATTCAGATACTACAGGACCGTATTAGAA

TGTATTTTAACACTAATTAGAGACTTAAGATTTGACTTAAAACTTGATAATTAATAATATAACTCGTTTT

TATATGTGGCTATTTCAACGTCTAATGTATTAGTTAAATATTAAAACTTACCACGTAAAACTTAAAATTT

AAAATGGTATTTCATTGACAGATCATACATTATGAAGTTTCAAGGACTTGTGTTAATTGACAATTGCAAA

AATCAATGGGTCGTTGGACCATTAATAGGAAAAGGTGGATTCGGTAGTATTTATACTACTAATGACAATA

ATTATGTAGTAAAAATAGAGCCCAAAGCTAACGGATCATTATTTACCGAACAGGCATTTTATACTAGAGT

ACTTAAACCATCCGTTATCGAAGAATGGAAAAAATCTCACAATATAAAGCACGTAGGTCTTATCACGTGC

AAGGCATTTGGTTTATACAAATCCATTAATGTGGAATATCGATTCTTGGTAATTAATAGATTAGGTGCAG

ATCTAGATGCGGTGATCAGAGCCAATAATAATAGACTACCAGAAAGGTCGGTGATGTTGATCGGAATCGA

AATCTTAAATACCATACAATTTATGCACGAGCAAGGATATTCTCACGGAGATATTAAAGCGAGTAATATA

GTCTTGGATCAAATAGATAAGAATAAATTATATCTAGTGGATTACGGATTGGTTTCTAAATTCATGTCTA

ACGGCGAACATGTTCCATTTATAAGAAATCCAAATAAAATGGATAACGGTACTCTAGAATTTACACCTAT

AGATTCGCATAAAGGATACGTTGTATCTAGACGTGGTGATCTAGAAACACTTGGATATTGTATGATTAGA

TGGTTGGGAGGTATCTTGCCATGGACTAAGATATCTGAAACAAAGAATTCTGCATTAGTAAGTGCTGCAA

AACAGAAATATGTTAACAATACTGCGACTTTGTTAATGACCAGTTTGCAATATGCACCTAGAGAATTGCT

GCAATATATTACCATGGTAAACTCTTTGACATATTTTGAGGAACCCAATTACGACGAGTTTCGTCGAGTA

TTAATGAATGGAGTTATGTAAAATTTTTGTTGATAAAAAAATTAAAAAAATAACTTAGTTATTATCACTC

TCGCGAGTGCAATAGAAACACGGCGATGTTTTACGCACACGCTTTCGGTGGGTACGACGAGAACCTTCAT

GCATTTCCTGGAATATCATCGACGGTTGCCAATGATGTCAGGAAATATTCTGTTGTGTCAGTTTATAATA

AAAAGTATAACATTGTAAAAAACAAATATATGTGGTGTAACAGTCAAGTGAACAAGAGATATATTGGAGC

ACTACTGCCTATGTTTGAGTGCAATGAATATCTACAAATTGGAGATCCAATCCATGATCTAGAAGGAAAT

CAAATCTCTATTGTCACATATCGCCACAAAAACTACTATGCTCTAAGTGGAATTGGGTACGAGAGTCTAG

ACTTGTGTTTGGAAGGAGTAGGGATTCATCATCACGTACTTGAAACAGGAAACGCGGTATATGGAAAAGT

TCAACATGAGTATTCTACTATCAAAGAGAAGGCCAAAGAAATGAATGCACTCAAACCAGGACCTATCATC

GATTACCACGTCTGGATAGGAGATTGTGTCTGCCAAGTTACTACTGTAGACGTGCATGGAAAGGAAATTA

TGAGAATGAGATTCAAAAGGGGTGCGGTGCTTCCGATTCCAAATCTGGTAAAAGTTAAAGTTGGGGAGGA

AAATGATACAATAAATCTTTCCACTTCCATATCAGCTCTCCTGAATTCCGGTGGCGGCACCATCGAGGTA

ACATCTAAGGAAGAACGTGTGGATTATGTACTCATGAAACGTTTGGAATCTATACATCATCTGTGGTCTG

TAGTGTATGATCATCTTAATGTTGTGAATGGCGAAGAACGATGCTATATACATATGCATTCATCTCATCA

AAGTCCTATGCTGAGTACTGTAAAAACAAATTTGTATATGAAGACTATGGGAGCATGTCTTCAAATGGAC

TCCATGGAAGCTCTAGAGTATCTTAGTGAACTGAAGGAATCAGGTGGGCGGAGTCCCAGACCAGAATTGC

AGAAATTTGAATATCCAGATGGAGTGAAAGACACTGAATCAATTGAGAGATTGGCAGAGGAGTTCTTCAA

TAGATCAGAACTTCAGGCTGGTGAATCAGTCAAATTTGGTAATTCTATTAATGTTAAACATACATCTGTT

TCAGCTAAGCAACTAAGAACACGTATACGGCAGCAGCTTCCTTCTATACTCTCATCTTTTGCCAACACAA

AGGGTGGATATTTGTTCATTGGAGTTGATAATAATACACACAAAGTAATTGGATTCACGGTGGGTCACGA

CTACCTCAAACTGGTAGAGAGAGATATAGAAAAGTATATCCAAAAACTTCCTGTTGTGCATTTCTGCAAG

AAAAAAGAGGACATCAAGTACGCATGTAGATTCATCAAGGTGTATAAACCTGGTGATGAGACTACCTCGA

CATATGTGTGCGCAATCAAAGTGGAAAGATGCTGCTGTGCTGTGTTTGCGGATTGGCCAGAATCATGGTA

CATGGATACTAGTGGTAGTATGAAGAAGTATTCTCCAGATGAATGGGTGTCACATATAAAATTTTAATTA

GGGTAAGGTAAAACTATATATAATAACTAACAATTTGTGTATCATATAGACAATTAATTAGGTAACTGTT

ATCTCTTTTTAACTAACTAACTAACTCTTATATACTATTA

ATAATACATCTATTAATCATTGATTAGTTTATTGCTTTAATTGTTTTTGTAAACTAACACTGTTCATTGA

AAAGGGATAACATGTTACAGAATATAAATTATATATGGATTTTTTTAAAAAGGAAATACTTGACTGGAGT

ATATATTTATTTCTTCATTACATAACACGTCTGTGTTCTAATTCTTCCAATTCTTCCACATCTCATATAA

TACAGGAATATAATCTTGTTCGAAAATACGAGAAAGTGGATAAAACAATAGTTGATTTTTTATCTAGGTG

GCCAAATTTATTCCATATTTTAGAATATGGGGAAAATATTCTACATATTTATTTTATAGATGCTGCTAAT

ACGAATATTATGATTTTTTTTCTAGATAGAGTATTAAATATTAATAAGAACCGTGGGTCATTTATACATA

ATCTCGGGTTATCATCCATTAATATAAAAGAATATGTATATCAATTAGTTAATAATGATCATCTAGATAA

TAGTATAAGACTAATGCTTGAAAATGGACGTAGAACAAGACATTTTTTGTCTTATATATTGGATACAGTT

AATATCTATATAAGTATTTTAATAAATCATAGATTTTATATAGATGCCGAAGACAGTTACGGTTGTACAT

TATTACATAGATGTATATATAACTATAGGAAATCAGAATCAGAATCATATAATGAATTAATTAAGATATT

GTTAAATAATGGATCAGATGTAGATAAAAAAGATACGTACGGAAACACACCGTTTATCCTATTATGTAAA

CACGATATCGACAACGCGGAATTGTTTGAGATATGTTTAGAGAATGCTAATATAGACTCTGTAGACTTTA

ATGGATATACACCTCTTCATTATGTCTCATGTCGTAATAAATATGATTTTGTAAAGTTATTAATTTCTAA

AGGAGCAAATGTTAATGCACGTAATAGATTCGGAACTACTCCATTTTATTGTGGAATTATACACGGTATC

TCGCTTATAAAACTATATTTGGAATCAGACACAGAGTTAGAAATAGATAATGAACATATAGTTCGTCATT

TAATAATTTTTGATGCTGTTGAATCTTTAGATTATCTATTGTCCAGAGGAGTTATTGATATTAACTATCG

TACTATATACAACGAAACATCTATTTACGACGCTGTCAGTTATAATGCGTATAATACGTTAGTCTATCTA

TTAAACAGAAATGGTGATTTTGAGACGATTACTACTAGTGGATGTACATGTATTTCGGAAGCAGTCGCGA

ACAACAACAAAATAATAATGGATATACTATTGTCTAAACGACCATCTTTGAAAATTATGATACCATCTAT

GATAGCAATTACTAAACATAAACAACATAATGCAGATTTATTGAAAATGTGTATAAAATATACTGCGTGT

ATGACCGATTATGATACTCTTATAGATGTACAATCGCTACATCAATATAAATGGTATATTTTAAAATGTT

TTGATGAAATAGATATCATGAAGAGATGTTATATAAAAAATAAAACTGTATTCCAATTAGTTTTTTGTAT

CAAAGACATTAATACTTTAATGAGATACGGTAGACATCCTTCTTTCGTGAAATGTAATATTCTCGACGTA

TACGGAAGTCATGTACGTAATATCATAGCATCTATTAGATATCGTCAGAGATTAATTAGTCTATTATCCA

AGAAGCTGGATGCTGGAGATAAATGGTCGTGTTTTCCTAACGAAATAAAATATAAAATATTGGAAAACTT

TAACGATAACGAACTGACCACATATCTAAAAATCTTATAAACACTATTAAAATATAAAATCTAAGTAGGA

TAAAATCACACTACATCATTGTTTCCTTTTAGTGCTCGACAGTGTATACTATTTTTAACACTCATAAATA

AAAATGAAAACGATTTCCGTTGTTACGTTGTTATGCGTACTACCTGCTGTTGTTTATTCAACATGTACTG

TACCCACTATGAATAACGCTAAATTAACGTCTACCGAAACATCGTTTAATGATAAACAGAAAGTTACGTT

TACATGTGATTCAGGATATCATTCTTTGGATCCAAATGCTGTCTGCGAAACAGATAAATGGAAATACGAA

AATCCATGCAAGAAAATGTGCACAGTTTCTGATTATGTCTCTGAACTATATGATAAGCCATTATACGAAG

TGAATTCCACCATGACACTAAGTTGCAACGGTGAAACAAAATATTTTCGTTGTGAAGAAAAAAATGGAAA

TACTTCTTGGAATGATACTGTCACGTGTCCTAATGCGGAATGTCAACCTCTTCAATTAGAACACGGATCG

TGTCAACCAGTTAAAGAAAAATACTCATTTGGGGAATATATGACTATCAACTGTGATGTTGGATATGAGG

TTATTGGTGTTTCGTATATAAGTTGTACGGCTAATTCTTGGAATGTTATTCCATCATGTCAACAAAAATG

TGATATACCGTCTCTATCTAATGGATTAATTTCCGGATCTACATTTTCTATCGGTGGCGTTATACATCTT

AGTTGTAAAAGTGGTTTTACACTAACGGGGTCTCCATCATCCACATGTATCGACGGTAAATGGAATCCCA

TACTCCCAACATGTGTACGATCTAACGAAGAATTTGATCCAGTGGATGATGGTCCCGACGATGAGACAGA

TCTGAGCAAACTCTCGAAAGACGTTGTACAATATGAACAAGAAATAGAATCGTTAGAAGCAACTTATCAT

ATAATCATAATGGCGTTGACAATTATGGGTGTCATATTTCTAATCTCTATTATAGTATTAGTTTGTTCCT

GTGACAAAAATAATGACCAATATAAGTTCCATAAATTGCTACCGTGAATATAAATCCGTTAAAATAATTA

ATAATTAATAATTAATAATTAATAACGAACAAGTATCAAAAGATTAAAGAATTAGCTAGAATCAATTAGA

TGTCTTCTTCAGTGGATGTTGATATCTACGATGCTGTTAGAGCATTTTTACTCAGGCACTATTATGACAA

GAGATTTATTGTGTATGGAAGAAGTAACACCATATTACATAATATATACAGGCTATTTACAAGATGCACC

GTTATACAGTTCGATGATATAGTACGTACTATGCCAAATGAATCACGTGTTAAACAATGGGTGATGGATA

CACTTAATGGTATAATGATGAATGAATGCGATACTGTATGTGTGGGTACCGGACTACGATTCATGGAAAT

GTTTTTCGATTACAATAAAAATAATCCCAAAAATAGCATCAACAATCAAATAATGTATGATATAATTAAT

AGCGTAGCCATAATTCTAGCTAATGAGAGATATAGAAGCGCGTTTAACGACGATAGAATATACATCCGTA

GAACTATGATGGACAAATTGTACGAATACGCATCTCTAACTACTATTGGTACGATCACTGGAGGTGTTTG

TTATTATCTGTTGATGCATCTAGTTAGTTTGTATAAATAATTATTTCGATATACTAGTAAAATTTTAAGA

TTTTAAATGTATAAAAAACTAATAACGTTTTTATTTGTAATAGGTGCAGTTGCATCCTATTCGAATAATG

AGTACACTCCGTTTAATAAACTGAGTGTAAAACTCTATATAGATGGAGTAGATAATATAGAAAATTCATA

TACTGATGATAATAATGAATTGGTGTTAAATTTTAAAGAGTACACAATTTCTATTATTACAGAGTCATGC

GACGTCGGATTTGATTCCATAGATATAGATGTTATAAACGACTATAAAATTATTGATATGTATACCATTG

ACTCGTCTACTATTCAACGCAGAGAACATACGTGTAGAATATCTACCAAATTATCATGCCATTATGATAA

GTACCCTTATATCCACAAATATGAGGGTGATGAACGACAATATTCTATTACTGCAGAGGGAAAATGCTAT

AAAGGAATAAAATATGAAATAAGTATGATGAACGATGATACTCTATTGAGAAAACATACTCTTAAAATTG

GATTTACTTATATATTCGATCGTCATGGGCATAGTAATACATATTATTCAAAATATGATTTTTAAAAATT

TAAAATATATTATCACTTCAGTGACAGTAGTCAAATAACAAACAACACCATGAGATATATTATAATTCTC

GCAGTTTTGTTCATTAATAGTATACATGCTAAAATAACTAGTTATAAGTTTGAATCCGTCAATTTTGATT

CCAAAATTGAATGGACTGGGGATGGTCTATACAATATATCCCTTAAAAATTATGGCATCAAGACGTGGCA

AACAATGTATACAAATGTACCAGAAGGAACATACGACATATCCGGATTTCCAAAGAATGATTTCGTATCT

TTCTGGGTTAAATTTGAACAAGGCGACTATAAAGTGGAAGAGTATTGTACGGGACTATGTGTCGAAGTAA

AAATTGGACCACCAACTGTAAGATTGACTGAATATGACGATCATATCAATTTGTTCATCGAGCATCCGTA

TGCTACTAGAGGTAGCAAGAAGATTCCTATTTACAAACGCGGTGACATGTGTGATATCTACTTGTTGTAT

ACGGCTAACTTCACATTCGGAGATTCTGAAGAACCAGTAACATATGATATCGATGACTACGATTGCACGT

CTACAGGTTGCAGCATAGACTTTGCCACAACAGAAAAAGTGTGTGTGACAGCACAGGGAGCCACAGAAGG

GTTTCTCGAAAAAATTACTCCATGGAGTTCGGAAGTATGTCTGACACCTAAAAAGAATGTATATACGTGC

GCAATTAGATCTAAAGAAGATGTTCCCAATTTCAAGGACAAAATAGCCAGAGTTATCACGAGAAAATTTA

ATAAACAGTCTCAATCTTATTTGACTAAATTTCTCGGTAGCACATCGAATGATGTTACAACTTTTCTTAG

CATTCTTGACTAAATATTCATAACTAATTTTTATTAATGATACAAAAATGAAATAAAACTGCATATTATA

CACTGGTTAACGCCCTTGGCTCTAACCATTTTTTAAGATGAGGTCCCTGATTATAGTCCTTCTGTTCCCC

TCTATCATCTACTCCATGTCTATTAGACGATGCGAGAAGACTGAAGAGGAAACATGGGGATTAAAAATAG

GGTTGTGTATAATTGCCAAAGATTTCTATCCCGAAAGAACTGATTGCAGTGTTCATCGCCCAACTGCAAG

TGGAGGATTGATAACTGAAGGCAATGGATTCAGAGTAGTTATATATGATCAATGTACAGAACCACATGAC

TTTATTATCACCGATACTCAACAAACACGTCTTGGATCATCTCATACATATATTAAATTCAGTAACATGA

ATACAGGTGTCCCATCTAGTATTCCAAAATGTTCCAGAACTCTCTCTATTTCTGTATATTGTGATCAAGA

GGCGGGAGACATAAAATTTGAGGAGTATACTCAAGAATCAAGTGATATCAGTATTAGAGTTAAGTATGAT

TCATCATGTATTGATTATCTGGGTATTAATCAAAGTTTCATGAATGAATGTATTCGAAGAATTACAACAT

GGGATAGAGAATCATGTGTCAGAATTGATACACAGACTATAAATAAATATCTTAAGTCTTGCACCAACAC

AAAATTCGACCGTAATGTCTACAAAAGGTACATACTGAAGAGTAAAGCACTCCATGCTAAAACAGAGTTG

TAATAGATATAAAATACTTTTTATAATAATTAGGCTAGAAAAATCTCACTCACATGTAATCTTAAAAAAA

TGATATGATAGTTCTTACAAGTAGAGATTGAGTTTTAAATGGATTCTATTAATTACCGGGGAACTTAACA

ATTCGTTCTGATCTACAGACATTGGTTAATAAATCATCTTATTTTGCCGATATATTAAAATGTGGAAACT

CCACTAATAATATTACATTGTGTGACTTTCAAGATGATGCGATATATAGGGTTATACAGTTTTAACAATT

ATATAATAGAGATAGAAAGTACAAAAGATGTAGAATCAATGATATGGCACGCTAAACAGTTGGGTGTGGA

ATCATTGCTAAAAGAATGTCAAAATTATTTGCTTAGAATATTACGTATATAATTGTTTAGAAATTTATAG

AATAACTAATATTAATACATTATCGTATATCTACAACGATGTAAGAAACTTCATATTGGATAATATTACT

ATTAATATATAAGGATCCAGATTTTATATATTTGCCTAAATACATTATTATAGATTTACTAGGACAATCA

CCTAAATGTTTTTAACGAAGATAATGTGGTAAAGATTATATACACTTATATATCTTCCGATATCTACAAG

GATATTCCATATCATCATTGTGTAAACTAAATAACGTTTTCTATGGCATTTAATAAGGACATTGGATAT

GTGGAAAAGATGATGGTGTATGGAAGTTAGTACATTATCAACTTCTCCTTATTGATTGAAAATGAAAATA

TAAATAGTTTTTATATATAGCGGTATCTACCCTATAGTTTTATTGCTTACTACTAACATGGATACAGATA

CAGATACAGATACAGATACAGATACAGATACAGATACAGATACcagatacagatacagatacagatacagatacagatacagatacagatacagatacagataagatacagatacagatacagatacagatgtagaagatatcatgaacgaaaTAGATAGAGAGAAAGAAGAAATACTAA

AAAATGTAGAAATTGAAAATAATAAAAACATTAACAAGAATCATCCCAGTGAATATATTAGAGAAGCACT

TGTTATTAATACCAGTAGTAATAGTGATTCCATTGATAAAGAAGTTATAGAATATATCAGTCACGATGTA

GGAATATAGATCATATCTACTAATTTTTATAATCGATACAAAACATAAAAACAACTCGTTATTACATAGC

AGGCATGGAATCCTTCAAGTATTGTTTTGATAACGATGGTAAGAAATGGATTATCGGAAATACTTTATAT

TCTGGTAATTCAATACTCTATAAGGTCAGAAAAAATTTCACTAGTTCGTTCTACAATTACGTAATGAAGA

TAGATCATAAATCACACAAGCCATTGTTGTCCGAAATACGATTCTATATATCTGTATTGGATCCTTTGAC

TATCGACAACTGGACACGGGAACGTGGTATAAAGTATTTGGCTATTCCAGATCTGTATGGAATTGGAGAA

ACCGATGATTATATGTTCTTCGTTATAAAGAATTTGGGAAGAGTATTCGCCCCAAAGGATAGTGAATCAG

TTTTCGAAGCATGTGTCACTATGATAAACACGTTAGAGTTTATACACTCTCGAGGATTTACTCATGGAAA

AATAGAACCGATGAATATACTGATTAGAAATAAACGTATTTCACTAATTGACTATTCTAGAACTAACAAA

CTATACAAAAGTGGAACACATATAGATTACAACGAGGACATGATAACTTCAGGAAATATCAATTATATGT

GTGTAGACAATCATCTTGGAGCAACAGTTTCAAGACGAGGAGATTTAGAAATGTTGGGATATTGCATGAT

AGAATGGTTCGGTGGTAAACTTCCATGGAAAAACGAAAGTAGTATAAAAGTAATAAAACAAAAAAAAGAA

TATAAACAATTTATAGCTACTTTTTTTGAGGACTGTTTTCCTGAAGGAAATGAACCTCTGGAATTAGTTA

GATATATAGAATTAGTATACATGTTAGATTATTCTCAAACTCCTAATTATGACAGACTACGTAGACTGTT

TATACAAGATTGAAATTATATTCTTTTTTTTATAGAGTGTGGGGTAGTGTTACGGATATCTGA

TATCTAATATTAATATTAGACTATCTCTATCGCGCTACACGACCAATATCGATTACTATGGATATCTTTA

GGGAAATCGCATCTTCTATGAAAGGAAAGAATGTATTCATTTCTCCAGCGTCAATCTCGTCAGTATTGAC

AATACTGTATTATGGAGCTAATGGATCCACTGCTGAACAGCTATCAAAATATGTAGAAAAGGAGGAGAAC

ATGGATAAGGTTAGCGCTCAGAATATCTCATTCAAATCCATGAATAAAGTATATGGGCGATATTCTGCCG

TGTTTAAAGATTCCTTTTTGGGAAAAATTGGCGATAAGTTTCAAACTGTTGACTTCACTGATTGTCGCAC

TATAGATGCAATCAATAAGTGTGTAGATATCTTTACTGAGGGAAAAATCAATCCACTATTGGATGAACCA

TTGTCTCCTGATACCTGTCTCCTAGCAATTAGTGCCGTATACTTTAAAGCAAAATGGTTGATGCCATTCG

AAAAGGAATTTACCAGTGATTATCCCTTTTACGTATCTCCAACGGAAATGGTAGATGTAAGTATGATGTC

TATTTACGGCGAGCCATTTAATCACGCATCTGTAAAAGAATCATTCGGTAACTTTTCAATCATAGAACTG

CCATATGTTGGAGATACTAGTATGATGGTCATTCTTCCAAACAAGATTGATGGATTAGAATCCATAGAAC

AAAATCTAACAGATACAAATTTTAAGAAATGGTGTAACTCTCTGGAAGCTACGTTTATCGATGTGCACAT

TCCTAAGTTTAAGGTAATAGGTTCGTATAATCTTGTGGATACGCTAATAAAGTTGGGACTGACAGATGTG

TTCTATTCAACTGGTGATTATATCAATATGTGTAATTCAGATGTGAGTGTTGACGCTATGATTCACAAAA

CGTATATAGATGTCAATGAAGAGTATACAGAAGCAGCTGCAGCAACTTCTGTACTAGTGGCAGACTGTGC

ATCAACAGTTACAAATGAGTTCTGTGCAGATCATCCGTTCATCTATGTGATTAGACATGTCGATGGTAAA

ATTCTTTTCGTTGGTAGATATTGCTCTCCAACAACTAATTAAGCACATTCTTAATATTAGAATATTATAT

AGTTAAGATTTTACTAACAGGTTAACATTTTTTTTAAAAAATAGAAAAAACATGTGGTATTAGTGCAGGT

CGTTATTCTTCCAATTGCAATTGGTAAGATGACGGCCAACTTTAGTACCCACGTCTTTTCACCACAGCAC

TGTGGATGTGACAGACTGACCAGTATTGATGACGTCAGACAATGTTTGACTGAATATATTTATTGGTCGT

CGTATGCATACCGCAACAGGCAATGCGCTGGACAACTGTATGGCACACTCCTCTCTTTTAAAGATGATGC

GGAATCAGTGTTCATCGACGTTCGTGAGCTGGTAAAAAATATGCCGTGGGATAATGTTAAGGATTGTACA

GAGATCATCCGTTGTTATATACCGGATGAGCAAAAAACCATCAGAGAGATTTCGGCCATCATTGGACTTT

GTGCATATGCTGCTACTTACTGGGGAGGTGAAGACCATCCCACTAGTAACAGTCTGAACGCATTGTTTGT

GATGCTTGGGATGCTCAATTACATGGATTATACCATCATATTCTGGCGTATGAATTGATGAGTTACAGCT

TGACATTTCTTCTTTCCTCCCTCTTCTTCTACTTTTCCCAGAAACAAACTTTTTTTACCTACTATAAAAT

AAAATGAGTATACTACCTGTTATATTTCTTCCTATATTTTTTTATTCTCCATTCGTTCAGACTTTTAACG

TGCCTGAATGTATCGACAAAGGGCAATATTTTGCATCATTCATGGAGTTAGAAAACGAGCCAGTAATCTT

ACCATGTCCTCAAATAAATACGCTATCATCCGGATATAATATATTAGATATTTTATGGGAAAAACGAGGA

GCGGATAATGATAGAATTATACAGATAGATAATGGTAGCAATATGCTAATTCTGAACCCGACACAATCAG

ACTCTGGTATTTATATATGCATTACCACGAACGAAACCTACTGTGACATGATGTCGTTAAATTTGACAAT

CGTGTCTGTCTCAGAATCAAATATAGATCTTATCTCGTATCCACAAATAGTAAATGAGAGATCTACTGGT

GAAATGGTATGTCCCAATATTAATGCATTTATTTCTAGTAACGTAAACGCAGATATTATATGGAGCGGAC

ATCGACGCCTTAGAAATAAGAGACTTAAACAACGGACACCTGGAATTATTACCATAGAAGATGTTAGAAA

AAATGATGCTGGTTATTATACATGTGTTTTAGAATATATATATATGGGCAAAACATATAACGTAACCAGA

ATTATAAAATTAGAGGTACGTGATAGAATAATACCTCCTACTATGAAATTACCAGAAGGAGTAGTAACTT

CAATAGGTAGTAATTTGACTATTACATGCAGAGTATCGTTGAGACTTCCCACAACGGACGCTGACGTCTT

TTGGATAAGTAATGGTATGTATTACGAAGAAGAAGACGAGGACGGAGACGGTAGAATAAGTGTAGCAAAT

AAAATCTATATGACTGATAAGAGACGTGTTATTACATCCTGGTTAAACATTAATCCTGTCAAGGAAGAAG

ATGCTACAACGTTTACGTGTATGGCGTTTACTATTCCTAGCATCAGCAAAACAGTTACTGTTAGTATAAC

GTGAATGTATGTTGTTACATTTCCATATCAATTGAGTTTATAAGAATTTTTATACATTATCTTCCAACAA

ACAATTGACGAACGTATTGCTATGATTAACTCCCACAATACTATGCATATTATTAATCATTAACTTGCAG

ACTATACCTAGTAGTGCTATTTTGACATACTCATGTTCTTGTGTAATTGCAGTATCTATATTATTAAAGT

ACGTAAATCTAGCTATAGTTTTATTATTTAATTTTAGATAATATACTGTCTCCGTATTTTTAAAAAATTG

CCACATCCTTTATTAAATCATGAATGGGAATTTCTGTGTCATCGTTAGTATATTGTGAACAACAAGAGCT

GATATCTATAGGAAAGGGTGGAATGCGATACATTGATCTATGTAGTTTTAAAACATAAGCGAACTTTGAA

GAATTTATATAAATCATCTCACGAGATATTGTTCTCTGTCATATTCATACACCTGCATAAACTTTCTATA

CATCTTACAATGTGTTATTTTATGATCATATTTACATATTTACTGGTATATCAAAGATGTTAGATTAGTT

AATGGGAATCGTCTATAATAATGAATATTAAACAATTATAGGAGGAATTTATACCTACAAAAACATCATA

AAAATGAGTCATCGTCCGATTTATGTTTTAAATATACTAACATTACTACCTTCAGAAATTATATACGAAA

TATTATACATGCTGACAATTAACGATCTTTATAATATATAGTATCCACCTACCAAAGTATAATTGTATTT

TTTCTCATGTGATGTGTGTAAAAAACTGATATTATATAATTATCTTAGTACCTATGATGAAGATGAAGAT

GATGGTCCGTATATATTTTGTATCATTATCGTTATTGCTATTCCATAGTTACGCCATAGACATCGAAAAT

GAAATCACCGAATTCTTCAATAAAATGAGAGATACTCTACCAGCTAAAGACTCTAAATGGTTGAATCCAG

TATGTATGTTTGGAGGCACAATGAATGATATGGCCGCTCTAGGAGAGCCATTCAGTGCAAAGTGTCCTCC

TATTGAAGACAGTCTTTTATCGCATAGATATAAAGACTATGTGGTTAAATGGGAAAGGCTAGAAAAGAAT

AGACGGCGACAGGTTTCTAATAAACGTGTCAAACATGGTGATTTATGGATAGCCAACTATACATCTAAAT

TCAGTAACCGTAGGTATTTATGTACCGTAACCACAAAGAATGGTGACTGTGTTCAGGGTGTAGTTAGATC

TCATGTGTGGAAACCTTCTTCATGCATTCCAAAAACATATGAACTAGGTACTTATGATAAGTATGGCATA

GACTTATACTGTGGAATTCTTTATGCGAAACATTATAATAATATAACTTGGTATAAAGATAATAAGGAAA

TTAATATCGACGATTTTAAGTATTCACAAGCGGGAAAGGAATTAATTATTCATAATCCAGAGTTAGAAGA

TAGTGGAAGATACGACTGTTACGTTCATTACGACGACGTTAGAATCAAGAATGATATCGTAGTATCAAGA

TGTAAAATACTTACGGTTATACCGTCACAAGACCACAGGTTTAAACTAATACTAGATCCGAAAATCAACG

TAACGATAGGAGAACCTGCCAATATAACATGCAGTGCTGTGTCAACGTCATTATTTGTCGACGATGTACT

GATTGAATGGGAAAATCCATCCGGATGGATTATAGGATTAGATTTTGGTGTATACTCTATTTTAACTAGT

AGAGGCGGTATCACCGAGGCGACTTTGTATTTTGAAAATGTTACTGAAGAATATATAGGCAATACATATA

CATGTCGTGGACACAACTATTATTTTGATAAAACTCTTACAACTACAGTAGTATTGGAGTAAATACACAA

TGCATTTTTATATACATTACTGAATTATTATTATTAATTATATCGTATTTGTGCTATAGAATGGATGAAG

ATACGCGACTATCTAGGTATTTGTATCTCACCGATAGAGAACATATAAATGTAGACTCTATTAAACAGTT

GTGTAAAATATCAGATCCTAATGCATGTTATAGATGTGGATGTACGGCTTTACATGAGTACTTTTATAAT

TATAGATCAGTCAACGGAAAATACAAGTATAGATACAACGGTTACTATCAATATTATTCATCTAGCGATT

ATGAAAATTATAATGAATATTATTATGATGATTATGATAGAACTGGTATGAACAGTGAGAGTGATAATAT

ATCAATCAAAACAGAATATGAGAATGAATATGAATTCTATGATGAAACACAAGATCAAAGTACACAACTA

GTAGATTACGACATTAAACTCAAAACCAATGAGGATGATTTTGTTGATGAATTCTATGGTTATGATAGAT

CAGTGGGTGTCCATGATTATATAGATGTATCAATTAATAAAGTAGTATATGGAAGAGAGTCTCACGTAAG

ATGGCGGGATATATGGCAAGAACATAATGATGGCGTATACAGTATAGGAAAGGAGTGCATAGATAATATA

TACGAAGACAGACATACCGTAGACGAATTCTACAAGATAGACAGCGTATCAGATGTAGATGACGCAGAAC

ATATATCTCAGATAACTAATGATGTATCTACACAAACATGGGAAAAGAAATCAGAGTTAGATAGATACAT

GGAAATGTATCCTCGTCATAGATATGGTAAGCATTCTGTCTTTAAGGGATTTTCTGACAAAGTTAGAAAA

AATGATTTAGACATGAATGTGGTAAAAGAATTACTTTCTAACGGTGCATCTCTAACAATCAAGGATAGCA

GTAATAAGGATCCAATTGCTGTTTATTTTAGAAGAACAATAATGAATTTAGAAATGATTGATATCATTAA

CAAACATACAACTATCTATGAACGCAGGTATATAGTACACTCCTATCTAAAAAATTATAGAAATTTCGAT

TATCCATTTTTCAGAAAGTTAGTTTTGACTAATAAACATTGTCTCAACAATTATTATAATATAAGCGACA

GCAAATATGGAACACCACTACATATATTAGCATCTAATAAAAAAATAATAACTCCTAATTACATGAAGTT

ATTAGTGTATAACGGAAATGATATAAACGCACGAGGTGAAGATACACAAATGCGAACTCCATTACACAAA

TATTTGTGTAAATTTGTATATCATAATATTGAATATGGTATCCGATACTATAATGAAAAGATTATAGACG

CATTTATAGAGTTAGGAGCCGATCTAACTATTCCAAATGACGATGGAATGATACCAGTAGTTTACTGTAT

ACACTCAAATGCCGAATATGGTTATAACAATATTACTAACATAAAGATAATACGTAAACTACTTAATCTT

AGTAGACATGCGTCACATAATCTATTTAGAGATCGAGTCATGCACGATTATATAAGTAATACATATATTG

ATCTTGAGTGTTTAGATATCATTAGATCACTGGATGGGTACGATATTAATTGTTACTTTGAAGGACGTAC

ACCACTTCATTGCGCTATACAATATAACTTCACTCAGATTGCTGAGTACTTATTAGATCGAGGAGCTGAT

ATATCATTAAAGACAGACGATGGTAAAACTGTATTTGATTTATCGTTATGTAGTTACATTCCTCTTAAAT

GGACTAGCTTTTTGATTAGTCGTCTACCGCCTAAAAGTGTCATATGCTCACTGACTAACCATATAATAGA

TTATGTTCTTACGAACAATAGACGTATTATTTGGCAGAGTCAAATGATTAATAAGTACGTACTGTTACTG

GACCCATCCTTTTATTATAGATTCAGAAATGCTATCGAAAACAAATTAGACCAATACAATAATCGTTATA

ATATGTTCGAACACGATAGGGACGTTAATGAAAAGTATGGCAAAGTCTTACATGACCTCGATACATATAT

CAAGGATGTACAAGTATTAAAATCTACTTCCATCACTAATAATATAACACTATACGACACTATTATAAAT

AATAAGTCAGAGTTTCCTATACGTCGTGCAAACGACAAACAATTAATTAATCTCATAAAATCCAATACAT

ATCATAATCTTATCGAAAAAGTTATTAAAAATACATTAGAGAAATATACTTTAACTAATATAGTCCTCGA

GTATATGATCTCATCTCAATCTCAATCTCAATCATCTTATTTGAGTCGTATTCCTAATGAGATATTACTC

GAAATATTATATAAACTCGACATGTACGATTTACGTAATCTATATACAAGATATATGAGAGAGAATGATA

TCACAGAGTATCATATAGAGAATACGAGGTCTGTTTCTACACAGACATGAATAATGAATACACATACAAC

GTTTTTTTTTAATCTTAGATATAACACTAATTACATCAAGATTATATATTGAAATCGTAATTTGAGTTG

TCTGATCATCATGGATATCGAAAATGATATACGTAACATTAGCAATCTTTTTTAGATGATTGATATATTA

TTATGCGATGTAATCATAACTATCGGAGATGTAGAAATTAAAGCGCATAAAACTATTTTGGCTGCCGGAT

CTACGTATTTTAAAACAATGTTCACAACACCTATGATAGCGAGAGATCTAGCAACTAGAGTAAATCTACA

GATGTTCGATAAGATGCCGTCAAAAATATTGTACAGTACTTATACAATAGGTATATAAGTTCTATGAATG

TGATAGACGTATTAAAATGCACCGACTAAGAACGTAGAACGAACTATAGAATGTTATACAATGGGTGATG

ATAAGTAGAAGATGTTACCCGATATACCCATAGCATTATCTAGTTATGGCATGTGTGTATTAGATCAATA

CATATACATTATAGGCGGTCGTACCCAACACTGATTATACATCGGTACATACAGTAAATAGCATAGATAT

GGAGGAGGATACAAATATTTCAAATAAAGTTATGAGATACGCACTGTCAATAATATATGGAAGACATTAC

CTAACTTCTGAACTGGAACTATAAATCCAGGCTCTTGCATAAAGATGAATATATATGTTGTATGCGACAT

CAAAGATGAAAAAATGTTAAGACTTATATATTTAGATATAACACGAATATGTATAACGGATGGGAATTGG

TAACGATGACAGAAAGCAGATTGTCAGCTCTGCATACTATTCTTCATGACAATACCATAATGATGTTACA

TTGTTATGAAACGTATATGTTACAAGATACATTTAATGTGCTTACGGAACATATATTTAGAAACATCTAC

TAACGATTTTTTATGCTTGTATTATTAATGGTATGTAATATGATTTAATTGATTGTGTACACGATACCAA

TTTGTCAAGTATGAATACGGAGTACAAACATAAACTGAAGTTTAACATTATTTATTTATGATATACATTATATACATTATATACATTATATACATTATATACATTATATacattatatacattatatacattatatacattatatacattatatacattatatacattatatACATTATATACATTATATACATTATATACAT

TATATACATTATATACATTATATACATTATATACATTATATACATTATATACATTATATACATTATATAT

CGTTATATATCGTTATATATCGTTATTGTTTGGTCTATGCCATGGATATCTTTAAAGAACTAATCTTAAA

ACATACGGATGAAAATGTTTTGATTTCTCCAGTTTCCATTTTATCTACTTTATCTATTCTGAATCATGGA

GTAGCTGGTTCTACAGCTGAACAACTATCAAAATATATAGAGAATATGAATGAGAATACACCCGATGATA

AGAAGGATGACAATAATGACATGGACGTAGATATTCCGTATTGTGCGACACTAGCTACCGCAAATAAAAT

ATACGGTAGTGATAGTATCGAGTTCCATGCCTCCTTCCTACAAAAAATAAAAGACGATTTTCAAACTGTA

AACTTTAATAATGCTAACCAAACAAAGGAACTAATCAACGAATGGGTTAAGACAATGACAAATGGTAAAA

TTAATTCCTTATTGACTAGTCCGCTATCCATTAATACTCGTATGACAGTTATTAGCGCCGTCCATTTTAA

AGCAATGTGGAAATATCCATTTTCTAAACATCTTACATATACAGACAAGTTTTATATTTCTAAGAATATA

GTTACCAGTGTTGATATGATGGTGGGTACCGAGAATGACTTGCAATATGTACATATTAATGAATTATTCG

GAGGATTCTCTATTATCGATATTCCATACGAGGGAAACTCTAGTATGGTGATTATACTGCCGGACGACAT

AGAAGGTATATATAACATAGAAAAAAATATAACAGATGAAAAATTTAAAAAATGGTGTGGTATGTTATCT

ACTAAAAGTATAGACTTGTATATGCCAAAGTTTAAAGTGGAAATGACGGAACCGTATAATCTGGTACCGA

TTCTAGAAAATTTAGGACTTACTAATATATTTGGATATTATGCAGATTTTAGTAAGATGTGTAATGAAAC

TATCACTGTAGAAAAATTTCTACATACGGCGTTTATAGATGTTAATGAGGAGTATACAGAAGCATCGGCC

GTTACAGGAGTATTCATGACTAACTTTTCGATGGTATATCGTATGAAGGTCTACATAAACCATCCATTCA

TATACATGATTAAAGATAACACCGGACATACACTTTTTATAGGGAAATACTGCTATCCGCAATAAATATA

AACAATAGACTTTTATCACGTTATCTCATGTATAAAATATTACAAATAGTATAGCATAAACTAAAGTCGA

TACATACATTAAAACTTAAAAAATAATGTAATTTACAATTAATAGTATAAACTAAAAAAATTAAAAAATT

AAAAACAATATCATTATTATAAGTAATATCAAAATGACGATATACGGATTAATAGCGTATCTTGTATTCG

TGACTTCATCCATCGCTAGTCCATTTTACATTCCCGTTATTCCGCCCATTTCGGAAGATAAATCGTTCAA

TAGTGTAGAGGTATTAGTTTCTTTGTTTCCCGATGACCAAAAAGACTATACAGTAACTTCTCAGTTCAAT

AACTACACTATCGGTACCAAAGACTGGACTATCAACGTACTATCCACACCTGATGGTCTGGACATACCAT

TGACTAATATAACTTATTGGTCACGGTTTACTATAGGTCGTGCATTGTTCAAATCAGAGTCTGAGGATAT

TTTCCAAAAGAAAATGAGTATTCTAGGTGTTTCTATAGAATGTAAGAAGCCGTCGACATTACTTACTTTT

TTAACCGTGCGTAAAATGACTCGAGTATTTAATAGATTTCCAGATATGGCTTATTATCGAGGAGACTGTC

TAGAAGCCGTTTATGTAACAATGACTTATAAAAATACTAAAACTGGAGAGACTGATTACACGTACCTCTC

TAATGGGGGGTTGCCTGCATACTATCGTAATGGGGTCGATGGTTGATTATTGATTAGTATATTCCTTATT

CTTTTTATTCACACAAAAAGAACATTTTTATAAACATGAAACCACTGTCTAAATGTAATTATGATCTTGA

TTTATAGATGATGATCAGCCTTCAGAGGATTTTGACCAGCATGTTTAATATGAAAAAAAACATAACTATT

AAGCGCTATTGCGCTATTGTGCTTAATTATTTTGCTCTATAAACTGAATATATATAGCCACAATTATTGA

CGGGCTTGTTTGTGACCGGTAATCATGAATTTACAGAAATTATCTCTGGCTATATATCTTACGGTGACAT

GTTCGTGGTGTTATGAAACATGTATGAGAAAAACTGCGTTGTATCATGACATTCAATTGGAGCATGTAGA

AGACAATAAAGATAGTGTAGCATCGCTACCGTACAAGTATCTACAAGTAGTCAAACAAAGAGAACGTAGT

AGATTGTTGGCTACATTTAATTGGACGGATATAGCTGAGGGTGTTAGAAATGAGTTCATTAAAATATGTG

ATATCAACGGAACATATCTATATAATTATACTATTGATGTTAGTATAATTATTGATTCCACGGAAGAACT

ACCAACAGTTACTCCAATTACAACATATGAACCTTCTATATATAATTATACTATCGATTATAGCACTGTT

ATTACTACTGAAGAACTACAAGTGACTCCAACATATGCGCCTGTAACAACTCCTCTTCCAACATCAGCAG

TTCCTTATGATCAACGATCGAATAACAATGTAAGTACTATATCTATTCAGATACTGAGTAAAATATTGGG

AGTCAATGAAACAGAATTAACTAATTATCTTATTATGCATAAAAATGACACTGTTGACAATAACACCATG

GTTGATGATGAGACATCTGACAATAACACATTGCATGGTAATATAGGATTTTTGGAAATAAATAATTGTT

ACAATGTTTCTGTGTCAGATGCTAGTTTTAGAATAACATTAGTAAACGATACTTCTGAAGAAATTTTGCT

AATGCTAACAGGAACTAGTTCATCCGACACCTTCATATCTTCCACCAATATCACTGAATGTTTGAAAACA

TTAATCAATAATGTGTCGATTAATGATGTACTTATAACACAAAATATGAATGTAACATCTAATTGTGATA

AATGCTCAATGAATTTGATGGCATCCGTTATTCCTGCAGTTAATGAATTTAACAATACGTTGATGAAAAT

TGGTGTAAAAGATGATGAAAACAATACGGTATATAACTATTATATTTGTAAACTAACTACAAATTCTACA

TGTGATGAGTTAATCAATTTAGATGAAGTCATTAACAACATAACTCTGACAAATATTATACGCAATAGTG

TTTCGACAACTAACAGCAGAAAAAGACGAGATCTGAATGGTGAGTTTGAATTTTCCACTTCCAAGGAATT

AGATTGTCTTTACGAATCATATGGTGTAAACGATGATATAAGTCATTGTTTTGCATCACCTAGACGTAGA

CGATCTGACGACAAAAAGGAGTACATGGACATGAAATTATTCGACCACGCGAAAAAAGATTTAGGAATAG

ACAGTGTTATTCCTAGAGGTACAACCCATTTCCAAGTAGGTGCATCTGGTGCAAGTGGTGGTGTTGTAGG

AGATAGTTTCCCATTTCAAAATGTTAAATCGCGTGCCAGTCTATTGGCGGAAAAAATAATGCCTAGAGTA

CCTATTACTGCTACCGAAGCTGATCTATATGCAACTGTAAATAGACAACCCAAGTTACCAGCAGGTGTTA

AAAGTACTCCGTTTACAGAGGCGCTTGCGTCTACGATAAACCAAAAGCTTTCTAATGTTAGAGAGGTAAC

TTATGCTTCGCTCAATCTGCCAGGATCAAGTGGCTATGTTCATAGACCATCTGATTCTGTTATTTACAGC

AGTATAAGACGGTCACGTTTACCTAGTGATAGCGATAGTGATTATGAGGATATACAAACTGTTGTTAAGG

AATATAATGAAAGATATGGTAGATCAGTCAGTAGAACACAGTCATCAAGTAGTGAAAGCGATTTTGAAGA

TATAGATACTGTTGTTAGGGAATATAGACAAAAATATGGCAATGCAATGGCAAAAGGACGTAGTAGTTCC

CCTAAACCTGATCCATTATATAGTACTGTTAAGAAAACAACTAAAAGTCTATCTACTGGTGTAGACATAG

TTACAAAACAATCAGACTATTCTCTATTACCTGACGTTAATACTGGCAGTTCTATTGTGTCACCTCTCAC

CAGAAAAGGAGCTACTAGACGACGACCTAGACGCCCTACAAATGATGGTCTACAGAGTCCAAATCCTCCT

CTCCGTAATCCACTTCCTCAACATGATGATTATTCTCCTCCACAAGTACACAGACCTCCGCCACTTCCTC

CTAAACCAGTCCAAAATCCGCCACAACTTCCCCCTAGACCAGTAGGTCAATTACCTCCTCCTATAGATCA

ACCAGATAAAGGATTTAGTAAGTTTGTATCACCTAGACGGTGTAGAAGAGCAAGCTCTGGAGTCATATGT

GGTATGATACAATCAAAACCAAACGATGATACCTATTCACTTCTTCAACGGCCAAAAATTGAACCAGAAT

ATGCGGAGGTTGGTAATGGTATACCCAAGAACAATGTTCCTGTAATAGGTAATAAACATAGTAAAAAATA

TACATCGACGATGTCAAAAATATCAACAAAATTTGATAAATCTACGGCATTTGGAGCAGCAATGTTACTA

ACTGGTCAGCAGGCCATTAGCCAACAGACTAGATCAACTACGTTGAGTAGAAAAGATCAGATGAGCAAGG

AAGAAAAGATATTCGAAGCAGTTACAATGAGTCTATCAACTATAGGTTCAACGTTGACGTCTGCAGGTAT

GACGGGTGGTCCAAAACTAATGATTGCAGGAATGGCTATAACGGCTATAACTGGTATAATAGATACGATA

AAAGATATATATTACATGTTTTCAGGACAGGAGAGGCCAGTAGATCCTGTTATTAAATTATTTAATAAGT

ACGCTGGCTTAATGTCCGATAATAATAAAATGGGTGTAAGAAAATGTTTGACACCCGGTGACGACACACT

TATTTATATCGCATACAGAAACGATACCAGTTTTAAACAGAATACGGATGCGATGGCTTTGTATTTCTTA

GATGTTATCGACTCAGAGATCCTATATCTAAACACATCAAATTTAGTTCTAGAGTATCAACTAAAGGTGG

CTTGCCCCATAGGAACATTAAGATCTGTAGATGTGGACATAACTGCGTATACAATATTATATGATACAGC

GGATAATATTAAGAAATACAAGTTTATCAGAATGGCAACGCTACTATCCAAACATCCAGTTATTAGATTG

ACATGTGGTTTAGCAGCAACATTGGTGATTAAACCGTACGAGGTACCCATCAGTGATATGCAACTACTAA

AAATGGCGACGCCTGGTGAACCAGAATCCACTAAATCTATACCATCCGATGTCTGTGATAGGTATCCTCT

AAAGAAATTCTATCTTTTAGCTGGTGGTTGTCCCTATGATACATCTCAAACTTTTATTGTACATACTACT

TGCAGTATTCTACTAAGAACAGCTACACGGGATCAGTTTAGAAACAGATGGGTGTTACAAAATCCATTTA

GACAAGAAGGGACATATAAGCAACTGTTTACCTTTAGCAAATACGATTTTAACGACACCATAATCGATCC

TAATGGTGTGGTGGGTCATGCTAGCTTTTGTACCAATAGAAGCAGCAACCAATGTTTCTGGTCCGAACCT

ATGATATTGGAAGATGTATCATCGTGTAGTTCTAGAACTAGAAAAATATACGTAAAACTGGGAATATTTA

ATGCCGAAGGTTTTAATAGTTTTGTACTAAATTGTCCAACTGGGTCTACACCTACATACATCAAACATAA

AAATGCGGACAGTAACAATGTTATCATAGAGCTACCTGTAGGTGATTACGGCACAGCCAAATTGTATTCA

GCAACAAAACCATCGAGGATAGCTGTGTTCTGCACACATAACTATGATAAACGATTCAAATCAGATATTA

TAGTTCTAATGTTTAATAAAAACAGCGGTATTCCATTTTGGAGCATGTACACAGGAAGTGTAACTAGTAA

AAATAGAATGTTTACCACATTGGCTAGAGGAATGCCGTTTAGATCAACGTATTGCGATAACAGACGACGA

TCAGGTTGTTATTATGCAGGAATACCATTTCATGAAGATAGTGTAGAAACAGATATACATTATGGACCAG

AAATAATGTTAAAGGAAACATATGACATAAACAGTATTGACCCACGAGTTATAACAAAGTCAAAGACCCA

TTTTCCTGCTCCATTGAGTGTAAAATTCATGGTTGACAATTTAGGAAATGGATATGACAACCCTAATTCA

TTTTGGGAAGATGCTAAAACTAAGAAACGGACATATAGTGCAATGACGATAAAAGTCCTACCATGTACAG

TGAGAAATAAAAATATAGACTTTGGATATAACTATGGAGATATTATTTCTAATATGGTTTATCTACAATC

TACTAGTCAGGATTATGGAGATGGTACCAAATATACATTTAAATCCGTAACTAGATCAGATCATGAGTGT

GAATCTAGCTTAGATCTGACGTCTAAGGAAGTAACTGTGACATGTCCTGCGTTTAGTATACCAAGAAATA

TATCAACATATGAAGGTCTATGCTTTAGTGTTACTACATCTAAAGATCATTGTGCTACAGGTATTGGTTG

GTTAAAATCTAGTGGCTATGGGAAGGAAGATGCTGATAAACCACGTGCTTGTTTTCATCATTGGAATTAT

TACACACTGTCGTTGGATTATTACTGTTCATACGAAGATATTTGGAGAAGCACCTGGCCTGACTATGATC

CATGTAAGTCATATATCCATATAGAGTATAGAGATACATGGATAGAATCTAACGTGTTACAGCAACCTCC

TTACACATTCGAATTCATTCATGACAATTCTAACGAATATGTGGATAAAGAAATTAGTAACAAATTAAAT

GATCTGTACAATGAATACAAGAAGATTATGGAATATAGCGACGGATCATTGCCGGCGTCTATAAACAGAT

TAGCAAAGGCATTGACTTCAGAGGGTAGAGAAATAGCAAGTGTTAATATAGATGGTAATCTGTTAGATAT

CGCATATCAAGCAGATAAGGAAAAGATGGCCGACATACAGACAAGAATAAATGATATTATTAGAGATTTG

TTTATACACACTCTATCAGACAAAGATATAAAAGACATTATAGAATCCGAAGAAGGTAAGAGATGTTGTA

TAATAGATGTTAAGAACAATCGTGTTAAAAAGTACTATTCTATTGATAATTATCTATGTGGTACTTTAGA

TGATTATATATACACCTCTGTAGAATATAACAAATCCTATGTGTTAGTAAACGATACTTATATGAGCTAT

GACTATCTTGAATCATCAGGTGTAGTTGTTCTATCATGTTATGAAATGACTATAATCTCCTTGGATACAA

AAGACGCCAAAGATGCTATAGAAGATGTGATAGTAGCAAGTGCGGTAGCCGAAGCATTGAATGACATGTT

TAAGGAATTTGATAAAAACGTAAGTGCTATTATAATAAAAGAAGAAGATAATTATCTAAACAGTTCGCCC

GATATCTACCATATAATATATATCATAGGTGGCACTATTCTGCTATTGTTAGTCATTATTTTAATATTGG

CAATTTATATAGCGCGCAATAAATACAGAACCAGGAAATATGAAATAATGAAATATGACAATATGAGCAT

TAAATCTGATCATCATGATAGTCTTGAAACAGTGTCTATGGAAATTATTGATAATCGGTACTAATAAAAT

AGTTTAACTCTTTTAGAACCAGTTTGGTACTGTAATTTCAGTTCATTACTCGTTGAGAATATTGATGATT

TTTTTTAAAATGAGTATCGGTAGTTACATATTACCATATCATCCATTATATAATCGATGATGCATGTATT

AAAATACTTTCCGAATAAGTCTTCTAAATATTGTATTAATTATGAAAAACTATGCTATGTGAGATGATT

CAAAGATGTTTAATGATACGATACTAGATTTTATCTCTAGCGAGATTGTTTAGAATCATTTATCATAACT

ATGTTTAATAAATTCATCAACGAATATCGATAAAGACCTCTTGTAATTCGAGTATAGGAAGCAGTATTAC

CATATCAACTTCCGAGTTAACAATTACTCTAAAACATGAGGATTGTACTCCTGTCTTTATTGGAGATTAC

TATTCAGTCGTTGATAAACTAGTAACCTCAGGTTTCTTTACAAACGATAAAGTACAACATCAAGACCTCA

CAACGCAGTGCAAGATTAATCTAGAAATCAAATGTAATTATGGAGGAGAATCTAGACAACTAACACCCAC

GGCGAAGTATACTTTATGCCTCATTCAGAAACGGTAACTGTAGTAGGAGACTGTCTCTCTAATCTCGATG

TATATATAATATATACCAATACGGACGCGATATATTCCGACATGAATGGCGTCGCTTATCATATGTTATA

TCCTAAATGTTGATCATATTCCACAAATGATTGTGAACGAGATTAAATCATCTAACAAATAATTAGTTTT

TATGACATTAACATATAATAAATAAATTAATCATTATTGACTTAACGATGACGAAAGTTATCATCATCTT

AGGATTCTTGATTATTAATACAAATTCGTTGTGTCTATGAAATGTGAACAATGTGTCTCATATTATAATA

CACAAGAATTAAAGTGTTGTAAACTATCTAAGCCAGGAACATATTCAGATCATCGATGTGATAAATACAG

CGATACCATCTGTGGACATTGTCCAAGTGACACATTCACGTCAATATATAATCGTTCTCCTCGGTGTCAT

AGTTGTAGAGGTCACACCTTGTACACCTACCACAAATAGAATCTGTCATTGTGACTCGAATAGTTATCGT

CTCCTTAAAGCTTCTGATGGTAACTGTGTTACATGTGCTCCTAAAACAAAATATGGTCGTGTGTACGGAA

AGAAAGGAGAAAATGATATGGAATACCATTTGTAAGAAATGTCGGAAGGGTACTTATTCAGATATTGTAT

CTGACTCTGATCAATGTAAACCTATGACAAGATAAGACTTACTCGCATCTACTGGATAGACATAAATATC

CTCCTCGTAATAATGAAATATAATATAATATACACTAATTATTAATATCAATCGAGTATTAACATATAAG

TTATTTTTAAACCCCTTTTGGGTTCCGTCCTAAACGGCGTTTCGGTCTGTGTCGCCACCATGGTCACACC

GAGCCTCTGCGTGCTCCTCCATCGAGGACGACTTCAACTATGACAGCTCGGTGGCGTCTGCCAGCGTGCA

CATACGAATGGCATTTCTAAGAAAAGTCTACGGTATCCTTGTCTACAATTTCCTTTAACAACGGCAACAG

CTGCAGTATTTTTATACTTTGAATGCATTCGGACATTTATACAAGGGAGTCCTGTTCTAATATTGGCATC

AATGTTCGGATCTATAGGCTTGATTTTCGCATTGACTTTACACAGACATAAACATCCCCTGAATCTGTAC

CTACTTTGTGGATTTACACTGTTAGAATCTCTAACGCTGGCCTCTGTTGTTACTTTCTATGATGCGCGTA

TCGTTATACAAGCTTTCATGTTGACTACTGCAGTGTTTCTTGCTCTGACTACATGTACTCTACAATCAAA

GAGAGATTTCAGTAAACTTGTAACAGGATTGTTTGCTGCTTTCTGGATTTTAATTTTGTCAGGAGTCTTG

AGGATAAAGTTTAAAATAGAATTAATAAAGAACATATAGGTCATTTTTTAAACATGGATAGAAACCAAGG

TTGTTAGTTAATAATATACAAGATATTTTTTCTCACTCTGATCCATGTAAACCAAGGACGAGAGACACTC

TCATTCCTCATTCACGACACCATTAAAAATGGAAATTAAAGCCCTCTATTAAGCACAGACGGCTACAGGT

CTACCATCAGGTTAACCTTCGTCTACCTTCACAATGGCCTTTCCTTGTGCCCAGTTCAGTCCCTGTCATT

GCCACGCTACTAAGGACTCCCTGAATACCGTGACTGACGTCAGACATTGTCTGACTGAATACATCCTGTG

GGTTTCTCATAGATGGACCCATAGAGAAAGCGCAGGGCCTCTCTACAGGCTTCTCATCTCTTTCAGAATT

GATGCAATGGAGCTATTTGGTAGCGAGTTGAAGGAGTTCTCGGATTCACTTCCGTGGGACAATATCGACA

ATTGCGTGGAGATCATTAAATGTTTCATCAGAAATGACTCCATGAAAACCGCCAAAGAACTTTGTGCAAT

AATTGGACTTTGTACTCAATCAGCTATTGTCACTGGAAGAGTCTTCAATGATAAGTATATCGACATACTA

CTTATGCTGCGAAAGATTCTGAATGAGAACGACTATCTCACCCTCTTGGATCATATCCTCACTGCTAAAT

ACTAAATCTCCTTCATGCTCTCTCACTAATACTCTTACTCACTACACTTTTTATCATCTTATGATGAATG

ATTGCCTTCATCATTTTTCGTGGAATATAATATAGGAATAATTAGCACCAGAATAGCTATGGATATCTCG

TTAAGAATATTCTCGATAAGAGACATAATGTAGACATAGTTATTATATCCTTCTTAGATAAGTGTTACGC

TACTGGAAAGTTTCCATCGTTATTATTACGTGAAGATGATATCATTAAACCAACATTGAGATTGGCTCTT

ATGTTAGCTGGATTGAATTACTGTAATAAATGCATCGAGTATAGAGGGATATAGCAATTCTCGATAATAG

TCATGCAATATTTGAATGAGACTGATAATTTAGGTAATACAGTACTACACACACATATCTTTCTAGATTA

TATATCGTTAAAAATCTGTAAGAGGTATATTTCTCATAAGTATCCACTGTGTAATATTATTAATGGATAT

ATAGATAACACAATAGGGACTAATAGTATTGTAAAAGATATAATCGACTATTTGTACATATCCAGATATC

TATATTCCTACTAGTTTGCTGCGTAGTTGCATCATTGATATGCATGATTTATCAGGATTCAGAGATGAAT

TACTAAGTAAACTACAATCCCACAATAAGTAAGAATCAAATATCAAAAACTCACTTTTGATTTTTCTAGT

CTTAAGTAATACATATATTTATTAATAGACCTATGAAATAAAAAAGTTAACAATGGATTCGCGTATAGCT

ATTTACGTATTAGTATCGGCATCTCTTTTGTATCTTGTTAATTGTCACAAACTAGTACATTACTTCAATC

TGAAAATAAATGGAAGTGATATAACTAATACAGCAGATATATTGCTGGACAATTATCCAATTATGACCTT

TGATGGAAAGGATATTTATCCATCTATCGCGTTCATGGTCGGTAATAAACTTTTCCTAGATCTTTATAAA

AATATCTTTGTAGAATTTTTCAGACTATTTCGAGTATCTGTAAGTAGTCAATACGAGGAATTAGAATATT

ATTATTCATGTGATTATACTAACAACCGTCCTACAATTAAACAACATTACTTTTATAACGGCGAAGAATA

TACTGAAATTGATAGATCGAAAAAAGCCACTAATAAAAACAGTTGGTTAATTACTTCAGGCTTTAGACTA

CAAAAATGGTTCGATAGCGAAGATTGTATAATTTATCTCAGATCTTTAGTTAGAAGAATGGAAGACAGTA

ACAAAAACAGTAAAAAAACTTAGTACTTAGATATCGAAAAAAAATATATTTTTGTAGACTCTTGAGAATA

GAAGGAAAACATGTACATAATTATAAAAAATGAAAATCAATGGCGAATAAGACAGTGCGATTCGCGCCAT

GGAGTCGGCAGATTTCATGGCTGTCGATGAGCAGTTTCACGACGACCTCGATCTTTGGTCATTATCTTTG

GTAGATGACTATAAAAAACATGGATTAGGTGTTGACTGTTATGTTCTAGAACCAGTTGTTGACAGGAAAA

TATTTGATAGATTTCTCCTTGAACCAATTTGTGATCCTGTAGATGTTCTGTATGATTATTTTAGGATTCA

TAGAGATAATATTGATCAGTATATAGTAGATAGACTGTTTGCATATATTACATATAAAGATATTATATCT

GCATTAGTGTCAAAGAATTATATGGAAGATATTTTCTCTATAATTATTAAGAATTGTAATTCTGTACAAG

ATCTCTTACTTTACTATCTATCTAATGCATATGTAGAAATAGACATTGTTGATTTTATGGTAGATCATGG

GGCTGTAATATATAAAATAGAATGCTTGAATGCCTATTTTAGGGGAATATGTAAAAAGGAAAGTAGTGTT

GTTGAGTTTATTTTGAATTGTGGTATCCCAGATGAAAATGATGTTAAATTAGATCTATATAAAATAATTC

AGTATACTAGGGGATTCCTTGTAGATGAACCCACAGTATTAGAAATTTATAAGCTTTGTATCCCATATAT

TGAAGATATCAATCAACTAGATGCTGGTGGAAGGACCTTGCTTTATCGCGCTATCTATGCAGGTTATATA

GATTTAGTATCATGGCTATTAGAAAATGGAGCAAATGTCAACGCAGTAATGAGTAATGGATATACATGTC

TTGACGTGGCCGTGGATAGGGGATCTGTCATCGCCCGTAGGGAAGCACATCTTAAAATATTAGAAATATT

GCTTAGAGAACCATTGTCTATTGACTGTATAAAATTAGCTATACTTAATAATACAATTGAAAACCATGAT

GTGATAAAGCTCTGTATCAAGTATTTTATGATGGTAGATTATTCACTTTGTAATGTGTATGCATCATCAC

TCTTTGATTATATAATTGATTGTAAACAAGAATTGGAGTACATTAGGCAGATGAAAATTCATAATACAAC

CATGTATGAGTTAATCTATAATAGAGACAAAAACAAGCATGCTTCCCATATTCTACATAGGTATTCTAAA

CATCCAGTTTTGACACAGTGTATCACTAAAGGATTCAAGATTTACACAGAAGTAACCGAGCAGGTCACTA

AAGCTCTAAACAGACGTGCTCTAATAGATGAGATAATAAACAATGTATCAACTGATGACAATCTCCTATC

AAAACTTCCATTAGAAATTAGGGATCTAATTGTTTCACAAGCTGTCATATAGAGTTCTCTCCACCCACCT

TTCTTGAAATGAGTTAATAGTCATAAGTTAGTTAAGTCATAAGTTAGTTTTATAAGTTAGTTTATAGTCT

AACACTTCTAATTTTTATACCTTGATCTTTTTCTCTAATTATGAAAAAGTAAATCATTATGAAGATGGAT

GAAAATGGACGAGATTGTGCGCATCGTTAACGATAGTATGTGGTACGTACCTAACGCATTTATGGACGAC

GGTGATAATGAAGGTCACATTTCTGTCAATAATGTCTGTCATATGTATCTCGCATTCTTTGATGTGGATA

TATCATCTCATCTGTTTAAATTAGTTATTAAACACTGCGATCTGAATAAACGACTAAAATGTGGTAACTC

TCCATTACATTGCTATACGATGAATACACGATTTAATCCATCTGTATTAAAGATATTGTTACGCCACGGC

ATGCGTAACTTTGATAGCAAGGATAAAAAAGGACATATTCCTCTACACCACTATCTGATTCATTCACTAT

CAATCGATAACAAGATCTTTGATATACTAACGGACCCCATTGATGACTTTAGTAAATCATCCGATCTATT

GCTGTGTTATCTTAGATATAAATTCAATGGGAGCTTAAACTATTACGTTCTGTACAAATTATTGACTAAA

GGATCTGACCCTAATTGCGTCGATGAGGATGGACTCACTTCTCTTCATTACTACTGTAAACACATATCCG

TGTTCCACGAAAGCAATTATTACAAGTCAAAGAGTCACACTAAGATGCGAGCTGAGAAGCGATTCATCTA

CGCGATAATAGATCATGGAGCAAACATTAACGCGGTTACGAAAATCGGAAATACGCCGTTACACACTTAC

CTTCAACAGTATACCAAACATAGTCCTCGTGTGGTGTATGCTCTTTTATCTCGAGGAGCCGATACGAGGA

TACGTAATAATCTTGATTGTACACCCATCATGGAATACATAAAGAACGATTGTGCAACAGGTCATATTCT

CATAATGTTACTCAATTGGCACGAACAAAAATACGGGAAATTACAAAAGGAAGAAGGACAACATCTACTT

TATCTATTCATAAAACATAATCAAGGATATGGAAGTCGCTCTCTCAATATACTACGGTATCTACTAGATA

GATTCGACATTCAGAAAGACGAATACTATAATACAATGACTCCTCTTCATACCGCCTTCCAGAATTGCAA

TAACAATGTTGCCTCATACCTCGTATACATCGGATACGACATCAACCTTCCGACTAAAGACGATAAGACA

GTATTCGACTTGGTGTTTGAAAACAGAAACATCATATACAAGGCGGATGTCGTTAATGACATCATCCACC

ACAGACTGAAAGTATCTCTACCTATGATTAAATCGTTGTTCTACAAGATGTCGGAGTTCTCTCCCTACGA

CGATCACTACGTAAAGAAGATAATAGCCTACTGCCTATTAAGGGACGAGTCATTTGCGGAACTACATACT

AAATTCTGTTTAAACGAGGACTATAAAAGTGTATTTATGAAAAATATATCATTCGATAAGATAGATTCCA

TCATCGAAAAATGTAGTCGTGACATAAGTCTCCTCAAAGAGATTCGAATCTCAGACACCAACTTGTATAC

GGTATTGAGAACAGAAGACATCCGGTATCACACCTATCTCGAAGCCATACATTCAGACAAACGCATTTCA

TTTCCCATGTACGACGATCTCATAGAACAGTGTCATCTATCGATGGAGCATAAAAGTAAACTCGTCGACA

AAGCACTCAATAAATTAGAGTCTACCATCGATAGTCAATCTAGACTATCGTATTTGCCTCCGGAAATTAT

GCGCAATATCATAACCAAGCTAAGCGACTACCATCTAAACAGTATGTTGTACGGAAAGAACCATTACAAA

TATTATCCATGATAGAAAGAAAATATTTAAAAAATAATCTATATGATTGGAGAAGTAGGAAACAAACAGT

AACAAGACGACGATTACTACATTATTAAATCATGAGGTCCGTATTATACTCGTATATATTGTTTCTCTCA

TGTATAATAATAAACGGAAGAGATATAGCACCACATGCACCATCCAATGGAAAATGTAAAGACAACGAAT

ACAGAAGCCGTAATCTATGTTGTCTATCGTGTCCTCCGGGAACTTACGCTTCCAGATTATGTGATAGCAA

GACTAATACACAATGTACGCCGTGTGGTTCGGATACCTTTACATCTCACAATAATCATTTACAGGCTTGT

CTAAGTTGTAACGGAAGATGTGATAGTAATCAGGTAGAGACGCGATCGTGTAACACGACTCACAATAGAA

TCTGTGAATGCTCTCCAGGATATTATTGTCTTCTCAAAGGATCATCAGGGTGTAGAACATGTATTTCTAA

AACAAAGTGTGGAATAGGATACGGAGTATCCGGATACACGTCTACCGGAGACGTCATCTGTTCTCCGTGT

GGTCCCGGAACATATTCTCACACCGTCTCTTCCACAGATAAATGCGAACCCGTAACCAGCAATACATTTA

ACTATATCGATGTGGAAATTAACCTGTATCCAGTCAACGACACATCGTGTACTCGGACGACCACTACCGG

TCTCAGCGAATCCATCTCAACGTCGGAACTAACTATTACCATGAATCATAAAGATTGTGATCCCGTCTTT

CGTGCAGAATACTTCTCTGTCCTTAATAATGTAGCAACTTCAGGATTCTTTACAGGAGAAAATAGATATC

AGAATACTTCAAAGATATGTACTCTGAATTTCGAGATTAAATGTAACAACAAAGATTCATCTTCCAAACA

GTTAACGAAAACAAAGAATGATACTATCATGCCGCATTCAGAGACGGTAACTCTAGTGGGCGACTGTCTA

TCTAGCGTCGACATCTACATACTATATAGTAATACCAATACTCAAGACTACGAAACGGATACAATCTCTT

ATCATATGGGTAATGTTCTCGATGTCAATAGCCATATGCCCGCTAGTTGCGATATACATAAACTGATCAC

TAATTCCCAGAATCCCACCCACTTATAGTAAGTTTTTTTTACCCATAAATAATAAATACAATAATTAAT

TTCTCGTAAAAGTAGAAAATATATTCTAATTTATTATATGGTAAGAAAGTAGAATCATCTAGAACAGTAA

TCAATCAATAGCAATCATGAAACAATATATTGTCCTGGCATGCATGTGCCTAGTGGCAGCTGCTATGCCT

ACTAGTCTTCAACAATCCTCATCCTCGTGTACTGAAGAAGAAAACAAACATCATATGGGAATCGATGTTA

TTATCAAAGTCACAAAGCAAGACCAAACACCGACCAATGATAAGATTTGTCAATCCGTAACGGAAGTTAC

AGAGACCGAAGATGATGAGGTATCCGAAGAAGTTGTAAAAGGAGATCCCACCACTTATTACACTATCGTC

GGCGCGGGTCTTAACATGAACTTTGGATTCACCAAATGCCCAAAGATTTCATCCATCTCCGAATCCTCTG

ATGGAAACACTGTGAATACTAGATTGTCCAGCGTGTCACCAGGACAAGGTAAGGACTCTCCCGCGATCAC

GCGTGAAGAAGCTCTGGCTATGATCAAAGACTGTGAGATGTCTATCGACATCAGATGTAGCGAAGAAGAG

AAAGACAGCGACATCAAGACCCATCCAGTACTTGGGTCTAACATCTCACATAAGAAAGTGAGTTACAAAG

ATATCATCGGTTCAACGATCGTTGATACAAAATGTGTTAAGAACCTAGAGTTTAGCGTACGTATCGGAGA

CATGTGTGAGGAATCATCTGAACTTGAAGTCAAGGATGGATTCAAGTATGTCGACGGATCGGCATCTGAA

GGTGCAACCGATGATACTTCACTCATCGATTCAACAAAACTCAAAGCATGTGTCTGAATCGATAACTCTA

TTCATCTGAAAATGGATGAGTTGGGTTAATCGAACGATTCAGACACCGCACCACGAATTAAAAAAGTGTA

CCGGGTACTATATTCCGGTTTGCAAAACAAAAATGTTTAACTACATTCACAAAAAGTTACCTCTCGTTAC

TTCTTCTTTCTGTTTCAATATGTGATACGATATGATCACTATTCGTATTCTCTTTCTCTCTTGTCTCATAAAAAAGTTTTACAAAAAAATATTTTTATTCTCTTTCTCTCTTCGATGGTCTCACAAAAATATTAAACCTCT

TTCTGATGTCTCAACTATTTCGTAAACGATAACGTCCAACAATATATTCTCGTAGAGCTTATCAATATCC

TTATGCCAATGGTTGTCAGACAATTGCATCATAAAATAATGTTTATAATTTACACGTTAACATCATA

TAATAAACGTATATAGTTAATATTTTTGGAATATAAATGATCTGTAAAATCCATGTAGGGGACACTGCTC

ACGTTTTTTCTCTAGTACATAATTTCACACAAGTTTTTATACAGACAAATTAATTCTCGTCCATATATTT

TAAAACATTGACTTTTGTACTAAGAAAAATATCTTGACTAACCATCTctgatggagtcgtaaaaagtttttatcCTTTCTCTCGTCGATGGGTCTCA

CAAAAATATTAAACCTCTTTctttctctcttcgatgggtctcacaaaaatattaaacctctttCTGATGGAGTCGTAAAAAGTTTTTATCCTTTCTCTCGTCGATGGGTCTCACAAAAATATTAAACCTCTTTCTGATGGTCTCTATAAACGATTGATTTTTCTTACCCTCTAGAGTTTCCTACGGTCGTGGGTCACACATTTTTTTCTAGACACTAAATAAAATAGTAAAATTAAATTAATTATAAAATTATGTATATAATTTACTAAC
