# Supplementary material for: Exploring the key genomic variation in monkeypox virus during the 2022 outbreak
Source: BMC Genom Data. 2023 Nov 16;24:67. doi: 10.1186/s12863-023-01171-0 (PMC10652487; doi:10.1186/s12863-023-01171-0)
Supplement: Supplementary file 6 — Additional file 6. Full length sequence of RS3. [file 12863_2023_1171_MOESM6_ESM.docx]

**Additional file6. Full length sequence of RS3.**

GTTAGTAAATTATATACATAATTTTATAATTAATTTAATTTTACTATTTTATTTAGTGTCTAGAAAAAAATGTGTGACCCACGACCGTAGGAAACTCTAGAGGGTAAGAAAAATCAATCGTTTATAGAGACCATCAGAAAGAGGTTTAATATTTTTGTGAGACCTATCGAAGAGAGAAAGGATAAAAACTTTTTACGACTCCATCAGAAAGAGGTTTAATATTTTTGTGAGACCCATCGAAGAGAGAAAGAGATGGTTAGTCAAGATATTTTTCTTAGTACAAAAGTCAATGTTTTAAAATATATGGACGAGAATTAATTTGTCTGTATAAAAACTTGTGTGAAATTATGTACTAGAGAAAAAACGTGAGCAGTGTCCCCTACATGGATTTTACAGATCATTTATATTCCAAAAATATTAACTATATACGTTTATTATATGATGTTAACGTGTAAATTATAAACATTATTTTATGATGCAATTGTCTGACAACCTAGATTGGTATAAGGATGTTGATAAGCTCTACGAGAATATATTGTTGGACGTTATCGTTTACGAAATAGTTGAGACATCAGAAAGAGGTTTAATATTTTTGTGAGACCATCGAAGAGAGAAATAGAATAAAAATATTTTTTTTTTGTAAAACTTTTTTATGAGACCAAGAGAATACGAATAGTGATCATATCGTATCACATATTGAAACAGAAAGAAGAAGTAACGAGAGGTAACTTTTTGTAAATGTAGTTAAATATTTTTGTTTTGCAAACCGGAATATAGTGCCCGGTCTTTTTTAATTCGTGGTGCGGTGTCTGAATCGTTCGATTAACCCAACTCATCCATTTTCAGATGAATAGAGTTATCGATTCAGACACATGCTTTGAGTTTTGTTGAATCGATGAGTGAAGTATCATCGGTTGCACCTTCAGATGCCGATCCGTCGACATACTTGAATCCATCCTTGACTTCAAGTTCAGATGATTCCTCACACATGTCTCCGATACGTACGCTAAACTCTAGGTTCTTGACACATTTTGTATCAACGATCGTTGAACCGATGATATCTTTGTAACTCACTTTCTTATGTGAGATGTTAGACCCAAGTACTGGATGGGTCTTGATGTCACTGTCTTTCTCTTCTTCGCTACATCTGATGTCGATAGACATCTCACAGTCTTTGATCATAGCCAGAGCTTCTTCACGCGTGATCGCGGGAGAGTCCTTACCTTGTCCCGGTGACACGCTGGACAATCTAGTATTCACAGTGTTTCCATCAGAGGATTCGGAGATGGATGAAATCTTTGGGCATTTGGTGAATCCAAAGTTCATGTTAAGACCCGCACCGACGATAGTGTAATAAGTGGTGGGATCTCCTTTTACAACTTCTTCGGATACCTCATCATCTTCGGATACCTCATCATCTTCGGTCTCTGTAACTTCCGTTACGGATTGACAAATCTTATCATTGGTCGGTGTTTGGTCTTGCTTTGTGACTTTGATAATAACATCGATTCCCATATGATGTTTGTTTTCTTCTTCAGTACACGAGGATGAAGATTGTTGAAGACTAGTAGGCATAGCAGCTGCCACTAGGCACATGCATGCCAGGACAATATATTGTTTCATGATTGCTATTGATTGATTACTGTTCTAGATGATTCTACTTTCTTACCATATAATAAATTAGAATATATTTTCTACTTTTACGAGAAATTAATTATTGTATTTATTATTTATGGGTAAAAAAACTTACTATAAGTGGGTGGGATTCTGGGAATTAGTGATCAGTTTATGTATATCGCAACTAGCGGGCATATGGCTATTGACATCGAGAACATTACCCATATGATAAGAGATTGTATCCGTTTCGTAGTCTTGAGTATTGGTATTACTATATAGTATGTAGATGTCGACGCTAGATAGACAGTCGCCCACTAGAGTTACCGTCTCTGAATGCGGCATGATAGTATCATTCTTTGTTTTCGTTAACTGTTTGGAAGATGAATCTTTGTTGTTACATTTAATCTCGAAATTCAGAGTACATATCTTTGAAGTATTCTGATATCTATTTTCTCCTGTAAAGAATCCTGAAGTTGCTACATTATTAAGGACAGAGAAGTATTCTGCACGAAAG

ACTGGATCACAATCTTTATGATTCATGGTAATAGTTAGTTCCGACGTTGAGATGGATTCGCTGAGACCGGTAGTGGTCGTCCGAGTACACGATGTGTCGTTGACTGGATACAGGTTAATTTCCACATCGATATAGTTAAATGTATTGCTGGTTACGACGGGTTCGCATTTATCTGTGGAAGAGACGGTGTGAGAATATGTTCCGGGACCACACGGAGAACAGATGACGTCTCCGGTAGACGTGTATCCGGATACTCCGTATCCTATTCCACACTTTGTTTTAGAAATACATGTTCTACACCCTGATGCTCCTTTGAGAAGACAATAATATCCTGGAGAGCATTCACAGATTCTATTGTGAGTCGTGTTACACGATCGCGTCTCTACCTGATTACTATCACATCTTCCGTTACAACTTAGACAAGCCTGTAAATGATTATTGTGAGATGTAAAGGTATCCGAACCACACGGTGTACATTGTGTATTAGTCT

TGCTATCACATAATCTGGAAGCGTAAGTTCCCGGAGGACACGATAGACAACATAGATTACGGCTTCTGTA

TTCGTTGTCTTTACACTTTCCATTGGATGGTGCATGTGGTGCTATATCTCTTCCGTTTATTATTATACAT

GAGAGAAACAATATATACGAGTATAATACGGACCTCATGATTTAATAATGTAGTAATCGTCGTCTTGTTA

CTGTTTGTTTCCTACTTCTCCAATCATATAGATTATTTTTTAAATATTTTCTTTCTATCATGGATAATAT

TTGTAATGGTTCTTTCCGTACAACATACTGTTTAGATGGTAGTCGCTTAGCTTGGTTATGATATTGCGCA

TAATTTCCGGAGGCAAATACGATAGTCTAGATTGATTATCGATGGTAGACTCTAATTTATTGAGTGCTTT

GTCGACGAGTTTACTTTTATGCTCCATCGATAGATGACACTGTTCTATGAGATCGTCGTACATGGGAAAT

GAAATGCGTTTGTCTGAATGTATGGCTTCGAGATAGGTGTGATACCGGATGTCTTCTGTTCTCAATACCG

TATACAAGTCGGTGTCTGAGATTCGAATCTCTTTGAGGAGACTTATGTCACGACTACATTTTTCGATGAT

GGAATCTATCTTATCGAATGATATATTTTTCATAAATACACTTTTATAGTCCTCGTTTAAACAGAATTTA

GTATGTAGTTCCGCAAATGACTCGTCCCTTAATAGGCAGTAGGCTATTATCTTCTTTACGTAGTGATCGT

CGTAGGGAGAGAACTCCGACATCTTGTAGAACAACGATTTAATCATAGGTAGAGATACTTTCAGTCTGTG

GTGGATGATGTCATTAACGACATCCGCCTTGTATATGATGTTTCTGTTTTCAAACACCAAGTCGAATACT

GTCTTATCGTCTTTAGTCGGAAGGTTGATGTCGTATCCGATGTATACGAGGTATGAGGCAACATTGTTAT

TGCAATTCTGGAAGGCGGTATGAAGAGGAGTCATTGTATTATAGTATTCGTCTTTCTGAATGTCGAATCT

ATCTAGTAGATACCGTAGTATATTGAGAGAGCGACTTCCATATCCTTGATTATGTTTTATGAATAGATAA

AGTAGATGTTGTCCTTCTTCCTTTTGTAATTTCCCGTATTTTTGTTCGTGCCAATTGAGTAACATTATGA

GAATATGACCTGTTGCACAATCGTTCTTTATGTATTCCATGATGGGTGTACAATCAAGATTATTACGTAT

CCTCGTATCAGCTCCTCGAGATAAAAGAGCATACACCACACGAGGACTATGTTTGGTATACTGTTGAAGG

TAAGTGTGTAACGGCGTATTTCCGATTTTCGTAACCGCGTTAATGTTTGCTCCATGATCTATTATCGCGT

AGATGAATCGCTTCTCAGCTCGCATCTTAGTGTGACTCTTTGACTTGTAATAATTGCTTTCGTGGAACGC

GGATATGTGTTTACAGTAGTAATGAAGAGAAGTGAGTCCATCCGCATCGACGCAATTAGGGTCAGATCCT

TTAGTCAATAATTTGTACAGAACGTAATAGTTTAAGCTCCCATTGAATTTATATCTAAGATAACACAGCA

ATAGATCGGATGATTTACTAAAGTCATCAATGGGGTCCGTTAGTATATCAAAGATCTTGTTATCGATTGA

TAGTGAATGAATCAGATAGTGGTGTAGAGGAATATGTCCTTTTTTATCCTTGCTATCAAAGTTACGCATG

CCGTGGCGTAACAATATCTTTAATACAGATGGATTAAATCGTGTATTCATCGTATAGCAATGTAATGGAG

AGTTACCACATTTTAGTCGTTTATTCAGATCGCAGTGTTTAATAACTAATTTAAACAGATGAGATGATAT

ATCCACATCAAAGAATGCGAGATACATATGACAGACATTATTGACAGAAATGTGACCTTCATTATCACCG

TCGTCCATAAATGCGTTAGGTACGTACCACATACTATCGTTAACGATGCGCACAATCTCGTCCATTTCAT

CCATCTTCATAATGATTTACTTTTTCATAATTAGAGAAAAAGATCAAGGTATAAAAATTAGAAGTGTTAG

ACTATAAACTAACTTATAAAACTAACTTATGACTTAACTAACTTACTTATAAAACTAACTTATGACTTAA

CTAACTTATGACTTAACTAACTTATGACTTAACTAACTTATGACTATTAACTCATTTCAAGAAAGGTGGG

TGGATAGAACTCTATATGACAGCTTGTGAAACGATTAGATCCCTAATTTCTAATGGAAGTTTTGATAGGA

GATTGTCATCAGTTGATACATTGTTTATTATCTCATCTATTAGAGCACGTCTGTTTAGAGCTTTAGTGAC

CTGCTCGGTTACTTCTGTGTAAATCTTGAATCCTTTAGTGATACACTGTGTCAAAACTGGATGTTTAGAA

TACCTATGTAGAATATGGGAAGCATGCTTGTTTTTGTCTCTATTATAGATTAACTCATACATGGTTGTAT

TATGAATTTTCATCTGCCTAATGTACTCCAATTCTTGTTTACAATCAATTATATAATCAAAGAGTGATGA

TGCATACACATTACAAAGTGAATAATCTACCATCATAAAATACTTGATACAGAGCTTTATCACATCATGG

TTTTCAATTGTATTATTAAGTATAGCTAATTTTATACAGTCAATAGACAATGGTTCTCTAAGCAATATTT

CTAATATTTTAAGATGTGCTTCCCTACGGGCGATGACAGATCCCCTATCCACGGCCACGTCAAGACATGT

ATATCCATTACTCATTACTGCGTTGACATTTGCTCCATTTTCTAATAGCAATGATACTAAATCTATATAA

CCTGCATAGATAGCGCGATAAAGCAAGGTCCTTCCACCAGCATCTAGTTGATTGATATCTTCAATATATG

GGATACAAAGCTTATAAATTTCTAATACTGTGGGTTCATCTACAAGGAATCCCCTAGTATACTGAATTAT

TTTATATAGATCTAATTTAACATCATTTTCATCTGGGATACCACAATTCAAAATAAACTCAACAACACTA

CTTTCCTTTTTACATATTCCCCTAAAATAGGCATTCAAGCATTCTATTTTATATATTACAGCCCCATGAT

CTACCATAAGATCAACAATGTCTATTTCTACATATGCATTAGATAGATAGTAAAGTAAGAGATCTTGCAC

AGAATTACAATTCTTAATAATTATAGAGAAAATATCTTCCATATAATTCTTTGACACTAATGCAGATATA

ATATCTTTATATGTAATATATGCAAACAGTCTATCTACTATATACTGATCAATATTATCTCTATGAATCC

TAAAATAATCATACAGAACATCTACAGGATCACAAATTGGTTCAAGGAGAAATCTATCAAATATTTTCCT

GTCAACAACTGGTTCTAGAACATAACAGTCAACACCTAATCCATGTTTTTTATAATCATCTACCAAAGAT

AATGACCAAAGATCGAGGTCGTCGTGAAACTGCTCATCGACAGCCATGAAATCTACCGACTCCATGGTGC

GAATCGCACTGTCTTATTCGCCATTGATTTTCATTTTTTATAATTATGTACATGTTTTCCTTCTATTCTC

AAGAGTCTACAAAAATATATTTTTTTCGATATCTAAGTACTAAGTTTTTTTACTGTTTTTGTTACTGTCT

TCCATTCTTCTAACTAAAGATCTGAGATAAATTATACAATCTTCGCTATCGAACCATTTTTGTAGTCTAA

AGCCTGAAGTAATTAACCAACTGTTTTTATTAGTGGCTTTTTTCGATCTATCAATTTCAGTATATTCTTC

GCCGTTATAAAAGTAATGTTGTTTAATTGTAGGACGGTTGTTAGTATAATCACATGAATAATAATATTCT

AATTCCTCGTATTGACTACTTACAGATACTCGAAATAGTCTGAAAAATTCTTCAAAGATATTTTTATAAA

GATCTAGGAAAAGTTTATTACCGACCATGAACGAGATAGATGGATAAATATCCTTTCCATCAAAGGTCAT

AATTGGATAATTGTCCAGCAATATATCTGCTGTATTAGTTATATCACTTCCATTTATTTTCAGATTGAAG

TAATGTACTAGTTTGTGACAATTAACAAGATACAAAAGAGATGCCGATACTAATACGTAAATAGCTATAC

GCGAATCCATTGTTACCTTTTTTTATTTCATAGGTCTATTAATAAATATATGTATTACTTAAGACTAGAA

AAATCAAAAGTAAGTTTTTGATATTTGATTCTTACTTATTGTGGGATTGTAGTTTACTTAGTAATTCATC

TCTGAATCCTGATAAATCATGCATATCAATGATGCAACTACGCAGCAAACTAGTAGGAATATAGATATCT

GGATATGTACGTAAATAGTCGATTATATCTTTTACAATACTATTAGTCCCTATTGCGTTATCTATATATC

CATTAATAATATTACACAGTGGATACTTATGAGAAATATACCTCTTACAGATTTTTAACGATATATAATCTA

GAAAGATATGTGTGTAGTACTGTATTACCTAAATTATCAGTCTCATTCAAATATTGCATGACTATTATCG

AGAATTGCTATATCCCTCTATACTCGATGCATTTATTACAGTAATTCAATCCAGCTAACATAAGAGCCAA

TCTCAATGTTGGTTTAATTATATCATCTTCATGTAATAATAACGATGGAAACTTTCCAGTAGCGTAACAC

TTATCTAAGAAGGATATAATAACTATGTCTACATTATGTCTCTTATCGAGAATATTCTTAACGAGATATC

CATAGCTATTCTGGTGCTAATTATTCCTATATTATATTCCACGAAAAATGATGAAGGCCATTCATCATAA

GATGATAAAAAGTGTAGTGAGTAAGAGTATTAGTGAGAGAGCATGAAGGAGATTTAGTATTTAGCAGTGA

GGATATGATCCAAGAGGGTGAGATAGTCGTTCTCGTTCAGAATCTTTCGCAGCATAAGTAGTATGTCGAT

ATACTTATCATTGAAGACTCTTCCGGTGACAATAGCTGATTGAGTACAAAGTCCAATTATTGCACAAAGT

TCTTTGGCGGTTTTCATGGAGTCATTTCTGATGAAACATTTAATGATCTCCACGCAATTGTCGATATTGT

CCCACGGAAGTGAATCCGAGAACTCCTTCAACTCGCTACCAAATAGCTCCATTGCATCAATTCTGAAAGA

GATGAGAAGCCTGTAGAGAGGCCCTGCGCTTTCTCTATGGGTCCATCTATGAGAAACCCACAGGATGTAT

TCAGTCAGACAATGTCTGACATCAGTCACGGTATTCAGGGAGTCCTTAGTAGCGTGGCAATGACAGGGAC

TGAACTGGGCACAAGGAGAGGCCATTGTGAAGGTAGACGAAGGTAACCTGATGGTAGACCTGTAGCCGTC

TGTGCTTAATAGAGGGCTTTAATTTCCATTTTTAATGGTGTCGTGAATGAGGAATGAGAGTGTCTCTCGT

CCTTGGTTTACATGGATCAGAGTGAGAAAAAATATCTTGTATATTATTAACTAACAACCTTGGTTTCTAT

CCATGTTTAAAAAATGACCTATATGTTCTTTATTAATTCTATTTTAAACTTTATCCTCAAGACTCCTGAC

AAAATTAAAATCCAGAAAGCAGCAAACAATCCTGTTACAAGTTTACTGAAATCTCTCTTTGATTGTAGAG

TATATGTAGTCAGAGCAAGAAACACTGCAGTAGTCAACATGAAAGCTTGCATAACGATACGTGCATCATA

GAAAGTAACAACAGAGGCCAGCGTTAGAGATTCTAACAGTGTAAATCCACAAAGTATGTACAGATTCAGG

GGATGTTCATGTCTGTGTAAAGTCAATGCGAAAATCAAGCCTATAGATCCGAACATTGATGCCAATATTA

GAACAGGACTCCCTTGTATAAATGTCCGATGCATTCAAAGTATAAAAATACTGCAGCTGTTGCCGTTGTT

AAAGGAAATTGTAGAAAGGATACCGTAGACTTTTCTTAGAAATGCCATTCGTATGTACACGCTGGCAGAC

GCCACCGAGCTGTCATAGTTGAAGTCGTCCTCGATAGTTTTTGTCTTTTACAGTATATACGTTATTGTCA

AAATCTAAACAAATATTAGCATAATACATCTATCTATAAGATCAGGATCCATGTTCGAGCATACTAGCCA

TATATATTTGTAACTTCGTCGTACAGCGTTAGATCAATAGAATAAACAATCGTGTGACGCAACTTTTTTA

CGATCTAGTTGTATGAGTTTATCGTTTACATAAGCAATTAACGGCTTTAACAGATGATCTGAGTAATAAT

ATACCTCTGTTATACGTTTAATGTTCACGGTCTTAGTATTTTTAGATATCAATTGTGATTTACACCATAT

TCGACTCCCTTGTGTGTAACGTTAGAAATTCTAAATCTATAGTATTATCTATTACAGCGTAAAACACATT

CAATATTGTATTGTTATTTTTATATTATTTACACAATTAACAATGTATTATTAGTTTATATTACTGAATT

AATAATATAAAATTCCCAATCTTGTCATAAACACAAAATCCATTAAAATGTCGATAAAATATCTGATGTT

GTTGTTCGCTGCTATGATAATCAGATCATTAGCCGATAGTGGTAATGCTATCGAAACAACATCGCCAGAA

ATTACAAACGCTACAACAGATATTCCAGCTATCAGATTATGTGGTCCAGAGGGAGATGGATATTGTTTAC

ACGGTGACTGTATCCACGCTAGAGATATCGACGGTATGTATTGTAGATGCTCTCATGGTTATACAGGTAT

TAGATGTCAGCATGTAGTATTAGTAGACTATCAACGTTCAGGAAAACCAGATACTACAACATCATATATC

CCATCTCTAGGTATTGTGCTTGTATTAGTAGGCATTATTATTACATGTTGTCTATTATCTGTTTATAGGT

TCACTCGAAGAACTAAACTACCTATACAAGATATGGTTGTGTTATATTTTTTATAAAATTTTCTTATGAG

TATTTTTACAAAAATGTATATGTATAAAAAAATACTAAGTATACGATGTATCCTGTATTATTTGTATTTA

TCTAAACAATACTTCTGCCTCTAGATGGGATACAAAAATTTTTTATTTCGGCATATTAAAGTAAAATCTA

GTTACCTTGAAAATGAATACAGTGGGTGGTTCCGTATCACCAGTAAGAACATAATAGTCGAATACAGTAT

CCGATTGAGATTTTGCATACAATACTAGTCTAGAAAGAAATTTGTAATCATCTTCTGTGACAGGAGTCCA

TATATCTGTATCATCGTCTAGTTTTTATCAGTGTCCTATGCTATATTCCTGTTATCATCATTAGTTAATG

AAAATAACTCTCGTGCTTCAGAAAAGTCAAATATTGTATCCATACATACATCTCCAAAACTATCACTTAT

ACGTTTATCTTTAACGAACATATACCTAGATGGTTATTTACTAACAGACATTTTTCAAGATCTATTGACA

ATAACTCCTATAGTTTCCACATCAACCAAGTAATGATCATCTATTGTTATATAACAATAACATAACTCTT

TTCCATTTTTATCAGTATCTATATCAACGTCGTTGTAGTGAATAGTAGTCATTGATCTATTATATGAAAC

GGATATGTCTAGTTAATATTTTCTTTGATTTAAAGTCTATAGTCTTTACAAACATAATATCCTTATCCGA

CTTTATATTTCCTGTAGGGTGGCATAATTTTATTCTGCCTCCACAATCAATGTTTCCAAATATATTACTG

GACAATATTCCATATAGTTATTAGTTAAGGGACCCAATTAGAACACATACGCGCTTATTCATCATTTGGA

TCGTATTTCATAAAAGTTATTATGTTATCGATGTCAACACATTCTACATTTTTTTAATTGTCTATATAGT

ATTTTTCTGATATTTTCTATAATATCAGAATTGTCTTCCATAGGAAGTTGTATACTATCGGAATCAGTTA

CATGTTTAAATAATTCTCTGATGTCATTCCTTATACAATCAAATTCATCATTAAACAGTTTAATAGTCTG

TAGACCTTTATCGTCGTACATATCCATTGTCTTATTAGTTACGCTTATTTTTATGGGTTTTACGTTGCTT

TATTATATTTTATAATAATGATTGTTTGACAATGTCGTAGTATAGATATATTATTAGAGGAGGTATAATT

ATAAAAAGTTTCTGAGTACGATGTTATAAGAGGAGAGGACACATTAACATCATACATCAATTAACTACAT

TCTTATAACATTGTAATCAAAAGAATTGCAATTTTGATGTATAACAACTGTCAATGGAATTGTATATTAC

AAATTACGGTATGTTGTAACAACAAATACCGATCGGTAATTGTCTCTGCCGCTGTAATAGAATTAATTAT

ATATCTATTACACCGGCCTTGTATCATAATAAAGTTGTGGTAGTATGATCTCCATATTTATAATTTAGTA

CTTTGTATTTAGTTTTTTTGGAATCATAAAAAAAGTTTTACTAATTTAAAATTTAAAAAGTATTTACATT

TTTCACTGTTTAGTCGCGGATATGGAATTCGATCCTGTCAAAATCAATACATCATCTATAGATCATGTAA

CAATATTACAATACATAGATGAACCAAATGATATAAGACTAACAGTATGCATTATCCGAAATATTAATAA

CATTACATATTATATCAATATCACAAAAATAAATCCACATTTGGCTAATCGATTTCGGGCTTGGAAAAAA

CGTATCGCCGGAAGGGACTATATGACTAACTTATCTAGAGATACAGGAATACAACAATCAAAACTTACTG

AAACTATACGTAACTGTCAAAAAAATAAAAACATATATGGTCTATATATACACTACAATTTAGTTATTAA

TGTGGTTATTGATTGGATAACCGATGTGATTGTTCAATCAATATTAAGAGGGTTGGTAAATTGGTACATA

GCTAATAATACATATACTCCAAATACACCCAATAATACTACAACCATTTCTGAGTTGGATATCATCAAAA

TACTGGATAAATACGAGGACATGTATAGAGTAAGTAAAGAAAAAGAATGTGGAATTTGCTATGAAGTTGT

TTACTCAAAACGATTAGAAAACGATAGATACTTTGGTTTATTGGATTCGTGTAATCATATATTTTGCATA

ACATGTATCAATATATGGCATAGAACACGAAGAGAAACCGGTGCGTCAGATAATTGTCCTATATGCCGTA

CCCGTTTTAGAAAAATAACAATGAGCAAGTTCTATAAGCTAGTTAACTAATAAATAAAAAGTTTAATTAT

CGACGATATATGTCGTTATTTTTCTCTCATATGAAAGATTAATTTGATTCTAATATAATCTTCAGTATTG

GATGAATCTCAATTCAAATTAATTCCATTAGATTAGATTAGATTAGATTAGATTAGATTAGATTAGATTA

GATTAGATTAGATTAGATCATAAATAAAAATAGTAGCACGCACTACTTCAGCCAAATATTCTTTTTTGAA

ACGCCATCTAGCGTAATGAGAACACAAGTGAACCTATAATGAGCAAATTTATTAGTATCGGTTACATGAA

GGACTTTACGTAGAGTGGTGATTCCTCCATCTGTGGTACGAACGGTTTCATCTTCTTTGATGCCATCACC

CAGATGTTCTATAAACTTGGTATCCTCGTCCGATTTCATATCATTTGCCAACCAATACATATAGCTAAAC

CCAGGCATACGTTCCACACATCCGGAACAATGAAATTCTCCAGAAGATGTTACAATGGCTAGATTTGGAC

ATTTGGTTTCAACCGCGTTAACATATGAGTGAACACACCCATACATGAAAGCGATGAGAAATAGGATTCT

CATCTTGCCAAAATATCACTATAAAAAATTTATTTATCAATTTTAAAGGTATAAAAAAATACTTATTGTT

GCTCGAATATTTTGTATTTGATGGTATACGGAAGATTAGAAATGTAGGTATTATCATCAACTGATTTTAT

GATAGTTTTATGAATTTTATTATGCTTCACTATTGCATCGGAAATAATATCATATGCTTCCACGTATATT

TTATTTTGTTTTGACTCATAATACGCACGTAATTCTGGATTATTGGCATATCGATGAATAATTTTAGCTC

CATGCTCAGTAAATATTAATGAGAACATAGTGTTGCCTCCTACCATTATTTTTTTCATCTCATTCAATTC

TTGATTGCAGAGATCTATATAATCATTATAGCGTTGACTTATGGACTCTGGAATCTTAGACGATGTACAG

TCATCTATAATCATGGTATATTTAATACATTGTTTTATAGCATAGGCATTATCTACGATATTAGATACTT

CACTCAATGAATCAATCACGCAATCTAATGTAGGTTTATGACATAATAGCATTTTCAGCAGTTCAATGTT

TCTAGATTCGTTGATGGCAATGGCTATACATGTATATCCGTTATTTGATCTAATGTTGACATCTGAACCG

GATTCTAGCAGTAAAGATACTAGAGATTGTTTATTATATCTAACAGCCTTGTGAAGAAGTGTTTCTCCTC

GTTTGTCAATCATGTTAATGTCTTTAAGATAAGGTAGGCAAATGTTTATAGTACTAAGAATTGGGCAAGC

ATAAGACATGTCACAAAGACCCTTTTTGTATGTATAAGTGTAGAAATTATAACATCCATAGTTGGATTCA

CATAGGTGTCCAATCGGGATCTCTCCATCATCGAGATGATTGACGGCATTTCCCCCTTCCTTTTTTAGTA

GATATTTCATCGTGTAAGAATCAATATTAATATTTCTAAAGTATCTGTGTATAGCCTCTTTATTTACCAC

AGCTCCATATTCCAACATGCATTCCACTAGAGGGATATCGATATCGCCGAATGTCATATACTCAATTAGT

ATATGTTGGAGGACATCCGAGTTCATTGTTTTCAATATCAAAGAGATGGTTTCCTTATCATTTCTCCATA

GTGGTACAATACTACACATTATTCCGTACGGCTTTCCATTCTCCAAAAACAATTTTACCAAATCTAAATC

TACATCTTTATTGTATCTATAATCACTATTTAGATAATCAGCCATAATTCCTCGAGTGCAACATGTTAGA

TCGTCTATATATGAATAAGCCGTGTTATCTATTCCTTTCATTAACAATTTAACGATGTCTATATCTATAT

GAGATGACTTAATATAATATTGAAGAGCTGTACAATAGTTTTTATCTATAGAAGACGGCTTGATTCCGTG

ATTAATTAGACATTTAACAACTTCCGGACGCACATATGCTCTCGTATCCGACTCTGAATACAGATGAGCG

ATGATATACAGATGCAATACGGTACCACAATTTCGTGGTTGATAATCATCATACACGTATCCGTACTCGT

CATCCTCATAAAGAACACTGCATCCATTTTCTATGAACAAATCAATAATTTCAGGAACAGGATCATCTGT

CATTACATAATTTTCTATAACTGAACGATGGTTTTCACATTTAACACTCAAGTCAAATCCATGTTCTACC

AACACCTTTATCAAGTCAACGTCTACATTTTTTGATTTCATATAGCTGAATATATTAAAGTCATTTATGT

TGCTATATCCAGTAGCTTCTAGTAGAGCCATCGCTATATCCTTATTGACTTTAACATGTCTACTATTTGT

GTATTCTTCTATTGGGGTAAACTGTCTCCAATTTTTGTGTAATGGATTAGTGCCACTGTCTAGTAGTAGT

TTGACGACCTCAACATTATTACAATGCTCATTGAAAAGGTATGCGTGTAAAGCATTATTCTTGAATTGGT

TCCTGGTATCATTAGGATCTCTGTCTCTCAACATCTGTTTAAGTTCATCGAGAACCACCTCCTCATTTTC

CAGATAGTCAAACATTTTGACTGAATAGAAGTGAATGAGCTACTGTGAACTCTATACACCCACACAACTA

ATGTCATTAAATATCATTTTTGAATGTATATTTATACCATGTCAAAAACTTGTACAATTATTAATAAAAA

TAATTAGTGTTTAAATTTTACCAGTTCCAGATTTTACACCTCCGTTAACACCTCCATTAACCCCACTTTT

TACACCACTGGACGATCCTCCTCCCCACATTCCACTGCCACTAGATGTATAAGTTTTAGATCCTTTATTA

CTACCATCATGTCCATGGATAAAGACACTCCACATGCCGCCACTACTACCCCCTTTAGACGACATATTAA

TAAGACAAGTTTAACAATAAAATTAATCACGAGTACCCTACTACCAACCACTATTATATGATTACAGTAC

CTTGACTAAAGTCTCTAGTCACAAGATCAATACTACCAACCTACGCTATTATATGATTATAGTTTCTATT

TTTATAGGAACGCGTACGAGAAAATCAAATGTCTAAGTTCTAACGGTAGTGTTGATAAACGATTGTTATC

CGCGGATACCTCATCTATCATGTTGTCTATTTTCTTACTTTGTTCTATTAACCTATTAGCATTATATATT

ATTTGATTATAAAACTTATATTGCTTATTAGCCCAATCTGTAAATATCGGATTATTAACATATCGTTTCT

TTGTAGGTTTATTTAACTTGTACATCACTGTAAGCATGTCCGTACCATTTATTTTAATTTGACACATATC

AGCAATTTCTTTTTCGCAGTCGGTTATATATTCTATATAAGATGGATACGTATCATATATGTACTTATAG

TCTACTAATATGAAGTACTTAATACATATTTTCAGTAACGATTTAGCCTTATTACCTATTAATAAGTGCC

TGTCGTTGGATAGGTAATCAACTGTTTTCTTAATACATTCGATGGTTGGTAATTTACTCAAAATAATTTC

CAATATCTTAATATATATTTCTGCTATTTCTGGTATACATGCATGTGCCATTATAACACAAATACCAATA

CATGTAGACCCATATGTTGTTGTTATATTAATATCTGCGCCATTATCTATTAACCATTCTATTAGGGCAA

CACTATGCGACTCGATACAATAATAAAGTATACTACGTCCATGTTTATCTATTTTGTTTATATCATCGAT

ATACGGCTTACAAATTTCTAGTATCGATAACACTTCTGACTCGTGAATAAATAAGGTAGGGAATAACGGC

ATAATATTTATTATGTTATCATCATTAACAACTACGTTTCCATTTTTTAAAATATACTCTACAACTTTAG

GATCCCTATTGTCAAATCTTTTAAAATATTTATTTATATGCTTAAATCTATATAATATAGCTCCTTCCCT

AATCATACATTTGATAACATTGATGTACACTGTATGATAAGATACATATTCTGACAATAGATCTTGTATA

GAATCTGTATATCTTTTAAGAATTGAGGATATTATGACATTATTACGTAAACTATTACACAATTCTAAAA

TATAAAATGTATCATGGGCAGATAATAGTTTATCCACTATATAATTATCTATTTTATGATTTTTCTTCCT

ATATTGTTTACGTAAATAGATAGATAGAATATGCATTAGTTCATTACCGCTATAGTTACTATCGAATAAC

ACGTCAAATATTTCCCGTTTAATATCGCATTTGTCAACATAATAATAGAGTATGGTACGTTCACGATAAG

TATAATGACACATCTCGTTTTCGTGCGAAATTAAATAGTTTATCACGTCCAAAGATGTCACATAACCATC

TTGTGACCTAGTAATAATATAATAATAGAGAACTGTTTTACCCATTCTATTATCATAATCAGTGGTGTAG

TCATAATCTAAATAATCAAACTCGTCATCCCAATTAAAATAAATATAATCAGTACATTGAATGGGTATGA

TATTGTACCCATACTGTATGTTGCTACATGTAGGTATTCCTTTATCCAATAATAGTTTAAATACATCTAT

ATTAGGATTTGATGTTGTCGCGTATTTCTCTACAATATTAATACCATTTTTGATACTATTTATTTCTATA

CCTTTCGAAATTAGTAATTTCAATAAGTCTATATCGATGTTATCAGAACATAGATATTCAAATATATCAA

AATCATTGATATTTTTATAGTCGACTGATGACAATAACAAAATCACAACATCGTTTTTGATATTATTATT

TTTTTTGGTAACGTATGCCTTTAATGGAGTTTCACCATCATACTCATATAATGGATTTGCACCACTTTCT

ATTAATGATTGTGCACTACTGGCATCGATGTTAAATGTTTTACAACTATCATAGAGTATCTTATCGTTAA

CCATGATTGGTTGTTGATGTTATCACATTTTTTGGTTTCTTTCATTTCAGTTATGTATGGATTTAGCACG

TTTGGGAAGCATGAGCTCATATGATTTCAGTACTGTAGTGTCAGTACTATTAGTTTCGATCAGATCAATG

TCTAGATCTATAGAATCAAAACACGATAGGTCAGAAGATAATGAATATCTGTACGCTTCTTCTTGTACTG

TAACTTCTGGTTTTGTTAGATGGTTGCATCGTGCTTTAACGTCAATGGTACAAATTTTATCCTCGCTTTG

TGTATATCATATTCGTCTCTAGTATAAAATTCTATATTCAAATTATCATGCGATGTGTGTACGCTAACGG

TATCAATAAACGGAGCACAGCATTTAGTCAACAGTAATCCAAAATTTTTTAAAGTATATCTTAACGAAAG

AAGTTGTCATCGTTAGAGTGTGGTAAATCATTGTCTACGGTACTAGATCCTCATAAGTGTATATATCTAGAG

TAATATTTAATTTATCAAATGGTTGATAATATGGATGTTGTGGCAATTTCCTAATACGGAAATAAGACAT

AAACACGCAATAAATCTAATTGCGGACATGTTACACTCCTTAAAAAATACGAATAAACACTTTGGCTTTT

AGTAAGTGTCATTTAACACTATACTCATATTAATCCATGGACTCATAATCTCTATACGGGATTAACGGAT

GTTCTATATACGGGGATGAGTAGTTCTCTTCTTTAACTTTATACTTTTTACTAATCATATTTAGACTGAT

GTATGGGTAATAGTGTTTGAAGAGCTCGTTCTCATCATCAGAATAAATCAATATCTCTGTTTTTTTGTTA

TACAGATGTATTACAGCCTCATATATTACGTAATAGAACGTGTAATCTACCTTATTAACTTTCACCGCAT

AGTTGTTTGCAAATACGGTTAATCCTTTGACCTCGTCGATTTCCGACCAATCTGGGCGTATAATGAATCT

TAACTTTAATTTCTTGTAATCATTCGAAATAATTTTTAGTTTGCATCCGTAGTTATCTCCTCTATGTAAC

TGTAAATTTCTCAACGCGATATCTCCATTAATAATGATGTCGAATTCGTGTTGTATACCCATACTGAATT

GATGAACTAATACCGACGGTATTAATAGTAATTTACTTTTCATCTTTACATACTTGGTAATAGTTTTACT

ATCATAAGTTTATAAATTCCACAAGCTACTATGGAATATACCAACCATCTTAGTATAGAACACATGTCTT

AAAGTTATTAATTAATTACATGTTGTTTTATATATCGCTACGAATTTAAACAGAGGAATCAGTTAGGAAG

AAAAAATTATCTGTCATCATCATCATCATCTATTGGATAACATCTCTGTATTCTACGATAGAGTGCTATT

TTAAGATGTGACAGATCCGTGTCATCAAATATATACTCCATTAAAATGATTATTCCGGCAGCGAACTTGA

TATTGGATACATCACGACCTTTGTTAATATCCACGACAATAGACAGCAATCCCATGGTTCCATAAACAGT

GAGTTTATCTTTCTTTGAAGTGATATTTTGTAGAGATCTTATAAAACTGTCGAATGACATCGTATTTATA

TCTTTAGCTAAATCATATATGTTACCATCATAATATCTAACAGCATCTATCTTAAACGTTTCCATCGCTG

TAAAGACGTTTCCGATAGATGGTCTCGTTTCATCAGTCATACTGAGCCAACAAATGTAATCGTGTATAAC

ATCTTTGATAGAATCAGACTCTAAAGAAAAGGAATCGGCTTTATTATACACATTCATGATAAACTTAATG

AAAAATGTTTTTCGTTGTTTAAGTTGGATGAATAGTATGTCTTAATAATTGTTATTATTTCACTAATTAA

TATTTAGTAACGAGTACACTCTATAAAAACGAGAATGACATAACTAATCATAACTAGTTATCAAAGAATG

TCTAGGACGCGTAATTTTTTATGGTATAGATCCTGTAAGCGTTGTCTGTATTCTGGAGCTATTTTCTCTA

TCGCATTAGTGAGTTCAGAATATGTTATAAATTTAAATCGAATAACGAACATAACTTTAGTAAAGTCGTC

TATATTAACTCTTTTATTTTCTAGCCATCGTAATACCATGTTTAAGATAGTATATTCTCTAGTTACTACG

ATCTCATCGTTGTCTAGAATATCGCATACTGAATCTACATCCAATTTTAGAAATTGGTCTGTGCTACATA

TCTCTTCTATATTATTGTTGATGTATTGTCGTAGAAAACTATTACGTAGACCATTTTCTTTATAAAACGA

ATATATAGTACTCCAATTATCTTTACCGATATATTTGCATACATAATCCATTCTCTCAATCACTACATCT

TTAAGAGTTTGGTTGTTAAGATATTTGGCTAAACTATATAATTCTATTAGATCATCAACAGAATCAGTAT

ATATTTTTCTAGATCCAAAGATGAACTCTTTGGCATCCTCTATAATATTATCAGAAAAGATATTTTCGTG

TTTTAGTTTATCAAGATTTAACCTGTTCATATCCATGATTAACGACGTCATATAACCACATAAAATAAAA

ATCCATTTTCATTTTTAGCACAATACTATTCATAATTGATATTGATGTAATATTTTGTTACTTTGAACGT

AAAGACAGTACACGGGTCCGTATCTCCAACAAGCACGTAGTAATCAAATTTGGTGTTGTTAAACTTCGCA

ATATTCATCAATTTAGATAGAAACTTATACTCATCATCTGTTTTAGGAATCCATGTATTATTACCACTTT

CCAACTTATCATTATCCCAGGCTATGTTTCGCCCATCATCGTTGCATAGAGTGAATAATTCTTTTGTATT

CGGTAGTTCAAATATATGATCCATGCATATATCGACAAAGCTATTGTAGATGTGATTTTTCCTAAATCTA

ATATAAAACTCGTTTACTAGCAAACATTTTCCTGATTTATCGACCAAGACACACATGGTTTCTAAATCTA

TCAAGTGGTGGGGATCCATAGTTATAACGCAGTAACATAGATTATTACCTTCTTGACTGTCGCTAATATC

TATATACTTATTGTTATCGTATTGGATTCTACATATAGATGGCTTGTATATCAAAGATATAGAACACATA

ACCAATTTATATTCTCGCTTTGTATTTTCGAATCTAAAGTTAAGAGATTTAGAAAACATTATATCATCGG

ATGATGATATCACTGTTTCCAGAGTAGGATATATTAAAGTCTTTAAAGATTTTGTCCGATTCAAATAAAT

CACTAAATAATATCCCATATTATCATCTGTTATAGTCGTGTCATTAAATCTATTATATTTTATGAAAGAT

ATATCACTGCTCACCTCTATATTTCGTACATTTTTAAACTGTTTGTATAATATCTCTCTAATACAATCAG

ATATATCTATTGTGTCGGTAGACGATACCGTTACATTTGAATTAATGGTGTTCCATTTTACAACTTTTAA

CAAGTTGACCAATTCATTTCTAATAGTATCAAACTCTCCATGATTAAATATTTTAATAGTATCCATTTTA

TATCACTACGGACATAAACCATTGTATAATTTTTATGTTTATTAGTGTACACATTTTGGAAGTAAGTTCC

TGGATCGGATGTCACCGCAGTAATATTGTTGATTATTTCTGACATCGACGTATTATATAGTTTTTTAATT

CCATATCTTTTAGAAAAGTTAAACATCCTTATACAATTTGTGGAATTAATATTATGAATCATGGTTTTTA

CACATAGATCTACTACAGGCGGAACATCAATTATTATGGCAGCAACTAGTATCATTTTCTACATTGTTTA

TGGTGATGTTTATCTTCTTCCAGCGCATATAGTCTAATATCGATTCAAACGCGAGATAGTTTATACCATT

CAATATAATCGCTTCATCCTTTAGATGGTGATCCTGAATGTGTTTAAAAAATTATACGGAGACGCCGTAA

TAATTTCCCCATTGATAGAAAATATCACGCTTTCCATTTTCTTGAAGTACTATAAGTAATTATAATATAA

TGAATGTAAAAAGGTTTATATATTCAATATTTTTTATAAAAAAAAAATCATTTCGACATTAATTCCTTTT

TAAATTTCAGTCTATCATCTATAGAAACATATTCTATGAATTTATAAAATGCTTTTACGTATCCTATCGT

AGGTGATAGAACCGCTAAAAAACCTATCGAATTTCTACAAAAGAATCTGTTATATGGTATAGGGAGAGTA

TAAAACATTAAATGTCCGTACTTATTAAAGTATTCAGTAGCCAATCCTAACTCTTTCGAATAATTATTAA

TGGCTCTTATTCTGTACGAATCTATTTTTTTGAACAATGGACCTAGTGGTATATCTTGTTCTATGTATCT

AAAATAATGTCTGACTAGATCCGTTAGTTTAATATCCGCAGTCATCTTGTCTAGAATGGCAAATCTAACT

GCGGGTTTAGGCGTAGGCGTTAGTTTAGTTTCTATATCTACATCTATGTCTTTATCTAACACCAAAAATA

TAATAGCTAATATTTTATTACAATCATCCGGATATTCTTCTACGATCTCACTAACTAATGTTTCTTTGGT

TATACTAGTATAGTCACGATCAGACAAATAAAGAAAATCAGATGATCGATGAATAATACATTTAAATTCA

TCATCTGTAAGATTTTTGAGATGTCTCATTAAAATATTATTAGTGTCAGTTCTCATTATCATATATTGAC

AGCAGCTATTACACTTATTTTATTTTTCTGTATTTTATTACTTTTCACCATATAGATCAGTCATTAGATC

ATCAAAATACTTTTCAATCATCCTAAAGAGTATGGTGAACGAATCTTCCCATCTAATTTCTGAACGTCTA

CCAATGTCTCTAGCCACTTTGGCACTAATAGCGATCATTCGCTTAACATCTTCTACATTATTAACTGGTT

GATTCAATCTATCTAGCAATGGACCGTCGGATAGCGTCATTCTCATGTTCTTAATCAATGTACATACATC

GTCATCATCTACCAATTCATCAAACAATATAAGCTTTTTAAAATCATCATTATAATAGGATGGATCGCTG

TCATTTCTCCAAAGAATATATCTAATAAGTAGAGTCCTCATGCTTAGTAATTTAACTATTTTAGTTAACA

ACTATTTTTTATGTTAAATCAATTAGTAACACCGCTATGTTTAATACTTATTCATATTTTAGTTTTAGGA

TCGAGAATCAATACAAAAATTAATACATCAATTTTGGAAATACTTAGTTTCCACGTAGTCAATGAAACAT

TTGAGCTCATCGTAAAGGACGTTCTCGTACAAGACGTAACTATAAATTGGTTTATATTTGTTCAAGATAG

ATACAAATCCGATAACTTTTTTGACGAATTCTACGGGATTCACTTTAAAAGTGTCATACCGGGTTCTTTT

TATTCTTTTAAACAGATCGATTGTGTGATGTTGATTAGGTCTTTTACGAATTTGATACAGAATAGCGTTT

ACATATCCACCATAGTAATCAATAGCCATTTGTTCGTAtgtcataaattctttaattATATGACACTGTG

TATTATTTAGTTCGTCCTTGTTCATCATTAGGAATCTATCCAATATGGCAATTATATTAGAACTATAACT

GCGTTGTATGCGCATGTTGATGTGTCTGTTTATACAATCAATTATACTAGGATCCATACCACTACATTCG

GGTAAAATTGTAGCATCATATACCATTTCTAGTACTTTAGATTCATTGTTATCCATTGCAGAGGACGTCA

TGATCGAATCCAAAAAAATATATTATTTTTATGTTATTTTGTTAAAATAATCATCAAATACTTCGTAAGA

TACTCCTTCATGAACATAATCAGTTACAAAACGTTTATATGAAGTAAAGTATCTACAATTTTTACAAAAG

TCAGGATGCATAAGTACAAAGTACGCGATAAACGGAATAATAATAGATTTATCTAGTTTATCTTTTTCTA

TCTCTTTCATAGTTATATACATGGTCTCAGAAGTCGGATTATGTAACATCAGCTTCGATAAAATGACTGG

GTTATTTAGTCTTACACATTCGCTCATACATGTATGACCGTTAACTATAGAGTCTACACTAAAATGATTG

AATAATAGATAGTCTACCATTGTTTCGTATTCAGATAGTACAGCGTAGTACATGGCATCTTCACAAATTA

TATCATTATCTAATAGATATTTGACGCATCTTATGGATCCCACTTCAACAGCCATCTTAAAATCGGTAGA

ATCATATTGCTTTCCTTTATCGTTAATAATTTCTAGAACATCATCTCTATCATAAAAGATACAAATATTA

ACTGTTTGATCAGTAATAACATTGCTAGTCGATATCAATTTGTTAATAAGATGCGCTGGGCTCAATGTCT

TAATAAGAAGTGTAAGAGGACTATCTCCGAATTTGTTTTGTTTATTAACATCCGTTGATGGAAGTAAAAG

ATTTATAATGTCTACATACTTGACTGTTTTAGAGCATACAATATGGAGAGGCGTATTTCCATCATGATCT

GGTTTTGAGGGACTAATTCCTAGTTTCATCATCCATGAGATTGTAGAAGCTTTTGGATTGTCTGACATAA

GATGTCTATGAATATGATTTTTGCCAAATTTATCCACTATCCTGGCTTCGAATCCGATAGACATTATTTT

TTTAAACACTCTTTCTGAAGGATCTGTACACGCCAACAACGGACCACATCCTTCTTCATCAACCGAGTTG

TTAATCTTGGCTCCATACTGTACCAATAAATTTATTCTCTCTATGACTTCATCATCTGTTCCCGAGAGAT

AATATAGAGGTGTTTTATTATGTTTATCACATGCGTTTGGATCTGCGCCGTGCACCAGCAGCATCGCGAC

TATTCTATTATTATTAATTTTAGAAGCTATATGCAATGGATAATTTCCATCATCATCCGTCTCATTTGGA

GAGTATCCTCTATGAAGAAGTTCTTCTATAAATCGTTCATCTAGTCCTTTAATACCACAATACGCATGTA

GAATGTGATAATTTCCAGAGGGTTCGATAACTTGTAGCATATTCCTAAATACATCTAAATTTTTACTATT

ATATTTGGCATAAAGAGATAGATAATACTCGACCGACATAATGTTGTGTTGTCCATTATAGTATAAAAAT

TAATATTTCTATTTCTATTTCTATATATTTGCAACAATTTACTCTCTATAACAAATATCATAACTTAGTT

CTTTTATGTCAAGAAGGCACTGGTTTAATTCATCTATAAATGTCACGCCATAACTACCACGCATACTATA

CTCAGAATTATGATAAAGATATTTATTCTTGGGGTGTAAGTAATGGGGATTAATCTTTGTTGGATCAGTC

TCTAAGTTAACACATGTCACACATGATCCATTTATAGTTATATCACACGATGATGATTTATGAATTGATT

CCGGAAGATCGCTATTGTATTTTGTAGTTCCACAATTCATTTCCATACATGTTATTGTCACACTAATATT

ATGATGAACTTTATCTAGCCGCTGAGTGGTAAACAACAGAACAGATAGTTTATTATCTTTACCAACACCC

TCAGCCGCTGCCACAAATCTCTGATCCGTATCCATGATGGTCATGTTTACTTTTAGTCCGTATCCAGTCA

ACACTATGTTAGCATTTCTGTCGATATAGCTTTCACTCATATGACACTCACCAATAATTGTAGAATTAAT

GTCGTAATTTACACCAATAGTGAGTTCGGCGACAAAGTACCAGTACCGGTAATCTTGTCGATGAGGACAT

ATAGTATTCTTGTATTCTACCGAATACCCGAGAGATGCGATACAAAAGAGTAAGACTAATTTGTAAACCA

TCTTACTCAAAAATATGCGACAATAGGAAATTTATCTTATACACATAATTATTCTATCAATTTTACCAAT

TAGTTAGTGTAATGTTAACAAAAATGTGGGATAATTTAATAGTTTTTCCTTACACAATTGACATACATGA

GTCTGAGTTCCTCGTTTTTGCTAATTATTTCGTCCAATTTATTATTCTTGACATCGTCAAGATCTTTTGT

ATAGGAGTCAGACTTGTATTCAACATGTTTTTCTATAATCATCTTAGCTATTTCGGCATCATCCAATAGT

ACATTTTCCAGATTAACAGAATAGATATTAATGTCGTATTTGAACAGAGCCTGTAACATCTCAATGTCTT

TATTATCTATAGCCAATTTGATGTCCGGAATGAAGAGAAGGGAATTGGTGTTTGTCGACGTCATATAGTC

GAGCAAGAGAATCATCATATCCACGTGTCCATTTTTTATAGTGGTGTGAATACAACTAAGGAGAATAGCC

AGATCAAAAGGAGATGGTATCTCTGAAAGAAAGTAGGAAACAATACTTACATCATTAAGCATGACAGCAT

GATAAAATGAAGTTTTCCATCCAGTTTTTCCATAGAACATCAGTCTCCAATTTTTCTTAACAAACAGTTT

TACCGTTTGCATGTTACCACTATCAACCGCATAATACAATGCGGTGTTTCCTTTGTCATCAAATTGTGAA

TCATCCATTCCACTGAATAGCAAAATCTTTACTATTTTGGTATCTTCTAATGTGGCTGCCTGATGTAATG

GAAATTCATTATCTAGAAGATTTTTCAATGCTCCAGCATTCAACAACGTACATACTAGACGCACGTTATT

ATCAGCTATTGCATAATACAAGACACTATGACCATTGATATCCGCCTTAAATGCATCTTTGCTAGAGAGA

AAGCTTTTCAGTTGCTTAGACTTCCAAGTATTAATTCGTGACAGATCCATGTCTGAAACGAGACGCTAAT

TAGTGTATATTTTTTCATTTTTTATAATTTTGTCATATTGTACCAGAATTAATAATATCTCTAATAGATC

TGATTAGTAGATACATGGCTATCAAACAACATATACACATTTAATAAAAATAATATTCATTAAGAAGATT

CAGATTCCACTGTACCCATCAATATAAATAAAATAATTATTCCTTACATCGTACCCATCAATATAAATAA

AATAATTATTCCTTACATCGTACCCATAAACAATATATTAAGTAGATTCCACCTTACCCATAAACAATAT

AAATCCAGTAATATCATGTCTAATGATGAACACAAATGGTGTATTAAATTCCAGTTCTTCAGGAGATGAT

CTCGCCGTAGCTACCATGATAGTAGATGCCTCCGCTACAGTTCCTTGTTCGTCTACATCTATCTTTACAT

TCTGAAACATTTTATAAATATATAATGGGTCCCTAGTCATATGTTTAAACGACGCCTTATCTGGATTAAA

CATACTAGGAGCCATCATTTCGGCTATCGACTTAATATCCCTCTTGTTTTCGATAGAAAATCTAGGGAGT

TTAAGATTGTACATTTTATTCCCTAATTGAGATGACCAATATTCTAATTTTGCAGCCGTGATAGAATCTG

TGAAATGGGTCATATTATCACCTATTGCCAGGTACATACTAATATTAGCATCCTTATACAGAAGGCGCAC

CATATCATATTCTTCGTCATCGATTGTGATTGTATTTCCTTGCAATTTAGTAACTACGTTCATCATGGGA

ACCGTTTTCGTACCGTACTTATTAGTAAAACTAGCATTGTGTGTTTTAGTGATATCAAACGGATATTGCC

ACGTACCTTTAAAATATATAGTATTAATGATTGTCCATAGAGTATTATCGTCGAGCATAGTAGAATCAAC

TACATTAGACATACCAGATCTACGTTCTACTATAGAATTAATTTTATTAACCGCATCTCGTCTAAAGTTT

AATCTATATAGGCCGAATCTATGATATTGTTGATAATACGACGGTTTAATACACACAGTACTATCGACGA

AACTTTGATACGTTAGATCGGTGTACGTATATTTAGATGTTTTCATCTTAGCTAATCCTGATATTAATTC

TGTAAATGCTGGACCCAGATCTCTTTTTCTCAAATTCATAGTATTCAATAATTCTACTCTAGTATTACCT

GATGCAGACAATAGCGACATAAACATAGAAAACGAATACCCAAACGGTGAGAAGACAATATTATCATTAT

CATCATCCCCATTTTGAATATTTTTATACGCTAATATACCGGCATTGATAAATCCCTGCAGACGATATGC

GGATACTGAACACGCTAATGATAGTATCAATAACGCAATCATGATTTTTATGGTATTAATAATTAACCTT

ATTTTTATGTTTGGTATAAAAATTATTGATGTCTACACATCCTTTATAATCAACTCTAATCACTTTAACT

TTTACAGTTTTCCCTACAAGTTTATCCCTATATTCAACATATCTATCCATATGCATCTCTTAACACTCTG

CCAAGATAGCTTCAGAGTGAGGATAGTCAAAAAGATAAATATATAGAGTATAATCATTCTCGTATACTCT

GCCCTTTATTACATCGCCCGCATTGGGCAACGAATAACAAAATGCAAGCATCTTGTTAACGGGCTCGTAA

ATTGGGATAAAATTATGTTTTTATTGTTTATCTATTTTATTCAAGAGAATATTCAGGAAGTTCCTTTTCT

GGTTGTATCTCGTCGCAGTATATATCATTTGTACATTGTTTCATATTTTTTAATAGTCTACACCTTTTAG

TAGGACTAGTATCGTACAATTCATAGCTGTATTTTGAATTCCAATCACGTATAAAAATATCTTCCAATTG

TTGACGAAGACCTAATCCATCATCCGGTGTAATATTAATAGATGCTCCACATATATCCGTAAAGTAATTT

CCTGTCCAATTTGATGTACCTATATACGCCGTTTTATCGGTTACCATATATTTTGCATGGTTTACCCTAG

AATACGGAATGGGAGGATCAGCATCTGGTACAATAAATAGCTTTACTTCTATATCTATGTTTTTAGATTT

TAGCATAGCTATAGATCTTAAAAAGTTTCTCATGATAAACGAAGATCGTTGCCAGCAACTAATCAATAGC

TTAACGGATACTTGTCTGTCTATAGCGGATCTTCTTAATTCATCTTCTATATAAGGCCAAAACAAAATTT

TACCCGCCTTTGAATAAATAATAGGAATAAAGTTCATAACAGATACATAAACGAATTTACTCGCATTTCC

GATACATGACAATAAAGCGGTTAAATCATTGGTTCTTTCCATAGTACATAATTGTTGTGGTGCAGAAGCA

ATAAATACAGAGTGTGGAACACCGCTTACGTTAATACTAAGAGGATGATCTGTATTATAATACGACGGAT

AAAAGTTTTTCCAATTATATGGTAGATTGTTAACTCCAAGATACCAGTATACCTCAAAAATTTGAGTGAG

ATCCGCTGCCAAGTTCCTATTATTGAAGATCGCAATACCCAATTCCTTGACCTGAGTTAGTGATCTCCAA

TCCATGTTAGCGCTTCCTAAATAAATATGTGTATTATCAGATATCCAAAATTTTGTATGAAGAACTCCTC

CTAGGATATTTGTAATATCTATGTATCGTACTTCAACTCCGGCCATTTGTAGTCTTTCAACATCCTTTAA

TGGTTTGTTGGATTTATTGACGGCTACTCTAACTCTTACTCCTCTTTTGGGTAATTGTACAATCTCGTTT

AATATTACCGTGCCGAAATTCGTACCCACTTCATCCGATAAACTCCAATAAAAAGATGATATATCTAGTG

TTTTTATGGTATTGGATAGAATTTCCCTCCACATGTTAAATGTAGTCAAATATACTTTATCAAATTGCAT

ACCTATAGGAATAGTCTCTGTAATCACTGCGATTGTATTATCCGGATTCATTTTATTTGTTAAAAAAATA

ATCCTATATCACTTCACTCTATTAAAAATCCAAGTTTCTATTTCTTTCATGACTGATTTTTTAACTTCAT

CCGTTTCCTTATGAAGATGATGTTTGGCACCTTCATAAATTTTTATTTCCCTATTACAATTTGCATGTTG

CATGAAATAATATGCACCTGAAACATCGCTAATCTCATTGTTTGTTCCCTGGAGTATGAGAGTCGGGGTG

TTAATCTTGGGAATTATTTTTCTAACCTTGTTGGTAGCCTTCAAGACCTGACTAGCAAATCCAGCCTTAA

TTTTTTCATGATTGACTAATGGATCGTATTGGTATTTATAAACTTCATCCATATCTCTAGATACTGATTC

TGGACATAGCTTTCCGACTGACGCATTTGGTGTAATGGTTCCCATAAGTTTTGCAGCTAGCAGATTCAGT

CTTGGAACAGCGTCTGCATTAACTAGAGGAGACATTAGAATCATTGCTGTAAACAAGTTTGGATTATCGC

AAGCAGCTAGTATAGAAATTGTTGCTCCCATGGAATGACCCAATAAGAAGACTGGAACTCCTGGATAAGT

AGATTTAATAGTCACCACGTGCTGTACCACATCTCTAACATACTTACCAAAGTCATCAATCATCATTTTT

TCACCATTACTTCTTCCATGGCCAATATGATCATGTGAGAATACTAAAATTCCTAACGATGATATGTTTT

CAGCTAGTTCGTCATAACGTCCAGAATGTTCACCAGCTCCATGACTTATGAATACTAATGCCTTAGGATA

TGTAATAGGTTTCCAATATTTACAATATATGTAATCATTGTCCAGATTGAACATACAGTTTGTACTCATG

ATTCACTATATAACTATCAATATTAACAGTTCGTTTAATGATCATATTATTTTTATGTTTTATTGATAAT

TGTAAAAATATACAATTAAATCAATATAGAGGAAGGAGACGGTACTGTATTTTGTGAGATAGTCATGGAG

ACTAAATCAGATTATGAGGATGCTGTTTTTTACTTTGTGGATGATGATGAAATATGTAGTCGCGACTCCA

TCATTGATCTAATAGATGAATATATCACGTGGAGAAATCATGTTATAGTGTTTAATAAAGATATTACCAG

TTGTGGAAGACTGTACAAGGAATTGATAAAGTTCGATGATGCCGCTATACGGTACTATGGTATTGATAAA

ATTAATGAGATTGTCGAGGCTATGAGTGAAGGAGACCACTACATCAATCTTACAGAAGTCCATGATCAGG

AAAGTCTATTCGCTACTATAGGAATATGTGCTAAAATCACTGAACATTGGGGATACAAAAAGATTTCAGA

ATCTAAATTCCAATCATTGGGAAACATTACAGATCTGATGACCGACGATAATATAAACATCTTGATACTT

TTTCTAGAAAAAAAAATGAATTGATGATATAAGTGTCTTCATAACGCATTATTACGTTAGCATTCTATTA

TCCAGTGTTAAAAAAATTATCCTATCATGTATTTGAGAGTCTTATATGTAGCAAACATGATAACTGCAAT

ACCCATAATCTTTAGATATTCACGCGTGCTATGGATGGCATTATCCCGCGGTGCGGAAATGTACGTTATA

TAATCTACAAAATAATCATCGCATATAGTATGAGATAGTAGAGTAAACATTTTTATCGTTTCTACTGGGT

TCATACATCGTCTACCCAATTCGGTAATGAATGAAATTGTCGCCAATCTTACACCCAAACCCTTGTTGTT

CATTAGTATAGTATTAACTTCATTATTTATGTCATAAACTGTAAATGATTCTGTAGATGCCATATCACAC

ATGATATTCATGTCACTATTATAATCATTATTAACTTTATCACAATACGTGTTGATAATATCTACATATG

ATCTAGTTTTTGTGGGTAATTGTCTATACAAGTCGTCTAAACGTTGTTTACTCATATAGTATTGAACAGC

CATCATTACATGGTCCCGTTCCGTTGATAGATAATCGAGTATGTTAGTAGACTTGTCAAATCTATATACC

ATATTTTCTGGAAGCGGATATACATAGTCGCGATCATCATTATCACTAGCCTCATCATCTATATCATGTA

CATGTACATAATCTATGATATTATTATACATAAACATCGACAACATACTATTGTCTATTATCTAAGTCCT

GTTGATCCAAACCCTTGATCTCCTCTATCTGTACTATCTAGAGATTGTACTTCTTCAAGTTCTGGATAAT

ATATACGTTGATAGATTAGCTGAGCTATTCTATCTCCAGTATTTACATTAAACGTACATTTTCCATTATT

AATAAGAATGACTCCTATGTTTCCCCTATAATCTTCGTCTATTACACCGCCTCCTATATCAATGCCTTTT

AGGGACAGACCAGACCTAGGAGCTATTCTACCATAGCAGAACTTAGGCATGGACATACTAATATCTGTCT

TAATTAACTGTCGTTCTCCAGGAGGGATAGTATAATCGTAAGCGCTATACAAATCATATCCGGCAGCACC

CGGCGATTGCCTAGTAGGCGATTTAGCTCTGTTAGTTTCCTTAACAAATCTAACTGGTGAGTTAATATTC

ATGTTGAACATAAAAAATATCATTTTATTTCAAAATTATTTACCATTCCATTCCATTCCATTCCATTCCA

TTCCATTCCATTCCATTCCATCCCATATATTCCATGAATAAGTGCGATTATTGTACACTTCTATA

GTATCTATATACGATCCACGATAAAATCCTCCTATCAATAGCAGTTTATTATCCACTATGATCAATTCTG

GATTATCCCTCGGATAAATAGGATCATCTATCAGAGTCCATGTATTGCTGGATTCACAATAAAATTCCGC

ATTTCTACCAACCAAGAATAACCTTCTACCAAACACTAACGCACATGATTTATAATGAGGATAATAAGTG

GATGGTCCAAACTGCCACTGATCATGATTGGGTAGCAAATATTCTGTAGTTGTATCAGTTTCAGAATGTC

CTCCCATTACGTATATAACATTGTTTATGGATGCCACTGCTGGATTACATCTAGGTTTCAGAAGACTCGG

CATATTAACCCAAGCAGCATCCCCGTGGAACCAACGCTCAACAGATGTGGGATTTGGTAGACCTCCTACT

ACGTATAATTTATTGTTAGCGGGTATCCCGCTAGCATACAGTCTGGGGCTATTCATCGGAGGAATTGGAA

TCCAATTGTTTGATATATAATTTACCGCTATAGCATTGTTATGTATTTCATTGTTCATCCATCCACCGAT

GAGATATACTACTTCTCCAACATGAGTACTTGTACACATATGGAATATATCTATAATTTGATCCATGTTC

ATAGGATACTCTATGAATGGATACTTGTATGATTTGCGTGGTTGTTTATCACAATGAAATATTTTGTTAC

AGTTTAGTATCCATTTTACATTATGTATACCTCTGGGAGAAAGATAATTTGACCTGATTACATTTTTGAT

AAGAAGTAGCAGATTTCCTAATCTATTTCTTCGCCTCATATACCACTTAATGACAAAATCAACTACATAA

TCCTCATCTGGAACATTTAGTTCGTCGCTTTCTAGAATAAGTTTCATAGATATATAATCAAAATTGTCTA

TGATGTCATCTTCCAGTTCCAAAAAGTGTTTGGTAATAAAGTCTTTAGTATGACATAAGAGATTGGATAG

TCCGTATTCTATACCCATCATGTAACACTCGATACAATATTCCTTTCTAAAATCTCGTAGGATAAAGTTT

ATACAAGTGTAGATGATAAATTCTACAGATGTTAATATAGAAGCACGTAATAAATTGACGACGTTATGAC

TATCTATATATACCTTTCCAGTATATGAGTAAATAACTATAGAAGTTAGACTGTGAATGTCAAGGTCTAG

ACAAACCCTCGTAACTGGATCTTTATTTTTTGTGTATTTTTGACGTAAATGTGTGCGAAAGTATGGAGAT

AACTTTTTCAATATCGTAGAATTGACTATTATATTACCTCCTATAGCTTCAATAATTGTTTTGAATTTCT

TAGTCGTGTACAATGCTAATATATTCTTACAGTACACAGTATTGACAAATATCGGCATTTATGTTTCTTT

AAAAGTCAACATCTAAAGAAAAATGATTGTCTTCTTGAGACATAACTCCCATTTTTTGGTATTCACCCAC

ACGTTTTTCGAAAAAATTAGTTTTTCCTTCCAATGATATATTTTCCATGAAATCAAACGGATTGGTAACA

TTGTAAATTTTTTTAAATCCCAATTCAGAAATCAATCTATCTGCGACGAATTCTATATATGTTTTCATCA

TTTCACAATTCATTCCTATGAGTTTAACTGGAAGAGCAACAGTAAGAAATTCTTGTTCAATGGATACCGC

ATTTGTTATAATAAATCTAACGGTTTCTTCACTCGGTGGATGCAATAAATGTTTAAACATCAAACATGCG

AAATCGCAGTGCAGACCCTCGTCTCTACTAATTAATTCGTTAGAAAACGTGAGTCCGGGCATTAGGCCAC

GCTTTTTAAGCCAAAATATGGAAGCGAATGATCCGGAAAAGAAGATTCCTTCTACTGCAGCAAAGGCAAT

AAGTCTCTCTCCATAACCGGCGCTGTCATGTATCCACTTTTGAGCCCAATCGGCCTTCTTTTTTACACAA

GGCATCGTTTCTATGGCATTAAAGAGATAGTTTTTTTCATTACTATCTTTAACATAAGTATCGATCAAAA

GACTATACATTTCCGAATGAATGTTTTCAATGGCCATCTGAAATCCGTAGAAACATCTAGCCTCGGTAAT

CTGCACTTCTGTACAAAATCGTTCTGCTAAATTTTCATTCACTATTCCGTCACTGGCTGCAAAAAACGCC

AATACATGTTTTATAAAATATTTTTCGTCTGGTGTTAGTTTATTCCAGTCATTGATATCTTTAGATATAT

CCACTTCTTCCACTGTCCAAAATGATGCCTCTGCCTTTTTATACATATTCCAGATGTCATGATATTGGAT

TGGGAAAATAACAAATCTATTTGGATTTGGTGCAAGGATAGGTTCCATAACTAAATTAACAATAGTAGTA

ATTTTTTTTCAGTTATCTGTATGACTGTACTTGGATCTTTTGTATATCGCTATCGCCGCAATCACTACAA

TAATTACAAGTATTATTGATAGCATTGTTATTACTACTATCATAATTAAATTATCGACATTCATGGATGT

TGAATAATCGTTATCATCATTTTGTAATTGTGACATCATACTAGATAAATCATTTGTGAGATTGTTGTGG

GAAGCGGGCACGGAAGATGCATTATCATTATTATTTAACGCCTCCCATTCGGATTCACAAATGTTACGCA

CGTTCAACGTTTTATGGAAACTATAATTTTGTGAAAACAGATAACAAGAAAACTCGTCATCGTTCAAATT

TTTAACGATAGTAAACCGATTAAACGTCGAGCTAATTTCTAACGCTAGCGACTCTGTTGGATATGGGTTT

CCAGATATATATCTTTTCAGTTCCCCTACGTATCTATAATCATCTGTAGGAAATGGAAGATATTTCCATT

TATCTACTGTTCCTAATATCATATGCGGTGGTGTAGAACCATTAAGCGCGAAAGATGTTATTTCGCATCG

TATTTTAACTTCGCAATAATTTCTGGTTAGATAACGCACTCTACCAGTCAAGTCAATGATATTAGCCTTT

ACAGATATATTCATAGTAGTCGTAACGATGACTCCATCTTTTAGATGTGATACTCCTTTGTATGTACCAG

AATCTTCGTACCTCAAACTCGATATATTTAAACAAGTTAATGATATATTAACGCGTTTTATGAATGATGA

TATATAACCAGAAGTTTTATCCTCTGTGGCTAGCGCTATAACCTTATCATTATAATACCAACTAGTGTAA

TTAATATGTGACACGACAGTGTGGGTACAAATATGTACATTATCGTCTACGTCGTATTTGATACATCCGC

ATTCAGCCAACAAATATAAAATTACAAAAACTCTAACGACGTTCGTACACATCTTGATGCGGTTTAATAA

ATGTTTTGATTTCAATTTATTGTAAAAAAGATTCGGTTTTATACTGTTCGATATTCTCATTGCTTATATT

CTCATCTATCATCTCCACACAGTCAAATCCATGGTTAACATGTACCTCATCAACCGGTAAAAGACTATCG

GATTCTTCTATCATCATAACTCGAGAATATTTAATTTGGTGGTCATTATTAATCAAGTCAATTATCTTAT

TTTTAACAAACGTAAGTATTTTACTCATTTTTTATAAAAACTTTTAGAAATATACAGACTCTATCGTGTG

TCTATATCTTCTTTTTATATCCAATGTATTTATGTCTGATTTTTCTTCATTTATCATATATAATGGTCCA

AATTCTACACGTGCTTCGGATTCATCCAGATCATTAAGGTTCTTATAATCGCAACATCCTTCTCTTCCAT

CTTCTACATCTTCCTTCTTATTCTTAGCGTCACAGAATCTACCACAGCAGGATCCCATGACGAGAGTCAC

ATTAAACTAATTCATTTTCAATTATAATATACTGATTAGTAATGACAATTAAAATAAAAATATTCTTCAT

AACCGGTAAGAAAGTAAAAAGTTCACATTGAAACTATGTCAGTAGTTATACATCATGAGATGATATACTC

TATTTTGGTGGAGGATTATATGATATAATTCGTGGATAATCATTCTTAAGACACATTTCTTCATTCGTAA

ATCTTTTCACATTAAATGAGTGTCCATATTTTGCAATTTCTTCATATGATGGCGGTGTACGTGGACGAGG

CTGCTCCTGTTCTTGTAGTCGCTGACTGTCGTGTTTGCGTTTAGATCCCTCCATTATCGCGATTGCGTAG

TGAGTACTATTTATACCTTGTAATTAAATTTTTTTATTAATTAAACGTATAAAAACGTTCCGTATCTGTA

TTTAAGAGCCAGATTTCGTCTAATAGAACAAATAGCTACAGTAAAAATAACTAGAATAATCGCTACACCC

ACTAGAAACCACGGATCGTAATACGGCAATCGGTTTTCGATAATAGGTGGAACGTATATTTTATTTAAGG

ACTTAACAATTGTCTGTAAACCACAATTTGCTTCCGCCGATCCTGTATTAACTATCTGTAAAAGCATATG

TTGGCCGGGAGGAGCCGAACATTCTCCGATATTCAATTTTTGTATATCTATAATGTTATTAACCTCCGCA

TACGCATTACAGTTCTTTTCTAGCTTGGATACTACACTAGGTACATCATCTAAATCTATTCCTATTTCCT

CAGCGATAGCTCTTCTATCCTTTTCCGGAAGTAATGAAATCACTTCAATAAATGATTCAACCATGAGTGT

GAAACTAAGTCGAGAATTACTCATGCATTTGTTAGTTATTCGGAGCGCGCAATTTTTAAACTGTCCTATA

ACCTCTCCTATATGAATAGCACAAGTGACATTAGTAGGGATAGAATGTTGAGCTAATTTTTGTAAATAAC

TATCTATAAAAAGATTATACAAAGTTTTAAACTCTTTAGTTTCCGCCATTTATCCAGTCTGAGAAAATGT

CTCTCATAATAAATTTTTCCAAGAAACTAATTGGGTGAAGAATGGAAACCTTTAATCTATATTTATCACA

GTCTGTTTTGGTACACATGATGAATTCTTCTAATGCTGTACTAAATTCGATATCTTTTTCGATTTCTGGA

TATGTTTTTAATAAAGTATGAACAAAGAAATGGAAATCGTAATACCAGTTATGTTCAACTTTGAAATTGT

TTTTTATTTTCTTGTTAATGATTCCAGCCACTTGGGAAAAGTCAAAGTCGTTTAATGCCGATTTAATACG

TTCATTAAAAACAAACTTTTTATTCTTTAGATGAATTATTATTGGTTCATTGGAATCAAAAAGTAAGATA

TTATCGGGTTTAAGATCTGCATGTAAAAAGTTGTCACAACAGGGTAGTTCGTAGATTTTAATGTATAACA

GAGACATCTGTAAAAAGATAAACTTTATGTATTGTACCAAAGATTTAAATCCTAATTTGATAGCTAACTC

GGTATCTACTTTATCTGCCGAATACAGTGCTAGGGGAAAAATTATAATATTTCCTCTTTCGTATTCGTAA

TTAGTTCTCTTTTCATGTTCGAAAAAGTGAAACATGCGGTTAAAATAGTTTATAACATTAATATTACTGT

TAATAACTGCCGGATAAAAGTGGGATAGTAATTTCACGAATTTGATACTGTCCTTTCTCTCGTTAAACGC

CTTTAGAAAAACTTTAGAAGAATATCTCAATGAGAGTTCCTGACCATCCATAGTTTGTATCAATAATAGC

AACATATGAAGAACCCGCTTATACAGAGTATGTAAAAATGTTAATTTATAGTTTAATCCCATGGCCCACG

CACACACGATTAATTTTTTTTCATCTCCCTTTAGATTGTTGTATAGAAATTTGGGTACTGTAAACTCCGC

CGTAGTTTCCATGGGACTATATAATTTTGTGGCCTCGAATACAAATTTTACTACATAGTTATCTATCTTA

AAGACTATACCATATCCTCCTGTAGATATGTGATAAAAATCGTCGTTTATAGGATAAAATCGTTTATCTT

TTTGTTGGAAAAAGGATGAATTAATGTAATCATTCTCTTCTATCTTTAGTAGTGTTTCCTTATTAAAATT

CTTAAAATAATTTAACAATCTAACTGATGGAGCCCAATTTTGGTGTAAATCTAATTGGGACATTATGTTG

TTAAAATATAAACAGTCTCCTAATATAACAGTATCTGATAATCTATGGGGAGACATCCATTGATATTCAG

GGGATGAATCATTGGCAACACCCATTTATTGTACAAAAAGCCCCAATTTACAAACGAAAGTCCAGGTTTG

ATAGAGATAAACTATTAACTATTTTGTCTCTGTTTTTAACACCTCCACAGTTTTTAATTTCTTTGGTAAT

GAAATTATTCACAATATCAGTATCTTCTTTATCTACCAGAGATTTTACTAACTTGATAACCTTGGCTGTC

TCATTCAATAGGGTAGTGATATTTGTATGTATGATATTGATATCTTTTTGAATTGTTTCTTTTAGAAGTG

ATTCTTTGATGGTATCAGCATACGAATTACAATAATGCAGAAACTCAGTTAACATGCAGGAATTATAGTA

AGCCAATTCCAATTGTTGCCTGTATTGTATTAGAGTATTAATATGCGCAATGATGTCCTTGCGTTTCTCT

GATAGAATGCGAGCAGCGATTTTGGCGTTATCATTTGACGATATTTCTGGAATGACGAATCCTGTTTCTA

CTAACTTCTTGGTAGGACAAAGTGAAACAATCAAGAAAATAGCTTCTCCTCCTATTTGTGGAAGAAATTG

AACTCCTCTAGATGATCTACTGACGATAGTATCTCCTTGACAGATATTGGACCGAACTACGGAAGTACCT

GGAATGTAAAGCCCTGAAACCCCCTCAttttttaAGCAGATTGTTGCCGTAAATCCTGCACTATGCCCAA

GatagagAGCTCCTTTGGTGAATCCATCACTATGTTTCAGTTTAACCAAGAAACAGTCAGTTGGTCTAAA

ATTTCCATCTCTATCTAATACAGAATCCAACTTGATGTCAGGGACTATGACCGGTTTAATGTTATATGTA

ACATTGAGTAAATCCTTAAGTTCATAATCATCGTTGTCATCAGTTATGTACGATCCAAACAATGTTTCTA

CCGGCATGGTGGATACGAAGATGCTATCCATCAGAATGTTTCCCTGATTAGTATTTTCTATATAGCTATT

CTTCTTTAAACGATTTTCCGAATCAGTAACTATGTTCATTTTTTTAGGAGTAGGACGTCTAGCCAGTATG

GAAGAGGATTTTCTAGATACTCTCTTCAACATCTTTGATCTCAATGGAATGCAAAACCCCATGGTGTAAC

AACCAACGATAAAAATAATATTGTTTTTTCACTTTTTATAATTTTACCATCTGACTCATGGATTCATTAA

TATCTTTACAAGAGCTACTAACGTATAATTCTTTATAACTGAACTGAGATATATACACCGGATCTATGGT

TTCCATAATTGAGTAAATGAATGCTCGGCAATAACTAATGGCAAATGTATAGAACAACGAAATTATACTA

GAGTTGTTAAAGTTAATATTTTCTATGAGTTGTTCCAATAAATTATTTGTTGTGACTGCGTTCAAGTCAT

AAATTATCTTGATACTATCCAGTAAACAGTCTTTAAGTTCTGGAATATTATCATCCCATTGTAAAGCCCC

TAGTTCGACTATCGAATATCCTGCTCTGATAGCAGTTTCAATATCGACGGACGTCAATACTGTAATAAAG

GTGGTAGTATTGTCATCATCGTGATAAACTACGGGAATATGGTCGTTAGTAGGTACCGTGACTTTACACA

ACGCGATATATAACTTTCCTTTTGTACCATTTTTAACGTAGTTGGGACGTCCTGCAGGGTATTGTTTTGA

AGAAATGATATCGAGAACAGATTTGATACGATATTTGTTGGATTCCTGATTATTCACTATAATATAATCT

AGACAGATAGATGATTCGATAAATAGAGAAGGTATATCGTTGGTAGGATAATACATCCCCATTCCAGTAT

TCTCGGATACTCTATTGATGACACTAGTTAAGAACATGTCTTCTATTCTAGAAAACGAAAACATCCTACA

TGGACTCATTAAAACTTCTAACGCTCCTGATTGTGTTTCGAATGCCTCGTACAAGGATTTCAAGGATGCC

ATAGATTCTTTGACCAACGATTTAGTATTGCGTTTAGCATCTGATTTTTTTATTAAATCAAATGGTCGGC

TCTCTGGTTTACTACCCCAATGATAACAATAGTCTTGTAAAGATAAACCGCAAGAAAATTTATACGCATC

CATCCAAATAACCCTAGCACCGTCGGATGATATTAATGTATTATTATAGATTTTCCATCCACAGTTATTG

GGCCAGTATACTGTTAGCAACGGTATATCGAATAGATTACTCATGTAACCTACTAGAATGATAGTTCGTG

TACTAGTCATAATATCTTTAATCCAATCTAAGAAATCTAAAATTAGATCTTTTACACTATTAAAGTTAAC

AAAGGTATTACCCGGGTACGTGGATATCATATATGGTATTGGTCCATTATCAGTAATGGCTCCATAAACT

GATACGGCGATGGTTTTTATATGTGTTTGAACTAATGAGGACGAAATTCGCGCCCACAATTCATCTCTAG

ATATGTATTTAATATCGAACGGTAACACATCAATCTCGGGACGCGTATATGTTTCTAAATTCTTAATCCA

AATATAATGATGACCTATATGCCCTATTATCATACTGTCAACTATAGTATACCTAGAGAACTTTCGATAC

ATCTGCTGTTTCCTGTAATCGTTAAATTTTACAAATCTATAACATGCTAAACCTTTTGACGACAGCCATT

CATTAATTTCTGATATGGAATCTGTATTCTCAATACCGTATCGTTCTAAAGCCAGTGCTATATCTCCCTG

TTCGTGGGAACGCTTTCGTATAATATCGATCAATGGATAATATGAAGTTTTTGGAGAATAATATGATTCA

TGATCTATTTCGTCCATAAACAATCTAGACATAGGAATTGGAGGCGATGATCTTAATTTTGTGCAATGGG

TCAATCCTATAACTTCTAATATTGTAATATTCATCATCGACATAACACTATCTATGTTATCATCGTATAT

TAGTATACCACGACCTTCTTCATTTCGTGCCAAAATGATATACAGTCTTAAATAATTACGCAATATCTCA

ATAGTTTCATAATTGTTAGCTGTTTTCATCAAGGTTTGTATCCTGTTTAACATGATGGCGTTCTATAACG

TCTCTATTTTCTATTTTTAATTTTTTTAAATTTTTAACGATTTACTGTGGCTAGATACCCAATCTCTCTC

AAATATTTTTTTAGCCTCGCTTACAAGCTGTTTATCTATACTATTAAAACTGACGAATCCGTGATTTTGG

TAATGGGTTCCGTCGAAATTTGCCGAAGTGATATGAACATATTCGTCGTCGACTATTAACAATTTTGTAT

TATTCTGAATAGTGAAAACCTTCACAGATAGATCATTTTGAACACACAACGCATCTAGACTTCTGGCGGT

TGCCATAGAATATACGTCGTTCTTATCCCAATTACCAACTAGAAGTCTGATCTTAACTCCTCTATTAATG

GCTGCTTCTATAATGGAGTTGTAAATGTCAGGCCAATAGTAGCTATTACCGTCGACACGTGTAGTGGGAA

CTATGGCCAAATGTTCAATATCTATACTAGTCTTAGCCGACTTGAGTTTATCAATAACTACATCAGTGTC

TAGATCTCTAGAATATCCCAATAGGTGTTCTGGAGAATCAGTAAAGAACACTCCACCTATAGGATTCTTA

ATATGATACGCAGTGCTAACTGGCAGACAACAAGCCGCAGAGCATAAATTCAACCATGAATTTTTTGCGC

TATTAAAGGCTTTAAAAGTATCAAATCTTCTACGAAGATCTGTGGCCAGCGGAGGATAATCAGAATATAC

GCCTAACGTTTTAATCGTATGTATAGATCCTCCAGTAAATGACGCGTTTCCTACATAACATCTTTCATCA

TCAGACACCCAAAAACAACCGAGTAGTAGTCCCACATTATTTTTTTTATCTATATTAACGGTTATAAAAT

TTATATCCGGGGAGTGACTTTGTAGCTCTCCCAGATTTCTTTTCCCTCGTTCATCTAGCAAAACTATTAT

TTTAATCCCTTTTTCAGATACCTCTTTTAGTTTATCAAAAATAAGCGCTCCCCTAGTAGTACTCAGAGGA

TTACAACAAAAAGATGCTATGTATATATATTTCTTAGCTAGAGTGATAATTTCGTTAAAACATTCAAATG

TTGTCAAATGATCGGATCTAAAATCCATATTTTCTGGTAGTGTTTCTACCAGCCTACATTTTGCTCCCGC

AGGTACCGATGCAAATGGCCACATTTAGTTAACATAAAAACTTATATATCCTGTTCTATCAACGATTCTA

GAATATCATCGGCTATATCGCTAAAATTTTCATCAAAGTCGACATCACAACCTAACTCAGTCAATATATT

AAGAAGTTCCATGATGTCATCTTCGTCTATTTCTATATCCGTATCCATTGTAGATTGTTGACCGATTATC

GAGTTTAAATCATTACTAATACTCAATCCTTCAGAATACAATCTGTGTTTCATTGTAAATTTATAGGCGG

TGTATTTAAGTTGGTAGATTTTCAATTATGTATCAATATAGCAACAGTAGTTCTTGCTCCTCCTTGATTT

TAGCATCCTCTTCATTATTTTCTTCTACGTACATAATCATGTCTAATACGTTAGACAACACACCGACGAT

GGTGGCCGCCACAGACACGAATATGACTAGACCGATGACCATTTAAAAAATACTCTCTAGCTTTAACTTA

AACTGTATCGATCATTCTTTTAGCACATGTATAATATAAAAACATTATTCTATTTCGAATTTAGGCTTCC

AAAAATTTTTCATCCGTAAACCGATAATAATATATATAGACTTGTTAATAGTCGGAATAAATATATTAAT

GCTTAAACTATCATCATCTCCACGATTAGAGATACAATATTTACATTCTTTTTGCTGTTTCGAAACTTTA

TCAATACACGTTAATACAAACCCAGGAAGGAGATATTGAAACTGAGGCTGTTGAAAATGAAACGGCGAAT

ACAATAATTCAGATAATGTAAAATCATGATTCCGTATTCTGATGATATTAGAACTGCTAATGGATGTCGA

TGGTATGTATCTAGGAGTATCTATTTTAACAAAGCATCGATTTGCTAATATACAATTATCCTTTTGATTA

ATTGTTATTTTATTCATATTCTTAAAAGGTTTCATATTTATCAATTCTTCTACATTAAAAATTTCCATTT

TTAATTTATCTAGCCCCGCAATACTCCTCATTACGTTTCATTTTTTGTCTAGAATACCCATTTTGTTCAT

CTTGGTACATAGATTATCCAATTGAGAAGCGCATTTAGTAGTTTTGTACATTTTAAGTTTATTAACGAAT

CGTCGAAAACTAGTTATAGTTAACATTTTATTATTTGATACCCTGATATTAATACCCCTGCCGTTACTAT

TATTTATAACTGATGTAACCCACGTAACATTGGAATTAATTATCGATAGTAATGCATCGACACTTCCAAA

ATTGTCTATTATAAACTCACCGATAATTTTTTTATTGCATGTTTTCATATTCATTAGGATTATCAAATCT

TTAATCTTATTACGATTGTATGCGTTGATATTACAAGACGTCATTCTAAAAGACGGAGGATTTCCATCAA

ATGCCAGACAATCACGTACAAAGTACATGGAAATAGGTTTTGTTCTATTACGCATCATAGATTCATATAA

AACACCCGTAGAAATACTAATTTGTTTTACTCTATAAAATACTATTGCATCTATTTCATCGTTTTGTATA

ACGTCTTTCCAAGTGTCAAATTCCAATTTTTTTTCATTGATAGTACCAAATTCTTCTATCTCTTTAACTA

CTTGCATAGATAGGTAATTACAGTGATGCCTACATGCCGTTTTTTGAAACTGAATAGATGCATCTAGAAG

CGATGCTACACTAGTCACGATCACCACTTTCATATTTAGAATATATGTATGTAAAAATATAGTAGAATTT

CATTTTGTTTTTTTCTATGCTATAAATGAATTCTCATTTTGTATCCGCACATACTCCGTTTTATATCAAT

ACCAAAGAAGGAAGATATCTGGTTCTAAAAGCCGTTAAAGTATGCGATGTTAGAACTGTAGAATGCGAAG

GAAGTAAAGCTTCCTGCGTACTCAAAGTAGATAAACCCTCATCACCCACGTGTGAGAGAAGACCTTCGTC

cccgtccagatgcgagagaatGAATAACCCTGGAAAACAAGTCCCGTTTATGAGAACGGACATGTTACAA

AATATGTTTGCTGCTAATCGCGACAACGTAACGTCAAGACTTTTGAACTAAAATACAATTATATCTTTTT

CGATATTAATAAATCCGTGTCTCCCGGGTTTTTTATCTCTTTCAGTATGTGAATAGATAGGTATTTTATC

TCTATTCATCATCGAATTTAAGAGATCCGATAAACATTGTTTGTATTCTCCAGATGTCAGCATCTGATAC

AACAATATATGTGCGCATAAACCTCTGGCACTTATTTCATGTACCTTCCCCTTATCACTAAGGAGAATAG

TATTTGAGAAATATGTATACATGATATTATCATGTATTAGATATACAGAATTTGTAACACTCTCGAAATC

ACACGATGTGTCGGCGTTAAGATCTAATATATCACTCGATAACACATTTTCATCTAGATACACTAGACAT

TTTTTAAAGCTAAAATAGTCTTTAGTAGTAACAGTAACTATGCGATTATTTTCATCGATGATACATTTCA

TCGGCATATTATTACGCGTACCATCAAAGACTATACCATGTGTATATCTAACGTATTCTAGCATAGTTGC

CATACGTACATTAAACTTTTCAGGATCTTTGGATAGATCTTCCAATCTATCTATTTGAGAAAACATTTTT

ATCATGTTCAATAGTTGAAACGTCGGATCCACTATATAGATATTATCTATAAAGATTTTAGGAACTATGT

TCATGGTATCCTGGCGAATATTAAAACTATCAATGATATGATTATCGTTTTCATCTTTTATCACCATATA

GTTTCTAAGATATGGGATTTTACTTAATATAATATTATTTCCCGTAATAAATTTTATTAGAAATGCCAAA

TCTATAAGAAAAGTCCTAGAATTAGTCTGAAGAATATCTATATCACCGTACCGTATATTTGGATTAATTA

GATATAGAGAATATGATCCGTAACATATACAACTTTTATTATGACGTCTAAGATATTCTTCCATCAACTT

ATTAACATTTTTGACTAGGGAAGATACATTATGACGTCCCATTACTTTTGCCTTGTCTATTATAGCGACG

TTCATAGAATTTAGCATATCTCTTGCCAATTCTTCCATTGATGTTACATTATAAGAAATTTTAGATGAAA

TTACATTTGGAGCTTTAATAGTAAGAACTCCTAATATATCCGTGTATGTGGTCACTAATACAGATTGTAG

TTCTATAATCGTAAATAATTTACCTATATTATATGTTTGAGTTTGTTTAGAAAAGTAGCTAAGTATACGA

TCTTTTATTTCTGATGCCGATGTATCAACATCGAAAAAAAATCTTTTTTTATTCTTTTTTACTAACGATA

CGAATATGTCTTTGTTAAAAACAGTTATTTTCTGAATATTTCTAGCTTGTAATTTTAACATATGATATTC

GTTCACACTAGGTACTCTGCCTAAATAGGTTTCTATAATCTTTAATGTAATATTAGGAAGAGTATTCTGA

TCAGGATTCCTATTCATTTTGAGGATTTAAAACTCTGATTATTGTCTAATATGGTCTCAACACAAACTTT

TTCACAGAGTGATAGAGTTTTTGATAACTCGTTTTTCTTAAGAAATATAAAACTACTGTCTCCAGAGCTC

GCTCTATCTTTTATTTTATCTAATTCGATACAAACTCCTGATACTGGTTCAGAAAGTAATTCATTAATTT

TCAGTCCTTTATAGAAGATATTTAATATAGATAATACAAAATCTTCAGTTCTTGATATCGATCTGATTGA

TCCTAGAACTAGATATATTAATAACGTGCTCATTAGGCAGTTTATGGCAGCTTGATAATTAGATATAGTA

TATTCCAGTTCATATTTATTAGATACCGCATTGCCCAGATTTTGATATTCTATGAATTCCTCTGAAAATA

AATCCAAAATAACTAGACATTCTATTTTTTGTGGATTAGTGTACTCTCTTCCCTCTATCATGTTCACTAC

TGGTGTCCACAATGATAAATATCTAGAGGGAATATAATATAGTCCATATGATGCCAATCTAGCAATGTCG

AATAACTGTAATTTTATTCTTCGCTCTTCATTATGAATTGAATCTTGAGGTATAAACCTAACACAAATTA

TATCATTAGACTTTTCGTATGTAATGTCTTTCATGTTATAAGTTTTTAATCCTGGAATAGAATCTATTTT

AATGAGGCTTTTAAACGCAGCGTTCTCCAACGAGTCAAAGCATAATACTCTGTTGGTTTTCTTATATTCA

ATATTACGATTTTCTTCTTTGAATGGAATAGGTTTTTGAATTAGTTTATAATTACAACATAATAGATAAG

GAAGTGTGTAAATAGTACGCGGAAAAAACATAATAGCTCCCCTGTTTTCATCCATGGTTTTAAGTAAATG

ATCACTGGCTTCTTTAGTCAATGGATATTCGAACATTAACCGTTTCATCATCATTGGACAGAATCCATAT

TTCTTAATGTAAAGAGTGATCAAATCATTGTGTTTATTGTACCATCTTGTTGTAAATGTGTATTCGGTTA

TCGGATCTGCTCCTTTTTCTATTAAAGTATCGATATCGATCTCGTCTAAGAATTCAACTATATCGACATA

TTTCATTTGTATACACATAACCATTACTAACGTAGAATGTATAGGAAGAGATGTAACGGGAACAGGGTTT

GTTGATTCGCAAACTATTCTAATACATAATTCTTCTGTTAATACGTCTTGCACGTAATCTATTATAGATG

CCAAGATATCTATATAATTATTTTGTAAGATGATGTTAACAATGTGATCTATATAAGTAGTGTAATAATT

CATGTATTTCGATATATGTTCCAACTCTGTCTTTGTGATGTCTAGTTTCGTAATATCTATAGCGTCCTCA

AAAAATATATTCGCATATATTCCCAAGTCTTCAGTTCTATCTTCTAAAAAATCTTCAACGTATGGAATAT

AATAATCTATTTTACCTCTTCTGATGTCATTAATGATATAGTTTTTGACACTATTTTCCGTCAATTGATT

CTTATTCACTATGTCTAAAAACCGGATAGCGTCCCTAGGACGAACTACTGCCATTAATATCTCTATTATA

GCTTCTGGACATAAATCATCTATTATACCAGAATTAATGGGAACTATTCCGTATCTATCTAACATAGTTT

TAAGAAAGTCAGAATCTAAGACCTGATGTTCATATATTGGTTCATACATGAAATGATCTCTATTGATGAT

AGTGACTATTTCATTCTCTGAAAATTGGTAACTCATTCTATACACGCTTTCCTTGTTGATAAAGGATAGT

ATATACTCAATGGAATTTGTACCAACAAACTGTTCTCTTATGAATCGTATATCATCATCTGAAATGATCA

TGTAAGGCATACATTTAACAATAAGAGACTTGTCTCCTGTTATCAATATACTATTCTTGTGATAATTTAT

GTGTGTGGCAAATTTGTCCACGTTCTTTAATTTTGTTATAGTAGATATCAAATCCAATGGAGATACAGTT

CTTGGCTTAAACAGATATAGTTTTTCTGGAACGAATTCTACAACATTATTATAAAGGACTTTGGGTATAT

AAGTGGGATGAAATCCTATTTTAATTAATGCGATAGCCTTGTCCTCGTGCAGATATCCAAACGCTTTTGT

GATAGTATGGCATTCATTGTCTAGAAACGCTCTACGAATATCTGTAACAGATATCATCTTTAGAGAATAC

TAGTCGCGTTAATAGTACTAAAATTTGTATTTTTTAATCTATCTCAATAAAAAATTAATATGTATGATTC

AATGTATAACTAAACTACTAACTGTTATTGATAACTAGAATCAGAATCTAATGATGACATAACTAAGAAG

TTTATCTACAGCCAATTTAGCTGCATTATTTTTAGCATCTCGTTTAGATTTTCCATCTGCCTTATCGAAT

ACTCTTCCGTCAATGTCTACACAGGCATAAAATGTAGGAGAGTTACTAGGCCCCACTGATTCAATACGAA

AAGACCAATCTCTCCTAGTTATTTGACAGTACTCATTAATAACGGTGACAGGGTTAACACCTTTCCAATA

AATAATTTTTTTAACCGGAATAACATCATCAAAAGACTTATGATCCTCTCTCATTGATTTTTCGCGGGAT

ACATCATCTATTATAGCATCAGCATCAGAATCTGTAGGCCGTGTATCAGCATCCATTGTCGTAGACCAAC

GAGGAGGAGTATCGTTGGAGCTGTAAACCATAGCACTACGTTGAAGATCATACAGAGCTTTATTAACTTC

TCGCTTCTCCATATTAAGTTGTTTAGTTAGTTGTACAGCAGTAGCTCCTTAGTCCAATGTTTTTAATAAC

CGCACACAATCTCTGTGTCAGAACGCTCGTCAATATAGATCTTAGAAATTTTTTTAGAGAGAACTAACGC

AACTAGCAATAAAACTGATCTTATTTTATCATTTTTTTTATTCATCATCCTCTGGTGGTTCGTCGTTCCT

ATCGAATGTAGCTCTGATTAACCCGTCATCTATAGGTGATGCTGGTTCTGGAGATTCTGGAGGAGATGGA

TTATTATCTGGAAGAATCTCTGTTATTTCCTTGTTTTCATGTATCGATTGCGTTGTAACATTAAGATTGC

GAAATGCTCTAAATTTGGGAGGCTTAAAGTGTTGTTTACAATCTCTACACGCGTGTCTAACTAATGGAGG

TTCGTCAGCGGCTCTAGTTTGAATCATCATCGGTGTAGTATTCCTACTTTTACAGTTAGGACACGGTGTA

TTGTATTTCTCGTCGAGAACGTTAAAATAATCGTTGTAACTCACATCCTTTATTTTATCTATATTGTATT

CTACTCCTTTCTTAATGCATTTTATACCGAACAAGAGATAGCGAAGGAATTCTTTTTCGGTACCGCTAGT

ACCCTTAATCATATCACATAGTGTTTTATATTCTAAATGTGTGGCAATGGACGGTTTATTTCTATACGAT

AGTTTGTTTTTGGAATCCTTTGAGTATTCTATACCAATATTATTCTTTGATTCGAATTTAGTTTCTTCGA

TATTAGATTTTGTATTACCTATATTCTTGATGTAGTACTTTGATGATTTTTCCATGGCCCATTCTATTAA

GTTTTCCAAGTTGGCATCATCCACATATTGTGATAGTAATTCTCGGATATCAGTAGTGACTACCGCCATT

GATATTTGTTCATTTGATGAGTAACTACTAATGTATACATTTTCCATTTATAACACTTATGTATTAACTT

TGTTTATTTATATTTTTTCATTATTATGTTGATATTAATAATCGTATTGTGGTTATATGGCTACAATTTC

ATAATGAGTTGAAGTCAGTGTCCTATGATCAATGACGATAGCTTTACTCTGAAAAGAAAGTATCAAATCG

ATAGTGCAGAGTCAACAATGAAAATGGATAAGACGATGACAAAGTTTCAGAATAGAGTCAAAATGGTAAA

AGAAATAAATCAGACGATAAGAGCAGCACAAACTCATTACGAGACATTGAAACTAGGATATATAAAATTT

AAGGGAATGATTAGGACTACTACTCTAGAAGATATAGCACCATCTATTCCAAATAATCAGAAAACTTATA

AACTATTCTCGTACATTTCAGTCATTGGCAAAGCATCACAGAATCCGAGTAAGATGATATATGCTCGCTG

CTTTACATGTTTCCCAATTTGTTTGGAGATGACCATAGATTCATTTGTTATAGAATGCATCCAACATTGT

TCATGATATAGTTGAATCATGTATGCCTGTTCGTATGCCTGTGGCTAAGATACTGTGTAAAGAAATGGTA

AATAAATACTTTGAGAATCTTTAAGAGTGCATTGACTTTGTTAGTGAATAGGCATTCCATCTTTCTCCAA

TACTAATTCAAATTGTTAAATTAATAATGGAATAGTATAAATAGTTATTAGTGATAAGATAGTAAAAATA

ATTATTAGAATAGTAGTGTAGTATCATAGATAACTCTCTTCTATAAAAAATGGATTTTATTCGTAGAAAG

TATCTTATATACACAGTAGAAAATAATATAGATTTTTTAAAGGATGATACATTAAGTAAAGTAAACAATT

TTACCCTCAATCATGTACTAGCTCTCAAGTATCTAGTTAGCAATTTTCCTCAACATGTTATTACTAAGGA

TGTATTAGCTAATACCAATTTTTTTGTTTTCATACATATGGTACGATGCTGTAAAGTATACGAAGCGGTT

TTACGACACGCATTTGATGCACCCACGTTGTACGTTAAAGCATTGACTAAGAATTATTTATCGTTTAGTA

ACACAATACAGTCGTACAAGGAAACCGTGCATAAACTAACACAAGATGAAAAATTTTTAGAGGTTGCCGA

ATACATGGACGAATTAGGAGAACTTATAGGCGTAAATTATGACTTAGTTCTTAATCCATTATTTCACGGA

GGGGAACCCATCAAAGATATGGAAATCATTTTTTTAAAACTGTTTAAGAAAACAGACTTCAAAGTTGTTA

AAAAATTAAGTGTTATAAGATTACTTATTTGGGCATACCTAAGCAAGAAAGATACAGGCATAGAGTTTGC

GGATAATGATAGACAAGATATATATACTCTATTTCAACAAACTGGTAGAATAGTCCATAGCAATCTAACA

GAAACGTTTAGGGATTATATCTTTCCCGGAGATAAGACTAGCTATTGGGTGTGGTTAAACGAAAGTATAG

CTAATGATGCGGATATCGTTATTAATAGACCCGCCATTACCATGTATGATAAAATTCTTAGTTATATATA

CTCTGAGATAAAACAGGGACGCGTTAATAAAAACATGCTTAAGTTAGTTTATATCTTTGAGCCTGAAAAA

GATATCAGAGAACTTCTGCTAGAAATCATATATGATATTCCTGGAGATATCCTATCTATTATTGATGCAA

AAAACGACGATTGGAAAAAATATTTTATTAGTTTTTACAAAGCTAATTTTATTAACGGTAATACATTTAT

TAGTGATAGAACGTTTAACGATGACTTATTCAGAGTTGTTGTTCAAATAGATCCCGAATATTTCGATAAT

GAACGAATTATGTCTTTATTCTATACGAGTGCTGCGGACATTAAACGATTTGATGAGTTAGATATTAATA

ACAGTTATATATCTAATATAATTTATGAGGTGAACGATATCACATTAGATACAATGGATGATATGAAGAA

GTGTCAAATCTTTAACGAGGATACGTTGTATTATGTTAAGGAATACAATACATACCTGTTTTTGCACGAG

TCGGATCCCATGGTCATAGAGAACGGAATACTAAAGAAACTGTCATCTATAAAATCCAAGAGTAGACGGC

TGAACTTGTTTAGCAAAAACATTTTAAAATATTATTTAGACGGACAATTGGCTCGTCTAGGTCTTGTGTT

AGATGATTATAAAGGAGACTTATTAGTTAAAATGATAAACCATCTCAAATCTGTGGAGGATGTATCCGCA

TTCGTTAGATTTTCTACAGATAAAAACCCTAGTATTCTTCCATCGCTAATCAAAACTATTTTAGCTAGTT

ATAATATTTCCATCATCGTCTTATTTCAAAGGTTTTTAAGAGATAATCTATATCATGTAGAAGAATTCTT

GGATAAAAGCATCCATCTAACCAAGACGGATAAGAAATATATACTTCAATTGATAAGGCACGGTAGATCA

TAGAACAAACCAAATATATTATTAATAATTTGTATATACATAGATATAATTATCACATATTAAAAAATAA

CACATTTTTGATAAATGGAAACCGTTGCAACAATTCAGACTCCCACCAAATTAATGAATAAAGAAAATGC

AGAAATGATTTTGGAAAAAATTGTTAATCATATAGCTATGTATATTAGTGACGAATCAATATATTCAGAA

AATAATCCTGAATATATTGATTTTCGTAACAGATACGGAGACTATAGATCTCTCATTATAAAAAGTGATC

ACGAGTTTGTAAAGCTATGTAAAGATCATGCAGAGAAAAGTTCTCCAGAAACGCAACAAATGATTATCAA

ACACATATACGAACAATATCTTATTCCAGTATCTGAAGTACTATTAAAACCTATAATGTCCATGGGTGAC

ATATTTACATATAACGGATGTAAAGACAATGAATGGATGCTAGAACAACTCTCTACCCTAAACTTTAACA

ATCTctacACATGGAACTCATGTAGCATAGGCAATGTAACGCGTCTGTTTTATACATTTTTTAGTTATCT

GATGAAAGATAAACTAAATATATAAGTATAATACCATTCTAATACTTTAACCTGATGTATTATTACCTGC

ATCTTATTAGAATATTAACCTAACTAAAAGACATAAAAAGTGGTAGGATATAAATATTATGGCCGCAACC

GTTCCGCGTTTTGACGATGTGTACAAAAATGCACAAAGAAGAATTCTAGATCAAGAAACATTTTTTAGTA

GAGGTCTAAGTAGACCGTTAATGAAAAACACATATCTATTTGATAATTACGCGTATGGATGGATACCAGA

AACTGCAATTTGGAGTAGTAGATACGCAAACCTAGATGCTAGTGACTATTATCCCATTTCGTTGGGATTA

CTTAAAAAGTTTGAATTTCTCATGTCTCTATATAAAGGTCCTATTCCCGTATATGAAGAAAAAGTAAATA

CTGAATTCATTGCTAATGGATCTTTCTCCGGTAGATACGTATCATATCTTAGAAAGTTTTCTGCCCTTCC

AACAAACGAGTTTATTAGTTTTTTATTATTGACCTCCATCCCTATCTATAATATCTTATTCTGGTTTAAA

AACACACAGTTTGATATTACTAAACACACATTATTCAGATACGTCTATACAGATAATACCAAACACCTTG

CGTTGGCTAGGTATATACATCAAACAGGAGACTATAAGCCTTTGTTTAGTCGTCTCAAAGAGAATTATAT

ATTTACCGGTCCCGTTCCAATAGGTATCAAAGATATAGATCACCCTAATCTTAGTAGAGCAAGAAGTCCA

TCCGATTATGAGACATTAGCTAATATTAGTACTATATTGTACTTTACCAAGTATGATCCAGTATTAATGT

TTTTATTGTTTTACGTACCTGGGTATTCAATTACTACAAAAATTACTCCAGCCGTAGAATATCTAATGGA

TAAACTGAATCTAACAAAGAGCGACGTACAACTGTTGTAAATTATTTTATGCTTCGTAAAATGTAGGTCT

TGAACCAAACATTCTTTGAAAAAATGAGATGCATAAAACTTTATTATCCAATAGATTAACTATTTCAGAC

GTCAATCGTTTAAAGTAAACTTCGTAAAATATTCTTTGATTGCTGCCGAGTTTAAAACTTCTATCGATAA

TTGTTTCATATGTTTTAATATTTACAAGTTTTTTGGTCCATGGTACATTAGCTGGACAGATATATGCAAA

ATAATATCGTTCTCCAAGTTCTATAGTCTCTGGATTGTTTTTATTATATTCAGTAACCAAATACATATTA

GGGTTATCTGCGGATTTATAATTTGAGTGATGCATTCGACTCAACATAAATAATTCTAGAGGAGACGATC

TACTATCAAATTCGGATCGTAAATCTGTTTCTAAAGAACGGAGAATATCTATACATACCTGATTAGAATT

CATCCGTCCTTCAGACAACATCTCAGACAGTCTGGTCTTGTATGTCTTAATCATATTCTTATGAAACTTG

GAAACATCTCTTCTAGTTTCACTAGTACCTTTATTAATTCTCTCAGGTACAGATTTTGAATTCGACGATG

CCGAGTATTTCATCGTTGTATATTTCTTCTTCGATTGCATAATCAAATTCTTATATACCGCCTCAAACTC

TATTTTAAAATTATTAAACAATACTCTACTATTAATCAGTCGTTCTAACTCCTTTGCTATTTCTATGGAC

TTATCTACATCTTGACTGTCTATCTCTGTAAACACGGAGTCGGTATCTCCATACACGCTACGAAAACGAA

ATCTATAATCTATAGGCAACGATGTTTTCACAATCGGATTAATATCTCTATCGTCCATATAAAATGGATT

ACTTAATGTATTGGCAAACCGTAACATACCGTTGGATAACTCTGCTCCATTTAGTACCGATTCTAGATAC

AATATCATTCTACGTCCTATGGATGTGCAACTCTTAGCCGAAGCGTATGAGTATAGAGCACTATTTCTAA

ATCCCATCAGACCATATACTGAGTTGGCTACTATCTTGTACGTATATTGCATGGAATCATAGATGGCCTT

TTCAGTTGAACTGGTAGCCTGTTTTAACATCTTTTTATATCTGGCTCTCTCTGCCAAAAATGTTCTTAAT

AGTCTAGGAATGGTTCCTTCTATTGATCTATCGAAAATTGCTATTTCAGAGATGAGGTTCGGTAGTCTAG

GTTCACAATGAACCGTAATATATCTAGGAGGTGGATATTTCTGAAGCAAGAGTTGATTATTTATTTCTTC

TTCCAATCTATTGGTACTAACAACGACACCGACTAATGTTTCCGGAGATAGATTTCCAAAGATACACACA

TTAGGATACAGACTGTTATAATCAAAGATTAATACATTATTACTAAACATTTTTTGTTTTGGAGCAAATA

CCTTACCGCCTTCATAAGGAAACTTTTGTTTTGTTTCTGATCTGACTAAGATAGTTTTAGTTTCCAACAA

TAGCTTTAACAGTGGACCCTTGATGATTGTACTCGCTCTATATTCGAATACCATGGATTGAGGAAGCACA

TATGTTGCCGCACCAGCGTCTGTTTTTGTTTCTACTCCATAATACTCCCACAAATACTGACACAAACAAG

CATCATGAATACAGTATCTAGCCATATCTAAAGCTATGTTTAGATTATAATCCTTATACATCTGAGCTAA

ATCAATGTCATCCTTTCCGAAAGATAATTTATATATATCATTAGGTAAAGTAGGACATGATAGTACGACT

TTAAATCCATTTTCCCAAATATCTTTACGAATTACTTTACATATAATATCCTCATCAACAGTCACGTAAT

TACCTGTGGTTAAAACCTTTGCAAATGTATCGGCTTTGCCTTTCGCGTCCGTAGTATCGTCACCGATGAA

CGTCATTTCTCTAACTCCTCTATTTAATACTTTACCCATGCAACTGAACGCGTTCTTGGATATAGAATCC

AATTTGTACGAATCCAATTTTTCAGATTTTTGAATGAATGAATATAGATCGAAAAATATAGTTCCATTAT

TGTTATTAACGTGAAACGTAGTATTGGCCATGCCGCATACTCCCTTATGACTAGACTGATTTCTCTCATA

AATACAGAGATGTACAGCTTCCTTTTTGTCTGGAGATCTAAAGATAATCTTCTCTCCTGTTAATAACTCT

AGACGATTAGTAATATATCTCAGATCAAAGTTATGTCCGTTAAAGGTAACGACGTAGTCGAACGTTAGTT

CCAACAATTGTTTAGCTATTCGTAACAAAACTATTTCAGAACATAGAACTAGTTCTCGTTCGTAATCCAT

TTCCATTAGCGACTGTATCCTCAAACATCCTCTATCGACGGCTTCTTGTATTTCCTGTTCCGTTAACATC

TCTTCATTAATGAGCGTAAACAGTAATCGTTTACCACTTAAATCGATATAACAGTAACTTGTATGCGAGA

TTGGGTTAATAAATACAGAAGGAAACTTCTTATCGAAGTGACACTCTATATCTAGAAATAAGTACGATCT

TGGGATATCGAATCTAGGTATTTCTTTAGCGAAACAGTTACGTGGATCGTCGCAATGATAACATCCATTG

TTAATCTTTGTCAAATATTGCTCGTCCAACGAGTAACATCCGTCTGGAGATATCCCGTTAGAAATATAAA

ACCAACTAATATTGAGAAATTCATCCATGGTGGCATTTTGTATGCTGCGTTTCTTTGGCTCTTCTATCAA

CCACATATCTGCGACGGAGCATTTTCTATCTTTAATATCTAGATTATAACTTATTGTCTCGTCAATGTCT

ATAGTTCTCATCTTTCCCATCGGCCTCGCATTAAATGGAGGAGGAGATAATGACTGATATATTTCGTCCG

TCACTACGTAATAAAAGTAATGAGGAAATCGTATAAATACGGTCTCGCCATTTCGACATCTGGATTTCAG

ATATAAAAATCTGTTTTCACCGTGACTTTCAAACCAATTAATACACCTAACATCCATTTCTAGAATTTAG

AAATATATTTTCATTTAAATGAATCCCAAACATTGGGGAAGAGCCGTATGGACCATTATTTTTATAGTAC

TTTCGCAAGCGGGTTTAGACGGCAACATAGAAGCGTGTAAACGAAAACTATATACTATAGTCAGCACTCT

TCCATGTCCTGCATGTAGACGACACGCGACTATCGCTATAGAGGACAATAATGTCATGTCTAGCGATGAT

CTGAATTATATTTATTATTTTTTCATCAGATTATTTAACAATTTGGCATTTGATCCCAAATACGCAATCG

ATGTGTCAAAGGTTAAACCTTTATAAACTTAACCCATTATAAAACTTATGATTAGTCACGACTGAAATAA

CCGCGTGATTATTTTTTGGTATAATTCTACACGGCATGGTTTCTGTGACTATGAATTCAACACCTGTTAT

CTTAGTGAAATCTTTAACAAACAGCAAGGGTTCGTCAAAGACATAAAACTCATTGTTTACGATCGAAATA

GACCCCCTATCACACTTAAAATAAAAAATATCCTTATCCTTTACCACCAAATAAAATTCTGATTGGTCAA

TGTGAATGTATTCACTTAACAGTTCCACAAATTTATTTATTAACTCCGAGGCACATACATCGTCGGTATT

TTTTATGACAAACTTTACTCTTCCAGCATCCGTTTCTAAAAAAATATTAACGAGTTCCATTTATATCATC

CAATATTATTGAAATGACGTTGATGGACAGATGATATAAATAAGAAGGTACAGTACCTTTGTCAACCATC

TCCTCCAATTCATACTCTATTTTGTCATTAACTTTAATGTGTGAAAACAGTACGCCACATGCTTCCATGA

CAGTGTGTAACACTTTGGATACAAAATGTTTGACATTAGTATAATTGTCCAAGACTGTCAATCTATAATA

GATAGTAGCTATAATATATTCTATGATGGTATTGAAGAAGATGACAACCTTGGCATATTGATCATTTAAC

ACAGACATGGTATCAACAAATAGCTTAAATGAAAGAGAATCAGTAATTGGAATAAGCGTCTTCTCGATGT

AGTGTCCGTATACCAACATGTCTGATATTTTGATGTATTCCATTAAATTATTTAGTTTTTTCTTTTTATT

CTCGTTAAACAGAATTTCTGTCAATGGACCCCAACATCGTTGACCTATTAAGTTTTGATTGATTTTTCCG

TGTAAGGCGTATCTAGTCAGATCGTATAGCCTATCCAATAATCCATCGTCTGTGCGTAGATCACATCGTA

CACTTTTTAATTTTCTATAGAAGAGTGACAGACATCTGAAGCAATTACAGACAGCAATTTCTTTATTCTC

TACAGATGTAAGATACTTGAAGACATTCCTATGATGATGCAGAATTTTGGATAACACGGTATTGATGGTA

TCTGTTACCATAATTCCTTTGACTGATAGTGTCAAAGTACAAGATTTCCAATCTTTTGCAATTTTCAGTA

CCATTATCTTTGTTTTGATATCTATATCAGACAGCATGGTACGTCTGACAACACAGGGATTAAGACGGAA

AGATGAAATGATTCTCTCAACATCTTCAATAGATACCTTGCTATTTTTTTTGGCATTATCTATATGTGAG

AGAATATCCTCTAGAGAATCAGTATCCTTTTTGATGATAGTGGATCTCAATGACATGGGACGTCTAAACC

TTCTTATTCTATCACCAGATTGCATGGTGATTTGTCTTCTTTCTTTTATCATGATGTAATCTCTAAATTC

ATCGGCAAATTGTCTATATCTAAAATCATAATATGAGATGTTTACCTCTACAAATATCTGTTCGTCCAAT

GTTAGAGTATCTATATCAGTTTTGTATTCCAAATTAAACATGGCAACGGATTTAATTTTATATTCCTCTA

TTAAGTCCTCGTCGATAATAACAGAATGTAGATAATCATTTAATCCATCGTACATGGTTGGAAGATGCTC

GTTGACAAAATCTTTAATTGTCTTGATGAAGGTGGGACTATATCTAACATCTTGATTAATAAAATTTATA

ACATTGTCCATAGGATACTTTGTAACTAGTTTTATACACATCTCTTCATTGGTAAGTTTAGACAGAATAT

CGTGAACAGGTGGTATATTATATTCATCAGATATACGAAGAATAATGTCCAAATCTATATTGTTTAATAT

ATTATATAGATGTAGTGTAGCTCCTACAGGAATATCTTTAACTAAGTCAATGATTTCATCAACAGTTAGA

TCTATTTTAAAGTTAATCATATAGGCATTGATTTTTAAAAGGTATGTAGCCTTGACTACATTCTCATTAA

TTAACCATTCCAAGTCACTGTGTGTAAGAAGATTATATTCTATCATAAGCTTGACTACATTTGGTCCCGA

TACCATTAAAGAATTCTTATGATATAAGGAAACAGCTTTTAGGTACTCATCTACTCTACAAGAATTTTGG

AGAGCCTTAACGATATCAGTGACGTTTATTATTTCAGGAGGAAAGAACCTAACATTGAGAATATCTGAAT

TAATAGCTTCCAGATACAGTGATTTTGGCAATAGTCCGTGTAATCCATAATCCAGTAACACGAGCTGGTG

CTTGCTAGACACCTTTTCAATGTTTAATTTTTTTGAAATAAGCTTTGATAAAGCCTTCCTCGCAAATTCC

GGATACATGAACATGTCGCCAACATGATTAAGTATTGTTTTTCATTATTTTTATATTTTCTCAACAAGTT

CTCAATACCCCAATAGATAATAGAATATCACCCAATGCGTCCATGTTGTCTATTTCCAACAGGTCGCTAT

ATCCACCAATAGAAGTTTTCCCAAAAAAGATTCTAGGAACAGTTCTACCACCAGTAATTTGTTCAAAATA

GTCACGCAATTCATTTTCGGGTTTAAATTCTTTAATATCTACAATTTCATACGCTCCTCTTTTGAAACTA

AACTTATTTAGAATATCCAGTGCGTTTCTACAAAAAGGACATGTAAACTTGACAAAAATTGTCACTTTGT

TATTGGCCAACCTTTGTTGTACAAATTCCTCGGCCATTTTTAATATTTAAGTGATACAAAACTATCTCGA

CTTATTTAACTCTTTAGTCGAGATATATGGACACAGATAGCTATATGATAACCAACTACAGAAGACAAAC

GCTATAAAAAACATAATTACGACGAGCATATTTATAAATATTTTTATTCAGTATTACTTGATATAGTAAT

ATTAGGCACAGTCAAACATTCAACCACTCTAGATACATTAACTCTCTCATTTTCTTTAACAAATTCTGCA

ATATCTTCGTAAAAAGATTCTTGAAACTTTTTAGAATATCTATCGACTCTAGATGAAATAGCGTTCGTCA

ACATACTATGTTTTGTATACATAAAGGCGCCCATTTTAACAGTTTCTAGTGACAAAATGCTAGCGATCCT

AGGATCCTTTAGAATCACATAGATTGACGATTCGTCTCTCTTAGTAACTCTAGTAAAATAATCATACAAT

CTAGTACGCGAAATAATATTATCCTTGACTTGAGGAGATCTAAACAATCTAGTTTTGAGAACATCGATAA

GTTCATCGGGAATTACATACATACTATCTTTAATAGAACTCTTTTCATCCAGTTGAATGGATTCGTCCTT

AACCAACTGATTAATGAGATCTTCTATTTTATCATTTTCTAGATGATATGTATGTCCATTAAAGTTAAAT

TGTGTAGCGCTTCTTTTTAGCCTAGCAGCCAATACTTTAACATCACTAATATCGATATACAAAGGAGATG

ATTTATCGATGGTATTAAGAATTCGTTTTTCGACATCCGTCAAAACCAATTCCTTTTTGCCTGTATCATC

CAGTTTGCCATTCTTTGTAAAGAAATTATTTTCTACTAGACTATTAATAAGACTGATAAGGATTCCTCCA

TAATTGCACAATCCAAACTTTTTCACAAAACTAGACTTTACGAGATCTACAGGAATGCGTACTTCAGGTT

TCTTAGCTTGTGATTTTTTCTTTTGCGGACATTTTCTAGTTACCAACTCATCTACCATTTCATTGATTTT

AGCAGTGAAATAAGCTTTCAATGCACGGGCACTGATACTATTGAAAACGAGTTGATCTTCAAATTCCGCC

ATTTAAGTTCACCAAACAACTTTTAAATACAAATATATCAATAGTAGTAGAATAAGAACTATAAAAAAAA

TAATAATTAACCAATACCAACCCCAACAACCTGTATTATTAGTTGATGTGACAGTTTTCTCATCACTTAG

AACAGATTTAACAATTTCTATAAAGTCTGTCAAATCATCTTCCTGAGAACCCATAAATACACCAAATATA

GCAGCGTACAACTTATCCATTTATACATTGAATATTGGCTTTTCTTTATCGCTATCTTCATCATATTCAT

CATCAATATCAACAAGTCCCAGATTACGAACCAGATCTTCTTCTACATTTTCAGTCATTGATACGCGTTC

ACTATCTCCAGAGAGTCCGATAACGTTAGCCACTACTTCTCTATCAATGATTAGTTTCTTGAGCGCGAAT

GTAATTTTTGTTTCCGTTCCGGATCTATAGAAAACTACAGGTGTAATAATTGCCTTGGCTAATTGTCTTT

CTCTTTTACTGAGTGATTCTAGTTCACCTTCTATAGATCTGAGAATGGATGATTCTCCAGTCGAAACATA

TTCTACCATGGCTCCGTTTAATTTGTTGATGAAGATGGATTCATCCTTAAATGTTTTCTCTGTAATAGTT

TCCGCCGAAAGACTATGCAAAGAATTTGGAATGCGTTCCTTGTGCGTAATGTTTCCATAGACAGCTTCTA

GAAGTTGATACAACATAGGACTAGCCGCGGTAACTTTTATTTTTAGAAAGTATCCATCGCTTCTATCTTG

TTTAGATTTATTTTTATAAAGTTTAGTCTCTCCTTCCAACATAATAAAAGTGGAAGTCATCTGACTAGAT

AAACTATCAGTAAGTTTTATAGAGATAGATGAACAATTAGCGTATTGAGAAGCATTTAGTGTAACGCATT

CGATACATTTTGCATTAGATTTACTAATCGATTTTGCATACTCTATAACACCCGCACAAGTCTGTAGAGA

ATCGCTAGATGCTGTAGGTCTTGGTGAAGTTTCAACTCTCTTCTTGATTACCTTACTCATGATTAAACCT

AAATAATTGTACTTTGTAATATAATGATATATATTTTCACTTTATCTCATTTGAGAATAAAAATGTTTTT

GTTAACCACTGCATGATGTACAGATTTCGGAATCGCAAACCACTTGTGGTTTTATTTTATCCTTGTCCAA

TGTGAATTGAATGGGAGCGGATGCGGGTTTCGTACGTAGATAGTACATTCCCGTTTTTAGACCGAGACTC

CATCCGTAAAAATGCATACTCGTTAGTTTGGAATAACTCGGATCTGCTATATGGATATTCATAGATTGAC

TTTGATCGATGAAGGCTCCCCTGTCTGCAGCCATTTTTATGATCGTCTTTTGTGGAATTTCCCAAATAGT

TTTATAAACTCGCTTAATATCTTCTGGAAGGTTTGTATTCTGAATGGATCCACCATCTACCATAATCCTA

TTCTTGATCTCATCATTCCATAATTTTCTCTCGGTTAAAACTCTAAGGAGATGCGGGTTAACTACTTGGA

ATTCTCCAGACAATACTCTCCGAGTGTAAATATTACTGGTATACGGTTCCACCGACTCATTATTTCCCAA

AATTTGAGCAGTTGATGCAGTCGGCATAGGTGCCACCAATAAACTATTTCTAAGACCGTATGTTCTGATT

TTATCTTTTAGAGGTTCCCAATTCCAAAGATCCGACGGTACAACATTCCAAAGATCATATTGTAGAATAC

CGTTACTGGCGTACGATCCTACATATGTATCATATGGTCCTTCCTTCTCAGCTAGTTTACAACTCGCCTC

TAATGCACCGTAATAAATGGTTTCAAAGATCTTCTTATTTAGATCTTGTGCTTCCAGGCTATCAAATGGA

TAATTTAAGAGAATAAACGCGTCCGCTAATCCTTGAACACCAATACCGATAGGTCTATGTCTCTTATTAG

AGATTTCAGCTTCTGGAATAGGATAATAATTAATATCTATAATTTTATTGAGATTTCTGACAATTACTTT

GACCACATCCTTCAGTTTGAGAAAATCAAATCGCCCATCTATTACAAACATGTTCAATGCAACAGATGCC

AGATTACACACGGCTACCTCATTAGCATCCGCATATTGTATTATCTCAGTGCAAAGATTACTACACTTGA

TGGTTCCTAAATTTTGTTGATTACTCTTTTTGTTACACGCATCCTTATAAAGAATGAATGGAGTACCAGT

TTCAATCTGAGATTCTATAATCGCTTTCCAGACGACTCGAGCCTTTATTATACATTTGTATCTCCTTTCT

CTTTCGTATAGTGTATACAATCGTTCGAACTCGTCTCCCCAAACATTGTCCAATCCAGGACATTCATCCG

GACACATCAACGACCACTCTCCGTCATCCTTCACTCGTTTCATAAAGAGATCAGGAATCCAAAGAGCTAT

AAATAGATCTCTTGTTCTATGTTCATCGTTTCCTGTATTCTTTTTAAGATCGAGGAACGCCATAATATCA

GAATGCCACGGTTCCAAGTATATGGCCATAACTCCAGGCCGTTTGTTTCCTCCCTGATCTATGTATCTAG

CGGTGTTATTATAAACTCTCAACATTGGAATAATACCGTTTGATATACCATTGGTACCGGAGATATAGCT

TCCACTGGCACGAATATTACTAATTGATAGACCTATTCCCCCTGCCATTTTAGAGATTAATGCGCATCGT

TTTAACGTGTCATAGATGCCTTCTATGCTATCATCGATCATGTTAAGTAGAAAACAGCTAGACATTTGGT

GACGAGTAGTTCCCGCATTAAATAAGGTAGGAGAAGCGTGCGTAAACCATTTTTCAGAAAGTAGATTGTA

CGTCTCAATAGCTGAGTCTATATCCCATTGATGAATTCCTACTGCGACACGCATTAACATGTGCTGAGGT

CTTTCAACAATTTTGTTGTTTATTTTCAACAAGTAGGATTTTTCCAAAGTTTTAAAACCAAAATAGTTGT

ATGAAAAGTCTCGTTCGTAAATAATAACCGAATTGAGCTTATCCTTATATTTGTTAACTATATCCATGGT

AATACTTGAAATAATCGGAGAATGTTTCCCATTTTTAGGATTAACATAGTTGAATAAATCCTCCATCACT

TCACTAAATAGTTTTTTTGTTTCCTTGTGTAGATTTGATATGGCTATTCTGGCGGCTAGAATGGCATAAT

CCGGATGTTGTGTAGTACAAGTGGCTGCTATTTCGGCTGCCAGAGTGTCCAATTCTACCGTTGTTACTCC

ATTATATATTCCTTGAATAACCTTCATAGCTATTTTAATAGGATCTATATGATCAGTGTTTAAGCCATAG

CACAATTTTCTAATACGAGACGTGATTTTATCAAACATGACATTTTCCTTGTATCCATTTCGTTTAATGA

CAAACATTTTTGTTGGTGTAATAAAAAAAAATTATTTAATTTTTCATTAATAGGGATTTGACGTATGTAG

CGTACAAAATTATCGTTCCTGGTATATAGATAAAGAGTCCTATATATTTGAAAATCGTTACGGTTCGATT

AAACTTTAATGATTGCATTGTGAATATATCATTAGGATTTAACTCCTTGACTATCATGGCGGTGCCAGAA

ATTACCATCAAAAGCATTAATACAGTTATGCAGATCGCAGTTAGAACGGTTATAGCATCCACCATTTATA

TCTAAAAATTAGATCAAAGAATATGTGACAACGTCCTAGTTGTATACTGAGAATTGACGAAACAATGTTT

CTTACATATTTTTTTCTTATTAGTAACCGACTTAATAGTAGGAACTGGAAAACTAGACTTGATTATTCTA

TAAGTATAGATACCCTTCCAAATAATGTTCTCTTTGATAAAAGTTCCAGAAAATGTAGAATTTTTTAAAA

AGTTATCTTTTGCTATTACTAATATCGTGGTTAGACGCTTATTATTAATATGAGTGATGAAATCCACACC

GCTTCTAGATATCGCTTTTATTTCCACATTAGATGGTAAATCCAATAGTGAAACTATCTTTTTAGGAATG

TATGGACTCGCGTTTAGAGGAGTGAACGTCTTCGGAGTAGTAAAGGATGATTCGTCAAATGAATAAACAA

TTTCACAAATGGATGTTAATGTATTAGTAGGAAATTTTTTGACGCTAGTGGAATTGAAGATTCTAATGGA

TGATGTTCTACCTATTTCATCCGATAACATGTTAATTTCCAATACCAACGGTTTTAATATTTCGATGATA

TACGGTAGTCTCTCTTTCGGACTTATATAGCTTATTCCACAATACGAGTCATTATATACTCCAAAAAACA

AAATAACTAGTATAAAATCTGTATCGAATGGGAAAAACGAAATTATCGATATAGGTATAGAATCCGGAAC

ATTGAACGTATTAATACTTAATTCTTTTTCAGTGGTAAGAACCGATAGGTTATTGACATTGTATGGTTTT

AAATATTCTATAACTTGAGACTTGATAGATATTAATGACGAATTGAAAATTATTTTTATCACCACGTGTG

TTTCAGGATCATCGTCGACGCCAGTTAACCAACCGAATGGAGTAAAATAAATATCATTAATATATGCTCT

AGATATTAGTATTTTTATTAATCCTTTGATTATCATCTTCTCGTACGCGAATGATTCCATGATCAAGAGT

GATTTGAGAACATCCTCCGGAGTATTAATGGGTTTAGTAAACAGTCCATCGTTGCAATAATAAAAGTTGT

CCAAGTTAAAGGATATTATGCATTCGTTTAAAGATATCACCTCATCTAACGGAGACAATTTTTTGGTAGG

TTTTAGAGACTTTGAAGCTACTTGTTTAACAAAGTTATTCATCGTCGTCTACTATTCTATTTAATTTTGT

AGTTAATTTATCACATATCACATTAATTGACTTTTTGGTCCACTTTTCCATACGTTTATATTCTTTTAAT

CCTGCGTTATCCGTTTCCGTTATATACAGGGATAGATCTTGCAAGTTAAATAGAATGCTCTTAAATAATG

TCATTTTTTTATCCGCTAAAAATTTAAAGAATGTATAAACTTTTTTCAAAGATTTAAAACTTTTAGGTGG

AGTTCTGGTACACAATATCATAAACAAACTAATAAACATCCCACATTCAGATTCCAACAATTGATTAACT

TCCACATTAATACAGCCTATTTTCGCTCCAAATGTACATTCGAAAAATCTGAATAAAACATCAATATCGC

AATTTGTATTATCCAATACAGAATGTCTGTGATTCGTGTTAAAACCATCGGAAAAAGAATAGAAATAAAA

ATTATTATAATGGTGGAATTCAGTTGGAATATTGCCTCCGGAGTCATAAAAGGATACTAAACATTGTTTT

TTATCGTAAATTACACATTTCCAATGAGACAAATAACAAAATCCAAACATTACAAATCTAGAGGTAGAAC

TTTTAATTTTGTCTTTAAGTATATACGATAAGATATGTTTATTCATAAACGCGTCAAATTTTTCATGAAT

AGCTAAGGAGTTTAAGAATCTCATGTCAAATTGTCCTATATAATCCACTTCGGATCCATAAGCAAACTGA

GAGACTAAGTTCTTAATACTTCGATTGCTCATCCAGGCTCCTCTCTCAGGCTCTATTTTCATCTTGACGA

CCTTTGGATTTTCACCAGTATGTATTCCTTTACGTGATAAATCATCGATTTTCAAATCCATTTGTGAGAA

GTCTATCGCCTTAGATACTTTTTCCCGTAGTTGAGGTTTAAAGAAATACGCTAACGGTATACTAGTAGGT

AACTCAAAGACATCATATATAGAATGGTAACGCGTCGTTAACTCGTCGGTTAACTCTTTCTTTTGATCGA

GTTCATCGCTACTATTGGGTCTGCTCAGGTGCCCCGACTCTACTAGTTCCAACATCATACCGATAGGAAT

ACAAGACACTTTGCCAGCGGTTGTAGATTTATCATATTTCTCCACCACATATCCGTTACAATTTGTTAAG

AATTTAGATACATCTATATTGCTACATAATCCAGCTAGTGAATATATATGACATAATAAATTGGTAAATC

CTAGTTCTGGTATTTTACTAATTACTAAATCTGTATATCTTTCCATTTATCATGGAAAAGAATTTACCAG

ATATCTTCTTTTTTCCAAACTGCGTTAATGTATTCTCTTACAAATATTCACAAGATGAATTCAGTAATAT

GAGTAAAACGGAACGTGATAATTTCTCATTGGCTGTGTTTCCAGTGATAAAACATAGATGGCATAACGCA

CACGTTGTAAAACATAAAGGAATATACAAAGTTAGTACAGAAGCACGTGGAAAAAAAGTATCTCCTCCAT

CACTAGGAAAACCCGCACATATAAACCTAATGTCGAAGCAATATATATATAGTGAGTATGCAATAAGCTT

TGAATGTTATAGTTTTCTAAAATGTATAACAAATACAGAAATCAATTCGTTCGATGAGTATATATTAAGA

GGACTATTAGAAGCTGGTAATAGTTTACAGATATTTTCCAATTCCGTAGGTAAACGAATAGATACTATAG

GTGTACTAGGGAATAAGTATCCATTTAGCAAAATTCCATTGGCCTCATTAACTCCTAAAGCACAACGAGA

GATATTTTTAGCGTGGATTTCTCATAGACCTGTAGTTTTAACTGGAGGAACCGGAGTGGGTAAGACGTCA

CAGGTACCCAAGTTATTGCTTTGGTTTAATTATTTATTTGGTGGATTCTCTTCTCTAGATAAAATCACTG

ACTTTCACGAAAGACCAGTCATTCTATCTCTTCCTAGGATAGCTTTAGTTAGATTGCATAGCAATACCAT

TTTAAAATCATTGGGATTTAAGGTACTAGATGGATCTCCTATCTCTTTACGGTACGGATCTATACCGGAA

GAATTAATAAACAAACAACCAAAAAAATATGGAATTGTATTTTCTACCCATAAGTTATCTCTAACAAAAC

TATTTAGTTATGGCACTATTATTATAGACGAAGTTCATGAGCATGATCAAATAGGAGATATTATTATAGC

AGTAGCGAGAAAACATCATACGAAAATAGATTCTATGTTTTTAATGACTGCCACGTTAGAGGATGACAGG

GAACGTCTAAAAATATTTTTACCTAATCCCGCATTTATACATATTCCTGGAGATACACTGTTTAAAATTA

GCGAGGTATTTATTCATAATAAGATAAATCCATCTTCCAGAATGGCATATATAGAAGAAGAAAAGAGAAA

TTTAGTTACTGCTATACAGATGTATACTCCTCCTGATGGATCATCCGGTATAGTCTTTGTGGCATCCGTT

GCACAGTGTCACGAATATAAATCATATTTAGAAAAAAGATTACCGTATGATATGTATATTATTCATGGTA

AGGTCTTAGATATAGACGAAATATTAGAAAAAGTGTATTCATCACCTAATGTATCGATAATTATTTCTAC

TCCTTATTTGGAATCCAGCGTTACTATACGCAATGTTACACACATTTATGATATGGGTAGAGTTTTTGTC

CCCGCTCCTTTTGGAGGATCACAACAATTTATTTCTAAATCTATGAGAGATCAACGAAAAGGAAGAGTAG

GAAGAGTTAATCCTGGAACATACGTATATTTCTATGATCTGTCTTATATGAAATCTATACAGCGAATAGA

TTCAGAATTTCTACATAATTATATATTGTACGCTAATAAGTTTAATCTAACACTCCCCGAAGATTTGTTT

ATAATCCCTACAAATTTGGATATTCTATGGCGTACAAAGGAATATATAGACTCGTTCGATATTAGTACAG

AAACATGGAATAAATTATTATCCAATTATTATATGAAGATGATAGAGTATGCTAAACTTTATGTACTAAG

TCCTATTCTCGCTGAGGAGTTGGATAACTTTGAGAGGACGGGAGAATTAACTAGTATTGTACAAGAAGCC

ATTTTATCTCTAAATTTACGAATTAAGATTTTAAAATTTAAACATAAAGATGATGATACGTATATACACT

TTTGTAGAATATTATTCGGTGTCTATAACGGAACAAACGCTACTATATATTATCATAGACCTCTAACGGG

ATATATGAATATGATTTCAGATACTATATTTGTTCCTGTAGATAATAACTAAAAATCAAAATCTAATGAC

CACATCTTTTTTTAGAGATGAAAAATTTTCCACATCTCCTTTTGTAGACACGACTAAACATTTTGCAGAA

AAAAGTTTATTATTATTTAGATAATCGTATACTTCATCAGTGTAGATAGTAAATGTGAACAGATAAAAGG

TATTCTTGCTCAATAGATTGGTAAATTCCATAGAATATATTAATCCTTTCTTCTTGAGATCCCACATCAT

TTCAACCAAAGACGTTTTATCCAATGATTTACCTCGTACTATACCACATACAAAACTAGATTTTGCAGTG

ATGTCGTACCTGGTATTCCTACCAAACAAAATTTTACTTTTAGTTCTTTTAGAAAATTCTAAGGTAGAAT

CTCTATTTGTCAATATGTCATCTATGGAATTACCACTAGCAAAAAATGATAGAAATATATATTGATACAT

CGCAGCTGGTTTTGATCTACTATACTTTAAAAACGAATCAGATTCCATAATTGCTTGTATATCATCAGCT

GAAAAACTATGTTTTACACGTATTCCTTCGGCATTTCTTTTTAATGATATATCTTGTTTAGACAATGATA

AAGTTATCATGTCCATGAGAGACGCGTCTCCGTATCGTATAAATATTTCATTAGATGTTAGACGCTTCAT

TAGGGGTATACTTCTATAAGGTTTCTTAATTAGTCCATCATTGGTTGCGTCAAGAACTACTATCTGATGT

TGTTGGGTATCTCTAGTGTTACACATGGCCTTACTAAAGTTTGGGTAAATAACTATGATATCTCTATTAA

TTATAGATGTATATATTTCATTCGTCAAGGATATTAATATCGACTTACTATCGTCATTAATACGTGTAAT

GTAATCATATAAATCATGCGATAGCCAAGGAAAATTCAAATAGATGTTCATCATATAATCGTCGCTATAA

TTCATATTAATACTTTGACATTGACTAATTTGTAATATAGCCTCGCCACGAAGAAAGCTCTCGTATTCAG

TTTCATCGATAAAGGATACCGTTAAATATAACTGGTTGCCGATAGTCTCATAGTCTATTAAGTGGTAAGT

TTCGTACAAATACAGAATCCCTAAAATATTATCTAATGTGGGATTAATCCTTACCATAACTGTATAAAAT

GGAGCCGGAGTCATAACTATTTTACCGTTTGTACTTACTGGAATAGATGAAGGAATAATCTCCGGACATG

ATGGTAAAGACCCAAATGTCTGTTTGAAGAAATCCAATGTTCCAGGTCCTAATCTCTTGACAAAAATTAC

GATATTCGATCCCGATATCCTTTGCATTCTATTTACCAGCATATCACGAACTATATTAAGATTATCTATC

ATGTCTATTCTCCCACCGTTATATAAATCGCCTCCGCTAAGAAACGTTAGTATATCCATACAATGGAATA

CTTCATTTCTAAAATAGTATTCGTTTTCTAATTCTTTAATGTGAAATCGTATACTAGAAAGGGAAAAATT

ATCTTTGAGTTTTCCATTAGAAAAGAACCACGAAACTAATGTTCTGATTGCGTCTGACTCCGTCGCTGAA

TTAATAGATTTACACCAAAAACTCATATAACTTCTAGATGTAGAAGCATTCGCTAAAAAATTAGTAGAAT

CAAAGGATATAAGTAGATGTTCCAACAAGTGAGCAATTCCCAAGATTTCATCTATATCATTCTCGAATCC

GAAATTAGAAATTCCCAAGTAGATATCCTTTTTCATCCGATCATTGATGAAAATACGAACTTTATTCGGT

AAGACGATCATTTACTAAGGAGTAAAATAGGAAGTAACGTTCGTATATCGTTATCGTCGTATAAATTAAA

GGTGTGTTTTTTGCCATTAAGAGACATTATAATTTTACCAATATTGGAATTATAATATAGGTGTATTTGA

GCACTAGAAACGGTCGATGCATCGGTAAATATAGCTGTATCTAATGTTCTAGTCGGTATTTCTTCATTTC

GCTGTCTAATGATAGCGTTTTCTCTATCTGTTTCCATTACAGCTGCCTGAAGTTTATTGGTCGGATAATA

TGTAAAATAATAAGAAATACATACGAATAACAAAAATAAAATAAGATATAATAAAGATGCCATTTAGAGA

TCTAATTTTGTTCAACTTGTCCAAATTCCTACTTACAGAAGATGAGGAATCGTTGGAGATAGTATCTTCC

TTATGTAGAGGATTTGAAATATCTTACGATGACTTAATATCGTACTTTCCAGATAGGAAATACCATAAAT

ATATTTCTAAGGTATTTGAACATGTAGATTTATCGGAGGAATTAAGTATGGAATTCCATGATACAACTCT

GAGAGATTTAGTATATCTTAGATTGTACAAGTATTCCAAGTATATACGGCCGTGTTATAAATTAGGAGAT

AATCTAAAAGGTATAGTTGTTATAAAGGACAGAAATATATATATTAGAGAAGCAAATGATGACTTGATAG

AATATCTCCTCAAGGAATACACTCCTCAGATTTATACATATTCTAATGAGCGAGTTCCCATAGCTGGTTC

AAAATTAATTCTTTGTGGATTTTCTCAAGTTACATTTATGGCGTATACAACGTCGCATATAACAACAAAT

AAAAAGGTAGATGTTCTCGTTTCCAAAAAATGTATAGATGAACTAGTCGATCCAATAAATTATCAAATAC

TTCAAAATTTATTTGATAAAGGAAGCGGAACAATAAACAAAATACTCAGGAAGATATTTTATTCGGTAAC

AGGTGGCCAAACTCCATAGGTAGCTTTTTCTATTTCGGATTTTAGAATTTCCAAATTCACCAGCGATTTA

TCGGTTTTGGTGAAATCCAAGGATTTATTAATGTCCACAAATGCCATTTGTTTTGTCTGTGGATTGTATT

TGAAAATGGAAACGATGTAGTTAGATAGATGCGCGGCGAAGTTTCCTATTAGGGTTCCGCGCTTCACGTC

ACCCAACATACTTGAATCACCATCCTTTAAAAAAAATGATAAGATATCAACATGGAGTATATCATACTCG

GATTTTAATTCTTCTACTGCCTCACTGACATTTTCACAAATACTACAATACGGTTTACCGAAAATAATCA

GCACGTTCTTCATTTATGGGTATCAAAAACTTAAAATCGTTACTGCTGGAAAATAAATCACTGACGATAT

TAGATGATAATTTATACAAAGTATACAATGGAATATTTGTGGATACAATGAGTATTTATATAGCCGTCGC

CAATTGTGTCAGAAACTTAGAAGAGTTAACTACGGTATTCATAAAATACGTAAACGGATGGGTAAAAAAG

GGAGGACATGTAACCCTTTTTATCGATAGAGGAAGTATAAAAATTAAACAAGACGTTAGAGACAAGAGAC

GTAAATATTCTAAATTAACCAAGGACAGAAAAATGTTAGAATTAGAAAAGTGTACATCCGAAATACAAAA

TGTTACCGGATTTATGGAAGAAGAAATAAAGGCAGAAATGCAATTAAAAATCGATAAACTCACATTTCAA

ATATATTTATCTGATTATGATAACATAAAAATATCATTGAATGAGATACTAACACATTTCAACAATAATG

AGAATGTTACATTATTTTATTGTGATGAACGAGACGCAGAATTCGTTATGTGTCTAGCGGCTAAAACACA

GTTCTCTACCACAGGAGAATGGCCGTTAATAATAAGTACCGATCAGGATACTATGCTATTCGCGTCTGCT

GATAATCATCCTAAGATGATAAAAAACTTAACTCAACTGTTTAAATTTGTTCCCTCGGCAGAGGATAACT

ATTTAGCAAAATTAACTGCATTAGTGAATGGATGTGATTTCTTTCCTGGACTCTATGGGGCATCTATAAC

ACCCAACAACTTAAACAAAATACAATTGTTTAGTGATTTTACAATCGATAATATAGTCACTAGTTTGGCA

ATTAAAAATTATTATAGAAAGACTAACTCTACCGTAGACGTGCGTAATATTGTTACGTTTATAAACGATT

ACGCTAATTTAGACGATGTCTACTCGTATATTCCTCCTTGTCAATGCACTGTTCAAGAATTTATATTCTC

CGCATTAGATGAAAAATGGAATGAATTTAAATCATCTTATTTAGAGAGCGTGCCGTTACCCTGCCAATTA

ATGTACGCATTAGAACCACGTAAGGAGATTGATGTTTCAGAAGTTAAAACTTTATCATCTTATATAGATT

TCGAAAATACTAAATCAGATATCGATGTTATAAAATCTATATCCTCGATTTTTGGATATTCTAACGAAAA

CTGTAACACCATAGTGTTCGGCATCTATAAGGATAATTTACTACTGAGTATAAATAATTCATTTTACTTT

AACGATAGTCTGTTAATAACCAATACTAAAAGTGATAATATAATAAATATAGGTTACTAGATTAAAAAAT

GGTGTTCCAGCTCGTGTGTTCTACATGCGGCAAAGATATTTCTCACGAACGATATAAATTGATTATACGA

AAAAAATCATTAAAGGATGTACTAGTCAGTGTAAAGAACGAATGTTGTAGGTTAAAATTATCTACACAAA

TAGAACCTCAACGTAACTTAACAGTGCAACCTCTATTGGATATAAACTAATGGATCCGGTTAATTTTATC

AAGACATATGCGCCTAGAGGTTCTATTATTTTTATTAATTATGCCATGTCATTAACTAGTCATTTGAATC

CATCGATAGAAAAACATGTGGGTATTTATTATGGTACGTTATTATCGGAACACTTGGTAGTTGAATCTAC

CTATAGAAAAGGAGTTAGAATAGTCCCATTGGATAGATTTTTTGAAGGATATCTTAGTGCAAAAGTATAC

ATGTTAGAGAATATTCAAGTTATGAAAATAGCAGCTGATATGTCGTTAACTTTACTAGGTATTCCATATG

GATTTGGTCATGATAGAATGTATTGTTTTAAATTGGTAGCTGAATGTTATAAAAATGCCGGTATTGATAC

ATCGTCTAAACGAATATTAGGTAAAGATATTTTTCTGAGCCAAAACTTTACAGATGATAATAGATGGATA

AAGATATATGATTCTAATAATTTAACATTTTGGCAAATTGATTACCTTAAAGGGTGAGTTAATATGCATA

ACTACTCCTCCGTTGTTTTTTCCCTCGTTCTTTTTCTTAACGTTGTTTGCCATCACTCTCATAATGTAAA

GATATTCTAAAATGGTAAACTTTTGCATATCGGATGCAGAAATTGGTATAAATGTTGTAATTGTATTATT

TCCCGTCAATGGACTAGTCACAGCTCCATCAGTTTTATATCCTTTAGAGTATTTCTCACTCGTGTCTAGC

ATTCTAGAGCATTCCATGATCTGTTTATCGTTGATATTGGCCGGAAAGATAGATTTTTTATTTTTTATTA

TATTACTATTGGCAATTGTAGATATAACTTCTGGTAAATATTTTTCTACCTTTTCAATCTCTTCTATTTT

CAAGCCGGCTATATATTCTGCTATATTGTTACTAGTATCAATACCTTTTCTGGCTAAGAAGTCATATGTG

GTATTCACTATATCAGTTTTAACTGGTAGTTCCATTAGCCTTTCCACTTCTGCAGAATAATCAGAAATTG

GTTCTTTACCAGAAAATCCAGCTACTATAATAGGCTCACCGATGATCATTGGCAAAATCCTATATTGTAC

CAGATTAATGAGAGCATATTTCATTTCCAATAATTCTGCTAGTTCTTGAGACATTGATTTATTTGATGAA

TCTATTTGGTTCTCTAGATACTCTACCATTTCTGCCGCATACAATAACTTGTTAGATAAAATCAGGGTTA

TCAAAGTGTTTAGTGTGGCTAGAATAGTGGGCTTGCACGTATTAAAGAATGCTGTAGTATGAGTAAACCG

TTTTAACGAATTATATAGTCTCCAGAAATCTGTGGCGTTGCATACATGAACTGAATGACATCGAAGATTG

TCCAATATTTTTAATAGCTGCTCTTTGTCCATTATTTCTATATTTGACTCGCAACAATTGTAGATACCAT

TAATCACTGATTCCTTTTTCGATGCCGGACAATAGCACAATTGTTTAGCTTTGGACTCTATGTATTCAGA

ATTAATAGATATATCTCTCAATACAGATTTCACTATACATTTTGAAACTATGTCAAAAATTGTAGAACGA

CGCTGTTCTGTAGCCATTTAACTTTAAATAATTTACAAAAATTTAAAATGAGCATCCGTATAAAAATCGA

TAAATTGCGCCAAATTGTGGCATATTTTTCAGAGTTCAGCGAAGAAGTGTCTATAAATGTAGACTCGACG

GATGAATTAATGTATATTTTTGCCGCCTTGGGCGGATCTGTAAACATTTGGGCCATTATACCTCTCAGTG

CATCAGTGTTCTACCGCGGAGCCGAAAATATTGTGTTTAACCTTCCAGTGTCCAAGGTAAAATCGTGTTT

GTGTAGTTTTCACAATGATGCTATCATAGATATAGAACCTGATCTGGAAAATAATCTAGTAAAACTTTCT

AGTTATCATGTAGTAAGTGTCGATTGTAACAAGGAACTGATGCCTATTAGGACAGATACTACTATTTGTC

TAAGTATAGATCAAAAGAAATCTTACGTATTTAATTTTCACAAGTATGAAGAAAAATGTTGTGGTAGAAC

CGTCATTCATCTAGAATGGTTGTTGGGCTTTATCAAGTGTATTAGTCAGCATCAGCATTTGGCTATTATG

TTTAAAGATGACAATATTATTATGAAGACTCCTGGTAATACTGATGCGTTTTCCAGGGAATATTCTATGA

CTGAATGTTCTCAAGAACTACAAAAGTTTTCTTTCAAAATAGCTATCTCGTCTCTCAACAAACTACGAGG

ATTCAAAAAGAGAGTCAATGTTTTTGAAACTAGAATCGTAATGGATAATGACGATAACATTCTAGGAATG

TTGTTTTCGGATAGAGTTCAATCCTTTAAGATTAACATCTTTATGGCGTTTTTAGACTAATACTTTCAAT

GAGATAAATATGGGTGGCGGAGTAAGTGTTGAGCTCCCTAAACGGGATCCGCCTCCGGGAGTACCCACTG

ATGAGATGTTATTAAACGTGGATAAAATGCATGACGTGATAGCTCCCGCTAAGCTTTTAGAATATGTGCA

TATAGGACCACTAACAAAAGATAAAGAGGATAAAGTAAAGAAAAGATATCCAGAGTTTAGATTAGTCAAC

ACAGGACCCGGTGGTCTTTCGGCATTATTAAGACAATCATATAATGGAACCGCACCCAATTGCTGTCGCA

CTTTTAATCGTACTCATTATTGGAAGAAGGATGGAAAGATATCAGATAAGTATGAAGAGGGTGCAGTATT

AGAATCGTGTTGGCCCGACGTCCACGACACTGGAAAATGCGATGTTGATTTATTCGACTGGTGTCAGGGG

GATACGTTCGATATGAACATATGCCATCAGTGGATCGGTTCAGCCTTTAATAGGAGTGATAGAACTGTAG

AGGGTCGACAATCGTTAATAAATCTGTATAATAAGATGCAAACATTATGTAGTAAAGATGCTAGTGTACC

AATATGTGAATTATTTTTGCATCATTTACGCGCACACAATACAGAAGATAGCAAAGAGATGATCGATTAT

ATTCTAAGACAACAGTCGGCGGACTTTAAACAGAAATATATGAGATGTAGTTATCCCACTAGAGATAAGT

TAGAAGAGTCATTAAAATATGCGGAACCTCGAGAATGTTGGGATCCAGAGTGTTCGAATGCCAATGTTAA

TTTCTTACTAACACGTAATTATAATAATTTAGGACTTTGCAATATTGTACGATGTAATACGAGCGTGAAT

AACTTACAGATGGATAAAACTTCCTCATTAAGATTATCATGTGGATTAAGCAATAGTGATAGATTTTCTA

CTGTTCCCGTCAATAGAGCAAAAGTAGTTCAACATAATATTAAACATTCGTTCGACCTAAAATTGCATTT

GATCAGTTTATTATCTCTCTTGGTAATATGGATACTAATTGTAGCTATTTAAATGGGTGCCGCAGCAAGC

ATACAGACGACTGTGAATACACTCAGTGAACGTATCTCGTCTAAATTAGAACAAGAAGCGAACGCTAGTG

CTCAAACAAAATGTGATATAGAAATCGGAAATTTTTATATCCGACAAAACCATGGATGTAACATCACTGT

TAAAAATATGTGCTCTGCGGACGCGGATGCTCAGTTGGATGCTGTGTTATCAGCCGCTACAGAAACATAT

AGTGGATTAACACCGGAACAAAAAGCATACGTACCAGCTATGTTTACTGCTGCGTTAAACATTCAGACGA

GTGTAAACACTGTTGTTAGAGATTTTGAAAATTATGTGAAACAGACTTGTAATTCTAGCGCTGTTGTCGA

TAACAAATTAAAGATACAAAACGTAATTATAGATGAATGTTACGGAGCCCCAGGATCTCCAACAAATTTG

GAATTTATTAATACAGGATCTAGCAAAGGAAATTGTGCCATTAAGGCGTTGATGCAATTGACTACTAAGG

CCACTACTCAAATAGCACCTAGACAAGTTGCTGGTACAGGAGTTCAGTTTTATATGATTGTTATCGGTGT

TATAATATTGGCAGCGTTGTTTATGTACTATGCCAAGCGTATGCTGTTCACATCCACCAATGATAAAATC

AAACTTATTTTAGCCAATAAGGAAAACGTCCATTGGACTACTTACATGGACACATTCTTTAGAACTTCTC

CGATGATTATTGCTACCACGGATATACAAAACTGAAAATATATTGATAATATTTTAATAGATTAACATGG

AAGTTATCGCTGATCGTCTAGACGATATAGTGAAACAAAATATAGCGGATGAAAAATTTGTAGATTTTGT

TATACACGGTCTAGAGCATCAATGTCCTGCTATACTTCGACCATTAATTAGGTTGTTTATTGATATACTA

TTATTTGTTATAGTAATTTATATTTTTACGGTACGTCTAGTAAGTAGAAATTATCAAATATTGTTGGTGT

TGGTGGCGCTAGTCATCACATTAACTATTTTTTTATTACTTTATACTATAATAGTACTAGACTGACTTCT

AACAAACATCTCACCTGCCATAAATAAATGCTTGATATTAAAGTCTTCTATTTCTAACACTATTCCATCT

GTGGAAAATAATACTCTGACATTATCGCTAATTGATACATCGGTAAGTGATATGCCTATAAAGTAATAAT

CTTCTTTGGGCACATATACCAGTGTACCAGGTTCTAACAACCTATTTACTGGTGCTCCTGTAGCATACTT

TTTTTTTACCTTGAGAATATCCATTGTTTGCTTGGTCAATAGTGATATGTGATTTTTTATCAACCACTCA

AAAAAGTAATTGGAGTGTTCATATCCTCTACGGGCTATTGTCTCATGACCGTGTATGAAATTTAAGTAAC

ACGACTGTGGTAGATTTGTTCTATAGAGCCGGTTGCCGCAAATAGATAGAACTACCAATATGTCTGTACA

AATGTTAAACATTAATTGATTAACAGAAAAAACAATGTTCGTTCTGGGAATAGAAACCAGATTAAAACAA

AATTCATTAGAATATATGCCACGTTTATACATAGAATATAAAATAACTACAGTTTGAAAAATAACAGTAT

CATTTAAACATTTAACTTGCGGGGTTAATCTCACAACTTTACTGTTTTTGAACTGTTCAAAATATAGCAT

AGATCCATGAGAAATACGTTTAGCCGCCTTTAATAGAGGAAATCCAACCGCCTTTCTGGATCTCACCAAC

GACGATAGTTCTGACCAGCAACTCATTTCTTCATCATCCACCTGTTTTAACATATAATAGGCAGGAGATA

GATATCCATCATTGCAATATTCCTTCTCGTAGGCACACAATCTAATATTGATAAAATCTCCATTCTCTTC

TCTGTATTTATTATCTTGTCTCGGTGGCTGATTAGGCTGTGGTCTATCGTTGTTGAATCTATTTTGGTCA

TTAAATCTTTCATTTCTTCCTGGTATATTTCTATCACCTCGTTTGGTTGGATTTTTGTCTATATTATCGT

TTGTAACATCGGTACGGGTATTCATTTATCACAAAAAAAACTTCTCTAAATGAGTCTACTACTAGAAAAC

CTCATCGAAGAAGATACCATATTTTTTGCAGGAAGTATATCTGAGTATGATGATTTACAAATGGTTATTG

CTGGTGCAAAATCCAAATTTCCAAGATCTATGCTTTCTATTTTTAATATAGTACCTAGAACGATGTCAAA

ATATGAGTTGGAGTTGATTCATAACGAGAATATCACAGGGGCAATGTTTACCACAATGTATAATATAAGA

AACAATTTGGGTCTAGGCGATGATAAACTAACTATTGAAGCCATTGAAAACTATTTCTTGGATCCTAACA

ATGAGGTTATGCCTCTTATCATTAATAATACGGATATGACTACCGTCATTCCTAAAAAAAGTGGTAGGAG

AAAGAATAAGAACATGGTTATCTTCCGTCAAGGATCATCACCTATCTTGTGTATTTTCGAAACTCGTAAA

AAGATTAATATTTATAAAGAAAATATGGAATCCGTATCGACTGAGTATACACCTATCGGAGACAACAAGG

CTTTGATATCTAAATATGCGGGAATTAATATCCTGAATGTATATTCTCCTTCCACGTCCATGAGATTGAA

TGCCATTTACGGATTCACCAATAAAAATAAACTAGAGAAACTTAGTACTAATAAGGAACTAGAATCGTAT

AGTTCTAGCCCTCTTCAAGAACCCATTAGGTTAAATGATTTTCTGGGACTATTGGAATGTGTTAAAAAGA

ATATTCCTCTAACAGATATTCCGACAAAGGATTGATTACTATAAATGGAGAATGTTCCTAATGTATACTT

TAATCCTGTGTTTATAGAGCCCACGTTTAAACATTCTTTATTAAGTGTTTATAAACACAGATTAATAGTT

TTATTTGAAGTATTCGTTGTATTCATTCTAATATATGTATTTTTTAGATCTGAATTAAATATGTTCTTCA

TGCCTAAACGAAAAATACCCGATCCTATTGATAGATTACGACGTGCTAATCTAGCGTGTGAAGACGATAA

ATTAATGATCTATGGATTACCATGGATAACAACTCAAACATCTGCGTTATCAATAAATAGTAAACCGATA

GTGTATAAAGATTGTGCAAAGCTTTTGCGATCAATAAATGGATCACAACCAGTATCTCTTAACGATGTTC

TTCGCAGATGATGATTCATTTTTTAAGTATTTTGCTAGTCAAGATGATGAATCTTCATTATCTGATATAT

TGCAAATCACTCAATATCTAGACTTTCTGTTATTATTATTGATCCAATCAAAAAATAAATTAGAAGCTGT

GGGTCATTGTTATGAATCTCTTTCAGAGGAATACAGACAATTGACAAAATTCACAGACTCTCAAGATTTT

AAAAAACTGTTTAACAAGGTCCCTATTGTTACAGATGGAAGGGTCAAACTTAATAAAGGATATTTGTTCG

ACTTTGTGATTAGTTTGATGCGATTCAAAAAAGAATCAGCTCTAGCTACCACCGCAATAGATCCTGTTAG

ATACATAGATCCTCGTCGTGATATCGCATTTTCTAACGTGATGGATATATTAAAGTCGAATAAAGTTGAA

AAATAATTAATTCTTTATTGTTATCATGAACGGCGGACATATTCAGTTGATAATCGGCCCCATGTTTTCA

GGTAAAAGTACAGAATTAATTAGACGAGTTAGACGTTATCAAATAGCTCAATATAAATGTGTGACTATAA

AATATTCTAACGATAATAGATACGGAACGGGACTATGGACACATGATAAGAATAATTTTGCAGCATTGGA

AGTAACTAAACTATGTGATGTCTTGGAAGCAATTACAGATTTCTCCGTGATAGGTATCGATGAAGGACAG

TTCTTTCCAGACATTGTTGAATTCTGTGAGCGTATGGCAAACGAAGGAAAAATAGTTATAGTAGCCGCGC

TCGATGGGACATTTCAACGTAGACCGTTTAATAATATTTTGAATCTTATTCCATTATCTGAAATGGTGGT

AAAACTAACTGCAGTGTGTATGAAATGCTTTAAGGAGGCTTCCTTTTCTAAACGATTAGGTACAGAAACC

GAGATAGAAATAATAGGAGGTAATGATATGTATCAATCTGTGTGTAGAAAGTGTTACATCGACTCATAAT

ATTATATTTTTTATCTAAAAAACTAAAAATAAACATTGATTAAATTTTAATATAATACTTAAAAATGGAT

GTTGTGTCGTTAGATAAACCGTTTATGTATTTTGAGGAAATTGATAATGAGTTAGATTACGAACCAGAAA

GTGCAAATGAGGTCGCAAAAAAACTGCCGTATCAAGGACAGTTAAAACTATTACTAGGAGAATTATTTTT

TCTTAGTAAGTTACAGCGACACGGTATATTAGATGGCGCCACCGTAGTGTATATAGGATCTGCTCCAGGT

ACACATATACGTTATTTGAGAGATCATTTCTATAATTTAGGAGTGATCATCAAATGGATGCTAATTGACG

GCCGCCATCATGATCCTATTCTAAATGGATTGCGTGATGTGACTCTAGTGACTCGGTTTGTTGATGAGGA

ATATCTACGATCCATCAAAAAACAACTACATCCTTCTAAGATTATTTTAATTTCTGATGTGCGATCCAAA

CGAGGAGGAAATGAACCTAGTACTGCGGATTTACTAAGTAATTATGCTCTACAAAATGTCATGATTAGTA

TTTTAAACCCCGTGGCGTCTAGTCTTAAATGGAGATGCCCGTTTCCAGATCAATGGATCAAGGACTTTTA

TATCCCATACGGTAATAAAATGTTACAACCTTTTGCTCCTTCATATTCAGCTGAAATGAGATTATTAAGT

ATTTATACCGGTGAGAATATGAGACTGACTCGAGTTACCAAATCAGACGCTGTAAATTATGAAAAAAAGA

TGTATTACCTTAATAAGATAGTCCGCAACAAAGTAGTTATTAACTTTGATTATCCTAATCAGGAATATGA

CTATTTTCACATGTACTTTATGTTGAGGACCGTATACTGCAATAAAACATTTCCTACTACTAAAGCAAAG

ATACTATTTCTACAACAATCTATATTTCGTTTCTTAAATATTCCAACGACATCAACTGAAAAAGTTAGTC

ATGAACCAATACAACGTAAAATATCTAGCAAAGATTCTATGTCTAAAAACAGAAATAGCAAGAGATCCGT

ACGCGGTAATAAATAGAAACGTACTACTGAGATATACTACCGATATAGAGTATAATGATTTAGTTACTTT

AATAACCGTTAGACATAAAATTGATTCTATGAAAACTGTGTTTCAGGTATTTAACGAATCATCCATAAAT

TATACTCCGGTTGATGATGATTATGGAGAACCAATCATTATAACATCGTATCTTCAAAAAGGTCATAACA

AGTTTCCTGTAAATTTTCTATACATAGATGTGGTAATATCTGACTTATTTCCTAGCTTTGTTAGACTAGA

TACTACAGAAACTAATATAGTTAATAGTGTACTACAAACAGGCGATGGTAAAAAGACTCTTCGTCTTCCT

AAAATGTTAGAGACTGAAATAGTTGTCAAGATTCTCTATCGTCCTAATATACCATTAAAAATTGTTAGAT

TTTTCCGCAATAACATGGTAACTGGAGTAGAGATAGCCGATAGATCTGTTATTTCAGTCGCTGATTAATC

AATTAGTAGAGATGAGAGATAAGAACATTATAATAATCAATAATATATCTTATATATCTGTTTAGAAAAA

TGCTAATATTAAAATAGCTAACGCTAGTAATCCAATCGGAAGCCATTTGATATCTATAATAGGGTATCTA

ATTTCCTGATTCAGATAGCGTACGGCTATATTCTCGGTAGCTACTCGTTTGGAATCACAAACATTATTTA

CATCTAATTTACTATCTGTAATGGAAACGTTTCCCAATGAAATGGTACAATCAGATACATTACATCTTGA

TATATTTTTTTTTAAAGAGGCTGGTAACAACGCATCGCTTCGTTTACATGGCTCGTACCAACAATAATAG

GGTAATCTTGTATCTATTCCTATCCGTACTATACTTTTATCAGGATAAATACATTTACATCGTATATCGT

CTTTGTTAGCATCACAGAATGCATAAATTTGTTCGTCCGTCATGATAAAAATTTAAAGTGTAAATATAAC

TATTATTTTTATAGTTGTAATAAAAAGGGAAATTTGATTGTATACCTTCGGTTCTTTAAAAGAAACTGAC

TTGATAAAAATGGCTGTAATCTCTAAGGTTACGTATAGTCTATACGATCAAAAAGAGATTAATGCTACAG

ATATTATCATTAGTCATATTAAAAATGACGACGATATCGGTACCGTTAAAGATGGTAGACTAGGTGCTAT

GGATGGGGCATTATGTAAGACTTGTGGGAAAACGGAATTGGAATGTTTCGGTCACTGGGGTAAAGTAAGT

ATTTATAAAACTCATATAGTTAAGCCTGAATTTATTTCAGAAATTATTCGTTTACTGAATCATATATGTA

TTCATTGCGGATTATTGCGTTCACGAGAACCGTATTCCGACGATATTAACCTAAAAGCGTTATCGGGACA

CGCTCTTAGGAGATTAAAGGATAAAATATTATCCAAGAAAAAGTCATGTTGGAACAGCGAATGTATGCAA

CCGTATCAAAAAATTACTTTTTCAAAGAAAAAGGTTTGTTTCGTCAACAAGTTGGATGATATTAACGTTC

CTAATTCTCTCATCTATCAAAAGTTAATTTCTATTCATGAAAAGTTTTGGCCATTATTAGAAATTCATCA

ATATCCAGCTAACTTATTTTATACAGACTACTTTCCCATCCCTCCGTTGATTATTAGACCGGCTATTAGT

TTTTGGATAGATAGTATACCCAAAGAGACAAATGAATTAACTTACTTATTAGGTATGATCGTTAAGAATT

GTAACTTGAATGCTGATGAACAGGTTATCCAGAAGGCGGTAATAGAATACGATGATATTAAAATTATTTC

TAATAACACTACCAGTATCAATTTATCATATATCACATCCGGCAAAAATAATATGATTAGAAGTTATATC

GTCGCTCGGCGAAAAGATCAGACCGCTAGATCCGTAATTGGTCCCAGTACATCTATCACCGTTAATGAGG

TAGGAATGCCCACATATATTAGAAATACACTTACAGAAAAGATATTTGTTAATGCCTTTACAGTGGATAA

AGTTAAACAACTATTAGCATCAAACCAAGTTAAATTTTACTTTAATAAACGATTAAACCAATTAACAAGA

ATACGTCAAGGAAAGTTTATCAAAAATAAAATACATTTATTGCCTGGTGATTGGGTAGAAGTAGCTGTTC

AAGAATATACAAGTATTATTTTTGGAAGACAACCGTCTCTACATAGATACAACGTCATCGCTTCATCTAT

CAGAGCTACCGAAGGAGATACTATCAAAATATCTCCCGGAATTGCCAACTCTCAAAATGCTGATTTCGAC

GGAGATGAAGAATGGATGATATTGGAGCAAAATCCTAAAGCCGTAGTTGAACAAAGTATTCTTATGTATC

CGACGACGTTACTCAAACACGATATTCATGGAGCCCCCGTTTATGGATCTATTCAAGATGAAATCGTAGC

AGCGTATTCATTGTTTAGGATACAAGATCTTTGTTTAGATGAAGTATTGAACATCTTGGGGAAATATGGA

AGAGAGTTCGATCCTAAAGGTAAATGTAAATTCAGCGGTAAAGATATCTATACTTACTTGATAGGTGAAA

AGATTAATTATCCGGGTCTCTTAAAGGATGGTGAAATTATTGCAAACGACGTAGATAGTAATTTTGTTGT

AGCTATGAGGCATCTGTCATTGGCTGGACTCTTATCCGATCATAAATCGAACGTGGAAGGTATCAACTTT

ATTATCAAGTCATCTTATGTTTTTAAGAGATATCTATCTATTTACGGTTTTGGGGTGACATTCAAAGATC

TGAGACCAAATTCGACGTTCACTAATAAATTGGAGGCTATCAACGTAGAAAAAATAGAACTTATCAAAGA

AGCATACGCCAAATATCTCAAAGATGTAAGAGACGGGAAAATAGTTCCATTATCTAAAGCTTTAGAGGCG

GACTACTTGGAATCCATGTTATCCAACTTGACAAATCTTAATATCAGAGAGATAGAAGAACATATGAGAC

AAACGCTGATAGATGATCCAGATAATAACCTCCTGAAAATGGCCAAAGCGGGTTATAAAGTAAATCCCAC

AGAACTAATGTATATTCTAGGTACTTATGGACAACAGAGGATAGATGGCGAACCAGCAGAGACTCGAGTA

TTGGGTAGAGTCTTACCTTACTATCTTCCAGACTCTAAGGATCCAGAAGGAAGAGGTTATATTCTTAATT

CTTTAACAAAAGGATTAACGGGTTCTCAATATTACTTTTCGATGCTGGTTGCAAGATCTCAATCTACTGA

TATTGTCTGTGAAACATCACGTACCGGAACACTGGCTAGAAAAATCATTAAAAAGATGGAGGATATGGTG

GTCGACGGATACGGACAAGTAGTTATAGGTAATACGCTCATCAAGTACGCAGCCAATTATACCAAAATTC

TAGGCTCAGTATGTAAACCTGTAGATCTTATCTATCCAGATGAGTCCATGACTTGGTATTTGGAAATTAG

TGCTTTGTGGAATAAAATAAAACAGGGATTCGTTTACTCTCAGAAACAGAAACTTGCAAAGAAGACATTG

GCGCCGTTTAATTTCCTAGTATTCGTCAAACCCACCACTGAGGATAATGCTATTAAGGTTAAGGATCTGT

ACGATATGATTCATAACGTCATTGATGATGTGAGAGAGAAATACTTCTTTACGGTATCTAATATAGATTT

TATGGAGTATATATTCTTGACGCATCTTAATCCTTCTAGAATTAGAATTACAAAAGAAACGGCTATTACT

ATCTTTGAAAAGTTCTATGAAAAACTCAATTATACTCTAGGTGGTGGAACTCCTATTGGAATTATTTCTG

CACAGGTATTGTCTGAGAAGTTTACACAACAAGCCCTGTCCAGTTTTCACACTACTGAAAAGAGTGGTGC

TGTAAAACAAAAACTTGGTTTCAACGAGTTTAATAACTTGACTAATTTGAGTAAGAATAAGACCGAAATT

ATCACTCTGGTATCCGATGATATCTCTAAACTTCAATCTGTTAAGATTAATTTCGAATTTGTATGTTTGG

GAGAATTAAATCCAGACATCACTCTTCGAAAAGAAACAGATAGATATGTAGTAGACATAATAGTCAATAG

ATTATACATCAAGAGAGCAGAAATAACCGAATTAGTCGTCGAATATATGATTGAACGATTTATCTCCTTT

AGCGTCATTGTAAAGGAATGGGGTATGGAGACATTCATTGAGGACGAGGATAATATTAGATTTACTGTCT

ACCTAAATTTCGTTGAACCGGAGGAATTGAATCTTAATAAGTTTATGATGGTTCTTCCAGGTGCCGCCAA

CAAGGGCAAGATTAGTAAATTCAAGATTCCTATCTCTGACTATACGGGATATAACGACTTCAATCAAACA

AAAAAGCTCAATAAGATGACTGTAGAACTCATGAATCTAAAAGAATTGGGTTCTTTCGATTTGGAGAACG

TCAACGTGTATCCTGGAGTATGGAATACATACGATATCTTCGGTATTGAGGCCGCTCGTGGATACTTGTG

CGAAGCCATGTTAAACACCTATGGAGAAGGTTTCGATTATCTGTACCAGCCTTGTGATCTTCTCGCTAGT

TTACTATGTGCTAGTTACGAACCAGAATCAGTTAATAAATTCAAGTTCGGTGCAGCTAGTACTCTTAAGA

GAGCTACGTTCGGAGATAATAAAGCATTGTTAAACGCGGCTCTTCATAAAAAGTCAGAACCTATTAACGA

TAATAGTAGCTGCCACTTTTTTAGCAAGGTCCCTAATATAGGAACTGGATATTACAAATACTTTATCGAC

TTGGGTCTTCTCATGAGAATGGAAAGGAAACTATCTGATAAGATATCTTCTCAAAAGATCAAGGAGATAG

AAGAAACAGAAGACTTTTAATTCTTATCAATAACATATTTTTCTATGATCTGTCTTTTAAACGATGGATT

TTCCACAAATGCGCCTCTCAAGTCCCTCATAGAATGATACACGTATAAAAAATATAGCATAGGTGATGAC

TCCTTATTTTTAGACATTAGATATGCCAAAATCATAGCTCCGCTTCTATTTACTCCCGCAACACAATGAA

CCAACACGGGCTCGTTTCGTTGATCACATTTAGATAAGAAGGCGGTCACGTCGTCAAAATATTTACTAAT

ATCAGTAGTTGTATCATCTACCAACGGTATATGAATAATATTAATATTAGAGTTAGGTAATGTATATTTA

TCCATCGTCAAATTTAAAACATATTTGAACTTAACTTCAGATGATGGTGCATCCATAGCATTTTTATAAT

TTCCCAAATACACATTATTTGTTACTCTTGTCATTATAGTGGGAGATTTGGCTCTGTGCATATCTCCAGT

TGAACGTAGTAGTAAGTATTTATACAAACTTTTCTTATCCATTTATAACGTACAAATGGATAAAACTACT

TTATCAGTAAACGCATGCAATTTAGAATACGTTAGAGAAAAGGCTATAGTAGGCGTACAAGCAGCCAAGA

CATCAACACTTATATTTTTTGTTATTATATTGGCAATTAGTGCGCTATTACTCTGGTTTCAGACATCTGA

TAATCCAGTCTTTAATGAATTAACGAGATATATGCGAATTAAAAATACGGTTAACGATTGGAAATCATTA

ACGGATAGCAAAACAAAATTAGAAAGCGATAGAGGTAGACTTCTAGCCGCTGGTAAGGATGATATATTCG

AATTCAAATGTGTGGATTTCGGCGCCTATTTTATAGCTATGCGATTGGATAAGAAAACATATCTGCCGCA

AGCTATTAGGCGAGGTACTGGAGACGCGTGGATGGTTAAAAAGGCGGCAAAAGTCGATCCATCTGCTCAA

CAATTTTGTCAGTATTTGATAGAACACAAGTCTAATAATGTTATTACTTGTGGTAATGAGATGTTAAATG

AATTAGGTTATAGCGGTTATTTTATGTCACCGCATTGGTGTTCCGATCTTAGTAATATGGAATAAGTGTT

AGATAAATGCGGTAACAAATGTTCCTGTAAGGAACCATAACAGTTTAGATTTAACATTAAAGATGAGCAT

AAACATAATAAACAAAATTACAATCAAACCTATAACATTAATATCAAACAATCCAAAAAATGAAATCAGT

GGAGTAGTAAACGTGTACATAACTCCTGGATAACGTTTAGCAGCTACCGTTCCTATTCTAGACCAAAAAT

TTGGTTTCATGGTTTCGAAGCGGTGTTCTGCAACAAGACGAGGATCGTGTTCTACATATTTGGCAGAGTT

ATCCATTATTTGCCTGTTAATCTTCATTTCGTTTTCGATTCTGGCTATTTCAAAATAAAATCCCGATGAT

AGACCTCCAGACTTTATAATTTCATCTACGATGTTCAGCGCCGTAGTAACTCTAATAATATAGGCGGATA

AGCTAACATCATACCCTCCTGTATATGTGAATATGGCATGATCTTTGTCTATTACAAGCTCGGTTTTAAC

TTTATTTCCTGTAATAATTTCTCTCATCTGTAGGATATCTATTTTCTTGTCATGTATTGCCTTCAAGACG

GGACGAAGAAACGTAATATCCTCAATAACGTTATCGTTTTCTATAATAACTACATATTCTACATTTTTAT

TTTCTAGCTCGGTAAAAAATTTAGAATCCCATAGGGCTAAATGTCTAGCGATATTTCTTTTCGTTTCCTC

TGTACACATAGTGTTACAAAACCCTGAAAAGAAGTGAGTATACTTGTCATCATCTCTAATATTTCCTCCA

GTCCATTGTATAAACACATAATCCTTGTAATGATCTGGATCATCATTGACTATCACAACGTCTCTTTTTT

CTTGCATAACTTCATTGTCCTTCACATCATCGAACTTCTGATCATTAATATGCTCATGAACATTAGGAAA

TGTTTCTGATGGAGGTCTATCAATAACTGGCACAACAATAACAGGAGTTTTCGCCGCCACCATTTAGTTA

TTGAAATTAATCATATACAACTCTCTAATACGAGTTATATTTTCGTCTATCCATTGTTTCACATTGACAT

ATTTCGACAAAAAGATATAAAATGCGTATTCCAATGCTTCTCTGTTTAATGAATTACTAAAATATACAAA

CACGTCACTGTCTGGTAATAAATGATATCTTAGAATATTGTAACAATTTATTTTGTATTGCACATGTTCG

TGATCTATGAGTTCTTCTTCGAATGGCATAGGATCTCCGAATCTGAAAACGTATAAATAGGAGTTAGAAT

AATAATATTTGAGAGTATTGGTAATGTATAAACTCTTTAGCGGTATAATTAGTTTTTTTCTCTCGATTTC

TATTTTTAGATGTGATGGAAAAATGACTAATTTTGTAGCATTAGTATCATGAACTCTAATCAAAATCTTA

ATATCTTCGTCACACGTTAGCTCTTTGAAGTTTTTAAGAGATGCATCAGTTGGTTTTACAGATGGAGTAG

GTGCAACAATTTTTTGTTTAATGCATGCATGTATTGGAGCCATTGTCTTAACTATAATGGTGCTTGTATC

GAAAAACTTTAATGCGGATAACGGAAGCTCTTCGCCGCGACTTTCTACGTCGTAATTGGGTTCTAATGCC

GATCTCTGAATGGATACTAGTTTTCTAAGTTCTAATGTGATTCTCTGAAAATGTAAATCCAATTCCTCCG

GCATTATAGATGTGTATACATCGGTAAATAAAACTATAGTATCCAACGATCCCTTCTCGCAAATTCTAGT

CTTAACCAAGAAATCGTATATAACTACGGAGATGGCGTATTTAAGAGTGGATTCTTCTACCGTTTTGTTC

TTGGATTTCATATAAGAAACTATAAAGTCCGCACTACTGTTAAGAATGATCACTAACGCAACTATATAGT

TCAAATTAAGCATCTTGGAAACATAAAATAACTCTGTAGATGATACTTGACTTTCGAATAAGTTTGCAGA

CAAACGAAGAAAGAACAGACCTCTCTTAATTTCAGAAGAAAACTTTTTTTCGTATTCCTGACGTCTAGAG

TTTATATCAATAAGAAAGTTAAGAATTAGTCGGTTAATGTTGTATTTCATTACCCAAGTTTGAGATTTCA

TAATATTGTCAAAAGACATGATAATATTAAAGATAAAGCGCTGACTATGAACGAAATAGCTATATGGTTC

GCTCAAGAATATAGTCTTGTTAAACGTGGAAACGATAACTGTATTTTTAATCACGTCAGCGGCATCTAAA

TTAAATATAGGTATATTTATTCCACACACTCTACAATATGCCACACCATCTTCATAATAAATAAATTCGT

TAGCAAAATTATTAATTTTAGTGAAATAGTTAGCGTCAACTTTCATAGCTTCCTTCAATCTAATTTGATG

CTCACATGGCGCGAATTCTACTCTAACATCCCTTTTCCATGCCTCAGGTTCATCGATCTCTATAATATCT

AGTTTCTTGCGTTTCACAAACACTGGCTCGTCTCTCGCGATGAGATCTGTATAGTAACTATGTAAATGAT

AACTAGATAGAAAGATGTAGCTATATAGATGACGATCCTTTAAGAGAGGTATAATAACTTTACCCCAATC

AGATAGACTGTTGTTATGGTCTTCGGAAAAAGAATTTTTATAAATTTTTCCAGTATTTTCTAAATATACG

TACTTGATATCTAAGAAATCCTTAATAATAATAGGAATGGATAATCCGTCTATTTTATAAAGAAATACAT

ATCGCATATTATACTTTTTTTTGGAAATTGGAATACCGATGTGTCTACATAAATACGCAAAGTCTAAATA

TTTTTTAGAGAATCTTAGTTGGTCCAAATTCTTTTCCAAGTACGGTAATAGATTTTTCATATTGAACGGT

ATCTTCTTGATCTCTGGTTCTAATTCCGCATTAAATGATGAAACTAAGTCACTATTTTTATAACTAACGA

TTACATCACCTCTAACATCATCATTTACCAGGATACTGATCTTCTTTTGTCGTAAATACATGTCTAATGT

GTTAAAAAAAAGATCATACAAGTTATACGTCATTTCATCTGTAGTATTCTTGTCATTGAAGGATAAACTC

GTACTAATCTCTTCTTTAACAGTCTGTTCAAATTTATATCCTATATACGAAAAAATAGCAACCAGTGTTT

GATCATCCGCGTCAATATTCTGTTCTATCGTAGTGTATAACAATCTTATATCTTCTTCTGTGATAGTCGA

TACGTTATAAAGGTTGATAACGAAAATATTTTTATTTCGTGAAATAAAGTCATTGTAGGATTTTGGACTT

ATATTCGTGTCTAGTAGATATGATTTTATTTTTGGAATGATCTCAATTAAAATAGTCTCTTTAGAGTCCA

TTTAAAGTTACAAACAACTAGGAAATTGGTTTATGATGTATAATTTTTTTAGTTTTTATAGATTCTTTAT

TCTATACTTAAAAAATGAAAATAAATACAAAGGTTCTTGAGGGTTGTGTTAATTGAAAGCGAGAAATAAT

CATAAATTATTTCATTATCGTGATATCCGTTAAGTTTGTATCGTAATGGCGTGGTCAATTACGAATAAAG

CGGATACTAGTAGTTTCACAAAGATGGCTGAAATCAGAGCTCATCTAAGAAATAGCGCTGAAAATAAAGA

TAAAAACGAGGATATTTTCCCGGAAGATGTAATAATTCCATCTACTAAGCCCAAAACCAAACGAACCACT

ACTCCTCGTAAACCAGCGGCTACTAAAAGATCAACCAAAAAGGATAAAGAAAAGGAGGAAGTGGAAGAAG

TAATTATAGAGGAATATCATCAAACAACTGAAGAAAATTCTCCACCTCCGTCATCATCTCCTGGAGTCGG

CGACATTGTAGAAAGCGTGGCCGCTGTAGAGCTCGATGATAGCGACGGGGATGATGAACCTATGGTACAA

GTTGAAGCTGGTAAAGTAAATCATAGTACTAGAAGCGATCTCTCTGACCTAAAGGTGGCTACCGACAATA

TCGTTAAAGATCTTAAGAAAATTATTACTAGAATCTCTGCAGTATCGACTGTTCTAGAGGATGTTCAAGC

AGCTGGTATCTCTAGACAATTTACTTCTATGACTAAAGCTATTACAACACTATCTGATCTAGTCACCGAG

GGAAAATCTAAAGTTGTTCGTAAAAAAGTTAAAACTTGTAAGAAGTAAATGCGTGCACTTTTTTATAAAG

ATGGTAAACTGTTTACCGATAATAATTTTTTAAATCCTGTATCAGACGATAATCCAGCGTATGAGGTTTT

GCAACATGTTAAAATTCCTACTCATTTAACAGATGTAGTAGTATATGAACAAACGTGGGAAGAGGCATTA

ACTAGATTAATTTTTGTGGGAAGTGATTCAAAAGGACGTAGACAATACTTTTACGGAAAAATGCATATAC

AGAATCGCAATGCTAAAAGAGATCGTATTTTTGTTAGAGTATATAACGTTATGAAACGAATTAATTGTTT

TATAAACAAAAATATAAAGAAATCGTCCACAGATTCCAATTATCAGTTGGCGGTTTTTATGTTAATGGAA

ACTATGTTTTTTATTAGATTTGGTAAAATGAAATATCTTAAGGAGAATGAAACAGTAGGGTTATTAACAC

TAAAAAATAAACACATAGAAATAAGTCCCGATGAAATAGTTATCAAGTTTGTAGGAAAGGACAAAGTTTC

ACATGAATTTGTTGTTCATAAGTCTAATAGACTATATAAACCGCTATTGAAACTGACTGATGATTCTAGT

CCCGAAGAATTTCTGTTCAACAAACTAAGTGAACGAAAGGTATATGAATGTATCAAACAGTTTGGTATTA

GAATCAAGGATCTCCGAACGTATGGAGTCAATTATACGTTTTTATATAATTTTTGGACAAATGTAAAGTC

CGTATCTCCTCTTCCATCACCAAAAAAGTTGATAGCGTTAACTATCAAACAAACTGCTGAAGTGGTAGGT

CATACTCCATCAATTTCAAAAAGAGCTTATATGGCAACGACTATTTTAGAAATGGTAAAGGATAAAAATT

TTTTAGACGTAGTATCTAAAACTACGTTCGATGAATTCCTATCTATAGTCGTAGATCACGTTAAATCATC

TACGGATGGATGATAATAGATCTTTACACAAATAATTACAAGACCGATAAATGGATAAACGGATGAAATC

TCTCGCTATGACAGCTTTCTTCGGAGAGCTAAACACGTTAGATATTATGGCATTGATAATGTCTATATTT

AAACACCATCCAAACAATACCATTTTTTCAGTGGATAAGGATGGTCAATTTATGATTGATTTCGAATACG

ATAATTATAAGGCTTCTCAATATTTGGATCTGACCCTCACTCCGATATCTGGAAATGAATGCAAGACTCA

CGCATCTAGTATAGCCGAACAATTGGCGTGTGTGGATATTATTAAAGAGGATATTAGCGAATATATCAAA

ACTACTCCCCGTCTTAAACGATTTATAAAAAAATACCGCAATAGATCATATACTCGTATCAGTCGAGATA

CAGAAAAGCTTAAAATAGCTCTAGCTAAAGGCATAGATTACGAATATATAAAAGACGCTTGTTAATAAGT

AAATGAAAAAAAACTAGTCGTTTATAATAAAACACGATATGGATACCAACATAGTATCATCTTCTACTAT

TGCGACGTATATAGACGCTTTAGCGAAGAATGCTTCAGAATTAGAACAGAGGTCTACCGCATACGAAATA

AATAATGAATTGGAACTAGTATTTATTAAACCGCCATTGATTACGTTGACAAATGTAGTAAATATCTCCA

CGATTCAGGAATCGTTTATTCGATTTACCGTTACTAATAAGGAAGGTATCAAAATTAGAACTAAGATTCC

ATTATCTAAGGTACATGGTCTAGATGTAAAAAATGTACAGTTGGTAGATGCTATAGATAACATAGTTTGG

GAAAAGAAATCATTAGTGACGGAAAATCGTCTTCACAAAGAATGCTTGTTGAGACTATCGACAGAGGAAC

GTCATATATTTTTGGATTACAAGAAATATGGATCCTCTATCCGACTAGAATTAGTCAATCTTATTCAAGC

AAAAACAAAAAACTTTACGATAGACTTTAAGCTAAAATATTTTCTAGGATCTGGTGCTCAATCTAAAAGT

TCTTTATTGCACGCTATTAATCATCCAAAGTCAAGGCCTAATACATCTCTGGAAATAGAATTTACACCTA

GAGACAATGAAACAGTTCCATATGATGAACTAATAAAGGAATTGACGACTCTCTCGCGTCATATATTTAT

GGCTTCTCCAGAGAATGTAATTCTTTCTCCACCTATTAACGCACCTATAAAGACTTTTATGTTGCCTAAA

CAAGATATAGTAGGTCTGGATCTGGAAAATCTATATGCCGTAACTAAGACTGATGGCATTCCTATAACTA

TCAGAGTTACATCAAAAGGGTTGTATTGTTATTTTACACATCTTGGTTATATTATTAGATATCCAGTTAA

GAGAACAATAGATTCCGAAGTAGTAGTCTTTGGTGAGGCAGTTAAGGATAAGAACTGGACCGTATATCTC

ATTAAGCTAATAGAGCCCGTAAATGCAATCAGTGATAGACTAGAAGAAAGTAAGTATGTTGAATCTAAAC

TAGTGGATATTTGTGATCGGATAGTATTCAAGTCAAAGAAATACGAAGGTCCGTTTACTACAACTAGTGA

AGTCGTCGATATGTTATCTACATATTTACCAAAGCAACCAGAAGGTGTTATTCTGTTCTATTCAAAGGGA

CCTAAATCTAACATTGATTTTAAAATCAAAAAGGAGAATACTATAGACCAAACTGCAAATGTAGTATTTA

GGTACATGTCCAGTGAACCAATTATCTTTGGAGAGTCGTCTATCTTTATAGAGTATAAGAAATTTACCAA

CGATAAAGGCTTTCCTAAAGAATATGGTTCTGGTAAGATTGTGTTATATAACGGCGTTAATTATCTAAAT

AATATCTATTGTTTGGAATATATTAATACACATAATGAAGTGGGTATTAAGTCCGTTGTTGTACCTATTA

AGTTTATAGCAGAATTCTTAGTCAATGGAGAAATACTTAAACCTAGAATCGATAAAACCATGAAATATAT

TAACTCAGAAGACTATTATGGAAATCAACATAATATCATAGTCGAACATTTAAGAGATCAAAGCATCAAA

ATAGGAGATGTCTTTAACGAGGATAAACTATCGGATGTTGGACATCAATACGCTACCAACAACGATAAAT

TTAGATTAAATCCAGAAGTTAGTTATTTTACTAATAAACGAACTAGAGGACCGTTGGGAATTTTATCAAA

CTACGTCAAGACTCTTCTTATTTCTATGTATTGTTCCAAAACATTTTTAGACGATTCCAACAAACGAAAG

GTATTAGCGATTGATTTTGGAAACGGTGCTGACCTGGAAAAATACTTTTATGGAGAGATTGCGTTATTGG

TAGCGACGGATCCGGATGCTGATGCTATAGCTAGAGGAAATGAAAGATACAACAAATTAAATTCTGGAAT

TAAAACCAAGTACTACAAATTTGACTACATTCAGGAAACTATTCGATCCGATACATTTGTCTCTAGTGTC

AGAGAAGTATTCTATTTTGGAAAGTTTAATATCATTGACTGGCAGTTCGCTATTCATTATTCTTTTCATC

CAAGACATTATGCTACAGTCATGAATAACTTATCCGAACTAACTGCTTCTGGAGGCAAGGTATTAATTAC

TACCATGGATGGAGACAAATTATCAAAATTAACCGATAAAAAGACTTTTATAATTCATAAGAATCTACCT

AGTAGCGAAAACTATATGTCTGTAGAAAAAATAGCTGATGATAGAATAGTGGTATATAATCCATCAACAA

TGTCTACTCCAATGACTGAATACATTATCAAAAAGAACGATATAGTCAGAGTGTTTAACGAATACGGATT

TGTTCTTGTAGATAATGTTGATTTCGCTACAATTATAGAACGAAGTAAAAAGTTTATTAATGGCGCATCT

ACAATGGAAGATAGACCGTCTACAAGAAACTTTTTCGAACTAAATAGAGGAGCCATTAAATGTGAAGGTT

TAGATGTCGAAGACTTACTTAGTTACTATGTTGTTTATGTCTTTTCTAAGCGGTAAATAATAATATGGTA

TGGGTTCTGATATCCCCGTTCTAAATGCATTAAATAATTCCAATAGAGCGATTTTTGTTCCTATAGGACC

TTCCAACTGTGGATACTCTGTATTATTAATAGATATATTAATACTTTTGTATGGTAACAGAGGTTCTACG

TCTTCTAAAAATAAAAGTTTTATAACATCTGGCCTGTTCATAAATAAAAACTTGGCGATTCTATATATAC

TCTTATTATCAAATCTAGCCATTGTCTTATAGATGTGAGCTACTGTAGGTGTACCATTTGATTTTCTTTC

TAATACTATATATTTCTCTCGAAGAAGTTCTTGCAGATCATCTGGGAATAAAATACTACTGTTGAGTAAA

TCAGTTATTTTTTTTATATCGATATTGATGGACATTTTTATAGTTAAGGATAATAAGTATCCCAAAGTAG

ATAACGACGATAACGAAGTATTTATACTTTTAGGAAATCACAATGACTTTATCAGATCAAAATTAACAAA

ATTAAAGGAGCATGTATTTTTTTCTGAATATATTGTGACTCCAGATACATATGGATCTTTATGCGTCGAA

TTAAATGGGTCTAGTTTTCAGCACGGTGGTAGATATATAGAGGTGGAGGAATTTATAGATGCTGGAAGAC

AAGTTAGATGGTGTTCTACATCCAATCATATATCTGAAGATATACACACTGATAAATTTGTCATTTATGA

TATTTATACGTTTGATTCGTTCAAGAATAAACGATTGGTATTTGTACAGGTGCCTCCATCATTAGGAGAT

GATAGCTATTTAACTAATCCGTTATTGTCTCCGTATTATCGTAATTCAGTAGCCAGACAAATGGTCAATG

ATATGATTTTTAATCAAGATTCATTTTTAAAATATTTATTAGAACATCTGATTAGAAGCCACTATAGAGT

TTCTAAACATATAACAATAGTTAGATACAAGGATACCGAAGAATTAAATCTAACAAGAATATGTTATAAT

AGAGATAAGTTTAAGGCGTTTGTATTCGCTTGGTTTAACGGCGTTTCGGAAAATGAAAAGGTACTAGATA

CGTATAAAAAGGTATCTGATTTGATATAATGAATTCAGTGACTATATCACACGCACCATATACTATTACT

TATCACGATGATTGGGAACCAGTAATGAGTCAATTGGTAGAGTTTTATAACGAAGTAGCCAGTTGGTTGC

TACGAGACGAGACGTCGCCTATTCCTGATAAGTTCTTTATACAATTGAAACAGCCGCTTAGAAATAAACG

AGTATGTGTGTGTGGTATAGATCCGTATCCAAAAGATGGAACTGGTGTACCGTTCGAATCACCAAATTTT

ACAAAAAAATCAATTAAGGAGATAGCTTCATCTATATCTAGATTAACCGGAGTAATTGATTATAAAGGTT

ATAACCTTAATATAATAGACGGGGTTATACCCTGGAATTATTACTTAAGTTGTAAATTAGGAGAAACAAA

AAGTCACGCGATTTACTGGGATAAGATTTCCAAGTTACTGCTGCAGCATATAACTAAACACGTTAGTGTT

CTTTATTGTTTGGGTAAAACAGATTTCTCGAATATACGGGCAAAGTTAGAATCCCCGGTAACTACCATAG

TGGGATATCATCCAGCGGCCAGAGACCACCAATTCGAGAAAGATCGATCATTTGAAATTATCAACGTTTT

ACTGGAATTAGACAACAAGACACCTATAAATTGGGCTCAAGGGTTTATTTATTAATGCTTTAGTGAAATT

TTAACTTGTGTTCTAAATGGATGCGGCTATTAGAGGTAATGATGTTATCTTTGTTCTTAAGACTATAGGT

GTCCCGTCAGCATGCAGACAAAATGAAGATCCAAGATTCGTAGAAGCATTTAAATGCGACGAGTTAGAAA

GATATATTGATAATAATCCAGAATGTACACTATTCGAAAGTCTTAGGGATGAGGAAGCATACTCTATAGT

CAGAATTTTCATGGATGTAGATTTAGACGCGTGTCTAGACGAAATAGATTATTTAACGGCTATTCAAGAT

TTTATTATCGAGGTGTCAAACTGTGTAGCTAGATTCGCATTTACAGAATGCGGTGCCATTCATGAAAATG

TAATAAAATCCATGAGATCTAATTTTTCATTGACTAAGTCTACAAATAGAGATAAAACAAGTTTTCATAT

TATCTTTTTAGACACGTATACCACTATGGATACATTGATAGCTATGAAACGAACACTATTAGAATTAAGT

AGATCATCTGAAAATCCACTAACAAGATCGATAGACACTGCCGTATATAGGAGAAAAACAACTCTTCGGG

TTGTAGGTACTAGGAAAAATCCAAATTGCGACACTATTCATGTAATGCAACCACCTCACGATAATATAGA

AGATTACCTATTCACTTACGTGGATATGAACAACAATAGTTATTACTTTTCTCTACAACGACGATTGGAG

GATTTAGTTCCTGATAAGTTATGGGAACCAGGGTTTATTTCGTTCGAAGACGCTATAAAAAGAGTTTCAA

AAATATTCATTAATTCTATAATAAACTTTAATGATCTCGATGAAAATAATTTTACAACGGTACCACTGGT

CATAGATTATGTAACACCTTGTGCATTATGTAAAAAACGATCGCATAAACATCCGCATCAACTATCGTTG

GAAAATGGTGCTATTAGAATTTACAAAACTGGTAATCCACATAGTTGTAAAGTTAAAATTGTTCCGTTGG

ATGGTAATAAACTGTTTAATATTGCACAAAGAATTTTAGACACTAACTCTGTTTTATTAACCGAACGAGG

AGACCATATAGTTTGGATTAATAATTCATGGAAATTTAACAGCGAAGAACCCTTGATAACAAAACTAATT

CTATCAATAAGACATCAACTACCTAAGGAATATTCAAGCGAATTACTCTGTCCGAGGAAACGAAAGACTG

TAGAAGCTAACATACGAGACATGTTAGTAGATTCAGTAGAGACCGATACCTATCCGGATAAACTTCCGTT

TAAAAATGGTGTATTGGACCTGGTAGACGGAATGTTTTACTCTGGAGATGATGCTAAAAAATATACGTGT

ACTGTATCAACCGGATTTAAATTTGACGATACAAAATTCGTCGAAGACAGTCCAGAAATGGAAGAGTTAA

TGAATATCATTAACGATATCCAACCATTAACGGATGAAAATAAGAAAAATAGAGAGCTGTATGAAAAAAC

ATTATCTAGTTGTTTATGTGGTGCTACCAAAGGATGTTTAACATTCTTTTTTGGAGAAACCGCAACTGGG

AAGTCGACAACCAAACGTTTGTTAAAGTCTGCTATCGGTGACCTGTTTGTCGAGACGGGTCAAACAATTT

TAACAGATGTATTGGATAAAGGACCTAATCCATTTATCGCTAATATGCATTTAAAAAGATCTGTATTCTG

TAGCGAACTACCTGATTTTGCATGTAGTGGATCAAAGAAAATTAGATCTGATAATATTAAAAAGTTGACA

GAACCTTGTGTCATTGGAAGACCGTGTTTCTCCAATAAAATTAATAATAGAAACCATGCGACAATCATTA

TCGATACTAATTACAAACCTGTCTTTGATAGGATAGATAACGCATTAATGAGAAGAATCGCCGTCGTGCG

ATTCAGAACACACTTTTCTCAACCTTCTGGTAGAGAGGCTGCTGAAAATAATGACGCGTACGATAAAGTC

AAACTATTAGACGAGGGATTAGATGGTAAAATACAGAATAATAGATATAGATTCGCATTTCTATACTTGT

TGGTTAAATGGTACAAAAAATATCATATTCCTATTATGAAACTATATCCTACACCGGAAGAGATTCCGGA

CTTTGCATTCTATCTCAAAATAGGTACTCTGTTGGTATCTAGCTCTGTAAAGCATATTCCATTAATGACG

GACCTCTCCAAAAAGGGATATATATTGTACGATAATGTGGTTACTCTTCCGTTGACTACTTTCCAACAGA

AAATATCCAAGTATTTTAATTCTAGACTATTTGGACACGATATAGAGAGCTTCATCAATAGACATAAGAA

ATTTGCCAATGTTAGTGATGAATATCTGCAATATATATTCATAGAGGATATTTCATCTCCGTAAATATAT

GCCATATATTTATAGAAGATATCACATATCTAAATGAATACCGGAATCATAGATTTATTTGATAATCATG

TTGATAGTATACCAACTATATTACCTCATCAGTTAGCTACTTTAGATTATCTAGTTAGAACTATCATAGA

TGAGAACAGAAGCGTGTTATTGTTCCATATTATGGGATCGGGTAAAACAATAATCGCTTTGTTGTTCGCC

TTGGTAGCTTCCAGATTTAAAAAGGTTTACATTTTAGTACCGAACATCAACATCTTAAAAATTTTCAATT

ATAATATGGGTGTAGCTATGAACTTGTTTAATGACGAATTCATAGCTGAGAATATCTTTATTCATTCCAC

AACAAGTTTTTATTCTCTTAATTATAACGATAACGTCATTAATTATAACGGATTAAGTCGCTACAATAAC

TCTATTTTTATCGTTGATGAGGCACATAATATTTTTGGGAATAATACTGGAGAACTTATGACCGTGATAA

AAAATAAAAACAAGATTCCTTTTCTACTATTGTCTGGATCTCCCATTACTAACACACCTAATACGCTGGG

TCATATTATAGATTTAATGTCCGAAGAGACGATAGATTTTGGTGAGATTATTAGTCGTGGTAAGAAAGTA

ATTCAGACACTTCTTAACGAACGCGGAGTGAATGTACTCAAGGATTTGCTTAAAGGAAGAATATCATATT

ACGAAATGCCGGACAAAGATCTACCAACAATAAGATATCACGGACGTAAATTTCTAGATACTCGAGTAGT

ATATTGTCACATGTCTAAACTTCAAGAGAGAGATTATATGATTACTAGACGGCAGCTATGTTATCATGAA

ATGTTTGATAAAAATATGTATAACGTGTCAATGGTAGTATTGGGACAACTTAATCTGATGAATAATTTAG

ATACGTTATTTCAGGAACAGGATAAGGAATTGTACCCAAATCTGAAAATAAATAATGGAGTGTTATACGG

TGAAGAATTGGTAACGTTAAACATTAGTTCCAAATTTAAGTACTTTATCAATCGGATACAGACACTCAAG

GGAAAACACTTTATATACTTCTCTAATTCTACATATGGTGGATTGGTAATTAAATATATCATGCTCAGTA

ATGGATATTCTGAATATAATGGTTCTCAGGGAACTAATCCACATATGATAAACGACAAACCAAAAACATT

TGCTATCGTTACTAGTAAAATGAAATCGTCTTTAGAGGATCTATTAGATGTGTATAATTCTCCTGAAAAC

GATGATGGCAGTCAATTGATGTTTTTGTTTTCGTCAAACATTATGTCTGAATCCTATACTCTGAAAGAGG

TAAGGCATATTTGGTTTATGACTATCCCGGATACTTTTTCTCAATACAACCAAATTCTTGGACGATCTAT

TAGAAAATTCTCTTACGCCGATATTTCTGAACCCGTTAATGTATATCTTTTAGCAGCCGTATATTCAGAT

TTCAATGACGAAGTGACGTCATTAAACGATTATACACAGGATGAATTGATTAATGTTTTACCCTTTGACA

TCAAAAAGCTGTTGTATCTAAAATTTAAGACTAAAGAAACGAATAGAATATACTCTATTCTTCAAGAGAT

GTCTGAAACGTATTCTCTTCCACCACATCCATCAATTGTAAAAGTTTTATTGGGAGAATTGGTCAGACAA

TTTTTTTATAATAATTCTCGTATTAAGTATAACGACTCCAAGTTACTTAAAATGGTTACATCAGTTATAA

AAAATAAAGAAGACGCTAGGAATTACATAGATGATATTGTAAACGGTCACTTCTTTGTATCGAATAAAGT

ATTTGATAAATCTCTTTTATACAAATACGAAAACGATATTATTACAGTACCGTTTAGACTTTCCTACGAA

CCATTTGTTTGGGGAGTTAACTTTCGTAAAGAATATAATGTGGTATCTTCTCCATAAAACTGATGAGATA

TATAAAGAAATAAATGTCGAGCTTTGTTACCAATGGATATCTTCCAGTTACATTGGAACCACATGAGTTG

ACGTTAGACATAAAAACTAATATTAGGAATGCCGTATATAAGGCGTATCTCCATAGAGAAATTAGTGGTA

AAATGGCCAAGAAAATAGAAATTCGTGAAGACGTGGAATTACCTCTCGGCGAAATAGTTAATAATTCTGT

AGTTATAAACGTTCCGTGTGTAATAACCTACGCATATTATCACGTTGGGGATATAGTCAGAGGAACATTA

AACATCGAAGATGAATCAAATGTAACTATTCAATGTGGAGATTTAATCTGTAAACTAAGTAGAGATTCGG

GTACTGTATCATTTAGCGATTCAAAGTACTGCTTTTTTCGAAATGGTAATGCGTATGATAACGGCATCGA

AGTCTCCGCCGTTCTAATGGAGGCTCAACAAGGTACCGAATCTAGTTTTGTTTTTCTCGCGAATATCGTT

GACTCATAAGAAAGAGAATAGCGGTGAGTATAAATACGAATACTATGGCAATAATTGCGAATGTTTTATT

CCCTTCGATATATTTTTGATAATATGAAAAACATACCTCTCTCAAATCAGACAACCATTTCATAAAATAG

TTCTCTCGCACTGGTGAGGTGGTTGCAGCTCGTATAATCTCCCCAGAATAATATACTTGCGTGTCGTCGT

TCAATTTATACGGATTTCTATAATTCTCTGTTATATAATGAGGTTTACCCTCATGATTAGACGACGACAA

TAGTGTTCTGAATTTAGATAGTTGATCAGAATGAATGTTTATTGGTGTTGGAAAAATTATCCATGCTGCG

TCTGCAGAGTGGTTGATAGTTGTTCCTAGATATGTAAAATAATCCAACGTACTAGGTAGCAAATTGTCTA

GATAAAATACTGAATCAAATGGCGCAGACATATTAGCGGATCTAATGGAATCCAATTGATTGACTATCTT

TTGAAAATATACATTTTTATGATCTGATACTTGTAAGAATATAGCAATAATGATAATTCCATCATCGTGT

TTTTTTGCCTCTTCATAAGAACTATATTTTTTCTTATTCCAATGAACCAGATTAATCTCTCCAGAGTATT

TGTATACATCTATCAAGTGATTGGATCCATAATCGTCTTCCTTTCCCCAATATATATGTATTGTTGATAA

CACATATTCATTGGGGAGAAACCCTCCACTTATATATCCTCCTTTAAAATTAATCCTTACTAGTTTTCCA

GTATTCTGGATAGTGGTTGGTTTCGACTCATTATAATGTATGTCTAACGTCTTCAATCGCGCGTCAGAAA

TTGCTTTTTTAGTTTCTATATTAATAGGAGATAGTTGTTGAGGCATAGTAAAAATGAAATGATAACTGTC

TAGAAATAGCTCTTAGTATGGGATTTACAATGGATGAGGAAGTGATATTTGAAACTCCTAGAGAATTAAT

ATCTATTAAACGAATAAAAGATATTCCAAGATCAAAAGACACGCACGTGTTTGCTGCGTGTATAACAAGT

GACGGATATCCGTTAATAGGAGCTAGAAGAACTTCATTCGCATTCCAGGCGATATTATCTCAACAAAATT

CAGATTCTATCTTTAGAGTATCCACTAAACTATTACGGTTTATGTACTACAATGAACTAAGAGAAATCTT

TAGACGGTTGAGAAAAGGTTCTATCAACAATATCGATCCTCACTTCGAAGAGTTAATATTATTGGGTGGT

AAACTAGATAAAAAGGAATCTATTAAAGATTGTTTAAGAAGAGAATTAAAAGAGGAAAGTGATGAACATA

TAACAGTAAAAGAATTCGGAAATGTAATTCTAAAACTTACAACGAGTGATAAATTATTTAATAAAGTATA

TATAGGTTATTGCATGGCATGTTTTATTAATCAATCGTTGGAGGATTTATCACATACTAGTATTTACAAT

GTAGAAATTAGAAAGATTAAATCGTTAAATGATTGTATTAACGACGATAAATACGAATATCTGTCTTATA

TTTATAATATACTAATTAATAGTAAATGAGCTTTTACAGATCTAGTATAATTAGTCAGATTATTAAGTAT

AATAGACGACTAGCTAAGTCTATTATTTGCGAGGATGACTCTCAAATTATTACACTCACGGCATTCGTTA

ACCAATGCCTATGGTGTCATAAACGAGTATCCGTGTCCGCTATTTTATTAACTACTGATAACAAAATATT

AGTATGTAACAGACGAGATAGTTTTCTCTATTCTGAAATAATTAGAACTAGAAACATGTATAGAAAGAAA

CGATTATTTCTGAATTATTCCAATTATTTGAACAAACAGGAAAGAAGTATACTATCGTCATTTTTTTCTC

TAGATCCAGCTACTGCTGATAATGATAGAATAAACGCTATTTATCCGGGTGGTATACCCAAAAGGGGTGA

GAACGTTCCAGAGTGTTTATCCAGGGAAATCAAAGAAGAAGTTAATATAGACAATTCTTTTGTATTCATA

GACACTCGTTTTTTTATTCATGGTATCATAGAAGATACCATTATTAACAAATTTTTTGAGGTAATTTTCT

TTGTTGGACGAATATCTCTAACGAGTGATCAAATTATTGATACCTTTAAAAGTAATCATGAAATAAAGGA

TCTAATATTTTTAGATCCAAATTCAGGTAATGGACTCCAATACGAAATTGCAAAATATGCTCTAGATACT

GCAAAACTTAAATGTTACGGTCATAGAGGATGTTATTATGAATCATTAAAAAAATTAACTGAGGATGATT

GATTAGAAAATATAAATTAATTTACCATCGTGTATTTTTATAACGGGATTGTCTGGCATATCATGTAGAT

AGTTACCGTCTACATCGTATACTCTACCATCTACGCCTTTAAATCCTCTATTTATTGATATTAATCTATT

AGAATTGGAATACCAAATATTAGTACCCTCAATTAGTTTATTGGTAATATTTTTTTTAGACGATAGATCG

ATGGCTCTTGAAACCAAGGTTTTCCAACCGGACTCATTGTCTATCGGTGAGAAGTCTTTTTCATTAGCAT

GAATCCATTCTAATGATGTATGTTTAAACACTCTAAACAATTGTACAAATTCTTTTGATTTGTTTTGAAT

GATTTCAAATAGGTCTTCGTCTACAGTAGGCATACCATTAGATAATCTAGCCATTATAAAGTGCACGTTT

ACATATCTACGTTCTGGAGGAGTAAGAACGTGACTATTGAGACGAATGGCTCTTCCTACTATCTGACGAA

GAGACGCCTCGTTCCATGTCATATCTAAAATGAAGATATCATTGATTGAGAAGAAACTAATACCCTCGCC

TCCGCTAGAAGAGAATACGCATGTTTTAATGCATTCTCCGTTAGTGTTTGATTCTTGGTTAAACTCAGCC

ACCGCCTTGATTCTAGTATCTTTTGTTCTAGATGAGAACTCTATATTAGAGATACCAAAGACTTTGAAAT

ATAGTAATAAGATTTCTATTCCTGACTGATTAACAAATGGTTCAAAGACTAGACATTTACCATGGGATGC

TAATATTCCCAAACATACATCTATAAATTTGACGCTTTTCTCTTTTAATTCAGTAAATAGAGAGATATCA

GCCGCAATAGCATCCCCTCCCAATAGTTCTCCCTTTTTAAAGGTGTCTAATGCGGATTTAGAAAATTCTC

TATCTCTTAATGAATTTTTAAAATCATTATATAGGGTTGCTATCTCTTGTGCGTATTCTCCCGGATCACG

ATTTTGTCTTTCAGGAAAGCTATCGAATGTAAACGTAGTAGCCATACGTCTCAGAATTCTAAATGATGAT

ATACCAGTTTTTATTTCTGCGAGTTTAGCCTTTTGATAAATCTCTTCTTGCTTTTTTGACATATTAACGT

ATCGCATTAATACTGTTTTCTTAGCGAATGATGCAGACCCTTCCACATCATCAAAAATAGAAAACTCGTT

ATTAACTATGTACGAACATAGGCCTCCTAGTTTGGAGACTAATTCTTTTTCATCGACTAGACGTTTATTC

TCAAATAGCGATTGGTGTTGTAAGGATCCTGGTCGCAGTAAGTTAACCAACATGGTGAATTCTTGCACAC

TATTAACGATAGGTGTAGCCGATAAACAAATCATCTTATGGTTTTTTAACGCAATGGTCTTAGATAAAAA

ATTATATACTGACCGAGTAGGACGGATCTTACCATCTTCTTTGATTAATGATTTAGAAATGAAGTTATGA

CATTCATCAATGATGACGCATATTCTACTCTTGGAATTAATAGTTTTGATATTAGTAAAAAATTTATTTC

TAAAATTTTGATCATCGTAATTAATAAAAATACAATCCTTCGTTATCTCTGGAGCGTATCTGAGTATAGT

GTTTATCCAAGGATCTTCTATCAAAGCCTTTTTTACCAATAAGATAATTGCCCAATTCGTATAAATATCC

TTAAGATGTTTGAGAATATATACAGTAGTCATTGTTTTACCGACACCTGTTTCATGGAACAATAAAAGAG

AATGCATACTGTCTAATCCTAAGAAAACTCTTGCTACAAAATGTTGATAATCCTTGAGGCGTGCTACGTC

TGACCCCATCATTTCAACGGGCATATTAGTAGTTCTGCGTAAGGCATAATCGATATAGGCCGCGTGTGAT

TTACTCATTTATGAGTGATAAGTAATAACTATGTTTTAAAAATCACAGCAGTAGTTTAACTAGTCTTCTC

TGATGTTTGTTTTCGATACTTTTTGAATCAGAAGTCATACTAGAATAAAGCAGCGAGTGAACGTAATAGA

GAGCTTCGTATACTCTATTCGAAAACTCTAAGAACTTATTAATGAATTCCGTATCCACTGGATCGTTTAA

AATACTAAATTGAACAGTGTTCACATCCTTCCAAGACGAAGACTTAGTGACGGACTTAACATGAGACATA

AATAAATCCAAATTTTTTTTATAAACATCACTAGCCACCATAATGGCGCTATCTTTCAACCAACTATCGC

TTACGCATTTTAACAGTCTAACATTTTTAAAGAGACTACAATATATTCTCATAGTATCGATTACACCTCT

ACCGAATAGAGTGGGAAGTTTAATAATACAATATTTTTCGTTTACAAAATCAAATAATGGTCGAAACACG

TCGAAGGTTAACATCTTATAATCGCTAATGTATAGATTGTTTTCAGTGAGATGATTATTAGATTTAATAG

CATCTCGTTCACGTTTGAACAGTTTATTGCGTGCGCTGAGGTCGGCAACTACGGCATCCGCTCTAGTACT

CCTCCCATAATACTTTACGCTATTAATCTTTAAAATTTCATAGACTTTATCTAGATCGCTTTCTGGTAAC

ATGATATCATGTGTAAAAAGTTTTAACATGTCGGTCGGCATTCTATTTAGATCATTAACTCTAGAAATCT

GAAGAAAGTAATTAGCTCCATATTCCAGACTAGGTAATGGGCTTTTACCTAAAGACAAGTTAAGTTCTGG

CAATGTTTCATAAAATGGAAGAAGGACATGTGTCCCCTCCCGGATATTTTTTACAATTTCATCCATTTAC

AACTCTATAGTTTGTTTTCATTATTATTAGTTATTATCTCCCATAATCTTGGTAATACTTACACCTTGAT

CATAAGATACCTTATACAGGTCATTACATACAACTACCAATTGTTTTTGTACATAATAGATTGGATGATT

GACATCCATGGTGGAATAAACTACTCGAACAGATAGTTTATCTTTCCCCCTAGATACATTGGCCGTAATA

GTTGTCGGCCTAAAGAATATCTTTGGTGTAAAGTTAAAAGTTAGGGTTCTTGTTCCATTATTGCTTTTTG

TCAGTAGTTCGTTATAAATTCTCGAGATGGGCCCGTTCTCTGAATATAGAACATCATTTCCAAATCTAAC

TTCTAGTCTAGAAATAATATCGGTCTTATTTTTAAAATCTATTCCCTTGATGAATGGATCGTTAATAAAC

AAATCCTTGGCCTTTGATTCGGCTGATCTATTATCTCCGTTATAGACGTTACGTTGACTAGTCCAAATAC

TTACAGGAATAGATGTATCGATGATGTTGATAGTATGTGATATGTGAGCAAAGACTGTTCTCTTGGTGGC

GTCGCTATATGTTCCAGTAATGGCGGAAAACTTTTTAGAAATGTTATATATAAAAGAATTTTTTCGGGTT

CCAAACATTAACAGATTAGTATGAAGATAAACACTCATATTATCAGGAACATTATCAATTTTTACATAAA

CATCGGCATCTTGAATAGAAACAACACCATCTTCTGGAACCTCTACGATCTCGGCAGATTCCGGATAACC

AGTCGGTGGACCATCACTAACAATAACTAGATCATCCAACAATCTACTCACATATGCGTCTATATAATCT

TTTTCATCTTGTGAGTACCCTGGATACGAAATAAATTTGTTATCAGTATTTCCATAATAAGGTTTAGTAT

AAACAGAGAGAGATGTTGCTGCATGAACTTCGGTTACTGTCGCCGTTGGTTGGTTTATTTGACCTATTAC

TCTCCTAGGTTTCTCTATAAATGATGGTTTAATTTGTACATTCTTAACCATATATCCAATAAAGCTCAAT

TCAGGAACATAAACAAATTCTTTGTTGAACGTTTCAAAGTCGAACGAAGAGTCACGAATAACGATATCGG

ATACTGGATTGAAGGTCACCGTTACGGTAATTTTTGAATCGGATAGTTTAAGACTACTGAATGTATCTTC

CACATCAAACGGAGTTTTAATATAAACGTATACTGTAGATGGTTCTTTAATAGTGTCATTAGGAGTTAGA

CCAATAGAAATATCATTAAGTTCACTAGAATATCCAGAATGTTTCAAAGCAATTGTATTATTGATACAAT

TATTATATAATTCTTCGCCCTCAATTTCCCAAATAACACCGTTACACGAAGAGACAGATACATTATTAAT

ACATTTATATCCAACATATGGTACGTAACCGAATCTTCCCATACCTTTAACTTCTGGAAGTTCCAAACTC

AGAACCAAATGATTAAGCGCAGTAATATACTGATCCCTAATTTCGAAGCTAGCGATAGCCTGATTGTCTG

GCCCATCGTTTGTCATAACTCCGGATAGAGAAATATATTGCGGCATATATAAAGTTGGAATTTGACTATC

AACTGCGAAGACATTAGACCGTTTAATAAAGTCATCCCCACCGATCAAAGAATTAATGATAGTATTATTC

ATTTTCTATTTAAAATGGAAAAAGCTTACAATAAACTCCGTAGAGAAATATCTATAATTTGTGAGTTTTC

CTTAAAGTAACAGCTTCCGTAAACACCGTCTTTATCTCTTAGTAAGTTTATTGTATTTATGACCTTTTCC

TTATCTTCATAGAATACTAAAGGCAATAAAGAAATTTTTGGTTCTTCTCTAAGAGCTACGTGAGACTTAA

CCATAGACGCCAACGAATCCCTACATATTTTAGAACAGAAATACCCAACTTCACCACCCTTGAATGTCTC

AATACTAATAGGTCTAAAAACCAAATCTTGATTACAAAACCAACACTTATCAATTACACTATTTGTCTTA

ATAGACATATCTGCCATAGATTTATAATACTTTGGTAGTATACAAGCGAGTGCTTCTTCTTTAGCGGGCT

TAAAGACTGCTTTAGGTGCTGAAATAACCACATCTGGAAGACTTACTCGCTTAGCCATTTAATTACGGAA

CTATTTTTTTATACTTCTAATGAACAAGTAGAAAACCTCTCATCTACAAAAACATACTCGTGTCCATAAT

CCTCTACCATAGTAACACGTTTTTTAGATCTCATATGTGCTAAAAAGTTTTCCCATACTAATTGGTTACT

ATTATTTTTCGTATAATTTTTAACAGTTTGAGGTTTTAGATTTTTAGTTACAGAAGTGATATCGAATATT

TTATCCAAAAAGAATGAGTAATTAATTGTCTTAGAAGGAGTGTTTTCTTGGCAAAAGAATACCAAGTGCT

TAAATATTTCTACTACTTCATTAATCTTTTCTGTACTCAGATTCAGTTTCTCATCTTTTACTTGATTGAT

TATTTCAAAGACTAACTTATAATCCTTTTTATTTATTCTCTCGTTAGCCTTAAGAAAACTAGATACAAAA

TTTGCATCTACATCATCCGTGGATATTTGATTTTTTTCCATGATATCCAATAGTTCCGAGATAATTTCTC

CAGAACATTGATGAGACAATAATCTCCGCAATACATTTCTCAAATGAATAAGTTTATTAGACACGTGGAA

GTTTGACTTTTTTTGTACCTTTGTACATTTTTGAAATACAGACTCGCAAAAAATACAATATTCATATCCT

TGTTCAGATACTATACCGTTATGTCTACAACAGCTACATAATCGTAGATTCATGTTAACACTCTACGTAT

CTCGTCGTCCAATATTTTATATAAAAACATTTTATTTCTAGACGTTGTCAGAAAATCCTGTAATATTTTT

AGTTTTTTTGGTTGTGAATAAAGTATCGCCCTAATAATATTGGTACCGTCTTCCGACAATATAGTAGTTA

AATTATCCGAGCATGTAGAAGAACACCGCTTAGGCGGATTCAGTACAATGTTATATTTTTCGTACCAACT

CATTTAAATATCATAATCTAAAATAGTTCTGTAATATGTCTAGCGCTAATATATTGATCATAATCCTGTG

CATAAATTAAGATACAACAATGTCTTGAAATCATCGACATGGCTTCTTCCATAGTTAGAAGATCATCGTC

AAAGTTAGCAACGTGATTCATCAACATTTGCTGTTTTGAGGCAGCAAATACTGAACCATCACCATTCAAC

CATTCATAAAAACCATCGTCTGAATCCATTGATAATTTCTTGTACTGGTTTTTGAGAGCTCGCATCAATC

TAGCATTTCTAGCTCCCGGATTGAAAACAGAAAGAGGATCGTACATCCAGGGTCCATTTTCTGTAAATAG

AATCGTATAATGTCCCTTCAAGAAGATATCAGACGATCCACAATCAAAGAATTGGTCTCCGAGTTTGTAA

CAGACTGCGGACTTTAACCTATACATGATACCGTTTAGCATGATTTCTGGTGATACGTCAATCGGAGTAT

CATCTATTAGAGATCTAAAGCCGGTGTAACATTCTCCACCAAACATATTCTTATTCTGACGTCGTTCTAC

ATAAAACATCATTGCTCCATTAACGATAACAGGTGAATGAACAGCACTACCCATCACATTAGTTCCCAAT

GGATCAATGTGTGTAACTCCAGAACATCTTCCATAGCCTATGTTAGGAGGAGCGAACACCACTCTTCCAC

TATTGCCATCGAATGCCATAGAATAAATATCCTTGGAATTGATAGAAATCGGACTGTCGGATGTTGTTAT

CATCTTCATAGGATTAACAACGATGTATGGTGCAGCCTGAAGTTTCATATCGTAACTGATGCCGTTCATA

GGTCTAGCCACAGAAACCAACGTAGGTCTAAATCCAACTATAGACAAAATAGAAGCTAATATCTGTTCCT

CATCTGTCATAACTTGAGAGCATCCAGTATGAATAATCTTCATTAGATGGGGATCTACCGCATCATCATC

GTTACAATAAAAAATTCCCATTCTAATGTTCATAATTGCTTTTCTAATCATGGTATGAATGTTTGCTCTC

TGAATCTCTGTGGAAATTAGATCTGATACACCTGTAATCACTATCGGATTATCCTCCGTAAGACGATTAA

CCAACAACATATAATTATAAGACTTTACTCTTCTAAATTCATAAAGTTGCTGGATTAGACTATATGTGTC

TCCATGTACATACGCGTTCTCGAGCGCAGGAAGTTTAATACCGAATAGTGCCATCAGAATAGGATGAATG

TAGTAATTAGTTTCTGGTTTTCTATAAATAAAAGACAAATCTTGTGAACTAGACATATCGGTAAAATGCA

TGGATTGGAATCGTGTAGTCGACAGAAGAATATGATGATTAGATGGAGAGTATATTTTATCTAACTCTTT

GAGTTGGTCACCGATTCTAGGACTAGCTCGAGAATGAATAAGTACTAAGGGATGAGTACATTTCACAGAA

ACACTGGCGTTGTTCAACGTACTCTTTACATGGGAAAGGAGTTGAAATAGCTCGTTTCTATTTGTCCTGA

CAATATTTAGTTTATTCATAATATTAAGCATATCCTGAATAGTAAAGTTAGATGTGTCATACTTGTTAGT

AGTTAGATATTTAGCAATTGCATTCCCATCATTTCTCAATCTCGTACTCCAATCATGTGTGGATGCTACT

TCGTCGATGGAAACCATACAATCCTTTTTGATAGGCTGTTGAGATTGATCATTTCCTGTACGTTTAGGTT

TGGTACGTTGATTTCTAGCCCCTGCTGATATAAAGTCATCGTCTACAATTTGGGATAATGAATTACATAC

ACTACAAGACAAAGATTTATCAGAAGTGTGAATATGATCTTCATCTACCAAAGAAAGAGTTTGATTAGTA

TAACTAGATTTTAGTCCCGCGTTAGATGTTAAAAAAACATCGCTATTGACCACGGCTTCCATTATTTATA

TTCGTAGTTTTTACTCGAAAGCGTGATTTTAATATCCAATCTTATTACTTTTGGAATCGTTCAAAACCTT

TGACTAGTTGTAGAATTTGATCTATTGCCCTACGCGTATACTCCCTTGCATCATATACGTTCGTCACCAG

ATCGTTTGTTTCGGCCTGAAGTTGACGCATATCTTTTTCAACACTCGACATGAGATCCTTAAGGGTCATA

TCGTCTAGATTTTGTTGAGATGCTGCTCCTGGATTTGGATTTTGTTGTGCTGTTGTACATACTGTACCAC

CAGTAGGTGTAGGAGTACATACAGTGGCCACAATAGGAGGTTGAAGAGGTGTAACCGTTGGAGTAGTACA

AGAAATACTTCCATCCGATTGTTGTGTACATGTGGTTGTTGGTAACGTCTGAGAAGGTTGGGTAGATGGC

GGTGTCGTCATCTTTTGATCTTTATTAAATTTAGAGATAATATCCTGAACAGTATTGCTCGGCGTCAACG

CTGGAAGGAGTGTACTCGCCGGCGCATCAGTATCTGTAGACAACCAATCAAAAAGATTAGACATATCAGA

TGATGTATTAGTTTGTTGACGTGGTTTTAGTACAGGAGCAGTACTACTAGGTAGAAGAATAGGAGCCGGT

GTAGGTGTCGGAACCGGCTGTGGAGTTATATGAATAGTTGGTTGTAGCGGTTGGGTAGGCTGTCTGCTGG

CGGCCATCATATTATCTCTAGCTAGTTGTTCTCGCAACTGTCTTTGATAATACGACTCTTGAGACTTTAG

TCCTATTTCAATCGCTTCATCCTTTTTCGTATCCGGATCCTTTTCTTCAGAATAATAGATTGACGACTTT

GGTGTAGAGGATTCTGCCAGCCCCTGTGAGAACTTGTTAAAGAAGTCCATTTAAGGCTTTAAAATTGAAT

TGCGATTATAAGATTAAATGGCAGACACGGACGATATTATCGACTATGAATCCGATGATCTCACCGAATA

CGAGGATGATGAAGAAGATGGAGAGTCACTAGAAACTAGTGATATAGATCCCAAATCTTCTTATAAGATT

GTAGAATCAACATCCACTCATATAGAAGATGCGCATTCCAATCTTAAACATATAGGGAATCATATATCTG

CTCTTAAACGACGCTATACTAGACGTATAAGTCTATTTGAAATAGCGGGTATAATAGCAGAAAGCTATAA

CTTGCTTCAACGAGGAAGATTACCTCTAGTTTCAGAATTTTCTGACGAAACGATGAAGCAAAATATGCTA

CATGTAATTATACAAGAGATAGAGGAGGGTTCTTGTCCTATAGTCATCGAAAAGAACGGAGAATTGTTGT

CGGTAAACGATTTTGATAAAGATGGTCTAAAATTCCATCTAGACTATATTATCAAAATTTGGAAACTTCA

AAAACGATATTAGAATTTATACGAATATCGTTCTCTAAATGTCACAATCAAGTCTCTCATATTCAGCAGT

TTATTGTCGTACTTTATATCGTGTTCATTAACGATATTTTGCAAAATAGTAATGATTCTATCTTCCTTCG

ATAGATATTCTTCAGAGATTATTGTCTTATATTCTTTCTTGTTATCCGATATGAATTTGATAAGACTTTG

AACATTATTAATACCCGTCTGTTTAATTTTTTCTATAGATATTTTAGTTTTGGTAGATTCTATGGTGTCT

GTTAATAGGCATCCAACATCGACATTCGACGTCAATTGTCTATAAATCAGAGTATAAATTTTAGAAATAA

CATTAGCAAATTGTTGTGCGTTGATGTCGTTATTCTGAAACAGTATGATTTTAGGTAGCATTTTCTTAAC

AAAGAGAACGTATTTATTGTTACTCAGTTGAACAGATGATATATCCAGATTACTAACGCATCTGATTCCA

TATACCAAACTTTCAGAAGAAATGGTGTACAATTGTTTGTATTCATTCAATGTCTCCTTTTCAGAAATTA

GTTTAGAGTCGAATACTGCAATAATTTTCAAGAGATAGTTTTCATCAGATAAGATTTTATTTAGTGTAGA

TATGATAAAACTATTGTTTTGTTGGAGAACTTGATACGCCGCATTCTCTGTAGTCGACGCTCTCAAATGG

GAAACAATCTCTATTATTTTTTTGGAATCGGATACTATATCTTCGGTATCTTGACGCAGTCTAGTATACA

TAGAGTTAAGAGAAATTAGAGTTTGTACATTAAGCAACATGTCTCTAAATGTGGCTACAAACTTTTCTTT

TTCCACATCATCTAGTTTATTATATACCGATTTCACAACGGCACCAGATTTAAGGAACCAGAATGAAAAA

CTCTGATAACTACAATATTTCATCATAGTTACGATTTTATCATCTTCTATAGTTGGTGTGATAACACATA

CCTTTTTCTCCAAGACTGGAACCAACGTCATAAAAATGTTTAAATCAAAATCCATATCAACATCTGATGC

GCTAAGACCAGTCTCGCGTTCAAGATTATCTTTACTAATGGTGACGAACTCATCGTATAGAACTCTAAGT

TTGTCCATTATTTATTTACAGATTTAGTTGTTTAATTTATTTGTGCTCTTCCAGAGTTGGGATAGTATTT

TTCTAACGTCGGTATTATATTATTAGGATCTACGTTCATATGTATCATAATATTAATCATCCACGTTTTG

ATAAATCTATCTTTAGCTTCTGAAATAACGTATTTAAACAAAGGAGAAAAATATTTAGTTACGGCATCAG

ACGCGATAACATTTTTTGTAAATGTAACGTATTTAGACGACAGATCTTCGTTAAAAAGTTTTCCATCTAT

GTAGAATCCATCGGTTGTTAACACCATTCCCGCGTCAGAGTGAATAGGAGTTTGAATAGTTTGTTTTGGA

AATAGATCCTTCAATAACTTATAGTTGGGTGGGAAAAAATCGATTTTATCACTAGACTCTTTCTTTTTTA

CTATCATTACCTCATGAACTATTTCTTGAATGAGTATATGTATTTTCTTTCCTATATCGGTCGCGTTCAT

TGGAAAATATATCATGTCGTTAACTATAAGAATATTTTTATCCTCGTTTACAAACTGAATAATATCAGAT

ATAGTTCGTAAACGAACTATATCATCACCAGCACAACATCTAACTATATGATATCCACTAGTTTCCTTTA

GCCGTTTATTATCTTGTTCCATATTAGCAGTCATTCCATCATTTAAGAAGGCGTCAAAGATAATAGGGAG

AAATGACATTTTGGATTCTGTTACGACTTTACCAAAATTAAGGATATACGGACTTACTATCTTTTTCTCA

ACGTCGATTTGATGAACACACGATGAAAATGTACTTCGATGAGATTGATCATGTAGAAAACAACAAGGGA

TACAATATTTCCGCATATCATGAAATATATTAAGAAATCCCACTTTATTATATTTCCCCAAAGGATCAAT

GCATGTAAACATTATACCGTTATCATTAATAAAGACTTCTTTCTCATCGGATCTGTAAAAGTTGTTACTG

ATTTTTTTCATTCCAGGATCTAGATAATTAATAATAATGGGTTTTCTATTCTTATTCTTTGTATTTTGAC

ATATCCTAGACCAGTAAACAGTTTCCACTTTGGTAAAATCAGAAGACTTTTGAACGCTATTAAACATGGC

ATTAATGGCAATAACTAAAAATGTAAAATATTTTTCTATGTTAGGAATATGGTTTTTCACTTTAATAGAT

ATATGGTTTTTTGCCAAAATGATAGATATTTTTTTATCCGATGATAGTAAAATATTATTAGTCGCCGTCT

CTATAAAAATGAAGCTAGTCTCGATATCCAATTTTATTCTAGAATTGATAGGAGTCGCCAAATGTACCTT

ATACGTTATATCTCCCTTGATGCGTTCCATTTGTGTATCTATATCGGACACAAGATCTGTAAATAGTTTT

ACGTTATTAATCATCACGGTATCGCCATCGCTAGATAATGCTAATGTACTATCCAAGTCCCAAATGGAGA

GATTTAACTGTTCATCGTTTAGAATAAAATGATTACCTGTCATATTAATAAAGTGTTCATCGTATCTAGA

TAACAACGACTTATAATTAATGTCCAAGTCTTGAACTCGCTGAATGATCTTTTTTAACCCAGTTAGTTTT

AGATTGGTACGAAATATATTGTTAAACTTTGATTCTACAGTAATGTCCAAATCTAGTTGTGGAAATACTT

CCATCAACATTGTTTCAAACTTGATAATATTATTATCTACATCTTCGTACGATCCAAATTCCGGAATAGA

TGTATCGCACGCTCTGGCCACCCAGATAACCAAAAAGTCACACGCTCCAGAATATACATTGTATAAAAAG

CTATCGTTTTTTAGTAGTGTTTTTTTCTGAGTATATACGAAAGGATTAAAAATAGTATTATCAACGTAAC

TATATTCCAAATTATTCTTATGAGAATAGATAATAATATCGTCCTTAATATCTAACAAATTTCCTAAATA

TCCCTTTAATTGAGTCATTCGAAGCGTTAATAAAATATGTCTCTTAACTATTTCCGGCCGTTGTATATTT

AAATGACTTCGTAAGAAATAATATATAGGCGACTTCTCATCTATGTAATCATATGGAGTGAGATATAGGG

CTCGTTCTACCTCCTGCCCCTTACCCACCTGTAATACCAATTGCGGACTCACTATATATCGCATATTTAT

ATCGTGGGGTAAAGTGAAAATCTACTACCGATGATGTAAGTCTTACAATGTTCGAACCAGTACCAGATCT

TAATTTGGAGGCCTCCGTAGAACTAGGGGAGGTAAATATAGATCAAACAACACCTATGATAAAGGAGAAT

AGCGGTTTTATATCCCGTAGTAGACGTCTATTCGTCCATAGATCTAAGGATGATGAGAGAAAACTAGCAC

TACGATTCTTTTTACAAAGACTTTATTTTTTAGATCATAGAGAGATTCATTATTTGTTCAGATGCGTCGA

CGCTGTAAAAGACGTCACTATTACCAAAAAAAATAACATTATCGTGGCGCCTTATATAGCACTTTTAACT

ATCGCATCAAAAGGATGCAAACTTACAGAAACAATGATTGAAGCATTCTTTCCAGAACTATATAATGAAC

ATAGTAAGAAATTCAAATTCAACTCTCAAGTATCCATCATCCAAGAAAAACTCGGATACCAGTCTGGAAA

CTATCACGTTTATGATTTTGAACCGTATTACTCTACAGTAGCTCTGGCTATTCGAGATGAACATTCATCT

GGCATTTTTAATATCCGTCAAGAGAGTTATCTTGTAAGTTCATTATCTGAAATAACATATAGATTTTATC

TAATTAATCTAAAATCTGATCTTGTTCAATGGAGTGCTAGTACGGGCGCTGTAATTAATCAAATGGTAAA

TACTGTATTGATTACAGTGTATGAAAAATTACAACTGGCCATAGAAAATGATTCACAATTTACATGTTCA

TTGGCTGTGGAATCAGAACTTCCAATAAAATTACTTAAAGATAGAAATGAATTATTTACAAAATTCATTA

ACGAGTTAAAAAAGACCAGTTCATTCAAGATAAGCAAACGTGATAAGGATACGCTATTAAAACATTTTAC

TTATGACTGGAGTTAGAATTTATAGACGACGCATTTCGTTTATCATTGTTACTATTACTATTACTATTAC

TATTACTATCATTATTAGTATTCTTCTTGTCATCTTGTTCAGAAATATACAGCAATGCTATACCTAATAC

TAAATACATTATCATGCTTGCAATGGCTCTAACAACAACGAACCAAAATGAATTTGGTCGTAGCTTTTGT

TCACAAAAATACATAAAGAAATGTCTACATAAATCTATGGCGCCATTGGCTACTTGAAATAGCGCCAGTC

CTCCTACAGATTTTAATATAGCTGTATAACATGACATTTATTCATCATCAAAAGAGACAGAGTCACCATC

TGTCATATTTAGATTTTTTTTCATGTGTTCAAAGTATCCTCTACTCATTTCATTATAATAGTTTATCATG

CTTAGAATTTTAGGACGGATCAATGAGTAAGACTTGACTAGATCGTCAGTAGTAATTTGTGCATCATCTA

TTCTGCATCCGCTTCGTCGAATAATGTATAGCATCGCTTTGAGATTCTCCATAGCTATCAAGTCTTTATA

TAATGACATGGAAATATCTGTGAATGCTTTATACTTCTCCAACATCGATGCCTTAACATCATCACATACT

TTAGCATTGAAAATACGTTCTATTGTGTAGATGGATGTAGCAAGATTTTTAAACAACAATGCCATCTTAC

ATGATGATTGTCTCAAGTCTCCAATCGTTTGTTTAGAACGATTAGCTACAGAGTCCAATGCTTGGCTAAC

TAGCATATTATTATCTTTAGAAATTGTATTCTTCAATGAGGCGTTTATCATATCTGTGATTTCGTTAGTC

ATATTACAGTCTGACTGGGTTGTAATGTTATCCAACATATCACCTATGGATACGGTACACGTACCAGCAT

TTGTAATAATCCTATCTAAGATGTTGTATGGCATTGCGCAGAAAATATCTTCTCCTGTAATATCTCCACT

CTCGATAAATCTACTCAGATTATTCTTAAATGCCTTATTCTCTGGAGAAAAGATATCAGTGTCCATCATT

TCATTAATAGTATACGCAGAAAAGATACCACGAGTATCAATTCTATCCAAGATACTTATCGGTTCCGAGT

CACAGATAATTGTTTCCTCTCCTTCGGGAGATCCTGCATAGAAATATCTAGGACAATAGTTTCTATACTG

TCTGTAACTCTGATAATCTCTAAAGTCACTAACTGATACCATGAAATTGAGAAGATCAAACGCTGAAGTA

ATCAATTTTTCTGCCTCGTTTTTACTACAACTAGTTTTCATCAATGTAGTGACGATGTATTGTTTAGTTA

CTCTTGGTCTAATACTGATGATAGAGATATTATTGCTTCCCATAATGGATCTTCTAGTAGTCACCTTAAA

GCCCATTGATGCGAATAGCAGATAGATAAAGTCTTGGTATGACTCCTTTCTAATATAGTACGGACTACCT

TTGTCACCCAACTTTATACCCACATAAGCCATAACAACCTCTTTAATAGCCGTTTCATGAGGTTTATCAG

CCATGAGCCTGAGTAGTTGAAAGAATCGCATGAATCCCGTCTCAGAAAGTCCTATATGCATGATAGATTT

ATCTTTCCTGGGAAACTCTCGTATAGTTATAGATGAAATACTCTTCAAAGTTTCTGAAATAAGATTAGTA

ACAGTCTTACCTCCGACTACTCTGGGTAACAAACATACTCTAATAGGTGTTTTCTCTGCGGAGATAATAT

CAGAAAGGATAGAGCAATAAGTAGTATTATTGTGATTATAAAGACCGAATACATAACAGGTAGAATTTAT

AAACATCATGTCCTGAAGGGTTTTAGACTTGTATTCCTCGTAATCTATACCGTCCCAAAACATGGATTTG

GTAACTTTGATAGCCGTAGATCTTTGTTCCTTCGCTAACAGGTTAAAGAAATTAATAAAGAATTTGTTGT

TTCTATTTATGTCCACAAATTGCACGTTTGGAAGCGCCACGGTTACATTCACTGCAGCATTTTGAGGATC

GCGAGTATGAAGTACGATGTTATTGTTTACTGGTATATCTGGAAAGAAATCTACCAGTCTAGGAATAAGA

GATTGATATCGCATAGAAATAGTAAAGTTTATAATCTCATCATTGAAGATTACTCTGTTACCATTGTAAT

AAATTGGTACTCTATCATAATCATCGACAAAGTACTGTTCATACATGATGAGATGTTTATATGTTGGCAT

AGTAGTGAGATCGACGTTTGGTAATGGCAATGTATTAAGATTAACTCCATAATGTCTAGCAGCATCTGCG

ATGTTATAAGTGATGTCAAAGCGGGGTTGATCTTGTGCTGTTATATATTGTCTAACACCTATAAGATTAT

CAAAATCTTGTCTGCTTAATACACCGTTAACAATTTTTGCCTTGAATTCTTTTATTGGTGCATTAATAAC

ATCCTTATAGAGGATGTTAAACAAATAAGTATTATCAAAGTTAAGATCTGGGTATTTCTTTTCTGCTAGA

ACATCCATTGAGTCGGAGCCATCTGGTTTAATATAACCACCGATAAATCTAGCTCTGTATTCTGTATCCG

TCAATCTAATATTAAGAAGGTGTTGAGTGAAAGGTGGAAGATCGTAAAAGCTGTGAGTATTAATAATAGG

GTTAGTTTCCGAACTAATGTTAATTGGATGATTAATAATATCTATATTTCCAGCGTTAAGTGTAACATTA

AACAGTTTTAATTCACGTGACGTGGTATCAATTAAATAATTAATGCCCAATTTGGATATAGTAGCCTGAA

GCTCATCTTGTTTAGTTACGGATCCTAATGAGTTATTAAGAAATACATCGAACGGATGAACGAAGGTTGT

TTTAAGTTGGTCACATACTTTGTAATCTAGACATAGATGTGGAAGAACGGTAGAAACTATACGAAATAGA

TATTCAGAGTCCTCTAATTGATCAAGAGTAACTATTGACTTAATAGGCATCATTTATTTAGTATTAAATG

ACGACCGTACCAGTGACAGATATACAAAACGACTTAATTACAGAGTTTTCAGAAGATAATTATCCATCTA

ACAAAAATTATGAAATAACTCTTCGCCAAATGTCTATTCTAACTCACGTTAACAACGTGGTAGATAGAGA

ACATAATGCCGCCGTAGTGTCATCTCCAGAGGAAATATCATCACAACTTAATGAAGATCTATTTCCAGAT

GATGATTCACCGGCCACTATTATCGAACGAGTACAACCTCATACTACTATTATTGACGATACGCCACCTC

CTACTTTTCGTAGAGAGTTATTGATATCGGAACAACGTCAACAACGAGAAAAAAGATTTAATATTACAGT

ATCAAAAAATTCTGAAGCAATAATGGAATCTAGATCTATGATAACTTCTATGCCAACACAAACACCATCC

TTGGGAGTAGTTTATGATAAAGATAAAAGAATTCAGATGCTAGAGGATGAAGTGGTTAATCTTAGAAATC

AACGATCTAATACAAAATCATCTGATAATTTAGATAATTTTACCAGAATACTATTTGGTAAGACTCCGTA

TAAATCAACCGAAGTTAATAAGCGTATAGCCATCGTTAATTATGCAAATTTGAACGGGTCCCCCTTATCA

GTCGAGGACTTGGATGTTTGTTCGGAGGATGAAATAGATAGAATCTATAAAACGATTAAACAATATCACG

AAAGTAGAAAACGAAAAATTATCGTCACTAACGTGATTATTATTGTCATAAACATTATTGAGCAGGCATT

GCTAAAACTCGGATTTGAAGAAATCAAAGGACTGAGTACCGATATCACTTCAGAAATTATCGATGTGGAG

ATCGGAGATGACTGCGATGCTGTAGCATCAAAACTAGGAATCGGTAACAGTCCGGTTCTTAATATTGTAT

TGTTTATACTCAAGATATTCGTTAAACGAATTAAAATTATTTAATTTAATACATTCCCATATCCAGACAA

CAATCGTCTGGATTAATCTGTTCCTGTCGTCTCATACCGGACGACATATTAATCTTTTTATTAGTGGGCA

TCTTTTTAGATGGTTTCTTTTTCCCAGCATTAACTGATTCGATACCTAGAAGATCGTGATTGATTTCTCC

GACCATTCCACGAACTTCTAATTGGCCGTCTCTAACGGTACCATAAACTATTTTACCAGCATTAGTAACA

GCTTGGACAATCTGACCATCCATTGCGTTGAATGATGTAGTTGCTGTTGTTCTACGTCTAGGAGCACCAG

AGGTATTTTTAGAGCTCTTGGATGTTGATGTAGAAGACGAGGATTTTGATTTTGGTTTACATGTAATACA

TTTTGAACTCTTTGATTTTGTATCACATGCGCCGGCAGTCACATCTGTTTGAGAATTAAGATTATTGTTG

CCTCCTTTGACGGCTGCATCTCCACCGATCTGCGCTAGTAGATTTTTAAGCTGTGGTGTAATCTTATTAA

CTGTTTCAATATAATCATCGTAACTACTTCTAACGGCTAAATTTTTTTTATCCGCCATTTAGAAGCTAAA

AATATTTTTATTTATGCAGAAGATTTAACTAGATTATACAATGAACTAATATGATCCTTTTCTAGATTAT

TTACGAACTTGGTATTTCTTGTTTCTGGAGGAGGAGAATTTAAATTCGGACTTGGATTCGGATTTTGTGG

GTTCTTGATCTTATTATACAGCGTGTATAGGATGGCGACGGTAACTGCTACACAAATACCGATCAACAGA

AGAATACCAATCATTTATTGACAATAACTTCACTATGATCAAGTATGTAATAATCATCTTTTCACTAAGT

AAGTAGTAATAATGATTCAACAATGACACGATATATGGACGATAATAATTTAGTTCATGGAAATATCGCT

ATGATTGGTGTGAATGACTCCGCTAACTCTGTTGGGTGCACAGTGCTTTCCCCACATAGAATAAATTAGC

ATTCCGACTGTGATAATAATACCAAGTATAAACGCCATAATACTCAATACTTTCCATGTACGAGTGGGAC

TGGTAGACTTACTAAAGTCAATAAAGGCGAAGATACACGAAAGAATCAAAAGAATGATTCCAGCGATTAG

CACGCCAGAAAAATAATTTCCAATCATAAGCATCATGTCCATTTAACTAATAAAAATTTTAAATCGCCGA

ATAAACAAAGTGGAATATAAACCATATAAAAACAATAGTTTGTACTGCAAAAATAATATCTATTTTTGTT

TTCGAAGATATGGTAAAATTAAATAGTAGTACACAGCATGTTATAACTAACAGCAGCAACGGCTCGTAAT

TACTTATCATTTACTAGACGAAAAGGTGGTGGGATATTTTCTTGCTCAAATAATACGAATATATCACCCA

TCCATTTTATACGATGTTTATATACTCTAATCTTTAATAGATCTATAGATGACGGGTTTACCAATAATAT

AGATTTTATCGATTCATCTAATTTAAACCCTTCCTTAAACGTGAATGATCTATTATCTGGCATAATGATG

ACCCTACCTGATGAATCTGACAATGTACTGGGCCATGTAGAATAAATTATCAACGAATTATCGTCTACGA

ACATTTATATCATTTGTTTTAATTTTAGGACGTGAATAAATAGATATAAAATAGAAAATAACAGATATTA

CAACCAGTGTTATGGACGCACCCAACCATGTAGGCAGTTTTATTTTATCGTTTACTACAGGTTCTCCTGG

ATGTACGTCACCAACTGCAGACGTAGTTCTAGTACAATTAGACGTAAGTTCCGCTTGGGAATTTTTTAAC

GCTAAAGAGTTAACGTTGATCGTACACCCAACGTATTTACATCTAGTTCTTTGAACATCTTGATTATAAT

ATAACCATTTTCTATCTCTAGATTCGTCAGTGCACTCATGTAACCAACATACCCTAGGTCCTAAATATTT

ATCTCCGGAATTAGATTTTGGATAATTCGCGCACCAACAATTTCTATTTCCTTTATGGTCGTTACAAAAG

ACGTATAATGCCGTATCCCCAAAAGTAAAATAATCAGGACGAATAATTCTAATAAACTCAGAACAATATC

TCGCATCCATATGTTTGGAGCAAATATCGGAATAAGTAGACATAGCCGGTTTCCGTTTTACACGTAACCA

TTCTAAACAATTGGGGTTTCCAGGATCGTTTCTACAAAAACCAGTCATGAAATCGTCACAATGTTCTGTC

TTGTAATTATTATTAAATATTTTTGGACAGTGTTTGGTATTTGTCTTAGAACAACATTTTGCCACGCTAT

CACTATCACCCAGGAGATAATCCTTTTTTATAAAATGACATCGTTGCCCGGATGCTATATAATCAGTAGC

ATATTTTAAATCCTTAATATATTCAGGAGTTACCTCGTTCTGATAATAGATTAATGATCCAGGACGAAAT

TTGAAAGAACTACATGGTTCTCCATGAATTAATACATATTGTTTAGCAAATTCAGGAACTATAAAACTAC

TACAATGATCTATCGACATACCATCTATCAAACAAAATTTGGGTTTAATTTCTCCTGGAGACGTTTCATA

ATAATACATATAACTTTCTTCGGCAAACCTAACAGCTCTATTATATTCAGGATAATTAAAATCTAATACC

ATATATTTGTCTCGTATATCTGCTATTCCTGTCTCTATTTTGATTCTATTAAGAGTAACAGCTGCCCCCA

TTCTTAATAATCATCAGTATTTAAACTGTTAAATGTTGGTATATCAACATCTATCTTATTTCCCGCAGTA

TAAGGTTTGTTGCAGGTATACTGTTCAGGAATGGGTACATTTATACTTCTTTTATAGTCCTGTCTTTCGA

TGTTCATCACAAATGCAAAGAACAGAATAAACAAAATAATGTAAGAAATAATATTAAATATCTGTGAATT

CGTAAATACATTGATTGCCATAATAATTACAGCAGCTACAATACACACAATAGACATTCCCACAGTGTTG

CCATTACCTCCACGATACATTTGAGTTACTAAGCAATAGGTAATAACTAAGCTAGTAAGAGGCAATAGAA

AAGATGAGATAAATATCATCAATATAGAGATTAGAGGAGGGCTATATAGAGCCAAGACGAACAAAATCAA

ACCGAGTAACGTTCTAACATCATTATTTTTGAAGATTCCCAAATAATCATTCATTATTCCTCCATAATCG

TTTTGCATCATACCCCCATCTTTAGGCATAAACGATTGCTGCTGTTCCTCTGTAAATAAATCTTTATCAA

GCACTCCAGCACCCGCAGAGAAGTCATCAAGCATATTGTAATATCTTAAATAACTCATTTATATATTAAA

AAATGTCACTATTAAAGATGGAGTATAATCTTTATGCCGAACTAAAAAAAATGACTTGTGGTCAGACCAT

AAGTCTTTTTAATGAAGACGGCGATTTCGTAGAAGTTGAACCAGGATCATCCTTTAAGTTTCTAATACCT

AAGGGATTTTACTCCTCTCCTTGTGTAAAGACGAGTCTAGTATTCGAGACATTAACAACGACCGATAATA

AAATTACTAGTATCAATCCAACAAATGCGCCAAAGTTATATCCTCTTCAACGCAAAGTCGTATCTGAAGT

AGTTTCTAATATGAGGAAAATGATCGAATTAAAACGTCCTCTATACATCACTCTTCACTTGGCATGTGGA

TTTGGTAAGACTATTACCACGTGTTATCTTATGACCACACACGGCAGAAAAACCATCATTTGCGTACCCA

ATAAAATGTTAATACATCAATGGAAGACACAGGTAGAGGCAGTCGGATTGGAACATAAGATATCTATAGA

TGGAGTTAGTAGTCTATTAAAGGAACTAAAGACTCAAAGTCCGGATGTATTAATCGTAGTCAGTAGACAT

CTGACAAACGATGCATTTTGTAAATATATCAATAAGCATTATGATTTGTTTATCTTGGATGAATCACATA

CGTATAATCTGATGAACAATACAGCAGTTACAAGATTTTTAGCGTATTATCCTCCGATGATGTGTTATTT

TTTAACTGCTACACCTAGACCAGCTAACCGAATTTATTGTAATAGTATTATTAATATTGCCAAGTTATCC

GATCTAAAAAAAACTATCTATACAGTAGATAGTTTTTTTGAGCCATATTCCACAGACAATATTAGAAATA

TGGTAAAACGACTAGATGGACCATCTAATAAATATCATATATATACCGAGAAGTTATTATCTGTAGACGA

GCCTAGAAACCAACTTATTCTTGATACCCTGGTAGAAGAATTCAAGTCAGGAACTATTAATAGAATTTTA

GTTATTACTAAACTACGTGAACATATGGTATTCTTCTACAAACGATTATTAGATCTTTTCGGACCAGAGG

TTGTATTTATAGGAGACGCCCAAAATAGACGTACTCCAGATATGGTCAAATCGATTAAGGAACTAAATAG

ATTTATATTCGTATCCACCTTATTTTATTCCGGCACTGGTTTAGATATTCCAAGTTTGGATTCTTTGTTC

ATTTGCTCGGCAGTAATCAACAATATGCAAATAGAGCAATTACTAGGGAGGGTATGTCGAGAAACAGAAC

TATTAGATAGGACGGTATATGTATTTCCTAACACATCCATCAAAGAAATAAAGTACATGATAGGAAATTT

CGTGCAACGAATTATTAGTCTGTCTGTAGATAAACTCGGATTTAAACAAGAAAGTTATCAGAAACATCAG

GAATCTGAACCCGCTTCCGTACCAACATCCTCCAGAGAAGAACGTGTATTAAATAGAATATTTAACTCGC

AAAATCGTTAAGAAGTTTAAGAGACGATCCACATGCTGAGCAGGCCAGTGTATTACCCCTCATAGTATTA

ATATAATCCAATGATACTTTTGTGATGTCTGAAATCTTAACCAATTTAGACTGACAGGCAGAACACGTCA

TACAATCATCATCGTCATCGATAACTGTAGTCTTGGGCTTCTTTTTGCGACTCTTCATTCCGGAACGCAT

ATTGGTGCTATCCATTTAGGTAGTAAAAAATAAGTCAGAATATGCCCTATAACACGATCGTGCAAAACCT

GGTATATCGTCTCTATCTTTATCACAATATAGTGTATCAACATCTTTATTATTGACCTCGTTTATCTTGG

AACATGGAATGGGAACATTTTTGTTAACGGCCACCTTTGCCTTAATTCCAGATGTTGTAAAATTATAACT

AAACAGTCTATCATCGACACAAATGAAATTCTTGTTTAGACGTTTGTAGTTTACGTATGCGGCTCGTTCT

CGTCTCATTTTTTCAGATATTGCAGGTACTATAATATTAAAAATAAGAATGAAATAACATAGGATTAAAA

ATAAAGTTATCATGACTTCTAGTGCTGATTTAACTAACTTAAAAGAATTACTTAGTCTGTACAAAAGTTT

GAGATTTTCAGATTCTGTGGCTATAGAGAAGTATAATTCTTTGGTAGAATGGGGAACATCTACTTACTGG

AAAATAGGCGTACAAAAGGTAACTAATGTCGAGACGTCCATATCTGATTATTATGATGAGGTAAAAAATA

AACCGTTTAATATTGATCCGGGGTATTATATTTTCTTACCAGTATATTTTGGAAGCGTCTTTATTTATTC

GAAGGGTAAAAATATGGTAGAACTTGGATCTGGAAACTCTTTTCAAATACCGGATGAGATTCGAAGTGCG

TGTAACAAAGTATTAGATAGTGATAACGGAATAGACTTTCTGAGATTTGTTTTGTTAAACAATAGATGGA

TAATGGAAGACGCTATATCAAAATACCAGTCTCCAGTTAATATATTTAAACTAGCTAGTGAGTACGGATT

AAACATACCCAACTATTTAGAAATTGAAATAGAGGAAGACACATTATTTGACGATGAGTTATACTCTATT

ATGGAACGCTCTTTCGATGATACATTTCCAAAAATATCTATATCGTATATTAAGTTGGGAGAACTTAAGC

GGCAAGTTGTAGACTTTTTCAAATTCTCATTCATGTATATTGAGTCAATCAAGGTAGATCGTATAGGAGA

TAATATTTTTATTCCTAGCGTTATAACAAAATCAGGAAAAAAGATATTAGTAAAAGATGTAGACCATTTA

ATACGATCCAAGGTTAGAGAACATACATTTGTAAAAGTAAAAAAGAAAAACACATTTTCCATTTTATACG

ACTATGATGGGAACGGAACAGAAACTAGAGGAGAAGTAATAAAACGAATTATAGACACTATAGGACGAGA

CTATTATGTTAATGGAAAGTATTTCTCTAAGGTTGGTATTGCAGGCTTAAAGCAATTGACTAATAAATTA

GATATTAATGAGTGTGCAACTGTCGATGAGTTAGTTGATGAGATTAATAAATCCGGAACTGTAAAACGAA

AAATAAAAAACCAATCAGTATTTGATTTAAGCAGAGAATGTTTGGGATATCCAGAAGCGGATTTTATAAC

GTTAGTTAATAACATGCGGTTCAAAATAGAAAATTGTAAGGTTGTAAATTTCAATATTGAAAATACTAAT

TGTTTAAATAACCCGAGTATTGAAACTATATATGGAAACTTCAACCAGTTCGTCTCAATCTTTAATACCG

TTACCGATGTCAAAAAAAGATTATTCGAGTGAAATAATATGCGCCTTTGATATAGGTGCAAAAAATCCTG

CCAGAACTGTTTTAGAAGTCAAGGATAACTCCGTTAGGGTATTGGATATATCAAAATTAGACTGGAGTTC

TGATTGGGAAAGGCGCATAGCTCAAGATTTGTCACAATATGAATACACTACAGTTCTTCTAGAACGTCAG

CCTAGAAGGTCACCGTACGTCAAATTTATCTATTTTATTAAAGGCTTTTTATATCATACATCTGCTGCCA

AAGTTATTTGCGTCTCACCTGTCATGTCTGGTAATTCATATAGAGATCGAAAAAAGAGATCTGTTGAAGC

ATTTCTTGATTGGATGGACACATTCGGATTGCGAGACTCCGTTCCGGATAGACGCAAATTAGACGATGTA

GCGGATAGTTTCAATTTGGCTATGAGATACGTATTAGATAAATGGAATACTAATTATACACCTTATAATA

GGTGTAAATATAGAAATTACATAAAAAAAATGTAATAACGTTAGTAACGCCATTATGGATAATCTATTTA

CCTTTCTACATGAAATAGAAGATAGATATGCCAGAACTATTTTTAACTTTCATCTAATAAGTTGTGATGA

AATAGGAGATATATATGGTCTTATGAAAGAACGCATTTCCTCAGAGGATATGTTTGACAATATAGTATAT

AATAAAGATATACATCCTGCCATTAAGAAACTAGTTTATTGCGACATCCAACTTACTAAACATATTATTA

ATCAGAATACGTATCCGGTATTTAACGATTCTTCACAAGTGAAATGTTGTCATTATTTCGATATAAACTC

AGATAATAGCAATATTAGTTCTCGTACAGTAGAGATATTTGAGAGTGAAAAGTCATCTCTTGTATCATAT

ATTAAAACTACCAATAAGAAGAGAAAGGTCAATTACGGCGAAATAAAGAAAACTGTACATGGAGGCACTA

ATGCAAATTACTTTTCCGGTAAAAAGTCTGATGAGTATCTGAGCACTACAGTCAGGTCCAACATTAATCA

ACCTTGGATCAAAACCATTTCTAAGAGAATGAGAGTAGATATCATTAATCACTCTATAGTAACGCGTGGA

AAAAGCTCTATATTACAAACTATAGAAATTATTTTTACTAATAGAACATGTGTGAAAATATTCAAGGATT

CTACTATGCACATTATTCTATCCAAGGACAAGGATGAAAAGGGATGTATAAACATGATTGATAAATTATT

CTATGTATATTATAATTTATTTCTGTTGTTCGAGGATATCATCCAAAACGATTACTTTAAAGAAGTAGCT

AATGTTGTAAACCATGTACTCATGGCTACGGCATTAGATGAGAAATTATTCCTAATTAAGAAAATGGCTG

AACACGATGTTTATGGAGTTAGCAATTTCAAAATAGGGATGTTTAACCTGACATTTATTAAGTCGTTGGA

TCATACCGTTTTCCCCTCTCTGTTAGATGAGGATAGCAAAATAAAGTTTTTTAAGGGGAAAAAGCTCAAT

ATTGTAGCATTACGATCTCTGGAGGATTGTACAAATTACGTGACTAAATCCGAGAATATGATAGAAATGA

TGAAGGAAAGATCGACTATTTTAAATAGCATAGATATAGAAACGGAATCGGTAGATCGTCTAAAAGAATT

GCTTCTAAAATGAAAAAAAACACTGATTCAGAAATGGATCAACGACTCGGGTATAAGTTTTTGGTGCCTG

ATCCTAAAGCCGGAGTTTTTTATAGACCGTTACATTTCCAATATGTATCGTATTCTAATTTTATATTGCA

TCGATTGCATGAAATCTTGACCGTCAAGCGGCCACTCTTATCGTTTAAGAATAATACAGAACGAATTATG

ATAGAAATTAGCAATGTTAAAGTGACTCCTCCAGATTACTCACCTATAATTGCGAGTATTAAAGGTAAGA

GTTATGACGCATTAGCCACGTTCACTGTAAATATCTTTAAAGAGGTAATGACCAAAGAGGGTATATCCAT

CACTAAAATAAGTAGTTATGAGGGAAAAGATTCTCATTTGATAAAAATTCCGCTACTAATAGGATATGGG

AATAAAAATCCACTTGATACAGCCAAGTATCTTGTTCCTAATGTCATAGGTGGAGTCTTTATCAATAAAC

AATCTGTCGAAAAAGTAGGAATTAATCTAGTAGAAAAGATTACAACATGGCCAAAATTTAGGGTTGTTAA

GCCAAACTCATTCACTTTCTCGTTTTCCTCCGTATCCCCTCCTAATGTATTACCGACAAGATATCGCCAT

TACAAGATATCTCTGGATATATCACAATTGGAAGCGTCGAATATATCATCGACAAAGACATTTATAACGG

TCAATATTGTTTTGCTGTCTCAATATTTATCTAGAGTGAGTCTAGAATTCATTAGACGTAGTTTATCATA

CGATATGCCTCCAGAAGTTGTCTATCTAGTAAACGCGATAATAGATAGTGCTAAACGACTTACCGAATCT

ATTACTGACTTTAATATTGATACATACATTAATGACCTGGTGGAAGCTGAACACATTAAACAAAAATCTC

AGTTAACGATTAACGAGTTTAAATATGAAATGCTGCATAACTTTTTACCTCATATGAACTATACACCCGA

TCAACTAAAGGGATTTTATATGATATCTTTACTAAGAAAGTTTCTCTACTGTATCTACCACACTTCTAGA

TATCCAGATAGAGATTCGATGGTTTGTCATCGCATCCTAACGTACGGCAAATATTTTGAGACGTTAGCAC

ATGATGAATTAGAGAATTACATAGGTAACATCCGAAACGATATCATGAACAATCACAAGAACAGAGGCAC

TTACGCAGTAAACATTCATGTACTAACAACTCCTGGACTTAATCATGCATTTTCTAGTCTATTGAGTGGA

AAGTTCAAAAAGTCAGACGGTAGTTATCGAACACATCCTCACTATTCATGGATGCAGAATATTTCTATTC

CTAGAAGTGTTGGATTTTATCCGGATCAAGTAAAGATTTCAAAGATGTTTTCTGTCAGAAAATACCATCC

AAGCCAATATCTTTACTTTTGTTCATCAGACGTTCCGGAAAGAGGTCCTCAGGTAGGTTTAGTATCTCAA

TTGTCTGTCTTGAGTTCCATTACAAATATACTAACGTCTGAGTATTTGGATTTGGAAAAGAAAATTTGTG

AGTATATCAGATCATATTATAAAGATGATATAAGTTACTTTGAAACAGGATTTCCAATCACTATAGAAAA

TGCTCTAGTCGCATCTCTTAATCCAAATATGATATGTGATTTTGTAACTGACTTTAGACGTAGAAAACGG

ATGGGATTCTTCGGTAACTTGGAGGTAGGTATTACTTTAGTTAGGGATCACATGAATGAAATTCGCATTA

ATATTGGAGCAGGAAGATTAGTCAGACCATTCTTGGTTGTGGATAACGGAGAGCTCATGATGGATGTGTG

TCCGGAGTTAGAAAGCAGATTAGACGACATGACATTCTCTGACATTCAGAAAGAGTTTCCACATGTCATC

GAAATGGTAGATATAGAACAATTTACTTTTAGTAACGTATGCGAATCGGTTCAAAAATTTAGAATGATGT

CAAAGGATGAAAGAAAGCAATACGATTTATGTGACTTTCCTGCCGAATTTAGAGATGGATATGTAGCATC

TTCACTAGTGGGAATCAATCACAATTCTGGACCCAGAGCTATTCTTGGATGTGCTCAAGCTAAACAAGCT

ATCTCTTGTCTGAGTTCGGATATACGAAATAAAATAGACAATGGAATTCATTTGATGTATCCAGAGAGGC

CAATTGTGATTAGTAAGGCTTTAGAAACTTCAAAGATTGCGGCTAATTGCTTCGGACAACATGTTACTAT

AGCATTAATGTCGTACAAAGGTATCAATCAAGAGGATGGAATTATCATCAAAAAACAATTTATTCAGAGA

GGCGGTCTCGATATTGTTACAGCCAAGAAACATCAAGTAGAAATTCCATTGGAAAACTTTAATAACAAAG

AAAGAGATAGGTCTAACGCCTATTCGAAATTAGAAAGTAATGGATTAGTTAGACTGAATGCTTTCTTGGA

ATCCGGAGACGCTATGGCAAGAAATATCTCATCAAGAACTCTTGAAGATGATTTTGCTAGAGATAATCAG

ATTAGCTTTGATGTTTCCGAGAAATATACAGATATGTACAAATCTCGCGTTGAACGAGTACAAGTAGAAC

TTACTGACAAAGTTAAGGTGCGAGTATTAACCATGAAAGAAAGGAGACCCATTCTAGGAGACAAATTTAC

TACTAGAACGAGTCAAAAGGGAACAGTCGCGTATATCGCAGATGAAACGGAACTTCCGTACGACGAAAAT

GGTATCACACCAGATGTCATTATTAATTCTACATCCATCTTCTCTAGAAAAACTATATCTATGTTGATAG

AAGTTATTTTAACAGCCGCATATTCTACTAAGCCGTACAACAATAAGGGAGAAAACCGACCTGTCTGTTT

TCCTAGTAGTAACGAAACATCTATCGATGCATATATGCAATTCGCTAAACAATGTTATGAGTATTCAAAT

CCGAAATTGTCCGAGGAAGAATTATCGGATAAAATCTTTTGTGAAAAGATTCTCTATGATCCTGAAACGG

ATAAGCCTTATGAATCCAAAGTATTTTTTGGACCAATTTATTACTTGCGTCTGAGACATTTAACTCAGGA

CAAGGCAACCGTTAGATGTAGAGGTAAAAAGACGAAGCTCATTAGACAAGCGAATGAGGGACGAAAACGT

GGAGGAGGTATCAAGTTTGGAGAAATGGAGAGAGACTGTTTAATAGCACATGGTGCAGCCAATACTATTA

CAGAAGTTTTAAAAGACTCAGAAGAGGATTATCAAGATGTGTATATTTGTGAAAATTGTGGAGACATAGC

AGCACAAATCAAAAGTATTAATACATGTCTTAGATGTTCAAAACTTAATCTCTCTCCTCTCTTAACAAAA

ATTGATACCACGCACGTATCTAAAGTATTTCTTACTCAAATGAACGCCAGAGGCGTAAAAGTTAAATTAG

ATTTCGAACGAAGGCCTCCTTCGTTTTATAAACCATTAGATAAAGTTGATCTTAAACCGTCTTTTCTGGT

ATAATATTGTTTAGTAGATACTCATCAAGATTATCAAGATAAGCTAATTCACTAAACATATTATCGGATT

CGGTATTGTTACTCGAGAATAGAGTTCGTTATGCTCCTGATATTCGGAAATCTGTGGAGTTTCAGGTTTT

GGTGGAAGTGTAACTGCTACTTGGTGGGATACTGAAGGATATTTCAGAGAGTTGTGGATGTTCGGGTTCG

ACATCCACCGATGGTGTCACGCCACTAATCGGTTCGGTAACGTCTGTGGATGGAGGTGCTACTTCTACAG

AACCTGTAGCCTCAGTTGTCAACGGAGATACATCTTCAATGCGCGGAAATGTATAATTTGGTAATGGTTT

CTCATGTGGATCTTAAGAAGAAGAGGTAAGATATCTACGAAAGATACCGATCACGTTCTAGTTCTCTTTT

GTAGAACTTTAACTTTTTCTTTCTCAGCATCTAGTTGATATCCCGACCTCTTCACGTTTCGCATGGGTTA

CCTCCGCAGTTTTTACAAGCGATTTCACGTTCCAGATCACGTTCAGCCTTCATACGTCTCTCCCTCTCTC

TATCGAGTTTATCAGAGCAGTCTTTCTGAAGGCGATCGAACTCCATAAATTTCTCCAACGCTTTGATTGT

TTCCATAGATTTCCGAAGTTTAGCTTCTAGGACGGCGATTCTTTTTTTTCTTTCGAATTCACGGGGTACA

ACCGTTTCCATTACCACCATCTCTACGTTTCTTTTTCTAGATCGACAATCTTTCTCAACAtttcatccccatgcctTTTCATTCCT

CGAGTCTATTGTCGTCGAAATATCGTTCCAGCTCCTTTTCAACCTCAATAACTTTAGCACGTTGTTTCAT

CAAGCTCTCTCTTGTAGTACTATCATTTTTATCTGATTCCCTGACACGTTTAAGATCTTCATGTAATTGA

GTCAGCTCTTGACGCAATCTCTTAACTAACTTCCTCTCTTGCTTCTTCGTCATAGTACTTACAATCACTA

TGGATCCATTGTTACCACGTCTGTACTCGACGAGCTCACGTTTAAGAGATTCAATTTCCAGTTTGTATCG

GTCCATGTCTCCATTGCTACACCATCATTAGATTTACAGGCTGTTAGTTGTCGTTCGAGATCAGAAATAC

GTGTTTTCTTGGAATGGATTTCGTCGATGTACTTGTCATGATTGGCATCGAAACACTTATTAAGTTCTTT

TTTTCAATTCTACGATTTTATTTCTTTCGCGAGTCAATTCCCTCCTATAGTAACTATCAGTTTTGTCAGA

TTCACGCTCTCTACGTAGACTTTCTTGCAAGTTACTAATTTGTTCCCTGGCATTACCGAGTTCAGTTTTA

TATGCCGAATAGAGTTCTGATTCATCCTTTGAGAAGATCTCTAGCGATCGTTCAAGATCCCTGATTCTAG

TCTTTAGCCTATTTACCTCCTCAGAAGATGCTCCGTTACCGTTTTTACAATCGTTAAGATGTCTATCAAG

ATCCATGATTCTATCTCTTTTCCATATCAGCATTGATTTCATTATTACGTCCGCAGTCGTTCAACTGTAT

TTCAAGATCTGAGATTCTAGATTGTAATCTCTGTAGCATTTCCACGGCATTCACTCAGTTGTCTTTCAAG

ATCTGAGATTCTAGATTGGAGTCTGCTAATCTCTGTAAGATTTCCTCCTCCGCTCTCGATGCAGTCGGTC

AACTTATTCTCTAGTTCTCTAATACGCGAACGCAGTGCATCAACTTCTTGTGTGTCTTCTTGATTGCGTG

TGCATTCATCGAGTCTAGATTCGAGATCTCTAACGTGACGTCGTTCTTCCTCAAGTTCTCTGTGTACTAC

AGAAAGCGTGTCCCTATCTTGTTGATATTTAGCAATTTCTGATTCTAGAGTACTGATTCTACTCACGTAT

GTACTAATAGTTGTCTTAGCCTTATCAAGATCCTCCTTGTATTTGTCACATTCCTTGATATCCATACGAA

GTCTGGACAGTTCCCATTCGACATTACGACGTTTATCGATTTCAGCTCGGAGATCGTCGTCGCGTTGTTT

TAGCCACATACGACTAAGTTCAAGTTCTCGTTGACAAGATCCATCTACTTTTCCATCCCTAATAGTATCC

AGTTCCTTTTCTAGTTCTGACCGCATTTCTCGTTCCATATCAAGAGATTCTCTCAATTCTCGTATAGTCT

TCTTATCAATTTCTGATGAATCTGAACCATCATCTGTCCCATTTTGTTGCATATCCCTGAGTTCTTTGAT

CTCTGTTGTAAGTCTGTCGATTCTTTCGGTTTTATAAACAGAATCCCTTTCCAAAGTCCTAATCTTACTG

AGTTTATCATTAAGTTCTTCATTCAATTCAGTGAGTTTTCTCTTGGCTTCTTCCAAGTCTGTTTTAAACT

CTCCATCATTTCCGCATTCTTCCTCGCATTTATCTAACCATTCAATTAGTTTATTAATGACTAGTTGGTA

ATCAGCGATTCCTATAGCCGTTCTTGTATTTGTGGGAACATAATTAGGATCTTCTAATGGATTGTATGGC

TTGATAGCATCATCTTTATCATTATTAGGTGGGGGATGGACAACCTTAATTGGTTGGTCCTCCTTATCTC

CTCCAGTAGCATGTGGTTCTTCAATACCAGTATTAGTAATAGGCTTAGACAAATGCTTGTCGTACGCGGG

CACTTCCTCATCCATCAAGTATTTATAATCGGGTTCTGTTTCAGAATATTCTTTTCTAAGAGACGCGACT

TCAGGAGTTAGTAGAAGAACTCTGTTTCTGTATCTATCAACGCTGGAATCGATACTCAAGTTAAGGATAG

CGAATACCTCATCGTCATCATCCGTATCTTCTGAAACGCCATCATATGACATTTCATGAAGTCTAACGTA

TTGATAAACAGAATCAGATTTAGTATTAAACAGATCCTTGACCTTTTTAGTAAATACATATGTATATTTT

AGATCTCCAGATTTCATAATATGATCGCATGCCTTAAATGTCAATGCTTCCATGATATAGTCTGGAACAC

TAATGGGTGACGAAAAAGATACAGCACCATATGCTACGTTGATAAATAGATCTGAACCACTAAGTAGATA

ATGATTAATGTTAAGGAAGAGGAAATATTCAGTATATAGATATGCCTTAGCATCATATCTTGTACTAAAC

ACGCTAAACAGTTTATTGATGTGATCAATTTCCAACAGAACAATTAGAGCGGCAGGAATACCAACAAACA

TATTACCACATCCGTATTTTCTATGAATATCACATATCATGTTAAAAAATCTTGATAGAAGAGCGAATAT

CTCGTCTGACTTAATGAGATGTAGTTCAGCAGCATAAGTCATAACTGTAAATAGAACATACTTTCCTGTA

GTGTTGATTCTAGACTCCACATCAACACCATTATTAAAAATAGTTTTATATACATCTTTAATCTGCTCTC

CGTTAATCGTCGAACGTTCTAGTATACGGAAACACTTTGATTTCTTATCTGTAGTTAATGACTTAGTGAT

ATCACGAAGAATATTACGAATTACATTTCTTGTTTTTCTTGAGAGACCTGATTCAGAACTCAACTCATCG

TTCCATAGTTTTTCTACCTCAGTGGCGAAATCTTTGGAGTGTTTGGTACATTTTTTAATAAGGTTCGTGA

CCTCCATTTATTATAAAAAATTTTTATTCAAAACTTAACTACAATCGGGTAATTATAAGATCGTAGATCT

CCCATGTGGTGGAATACTACCATCTATCGCATGTTGATGGACAGTAGGTAATGGCCATGGGAACAGTAAT

GTTTGCATATTTATCTTTCTTGCTAGTATTACTGTATATTGTCCCAATGTTTCAATGTGATGTTCTAACC

TATCAACTGCCACTGTATCACAACAATAATGTCCGATGGAATTAAGATTATGATCCAATGTGTTTAATAT

ATGATTATCAAGTCTTATACGATCCGCGTCTTTTTTGACAGGATCAGGCTCTTCTACAGGAAGAAGTTTC

GGCCTCTTATAATAGTCATGTCTGGGAAATGGTGGTCTAGGATGAGGATCAGGTATCGGAGTGGGTTTTG

GATTATAATCATCATCATCATCATCATCATCATCATCATCATCATCATCATCATCATCatcatcGATATT

TATTTTGCTATCTTGATAATGTCCTGTATCAGTTGCATTTTCAGCACTCGACTGAATATTAGCACATTCA

TTGTCTATTATTAACGTATTTCTAAACCCAAAATGTATGTGTTGAACATCACTACTATAGTTGATGAGTC

TTATAGCATGAATTCGCTTATCGTTATCGGGTTTATCTTCTGTCACCTTAACAATTCCTTTTTTATTAAA

CTCTGCATAATCATAACCATTTCTATTGTTTGTTCTAATATAAACGAGTATAGCATCATTGCTAAATTTT

TCAATAGTATCGAAAACAGAATATCCTAAACCATATAATATATATTCAGGAACACTCAAACTAAATGTCC

AGGATTCTCCTAAATACGTAAACTTTAATAGTGCTAAATCATTCAAAAATCTACCGCTTATAGATAGATA

GTACATGAATGCGTATAGTAGTCTACCTATCTCTTTATTATGAAAACCGACATTACGATCATATATTTCG

TGATATACATGTGACCCGTTTACGTTAAACCATAAATACATGGGTGATCCTATAAACATGAATTTATTTC

TAATTCTCAGAGCCATAGTTAATTGACCGTGTAATATTTGTTTACATGCATACTTGATACGATCATTAAT

AAGATTTTTATCATTGCTCGTTATTTCAGAATCGTATATATAAGGAGTACCATCATGATTCTTACCAGAT

ATTATACAAAATACTATATATAAAATATATTGACCCACGTTAGTAATCATGTAAATGTTTAATGTTTTAA

ATTTTGTATTTAATGATCCATCATCATATGCTAGCATGGTCTTGTGATATTCATTCTTTAAAATATAATA

TTGTGTTAGCCATTGCATTGGAGCTCCTAATGGAGATTTTCTATTCTCGTCCATTTTAGGATATGCTTTC

ATAAAGTCCCTAATAACTTCGTGAATGATGTTTCTATGTTTTCTACTGATGCATGTATTTGCTTCGATTT

TTTTATCCCATGTTTCATCTATCATAGATTTAAACGCAGTAATGCTCGCAACATTAACATCTTGAACCGT

TGGTACAATTCCGTTCCATAAATTTATAATGTTCGCCATTTATATAACTCATTTTTTGAATATACTTTTA

ATTGAACAAAAGAGTTAAGTTACTCATATGGATGCCGTCCAGTCTGTACATCAATCTTTTTAGCCAGAGA

TATCATAGCCGCTCTTAGAGTTTCAGCGTGATTTTCCAACCTAAATAGAACTTCATCGTTGCGTTTACAA

CACTTTTCTATTTGTTCAAACTTTGTTGTTATATTAGTAATCTTTTTTTCCAAATTAGTTAGCCGTTGTT

TGAGAGTTTCCTCATTATCGTCTCCATAGGCTTTAACAATTGCTTCGCGTTTAGTCTCTGGATTTTTAGC

AGCCTTTGTAGAGAAAAATTCAGTTGCTGGAATTGCAAGATCGTCATCTCCGGGGAAAAGAGTTCCGTCC

ATTTAAAGTACAGATTTTAGAAACTGACACTCTGTGTTATTTATATTTGGCGCAATACATGGATTATAAA

TATCGATGTTAATAACATCAGAAAATGTAAAGTCTATACATTGTCGCATCGTGTTAAATTTTCTAATGGA

TCTAGTATTATTGGGTCCAACTTCTGCCTGAAATCCAAATATGGAAGCGGATACAAAACCGTTTCCTGGA

TAAACCACACATCTCCACTTTTGCTTTACATCAGAAATTGTGTCATTGACATCTTGAACTCTCCTATCTA

ATGCCGGTGTTCCACCTATAGATTTTGAATACTCGAATGCTGCATGAGTAGCATTGAATTCCTTAATATT

GCCATAATTTTCATATATTGAGTAACTCTGGATAAAAAGTAAACACACCGCAGCCGTCGCTACTACAATA

AAAAAAATTGATAGAGAGTTCATTTATAATCTATTAGAAGCTGATAAAATTTTTTTACACGCGTCAGACA

ATGCTTTAATAAATAGTTCAACATCTACTTTTGTCATATCGAACCGATGGTATGATTCTAACCTAGAATT

ACATCCGAAAAAGTTGACTATGTTCATAGTCATTAAGTCATTAACGAACAACATTCCAGACTCTGGATTA

TAAGACGATACTGTTTCGTCACAATCACCCACCTTAATCATGTGATTATGAATATTGGCTATTAGAGTAC

CTTCTAAGAAATCTATAATATCTTTGAAACACGATTTAAAATCAAACCACGAATATACTTCTACGAAGAA

AGTTAGTTTACCCATAGGAGAGATAACTATAAATGGAGATCTAGATACAAAATCCGGATCTATGATAGTT

TTAACATTATTATATTCTCTATTAAATACCTCCACATCTAAAAATGTTAATTTTGAAACTATGTCTTCGT

TTATTACCGTACCTGAACTAAACGCTATAAGCTCTATTGTTTGAGAACTCTTTAAACGATATTCTTGAAA

TACATGTAACAAAGTTTCCTTTAACTCGGTCGGTTTATCTACCATAGTTACAGAATTTGTATCCTTATCT

ATAATATAATAATCAAAATCGTATAAAGTTATATAATTATCGTGTTCAGATTGTGATCTTTTCAAATAGA

CTAAAAACCCCATTTCTCTAGTAAGTATCTTATGTATATGTTTGTAAAATATCTTCATGGTGGGAATATG

CTCTACAGCAGTTAGCCATTCCTCATTGACAGCTGTAGATGTATTATACAAAACTACTCCAATGTTTAAC

AAGGGCCATTTTACGAGATTATTAAATCCTTGTTTGATAAATGTAGCCAATGCGGGTTCGAGTTCAACGA

CGATTGAATTCTCTTCCCGTGGATGCTGCATGATGAACGACGGGATGTTGTTGTTCTATTGATTTGGAAT

TCTTTTTCGACTTTTTGTTTATATTAAATATTTTAAAATTTATGGCTGATAGTAATTCATGTACTACGGA

TAATGTAGACGTGTATTGCATATCGATATCTTTATTATTAGATAAATTTATCAATAAATGTGAGAAGTTT

GCCTCGTTAAGGTCTTCCATTTAAATATTATATAAATATTTGTGTTTGTATTTTATTCGTCTTTTATGGG

ATAGTTTTTAACTAGTAAAGCTGTAATTACATACTTTGTCCGTAAAACATAAATATAAATACCCGCTTTT

ATCAAACGTTCCAAAAAGTCGGCAGCTGACATTTTTAACATGACATCTATTTTAAATACACTTAGGTTTT

TAGAAAAAACATCATTTTATAATTGTAACGATTCAATAACTAAAGAAAAGATTAAGATTAAACATAAGGG

AATGTTATTTGTATTTTATAAGCCAAAGCATTCTACCGTTGTTAAATACTTGTCTGGAGGAGGTATATAT

CATGATGATTTGGTTGTATTGGGGAAGGTAACAATTAATGATCTAAAGATGATGCTATTTTACATGGATT

TATCATATCATGGAGTGACAAGTAGTGGAGCAATTTACAAATTGGGATCGTCTATCGATAGACTTTCTCT

AAATAGGACTATTGTTACAAAAGTTAATAACAATTATAACAATTATAACAATTATAATTGTTATAATAAT

TATAATTGTTATAATTATGATGATACATTTTTTGACGATGATGATTGATCACTATTACACAATTTTGTTT

TTGTACTTTCTAATATAGTGTTTAGGTTCTTTTTCATATGAGAATATTGACTTACTAAAATATCTATGTT

TAACTTTTGTTCTATAACGTCCTTATCGGCGGTATCGGTACATATACGTAATTCACCTTCACAAAATACG

GAGTCTTCGATAATAATAGCCAATCGATTATTGGATCTAGCTGTCTGTATCATATTCAACATGTTTAATA

TATCCTTTCGTTTCCCCTTTACAGGCATCGATCGTAGCATATTTTCCGCGTCTGAGATGGAAATGTTAAA

ACTGCAAAAATGCGTAATGTTAGCCCGTCCTAATATTGGTACGTGTCTATAAGTTTGGCATAGTAGAATA

ATAGACGTGTTTAAATGCCTTCCAAAGTTTAAGAATTCTATTAGAGTATTACATTTTGATAGTTTATCAC

CTACATCATCAAAAATAAGTAAAAAGTGTGCTGATTTTTTATGATTTTGTGCGACAGCAATACATTTTTC

TATGTTACTTTTAGTTCGTATCAGATTATATTCTAGAGCTTCCTGACTACTAACAAAATTAATATGATTT

GGCCAAATGTATCCATCATAATCTGGGTTATAAACGGGTGTAAACAAGAATATATGTTTATATTTTTTAA

CTAGTGTAGAAAACAGAGATAGTAAATAGATAGTTTTTCCAGATCCAGATCCTCCTGTTAAAACCATTCT

AAACGGCATTTTTAATAAATTTTCTCTTGAAAATTGTTTTTCTTGAAAACAATTCATAATTATATTTACA

GTTACTAAATTAATTTGATAATAAATCAAAATATGGAAAACTAAGGTCGTTAGTAGGGAGGAGAACAACG

AAGGCATATCGTGATATAAATAACATTTATTATCATGATGACACCAGAAAACGACGAAGAGCAGACATCT

GTGTTCTCCGCTACTGTTTACGGAGACAAAATTCAGGGAAAGAATAAACGCAAACGCGTGATTGGTCTAT

GTATTAGAATATCTATGGTTATTTCACTACTATCTATGATTACCATGTCCGCGTTTCTCATAGTGCGCCT

AAATCAATGCATGTCTGCTAACGAGGCTGCTATTACTGACTCCGCTGTTGCCGTTGCTGCGGCATCATCT

ACTCATAGAAAGGTTGCGTCTAGCACTACACAATATGATCACAAAGAAAGCTGTAATGGTTTATATTACC

AGGGTTCTTGTTATATATTACATTCAGACTATAAGTCATTCGAGGATGCTAAAGCAAACTGCGCTGCGGA

ATCATCAACACTACCCAATAAATCCGATGTCTTGACTACCTGGCTCATTGATTATGTTGAGGATACATGG

GGATCTGATGGTAATCCAATTACAAAAACTACATCCGATTATCAAGATTCTGATGTATCACAAGAAGTTA

GAAAGTATTTTTGTACATAAATAAATGAAATCGCTTAATAGACAAACTGTAAGTAGGTTTAGGAAGTTGT

CGGTGCCGGCCGCTATAATGATGTTACTCTCAACCATTATTAGCGGCATAGGAACATTTCTGCATTACAG

AGAAGAACTGATGCCTAGTGCTTGCGCCAATGGATGGATACAATACGATAAACATTGTTATCTGGATACC

AACATTAAAATGTCTACGGATAATGCAGTTTATCAGTGTCGCAAATTACGAGCTAGATTGCCTAGACCTG

ATACTAGACATCTGAGAGTATTGTTTAGTATTTTTTATAAAGATTATTGGGTAAGTTTAAAAAAGACCAA

TGATAAATGGTTAGATATTAATAATGATAAAGATATAGATATTAGTAAATTAACAAATTTTAAGCAACTA

AACAGCACGACGGATTCTGAGGCGTGTTATATATACAAGTCTGGAAAACTGGTTAAAACAGTATGTAAAA

GTACTCAATCTGTACTATGCGTTAAAAGATTCTACAAGTGACAACAAAAAATGAATTAATAGTAAGTCGT

TAACGTACGCCGCCATGGACGCCGCGTTTGTTATTACTCCAATGGGTGTGTTGACTATAACAGATACATT

GTATGATGATCTCGATATCTCAATCATGGACTTTATAGGACCATACATTATAGGTAACATAAAAATTGTC

CAAATAGATATACGGGATATAAAATATTCCGACATGCAAAAATGCTACTTTAGCTATAAGGGTAAAATAG

TTCCTCAGGATTCTAATGATTTGGCTAGATTCAACATTTATAGTATTTGTACCGCATACAGATCAAAAAA

TACCATCATCATAGCATGCGACTATGATATCATGTTAGATGTAGAAGGTAAACATCAACCATTTTATCTA

TTCCCATCTATTGATGTTTTTAACGCTACAATCATAGAAGCGTATAATCTGTATACAGCTGGAGATTATC

ATCTGATCATCAATCCTTCAGATAATCTGAAAATGAAATTGTCGTTTAATTCTTCATTTTGTATATCAGA

CGGCAATGGATGGATTATAATTGATGGGAAATGTAATAGTAATTTTTTATCATAAAAGTTGTAAAGTAAA

TAATAAAACAATAAATATTGAACTAGTAGTATGTTGTATATTGAGCAATCAGAGATGATGCTGGTACCTC

TTATCACGGTGACCGTAGTTGCGGGAACAATATTAGTATGTTATATATTATATATTTGTAGGAAAAAGAT

ACGTACTGTCTATAATGACAATAAAATTATCATGACAAAATTAAAAAAGATAAAGAGTCCTAATTCCAGC

AAATCTAGTAAATCAACTGATAGCGAATCAGACTGGGAGGATCACTGTAGTGCTATGGAACAAAACAATG

ACGTAGATAATATTTCTAGAAATGAGATATTGAACGATGATAGCTTCGCTGGTAGTTTAATATGGGATAA

CGAATCCAATATCATGGCGCCTAGCACAGAACACATTTACGATAGTGTTGCTGGAAGCACGCTGCTAATA

AATAATGATCGTAATGAACAGACTATTTATCAGAATACTACAGTAGTAATTAATGATACAGAGACTGTTG

AAATACTTAATGAAGATACCAAACAGATTCCTAGCTATTCTTCCAATCCTTTCGTAAATTATAATAAAAC

CAGTATTTGTAGCAAGTCAAATCCGTTCATTGCAGAACTCAACAATAAATTTAGTGATAATAATCCGTTT

AGGAGAGCACATAGTGACGATTATCTTAATAAGCAACAAGATCATGAACACGATGATATAGAATCATCGG

TTGTATCATTGGTCTGATTAGTTTCCTTTTTATAAAATTGAAGTAATATTTAGTATTAATTACCGCCGAT

GCATTATACAAATATGGAGATATTCCCTGTATTCGGCATTTCTAAAATTAGCAATTTTATTGCTAATAAT

GACTGTAGATATTATATAGATGTAGAGCATCAAAAAATTATATCTGATGAGATCAATAGACAGATGGATG

AAACGGTACTTCTTACCAACATCTTAAGCGTAGAAGTTGTAAATGACAATGAGATGTACCATCTTATTCC

CCATAGACTATCGACTATTATACTCTGTATTAGTTCTGTTGGAGGATGTGTTATCTCTATAGATAATGAC

GTCAATGACAAAAATATTCTAACATTTCCCATTGATCATGCTGTAATCATATCCCCACTGAGTAAATGTG

TCGTAGTTAGCAAGGGCCCTACAACCATACTGGTTGTTAAAGCGGATATACCCAGCAAACGATTGGTAAC

ATCGTTTACAAACGACATACTGTATGTAAACAATCTATCACTGATTAATTATTTACCGTCGTCTGTATTC

ATTATTAGACGAGTCACCGACTATTTGGATAGACACATATGTGATCAGATATTTGCTAATAATAAGTGGT

ATTCCATTATAACTATCGACGATAAGCAATATCCTATTCCATCAAATTGTATAGGTATGTCTTCTGCCAA

GTACATAAATTCGAGCATCGAGCAAGATATTTTGATCCATGTTTGTAACCTCGAGCATCCATTCGACTCA

GTCTACAAAAAAATGCAGTCGTACAATTCTCTACCTATCAAGGAACAAATATTGTATGGTAGAATTGATA

ATATAAATATGAGCATTAGTATTTCGGTGGATTAATAGATTTCTCTAGTATGGGATCATTAATCATCTCT

AAATACATCATAAAAAAGCTATTATCAAATACTGTACTGAATGGATTCATTCTTTTCTCTTTTTATGAAA

CTCTGTTGTATATCTACGGATAAAACTAGAAGCAAAAAATCTGATAGGAAGAATAATGATTATATGGAGG

AACACGATTATTATAAAATAACAATAGTTCCTGGTTCCTCTTCCACGTCTACTAGCTCGTGGTATTATAC

ACATGCCTAGTAATAGTCTCTTTGCGTTGACGGAAAGCAGACTAGAAATAACAGGCCAAAATGTTCAGAC

ACCATAATAGTTCCCAACCCAGATAATAACAGAGTTCCATCAACACATTCCTTTAAACTCAATCCCAAAC

CCAAAACCGTTAAAATGTATCCAGCCAATTGATAGTAGATAATGAGGTGTACAGCACATGATAATTTACA

CAGTAACCAAAATGAAAACACTTTAGTAATTATAAGAAATATAGACGGTAATGTCATCATCAACAATCCA

ATAATATGCCTGAGAGTAAACATTGACGGATAAAACAAAAATGCCCCGCATAACTCTATCATGGCAATAA

CGCAACCAAACACTTGTAAAATTCCTAAATTAGTAGAAAATACAACTGATATCGATGTATAAGCGATTTC

GAGGAATAATAAGAACAAAGTAATTCCCGTAAAGATAAACATCAACATTGTTTGGTAATCATTAAACCAA

TTAGTATGACGTTGAATTAATTTCACAGTATATTTTATTCCAGTATTATCCCCGCATGTATACGTACCTG

GTAAGATATCTTTATATTCCATAATCAATGAGACATCACTATCCGATAACGAATGAAGTCTAGCACTAGT

ATGCCATTTACTTAATATGGTCGTCTTGGAAGTTTTATTATAAGTTAAAATATCATGATTGTCCAATTTC

CATCTAATATACTTTGTCGGATTATCTATAATACACGGAATAATGATGGTATCATTACATGCTGTATATT

CTATAGTCTTTGTAGATGTTATAACCACAAAAGTACAGAGGTATATCAACAATATTCTAACTCTTAACAT

TTTTATTTATTTAAAATGATACCTTTGTTATTTATTTTATTCTTATTTTGCTAACGGTATCGAATGGCAT

AAGTTTGAAACGAGTGAAGAAATAATTTCTACTTACTTAATAGATTATGTGGTAACTAACGGGTGTTATT

AATGGGGATGTATATACATTTTCAAATAATGAACTAAACAAAACTGGGTTAACTAATAACAATAATTATA

TCACAACATCTATAAAAGTAGAGGATAAGGATACATTAGTAGTATGCGGAACCAATAACGGAAATCCCAA

ATGTTGGAAAATAGACGGTTCATACTACCCAAAACATATAGGTAGAGGATACGATCATCAAAATAGCAAA

GTAACGATAATCAGTCACAATGAATGTGTACTATCCGACATAAACATATCAAAAGAAGGAATTAAACGAT

GGAGAAGATTTGACGGACCATGTGGTTATGATTAAACGAGTTAAGTTTTTTAAGAAGCCTTAGAAGAGAG

GCTATTGGGTATGAGAATCCGAAATATTAAACCAGACAACCCCATATAATTTTATAGCTAAGAATGCCGC

GAAGAATGGAACTAATAAAAACGGAAATATTTGTAGCACAACGAATAACTCCCAAACTGCATTCATGTTA

CACTATATAACAATTACAATACATTTTTATCATAACACTACTTCGGTTAGATGTTTTAGAAAAAAATAAA

TATCACCGTACCGTTTTGTTGTATAAAAATAACAATTAACAATTATCAATTTTTTTCTTTAATATTTTAC

GTGGTTGACCATTCTTGGTGGTAAAATAATCTCTTAGTGTTGGAATGGAATGCTGTTTAATGTTTCCACA

CTCATCGTATATTTTGACGTATGCAGTCACATCGTTTACGCAATAGTCAGACTGTAGTTCTATCATGCTT

CCTACGTTAGAAGGAGGAACAGTTTTAAAGTCTCTTGGTTTTAATCTATTGTCATTAGTTTTCATGAAAT

CCTTTGTTTTATCCACTTCACATTTTAAATAAATGTCCACTATACATTCTTCTGTTAATTTTACTAGATC

ATCATGAGTCATAGAATTCATAGGTTCCGTAGTCCATGGATCCAAACTAGCAAACTTCGCGTATACGGTA

TCGCGATTAGTGTATACACCAACTGTATGAAAATTAAGAAAACAGTTTAATAAATCTACAGAAATATTTA

ATCCTCCGTTTGATACAGATGCGCCATATTTATGGATTTCGGATTCACACGTTGTTTGTCTAAGGGGTTC

GTCTAGTGTTGCTTCTACATAGACTTCGATTCCCATATATTCTTTATTGTCAGAATCACATACCGATTTA

TCATACGCTGGTTCACTTGTTTGAAAACTAAATGGTAGTAGATACATCAAAATAATAAATAATAAGTACA

TTCTGCAATATTGTTATCGTAATTGGAAAATTGGTATTCAAGTGAGCTGGATTATGTGAGTATTGGATTG

TATATTTTATTTTATATTTTATATTTTATATTTTATTTTATATTTTATATTTTATTTTATATTTTGTAGT

AAGAATAGAATGCTAATGTCAAGTTTATTCGAATAGATGTCTTATTAAAAAACATATATAATAAATAACA

ATGGCTGAATGGCATAAAATTATCGAGGATATCTCAAAAAATAATAAGTTCGAGGATGCCGCCATCGTTG

ATTACAAGACTACAAAGAATGTTCTAGCGGCTATTCCTAACAGAACATTTGCAAAGATTAATCCGGGTGA

AGTTATTCCCCTCATCACTAATCATAATATTCTAAAACCTCTTATTGGTCAGAAATTTTGTATTGTATAT

ACTAACTCTCTAATGGATGAGAACACGTATGCTATGGAGTTGCTTACTGGGTACGCCCCTGTATCTCCGA

TCGTTATAGCGAGAACTCATACCGCACTTATATTTTTGATGGGTAAGCCAACAACATCCAGACGTGATGT

GTATAGAACATGTAGAGATCACGCTACCCGTGTACGTGCAACTGGTAATTAAAATAAAAAGTAATATTCA

TATGTAGTGTCAATTTTAAATGATGATGAAATGGATAATATCCATATTGACGATGTCAATAATGCCGGTA

TTGACATACAGCTCATCGATTTTTAGATTTCATTCAGAGGATATTGAATTATGTTATGGGAATTTGTATT

TTGATAGGATCTATAATAATGTAGTAAATATAAAATATATTCCTGAGCATATTCCATATAGATATAATTT

TATTAATCGTACGTTCTCCGTAGATGAACTAGATGATAATGTCTTTTTTACACATGGTTATTTTTTAAAA

CACAAATATGGTTGTTCACTTAATCCTAGTTTGATTGTCTCATTATCAGGAAACTTAAAATATAATGATA

TACAATGCTCAGTAAATGTATCGTGTCTCATTAAAAATTTGGCAACGAGTACATCTACTATATTAACATC

TAAACATAAGACTTATTCTCTATATCGGTCCACGTGTATTGCTATAATAGGATACGATTCTATTATATGG

TATAAATATATAAATGACAGGTATAATGTCATCTATGATTTTACTGCAATATGTATGCTAATAGCGTCTA

CATTGATAGTGATCATATACGTGTTTAAAAAAATAAAAATGAACTCTTAATTATGTTATACTATTAGAAA

TGGATAAAATCAAAATTACGATTGATTCAAAAATTGGTAATGTTGTTACCATATCGTATAACTTGGAAAA

GATAACTATTGATGTCGCACCAAAAAAGAAAAAAGAAAAGGATGTATTATTAGCGCAATCAGTTGCTGTC

GAAGAGGCAAAAGATGTCAAGGTGGAAGAAAAAAATATTATCGATATTGAAGATGACGATGATATGGATA

TAGAAAACACGTAATACGATCTATAAAAATAAGTATTAAATACTTTTTATTTACGGTACTCTTGTAGTGG

TGATACCACTAATCGATTATTTTTTTTAAAAAAAATACTTATTCTGATTCTTCTAGCCATTTCCGTGTTC

GTTCGAATGCCACATCGACGTCAAAGATAGGGGAGTAGTTGAAATCTAGTTCTGCATTGTTGGTACGCAC

CTCAAATGTAGTGTTGGATATCTTCAACGTATAGTTGTTGAGTATTGATGGTTTTCTAAATAGAATTCTC

TTCATATCATTCTTGTACGCGTACATTTTTAGCATCCATCTTGGAATCCTAGATCCTTGTTCTATTCCCA

ATGGTTTCATCAATAGAAGATTAAACATATCGTAAGAACACGATGGAGAGTAATCGTAGCAAAAGTAAGC

ATTTCCTTTAATCGCAGATCCCGGATACTGGATATATTTTGCAGCCAACACGTGCATCCATGCAACATTT

CCTACATATACCCGGCTATGCACAGCGTCATCATCGACTGTACGATACATAATGTTACCGTGTTGCTTAC

ATTGCTCGTAAAAGACTTTCGTCAATTTGTCTCCTTCTCCGTAAATTCCAGTGGGTCTTAGGCAACAAGT

ATACAATTTTGCGCCATTCATGATTACGGAATTATTGGCTTTCATAACCAGTTGCTCGGCCATACGTTTA

CTTTTTGCGTATACATGTCCTGGTGATATATCATAAAGGGTATGCTCATGACCGATGAATGGATTACCGT

GTTTATTTGGTCCTATTGCTTCCATGCTACTAGTATAGATCAAATACTTGATTCCTAGGTCCACACAAGC

TGCCAATATAGTCTGTGTTCCATAATAGTTTACTTTCATGATTTCATTATCAGTGTATTTTCCAAATACA

TCCACTAGAGCAGCCGTATGAATAATCAGATTTACCCCATCTAGCGCTTCTCTCACCTTATCAAAGTCGT

TTATATCACATTGTATATAGTTTATAACCTTAACTTTCGAGGTTATTGGTTGTGGATCTTCTACAATATC

TATGACTCTTATTTCTTGAACATCATCTGCGCTAATTAAAAGTTTTACTATATACCTGCCTAGAAATCCG

GCACCGCCAGTAACCGCGTACACGGCCATTGCTGCCACTCATAATATCAGACTACTTATTCTATTTTACT

AAATAATGGCTGTTTGTATAATAGACCACGATAATATCAGAGGAGTTATTTACGTTGAACAAGTCCATGG

AAAAGATAAAGTTTTAGGATCAGTTATTGGATTAAAATCCGGAACGTATAGTTTGATAATTCATCGTTAC

GGAGATATTAGTCGAGGATGTGATTCCATAGGCAGTCCAGAAATATTTATCGGTAACATCTTTGTAAACA

GATATGGTGTAGCATATGTTTATTTAGATACAGATGTAAATATATCTACAATTATTGGAAAGGCGTTATC

TATTTCAAAAAATGATCAGAGATTAGCGTGTGGAGTTATTGGTATTTCTTACATAAATGAAAAGATAATA

CATTTTCTTACAATTAACGAGAATGGCGTTTGATATATCAGTTAATGCGTCTAAAACAATAAATGCATTA

GTTTACTTTTCTACTCAGCAAAATAAATTAGTCATACGTAATGAAGTTAATGATATACACTACACTGTCG

AATTTGATAGGGACAAAGTAGTTGATACGTTTATTTCATATAATAGACATAATGACTCCATAGAGATAAG

AGGGGTGCTTCCAGAGGAAACTAATATTGGTCGCGTGGTTAATACGCCGGTTAGTATGACTTACTTGTAT

AATAAGTATAGTTTTAAACCGATTTTAGCAGAATATATAAGACACAGAAATACTATATCCGGCAACATTT

ATTCGGCATTGATGACGCTAGATGATTTGGTTATTAAACAGTATGGAGACATTGATCTATTATTTAATGA

GAAACTTAAAGTAGACTCCGATTCGGGACTATTTGACTTTGTCAACTTTGTAAAGGATATGATATGTTGT

GATTCTAGAATAGTAGTAGCTCTATCTAGTCTAGTATCTAAACATTGGGAATTGACAAATAAAAAGTATA

GGTGTATGGCATTAGCCGAACATATAGCTGATAGTATTCCAATATCTGAGCTATCTAGACTACGATACAA

TCTATGTAAGTATCTACGCGGACACACAGATAGCATAGAGGATGAATTTGATCATTTTGAAGACGATGAT

TTGTCTACATGTTCTGCCGTAACCGATAGGGAAACGGATGTATAATTTTTTTTTATAGTATGATGGATA

TGATGGATATGATGGATATGATGGATATGATAAAAAAATATAATTGTTGTATCCATTCCCATTCAAATCA

CCTTATATGATTCTGTAACACAATGAAGGAGTCTCATAGATATATAGAGGTCAGATACTGGTTTGATAAA

CTTTTTATTCCACATGAGTATGTTTGACTTATGGTTAGACACACATACTTTAACAAATCACTGAAAATTG

GAGTTAGGTATTCCTCTCAGAATCAGTTGCCGTTCTGGAACATTAAATGTATTTTTTATGATATACTCCA

ACGCATTTATGTGGGTATACAACAAGTCATTAATAATGAGTATTTCCAAGAGTTTTAGTTGTCTAGTATT

TAACAAGAGAAGAGATTTCAACAGACTGTTTATGAACTCGAATACCGCCTCATTGTCGCTTATATTGATG

ACATGACATCGAATTCCCAATATCAATCTCATCAGTGATGAGTAGCTCAATCTTGTTATCGGGATCCAAT

TTCTAAAGATGTCATTAAACCCTCGATCGTGAATGGATTTATCATCATCGTTTTTATGTTGGACATGAGC

TTAGTCCGTTTGTCCACATCTATATACGATGATTTCTGAATTATTTCATATATCTCTCGTTAACTCCAGG

AACTTGTCAGGGATCTAACTTTAATAtgttctCGTCTAAGAGATGAAAATCTTTGGATGGTTGCATGTGA

CTTTTCTCTAAAGGATGATGTTACCCGATCCTCTCTTAAATGACTCCATCTTATCCTTGGACAAGATGGA

CAGTCTATTTTCCTTAGATGGTTTAATATTTTTTACCCATGATCTATAAAGGTAGACAGACCTAATCGTC

TCGGATGACCATATATTATTTTCAGTTTTATTATACGCATAAATTGTAAAAAATATGTTAGGTTTACTAA

AATGTCTCGTGGGGCATTAATCGTTTTTGAAGGATTGGACAAATCTGGAAAAACAACACAATGTATGAAC

ATCATGGAATCTATACCGGCAAACACGATAAAATATCTTAACTTTCCTCAGCGATCCACAGTCACTGGAA

AGATGATAGATGACTATCTAACTCGTAAAAAAACCTATAATGATCATATAGTTAATCTATTATTTTGTGC

AAATAGATGGGAGTTTGCATCTTTTATACAAGAACAACTAGAACAGGGAATTACTTTAATAGTTGACAGA

TACGCGTTCTCTGGAGTAGCGTATGCCACCGCTAAAGGCGCGTCAATGACTCTCAGTAAGAGTTATGAAT

CTGGATTGCCTAAACCCGACTTAGTTATATTCTTGGAATCTGGTAGCAAAGAAATTAATAGAAACATCGG

CGAGGAAATTTATGAAGATGTTGAATTCCAACAAAAGGTATTACAAGAATATAAAAAAATGATTGAAGAA

GGAGATATTCATTGGCAAATTATTTCTTCTGAATTCGAGGAAGATGTAAAGAAGGAGTTGATTAAGAATA

TAGTTATAGAGGCTATACACACGGTTACTGGACCAGTGGGGCAACTGTGGATGTAATAAAATGAAATTAC

ATTTTTATAAATAGATGTTAGTACAGTGTTATAAATGGATGAAGCATATTACTCTGGCAACTTGGAATCA

GTACTCGGGGATACGTGTCCGATATGCATACCGAACTCGCATCAATATCTCAATTAGTTATTGCCAAGAT

AGAAACTATAGATAATGATTATTAAACAAGGACATTGTAAATTTTATCATGTGTAGATCAAACTTGGATA

ATCCATTTATCTCTTTCCTAGATACTGCATATACTATCATAGATCAAGAGATCTATCAGAACGAGTTGAT

TAATTCATTAGACGATAATGAAATTATCGATTGTATAGTTAACAAGTTTATGAGCTTTTATAAGGATAAC

CTAGAAAATATGGTAGATGCTATCATTACTCTAAAATATTATAATTAATAATCCAGATTTTAAAACTACG

TATGTGGAAGTACTCGGTTCCAGAATAGCTGATATAGATATTAAACAAGTGATACGTAAGAATATAATAC

AATTGTCTAATGATCCGCGAACGATATTTGTGAAAATATTAAAAAAAAATACTTTTTTTATTAAATGACG

TCTCTTCGCGAATTTAGAAAATTATGCTGTGATATATATCACGCATCAGGATATAAAGAAAAATCTAAAT

TAATTAGAGACTTTATAACAGATAGAGATGATACCGATACATATTTGATCATTAAGCTATTGCTTCCCGG

ATTAGACGATAGAATGTATAACATGAACGATAAACAAATTATAAAATTATATAGTATAATATTTAAACAA

TCTCAGGAAGATATGCTACAAGATTTAGGATACGGATATATAGGAGACACTATTAGGACTTTCTTCAAAG

AGAACACGGAAATCCGTCCACGAGATAAAAGCATTTTAACTTTAGAAGAAGTGGATAGTTTTTTAACTAC

GTTATCATCAGTAACTAAAGAATCACATCAAATAAAATTATTGACTGATATAGCATCTGTTTGTACATGT

AATGATTTAAAATGTGTAGTCATGCTTATTGATAAAGATCTAAAAATTAAAGCGGGTCCTCGGTACGTGC

TTAACGCTATTAGTCCTCATGCATATGATGTTTTTAGAAAATCTAATAACTTGAAAGAGATAATAGAAAA

TGCAGCTAAACAAAATCTAGACTCTATATCTATTTCTGTTATGACTCCAATTAATCCCATGTTAGCGGAA

TCATGTGATTCTGTCAATAAGGCGTTTAAAAAATTTCCATCAGGAATGTTTGCGGAAGTCAAATACGATG

GTGAAAGAGTACAAGTTCATAAAAAAAATAACGAGTTTGCATTCTTTAGTAGAAACATGAAACCAGTACT

CTCTCATAAAGTGGATTATCTCAAAGAATACATACCGAAAGCATTTAAAAAAGCTACGTCTATCGTATTG

GATTCTGAAATTGTTCTTGTAGACGAACATAATGTACCGCTACCGTTTGGAAGTTTAGGTATACACAAAA

AGAAAGAATATAAAAACTCTAACATGTGTTTGTTCGTATTTGACTGTTTATACTTTGATGGATTCGATAT

GACAGACATTCCATTGTATGAACGAAGATCTTTTCTCAAAGATGTTATGGTCGAAATACCCAATAGAATA

GTATTCTCAGAGTTGACGAATATTAGTAACGAGTCTCAGTTAACTGATGTATTAGATGATGCACTAACGA

GAAAATTAGAAGGATTGGTCTTAAAAGATATTAATGGCGTATACGAACCGGGAAAGAGAAGATGGTTAAA

AATAAAACGAGACTATTTGAACGAGGGTTCCATGGCAGATTCTGCCGATTTAGTAGTACTAGGTGCCTAC

TATGGTAAAGGAGGAAAGGGTGGTATCATGGCAGTCTTTCTAATGGGTTGTTACGACGACGAATCCGGTA

AATGGAAGACGGTAACTAAATGTTCCGGTCACGATGATAATACGTTAAGGGTTTTGCAAGACCAATTAAC

GATGGTTAAAATTAACAAGGATCCCAAAAAAATTCCAGAGTGGTTGGTAGTTAATAAAATCTATATTCCC

GATTTTGTAGTAGATGATCCGAAACAATCTCAGATATGGGAAATTTCAGGAGCAGAGTTTACATCTTCCA

AGTCACATACAGCGAATGGAATATCGATTAGATTTCCTAGATTTACTAGGATTAGAGAAGATAAAACGTG

GAAAGAATCTACTCATCTAAACGATTTAGTAAACTTGACTAAATCTCTTAATAGTTACATATAAACTGAA

AAATAAAATAACACTATTTTAGTTGGTGGTCGCCATGGATGGTGTTATCGTATACTGTCTAAATGCGTTA

GTAAAACATGGCGAGGAAATAAATCATATAAAAAATGATTTCATGATTAAACCATGTTGTGAAAGAGTTT

GTGAAAAAGTCAAGAACGTTCACATCGGCGGACAATCTAAAAACAATACAGTGATTGCAGATTTGCCATA

TCTGGATAATGCTGTATCAGATGTATGCAAATCAATATATAAAAAGAATGTATCAAGAATATCCAGATTT

GCTAATTTGATAAAAATAGATGACGATGACAAGACTCCTACCGGCGTATATAATTATTTTAAACCTAAAG

ATGCTATTCCTGTTATTATATCCATAGGAAAGGATAAAGATGTCTGTGAACTATTAATCTCATCTGATAA

AGCGTGTGTGTGTATAAAGTTAAATTTATATAAAGTAGCCATTCTTCCCATGGATGTTTCCTTTTTTACC

AAAGGAAATGCATCATTGATTATTCTCCTGTTTGATTTCTCTATCGATGCGGCACCTCTCTTAAGAAGTG

TAACCGATAATAATGTTATTATATCTAGACACCAGCGCCTACATGACGAGCTTCCGAGTTCCAATTGGTT

CAAGTTTTACATAAGTATAAAGTCCGACTATTGTTCTATATTATATATGGTTGTTGATGGATCTATGATG

TATGCGATAGCTGATAATAGAACTCACGCAATTATTAGCAAAAATATATTAGACAATACTACGATTAACG

ATGAGTGTAGATGCTGTTATTCTGAACCACAGATTAGGATTCTTGATAGAGATGAGATGCTCAATGGATC

ATCGTGTTATATGAACAGACATTGTATTATGATGAATTTACCTGATGTAGGCGAATTTGGATCTAGTATG

TTGGGGAAATATGAACCTGACATGATTAAGATTGCTCTTTCGGTGGCTGGTAATTTAATAAGAAATCGAG

ACTACATTCCCGGGAGACGAGGCTATAGCTACTACGTTTACGGTATAGCCTCTAGATAATTTTTTTTAAG

CACGAAATAAAAACATAATTTTAAACAATCTATTTCATACTATTTTGTGTGCTCACCATGAACATAAAGA

TAGATATATTAGTATTTCTGGTGATAAATTTACGGCGACTGCTAGGAGGGAAAATGAAGAAAGAAAAAAT

ATCTACCTCTCCAAAAAGAAAAACTACTGATGTTATCAAACCTGATTATCTTGAGTACAATGACTTGTTA

GATAGAGATGAGATGTCTACTATTCTAGAGGAATATTAGGCCTTAGAATAAAATATGGACGACTCTTAAC

GAAATTAGAAAATTCGATAATGATGTTGAAGAACAATTCGGTACTATAGAAGAACTCAAGCAGAAGCTTA

GATTAAATTCTGAAGAGGGAGCAGATAATTTTATAGATTATATAAAGGTACAAAAACAGGATATCATCAA

ACTTACTGTATACGATTGCATATATCTATGATAGGATTGTGTGCGTGCGTGGTAGATGTTTGGAGAAATG

AGAAACTGTTTTCTAGATGGAAATATTGGTTACAAGCGATTAAACTGTTTATTGATGATCACATGCTTGA

TAAGATAAAATCTATTGTAGAATAGACTAGTGTATATGGAAATGTCATAGAAAGTTAAAAGTTAATGAGA

GCAAAAATATATAAGGTTGTATTTCATATTTGTTATTTTTTTCTGTAATAGTTAGAAAATACATTCGATG

GTCTATCTACCAGATTATTATGTGTTATAAGGTAATTTTCTCATAATAAACTAGAGTATGAGTAAGATAG

TGTTTTTCAAAAACATATAAATCTAAAATTGATGGATGAGATATACAGCTATTAATTTCGAAAATATATT

TTAATCTGATAACTTTAAACATGGATTTTTGATGGTGGTTTAAGTTTAAAAAAGATTTTGTTATTGTAGT

ATGATAATATCAAAAAGATGGATATAAAGAATTTACTGACTACATGTACTATTTTACATTACTACATTGG

CTACGGTATATACCTACTTCGTCACTTCCACACGCTCCAGAAAACGGGTGTCATGTGACGAGGGAGAATC

TTGATAAGAGGCATAATCAATGTTGTAATCCGGTGTCCACCTGGAGAATTTGCCAAGGTCAGATGTAGTT

GGTAGTGATAACACAAAATGTGAACACTGCCCACCTCATACATATACCGCAATCCCCAATTATTCTAATA

GATGTCATCAATGTATAAAATGCCCAACAGGATCATTTGATAAGGTAAAGTGTACCGGAACACAGAACAA

ATGTTCGTGTCATCCTGGTTGGTATACGCTACTGATTCTTCACAGACTGAAGATTGTCGAGATTTGTGTA

CCAAAAAAGGAGATGTCCATGCGGATACTTTGGTGGAATAGATGAAGGAAATCCTATTTGTAAATCGTGT

TGTGTTGGTGAATATTGCGACTACCTACGTAATTATAGACTTGATCCATTTCCTCCATGCAAACTATCTA

TCTAAATGTAATTAATTATGATTTTGATGATAATGTTACCATACATTATATCGCTACTTGGTTAGTGTGT

ATTATTCAGTATGGAAGACCTATTAATAATTACTTATCTTTTGACGATCTTGTTATAATTATAATATAAA

AATACTTATGACATAGTAACAGTAACTCATAATTGCTGACGCGATAAATTCGTAATAATCTGTTTTGTTC

AAATTTTTATAAGGAATCTACAGGCATAAAAATAAAAATATAATCTATAATATACTCTTACAACGCCATC

ATGAATAGCAGTGAATTAATTGCTGTTATTATGGATTTAGAAATAGTGGACGATTTTGTGATATTAATAT

AGTTATTAATGATGAAAGGATAAACGCTCATAGATTCATCCTATCTGGAGCCTCCGAATATTTTTTCCAT

TCTGTTTTCCAATAATTTTATCGATTCTAATGAATACGAAGTTAATCTAAGTCATTTAGATTATCAAAGT

GTTAACTATTTGATCGATTACATTTATGGGATACCTTTGAGCCTAACTAACGATAACGTGAAATATATTC

TTTCAACCGCTGATTTTTTTACAAATTGGATCTGCCATTACTGAGTGCGAAAAATACATACTTAAAAATC

TTTGTTTTAGAAACTGTATCGATTTCTACATATACGCTGATAAATATAATAACAAGAAAATAGAATTAGC

ATCGTTTAACACAATATTACGAAATATTTTGAGACTCATCAACAATGAAAACTTTAAATACTTAACAGAG

GAATCAATGATATGTTATATATAAAAAATGAGGATTTCACCCCACTGATTCTCATTAAATGGTTAGAGAG

TACACCAACCATGTACCGTCGAGTTACTTAGATGCCTCAGAATATCATTTCTTTCCCCACAAGTTATAAA

ATCACTTTATAGTCATCGACTGGTTAGTTCAATCTACGAATGTATAACATTCTTAAACAATATAGCATTC

TTGGATAAATCATTTCCTAGATACCATATCATCGAGTTGATATCTATCGGTATAAGTAATTCACATGATA

AGATTTCCATAAACTGCTACAATCATAAAAAAATTCATGGGAAATGATATCTTCACGTAGATGGTGTAGT

TTCGCAGTGACCGTCCTGGATAATATTATCTATATGATGGGTGGATATGATCAGTCCCTGTATAGAAGTT

CAAAGGTTATAGCGTACAATACATGTACTAATTCTTGGATATATGATATACCAGAGCTAAAATATCCTCG

TTCTAATTGCGGAGGAGTTGCCAATGACGAATACATTTATTGTATAGGCGGTATACGCGATCAGGAGTCA

TCGTTGATATCTAGTATCGATAGATGGAAGCCATCAAAACCATATTGGCAGAAGTATGCTAAAATGTGCG

AACCAAAATGTGATATGGGGTTGCGATTTTAAACGGATTAATATATGTCATAGGTGGAGTCGTTAAAGGT

GACACACATGTACCGACGCACTAGAGAGTTTATCAGAAGATGGATGGATGAATCATCAACGTCTTCCAAT

AAAAATGTCCAATATGTCGACGATTGTTCATGCTGGAAAGATTTATATATCTGGAGGTTACAACAATAGT

AGTGTAGTTAATGTAATATCGAATCTAGAGTCCTTAGCTATAATCCGATATATGATGAATGGACCAAATT

ATCATCATTAAATATTCCTAGAATTAATCCTGCTCTATGGTCAGTGTATAATAAATTATATGTAGGAGGA

GGAATATCTGATGATGTTCAAACTAATACATCTGAAACATACGATAAAGAAAAAGATTGTTGGACATTGG

ATAATGGTCACTTGTTACCACATAATTATATAATGTATAAATGCGAACCGTTTAAACATAGATATCCATT

GGAAAAAACACAGTACACGAATGATTTTCTAAAGTATTTGGAAAGTTTTATAGGTAGTTGATAGAACAAA

ATACATAATTTTGTAAAAATAAATCACTTTTTATACTAATATGACACAATTACCAATACTTTTGTTACTA

ATATCATTAGTATACGCTACACCTTCTCCTCAGATATCTAAAAAAATAGGTGATGATGCAACTATATCAT

GTAGTCGAAATAATACAAATTACTACGTTGTTATGAGTGCTTGGTATAAGGAGCCCAATTCCATTATTCT

CTTAGCTGCCAAAAGCGACGTCTTGTATTTTGATAATTATACCAAGGATAAAATATCTTACGACTCTCCA

TACGATGATCTAGTTACAACTATCACAATTAAATCATTGACTGCTGGAGATGCCGGTACTTATATATGTG

CATTCTTTATGACATCGACTACAAATGATACTGATAAAGTAGATTATGAAGAATACTCCATAGAGTTGAT

TGTAAATACAGATAGTGAATCGACTATAGACATAATACTATCTGGATCTACACCAGAAACTATTTCTGAG

AAACCAGAGGATATAGATAATTCTAATTGCTCGTCTGTATTCGAAATCGCGACTCCGGAACCAATTACTG

ATAATGTAGAAGACCATACAGACACCGTCACATACACTAGTGATAGCATTAATACAGTAAATGCATCATC

TGGAGAATCCACAACAGACGAGATTCCGGAACCAATTACTGATAAAGAAGAAGATCATACAGTAACAGAC

ACTGTCTCATACACTACAGTAAGTACATCATCTGGAATTGTCACTACTAAATCAACCACCGATGATGCGG

ATCTTTATGATACATACAATGATAATGATACAGTACCGCCAACTACTGTAGGTGGTAGTACAACCTCTAT

TAGCAATTATAAAACCAAGGACTTCGTAGAAATATTTGGTATTACCACATTAATTATATTGTCAGCAGTG

GCGATTTTCTGTATTACGTATTATATATGTAATAAACACCCACGTAAATACAAAACAGAGAACAAAGTCT

AGATTTTTGACTTACATAAATATCTGGGATAATAAAATCTATCATATTGAGAGGACCATCTGGTTCAGGA

AAGACAGCCATAACCAAAAGACTGTTAAAAGACTATGTGAATATATTTGGATTTGTGGTGTCCCATACCA

CTAGATTTCCTCGTCCTATGGAACGAGAAGGTGTTGATTACCTTACGTTAACAGAGAGGCCATCTGGAAG

GGAATAGCCGCCGGAAACTTTCTAGAACATACTGAGTTTTTAGGAAATATTTATGGAACTTCTAAAACAG

CTGTAAATACAGCGGCTATTAATAATCGTATTTGCGCGATGGATTTAAACATCAACGGTGTTAGAAGTCT

TAAAAATACTTACCTAATGCATTACTTGGGTATATAAGACCTACCTCTCTTAAAATGGTTGAGAGCAATC

TTCGTCGTAGAAACACTGAAGCGGACGACGAATCTCATCGTCGCGTGATGTTGGCAAAAAACGGATATGG

ATGAGGTCAACGAAGCAGGTCTATTCGACACTATTATTATTGAAGATGATGTGAATTTAGCACATATAGT

AAGTGTTAATTCAGATACTACAGGACCGTATTAGAATGTATTTTAACACTAATTAGAGACTTAAGATTTG

ACTTAAAACTTGATAATTAATAATATAACTCGTTTTTATATGTGGCTATTTCAACGTCTAATGTATTAGT

TAAATATTAAAACTTACCACGTAAAACTTAAAATTTAAAATGGTATTTCATTGACAGATCATACATTATG

AAGTTTCAAGGACTTGTGTTAATTGACAATTGCAAAAATCAATGGGTCGTTGGACCATTAATAGGAAAAG

GTGGATTCGGTAGTATTTATACTACTAATGACAATAATTATGTAGTAAAAATAGAGCCCAAAGCTAACGG

ATCATTATTTACCGAACAGGCATTTTATACTAGAGTACTTAAACCATCCGTTATCGAAGAATGGAAAAAA

TCTCACAATATAAAGCACGTAGGTCTTATCACGTGCAAGGCATTTGGTTTATACAAATCCATTAATGTGG

AATATCGATTCTTGGTAATAAATAGATTAGGTGCAGATCTAGATGCGGTGATCAGAGCCAATAATAATAG

ACTACCAGAAAGGTCGGTGATGTTGATCGGAATCGAAATCTTAAATACCATACAATTTATGCACGAGCAA

GGATATTCTCACGGAGATATTAAAGCGAGTAATATAGTCTTGGATCAAATAGATAAGAATAAATTATATC

TAGTGGATTACGGATTGGTTTCTAAATTCATGTCTAACGGCGAACATGTTCCATTTATAAGAAATCCAAA

TAAAATGGATAACGGTACTCTAGAATTTACACCTATAGATTCGCATAAAGGATACGTTGTATCTAGACGT

GGTGATCTAGAAACACTTGGATATTGTATGATTAGATGGTTGGGAGGTATCTTGCCATGGACTAAGATAT

CTGAAACAAAGAATTCTGCATTAGTAAGTGCCGCAAAACAGAAATATGTTAACAATACTGCGACTTTGTT

AATGACCAGTTTGCAATATGCACCTAGAGAATTGCTGCAATATATTACCATGGTAAACTCTTTGACATAT

TTTGAGGAACCCAATTACGACGAGTTTCGTCGAGTATTAATGAATGGAGTTATGAAAAATTTTTGTTGAT

AAAAAATTAAAAAAATAACTTAGTTATTATCACTCTCGCGAGTACAATAGAAACATGGCGATGTTTTACG

CACACGCTTTCGGTGGGTACGACGAGAACCTTCATGCATTTCCTGGAATATCATCGACGGTTGCCAATGA

TGTCAGGAAATATTCTGTTGTGTCAGTTTATAATAAAAAGTATAACATTGTAAAAAACAAATATATGTGG

TGTAACAGTCAAGTAAACAAGAGATATATTGGAGCACTACTGCCTATGTTTGAGTGCAATGAATATCTAC

AAATTGGAGATCCAATCCATGATCTAGAAGGAAATCAAATCTCTATTGTCACATATCGCCACAAAAACTA

CTATGCTCTAAGTGGAATTGGGTACGAGAGTCTAGACTTGTGTTTGGAAGGAGTAGGGATTCATCATCAC

GTACTTGAAACAGGAAACGCGGTATATGGAAAAGTTCAACATGAGTATTCTACTATCAAAGAGAAGGCCA

AAGAAATGAATGCACTCAAACCAGGACCTATCATCGATTACCACGTCTGGATAGGAGATTGTGTCTGCCA

AGTTACTACTGTAGACGTGCATGGAAAGGAAATTATGAGAATGAGATTCAAAAGGGGTGCGGTGCTTCCG

ATTCCAAATCTGGTAAAAGTTAAAGTTGGGGAGGAAAATGATACAATAAATCTTTCCACTTCCATATCAG

CTCTCCTGAATTCCGGTGGCGGCACCATCGAGGTAACATCTAAGGAAGAACGTGTGGATTATGTACTCAT

GAAACGTTTGGAATCTATACATCATCTGTGGTCTGTAGTGTATGATCATCTTAATGTTGTGAATGGCGAA

GAACGATGTTATGTACATATGCATTCATCTCATCAAAGTCCTATGCTGAGTACTGTAAAAACAAATTTGT

ACATGAAGACTATGGGAGCATGTCTTCAAATGGACTCCATGGAAGCTCTAGAGTATCTTAGTGAACTGAA

GGAATCAGGTGGGCGGAGTCCCAGACCAGAATTGCAGAAATTTGAATATCCAGATGGAGTGAAAGACACT

GAATCAATTGAGAGATTGGCAGAGGAGTTCTTCAATAGATCAGAACTTCAGGCCGGTGAATCAGTCAAAT

TTGGTAATTCTATTAATGTTAAACATACATCTGTTTCAGCTAAGCAACTAAGAACACGTATACGACAGCA

GCTTCCTTCTATACTCTCATCTTTTGCCAACACAAAGGGTGGATATTTGTTCATTGGAGTTGATAATAAT

ACACACAAAGTAATTGGATTCACGGTGGGTCACGACTACCTCAAACTGGTAGAGAGTGATATAGAAAAGT

ATATCCAAAAACTTCCTGTTGTGCATTTCTGCAAGAAAAAAGAGGACATCAAGTACGCATGTAGATTCAT

CAAGGTGTATAAACCTGGTGATGAGACTACCTCGACATATGTGTGCGCAATCAAAGTGGAAAGATGCTGC

TGTGCTGTGTTTGCGGATTGGCCAGAATCATGGTACATGGATACTAGTGGTAGTATGAAGAAGTATTCTC

CAGATGAATGGGTGTCACATATAAAATTTTAATTAGGGTAAGGTAAAACTATATATAATAACTAACAATT

TGTGTATCATATAGACAATTAATTAGGTAACTGTTATCTCTTTTTAACTAACTAACTAACTCTTATATAC

TATTAATAATACATCTATTAATCATTGATTAGCTTATTGCTTTAATTGTTTTTGTAAACTAACACTGTTC

ATTGAAAAGGGATAACATGTTACAGAATATAAATTATATATGGATTTTTTTAAAAAGGAAATACTTGACT

GGAGTATATATTTATTTCTTCATTACATAACACGTCTGTGTTCTAATTCTTCCAATTCTTCCACATCTCA

TATAATACAGGAATATAATCTTGTTCGAAAATACGAGAAAGTGGATAAAACAATAGTTGATTTTTTATCT

AGGTGGCCAAATTTATTCCATATTTTAGAATATGGGGAAAATATTCTACATATTTATTTTATAGATGCTG

CTAATACGAATATTATGATTTTTTTTCTAGATAGAGTATTAAATATTAATAAGAACCGTGGGTCATTTAT

ACATAATCTCGGGTTATCATCCATTAATATAAAAGAATATGTATATCAATTAGTTAATAATGATCATCTA

GATAATAGTATAAGACTAATGCTTGAAAATGGACGTAGAACAAGACATTTTTTGTCTTATATATTGGATA

CAGTTAATATCTATATAAGTATTTTAATAAATCATAGATTTTATATAGATGCCGAAGACAGTTACGGTTG

TACATTATTACATAGATGTATATATAACTATAAGAAATCAGAATCAGAATCATATAATGAATTAATTAAG

ATATTGTTAAATAATGGATCAGATGTAGATAAAAAAGATACGTACGGAAACACACCGTTTATCCTATTAT

GTAAACACGATATCGACAACGCGGAATTGTTTGAGATATGTTTAGAGAATGCTAATATAGACTCTGTAGA

CTTTAATGGATATACACCTCTTCATTATGTCTCATGTCGTAATAAATATGATTTTGTAAAGTTATTAATT

TCTAAAGGAGCAAATGTTAATGCACGTAATAGATTCGGAACTACTCCATTTTATTGTGGAATTATACACG

GTATCTCGCTTATAAAACTATATTTGGAATCAGACACAGAGTTAGAAATAGATAATGAACATATAGTTCG

TCATTTAATAATTTTTGATGCTGTTGAATCTTTAGATTATCTATTGTCCAGAGGAGTTATTGATATTAAC

TATCGTACTATATACAACGAAACATCTATTTACGACGCTGTCAGTTATAATGCGTATAATACGTTAGTCT

ATCTATTAAACAGAAATGGTGATTTTGAGACGATTACTACTAGTGGATGTACATGTATTTCGGAAGCAGT

CGCGAACAACAACAAAATAATAATGGATATACTATTGTCTAAACGACCATCTTTGAAAATTATGATACCA

TCTATGATAGCAATTACTAAACATAAACAACATAATGCAGATTTATTGAAAATGTGTATAAAATATACTG

CGTGTATGACCGATTATGATACTCTTATAGATGTACAATCGCTACATCAATATAAATGGTATATTTTAAA

ATGTTTTGATGAAATAGATATCATGAAGAGATGTTATATAAAAAATAAAACTGTATTCCAATTAGTTTTT

TGTATCAAAGACATTAATACTTTAATGAGATACGGTAGACATCCTTCTTTCGTGAAATGTAATATTCTCG

ACGTATACGGAAGTCGTGTACGTAATATCATAGCATCTATTAGATATCGTCAGAGATTAATTAGTCTATT

ATCCAAGAAGCTGGATGCTGGAGATAAATGGTCGTGTTTTCCTAACGAAATAAAATATAAAATATTGGAA

AACTTTAACGATAACGAACTGACCACATATCTGAAAATCTTATAAACACTATTAAAATATAAAATCTAAG

TAGGATAAAATCACACTACATCATTGTTTCCTTTTAGTGCTCGACAGTGTATACTATTTTTAACACTCAT

AAATAAAAATGAAAACGATTTCCGTTGTTACGTTGTTATGCGTACTACCTGCTGTTGTTTATTCAACATG

TACTGTACCCACTATGAATAACGCTAAATTAACGTCTACCGAAACATCGTTTAATGATAAACAGAAAGTT

ACGTTTACATGTGATTCAGGATATCATTCTTTGGATCCAAATGCTGTCTGCGAAACAGATAAATGGAAAT

ACGAAAATCCATGCAAGAAAATGTGCACAGTTTCTGATTATGTCTCTGAACTATATGATAAGCCATTATA

CGAAGTGAATTCCACCATGACACTAAGTTGCAACGGTGAAACAAAATATTTTCGTTGTGAAGAAAAAAAT

GGAAATACTTCTTGGAATGATACTGTCACGTGTCCTAATGCGGAATGTCAACCTCTTCAATTAGAACACG

GATCGTGTCAACCAGTTAAAGAAAAATACTCATTTGGGGAATATATGACTATCAACTGTGATGTTGGATA

TGAGGTTATTGGTGTTTCGTATATAAGTTGTACGGCTAATTCTTGGAATGTTATTCCATCATGTCAACAA

AAATGTGATATACCGTCCCTATCTAATGGATTAATTTCCGGATCTACATTTTCTATCGGTGGCGTTATAC

ATCTTAGTTGTAAAAGTGGTTTTACACTAACGGGGTCTCCATCATCCACATGTATCGACGGTAAATGGAA

TCCCATACTCCCAACATGTGTACGATCTAACGAAGAATTTGATCCAGTGGATGATGGTCCCGACGATGAG

ACAGATCTGAGCAAACTCTCGAAAGACGTTGTACAATATGAACAAGAAATAGAATCGTTAGAAGCAACTT

ATCATATAATCATAATGGCGTTGACAATTATGGGTGTCATATTTCTAATCTCCATTATAGTATTAGTTTG

TTCCTGTGACAAAAATAATGACCAATATAAGTTCCATAAATTGCTACCGTGAATATAAATCCGTTAAAAT

AATTAATAACGAACAAGTATCAAAAGATTAAAGAATTAGCTAGAATCAATTAGATGTCTTCTTCAGTGG

ATGTTGATATCTACGATGCCGTTAGAGCATTTTTACTCAGGCACTATTATGACAAGAGATTTATTGTGTA

TGGAAGAAGTAACACCATATTACATAATATATACAGGCTATTTACAAGATGCACCGTTATACCGTTCGAT

GATATAGTACGTACTATGCCAAATGAATCACGTGTTAAACAATGGGTGATGGATACACTTAATGGTATAA

TGATGAATGAATGCGATACTGTATGTGTGGGTACCGGACTACGATTCATGGAAATGTTTTTCGATTACAA

TAAAAATAATCCCAAAAATAGCATCAACAATCAAATAATGTATGATATAATTAATAGCGTAGCCATAATT

CTAGCTAATGAGAGATATAGAAGCGCGTTTAACGACGATAGAATATACATCCGTAGAACTATGATGGACA

AATTGTACGAATACGCATCTCTAACTACTATTGGTACGATCACTGGAGGTGTTTGTTATTATCTGTTGAT

GCATCTAGTTAGTTTGTATAAATAATTATTTCGATATACTAGTTAAAATTTTAAGATTTTAAATGTATAA

AAAACTAATAACGTTTTTATTTGTAATAGGTGCAGTTGCATCCTATTCGAATAATGAGTACACTCCGTTT

AATAAACTGAGTGTAAAACTCTATATAGATGGAGTAGATAATATAGAAAATTCATATACTGATGATAATA

ATGAATTGGTGTTAAATTTTAAAGAGTACACAATTTCTATTATTACAGAGTCATGTGACGTCGGATTTGA

TTCCATAGATATAGATGTTATAAACGACTATAAAATTATTGATATGTATACCATTGACTCGTCTACTATT

CAACGCAGAGGACATACGTGTAGAATATCTACCAAATTATCATGCCATTATGATAAGTACCCTTATATCC

ACAAATATGAGGGTGATGAACGACAATATTCTATTACTGCAGAGGGAAAATGCTATAAAGGAATAAAATA

TGAAATAAGTATGATGAACGATGATACTCTATTGAGAAAACATACTCTTAAAATTGGATTTACTTATATA

TTCGATCGTCATGGGCATAGTAATACATATTATTCAAAATATGATTTTTAAAAATTTAAAATATATTATC

ACTTCAGTGACAGTAGTCAAATAACAAACAACACCATGAGATATATTATAATTCTCGCAGTTTTGTTCAT

TAATAGTATACATGCTAAAATAACTAGTTATAAGTTTGAATCCGTCAATTTTGATTCCAAAATTGAATGG

ACTGGGGATGGTCTATACAATATATCCCTTAAAAATTATGGCATCAAGACGTGGCAAACAATGTATACAA

ATGTACCAGAAGGAACATACGACATATCCGGATTTCCAAAGAATGATTTCGTATCTTTCTGGGTTAAATT

TGAACAAGGCGACTATAAAGTGGAAGAGTATTGTACGGGACTATGTGTCGAAGTAAAAATTGGACCACCA

ACTGTAAGATTGACTGAATATGACGATCATATCAATTTGTTCATCGAGCATCCGTATGCTACTAGAGGTA

GCAAGAAGATTCCTATTTACAAACGCGGTGACATGTGTGATATCTACTTGTTGTATACGGCTAACTTCAC

ATTCGGAGATTCTGAAGAACCAGTAACATATGATATCGATGACTACGATTGCACGTCTACAGGTTGCAGC

ATAGACTTTGCCACAACAGAAAAAGTGTGTGTGACAGCACAGGGAGCCACAGAAGGGTTTCTCGAAAAAA

TTACTCCATGGAGTTCGGAAGTATGTCTGACACCTAAAAAGAATGTATATACGTGCGCAATTAGATCTAA

AGAAGATGTTCCCAATTTCAAGGACAAAATAGCCAGAGTTATCACGAGAAAATTTAATAAACAGTCTCAA

TCTTATTTGACTAAATTTCTCGGTAGCACATCGAATGATGTTACAACTTTTCTTAGCATTCTTGACTAAA

TATTCATAACTAATTTTTATTAATGATACAAAAATGAAATAAAACTGTATATTATACACTGGTTAACGCC

CTTGGCTCTAACCATTTTCAAGATGAGGTCCCTGATTATAGTCCTTCTGTTCCCCTCTATCATCTACTCC

ATGTCTATTAGACGATGCGAGAAGACTGAAGAGGAAACATGGGGATTAAAAATAGGGTTGTGTATAATTG

CCAAAGATTTCTATCCCGAAAGAACTGATTGCAGTGTTCATCGCCCAACTGCAAGTGGAGGATTGATAAC

TGAAGGCAATGGATTCAGAGTAGTTATATGATCAATGTACAGAACCCCATGACTTTATTATCACCGATAC

TCAACAAACACGTCTTGGATCATCTCATACATATATTAAATTCAGTAACATGAATACAGGTGTCCCATCT

AGTATTCCAAAATGTTCCAGAACTCTCTCTATTTCTGTATATTGTGATCAAGAGGCGGGAGACATAAAAT

TTGAGGAGTATACTCAAGAATCAAGTGATATCAGTATTAGAGTTAAGTATGATTCATCATGTATTGATTA

TCTGGGTATTAATCAAAGTTTCATGAATGAATGTATTCGAAGAATTACAACATGGGATAGAGAATCATGC

GTCAGAATTGATACACAGACTATAAATAAATATCTTAAGTCTTGCACCAACACAAAATTCGACCGTAATG

TCTACAAAAGGTACATACTGAAGAGTAAAGCACTCCATGCTAAAACAGAGTTGTAATAGATATAAAATAC

TTTTTATAATAATTAGGCTAGAAAAATCTCACTCACATGTAATCTTAAAAAAAATGATATGATAGTTCTT

ACAAGTAGCGATTGAGTTTTAAATGGATTCTATTAATTACCGGGGAACTTAACAATTCGTTCTGATCTAC

AGACATTGGTTAATAAATCATCTTATTTTGCCAATATATTAAAATGTGGAAACTCCACTAATAATATTAC

ATTGTGCGACTTTCAAGATGATGTGATATATAGGGTTATACAGTTTTAACAATTATATAATAGAGATAGA

AAGTACAAAAGATGTAGAATCAATGATATGGCACGCTAAACAGTTGGGTGTGGAATCATTGCTAAAAGAA

TGTCAAAATTATTTGCTTAGAATATTACGTATATAATTGTTTAGAAATTTATAGAATAACTAATATTAAT

ACATTATCGTATATCTACAACGATATAAGAAACTTCATATTGGATAATATTACTATTAATATATAAGGAT

CCAGATTTTATATATTTGCCTAAATACATTATTATAGATTTACTAGGACAATCACCTAAATGTTTTTAAC

GAAGATAATGTGGTAAAGATTATATACACTTATATATCTTCCGATATCTACAAGGATATTCCATATCATC

ATTGTGTAAACTAAATAACGTTTTCTATGGCATTTGATAAGGACATTGGATATGTGGAAAAGATGATGGT

GTATGGAAGTTAGTACATTATCAACTTCTCCTTATTGATTGAAAATGAAAATATAAATAGTTTTTATGTA

TAGCGGTATCTACCCTATAGTTTTATTGCTTACTACTAACATGGATTCAGAT

ACAGATACAGATACAGATACAGATACAGATACAGATACAGATACAGATACAGATGTTACAAATGTAGAAGATATCA

TGAATGAAATAGATAGAGAGAAAGAAATACTAAAAAATGTAGAAATTGAAAATAATAAAAACATTAACAA

GAATCATCCCAGTGAATATATTAGAGAAGCACTTGTTATTAATACCAGTAGTAATAGTGATTCCATTGAT

AAAGAAGTTATAGAATATATCAGTCACGATGTAGGAATATAGATCATATCTACTAATTTTTATAATCGAT

ACAAAACATAAAAACAACTCGTTATTACATAGCAGGTATGGAATCCTTCAAGTATTGTTTTGATAACGAT

GGTAAGAAATGGATTATCGGAAATACTTTATATTCTGGTAATTCAATACTCTATAAGGTCAGAAAAAATT

TCACTAGTTCGTTCTACAATTACGTAATGAAGATAGATCATAAATCACACAAGCCATTGTTGTCCGAAAT

ACGATTCTATATATCTGTATTGGATCCTTTGACTATCGACAACTGGACACGGGAACGTGGTATAAAGTAT

TTAGCTATTCCAGATCTGTATGGAATTGGAGAAACCGATGATTATATGTTCTTCATTATAAAGAATTTGG

GAAGAGTATTCGCCCCAAAGGATAGTGAATCAGTTTTCGAAGCATGTGTCACTATGATAAACACGTTAGA

GTTTATACACTCTCGAGGATTTACTCATGGAAAAATAGAACCGATGAATATACTGATTAGAAATAAACGT

ATTTCACTAATTGACTATTCTAGAACTAACAAACTATACAAAAGTGGAACACATATAGATTACAACGAGG

ACATGATAACTTCAGGAAATATCAATTATATGTGTGTAGACAATCATCTTGGAGCAACAGTTTCAAGACG

AGGAGATTTAGAAATGTTGGGATATTGCATGATAGAATGGTTCGGTGGTAAACTTCCATGGAAAAACGAA

AGTAGTATAAAAGTAATAAAACAAAAAAAAGAATATAAACAATTTATAGCTACTTTTTTTGAGGACTGTT

TTCCTGAAGGAAATGAACCTCTGGAATTAGTTAGATATATAGAATTAGTATACATGTTAGATTATTCTCA

AACTCCTAATTATGACAGACTACGTAGACTGTTTATACAAGATTGAAATTATATTCTTTTTTTATAGAGT

GTGGGGTAGTGTTACGGATATCTAATATTAATATTAGACTATCTCTATCGCGCTACACGACCAATATCGA

TTACTATGGATATCTTCAGGGAAATCGCATCTTCTATGAAAGGAAAGAATGTATTCATTTCTCCAGCGTC

AATCTCGTCAGTATTAACAATACTGTATTATGGAGCTAATGGATCCACTGCTGAACAGCTATCAAAATAT

GTAGAAAAGGAGGAGAACATGGATAAGGTTAGCGCTCAGAATATCTCATTCAAATCCATGAATAAAGTAT

ATGGGCGATATTCTGCCGTGTTTAAAGATTCCTTTTTGGGAAAAATTGGCGATAAGTTTCAAACTGTTGA

CTTCACTGATTGTCGCACTATAGATGCAATCAATAAGTGTGTAGATATCTTTACTGAGGGAAAAATCAAT

CCACTATTGGATGAACCATTGTCTCCTGATACCTGTCTCCTAGCAATTAGTGCCGTATACTTTAAAGCAA

AATGGTTGATGCCATTCGAAAAGGAATTTACCAGTGATTATCCCTTTTACGTATCTCCAACGGAAATGGT

AGATGTAAGTATGATGTCTATTTACGGCGAGCCATTTAATCACGCATCTGTAAAAGAATCATTCGGTAAC

TTTTCAATCATAGAACTGCCATATGTTGGAGATACTAGTATGATGGTCATTCTTCCAAACAAGATTGATG

GATTAGAATCCATAGAACAAAATCTAACAGATACAAATTTTAAGAAATGGTGTAACTCTCTGGAAGCTAC

GTTTATCGATGTGCACATTCCTAAGTTTAAGGTAATAGGTTCGTATAATCTTGTGGATACGCTAATAAAG

TTGGGACTGACAGATGTGTTCTATTCAACTGGTGATTATATCAATATGTGTAATTCAGATGTGAGTGTTG

ACGCTATGATTCACAAAACGTATATAGATGTCAATGAAGAGTATACAGAAGCAGCTGCAGCAACTTCTGT

ACTAGTGGCAGACTGTGCATCAACAGTTACAAATGAGTTCTGTGCAGATCATCCGTTCATCTATGTGATT

AGACATGTCGATGGTAAAATTCTTTTCGTTGGTAGATATTGCTCTCCAACAACTAATTAAGCACATTCTT

AATATTAGAATATTATATAGTTAAGATTTTTACTAACAGGTTAACATTTTTTTTAAAAATAGAAAAAACA

TGTGGTATTAGTGCAGGTCGTTATTCTTCCAATTGCAATTGGTAAGATGACGGCCAACTTTAGTACCCAC

GTCTTTTCACCACAGCACTGTGGATGTGACAGACTGACCAGTATTGATGACGTCAGACAATGTTTGACTG

AATATATTTATTGGTCGTCGTATGCATACCGCAACAGGCAATGCGCTGGACAACTGTATGACACACTCCT

CTCTTTTAAAGATGATGCGGAATCAGTGTTCATCGACGTTCGTGAGCTGGTAAAAAATATGCCGTGGGAT

AATGTTAAGGATTGTACAGAGATCATCCGTTGTTATATACCGGATGAGCAAAAAACCATCAGAGAGATTT

CGGCCATCATTGGACTTTGTGCATATGCTGCTACTTACTGGGGAGGTGAAGACCATCCCACTAGTAACAG

CCTGAACGCATTGTTTGTGATGCTTGAGATGCTCAATTACATGGATTATACCATCATATTCTGGCGTATG

AATTGATGAGTTACAGCTTGACATTTCTTCTTTCCTCCCTCTTCTTCTACCTTTCCCAGAAACAAACTTT

TTTACCCACTATAAAATAAAATGAGTATACTACCTGTTATATTTCTTCCTATATTTTTTTTATTCTCCAT

TCGTTCAGACTTTTAACGTGCCTGAATGTATCGACAAAGGGCAATATTTTGCATCATTCATGGAGTTAGA

AAACGAGCCAGTAATCTTACCATGTCCTCAAATAAATACGCTATCATCCGGATATAATATATTAGATATT

TTATGGGAAAAACGAGGAGCGGATAATGATAGAATTATACAGATAGATAATGGTAGCAATATGCTAATTC

TGAACCCGACACAATCAGACTCTGGTATTTATATATGCATTACCACGAACGAAACCTACTGTGACATGAT

GTCGTTAAATTTGACAATCGTGTCTGTCTCAGAATCAAATATAGATCTTATCTCGTATCCACAAATAGTA

AATGAGAGATCTACTGGTAAAATGGTATGTCCCAATATTAATGCATTTATTTCTAGTAACGTAAACGCAG

ATATTATATGGAGCGGACATCGACGCCTTAGAAATAAGAGACTTAAACAACGGACACCTGGAATTATTAC

CATAGAAGATGTTAGAAAAAATGATGCTGGTTATTATACATGTGTTTTAGAATATATATAGGCAAAACAT

ATAACGTAACCAGAATTATAAAATTAGAGGTACGGGATAGAATAATACCTCCTACTATGAAATTACCAGA

AGGAGTAGTAACTTCAATAGGTAGTAATTTGACTATTGCATGCAGAGTATCGTTGAGACTTCCCACAACG

GACACCGACGTCTTTTGGATAAGTAATGGTATGTATTACGAAGAAGAAGACGAGGACGGAG

ACGGTAGAATAAATGTAGCAAATAAAATCTATATGACCGATAAGAGACGTGTTATTACATCCTGGTTAAA

CATTAATCCTGTCAAGGAAGAAGATGCTACAACGTTTACATGTATGGCGTTTACTATTCCTAGCATCAGC

AAAACAGTTACTGTTAGTATAACGTGAATGTATGTTGTTACATTTCCATATCAATTGAGTTTATAAGAAT

TTTTATACATTATCTTCCAACAAACAATTGACGAACGTATTGCTATGATTAACTCCCACAATACTATGCA

TATTATTAATCATTAACTTGCAGACTATACCTAGTAGTGCTATTTTGACATACTCATGTTCTTGTGTAAT

TGCAGTATCTATATTATTAAAGTACGTAAATCTAGCTATAGTTTTATTATTTAATTTTAGATAATATACT

GTCTCCGTATTTTTAAAAAATTACCACATCCTTTATTAAATCATGAATGGGAATTTCTGTGTCATCGTTA

GTATATTGTGAACAACAAGAGCAGATATCTATAGGAAAGGGTGGAATGCGATACATTGATCTATGTAGTT

TTAAAACATACGCGAACTTTGAAGAATTTATATAAATCATCTCACGAGATATTGCTCTCTGTCATATTCA

TACACCTGCATAAACTTTCTAGACATCTTACAATGTGTTATTTTATGATCATATTTACATATTTACTGGT

ATATCAAAGATGTTAGATTAGTTAATGGGAATCGTCTATAATAATGAATATTAAACAATTATAGGAGGAG

TTTATACCTACAAAAACATCATAAAAATGAGTCATCGTCCGATTTATGTTTTAAATATACTAACATTACT

ACCTTCAGAAATTATATACGAAATATTATACATGCTGACAATTAACGATCTTTATAATATATAGTATCCA

CCTACCAAAGTATAATTGTATTTTTTCTCATGTGATGTGTGTAAAAAACTGATATTATATAATTATCTTA

GTACCTATGATGAAGATGAAGATGATGGTCCGTATATATTTTGTATCATTATCGTTATTGCTATTCCATA

GTTACGCCATAGACATCGAAAATGAAATCACCGAATTCTTCAATAAAATGAGAGATACTCTACCAGCTAA

AGACTCTAAATGGTTGAATCCAGTATGTATGTTTGGAGGCACAATGAATGATATGGCCGCTCTAGGAGAG

CCATTCAGTGCAAAGTGTCCTCCTATTGAAGACAGTCTTTTATCGCATAGATATAAAGACTATGTGGTTA

AATGGGAAAGGCTAGAAAAGAATAGACGGCGACAGGTTTCTAATAAACGTGTTAAACATGGTGATTTATG

GATAGCCAACTATACATCTAAATTCAGTAACCGTAGGTATTTATGTACCGTAACCACAAAGAATGGTGAC

TGTGTTCAGGGTGTAGTTAGATCTCATGTGTGGAAACCTTCTTCATGCATTCCAAAAACATATGAACTAG

GTACTTATGATAAGTATGGCATAGACTTATACTGTGGAATTCTTTATGCGAAACATTATAATAATATAAC

TTGGTATAAAGATAATAAGGAAATTAATATCGACGATTTTAAGTATTCACAAGCGGGAAAGGAATTAATT

ATTCATAATCCAGAGTTAGAAGATAGTGGAAGATACGACTGTTACGTTCATTACGACGACGTTAGAATCA

AGAATGATATCGTAGTATCAAGATGTAAAATACTTACGGTTATACCGTCACAAGACCACAGGTTTAAACT

AATACTAGATCCGAAAATCAACGTAACGATAGGAGAACCTGCCAATATAACATGCAGTGCTGTGTCAACG

TCATTATTTGTCGACGATGTACTGATTGAATGGAAAAATCCATCCGGATGGATTATAGGATTAGATTTTG

GTGTATACTCTATTTTAACTAGTAGAGGCGGTATCACCGAGGCGACTTTGTATTTTGAAAATGTTACTGA

AGAATATATAGGCAATACATATACATGTCGTGGACACAACTATTATTTTGATAAAACTCTTACAACTACA

GTAGTATTGGAGTAAATACACAATGCATTTTTATATACATTACTGAATTATTATTATTAATTATATCGTA

TTTGTGCTATAGAATGGATGAAGATACGCGACTATCTAGGTATTTGTATCTCACCGATAGAGAACATATA

AATGTAGACTCTATTAAACAGTTGTGTAAAATATCAGATCCTAATGCATGTTATAGATGTGGATGTACGG

CTTTACATGAGTACTTTTATAATTATAGATCAGTCAACGGAAAATACAAGTATAGATACAACGGTTACTA

TCAATATTATTCATCTAGCGATTATGAAAATTATAATGAATATTATTATGATGATTATGATAGAACTGGT

ATGAACAGTGAGAGTGATAATATATCAATCAAAACAGAATACGAGAATGAATATGAATTCTATGATGAAA

CACAAGATCAAAGTACACAACTAGTAGATTACGACATTAAACTCAAAACCAATGAGGATGATTTTGTTGA

TGAATTCTATGGTTATGATAGATCAGTGGGTGTCCATGATTATATAGATGTATCAATTAATAAAGTAGTA

TATGGAAGAGAGTCTCACGTAAGATGGCGGGATATATGGCAAGAACATAATGATGGCGTATACAGTATAG

GAAAGGAGTGCATAGATAATATATACGAAGACAGACATACCGTAGACGAATTCTACAAGATAGACAGCGT

ATCAGATGTAGATGACGCAGAACATATATCTCAGATAACTAATGATGTATCTACACAAACATGGGAAAAG

AAATCAGAGTTAGATAGATACATGGAAATGTATCCTCGTCATAGATATGGTAAGCATTCTGTCTTTAAGG

GATTTTCTGACAAAGTTAGAAAAAATGATTTAGACATGAACGTGGTAAAAGAATTACTTTCTAACGGTGC

ATCTCTAACAATCAAGGATAGCAGTAATAAGGATCCAATTGCTGTTTATTTTAGAAGAACAATAATGAAT

TTAGAAATGATTGATATCATTAACAAACATACAACTATCTATGAACGCAGGTATATAGTACACTCCTATC

TAAAAAATTATAGAAATTTCGATTATCCATTTTTCAGAAAGTTAGTTTTGACTAATAAACATTGTCTCAA

CAATTATTGTAATATAAGCGACGGCAAATATGGAACACCACTACATATATTAGCATCTAATAAAAAAATA

ATAACTCCTAATTACATGAAGTTATTAGTGTATAACGGAAATGATATAAACGCACGAGGTGAAGATACAC

AAATGCGAACTCCATTACACAAATATTTGTGTAAATTTGTATATCATAATATTGAATATGGTATCCGATA

CTATAATGAAAAGATTATAGACGCATTTATAGAGTTAGGAGCCGATCTAACTATTCCAAATGACGATGGA

ATGATACCAGTAGTTTACTGTATACACTCAAATGCCGAATATGGTTATAACAATATTACTAACATAAAGA

TAATACGTAAACTACTTAATCTTAGTAGACGTGCGTCACATAATCTATTTAGAGATCGAGTCATGCACGA

TTATATAAGTAATACATATATTGATCTTGAGTGTTTAGATATCATTAGATCACTGGATGGGTACGATATT

AATTGTTACTTTGAAGGACGTACACCACTTCATTGCGCTATACAATATAACTTCACTCAGATTGCTGAGT

ACTTATTAGATCGAGGAGCTGATATATCATTAAAGACAGACGATGGTAAAACTGTATTTGATTTATCGTT

ATGTAGTTACATTCCTCTTAAATGGACTAGCTTTTTGATTAGTCGTCTACCGCCTAAAAGTGTCATATGC

TCACTGACTAACCATATAATAGATTATGTTCTTACGAACAATAGACGTATTATTTGGCAGAGTCAAATGA

TTAATAAGTACGTACTGTTACTGGACCCATCCTTTTATTATAGATTCAGAAATGTTATCGAAAACAAATT

AGACCAATACAATAATCGTTATAATATGTTCGAACACGATAGGGACGTTAATGAAAAGTATGGCAAAGTC

TTACATGACCTCGATACATATATCAAGGATGTACAAGTATTAAAATCTACTTCCATCACTAATAATATAA

CACTATACGACACTATTATAAATAATAAGTCAGAGTTTCCTATACGTCGTGTAAACGACAAACAATTAAT

TAATCTCATAAAATCCAATACATATCATAATCTTATCGAAAAAGTTATTAAAAATACATTAGAGAAATAT

ACTTTAACTAATATAGTCCTCGAGTATATGATCTCATCTCGATCTCAATCATCTTATTTGAGTCGTATTC

CTAATGAGATATTACTCGAAATATTATATAAACTCGACATGTACGATTTACGTAATCTATATACAAGATA

TATGAGAGAGAATGATATCACAGAGTATCATATAGAGAATACGAGGTCTGTTTCTACACAGACATGAATA

ATGAATACACATACAACGTTTTTTTTTTTTAATCTTAGATATAACACTAATTACATCAAGATTATATATT

GAAATCGTAATTTGAGTTGTCTGATCATCATGGATATCGAAAATGATATACGTAACATTAGCAATCTTTT

AGATGATTGATATATTATTATGCGATGTAATCATAACTATCGGAGATGTAGAAATTAAAGCGCATAAAAC

TATTTTGGTTGCCGGATCTACGTATTTTAAAACAATGTTCACAACACCTATGATAGTGAGAGATCTAGCA

ACTAGAGTAAATATACAGATGTTCGATAAAGATGCCGTCAAAAATATTGTACAGTACTTATACAATAGGT

ATATAAGTTCTATGAATGTGATAGACGTATTAAAATGCACCGACTAAGAACGTAGAACGAACTATAGAAT

GTTATACAATGGGTGATGATAAGTAGAAGATGTTACCCGATATACCCATAGCATTATCTAGTTATGGCAT

GTGTGTATTAGATCAATACATATACATTATAGGCGGTCGTACCCAACACTGATTATACATCGGTACATAC

AGTAAATAGCATAGATATGGAGGAGGATACAAATATTTCAAATAAAGTTATGAGATACGCGCTGTCAATA

ATATATGGAAGACATTACCTAACTTCTGAACTGGAACTATAAATCCAGGCTCTCGCATAAAGATGAATAT

ATATGTTGTATGCGACATCAAAGATGAAAAAATGTTAAGACTTATATATTTAGATATAACACGAATATGT

ATGACGGATGGGAATTGGTAACGATGACAGAAAGCAGATTGTCAGCTCTGCATACTATTCTTCATGACAA

TACCATAATGATGTTACATTGTTATGAAACGTATATGTTACAAGATACATTTAATGTGCTTACGGAACAT

ATATTTAGAAACATCTACTAACGATTTTTTATGCTTGTATTATTAATGGTATGTAATATGATTTAATTGA

TTGTGTACACGATACCAATTTGTCGAGTATGAATACGGAGTACAAACATAAACTGAAGTTTAACATTATA

TACATTATATACATTATATACATTATATACATTATATACATTatatacattatatacattatatacattatatacattatatacattatatacattatatacattatatacattatatacattatatacattatatacattatatacattatataCATTATATAtcgttattgTTTGGTCTATGCCA

TGGATATCTTTAAAGAACTAATCTTAAAACATACGGATGAAAATGTTTTGATTTCTCCAGTTTCCATTTT

ATCTACTTTATCTATTCTGAATCATGGAGCAGCTGGTTCTACAGCTGAACAACTATCAAAATATATAGAG

AATATGAATGAGAATATACCCGATGATAAGAAGGATGACAATAATGACATGGACGTAGATATTCCGTATT

GCGCGACACTAGCTACCGCAAATAAAATATACGGTAGTGATAGTATCGAGTTCCATGCCTCATTCCTACA

AAAAATAAAAGACGATTTTCAAACTGTAAACTTTAATAATGCGAACCAAACAAAGGAACTAATCAACGAA

TGGGTTAAGACAATGACAAATGGTAAAATTAATTCCTTATTGACTAGTCCGCTATCCATTAATACTCGTA

TGATAGTTATTAGCGCCGTCCATTTTAAAGCAATGTGGAAATATCCATTTTCTAAACATCTTACATATAC

AGACAAGTTTTATATTTCTAAGAATATAGTTACCAGTGTTGATATGATGGTGGGTACCGAGAATGACTTG

CAATATGTACATATTAATGAATTATTCGGAGGATTCTCTATTATCGATATTCCATACGAGGGAAACTCTA

GTATGGTGATTATACTGCCGGACGACATAGAAGGTATATATAACATAGAAAAAAATATAACAGATGAAAA

ATTTAAAAAATGGTGTGGTATGTTATCTACTAAAAGTATAGACTTGTATATGCCAAAGTTTAAAGTGGAA

ATGACGGAACCGTATAATCTGGTACCGATTCTAGAAAATTTAGGACTTACTAATATATTTGGATATTATG

CAGATTTTAGTAAGATGTGTAATGAAACTATCACTGTAGAAAAATTTCTACATACGACGTTTATAGATGT

TAATGAGGAGTATACAGAAGTATCGGCCGTTACAGGAGTATTCATGACTAACTTTTCGATGGTATATCGT

ATGAAGGTCTACATAAACCATCCATTCATATACATGATTAAAGATAACACCGGACATACACTTTTTATAG

GGAAATACTGCTATCCGCAATAAATATAAACAATAGACTTTTATCACGTTATCTCATGTATAAAATATTA

CAAATAGTATAGCATAAACTAAAGTCGATACATACATTAAAACTTAAATAATAATGTAATTTACAATTAA

TAGTATAAACTAAAAAAAATTAAAAACAATATCATTATTATAAGTAATATCAAAATGACGATATACGGAT

TAATAGCGTATCTTATATTCGTGACTTCATCCATCGCTAGTCCACTTTACATTCCCGTTATTCCGCCCAT

TTCGGAAGATAAATCGTTCAATAGTGTAGAGGTATTAGTTTCTTTGTTTCCCGATGACCAAAAAGACTAT

ACAGTAACTTCTCAGTTCAATAACTACACTATCGGTACCAAAGACTGGACTATCAACGTACTATCCACAC

CTGATGGTCTGGACATACCATTGACTAATATAACTTATTGGTCACGGTTTACTATAGGTCGTGCATTGTT

CAAATCAGAGTCTGAGGATATTTTCCAAAAGAAAATGAGTATTCTAGGTGTTTCTATAGAATGTAAGAAG

CCGTCGACATTACTTACTTTTTTAACCGTGCGTAAAATGACTCGAGTATTTAATAGATTTCCAGATATGG

CTTATTATCGAGGAGACTGTCTAGAAGCCGTTTATGTAACAATGACTTATAAAAATACTAAAACTGGAGA

GACTGATTACACGTACCTCTCTAATGGGGGGTTGCCTGCATACTATCGTAATAGGGTCGATGGTTGATTA

TTGATTAGTATATTCCTTATTCTTTTTATTCACACAAAAAGAACATTTTTATAAACATGAAACCACTGTC

TAAATGTAATTATGATCTTGATTTATAGATGATGATCAGCCTTCAGAGGATTTTGACCAGTATGTTTAAT

ATGAAAAAAAACATAACTATTAAGCGCTATTGCGCTATTGTGCTTAATTATTTTGCTCTATAAACTGAAT

ATATAGCCACAATTATTGACGGGCTTGTTTGTGACCGGCAATCATGAATTTTCAGAAATTATCTCTGGCT

ATATATCTTACGGTGACATGTTCGTGGTGTTATGAAACATGTATGAGAAAAACTGCGTTGTATCATGACA

TTCAATTGGAGCATGTAGAAGACAATAAAGATAGTGTAGCATCGCTACCGTACAAGTATCTACAAGTAGT

CAAACAAAGAGAACGTAGTAGATTGTTGGCTACATTTAATTGGACGGATATAGCTGAGGGTGTTAGAAAT

GAGTTCATTAAAATATGTGATATCAACGGAACATATCTATATAATTATACTATTGCTGTTAGTATAATTA

TTGATTCCACGGAAGAACTACCAACAGTTACTCCAATTACAACATATGAACCTTCTATATATAATTATAC

TATCGATTATAGCACTGTTATTACTACTGAAGAACTACAAGTGACTCCAACATATGCACCTGTAACAACT

CCTCTTCCAACATCAGCAGTTCCTTATGATCAACGATCGAATAACAATGTAAGTACTATATCTATTCAGG

TACTGAGTAAAATATTGGGAGTCAATGAAACAGAATTAACTAATTATCTTATTATGCATAAAAATGACAC

TGTTGACAATAACACCATGGTTGATGATGAGACATCTGACAATAACACATTGCATGGTAATATAGGATTT

TTGGAAATAAATAATTGTTATAATGTTTCTGTGTCAGATGCTAGTTTTAGAATAACATTAGTAAACGATA

CTTCTGAAGAAATTTTGCTAATGCTAACAGGAACTAGTTCATCCGACACCTTCATATCTTCCACCAATAT

CACTGAATGTTTGAAAACATTAATCAATAATGTGTCGATTAATGATGTACTTATAACACAAAATATGAAT

GTAACATCTAATTGTGATAAATGCTCAATGAATTTGATGGCATCCGTTATTCCTGCAGTTAATGAATTTA

ACAATACGTTGATGAAAATTGGTGTAAAAGATGATGAAAACAATACGGTATATAAATATTATAATTGTAA

ACTAACTACAAATTCTACATGTGATGAGTTAATCAATTTAGATGAAGTAATTAACAACATAACTCTGACA

AATATTATACGCAATAGTGTTTCGACAACTAACAGCAGAAAAAGACGAGATCTGAATGGTGAGTTTGAAT

TTTCCACTTCCAAGGAATTAGATTGTCTTTACGAATCATATGGTGTAAACGATGATATAAGTCATTGTTT

TGCATCACCTAGACGTAGACGATCTGATGACAAAAAGGAGTACATGGACATGAAATTATTCGACCACGCG

AAAAAAGATTTAGGAATAGACAGTGTTATTCCTAGAGGTACAACCCATTTCCAAGTAGGTGCATCTGGTG

CAAGTGGTGGTGTTGTAGGAGATAGTTTCCCATTTCAAAATGTTAAATCGCGTGCCAGTCTATTGGCGGA

AAAAATAATGCCTAGAGTACCTATTACTGCTACCGAAGCTGATCTATATGCAACTGTAAATAGACAACCC

AAGTTACCAGCAGGTGTTAAAAGTACTCCGTTTACAGAGGCGCTTGTGTCTACGATAAACCAAAAGCTTT

CTAATGTTAGAGAGGTAACTTATGCTTCGCTCAATCTGCCAGGATCAAGTGGCTATGTTCATAGACCATC

TGATTCTGTTATTTACAGCAGTATAAGACGGTCACGTTTACCTAGTGATAGCGATAGTGATTATGAGGAT

ATACAAACTGTTGTTAAGGAATATAATGAAAGATATGGTAGATCAGTCAGTAGAACACAGTCATCAAGTA

GTGAAAGCGATTTTGAAGATATAGATACTGTTGTTAGGGAATATAGACAAAAATATGGCAATGCAATGGC

AAAAGGACGTAGTAGTTCCCCTAAACCTGATCCATTATATAGTACTGTTAAGAAAACAACTAAAAGTCTA

TCTACTGGTGTAGACATAGTTACAAAACAATCAGACTATTCTCTATTACCTGACGTTAATACTGGCAGTT

CTATTGTGTCACCTCTCACCAGAAAAGGAGCTACTAGACGACGACCTAGACGCCCTACAAATGATGGTCT

ACAGAGTCCAAATCCTCCTCTCCGTAATCCACTTCCTCAACATGATGATTATTATCCTCCACAAGTACAC

AGACCTCCACCACTTCCTCCTAAACCAGTCCAAAATCCGCCACAACTTCCCCCTAGACCAGTAGGTCAAT

TACTACCTCCTCCTATAGATCAACCAGATAAAGGATTTAGTAAGTTTGTATCACCTAGACGGTGTAGAAG

AGCAAGCTCTGGAGTCATATGTGGTATGATACAATCAAAACCAAACGATGATACCTATTCACTTCTTCAA

CGGCCAAAAATTGAACCAGAATATGTGGAGGTTGGTAATGGTATACCCAAGAACAATGTTCCTGTAATAG

GTAATAAACATAGTAAAAAATATACATCGACGATGTCAAAAATATCAACAAAATTTGATAAATCTACGGC

ATTTGGAGCAGCAATGTTACTAACTGGTCAGCAGGCCATTAGCCAACAGACTAGATCAACTACGTTGAGT

AGAAAAGATCAGATGAGTAAGGAAGAAAAGATATTCGAAGCAGTTACAATGAGTCTATCAACTATAGGTT

CAACGTTGACGTCTGCAGGTATGACGGGTGGTCCAAAACTAATGATTGCAGGAATGGCTATAACGGCTAT

AACTGGTATAATAGATACGATAAAAGATATATATTACATGTTTTCAGGACAGGAGAGGCCAGTAGATCCT

GTTATTAAATTATTTAATAAGTACGCTGGCTTAATGTCCGATAATAATAAAATGGGTGTAAGAAAATGTT

TGACACCCGGTGACGACACACTTATTTATATCGCATACAGAAACGATACCAGTTTTAAACAGAATACGGA

TGCGATGGCTTTGTATTTCTTAGATGTTATCGATTCAGAGATCCTATATCTAAACACATCAAATTTAGTT

CTAGAGTATCAACTAAAGGTGGCTTGCCCCATAGGAACATTAAGATCTGTAGATGTGGACATAACTGCGT

ATACAATATTATATGATACAGCGGATAATATTAAGAAATACAAGTTTATCAGAATGGCAACGCTACTATC

CAAACATCCAGTTATTAGATTGACATGTGGTTTAGCAGCAACATTGGTGATTAAACCGTACGAGGTACCC

ATCAGTGATATGCAACTACTAAAAATGGCGACGCCTGGTGAACCAGAATCCACTAAATCTATACCATCCG

ATGTCTGTGATAGGTATCCTCTAAAGAAATTCTATCTTTTAGCTGGTGGTTGTCCCTATGATACATCTCA

AACTTTTATTGTACATACTACTTGCAGTATTCTACTAAGAACAGCTACACGGGATCAGTTTAGAAACAGA

TGGGTGTTACAAAATCCATTTAGACAAGAAGGGACATATAAGCAACTGTTTACCTTTAGCAAATACGATT

TTAACGACACCATAATCGATCCTAATGGTGTGGTGGGTCATGCTAGCTTTTGTACCAATAGAAGCAGCAA

CCAATGTTTCTGGTCCGAACCTATGATATTGGAAGATGTATCATCGTGTAGTTCTAGAACTAGAAAAATA

TACGTAAAACTGGGAATATTTAATGCTGAAGGTTTTAATAGTTTTGTACTAAATTGTCCAACTGGGTCTA

CACCTACATACATCAAACATAAAAATGCGGACAGTAACAATGTTATCATAGAGCTACCTGTAGGTGATTA

CGGCACAGCCAAATTGTATTCAGCAACAAAACCATCGAGGATAGCTGTGTTCTGCACACATAACTATGAT

AAACGATTCAAATCAGATATTATAGTTCTAATGTTTAATAAAAACAGCGGTATTCCATTTTGGAGCATGT

ACACAGGAAGTGTAACTAGTAAAAATAGAATGTTTGCCACATTGGCTAGAGGAATGCCGTTTAGATCAAC

GTATTGCGATAACAGACGACGATCAGGTTGTTATTATGCAGGAATACCATTTCATGAAGATAGTGTAGAA

ACAGATATACATTATGGACCAGAAATAATGTTAAAGGAAACATATGACATAAACAGTATTGACCCACGAG

TTATAACAAAGTCAAAGACCCATTTTCCTGCTCCATTGAGTGTAAAATTCATGGTTGACAATTTAGGAAA

TGGATATGACAACCCTAATTCATTTTGGGAAGATGCTAAAACTAAGAAACGGACATATAGTGCAATGACG

ATAAAAGTCCTACCATGTACAGTGAGAAATAAAAATATAGACTTTGGATATAACTATGGAGATATTATTT

CTAATATGGTTTATCTACAATCTACTAGTCAGGATTATGGAGATGGTACCAAATATACATTTAAATCCGT

AACTAGATCAGATCATGAGTGTGAATCTAGCTTAGATCTAACGTCTAAGGAAGTAACTGTGACATGTCCT

GCGTTTAGTATACCAAGAAATATATCAACATATGAAGGTCTATGCTTTAGTGTTACTACATCTAAAGATC

ATTGTGCTACAGGTATTGGTTGGTTAAAATCTAGTGGTTATGGGAAGGAAGATGCTGATAAACCACGTGC

TTGTTTTCATCATTGGAATTATTACACACTGTCGTTGGATTATTACTGTTCATACGAAGATATTTGGAGA

AGCACCTGGCCTGACTATGATCCATGTAAGTCATATATCCATATAGAGTATAGAGATACATGGATAGAAT

CTAATGTGTTACAGCAACCTCCTTACACATTCGAATTCATTCATGACAATTCTAACGAATATGTGGATAA

AGAAATTAGTAACAAATTAAATGATCTGTACAATGAATACAAGAAGATTATGGAATATAGCGACGGATCA

TTGCCGGCGTCTATAAACAGATTAGCAAAGGCATTGACTTCAGAGGGTAGAGAAATAGCAAGTGTTAATA

TAGATGGTAATCTGTTAGATATCGCATATCAAGCAGATAAGGAAAAGATGGCCGACATACAGACAAGAAT

AAATGATATTATTAGAGATTTGTTTATACACACTCTATCAGACAAAGATATAAAAGACATTATAGAATCC

GAAGAAGGTAAGAGATGTTGTATAATAGATGTTAAGAACAATCTTGTTAAAAAGTACTATTCTATTGATA

ATTATCTATGTGATACTTTAGATGATTATATATACACCTCTGTAGAATATAACAAATCCTATGTGTTAGT

AAACGATACTTATATGAGCTATGACTATCTTGAATCATCAGGTGTAGTTGTTCTATCATGTTATGAAATG

ACTATAATCTCCTTGGATACAAAAGACGCCAAAGATGCTATAGAAGATGTGATAGTAGCAAGTGCGGTAG

CCGAAGCATTGAATGACATGTTTAAGGAATTTGATAAAAACGTAAGTGCTATTATAATAAAAGAAGAAGA

TAATTATCTAAACAGTTCGCCCGATATCTACCATATAATATATATCATAGGTGGCACTATTCTGCTACTG

TTAGTCATTATTTTAATATTGGCAATTTATATAGCGCGCAATAAATACAGAACCAGGAAATATGAAATAA

TGAAATATGACAATATGAGCATTAAATCTGAGCATCATGATAGTCTTGAAACAGTGTCTATGGAAATTAT

TGATAATCGGTACTAATAAAATAGTTTAACTCTTTTAGAACCAGTTTGGTACTGTAATTTCAGTTCATTA

CTCGTTGAGAATATTGATGATTTTTTTTAAATGAGTATCGGTAGTTACATATTACCATATCATCCATTAT

ATAATCGATGATGCATGTATTAGAATACTTTCCGAATAAGTCTTCTAAATATTGTATTAATTATGAAAAA

CTATGCTATGTGAGTATGATTCAAAGATGTTTAATGATACGATACTAGATTTTATCTCTAGCGAGATTGT

TTAGAATCATTTATCATAACTATGTTTAATAAATTCATCAACGAATATCGATAAAGACCTCTTGTAATTC

GAGTATAGGAAGTAGTATTACCATATCAACTTCCGAGTTAACAATTACTCTAAAACATGAGGATTTCCTG

TCTTTATTGGAGATTACTATTCAGTCGTTGATAAACTAGTAACCTCAGGTTTCTTTACAAACGATAAAGT

ACAACATCAAGACCTCACAACACAGTGCAAGATTAATCTAGAAATCAAATGTAATTCTGGAGGAGAATCT

AGACAACTAACACCCACGGCGAAGTATACTTTATGCCTCATTCAGAAACGGTAACTGTAGTAGGAGACTG

TCTCTCTAATCTCGATGTATATATAATATATACCAATACGGACGCGATATATTCCGACATGAATGGCGTC

GCTTATCATATGTTATATCCTAAATGTTGATCATATTCCACAAATGATTGTGAACGAGATTAAATCATCT

AACAAATAATTAGTTTTTTATGACATTAACATATAATAAATAAATTAATCATTATTGACTTAACGATGAC

GAAAGTTATCATTATCTTAGGATTCTTGATTATTAATACAAATTCGTTGTGTCTATGAAATGTGAACAAG

GTGTCTCATATTATAATGCACAAGAATTAAAGTGTTGTAAACTATCTAGCCAGGAACATATTCAGATTAT

CGATGTGATAAATACAGCGATACCATCTGTGGACATTGTCCAAGTGACACATTCACGTCAATATATAATC

GTTCTCCTCGGTGTCATAGTTGTAGAGGTCACACCTTGTACACCTACCACAAATAGAATCTGTCATTGTG

ACTCGAATAGTTATCGTCTCCTTAAAGCTTCTGATGGTAACTGTGTTACATGTGCTCCTAAAACAAAATA

TGGTCGTGTGTACGGAAAGAAAGGAGAAAATGATATGGAATACCATTTGTAAGAAATGTCGGAAGGGTAC

TTATTCAGATATTGTATCTGACTCTGATCAATGTAAACCTATGACAAGATAAGACTTACTCGCATCTACT

GGATAGACATAAATATCCTCCTCGTAATAATGAAATATAATATACACTAATTATTAATATCAATCGAGTA

TTAACATATAAGTTATTTTTAAACCCCTTTTGTGTTCCGTCCTAAACGGCGTTTCGGTCTGTGTCGCCAC

TATGGTCACACCGAGCCTCTGCGTGCTCCTCCATCGAGGACGACTTCAACTATGACAGCTCGGTGGCGTC

TGCCAGCGTGTACATACGAATGGCATTTCTAAGAAAAGTCTACGGTATCCTTTCTACAATTTCCTTTAAC

AACGGCAACAGCTGCAGTATTTTTATACTTTGAATGCATCGGACATTTATACAAGGGAGTCCTGTTCTAA

TATTGGCATCAATGTTCGGATCTATAGGCTTGATTTTCGCATTGACTTTACACAGACATGAACATCCCCT

GAATCTGTACATACTTTGTGGATTTACACTGTTAGAATCTCTAACGCTGGCCTCTGTTGTTACTTTCTAT

GATGCACGTATCGTTATGCAAGCTTTCATGTTGACTACTGCAGTGTTTCTTGCTCTGACTACATATACTC

TACAATCAAAGAGAGATTTCAGTAAACTTGTAACAGGATTGTTTGCTGCTTTCTGGATTTTAATTTTGTC

AGGAGTCTTGAGGATAAAGTTTAAAATAGAATTAATAAAGAACATATAGGTCATTTTTTAAACATGGATA

GAAACCAAGGTTGTTAGTTAATAATATACAAGATATTTTTTCTCACTCTGATCCATGTAAACCAAGGACG

AGAGACACTCTCATTCCTCATTCACGACACCATTAAAAATGGAAATTAAAGCCCTCTATTAAGCACAGAC

GGCTACAGGTCTACCATCAGGTTACCTTCGTCTACCTTCACAATGGCCTCTCCTTGTGCCCAGTTCAGTC

CCTGTCATTGCCACGCTACTAAGGACTCCCTGAATACCGTGACTGATGTCAGACATTGTCTGACTGAATA

CATCCTGTGGGTTTCTCATAGATGGACCCATAGAGAAAGCGCAGGGCCTCTCTACAGGCTTCTCATCTCT

TTCAGAATTGATGCAATGGAGCTATTTGGTAGCGAGTTGAAGGAGTTCTCGGATTCACTTCCGTGGGACA

ATATCGACAATTGCGTGGAGATCATTAAATGTTTCATCAGAAATGACTCCATGAAAACCGCCAAAGAACT

TTGTGCAATAATTGGACTTTGTACTCAATCAGCTATTGTCACCGGAAGAGTCTTCAATGATAAGTATATC

GACATACTACTTATGCTGCGAAAGATTCTGAACGAGAACGACTATCTCACCCTCTTGGATCATATCCTCA

CTGCTAAATACTAAATCTCCTTCATGCTCTCTCACTAATACTCTTACTCACTACACTTTTTATCATCTTA

TGATGAATGGCCTTCATCATTTTTCGTGGAATATAATATAGGAATAATTAGCACCAGAATAGCTATGGAT

ATCTCGTTAAGAATATTCTCGATAAGAGACATAATGTAGACATAGTTATTATATCCTTCTTAGATAAGTG

TTACGCTACTGGAAAGTTTCCATCGTTATTATTACATGAAGATGATATAATTAAACCAACATTGAGATTG

GCTCTTATGTTAGCTGGATTGAATTACTGTAATAAATGCATCGAGTATAGAGGGATATAGCAATTCTCGA

TAATAGTCATGCAATATTTGAATGAGACTGATAATTTAGGTAATACAGTACTACACACATATCTTTCTAG

ATTATATATCGTTAAAAATCTGTAAGAGGTATATTTCTCATAAGTATCCACTGTGTAATATTATTAATGGAT

ATATAGATAACGCAATAGGGACTAATAGTATTGTAAAAGATATAATCGACTATTTACGTACATATCCAGA

TATCTATATTCCTACTAGTTTGCTGCGTAGTTGCATCATTGATATGCATGATTTATCAGGATTCAGAGAT

GAATTACTAAGTAAACTACAATCCCACAATAAGTAAGAATCAAATATCAAAAACTTACTTTTGATTTTTC

TAGTCTTAAGTAATACATATATTTATTAATAGACCTATGAAATAAAAAAAGGTAACAATGGATTCGCGTA

TAGCTATTTACGTATTAGTATCGGCATCTCTTTTGTATCTTGTTAATTGTCACAAACTAGTACATTACTT

CAATCTGAAAATAAATGGAAGTGATATAACTAATACAGCAGATATATTGCTGGACAATTATCCAATTATG

ACCTTTGATGGAAAGGATATTTATCCATCTATCTCGTTCATGGTCGGTAATAAACTTTTCCTAGATCTTT

ATAAAAATATCTTTGAAGAATTTTTCAGACTATTTCGAGTATCTGTAAGTAGTCAATACGAGGAATTAGA

ATATTATTATTCATGTGATTATACTAACAACCGTCCTACAATTAAACAACATTACTTTTATAACGGCGAA

GAATATACTGAAATTGATAGATCGAAAAAAGCCACTAATAAAAACAGTTGGTTAATTACTTCAGGCTTTA

GACTACAAAAATGGTTCGATAGCGAAGATTGTATAATTTATCTCAGATCTTTAGTTAGAAGAATGGAAGA

CAGTAACAAAAACAGTAAAAAAACTTAGTACTTAGATATCGAAAAAAATATATTTTTGTAGACTCTTGAG

AATAGAAGGAAAACATGTACATAATTATAAAAAATGAAAATCAATGGCGAATAAGACAGTGCGATTCGCA

CCATGGAGTCGGTAGATTTCATGGCTGTCGATGAGCAGTTTCACGACGACCTCGATCTTTGGTCATTATC

TTTGGTAGATGATTATAAAAAACATGGATTAGGTGTTGACTGTTATGTTCTAGAACCAGTTGTTGACAGG

AAAATATTTGATAGATTTCTCCTTGAACCAATTTGTGATCCTGTAGATGTTCTGTATGATTATTTTAGGA

TTCATAGAGATAATATTGATCAGTATATAGTAGATAGACTGTTTGCATATATTACATATAAAGATATTAT

ATCTGCATTAGTGTCAAAGAATTATATGGAAGATATTTTCTCTATAATTATTAAGAATTGTAATTCTGTG

CAAGATCTCTTACTTTACTATCTATCTAATGCATATGTAGAAATAGACATTGTTGATCTTATGGTAGATC

ATGGGGCTGTAATATATAAAATAGAATGCTTGAATGCCTATTTTAGGGGAATATGTAAAAAGGAAAGTAG

TGTTGTTGAGTTTATTTTGAATTGTGGTATCCCAGATGAAAATGATGTTAAATTAGATCTATATAAAATA

ATTCAGTATACTAGGGGATTCCTTGTAGATGAACCCACAGTATTAGAAATTTATAAGCTTTGTATCCCAT

ATATTGAAGATATCAATCAACTAGATGCTGGTGGAAGGACCTTGCTTTATCGCGCTATCTATGCAGGTTA

TATAGATTTAGTATCATTGCTATTAGAAAATGGAGCAAATGTCAACGCAGTAATGAGTAATGGATATACA

TGTCTTGACGTGGCCGTGGATAGGGGATCTGTCATCGCCCGTAGGGAAGCACATCTTAAAATATTAGAAA

TATTGCTTAGAGAACCATTGTCTATTGACTGTATAAAATTAGCTATACTTAATAATACAATTGAAAACCA

TGATGTGATAAAGCTCTGTATCAAGTATTTTATGATGGTAGATTATTCACTTTGTAATGTGTATGCATCA

TCACTCTTTGATTATATAATTGATTGTAAACAAGAATTGGAGTACATTAGGCAGATGAAAATTCATAATA

CAACCATGTATGAGTTAATCTATAATAGAGACAAAAACAAGCATGCTTCCCATATTCTACATAGGTATTC

TAAACATCCAGTTTTGACACAGTGTATCACTAAAGGATTCAAGATTTACACAGAAGTAACCGAGCAGGTC

ACTAAAGCTCTAAACAGACGTGCTCTAATAGATGAGATAATAAACAATGTATCAACTGATGACAATCTCC

TATCAAAACTTCCATTAGAAATTAGGGATCTAATCGTTTCACAAGCTGTCATATAGAGTTCTATCCACCC

ACCTTTCTTGAAATGAGTTAATagtcataagttagttaAGTCATAAGTTAGTTAAGTCATAAGTTAGTTAAGTCATAAGTTAGTTA

AGTCATAAGTTAGTTTTATAAGTAAGTTAGTTAAGTCATAAGTTAGTTTTATAAGTTAGTTTATAGTCTA

ACACTTCTAATTTTTATACCTTGATCTTTTTCTCTAATTATGAAAAAGTAAATCATTATGAAGATGGATG

AAATGGACGAGATTGTGCGCATCGTTAACGATAGTATGTGGTACGTACCTAACGCATTTATGGACGACGG

TGATAATGAAGGTCACATTTCTGTCAATAATGTCTGTCATATGTATCTCGCATTCTTTGATGTGGATATA

TCATCTCATCTGTTTAAATTAGTTATTAAACACTGCGATCTGAATAAACGACTAAAATGTGGTAACTCTC

CATTACATTGCTATACGATGAATACACGATTTAATCCATCTGTATTAAAGATATTGTTACGCCACGGCAT

GCGTAACTTTGATAGCAAGGATAAAAAAGGACATATTCCTCTACACCACTATCTGATTCATTCACTATCA

ATCGATAACAAGATCTTTGATATACTAACGGACCCCATTGATGACTTTAGTAAATCATCCGATCTATTGC

TGTGTTATCTTAGATATAAATTCAATGGGAGCTTAAACTATTACGTTCTGTACAAATTATTGACTAAAGG

ATCTGACCCTAATTGCGTCGATGCGGATGGACTCACTTCTCTTCATTACTACTGTAAACACATATCCGCG

TTCCACGAAAGCAATTATTACAAGTCAAAGAGTCACACTAAGATGCGAGCTGAGAAGCGATTCATCTACG

CGATAATAGATCATGGAGCAAACATTAACGCGGTTACGAAAATCGGAAATACGCCGTTACACACTTACCT

TCAACAGTATACCAAACATAGTCCTCGTGTGGTGTATGCTCTTTTATCTCGAGGAGCTGATACGAGGATA

CGTAATAATCTTGATTGTACACCCATCATGGAATACATAAAGAACGATTGTGCAACAGGTCATATTCTCA

TAATGTTACTCAATTGGCACGAACAAAAATACGGGAAATTACAAAAGGAAGAAGGACAACATCTACTTTA

TCTATTCATAAAACATAATCAAGGATATGGAAGTCGCTCTCTCAATATACTACGGTATCTACTAGATAGA

TTCGACATTCAGAAAGACGAATACTATAATACAATGACTCCTCTTCATACCGCCTTCCAGAATTGCAATA

ACAATGTTGCCTCATACCTCGTATACATCGGATACGACATCAACCTTCCGACTAAAGACGATAAGACAGT

ATTCGACTTGGTGTTTGAAAACAGAAACATCATATACAAGGCGGATGTCGTTAATGACATCATCCACCAC

AGACTGAAAGTATCTCTACCTATGATTAAATCGTTGTTCTACAAGATGTCGGAGTTCTCTCCCTACGACG

ATCACTACGTAAAGAAGATAATAGCCTACTGCCTATTAAGGGACGAGTCATTTGCGGAACTACATACTAA

ATTCTGTTTAAACGAGGACTATAAAAGTGTATTTATGAAAAATATATCATTCGATAAGATAGATTCCATC

ATCGAAAAATGTAGTCGTGACATAAGTCTCCTCAAAGAGATTCGAATCTCAGACACCGACTTGTATACGG

TATTGAGAACAGAAGACATCCGGTATCACACCTATCTCGAAGCCATACATTCAGACAAACGCATTTCATT

TCCCATGTACGACGATCTCATAGAACAGTGTCATCTATCGATGGAGCATAAAAGTAAACTCGTCGACAAA

GCACTCAATAAATTAGAGTCTACCATCGATAATCAATCTAGACTATCGTATTTGCCTCCGGAAATTATGC

GCAATATCATAACCAAGCTAAGCGACTACCATCTAAACAGTATGTTGTACGGAAAGAACCATTACAAATA

TTATCCATGATAGAAAGAAAATATTTAAAAAATAATCTATATGATTGGAGAAGTAGGAAACAAACAGTAA

CAAGACGACGATTACTACATTATTAAATCATGAGGTCCGTATTATACTCGTATATATTGTTTCTCTCATG

TATAATAATAAACGGAAGAGATATAGCACCACATGCACCATCCAATGGAAAGTGTAAAGACAACGAATAC

AGAAGCCGTAATCTATGTTGTCTATCGTGTCCTCCGGGAACTTACGCTTCCAGATTATGTGATAGCAAGA

CTAATACACAATGTACACCGTGTGGTTCGGATACCTTTACATCTCACAATAATCATTTACAGGCTTGTCT

AAGTTGTAACGGAAGATGTGATAGTAATCAGGTAGAGACGCGATCGTGTAACACGACTCACAATAGAATC

TGTGAATGCTCTCCAGGATATTATTGTCTTCTCAAAGGAGCATCAGGGTGTAGAACATGTATTTCTAAAA

CAAAGTGTGGAATAGGATACGGAGTATCCGGATACACGTCTACCGGAGACGTCATCTGTTCTCCGTGTGG

TCCCGGAACATATTCTCACACCGTCTCTTCCACAGATAAATGCGAACCCGTCGTAACCAGCAATACATTT

AACTATATCGATGTGGAAATTAACCTGTATCCAGTCAACGACACATCGTGTACTCGGACGACCACTACCG

GTCTCAGCGAATCCATCTCAACGTCGGAACTAACTATTACCATGAATCATAAAGATTGTGATCCAGTCTT

TCGTGCAGAATACTTCTCTGTCCTTAATAATGTAGCAACTTCAGGATTCTTTACAGGAGAAAATAGATAT

CAGAATACTTCAAAGATATGTACTCTGAATTTCGAGATTAAATGTAACAACAAAGATTCATCTTCCAAAC

AGTTAACGAAAACAAAGAATGATACTATCATGCCGCATTCAGAGACGGTAACTCTAGTGGGCGACTGTCT

ATCTAGCGTCGACATCTACATACTATATAGTAATACCAATACTCAAGACTACGAAACGGATACAATCTCT

TATCATATGGGTAATGTTCTCGATGTCAATAGCCATATGCCCGCTAGTTGCGATATACATAAACTGATCA

CTAATTCCCAGAATCCCACCCACTTATAGTAAGTTTTTTTACCCATAAATAATAAATACAATAATTAATT

TCTCGTAAAAGTAGAAAATATATTCTAATTTATTATATGGTAAGAAAGTAGAATCATCTAGAACAGTAAT

CAATCAATAGCAATCATGAAACAATATATTGTCCTGGCATGCATGTGCCTAGTGGCAGCTGCTATGCCTA

CTAGTCTTCAACAATCTTCATCCTCGTGTACTGAAGAAGAAAACAAACATCATATGGGAATCGATGTTAT

TATCAAAGTCACAAAGCAAGACCAAACACCGACCAATGATAAGATTTGTCAATCCGTAACGGAAGTTACA

GAGACCGAAGATGATGAGGTATCCGAAGATGATGAGGTATCCGAAGAAGTTGTAAAAGGAGATCCCACCA

CTTATTACACTATCGTCGGTGCGGGTCTTAACATGAACTTTGGATTCACCAAATGCCCAAAGATTTCATC

CATCTCCGAATCCTCTGATGGAAACACTGTGAATACTAGATTGTCCAGCGTGTCACCGGGACAAGGTAAG

GACTCTCCCGCGATCACGCGTGAAGAAGCTCTGGCTATGATCAAAGACTGTGAGATGTCTATCGACATCA

GATGTAGCGAAGAAGAGAAAGACAGTGACATCAAGACCCATCCAGTACTTGGGTCTAACATCTCACATAA

GAAAGTGAGTTACAAAGATATCATCGGTTCAACGATCGTTGATACAAAATGTGTCAAGAACCTAGAGTTT

AGCGTACGTATCGGAGACATGTGTGAGGAATCATCTGAACTTGAAGTCAAGGATGGATTCAAGTATGTCG

ACGGATCGGCATCTGAAGGTGCAACCGATGATACTTCACTCATCGATTCAACAAAACTCAAAGCATGTGT

CTGAATCGATAACTCTATTCATCTGAAAATGGATGAGTTGGGTTAATCGAACGATTCAGACACCGCACCA

CGAATTAAAAAAGACCGGGCACTATATTCCGGTTTGCAAAACAAAAATATTTAACTACATTTACAAAAAG

TTACCTCTCGTTACTTCTTCTTTCTGTTTCAATATGTGATACGATATGATCACTATTCGTATTCTCTTGG

TCTCATAAAAAAGTTTTACAAAAAAAAAATATTTTTATTCTATTTCTCTCTTCGATGGTCTCACAAAAAT

ATTAAACCTCTTTCTGATGTCTCAACTATTTCGTAAACGATAACGTCCAACAATATATTCTCGTAGAGCT

TATCAACATCCTTATACCAATCTAGGTTGTCAGACAATTGCATCATAAAATAATGTTTATAATTTACACG

TTAACATCATATAATAAACGTATATAGTTAATATTTTTGGAATATAAATGATCTGTAAAATCCATGTAGG

GGACACTGCTCACGTTTTTTCTCTAGTACATAATTTCACACAAGTTTTTATACAGACAAATTAATTCTCG

TCCATATATTTTAAAACATTGACTTTTGTACTAAGAAAAATATCTTGACTAACCATCTctttctctcttcgatgggtctcacaaaaatattaaacctctttctgatggagtcgtaaaaagtttttatcctttctctcttcgatgggt

CTCACAAAAATATTAAACCTCTTTCTGATG

GTCTCTATAAACGATTGATTTTTCTTACCCTCTAGAGTTTCCTACGGTCGTGGGTCACACATTTTTTTCTAGACACTAAATAAAATAGTA

AAATTAAATTAATTATAAAATTATGTATATAATTTACTAAC
