# Supplementary material for: Exploring the key genomic variation in monkeypox virus during the 2022 outbreak
Source: BMC Genom Data. 2023 Nov 16;24:67. doi: 10.1186/s12863-023-01171-0 (PMC10652487; doi:10.1186/s12863-023-01171-0)
Supplement: Supplementary file 7 — Additional file 7. Full length sequence of RS4. [file 12863_2023_1171_MOESM7_ESM.docx]

**Additional file7. Full length sequence of RS4.**

GTTAGTAAATTATATACATAATTTTATAATTAATTTAATTTTACTATTTTATTTAGTGTCTAGAAAAAAATGTGTGACCCACGACCGTAGGAAACTCTAGAGGGTAAGAAAAATCAATCGTTTATAGAGACCATCAGAAAGAGGTTTAATATTTTT

GTGAGACCTATCGAAGAGAGAAAGGATAAAAACTTTTTACGACTCCATCAGAAAGAGGTTTAATATTTTT

GTGAGACCCATCGAAGAGAGAAAGAGATGGTTAGTCAAGATATTTTTCTTAGTACAAAAGTCAATGTTTT

AAAATATATGGACGAGAATTAATTTGTCTGTATAAAAACTTGTGTGAAATTATGTACTAGAGAAAAAACG

TGAGCAGTGTCCCCTACATGGATTTTACAGATCATTTATATTCCAAAAATATTAACTATATACGTTTATT

ATATGATGTTAACGTGTAAATTATAAACATTATTTTATGATGCAATTGTCTGACAACCTAGATTGGTATA

AGGATGTTGATAAGCTCTACGAGAATATATTGTTGGACGTTATCGTTTACGAAATAGTTGAGACATCAGA

AAGAGGTTTAATATTTTTGTGAGACCATCGAAGAGAGAAAGAGAATAAAAATATTTTTTTTTTTTTTTTT

TTTTTGTAAAACTTTTTTATGAGACCAAGAGAATACGAATAGTGATCATATCGTATCACATATTGAAACA

GAAAGAAGAAGTAACGAGAGGTAACTTTTTGTGAATGTAGTTAAATATTTTTGTTTTGCAAACCGGAATA

TAGTGCCCGGTCTTTTTTAATTCGTGGTGCGGTGTCTGAATCGTTCGATTAACCCAACTCATCCATTTTC

AGATGAATAGAGTTATCGATTCAGACACATGCTTTGAGTTTTGTTGAATCGATGAGTGAAGTATCATCGG

TTGCACCTTCAGATGCCGATCCGTCGACATACTTGAATCCATCCTTGACTTCAAGTTCAGATGATTCCTC

ACACATGTCTCCGATACGTACGCTAAACTCTAGGTTCTTGACACATTTTGTATCAACGATCGTTGAACCG

ATGATATCTTTGTAACTCACTTTCTTATGTGAGATGTTAGACCCAAGTACTGGATGGGTCTTGATGTCAC

TGTCTTTCTCTTCTTCGCTACATCTGATGTCGATAGACATCTCACAGTCTTTGATCATAGCCAGAGCTTC

TTCACGCGTGATCGCGGGAGAGTCCTTACCTTGTCCCGGTGACACGCTGGACAATCTAGTATTCACAGTG

TTTCCATCAGAGGATTCGGAGATGGATGAAATCTTTGGGCATTTGGTGAATCCAAAGTTCATGTTAAGAC

CCGCACCGACGATAGTGTAATAAGTGGTGGGATCTCCTTTTACAACTTCTTCGGATACCTCATCATCTTC

GGTCTCTGTAACTTCCGTTACGGATTGACAAATCTTATCATTGGTCGGTGTTTGGTCTTGCTTTGTGACT

TTGATAATAACATCGATTCCCATATGATGTTTGTTTTCTTCTTCAGTACACGAGGATGAAGATTGTTGAA

GACTAGTAGGCATAGCAGCTGCCACTAGGCACATGCATGCCAGGACAATATATTGTTTCATGATTGCTAT

TGATTGATTACTGTTCTAGATGATTCTACTTTCTTACCATATAATAAATTAGAATATATTTTCTACTTTT

ACGAGAAATTAATTATTGTATTTATTATTTATAGGTAAAAAAAACTTACTATAAGTGGGTGGGATTCTGG

GAATTAGTGATCAGTTTATGTATATCGCAACTAGCGGGCATATGGCTATTGACATCGAGAACATTACCCA

TATGATAAGAGATTGTATCATTTTCGTAGTCTTGAGTATTGGTATTACTATATAGTATGTAGATGTCGAC

GCTAGATAGACAGTCGCCCACTAGAGTTACCGTCTCTGAATGCGGCATGATAGTATCATTCTTTGTTTTC

GTTAACTGTTTGGAAGATGAATCTTTGTTGTTACATTTAATCTCGAAATTCAGAGTACATATCTTTGAAG

TATTCTGATATCTATTTTCTCCTGTAAAGAATCCTGAAGTTGCTACATTATTAAGGACAGAGAAGTATTC

TGCACGAAAGACTGGATCACAATCTTTATGATTCATGGTAATAGTTAGTTCCGACGTTGAGATGGATTCG

CTGAGACCGGTAGTGGTCGTCCGAGTACACGATGTGTCGTTGACTGGATACAGGTTAATTTCCACATCGA

TATAGTTAAATGTATTGCTGGTTACGACGGGTTCGCATTTATCTGTGGAAGAGACGGTGTGAGAATATGT

TCCGGGACCACACGGAGAACAGATGACGTCTCCGGTAGACGTGTATCCGGATACTCCGTATCCTATTCCA

CACTTTGTTTTAGAAATACATGTTCTACACCCTGATGCTCCTTTGAGAAGACAATAATATCCTGGAGAGC

ATTCACAGATTCTATTGTGAGTCGTGTTACACGATCGCGTCTCTACCTGATTACTATCACATCTTCCGTT

ACAACTTAGACAAGCCTGTAAATGATTATTGTGAGATGTAAAGGTATCCGAACCACACGGTGTACATTGT

GTATTAGTCTTGCTATCACATAATCTGGAAGCGTAAGTTCCCGGAGGACACGATAGACAACATAGATTAC

GGCTTCTGTATTCGTTGTCTTTACACTTTCCATTGGATGGTGCATGTGGTGCTATATCTCTTCCGTTTAT

TATTATACATGAGAGAAACAATATATACGAGTATAATACGGACCTCATGATTTAATAATGTAGTAATCGT

CGTCTTGTTACTGTTTGTTTCCTACTTCTCCAATCATATAGATTATTTTTTAAATATTTTCTTTCTATCA

TGGATAATATTTGTAATGGTTCTTTCCGTACAACATACTGTTTAGATGGTAGTCGCTTAGCTTGGTTATG

ATATTGCGCATAATTTCCGGAGGCAAATACGATAGTCTAGATTGACTATCGATGGTAGACTCTAATTTAT

TGAGTGCTTTGTCGACGAGTTTACTTTTATGCTCCATCGATAGATGACACTGTTCTATGAGATCGTCGTA

CATGGGAAATGAAATGCGTTTGTCTGAATGTATGGCTTCGAGATAGGTGTGATACCGGATGTCTTCTGTT

CTCAATACCGTATACAAGTCGGTGTCTGAGATTCGAATCTCTTTGAGGAGACTTATGTCACGACTACATT

TTTCGATGATGGAATCTATCTTATCGAATGATATATTTTTCATAAATACACTTTTATAGTCCTCGTTTAA

ACAGAATTTAGTATGTAGTTCCGCAAATGACTCGTCCCTTAATAGGCAGTAGGCTATTATCTTCTTTACG

TAGTGATCGTCGTAGGGAGAGAACTCCGACATCTTGTAGAACAACGATTTAATCATAGGTAGAGATACTT

TCAGTCTGTGGTGGATGATGTCATTAACGACATCCGCCTTGTATATGATGTTTCTGTTTTCAAACACCAA

GTCGAATACTGTCTTATCGTCTTTAGTCGGAAGGTTGATGTCGTATCCGATGTATACGAGGTATGAGGCA

ACATTGTTATTGCAATTCTGGAAGGCGGTATGAAGAGGAGTCATTGTATTATAGTATTCGTCTTTCTGAA

TGTCGAATCTATCTAGTAGATACCGTAGTATATTGAGAGAGCGACTTCCATATCCTTGATTATGTTTTAT

GAATAGATAAAGTAGATGTTGTCCTTCTTCCTTTTGTAATTTCCCGTATTTTTGTTCGTGCCAATTGAGT

AACATTATGAGAATATGACCTGTTGCACAATCGTTCTTTATGTATTCCATGATGGGTGTACAATCAAGAT

TATTACGTATCCTCGTATCGGCTCCTCGAGATAAAAGAGCATACACCACACGAGGACTATGTTTGGTATA

CTGTTGAAGGTAAGTGTGTAACGGCGTATTTCCGATTTTCGTAACCGCGTTAATGTTTGCTCCATGATCT

ATTATCGCGTAGATGAATCGCTTCTCAGCTCGCATCTTAGTGTGACTCTTTGACTTGTAATAATTGCTTT

CGTGGAACGCGGATATGTGTTTACAGTAGTAATGAAGAGAAGTGAGTCCATCCTCATCGACGCAATTAGG

GTCAGATCCTTTAGTCAATAATTTGTACAGAACGTAATAGTTTAAGCTCCCATTGAATTTATATCTAAGA

TAACACAGCAATAGATCGGATGATTTACTAAAGTCATCAATGGGGTCCGTTAGTATATCAAAGATCTTGT

TATCGATTGATAGTGAATGAATCAGATAGTGGTGTAGAGGAATATGTCCTTTTTTATCCTTGCTATCAAA

GTTACGCATGCCGTGGCGTAACAATATCTTTAATACAGATGGATTAAATCGTGTATTCATCGTATAGCAA

TGTAATGGAGAGTTACCACATTTTAGTCGTTTATTCAGATCGCAGTGTTTAATAACTAATTTAAACAGAT

GAGATGATATATCCACATCAAAGAATGCGAGATACATATGACAGACATTATTGACAGAAATGTGACCTTC

ATTATCACCGTCGTCCATAAATGCGTTAGGTACGTACCACATACTATCGTTAACGATGCGCACAATCTCG

TCCATTTCATCCATCTTCATAATGATTTACTTTTTCATAATTAGAGAAAAAGATCAAGGTATAAAAATTA

GAAGTGTTAGACTATAAACTAACTTATAAAACTAACTTATGACTTAACTAACTTACTTATAAAACTAACT

TATGACTTAACTAACTTATGACTTAACTAACTTATGACTTAACTAACTTATGACTATTAACTCATTTCAA

GAAAGGTGGGTGGATAGAACTCTATATGACAGCTTGTGAAACAATTAGATCCCTAATTTCTAATGGAAGT

TTTGATAGGAGATTGTCATCAGTTGATACATTGTTTATTATCTCATCTATTAGAGCACGTCTGTTTAGAG

CTTTAGTGACCTGCTCGGTTACTTCTGTGTAAATCTTGAATCCTTTAGTGATACACTGTGTCAAAACTGG

ATGTTTAGAATACCTATGTAGAATATGGGAAGCATGCTTGTTTTTGTCTCTATTATAGATTAACTCATAC

ATGGTTGTATTATGAATTTTCATCTGCCTAATGTACTCCAATTCTTGTTTACAATCAATTATATAATCAA

AGAGTGATGATGCATACACATTACAAAGTGAATAATCTACCATCATAAAATACTTGATACAGAGCTTTAT

CACATCATGGTTTTCAATTGTATTATTAAGTATAGCTAATTTTATACAGTCAATAGACAATGGTTCTCTA

AGCAATATTTCTAATATTTTAAGATGTGCTTCCCTACGGGCGATGACAGATCCCCTATCCACGGCCACGT

CAAGACATGTATATCCATTACTCATTACTGCGTTGACATTTGCTCCATTTTCTAATAGCCATGATACTAA

ATCTATATAACCTGCATAGATAGCGCGATAAAGCAAGGTCCTTCCACCAGCATCTAGTTGATTGATATCT

TCAATATATGGGATACAAAGCTTATAAATTTCTAATACTGTGGGTTCATCTACAAGGAATCCCCTAGTAT

ACTGAATTATTTTATATAGATCTAATTTAACATCATTTTCATCTGGGATACCACAATTCAAAATAAACTC

AACAACACTACTTTCCTTTTTACATATTCCCCTAAAATAGGCATTCAAGCATTCTATTTTATATATTACA

GCCCCATGATCTACCATAAGATCAACAATGTCTATTTCTACATATGCATTAGATAGATAGTAAAGTAAGA

GATCTTGCACAGAATTACAATTCTTAATAATTATAGAGAAAATATCTTCCATATAATTCTTTGACACTAA

TGCAGATATAATATCTTTATATGTAATATATGCAAACAGTCTATCTACTATATACTGATCAATATTATCT

CTATGAATCCTAAAATAATCATACAGAACATCTACAGGATCACAAATTGGTTCAAGGAGAAATCTATCAA

ATATTTTCCTGTCAACAACTGGTTCTAGAACATAACAGTCAACACCTAATCCATGTTTTTTATAATCATC

TACCAAAGATAATGACCAAAGATCGAGGTCGTCGTGAAACTGCTCATCGACAGCCATGAAATCTACCGAC

TCCATGGTGCGAATCGCACTGTCTTATTCGCCATTGATTTTCATTTTTTATAATTATGTACATGTTTTCC

TTCTATTCTCAAGAGTCTACAAAAATATATTTTTTTCGATATCTAAGTACTAAGTTTTTTACTGTTTTTG

TTACTGTCTTCCATTCTTCTAACTAAAGATCTGAGATAAATTATACAATCTTCGCTATCGAACCATTTTT

GTAGTCTAAAGCCTGAAGTAATTAACCAACTGTTTTTATTAGTGGCTTTTTTCGATCTATCCTCGTCCTC

TCATCATCCTTATATTATTATCATTATTATCATAGTCTATTAAACACAAATCATCTACGTTTATAACAAC

ATTCTCATTATTAATTAGTTCTGTAGAATATCTTTAATAATTTGGCTATACATCTGTTCAATACTATCTA

TTGATGATTTCTTTTTTAAGACTTAAACTAGTTATGGTAATGACGATGAAATCGAGTAGTAACTTCTAAT

AAAGACTTGATATCATTATCATATGTTTGATCGTCATAGTTAATAGTGTGGCTAAATGGTACTGTTAATA

AGTTTATAGACAATATCATAGTATTTTCTTTCCAGAATTAGATTATTTTTTTAAATACTGATCCTCACAA

TTCCGTGATGTAGCAGTAGTTGGTGCATGGTCTATATCGTTAAAATGTATCATATATAATAGTTTTCTGA

CGTGGAGTACAGAATTTTCGATTAATGAGTTCATGGTAAGGAAGGGCAAATGCCTGTATATAATATACAT

AAGTTAATAGTTTTTTATCATATTTTCTAATACCATAATAAAAATTATCATTATTGCGTTTGGTAGTTCT

GCCCTATCATCTATATCACTGTCACTCTCGCTCTCACTATATCTTCTAAAATTACAAACAACTGGATATT

CGATAACAGCATTTGTGTAGTTTTTGTCTTTTACAGTATATACGTTATTGTCAAAATCTAAACAAATATT

AGCATAATACATCTATCTATAAGATCAGGATCCATGTTCGAGCATACTAGCCATGTATATTTGTAACTTC

GTCGTACAGCGTTAGATCAATAGAATAAACAATCGTGTGACGCAACTTTTTTACGATCTAGTTGTATGAG

TTTATCGTTTACATAAGCAATTAACGGCTTTAACAGATGATCTGAGTAATAATATACCTCTGTTATACGT

TTAATGTTCACGGTCTTAGTATTTTTAGATATCAATTGTGATTTACACCATATTCGACTCCCTTGTGTGT

AACGTTAGAAATTCTAAATCTATATTATCTATTACAGCGTAAAACACATTCAATATTGTATTGTTATTTT

TATATTATTTACACAATTAACAATGTATTATTAGTTTATATTACTGAATTAATAATATAAAATTCCCAAT

CTTGTCATAAACACAAAATCCATTAAAAATGTCGATAAAATATCTGATGTTGTTGTTCGCTGCTATGATA

ATCAGATCATTAGCCGATAGTGGTAATGCTATCGAAACAACATCGCCAGAAATTACAAACGCTACAACAG

ATATTCCAGCTATCAGATTATGTGGTCCAGAGGGAGATGGATATTGTTTACACGGTGACTGTATCCACGC

TAGAGATATCGACGGTATGTATTGTAGATGCTCTCATGGTTATACAGGTATTAGATGTCAGCATGTAGTA

TTAGTAGACTATCAACGTTCAGGAAAACCAGACACTACAACATCATATATCCCATCTCTAGGTATTGTGC

TTGTATTAGTAGGCATTATTATTACGTGTTGTCTATTATCTGTTTATATGTTCACTCGAAGAACTAAACT

ACCTATACAAGATATGGTTGTGTTATATTTTTTATAAAATTTTTTTATGAGTATTTTTACAAAAATGTAT

ATGTATAAAAAAAATACTAAGTATACGATGTATCCTGTATTATTTGTATTTATCTAAACAATACTTCTGC

CTCTAGATGGGATACAAAAATTTTTTATTTCAGCATATTAAAGTAAAATCTAGTTACCTTGAAAATGAAT

ACAGTGGGTGGTTCCGTATCACCAGTAAGAACATAATAGTCGAATACAGTATCCGATTGAGATTTTGCAT

ACAATACTAGTCTAGAAAGAAATTTGTAATCATCCTCTGTGACAGGAGTCCATATATCTGTATCATCGTC

TAGTTTTTATCAGTGTCCTATGCTATATTCCTGTTATCATCATTAGTTAATGAAAATAACTCTCGTGCTT

CAGAAAAGTCAAATATTGTATCCATACATATATCTCCAAAACTATCACTTATACGTTTATCTTTAACGAA

CATATACCTAGATGGTTATTTACTAACAGACATTTTTTCAAGATCTATTGACAATAACTCCTATAGTTTC

CACATCAACCAAGTAATGATCATCTATTGTTATATAACAATAACATAACTCTTTTCCATTTTTATCAGTA

TCTATATCAACGTCGTTGTAGTGAATAGTAGTCATTGATCTATTATATGAAACGGATATGTCTAGTTAAT

ATTTTCTTTGATTTAAAGTCTATAGTCTTTACAAACATAATATCCTTATCCGACTTTATATTTCCTGTAG

GGTGGCATAATTTTATTCTGCCTCCACAATCAGTGTTTCCAAATATATTACTAGACAATATTCCATATAG

TTATTAGTTAAGGGACCCAATTAGAACACGTACGCGCTTATTCATCATTTGGATCGTATTTCATAAAAGT

TATTATGTTATCGATGTCAACACATTCTACATTTTTTTTAATTGTCTATATAGTATTTTTCTGATATTTT

CTATAATATCAGAATTGTCTTCCATAGGAAGTTGTATACTATTCGGAATCAGTTACATGTTTAAATAATT

CTCTGATGTCATTCCTTATACAATCAAATTCATTATTAAACAGTTTAATAGTCTGTAGACCTTTATCGTC

GTACATATCCATTGTCTTATTAGTTACGCTTATTTTTATGGGTTTTACGTTGCTTTATTATATTTTATAA

TAATGATTGTTTGACAATGTCGTAGTATAGATATATTATTAGAGGAGGTATAATTATAAAAAGTTTCTGA

GTACGATGTTATAAGAGGAGAGGACACATTAACATCATACATCAATTAACTACATTCTTATAACATTGTA

ATCAAAAGAATTGCAATTTTGATGTATAACAACTGTCAATGGAATTGTATATTACAAATTACGGTATGTT

GTAACAACAAATACCGATCGGTAATTGTCTCTGCCGCTGTAATAGAATTAATTATATATCTATTACACCG

GCCTTGTATCATAATAAAGTTGTGGTAGTATGATCTCCATATTTATAATTTAGTACTTTGTATTTAGTTT

TTTTGGAATCATAAAAAAAGTTTTACTAATTTAAAATTTAAAAAGTATTTACATTTTTCACTGTTTAGTC

GCGGATATGGAATTCGATCCTGTCAAAATCAATACATCATCTATAGATCATGTAACAATATTACAATACA

TAGATGAACCAAATGATATAAGACTAACAGTATGCATTATCCGAAATGTTAATAACATTACATATTATAT

CAATATCACAAAAATAAATCCACATTTGGCTAATCGATTTCGGGCTTGGAAAAAACGTATCGCCGGAAGG

GACTATATGACTAACTTATCTAGAGATACAGGAATACAACAATCAAAACTTACTGAAACTATACGTAACT

GTCAAAAAAATAAAAACATATATGGTCTATATATACACTACAATTTAGTTATTAATGTGGTTATTGATTG

GATAACCGATGTGATTGTTCAATCAATATTAAGAGGGTTGGTAAATTGGTACATAGCTAATAATACATAT

ACTCCAAATACACCCAATAATACTACAACCATTTCTGAGTTGGATATCATCAAAATACTGGATAAATACG

AGGACATGTATAGAGTAAGTAAAGAAAAAGAATGTGGAATTTGCTATGAAGTTGTTTACTCAAAACGATT

AGAAAACGATAGATACTTTGGTTTATTGGATTCGTGTAATCATATATTTTGCATAACATGTATCAATATA

TGGCATAGAACACGAAGAGAAACCGGTGCGTCAGATAATTGTCCTATATGCCGTACACGTTTTAAAAAAA

TAACAATGAGCAAGTTCTATAAGCTAGTTAACTAATAAATAAAAAGTTTAATTATCGACGATATATGTCG

TTATTTTTCTCTCATATGAAAGATTAATTTGATTCTAATATAATCTTCAGTATTGGATGAATCTCAATTC

AAATTAATTCCATTAGATTAGATTAGATTAGATCATAAATAAAAATAGTAGCACGCACTACTTCAGCCAA

ATATTCTTTTTTGAAACGCCATCTAGCGTAATGAGAACACAAGTGAACCTATAATGAGCAAATTTATTAG

TATCGGTTACATGAAGGACTTTACGTAGAGTGGTGATTCCTCCATCTGTGGTACGAACGGTTTCATCTTC

TTTGATGCCACCACCCAGATGTTCTATAAACTTGGTATCCTCGTCCGATTTCATATCATTTGCCAACCAA

TACATATAGCTAAACCCAGGCATACGTTCCACACATCCGGAACAATGAAATTCTCCAGAAGATGTTACAA

TGGCTAGATTTGGACATTTGGTTTCAACCGCGTTAACATATGAGTGAACACACCCATACATGAAAGCGAT

GAGAAATAGGATTCTCATCTTGCCAAAATATCACTATAAAAAATTTATTTATCAATTTTAAAGGTATAAA

AAAATACTTATTGTTGCTCGAATATTTTGTATTTGATGGTATACGGAAGATTAGAAATGTAGGTATTATC

ATCAACTGATTTTATGATGGTTTTATGAATTTTATTATGCTTCACTATTGCATCGGAAATAATATCATAT

GCTTCCACGTATATTTTATTTTGTTTTGACTCATAATACGCACGTAATTCTGGATTATTGGCATATCGAT

GAATAATTTTAGCTCCATGCTCAGTAAATATTAATGAGAACATAGTGTTGCCTCCTACCATTATTTTTTT

CATCTCATTCAATTCTTGATTGCAGAGATCTATATAATCATTATAGCGTTGACTTATGGACTCTGGAATC

TTAGACGATGTACAGTCATCTATAATCATGGTATATTTAATACATTGTTTTATAGCATAGGCATTATCTA

CGATATTAGATACTTCACTCAATGAATCAATCACACAATCTAATGTAGGTTTATGACATAATAGCATTTT

CAGCAGTTCAATGTTTCTAGATTCGTTGATGGCAATGGTTATACATGTATATCCGTTATTTGATCTAATG

TTGACATCTGAACCGGATTCTAGCAGTAAAGATACTAGAGATTGTTTATTATATCTAACAGCCTTGTGAA

GAAGTGTTTCTCCTCGTTTGTCAATCATGTTAATGTCTTTAAGATAAGGTAGACAAATGTTTATAGTACT

AAGAATTGGGCAAACATAAGACATGTCACAAAGACCCTTTTTGTATGTATAAGTGTAGAAATTATAACAT

CCATAGTTGGATTCACATAGGTGTCCAATCGGGATCTCTCCATCATCGAGATGATTGACGGCATCTCCCC

CTTCCTTTTTTAGTAGATATTTCATCGTGTAAGAATCAATATTAATATTTCTAAAGTATCTGTGTATAGC

CTCTTTATTTACCACAGCTCCATATTCCAACATGCATTCCACTAGAGGGATATCGATATCGCCGAATGTC

ATATACTCAATTAGTATATGTTGGAGGACATCCGAGTTCATTGTTTTCAATATCAAAGAGATGGTTTCCT

TATCATTTCTCCATAGTGGTACAATACTACACATTATTCCGTACGGCTTTCCATTCTCCAAAAACAATTT

TACCAAATCTAAATCTACATCTTTATTGTATCTATAATCACTATTTAGATAATCAGCCATAATTCCTCGA

GTGCAACATGTTAGATCGTCTATATATGAATAAGCCGTGTTATCTATTCCTTTCATTAACAATTTAACGA

TGTCTATATCTATATGAGATGACTTAATATAATATTGAAGAGCTGTACAATAGTTTTTATCTATAGAAGA

CGGCTTGATTCCGTGATTAATTAGACATTTAACAACTTCCGGACGCACATATGCTCTCGTATCCGACTCT

GAATACAGATGAGCGATGATATACAGATGCAATACGGTACCACAATTTCGTGGTTGATAATCATCATACA

CGTATCCGTACTCGTCATCCTCATAAAGAACACTGCATCCATTTTCTATGAACAAATCAATAATTTCAGG

AACAGGATCATCTGTCATTACATAATTTTCTATAACTGAACGATGGTTTTCACATTTAACACTCAAGTCA

AATCCATGTTCTACCAACACCTTTATCAAGTCAACGTCTACATTTTTTGATTTCATATAGCTGAATATAT

TAAAGTCATTTATGTTGCTATATCCAGTAGCTTCTAGTAGAGCCATCGCTATATCCTTATTGACTTTAAC

ATGTCTACTATTTGTGTATTCTTCTATTGGGGTAAACTGTCTCCAATTTTTATGTAATGGATTAGTGCCA

CTATCTAGTAGTAGTTTGACGACCTCAACATTATTACAATGCTCATTGAAAAGGTATGCGTGTAAAGCAT

TATTCTTGAATTGGTTCCTGGTATCATTAGGATCTCTGTCTCTCAACATCTGTTTAAGTTCATCGAGAAC

CACCTCCTCATTTTCCAGATAGTCAAACATTTTGACTGAATAGAAGTGAATGAGCTACTGTGAACTCTAT

ACACCCGCACAACTAATGTCATTAAATATCATTTTTGAATGTATTTATACCATGTCAAAAACTTGTACAA

TTATTAATAAAAATAATTAGTGTTTAAATTTTACCAGTTCCAGATTTTACACCTCCGTTAACACCTCCAT

TAACCCCACTTTTTACACCACTGGACGATCCTCCTCCCCACATTCCACTGCCACTAGATGTATAAGTTTT

AGATCCTTTATTACTACCATCATGTCCATGGATAAAGACACTCCACATGCCGCCACTACTACCCCCTTTA

GACGACATATTAATAAGACAAGTTTAACAATAAAATTAATCACGAGTACCCTACTACCAACCACTATTAT

ATGATTACAGTACCTTGACTAAAGTCTCTAGTCACAAGATCAATACTACCAACCTACGCTATTATATGAT

TATAGTTTCTATTTTTATAGGAACGCGTACGAGAAAATCAAATGTCTAAGTTCTAACGGTAGTGTTGATA

AACGATTGTTATCCGCGGATACCTCATCTATCATGTTGTCTATTTTCTTACTTTGTTCTATTAACCTATT

AGCATTATATATTATTTGATTATAAAACTTATATTGCTTATTAGCCCAATCTGTAAATATCGGATTATTA

ACATATCGTTTCTTTGTAGGTTTATTTAACTTGTACATCACTGTAAGCATGTCCGTACCATTTATTTTAA

TTTGACACATATCAGCAATTTCTTTTTCGCAGTCGGTTATATATTCTATATAAGATGGATACGTATCACA

TATGTACTTATAGTCTACTAATATGAAGTACTTAATACATATTTTCAGTAACGATTTAGCCTTATTACCT

ATTAATAAGTGCCTGTCGTTGGATAGGTAATCAACTGTTTTCTTAATACATTCGATGGTTGGTAATTTAC

TCAAAATAATTTCCAATATCTTAATATATATTTCTGCTATTTCTGGTATACATGCATGTGCCATTATAAC

ACAAATACCAATACATGTAGACCCATATGTTGTTGTTATATTAATATCTGCGCCATTATCTATTAACCAT

TCTATTAGGGCAACACTATGCGACTCGATACAATAATAAAGTATACTACGTCCATGTTTATCTATTTTGT

TTATATCATCGATATACGGCTTACAAATTTCTAGTATCGATAACACTTCTGACTCGTGAATAAATAAGGT

AGGGAATAACGGCATAATATTTATTATGTTATCATCATTAACAACTACGTTTCCATTTTTTAAAATATAC

TCTACAACTTTAGGATCCCTATTGTCAAATCTTTTAAAATATTTATTTATATGCTTAAATCTATATAATA

TAGCTCCTTCCCTAATCATACATTTGATAACATTGATGTACACTGTATGATAAGATACATATTCTGACAA

TAGATCTTGTATAGAATCTGTATATCTTTTAAGAATTGAGGATATTATGACATTATTACGTAAACTATTA

CACAATTCTAAAATATAAAATGTATCATGGGCAGATAATAGTTTATCCACTATATAATTATCTATTTTAT

GATTTTTCTTCCTATATTGTTTACGTAAATAGATAGATAGAATATGCATTAGTTCATTACCGCTATAGTT

ACTATCGAATAACACGTCAAATATTTCCCGTTTAATATCGCATTTGTCAACATAATAATAGAGTATGGTA

CGTTCACGATAAGTATAATGACACATCTCGTTTTCGTGCGAAATTAAATAGTTTATCACGTCCAAAGATG

TCACATAACCATCTTGTGACCTAGTAATAATATAATAATAGAGAACTGTTTTACCCATTCTATTATCATA

ATCAGTGGTGTAGTCATAATCTAAATAATCAAACTCGTCATCCCAATTAAAATAAATATAATCAGTACAT

TGAATGGGTATGATATTGTACCCATACTGTATGTTGCTACATGTAGGTATTCCTTTATCCAATAATAGTT

TAAATACATCTATATTAGGATTTGATGTTGTCGCGTATTTCTCTACAATATTAATACCATTTTTGATACT

ATTTATTTCTATACCTTTCGAAATTAGTAATTTCAATAAGTCTATATCGATGTTATCAGAACATAGATAT

TCAAATATATCAAAATCATTGATATTTTTATAGTCGACTGACGACAATAACAAAATCACAACATCGTTTT

TGATATTATTATTTTTTTTGGTAACGTATGCCTTTAATGGAGTTTCACCATCATACTCATATAATGGATT

TGCACCACTTTCTATTAATGATTGTGCACTACTGGCATCGATGTTAAATGTTTTACAACTATCATAGAGT

ATCTTATCGTTAACCATGATTGGTTGTTGATGTTATCACATTTTTTGGTTTCTTTCATTTCAGTTATGTA

TGGATTTAGCACGTTTGGGAAGCATGAGCTCATATGATTTCAGTACTGTAGTGTCAGTACTATTAGTTTC

GATCAGATCAATGTCTAGATCTATAGAATCAAAACACGATAGGTCAGAAGATAATGAATATCTGTACGCT

TCTTCTTGTACTGTAACTTCTGGTTTTGTTAGATGGTTGCATCGTGCTTTAACGTCAATGGTACAAATTT

TATCCTCGCTTTGTGTATATCATATTCGTCTCTAGTATAAAATTCTATATTCAAATTATCATGCGATGTG

TGTACGCTAACGGTATCAATAAACGGAGCACAGCATTTAGTCAACAGTAATCCAAAATTTTTTAAAGTAT

ATCTTAACGAAAGAAGTTGTCATCGTTAGAGTGTGGTAAATCATTGTCTACGGTACTAGATCCTCATAAG

TGTATATATCTAGAGTAATATTTAATTTATCAAATGGTTGATAATATGGATGTTGTGGCAATTTCCTAAT

ACGGAAATAAGACATAAACACGCAATAAATCTAATTGCGGACATGTTACACTCCTTAAAAAATACGAATA

AACACTTTGGCTTTTAGTAAGTGTCATTTAACACTATACTCATATTAATCCATGGACTCATAATCTCTAT

ACGGGATTAACGGATGTTCTATATACGGGGATGAGTAGTTCTCTTCTTTAACTTTATACTTTTTACTAAT

CATATTTAGACTGATGTATGGGTAATAGTGTTTGAAGAGCTCGTTCTCATCATCAGAATAAATCAATATC

TCTGTTTTTTTGTTATACAGATGTATTACAGCCTCATATATTACGTAATAGAACGTGTAATCTACCTTAT

TAACTTTCACCGCATAGTTGTTTGCAAATACGGTTAATCCTTTGACCTCGTCGATTTCCGACCAATCTGG

GCGTATAATGAATCTTAACTTTAATTTCTTGTAATCATTCGAAATAATTTTTAGTTTGCATCCGTAGTTA

TCTCCTCTATGTAACTGTAAATTTCTCAACGCGATATCTCCATTAATAATGATGTCGAATTCGTGTTGTA

TACCCATACTGAATTGATGAACTAATACCGACGGTATTAATAGTAATTTACTTTTCATCTTTACATACTT

GGTAATAGTTTTACTATCATAAGTTTATAAATTCCACAAGCTACTATGGAATATACCAACCATCTTAGTA

TAGAACACATGTCTTAAAGTTATTAATTAATTACATGTTGTTTTATATATCGCTACGAATTTAAACAGAG

GAATCAGTTAGGAAGAAAAAATTATCTGTCATCATCATCATCATCATCATCTATTGGATAACATCTCTGT

ATTCTACGATAGAGTGCTATTTTAAGATGTGACAGATCCGTGTCATCAAATATATACTCCATTAAAATGA

TTATTCCGGCAGCGAACTTGATATTGGATACATCACGACCTTTGTTAATATCCACGACAATAGACAGCAA

TCCCATTGTTCCATAAACAGTGAGTTTATCTTTCTTTGAAGTGATATTTTGTAGAGATCTTATAAAACTG

TCGAATGACATCGTATTTATATCTTTAGCTAAATCATATATGTTACCATCATAATATCTAACAGCATCTA

TCTTAAACGTTTCCATCGCTGTAAAGACGTTTCCGATAGATGGTCTCGTTTCATCAGTCATACTGAGCCA

ACAAATGTAATCGTGTATAACATCTTTGATAGAATCAGACTCTAAAGAAAAGGAATCGGCTTTATTATAC

ACATTCATGATAAACTTAATGAAAAATGTTTTTCGTTGTTTAAGTTGGATGAATAGTATGTCTTAATAAT

TGTTATTATTTCACTAATTAATATTTAGTAACGAGTACACTCTATAAAAACGAGAATGACATAACTAATC

ATAACTAGTTATCAAAGAATGTCTAGGACGCGTAATTTTTTATGGTATAGATCCTGTAAGCGTTGTCTGT

ATTCTGGAGCTATTTTCTCTATCGCATTAGTGAGTTCAGAATATGTTATAAATTTAAATCGAATAACGAA

CATAACTTTAGTAAAGTCGTCTATATTAACTCTTTTATTTTCTAGCCATCGTAATACCATGTTTAAGATA

GTATATTCTCTAGTTACTACGATCTCATCGTTGTCTAGAATATCGCATACTGAATCTACATCCAATTTTA

GAAATTGGTCTGTGCTACATATCTCTTCTATATTATTGTTGATGTATTGTCGTAGAAAACTATTACGTAG

ACCATTTTCTTTATAAAACGAATATATAGTACTCCAATTATCTTTACCGATATATTTGCATACATAATCC

ATTCTCTCAATCACTACATCTTTAAGAGTTTGGTTGTTAAGATATTTGGCTAAACTATATAATTCTATTA

GATCATCAACAGAATCAGTATATATTTTTCTAGATCCAAAGATGAACTCTTTGGCATCCTCTATAATATT

ATCAGAAAAGATATTTTCGTGTTTTAGTTTATCAAGATTTAACCTGTTCATATCCATGATTAACGACGTC

ATATAACCACATAAAATAAAAATCCATTTTCATTTTTAGCACAATACTATTCATAATTGATATTGATGTA

ATATTTTGTTACTTTGAACGTAAAGACAGTACACGGGTCCGTATCTCCAACAAGCACGTAGTAATCAAAT

TTGGTGTTGTTAAACTTCGCAATATTCATCAATTTAGATAGAAACATATACTCATCATCTGTTTTAGGAA

TCCATGTAGTATTACCACTTTCCAACTTATCATTATCCCAGGCTATGTTTCGCCCATCATCGTTGCACAG

AGTGAATAATTCTTTTGTATTCGGTAGTTCAAATATATGATCCATGCATATATCGACAAAGCTATTGTAG

ATGTGATTTTTCCTAAATCTAATATAAAACTCGTTTACTAGCAAACATTTTCCTGATTTATCGACCAAGA

CACACATGGTTTCTAAATCTATCAAGTGGTGGGGATCCATAGTTATAACGCAGTAACATAGATTATTACC

TTCTTGACTGTCGCTAATATCTATATACTTATTGTTATCGTATTGGATTCTACATATAGATGGCTTGTAT

ATCAAAGATATAGAACACATAACCAATTTATATTCTCGCTTTGTATTTTCGAATCTAAAGTTAAGAGATT

TAGAAAACATTATATCATCGGATGATGATATCACTGTTTCCAGAGTAGGATATATTAAAGTCTTTAAAGA

TTTTGTCCGATTCAAATAAATCACTAAATAATATCCCATATTATCATCTGTTATAGTCGTGTCATTAAAT

CTATTATATTTTATGAAAGATATATCACTGCTCACCTCTATATTTCGTACATTTTTAAACTGTTTGTATA

ATATCTCTCTAATACAATCAGATATATCTATTGTGTCGGTAGACGATACCGTTACATTTGAATTAATGGT

GTTCCATTTTACAACTTTTAACAAGTTGACCAATTCATTTCTAATAGTATCAAACTCTCCATGATTAAAT

ATTTTAATAGTATCCATTTTATATCACTACGGACATAAACCATTGTATATTTTTTATGTTTATTAGTGTA

CACATTTTGGAAGTAAGTTCCTGGATCGGATGTCACCGCAGTAATATTGTTGATTATTTCTGACATCGAC

GTATTATATAGTTTTTTAATTCCATATCTTTTAGAAAAGTTAAACATCCTTATACAATTTGTGGAATTAA

TATTATGAATCATGGTTTTTACACATAGATCTATTACAGGCGGAACATCAATTATTATGGCAGCAACTAG

TATCATTTCTACATTGTTTATGGTGATGTTTATCTTCTTCCAGCGCATATAGTCTAATATCGATTCAAAC

GCGTGATAGTTTATACCATTCAATATAATCGCTTCATCCTTTAGATGGTGATCCTGAATGTGTTTAAAAA

ATTATACGGAGACGCCGTAATAATTTCCCCATTGATAGAAAATATCACGCTTTCCATTTTCTTGAAGTAC

TAAAAGTAATTATAATATAATGTAAAGGTTTATATATTCAATATTTTTTATAAAAAAAATCATTTCGACA

TTAATTCCTTTTTAAATTTCAGTCTATCATCTATAGAAACATATTCTATGAATTTATAAAATGCTTTTAC

GTATCCTATCGTAGGTGATAGAACCGCTAAAAAACCTATCGAATTTCTACAAAAGAATCTGTTATATGGT

ATAGGGAGAGTATAAAACATTAAATGTCCGTACTTATTAAAGTATTCAGTAGCCAATCCTAACTCTTTCG

AATAATTATTAATGGCTCTTATTCTGTACGAATCTATTTTTTTGAACAATGGACCTAGTGGTATATCTTG

TTCTATGTATCTAAAATAATGTCTGACTAGATCCGTTAGTTTAATATCCGCAGTCATCTTGTCTAGAATG

GCAAATCTAACTGCGGGTTTAGGCGTAGGCGTTAGTTTAGTTTCTATATCTACATCTATGTCTTTATCTA

ACACCAAAAATATAATAGCTAATATTTTATTACAATCATCCGGATATTCTTCTACGATCTCACTAACTAA

TGTTTCTTTGGTTATACTAGTATAGTCACGATCAGACAAATAAAGAAAATCAGATGATCGATGAATAATA

CATTTAAATTCATCATCTGTAAGATTTTTGAGATGTCTCATTAAAATATTATTAGTGTCAGTTCTCATTA

TCATATATTGACAGCAGCTATTACACTTATTTTATTTTTCTGTATTTTTATTACTTTTCACCATATAGAT

CAGTCATTAGATCATCAAAATACTTTTCAATCATCCTAAAGAGTATGGTGAACGAATCTTCCCATCTAAT

TTCTGAACGTCTACCAATGTCTCTAGCCACTTTGGCACTAATAGCGATCATTCGCTTAACATCTTCTACA

TTATTAACTGGTTGATTCAATCTATCTAGCAATGGACCGTCGGATAGCGTCATTCTCATGTTCTTAATCA

ATGTACATACATCGTCATCATCTACCAATTCATCAAACAATATAAGCTTTTTAAAATCATCATTATAATA

GGATGGATCGCCGTCATTTCTCCAAAGAATATATCTAATAAGTAGAGTCCTCATGCTTAGTAATTTAACT

ATTTTAGTTAACAACTATTTTTTATGTTAAATCAATTAGTAACACCGCTATGTTTAATACTTATTCATAT

TTTAGTTTTAGGATCGAGAATCAATACAAAAATTAATACATCAATTTTGGAAATACTTAGTTTCCACGTA

GTCAATGAAACATTTGAGCTCATCGTAAAGGACGTTCTCGTACAAGACGTAACTATAAATTGGTTTATAT

TTGTTCAAGATAGATACAAATCCGATAACTTTTTTGACGAATTCTACGGGATTCACTTTAAAAGTGTCAT

ACCGGGTTCTTTTTATTCTTTTAAACAGATCGATTGTGTGATGTTGATTAGGTCTTTTACGAATTTGATA

CAGAATAGCGTTTACATATCCACCATAGTAATCAATAGCCATTTGTTCGTATGTCATAAATTCTTTAATT

ATATGACACTGTGTATTATTTAGTTCGTCCTTGTTCATCATTAGGAATCTATCCAATATGGCAATTATAT

TAGAACTATAACTGCGTTGTATGCGCATGTTGATGTGTCTGTTTATACAATCAATTATACTAGGATCCAT

ACCACTACAATCGGGTAAAATTGTAGCATCATATACCATTTCTAGTACTTTAGATTCATTGTTATCCATT

GCAGAGGACGTCATGATCGAATCCAAAAAAATATATTATTTTTATGTTATTTTGTTAAAAATAATCATCA

AATACTTCGTAAGATACTCCTTCATGAACATAATCAGTTACAAAACGTTTATATGAAGTAAAGTATCTAC

AATTTTTACAAAAGTCAGGATGCATAAGTACAAAGTACGCGATAAACGGAATAATAATAGATTTATCTAG

TTTATCTTTTTCTATCTCTTTCATAGTTATATACATGGTCTCAGAAGTCGGATTATGTAACATCAGCTTC

GATAAAATGACTGGGTTATTTAGTCTTACACATTCGCTCATACATGTATGACCGTTAACTATAGAGTCTA

CACTAAAATGATTGAATAATAGATAGTCTACCATTGTTTCGTATTCAGATAGTACAGCGTAGTACATGGC

ATCTTCACAAATTATATCATTATCTAATAGATATTTGACGCATCTTATGGATCCCACTTCAACAGCCATC

TTAAAATCGGTAGAATCATATTGCTTTCCTTTATCGTTAATAATTTCTAGAACATCATCTCTATCATAAA

AGATACAAATATTAACTGTTTGATCAGTAATAACATTGCTAGTCGATATCAATTTGTTAATAAGATGCGC

TGGGCTCAATGTCTTAATAAGAAGTGTAAGAGGACTATCTCCGAATTTGTTTTGTTTATTAACATCCGTT

GATGGAAGTAAAAGATTTATAATGTCTACATACTTGACTGTTTTAGAGCATACAATATGGAGAGGCGTAT

TTCCATCATGATCTGGTTTTGAGGGACTAATTCCTAGTTTCATCATCCATGAGATTGTAGAAGCTTTTGG

ATTGTCTGACATAAGATGTCTATGAATATGATTTTTGCCAAATTTATCCACTATCCTGGCTTCGAATCCG

ATAGACATTATTTTTTTAAACACTCTTTCTGAAGGATCTGTACACGCCAACAACGGACCACATCCTTCTT

CATCAACCGAGTTGTTAATCTTGGCTCCATACTGTACCAATAAATTTATTCTCTCTATGACTTCATCATC

TGTTCCCGAGAGATAATATAGAGGTGTTTTATTATGTTTATCACATGCGTTTGGATCTGCGCCGTGCACC

AGCAGCATCGCGACTATTCTATTATTATTAATTTTAGAAGCTATATGCAATGGATAATTTCCATCATCAT

CCGTCTCATTTGGAGAGTATCCTCTATGAAGAAGTTCTTCTATAAATCGTTCATCTAGTCCTTTAATGCC

ACAATACGCATGTAGAATGTGATAATTTCCAGAGGGTTCGATAACTTGTAGCATATTCCTAAATACATCT

AAATTTTTACTATTATATTTGGCATAAAGAGATAGATAATACTCGACCGACATAATGTTGTGTTGTCCAT

TATAGTATAAAAATTAATATTTCTATTTCTATATATTTGCAACAATTTACTCTCTATAACAAATATCATA

ACTTAGTTCTTTTATGTCAAGAAGGCACTGGTTTAATTCATCTATAAATGTCACTCCATAACTACCACGC

ATACTATACTCAGAATTATGATAAAGATATTTATTCTTGGGGTGTAAGTAATGGGGATTAATCTTTGTTG

GATCAGTCTCTAAGTTAACACATGTCACACATGATCCATTTATAGTTATATCACACGATGATGATTTATG

AATTGATTCCGGAAGATCGCTATTGTATTTTGTAGTTCCACAATTCATTTCCATACATGTTATTGTCACA

CTAATATTATGATGAACTTTATCTAGCCGCTGAGTGGTAAACAACAGAACAGATAGTTTATTATCTTTAC

CAACACCCTCAGCCGCTGCCACAAATCTCTGATCCGTATCCATGATGGTCATGTTTACTTTTAGTCCGTA

TCCAGTCAACACTATGTTAGCATTTCTGTCGATATAGCTTTCACTCATATGACACTCACCAATAATTGTA

GAATTAATGTCGTAATTTACACCAATAGTGAGTTCGGCGACAAAGTACCAGTACCGGTAATCTTGTCGAT

GAGGACATATAGTATTCTTGTATTCTACCGAATACCCGAGAGATGCGATACAAAAGAGTAAGACTAATTT

GTAAACCATCTTACTCAAAAATATGCGACAATAGGAAATCTATCTTATACACATAATTATTCTATCAATT

TTACCAATTAGTTAGTGTAATGTTAACAAAAATGTGGGATAATTTAATAGTTTTTCCTTACACAATTGAC

ATACATGAGTCTGAGTTCCTCGTTTTTGCTAATTATTTCGTCCAATTTATTATTCTTGACATCGTCAAGA

TCTTTTGTATAGGAGTCAGACTTGTATTCAACATGTTTTTCTATAATCATCTTAGCTATTTCGGCATCAT

CCAATAGTACATTTTCCAGATTAACAGAATAGATATTAATGTCGTATTTGAACAGAGCCTGTAACATCTC

AATGTCTTTATTATCTATAGCCAATTTGATGTCCGGAATGAAGAGAAGGGAATTGGTGTTTGTCGACGTC

ATATAGTCGAGCAAGAGAATCATCATATCCACGTGTCCATTTTTTATAGTGGTGTGAATACAACTAAGGA

GAATAGCCAGATCAAAAGGAGATGGTATCTCTGAAAGAAAGTAGGAAACAATACTTACATCATTAAGCAT

GACAGCATGATAAAATGAAGTTTTCCATCCAGTTTTCCCATAGAACATCAGTCTCCAATTTTTCTTAACA

AACAGTTTTACCGTTTGCATGTTACCACTATCAACCGCATAATACAATGCGGTGTTTCCTTTGTCATCAA

ATTGTGAATCATCCATTCCACTGAATAGCAAAATCTTTACTATTTTGGTATCTTCTAATGTGGCTGCCTG

ATGTAATGGAAATTCATTATCTAGAAGATTTTTCAATGCTCCAGCGTTCAACAACGTACATACTAGACGC

ACGTTATTATCAGCTATTGCATAATACAAGACACTATGACCATTGATATCCGCCTTAAATGCATCTTTGC

TAGAGAGAAAGCTTTTCAGTTGCTTAGACTTCCAAGTATTAATTCGTGACAGATCCATGTCTGAAACGAG

ACGCTAATTAGTGTATATTTTTTCATTTTTTATAATTTTGTCATATTGTACCAGAATTAATAATATCTCT

AATAGATCTGATTAGTAGATATATGGCTATCGCAAAACAACATATACACATTTAATAAAAATAATATTCA

TTAAGAAGATTCAGATTCCACTGTACCCATCAATATAAAATAAAATAATTATTCCTTACATCGTACCCAT

AAACAATATATTAAGTATATTCCACCTTACCCATAAACAATATAAATCCAGTAATATCATGTCTAATGAT

GAACACAAATGGTGTATTAAATTCCAGTTCTTCAGGAGATGATCTCGCCGTAGCTACCATGATAGTAGAT

GCCTCCGCTACAGTTCCTTGTTCGTCTACATCTATCTTTACATTCTGAAACATTTTATAAATATATAATG

GGTCCCTAGTCATATGTTTAAACGACGCCTTATCTGGATTAAACATACTAGGAGCCATCATTTCGGCTAT

CGACTTAATATCCCTCTTGTTTTCGATAGAAAATCTAGGGAGTTTAAGATTGTACATTTTATTCCCTAAT

TGAGATGACCAATATTCTAATTTTGCAGGCGTGATAGAATCTGTGAAATGGGTCATATTATCACCTATTG

CCAGGTACATACTAATATTAGCATCCTTATACAGAAGGCGCACCATATCATATTCTTCGTCATCGATTGT

GATTGTATTTCCTTGCAATTTAGTAACTACGTTCATCATGGGAACCGTTTTCGTACCGTACTTATTAGTA

AAACTAGCATTGTGTGTTTTAGTGATATCAAACGGATATTGCCACGTACCTTTAAAATATATAGTATTAA

TGATTGCCCATAGAGTATTATCGTCGAGCATAGTAGAATCAACTACATTAGACATACCAGATCTACGTTC

TACTATAGAATTAATTTTATTAACCGCATCTCGTCTAAAGTTTAATCTATATAGGCCGAATCTATGATAT

TGTTGATAATACGACGGTTTAATACACACAGTACTATCGACGAAACTTTGATACGTTAGATCTGTGTACG

TATATTTAGATGTTTTCATCTTAGCTAATCCGGATATTAATTCTGTAAATGCTGGACCCAGATCTCTTTT

TCTCAAATTCATAGTATTCAATAATTCTACTCTAGTATTACCTGATGCAGACAATAGCGACATAAACATA

GAAAACGAATACCCAAACGGTGAGAAGACAATATTATCATTATCATCCTCATCCCCATTTTGAATATTTT

TATACGCTAATATACCAGCATTGATAAATCCCTGCAGACGATATGCGGATACTGAACACGCTAATGATAG

TATCAATAACGCAATCATGATTTTTATGGTATTAATAATTAACCTTATTTTTATGTTTGGTATAAAAATT

ATTGATGTCTACACATCCTTTATAATCAACTCTAATCACTTTAACTTTTACAGTTTTCCCTACAAGTTTA

TCCCTATATTCAACATATCTATCCATATGCATCTCTTAACACTCTGCCAAGATAGCTTCAGAGTGAGGAT

AGTCAAAAAGATAAATATATAGAGTATAATCATTCTCGTATACTCTGCCCTTTATTACATCGCCCGCATT

GGGCAACGAATAACAAAATGCAAGCATCGTGTTAACGGGCTCGTAAATTGGGATAAAATTATGTTTTTAT

TGTTTATCTATTTTATTCAAGAGAATATTCAGGAAGTTCCTTTTCTGGTTGTATCTCGTCGCAGTATATA

TCATTTGTACATTGTTTCATATTTTTTAATAGTCTACACCTTTTAGTAGGACTAGTATCGTACAATTCAT

AGCTGTATTTTGAATTCCAATCACGTATAAAAATATCTTCCAATTGTTGACGAAGACCTAATCCATCATC

CGGTGTAATATTAATAGATGCTCCACATATATCCGTAAAGTAATTTCCTGTCCAATTTGATGTACCTATA

TACGCCGTTTTATCGGTTACCATATATTTTGCATGGTTTACCCTAGAATACGGAATGGGAGGATCAGCAT

CTGGTACAATAAATAGCTTTACTTCTATATCTATGTTTTTAGATTTTAGCATAGCTATAGATCTTAAAAA

GTTTCTCATGATAAACGAAGATCGTTGCCAGCAACTAATCAATAGCTTAACGGATACTTGTCTGTCTATA

GCGGATCTTCTTAATTCATCTTCTATATAAGGCCAAAACAAAATTTTACCCGCCTTTGAATAAATAATAG

GAATAAAGTTCATAACAGATACATAAACGAATTTACTCGCATTTCCGATACATGACAATAAAGCGGTTAA

ATCATTGGTTCTTTCCATAGTACATAATTGTTGTGGTGCAGAAGCAATAAATACAGAGTGTGGAACACCG

CTTACGTTAATACTAAGAGGATGATCTGTATTATAATACGACGGATAAAAGTTTTTCCAATTATATGGTA

GATTGTTAACTCCAAGATACCAGTATACCTCAAAAATTTGAGTGAGATCCGCTGCCAAGTTCCTATTATT

GAAGATCGCAATACCCAATTCCTTGACCTGAGTTAGTGATCTCCAATCCATGTTAGCGCTTCCTAAATAA

ATATGTGTATTATCAGATATCCAAAATTTTGTATGAAGAACTCCTCCTAGGATATTTGTAATATCTATGT

ATCGTACTTCAACTCCGGCCATTTGTAGTCTTTCAACATCCTTTAATGGTTTGTTGGATTTATTGACGGC

TACTCTAACTCTTACTCCTCTTTTGGGTAATTGTACAATCTCGTTTAATATTACCGTGCCGAAATTCGTA

CCCACTTCATCCGATAAACTCCAATAAAAAGATGATATATCTAGTGTTTTTATGGTATTGGATAGAATTT

CCCTCCACATGTTAAATGTAGTCAAATATACTTTATCAAATTGCATACCTATAGGAATAGTCTCTGTAAT

CACTGCGATTGTATTATCCGGATTCATTTTATTTGTTAAAAAAATAATCCTATATCACTTCACTCTATTA

AAAATCCAAGTTTCTATTTCTTTCATGACTGATTTTTTAACTTCATCCGTTTCCTTATGAAGATGATGTT

TGGCGCCTTCATAAATTTTTATTTCCCTATTACAATTTGCATGTTGCATGAAATAATATGCACCTGAAAC

ATCGCTAATCTCATTGTTTGTTCCCTGGAGTATGAGAGTCGGGGTGTTAATCTTGGGAATTATTTTTCTA

ACCTTGTTGGTAGCCTTCAAGACCTGACTAGCAAATCCAGCCTTAATTTTTTCATGATTGACTAATGGAT

CGTATTGGTATTTATAAACTTCATCCATATCTCTAGATACTGATTCTGGACATAGCTTTCCGACTGACGC

ATTTGGTGTAATGGTTCCCATAAGTTTTGCAGCTAGCAGATTCAGTCTTGGAACAGCGTCTGCATTAACT

AGAGGAGACATTAGAATCATTGCTGTAAACAAGTTTGGATTATCGCAAGCAGCTAGTATAGAAATTGTTG

CTCCCATGGAATGACCCAATAAGAAGACTGGAACTCCTGGATAAGTAGATTTAATAGTCACCACGTGCTG

TACCACATCTCTAACATACTTACCAAAGTCATCAATCATCATTTTTTCACCATTACTTCTTCCATGGCCA

ATATGATCATGTGAGAATACTAAAATTCCTAACGATGATATGTTTTCAGCTAGTTCGTCATAACGTCCAG

AATGTTCACCAGCTCCATGACTTATGAATACTAATGCCTTAGGATATGTAATAGGTTTCCAATATTTACA

ATATATGTAATCATTGTCCAGATTGAACATACAGTTTGTACTCATGATTCACTATATAACTATCAATATT

AACAGTTCGTTTAATGATCATATTATTTTTATGTTTTATTGATAATTGTAAAAATATACAATTAAATCAA

TATAGAGGAAGGAGACGGTACTGTATTTTGTGAGATAGTAATGGAGACTAAATCAGATTATGAGGATGCT

GTTTTTTACTTTGTGGATGATGATGAAATATGTAGTCGCGACTCCATCATTGATCTAATAGATGAATATA

TCACGTGGAGAAATCATGTTATAGTGTTTAATAAAGATATTACCAGTTGTGGAAGACTGTACAAGGAATT

GATAAAGTTCGATGATGTCGCTATACGGTACTATGGTATTGATAAAATTAATGAGATTGTCGAGGCTATG

AGTGAAGGAGACCACTACATCAATCTTACAGAAGTCCATGATCAGGAAAGTCTATTCGCTACCATAGGAA

TATGTGCTAAAATCACTGAACATTGGGGATACAAAAAGATTTCAGAATCTAAATTCCAATCATTGGGAAA

CATTACAGATCTGATGACCGACGATAATATAAACATCTTGATACTTTTTCTAGAAAAAAAAATGAATTGA

TGATATAAGTGTCTTCATAACGCATTATTACGTTAGCATTCTATTATCCAGTGTTAAAAAAATTATCCTA

TCATGTATTTGAGAGTCTTATATGTAGCAAACATGATAACTGCAATACCCATAATCTTTAGATATTCACG

CGTGCTATGGATGGCATTATCCCGCGGTGCGGAAATGTACGTTATATAATCTACAAAATAATCATCGCAT

ATAGTATGAGATAGTAGAGTAAACATTTTTATCGTTTCTACTGGGTTCATACATCGTCTACCCAATTCGG

TAATGAATGAAATTGTCGCCAATCTTACACCCAAACCCTTGTTGTTCATTAGTATAGTATTAACTTCATT

ATTTATGTCATAAACTGTAAATGATTCTGTAGATGCCATATCACACATGATATTCATGTCACTATTATAA

TCATTATTAACTTTATCACAATACGTGTTGATAATATCTACATATGATCTAGTTTTTGTGGGTAATTGTC

TATACAAGTCGTCTAAACGTTGTTTACTCATATAGTATTGAACAGCCATCATTACATGGTCCCGTTCCGT

TGATAGATAATCGAGTATGTTAGTAGACTTGTCAAATCTATATACCATATTTTCTGGAAGCGGATATACA

TAGTCGCGATCATCATTATCACTAGCCTCATCCTCTATATCATGTACATGTACATAATCTATGATATTAT

TATACATAAACATCGACAACATACTATTGTCTATTATCTAAGTCCTGTTGATCCAAACCCTTGATCTCCT

CTATCTGTACTATCTAGAGATTGTACTTCTTCAAGTTCTGGATAATATATACGTTGATAGATTAGCTGAG

CTATTCTATCTCCAGTATTTACATTAAACGTACATTTTCCATTATTAATAAGAATGACTCCTATGCTTCC

CCTATAATCTTCGTCTATTACACCGCCTCCTATATCAATGCCTTTTAGGGACAGACCAGACCTAGGAGCT

ATTCTACCATAGCAGAACTTAGGCATGGACATACTAATATCTGTCTTAATTAACTGTCGTTCTCCAGGAG

GGATAGTATAATCGTAAGCGCTATACAAATCATATCCGGCAGCACCCGGCGATTGCCTAGTAGGTGATTT

AGCTCTGTTAGTTTCCTTAACAAATCTAACTGGTGAGTTAATATTCATGTTGAACATAAAAAATATCATT

TTATTTCAAAATTATTTACCATTCCATTCCATTCCATTCCATCCCATATATTCCATGAATAAGTGCGATT

ATTGTACACTTCTATAGTATCTATATACGATCCACGATAAAATCCTCCTATCAATAGCAGTTTATTATCC

ACTATGATCAATTCTGGATTATCCCTCGGATAAATAGGATCATCTATCAGAGTCCATGTATTGCTGGATT

CACAATAAAATTCCGCATTTCTACCAACCAAGAATAACCTTCTACCAAACACTAACGCACATGATTTATA

ATGAGGATAATAAGTGGATGGTCCAAACTGCCACTGATCATGATTGGGTAGCAAATATTCTGTAGTTGTA

TCAGTTTCAGAATGTCCTCCCATTACGTATATAACATTGTTTATGGATGCCACTGCTGGATTACATCTAG

GTTTCAGAAGACTCGGCATATTAACCCAAGCAGCATCCCCGTGGAACCAACGCTCAACAGATGTGGGATT

TGGTAGACCTCCTACTACGTATAATTTATTGTTAGCGGGTATCCCGCTAGCATACAGTCTGGGGCTATTC

ATCGGAGGAATTGGAATCCAATTGTTTGATATATAATTTACCGCTATAGCATTGTTATGTATTTCATTGT

TCATCCATCCACCGATGAGATATACTACTTCTCCAACATGAGTACTTGTACACATATGGAATATATCTAT

AATTTGATCCATGTTCATAGGATACTCTATGAATGGATACTTGTATGATTTGCGTGGTTGTTTATCACAA

TGAAATATTATGTTACAGTCTAGTATCCATTTTACATTATGTATACCTCTGGGAGAAAGATAATTTGACC

TGATTACATTTTTGATAAGAAGTAGCAGATTTCCTAATCTATTTCTTCGCCTCATATACCACTTAATGAC

AAAATCAACTACATAATCCTCATCTGGAACATTTAGTTCGTCGCTTTCTAGAATAAGTTTCATAGATAGA

TAATCAAAATTGTCTATGATGTCATCTTCCAGTTCCAAAAAGTGTTTGGTAATAAAGTCTTTAGTATGAC

ATAAGAGATTGGATAGTCCGTATTCTATACCCATCATGTAACACTCGATACAATATTCCTTTCTAAAATC

TCGTAGGATAAAGTTTATACAAGTGTAGATGATAAATTCTACAGATGTTAATATAGAAGCACGTAATAAA

TTGACGACGTTATGACTATCTATATATACCTTTCCAGTATATGAGTAAATAACTATAGAAGTTAGACTGT

GAATGTCAAGGTCTAGACAAACCCTCGTAACTGGATCTTTATTTTTTGTGTATTTTTGGCGTAAATGTGT

GCGAAAGTATGGAGATAACTTTTTCAATATCGTAGAATTGACTATTATATTACCTCCTATAGCTTCAATA

ATTGTTTTGAATTTCTTAGTCGTGTACAATGCTAATATATTCTTACAGTACACAGTATTGACAAATATCG

GCATTTATGTTTCTTTAAAAGTCAACATCTAAAGAAAAATGATTGTCTTCTTGAGACATAACTCCCATTT

TTTGGTATTCACCCACACGTTTTTCGAAAAAATTAGTTTTTCCTTCCAATGATATATTTTCCATGAAATC

AAACGGATTGGTAACATTGTAAATTTTTTTAAATCCCAATTCAGAAATCAATCTATCTGCGACGAATTCT

ATATATGTTTTCATCATTTCACAATTCATTCCTATGAGTTTAACTGGAAGAGCCACAGTAAGAAATTCTT

GTTCAATGGATACCGCATTTGTTATAATAAATCTAACGGTTTCTTCACTCGGTGGATGCAATAAATGTTT

AAACATCAAACATGCGAAATCGCAGTGCAGACCCTCGTCTCTACTAATTAATTCGTTAGAAAACGTGAGT

CCGGGCATTAGGCCACGCTTTTTAAGCCAAAATATGGAAGCGAATGATCCGGAAAAGAAGATTCCTTCTA

CTGCAGCAAAGGCAATAAGTCTCTCTCCATAACCGGCGCTGTCATGTATCCACTTTTGAGCCCAATCGGC

CTTCTTTTTTACACAAGGCATCGTTTCTATGGCATTAAAGAGGTAGTTTTTTTCATTACTATCTTTAACA

TAAGTATCGATCAAAAGACTATACATTTCCGAATGAATGTTTTCAATGGCCATCTGAAATCCGTAGAAAC

ATCTAGCCTCGGTAATCTGCACTTCTGTACAAAATCGTTCTGCTAAATTTTCATTCACTATTCCGTCACT

GGCTGCAAAAAACGCCAATACATGTTTTATAAAATATTTTTCGTCTGGTGTTAGTTTATTCCAGTCATTG

ATATCTTTAGATATATCCACTTCTTCCACTGTCCAAAATGATGCCTCTGCCTTTTTATACATATTCCAGA

TGTCATGATATTGGATTGGGAAAATAACAAATCTATTTGGATTTGGTGCAAGGATAGGTTCCATAACTAA

ATTAACAATAGTAGTAATTTTTTTTCAGTTATCTGTATGACTGTACTTGGATCTTTTGTATATCGCTATC

GCCGCAATCACTACAATAATTACAAGTATTATTGATAGCATTGTTATTACTACTATCATAATTAAATTAT

CGACATTCATGGGTGTTGAATAATCGTTATCATCATTTTGTAATTGTGACGTCATACTAGATAAATCATT

TGTGAGATTGTTGTGGGAAGCGGGCACGGAAGATGCATTATCATTATTATTTAACGCCTCCCATTCGGAT

TCACAAATGTTACGCACGTTCAAAGTTTTATGGAAACTATAATTTTGTGAAAACAGATAACAAGAAAACT

CGTCATCGTTCAAATTTTTAACGATAGTAAACCGATTAAACGTCGAGCTAATTTCTAACGCTAGCGACTC

TGTTGGATATGGGTTTCCAGATATATATCTTTTCAGTTCCCCTACGTATCTATAATCATCTGTAGGAAAT

GGAAGATATTTCCATTTATCTACTGTTCCTAATATCATATGCGGTGGTGTAGAACCATTAAGCGCGAAAG

ATGTTATTTCGCATCGTATTTTAACTTCGCAATAATTTCTGGTTAGATAACGCACTCTACCAGTCAAGTC

AATGATATTAGCCTTTACAGATATATTCATAGTAGTCGTAACGATGACTCCATCTTTTAGATGTGATACT

CCTTTGTATGTACCAGAATCTTCGTACCTCAAACTCGATATATTTAAACAAGTTAATGATATATTAACGC

GTTTTATGAATGATGATATATAACCAGAAGTTTTATCCTCTGTGGCTAGCGCTATAACCTTATCATTATA

ATACCAACTAGTGTAATTAATATGTGACACGACAGTGTGGGTACAAATATGTACATTATCGTCTACGTCG

TATTTGATACATCCGCATTCAGCCAACAAATATAAAATTACAAAAACTCTAACGACGTTCGTGCACATCT

TGATGCGGTTTAATAAATGTTTTGATTTCAATTTATTGTAAAAAAGATTCGGTTTTATACTGTTCGATAT

TCTCATTGCTTATATTCTCATCTATCATCTCCACACAGTCAAATCCATGGTTAACATGTACCTCATCAAC

CGGTAAAAGACTATCGGATTCTTCTATCATCATAACTCGAGAATATTTAATTTGGTGGTCATTATTAATC

AAGTCAATTATCTTATTTTTAACAAACGTAAGTATTTTACTCATTTTTTATAAAAACTTTTAGAAATATA

CAGACTCTATCGTGTGTCTATATCTTCTTTTTATATCCAATGTATTTATGTCTGATTTTTCTTCATTTAT

CATATATAATGGTCCAAATTCTACACGTGCTTCGGATTCATCCAGATCATTAAGGTTCTTATAATCGCAA

CATCCTTCTCTTCCATCTTCTACATCTTCCTTCTTATTCTTAGCGTCACAGAATCTACCACAGCAGGATC

CCATGACGAGAGTCACATTAAACTAATTCATTTTCAATTATAATATACTGATTAGTAATGACAATTAAAA

TAAAAATATTCTTCATAACCGGTAAGAAAGTAAAAAGTTCACATTGAAACTATGTCAGTAGTTATACATC

ATGAGATGATATACTCTATTTTGGTGGAGGATTATATGATATAATTCGTGGATAATCATTCTTAAGACAC

ATTTCTTCATTCGTAAATCTTTTCACATTAAATGAGTGTCCATATTTTGCAATTTCTTCATATGATGGCG

GTGTACGTGGACGAGGCTGCTCCTGTTCTTGTAGTCGCCGACTGTCGTGTTTGCGTTTAGATCCCTCCAT

TATCGCGATTGCGTAGTGAGTACTATTTATACCTTGTAATTAAATTTTTTTATTAATTAAACGTATAAAA

ACGTTCCGTATCTGTATTTAAGAGCCAGATTTCGTCTAATAGAACAAATAGCTACAGTAAAAATAACTAG

AATAATCGCTACACCCACTAGAAACCACGGATCGTAATACGGCAATCGGTTTTCGATAATAGGTGGAACG

TATATTTTATTTAAGGACTTAACAATTGTCTGTAAACCACAATTTGCTTCCGCCGATCCTGTATTAACTA

TCTGTAAAAGCATATGTTGGCCGGGAGGAGCCGAACATTCTCCGATATTCAATTTTTGTATATCTATAAT

GTTATTAACCTCCGCATACGCATTACAGTTCTTTTCTAGCTTGGATACTACACTAGGTACATCATCTAAA

TCTATTCCTATTTCCTCAGCGATAGCTCTTCTATCCTTTTCCGGAAGTAATGAAATCACTTCAATAAATG

ATTCAACCATGAGTGTGAAACTAAGTCGAGAATTACTCATGCATTTGTTAGTTATTCGGAGCGCGCAATT

TTTAAACTGTCCTATAACCTCTCCTATATGAATAGCACAAGTGACATTAGTAGGGATAGAATGTTGAGCT

AATTTTTGTAAATAACTATCTATAAAAAGATTATACAAAGTTTTAAACTCTTTAGTTTCCGCCATTTATC

CAGTCTGAGAAAATGTCTCTCATAATAAATTTTTCCAAGAAACTAATTGGGTGAAGAATGGAAACCTTTA

ATCTATATTTATCACAGTCTGTTTTGGTACACATGATGAATTCTTCTAATGCTGTACTAAATTCGATATC

TTTTTCGATTTCTGGATATGTTTTTAATAAAGTATGAACAAAGAAATGGAAATCGTAATACCAGTTATGT

TCAACTTTGAAATTGTTTTTTATTTTCTTGTTAATGATTCCAGCCACTTGGGAAAAGTCAAAGTCGTTTA

ATGCCGATTTAATACGTTCATTAAAAACAAACTTTTTATTCTTTAGATGAATTATTATTGGTTCATTGGA

ATCAAAAAGTAAGATATTATCGGGTTTAAGATCTGCATGTAAAAAGTTGTCACAACAGGGTAGTTCGTAG

ATTTTAATGTATAACAGAGACATCTGTAAAAAGATAAACTTTATGTATTGTACCAAAGATTTAAATCCTA

ATTTGATAGCTAACTCGGTATCTACTTTATCTGCCGAATACAGTGCTAGGGGAAAAATTATAATATTTCC

TCTTTCGTATTCGTAATTAGTTCTCTTTTCATGTTCGAAAAAGTGAAACATGCGGTTAAAATAGTTTATA

ACATTAATATTACTGTTAATAACTGCCGGATAAAAGTGGGATAGTAATTTCACGAATTTGATACTGTCCT

TTCTCTCGTTAAACGCCTTTAGAAAAACTTTAGAAGAATATCTCAATGAGAGTTCCTGACCATCCATAGT

TTGTATCAATAATAGCAACATATGAAGAACCCGCTTATACAGAGTATGTAAAAATGTTAATTTATAGTTT

AATCCCATGGCCCACGCACACACGATTAATTTTTTTTCATCTCCCTTTAGATTGTTGTATAGAAATTTGG

GTACTGTAAACTCCGCCGTAGTTTCCATGGGACTATATAATTTTGTGGCCTCGAATACAAATTTTACTAC

ATAGTTATCTATCTTAAAGACTATACCATATCCTCCTGTAGATATGTGATAAAAATCGTCGTTTATAGGA

TAAAATCGTTTATCTTTTTGTTGGAAAAAGGATGAATTAATGTAATCATTCTCTTCTATCTTTAGTAGTG

TTTCCTTATTAAAATTCTTAAAATAATTTAACAATCTAACTGATGGAGCCCAATTTTGGTGTAAATCTAA

TTGGGACATTATGTTGTTAAAATATAAACAGTCTCCTAATATAACAGTATCTGATAATCTATGGGGAGAC

ATCCATTGATATTCAGGGGATGAATCATTGGCAACACCCATTTATTGTACAAAAAGCCCCAATTTACAAA

CGAAAGTCCAGGTTTGATAGAGATAAACTATTAACTATTTTGTCTCTGTTTTTAACACCTCCACAGTTTT

TAATTTCTTTGGTAATGAAATTATTCACAATATCAGTATCTTCTTTATCTACCAGAGATTTTACTAACTT

GATAACCTTGGCTGTCTCATTCAATAGGGTAGTGATATTTGTATGTATGATATTGATATCTTTTTGAATT

GTTTCTTTTAGAAGTGATTCTTTGATGGTATCAGCATACGAATTACAATAATGCAGAAACTCAGTTAACA

TGCAGGAATTATAGTAAGCCAATTCCAATTGTTGCCTGTATTGTATTAGAGTATTAATATGCGCAATGAT

GTCCTTGCGTTTCTCTGATAGAATGCGAGCAGCGATTTTGGCGTTATCATTTGACGATATTTCTGGAATG

ACGAATCCTGTTTCTACTAACTTCTTGGTAGGACAAAGTGAAACAATCAAGAAAATAGCTTCTCCTCCTA

TTTGTGGAAGAAATTGAACTCCTCTAGATGATCTACTGACGATAGTATCTCCTTGACAGATATTGGACCG

AACTACGGAAGTACCTGGAATGTAAAGCCCTGAAACCCCCTCATTTTTTAAGCAGATTGTTGCCGTAAAT

CCTGCACTATGCCCAAGATAGAGAGCTCCTTTGGTGAATCCATCACTATGTTTCAGTTTAACCAAGAAAC

AGTCAGTTGGTCTAAAATTTCCATCTCTATCTAATACAGAATCCAACTTGATGTCAGGGACTATGACCGG

TTTAATGTTATATGTAACATTGAGTAAATCCTTAAGTTCATAATCATCGTTGTCATCAGTTATGTACGAT

CCAAACAATGTTTCTACCGGCATGGTGGATACGAAGATGCTATCCATCAGAATGTTTCCCTGATTAGTAT

TTTCTATATAGCTATTCTTCTTTAAACGATTTTCCGAATCAGTAACTATGTTCATTTTTTTAGGAGTAGG

ACGTCTAGCCAGTATGGAAGAGGATTTTCTAGATACTCTCTTCAACATCTTTGATCTCAATGGAATGCAA

AACCCCATGGTGTAACAACCAACGATAAAAATAATATTGTTTTTTCACTTTTTATAATTTTACCATCTGA

CTCATGGATTCATTAATATCTTTACAAGAGCTACTAACGTATAATTCTTTATAACTGAACTGAGATATAT

ACACCGGATCTATGGTTTCCATAATTGAGTAAATGAATGCTCGGCAATAACTAATGGCAAATGTATAGAA

CAACGAAATTATACTAGAGTTGTTAAAGTTAATATTTTCTATGAGTTGTTCCAATAAATTATTTGTTGTG

ACTGCGTTCAAGTCATAAATTATCTTGATACTATCCAGTAAACAGTCTTTAAGTTCTGGAATATTATCAT

CCCATTGTAAAGCCCCTAGTTCGACTATCGAATATCCTGCTCTGATAGCAGTTTCAATATCGACGGACGT

CAATACTGTAATAAAGGTGGTAGTATTGTCATCATCGTGATAAACTACGGGAATATGGTCGTTAGTAGGT

ACCGTGACTTTACACAACGCGATATATAACTTTCCTTTTGTACCATTTTTAACGTAGTTGGGACGTCCTG

CAGGGTATTGTTTTGAAGAAATGATATCGAGAACAGATTTGATACGATATTTGTTGGATTCCTGATTATT

CACTATAATATAATCTAGACAGATAGATGATTCGATAAATAGAGAAGGTATATCGTTGGTAGGATAATAC

ATCCCCATTCCAGTATTCTCGGATACTCTATTGATGACACTAGTTAAGAACATGTCTTCTATTCTAGAAA

ACGAAAACATCCTACATGGACTCATTAAAACTTCTAACGCTCCTGATTGTGTTTCGAATGCCTCGTACAA

GGATTTCAAGGATGCCATAGATTCTTTGACCAACGATTTAGTATTGCGTTTAGCATCTGATTTTTTTATT

AAATCAAATGGTCGGCTCTCTGGTTTACTACCCCAATGATAACAATAGTCTTGTAAAGATAAACCGCAAG

AAAATTTATACGCATCCATCCAAATAACCCTAGCACCGTCGGATGATATTAATGTATTATTATAGATTTT

CCATCCACAGTTATTGGGCCAGTATACTGTTAGCAACGGTATATCGAATAGATTACTCATGTAACCTACT

AGAATGATAGTTCGTGTACTAGTCATAATATCTTTAATCCAATCTAAGAAATCTAAAATTAGATCTTTTA

CACTATTAAAGTTAACAAAGGTATTACCCGGGTACGTGGATATCATATATGGTATTGGTCCATTATCAGT

AATGGCTCCATAAACTGATACGGCGATGGTTTTTATATGTGTTTGATCTAATGAGGACGAAATTCGCGCC

CACAATTCATCTCTAGATATGCATTTAATATCGAACGGTAACACATCAATCTCGGGACGCGTATATGTTT

CTAAATTCTTAATCCAAATATAATGATGACCTATATGCCCTATTATCATACTGTCAACTATAGTATACCT

AGAGAACTTTCGATACATCTGCTGTTTCCTGTAATCGTTAAATTTTACAAATCTATAACATGCTAAACCT

TTTGACGACAGCCATTCATTAATTTCTGATATGGAATCTGTATTCTCAATACCGTATCGTTCTAAAGCCA

GTGCTATATCTCCCTGTTCGTGGGAACGCTTTCGTATAATATCGATCAATGGATAATATGAAGTTTTTGG

AGAATAATATGATTCATGATCTATTTCGTCCATAAACAATCTAGACATAGGAATTGGAGGCGATGATCTT

AATTTTGTGCAATGGGTCAATCCTATAACTTCTAATATTGTAATATTCATCATCGACATAACACTATCTA

TGTTATCATCGTATATTAGTATACCACGACCTTCTTCATTTCGTGCCAAAATGATATACAGTCTTAAATA

ATTACGCAATATCTCAATAGTTTCATAATTGTTAGCTGTTTTCATCAAGGTTTGTATCCTGTTTAACATG

ATGGCGTTCTATAACGTCTCTATTTTCTATTTTTAATTTTTTTAAATTTTTAACGATTTACTGTGGCTAG

ATACCCAATCTCTCTCAAATATTTTTTTAGCCTCGCTTACAAGCTGTTTATCTATACTATTAAAACTGAC

GAATCCGTGATTTTGGTAATGGGTTCCGTCGAAATTTGCCGAAGTGATATGAACATATTCGTCGTCGACT

ATTAACAATTTTGTATTATTCTGAATAGTGAAAACCTTCACAGATAGATCATTTTGAACACACAACGCAT

CTAGACTTCTGGCGGTTGCCATAGAATATACGTCGTTCTTATCCCAATTACCAACTAGAAGTCTGATCTT

AACTCCTCTATTAATGGCTGCTTCTATAATGGAGTTGTAAATGTCAGGCCAATAGTAGCTATTACCGTCG

ACACGTGTAGTGGGAACTATGGCCAAATGTTCAATATCTATACTAGTCTTAGCCGACTTGAGTTTATCAA

TAACTACATCTGTGTCTAGATCTCTAGAATATCCCAATAGGTGTTCTGGAGAATCAGTAAAGAACACTCC

ACCTATAGGATTCTTAATATGATACGCAGTGCTAACTGGCAGACAACAAGCCGCAGAGCATAAATTCAAC

CATGAATTTTTTGCGCTATTAAAGGCTTTAAAAGTATCAAATCTTCTACGAAGATCTGTGGCCAGCGGAG

GATAATCAGAATATACGCCTAACGTTTTAATCGTATGTATAGATCCTCCAGTAAATGACGCGTTTCCTAC

ATAACATCTTTCATCATCAGACACCCAAAAACAACCGAGTAGTAGTCCCACATTATTTTTTTTATCTATA

TTAACGGTTATAAAATTTATATCCGGGGAGTGACTTTGTAGCTCTCCCAGATTTCTTTTCCCTCGTTCAT

CTAGCAAAACTATTATTTTAATCCCTTTTTCAGATACCTCTTTTAGTTTATCAAAAATAAGCGCTCCCCT

AGTAGTACTCAGAGGATTACAACAAAAAGATGCTATGTATATATATTTCTTAGCTAGAGTGATAATTTCG

TTAAAACATTCAAATGTTGTCAAATGATCGGATCTAAAATCCATATTTTCTGGTAGTGTTTCTACCAGCC

TACATTTTGCTCCCGCAGGTACCGATGCAAATGGCCACATTTAGTTAACATAAAAACTTATATATCCTGT

TCTATCAACGATTCTAGAATATCATCGGCTATATCGCTAAAATTTTCATCAAAGTCGACATCACAACCTA

ACTCAGTCAATATATTAAGAAGTTCCATGATGTCATCTTCGTCTATTTCTATATCCGTATCCATTGTAGA

TTGTTGACCGATTATCGAGTTTAAATCATTACTAATACTCAATCCTTCAGAATACAATCTGTGTTTCATT

GTAAATTTATAGGCGGTGTATTTAAGTTGGTAGATTTTCAATTATGTATCAATATAGCAACAGTAGTTCT

TGCTCCTCCTTGATTTTAGCATCCTCTTCATTATTTTCTTCTACGTACATAATCATGTCTAATACGTTAG

ACAACACACCGACGATGGTGGCCGCCACAGACACGAATATGACTAGACCGATGACCATTTAAAAAATACT

CTCTAGCTTTAACTTAAACTGTATCGATCATTCTTTTAGCACATGTATAATATAAAAACATTATTCTATT

TCGAATTTAGGCTTCCAAAAATTTTTCATCCGTAAACCGATAATAATATATATAGACTTGTTAATAGTCG

GAATAAATATATTAATGCTTAAACTATCATCATCTCCACGATTAGAGATACAATATTTACATTCTTTTTG

CTGTTTCGAAACTTTATCAATACACGTTAATACAAACCCAGGAAGGAGATATTGAAACTGAGGCTGTTGA

AAATGAAACGGCGAATACAATAATTCAGATAATGTAAAATCATGATTCCGTATTCTGATGATATTAGAAC

TGCTAATGGATGTCGATGGTATGTATCTAGGAGTATCTATTTTAACAAAGCATCGATTTGCTAATATACA

ATTATCCTTTTGATTAATTGTTATTTTATTCATATTCTTAAAAGGTTTCATATTTATCAATTCTTCTACA

TTAAAAATTTCCATTTTTAATTTATCTAGCCCCGCAATACTCCTCATTACGTTTCATTTTTTGTCTAGAA

TACCCATTTTGTTCATCTTGGTACATAGATTATCCAATTGAGAAGCGCATTTAGTAGTTTTGTACATTTT

AAGTTTATTAACGAATCGTCGAAAACTAGTTATAGTTAACATTTTATTATTTGATACCCTGATATTAATA

CCCCTGCCGTTACTATTATTTATAACTGATGTAACCCACGTAACATTGGAATTAATTATCGATAGTAATG

CATCGACACTTCCAAAATTGTCTATTATAAACTCACCGATAATTTTTTTATTGCATGTTTTCATATTCAT

TAGGATTATCAAATCTTTAATCTTATTACGATTGTATGCGTTGATATTACAAGACGTCATTCTAAAAGAC

GGAGGATTTCCATCAAATGCCAGACAATCACGTACAAAGTACATGGAAATAGGTTTTGTTCTATTACGCA

TCATAGATTCATATAAAACACCCGTAGAAATACTAATTTGTTTTACTCTATAAAATACTATTGCATCTAT

TTCATCGTTTTGTATAACGTCTTTCCAAGTGTCAAATTCCAATTTTTTTTCATTGATAGTACCAAATTCT

TCTATCTCTTTAACTACTTGCATAGATAGGTAATTACAGTGATGCCTACATGCCGTTTTTTGAAACTGAA

TAGATGCATCTAGAAGCGATGCTACACTAGTCACGATCACCACTTTCATATTTAGAATATATGTATGTAA

AAATATAGTAGAATTTCATTTTGTTTTTTTCTATGCTATAAATGAATTCTCATTTTGTATCCGCACATAC

TCCGTTTTATATCAATACCAAAGAAGGAAGATATCTGGTTCTAAAAGCCGTTAAAGTATGCGATGTTAGA

ACTGTAGAATGCGAAGGAAGTAAAGCTTCCTGCGTACTCAAAGTAGATAAACCCTCATCACCCACGTGTG

AGAGAAGACCTTCGTCCCCGTCCAGATGCGAGAGAATGAATAACCCTGGAAAACAAGTCCCGTTTATGAG

AACGGACATGTTACAAAATATGTTTGCTGCTAATCGCGACAACGTAACGTCAAGACTTTTGAACTAAAAT

ACAATTATATCTTTTTCGATATTAATAAATCCGTGTCTCCCGGGTTTTTTATCTCTTTCAGTATGTGAAT

AGATAGGTATTTTATCTCTATTCATCATCGAATTTAAGAGATCCGATAAACATTGTTTGTATTCTCCAGA

TGTCAGCATCTGATACAACAATATATGTGCACATAAACCTCTGGCACTTATTTCATGTACCTTCCCCTTA

TCACTAAGGAGAATAGTATTTGAGAAATATGTATACATGATATTATCATGTATTAGATATACAGAATTTG

TAACACTCTCGAAATCACACGATGTGTCGGCGTTAAGATCTAATATATCACTCGATAACACATTTTCATC

TAGATACACTAGACATTTTTTAAAGCTAAAATAGTCTTTAGTAGTAACAGTAACTATGCGATTATTTTCA

TCGATGATACATTTCATCGGCATATTATTACGCGTACCATCAAAGACTATACCATGTGTATATCTAACGT

ATTCTAGCATAGTTGCCATACGTACATTAAACTTTTCAGGATCTTTGGATAGATCTTCCAATCTATCTAT

TTGAGAAAACATTTTTATCATGTTCAATAGTTGAAACGTCGGATCCACTATATAGATATTATCTATAAAG

ATTTTAGGAACTATGTTCATGGTATCCTGGCGAATATTAAAACTATCAATGATATGATTATCGTTTTCAT

CTTTTATCACCATATAGTTTCTAAGATATGGGATTTTACTTAATATAATATTATTTCCCGTAATAAATTT

TATTAGAAATGCCAAATCTATAAGAAAAGTCCTAGAATTAGTCTGAAGAATATCTATATCACCGTACCGT

ATATTTGGATTAATTAGATATAGAGAATATGATCCGTAACATATACAACTTTTATTATGACGTCTAAGAT

ATTCTTCCATCAACTTATTAACATTTTTGACTAGGGAAGATACATTATGACGTCCCATTACTTTTGCCTT

GTCTATTACAGCGACGTTCATAGAATTTAGCATATCTCTTGCCAATTCTTCCATTGATGTTACATTATAA

GAAATTTTAGATGAAATTACATTTGGAGCTTTAATAGTAAGAACTCCTAATATATCCGTGTATGTGGTCA

CTAATACAGATTGTAGTTCTATAATCGTAAATAATTTACCTATATTATATGTTTGAGTTTGTTTAGAAAA

GTAGCTAAGTATACGATCTTTTATTTCTGATGCCGATGTATCAACATCGAAAAAAAATCTTTTTTTATTC

TTTTTTACTAACGATACGAATATGTCTTTGTTAAAAACAGTTATTTTCTGAATATTTCTAGCTTGTAATT

TTAACATATGATATTCGTTCACACTAGGTACTCTGCCTAAATAGGTTTCTATAATCTTTAATGTAATATT

AGGAAGAGTATTCTGATCAGGATTCCTATTCATTTTGAGGATTTAAAACTCTGATTATTGTCTAATATGG

TCTCAACACAAACTTTTTCACAGAGTGATAGAGTTTTTGATAACTCGTTTTTCTTAAGAAATATAAAACT

ACTGTCTCCAGAGCTCGCTCTATCTTTTATTTTATCTAATTCGATACAAACTCCTGATACTGGTTCAGAA

AGTAATTCATTAATTTTCAGTCCTTTATAGAAGATATTTAATATAGATAATACAAAATCTTCAGTTCTTG

ATATCGATCTGATTGATCCTAGAACTAGATATATTAATAACGTGCTCATTAGGCAGTTTATGGCAGCTTG

ATAATTAGATATAGTATATTCCAGTTCATATTTATTAGATACCGCATTGCCCAGATTTTGATATTCTATG

AATTCCTCTGAAAATAAATCCAAAATAACTAGACATTCTATTTTTTGTGGATTAGTGTACTCTCTTCCCT

CTATCATGTTCACTACTGGTGTCCACAATGATAAATATCTAGAGGGAATATAATATAGTCCATATGATGC

CAATCTAGCAATGTCGAATAACTGTAATTTTATTCTTCGCTCTTCATTATGAATTGAATCTTGAGGTATA

AACCTAACACAAATTATATCATTAGACTTTTCGTATGTAATGTCTTTCATGTTATAAGTTTTTAATCCTG

GAATAGAATCTATTTTAATGAGGCTTTTAAACGCAGCGTTCTCCAACGAGTCAAAGCATAATACTCTGTT

GGTTTTCTTATATTCAATATTACGATTTTCTTCTTTGAATGGAATAGGTTTTTGAATTAGTTTATAATTA

CAACATAATAGATAAGGAAGTGTGTAAATAGTACGCGGAAAAAACATAATAGCTCCCCTGTTTTCATCCA

TGGTTTTAAGTAAATGATCACTGGCTTCTTTAGTCAATGGATATTCGAACATTAACCGTTTCATCATCAT

TGGACAGAATCCATATTTCTTAATGTAAAGAGTGATCAAATCATTGTGTTTATTGTACCATCTTGTTGTA

AATGTGTATTCGGTTATCGGATCTGCTCCTTTTTCTATTAAAGTATCGATATCGATCTCGTCTAAGAATT

CAACTATATCGACATATTTCATTTGTATACACATAACCATTACTAACGTAGAATGTATAGGAAGAGATGT

AACGGGAACAGGGTTTGTTGATTCGCAAACTATTCTAATACATAATTCTTCTGTTAATACGTCTTGCACG

TAATCTATTATAGATGCCAAGATATCTATATAATTATTTTGTAAGATGATGTTAACAATGTGATCTATAT

AAGTAGTGTAATAATTCATGTATTTCGATATATGTTCCAACTCTGTCTTTGTGATGTCTAGTTTCGTAAT

ATCTATAGCGTCCTCAAAAAATATATTCGCATATATTCCCAAGTCTTCAGTTCTATCTTCTAAAAAATCT

TCAACGTATGGAATATAATAATCTATTTTACCTCTTCTGATGTCATTAATGATATAGTTTTTGACACTAT

TTTCCGTCAATTGATTCTTATTCACTATGTCTAAAAACCGGATAGCGTCCCTAGGACGAACTACTGCCAT

TAATATCTCTATTATAGCTTCTGGACATAAATCATCTATTATACCAGAATTAATGGGAACTATTCCGTAT

CTATCTAACATAGTTTTAAGAAAGTCAGAATCTAAGACCTGATGTTCATATATTGGTTCATACATGAAAT

GATCTCTATTGATGATAGTGACTATTTCATTCTCTGAAAATTGGTAACTCATTCTATACACGCTTTCCTT

GTTGATAAAGGATAGTATATACTCAATGGAATTTGTACCAACAAACTGTTCTCTTATGAATCGTATATCA

TCATCTGAAATGATCATGTAAGGCATACATTTAACAATAAGAGACTTGTCTCCTGTTATCAATATACTAT

TCTTGTGATAATTTATGTGTGCGGCAAATTTGTCCACGTTCTTTAATTTTGTTATAGTAGATATCAAATC

CAATGGAGATACAGTTCTTGGCTTAAACAGATATAGTTTTTCTGGAACGAATTCTACAACATTATTATAA

AGGACTTTGGGTATATAAGTGGGATGAAATCCTATTTTAATTAATGCGATAGCCTTGTCCTCGTGCAGAT

ATCCAAACGCTTTTGTGATAGTATGGCATTCATTGTCTAGAAACGCTCTACGAATATCTGTTACAGATAT

CATCTTTAGAGAATACTAGTCGCGTTAATAGTACTAAAATTTGTATTTTTTAATCTATCTCAATAAAAAA

TTAATATGTATGATTCAATGTATAACTAAACTACTAACTGTTATTGATAACTAGAATCAGAATCTAATGA

TGACATAACTAAGAAGTTTATCTACAGCCAATTTAGCTGCATTATTTTTAGCATCTCGTTTAGATTTTCC

ATCTGCCTTATCGAATACTCTTCCGTCAATGTCTACACAGGCATAAAATGTAGGAGAGTTACTAGGCCCC

ACTGATTCAATACGAAAAGACCAATCTCTCCTAGTTATTTGACAGTACTCATTAATAACGGTGACAGGGT

TAACACCTTTCCAATAAATAATTTTTTTAACCGGAATAACATCATCAAAAGACTTATGATCCTCTCTCAT

TGATTTTTCGCGGGATACATCATCTATTATAGCATCAGCATCAGAATCTGTAGGCCGTGTATCAGCATCC

ATTGTCGTAGACCAACGAGGAGGAGTATCGTTGGAGCTGTAAACCATAGCACTACGTTGAAGATCATACA

GAGCTTTATTAACTTCTCGCTTCTCCATATTAAGTTGTTTAGTTAGTTGTACAGCAGTAGCTCCTTAGTC

CAATGTTTTTAATAACCGCACACAATCTCTGTGTCAGAACGCTCGTCAATATAGATCTTAGAAATTTTTT

TAGAGAGAACTAACGCAACTAGCAATAAAACTGATCTTATTTTATCATTTTTTTTTATTCATCATCCTCT

GGTGGTTCGTCGTTCCTATCGAATGTAGCTCTGATTAACCCGTCATCTATAGGTGATGCTGGTTCTGGAG

ATTCTGGAGGAGATGGATTATTATCTGGAAGAATCTCTGTTATTTCCTTGTTTTCATGTATCGATTGCGT

TGTAACATTAAGATTGCGAAATGCTCTAAATTTGGGAGGCTTAAAGTGTTGTTTACAATCTCTACACGCG

TGTCTAACTAATGGAGGTTCGTCAGCGGCTCTAGTTTGAATCATCATCGGTGTAGTATTCCTACTTTTAC

AGTTAGGACACGGTGTATTGTATTTCTCGTCGAGAACGTTAAAATAATCGTTGTAACTCACATCCTTTAT

TTTATCTATATTGTATTCTACTCCTTTCTTAATGCATTTTATACCGAACAAGAGATAGCGAAGGAATTCT

TTTTCGGTACCGCTAGTACCCTTAATCATATCACATAGTGTTTTATATTCTAAATGTGTGGCAATGGACG

GTTTATTTCTATACGATAGTTTGTTTTTGGAATCCTTTGAGTATTCTATACCAATATTATTCTTTGATTC

GAATTTAGTTTCTTCGATATTAGATTTTGTATTACCTATATTCTTGATGTAGTACTTTGATGATTTTTCC

ATGGCCCATTCTATTAAGTTTTCCAAGTTGGCATCATCCACATATTGTGATAGTAATTCTCGGATATCAG

TAGTGACTACAGCCATTGATATTTGTTCATTTGATGAGTAACTACTAATGTATACATTTTCCATTTATAA

CACTTATGTATTAACTTTGTTTATTTATATTTTTTCATTATTATGTTGATATTAATAATCGTATTGTGGT

TATATGGCTACAATTTCATAATGAGTTGAAGTCAGTGTCCTATGATCAATGACGATAGCTTTACTCTGAA

AAGAAAGTATCAAATCGATAGTGCAGAGTCAACAATGAAAATGGATAAGACGATGACAAAGTTTCAGAAT

AGAATCAAAATGGTAAAAGAAATAAATCAGACGATAAGAGCAGCACAAACTCATTACGAGACATTGAAAC

TAGGATATATAAAATTTAAGGGAATGATTAGGACTACTACTCTAGAAGATATAGCACCATCTATTCCAAA

TAATCAGAAAACTTATAAACTATTCTCGGACATTTCAGTCATTGGCAAAGCATCACAGAATCCGAGTAAG

ATGATATATGCTCGCTGCTTTACATGTTTCCCAATTTGTTTGGAGATGACCATAGATTCATTTGTTATAG

AATGCATCCAACATTGTTCATGATATAGTTGAATCATGTATGCCTGTTCGTATGCCTGTGGCTAAGATAC

TGTGTAAAGAAATGGTAAATAAATACTTTGAGAATCTTTAAGAGTGCATTGACTTTGTTAGTGAATAGGC

ATTCCATCTTTCTCCAATACTAATTCAAATTGTTAAATTAATAATGGAATAGTATAAATAGTTATTAGTG

ATAAGATAGTAAACATAATTATTAGAATAGTAGTGTAGTATCATAGATAACTCTCTTCTATAAAAAATGG

ATTTTATTCGTAGAAAGTATCTTATATACACAGTAGAAAATAATATAGATTTTTTAAAGGATGATACATT

AAGTAAAGTAAACAATTTTACCCTCAATCATGTACTAGCTCTCAAGTATCTAGTTAGCAATTTTCCTCAA

CATGTTATTACTAAGGATGTATTAGCTAATACCAATTTTTTTGTTTTCATACATATGGTACGATGCTGTA

AAGTATACGAAGCGGTTTTACGACACGCATTTGATGCACCCACGTTGTACGTTAAAGCATTGACTAAGAA

TTATTTATCGTTTAGTAACACAATACAGTCGTACAAGGAAACCGTGCATAAACTAACACAAGATGAAAAA

TTTTTAGAGGTTGCCGAATACATGGACGAATTAGGAGAACTTATAGGCGTAAATTATGACTTAGTTCTTA

ATCCATTATTTCACGGAGGGGAACCCATCAAAGATATGGAAATCATTTTTTTAAAACTGTTTAAGAAAAC

AGACTTCAAAGTTGTTAAAAAATTAAGTGTTATAAGATTACTTATTTGGGCATACCTAAGCAAGAAAGAT

ACAGGCATAGAGTTTGCGGATAATGATAGACAAGATATATATACTCTATTTCAACAAACTGGTAGAATAG

TCCATAGCAATCTAACAGAAACGTTTAGGGATTATATCTTTCCCGGAGATAAGACTAGCTATTGGGTGTG

GTTAAACGAAAGTATAGCTAATGATGCGGATATCGTTATTAATAGACCCGCCATTACCATGTATGATAAA

ATTCTTAGTTATATATACTCTGAGATAAAACAGGGACGCGTTAATAAAAACATGCTTAAGTTAGTTTATA

TCTTTGAGCCTGAAAAAGATATCAGAGAACTTCTGCTAGAAATCATATATGATATTCCTGGAGATATCCT

ATCTATTATTGATGCAAAAAACGACGATTGGAAAAAATATTTTATTAGTTTTTACAAAGCTAATTTTATT

AACGGTAATACATTTATTAGTGATAGAACGTTTAACGATGACTTATTCAGAGTTGTTGTTCAAATAGATC

CCGAATATTTCGATAATGAACGAATTATGTCTTTATTCTATACGAGTGCTGCGGACATTAAACGATTTGA

TGAGTTAGATATTAATAACAGTTATATATCTAATATAATTTATGAGGTGAACGATATCACATTAGATACA

ATGGATGATATGAAGAAGTGTCAAATCTTTAACGAGGATACGTTGTATTATGTTAAGGAATACAATACAT

ACCTGTTTTTGCACGAGTCGGATCCCATGGTCATAGAGAACGGAATACTAAAGAAACTGTCATCTATAAA

ATCCAAGAGTAGACGGCTGAACTTGTTTAGCAAAAACATTTTAAAATATTATTTAGACGGACAATTGGCT

CGTCTAGGTCTTGTGTTAGATGATTATAAAGGAGACTTATTAGTTAAAATGATAAACCATCTCAAATCTG

TGGAGGATGTATCCGCATTCGTTAGATTTTCTACAGATAAAAACCCTAGTATTCTTCCATCGCTAATCAA

AACTATTTTAGCTAGTTATAATATTTCCATCATCGTCTTATTTCAAAGGTTTTTAAGAGATAATCTATAT

CATGTAGAAGAATTCTTGGATAAAAGCATCCATCTAACCAAGACGGATAAGAAATATATACTTCAATTGA

TAAGGCACGGTAGATCATAGAACAAACCAAATATATTATTAATAATTTGTATATACATAGATATAATTAT

CACATATTAAAAAATAACACATTTTTGATAAATGGAAACCGTTGCAACAATTCAGACTCCCACCAAATTA

ATGAATAAAGAAAATGCAGAAATGATTTTGGAAAAAATTGTTAATCATATAGCTATGTATATTAGTGACG

AATCAATATATTCAGAAAATAATCCTGAATATATTGATTTTCGTAACAGATACGGAGACTATAGATCTCT

CATTATAAAAAGTGATCACGAGTTTGTAAAGCTATGTAAAGATCATGCAGAGAAAAGTTCTCCAGAAACG

CAACAAATGATTATCAAACACATATACGAACAATATCTTATTCCAGTATCTGAAGTACTATTAAAACCTA

TAATGTCCATGGGTGACATATTTACATATAACGGATGTAAAGACAATGAATGGATGCTAGAACAACTCTC

TACCCTAAACTTTAACAATCTCTACACATGGAACTCATGTAGCATAGGCAATGTAACGCGTCTGTTTTAT

ACATTTTTTAGTTATCTGATGAAAGATAAACTAAATATATAAGTATAATCCCATTCTAATACTTTAACCT

GATGTATTATTACCTGCATCTTATTAGAATATTAACCTAACTAAAAGACATAAAAAGCGGTAGGATATAA

ATATTATGGCCGCAACCGTTCCGCGTTTTGACGATGTGTACAAAAATGCACAAAGAAGAATTCTAGATCA

AGAAACATTTTTTAGTAGAGGTCTAAGTAGACCGTTAATGAAAAACACATATCTATTTGATAATTACGCG

TATGGATGGATACCAGAAACTGCAATTTGGAGTAGTAGATACGCAAACCTAGATGCTAGTGACTATTATC

CCATTTCGTTGGGATTACTTAAAAAGTTTGAATTTCTCATGTCTCTATATAAAGGTCCTATTCCCGTATA

TGAAGAAAAAGTAAATACTGAATTCATTGCTAATGGATCTTTCTCCGGTAGATACGTATCATATCTTAGA

AAGTTTTCTGCCCTTCCAACAAACGAGTTTATTAGTTTTTTATTATTGACCTCCATCCCTATCTATAATA

TCTTATTCTGGTTTAAAAACACACAGTTTGATATTACTAAACACACATTATTCAGATACGTCTATACAGA

TAATACCAAACACCTTGCGTTGGCTAGGTATATACATCAAACAGGAGACTATAAGCCTTTGTTTAGTCGT

CTCAAAGAGAATTATATATTTACTGGTCCCGTTCCAATAGGTATCAAAGATATAGATCACCCTAATCTTA

GTAGAGCAAGAAGTCCATCCGATTATGAGACATTAGCTAATATTAGTACTATATTGTACTTTACCAAGTA

TGATCCAGTATTAATGTTTTTATTGTTTTACGTACCTGGGTATTCAATTACTACAAAAATTACTCCAGCC

GTAGAATATCTAATGGATAAACTGAATCTAACAAAGAGCGACGTACAACTGTTGTAAATTATTTTATGCT

TCGTAAAATGTAGGTCTTGAACCAAACATTCTTTGAAAAAATGAGATGCATAAAACTTTATTATCCAATA

GATTAACTATTTCAGACGTCAATCGTTTAAAGTAAACTTCGTAAAATATTCTTTGATTGCTGCCGAGTTT

AAAACTTCTATCGATAATTGTTTCATATGTTTTAATATTTACAAGTTTTTTGGTCCATGGTACATTAGCT

GGACAGATATATGCAAAATAATATCGTTCTCCAAGTTCTATAGTCTCTGGATTGTTTTTATTATATTCAG

TAACCAAATACATATTAGGGTTATCTGCGGATTTATAATTTGAGTGATGCATTCGACTCAACATAAATAA

TTCTAGAGGAGACGATCTACTATCAAATTCGGATCGTAAATCTGTTTCTAAAGAACGGAGAATATCTATA

CATACCTGATTAGAATTCATCCGTCCTTCAGACAACATCTCAGACAGTCTGGTCTTGTATGTCTTAATCA

TATTCTTATGAAACTTGGAAACATCTCTTCTAGTTTCACTAGTACCTTTATTAATTCTCTCAGGTACAGA

TTTTGAATTCGACGATGCCGAGTATTTCATCGTTGTATATTTCTTCTTCGATTGCATAATCAAATTCTTA

TATACCGCCTCAAACTCTATTTTAAAATTATTAAACAATACTCTACTATTAATCAGTCGTTCTAACTCCT

TTGCTATTTCTATGGACTTATCTACATCTTGACTGTCTATCTCTGTAAACACGGAGTCGGTATCTCCATA

CACGCTACGAAAACGAAATCTATAATCTATAGGCAACGATGTTTTCACAATCGGATTAATATCTCTATCG

TCCATATAAAATGGATTACTTAATGTATTGGCAAACCGTAACATACCGTTGGATAACTCTGCTCCATTTA

GTACCGATTCTAGATACAATATCATTCTACGTCCTATGGATGTGCAACTCTTAGCCGAAGCGTATGAGTA

TAGAGCACTATTTCTAAATCCCATCAGACCATATACTGAGTTGGCTACTATCTTGTACGTATATTGCATG

GAATCATAGATGGCCTTTTCAGTTGAACTGGTAGCCTGTTTTAACATCTTTTTATATCTGGCTCTCTCTG

CCAAAAATGTTCTTAATAGTCTAGGAATGGTTCCTTCTATTGATCTATCGAAAATTGCTATTTCAGAGAT

GAGGTTCGGTAGTCTAGGTTCACAATGAACCGTAATATATCTAGGAGGTGGATATTTCTGAAGCAAGAGT

TGATTATTTATTTCTTCTTCCAATCTATTGGTACTAACAACGACACCGACTAATGTTTCCGGAGATAGAT

TTCCAAAGATACACACATTAGGATACAGACTGTTATAATCAAAGATTAATACATTATTACTAAACATTTT

TTGTTTTGGAGCAAATACCTTACCGCCTTCATAAGGAAACTTTTGTTTTGTTTCTGATCTGACTAAGATA

GTTTTAGTTTCCAACAATAGCTTTAACAGTGGACCCTTGATGATTGTACTCGCTCTATATTCGAATACCA

TGGATTGAGGAAGCACATATGTTGCCGCACCAGCGTCTGTTTTTGTTTCTACTCCATAATACTCCCACAA

ATACTGACACAAACAAGCATCATGAATACAGTATCTAGCCATATCTAAAGCTATGTTTAGATTATAATCC

TTATACATCTGAGCTAAATCAATGTCATCCTTTCCGAAAGATAATTTATATATATCATTAGGTAAAGTAG

GACATGATAGTACGACTTTAAATCCATTTTCCCAAATATCTTTACGAATTACTTTACATATAATATCCTC

ATCAACAGTCACGTAATTACCTGTGGTTAAAACCTTTGCAAATGTATCGGCTTTGCCTTTCGCGTCCGTA

GTATCGTCACCGATGAACGTCATTTCTCTAACTCCTCTATTTAATACTTTACCCATGCAACTGAACGCGT

TCTTGGATATAGAATCCAATTTGTACGAATCCAATTTTTCAGATTTTTGAATGAATGAATATAGATCGAA

AAATATAGTTCCATTATTGTTATTAACGTGAAACGTAGTATTGGCCATGCCGCATACTCCCTTATGACTA

GACTGATTTCTCTCATAAATACAGAGATGTACAGCTTCCTTTTTGTCTGGAGATCTAAAGATAATCTTCT

CTCCTGTTAATAACTCTAGACGATTAGTAATATATCTCAGATCAAAGTTATGTCCGTTAAAGGTAACGAC

GTAGTCGAACGTTAGTTCCAACAATTGTTTAGCTATTCGTAACAAAACTATTTCAGAACATAGAACTAGT

TCTCGTTCGTAATCCATTTCCATTAGCGACTGTATCCTCAAACATCCTCTATCGACGGCTTCTTGTATTT

CCTGTTCCGTTAACATCTCTTCATTAATGAGCGTAAACAGTAATCGTTTACCACTTAAATCGATATAACA

GTAACTTGTATGCGAGATTGGGTTAATAAATACAGAAGGAAACTTCTTATCGAAGTGACACTCTATATCT

AGAAATAAGTACGATCTTGGGATATCGAATCTAGGTATTTCTTTAGCGAAACAGTTACGTGGATCGTCAC

AATGATAACATCCATTGTTAATCTTTGTCAAATATTGCTCGTCCAACGAGTAACATCCGTCTGGAGATAT

CCCGTTAGAAATATAAAACCAACTAATATTGAGAAATTCATCCATGGTGGCATTTTGTATGCTGCGTTTC

TTTGGCTCTTCTATCAACCACATATCTGCGACGGAGCATTTTCTATCTTTAATATCTAGATTATAACTTA

TTGTCTCGTCAATGTCTATAGTTCTCATCTTTCCCATCGGCCTCGCATTAAATGGAGGAGGAGATAATGA

CTGATATATTTCGTCCGTCACTACGTAATAAAAGTAATGAGGAAATCGTATAAATACGGTCTCGCCATTT

CGACATCTGGATTTCAGATATAAAAATCTGTTTTCACCGTGACTTTCAAACCAATTAATACACCTAACAT

CCATTTCTAGAATTTAGAAATATATTTTCATTTAAATGAATCCCAAACATTGGGGAAGAGCCGTATGGAC

CATTATTTTTATAGTACTTTCGCAAGCGGGTTTAGACGGCAACATAGAAGCGTGTAAACGAAAACTATAT

ACTATAGTCAGCACTCTTCCATGTCCTGCATGTAGACGACACGCGACTATCGCTATAGAGGACAATAATG

TCATGTCTAGCGATGATCTGAATTATATTTATTATTTTTTCATCAGATTATTTAACAATTTGGCATTTGA

TCCCAAATACGCAATCGATGTGTCAAAGGTTAAACCTTTATAAACTTAACCCATTATAAAACTTATGATT

AGTCACGACTGAAATAACCGCGTGATTATTTTTTGGTATAATTCTACACGGCATGGTTTCTGTGACTATG

AATTCAACACCTGTTATCTTAGTGAAATCTTTAACAAACAGCAAGGGTTCGTCAAAGACATAAAACTCAT

TGTTTACGATCGAAATAGACCCCCTATCACACTTAAAATAAAAAATATCCTTATCCTTTACCACCAAATA

AAATTCTGATTGGTCAATGTGAATGTATTCACTTAACAGTTCCACAAATTTATTTATTAACTCCGAGGCA

CATACATCGTCGGTATTTTTTATGACAAACTTTACTCTTCCAGCATCCGTTTCTAAAAAAATATTAACGA

GTTCCATTTATATCATCCAATATTATTGAAATGACGTTGATGGACAGATGATATAAATAAGAAGGTACAG

TACCTTTGTCCACCATCTCCTCCAATTCATACTCTATTTTGTCATTAACTTTAATGTGTGAAAACAGTAC

GCCACATGCTTCCATGACAGTGTGTAACACTTTGGATACAAAATGTTTGACATTAGTATAATTGTCCAAG

ACTGTCAATCTATAATAGATAGTAGCTATAATATATTCTATGATGGTATTGAAGAAGATGACAACCTTGG

CATATTGATCATTTAACACAGACATGGTATCAACAAATAGCTTAAATGAAAGAGAATCAGTAATTGGAAT

AAGCGTCTTCTCGATGTAGTGTCCGTATACCAACATGTCTGATATTTTGATGTATTCCATTAAATTATTT

AGTTTTTTCTTTTTATTCTCGTTAAACAGAATTTCTGTCAATGGACCCCAACATCGTTGACCTATTAAGT

TTTGATTGATTTTTCCGTGTAAGGCGTATCTAGTCAGATCGTATAGCCTATCCAATAATCCATCGTCTGT

GCGTAGATCACATCGTACACTTTTTAATTTTCTATAGAAGAGTGACAGACATCTGAAGCAATTACAGACA

GCAATTTCTTTATTCTCTACAGATGTAAGATACTTGAAGACATTCCTATGATGATGCAGAATTTTGGATA

ACACGGTATTGATGGTATCTGTTACCATAATTCCTTTGACTGATAGTGTCAAAGTACAAGATTTCCAATC

TTTTGCAATTTTCAGTACCATTATCTTTGTTTTGATATCTATATCAGACAGCATGGTACGTCTGACAACA

CAGGGATTAAGACGGAAAGATGAAATGATTCTCTCAACATCTTCAATAGATACCTTGCTATTTTTTTTGG

CATTATCTATATGTGAGAGAATATCCTCTAGAGAATCAGTATCCTTTTTGATGATAGTGGATCTCAATGA

CATGGGACGTCTAAACCTTCTTATTCTATCACCAGATTGCATGGTGATTTGTCTTCTTTCTTTTATCATG

ATGTAATCTCTAAATTCATTGGCAAATTGTCTATATCTAAAATCATAATATGAGATGTTTACCTCTACAA

ATATCTGTTCGTCCAATGTTAGAGTATCTATATCAGTTTTGTATTCCAAATTAAACATGGCAACGGATTT

AATTTTATATTCCTCTATTAAGTCCTCGTCGATAATAACAGAATGTAGATAATCATTTAATCCATCGTAC

ATGGTTGGAAGATGCTCGTTGACAAAATCTTTAATTGTCTTGATGAAGGTGGGACTATATCTAACATCTT

GATTAATAAAATTTATAACATTGTCCATAGGATACTTTGTAACTAGTTTTATACACATCTCTTCATTGGT

AAGTTTAGACAGAATATCGTGAACAGGTGGTATATTATATTCATCAGATATACGAAGAATAATGTCCAAA

TCTATATTGTTTAATATATTATATAGATGTAGTGTAGCTCCTACAGGAATATCTTTAACTAAGTCAATGA

TTTCATCAACAGTTAGATCTATTTTAAAGTTAATCATATAGGCATTGATTTTTAAAAGGTATGTAGCCTT

GACTACATTCTCATTAATTAACCATTCCAAGTCACTGTGTGTAAGAAGATTATATTCTATCATAAGCTTG

ACTACATTTGGTCCCGATACCATTAAAGAATTCTTATGATATAAGGAAACAGCTTTTAGGTACTCATCTA

CTCTACAAGAATTTTGGAGAGCCTTAACGATATCAGTGACGTTTATTATTTCAGGAGGAAAGAACCTAAC

ATTGAGAATATCTGAATTAATAGCTTCCAGATACAGTGATTTTGGCAATAGTCCGTGTAATCCATAATCC

AGTAACACGAGCTGGTGCTTGCTAGACACCTTTTCAATGTTTAATTTTTTTGAAATAAGCTTTGATAAAG

CCTTCCTCGCAAATTCCGGATACATGAACATGTCGCCAACATGATTAAGTATTGTTTTTCATTATTTTTA

TATTTTCTCAACAAGTTCTCAATACCCCAATAGATAATAGAATATCACCCAATGCGTCCATGTTGTCTAT

TTCCAACAGGTCGCTATATCCACCAATAGAAGTTTTCCCAAAAAAGATTCTAGGAACAGTTCTACCACCA

GTAATTTGTTCAAAATAGTCACGCAATTCATTTTCGGGTTTAAATTCTTTAATATCTACAATTTCATACG

CTCCTCTTTTGAAACTAAACTTATTTAGAATATCCAGTGCGTTTCTACAAAAAGGACATGTAAACTTGAC

AAAAATTGTCACTTTGTTATTGGCCAACCTTTGTTGTACAAATTCCTCGGCCATTTTTAATATTTAAGTG

ATACAAAACTATCTCGACTTATTTAACTCTTTAGTCGAGATATATGGACACAGATAGCTATATGATAACC

AACTACAGAAGACAAACGCTATAAAAAACATAATTACGACGAGCATATTTATAAATATTTTTATTCAGTA

TTACTTGATATAGTAATATTAGGCACAGTCAAACATTCAACCACTCTAGATACATTAACTCTCTCATTTT

CTTTAACAAATTCTGCAATATCTTCGTAAAAAGATTCTTGAAACTTTTTAGAATATCTATCGACTCTAGA

TGAAATAGCGTTCGTCAACATACTATGTTTTGTATACATAAAGGCGCCCATTTTAACAGTTTCTAGTGAC

AAAATGCTAGCGATCCTAGGATCCTTTAGAATCACATAGATTGACGATTCGTCTCTCTTAGTAACTCTAG

TAAAATAATCATACAATCTAGTACGCGAAATAATATTATCCTTGACTTGAGGAGATCTAAACAATCTAGT

TTTGAGAACATCGATAAGTTCATCGGGAATTACATACATACTATCTTTAATAGAACTCTTTTCATCCAGT

TGAATGGATTCGTCCTTAACCAACTGATTAATGAGATCTTCTATTTTATCATTTTCTAGATGATATGTAT

GTCCATTAAAGTTAAATTGTGTAGCGCTTCTTTTTAGCCTAGCAGCCAATACTTTAACATCACTAATATC

GATATACAAAGGAGATGATTTATCGATGGTATTAAGAATTCGTTTTTCGACATCCGTCAAAACCAATTCC

TTTTTGCCTGTATCATCCAGTTTGCCATTCTTTGTAAAGAAATTATTTTCTACTAGACTATTAATAAGAC

TGATAAGGATTCCTCCATAATTGCACAATCCAAACTTTTTCACAAAACTAGATTTTACGAGATCTACAGG

AATGCGTACTTCAGGTTTCTTAGCTTGTGATTTTTTCTTTTGCGGACATTTTCTAGTTACCAACTCATCT

ACCATTTCATTGATTTTAGCAGTGAAATAAGCTTTCAATGCACGGGCACTGATACTATTGAAAACGAGTT

GATCTTCAAATTCCGCCATTTAAGTTCACCAAACAACTTTTAAATACAAATATATCAATAGTAGTAGAAT

AAGAACTATAAAAAAAATAATAATTAACCAATACCAACCCCAACAACCTGTATTATTAGTTGATGTGACA

GTTTTCTCATCACTTAGAACAGATTTAACAATTTCTATAAAGTCTGTCAAATCATCTTCCTGAGAACCCA

TAAATACACCAAATATAGCAGCGTACAACTTATCCATTTATACATTGAATATTGGCTTTTCTTTATCGCT

ATCTTCATCATATTCATCATCAATATCAACAAGTCCCAGATTACGAACCAGATCTTCTTCTACATTTTCA

GTCATTGATACGCGTTCACTATCTCCAGAGAGTCCGATAACGTTAGCCACTACTTCTCTATCAATGATTA

GTTTCTTGAGCGCGAATGTAATTTTTGTTTCCGTTCCGGATCTATAGAAAACTACAGGTGTAATAATTGC

CTTGGCTAATTGTCTTTCTCTTTTACTGAGTGATTCTAGTTCACCTTCTATAGATCTGAGAATGGATGAT

TCTCCAGTCGAAACATATTCTACCATGGCTCCGTTTAATTTGTTGATGAAGATGGATTCATCCTTAAATG

TTTTCTCTGTAATAGTTTCCGCCGAAAGACTATGCAAAGAATTTGGAATGCGTTCCTTGTGTGTAATGTT

TCCATAGACAGCTTCTAGAAGTTGATACAACATAGGACTAGCCGCGGTAACTTTTATTTTTAGAAAGTAT

CCATCGCTTCTATCTTGTTTAGATTTATTTTTATAAAGTTTAGTCTCTCCTTCCAACATAATAAAAGTGG

AAGTCATCTGACTAGATAAACTATCAGTAAGTTTTATAGAGATAGATGAACAATTAGCGTATTGAGAAGC

ATTTAGTGTAACGCATTCGATACATTTTGCATTAGATTTACTAATCGATTTTGCATACTCTATAACACCC

GCACAAGTCTGTAGAGAATCGCTAGATGCTGTAGGTCTTGGTGAAGTTTCAACTCTCTTCTTGATTACCT

TACTCATGATTAAACCTAAATAATTGTACTTTGTAATATAATGATATATATTTTCACTTTATCTCATTTG

AGAATAAAAATGTTTTTGTTAACCACTGCATGATGTACAGATTTCGGAATCGCAAACCACTTGTGGTTTT

ATTTTATCCTTGTCCAATGTGAATTGAATGGGAGCGGATGCGGGTTTCGTACGTAGATAGTACATTCCCG

TTTTTAGACCGAGACTCCATCCGTAAAAATGCATACTCGTTAGTTTGGAATAACTCGGATCTGCTATATG

GATATTCATAGATTGACTTTGATCGATGAAGGCTCCCCTGTCTGCAGCCATTTTTATGATCGTCTTTTGT

GGAATTTCCCAAATAGTTTTATAAACTCGCTTAATATCTTCTGGAAGGTTTGTATTCTGAATGGATCCAC

CATCTACCATAATCCTATTCTTGATCTCATCATTCCATAATTTTCTCTCGGTTAAAACTCTAAGGAGATG

CGGGTTAACTACTTGGAATTCTCCAGACAATACTCTCCGAGTGTAAATATTACTGGTATACGGTTCCACC

GACTCATTATTTCCCAAAATTTGAGCAGTTGATGCAGTCGGCATAGGTGCCACCAATAAACTATTTCTAA

GACCGTATGTTCTGATTTTATCTTTTAGAGGTTCCCAATTCCAAAGATCCGACGGTACAACATTCCAAAG

ATCATATTGTAGAATACCGTTACTGGCGTACGATCCTACATATGTATCATATGGTCCTTCCTTCTCAGCT

AGTTTACAACTCGCCTCTAATGCACCGTAATAAATGGTTTCAAAGATCTTCTTATTTAGATCTTGTGCTT

CCAGGCTATCAAATGGATAATTTAAGAGAATAAACGCGTCCGCTAATCCTTGAACACCAATACCGATAGG

TCTATGTCTCTTATTAGAGATTTCAGCTTCTGGAATAGGATAATAATTAATATCTATAATTTTATTGAGA

TTTCTGACAATTACTTTGACCACATCCTTCAGTTTGAGAAAATCAAATCGCCCATCTATTACAAACATGT

TCAATGCAACAGATGCCAGATTACACACGGCTACCTCATTAGCATCCGCATATTGTATTATCTCAGTGCA

AAGATTACTACACTTGATGGTTCCTAAATTTTGTTGATTACTCTTTTTGTTACACGCATCCTTATAAAGA

ATGAATGGAGTACCAGTTTCAATCTGAGATTCTATAATCGCTTTCCAGACGACTCGAGCCTTTATTATAC

ATTTGTATCTCCTTTCTCTTTCGTATAGTGTATACAATCGTTCGAACTCGTCTCCCCAAACATTGTCCAA

TCCAGGACATTCATCCGGACACATCAACGACCACTCTCCGTCATCCTTCACTCGTTTCATAAAGAGATCA

GGAATCCAAAGAGCTATAAATAGATCTCTTGTTCTATGTTCATCGTTTCCTGTATTCTTTTTAAGATCGA

GGAACGCCATAATATCAGAATGCCACGGTTCCAAGTATATGGCCATAACTCCAGGCCGTTTGTTTCCTCC

CTGATCTATGTATCTAGCGGTGTTATTATAAACTCTCAACATTGGAATAATACCGTTTGATATACCATTG

GTACCGGAGATATAGCTTCCACTGGCACGAATATTACTAATTGATAGACCTATTCCCCCTGCCATTTTAG

AGATTAATGCGCATCGTTTTAACGTGTCATAGATGCCTTCTATGCTATCATCGATCATGTTAAGTAGAAA

ACAGCTAGACATTTGGTGACGAGTAGTTCCCGCATTAAATAAGGTAGGAGAAGCGTGCGTAAACCATTTT

TCAGAAAGTAGATTGTACGTCTCAATAGCTGAGTCTATATCCCATTGATGAATTCCTACTGCGACACGCA

TTAACATGTGCTGAGGTCTTTCAACAATTTTGTTGTTTATTTTCAACAAGTAGGATTTTTCCAAAGTTTT

AAAACCAAAATAGTTGTATGAAAAGTCTCGTTCGTAAATAATAACCGAATTGAGCTTATCCTTATATTTG

TTAACTATATCCATGGTAATACTTGAAATAATCGGAGAATGTTTCCCATTTTTAGGATTAACATAGTTGA

ATAAATCCTCCATCACTTCACTAAATAGTTTTTTTGTTTCCTTGTGTAGATTTGATATGGCTATTCTGGC

GGCTAGAATGGCATAATCCGGATGTTGTGTAGTACAAGTGGCTGCTATTTCGGCTGCCAGAGTGTCCAAT

TCTACCGTTGTTACTCCATTATATATTCCTTGAATAACCTTCATAGCTATTTTAATAGGATCTATATGAT

CAGTGTTTAAGCCATAGCACAATTTTCTAATACGAGACGTGATTTTATCAAACATGACATTTTCCTTGTA

TCCATTTCGTTTAATGACAAACATTTTTGTTGGTGTAATAAAAAAAAATTATTTAATTTTTCATTAATAG

GGATTTGACGTATGTAGCGTACAAAATTATCGTTCCTGGTATATAGATAAAGAGTCCTATATATTTGAAA

ATCGTTACGGTTCGATTAAACTTTAATGATTGCATTGTGAATATATCATTAGGATTTAACTCCTTGACTA

TCATGGCGGTGCCAGAAATTACCATCAAAAGCATTAATACAGTTATGCAGATCGCAGTTAGAACGGTTAT

AGCATCCACCATTTATATCTAAAAATTAGATCAAAGAATATGTGACAACGTCCTAGTTGTATACTGAGAA

TTGACGAAACAATGTTTCTTACATATTTTTTTCTTATTAGTAACCGACTTAATAGTAGGAACTGGAAAAC

TAGACTTGATTATTCTATAAGTATAGATACCCTTCCAAATAATGTTCTCTTTGATAAAAGTTCCAGAAAA

TGTAGAATTTTTTAAAAAGTTATCTTTTGCTATTACTAATATCGTGGTTAGACGCTTATTATTAATATGA

GTGATGAAATCCACACCGCTTCTAGATATCGCTTTTATTTCCACATTAGATGGTAAATCCAATAGTGAAA

CTATCTTTTTAGGAATGTATGGACTCGCGTTTAGAGGAGTGAACGTCTTCGGAGTAGTAAAGGATGATTC

GTCAAATGAATAAACAATTTCACAAATGGATGTTAATGTATTAGTAGGAAATTTTTTGACGCTAGTGGAA

TTGAAGATTCTAATGGATGATGTTCTACCTATTTCATCCGATAACATGTTAATTTCCAATACCAACGGTT

TTAATATTTCGATGATATACGGTAGTCTCTCTTTCGGACTTATATAGCTTATTCCACAATACGAGTCATT

ATATACTCCAAAAAACAAAATAACTAGTATAAAATCTGTATCGAATGGGAAAAACGAAATTATCGATATA

GGTATAGAATCCGGAACATTGAACGTATTAATACTTAATTCTTTTTCAGTGGTAAGAACCGATAGGTTAT

TGACATTGTATGGTTTTAAATATTCTATAACTTGAGACTTGATAGATATTAATGACGAATTGAAAATTAT

TTTTATCACCACGTGTGTTTCAGGATCATCGTCGACGCCAGTTAACCAACCGAATGGAGTAAAATAAATA

TCATTAATATATGCTCTAGATATTAGTATTTTTATTAATCCTTTGATTATCATCTTCTCGTACGCGAATG

ATTCCATGATCAAGAGTGATTTGAGAACATCCTCCGGAGTATTAATGGGTTTAGTAAACAGTCCATCGTT

GCAATAATAAAAGTTGTCCAAGTTAAAGGATATTATGCATTCGTTTAAAGATATCACCTCATCTAACGGA

GACAATTTTTTGGTAGGTTTTAGAGACTTTGAAGCTACTTGTTTAACAAAGTTATTCATCGTCGTCTACT

ATTCTATTTAATTTTGTAGTTAATTTATCACATATCACATTAATTGACTTTTTGGTCCACTTTTCCATAC

GTTTATATTCTTTTAATCCTGCGTTATCCGTTTCCGTTATATACAGGGATAGATCTTGCAAGTTAAATAG

AATGCTCTTAAATAATGTCATTTTTTTATCCGCTAAAAATTTAAAGAATGTATAAACTTTTTTCAAAGAT

TTAAAACTTTTAGGTGGAGTTCTGGTACACAATATCATAAACAAACTAATAAACATCCCACATTCAGATT

CCAACAATTGATTAACTTCCACATTAATACAGCCTATTTTCGCTCCAAATGTACATTCGAAAAATCTGAA

TAAAACATCAATATCGCAATTTGTATTATCCAATACAGAATGTCTGTGATTCGTGTTAAAACCATCGGAA

AAAGAATAGAAATAAAAATTATTATAATGGTGGAATTCAGTTGGAATATTGCCTCCGGAGTCATAAAAGG

ATACTAAACATTGTTTTTTATCGTAAATTACACATTTCCAATGAGACAAATAACAAAATCCAAACATTAC

AAATCTAGAGGTAGAACTTTTAATTTTGTCTTTAAGTATATACGATAAGATATGTTTATTCATAAACGCG

TCAAATTTTTCATGAATAGCTAAGGAGTTTAAGAATCTCATGTCAAATTGTCCTATATAATCCACTTCGG

ATCCATAAGCAAACTGAGAGACTAAGTTCTTAATACTTCGATTGCTCATCCAGGCTCCTCTCTCAGGCTC

TATTTTCATCTTGACGACCTTTGGATTTTCACCAGTATGTATTCCTTTACGTGATAAATCATCGATTTTC

AAATCCATTTGTGAGAAGTCTATCGCCTTAGATACTTTTTCCCGTAGTTGAGGTTTAAAGAAATACGCTA

ACGGTATACTAGTAGGTAACTCAAAGACATCATATATAGAATGGTAACGCGTCGTTAACTCGTCGGTTAA

CTCTTTCTTTTGATCGAGTTCATCGCTACTATTGGGTCTGCTCAGGTGCCCCGACTCTACTAGTTCCAAC

ATCATACCGATAGGAATACAAGACACTTTGCCAGCGGTTGTAGATTTATCATATTTCTCCACCACATATC

CGTTACAATTTGTTAAGAATTTAGATACATCTATATTGCTACATAATCCAGCTAGTGAATATATATGACA

TAATAAATTGGTAAATCCTAGTTCTGGTATTTTACTAATTACTAAATCTGTATATCTTTCCATTTATCAT

GGAAAAGAATTTACCAGATATCTTCTTTTTTCCAAACTGCGTTAATGTATTCTCTTACAAATATTCACAA

GATGAATTCAGTAATATGAGTAAAACGGAACGTGATAATTTCTCATTGGCTGTGTTTCCAGTGATAAAAC

ATAGATGGCATAACGCACACGTTGTAAAACATAAAGGAATATACAAAGTTAGTACAGAAGCACGTGGAAA

AAAAGTATCTCCTCCATCACTAGGAAAACCCGCACATATAAACCTAATGTCGAAGCAATATATATATAGT

GAGTATGCAATAAGCTTTGAATGTTATAGTTTTCTAAAATGTATAACAAATACAGAAATCAATTCGTTCG

ATGAGTATATATTAAGAGGACTATTAGAAGCTGGTAATAGTTTACAGATATTTTCCAATTCCGTAGGTAA

ACGAATAGATACTATAGGTGTACTAGGGAATAAGTATCCATTTAGCAAAATTCCATTGGCCTCATTAACT

CCTAAAGCACAACGAGAGATATTTTTAGCGTGGATTTCTCATAGACCTGTAGTTTTAACTGGAGGAACCG

GAGTGGGTAAGACGTCACAGGTACCCAAGTTATTGCTTTGGTTTAATTATTTATTTGGTGGATTCTCTTC

TCTAGATAAAATCACTGACTTTCACGAAAGACCAGTCATTCTATCTCTTCCTAGGATAGCTTTAGTTAGA

TTGCATAGCAATACCATTTTAAAATCATTGGGATTTAAGGTACTAGATGGATCTCCTATCTCTTTACGGT

ACGGATCTATACCGGAAGAATTAATAAACAAACAACCAAAAAAATATGGAATTGTATTTTCTACCCATAA

GTTATCTCTAACAAAACTATTTAGTTATGGCACTATTATTATAGACGAAGTTCATGAGCATGATCAAATA

GGAGATATTATTATAGCAGTAGCGAGAAAACATCATACGAAAATAGATTCTATGTTTTTAATGACTGCCA

CGTTAGAGGATGACAGGGAACGTCTAAAAATATTTTTACCTAATCCCGCATTTATACATATTCCTGGAGA

TACACTGTTTAAAATTAGCGAGGTATTTATTCATAATAAGATAAATCCATCTTCCAGAATGGCATATATA

GAAGAAGAAAAGAGAAATTTAGTTACTGCTATACAGATGTATACTCCTCCTGATGGATCATCCGGTATAG

TCTTTGTGGCATCCGTTGCACAGTGTCACGAATATAAATCATATTTAGAAAAAAGATTACCGTATGATAT

GTATATTATTCATGGTAAGGTCTTAGATATAGACGAAATATTAGAAAAAGTGTATTCATCACCTAATGTA

TCGATAATTATTTCTACTCCTTATTTGGAATCCAGCGTTACTATACGCAATGTTACACACATTTATGATA

TGGGTAGAGTTTTTGTCCCCGCTCCTTTTGGAGGATCACAACAATTTATTTCTAAATCTATGAGAGATCA

ACGAAAAGGAAGAGTAGGAAGAGTTAATCCTGGAACATACGTATATTTCTATGATCTGTCTTATATGAAA

TCTATACAGCGAATAGATTCAGAATTTCTACATAATTATATATTGTACGCTAATAAGTTTAATCTAACAC

TCCCCGAAGATTTGTTTATAATCCCTACAAATTTGGATATTCTATGGCGTACAAAGGAATATATAGACTC

GTTCGATATTAGTACAGAAACATGGAATAAATTATTATCCAATTATTATATGAAGATGATAGAGTATGCT

AAACTTTATGTACTAAGTCCTATTCTCGCTGAGGAGTTGGATAACTTTGAGAGGACGGGAGAATTAACTA

GTATTGTACAAGAAGCCATTTTATCTCTAAATTTACGAATTAAGATTTTAAAATTTAAACATAAAGATGA

TGATACGTATATACACTTTTGTAGAATATTATTCGGTGTCTATAACGGAACAAACGCTACTATATATTAT

CATAGACCTCTAACGGGATATATGAATATGATTTCAGATACTATATTTGTTCCTGTAGATAATAACTAAA

AATCAAAATCTAATGACCACATCTTTTTTTAGAGATGAAAAATTTTCCACATCTCCTTTTGTAGACACGA

CTAAACATTTTGCAGAAAAAAGTTTATTATTATTTAGATAATCGTATACTTCATCAGTGTAGATAGTAAA

TGTGAACAGATAAAAGGTATTCTTGCTCAATAGATTGGTAAATTCCATAGAATATATTAATCCTTTCTTC

TTGAGATCCCACATCATTTCAACCAAAGACGTTTTATCCAATGATTTACCTCGTACTATACCACATACAA

AACTAGATTTTGCAGTGATGTCGTACCTGGTATTCCTACCAAACAAAATTTTACTTTTAGTTCTTTTAGA

AAATTCTAAGGTAGAATCTCTATTTGTCAATATGTCATCTATGGAATTACCACTAGCAAAAAATGATAGA

AATATATATTGATACATCGCAGCTGGTTTTGATCTACTATACTTTAAAAACGAATCAGATTCCATAATTG

CTTGTATATCATCAGCTGAAAAACTATGTTTTACACGTATTCCTTCGGCATTTCTTTTTAATGATATATC

TTGTTTAGACAATGATAAAGTTATCATGTCCATGAGAGACGCGTCTCCGTATCGTATAAATATTTCATTA

GATGTTAGACGCTTCATTAGGGGTATACTTCTATAAGGTTTCTTAATTAGTCCATCATTGGTTGCGTCAA

GAACTACTATCTGATGTTGTTGGGTATCTCTAGTGTTACACATGGCCTTACTAAAGTTTGGGTAAATAAC

TATGATATCTCTATTAATTATAGATGTATATATTTCATTCGTCAAGGATATTAATATCGACTTACTATCG

TCATTAATACGTGTAATGTAATCATATAAATCATGCGATAGCCAAGGAAAATTCAAATAGATGTTCATCA

TATAATCGTCGCTATAATTCATATTAATACTTTGACATTGACTAATTTGTAATATAGCCTCGCCACGAAG

AAAGCTCTCGTATTCAGTTTCATCGATAAAGGATACCGTTAAATATAACTGGTTGCCGATAGTCTCATAG

TCTATTAAGTGGTAAGTTTCGTATAAATACAGAATCCCTAAAATATTATCTAATGTGGGATTAATCCTTA

CCATAACTGTATAAAATGGAGCCGGAGTCATAACTATTTTACCGTTTGTACTTACTGGAATAGATGAAGG

AATAATCTCCGGACATGATGGTAAAGACCCAAATGTCTGTTTGAAGAAATCCAATGTTCCAGGTCCTAAT

CTCTTGACAAAAATTACGATATTCGATCCCGATATCCTTTGCATTCTATTTACCAGCATATCACGAACTA

TATTAAGATTATCTATCATGTCTATTCTCCCACCGTTATATAAATCGCCTCCGCTAAGAAACGTTAGTAT

ATCCATACAATGGAATACTTCATTTCTAAAATAGTATTCGTTTTCTAATTCTTTAATGTGAAATCGTATA

CTAGAAAGGGAAAAATTATCTTTGAGTTTTCCATTAGAAAAGAACCACGAAACTAATGTTCTGATTGCGT

CTGACTCCGTCGCTGAATTAATAGATTTACACCAAAAACTCATATAACTTCTAGATGTAGAAGCATTCGC

TAAAAAATTAGTAGAATCAAAGGATATAAGTAGATGTTCCAACAAGTGAGCAATTCCCAAGATTTCATCT

ATATCATTCTCGAATCCGAAATTAGAAATTCCCAAGTAGATATCCTTTTTCATCCGATCATTGATGAAAA

TACGAACTTTATTCGGTAAGACGATCATTTACTAAGGAGTAAAATAGGAAGTAACGTTCGTATATCGTTA

TCGTCGTATAAATTAAAGGTGTGTTTTTTGCCATTAAGAGACATTATAATTTTACCAATATTGGAATTAT

AATATAGGTGTATTTGAGCACTAGAAACGGTCGATGCATCGGTAAATATAGCTGTATCTAATGTTCTAGT

CGGTATTTCTTCATTTCGCTGTCTAATGATAGCGTTTTCTCTATCTGTTTCCATTACAGCTGCCTGAAGT

TTATTGGTCGGATAATATGTAAAATAATAAGAAATACATACGAATAACAAAAATAAAATAAGATATAATA

AAGATGCCATTTAGAGATCTAATTTTGTTCAACTTGTCCAAATTCCTACTTACAGAAGATGAGGAATCGT

TGGAGATAGTATCTTCCTTATGTAGAGGATTTGAAATATCTTACGATGACTTAATATCGTACTTTCCAGA

TAGGAAATACCATAAATATATTTCTAAGGTATTTGAACATGTAGATTTATCGGAGGAATTAAGTATGGAA

TTCCATGATACAACTCTGAGAGATTTAGTATATCTTAGATTGTACAAGTATTCCAAGTATATACGGCCGT

GTTATAAATTAGGAGATAATCTAAAAGGTATAGTTGTTATAAAGGACAGAAATATATATATTAGAGAAGC

AAATGATGACTTGATAGAATATCTCCTCAAGGAATACACTCCTCAGATTTATACATATTCTAATGAGCGA

GTTCCCATAGCTGGTTCAAAATTAATTCTTTGTGGATTTTCTCAAGTTACATTTATGGCGTATACAACGT

CGCATATAACAACAAATAAAAAGGTAGATGTTCTCGTTTCCAAAAAATGTATAGATGAACTAGTCGATCC

AATAAATTATCAAATACTTCAAAATTTATTTGATAAAGGAAGCGGAACAATAAACAAAATACTCAGGAAG

ATATTTTATTCGGTAACAGGTGGCCAAACTCCATAGGTAGCTTTTTCTATTTCGGATTTTAGAATTTCCA

AATTCACCAGCGATTTATCGGTTTTGGTGAAATCCAAGGATTTATTAATGTCCACAAATGCCATTTGTTT

TGTCTGTGGATTGTATTTGAAAATGGAAACGATGTAGTTAGATAGATGCGCGGCGAAGTTTCCTATTAGG

GTTCCGCGCTTCACGTCACCCAACATACTTGAATCACCATCCTTTAAAAAAAATGATAAGATATCAACAT

GGAGTATATCATACTCGGATTTTAATTCTTCTACTGCCTCACTGACATTTTCACAAATACTACAATACGG

TTTACCGAAAATAATCAGTACGTTCTTCATTTATGGGTATCAAAAACTTAAAATCGTTACTGCTGGAAAA

TAAATCACTGACGATATTAGATGATAATTTATACAAAGTATACAATGGAATATTTGTGGATACAATGAGT

ATTTATATAGCCGTCGCCAATTGTGTCAGAAACTTAGAAGAGTTAACTACGGTATTCATAAAATACGTAA

ACGGATGGGTAAAAAAGGGAGGACATGTAACCCTTTTTATCGATAGAGGAAGTATAAAAATTAAACAAGA

CGTTAGAGACAAGAGACGTAAATATTCTAAATTAACCAAGGACAGAAAAATGTTAGAATTAGAAAAGTGT

ACATCCGAAATACAAAATGTTACCGGATTTATGGAAGAAGAAATAAAGGCAGAAATGCAATTAAAAATCG

ATAAACTCACATTTCAAATATATTTATCTGATTATGATAACATAAAAATATCATTGAATGAGATACTAAC

ACATTTCAACAATAATGAGAATGTTACATTATTTTATTGTGATGAACGAGACGCAGAATTCGTTATGTGT

CTAGCGGCTAAAACACAGTTCTCTACCACAGGAGAATGGCCGTTAATAATAAGTACCGATCAGGATACTA

TGCTATTCGCGTCTGCTGATAATCATCCTAAGATGATAAAAAACTTAACTCAACTGTTTAAATTTGTTCC

CTCGGCAGAGGATAACTATTTAGCAAAATTAACTGCATTAGTGAATGGATGTGATTTCTTTCCTGGACTC

TATGGGGCATCTATAACACCCAACAACTTAAACAAAATACAATTGTTTAGTGATTTTACAATCGATAATA

TAGTCACTAGTTTGGCAATTAAAAATTATTATAGAAAGACTAACTCTACCGTAGACGTGCGTAATATTGT

TACGTTTATAAACGATTACGCTAATTTAGACGATGTCTACTCGTATATTCCTCCTTGTCAATGCACTGTT

CAAGAATTTATATTCTCCGCATTAGATGAAAAATGGAATGAATTTAAATCATCTTATTTAGAGAGCGTGC

CGTTACCCTGCCAATTAATGTACGCATTAGAACCACGTAAGGAGATTGATGTTTCAGAAGTTAAAACTTT

ATCATCTTATATAGATTTCGAAAATACTAAATCAGATATCGATGTTATAAAATCTATATCCTCGATTTTT

GGATATTCTAACGAAAACTGTAACACCATAGTGTTCGGCATCTATAAGGATAATTTACTACTGAGTATAA

ATAATTCATTTTACTTTAACGATAGTCTGTTAATAACCAATACTAAAAGTGATAATATAATAAATATAGG

TTACTAGATTAAAAAATGGTGTTCCAGCTCGTGTGTTCTACATGCGGCAAAGATATTTCTCACGAACGAT

ATAAATTGATTATACGAAAAAAATCATTAAAGGATGTACTAGTCAGTGTAAAGAACGAATGTTGTAGGTT

AAAATTATCTACACAAATAGAACCTCAACGTAACTTAACAGTGCAACCTCTATTGGATATAAACTAATGG

ATCCGGTTAATTTTATCAAGACATATGCGCCTAGAGGTTCTATTATTTTTATTAATTATGCCATGTCATT

AACTAGTCATTTGAATCCATCGATAGAAAAACATGTGGGTATTTATTATGGTACGTTATTATCGGAACAC

TTGGTAGTTGAATCTACCTATAGAAAAGGAGTTAGAATAGTCCCATTGGATAGATTTTTTGAAGGATATC

TTAGTGCAAAAGTATACATGTTAGAGAATATTCAAGTTATGAAAATAGCAGCTGATATGTCGTTAACTTT

ACTAGGTATTCCATATGGATTTGGTCATGATAGAATGTATTGTTTTAAATTGGTAGCTGAATGTTATAAA

AATGCCGGTATTGATACATCGTCTAAACGAATATTAGGTAAAGATATTTTTCTGAGCCAAAACTTTACAG

ATGATAATAGATGGATAAAGATATATGATTCTAATAATTTAACATTTTGGCAAATTGATTACCTTAAAGG

GTGAGTTAATATGCATAACTACTCCTCCGTTGTTTTTTCCCTCGTTCTTTTTCTTAACGTTGTTTGCCAT

CACTCTCATAATGTAAAGATATTCTAAAATGGTAAACTTTTGCATATCGGATGCAGAAATTGGTATAAAT

GTTGTAATTGTATTATTTCCCGTCAATGGACTAGTCACAGCTCCATCAGTTTTATATCCTTTAGAGTATT

TCTCACTCGTGTCTAGCATTCTAGAGCATTCCATGATCTGTTTATCGTTGATATTGGCCGGAAAGATAGA

TTTTTTATTTTTTATTATATTACTATTGGCAATTGTAGATATAACTTCTGGTAAATATTTTTCTACCTTT

TCAATCTCTTCTATTTTCAAGCCGGCTATATATTCTGCTATATTGTTACTAGTATCAATACCTTTTCTGG

CTAAGAAGTCATATGTGGTATTCACTATATCAGTTTTAACTGGTAGTTCCATTAGCCTTTCCACTTCTGC

AGAATAATCAGAAATTGGTTCTTTACCAGAAAATCCAGCTACTATAATAGGCTCACCGATGATCATTGGC

AAAATCCTATATTGTACCAGATTAATGAGAGCATATTTCATTTCCAATAATTCTGCTAGTTCTTGAGACA

TTGATTTATTTGATGAATCTATTTGGTTCTCTAGATACTCTACCATTTCTGCCGCATACAATAACTTGTT

AGATAAAATCAGGGTTATCAAAGTGTTTAGTGTGGCTAGAATAGTGGGCTTGCACGTATTAAAGAATGCT

GTAGTATGAGTAAACCGTTTTAACGAATTATATAGTCTCCAGAAATCTGTGGCGTTGCATACATGAACTG

AATGACATCGAAGATTGTCCAATATTTTTAATAGCTGCTCTTTGTCCATTATTTCTATATTTGACTCGCA

ACAATTGTAGATACCATTAATCACTGATTCCTTTTTCGATGCCGGACAATAGCACAATTGTTTAGCTTTG

GACTCTATGTATTCAGAATTAATAGATATATCTCTCAATACAGATTGCACTATACATTTTGAAACTATGT

CAAAAATTGTAGAACGACGCTGTTCTGTAGCCATTTAACTTTAAATAATTTACAAAAATTTAAAATGAGC

ATCCGTATAAAAATCGATAAATTGCGCCAAATTGTGGCATATTTTTCAGAGTTCAGCGAAGAAGTGTCTA

TAAATGTAGACTCGACGGATGAATTAATGTATATTTTTGCCGCCTTGGGCGGATCTGTAAACATTTGGGC

CATTATACCTCTCAGTGCATCAGTGTTCTACCGCGGAGCCGAAAATATTGTGTTTAACCTTCCAGTGTCC

AAGGTAAAATCGTGTTTGTGTAGTTTTCACAATGATGCTATCATAGATATAGAACCTGATCTGGAAAATA

ATCTAGTAAAACTTTCTAGTTATCATGTAGTAAGTGTCGATTGTAACAAGGAACTGATGCCTATTAGGAC

AGATACTACTATTTGTCTAAGTATAGATCAAAAGAAATCTTACGTATTTAATTTTCACAAGTATGAAGAA

AAATGTTGTGGTAGAACCGTCATTCATCTAGAATGGTTGTTGGGCTTTATCAAGTGTATTAGTCAGCATC

AGCATTTGGCTATTATGTTTAAAGATGACAATATTATTATGAAGACTCCTGGTAATACTGATGCGTTTTC

CAGGGAATATTCTATGACTGAATGTTCTCAAGAACTACAAAAGTTTTCTTTCAAAATAGCTATCTCGTCT

CTCAACAAACTACGAGGATTCAAAAAGAGAGTCAATGTTTTTGAAACTAGAATCGTAATGGATAATGACG

ATAACATTCTAGGAATGTTGTTTTCGGATAGAGTTCAATCCTTTAAGATTAACATCTTTATGGCGTTTTT

AGACTAATACTTTCAATGAGATAAATATGGGTGGCGGAGTAAGTGTTGAGCTCCCTAAACGGGATCCACC

TCCGGGAGTACCCACTGATGAGATGTTATTAAACGTGGATAAAATGCATGACGTGATAGCTCCCGCTAAG

CTTTTAGAATATGTGCATATAGGACCACTAACAAAAGATAAAGAGGATAAAGTAAAGAAAAGATATCCAG

AGTTTAGATTAGTCAACACAGGACCCGGTGGTCTTTCGGCATTATTAAGACAATCATATAATGGAACCGC

ACCCAATTGCTGTCGCACTTTTAATCGTACTCATTATTGGAAGAAGGATGGAAAGATATCAGATAAGTAT

GAAGAGGGTGCAGTATTAGAATCGTGTTGGCCCGACGTCCACGACACTGGAAAATGCGATGTTGATTTAT

TCGACTGGTGTCAGGGGGATACGTTCGATATGAACATATGCCATCAGTGGATCGGTTCAGCCTTTAATAG

GAGTGATAGAACTGTAGAGGGTCGACAATCGTTAATAAATCTGTATAATAAGATGCAAAGATTATGTAGT

AAAGATGCTAGTGTACCAATATGTGAATTATTTTTGCATCATTTACGCGCACACAATACAGAAGATAGTA

AAGAGATGATCGATTATATTCTAAGACAACAGTCGGCGGACTTTAAACAGAAATATATGAGATGTAGTTA

TCCCACTAGAGATAAGTTAGAAGAGTCATTAAAATATGCGGAACCTCGAGAATGTTGGGATCCAGAGTGT

TCGAATGCCAATGTTAATTTCTTACTAACACGTAATTATAATAATTTAGGACTTTGCAATATTGTACGAT

GTAATACGAGCGTGAATAACTTACAGATGGATAAAACTTCCTCATTAAGATTATCATGTGGATTAAGCAA

TAGTGATAGATTTTCTACTGTTCCCGTCAATAGAGCAAAAGTAGTTCAACATAATATTAAACATTCGTTC

GACCTAAAATTGCATTTGATCAGTTTATTATCTCTCTTGGTAATATGGATACTAATTGTAGCTATTTAAA

TGGGTGCCGCAGCAAGCATACAGACGACTGTGAATACACTCAGTGAACGTATCTCGTCTAAATTAGAACA

AGAAGCGAACGCTAGTGCTCAAACAAAATGTGATATAGAAATCGGAAATTTTTATATCCGACAAAACCAT

GGATGTAACATCACTGTTAAAAATATGTGCTCTGCGGACGCGGATGCTCAGTTGGATGCTGTGTTATCAG

CCGCTACAGAAACATATAGTGGATTAACACCGGAACAAAAAGCATACGTACCAGCTATGTTTACTGCTGC

GTTAAACATTCAGACGAGTGTAAACACTGTTGTTAGAGATTTTGAAAATTATGTGAAACAGACTTGTAAT

TCTAGCGCTGTTGTCGATAACAAATTAAAGATACAAAACGTAATTATAGATGAATGTTACGGAGCCCCAG

GATCTCCAACAAATTTGGAATTTATTAATACAGGATCTAGCAAAGGAAATTGTGCCATTAAGGCGTTGAT

GCAATTGACTACTAAGGCCACTACTCAAATAGCACCTAGACAAGTTGCTGGTACAGGAGTTCAGTTTTAT

ATGATTGTTATCGGTGTTATAATATTGGCAGCGTTGTTTATGTACTATGCCAAGCGTATGCTGTTCACAT

CCACCAATGATAAAATCAAACTTATTTTAGCCAATAAGGAAAACGTCCATTGGACTACTTACATGGACAC

ATTCTTTAGAACTTCTCCGATGATTATTGCTACCACGGATATACAAAACTGAAAATATATTGATAATATT

TTAATAGATTAACATGGAAGTTATCGCTGATCGTCTAGACGATATAGTGAAACAAAATATAGCGGATGAA

AAATTTGTAGATTTTGTTATACACGGTCTAGAGCATCAATGTCCTGCTATACTTCGACCATTAATTAGGT

TGTTTATTGATATACTATTATTTGTTATAGTAATTTATATTTTTACGGTACGTCTAGTAAGTAGAAATTA

TCAAATATTGTTGGTGTTGGTGGCGCTAGTCATCACATTAACTATTTTTTTATTACTTTATACTATAATA

GTACTAGACTGACTTCTAACAAACATCTCACCTGCCATAAATAAATGCTTGATATTAAAGTCTTCTATTT

CTAACACTATTCCATCTGTGGAAAATAATACTCTGACATTATCGCTAATTGATACATCGGTAAGTGATAT

GCCTATAAAGTAATAATCTTCTTTGGGCACATATACCAGTGTACCAGGTTCTAACAACCTATTTACTGGT

GCTCCTGTAGCATACTTTTTTTTTACCTTGAGAATATCCATTGTTTGCTTGGTCAATAGTGATATGTGAT

TTTTTATCAACCACTCAAAAAAGTAATTGGAGTGTTCATATCCTCTACGGGCTATTGTCTCATGACCGTG

TATGAAATTTAAGTAACACGACTGTGGTAGATTTGTTCTATAGAGCCGGTTGCCGCAAATAGATAGAACT

ACCAATATGTCTGTACAAATGTTAAACATTAATTGATTAACAGAAAAAACAATGTTCGTTCTGGGAATAG

AAACCAGATTAAAACAAAATTCATTAGAATATATGCCACGTTTATACATGGAATATAAAATAACTACAGT

TTGAAAAATAACAGTATCATTTAAACATTTAACTTGCGGGGTTAATCTCACAACTTTACTGTTTTTGAAC

TGTTCAAAATATAGCATAGATCCATGAGAAATACGTTTAGCCGCCTTTAATAGAGGAAATCCAACCGCCT

TTCTGGATCTCACCAACGACGATAGTTCTGACCAGCAACTCATTTCTTCATCATCCACCTGTTTTAACAT

ATAATAGGCAGGAGATAGATATCCATCATTGCAATATTCCTTCTCGTAGGCACACAATCTAATATTGATA

AAATCTCCATTCTCTTCTCTGTATTTATTATCTTGTCTCGGTGGCTGATTAGGCTGTGGTCTATCGTTGT

TGAATCTATTTTGGTCATTAAATCTTTCATTTCTTCCTGGTATATTTCTATCACCTCGTTTGGTTGGATT

TTTGTCTATATTATCGTTTGTAACATCGGTACGGGTATTCATTTATCACAAAAAAAACTTCTCTAAATGA

GTCTACTACTAGAAAACCTCATCGAAGAAGATACCATATTTTTTGCAGGAAGTATATCTGAGTATGATGA

TTTACAAATGGTTATTGCTGGTGCAAAATCCAAATTTCCAAGATCTATGCTTTCTATTTTTAATATAGTA

CCTAGAACGATGTCAAAATATGAGTTGGAGTTGATTCATAACGAGAATATCACAGGGGCAATGTTTACCA

CAATGTATAATATAAGAAACAATTTGGGTCTAGGCGATGATAAACTAACTATTGAAGCCATTGAAAACTA

TTTCTTGGATCCTAACAATGAGGTTATGCCTCTTATCATTAATAATACGGATATGACTACCGTCATTCCT

AAAAAAAGTGGTAGGAGAAAGAATAAGAACATGGTTATCTTCCGTCAAGGATCATCACCTATCTTGTGTA

TTTTCGAAACTCGTAAAAAGATTAATATTTATAAAGAAAATATGGAATCCGTATCGACTGAGTATACACC

TATCGGAGACAACAAGGCTTTGATATCTAAATATGCGGGAATTAATATCCTGAATGTGTATTCTCCTTCC

ACGTCCATGAGATTGAATGCCATTTACGGATTCACCAATAAAAATAAACTAGAGAAACTTAGTACTAATA

AGGAACTAGAATCGTATAGTTCTAGCCCTCTTCAAGAACCCATTAGGTTAAATGATTTTCTGGGACTATT

GGAATGTGTTAAAAAGAATATTCCTCTAACAGATATTCCGACAAAGGATTGATTACTATAAATGGAGAAT

GTTCCTAATGTATACTTTAATCCTGTGTTTATAGAGCCCACGTTTAAACATTCTTTATTAAGTGTTTATA

AACACAGATTAATAGTTTTATTTGAAGTATTCGTTGTATTCATTCTAATATATGTATTTTTTAGATCTGA

ATTAAATATGTTCTTCATGCCTAAACGAAAAATACCCGATCCTATTGATAGATTACGACGTGCTAATCTA

GCGTGTGAAGACGATAAATTAATGATCTATGGATTACCATGGATAACAACTCAAACATCTGCGTTATCAA

TAAATAGTAAACCGATAGTGTATAAAGATTGTGCAAAGCTTTTGCGATCAATAAATGGATCACAACCAGT

ATCTCTTAACGATGTTCTTCGCAGATGATGATTCATTTTTTAAGTATTTTGCTAGTCAAGATGATGAATC

TTCATTATCTGATATATTGCAAATCACTCAATATCTAGACTTTCTGTTATTATTATTGATCCAATCAAAA

AATAAATTAGAAGCTGTGGGTCATTGTTATGAATCTCTTTCAGAGGAATACAGACAATTGACAAAATTCA

CAGACTCTCAAGATTTTAAAAAACTGTTTAACAAGGTCCCTATTGTTACAGATGGAAGGGTCAAACTTAA

TAAAGGATATTTGTTCGACTTTGTGATTAGTTTGATGCGATTCAAAAAAGAATCAGCTCTAGCTACCACC

GCAATAGATCCTGTTAGATACATAGATCCTCGTCGTGATATCGCATTTTCTAACGTGATGGATATATTAA

AGTCGAATAAAGTTGAAAAATAATTAATTCTTTATTGTTATCATGAACGGCGGACATATTCAGTTGATAA

TCGGCCCCATGTTTTCAGGTAAAAGTACAGAATTAATTAGACGAGTTAGACGTTATCAAATAGCTCAATA

TAAATGTGTGACTATAAAATATTCTAACGATAATAGATACGGAACGGGACTATGGACACATGATAAGAAT

AATTTTGCAGCATTGGAAGTAACTAAACTATGTGATGTCTTGGAAGCAATTACAGATTTCTCCGTGATAG

GTATAGATGAAGGACAGTTCTTTCCAGACATTGTTGAATTCTGTGAGCGTATGGCAAACGAAGGAAAAAT

AGTTATAGTAGCCGCGCTCGATGGGACATTTCAACGTAGACCGTTTAATAATATTTTGAATCTTATTCCA

TTATCTGAAATGGTGGTAAAACTAACTGCAGTGTGTATGAAATGCTTTAAGGAGGCTTCCTTTTCTAAAC

GATTAGGTACAGAAACCGAGATAGAAATAATAGGAGGTAATGATATGTATCAATCTGTGTGTAGAAAGTG

TTACATCGACTCATAATATTATATTTTTTATCTAAAAAACTAAAAATAAACATTGATTAAATTTTAATAT

AATACTTAAAAATGGATGTTGTGTCGTTAGATAAACCGTTTATGTATTTTGAGGAAATTGATAATGAGTT

AGATTACGAACCAGAAAGTGCAAATGAGGTCGCAAAAAAACTGCCGTATCAAGGACAGTTAAAACTATTA

CTAGGAGAATTATTTTTTCTTAGTAAGTTACAGCGACACGGTATATTAGATGGCGCCACCGTAGTGTATA

TAGGATCTGCTCCAGGTACACATATACGTTATTTGAGAGATCATTTCTATAATTTAGGAGTGATCATCAA

ATGGATGCTAATTGACGGCCGCCATCATGATCCTATTCTAAATGGATTGCGTGATGTGACTCTAGTGACT

CGGTTTGTTGATGAGGAATATCTACGATCCATCAAAAAACAACTACATCCTTCTAAGATTATTTTAATTT

CTGATGTGCGATCCAAACGAGGAGGAAATGAACCTAGTACTGCGGATTTACTAAGTAATTATGCTCTACA

AAATGTCATGATTAGTATTTTAAACCCCGTGGCGTCTAGTCTTAAATGGAGATGCCCGTTTCCAGATCAA

TGGATCAAGGACTTTTATATACCACACGGTAATAAAATGTTACAACCTTTTGCTCCTTCATATTCAGCTG

AAATGAGATTATTAAGTATTTATACCGGTGAGAATATGAGACTGACTCGAGTTACCAAATCAGACGCTGT

AAATTATGAAAAAAAGATGTATTACCTTAATAAGATAGTCCGCAACAAAGTAGTTATTAACTTTGATTAT

CCTAATCAGGAATATGACTATTTTCACATGTACTTTATGTTGAGGACCGTATACTGCAATAAAACATTTC

CTACTACTAAAGCAAAGATACTATTTCTACAACAATCTATATTTCGTTTCTTAAATATTCCAACGACATC

AACTGAAAAAGTTAGTCATGAACCAATACAACGTAAAATATCTAGCAAAGATTCTATGTCTAAAAACAGA

AATAGCAAGAGATCCGTACGCGGTAATAAATAGAAACGTACTACTGAGATATACTACCGATATAGAGTAT

AATGATTTAGTTACTTTAATAACCGTTAGACATAAAATTGATTCTATGAAAACTGTGTTTCAGGTATTTA

ACGAATCATCCATAAATTATACTCCGGTTGATGATGATTATGGAGAACCAATCATTATAACATCGTATCT

TCAAAAAGGTCATAACAAGTTTCCTGTAAATTTTCTATACATAGATGTGGTAATATCTGACTTATTTCCT

AGCTTTGTTAGACTAGATACCACAGAAACTAATATAGTTAATAGTGTACTACAAACAGGCGATGGTAAAA

AGACTCTTCGTCTTCCTAAAATGTTAGAGACGGAAATAGTTGTCAAGATTCTCTATCGTCCTAATATACC

ATTAAAAATTGTTAGATTTTTCCGCAATAACATGGTAACTGGAGTAGAGATAGCCGATAGATCTGTTATT

TCAGTCGCTGATTAATCAATTAGTAGAGATGAGATAAGAACATTATAATAATCAATAATATATCTTATAT

CTGTTTAGAAAAATGCTAATATTAAAATAGCTAACGCTAGTAATCCAATCGGAAGCCATTTGATATCTAT

AATAGGGTATCTAATTTCCTGATTCAGATAGCGTACGGCTATATTCTCGGTAGCTACTCGTTTGGAATCA

CAGACATTATTTACATCTAATTTACTATCTGTAATGGAAACGTTTCCCAATGAAATGGTACAATCAGATA

CATTACATCTTGATATATTTTTTTTTAAAGAGGCTGGTAACAACGCATCGCTTCGTTTACATGGCTCGTA

CCAACAATAATAGGGTAATCTTGTATCTATTCCTATCCGTACTATACTTTTATCAGGATAAATACATTTA

CATCGTATATCGTCTTTGTTAGCATCACAGAATGCATAAATTTGTTCGTCCGTCATGATAAAAATTTAAA

GTGTAAATATAACTATTATTTTTATAGTTATAATAAAAAGGGAAATTTGATTGTATACCTTCGGTTCTTT

AAAAGAAACTGACTTGATAAAAATGGCTGTAATCTCTAAGGTTACGTATAGTCTATACGATCAAAAAGAG

ATTAATGCCACAGATATTATCATTAGTCATATTAAAAATGACGACGATATCGGTACCGTTAAAGATGGTA

GACTAGGTGCTATGGATGGGGCATTATGTAAGACTTGTGGGAAAACGGAATTGGAATGTTTCGGTCACTG

GGGTAAAGTAAGTATTTATAAAACTCATATAGTTAAGCCTGAATTTATTTCAGAAATTATTCGTTTACTG

AATCATATATGTATTCATTGCGGATTATTGCGTTCACGAGAACCGTATTCCGACGATATTAACCTAAAAG

AGTTATCGGTACACGCTCTTAGGAGATTAAAGGATAAAATATTATCCAAGAAAAAGTCATGTTGGAACAG

CGAATGTATGCAACCGTATCAAAAAATTACTTTTTCAAAGAAAAAGGTTTGTTTCGTCAACAAGTTGGAT

GATATTAACGTTCCTAATTCTCTCATCTATCAAAAGTTAATTTCTATTCATGAAAAGTTTTGGCCATTAT

TAGAAATTCATCAATATCCAGCTAACTTATTTTATACAGACTACTTTCCCATCCCTCCGTTGATTATTAG

ACCGGCTATTAGTTTTTGGATAGATAGTATACCCAAAGAGACAAATGAATTAACTTACTTATTAGGTATG

ATCGTTAAGAATTGTAACTTGAATGCTGATGAACAGGTTATCCAGAAGGCGGTAATAGAATACGATGATA

TTAAAATTATTTCTAATAACACTACCAGTATCAATTTATCATATATCACATCCGGCAAAAATAATATGAT

TAGAAGTTATATCGTCGCTCGGCGAAAAGATCAGACCGCTAGATCCGTAATTGGTCCCAGTACATCTATC

ACCGTTAATGAGGTAGGAATGCCCACATATATTAGAAATACACTTACAGAAAAGATATTTGTTAATGCCT

TTACAGTGGATAAAGTTAAACAACTATTAGCATCAAACCAAGTTAAATTTTACTTTAATAAACGATTAAA

CCAATTAACAAGAATACGTCAAGGAAAGTTTATCAAAAATAAAATACATTTATTGCCTGGTGATTGGGTA

GAAGTAGCTGTTCAAGAATATACAAGTATTATTTTTGGAAGACAACCGTCTCTACATAGATACAACGTCA

TCGCTTCATCTATCAGAGCTACCGAAGGAGATACTATCAAAATATCTCCCGGAATTGCCAACTCTCAAAA

TGCTGATTTCGACGGAGATGAAGAATGGATGATATTGGAGCAAAATCCTAAAGCCGTAGTTGAACAAAGT

ATTCTTATGTATCCGACGACGTTACTCAAACACGATATTCATGGAGCCCCCGTTTATGGATCTATTCAAG

ATGAAATCGTAGCAGCGTATTCATTGTTTAGGATACAAGATCTTTGTTTAGATGAAGTATTGAACATCTT

GGGGAAATATGGAAGAGAGTTCGATCCTAAAGGTAAATGTAAATTCAGCGGTAAAGATATCTATACTTAC

TTGATAGGTGAAAAGATTAATTATCCGGGTCTCTTAAAGGATGGTGAAATTATTGCAAACGACGTAGATA

GTAATTTTGTTGTAGCTATGAGGCATCTGTCATTGGCTGGACTCTTATCCGATCATAAATCGAACGTGGA

AGGTATCAACTTTATTATCAAGTCATCTTATGTTTTTAAGAGATATCTATCTATATACGGTTTTGGGGTG

ACATTCAAAGATCTGAGACCAAATTCGACGTTCACTAATAAATTGGAGGCTATCAACGTAGAAAAAATAG

AACTTATCAAAGAAGCATACGCCAAATATCTCAAAGATGTAAGAGACGGGAAAATAGTTCCATTATCTAA

AGCTTTAGAGGCGGACTACTTGGAATCCATGTTATCCAACTTGACAAATCTTAATATCAGAGAGATAGAA

GAACATATGAGACAAACGCTGATAGATGATCCAGATAATAACCTCCTGAAAATGGCCAAAGCGGGTTATA

AAGTAAATCCCACAGAACTAATGTATATTCTAGGTACTTATGGACAACAGAGGATAGATGGCGAACCAGC

AGAGACTCGAGTATTGGGTAGAGTCTTACCTTACTATCTTCCAGACTCTAAGGATCCAGAAGGAAGAGGT

TATATTCTTAATTCTTTAACAAAAGGATTAACGGGTTCTCAATATTACTTTTCGATGCTGGTTGCAAGAT

CTCAATCTACTGATATTGTCTGTGAAACATCACGTACCGGAACACTGGCTAGAAAAATCATTAAAAAGAT

GGAGGATATGGTGGTCGACGGATACGGACAAGTAGTTATAGGTAATACGCTCATCAAGTACGCAGCCAAT

TATACCAAAATTCTAGGCTCAGTATGTAAACCTGTAGATCTTATCTATCCAGATGAGTCCATGACTTGGT

ATTTGGAAATTAGTGCTTTGTGGAATAAAATAAAACAGGGATTCGTTTACTCTCAGAAACAGAAACTTGC

AAAGAAGACATTGGCGCCGTTTAATTTCCTAGTATTCGTCAAACCCACCACTGAGGATAATGCTATTAAG

GTTAAGGATCTGTACGATATGATTCATAACGTCATTGATGATGTGAGAGAGAAATACTTCTTTACGGTAT

CTAATATAGATTTTATGGAGTATATATTCTTGACGCATCTTAATCCTTCTAGAATTAGAATTACAAAAGA

AACGGCTATTACTATCTTTGAAAAGTTCTATGAAAAACTCAATTATACTCTAGGTGGTGGAACTCCTATT

GGAATTATTTCTGCACAGGTATTGTCTGAGAAGTTTACACAACAAGCCCTGTCCAGTTTTCACACTACTG

AAAAGAGTGGTGCTGTAAAACAAAAACTTGGTTTCAACGAGTTTAATAACTTGACTAATTTGAGTAAGAA

TAAGACCGAAATTATCACTCTGGTATCCGATGATATCTCTAAACTTCAATCTGTTAAGATTAATTTCGAA

TTTGTATGTTTGGGAGAATTAAATCCAGACATCACTCTTCGAAAAGAAACAGATAGATATGTAGTAGACA

TAATAGTCAATAGATTATACATCAAGAGAGCAGAAATAACCGAATTAGTCGTCGAATATATGATTGAACG

ATTTATCTCCTTTAGCGTCATTGTAAAGGAATGGGGTATGGAGACATTCATTGAGGACGAGGATAATATT

AGATTTACTATCTACCTAAATTTCGTTGAACCGGAGGAATTGAATCTTAGTAAGTTTATGATGGTTCTTC

CAGGTGCCGCCAACAAGGGCAAGATTAGTAAATTCAAGATTCCTATCTCTGACTATACGGGATATAACGA

CTTCAATCAAACAAAAAAGCTCAATAAGATGACTGTAGAACTCATGAATCTAAAAGAATTGGGTTCTTTC

GATTTGGAGAACGTCAACGTGTATCCTGGAGTATGGAATACATACGATATCTTCGGTATTGAGGCCGCTC

GTGGATACTTGTGCGAAGCCATGTTAAACACCTATGGAGAAGGTTTCGATTATCTGTACCAGCCTTGTGA

TCTTCTCGCTAGTTTACTATGTGCTAGTTACGAACCAGAATCAGTTAATAAATTCAAGTTCGGTGCAGCT

AGTACTCTTAAGAGAGCTACGTTCGGAGATAATAAAGCATTGTTAAACGCGGCTCTTCATAAAAAGTCAG

AACCTATTAACGATAATAGTAGCTGCCACTTTTTTAGCAAGGTCCCTAATATAGGAACTGGATATTACAA

ATACTTTATCGACTTGGGTCTTCTCATGAGAATGGAAAGGAAACTATCTGATAAGATATCTTCTCAAAAG

ATCAAGGAGATAGAAGAAACAGAAGACTTTTAATTCTTATCAATAACATATTTTTCTATGATCTGTCTTT

TAAACGATGGATTTTCCACAAATGCGCCTCTCAAGTCCCTCATAGAATGATACACGTATAAAAAATATAG

CATAGGTGATGACTCCTTATTTTTAGACATTAGATATGCCAAAATCATAGCCCCGCTTCTATTTACTCCT

GCAACACAATGAACCAACACGGGCTCGTTTCGTTGATCACATTTAGATAAGAAGGCGGTCACGTCGTCAA

AATATTTACTAATATCAGTAGTTGTATCATCTACCAACGGTATATGAATAATATTAATATTAGAGTTAGG

TAATGTATATTTATCCATCGTCAAATTTAAAACATATTTGAACTTAACTTCAGATGATGGTGCATCCATA

GCATTTTTATAATTTCCCAAATACACATTATTTGTTACTCTTGTCATTATAGTGGGAGATTTGGCTCTGT

GCATATCTCCAGTTGAACGTAGTAGTAAGTATTTATACAAACTTTTCTTATCCATTTATAACGTACAAAT

GGATAAAACTACTTTATCAGTAAACGCATGCAATTTAGAATACGTTAGAGAAAAGGCTATAGTAGGCGTA

CAAGCAGCCAAGACATCAACACTTATATTTTTTGTTATTATATTGGCAATTAGTGCGCTATTACTCTGGT

TTCAGACGTCTGATAATCCAGTCTTTAATGAATTAACGAGATATATGCGAATTAAAAATACGGTTAACGA

TTGGAAATCATTAACGGATAGCAAAACAAAATTAGAAAGCGATAGAGGTAGACTTCTAGCCGCTGGTAAG

GATGATATATTCGAATTCAAATGTGTGGATTTCGGTGCCTATTTTATAGCTATGCGATTGGATAAGAAAA

CATATCTGCCGCAAGCTATTAGGCGAGGTACTGGAGACGCGTGGATGGTTAAAAAGGCGGCAAAAGTCGA

TCCATCTGCTCAACAATTTTGTCAGTATTTGATAAAACACAAGTCTAATAATGTTATTACTTGTGGTAAT

GAGATGTTAAATGAATTAGGTTATAGCGGTTATTTTATGTCACCGCATTGGTGTTCCGATCTTAGTAATA

TGGAATAAGTGTTAGATAAATGCGGTAACAAATGTTCCTGTAAGGAACCATAACAGTTTAGATTTAACAT

TAAAGATGAGCATAAACATAATAAACAAAATTACAATCAAACCTATAACATTAATATCAAACAATCCAAA

AAATGAAATCAATGGAGTAGTAAACGTGTACATGACTCCTGGATAACGTTTAGCAGCTACCGTTCCTATT

CTAGACCAAAAATTTGGTTTCATGGTTTCGAAGCGGTGTTCTGCAACAAGACGAGGATCGTGTTCTACAT

ATTTGGCAGAGTTATCCATTATTTGCCTGTTAATCTTCATTTCGTTTTCGATTCTGGCTATTTCAAAATA

AAATCCCGATGATAGACCTCCAGACTTTATAATTTCATCTACGATGTTCAGCGCCGTAGTAACTCTAATA

ATATAGGCGGATAAGCTAACATCATACCCTCCTGTATATGTAAATATGGCATGATCTTTGTCTATTACAA

GCTCGGTTTTAACTTTATTTCCTGTAATAATTTCTCTCATCTGTAGGATATCTATTTTCTTGTCATGTAT

TGCCTTCAAGACGGGACGAAGAAACGTAATATCCTCAATAACGTTATCGTTTTCTATAATAACTACATAT

TCTACATTTTTATTTTCTAGCTCGGTAAAAAATTTAGAATCCCATAGGGCTAAATGTCTAGCGATATTTC

TTTTCGTTTCCTCTGTACACATAGTGTTACAAAACCCTGAAAAGAAGTGAGTATACTTGTCATCATCTCT

AATATTTCCTCCAGTCCATTGTATAAACACATAATCCTTGTAATGATCTGGATCATCATTGACTATCACA

ACATCTCTTTTTTCTTGCATAACTTCATTGTCCTTCACATCATCGAACTTCTGATCATTAATATGCTCAT

GAACATTAGGAAATGTTTCTGATGGAGGTCTATCAATAACTGGCACAACAATAACAGGAGTTTTCACCGC

CGCCATTTAGTTATTGAAATTAATCATATACAACTCTCTAATACGAGTTATATTTTCGTCTATCCATTGT

TTCACATTGACATATTTCGACAAAAAGATATAAAATGCGTATTCCAATGCTTCTCTGTTTAATGAATTAC

TAAAATATACAAACACGTCACTGTCTGGTAATAAATGATATCTTAGAATATTGTAACAATTTATTTTGTA

TTGCACATGTTCGTGATCTATGAGTTCTTCTTCGAATGGCATAGGATCTCCGAATCTGAAAACGTATAAA

TAGGAGTTAGAATAATAATATTTGAGAGTATTGGTAATGTATAAACTCTTTAGCGGTATAATTAGTTTTT

TTCTCTCGATTTCTATTTTTAGATGTGATGGAAAAATGACTAATTTTGTAGCATTAGTATCATGAACTCT

AATCAAAATCTTAATATCTTCGTCACATGTTAGCTCTTTGAAGTTTTTAAGAGATGCATCAGTTGGTTTT

ACAGATGGAGTAGGTGCAACAATTTTTTGTTTAATGCATGCATGTATTGGAGCCATTGTCTTAACTATAA

TGGTGCTTGTATCGAAAAACTTTAATGCGGATAACGGAAGCTCTTCGCCGCGACTTTCTACGTCGTAATT

GGGTTCTAATGCCGATCTCTGAATGGATACTAGTTTTCTAAGTTCTAATGTAATTCTCTGAAAATGTAAA

TCCAATTCCTCCGGCATTATAGATGTGTATACATCGGTAAATAAAACTATAGTATCCAACGATCCCTTCT

CGCAAATTCTAGTCTTAACCAAGAAATCGTATATAACTACGGAGATGGCGTATTTAAGAGTGGATTCTTC

TACCGTTTTGTTCTTGGATTTCATATAAGAAACTATAAAGTCCGCACTACTGTTAAGAATGATCACTAAC

GCAACTATATAGTTCAAATTAAGCATCTTGGAAACATAAAATAACTCTGTAGATGATACTTGACTTTCGA

ATAAGTTTGCAGACAAACGAAGAAAGAACAGACCTCTCTTAATTTCAGAAGAAAACTTTTTTTCGTATTC

CTGACGTCTAGAGTTTATATCAATAAGAAAGTTAAGAATTAGTCGGTTAATGTTGTATTTCATTACCCAA

GTTTGAGATTTCATAATATTGTCAAAAGACATGATAATATTAAAGATAAAGCGCTGACTATGAACGAAAT

AGCTATATGGTTCGCTCAAGAATATAGTCTTGTTAAACGTGGAAACGATAACTGTATTTTTAATCACGTC

AGCGGCATCTAAATTAAATATAGGTATATTTATTCCACACACTCTACAATATGCCACACCATCTTCATAA

TAAATAAATTCGTTAGCAAAATTATTAATTTTAGTGAAATAGTTAGCGTCAACTTTCATAGCTTCCTTCA

ATCTAATTTGATGCTCACATGGCGCGAATTCTACTCTAACATCCCTTTTCCATGCCTCAGGTTCATCGAT

CTCTATAATATCTAGTTTCTTGCGTTTCACAAACACAGGCTCGTCTCTCGCGATGAGATCTGTATAGTAA

CTATGTAAATGATAACTAGATAGAAAGATGTAGCTATATAGATGACGATCCTTTAAGAGAGGTATAATAA

CTTTACCCCAATCAGATAGACTGTTGTTATGGTCTTCGGAAAAAGAATTTTTATAAATTTTTCCAGTATT

TTCTAAATATACGTACTTGATATCTAAGAAATCCTTAATAATAATAGGAATGGATAATCCGTCTATTTTA

TAAAGAAATACATATCGCATATTATACTTTTTTTTGGAAATTGGAATACCGATGTGTCTACATAAATACG

CAAAGTCTAAATATTTTTTAGAGAATCTTAGTTGGTCCAAATTCTTTTCCAAGTACGGTAATAGATTTTT

CATATTGAACGGTATCTTCTTGATCTCTGGTTCTAATTCCGCATTAAATGATGAAACTAAGTCACTATTT

TTATAACTAACGATTACATCACCTCTAACATCATCATTTACCAGGATACTGATCTTCTTTTGTCGTAAAT

ACATGTCTAATGTGTTAAAAAAAAGATCATACAAGTTATACGTCATTTCATCTGTAGTATTCTTGTCATT

GAAGGATAAACTCGTACTAATCTCTTCTTTAACAGTCTGTTCAAATTTATATCCTATATATGAAAAAATA

GCAACCAGTGTTTGATCATCCGCGTCAATATTCTGTTCTATCGTAGTGTATAACAATCTTATATCTTCTT

CTGTGATAGTCGATACGTTATAAAGGTTGATAACGAAAATATTTTTATTTCGTGAAATAAAGTCATTGTA

GGATTTTGGACTTATATTCGTGTCTAGTAGATATGATTTTATTTTTGGAATGATCTCAATTAAAATAGTC

TCTTTAGAGTCCATTTAAAGTTACAAACAACTAGGAAATTGGTTTATGATGTATAATTTTTTTAGTTTTT

ATAGATTCTTTATTCTATACTTAAAAAATGAAAATAAATACAAAGGTTCTTGAGGGTTGTGTTAATTGAA

AGCGATAAATAATCATAAATTATTTCATTATCGCGATATCCGTTAAGTTTGTATCGTAATGGCGTGGTCA

ATTACGAATAAAGCGGATACTAGTAGTTTCACAAAGATGGCTGAAATCAGAGCTCATCTAAGAAATAGCG

CTGAAAATAAAGATAAAAACGAGGATATTTTCCCGGAAGATGTAATAATTCCATCTACTAAGCCCAAAAC

CAAACGAACCACTACTCCTCGTAAACCAGCGGCTACTAAAAGATCAACCAAAAAGGATAAAGAAAAGGAG

GAAGTGGAAGAAGTAGTTATAGAGGAATATCATCAAACAACTGAAGAAAATTCTCCACCTCCGTCATCAT

CTCCTGGAGTCGGCGACATTGTAGAAAGCGTGGCCGCTGTAGAGCTCGATGATAGCGACGGGGATGATGA

ACCTATGGTACAAGTTGAAGCTGGTAAAGTAAATCATAGTGCTAGAAGCGATCTCTCTGACCTAAAGGTG

GCTACCGACAATATCGTTAAAGATCTTAAGAAAATTATTACTAGAATCTCTGCAGTATCGACTGTTCTAG

AGGATGTTCAAGCAGCTGGTATCTCTAGACAATTTACTTCTATGACTAAAGCTATTACAACACTATCTGA

TCTAGTCACCGAGGGAAAATCTAAAGTTGTTCGTAAAAAAGTTAAAACTTGTAAGAAGTAAATGCGTGCA

CTTTTTTATAAAGATGGTAAACTGTTTACCGATAATAATTTTTTAAATCCTGTATCAGACGATAATCCAG

CGTATGAGGTTTTGCAACATGTTAAAATTCCTACTCATTTAACAGATGTAGTAGTATATGAACAAACGTG

GGAAGAGGCATTAACTAGATTAATTTTTGTGGGAAGTGATTCAAAAGGACGTAGACAATACTTTTACGGA

AAAATGCATATACAGAATCGCAATGCTAAAAGAGATCGTATTTTTGTTAGAGTATATAACGTTATGAAAC

GAATTAATTGTTTTATAAACAAAAATATAAAGAAATCGTCCACAGATTCCAATTATCAGTTGGCGGTTTT

TATGTTAATGGAAACTATGTTTTTTATTAGATTTGGTAAAATGAAATATCTTAAGGAGAATGAAACAGTA

GGGTTATTAACACTAAAAAATAAACACATAGAAATAAGTCCCGATGAAATAGTTATCAAGTTTGTAGGAA

AGGACAAAGTTTCACATGAATTTGTTGTTCATAAGTCTAATAGACTATATAAACCGCTATTGAAACTGAC

TGATGATTCTAGTCCCGAAGAATTTCTGTTCAACAAACTAAGTGAACGAAAGGTATATGAATGTATCAAA

CAGTTTGGTATTAGAATCAAGGATCTCCGAACGTATGGAGTCAATTATACGTTTTTATATAATTTTTGGA

CAAATGTAAAGTCCGTATCTCCTCTTCCATCACCAAAAAAGTTGATAGCATTAACTATCAAACAAACTGC

TGAAGTGGTAGGTCATACTCCATCAATTTCAAAAAGAGCTTATATGGCAACGACTATTTTAGAAATGGTA

AAGGATAAAAATTTTTTAGACGTAGTATCTAAAACTACGTTCGATGAATTCCTATCTATAGTCGTAGATC

ACGTTAAATCATCTACGGATGGATGATAATAGATCTTTACACAAATAATTACAAGACCGATAAATGGAAA

TGGATAAACGGATGAAATCTCTCGCTATGACAGCTTTCTTCGGAGAGCTAAACACGTTAGATATTATGGC

ATTGATAATGTCTATATTTAAACACCATCCAAACAATACCATTTTTTCAGTGGATAAGGATGGTCAATTT

ATGATTGATTTCGAATACGATAATTATAAGGCTTCTCAATATTTGGATCTGACCCTCACTCCGATATCTG

GAAATGAATGCAAGACTCACGCATCTAGTATAGCCGAACAATTGGCGTGTGTGGATATTATTAAAGAGGA

TATTAGCGAATATATCAAAACTACTCCCCGTCTTAAACGATTTATAAAAAAATACCGCAATAGATCATAT

ACTCGTATCAGTCGAGATACAGAAAAGCTTAAAATAGCTCTAGCTAAAGGCATAGATTACGAATATATAA

AAGACGCTTGTTAATAAGTAAATGAAAAAAAACTAGTCGTTTATAATAAAACACGATATGGATGCCAACA

TAGTATCATCTTCTACTATTGCGACGTATATAGACGCTTTAGCGAAGAATGCTTCAGAATTAGAACAGAG

GTCTACCGCATACGAAATAAATAATGAATTGGAACTAGTATTTATTAAACCGCCATTGATTACGTTGACA

AATGTAGTAAATATCTCCACGATTCAGGAATCGTTTATTCGATTTACCGTTACTAATAAGGAAGGTATCA

AAATTAGAACTAAGATTCCATTATCTAAGGTACATGGTCTAGATGTAAAAAATGTGCAGTTGGTAGATGC

TATAGATAACATAGTTTGGGAAAAGAAATCATTAGTGACGGAAAATCGTCTTCACAAAGAATGCTTGTTG

AGACTATCAACAGAGGAACGTCATATATTTTTGGATTACAAGAAATATGGATCCTCTATCCGACTAGAAT

TAGTCAATCTTATTCAAGCAAAAACAAAAAACTTTACGATAGACTTTAAGCTAAAATATTTTCTAGGATC

TGGCGCTCAATCTAAAAGTTCTTTATTGCACGCTATTAATCATCCAAAGTCAAGGCCTAATACATCTCTG

GAAATAGAATTTACACCTAGAGACAATGAAACAGTTCCATATGATGAACTAATAAAGGAATTGACGACTC

TCTCGCGTCATATATTTATGGCTTCTCCAGAGAATGTAATTCTTTCTCCACCTATTAACGCACCTATAAA

GACTTTTATGTTGCCTAAACAAGATATAGTAGGTCTGGATCTGGAAAATCTATATGCCGTAACTAAGACT

GATGGCATTCCTATAACTATCAGAGTTACATCAAAAGGGTTGTATTGTTATTTTACACATCTTGGTTATA

TTATTAGATATCCAGTTAAGAGAACAATAGATTCCGAAGTAGTAGTCTTTGGTGAGGCAGTTAAGGATAA

GAACTGGACCGTATATCTCATTAAGCTAATAGAGCCCGTAAATGCAATCAGTGATAGACTAGAAGAAAGT

AAGTATGTTGAATCTAAACTAGTGGATATTTGTGATCGGATAGTATTCAAGTCAAAGAAATACGAAGGTC

CGTTTACTACAACTAGTGAAGTCGTCGATATGTTATCTACATATTTACCAAAGCAACCAGAAGGTGTTAT

TCTGTTCTATTCAAAGGGACCTAAATCTAACATTGATTTTAAAATCAAAAAGGAGAATACTATAGACCAA

ACTGCAAATGTAGTATTTAGGTACATGTCCAGTGAACCAATTATCTTTGGAGAGTCGTCTATCTTTATAG

AGTATAAGAAATTTACCAACGATAAAGGCTTTCCTAAAGAATATGGTTCTGGTAAGATTGTGTTATATAA

CGGCGTTAATTATCTAAATAATATCTATTGTTTGGAATATATTAATACACATAATGAAGTGGGTATTAAG

TCCGTTGTTGTACCTATTAAGTTTATAGCAGAATTCTTAGTCAATGGAGAAATACTTAAACCTAGAATCG

ATAAAACCATGAAATATATTAACTCAGAAGACTATTATGGAAATCAACATAATATCATAGTCGAACATTT

AAGAGATCAAAGCATCAAAATAGGAGATGTCTTTAACGAGGATAAACTATCGGATGTTGGACATCAATAC

GCTGCCAACAACGATAAATTTAGATTAAATCCAGAAGTTAGTTATTTTACTAATAAACGAACTAGAGGGC

CGTTGGGAATTTTATCAAACTACGTCAAGACTCTTCTTATTTCTATGTATTGTTCCAAAACATTTTTAGA

CGATTCCAACAAACGAAAGGTATTAGCGATTGATTTTGGAAACGGTGCTGACCTGGAAAAATACTTTTAT

GGAGAGATTGCGTTATTGGTAGCGACGGATCCGGATGCTGATGCTATAGCTAGAGGAAATGAAAGATACA

ACAAATTAAATTCTGGAATTAAAACCAAGTACTACAAATTTGACTACATTCAGGAAACTATTCGATCCGA

TACATTTGTCTCTAGTGTCAGAGAAGTATTCTATTTTGGAAAGTTTAATATCATTGACTGGCAGTTCGCT

ATTCATTATTCTTTTCATCCAAGACATTATGCTACAGTCATGAATAACTTATCCGAACTAACTGCTTCTG

GAGGCAAGGTATTAATTACTACCATGGATGGAGACAAATTATCAAAATTAACCGATAAAAAGACTTTTAT

AATTCATAAGAATCTACCTAGTAGCGAAAACTATATGTCTGTAGAAAAAATAGCTGATGATAGAATAGTG

GTATATAATCCATCAACAATGTCTACTCCAATGACTGAATACATTATCAAAAAGAACGATATAGTCAGAG

TGTTTAACGAATACGGATTTGTTCTTGTAGATAATGTTGATTTCGCTACAATTATAGAACGAAGTAAAAA

GTTTATTAATGGCGCATCTACAATGGAAGATAGACCGTCTACAAGAAACTTTTTCGAACTAAATAGAGGA

GCCATTAAATGTGAAGGTTTAGATGTCGAAGACTTACTTAGTTACTATGTTGTTTATGTCTTTTCTAAGC

GGTAAATAATAATATGGTATGGGTTCTGATATCCCCGTTCTAAATGCATTAAATAATTCCAATAGAGCGA

TTTTTGTTCCTATAGGACCTTCCAACTGTGGATACTCTGTATTATTAATAGATATATTAATACTTTTGTA

GGGTAACAGAGGTTCTACGTCTTCTAAAAATAAAAGTTTTATAACATCTGGCCTGTTCATAAATAAAAAC

TTGGCGATTCTATATATACTCTTATTATCAAATCTAGCCATTGTCTTATAGATGTGAGCTACTGTAGGTG

TACCATTTGATTTTCTTTCTAATACTATATATTTCTCTCGAAGAAGTTCTTGCAGATCATCTGGGAATAA

AATACTACTGTTGAGTAAATCAGTTATTTTTTTTATATCGATATTGATGGACATTTTTATAGTTAAGGAT

AATAAGTATCCCAAAGTAGATAACGACGATAACGAAGTATTTATACTTTTAGGAAATCACAATGACTTTA

TCAGATCAAAATTAACAAAATTAAAGGAGCATGTATTTTTTTCTGAATATATTGTGACTCCAGATACATA

TGGATCTTTATGCGTCGAATTAAATGGGTCTAGTTTTCAGCACGGTGGTAGATATATAGAGGTGGAGGAA

TTTATAGATGCTGGAAGACAAGTTAGATGGTGTTCTACATCAAATCATATATCTGAAGATATACACACTG

ATAAATTTGTCATTTATGATATTTATACGTTTGATTCGTTCAAGAATAAACGATTGGTATTTGTACAGGT

GCCTCCATCATTAGGAGATGATAGCTATTTAACTAATCCGTTATTGTCTCCGTATTATCGTAATTCAGTA

GCCAGACAAATGGTCAATGATATGATTTTTAATCAAGATTCATTTTTAAAATATTTATTAGAACATCTGA

TTAGAAGCCACTATAGAGTTTCTAAACATATAACAATAGTTAGATACAAGGATACCGAAGAATTAAATCT

AACAAGAATATGTTATAATAGAGATAAGTTTAAGGCGTTTGTATTCGCTTGGTTTAACGGCGTTTCGGAA

AATGAAAAGGTACTAGATACGTATAAAAAGGTATCTGATTTGATATAATGAATTCAGTGACTATATCACA

CGCACCATATACTATTACTTATCACGATGATTGGGAACCAGTAATGAGTCAATTGGTAGAGTTTTATAAC

GAAGTAGCCAGTTGGTTGCTACGCGACGAGACGTCGCCTATTCCTGATAAGTTCTTTATACAATTGAAAC

AGCCGCTTAGAAATAAACGAGTATGTGTGTGTGGTATAGATCCGTATCCAAAAGATGGAACTGGTGTACC

GTTCGAATCACCAAATTTTACAAAAAAATCAATTAAGGAGATAGCTTCATCTATATCTAGATTAACCGGA

GTAATTGATTATAAAGGTTATAACCTTAATATAATAGACGGGGTTATACCCTGGAATTATTACTTAAGTT

GTAAATTAGGAGAAACAAAAAGTCACGCGATTTACTGGGATAAGATTTCCAAGTTACTGCTACAGCATAT

AACTAAACACGTTAGTGTTCTTTATTGTTTGGGTAAAACAGATTTCTCGAATATACGGGCAAAGTTAGAA

TCCCCGGTAACTACCATAGTGGGATATCATCCAGCGGCCAGAGACCACCAATTCGAGAAAGATCGATCAT

TTGAAATTATCAACGTTTTACTGGAATTAGACAACAAGACACCTATAAATTGGGCTCAAGGGTTTATTTA

TTAATGCTTTAGTGAAATTTTAACTTGTGTTCTAAATGGATGCGGCTATTAGAGGTAATGATGTTATCTT

TGTTCTTAAGACTATAGGTGTCCCGTCAGCATGCAGACAAAATGAAGATCCAAGATTCGTAGAAGCATTT

AAATGCGACGAGTTAGAAAGATATATTGATAATAATCCAGAATGTACACTATTCGAAAGTCTTAGGGATG

AGGAAGCATACTCTATAGTCAGAATTTTCATGGATGTAGATTTAGACGCGTGTCTAGACGAAATAGATTA

TTTAACGGCTATTCAAGATTTTATTATCGAGGTGTCAAACTGTGTAGCTAGATTCGCATTTACAGAATGC

GGTGCCATTCATGAAAATGTAATAAAATCCATGAGATCTAATTTTTCATTGACTAAGTCTACAAATAGAG

ATAAAACAAGTTTTCATATTATCTTTTTAGACACGTATACCACTATGGATACATTGATAGCTATGAAACG

AACACTATTAGAATTAAGTAGATCATCTGAAAATCCACTAACAAGATCGATAGACACTGCCGTATATAGG

AGAAAAACAACTCTTCGGGTTGTAGGTACTAGGAAAAATCCAAATTGCGACACTATTCATGTAATGCAGC

CACCTCACGATAATATAGAAGATTACCTATTCACTTACGTGGATATGAACAACAATAGTTATTACTTTTC

TCTACAACGACGATTGGAGGATTTAGTTCCTGATAAGTTATGGGAACCAGGGTTTATTTCGTTCGAAGAC

GCTATAAAAAGAGTTTCAAAAATATTCATTAATTCTATAATAAACTTTAATGATCTCGATGAAAATAATT

TTACAACGGTACCACTGGTCATAGATTATGTAACACCTTGTGCATTATGTAAAAAACGATCGCATAAACA

TCCGCATCAACTATCGTTGGAAAATGGTGCTATTAGAATTTACAAAACTGGTAATCCACATAGTTGTAAA

GTTAAAATTGTTCCGTTGGATGGTAATAAACTGTTTAATATTGCACAAAGAATTTTAGACACTAACTCTG

TTTTATTAACCGAACGAGGAGACCATATAGTTTGGATTAATAATTCATGGAAATTTAACAGCGAAGAACC

CTTGATAACAAAACTAATTCTATCAATAAGACATCAACTACCTAAGGAATATTCAAGCGAATTACTCTGT

CCGAGGAAACGAAAGACTGTAGAAGCTAACATACGAGACATGTTAGTAGATTCAGTAGAGACCGATACCT

ATCCGGATAAACTTCCGTTTAAAAATGGTGTATTGGACCTGGTAGACGGAATGTTTTACTCTGGAGATGA

TGCTAAAAAATATACGTGTACTGTATCGACCGGATTTAAATTTGACGATACAAAATTCGTCGAAGACAGT

CCAGAAATGGAAGAGTTAATGAATATCATTAACGATATCCAACCATTAACGGATGAAAATAAGAAAAATA

GAGAGCTGTATGAAAAAACATTATCTAGTTGTTTATGTGGTGCTACCAAAGGATGTTTAACATTCTTTTT

TGGAGAAACCGCAACTGGGAAGTCGACAACCAAACGTTTGTTAAAGTCTGCTATCGGTGACCTGTTTGTC

GAGACGGGTCAAACAATTTTAACAGATGTATTGGATAAAGGACCTAATCCATTTATCGCTAATATGCATT

TAAAAAGATCTGTATTCTGTAGCGAACTACCTGATTTTGCATGTAGTGGATCAAAGAAAATTAGATCTGA

TAATATTAAAAAGTTGACAGAACCTTGTGTCATTGGAAGACCGTGTTTCTCCAATAAAATTAATAATAGA

AACCATGCGACAATCATTATCGATACTAATTACAAACCTGTCTTTGATAGGATAGATAACGCATTAATGA

GAAGAATTGCCGTCGTGCGATTCAGAACACACTTTTCTCAACCTTCTGGTAGAGAGGCTGCTGAAAATAA

TGACGCGTACGATAAAGTCAAACTATTAGACGAGGGATTAGATGGTAAAATACAGAATAATAGATATAGA

TTCGCATTTCTATACTTGTTGGTTAAATGGTACAAAAAATATCATATTCCTATTATGAAACTATATCCTA

CACCGGAAGAGATTCCGGACTTTGCATTCTATCTCAAAATAGGTACTCTGTTGGTATCTAGCTCTGTAAA

GCATATTCCATTAATGACGGACCTCTCCAAAAAGGGATATATATTGTACGATAATGTGGTTACTCTTCCG

TTGACTACTTTCCAACAGAAAATATCCAAGTATTTTAATTCTAGACTATTTGGACACGATATAGAGAGCT

TCATCAATAGACATAAGAAATTTGCCAATGTTAGTGATGAATATCTGCAATATATATTCATAGAGGATAT

TTCATCTCCGTAAATATATGCCATATATTTATAGAATATATCACATATCTAAATGAATACCGGAATCATA

GATTTATTTGATAATCATGTTGATAGTATACCAACTATATTACCTCATCAGTTAGCTACTTTAGATTATC

TAGTTAGAACTATCATAGATGAGAACAGAAGCGTGTTATTGTTCCATATTATGGGATCGGGTAAAACAAT

AATCGCTTTGTTGTTCGCCTTGGTAGCTTCCAGATTTAAAAAGGTTTACATTTTAGTACCGAACATCAAC

ATCTTAAAAATTTTCAATTATAATATGGGTGTAGCTATGAACTTGTTTAATGACGAATTCATAGCTGAGA

ATATCTTTATTCATTCCACAACAAGTTTTTATTCTCTTAATTATAACGATAACGTCATTAATTATAACGG

ATTAAGTCGCTACAATAACTCTATTTTTATCGTTGATGAGGCACATAATATTTTTGGGAATAATACTGGA

GAACTTATGACCGTGATAAAAAATAAAAACAAGATTCCTTTTCTACTATTGTCTGGATCTCCCATTACTA

ACACACCTAATACGCTGGGTCATATTATAGATTTAATGTCCGAAGAGACGATAGATTTTGGTGAGATTAT

TAGTCGTGGTAAGAAAGTAATTCAGACACTTCTTAACGAACGCGGAGTGAATGTACTCAAGGATTTGCTT

AAAGGAAGAATATCATATTACGAAATGCCGGACAAAGATCTACCAACAATAAGATATCACGGACGTAAAT

TTCTAGATACTCGAGTAGTATATTGTCACATGTCTAAACTTCAAGAGAGAGATTATATGATTACTAGACG

GCAGCTATGTTATCATGAAATGTTTGATAAAAATATGTATAACGTGTCAATGGCAGTATTGGGACAACTT

AATCTGATGAATAATTTAGATACGTTATTTCAGGAACAGGATAAGGAATTGTACCCAAATCTGAAAATAA

ATAATGGAGTGTTATACGGTGAAGAATTGGTAACGTTAAACATTAGTTCCAAATTTAAGTACTTTATCAA

TCGGATACAGACACTCAAGGGAAAACACTTTATATACTTCTCTAATTCTACATATGGTGGATTGGTAATT

AAATATATCATGCTCAGTAATGGATATTCTGAATATAATGGTTCTCAGGGAACTAATCCACATATGATAA

ACGGCAAACCAAAAACATTTGCTATCGTTACTAGTAAAATGAAATCGTCTTTAGAGGATCTATTAGATGT

GTATAATTCTCCTGAAAACGATGATGGCAGTCAATTGATGTTTTTGTTTTCGTCAAACATTATGTCTGAA

TCCTATACTCTGAAAGAGGTAAGGCATATTTGGTTTATGACTATCCCGGATACTTTTTCTCAATACAACC

AAATTCTTGGACGATCTATTAGAAAATTCTCTTACGTCGATATTTCTGAACCCGTTAATGTATATCTTTT

AGCAGCCGTATATTCAGATTTCAATGACGAAGTGACGTCATTAAACGATTATACACAGGATGAATTGATT

AATGTTTTACCCTTTGACATCAAAAAGCTGTTGTATCTAAAATTTAAGACTAAAGAAACGAATAGAATAT

ACTCTATTCTTCAAGAGATGTCTGAAACGTATTCTCTTCCACCACATCCATCAATTGTAAAAGTTTTATT

GGGAGAATTGGTCAGACAATTTTTTTATAATAATTCTCGTATTAAGTATAACGACTCCAAGTTACTTAAA

ATGGTTACATCAGTTATAAAAAATAAAGAAGACGCTAGGAATTACATAGATGATATTGTAAACGGTCACT

TCTTTGTATCGAATAAAGTATTTGATAAATCTCTTTTATACAAATACGAAAACGATATTATTACAGTACC

GTTTAGACTTTCCTACGAACCATTTGTTTGGGGAGTTAACTTTCGTAAAGAATATAATGTGGTATCTTCT

CCATAAAACTGATGAGATATATAAAGAAATAAATGTCGAGCTTTGTTACCAATGGATATCTTCCAGTTAC

ATTGGAACCACATGAGTTGACGTTAGACATAAAAACTAATATTAGGAATGCCGTATATAAGGCGTATCTC

CATAGAGAAATTAGTGGTAAAATGGCCAAGAAAATAGAAATTCGTGAAGACGTGGAATTACCTCTCGGCG

AAATAGTTAATAATTCTGTAGTTATAAACGTTCCGTGTGTAATAACCTACGCATATTATCACGTTGGGGA

TATAGTCAGAGGAACATTAAACATCGAAGATGAATCAAATGTAACTATTCAATGTGGAGATTTAATCTGT

AAACTAAGTAGAGATTCGGGTACTGTATCATTTAGCGATTCAAAGTACTGCTTTTTTCGAAATGGTAATG

CGTATGATAACGGCATCGAAGTCTCCGCCGTTCTAATGGAGGCTCAACAAGGTACCGAATCTAGTTTTGT

TTTTCTCGCGAATATCGTTGACTCATAAGAAAGAGAATAGCGGTGAGTATAAATACGAATACTATGGCAA

TAATTGCGAATGTTTTATTCCCTTCGATATATTTTTGATAATATGAAAAACATGCCTCTCTCAAATCAGA

CAACCATTTCATAAAATAGTTCTCTCGCACTGGTGAGGTGGTTGCAGCTCGTATAATCTCCCCAGAATAA

TATACTTGCGTGTCGTCGTTCAATTTATACGGATTTCTATAATTCTCTGTTATATAATGAGGTTTACCCT

CATGATTAGACGACGACAATAGTGTTCTGAATTTAGATAGTTGATCAGAATGAATGTTTATTGGTGTTGG

AAAAATTATCCATGCTGCGTCTGCAGAGTGGTTGATAGTTGTTCCTAGATATGTAAAATAATCCAACGTA

CTAGGTAGCAAATTGTCTAGATAAAATACTGAATCAAATGGCGCAGACATATTAGCGGATCTAATGGAAT

CCAATTGATTGACTATCTTTTGAAAATATACATTTTTATGATCTGATACTTGTAAGAATATAGCAATAAT

GATAATTCCATCATCGTGTTTTTTTGCCTCTTCATAAGAACTATATTTTTTCTTATTCCAATGAACCAGA

TTAATCTCTCCAGAGTATTTGTATACATCTATCAAGTGATTGGATCCATAATCGTCTTCCTTTCCCCAAT

ATATATGTATTGTTGATAACACATATTCATTGGGGAGAAACCCTCCACTTATATATCCTCCTTTAAAATT

AATCCTTACTAGTTTTCCAGTATTCTGGATAGTGGTTGGTTTCGACTCATTATAATGTATGTCTAACGTC

TTCAATCGCGCGTCAGAAATTGCTTTTTTAGTTTCTATATTAATAGGAGATAGTTGTTGAGGCATAGTAA

AAATGAAATGATAACTGTCTAGAAATAGCTCTTAGTATGGGATTTACAATGGATGAGGAAGTGATATTTG

AAACTCCTAGAGAATTAATATCTATTAAACGAATAAAAGATATTCCAAGATCAAAAGACACGCACGTGTT

TGCTGCGTGTATAACAAGTGACGGATATCCGTTAATAGGAGCTAGAAGAACTTCATTCGCATTCCAGGCG

ATATTATCTCAACAAAATTCAGATTCTATCTTTAGAGTATCCACTAAACTATTACGGTTTATGTACTACA

ATGAACTAAGAGAAATCTTTAGACGGTTGAGAAAAGGTTCTATCAACAATATCGATCCTCACTTCGAAGA

GTTAATATTATTGGGTGGTAAACTAGATAAAAAGGAATCTATTAAAGATTGTTTAAGAAGAGAATTAAAA

GAGGAAAGTGATGAACATATAACAGTAAAAGAATTCGGAAATGTAATTCTAAAACTTACAACGAGTGATA

AATTATTTAATAAAGTATATATAGGTTATTGCATGGCATGTTTTATTAATCAATCGTTGGAGGATTTATC

ACATACTAGTATTTACAATGTAGAAATTAGAAAGATTAAATCGTTAAATGATTGTATTAACGACGATAAA

TACGAATATCTGTCTTATATTTATAATATACTAATTAATAGTAAATGAGCTTTTACAGATCTAGTATAAT

TAGTCAGATTATTAAGTATAATAGACGACTAGCTAAGTCTATTATTTGCGAGGATGACTCTCAAATTATT

ACACTCACGGCATTCGTTAACCAATGCCTATGGTGTCATAAACGAGTATCCGTGTCCGCTATTTTATTAA

CTACTGATAACAAAATATTAGTATGTAACAGACGAGATAGTTTTCTCTATTCTGAAATAATTAGAACTAG

AAACATGTATAGAAAGAAACGATTATTTCTGAATTATTCCAATTATTTGAACAAACAGGAAAGAAGTATA

CTATCGTCATTTTTTTCTCTAGATCCAGCTACTGCTGATAATGATAGAATAAACGCTATTTATCCGGGTG

GTATACCCAAAAGGGGTGAGAACGTTCCAGAGTGTTTATCCAGGGAAATCAAAGAAGAAGTTAATATAGA

CAATTCTTTTGTATTCATAGACACTCGTTTTTTTATTCATGGTATCATAGAAGATACCATTATTAACAAA

TTTTTTGAGGTAATTTTCTTTGTTGGACGAATATCTCTAACGAGTGATCAAATTATTGATACCTTTAAAA

GTAATCATGAAATAAAGGATCTAATATTTTTAGATCCAAATTCAGGTAATGGACTCCAATACGAAATTGC

AAAATATGCTCTAGATACTGCAAAACTTAAATGTTACGGTCATAGAGGATGTTATTATGAATCATTAAAA

AAATTAACTGAGGATGATTGATTAGAAAATATAAATTAATTTACCATCGTGTATTTTTATAACGGGATTG

TCTGGCATATCATGTAGATAGTTACCGTCTACATCGTATACTCTACCATCTACGCCTTTAAATCCTCTAT

TTATTGATATTAATCTATTAGAATTGGAATACCAAATATTAGTACCCTCAATTAGTTTATTGGTAATATT

TTTTTTAGACGATAGATCGATGGCTCTTGAAACCAAGGTTTTCCAACCGGACTCATTGTCTATCGGTGAG

AAGTCTTTTTCATTAGCATGAATCCATTCTAATGATGTATGTTTAAACACTCTAAACAATTGTACAAATT

CTTTTGATTTGTTTTGAATGATTTCAAATAGGTCTTCGTCTACAGTAGGCATACCATTAGATAATCTAGC

CATTATAAAGTGCACGTTTACATATCTACGTTCTGGAGGAGTAAGAACGTGACTATTGAGACGAATGGCT

CTTCCTACTATCTGACGAAGAGACGCCTCGTTCCATGTCATATCTAAAATGAAGATATCATTGATTGAGA

AGAAACTAATACCCTCGCCTCCGCTAGAAGAGAATACGCATGTTTTAATGCATTCTCCGTTAGTGTTTGA

TTCTTGGTTAAACTCAGCCACCGCCTTGATTCTAGTATCTTTTGTTCTAGATGAGAACTCTATATTAGAG

ATACCAAAGACTTTGAAATATAGTAATAAGATTTCTATTCCTGACTGATTAACAAATGGTTCAAAGACTA

GACATTTACCATGGGATGCTAATATTCCCAAACATACATCTATAAATTTGACGCTTTTCTCTTTTAATTC

AGTAAATAGAGAGATATCAGCCGCAATAGCATCCCCTCCCAATAGTTCTCCCTTTTTAAAGGTGTCTAAT

GCGGATTTAGAAAATTCTCTATCTCTTAATGAATTTTTAAAATCATTATATAGGGTTGCTATCTCTTGTG

CGTATTCTCCCGGATCACGATTTTGTCTTTCAGGAAAGCTATCGAATGTAAACGTAGTAGCCATACGTCT

CAGAATTCTAAATGATGATATACCAGTTTTTATTTCTGCGAGTTTAGCCTTTTGATAAATCTCTTCTTGC

TTTTTTGACATATTAACGTATCGCATTAATACTGTTTTCTTAGCGAATGATGCAGACCCTTCCACATCAT

CAAAAATAGAAAACTCGTTATTAACTATGTACGAACATAGGCCTCCTAGTTTGGAGACTAATTCTTTTTC

ATCGACTAGACGTTTATTCTCAAATAGCGATTGGTGTTGTAAGGATCCTGGTCGCAGTAAGTTAACCAAC

ATGGTGAATTCTTGCACACTATTAACGATAGGTGTAGCCGATAAACAAATCATCTTATGGTTTTTTAACG

CAATGGTCTTAGATAAAAAATTATATACTGACCGAGTAGGACGGATCTTACCATCTTCTTTGATTAATGA

TTTAGAAATGAAGTTATGACATTCATCAATGATGACGCATATTCTACTCTTGGAATTAATAGTTTTGATA

TTAGTAAAAAATTTATTTCTAAAATTTTGATCATCGTAATTAATAAAAATACAATCCTTCGTTATCTCTG

GAGCGTATCTGAGTATAGTGTTTATCCAAGGATCTTCTATCAAAGCCTTTTTTACCAATAAGATAATTGC

CCAATTCGTATAAATATCCTTAAGATGTTTGAGAATATATACAGTAGTCATTGTTTTACCGACACCTGTT

TCATGGAACAATAAAAGAGAATGCATACTGTCTAATCCTAAGAAAACTCTTGCTACAAAATGTTGATAAT

CCTTGAGGCGTGCTACGTCTGACCCCATCATTTCAACGGGCATATTAGTAGTTCTGCGTAAGGCATAATC

GATATAGGCCGCGTGTGATTTACTCATTTATGAGTGATAAGTAATAACTATGTTTTAAAAATCACAGCAG

TAGTTTAACTAGTCTTCTCTGATGTTTGTTTTCGATACTTTTTGAATCAGAAGTCATACTAGAATAAAGC

AGCGAGTGAACGTAATAGAGAGCTTCGTATACTCTATTCGAAAACTCTAAGAACTTATTAATGAATTCCG

TATCCACTGGATCGTTTAAAATACTAAATTGAACAGTGTTCACATCCTTCCAAGACGAAGACTTAGTGAC

GGACTTAACATGAGACATAAATAAATCCAAATTTTTTTTATAAACATCACTAGCCACCATAATGGCGCTA

TCTTTCAACCAACTATCGCTTACGCATTTTAACAGTCTAACATTTTTAAAGAGACTACAATATATTCTCA

TAGTATCGATTACACCTCTACCGAATAGAGTGGGAAGTTTAATAATACAATATTTTTCGTTTACAAAATC

AAATAATGGTCGAAACACGTCGAAGGTTAACATCTTATAATCGCTAATGTATAGATTGTTTTCAGTGAGA

TGATTATTAGATTTAATAGCATCTCGTTCACGTTTGAACAGTTTATTGCGTGCGCTGAGGTCGGCAACTA

CGGCATCCGCTCTAGTACTCCTCCCATAATACTTTACGCTATTAATCTTTAAAATTTCATAGACTTTATC

TAGATCGCTTTCTGGTAACATGATATCATGTGTAAAAAGTTTTAACATGTCGGTCGGCATTCTATTTAGA

TCATTAACTCTAGAAATCTGAAGAAAGTAATTAGCTCCATATTCCAGACTAGGTAATGGGCTTTTACCTA

AAGACAAGTTAAGTTCTGGCAATGTTTCATAAAATGGAAGAAGGACATGTGTCCCCTCCCGGATATTTTT

TACAATTTCATCCATTTACAACTCTATAGTTTGTTTTCATTATTATTAGTTATTATCTCCCATAATCTTG

GTAATACTTACACCTTGATCATAAGATACCTTATACAGGTCATTACATACAACTACCAATTGTTTTTGTA

CATAATAGATTGGATGATTGACATCCATGGTGGAATAAACTACTCGAACAGATAGTTTATCTTTCCCCCT

AGATACATTGGCCGTAATAGTTGTCGGCCTAAAGAATATCTTTGGTGTAAAGTTAAAAGTTAGGGTTCTT

GTTCCATTATTGCTTTTTGTCAGTAGTTCGTTATAAATTCTCGAGATGGGCCCGTTCTCTGAATATAGAA

CATCATTTCCAAATCTAACTTCTAGTCTAGAAATAATATCGGTCTTATTTTTAAAATCTATTCCCTTGAT

GAATGGATCGTTAATAAACAAATCCTTGGCCTTTGATTCGGCTGATCTATTATCTCCGTTATAGACGTTA

CGTTGACTAGTCCAAAGACTTACAGGAATAGATGTATCGATGATGTTGATAGTATGTGATATGTGAGCAA

AGACTGTTCTCTTGGTGGCGTCGCTATATGTTCCAGTAATGGCGGAAAACTTTTTAGAAATGTTATATAT

AAAAGAATTTTTTCGGGTTCCAAACATTAACAGATTAGTATGAAGATAAACACTCATATTATCAGGAACA

TTATCAATTTTTACATAAACATCGGCATCTTGAATAGAAACAACACCATCTTCTGGAACCTCTACGATCT

CGGCAGATTCCGGATAACCAGTCGGTGGACCATCACTAACAATAACTAGATCATCCAACAATCTACTCAC

ATATGCGTCTATATAATCTTTTTCATCTTGTGAGTACCCTGGATACGAAATAAATTTGTTATCAGTATTT

CCATAATAAGGTTTAGTATAAACAGAGAGAGATGTTGCTGCATGAACTTCGGTTACTGTCGCCGTTGGTT

GGTTTATTTGACCTATTACTCTCCTAGGTTTCTCTATAAATGATGGTTTAATTTGTACATTCTTAACCAT

ATATCCAATAAAGCTCAATTCAGGAACATAAACAAATTCTTTGTTGAACGTTTCAAAGTCGAACGAAGAG

TCACGAATAACGATATCGGATACTGGATTGAAGGTCACCGTTACGGTAATTTTTGAATCGGATAGTTTAA

GACTACTGAATGTATCTTCCACATCAAACGGAGTTTTAATATAAACGTATACTGTAGATGGTTCTTTAAT

AGTGTCATTAGGAGTTAGACCAATAGAAATATCATTAAGTTCACTAGAATATCCAGAATGTTTCAAAGCA

ATTGTATTATTGATACAATTATTATATAATTCTTCGCCCTCAATTTCCCAAATAACACCGTTACACGAAG

AGACAGATACATTATTAATACATTTATATCCAACATATGGTACGTAACCGAATCTTCCCATACCTTTAAC

TTCTGGAAGTTCCAAACTCAGAACCAAATGATTAAGCGCAGTAATATACTGATCCCTAATTTCGAAGCTA

GCGATAGCCTGATTGTCTGGCCCATCGTTTGTCATAACTCCGGATAGAGAAATATATTGCGGCATATATA

AAGTTGGAATTTGACTATCAACTGCGAAGACATTAGACCGTTTAATAAAGTCATCCCCACCGATCAAAGA

ATTAATGATAGTATTATTCATTTTCTATTTAAAATGGAAAAAGCTTACAATAAACTCCGTAGAGAAATAT

CTATAATTTGTGAGTTTTCCTTAAAGTAACAGCTTCCGTAAACACCGTCTTTATCTCTTAGTAAGTTTAT

TGTATTTATGACCTTTTCCTTATCTTCATAGAATACTAAAGGCAATAAAGAAATTTTTGGTTCTTCTCTA

AGAGCTACGTGAGACTTAACCATAGACGCCAACGAATCCCTACATATTTTAGAACAGAAATACCCAACTT

CACCACCCTTGAATGTCTCAATACTAATAGGTCTAAAAACCAAATCTTGATTACAAAACCAACACTTATC

AATTACACTATTTGTCTTAATAGACATATCTGCCATAGATTTATAATACTTTGGTAGTATACAAGCGAGT

GCTTCTTCTTTAGCGGGCTTAAAGACTGCTTTAGGTGCTGAAATAACCACATCTGGAAGACTTACTCGCT

TAGCCATTTAATTACGGAACTATTTTTTTATACTTCTAATGAACAAGTAGAAAACCTCTCATCTACAAAA

ACATACTCGTGTCCATAATCCTCTACCATAGTAACACGTTTTTTAGATCTCATATGTGCTAAAAAGTTTT

CCCATACTAATTGGTTACTATTATTTTTCGTATAATTTTTAACAGTTTGAGGTTTTAGATTTTTAGTTAC

AGAAGTGATATCGAATATTTTATCCAAAAAGAATGAGTAATTAATTGTCTTAGAAGGAGTGTTTTCTTGG

CAAAAGAATACCAAGTGCTTAAATATTTCTACTACTTCATTAATCTTTTCTGTACTCAGATTCAGTTTCT

CATCTTTTACTTGATTGATTATTTCAAAGACTAACTTATAATCCTTTTTATTTATTCTCTCGTTAGCCTT

AAGAAAACTAGATACAAAATTTGCATCTACATCATCCGTGGATATTTGATTTTTTTCCATGATATCCAAT

AGTTCCGAGATAATTTCTCCAGAACATTGATGAGACAATAATCTCCGCAATACATTTCTCAAATGAATAA

GTTTATTAGACACGTGGAAGTTTGACTTTTTTTGTACCTTTGTACATTTTTGAAATACAGACTCGCAAAA

AATACAATATTCATATCCTTGTTCAGATACTATACCGTTATGTCTACAACAGCTACATAATCGTAGATTC

ATGTTAACACTCTACGTATCTCGTCGTCCAATATTTTATATAAAAACATTTTATTTCTAGACGTTGTCAG

AAAATCCTGTAATATTTTTAGTTTTTTTGGTTGTGAATAAAGTATCGCCCTAATAATATTGGTACCGTCT

TCCGACAATATAGTAGTTAAATTATCCGAGCATGTAGAAGAACACCGCTTAGGCGGATTCAGTACAATGT

TATATTTTTCGTACCAACTCATTTAAATATCATAATCTAAAATAGTTCTGTAATATGTCTAGCGCTAATA

TATTGATCATAATCCTGTGCATAAATTAAGATACAACAATGTCTTGAAATCATCGACATGGCTTCTTCCA

TAGTTAGAAGATCATCGTCAAAGTTAGCAACGTGATTCATCAACATTTGCTGTTTTGAGGCAGCAAATAC

TGAACCATCACCATTCAACCATTCATAAAAACCATCGTCTGAATCCATTGATAATTTCTTGTACTGGTTT

TTGAGAGCTCGCATCAATCTAGCATTTCTAGCTCCCGGATTGAAAACAGAAAGAGGATCGTACATCCAGG

GTCCATTTTCTGTAAATAGAATCGTATAATGTCCCTTCAAGAAGATATCAGACGATCCACAATCAAAGAA

TTGGTCTCCGAGTTTGTAACAGACTGCGGACTTTAACCTATACATGATACCGTTTAGCATGATTTCTGGT

GATACGTCAATCGGAGTATCATCTATTAGAGATCTAAAGCCGGTGTAACATTCTCCACCAAACATATTCT

TATTCTGACGTCGTTCTACATAAAACATCATTGCTCCATTAACGATAACAGGTGAATGAACAGCACTACC

CATCACATTAGTTCCCAATGGATCAATGTGTGTAACTCCAGAACATCTTCCATAGCCTATGTTAGGAGGA

GCGAACACCACTCTTCCACTATTGCCATCGAATGCCATAGAATAAATATCCTTGGAATTGATAGAAATCG

GACTGTCGGATGTTGTTATCATCTTCATAGGATTAACAACGATGTATGGTGCAGCCTGAAGTTTCATATC

GTAACTGATGCCGTTCATAGGTCTAGCCACAGAAACCAACGTAGGTCTAAATCCAACTATAGACAAAATA

GAAGCTAATATCTGTTCCTCATCTGTCATAACTTGAGAGCATCCAGTATGAATAATCTTCATTAGATGGG

GATCTACCGCATCATCATCGTTACAATAAAAAATTCCCATTCTAATGTTCATAATTGCTTTTCTAATCAT

GGTATGAATGTTTGCTCTCTGAATCTCTGTGGAAATTAGATCTGATACACCTGTAATCACTATCGGATTA

TCCTCCGTAAGACGATTAACCAACAACATATAATTATAAGACTTTACTCTTCTAAATTCATAAAGTTGCT

GGATTAGACTATATGTGTCTCCATGTACATACGCGTTCTCGAGCGCAGGAAGTTTAATACCGAATAGTGC

CATCAGAATAGGATGAATGTAGTAATTAGTTTCTGGTTTTCTATAAATAAAAGACAAATCTTGTGAACTA

GACATATCGGTAAAATGCATGGATTGGAATCGTGTAGTCGACAGAAGAATATGATGATTAGATGGAGAGT

ATATTTTATCTAACTCTTTGAGTTGGTCACCGATTCTAGGACTAGCTCGAGAATGAATAAGTACTAAGGG

ATGAGTACATTTCACAGAAACACTGGCGTTGTTCAACGTACTCTTTACATGGGAAAGGAGTTGAAATAGC

TCGTTTCTATTTGTCCTGACAATATTTAGTTTATTCATAATATTAAGCATATCCTGAATAGTAAAGTTAG

ATGTGTCATACTTGTTAGTAGTTAGATATTTAGCAATTGCATTCCCATCATTTCTCAATCTCGTACTCCA

ATCATGTGTGGATGCTACTTCGTCGATGGAAACCATACAATCCTTTTTGATAGGCTGTTGAGATTGATCA

TTTCCTGTACGTTTAGGTTTGGTACGTTGATTTCTAGCCCCTGCTGATATAAAGTCATCGTCTACAATTT

GGGATAATGAATTACATACACTACAAGACAAAGATTTATCAGAAGTGTGAATATGATCTTCATCTACCAA

AGAAAGAGTTTGATTAGTATAACTAGATTTTAGTCCCGCGTTAGATGTTAAAAAAACATCGCTATTGACC

ACGGCTTCCATTATTTATATTCGTAGTTTTTACTCGAAAGCGTGATTTTAATATCCAATCTTATTACTTT

TGGAATCGTTCAAAACCTTTGACTAGTTGTAGAATTTGATCTATTGCCCTACGCGTATACTCCCTTGCAT

CATATACGTTCGTCACCAGATCGTTTGTTTCGGCCTGAAGTTGACGCATATCTTTTTCAACACTCGACAT

GAGATCCTTAAGGGTCATATCGTCTAGATTTTGTTGAGATGCTGCTCCTGGATTTGGATTTTGTTGTGCT

GTTGTACATACTGTACCACCAGTAGGTGTAGGAGTACATACAGTGGCCACAATAGGAGGTTGAAGAGGTG

TAACCGTTGGAGTAGTACAAGAAATACTTCCATCCGATTGTTGTGTACATGTGGTTGTTGGTAACGTCTG

AGAAGGTTGGGTAGATGGTGGTGTCGTCATCTTTTGATCTTTATTAAATTTAGAGATAATATCCTGAACA

GTATTGCTCGGCGTCAACGCTGGAAGGAGTGTACTCGCCGGCGCATCAGTATCTGTAGACAACCAATCAA

AAAGATTAGACATATCAGATGATGTATTAGTTTGTTGACGTGGTTTTAGTACAGGAGCAGTACTACTAGG

TAGAAGAATAGGAGCCGGTGTAGGTGTCGGAACCGGCTGTGGAGTTATATGAATAGTTGGTTGTAGCGGT

TGGGTAGGCTGTCTGCTGGCGGTCATCATATTATCTCTAGCTAGTTGTTCTCGCAACTGTCTTTGATAAT

ACGACTCTTGAGACTTTAGTCCTATTTCAATCGCTTCATCCTTTTTCGTATCCGGATCCTTTTCTTCAGA

ATAATAGATTGACGACTTTGGTGTAGAGGATTCTGCCAGCCCCTGTGAGAACTTGTTAAAGAAGTCCATT

TAAGGCTTTAAAATTGAATTGCGATTATAAGATTAAATGGCAGACACGGACGATATTATCGACTATGAAT

CCGATGATCTCACCGAATACGAGGATGATGAAGAAGATGGAGAGTCACTAGAAACTAGTGATATAGATCC

CAAATCTTCTTATAAGATTGTAGAATCAACATCCACTCATATAGAAGATGCGCATTCCAATCTTAAACAT

ATAGGGAATCATATATCTGCTCTTAAACGACGCTATACTAGACGTATAAGTCTATTTGAAATAGCGGGTA

TAATAGCAGAAAGCTATAACTTGCTTCAACGAGGAAGATTACCTCTAGTTTCAGAATTTTCTGACGAAAC

GATGAAGCAAAATATGCTACATGTAATTATACAAGAGATAGAGGAGGGTTCTTGTCCTATAGTCATCGAA

AAGAACGGAGAATTGTTGTCGGTAAACGATTTTGATAAAGATGGTCTAAAATTCCATCTAGACTATATTA

TCAAAATTTGGAAACTTCAAAAACGATATTAGAATTTATACGAATATCGTTCTCTAAATGTCACAATCAA

GTCTCTCATATTCAGCAGTTTATTGTCGTACTTTATATCGTGTTCATTAACGATATTTTGCAAAATAGTA

ATGATTCTATCTTCCTTCGATAGATATTCTTCAGAGATTATTGTCTTATATTCTTTCTTGTTATCCGATA

TGAATTTGATAAGACTTTGAACATTATTAATACCCGTCTGTTTAATTTTTTCTATAGATATTTTAGTTTT

GGTAGATTCTATGGTGTCTGTTAATAGGCATCCAACATCGACATTCGACGTCAATTGTCTATAAATCAGA

GTATAAATTTTAGAAATAACATTAGCAAATTGTTGTGCGTTGATGTCGTTATTCTGAAACAGTATGATTT

TAGGTAGCATTTTCTTAACAAAGAGAACGTATTTATTGTTACTCAGTTGAACAGATGATATATCCAGATT

ACTAACGCATCTGATTCCATATACCAAACTTTCAGAAGAAATGGTGTACAATTGTTTGTATTCATTCAAT

GTCTCCTTTTCAGAAATTAGTTTAGAGTCGAATACTGCAATAATTTTCAAGAGATAGTTTTCATCAGATA

AGATTTTATTTAGTGTAGATATGATAAAACTATTGTTTTGTTGGAGAACTTGATACGCCGCATTCTCTGT

AGTCGACGCTCTCAAATGGGAAACAATCTCTATTATTTTTTTGGAATCGGATACTATATCTTCGGTATCT

TGACGCAGTCTAGTATACATAGAGTTAAGAGAAATTAGAGTTTGTACATTAAGCAACATGTCTCTAAATG

TGGCTACAAACTTTTCTTTTTCCACATCATCTAGTTTATTATATACCGATTTCACAACGGCACCAGATTT

AAGGAACCAGAATGAAAAACTCTGATAACTACAATATTTCATCATAGTTACGATTTTATCATCTTCTATA

GTTGGTGTGATAACACATACCTTTTTCTCCAAGACTGGAACCAACGTCATAAAAATGTTTAAATCAAAAT

CCATATCAACATCTGATGCGCTAAGACCAGTCTCGCGTTCAAGATTATCTTTACTAATGGTGACGAACTC

ATCGTATAGAACTCTAAGTTTGTCCATTATTTATTTACAGATTTAGTTGTTTAATTTATTTGTGCTCTTC

CAGAGTTGGGATAGTATTTTTCTAACGTCGGTATTATATTATTAGGATCTACGTTCATATGTATCATAAT

ATTAATCATCCACGTTTTGATAAATCTATCTTTAGCTTCTGAAATAACGTATTTAAACAAAGGAGAAAAA

TATTTAGTTACGGCATCAGACGCGATAACATTTTTTGTAAATGTAACGTATTTAGACGACAGATCTTCGT

TAAAAAGTTTTCCATCTATGTAGAATCCATCGGTTGTTAACACCATTCCCGCGTCAGAGTGAATAGGAGT

TTGAATAGTTTGTTTTGGAAATAGATCCTTCAATAACTTATAGTTGGGTGGGAAAAAATCGATTTTATCA

CTAGACTCTTTCTTTTTTACTATCATTACCTCATGAACTATTTCTTGAATGAGTATATGTATTTTCTTTC

CTATATCGGTCGCGTTCATTGGAAAATATATCATGTCGTTAACTATAAGAATATTTTTATCCTCGTTTAC

AAACTGAATAATATCAGATATAGTTCGTAAACGAACTATATCATCACCAGCACAACATCTAACTATATGA

TATCCACTAGTTTCCTTTAGCCGTTTATTATCTTGTTCCATATTAGCAGTCATTCCATCATTTAAGAAGG

CGTCAAAGATAATAGGGAGAAATGACATTTTGGATTCTGTTACGACTTTACCAAAATTAAGGATATACGG

ACTTACTATCTTTTTCTCAACGTCGATTTGATGAACACACGATGAAAATGTACTTCGATGAGATTGATCA

TGTAGAAAACAACAAGGGATACAATATTTCCGCATATCATGAAATATATTAAGAAATCCCACTTTATTAT

ATTTCCCCAAAGGATCAATGCATGTAAACATTATACCGTTATCATTAATAAAGACTTCTTTCTCATCGGA

TCTGTAAAAGTTGTTACTGATTTTTTTCATTCCAGGATCTAGATAATTAATAATAATGGGTTTTCTATTC

TTATTCTTTGTATTTTGACATATCCTAGACCAGTAAACAGTTTCCACTTTGGTAAAATCAGAAGACTTTT

GAACGCTATTAAACATGGCATTAATGGCAATAACTAAAAATGTAAAATATTTTTCTATGTTAGGAATATG

GTTTTTCACTTTAATAGATATATGGTTTTTTGCCAAAATGATAGATATTTTTTTATCCGATGATAGTAAA

ATATTATTAGTCGCCGTCTCTATAAAAATGAAGCTAGTCTCGATATCCAATTTTATTCTAGAATTGATAG

GAGTCGCCAAATGTACCTTATACGTTATATCTCCCTTGATGCGTTCCATTTGTGTATCTATATCGGACAC

AAGATCTGTAAATAGTTTTACGTTATTAATCATCACGGTATCGCCATCGCTAGATAATGCTAATGTACTA

TCCAAGTCCCAAATGGAGAGATTTAACTGTTCATCGTTTAGAATAAAATGATTACCTGTCATATTAATAA

AGTGTTCATCGTATCTAGATAACAACGACTTATAATTAATGTCCAAGTCTTGAACTCGCTGAATGATCTT

TTTTAACCCAGTTAGTTTTAGATTGGTACGAAATATATTGTTAAACTTTGATTCTACAGTAATGTCCAAA

TCTAGTTGTGGAAATACTTCCATCAACATTGTTTCAAACTTGATAATATTATTATCTACATCTTCATACG

ATCCAAATTCCGGAATAGATGTATCGCACGCTCTGGCCACCCAGATAACCAAAAAGTCACACGCTCCAGA

ATATACATTGTATAAAAAGCTATCGTTTTTTAGTAGTGTTTTTTTCTGAGTATATACGAAAGGATTAAAA

ATAGTATTATCAACGTAACTATATTCCAAATTATTCTTATGAGAATAGATAATAATATCGTCCTTAATAT

CTAACAAATTTCCTAAATATCCCTTTAATTGAGTCATTCGAAGCGTTAATAAAATATGTCTCTTAACTAT

TTCCGGCCGTTGTATATTTAAATGACTTCGTAAGAAATAATATATAGGCGACTTCTCATCTATGTAATCA

TATGGAGTGAGATATAGGGCTCGTTCTACCTCCTGCCCCTTACCCACCTGTAATACCAATTGCGGACTCA

CTATATATCGCATATTTATATCGTGGGGTAAAGTGAAAATCTACTACCGATGATGTAAGTCTTACAATGT

TCGAACCAGTACCAGATCTTAATTTGGAGGCCTCCGTAGAACTAGGGGAGGTAAATATAGATCAAACAAC

ACCTATGATAAAGGAGAATAGCGGTTTTATATCCCGTAGTAGACGTCTATTCGCCCATAGATCTAAGGAT

GATGAGAGAAAACTAGCACTACGATTCTTTTTACAAAGACTTTATTTTTTAGATCATAGAGAGATTCATT

ATTTGTTCAGATGCGTCGACGCTGTAAAAGACGTCACTATTACCAAAAAAAATAACATTATCGTGGCGCC

TTATATAGCACTTTTAACTATCGCATCAAAAGGATGCAAACTTACAGAAACAATGATTGAAGCATTCTTT

CCAGAACTATATAATGAACATAGTAAGAAATTCAAATTCAACTCTCAAGTATCCATCATCCAAGAAAAAC

TCGGATACCAGTCTGGAAACTATCACGTTTATGATTTTGAACCGTATTACTCTACAGTAGCTCTGGCTAT

TCGAGATGAACATTCATCTGGCATTTTTAATATCCGTCAAGAGAGTTATCTTGTAAGTTCATTATCTGAA

ATAACATATAGATTTTATCTAATTAATCTAAAATCTGATCTTGTTCAATGGAGTGCTAGTACGGGCGCTG

TAATTAATCAAATGGTAAATACTGTATTGATTACAGTGTATGAAAAATTACAACTGGCCATAGAAAATGA

TTCACAATTTACATGTTCATTGGCTGTGGAATCAGAACTTCCAATAAAATTACTTAAAGATAGAAATGAA

TTATTTACAAAATTCATTAACGAGTTAAAAAAGACCAGTTCATTCAAGATAAGCAAACGTGATAAGGATA

CGCTATTAAAACATTTTACTTATGACTGGAGTTAGAATTTATAGACGACACATTTCGTTTATCATTGTTA

CTATTACTATCATTATTAGTATTCTTCTTGTCATCTTGTTCAGAAATATACAGCAATGCTATACCTAATA

CTAAATACATTATCATGCTTGCAATGGCTCTAACAACAACGAACCAAAATGAATTTGGTCGTAGCTTTTG

TTCACAAAAATACATAAAGAAATGTCTACATAAATCTATGGCGCCATTGGCTACTTGAAATAGCGCCAGT

CCTCCTACAGATTTTAATATAGCTGTATAACATGACATTTATTCATCATCAAAAGAGACAGAGTCACCAT

CTGTCATATTTAGATTTTTTTTCATGTGTTCAAAGTATCCTCTACTCATTTCATTATAATAGTTTATCAT

GCTTAGAATTTTAGGACGGATCAATGAGTAAGACTTGACTAGATCGTCAGTAGTAATTTGTGCATCATCT

ATTCTGCATCCGCTTCGTCGAATAATGTATAGCATCGCTTTGAGATTCTCCATAGCTATCAAGTCTTTAT

ATAATGACATGGAAATATCTGTGAATGCTTTATACTTCTCCAACATCGATGCCTTAACATCATCACATAC

TTTAGCATTGAAAATACGTTCTATTGTGTAGATGGATGTAGCAAGATTTTTAAACAACAATGCCATCTTA

CATGATGATTGTCTCAAGTCTCCAATCGTTTGTTTAGAACGATTAGCTACAGAGTCCAATGCTTGGCTAA

CTAGCATATTATTATCTTTAGAAATTGTATTCTTCAATGAGGCGTTTATCATATCTGTGATTTCGTTAGT

CATATTACAGTCTGACTGGGTTGTAATGTTATCCAACATATCACCTATGGATACGGTACACGTACCAGCA

TTTGTAATAATCCTATCTAAGATGTTGTATGGCATTGCGCAGAAAATATCTTCTCCTGTAATATCTCCAC

TCTCGATAAATCTACTCAGATTATTCTTAAATGCCTTATTCTCTGGAGAAAAGATATCAGTGTCCATCAT

TTCATTAATAGTATACGCAGAAAAGATACCACGAGTATCAATTCTATCCAAGATACTTATCGGTTCCGAG

TCACAGATAATTGTTTCCTCTCCTTCGGGAGATCCTGCATAGAAATATCTAGGACAATAGTTTCTATACT

GTCTGTAACTCTGATAATCTCTAAAGTCACTAACTGATACCATGAAATTGAGAAGATCAAACGCTGAAGT

AATCAATTTTTCTGCCTCGTTTTTACTACAACTAGTTTTCATCAATGTAGTGACGATGTATTGTTTAGTT

ACTCTTGGTCTAATACTGATGATAGAGATATTATTGCTTCCCATAATGGATCTTCTAGTAGTCACCTTAA

AGCCCATTGATGCGAATAGCAGATAGATAAAGTCTTGGTATGACTCCTTTCTAATATAGTACGGACTACC

TTTGTCACCCAACTTTATACCCACATAAGCCATAACAACCTCTTTAATAGCCGTTTCATGAGGTTTATCA

GCCATGAGCCTGAGTAGTTGAAAGAATCGCATGAATCCCGTCTCAGAAAGTCCTATATGCATGATAGATT

TATCTTTCCTGGGAAACTCTCGTATAGTTATAGATGAAATACTCTTCAAAGTTTCTGAAATAAGATTAGT

AACAGTCTTACCTCCGACTACTCTGGGTAACAAACATACTCTAATAGGTGTTTTCTCTGCGGAGATAATA

TCAGAAAGGATAGAGCAATAAGTAGTATTATTGTGATTATAAAGACCGAATACATAACAGGTAGAATTTA

TAAACATCATGTCCTGAAGGGTTTTAGACTTGTATTCCTCGTAATCTATACCGTCCCAAAACATGGATTT

GGTAACTTTGATAGCCGTAGATCTTTGTTCCTTCGCTAACAGGTTAAAGAAATTAATAAAGAATTTGTTG

TTTCTATTTATGTCCACAAATTGCACGTTTGGAAGCGCCACGGTTACATTCACTGCAGCATTTTGAGGAT

CGCGAGTATGAAGTACGATGTTATTGTTTACTGGTATATCTGGAAAGAAATCTACCAGTCTAGGAATAAG

AGATTGATATCGCATAGAAATAGTAAAGTTTATAATCTCATCATTGAAGATTACTCTGTTACCATTGTAA

TAAATTGGTACTCTATCATAATCATCGACAAAGTACTGTTCATACATGATGAGATGTTTATATGTTGGCA

TAGTAGTGAGATCGACGTTTGGTAATGGCAATGTATTAAGATTAACTCCATAATGTCTAGCAGCATCTGC

GATGTTATAAGTGATGTCAAAGCGGGGTTGATCTTGTGCTGTTATATATTGTCTAACACCTATAAGATTA

TCAAAATCTTGTCTGCTTAATACACCGTTAACAATTTTTGCCTTGAATTCTTTTATTGGTGCATTAATAA

CATCCTTATAGAGGATGTTAAACAAATAAGTATTATCAAAGTTAAGATCTGGGTATTTCTTTTCTGCTAG

AACATCCATTGAGTCGGAGCCATCTGGTTTAATATAACCACCGATAAATCTAGCTCTGTATTCTGTATCC

GTCAATCTAATATTAAGAAGGTGTTGAGTGAAAGGTGGAAGATCGTAAAAGCTGTGAGTATTAATAATAG

GGTTAGTTTCCGAACTAATGTTAATTGGATGATTAATAATATCTATATTTCCAGCGTTAAGTGTAACATT

AAACAGTTTTAATTCACGTGACGTGGTATCAATTAAATAATTAATGCCCAATTTGGATATAGTAGCCTGA

AGCTCATCTTGTTTAGTTACGGATCCTAATGAGTTATTAAGAAATACATCGAACGGATGAACGAAGGTTG

TTTTAAGTTGGTCACATACTTTGTAATCTAGACATAGATGTGGAAGAACGGTAGAAACTATACGAAATAG

ATATTCAGAGTCCTCTAATTGATCAAGAGTAACTATTGACTTAATAGGCATCATTTATTTAGTATTAAAT

GACGACCGTACCAGTGACAGATATACAAAACGACTTAATTACAGAGTTTTCAGAAGATAATTATCCATCT

AACAAAAATTATGAAATAACTCTTCGCCAAATGTCTATTCTAACTCACGTTAACAACGTGGTAGATAGAG

AACATAATGCCGCCGTAGTGTCATCTCCAGAGGAAATATCATCACAACTTAATGAAGATCTATTTCCAGA

TGATGATTCACCGGCCACTATTATCGAACGAGTACAACCTCATACTACTATTATTGACGATACGCCACCT

CCTACTTTTCGTAGAGAGTTATTGATATCGGAACAACGTCAACAACGAGAAAAAAGATTTAATATTACAG

TATCAAAAAATTCTGAAGCAATAATGGAATCTAGATCTATGATAACTTCTATGCCAACACAAACACCATC

CTTGGGAGTAGTTTATGATAAAGATAAAAGAATTCAGATGCTAGAGGATGAAGTGGTTAATCTTAGAAAT

CAACGATCTAATACAAAATCATCTGATAATTTAGATAATTTTACCAGAATACTATTTGGTAAGACTCCGT

ATAAATCAACCGAAGTTAATAAGCGTATAGCCATCGTTAATTATGCAAATTTGAACGGGTCCCCCTTATC

AGTCGAGGACTTGGATGTCTGTTCGGAGGATGAAATAGATAGAATCTATAAAACGATTAAACAATATCAC

GAAAGTAGAAAACGAAAAATTATCGTCACTAACGTGATTATTATTGTCATAAACATTATTGAGCAGGCAT

TGCTAAAACTCGGATTTGAAGAAATCAAAGGACTGAGTACCGATATCACTTCAGAAATTATCGATGTGGA

GATCGGAGATGACTGCGATGCTGTAGCATCAAAACTAGGAATCGGTAACAGTCCGGTTCTTAATATTGTA

TTGTTTATACTCAAGATATTCGTTAAACGAATTAAAATTATTTAATTTAATACATTCCCATATCCAGACA

ACAATCGTCTGGATTAATCTGTTCCTGTCGTCTCATACCGGACGACATATTAATCTTTTTATTAGTGGGC

ATCTTTTTAGATGGTTTCTTTTTCCCAGCATTAACTGATTCGATACCTAGAAGATCGTGATTGATTTCTC

CGACCATTCCACGAACTTCTAATTGGCCGTCTCTAACGGTACCATAAACTATTTTACCAGCATTAGTAAC

AGCTTGGACAATCTGACCATCCATTGCGTTGAATGATGTAGTTGCTGTTGTTCTACGTCTAGGAGCACCA

GAGGTATTTTTAGAGCTCTTGGATGTTGATGTAGAAGACGAGGATTTTGATTTTGGTTTACATGTAATAC

ATTTTGAACTCTTTGATTTTGTATCACATGCACCGGCAGTCACATCTGTTTGAGAATTAAGATTATTGTT

GCCTCCTTTGACGGCTGCATCTCCACCGATCTGCGCTAGTAGATTTTTAAGCTGTGGTGTAATCTTATTA

ACTGTTTCAATATAATCATCGTAACTACTTCTAACGGCTAAATTTTTTTTATCCGCCATTTAGAAGCTAA

AAATATTTTTATTTATGCAGAAGATTTAACTAGATTATACAATGAACTAATATGATCCTTTTCTAGATTA

TTTACGAACTTGGTATTTCTTGTTTCTGGAGGAGGAGAATTTAAATTCGGACTTGGATTCGGATTTTGTG

GGTTCTTGATCTTATTATACAGCGTGTATAGGATGGCGACGGTAACTGCTACACAAATACCGATCAACAG

AAGAATACCAATCATTTATTGACAATAACTTCACTATGATCAAGTATGTAATAATCATCTTTTCACTAAG

TAAGTAGTAATAATGATTCAACAATGACACGATATATGGACGATAATAATTTAGTTCATGGAAATATCGC

TATGATTGGTGTGAATGACTCCGCTAACTCTGTGGGGTGCACAGTGCTTTCCCCACATAGAATAAATTAG

CATTCCGACTGTGATAATAATACCAAGTATAAACGCCATAATACTCAATACTTTCCATGTACGAGTGGGA

CTGGTAGACTTACTAAAGTCAATAAAGGCGAAGATACACGAAAGAATCAAAAGAATGATTCCAGCGATTA

GCACGCCAGAAAAATAATTTCCAATCATAAGCATCATGTCCATTTAACTAATAAAAATTTTAAATCGCCG

AATAAACAAAGTGGAATATAAACCATATAAAAACAATAGTTTGTACTGCAAAAATAATATCTATTTTTGT

TTTCGAAGATATGGTAAAATTAAATAGTAGTACACAGCATGTTATAACTAACAGCAGCAACGGCTCGTAA

TTACTTATCATTTACTAGACGAAAAGGTGGTGGGATATTTTCTTGCTCAAATAATACGAATATATCACCC

ATCCATTTTATACGATGTTTATATACTCTAATCTTTAATAGATCTATAGATGACGGGTTTACCAATAATA

TAGATTTTATCGATTCATCTAATTTAAACCCTTCCTTAAACGTGAATGATCTATTATCTGGCATAATGAT

GACCCTACCTGATGAATCTGACAATGTACTGGGCCATGTAGAATAAATTATCAACGAATTATCGTCTACG

AACATTTATATCATTTGTTTTAATTTTAGGACGTGAATAAATAGATATAAAATAGAAAATAACAGATATT

ACAACCAGTGTTATGGACGCACCCAACCATGTAGGCAGTTTTATTTTATCGTTTACTACAGGTTCTCCTG

GATGTACGTCACCAACTGCAGACGTAGTTCTAGTACAATTAGACGTAAGTTCCGCTTGGGAATTTTTTAA

CGCTAAAGAGTTAACGTTGATCGTACACCCAACGTATTTACATCTAGTTCTTTGAACATCTTGATTATAA

TATAACCATTTTCTATCTCTAGATTCGTCAGTGCACTCATGTAACCAACATACCCTAGGTCCTAAATATT

TATCTCCGGAATTAGATTTTGGATAATTCGCGCACCAACAATTTCTATTTCCTTTATGGTCGTTACAAAA

GACGTATAATGCCGTATCCCCAAAAGTAAAATAATCAGGACGAATAATTCTAATAAACTCAGAACAATAT

CTCGCATCCATATGTTTGGAGCAAATATCGGAATAAGTAGACATAGCCGGTTTCCGTTTTACACGTAACC

ATTCTAAACAATTGGGGTTTCCAGGATCGTTTCTACAAAAACCAGTCATGAAATCGTCACAATGTTCTGT

CTTGTAATTATTATTAAATATTTTTGGACAGTGTTTGGTATTTGTCTTAGAACAACATTTTGCCACGCTA

TCACTATCACCCAGGAGATAATCCTTTTTTATAAAATGACATCGTTGCCCGGATGCTATATAATCAGTAG

CATATTTTAAATCCTTAATATATTCAGGAGTTACCTCGTTCTGATAATAGATTAATGATCCAGGACGAAA

TTTGAAAGAACTACATGGTTCTCCATGAATTAATACATATTGTTTAGCAAATTCAGGAACTATAAAACTA

CTACAATGATCTATCGACATACCATCTATCAAACAAAATTTGGGTTTAATTTCTCCTGGAGACGTTTCAT

AATAATACATATAACTTTCTTCGGCAAACCTAACAGCTCTATTATATTCAGGATAATTAAAATCTAATAC

CATATATTTGTCTCGTATATCTGCTATTCCTGTCTCTATTTTGATTCTATTAAGAGTAACAGCTGCCCCC

ATTCTTAATAATCATCAGTATTTAAACTGTTAAATGTTGGTATATCAACATCTATCTTATTTCCCGCAGT

ATAAGGTTTGTTGCAGGTATACTGTTCAGGAATGGGTACATTTATACTTCTTTTATAGTCCTGTCTTTCG

ATGTTCATCACAAATGCAAAGAACAGAATAAACAAAATAATGTAAGAAATAATATTAAATATCTGTGAAT

TCGTAAATACATTGATTGCCATAATAATTACAGCAGCTACAATACACACAATAGACATTCCCACAGTGTT

GCCATTACCTCCACGATACATTTGAGTTACTAAGCAATAGGTAATAACTAAGCTAGTAAGAGGCAATAGA

AAAGATGAGATAAATATCATCAATATAGAGATTAGAGGAGGGCTATATAGAGCCAAGACGAACAAAATCA

AACCGAGTAACGTTCTAACATCATTATTTTTGAAGATTCCCAAATAATCATTCATTATTCCTCCATAATC

GTTTTGCATCATACCCCCATCTTTAGGCATAAACGATTGCTGCTGTTCCTCTGTAAATAAATCTTTATCA

AGCACTCCAGCACCCGCAGAGAAGTCATCAAGCATATTGTAATATCTTAAATAACTCATTTATATATTAA

AAAATGTCACTATTAAAGATGGACTATAATCTTTATGCCGAACTAAAAAAAATGACTTGTGGTCAGACCA

TAAGTCTTTTTAATGAAGACGGCGATTTCGTAGAAGTTGAACCAGGATCATCCTTTAAGTTTCTAATACC

TAAGGGATTTTACTCCTCTCCTTGTGTAAAGACGAGTCTAGTATTCGAGACATTAACAACGACCGATAAT

AAAATTACTAGTATCAATCCAACAAATGCGCCAAAGTTATATCCTCTTCAACGCAAAGTCGTATCTGAAG

TAGTTTCTAATATGAGGAAAATGATCGAATTAAAACGTCCTCTATACATCACTCTTCACTTGGCATGTGG

ATTTGGTAAGACTATTACCACGTGTTATCTTATGACCACACACGGTAGAAAAACCATCATTTGCGTACCC

AATAAAATGTTAATACATCAATGGAAGACACAGGTAGAGGCAGTCGGATTGGAACATAAGATATCTATAG

ATGGAGTTAGTAGTCTATTAAAGGAACTAAAGACTCAAAGTCCGGATGTATTAATCGTAGTCAGTAGACA

TCTGACAAACGATGCATTTTGTAAATATATCAATAAGCATTATGATTTGTTTATCTTGGATGAATCACAT

ACGTATAATCTGATGAACAATACAGCAGTTACAAGATTTTTAGCGTATTATCCTCCGATGATGTGTTATT

TTTTAACTGCTACACCTAGACCAGCTAACCGAATTTATTGTAATAGTATTATTAATATTGCCAAGTTATC

CGATCTAAAAAAAACTATCTATACAGTAGATAGTTTTTTTGAGCCATATTCCACAGACAATATTAGAAAT

ATGGTAAAACGACTAGATGGACCATCTAATAAATATCATATATATACCGAGAAGTTATTATCTGTAGACG

AGCCTAGAAACCAACTTATTCTTGATACCCTGGTAGAAGAATTCAAGTCAGGAACTATTAATAGAATTTT

AGTTATTACTAAACTACGTGAACATATGGTATTCTTCTACAAACGATTATTAGATCTTTTCGGACCAGAG

GTTGTATTTATAGGAGACGCCCAAAATAGACGTACTCCAGATATGGTCAAATCGATTAAGGAACTAAATA

GATTTATATTCGTATCCACCTTATTTTATTCCGGCACTGGTTTAGATATTCCGAGTTTGGATTCTTTGTT

CATTTGCTCGGCAGTAATCAACAATATGCAAATAGAGCAATTACTAGGGAGGGTATGTCGAGAAACAGAA

CTATTAGATAGGACGGTATATGTATTTCCTAACACATCCATCAAAGAAATAAAGTACATGATAGGAAATT

TCGTGCAACGAATTATTAGTCTGTCTGTAGATAAACTCGGATTTAAACAAGAAAGTTATCAGAAACATCA

GGAATCTGAACCCGCTTCCGTACCAACATCCTCCAGAGAAGAACGTGTATTAAATAGAATATTTAACTCG

CAAAATCGTTAAGAAGTTTAAGAGACGATCCACATGCTGAGCAGGCCAGTGTATTACCCCTCATAGTATT

AATATAATCCAATGATACTTTTGTGATGTCTGAAATCTTAACCAATTTAGACTGACAGGCAGAACACGTC

ATACAATCATCATCGTCATCGATAACTGTAGTCTTGGGCTTCTTTTTGCGACTCTTCATTCCGGAACGCA

TATTGGTGCTATCCATTTAGGTAGTAAAAAATAAGTCAGAATATGCCCTATAACACGATCGTGCAAAACC

TGGTATATCGTCTCTATCTTTATCACAATATAGTGTATCAACATCTTTATTATTGACCTCGTTTATCTTG

GAACATGGAATGGGAACATTTTTGTTAACGGCCACCTTTGCCTTAATTCCAGATGTTGTAAAATTATAAC

TAAACAGTCTATCATCGACACAAATGAAATTCTTGTTTAGACGTTTGTAGTTTACGTATGCGGCTCGTTC

TCGTCTCATTTTTTCAGATATTGCAGGTACTATAATATTAAAAATAAGAATGAAATAACATAGGATTAAA

AATAAAGTTATCATGACTTCTAGTGCTGATTTAACTAACTTAAAAGAATTACTTAGTCTGTACAAAAGTT

TGAGATTTTCAGATTCTGTGGCTATAGAGAAGTATAATTCTTTGGTAGAATGGGGAACATCTACTTACTG

GAAAATAGGCGTACAAAAGGTAACTAATGTCGAGACGTCCATATCTGATTATTATGATGAGGTAAAAAAT

AAACCGTTTAATATTGATCCGGGGTATTATATTTTCTTACCAGTATATTTTGGAAGCGTCTTTATTTATT

CGAAGGGTAAAAATATGGTAGAACTTGGATCTGGAAACTCTTTTCAAATACCGGATGAGATTCGAAGTGC

GTGTAACAAAGTATTAGATAGTGATAACGGAATAGACTTTCTGAGATTTGTTTTGTTAAACAATAGATGG

ATAATGGAAGACGCTATATCAAAATACCAGTCTCCAGTTAATATATTTAAACTAGCTAGTGAGTACGGAT

TAAACATACCCAACTATTTAGAAATTGAAATAGAGGAAGACACATTATTTGACGATGAGTTATACTCTAT

TATGGAACGCTCTTTCGATGATACATTTCCAAAAATATCTATATCGTATATTAAGTTGGGAGAACTTAAG

CGGCAAGTTGTAGACTTTTTCAAATTCTCATTCATGTATATTGAGTCAATCAAGGTAGATCGTATAGGAG

ATAATATTTTTATTCCTAGCGTTATAACAAAATCAGGAAAAAAGATATTAGTAAAAGATGTAGACCATTT

AATACGATCCAAGGTTAGAGAACATACATTTGTAAAAGTAAAAAAGAAAAACACATTTTCCATTTTATAC

GACTATGATGGGAACGGAACAGAAACTAGAGGAGAAGTAATAAAACGAATTATAGACACTATAGGACGAG

ACTATTATGTTAATGGAAAGTATTTCTCTAAGGTTGGTATTGCAGGCTTAAAGCAATTGACTAATAAATT

AGATATTAATGAGTGTGCAACTGTCGATGAGTTAGTTGATGAGATTAATAAATCCGGAACTGTAAAACGA

AAAATAAAAAACCAATCAGTATTTGATTTAAGCAGAGAATGTTTGGGATATCCAGAAGCGGATTTTATAA

CGTTAGTTAATAACATGCGGTTCAAAATAGAAAATTGTAAGGTTGTAAATTTCAATATTGAAAATACTAA

TTGTTTAAATAACCCGAGTATTGAAACTATATATGGAAACTTCAACCAGTTCGTCTCAATCTTTAATACC

GTTACCGATGTCAAAAAAAGATTATTCGAGTGAAATAATATGCGCCTTTGATATAGGTGCAAAAAATCCT

GCCAGAACTGTTTTAGAAGTCAAGGATAACTCCGTTAGGGTATTGGATATATCAAAATTAGACTGGAGTT

CTGATTGGGAAAGGCGCATAGCTCAAGATTTGTCACAATATGAATACACTACAGTTCTTCTAGAACGTCA

GCCTAGAAGGTCACCGTACGTCAAATTTATCTATTTTATTAAAGGCTTTTTATATCATACATCTGCTGCC

AAAGTTATTTGCGTCTCACCTGTCATGTCTGGTAATTCATATAGAGATCGAAAAAAGAGATCTGTTGAAG

CATTTCTTGATTGGATGGACACATTCGGATTGCGAGACTCCGTTCCGGATAGACGCAAATTAGACGATGT

AGCGGATAGTTTCAATTTGGCTATGAGATACGTATTAGATAAATGGAATACTAATTATACACCTTATAAT

AGGTGTAAATATAGAAATTACATAAAAAAAATGTAATAACGTTAGTAACGCCATTATGGATAATCTATTT

ACCTTTCTACATGAAATAGAAGATAGATATGCCAGAACTATTTTTAACTTTCATCTAATAAGTTGTGATG

AAATAGGAGATATATATGGTCTTATGAAAGAACGCATTTCCTCAGAGGATATGTTTGACAATATAGTATA

TAATAAAGATATACATCCTGCCATTAAGAAACTAGTTTATTGCGACATCCAACTTACTAAACATATTATT

AATCAGAATACGTATCCGGTATTTAACGATTCTTCACAAGTGAAATGTTGTCATTATTTCGATATAAACT

CAGATAATAGCAATATTAGCTCTCGTACAGTAGAGATATTTGAGAGTGAAAAGTCATCTCTTGTATCATA

TATTAAAACTACCAATAAGAAGAGAAAGGTCAATTACGGCGAAATAAAGAAAACTGTACATGGAGGCACT

AATGCAAATTACTTTTCCGGTAAAAAGTCTGATGAGTATCTGAGCACTACAGTCAGGTCCAACATTAATC

AACCTTGGATCAAAACCATTTCTAAGAGAATGAGAGTAGATATCATTAATCACTCTATAGTAACGCGTGG

AAAAAGCTCTATATTACAAACTATAGAAATTATTTTTACTAATAGAACATGTGTGAAAATATTCAAGGAT

TCTACTATGCACATTATTCTATCCAAGGACAAGGATGAAAAGGGATGTATAAACATGATTGATAAATTAT

TCTATGTATATTATAATTTATTTCTGTTGTTCGAGGATATCATCCAAAACGATTACTTTAAAGAAGTAGC

TAATGTTGTAAACCATGTACTCATGGCTACGGCATTAGATGAGAAATTATTCCTAATTAAGAAAATGGCT

GAACACGATGTTTATGGAGTTAGCAATTTCAAAATAGGGATGTTTAACCTGACATTTATTAAGTCGTTGG

ATCATACCGTTTTCCCCTCTCTGTTAGATGAGGATAGCAAAATAAAGTTTTTTAAGGGGAAAAAGCTCAA

TATTGTAGCATTACGATCTCTGGAGGATTGTACAAATTACGTGACTAAATCCGAGAATATGATAGAAATG

ATGAAGGAAAGATCGACTATTTTAAATAGCATAGATATAGAAACGGAATCGGTAGATCGTCTAAAAGAAT

TGCTTCTAAAATGAAAAAAAACACTGATTCAGAAATGGATCAACGACTCGGGTATAAGTTTTTGGTGCCT

GATCCTAAAGCCGGAGTTTTTTATAGACCGTTACATTTCCAATATGTATCGTATTCTAATTTTATATTGC

ATCGATTGCATGAAATCTTGACCGTCAAGCGGCCACTCTTATCGTTTAAGAATAATACAGAACGAATTAT

GATAGAAATTAGCAATGTTAAAGTGACTCCTCCAGATTACTCACCTATAATTGCGAGTATTAAAGGTAAG

AGTTATGACGCATTAGCCACGTTCACTGTAAATATCTTTAAAGAGGTAATGACCAAAGAGGGTATATCCA

TCACTAAAATAAGTAGTTATGAGGGAAAAGATTCTCATTTGATAAAAATTCCGCTACTAATAGGATATGG

GAATAAAAATCCACTTGATACAGCCAAGTATCTTGTTCCTAATGTCATAGGTGGAGTCTTTATCAATAAA

CAATCTGTCGAAAAAGTAGGAATTAATCTAGTAGAAAAGATTACAACATGGCCAAAATTTAGGGTTGTTA

AGCCAAACTCATTCACTTTCTCGTTTTCCTCCGTATCCCCTCCTAATGTATTACCGACAAGATATCGCCA

TTACAAGATATCTCTGGATATATCACAATTGGAAGCGTCGAATATATCATCGACAAAGACATTTATAACG

GTCAATATTGTTTTGCTGTCTCAATATTTATCTAGAGTGAGTCTAGAATTCATTAGACGTAGTTTATCAT

ACGATATGCCTCCAGAAGTTGTCTATCTAGTAAACGCGATAATAGATAGTGCTAAACGACTTACCGAATC

TATTACTGACTTTAATATTGATACATACATTAATGACCTGGTGGAAGCTGAACACATTAAACAAAAATCT

CAGTTAACGATTAACGAGTTTAAATATGAAATGCTGCATAACTTTTTACCTCATATGAACTATACACCCG

ATCAACTAAAGGGATTTTATATGATATCTTTACTAAGAAAGTTTCTCTACTGTATCTACCACACTTCTAG

ATATCCAGATAGAGATTCGATGGTTTGTCATCGCATCCTAACGTACGGCAAATATTTTGAGACGTTAGCA

CATGATGAATTAGAGAATTACATAGGTAACATCCGAAACGATATCATGAACAATCACAAGAACAGAGGCA

CTTACGCAGTAAACATTCATGTACTAACAACTCCTGGACTTAATCATGCATTTTCTAGTCTATTGAGTGG

AAAGTTCAAAAAGTCAGACGGTAGTTATCGAACACATCCTCACTATTCATGGATGCAGAATATTTCTATT

CCTAGAAGTGTTGGATTTTATCCGGATCAAGTAAAGATTTCAAAGATGTTTTCTGTCAGAAAATACCATC

CAAGCCAATATCTTTACTTTTGTTCATCAGACGTTCCGGAAAGAGGTCCTCAGGTAGGTTTAGTATCTCA

ATTGTCTGTCTTGAGTTCCATTACAAATATACTAACGTCTGAGTATTTGGATTTGGAAAAGAAAATTTGT

GAGTATATCAGATCATATTATAAAGATGATATAAGTTACTTTGAAACAGGATTTCCAATCACTATAGAAA

ATGCTCTAGTCGCATCTCTTAATCCAAATATGATATGTGATTTTGTAACTGACTTTAGACGTAGAAAACG

GATGGGATTCTTCGGTAACTTGGAGGTAGGTATTACTTTAGTTAGGGATCACATGAATGAAATTCGCATT

AATATTGGAGCAGGAAGATTAGTCAGACCATTCTTGGTTGTGGATAACGGAGAGCTCATGATGGATGTGT

GTCCGGAGTTAGAAAGCAGATTAGACGACATGACATTCTCTGACATTCAGAAAGAGTTTCCACATGTCAT

CGAAATGGTAGATATAGAACAATTTACTTTTAGTAACGTATGCGAATCGGTTCAAAAATTTAGAATGATG

TCAAAGGATGAAAGAAAGCAATACGATTTATGTGACTTTCCTGCCGAATTTAGAGATGGATATGTAGCAT

CTTCACTAGTGGGAATCAATCACAATTCTGGACCCAGAGCTATTCTTGGATGTGCTCAAGCTAAACAAGC

TATCTCTTGTCTGAGTTCGGATATACGAAATAAAATAGACAATGGAATTCATTTGATGTATCCAGAGAGG

CCAATTGTGATTAGTAAGGCTTTAGAAACTTCAAAGATTGCGGCTAATTGCTTCGGACAACATGTTACTA

TAGCATTAATGTCGTACAAAGGTATCAATCAAGAGGATGGAATTATCATCAAAAAACAATTTATTCAGAG

AGGCGGTCTCGATATTGTTACAGCCAAGAAACATCAAGTAGAAATTCCATTGGAAAACTTTAATAACAAA

GAAAGAGATAGGTCTAACGCCTATTCGAAATTAGAAAGTAATGGATTAGTTAGACTGAATGCTTTCTTGG

AATCCGGAGACGCTATGGCAAGAAATATCTCATCAAGAACTCTTGAAGATGATTTTGCTAGAGATAATCA

GATTAGCTTTGATGTTTCCGAGAAATATACAGATATGTACAAATCTCGCGTTGAACGAGTACAAGTAGAA

CTTACTGACAAAGTTAAGGTGCGAGTATTAACCATGAAAGAAAGGAGACCCATTCTAGGAGACAAATTTA

CTACTAGAACGAGTCAAAAGGGAACAGTCGCGTATATCGCAGATGAAACGGAACTTCCGTACGACGAAAA

TGGTATCACACCAGATGTCATTATTAATTCTACATCCATCTTCTCTAGAAAAACTATATCTATGTTGATA

GAAGTTATTTTAACAGCCGCATATTCTACTAAGCCGTACAACAATAAGGGAGAAAACCGACCTGTCTGTT

TTCCTAGTAGTAACGAAACATCTATCGATGCATATATGCAATTCGCTAAACAATGTTATGAGTATTCAAA

TCCGAAATTGTCCGAGGAAGAATTATCGGATAAAATCTTTTGTGAAAAGATTCTCTATGATCCTGAAACG

GATAAGCCTTATCAATCCAAAGTATTTTTTGGACCAATTTATTACTTGCGTCTGAGACATTTAACTCAGG

ACAAGGCAACCGTTAGATGTAGAGGTAAAAAGACGAAGCTCATTAGACAAGCGAATGAGGGACGAAAACG

TGGAGGAGGTATCAAGTTTGGAGAAATGGAGAGAGACTGTTTAATAGCACATGGTGCAGCCAATACTATT

ACAGAAGTTTTAAAAGACTCAGAAGAGGATTATCAAGATGTGTATATTTGTGAAAATTGTGGAGACATAG

CAGCACAAATCAAAAGTATTAATACATGTCTTAGATGTTCAAAACTTAATCTCTCTCCTCTCTTAACAAA

AATTGATACCACGCACGTATCTAAAGTATTTCTTACTCAAATGAACGCCAGAGGCGTAAAAGTTAAATTA

GATTTCGAACGAAGGCCTCCTTCGTTTTATAAACCATTAGATAAAGTTGATCTTAAACCGTCTTTTCTGG

TATAATATTGTTTAGTAGATACTCATCAAGATTATCAAGATAAGCTAATTCACTAAACATATTATCGGAT

TCGGTATTGTTACTCGAGAATAGAGTTCGTTATGCTCCTGATATTCGGAAATCTGTGGAGTTTCAGGTTT

TGGTGGAAGTGTAACTGCTACTTGGTGGGATACTGAAGGATATTTCAGAGAGTTGTGGATGTTCGGGTTC

GACATCCACCGATGGTGTCACGCCACTAATCGGTTCGGTAACGTCTGTGGATGGAGGTGCTACTTCTACA

GAACCTGTAGCCTCAGTTGTCAACGGAGATACATCTTCAATGCGCGGAAATGTATAATTTGGTAATGGTT

TCTCATGTGGATCTTAAGAAGAAGAGGTAAGATATCTACGAAAGATACCGATCACGTTCTAGTTCTCTTT

TGTAGAACTTTAACTTTTTCTTTCTCAGCATCTAGTTGATATTCCGACCTCTTCACGTTTCGCATGGGTT

ACCTCCGCAGTTTTTACAAGCGATTTCACGTTCCAGATCACGTTCAGCCTTCATACGTCTCTCCCTCTCT

CTATCGAGTTTATCAGAGCAGTCTTTCTGAAGGCGATCGAACTCCATAAATTTCTCCAACGCTTTGATTG

TTTCCATAGATTTCCGAAGTTTAGCTTCTAGGACGGCGATTCTTTTTTTTCTTTCGAATTCACTGGGTAC

AACCGTTTCCATTACCACCATCTCTACGTTTCTTTTCTAGATCGGCAATCTTTCTCAACATTTCATCCCC

ATGCCTTTTCATTCCTCGAGTCTATTGTCGTCGAAATATCGTTCCAGCTCCTTTTCGACCTCAATAACTT

TAGCACGTTGTTTCATCAAGCTCTCTCTTGTAGTACTATCATTTTTATCTGATTCCCTGACACGTTTAAG

ATCTTCATGTAATTGAGTCAGCTCTTGACGCAATCTCTTAACTAACTTCCTCTCTTGCTTCTTCGTCATA

GTACTTACAATCACTATGGGATCCATTGTTACCACGTCTGTACTCGACGAGCTCACGTTTAAGAGATTCA

ATTTCCAGTTTGTATCGGTCCATGTCTCCATTGCTACACCACCATTAGATTTACAGGCTGTTAGTTGTCG

TTCGAGATCAGAAATACGTGTTTTCTTGGAATGGATTTCGTCGATGTACTTGTCATGATTGGCATCGAAA

CACTTATTAAGTTCTTTTTTTCAATTCTACGATTTTATTTCTTTCGCGAGTCAATTCCCTCCTATAGTAA

CTATCAGTTTTGTCAGATTCACGCTCTCTACGTAGACTTTCTTGCAAGTTACTAATTTGTTCCCTGGCAT

TACCGAGTTCAGTTTTATATGCCGAATAGAGTTCTGATTCATCCTTTGAGAAGATCTCTAGCGATCGTTC

AAGATCCCTGATTCTAGTCTTTAGCCTATTTACCTCCTCAGAAGATGCTCCGTTACCGTTTTTTACAATC

GTTAAGATGTCTATCAAGATCCATGATTCTATCTCTTTTCCATATCAGCATTGATTTCATTATTACGTCC

GCAGTCGTTCAACTGTATTTCAAGATCTGAGATTCTAGATTGTAATCTCTGTAGCATTTCCACGGCATTC

ACTCAGTTGTCTTTCAAGATCTGAGATTCTAGATTGGAGTCTGCTAATCTCTGTAAGATTTCCTCCTCCG

CTCTCGATGCAGTCGGTCAACTTATTCTCTAGTTCTCTAATACGCGAACGCAGTGCATCAACTTCTTGTG

TGTCTTCTTGATTGCGTGTGCATTCATCGAGTCTAGATTCGAGATCTCTAACGTGACGTCGTTCTTCCTC

AAGTTCTCTGTGTACTACAGAAAGCGTGTCCCTATCTTGTTGATATTTAGCAATTTCTGATTCTAGAGTA

CTGATTCTACTCACGTATGTACTAATAGTTGTCTTAGCCTTATCAAGATCCTCCTTGTATTTGTCACATT

CCTTGATATCCATACGAAGTCTGGACAGTTCCCATTCGACATTACGACGTTTATCGATTTCAGCTCGGAG

ATCGTCGTCGCGTTGTTTTAGCCACATACGACTAAGTTCAAGTTCTCGTTGACAAGATCCATCTACTTTT

CCATCCCTAATAGTATCCAGTTCCTTTTCTAGTTCTGACCGCATTTCTCGTTCCATATCAAGAGATTCTC

TCAATTCTCGTATAGTCTTCTTATCAATTTCTGATGAATCTGAACCATCATCTGTCCCATTTTGTTGCAT

ATCCCTGAGTTCTTTGATCTCTGTTGTAAGTCTGTCGATTCTTTCGGTTTTATAAACAGAATCCCTTTCC

AAAGTCCTAATCTTACTGAGTTTATCATTAAGTTCTTCATTCAATTCAGTGAGTTTTCTCTTGGCTTCTT

CCAAGTCTGTTTTAAACTCTCCATCATTTCCGCATTCTTCCTCGCATTTATCTAACCATTCAATTAGTTT

ATTAATGACTAGTTGGTAATCAGCGATTCCTATAGCCGTTCTTGTATTTGTGGGAACATAATTAGGATCT

TCTAATGGATTGTATGGCTTGATAGCATCATCTTTATCATTATTAGGTGGGGGATGGACAACCTTAATTG

GTTGGTCCTCCTTATCTCCTCCAGTAGCATGTGGTTCTTCAATACCAGTATTAGTAATAGGCTTAGACAA

ATGCTTGTCGTACGCGGGCACTTCCTCATCCATCAAGTATTTATAATCGGGTTCTGTTTCAGAATATTCT

TTTCTAAGAGACGCGACTTCAGGAGTTAGTAGAAGAACTCTGTTTCTGTATCTATCAACGCTGGAATCGA

TACTCAAGTTAAGGATAGCGAATACCTCATCGTCATCATCCGTATCTTCTGAAACGCCATCATATGACAT

TTCATGAAGTCTAACGTATTGATAAACAGAATCAGATTTAGTATTAAACAGATCCTTGACCTTTTTAGTA

AATGCATATGTATATTTTAGATCTCCAGATTTCATAATATGATCGCATGCCTTAAATGTCAATGCTTCCA

TGATATAGTCTGGAACACTAATGGGTGACGAAAAAGATACAGCACCATATGCTACGTTGATAAATAGATC

TGAACCACTAAGTAGATAATGATTAATGTTAAGGAAGAGGAAATATTCAGTATATAGATATGCCTTAGCA

TCATATCTTGTACTAAACACGCTAAACAGTTTATTGATGTGATCAATTTCCAACAGAACAATTAGAGCGG

CAGGAATACCAACAAACATATTACCACATCCGTATTTTCTATGAATATCACATATCATGTTAAAAAATCT

TGATAGAAGAGCGAATATCTCGTCTGACTTAATGAGATGTAGTTCAGCAGCATAAGTCATAACTGTAAAT

AGAACATACTTTCCTGTAGTGTTGATTCTAGACTCCACATCAACACCATTATTAAAAATAGTTTTATATA

CATCTTTAATCTGCTCTCCGTTAATCGTCGAACGTTCTAGTATACGGAAACACTTTGATTTCTTATCTGT

AGTTAATGACTTAGTGATATCACGAAGAATATTACGAATTACATTTCTTGTTTTTCTTGAGAGACCTGAT

TCAGAACTCAACTCATCGTTCCATAGTTTTTCTACCTCAGTGGCGAAATCTTTGGAGTGTTTGGTACATT

TTTTAATAAGGTTCGTGACCTCCATTTATTATAAAAAATTTTTATTCAAAACTTAACTACAATCGGGTAA

TTATAAGATCGTAGATCTCCCATGTGGTGGAATACTACCATCTATCGCATGTTGATGGACAGTAGGTAAT

GGCCATGGGAACAGTAATGTTTGCATATTTATCTTTCTTGCTAGTATTACTGTATATTGTCCCAATGTTT

CAATGTGATGTTCTAACCTATCAACTGCCACTGTATCACAACAATAATGTCCGATGGAATTAAGATTATG

ATCCAATGTGTTTAATATATGATTATCAAGTCTTATACGATCCGCGTCTTTTTTGACAGGATCAGGCTCT

TCTACAGGAAGAAGTTTCGGCCTCTTATAATAGTCATGTCTGGGAAATGGTGGTCTAGGATGAGGATCAG

GTATCGGAGTGGGTTTTGGATTATAATCATCATCATCATCATCATCATCATCATCATCATCATCATCATC

ATCATCATCATCATCTTCGATATTTATTTTGCTATCTTGATAATGTCCTGTATCAGTTGCATTTTCAGCA

CTCGACTGAATATTAGCACATTCATTGTCTATTATTAACGTATTTCTAAACCCAAAATGTATGTGTTGAA

CATCACTACTATAGTTGATGAGTCTTATAGCATGAATTCGCTTATCGTTATCGGGTTTATCTTCTGTCAC

CTTAACAATTCCTTTTTTATTAAACTCTGCATAATCATAACCATTTCTATTGTTTGTTCTAATATAAACG

AGTATAGCATCATTGCTAAATTTTTCAATAGTATCGAAAACAGAATATCCTAAACCATATAATATATATT

CAGGAACACTCAAACTAAATGTCCAGGATTCTCCTAAATACGTAAACTTTAATAGTGTTAAATCATTCAA

AAATCTACCGCTTATAGATAGATAGTACATGAATGCGTATAGTAGTCTACCTATCTCTTTATTATGAAAA

CCGACATTACGATCATATATTTCGTGATATACATGTGACCCGTTTACGTTAAACCATAAATACATGGGTG

ATCCTATAAACATGAATTTATTTCTAATTCTCAGAGCCATAGTTAATTGACCGTGTAATATTTGTTTACA

TGCATACTTGATACGATCATTAATAAGATTTTTATCATTGCTCGTTATTTCAGAATCGTATATATAAGGA

GTACCATCATGATTCTTACCAGATATTATACAAAATACTATATATAAAATATATTGACCCACGTTAGTAA

TCATGTAAATGTTTAATGTTTTAAATTTTGTATTTAATGATCCATCATCATATGCTAGCATGGTCTTGTG

ATATTCATTCTTTAAAATATAATATTGTGTTAGCCATTGCATTGGAGCTCCTAATGGAGATTTTCTATTC

TCGTCCATTTTAGGATATGCTTTCATAAAGTCCCTAATAACTTCGTGAATGATGTTTCTATGTTTTCTAC

TGATGCATGTATTTGCTTCGATTTTTTTATCCCATGTTTCATCTATCATAGATTTAAACGCAGTAATGCT

CGCAACATTAACATCTTGAACCGTTGGTACAATTCCGTTCCATAAATTTATAATGTTCGCCATTTATATA

ACTCATTTTTTGAATATACTTTTAATTGAACAAAAGAGTTAAGTTACTCATATGGATGCCGTCCAGTCTG

TACATCAATCTTTTTAGCCAGAGATATCATAGCCGCTCTTAGAGTTTCAGCGTGATTTTCCAACCTAAAT

AGAACTTCATCGTTGCGTTTACAACACTTTTCTATTTGTTCAAACTTTGTTGTTATATTAGTAATCTTTT

TTTCCAAATTAGTTAGCCGTTGTTTGAGAGTTTCCTCATTATCGTCTCCATAGGCTTTAACAATTGCTTC

GCGTTTAGTCTCTGGATTTTTAGCAGCCTTTGTAGAGAAAAATTCAGTTGCTGGAATTGCAAGATCGTCA

TCTCCGGGGAAAAGAGTTCCGTCCATTTAAAGTACAGATTTTAGAAACTGACACTCTGTGTTATTTATAT

TTGGCGCAATACATGGATTATAAATATCGATGTTAATAACATCAGAAAATGTAAAGTCTATACATTGTCG

CATCGTGTTAAATTTTCTAATGGATCTAGTATTATTGGGTCCAACTTCTGCCTGAAATCCAAATATGGAA

GCGGATACAAAACCGTTTCCTGGATAAACCACACATCTCCACTTTTGCTTTACATCAGAAATTGTGTCAT

TGACATCTTGAACTCTCCTATCTAATGCCGGTGTTCCACCTATAGATTTTGAATACTCGAATGCTGCATG

AGTAGCATTGAATTCCTTAATATTGCCATAATTTTCATATATTGAGTAACTCTGGATAAAAAGTAAACAC

ACCGCAGCCGTCGCTACTACAATAAAAAAAATTGATAGAGAGTTCATTTATAATCTATTAGAAGCTGATA

AAATTTTTTTACACGCGTCAGACAATGCTTTAATAAATAGTTCAACATCTACTTTTGTCATATCGAACCG

ATGGTATGATTCTAACCTAGAATTACATCCGAAAAAGTTGACTATGTTCATAGTCATTAAGTCATTAACG

AACAACATTCCAGACTCTGGATTATAAGACGATACTGTTTCGTCACAATCACCCACCTTAATCATGTGAT

TATGAATATTGGCTATTAGAGTACCTTCTAAGAAATCTATAATATCTTTGAAACACGATTTAAAATCAAA

CCACGAATATACTTCTACGAAGAAAGTTAGTTTACCCATAGGAGAGATAACTATAAATGGAGATCTAGAT

ACAAAATCCGGATCTATGATAGTTTTAACATTATTATATTCTCTATTAAATACCTCCACATCTAAAAATG

TTAATTTTGAAACTATGTCTTCGTTTATTACCGTACCTGAACTAAACGCTATAAGCTCTATTGTTTGAGA

ACTCTTTAAACGATATTCTTGAAATACATGTAACAAAGTTTCCTTTAACTCGGTCGGTTTATCTACCATA

GTTACAGAATTTGTATCCTTATCTATAATATAATAATCAAAATCGTATAAAGTTATATAATTATCGTGTT

CAGATTGTGATCTTTTCAAATAGACTAAAAACCCCATTTCTCTAGTAAGTATCTTATGTATATGTTTGTA

AAATATCTTCATGGTGGGAATATGCTCTACAGCAGTTAGCCATTCCTCATTGACAGCTGTAGATGTATTA

TACAAAACTACTCCAATGTTTAACAAGGGCCATTTTACGAGATTATTAAATCCTTGTTTGATAAATGTAG

CCAATGCGGGTTCGAGTTCAACGACGATTGAATTCTCTTCCCGTGGATGCTGCATGATGAACGACGGGAT

GTTGTTGTTCTATTGATTTGGAATTCTTTTTCGACTTTTTGTTTATATTAAATATTTTAAAATTTATGGC

TGATAGTAATTCATGTACTACGGATAATGTAGACGTGTATTGCATATCGATATCTTTATTATTAGATAAA

TTTATCAATAAATGTGAGAAGTTTGCCTCGTTAAGGTCTTCCATTTAAATATTATATAAATATTTGTGTT

TGTATTTTATTCGTCTTTTATGGGATAGTTTTTAACTAGTAAAGCTGTAATTACATACTTTGTCCGTAAA

ACATAAATATAAATACCCGCTTTTATCAAACGTTCCAAAAAGTCGGCAGCTGACATTTTTAACATGACAT

CTATTTTAAATACACTTAGGTTTTTAGAAAAAACATCATTTTATAATTGTAACGATTCAATAACTAAAGA

AAAGATTAAGATTAAACATAAGGGAATGTTATTTGTATTTTATAAGCCAAAGCATTCTACCGTTGTTAAA

TACTTGTCTGGAGGAGGTATATATCATGATGATTTGGTTGTATTGGGGAAGGTAACAATTAATGATCTAA

AGATGATGCTATTTTACATGGATTTATCATATCATGGAGTGACAAGTAGTGGAGCAATTTACAAATTGGG

ATCGTCTATCGATAGACTTTCTCTAAATAGGACTATTGTTACAAAAGTTAATAACAATTATAACAATTAT

AATAATTATAATTGTTATAATAATTATAATTGTTATAATTATGATGATACATTTTTTGACGATGATGATT

GATCACTATTACACAATTTTGTTTTTGTACTTTCTAATATAGTGTTTAGGTTCTTTTTCATATGAGAATA

TTGACTTACTAAAATATCTATGTTTAACTTTTGTTCTATAACGTCCTTATCGGCGGTATCGGTACATATA

CGTAATTCACCTTCACAAAATACGGAGTCTTCGATAATAATAGCCAATCGATTATTGGATCTAGCTGTCT

GTATCATATTCAACATGTTTAATATATCCTTTCGTTTCCCCTTTACAGGCATCGATCGTAGCATATTTTC

CGCGTCTGAGATGGAAATGTTAAAACTGCAAAAATGCGTAATGTTAGCCCGTCCTAATATTGGTACGTGT

CTATAAGTTTGGCATAGTAGAATAATAGACGTGTTTAAATGCCTTCCAAAGTTTAAGAATTCTATTAGAG

TATTACATTTTGATAGTTTATCACCTACATCATCAAAAATAAGTAAAAAGTGTGCTGATTTTTTATGATT

TTGTGCGACAGCAATACATTTTTCTATGTTACTTTTAGTTCGTATCAGATTATATTCTAGAGCTTCCTGA

CTACTAACAAAATTAATATGATTTGGCCAAATGTATCCATCATAATCTGGGTTATAAACGGGTGTAAACA

AGAATATATGTTTATATTTTTTAACTAGTGTAGAAAACAGAGATAGTAAATAGATAGTTTTTCCAGATCC

AGATCCTCCTGTTAAAACCATTCTAAACGGCATTTTTAATAAATTTTCTCTTGAAAATTGTTTTTCTTGA

AAACAATTCATAATTATATTTACAGTTACTAAATTAATTTGATAATAAATCAAAATATGGAAAACTAAGG

TCGTTAGTAGGGAGGAGAACAACGAAGGCATATCGTGATATAAATAACATTTATTATCATGATGACACCA

GAAAACGACGAAGAGCAGACATCTGTGTTCTCCGCTACTGTTTACGGAGACAAAATTCAGGGAAAGAATA

AACGCAAACGCGTGATTGGTCTATGTATTAGAATATCTATGGTTATTTCACTACTATCTATGATTACCAT

GTCCGCGTTTCTCATAGTGCGCCTAAATCAATGCATGTCTGCTAACGAGGCTGCTATTACTGACTCCGCT

GTTGCCGTTGCTGCGGCATCATCTACTCATAGAAAGGTTGCGTCTAGCACTACACAATATGATCACAAAG

AAAGCTGTAATGGTTTATATTACCAGGGTTCTTGTTATATATTACATTCAGACTATAAGTCATTCGAGGA

TGCTAAAGCAAACTGCGCTGCGGAATCATCAACACTACCCAATAAATCCGATGTCTTGACTACCTGGCTC

ATTGATTATGTTGAGGATACATGGGGATCTGATGGTAATCCAATTACAAAAACTACATCCGATTATCAAG

ATTCTGATGTATCACAAGAAGTTAGAAAGTATTTTTGTACATAAATAAATGAAATCGCTTAATAGACAAA

CTGTAAGTAGGTTTAGGAAGTTGTCGGTGCCGGCCGCTATAATGATGTTACTCTCAACCATTATTAGCGG

CATAGGAACATTTCTGCATTACAGAGAAGAACTGATGCCTAGTGCTTGCGCCAATGGATGGATACAATAC

GATAAACATTGTTATCTGGATACCAACATTAAAATGTCTACGGATAATGCAGTTTATCAGTGTCGCAAAT

TACGAGCTAGATTGCCTAGACCTGATACTAGACATCTGAGAGTATTGTTTAGTATTTTTTATAAAGATTA

TTGGGTAAGTTTAAAAAAGACCAATGATAAATGGTTAGATATTAATAATGATAAAGATATAGATATTAGT

AAATTAACAAATTTTAAGCAACTAAACAGCACGACGGATTCTGAGGCGTGTTATATATACAAGTCTGGAA

AACTGGTTAAAACAGTATGTAAAAGTACTCAATCTGTACTATGCGTTAAAAGATTCTACAAGTGACAACA

AAAAATGAATTAATAGTAAGTCGTTAACGTACGCCGCCATGGACGCCGCGTTTGTTATTACTCCAATGGG

TGTGTTGACTATAACAGATACATTGTATGATGATCTCGATATCTCAATCATGGACTTTATAGGACCATAC

ATTATAGGTAACATAAAAATTGTCCAAATAGATATACGGGATATAAAATATTCCGACATGCAAAAATGCT

ACTTTAGCTATAAGGGTAAAATAGTTCCTCAGGATTCTAATGATTTGGCTAGATTCAACATTTATAGTAT

TTGTACCGCATACAGATCAAAAAATACCATCATCATAGCATGCGACTATGATATCATGTTAGATATAGAA

GGTAAACATCAACCATTTTATCTATTCCCATCTATTGATGTTTTTAACGCTACAATCATAGAAGCGTATA

ATCTGTATACAGCTGGAGATTATCATCTGATCATCAATCCTTCAGATAATCTGAAAATGAAATTGTCGTT

TAATTCTTCATTTTGTATATCAGACGGCAATGGATGGATTATAATTGATGGGAAATGTAATAGTAATTTT

TTATCATAAAAGTTGTAAAGTAAATAATAAAACAATAAATATTGAACTAGTAGTATGTTGTATATTGAGC

AATCAGAGATGATGCTGGTACCTCTTATCACGGTGACCGTAGTTGCGGGAACAATATTAGTATGTTATAT

ATTATATATTTGTAGGAAAAAGATACGTACTGTCTATAATGACAATAAAATTATCATGACAAAATTAAAA

AAGATAAAGAGTCCTAATTCCAGCAAATCTAGTAAATCAACTGATAGCGAATCAGACTGGGAGGATCACT

GTAGTGCTATGGAACAAAACAATGACGTAGATAATATTTCTAGAAATGAGATATTGAACGATGATAGCTT

CGCTGGTAGTTTAATATGGGATAACGAATCCAATATCATGGCGCCTAGCACAGAACACATTTACGATAGT

GTTGCTGGAAGCACGCTGCTAATAAATAATGATCGTAATGAACAGACTATTTATCAGAATACTACAGTAG

TAATTAATGATACAGAGACTGTTGAAATACTTAATGAAGATACCAAACAGATTCCTAGCTATTCTTCCAA

TCCTTTCGTAAATTATAATAAAACCAGTATTTGTAGCAAGTCAAATCCGTTCATTGCAGAACTCAACAAT

AAATTTAGTGATAATAATCCGTTTAGGAGAGCACATAGTGACGATTATCTTAATAAGCAACAAGATCATG

AATACGATGATATAGAATCATCGGTTGTATCATTGGTCTGATTAGTTTCCTTTTTATAAAATTGAAGTAA

TATTTAGTATTAATTACCGCCGATGCATTATACAAATATGGAGATATTCCCTGTATTCGGCATTTCTAAA

ATTAGCAATTTTATTGCTAATAATGACTGTAGATATTATATAGATGTAGAGCATCAAAAAATTATATCTG

ATGAGATCAATAGACAGATGGATGAAACGGTACTTCTTACCAACATCTTAAGCGTAGAAGTTGTAAATGA

CAATGAGATGTACCATCTTATTCCCCATAGACTATCGACTATTATACTCTGTATTAGTTCTGTTGGAGGA

TGTGTTATCTCTATAGATAATGACGTCAATGACAAAAATATTCTAACATTTCCCATTGATCATGCTGTAA

TCATATCCCCACTGAGTAAATGTGTCGTAGTTAGCAAGGGCCCTACAACCATACTGGTTGTTAAAGCGGA

TATACCCAGCAAACGATTGGTAACATCGTTTACAAACGACATACTGTATGTAAACAATCTATCACTGATT

AATTATTTACCGTCGTCTGTATTCATTATTAGACGAGTCACCGACTATTTGGATAGACACATATGTGATC

AGATATTTGCTAATAATAAGTGGTATTCCATTATAACTATCGACGATAAGCAATATCCTATTCCATCAAA

TTGTATAGGTATGTCTTCTGCCAAGTACATAAATTCGAGCATCGAGCAAGATATTTTGATCCATGTTTGT

AACCTCGAGCATCCATTCGACTCAGTCTACAAAAAAATGCAGTCGTACAATTCTCTACCTATCAAGGAAC

AAATATTGTATGGTAGAATTGATAATATAAATATGAGCATTAGTATTTCGGTGGATTAATAGATTTCTCT

AGTATGGGATCATTAATCATCTCTAAATACATCATAAAAAAGCTATTATCAAATACTGTACTGAATGGAT

TCATTCTTTTCTCTTTTTATGAAACTCTGTTGTATATCTACGGATAAAACTAGAAGCAAAAAATCTGATA

GGAAGAATAATGATTATATGGAGGAACACGATTATTATAAAATAACAATAGTTCCTGGTTCCTCTTCCAC

GTCTACTAGCTCGTGGTATTATACACATGCCTAGTAATAGTCTCTTTGCGTTGACGGAAAGCAGACTAGA

AATAACAGGCCAAAATGTTCAGACACCATAATAGTTCCCAACCCAGATAATAACAGAGTTCCATCAACAC

ATTCCTTTAAACTCAATCCCAAACCCAAAACCGTTAAAATGTATCCAGCCAATTGATAGTAGATAATGAG

GTGTACAGCACATGATAATTTACACAGTAACCAAAATGAAAACACTTTAGTAATTATAAGAAATATAGAC

GGTAATGTCATCATCAACAATCCAATAATATGCCTGAGAGTAAACATTGACGGATAAAACAAAAATGCCC

CGCATAACTCTATCATGGCAATAACGCAACCAAACACTTGTAAAATTCCTAAATTAGTAGAAAATACAAC

TGATATCGATGTATAAGCGATTTCGAGGAATAATAAGAACAAAGTAATTCCCGTAAAGATAAACATCAAC

ATTGTTTGGTAATCATTAAACCAATTAGTATGACGTTGAATTAATTTCACAGTATATTTTATTCCAGTAT

TATCCCCGCATGTATACGTACCTGGTAAGATATCTTTATATTCCATAATCAATGAGACATCACTATCCGA

TAACGAATGAAGTCTAGCACTAGTATGCCATTTACTTAATATGGTCGTCTTGGAAGTTTTATTATAAGTT

AAAATATCATGATTGTCCAATTTCCATCTAATATACTTTGTCGGATTATCTATAATACACGGAATAATGA

TGGTATCATTACATGCTGTATATTCTATAGTCTTTGTAGATGTTATAACCACAAAAGTACAGAGGTATAT

CAACAATATTCTAACTCTTAACATTTTTATTTATTTAAAATGATACCTTTGTTATTTATTTTATTCTTAT

TTTGCTAACGGTATCGAATGGCATAAGTTTGAAACGAGTGAAGAAATAATTTCTACTTACTTAATAGATT

ATGTGGTAACGGGTGTTATTAATGGGGATGTATATACATTTTCAAATAATGAACTAAACAAAACTGGGTT

AACTAATAACAATAATTATATCACAACATCTATAAAAGTAGAGGATAAGGATACATTAGTAGTATGCGGA

ACCAATAACGGAAATCCCAAATGTTGGAAAATAGACGGTTCATACTACCCAAAACATATAGGTAGAGGAT

ACGATCATCAAAATAGCAAAGTAACGATAATCAGTCACAATGAATGTGTACTATCCGACATAAACATATA

AAAAGAAGGAATTAAACGATGGAGAAGATTTGACGGACCATGTGGTTATGATTAAACGAGTTAAGTTTTT

TAAGAAGCCTTAGAAGAGAGGCTATTGGGTATGAGAATCCGAAATATTAAACCAGACAACCCCATATAAT

TTTATAGCTAAGAATGCCGCGAAGAATGGAACTAATAAAAACGGAAATATTTGTAGCACAACGAATAACT

CCCAAACTGCATTCATGTTACACTATATAACACTACTTCGGTTAGATGTTTTAGAAAAAATAAATATCAC

CGTACCGTTTTGTTGTATAAAAATAACAATTAACAATTATCAATTTTTTTCTTTAATATTTTACGTGGTT

GACCATTCTTGGTGGTAAAATAATCTCTTAGTGTTGGAATGGAATGCTGTTTAATGTTTCCACACTCATC

GTATATTTTGACGTATGCAGTCACATCGTTTACGCAATAGTCAGACTGTAGTTCTATCATGCTTCCTACG

TTAGAAGGAGGAACAGTTTTAAAGTCTCTTGGTTTTAATCTATTGTCATTAGTTTTCATGAAATCCTTTG

TTTTATCCACTTCACATTTTAAATAAATGTCCACTATACATTCTTCTGTTAATTTTACTAGATCATCATG

AGTCATAGAATTCATAGGTTCCGTAGTCCATGGATCCAAACTAGCAAACTTCGCGTATACGGTATCGCGA

TTAGTGTATACACCAACTGTATGAAAATTAAGAAAACAGTTTAATAAATCTACAGAAATATTTAATCCTC

CGTTTGATACAGATGCGCCATATTTATGGATTTCGGATTCACACGTTGTTTGTCTAAGGGGTTCGTCTAG

TGTTGCTTCTACATAGACTTCGATTCCCATATATTCTTTATTGTCAGAATCACATACCGATTTATCATAC

GCTGGTTCACTTGTTTGAAAACTAAATGGTAGTAGATACATCAAAATAATAAATAATAAGTACATTCTGC

AATATTGTTATCGTAATTGGAAAATTGGTATTCAAGTGAGCTGGATTATGTGAGTATTGGATTGTATATT

TTATTTTATATTTTATATTTTATATTTTATATTTTATATTTTATTTTATATTTTATATTTTATTTTATAT

TTTGTAGTAAGAATAGAATGCTAATGTCAAGTTTATTCGAATAGATGTCTTATTAAAAAACATATATAAT

AAATAACAATGGCTGAATGGCATAAAATTATCGAGGATATCTCAAAAAATAATAAGTTCGAGGATGCCGC

CATCGTTGATTACAAGACTACAAAGAATGTTCTAGCGGCTATTCCTAACAGAACATTTGCAAAGATTAAT

CCGGGTGAAGTTATTCCCCTCATCACTAATCATAATATTCTAAAACCTCTTATTGGTCAGAAATTTTGTA

TTGTATATACTAACTCTCTAATGGATGAGAACACGTATGCTATGGAGTTGCTTACTGGGTACGCCCCTGT

ATCTCCGATCGTTATAGCGAGAACTCATACCGCACTTATATTTTTGATGGGTAAGCCAACAACATCCAGA

CGTGATGTGTATAGAACATGTAGAGATCACGCTACCCATGTACGTGCAACTGGTAATTAAAATAAAAAGT

AATATTCATATGTAGTGTCAATTTTAAATGATGATGAAATGGATAATATCCATATTGACGATGTCAATAA

TGCCGGTATTGACATACAGCTCATCGATTTTTAGATTTCATTCAGAGGATATTGAATTATGTTATGGGAA

TTTGTATTTTGATAGGATCTATAATAATGTAGTAAATATAAAATATATTCCTGAGCATATTCCATATAGA

TATAATTTTATTAATCGTACGTTCTCCGTAGATGAACTAGATGATAATGTCTTTTTTACACATGGTTATT

TTTTAAAACACAAATATGGTTGTTCACTTAATCCTAGTTTGATTGTCTCATTATCAGGAAACTTAAAATA

TAATGATATACAATGCTCAGTAAATGTATCGTGTCTCATTAAAAATTTGGCAACGAGTACATCTACTATA

TTAACATCTAAACATAAGACTTATTCTCTATATCGGTCCACGTGTATTGCTATAATAGGATACGATTCTA

TTATATGGTATAAATATATAAATGACAGGTATAATGTCATCTATGATTTTACTGCAATATGTATGCTAAT

AGCGTCTACATTGATAGTGATCATATACGTGTTTAAAAAAATAAAAATGAACTCTTAATTATGTTATACT

ATTAGAAATGGATAAAATCAAAATTACGATTGATTCAAAAATTGGTAATGTTGTTACCATATCGTATAAC

TTGGAAAAGATAACTATTGATGTCGCACCAAAAAAGAAAAAAGAAAAGGATGTATTATTAGCGCAATCAG

TTGCTGTCGAAGAGGCAAAAGATGTCAAGGTGGAAGAAAAAAATATTATCGATATTGAAGATGACGATGA

TATGGATATAGAAAACACGTAATACGATCTATAAAAATAAGTATTAAATACTTTTTATTTACGGTACTCT

TGTAGTGGTGATACCACTAATCGATTATTTTTTTTAAAAAAAATACTTATTCTGATTCTTCTAGCCATTT

CCGTGTTCGTTCGAATGCCACATCGACGTCAAAGATAGGGGAGTAGTTGAAATCTAGTTCTGCATTGTTG

GTACGCACCTCAAATGTAGTGTTGGATATCTTCAACGTATAGTTGTTGAGTATTGATGGTTTTCTAAATA

GAATTCTCTTCATATCATTCTTGTACGCGTACATTTTTAGCATCCATCTTGGAATCCTAGATCCTTGTTC

TATTCCCAATGGTTTCATCAATAGAAGATTAAACATATCGTAAGAACACGATGGAGAGTAATCGTAGCAA

AAGTAAGCATTTCCTTTAATCGCAGATCCCGGATACTGGATATATTTTGCAGCCAACACGTGCATCCATG

CAACATTTCCTACATATACCCGGCTATGCACAGCGTCATCATCGACTGTACGATACATAATGTTACCGTG

TTGCTTACATTGCTCGTAAAAGACTTTCGTCAATTTGTCTCCTTCTCCGTAAATTCCAGTGGGTCTTAGG

CAACAAGTATACAATTTTGCGCCATTCATGATTACGGAATTATTGGCTTTCATAACCAGTTGCTCGGCCA

TACGTTTACTTTTTGCGTATACATGTCCTGGTGATATATCATAAAGGGTATGCTCATGACCGATGAATGG

ATTACCGTGTTTATTTGGTCCTATTGCTTCCATGCTACTAGTATAGATCAAATACTTGATTCCTAGGTCC

ACACAAGCTGCCAATATAGTCTGTGTTCCATAATAGTTTACTTTCATGATTTCATTATCAGTGTATTTTC

CAAATACATCCACTAGAGCAGCCGTATGAATAATCAGATTTACCCCATCTAGCGCTTCTCTCACCTTATC

AAAGTCGTTTATATCACATTGTATATAGTTTATAACCTTAACTTTCGAGGTTATTGGTTGTGGATCTTCT

ACAATATCTATGACTCTTATTTCTTGAACATCATCTGCGCTAATTAAAAGTTTTACTATATACCTGCCTA

GAAATCCGGCACCGCCAGTAACCGCGTACACGGCCATTGCTGCCACTCATAATATCAGACTACTTATTCT

ATTTTACTAAATAATGGCTGTTTGTATAATAGACCACGATAATATCAGAGGAGTTATTTACGTTGAACAA

GTCCATGGAAAAGATAAAGTTTTAGGATCAGTTATTGGATTAAAATCCGGAACGTATAGTTTGATAATTC

ATCGTTACGGAGATATTAGTCGAGGATGTGATTCCATAGGCAGTCCAGAAATATTTATCGGTAACATCTT

TGTAAACAGATATGGTGTAGCATATGTTTATTTAGATACAGATGTAAATATATCTACAATTATTGGAAAG

GCGTTATCTATTTCAAAAAATGATCAGAGATTAGCGTGTGGAGTTATTGGTATTTCGTACATAAATGAAA

AGATAATACATTTTCTTACAATTAACGAGAATGGCGTTTGATATATCAGTTAATGCGTCTAAAACAATAA

ATGCATTAGTTTACTTTTCTACTCAGCAAAATAAATTAGTCATACGTAATGAAGTTAATGATATACACTA

CACTGTCGAATTTGATAGGGACAAAGTAGTTGATACGTTTATTTCATATAATAGACATAATGACTCCATA

GAGATAAGAGGGGTGCTTCCAGAGGAAACTAATATTGGTCGCGTGGTTAATACGCCGGTTAGTATGACTT

ACTTGTATAATAAGTATAGTTTTAAACCGATTTTAGCAGAATATATAAGACACAGAAATACTATATCCGG

CAACATTTATTCGGCATTGATGACGCTAGATGATTTGGTTATTAAACAGTATGGAGACATTGATCTATTA

TTTAATGAGAAACTTAAAGTAGACTCCGATTCGGGACTATTTGACTTTGTCAACTTTGTAAAGGATATGA

TATGTTGTGATTCTAGAATAGTAGTAGCTCTATCTAGTCTAGTATCTAAACATTGGGAATTGACAAATAA

AAAGTATAGGTGTATGGCATTAGCCGAACATATAGCTGATAGTATTCCAATATCTGAGCTATCTAGACTA

CGATACAATCTATGTAAGTATCTACGCGGACACACTGATAGCATAGAGGATGAATTTGATCATTTTGAAG

ACGATGATTTGTCTACATGTTCTGCCGTAACCGATAGGGAAACGGATGTATAATTTTTTTTATAGTATGA

AGGATATGATGGATATGATGATATGATGGATATGATGATATGATGGATATGATGGATATGATGGATATGA

TGGATATGATGGATATGATAAAAAAATATAATTGTTGTATCCATTCCCATTCAAATCACCTTATATGATT

CTGTAACACAATGAAGGAGTCTCATAGATATATAGAGGTCAGATACTGGTTTGATAAACTTTTTATTCCA

CATGAGCATGTTTGACTTATGGTTAGACACACATACTTTAACAAATCACTGAAAATTGGAGTTAGGTATT

CCTCTCAGAATCAGTTGCCGTTCTGGAACATTAAATGTATTTTTTATGATATACTCCAACGCATTTATGT

GGGTATACAACAAGTCATTAATAATGAGTATTTCCAAGAGTTTTAGTTGTCTAGTATTTAACAAGAGAAG

AGATTTCAACAGACTGTTTATGAACTCGAATACCGCCTCATTGTCGCTTATATTGATGACATGACATCGA

ATTCCCAATATCAATCTCATCAGTGATGAGTAGCTCAATCTTGTTATCGGGATCCAATTTCTAAAGATGT

CATTAAACCCTCGATCGTGAATGGATTTATCATCATCGTTTTTATGTTGGACATGAGCTTAGTCCGTTTG

TCCACATCTATATACGATGATTTCTGAATTATTTCATATATCTCTCGTTAACTCCAGGAACTTGTCAGGG

ATCTAACTTTAATATGTTCTCGTCTAAGAGATGAAAATCTTTGGATGGTTGCATGTGACTTTTCTCTAAA

GGATGATGTTACCCGATCCTCTCTTAAATGACTCCATCTTATCCTTGGACAAGATGGACAGTCTATTTTC

CTTAGATGGTTTAATATTTTTTACCCATGATCTATAAAGGTAGACAGACCTAATCGTCTCGGATGACCAT

ATATTATTTTCAGTTTTATTATACGCATAAATTGTAAAAAATATGTTAGGTTTACGAAAATGTCTCGTGG

GGCATTAATCGTTTTTGAAGGATTGGACAAATCTGGAAAAACAACACAATGTATGAACATCATGGAATCT

ATACCGGCAAACACGATAAAATATCTTAACTTTCCTCAGCGATCCACAGTCACTGGAAAGATGATAGATG

ACTATCTAACTCGTAAAAAAACCTATAATGATCATATAGTTAATCTATTATTTTGTGCAAATAGATGGGA

GTTTGCATCTTTTATACAAGAACAACTAGAACAGGGAATTACTTTAATAGTTGACAGATACGCGTTCTCT

GGAGTAGCGTATGCCACCGCTAAAGGCGCGTCAATGACTCTCAGTAAGAGTTATGAATCTGGATTGCCTA

AACCCGACTTAGTTATATTCTTGGAATCTGGTAGCAAAGAAATTAATAGAAACATCGGCGAGGAAATTTA

TGAAGATGTTGAATTCCAACAAAAGGTATTACAAGAATATAAAAAAATGATTGAAGAAGGAGATATTCAT

TGGCAAATTATTTCTTCTGAATTCGAGGAAGATGTAAAGAAGGAGTTGATTAAGAATATAGTTATAGAGG

CTATACACACGGTTACTGGACCAGTGGGGCAACTGTGGATGTAATAAAATGAAATTACATTTTTATAAAT

AGATGTTAGTACAGTGTTATAAATGGATGAAGCATATTACTCTGGCAACTTGGAATCAGTACTCGGGGAT

ACGTGTCCGATATGCATACCGAACTCGCATCAATATCTCAATTAGTTATTGCCAAGATAGAAACTATAGA

TAATGATTATTAAACAAGGACATTGTAAATTTTATCATGTGTAGATCAAACTTGGATAATCCATTTATCT

CTTTCCTAGATACTGCATATACTATCATAGATCAAGAGATCTATCAGAACGAGTTGATTAATTCATTAGA

CGATAATGAAATTATCGATTGTATAGTTAACAAGTTTATGAGCTTTTATAAGGATAACCTAGAAAATATG

GTAGATGCTATCATTACTCTAAAATATTATAATTAATAATCCAGATTTTAAAACTACGTATGTGGAAGTA

CTCGGTTCCAGAATAGCTGATATAGATATTAAACAAGTGATACGTAAGAATATAATACAATTGTCTAATG

ATCCGCGAACGATATTTGTGAAAATATTAAAAAAAAATACTTTTTTTATTAAATGACGTCTCTTCGCGAA

TTTAGAAAATTATGCTGTGATATATATCACGCATCAGGATATAAAGAAAAATCTAAATTAATTAGAGACT

TTATAACAGATAGAGATGATACCGATACATATTTGATCATTAAGCTATTGCTTCCCGGATTAGACGATAG

AATGTATAACATGAACGATAAACAAATTATAAAATTATATAGTATAATATTTAAACAATCTCAGGAAGAT

ATGCTACAAGATTTAGGATACGGATATATAGGAGACACTATTAGGACTTTCTTCAAAGAGAACACGGAAA

TCCGTCCACGAGATAAAAGCATTTTAACTTTAGAAGAAGTGGATAGTTTTTTAACTACGTTATCATCAGT

AACTAAAGAATCACATCAAATAAAATTATTGACTGATATAGCATCTGTTTGTACATGTAATGATTTAAAA

TGTGTAGTCATGCTTATTGATAAAGATCTAAAAATTAAAGCGGGTCCTCGGTACGTGCTTAACGCTATTA

GTCCTCATGCATATGATGTTTTTAGAAAATCTAATAACTTGAAAGAGATAATAGAAAATGCAGCTAAACA

AAATCTAGACTCTATATCTATTTCTGTTATGACTCCAATTAATCCCATGTTAGCGGAATCATGTGATTCT

GTCAATAAGGCGTTTAAAAAATTTCCATCAGGAATGTTTGCGGAAGTCAAATACGATGGTGAAAGAGTAC

AAGTTCATAAAAAAAATAACGAGTTTGCATTCTTTAGTAGAAACATGAAACCAGTACTCTCTCATAAAGT

GGATTATCTCAAAGAATACATACCGAAAGCATTTAAAAAAGCTACGTCTATCGTATTGGATTCTGAAATT

GTTCTTGTAGACGAACATAATGTACCGCTACCGTTTGGAAGTTTAGGTATACACAAAAAGAAAGAATATA

AAAACTCTAACATGTGTTTGTTCGTATTTGACTGTTTATACTTTGATGGATTCGATATGACAGACATTCC

ATTGTATGAACGAAGATCTTTTCTCAAAGATGTTATGGTCGAAATACCCAATAGAATAGTATTCTCAGAG

TTGACGAATATTAGTAACGAGTCTCAGTTAACTGATGTATTAGATGATGCACTAACGAGAAAATTAGAAG

GATTGGTCTTAAAAGATATTAATGGCGTATACGAACCGGGAAAGAGAAGATGGTTAAAAATAAAGCGAGA

CTATTTGAACGAGGGTTCCATGGCAGATTCTGCCGATTTAGTAGTACTAGGTGCCTACTATGGTAAAGGA

GGAAAGGGTGGTATCATGGCAGTCTTTCTAATGGGTTGTTACGACGATGAATCCGGTAAATGGAAGACGG

TAACTAAATGTTCCGGTCACGATGATAATACGTTAAGGGTTTTGCAAGACCAATTAACGATGGTTAAAAT

TAACAAGGATCCCAAAAAAATTCCAGAGTGGTTGGTAGTTAATAAAATCTATATTCCCGATTTTGTAGTA

GATGATCCGAAACAATCTCAGATATGGGAAATTTCAGGAGCAGAGTTTACATCTTCCAAGTCACATACAG

CGAATGGAATATCGATTAGATTTCCTAGATTTACTAGGATTAGAGAAGATAAAACGTGGAAAGAATCTAC

TCATCTAAACGATTTAGTAAACTTGACTAAATCTCTTAATAGTTACATATAAACTGAAAAATAAAATAAC

ACTATTTTAGTTGGTGGTCGCCATGGATGGTGTTATCGTATACTGTCTAAATGCGTTAGTAAAACATGGC

GAGGAAATAAATCATATAAAAAATGATTTCATGATTAAACCATGTTGTGAAAGAGTTTGTGAAAAAGTCA

AGAACGTTCACATCGGCGGACAATCTAAAAACAATACAGTGATTGCAGATTTGCCATATCTGGATAATGC

TGTATCAGATGTATGCAAATCAATATATAAAAAGAATGTATCAAGAATATCCAGATTTGCTAATTTGATA

AAAATAGATGACGATGACAAGACTCCTACCGGCGTATATAATTATTTTAAACCTAAAGATGCTATTCCTG

TTATTATATCCATAGGAAAGGATAAAGATGTCTGTGAACTATTAATCTCATCTGATAAAGCGTGTGCGTG

TATAAAGTTAAATTTATATAAAGTAGCCATTCTTCCCATGGATGTTTCCTTTTTTACCAAAGGAAATGCA

TCATTGATTATTCTCCTGTTTGATTTCTCTATCGATGCGGCACCTCTCTTAAGAAGTGTAACCGATAATA

ATGTTATTATATCTAGACACCAGCGCCTACATGACGAGCTTCCGAGTTCCAATTGGTTCAAGTTTTACAT

AAGTATAAAGTCCGACTATTGTTCTATATTATATATGGTTGTTGATGGATCTATGATGTATGCGATAGCT

GATAATAGAACTCACGCAATTATTAGCAAAAATATATTAGACAATACTACGATTAACGATGAGTGTAGAT

GCTGTTATTCTGAACCACAGATTAGGATTCTTGATAGAGATGAGATGCTCAATGGATCATCGTGTTATAT

GAACAGACATTGTATTATGATGAATTTACCTGATGTAGGCGAATTTGGATCTAGTATGTTGGGGAAATAT

GAACCTGACATGATTAAGATTGCTCTTTCGGTGGCTGGTAATTTAATAAGAAATCGAGACTACATTCCCG

GGAGACGAGGCTATAGCTACTACGTTTACGGTATAGCCTCTAGATAATTTTTTTAAGCACGAAATAAAAA

CATAATTTTAAACAATCTATTTCATACTATTTTGTGTGCTCACCATGAACATAAAGATAGATATATTAGT

ATTTCTGGTGATAAATTTACGGCGACTGCTAGGAGGGAAAATGAAGAAAGAAAAAATATCTACCTCTCCA

AAAAGAAAAACTACTGATGTTATCAAACCTGATTATCTTGAGTACAATGACTTGTTAGATAGAGATGAGA

TGTCTACTATTCTAGAGGAATATTAGGCCTTAGAATAAAATATGGACGACTCTTAACGAAATTAGAAAAT

TCGATAATGATGTTGAAGAACAATTCGGTACTATAGAAGAACTCAAGCAGAAGCTTAGATTAAATTCTGA

AGAGGGAGCAGATAATTTTATAGATTATATAAAGGTACAAAAACAGGATATCATCAAACTTACTGTATAC

GATTGCATATATCTATGATAGGATTGTGTGCGTGTGTGGTAGATGTTTGGAGAAATGAGAAACTGTTTTC

TAGATGGAAATATTGGTTACAAGCGATTAAACTGTTTATTGATGATCACATGCTTGATAAGATAAAATCT

ATTGTAGAATAGACTAGTGTATGTGGAAATGTCATAGAAAGTTAAAAGTTAATGAGAGCAAAAATATATA

AGGTTGTATTTCATATTTGTTATTTTTTTCTGTAATAGTTAGAAAATACATTCGATGGTCTATCTACCAG

ATTATTATGTGTTATAAGGTAATTTTCTCATAATAAACTAGAGTATGAGTAAGATAGTGTTTTTCAAAAA

CATATAAATCTAAAATTGATGGATGAGATATACAGCTATTAATTTCGAAAATATATTTTAATCTGATAAC

TTTAAACATGGATTTTTGATGGTGGTTTAAGTTTAAAAAAGATTTTGTTATTGTAGTATGATAATATCAA

AAAGATGGATATAAAGAATTTACTGACTACATGTTGTACTATTTTACATTACTACATTGGCTACGGTATA

TACCTACTTCGTCACTTCCACACGCTCCAGTAAACGGGTGTCATGTGACGAGGGAGAATCTTGATAAGAG

GCATAATCAATGTTGTAATCCGGTGTCCACCTGGAGAATTTGCCAAGGTCATATGTAGTTGGTAGTGATA

ACACAAAATGTGAACACTGTCCACCTCATACATATACCGCAATCCCCAATTATTCTAATAGATGTCATCA

ATGTAGAAAATGCCTAACAGGATCATTTGATAAGGTAAAGTGTACCGGAACACAGAACAAATGTTCGTGT

CATCCTGGTTGGTATACGCTACTGATTCTTCACAGACTGAAGATTGTCGAGATTTGTGTACCAAAAAAGG

AGATGTCCATGCGGATACTTTGGTGGAATAGATGAAGGAAATCCTATTTGTAAATCGTGTTGTGTTGGTG

AATATTGCGACTACCTACGTAATTATAGACTTGATCCATTTCCTCCATGCAAACTATCTATCTAAATGTA

ATTAATTATGATTTTGATGATAATGTTACCATACATTATATCGCTACTTGGTTAGTGTGTATTATTCAGT

ATGGAAGACCTATTAATAATTACTTATCTTTTGACGATCTTGTTATAATTATAATATAAAAATACTTATG

ACATAGTAACAGTAACTCATAATTGCTGACGCGATAAATTCGTAATAATCTGTTTTGTTCAAATTTTTAT

AAGGAATCTACAGGCATAAAAATAAAAATATAATCTATAATATACTCTTACAACGCCATCATGAATAGCA

GTGAATTAATTGCTGTTATTATGGATTTAGAAATAGTGGACGATTTTGTGATATTAATATAGTTATTAAT

GATGAAAGGATAAACGCTCATAGATTCATCCTATCTGGAGCCTCCGAATATTTTTTCCATTCTGTTTTCC

AATAATTTTATCGATTCTAATGAATACGAAGTTAATCTAAGTCATTTAGATTATCAAAGTGTTAACTATT

TGATCGATTACATTTATGGGATACCTTTGAGCCTAACTAACGATAACGTGAAATATATTCTTTCAACCGC

TGATTTTTTTTTACAAATTGGATCTGCCATTACTGAGTGCGAAAAATACATACTTAAAAATCTTTGTTTT

AGAAACTGTATCGATTTCTACATATACGCTGATAAATATAATAACAAGAAAATAGAATTAGCATCGTTTA

ACACAATATTACGAAATATTTTGAGACTCATCAACAATGAAAACTTTAAATACTTAACAGAGGAATCAAT

GATATGTTATATATAAAAAATGAGGATTTCACCCCACTGATTCTCATTAAATGGTTAGAGAGTACACCAA

CCATGTACCGTCGAGTTACTTAGATGCCTCAGAATATCATTTCTTTCCCCACAAGTTATAAAATCACTTT

ATAGTCATCGACTGGTTAGTTCAATCTACGAATGTATAACATTCTTAAACAATATAGCATTCTGGGATAA

ATCATTTCCTAGATACCATATCATCGAGTTGATATCTATCGGTATAAGTAATTCACATGATAAGATTTCC

ATAAACTGCTACAATCATAAAAAAATTCATGGGAAATGATATCTTCACGTAGATGGTGTAGTTTCGCAGT

GACCGTCCTGGATAATATTATCTATATGATGGGTGGATATGATCAGTCCCTGTATAGAAGTTCAAAGGTT

ATAGCGTACAATACATGTACTAATTCTTGGATATATGATATACCAGAGCTAAAATATCCTCGTTCTAATT

GCGGAGGAGTTGCCAATGACGAATACATTTATTGTATAGGCGGTATACGCGATCAGGAGTCATCGTTGAT

ATCTAGTATCGATAGATGGAAGCCATCAAAACCATATTGGCAGAAGTATGCTAAAATGTGCGAACCAAAA

TGTGATATGGGGTTGCGATTTTAAACGGATTAATATATGTCATAGGTGGAGTCGTTAAAGGTGACACACA

TGTACCGACGCACTAGAGAGTTTATCAGAAGATGGATGGATGAATCATCAACGTCTTCCAATAAAAATGT

CCAATATGTCGACGATTGTTCATGCTGGAAAGATTTATATATCTGGAGGTTACAACAATAGTAGTGTAGT

TAATGTAATATCGAATCTAGAGTCCTTAGCTATAATCCGATATATGATGAATGGACCAAATTATCATCAT

TAAATATTCCTAGAATTAATCCTGCTCTATGGTCAGTGTATAATAAATTATATGTAGGAGGAGGAATATC

TGATGATGTTCAAACTAATACATCTGAAACATACGATAAAGAAAAAGATTGTTGGACATTGGATAATGGT

CACTTGTTACCACATAATTATATAATGTATAAATGCGAACCGTTTAAACATAGATATCCATTGGAAAAAA

CACAGTACACGAATGATTTTCTAAAGTATTTGGAAAGTTTTATAGGTAGTTGATAGAACAAAATACATAA

TTTTGTAAAAATAAATCACTTTTTATACTAATATGACACAATTACCAATAGTTTTGTTACTAATATCATT

AGTATACGCTACACCTTCTCCTCAGATATCTAAAAAAATAGGTGATGATGCAACTATATCATGTAGTCGA

AATAATACAAATTACTACGTTGTTATGAGTGCTTGGTATAAGGAGCCCAATTCCATTATTCTCTTAGCTG

CCAAAAGCGACGTCTTGTATTTTGATAATTATACCAAGGATAAAATATCTTACGACTCTCCATACGATGA

TCTAGTTACAACTATCACAATTACATCATTGACTGCTGGAGATGCCGGTACTTATATATGTGCATTCTTT

ATGACATCGACTACAAATGATACTGATAAAGTAGATTATGAAGAATACTCCATAGAGTTGATTGTAAATA

CAGATAGTGAATCGACTATAGACATAATACTATCTGGATCTACACCAGAAACTATTTCTGAGAAACCAGA

GGATATAGATAATTCTAATTGCTCGTCTGTATTCGAAATCGCGACTCCGGAACCAATTACTGATAATGTA

GAAGACCATACAGACACCGTCACATACACTAGTGATAGCATTAATACAGTAAATGTATCATCTGGAGAAT

CCACAACAGACGAGATTCCGGAACCAATTATTGATAAAGAAGAAGATCATACAGTAACAGACACTGTCTC

ATACACTACAGTAAGTACATCATCTGGAATTGTCACTACTAAATCAACCACCGATGATGCGGATCTTTAT

GATACATACAATGATAATGATACAGTACCGCCAACTACTGTAGGTGGTAGTACAACCTCTATTAGCAATT

ATAAAACCAAGGACTTCGTAGAAATATTTGGTATTACCACATTAATTATATTGTCAGCAGTGGCGATTTT

CTGTATTACGTATTATATATGTAATAAACACCCACGTAAATACAAAACAGAGAACAAAGTCTAGATTTTT

GACTTACATAAATATCTGGGATAATAAAATCTATCATATTGAGAGGACCATCTGGTTCAGGAAAGACAGC

CATAACCAAAAGACTGTTAAAAGACTATGTGAATATATTTGGATTTGTGGTGTCCCATACCACTAGATTT

CCTCGTCCTATGGAACGAGAAGGTGTTGATTACCTTACGTTAACAGAGAGGCCATCTGGAAGGGAATAGC

CGCCGGAAACTTTCTAGAACATACTGAGTTTTTAGGAAATATTTATGGAACTTCTAAAACAGCTGTAAAT

ACAGCGGCTATTAATAATCGTATTTGCGCGATGGATTTAAACATCAACGGTGTTAGAAGTCTTAAAAATA

CTTACCTAATGCATTACTTGGGTATATAAGACCTACCTCTCTTAAAATGGTTGAGACCAATCTTCGTCGT

AGAAACACTGAAGCGGACGACGAATCTCATCGTCGCGTGATGTTGGCAAAAAACGGATATGGATGAGGTC

AACGAAGCAGGTCTATTCGACACTATTATTATTGAAGATGATGTGAATTTAGCATATAGTAAGTGTTAAT

TCAGATACTACAGGACCGTATTAGAATGTATTTTAACACTAATTAGAGACTTAAGATTTGACTTAAAACT

TGATAATTAATAATATAACTCGTTTTTATATGTGGCTATTTCAACGTCTAATGTATTAGTTAAATATTAA

AACTTACCACGTAAAACTTAAAATTTAAAATGGTATTTCATTGACAGATCATACATTATGAAGTTTCAAG

GACTTGTGTTAATTGACAATTGCAAAAATCAATGGGTCATTGGACCATTAATAGGAAAAGGTGGATTCGG

TAGTATTTATACTACTAATGACAATAATTATGTAGTAAAAATAGAGCCCAAAGCTAACGGATCATTATTT

ACCGAGCAGGCATTTTATACTAGAGTACTTAAACCATCCGTTATCGAAGAATGGAAAAAATCTCACAATA

TAAAGCACGTAGGTCTTATCACGTGCAAGGCATTTGGTTTATACAAATCCATTAATGTGGAATATCGATT

CTTGGTAATAAATAGATTAGGTGCAGATCTAGATGCGGTGATCAGAGCCAATAATAATAGACTACCAGAA

AGGTCGGTGATGTTGATCGGAATCGAAATCTTAAATACCATACAATTTATGCACGAGCAAGGATATTCTC

ACGGAGATATTAAAGCGAGTAATATAGTCTTGGATCAAATAGATAAGAATAAATTATATCTAGTGGATTA

CGGATTGGTTTCTAAATTCATGTCTAACGGCGAACATGTTCCATTTATAAGAAATCCAAATAAAATGGAT

AACGGTACTCTAGAATTTACACCTATAGATTCGCATAAAGGATACGTTGTATCTAGACGTGGTGATCTAG

AAACACTTGGATATTGTATGATTAGATGGTTGGGAGGTATCTTGCCATGGACTAAGATATCTGAAACAAA

GAATTCTGCATTAGTAAGTGCCGCAAAACAGAAATATGTTAACAATACTGCGACTTTGTTAATGACCAGT

TTGCAATATGCACCTAGAGAATTGCTGCAATATATTACCATGGTAAACTCTTTGACATATTTTGAGGAAC

CCAATTACGACGAGTTTCGTCGAGTATTAATGAATGGAGTTATGAAAAATTTTTGTTGATAAAAAAATTA

AAAAAATAACTTAGTTATTATCACTCTCGCGAGTACAATAGAAACATGGCGATGTTTTACGCACACGCTT

TCGGTGGGTACGACGAGAACCTTCATGCATTTCCTGGAATATCATCGACGGTTGCCAATGATGTCAGGAA

ATATTCTGTTGTGTCAGTTTATAATAAAAAGTATAACATTGTAAAAAACAAATATATGTGGTGTAACAGT

CAAGTAAACAAGAGATATATTGGAGCACTACTGCCTATGTTTGAGTGCAATGAATATCTACAAATTGGAG

ATCCAATCCATGATCTAGAAGGAAATCAAATCTCTATTGTCACATATCGCCACAAAAACTACTATGCTCT

AAGTGGAATTGGGTACGAGAGTCTAGACTTGTGTTTGGAAGGAGTAGGGATTCATCATCACGTACTTGAA

ACAGGAAACGCGGTATATGGAAAAGTTCAACATGAGTATTCTACTATCAAAGAGAAGGCCAAAGAAATGA

ATGCACTCAAACCAGGACCTATCATCGATTACCACGTCTGGATAGGAGATTGTGTCTGCCAAGTTACTAC

TGTAGACGTGCATGGAAAGGAAATTATGAGAATGAGATTCAAAAGGGGTGCGGTGCTTCCGATTCCAAAT

CTGGTAAAAGTTAAAGTTGGGGAGGAAAATGATACAATAAATCTTTCCACTTCCATATCAGCTCTCCTGA

ATTCCGGTGGCGGCACCATCGAGGTAACATCTAAGGAAGAACGTGTGGATTATGTACTCATGAAACGTTT

GGAATCTATACATCATCTGTGGTCTGTAGTGTATGATCATCTTAATGTTGTGAATGGCGAAGAACGATGT

TATGTACATATGCATTCATCTCATCAAAGTCCTATGCTGAATACTGTAAAAACAAATTTGTACATGAAGA

CTATGGGAGCATGTCTTCAAATGGACTCCATGGAAGCTCTAGAGTATCTTAGTGAACTGAAGGAATCAGG

TGGGCGGAGTCCCAGACCAGAATTGCAGAAATTTGAATATCCAGATGGAGTGAAAGACACTGAATCAATT

GAGAGATTGGCAGAGGAGTTCTTCAATAGATCAGAACTTCAGGCCGGTGAATCAGTCAAATTTGGTAATT

CTATTAATGTTAAACATACATCTGTTTCAGCTAAGCAACTAAGAACACGTATACGACAGCAGCTTCCTTC

TATACTCTCATCTTTTGCCAACACAAAGGGTGGATATTTGTTCATTGGAGTTGATAATAATACACACAAA

GTAATTGGATTCACGGTGGGTCACGACTACCTCAAACTGGTAGAGAGTGATATAGAAAAGTATATCCAAA

AACTTCCTGTTGTGCATTTCTGCAAGAAAAAAGAGGACATCAAGTACGCATGTAGATTCATCAAGGTGTA

TAAACCTGGTGATGCGACTACCTCGACATATGTGTGCGCAATCAAAGTGGAAAGATGCTGCTGTGCTGTG

TTTGCGGATTGGCCAGAATCATGGTACATGGATACTAGTGGTAGTATGAAGAAGTATTCTCCAGATGAAT

GGGTGTCACATATAAAATTTTAATTAGGGTAAGGTAAAACTATATATAATAACTAACAATTTGTGTATCA

TATAGACAATTAATTAGGTAACTGTTATCTCTTTTTAACTAACTAACTAACTCTTATATACTATTAATAA

TACATCTATTAATCATTGATTAGCTTATTGCTTTAATTGTTTTTGTAAACTAACACTGTTCATTGAAAAG

GGATAACATGTTACAGAATATAAATTATATATGGATTTTTTTAAAAAGGAAATACTTGACTGGAGTATAT

ATTTATTTCTTCATTACATAACACGTCTGTGTTCTAATTCTTCCACATCTCATATAATACAGGAATATAA

TCTTGTTCGAAAATACGAGAAAGTGGATAAAACAATAGTTGATTTTTTATCTAGGTGGCCAAATTTATTC

CATATTTTAGAATATGGGGAAAATATTCTACATATTTATTTTATAGATGCTGCTAATACGAATATTATGA

TTTTTTTTCTAGATAGAGTATTAAATATTAATAAGAACCGTGGGTCATTTATACATAATCTCGGGTTATC

ATCCATTAATATAAAAGAATATGTATATCAATTAGTTAATAATGATCATCTAGATAATAGTATAAGACTA

ATGCTTGAAAATGGACGTAGAACAAGACATTTTTTGTCTTATATATTGGATACAGTTAATATCTATATAA

GTATTTTAATAAATCATAGATTTTATATAGATGCCGAAGACAGTTACGGTTGTACATTATTACATAGATG

TATATATAACTATAAGAAATCAGAATCAGAATCATATAATGAATTAATTAAGATATTGTTAAATAATGGA

TCAGATGTAGATAAAAAAGATACGTACGGAAACACACCGTTTATCCTATTATGTAAACACGATATCGACA

ACGCGGAATTGTTTGAGATATGTTTAGAGAATGCTAATATAGACTCTGTAGACTTTAATGGATATACACC

TCTTCATTATGTCTCATGTCGTAATAAATATGATTTTGTAAAGTTATTAATTTCTAAAGGAGCAAATGTT

AATGCACGTAATAGATTCGGAACTACTCCATTTTATTGTGGAATTATACACGGTATCTCGCTTATAAAAC

TATATTTGGAATCAGACACAGAGTTAGAAATAGATAATGAACATATAGTTCGTCATTTAATAATTTTTGA

TGCTGTTGAATCTTTAGATTATCTATTGTCCAGAGGAGTTATTGATATTAACTATCGTACTATATACAAC

GAAACATCTATTTACGACGCTGTCAGTTATAATGCGTATAATACGTTAGTCTATCTATTAAACAGAAATG

GTGATTTTGAGACGATTACTACTAGTGGATGTACATGTATTTCGGAAGCAGTCGCGAACAACAACAAAAT

AATAATGGATATACTATTGTCTAAACGACCATCTTTGAAAATTATGATACCATCTATGATAGCAATTACT

AAACATAAACAACATAATGCAGATTTATTGAAAATGTGTATAAAATATACTGCGTGTATGACCGATTATG

ATACTCTTATAGATGTACAATCGCTACATCAATATAAATGGTATATTTTAAAATGTTTTGATGAAATAGA

TATCATGAAGAGATGTTATATAAAAAATAAAACTGTATTCCAATTAGTTTTTTGTATCAAAGACATTAAT

ACTTTAATGAGATACGGTAGACATCCTTCTTTCGTGAAATGTAATATTCTCGACGTATACGGAAGTCGTG

TACGTAATATCATAGCATCTATTAGATATCGTCAGAGATTAATTAGTCTATTATCCAAGAAGCTGGATGC

TGGAGATAAATGGTCGTGTTTTCCTAACGAAATAAAATATAAAATATTGGAAAACTTTAACGATAACGAA

CTGACCACATATCTGAAAATCTTATAAACACTATTAAAATATAAAATCTAAGTAGGATAAAATCACACTA

CATCATTGTTTCCTTTTAGTGCTCGACAGTGTATACTATTTTTAACACTCATAAATAAAAATGAAAACGA

TTTCCGTTGTTACGTTGTTATGCGTACTACCTGCTGTTGTTTATTCAACATGTACTGTACCCACTATGAA

TAACGCTAAATTAACGTCTACCGAAACATCGTTTAATGATAAACAGAAAGTTACGTTTACATGTGATTCA

GGATATCATTCTTTGGATCCAAATGCTGTCTGCGAAACAGATAAATGGAAATACGAAAATCCATGCAAGA

AAATGTGCACAGTTTCTGATTATGTCTCTGAACTATATGATAAGCCATTATACGAAGTGAATTCCACCAT

GACACTAAGTTGCAACGGTGAAACAAAATATTTTCGTTGTGAAGAAAAAAATGGAAATACTTCTTGGAAT

GATACTGTCACGTGTCCTAATGCGGAATGTCAACCTCTTCAATTAGAACACGGATCGTGTCAACCAGTTA

AAGAAAAATACTCATTTGGGGAATATATGACTATCAACTGTGATGTTGGATATGAGGTTATTGGTGTTTC

GTATATAAGTTGTACGGCTAATTCTTGGAATGTTATTCCATCATGTCAACAAAAATGTGATATACCGTCC

CTATCTAATGGATTAATTTCCGGATCTACATTTTCTATCGGTGGCGTTATACATCTTAGTTGTAAAAGTG

GTTTTACACTAACGGGGTCTCCATCATCCACATGTATCGACGGTAAATGGAATCCCATACTCCCAACATG

TGTACGATCTAACGAAGAATTTGATCCAGTGGATGATGGTCCCGACGATGAGACAGATCTGAGCAAACTC

TCGAAAGACGTTGTACAATATGAACAAGAAATAGAATCGTTAGAAGCAACTTATCATATAATCATAATGG

CGTTGACAATTATGGGTGTCATATTTCTAATCTCCATTATAGTATTAGTTTGTTCCTGTGACAAAAATAA

TGACCAATATAAGTTCCATAAATTGCTACCGTGAATATAAATCCGTTAAAATAATTAATAACGAACAAGT

ATCAAAAGATTAAAGAATTAGCTAAAATCAATTAGATGTCTTCTTCAGTGGATGTTGATATCTACGATGC

CGTTAGAGCATTTTTACTCAGGCACTATTATGACAAGAGATTTATTGTGTATGGAAGAAGTAACACCATA

TTACATAATATATACAGGCTATTTACAAGATGCACCGTTATACCGTTCGATGATATAGTACGTACTATGC

CAAATGAATCACGTGTTAAACAATGGGTGATGGATACACTTAATGGTATAATGATGAATGAATGCGATAC

TGTATGTGTGGGTACCGGACTACGATTCATGGAAATGTTTTTCGATTACAATAAAAATAATCCCAAAAAT

AGCATCAACAATCAAATAATGTATGATATAATTAATAGCGTAGCCATAATTCTAGCTAATGAGAGATATA

GAAGCGCGTTTAACGACGATAGAATATACATCCGTAGAACTATGATGGACAAATTGTACGAATACGCATC

TCTAACTACTATTGGTACGATCACTGGAGGTGTTTGTTATTATCTATTGATGCATCTAGTTAGTTTGTAT

AAATAATTATTTCGATATACTAGTTAAAATTTTAAGATTTTAAATGTATAAAAAACTAATAACGTTTTTA

TTTGTAATAGGTGCAGTTGCATCCTATTCGAATAATGAGTACACTCCGTTTAATAAACTGAGTGTAAAAC

TCTATATAGATGGAGTAGATAATATAGAAAATTCATATACTGATGATAATAATGAATTGGTGTTAAATTT

TAAAGAGTACACAATTTCTATTATTACAGAGTCATGTGACGTCGGATTTGATTCCATAGATATAGATGTT

ATAAACGACTATAAAATTATTGATATGTATACCATTGACTCGTCTACTATTCAACGCAGAGGACATACGT

GTAGAATATCTACCAAATTATCATGCCATTATGATAAGTACCCTTATATCCACAAATATGAGGGTGATGA

ACGACAATATTCTATTACTGCAGAGGGAAAATGCTATAAAGGAATAAAATATGAAATAAGTATGATGAAC

GATGATACTCTATTGAGAAAACATACTCTTAAAATTGGATTTACTTATATATTCGATCGTCATGGGCATA

GTAATACATATTATTCAAAATATGATTTTTAAAAATTTAAAATATATTATCACTTCAGTGACAGTAGTCA

AATAACAAACAACACCATGAGATATATTATAATTCTCGCAGTTTTGTTCATTAATAGTATACATGCTAAA

ATAACTAGTTATAAGTTTGAATCCGTCAATTTTGATTCCAAAATTGAATGGACTGGGGATGGTCTATACA

ATATATCCCTTAAAAATTATGGCATCAAGACGTGGCAAACAATGTATACAAATGTACCAGAAGGAACATA

CGACATATCCGGATTTCCAAAGAATGATTTCGTATCTTTCTGGGTTAAATTTGAACAAGGCGACTATAAA

GTGGAAGAGTATTGTACGGGACTATGTGTCGAAGTAAAAATTGGACCACCAACTGTAAGATTGACTGAAT

ATGACGATCATATCAATTTGTTCATCGAGCATCCGTATGCTACTAGAGGTAGCAAGAAGATTCCTATTTA

CAAACGCGGTGACATGTGTGATATCTACTTGTTGTATACGGCTAACTTCACATTCGGAGATTCTGAAGAA

CCAGTAACATATGATATCGATGACTACGATTGCACGTCTACAGGTTGCAGCATAGACTTTGCCACAACAG

AAAAAGTGTGTGTGACAGCACAGGGAGCCACAGAAGGGTTTCTCGAAAAAATTACTCCATGGAGTTCGGA

AGTATGTCTGACACCTAAAAAGAATGTATATACGTGCGCAATTAGATCTAAAGAAGATGTTCCCAATTTC

AAGGACAAAATAGCCAGAGTTATCACGAGAAAATTTAATAAACAGTCTCAATCTTATTTGACTAAATTTC

TCGGTAGCACATCGAATGATGTTACAACTTTTCTTAGCATTCTTGACTAAATATTCATAACTAATTTTTA

TTAATGATACAAAAATGAAATAAAACTGTATATTATACACTGGTTAACGCCCTTGGCTCTAACCATTTTC

AAGATGAGGTCCCTGATTATAGTCCTTCTGTTCCCCTCTATCATCTACTCCATGTCTATTAGACGATGCG

AGAAGACTGAAGAGGAAACATGGGGATTAAAAATAGGGTTGTGTATAATTGCCAAAGATTTCTATCCCGA

AAGAACTGATTGCAGTGTTCATCGCCCAACTGCAAGTGGAGGATTGATAACTGAAGGCAATGGATTCAGA

GTAGTTATATGATCAATGTACAGAACCCCATGACTTTATTATCACCGATACTCAACAAACACGTCTTGGA

TCATCTCATACATATATTAAATTCAGTAACATGAATACAGGTGTCCCATCTAGTATTCCAAAATGTTCCA

GAACTCTCTCTATTTCTGTATATTGTGATCAAGAGGCGGGAGACATAAAATTTGAGGAGTATACTCAAGA

ATCAAGTGATATCAGTATTAGAGTTAAGTATGATTCATCATGTATTGATTATCTGGGTATTAATCAAAGT

TTCATGAATGAATGTATTCGAAGAATTACAACATGGGATAGAGAATCATGCGTCAGAATTGATACACAGA

CTATAAATAAATATCTTAAGTCTTGCACCAACACAAAATTCGACCGTAATGTCTACAAAAGGTACATACT

GAAGAGTAAAGCACTCCATGCTAAAACAGAGTTGTAATAGATATAAAATACTTTTTATAATAATTAGGCT

AGAAAAATCTCACTCACATGTAATCTTAAAAAAATGATATGATAGTTCTTACAAGTAGCGATTGAGTTTT

AAATGGATTCTATTAATTACCGGGGAACTTAACAATTCGTTCTGATCTACAGACATTGGTTAATAAATCA

TCTTATTTTGCCAATATATTAAAATGTGGAAACTCCACTAATAATATTACATTGTGCGACTTTCAAGATG

ATGTGATATATAGGGTTATACAGTTTTAACAATTATATAATAGAGATAGAAAGTACAAAAGATGTAGAAT

CAATGATATGGCACGCTAAACAGTTGGGTGTGGAATCATTGCTAAAAGAATGTCAAAATTATTTGCTTAG

AATATTACGTATATAATTGTTTAGAAATTTATAGAATAACTAATATTAATACACTATCGTATATCTACAA

CGATATAAGAAACTTCATATTGGATAATATTACTATTAATATATAAGGATCCAGATTTTATATATTTGCC

TAAATACATTATTATAGATTTACTAGGACAATCACCTAAATGTTTTTAACGAAGATAATGTGGTAAAGAT

TATATACATTTATATATCTTCCGATATCTACAAGGATATTCCATATCATCATTGTGTAAACTAAATAACG

TTTTCTATGGCATTTGATAAGGACATTGGATATGTGGAAAAGATGATGGTGTATGGAAGTTAGTACATTA

TCAACTTCTCCTTATTGATTGAAAATGAAAATATAAATAGTTTTTATGTATAGCGGTATCTACCCTATAG

TTTTATTGCTTACTACTAACATGGATTCAGATACAGATACAGATACAGATACAGATACAGATACAGATAC

AGATACAGATACAGATGTTACAAATGTAGAAGATATCATGAATGAAATAGATAGAGAGAAAGAAATACTA

AAAAATGTAGAAATTGAAAATAATAAAAACATTAACAAGAATCATCCCAGTGAATATATTAGAGAAGCAC

TTGTTATTAATACCAGTAGTAATAGTGATTCCATTGATAAAGAAGTTATAGAATATATCAGTCACGATGT

AGGAATATAGATCATATCTACTAATTTTTATAATCGATACAAAACATAAAAACAACTCGTTATTACATAG

CAGGTATGGAATCCTTCAAGTATTGTTTTGATAACGATGGTAAGAAATGGATTATCGGAAATACTTTATA

TTCTGGTAATTCAATACTCTATAAGGTCAGAAAAAATTTCACTAGTTCGTTCTACAATTACGTAATGAAG

ATAGATCATAAATCACACAAGCCATTGTTGTCCGAAATACGATTCTATATATCTGTATTGGATCCTTTGA

CTATCGACAACTGGACACGGGAACGTGGTATAAAGTATTTAGCTATTCCAGATCTGTATGGAATTGGAGA

AACCGATGATTATATGTTCTTCATTATAAAGAATTTGGGAAGAGTATTCGCCCCAAAGGATAGTGAATCA

GTTTTCGAAGCATGTGTCACTATGATAAACACGTTAGAGTTTATACACTCTCGAGGATTTACTCATGGAA

AAATAGAACCGATGAATATACTGATTAGAAATAAACGTATTTCACTAATTGACTATTCTAGAACTAACAA

ACTATACAAAAGTGGAACACATATAGATTACAACGAGGACATGATAACTTCAGGAAATATCAATTATATG

TGTGTAGACAATCATCTTGGAGCAACAGTTTCAAGACGAGGAGATTTAGAAATGTTGGGATATTGCATGA

TAGAATGGTTCGGTGGTAAACTTCCATGGAAAAACGAAAGTAGTATAAAAGTAATAAAACAAAAAAAAGA

ATATAAACAATTTATAGCTACTTTTTTTGAGGACTGTTTTCCTGAAGGAAATGAACCTCTGGAATTAGTT

AGATATATAGAATTAGTATACATGTTAGATTATTCTCAAACTCCTAATTATGACAGACTACGTAGACTGT

TTATACAAGATTGAAATTATATTCTTTTTTTTATAGAGTGTGGGGTAGTGTTACGGATATCTAATATATT

AATATTAGACTATCTCTATCGTGCTACACGACCAATATCGATTACTATGGATATCTTCAGGGAAATCGCA

TCTTCTATGAAAGGAAAGAATGTATTCATTTCTCCAGCGTCAATCTCGTCAGTATTAACAATACTGTATT

ATGGAGCTAATGGATCCACTGCTGAACAGCTATCAAAATATGTAGAAAAGGAGGAGAACATGGATAAGGT

TAGCGCTCAGAATATCTCATTCAAATCCATGAATAAAGTATATGGGCGATATTCTGCCGTGTTTAAAGAT

TCCTTTTTGGGAAAAATTGGCGATAAGTTTCAAACTGTTGACTTCACTGATTGTCGCACTATAGATGAAA

TCAATAAGTGTGTAGATATCTTTACTGAGGGAAAAATCAATCCACTATTGGATGAACCATTGTCTCCTGA

TACCTGTCTCCTAGCAATTAGTGCCGTATACTTTAAAGCAAAATGGTTGATGCCATTCGAAAAGGAATTT

ACCAGTGATTATCCCTTTTACGTATCTCCAACGGAAATGGTAGATGTAAGTATGATGTCTATTTACGGCG

AGCCATTTAATCACGCATCTGTAAAAGAATCATTCGGTAACTTTTCAATCATAGAACTGCCATATGTTGG

AGATACTAGTATGATGGTCATTCTTCCAAACAAGATTGATGGATTAGAATCCATAGAACAAAATCTAACA

GATACAAATTTTAAGAAATGGTGTAACTCTCTGGAAGCTACGTTTATCGATGTGCACATTCCTAAGTTTA

AGGTAATAGGTTCGTATAATCTTGTGGATACGCTAATAAAGTTGGGACTGACAGATGTGTTCTATTCAAC

TGGTGATTATATCAATATGTGTAATTCAGATGTGAGTGTTGACGCTATGATTCACAAAACGTATATAGAT

GTCAATGAAGAGTATACAGAAGCAGCTGCAGCAACTTCTGTACTAGTGGCAGACTGTGCATCAACAGTTA

CAAATGAGTTCTGTGCAGATCATCCGTTCATCTATGTGATTAGACATGTCGATGGTAAAATTCTTTTCGT

TGGTAGATATTGCTCTCCAACAACTAATTAAGCACATTCTTAATATTAGAATATTATATAGTTAAGATTT

TTACTAACAGGTTAACATTTTTTTAAAAATAGAAAAAACATGTGGTATTAGTGCAGGTCGTTATTCTTCC

AATTGCAATTGGTAAGATGACGGCCAACTTTAGTACCCACGTCTTTTCACCACAGCACTGTGGATGTGAC

AGACTGACCAGTATTGATGACGTCAGACAATGTTTGACTGAATATATTTATTGGTCGTCGTATGCATACC

GCAACAGGCAATGCGCTGGACAACTGTATGACACACTCCTCTCTTTTAAAGATGATGCGGAATCAGTGTT

CATCGACGTTCGTGAGCTGGTAAAAAATATGCCGTGGGATAATGTTAAGGATTGTACAGAGATCATCCGT

TGTTATATACCGGATGAGCAAAAAACCATCAGAGAGATTTCGGCCATCATTGGACTTTGTGCATATGCTG

CTACTTACTGGGGAGGTGAAGACCATCCCACTAGTAACAGCCTGAACGCATTGTTTGTGATGCTTGAGAT

GCTCAATTACATGGATTATACCATCATATTCTGGCGTATGAATTGATGAGTTACAGCTTGACATTTCTTC

TTTCCTCCCTCTTCTTCTACCTTTCCCAGAAACAAACTTTTTTTACCCACTATAAAATAAAATGAGTATA

CTACCTGTTATATTTCTTCCTATATTTTTTTTATTCTCCATTCGTTCAGACTTTTAACGTGCCTGAATGT

ATCGACAAAGGGCAATATTTTGCATCATTCATGGAGTTAGAAAACGAGCCAGTAATCTTACCATGTCCTC

AAATAAATACGCTATCATCCGGATATAATATATTAGATATTTTATGGGAAAAACGAGGAGCGTATAATGA

TAGAATTATACAGATAGATAATGGTAGCAATATGCTAATTCTGAACCCGACACAATCAGACGCTGGTATT

TATGTATGCATTACCACGAACGAAACCTACTGTGACATGAATGTCGTTAAATTTGACAATCGTGTCTGTC

TCAGAATCAAATATAGATCTTATCTCGTATCCACAAATAGTAAATGAGAGATCTACTGGTAAAATGGTAT

GTCTCAATATTAATGCATTTATTTCTAGTAACGTAAACGCAGATATTATATGGAGCGGACATCGACGCCT

TAGAAATAAGAGACTTAAACAACGGACACCTGGAATTATTACCATAGAAGATGTTAGAAAAAATGATGCT

GGTTATTATACATGTGTTTTAGAATATATATATATATATATATATATATATATATATATATATATATATA

GGCAAAACATATAACGTAACCAGAATTATAAAATTAGAGGTACGGGATAGAATAATACCTCCTACTATGA

AATTACCAGAAGGAGTAGTAACTTCAATAGGTAGTAATTTGACTATTGCATGCAGAGTATCGTTGAGACT

TCCCACAACGGACACCGACGTCTTTTGGATAAGTAATGGTATGTATTACGAAGAAGAAGACGAGGACGGA

GACGGTAGAATAAATGTAGCAAATAAAATCTATATGACCGATAAGAGACGTGTTATTACATCCTGGTTAA

ACATTAATCCTGTCAAGGAAGAAGATGCTACAACGTTTACATGTATGGCGTTTACTATTCCTAGCATCAG

CAAAACAGTTACTGTTAGTATAACGTGAATGTATGTTGTTACATTTCCATATCAATTGAGTTTATAAGAA

TTTTTATACATTATCTTCCAACAAACAATTGACGAACGTATTGCTATGATTAACTCCCACAATACTATGC

ATATTATTAATCATTAACTTGCAGACTATACCTAGTAGTGCTATTTTGACATACTCATGTTCTTGTGTAA

TTGCAGTATCTATATTATTAAAGTACGTAAATCTAGCTATAGTTTTATTATTTAATTTTAGATAATATAC

TGTCTCCGTATTTTTAAAAAATTACCACATCCTTTATTAAATCATGAATGGGAATTTCTGTGTCATCGTT

AGTATATTGTGAACAACAAGAGCAGATATCTATAGGAAAGGGTGGAATGCGATACATTGATCTATGTAGT

TTTAAAACATACGCGAACTTTGAAGAATTTATATAAATCATCTCACGAGATATTGCTCTCTGTCATATTC

ATACACCTGCATAAACTTTCTAGACATCTTACAATGTGTTATTTTATGATCATATTTACATATTTACATA

TTTACTGGTATATCAAAGATGTTAGATTAGTTAATGGGAATCGTCTATAATAATGAATATTAAACAATTA

TAGGAGGAGTTTATACCTACAAAAACATCATAAAAATGAGTCATCGTCCGATTTATGTTTTAAATATACT

AACATTACTACCTTCAGAAATTATATACGAAATATTATACATGCTGACAATTAACGATCTTTATAATATA

TAGTATCCACCTACCAAAGTATAATTGTATTTTTTCTCATGTGATGTGTGTAAAAAACTGATATTATATA

ATTATCTTAGTACCTATGATGAAGATGAAGATGATGGTCCGTATATATTTTGTATCATTATCGTTATTGC

TATTCCATAGTTACGCCATAGACATCGAAAATGAAATCACCGAATTCTTCAATAAAATGAGAGATACTCT

ACCAGCTAAAGACTCTAAATGGTTGAATCCAGTATGTATGTTTGGAGGCACAATGAATGATATGGCCGCT

CTAGGAGAGCCATTCAGTGCAAAGTGTCCTCCTATTGAAGACAGTCTTTTATCGCATAGATATAAAGACT

ATGTGGTTAAATGGGAAAGGCTAGAAAAGAATAGACGGCGACAGGTTTCTAATAAACGTGTTAAACATGG

TGATTTATGGATAGCCAACTATACATCTAAATTCAGTAACCGTAGGTATTTATGTACCGTAACCACAAAG

AATGGTGACTGTGTTCAGGGTGTAGTTAGATCTCATGTGTGGAAACCTTCTTCATGCATTCCAAAAACAT

ATGAACTAGGTACTTATGATAAGTATGGCATAGACTTATACTGTGGAATTCTTTATGCGAAACATTATAA

TAATATAACTTGGTATAAAGATAATAAGGAAATTAATATCGACGATTTTAAGTATTCACAAGCGGGAAAG

GAATTAATTATTCATAATCCAGAGTTAGAAGATAGTGGAAGATACGACTGTTACGTTCATTACGACGACG

TTAGAATCAAGAATGATATCGTAGTATCAAGATGTAAAATACTTACGGTTATACCGTCACAAGACCACAG

GTTTAAACTAATACTAGATCCGAAAATCAACGTAACGATAGGAGAACCTGCCAATATAACATGCAGTGCT

GTGTCAACGTCATTATTTGTCGACGATGTACTGATTGAATGGAAAAATCCATCCGGATGGATTATAGGAT

TAGATTTTGGTGTATACTCTATTTTAACTAGTAGAGGCGGTATCACCGAGGCGACTTTGTATTTTGAAAA

TGTTACTGAAGAATATATAGGCAATACATATACATGTCGTGGACACAACTATTATTTTGATAAAACTCTT

ACAACTACAGTAGTATTGGAGTAAATACACAATGCATTTTTATATACATTACTGAATTATTATTATTAAT

TATATCGTATGTGTGCTATAGAATGGATGAAGATACGCGACTATCTAGGTATTTGTATCTCACCGATAGA

GAACATATAAATGTAGACTCTATTAAACAGTTGTGTAAAATATCAGATCCTAATGCATGTTATAGATGTG

GATGTACGGCTTTACATGAGTACTTTTATAATTATAGATCAGTCAACGGAAAATACAAGTATAGATACAA

CGGTTACTATCAATATTATTCATCTAGCGATTATGAAAATTATAATGAATATTATTATGATGATTATGAT

AGAACTGGTATGAACAGTGAGAGTGATAATATATCAATCAAAACAGAATACGAGAATGAATATGAATTCT

ATGATGAAACACAAGATCAAAGTACACAACTAGTAGATTACGACATTAAACTCAAAACCAATGAGGATGA

TTTTGTTGATGAATTCTATGGTTATGATAGATCAGTGGGTGTCCATGATTATATAGATGTATCAATTAAT

AAAGTAGTATATGGAAGAGAGTCTCACGTAAGATGGCGGGATATATGGCAAGAACATAATGATGGCGTAT

ACAGTATAGGAAAGGAGTGCATAGATAATATATACGAAGACAGACATACAGTAGACGAATTCTACAAGAT

AGACAGCGTATCAGATGTAGATGACGCAGAACATATATCTCAGATAACTAATGATGTATCTACACAAACA

TGGGAAAAGAAATCAGAGTTAGATAGATACATGGAAATGTATCCTCGTCATAGATATGGTAAGCATTCTG

TCTTTAAGGGATTTTCTGACAAAGTTAGAAAAAATGATTTAGACATGAACGTGGTAAAAGAATTACTTTC

TAACGGTGCATCTCTAACAATCAAGGATAGCAGTAATAAGGATCCAATTGCTGTTTATTTTAGAAGAACA

ATAATGAATTTAGAAATGATTGATATCATTAACAAACATACAACTATCTATGAACGCAGGTATATAGTAC

ACTCCTATCTAAAAAATTATAGAAATTTCGATTATCCATTTTTCAGAAAGTTAGTTTTGACTAATAAACA

TTGTCTCAACAATTATTGTAATATAAGCGACGGCAAATATGGAACACCACTACATATATTAGCATCTAAT

AAAAAAATAATAACTCCTAATTACATGAAGTTATTAGTGTATAACGGAAATGATATAAACGCACGAGGTG

AAGATACACAAATGCGAACTCCATTACACAAATATTTGTGTAAATTTGTATATCATAATATTGAATATGG

TATCCGATACTATAATGAAAAGATTATAGACGCATTTATAGAGTTAGGAGCCGATCTAACTATTCCAAAT

GACGATGGAATGATACCAGTAGTTTACTGTATACACTCAAATGCCGAATATGGTTATAACAATATTACTA

ACATAAAGATAATACGTAAACTACTTAATCTTAGTAGACGTGCGTCACATAATCTATTTAGAGATCGAGT

CATGCACGATTATATAAGTAATACATATATTGATCTTGAGTGTTTAGATATCATTAGATCACTGGATGGG

TACGATATTAATTGTTACTTTGAAGGACGTACACCACTTCATTGCGCTATACAATATAACTTCACTCAGA

TTGCTGAGTACTTATTAGATCGAGGAGCTGATATATCATTAAAGACAGACGATGGTAAAACTGTATTTGA

TTTATCGTTATGTAGTTACATTCCTCTTAAATGGACTAGCTTTTTGATTAGTCGTCTACCGCCTAAAAGT

GTCATATGCTCACTGACTAACCATATAATAGATTATGTTCTTACGAACAATAGACGTATTATTTGGCAGA

GTCAAATGATTAATAAGTACGTACTGTTACTGGACCCATCCTTTTATTATAGATTCAGAAATGTTATCGA

AAACAAATTAGACCAATACAATAATCGTTATAATATGTTCGAACACGATAGGGACGTTAATGAAAAGTAT

GGCAAAGTCTTACATGACCTCGATACATATATCAAGGATGTACAAGTATTAAAATCTACTTCCATCACTA

ATAATATAACACTATACGACACTATTATAAATAATAAGTCAGAGTTTCCTATACGTCGTGTAAACGACAA

ACAATTAATTAATCTCATAAAATCCAATACATATCATAATCTTATCGAAAAAGTTATTAAAAATACATTA

GAGAAATATACTTTAACTAATATAGTCCTCGAGTATATGATCTCATCTCGATCTCAATCATCTTATTTGA

GTCGTATTCCTAATGAGATATTACTCGAAATATTATATAAACTCGACATGTACGATTTACGTAATCTATA

TACAAGATATATGAGAGAGAATGATATCACAGAGTATCATATAGAGAATACGAGGTCTGTTTCTACACAG

ACATGAATAATGAATACACATACAACGTTTTTTTTTAATCTTAGATATAACACTAATTACATCAAGATTA

TATATTGAAATCGTAATTTGAGTTGTCTGATCATCATGGATATCGAAAATGATATACGTAACATTAGCAA

TCTTTTAGATGATTGATATATTATTATGCGATGTAATCATAACTATCGGAGATGTAGAAATTAAAGCGCA

TAAAACTATTTTGGTTGCCGGATCTACGTATTTTAAAACAATGTTCACAACACCTATGATAGTGAGAGAT

CTAGCAACTAGAGTAAATATACAGATGTTCGATAAAGATGCCGTCAAAAATATTGTACAGTACTTATACA

ATAGGTATATAAGTTCTATGAATGTGATAGACGTATTAAAATGCACCGACTAAGAACGTAGAACGAACTA

TAGAATGTTATACAATGGGTGATGATAAGTAGAAGATGTTACCCGATATACCCATAGCATTATCTAGTTA

TGGCATGTGTGTATTAGATCAATACATATACATTATAGGCGGTCGTACACAACACTGATTATACATCGGT

ACATACAGTAAATAGCATAGATATGGAGGAGGATACAAATATTTCAAATAAAGTTATGAGATACGCGCTG

TCAATAATATATGGAAGACATTACCTAACTTCTGAACTGGAACTATAAATCCAGGCTCTCGCATAAAGAT

GAATATATATGTTGTATGCGACATCAAAGATGAAAAAATGTTAAGACTTATATATTTAGATATAACACGA

ATATGTATGACGGATGGGAATTGGTAACGATGACAGAAAGCAGATTGTCAGCTCTGCATACTATTCTTCA

TGACAATACCATAATGATGTTACATTGTTATGAAACGTATATGTTACAAGATACATTTAATGTGCTTACG

GAACATATATTTAGAAACATCTACTAACGATTTTTTATGCTTGTATTATTAATGGTATGTAATATGATTT

AATTGATTGTGTACACGATACCAATTTGTCGAGTATGAATACGGAGTACAAACATAAACTGAAGTTTAAC

ATTATATACATTATATACATTATATACATTATATACATTATATACATTATATACATTATATGCATTATAT

ACATTATATACATTATATACATTATATACATTATATACATTATATACATTATATACATTATATACATTAT

ATACATTATATACGTTATATACATTATATACATTATATACATTATATACATTATATACATTATATACATT

ATATATCGTTATTGTTTGGTCTATGCCATGGATATCTTTAAAGAACTAATCTTAAAACATACGGATGAAA

ATGTTTTGATTTCTCCAGTTTCCATTTTATCTACTTTATCTATTCTGAATCATGGAGCAGCTGGTTCTAC

AGCTGAACAACTATCAAAATATATAGAGAATATGAATGAGAATATACCCGATGATAAGAAGGATGACAAT

AATGACATGGACGTAGATATTCCGTATTGCGCGACACTAGCTACCGCAAATAAAATATACGGTAGTGATA

GTATCGAGTTCCATGCCTCATTCCTACAAAAAATAAAAGACGATTTTCAAACTGTAAACTTTAATAATGC

GAACCAAACAAAGGAACTAATCAACGAATGGGTTAAGACAATGACAAATGGTAAAATTAATTCCTTATTG

ACTAGTCCGCTATCCATTAATACTCGTATGATAGTTATTAGCGCCGTCCATTTTAAAGCAATGTGGAAAT

ATCCATTTTCTAAACATCTTACATATACAGACAAGTTTTATATTTCTAAGAATATAGTTACCAGTGTTGA

TATGATGGTGGGTACCGAGAATGACTTGCAATATGTACATATTAATGAATTATTCGGAGGATTCTCTATT

ATCGATATTCCATACGAGGGAAACTCTAGTATGGTGATTATACTGCCGGACGACATAGAAGGTATATATA

ACATAGAAAAAAATATAACAGATGAAAAATTTAAAAAATGGTGTGGTATGTTATCTACTAAAAGTATAGA

CTTGTATATGCCAAAGTTTAAAGTGGAAATGACGGAACCGTATAATCTGGTACCGATTCTAGAAAATTTA

GGACTTACTAATATATTTGGATATTATGCAGATTTTAGTAAGATGTGTAATGAAACTATCACTGTAGAAA

AATTTCTACATACGACGTTTATAGATGTTAATGAGGAGTATACAGAAGTATCGGCCGTTACAGGAGTATT

CATGACTAACTTTTCGATGGTATATCGTATGAAGGTCTACATAAACCATCCATTCATATACATGATTAAA

GATAACACCGGACATACACTTTTTATAGGGAAATACTGCTATCCGCAATAAATATAAACAATAGACTTTT

ATCACGTTATCTCATGTATAAAATATTACAAATAGTATAGCATAAACTAAAGTCGATACATACATTAAAA

CTTAAATAATAATGTAATTTACAATTAATAGTATAAACTAAAAAAATTAAAAACAATATCATTATTATAA

GTAATATCAAAATGACGATATACGGATTAATAGCGTATCTTATATTCGTGACTTCATCCATCGCTAGTCC

ACTTTACATTCCCGTTATTCCGCCCATTTCGGAAGATAAATCGTTCAATAGTGTAGAGGTATTAGTTTCT

TTGTTTCCCGATGACCAAAAAGACTATACAGTAACTTCTCAGTTCAATAACTACACTATCGGTACCAAAG

ACTGGACTATCAACGTACTATCCACACCTGATGGTCTGGACATACCATTGACTAATATAACTTATTGGTC

ACGGTTTACTATAGGTCGTGCATTGTTCAAATCAGAGTCTGAGGATATTTTCCAAAAGAAAATGAGTATT

CTAGGTGTTTCTATAGAATGTAAGAAGCCGTCGACATTACTTACTTTTTTAACCGTGCGTAAAATGACTC

GAGTATTTAATAGATTTCCAGATATGGCTTATTATCGAGGAGACTGTCTAGAAGCCGTTTATGTAACAAT

GACTTATAAAAATACTAAAACTGGAGAGACTGATTACACGTACCTCTCTAATGGGGGGTTGCCTGCATAC

TATCGTAATAGGGTCGATGGTTGATTATTGATTAGTATATTCCTTATTCTTTTTATTCACACAAAAAGAA

CATTTTTATAAACATGAAACCACTGTCTAAATGTAATTATGATCTTGATTTATAGATGATGATCAGCCTT

CAGAGGATTTTGACCAGTATGTTTAATATGAAAAAAACATAACTATTAAGCGCTATTGCGCTATTGTGCT

TAATTATTTTGCTCTATAAACTGAATATATAGCCACAATTATTGACGGGCTTGTTTGTGACCGGCAATCA

TGAATTTTCAGAAATTATCTCTGGCTATATATCTTACGGTGACATGTTCGTGGTGTTATGAAACATGTAT

GAGAAAAACTGCGTTGTATCATGACATTCAATTGGAGCATGTAGAAGACAATAAAGATAGTGTAGCATCG

CTACCGTACAAGTATCTACAAGTAGTCAAACAAAGAGAACGTAGTAGATTGTTGGCTACATTTAATTGGA

CGGATATAGCTGAGGGTGTTAGAAATGAGTTCATTAAAATATGTGATATCAACGGAACATATCTATATAA

TTATACTATTGCTGTTAGTATAATTATTGATTCCACGGAAGAACTACCAACAGTTACTCCAATTACAACA

TATGAACCTTCTATATATAATTATACTATCGATTATAGCACTGTTATTACTACTGAAGAACTACAAGTGA

CTCCAACATATGCACCTGTAACAACTCCTCTTCCAACATCAGCAGTTCCTTATGATCAACGATCGAATAA

CAATGTAAGTACTATATCTATTCAGGTACTGAGTAAAATATTGGGAGTCAATGAAACAGAATTAACTAAT

TATCTTATTATGCATAAAAATGACACTGTTGACAATAACACCATGGTTGATGATGAGACATCTGACAATA

ACACATTGCATGGTAATATAGGATTTTTGGAAATAAATAATTGTTATAATGTTTCTGTGTCAGATGCTAG

TTTTAGAATAACATTAGTAAACGATACTTCTGAAGAAATTTTGCTAATGCTAACAGGAACTAGTTCATCC

GACACCTTCATATCTTCCACCAATATCACTGAATGTTTGAAAACATTAATCAATAATGTGTCGATTAATG

ATGTACTTATAACACAAAATATGAATGTAACATCTAATTGTGATAAATGCTCAATGAATTTGATGGCATC

CGTTATTCCTGCAGTTAATGAATTTAACAATACGTTGATGAAAATTGGTGTAAAAGATGATGAAAACAAT

ACGGTATATAAATATTATAATTGTAAACTAACTACAAATTCTACATGTGATGAGTTAATCAATTTAGATG

AAGTAATTAACAACATAACTCTGACAAATATTATACGCAATAGTGTTTCGACAACTAACAGCAGAAAAAG

ACGAGATCTGAATGGTGAGTTTGAATTTTCCACTTCCAAGGAATTAGATTGTCTTTACGAATCATATGGT

GTAAACGATGATATAAGTCATTGTTTTGCATCACCTAGACGTAGACGATCTGATGACAAAAAGGAGTACA

TGGACATGAAATTATTCGACCACGCGAAAAAAGATTTAGGAATAGACAGTGTTATTCCTAGAGGTACAAC

CCATTTCCAAGTAGGTGCATCTGGTGCAAGTGGTGGTGTTGTAGGAGATAGTTTCCCATTTCAAAATGTT

AAATCGCGTGCCAGTCTATTGGCGGAAAAAATAATGCCTAGAGTACCTATTACTGCTACCGAAGCTGATC

TATATGCAACTGTAAATAGACAACCCAAGTTACCAGCAGGTGTTAAAAGTACTCCGTTTACAGAGGCGCT

TGTGTCTACGATAAACCAAAAGCTTTCTAATGTTAGAGAGGTAACTTATGCTTCGCTCAATCTGCCAGGA

TCAAGTGGCTATGTTCATAGACCATCTGATTCTGTTATTTACAGCAGTATAAGACGGTCACGTTTACCTA

GTGATAGCGATAGTGATTATGAGGATATACAAACTGTTGTTAAGGAATATAATGAAAGATATGGTAGATC

AGTCAGTAGAACACAGTCATCAAGTAGTGAAAGCGATTTTGAAGATATAGATACTGTTGTTAGGGAATAT

AGACAAAAATATGGCAATGCAATGGCAAAAGGACGTAGTAGTTCCCCTAAACCTGATCCATTATATAGTA

CTGTTAAGAAAACAACTAAAAGTCTATCTACTGGTGTAGACATAGTTACAAAACAATCAGACTATTCTCT

ATTACCTGACGTTAATACTGGCAGTTCTATTGTGTCACCTCTCACCAGAAAAGGAGCTACTAGACGACGA

CCTAGACGCCCTACAAATGATGGTCTACAGAGTCCAAATCCTCCTCTCCGTAATCCACTTCCTCAACATG

ATGATTATTCTCCTCCACAAGTACACAGACCTCCACCACTTCCTCCTAAACCAGTCCAAAATCCGCCACA

ACTTCCCCCTAGACCAGTAGGTCAATTACTACCTCCTCCTATAGATCAACCAGATAAAGGATTTAGTAAG

TTTGTATCACCTAGACGGTGTAGAAGAGCAAGCTCTGGAGTCATATGTGGTATGATACAATCAAAACCAA

ACGATGATACCTATTCACTTCTTCAACGGCCAAAAATTGAACCAGAATATGTGGAGGTTGGTAATGGTAT

ACCCAAGAACAATGTTCCTGTAATAGGTAATAAACATAGTAAAAAATATACATCGACGATGTCAAAAATA

TCAACAAAATTTGATAAATCTACGGCATTTGGAGCAGCAATGTTACTAACTGGTCAGCAGGCCATTAGCC

AACAGACTAGATCAACTACGTTGAGTAGAAAAGATCAGATGAGTAAGGAAGAAAAGATATTCGAAGCAGT

TACAATGAGTCTATCAACTATAGGTTCAACGTTGACGTCTGCAGGTATGACGGGTGGTCCAAAACTAATG

ATTGCAGGAATGGCTATAACGGCTATAACTGGTATAATAGATACGATAAAAGATATATATTACATGTTTT

CAGGACAGGAGAGGCCAGTAGATCCTGTTATTAAATTATTTAATAAGTACGCTGGCTTAATGTCCGATAA

TAATAAAATGGGTGTAAGAAAATGTTTGACACCCGGTGACGACACACTTATTTATATCGCATACAGAAAC

GATACCAGTTTTAAACAGAATACGGATGCGATGGCTTTGTATTTCTTAGATGTTATCGATTCAGAGATCC

TATATCTAAACACATCAAATTTAGTTCTAGAGTATCAACTAAAGGTGGCTTGCCCCATAGGAACATTAAG

ATCTGTAGATGTGGACATAACTGCGTATACAATATTATATGATACAGCGGATAATATTAAGAAATACAAG

TTTATCAGAATGGCAACGCTACTATCCAAACATCCAGTTATTAGATTGACATGTGGTTTAGCAGCAACAT

TGGTGATTAAACCGTACGAGGTACCCATCAGTGATATGCAACTACTAAAAATGGCGACGCCTGGTGAACC

AGAATCCACTAAATCTATACCATCCGATGTCTGTGATAGGTATCCTCTAAAGAAATTCTATCTTTTAGCT

GGTGGTTGTCCCTATGATACATCTCAAACTTTTATTGTACATACTACTTGCAGTATTCTACTAAGAACAG

CTACACGGGATCAGTTTAGAAACAGATGGGTGTTACAAAATCCATTTAGACAAGAAGGGACATATAAGCA

ACTGTTTACCTTTAGCAAATACGATTTTAACGACACCATAATCGATCCTAATGGTGTGGTGGGTCATGCT

AGCTTTTGTACCAATAGAAGCAGCAACCAATGTTTCTGGTCCGAACCTATGATATTGGAAGATGTATCAT

CGTGTAGTTCTAGAACTAGAAAAATATACGTAAAACTGGGAATATTTAATGCTGAAGGTTTTAATAGTTT

TGTACTAAATTGTCCAACTGGGTCTACACCTACATACATCAAACATAAAAATGCGGACAGTAACAATGTT

ATCATAGAGCTACCTGTAGGTGATTACGGCACAGCCAAATTGTATTCAGCAACAAAACCATCGAGGATAG

CTGTGTTCTGCACACATAACTATGATAAACGATTCAAATCAGATATTATAGTTCTAATGTTTAATAAAAA

CAGCGGTATTCCATTTTGGAGCATGTACACAGGAAGTGTAACTAGTAAAAATAGAATGTTTGCCACATTG

GCTAGAGGAATGCCGTTTAGATCAACGTATTGCGATAACAGACGACGATCAGGTTGTTATTATGCAGGAA

TACCATTTCATGAAGATAGTGTAGAAACAGATATACATTATGGACCAGAAATAATGTTAAAGGAAACATA

TGACATAAACAGTATTGACCCACGAGTTATAACAAAGTCAAAGACCCATTTTCCTGCTCCATTGAGTGTA

AAATTCATGGTTGACAATTTAGGAAATGGATATGACAACCCTAATTCATTTTGGGAAGATGCTAAAACTA

AGAAACGGACATATAGTGCAATGACGATAAAAGTCCTACCATGTACAGTGAGAAATAAAAATATAGACTT

TGGATATAACTATGGAGATATTATTTCTAATATGGTTTATCTACAATCTACTAGTCAGGATTATGGAGAT

GGTACCAAATATACATTTAAATCCGTAACTAGATCAGATCATGAGTGTGAATCTAGCTTAGATCTAACGT

CTAAGGAAGTAACTGTGACATGTCCTGCGTTTAGTATACCAAGAAATATATCAACATATGAAGGTCTATG

CTTTAGTGTTACTACATCTAAAGATCATTGTGCTACAGGTATTGGTTGGTTAAAATCTAGTGGTTATGGG

AAGGAAGATGCTGATAAACCACGTGCTTGTTTTCATCATTGGAATTATTACACACTGTCGTTGGATTATT

ACTGTTCATACGAAGATATTTGGAGAAGCACCTGGCCTGACTATGATCCATGTAAGTCATATATCCATAT

AGAGTATAGAGATACATGGATAGAATCTAATGTGTTACAGCAACCTCCTTACACATTCGAATTCATTCAT

GACAATTCTAACGAATATGTGGATAAAGAAATTAGTAACAAATTAAATGATCTGTACAATGAATACAAGA

AGATTATGGAATATAGCGACGGATCATTGCCGGCGTCTATAAACAGATTAGCAAAGGCATTGACTTCAGA

GGGTAGAGAAATAGCAAGTGTTAATATAGATGGTAATCTGTTAGATATCGCATATCAAGCAGATAAGGAA

AAGATGGCCGACATACAGACAAGAATAAATGATATTATTAGAGATTTGTTTATACACACTCTATCAGACA

AAGATATAAAAGACATTATAGAATCCGAAGAAGGTAAGAGATGTTGTATAATAGATGTTAAGAACAATCT

TGTTAAAAAGTACTATTCTATTGATAATTATCTATGTGATACTTTAGATGATTATATATACACCTCTGTA

GAATATAACAAATCCTATGTGTTAGTAAACGATACTTATATGAGCTATGACTATCTTGAATCATCAGGTG

TAGTTGTTCTATCATGTTATGAAATGACTATAATCTCCTTGGATACAAAAGACGCCAAAGATGCTATAGA

AGATGTGATAGTAGCAAGTGCGGTAGCCGAAGCATTGAATGACATGTTTAAGGAATTTGATAAAAACGTA

AGTGCTATTATAATAAAAGAAGAAGATAATTATCTAAACAGTTCGCCCGATATCTACCATATAATATATA

TCATAGGTGGCACTATTCTGCTACTGTTAGTCATTATTTTAATATTGGCAATTTATATAGCGCGCAATAA

ATACAGAACCAGGAAATATGAAATAATGAAATATGACAATATGAGCATTAAATCTGAGCATCATGATAGT

CTTGAAACAGTGTCTATGGAAATTATTGATAATCGGTACTAATAAAATAGTTTAACTCTTTTAGAACCAG

TTTGGTACTGTAATTTCAGTTCATTACTCGTTGAGAATATTGATGATTTTTTTTAAATGAGTATCGGTAG

TTACATATTACCATATCATCCATTATATAATCGATGATGCATGTATTAGAATACTTTCCGAATAAGTCTT

CTAAATATTGTATTAATTATGAAAAACTATGCTATGTGAGTATGATTCAAAGATGTTTAATGATACGATA

CTAGATTTTATCTCTAGCGAGATTGTTTAGAATCATTTATCATAACTATGTTTAATAAATTCATCAACGA

ATATCGATAAAGACCTCTTGTAATTCGAGTATAGGAAGTAGTATTACCATATCAACTTCCGAGTTAACAA

TTACTCTAAAACATGAGGATTGTACTCCTGTCTTTATTGGAGATCACTATTCAGTCGTTGATAAACTAGT

AACCTCAGGTTTCTTTACAAACGATAAAGTACAACATCAAGACCTCACAACACAGTGCAAGATTAATCTA

GAAATCAAATGTAATTCTGGAGGAGAATCTAGACAACTAACACCCACGGCGAAGTATACTTTATGCCTCA

TTCAGAAACGGTAACTGTAGTAGGAGACTGTCTCTCTAATCTCGATGTATATATAATATATACCAATACG

GACGCGATATATTCCGACATGAATGGCGTCGCTTATCATATGTTATATCCTAAATGTTGATCATATTCCA

CAAATGATTGTGAACGAGATTAAATCATCTAACAAATAATTAGTTTTTTTATGACATTAACATATAATAA

ATAAATTAATCATTATTGACTTAACGATGACGAAAGTTATCATTATCTTAGGATTCTTGATTATTAATAC

AAATTCGTTGTGTCTATGAAATGTGAACAAGGTGTCTCATATTATAATGCACAAGAATTAAAGTGTTGTA

AACTATCTAGCCAGGAACATATTCAGATTATCGATGTGATAAATACAGCGATACCATCTGTGGACATTGT

CCAAGTGATACATTCACGTCAATATATAATCGTTCTCCTCGGTGTCATAGTTGTAGAGGTCACACCTTGT

ACACCTACCACAAATAGAATATGTCATTGTGACTCGAATAGTTATCGTCTCCTTAAAGCTTCTGATGGTA

ACTGTGTTACATGTGCTCCTAAAACAAAATATGGTCGTGTGTACGGAAAGAAAGGAGAAAATGATATGGA

ATACCATTTGTAAGAAATGTCGGAAGGGTACTTATTCAGATATTGTATCTGACTCTGATCAATGTAAACC

TATGACAAGATAAGACTTACTCGCATCTACTGGATAGACATAAATATCCTCCTCGTAATAATGAAATATA

ATATACACTAATTATTAATATCAATCGAGTATTAACATATAAGTTATTTTTAAACCCCTTTTGGGTTCCG

TCCTAAACGGCGTTTCGGTCTGTGTCGCCACCATGGTCACACCGAGCCTCTGCGTGCTCCTCCATCGAGG

ACGACTTCAACTATGACAGCTCGGTGGCGTCTGCCAGCGTGTACATACGAATGGCATTTCTAAGAAAAGT

CTACGGTATCCTTTCTACAATTTCCTTTAACAACGGCAACAGCTGCAGTATTTTTATACTTTGAATGCAT

CGGACATTTATACAAGGGAGTCCTGTTCTAATATTGGCATCAATGTTCGGATCTATAGGCTTGATTTTCG

CATTGACTTTACACAGACATGAACATCCCCTGAATCTGTACATACTTTGTGGATTTACACTGTTAGAATC

TCTAACGCTGGCCTCTGTTGTTACTTTCTATGATGCACGTATCGTTATGCAAGCTTTCATGTTGACTACT

GCAGTGTTTCTTGCTCTGACTACATATACTCTACAATCAAAGAGAGATTTCAGTAAACTTGTAACAGGAT

TGTTTGCTGCTTTCTGGATTTTAATTTTGTCAGGAGTCTTGAGGATAAAGTTTAAAATAGAATTAATAAA

GAACATATAGGTCATTTTTTAAACATGGATAGAAACCAAGGTTGTTAGTTAATAATATACAAGATATTTT

TTCTCACTCTGATCCATGTAAACCAAGGACGAGAGACACTCTCATTCCTCATTCACGACACCATTAAAAA

TGGAAATTAAAGCCCTCTATTAAGCACAGACGGCTACAGGTCTACCATCAGGTTACCTTCGTCTACCTTC

ACAATGGCCTCTCCTTGTGCCCAGTTCAGTCCCTGTCATTGCCACGCTACTAAGGACTCCCTGAATACCG

TGACTGATGTCAGACATTGTCTGACTGAATACATCCTGTGGGTTTCTCATAGATGGACCCATAGAGAAAG

CGCAGGGCCTCTCTACAGGCTTCTCATCTCTTTCAGAATTGATGCAATGGAGCTATTTGGTAGCGAGTTG

AAGGAGTTCTCGGATTCACTTCCGTGGGACAATATCGACAATTGCGTGGAGATCATTAAATGTTTCATCA

GAAATGACTCCATGAAAACCGCCAAAGAACTTTGTGCAATAATTGGACTTTGTACTCAATCAGCTATTGT

CACTGGAAGAGTCTTCAATGATAAGTATATCGACATACTACTTATGCTGCGAAAGATTCTGAACGAGAAC

GACTATCTCACCCTCTTGGATCATATCCTCACTGCTAAATACTAAATCTCCTTCATGCTCTCTCACTAAT

ACTCTTACTCACTACACTTTTTATCATCTTATGATGAATGATTGCCTTCATCATTTTTCGTGGAATATAA

TATAGGAATAATTAGCACCAGAATAGCTATGGATATCTCGTTAAGAATATTCTCGATAAGAGACATAATG

TAGACATAGTTATTATATCCTTCTTAGATAAGTGTTACGCTACTGGAAAGTTTCCATCGTTATTATTACA

TGAAGATGATATAATTAAACCAACATTGAGATTGGCTCTTATGTTAGCTGGATTGAATTACTGTAATAAA

TGCATCGAGTATAGAGGGATATAGCAATTCTCGATAATAGTCATGCAATATTTGAATGAGACTGATAATT

TAGGTAATACAGTACTACACACATATCTTTCTAGATTATATATCGTTAAAAATCTGTAAGAGGTATATTT

CTCATAAGTATCCACTGTGTAATATTATTAATGGATATATAGATAACGCAATAGGGACTAATAGTATTGT

AAAAGATATAATCGACTATTTACGTACATATCCAGATATCTATATTCCTACTAGTTTGCTGCGTAGTTGC

ATCATTGATATGCATGATTTATCAGGATTCAGAGATGAATTACTAAGTAAACTACAATCCCACAATAAGT

AAGAATCAAATATCAAAAACTCACTTTTGATTTTTCTAGTCTTAAGTAATACATATATTTATTAATAGAC

CTATGAAATAAAAAAAGGTAACAATGGATTCGCGTATAGCTATTTACGTATTAGTATCGGCATCTCTTTT

GTATCTTGTTAATTGTCACAAACTAGTACATTACTTCAATCTGAAAATAAATGGAAGTGATATAACTAAT

ACAGCAGATATATTGCTGGACAATTATCCAATTATGACCTTTGATGGAAAGGATATTTATCCATCTATCT

CGTTCATGGTCGGTAATAAACTTTTCCTAGATCTTTATAAAAATATCTTTGAAGAATTTTTCAGACTATT

TCGAGTATCTGTAAGTAGTCAATACGAGGAATTAGAATATTATTATTCATGTGATTATACTAACAACCGT

CCTACAATTAAACAACATTACTTTTATAACGGCGAAGAATATACTGAAATTGATAGATCGAAAAAAGCCA

CTAATAAAAACAGTTGGTTAATTACTTCAGGCTTTAGACTACAAAAATGGTTCGATAGCGAAGATTGTAT

AATTTATCTCAGATCTTTAGTTAGAAGAATGGAAGACAGTAACAAAAACAGTAAAAAACTTAGTACTTAG

ATATCGAAAAAAATATATTTTTGTAGACTCTTGAGAATAGAAGGAAAACATGTACATAATTATAAAAAAT

GAAAATCAATGGCGAATAAGACAGTGCGATTCGCACCATGGAGTCGGTAGATTTCATGGCTGTCGATGAG

CAGTTTCACGACGACCTCGATCTTTGGTCATTATCTTTGGTAGATGATTATAAAAAACATGGATTAGGTG

TTGACTGTTATGTTCTAGAACCAGTTGTTGACAGGAAAATATTTGATAGATTTCTCCTTGAACCAATTTG

TGATCCTGTAGATGTTCTGTATGATTATTTTAGGATTCATAGAGATAATATTGATCAGTATATAGTAGAT

AGACTGTTTGCATATATTACATATAAAGATATTATATCTGCATTAGTGTCAAAGAATTATATGGAAGATA

TTTTCTCTATAATTATTAAGAATTGTAATTCTGTGCAAGATCTCTTACTTTACTATCTATCTAATGCATA

TGTAGAAATAGACATTGTTGATCTTATGGTAGATCATGGGGCTGTAATATATAAAATAGAATGCTTGAAT

GCCTATTTTAGGGGAATATGTAAAAAGGAAAGTAGTGTTGTTGAGTTTATTTTGAATTGTGGTATCCCAG

ATGAAAATGATGTTAAATTAGATCTATATAAAATAATTCAGTATACTAGGGGATTCCTTGTAGATGAACC

CACAGTATTAGAAATTTATAAGCTTTGTATCCCATATATTGAAGATATCAATCAACTAGATGCTGGTGGA

AGGACCTTGCTTTATCGCGCTATCTATGCAGGTTATATAGATTTAGTATCATGGCTATTAGAAAATGGAG

CAAATGTCAACGCAGTAATGAGTAATGGATATACATGTCTTGACGTGGCCGTGGATAGGGGATCTGTCAT

CGCCCGTAGGGAAGCACATCTTAAAATATTAGAAATATTGCTTAGAGAACCATTGTCTATTGACTGTATA

AAATTAGCTATACTTAATAATACAATTGAAAACCATGATGTGATAAAGCTCTGTATCAAGTATTTTATGA

TGGTAGATTATTCACTTTGTAATGTGTATGCATCATCACTCTTTGATTATATAATTGATTGTAAACAAGA

ATTGGAGTACATTAGGCAGATGAAAATTCATAATACAACCATGTATGAGTTAATCTATAATAGAGACAAA

AACAAGCATGCTTCCCATATTCTACATAGGTATTCTAAACATCCAGTTTTGACACAGTGTATCACTAAAG

GATTCAAGATTTACACAGAAGTAACCGAGCAGGTCACTAAAGCTCTAAACAGACGTGCTCTAATAGATGA

GATAATAAACAATGTATCAACTGATGACAATCTCCTATCAAAACTTCCATTAGAAATTAGGGATCTAATT

GTTTCACAAGCTGTCATATAGAGTTCTATCCACCCACCTTTCTTGAAATGAGTTAATAGTCATAAGTTAG

TTAAGTCATAAGTTAGTTAAGTCATAAGTTAGTTAAGTCATAAGTTAGTTTTATAAGTAAGTTAGTTAAG

TCATAAGTTAGTTTTATAAGTTAGTTTATAGTCTAACACTTCTAATTTTTATACCTTGATCTTTTTCTCT

AATTATGAAAAAGTAAATCATTATGAAGATGGATGAAATGGACGAGATTGTGCGCATCGTTAACGATAGT

ATGTGGTACGTACCTAACGCATTTATGGACGACGGTGATAATGAAGGTCACATTTCTGTCAATAATGTCT

GTCATATGTATCTCGCATTCTTTGATGTGGATATATCATCTCATCTGTTTAAATTAGTTATTAAACACTG

CGATCTGAATAAACGACTAAAATGTGGTAACTCTCCATTACATTGCTATACGATGAATACACGATTTAAT

CCATCTGTATTAAAGATATTGTTACGCCACGGCATGCGTAACTTTGATAGCAAGGATAAAAAAGGACATA

TTCCTCTACACCACTATCTGATTCATTCACTATCAATCGATAACAAGATCTTTGATATACTAACGGACCC

CATTGATGACTTTAGTAAATCATCCGATCTATTGCTGTGTTATCTTAGATATAAATTCAATGGGAGCTTA

AACTATTACGTTCTGTACAAATTATTGACTAAAGGATCTGACCCTAATTGCGTCGATGAGGATGGACTCA

CTTCTCTTCATTACTACTGTAAACACATATCCGCGTTCCACGAAAGCAATTATTACAAGTCAAAGAGTCA

CACTAAGATGCGAGCTGAGAAGCGATTCATCTACGCGATAATAGATCATGGAGCAAACATTAACGCGGTT

ACGAAAATCGGAAATACGCCGTTACACACTTACCTTCAACAGTATACCAAACATAGTCCTCGTGTGGTGT

ATGCTCTTTTATCTCGAGGAGCCGATACGAGGATACGTAATAATCTTGATTGTACACCCATCATGGAATA

CATAAAGAACGATTGTGCAACAGGTCATATTCTCATAATGTTACTCAATTGGCACGAACAAAAATACGGG

AAATTACAAAAGGAAGAAGGACAACATCTACTTTATCTATTCATAAAACATAATCAAGGATATGGAAGTC

GCTCTCTCAATATACTACGGTATCTACTAGATAGATTCGACATTCAGAAAGACGAATACTATAATACAAT

GACTCCTCTTCATACCGCCTTCCAGAATTGCAATAACAATGTTGCCTCATACCTCGTATACATCGGATAC

GACATCAACCTTCCGACTAAAGACGATAAGACAGTATTCGACTTGGTGTTTGAAAACAGAAACATCATAT

ACAAGGCGGATGTCGTTAATGACATCATCCACCACAGACTGAAAGTATCTCTACCTATGATTAAATCGTT

GTTCTACAAGATGTCGGAGTTCTCTCCCTACGACGATCACTACGTAAAGAAGATAATAGCCTACTGCCTA

TTAAGGGACGAGTCATTTGCGGAACTACATACTAAATTCTGTTTAAACGAGGACTATAAAAGTGTATTTA

TGAAAAATATATCATTCGATAAGATAGATTCCATCATCGAAAAATGTAGTCGTGACATAAGTCTCCTCAA

AGAGATTCGAATCTCAGACACCGACTTGTATACGGTATTGAGAACAGAAGACATCCGGTATCACACCTAT

CTCGAAGCCATACATTCAGACAAACGCATTTCATTTCCCATGTACGACGATCTCATAGAACAGTGTCATC

TATCGATGGAGCATAAAAGTAAACTCGTCGACAAAGCACTCAATAAATTAGAGTCTACCATCGATAGTCA

ATCTAGACTATCGTATTTGCCTCCGGAAATTATGCGCAATATCATAACCAAGCTAAGCGACTACCATCTA

AACAGTATGTTGTACGGAAAGAACCATTACAAATATTATCCATGATAGAAAGAAAATATTTAAAAAATAA

TCTATATGATTGGAGAAGTAGGAAACAAACAGTAACAAGACGACGATTACTACATTATTAAATCATGAGG

TCCGTATTATACTCGTATATATTGTTTCTCTCATGTATAATAATAAACGGAAGAGATATAGCACCACATG

CACCATCCAATGGAAAGTGTAAAGACAACGAATACAGAAGCCGTAATCTATGTTGTCTATCGTGTCCTCC

GGGAACTTACGCTTCCAGATTATGTGATAGCAAGACTAATACACAATGTACACCGTGTGGTTCGGATACC

TTTACATCTCACAATAATCATTTACAGGCTTGTCTAAGTTGTAACGGAAGATGTGATAGTAATCAGGTAG

AGACGCGATCGTGTAACACGACTCACAATAGAATCTGTGAATGCTCTCCAGGATATTATTGTCTTCTCAA

AGGAGCATCAGGGTGTAGAACATGTATTTCTAAAACAAAGTGTGGAATAGGATACGGAGTATCCGGATAC

ACGTCTACCGGAGACGTCATCTGTTCTCCGTGTGGTCCCGGAACATATTCTCACACCGTCTCTTCCACAG

ATAAATGCGAACCCGTCGTAACCAGCAATACATTTAACTATATCGATGTGGAAATTAACCTGTATCCAGT

CAACGACACATCGTGTACTCGGACGACCACTACCGGTCTCAGCGAATCCATCTCAACGTCGGAACTAACT

ATTACCATGAATCATAAAGATTGTGATCCAGTCTTTCGTGCAGAATACTTCTCTGTCCTTAATAATGTAG

CAACTTCAGGATTCTTTACAGGAGAAAATAGATATCAGAATACTTCAAAGATATGTACTCTGAATTTCGA

GATTAAATGTAACAACAAAGATTCATCTTCCAAACAGTTAACGAAAACAAAGAATGATACTATCATGCCG

CATTCAGAGACGGTAACTCTAGTGGGCGACTGTCTATCTAGCGTCGACATCTACATACTATATAGTAATA

CCAATACTCAAGACTACGAAAATGATACAATCTCTTATCATATGGGTAATGTTCTCGATGTCAATAGCCA

TATGCCCGCTAGTTGCGATATACATAAACTGATCACTAATTCCCAGAATCCCACCCACTTATAGTAAGTT

TTTTTTACCTATAAATAATAAATACAATAATTAATTTCTCGTAAAAGTAGAAAATATATTCTAATTTATT

ATATGGTAAGAAAGTAGAATCATCTAGAACAGTAATCAATCAATAGCAATCATGAAACAATATATTGTCC

TGGCATGCATGTGCCTAGTGGCAGCTGCTATGCCTACTAGTCTTCAACAATCTTCATCCTCGTGTACTGA

AGAAGAAAACAAACATCATATGGGAATCGATGTTATTATCAAAGTCACAAAGCAAGACCAAACACCGACC

AATGATAAGATTTGTCAATCCGTAACGGAAGTTACAGAGACCGAAGATGATGAGGTATCCGAAGAAGTTG

TAAAAGGAGATCCCACCACTTATTACACTATCGTCGGTGCGGGTCTTAACATGAACTTTGGATTCACCAA

ATGCCCAAAGATTTCATCCATCTCCGAATCCTCTGATGGAAACACTGTGAATACTAGATTGTCCAGCGTG

TCACCGGGACAAGGTAAGGACTCTCCCGCGATCACGCGTGAAGAAGCTCTGGCTATGATCAAAGACTGTG

AGATGTCTATCGACATCAGATGTAGCGAAGAAGAGAAAGACAGTGACATCAAGACCCATCCAGTACTTGG

GTCTAACATCTCACATAAGAAAGTGAGTTACAAAGATATCATCGGTTCAACGATCGTTGATACAAAATGT

GTCAAGAACCTAGAGTTTAGCGTACGTATCGGAGACATGTGTGAGGAATCATCTGAACTTGAAGTCAAGG

ATGGATTCAAGTATGTCGACGGATCGGCATCTGAAGGTGCAACCGATGATACTTCACTCATCGATTCAAC

AAAACTCAAAGCATGTGTCTGAATCGATAACTCTATTCATCTGAAAATGGATGAGTTGGGTTAATCGAAC

GATTCAGACACCGCACCACGAATTAAAAAAGACCGGGCACTATATTCCGGTTTGCAAAACAAAAATATTT

AACTACATTCACAAAAAGTTACCTCTCGTTACTTCTTCTTTCTGTTTCAATATGTGATACGATATGATCA

CTATTCGTATTCTCTTGGTCTCATAAAAAAGTTTTACAAAAAAAAAAAAAAAAAAAAAATATTTTTATTC

TCTTTCTCTCTTCGATGGTCTCACAAAAATATTAAACCTCTTTCTGATGTCTCAACTATTTCGTAAACGA

TAACGTCCAACAATATATTCTCGTAGAGCTTATCAACATCCTTATACCAATCTAGGTTGTCAGACAATTG

CATCATAAAATAATGTTTATAATTTACACGTTAACATCATATAATAAACGTATATAGTTAATATTTTTGG

AATATAAATGATCTGTAAAATCCATGTAGGGGACACTGCTCACGTTTTTTCTCTAGTACATAATTTCACA

CAAGTTTTTATACAGACAAATTAATTCTCGTCCATATATTTTAAAACATTGACTTTTGTACTAAGAAAAA

TATCTTGACTAACCATCTCTTTCTCTCTTCGATGGGTCTCACAAAAATATTAAACCTCTTTCTGATGGAG

TCGTAAAAAGTTTTTATCCTTTCTCTCTTCGATAGGTCTCACAAAAATATTAAACCTCTTTCTGATGGTC

TCTATAAACGATTGATTTTTCTTACCCTCTAGAGTTTCCTACGGTCGTGGGTCACACATTTTTTTCTAGA

CACTAAATAAAATAGTAAAATTAAATTAATTATAAAATTATGTATATAATTTACTAAC
