# Supplementary material for: Exploring the key genomic variation in monkeypox virus during the 2022 outbreak
Source: BMC Genom Data. 2023 Nov 16;24:67. doi: 10.1186/s12863-023-01171-0 (PMC10652487; doi:10.1186/s12863-023-01171-0)
Supplement: Supplementary file 8 — Additional file 8. Full length sequence of RS5. [file 12863_2023_1171_MOESM8_ESM.docx]

**Additional file8. Full length sequence of RS5.**

CGAAGAGAGAAAGAGATGGTTAGTCAAGATATTTTTCTTAGTACAAAAGTCAATGTTTTAAAATATATGG

ACGAGAATTAATTTGTCTGTATAAAAACTTGTGTGAAATTATGTACTAGAGAAAAAACGTGAGCAGTGTC

CCCTACATGGATTTTACAGATCATTTATATTCCAAAAATATTAACTATATACGTTTATTATATGATGTTA

ACGTGTAAATTATAAACATTATTTTATGATGCAATTGTCTGACAACCTAGATTGGTATAAGGATGTTGAT

AAGCTCTACGAGAATATATTGTTGGACGTTATCGTTTACGAAATAGTTGAGACATCAGAAAGAGGTTTAA

TATTTTTGTGAGACCATCGAAGAGAGAAAGAGAATAAAAATATTTTTTTTTTTTTTGTAAAACTTTTTTA

TGAGACCAAGAGAATACGAATAGTGATCATATCGTATCACATATTGAAACAGAAAGAAGAAGTAACGAGA

GGTAACTTTTTGTGAATGTAGTTAAATATTTTTGTTTTGCAAACCGGAATATAGTGCCCGGTCTTTTTTA

ATTCGTGGTGCGGTGTCTGAATCGTTCGATTAACCCAACTCATCCATTTTCAGATGAATAGAGTTATCGA

TTCAGACACATGCTTTGAGTTTTGTTGAATCGATGAGTGAAGTATCATCGGTTGCACCTTCAGATGCCGA

TCCGTCGACATACTTGAATCCATCCTTGACTTCAAGTTCAGATGATTCCTCACACATGTCTCCGATACGT

ACGCTAAACTCTAGGTTCTTGACACATTTTGTATCAACGATCGTTGAACCGATGATATCTTTGTAACTCA

CTTTCTTATGTGAGATGTTAGACCCAAGTACTGGATGGGTCTTGATGTCACTGTCTTTCTCTTCTTCGCT

ACATCTGATGTCGATAGACATCTCACAGTCTTTGATCATAGCCAGAGCTTCTTCACGCGTGATCGCGGGA

GAGTCCTTACCTTGTCCCGGTGACACGCTGGACAATCTAGTATTCACAGTGTTTCCATCAGAGGATTCGG

AGATGGATGAAATCTTTGGGCATTTGGTGAATCCAAAGTTCATGTTAAGACCCGCACCGACGATAGTGTA

ATAAGTGGTGGGATCTCCTTTTACAACTTCTTCGGATACCTCATCATCTTCGGTCTCTGTAACTTCCGTT

ACGGATTGACAAATCTTATCATTGGTCGGTGTTTGGTCTTGCTTTGTGACTTTGATAATAACATCGATTC

CCATATGATGTTTGTTTTCTTCTTCAGTACACGAGGATGAAGATTGTTGAAGACTAGTAGGCATAGCAGC

TGCCACTAGGCACATGCATGCCAGGACAATATATTGTTTCATGATTGCTATTGATTGATTACTGTTCTAG

ATGATTCTACTTTCTTACCATATAATAAATTAGAATATATTTTCTACTTTTACGAGAAATTAATTATTGT

ATTTATTATTTATAGGTAAAAAAAACTTACTATAAGTGGGTGGGATTCTGGGAATTAGTGATCAGTTTAT

GTATATCGCAACTAGCGGGCATATGGCTATTGACATCGAGAACATTACCCATATGATAAGAGATTGTATC

ATTTTCGTAGTCTTGAGTATTGGTATTACTATATAGTATGTAGATGTCGACGCTAGATAGACAGTCGCCC

ACTAGAGTTACCGTCTCTGAATGCGGCATGATAGTATCATTCTTTGTTTTCGTTAACTGTTTGGAAGATG

AATCTTTGTTGTTACATTTAATCTCGAAATTCAGAGTACATATCTTTGAAGTATTCTGATATCTATTTTC

TCCTGTAAAGAATCCTGAAGTTGCTACATTATTAAGGACAGAGAAGTATTCTGCACGAAAGACTGGATCA

CAATCTTTATGATTCATGGTAATAGTTAGTTCCGACGTTGAGATGGATTCGCTGAGACCGGTAGTGGTCG

TCCGAGTACACGATGTGTCGTTGACTGGATACAGGTTAATTTCCACATCGATATAGTTAAATGTATTGCT

GGTTACGACGGGTTCGCATTTATCTGTGGAAGAGACGGTGTGAGAATATGTTCCGGGACCACACGGAGAA

CAGATGACGTCTCCGGTAGACGTGTATCCGGATACTCCGTATCCTATTCCACACTTTGTTTTAGAAATAC

ATGTTCTACACCCTGATGCTCCTTTGAGAAGACAATAATATCCTGGAGAGCATTCACAGATTCTATTGTG

AGTCGTGTTACACGATCGCGTCTCTACCTGATTACTATCACATCTTCCGTTACAACTTAGACAAGCCTGT

AAATGATTATTGTGAGATGTAAAGGTATCCGAACCACACGGTGTACATTGTGTATTAGTCTTGCTATCAC

ATAATCTGGAAGCGTAAGTTCCCGGAGGACACGATAGACAACATAGATTACGGCTTCTGTATTCGTTGTC

TTTACACTTTCCATTGGATGGTGCATGTGGTGCTATATCTCTTCCGTTTATTATTATACATGAGAGAAAC

AATATATACGAGTATAATACGGACCTCATGATTTAATAATGTAGTAATCGTCGTCTTGTTACTGTTTGTT

TCCTACTTCTCCAATCATATAGATTATTTTTTAAATATTTTCTTTCTATCATGGATAATATTTGTAATGG

TTCTTTCCGTACAACATACTGTTTAGATGGTAGTCGCTTAGCTTGGTTATGATATTGCGCATAATTTCCG

GAGGCAAATACGATAGTCTAGATTGACTATCGATGGTAGACTCTAATTTATTGAGTGCTTTGTCGACGAG

TTTACTTTTATGCTCCATCGATAGATGACACTGTTCTATGAGATCGTCGTACATGGGAAATGAAATGCGT

TTGTCTGAATGTATGGCTTCGAGATATGTGTGATACCGGATGTCTTCTGTTCTCAATACCGTATACAAGT

CGGTGTCTGAGATTCGAATCTCTTTGAGGAGACTTATGTCACGACTACATTTTTCGATGATGGAATCTAT

CTTATCGAATGATATATTTTTCATAAATACACTTTTATAGTCCTCGTTTAAACAGAATTTAGTATGTAGT

TCCGCAAATGACTCGTCCCTTAATAGGCAGTAGGCTATTATCTTCTTTACGTAGTGATCGTCGTAGGGAG

AGAACTCCGACATCTTGTAGAACAACGATTTAATCATAGGTAGAGATACTTTCAGTCTGTGGTGGATAAT

GTCATTAACGACATCCGCCTTGTATATGATGTTTCTGTTTTCAAACACCAAGTCGAATACTGTCTTATCG

TCTTTAGTCGGAAGGTTGATGTCGTATCCGATGTATACGAGGTATGAGGCAACATTGTTATTGCAATTCT

GGAAGGCGGTATGAAGAGGAGTCATTGTATTATAGTATTCGTCTTTCTGAATGTCGAATCTATCTAGTAG

ATACCGTAGTATATTGAGAGAGCGACTTCCATATCCTTGATTATGTTTTATGAATAGATAAAGTAGATGT

TGTCCTTCTTCCTTTTGTAATTTCCCGTATTTTTGTTCGTGCCAATTGAGTAACATTATGAGAATATGAC

CTGTTGCACAATCGTTCTTTATGTATTCCATGATGGGTGTACAATCAAGATTATTACGTATCCTCGTATC

GGCTCCTCGAGATAAAAGAGCATACACCACACGAGGACTATGTTTGGTATACTGTTGAAGGTAAGTGTGT

AACGGCGTATTTCCGATTTTCGTAACCGCGTTAATGTTTGCTCCATGATCTATTATCGCGTAGATGAATC

GCTTCTCAGCTCGCATCTTAGTGTGACTCTTTGACTTGTAATAATTGCTTTCGTGGAACGCGGATATGTG

TTTACAGTAGTAATGAAGAGAAGTGAGTCCATCCTCATCGACGCAATTAGGGTCAGATCCTTTAGTCAAT

AATTTGTACAGAACGTAATAGTTTAAGCTCCCATTGAATTTATATCTAAGATAACACAGCAATAGATCGG

ATGATTTACTAAAGTCATCAATGGGGTCCGTTAGTATATCAAAGATCTTGTTATCGATTGATAGTGAATG

AATCAGATAGTGGTGTAGAGGAATATGTCCTTTTTTATCCTTGCTATCAAAGTTACGCATGCCGTGGCGT

AACAATATCTTTAATACAGATGGATTAAATCGTGTATTCATCGTATAGCAATGTAATGGAGAGTTACCAC

ATTTTAGTCGTTTATTCAGATCGCAGTGTTTAATAACTAATTTAAACAGATGAGATGATATATCCACATC

AAAGAATGCGAGATACATATGACAGACATTATTGACAGAAATGTGACCTTCATTATCACCGTCGTCCATA

AATGCGTTAGGTACGTACCACATACTATCGTTAACGATGCGCACAATCTCGTCCATTTCATCCATCTTCA

TAATGATTTACTTTTTCATAATTAGAGAAAAAGATCAAGGTATAAAAATTAGAAGTGTTAGACTATAAAC

TAACTTATGACTTAACTAACTTATGACTTAACTAACTTATGACTTAACTAACTTATGACTTAACTAACTT

ATGACTTAACTAACTTATGACTTAACTAACTTATGACTTAACTAACTTATGACTTAACTAACTTATGACT

TAACTAACTTATGACTTAACTAACTTATGACTTAACTAACTTATGACTTAACTAACTTATGACTTAACTA

ACTTATGACTTAACTAACTTATGACTTAACTAACTTATGACTTAACTTATGACTTAACTAACTTATGACT

TAACTAACTTATGACTTAACTAACTTATGACTTAACTAACTTATGACTTAACTAACTTATGACTTAACTA

ACTTATGACTTAACTAACTTATGACTTAACTAACTTATGACTTAACTAACTTATGACTTAACTAACTTAT

GACTTAACTAACTTATGACTTAACTAACTTATGACTTAACTAACTTATGACTTAACTAACTTATGACTAT

TAACTCATTTCAAGAAAGGTGGGTGGATAGAACTCTATATGACAGCTTGTGAAACAATTAGATCCCTAAT

TTCTAATGGAAGTTTTGATAGGAGATTGTCATCAGTTGATACATTGTTTATTATCTCATCTATTAGAGCA

CGTCTGTTTAGAGCTTTAGTGACCTGCTCGGTTACTTCTGTGTAAATCTTGAATCCTTTAGTGATACACT

GTGTCAAAACTGGATGTTTAGAATACCTATGTAGAATATGGGAAGCATGCTTGTTTTTGTCTCTATTATA

GATTAACTCATACATGGTTGTATTATGAATTTTCATCTGCCTAATGTACTCCAATTCTTGTTTACAATCA

ATTATATAATCAAAGAGTGATGATGCATACACATTACAAAGTGAATAATCTACCATCATAAAATACTTGA

TACAGAGCTTTATCACATCATGGTTTTCAATTGTATTATTAAGTATAGCTAATTTTATACAGTCAATAGA

CAATGGTTCTCTAAGCAATATTTCTAATATTTTAAGATGTGCTTCCCTACGGGCGATGACAGATCCCCTA

TCCACGGCCACGTCAAGACATGTATATCCATTACTCATTACTGCGTTGACATTTGCTCCATTTTCTAATA

GCCATGATACTAAATCTATATAACCTGCATAGATAGCGCGATAAAGCAAGGTCCTTCCACCAGCATCTAG

TTGATTGATATCTTCAATATATGGGATACAAAGCTTATAAATTTCTAATACTGTGGGTTCATCTACAAGG

AATCCCCTAGTATACTGAATTATTTTATATAGATCTAATTTAACATCATTTTCATCTGGGATACCACAAT

TCAAAATAAACTCAACAACACTACTTTCCTTTTTACATATTCCCCTAAAATAGGCATTCAAGCATTCTAT

TTTATATATTACAGCCCCATGATCTACCATAAGATCAACAATGTCTATTTCTACATATGCATTAGATAGA

TAGTAAAGTAAGAGATCTTGCACAGAATTACAATTCTTAATAATTATAGAGAAAATATCTTCCATATAAT

TCTTTGACACTAATGCAGATATAATATCTTTATATGTAATATATGCAAACAGTCTATCTACTATATACTG

ATCAATATTATCTCTATGAATCCTAAAATAATCATACAGAACATCTACAGGATCACAAATTGGTTCAAGG

AGAAATCTATCAAATATTTTCCTGTCAACAACTGGTTCTAGAACATAACAGTCAACACCTAATCCATGTT

TTTTATAATCATCTACCAAAGATAATGACCAAAGATCGAGGTCGTCGTGAAACTGCTCATCGACAGCCAT

GAAATCTACCGACTCCATGGTGCGAATCGCACTGTCTTATTCGCCATTGATTTTCATTTTTTATAATTAT

GTACATGTTTTCCTTCTATTCTCAAGAGTCTACAAAAATATATTTTTTCGATATCTAAGTACTAAGTTTT

TTTACTGTTTTTGTTACTGTCTTCCATTCTTCTAACTAAAGATCTGAGATAAATTATACAATCTTCGCTA

TCGAACCATTTTTGTAGTCTAAAGCCTGAAGTAATTAACCAACTGTTTTTATTAGTGGCTTTTTTCGATC

TATCCTCGTCCTCTCATCATCCTTATATTATTATCATTATTATCATAGTCTATTAAACACAAATCATCTA

CGTTTATAACAACATTCTCATTATTAATTAGTTCTGTAGAATATCTTTAATAATTTGGCTATACATCTGT

TCAATACTATCTATTGATGATTTCTTTTTTAAGACTTAAACTAGTTATGGTAATGACGATGAAATCGAGT

AGTAACTTCTAATAAAGACTTGATATCATTATCATATGTTTGATCGTCATAGTTAATAGTGTGGCTAAAT

GGTACTGTTAATAAGTTTATAGACAATATCATAGTATTTTCTTTCCAGAATTAGATTATTTTTTTAAATA

CTGATCCTCACAATTCCGTGATGTAGCAGTAGTTGGTGCATGGTCTATATCGTTAAAATGTATCATATAT

AATAGTTTTCTGACGTGGAGTACAGAATTTTCGATTAATGAGTTCATGGTAAGGAAGGGCAAATGCCTGT

ATATAATATACATAAGTTAATAGTTTTTTATCATATTTTCTAATACCATAATAAAAATTATCATTATTGC

GTTTGGTAGTTCTGCCCTATCATCTATATCACTGTCACTCTCGCTCTCACTATATCTTCTAAAATTACAA

ACAACTGGATATTCGATAACAGCATTTGTGTAGTTTTTGTCTTTTACAGTATATACGTTATTGTCAAAAT

CTAAACAAATATTAGCATAATACATCTATCTATAAGATCAGGATCCATGTTCGAGCATACTAGCCATGTA

TATTTGTAACTTCGTCGTACAGCGTTAGATCAATAGAATAAACAATCGTGTGACGCAACTTTTTTACGAT

CTAGTTGTATGAGTTTATCGTTTACATAAGCAATTAACGGCTTTAACAGATGATCTGAGTAATAATATAC

CTCTGTTATACGTTTAATGTTCACGGTCTTAGTATTTTTAGATATCAATTGTGATTTACACCATATTCGA

CTCCCTTGTGTGTAACGTTAGAAATTCTAAATCTATATTATCTATTACAGCGTAAAACACATTCAATATT

GTATTGTTATTTTTATATTATTTACACAATTAACAATGTATTATTAGTTTATATTACTGAATTAATAATA

TAAAATTCCCAATCTTGTCATAAACACAAAATCCATTAAAAATGTCGATAAAATATCTGATGTTGTTGTT

CGCTGCTATGATAATCAGATCATTAGCCGATAGTGGTAATGCTATCGAAACAACATCGCCAGAAATTACA

AACGCTACAACAGATATTCCAGCTATCAGATTATGTGGTCCAGAGGGAGATGGATATTGTTTACACGGTG

ACTGTATCCACGCTAGAGATATCGACGGTATGTATTGTAGATGCTCTCATGGTTATACAGGTATTAGATG

TCAGCATGTAGTATTAGTAGACTATCAACGTTCAGGAAAACCAGACACTACAACATCATATATCCCATCT

CTAGGTATTGTGCTTGTATTAGTAGGCATTATTATTACGTGTTGTCTATTATCTGTTTATATGTTCACTC

GAAGAACTAAACTACCTATACAAGATATGGTTGTGTTATATTTTTTATAAAATTTTTTTATGAGTATTTT

TACAAAAATGTATATGTATAAAAAAAATACTAAGTATACGATGTATCCTGTATTATTTGTATTTATCTAA

ACAATACTTCTGCCTCTAGATGGGATACAAAAATTTTTTATTTCAGCATATTAAAGTAAAATCTAGTTAC

CTTGAAAATGAATACAGTGGGTGGTTCCGTATCACCAGTAAGAACATAATAGTCGAATACAGTATCCGAT

TGAGATTTTGCATACAATACTAGTCTAGAAAGAAATTTGTAATCATCCTCTGTGACAGGAGTCCATATAT

CTGTATCATCGTCTAGTTTTTATCAGTGTCCTATGCTATATTCCTGTTATCATCATTAGTTAATGAAAAT

AACTCTCGTGCTTCAGAAAAGTCAAATATTGTATCCATACATATATCTCCAAAACTATCACTTATACGTT

TATCTTTAACGAACATATACCTAGATGGTTATTTACTAACAGACATTTTTTCAAGATCTATTGACAATAA

CTCCTATAGTTTCCACATCAACCAAGTAATGATCATCTATTGTTATATAACAATAACATAACTCTTTTCC

ATTTTTATCAGTATCTATATCAACGTCGTTGTAGTGAATAGTAGTCATTGATCTATTATATGAAACGGAT

ATGTCTAGTTAATATTTTCTTTGATTTAAAGTCTATAGTCTTTACAAACATAATATCCTTATCCGACTTT

ATATTTCCTGTAGGGTGGCATAATTTTATTCTGCCTCCACAATCAGTGTTTCCAAATATATTACTAGACA

ATATTCCATATAGTTATTAGTTAAGGGACCCAATTAGAACACGTACGCGCTTATTCATCATTTGGATCGT

ATTTCATAAAAGTTATTATGTTATCGATGTCAACACATTCTACATTTTTTTAATTGTCTATATAGTATTT

TTCTGATATTTTCTATAATATCAGAATTGTCTTCCATAGGAAGTTGTATACTATTCGGAATCAGTTACAT

GTTTAAATAATTCTCTGATGTCATTCCTTATACAATCAAATTCATTATTAAACAGTTTAATAGTCTGTAG

ACCTTTATCGTCGTACATATCCATTGTCTTATTAGTTACGCTTATTTTTATGGGTTTTACGTTGCTTTAT

TATATTTTATAATAATGATTGTTTGACAATGTCGTAGTATAGATATATTATTAGAGGAGGTATAATTATA

AAAAGTTTCTGAGTACGATGTTATAAGAGGAGAGGACACATTAACATCATACATCAATTAACTACATTCT

TATAACATTGTAATCAAAAGAATTGCAATTTTGATGTATAACAACTGTCAATGGAATTGTATATTACAAA

TTACGGTATGTTGTAACAACAAATACCGATCGGTAATTGTCTCTGCCGCTGTAATAGAATTAATTATATA

TCTATTACACCGGCCTTGTATCATAATAAAGTTGTGGTAGTATGATCTCCATATTTATAATTTAGTACTT

TGTATTTAGTTTTTTTGGAATCATAAAAAAAGTTTTACTAATTTAAAATTTAAAAAGTATTTACATTTTT

CACTGTTTAGTCGCGGATATGGAATTCGATCCTGTCAAAATCAATACATCATCTATAGATCATGTAACAA

TATTACAATACATAGATGAACCAAATGATATAAGACTAACAGTATGCATTATCCGAAATGTTAATAACAT

TACATATTATATCAATATCACAAAAATAAATCCACATTTGGCTAATCGATTTCGGGCTTGGAAAAAACGT

ATCGCCGGAAGGGACTATATGACTAACTTATCTAGAGATACAGGAATACAACAATCAAAACTTACTGAAA

CTATACGTAACTGTCAAAAAAATAAAAACATATATGGTCTATATATACACTACAATTTAGTTATTAATGT

GGTTATTGATTGGATAACCGATGTGATTGTTCAATCAATATTAAGAGGGTTGGTAAATTGGTACATAGCT

AATAATACATATACTCCAAATACACCCAATAATACTACAACCATTTCTGAGTTGGATATCATCAAAATAC

TGGATAAATACGAGGACATGTATAGAGTAAGTAAAGAAAAAGAATGTGGAATTTGCTATGAAGTTGTTTA

CTCAAAACGATTAGAAAACGATAGATACTTTGGTTTATTGGATTCGTGTAATCATATATTTTGCATAACA

TGTATCAATATATGGCATAGAACACGAAGAGAAACCGGTGCGTCAGATAATTGTCCTATATGCCGTACAC

GTTTTAAAAAAATAACAATGAGCAAGTTCTATAAGCTAGTTAACTAATAAATAAAAAGTTTAATTATCGA

CGATATATGTCGTTATTTTTCTCTCATATGAAAGATTAATTTGATTCTAATATAATCTTCAGTATTGGAT

GAATCTCAATTCAAATTAATTCCATTAGATTAGATTAGATTAGATCATAAATAAAAATAGTAGCACGCAC

TACTTCAGCCAAATATTCTTTTTTGAAACGCCATCTAGCGTAATGAGAACACAAGTGAACCTATAATGAG

CAAATTTATTAGTATCGGTTACATGAAGGACTTTACGTAGAGTGGTGATTCCTCCATCTGTGGTACGAAC

GGTTTCATCTTCTTTGATGCCACCACCCAGATGTTCTATAAACTTGGTATCCTCGTCCGATTTCATATCA

TTTGCCAACCAATACATATAGCTAAACCCAGGCATACGTTCCACACATCCGGAACAATGAAATTCTCCAG

AAGATGTTACAATGGCTAGATTTGGACATTTGGTTTCAACCGCGTTAACATATGAGTGAACACACCCATA

CATGAAAGCGATGAGAAATAGGATTCTCATCTTGCCAAAATATCACTATAAAAAATTTATTTATCAATTT

TAAAGGTATAAAAAAATACTTATTGTTGCTCGAATATTTTGTATTTGATGGTATACGGAAGATTAGAAAT

GTAGGTATTATCATCAACTGATTTTATGATGGTTTTATGAATTTTATTATGCTTCACTATTGCATCGGAA

ATAATATCATATGCTTCCACGTATATTTTATTTTGTTTTGACTCATAATACGCACGTAATTCTGGATTAT

TGGCATATCGATGAATAATTTTAGCTCCATGCTCAGTAAATATTAATGAGAACATAGTGTTGCCTCCTAC

CATTATTTTTTTCATCTCATTCAATTCTTGATTGCAGAGATCTATATAATCATTATAGCGTTGACTTATG

GACTCTGGAATCTTAGACGATGTACAGTCATCTATAATCATGGTATATTTAATACATTGTTTTATAGCAT

AGGCATTATCTACGATATTAGATACTTCACTCAATGAATCAATCACACAATCTAATGTAGGTTTATGACA

TAATAGCATTTTCAGCAGTTCAATGTTTCTAGATTCGTTGATGGCAATGGTTATACATGTATATCCGTTA

TTTGATCTAATGTTGACATCTGAACCGGATTCTAGCAGTAAAGATACTAGAGATTGTTTATTATATCTAA

CAGCCTTGTGAAGAAGTGTTTCTCCTCGTTTGTCAATCATGTTAATGTCTTTAAGATAAGGTAGACAAAT

GTTTATAGTACTAAGAATTGGGCAAACATAAGACATGTCACAAAGACCCTTTTTGTATGTATAAGTGTAG

AAATTATAACATCCATAGTTGGATTCACATAGGTGTCCAATCGGGATCTCTCCATCATCGAGATGATTGA

CGGCATCTCCCCCTTCCTTTTTTAGTAGATATTTCATCGTGTAAGAATCAATATTAATATTTCTAAAGTA

TCTGTGTATAGCCTCTTTATTTACCACAGCTCCATATTCCAACATGCATTCCACTAGAGGGATATCGATA

TCGCCGAATGTCATATACTCAATTAGTATATGTTGGAGGACATCCGAGTTCATTGTTTTCAATATCAAAG

AGATGGTTTCCTTATCATTTCTCCATAGTGGTACAATACTACACATTATTCCGTACGGCTTTCCATTCTC

CAAAAACAATTTTACCAAATCTAAATCTACATCTTTATTGTATCTATAATCACTATTTAGATAATCAGCC

ATAATTCCTCGAGTGCAACATGTTAGATCGTCTATATATGAATAAGCCGTGTTATCTATTCCTTTCATTA

ACAATTTAACGATGTCTATATCTATATGAGATGACTTAATATAATATTGAAGAGCTGTACAATAGTTTTT

ATCTATAGAAGACGGCTTGATTCCGTGATTAATTAGACATTTAACAACTTCCGGACGCACATATGCTCTC

GTATCCGACTCTGAATACAGATGAGCGATGATATACAGATGCAATACGGTACCACAATTTCGTGGTTGAT

AATCATCATACACGTATCCGTACTCGTCATCCTCATAAAGAACACTGCATCCATTTTCTATGAACAAATC

AATAATTTCAGGAACAGGATCATCTGTCATTACATAATTTTCTATAACTGAACGATGGTTTTCACATTTA

ACACTCAAGTCAAATCCATGTTCTACCAACACCTTTATCAAGTCAACGTCTACATTTTTTGATTTCATAT

AGCTGAATATATTAAAGTCATTTATGTTGCTATATCCAGTAGCTTCTAGTAGAGCCATCGCTATATCCTT

ATTGACTTTAACATGTCTACTATTTGTGTATTCTTCTATTGGGGTAAACTGTCTCCAATTTTTATGTAAT

GGATTAGTGCCACTATCTAGTAGTAGTTTGACGACCTCAACATTATTACAATGCTCATTGAAAAGGTATG

CGTGTAAAGCATTATTCTTGAATTGGTTCCTGGTATCATTAGGATCTCTGTCTCTCAACATCTGTTTAAG

TTCATCGAGAACCACCTCCTCATTTTCCAGATAGTCAAACATTTTGACTGAATAGAAGTGAATGAGCTAC

TGTGAACTCTATACACCCGCACAACTAATGTCATTAAATATCATTTTTGAATGTATTTATACCATGTCAA

AAACTTGTACAATTATTAATAAAAATAATTAGTGTTTAAATTTTACCAGTTCCAGATTTTACACCTCCGT

TAACACCTCCATTAACCCCACTTTTTACACCACTGGACGATCCTCCTCCCCACATTCCACTGCCACTAGA

TGTATAAGTTTTAGATCCTTTATTACTACCATCATGTCCATGGATAAAGACACTCCACATGCCGCCACTA

CTACCCCCTTTAGACGACATATTAATAAGACAAGTTTAACAATAAAATTAATCACGAGTACCCTACTACC

AACCACTATTATATGATTACAGTACCTTGACTAAAGTCTCTAGTCACAAGATCAATACTACCAACCTACG

CTATTATATGATTATAGTTTCTATTTTTATAGGAACGCGTACGAGAAAATCAAATGTCTAAGTTCTAACG

GTAGTGTTGATAAACGATTGTTATCCGCGGATACCTCATCTATCATGTTGTCTATTTTCTTACTTTGTTC

TATTAACCTATTAGCATTATATATTATTTGATTATAAAACTTATATTGCTTATTAGCCCAATCTGTAAAT

ATCGGATTATTAACATATCGTTTCTTTGTAGGTTTATTTAACTTGTACATCACTGTAAGCATGTCCATAC

CATTTATTTTAATTTGACACATATCAGCAATTTCTTTTTCGCAGTCGGTTATATATTCTATATAAGATGG

ATACGTATCACATATGTACTTATAGTCTACTAATATGAAGTACTTAATACATATTTTCAGTAACGATTTA

GCCTTATTACCTATTAATAAGTGCCTGTCGTTGGATAGGTAATCAACTGTTTTCTTAATACATTCGATGG

TTGGTAATTTACTCAAAATAATTTCCAATATCTTAATATATATTTCTGCTATTTCTGGTATACATGCATG

TGCCATTATAACACAAATACCAATACATGTAGACCCATATGTTGTTGTTATATTAATATCTGCGCCATTA

TCTATTAACCATTCTATTAGGGCAACACTATGCGACTCGATACAATAATAAAGTATACTACGTCCATGTT

TATCTATTTTGTTTATATCATCGATATACGGCTTACAAATTTCTAGTATCGATAACACTTCTGACTCGTG

AATAAATAAGGTAGGGAATAACGGCATAATATTTATTATGTTATCATCATTAACAACTACGTTTCCATTT

TTTAAAATATACTCTACAACTTTAGGATCCCTATTGTCAAATCTTTTAAAATATTTATTTATATGCTTAA

ATCTATATAATATAGCTCCTTCCCTAATCATACATTTGATAACATTGATGTACACTGTATGATAAGATAC

ATATTCTGACAATAGATCTTGTATAGAATCTGTATATCTTTTAAGAATTGAGGATATTATGACATTATTA

CGTAAACTATTACACAATTCTAAAATATAAAATGTATCATGGGCAGATAATAGTTTATCCACTATATAAT

TATCTATTTTATGATTTTTCTTCCTATATTGTTTACGTAAATAGATAGATAGAATATGCATTAGTTCATT

ACCGCTATAGTTACTATCGAATAACACGTCAAATATTTCCCGTTTAATATCGCATTTGTCAACATAATAA

TAGAGTATGGTACGTTCACGATAAGTATAATGACACATCTCGTTTTCGTGCGAAATTAAATAGTTTATCA

CGTCCAAAGATGTCACATAACCATCTTGTGACCTAGTAATAATATAATAATAGAGAACTGTTTTACCCAT

TCTATTATCATAATCAGTGGTGTAGTCATAATCTAAATAATCAAACTCGTCATCCCAATTAAAATAAATA

TAATCAGTACATTGAATGGGTATGATATTGTACCCATACTGTATGTTGCTACATGTAGGTATTCCTTTAT

CCAATAATAGTTTAAATACATCTATATTAGGATTTGATGTTGTCGCGTATTTCTCTACAATATTAATACC

ATTTTTGATACTATTTATTTCTATACCTTTCGAAATTAGTAATTTCAATAAGTCTATATCGATGTTATCA

GAACATAGATATTCAAATATATCAAAATCATTGATATTTTTATAGTCGACTGACGACAATAACAAAATCA

CAACATCGTTTTTGATATTATTATTTTTTTTGGTAACGTATGCCTTTAATGGAGTTTCACCATCATACTC

ATATAATGGATTTGCACCACTTTCTATTAATGATTGTGCACTACTGGCATCGATGTTAAATGTTTTACAA

CTATCATAGAGTATCTTATCGTTAACCATGATTGGTTGTTGATGTTATCACATTTTTTGGTTTCTTTCAT

TTCAGTTATGTATGGATTTAGCACGTTTGGGAAGCATGAGCTCATATGATTTCAGTACTGTAGTGTCAGT

ACTATTAGTTTCGATCAGATCAATGTCTAGATCTATAGAATCAAAACACGATAGGTCAGAAGATAATGAA

TATCTGTACGCTTCTTCTTGTACTGTAACTTCTGGTTTTGTTAGATGGTTGCATCGTGCTTTAACGTCAA

TGGTACAAATTTTATCCTCGCTTTGTGTATATCATATTCGTCTCTAGTATAAAATTCTATATTCAAATTA

TCATGCGATGTGTGTACGCTAACGGTATCAATAAACGGAGCACAGCATTTAGTCAACAGTAATCCAAAAT

TTTTTAAAGTATATCTTAACGAAAGAAGTTGTCATCGTTAGAGTGTGGTAAATCATTGTCTACGGTACTA

GATCCTCATAAGTGTATATATCTAGAGTAATATTTAATTTATCAAATGGTTGATAATATGGATGTTGTGG

CAATTTCCTAATACGGAAATAAGACATAAACACGCAATAAATCTAATTGCGGACATGTTACACTCCTTAA

AAAATACGAATAAACACTTTGGCTTTTAGTAAGTGTCATTTAACACTATACTCATATTAATCCATGGACT

CATAATCTCTATACGGGATTAACGGATGTTCTATATACGGGGATGAGTAGTTCTCTTCTTTAACTTTATA

CTTTTTACTAATCATATTTAGACTGATGTATGGGTAATAGTGTTTGAAGAGCTCGTTCTCATCATCAGAA

TAAATCAATATCTCTGTTTTTTTGTTATACAGATGTATTACAGCCTCATATATTACGTAATAGAACGTGT

AATCTACCTTATTAACTTTCACCGCATAGTTGTTTGCAAATACGGTTAATCCTTTGACCTCGTCGATTTC

CGACCAATCTGGGCGTATAATGAATCTTAACTTTAATTTCTTGTAATCATTCGAAATAATTTTTAGTTTG

CATCCGTAGTTATCTCCTCTATGTAACTGTAAATTTCTCAACGCGATATCTCCATTAATAATGATGTCGA

ATTCGTGTTGTATACCCATACTGAATTGATGAACTAATACCGACGGTATTAATAGTAATTTACTTTTCAT

CTTTACATACTTGGTAATAGTTTTACTATCATAAGTTTATAAATTCCACAAGCTACTATGGAATATACCA

ACCATCTTAGTATAGAACACATGTCTTAAAGTTATTAATTAATTACATGTTGTTTTATATATCGCTACGA

ATTTAAACAGAGGAATCAGTTAGGAAGAAAAAATTATCTGTCATCATCATCATCATCATCTATTGGATAA

CATCTCTGTATTCTACGATAGAGTGCTATTTTAAGATGTGACAGATCCGTGTCATCAAATATATACTCCA

TTAAAATGATTATTCCGGCAGCGAACTTGATATTGGATACATCACGACCTTTGTTAATATCCACGACAAT

AGACAGCAATCCCATTGTTCCATAAACAGTGAGTTTATCTTTCTTTGAAGTGATATTTTGTAGAGATCTT

ATAAAACTGTCGAATGACATCGTATTTATATCTTTAGCTAAATCATATATGTTACCATCATAATATCTAA

CAGCATCTATCTTAAACGTTTCCATCGCTGTAAAGACGTTTCCGATAGATGGTCTCGTTTCATCAGTCAT

ACTGAGCCAACAAATGTAATCGTGTATAACATCTTTGATAGAATCAGACTCTAAAGAAAAGGAATCGGCT

TTATTATACACATTCATGATAAACTTAATGAAAAATGTTTTTCGTTGTTTAAGTTGGATGAATAGTATGT

CTTAATAATTGTTATTATTTCACTAATTAATATTTAGTAACGAGTACACTCTATAAAAACGAGAATGACA

TAACTAATCATAACTAGTTATCAAAGAATGTCTAGGACGCGTAATTTTTTATGGTATAGATCCTGTAAGC

GTTGTCTGTATTCTGGAGCTATTTTCTCTATCGCATTAGTGAGTTCAGAATATGTTATAAATTTAAATCG

AATAACGAACATAACTTTAGTAAAGTCGTCTATATTAACTCTTTTATTTTCTAGCCATCGTAATACCATG

TTTAAGATAGTATATTCTCTAGTTACTACGATCTCATCGTTGTCTAGAATATCGCATACTGAATCTACAT

CCAATTTTAGAAATTGGTCTGTGCTACATATCTCTTCTATATTATTGTTGATGTATTGTCGTAGAAAACT

ATTACGTAGACCATTTTCTTTATAAAACGAATATATAGTACTCCAATTATCTTTACCGATATATTTGCAT

ACATAATCCATTCTCTCAATCACTACATCTTTAAGAGTTTGGTTGTTAAGATATTTGGCTAAACTATATA

ATTCTATTAGATCATCAACAGAATCAGTATATATTTTTCTAGATCCAAAGATGAACTCTTTGGCATCCTC

TATAATATTATCAGAAAAGATATTTTCGTGTTTTAGTTTATCAAGATTTAACCTGTTCATATCCATGATT

AACGACGTCATATAACCACATAAAATAAAAATCCATTTTCATTTTTAGCACAATACTATTCATAATTGAT

ATTGATGTAATATTTTGTTACTTTGAACGTAAAGACAGTACACGGGTCCGTATCTCCAACAAGCACGTAG

TAATCAAATTTGGTGTTGTTAAACTTCGCAATATTCATCAATTTAGATAGAAACATATACTCATCATCTG

TTTTAGGAATCCATGTATTATTACCACTTTCCAACTTATCATTATCCCAGGCTATGTTTCGCCCATCATC

GTTGCACAGAGTGAATAATTCTTTTGTATTCGGTAGTTCAAATATATGATCCATGCATATATCGACAAAG

CTATTGTAGATGTGATTTTTCCTAAATCTAATATAAAACTCGTTTACTAGCAAACATTTTCCTGATTTAT

CGACCAAGACACACATGGTTTCTAAATCTATCAAGTGGTGGGGATCCATAGTTATAACGCAGTAACATAG

ATTATTACCTTCTTGACTGTCGCTAATATCTATATACTTATTGTTATCGTATTGGATTCTACATATAGAT

GGCTTGTATATCAAAGATATAGAACACATAACCAATTTATATTCTCGCTTTGTATTTTCGAATCTAAAGT

TAAGAGATTTAGAAAACATTATATCATCGGATGATGATATCACTGTTTCCAGAGTAGGATATATTAAAGT

CTTTAAAGATTTTGTCCGATTCAAATAAATCACTAAATAATATCCCATATTATCATCTGTTATAGTCGTG

TCATTAAATCTATTATATTTTATGAAAGATATATCACTGCTCACCTCTATATTTCGTACATTTTTAAACT

GTTTGTATAATATCTCTCTAATACAATCAGATATATCTATTGTGTCGGTAGACGATACCGTTACATTTGA

ATTAATGGTGTTCCATTTTACAACTTTTAACAAGTTGACCAATTCATTTCTAATAGTATCAAACTCTCCA

TGATTAAATATTTTAATAGTATCCATTTTATATCACTACGGACATAAACCATTGTATATTTTTTATGTTT

ATTAGTGTACACATTTTGGAAGTAAGTTCCTGGATCGGATGTCACCGCAGTAATATTGTTGATTATTTCT

GACATCGACGTATTATATAGTTTTTTAATTCCATATCTTTTAGAAAAGTTAAACATCCTTATACAATTTG

TGGAATTAATATTATGAATCATGGTTTTTACACATAGATCTATTACAGGCGGAACATCAATTATTATGGC

AGCAACTAGTATCATTTCTACATTGTTTATGGTGATGTTTATCTTCTTCCAGCGCATATAGTCTAATATC

GATTCAAACGCGTGATAGTTTATACCATTCAATATAATCGCTTCATCCTTTAGATGGTGATCCTGAATGT

GTTTAAAAAATTATACGGAGACGCCGTAATAATTTCCCCATTGATAGAAAATATCACGCTTTCCATTTTC

TTGAAGTACTAAAAGTAATTATAATATAATGTAAAGGTTTATATATTCAATATTTTTTATAAAAAAAATC

ATTTCGACATTAATTCCTTTTTAAATTTCAGTCTATCATCTATAGAAACATATTCTATGAATTTATAAAA

TGCTTTTACGTATCCTATCGTAGGTGATAGAACCGCTAAAAAACCTATCGAATTTCTACAAAAGAATCTG

TTATATGGTATAGGGAGAGTATAAAACATTAAATGTCCGTACTTATTAAAGTATTCAGTAGCCAATCCTA

ACTCTTTCGAATAATTATTAATGGCTCTTATTCTGTACGAATCTATTTTTTTGAACAATGGACCTAGTGG

TATATCTTGTTCTATGTATCTAAAATAATGTCTGACTAGATCCGTTAGTTTAATATCCGCAGTCATCTTG

TCTAGAATGGCAAATCTAACTGCGGGTTTAGGCGTAGGCGTTAGTTTAGTTTCTATATCTACATCTATGT

CTTTATCTAACACCAAAAATATAATAGCTAATATTTTATTACAATCATCCGGATATTCTTCTACGATCTC

ACTAACTAATGTTTCTTTGGTTATACTAGTATAGTCACGATCAGACAAATAAAGAAAATCAGATGATCGA

TGAATAATACATTTAAATTCATCATCTGTAAGATTTTTGAGATGTCTCATTAAAATATTATTAGTGTCAG

TTCTCATTATCATATATTGACAGCAGCTATTACACTTATTTTATTTTTCTGTATTTTATTACTTTTCACC

ATATAGATCAGTCATTAGATCATCAAAATACTTTTCAATCATCCTAAAGAGTATGGTGAACGAATCTTCC

CATCTAATTTCTGAACGTCTACCAATGTCTCTAGCCACTTTGGCACTAATAGCGATCATTCGCTTAACAT

CTTCTACATTATTAACTGGTTGATTCAATCTATCTAGCAATGGACCGTCGGATAGCGTCATTCTCATGTT

CTTAATCAATGTACATACATCGTCATCATCTACCAATTCATCAAACAATATAAGCTTTTTAAAATCATCA

TTATAATAGGATGGATCGCCGTCATTTCTCCAAAGAATATATCTAATAAGTAGAGTCCTCATGCTTAGTA

ATTTAACTATTTTAGTTAACAACTATTTTTTATGTTAAATCAATTAGTAACACCGCTATGTTTAATACTT

ATTCATATTTTAGTTTTAGGATCGAGAATCAATACAAAAATTAATACATCAATTTTGGAAATACTTAGTT

TCCACGTAGTCAATGAAACATTTGAGCTCATCGTAAAGGACGTTCTCGTACAAGACGTAACTATAAATTG

GTTTATATTTGTTCAAGATAGATACAAATCCGATAACTTTTTTGACGAATTCTACGGGATTCACTTTAAA

AGTGTCATACCGGGTTCTTTTTATTCTTTTAAACAGATCGATTGTGTGATGTTGATTAGGTCTTTTACGA

ATTTGATACAGAATAGCGTTTACATATCCACCATAGTAATCAATAGCCATTTGTTCGTATGTCATAAATT

CTTTAATTATATGACACTGTGTATTATTTAGTTCGTCCTTGTTCATCATTAGGAATCTATCCAATATGGC

AATTATATTAGAACTATAACTGCGTTGTATGCGCATGTTGATGTGTCTGTTTATACAATCAATTATACTA

GGATCCATACCACTACAATCGGGTAAAATTGTAGCATCATATACCATTTCTAGTACTTTAGATTCATTGT

TATCCATTGCAGAGGACGTCATGATCGAATCCAAAAAAATATATTATTTTTATGTTATTTTGTTAAAAAT

AATCATCAAATACTTCGTAAGATACTCCTTCATGAACATAATCAGTTACAAAACGTTTATATGAAGTAAA

GTATCTACAATTTTTACAAAAGTCAGGATGCATAAGTACAAAGTACGCGATAAACGGAATAATAATAGAT

TTATCTAGTTTATCTTTTTCTATCTCTTTCATAGTTATATACATGGTCTCAGAAGTCGGATTATGTAACA

TCAGCTTCGATAAAATGACTGGGTTATTTAGTCTTACACATTCGCTCATACATGTATGACCGTTAACTAT

AGAGTCTACACTAAAATGATTGAATAATAGATAGTCTACCATTGTTTCGTATTCAGATAGTACAGCGTAG

TACATGGCATCTTCACAAATTATATCATTATCTAATAGATATTTGACGCATCTTATGGATCCCACTTCAA

CAGCCATCTTAAAATCGGTAGAATCATATTGCTTTCCTTTATCGTTAATAATTTCTAGAACATCATCTCT

ATCATAAAAGATACAAATATTAACTGTTTGATCAGTAATAACATTGCTAGTCGATATCAATTTGTTAATA

AGATGCGCTGGGCTCAATGTCTTAATAAGAAGTGTAAGAGGACTATCTCCGAATTTGTTTTGTTTATTAA

CATCCGTTGATGGAAGTAAAAGATTTATAATGTCTACATACTTGACTGTTTTAGAGCATACAATATGGAG

AGGCGTATTTCCATCATGATCTGGTTTTGAGGGACTAATTCCTAGTTTCATCATCCATGAGATTGTAGAA

GCTTTTGGATTGTCTGACATAAGATGTCTATGAATATGATTTTTGCCAAATTTATCCACTATCCTGGCTT

CGAATCCGATAGACATTATTTTTTTAAACACTCTTTCTGAAGGATCTGTACACGCCAACAACGGACCACA

TCCTTCTTCATCAACCGAGTTGTTAATCTTGGCTCCATACTGTACCAATAAATTTATTCTCTCTATGACT

TCATCATCTGTTCCCGAGAGATAATATAGAGGTGTTTTATTATGTTTATCACATGCGTTTGGATCTGCGC

CGTGCACCAGCAGCATCGCGACTATTCTATTATTATTAATTTTAGAAGCTATATGCAATGGATAATTTCC

ATCATCATCCGTCTCATTTGGAGAGTATCCTCTATGAAGAAGTTCTTCTATAAATCGTTCATCTAGTCCT

TTAATGCCACAATACGCATGTAGAATGTGATAATTTCCAGAGGGTTCGATAACTTGTAGCATATTCCTAA

ATACATCTAAATTTTTACTATTATATTTGGCATAAAGAGATAGATAATACTCGACCGACATAATGTTGTG

TTGTCCATTATAGTATAAAAATTAATATTTCTATTTCTATATATTTGCAACAATTTACTCTCTATAACAA

ATATCATAACTTAGTTCTTTTATGTCAAGAAGGCACTGGTTTAATTCATCTATAAATGTCACTCCATAAC

TACCACGCATACTATACTCAGAATTATGATAAAGATATTTATTCTTGGGGTGTAAGTAATGGGGATTAAT

CTTTGTTGGATCAGTCTCTAAGTTAACACATGTCACACATGATCCATTTATAGTTATATCACACGATGAT

GATTTATGAATTGATTCCGGAAGATCGCTATTGTATTTTGTAGTTCCACAATTCATTTCCATACATGTTA

TTGTCACACTAATATTATGATGAACTTTATCTAGCCGCTGAGTGGTAAACAACAGAACAGATAGTTTATT

ATCTTTACCAACACCCTCAGCCGCTGCCACAAATCTCTGATCCGTATCCATGATGGTCATGTTTACTTTT

AGTCCGTATCCAGTCAACACTATGTTAGCATTTCTGTCGATATAGCTTTCACTCATATGACACTCACCAA

TAATTGTAGAATTAATGTCGTAATTTACACCAATAGTGAGTTCGGCGACAAAGTACCAGTACCGGTAATC

TTGTCGATGAGGACATATAGTATTCTTGTATTCTACCGAATACCCGAGAGATGCGATACAAAAGAGTAAG

ACTAATTTGTAAACCATCTTACTCAAAAATATGCGACAATAGGAAATCTATCTTATACACATAATTATTC

TATCAATTTTACCAATTAGTTAGTGTAATGTTAACAAAAATGTGGGATAATTTAATAGTTTTTCCTTACA

CAATTGACATACATGAGTCTGAGTTCCTCGTTTTTGCTAATTATTTCGTCCAATTTATTATTCTTGACAT

CGTCAAGATCTTTTGTATAGGAGTCAGACTTGTATTCAACATGTTTTTCTATAATCATCTTAGCTATTTC

GGCATCATCCAATAGTACATTTTCCAGATTAACAGAATAGATATTAATGTCGTATTTGAACAGAGCCTGT

AACATCTCAATGTCTTTATTATCTATAGCCAATTTGATGTCCGGAATGAAGAGAAGGGAATTGGTGTTTG

TCGACGTCATATAGTCGAGCAAGAGAATCATCATATCCACGTGTCCATTTTTTATAGTGGTGTGAATACA

ACTAAGGAGAATAGCCAGATCAAAAGGAGATGGTATCTCTGAAAGAAAGTAGGAAACAATACTTACATCA

TTAAGCATGACAGCATGATAAAATGAAGTTTTCCATCCAGTTTTCCCATAGAACATCAGTCTCCAATTTT

TCTTAACAAACAGTTTTACCGTTTGCATGTTACCACTATCAACCGCATAATACAATGCGGTGTTTCCTTT

GTCATCAAATTGTGAATCATCCATTCCACTGAATAGCAAAATCTTTACTATTTTGGTATCTTCTAATGTG

GCTGCCTGATGTAATGGAAATTCATTATCTAGAAGATTTTTCAATGCTCCAGCGTTCAACAACGTACATA

CTAGACGCACGTTATTATCAGCTATTGCATAATACAAGACACTATGACCATTGATATCCGCCTTAAATGC

ATCTTTGCTAGAGAGAAAGCTTTTCAGTTGCTTAGACTTCCAAGTATTAATTCGTGACAGATCCATGTCT

GAAACGAGACGCTAATTAGTGTATATTTTTTCATTTTTTATAATTTTGTCATATTGTACCAGAATTAATA

ATATCTCTAATAGATCTGATTAGTAGATATATGGCTATCGCAAAACAACATATACACATTTAATAAAAAT

AATATTCATTAAGAAGATTCAGATTCCACTGTACCCATCAATATAAAATAAAATAATTATTCCTTACATC

GTACCCATAAACAATATATTAAGTATATTCCACCTTACCCATAAACAATATAAATCCAGTAATATCATGT

CTAATGATGAACACAAATGGTGTATTAAATTCCAGTTCTTCAGGAGATGATCTCGCCGTAGCTACCATGA

TAGTAGATGCCTCCGCTACAGTTCCTTGTTCGTCTACATCTATCTTTACATTCTGAAACATTTTATAAAT

ATATAATGGGTCCCTAGTCATATGTTTAAACGACGCCTTATCTGGATTAAACATACTAGGAGCCATCATT

TCGGCTATCGACTTAATATCCCTCTTGTTTTCGATAGAAAATCTAGGGAGTTTAAGATTGTACATTTTAT

TCCCTAATTGAGATGACCAATATTCTAATTTTGCAGGCGTGATAGAATCTGTGAAATGGGTCATATTATC

ACCTATTGCCAGGTACATACTAATATTAGCATCCTTATACAGAAGGCGCACCATATCATATTCTTCGTCA

TCGATTGTGATTGTATTTCCTTGCAATTTAGTAACTACGTTCATCATGGGAACCGTTTTCGTACCGTACT

TATTAGTAAAACTAGCATTGTGTGTTTTAGTGATATCAAACGGATATTGCCACGTACCTTTAAAATATAT

AGTATTAATGATTGCCCATAGAGTATTATCGTCGAGCATAGTAGAATCAACTACATTAGACATACCAGAT

CTACGTTCTACTATAGAATTAATTTTATTAACCGCATCTCGTCTAAAGTTTAATCTATATAGGCCGAATC

TATGATATTGTTGATAATACGACGGTTTAATACACACAGTACTATCGACGAAACTTTGATACGTTAGATC

TGTGTACGTATATTTAGATGTTTTCATCTTAGCTAATCCGGATATTAATTCTGTAAATGCTGGACCCAGA

TCTCTTTTTCTCAAATTCATAGTATTCAATAATTCTACTCTAGTATTACCTGATGCAGACAATAGCGACA

TAAACATAGAAAACGAATACCCAAACGGTGAGAAGACAATATTATCATTATCATCCTCATCCCCATTTTG

AATATTTTTATACGCTAATATACCAGCATTGATAAATCCCTGCAGACGATATGCGGATACTGAACACGCT

AATGATAGTATCAATAACGCAATCATGATTTTTATGGTATTAATAATTAACCTTATTTTTATGTTTGGTA

TAAAAATTATTGATGTCTACACATCCTTTATAATCAACTCTAATCACTTTAACTTTTACAGTTTTCCCTA

CAAGTTTATCCCTATATTCAACATATCTATCCATATGCATCTCTTAACACTCTGCCAAGATAGCTTCAGA

GTGAGGATAGTCAAAAAGATAAATATATAGAGTATAATCATTCTCGTATACTCTGCCCTTTATTACATCG

CCCGCATTGGGCAACGAATAACAAAATGCAAGCATCGTGTTAACGGGCTCGTAAATTGGGATAAAATTAT

GTTTTTATTGTTTATCTATTTTATTCAAGAGAATATTCAGGAAGTTCCTTTTCTGGTTGTATCTCGTCGC

AGTATATATCATTTGTACATTGTTTCATATTTTTTAATAGTCTACACCTTTTAGTAGGACTAGTATCGTA

CAATTCATAGCTGTATTTTGAATTCCAATCACGTATAAAAATATCTTCCAATTGTTGACGAAGACCTAAT

CCATCATCCGGTGTAATATTAATAGATGCTCCACATATATCCGTAAAGTAATTTCCTGTCCAATTTGATG

TACCTATATACGCCGTTTTATCGGTTACCATATATTTTGCATGGTTTACCCTAGAATACGGAATGGGAGG

ATCAGCATCTGGTACAATAAATAGCTTTACTTCTATATCTATGTTTTTAGATTTTAGCATAGCTATAGAT

CTTAAAAAGTTTCTCATGATAAACGAAGATCGTTGCCAGCAACTAATCAATAGCTTAACGGATACTTGTC

TGTCTATAGCGGATCTTCTTAATTCATCTTCTATATAAGGCCAAAACAAAATTTTACCCGCCTTTGAATA

AATAATAGGAATAAAGTTCATAACAGATACATAAACGAATTTACTCGCATTTCCGATACATGACAATAAA

GCGGTTAAATCATTGGTTCTTTCCATAGTACATAATTGTTGTGGTGCAGAAGCAATAAATACAGAGTGTG

GAACACCGCTTACGTTAATACTAAGAGGATGATCTGTATTATAATACGACGGATAAAAGTTTTTCCAATT

ATATGGTAGATTGTTAACTCCAAGATACCAGTATACCTCAAAAATTTGAGTGAGATCCGCTGCCAAGTTC

CTATTATTGAAGATCGCAATACCCAATTCCTTGACCTGAGTTAGTGATCTCCAATCCATGTTAGCGCTTC

CTAAATAAATATGTGTATTATCAGATATCCAAAATTTTGTATGAAGAACTCCTCCTAGGATATTTGTAAT

ATCTATGTATCGTACTTCAACTCCGGCCATTTGTAGTCTTTCAACATCCTTTAATGGTTTGTTGGATTTA

TTGACGGCTACTCTAACTCTTACTCCTCTTTTGGGTAATTGTACAATCTCGTTTAATATTACCGTGCCGA

AATTCGTACCCACTTCATCCGATAAACTCCAATAAAAAGATGATATATCTAGTGTTTTTATGGTATTGGA

TAGAATTTCCCTCCACATGTTAAATGTAGTCAAATATACTTTATCAAATTGCATACCTATAGGAATAGTC

TCTGTAATCACTGCGATTGTATTATCCGGATTCATTTTATTTGTTAAAAAAATAATCCTATATCACTTCA

CTCTATTAAAAATCCAAGTTTCTATTTCTTTCATGACTGATTTTTTAACTTCATCCGTTTCCTTATGAAG

ATGATGTTTGGCGCCTTCATAAATTTTTATTTCCCTATTACAATTTGCATGTTGCATGAAATAATATGCA

CCTGAAACATCGCTAATCTCATTGTTTGTTCCCTGGAGTATGAGAGTCGGGGTGTTAATCTTGGGAATTA

TTTTTCTAACCTTGTTGGTAGCCTTCAAGACCTGACTAGCAAATCCAGCCTTAATTTTTTCATGATTGAC

TAATGGATCGTATTGGTATTTATAAACTTCATCCATATCTCTAGATACTGATTCTGGACATAGCTTTCCG

ACTGACGCATTTGGTGTAATGGTTCCCATAAGTTTTGCAGCTAGCAGATTCAGTCTTGGAACAGCGTCTG

CATTAACTAGAGGAGACATTAGAATCATTGCTGTAAACAAGTTTGGATTATCGCAAGCAGCTAGTATAGA

AATTGTTGCTCCCATGGAATGACCCAATAAGAAGACTGGAACTCCTGGATAAGTAGATTTAATAGTCACC

ACGTGCTGTACCACATCTCTAACATACTTACCAAAGTCATCAATCATCATTTTTTCACCATTACTTCTTC

CATGGCCAATATGATCATGTGAGAATACTAAAATTCCTAACGATGATATGTTTTCAGCTAGTTCGTCATA

ACGTCCAGAATGTTCACCAGCTCCATGACTTATGAATACTAATGCCTTAGGATATGTAATAGGTTTCCAA

TATTTACAATATATGTAATCATTGTCCAGATTGAACATACAGTTTGTACTCATGATTCACTATATAACTA

TCAATATTAACAGTTCGTTTAATGATCATATTATTTTTATGTTTTATTGATAATTGTAAAAATATACAAT

TAAATCAATATAGAGGAAGGAGACGGTACTGTATTTTGTGAGATAGTAATGGAGACTAAATCAGATTATG

AGGATGCTGTTTTTTACTTTGTGGATGATGATGAAATATGTAGTCGCGACTCCATCATTGATCTAATAGA

TGAATATATCACGTGGAGAAATCATGTTATAGTGTTTAATAAAGATATTACCAGTTGTGGAAGACTGTAC

AAGGAATTGATAAAGTTCGATGATGTCGCTATACGGTACTATGGTATTGATAAAATTAATGAGATTGTCG

AGGCTATGAGTGAAGGAGACCACTACATCAATCTTACAGAAGTCCATGATCAGGAAAGTCTATTCGCTAC

CATAGGAATATGTGCTAAAATCACTGAACATTGGGGATACAAAAAGATTTCAGAATCTAAATTCCAATCA

TTGGGAAACATTACAGATCTGATGACCGACGATAATATAAACATCTTGATACTTTTTCTAGAAAAAAAAA

TGAATTGATGATATAAGTGTCTTCATAACGCATTATTACGTTAGCATTCTATTATCCAGTGTTAAAAAAA

TTATCCTATCATGTATTTGAGAGTCTTATATGTAGCAAACATGATAACTGCAATACCCATAATCTTTAGA

TATTCACGCGTGCTATGGATGGCATTATCCCGCGGTGCGGAAATGTACGTTATATAATCTACAAAATAAT

CATCGCATATAGTATGAGATAGTAGAGTAAACATTTTTATCGTTTCTACTGGGTTCATACATCGTCTACC

CAATTCGGTAATGAATGAAATTGTCGCCAATCTTACACCCAAACCCTTGTTGTTCATTAGTATAGTATTA

ACTTCATTATTTATGTCATAAACTGTAAATGATTCTGTAGATGCCATATCACACATGATATTCATGTCAC

TATTATAATCATTATTAACTTTATCACAATACGTGTTGATAATATCTACATATGATCTAGTTTTTGTGGG

TAATTGTCTATACAAGTCGTCTAAACGTTGTTTACTCATATAGTATTGAACAGCCATCATTACATGGTCC

CGTTCCGTTGATAGATAATCGAGTATGTTAGTAGACTTGTCAAATCTATATACCATATTTTCTGGAAGCG

GATATACATAGTCGCGATCATCATTATCACTAGCCTCATCCTCTATATCATGTACATGTACATAATCTAT

GATATTATTATACATAAACATCGACAACATACTATTGTCTATTATCTAAGTCCTGTTGATCCAAACCCTT

GATCTCCTCTATCTGTACTATCTAGAGATTGTACTTCTTCAAGTTCTGGATAATATATACGTTGATAGAT

TAGCTGAGCTATTCTATCTCCAGTATTTACATTAAACGTACATTTTCCATTATTAATAAGAATGACTCCT

ATGCTTCCCCTATAATCTTCGTCTATTACACCGCCTCCTATATCAATGCCTTTTAGGGACAGACCAGACC

TAGGAGCTATTCTACCATAGCAGAACTTAGGCATGGACATACTAATATCTGTCTTAATTAACTGTCGTTC

TCCAGGAGGGATAGTATAATCGTAAGCGCTATACAAATCATATCCGGCAGCACCCGGCGATTGCCTAGTA

GGTGATTTAGCTCTGTTAGTTTCCTTAACAAATCTAACTGGTGAGTTAATATTCATGTTGAACATAAAAA

ATATCATTTTATTTCAAAATTATTTACCATTCCATTCCATTCCATCCCATATATTCCATGAATAAGTGCG

ATTATTGTACACTTCTATAGTATCTATATACGATCCACGATAAAATCCTCCTATCAATAGCAGTTTATTA

TCCACTATGATCAATTCTGGATTATCCCTCGGATAAATAGGATCATCTATCAGAGTCCATGTATTGCTGG

ATTCACAATAAAATTCCGCATTTCTACCAACCAAGAATAACCTTCTACCAAACACTAACGCACATGATTT

ATAATGAGGATAATAAGTGGATGGTCCAAACTGCCACTGATCATGATTGGGTAGCAAATATTCTGTAGTT

GTATCAGTTTCAGAATGTCCTCCCATTACGTATATAACATTGTTTATGGATGCCACTGCTGGATTACATC

TAGGTTTCAGAAGACTCGGCATATTAACCCAAGCAGCATCCCCGTGGAACCAACGCTCAACAGATGTGGG

ATTTGGTAGACCTCCTACTACGTATAATTTATTGTTAGCGGGTATCCCGCTAGCATACAGTCTGGGGCTA

TTCATCGGAGGAATTGGAATCCAATTGTTTGATATATAATTTACCGCTATAGCATTGTTATGTATTTCAT

TGTTCATCCATCCACCGATGAGATATACTACTTCTCCAACATGAGTACTTGTACACATATGGAATATATC

TATAATTTGATCCATGTTCATAGGATACTCTATGAATGGATACTTGTATGATTTGCGTGGTTGTTTATCA

CAATGAAATATTATGTTACAGTCTAGTATCCATTTTACATTATGTATACCTCTGGGAGAAAGATAATTTG

ACCTGATTACATTTTTGATAAGAAGTAGCAGATTTCCTAATCTATTTCTTCGCCTCATATACCACTTAAT

GACAAAATCAACTACATAATCCTCATCTGGAACATTTAGTTCGTCGCTTTCTAGAATAAGTTTCATAGAT

AGATAATCAAAATTGTCTATGATGTCATCTTCCAGTTCCAAAAAGTGTTTGGTAATAAAGTCTTTAGTAT

GACATAAGAGATTGGATAGTCCGTATTCTATACCCATCATGTAACACTCGATACAATATTCCTTTCTAAA

ATCTCGTAGGATAAAGTTTATACAAGTGTAGATGATAAATTCTACAGATGTTAATATAGAAGCACGTAAT

AAATTGACGACGTTATGACTATCTATATATACCTTTCCAGTATATGAGTAAATAACTATAGAAGTTAGAC

TGTGAATGTCAAGGTCTAGACAAACCCTCGTAACTGGATCTTTATTTTTTGTGTATTTTTGGCGTAAATG

TGTGCGAAAGTATGGAGATAACTTTTTCAATATCGTAGAATTGACTATTATATTACCTCCTATAGCTTCA

ATAATTGTTTTGAATTTCTTAGTCGTGTACAATGCTAATATATTCTTACAGTACACAGTATTGACAAATA

TCGGCATTTATGTTTCTTTAAAAGTCAACATCTAAAGAAAAATGATTGTCTTCTTGAGACATAACTCCCA

TTTTTTGGTATTCACCCACACGTTTTTCGAAAAAATTAGTTTTTCCTTCCAATGATATATTTTCCATGAA

ATCAAACGGATTGGTAACATTGTAAATTTTTTTAAATCCCAATTCAGAAATCAATCTATCTGCGACGAAT

TCTATATATGTTTTCATCATTTCACAATTCATTCCTATGAGTTTAACTGGAAGAGCCACAGTAAGAAATT

CTTGTTCAATGGATACCGCATTTGTTATAATAAATCTAACGGTTTCTTCACTCGGTGGATGCAATAAATG

TTTAAACATCAAACATGCGAAATCGCAGTGCAGACCCTCGTCTCTACTAATTAATTCGTTAGAAAACGTG

AGTCCGGGCATTAGGCCACGCTTTTTAAGCCAAAATATGGAAGCGAATGATCCGGAAAAGAAGATTCCTT

CTACTGCAGCAAAGGCAATAAGTCTCTCTCCATAACCGGCGCTGTCATGTATCCACTTTTGAGCCCAATC

GGCCTTCTTTTTTACACAAGGCATCGTTTCTATGGCATTAAAGAGGTAGTTTTTTTCATTACTATCTTTA

ACATAAGTATCGATCAAAAGACTATACATTTCCGAATGAATGTTTTCAATGGCCATCTGAAATCCGTAGA

AACATCTAGCCTCGGTAATCTGCACTTCTGTACAAAATCGTTCTGCTAAATTTTCATTCACTATTCCGTC

ACTGGCTGCAAAAAACGCCAATACATGTTTTATAAAATATTTTTCGTCTGGTGTTAGTTTATTCCAGTCA

TTGATATCTTTAGATATATCCACTTCTTCCACTGTCCAAAATGATGCCTCTGCCTTTTTATACATATTCC

AGATGTCATGATATTGGATTGGGAAAATAACAAATCTATTTGGATTTGGTGCAAGGATAGGTTCCATAAC

TAAATTAACAATAGTAGTAATTTTTTTTCAGTTATCTGTATGACTGTACTTGGATCTTTTGTATATCGCT

ATCGCCGCAATCACTACAATAATTACAAGTATTATTGATAGCATTGTTATTACTACTATCATAATTAAAT

TATCGACATTCATGGGTGTTGAATAATCGTTATCATCATTTTGTAATTGTGACGTCATACTAGATAAATC

ATTTGTGAGATTGTTGTGGGAAGCGGGCACGGAAGATGCATTATCATTATTATTTAACGCCTCCCATTCG

GATTCACAAATGTTACGCACGTTCAAAGTTTTATGGAAACTATAATTTTGTGAAAACAGATAACAAGAAA

ACTCGTCATCGTTCAAATTTTTAACGATAGTAAACCGATTAAACGTCGAGCTAATTTCTAACGCTAGCGA

CTCTGTTGGATATGGGTTTCCAGATATATATCTTTTCAGTTCCCCTACGTATCTATAATCATCTGTAGGA

AATGGAAGATATTTCCATTTATCTACTGTTCCTAATATCATATGCGGTGGTGTAGAACCATTAAGCGCGA

AAGATGTTATTTCGCATCGTATTTTAACTTCGCAATAATTTCTGGTTAGATAACGCACTCTACCAGTCAA

GTCAATGATATTAGCCTTTACAGATATATTCATAGTAGTCGTAACGATGACTCCATCTTTTAGATGTGAT

ACTCCTTTGTATGTACCAGAATCTTCGTACCTCAAACTCGATATATTTAAACAAGTTAATGATATATTAA

CGCGTTTTATGAATGATGATATATAACCAGAAGTTTTATCCTCTGTGGCTAGCGCTATAACCTTATCATT

ATAATACCAACTAGTGTAATTAATATGTGACATGACAGTGTGGGTACAAATATGTACATTATCGTCTACG

TCGTATTTGATACATCCGCATTCAGCCAACAAATATAAAATTACAAAAACTCTAACGACGTTCGTACACA

TCTTGATGCGGTTTAATAAATGTTTTGATTTCAATTTATTGTAAAAAAAGATTCGGTTTTATACTGTTCG

ATATTCTCATTGCTTATATTCTCATCTATCATCTCCACACAGTCAAATCCATGGTTAACATGTACCTCAT

CAACCGGTAAAAGACTATCGGATTCTTCTATCATCATAACTCGAGAATATTTAATTTGGTGGTCATTATT

AATCAAGTCAATTATCTTATTTTTAACAAACGTAAGTATTTTACTCATTTTTTTATAAAAACTTTTAGAA

ATATACAGACTCTATCGTGTGTCTATATCTTCTTTTTATATCCAATGTATTTATGTCTGATTTTTCTTCA

TTTATCATATATAATGGTCCAAATTCTACACGTGCTTCGGATTCATCCAGATCATTAAGGTTCTTATAAT

CGCAACATCCTTCTCTTCCATCTTCTACATCTTCCTTCTTATTCTTAGCGTCACAGAATCTACCACAGCA

GGATCCCATGACGAGAGTCACATTAAACTAATTCATTTTCAATTATAATATACTGATTAGTAATGACAAT

TAAAATAAAAATATTCTTCATAACCGGTAAGAAAGTAAAAAGTTCACATTGAAACTATGTCAGTAGTTAT

ACATCATGAGATGATATACTCTATTTTGGTGGAGGATTATATGATATAATTCGTGGATAATCATTCTTAA

GACACATTTCTTCATTCGTAAATCTTTTCACATTAAATGAGTGTCCATATTTTGCAATTTCTTCATATGA

TGGCGGTGTACGTGGACGAGGCTGCTCCTGTTCTTGTAGTCGTCGACTGTCGTGTTTGCGTTTAGATCCC

TCCATTATCGCGATCGCGTAGTGAGTACTATTTATACCTTGTAATTAAATTTTTTTATTAATTAAACGTA

TAAAAACGTTCCGTATCTGTATTTAAGAGCCAGATTTCGTCTAATAGAACAAATAGCTACAGTAAAAATA

ACTAGAATAATCGCTACACCCACTAGAAACCACGGATCGTAATACGGCAATCGGTTTTCGATAATAGGTG

GAACGTATATTTTATTTAAGGACTTAACAATTGTCTGTAAACCACAATTTGCTTCCGCCGATCCTGTATT

AACTATCTGTAAAAGCATATGTTGACCGGGAGGAGCCGAACATTCTCCGATATTCAATTTTTGTATATCT

ATAATGTTATTAACCTCCGCATACGCATTACAGTTCTTTTCTAGCTTGGATACTACACTAGGTACATCAT

CTAAATCTATTCCTATTTCCTCAGCGATAGCTCTTCTATCCTTTTCCGGAAGTAATGAAATCACTTCAAT

AAATGATTCAACCATGAGTGTGAAACTAAGTCGAGAATTACTCATGCATTTGTTAGTTATTCGGAGCGCG

CAATTTTTAAACTGTCCTATAACCTCTCCTATATGAATAGCACAAGTGACATTAGTAGGGATAGAATGTT

GAGCTAATTTTTGTAAATAACTATCTATAAAAAGATTATACAAAGTTTTAAACTCTTTAGTTTCTGCCAT

TTATCCAGTCTGAGAAAATGTCTCTCATAATAAATTTTTCCAAGAAACTAATTGGGTGAAGAATGGAAAC

CTTTAATCTATATTTATCACAGTCTGTTTTGGTACACATGATGAATTCTTCTAATGCTGTACTAAATTCG

ATATCTTTTTCGATTTCTGGATATGTTTTTAATAAAGTATGAACAAAGAAATGGAAATCGTAATACCAGT

TATGTTCAACTTTGAAATTGTTTTTTATTTTCTTGTTAATGATTCCAGCCACTTGGGAAAAGTCAAAGTC

GTTTAATGCCGATTTAATACGTTCATTAAAAACAAACTTTTTATTCTTTAGATGAATTATTATTGGTTCA

TTGGAATCAAAAAGTAAGATATTATCGGGTTTAAGATCTGCATGTAAAAAGTTGTCACAACAGGGTAGTT

CGTAGATTTTAATGTATAACAGAGACATCTGTAAAAAGATAAACTTTATGTATTGTACCAAAGATTTAAA

TCCTAATTTGATAGCTAACTCGGTATCTACTTTATCTGCCGAATACAGTGCTAGGGGAAAAATTATAATA

TTTCCTCTTTCGTATTCGTAATTAGTTCTCTTTTCATGTTCGAAAAAGTGAAACATGCGGTTAAAATAGT

TTATAACATTAATATTACTGTTAATAACTGCCGGATAAAAGTGGGATAGTAATTTCACGAATTTGATACT

GTCCTTTCTCTCGTTAAACGCCTTTAGAAAAACTTTAGAAGAATATCTCAATGAGAGTTCCTGACCATCC

ATAGTTTGTATCAATAATAGCAACATATGAAGAACCCGCTTATACAGAGTATGTAAAAATGTTAATTTAT

AGTTTAATCCCATGGCCCACGCACACACGATTAATTTTTTTTCATCTCCCTTTAGATTGTTGTATAGAAA

TTTGGGTACTGTAAACTCCGCCGTAGTTTCCATGGGACTATATAATTTTGTGGCCTCGAATACAAATTTT

ACTACATAGTTATCTATCTTAAAGACTATACCATATCCTCCTGTAGATATGTGATAAAAATCGTCGTTTA

TAGGATAAAATCGTTTATCTTTTTGTTGGAAAAAGGATGAATTAATGTAATCATTCTCTTCTATCTTTAG

TAGTGTTTCCTTATTAAAATTCTTAAAATAATTTAACAATCTAACTGACGGAGCCCAATTTTGGTGTAAA

TCTAATTGGGACATTATGTTGTTAAAATATAAACAGTCTCCTAATATAACAGTATCTGATAATCTATGGG

GAGACATCCATTGATATTCAGGGGATGAATCATTGGCAACACCCATTTATTGTACAAAAAGCCCCAATTT

ACAAACGAAAGTCCAGGTTTGATAGAGATAAACTATTAACTATTTTGTCTCTGTTTTTAACACCTCCACA

GTTTTTAATTTCTTTGGTAATGAAATTATTCACAATATCAGTATCTTCTTTATCTACCAGAGATTTTACT

AACTTGATAACCTTGGCTGTCTCATTCAATAGGGTAGTGATATTTGTATGTATGATATTGATATCTTTTT

GAATTGTTTCTTTTAGAAGTGATTCTTTGATGGTATCAGCATACGAATTACAATAATGCAGAAACTCAGT

TAACATGCAGGAATTATAGTAAGCCAATTCCAATTGTTGCCTGTATTGTATTAGAGTATTAATATGCGCA

ATGATGTCCTTGCGTTTCTCTGATAGAATGCGAGCAGCGATTTTGGCGTTATCATTTGACGATATTTCTG

GAATGACGAATCCTGTTTCTACTAACTTCTTGGTAGGACAAAGTGAAACAATCAAGAAAATAGCTTCTCC

TCCTATTTGTGGAAGAAATTGAACTCCTCTAGATGATCTACTGACGATAGTATCTCCTTGACAGATATTG

GACCGAACTACGGAAGTACCTGGAATGTAAAGCCCTGAAACCCCCTCATTTTTTAAGCAGATTGTTGCCG

TAAATCCTGCACTATGCCCAAGATAGAGAGCTCCTTTGGTGAATCCATCACTATGTTTCAGTTTAACCAA

GAAACAGTCAGCTGGTCTAAAATTTCCATCTCTATCTAATACAGAATCCAACTTGATGTCAGGGACTATG

ACCGGTTTAATGTTATATGTAACATTGAGTAAATCCTTAAGTTCATAATCATCGTTGTCATCAGTTATGT

ACGATCCAAACAATGTTTCTACCGGCATGGTGGATACGAAGATGCTATCCATCAGAATGTTTCCCTGATT

AGTATTTTCTATATAGCTATTCTTCTTTAAACGATTTTCCGAATCAGTAACTATGTTCATTTTTTTAGGA

GTAGGACGTCTAGCCAGTATGGAAGAGGATTTTCTAGATACTCTCTTCAACATCTTTGATCTCAATGGAA

TGCAAAACCCCATGGTGTAACAACCAACGATAAAAATAATATTGTTTTTTCACTTTTTATAATTTTACCA

TCTGACTCATGGATTCATTAATATCTTTACAAGAGCTACTAACGTATAATTCTTTATAACTGAACTGAGA

TATATACACCGGATCTATGGTTTCCATAATTGAGTAAATGAATGCTCGGCAATAACTAATGGCAAATGTA

TAGAACAACGAAATTATACTAGAGTTGTTAAAGTTAATATTTTCTATGAGTTGTTCCAATAAATTATTTG

TTGTGACTGCGTTCAAGTCATAAATTATCTTGATACTATCCAGTAAACAGTCTTTAAGTTCTGGAATATT

ATCATCCCATTGTAAAGCCCCTAGTTCGACTATCGAATATCCTGCTCTGATAGCAGTTTCAATATCGACG

GACGTCAATACTGTAATAAAGGTGGTAGTATTGTCATCATCGTGATAAACTACGGGAATATGGTCGTTAG

TAGGTACCGTGACTTTACACAACGCGATATATAACTTTCCTTTTGTACCATTTTTAACGTAGTTGGGACG

TCCTGCAGGGTATTGTTTTGAAGAAATGATATCGAGAACAGATTTGATACGATATTTGTTGGATTCCTGA

TTATTCACTATAATATAATCTAGACAGATAGATGATTCGATAAATAGAGAAGGTATATCGTTGGTAGGAT

AATACATCCCCATTCCAGTATTCTCGGATACTCTATTGATGACACTAGTTAAGAACATGTCTTCTATTCT

AGAAAACGAAAACATCCTACATGGACTCATTAAAACTTCTAACGCTCCTGATTGTGTTTCGAATGCCTCG

TACAAGGATTTCAAGGATGCCATAGATTCTTTGACCAACGATTTAGTATTGCGTTTAGCATCTGATTTTT

TTATTAAATCAAATGGTCGGCTCTCTGGTTTACTACCCCAATGATAACAATAGTCTTGTAAAGATAAACC

GCAAGAAAATTTATACGCATCCATCCAAATAACCCTAGCACCGTCGGATGATATTAATGTATTATTATAG

ATTTTCCATCCACAGTTATTGGGCCAGTATACTGTTAGCAACGGTATATCGAATAGATTACTCATGTAAC

CTACTAGAATGATAGTTCGTGTACTAGTCATAATATCTTTAATCCAATCTAAGAAATCTAAAATTAGATC

TTTTACACTATTAAAGTTAACAAAGGTATTACCCGGGTACGTGGATATCATATATGGTATTGGTCCATTA

TCAGTAATGGCTCCATAAACTGATACGGCGATGGTTTTTATATGTGTTTGATCTAATGAGGACGAAATTC

GCGCCCACAATTCATCTCTAGATATGCATTTAATATCGAACGGTAACACATCAATCTCGGGACGCGTATA

TGTTTCTAAATTCTTAATCCAAATATAATGATGACCTATATGCCCTATTATCATACTGTCAACTATAGTA

TACCTAGAGAACTTTCGATACATCTGCTGTTTCCTGTAATCGTTAAATTTTACAAATCTATAACATGCTA

AACCTTTTGACGACAGCCATTCATTAATTTCTGATATGGAATCTGTATTCTCAATACCGTATCGTTCTAA

AGCCAGTGCTATATCTCCCTGTTCGTGGGAACGCTTTCGTATAATATCGATCAATGGATAATATGAAGTT

TTTGGAGAATAATATGATTCATGATCTATTTCGTCCATAAACAATCTAGACATAGGAATTGGAGGCGATG

ATCTTAATTTTGTGCAATGGGTCAATCCTATAACTTCTAATATTGTAATATTCATCATCGACATAACACT

ATCTATGTTATCATCGTATATTAGTATACCACGACCTTCTTCATTTCGTGCCAAAATGATATACAGTCTT

AAATAATTACGCAATATCTCAATAGTTTCATAATTGTTAGCTGTTTTCATCAAGGTTTGTATCCTGTTTA

ACATGATGGCGTTCTATAACGTCTCTATTTTCTATTTTTAATTTTTTTAAATTTTTAACGATTTACTGTG

GCTAGATACCCAATCTCTCTCAAATATTTTTTTAGCCTCGCTTACAAGCTGTTTATCTATACTATTAAAA

CTGACGAATCCGTGATTTTGGTAATGGGTTCCGTCGAAATTTGCCGAAGTGATATGAACATATTCGTCGT

CGACTATTAACAATTTTGTATTATTCTGAATAGTGAAAACCTTCACAGATAGATCATTTTGAACACACAA

CGCATCTAGACTTCTGGCGGTTGCCATAGAATATACGTCGTTCTTATCCCAATTACCAACTAGAAGTCTG

ATCTTAACTCCTCTATTAATGGCTGCTTCTATAATGGAGTTGTAAATGTCAGGCCAATAGTAGCTATTAC

CGTCGACACGTGTAGTGGGAACTATGGCCAAATGTTCAATATCTATACTAGTCTTAGCCGACTTGAGTTT

ATCAATAACTACATCAGTGTCTAGATCTCTAGAATATCCCAATAGGTGTTCTGGAGAATCAGTAAAGAAC

ACTCCACCTATAGGATTCTTAATATGATACGCAGTGCTAACTGGCAGACAACAAGCCGCAGAGCATAAAT

TCAACCATGAATTTTTTGCGCTATTAAAGGCTTTAAAAGTATCAAATCTTCTACGAAGATCTGTGGCCAG

CGGAGGATAATCAGAATATACGCCTAACGTTTTAATCGTATGTATAGATCCTCCAGTAAATGACGCGTTT

CCTACATAACATCTTTCATCATCAGACACCCAAAAACAACCGAGTAGTAGTCCCACATTATTTTTTTTAT

CTATATTAACGGTTATAAAATTTATATCCGGGGAGTGACTTTGTAGCTCTCCCAGATTTCTTTTCCCTCG

TTCATCTAGCAAAACTATTATTTTAATCCCTTTTTCAGATACCTCTTTTAGTTTATCAAAAATAAGCGCT

CCCCTAGTAGTACTCAGAGGATTACAACAAAAAGATGCTATGTATATATATTTCTTAGCTAGAGTGATAA

TTTCGTTAAAACATTCAAATGTTGTCAAATGATCGGATCTAAAATCCATATTTTCTGGTAGTGTTTCTAC

CAGCCTACATTTTGCTCCCGCAGGTACCGATGCAAATGGCCACATTTAGTTAACATAAAAACTTATATAT

CCTGTTCTATCAACGATTCTAGAATATCATCGGCTATATCGCTAAAATTTTCATCAAAGTCGACATCACA

ACCTAACTCAGTCAATATATTAAGAAGTTCCATGATGTCATCTTCGTCTATTTCTATATCCGTATCCATT

GTAGATTGTTGACCGATTATCGAGTTTAAATCATTACTAATACTCAATCCTTCAGAATACAATCTGTGTT

TCATTGTAAATTTATAGGCGGTGTATTTAAGTTGGTAGATTTTCAATTATGTATCAATATAGCAACAGTA

GTTCTTGCTCCTCCTTGATTTTAGCATCCTCTTCATTATTTTCTTCTACGTACATAATCATGTCTAATAC

GTTAGACAACACACCGACGATGGTGGCCGCCACAGACACGAATATGACTAGACCGATGACCATTTAAAAA

ATACTCTCTAGCTTTAACTTAAACTGTATCGATCATTCTTTTAGCACATGTATAATATAAAAACATTATT

CTATTTCGAATTTAGGCTTCCAAAAATTTTTCATCCGTAAACCGATAATAATATATATAGACTTGTTAAT

AGTCGGAATAAATATATTAATGCTTAAACTATCATCATCTCCACGATTAGAGATACAATATTTACATTCT

TTTTGCTGTTTCGAAACTTTATCAATACACGTTAATACAAACCCAGGAAGGAGATATTGAAACTGAGGCT

GTTGAAAATGAAACGGCGAATACAATAATTCAGATAATGTAAAATCATGATTCCGTATTCTGATGATATT

AGAACTGCTAATGGATGTCGATGGTATGTATCTAGGAGTATCTATTTTAACAAAGCATCGATTTGCTAAT

ATACAATTATCCTTTTGATTAATTGTTATTTTATTCATATTCTTAAAAGGTTTCATATTTATCAATTCTT

CTACATTAAAAATTTCCATTTTTAATTTATCTAGCCCCGCAATACTCCTCATTACGTTTCATTTTTTGTC

TAGAATACCCATTTTGTTCATCTTGGTACATAGATTATCCAATTGAGAAGCGCATTTAGTAGTTTTGTAC

ATTTTAAGTTTATTAACGAATCGTCGAAAACTAGTTATAGTTAACATTTTATTATTTGATACCCTGATAT

TAATACCCCTGCCGTTACTATTATTTATAACTGATGTAACCCACGTAACATTGGAATTAATTATCGATAG

TAATGCATCGACACTTCCAAAATTGTCTATTATAAACTCACCGATAATTTTTTTATTGCATGTTTTCATA

TTCATTAGGATTATCAAATCTTTAATCTTATTACGATTGTATGCGTTGATATTACAAGACGTCATTCTAA

AAGACGGAGGATTTCCATCAAATGCCAGACAATCACGTACAAAGTACATGGAAATAGGTTTTGTTCTATT

ACGCATCATAGATTCATATAAAACACCCGTAGAAATACTAATTTGTTTTACTCTATAAAATACTATTGCA

TCTATTTCATCGTTTTGTATAACGTCTTTCCAAGTGTCAAATTCCAATTTTTTTTCATTGATAGTACCAA

ATTCTTCTATCTCTTTAACTACTTGCATAGATAGGTAATTACAGTGATGCCTACATGCCGTTTTTTGAAA

CTGAATAGATGCATCTAGAAGCGATGCTACACTAGTCACGATCACCACTTTCATATTTAGAATATATGTA

TGTAAAAATATAGTAGAATTTCATTTTGTTTTTTTCTATGCTATAAATGAATTCTCATTTTGTATCCGCA

CATACTCCGTTTTATATCAATACCAAAGAAGGAAGATATCTGGTTCTAAAAGCCGTTAAAGTATGCGATG

TTAGAACTGTAGAATGCGAAGGAAGTAAAGCTTCCTGCGTACTCAAAGTAGATAAACCCTCATCACCCAC

GTGTGAGAGAAGACCTTCGTCCCCGTCCAGATGCGAGAGAATGAATAACCCTGGAAAACAAGTCCCGTTT

ATGAGAACGGACATGTTACAAAATATGTTTGCTGCTAATCGCGACAACGTAACGTCAAGACTTTTGAACT

AAAATACAATTATATCTTTTTCGATATTAATAAATCCGTGTCTCCCGGGTTTTTTATCTCTTTCAGTATG

TGAATAGATAGGTATTTTATCTCTATTCATCATCGAATTTAAGAGATCCGATAAACATTGTTTGTATTCT

CCAGATGTCAGCATCTGATACAACAATATATGTGCACATAAACCTCTGGCACTTATTTCATGTACCTTCC

CCTTATCACTAAGGAGAATAGTATTTGAGAAATATGTATACATGATATTATCATGTATTAGATATACAGA

ATTTGTAACACTCTCGAAATCACACGATGTGTCGGCGTTAAGATCTAATATATCACTCGATAACACATTT

TCATCTAGATACACTAGACATTTTTTAAAGCTAAAATAGTCTTTAGTAGTAACAGTAACTATGCGATTAT

TTTCATCGATGATACATTTCATCGGCATATTATTACGCGTACCATCAAAGACTATACCATGTGTATATCT

AACGTATTCTAGCATAGTTGCCATACGTACATTAAACTTTTCAGGATCTTTGGATAGATCTTCCAATCTA

TCTATTTGAGAAAACATTTTTATCATGTTCAATAGTTGAAACGTCGGATCCACTATATAGATATTATCTA

TAAAGATTTTAGGAACTATGTTCATGGTATCCTGGCGAATATTAAAACTATCAATGATATGATTATCGTT

TTCATCTTTTATCACCATATAGTTTCTAAGATATGGGATTTTACTTAATATAATATTATTTCCCGTAATA

AATTTTATTAGAAATGCCAAATCTATAAGAAAAGTCCTAGAATTAGTCTGAAGAATATCTATATCACCGT

ACCGTATATTTGGATTAATTAGATATAGAGAATATGATCCGTAACATATACAACTTTTATTATGACGTCT

AAGATATTCTTCCATCAACTTATTAACATTTTTGACTAGGGAAGATACATTATGACGTCCCATTACTTTT

GCCTTGTCTATTACAGCGACGTTCATAGAATTTAGCATATCTCTTGCCAATTCTTCCATTGATGTTACAT

TATAAGAAATTTTAGATGAAATTACATTTGGAGCTTTAATAGTAAGAACTCCTAATATATCCGTGTATGT

GGTCACTAATACAGATTGTAGTTCTATAATCGTAAATAATTTACCTATATTATATGTTTGAGTTTGTTTA

GAAAAGTAGCTAAGTATACGATCTTTTATTTCTGATGCCGATGTATCAACATCGAAAAAAAATCTTTTTT

TATTCTTTTTTACTAACGATACGAATATGTCTTTGTTAAAAACAGTTATTTTCTGAATATTTCTAGCTTG

TAATTTTAACATATGATATTCGTTCACACTAGGTACTCTGCCTAAATAGGTTTCTATAATCTTTAATGTA

ATATTAGGAAGAGTATTCTGATCAGGATTCCTATTCATTTTGAGGATTTAAAACTCTGATTATTGTCTAA

TATGGTCTCAACACAAACTTTTTCACAGAGTGATAGAGTTTTTGATAACTCGTTTTTCTTAAGAAATATA

AAACTACTGTCTCCAGAGCTCGCTCTATCTTTTATTTTATCTAATTCGATACAAACTCCTGATACTGGTT

CAGAAAGTAATTCATTAATTTTCAGTCCTTTATAGAAGATATTTAATATAGATAATACAAAATCTTCAGT

TCTTGATATCGATCTGATTGATCCTAGAACTAGATATATTAATAACGTGCTCATTAGGCAGTTTATGGCA

GCTTGATAATTAGATATAGTATATTCCAGTTCATATTTATTAGATACCGCATTGCCCAGATTTTGATATT

CTATGAATTCCTCTGAAAATAAATCCAAAATAACTAGACATTCTATTTTTTGTGGATTAGTGTACTCTCT

TCCCTCTATCATGTTCACTACTGGTGTCCACAATGATAAATATCTAGAGGGAATATAATATAGTCCATAT

GATGCCAATCTAGCAATGTCGAATAACTGTAATTTTATTCTTCGCTCTTCATTATGAATTGAATCTTGAG

GTATAAACCTAACACAAATTATATCATTAGACTTTTCGTATGTAATGTCTTTCATGTTATAAGTTTTTAA

TCCTGGAATAGAATCTATTTTAATGAGGCTTTTAAACGCAGCGTTCTCCAACGAGTCAAAGCATAATACT

CTGTTGGTTTTCTTATATTCAATATTACGATTTTCTTCTTTGAATGGAATAGGTTTTTGAATTAGTTTAT

AATTACAACATAATAGATAAGGAAGTGTGTAAATAGTACGCGGAAAAAACATAATAGCTCCCCTGTTTTC

ATCCATGGTTTTAAGTAAATGATCACTGGCTTCTTTAGTCAATGGATATTCGAACATTAACCGTTTCATC

ATCATTGGACAGAATCCATATTTCTTAATGTAAAGAGTGATCAAATCATTGTGTTTATTGTACCATCTTG

TTGTAAATGTGTATTCGGTTATCGGATCTGCTCCTTTTTCTATTAAAGTATCGATATCGATCTCGTCTAA

GAATTCAACTATATCGACATATTTCATTTGTATACACATAACCATTACTAACGTAGAATGTATAGGAAGA

GATGTAACGGGAACAGGGTTTGTTGATTCGCAAACTATTCTAATACATAATTCTTCTGTTAATACGTCTT

GCACGTAATCTATTATAGATGCCAAGATATCTATATAATTATTTTGTAAGATGATGTTAACAATGTGATC

TATATAAGTAGTGTAATAATTCATGTATTTCGATATATGTTCCAACTCTGTCTTTGTGATGTCTAGTTTC

GTAATATCTATAGCGTCCTCAAAAAATATATTCGCATATATTCCCAAGTCTTCAGTTCTATCTTCTAAAA

AATCTTCAACGTATGGAATATAATAATCTATTTTACCTCTTCTGATGTCATTAATGATATAGTTTTTGAC

ACTATTTTCCGTCAATTGATTCTTATTCACTATGTCTAAAAACCGGATAGCGTCCCTAGGACGAACTACT

GCCATTAATATCTCTATTATAGCTTCTGGACATAAATCATCTATTATACCAGAATTAATGGGAACTATTC

CGTATCTATCTAACATAGTTTTAAGAAAGTCAGAATCTAAGACCTGATGTTCATATATTGGTTCATACAT

GAAATGATCTCTATTGATGATAGTGACTATTTCATTCTCTGAAAATTGGTAACTCATTCTATACACGCTT

TCCTTGTTGATAAAGGATAGTATATACTCAATGGAATTTGTACCAACAAACTGTTCTCTTATGAATCGTA

TATCATCATCTGAAATGATCATGTAAGGCATACATTTAACAATAAGAGACTTGTCTCCTGTTATCAATAT

ACTATTCTTGTGATAATTTATGTGTGCGGCAAATTTGTCCACGTTCTTTAATTTTGTTATAGTAGATATC

AAATCCAATGGAGATACAGTTCTTGGCTTAAACAGATATAGTTTTTCTGGAACGAATTCTACAACATTAT

TATAAAGGACTTTGGGTATATAAGTGGGATGAAATCCTATTTTAATTAATGCGATAGCCTTGTCCTCGTG

CAGATATCCAAACGCTTTTGTGATAGTATGGCATTCATTGTCTAGAAACGCTCTACGAATATCTGTAACA

GATATCATCTTTAGAGAATACTAGTCGCGTTAATAGTACTAAAATTTGTATTTTTTAATCTATCTCAATA

AAAAATTAATATGTATGATTCAATGTATAACTAAACTACTAACTGTTATTGATAACTAGAATCAGAATCT

AATGATGACATAACTAAGAAGTTTATCTACAGCCAATTTAGCTGCATTATTTTTAGCATCTCGTTTAGAT

TTTCCATCTGCCTTATCGAATACTCTTCCGTCAATGTCTACACAGGCATAAAATGTAGGAGAGTTACTAG

GCCCCACTGATTCAATACGAAAAGACCAATCTCTCCTAGTTATTTGACAGTACTCATTAATAACGGTGAC

AGGGTTAACACCTTTCCAATAAATAATTTTTTTAACCGGAATAACATCATCAAAAGACTTATGATCCTCT

CTCATTGATTTTTCGCGGGATACATCATCTATTATAGCATCAGCATCAGAATCTGTAGGCCGTGTATCAG

CATCCATTGTCGTAGACCAACGAGGAGGAGTATCGTTGGAGCTGTAAACCATAGCACTACGTTGAAGATC

ATACAGAGCTTTATTAACTTCTCGCTTCTCCATATTAAGTTGTTTAGTTAGTTGTACAGCAGTAGCTCCT

TAGTCCAATGTTTTTAATAACCGCACACAATCTCTGTGTCAGAACGCTCGTCAATATAGATCTTAGAAAT

TTTTTTAGAGAGAACTAACGCAACTAGCAATAAAACTGATCTTATTTTATCATTTTTTTTATTCATCATC

CTCTGGTGGTTCGTCGTTCCTATCGAATGTAGCTCTGATTAACCCGTCATCTATAGGTGATGCTGGTTCT

GGAGATTCTGGAGGAGATGGATTATTATCTGGAAGAATCTCTGTTATTTCCTTGTTTTCATGTATCGATT

GCGTTGTAACATTAAGATTGCGAAATGCTCTAAATTTGGGAGGCTTAAAGTGTTGTTTACAATCTCTACA

CGCGTGTCTAACTAATGGAGGTTCGTCAGCGGCTCTAGTTTGAATCATCATCGGTGTAGTATTCCTACTT

TTACAGTTAGGACACGGTGTATTGTATTTCTCGTCGAGAACGTTAAAATAATCGTTGTAACTCACATCCT

TTATTTTATCTATATTGTATTCTACTCCTTTCTTAATGCATTTTATACCGAACAAGAGATAGCGAAGGAA

TTCTTTTTCGGTACCGCTAGTACCCTTAATCATATCACATAGTGTTTTATATTCTAAATGTGTGGCAATG

GACGGTTTATTTCTATACGATAGTTTGTTTTTGGAATCCTTTGAGTATTCTATACCAATATTATTCTTTG

ATTCGAATTTAGTTTCTTCGATATTAGATTTTGTATTACCTATATTCTTGATGTAGTACTTTGATGATTT

TTCCATGGCCCATTCTATTAAGTTTTCCAAGTTGGCATCATCCACATATTGTGATAGTAATTCTCGGATA

TCAGTAGTGACTACCGCCATTGATATTTGTTCATTTGATGAGTAACTACTAATGTATACATTTTCCATTT

ATAACACTTATGTATTAACTTTGTTTATTTATATTTTTTCATTATTATGTTGATATTAATAATCGTATTG

TGGTTATATGGCTACAATTTCATAATGAGTTGAAGTCAGTGTCCTATGATCAATGACGATAGCTTTACTC

TGAAAAGAAAGTATCAAATCGATAGTGCAGAGTCAACAATGAAAATGGATAAGACGATGACAAAGTTTCA

GAATAGAGTCAAAATGGTAAAAGAAATAAATCAGACGATAAGAGCAGCACAAACTCATTACGAGACATTG

AAACTAGGATATATAAAATTTAAGGGAATGATTAGGACTACTACTCTAGAAGATATAGCACCATCTATTC

CAAATAATCAGAAAACTTATAAACTATTCTCGGACATTTCAGTCATTGGCAAAGCATCACAGAATCCGAG

TAAGATGATATATGCTCGCTGCTTTACATGTTTCCCAATTTGTTTGGAGATGACCATAGATTCATTTGTT

ATAGAATGCATCCAACATTGTTCATGATATAGTTGAATCATGTATGCCTGTTCGTATGCCTGTGGCTAAG

ATACTGTGTAAAGAAATGGTAAATAAATACTTTGAGAATCTTTAAGAGTGCATTGACTTTGTTAGTGAAT

AGGCATTCCATCTTTCTCCAATACTAATTCAAATTGTTAAATTAATAATGGAATAGTATAAATAGTTATT

AGTGATAAGATAGTAAACATAATTATTAGAATAGTAGTGTAGTATCATAGATAACTCTCTTCTATAAAAA

ATGGATTTTATTCGTAGAAAGTATCTTATATACACAGTAGAAAATAATATAGATTTTTTAAAGGATGATA

CATTAAGTAAAGTAAACAATTTTACCCTCAATCATGTACTAGCTCTCAAGTATCTAGTTAGCAATTTTCC

TCAACATGTTATTACTAAGGATGTATTAGCTAATACCAATTTTTTTGTTTTCATACATATGGTACGATGC

TGTAAAGTATACGAAGCGGTTTTACGACACGCATTTGATGCACCCACGTTGTACGTTAAAGCATTGACTA

AGAATTATTTATCGTTTAGTAACACAATACAGTCGTACAAGGAAACCGTGCATAAACTAACACAAGATGA

AAAATTTTTAGAGGTTGCCGAATACATGGACGAATTAGGAGAACTTATAGGCGTAAATTATGACTTAGTT

CTTAATCCATTATTTCACGGAGGGGAACCCATCAAAGATATGGAAATCATTTTTTTAAAACTGTTTAAGA

AAACAGACTTCAAAGTTGTTAAAAAATTAAGTGTTATAAGATTACTTATTTGGGCATACCTAAGCAAGAA

AGATACAGGCATAGAGTTTGCGGATAATGATAGACAAGATATATATACTCTATTTCAACAAACTGGTAGA

ATAGTCCATAGCAATCTAACAGAAACGTTTAGGGATTATATCTTTCCCGGAGATAAGACTAGCTATTGGG

TGTGGTTAAACGAAAGTATAGCTAATGATGCGGATATCGTTATTAATAGACCCGCCATTACCATGTATGA

TAAAATTCTTAGTTATATATACTCTGAGATAAAACAGGGACGCGTTAATAAAAACATGCTTAAGTTAGTT

TATATCTTTGAGCCTGAAAAAGATATCAGAGAACTTCTGCTAGAAATCATATATGATATTCCTGGAGATA

TCCTATCTATTATTGATGCAAAAAACGACGATTGGAAAAAATATTTTATTAGTTTTTACAAAGCTAATTT

TATTAACGGTAATACATTTATTAGTGATAGAACGTTTAACGATGACTTATTCAGAGTTGTTGTTCAAATA

GATCCCGAATATTTCGATAATGAACGAATTATGTCTTTATTCTATACGAGTGCTGCGGACATTAAACGAT

TTGATGAGTTAGATATTAATAACAGTTATATATCTAATATAATTTATGAGGTGAACGATATCACATTAGA

TACAATGGATGATATGAAGAAGTGTCAAATCTTTAACGAGGATACGTTGTATTATGTTAAGGAATACAAT

ACATACCTGTTTTTGCACGAGTCGGATCCCATGGTCATAGAGAACGGAATACTAAAGAAACTGTCATCTA

TAAAATCCAAGAGTAGACGGCTGAACTTGTTTAGCAAAAACATTTTAAAATATTATTTAGACGGACAATT

GGCTCGTCTAGGTCTTGTGTTAGATGATTATAAAGGAGACTTATTAGTTAAAATGATAAACCATCTCAAA

TCTGTGGAGGATGTATCCGCATTCGTTAGATTTTCTACAGATAAAAACCCTAGTATTCTTCCATCGCTAA

TCAAAACTATTTTAGCTAGTTATAATATTTCCATCATCGTCTTATTTCAAAGGTTTTTAAGAGATAATCT

ATATCATGTAGAAGAATTCTTGGATAAAAGCATCCATCTAACCAAGACGGATAAGAAATATATACTTCAA

TTGATAAGGCACGGTAGATCATAGAACAAACCAAATATATTATTAATAATTTGTATATACATAGATATAA

TTATCACATATTAAAAAATAACACATTTTTGATAAATGGAAACCGTTGCAACAATTCAGACTCCCACCAA

ATTAATGAATAAAGAAAATGCAGAAATGATTTTGGAAAAAATTGTTAATCATATAGCTATGTATATTAGT

GACGAATCAATATATTCAGAAAATAATCCTGAATATATTGATTTTCGTAACAGATACGGAGACTATAGAT

CTCTCATTATAAAAAGTGATCACGAGTTTGTAAAGCTATGTAAAGATCATGCAGAGAAAAGTTCTCCAGA

AACGCAACAAATGATTATCAAACACATATACGAACAATATCTTATTCCAGTATCTGAAGTACTATTAAAA

CCTATAATGTCCATGGGTGACATATTTACATATAACGGATGTAAAGACAATGAATGGATGCTAGAACAAC

TCTCTACCCTAAACTTTAACAATCTCTACACATGGAACTCATGTAGCATAGGCAATGTAACGCGTCTGTT

TTATACATTTTTTAGTTATCTGATGAAAGATAAACTAAATATATAAGTATAATCCCATTCTAATACTTTA

ACCTGATGTATTATTACCTGCATCTTATTAGAATATTAACCTAACTAAAAGACATAAAAAGCGGTAGGAT

ATAAATATTATGGCCGCAACCGTTCCGCGTTTTGACGATGTGTACAAAAATGCACAAAGAAGAATTCTAG

ATCAAGAAACATTTTTTAGTAGAGGTCTAAGTAGACCGTTAATGAAAAACACATATCTATTTGATAATTA

CGCGTATGGATGGATACCAGAAACTGCAATTTGGAGTAGTAGATACGCAAACCTAGATGCTAGTGACTAT

TATCCCATTTCGTTGGGATTACTTAAAAAGTTTGAATTTCTCATGTCTCTATATAAAGGTCCTATTCCCG

TATATGAAGAAAAAGTAAATACTGAATTCATTGCTAATGGATCTTTCTCCGGTAGATACGTATCATATCT

TAGAAAGTTTTCTGCCCTTCCAACAAACGAGTTTATTAGTTTTTTATTATTGACCTCCATCCCTATCTAT

AATATCTTATTCTGGTTTAAAAACACACAGTTTGATATTACTAAACACACATTATTCAGATACGTCTATA

CAGATAATACCAAACACCTTGCGTTGGCTAGGTATATACATCAAACAGGAGACTATAAGCCTTTGTTTAG

TCGTCTCAAAGAGAATTATATATTTACTGGTCCCGTTCCAATAGGTATCAAAGATATAGATCACCCTAAT

CTTAGTAGAGCAAGAAGTCCATCCGATTATGAGACATTAGCTAATATTAGTACTATATTGTACTTTACCA

AGTATGATCCAGTATTAATGTTTTTATTGTTTTACGTACCTGGGTATTCAATTACTACAAAAATTACTCC

AGCCGTAGAATATCTAATGGATAAACTGAATCTAACAAAGAGCGACGTACAACTGTTGTAAATTATTTTA

TGCTTCGTAAAATGTAGGTCTTGAACCAAACATTCTTTGAAAAAATGAGATGCATAAAACTTTATTATCC

AATAGATTAACTATTTCAGACGTCAATCGTTTAAAGTAAACTTCGTAAAATATTCTTTGATTGCTGCCGA

GTTTAAAACTTCTATCGATAATTGTTTCATATGTTTTAATATTTACAAGTTTTTTGGTCCATGGTACATT

AGCTGGACAGATATATGCAAAATAATATCGTTCTCCAAGTTCTATAGTCTCTGGATTGTTTTTATTATAT

TCAGTAACCAAATACATATTAGGGTTATCTGCGGATTTATAATTTGAGTGATGCATTCGACTCAACATAA

ATAATTCTAGAGGAGACGATCTACTATCAAATTCGGATCGTAAATCTGTTTCTAAAGAACGGAGAATATC

TATACATACCTGATTAGAATTCATCCGTCCTTCAGACAACATCTCAGACAGTCTGGTCTTGTATGTCTTA

ATCATATTCTTATGAAACTTGGAAACATCTCTTCTAGTTTCACTAGTACCTTTATTAATTCTCTCAGGTA

CAGATTTTGAATTCGACGATGCCGAGTATTTCATCGTTGTATATTTCTTCTTCGATTGCATAATCAAATT

CTTATATACCGCCTCAAACTCTATTTTAAAATTATTAAACAATACTCTACTATTAATCAGTCGTTCTAAC

TCCTTTGCTATTTCTATGGACTTATCTACATCTTGACTGTCTATCTCTGTAAACACGGAGTCGGTATCTC

CATACACGCTACGAAAACGAAATCTATAATCTATAGGCAACGATGTTTTCACAATCGGATTAATATCTCT

ATCGTCCATATAAAATGGATTACTTAATGTATTGGCAAACCGTAACATACCGTTGGATAACTCTGCTCCA

TTTAGTACCGATTCTAGATACAATATCATTCTACGTCCTATGGATGTGCAACTCTTAGCCGAAGCGTATG

AGTATAGAGCACTATTTCTAAATCCCATCAGACCATATACTGAGTTGGCTACTATCTTGTACGTATATTG

CATGGAATCATAGATGGCCTTTTCAGTTGAACTGGTAGCCTGTTTTAACATCTTTTTATATCTGGCTCTC

TCTGCCAAAAATGTTCTTAATAGTCTAGGAATGGTTCCTTCTATTGATCTATCGAAAATTGCTATTTCAG

AGATGAGGTTCGGTAGTCTAGGTTCACAATGAACCGTAATATATCTAGGAGGTGGATATTTCTGAAGCAA

GAGTTGATTATTTATTTCTTCTTCCAATCTATTGGTACTAACAACGACACCGACTAATGTTTCCGGAGAT

AGATTTCCAAAGATACACACATTAGGATACAGACTGTTATAATCAAAGATTAATACATTATTACTAAACA

TTTTTTGTTTTGGAGCAAATACCTTACCGCCTTCATAAGGAAACTTTTGTTTTGTTTCTGATCTGACTAA

GATAGTTTTAGTTTCCAACAATAGCTTTAACAGTGGACCCTTGATGATTGTACTCGCTCTATATTCGAAT

ACCATGGATTGAGGAAGCACATATGTTGCCGCACCAGCGTCTGTTTTTGTTTCTACTCCATAATACTCCC

ACAAATACTGACACAAACAAGCATCATGAATACAGTATCTAGCCATATCTAAAGCTATGTTTAGATTATA

ATCCTTATACATCTGAGCTAAATCAATGTCATCCTTTCCGAAAGATAATTTATATATATCATTAGGTAAA

GTAGGACATGATAGTACGACTTTAAATCCATTTTCCCAAATATCTTTACGAATTACTTTACATATAATAT

CCTCATCAACAGTCACGTAATTACCTGTGGTTAAAACCTTTGCAAATGTATCGGCTTTGCCTTTCGCGTC

CGTAGTATCGTCACCGATGAACGTCATTTCTCTAACTCCTCTATTTAATACTTTACCCATGCAACTGAAC

GCGTTCTTGGATATAGAATCCAATTTGTACGAATCCAATTTTTCAGATTTTTGAATGAATGAATATAGAT

CGAAAAATATAGTTCCATTATTGTTATTAACGTGAAACGTAGTATTGGCCATGCCGCATACTCCCTTATG

ACTAGACTGATTTCTCTCATAAATACAGAGATGTACAGCTTCCTTTTTGTCTGGAGATCTAAAGATAATC

TTCTCTCCTGTTAATAACTCTAGACGATTAGTAATATATCTCAGATCAAAGTTATGTCCGTTAAAGGTAA

CGACGTAGTCGAACGTTAGTTCCAACAATTGTTTAGCTATTCGTAACAAAACTATTTCAGAACATAGAAC

TAGTTCTCGTTCGTAATCCATTTCCATTAGCGACTGTATCCTCAAACATCCTCTATCGACGGCTTCTTGT

ATTTCCTGTTCCGTTAACATCTCTTCATTAATGAGCGTAAACAGTAATCGTTTACCACTTAAATCGATAT

AACAGTAACTTGTATGCGAGATTGGGTTAATAAATACAGAAGGAAACTTCTTATCGAAGTGACACTCTAT

ATCTAGAAATAAGTACGATCTTGGGATATCGAATCTAGGTATTTCTTTAGCGAAACAGTTACGTGGATCG

TCACAATGATAACATCCATTGTTAATCTTTGTCAAATATTGCTCGTCCAACGAGTAACATCCGTCTGGAG

ATATCCCGTTAGAAATATAAAACCAACTAATATTGAGAAATTCATCCATGGTGGCATTTTGTATGCTGCG

TTTCTTTGGCTCTTCTATCAACCACATATCTGCGACGGAGCATTTTCTATCTTTAATATCTAGATTATAA

CTTATTGTCTCGTCAATGTCTATAGTTCTCATCTTTCCCATCGGCCTCGCATTAAATGGAGGAGGAGATA

ATGACTGATATATTTCGTCCGTCACTACGTAATAAAAGTAATGAGGAAATCGTATAAATACGGTCTCGCC

ATTTCGACATCTGGATTTCAGATATAAAAATCTGTTTTCACCGTGACTTTCAAACCAATTAATACACCTA

ACATCCATTTCTAGAATTTAGAAATATATTTTCATTTAAATGAATCCCAAACATTGGGGAAGAGCCGTAT

GGACCATTATTTTTATAGTACTTTCGCAAGCGGGTTTAGACGGCAACATAGAAGCGTGTAAACGAAAACT

ATATACTATAGTCAGCACTCTTCCATGTCCTGCATGTAGACGACACGCGACTATCGCTATAGAGGACAAT

AATGTCATGTCTAGCGATGATCTGAATTATATTTATTATTTTTTCATCAGATTATTTAACAATTTGGCAT

TTGATCCCAAATACGCAATCGATGTGTCAAAGGTTAAACCTTTATAAACTTAACCCATTATAAAACTTAT

GATTAGTCACGACTGAAATAACCGCGTGATTATTTTTTGGTATAATTCTACACGGCATGGTTTCTGTGAC

TATGAATTCAACACCTGTTATCTTAGTGAAATCTTTAACAAACAGCAAGGGTTCGTCAAAGACATAAAAC

TCATTGTTTACGATCGAAATAGACCCCCTATCACACTTAAAATAAAAAATATCCTTATCCTTTACCACCA

AATAAAATTCTGATTGGTCAATGTGAATGTATTCACTTAACAGTTCCACAAATTTATTTATTAACTCCGA

GGCACATACATCGTCGGTATTTTTTATGACAAACTTTACTCTTCCAGCATCCGTTTCTAAAAAAATATTA

ACGAGTTCCATTTATATCATCCAATATTATTGAAATGACGTTGATGGACAGATGATATAAATAAGAAGGT

ACAGTACCTTTGTCCACCATCTCCTCCAATTCATACTCTATTTTGTCATTAACTTTAATGTGTGAAAACA

GTACGCCACATGCTTCCATGACAGTGTGTAACACTTTGGATACAAAATGTTTGACATTAGTATAATTGTC

CAAGACTGTCAATCTATAATAGATAGTAGCTATAATATATTCTATGATGGTATTGAAGAAGATGACAACC

TTGGCATATTGATCATTTAACACAGACATGGTATCAACAAATAGCTTAAATGAAAGAGAATCAGTAATTG

GAATAAGCGTCTTCTCGATGTAGTGTCCGTATACCAACATGTCTGATATTTTGATGTATTCCATTAAATT

ATTTAGTTTTTTCTTTTTATTCTCGTTAAACAGAATTTCTGTCAATGGACCCCAACATCGTTGACCTATT

AAGTTTTGATTGATTTTTCCGTGTAAGGCGTATCTAGTCAGATCGTATAGCCTATCCAATAATCCATCGT

CTGTGCGTAGATCACATCGTACACTTTTTAATTTTCTATAGAAGAGTGACAGACATCTGGAGCAATTACA

GACAGCAATTTCTTTATTCTCTACAGATGTAAGATACTTGAAGATATTCCTATGATGATGCAGAATTTTG

GATAACACGGTATTGATGGTATCTGTTACCATAATTCCTTTGACTGATAGTGTCAAAGTACAAGATTTCC

AATCTTTTGCAATTTTCAGTACCATTATCTTTGTTTTGATATCTATATCAGACAGCATGGTACGTCTGAC

AACACAGGGATTAAGACGGAAAGATGAAATGATTCTCTCAACATCTTCAATAGATACCTTGCTATTTTTT

TTGGCATTATCTATATGTGAGAGAATATCCTCTAGAGAATCAGTATCCTTTTTGATGATAGTGGATCTCA

ATGACATGGGACGTCTAAACCTTCTTATTCTATCACCAGATTGCATGGTGATTTGTCTTCTTTCTTTTAT

CATGATGTAATCTCTAAATTCATCGGCAAATTGTCTATATCTAAAATCATAATATGAGATGTTTACCTCT

ACAAATATCTGTTCGTCCAATGTTAGAGTATCTATATCAGTTTTGTATTCCAAATTAAACATGGCAACGG

ATTTAATTTTATATTCCTCTATTAAGTCCTCGTCGATAATAACAGAATGTAGATAATCATTTAATCCATC

GTACATGGTTGGAAGATGCTCGTTGACAAAATCTTTAATTGTCTTGATGAAGGTGGGACTATATCTAACA

TCTTGATTAATAAAATTTATAACATTGTCCATAGGATACTTTGTAACTAGTTTTATACACATCTCTTCAT

TGGTAAGTTTAGACAGAATATCGTGAACAGGTGGTATATTATATTCATCAGATATACGAAGAATAATGTC

CAAATCTATATTGTTTAATATATTATATAGATGTAGTGTAGCTCCTACAGGAATATCTTTAACTAAGTCA

ATGATTTCATCAACAGTTAGATCTATTTTAAAGTTAATCATATAGGCATTGATTTTTAAAAGGTATGTAG

CCTTGACTACATTCTCATTAATTAACCATTCCAAGTCACTGTGTGTAAGAAGATTATATTCTATCATAAG

CTTGACTACATTTGGTCCCGATACCATTAAAGAATTCTTATGATATAAGGAAACAGCTTTTAGGTACTCA

TCTACTCTACAAGAATTTTGGAGAGCCTTAACGATATCAGTGACGTTTATTATTTCAGGAGGAAAGAACC

TAACATTGAGAATATCTGAATTAATAGCTTCCAGATACAGTGATTTTGGCAATAGTCCGTGTAATCCATA

ATCCAGTAACACGAGCTGGTGCTTGCTAGACACCTTTTCAATGTTTAATTTTTTTGAAATAAGCTTTGAT

AAAGCCTTCCTCGCAAATTCCGGATACATGAACATGTCGCCAACATGATTAAGTATTGTTTTTCATTATT

TTTATATTTTCTCAACAAGTTCTCAATACCCCAATAGATAATAGAATATCACCCAATGCGTCCATGTTGT

CTATTTCCAACAGGTCGCTATATCCACCAATAGAAGTTTTCCCAAAAAAGATTCTAGGAACAGTTCTACC

ACCAGTAATTTGTTCAAAATAGTCACGCAATTCATTTTCGGGTTTAAATTCTTTAATATCTACAATTTCA

TACGCTCCTCTTTTGAAACTAAACTTATTTAGAATATCCAGTGCGTTTCTACAAAAAGGACATGTAAACT

TGACAAAAATTGTCACTTTGTTATTGGCCAACCTTTGTTGTACAAATTCCTCGGCCATTTTTAATATTTA

AGTGATACAAAACTATCTCGACTTATTTAACTCTTTAGTCGAGATATATGGACACAGATAGCTATATGAT

AACCAACTACAGAAGACAAACGCTATAAAAAACATAATTACGACGAGCATATTTATAAATATTTTTATTC

AGTATTACTTGATATAGTAATATTAGGCACAGTCAAACATTCAACCACTCTAGATACATTAACTCTCTCA

TTTTCTTTAACAAATTCTGCAATATCTTCGTAAAAAGATTCTTGAAACTTTTTAGAATATCTATCGACTC

TAGATGAAATAGCGTTCGTCAACATACTATGTTTTGTATACATAAAGGCGCCCATTTTAACAGTTTCTAG

TGACAAAATGCTAGCGATCCTAGGATCCTTTAGAATCACATAGATTGACGATTCGTCTCTCTTAGTAACT

CTAGTAAAATAATCATACAATCTAGTACGCGAAATAATATTATCCTTGACTTGAGGAGATCTAAACAATC

TAGTTTTGAGAACATCGATAAGTTCATCGGGAATTACATACATACTATCTTTAATAGAACTCTTTTCATC

CAGTTGAATGGATTCGTCCTTAACCAACTGATTAATGAGATCTTCTATTTTATCATTTTCTAGATGATAT

GTATGTCCATTAAAGTTAAATTGTGTAGCGCTTCTTTTTAGCCTAGCAGCCAATACTTTAACATCACTAA

TATCGATATACAAAGGAGATGATTTATCGATGGTATTAAGAATTCGTTTTTCGACATCCGTCAAAACCAA

TTCCTTTTTGCCTGTATCATCCAGTTTGCCATTCTTTGTAAAGAAATTATTTTCTACTAGACTATTAATA

AGACTGATAAGGATTCCTCCATAATTGCACAATCCAAACTTTTTCACAAAACTAGACTTTACGAGATCTA

CAGGAATGCGTACTTCAGGTTTCTTAGCTTGTGATTTTTTCTTTTGTGGACATTTTCTAGTGACCAACTC

ATCTACCATTTCATTGATTTTAGCAGTGAAATAAGCTTTCAATGCACGGGCACTGATACTATTGAAAACG

AGTTGATCTTCAAATTCCGCCATTTAAGTTCACCAAACAACTTTTAAATACAAATATATCAATAGTAGTA

GAATAAGAACTATAAAAAAAATAATAATTAACCAATACCAACCCCAACAACCTGTATTATTAGTTGATGT

GACAGTTTTCTCATCACTTAGAACAGATTTAACAATTTCTATAAAGTCTGTCAAATCATCTTCCTGAGAA

CCCATAAATACACCAAATATAGCAGCGTACAACTTATCCATTTATACATTGAATATTGGCTTTTCTTTAT

CGCTATCTTCATCATATTCATCATCAATATCAACAAGTCCCAGATTACGAACCAGATCTTCTTCTACATT

TTCAGTCATTGATACGCGTTCACTATCTCCAGAGAGTCCGATAACGTTAGCCACTACTTCTCTATCAATG

ATTAGTTTCTTGAGCGCGAATGTAATTTTTGTTTCCGTTCCGGATCTATAGAAAACTACAGGTGTAATAA

TTGCCTTGGCTAATTGTCTTTCTCTTTTACTGAGTGATTCTAGTTCACCTTCTATAGATCTGAGAATGGA

TGATTCTCCAGTCGAAACATATTCTACCATGGCTCCGTTTAATTTGTTGATGAAGATGGATTCATCCTTA

AATGTTTTCTCTGTAATAGTTTCCGCCGAAAGACTATGCAAAGAATTTGGAATGCGTTCCTTGTGTGTAA

TGTTTCCATAGACAGCTTCTAGAAGTTGATACAACATAGGACTAGCCGCGGTAACTTTTATTTTTAGAAA

GTATCCATCGCTTCTATCTTGTTTAGATTTATTTTTATAAAGTTTAGTCTCTCCTTCCAACATAATAAAA

GTGGAAGTCATCTGACTAGATAAACTATCAGTAAGTTTTATAGAGATAGATGAACAATTAGCGTATTGAG

AAGCATTTAGTGTAACGCATTCGATACATTTTGCATTAGATTTACTAATCGATTTTGCATACTCTATAAC

ACCCGCACAAGTCTGTAGAGAATCGCTAGATGCTGTAGGTCTTGGTGAAGTTTCAACTCTCTTCTTGATT

ACCTTACTCATGATTAAACCTAAATAATTGTACTTTGTAATATAATGATATATATTTTCACTTTATCTCA

TTTGAGAATAAAAATGTTTTTGTTAACCACTGCATGATGTACAGATTTCGGAATCGCAAACCACTTGTGG

TTTTATTTTATCCTTGTCCAATGTGAATTGAATGGGAGCGGATGCGGGTTTCGTACGTAGATAGTACATT

CCCGTTTTTAGACCGAGACTCCATCCGTAAAAATGCATACTCGTTAGTTTGGAATAACTCGGATCTGCTA

TATGGATATTCATAGATTGACTTTGATCGATGAAGGCTCCCCTGTCTGCAGCCATTTTTATGATCGTCTT

TTGTGGAATTTCCCAAATAGTTTTATAAACTCGCTTAATATCTTCTGGAAGGTTTGTATTCTGAATGGAT

CCACCATCTACCATAATCCTATTCTTGATCTCATCATTCCATAATTTTCTCTCGGTTAAAACTCTAAGGA

GATGCGGGTTAACTACTTGGAATTCTCCAGACAATACTCTCCGAGTGTAAATATTACTGGTATACGGTTC

CACCGACTCATTATTTCCCAAAATTTGAGCAGTTGATGCAGTCGGCATAGGTGCCACCAATAAACTATTT

CTAAGACCGTATGTTCTGATTTTATCTTTTAGAGGTTCCCAATTCCAAAGATCCGACGGTACAACATTCC

AAAGATCATATTGTAGAATACCGTTACTGGCGTACGATCCTACATATGTATCATATGGTCCTTCCTTCTC

AGCTAGTTTACAACTCGCCTCTAATGCACCGTAATAAATGGTTTCAAAGATCTTCTTATTTAGATCTTGT

GCTTCCAGGCTATCAAATGGATAATTTAAGAGAATAAACGCGTCCGCTAATCCTTGAACACCAATACCGA

TAGGTCTATGTCTCTTATTAGAGATTTCAGCTTCTGGAATAGGATAATAATTAATATCTATAATTTTATT

GAGATTTCTGACAATTACTTTGACCACATCCTTCAGTTTGAGAAAATCAAATCGCCCATCTATTACAAAC

ATGTTCAATGCAACAGATGCCAGATTACACACGGCTACCTCATTAGCATCCGCATATTGTATTATCTCAG

TGCAAAGATTACTACACTTGATGGTTCCTAAATTTTGTTGATTACTCTTTTTGTTACACGCATCCTTATA

AAGAATGAATGGAGTACCAGTTTCAATCTGAGATTCTATAATCGCTTTCCAGACGACTCGAGCCTTTATT

ATACATTTGTATCTCCTTTCTCTTTCGTATAGTGTATACAATCGTTCGAACTCGTCTCCCCAAACATTGT

CCAATCCAGGACATTCATCCGGACACATCAACGACCACTCTCCGTCATCCTTCACTCGTTTCATAAAGAG

ATCAGGAATCCAAAGAGCTATAAATAGATCTCTTGTTCTATGTTCATCGTTTCCTGTATTCTTTTTAAGA

TCGAGGAACGCCATAATATCAGAATGCCACGGTTCCAAGTATATGGCCATAACTCCAGGCCGTTTGTTTC

CTCCCTGATCTATGTATCTAGCGGTGTTATTATAAACTCTCAACATTGGAATAATACCGTTTGATATACC

ATTGGTACCGGAGATATAGCTTCCACTGGCACGAATATTACTAATTGATAGACCTATTCCCCCTGCCATT

TTAGAGATTAATGCGCATCGTTTTAACGTGTCATAGATGCCTTCTATGCTATCATCGATCATGTTAAGTA

GAAAACAGCTAGACATTTGGTGACGAGTAGTTCCCGCATTAAATAAGGTAGGAGAAGCGTGCGTAAACCA

TTTTTCAGAAAGTAGATTGTACGTCTCAATAGCTGAGTCTATATCCCATTGATGAATTCCTACTGCGACA

CGCATTAACATGTGCTGAGGTCTTTCAACAATTTTGTTGTTTATTTTCAACAAGTAGGATTTTTCCAAAG

TTTTAAAACCAAAATAGTTGTATGAAAAGTCTCGTTCGTAAATAATAACCGAATTGAGCTTATCCTTATA

TTTGTTAACTATATCCATGGTAATACTTGAAATAATCGGAGAATGTTTCCCATTTTTAGGATTAACATAG

TTGAATAAATCCTCCATCACTTCACTAAATAGTTTTTTTGTTTCCTTGTGTAGATTTGATATGGCTATTC

TGGCGGCTAGAATGGCATAATCCGGATGTTGTGTAGTACAAGTGGCTGCTATTTCGGCTGCCAGAGTGTC

CAATTCTACCGTTGTTACTCCATTATATATTCCTTGAATAACCTTCATAGCTATTTTAATAGGATCTATA

TGATCAGTGTTTAAGCCATAGCACAATTTTCTAATACGAGACGTGATTTTATCAAACATGACATTTTCCT

TGTATCCATTTCGTTTAATGACAAACATTTTTGTTGGTGTAATAAAAAAAATTATTTAATTTTTCATTAA

TAGGGATTTGACGTATGTAGCGTACAAAATTATCGTTCCTGGTATATAGATAAAGAGTCCTATATATTTG

AAAATCGTTACGGTTCGATTAAACTTTAATGATTGCATTGTGAATATATCATTAGGATTTAACTCCTTGA

CTATCATGGCGGTGCCAGAAATTACCATCAAAAGCATTAATACAGTTATGCAGATCGCAGTTAGAACGGT

TATAGCATCCACCATTTATATCTAAAAATTAGATCAAAGAATATGTGACAACGTCCTAGTTGTATACTGA

GAATTGACGAAACAATGTTTCTTACATATTTTTTTCTTATTAGTAACCGACTTAATAGTAGGAACTGGAA

AACTAGACTTGATTATTCTATAAGTATAGATACCCTTCCAAATAATGTTCTCTTTGATAAAAGTTCCAGA

AAATGTAGAATTTTTTAAAAAGTTATCTTTTGCTATTACTAATATCGTGGTTAGACGCTTATTATTAATA

TGAGTGATGAAATCCACACCGCTTCTAGATATCGCTTTTATTTCCACATTAGATGGTAAATCCAATAGTG

AAACTATCTTTTTAGGAATGTATGGACTCGCGTTTAGAGGAGTGAACGTCTTCGGAGTAGTAAAGGATGA

TTCGTCAAATGAATAAACAATTTCACAAATGGATGTTAATGTATTAGTAGGAAATTTTTTGACGCTAGTG

GAATTGAAGATTCTAATGGATGATGTTCTACCTATTTCATCCGATAACATGTTAATTTCCAATACCAACG

GTTTTAATATTTCGATGATATACGGTAGTCTCTCTTTCGGACTTATATAGCTTATTCCACAATACGAGTC

ATTATATACTCCAAAAAACAAAATAACTAGTATAAAATCTGTATCGAATGGGAAAAACGAAATTATCGAT

ATAGGTATAGAATCCGGAACATTGAACGTATTAATACTTAATTCTTTTTCAGTGGTAAGAACCGATAGGT

TATTGACATTGTATGGTTTTAAATATTCTATAACTTGAGACTTGATAGATATTAATGACGAATTGAAAAT

TATTTTTATCACCACGTGTGTTTCAGGATCATCGTCGACGCCAGTTAACCAACCGAATGGAGTAAAATAA

ATATCATTAATATATGCTCTAGATATTAGTATTTTTATTAATCCTTTGATTATCATCTTCTCGTACGCGA

ATGATTCCATGATCAAGAGTGATTTGAGAACATCCTCCGGAGTATTAATGGGTTTAGTAAACAGTCCATC

GTTGCAATAATAAAAGTTGTCCAAGTTAAAGGATATTATGCATTCGTTTAAAGATATCACCTCATCTAAC

GGAGACAATTTTTTGGTAGGTTTTAGAGACTTTGAAGCTACTTGTTTAACAAAGTTATTCATCGTCGTCT

ACTATTCTATTTAATTTTGTAGTTAATTTATCACATATCACATTAATTGACTTTTTGGTCCACTTTTCCA

TACGTTTATATTCTTTTAATCCTGCGTTATCCGTTTCCGTTATATACAGGGATAGATCTTGCAAGTTAAA

TAGAATGCTCTTAAATAATGTCATTTTTTTATCCGCTAAAAATTTAAAGAATGTATAAACTTTTTTCAAA

GATTTAAAACTTTTAGGTGGAGTTCTGGTACACAATATCATAAACAAACTAATAAACATCCCACATTCAG

ATTCCAACAATTGATTAACTTCCACATTAATACAGCCTATTTTCGCTCCAAATGTACATTCGAAAAATCT

GAATAAAACATCAATATCGCAATTTGTATTATCCAATACAGAATGTCTGTGATTCGTGTTAAAACCATCG

GAAAAAGAATAGAAATAAAAATTATTATAATGGTGGAATTCAGTTGGAATATTGCCTCCGGAGTCATAAA

AGGATACTAAACATTGTTTTTTATCGTAAATTACACATTTCCAATGAGACAAATAACAAAATCCAAACAT

TACAAATCTAGAGGTAGAACTTTTAATTTTGTCTTTAAGTATATACGATAAGATATGTTTATTCATAAAC

GCGTCAAATTTTTCATGAATAGCTAAGGAGTTTAAGAATCTCATGTCAAATTGTCCTATATAATCCACTT

CGGATCCATAAGCAAACTGAGAGACTAAGTTCTTAATACTTCGATTGCTCATCCAGGCTCCTCTCTCAGG

CTCTATTTTCATCTTGACGACCTTTGGATTTTCACCAGTATGTATTCCTTTACGTGATAAATCATCGATT

TTCAAATCCATTTGTGAGAAGTCTATCGCCTTAGATACTTTTTCCCGTAGTTGAGGTTTAAAGAAATACG

CTAACGGTATACTAGTAGGTAACTCAAAGACATCATATATAGAATGGTAACGCGTCGTTAACTCGTCGGT

TAACTCTTTCTTTTGATCGAGTTCATCGCTACTATTGGGTCTGCTCAGGTGCCCCGACTCTACTAGTTCC

AACATCATACCGATAGGAATACAAGACACTTTGCCAGCGGTTGTAGATTTATCATATTTCTCCACCACAT

ATCCGTTACAATTTGTTAAGAATTTAGATACATCTATATTGCTACATAATCCAGCTAGTGAATATATATG

ACATAATAAATTGGTAAATCCTAGTTCTGGTATTTTACTAATTACTAAATCTGTATATCTTTCCATTTAT

CATGGAAAAGAATTTACCAGATATCTTCTTTTTTCCAAACTGCGTTAATGTATTCTCTTACAAATATTCA

CAAGATGAATTCAGTAATATGAGTAAAACGGAACGTGATAATTTCTCATTGGCTGTGTTTCCAGTGATAA

AACATAGATGGCATAACGCACACGTTGTAAAACATAAAGGAATATACAAAGTTAGTACAGAAGCACGTGG

AAAAAAAGTATCTCCTCCATCACTAGGAAAACCCGCACATATAAACCTAATGTCGAAGCAATATATATAT

AGTGAGTATGCAATAAGCTTTGAATGTTATAGTTTTCTAAAATGTATAACAAATACAGAAATCAATTCGT

TCGATGAGTATATATTAAGAGGACTATTAGAAGCTGGTAATAGTTTACAGATATTTTCCAATTCCGTAGG

TAAACGAATAGATACTATAGGTGTACTAGGGAATAAGTATCCATTTAGCAAAATTCCATTGGCCTCATTA

ACTCCTAAAGCACAACGAGAGATATTTTTAGCGTGGATTTCTCATAGACCTGTAGTTTTAACTGGAGGAA

CCGGAGTGGGTAAGACGTCACAGGTACCCAAGTTATTGCTTTGGTTTAATTATTTATTTGGTGGATTCTC

TTCTCTAGATAAAATCACTGACTTTCACGAAAGACCAGTCATTCTATCTCTTCCTAGGATAGCTTTAGTT

AGATTGCATAGCAATACCATTTTAAAATCATTGGGATTTAAGGTACTAGATGGATCTCCTATCTCTTTAC

GGTACGGATCTATACCGGAAGAATTAATAAACAAACAACCAAAAAAATATGGAATTGTATTTTCTACCCA

TAAGTTATCTCTAACAAAACTATTTAGTTATGGCACTATTATTATAGACGAAGTTCATGAGCATGATCAA

ATAGGAGATATTATTATAGCAGTAGCGAGAAAACATCATACGAAAATAGATTCTATGTTTTTAATGACTG

CCACGTTAGAGGATGACAGGGAACGTCTAAAAATATTTTTACCTAATCCCGCATTTATACATATTCCTGG

AGATACACTGTTTAAAATTAGCGAGGTATTTATTCATAATAAGATAAATCCATCTTCCAGAATGGCATAT

ATAGAAGAAGAAAAGAGAAATTTAGTTACTGCTATACAGATGTATACTCCTCCTGATGGATCATCCGGTA

TAGTCTTTGTGGCATCCGTTGCACAGTGTCACGAATATAAATCATATTTAGAAAAAAGATTACCGTATGA

TATGTATATTATTCATGGTAAGGTCTTAGATATAGACGAAATATTAGAAAAAGTGTATTCATCACCTAAT

GTATCGATAATTATTTCTACTCCTTATTTGGAATCCAGCGTTACTATACGCAATGTTACACACATTTATG

ATATGGGTAGAGTTTTTGTCCCCGCTCCTTTTGGAGGATCACAACAATTTATTTCTAAATCTATGAGAGA

TCAACGAAAAGGAAGAGTAGGAAGAGTTAATCCTGGAACATACGTATATTTCTATGATCTGTCTTATATG

AAATCTATACAGCGAATAGATTCAGAATTTCTACATAATTATATATTGTACGCTAATAAGTTTAATCTAA

CACTCCCCGAAGATTTGTTTATAATCCCTACAAATTTGGATATTCTATGGCGTACAAAGGAATATATAGA

CTCGTTCGATATTAGTACAGAAACATGGAATAAATTATTATCCAATTATTATATGAAGATGATAGAGTAT

GCTAAACTTTATGTACTAAGTCCTATTCTCGCTGAGGAGTTGGATAACTTTGAGAGGACGGGAGAATTAA

CTAGTATTGTACAAGAAGCCATTTTATCTCTAAATTTACGAATTAAGATTTTAAAATTTAAACATAAAGA

TGATGATACGTATATACACTTTTGTAGAATATTATTCGGTGTCTATAACGGAACAAACGCTACTATATAT

TATCATAGACCTCTAACGGGATATATGAATATGATTTCAGATACTATATTTGTTCCTGTAGATAATAACT

AAAAATCAAAATCTAATGACCACATCTTTTTTTAGAGATGAAAAATTTTCCACATCTCCTTTTGTAGACA

CGACTAAACATTTTGCAGAAAAAAGTTTATTATTATTTAGATAATCGTATACTTCATCAGTGTAGATAGT

AAATGTGAACAGATAAAAGGTATTCTTGCTCAATAGATTGGTAAATTCCATAGAATATATTAATCCTTTC

TTCTTGAGATCCCACATCATTTCAACCAAAGACGTTTTATCCAATGATTTACCTCGTACTATACCACATA

CAAAACTAGATTTTGCAGTGATGTCGTACCTGGTATTCCTACCAAACAAAATTTTACTTTTAGTTCTTTT

AGAAAATTCTAAGGTAGAATCTCTATTTGTCAATATGTCATCTATGGAATTACCACTAGCAAAAAATGAT

AGAAATATATATTGATACATCGCAGCTGGTTTTGATCTACTATACTTTAAAAACGAATCAGATTCCATAA

TTGCTTGTATATCATCAGCTGAAAAACTATGTTTTACACGTATTCCTTCGGCATTTCTTTTTAATGATAT

ATCTTGTTTAGACAATGATAAAGTTATCATGTCCATGAGAGACGCGTCTCCGTATCGTATAAATATTTCA

TTAGATGTTAGACGCTTCATTAGGGGTATACTTCTATAAGGTTTCTTAATTAGTCCATCATTGGTTGCGT

CAAGAACTACTATCTGATGTTGTTGGGTATCTCTAGTGTTACACATGGCCTTACTAAAGTTTGGGTAAAT

AACTATGATATCTCTATTAATTATAGATGTATATATTTCATTCGTCAAGGATATTAATATCGACTTACTA

TCGTCATTAATACGTGTAATGTAATCATATAAATCATGCGATAGCCAAGGAAAATTCAAATAGATGTTCA

TCATATAATCGTCGCTATAATTCATATTAATACTTTGACATTGACTAATTTGTAATATAGCCTCGCCACG

AAGAAAGCTCTCGTATTCAGTTTCATCGATAAAGGATACCGTTAAATATAACTGGTTGCCGATAGTCTCA

TAGTCTATTAAGTGGTAAGTTTCGTATAAATACAGAATCCCTAAAATATTATCTAATGTGGGATTAATCC

TTACCATAACTGTATAAAATGGAGCCGGAGTCATAACTATTTTACCGTTTGTACTTACTGGAATAGATGA

AGGAATAATCTCCGGACATGATGGTAAAGACCCAAATGTCTGTTTGAAGAAATCCAATGTTCCAGGTCCT

AATCTCTTGACAAAAATTACGATATTCGATCCCGATATCCTTTGCATTCTATTTACCAGCATATCACGAA

CTATATTAAGATTATCTATCATGTCTATTCTCCCACCGTTATATAAATCGCCTCCGCTAAGAAACGTTAG

TATATCCATACAATGGAATACTTCATTTCTAAAATAGTATTCGTTTTCTAATTCTTTAATGTGAAATCGT

ATACTAGAAAGGGAAAAATTATCTTTGAGTTTTCCATTAGAAAAGAACCACGAAACTAATGTTCTGATTG

CGTCTGACTCCGTCGCTGAATTAATAGATTTACACCAAAAACTCATATAACTTCTAGATGTAGAAGCATT

CGCTAAAAAATTAGTAGAATCAAAGGATATAAGTAGATGTTCCAACAAGTGAGCAATTCCCAAGATTTCA

TCTATATCATTCTCGAATCCGAAATTAGAAATTCCCAAGTAGATATCCTTTTTCATCCGATCATTGATGA

AAATACGAACTTTATTCGGTAAGACGATCATTTACTAAGGAGTAAAATAGGAAGTAACGTTCGTATATCG

TTATCGTCGTATAAATTAAAGGTGTGTTTTTTGCCATTAAGAGACATTATAATTTTACCAATATTGGAAT

TATAATATAGGTGTATTTGAGCACTAGAAACGGTCGATGCATCGGTAAATATAGCTGTATCTAATGTTCT

AGTCGGTATTTCTTCATTTCGCTGTCTAATGATAGCGTTTTCTCTATCTGTTTCCATTACAGCTGCCTGA

AGTTTATTGGTCGGATAATATGTAAAATAATAAGAAATACATACGAATAACAAAAATAAAATAAGATATA

ATAAAGATGCCATTTAGAGATCTAATTTTGTTCAACTTGTCCAAATTCCTACTTACAGAAGATGAGGAAT

CGTTGGAGATAGTATCTTCCTTATGTAGAGGATTTGAAATATCTTACGATGACTTAATATCGTACTTTCC

AGATAGGAAATACCATAAATATATTTCTAAGGTATTTGAACATGTAGATTTATCGGAGGAATTAAGTATG

GAATTCCATGATACAACTCTGAGAGATTTAGTATATCTTAGATTGTACAAGTATTCCAAGTATATACGGC

CGTGTTATAAATTAGGAGATAATCTAAAAGGTATAGTTGTTATAAAGGACAGAAATATATATATTAGAGA

AGCAAATGATGACTTGATAGAATATCTCCTCAAGGAATACACTCCTCAGATTTATACATATTCTAATGAG

CGAGTTCCCATAGCTGGTTCAAAATTAATTCTTTGTGGATTTTCTCAAGTTACATTTATGGCGTATACAA

CGTCGCATATAACAACAAATAAAAAGGTAGATGTTCTCGTTTCCAAAAAATGTATAGATGAACTAGTCGA

TCCAATAAATTATCAAATACTTCAAAATTTATTTGATAAAGGAAGCGGAACAATAAACAAAATACTCAGG

AAGATATTTTATTCGGTAACAGGTGGCCAAACTCCATAGGTAGCTTTTTCTATTTCGGATTTTAGAATTT

CCAAATTCACCAGCGATTTATCGGTTTTGGTGAAATCCAAGGATTTATTAATGTCCACAAATGCCATTTG

TTTTGTCTGTGGATTGTATTTGAAAATGGAAACGATGTAGTTAGATAGATGCGCGGCGAAGTTTCCTATT

AGGGTTCCGCGCTTCACGTCACCCAACATACTTGAATCACCATCCTTTAAAAAAAATGATAAGATATCAA

CATGGAGTATATCATACTCGGATTTTAATTCTTCTACTGCCTCACTGACATTTTCACAAATACTACAATA

CGGTTTACCGAAAATAATCAGTACGTTCTTCATTTATGGGTATCAAAAACTTAAAATCGTTACTGCTGGA

AAATAAATCACTGACGATATTAGATGATAATTTATACAAAGTATACAATGGAATATTTGTGGATACAATG

AGTATTTATATAGCCGTCGCCAATTGTGTCAGAAACTTAGAAGAGTTAACTACGGTATTCATAAAATACG

TAAACGGATGGGTAAAAAAGGGAGGACATGTAACCCTTTTTATCGATAGAGGAAGTATAAAAATTAAACA

AGACGTTAGAGACAAGAGACGTAAATATTCTAAATTAACCAAGGACAGAAAAATGTTAGAATTAGAAAAG

TGTACATCCGAAATACAAAATGTTACCGGATTTATGGAAGAAGAAATAAAGGCAGAAATGCAATTAAAAA

TCGATAAACTCACATTTCAAATATATTTATCTGATTATGATAACATAAAAATATCATTGAATGAGATACT

AACACATTTCAACAATAATGAGAATGTTACATTATTTTATTGTGATGAACGAGACGCAGAATTCGTTATG

TGTCTAGAGGCTAAAACACAGTTCTCTACCACAGGAGAATGGCCGTTAATAATAAGTACCGATCAGGATA

CTATGCTATTCGCGTCTGCTGATAATCATCCTAAGATGATAAAAAACTTAACTCAACTGTTTAAATTTGT

TCCCTCGGCAGAGGATAACTATTTAGCAAAATTAACTGCATTAGTGAATGGATGTGATTTCTTTCCTGGA

CTCTATGGGGCATCTATAACACCCAACAACTTAAACAAAATACAATTGTTTAGTGATTTTACAATCGATA

ATATAGTCACTAGTTTGGCAATTAAAAATTATTATAGAAAGACTAACTCTACCGTAGACGTGCGTAATAT

TGTTACGTTTATAAACGATTACGCTAATTTAGACGATGTCTACTCGTATATTCCTCCTTGTCAATGCACT

GTTCAAGAATTTATATTCTCCGCATTAGATGAAAAATGGAATGAATTTAAATCATCTTATTTAGAGAGCG

TGCCGTTACCCTGCCAATTAATGTACGCATTAGAACCACGTAAGGAGATTGATGTTTCAGAAGTTAAAAC

TTTATCATCTTATATAGATTTCGAAAATACTAAATCAGATATCGATGTTATAAAATCTATATCCTCGATT

TTTGGATATTCTAACGAAAACTGTAACACCATAGTGTTCGGCATCTATAAGGATAATTTACTACTGAGTA

TAAATAATTCATTTTACTTTAACGATAGTCTGTTAATAACCAATACTAAAAGTGATAATATAATAAATAT

AGGTTACTAGATTAAAAAATGGTGTTCCAGCTCGTGTGTTCTACATGCGGCAAAGATATTTCTCACGAAC

GATATAAATTGATTATACGAAAAAAATCATTAAAGGATGTACTAGTCAGTGTAAAGAACGAATGTTGTAG

GTTAAAATTATCTACACAAATAGAACCTCAACGTAACTTAACAGTGCAACCTCTATTGGATATAAACTAA

TGGATCCGGTTAATTTTATCAAGACATATGCGCCTAGAGGTTCTATTATTTTTATTAATTATGCCATGTC

ATTAACTAGTCATTTGAATCCATCGATAGAAAAACATGTGGGTATTTATTATGGTACGTTATTATCGGAA

CACTTGGTAGTTGAATCTACCTATAGAAAAGGAGTTAGAATAGTCCCATTGGATAGATTTTTTGAAGGAT

ATCTTAGTGCAAAAGTATACATGTTAGAGAATATTCAAGTTATGAAAATAGCAGCTGATATGTCGTTAAC

TTTACTAGGTATTCCATATGGATTTGGTCATGATAGAATGTATTGTTTTAAATTGGTAGCTGAATGTTAT

AAAAATGCCGGTATTGATACATCGTCTAAACGAATATTAGGTAAAGATATTTTTCTGAGCCAAAACTTTA

CAGATGATAATAGATGGATAAAGATATATGATTCTAATAATTTAACATTTTGGCAAATTGATTACCTTAA

AGGGTGAGTTAATATGCATAACTACTCCTCCGTTGTTTTTTCCCTCGTTCTTTTTCTTAACGTTGTTTGC

CATCACTCTCATAATGTAAAGATATTCTAAAATGGTAAACTTTTGCATATCGGATGCAGAAATTGGTATA

AATGTTGTAATTGTATTATTTCCCGTCAATGGACTAGTCACAGCTCCATCAGTTTTATATCCTTTAGAGT

ATTTCTCACTCGTGTCTAGCATTCTAGAGCATTCCATGATCTGTTTATCGTTGATATTGGCCGGAAAGAT

AGATTTTTTATTTTTTATTATATTACTATTGGCAATTGTAGATATAACTTCTGGTAAATATTTTTCTACC

TTTTCAATCTCTTCTATTTTCAAGCCGGCTATATATTCTGCTATATTGTTACTAGTATCAATACCTTTTC

TGGCTAAGAAGTCATATGTGGTATTCACTATATCAGTTTTAACTGGTAGTTCCATTAGCCTTTCCACTTC

TGCAGAATAATCAGAAATTGGTTCTTTACCAGAAAATCCAGCTACTATAATAGGCTCACCGATGATCATT

GGCAAAATCCTATATTGTACCAGATTAATGAGAGCATATTTCATTTCCAATAATTCTGCTAGTTCTTGAG

ACATTGATTTATTTGATGAATCTATTTGGTTCTCTAGATACTCTACCATTTCTGCCGCATACAATAACTT

GTTAGATAAAATCAGGGTTATCAAAGTGTTTAGTGTGGCTAGAATAGTGGGCTTGCACGTATTAAAGAAT

GCTGTAGTATGAGTAAACCGTTTTAACGAATTATATAGTCTCCAGAAATCTGTGGCGTTGCATACATGAA

CTGAATGACATCGAAGATTGTCCAATATTTTTAATAGCTGCTCTTTGTCCATTATTTCTATATTTGACTC

GCAACAATTGTAGATACCATTAATCACTGATTCCTTTTTCGATGCCGGACAATAGCACAATTGTTTAGCT

TTGGACTCTATGTATTCAGAATTAATAGATATATCTCTCAATACAGATTGCACTATACATTTTGAAACTA

TGTCAAAAATTGTAGAACGACGCTGTTCTGTAGCCATTTAACTTTAAATAATTTACAAAAATTTAAAATG

AGCATCCGTATAAAAATCGATAAATTGCGCCAAATTGTGGCATATTTTTCAGAGTTCAGCGAAGAAGTGT

CTATAAATGTAGACTCGACGGATGAATTAATGTATATTTTTGCCGCCTTGGGCGGATCTGTAAACATTTG

GGCCATTATACCTCTCAGTGCATCAGTGTTCTACCGCGGAGCCGAAAATATTGTGTTTAACCTTCCAGTG

TCCAAGGTAAAATCGTGTTTGTGTAGTTTTCACAATGATGCTATCATAGATATAGAACCTGATCTGGAAA

ATAATCTAGTAAAACTTTCTAGTTATCATGTAGTAAGTGTCGATTGTAACAAGGAACTGATGCCTATTAG

GACAGATACTACTATTTGTCTAAGTATAGATCAAAAGAAATCTTACGTATTTAATTTTCACAAGTATGAA

GAAAAATGTTGTGGTAGAACCGTCATTCATCTAGAATGGTTGTTGGGCTTTATCAAGTGTATTAGTCAGC

ATCAGCATTTGGCTATTATGTTTAAAGATGACAATATTATTATGAAGACTCCTGGTAATACTGATGCGTT

TTCCAGGGAATATTCTATGACTGAATGTTCTCAAGAACTACAAAAGTTTTCTTTCAAAATAGCTATCTCG

TCTCTCAACAAACTACGAGGATTCAAAAAGAGAGTCAATGTTTTTGAAACTAGAATCGTAATGGATAATG

ACGATAACATTCTAGGAATGTTGTTTTCGGATAGAGTTCAATCCTTTAAGATTAACATCTTTATGGCGTT

TTTAGACTAATACTTTCAATGAGATAAATATGGGTGGCGGAGTAAGTGTTGAGCTCCCTAAACGGGATCC

ACCTCCGGGAGTACCCACTGATGAGATGTTATTAAACGTGGATAAAATGCATGACGTGATAGCTCCCGCT

AAGCTTTTAGAATATGTGCATATAGGACCACTAACAAAAGATAAAGAGGATAAAGTAAAGAAAAGATATC

CAGAGTTTAGATTAGTCAACACAGGACCCGGTGGTCTTTCGGCATTATTAAGACAATCATATAATGGAAC

CGCACCCAATTGCTGTCGCACTTTTAATCGTACTCATTATTGGAAGAAGGATGGAAAGATATCAGATAAG

TATGAAGAGGGTGCAGTATTAGAATCGTGTTGGCCCGACGTCCACGACACTGGAAAATGCGATGTTGATT

TATTCGACTGGTGTCAGGGGGATACGTTCGATATGAACATATGCCATCAGTGGATCGGTTCAGCCTTTAA

TAGGAGTGATAGAACTGTAGAGGGTCGACAATCGTTAATAAATCTGTATAATAAGATGCAAAGATTATGT

AGTAAAGATGCTAGTGTACCAATATGTGAATTATTTTTGCATCATTTACGCGCACACAATACAGAAGATA

GTAAAGAGATGATCGATTATATTCTAAGACAACAGTCGGCGGACTTTAAACAGAAATATATGAGATGTAG

TTATCCCACTAGAGATAAGTTAGAAGAGTCATTAAAATATGCGGAACCTCGAGAATGTTGGGATCCAGAG

TGTTCGAATGCCAATGTTAATTTCTTACTAACACGTAATTATAATAATTTAGGACTTTGCAATATTGTAC

GATGTAATACGAGCGTGAATAACTTACAGATGGATAAAACTTCCTCATTAAGATTATCATGTGGATTAAG

CAATAGTGATAGATTTTCTACTGTTCCCGTCAATAGAGCAAAAGTAGTTCAACATAATATTAAACATTCG

TTCGACCTAAAATTGCATTTGATCAGTTTATTATCTCTCTTGGTAATATGGATACTAATTGTAGCTATTT

AAATGGGTGCCGCAGCAAGCATACAGACGACTGTGAATACACTCAGTGAACGTATCTCGTCTAAATTAGA

ACAAGAAGCGAACGCTAGTGCTCAAACAAAATGTGATATAGAAATCGGAAATTTTTATATCCGACAAAAC

CATGGATGTAACATCACTGTTAAAAATATGTGCTCTGCGGACGCGGATGCTCAGTTGGATGCTGTGTTAT

CAGCCGCTACAGAAACATATAGTGGATTAACACCGGAACAAAAAGCATACGTACCAGCTATGTTTACTGC

TGCGTTAAACATTCAGACGAGTGTAAACACTGTTGTTAGAGATTTTGAAAATTATGTGAAACAGACTTGT

AATTCTAGCGCTGTTGTCGATAACAAATTAAAGATACAAAACGTAATTATAGATGAATGTTACGGAGCCC

CAGGATCTCCAACAAATTTGGAATTTATTAATACAGGATCTAGCAAAGGAAATTGTGCCATTAAGGCGTT

GATGCAATTGACTACTAAGGCCACTACTCAAATAGCACCTAGACAAGTTGCTGGTACAGGAGTTCAGTTT

TATATGATTGTTATCGGTGTTATAATATTGGCAGCGTTGTTTATGTACTATGCCAAGCGTATGCTGTTCA

CATCCACCAATGATAAAATCAAACTTATTTTAGCCAATAAGGAAAACGTCCATTGGACTACTTACATGGA

CACATTCTTTAGAACTTCTCCGATGATTATTGCTACCACGGATATACAAAACTGAAAATATATTGATAAT

ATTTTAATAGATTAACATGGAAGTTATCGCTGATCGTCTAGACGATATAGTGAAACAAAATATAGCGGAT

GAAAAATTTGTAGATTTTGTTATACACGGTCTAGAGCATCAATGTCCTGCTATACTTCGACCATTAATTA

GGTTGTTTATTGATATACTATTATTTGTTATAGTAATTTATATTTTTACGGTACGTCTAGTAAGTAGAAA

TTATCAAATATTGTTGGTGTTGGTGGCGCTAGTCATCACATTAACTATTTTTTTATTACTTTATACTATA

ATAGTACTAGACTGACTTCTAACAAACATCTCACCTGCCATAAATAAATGCTTGATATTAAAGTCTTCTA

TTTCTAACACTATTCCATCTGTGGAAAATAATACTCTGACATTATCGCTAATTGATACATCGGTAAGTGA

TATGCCTATAAAGTAATAATCTTCTTTGGGCACATATACCAGTGTACCAGGTTCTAACAACCTATTTACT

GGTGCTCCTGTAGCATACTTTTTTTTTACCTTGAGAATATCCATTGTTTGCTTGGTCAATAGTGATATGT

GATTTTTTATCAACCACTCAAAAAAGTAATTGGAGTGTTCATATCCTCTACGGGCTATTGTCTCATGACC

GTGTATGAAATTTAAGTAACACGACTGTGGTAGATTTGTTCTATAGAGCCGGTTGCCGCAAATAGATAGA

ACTACCAATATGTCTGTACAAATGTTAAACATTAATTGATTAACAGAAAAAACAATGTTCGTTCTGGGAA

TAGAAACCAGATTAAAACAAAATTCATTAGAATATATGCCACGTTTATACATGGAATATAAAATAACTAC

AGTTTGAAAAATAACAGTATCATTTAAACATTTAACTTGCGGGGTTAATCTCACAACTTTACTGTTTTTG

AACTGTTCAAAATATAGCATAGATCCATGAGAAATACGTTTAGCCGCCTTTAATAGAGGAAATCCAACCG

CCTTTCTGGATCTCACCAACGACGATAGTTCTGACCAGCAACTCATTTCTTCATCATCCACCTGTTTTAA

CATATAATAGGCAGGAGATAGATATCCATCATTGCAATATTCCTTCTCGTAGGCACACAATCTAATATTG

ATAAAATCTCCATTCTCTTCTCTGTATTTATTATCTTGTCTCGGTGGCTGATTAGGCTGTGGTCTATCGT

TGTTGAATCTATTTTGGTCATTAAATCTTTCATTTCTTCCTGGTATATTTCTATCACCTCGTTTGGTTGG

ATTTTTGTCTATATTATCGTTTGTAACATCGGTACGGGTATTCATTTATCACAAAAAAAACTTCTCTAAA

TGAGTCTACTACTAGAAAACCTCATCGAAGAAGATACCATATTTTTTGCAGGAAGTATATCTGAGTATGA

TGATTTACAAATGGTTATTGCTGGTGCAAAATCCAAATTTCCAAGATCTATGCTTTCTATTTTTAATATA

GTACCTAGAACGATGTCAAAATATGAGTTGGAGTTGATTCATAACGAGAATATCACAGGGGCAATGTTTA

CCACAATGTATAATATAAGAAACAATTTGGGTCTAGGCGATGATAAACTAACTATTGAAGCCATTGAAAA

CTATTTCTTGGATCCTAACAATGAGGTTATGCCTCTTATCATTAATAATACGGATATGACTACCGTCATT

CCTAAAAAAAGTGGTAGGAGAAAGAATAAGAACATGGTTATCTTCCGTCAAGGATCATCACCTATCTTGT

GTATTTTCGAAACTCGTAAAAAGATTAATATTTATAAAGAAAATATGGAATCCGTATCGACTGAGTATAC

ACCTATCGGAGACAACAAGGCTTTGATATCTAAATATGCGGGAATTAATATCCTGAATGTGTATTCTCCT

TCCACGTCCATGAGATTGAATGCCATTTACGGATTCACCAATAAAAATAAACTAGAGAAACTTAGTACTA

ATAAGGAACTAGAATCGTATAGTTCTAGCCCTCTTCAAGAACCCATTAGGTTAAATGATTTTCTGGGACT

ATTGGAATGTGTTAAAAAGAATATTCCTCTAACAGATATTCCGACAAAGGATTGATTACTATAAATGGAG

AATGTTCCTAATGTATACTTTAATCCTGTGTTTATAGAGCCCACGTTTAAACATTCTTTATTAAGTGTTT

ATAAACACAGATTAATAGTTTTATTTGAAGTATTCGTTGTATTCATTCTAATATATGTATTTTTTAGATC

TGAATTAAATATGTTCTTCATGCCTAAACGAAAAATACCCGATCCTATTGATAGATTACGACGTGCTAAT

CTAGCGTGTGAAGACGATAAATTAATGATCTATGGATTACCATGGATAACAACTCAAACATCTGCGTTAT

CAATAAATAGTAAACCGATAGTGTATAAAGATTGTGCAAAGCTTTTGCGATCAATAAATGGATCACAACC

AGTATCTCTTAACGATGTTCTTCGCAGATGATGATTCATTTTTTAAGTATTTTGCTAGTCAAGATGATGA

ATCTTCATTATCTGATATATTGCAAATCACTCAATATCTAGACTTTCTGTTATTATTATTGATCCAATCA

AAAAATAAATTAGAAGCTGTGGGTCATTGTTATGAATCTCTTTCAGAGGAATACAGACAATTGACAAAAT

TCACAGACTCTCAAGATTTTAAAAAACTGTTTAACAAGGTCCCTATTGTTACAGATGGAAGGGTCAAACT

TAATAAAGGATATTTGTTCGACTTTGTGATTAGTTTGATGCGATTCAAAAAAGAATCAGCTCTAGCTACC

ACCGCAATAGATCCTGTTAGATACATAGATCCTCGTCGTGATATCGCATTTTCTAACGTGATGGATATAT

TAAAGTCGAATAAAGTTGAAAAATAATTAATTCTTTATTGTTATCATGAACGGCGGACATATTCAGTTGA

TAATCGGCCCCATGTTTTCAGGTAAAAGTACAGAATTAATTAGACGAGTTAGACGTTATCAAATAGCTCA

ATATAAATGTGTGACTATAAAATATTCTAACGATAATAGATACGGAACGGGACTATGGACACATGATAAG

AATAATTTTGCAGCATTGGAAGTAACTAAACTATGTGATGTCTTGGAAGCAATTACAGATTTCTCCGTGA

TAGGTATAGATGAAGGACAGTTCTTTCCAGACATTGTTGAATTCTGTGAGCGTATGGCAAACGAAGGAAA

AATAGTTATAGTAGCCGCGCTCGATGGGACATTTCAACGTAGACCGTTTAATAATATTTTGAATCTTATT

CCATTATCTGAAATGGTGGTAAAACTAACTGCAGTGTGTATGAAATGCTTTAAGGAGGCTTCCTTTTCTA

AACGATTAGGTACAGAAACCGAGATAGAAATAATAGGAGGTAATGATATGTATCAATCTGTGTGTAGAAA

GTGTTACATCGACTCATAATATTATATTTTTTATCTAAAAAACTAAAAATAAACATTGATTAAATTTTAA

TATAATACTTAAAAATGGATGTTGTGTCGTTAGATAAACCGTTTATGTATTTTGAGGAAATTGATAATGA

GTTAGATTACGAACCAGAAAGTGCAAATGAGGTCGCAAAAAAACTGCCGTATCAAGGACAGTTAAAACTA

TTACTAGGAGAATTATTTTTTCTTAGTAAGTTACAGCGACACGGTATATTAGATGGCGCCACCGTAGTGT

ATATAGGATCTGCTCCAGGTACACATATACGTTATTTGAGAGATCATTTCTATAATTTAGGAGTGATCAT

CAAATGGATGCTAATTGACGGCCGCCATCATGATCCTATTCTAAATGGATTGCGTGATGTGACTCTAGTG

ACTCGGTTTGTTGATGAGGAATATCTACGATCCATCAAAAAACAACTACATCCTTCTAAGATTATTTTAA

TTTCTGATGTGCGATCCAAACGAGGAGGAAATGAACCTAGTACTGCGGATTTACTAAGTAATTATGCTCT

ACAAAATGTCATGATTAGTATTTTAAACCCCGTGGCGTCTAGTCTTAAATGGAGATGCCCGTTTCCAGAT

CAATGGATCAAGGACTTTTATATCCCACACGGTAATAAAATGTTACAACCTTTTGCTCCTTCATATTCAG

CTGAAATGAGATTATTAAGTATTTATACCGGTGAGAATATGAGACTGACTCGAGTTACCAAATCAGACGC

TGTAAATTATGAAAAAAAGATGTATTACCTTAATAAGATAGTCCGCAACAAAGTAGTTATTAACTTTGAT

TATCCTAATCAGGAATATGACTATTTTCACATGTACTTTATGTTGAGGACCGTATACTGCAATAAAACAT

TTCCTACTACTAAAGCAAAGATACTATTTCTACAACAATCTATATTTCGTTTCTTAAATATTCCAACGAC

ATCAACTGAAAAAGTTAGTCATGAACCAATACAACGTAAAATATCTAGCAAAGATTCTATGTCTAAAAAC

AGAAATAGCAAGAGATCCGTACGCGGTAATAAATAGAAACGTACTACTGAGATATACTACCGATATAGAG

TATAATGATTTAGTTACTTTAATAACCGTTAGACATAAAATTGATTCTATGAAAACTGTGTTTCAGGTAT

TTAACGAATCATCCATAAATTATACTCCGGTTGATGATGATTATGGAGAACCAATCATTATAACATCGTA

TCTTCAAAAAGGTCATAACAAGTTTCCTGTAAATTTTCTATACATAGATGTGGTAATATCTGACTTATTT

CCTAGCTTTGTTAGACTAGATACTACAGAAACTAATATAGTTAATAGTGTACTACAAACAGGCGATGGTA

AAAAGACTCTTCGTCTTCCTAAAATGTTAGAGACGGAAATAGTTGTCAAGATTCTCTATCGTCCTAATAT

ACCATTAAAAATTGTTAGATTTTTCCGCAATAACATGGTAACTGGAGTAGAGATAGCCGATAGATCTGTT

ATTTCAGTCGCTGATTAATCAATTAGTAGAGATGAGATAAGAACATTATAATAATCAATAATATATCTTA

TATCTGTTTAGAAAAATGCTAATATTAAAATAGCTAACGCTAGTAATCCAATCGGAAGCCATTTGATATC

TATAATAGGGTATCTAATTTCCTGATTCAGATAGCGTACGGCTATATTCTCGGTAGCTACTCGTTTGGAA

TCACAGACATTATTTACATCTAATTTACTATCTGTAATGGAAACGTTTCCCAATGAAATGGTACAATCAG

ATACATTACATCTTGATATATTTTTTTTTAAAGAGGCTGGTAACAACGCATCGCTTCGTTTACATGGCTC

GTACCAACAATAATAGGGTAATCTTGTATCTATTCCTATCCGTACTATACTTTTATCAGGATAAATACAT

TTACATCGTATATCGTCTTTGTTAGCATCACAGAATGCATAAATTTGTTCGTCCGTCATGATAAAAATTT

AAAGTGTAAATATAACTATTATTTTTATAGTTATAATAAAAAGGGAAATTTGATTGTATACCTTCGGTTC

TTTAAAAGAAACTGACTTGATAAAAATGGCTGTAATCTCTAAGGTTACGTATAGTCTATACGATCAAAAA

GAGATTAATGCCACAGATATTATCATTAGTCATATTAAAAATGACGACGATATCGGTACCGTTAAAGATG

GTAGACTAGGTGCTATGGATGGGGCATTATGTAAGACTTGTGGGAAAACGGAATTGGAATGTTTCGGTCA

CTGGGGTAAAGTAAGTATTTATAAAACTCATATAGTTAAGCCTGAATTTATTTCAGAAATTATTCGTTTA

CTGAATCATATATGTATTCATTGCGGATTATTGCGTTCACGAGAACCGTATTCCGACGATATTAACCTAA

AAGAGTTATCGGTACACGCTCTTAGGAGATTAAAGGATAAAATATTATCCAAGAAAAAGTCATGTTGGAA

CAGCGAATGTATGCAACCGTATCAAAAAATTACTTTTTCAAAGAAAAAGGTTTGTTTCGTCAACAAGTTG

GATGATATTAACGTTCCTAATTCTCTCATCTATCAAAAGTTAATTTCTATTCATGAAAAGTTTTGGCCAT

TATTAGAAATTCATCAATATCCAGCTAACTTATTTTATACAGACTACTTTCCCATCCCTCCGTTGATTAT

TAGACCGGCTATTAGTTTTTGGATAGATAGTATACCCAAAGAGACAAATGAATTAACTTACTTATTAGGT

ATGATCGTTAAGAATTGTAACTTGAATGCTGATGAACAGGTTATCCAGAAGGCGGTAATAGAATACGATG

ATATTAAAATTATTTCTAATAACACTACCAGTATCAATTTATCATATATCACATCCGGCAAAAATAATAT

GATTAGAAGTTATATCGTCGCTCGGCGAAAAGATCAGACCGCTAGATCCGTAATTGGTCCCAGTACATCT

ATCACCGTTAATGAGGTAGGAATGCCCACATATATTAGAAATACACTTACAGAAAAGATATTTGTTAATG

CCTTTACAGTGGATAAAGTTAAACAACTATTAGCATCAAACCAAGTTAAATTTTACTTTAATAAACGATT

AAACCAATTAACAAGAATACGTCAAGGAAAGTTTATCAAAAATAAAATACATTTATTGCCTGGTGATTGG

GTAGAAGTAGCTGTTCAAGAATATACAAGTATTATTTTTGGAAGACAACCGTCTCTACATAGATACAACG

TCATCGCTTCATCTATCAGAGCTACCGAAGGAGATACTATCAAAATATCTCCCGGAATTGCCAACTCTCA

AAATGCTGATTTCGACGGAGATGAAGAATGGATGATATTGGAGCAAAATCCTAAAGCCGTAGTTGAACAA

AGTATTCTTATGTATCCGACGACGTTACTCAAACACGATATTCATGGAGCCCCCGTTTATGGATCTATTC

AAGATGAAATCGTAGCAGCGTATTCATTGTTTAGGATACAAGATCTTTGTTTAGATGAAGTATTGAACAT

CTTGGGGAAATATGGAAGAGAGTTCGATCCTAAAGGTAAATGTAAATTCAGCGGTAAAGATATCTATACT

TACTTGATAGGTGAAAAGATTAATTATCCGGGTCTCTTAAAGGATGGTGAAATTATTGCAAACGACGTAG

ATAGTAATTTTGTTGTAGCTATGAGGCATCTGTCATTGGCTGGACTCTTATCCGATCATAAATCGAACGT

GGAAGGTATCAACTTTATTATCAAGTCATCTTATGTTTTTAAGAGATATCTATCTATATACGGTTTTGGG

GTGACATTCAAAGATCTGAGACCAAATTCGACGTTCACTAATAAATTGGAGGCTATCAACGTAGAAAAAA

TAGAACTTATCAAAGAAGCATACGCCAAATATCTCAAAGATGTAAGAGACGGGAAAATAGTTCCATTATC

TAAAGCTTTAGAGGCGGACTACTTGGAATCCATGTTATCCAACTTGACAAATCTTAATATCAGAGAGATA

GAAGAACATATGAGACAAACGCTGATAGATGATCCAGATAATAACCTCCTGAAAATGGCCAAAGCGGGTT

ATAAAGTAAATCCCACAGAACTAATGTATATTCTAGGTACTTATGGACAACAGAGGATAGATGGCGAACC

AGCAGAGACTCGAGTATTGGGTAGAGTCTTACCTTACTATCTTCCAGACTCTAAGGATCCAGAAGGAAGA

GGTTATATTCTTAATTCTTTAACAAAAGGATTAACGGGTTCTCAATATTACTTTTCGATGCTGGTTGCAA

GATCTCAATCTACTGATATTGTCTGTGAAACATCACGTACCGGAACACTGGCTAGAAAAATCATTAAAAA

GATGGAGGATATGGTGGTCGACGGATACGGACAAGTAGTTATAGGTAATACGCTCATCAAGTACGCAGCC

AATTATACCAAAATTCTAGGCTCAGTATGTAAACCTGTAGATCTTATCTATCCAGATGAGTCCATGACTT

GGTATTTGGAAATTAGTGCTTTGTGGAATAAAATAAAACAGGGATTCGTTTACTCTCAGAAACAGAAACT

TGCAAAGAAGACATTGGCGCCGTTTAATTTCCTAGTATTCGTCAAACCCACCACTGAGGATAATGCTATT

AAGGTTAAGGATCTGTACGATATGATTCATAACGTCATTGATGATGTGAGAGAGAAATACTTCTTTACGG

TATCTAATATAGATTTTATGGAGTATATATTCTTGACGCATCTTAATCCTTCTAGAATTAGAATTACAAA

AGAAACGGCTATTACTATCTTTGAAAAGTTCTATGAAAAACTCAATTATACTCTAGGTGGTGGAACTCCT

ATTGGAATTATTTCTGCACAGGTATTGTCTGAGAAGTTTACACAACAAGCCCTGTCCAGTTTTCACACTA

CTGAAAAGAGTGGTGCTGTAAAACAAAAACTTGGTTTCAACGAGTTTAATAACTTGACTAATTTGAGTAA

GAATAAGACCGAAATTATCACTCTGGTATCCGATGATATCTCTAAACTTCAATCTGTTAAGATTAATTTC

GAATTTGTATGTTTGGGAGAATTAAATCCAGACATCACTCTTCGAAAAGAAACAGATAGATATGTAGTAG

ACATAATAGTCAATAGATTATACATCAAGAGAGCAGAAATAACCGAATTAGTCGTCGAATATATGATTGA

ACGATTTATCTCCTTTAGCGTCATTGTAAAGGAATGGGGTATGGAGACATTCATTGAGGACGAGGATAAT

ATTAGATTTACTATCTACCTAAATTTCGTTGAACCGGAGGAATTGAATCTTAGTAAGTTTATGATGGTTC

TTCCAGGTGCCGCCAACAAGGGCAAGATTAGTAAATTCAAGATTCCTATCTCTGACTATACGGGATATAA

CGACTTCAATCAAACAAAAAAGCTCAATAAGATGACTGTAGAACTCATGAATCTAAAAGAATTGGGTTCT

TTCGATTTGGAGAACGTCAACGTGTATCCTGGAGTATGGAATACATACGATATCTTCGGTATTGAGGCCG

CTCGTGGATACTTGTGCGAAGCCATGTTAAACACCTATGGAGAAGGTTTCGATTATCTGTACCAGCCTTG

TGATCTTCTCGCTAGTTTACTATGTGCTAGTTACGAACCAGAATCAGTTAATAAATTCAAGTTCGGTGCA

GCTAGTACTCTTAAGAGAGCTACGTTCGGAGATAATAAAGCATTGTTAAACGCGGCTCTTCATAAAAAGT

CAGAACCTATTAACGATAATAGTAGCTGCCACTTTTTTAGCAAGGTCCCTAATATAGGAACTGGATATTA

CAAATACTTTATCGACTTGGGTCTTCTCATGAGAATGGAAAGGAAACTATCTGATAAGATATCTTCTCAA

AAGATCAAGGAGATAGAAGAAACAGAAGACTTTTAATTCTTATCAATAACATATTTTTCTATGATCTGTC

TTTTAAACGATGGATTTTCCACAAATGCGCCTCTCAAGTCCCTCATAGAATGATACACGTATAAAAAATA

TAGCATAGGTGATGACTCCTTATTTTTAGACATTAGATATGCCAAAATCATAGCCCCGCTTCTATTTACT

CCTGCAACACAATGAACCAACACGGGCTCGTTTCGTTGATCACATTTAGATAAGAAGGCGGTCACGTCGT

CAAAATATTTACTAATATCAGTAGTTGTATCATCTACCAACGGTATATGAATAATATTAATATTAGAGTT

AGGTAATGTATATTTATCCATCGTCAAATTTAAAACATATTTGAACTTAACTTCAGATGATGGTGCATCC

ATAGCATTTTTATAATTTCCCAAATACACATTATTTGTTACTCTTGTCATTATAGTGGGAGATTTGGCTC

TGTGCATATCTCCAGTTGAACGTAGTAGTAAGTATTTATACAAACTTTTCTTATCCATTTATAACGTACA

AATGGATAAAACTACTTTATCAGTAAACGCATGCAATTTAGAATACGTTAGAGAAAAGGCTATAGTAGGC

GTACAAGCAGCCAAGACATCAACACTTATATTTTTTGTTATTATATTGGCAATTAGTGCGCTATTACTCT

GGTTTCAGACGTCTGATAATCCAGTCTTTAATGAATTAACGAGATATATGCGAATTAAAAATACGGTTAA

CGATTGGAAATCATTAACGGATAGCAAAACAAAATTAGAAAGCGATAGAGGTAGACTTCTAGCCGCTGGT

AAGGATGATATATTCGAATTCAAATGTGTGGATTTCGGCGCCTATTTTATAGCTATGCGATTGGATAAGA

AAACATATCTGCCGCAAGCTATTAGGCGAGGTACTGGAGACGCGTGGATGGTTAAAAAGGCGGCAAAAGT

CGATCCATCTGCTCAACAATTTTGTCAGTATTTGATAAAACACAAGTCTAATAATGTTATTACTTGTGGT

AATGAGATGTTAAATGAATTAGGTTATAGCGGTTATTTTATGTCACCGCATTGGTGTTCCGATCTTAGTA

ATATGGAATAAGTGTTAGATAAATGCGGTAACAAATGTTCCTGTAAGGAACCATAACAGTTTAGATTTAA

CATTAAAGATGAGCATAAACATAATAAACAAAATTACAATCAAACCTATAACATTAATATCAAACAATCC

AAAAAATGAAATCAATGGAGTAGTAAACGTGTACATAACTCCTGGATAACGTTTAGCAGCTACCGTTCCT

ATTCTAGACCAAAAATTTGGTTTCATGGTTTCGAAGCGGTGTTCTGCAACAAGACGAGGATCGTGTTCTA

CATATTTGGCAGAGTTATCCATTATTTGCCTGTTAATCTTCATTTCGTTTTCGATTCTGGCTATTTCAAA

ATAAAATCCCGATGATAGACCTCCAGACTTTATAATTTCATCTACGATGTTCAGCGCCGTAGTAACTCTA

ATAATATAGGCGGATAAGCTAACATCATACCCTCCTGTATATGTAAATATGGCATGATCTTTGTCTATTA

CAAGCTCGGTTTTAACTTTATTTCCTGTAATAATTTCTCTCATCTGTAGGATATCTATTTTCTTGTCATG

TATTGCCTTCAAGACGGGACGAAGAAACGTAATATCCTCAATAACGTTATCGTTTTCTATAATAACTACA

TATTCTACATTTTTATTTTCTAGCTCGGTAAAAAATTTAGAATCCCATAGGGCTAAATGTCTAGCGATAT

TTCTTTTCGTTTCCTCTGTACACATAGTGTTACAAAACCCTGAAAAGAAGTGAGTATACTTGTCATCATC

TCTAATATTTCCTCCAGTCCATTGTATAAACACATAATCCTTGTAATGATCTGGATCATCATTGACTATC

ACAACATCTCTTTTTTCTTGCATAACTTCATTGTCCTTCACATCATCGAACTTCTGATCATTAATATGCT

CATGAACATTAGGAAATGTTTCTGATGGAGGTCTATCAATAACTGGCACAACAATAACAGGAGTTTTCAC

CGCCGCCATTTAGTTATTGAAATTAATCATATACAACTCTCTAATACGAGTTATATTTTCGTCTATCCAT

TGTTTCACATTGACATATTTCGACAAAAAGATATAAAATGCGTATTCCAATGCTTCTCTGTTTAATGAAT

TACTAAAATATACAAACACGTCACTGTCTGGTAATAAATGATATCTTAGAATATTGTAACAATTTATTTT

GTATTGCACATGTTCGTGATCTATGAGTTCTTCTTCGAATGGCATAGGATCTCCGAATCTGAAAACGTAT

AAATAGGAGTTAGAATAATAATATTTGAGAGTATTGGTAATGTATAAACTCTTTAGCGGTATAATTAGTT

TTTTTCTCTCGATTTCTATTTTTAGATGTGATGGAAAAATGACTAATTTTGTAGCATTAGTATCATGAAC

TCTAATCAAAATCTTAATATCTTCGTCACATGTTAGCTCTTTGAAGTTTTTAAGAGATGCATCAGTTGGT

TTTACAGATGGAGTAGGTGCAACAATTTTTTGTTTAATGCATGCATGTATTGGAGCCATTGTCTTAACTA

TAATGGTGCTTGTATCGAAAAACTTTAATGCGGATAACGGAAGCTCTTCGCCGCGACTTTCTACGTCGTA

ATTGGGTTCTAATGCCGATCTCTGAATGGATACTAGTTTTCTAAGTTCTAATGTAATTCTCTGAAAATGT

AAATCCAATTCCTCCGGCATTATAGATGTGTATACATCGGTAAATAAAACTATAGTATCCAACGATCCCT

TCTCGCAAATTCTAGTCTTAACCAAGAAATCGTATATAACTACGGAGATGGCGTATTTAAGAGTGGATTC

TTCTACCGTTTTGTTCTTGGATTTCATATAAGAAACTATAAAGTCCGCACTACTGTTAAGAATGATCACT

AACGCAACTATATAGTTCAAATTAAGCATCTTGGAAACATAAAATAACTCTGTAGATGATACTTGACTTT

CGAATAAGTTTGCAGACAAACGAAGAAAGAACAGACCTCTCTTAATTTCAGAAGAAAACTTTTTTTCGTA

TTCCTGACGTCTAGAGTTTATATCAATAAGAAAGTTAAGAATTAGTCGGTTAATGTTGTATTTCATTACC

CAAGTTTGAGATTTCATAATATTGTCAAAAGACATGATAATATTAAAGATAAAGCGCTGACTATGAACGA

AATAGCTATATGGTTCGCTCAAGAATATAGTCTTGTTAAACGTGGAAACGATAACTGTATTTTTAATCAC

GTCAGCGGCATCTAAATTAAATATAGGTATATTTATTCCACACACTCTACAATATGCCACACCATCTTCA

TAATAAATAAATTCGTTAGCAAAATTATTAATTTTAGTGAAATAGTTAGCGTCAACTTTCATAGCTTCCT

TCAATCTAATTTGATGCTCACATGGCGCGAATTCTACTCTAACATCCCTTTTCCATGCCTCAGGTTCATC

GATCTCTATAATATCTAGTTTCTTGCGTTTCACAAACACAGGCTCGTCTCTCGCGATGAGATCTGTATAG

TAACTATGTAAATGATAACTAGATAGAAAGATGTAGCTATATAGATGACGATCCTTTAAGAGAGGTATAA

TAACTTTACCCCAATCAGATAGACTGTTGTTATGGTCTTCGGAAAAAGAATTTTTATAAATTTTTCCAGT

ATTTTCTAAATATACGTACTTGATATCTAAGAAATCCTTAATAATAATAGGAATGGATAATCCGTCTATT

TTATAAAGAAATACATATCGCATATTATACTTTTTTTTGGAAATTGGAATACCGATGTGTCTACATAAAT

ACGCAAAGTCTAAATATTTTTTAGAGAATCTTAGTTGGTCCAAATTCTTTTCCAAGTACGGTAATAGATT

TTTCATATTGAACGGTATCTTCTTGATCTCTGGTTCTAATTCCGCATTAAATGATGAAACTAAGTCACTA

TTTTTATAACTAACGATTACATCACCTCTAACATCATCATTTACCAGGATACTGATCTTCTTTTGTCGTA

AATACATGTCTAATGTGTTAAAAAAAAGATCATACAAGTTATACGTCATTTCATCTGTAGTATTCTTGTC

ATTGAAGGATAAACTCGTACTAATCTCTTCTTTAACAGTCTGTTCAAATTTATATCCTATATATGAAAAA

ATAGCAACCAGTGTTTGATCATCCGCGTCAATATTCTGTTCTATCGTAGTGTATAACAATCTTATATCTT

CTTCTGTGATAGTCGATACGTTATAAAGGTTGATAACGAAAATATTTTTATTTCGTGAAATAAAGTCATT

GTAGGATTTTGGACTTATATTCGTGTCTAGTAGATATGATTTTATTTTTGGAATGATCTCAATTAAAATA

GTCTCTTTAGAGTCCATTTAAAGTTACAAACAACTAGGAAATTGGTTTATGATGTATAATTTTTTTAGTT

TTTATAGATTCTTTATTCTATACTTAAAAAATGAAAATAAATACAAAGGTTCTTGAGGGTTGTGTTAATT

GAAAGCGATAAATAATCATAAATTATTTCATTATCGCGATATCCGTTAAGTTTGTATCGTAATGGCGTGG

TCAATTACGAATAAAGCGGATACTAGTAGTTTCACAAAGATGGCTGAAATCAGAGCTCATCTAAGAAATA

GCGCTGAAAATAAAGATAAAAACGAGGATATTTTCCCGGAAGATGTAATAATTCCATCTACTAAGCCCAA

AACCAAACGAACCACTACTCCTCGTAAACCAGCGGCTACTAAAAGATCAACCAAAAAGGATAAAGAAAAG

GAGGAAGTGGAAGAAGTAGTTATAGAGGAATATCATCAAACAACTGAAGAAAATTCTCCACCTCCGTCAT

CATCTCCTGGAGTCGGCGACATTGTAGAAAGCGTGGCCGCTGTAGAGCTCGATGATAGCGACGGGGATGA

TGAACCTATGGTACAAGTTGAAGCTGGTAAAGTAAATCATAGTGCTAGAAGCGATCTCTCTGACCTAAAG

GTGGCTACCGACAATATCGTTAAAGATCTTAAGAAAATTATTACTAGAATCTCTGCAGTATCGACTGTTC

TAGAGGATGTTCAAGCAGCTGGTATCTCTAGACAATTTACTTCTATGACTAAAGCTATTACAACACTATC

TGATCTAGTCACCGAGGGAAAATCTAAAGTTGTTCGTAAAAAAGTTAAAACTTGTAAGAAGTAAATGCGT

GCACTTTTTTATAAAGATGGTAAACTGTTTACCGATAATAATTTTTTAAATCCTGTATCAGACGATAATC

CAGCGTATGAGGTTTTGCAACATGTTAAAATTCCTACTCATTTAACAGATGTAGTAGTATATGAACAAAC

GTGGGAAGAGGCATTAACTAGATTAATTTTTGTGGGAAGTGATTCAAAAGGACGTAGACAATACTTTTAC

GGAAAAATGCATATACAGAATCGCAATGCTAAAAGAGATCGTATTTTTGTTAGAGTATATAACGTTATGA

AACGAATTAATTGTTTTATAAACAAAAATATAAAGAAATCGTCCACAGATTCCAATTATCAGTTGGCGGT

TTTTATGTTAATGGAAACTATGTTTTTTATTAGATTTGGTAAAATGAAATATCTTAAGGAGAATGAAACA

GTAGGGTTATTAACACTAAAAAATAAACACATAGAAATAAGTCCCGATGAAATAGTTATCAAGTTTGTAG

GAAAGGACAAAGTTTCACATGAATTTGTTGTTCATAAGTCTAATAGACTATATAAACCGCTATTGAAACT

GACTGATGATTCTAGTCCCGAAGAATTTCTGTTCAACAAACTAAGTGAACGAAAGGTATATGAATGTATC

AAACAGTTTGGTATTAGAATCAAGGATCTCCGAACGTATGGAGTCAATTATACGTTTTTATATAATTTTT

GGACAAATGTAAAGTCCGTATCTCCTCTTCCATCACCAAAAAAGTTGATAGCATTAACTATCAAACAAAC

TGCTGAAGTGGTAGGTCATACTCCATCAATTTCAAAAAGAGCTTATATGGCAACGACTATTTTAGAAATG

GTAAAGGATAAAAATTTTTTAGACGTAGTATCTAAAACTACGTTCGATGAATTCCTATCTATAGTCGTAG

ATCACGTTAAATCATCTACGGATGGATGATAATAGATCTTTACACAAATAATTACAAGACCGATAAATGG

AAATGGATAAACGGATGAAATCTCTCGCTATGACAGCTTTCTTCGGAGAGCTAAACACGTTAGATATTAT

GGCATTGATAATGTCTATATTTAAACACCATCCAAACAATACCATTTTTTCAGTGGATAAGGATGGTCAA

TTTATGATTGATTTCGAATACGATAATTATAAGGCTTCTCAATATTTGGATCTGACCCTCACTCCGATAT

CTGGAAATGAATGCAAGACTCACGCATCTAGTATAGCCGAACAATTGGCGTGTGTGGATATTATTAAAGA

GGATATTAGCGAATATATCAAAACTACTCCCCGTCTTAAACGATTTATAAAAAAATACCGCAATAGATCA

TATACTCGTATCAGTCGAGATACAGAAAAGCTTAAAATAGCTCTAGCTAAAGGCATAGATTACGAATATA

TAAAAGACGCTTGTTAATAAGTAAATGAAAAAAAACTAGTCGTTTATAATAAAACACGATATGGATGCCA

ACATAGTATCATCTTCTACTATTGCGACGTATATAGACGCTTTAGCGAAGAATGCTTCAGAATTAGAACA

GAGGTCTACCGCATACGAAATAAATAATGAATTGGAACTAGTATTTATTAAACCGCCATTGATTACGTTG

ACAAATGTAGTAAATATCTCCACGATTCAGGAATCGTTTATTCGATTTACCGTTACTAATAAGGAAGGTA

TCAAAATTAGAACTAAGATTCCATTATCTAAGGTACATGGTCTAGATGTAAAAAATGTGCAGTTGGTAGA

TGCTATAGATAACATAGTTTGGGAAAAGAAATCATTAGTGACGGAAAATCGTCTTCACAAAGAATGCTTG

TTGAGACTATCAACAGAGGAACGTCATATATTTTTGGATTACAAGAAATATGGATCCTCTATCCGACTAG

AATTAGTCAATCTTATTCAAGCAAAAACAAAAAACTTTACGATAGACTTTAAGCTAAAATATTTTCTAGG

ATCTGGCGCTCAATCTAAAAGTTCTTTATTGCACGCTATTAATCATCCAAAGTCAAGGCCTAATACATCT

CTGGAAATAGAATTTACACCTAGAGACAATGAAACAGTTCCATATGATGAACTAATAAAGGAATTGACGA

CTCTCTCGCGTCATATATTTATGGCTTCTCCAGAGAATGTAATTCTTTCTCCACCTATTAACGCACCTAT

AAAGACTTTTATGTTGCCTAAACAAGATATAGTAGGTCTGGATCTGGAAAATCTATATGCCGTAACTAAG

ACTGATGGCATTCCTATAACTATCAGAGTTACATCAAAAGGGTTGTATTGTTATTTTACACATCTTGGTT

ATATTATTAGATATCCAGTTAAGAGAACAATAGATTCCGAAGTAGTAGTCTTTGGTGAGGCAGTTAAGGA

TAAGAACTGGACCGTATATCTCATTAAGCTAATAGAGCCCGTAAATGCAATCAGTGATAGACTAGAAGAA

AGTAAGTATGTTGAATCTAAACTAGTGGATATTTGTGATCGGATAGTATTCAAGTCAAAGAAATACGAAG

GTCCGTTTACTACAACTAGTGAAGTCGTCGATATGTTATCTACATATTTACCAAAGCAACCAGAAGGTGT

TATTCTGTTCTATTCAAAGGGACCTAAATCTAACATTGATTTTAAAATCAAAAAGGAGAATACTATAGAC

CAAACTGCAAATGTAGTATTTAGGTACATGTCCAGTGAACCAATTATCTTTGGAGAGTCGTCTATCTTTA

TAGAGTATAAGAAATTTACCAACGATAAAGGCTTTCCTAAAGAATATGGTTCTGGTAAGATTGTGTTATA

TAACGGCGTTAATTATCTAAATAATATCTATTGTTTGGAATATATTAATACACATAATGAAGTGGGTATT

AAGTCCGTTGTTGTACCTATTAAGTTTATAGCAGAATTCTTAGTCAATGGAGAAATACTTAAACCTAGAA

TCGATAAAACCATGAAATATATTAACTCAGAAGACTATTATGGAAATCAACATAATATCATAGTCGAACA

TTTAAGAGATCAAAGCATCAAAATAGGAGATGTCTTTAACGAGGATAAACTATCGGATGTTGGACATCAA

TACGCTGCCAACAACGATAAATTTAGATTAAATCCAGAAGTTAGTTATTTTACTAATAAACGAACTAGAG

GGCCGTTGGGAATTTTATCAAACTACGTCAAGACTCTTCTTATTTCTATGTATTGTTCCAAAACATTTTT

AGACGATTCCAACAAACGAAAGGTATTAGCGATTGATTTTGGAAACGGTGCTGACCTGGAAAAATACTTT

TATGGAGAGATTGCGTTATTGGTAGCGACGGATCCGGATGCTGATGCTATAGCTAGAGGAAATGAAAGAT

ACAACAAATTAAATTCTGGAATTAAAACCAAGTACTACAAATTTGACTACATTCAGGAAACTATTCGATC

CGATACATTTGTCTCTAGTGTCAGAGAAGTATTCTATTTTGGAAAGTTTAATATCATTGACTGGCAGTTC

GCTATTCATTATTCTTTTCATCCAAGACATTATGCTACAGTCATGAATAACTTATCCGAACTAACTGCTT

CTGGAGGCAAGGTATTAATTACTACCATGGATGGAGACAAATTATCAAAATTAACCGATAAAAAGACTTT

TATAATTCATAAGAATCTACCTAGTAGCGAAAACTATATGTCTGTAGAAAAAATAGCTGATGATAGAATA

GTGGTATATAATCCATCAACAATGTCTACTCCAATGACTGAATACATTATCAAAAAGAACGATATAGTCA

GAGTGTTTAACGAATACGGATTTGTTCTTGTAGATAATGTTGATTTCGCTACAATTATAGAACGAAGTAA

AAAGTTTATTAATGGCGCATCTACAATGGAAGATAGACCGTCTACAAGAAACTTTTTCGAACTAAATAGA

GGAGCCATTAAATGTGAAGGTTTAGATGTCGAAGACTTACTTAGTTACTATGTTGTTTATGTCTTTTCTA

AGCGGTAAATAATAATATGGTATGGGTTCTGATATCCCCGTTCTAAATGCATTAAATAATTCCAATAGAG

CGATTTTTGTTCCTATAGGACCTTCCAACTGTGGATACTCTGTATTATTAATAGATATATTAATACTTTT

GTAGGGTAACAGAGGTTCTACGTCTTCTAAAAATAAAAGTTTTATAACATCTGGCCTGTTCATAAATAAA

AACTTGGCGATTCTATATATACTCTTATTATCAAATCTAGCCATTGTCTTATAGATGTGAGCTACTGTAG

GTGTACCATTTGATTTTCTTTCTAATACTATATATTTCTCTCGAAGAAGTTCTTGCAGATCATCTGGGAA

TAAAATACTACTGTTGAGTAAATCAGTTATTTTTTTTATATCGATATTGATGGACATTTTTATAGTTAAG

GATAATAAGTATCCCAAAGTAGATAACGACGATAACGAAGTATTTATACTTTTAGGAAATCACAATGACT

TTATCAGATCAAAATTAACAAAATTAAAGGAGCATGTATTTTTTTCTGAATATATTGTGACTCCAGATAC

ATATGGATCTTTATGCGTCGAATTAAATGGGTCTAGTTTTCAGCACGGTGGTAGATATATAGAGGTGGAG

GAATTTATAGATGCTGGAAGACAAGTTAGATGGTGTTCTACATCCAATCATATATCTGAAGATATACACA

CTGATAAATTTGTCATTTATGATATTTATACGTTTGATTCGTTCAAGAATAAACGATTGGTATTTGTACA

GGTGCCTCCATCATTAGGAGATGATAGCTATTTAACTAATCCGTTATTGTCTCCGTATTATCGTAATTCA

GTAGCCAGACAAATGGTCAATGATATGATTTTTAATCAAGATTCATTTTTAAAATATTTATTAGAACATC

TGATTAGAAGCCACTATAGAGTTTCTAAACATATAACAATAGTTAGATACAAGGATACCGAAGAATTAAA

TCTAACAAGAATATGTTATAATAGAGATAAGTTTAAGGCGTTTGTATTCGCTTGGTTTAACGGCGTTTCG

GAAAATGAAAAGGTACTAGATACGTATAAAAAGGTATCTGATTTGATATAATGAATTCAGTGACTATATC

ACACGCACCATATACTATTACTTATCACGATGATTGGGAACCAGTAATGAGTCAATTGGTAGAGTTTTAT

AACGAAGTAGCCAGTTGGTTGCTACGCGACGAGACGTCGCCTATTCCTGATAAGTTCTTTATACAATTGA

AACAGCCGCTTAGAAATAAACGAGTATGTGTGTGTGGTATAGATCCGTATCCAAAAGATGGAACTGGTGT

ACCGTTCGAATCACCAAATTTTACAAAAAAATCAATTAAGGAGATAGCTTCATCTATATCTAGATTAACC

GGAGTAATTGATTATAAAGGTTATAACCTTAATATAATAGACGGGGTTATACCCTGGAATTATTACTTAA

GTTGTAAATTAGGAGAAACAAAAAGTCACGCGATTTACTGGGATAAGATTTCCAAGTTACTGCTACAGCA

TATAACTAAACACGTTAGTGTTCTTTATTGTTTGGGTAAAACAGATTTCTCGAATATACGGGCAAAGTTA

GAATCCCCGGTAACTACCATAGTGGGATATCATCCAGCGGCCAGAGACCACCAATTCGAGAAAGATCGAT

CATTTGAAATTATCAACGTTTTACTGGAATTAGACAACAAGACACCTATAAATTGGGCTCAAGGGTTTAT

TTATTAATGCTTTAGTGAAATTTTAACTTGTGTTCTAAATGGATGCGGCTATTAGAGGTAATGATGTTAT

CTTTGTTCTTAAGACTATAGGTGTCCCGTCAGCATGCAGACAAAATGAAGATCCAAGATTCGTAGAAGCA

TTTAAATGCGACGAGTTAGAAAGATATATTGATAATAATCCAGAATGTACACTATTCGAAAGTCTTAGGG

ATGAGGAAGCATACTCTATAGTCAGAATTTTCATGGATGTAGATTTAGACGCGTGTCTAGACGAAATAGA

TTATTTAACGGCTATTCAAGATTTTATTATCGAGGTGTCAAACTGTGTAGCTAGATTCGCATTTACAGAA

TGCGGTGCCATTCATGAAAATGTAATAAAATCCATGAGATCTAATTTTTCATTGACTAAGTCTACAAATA

GAGATAAAACAAGTTTTCATATTATCTTTTTAGACACGTATACCACTATGGATACATTGATAGCTATGAA

ACGAACACTATTAGAATTAAGTAGATCATCTGAAAATCCACTAACAAGATCGATAGACACTGCCGTATAT

AGGAGAAAAACAACTCTTCGGGTTGTAGGTACTAGGAAAAATCCAAATTGCGACACTATTCATGTAATGC

AACCACCTCACGATAATATAGAAGATTACCTATTCACTTACGTGGATATGAACAACAATAGTTATTACTT

TTCTCTACAACGACGATTGGAGGATTTAGTTCCTGATAAGTTATGGGAACCAGGGTTTATTTCGTTCGAA

GACGCTATAAAAAGAGTTTCAAAAATATTCATTAATTCTATAATAAACTTTAATGATCTCGATGAAAATA

ATTTTACAACGGTACCACTGGTCATAGATTATGTAACACCTTGTGCATTATGTAAAAAACGATCGCATAA

ACATCCGCATCAACTATCGTTGGAAAATGGTGCTATTAGAATTTACAAAACTGGTAATCCACATAGTTGT

AAAGTTAAAATTGTTCCGTTGGATGGTAATAAACTGTTTAATATTGCACAAAGAATTTTAGACACTAACT

CTGTTTTATTAACCGAACGAGGAGACCATATAGTTTGGATTAATAATTCATGGAAATTTAACAGCGAAGA

ACCCTTGATAACAAAACTAATTCTATCAATAAGACATCAACTACCTAAGGAATATTCAAGCGAATTACTC

TGTCCGAGGAAACGAAAGACTGTAGAAGCTAACATACGAGACATGTTAGTAGATTCAGTAGAGACCGATA

CCTATCCGGATAAACTTCCGTTTAAAAATGGTGTATTGGACCTGGTAGACGGAATGTTTTACTCTGGAGA

TGATGCTAAAAAATATACGTGTACTGTATCGACCGGATTTAAATTTGACGATACAAAATTCGTCGAAGAC

AGTCCAGAAATGGAAGAGTTAATGAATATCATTAACGATATCCAACCATTAACGGATGAAAATAAGAAAA

ATAGAGAGCTGTATGAAAAAACATTATCTAGTTGTTTATGTGGTGCTACCAAAGGATGTTTAACATTCTT

TTTTGGAGAAACCGCAACTGGGAAGTCGACAACCAAACGTTTGTTAAAGTCTGCTATCGGTGACCTGTTT

GTCGAGACGGGTCAAACAATTTTAACAGATGTATTGGATAAAGGACCTAATCCATTTATCGCTAATATGC

ATTTAAAAAGATCTGTATTCTGTAGCGAACTACCTGATTTTGCATGTAGTGGATCAAAGAAAATTAGATC

TGATAATATTAAAAAGTTGACAGAACCTTGTGTCATTGGAAGACCGTGTTTCTCCAATAAAATTAATAAT

AGAAACCATGCGACAATCATTATCGATACTAATTACAAACCTGTCTTTGATAGGATAGATAACGCATTAA

TGAGAAGAATTGCCGTCGTGCGATTCAGAACACACTTTTCTCAACCTTCTGGTAGAGAGGCTGCTGAAAA

TAATGACGCGTACGATAAAGTCAAACTATTAGACGAGGGATTAGATGGTAAAATACAGAATAATAGATAT

AGATTCGCATTTCTATACTTGTTGGTTAAATGGTACAAAAAATATCATATTCCTATTATGAAACTATATC

CTACACCGGAAGAGATTCCGGACTTTGCATTCTATCTCAAAATAGGTACTCTGTTGGTATCTAGCTCTGT

AAAGCATATTCCATTAATGACGGACCTCTCCAAAAAGGGATATATATTGTACGATAATGTGGTTACTCTT

CCGTTGACTACTTTCCAACAGAAAATATCCAAGTATTTTAATTCTAGACTATTTGGACACGATATAGAGA

GCTTCATCAATAGACATAAGAAATTTGCCAATGTTAGTGATGAATATCTGCAATATATATTCATAGAGGA

TATTTCATCTCCGTAAATATATGCCATATATTTATAGAATATATCACATATCTAAATGAATACCGGAATC

ATAGATTTATTTGATAATCATGTTGATATATACCAACTATATTACCTCATCAGTTAGCTACTTTAGATTA

TCTAGTTAGAACTATCATAGATGAGAACAGAAGCGTGTTATTGTTCCATATTATGGGATCGGGTAAAACA

ATAATCGCTTTGTTGTTCGCCTTGGTAGCTTCCAGATTTAAAAAGGTTTACATTTTAGTACCGAACATCA

ACATCTTAAAAATTTTCAATTATAATATGGGTGTAGCTATGAACTTGTTTAATGATGAATTCATAGCTGA

GAATATCTTTATTCATTCCACAACAAGTTTTTATTCTCTTAATTATAACGATAACGTCATTAATTATAAC

GGATTAAGTCGCTACAATAACTCTATTTTTATCGTTGATGAGGCACATAATATTTTTGGGAATAATACTG

GAGAACTTATGACCGTGATAAAAAATAAAAACAAGATTCCTTTTCTACTATTGTCTGGATCTCCCATTAC

TAACACACCTAATACGCTGGGTCATATTATAGATTTAATGTCCGAAGAGACGATAGATTTTGGTGAGATT

ATTAGTCGTGGTAAGAAAGTAATTCAGACACTTCTTAACGAACGCGGAGTGAATGTACTCAAGGATTTGC

TTAAAGGAAGAATATCATATTACGAAATGCCGGACAAAGATCTACCAACAATAAGATATCACGGACGTAA

ATTTCTAGATACTCGAGTAGTATATTGTCACATGTCTAAACTTCAAGAGAAAGATTATATGATTACTAGA

CGGCAGCTATGTTATCATGAAATGTTTGATAAAAATATGTATAACGTGTCAATGGCAGTATTGGGACAAC

TTAATCTGATGAATAATTTAGATACGTTATTTCAGGAACAGGATAAGGAATTGTACCCAAATCTGAAAAT

AAATAATGGAGTGTTATACGGTGAAGAATTGGTAACGTTAAACATTAGTTCCAAATTTAAGTACTTTATC

AATCGGATACAGACACTCAAGGGAAAACACTTTATATACTTCTCTAATTCTACATATGGTGGATTGGTAA

TTAAATATATCATGCTCAGTAATGGATATTCTGAATATAATGGTTCTCAGGGAACTAATCCACATATGAT

AAACGGCAAACCAAAAACATTTGCTATCGTTACTAGTAAAATGAAATCGTCTTTAGAGGATCTATTAGAT

GTGTATAATTCTCCTGAAAACGATGATGGCAATCAATTGATGTTTTTGTTTTCGTCAAACATTATGTCTG

AATCCTATACTCTGAAAGAGGTAAGGCATATTTGGTTTATGACTATCCCGGATACTTTTTCTCAATACAA

CCAAATTCTTGGACGATCTATTAGAAAATTCTCTTACGTCGATATTTCTGAACCCGTTAATGTATATCTT

TTAGCAGCCGTATATTCAGATTTCAATGACGAAGTGACGTCATTAAACGATTATACACAGGATGAATTGA

TTAATGTTTTACCCTTTGACATCAAAAAGCTGTTGTATCTAAAATTTAAGACTAAAGAAACGAATAGAAT

ATACTCTATTCTTCAAGAGATGTCTGAAACGTATTCTCTTCCACCACATCCATCAATTGTAAAAGTTTTA

TTGGGAGAATTGGTCAGACAATTTTTTTATAATAATTCTCGTATTAAGTATAACGACTCCAAGTTACTTA

AAATGGTTACATCAGTTATAAAAAATAAAGAAGACGCTAGGAATTACATAGATGATATTGTAAACGGTCA

CTTCTTTGTATCGAATAAAGTATTTGATAAATCTCTTTTATACAAATACGAAAACGATATTATTACAGTA

CCGTTTAGACTTTCCTACGAACCATTTGTTTGGGGAGTTAACTTTCGTAAAGAATATAATGTGGTATCTT

CTCCATAAAACTGATGAGATATATAAAGAAATAAATGTCGAGCTTTGTTACCAATGGATATCTTCCAGTT

ACATTGGAACCACATGAGTTGACGTTAGACATAAAAACTAATATTAGGAATGCCGTATATAAGGCGTATC

TCCATAGAGAAATTAGTGGTAAAATGGCCAAGAAAATAGAAATTCGTGAAGACGTGGAATTACCTCTCGG

CGAAATAGTTAATAATTCTGTAGTTATAAACGTTCCGTGTGTAATAACCTACGCATATTATCACGTTGGG

GATATAGTCAGAGGAACATTAAACATCGAAGATGAATCAAATGTAACTATTCAATGTGGAGATTTAATCT

GTAAACTAAGTAGAGATTCGGGTACTGTATCATTTAGCGATTCAAAGTACTGCTTTTTTCGAAATGGTAA

TGCGTATGATAACGGCATCGAAGTCTCCGCCGTTCTAATGGAGGCTCAACAAGGTACCGAATCTAGTTTT

GTTTTTCTCGCGAATATCGTTGACTCATAAGAAAGAGAATAGCGGTGAGTATAAATACGAATACTATGGC

AATAATTGCGAATGTTTTATTCCCTTCGATATATTTTTGATAATATGAAAAACATGCCTCTCTCAAATCA

GACAACCATTTCATAAAATAGTTCTCTCGCACTGGTGAGGTGGTTGCAGCTCGTATAATCTCCCCAGAAT

AATATACTTGCGTGTCGTCGTTCAATTTATACGGATTTCTATAATTCTCTGTTATATAATGAGGTTTACC

CTCATGATTAGACGACGACAATAGTGTTCTGAATTTAGATAGTTGATCAGAATGAATGTTTATTGGTGTT

GGAAAAATTATCCATGCTGCGTCTGCAGAGTGGTTGATAGTTGTTCCTAGATATGTAAAATAATCCAACG

TACTAGGTAGCAAATTGTCTAGATAAAATACTGAATCAAATGGCGCAGACATATTAGCGGATCTAATGGA

ATCCAATTGATTGACTATCTTTTGAAAATATACATTTTTATGATCTGATACTTGTAAGAATATAGCAATA

ATGATAATTCCATCATCGTGTTTTTTTGCCTCTTCATAAGAACTATATTTTTTCTTATTCCAATGAACCA

GATTAATCTCTCCAGAGTATTTGTATACATCTATCAAGTGATTGGATCCATAATCGTCTTCCTTTCCCCA

ATATATATGTATTGTTGATAACACATATTCATTGGGGAGAAACCCTCCACTTATATATCCTCCTTTAAAA

TTAATCCTTACTAGTTTTCCAGTATTCTGGATAGTGGTTGGTTTCGACTCATTATAATGTATGTCTAACG

TCTTCAATCGCGCGTCAGAAATTGCTTTTTTAGTTTCTATATTAATAGGAGATAGTTGTTGAGGCATAGT

AAAAATGAAATGATAACTGTCTAGAAATAGCTCTTAGTATGGGATTTACAATGGATGAGGAAGTGATATT

TGAAACTCCTAGAGAATTAATATCTATTAAACGAATAAAAGATATTCCAAGATCAAAAGACACGCACGTG

TTTGCTGCGTGTATAACAAGTGACGGATATCCGTTAATAGGAGCTAGAAGAACTTCATTCGCATTCCAGG

CGATATTATCTCAACAAAATTCAGATTCTATCTTTAGAGTATCCACTAAACTATTACGGTTTATGTACTA

CAATGAACTAAGAGAAATCTTTAGACGGTTGAGAAAAGGTTCTATCAACAATATCGATCCTCACTTCGAA

GAGTTAATATTATTGGGTGGTAAACTAGATAAAAAGGAATCTATTAAAGATTGTTTAAGAAGAGAATTAA

AAGAGGAAAGTGATGAACATATAACAGTAAAAGAATTCGGAAATGTAATTCTAAAACTTACAACGAGTGA

TAAATTATTTAATAAAGTATATATAGGTTATTGCATGGCATGTTTTATTAATCAATCGTTGGAGGATTTA

TCACATACTAGTATTTACAATGTAGAAATTAGAAAGATTAAATCGTTAAATGATTGTATTAACGACGATA

AATACGAATATCTGTCTTATATTTATAATATACTAATTAATAGTAAATGAGCTTTTACAGATCTAGTATA

ATTAGTCAGATTATTAAGTATAATAGACGACTAGCTAAGTCTATTATTTGCGAGGATGACTCTCAAATTA

TTACACTCACGGCATTCGTTAACCAATGCCTATGGTGTCATAAACGAGTATCCGTGTCCGCTATTTTATT

AACTACTGATAACAAAATATTAGTATGTAACAGACGAGATAGTTTTCTCTATTCTGAAATAATTAGAACT

AGAAACATGTATAGAAAGAAACGATTATTTCTGAATTATTCCAATTATTTGAACAAACAGGAAAGAAGTA

TACTATCGTCATTTTTTTCTCTAGATCCAGCTACTGCTGATAATGATAGAATAAACGCTATTTATCCGGG

TGGTATACCCAAAAGGGGTGAGAACGTTCCAGAGTGTTTATCCAGGGAAATCAAAGAAGAAGTTAATATA

GACAATTCTTTTGTATTCATAGACACTCGTTTTTTTATTCATGGTATCATAGAAGATACCATTATTAACA

AATTTTTTGAGGTAATTTTCTTTGTTGGACGAATATCTCTAACGAGTGATCAAATTATTGATACCTTTAA

AAGTAATCATGAAATAAAGGATCTAATATTTTTAGATCCAAATTCAGGTAATGGACTCCAATACGAAATT

GCAAAATATGCTCTAGATACTGCAAAACTTAAATGTTACGGTCATAGAGGATGTTATTATGAATCATTAA

AAAAATTAACTGAGGATGATTGATTAGAAAATATAAATTAATTTACCATCGTGTATTTTTATAACGGGAT

TGTCTGGCATATCATGTAGATAGTTACCGTCTACATCGTATACTCTACCATCTACGCCTTTAAATCCTCT

ATTTATTGATATTAATCTATTAGAATTGGAATACCAAATATTAGTACCCTCAATTAGTTTATTGGTAATA

TTTTTTTTAGACGATAGATCGATGGCTCTTGAAACCAAGGTTTTCCAACCGGACTCATTGTCTATCGGTG

AGAAGTCTTTTTCATTAGCATGAATCCATTCTAATGATGTATGTTTAAACACTCTAAACAATTGTACAAA

TTCTTTTGATTTGTTTTGAATGATTTCAAATAGGTCTTCGTCTACAGTAGGCATACCATTAGATAATCTA

GCCATTATAAAGTGCACGTTTACATATCTACGTTCTGGAGGAGTAAGAACGTGACTATTGAGACGAATGG

CTCTTCCTACTATCTGACGAAGAGACGCCTCGTTCCATGTCATATCTAAAATGAAGATATCATTGATTGA

GAAGAAACTAATACCCTCGCCTCCGCTAGAAGAGAATACGCATGTTTTAATGCATTCTCCGTTAGTGTTT

GATTCTTGGTTAAACTCAGCCACCGCCTTGATTCTAGTATCTTTTGTTCTAGATGAGAACTCTATATTAG

AGATACCAAAGACTTTGAAATATAGTAATAAGATTTCTATTCCTGACTGATTAACAAATGGTTCAAAGAC

TAGACATTTACCATGGGATGCTAATATTCCCAAACATACATCTATAAATTTGACGCTTTTCTCTTTTAAT

TCAGTAAATAGAGAGATATCAGCCGCAATAGCATCCCCTCCCAATAGTTCTCCCTTTTTAAAGGTGTCTA

ATGCGGATTTAGAAAATTCTCTATCTCTTAATGAATTTTTAAAATCATTATATAGGGTTGCTATCTCTTG

TGCGTATTCTCCCGGATCACGATTTTGTCTTTCAGGAAAGCTATCGAATGTAAACGTAGTAGCCATACGT

CTCAGAATTCTAAATGATGATATACCAGTTTTTATTTCTGCGAGTTTAGCCTTTTGATAAATCTCTTCTT

GCTTTTTTGACATATTAACGTATCGCATTAATACTGTTTTCTTAGCGAATGATGCAGACCCTTCCACATC

ATCAAAAATAGAAAACTCGTTATTAACTATGTACGAACATAGGCCTCCTAGTTTGGAGACTAATTCTTTT

TCATCGACTAGACGTTTATTCTCAAATAGCGATTGGTGTTGTAAGGATCCTGGTCGCAGTAAGTTAACCA

ACATGGTGAATTCTTGCACACTATTAACGATAGGTGTAGCCGATAAACAAATCATCTTATGGTTTTTTAA

CGCAATGGTCTTAGATAAAAAATTATATACTGACCGAGTAGGACGGATCTTACCATCTTCTTTGATTAAT

GATTTAGAAATGAAGTTATGACATTCATCAATGATGACGCATATTCTACTCTTGGAATTAATAGTTTTGA

TATTAGTAAAAAATTTATTTCTAAAATTTTGATCATCGTAATTAATAAAAATACAATCCTTCGTTATCTC

TGGAGCGTATCTGAGTATAGTGTTTATCCAAGGATCTTCTATCAAAGCCTTTTTTACCAATAAGATAATT

GCCCAATTCGTATAAATATCCTTAAGATGTTTGAGAATATATACAGTAGTCATTGTTTTACCGACACCTG

TTTCATGGAACAATAAAAGAGAATGCATACTGTCTAATCCTAAGAAAACTCTTGCTACAAAATGTTGATA

ATCCTTGAGGCGTACTACGTCTGACCCCATCATTTCAACGGGCATATTAGTAGTTCTGCGTAAGGCATAA

TCGATATAGGCCGCGTGTGATTTACTCATTTATGAGTGATAAGTAATAACTATGTTTTAAAAATCACAGC

AGTAGTTTAACTAGTCTTCTCTGATGTTTGTTTTCGATACTTTTTGAATCAGAAGTCATACTAGAATAAA

GCAGCGAGTGAACGTAATAGAGAGCTTCGTATACTCTATTCGAAAACTCTAAGAACTTATTAATGAATTC

CGTATCCACTGGATCGTTTAAAATACTAAATTGAACAGTGTTCACATCCTTCCAAGACGAAGACTTAGTG

ACGGACTTAACATGAGACATAAATAAATCCAAATTTTTTTTATAAACATCACTAGCCACCATAATGGCGC

TATCTTTCAACCAACTATCGCTTACGCATTTTAACAGTCTAACATTTTTAAAGAGACTACAATATATTCT

CATAGTATCGATTACACCTCTACCGAATAGAGTGGGAAGTTTAATAATACAATATTTTTCGTTTACAAAA

TCAAATAATGGTCGAAACACGTCGAAGGTTAACATCTTATAATCGCTAATGTATAGATTGTTTTCAGTGA

GATGATTATTAGATTTAATAGCATCTCGTTCACGTTTGAACAGTTTATTGCGTGCGCTGAGGTCGGCAAC

TACGGCATCCGCTCTAGTACTCCTCCCATAATACTTTACGCTATTAATCTTTAAAATTTCATAGACTTTA

TCTAGATCGCTTTCTGGTAACATGATATCATGTGTAAAAAGTTTTAACATGTCGGTCGGCATTCTATTTA

GATCATTAACTCTAGAAATCTGAAGAAAGTAATTAGCTCCATATTCCAGACTAGGTAATGGGCTTTTACC

TAAAGACAAGTTAAGTTCTGGCAATGTTTCATAAAATGGAAGAAGGACATGTGTCCCCTCCCGGATATTT

TTTACAATTTCATCCATTTACAACTCTATAGTTTGTTTTCATTATTATTAGTTATTATCTCCCATAATCT

TGGTAATACTTACACCTTGATCATAAGATACCTTATACAGGTCATTACATACAACTACCAATTGTTTTTG

TACATAATAGATTGGATGATTGATATCCATGGTGGAATAAACTACTCGAACAGATAGTTTATCTTTCCCC

CTAGATACATTGGCCGTAATAGTTGTCGGCCTAAAGAATATCTTTGGTGTAAAGTTAAAAGTTAGGGTTC

TTGTTCCATTATTGCTTTTTGTCAGTAGTTCGTTATAAATTCTCGAGATGGGCCCGTTCTCTGAATATAG

AACATCATTTCCAAATCTAACTTCTAGTCTAGAAATAATATCGGTCTTATTTTTAAAATCTATTCCCTTG

ATGAATGGATCGTTAATAAACAAATCCTTGGCCTTTGATTCGGCTGATCTATTATCTCCGTTATAGACGT

TACGTTGACTAGTCCAAAGACTTACAGGAATAGATGTATCGATGATGTTGATAGTATGTGATATGTGAGC

AAAGACTGTTCTCTTGGTGGCGTCGCTATATGTTCCAGTAATGGCGGAAAACTTTTTAGAAATGTTATAT

ATAAAAGAATTTTTTCGGGTTCCAAACATTAACAGATTAGTATGAAGATAAACACTCATATTATCAGGAA

CATTATCAATTTTTACATAAACATCGGCATCTTGAATAGAAACAACACCATCTTCTGGAACCTCTACGAT

CTCGGCAGATTCCGGATAACCAGTCGGTGGACCATCACTAACAATAACTAGATCATCCAACAATCTACTC

ACATATGCGTCTATATAATCTTTTTCATCTTGTGAGTACCCTGGATACGAAATAAATTTGTTATCAGTAT

TTCCATAATAAGGTTTAGTATAAACAGAGAGAGATGTTGCTGCATGAACTTCGGTTACTGTCGCCGTTGG

TTGGTTTATTTGACCTATTACTCTCCTAGGTTTCTCTATAAATGATGGTTTAATTTGTACATTCTTAACC

ATATATCCAATAAAGCTCAATTCAGGAACATAAACAAATTCTTTGTTGAACGTTTCAAAGTCGAACGAAG

AGTCACGAATAACGATATCGGATACTGGATTGAAGGTCACCGTTACGGTAATTTTTGAATCGGATAGTTT

AAGACTACTGAATGTATCTTCCACATCAAACGGAGTTTTAATATAAACGTATACTGTAGATGGTTCTTTA

ATAGTGTCATTAGGAGTTAGGCCAATAGAAATATCATTAAGTTCACTAGAATATCCAGAATGTTTCAAAG

CAATTGTATTATTGATACAATTATTATATAATTCTTCGCCCTCAATTTCCCAAATAACACCGTTACACGA

AGAGACAGATACATGATTAATACATTTATATCCAACATATGGCACGTAACCGAATCTTCCCATACCTTTA

ACTTCTGGAAGTTCCAAACTCAGAACCAAATGATTAAGCGCAGTAATATACTGATCCCTAATTTCGAAGC

TAGCGATAGCCTGATTGTCTGGCCCATCGTTTGTCATAACTCCGGATAGAGAAATATATTGCGGCATATA

TAAAGTTGGAATTTGACTATCAACTGCGAAGACATTAGACCGTTTAATAAAGTCATCCCCACCGATCAAA

GAATTAATGATAGTATTATTCATTTTCTATTTAAAATGGAAAAAGCTTACAATAAACTCCGTAGAGAAAT

ATCTATAATTTGTGAGTTTTCCTTAAAGTAACAGCTTCCGTAAACACCGTCTTTATCTCTTAGTAAGTTT

ATTGTATTTATGACCTTTTCCTTATCTTCATAGAATACTAAAGGCAATAAAGAAATTTTTGGTTCTTCTC

TAAGAGCTACGTGAGACTTAACCATAGACGCCAACGAATCCCTACATATTTTAGAACAGAAATACCCAAC

TTCACCACCCTTGAATGTCTCAATACTAATAGGTCTAAAAACCAAATCTTGATTACAAAACCAACACTTA

TCAATTACACTATTTGTCTTAATAGACATATCTGCCATAGATTTATAATACTTTGGTAGTATACAAGCGA

GTGCTTCTTCTTTAGCGGGCTTAAAGACTGCTTTAGGTGCTGAAATAACCACATCTGGAAGACTTACTCG

CTTAGCCATTTAATTACGGAACTATTTTTTTATACTTCTAATGAACAAGTAGAAAACCTCTCATCTACAA

AAACATACTCGTGTCCATAATCCTCTACCATAGTAACACGTTTTTTAGATCTCATATGTGCTAAAAAGTT

TTCCCATACTAATTGGTTACTATTATTTTTCGTATAATTTTTAACAGTTTGAGGTTTTAGATTTTTAGTT

ACAGAAGTGATATCGAATATTTTATCCAAAAAGAATGAGTAATTAATTGTCTTAGAAGGAGTGTTTTCTT

GGCAAAAGAATACCAAGTGCTTAAATATTTCTACTACTTCATTAATCTTTTCTGTACTCAGATTCAGTTT

CTCATCTTTTACTTGATTGATTATTTCAAAGACTAACTTATAATCCTTTTTATTTATTCTCTCGTTAGCC

TTAAGAAAACTAGATACAAAATTTGCATCTACATCATCCGTGGATATTTGATTTTTTTCCATGATATCCA

ATAGTTCCGAGATAATTTCTCCAGAACATTGATGAGACAATAATCTCCGCAATACATTTCTCAAATGAAT

AAGTTTATTAGACACGTGGAAGTTTGACTTTTTTTGTACCTTTGTACATTTTTGAAATACAGACTCGCAA

AAAATACAATATTCATATCCTTGTTCAGATACTATACCGTTATGTCTACAACAGCTACATAATCGTAGAT

TCATGTTAACACTCTACGTATCTCGTCGTCCAATATTTTATATAAAAACATTTTATTTCTAGACGTTGTC

AGAAAATCCTGTAATATTTTTAGTTTTTTTGGTTGTGAATAAAGTATCGCCCTAATAATATTGGTACCGT

CTTCCGACAATATAGTAGTTAAATTATCCGAGCATGTAGAAGAACACCGCTTAGGCGGATTCAGTACAAT

GTTATATTTTTCGTACCAACTCATTTAAATATCATAATCTAAAATAGTTCTGTAATATGTCTAGCGCTAA

TATATTGATCATAATCCTGTGCATAAATTAAGATACAACAATGTCTTGAAATCATCGACATGGCTTCTTC

CATAGTTAGAAGATCATCGTCAAAGTTAGCAACGTGATTCATCAACATTTGCTGTTTTGAGGCAGCAAAT

ACTGAACCATCACCATTCAACCATTCATAAAAACCATCGTCTGAATCCATTGATAATTTCTTGTACTGGT

TTTTGAGAGCTCGCATCAATCTAGCATTTCTAGCTCCCGGATTGAAAACAGAAAGAGGATCGTACATCCA

AGGTCCATTTTCTGTAAATAGAATCGTATAATGTCCCTTCAAGAAGATATCAGACGATCCACAATCAAAG

AATTGGTCTCCGAGTTTGTAACAGACTGCGGACTTTAACCTATACATGATACCGTTTAGCATGATTTCTG

GTGATACGTCAATCGGAGTATCATCTATTAGAGATCTAAAGCCGGTGTAACATTCTCCGCCAAACATATT

CTTATTCTGACGTCGTTCTACATAAAACATCATTGCTCCATTAACGATAACAGGTGAATGAACAGCACTA

CCCATCACATTAGTTCCCAATGGATCAATGTGTGTAACTCCAGAACATCTTCCATAGCCTATGTTAGGAG

GAGCGAACACCACTCTTCCACTATTGCCATCGAATGCCATAGAATAAATATCCTTGGAATTGATAGAAAT

CGGACTGTCGGATGTTGTTATCATCTTCATAGGATTAACAACGATGTATGGTGCAGCCTGAAGTTTCATA

TCGTAACTGATGCCGTTCATAGGTCTAGCCACAGAAACCAACGTAGGTCTAAATCCAACTATAGACAAAA

TAGAAGCTAATATCTGTTCCTCATCTGTCATAACTTGAGAGCATCCAGTATGAATAATCTTCATTAGATG

GGGATCTACCGCATCATCATCGTTACAATAAAAAATTCCCATTCTAATGTTCATAATTGCTTTTCTAATC

ATGGTATGAATGTTTGCTCTCTGAATCTCTGTGGAAATTAGATCTGATACACCTGTAATCACTATCGGAT

TATCCTCCGTAAGACGATTAACCAACAACATATAATTATAAGACTTTACTCTTCTAAATTCATAAAGTTG

CTGGATTAGACTATATGTGTCTCCATGTACATACGCGTTCTCGAGCGCAGGAAGTTTAATACCGAATAGT

GCCATCAGAATAGGATGAATGTAGTAATTAGTTTCTGGTTTTCTATAAATAAAAGACAAATCTTGTGAAC

TAGACATATCGGTAAAATGCATGGATTGGAATCGTGTAGTCGACAGAAGAATATGATGATTAGATGGAGA

GTATATTTTATCTAACTCTTTGAGTTGGTCACCGATTCTAGGACTAGCTCGAGAATGAATAAGTACTAAG

GGATGAGTACATTTCACAGAAACACTGGCGTTGTTCAACGTACTCTTTACATGGGAAAGGAGTTGAAATA

GCTCGTTTCTATTTGTCCTGACAATATTTAGTTTATTCATAATATTAAGCATATCCTGAATAGTAAAGTT

AGATGTGTCATACTTGTTAGTAGTTAGATATTTAGCAATTGCATTCCCATCATTTCTCAATCTCGTACTC

CAATCATGTGTGGATGCTACTTCGTCGATGGAAACCATACAATCCTTTTTGATAGGCTGTTGAGATTGAT

CATTTCCTGTACGTTTAGGTTTGGTACGTTGATTTCTAGCCCCTGCTGATATAAAGTCATCGTCTACAAT

TTGGGATAATGAATTACATACACTACAAGACAAAGATTTATCAGAAGTGTGAATATGATCTTCATCTACC

AAAGAAAGAGTTTGATTAGTATAACTAGATTTTAGTCCCGCGTTAGATGTTAAAAAAACATCGCTATTGA

CCACGGCTTCCATTATTTATATTCGTAGTTTTTACTCGAAAGCGTGATTTTAATATCCAATCTTATTACT

TTTGGAATCGTTCAAAACCTTTGACTAGTTGTAGAATTTGATCTATTGCCCTACGCGTATACTCCCTTGC

ATCATATACGTTCGTCACCAGATCGTTTGTTTCGGCCTGAAGTTGACGCATATCTTTTTCAACACTCGAC

ATGAGATCCTTAAGGGTCATATCGTCTAGATTTTGTTGAGATGCTGCTCCTGGATTTGGATTTTGTTGTG

CTGTTGTACATACTGTACCACCAGTAGGTGTAGGAGTACATACAGTGGCCACAATAGGAGGTTGAAGAGG

TGTAACCGTTGGAGTAGTACAAGAAATACTTCCATCCGATTGTTGTGTACATGTGGTTGTTGGTAACGTC

TGAGAAGGTTGGGTAGATGGCGGTGTCGTCATCTTTTGATCTTTATTAAATTTAGAGATAATATCCTGAA

CAGTATTGCTCGGCGTCAACGCTGGAAGGAGTGTACTCGCCGGCGCATCAGTATCTGTAGACAACCAATC

AAAAAGATTAGACATATCAGATGATGTATTAGTTTGTTGACGTGGTTTTAGTACAGGAGCAGTACTACTA

GGTAGAAGAATAGGAGCCGGTGTAGGTGTCGGAACCGGCTGTGGAGTTATATGAATAGTTGGTTGTAGCG

GTTGGGTAGGCTGTCTGCTGGCGGTCATCATATTATCTCTAGCTAGTTGTTCTCGCAACTGTCTTTGATA

ATACGACTCTTGAGACTTTAGTCCTATTTCAATCGCTTCATCCTTTTTCGTATCCGGATCCTTTTCTTCA

GAATAATAGATTGACGACTTTGGTGTAGAGGATTCTGCCAGCCCCTGTGAGAACTTGTTAAAGAAGTCCA

TTTAAGGCTTTAAAATTGAATTGCGATTATAAGATTAAATGGCAGACACGGACGATATTATCGACTATGA

ATCCGATGATCTCACCGAATACGAGGATGATGAAGAAGATGGAGAGTCACTAGAAACTAGTGATATAGAT

CCCAAATCTTCTTATAAGATTGTAGAATCAACATCCACTCATATAGAAGATGCGCATTCCAATCTTAAAC

ATATAGGGAATCATATATCTGCTCTTAAACGACGCTATACTAGACGTATAAGTCTATTTGAAATAGCGGG

TATAATAGCAGAAAGCTATAACTTGCTTCAACGAGGAAGATTACCTCTAGTTTCAGAATTTTCTGACGAA

ACGATGAAGCAAAATATGCTACATGTAATTATACAAGAGATAGAGGAGGGTTCTTGTCCTATAGTCATCG

AAAAGAACGGAGAATTGTTGTCGGTAAACGATTTTGATAAAGATGGTCTAAAATTCCATCTAGACTATAT

TATCAAAATTTGGAAACTTCAAAAACGATATTAGAATTTATACGAATATCGTTCTCTAAATGTCACAATC

AAGTCTCTCATATTCAGCAGTTTATTGTCGTACTTTATATCGTGTTCATTAACGATATTTTGCAAAATAG

TAATGATTCTATCTTCCTTCGATAGATATTCTTCAGAGATTATTGTCTTATATTCTTTCTTGTTATCCGA

TATGAATTTGATAAGACTTTGAACATTATTAATACCCGTCTGTTTAATTTTTTCTATAGATATTTTAGTT

TTGGTAGATTCTATGGTGTCTGTTAATAGGCATCCAACATCGACATTCGACGTCAATTGTCTATAAATCA

GAGTATAAATTTTAGAAATAACATTAGCAAATTGTTGTGCGTTGATGTCGTTATTCTGAAACAGTATGAT

TTTAGGTAGCATTTTCTTAACAAAGAGAACGTATTTATTGTTACTCAGTTGAACAGATGATATATCCAGA

TTACTAACGCATCTGATTCCATATACCAAACTTTCAGAAGAAATGGTGTACAATTGTTTGTATTCATTCA

ATGTCTCCTTTTCAGAAATTAGTTTAGAGTCGAATACTGCAATAATTTTCAAGAGATAGTTTTCATCAGA

TAAGATTTTATTTAGTGTAGATATGATAAAACTATTGTTTTGTTGGAGAACTTGATACGCCGCATTCTCT

GTAGTCGACGCTCTCAAATGGGAAACAATCTCTATTATTTTTTTGGAATCGGATACTATATCTTCGGTAT

CTTGACGCAGTCTAGTATACATAGAGTTAAGAGAAATTAGAGTTTGTACATTAAGCAACATGTCTCTAAA

TGTGGCTACAAACTTTTCTTTTTCCACATCATCTAGTTTATTATATACCGATTTCACAACGGCACCAGAT

TTAAGGAACCAGAATGAAAAACTCTGATAACTACAATATTTCATCATAGTTACGATTTTATCATCTTCTA

TAGTTGGTGTGATAACACATACCTTTTTCTCCAAGACTGGAACCAACGTCATAAAAATGTTTAAATCAAA

ATCCATATCAACATCTGATGCGCTAAGACCAGTCTCGCGTTCAAGATTATCTTTACTAATGGTGACGAAC

TCATCGTATAGAACTCTAAGTTTGTCCATTATTTATTTACAGATTTAGTTGTTTAATTTATTTGTGCTCT

TCCAGAGTTGGGATAGTATTTTTCTAACGTCGGTATTATATTATTAGGATCTACGTTCATATGTATCATA

ATATTAATCATCCACGTTTTGATAAATCTATCTTTAGCTTCTGAAATAACGTATTTAAACAAAGGAGAAA

AATATTTAGTTACGGCATCAGACGCGATAACATTTTTTGTAAATGTAACGTATTTAGACGACAGATCTTC

GTTAAAAAGTTTTCCATCTATGTAGAATCCATCGGTTGTTAACACCATTCCCGCGTCAGAGTGAATAGGA

GTTTGAATAGTTTGTTTTGGAAATAGATCCTTCAATAACTTATAGTTGGGTGGGAAAAAATCGATTTTAT

CACTAGACTCTTTCTTTTTTACTATCATTACCTCATGAACTATTTCTTGAATGAGTATATGTATTTTCTT

TCCTATATCGGTCGCGTTCATTGGAAAATATATCATGTCGTTAACTATAAGAATATTTTTATCCTCGTTT

ACAAACTGAATAATATCAGATATAGTTCGTAAACGAACTATATCATCACCAGCACAACATCTAACTATAT

GATATCCACTAGTTTCCTTTAGCCGTTTATTATCTTGTTCCATATTAGCAGTCATTCCATCATTTAAGAA

GGCGTCAAAGATAATAGGGAGAAATGACATTTTGGATTCTGTTACGACTTTACCAAAATTAAGGATATAC

GGACTTACTATCTTTTTCTCAACGTCGATTTGATGAACACACGATGAAAATGTACTTCGATGAGATTGAT

CATGTAGAAAACAACAAGGGATACAATATTTCCGCATATCATGAAATATATTAAGAAATCCCACTTTATT

ATATTTCCCCAAAGGATCAATGCATGTAAACATTATACCGTTATCATTAATAAAGACTTCTTTCTCATCG

GATCTGTAAAAGTTGTTACTGATTTTTTTCATTCCAGGATCTAGATAATTAATAATAATGGGTTTTCTAT

TCTTATTCTTTGTATTTTGACATATCCTAGACCAGTAAACAGTTTCCACTTTGGTAAAATCAGAAGACTT

TTGAACGCTATTAAACATGGCATTAATGGCAATAACTAAAAATGTAAAATATTTTTCTATGTTAGGAATA

TGGTTTTTCACTTTAATAGATATATGGTTTTTTGCCAAAATGATAGATATTTTTTTATCCGATGATAGTA

AAATATTATTAGTCGCCGTCTCTATAAAAATGAAGCTAGTCTCGATATCCAATTTTATTCTAGAATTGAT

AGGAGTCGCCAAATGTACCTTATACGTTATATCTCCCTTGATGCGTTCCATTTGTGTATCTATATCGGAC

ACAAGATCTGTAAATAGTTTTACGTTATTAATCATCACGGTATCGCCATCGCTAGATAATGCTAATGTAC

TATCCAAGTCCCAAATGGAGAGATTTAACTGTTCATCGTTTAGAATAAAATGATTACCTGTCATATTAAT

AAAGTGTTCATCGTATCTAGATAACAACGACTTATAATTAATGTCCAAGTCTTGAACTCGCTGAATGATC

TTTTTTAACCCAGTTAGTTTTAGATTGGTACGAAATATATTGTTAAACTTTGATTCTACAGTAATGTCCA

AATCTAGTTGTGGAAATACTTCCATCAACATTGTTTCAAACTTGATAATATTATTATCTACATCTTCGTA

CGATCCAAATTCCGGAATAGATGTATCGCACGCTCTGGCCACCCAGATAACCAAAAAGTCACACGCTCCA

GAATATACATTGTATAAAAAGCTATCGTTTTTTAGTAGTGTTTTTTTCTGAGTATATACGAAAGGATTAA

AAATAGTATTATCAACGTAACTATATTCCAAATTATTCTTATGAGAATAGATAATAATATCGTCCTTAAT

ATCTAACAAATTTCCTAAATATCCCTTTAATTGAGTCATTCGAAGCGTTAATAAAATATGTCTCTTAACT

ATTTCCGGCCGTTGTATATTTAAATGACTTCGTAAGAAATAATATATAGGCGACTTCTCATCTATGTAAT

CATATGGAGTGAGATATAGGGCTCGTTCTACCTCCTGCCCCTTACCCACCTGTAATACCAATTGCGGACT

CACTATATATCGCATATTTATATCGTGGGGTAAAGTGAAAATCTACTACCGATGATGTAAGTCTTACAAT

GTTCGAACCAGTACCAGATCTTAATTTGGAGGCCTCCGTAGAACTAGGGGAGGTAAATATAGATCAAACA

ACACCTATGATAAAGGAGAATAGCGGTTTTATATCCCGTAGTAGACGTCTATTCGCCCATAGATCTAAGG

ATGATGAGAGAAAACTAGCACTACGATTCTTTTTACAAAGACTTTATTTTTTAGATCATAGAGAGATTCA

TTATTTGTTCAGATGCGTCGACGCTGTAAAAGACGTCACTATTACCAAAAAAAATAACATTATCGTGGCG

CCTTATATAGCACTTTTAACTATCGCATCAAAAGGATGCAAACTTACAGAAACAATGATTGAAGCATTCT

TTCCAGAACTATATAATGAACATAGTAAGAAATTCAAATTCAACTCTCAAGTATCCATCATCCAAGAAAA

ACTCGGATACCAGTCTGGAAACTATCACGTTTATGATTTTGAACCGTATTACTCTACAGTAGCTCTGGCT

ATTCGAGATGAACATTCATCTGGCATTTTTAATATCCGTCAAGAGAGTTATCTTGTAAGTTCATTATCTG

AAATAACATATAGATTTTATCTAATTAATCTAAAATCTGATCTTGTTCAATGGAGTGCTAGTACGGGCGC

TGTAATTAATCAAATGGTAAATACTGTATTGATTACAGTGTATGAAAAATTACAACTGGCCATAGAAAAT

GATTCACAATTTACATGTTCATTGGCTGTGGAATCAGAACTTCCAATAAAATTACTTAAAGATAGAAATG

AATTATTTACAAAATTCATTAACGAGTTAAAAAAGACCAGTTCATTCAAGATAAGCAAACGTGATAAGGA

TACGCTATTAAAACATTTTACTTATGACTGGAGTTAGAATTTATAGACGACACATTTCGTTTATCATTGT

TACTATTACTATCATTATTAGTATTCTTCTTGTCATCTTGTTCAGAAATATACAGCAATGCTATACCTAA

TACTAAATACATTATCATGCTTGCAATGGCTCTAACAACAACGAACCAAAATGAATTTGGTCGTAGCTTT

TGTTCACAAAAATACATAAAGAAATGTCTACATAAATCTATGGCGCCATTGGCTACTTGAAATAGCGCCA

GTCCTCCTACAGATTTTAATATAGCTGTATAACATGACATTTATTCATCATCAAAAGAGACAGAGTCACC

ATCTGTCATATTTAGATTTTTTTTCATGTGTTCAAAGTATCCTCTACTCATTTCATTATAATAGTTTATC

ATGCTTAGAATTTTAGGACGGATCAATGAGTAAGACTTGACTAGATCGTCAGTAGTAATTTGTGCATCAT

CTATTCTGCATCCGCTTCGTCGAATAATGTATAGCATCGCTTTGAGATTCTCCATAGCTATCAAGTCTTT

ATATAATGACATGGAAATATCTGTGAATGCTTTATACTTCTCCAACATCGATGCCTTAACATCATCACAT

ACTTTAGCATTGAAAATACGTTCTATTGTGTAGATGGATGTAGCAAGATTTTTAAACAACAATGCCATCT

TACATGATGATTGTCTCAAGTCTCCAATCGTTTGTTTAGAACGATTAGCTACAGAGTCCAATGCTTGGCT

AACTAGCATATTATTATCTTTAGAAATTGTATTCTTCAATGAGGCGTTTATCATATCTGTGATTTCGTTA

GTCATATTACAGTCTGACTGGGTTGTAATGTTATCCAACATATCACCTATGGATACGGTACACGTACCAG

CATTTGTAATAATCCTATCTAAGATGTTGTATGGCATTGCGCAGAAAATATCTTCTCCTGTAATATCTCC

ACTCTCGATAAATCTACTCAGATTATTCTTAAATGCCTTATTCTCTGGAGAAAAGATATCAGTGTCCATC

ATTTCATTAATAGTATACGCAGAAAAGATACCACGAGTATCAATTCTATCCAAGATACTTATCGGTTCCG

AGTCACAGATAATTGTTTCCTCTCCTTCGGGAGATCCTGCATAGAAATATCTAGGACAATAGTTTCTATA

CTGTCTGTAACTCTGATAATCTCTAAAGTCACTAACTGATACCATGAAATTGAGAAGATCAAACGCTGAA

GTAATCAATTTTTCTGCCTCGTTTTTACTACAACTAGTTTTCATCAATGTAGTGACGATGTATTGTTTAG

TTACTCTTGGTCTAATACTGATGATAGAGATATTATTGCTTCCCATAATGGATCTTCTAGTAGTCACCTT

AAAGCCCATTGATGCGAATAGCAGATAGATAAAGTCTTGGTATGACTCCTTTCTAATATAGTACGGACTA

CCTTTGTCACCCAACTTTATACCCACATAAGCCATAACAACCTCTTTAATAGCCGTTTCATGAGGTTTAT

CAGCCATGAGCCTGAGTAGTTGAAAGAATCGCATGAATCCCGTCTCAGAAAGTCCTATATGCATGATAGA

TTTATCTTTCCTGGGAAACTCTCGTATAGTTATAGATGAAATACTCTTCAAAGTTTCTGAAATAAGATTA

GTAACAGTCTTACCTCCGACTACTCTGGGTAACAAACATACTCTAATAGGTGTTTTCTCTGCGGAGATAA

TATCAGAAAGGATAGAGCAATAAGTAGTATTATTGTGATTATAAAGACCGAATACATAACAGGTAGAATT

TATAAACATCATGTCCTGAAGGGTTTTAGACTTGTATTCCTCGTAATCTATACCGTCCCAAAACATGGAT

TTGGTAACTTTGATAGCCGTAGATCTTTGTTCCTTCGCTAACAGGTTAAAGAAATTAATAAAGAATTTGT

TGTTTCTATTTATGTCCACAAATTGCACGTTTGGAAGCGCCACGGTTACATTCACTGCAGCATTTTGAGG

ATCGCGAGTATGAAGTACGATGTTATTGTTTACTGGTATATCTGGAAAGAAATCTACCAGTCTAGGAATA

AGAGATTGATATCGCATAGAAATAGTAAAGTTTATAATCTCATCATTGAAGATTACTCTGTTACCATTGT

AATAAATTGGTACTCTATCATAATCATCGACAAAGTACTGTTCATACATGATGAGATGTTTATATGTTGG

CATAGTAGTGAGATCGACGTTTGGTAATGGCAATGTATTAAGATTAACTCCATAATGTCTAGCAGCATCT

GCGATGTTATAAGTGATGTCAAAGCGGGGTTGATCTTGTGCTGTTATATATTGTCTAACACCTATAAGAT

TATCAAAATCTTGTCTGCTTAATACACCGTTAACAATTTTTGCCTTGAATTCTTTTATTGGTGCATTAAT

AACATCCTTATAGAGGATGTTAAACAAATAAGTATTATCAAAGTTAAGATCTGGGTATTTCTTTTCTGCT

AGAACATCCATTGAGTCGGAGCCATCTGGTTTAATATAACCACCGATAAATCTAGCTCTGTATTCTGTAT

CCGTCAATCTAATATTAAGAAGGTGTTGAGTGAAAGGTGGAAGATCGTAAAAGCTGTGAGTATTAATAAT

AGGGTTAGTTTCCGAACTAATGTTAATTGGATGATTAATAATATCTATATTTCCAGCGTTAAGTGTAACA

TTAAACAGTTTTAATTCACGTGACGTGGTATCAATTAAATAATTAATGCCCAATTTGGATATAGTAGCCT

GAAGCTCATCTTGTTTAGTTACGGATCCTAATGAGTTATTAAGAAATACATCGAACGGATGAACGAAGGT

TGTTTTAAGTTGGTCACATACTTTGTAATCTAGACATAGATGTGGAAGAACGGTAGAAACTATACGAAAT

AGATATTCAGAGTCCTCTAATTGATCAAGAGTAACTATTGACTTAATAGGCATCATTTATTTAGTATTAA

ATGACGACCGTACCAGTGACAGATATACAAAACGACTTAATTACAGAGTTTTCAGAAGATAATTATCCAT

CTAACAAAAATTATGAAATAACTCTTCGCCAAATGTCTATTCTAACCCACGTTAACAACGTGGTAGATAG

AGAACATAATGCCGCCGTAGTGTCATCTCCAGAGGAAATATCATCACAACTTAATGAAGATCTATTTCCA

GATGATGATTCACCGGCCACTATTATCGAACGAGTACAACCTCATACTACTATTATTGACGATACGCCAC

CTCCTACTTTTCGTAGAGAGTTATTGATATCGGAACAACGTCAACAACGAGAAAAAAGATTTAATATTAC

AGTATCAAAAAATTCTGAAGCAATAATGGAATCTAGATCTATGATAACTTCTATGCCAACACAAACACCA

TCCTTGGGAGTAGTTTATGATAAAGATAAAAGAATTCAGATGCTAGAGGATGAAGTGGTTAATCTTAGAA

ATCAACGATCTAATACAAAATCATCTGATAATTTAGATAATTTTACCAGAATACTATTTGGTAAGACTCC

GTATAAATCAACCGAAGTTAATAAGCGTATAGCCATCGTTAATTATGCAAATTTGAACGGGTCCCCCTTA

TCAGTCGAGGACTTGGATGTCTGTTCGGAGGATGAAATAGATAGAATCTATAAAACGATTAAACAATATC

ACGAAAGTAGAAAACGAAAAATTATCGTCACTAACGTGATTATTATTGTCATAAACATTATTGAGCAGGC

ATTGCTAAAACTCGGATTTGAAGAAATCAAAGGACTGAGTACCGATATCACTTCAGAAATTATCGATGTG

GAGATCGGAGATGACTGCGATGCTGTAGCATCAAAACTAGGAATCGGTAACAGTCCGGTTCTTAATATTG

TATTGTTTATACTCAAGATATTCGTTAAACGAATTAAAATTATTTAATTTAATACATTCCCATATCCAGA

CAACAATCGTCTGGATTAATCTGTTCCTGTCGTCTCATACCGGACGACATATTAATCTTTTTATTAGTGG

GCATCTTTTTAGATGGTTTCTTTTTCCCAGCATTAACTGATTCGATACCTAGAAGATCGTGATTGATTTC

TCCGACCATTCCACGAACTTCTAATTGGCCGTCTCTAACGGTACCATAAACTATTTTACCAGCATTAGTA

ACAGCTTGGACAATCTGACCATCCATTGCGTTGAATGATGTAGTTGCTGTTGTTCTACGTCTAGGAGCAC

CAGAGGTATTTTTAGAGCTCTTGGATGTTGATGTAGAAGACGAGGATTTTGATTTTGGTTTACATGTAAT

ACATTTTGAACTCTTTGATTTTGTATCACATGCACCGGCAGTCACATCTGTTTGAGAATTAAGATTATTG

TTGCCTCCTTTGACGGCTGCATCTCCACCGATCTGCGCTAGTAGATTTTTAAGCTGTGGTGTAATCTTAT

TAACTGTTTCAATATAATCATCGTAACTACTTCTAACGGCTAAATTTTTTTTATCCGCCATTTAGAAGCT

AAAAATATTTTTATTTATGCAGAAGATTTAACTAGATTATACAATGAACTAATATGATCCTTTTCTAGAT

TATTTACGAACTTGGTATTTCTTGTTTCTGGAGGAGGAGAATTTAAATTCGGACTTGGATTCGGATTTTG

TGGGTTCTTGATCTTATTATACAGCGTGTATAGGATGGCGACGGTAACTGCTACACAAATACCGATCAAC

AGAAGAATACCAATCATTTATTGACAATAACTTCACTATGATCAAGTATGTAATAATCATCTTTTCACTA

AGTAAGTAGTAATAATGATTCAACAATGACACGATATATGGACGATAATAATTTAGTTCATGGAAATATC

GCTATGATTGGTGTGAATGACTCCGCTAACTCTGTGGGGTGCACAGTGCTTTCCCCACATAGAATAAATT

AGCATTCCGACTGTGATAATAATACCAAGTATAAACGCCATAATACTCAATACTTTCCATGTACGAGTGG

GACTGGTAGACTTACTAAAGTCAATAAAGGCGAAGATACACGAAAGAATCAAAAGAATGATTCCAGCGAT

TAGCACGCCAGAAAAATAATTTCCAATCATAAGCATCATGTCCATTTAACTAATAAAAATTTTAAATCGC

CGAATAAACAAAGTGGAATATAAACCATATAAAAACAATAGTTTGTACTGCAAAAATAATATCTATTTTT

GTTTTCGAAGATATGGTAAAATTAAATAGTAGTACACAGCATGTTATAACTAACAGCAGCAACGGCTCGT

AATTACTTATCATTTACTAGACGAAAAGGTGGTGGGATATTTTCTTGCTCAAATAATACGAATATATCAC

CCATCCATTTTATACGATGTTTATATACTCTAATCTTTAATAGATCTATAGATGACGGGTTTACCAATAA

TATAGATTTTATCGATTCATCTAATTTAAACCCTTCCTTAAACGTGAATGATCTATTATCTGGCATAATG

ATGACCCTACCTGATGAATCTGACAATGTACTGGGCCATGTAGAATAAATTATCAACGAATTATCGTCTA

CGAACATTTATATCATTTGTTTTAATTTTAGGACGTGAATAAATAGATATAAAATAGAAAATAACAGATA

TTACAACCAGTGTTATGGACGCACCCAACCATGTAGGCAGTTTTATTTTATCGTTTACTACAGGTTCTCC

TGGATGTACGTCACCAACTGCAGACGTAGTTCTAGTACAATTAGACGTAAGTTCCGCTTGGGAATTTTTT

AACGCTAAAGAGTTAACGTTGATCGTACACCCAACGTATTTACATCTAGTTCTTTGAACATCTTGATTAT

AATATAACCATTTTCTATCTCTAGATTCGTCAGTGCACTCATGTAACCAACATACCCTAGGTCCTAAATA

TTTATCTCCGGAATTAGATTTTGGATAATTCGCGCACCAACAATTTCTATTTCCTTTATGGTCGTTACAA

AAGACGTATAATGCCGTATCCCCAAAAGTAAAATAATCAGGACGAATAATTCTAATAAACTCAGAACAAT

ATCTCGCATCCATATGTTTGGAGCAAATATCGGAATAAGTAGACATAGCCGGTTTCCGTTTTACACGTAA

CCATTCTAAACAATTGGGGTTTCCAGGATCGTTTCTACAAAAACCAGTCATGAAATCGTCACAATGTTCT

GTCTTGTAATTATTATTAAATATTTTTGGACAGTGTTTGGTATTTGTCTTAGAACAACATTTTGCCACGC

TATCACTATCACCCAGGAGATAATCCTTTTTTATAAAATGACATCGTTGCCCGGATGCTATATAATCAGT

AGCATATTTTAAATCCTTAATATATTCAGGAGTTACCTCGTTCTGATAATAGATTAATGATCCAGGACGA

AATTTGAAAGAACTACATGGTTCTCCATGAATTAATACATATTGTTTAGCAAATTCAGGAACTATAAAAC

TACTACAATGATCTATCGACATACCATCTATCAAACAAAATTTGGGTTTAATTTCTCCTGGAGACGTTTC

ATAATAATACATATAACTTTCTTCGGCAAACCTAACAGCTCTATTATATTCAGGATAATTAAAATCTAAT

ACCATATATTTGTCTCGTATATCTGCTATTCCTGTCTCTATTTTGATTCTATTAAGAGTAACAGCTGCCC

CCATTCTTAATAATCATCAGTATTTAAACTGTTAAATGTTGGTATATCAACATCTATCTTATTTCCCGCA

GTATAAGGTTTGTTGCAGGTATACTGTTCAGGAATGGGTACATTTATACTTCTTTTATAGTCCTGTCTTT

CGATGTTCATCACAAATGCAAAGAACAGAATAAACAAAATAATGTAAGAAATAATATTAAATATCTGTGA

ATTCGTAAATACATTGATTGCCATAATAATTACAGCAGCTACAATACACACAATAGACATTCCCACAGTG

TTGCCATTACCTCCACGATACATTTGAGTTACTAAGCAATAGGTAATAACTAAGCTAGTAAGAGGCAATA

GAAAAGATGAGATAAATATCATCAATATAGAGATTAGAGGAGGGCTATATAGAGCCAAGACGAACAAAAT

CAAACCGAGTAACGTTCTAACATCATTATTTTTGAAGATTCCCAAATAATCATTCATTATTCCTCCATAA

TCGTTTTGCATCATACCCCCATCTTTAGGCATAAACGATTGCTGCTGTTCCTCTGTAAATAAATCTTTAT

CAAGCACTCCAGCACCCGCAGAGAAGTCATCAAGCATATTGTAATATCTTAAATAACTCATTTATATATT

AAAAAATGTCACTATTAAAGATGGAGTATAATCTTTATGCCGAACTAAAAAAAATGACTTGTGGTCAGAC

CATAAGTCTTTTTAATGAAGACGGCGATTTCGTAGAAGTTGAACCAGGATCATCCTTTAAGTTTCTAATA

CCTAAGGGATTTTACTCCTCTCCTTGTGTAAAGACGAGTCTAGTATTCGAGACATTAACAACGACCGATA

ATAAAATTACTAGTATCAATCCAACAAATGCGCCAAAGTTATATCCTCTTCAACGCAAAGTCGTATCTGA

AGTAGTTTCTAATATGAGGAAAATGATCGAATTAAAACGTCCTCTATACATCACTCTTCACTTGGCATGT

GGATTTGGTAAGACTATTACCACGTGTTATCTTATGACCACACACGGCAGAAAAACCATCATTTGCGTAC

CCAATAAAATGTTAATACATCAATGGAAGACACAGGTAGAGGCAGTCGGATTGGAACATAAGATATCTAT

AGATGGAGTTAGTAGTCTATTAAAGGAACTAAAGACTCAAAGTCCGGATGTATTAATCGTAGTCAGTAGA

CATCTGACAAACGATGCATTTTGTAAATATATCAATAAGCATTATGATTTGTTTATCTTGGATGAATCAC

ATACGTATAATCTGATGAACAATACAGCAGTTACAAGATTTTTAGCGTATTATCCTCCGATGATGTGTTA

TTTTTTAACTGCTACACCTAGACCAGCTAACCGAATTTATTGTAATAGTATTATTAATATTGCCAAGTTA

TCCGATCTAAAAAAAACTATCTATATAGTAGATAGTTTTTTTGAGCCATATTCCACAGACAATATTAGAA

ATATGGTAAAACGACTAGATGGACCATCTAATAAATATCATATATATACCGAGAAGTTATTATCTGTAGA

CGAGCCTAGAAACCAACTTATTCTTGATACCCTGGTAGAAGAATTCAAGTCAGGAACTATTAATAGAATT

TTAGTTATTACTAAACTACGTGAACATATGGTATTCTTCTACAAACGATTATTAGATCTTTTCGGAGCAG

AGGTTGTATTTATAGGAGACGCCCAAAATAGACGTACTCCAGATATGGTCAAATCGATTAAGGAACTAAA

TAGATTTATATTCGTATCCACCTTATTTTATTCCGGCACTGGTTTAGATATTCCGAGTTTGGATTCTTTG

TTCATTTGCTCGGCAGTAATCAACAATATGCAAATAGAGCAATTACTAGGGAGGGTATGTCGAGAAACAG

AACTATTAGATAGGACGGTATATGTATTTCCTAACACATCCATCAAAGAAATAAAGTACATGATAGGAAA

TTTCGTGCAACGAATTATTAGTCTGTCTGTAGATAAACTCGGATTTAAACAAGAAAGTTATCAGAAACAT

CAGGAATCTGAACCCGCTTCCGTACCAACATCCTCCAGAGAAGAACGTGTATTAAATAGAATATTTAACT

CGCAAAATCGTTAAGAAGTTTAAGAGACGATCCACATGCTGAGCAGGCCAGTGTATTACCCCTCATAGTA

TTAATATAATCCAATGATACTTTTGTGATGTCGGAAATCTTAACCAATTTAGACTGACAGGCAGAACACG

TCATACAATCATCATCGTCATCGATAACTGTAGTCTTGGGCTTCTTTTTGCGACTCTTCATTCCGGAACG

CATATTGGTGCTATCCATTTAGGTAGTAAAAAATAAGTCAGAATATGCCCTATAACACGATCGTGCAAAA

CCTGGTATATCGTCTCTATCTTTATCACAATATAGTGTATCAACATCTTTATTATTGACCTCGTTTATCT

TGGAACATGGAATGGGAACATTTTTGTTAACGGCCACCTTTGCCTTAATTCCAGATGTTGTAAAATTATA

ACTAAACAGTCTATCATCGACACAAATGAAATTCTTGTTTAGACGTTTGTAGTTTACGTATGCGGCTCGT

TCTCGTCTCATTTTTTCAGATATTGCAGGTACTATAATATTAAAAATAAGAATGAAATAACATAGGATTA

AAAATAAAGTTATCATGACTTCTAGTGCTGATTTAACTAACTTAAAAGAATTACTTAGTCTGTACAAAAG

TTTGAGATTTTCAGATTCTGTGGCTATAGAGAAGTATAATTCTTTGGTAGAATGGGGAACATCTACTTAC

TGGAAAATAGGCGTACAAAAGGTAACTAATGTCGAGACGTCCATATCTGATTATTATGATGAGGTAAAAA

ATAAACCGTTTAATATTGATCCGGGGTATTATATTTTCTTACCAGTATATTTTGGAAGCGTCTTTATTTA

TTCAAAGGGTAAAAATATGGTAGAACTTGGATCTGGAAACTCTTTTCAAATACCGGATGAGATTCGAAGT

GCGTGTAACAAAGTATTAGATAGTGATAACGGAATAGACTTTCTGAGATTTGTTTTGTTAAACAATAGAT

GGATAATGGAAGACGCTATATCAAAATACCAGTCTCCAGTTAATATATTTAAACTAGCTAGTGAGTACGG

ATTAAACATACCCAACTATTTAGAAATTGAAATAGAGGAAGACACATTATTTGACGATGAGTTATACTCT

ATTATGGAACGCTCTTTCGATGATACATTTCCAAAAATATCTATATCGTATATTAAGTTGGGAGAACTTA

AGCGGCAAGTTGTAGACTTTTTCAAATTCTCATTCATGTATATTGAGTCAATCAAGGTAGATCGTATAGG

AGATAATATTTTTATTCCTAGCGTTATAACAAAATCAGGAAAAAAGATATTAGTAAAAGATGTAGACCAT

TTAATACGATCCAAGGTTAGAGAACATACATTTGTAAAAGTAAAAAAGAAAAACACATTTTCCATTTTAT

ACGACTATGATGGGAACGGAACAGAAACTAGAGGAGAAGTAATAAAACGAATTATAGACACTATAGGACG

AGACTATTATGTTAATGGAAAGTATTTCTCTAAGGTTGGTATTGCAGGCTTAAAGCAATTGACTAATAAA

TTAGATATTAATGAGTGTGCAACTGTCGATGAGTTAGTTGATGAGATTAATAAATCCGGAACTGTAAAAC

GAAAAATAAAAAACCAATCAGTATTTGATTTAAGCAGAGAATGTTTGGGATATCCAGAAGCGGATTTTAT

AACGTTAGTTAATAACATGCGGTTCAAAATAGAAAATTGTAAGGTTGTAAATTTCAATATTGAAAATACT

AATTGTTTAAATAACCCGAGTATTGAAACTATATATGGAAACTTCAACCAGTTCGTCTCAATCTTTAATA

CCGTTACCGATGTCAAAAAAAGATTATTCGAGTGAAATAATATGCGCCTTTGATATAGGTGCAAAAAATC

CTGCCAGAACTGTTTTAGAAGTCAAGGATAACTCCGTTAGGGTATTGGATATATCAAAATTAGACTGGAG

TTCTGATTGGGAAAGGCGCATAGCTCAAGATTTGTCACAATATGAATACACTACAGTTCTTCTAGAACGT

CAGCCTAGAAGGTCACCGTACGTCAAATTTATCTATTTTATTAAAGGCTTTTTATATCATACATCTGCTG

CCAAAGTTATTTGCGTCTCACCTGTCATGTCTGGTAATTCATATAGAGATCGAAAAAAGAGATCTGTTGA

AGCATTTCTTGATTGGATGGACACATTCGGATTGCGAGACTCCGTTCCGGATAGACGCAAATTAGACGAT

GTAGCGGATAGTTTCAATTTGGCTATGAGATACGTATTAGATAAATGGAATACTAATTATACACCTTATA

ATAGGTGTAAATATAGAAATTACATAAAAAAAATGTAATAACGTTAGTAACGCCATTATGGATAATCTAT

TTACCTTTCTACATGAAATAGAAGATAGATATGCCAGAACTATTTTTAACTTTCATCTAATAAGTTGTGA

TGAAATAGGAGATATATATGGTCTTATGAAAGAACGCATTTCCTCAGAGGATATGTTTGACAATATAGTA

TATAATAAAGATATACATCCTGCCATTAAGAAACTAGTTTATTGCGACATCCAACTTACTAAACATATTA

TTAATCAGAATACGTATCCGGTATTTAACGATTCTTCACAAGTGAAATGTTGTCATTATTTCGATATAAA

CTCAGATAATAGCAATATTAGCTCTCGTACAGTAGAGATATTTGAGAGTGAAAAGTCATCTCTTGTATCA

TATATTAAAACTACCAATAAGAAGAGAAAGGTCAATTACGGCGAAATAAAGAAAACTGTACATGGAGGCA

CTAATGCAAATTACTTTTCCGGTAAAAAGTCTGATGAGTATCTGAGCACTACAGTCAGGTCCAACATTAA

TCAACCTTGGATCAAAACCATTTCTAAGAGAATGAGAGTAGATATCATTAATCACTCTATAGTAACGCGT

GGAAAAAGCTCTATATTACAAACTATAGAAATTATTTTTACTAATAGAACATGTGTGAAAATATTCAAGG

ATTCTACTATGCACATTATTCTATCCAAGGACAAGGATGAAAAGGGATGTATAAACATGATTGATAAATT

ATTCTATGTATATTATAATTTATTTCTGTTGTTCGAGGATATCATCCAAAACGATTACTTTAAAGAAGTA

GCTAATGTTGTAAACCATGTACTCATGGCTACGGCATTAGATGAGAAATTATTCCTAATTAAGAAAATGG

CTGAACACGATGTTTATGGAGTTAGCAATTTCAAAATAGGGATGTTTAACCTGACATTTATTAAGTCGTT

GGATCATACCGTTTTCCCCTCTCTGTTAGATGAGGATAGCAAAATAAAGTTTTTTAAGGGGAAAAAGCTC

AATATTGTAGCATTACGATCTCTGGAGGATTGTACAAATTACGTGACTAAATCCGAGAATATGATAGAAA

TGATGAAGGAAAGATCGACTATTTTAAATAGCATAGATATAGAAACGGAATCGGTAGATCGTCTAAAAGA

ATTGCTTCTAAAATGAAAAAAAACACTGATTCAGAAATGGATCAACGACTCGGGTATAAGTTTTTGGTGC

CTGATCCTAAAGCCGGAGTTTTTTATAGACCGTTACATTTCCAATATGTATCGTATTCTAATTTTATATT

GCATCGATTGCATGAAATCTTGACCGTCAAGCGGCCACTCTTATCGTTTAAGAATAATACAGAACGAATT

ATGATAGAAATTAGCAATGTTAAAGTGACTCCTCCAGATTACTCACCTATAATTGCGAGTATTAAAGGTA

AGAGTTATGACGCATTAGCCACGTTCACTGTAAATATCTTTAAAGAGGTAATGACCAAAGAGGGTATATC

CATCACTAAAATAAGTAGTTATGAGGGAAAAGATTCTCATTTGATAAAAATTCCGCTACTAATAGGATAT

GGGAATAAAAATCCACTTGATACAGCCAAGTATCTTGTTCCTAATGTCATAGGTGGAGTCTTTATCAATA

AACAATCTGTCGAAAAAGTAGGAATTAATCTAGTAGAAAAGATTACAACATGGCCAAAATTTAGGGTTGT

TAAGCCAAACTCATTCACTTTCTCGTTTTCCTCCGTATCCCCTCCTAATGTATTACCGACAAGATATCGC

CATTACAAGATATCTCTGGATATATCACAATTGGAAGCGTCGAATATATCATCGACAAAGACATTTATAA

CGGTCAATATTGTTTTGCTGTCTCAATATTTATCTAGAGTGAGTCTAGAATTCATTAGACGTAGTTTATC

ATACGATATGCCTCCAGAAGTTGTCTATCTAGTAAACGCGATAATAGATAGTGCTAAACGACTTACCGAA

TCTATTACTGGCTTTAATATTGATACATACATTAATGACCTGGTGGAAGCTGAACACATTAAACAAAAAT

CTCAGTTAACGATTAACGAGTTTAAATATGAAATGCTGCATAACTTTTTACCTCATATGAACTATACACC

CGATCAACTAAAGGGATTTTATATGATATCTTTACTAAGAAAGTTTCTCTACTGTATCTACCACACTTCT

AGATATCCAGATAGAGATTCGATGGTTTGTCATCGCATCCTAACGTACGGCAAATATTTTGAGACGTTAG

CACATGATGAATTAGAGAATTACATAGGTAACATCCGAAACGATATCATGAACAATCACAAGAACAGAGG

CACTTACGCAGTAAACATTCATGTACTAACAACTCCTGGACTTAATCATGCATTTTCTAGTCTATTGAGT

GGAAAGTTCAAAAAGTCAGACGGTAGTTATCGAACACATCCTCACTATTCATGGATGCAGAATATTTCTA

TTCCTAGAAGTGTTGGATTTTATCCGGATCAAGTAAAGATTTCAAAGATGTTTTCTGTCAGAAAATACCA

TCCAAGCCAATATCTTTACTTTTGTTCATCAGACGTTCCGGAAAGAGGTCCTCAGGTAGGTTTAGTATCT

CAATTGTCTGTCTTGAGTTCCATTACAAATATACTAACGTCTGAGTATTTGGATTTGGAAAAGAAAATTT

GTGAGTATATCAGATCATATTATAAAGATGATATAAGTTACTTTGAAACAGGATTTCCAATCACTATAGA

AAATGCTCTAGTCGCATCTCTTAATCCAAATATGATATGTGATTTTGTAACTGACTTTAGACGTAGAAAA

CGGATGGGATTCTTCGGTAACTTGGAGGTAGGTATTACTTTAGTTAGGGATCACATGAATGAAATTCGCA

TTAATATTGGAGCAGGAAGATTAGTCAGACCATTCTTGGTTGTGGATAACGGAGAGCTCATGATGGATGT

GTGTCCGGAGTTAGAAAGCAGATTAGACGACATGACATTCTCTGACATTCAGAAAGAGTTTCCACATGTC

ATCGAAATGGTAGATATAGAACAATTTACTTTTAGTAACGTATGTGAATCGGTTCAAAAATTTAGAATGA

TGTCAAAGGATGAAAGAAAGCAATACGATTTATGTGACTTTCCTGCCGAATTTAGAGATGGATATGTAGC

ATCTTCACTAGTGGGAATCAATCACAATTCTGGACCCAGAGCTATTCTTGGATGTGCTCAAGCTAAACAA

GCTATCTCTTGTCTGAGTTCGGATATACGAAATAAAATAGACAATGGAATTCATTTGATGTATCCAGAGA

GGCCAATTGTGATTAGTAAGGCTTTAGAAACTTCAAAGATTGCGGCTAATTGCTTCGGACAACATGTTAC

TATAGCATTAATGTCGTACAAAGGTATCAATCAAGAGGATGGAATTATCATCAAAAAACAATTTATTCAG

AGAGGCGGTCTCGATATTGTTACAGCCAAGAAACATCAAGTAGAAATTCCATTGGAAAACTTTAATAACA

AAGAAAGAGATAGGTCTAACGCCTATTCGAAATTAGAAAGTAATGGATTAGTTAGACTGAATGCTTTCTT

GGAATCCGGAGACGCTATGGCAAGAAATATCTCATCAAGAACTCTTGAAGATGATTTTGCTAGAGATAAT

CAGATTAGCTTTGATGTTTCCGAGAAATATACAGATATGTACAAATCTCGCGTTGAACGAGTACAAGTAG

AACTTACTGACAAAGTTAAGGTGCGAGTATTAACCATGAAAGAAAGAAGACCCATTCTAGGAGACAAATT

TACTACTAGAACGAGTCAAAAGGGAACAGTCGCGTATATCGCAGATGAAACGGAACTTCCGTACGACGAA

AATGGTATCACACCAGATGTCATTATTAATTCTACATCCATCTTCTCTAGAAAAACTATATCTATGTTGA

TAGAAGTTATTTTAACAGCCGCATATTCTACTAAGCCGTACAACAATAAGGGAGAAAACCGACCTGTCTG

TTTTCCTAGTAGTAACGAAACATCTATCGATGCATATATGCAATTCGCTAAACAATGTTATGAGTATTCA

AATCCGAAATTGTCCGAGGAAGAATTATCGGATAAAATCTTTTGTGAAAAGATTCTCTATGATCCTGAAA

CGGATAAGCCTTATGAATCCAAAGTATTTTTTGGACCAATTTATTACTTGCGTCTGAGACATTTAACTCA

GGACAAGGCAACCGTTAGATGTAGAGGTAAAAAGACGAAGCTCATTAGACAAGCGAATGAGGGACGAAAA

CGTGGAGGAGGTATCAAGTTTGGAGAAATGGAGAGAGACTGTTTAATAGCACATGGTGCAGCCAATACTA

TTACAGAAGTTTTAAAAGACTCAGAAGAGGATTATCAAGATGTGTATATTTGTGAAAATTGTGGAGACAT

AGCAGCACAAATCAAAAGTATTAATACATGTCTTAGATGTTCAAAACTTAATCTCTCTCCTCTCTTAACA

AAAATTGATACCACGCACGTATCTAAAGTATTTCTTACTCAAATGAACGCCAGAGGCGTAAAAGTTAAAT

TAGATTTCGAACGAAGGCCTCCTTCGTTTTATAAACCATTAGATAAAGTTGATCTTAAACCGTCTTTTCT

GGTATAATATTGTTTAGTAGATACTCATCAAGATAAGCTAATTCACTAAACATATTATCGGATTCGGTAT

TGTTACTCGAGAATAGAGTTCGTTATGCTCCTGATATTCGGAAATCTGTGGAGTTTCAGGTTTTGGTGGA

AGTGTAACTGCTACTTGGTGGGATACTGAAGGATATTTCAGAGAGTTGTGGATGTTCGGGTTCGACATCC

ACCGATGGTGTCACGCCACTAATCGGTTCGGTAACGTCTGTGGATGGAGGTGCTACTTCTACAGAACCTG

TAGCCTCAGTTGTCAACGGAGATACATATTCAATGCGCGGAAATGTATAATTTGGTAATGGTTTCTCATG

TGGATCTTAAGAAGAAGAGGTAAGATATCTACGAAAGATACCGATCACGTTTCTAGTTCTCTTTTGTAGA

ACTTTAACTTTTTCTTTCTCAGCATCTAGTTGATATTCCGACCTCTTCACGTTTCGCATGGGTTACCTCC

GCAGTTTTTACAAGCGATTTCACGTTCCAGATCACGTTCAGCCTTCATACGTCTCTCCCTCTCTCTATCG

AGTTTATCAGAGCAGTCTTTCTGAAGGCGATCGAACTCCATAAATTTCTCCAACGCTTTGATTGTTTCCA

TAGATTTCCGAAGTTTAGCTTCTAGGACGGCGATTCTTTTTTTTTTTTTTTTTTTTTTTTTCTTTCGAAT

TCACGGGGTACAACCGTTTCCATTACCACCATCTCTACGTTTCTTTTCTAGATCGGCAATCTTTCTCAAT

CTTTCTCAACATTTCATCCCCATACCTTTTCATTCCTCGAGTCTATTGTCGTCGAAATATCGTTCCAGCT

CCTTTTCGACCTCAATAACTTTAGCACGTTGTTTCATCAAGCTCTCTCTTGTAGTACTATCATTTTTATC

TGATTCCCTGACACGTTTAAGATCTTCATGTAATTGAGTCAGCTCTTGACGCAATCTCTTAACTAACTTC

CTCTCTTGCTTCTTCGTCATAGTACTTACAATCACTATGGGATCCATTGTTACCACGTCTGTACTCGACG

AGCTCACGTTTAAGAGATTCAATTTCCAGTTTGTATCGGTCCATGTCTCCATTGCTACACCACCATTAGA

TTTACAGGCTGCTAGTTGTCGTTCGAGATCAGAAATACGTGTTTTCTTGGAATGGATTTCGTCGATGTAC

TTGTCATGATTGGCATCGAAACACTTATTAAGTTCTTTTTTTCAATTCTACGATTTTATTTCTTTCGCGA

GTCAATTCCCTCCTGTAGTAACTATCAGTTTTGTCAGATTCACGCTCTCTACGTAGACTTTCTTGTAAGT

TACTAATTTGTTCCCTGGCATTACCGAGTTCAGTTTTATATGCCGAATAGAGTTCTGATTCATCCTTTGA

GAAGATCTCTAGCGATCGTTCAAGATCCCTGATTCTAGTCTTTAGCCTATTTACCTCCTCAGAAGATGCT

CCGTTACCGTTTTTACAATCGTTAAGATGTCTATCAAGATCCATGATTCTATCTCTTTTCCATATCAGCA

TTGATTTCATTATTACGTTCGCAGTCGTTCAACTGTATTTCAAGATCTGAGATTCTAGATTGTAATCTCT

GTAGCATTTCCACGGCATTCACTCAGTTGTCTTTCAAGATCTGAGATTCTAGATTGGAGTCTGCTAATCT

CTGTAAGATTTCCTCCTCCGCTCTCGATGCAGTCGGTCAACTTATTCTCTAGTTCTCTAATACGCGAACG

CAGTGCATCAACTTCTTGTGTGTCTTCTTGATTGCGTGTGCATTCATCGAGTCTAGATTCGAGATCTCTA

ACGTGACGTCGTTCTTCCTCAAGTTCTCTGTGTACTACAGAAAGCGTGTCCCTATCTTGTTGATATTTAG

CAATTTCTGATTCTAGAGTACTGATTCTACTCACGTATGTACTAATAGTTGTCTTAGCCTTATCAAGATC

CTCCTTGTATTTGTCACATTCCTTGATATCCATACGAAGTCTGGACAGTTCCCATTCGACATTACGACGT

TTATCGATTTCAGCTCGGAGATCGTCGTCGCGTTGTTTTAGCCACATACGACTAAGTTCAAGTTCTCGTT

GACAAGATCCATCTACTTTTCCATCCCTAATAGTATCCAGTTCCTTTTCTAGTTCTGACCGCATTTCTCG

TTCCATATCAAGAGATTCTCTCAATTCTCGTATAGTCTTCTTATCAATTTCTGATGAATCTGAACCATCA

TCTGTCCCATTTTGTTGCATATCCCTGAGTTCTTTGATCTCTGTTGTAAGTCTGTCGATTCTTTCGGTTT

TATAAACAGAATCCCTTTCCAAAGTCCTAATCTTACTGAGTTTATCATTAAGTTCTTCATTCAATTCAGT

GAGTTTTCTCTTGGCTTCTTCCAAGTCTGTTTTAAACTCTCCATCATTTCCGCATTCTTCCTCGCATTTA

TCTAACCATTCAATTAGTTTATTAATAACTAGTTGGTAATCAGCGATTCCTATAGCCGTTCTTGTATTTG

TGGGAACATAATTAGGATCTTCTAATGGATTGTATGGCTTGATAGCATCATCTTTATCATTATTAGGTGG

GGGATGGACAACCTTAATTGGTTGGTCCTCCTTATCTCCTCCAGTAGCATGTGGTTCTTCAATACCAGTA

TTAGTAATAGGCTTAGACAAATGCTTGTCGTACGCGGGCACTTCCTCATCCATCAAGTATTTATAATCGG

GTTCTGTTTCAGAATATTCTTTTCTAAGAGACGCGACTTCAGGAGTTAGTAGAAGAACTCTGTTTCTGTA

TCTATCAACGCTGGAATCGATACTCAAGTTAAGGATAGCGAATACCTCATCGTCATCATCCGTATCTTCT

GAAACGCCATCATATGACATTTCATGAAGTCTAACGTATTGATAAACAGAATCAGATTTAGTATTAAACA

GATCCTTGACCTTTTTAGTAAATGCATATGTATATTTTAGATCTCCAGATTTCATAATATGATCGCATGC

CTTAAATGTCAATGCTTCCATGATATAGTCTGGAACACTAATGGGTGACGAAAAAGATACAGCACCATAT

GCTACGTTGATAAATAGATCTGAACCACTAAGTAGATAATGATTAATGTTAAGGAAGAGGAAATATTCAG

TATATAGATATGCCTTAGCATCATATCTTGTACTAAACACGCTAAACAGTTTATTGATGTGATCAATTTC

CAACAGAACAATTAGAGCGGCAGGAATACCAACAAACATATTACCACATCCGTATTTTCTATGAATATCA

CATATCATATTAAAAAATCTTGATAGAAGAGCGAATATCTCGTCTGACTTAATGAGATGTAGTTCAGCAG

CATAAGTCATAACTGTAAATAGAACATACTTTCCTGTAGTGTTGATTCTAGACTCCACATCAACACCATT

ATTAAAAATAGTTTTATATACATCTTTAATCTGCTCTCCGTTAATCGTCGAACGTTCTAGTATACGGAAA

CACTTTGATTTCTTATCTGTAGTTAATGACTTAGTGATATCACGAAGAATATTACGAATTACATTTCTTG

TTTTTCTTGAGAGACCTGATTCAGAACTCAACTCATCGTTCCATAGTTTTTCTACCTCAGTGGCGAAATC

TTTGGAGTGTTTGGTACATTTTTTAATAAGGTTCGTGACCTCCATTTATTATAAAAAATTTTTATTCAAA

ACTTAACTACAATCGGGTAATTATAAGATCGTAGATCTCCCATGTGGTGGAATACTACCATCTATCGCAT

GTTGATGGACAGTAGGTAATGGCCATGGGAACAGTAATGTTTGCATATTTATCTTTCTTGCTAGTATTAC

TGTATATTGTCCCAATGTTTCAATGTGATGTTCTAACCTATCAACTGCCACTGTATCACAACAATAATGT

CCGATGGAATTAAGATTATGATCCAATGTGTTTAATATATGATTATCAAGTCTTATACGATCCGCGTCTT

TTTTGACAGGATCAGGCTCTTCTACAGGAAGAAGTTTCGGCCTCTTATGATAGTCATGTCTGGGAAATGG

TGGTCTAGGATGAGGATCAGGTATCGGAGTAGGTTTTGGATTATAATCATCATCATCATCATCATCATCA

TCATCATCATCATCATCRTCATCATSATCATCATCATCATCTTCGATATTTATTTTGCTATCTTGATAAT

GTCCTATATCAGTTGCATTTTCAGCACTCGACTGAATATTAGTACATTCATTGTCTATTATTAACGTATT

TCTAAACCCAAAATGTATATGTTGAACATCACTACTATAGTTGATGAGTCTTATAGCATGAATTCGCTTA

TCGTTATCGGGTTTATCTTCTGTCACCTTAACAATTCCTTTTTTATTAAACTCTGCATAATCATAACCAT

TTCTATTGTTTGTTCTAATATAAACGAGTATAGCATCATTGCTAAATTTTTCAATAGTATCAAAAACAGA

ATATCCTAAACCATATAATATATATTCAGGAACACTCAAACTAAATGTCCAGGATTCTCCTAAATACGTA

AACTTTAATAGTGCTAAATCATTCAAAAATCTACCGCTTATAGATAGATAGTACATGAATGCGTATAGTA

GTCTACCTATCTCTTTATTATGAAAACCGACATTACGATCATATATTTCGTGATATACATGTGACCCGTT

TACGTTAAACCATAAATACATGGGTGATCCTATAAACATGAATTTATTTCTAATTCTCAGAGCCATAGTT

AATTGACCGTGTAATATTTGTTTACATGCATACTTGATACGATCATTAATAAGATTTTTATCATTGCTCG

TTATTTCAGAATCGTATATATAAGGAGTACCATCATGATTCTTACCAGATATTATACAAAATACTATATA

TAAAATATATTGACCCACGTTAGTAATCATGTAAATGTTTAATGTTTTAAATTTTGTATTTAATGATCCA

TCATCATATGCTAGCATGGTCTTGTGATATTCATTCTTTAAAATATAATATTGTGTTAGCCATTGCATTG

GAGCTCCTAATGGAGATTTTCTATTCTCGTCCATTTTAGGATATGCTTTCATAAAGTCCCTAATAACTTC

GTGAATAATGTTTCTATGTTTTCTACTGATGCATGTATTTGCTTCGATTTTTTTATCCCATGTTTCATCT

ATCATAGATTTAAACGCAGTAATGCTCGCAACATTAACATCTTGAACCGTTGGTACAATTCCGTTCCATA

AATTTATAATGTTCGCCATTTATATAACTCATTTTTTGAATATACTTTTAATTGAACAAAAGAGTTAAGT

TACTCATATGGATGCCGTCCAGTCTGTACATCAATCTTTTTAGCCAGAGATATCATAGCCGCTCTTAGAG

TTTCAGCGTGATTTTCCAACCTAAATAGAACTTCATCGTTGCGTTTACAACACTTTTCTATTTGTTCAAA

CTTTGTTGTTATATTAGTAATCTTTTTTTCCAAATTAGTTAGCCGTTGTTTGAGAGTTTCCTCATTATCG

TCTCCATAGGCTTTAACAATTGCTTCGCGTTTAGTCTCTGGATTTTTAGCAGCCTTTGTAGAGAAAAATT

CAGTTGCTGGAATTGCAAGATCGTCATCTCCGGGGAAAAGAGTTCCGTCCATTTAAAGTACAGATTTTAG

AAACTGACACTCTGTGTTATTTATATTTGGCGCAATACATGGATTATAAATATCGATGTTAATAACATCA

GAAAATGTAAAGTCTATACATTGTCGCATCGTGTTAAATTTTCTAATGGATCTAGTATTATTGGGTCCAA

CTTCTGCCTGAAATCCAAATATGGAAGCGGATACAAAACCGTTTCCTGGATAAACCACACATCTCCACTT

TTGCTTTACATCAGAAATTGTGTCATTGACATCTTGAACTCTCCTATCTAATGCCGGTGTTCCACCTATA

GATTTTGAATACTCGAATGCTGCATGAGTAGCATTGAATTCCTTAATATTGCCATAATTTTCATATATTG

AGTAACTCTGGATAAAAAGTAAACACACCGCAGCCGTCGCTACTACAATAAAAAAAATTGATAGAGAGTT

CATTTATAATCTATTAGAAGCTGATAAAATTTTTTTACACGCGTCAGACAATGCTTTAATAAATAGTTCA

ACATCTACTTTTGTCATATCGAACCGATGGTATGATTCTAACCTAGAATTACATCCGAAAAAGTTGACTA

TGTTCATAGTCATTAAGTCATTAACGAACAACATTCCAGACTCTGGATTATAAGACGATACTGTTTCGTC

ACAATCACCCACCTTAATCATGTGATTATGAATATTGGCTATTAGAGTACCTTCTAAGAAATCTATAATA

TCTTTGAAACACGATTTAAAATCAAACCACGAATATACTTCTACGAAGAAAGTTAGTTTACCCATAGGAG

AGATAACTATAAATGGAGATCTAGATACAAAATCCGGATCTATGATAGTTTTAACATTATTATATTCTCT

ATTAAATACCTCCACATCTAAAAATGTTAATTTTGAAACTATGTCTTCGTTTATTACCGTACCTGAACTA

AACGCTATAAGCTCTATTGTTTGAGAACTCTTTAAACGATATTCTTGAAATACATGTAACAAAGTTTCCT

TTAACTCGGTCGGTTTATCTACCATAGTTACAGAATTTGTATCCTTATCTATAATATAATAATCAAAATC

GTATAAAGTTATATAATTATCGTGTTCAGATTGTGATCTTTTCAAATAGACTAAAAACCCCATTTCTCTA

GTAAGTATCTTATGTATATGTTTGTAAAATATCTTCATGGTGGGAATATGCTCTACAGCAGTTAGCCATT

CCTCATTGACAGCTGTAGATGTATTATACAAAACTACTCCAATGTTTAACAAGGGCCATTTTACGAGATT

ATTAAATCCTTGTTTGATAAATGTAGCCAATGCGGGTTCGAGTTCAACGACGATTGAATTCTCTTCCCGT

GGATGCTGCATGATGAACGACGGGATGTTGTTGTTCTATTGATTTGGAATTCTTTTTCGACTTTTTGTTT

ATATTAAATATTTTAAAATTTATGGCTGATAGTAATTCATGTACTACGGATAATGTAGACGTGTATTGCA

TATCGATATCTTTATTATTAGATAAATTTATCAATAAATGTGAGAAGTTTGCCTCGTTAAGGTCTTCCAT

TTAAATATTATATAAATATTTGTGTTTGTATTTTATTCGTCTTTTATGGGATAGTTTTTAACTAGTAAAG

CTGTAATTACATACTTTGTCCGTAAAACATAAATATAAATACCCGCTTTTATCAAACGTTCCAAAAAGTC

GGCAGCTGACATTTTTAACATGACATCTATTTTAAATACACTTAGGTTTTTAGAAAAAACATCATTTTAT

AATTGTAACGATTCAATAACTAAAGAAAAGATTAAGATTAAACATAAGGGAATGTTATTTGTATTTTATA

AGCCAAAGCATTCTACCGTTGTTAAATACTTGTCTGGAGGAGGTATATATCATGATGATTTGGTTGTATT

GGGGAAGGTAACAATTAATGATCTAAAGATGATGCTATTTTACATGGATTTATCATATCATGGAGTGACA

AGTAGTGGAGCAATTTACAAATTGGGATCGTCTATCGATAGACTTTCTCTAAATAGGACTATTGTTACAA

AAGTTAATAACAATTATAACAATTATAACAATTATAATTGTTATAATAATTATAATTGTTATAATTATGA

TGATACATTTTTTGACGATGATGATTGATCACTATTACACAATTTTGTTTTTGTACTTTCTAATATAGTG

TTTAGGTTCTTTTTCATATGAGAATATTGACTTACTAAAATATCTATGTTTAACTTTTGTTCTATAACGT

CCTTATCGGCGGTATCGGTACATATACGTAATTCACCTTCACAAAATACGGAGTCTTCGATAATAATAGC

CAATCGATTATTGGATCTAGCTGTCTGTATCATATTCAACATGTTTAATATATCCTTTCGTTTCCCCTTT

ACAGGCATCGATCGTAGCATATTTTCCGCGTCTGAGATGGAAATGTTAAAACTGCAAAAATGCGTAATGT

TAGCCCGTCCTAATATTGGTACGTGTCTATAAGTTTGGCATAGTAGAATAATAGACGTGTTTAAATGCCT

TCCAAAGTTTAAGAATTCTATTAGAGTATTACATTTTGATAGTTTATCACCTACATCATCAAAAATAAGT

AAAAAGTGTGCTGATTTTTTATGATTTTGTGCGACAGCAATACATTTTTCTATGTTACTTTTAGTTCGTA

TCAGATTATATTCTAGAGCTTCCTGACTACTAACGAAATTAATATGATTTGGCCAAATGTATCCATCATA

ATCTGGGTTATAAACGGGTGTAAACAAGAATATATGTTTATATTTTTTAACTAGTGTAGAAAACAGAGAT

AGTAAATAGATAGTTTTTCCAGATCCAGATCCTCCTGTTAAAACCATTCTAAACGGCATTTTTAATAAAT

TTTCTCTTGAAAATTGTTTTTCTTGAAAACAATTCATAATTATATTTACAGTTACTAAATTAATTTGATA

ATAAATCAAAATATGGAAAACTAAGGTCGTTAGTAGGGAGGAGAACAACGAAGGCATATCGTGATATAAA

TAACATTTATTATCATGATGACACCAGAAAACGACGAAGAGCAGACATCTGTGTTCTCCGCTACTGTTTA

CGGAGACAAAATTCAGGGAAAGAATAAACGCAAACGCGTGATTGGTCTATGTATTAGAATATCTATGGTT

ATTTCACTACTATCTATGATTACCATGTCCGCGTTTCTCATAGTGCGCCTAAATCAATGCATGTCTGCTA

ACGAGGCTGCTATTACTGACTCCGCTGTTGCCGTTGCTGCGGCATCATCTACTCATAGAAAGGTTGCGTC

TAGCACTACACAATATGATCACAAAGAAAGCTGTAATGGTTTATATTACCAGGGTTCTTGTTATATATTA

CATTCAGACTATAAGTCATTCGAGGATGCTAAAGCAAACTGCGCTGCGGAATCATCAACACTACCCAATA

AATCCGATGTCTTGACTACCTGGCTCATTGATTATGTTGAGGATACATGGGGATCTGATGGTAATCCAAT

TACAAAAACTACATCCGATTATCAAGATTCTGATGTATCACAAGAAGTTAGAAAGTATTTTTGTACATAA

ATAAATGAAATCGCTTAATAGACAAACTGTAAGTAGGTTTAGGAAGTTGTCGGTGCCGGCCGCTATAATG

ATGTTACTCTCAACCATTATTAGCGGCATAGGAACATTTCTGCATTACAGAGAAGAACTGATGCCTAGTG

CTTGCGCCAATGGATGGATACAATACGATAAACATTGTTATCTGGATACCAACATTAAAATGTCTACGGA

TAATGCAGTTTATCAGTGTCGCAAATTACGAGCTAGATTGCCTAGACCTGATACTAGACATCTGAGAGTA

TTGTTTAGTATTTTTTATAAAGATTATTGGGTAAGTTTAAAAAAGACCAATGATAAATGGTTAGATATTA

ATAATGATAAAGATATAGATATTAGTAAATTAACAAATTTTAAGCAACTAAACAGCACGACGGATTCTGA

GGCGTGTTATATATACAAGTCTGGAAAACTGGTTAAAACAGTATGTAAAAGTACTCAATCTGTACTATGC

GTTAAAAGATTCTACAAGTGACAACAAAAAATGAATTAATAGTAAGTCGTTAACGTACGCCGCCATGGAC

GCCGCGTTTGTTATTACTCCAATGGGTGTGTTGACTATAACAGATACATTGTATGATGATCTCGATATCT

CAATCATGGACTTTATAGGACCATACATTATAGGTAACATAAAAATTGTCCAAATAGATGTACGGGATAT

AAAATATTCCGACATGCAAAAATGCTACTTTAGCTATAAGGGTAAAATAGTTCCTCAGGATTCTAATGAT

TTGGCTAGATTCAACATTTATAGTATTTGTACCGCATACAGATCAAAAAATACCATCATCATAGCATGCG

ACTATGATATCATGTTAGATATAGAAGGTAAACATCAACCATTTTATCTATTCCCATCTATTGATGTTTT

TAACGCTACAATCATAGAAGCGTATAATCTGTATACAGCTGGAGATTATCATCTGATCATCAATCCTTCA

GATAATCTGAAAATGAAATTGTCGTTTAATTCTTCATTTTGTATATCAGACGGCAATGGATGGATTATAA

TTGATGGGAAATGTAATAGTAATTTTTTATCATAAAAGTTGTAAAGTAAATAATAAAACAATAAATATTG

AACTAGTAGTATGTTGTATATTGAGCAATCAGAGATGATGCTGGTACCTCTTATCACGGTGACCGTAGTT

GCGGGAACAATATTAGTATGTTATATATTATATATTTGTAGGAAAAAGATACGTACTGTCTATAATGACA

ATAAAATTATCATGACAAAATTAAAAAAGATAAAGAGTCCTAATTCCAGCAAATCTAGTAAATCAACTGA

TAGCGAATCAGACTGGGAGGATCACTGTAGTGCTATGGAACAAAACAATGACGTAGATAATATTTCTAGA

AATGAGATATTGAACGATGATAGCTTCGCTGGTAGTTTAATATGGGATAACGAATCCAATATCATGGCGC

CTAGCACAGAACACATTTACGATAGTGTTGCTGGAAGCACGCTGCTAATAAATAATGATCGTAATGAACA

GACTATTTATCAGAATACTACAGTAGTAATTAATGATACAGAGACTGTTGAAATACTTAATGAAGATACC

AAACAGATTCCTAGCTATTCTTCCAATCCTTTCGTAAATTATAATAAAACCAGTATTTGTAGCAAGTCAA

ATCCGTTCATTGCAGAACTCAACAATAAATTTAGTGATAATAATCCGTTTAGGAGAGCACATAGTGACGA

TTATCTTAATAAGCAACAAGATCATGAATACGATGATATAGAATCATCGGTTGTATCATTGGTCTGATTA

GTTTCCTTTTTATAAAATTGAAGTAATATTTAGTATTAATTACCGCCGATGCATTATACAAATATGGAGA

TATTCCCTGTATTCGGCATTTCTAAAATTAGCAATTTTATTGCTAATAATGACTGTAGATATTATATAGA

TGTAGAGCATCAAAAAATTATATCTGATGAGATCAATAGACAGATGGATGAAACGGTACTTCTTACCAAC

ATCTTAAGCGTAGAAGTTGTAAATGACAATGAGATGTACCATCTTATTCCCCATAGACTATCGACTATTA

TACTCTGTATTAGTTCTGTTGGAGGATGTGTTATCTCTATAGATAATGACGTCAATGACAAAAATATTCT

AACATTTCCCATTGATCATGCTGTAATCATATCCCCACTGAGTAAATGTGTCGTAGTTAGCAAGGGCCCT

ACAACCATACTGGTTGTTAAAGCGGATATACCCAGCAAACGATTGGTAACATCGTTTACAAACGACATAC

TGTATGTAAACAATCTATCACTGATTAATTATTTACCGTCGTCTGTATTCATTATTAGACGAGTCACCGA

CTATTTGGATAGACACATATGTGATCAGATATTTGCTAATAATAAGTGGTATTCCATTATAACTATCGAC

GATAAGCAATATCCTATTCCATCAAATTGTATAGGTATGTCTTCTGCCAAGTACATAAATTCGAGCATCG

AGCAAGATATTTTGATCCATGTTTGTAACCTCGAGCATCCATTCGACTCAGTCTACAAAAAAATGCAGTC

GTACAATTCTCTACCTATCAAGGAACAAATATTGTATGGTAGAATTGATAATATAAATATGAGCATTAGT

ATTTCGGTGGATTAATAGATTTCTCTAGTATGGGATCATTAATCATCTCTAAATACATCATAAAAAAGCT

ATTATCAAATACTGTACTGAATGGATTCATTCTTTTCTCTTTTTATGAAACTCTGTTGTATATCTACGGA

TAAAACTAGAAGCAAAAAATCTGATAGGAAGAATAATGATTATATGGAGGAACACGATTATTATAAAATA

ACAATAGTTCCTGGTTCCTCTTCCACGTCTACTAGCTCGTGGTATTATACACATGCCTAGTAATAGTCTC

TTTGCGTTGACGGAAAGCAGACTAGAAATAACAGGCCAAAATGTTCAGACACCATAATAGTTCCCAACCC

AGATAATAACAGAGTTCCATCAACACATTCCTTTAAACTCAATCCCAAACCCAAAACCGTTAAAATGTAT

CCAGCCAATTGATAGTAGATAATGAGGTGTACAGCACATGATAATTTACACAGTAACCAAAATGAAAACA

CTTTAGTAATTATAAGAAATATAGACGGTAATGTCATCATCAACAATCCAATAATATGCCTGAGAGTAAA

CATTGACGGATAAAACAAAAATGCCCCGCATAACTCTATCATGGCAATAACGCAACCAAACACTTGTAAA

ATTCCTAAATTAGTAGAAAATACAACTGATATCGATGTATAAGCGATTTCGAGGAATAATAAGAACAAAG

TAATTCCCGTAAAGATAAACATCAACATTGTTTGGTAATCATTAAACCAATTAGTATGACGTTGAATTAA

TTTCACAGTATATTTTATTCCAGTATTATCCCCGCATGTATACGTACCTGGTAAGATATCTTTATATTCC

ATAATCAATGAGACATCACTATCCGATAACGAATGAAGTCTAGCACTAGTATGCCATTTACTTAATATGG

TCGTCTTGGAAGTTTTATTATAAGTTAAAATATCATGATTGTCCAATTTCCATCTAATATACTTTGTCGG

ATTATCTATAATACACGGAATAATGATGGTATCATTACATGCTGTATATTCTATAGTCTTTGTAGATGTT

ATAACCACAAAAGTACAGAGGTATATCAACAATATTCTAACTCTTAACATTTTTATTTATTTAAAATGAT

ACCTTTGTTATTTATTTTATTCTTATTTTGCTAACGGTATCGAATGGCATAAGTTTGAAACGAGTGAAGA

AATAATTTCTACTTACTTAATAGATTATGTGGTAACGGGTGTTATTAATGGGGATGTATATACATTTTCA

AATAATGAACTAAACAAAACTGGGTTAACTAATAACAATAATTATATCACAACATCTATAAAAGTAGAGG

ATAAGGATACATTAGTAGTATGCGGAACCAATAACGGAAATCCCAAATGTTGGAAAATAGACGGTTCATA

CTACCCAAAACATATAGGTAGAGGATACGATCATCAAAATAGCAAAGTAACGATAATCAGTCACAATGAA

TGTGTACTATCCGACATAAACATATAAAAAGAAGGAATTAAACGATGGAGAAGATTTGACGGACCATGTG

GTTATGATTAAACGAGTTAAGTTTTTTAAGAAGCCTTAGAAGAGAGGCTATTGGGTATGAGAATCCGAAA

TATTAAACCAGACAACCCCATATAATTTTATAGCTAAGAATGCCGCGAAGAATGGAACTAATAAAAACGG

AAATATTTGTAGCACAACGAATAACTCCCAAACTGCATTCATGTTACACTATATAACACTACTTCGGTTA

GATGTTTTAGAAAAAATAAATATCACCGTACCGTTTTGTTGTATAAAAATAACAATTAACAATTATCAAT

TTTTTTCTTTAATATTTTACGTGGTTGACCATTCTTGGTGGTAAAATAATCTCTTAGTGTTGGAATGGAA

TGCTGTTTAATGTTTCCACACTCATCGTATATTTTGACGTATGCAGTCACATCGTTTACGCAATAGTCAG

ACTGTAGTTCTATCATGCTTCCTACGTTAGAAGGAGGAACAGTTTTAAAGTCTCTTGGTTTTAATCTATT

GTCATTAGTTTTCATGAAATCCTTTGTTTTATCCACTTCACATTTTAAATAAATGTCCACTATACATTCT

TCTGTTAATTTTACTAGATCATCATGAGTCATAGAATTCATAGGTTCCGTAGTCCATGGATCCAAACTAG

CAAACTTCGCGTATACGGTATCGCGATTAGTGTATACACCAACTGTATGAAAATTAAGAAAACAGTTTAA

TAAATCTACAGAAATATTTAATCCTCCGTTTGATACAGATGCGCCATATTTATGGATTTCGGATTCACAC

GTTGTTTGTCTAAGGGGTTCGTCTAGTGTTGCTTCTACATAGACTTCGATTCCCATATATTCTTTATTGT

CAGAATCACATACCGATTTATCATACGCTGGTTCACTTGTTTGAAAACTAAATGGTAGTAGATACATCAA

AATAATAAATAATAAGTACATTCTGCAATATTGTTATCGTAATTGGAAAATTGGTATTCAAGTGAGCTGG

ATTATGTGAGTATTGGATTGTATATTTTATTTTATATTTTATATTTTATATTTTATTTTATATTTTGTAG

TAAGAATAGAATGCTAATGTCAAGTTTATTCGAATAGATGTCTTATTAAAAAACATATATAATAAATAAC

AATGGCTGAATGGCATAAAATTATCGAGGATATCTCAAAAAATAATAAGTTCGAGGATGCCGCCATCGTT

GATTACAAGACTACAAAGAATGTTCTAGCGGCTATTCCTAACAGAACATTTGCAAAGATTAATCCGGGTG

AAGTTATTCCCCTCATCACTAATCATAATATTCTAAAACCTCTTATTGGTCAGAAATTTTGTATTGTATA

TACTAACTCTCTAATGGATGAGAACACGTATGCTATGGAGTTGCTTACTGGGTACGCCCCTGTATCTCCG

ATCGTTATAGCGAGAACTCATACCGCACTTATATTTTTGATGGGTAAGCCAACAACATCCAGACGTGATG

TGTATAGAACATGTAGAGATCACGCTACCCGTGTACGTGCAACTGGTAATTAAAATAAAAAGTAATATTC

ATATGTAGTGTCAATTTTAAATGATGATGAAATGGATAATATCCATATTGACGATGTCAATAATGCCGGT

ATTGACATACAGCTCATCGATTTTTAGATTTCATTCAGAGGATATTGAATTATGTTATGGGAATTTGTAT

TTTGATAGGATCTATAATAATGTAGTAAATATAAAATATATTCCTGAGCATATTCCATATAGATATAATT

TTATTAATCGTACGTTCTCCGTAGATGAACTAGATGATAATGTCTTTTTTACACATGGTTATTTTTTAAA

ACACAAATATGGTTGTTCACTTAATCCTAGTTTGATTGTCTCATTATCAGGAAACTTAAAATATAATGAT

ATACAATGCTCAGTAAATGTATCGTGTCTCATTAAAAATTTGGCAACGAGTACATCTACTATATTAACAT

CTAAACATAAGACTTATTCTCTATATCGGTCCATGTGTATTGCTATAATAGGATACGATTCTATTATATG

GTATAAATATATAAATGACAGGTATAATGACATCTATGATTTTACTGCAATATGTATGCTAATAGCGTCT

ACATTGATAGTGATCATATACGTGTTTAAAAAAATAAAAATGAACTCTTAATTATGTTATACTATTAGAA

ATGGATAAAATCAAAATTACGATTGATTCAAAAATTGGTAATGTTGTTACCATATCGTATAACTTGGAAA

AGATAACTATTGATGTCACGCCAAAAAAGAAAAAAGAAAAGGATGTATTATTAGCGCAATCAGTTGCTGT

CGAAGAGGCAAAAGATGTCAAGGTGGAAGAAAAAAATATTATCGATATTGAAGATGACGATGATATGGAT

ATAGAAAACACGTAATACGATCTATAAAAATAAGTATTAAATACTTTTTATTTACGGTACTCTTGTAGTG

GTGATACCACTAATCGATTATTTTTTTTAAAAAAATACTTATTCTGATTCTTCTAGCCATTTCCGTGTTC

GTTCGAATGCCACATCGACGTCAAAGATAGGGGAGTAGTTGAAATCTAGTTCTGCATTGTTGGTACGCAC

CTCAAATGTAGTGTTGGATATCTTCAACGTATAGTTGTTGAGTATTGATGGTTTTCTAAATAGAATTCTC

TTCATATCATTCTTGCACGCGTACATTTTTAGCATCCATCTTGGAATCCTAGATCCTTGTTCTATTCCCA

ATGGTTTCATCAATAGAAGATTAAACATATCGTAAGAACACGATGGAGAGTAATCGTAGCAAAAGTAAGC

ATTTCCTTTAATCGCAGATCCCGGATACTGGATATATTTTGCAGCCAACACGTGCATCCATGCAACATTT

CCTACATATACCCGGCTATGCACAGCGTCATCATCGACTGTACGATACATAATGTTACCGTGTTGCTTAC

ATTGCTCGTAAAAGACTTTCGTCAATTTGTCTCCTTCTCCGTAAATTCCAGTGGGTCTTAGGCAACAAGT

ATACAATTTTGCGCCATTCATGATTACGGAATTATTGGCTTTCATAACCAGTTGCTCGGCCATACGTTTA

CTTTTTGCGTATACATGTCCTGGTGATATATCATAAAGGGTATGCTCATGACCGATGAATGGATTACCGT

GTTTATTTGGTCCTATTGCTTCCATGCTACTAGTATAGATCAAATACTTGATTCCTAGGTCCACACAAGC

TGCCAATATAGTCTGTGTTCCATAATAGTTTACTTTCATGATTTCATTATCAGTGTATTTTCCAAATACA

TCCACTAGAGCAGCCGTATGAATAATCAGATTTACCCCATCTAGCGCTTCTCTCACCTTATCAAAGTCGT

TTATATCACATTGTATATAGTTTATAACCTTAACTTTCGAGGTTATTGGTTGTGGATCTTCTACAATATC

TATGACTCTTATTTCTTGAACATCATCTGCGCTAATTAAAAGTTTTACTATATACCTGCCTAGAAATCCG

GCACCGCCAGTAACCGCGTACACGGCCATTGCTGCCACTCATAATATCAGACTACTTATTCTATTTTACT

AAATAATGGCTGTTTGTATAATAGACCACGATAATATCAGAGGAGTTATTTACGTTGAACAAGTCCATGG

AAAAGATAAAGTTTTAGGATCAGTTATTGGATTAAAATCCGGAACGTATAGTTTGATAATTCATCGTTAC

GGAGATATTAGTCGAGGATGTGATTCCATAGGCAGTCCAGAAATATTTATCGGTAACATCTTTGTAAACA

GATATGGTGTAGCATATGTTTATTTAGATACAGATGTAAATATATCTACAATTATTGGAAAGGCGTTATC

TATTTCAAAAAATGATCAGAGATTAGCATGTGGAGTTATTGGTATTTCGTACATAAATGAAAAGATAATA

CATTTTCTTACAATTAACGAGAATGGCGTTTGATATATCAGTTAATGCGTCTAAAACAATAAATGCATTA

GTTTACTTTTCTACTCAGCAAAATAAATTAGTCATACGTAATGAAGTTAATGATATACACTACACTGTCG

AATTTGATAGGGACAAAGTAGTTGATACGTTTATTTCATATAATAGACATAATGACTCCATAGAGATAAG

AGGGGTGCTTCCAGAGGAAACTAATATTGGTCGCGTGGTTAATACGCCGGTTAGTATGACTTACTTGTAT

AATAAGTATAGTTTTAAACCGATTTTAGCAGAATATATAAGACACAGAAATACTATATCCGGCAACATTT

ATTCGGCATTGATGACGCTAGATGATTTGGTTATTAAACAGTATGGAGACATTGATCTATTATTTAATGA

GAAACTTAAAGTAGACTCCGATTCGGGACTATTTGACTTTGTCAACTTTGTAAAGGATATGATATGTTGT

GATTCTAGAATAGTAGTAGCTCTATCTAGTCTAGTATCTAAACATTGGGAATTGACAAATAAAAAGTATA

GGTGTATGGCATTAGCCGAACATATAGCTGATAGTATTCCAATATCTGAGCTATCTAGACTACGATACAA

TCTATGTAAGTATCTACGCGGACACACTGATAGCATAGAGGATGAATTTGATCATTTTGAAGACGATGAT

TTGTCTACATGTTCTGCCGTAACCGATAGGGAAACGGATGTATAATTTTTTTTATAGTATGAAGGATATG

ATGGATATGATGATATGATGGATATGATGATATGATGGATATGATGGATATGATGGATATGATGGATATG

ATAAAAAAATATAATTGTTGTATCCATTCCCATTCAAATCACCTTATATGATTCTGTAACACAATGAAGG

AGTCTCATAGATATATAGAGGTCAGATACTGGTTTGATAAACTTTTTATTCCACATGAGCATGTTTGACT

TATGGTTAGACACACATACTTTAACAAATCACTGAAAATTGGAGTTAGGTATTCCTCTCAGAATCAGTTG

CCGTTCTGGAACATTAAATGTATTTTTTATGATATACTCCAACGCATTTATGTGGGTATACAACAAGTCA

TTAATAATGAGTATTTCCAAGAGTTTTAGTTGTCTAGTATTTAACAAGAGAAGAGATTTCAACAGACTGT

TTATGAACTCGAATACCGCCTCATTGTCGCTTATATTGATGACATGACATCGAATTCCCAATATCAATCT

CATCAGTGATGAGTAGCTCAATCTTGTTATCGGGATCCAATTTCTAAAGATGTCATTAAACCCTCGATCG

TGAATGGATTTATCATCATCGTTTTTATGTTGGACATGAGCTTAGTCCGTTTGTCCACATCTATATACGA

TGATTTCTGAATTATTTCATATATCTCTCGTTAACTCCAGGAACTTGTCAGGGATCTAACTTTAATATGT

TCTCGTCTAAGAGATGAAAATCTTTGGATGGTTGCATGTGACTTTTCTCTAAAGGATGATGTTACCCGAT

CCTCTCTTAAATGACTCCATCTTATCCTTGGACAAGATGGACAGTCTATTTTCCTTAGATGGTTTAATAT

TTTTTACCCATGATCTATAAAGGTAGACAGACCTAATCGTCTCGGATGACCATATATTATTTTCAGTTTT

ATTATACGCATAAATTGTAAAAAATATGTTAGGTTTACGAAAATGTCTCGTGGGGCATTAATCGTTTTTG

AAGGATTGGACAAATCTGGAAAAACAACACAATGTATGAACATCATGGAATCTATACCGGCAAACACGAT

AAAATATCTTAACTTTCCTCAGCGATCCACAGTCACTGGAAAGATGATAGATGACTATCTAACTCGTAAA

AAAACCTATAATGATCATATAGTTAATCTATTATTTTGTGCAAATAGATGGGAGTTTGCATCTTTTATAC

AAGAACAACTAGAACAGGGAATTACTTTAATAGTTGACAGATACGCGTTCTCTGGAGTAGCGTATGCCAC

CGCTAAAGGCGCGTCAATGACTCTCAGTAAGAGTTATGAATCTGGATTGCCTAAACCCGACTTAGTTATA

TTCTTGGAATCTGGTAGCAAAGAAATTAATAGAAACATCGGCGAGGAAATTTATGAAGATGTTGAATTCC

AACAAAAGGTATTACAAGAATATAAAAAAATGATTGAAGAAGGAGATATTCATTGGCAAATTATTTCTTC

TGAATTCGAGGAAGATGTAAAGAAGGAGTTGATTAAGAATATAGTTATAGAGGCTATACACACGGTTACT

GGACCAGTGGGGCAACTGTGGATGTAATAAAATGAAATTACATTTTTATAAATAGATGTTAGTACAGTGT

TATAAATGGATGAAGCATATTACTCTGGCAACTTGGAATCAGTACTCGGGGATACGTGTCCGATATGCAT

ACCGAACTCGCATCAATATCTCAATTAGTTATTGCCAAGATAGAAACTATAGATAATGATTATTAAACAA

GGACATTGTAAATTTTATCATGTGTAGATCAAACTTGGATAATCCATTTATCTCTTTCCTAGATACTGCA

TATACTATCATAGATCAAGAGATCTATCAGAACGAGTTGATTAATTCATTAGACGATAATGAAATTATCG

ATTGTATAGTTAACAAGTTTATGAGCTTTTATAAGGATAACCTAGAAAATATGGTAGATGCTATCATTAC

TCTAAAATATTATAATTAATAATCCAGATTTTAAAACTACGTATGTGGAAGTACTCGGTTCCAGAATAGC

TGATATAGATATTAAACAAGTGATACGTAAGAATATAATACAATTGTCTAATGATCCGCGAACGATATTT

GTGAAAATATTAAAAAAAAATACTTTTTTTATTAAATGACGTCTCTTCGCGAATTTAGAAAATTATGCTG

TGATATATATCACGCATCAGGATATAAAGAAAAATCTAAATTAATTAGAGACTTTATAACAGATAGAGAT

GATACCGATACATATTTGATCATTAAGCTATTGCTTCCCGGATTAGACGATAGAATGTATAACATGAACG

ATAAACAAATTATAAAATTATATAGTATAATATTTAAACAATCTCAGGAAGATATGCTACAAGATTTAGG

ATACGGATATATAGGAGACACTATTAGGACTTTCTTCAAAGAGAACACGGAAATCCGTCCACGAGATAAA

AGCATTTTAACTTTAGAAGAAGTGGATAGTTTTTTAACTACGTTATCATCAGTAACTAAAGAATCACATC

AAATAAAATTATTGACTGATATAGCATCTGTTTGTACATGTAATGATTTAAAATGTGTAGTCATGCTTAT

TGATAAAGATCTAAAAATTAAAGCGGGTCCTCGGTACGTGCTTAACGCTATTAGTCCTCATGCCTATGAT

GTTTTTAGAAAATCTAATAACTTGAAAGAGATAATAGAAAATGCAGCTAAACAAAATCTAGACTCTATAT

CTATTTCTGTTATGACTCCAATTAATCCCATGTTAGCGGAATCATGTGATTCTGTCAATAAGGCGTTTAA

AAAATTTCCATCAGGAATGTTTGCGGAAGTCAAATACGATGGTGAAAGAGTACAAGTTCATAAAAAAAAT

AACGAGTTTGCATTCTTTAGTAGAAACATGAAACCAGTACTCTCTCATAAAGTGGATTATCTCAAAGAAT

ACATACCGAAAGCATTTAAAAAAGCTACGTCTATCGTATTGGATTCTGAAATTGTTCTTGTAGACGAACA

TAATGTACCGCTACCGTTTGGAAGTTTAGGTATACACAAAAAGAAAGAATATAAAAACTCTAACATGTGT

TTGTTCGTATTTGACTGTTTATACTTTGATGGATTCGATATGACAGACATTCCATTGTATGAACGAAGAT

CTTTTCTCAAAGATGTTATGGTCGAAATACCCAATAGAATAGTATTCTCAGAGTTGACGAATATTAGTAA

CGAGTCTCAGTTAACTGATGTATTAGATGATGCACTAACGAGAAAATTAGAAGGATTGGTCTTAAAAGAT

ATTAATGGCGTATACGAACCGGGAAAGAGAAGATGGTTAAAAATAAAGCGAGACTATTTGAACGAGGGTT

CCATGGCAGATTCTGCCGATTTAGTAGTACTAGGTGCCTACTATGGTAAAGGAGGAAAGGGTGGTATCAT

GGCAGTCTTTCTAATGGGTTGTTACGACGATGAATCCGGTAAATGGAAGACGGTAACTAAATGTTCCGGT

CACGATGATAATACGTTAAGGGTTTTGCAAGACCAATTAACGATGGTTAAAATTAACAAGGATCCCAAAA

AAATTCCAGAGTGGTTGGTAGTTAATAAAATCTATATTCCCGATTTTGTAGTAGATGATCCGAAACAATC

TCAGATATGGGAAATTTCAGGAGCAGAGTTTACATCTTCCAAGTCACATACAGCGAATGGAATATCGATT

AGATTTCCTAGATTTACTAGGATTAGAGAAGATAAAACGTGGAAAGAATCTACTCATCTAAACGATTTAG

TAAACTTGACTAAATCTCTTAATAGTTACATATAAACTGAAAAATAAAATAACACTATTTTAGTTGGTAG

TCGCCATGGATGGTGTTATCGTATACTGTCTAAATGCGTTAGTAAAACATGGCGAGGAAATAAATCATAT

AAAAAATGATTTCATGATTAAACCATGTTGTGAAAGAGTTTGTGAAAAAGTCAAGAACGTTCACATCGGC

GGACAATCTAAAAACAATACAGTGATTGCAGATTTGCCATATCTGGATAATGCTGTATCAGATGTATGCA

AATCAATATATAAAAAGAATGTATCAAGAATATCCAGATTTGCTAATTTGATAAAAATAGATGACGATGA

CAAGACTCCTACCGGCGTATATAATTATTTTAAACCTAAAGATGCTATTCCTGTTATTATATCCATAGGA

AAGGATAAAGATGTCTGTGAACTATTAATCTCATCTGATAAAGCGTGTGCGTGTATAAAGTTAAATTTAT

ATAAAGTAGCCATTCTTCCCATGGATGTTTCCTTTTTTACCAAAGGAAATGCATCATTGATTATTCTCCT

GTTTGATTTCTCTATCGATGCGGCACCTCTCTTAAGAAGTGTAACCGATAATAATGTTATTATATCTAGA

CACCAGCGCCTACATGACGAGCTTCCGAGTTCCAATTGGTTCAAGTTTTACATAAGTATAAAGTCCGACT

ATTGTTCTATATTATATATGGTTGTTGATGGATCTATGATGTATGCGATAGCTGATAATAGAACTCACGC

AATTATTAGCAAAAATATATTAGACAATACTACGATTAACGATGAGTGTAGATGCTGTTATTCTGAACCA

CAGATTAGGATTCTTGATAGAGATGAGATGCTCAATGGATCATCGTGTTATATGAACAGACATTGTATTA

TGATGAATTTACCTGATGTAGGCGAATTTGGATCTAGTATGTTGGGGAAATATGAACCTGACATGATTAA

GATTGCTCTTTCGGTGGCTGGTAATTTAATAAGAAATCGAGACTACATTCCCGGGAGACGAGGCTATAGC

TACTACGTTTACGGTATAGCCTCTAGATAATTTTTTTTAAGCACGAAATAAAAACATAATTTTAAACAAT

CTATTTCATACTATTTTGTGTGCTCACCATGAACATAAAGATAGATATATTAGTATTTCTGGTGATAAAT

TTACGGCGACTGCTAGGAGGGAAAATGAAGAAAGAAAAAATATCTACCTCTCCAAAAAGAAAAACTACTG

ATGTTATCAAACCTGATTATCTTGAGTACAATGACTTGTTAGATAGAGATGAGATGTCTACTATTCTAGA

GGAATATTAGGCCTTAGAATAAAATATGGACGACTCTTAACGAAATTAGAAAATTCGATAATGATGTTGA

AGAACAATTCGGTACTATAGAAGAACTCAAGCAGAAGCTTAGATTAAATTCTGAAGAGGGAGCAGATAAT

TTTATAGATTATATAAAGGTACAAAAACAGGATATCATCAAACTTACTGTATACGATTGCATATATCTAT

GATAGGATTGTGTGCGTGCGTGGTAGATGTTTGGAGAAATGAGAAACTGTTTTCTAGATGGAAATATTGG

TTACAAGCGATTAAACTGTTTATTGATGATCACATGCTTGATAAGATAAAATCTATTGTAGAATAGACTA

GTGTATGTGGAAATGTCATAGAAAGTTAAAAGTTAATGAGAGCAAAAATATATAAGGTTGTATTCCATAT

TTGTTATTTTTTTCTGTAATAGTTAGAAAATACATTCGATGGTCTATCTACCAGATTATTATGTGTTATA

AGGTACTTTTCTCATAATAAACTAGAGTATGAGTAAGATAGTGTTTTTCAAAAACATATAAATCTAAAAT

TGATGGATGAGATATACAGCTATTAATTTCGAAAATATATTTTAATCTGATAACTTTAAACATGGATTTT

TGATGGTGGTTTAAGTTTAAAAAAGATTTTGTTATTGTAGTATGATAATATCAAAAAGATGGATATAAAG

AATTTACTGACTACATGTACTATTTTACATTACTACATTGGCTACGGCATATATACCTATTTCGTCACTT

CCACACGCTCCGGTAAACGGGTGTCATGTGACGAGGGAGAATCTTGATAAGAGGCATAATCAATGTTGTA

ATCCGATGTCCACCTGGAGAATTTGCCAAGGTCAGATGTAGAGTTGGTAGTGATAACACAAAATGTGAAC

ACTGCCCACCTCATACATATACCGCAATCCCCAATTATTCTAATAGATGTCATCAATGTAGAAAATGCCC

AACAGGATCATTTGATAAGGTAAAGTGTACCGGAACACAGAACAAATGTTCGTGTCATCCTGGTTGGTAT

ACGCTACTGATTCTTCACAGACTGAAGATTGTCGAGATTTGTGTACCAAAAAAGGAGATGTCCATGCGGA

TACTTTGGTGGAATAGATGAAGGAAATCCTATTTGTAAATCGTGTTGTGTTGGTGAATATTGCGACTACC

TACGTAATTATAGACTTGATCCATTTCCTCCATGCAAACTATCTATCTAAATGTAATTAATTATGATTTT

GATGATAATGTTACCATACATTATATCACTACTTGGTTAGTGTGTATTATTTAGTATGGAAGACCTATTA

ATAATTACTTATCTTTTGACGATCTTGTTATAATTATAATATAAAAATACTTATGACATAGTAACTCATA

ATTGCTGACGCGATAAATTCGTAATAATCTGTTTTGTTCAAATTTTTATAAGGAATCTACAGGCATAAAA

ATAAAAATATAATCTATAATATACTCTTACAACGCCATCATGAATAGCAGTGAATTAATTGCTGTTATTA

TGGATTTAGAAATAGTGGACGATTTTGTGATATTAATATAGTTATTAATGATGAAAGGATAAACGCGCAT

AGACTCATCCTATCTGGAGCCTCCGAATATTTTTTCCATTCTGTTTTCCAATAATTTTATCGATTCTAAT

GAATACGAAGTTAATCTAAGTCATTTAGATTATCAAAGTGTTAACTATTTGATCGATTACATTTATGGGA

TACCTTTGAGCCTAACTAACAATAACGTGAAATATATTCTTTCAACCGCTGATTTTTTTACAAATTGGAT

CTGTCATTACTGAGTGCGAAAAATACATACTTAAAAATCTTTGTTTTAGAAACTGTATCGATTTCTACAT

ATACGCTGATAAATATAATAACAAGAAAATAGAATTAGCATCGTTTAACACAATATTACGAAATATTTTG

AGACTCATCAACAATGAAAACTTTAAATACTTAACAGAGGAATCAATGATAAAAATTTTAAGCGATGATA

TGTTATATATAAAAAATGAGGATTTCACCCCACTGATTCTCATTAAATGGTTAGAGAGTACACCAACCAT

GTACCGTCGAGTTACTTAGATGCCTCAGAATATCATTTCTTTCCCCACAAGTTATAAAATCACTTTATAG

TCATCGACTGGTTAGTTCAATCTACGAATGTATAACATTCTTAAACAATATAGCATTCTTGGATAAATCA

TTTCCTAGATACCATATCATCGAGTTGATATCTATCGGTATAAGTAATTCACATGATAAGATTTCCATAA

ACTGCTACAATCATAAAAAAATTCATGGGAAATGATATCTTCACGTAGATATAGGTGTAGTTTCGCAGTG

ACCGTCCTGGATAATATTATCTATATGATGGGTGGATATGATCAGTCCCTGTATAGAAGTTCAAAGGTTA

TAGCGTACAATACATGTACTAATTCTTGGATATATGATATACCAGAGCTAAAATATCATCGTTCTAATTG

CGGAGGAGTTGCCAATGACGAATACATTTATTGTATAGGCGGTATACGCGATCAGGAGTCATCGTTGATA

TCTAGTATCGATAGATGGAAGCCATCAAAACCATATTGGCAGAAGTATGCTAAAATGTGCGAACCAAAAT

GTGATATGGGGTTGCGATTTTAAACGGATTAATATATGTCATAGGTGGAGTCGTTAAAGGTGACACACAT

ATACCAACGCACTAGAGAGTTTATCAGAAGATGGATGGATGAATCATCAACGTCTTCCAATAAAAATGTC

CAATATGTCGACGATTGTTCATGCTGGAAAGATTTATATATCTAGAGGTTACAACAATAGTAGTGTAGTT

AATGTAATATCGAATCTAGTCCTTAGCTATAATCCGATATATGATGAATGGACCAAATTATCATCATTAA

ATATTCCTAGAATTAATCCTGCTCTATGGTCAGTGTATAATAAATTATATGTAGGAGGAGTAATATCTGA

TGATGTTCAAACTAATACATCTGAAACATACGATAAAGAAAAAGATTGTTGGACATTGGATAATGGTCAC

TTGTTACCACATAATTATATAATGTATAAATGCGAACCGTTTAAACATAGATATCCATTGGAAAAAACAC

AGTACACGAATGATTTTCTAAAGTATTTGGAAAGTTTTATAGGTAGTTGATAGAACAAAATACATAATTT

TGTAAAAATAAATCACTTTTTATACTAATATGACACAATTACCAATACTTTTGTTACTAATATCATTAGT

ATACGCTACACCTTCTCCTCAGACATCTAAAAAAATAGGTGATGATGCAACTATATCATGTAGTCGAAAT

AATACAAATTACTACGTTGTTATGAGTGCTTGGTATAAGGAGCCCAATTCCATTATTCTCTTAGCTGCCA

AAAGCGACGTCTTGTATTTTGATAATTATACCAAGGATAAAATATCTTACGACTCTCCATACGATGATCT

AGTTACAACTATCACAATTAAATCATTGACTGCTGGAGATGCCGGTACTTATATATGTGCATTCTTTATG

ACATCGACTACAAATGATACTGATAAAGTAGATTATGAAGAATACTCCATAGAGTTGATTGTAAATACAG

ATAGTGAATCGACTATAGACATAATACTATCTGGATCTACACCAGAAACTATTTCTGAGAAACCAGAGGA

TATAGATAATTCTAATTGCTCGTCTGTATTCGAAATCACGACTCCGGAACCAATTACTGATAATGTAGAC

GACCATACAGACACCGTCACATACACTAGTGATAGCATTAATACAGTAAATGCATCATCTGGAGAATCCA

CAACAGACGAGATTCCGGAACCAATTACTGATAAAGAAGAAGATCATACAGTAACAGACACTGTCTCATA

CACTACAGTAAGTACATCATCTGGAATTGTCACTACTAAATCAACCACCGATGATGCGGATCTTTATGAT

ACATACAATGATAATGATACAGTACCGCCAACTACTGTAGGTGGTAGTACAACCTCTATTAGCAATTATA

AAACCAAGGACTTCGTAGAAATATTTGGTATTACCACATTAATTATATTGTCAGCAGTGGCGATTTTCTG

TATTACGTATTATATATGTAATAAACACCCACGTAAATACAAAACAGAGAACAAAGTCTAGATTTTTGAC

TTACATAAATATCTGGGATAATAAAATCTATCATATTGAGAGGACCATCTGGTTCAGGAAAGACAGCCAT

AACCAAAAGACTGTTAAAAGACTATGGGAATATATTTGGATTTGTGGTGTCCCATACCACTAGATTTCCT

CGTCCTATGGAACGAGAAGGTGTTGATTACCTTACGTTAACAGAGAGGCCATCTGGAAGGGAATAGCCGC

CGGAAACTTTCTAGAACATACTGAGTTTTTAGGAAATATTTACGGAACTTCTAAAACAGCTGTAAATACA

GCGGTTATTAATAATCGTATTTGCGCTATGGATTTAAACATCAACGGTGTTAGAAGTCTTAAAAATACTT

ACCTAATGCATTACTTGGGTATATAAGACCTACCTCTCTTAAAATGGTTGAGACCAATCTTCGTCGTAGA

AACACTGAAGCGGACGACGAATCTCATCGTCGCGTGATGTTGGCAAAAAACGGATATGGATGAGGTCAAC

GAAGCAGGTCTATTCGACACTATTATTATTGAAGATGATGTGAATTTAGCATATAGTAAGTGTTAATTCA

GATACTACAGGACCGTATTAGAATGTATTTTAACACTAATTAGAGACTTAAGATTTGACTTAAAACTTGA

TAATTAATAATATAACTCGTTTTTATATGTGGCTATTTCAACGTCTAATGTATTAGTTAAATATTAAAAC

TTACCACGTAAAACTTAAAATTTAAAATGGTATTTCATTGACAGATCATACATTATGAAGTTTCAAGGAC

TTGTGTTAATTGACAATTGCAAAAATCAATGGGTCGTTGGACCATTAATAGGAAAAGGTGGATTCGGTAG

TATTTATACTACTAATGACAATAATTATGTAGTAAAAATAGAGCCCAAAGCTAACGGATCATTATTTACC

GAACAGGCATTTTATACTAGAGTACTTAAACCATCCGTTATCGAAGAATGGAAAAAATCTCACAATATAA

AGCATGTAGGTCTTATCACGTGCAAGGCATTTGGTTTATACAAATCCATTAATGTGGAATATCGATTCTT

GGTAATAAATAGATTAGGTGCAGATCTAGATGCGGTGATCAGAGCCAATAATAATAGACTACCAGAAAGG

TCGGTGATGTTGATCGGAATCGAAATCTTAAATACCATACAATTTATGCACGAGCAAGGATATTCTCACG

GAGATATTAAAGCGAGTAATATAGTCTTGGATCAAATAGATAAGAATAAATTATATCTAGTGGATTACGG

ATTGGTTTCTAAATTCATGTCTAACGGCGAACATGTTCCATTTATAAGAAATCCAAATAAAATGGATAAC

GGTACTCTAGAATTTACACCTATAGATTCGCATAAAGGATACGTTGTATCTAGACGTGGTGATCTAGAAA

CACTTGGATATTGTATGATTAGATGGTTGGGAGGTATCTTGCCATGGACTAAGATATCTGAAACAAAGAA

TTCTGCATTAGTAAGTGCCGCAAAACAGAAATATGTTAACAATACTGCGACTTTGTTAATGACCAGTTTG

CAATATGCACCTAGAGAATTGCTGCAATATATTACCATGGTAAACTCTTTGACATATTTTGAGGAACCCA

ATTACGACGAGTTTCGTCGAGTATTAATGAATGGAGTTATGAAAAATTTTTGTTGATAAAAAAATTAAAA

AAATAACTTAGTTATTATCACTCTCGTGAGTACAATAGAAACATGGCGATGTTTTACGCACACGCTTTCG

GTGGGTACGACGAGAACCTTCATGCATTTCCTGGAATATCATCGACGGTTGCCAATGATGTCAGGAAATA

TTCTGTTGTGTCAGTTTATAATAAAAAGTATAACATTGTAAAAAACAAATATATGTGGTGTAACAGTCAA

GTGAACAAGAGATATATTGGAGCACTACTGCCTATGTTTGAATGCAATGAATATCTACAAATTGGAGATC

CAATCCATGATCTAGAAGGAAATCAAATCTCTATTGTCACATATCGCCACAAAAACTACTATGCTCTAAG

TGGAATTGGGTACGAGAGTCTAGACTTGTGTTTGGAAGGAGTAGGGATTCATCATCACGTACTTGAAACA

GGAAACGCGGTATATGGAAAAGTTCAACATGAGTATTCTACTATCAAAGAGAAGGCCAAAGAAATGAATG

CACTCAAACCAGGACCTATCATCGATTACCACGTCTGGATAGGAGATTGTGTCTGCCAAGTTACTACTGT

AGACGTGCATGGAAAGGAAATTATGAGAATGAGATTCAAAAGGGGTGCGGTGCTTCCGATTCCAAATCTG

GTAAAAGTTAAAGTTGGGGAGGAAAATGATACAATAAATCTTTCCACTTCCATATCAGCTCTCCTGAATT

CCGGTGGCGGCACCATCGAGGTAACATCTAAGGAAGAACGTGTAGATTATGTACTCATGAAACGTTTGGA

ATCTATACATCATCTGTGGTCTGTAGTGTATGATCATCTTAATGTTGTGAATGGCGAAGAACGATGTTAT

GTACATATGCATTCATCTCATCAAAGTCCTATGCTGAGTACTGTAAAAACAAATTTGTACATGAAGACTA

TGGGAGCATGTCTTCAAATGGACTCCATGGAAGCTCTAGAGTATCTTAGTGAACTGAAGGAATCAGGTGG

GCGGAGTCCCAGACCAGAATTGCAGAAATTTGAATATCCAGATGGAGTGAAAGACACTGAATCAATTGAG

AGATTGGCAGAGGAGTTCTTCAATAGATCAGAACTTCAGGCCGGTGAATCAGTCAAATTTGGTAATTCTA

TTAATGTTAAACATACATCTGTTTCAGCTAAGCAACTAAGAACACGTATACGACAGCAGCTTCCTTCTAT

ACTCTCATCTTTTGCCAACACAAAGGGTGGATATTTGTTCATTGGAGTTGATAATAATACACACAAAGTA

ATTGGATTCACGGTGGGTCATGACTACCTCAAACTGGTAGAGAGTGATATAGAAAAGTATATCCAAAAAC

TTCCTGTTGTGCATTTCTGCAAGAAAAAAGAGGACATCAAGTACGCATGTAGATTCATCAAGGTGTATAA

ACCTGGTGATGAGACTACCTCGACATATGTGTGCGCAATCAAAGTGGAAAGATGCTGCTGTGCTGTGTTT

GCGGATTGGCCAGAATCATGGTACATGGATACTAGTGGTAGTATGAAGAAGTATTCTCCAGATGAATGGG

TGTCACATATAAAATTTTAATTAGGGTAAGGTAAAACTATATATAATAACTAACAATTTGTGTATCATAT

AGACAATTAATTAGGTAACTGTTATCTCTTTTTAACTAACTAACTAACTAACTAACTAACTCTTATATAC

TATTAATAATACATCTATTAATCATTGATTAGCTTATTGCTTTAATTGTTTTTGTAAACTAACACTGTTC

ATTGAAAAGGGATAACATGTTACAGAATATAAATTATATATGGATTTTTTTAAAAAGGAAATACTTGACT

GGAGTATATATTTATTTCTTCATTACATAACACGTCTGTGTTCTAATTCTTCCAATTCTTCCACATCTCA

TATAATACAGGAATATAATCTTGTTCGAAAATATGAGAAAGTGGATAAAACAATAGTTGATTTTTTATCT

AGGTGGCCAAATTTATTCCATATTTTAGAATATGGGGAAAATATTCTACATATTTATTTTATAGATGCTG

CTAATACGAATATTATGATTTTTTTTCTAGATAGAGTATTAAATATTAATAAGAACCGTGGGTCATTTAT

ACATAATCTCGGGTTATCATCCATTAATATAAAAGAATATGTATATCAATTAGTTAATAATGATCATCTA

GATAATAGTATAAGACTAATGCTTGAAAATGGACGTAGAACAAGACATTTTTTGTCTTATATATTGGATA

CAGTTAATATCTATATAAGTATTTTAATAAATCATAGATTTTATATAGATGCCGAAGACAGTTACGGTTG

TACATTATTACATAGATGTATATATAACTATAAGAAATCAGAATCAGAATCATATAATGAATTAATTAAG

ATATTGTTAAATAATGGATCAGATGTAGATAAAAAAGATACGTACGGAAACACACCGTTTATCCTATTAT

GTAAACACGATATCGACAACGCGGAATTGTTTGAGATATGTTTAGAGAATGCTAATATAGACTCTGTAGA

CTTTAATGGATATACACCTCTTCATTATGTCTCATGTCGTAATAAATATGATTTTGTAAAGTTATTAATT

TCTAAAGGAGCAAATGTTAATGCACGTAATAGATTCGGAACTACTCCATTTTATTGTGGAATTATACACG

GTATCTCGCTTATAAAACTATATTTGGAATCAGACACAGAGTTAGAAATAGATAATGAACATATAGTTCG

TCATTTAATAATTTTTGATGCTGTTGAATCTTTAGATTATCTATTGTCCAGAGGAGTTATTGATATTAAC

TATCGTACTATATACAACGAAACATCTATTTACGACGCTGTCAGTTATAATGCGTATAATACGTTAGTCT

ATCTATTAAACAGAAATGGTGATTTTGAGACGATTACTACTAGTGGATGTACATGTATTTCGGAAGCAGT

CGCGAACAACAACAAAATAATAATGGATATACTATTGTCTAAACGACCATCTTTGAAAATTATGATACCA

TCTATGATAGCAATTACTAAACATAAACAACATAATGCAGATTTATTGAAAATGTGTATAAAATATACTG

CGTGTATGACCGATTATGATACTCTTATAGATGTACAATCGCTACATCAATATAAATGGTATATTTTAAA

ATGTTTTGATGAAATAGATATCATGAAGAGATGTTATATAAAAAATAAAACTGTATTCCAATTAGTTTTT

TGTATCAAAGACATTAATACTTTAATGAGATACGGTAGACATCCTTCTTTCGTGAAATGTAATATTCTCG

ACGTATACGGAAGTTGTGTACGTAATATCATAGCATCTATTAGATATCGTCAGAGATTAATTAGTCTATT

ATCCAAGAAGCTGGATGCTGGAGATAAATGGTCGTGTTTTCCTAACGAAATAAAATATAAAATATTGGAA

AACTTTAACGATAACGAACTGACCACATATCTGAAAATCTTATAAACACTATTAAAATATAAAATCTAAG

TAGGATAAAATCACACTACATCATTGTTTCCTTTTAGTGCTCGACAGTGTATACTATTTTTAACACTCAT

AAATAAAAATGAAAACGATTTCCGTTGTTACGTTGTTATGCGTACTACCTGCTGTTGTTTATTCAACATG

TACTGTACCCACTATGAATAACGCTAAATTAACGTCTACCGAAACATCGTTTAATGATAAACAGAAAGTT

ACGTTTACATGTGATTCAGGATATCATTCTTTGGATCCAAATGCTGTCTGTGAAACAGATAAATGGAAAT

ACGAAAATCCATGCAAGAAAATGTGCACAGTTTCTGATTATGTCTCTGAACTATATGATAAGCCATTATA

CGAAGTGAATTCCACCATGACACTAAGTTGCAACGGTGAAACAAAATATTTTCGTTGTGAAGAAAAAAAT

GGAAATACTTCTTGGAATGATACTGTCACGTGTCCTAATGCGGAATGTCAACCTCTTCAATTAGAACACG

GATCGTGTCAACCAGTTAAAGAAAAATACTCATTTGGGGAATATATGACTATCAACTGTGATGTTGGATA

TGAGGTTATTGGTGTTTCGTATATAAGTTGTACGGCTAATTCTTGGAATGTTATTCCATCATGTCAACAA

AAATGTGATATACCGTCCCTATCTAATGGATTAATTTCCGGATCTACATTTTCTATCGGTGGCGTTATAC

ATCTTAGTTGTAAAAGTGGTTTTACACTAACGGGGTCTCCATCATCCACATGTATCGACGGTAAATGGAA

TCCCATACTCCCAACATGTGTACGATCTAACGAAGAATTTGATCCAGTGGATGATGGTCCCGACGATGAG

ACAGATCTGAGCAAACTCTCGAAAGACGTTGTACAATATGAACAAGAAATAGAATCGTTAGAAGCAACTT

ATCATATAATCATAATGGCGTTGACAATTATGGGTGTCATATTTCTAATCTCCATTATAGTATTAGTTTG

TTCCTGTGACAAAAATAATGACCAATATAAGTTCCATAAATTGCTACCGTGAATATAAATCCGTTAAAAT

AATTAATAATTAATAATTAATAACGAACAAGTATCAAAAGATTAAAGAATTAGCTAGAATCAATTAGATG

TCTTCTTCAGTGGATGTTGATATCTACGATGCCGTTAGAGCATTTTTACTCAGGCACTATTATGACAAGA

GATTTATTGTGTATGGAAGAAGTAACACCATATTACATAATATATACAGGCTATTTACAAGATGCACCGT

TATACCGTTCGATGATATAGTACGTACTATGCCAAATGAATCACGTGTTAAACAATGGGTGATGGATACA

CTTAATGGTATAATGATGAATGAATTCGATACTGTATGTGTGGGTACCGGACTACGATTCATGGAAATGT

TTTTCGATTACAATAAAAATAATCCCAAAAATAGCATCAACAATCAAATAATGTATGATATAATTAATAG

CGTAGCCATAATTCTAGCTAATGAGAGATATAGAAGCGCGTTTAACGACGATAGAATATACATCCGTAGA

ACTATGATGGACAAATTGTACGAATACGCATCTCTAACTACTATTGGTACGATCACTGGAGGTGTTTGTT

ATTATCTGTTGATGCATCTAGTTAGTTTGTATAAATAATTATTTCGATATACTAGTTAAAATTTTAAGAT

TTTAAATGTATAAAAAACTAATAACGTTTTTATTTGTAATAGGTGCAGTTGCATCCTATTCGAATAATGA

GTACACTCCGTTTAATAAACTGAGTGTAAAACTCTATATAGATGGAGTAGATAATATAGAAAATTCATAT

ACTGATGATAATAATGAATTGGTGTTAAATTTTAAAGAGTACACAATTTCTATTATTACAGAGTCATGTG

ACGTCGGATTTGATTCCATAGATATAGATGTTATAAACGACTATAAAATTATTGATATGTATACCATTGA

CTCGTCTACTATTCAACGCAGAGGACATACGTGTAGAATATCTACCAAATTATCATGCCATTATGATAAG

TACCCTTATATCCACAAATATGAGGGTGATGAACGACAATATTCTATTACCGCAGAGGGAAAATGCTATA

AAGGAATAAAATATGAAATAAGTATGATGAACGATGATACTCTATTGAGAAAACATACTCTTAAAATTGG

ATTTACTTATATATTCGATCGTCATGGGCATAGTAATACATATTATTCAAAATATGATTTTTAAAAATTT

AAAATATATTATCACTTCAGTGACAGTAGTCAAATAACAAACAACACCATGAGATATATTATAATTCTCG

CAGTTTTGTTCATTAATAGTATACATGCTAAAATAACTAGTTATAAGTTTGAATCCGTCAATTTTGATTC

CAAAATTGAATGGACTGGGGATGGTCTATACAATATATCCCTTAAAAATTATGGCATCAAGACGTGGCAA

ACAATGTATACAAATGTACCAGAAGGAACATACGACATATCCGGATTTCCAAAGAATGATTTCGTATCTT

TCTGGGTTAAATTTGAACAAGGCGACTATAAAGTGGAAGAGTATTGTACGGGACTATGTGTCGAAGTAAA

AATTGGACCACCAACTGTAAGATTGACTGAATATGACGATCATATCAATTTGTTCATCGAGCATCCGTAT

GCTACTAGAGGTAGCAAGAAGATTCCTATTTACAAACGCGGTGACATGTGTGATATCTACTTGTTGTATA

CGGCTAACTTCACATTCGGAGATTCTGAAGAACCAGTAACATATGATATCGATGACTACGATTGCACGTC

TACAGGTTGCAGTATAGACTTTGCCACAACAGAAAAAGTGTGTGTGACAGCACAGGGAGCCACAGAAGGG

TTTCTCGAAAAAATTACTCCATGGAGTTCGGAAGTATGTCTGACACCTAAAAAGAATGTATATACGTGCG

CAATTAGATCTAAAGAAGATGTTCCCAATTTCAAGGACAAAATAGCCAGAGTTATCACGAGAAAATTTAA

TAAACAGTCTCAATCTTATTTGACTAAATTTCTCGGTAGCACATCGAATGATGTTACAACTTTTCTTAGC

ATTCTTGACTAAATATTCATAACTAATTTTTATTAATGATACAAAAATGAAATAAACTGTATATTATACA

CTGGTTAACGCCCTTGGCTCTAACCATTTTCAAGATGAGGTCCCTGATTATAGTCCTTCTGTTCCCCTCT

ATCATCTACTCAATGTCTATTAGACGATGCGAGAAGACTGAAGAGGAAACATGGGGATTAAAAATAGGGT

TGTGTATAATTGCCAAAGATTTCTATCCCGAAAGAACTGATTGCAGTGTTCATCGCCCAACTGCAAGTGG

AGGATTGATAACTGAAGGCAATGGATTCAGAGTAGTTATATATGATCAATGTACAGAACCCCATGACTTT

ATTATCACCGATACTCAACAAACACGTCTTGGATCATCTCATACATATATTAAATTCAGTAACATGAATA

CAGGTGTCCCATCTAGTATTCCAAAATGTTCCAGAACTCTCTGTATTTCTGTATATTGTGATCAAGAGGC

GGGAGACATAAAATTTGAGGAGTATACTCAAGAATCAAGTGATATCAGTATTAGAGTTAAGTATGATTCA

TCATGTATTGATTATCTGGGTATTAATCAAAGTTTCATGAATGAATGTATTCGAAGAATTACAACATGGG

ATAGAGAATCATGCGTCAGAATTGATACACAGACTATAAATAAATATCTTAAGTCTTGCACCAACACAAA

ATTCGACCGTAATGTCTACAAAAGGTACATACTGAAGAGTAAAGCACTCCATGCTAAAACAGAGTTGTAA

TAGATATAAAATACTTTTTATAATAATTAGGCTAGAAAAATCTCACTCACATGTAATCTTAAAAAAATGA

TATGATAGTTCTTACAAGTAGCGATTGAGTTTTAAATGGATTCTATTAATTACCGGGGAACTTAACAATT

CGTTCTGATCTACAGACATTGGTTAATAAATCATCTTATTTTGCCAATATATTAAAATGTGGAAACTCCA

CTAATAATATTACATTGTGCGACTTTCAAGATGATGTGATATATAGGGTTATACAGTTTTAACAATTATA

TAATAGAGATAGAAAGTACAAAAGATGTAGAATCAATGATATGGCACGCTAAACAGTTGGGTGTGGAATC

ATTGCTAAAAGAATGTCAAAATTATTTGCTTAGAATATTACGTATATAATTGTTTAGAAATTTATAGAAT

AACTAATATTAATACATTATCGTATATCTACAACGATATAAGAAACTTCATATTGGATAATATTACTATT

AATATATAAGGATCCAGATTTTATATATTTGCCTAAATACATTATTATAGATTTACTAGGACAATCACCT

AAATGTTTTTAACGAAGATAATGTGGTAAAGATTATATACACTTATATATCTTCCGATATCTACAAGGAT

ATTCCATATCATCATTGTGTAAACTAAATAACGTTTTCTATGGCATTTAATAAGGACATTGGATATGTGG

AAAAGTGATGTATGGAAGTTAGTACATTATCAACTTCTCCTTATTGATTGAAAATGAAAATATAAATAGT

TTTTATGTATAGCGGTATCTACCCTATAGTTTTATTGCTTACTACTAACATGGATTCAGATACAGATACA

GATACAGATACAGATACAGATACAGATACAGATGTAGAAGATATCATGAATGAAATAGATAGAGAGAAAG

AAGAAATACTAAAAAATGTAGAAATTGAAAATAATAAAAACATTAACAAGAATCATCCCAGTGAATATAT

TAGAGAAGCACTTGTTATTAATACCAGTAGTAATAGTGATTCCATTGATAAAGAAGTTATAGAATATATC

AGTCACGATGTAGGAATATAGATCATATCTACTAATTTTTATAATCGATACAAAACATAAAAACAACTCG

TTATTACATAGCAGGTATGGAATCCTTCAAGTATTGTTTTGATAACGATGGTAAGAAATGGATTATCGGA

AATACTTTATATTCTGGTAATTCAATACTCTATAAGGTCAGAAAAAATTTCACTAGTTCGTTCTACAATT

ACGTAATGAAGATAGATCATAAATCACACAAGCCATTGTTGTCCGAAATACGATTCTATATATCTGTATT

GGATCCTTTGACTATCAACAACTGGACACGGGAACGTGGTATAAAGTATTTGGCTATTCCAGATCTGTAT

GGAATTGGAGAAACCGATGATTATATGTTCTTCATTATAAAGAATTTGGGAAGAGTATTCGCCCCAAAGG

ATAGTGAATCAGTTTTCGAAGCATGTGTCACTATGATAAACACGTTAGAGTTTATACACTCTCAAGGATT

TACTCATGGAAAAATAGAACCGATGAATATACTGATTAGAAATAAACGTATTTCACTAATTGACTATTCT

AGAACTAACAAACTATACAAAAGTGGAACACATATAGATTACAACGAGGACATGATAACTTCAGGAAATA

TCAATTATATGTGTGTAGACAATCATCTTGGAGCAACAGTTTCAAGACGAGGAGATTTAGAAATGTTGGG

ATATTGCATGATAGAATGGTTCGGTGGTAAACTTCCATGGAAAAACGAAAGTAGTATAAAAGTAATAAAA

CAAAAAAAAGAATATAAACAATTTATAGCTACTTTTTTTGAGGACTGTTTTCCTGAAGGAAATGAACCTC

TGGAATTAGTTAGATATATAGAATTAGTATACATGTTAGATTATTCTCAAACTCCTAATTATGACAGACT

ACGTAGACTGTTTATACAAGATTGAAATTATATTCTTTTTTTTATAGAGTGTGGGGGTAGTGTTACGGAT

ATCTAATATTAATATTAGACTATCTCTATCGCGCTACACGACCAATATCGATTACTATGGATATCTTCAG

GGAAATCGCATCTTCTATGAAAGGAAAGAATGTATTCATTTCTCCAGCGTCAATCTCGTCAGTATTGACA

ATACTGTATTATGGAGCTAATGGATCCACTGCTGAACAGCTATCAAAATATGTAGAAAAGGAGGAGAACA

TGGATAAGGTTAGCGCTCAGAATATCTCATTCAAATCCATGAATAAAGTATATGGGCGATATTCTGCCGT

GTTTAAAGATTCCTTTTTGGGAAAAATTGGCGATAAGTTTCAAACTGTTGACTTCACTGATTGTCGCACT

ATAGATGCAATCAATAAGTGTGTAGATATCTTTACTGAGGGAAAAATCAATCCACTATTGGATGAACCAT

TGTCTCCTGATACCTGTCTCCTAGCAATTAGTGCCGTATACTTTAAAGCAAAATGGTTGATGCCATTCGA

AAAGGAATTTACCAGTGATTATCCCTTTTACGTATCTCCAACGGAAATGGTAGATGTAAGTATGATGTCT

ATTTACGGCGAGCCATTTAATCACGCATCTGTAAAAGAATCATTCGGTAACTTTTCAATCATAGAACTGC

CATATGTTGGAGATACTAGTATGATGGTCATTCTTCCAAACAAGATTGATGGATTAGAATCCATAGAACA

AAATCTAACAGATACAAATTTTAAGAAATGGTGTAACTCTCTGGAAGCTACGTTTATCGATGTGCACATT

CCTAAGTTTAAGGTAATAGGTTCGTATAATCTTGTGGATACGCTAATAAAGTTGGGACTGACAGATGTGT

TCTATTCAACTGGTGATTATATCAATATGTGTAATTCAGATGTGAGTGTTGACGCTATGATTCACAAAAC

GTATATAGATGTCAATGAAGAGTATACAGAAGCAGCTGCAGCAACTTCTGTACTAGTGGCAGACTGTGCA

TCAACAGTTACAAATGAGTTCTGTGCAGATCATCCGTTCATCTATGTGATTAGACATGTCGATGGTAAAA

TTCTTTTCGTTGGTAGATATTGCTCTCCAACAACTAATTAAGCACATTCTTAATATTAGAATATTATATA

GTTAAGATTTTTACTAACAGGTTAACATTTTTTTTTAAAAATAGAAAAAACATGTGGTATTAGTGCAGGT

CGTTATTCTTCCAATTGCAATTGGTAAGATGACGGCCAACTTTAGTACCCACGTCTTTTCACCACAACAC

TGTGGATGTGACAGACTGACCAGTATTGATGACGTCAGACAATGTTTGACTGAATATATTTATTGGTCGT

CGTATGCATACCGCAACAGGCAATGCGCTGGACAACTGTATGACACACTCCTCTCTTTTAAAGATGATGC

GGAATCAGTGTTCATCGACGTTCGTGAGCTGGTAAAAAATATGCCGTGGGATAATGTTAAGGATTGTACA

GAGATCATCCGTTGTTATATACCGGATGAGCAAAAAACCATCAGAGAGATTTCGGCCATCATTGGACTTT

GTGCATATGCTGCTACTTACTGGGGAGGTGAAGACCATCCCACTAGTAACAGTCTGAACGCATTGTTTGT

GATGCTTGAGATGCTCAATTACATGGATTATACCATCATATTCTGGCGTATGAATTGATGAGTTACAGCT

TGACATTTCTTCTTTCCTCCCTCTTCTTCTACCTTTCCCAGAAACAAACTTTTTTTACCCACTATAAAAT

AAAATGAGTATACTACCTGTTATATTTCTTCCTATATTTTTTTATTCTCCATTCGTTCAGACTTTTAACG

TGCCTGAATGTATCGACAAAGGGCAATATTTTGCATCATTCATGGAGTTAGAAAACGAGCCAGTAATCTT

ACCATGTCCTCAAATAAATACGCTATCATCCGGATATAATATATTAGATATTTTATGGGAAAAACGAGGA

GCGGATAATGATAGAATTATACAGATAGATAATGGTAGCAATATGCTAATTCTGAACCCGACACAATCAG

ACTCTGGTATTTATATATACATTACCACGAACGAAACCTACTGTGACATGATGTCGTTAAATTTGACAAT

CGTGTCTGTCTCAGAATCAAATATAGATCTTATCTCGTATCCACAAATAGTAAATGAGAGATCTACTGGT

AAAATGGTATGTCCCAATATTAATGCATTTATTTCTAGTAACGTAAACACAGAATTATATGGAGCGGACA

TCGACGCCTTAGAAATAAGAGACTTAAACAACGGACACCTGGAATTATTACCATAGAAGATGTTAGAAAA

AATGATGCTGGTTATTATACATGTGTTTTAGAATATATATATATATATATATATATATATATATATATAT

ATATATATATATATATATATATATATATATATATATATATATGGGCAAAACATATAACGTAACCAGAATT

ATAAAATTAGAGGTACGGGATAGAATAATACCTCCTACTATGAAATTACCAGAAGGAGTAGTAACTTCAA

TAGGTAGTAATTTGACTATTGCATGTAGAGTATCGTTGAGACTTCCCACAACGGACACCGACGTCTTTTG

GATAAGTAATGGTATATGTATTACGAAGAAGAAGACGAGGACGGAGACGGTAGAATAAGTGTAGCAAATA

AAATCTATATGACCGATAAGAGACGTGTTATTACATCCTGGTTAAACATTAATCCTGTCAAGGAAGAAGA

TGCTACAACGTTTACGTGTATGGCGTTTACTATTCCTAGCATCAGCAAAACAGTTACTGTTAGTAAACGT

GAATGTATGTTGTTACATTTCCATATCAATTGAGTTTATAAGAATTTTTTATACATTATCTTCCAACAAA

CAATTGACGAACGTATTGCTATGATTAACTCCCACAATACTATATATATTATTAATCATTAACTTGCAGA

CTATACCTAGTAGTGCTATTTTGACATACTCATGTTCTTGTGTAATCGCAGTATCTATATTATTAAAGTA

CGTAAATCTAGCTATAGTTTTATTATTTAATTTTAGATAATATACTGTCTCCGTATTTTTAAAAAATTAC

CACATCCTTTATTAAATCATGAATGGGAATTTCTGTGTCATCGTTAGTATATTGTGAACAACAAGAGCAG

ATATCTATAGGAAAGGGTGGAATGCGATACATTGATCTATGTAGTTTTAAAACATACGCGAACTTTGAAG

AATTTATATAAATCATCTCACGAGATATTGCTCTCTGTCATATTCATACACCTGTATAAACTTTCTAGAC

ATCTTACAATGTGTTATTTTATGATCATATTTACATATTTACTGGTATATCAAAGATGTTAGATTAGTTA

ATGGGAATCGTCTATAATAATGAATATTAAACAATTATAGGAGGAGTTTATACCTACAAAAACATCATAA

AAATGAGTCATCGTCCGATTTATGTTTTAAATATACTAACATTACTACCTTCAGAAATTATATACGAAAT

ATTATACATGCTGACAATTAACGATCTTTATAATATATAGTATCCACCTACCAAAGTATAATTGTATTTT

TCTCATGTGATGTGTGTAAAAAACTGATATTATATAATTATCTTAGTACCTATGATGAAGATGAAGATGA

AGATGATGGTCCGTATATATTTTGTATCATTATCGTTATTGCTATTCCATAGTTACGCCATAGACATCGA

AAATGAAATCACCGAATTCTTCAATAAAATGAGAGATACTCTACCAGCTAAAGACTCTAAATGGTTGAAT

CCAGTATGTATGTTTGGAGGCACAATGAATGATATGGCCGCTCTAGGAGAGCCATTCAGTGCAAAGTGTC

CTCCTATTGAAGACAGTCTTTTATCGCATAGATATAAAGACTATGTGGTTAAATGGGAAAGGCTAGAAAA

GAATAGACGGCGACAGGTTTCTAATAAACGTGTTAAACATGGTGATTTATGGATAGCCAACTATACATCT

AAATTCAGTAACCGTAGGTATTTATGTACCGTAACCACAAAGAATGGTGACTGTGTTCAGGGTGTAGTTA

GATCTCATGTGTGGAAACCTTCTTCATGCATTCCAAAAACATATGAACTAGGTACTTATGATAAGTATGG

CATAGACTTATACTGTGGAATTCTTTATGCGAACCATTATAATAATATAACTTGGTATAAAGATAATAAG

GAAATTAATATCGACGATTTTAAGTATTCACAAGCGGGAAAGGAATTAATTATTCATAATCCAGAGTTAG

AAGATAGTGGAAGATACGACTGTTACGTTCATTACGACGACGTTAGAATCAAGAATGATATCGTAGTATC

AAGATGTAAAATACTTACGGTTATACCGTCACAAGACCACAGGTTTAAACTAATACTAGATCCGAAAATC

AACGTAACGATAGGAGAACCTGCCAATATAACATGCAGTGCTGTGTCAACGTCATTATTTGTCGACGATG

TACTGATTGAATGGGAAAATCCATCCGGATGGATTATAGGATTAGATTTTGGTGTATACTCTATTTTAAC

TAGTAGAGGCGGTATCACCGAGGCGACTTTGTATTTTGAAAATGTTACTGAAGAATATATAGGCAATACA

TATACATGTCGTGGACACAACTATTATTTTGATAAAACTCTTACAACTACAGTAGTATTGGAGTAAATAC

ACAATGCATTTTTATATACATTACTGAATTATTATTATTAATTATATCGTATTTGTGCTATAGAATGGAT

GAAGATACGCGACTATCTAGGTATTTGTATCTCACCGATAGAGAACATATAAATGTAGACTCTATTAAAC

AGTTGTGTAAAATATCAGATCCTAATGCATGTTATAGATGTGGATGTACGGCTTTACATGAGTACTTTTA

TAATTATAGATCAGTCAACGGAAAATACAAGTATAGATACAACGGTTACTATCAATATTATTCATCTAGC

GATTATGAAAATTATAATGAATATTATTATGATGATTATGATAGAACTGGTATGAACAGTGAGAGTGATA

ATATATCAATCAAAACAGAATACGAGAATGAATATGAATTCTATGATGAAACACAAGATCAAAGTACACA

ACTAGTAGATTACGACATTAAACTCAAAACCAATGAGGATGATTTTGTTGATGAATTCTATGGTTATGAT

AGATCAGTGGGTGTCCATGATTATATAGATGTATCAATTAATAAAGTAGTATATGGAAGAGAGTCTCACG

TAAGATGGTGGGATATATGGCAAGAACATAATGATGGCGTATACAGTATAGGAAAGGAGTGCATAGATAA

TATATACGAAGACAGACATACCGTAGACGAATTCTACAAGATAGACAGCGTATCAGATGTAGATGACGCA

GAACATATATCTCAGATAACTAATGATGTATCTACACAAACATGGGAAAAGAAATCAGAGTTAGATAGAT

ACATGGAAATGTATCCTCGTCATAGATATGGTAAGCATTCTGTCTTTAAGGGATTTTCTGACAAAGTTAG

AAAAAATGATTTAGACATGAACGTGGTAAAAGAATTACTTTCTAACGGTGCATCTCTAACAATCAAGGAT

AGCAGTAATAAGGATCCAATTGCTGTTTATTTTAGAAGAACAATAATGAATTTAGAAATGATTGATATCA

TTAACAAACATACAACTATCTATGAACGCAGGTATATAGTACACTCCTATCTAAAAAATTATAGAAATTT

CGATTATCCATTTTTCAGAAAGTTAGTTTTGACTAATAAACATTGTCTCAACAATTATTGTAATATAAGC

GACGGCAAATATGGAACACCACTACATATATTAGCATCTAATAAAAAAATAATAACTCCTAATTACATGA

AGTTATTAGTGTATAACGGAAATGATATAAACGCACGAGGTGAAGATACACAAATGCGAACTCCATTACA

CAAATATTTGTGTAAATTTGTATATCATAATATTGAATATGGTATCCGATACTATAATGAAAAGATTATA

GACGCATTTATAGAGTTAGGAGCCGATCTAACTATTCCAAATGACGATGGAATGATACCAGTAGTTTACT

GTATACACTCAAATGCCGAATATGGTTATAACAATATTACTAACATAAAGATAATACGTAAACTACTTAA

TCTTAGTAGACATGCGTCACATAATCTATTTAGAGATCGAGTCATGCACGATTATATAAGTAATACATAT

ATTGATCTTGAGTGTTTAGATATCATTAGATCACTTGATGGGTACGATATTAATTGTTACTTTGAAGGAC

GTACACCACTTCATTGCGCTATACAATATAACTTCACTCAGATTGCTGAGTACTTATTAGATCGAGGAGC

TGATATATCATTAAAGACAGACGATGGTAAAACTGTATTTGATTTATCGTTATGTAGTTACATTCCTCTT

AAATGGACTAGCTTTTTGATTAGTCGTCTACCGCCTAAAAGTGTCATATGCTCACTGACTAACCATATAA

TAGATTATGTTCTTACGAACAATAGACGTATTATTTGGCAGAGTCAAATGATTAATAAGTACGTACTGTT

ACTGGACCCATCCTTTTATTATAGATTCAGAAATGTTATCGAAAACAAATTAGACCAATACAATAATCGT

TATAATATGTTCGAACACGATAGGGACGTTAATGAAAAGTATGGCAAAGTCTTACATGACCTCGATACAT

ATATCAAGGATGTACAAGTATTAAAATCTACTTCCATCACTAATAATATAACACTATACGACACTATTAT

AAATAATAAGTCAGAGTTTCCTATACGTCGTGTAAACGACAAACAATTAATTAATCTCATAAAATCCAAT

ACATATCATAATCTTATCGAAAAAGTTATTAAAAATACATTAGAGAAATATACTTTAACTAATATAGTCC

TCGAGTATATGATCTCATCTCGATCTCAATCATCTTATTTGAGTCGTATTCCTAATGAGATATTACTCGA

AATATTATATAAACTCGACATGTACGATTTACGTAATCTATATACAAGATATATGAGAGAGAATGATATC

ACAGAGTATCATATAGAGAATACGAGGTCTGTTTCTACACAGACATGAATAATGAATACACATACAACGT

TTTTTTTTTTAATCTTAGATATAACACTAATTACATCAAGATTATATATTGAAATCGTAATTTGAGTTGT

CTGATCATCATGGATATCGAAAATGATATACGTAACATTAGCAATCTTTTAGATGATTGATATATTATTA

TGCGATGTAATCATAACTATCGGAGATGTAGAAATTAAAGCGCATAAAACTATTTTGGTTGCCGGATCTA

CGTATTTTAAAACAATGTTCACAACATCTATGATAGCGAGAGATCTAGCAACTAGAGTAAATATACAGAT

GTTCGATAAAGATGCCGTCAAAAATATTGTACAGTACTTATACAATAGGTATATAAGTTCTATGAATGTG

ATAGACATATTAAAATGCACCGACTAAGAACGTAGAACGAACTATAGAATGTTATACAATGGGTGATGAT

AAGTAGAAGATGTTACCCGATATACCCATAGCATTATCTAGTTATGGCATGTGTGTATTAGATCAATACA

TATACATTATAAGCGGTCGTACCCAACACTGATTATACATCGGTACATACAGTAAATAGCATAGATATGG

AGGAGGATACAAATATTTCAAATAAAGTTATGAGATACGCGCTGTCAATAATATATGGAAGACATTACCT

AACTTCTGAACTGGAACTATAAATCCAGGCTCTCGCATAAAGATGAATATATATGTTGTATGCGACATCA

AAGATGAAAAAATGTTAAGACTTATATATTTAGATATAACACGAATATGTATGACGGATGGGAATTGGTA

ACGATGACAGAAAGCAGATTGTCAGCTCTGCATACTATTCTTCATGACAATACCATAATGATGTTACATT

GTTATGAAGCGTATATGTTACAAGATACATTTAATGTGCTTACGGAACATATATTTAGAAACATCTACTA

ACGATTTTTTATGCTTGTATTATTAATGGTATGTAATATGATTTAATTGATTGTGTACACGATACCAATT

TGTCGAGTATGAATACGGAGTACAAACATAAACTGAAGTTTAACATTATTTATTTATGATATACATTATA

TACATTATATACATTATATACATTATATACATTATATACATTATATACATTATATACATTATATACATTA

TATACATTATATACATTATATACATTATATACATTATATACATTATATACATTATATACATTATATACAT

TATATACATTATATATCGTTATTGTTTGGTCTATGCCATGGATATCTTTAAAGAACTAATCTTAAAACAT

ACGGATGAAAATGTTTTGATTTCTCCAGTTTCCATTTTATCTACTTTATCTATTCTGAATCATGGAGCAG

CTGGTTCTACAGCTGAACAACTATCAAAATATATAGAGAATATGAATGAGAATACACCCGATGATAAGAA

GGATGACAATAATGACATGGACGTAGATATTCCGTATTGCGCGACACTAGCTACCGCAAATAAAATATAC

GGTAGTGATAGTATCGAGTTCCATGCCTCATTCCTACAAAAAATAAAAGACGATTTTCAAACTGTAAACT

TTAATAATGCGAACCAAACAAAGGAACTAATCAACGAATGGGTTAAGACAATGACAAATGGTAAAATTAA

TTCCTTATTGACTAGTCCGCTATCCATTAATACTCGTATGATAGTTATTAGCGCCGTCCATTTTAAAGCA

ATGTGGAAATATCCATTTTCTAAACATCTTACATATACAGACAAGTTTTATATTTCTAAGAATATAGTTA

CCAGTGTTGATATGATGGTGGGTACCGAGAATGACTTGCAATATGTACATATTAATGAATTATTCGGAGG

ATTCTCTATTATCGATATTCCATACGAGGGAAACTCTAGTATGGTGATTATACTGCCGGACGACATAGAA

GGTATATATAACATAGAAAAAAATATAACAGATGAAAAATTTAAAAAATGGTGTGGTATGTTATCTACTA

AAAGTATAGACTTGTATATGCCAAAGTTTAAAGTGGAAATGACGGAACCGTATAATCTGGTACCGATTCT

AGAAAATTTAGGACTTACTAATATATTTGGATATTATGCAGATTTTAGTAAGATGTGTAATGAAACTATC

ACTGTAGAAAAATTTCTACATACGACGTTTATAGATGTTAATGAGGAGTATACAGAAGTATCGGCCGTTA

CAGGAGTATTCATGACTAACTTTTCGATGGTATATCGTATGAAGGTCTACATAAACCATCCATTCATATA

CATGATTAAAGATAACACCGGACATACACTTTTTATAGGGAAATACTGCTATCCGCAATAAATATAAACA

ATAGACTTTTATCACGTTATCTCATGTATAAAATATTACAAATAGTATAGCATAAACTAAAGTCGATACA

TACATTAAAACTTAAATAATAATGTAATTTACAATTAATAGTATAAACTAAAAAAATTAAAAAATTAAAA

ACAATATCATTATTATAAGTAATATCAAAATGACGATATACGGATTAATAGCGTATCTTATATTCGTGAC

TTCATCCATCGCTAGTCCACTTTACATTCCCGTTATTCCGCCCATTTCGGAAGATAAATCGTTCAATAGT

GTAGAGGTATTAGTTTCTTTGTTTCCCGATGACCAAAAAGACTATACAGTAACTTCTCAGTTCAATAACT

ACACTATCGGTACCAAAGACTGGACTATCAACGTACTATCCACACCTGATGGTCTGGACATACCATTGAC

TAATATAACTTATTGGTCACGGTTTACTATAGGTCGTGCATTGTTCAAATCAGAGTCTGAGGATATTTTC

CAAAAGAAAATGAGTATTCTAGGTGTTTCTATAGAATGTAAGAAGCCGTCGACATTACTTACTTTTTTAA

CCGTGCGTAAAATGACTCGAGTATTTAATAGATTTCCAGATATGGCTTATTATCGAGGAGACTGTCTAGA

AGCCGTTTATGTAACAATGACTTATAAAAATACTAAAACTGGAGAGACTGATTACACGTACCTCTCTAAT

GGGGGGTTGCCTGCATACTATCGTAATGGGGTCGATGGTTGATTATTGATTAGTATATTCCTTATATTCC

TTATTCTTTTTATTCACACAAAAAGAACATTTTTATAAACATGAAACCACTGTCTAAATGTAATTATGAT

CTTGATTTATAGATGATGATCAGCCTTCAGAGGATTTTGACCAGTATGTTTAATATGAAAAAAACATAAC

TATTAAGCGCTATTGCGCTATTGTGCTTAATTATTTTGCTCTATAAACTGAATATATAGCCACAATTATT

GACGGGCTTGTTTGTGACCGACAATCATGAATTTTCAGAAATTATCTCTGGCTATATATCTTACGGTGAC

ATGTTCGTGGTGTTATGAAACATGTATGAGAAAAACTGCGTTGTATCATGACATTCAATTGGAGCATGTA

GAAGACAATAAAGATAGTGTAGCATCGCTACCGTACAAGTATCTACAAGTAGTCAAACAAAGAGAACGTA

GTAGATTGTTGGCTACATTTAATTGGACGGATATAGCTGAGGGTGTTAGAAATGAGTTCATTAAAATATG

TGATATCAACGGAACATATCTATATAATTATACTATTGCTGTTAGTATAATTATTGATTCCACGGAAGAA

CTACCAACAGTTACTCCAATTACAACATATGAACCTTCTATATATAATTATACTATCGATTATAGCACTG

TTATTACTACTGAAGAACTACAAGTGACTCCAACATATGCACCTGTAACAACTCCTCTTCCAACATCAGC

AGTTCCTTATGATCAACGATCGAATAACAATGTAAGTACTATATCTATTCAGGTACTGAGTAAAATATTG

GGAGTCAATGAAACAGAATTAACTAATTATCTTATTATGCATAAAAATGACACTGTTGACAATAACACCA

TGGTTGATGATGAGACATCTGACAATAACACATTACATGGTAATATAGGATTTTTGGAAATAAATAATTG

TTATAATGTTTCTGTGTCAGATGCTAGTTTTAGAATAACATTAGTAAACGATACTTCTGAAGAAATTTTG

CTAATGCTAACAGGAACTAGTTCATCCGACACCTTCATATCTTCCACCAATATCACTGAATGTTTGAAAA

CATTAATCAATAATGTGTCGATTAATGATGTACTTATAACACAAAATATGAATGTAACATCTAATTGTGA

TAAATGCTCAATGAATTTGATGGCATCCGTTATTCCTGCAGTTAATGAATTTAACAATACGTTGATGAAA

ATTGGTGTAAAAGATGATGAAAACAATACGGTATATAAATATTATAATTGTAAACTAACTACAAATTCTA

CATGTGATGAGTTAATCAATTTAGATGAAGTCATTAACAACATAACTCTGACAAATATTATACACAATAG

TGTTTCGACAACTAACAGCAGAAAAAGACGAGATCTGAATGATGAGTTTGAATTTTCCACTTCCAAGGAA

TTAGATTGTCTTTACGAATCATATGGTGTAAACGATGATATAAGTCATTGTTTTGCATCACCTAGACGTA

GACGATCTGACGACAAAAAGGAGTACATGGACATGAAATTATTCGACCACGCGAAAAAAGATTTAGGAAT

AGACAGTGTTATTCCTAGAGGTACAACCCATTTCCAAGTAGGTGCATCTGGTGCAAGTGGTGGTGTTGTA

GGAGATAGTTTCCCATTTCAAAATGTTAAATCGCGTGCCAGTCTATTGGCGGAAAAAATAATGCCTAGAG

TACCTATTACTGCTACCGAAGCTGATCTATATGCAACTGTAAATAGACAACCCAAGTTACCAGCAGGTGT

TAAAAGTACTCCGTTTACAGAGGCGCTTGTGTCTACGATAAACCAAAAGCTTTCTAATGTTAGAGAGGTA

ACTTATGCTTCGCTCAATCTGCCAGGATCAAGTGGCTATGTTCATAGACCATCTGATTCTGTTATTTATA

GCAGTATAAGACGGTCACGTTTACCTAGTGATAGCGATAGTGATTATGAGGATATACAAACTGTTGTTAA

GGAATATAATGAAAGATATGGTAGATCAGTCAGTAGAACACAGTCATCAAGTAGTGAAAGCGATTTTGAA

GATATAGATACTGTTGTTAGGGAATATAGACAAAAATATGGCAATGCAATGGCAAAAGGACGTAGTAGTT

CCCCTAAACCTGATCCATTATATAGTACTGTTAAGAAAACAACTAAAAGTCTATCTACTGGTGTAGACAT

AGTTACAAAACAATCAGACTATTCTCTATTACCTGACGTTAATACTGGCAGTTCTATTGTGTCACCTCTC

ACCAGAAAAGGAGCTACTAGACGACGACCTAGACGCCCTACAAATGATGGTCTACAGAGTCCAAATCCTC

CTCTCCGTAATCCACTTCCTCAACATGATGATTATTCTCCTCCACAAGTACACAGACCTCCACCACTTCC

TCCTAAACCAGTCCAAAATCCGCCACAACTTCCCCCTAGACCAGTAGGTCAATTACTACCTCCTCCTATA

GATCAACCAGATAAAGGATTTAGTAAGTTTGTATCACCTAGACGGTGTAGAAGAGCAAGCTCTGGAGTCA

TATGTGGTATGATACAATCAAAACCAAACGATGATACCTATTCACTTCTTCAACGATCAAAAATTGAACC

AGAATATGTGGAGGTTGGTAATGGTATACCCAAGAACAATGTTCCTGTAATAGGTAATAAACATAGTAAA

AAATATACATCGACGATGTCAAAAATATCAACAAAATTTGATAAATCTACGGCATTTGGAGCAGCAATGT

TACTAACTGGTCAGCAGGCCATTAGCCAACAGACTAGATCAACTACGTTGAGTAGAAAAGATCAGATGAG

TAAGGAAGAAAAGATATTCGAAGCAGTTACAATGAGTCTATCAACTATAGGTTCAACGTTGACGTCTGCA

GGTATGACGGGTGGTCCAAAACTAATGATTGCAGGAATGGCTATAACGGCTATAACTGGTATAATAGATA

CGATAAAAGATATATATTACATGTTTTCAGGACAGGAGAGGCCAGTAGATCCTGTTATTAAATTATTTAA

TAAGTACACTGGCTTAATGTCCGATAATAATAAAATGGGTGTAAGAAAATGTTTGACACCCGGTGACGAC

ACACTTATTTATATCGCATACAGAAACGATACCAGTTTTAAACAGAATACGGATGCGATGGCTTTGTATT

TCTTAGATGTTATCGATTCAGAGATCCTATATCTAAACACATCAAATTTAGTTCTAGAGTATCAACTAAA

GGTGGCTTGCCCCATAGGAACATTAAGATCTGTAGATGTGGACATAACTGCGTATACAATATTATATGAT

ACAGCGGATAATATTAAGAAATACAAGTTTATCAGAATGGCAACGCTACTATCCAAACATCCAGTTATTA

GATTGACATGTGGTTTAGCAGCAACATTGGTGATTAAACCGTACGAGGTACCCATCAGTGATATGCAACT

ACTAAAAATGGCGACGCCTGGTGAACCAGAATCCACTAAATCTATACCATCCGATGTCTGTGATAGGTAT

CCTCTAAAGAAATTCTATCTTTTAGCTGGTGGTTGTCCCTATGATACATCTCAAACTTTTATTGTACATA

CTACTTGCAGTATTCTACTAAGAACAGCTACACGGGATCAGTTTAGAAACAGATGGGTGTTACAAAATCC

ATTTAGACAAGAAGGGACATATAAGCAACTGTTTACCTTTAGCAAATACGATTTTAACGACACCATAATC

GATCCTAATGGTGTGGTGGGTCATGCTAGCTTTTGTACCAATAGAAGCAGCAACCAATGTTTCTGGTCCG

AACCTATGATATTGGAAGATGTATCATCGTGTAGTTCTAGAACTAGAAAAATATACGTAAAACTGGGAAT

ATTTAATGCTGAAGGTTTTAATAGTTTTGTACTAAATTGTCCAACTGGGTCTACACCTACATACATCAAA

CATAAAAATGCGGACAGTAACAATGTTATCATAGAGCTACCTGTAGGTGATTACGGCACAGCCAAATTGT

ATTCAGCAACAAAACCATCGAGGATAGCTGTGTTCTGCACACATAACTATGATAAACGATTCAAATCAGA

TATTATAGTTCTAATGTTTAATAAAAACAGCGGTATTCCATTTTGGAGCATGTACACAGGAAGTGTAACT

AGTAAAAATAGAATGTTTGCCACATTGGCTAGAGGAATGCCGTTTAGATCAACGTATTGCGATAACAGAC

GACGATCAGGTTGTTATTATGCAGGAATACCATTTCATGAAGATAGTGTAGAAACAGATATACATTATGG

ACCAGAAATAATGTTAAAGGAAACATATGACATAAACAGTATTGACCCACGAGTTATAACAAAGTCAAAG

ACCCATTTTCCTGCTCCATTGAGTGTAAAATTCATGGTTGACAATTTAGGAAATGGATATGACAACCCTA

ATTCATTTTGGGAAGATGCTAAAACTAAGAAACGGACATATAGTGCAATGACGATAAAAGTCCTACCATG

TACAGTGAGAAATAAAAATATAGACTTTGGATATAACTATGGAGATATTATTTCTAATATGGTTTATCTA

CAATCTACTAGTCAGGATTATGGAGATGGTACCAAATATACATTTAAATCCGTAACTAGATCAGATCATG

AGTGTGAATCTAGCTTAGATCTAACGTCTAAGGAAGTAACTGTGACATGTCCTGCGTTTAGTATACCAAG

AAATATATCAACATATGAAGGTCTATGCTTTAGTGTTACTACATCTAAAGATCATTGTGCTACAGGTATT

GGTTGGTTAAAATCTAGTGGTTATGGGAAGGAAGATGCTGATAAACCACGTGCTTGTTTTCATCATTGGA

ATTATTACACACTGTCGTTGGATTATTACTGTTCATACGAAGATATTTGGAGAAGCACCTGGCCTGACTA

TGATCCATGTAAGTCATATATCCATATAGAGTATAGAGATACATGGATAGAATCTAATGTGTTACAGCAA

CCTCCTTACACATTCGAATTCATTCATGACAATTCTAACGAATATGTGGATAAAGAAATTAGTAACAAAT

TAAATGATCTGTACAATGAATACAAGAAGATTATGGAATATAGCGACGGATCATTGCCGGCGTCTATAAA

CAGATTAGCAAAGGCATTGACTTCAGAGGGTAGAGAAATAGCAAGTGTTAATATAGATGGTAATCTGTTA

GATATCGCATATCAAGCAGATAAGGAAAAGATGGCCGACATACAGACAAGAATAAATGATATTATTAGAG

ATTTGTTTATACACACTCTATCAGACAAAGATATAAAAGACATTATAGAATCCGAAGAAGGTAAGAGATG

TTGTATAATAGATGTTAAGAACAATCTTGTTAAAAAGTACTATTCTATTGATAATTATCTATGTGATACT

TTAGATGATTATATATACACCTCTGTAGAATATAACAAATCCTATGTGTTAGTAAACGATACTTATATGA

GCTATGACTATCTTGAATCATCAGGTGTAGTTGTTCTATCATGTTATGAAATGACTATAATCTCCTTGGA

TACAAAAGACGCCAAAGATGCTATAGAAGATGTGATAGTAGCAAGTGCGGTAGCCGAAGCATTGAATGAC

ATGTTTAAGGAATTTGATAAAAACGTAAGTGCTATTATAATAAAAGAAGAAGATAATTATCTAAACAGTT

CGCCCGATATCTACCATATAATATATATCATAGGTGGCACTATTCTGCTACTGTTAGTCATTATTTTAAT

ATTGGCAATTTATATAGCGCGCAATAAATACAGAACCAGGAAATATGAAATAATGAAATATGACAATATG

AGCATTAAATCTGAGCATCATGATAGTCTTGAAACAGTGTCTATGGAAATTATTGATAATCGGTACTAAT

AAAATAGTTTAACTCTTTTAGAACCAGTTTGGTACTGTAATTTCAGTTCATTACTCGTTGAGAATATTGA

TGATTTTTTTAAATGAGTATCGGTAGTTACATATTACCATATCATCCATTATATAATCGATGATGCATGT

ATTAGAATACTTTCCGAATAAGTCTTCTAAATATTGTATTAATTATGAAAAACTATGCTATGTGAGTATG

ATTCAAAGATGTTTAATGATACGATACTAGATTTTATCTCTAGCGAGATTGTTTAGAATCATTTATCATA

ACTATGTTTAATAAATTCATCAACGAATATCGATAAAGACCTCTTGTAATTCGAGTATAGGAAGTAGTAT

TACCATATCAACTTCCGAGTTAACAATTACTCTAAAACATGAGGATTGTACTCCTGTCTTTATTGGAGAT

CACTATTCAGTCGTTGATAAACTAGTAACCTCAGGTTTCTTTACAAACGATAAAGTACAACATCAAGACC

TCACAACACAGTGCAAGATTAATCTAGAAATCAAATGTAATTCTGGAGGAGAATCTAGACAACTAACACC

CACGGCGAAGTATACTTTATGCCTCATTCAGAAACGGTAACTGTAGTAGGAGACTGTCTCTCTAATCTCG

ATGTATATATAATATATACCAATACGGACGCGATATATTCCGACATGAATGGCGTCGCTTATCATATGTT

ATATCCTAAATGTTGATCATATTCCACAAATGATTGTGAACGAGATTAAATCATCTAACAAATAATTAGT

TTTTTATGACATTAACATATAATAAATAAATTAATCATTATTGACTTAACGATGACGAAAGTTATCATTA

TCTTAGGATTCTTGATTATTAATACAAATTCGTTGTGTCTATGAAATGTGAACAAGGTGTCTCATATTAT

AATGCACAAGAATTAAAGTGTTGTAAACTATCTAGCCAGGAACATATTCAGATTATCGATGTGATAAATA

CAGCGATACCATCTGTGGACATTGTCCAAGTGACACATTCACGTCAATATATAATCGTTCTCCTCGGTGT

CATAGTTGTAGAGGTCACACCTTGTACACCTACCACAAATAGAATATGTCATTGTGACTCGAATAGTTAT

CGTCTCCTTAAAGCTTCTGATGGTAACTGTGTTACATGTGCTCCTAAAACAAAATATGGTCGTGTGTACG

GAAAGAAAGGAGAAAATGATATGGAATACCATTTGTAAGAAATGTCGGAAGGGTACTTATTCAGATATTG

TATCTGACTCTGATCAATGTAAACCTATGACAAGATAAGACTTACTCGCATCTACTGGATAGACATAAAT

ATCCTCCTCGTAATAATGAAATATAATATACACTAATTATTAATATCAATCGAGTATTAACATATAAGTT

ATTTTTAAACCCCTTTTGGGTTCCGTCCTAAACGGCGTTTCGGTCTGTGTCGCCACCATGGTCACACCGA

GCCTCTGCGTGCTCCTCCATCGAGGACGACTTCAACTATGACAGCTCGGTGGCGTCTGCCAGCGTGTACA

TACGAATGGCATTTCTAAGAAAAGTCTACGGTATCCTTTCTACAATTTCCTTTAACAACGGCAACAGCTG

CAGTATTTTTATACTTTGAATGCATCGGACATTTATACAAGGGAGTCCTGTTCTAATATTGGCATCAATG

TTCGGATCTATAGGCTTGATTTTCGCATTGACTTTACACAGACATGAACATCCCCTGAATCTGTACATAC

TTTGTGGATTTACACTGTTAGAATCTCTAACGCTGGCCTCTGTTGTTACTTTCTATGATGCACGTATCGT

TATGCAAGCTTTCATGTTGACTACTGCAGTGTTTCTTGCTCTGACTACATATACTCTACAATCAAAGAGA

GATTTCAGTAAACTTGTAACAGGATTGTTTGCTGCTTTCTGGATTTTAATTTTGTCAGGAGTCTTGAGGA

TAAAGTTTAAAATAGAATTAATAAAGAACATATAGGTCATTTTTTAAACATGGATAGAAACCAAGGTTGT

TAGTTAATAATATACAAGATATTTTTTCTCACTCTGATCCATGTAAACCAAGGACGAGAGACACTCTCAT

TCCTCATTCACGACACCATTAAAAATGGAAATTAAAGCCCTCTATTAAGCACAGACGGCTACAGGTCTAC

CATCAGGTTACCTTCGTCTACCTTCACAATGGCCTCTCCTTGTGCCCAGTTCAGTCCCTGTCATTGCCAC

GCTACTAAGGACTCCCTGAATACCGTGACTGACGTCAGACATTGTCTGACTGAATACATCCTGTGGGTTT

CTCATAGATGGACCCATAGAGAAAGCGCAGGGCCTCTCTACAGGCTTCTCATCTCTTTCAGAATTGATGC

AATGGAGCTATTTGGTAGCGAGTTGAAGGAGTTCTCGAATTCACTTCCGTGGGACAATATCGACAATTGC

GTGGAGATCATTAAATGTTTCATCAGAAATGACTCCATGAAAACCGCCAAAGAACTTTGTGCAATAATTG

GACTTTGTACTCAATCAGCTATTGTCACTGGAAGAGTCTTCAATGATAAGTATATCGACATACTACTTAT

GCTGCGAAAGATTCTGAACGAGAACGACTATCTCACCCTCTTGGATCATATCCTCACTGCTAAATACTAA

ATCTCCTTCATGCTCTCTCACTAATACTCTTACTCACTACACTTTTTATCATCTTATGATGAATGATTGC

CTTCATCATTTTTTCGTGGAATATAATATAGGAATAATTAGCACCAGAATAGCTATGGATATCTCGTTAA

GAATATTCTCTATAAGAGACATAATGTAGACATAGTTATTATATCCTTCTTAGATAAGTGTTACGCTACT

GGAAAGTTTCCATCGTTATTATTACATGAAGATGATATAATTAAACCAACATTGAGATTGGCTCTTATGT

TAGCTGGATTGAATTACTGTAATAAATGCATCGAGTATAGAGGGATATAGCAATTCTCGATAATAGTCAT

GCAATATTTGAATGAGACTGATAATTTAGGTAATACAGTACTACACACATATCTTTCTAGATTATATATC

GTTAAAAATCTGTAAGATGTATATTTCTCATAAGTATCCACTGTGTAATATTATTAATGGATATATAGAT

AACGCAATAGGGACTAATAGTATTGTAAAAGATATAATCGACTATTTACGTACATATCCAGATATCTATA

TTCCTACTAGTTTGCTGCGTAGTTGCATCATTGATATGCATGATTTATCAGGATTCAGAGATGAATTACT

AAGTAAACTACAATCCCACAATAAGTAAGAATCAAATATCAAAAACTCACTTTTGATTTTTCTAGTCTTA

AGTAATACATATATTTATTAATAGACCTATGAAATAAAAAAAGGTAACAATGGATTCGCGTATAGCTATT

TACGTATTAGTATCGGCATCTCTTTTGTATCTTGTTAATTGTCACAAACTAGTACATTACTTCAATCTGA

AAATAAATGGAAGTGATATAACTAATACAGCAGATATATTGCTGGACAATTATCCAATTATGACCTTTGA

TGGAAAGGATATTTATCCATCTATCTCGTTCATGGTCGGTAATAAACTTTTCCTAGATCTTTATAAAAAT

ATCTTTGAAGAATTTTTCAGACTATTTCGAGTATCTGTAAGTAGTCAATACGAGGAATTAGAATATTATT

ATTCATGTGATTATACTAACAACCGTCCTACAATTAAACAACATTACTTTTATAACGGCGACGAATATAC

TGAAATTGATAGATCGAAAAAAGCCACTAATAAAAACAGTTGGTTAATTACTTCAGGCTTTAGACTACAA

AAATGGTTCGATAGCGAAGATTGTATAATTTATCTCAGATCTTTAGTTAGAAGAATGGAAGACAGTAACA

AAAACAGTAAAAAAACTTAGTACTTAGATATCGAAAAAATATATTTTTGTAGACTCTTGAGAATAGAAGG

AAAACATGTACATAATTATAAAAAATGAAAATCAATGGCGAATAAGACAGTGCGATTCGCACCATGGAGT

CGGTAGATTTCATGGCTGTCGATGAGCAGTTTCACGACGACCTCGATCTTTGGTCATTATCTTTGGTAGA

TGATTATAAAAAACATGGATTAGGTGTTGACTGTTATGTTCTAGAACCAGTTGTTGACAGGAAAATATTT

GATAGATTTCTCCTTGAACCAATTTGTGATCCTGTAGATGTTCTGTATGATTATTTTAGGATTCATAGAG

ATAATATTGATCAGTATATAGTAGATAGACTGTTTGCATATATTACATATAAAGATATTATATCTGCATT

AGTGTCAAAGAATTATATGGAAGATATTTTCTCTATAATTATTAAGAATTGTAATTCTGTGCAAGATCTC

TTACTTTACTATCTATCTAATGCATATGTAGAAATAGACATTGTTGATCTTATGGTAGATCATGGGGCTG

TAATATATAAAATAGAATGCTTGAATGCCTATTTTAGGGGAATATGTAAAAAGGAAAGTAGTGTTGTTGA

GTTTATTTTGAATTGTGGTATCCCAGATGAAAATGATGTTAAATTAGATCTATATAAAATAATTCAGTAT

ACTAGGGGATTCCTTGTAGATGAACCCACAGTATTAGAAATTTATAAGCTTTGTATCCCATATATTGAAG

ATATCAATCAACTAGATGCTGGTGGAAGGACCTTGCTTTATCGCGCTATCTATGCAGGTTATATAGATTT

AGTATCATGGCTATTAGAAAATGGAGCAAATGTCAACGCAGTAATGAGTAATGGATATACATGTCTTGAC

GTGGCCGTGGATAGGGGATCTGTCATCGCCCGTAGGGAAGCACATCTTAAAATATTAGAAATATTGCTTA

GAGAACCATTGTCTATTGACTGTATAAAATTAGCTATACTTAATAATACAATTGAAAACCATGATGTGAT

AAAGCTCTGTATCAAGTATTTTATGATGGTAGATTATTCACTTTGTAATGTGTATGCATCATCACTCTTT

GATTATATAATTGATTGTAAACAAGAATTGGAGTACATTAGGCAGATGAAAATTCATAATACAACCATGT

ATGAGTTAATCTATAATAGAGACAAAAACAAGCATGCTTCCCATATTCTACATAGGTATTCTAAACATCC

AGTTTTGACACAGTGTATCACTAAAGGATTCAAGATTTACACAGAAGTAACCGAGCAGGTCACTAAAGCT

CTAAACAGACGTGCTCTAATAGATGAGATAATAAACAATGTATCAACTGATGACAATCTCCTATCAAAAC

TTCCATTAGAAATTAGGGATCTAATTGTTTCACAAGCTGTCATATAGAGTTCTATCCACCCACCTTTCTT

GAAATGAGTTAATAGTCATAAGTTAGTTAAGTCATAAGTTAGTTAAGTCATAAGTTAGTTAAGTCATAAG

TTAGTTAAGTCATAAGTTAGTTAAGTCATAAGTTAGTTAAGTCATAAGTTAGTTAAGTCATAAGTTAGTT

AAGTCATAAGTTAGTTAAGTCATAAGTTAGTTAAGTCATAAGTTAGTTAAGTCATAAGTTAGTTAAGTCA

TAAGTTAGTTAAGTCATAAGTTAGTTAAGTCATAAGTTAAGTCATAAGTTAGTTAAGTCATAAGTTAGTT

AAGTCATAAGTTAGTTAAGTCATAAGTTAGTTAAGTCATAAGTTAGTTAAGTCATAAGTTAGTTAAGTCA

TAAGTTAGTTAAGTCATAAGTTAGTTAAGTCATAAGTTAGTTAAGTCATAAGTTAGTTAAGTCATAAGTT

AGTTAAGTCATAAGTTAGTTAAGTCATAAGTTAGTTAAGTCATAAGTTAGTTAAGTCATAAGTTAGTTAA

GTCATAAGTTAGTTTATAGTCTAACACTTCTAATTTTTATACCTTGATCTTTTTCTCTAATTATGAAAAA

GTAAATCATTATGAAGATGGATGAAATGGACGAGATTGTGCGCATCGTTAACGATAGTATGTGGTACGTA

CCTAACGCATTTATGGACGACGGTGATAATGAAGGTCACATTTCTGTCAATAATGTCTGTCATATGTATC

TCGCATTCTTTGATGTGGATATATCATCTCATCTGTTTAAATTAGTTATTAAACACTGCGATCTGAATAA

ACGACTAAAATGTGGTAACTCTCCATTACATTGCTATACGATGAATACACGATTTAATCCATCTGTATTA

AAGATATTGTTACGCCACGGCATGCGTAACTTTGATAGCAAGGATAAAAAAGGACATATTCCTCTACACC

ACTATCTGATTCATTCACTATCAATCGATAACAAGATCTTTGATATACTAACGGACCCCATTGATGACTT

TAGTAAATCATCCGATCTATTGCTGTGTTATCTTAGATATAAATTCAATGGGAGCTTAAACTATTACGTT

CTGTACAAATTATTGACTAAAGGATCTGACCCTAATTGCGTCGATGAGGATGGACTCACTTCTCTTCATT

ACTACTGTAAACACATATCCGCGTTCCACGAAAGCAATTATTACAAGTCAAAGAGTCACACTAAGATGCG

AGCTGAGAAGCGATTCATCTACGCGATAATAGATCATGGAGCAAACATTAACGCGGTTACGAAAATCGGA

AATACGCCGTTACACACTTACCTTCAACAGTATACCAAACATAGTCCTCGTGTGGTGTATGCTCTTTTAT

CTCGAGGAGCCGATACGAGGATACGTAATAATCTTGATTGTACACCCATCATGGAATACATAAAGAACGA

TTGTGCAACAGGTCATATTCTCATAATGTTACTCAATTGGCACGAACAAAAATACGGGAAATTACAAAAG

GAAGAAGGACAACATCTACTTTATCTATTCATAAAACATAATCAAGGATATGGAAGTCGCTCTCTCAATA

TACTACGGTATCTACTAGATAGATTCGACATTCAGAAAGACGAATACTATAATACAATGACTCCTCTTCA

TACCGCCTTCCAGAATTGCAATAACAATGTTGCCTCATACCTCGTATACATCGGATACGACATCAACCTT

CCGACTAAAGACGATAAGACAGTATTCGACTTGGTGTTTGAAAACAGAAACATCATATACAAGGCGGATG

TCGTTAATGACATTATCCACCACAGACTGAAAGTATCTCTACCTATGATTAAATCGTTGTTCTACAAGAT

GTCGGAGTTCTCTCCCTACGACGATCACTACGTAAAGAAGATAATAGCCTACTGCCTATTAAGGGACGAG

TCATTTGCGGAACTACATACTAAATTCTGTTTAAACGAGGACTATAAAAGTGTATTTATGAAAAATATAT

CATTCGATAAGATAGATTCCATCATCGAAAAATGTAGTCGTGACATAAGTCTCCTCAAAGAGATTCGAAT

CTCAGACACCGACTTGTATACGGTATTGAGAACAGAAGACATCCGGTATCACACATATCTCGAAGCCATA

CATTCAGACAAACGCATTTCATTTCCCATGTACGACGATCTCATAGAACAGTGTCATCTATCGATGGAGC

ATAAAAGTAAACTCGTCGACAAAGCACTCAATAAATTAGAGTCTACCATCGATAGTCAATCTAGACTATC

GTATTTGCCTCCGGAAATTATGCGCAATATCATAACCAAGCTAAGCGACTACCATCTAAACAGTATGTTG

TACGGAAAGAACCATTACAAATATTATCCATGATAGAAAGAAAATATTTAAAAAATAATCTATATGATTG

GAGAAGTAGGAAACAAACAGTAACAAGACGACGATTACTACATTATTAAATCATGAGGTCCGTATTATAC

TCGTATATATTGTTTCTCTCATGTATAATAATAAACGGAAGAGATATAGCACCACATGCACCATCCAATG

GAAAGTGTAAAGACAACGAATACAGAAGCCGTAATCTATGTTGTCTATCGTGTCCTCCGGGAACTTACGC

TTCCAGATTATGTGATAGCAAGACTAATACACAATGTACACCGTGTGGTTCGGATACCTTTACATCTCAC

AATAATCATTTACAGGCTTGTCTAAGTTGTAACGGAAGATGTGATAGTAATCAGGTAGAGACGCGATCGT

GTAACACGACTCACAATAGAATCTGTGAATGCTCTCCAGGATATTATTGTCTTCTCAAAGGAGCATCAGG

GTGTAGAACATGTATTTCTAAAACAAAGTGTGGAATAGGATACGGAGTATCCGGATACACGTCTACCGGA

GACGTCATCTGTTCTCCGTGTGGTCCCGGAACATATTCTCACACCGTCTCTTCCACAGATAAATGCGAAC

CCGTCGTAACCAGCAATACATTTAACTATATCGATGTGGAAATTAACCTGTATCCAGTCAACGACACATC

GTGTACTCGGACGACCACTACCGGTCTCAGCGAATCCATCTCAACGTCGGAACTAACTATTACCATGAAT

CATAAAGATTGTGATCCAGTCTTTCGTGCAGAATACTTCTCTGTCCTTAATAATGTAGCAACTTCAGGAT

TCTTTACAGGAGAAAATAGATATCAGAATACTTCAAAGATATGTACTCTGAATTTCGAGATTAAATGTAA

CAACAAAGATTCATCTTCCAAACAGTTAACGAAAACAAAGAATGATACTATCATGCCGCATTCAGAGACG

GTAACTCTAGTGGGCGACTGTCTATCTAGCGTCGACATCTACATACTATATAGTAATACCAATACTCAAG

ACTACGAAAATGATACAATCTCTTATCATATGGGTAATGTTCTCGATGTCAATAGCCATATGCCCGCTAG

TTGCGATATACATAAACTGATCACTAATTCCCAGAATCCCACCCACTTATAGTAAGTTTTTTTTACCTAT

AAATAATAAATACAATAATTAATTTCTCGTAAAAGTAGAAAATATATTCTAATTTATTATATGGTAAGAA

AGTAGAATCATCTAGAACAGTAATCAATCAATAGCAATCATGAAACAATATATTGTCCTGGCATGCATGT

GCCTAGTGGCAGCTGCTATGCCTACTAGTCTTCAACAATCTTCATCCTCGTGTACTGAAGAAGAAAACAA

ACATCATATGGGAATCGATGTTATTATCAAAGTCACAAAGCAAGACCAAACACCGACCAATGATAAGATT

TGTCAATCCGTAACGGAAGTTACAGAGACCGAAGATGATGAGGTATCCGAAGAAGTTGTAAAAGGAGATC

CCACCACTTATTACACTATCGTCGGTGCGGGTCTTAACATGAACTTTGGATTCACCAAATGCCCAAAGAT

TTCATCCATCTCCGAATCCTCTGATGGAAACACTGTGAATACTAGATTGTCCAGCGTGTCACCGGGACAA

GGTAAGGACTCTCCCGCGATCACGCGTGAAGAAGCTCTGGCTATGATCAAAGACTGTGAGATGTCTATCG

ACATCAGATGTAGCGAAGAAGAGAAAGACAGTGACATCAAGACCCATCCAGTACTTGGGTCTAACATCTC

ACATAAGAAAGTGAGTTACAAAGATATCATCGGTTCAACGATCGTTGATACAAAATGTGTCAAGAACCTA

GAGTTTAGCGTACGTATCGGAGACATGTGTGAGGAATCATCTGAACTTGAAGTCAAGGATGGATTCAAGT

ATGTCGACGGATCGGCATCTGAAGGTGCAACCGATGATACTTCACTCATCGATTCAACAAAACTCAAAGC

ATGTGTCTGAATCGATAACTCTATTCATCTGAAAATGGATGAGTTGGGTTAATCGAACGATTCAGACACC

GCACCACGAATTAAAAAAGACCGGGCACTATATTCCGGTTTGCAAAACAAAAATATTTAACTACATTCAC

AAAAAGTTACCTCTCGTTACTTCTTCTTTCTGTTTCAATATGTGATACGATATGATCACTATTCGTATTC

TCTTGGTCTCATAAAAAAGTTTTACAAAAAAAAAAAAAATATTTTTATTCTCTTTCTCTCTTCGATGGTC

TCACAAAAATATTAAACCTCTTTCTGATGTCTCAACTATTTCGTAAACGATAACGTCCAACAATATATTC

TCGTAGAGCTTATCAACATCCTTATACCAATCTAGGTTGTCAGACAATTGCATCATAAAATAATGTTTAT

AATTTACACGTTAACATCATATAATAAACGTATATAGTTAATATTTTTGGAATATAAATGATCTGTAAAA

TCCATGTAGGGGACACTGCTCACGTTTTTTCTCTAGTACATAATTTCACACAAGTTTTTATACAGACAAA

TTAATTCTCGTCCATATATTTTAAAACATTGACTTTTGTACTAAGAAAAATATCTTGACTAACCATCTCT

TTCTCTCTTCG
