# Supplementary material for: Exploring the key genomic variation in monkeypox virus during the 2022 outbreak
Source: BMC Genom Data. 2023 Nov 16;24:67. doi: 10.1186/s12863-023-01171-0 (PMC10652487; doi:10.1186/s12863-023-01171-0)
Supplement: Supplementary file 10 — Additional file 10. Full length sequence of RS7. [file 12863_2023_1171_MOESM10_ESM.docx]

**Additional file10. Full length sequence of RS7.**

GTTAGTAAATTATATACATAATTTTATAATTAATTTAATTTTACTATTTTATTTAGTGTCTAGAAAAAAATGTGTGACCCACGACCGTAGGAAACTCTAGAGGGTAAGAAAAATCAATCGTTTATAGAGACCATCAGAAAGAGGTTTAATATTTTTGTGAGACCTATCGAAGAGAGAAAGGATAAAAACTTTTTACGACTCCATCAGAAAGAGGTTTAATATTTTTGTGAGACCCATCGAAGAGAGAAAGAGATGGTTAGTCAAGATATTTTTCTTAGTACAAAAGTCAATGTTTTAAAATATATGGACGAGAATTAATTTGTCTGTATAAAAACTTGTGTGAAATTATGTACTAGAGAAAAAACGTGAGCAGTGTCCCCTACATGGATTTTACAGATCATTTATATTCCAAAAATATTAACTATATACGTTTATTATATGATGTTAACGTGTAAATTATAAACATTATTTTATGATGCAATTGTCTGACAACCTAGATTGGTATAAGGATGTTGATAAGCTCTACGAGAATATATTGTTGGACGTTATCGTTTACGAAATAGTTGAGACATCAGAAAGAGGTTTAATATTTTTGTGAGACCATCGAAGAGAGAAAGAGAATAAAAATATTTTTTTTTTTTTTTTTGTAAAACTTTTTTATGAGACCAAGAGAATACGAATAGTGATCATATCGTATCACATATTGAAACAGAAAGAAGAAGTAACGAGAGGTAACTTTTTGTGAATGTAGTTAAATATTTTTGTTTTGCAAACCGGAATATAGTGCCCGGTCTTTTTTAATTCGTGGTGCGGTGTCTGAATCGTTCGATTAACCCAACTCATCCATTTTCAGATGAATAGAGTTATCGATTCAGACACATGCTTTGAGTTTTGTTGAATCGATGAGTGAAGTATCATCGGTTGCACCTTCAGATGCCGATCCGTCGACATACTTGAATCCATCCTTGACTTCAAGTTCAGATGATTCCTCACACATGTCTCCGATACGTACGCTAAACTCTAGGTTCTTGACACATTTTGTATCAACGATCGTTGAACCGATGATATCTTTGTAACTCACTTTCTTATGTGAGATGTTAGACCCAAGTACTGGATGGGTCTTGATGTCACTGTCTTTCTCTTCTTCGCTACATCTGATGTCGATAGACATCTCACAGTCTTTGATCATAGCCAGAGCTTCTTCACGCGTGATCGCGGGAGAGTCCTTACCTTGTCCCGGTGACACGCTGGACAATCTAGTATTCACAGTGTTTCCATCAGAGGATTCGGAGATGGATGAAATCTTTGGGCATTTGGTGAATCCAAAGTTCATGTTAAGACCCGCACCGACGATAGTGTAATAAGTGGTGGGATCTCCTTTTACAACTTCTTCGGATACCTCATCATCTTCGGTCTCTGTAACTTCCGTTACGGATTGACAAATCTTATCATTGGTCGGTGTTTGGTCTTGCTTTGTGACTTTGATAATAACATCGATTCCCATATGATGTTTGTTTTCTTCTTCAGTACACGAGGATGAAGATTGTTGAAGACTAGTAGGCATAGCAGCTGCCACTAGGCACATGCATGCCAGGACAATATATTGTTTCATGATTGCTATTGATTGATTACTGTTCTAGATGATTCTACTTTCTTACCATATAATAAATTAGAATATATTTTCTACTTTTACGAGAAATTAATTATTGTATTTATTATTTATAGGTAAAAAAACTTACTATAAGTGGGTGGGATTCTGGGAATTAGTGATCAGTTTATGTATATCGCAACTAGCGGGCATATGGCTATTGACATCGAGAACATTACCCATATGATAAGAGATTGTATCATTTTCGTAGTCTTGAGTATTGGTATTACTATATAGTATGTAGATGTCGACGCTAGATAGACAGTCGCCCACTAGAGTTACCGTCTCTGAATGCGGCATGATAGTATCATTCTTTGTTTTCGTTAACTGTTTGGAAGATGAATCTTTGTTGTTACATTTAATCTCGAAATTCAGAGTACATATCTTTGAAGTATTCTGATATCTATTTTCTCCTGTAAAGAATCCTGAAGTTGCTACATTATTAAGGACAGAGAAGTATTCTGCACGAAAGACTGGATCACAATCTTTATGATTCATGGTAATAGTTAGTTCCGACGTTGAGATGGATTCGCTGAGACCGGTAGTGGTCGTCCGAGTACACGATGTGTCGTTGACTGGATACAGGTTAATTTCCACATCGATATAGTTAAATGTATTGCTGGTTACGACGGGTTCGCATTTATCTGTGGAAGAGACGGTGTGAGAATATGTTCCGGGACCACACGGAGAACAGATGACGTCTCCGGTAGACGTGTATCCGGATACTCCGTATCCTATTCCACACTTTGTTTTAGAAATACATGTTCTACACCCTGATGCTCCTTTGAGAAGACAATAATATCCTGGAGAGCATTCACAGATTCTATTGTGAGTCGTGTTACACGATCGCGTCTCTACCTGATTACTATCACATCTTCCGTTACAACTTAGACAAGCCTGTAAATGATTATTGTGAGATGTAAAGGTATCCGAACCACACGGTGTACATTGTGTATTAGTCTTGCTATCACATAATCTGGAAGCGTAAGTTCCCGGAGGACACGATAGACAACATAGATTACGGCTTCTGTATTCGTTGTCTTTACACTTTCCATTGGATGGTGCATGTGGTGCTATATCTCTTCCGTTTATTATTATACATGAGAGAAACAATATATACGAGTATAATACGGACCTCATGATTTAATAATGTAGTAATCGTCGTCTTGTTACTGTTTGTTTCCTACTTCTCCAATCATATAGATTATTTTTTAAATATTTTCTTTCTATCATGGATAATATTTGTAATGGTTCTTTCCGTACAACATACTGTTTAGATGGTAGTCGCTTAGCTTGGTTATGATATTGCGCATAATTTCCGGAGGCAAATACGATAGTCTAGATTGACTATCGATGGTAGACTCTAATTTATTGAGTGCTTTGTCGACGAGTTTACTTTTATGCTCCATCGATAGATGACACTGTTCTATGAGATCGTCGTACATGGGAAATGAAATGCGTTTGTCTGAATGTATGGCTTCGAGATATGTGTGATACCGGATGTCTTCTGTTCTCAATACCGTATACAAGTCGGTGTCTGAGATTCGAATCTCTTTGAGGAGACTTATGTCACGACTACATTTTTCGATGATGGAATCTATCTTATCGAATGATATATTTTTCATAAATACACTTTTATAGTCCTCGTTTAAACAGAATTTAGTATGTAGTTCCGCAAATGACTCGTCCCTTAATAGGCAGTAGGCTATTATCTTCTTTACGTAGTGATCGTCGTAGGGAGAGAACTCCGACATCTTGTAGAACAACGATTTAATCATAGGTAGAGATACTTTCAGTCTGTGGTGGATAATGTCATTAACGACATCCGCCTTGTATATAATGTTTCTGTTTTCAAACACCAAGTCGAATACTGTCTTATCGTCTTTAGTCGGAAGGTTGATGTCGTATCCGATGTATACGAGGTATGAGGCAACATTGTTATTGCAATTCTGGAAGGCGGTATGAAGAGGAGTCATTGTATTATAGTATTCGTCTTTCTGAATGTCGAATCTATCTAGTAGATACCGTAGTATATTGAGAGAGCGACTTCCATATCCTTGATTATGTTTTATGAATAGATAAAGTAGATGTTGTCCTTCTTCCTTTTGTAATTTCCCGTATTTTTGTTCGTGCCAATTGAGTAACATTATGAGAATATGACCTGTTGCACAATCGTTCTTTATGTATTCCATGATGGGTGTACAATCAAGATTATTACGTATCCTCGTATCGGCTCCTCGAGATAAAAGAGCATACACCACACGAGGACTATGTTTGGTATACTGTTGAAGGTAAGTGTGTAACGGCGTATTTCCGATTTTCGTAACCGCGTTAATGTTTGCTCCATGATCTATTATCGCGTAGATGAATCGCTTCTCAGCTCGCATCTTAGTGTGACTCTTTGACTTGTAATAATTGCTTTCGTGGAACGCGGATATGTGTTTACAGTAGTAATGAAGAGAAGTGAGTCCATCCTCATCGACGCAATTAGGGTCAGATCCTTTAGTCAATAATTTGTACAGAACGTAATAGTTTAAGCTCCCATTGAATTTATATCTAAGATAACACAGCAATAGATCGGATGATTTACTAAAGTCATCAATGGGGTCCGTTAGTATATCAAAGATCTTGTTATCGATTGATAGTGAATGAATCAGATAGTGGTGTAGAGGAATATGTCCTTTTTTATCCTTGCTATCAAAGTTACGCATGCCGTGGCGTAACAATATCTTTAATACAGATGGATTAAATCGTGTATTCATCGTATAGCAATGTAATGGAGAGTTACCACATTTTAGTCGTTTATTCAGATCGCAGTGTTTAATAACTAATTTAAACAGATGAGATGATATATCCACATCAAAGAATGCGAGATACATATGACAGACATTATTGACAGAAATGTGACCTTCATTATCACCGTCGTCCATAAATGCGTTAGGTACGTACCACATACTATCGTTAACGATGCGCACAATCTCGTCCATTTCATCCATCTTCATAATGATTTACTTTTTCATAATTAGAGAAAAAGATCAAGGTATAAAAATTAGAAGTGTTAGACTATAAACTAACTTATGACTTAACTAACTTATGACTTAACTAACTTATGACTTAACTAACTTATGACTTAACTAACTTATGACTTAACTAACTTATGACTTAACTAACTTATGACTTAACTAACTTATGACTATTAACTCATTTCAAGAAAGGTGGGTGGATAGAACTCTATATGACAGCTTGTGAAACAATTAGATCCCTAATTTCTAATGGAAGTTTTGATAGGAGATTGTCATCAGTTGATACATTGTTTATTATCTCATCTATTAGAGCACGTCTGTTTAGAGCTTTAGTGACCTGCTCGGTTACTTCTGTGTAAATCTTGAATCCTTTAGTGATACACTGTGTCAAAACTGGATGTTTAGAATACCTATGTAGAATATGGGAAGCATGCTTGTTTTTGTCTCTATTATAGATTAACTCATACATGGTTGTATTATGAATTTTCATCTGCCTAATGTACTCCAATTCTTGTTTACAATCAATTATATAATCAAAGAGTGATGATGCATACACATTACAAAGTGAATAATCTACCATCATAAAATACTTGATACAGAGCTTTATCACATCATGGTTTTCAATTGTATTATTAAGTATAGCTAATTTTATACAGTCAATAGACAATGGTTCTCTAAGCAATATTTCTAATATTTTAAGATGTGCTTCCCTACGGGCGATGACAGATCCCCTATCCACGGCCACGTCAAGACATGTATATCCATTACTCATTACTGCGTTGACATTTGCTCCATTTTCTAATAGCCATGATACTAAATCTATATAACCTGCATAGATAGCGCGATAAAGCAAGGTCCTTCCACCAGCATCTAGTTGATTGATATCTTCAATATATGGGATACAAAGCTTATAAATTTCTAATACTGTGGGTTCATCTACAAGGAATCCCCTAGTATACTGAATTATTTTATATAGATCTAATTTAACATCATTTTCATCTGGGATACCACAATTCAAAATAAACTCAACAACACTACTTTCCTTTTTACATATTCCCCTAAAATAGGCATTCAAGCATTCTATTTTATATATTACAGCCCCATGATCTACCATAAGATCAACAATGTCTATTTCTACATATGCATTAGATAGATAGTAAAGTAAGAGATCTTGCACAGAATTACAATTCTTAATAATTATAGAGAAAATATCTTCCATATAATTCTTTGACACTAATGCAGATATAATATCTTTATATGTAATATATGCAAACAGTCTATCTACTATATACTGATCAATATTATCTCTATGAATCCTAAAATAATCATACAGAACATCTACAGGATCACAAATTGGTTCAAGGAGAAATCTATCAAATATTTTCCTGTCAACAACTGGTTCTAGAACATAACAGTCAACACCTAATCCATGTTTTTTATAATCATCTACCAAAGATAATGACCAAAGATCGAGGTCGTCGTGAAACTGCTCATCGACAGCCATGAAATCTACCGACTCCATGGTGCGAATCGCACTGTCTTATTCGCCATTGATTTTCATTTTTTATAATTATGTACATGTTTTCCTTCTATTCTCAAGAGTCTACAAAAATATATTTTTTCGATATCTAAGTACTAAGTTTTTTTACTGTTTTTGTTACTGTCTTCCATTCTTCTAACTAAAGATCTGAGATAAATTATACAATCTTCGCTATCGAACCATTTTTGTAGTCTAAAGCCTGAAGTAATTAACCAACTGTTTTTATTAGTGGCTTTTTTCGATCTATCCTCGTCCTCTCATCATCCTTATATTATTATCATTATTATCATAGTCTATTAAACACAAATCATCTACGTTTATAACAACATTCTCATTATTAATTAGTTCTGTAGAATATCTTTAATAATTTGGCTATACATCTGTTCAATACTATCTATTGATGATTTCTTTTTTAAGACTTAAACTAGTTATGGTAATGACGATGAAATCGAGTAGTAACTTCTAATAAAGACTTGATATCATTATCATATGTTTGATCGTCATAGTTAATAGTGTGGCTAAATGGTACTGTTAATAAGTTTATAGACAATATCATAGTATTTTCTTTCCAGAATTAGATTATTTTTTTAAATACTGATCCTCACAATTCCGTGATGTAGCAGTAGTTGGTGCATGGTCTATATCGTTAAAATGTATCATATATAATAGTTTTCTGACGTGGAGTACAGAATTTTCGATTAATGAGTTCATGGTAAGGAAGGGCAAATGCCTGTATATAATATACATAAGTTAATAGTTTTTTATCATATTTTCTAATACCATAATAAAAATTATCATTATTGCGTTTGGTAGTTCTGCCCTATCATCTATATCACTGTCACTCTCGCTCTCACTATATCTTCTAAAATTACAAACAACTGGATATTCGATAACAGCATTTGTGTAGTTTTTGTCTTTTACAGTATATACGTTATTGTCAAAATCTAAACAAATATTAGCATAATACATCTATCTATAAGATCAGGATCCATGTTCGAGCATACTAGCCATGTATATTTGTAACTTCGTCGTACAGCGTTAGATCAATAGAATAAACAATCGTGTGACGCAACTTTTTTACGATCTAGTTGTATGAGTTTATCGTTTACATAAGCAATTAACGGCTTTAACAGATGATCTGAGTAATAATATACCTCTGTTATACGTTTAATGTTCACGGTCTTAGTATTTTTAGATATCAATTGTGATTTACACCATATTCGACTCCCTTGTGTGTAACGTTAGAAATTCTAAATCTATATTATCTATTACAGCGTAAAACACATTCAATATTGTATTGTTATTTTTATATTATTTACACAATTAACAATGTATTATTAGTTTATATTACTGAATTAATAATATAAAATTCCCAATCTTGTCATAAACACAAAATCCATTAAAAATGTCGATAAAATATCTGATGTTGTTGTTCGCTGCTATGATAATCAGATCATTAGCCGATAGTGGTAATGCTATCGAAACAACATCGCCAGAAATTACAAACGCTACAACAGATATTCCAGCTATCAGATTATGTGGTCCAGAGGGAGATGGATATTGTTTACACGGTGACTGTATCCACGCTAGAGATATCGACGGTATGTATTGTAGATGCTCTCATGGTTATACAGGTATTAGATGTCAGCATGTAGTATTAGTAGACTATCAACGTTCAGGAAAACCAGACACTACAACATCATATATCCCATCTCTAGGTATTGTGCTTGTATTAGTAGGCATTATTATTACGTGTTGTCTATTATCTGTTTATATGTTCACTCGAAGAACTAAACTACCTATACAAGATATGGTTGTGTTATATTTTTTATAAAATTTTTTTATGAGTATTTTTACAAAAATGTATATGTATAAAAAAAATACTAAGTATACGATGTATCCTGTATTATTTGTATTTATCTAAACAATACTTCTGCCTCTAGATGGGATACAAAAATTTTTTATTTCAGCATATTAAAGTAAAATCTAGTTACCTTGAAAATGAATACAGTGGGTGGTTCCGTATCACCAGTAAGAACATAATAGTCGAATACAGTATCCGATTGAGATTTTGCATACAATACTAGTCTAGAAAGAAATTTGTAATCATCCTCTGTGACAGGAGTCCATATATCTGTATCATCGTCTAGTTTTTATCAGTGTCCTATGCTATATTCCTGTTATCATCATTAGTTAATGAAAATAACTCTCGTGCTTCAGAAAAGTCAAATATTGTATCCATACATATATCTCCAAAACTATCACTTATACGTTTATCTTTAACGAACATATACCTAGATGGTTATTTACTAACAGACATTTTTTCAAGATCTATTGACAATAACTCCTATAGTTTCCACATCAACCAAGTAATGATCATCTATTGTTATATAACAATAACATAACTCTTTTCCATTTTTATCAGTATCTATATCAACGTCGTTGTAGTGAATAGTAGTCATTGATCTATTATATGAAACGGATATGTCTAGTTAATATTTTCTTTGATTTAAAGTCTATAGTCTTTACAAACATAATATCCTTATCCGACTTTATATTTCCTGTAGGGTGGCATAATTTTATTCTGCCTCCACAATCAGTGTTTCCAAATATATTACTAGACAATATTCCATATAGTTATTAGTTAAGGGACCCAATTAGAACACGTACGCGCTTATTCATCATTTGGATCGTATTTCATAAAAGTTATTATGTTATAGATGTCAACACATTCTACATTTTTTTAATTGTCTATATAGTATTTTTCTGATATTTTCTATAATATCAGAATTGTCTTCCATAGGAAGTTGTATACTATTCGGAATCAGTTACATGTTTAAATAATTCTCTGATGTCATTCCTTATACAATCAAATTCATTATTAAACAGTTTAATAGTCTGTAGACCTTTATCGTCGTACATATCCATTGTCTTATTAGTTACGCTTATTTTTATGGGTTTTACGTTGCTTTATTATATTTTATAATAATGATTGTTTGACAATGTCGTAGTATAGATATATTATTAGAGGAGGTATAATTATAAAAAGTTTCTGAGTACGATGTTATAAGAGGAGAGGACACATTAACATCATACATCAATTAACTACATTCTTATAACATTGTAATCAAAAGAATTGCAATTTTGATGTATAACAACTGTCAATGGAATTGTATATTACAAATTACGGTATGTTGTAACAACAAATACCGATCGGTAATTGTCTCTGCCGCTGTAATAGAATTAATTATATATCTATTACACCGGCCTTGTATCATAATAAAGTTGTGGTAGTATGATCTCCATATTTATAATTTAGTACTTTGTATTTAGTTTTTTTGGAATCATAAAAAAAGTTTTACTAATTTAAAATTTAAAAAGTATTTACATTTTTCACTGTTTAGTCGCGGATATGGAATTCGATCCTGTCAAAATCAATACATCATCTATAGATCATGTAACAATATTACAATACATAGATGAACCAAATGATATAAGACTAACAGTATGCATTATCCGAAATGTTAATAACATTACATATTATATCAATATCACAAAAATAAATCCACATTTGGCTAATCGATTTCGGGCTTGGAAAAAACGTATCGCCGGAAGGGACTATATGACTAACTTATCTAGAGATACAGGAATACAACAATCAAAACTTACTGAAACTATACGTAACTGTCAAAAAAATAAAAACATATATGGTCTATATATACACTACAATTTAGTTATTAATGTGGTTATTGATTGGATAACCGATGTGATTGTTCAATCAATATTAAGAGGGTTGGTAAATTGGTACATAGCTAATAATACATATACTCCAAATACACCCAATAATACTACAACCATTTCTGAGTTGGATATCATCAAAATACTGGATAAATACGAGGACATGTATAGAGTAAGTAAAGAAAAAGAATGTGGAATTTGCTATGAAGTTGTTTACTCAAAACGATTAGAAAACGATAGATACTTTGGTTTATTGGATTCGTGTAATCATATATTTTGCATAACATGTATCAATATATGGCATAGAACACGAAGAGAAACCGGTGCGTCAGATAATTGTCCTATATGCCGTACACGTTTTAAAAAAATAACAATGAGCAAGTTCTATAAGCTAGTTAACTAATAAATAAAAAGTTTAATTATCGACGATATATGTCGTTATTTTTCTCTCATATGAAAGATTAATTTGATTCTAATATAATCTTCAGTATTGGATGAATCTCAATTCAAATTAATTCCATTAGATTAGATTAGATTAGATCATAAATAAAAATAGTAGCACGCACTACTTCAGCCAAATATTCTTTTTTGAAACGCCATCTAGCGTAATGAGAACACAAGTGAACCTATAATGAGCAAATTTATTAGTATCGGTTACATGAAGGACTTTACGTAGAGTGGTGATTCCTCCATCTGTGGTACGAACGGTTTCATCTTCTTTGATGCCACCACCCAGATGTTCTATAAACTTGGTATCCTCGTCCGATTTCATATCATTTGCCAACCAATACATATAGCTAAACCCAGGCATACGTTCCACACATCCGGAACAATGAAATTCTCCAGAAGATGTTACAATGGCTAGATTTGGACATTTGGTTTCAACCGCGTTAACATATGAGTGAACACACCCATACATGAAAGCGATGAGAAATAGGATTCTCATCTTGCCAAAATATCACTATAAAAAATTTATTTATCAATTTTAAAGGTATAAAAAAATACTTATTGTTGCTCGAATATTTTGTATTTGATGGTATACGGAAGATTAGAAATGTAGGTATTATCATCAACTGATTTTATGATGGTTTTATGAATTTTATTATGCTTCACTATTGCATCGGAAATAATATCATATGCTTCCACGTATATTTTATTTTGTTTTGACTCATAATACGCACGTAATTCTGGATTATTGGCATATCGATGAATAATTTTAGCTCCATGCTCAGTAAATATTAATGAGAACATAGTGTTGCCTCCTACCATTATTTTTTTCATCTCATTCAATTCTTGATTGCAGAGATCTATATAATCATTATAGCGTTGACTTATGGACTCTGGAATCTTAGACGATGTACAGTCATCTATAATCATGGTATATTTAATACATTGTTTTATAGCATAGGCATTATCTACGATATTAGATACTTCACTCAATGAATCAATCACACAATCTAATGTAGGTTTATGACATAATAGCATTTTCAGCAGTTCAATGTTTCTAGATTCGTTGATGGCAATGGTTATACATGTATATCCGTTATTTGATCTAATGTTGACATCTGAACCGGATTCTAGCAGTAAAGATACTAGAGATTGTTTATTATATCTAACAGCCTTGTGAAGAAGTGTTTCTCCTCGTTTGTCAATCATGTTAATGTCTTTAAGATAAGGTAGACAAATGTTTATAGTACTAAGAATTGGGCAAACATAAGACATGTCACAAAGACCCTTTTTGTATGTATAAGTGTAGAAATTATAACATCCATAGTTGGATTCACATAGGTGTCCAATCGGGATCTCTCCATCATCGAGATGATTGACGGCATCTCCCCCTTCCTTTTTTAGTAGATATTTCATCGTGTAAGAATCAATATTAATATTTCTAAAGTATCTGTGTATAGCCTCTTTATTTACCACAGCTCCATATTCCAACATGCATTCCACTAGAGGGATATCGATATCGCCGAATGTCATATACTCAATTAGTATATGTTGGAGGACATCCGAGTTCATTGTTTTCAATATCAAAGAGATGGTTTCCTTATCATTTCTCCATAGTGGTACAATACTACACATTATTCCGTACGGCTTTCCATTCTCCAAAAACAATTTTACCAAATCTAAATCTACATCTTTATTGTATCTATAATCACTATTTAGATAATCAGCCATAATTCCTCGAGTGCAACATGTTAGATCGTCTATATATGAATAAGCCGTGTTATCTATTCCTTTCATTAACAATTTAACGATGTCTATATCTATATGAGATGACTTAATATAATATTGAAGAGCTGTACAATAGTTTTTATCTATAGAAGACGGCTTGATTCCGTGATTAATTAGACATTTAACAACTTCCGGACGCACATATGCTCTCGTATCCGACTCTGAATACAGATGAGCGATGATATACAGATGCAATACGGTACCACAATTTCGTGGTTGATAATCATCATACACGTATCCGTACTCGTCATCCTCATAAAGAACACTGCATCCATTTTCTATGAACAAATCAATAATTTCAGGAACAGGATCATCTGTCATTACATAATTTTCTATAACTGAACGATGGTTTTCACATTTAACACTCAAGTCAAATCCATGTTCTACCAACACCTTTATCAAGTCAACGTCTACATTTTTTGATTTCATATAGCTGAATATATTAAAGTCATTTATGTTGCTATATCCAGTAGCTTCTAGTAGAGCCATCGCTATATCCTTATTGACTTTAACATGTCTACTATTTGTGTATTCTTCTATTGGGGTAAACTGTCTCCAATTTTTATGTAATGGATTAGTGCCACTATCTAGTAGTAGTTTGACGACCTCAACATTATTACAATGCTCATTGAAAAGGTATGCGTGTAAAGCATTATTCTTGAATTGGTTCCTGGTATCATTAGGATCTCTGTCTCTCAACATCTGTTTAAGTTCATCGAGAACCACCTCCTCATTTTCCAGATAGTCAAACATTTTGACTGAATAGAAGTGAATGAGCTACTGTGAACTCTATACACCCGCACAACTAATGTCATTAAATATCATTTTTGAATGTATTTATACCATGTCAAAAACTTGTACAATTATTAATAAAAATAATTAGTGTTTAAATTTTACCAGTTCCAGATTTTACACCTCCGTTAACACCTCCATTAACCCCACTTTTTACACCACTGGACGATCCTCCTCCCCACATTCCACTGCCACTAGATGTATAAGTTTTAGATCCTTTATTACTACCATCATGTCCATGGATAAAGACACTCCACATGCCGCCACTACTACCCCCTTTAGACGACATATTAATAAGACAAGTTTAACAATAAAATTAATCACGAGTACCCTACTACCAACCACTATTATATGATTATAGTACCTTGACTAAAGTCTCTAGTCACAAGATCAATACTACCAACCTACGCTATTATATGATTATAGTTTCTATTTTTATAGGAACGCGTACGAGAAAATCAAATGTCTAAGTTCTAACGGTAGTGTTGATAAACGATTGTTATCCGCGGATACCTCATCTATCATGTTGTCTATTTTCTTACTTTGTTCTATTAACCTATTAGCATTATATATTATTTGATTATAAAACTTATATTGCTTATTAGCCCAATCTGTAAATATCGGATTATTAACATATCGTTTCTTTGTAGGTTTATTTAACTTGTACATCACTGTAAGCATGTCCGTACCATTTATTTTAATTTGACACATATCAGCAATTTCTTTTTCGCAGTCGGTTATATATTCTATATAAGATGGATACGTATCACATATGTACTTATAGTCTACTAATATGAAGTACTTAATACATATTTTCAGTAACGATTTAGCCTTATTACCTATTAATAAGTGCCTGTCGTTGGATAGGTAATCAACTGTTTTCTTAATACATTCGATGGTTGGTAATTTACTCAAAATAATTTCCAATATCTTAATATATATTTCTGCTATTTCTGGTATACATGCATGTGCCATTATAACACAAATACCAATACATGTAGACCCATATGTTGTTGTTATATTAATATCTGCGCCATTATCTATTAACCATTCTATTAGGGCAACACTATGCGACTCGATACAATAATAAAGTATACTACGTCCATGTTTATCTATTTTGTTTATATCATCGATATACGGCTTACAAATTTCTAGTATCGATAACACTTCTGACTCGTGAATAAATAAGGTAGGGAATAACGGCATAATATTTATTATGTTATCATCATTAACAACTACGTTTCCATTTTTTAAAATATACTCTACAACTTTAGGATCCCTATTGTCAAATCTTTTAAAATATTTATTTATATGCTTAAATCTATATAATATAGCTCCTTCCCTAATCATACATTTGATAACATTGATGTACACTGTATGATAAGATACATATTCTGACAATAGATCTTGTATAGAATCTGTATATCTTTTAAGAATTGAGGATATTATGACATTATTACGTAAACTATTACACAATTCTAAAATATAAAATGTATCATGGGCAGATAATAGTTTATCCACTATATAATTATCTATTTTATGATTTTTCTTCCTATATTGTTTACGTAAATAGATAGATAGAATATGCATTAGTTCATTACCGCTATAGTTACTATCGAATAACACGTCAAATATTTCCCGTTTAATATCGCATTTGTCAACATAATAATAGAGTATGGTACGTTCACGATAAGTATAATGACACATCTCGTTTTCGTGCGAAATTAAATAGTTTATCACGTCCAAAGATGTCACATAACCATCTTGTGACCTAGTAATAATATAATAATAGAGAACTGTTTTACCCATTCTATTATCATAATCAGTGGTGTAGTCATAATCTAAATAATCAAACTCGTCATCCCAATTAAAATAAATATAATCAGTACATTGAATGGGTATGATATTGTACCCATACTGTATGTTGCTACATGTAGGTATTCCTTTATCCAATAATAGTTTAAATACATCTATATTAGGATTTGATGTTGTCGCGTATTTCTCTACAATATTAATACCATTTTTGATACTATTTATTTCTATACCTTTCGAAATTAGTAATTTCAATAAGTCTATATCGATGTTATCAGAACATAGATATTCAAATATATCAAAATCATTGATATTTTTATAGTCGACTGACGACAATAACAAAATCACAACATCGTTTTTGATATTATTATTTTTTTTGGTAACGTATGCCTTTAATGGAGTTTCACCATCATACTCATATAATGGATTTGCACCACTTTCTATTAATGATTGTGCACTACTGGCATCGATGTTAAATGTTTTACAACTATCATAGAGTATCTTATCGTTAACCATGATTGGTTGTTGATGTTATCACATTTTTTGGTTTCTTTCATTTCAGTTATGTATGGATTTAGCACGTTTGGGAAGCATGAGCTCATATGATTTCAGTACTGTAGTGTCAGTACTATTAGTTTCGATCAGATCAATGTCTAGATCTATAGAATCAAAACACGATAGGTCAGAAGATAATGAATATCTGTACGCTTCTTCTTGTACTGTAACTTCTGGTTTTGTTAGATGGTTGCATCGTGCTTTAACGTCAATGGTACAAATTTTATCCTCGCTTTGTGTATATCATATTCGTCTCTAGTATAAAATTCTATATTCAAATTATCATGCGATGTGTGTACGCTAACGGTATCAATAAACGGAGCACAGCATTTAGTCAACAGTAATCCAAAATTTTTTAAAGTATATCTTAACGAAAGAAGTTGTCATCGTTAGAGTGTGGTAAATCATTGTCTACGGTACTAGATCCTCATAAGTGTATATATCTAGAGTAATATTTAATTTATCAAATGGTTGATAATATGGATGTTGTGGCAATTTCCTAATACGGAAATAAGACATAAACACGCAATAAATCTAATTGCGGACATGTTACACTCCTTAAAAAATACGAATAAACACTTTGGCTTTTAGTAAGTGTCATTTAACACTATACTCATATTAATCCATGGACTCATAATCTCTATACGGGATTAACGGATGTTCTATATACGGGGATGAGTAGTTCTCTTCTTTAACTTTATACTTTTTACTAATCATATTTAGACTGATGTATGGGTAATAGTGTTTGAAGAGCTCGTTCTCATCATCAGAATAAATCAATATCTCTGTTTTTTTGTTATACAGATGTATTACAGCCTCATATATTACGTAATAGAACGTGTAATCTACCTTATTAACTTTCACCGCATAGTTGTTTGCAAATACGGTTAATCCTTTGACCTCGTCGATTTCCGACCAATCTGGGCGTATAATGAATCTTAACTTTAATTTCTTGTAATCATTCGAAATAATTTTTAGTTTGCATCCGTAGTTATCTCCTCTATGTAACTGTAAATTTCTCAACGCGATATCTCCATTAATAATGATGTCGAATTCGTGTTGTATACCCATACTGAATTGATGAACTAATACCGACGGTATTAATAGTAATTTACTTTTCATCTTTACATACTTGGTAATAGTTTTACTATCATAAGTTTATAAATTCCACAAGCTACTATGGAATATACCAACCATCTTAGTATAGAACACATGTCTTAAAGTTATTAATTAATTACATGTTGTTTTATATATCGCTACGAATTTAAACAGAGGAATCAGTTAGGAAGAAAAAATTATCTGTCATCATCATCATCTATTGGATAACATCTCTGTATTCTACGATAGAGTGCTATTTTAAGATGTGACAGATCCGTGTCATCAAATATATACTCCATTAAAATGATTATTCCGGCAGCGAACTTGATATTGGATACATCACGACCTTTGTTAATATCCACGACAATAGACAGCAATCCCATTGTTCCATAAACAGTGAGTTTATCTTTCTTTGAAGTGATATTTTGTAGAGATCTTATAAAACTGTCGAATGACATCGTATTTATATCTTTAGCTAAATCATATATGTTACCATCATAATATCTAACAGCATCTATCTTAAACGTTTCCATCGCTGTAAAGACGTTTCCGATAGATGGTCTCGTTTCATCAGTCATACTGAGCCAACAAATGTAATCGTGTATAACATCTTTGATAGAATCAGACTCTAAAGAAAAGGAATCGGCTTTATTATACACATTCATGATAAACTTAATGAAAAATGTTTTTCGTTGTTTAAGTTGGATGAATAGTATGTCTTAATAATTGTTATTATTTCACTAATTAATATTTAGTAACGAGTACACTCTATAAAAACGAGAATGACATAACTAATCATAACTAGTTATCAAAGAATGTCTAGGACGCGTAATTTTTTATGGTATAGATCCTGTAAGCGTTGTCTGTATTCTGGAGCTATTTTCTCTATCGCATTAGTGAGTTCAGAATATGTTATAAATTTAAATCGAATAACGAACATAACTTTAGTAAAGTCGTCTATATTAACTCTTTTATTTTCTAGCCATCGTAATACCATGTTTAAGATAGTATATTCTCTAGTTACTACGATCTCATCGTTGTCTAGAATATCGCATACTGAATCTACATCCAATTTTAGAAATTGGTCTGTGCTACATATCTCTTCTATATTATTGTTGATGTATTGTCGTAGAAAACTATTACGTAGACCATTTTCTTTATAAAACGAATATATAGTACTCCAATTATCTTTACCGATATATTTGCATACATAATCCATTCTCTCAATCACTACATCTTTAAGAGTTTGGTTGTTAAGATATTTGGCTAAACTATATAATTCTATTAGATCATCAACAGAATCAGTATATATTTTTCTAGATCCAAAGATGAACTCTTTGGCATCCTCTATAATATTATCAGAAAAGATATTTTCGTGTTTTAGTTTATCAAGATTTAACCTGTTCATATCCATGATTAACGACGTCATATAACCACATAAAATAAAAATCCATTTTCATTTTTAGCACAATACTATTCATAATTGATATTGATGTAATATTTTGTTACTTTGAACGTAAAGACAGTACACGGGTCCGTATCTCCAACAAGCACGTAGTAATCAAATTTGGTGTTGTTAAACTTCGCAATATTCATCAATTTAGATAGAAACATATACTCATCATCTGTTTTAGGAATCCATGTATTATTACCACTTTCCAACTTATCATTATCCCAGGCTATGTTTCGCCCATCATCGTTGCACAGAGTGAATAATTCTTTTGTATTCGGTAGTTCAAATATATGATCCATGCATATATCGACAAAGCTATTGTAGATGTGATTTTTCCTAAATCTAATATAAAACTCGTTTACTAGCAAACATTTTCCTGATTTATCGACCAAGACACACATGGTTTCTAAATCTATCAAGTGGTGGGGATCCATAGTTATAACGCAGTAACATAAATTATTACCTTCTTGACTGTCGCTAATATCTATATACTTATTGTTATCGTATTGGATTCTACATATAGATGGCTTGTATATCAAAGATATAGAACACATAACCAATTTATATTCTCGCTTTGTATTTTCGAATCTAAAGTTAAGAGATTTAGAAAACATTATATCATCGGATGATGATATCACTGTTTCCAGAGTAGGATATATTAAAGTCTTTAAAGATTTTGTCCGATTCAAATAAATCACTAAATAATATCCCATATTATCATCTGTTATAGTCGTGTCATTAAATCTATTATATTTTATGAAAGATATATCACTGCTCACCTCTATATTTCGTACATTTTTAAACTGTTTGTATAATATCTCTCTAATACAATCAGATATATCTATTGTGTCGGTAGACGATACCGTTACATTTGAATTAATGGTGTTCCATTTTACAACTTTTAACAAGTTGACCAATTCATTTCTAATAGTATTAAACTCTCCATGATTAAATATTTTAATAGTATCCATTTTATATCACTACGGACATAAACCATTGTATATTTTTTATGTTTATTAGTGTACACATTTTGGAAGTAAGTTCCTGGATCGGATGTCACCGCAGTAATATTGTTGATTATTTCTGACATCGACGTATTATATAGTTTTTTAATTCCATATCTTTTAGAAAAGTTAAACATCCTTATACAATTTGTGGAATTAATATTATGAATCATGGTTTTTACACATAGATCTATTACAGGCGGAACATCAATTATTATGGCAGCAACTAGTATCATTTCTACATTGTTTATGGTGATGTTTATCTTCTTCCAGCGCATATAGTCTAATATCGATTCAAACGCGTGATAGTTTATACCATTCAATATAATCGCTTCATCCTTTAGATGGTGATCCTGAATGTGTTTAAAAAATTATACGGAGACGCCGTAATAATTTCCCCATTGATAGAAAATATCACGCTTTCCATTTTCTTGAAGTACTAAAAGTAATTATAATATAATGTAAAGGTTTATATATTCAATATTTTTTATAAAAAAAATCATTTTGACATTAATTCCTTTTTAAATTTCAGTCTATCATCTATAGAAACATATTCTATGAATTTATAAAATGCTTTTACGTATCCTATCGTAGGTGATAGAACCGCTAAAAAACCTATCGAATTTCTACAAAAGAATCTGTTATATGGTATAGGGAGAGTATAAAACATTAAATGTCCGTACTTATTAAAGTATTCAGTAGCCAATCCTAACTCTTTCGAATAATTATTAATGGCTCTTATTCTGTACGAATCTATTTTTTTGAACAATGGACCTAGTGGTATATCTTGTTCTATGTATCTAAAATAATGTCTGACTAGATCCGTTAGTTTAATATCCGCAGTCATCTTGTCTAGAATGGCAAATCTAACTGCGGGTTTAGGCGTAGGCGTTAGTTTAGTTTCTATATCTACATCTATGTCTTTATCTAACACCAAAAATATAATAGCTAATATTTTATTACAATCATCCGGATATTCTTCTACGATCTCACTAACTAATGTTTCTTTGGTTATACTAGTATAGTCACGATCAGACAAATAAAGAAAATCAGATGATCGATGAATAATACATTTAAATTCATCATCTGTAAGATTTTTGAGATGTCTCATTAAAATATTATTAGTGTCAGTTCTCATTATCATATATTGACAGCAGCTATTACACTTATTTTATTTTTCTGTATTTTATTACTTTTCACCATATAGATCAGTCATTAGATCATCAAAATACTTTTCAATCATCCTAAAGAGTATGGTGAACGAATCTTCCCATCTAATTTCTGAACGTCTACCAATGTCTCTAGCCACTTTGGCACTAATAGCGATCATTCGCTTAACATCTTCTACATTATTAACTGGTTGATTCAATCTATCTAGCAATGGACCGTCGGATAGCGTCATTCTCATGTTCTTAATCAATGTACATACATCGTCATCATCTACCAATTCATCAAACAATATAAGCTTTTTAAAATCATCATTATAATAGGATGGATCGCCGTCATTTCTCCAAAGAATATATCTAATAAGTAGAGTCCTCATGCTTAGTAATTTAACTATTTTAGTTAACAACTATTTTTTATGTTAAATCAATTAGTAACACCGCTATGTTTAATACTTATTCATATTTTAGTTTTAGGATCGAGAATCAATACAAAAATTAATACATCAATTTTGGAAATACTTAGTTTCCACGTAGTCAATGAAACATTTGAGCTCATCGTAAAGGACGTTCTCGTACAAGACGTAACTATAAATTGGTTTATATTTGTTCAAGATAGATACAAATCCGATAACTTTTTTGACGAATTCTACGGGATTCACTTTAAAAGTGTCATACCGGGTTCTTTTTATTCTTTTAAACAGATCGATTGTGTGATGTTGATTAGGTCTTTTACGAATTTGATACAGAATAGCGTTTACATATCCACCATAGTAATCAATAGCCATTTGTTCGTATGTCATAAATTCTTTAATTATATGACACTGTGTATTATTTAGTTCGTCCTTGTTCATCATTAGGAATCTATCCAATATGGCAATTATATTAGAACTATAACTGCGTTGTATGCGCATGTTGATGTGTCTGTTTATACAATCAATTATACTAGGATCCATACCACTACAATCGGGTAAAATTGTAGCATCATATACCATTTCTAGTACTTTAGATTCATTGTTATCCATTGCAGAGGACGTCATGATCGAATCCAAAAAAATATATTATTTTTATGTTATTTTGTTAAAAATAATCATCAAATACTTCGTAAGATACTCCTTCATGAACATAATCAGTTACAAAACGTTTATATGAAGTAAAGTATCTACAATTTTTACAAAAGTCAGGATGCATAAGTACAAAGTACGCGATAAACGGAATAATAATAGATTTATCTAGTTTATCTTTTTCTATCTCTTTCATAGTTATATACATGGTCTCAGAAGTCGGATTATGTAACATCAGCTTCGATAAAATGACTGGGTTATTTAGTCTTACACATTCGCTCATACATGTATGACCGTTAACTATAGAGTCTACACTAAAATGATTGAATAATAGATAGTCTACCATTGTTTCGTATTCAGATAGTACAGCGTAGTACATGGCATCTTCACAAATTATATCATTATCTAATAGATATTTGACGCATCTTATGGATCCCACTTCAACAGCCATCTTAAAATCGGTAGAATCATATTGCTTTCCTTTATCGTTAATAATTTCTAGAACATCATCTCTATCATAAAAGATACAAATATTAACTGTTTGATCAGTAATAACATTGCTAGTCGATATCAATTTGTTAATAAGATGCGCTGGGCTCAATGTCTTAATAAGAAGTGTAAGAGGACTATCTCCGAATTTGTTTTGTTTATTAACATCCGTTGATGGAAGTAAAAGATTTATAATGTCTACATACTTGACTGTTTTAGAGCATACAATATGGAGAGGCGTATTTCCATCATGATCTGGTTTTGAGGGACTAATTCCTAGTTTCATCATCCATGAGATTGTAGAAGCTTTTGGATTGTCTGACATAAGATGTCTATGAATATGATTTTTGCCAAATTTATCCACTATCCTGGCTTCGAATCCGATAGACATTATTTTTTTAAACACTCTTTCTGAAGGATCTGTACACGCCAACAACGGACCACATCCTTCTTCATCAACCGAGTTGTTAATCTTGGCTCCATACTGTACCAATAAATTTATTCTCTCTATGACTTCATCATCTGTTCCCGAGAGATAATATAGAGGTGTTTTATTATGTTTATCACATGCGTTTGGATCTGCGCCGTGCACCAGCAGCATCGCGACTATTCTATTATTATTAATTTTAGAAGCTATATGCAATGGATAATTTCCATCATCATCCGTCTCATTTGGAGAGTATCCTCTATGAAGAAGTTCTTCTATAAATCGTTCATCTAGTCCTTTAATGCCACAATACGCATGTAGAATGTGATAATTTCCAGAGGGTTCGATAACTTGTAGCATATTCCTAAATACATCTAAATTTTTACTATTATATTTGGCATAAAGAGATAGATAATACTCGACCGACATAATGTTGTGTTGTCCATTATAGTATAAAAATTAATATTTCTATTTCTATATATTTGCAACAATTTACTCTCTATAACAAATATCATAACTTAGTTCTTTTATGTCAAGAAGGCACTGGTTTAATTCATCTATAAATGTCACTCCATAACTACCACGCATACTATACTCAGAATTATGATAAAGATATTTATTCTTGGGGTGTAAGTAATGGGGATTAATCTTTGTTGGATCAGTCTCTAAGTTAACACATGTCACACATGATCCATTTATAGTTATATCACACGATGATGATTTATGAATTGATTCCGGAAGATCGCTATTGTATTTTGTAGTTCCACAATTCATTTCCATACATGTTATTGTCACACTAATATTATGATGAACTTTATCTAGCCGCTGAGTGGTAAACAACAGAACAGATAGTTTATTATCTTTACCAACACCCTCAGCCGCTGCCACAAATCTCTGATCCGTATCCATGATGGTCATGTTTACTTTTAGTCCGTATCCAGTCAACACTATGTTAGCATTTCTGTCGATATAGCTTTCACTCATATGACACTCACCAATAATTGTAGAATTAATGTCGTAATTTACACCAATAGTGAGTTCGGCGACAAAGTACCAGTACCGGTAATCTTGTCGATGAGGACATATAGTATTCTTGTATTCTACCGAATACCCGAGAGATGCGATACAAAAGAGTAAGACTAATTTGTAAACCATCTTACTCAAAAATATGCGACAATAGGAAATCTATCTTATACACATAATTATTCTATCAATTTTACCAATTAGTTAGTGTAATGTTAACAAAAATGTGGGATAATTTAATAGTTTTTCCTTACACAATTGACATACATGAGTCTGAGTTCCTCGTTTTTGCTAATTATTTCGTCCAATTTATTATTCTTGACATCGTCAAGATCTTTTGTATAGGAGTCAGACTTGTATTCAACATGTTTTTCTATAATCATCTTAGCTATTTCGGCATCATCCAATAGTACATTTTCCAGATTAACAGAATAGATATTAATGTCGTATTTGAACAGAGCCTGTAACATCTCAATGTCTTTATTATCTATAGCCAATTTGATGTCCGGAATGAAGAGAAGGGAATTGGTGTTTGTCGACGTCATATAGTCGAGCAAGAGAATCATCATATCCACGTGTCCATTTTTTATAGTGGTGTGAATACAACTAAGGAGAATAGCCAGATCAAAAGGAGATGGTATCTCTGAAAGAAAGTAGGAAACAATACTTACATCATTAAGCATGACAGCATGATAAAATGAAGTTTTCCATCCAGTTTTCCCATAGAACATCAGTCTCCAATTTTTCTTAACAAACAGTTTTACCGTTTGCATGTTACCACTATCAACCGCATAATACAATGCGGTGTTTCCTTTGTCATCAAATTGTGAATCATCCATTCCACTGAATAGCAAAATCTTTACTATTTTGGTATCTTCTAATGTGGCTGCCTGATGTAATGGAAATTCATTATCTAGAAGATTTTTCAATGCTCCAGCGTTCAACAACGTACATACTAGACGCACGTTATTATCAGCTATTGCATAATACAAGACACTATGACCATTGATATCCGCCTTAAATGCATCTTTGCTAGAGAGAAAGCTTTTCAGTTGCTTAGACTTCCAAGTATTAATTCGTGACAGATCCATGTCTGAAACGAGACGCTAATTAGTGTATATTTTTTCATTTTTTATAATTTTGTCATATTGTACCAGAATTAATAATATCTCTAATAGATCTGATTAGTAGATATATGGCTATCGCAAAACAACATATACACATTTAATAAAAATAATATTCATTAAGAAGATTCAGATTCCACTGTACCCATCAATATAAAATAAAATAATTATTCCTTACATCGTACCCATAAACAATATATTAAGTATATTCCACCTTACCCATAAACAATATAAATCCAGTAATATCATGTCTAATGATGAACACAAATGGTGTATTAAATTCCAGTTCTTCAGGAGATGATCTCGCCGTAGCTACCATGATAGTAGATGCCTCCGCTACAGTTCCTTGTTCGTCTACATCTATCTTTACATTCTGAAACATTTTATAAATATATAATGGGTCCCTAGTCATATGTTTAAACGACGCCTTATCTGGATTAAACATACTAGGAGCCATCATTTCGGCTATCGACTTAATATCCCTCTTGTTTTCGATAGAAAATCTAGGGAGTTTAAGATTGTACATTTTATTCCCTAATTGAGATGACCAATATTCTAATTTTGCAGGCGTGATAGAATCTGTGAAATGGGTCATATTATCACCTATTGCCAGGTACATACTAATATTAGCATCCTTATACAGAAGGCGCACCATATCATATTCTTCGTCATCGATTGTGATTGTATTTCCTTGCAATTTAGTAACTACGTTCATCATGGGAACCGTTTTCGTACCGTACTTATTAGTAAAACTAGCATTGTGTGTTTTAGTGATATCAAACGGATATTGCCACGTACCTTTAAAATATATAGTATTAATGATTGCCCATAGAGTATTATCGTCGAGCATAGTAGAATCAACTACATTAGACATACCAGATCTACGTTCTACTATAGAATTAATTTTATTAACCGCATCTCGTCTAAAGTTTAATCTATATAGGCCGAATCTATGATATTGTTGATAATACGACGGTTTAATACACACAGTACTATCGACGAAACTTTGATACGTTAGATCTGTGTACGTATATTTAGATGTTTTCATCTTAGCTAATCCGGATATTAATTCTGTAAATGCTGGACCCAGATCTCTTTTTCTCAAATTCATAGTATTCAATAATTCTACTCTAGTATTACCTGATGCAGACAATAGCGACATAAACATAGAAAACGAATACCCAAACGGTGAGAAGACAATATTATCATTATCATCCTCATCCCCATTTTGAATATTTTTATACGCTAATATACCAGCATTGATAAATCCCTGCAGACGATATGCGGATACTGAACACGCTAATGATAGTATCAATAACGCAATCATGATTTTTATGGTATTAATAATTAACCTTATTTTTATGTTTGGTATAAAAATTATTGATGTCTACACATCCTTTATAATCAACTCTAATCACTTTAACTTTTACAGTTTTCCCTACAAGTTTATCCCTATATTCAACATATCTATCCATATGCATCTCTTAACACTCTGCCAAGATAGCTTCAGAGTGAGGATAGTCAAAAAGATAAATATATAGAGTATAATCATTCTCGTATACTCTGCCCTTTATTACATCGCCCGCATTGGGCAACGAATAACAAAATGCAAGCATCGTGTTAACGGGCTCGTAAATTGGGATAAAATTATGTTTTTATTGTTTATCTATTTTATTCAAGAGAATATTCAGGAAGTTCCTTTTCTGGTTGTATCTCGTCGCAGTATATATCATTTGTACATTGTTTCATATTTTTTAATAGTCTACACCTTTTAGTAGGACTAGTATCGTACAATTCATAGCTGTATTTTGAATTCCAATCACGTATAAAAATATCTTCCAATTGTTGACGAAGACCTAATCCATCATCCGGTGTAATATTAATAGATGCTCCACATATATCCGTAAAGTAATTTCCTGTCCAATTTGATGTACCTATATACGCCGTTTTATCGGTTACCATATATTTTGCATGGTTTACCCTAGAATACGGAATGGGAGGATCAGCATCTGGTACAATAAATAGCTTTACTTCTATATCTATGTTTTTAGATTTTAGCATAGCTATAGATCTTAAAAAGTTTCTCATGATAAACGAAGATCGTTGCCAGCAACTAATCAATAGCTTAACGGATACTTGTCTGTCTATAGCGGATCTTCTTAATTCATCTTCTATATAAGGCCAAAACAAAATTTTACCCGCCTTTGAATAAATAATAGGAATAAAGTTCATAACAGATACATAAACGAATTTACTCGCATTTCCGATACATGACAATAAAGCGGTTAAATCATTGGTTCTTTCCATAGTACATAATTGTTGTGGTGCAGAAGCAATAAATACAGAGTGTGGAACACCGCTTACGTTAATACTAAGAGGATGATCTGTATTATAATACGACGGATAAAAGTTTTTCCAATTATATGGTAGATTGTTAACTCCAAGATACCAGTATACCTCAAAAATTTGAGTGAGATCCGCTGCCAAGTTCCTATTATTGAAGATCGCAATACCCAATTCCTTGACCTGAGTTAGTGATCTCCAATCCATGTTAGCGCTTCCTAAATAAATATGTGTATTATCAGATATCCAAAATTTTGTATGAAGAACTCCTCCTAGGATATTTGTAATATCTATGTATCGTACTTCAACTCCGGCCATTTGTAGTCTTTCAACATCCTTTAATGGTTTGTTGGATTTATTGACGGCTACTCTAACTCTTACTCCTCTTTTGGGTAATTGTACAATCTCGTTTAATATTACCGTGCCGAAATTCGTACCCACTTCATCCGATAAACTCCAATAAAAAGATGATATATCTAGTGTTTTTATGGTATTGGATAGAATTTCCCTCCACATGTTAAATGTAGTCAAATATACTTTATCAAATTGCATACCTATAGGAATAGTCTCTGTAATCACTGCGATTGTATTATCCGGATTCATTTTATTTGTTAAAAAAATAATCCTATATCACTTCACTCTATTAAAAATCCAAGTTTCTATTTCTTTCATGACTGATTTTTTAACTTCATCCGTTTCCTTATGAAGATGATGTTTGGCGCCTTCATAAATTTTTATTTCCCTATTACAATTTGCATGTTGCATGAAATAATATGCACCTGAAACATCGCTAATCTCATTGTTTGTTCCCTGGAGTATGAGAGTCGGGGTGTTAATCTTGGGAATTATTTTTCTAACCTTGTTGGTAGCCTTCAAGACCTGACTAGCAAATCCAGCCTTAATTTTTTCATGATTGACTAATGGATCGTATTGGTATTTATAAACTTCATCCATATCTCTAGATACTGATTCTGGACATAGCTTTCCGACTGACGCATTTGGTGTAATGGTTCCCATAAGTTTTGCAGCTAGCAGATTCAGTCTTGGAACAGCGTCTGCATTAACTAGAGGAGACATTAGAATCATTGCTGTAAACAAGTTTGGATTATCGCAAGCAGCTAGTATAGAAATTGTTGCTCCCATGGAATGACCCAATAAGAAGACTGGAACTCCTGGATAAGTAGATTTAATAGTCACCACGTGCTGTACCACATCTCTAACATACTTACCAAAGTCATCAATCATCATTTTTTCACCATTACTTCTTCCATGGCCAATATGATCATGTGAGAATACTAAAATTCCTAACGATGATATGTTTTCAGCTAGTTCGTCATAACGTCCAGAATGTTCACCAGCTCCATGACTTATGAATACTAATGCCTTAGGATATGTAATAGGTTTCCAATATTTACAATATATGTAATCATTGTCCAGATTGAACATACAGTTTGTACTCATGATTCACTATATAACTATCAATATTAACAGTTCGTTTAATGATCATATTATTTTTATGTTTTATTGATAATTGTAAAAATATACAATTAAATCAATATAGAGGAAGGAGACGGTACTGTATTTTGTGAGATAGTAATGGAGACTAAATCAGATTATGAGGATGCTGTTTTTTACTTTGTGGATGATGATGAAATATGTAGTCGCGACTCCATCATTGATCTAATAGATGAATATATCACGTGGAGAAATCATGTTATAGTGTTTAATAAAGATATTACCAGTTGTGGAAGACTGTACAAGGAATTGATAAAGTTCGATGATGTCGCTATACGGTACTATGGTATTGATAAAATTAATGAGATTGTCGAGGCTATGAGTGAAGGAGACCACTACATCAATCTTACAGAAGTCCATGATCAGGAAAGTCTATTCGCTACCATAGGAATATGTGCTAAAATCACTGAACATTGGGGATACAAAAAGATTTCAGAATCTAAATTCCAATCATTGGGAAACATTACAGATCTGATGACCGACGATAATATAAACATCTTGATACTTTTTCTAGAAAAAAAAATGAATTGATGATATAAGTGTCTTCATAACGCATTATTACGTTAGCATTCTATTATCCAGTGTTAAAAAAATTATCCTATCATGTATTTGAGAGTCTTATATGTAGCAAACATGATAACTGCAATACCCATAATCTTTAGATATTCACGCGTGCTATGGATGGCATTATCCCGCGGTGCGGAAATGTACGTTATATAATCTACAAAATAATCATCGCATATAGTATGAGATAGTAGAGTAAACATTTTTATCGTTTCTACTGGGTTCATACATCGTCTACCCAATTCGGTAATGAATGAAATTGTCGCCAATCTTACACCCAAACCCTTGTTGTTCATTAGTATAGTATTAACTTCATTATTTATGTCATAAACTGTAAATGATTCTGTAGATGCCATATCACACATGATATTCATGTCACTATTATAATCATTATTAACTTTATCACAATACGTGTTGATAATATCTACATATGATCTAGTTTTTGTGGGTAATTGTCTATACAAGTCGTCTAAACGTTGTTTACTCATATAGTATTGAACAGCCATCATTACATGGTCCCGTTCCGTTGATAGATAATCGAGTATGTTAGTAGACTTGTCAAATCTATATACCATATTTTCTGGAAGCGGATATACATAGTCGCGATCATCATTATCACTAGCCTCATCCTCTATATCATGTACATGTACATAATCTATGATATTATTATACATAAACATCGACAACATACTATTGTCTATTATCTAAGTCCTGTTGATCCAAACCCTTGATCTCCTCTATCTGTACTATCTAGAGATTGTACTTCTTCAAGTTCTGGATAATATATACGTTGATAGATTAGCTGAGCTATTCTATCTCCAGTATTTACATTAAACGTACATTTTCCATTATTAATAAGAATGACTCCTATGCTTCCCCTATAATCTTCGTCTATTACACCGCCTCCTATATCAATGCCTTTTAGGGACAGACCAGACCTAGGAGCTATTCTACCATAGCAGAACTTAGGCATGGACATACTAATATCTGTCTTAATTAACTGTCGTTCTCCAGGAGGGATAGTATAATCGTAAGCGCTATACAAATCATATCCGGCAGCACCCGGCGATTGCCTAGTAGGTGATTTAGCTCTGTTAGTTTCCTTAACAAATCTAACTGGTGAGTTAATATTCATGTTGAACATAAAAAATATCATTTTATTTCAAAATTATTTACCATTCCATTCCATCCCATATATTCCATGAATAAGTGCGATTATTGTACACTTCTATAGTATCTATATACGATCCACGATAAAATCCTCCTATCAATAGCAGTTTATTATCCACTATGATCAATTCTGGATTATCCCTCGGATAAATAGGATCATCTATCAGAGTCCATGTATTGCTGGATTCACAATAAAATTCCGCATTTCTACCAACCAAGAATAACCTTCTACCAAACACTAACGCACATGATTTATAATGAGGATAATAAGTGGATGGTCCAAACTGCCACTGATCATGATTGGGTAGCAAATATTCTGTAGTTGTATCAGTTTCAGAATGTCCTCCCATTACGTATATAACATTGTTTATGGATGCCACTGCTGGATTACATCTAGGTTTCAGAAGACTCGGCATATTAACCCAAGCAGCATCCCCGTGGAACCAACGCTCAACAGATGTGGGATTTGGTAGACCTCCTACTACGTATAATTTATTGTTAGCGGGTATCCCGCTAGCATACAGTCTGGGGCTATTCATCGGAGGAATTGGAATCCAATTGTTTGATATATAATTTACCGCTATAGCATTGTTATGTATTTCATTGTTCATCCATCCACCGATGAGATATACTACTTCTCCAACATGAGTACTTGTACACATATGGAATATATCTATAATTTGATCCATGTTCATAGGATACTCTATGAATGGATACTTGTATGATTTGCGTGGTTGTTTATCACAATGAAATATTATGTTACAGTCTAGTATCCATTTTACATTATGTATACCTCTGGGAGAAAGATAATTTGACCTGATTACATTTTTGATAAGAAGTAGCAGATTTCCTAATCTATTTCTTCGCCTCATATACCACTTAATGACAAAATTAACTACATAATCCTCATCTGGAACATTTAGTTCGTCGCTTTCTAGAATAAGTTTCATAGATAGATAATCAAAATTGTCTATGATGTCATCTTCCAGTTCCAAAAAGTGTTTGGTAATAAAGTCTTTAGTATGACATAAGAGATTGGATAGTCCGTATTCTATACCCATCATGTAACACTCGATACAATATTCCTTTCTAAAATCTCGTAGGATAAAGTTTATACAAGTGTAGATGATAAATTCTACAGATGTTAATATAGAAGCACGTAATAAATTGACGACGTTATGACTATCTATATATACCTTTCCAGTATATGAGTAAATAACTATAGAAGTTAGACTGTGAATGTCAAGGTCTAGACAAACCCTCGTAACTGGATCTTTATTTTTTGTGTATTTTTGGCGTAAATGTGTGCGAAAGTATGGAGATAACTTTTTCAATATCGTAGAATTGACTATTATATTACCTCCTATAGCTTCAATAATTGTTTTGAATTTCTTAGTCGTGTACAATGCTAATATATTCTTACAGTACACAGTATTGACAAATATCGGCATTTATGTTTCTTTAAAAGTCAACATCTAAAGAAAAATGATTGTCTTCTTGAGACATAACTCCCATTTTTTGGTATTCACCCACACGTTTTTCGAAAAAATTAGTTTTTCCTTCCAATGATATATTTTCCATGAAATCAAACGGATTGGTAACATTGTAAATTTTTTTAAATCCCAATTCAGAAATCAATCTATCTGCGACGAATTCTATATATGTTTTCATCATTTCACAATTCATTCCTATGAGTTTAACTGGAAGAGCCACAGTAAGAAATTCTTGTTCAATGGATACCGCATTTGTTATAATAAATCTAACGGTTTCTTCACTCGGTGGATGCAATAAATGTTTAAACATCAAACATGCGAAATCGCAGTGCAGACCCTCGTCTCTACTAATTAATTCGTTAGAAAACGTGAGTCCGGGCATTAGGCCACGCTTTTTAAGCCAAAATATGGAAGCGAATGATCCGGAAAAGAAGATTCCTTCTACTGCAGCAAAGGCAATAAGTCTCTCTCCATAACCGGCGCTGTCATGTATCCACTTTTGAGCCCAATCGGCCTTCTTTTTTACACAAGGCATCGTTTCTATGGCATTAAAGAGGTAGTTTTTTTCATTACTATCTTTAACATAAGTATCGATCAAAAGACTATACATTTCCGAATGAATGTTTTCAATGGCCATCTGAAATCCGTAGAAACATCTAGCCTCGGTAATCTGCACTTCTGTACAAAATCGTTCTGCTAAATTTTCATTCACTATTCCGTCACTGGCTGCAAAAAACGCCAATACATGTTTTATAAAATATTTTTCGTCTGGTGTTAGTTTATTCCAGTCATTGATATCTTTAGATATATCCACTTCTTCCACTGTCCAAAATGATGCCTCTGCCTTTTTATACATATTCCAGATGTCATGATATTGGATTGGGAAAATAACAAATCTATTTGGATTTGGTGCAAGGATAGGTTCCATAACTAAATTAACAATAGTAGTAATTTTTTTTCAGTTATCTGTATGACGACTGTACTTGGATCTTTTGTATATCGCTATCGCCGCAATCACTACAATAATTACAAGTATTATTGATAGCATTGTTATTACTACTATCATAATTAAATTATCGACATTCATGGGTGTTGAATAATCGTTATCATCATTTTGTAATTGTGACGTCATACTAGATAAATCATTTGTGAGATTGTTGTGGGAAGCGGGCACGGAAGATGCATTATCATTATTATTTAACGCCTCCCATTCGGATTCACAAATGTTACGCACGTTCAAAGTTTTATGGAAACTATAATTTTGTGAAAACAGATAACAAGAAAACTCGTCATCGTTCAAATTTTTAACGATAGTAAACCGATTAAACGTCGAGCTAATTTCTAACGCTAGCGACTCTGTTGGATATGGGTTTCCAGATATATATCTTTTCAGTTCCCCTACGTATCTATAATCATCTGTAGGAAATGGAAGATATTTCCATTTATCTACTGTTCCTAATATCATATGCGGTGGTGTAGAACCATTAAGCGCGAAAGATGTTATTTCGCATCGTATTTTAACTTCGCAATAATTTCTGGTTAGATAACGCACTCTACCAGTCAAGTCAATGATATTAGCCTTTACAGATATATTCATAGTAGTCGTAACGATGACTCCATCTTTTAGATGTGATACTCCTTTGTATGTACCAGAATCTTCGTACCTCAAACTCGATATATTTAAACAAGTTAATGATATATTAACGCGTTTTATGAATGATGATATATAACCAGAAGTTTTATCCTCTGTGGCTAGCGCTATAACCTTATCATTATAATACCAACTAGTGTAATTAATATGTGACATGACAGTGTGGGTACAAATATGTACATTATCGTCTACGTCGTATTTGATACATCCGCATTCAGCCAACAAATATAAAATTACAAAAACTCTAACGACGTTTGTACACATCTTGATGCGGTTTAATAAATGTTTTGATTTCAATTTATTGTAAAAAAAGATTCGGTTTTATACTGTTCGATATTCTCATTGCTTATATTCTCATCTATCATCTCCACACAGTCAAATCCATGGTTAACATGTACCTCATCAACCGGTAAAAGACTATCGGATTCTTCTATCATCATAACTCGAGAATATTTAATTTGGTGGTCATTATTAATCAAGTCAATTATCTTATTTTTAACAAACGTAAGTATTTTACTCATTTTTTTATAAAAACTTTTAGAAATATACAGACTCTATCGTGTGTCTATATCTTCTTTTTATATCCAATGTATTTATGTCTGATTTTTCTTCATTTATCATATATAATGGTCCAAATTCTACACGTGCTTCGGATTCATCCAGATCATTAAGGTTCTTATAATCGCAACATCCTTCTCTTCCATCTTCTACATCTTCCTTCTTATTCTTAGCGTCACAGAATCTACCACAGCAGGATCCCATGACGAGAGTCACATTAAACTAATTCATTTTCAATTATAATATACTGATTAGTAATGACAATTAAAATAAAAATATTCTTCATAACCGGTAAGAAAGTAAAAAGTTCACATTGAAACTATGTCAGTAGTTATACATCATGAGATGATATACTCTATTTTGGTGGAGGATTATATGATATAATTCGTGGATAATCATTCTTAAGACACATTTCTTCATTCGTAAATCTTTTCACATTAAATGAGTGTCCATATTTTGCAATTTCTTCATATGATGGCGGTGTACGTGGACGAGGCTGCTCCTGTTCTTGTAGTCGTCGACTGTCGTGTTTGCGTTTAGATCCCTCCATTATCGCGATCGCGTAGTGAGTACTATTTATACCTTGTAATTAAATTTTTTTATTAATTAAACGTATAAAAACGTTCCGTATCTGTATTTAAGAGCCAGATTTCGTCTAATAGAACAAATAGCTACAGTAAAAATAACTAGAATAATCGCTACACCCACTAGAAACCACGGATCGTAATACGGCAATCGGTTTTCGATAATAGGTGGAACGTATATTTTATTTAAGGACTTAACAATTGTCTGTAAACCACAATTTGCTTCCGCCGATCCTGTATTAACTATCTGTAAAAGCATATGTTGACCGGGAGGAGCCGAACATTCTCCGATATTCAATTTTTGTATATCTATAATGTTATTAACCTCCGCATACGCATTACAGTTCTTTTCTAGCTTGGATACTACACTAGGTACATCATCTAAATCTATTCCTATTTCCTCAGCGATAGCTCTTCTATCCTTTTCCGGAAGTAATGAAATTACTTCAATAAATGATTCAACCATGAGTGTGAAACTAAGTCGAGAATTACTCATGCATTTGTTAGTTATTCGGAGCGCGCAATTTTTAAACTGTCCTATAACCTCTTCTATATGAATAGCACAAGTGACATTAGTAGGGATAGAATGTTGAGCTAATTTTTGTAAATAACTATCTATAAAAAGATTATACAAAGTTTTAAACTCTTTAGTTTCTGCCATTTATCCAGTCTGAGAAAATGTCTCTCATAATAAATTTTTCCAAGAAACTAATTGGGTGAAGAATGGAAACCTTTAATCTATATTTATCACAGTCTGTTTTGGTACACATGATGAATTCTTCTAATGCTGTACTAAATTCGATATCTTTTTCGATTTCTGGATATGTTTTTAATAAAGTATGAACAAAGAAATGGAAATCGTAATACCAGTTATGTTCAACTTTGAAATTGTTTTTTATTTTCTTGTTAATGATTCCAGCCACTTGGGAAAAGTCAAAGTCGTTTAATGCCGATTTAATACGTTCATTAAAAACAAACTTTTTATTCTTTAGATGAATTATTATTGGTTCATTGGAATCAAAAAGTAAGATATTATCGGGTTTAAGATCTGCATGTAAAAAGTTGTCACAACAGGGTAGTTCGTAGATTTTAATGTATAACAGAGACATCTGTAAAAAGATAAACTTTATGTATTGTACCAAAGATTTAAATCCTAATTTGATAGCTAACTCGGTATCTACTTTATCTGCCGAATACAGTGCTAGGGGAAAAATTATAATATTTCCTCTTTCGTATTCGTAATTAGTTCTCTTTTCATGTTCGAAAAAGTGAAACATGCGGTTAAAATAGTTTATAACATTAATATTACTGTTAATAACTGCCGGATAAAAGTGGGATAGTAATTTCACGAATTTGATACTGTCCTTTCTCTCGTTAAACGCCTTTAGAAAAACTTTAGAAGAATATCTCAATGAGAGTTCCTGACCATCCATAGTTTGTATCAATAATAGCAACATATGAAGAACCCGCTTATACAGAGTATGTAAAAATGTTAATTTATAGTTTAATCCCATGGCCCACGCACACACGATTAATTTTTTTTCATCTCCCTTTAGATTGTTGTATAGAAATTTGGGTACTGTAAACTCCGCCGTAGTTTCCATGGGACTATATAATTTTGTGGCCTCGAATACAAATTTTACTACATAGTTATCTATCTTAAAGACTATACCATATCCTCCTGTAGATATGTGATAAAAATCGTCGTTTATAGGATAAAATCGTTTATCTTTTTGTTGGAAAAAGGATGAATTAATGTAATCATTCTCTTCTATCTTTAGTAGTGTTTCCTTATTAAAATTCTTAAAATAATTTAACAATCTAACTGACGGAGCCCAATTTTGGTGTAAATCTAATTGGGACATTATGTTGTTAAAATATAAACAGTCTCCTAATATAACAGTATCTGATAATCTATGGGGAGACATCCATTGATATTCAGGGGATGAATCATTGGCAACACCCATTTATTGTACAAAAAGCCCCAATTTACAAACGAAAGTCCAGGTTTGATAGAGATAAACTATTAACTATTTTGTCTCTGTTTTTAACACCTCCACAGTTTTTAATTTCTTTGGTAATGAAATTATTCACAATATCAGTATCTTCTTTATCTACCAGAGATTTTACTAACTTGATAACCTTGGCTGTCTCATTCAATAGGGTAGTGATATTTGTATGTATGATATTGATATCTTTTTGAATTGTTTCTTTTAGAAGTGATTCTTTGATGGTATCAGCATACGAATTACAATAATGCAGAAACTCAGTTAACATGCAGGAATTATAGTAAGCCAATTCCAATTGTTGCCTGTATTGTATTAGAGTATTAATATGCGCAATGATGTCCTTGCGTTTCTCTGATAGAATGCGAGCAGCGATTTTGGCGTTATCATTTGACGATATTTCTGGAATGACGAATCCTGTTTCTACTAACTTCTTGGTAGGACAAAGTGAAACAATCAAGAAAATAGCTTCTCCTCCTATTTGTGGAAGAAATTGAACTCCTCTAGATGATCTACTGACGATAGTATCTCCTTGACAGATATTGGACCGAACTACGGAAGTACCTGGAATGTAAAGCCCTGAAACCCCCTCATTTTTTAAGCAGATTGTTGCCGTAAATCCTGCACTATGCCCAAGATAGAGAGCTCCTTTGGTGAATCCATCACTATGTTTCAGTTTAACCAAGAAACAGTCAGCTGGTCTAAAATTTCCATCTCTATCTAATACAGAATCCAACTTGATGTCAGGGACTATGACCGGTTTAATGTTATATGTAACATTGAGTAAATCCTTAAGTTCATAATCATCGTTGTCATCAGTTATGTACGATCCAAACAATGTTTCTACCGGCATGGTGGATACAAAGATGCTATCCATCAGAATGTTTCCCTGATTAGTATTTTCTATATAGCTATTCTTCTTTAAACGATTTTCCGAATCAGTAACTATGTTCATTTTTTTAGGAGTAGGACGTCTAGCCAGTATGGAAGAGGATTTTCTAGATACTCTCTTCAACATCTTTGATCTCAATGGAATGCAAAACCCCATGGTGTAACAACCAACGATAAAAATAATATTGTTTTTTCACTTTTTATAATTTTACCATCTGACTCATGGATTCATTAATATCTTTACAAGAGCTACTAACGTATAATTCTTTATAACTGAACTGAGATATATACACCGGATCTATGGTTTCCATAATTGAGTAAATGAATGCTCGGCAATAACTAATGGCAAATGTATAGAACAACGAAATTATACTAGAGTTGTTAAAGTTAATATTTTCTATGAGTTGTTCCAATAAATTATTTGTTGTGACTGCGTTCAAGTCATAAATTATCTTGATACTATCCAGTAAACAGTCTTTAAGTTCTGGAATATTATCATCCCATTGTAAAGCCCCTAGTTCGACTATCGAATATCCTGCTCTGATAGCAGTTTCAATATCGACGGACGTCAATACTGTAATAAAGGTGGTAGTATTGTCATCATCGTGATAAACTACGGGAATATGGTCGTTAGTAGGTACCGTGACTTTACACAACGCGATATATAACTTTCCTTTTGTACCATTTTTAACGTAGTTGGGACGTCCTGCAGGGTATTGTTTTGAAGAAATGATATCGAGAACAGATTTGATACGATATTTGTTGGATTCCTGATTATTCACTATAATATAATCTAGACAGATAGATGATTCGATAAATAGAGAAGGTATATCGTTGGTAGGATAATACATCCCCATTCCAGTATTCTCGGATACTCTATTGATGACACTAGTTAAGAACATGTCTTCTATTCTAGAAAACGAAAACATCCTACATGGACTCATTAAAACTTCTAACGCTCCTGATTGTGTTTCGAATGCCTCGTACAAGGATTTCAAGGATGCCATAGATTCTTTGACCAACGATTTAGTATTGCGTTTAGCATCTGATTTTTTTATTAAATCAAATGGTCGGCTCTCTGGTTTACTACCCCAATGATAACAATAGTCTTGTAAAGATAAACCGCAAGAAAATTTATACACATCCATCCAAATAACCCTAGCACCGTCGGATGATATTAATGTATTATTATAGATTTTCCATCCACAGTTATTGGGCCAGTATACTGTTAGCAACGGTATATCGAATAGATTACTCATGTAACCTACTAGAATGATAGTTCGTGTACTAGTCATAATATCTTTAATCCAATCTAAGAAATCTAAAATTAGATCTTTTACACTATTAAAGTTAACAAAGGTATTACCCGGGTACGTGGATATCATATATGGTATTGGTCCATTATCAGTAATGGCTCCATAAACTGATACGGCGATGGTTTTTATATGTGTTTGATCTAATGAGGACGAAATTCGCGCCCACAATTCATCTCTAGATATGCATTTAATATCGAACGGTAACACATCAATCTCGGGACGCGTATATGTTTCTAAATTCTTAATCCAAATATAATGATGACCTATATGCCCTATTATCATACTGTCAACTATAGTATACCTAGAGAACTTTCGATACATCTGCTGTTTCCTGTAATCGTTAAATTTTACAAATCTATAACATGCTAAACCTTTTGACGACAGCCATTCATTAATTTCTGATATGGAATCTGTATTCTCAATACCGTATCGTTCTAAAGCCAGTGCTATATCTCCCTGTTCGTGGGAACGCTTTCGTATAATATCGATCAATGGATAATATGAAGTTTTTGGAGAATAATATGATTCATGATCTATTTCGTCCATAAACAATCTAGACATAGGAATTGGAGGCGATGATCTTAATTTTGTGCAATGGGTCAATCCTATAACTTCTAATATTGTAATATTCATCATCGACATAACACTATCTATGTTATCATCGTATATTAGTATACCACGACCTTCTTCATTTCGTGCCAAAATGATATACAGTCTTAAATAATTACGCAATATCTCAATAGTTTCATAATTGTTAGCTGTTTTCATCAAGGTTTGTATCCTGTTTAACATGATGGCGTTCTATAACGTCTCTATTTTCTATTTTTAATTTTTTTAAATTTTTAACGATTTACTGTGGCTAGATACCCAATCTCTCTCAAATATTTTTTTAGCCTCGCTTACAAGCTGTTTATCTATACTATTAAAACTGACGAATCCGTGATTTTGGTAATGGGTTCCGTCGAAATTTGCCGAAGTGATATGAACATATTCGTCGTCGACTATTAACAATTTTGTATTATTCTGAATAGTGAAAACCTTCACAGATAGATCATTTTGAACACACAACGCATCTAGACTTCTGGCGGTTGCCATAGAATATACGTCGTTCTTATCCCAATTACCAACTAGAAGTCTGATCTTAACTCCTCTATTAATGGCTGCTTCTATAATGGAGTTGTAAATGTCAGGCCAATAGTAGCTATTACCGTCGACACGTGTAGTGGGAACTATGGCCAAATGTTCAATATCTATACTAGTCTTAGCCGACTTGAGTTTATCAATAACTACATCAGTGTCTAGATCTCTAGAATATCCCAATAGGTGTTCTGGAGAATCAGTAAAGAACACTCCACCTATAGGATTCTTAATATGATACGCAGTGCTAACTGGCAGACAACAAGCCGCAGAGCATAAATTCAACCATGAATTTTTTGCGCTATTAAAGGCTTTAAAAGTATCAAATCTTCTACGAAGATCTGTGGCCAGCGGAGGATAATCAGAATATACGCCTAACGTTTTAATCGTATGTATAGATCCTCCAGTAAATGACGCGTTTCCTACATAACATCTTTCATCATCAGACACCCAAAAACAACCGAGTAGTAGTCCCACATTATTTTTTTTATCTATATTAACGGTTATAAAATTTATATCCGGGGAGTGACTTTGTAGCTCTCCCAGATTTCTTTTCCCTCGTTCATCTAGCAAAACTATTATTTTAATCCCTTTTTCAGATACCTCTTTTAGTTTATCAAAAATAAGCGCTCCCCTAGTAGTACTCAGAGGATTACAACAAAAAGATGCTATGTATATATATTTCTTAGCTAGAGTGATAATTTCGTTAAAACATTCAAATGTTGTCAAATGATCGGATCTAAAATCCATATTTTCTGGTAGTGTTTCTACCAGCCTACATTTTGCTCCCGCAGGTACCGATGCAAATGGCCACATTTAGTTAACATAAAAACTTATATATCCTGTTCTATCAACGATTCTAGAATATCATCGGCTATATCGCTAAAATTTTCATCAAAGTCGACATCACAACCTAACTCAGTCAATATATTAAGAAGTTCCATGATGTCATCTTCGTCTATTTCTATATCCGTATCCATTGTAGATTGTTGACCGATTATCGAGTTTAAATCATTACTAATACTCAATCCTTCAGAATACAATCTGTGTTTCATTGTAAATTTATAGGCGGTGTATTTAAGTTGGTAGATTTTCAATTATGTATCAATATAGCAACAGTAGTTCTTGCTCCTCCTTGATTTTAGCATCCTCTTCATTATTTTCTTCTACGTACATAATCATGTCTAATACGTTAGACAACACACCGACGATGGTGGCCGCCACAGACACGAATATGACTAGACCGATGACCATTTAAAAAATACTCTCTAGCTTTAACTTAAACTGTATCGATCATTCTTTTAGCACATGTATAATATAAAAACATTATTCTATTTCGAATTTAGGCTTCCAAAAATTTTTCATCCGTAAACCGATAATAATATATATAGACTTGTTAATAGTCGGAATAAATATATTAATGCTTAAACTATCATCATCTCCACGATTAGAGATACAATATTTACATTCTTTTTGCTGTTTCGAAACTTTATCAATACACGTTAATACAAACCCAGGAAGGAGATATTGAAACTGAGGCTGTTGAAAATGAAACGGCGAATACAATAATTCAGATAATGTAAAATCATGATTCCGTATTCTGATGATATTAGAACTGCTAATGGATGTCGATGGTATGTATCTAGGAGTATCTATTTTAACAAAGCATCGATTTGCTAATATACAATTATCCTTTTGATTAATTGTTATTTTATTCATATTCTTAAAAGGTTTCATATTTATCAATTCTTCTACATTAAAAATTTCCATTTTTAATTTATCTAGCCCCGCAATACTCCTCATTACGTTTCATTTTTTGTCTAGAATACCCATTTTGTTCATCTTGGTACATAGATTATCCAATTGAGAAGCGCATTTAGTAGTTTTGTACATTTTAAGTTTATTAACGAATCGTCGAAAACTAGTTATAGTTAACATTTTATTATTTGATACCCTGATATTAATACCCCTGCCGTTACTATTATTTATAACTGATGTAACCCACGTAACATTGGAATTAATTATCGATAGTAATGCATCGACACTTCCAAAATTGTCTATTATAAACTCACCGATAATTTTTTTATTGCATGTTTTCATATTCATTAGGATTATCAAATCTTTAATCTTATTACGATTGTATGCGTTGATATTACAAGACGTCATTCTAAAAGACGGAGGATTTCCATCAAATGCCAGACAATCACGTACAAAGTACATGGAAATAGGTTTTGTTCTATTACGCATCATAGATTCATATAAAACACCCGTAGAAATACTAATTTGTTTTACTCTATAAAATACTATTGCATCTATTTCATCGTTTTGTATAACGTCTTTCCAAGTGTCAAATTCCAATTTTTTTTCATTGATAGTACCAAATTCTTCTATCTCTTTAACTACTTGCATAGATAGGTAATTACAGTGATGCCTACATGCCGTTTTTTGAAACTGAATAGATGCATCTAGAAGCGATGCTACACTAGTCACGATCACCACTTTCATATTTAGAATATATGTATGTAAAAATATAGTAGAATTTCATTTTGTTTTTTTCTATGCTATAAATGAATTCTCATTTTGTATCCGCACATACTCCGTTTTATATCAATACCAAAGAAGGAAGATATCTGGTTCTAAAAGCCGTTAAAGTATGCGATGTTAGAACTGTAGAATGCGAAGGAAGTAAAGCTTCCTGCGTACTCAAAGTAGATAAACCCTCATCACCCACGTGTGAGAGAAGACCTTCGTCCCCGTCCAGATGCGAGAGAATGAATAACCCTGGAAAACAAGTCCCGTTTATGAGAACGGACATGTTACAAAATATGTTTGCTGCTAATCGCGACAACGTAACGTCAAGACTTTTGAACTAAAATACAATTATATCTTTTTCGATATTAATAAATCCGTGTCTCCCGGGTTTTTTATCTCTTTCAGTATGTGAATAGATAGGTATTTTATCTCTATTCATCATCGAATTTAAGAGATCCGATAAACATTGTTTGTATTCTCCAGATGTCAGCATCTGATACAACAATATATGTGCACATAAACCTCTGGCACTTATTTCATGTACCTTCCCCTTATCACTAAGGAGAATAGTATTTGAGAAATATGTATACATGATATTATCATGTATTAGATATACAGAATTTGTAACACTCTCGAAATCACACGATGTGTCGGCGTTAAGATCTAATATATCACTCGATAACACATTTTCATCTAGATACACTAGACATTTTTTAAAGCTAAAATAGTCTTTAGTAGTAACAGTAACTATGCGATTATTTTCATCGATGATACATTTCATCGGCATATTATTACGCGTACCATCAAAGACTATACCATGTGTATATCTAACGTATTCTAGCATAGTTGCCATACGTACATTAAACTTTTCAGGATCTTTGGATAGATCTTCCAATCTATCTATTTGAGAAAACATTTTTATCATGTTCAATAGTTGAAACGTCGGATCCACTATATAGATATTATCTATAAAGATTTTAGGAACTATGTTCATGGTATCCTGGCGAATATTAAAACTATCAATGATATGATTATCGTTTTCATCTTTTATCACCATATAGTTTCTAAGATATGGGATTTTACTTAATATAATATTATTTCCCGTAATAAATTTTATTAGAAATGCCAAATCTATAAGAAAAGTCCTAGAATTAGTCTGAAGAATATCTATATCACCGTACCGTATATTTGGATTAATTAGATATAGAGAATATGATCCGTAACATATACAACTTTTATTATGACGTCTAAGATATTCTTCCATCAACTTATTAACATTTTTGACTAGGGAAGATACATTATGACGTCCCATTACTTTTGCCTTGTCTATTACAGCGACGTTCATAGAATTTAGCATATCTCTTGCCAATTCTTCCATTGATGTTACATTATAAGAAATTTTAGATGAAATTACATTTGGAGCTTTAATAGTAAGAACTCCTAATATATCCGTGTATGTGGTCACTAATACAGATTGTAGTTCTATAATCGTAAATAATTTACCTATATTATATGTTTGAGTTTGTTTAGAAAAGTAGCTAAGTATACGATCTTTTATTTCTGATGCCGATGTATCAACATCGAAAAAAAATCTTTTTTTATTCTTTTTTACTAACGATACGAATATGTCTTTGTTAAAAACAGTTATTTTCTGAATATTTCTAGCTTGTAATTTTAACATATGATATTCGTTCACACTAGGTACTCTGCCTAAATAGGTTTCTATAATCTTTAATGTAATATTAGGAAGAGTATTCTGATCAGGATTCCTATTCATTTTGAGGATTTAAAACTCTGATTATTGTCTAATATGGTCTCAACACAAACTTTTTCACAGAGTGATAGAGTTTTTGATAACTCGTTTTTCTTAAGAAATATAAAACTACTGTCTCCAGAGCTCGCTCTATCTTTTATTTTATCTAATTCGATACAAACTCCTGATACTGGTTCAGAAAGTAATTCATTAATTTTCAGTCCTTTATAGAAGATATTTAATATAGATAATACAAAATCTTCAGTTCTTGATATCGATCTGATTGATCCTAGAACTAGATATATTAATAACGTGCTCATTAGGCAGTTTATGGCAGCTTGATAATTAGATATAGTATATTCCAGTTCATATTTATTAGATACCGCATTGCCCAGATTTTGATATTCTATGAATTCCTCTGAAAATAAATCCAAAATAACTAGACATTCTATTTTTTGTGGATTAGTGTACTCTCTTCCCTCTATCATGTTCACTACTGGTGTCCACAATGATAAATATCTAGAGGGAATATAATATAGTCCATATGATGCCAATCTAGCAATGTCGAATAACTGTAATTTTATTCTTCGCTCTTCATTATGAATTGAATCTTGAGGTATAAACCTAACACAAATTATATCATTAGACTTTTCGTATGTAATGTCTTTCATGTTATAAGTTTTTAATCCTGGAATAGAATCTATTTTAATGAGGCTTTTAAACGCAGCGTTCTCCAACGAGTCAAAGCATAATACTCTGTTGGTTTTCTTATATTCAATATTACGATTTTCTTCTTTGAATGGAATAGGTTTTTGAATTAGTTTATAATTACAACATAATAGATAAGGAAGTGTGTAAATAGTACGCGGAAAAAACATAATAGCTCCCCTGTTTTCATCCATGGTTTTAAGTAAATGATCACTGGCTTCTTTAGTCAATGGATATTCGAACATTAACCGTTTCATCATCATTGGACAGAATCCATATTTCTTAATGTAAAGAGTGATCAAATCATTGTGTTTATTGTACCATCTTGTTGTAAATGTGTATTCGGTTATCGGATCTGCTCCTTTTTCTATTAAAGTATCGATATCGATCTCGTCTAAGAATTCAACTATATCGACATATTTCATTTGTATACACATAACCATTACTAACGTAGAATGTATAGGAAGAGATGTAACGGGAACAGGGTTTGTTGATTCGCAAACTATTCTAATACATAATTCTTCTGTTAATACGTCTTGCACGTAATCTATTATAGATGCCAAGATATCTATATAATTATTTTGTAAGATGATGTTAACAATGTGATCTATATAAGTAGTGTAATAATTCATGTATTTCGATATATGTTCCAACTCTGTCTTTGTGATGTCTAGTTTCGTAATATCTATAGCGTCCTCAAAAAATATATTCGCATATATTCCCAAGTCTTCAGTTCTATCTTCTAAAAAATCTTCAACGTATGGAATATAATAATCTATTTTACCTCTTCTGATGTCATTAATGATATAGTTTTTGACACTATTTTCCGTCAATTGATTCTTATTCACTATGTCTAAAAACCGGATAGCGTCCCTAGGACGAACTACTGCCATTAATATCTCTATTATAGCTTCTGGACATAAATCATCTATTATACCAGAATTAATGGGAACTATTCCGTATCTATCTAACATAGTTTTAAGAAAGTCAGAATCTAAGACCTGATGTTCATATATTGGTTCATACATGAAATGATCTCTATTGATGATAGTGACTATTTCATTCTCTGAAAATTGGTAACTCATTCTATACACGCTTTCCTTGTTGATAAAGGATAGTATATACTCAATGGAATTTGTACCAACAAACTGTTCTCTTATGAATCGTATATCATCATCTGAAATGATCATGTAAGGCATACATTTAACAATAAGAGACTTGTCTCCTGTTATCAATATACTATTCTTGTGATAATTTATGTGTGCGGCAAATTTGTCCACGTTCTTTAATTTTGTTATAGTAGATATCAAATCCAATGGAGATACAGTTCTTGGCTTAAACAGATATAGTTTTTCTGGAACGAATTCTACAACATTATTATAAAGGACTTTGGGTATATAAGTGGGATGAAATCCTATTTTAATTAATGCGATAGCCTTGTCCTCGTGCAGATATCCAAACGCTTTTGTGATAGTATGGCATTCATTGTCTAGAAACGCTCTACGAATATCTGTAACAGATATCATCTTTAGAGAATACTAGTCGCGTTAATAGTACTAAAATTTGTATTTTTTAATCTATCTCAATAAAAAATTAATATGTATGATTCAATGTATAACTAAACTACTAACTGTTATTGATAACTAGAATCAGAATCTAATGATGACATAACTAAGAAGTTTATCTACAGCCAATTTAGCTGCATTATTTTTAGCATCTCGTTTAGATTTTCCATCTGCCTTATCGAATACTCTTCCGTCAATGTCTACACAGGCATAAAATGTAGGAGAGTTACTAGGCCCCACTGATTCAATACGAAAAGACCAATCTCTCCTAGTTATTTGACAGTACTCATTAATAACGGTGACAGGGTTAACACCTTTCCAATAAATAATTTTTTTAACCGGAATAACATCATCAAAAGACTTATGATCCTCTCTCATTGATTTTTCGCGGGATACATCATCTATTATAGCATCAGCATCAGAATCTGTAGGCCGTGTATCAGCATCCATTGTCGTAGACCAACGAGGAGGAGTATCGTTGGAGCTGTAAACCATAGCACTACGTTGAAGATCATACAGAGCTTTATTAACTTCTCGCTTCTCCATATTAAGTTGTTTAGTTAGTTGTACAGCAGTAGCTCCTTAGTCCAATGTTTTTAATAACCGCACACAATCTCTGTGTCAGAACGCTCGTCAATATAGATCTTAGAAATTTTTTTAGAGAGAACTAACGCAACTAGCAATAAAACTGATCTTATTTTATCATTTTTTTTATTCATCATCCTCTGGTGGTTCGTCGTTCCTATCGAATGTAGCTCTGATTAACCCGTCATCTATAGGTGATGCTGGTTCTGGAGATTCTGGAGGAGATGGATTATTATCTGGAAGAATCTCTGTTATTTCCTTGTTTTCATGTATCGATTGCGTTGTAACATTAAGATTGCGAAATGCTCTAAATTTGGGAGGCTTAAAGTGTTGTTTACAATCTCTACACGCGTGTCTAACTAATGGAGGTTCATCAGCGGCTCTAGTTTGAATCATCATCGGTGTAGTATTCCTACTTTTACAGTTAGGACACGGTGTATTGTATTTCTCGTCGAGAACGTTAAAATAATCGTTGTAACTCACATCCTTTATTTTATCTATATTGTATTCTACTCCTTTCTTAATGCATTTTATACCGAACAAGAGATAGCGAAGGAATTCTTTTTCGGTACCGCTAGTACCCTTAATCATATCACATAGTGTTTTATATTCTAAATGTGTGGCAATGGACGGTTTATTTCTATACGATAGTTTGTTTTTGGAATCCTTTGAGTATTCTATACCAATATTATTCTTTGATTCGAATTTAGTTTCTTCGATATTAGATTTTGTATTACCTATATTCTTGATGTAGTACTTTGATGATTTTTCCATGGCCCATTCTATTAAGTTTTCCAAGTTGGCATCATCCACATATTGTGATAGTAATTCTCGGATATCAGTAGTGACTACCGCCATTGATATTTGTTCATTTGATGAGTAACTACTAATGTATACATTTTCCATTTATAACACTTATGTATTAACTTTGTTTATTTATATTTTTTCATTATTATGTTGATATTAATAATCGTATTGTGGTTATATGGCTACAATTTCATAATGAGTTGAAGTCAGTGTCCTATGATCAATGACGATAGCTTTACTCTGAAAAGAAAGTATCAAATCGATAGTGCAGAGTCAACAATGAAAATGGATAAGACGATGACAAAGTTTCAGAATAGAGTCAAAATGGTAAAAGAAATAAATCAGACGATAAGAGCAGCACAAACTCATTACGAGACATTGAAACTAGGATATATAAAATTTAAGGGAATGATTAGGACTACTACTCTAGAAGATATAGCACCATCTATTCCAAATAATCAGAAAACTTATAAACTATTCTCGGACATTTCAGTCATTGGCAAAGCATCACAGAATCCGAGTAAGATGATATATGCTCGCTGCTTTACATGTTTCCCAATTTGTTTGGAGATGACCATAGATTCATTTGTTATAGAATGCATCCAACATTGTTCATGATATAGTTGAATCATGTATGCCTGTTCGTATGCCTGTGGCTAAGATACTGTGTAAAGAAATGGTAAATAAATACTTTGAGAATCTTTAAGAGTGCATTGACTTTGTTAGTGAATAGGCATTCCATCTTTCTCCAATACTAATTCAAATTGTTAAATTAATAATGGAATAGTATAAATAGTTATTAGTGATAGGATAGTAAACATAATTATTAGAATAGTAGTGTAGTATCATAGATAACTCTCTTCTATAAAAAATGGATTTTATTCGTAGAAAGTATCTTATATACACAGTAGAAAATAATATAGATTTTTTAAAGGATGATACATTAAGTAAAGTAAACAATTTTACCCTCAATCATGTACTAGCTCTCAAGTATCTAGTTAGCAATTTTCCTCAACATGTTATTACTAAGGATGTATTAGCTAATACCAATTTTTTTGTTTTCATACATATGGTACGATGCTGTAAAGTATACGAAGCGGTTTTACGACACGCATTTGATGCACCCACGTTGTACGTTAAAGCATTGACTAAGAATTATTTATCGTTTAGTAACACAATACAGTCGTACAAGGAAACAGTGCATAAACTAACACAAGATGAAAAATTTTTAGAGGTTGCCGAATACATGGACGAATTAGGAGAACTTATAGGCGTAAATTATGACTTAGTTCTTAATCCATTATTTCACGGAGGGGAACCCATCAAAGATATGGAAATCATTTTTTTAAAACTGTTTAAGAAAACAGACTTCAAAGTTGTTAAAAAATTAAGTGTTATAAGATTACTTATTTGGGCATACCTAAGCAAGAAAGATACAGGCATAGAGTTTGCGGATAATGATAGACAAGATATATATACTCTATTTCAACAAACTGGTAGAATAGTCCATAGCAATCTAACAGAAACGTTTAGGGATTATATCTTTCCCGGAGATAAGACTAGCTATTGGGTGTGGTTAAACGAAAGTATAGCTAATGATGCGGATATCGTTATTAATAGACCCGCCATTACCATGTATGATAAAATTCTTAGTTATATATACTCTGAGATAAAACAGGGACGCGTTAATAAAAACATGCTTAAGTTAGTTTATATCTTTGAGCCTGAAAAAGATATCAGAGAACTTCTGCTAGAAATCATATATGATATTCCTGGAGATATCCTATCTATTATTGATGCAAAAAACGACGATTGGAAAAAATATTTTATTAGTTTTTACAAAGCTAATTTTATTAACGGTAATACATTTATTAGTGATAGAACGTTTAACGATGACTTATTCAGAGTTGTTGTTCAAATAGATCCCGAATATTTCGATAATGAACGAATTATGTCTTTATTCTATACGAGTGCTGCGGACATTAAACGATTTGATGAGTTAGATATTAATAACAGTTATATATCTAATATAATTTATGAGGTGAACGATATCACATTAGATACAATGGATGATATGAAGAAGTGTCAAATCTTTAACGAGGATACGTTGTATTATGTTAAGGAATACAATACATACCTGTTTTTGCACGAGTCGGATCCCATGGTCATAGAGAACGGAATACTAAAGAAACTGTCATCTATAAAATCCAAGAGTAGACGGCTGAACTTGTTTAGCAAAAACATTTTAAAATATTATTTAGACGGACAATTGGCTCGTCTAGGTCTTGTGTTAGATGATTATAAAGGAGACTTATTAGTTAAAATGATAAACCATCTCAAATCTGTGGAGGATGTATCCGCATTCGTTAGATTTTCTACAGATAAAAACCCTAGTATTCTTCCATCGCTAATCAAAACTATTTTAGCTAGTTATAATATTTCCATCATCGTCTTATTTCAAAGGTTTTTAAGAGATAATCTATATCATGTAGAAGAATTCTTGGATAAAAGCATCCATCTAACCAAGACGGATAAGAAATATATACTTCAATTGATAAGGCACGGTAGATCATAGAACAAACCAAATATATTATTAATAATTTGTATATACATAGATATAATTATCACATATTAAAAAATAACACATTTTTGATAAATGGAAACCGTTGCAACAATTCAGACTCCCACCAAATTAATGAATAAAGAAAATGCAGAAATGATTTTGGAAAAAATTGTTAATCATATAGCTATGTATATTAGTGACGAATCAATATATTCAGAAAATAATCCTGAATATATTGATTTTCGTAACAGATACGGAGACTATAGATCTCTCATTATAAAAAGTGATCACGAGTTTGTAAAGCTATGTAAAGATCATGCAGAGAAAAGTTCTCCAGAAACGCAACAAATGATTATCAAACACATATACGAACAATATCTTATTCCAGTATCTGAAGTACTATTAAAACCTATAATGTCCATGGGTGACATATTTACATATAACGGATGTAAAGACAATGAATGGATGCTAGAACAACTCTCTACCCTAAACTTTAACAATCTCTACACATGGAACTCATGTAGCATAGGCAATGTAACGCGTCTGTTTTATACATTTTTTAGTTATCTGATGAAAGATAAACTAAATATATAAGTATAATCCCATTCTAATACTTTAACCTGATGTATTATTACCTGCATCTTATTAGAATATTAACCTAACTAAAAGACATAAAAAGCGGTAGGATATAAATATTATGGCCGCAACCGTTCCGCGTTTTGACGATGTGTACAAAAATGCACAAAGAAGAATTCTAGATCAAGAAACATTTTTTAGTAGAGGTCTAAGTAGACCGTTAATGAAAAACACATATCTATTTGATAATTACGCGTATGGATGGATACCAGAAACTGCAATTTGGAGTAGTAGATACGCAAACCTAGATGCTAGTGACTATTATCCCATTTCGTTGGGATTACTTAAAAAGTTTGAATTTCTCATGTCTCTATATAAAGGTCCTATTCCCGTATATGAAGAAAAAGTAAATACTGAATTCATTGCTAATGGATCTTTCTCCGGTAGATACGTATCATATCTTAGAAAGTTTTCTGCCCTTCCAACAAACGAGTTTATTAGTTTTTTATTATTGACCTCCATCCCTATCTATAATATCTTATTCTGGTTTAAAAACACACAGTTTGATATTACTAAACACACATTATTCAGATACGTCTATACAGATAATACCAAACACCTTGCGTTGGCTAGGTATATACATCAAACAGGAGACTATAAGCCTTTGTTTAGTCGTCTCAAAGAGAATTATATATTTACTGGTCCCGTTCCAATAGGTATCAAAGATATAGATCACCCTAATCTTAGTAGAGCAAGAAGTCCATCCGATTATGAGACATTAGCTAATATTAGTACTATATTGTACTTTACCAAGTATGATCCAGTATTAATGTTTTTATTGTTTTACGTACCTGGGTATTCAATTACTACAAAAATTACTCCAGCCGTAGAATATCTAATGGATAAACTGAATCTAACAAAGAGCGACGTACAACTGTTGTAAATTATTTTATGCTTCGTAAAATGTAGGTCTTGAACCAAACATTCTTTGAAAAAATGAGATGCATAAAACTTTATTATCCAATAGATTAACTATTTCAGACGTCAATCGTTTAAAGTAAACTTCGTAAAATATTCTTTGATTGCTGCCGAGTTTAAAACTTCTATCGATAATTGTTTCATATGTTTTAATATTTACAAGTTTTTTGGTCCATGGTACATTAGCTGGACAGATATATGCAAAATAATATCGTTCTCCAAGTTCTATAGTCTCTGGATTGTTTTTATTATATTCAGTAACCAAATACATATTAGGGTTATCTGCGGATTTATAATTTGAGTGATGCATTCGACTCAACATAAATAATTCTAGAGGAGACGATCTACTATCAAATTCGGATCGTAAATCTGTTTCTAAAGAACGGAGAATATCTATACATACCTGATTAGAATTCATCCGTCCTTCAGACAACATCTCAGACAGTCTGGTCTTGTATGTCTTAATCATATTCTTATGAAACTTGGAAACATCTCTTCTAGTTTCACTAGTACCTTTATTAATTCTCTCAGGTACAGATTTTGAATTCGACGATGCCGAGTATTTCATCGTTGTATATTTCTTCTTCGATTGCATAATCAAATTCTTATATACCGCCTCAAACTCTATTTTAAAATTATTAAACAATACTCTACTATTAATCAGTCGTTCTAACTCCTTTGCTATTTCTATGGACTTATCTACATCTTGACTGTCTATCTCTGTAAACACGGAGTCGGTATCTCCATACACGCTACGAAAACGAAATCTATAATCTATAGGCAACGATGTTTTCACAATCGGATTAATATCTCTATCGTCCATATAAAATGGATTACTTAATGTATTGGCAAACCGTAACATACCGTTGGATAACTCTGCTCCATTTAGTACCGATTCTAGATACAATATCATTCTACGTCCTATGGATGTGCAACTCTTAGCCGAAGCGTATGAGTATAGAGCACTATTTCTAAATCCCATCAGACCATATACTGAGTTGGCTACTATCTTGTACGTATATTGCATGGAATCATAGATGGCCTTTTCAGTTGAACTGGTAGCCTGTTTTAACATCTTTTTATATCTGGCTCTCTCTGCCAAAAATGTTCTTAATAGTCTAGGAATGGTTCCTTCTATTGATCTATCGAAAATTGCTATTTCAGAGATGAGGTTCGGTAGTCTAGGTTCACAATGAACCGTAATATATCTAGGAGGTGGATATTTCTGAAGCAAGAGTTGATTATTTATTTCTTCTTCCAATCTATTGGTACTAACAACGACACCGACTAATGTTTCCGGAGATAGATTTCCAAAGATACACACATTAGGATACAGACTGTTATAATCAAAGATTAATACATTATTACTAAACATTTTTTGTTTTGGAGCAAATACCTTACCGCCTTCATAAGGAAACTTTTGTTTTGTTTCTGATCTGACTAAGATAGTTTTAGTTTCCAACAATAGCTTTAACAGTGGACCCTTGATGATTGTACTCGCTCTATATTCGAATACCATGGATTGAGGAAGCACATATGTTGCCGCACCAGCGTCTGTTTTTGTTTCTACTCCATAATACTCCCACAAATACTGACACAAACAAGCATCATGAATACAGTATCTAGCCATATCTAAAGCTATGTTTAGATTATAATCCTTATACATCTGAGCTAAATCAATGTCATCCTTTCCGAAAGATAATTTATATATATCATTAGGTAAAGTAGGACATGATAGTACGACTTTAAATCCATTTTCCAAAATATCTTTACGAATTACTTTACATATAATATCCTCATCAACAGTCACGTAATTACCTGTGGTTAAAACCTTTGCAAATGTATCGGCTTTGCCTTTCGCGTCCGTAGTATCGTCACCGATGAACGTCATTTCTCTAACTCCTCTATTTAATACTTTACCCATGCAACTGAACGCGTTCTTGGATATAGAATCCAATTTGTACGAATCCAATTTTTCAGATTTTTGAATGAATGAATATAGATCGAAAAATATAGTTCCATTATTGTTATTAACGTGAAACGTAGTATTGGCCATGCCGCATACTCCCTTATGACTAGACTGATTTCTCTCATAAATACAGAGATGTACAGCTTCCTTTTTGTCTGGAGATCTAAAGATAATCTTCTCTCCTGTTAATAACTCTAGACGATTAGTAATATATCTCAGATCAAAGTTATGTCCGTTAAAGGTAACGACGTAGTCGAACGTTAGTTCCAACAATTGTTTAGCTATTCGTAACAAAACTATTTCAGAACATAGAACTAGTTCTCGTTCGTAATCCATTTCCATTAGCGACTGTATCCTCAAACATCCTCTATCGACGGCTTCTTGTATTTCCTGTTCCGTTAACATCTCTTCATTAATGAGCGTAAACAGTAATCGTTTACCACTTAAATCGATATAACAGTAACTTGTATGCGAGATTGGGTTAATAAATACAGAAGGAAACTTCTTATCGAAGTGACACTCTATATCTAGAAATAAGTACGATCTTGGGATATCGAATCTAGGTATTTCTTTAGCGAAACAGTTACGTGGATCGTCACAATGATAACATCCATTGTTAATCTTTGTCAAATATTGCTCGTCCAACGAGTAACATCCGTCTGGAGATATCCCGTTAGAAATATAAAACCAACTAATATTGAGAAATTCATCCATGGTGGCATTTTGTATGCTGCGTTTCTTTGGCTCTTCTATCAACCACATATCTGCGACGGAGCATTTTCTATCTTTAATATCTAGATTATAACTTATTGTCTCGTCAATGTCTATAGTTCTCATCTTTCCCATCGGCCTCGCATTAAATGGAGGAGGAGATAATGACTGATATATTTCGTCCGTCACTACGTAATAAAAGTAATGAGGAAATCGTATAAATACGGTCTCGCCATTTCGACATCTGGATTTCAGATATAAAAATCTGTTTTCACCGTGACTTTCAAACCAATTAATACACCTAACATCCATTTCTAGAATTTAGAAATATATTTTCATTTAAATGAATCCCAAACATTGGGGAAGAGCCGTATGGACCATTATTTTTATAGTACTTTCGCAAGCGGGTTTAGACGGCAACATAGAAGCGTGTAAACGAAAACTATATACTATAGTCAGCACTCTTCCATGTCCTGCATGTAGACGACACGCGACTATCGCTATAGAGGACAATAATGTCATGTCTAGCGATGATCTGAATTATATTTATTATTTTTTCATCAGATTATTTAACAATTTGGCATTTGATCCCAAATACGCAATCGATGTGTCAAAGGTTAAACCTTTATAAACTTAACCCATTATAAAACTTATGATTAGTCACGACTGAAATAACCGCGTGATTATTTTTTGGTATAATTCTACACGGCATGGTTTCTGTGACTATGAATTCAACACCTGTTATCTTAGTGAAATCTTTAACAAACAGCAAGGGTTCGTCAAAGACATAAAACTCATTGTTTACGATCGAAATAGACCCCCTATCACACTTAAAATAAAAAATATCCTTATCCTTTACCACCAAATAAAATTCTGATTGGTCAATGTGAATGTATTCACTTAACAGTTCCACAAATTTATTTATTAACTCCGAGGCACATACATCGTCGGTATTTTTTATGACAAACTTTACTCTTCCAGCATCCGTTTCTAAAAAAATATTAACGAGTTCCATTTATATCATCCAATATTATTGAAATGACGTTGATGGACAGATGATATAAATAAGAAGGTACAGTACCTTTGTCCACCATCTCCTCCAATTCATACTCTATTTTGTCATTAACTTTAATGTGTGAAAACAGTACGCCACATGCTTCCATGACAGTGTGTAACACTTTGGATACAAAATGTTTGACATTAGTATAATTGTCCAAGACTGTCAATCTATAATAGATAGTAGCTATAATATATTCTATGATGGTATTGAAGAAGATGACAACCTTGGCATATTGATCATTTAACACAGACATGGTATCAACAAATAGCTTAAATGAAAGAGAATCAGTAATTGGAATAAGCGTCTTCTCGATGTAGTGTCCGTATACCAACATGTCTGATATTTTGATGTATTCCATTAAATTATTTAGTTTTTTCTTTTTATTCTCGTTAAACAGAATTTCTGTCAATGGACCCCAACATCGTTGACCTATTAAGTTTTGATTGATTTTTCCGTGTAAGGCGTATCTAGTCAGATCGTATAGCCTATCCAATAATCCATCGTCTGTGCGTAGATCACATCGTACACTTTTTAATTTTCTATAGAAGAGTGACAGACATCTGGAGCAATTACAGACAGCAATTTCTTTATTCTCTACAGATGTAAGATACTTGAAGATATTCCTATGATGATGCAGAATTTTGGATAACACGGTATTGATGGTATCTGTTACCATAATTCCTTTGACTGATAGTGTCAAAGTACAAGATTTCCAATCTTTTGCAATTTTCAGTACCATTATCTTTGTTTTGATATCTATATCAGACAGCATGGTACGTCTGACAACACAGGGATTAAGACGGAAAGATGAAATGATTCTCTCAACATCTTCAATAGATACCTTGCTATTTTTTTTGGCATTATCTATATGTGAGAGAATATCCTCTAGAGAATCAGTATCCTTTTTGATGATAGTGGATCTCAATGACATGGGACGTCTAAACCTTCTTATTCTATCACCAGATTGCATGGTGATTTGTCTTCTTTCTTTTATCATGATGTAATCTCTAAATTCATCGGCAAATTGTCTATATCTAAAATCATAATATGAGATGTTTACCTCTACAAATATCTGTTCGTCCAATGTTAGAGTATCTATATCAGTTTTGTATTCCAAATTAAACATGGCAACGGATTTAATTTTATATTCCTCTATTAAGTCCTCGTCGATAATAACAGAATGTAGATAATCATTTAATCCATCGTACATGGTTGGAAGATGCTCGTTGACAAAATCTTTAATTGTCTTGATGAAGGTGGGACTATATCTAACATCTTGATTAATAAAATTTATAACATTGTCCATAGGATACTTTGTAACTAGTTTTATACACATCTCTTCATTGGTAAGTTTAGACAGAATATCGTGAACAGGTGGTATATTATATTCATCAGATATACGAAGAATAATGTCCAAATCTATATTGTTTAATATATTATATAGATGTAGTGTAGCTCCTACAGGAATATCTTTAACTAAGTCAATGATTTCATCAACAGTTAGATCTATTTTAAAGTTAATCATATAGGCATTGATTTTTAAAAGGTATGTAGCCTTGACTACATTCTCATTAATTAACCATTCCAAGTCACTGTGTGTAAGAAGATTATATTCTATCATAAGCTTGACTACATTTGGTCCCGATACCATTAAAGAATTCTTATGATATAAGGAAACAGCTTTTAGGTACTCATCTACTCTACAAGAATTTTGGAGAGCCTTAACGATATCAGTGACGTTTATTATTTCAGGAGGAAAGAACCTAACATTGAGAATATCTGAATTAATAGCTTCCAGATACAGTGATTTTGGCAATAGTCCGTGTAATCCATAATCCAGTAACACGAGCTGGTGCTTGCTAGACACCTTTTCAATGTTTAATTTTTTTGAAATAAGCTTTGATAAAGCCTTCCTCGCAAATTCCGGATACATGAACATGTCGCCAACATGATTAAGTATTGTTTTTCATTATTTTTATATTTTCTCAACAAGTTCTCAATACCCCAATAGATAATAGAATATCACCCAATGCGTCCATGTTGTCTATTTCCAACAGGTCGCTATATCCACCAATAGAAGTTTTCCCAAAAAAGATTCTAGGAACAGTTCTACCACCAGTAATTTGTTCAAAATAGTCACGCAATTCATTTTCGGGTTTAAATTCTTTAATATCTACAATTTCATACGCTCCTCTTTTGAAACTAAACTTATTTAGAATATCCAGTGCGTTTCTACAAAAAGGACATGTAAACTTGACAAAAATTGTCACTTTGTTATTGGCCAACCTTTGTTGTACAAATTCCTCGGCCATTTTTAATATTTAAGTGATACAAAACTATCTCGACTTATTTAACTCTTTAGTCGAGATATATGGACACAGATAGCTATATGATAACCAACTACAGAAGACAAACGCTATAAAAAACATAATTACGACGAGCATATTTATAAATATTTTTATTCAGTATTACTTGATATAGTAATATTAGGCACAGTCAAACATTCAACCACTCTAGATACATTAACTCTCTCATTTTCTTTAACAAATTCTGCAATATCTTCGTAAAAAGATTCTTGAAACTTTTTAGAATATCTATCGACTCTAGATGAAATAGCGTTCGTCAACATACTATGTTTTGTATACATAAAGGCGCCCATTTTAACAGTTTCTAGTGACAAAATGCTAGCGATCCTAGGATCCTTTAGAATCACATAGATTGACGATTCGTCTCTCTTAGTAACTCTAGTAAAATAATCATACAATCTAGTACGCGAAATAATATTATCCTTGACTTGAGGAGATCTAAACAATCTAGTTTTGAGAACATCGATAAGTTCATCGGGAATTACATACATACTATCTTTAATAGAACTCTTTTCATCCAGTTGAATGGATTCGTCCTTAACCAACTGATTAATGAGATCTTCTATTTTATCATTTTCTAGATGATATGTATGTCCATTAAAGTTAAATTGTGTAGCGCTTCTTTTTAGCCTAGCAGCCAATACTTTAACATCACTAATATCGATATACAAAGGAGATGATTTATCGATGGTATTAAGAATTCGTTTTTCGACATCCGTCAAAACCAATTCCTTTTTGCCTGTATCATCCAGTTTGCCATTCTTTGTAAAGAAATTATTTTCTACTAGACTATTAATAAGACTGATAAGGATTCCTCCATAATTGCACAATCCAAACTTTTTCACAAAACTAGACTTTACGAGATCTACAGGAATGCGTACTTCAGGTTTCTTAGCTTGTGATTTTTTCTTTTGTGGACATTTTCTAGTGACCAACTCATCTACCATTTCATTGATTTTAGCAGTGAAATAAGCTTTCAATGCACGGGCACTGATACTATTGAAAACGAGTTGATCTTCAAATTCCGCCATTTAAGTTCACCAAACAACTTTTAAATACAAATATATCAATAGTAGTAGAATAAGAACTATAAAAAAAATAATAATTAACCAATACCAACCCCAACAACCTGTATTATTAGTTGATGTGACAGTTTTCTCATCACTTAGAACAGATTTAACAATTTCTATAAAGTCTGTCAAATCATCTTCCTGAGAACCCATAAATACACCAAATATAGCAGCGTACAACTTATCCATTTATACATTGAATATTGGCTTTTCTTTATCGCTATCTTCATCATATTCATCATCAATATCAACAAGTCCCAGATTACGAACCAGATCTTCTTCTACATTTTCAGTCATTGATACGCGTTCACTATCTCCAGAGAGTCCGATAACGTTAGCCACTACTTCTCTATCAATGATTAGTTTCTTGAGCGCGAATGTAATTTTTGTTTCCGTTCCGGATCTATAGAAAACTACAGGTGTAATAATTGCCTTGGCTAATTGTCTTTCTCTTTTACTGAGTGATTCTAGTTCACCTTCTATAGATCTGAGAATGGATGATTCTCCAGTCGAAACATATTCTACCATGGCTCCGTTTAATTTGTTGATGAAGATGGATTCATCCTTAAATGTTTTCTCTGTAATAGTTTCCGCCGAAAGACTATGCAAAGAATTTGGAATGCGTTCCTTGTGTGTAATGTTTCCATAGACAGCTTCTAGAAGTTGATACAACATAGGACTAGCCGCGGTAACTTTTATTTTTAGAAAGTATCCATCGCTTCTATCTTGTTTAGATTTATTTTTATAAAGTTTAGTCTCTCCTTCCAACATAATAAAAGTGGAAGTCATCTGACTAGATAAACTATCAGTAAGTTTTATAGAGATAGATGAACAATTAGCGTATTGAGAAGCATTTAGTGTAACGCATTCGATACATTTTGCATTAGATTTACTAATCGATTTTGCATACTCTATAACACCCGCACAAGTCTGTAGAGAATCGCTAGATGCTGTAGGTCTTGGTGAAGTTTCAACTCTCTTCTTGATTACCTTACTCATGATTAAACCTAAATAATTGTACTTTGTAATATAATGATATATATTTTCACTTTATCTCATTTGAGAATAAAAATGTTTTTGTTAACCACTGCATGATGTACAGATTTCGGAATCGCAAACCACTTGTGGTTTTATTTTATCCTTGTCCAATGTGAATTGAATGGGAGCGGATGCGGGTTTCGTACGTAGATAGTACATTCCCGTTTTTAGACCGAGACTCCATCCGTAAAAATGCATACTCGTTAGTTTGGAATAACTCGGATCTGCTATATGGATATTCATAGATTGACTTTGATCGATGAAGGCTCCCCTGTCTGCAGCCATTTTTATGATCGTCTTTTGTGGAATTTCCCAAATAGTTTTATAAACTCGCTTAATATCTTCTGGAAGGTTTGTATTCTGAATGGATCCACCATCTACCATAATCCTATTCTTGATCTCATCATTCCATAATTTTCTCTCGGTTAAAACTCTAAGGAGATGCGGGTTAACTACTTGGAATTCTCCAGACAATACTCTCCGAGTGTAAATATTACTGGTATACGGTTCCACCGACTCATTATTTCCCAAAATTTGAGCAGTTGATGCAGTCGGCATAGGTGCCACCAATAAACTATTTCTAAGACCGTATGTTCTGATTTTATCTTTTAGAGGTTCCCAATTCCAAAGATCCGACGGTACAACATTCCAAAGATCATATTGTAGAATACCGTTACTGGCGTACGATCCTACATATGTATCATATGGTCCTTCCTTCTCAGCTAGTTTACAACTCGCCTCTAATGCACCGTAATAAATGGTTTCAAAGATCTTCTTATTTAGATCTTGTGCTTCCAGGCTATCAAATGGATAATTTAAGAGAATAAACGCGTCCGCTAATCCTTGAACACCAATACCGATAGGTCTATGTCTCTTATTAGAGATTTCAGCTTCTGGAATAGGATAATAATTAATATCTATAATTTTATTGAGATTTCTGACAATTACTTTGACCACATCCTTCAGTTTGAGAAAATCAAATCGCCCATCTATTACAAACATGTTCAATGCAACAGATGCCAGATTACACACGGCTACCTCATTAGCATCCGCATATTGTATTATCTCAGTGCAAAGATTACTACACTTGATGGTTCCTAAATTTTGTTGATTACTCTTTTTGTTACACGCATCCTTATAAAGAATGAATGGAGTACCAGTTTCAATCTGAGATTCTATAATCGCTTTCCAGACGACTCGAGCCTTTATTATACATTTGTATCTCCTTTCTCTTTCGTATAGTGTATACAATCGTTCGAACTCGTCTCCCCAAACATTGTCCAATCCAGGACATTCATCCGGACACATCAACGACCACTCTCCGTCATCCTTCACTCGTTTCATAAAGAGATCAGGAATCCAAAGAGCTATAAATAGATCTCTTGTTCTATGTTCATCGTTTCCTGTATTCTTTTTAAGATCGAGGAACGCCATAATATCAGAATGCCACGGTTCCAAGTATATGGCCATAACTCCAGGCCGTTTGTTTCCTCCCTGATCTATGTATCTAGCGGTGTTATTATAAACTCTCAACATTGGAATAATACCGTTTGATATACCATTGGTACCGGAGATATAGCTTCCACTGGCACGAATATTACTAATTGATAGACCTATTCCCCCTGCCATTTTAGAGATTAATGCGCATCGTTTTAACGTGTCATAGATGCCTTCTATGCTATCATCGATCATGTTAAGTAGAAAACAGCTAGACATTTGGTGACGAGTAGTTCCCGCATTAAATAAGGTAGGAGAAGCGTGCGTAAACCATTTTTCAGAAAGTAGATTGTACGTCTCAATAGCTGAGTCTATATCCCATTGATGAATTCCTACTGCGACACGCATTAACATGTGCTGAGGTCTTTCAACAATTTTGTTGTTTATTTTCAACAAGTAGGATTTTTCCAAAGTTTTAAAACCAAAATAGTTGTATGAAAAGTCTCGTTCGTAAATAATAACCGAATTGAGCTTATCCTTATATTTGTTAACTATATCCATGGTAATACTTGAAATAATCGGAGAATGTTTCCCATTTTTAGGATTAACATAGTTGAATAAATCCTCCATCACTTCACTAAATAGTTTTTTTGTTTCCTTGTGTAGATTTGATATGGCTATTCTGGCGGCTAGAATGGCATAATCCGGATGTTGTGTAGTACAAGTGGCTGCTATTTCGGCTGCCAGAGTGTCCAATTCTACCGTTGTTACTCCATTATATATTCCTTGAATAACCTTCATAGCTATTTTAATAGGATCTATATGATCAGTGTTTAAGCCATAGCACAATTTTCTAATACGAGACGTGATTTTATCAAACATGACATTTTCCTTGTATCCATTTCGTTTAATGACAAACATTTTTGTTGGTGTAATAAAAAAAATTATTTAATTTTTCATTAATAGGGATTTGACGTATGTAGCGTACAAAATTATCGTTCCTGGTATATAGATAAAGAGTCCTATATATTTGAAAATCGTTACGGTTCGATTAAACTTTAATGATTGCATTGTGAATATATCATTAGGATTTAACTCCTTGACTATCATGGCGGTGCCAGAAATTACCATCAAAAGCATTAATACAGTTATGCAGATCGCAGTTAGAACGGTTATAGCATCCACCATTTATATCTAAAAATTAGATCAAAGAATATGTGACAACGTCCTAGTTGTATACTGAGAATTGACGAAACAATGTTTCTTACATATTTTTTTCTTATTAGTAACCGACTTAATAGTAGGAACTGGAAAACTAGACTTGATTATTCTATAAGTATAGATACCCTTCCAAATAATGTTCTCTTTGATAAAAGTTCCAGAAAATGTAGAATTTTTTAAAAAGTTATCTTTTGCTATTACTAATATCGTGGTTAGACGCTTATTATTAATATGAGTGATGAAATCCACACCGCTTCTAGATATCGCTTTTATTTCCACATTAGATGGTAAATCCAATAGTGAAACTATCTTTTTAGGAATGTATGGACTCGCGTTTAGAGGAGTGAACGTCTTCGGAGTAGTAAAGGATGATTCGTCAAATGAATAAACAATTTCACAAATGGATGTTAATGTATTAGTAGGAAATTTTTTGACGCTAGTGGAATTGAAGATTCTAATGGATGATGTTCTACCTATTTCATCCGATAACATGTTAATTTCCAATACCAACGGTTTTAATATTTCGATGATATACGGTAGTCTCTCTTTCGGACTTATATAGCTTATTCCACAATACGAGTCATTATATACTCCAAAAAACAAAATAACTAGTATAAAATCTGTATCGAATGGGAAAAACGAAATTATCGATATAGGTATAGAATCCGGAACATTGAACGTATTAATACTTAATTCTTTTTCAGTGGTAAGAACCGATAGGTTATTGACATTGTATGGTTTTAAATATTCTATAACTTGAGACTTGATAGATATTAATGACGAATTGAAAATTATTTTTATCACCACGTGTGTTTCAGGATCATCGTCGACGCCAGTTAACCAACCGAATGGAGTAAAATAAATATCATTAATATATGCTCTAGATATTAGTATTTTTATTAATCCTTTGATTATCATCTTCTCGTACGCGAATGATTCCATGATCAAGAGTGATTTGAGAACATCCTCCGGAGTATTAATGGGTTTAGTAAACAGTCCATCGTTGCAATAATAAAAGTTGTCCAAGTTAAAGGATATTATGCATTCGTTTAAAGATATCACCTCATCTAACGGAGACAATTTTTTGGTAGGTTTTAGAGACTTTGAAGCTACTTGTTTAACAAAGTTATTCATCGTCGTCTACTATTCTATTTAATTTTGTAGTTAATTTATCACATATCACATTAATTGACTTTTTGGTCCACTTTTCCATACGTTTATATTCTTTTAATCCTGCGTTATCCGTTTCCGTTATATACAGGGATAGATCTTGCAAGTTAAATAGAATGCTCTTAAATAATGTCATTTTTTTATCCGCTAAAAATTTAAAGAATGTATAAACTTTTTTCAAAGATTTAAAACTTTTAGGTGGAGTTCTGGTACACAATATCATAAACAAACTAATAAACATCCCACATTCAGATTCCAACAATTGATTAACTTCCACATTAATACAGCCTATTTTCGCTCCAAATGTACATTCGAAAAATCTGAATAAAACATCAATATCGCAATTTGTATTATCCAATACAGAATGTCTGTGATTCGTGTTAAAACCATCGGAAAAAGAATAGAAATAAAAATTATTATAATGGTGGAATTCAGTTGGAATATTGCCTCCGGAGTCATAAAAGGATACTAAACATTGTTTTTTATCGTAAATTACACATTTCCAATGAGACAAATAACAAAATCCAAACATTACAAATCTAGAGGTAGAACTTTTAATTTTGTCTTTAAGTATATACGATAAGATATGTTTATTCATAAACGCGTCAAATTTTTCATGAATAGCTAAGGAGTTTAAGAATCTCATGTCAAATTGTCCTATATAATCCACTTCGGATCCATAAGCAAACTGAGAGACTAAGTTCTTAATACTTCGATTGCTCATCCAGGCTCCTCTCTCAGGCTCTATTTTCATCTTGACGACCTTTGGATTTTCACCAGTATGTATTCCTTTACGTGATAAATCATCGATTTTCAAATCCATTTGTGAGAAGTCTATCGCCTTAGATACTTTTTCCCGTAGTTGAGGTTTAAAGAAATACGCTAACGGTATACTAGTAGGTAACTCAAAGACATCATATATAGAATGGTAACGCGTCGTTAACTCGTCGGTTAACTCTTTCTTTTGATCGAGTTCATCGCTACTATTGGGTCTGCTCAGGTGCCCCGACTCTACTAGTTCCAACATCATACCGATAGGAATACAAGACACTTTGCCAGCGGTTGTAGATTTATCATATTTCTCCACCACATATCCGTTACAATTTGTTAAGAATTTAGATACATCTATATTGCTACATAATCCAGCTAGTGAATATATATGACATAATAAATTGGTAAATCCTAGTTCTGGTATTTTACTAATTACTAAATCTGTATATCTTTCCATTTATCATGGAAAAGAATTTACCAGATATCTTCTTTTTTCCAAACTGCGTTAATGTATTCTCTTACAAATATTCACAAGATGAATTCAGTAATATGAGTAAAACGGAACGTGATAATTTCTCATTGGCTGTGTTTCCAGTGATAAAACATAGATGGCATAACGCACACGTTGTAAAACATAAAGGAATATACAAAGTTAGTACAGAAGCACGTGGAAAAAAAGTATCTCCTCCATCACTAGGAAAACCCGCACATATAAACCTAATGTCGAAGCAATATATATATAGTGAGTATGCAATAAGCTTTGAATGTTATAGTTTTCTAAAATGTATAACAAATACAGAAATCAATTCGTTCGATGAGTATATATTAAGAGGACTATTAGAAGCTGGTAATAGTTTACAGATATTTTCCAATTCCGTAGGTAAACGAATAGATACTATAGGTGTACTAGGGAATAAGTATCCATTTAGCAAAATTCCATTGGCCTCATTAACTCCTAAAGCACAACGAGAGATATTTTTAGCGTGGATTTCTCATAGACCTGTAGTTTTAACTGGAGGAACCGGAGTGGGTAAGACGTCACAGGTACCCAAGTTATTGCTTTGGTTTAATTATTTATTTGGTGGATTCTCTTCTCTAGATAAAATCACTGACTTTCACGAAAGACCAGTCATTCTATCTCTTCCTAGGATAGCTTTAGTTAGATTGCATAGCAATACCATTTTAAAATCATTGGGATTTAAGGTACTAGATGGATCTCCTATCTCTTTACGGTACGGATCTATACCGGAAGAATTAATAAACAAACAACCAAAAAAATATGGAATTGTATTTTCTACCCATAAGTTATCTCTAACAAAACTATTTAGTTATGGCACTATTATTATAGACGAAGTTCATGAGCATGATCAAATAGGAGATATTATTATAGCAGTAGCGAGAAAACATCATACGAAAATAGATTCTATGTTTTTAATGACTGCCACGTTAGAGGATGACAGGGAACGTCTAAAAATATTTTTACCTAATCCCGCATTTATACATATTCCTGGAGATACACTGTTTAAAATTAGCGAGGTATTTATTCATAATAAGATAAATCCATCTTCCAGAATGGCATATATAGAAGAAGAAAAGAGAAATTTAGTTACTGCTATACAGATGTATACTCCTCCTGATGGATCATCCGGTATAGTCTTTGTGGCATCCGTTGCACAGTGTCACGAATATAAATCATATTTAGAAAAAAGATTACCGTATGATATGTATATTATTCATGGTAAGGTCTTAGATATAGACGAAATATTAGAAAAAGTGTATTCATCACCTAATGTATCGATAATTATTTCTACTCCTTATTTGGAATCCAGCGTTACTATACGCAATGTTACACACATTTATGATATGGGTAGAGTTTTTGTCCCCGCTCCTTTTGGAGGATCACAACAATTTATTTCTAAATCTATGAGAGATCAACGAAAAGGAAGAGTAGGAAGAGTTAATCCTGGAACATACGTATATTTCTATGATCTGTCTTATATGAAATCTATACAGCGAATAGATTCAGAATTTCTACATAATTATATATTGTACGCTAATAAGTTTAATCTAACACTCCCCGAAGATTTGTTTATAATCCCTACAAATTTGGATATTCTATGGCGTACAAAGGAATATATAGACTCGTTCGATATTAGTACAGAAACATGGAATAAATTATTATCCAATTATTATATGAAGATGATAGAGTATGCTAAACTTTATGTACTAAGTCCTATTCTCGCTGAGGAGTTGGATAACTTTGAGAGGACGGGAGAATTAACTAGTATTGTACAAGAAGCCATTTTATCTCTAAATTTACAAATTAAGATTTTAAAATTTAAACATAAAGATGATGATACGTATATACACTTTTGTAGAATATTATTCGGTGTCTATAACGGAACAAACGCTACTATATATTATCATAGACCTCTAACGGGATATATGAATATGATTTCAGATACTATATTTGTTCCTGTAGATAATAACTAAAAATCAAAATCTAATGACCACATCTTTTTTTAGAGATGAAAAATTTTCCACATCTCCTTTTGTAGACACGACTAAACATTTTGCAGAAAAAAGTTTATTATTATTTAGATAATCGTATACTTCATCAGTGTAGATAGTAAATGTGAACAGATAAAAGGTATTCTTGCTCAATAGATTGGTAAATTCCATAGAATATATTAATCCTTTCTTCTTGAGATCCCACATCATTTCAACCAAAGACGTTTTATCCAATGATTTACCTCGTACTATACCACATACAAAACTAGATTTTGCAGTGATGTCGTACCTGGTATTCCTACCAAACAAAATTTTACTTTTAGTTCTTTTAGAAAATTCTAAGGTAGAATCTCTATTTGTCAATATGTCATCTATGGAATTACCACTAGCAAAAAATGATAGAAATATATATTGATACATCGCAGCTGGTTTTGATCTACTATACTTTAAAAACGAATCAGATTCCATAATTGCTTGTATATCATCAGCTGAAAAACTATGTTTTACACGTATTCCTTCGGCATTTCTTTTTAATGATATATCTTGTTTAGACAATGATAAAGTTATCATGTCCATGAGAGACGCGTCTCCGTATCGTATAAATATTTCATTAGATGTTAGACGCTTCATTAGGGGTATACTTCTATAAGGTTTCTTAATTAGTCCATCATTGGTTGCGTCAAGAACTACTATCTGATGTTGTTGGGTATCTCTAGTGTTACACATGGCCTTACTAAAGTTTGGGTAAATAACTATGATATCTCTATTAATTATAGATGTATATATTTCATTCGTCAAGGATATTAATATCGACTTACTATCGTCATTAATACGTGTAATGTAATCATATAAATCATGCGATAGCCAAGGAAAATTCAAATAGATGTTCATCATATAATCGTCGCTATAATTCATATTAATACTTTGACATTGACTAATTTGTAATATAGCCTCGCCACGAAGAAAGCTCTCGTATTCAGTTTCATCGATAAAGGATACCGTTAAATATAACTGGTTGCCGATAGTCTCATAGTCTATTAAGTGGTAAGTTTCGTATAAATACAGAATCCCTAAAATATTATCTAATGTGGGATTAATCCTTACCATAACTGTATAAAATGGAGCCGGAGTCATAACTATTTTACCGTTTGTACTTACTGGAATAGATGAAGGAATAATCTCCGGACATGATGGTAAAGACCCAAATGTCTGTTTGAAGAAATCCAATGTTCCAGGTCCTAATCTCTTGACAAAAATTACGATATTCGATCCCGATATCCTTTGCATTCTATTTACCAGCATATCACGAACTATATTAAGATTATCTATCATGTCTATTCTCCCACCGTTATATAAATCGCCTCCGCTAAGAAACGTTAGTATATCCATACAATGGAATACTTCATTTCTAAAATAGTATTCGTTTTCTAATTCTTTAATGTGAAATCGTATACTAGAAAGGGAAAAATTATCTTTGAGTTTTCCATTAGAAAAGAACCACGAAACTAATGTTCTGATTGCGTCTGACTCCGTCGCTGAATTAATAGATTTACACCAAAAACTCATATAACTTCTAGATGTAGAAGCATTCGCTAAAAAATTAGTAGAATCAAAGGATATAAGTAGATGTTCCAACAAGTGAGCAATTCCCAAGATTTCATCTATATCATTCTCGAATCCGAAATTAGAAATTCCCAAGTAGATATCCTTTTTCATCCGATCATTGATGAAAATACGAACTTTATTCGGTAAGACGATCATTTACTAAGGAGTAAAATAGGAAGTAACGTTCGTATATCGTTATCGTCGTATAAATTAAAGGTGTGTTTTTTGCCATTAAGAGACATTATAATTTTACCAATATTGGAATTATAATATAGGTGTATTTGAGCACTAGAAACGGTCGATGCATCGGTAAATATAGCTGTATCTAATGTTCTAGTCGGTATTTCTTCATTTCGCTGTCTAATGATAGCGTTTTCTCTATCTGTTTCCATTACAGCTGCCTGAAGTTTATTGGTCGGATAATATGTAAAATAATAAGAAATACATACGAATAACAAAAATAAAATAAGATATAATAAAGATGCCATTTAGAGATCTAATTTTGTTCAACTTGTCCAAATTCCTACTTACAGAAGATGAGGAATCGTTGGAGATAGTATCTTCCTTATGTAGAGGATTTGAAATATCTTACGATGACTTAATATCGTACTTTCCAGATAGGAAATACCATAAATATATTTCTAAGGTATTTGAACATGTAGATTTATCGGAGGAATTAAGTATGGAATTCCATGATACAACTCTGAGAGATTTAGTATATCTTAGATTGTACAAGTATTCCAAGTATATACGGCCGTGTTATAAATTAGGAGATAATCTAAAAGGTATAGTTGTTATAAAGGACAGAAATATATATATTAGAGAAGCAAATGATGACTTGATAGAATATCTCCTCAAGGAATACACTCCTCAGATTTATACATATTCTAATGAGCGAGTTCCCATAGCTGGTTCAAAATTAATTCTTTGTGGATTTTCTCAAGTTACATTTATGGCGTATACAACGTCGCATATAACAACAAATAAAAAGGTAGATGTTCTCGTTTCCAAAAAATGTATAGATGAACTAGTCGATCCAATAAATTATCAAATACTTCAAAATTTATTTGATAAAGGAAGCGGAACAATAAACAAAATACTCAGGAAGATATTTTATTCGGTAACAGGTGGCCAAACTCCATAGGTAGCTTTTTCTATTTCGGATTTTAGAATTTCCAAATTCACCAGCGATTTATCGGTTTTGGTGAAATCCAAGGATTTATTAATGTCCACAAATGCCATTTGTTTTGTCTGTGGATTGTATTTGAAAATGGAAACGATGTAGTTAGATAGATGCGCGGCGAAGTTTCCTATTAGGGTTCCGCGCTTCACGTCACCCAACATACTTGAATCACCATCCTTTAAAAAAAATGATAAGATATCAACATGGAGTATATCATACTCGGATTTTAATTCTTCTACTGCCTCACTGACATTTTCACAAATACTACAATACGGTTTACCGAAAATAATCAGTACGTTCTTCATTTATGGGTATCAAAAACTTAAAATCGTTACTGCTGGAAAATAAATCACTGACGATATTAGATGATAATTTATACAAAGTATACAATGGAATATTTGTGGATACAATGAGTATTTATATAGCCGTCGCCAATTGTGTCAGAAACTTAGAAGAGTTAACTACGGTATTCATAAAATACGTAAACGGATGGGTAAAAAAGGGAGGACATGTAACCCTTTTTATCGATAGAGGAAGTATAAAAATTAAACAAGACGTTAGAGACAAGAGACGTAAATATTCTAAATTAACCAAGGACAGAAAAATGTTAGAATTAGAAAAGTGTACATCCGAAATACAAAATGTTACCGGATTTATGGAAGAAGAAATAAAGGCAGAAATGCAATTAAAAATCGATAAACTCACATTTCAAATATATTTATCTGATTATGATAACATAAAAATATCATTGAATGAGATACTAACACATTTCAACAATAATGAGAATGTTACATTATTTTATTGTGATGAACGAGACGCAGAATTCGTTATGTGTCTAGAGGCTAAAACACAGTTCTCTACCACAGGAGAATGGCCGTTAATAATAAGTACCGATCAGGATACTATGCTATTCGCGTCTGCTGATAATCATCCTAAGATGATAAAAAACTTAACTCAACTGTTTAAATTTGTTCCCTCGGCAGAGGATAACTATTTAGCAAAATTAACTGCATTAGTGAATGGATGTGATTTCTTTCCTGGACTCTATGGGGCATCTATAACACCCAACAACTTAAACAAAATACAATTGTTTAGTGATTTTACAATCGATAATATAGTCACTAGTTTGGCAATTAAAAATTATTATAGAAAGACTAACTCTACCGTAGACGTGCGTAATATTGTTACGTTTATAAACGATTACGCTAATTTAGACGATGTCTACTCGTATATTCCTCCTTGTCAATGCACTGTTCAAGAATTTATATTCTCCGCATTAGATGAAAAATGGAATGAATTTAAATCATCTTATTTAGAGAGCGTGCCGTTACCCTGCCAATTAATGTACGCATTAGAACCACGTAAGGAGATTGATGTTTCAGAAGTTAAAACTTTATCATCTTATATAGATTTCGAAAATACTAAATCAGATATCGATGTTATAAAATCTATATCCTCGATTTTTGGATATTCTAACGAAAACTGTAACACCATAGTGTTCGGCATCTATAAGGATAATTTACTACTGAGTATAAATAATTCATTTTACTTTAACGATAGTCTGTTAATAACCAATACTAAAAGTGATAATATAATAAATATAGGTTACTAGATTAAAAAATGGTGTTCCAGCTCGTGTGTTCTACATGCGGCAAAGATATTTCTCACGAACGATATAAATTGATTATACGAAAAAAATCATTAAAGGATGTACTAGTCAGTGTAAAGAACGAATGTTGTAGGTTAAAATTATCTACACAAATAGAACCTCAACGTAACTTAACAGTGCAACCTCTATTGGATATAAACTAATGGATCCGGTTAATTTTATCAAGACATATGCGCCTAGAGGTTCTATTATTTTTATTAATTATGCCATGTCATTAACTAGTCATTTGAATCCATCGATAGAAAAACATGTGGGTATTTATTATGGTACGTTATTATCGGAACACTTGGTAGTTGAATCTACCTATAGAAAAGGAGTTAGAATAGTCCCATTGGATAGATTTTTTGAAGGATATCTTAGTGCAAAAGTATACATGTTAGAGAATATTCAAGTTATGAAAATAGCAGCTGATATGTCGTTAACTTTACTAGGTATTCCATATGGATTTGGTCATGATAGAATGTATTGTTTTAAATTGGTAGCTGAATGTTATAAAAATGCCGGTATTGATACATCGTCTAAACGAATATTAGGTAAAGATATTTTTCTGAGCCAAAACTTTACAGATGATAATAGATGGATAAAGATATATGATTCTAATAATTTAACATTTTGGCAAATTGATTACCTTAAAGGGTGAGTTAATATGCATAACTACTCCTCCGTTGTTTTTTCCCTCGTTCTTTTTCTTAACGTTGTTTGCCATCACTCTCATAATGTAAAGATATTCTAAAATGGTAAACTTTTGCATATCGGATGCAGAAATTGGTATAAATGTTGTAATTGTATTATTTCCCGTCAATGGACTAGTCACAGCTCCATCAGTTTTATATCCTTTAGAGTATTTCTCACTCGTGTCTAGCATTCTAGAGCATTCCATGATCTGTTTATCGTTGATATTGGCCGGAAAGATAGATTTTTTATTTTTTATTATATTACTATTGGCAATTGTAGATATAACTTCTGGTAAATATTTTTCTACCTTTTCAATCTCTTCTATTTTCAAGCCGGCTATATATTCTGCTATATTGTTACTAGTATCAATACCTTTTCTGGCTAAGAAGTCATATGTGGTATTCACTATATCAGTTTTAACTGGTAGTTCCATTAGCCTTTCCACTTCTGCAGAATAATCAGAAATTGGTTCTTTACCAGAAAATCCAGCTACTATAATAGGCTCACCGATGATCATTGGCAAAATCCTATATTGTACCAGATTAATGAGAGCATATTTCATTTCCAATAATTCTGCTAGTTCTTGAGACATTGATTTATTTGATGAATCTATTTGGTTCTCTAGATACTCTACCATTTCTGCCGCATACAATAACTTGTTAGATAAAATCAGGGTTATCAAAGTGTTTAGTGTGGCTAGAATAGTGGGCTTGCACGTATTAAAGAATGCTGTAGTATGAGTAAACCGTTTTAACGAATTATATAGTCTCCAGAAATCTGTGGCGTTGCATACATGAACTGAATGACATCGAAGATTGTCCAATATTTTTAATAGCTGCTCTTTGTCCATTATTTCTATATTTGACTCGCAACAATTGTAGATACCATTAATCACTGATTCCTTTTTCGATGCCGGACAATAGCACAATTGTTTAGCTTTGGACTCTATGTATTCAGAATTAATAGATATATCTCTCAATACAGATTGCACTATACATTTTGAAACTATGTCAAAAATTGTAGAACGACGCTGTTCTGTAGCCATTTAACTTTAAATAATTTACAAAAATTTAAAATGAGCATCCGTATAAAAATCGATAAATTGCGCCAAATTGTGGCATATTTTTCAGAGTTCAGCGAAGAAGTGTCTATAAATGTAGACTCGACGGATGAATTAATGTATATTTTTGCCGCCTTGGGCGGATCTGTAAACATTTGGGCCATTATACCTCTCAGTGCATCAGTGTTCTACCGCGGAGCCGAAAATATTGTGTTTAACCTTCCAGTGTCCAAGGTAAAATCGTGTTTGTGTAGTTTTCACAATGATGCTATCATAGATATAGAACCTGATCTGGAAAATAATCTAGTAAAACTTTCTAGTTATCATGTAGTAAGTGTCGATTGTAACAAGGAACTGATGCCTATTAGGACAGATACTACTATTTGTCTAAGTATAGATCAAAAGAAATCTTACGTATTTAATTTTCACAAGTATGAAGAAAAATGTTGTGGTAGAACCGTCATTCATCTAGAATGGTTGTTGGGCTTTATCAAGTGTATTAGTCAGCATCAGCATTTGGCTATTATGTTTAAAGATGACAATATTATTATGAAGACTCCTGGTAATACTGATGCGTTTTCCAGGGAATATTCTATGACTGAATGTTCTCAAGAACTACAAAAGTTTTCTTTCAAAATAGCTATCTCGTCTCTCAACAAACTACGAGGATTCAAAAAGAGAGTCAATGTTTTTGAAACTAGAATCGTAATGGATAATGACGATAACATTCTAGGAATGTTGTTTTCGGATAGAGTTCAATCCTTTAAGATTAACATCTTTATGGCGTTTTTAGACTAATACTTTCAATGAGATAAATATGGGTGGCGGAGTAAGTGTTGAGCTCCCTAAACGGGATCCACCTCCGGGAGTACCCACTGATGAGATGTTATTAAACGTGGATAAAATGCATGACGTGATAGCTCCCGCTAAGCTTTTAGAATATGTGCATATAGGACCACTAACAAAAGATAAAGAGGATAAAGTAAAGAAAAGATATCCAGAGTTTAGATTAGTCAACACAGGACCCGGTGGTCTTTCGGCATTATTAAGACAATCATATAATGGAACCGCACCCAATTGCTGTCGCACTTTTAATCGTACTCATTATTGGAAGAAGGATGGAAAGATATCAGATAAGTATGAAGAGGGTGCAGTATTAGAATCGTGTTGGCCCGACGTCCACGACACTGGAAAATGCGATGTTGATTTATTCGACTGGTGTCAGGGGGATACGTTCGATATGAACATATGCCATCAGTGGATCGGTTCAGCCTTTAATAGGAGTGATAGAACTGTAGAGGGTCGACAATCGTTAATAAATCTGTATAATAAGATGCAAAGATTATGTAGTAAAGATGCTAGTGTACCAATATGTGAATTATTTTTGCATCATTTACGCGCACACAATACAGAAGATAGTAAAGAGATGATCGATTATATTCTAAGACAACAGTCGGCGGACTTTAAACAGAAATATATGAGATGTAGTTATCCCACTAGAGATAAGTTAGAAGAGTCATTAAAATATGCGGAACCTCGAGAATGTTGGGATCCAGAGTGTTCGAATGCCAATGTTAATTTCTTACTAACACGTAATTATAATAATTTAGGACTTTGCAATATTGTACGATGTAATACGAGCGTGAATAACTTACAGATGGATAAAACTTCCTCATTAAGATTATCATGTGGATTAAGCAATAGTGATAGATTTTCTACTGTTCCCGTCAATAGAGCAAAAGTAGTTCAACATAATATTAAACATTCGTTCGACCTAAAATTGCATTTGATCAGTTTATTATCTCTCTTGGTAATATGGATACTAATTGTAGCTATTTAAATGGGTGCCGCAGCAAGCATACAGACGACTGTGAATACACTCAGTGAACGTATCTCGTCTAAATTAGAACAAGAAGCGAACGCTAGTGCTCAAACAAAATGTGATATAGAAATCGGAAATTTTTATATCCGACAAAACCATGGATGTAACATCACTGTTAAAAATATGTGCTCTGCGGACGCGGATGCTCAGTTGGATGCTGTGTTATCAGCCGCTACAGAAACATATAGTGGATTAACACCGGAACAAAAAGCATACGTACCAGCTATGTTTACTGCTGCGTTAAACATTCAGACGAGTGTAAACACTGTTGTTAGAGATTTTGAAAATTATGTGAAACAGACTTGTAATTCTAGCGCTGTTGTCGATAACAAATTAAAGATACAAAACGTAATTATAGATGAATGTTACGGAGCCCCAGGATCTCCAACAAATTTGGAATTTATTAATACAGGATCTAGCAAAGGAAATTGTGCCATTAAGGCGTTGATGCAATTGACTACTAAGGCCACTACTCAAATAGCACCTAGACAAGTTGCTGGTACAGGAGTTCAGTTTTATATGATTGTTATCGGTGTTATAATATTGGCAGCGTTGTTTATGTACTATGCCAAGCGTATGCTGTTCACATCCACCAATGATAAAATCAAACTTATTTTAGCCAATAAGGAAAACGTCCATTGGACTACTTACATGGACACATTCTTTAGAACTTCTCCGATGATTATTGCTACCACGGATATACAAAACTGAAAATATATTGATAATATTTTAATAGATTAACATGGAAGTTATCGCTGATCGTCTAGACGATATAGTGAAACAAAATATAGCGGATGAAAAATTTGTAGATTTTGTTATACACGGTCTAGAGCATCAATGTCCTGCTATACTTCGACCATTAATTAGGTTGTTTATTGATATACTATTATTTGTTATAGTAATTTATATTTTTACGGTACGTCTAGTAAGTAGAAATTATCAAATATTGTTGGTGTTGGTGGCGCTAGTCATCACATTAACTATTTTTTTATTACTTTATACTATAATAGTACTAGACTGACTTCTAACAAACATCTCACCTGCCATAAATAAATGCTTGATATTAAAGTCTTCTATTTCTAACACTATTCCATCTGTGGAAAATAATACTCTGACATTATCGCTAATTGATACATCGGTAAGTGATATGCCTATAAAGTAATAATCTTCTTTGGGCACATATACCAGTGTACCAGGTTCTAACAACCTATTTACTGGTGCTCCTGTAGCATACTTTTTTTTTACCTTGAGAATATCCATTGTTTGCTTGGTCAATAGTGATATGTGATTTTTTATCAACCACTCAAAAAAGTAATTGGAGTGTTCATATCCTCTACGGGCTATTGTCTCATGACCGTGTATGAAATTTAAGTAACACGACTGTGGTAGATTTGTTCTATAGAGCCGGTTGCCGCAAATAGATAGAACTACCAATATGTCTGTACAAATGTTAAACATTAATTGATTAACAGAAAAAACAATGTTCGTTCTGGGAATAGAAACCAGATTAAAACAAAATTCATTAGAATATATGCCACGTTTATACATGGAATATAAAATAACTACAGTTTGAAAAATAACAGTATCATTTAAACATTTAACTTGCGGGGTTAATCTCACAACTTTACTGTTTTTGAACTGTTCAAAATATAGCATAGATCCATGAGAAATACGTTTAGCCGCCTTTAATAGAGGAAATCCAACCGCCTTTCTGGATCTCACCAACGACGATAGTTCTGACCAGCAACTCATTTCTTCATCATCCACCTGTTTTAACATATAATAGGCAGGAGATAGATATCCATCATTGCAATATTCCTTCTCGTAGGCACACAATCTAATATTGATAAAATCTCCATTCTCTTCTCTGTATTTATTATCTTGTCTCGGTGGCTGATTAGGCTGTGGTCTATCGTTGTTGAATCTATTTTGGTCATTAAATCTTTCATTTCTTCCTGGTATATTTCTATCACCTCGTTTGGTTGGATTTTTGTCTATATTATCGTTTGTAACATCGGTACGGGTATTCATTTATCACAAAAAAAACTTCTCTAAATGAGTCTACTACTAGAAAACCTCATCGAAGAAGATACCATATTTTTTGCAGGAAGTATATCTGAGTATGATGATTTACAAATGGTTATTGCTGGTGCAAAATCCAAATTTCCAAGATCTATGCTTTCTATTTTTAATATAGTACCTAGAACGATGTCAAAATATGAGTTGGAGTTGATTCATAACGAGAATATCACAGGGGCAATGTTTACCACAATGTATAATATAAGAAACAATTTGGGTCTAGGCGATGATAAACTAACTATTGAAGCCATTGAAAACTATTTCTTGGATCCTAACAATGAGGTTATGCCTCTTATCATTAATAATACGGATATGACTACCGTCATTCCTAAAAAAAGTGGTAGGAGAAAGAATAAGAACATGGTTATCTTCCGTCAAGGATCATCACCTATCTTGTGTATTTTCGAAACTCGTAAAAAGATTAATATTTATAAAGAAAATATGGAATCCGTATCGACTGAGTATACACCTATCGGAGACAACAAGGCTTTGATATCTAAATATGCGGGAATTAATATCCTGAATGTGTATTCTCCTTCCACGTCCATGAGATTGAATGCCATTTACGGATTCACCAATAAAAATAAACTAGAGAAACTTAGTACTAATAAGGAACTAGAATCGTATAGTTCTAGCCCTCTTCAAGAACCCATTAGGTTAAATGATTTTCTGGGACTATTGGAATGTGTTAAAAAGAATATTCCTCTAACAGATATTCCGACAAAGGATTGATTACTATAAATGGAGAATGTTCCTAATGTATACTTTAATCCTGTGTTTATAGAGCCCACGTTTAAACATTCTTTATTAAGTGTTTATAAACACAGATTAATAGTTTTATTTGAAGTATTCGTTGTATTCATTCTAATATATGTATTTTTTAGATCTGAATTAAATATGTTCTTCATGCCTAAACGAAAAATACCCGATCCTATTGATAGATTACGACGTGCTAATCTAGCGTGTGAAGACGATAAATTAATGATCTATGGATTACCATGGATAACAACTCAAACATCTGCGTTATCAATAAATAGTAAACCGATAGTGTATAAAGATTGTGCAAAGCTTTTGCGATCAATAAATGGATCACAACCAGTATCTCTTAACGATGTTCTTCGCAGATGATGATTCATTTTTTAAGTATTTTGCTAGTCAAGATGATGAATCTTCATTATCTGATATATTGCAAATCACTCAATATCTAGACTTTCTGTTATTATTATTGATCCAATCAAAAAATAAATTAGAAGCTGTGGGTCATTGTTATGAATCTCTTTCAGAGGAATACAGACAATTGACAAAATTCACAGACTCTCAAGATTTTAAAAAACTGTTTAACAAGGTCCCTATTGTTACAGATGGAAGGGTCAAACTTAATAAAGGATATTTGTTCGACTTTGTGATTAGTTTGATGCGATTCAAAAAAGAATCAGCTCTAGCTACCACCGCAATAGATCCTGTTAGATACATAGATCCTCGTCGTGATATCGCATTTTCTAACGTGATGGATATATTAAAGTCGAATAAAGTTGAAAAATAATTAATTCTTTATTGTTATCATGAACGGCGGACATATTCAGTTGATAATCGGCCCCATGTTTTCAGGTAAAAGTACAGAATTAATTAGACGAGTTAGACGTTATCAAATAGCTCAATATAAATGTGTGACTATAAAATATTCTAACGATAATAGATACGGAACGGGACTATGGACACATGATAAGAATAATTTTGCAGCATTGGAAGTAACTAAACTATGTGATGTCTTGGAAGCAATTACAGATTTCTCCGTGATAGGTATAGATGAAGGACAGTTCTTTCCAGACATTGTTGAATTCTGTGAGCGTATGGCAAACGAAGGAAAAATAGTTATAGTAGCCGCGCTCGATGGGACATTTCAACGTAGACCGTTTAATAATATTTTGAATCTTATTCCATTATCTGAAATGGTGGTAAAACTAACTGCAGTGTGTATGAAATGCTTTAAGGAGGCTTCCTTTTCTAAACGATTAGGTACAGAAACCGAGATAGAAATAATAGGAGGTAATGATATGTATCAATCTGTGTGTAGAAAGTGTTACATCGACTCATAATATTATATTTTTTATCTAAAAAACTAAAAATAAACATTGATTAAATTTTAATATAATACTTAAAAATGGATGTTGTGTCGTTAGATAAACCGTTTATGTATTTTGAGGAAATTGATAATGAGTTAGATTACGAACCAGAAAGTGCAAATGAGGTCGCAAAAAAACTGCCGTATCAAGGACAGTTAAAACTATTACTAGGAGAATTATTTTTTCTTAGTAAGTTACAGCGACACGGTATATTAGATGGCGCCACCGTAGTGTATATAGGATCTGCTCCAGGTACACATATACGTTATTTGAGAGATCATTTCTATAATTTAGGAGTGATCATCAAATGGATGCTAATTGACGGCCGCCATCATGATCCTATTCTAAATGGATTGCGTGATGTGACTCTAGTGACTCGGTTTGTTGATGAGGAATATCTACGATCCATCAAAAAACAACTACATCCTTCTAAGATTATTTTAATTTCTGATGTGCGATCCAAACGAGGAGGAAATGAACCTAGTACTGCGGATTTACTAAGTAATTATGCTCTACAAAATGTCATGATTAGTATTTTAAACCCCGTGGCGTCTAGTCTTAAATGGAGATGCCCGTTTCCAGATCAATGGATCAAGGACTTTTATATCCCACACGGTAATAAAATGTTACAACCTTTTGCTCCTTCATATTCAGCTGAAATGAGATTATTAAGTATTTATACCGGTGAGAATATGAGACTGACTCGAGTTACCAAATCAGACGCTGTAAATTATGAAAAAAAGATGTATTACCTTAATAAGATAGTCCGCAACAAAGTAGTTATTAACTTTGATTATCCTAATCAGGAATATGACTATTTTCACATGTACTTTATGTTGAGGACCGTATACTGCAATAAAACATTTCCTACTACTAAAGCAAAGATACTATTTCTACAACAATCTATATTTCGTTTCTTAAATATTCCAACGACATCAACTGAAAAAGTTAGTCATGAACCAATACAACGTAAAATATCTAGCAAAGATTCTATGTCTAAAAACAGAAATAGCAAGAGATCCGTACGCGGTAATAAATAGAAACGTACTACTAAGATATACTACCGATATAGAGTATAATGATTTAGTTACTTTAATAACCGTTAGACATAAAATTGATTCTATGAAAACTGTGTTTCAGGTATTTAACGAATCATCCATAAATTATACTCCGGTTGATGATGATTATGGAGAACCAATCATTATAACATCGTATCTTCAAAAAGGTCATAACAAGTTTCCTGTAAATTTTCTATACATAGATGTGGTAATATCTGACTTATTTCCTAGCTTTGTTAGACTAGATACTACAGAAACTAATATAGTTAATAGTGTACTACAAACAGGCGATGGTAAAAAGACTCTTCGTCTTCCTAAAATGTTAGAGACGGAAATAGTTGTCAAGATTCTCTATCGTCCTAATATACCATTAAAAATTGTTAGATTTTTCCGCAATAACATGGTAACTGGAGTAGAGATAGCCGATAGATCTGTTATTTCAGTCGCTGATTAATCAATTAGTAGAGATGAGATAAGAACATTATAATAATCAATAATATATCTTATATCTGTTTAGAAAAATGCTAATATTAAAATAGCTAACGCTAGTAATCCAATCGGAAGCCATTTGATATCTATAATAGGGTATCTAATTTCCTGATTCAGATAGCGTACGGCTATATTCTCGGTAGCTACTCGTTTGGAATCACAGACATTATTTACATCTAATTTACTATCTGTAATGGAAACGTTTCCCAATGAAATGGTACAATCAGATACATTACATCTTGATATATTTTTTTTTAAAGAGGCTGGTAACAACGCATCGCTTCGTTTACATGGCTCGTACCAACAATAATAGGGTAATCTTGTATCTATTCCTATCCGTACTATACTTTTATCAGGATAAATACATTTACATCGTATATCGTCTTTGTTAGTATCACAGAATGCATAAATTTGTTCGTCCGTCATGATAAAAATTTAAAGTGTAAATATAACTATTATTTTTATAGTTATAATAAAAAGGGAAATTTGATTGTATACCTTCGGTTCTTTAAAAGAAACTGACTTGATAAAAATGGCTGTAATCTCTAAGGTTACGTATAGTCTATACGATCAAAAAGAGATTAATGCCACAGATATTATCATTAGTCATATTAAAAATGACGACGATATCGGTACCGTTAAAGATGGTAGACTAGGTGCTATGGATGGGGCATTATGTAAGACTTGTGGGAAAACGGAATTGGAATGTTTCGGTCACTGGGGTAAAGTAAGTATTTATAAAACTCATATAGTTAAGCCTGAATTTATTTCAGAAATTATTCGTTTACTGAATCATATATGTATTCATTGCGGATTATTGCGTTCACGAGAACCGTATTCCGACGATATTAACCTAAAAGAGTTATCGGTACACGCTCTTAGGAGATTAAAGGATAAAATATTATCCAAGAAAAAGTCATGTTGGAACAGCGAATGTATGCAACCGTATCAAAAAATTACTTTTTCAAAGAAAAAGGTTTGTTTCGTCAACAAGTTGGATGATATTAACGTTCCTAATTCTCTCATCTATCAAAAGTTAATTTCTATTCATGAAAAGTTTTGGCCATTATTAGAAATTCATCAATATCCAGCTAACTTATTTTATACAGACTACTTTCCCATCCCTCCGTTGATTATTAGACCGGCTATTAGTTTTTGGATAGATAGTATACCCAAAGAGACAAATGAATTAACTTACTTATTAGGTATGATCGTTAAGAATTGTAACTTGAATGCTGATGAACAGGTTATCCAGAAGGCGGTAATAGAATACGATGATATTAAAATTATTTCTAATAACACTACCAGTATCAATTTATCATATATCACATCCGGCAAAAATAATATGATTAGAAGTTATATCGTCGCTCGGCGAAAAGATCAGACCGCTAGATCCGTAATTGGTCCCAGTACATCTATCACCGTTAATGAGGTAGGAATGCCCACATATATTAGAAATACACTTACAGAAAAGATATTTGTTAATGCCTTTACAGTGGATAAAGTTAAACAACTATTAGCATCAAACCAAGTTAAATTTTACTTTAATAAACGATTAAACCAATTAACAAGAATACGTCAAGGAAAGTTTATCAAAAATAAAATACATTTATTGCCTGGTGATTGGGTAGAAGTAGCTGTTCAAGAATATACAAGTATTATTTTTGGAAGACAACCGTCTCTACATAGATACAACGTCATCGCTTCATCTATCAGAGCTACCGAAGGAGATACTATCAAAATATCTCCCGGAATTGCCAACTCTCAAAATGCTGATTTCGACGGAGATGAAGAATGGATGATATTGGAGCAAAATCCTAAAGCCGTAGTTGAACAAAGTATTCTTATGTATCCGACGACGTTACTCAAACACGATATTCATGGAGCCCCCGTTTATGGATCTATTCAAGATGAAATCGTAGCAGCGTATTCATTGTTTAGGATACAAGATCTTTGTTTAGATGAAGTATTGAACATCTTGGGGAAATATGGAAGAGAGTTCGATCCTAAAGGTAAATGTAAATTCAGCGGTAAAGATATCTATACTTACTTGATAGGTGAAAAGATTAATTATCCGGGTCTCTTAAAGGATGGTGAAATTATTGCAAACGACGTAGATAGTAATTTTGTTGTAGCTATGAGGCATCTGTCATTGGCTGGACTCTTATCCGATCATAAATCGAACGTGGAAGGTATCAACTTTATTATCAAGTCATCTTATGTTTTTAAGAGATATCTATCTATATACGGTTTTGGGGTGACATTCAAAGATCTGAGACCAAATTCGACGTTCACTAATAAATTGGAGGCTATCAACGTAGAAAAAATAGAACTTATCAAAGAAGCATACGCCAAATATCTCAAAGATGTAAGAGACGGGAAAATAGTTCCATTATCTAAAGCTTTAGAGGCGGACTACTTGGAATCCATGTTATCCAACTTGACAAATCTTAATATCAGAGAGATAGAAGAACATATGAGACAAACGCTGATAGATGATCCAGATAATAACCTCCTGAAAATGGCCAAAGCGGGTTATAAAGTAAATCCCACAGAACTAATGTATATTCTAGGTACTTATGGACAACAGAGGATAGATGGCGAACCAGCAGAGACTCGAGTATTGGGTAGAGTCTTACCTTACTATCTTCCAGACTCTAAGGATCCAGAAGGAAGAGGTTATATTCTTAATTCTTTAACAAAAGGATTAACGGGTTCTCAATATTACTTTTCGATGCTGGTTGCAAGATCTCAATCTACTGATATTGTCTGTGAAACATCACGTACCGGAACACTGGCTAGAAAAATCATTAAAAAGATGGAGGATATGGTGGTCGACGGATACGGACAAGTAGTTATAGGTAATACGCTCATCAAGTACGCAGCCAATTATACCAAAATTCTAGGCTCAGTATGTAAACCTGTAGATCTTATCTATCCAGATGAGTCCATGACTTGGTATTTGGAAATTAGTGCTTTGTGGAATAAAATAAAACAGGGATTCGTTTACTCTCAGAAACAGAAACTTGCAAAGAAGACATTGGCGCCGTTTAATTTCCTAGTATTCGTCAAACCCACCACTGAGGATAATGCTATTAAGGTTAAGGATCTGTACGATATGATTCATAACGTCATTGATGATGTGAGAGAGAAATACTTCTTTACGGTATCTAATATAGATTTTATGGAGTATATATTCTTGACGCATCTTAATCCTTCTAGAATTAGAATTACAAAAGAAACGGCTATTACTATCTTTGAAAAGTTCTATGAAAAACTCAATTATACTCTAGGTGGTGGAACTCCTATTGGAATTATTTCTGCACAGGTATTGTCTGAGAAGTTTACACAACAAGCCCTGTCCAGTTTTCACACTACTGAAAAGAGTGGTGCTGTAAAACAAAAACTTGGTTTCAACGAGTTTAATAACTTGACTAATTTGAGTAAGAATAAGACCGAAATTATCACTCTGGTATCCGATGATATCTCTAAACTTCAATCTGTTAAGATTAATTTCGAATTTGTATGTTTGGGAGAATTAAATCCAGACATCACTCTTCGAAAAGAAACAGATAGATATGTAGTAGACATAATAGTCAATAGATTATACATCAAGAGAGCAGAAATAACCGAATTAGTCGTCGAATATATGATTGAACGATTTATCTCCTTTAGCGTCATTGTAAAGGAATGGGGTATGGAGACATTCATTGAGGACGAGGATAATATTAGATTTACTATCTACCTAAATTTCGTTGAACCGGAGGAATTGAATCTTAGTAAGTTTATGATGGTTCTTCCAGGTGCCGCCAACAAGGGCAAGATTAGTAAATTCAAGATTCCTATCTCTGACTATACGGGATATAACGACTTCAATCAAACAAAAAAGCTCAATAAGATGACTGTAGAACTCATGAATCTAAAAGAATTGGGTTCTTTCGATTTGGAGAACGTCAACGTGTATCCTGGAGTATGGAATACATACGATATCTTCGGTATTGAGGCCGCTCGTGGATACTTGTGCGAAGCCATGTTAAACACCTATGGAGAAGGTTTCGATTATCTGTACCAGCCTTGTGATCTTCTCGCTAGTTTACTATGTGCTAGTTACGAACCAGAATCAGTTAATAAATTCAAGTTCGGTGCAGCTAGTACTCTTAAGAGAGCTACGTTCGGAGATAATAAAGCATTGTTAAACGCGGCTCTTCATAAAAAGTCAGAACCTATTAACGATAATAGTAGCTGCCACTTTTTTAGCAAGGTCCCTAATATAGGAACTGGATATTACAAATACTTTATCGACTTGGGTCTTCTCATGAGAATGGAAAGGAAACTATCTGATAAGATATCTTCTCAAAAGATCAAGGAGATAGAAGAAACAGAAGACTTTTAATTCTTATCAATAACATATTTTTCTATGATCTGTCTTTTAAACGATGGATTTTCCACAAATGCGCCTCTCAAGTCCCTCATAGAATGATACACGTATAAAAAATATAGCATAGGTGATGACTCCTTATTTTTAGACATTAGATATGCCAAAATCATAGCCCCGCTTCTATTTACTCCTGCAACACAATGAACCAACACGGGCTCGTTTCGTTGATCACATTTAGATAAGAAGGCGGTCACGTCGTCAAAATATTTACTAATATCAGTAGTTGTATCATCTACCAACGGTATATGAATAATATTAATATTAGAGTTAGGTAATGTATATTTATCCATCGTCAAATTTAAAACATATTTGAACTTAACTTCAGATGATGGTGCATCCATAGCATTTTTATAATTTCCCAAATACACATTATTTGTTACTCTTGTCATTATAGTGGGAGATTTGGCTCTGTGCATATCTCCAGTTGAACGTAGTAGTAAGTATTTATACAAACTTTTCTTATCCATTTATAACGTACAAATGGATAAAACTACTTTATCAGTAAACGCATGCAATTTAGAATACGTTAGAGAAAAGGCTATAGTAGGCGTACAAGCAGCCAAGACATCAACACTTATATTTTTTGTTATTATATTGGCAATTAGTGCGCTATTACTCTGGTTTCAGACGTCTGATAATCCAGTCTTTAATGAATTAACGAGATATATGCGAATTAAAAATACGGTTAACGATTGGAAATCATTAACGGATAGCAAAACAAAATTAGAAAGCGATAGAGGTAGACTTCTAGCCGCTGGTAAGGATGATATATTCGAATTCAAATGTGTGGATTTCGGCGCCTATTTTATAGCTATGCGATTGGATAAGAAAACATATCTGCCGCAAGCTATTAGGCGAGGTACTGGAGACGCGTGGATGGTTAAAAAGGCGGCAAAAGTCGATCCATCTGCTCAACAATTTTGTCAGTATTTGATAAAACACAAGTCTAATAATGTTATTACTTGTGGTAATGAGATGTTAAATGAATTAGGTTATAGCGGTTATTTTATGTCACCGCATTGGTGTTCCGATCTTAGTAATATGGAATAAGTGTTAGATAAATGCGGTAACAAATGTTCCTGTAAGGAACCATAACAGTTTAGATTTAACATTAAAGATGAGCATAAACATAATAAACAAAATTACAATCAAACCTATAACATTAATATCAAACAATCCAAAAAATGAAATCAATGGAGTAGTAAACGTGTACATAACTCCTGGATAACGTTTAGCAGCTACCGTTCCTATTCTAGACCAAAAATTTGGTTTCATGGTTTCGAAGCGGTGTTCTGCAACAAGACGAGGATCGTGTTCTACATATTTGGCAGAGTTATCCATTATTTGCCTGTTAATCTTCATTTCGTTTTCGATTCTGGCTATTTCAAAATAAAATCCCGATGATAGACCTCCAGACTTTATAATTTCATCTACGATGTTCAGCGCCGTAGTAACTCTAATAATATAGGCGGATAAGCTAACATCATACCCTCCTGTATATGTAAATATGGCATGATCTTTGTCTATTACAAGCTCGGTTTTAACTTTATTTCCTGTAATAATTTCTCTCATCTGTAGGATATCTATTTTCTTGTCATGTATTGCCTTCAAGACGGGACGAAGAAACGTAATATCCTCAATAACGTTATCGTTTTCTATAATAACTACATATTCTACATTTTTATTTTCTAGCTCGGTAAAAAATTTAGAATCCCATAGGGCTAAATGTCTAGCGATATTTCTTTTCGTTTCCTCTGTACACATAGTGTTACAAAACCCTGAAAAGAAGTGAGTATACTTGTCATCATCTCTAATATTTCCTCCAGTCCATTGTATAAACACATAATCCTTGTAATGATCTGGATCATCATTGACTATCACAACATCTCTTTTTTCTTGCATAACTTCATTGTCCTTCACATCATCGAACTTCTGATCATTAATATGCTCATGAACATTAGGAAATGTTTCTGATGGAGGTCTATCAATAACTGGCACAACAATAACAGGAGTTTTCACCGCCGCCATTTAGTTATTGAAATTAATCATATACAACTCTCTAATACGAGTTATATTTTCGTCTATCCATTGTTTCACATTGACATATTTCGACAAAAAGATATAAAATGCGTATTCCAATGCTTCTCTGTTTAATGAATTACTAAAATATACAAACACGTCACTGTCTGGTAATAAATGATATCTTAGAATATTGTAACAATTTATTTTGTATTGCACATGTTCGTGATCTATGAGTTCTTCTTCGAATGGCATAGGATCTCCGAATCTGAAAACGTATAAATAGGAGTTAGAATAATAATATTTGAGAGTATTGGTAATGTATAAACTCTTTAGCGGTATAATTAGTTTTTTTCTCTCGATTTCTATTTTTAGATGTGATGGAAAAATGACTAATTTTGTAGCATTAGTATCATGAACTCTAATCAAAATCTTAATATCTTCGTCACATGTTAGCTCTTTGAAGTTTTTAAGAGATGCATCAGTTGGTTTTACAGATGGAGTAGGTGCAACAATTTTTTGTTTAATGCATGCATGTATTGGAGCCATTGTCTTAACTATAATGGTGCTTGTATCGAAAAACTTTAATGCGGATAACGGAAGCTCTTCGCCGCGACTTTCTACGTCGTAATTGGGTTCTAATGCCGATCTCTGAATGGATACTAGTTTTCTAAGTTCTAATGTAATTCTCTGAAAATGTAAATCCAATTCCTCCGGCATTATAGATGTGTATACATCGGTAAATAAAACTATAGTATCCAACGATCCCTTCTCGCAAATTCTAGTCTTAACCAAGAAATCGTATATAACTACGGAGATGGCGTATTTAAGAGTGGATTCTTCTACCGTTTTGTTCTTGGATTTCATATAAGAAACTATAAAGTCCGCACTACTGTTAAGAATGATCACTAACGCAACTATATAGTTCAAATTAAGCATCTTGGAAACATAAAATAACTCTGTAGATGATACTTGACTTTCGAATAAGTTTGCAGACAAACGAAGAAAGAACAGACCTCTCTTAATTTCAGAAGAAAACTTTTTTTCGTATTCCTGACGTCTAGAGTTTATATCAATAAGAAAGTTAAGAATTAGTCGGTTAATGTTGTATTTCATTACCCAAGTTTGAGATTTCATAATATTGTCAAAAGACATGATAATATTAAAGATAAAGCGCTGACTATGAACGAAATAGCTATATGGTTCGCTCAAGAATATAGTCTTGTTAAACGTGGAAACGATAACTGTATTTTTAATCACGTCAGCGGCATCTAAATTAAATATAGGTATATTTATTCCACACACTCTACAATATGCCACACCATCTTCATAATAAATAAATTCGTTAGCAAAATTATTAATTTTAGTGAAATAGTTAGCGTCAACTTTCATAGCTTCCTTCAATCTAATTTGATGCTCACATGGCGCGAATTCTACTCTAACATCCCTTTTCCATGCCTCAGGTTCATCGATCTCTATAATATCTAGTTTCTTGCGTTTCACAAACACAGGCTCGTCTCTCGCGATGAGATCTGTATAGTAACTATGTAAATGATAACTAGATAGAAAGATGTAGCTATATAGATGACGATCCTTTAAGAGAGGTATAATAACTTTACCCCAATCAGATAGACTGTTGTTATGGTCTTCGGAAAAAGAATTTTTATAAATTTTTCCAGTATTTTCTAAATATACGTACTTGATATCTAAGAAATCCTTAATAATAATAGGAATGGATAATCCGTCTATTTTATAAAGAAATACATATCGCATATTATACTTTTTTTTGGAAATTGGAATACCGATGTGTCTACATAAATACGCAAAGTCTAAATATTTTTTAGAGAATCTTAGTTGGTCCAAATTCTTTTCCAAGTACGGTAATAGATTTTTCATATTGAACGGTATCTTCTTGATCTCTGGTTCTAATTCCGCATTAAATGATGAAACTAAGTCACTATTTTTATAACTAACGATTACATCACCTCTAACATCATCATTTACCAGGATACTGATCTTCTTTTGTCGTAAATACATGTCTAATGTGTTAAAAAAAAGATCATACAAGTTATACGTCATTTCATCTGTAGTATTCTTGTCATTGAAGGATAAACTCGTACTAATCTCTTCTTTAACAGTCTGTTCAAATTTATATCCTATATATGAAAAAATAGCAACCAGTGTTTGATCATCCGCGTCAATATTCTGTTCTATCGTAGTGTATAACAATCTTATATCTTCTTCTGTGATAGTCGATACGTTATAAAGGTTGATAACGAAAATATTTTTATTTCGTGAAATAAAGTCATTGTAGGATTTTGGACTTATATTCGTGTCTAGTAGATATGATTTTATTTTTGGAATGATCTCAATTAAAATAGTCTCTTTAGAGTCCATTTAAAGTTACAAACAACTAGGAAATTGGTTTATGATGTATAATTTTTTTAGTTTTTATAGATTCTTTATTCTATACTTAAAAAATGAAAATAAATACAAAGGTTCTTGAGGGTTGTGTTAATTGAAAGCGATAAATAATCATAAATTATTTCATTATCGCGATATCCGTTAAGTTTGTATCGTAATGGCGTGGTCAATTACGAATAAAGCGGATACTAGTAGTTTCACAAAGATGGCTGAAATCAGAGCTCATCTAAGAAATAGCGCTGAAAATAAAGATAAAAACGAGGATATTTTCCCGGAAGATGTAATAATTCCATCTACTAAGCCCAAAACCAAACGAACCACTACTCCTCGTAAACCAGCGGCTACTAAAAGATCAACCAAAAAGGATAAAGAAAAGGAGGAAGTGGAAGAAGTAGTTATAGAGGAATATCATCAAACAACTGAAGAAAATTCTCCACCTCCGTCATCATCTCCTGGAGTCGGCGACATTGTAGAAAGCGTGGCCGCTGTAGAGCTCGATGATAGCGACGGGGATGATGAACCTATGGTACAAGTTGAAGCTGGTAAAGTAAATCATAGTGCTAGAAGCGATCTCTCTGACCTAAAGGTGGCTACCGACAATATCGTTAAAGATCTTAAGAAAATTATTACTAGAATCTCTGCAGTATCGACTGTTCTAGAGGATGTTCAAGCAGCTGGTATCTCTAGACAATTTACTTCTATGACTAAAGCTATTACAACACTATCTGATCTAGTCACCGAGGGAAAATCTAAAGTTGTTCGTAAAAAAGTTAAAACTTGTAAGAAGTAAATGCGTGCACTTTTTTATAAAGATGGTAAACTGTTTACCGATAATAATTTTTTAAATCCTGTATCAGACGATAATCCAGCGTATGAGGTTTTGCAACATGTTAAAATTCCTACTCATTTAACAGATGTAGTAGTATATGAACAAACGTGGGAAGAGGCATTAACTAGATTAATTTTTGTGGGAAGTGATTCAAAAGGACGTAGACAATACTTTTACGGAAAAATGCATATACAGAATCGCAATGCTAAAAGAGATCGTATTTTTGTTAGAGTATATAACGTTATGAAACGAATTAATTGTTTTATAAACAAAAATATAAAGAAATCGTCCACAGATTCCAATTATCAGTTGGCGGTTTTTATGTTAATGGAAACTATGTTTTTTATTAGATTTGGTAAAATGAAATATCTTAAGGAGAATGAAACAGTAGGGTTATTAACACTAAAAAATAAACACATAGAAATAAGTCCCGATGAAATAGTTATCAAGTTTGTAGGAAAGGACAAAGTTTCACATGAATTTGTTGTTCATAAGTCTAATAGACTATATAAACCGCTATTGAAACTGACTGATGATTCTAGTCCCGAAGAATTTCTGTTCAACAAACTAAGTGAACGAAAGGTATATGAATGTATCAAACAGTTTGGTATTAGAATCAAGGATCTCCGAACGTATGGAGTCAATTATACGTTTTTATATAATTTTTGGACAAATGTAAAGTCCGTATCTCCTCTTCCATCACCAAAAAAGTTGATAGCATTAACTATCAAACAAACTGCTGAAGTGGTAGGTCATACTCCATCAATTTCAAAAAGAGCTTATATGGCAACGACTATTTTAGAAATGGTAAAGGATAAAAATTTTTTAGACGTAGTATCTAAAACTACGTTCGATGAATTCCTATCTATAGTCGTAGATCACGTTAAATCATCTACGGATGGATGATAATAGATCTTTACACAAATAATTACAAGACCGATAAATGGAAATGGATAAACGGATGAAATCTCTCGCTATGACAGCTTTCTTCGGAGAGCTAAACACGTTAGATATTATGGCATTGATAATGTCTATATTTAAACACCATCCAAACAATACCATTTTTTCAGTGGATAAGGATGGTCAATTTATGATTGATTTCGAATACGATAATTATAAGGCTTCTCAATATTTGGATCTGACCCTCACTCCGATATCTGGAAATGAATGCAAGACTCACGCATCTAGTATAGCCGAACAATTGGCGTGTGTGGATATTATTAAAGAGGATATTAGCGAATATATCAAAACTACTCCCCGTCTTAAACGATTTATAAAAAAATACCGCAATAGATCATATACTCGTATCAGTCGAGATACAGAAAAGCTTAAAATAGCTCTAGCTAAAGGCATAGATTACGAATATATAAAAGACGCTTGTTAATAAGTAAATGAAAAAAAACTAGTCGTTTATAATAAAACACGATATGGATGCCAACATAGTATCATCTTCTACTATTGCGACGTATATAGACGCTTTAGCGAAGAATGCTTCAGAATTAGAACAGAGGTCTACCGCATACGAAATAAATAATGAATTGGAACTAGTATTTATTAAACCGCCATTGATTACGTTGACAAATGTAGTAAATATCTCCACGATTCAGGAATCGTTTATTCGATTTACCGTTACTAATAAGGAAGGTATCAAAATTAGAACTAAGATTCCATTATCTAAGGTACATGGTCTAGATGTAAAAAATGTGCAGTTGGTAGATGCTATAGATAACATAGTTTGGGAAAAGAAATCATTAGTGACGGAAAATCGTCTTCACAAAGAATGCTTGTTGAGACTATCAACAGAGGAACGTCATATATTTTTGGATTACAAGAAATATGGATCCTCTATCCGACTAGAATTAGTCAATCTTATTCAAGCAAAAACAAAAAACTTTACGATAGACTTTAAGCTAAAATATTTTCTAGGATCTGGCGCTCAATCTAAAAGTTCTTTATTGCACGCTATTAATCATCCAAAGTCAAGGCCTAATACATCTCTGGAAATAGAATTTACACCTAGAGACAATGAAACAGTTCCATATGATGAACTAATAAAGGAATTGACGACTCTCTCGCGTCATATATTTATGGCTTCTCCAGAGAATGTAATTCTTTCTCCACCTATTAACGCACCTATAAAGACTTTTATGTTGCCTAAACAAGATATAGTAGGTCTGGATCTGGAAAATCTATATGCCGTAACTAAGACTGACGGCATTCCTATAACTATCAGAGTTACATCAAAAGGGTTGTATTGTTATTTTACACATCTTGGTTATATTATTAGATATCCAGTTAAGAGAACAATAGATTCCGAAGTAGTAGTCTTTGGTGAGGCAGTTAAGGATAAGAACTGGACCGTATATCTCATTAAGCTAATAGAGCCCGTAAATGCAATCAGTGATAGACTAGAAGAAAGTAAGTATGTTGAATCTAAACTAGTGGATATTTGTGATCGGATAGTATTCAAGTCAAAGAAATACGAAGGTCCGTTTACTACAACTAGTGAAGTCGTCGATATGTTATCTACATATTTACCAAAGCAACCAGAAGGTGTTATTCTGTTCTATTCAAAGGGACCTAAATCTAACATTGATTTTAAAATCAAAAAGGAGAATACTATAGACCAAACTGCAAATGTAGTATTTAGGTACATGTCCAGTGAACCAATTATCTTTGGAGAGTCGTCTATCTTTATAGAGTATAAGAAATTTACCAACGATAAAGGCTTTCCTAAAGAATATGGTTCTGGTAAGATTGTGTTATATAACGGCGTTAATTATCTAAATAATATCTATTGTTTGGAATATATTAATACACATAATGAAGTGGGTATTAAGTCCGTTGTTGTACCTATTAAGTTTATAGCAGAATTCTTAGTCAATGGAGAAATACTTAAACCTAGAATCGATAAAACCATGAAATATATTAACTCAGAAGACTATTATGGAAATCAACATAATATCATAGTCGAACATTTAAGAGATCAAAGCATCAAAATAGGAGATGTCTTTAACGAGGATAAACTATCGGATGTTGGACATCAATACGCTGCCAACAACGATAAATTTAGATTAAATCCAAAAGTTAGTTATTTTACTAATAAACGAACTAGAGGGCCGTTGGGAATTTTATCAAACTACGTCAAGACTCTTCTTATTTCTATGTATTGTTCCAAAACATTTTTAGACGATTCCAACAAACGAAAGGTATTAGCGATTGATTTTGGAAACGGTGCTGACCTGGAAAAATACTTTTATGGAGAGATTGCGTTATTGGTAGCGACGGATCCGGATGCTGATGCTATAGCTAGAGGAAATGAAAGATACAACAAATTAAATTCTGGAATTAAAACCAAGTACTACAAATTTGACTACATTCAGGAAACTATTCGATCCGATACATTTGTCTCTAGTGTCAGAGAAGTATTCTATTTTGGAAAGTTTAATATCATTGACTGGCAGTTCGCTATTCATTATTCTTTTCATCCAAGACATTATGCTACAGTCATGAATAACTTATCCGAACTAACTGCTTCTGGAGGCAAGGTATTAATTACTACCATGGATGGAGACAAATTATCAAAATTAACCGATAAAAAGACTTTTATAATTCATAAGAATCTACCTAGTAGCGAAAACTATATGTCTGTAGAAAAAATAGCTGATGATAGAATAGTGGTATATAATCCATCAACAATGTCTACTCCAATGACTGAATACATTATCAAAAAGAACGATATAGTCAGAGTGTTTAACGAATACGGATTTGTTCTTGTAGATAATGTTGATTTCGCTACAATTATAGAACGAAGTAAAAAGTTTATTAATGGCGCATCTACAATGGAAGATAGACCGTCTACAAGAAACTTTTTCGAACTAAATAGAGGAGCCATTAAATGTGAAGGTTTAGATGTCGAAGACTTACTTAGTTACTATGTTGTTTATGTCTTTTCTAAGCGGTAAATAATAATATGGTATGGGTTCTGATATCCCCGTTCTAAATGCATTAAATAATTCCAATAGAGCGATTTTTGTTCCTATAGGACCTTCCAACTGTGGATACTCTGTATTATTAATAGATATATTAATACTTTTGTAGGGTAACAGAGGTTCTACGTCTTCTAAAAATAAAAGTTTTATAACATCTGGCCTGTTCATAAATAAAAACTTGGCGATTCTATATATACTCTTATTATCAAATCTAGCCATTGTCTTATAGATGTGAGCTACTGTAGGTGTACCATTTGATTTTCTTTCTAATACTATATATTTCTCTCGAAGAAGTTCTTGCAGATCATCTGGGAATAAAATACTACTGTTGAGTAAATCAGTTATTTTTTTTATATCGATATTGATGGACATTTTTATAGTTAAGGATAATAAGTATCCCAAAGTAGATAACGACGATAACGAAGTATTTATACTTTTAGGAAATCACAATGACTTTATCAGATCAAAATTAACAAAATTAAAGGAGCATGTATTTTTTTCTGAATATATTGTGACTCCAGATACATATGGATCTTTATGCGTCGAATTAAATGGGTCTAGTTTTCAGCACGGTGGTAGATATATAGAGGTGGAGGAATTTATAGATGCTGGAAGACAAGTTAGATGGTGTTCTACATCCAATCATATATCTGAAGATATACACACTGATAAATTTGTCATTTATGATATTTATACGTTTGATTCGTTCAAGAATAAACGATTGGTATTTGTACAGGTGCCTCCATCATTAGGAGATGATAGCTATTTAACTAATCCGTTATTGTCTCCGTATTATCGTAATTCAGTAGCCAGACAAATGGTCAATGATATGATTTTTAATCAAGATTCATTTTTAAAATATTTATTAGAACATCTGATTAGAAGCCACTATAGAGTTTCTAAACATATAACAATAGTTAGATACAAGGATACCGAAGAATTAAATCTAACAAGAATATGTTATAATAGAGATAAGTTTAAGGCGTTTGTATTCGCTTGGTTTAACGGCGTTTCGGAAAATGAAAAGGTACTAGATACGTATAAAAAGGTATCTGATTTGATATAATGAATTCAGTGACTATATCACACGCACCATATACTATTACTTATCACGATGATTGGGAACCAGTAATGAGTCAATTGGTAGAGTTTTATAACGAAGTAGCCAGTTGGTTGCTACGCGACGAGACGTCGCCTATTCCTGATAAGTTCTTTATACAATTGAAACAGCCGCTTAGAAATAAACGAGTATGTGTGTGTGGTATAGATCCGTATCCAAAAGATGGAACTGGTGTACCGTTCGAATCACCAAATTTTACAAAAAAATCAATTAAGGAGATAGCTTCATCTATATCTAGATTAACCGGAGTAATTGATTATAAAGGTTATAACCTTAATATAATAGACGGGGTTATACCCTGGAATTATTACTTAAGTTGTAAATTAGGAGAAACAAAAAGTCACGCGATTTACTGGGATAAGATTTCCAAGTTACTGCTACAGCATATAACTAAACACGTTAGTGTTCTTTATTGTTTGGGTAAAACAGATTTCTCGAATATACGGGCAAAGTTAGAATCCCCGGTAACTACCATAGTGGGATATCATCCAGCGGCCAGAGACCACCAATTCGAGAAAGATCGATCATTTGAAATTATCAACGTTTTACTGGAATTAGACAACAAGACACCTATAAATTGGGCTCAAGGGTTTATTTATTAATGCTTTAGTGAAATTTTAACTTGTGTTCTAAATGGATGCGGCTATTAGAGGTAATGATGTTATCTTTGTTCTTAAGACTATAGGTGTCCCGTCAGCATGCAGACAAAATGAAGATCCAAGATTCGTAGAAGCATTTAAATGCGACGAGTTAGAAAGATATATTGATAATAATCCAGAATGTACACTATTCGAAAGTCTTAGGGATGAGGAAGCATACTCTATAGTCAGAATTTTCATGGATGTAGATTTAGACGCGTGTCTAGACGAAATAGATTATTTAACGGCTATTCAAGATTTTATTATCGAGGTGTCAAACTGTGTAGCTAGATTCGCATTTACAGAATGCGGTGCCATTCATGAAAATGTAATAAAATCCATGAGATCTAATTTTTCATTGACTAAGTCTACAAATAGAGATAAAACAAGTTTTCATATTATCTTTTTAGACACGTATACCACTATGGATACATTGATAGCTATGAAACGAACACTATTAGAATTAAGTAGATCATCTGAAAATCCACTAACAAGATCGATAGACACTGCCGTATATAGGAGAAAAACAACTCTTCGGGTTGTAGGTACTAGGAAAAATCCAAATTGCGACACTATTCATGTAATGCAACCACCTCACGATAATATAGAAGATTACCTATTCACTTACGTGGATATGAACAACAATAGTTATTACTTTTCTCTACAACGACGATTGGAGGATTTAGTTCCTGATAAGTTATGGGAACCAGGGTTTATTTCGTTCGAAGACGCTATAAAAAGAGTTTCAAAAATATTCATTAATTCTATAATAAACTTTAATGATCTCGATGAAAATAATTTTACAACGGTACCACTGGTCATAGATTATGTAACACCTTGTGCATTATGTAAAAAACGATCGCATAAACATCCGCATCAACTATCGTTGGAAAATGGTGCTATTAGAATTTACAAAACTGGTAATCCACATAGTTGTAAAGTTAAAATTGTTCCGTTGGATGGTAATAAACTGTTTAATATTGCACAAAGAATTTTAGACACTAACTCTGTTTTATTAACCGAACGAGGAGACCATATAGTTTGGATTAATAATTCATGGAAATTTAACAGCGAAGAACCCTTGATAACAAAACTAATTCTATCAATAAGACATCAACTACCTAAGGAATATTCAAGCGAATTACTCTGTCCGAGGAAACGAAAGACTGTAGAAGCTAACATACGAGACATGTTAGTAGATTCAGTAGAGACCGATACCTATCCGGATAAACTTCCGTTTAAAAATGGTGTATTGGACCTGGTAGACGGAATGTTTTACTCTGGAGATGATGCTAAAAAATATACGTGTACTGTATCGACCGGATTTAAATTTGACGATACAAAATTCGTCGAAGACAGTCCAGAAATGGAAGAGTTAATGAATATCATTAACGATATCCAACCATTAACGGATGAAAATAAGAAAAATAGAGAGCTGTATGAAAAAACATTATCTAGTTGTTTATGTGGTGCTACCAAAGGATGTTTAACATTCTTTTTTGGAGAAACCGCAACTGGGAAGTCGACAACCAAACGTTTGTTAAAGTCTGCTATCGGTGACCTGTTTGTCGAGACGGGTCAAACAATTTTAACAGATGTATTGGATAAAGGACCTAATCCATTTATCGCTAATATGCATTTAAAAAGATCTGTATTCTGTAGCGAACTACCTGATTTTGCATGTAGTGGATCAAAGAAAATTAGATCTGATAATATTAAAAAGTTGACAGAACCTTGTGTCATTGGAAGACCGTGTTTCTCCAATAAAATTAATAATAGAAACCATGCGACAATCATTATCGATACTAATTACAAACCTGTCTTTGATAGGATAGATAACGCATTAATGAGAAGAATTGCCGTCGTGCGATTCAGAACACACTTTTCTCAACCTTCTGGTAGAGAGGCTGCTGAAAATAATGACGCGTACGATAAAGTCAAACTATTAGACGAGGGATTAGATGGTAAAATACAGAATAATAGATATAGATTCGCATTTCTATACTTGTTGGTTAAATGGTACAAAAAATATCATATTCCTATTATGAAACTATATCCTACACCGGAAGAGATTCCGGACTTTGCATTCTATCTCAAAATAGGTACTCTGTTGGTATCTAGCTCTGTAAAGCATATTCCATTAATGACGGACCTCTCCAAAAAGGGATATATATTGTACGATAATGTGGTTACTCTTCCGTTGACTACTTTCCAACAGAAAATATCCAAGTATTTTAATTCTAGACTATTTGGACACGATATAGAGAGCTTCATCAATAGACATAAGAAATTTGCCAATGTTAGTGATGAATATCTGCAATATATATTCATAGAGGATATTTCATCTCCGTAAATATATGCCATATATTTATAGAATATATCACATATCTAAATGAATACCGGAATCATAGATTTATTTGATAATCATGTTGATAGTATACCAACTATATTACCTCATCAGTTAGCTACTTTAGATTATCTAGTTAGAACTATCATAGATGAGAACAGAAGCGTGTTATTGTTCCATATTATGGGATCGGGTAAAACAATAATCGCTTTGTTGTTCGCCTTGGTAGCTTCCAGATTTAAAAAGGTTTACATTTTAGTACCGAACATCAACATCTTAAAAATTTTCAATTATAATATGGGTGTAGCTATGAACTTGTTTAATGACGAATTCATAGCTGAGAATATCTTTATTCATTCCACAACAAGTTTTTATTCTCTTAATTATAACGATAACGTCATTAATTATAACGGATTAAGTCGCTACAATAACTCTATTTTTATCGTTGATGAGGCACATAATATTTTTGGGAATAATACTGGAGAACTTATGACCGTGATAAAAAATAAAAACAAGATTCCTTTTCTACTATTGTCTGGATCTCCCATTACTAACACACCTAATACGCTGGGTCATATTATAGATTTAATGTCCGAAGAGACGATAGATTTTGGTGAGATTATTAGTCGTGGTAAGAAAGTAATTCAGACACTTCTTAACGAACGCGGAGTGAATGTACTCAAGGATTTGCTTAAAGGAAGAATATCATATTACGAAATGCCGGACAAAGATCTACCAACAATAAGATATCACGGACGTAAATTTCTAGATACTCGAGTAGTATATTGTCACATGTCTAAACTTCAAGAGAAAGATTATATGATTACTAGACGGCAGCTATGTTATCATGAAATGTTTGATAAAAATATGTATAACGTGTCAATGGCAGTATTGGGACAACTTAATCTGATGAATAATTTAGATACGTTATTTCAGGAACAGGATAAGGAATTGTACCCAAATCTGAAAATAAATAATGGAGTGTTATACGGTGAAGAATTGGTAACGTTAAACATTAGTTCCAAATTTAAGTACTTTATCAATCGGATACAGACACTCAAGGGAAAACACTTTATATACTTCTCTAATTCTACATATGGTGGATTGGTAATTAAATATATCATGCTCAGTAATGGATATTCTGAATATAATGGTTCTCAGGGAACTAATCCACATATGATAAACGGCAAACCAAAAACATTTGCTATCGTTACTAGTAAAATGAAATCGTCTTTAGAGGATCTATTAGATGTGTATAATTCTCCTGAAAACGATGATGGCAATCAATTGATGTTTTTGTTTTCGTCAAACATTATGTCTGAATCCTATACTCTGAAAGAGGTAAGGCATATTTGGTTTATGACTATCCCGGATACTTTTTCTCAATACAACCAAATTCTTGGACGATCTATTAGAAAATTCTCTTACGTCGATATTTCTGAACCCGTTAATGTATATCTTTTAGCAGCCGTATATTCAGATTTCAATGACGAAGTGACGTCATTAAACGATTATACACAGGATGAATTGATTAATGTTTTACCCTTTGACATCAAAAAGCTGTTGTATCTAAAATTTAAGACTAAAGAAACGAATAGAATATACTCTATTCTTCAAGAGATGTCTGAAACGTATTCTCTTCCACCACATCCATCAATTGTAAAAGTTTTATTGGGAGAATTGGTCAGACAATTTTTTTATAATAATTCTCGTATTAAGTATAACGACTCCAAGTTACTTAAAATGGTTACATCAGTTATAAAAAATAAAGAAGACGCTAGGAATTACATAGATGATATTGTAAACGGTCACTTCTTTGTATCGAATAAAGTATTTGATAAATCTCTTTTATACAAATACGAAAACGATATTATTACAGTACCGTTTAGACTTTCCTACGAACCATTTGTTTGGGGAGTTAACTTTCGTAAAGAATATAATGTGGTATCTTCTCCATAAAACTGATGAGATATATAAAGAAATAAATGTCGAGCTTTGTTACCAATGGATATCTTCCAGTTACATTGGAACCACATGAGTTGACGTTAGACATAAAAACTAATATTAGGAATGCCGTATATAAGGCGTATCTCCATAGAGAAATTAGTGGTAAAATGGCCAAGAAAATAGAAATTCGTGAAGACGTGGAATTACCTCTCGGTGAAATAGTTAATAATTCTGTAGTTATAAACGTTCCGTGTGTAATAACCTACGCATATTATCACGTTGGGGATATAGTCAGAGGAACATTAAACATCGAAGATGAATCAAATGTAACTATTCAATGTGGAGATTTAATCTGTAAACTAAGTAGAGATTCGGGTACTGTATCATTTAGCGATTCAAAGTACTGCTTTTTTCGAAATGGTAATGCGTATGATAACGGCATCGAAGTCTCCGCCGTTCTAATGGAGGCTCAACAAGGTACCGAATCTAGTTTTGTTTTTCTCGCGAATATCGTTGACTCATAAGAAAGAGAATAGCGGTGAGTATAAATACGAATACTATGGCAATAATTGCGAATGTTTTATTCCCTTCGATATATTTTTGATAATATGAAAAACATGCCTCTCTCAAATCAGACAACCATTTCATAAAATAGTTCTCTCGCACTGGTGAGGTGGTTGCAGCTCGTATAATCTCCCCAGAATAATATACTTGCGTGTCGTCGTTCAATTTATACGGATTTCTATAATTCTCTGTTATATAATGAGGTTTACCCTCATGATTAGACGACGACAATAGTGTTCTGAATTTAGATAGTTGATCAGAATGAATGTTTATTGGTGTTGGAAAAATTATCCATGCTGCGTCTGCAGAGTGGTTGATAGTTGTTCCTAGATATGTAAAATAATCCAACGTACTAGGTAGCAAATTGTCTAGATAAAATACTGAATCAAATGGCGCAGACATATTAGCGGATCTAATGGAATCCAATTGATTGACTATCTTTTGAAAATATACATTTTTATGATCTGATACTTGTAAGAATATAGCAATAATGATAATTCCATCATCGTGTTTTTTTGCCTCTTCATAAGAACTATATTTTTTCTTATTCCAATGAACCAGATTAATCTCTCCAGAGTATTTGTATACATCTATCAAGTGATTGGATCCATAATCGTCTTCCTTTCCCCAATATATATGTATTGTTGATAACACATATTCATTGGGGAGAAACCCTCCACTTATATATCCTCCTTTAAAATTAATCCTTACTAGTTTTCCAGTATTCTGGATAGTGGTTGGTTTCGACTCATTATAATGTATGTCTAACGTCTTCAATCGCGCGTCAGAAATTGCTTTTTTAGTTTCTATATTAATAGGAGATAGTTGTTGAGGCATAGTAAAAATGAAATGATAACTGTCTAGAAATAGCTCTTAGTATGGGATTTACAATGGATGAGGAAGTGATATTTGAAACTCCTAGAGAATTAATATCTATTAAACGAATAAAAGATATTCCAAGATCAAAAGACACGCACGTGTTTGCTGCGTGTATAACAAGTGACGGATATCCGTTAATAGGAGCTAGAAGAACTTCATTCGCATTCCAGGCGATATTATCTCAACAAAATTCAGATTCTATCTTTAGAGTATCCACTAAACTATTACGGTTTATGTACTACAATGAACTAAGAGAAATCTTTAGACGGTTGAGAAAAGGTTCTATCAACAATATCGATCCTCACTTCGAAGAGTTAATATTATTGGGTGGTAAACTAGATAAAAAGGAATCTATTAAAGATTGTTTAAGAAGAGAATTAAAAGAGGAAAGTGATGAACATATAACAGTAAAAGAATTCGGAAATGTAATTCTAAAACTTACAACGAGTGATAAATTATTTAATAAAGTATATATAGGTTATTGCATGGCATGTTTTATTAATCAATCGTTGGAGGATTTATCACATACTAGTATTTACAATGTAGAAATTAGAAAGATTAAATCGTTAAATGATTGTATTAACGACGATAAATACGAATATCTGTCTTATATTTATAATATACTAATTAATAGTAAATGAGCTTTTACAGATCTAGTATAATTAGTCAGATTATTAAGTATAATAGACGACTAGCTAAGTCTATTATTTGCGAGGATGACTCTCAAATTATTACACTCACGGCATTCGTTAACCAATGCCTATGGTGTCATAAACGAGTATCCGTGTCCGCTATTTTATTAACTACTGATAACAAAATATTAGTATGTAACAGACGAGATAGTTTTCTCTATTCTGAAATAATTAGAACTAGAAACATGTATAGAAAGAAACGATTATTTCTGAATTATTCCAATTATTTGAACAAACAGGAAAGAAGTATACTATCGTCATTTTTTTCTCTAGATCCAGCTACTGCTGATAATGATAGAATAAACGCTATTTATCCGGGTGGTATACCCAAAAGGGGTGAGAACGTTCCAGAGTGTTTATCCAGGGAAATCAAAGAAGAAGTTAATATAGACAATTCTTTTGTATTCATAGACACTCGTTTTTTTATTCATGGTATCATAGAAGATACCATTATTAACAAATTTTTTGAGGTAATTTTCTTTGTTGGACGAATATCTCTAACGAGTGATCAAATTATTGATACCTTTAAAAGTAATCATGAAATAAAGGATCTAATATTTTTAGATCCAAATTCAGGTAATGGACTCCAATACGAAATTGCAAAATATGCTCTAGATACTGCAAAACTTAAATGTTACGGTCATAGAGGATGTTATTATGAATCATTAAAAAAATTAACTGAGGATGATTGATTAGAAAATATAAATTAATTTACCATCGTGTATTTTTATAACGGGATTGTCTGGCATATCATGTAGATAGTTACCGTCTACATCGTATACTCTACCATCTACGCCTTTAAATCCTCTATTTATTGATATTAATCTATTAGAATTGGAATACCAAATATTAGTACCCTCAATTAGTTTATTGGTAATATTTTTTTTAGACGATAGATCGATGGCTCTTGAAACCAAGGTTTTCCAACCGGACTCATTGTCTATCGGTGAGAAGTCTTTTTCATTAGCATGAATCCATTCTAATGATGTATGTTTAAACACTCTAAACAATTGTACAAATTCTTTTGATTTGTTTTGAATGATTTCAAATAGGTCTTCGTCTACAGTAGGCATACCATTAGATAATCTAGCCATTATAAAGTGCACGTTTACATATCTACGTTCTGGAGGAGTAAGAACGTGACTATTGAGACGAATGGCTCTTCCTACTATCTGACGAAGAGACGCCTCGTTCCATGTCATATCTAAAATGAAGATATCATTGATTGAGAAGAAACTAATACCCTCGCCTCCGCTAGAAGAGAATACGCATGTTTTAATGCATTCTCCGTTAGTGTTTGATTCTTGGTTAAACTCAGCCACCGCCTTGATTCTAGTATCTTTTGTTCTAGATGAGAACTCTATATTAGAGATACCAAAGACTTTGAAATATAGTAATAAGATTTCTATTCCTGACTGATTAACAAATGGTTCAAAGACTAGACATTTACCATGGGATGCTAATATTCCCAAACATACATCTATAAATTTGACGCTTTTCTCTTTTAATTCAGTAAATAGAGAGATATCAGCCGCAATAGCATCCCCTCCCAATAGTTCTCCCTTTTTAAAGGTGTCTAATGCGGATTTAGAAAATTCTCTATCTCTTAATGAATTTTTAAAATCATTATATAGGGTTGCTATCTCTTGTGCGTATTCTCCCGGATCACGATTTTGTCTTTCAGGAAAGCTATCGAATGTAAACGTAGTAGCCATACGTCTCAGAATTCTAAATGATGATATACCAGTTTTTATTTCTGCGAGTTTAGCCTTTTGATAAATCTCTTCTTGCTTTTTTGACATATTAACGTATCGCATTAATACTGTTTTCTTAGCGAATGATGCAGACCCTTCCACATCATCAAAAATAGAAAACTCGTTATTAACTATGTACGAACATAGGCCTCCTAGTTTGGAGACTAATTCTTTTTCATCGACTAGACGTTTATTCTCAAATAGCGATTGGTGTTGTAAGGATCCTGGTCGCAGTAAGTTAACCAACATGGTGAATTCTTGCACACTATTAACGATAGGTGTAGCCGATAAACAAATCATCTTATGGTTTTTTAACGCAATGGTCTTAGATAAAAAATTATATACTGACCGAGTAGGACGGATCTTACCATCTTCTTTGATTAATGATTTAGAAATGAAGTTATGACATTCATCAATGATGACGCATATTCTACTCTTGGAATTAATAGTTTTGATATTAGTAAAAAATTTATTTCTAAAATTTTGATCATCGTAATTAATAAAAATACAATCCTTCGTTATCTCTGGAGCGTATCTGAGTATAGTGTTTATCCAAGGATCTTCTATCAAAGCCTTTTTTACCAATAAGATAATTGCCCAATTCGTATAAATATCCTTAAGATGTTTGAGAATATATACAGTAGTCATTGTTTTACCGACACCTGTTTCATGGAACAATAAAAGAGAATGCATACTGTCTAATCCTAAGAAAACTCTTGCTACAAAATGTTGATAATCCTTGAGGCGTACTACGTCTGACCCCATCATTTCAACGGGCATATTAGTAGTTCTGCGTAAGGCATAATCGATATAGGCCGCGTGTGATTTACTCATTTATGAGTGATAAGTAATAACTATGTTTTAAAAATCACAGCAGTAGTTTAACTAGCCTTCTCTGATGTTTGTTTTCGATACTTTTTGAATCAGAAGTCATACTAGAATAAAGCAGCGAGTGAACGTAATAGAGAGCTTCGTATACTCTATTCGAAAACTCTAAGAACTTATTAATGAATTCCGTATCCACTGGATCGTTTAAAATACTAAATTGAACAGTGTTCACATCCTTCCAAGACGAAGACTTAGTGACGGACTTAACATGAGACATAAATAAATCCAAATTTTTTTTATAAACATCACTAGCCACCATAATGGCGCTATCTTTCAACCAACTATCGCTTACGCATTTTAACAGTCTAACATTTTTAAAGAGACTACAATATATTCTCATAGTATCGATTACACCTCTACCGAATAGAGTGGGAAGTTTAATAATACAATATTTTTCGTTTACAAAATCAAATAATGGTCGAAACACGTCGAAGGTTAACATCTTATAATCGCTAATGTATAGATTGTTTTCAGTGAGATGATTATTAGATTTAATAGCATCTCGTTCACGTTTGAACAGTTTATTGCGTGCGCTGAGGTCGGCAACTACGGCATCCGCTCTAGTACTCCTCCCATAATACTTTACGCTATTAATCTTTAAAATTTCATAGACTTTATCTAGATCGCTTTCTGGTAACATGATATCATGTGTAAAAAGTTTTAACATGTCGGTCGGCATTCTATTTAGATCATTAACTCTAGAAATCTGAAGAAAGTAATTAGCTCCATATTCCAGACTAGGTAATGGGCTTTTACCTAAAGACAAGTTAAGTTCTGGCAATGTTTCATAAAATGGAAGAAGGACATGTGTCCCCTCCCGGATATTTTTTACAATTTCATCCATTTACAACTCTATAGTTTGTTTTCATTATTATTAGTTATTATCTCCCATAATCTTGGTAATACTTACACCTTGATCATAAGATACCTTATACAGGTCATTACATACAACTACCAATTGTTTTTGTACATAATAGATTGGATGATTGATATCCATGGTGGAATAAACTACTCGAACAGATAGTTTATCTTTCCCCCTAGATACATTGGCCGTAATAGTTGTCGGCCTAAAGAATATCTTTGGTGTAAAGTTAAAAGTTAGGGTTCTTGTTCCATTATTGCTTTTTGTCAGTAGTTCGTTATAAATTCTCGAGATGGGCCCGTTCTCTGAATATAGAACATCATTTCCAAATCTAACTTCTAGTCTAGAAATAATATCGGTCTTATTTTTAAAATCTATTCCCTTGATGAATGGATCGTTAATAAACAAATCCTTGGCCTTTGATTCGGCTGATCTATTATCTCCGTTATAGACGTTACGTTGACTAGTCCAAAGACTTACAGGAATAGATGTATCGATGATGTTGATAGTATGTGATATGTGAGCAAAGACTGTTCTCTTGGTGGCGTCGCTATATGTTCCAGTAATGGCGGAAAACTTTTTAGAAATGTTATATATAAAAGAATTTTTTCGGGTTCCAAACATTAACAGATTAGTATGAAGATAAACACTCATATTATCAGGAACATTATCAATTTTTACATAAACATCGGCATCTTGAATAGAAACAACACCATCTTCTGGAACCTCTACGATCTCGGCAGATTCCGGATAACCAGTCGGTGGACCATCACTAACAATAACTAGATCATCCAACAATCTACTCACATATGCGTCTATATAATCTTTTTCATCTTGTGAGTACCCTGGATACGAAATAAATTTGTTATCAGTATTTCCATAATAAGGTTTAGTATAAACAGAGAGAGATGTTGCTGCATGAACTTCGGTTACTGTCGCCGTTGGTTGGTTTATTTGACCTATTACTCTCCTAGGTTTCTCTATAAATGATGGTTTAATTTGTACATTCTTAACCATATATCCAATAAAGCTCAATTCAGGAACATAAACAAATTCTTTGTTGAACGTTTCAAAGTCGAACGAAGAGTCACGAATAACGATATCGGATACTGGATTGAAGGTCACCGTTACGGTAATTTTTGAATCGGATAGTTTAAGACTACTGAATGTATCTTCCACATCAAACGGAGTTTTAATATAAACGTATACTGTAGATGGTTCTTTAATAGTGTCATTAGGAGTTAGGCCAATAGAAATATCATTAAGTTCACTAGAATATCCAGAATGTTTCAAAGCAATTGTATTATTGATACAATTATTATATAATTCTTCGCCCTCAATTTCCCAAATAACACCGTTACACGAAGAGACAGATACATGATTAATACATTTATATCCAACATATGGCACGTAACCGAATCTTCCCATACCTTTAACTTCTGGAAGTTCCAAACTCAGAACCAAATGATTAAGCGCAGTAATATACTGATCCCTAATTTCGAAGCTAGCGATAGCCTGATTGTCTGGCCCATCGTTTGTCATAACTCCGGATAGAGAAATATATTGCGGCATATATAAAGTTGGAATTTGACTATCAACTGCGAAGACATTAGACCGTTTAATAAAGTCATCCCCACCGATCAAAGAATTAATGATAGTATTATTCATTTTCTATTTAAAATGGAAAAAGCTTACAATAAACTCCGTAGAGAAATATCTATAATTTGTGAGTTTTCCTTAAAGTAACAGCTTCCGTAAACACCGTCTTTATCTCTTAGTAAGTTTATTGTATTTATGACCTTTTCCTTATCTTCATAGAATACTAAAGGCAATAAAGAAATTTTTGGTTCTTCTCTAAGAGCTACGTGAGACTTAACCATAGACGCCAACGAATCCCTACATATTTTAGAACAGAAATACCCAACTTCACCACCCTTGAATGTCTCAATACTAATAGGTCTAAAAACCAAATCTTGATTACAAAACCAACACTTATCAATTACACTATTTGTCTTAATAGACATATCTGCCATAGATTTATAATACTTTGGTAGTATACAAGCGAGTGCTTCTTCTTTAGCGGGCTTAAAGACTGCTTTAGGTGCTGAAATAACCACATCTGGAAGACTTACTCGCTTAGCCATTTAATTACGGAACTATTTTTTTATACTTCTAATGAACAAGTAGAAAACCTCTCATCTACAAAAACATACTCGTGTCCATAATCCTCTACCATAGTAACACGTTTTTTAGATCTCATATGTGCTAAAAAGTTTTCCCATACTAATTGGTTACTATTATTTTTCGTATAATTTTTAACAGTTTGAGGTTTTAGATTTTTAGTTACAGAAGTGATATCGAATATTTTATCCAAAAAGAATGAGTAATTAATTGTCTTAGAAGGAGTGTTTTCTTGGCAAAAGAATACCAAGTGCTTAAATATTTCTACTACTTCATTAATCTTTTCTGTACTCAGATTCAGTTTCTCATCTTTTACTTGATTGATTATTTCAAAGACTAACTTATAATCCTTTTTATTTATTCTCTCGTTAGCCTTAAGAAAACTAGATACAAAATTTGCATCTACATCATCCGTGGATATTTGATTTTTTTCCATGATATCCAATAGTTCCGAGATAATTTCTCCAGAACATTGATGAGACAATAATCTCCGCAATACATTTCTCAAATGAATAAGTTTATTAGACACGTGGAAGTTTGACTTTTTTTGTACCTTTGTACATTTTTGAAATACAGACTCGCAAAAAATACAATATTCATATCCTTGTTCAGATACTATACCGTTATGTCTACAACAGCTACATAATCGTAGATTCATGTTAACACTCTACGTATCTCGTCGTCCAATATTTTATATAAAAACATTTTATTTCTAGACGTTGTCAGAAAATCCTGTAATATTTTTAGTTTTTTTGGTTGTGAATAAAGTATCGCCCTAATAATATTGGTACCGTCTTCCGACAATATAGTAGTTAAATTATCCGAGCATGTAGAAGAACACCGCTTAGGCGGATTCAGTACAATGTTATATTTTTCGTACCAACTCATTTAAATATCATAATCTAAAATAGTTCTGTAATATGTCTAGCGCTAATATATTGATCATAATCCTGTGCATAAATTAAGATACAACAATGTCTTGAAATCATCGACATGGCTTCTTCCATAGTTAGAAGATCATCGTCAAAGTTAGCAACGTGATTCATCAACATTTGCTGTTTTGAGGCAGCAAATACTGAACCATCACCATTCAACCATTCATAAAAACCATCGTCTGAATCCATTGATAATTTCTTGTACTGGTTTTTGAGAGCTCGCATCAATCTAGCATTTCTAGCTCCCGGATTGAAAACAGAAAGAGGATCGTACATCCAAGGTCCATTTTCTGTAAATAGAATCGTATAATGTCCCTTCAAGAAGATATCAGACGATCCACAATCAAAGAATTGGTCTCCGAGTTTGTAACAGACTGCGGACTTTAACCTATACATGATACCGTTTAGCATGATTTCTGGTGATACGTCAATCGGAGTATCATCTATTAGAGATCTAAAGCCGGTGTAACATTCTCCGCCAAACATATTCTTATTCTGACGTCGTTCTACATAAAACATCATTGCTCCATTAACGATAACAGGTGAATGAACAGCACTACCCATCACATTAGTTCCCAATGGATCAATGTGTGTAACTCCAGAACATCTTCCATAGCCTATGTTAGGAGGAGCGAACACCACTCTTCCACTATTGCCATCGAATGCCATAGAATAAATATCCTTGGAATTGATAGAAATCGGACTGTCGGATGTTGTTATCATCTTCATAGGATTAACAACGATGTATGGTGCAGCCTGAAGTTTCATATCGTAACTGATGCCGTTCATAGGTCTAGCCACAGAAACCAACGTAGGTCTAAATCCAACTATAGACAAAATAGAAGCTAATATCTGTTCCTCATCTGTCATAACTTGAGAGCATCCAGTATGAATAATCTTCATTAGATGGGGATCTACCGCATCATCATCGTTACAATAAAAAATTCCCATTCTAATGTTCATAATTGCTTTTCTAATCATGGTATGAATGTTTGCTCTCTGAATCTCTGTGGAAATTAGATCTGATACACCTGTAATCACTATCGGATTATCCTCCGTAAGACGATTAACCAACAACATATAATTATAAGACTTTACTCTTCTAAATTCATAAAGTTGCTGGATTAGACTATATGTGTCTCCATGTACATACGCGTTCTCGAGCGCAGGAAGTTTAATACCGAATAGTGCCATCAGAATAGGATGAATGTAGTAATTAGTTTCTGGTTTTCTATAAATAAAAGACAAATCTTGTGAACTAGACATATCGGTAAAATGCATGGATTGGAATCGTGTAGTCGACAGAAGAATATGATGATTAGATGGAGAGTATATTTTATCTAACTCTTTGAGTTGGTCACCGATTCTAGGACTAGCTCGAGAATGAATAAGTACTAAGGGATGAGTACATTTCACAGAAACACTGGCGTTGTTCAACGTACTCTTTACATGGGAAAGGAGTTGAAATAGCTCGTTTCTATTTGTCCTGACAATATTTAGTTTATTCATAATATTAAGCATATCCTGAATAGTAAAGTTAGATGTGTCATACTTGTTAGTAGTTAGATATTTAGCAATTGCATTCCCATCATTTCTCAATCTCGTACTCCAATCATGTGTGGATGCTACTTCGTCGATGGAAACCATACAATCCTTTTTGATAGGCTGTTGAGATTGATCATTTCCTGTACGTTTAGGTTTGGTACGTTGATTTCTAGCCCCTGCTGATATAAAGTCATCGTCTACAATTTGGGATAATGAATTACATACACTACAAGACAAAGATTTATCAGAAGTGTGAATATGATCTTCATCTACCAAAGAAAGAGTTTGATTAGTATAACTAGATTTTAGTCCCGCGTTAGATGTTAAAAAAACATCGCTATTGACCACGGCTTCCATTATTTATATTCGTAGTTTTTACTCGAAAGCGTGATTTTAATATCCAATCTTATTACTTTTGGAATCGTTCAAAACCTTTGACTAGTTGTAGAATTTGATCTATTGCCCTACGCGTATACTCCCTTGCATCATATACGTTCGTCACCAGATCGTTTGTTTCGGCCTGAAGTTGACGCATATCTTTTTCAACACTCGACATGAGATCCTTAAGGGTCATATCGTCTAGATTTTGTTGAGATGCTGCTCCTGGATTTGGATTTTGTTGTGCTGTTGTACATACTGTACCACCAGTAGGTGTAGGAGTACATACAGTGGCCACAATAGGAGGTTGAAGAGGTGTAACCGTTGGAGTAGTACAAGAAATACTTCCATCCGATTGTTGTGTACATGTGGTTGTTGGTAACGTCTGAGAAGGTTGGGTAGATGGCGGTGTCGTCATCTTTTGATCTTTATTAAATTTAGAGATAATATCCTGAACAGTATTGCTCGGCGTCAACGCTGGAAGGAGTGTACTCGCCGGCGCATCAGTATCTGTAGACAACCAATCAAAAAGATTAGACATATCAGATGATGTATTAGTTTGTTGACGTGGTTTTAGTACAGGAGCAGTACTACTAGGTAGAAGAATAGGAGCCGGTGTAGGTGTCGGAACCGGCTGTGGAGTTATATGAATAGTTGGTTGTAGCGGTTGGGTAGGCTGTCTGCTGGCGGTCATCATATTATCTCTAGCTAGTTGTTCTCGCAACTGTCTTTGATAATACGACTCTTGAGACTTTAGTCCTATTTCAATCGCTTCATCCTTTTTCGTATCCGGATCCTTTTCTTCAGAATAATAGATTGACGACTTTGGTGTAGAGGATTCTGCCAGCCCCTGTGAGAACTTGTTAAAGAAGTCCATTTAAGGCTTTAAAATTGAATTGCGATTATAAGATTAAATGGCAGACACGGACGATATTATCGACTATGAATCCGATGATCTCACCGAATACGAGGATGATGAAGAAGATGGAGAGTCACTAGAAACTAGTGATATAGATCCCAAATCTTCTTATAAGATTGTAGAATCAACATCCACTCATATAGAAGATGCGCATTCCAATCTTAAACATATAGGGAATCATATATCTGCTCTTAAACGACGCTATACTAGACGTATAAGTCTATTTGAAATAGCGGGTATAATAGCAGAAAGCTATAACTTGCTTCAACGAGGAAGATTACCTCTAGTTTCAGAATTTTCTGACGAAACGATGAAGCAAAATATGCTACATGTAATTATACAAGAGATAGAGGAGGGTTCTTGTCCTATAGTCATCGAAAAGAACGGAGAATTGTTGTCGGTAAACGATTTTGATAAAGATGGTCTAAAATTCCATCTAGACTATATTATCAAAATTTGGAAACTTCAAAAACGATATTAGAATTTATACGAATATCGTTCTCTAAATGTCACAATCAAGTCTCTCATATTCAGCAGTTTATTGTCGTACTTTATATCGTGTTCATTAACGATATTTTGCAAAATAGTAATGATTCTATCTTCCTTCGATAGATATTCTTCAGAGATTATTGTCTTATATTCTTTCTTGTTATCCGATATGAATTTGATAAGACTTTGAACATTATTAATACCCGTCTGTTTAATTTTTTCTATAGATATTTTAGTTTTGGTAGATTCTATGGTGTCTGTTAATAGGCATCCAACATCGACATTCGACGTCAATTGTCTATAAATCAGAGTATAAATTTTAGAAATAACATTAGCAAATTGTTGTGCGTTGATGTCGTTATTCTGAAACAGTATGATTTTAGGTAGCATTTTCTTAACAAAGAGAACGTATTTATTGTTACTCAGTTGAACAGATGATATATCCAGATTACTAACGCATCTGATTCCATATACCAAACTTTCAGAAGAAATGGTGTACAATTGTTTGTATTCATTCAATGTCTCCTTTTCAGAAATTAGTTTAGAGTCGAATACTGCAATAATTTTCAAGAGATAGTTTTCATCAGATAAGATTTTATTTAGTGTAGATATGATAAAACTATTGTTTTGTTGGAGAACTTGATACGCCGCATTCTCTGTAGTCGACGCTCTCAAATGGGAAACAATCTCTATTATTTTTTTGGAATCGGATACTATATCTTCGGTATCTTGACGCAGTCTAGTATACATAGAGTTAAGAGAAATTAGAGTTTGTACATTAAGCAACATGTCTCTAAATGTGGCTACAAACTTTTCTTTTTCCACATCATCTAGTTTATTATATACCGATTTCACAACGGCACCAGATTTAAGGAACCAGAATGAAAAACTCTGATAACTACAATATTTCATCATAGTTACGATTTTATCATCTTCTATAGTTGGTGTGATAACACATACCTTTTTCTCCAAGACTGGAACCAACGTCATAAAAATGTTTAAATCAAAATCCATATCAACATCTGATGCGCTAAGACCAGTCTCGCGTTCAAGATTATCTTTACTAATGGTGACGAACTCATCGTATAGAACTCTAAGTTTGTCCATTATTTATTTACAGATTTAGTTGTTTAATTTATTTGTGCTCTTCCAGAGTTGGGATAGTATTTTTCTAACGTCGGTATTATATTATTAGGATCTACGTTCATATGTATCATAATATTAATCATCCACGTTTTGATAAATCTATCTTTAGCTTCTGAAATAACGTATTTAAACAAAGGAGAAAAATATTTAGTTACGGCATCAGACGCGATAACATTTTTTGTAAATGTAACGTATTTAGACGACAGATCTTCGTTAAAAAGTTTTCCATCTATGTAGAATCCATCGGTTGTTAACACCATTCCCGCGTCAGAGTGAATAGGAGTTTGAATAGTTTGTTTTGGAAATAGATCCTTCAATAACTTATAGTTGGGTGGGAAAAAATCGATTTTATCACTAGACTCTTTCTTTTTTACTATCATTACCTCATGAACTATTTCTTGAATGAGTATATGTATTTTCTTTCCTATATCGGTCGCGTTCATTGGAAAATATATCATGTCGTTAACTATAAGAATATTTTTATCCTCGTTTACAAACTGAATAATATCAGATATAGTTCGTAAACGAACTATATCATCACCAGCACAACATCTAACTATATGATATCCACTAGTTTCCTTTAGCCGTTTATTATCTTGTTCCATATTAGCAGTCATTCCATCATTTAAGAAGGCGTCAAAGATAATAGGGAGAAATGACATTTTGGATTCTGTTACGACTTTACCAAAATTAAGGATATACGGACTTACTATCTTTTTCTCAACGTCGATTTGATGAACACACGATGAAAATGTACTTCGATGAGATTGATCATGTAGAAAACAACAAGGGATACAATATTTCCGCATATCATGAAATATATTAAGAAATCCCACTTTATTATATTTCCCCAAAGGATCAATGCATGTAAACATTATACCGTTATCATTAATAAAGACTTCTTTCTCATCGGATCTGTAAAAGTTGTTACTGATTTTTTTCATTCCAGGATCTAGATAATTAATAATAATGGGTTTTCTATTCTTATTCTTTGTATTTTGACATATCCTAGACCAGTAAACAGTTTCCACTTTGGTAAAATCAGAAGACTTTTGAACGCTATTAAACATGGCATTAATAGCAATAACTAAAAATGTAAAATATTTTTCTATGTTAGGAATATGGTTTTTCACTTTAATAGATATATGGTTTTTTGCCAAAATGATAGATATTTTTTTATCCGATGATAGTAAAATATTATTAGTCGCCGTCTCTATAAAAATGAAGCTAGTCTCGATATCCAATTTTATTCTAGAATTGATAGGAGTCGCCAAATGTACCTTATACGTTATATCTCCCTTGATGCGTTCCATTTGTGTATCTATATCGGACACAAGATCTGTAAATAGTTTTACGTTATTAATCATCACGGTATCGCCATCGCTAGATAATGCTAATGTACTATCCAAGTCCCAAATGGAGAGATTTAACTGTTCATCGTTTAGAATAAAATGATTACCTGTCATATTAATAAAGTGTTCATCGTATCTAGATAACAACGACTTATAATTAATGTCCAAGTCTTGAACTCGCTGAATGATCTTTTTTAACCCAGTTAGTTTTAGATTGGTACGAAATATATTGTTAAACTTTGATTCTACAGTAATGTCCAAATCTAGTTGTGGAAATACTTCCATCAACATTGTTTCAAACTTGATAATATTATTATCTACATCTTCGTACGATCCAAATTCCGGAATAGATGTATCGCACGCTCTGGCCACCCAGATAACCAAAAAGTCACACGCTCCAGAATATACATTGTATAAAAAGCTATCGTTTTTTAGTAGTGTTTTTTTCTGAGTATATACGAAAGGATTAAAAATAGTATTATCAACGTAACTATATTCCAAATTATTCTTATGAGAATAGATAATAATATCGTCCTTAATATCTAACAAATTTCCTAAATATCCCTTTAATTGAGTCATTCGAAGCGTTAATAAAATATGTCTCTTAACTATTTCCGGCCGTTGTATATTTAAATGACTTCGTAAGAAATAATATATAGGCGACTTCTCATCTATGTAATCATATGGAGTGAGATATAGGGCTCGTTCTACCTCCTGCCCCTTACCCACCTGTAATACCAATTGCGGACTCACTATATATCGCATATTTATATCGTGGGGTAAAGTGAAAATCTACTACCGATGATGTAAGTCTTACAATGTTCGAACCAGTACCAGATCTTAATTTGGAGGCCTCCGTAGAACTAGGGGAGGTAAATATAGATCAAACAACACCTATGATAAAGGAGAATAGCGGTTTTATATCCCGTAGTAGACGTCTATTCGCCCATAGATCTAAGGATGATGAGAGAAAACTAGCACTACGATTCTTTTTACAAAGACTTTATTTTTTAGATCATAGAGAGATTCATTATTTGTTCAGATGCGTCGACGCTGTAAAAGACGTCACTATTACCAAAAAAAATAACATTATCGTGGCGCCTTATATAGCACTTTTAACTATCGCATCAAAAGGATGCAAACTTACAGAAACAATGATTGAAGCATTCTTTCCAGAACTATATAATGAACATAGTAAGAAATTCAAATTCAACTCTCAAGTATCCATCATCCAAGAAAAACTCGGATACCAGTCTGGAAACTATCACGTTTATGATTTTGAACCGTATTACTCTACAGTAGCTCTGGCTATTCGAGATGAACATTCATCTGGCATTTTTAATATCCGTCAAGAGAGTTATCTTGTAAGTTCATTATCTGAAATAACATATAGATTTTATCTAATTAATCTAAAATCTGATCTTGTTCAATGGAGTGCTAGTACGGGCGCTGTAATTAATCAAATGGTAAATACTGTATTGATTACAGTGTATGAAAAATTACAACTGGCCATAGAAAATGATTCACAATTTACATGTTCATTGGCTGTGGAATCAGAACTTCCAATAAAATTACTTAAAGATAGAAATGAATTATTTACAAAATTCATTAACGAGTTAAAAAAGACCAGTTCATTCAAGATAAGCAAACGTGATAAGGATACGCTATTAAAACATTTTACTTATGACTGGAGTTAGAATTTATAGACGACACATTTCGTTTATCATTGTTACTATTACTATCATTATTAGTATTCTTCTTGTCATCTTGTTCAGAAATATACAGCAATGCTATACCTAATACTAAATACATTATCATGCTTGCAATGGCTCTAACAACAACGAACCAAAATGAATTTGGTCGTAGCTTTTGTTCACAAAAATACATAAAGAAATGTCTACATAAATCTATGGCGCCATTGGCTACTTGAAATAGCGCCAGTCCTCCTACAGATTTTAATATAGCTGTATAACATGACATTTATTCATCATCAAAAGAGACAGAGTCACCATCTGTCATATTTAGATTTTTTTTCATGTGTTCAAAGTATCCTCTACTCATTTCATTATAATAGTTTATCATGCTTAGAATTTTAGGACGGATCAATGAGTAAGACTTGACTAGATCGTCAGTAGTAATTTGTGCATCATCTATTCTGCATCCGCTTCGTCGAATAATGTATAGCATCGCTTTGAGATTCTCCATAGCTATCAAGTCTTTATATAATGACATGGAAATATCTGTGAATGCTTTATACTTCTCCAACATCGATGCCTTAACATCATCACATACTTTAGCATTGAAAATACGTTCTATTGTGTAGATGGATGTAGCAAGATTTTTAAACAACAATGCCATCTTACATGATGATTGTCTCAAGTCTCCAATCGTTTGTTTAGAACGATTAGCTACAGAGTCCAATGCTTGGCTAACTAGCATATTATTATCTTTAGAAATTGTATTCTTCAATGAGGCGTTTATCATATCTGTGATTTCGTTAGTCATATTACAGTCTGACTGGGTTGTAATGTTATCCAACATATCACCTATGGATACGGTACACGTACCAGCATTTGTAATAATCCTATCTAAGATGTTGTATGGCATTGCGCAGAAAATATCTTCTCCTGTAATATCTCCACTCTCGATAAATCTACTCAGATTATTCTTAAATGCCTTATTCTCTGGAGAAAAGATATCAGTGTCCATCATTTCATTAATAGTATACGCAGAAAAGATACCACGAGTATCAATTCTATCCAAGATACTTATCGGTTCCGAGTCACAGATAATTGTTTCCTCTCCTTCGGGAGATCCTGCATAGAAATATCTAGGACAATAGTTTCTATACTGTCTGTAACTCTGATAATCTCTAAAGTCACTAACTGATACCATGAAATTGAGAAGATCAAACGCTGAAGTAATCAATTTTTCTGCCTCGTTTTTACTACAACTAGTTTTCATCAATGTAGTGACGATGTATTGTTTAGTTACTCTTGGTCTAATACTGATGATAGAGATATTATTGCTTCCCATAATGGATCTTCTAGTAGTCACCTTAAAGCCCATTGATGCGAATAGCAGATAGATAAAGTCTTGGTATGACTCCTTTCTAATATAGTACGGACTACCTTTGTCACCCAACTTTATACCCACATAAGCCATAACAACCTCTTTAATAGCCGTTTCATGAGGTTTATCAGCCATGAGCCTGAGTAGTTGAAAGAATCGCATGAATCCCGTCTCAGAAAGTCCTATATGCATGATAGATTTATCTTTCCTGGGAAACTCTCGTATAGTTATAGATGAAATACTCTTCAAAGTTTCTGAAATAAGATTAGTAACAGTCTTACCTCCGACTACTCTGGGTAACAAACATACTCTAATAGGTGTTTTCTCTGCGGAGATAATATCAGAAAGGATAGAGCAATAAGTAGTATTATTGTGATTATAAAGACCGAATACATAACAGGTAGAATTTATAAACATCATGTCCTGAAGGGTTTTAGACTTGTATTCCTCGTAATCTATACCGTCCCAAAACATGGATTTGGTAACTTTGATAGCCGTAGATCTTTGTTCCTTCGCTAACAGGTTAAAGAAATTAATAAAGAATTTGTTGTTTCTATTTATGTCCACAAATTGCACGTTTGGAAGCGCCACGGTTACATTCACTGCAGCATTTTGAGGATCGCGAGTATGAAGTACGATGTTATTGTTTACTGGTATATCTGGAAAGAAATCTACCAGTCTAGGAATAAGAGATTGATATCGCATAGAAATAGTAAAGTTTATAATCTCATCATTGAAGATTACTCTGTTACCATTGTAATAAATTGGTACTCTATCATAATCATCGACAAAGTACTGTTCATACATGATGAGATGTTTATATGTTGGCATAGTAGTGAGATCGACGTTTGGTAATGGCAATGTATTAAGATTAACTCCATAATGTCTAGCAGCATCTGCGATGTTATAAGTGATGTCAAAGCGGGGTTGATCTTGTGCTGTTATATATTGTCTAACACCTATAAGATTATCAAAATCTTGTCTGCTTAATACACCGTTAACAATTTTTGCCTTGAATTCTTTTATTGGTGCATTAATAACATCCTTATAGAGGATGTTAAACAAATAAGTATTATCAAAGTTAAGATCTGGGTATTTCTTTTCTGCTAGAACATCCATTGAGTCGGAGCCATCTGGTTTAATATAACCACCGATAAATCTAGCTCTGTATTCTGTATCCGTCAATCTAATATTAAGAAGGTGTTGAGTGAAAGGTGGAAGATCGTAAAAGCTGTGAGTATTAATAATAGGGTTAGTTTCCGAACTAATGTTAATTGGATGATTAATAATATCTATATTTCCAGCGTTAAGTGTAACATTAAACAGTTTTAATTCACGTGACGTGGTATCAATTAAATAATTAATGCCCAATTTGGATATAGTAGCCTGAAGCTCATCTTGTTTAGTTACGGATCCTAATGAGTTATTAAGAAATACATCGAACGGATGAACGAAGGTTGTTTTAAGTTGGTCACATACTTTGTAATCTAGACATAGATGTGGAAGAACGGTAGAAACTATACGAAATAGATATTCAGAGTCCTCTAATTGATCAAGAGTAACTATTGACTTAATAGGCATCATTTATTTAGTATTAAATGACGACCGTACCAGTGACAGATATACAAAACGACTTAATTACAGAGTTTTCAGAAGATAATTATCCATCTAACAAAAATTATGAAATAACTCTTCGCCAAATGTCTATTCTAACTCACGTTAACAACGTGGTAGATAGAGAACATAATGCCGCCGTAGTGTCATCTCCAGAGGAAATATCATCACAACTTAATGAAGATCTATTTCCAGATGATGATTCACCGGCCACTATTATCGAACGAGTACAACCTCATACTACTATTATTGACGATACGCCACCTCCTACTTTTCGTAGAGAGTTATTGATATCGGAACAACGTCAACAACGAGAAAAAAGATTTAATATTACAGTATCAAAAAATTCTGAAGCAATAATGGAATCTAGATCTATGATAACTTCTATGCCAACACAAACACCATCCTTGGGAGTAGTTTATGATAAAGATAAAAGAATTCAGATGCTAGAGGATGAAGTGGTTAATCTTAGAAATCAACGATCTAATACAAAATCATCTGATAATTTAGATAATTTTACCAGAATACTATTTGGTAAGACTCCGTATAAATCAACCGAAGTTAATAAGCGTATAGCCATCGTTAATTATGCAAATTTGAACGGGTCCCCCTTATCAGTCGAGGACTTGGATGTCTGTTCGGAGGATGAAATAGATAGAATCTATAAAACGATTAAACAATATCACGAAAGTAGAAAACGAAAAATTATCGTCACTAACGTGATTATTATTGTCATAAACATTATTGAGCAGGCATTGCTAAAACTCGGATTTGAAGAAATCAAAGGACTGAGTACCGATATCACTTCAGAAATTATCGATGTGGAGATCGGAGATGACTGCGATGCTGTAGCATCAAAACTAGGAATCGGTAACAGTCCGGTTCTTAATATTGTATTGTTTATACTCAAGATATTCGTTAAACGAATTAAAATTATTTAATTTAATACATTCCCATATCCAGACAACAATCGTCTGGATTAATCTGTTCCTGTCGTCTCATACCGGACGACATATTAATCTTTTTATTAGTGGGCATCTTTTTAGATGGTTTCTTTTTCCCAGCATTAACTGATTCGATACCTAGAAGATCGTGATTGATTTCTCCGACCATTCCACGAACTTCTAATTGGCCGTCTCTAACGGTACCATAAACTATTTTACCAGCATTAGTAACAGCTTGGACAATCTGACCATCCATTGCGTTGAATGATGTAGTTGCTGTTGTTCTACGTCTAGGAGCACCAGAGGTATTTTTAGAGCTCTTGGATGTTGATGTAGAAGACGAGGATTTTGATTTTGGTTTACATGTAATACATTTTGAACTCTTTGATTTTGTATCACATGCACCGGCAGTCACATCTGTTTGAGAATTAAGATTATTGTTGCCTCCTTTGACGGCTGCATCTCCACCGATCTGCGCTAGTAGATTTTTAAGCTGTGGTGTAATCTTATTAACTGTTTCAATATAATCATCGTAACTACTTCTAACGGCTAAATTTTTTTTATCCGCCATTTAGAAGCTAAAAATATTTTTATTTATGCAGAAGATTTAACTAGATTATACAATGAACTAATATGATCCTTTTCTAGATTATTTACGAACTTGGTATTTCTTGTTTCTGGAGGAGGAGAATTTAAATTCGGACTTGGATTCGGATTTTGTGGGTTCTTGATCTTATTATACAGCGTGTATAGGATGGCGACGGTAACTGCTACACAAATACCGATCAACAGAAGAATACCAATCATTTATTGACAATAACTTCACTATGATCAAGTATGTAATAATCATCTTTTCACTAAGTAAGTAGTAATAATGATTCAACAATGACACGATATATGGACGATAATAATTTAGTTCATGGAAATATCGCTATGATTGGTGTGAATGACTCCGCTAACTCTGTGGGGTGCACAGTGCTTTCCCCACATAGAATAAATTAGCATTCCGACTGTGATAATAATACCAAGTATAAACGCCATAATACTCAATACTTTCCATGTACGAGTGGGACTGGTAGACTTACTAAAGTCAATAAAGGCGAAGATACACGAAAGAATCAAAAGAATGATTCCAGCGATTAGCACGCCAGAAAAATAATTTCCAATCATAAGCATCATGTCCATTTAACTAATAAAAATTTTAAATCGCCGAATAAACAAAGTGGAATATAAACCATATAAAAACAATAGTTTGTACTGCAAAAATAATATCTATTTTTGTTTTCGAAGATATGGTAAAATTAAATAGTAGTACACAGCATGTTATAACTAACAGCAGCAACGGCTCGTAATTACTTATCATTTACTAGACGAAAAGGTGGTGGGATATTTTCTTGCTCAAATAATACGAATATATCACCCATCCATTTTATACGATGTTTATATACTCTAATCTTTAATAGATCTATAGATGACGGGTTTACCAATAATATAGATTTTATCGATTCATCTAATTTAAACCCTTCCTTAAACGTGAATGATCTATTATCTGGCATAATGATGACCCTACCTGATGAATCTGACAATGTACTGGGCCATGTAGAATAAATTATCAACGAATTATCGTCTACGAACATTTATATCATTTGTTTTAATTTTAGGACGTGAATAAATAGATATAAAATAGAAAATAACAGATATTACAACCAGTGTTATGGACGCACCCAACCATGTAGGCAGTTTTATTTTATCGTTTACTACAGGTTCTCCTGGATGTACGTCACCAACTGCAGACGTAGTTCTAGTACAATTAGACGTAAGTTCCGCTTGGGAATTTTTTAACGCTAAAGAGTTAACGTTGATCGTACACCCAACGTATTTACATCTAGTTCTTTGAACATCTTGATTATAATATAACCATTTTCTATCTCTAGATTCGTCAGTGCACTCATGTAACCAACATACCCTAGGTCCTAAATATTTATCTCCGGAATTAGATTTTGGATAATTCGCGCACCAACAATTTCTATTTCCTTTATGGTCGTTACAAAAGACGTATAATGCCGTATCCCCAAAAGTAAAATAATCAGGACGAATAATTCTAATAAACTCAGAACAATATCTCGCATCCATATGTTTGGAGCAAATATCGGAATAAGTAGACATAGCCGGTTTCCGTTTTACACGTAACCATTCTAAACAATTGGGGTTTCCAGGATCGTTTCTACAAAAACCAGTCATGAAATCGTCACAATGTTCTGTCTTGTAATTATTATTAAATATTTTTGGACAGTGTTTGGTATTTGTCTTAGAACAACATTTTGCCACGCTATCACTATCACCCAGGAGATAATCCTTTTTTATAAAATGACATCGTTGCCCGGATGCTATATAATCAGTAGCATATTTTAAATCCTTAATATATTCAGGAGTTACCTCGTTCTGATAATAGATTAATGATCCAGGACGAAATTTGAAAGAACTACATGGTTCTCCATGAATTAATACATATTGTTTAGCAAATTCAGGAACTATAAAACTACTACAATGATCTATCGACATACCATCTATCAAACAAAATTTGGGTTTAATTTCTCCTGGAGACGTTTCATAATAATACATATAACTTTCTTCGGCAAACCTAACAGCTCTATTATATTCAGGATAATTAAAATCTAATACCATATATTTGTCTCGTATATCTGCTATTCCTGTCTCTATTTTGATTCTATTAAGAGTAACAGCTGCCCCCATTCTTAATAATCATCAGTATTTAAACTGTTAAATGTTGGTATATCAACATCTATCTTATTTCCCGCAGTATAAGGTTTGTTGCAGGTATACTGTTCAGGAATGGGTACATTTATACTTCTTTTATAGTCCTGTCTTTCGATGTTCATCACAAATGCAAAGAACAGAATAAACAAAATAATGTAAGAAATAATATTAAATATCTGTGAATTCGTAAATACATTGATTGCCATAATAATTACAGCAGCTACAATACACACAATAGACATTCCCACAGTGTTGCCATTACCTCCACGATACATTTGAGTTACTAAGCAATAGGTAATAACTAAGCTAGTAAGAGGCAATAGAAAAGATGAGATAAATATCATCAATATAGAGATTAGAGGAGGGCTATATAGAGCCAAGACGAACAAAATCAAACCGAGTAACGTTCTAACATCATTATTTTTGAAGATTCCCAAATAATCATTCATTATTCCTCCATAATCGTTTTGCATCATACCCCCATCTTTAGGCATAAACGATTGCTGCTGTTCCTCTGTAAATAAATCTTTATCAAGCACTCCAGCACCCGCAGAGAAGTCATCAAGCATATTGTAATATCTTAAATAACTCATTTATATATTAAAAAATGTCACTATTAAAGATGGAGTATAATCTTTATGCCGAACTAAAAAAAATGACTTGTGGTCAGACCATAAGTCTTTTTAATGAAAACGGCGATTTCGTAGAAGTTGAACCAGGATCATCCTTTAAGTTTCTAATACCTAAGGGATTTTACTCCTCTCCTTGTGTAAAGACGAGTCTAGTATTCGAGACATTAACAACGACCGATAATAAAATTACTAGTATCAATCCAACAAATGCGCCAAAGTTATATCCTCTTCAACGCAAAGTCGTATCTGAAGTAGTTTCTAATATGAGGAAAATGATCGAATTAAAACGTCCTCTATACATCACTCTTCACTTGGCATGTGGATTTGGTAAGACTATTACCACGTGTTATCTTATGACCACACACGGCAGAAAAACCATCATTTGCGTACCCAATAAAATGTTAATACATCAATGGAAGACACAGGTAGAGGCAGTCGGATTGGAACATAAGATATCTATAGATGGAGTTAGTAGTCTATTAAAGGAACTAAAGACTCAAAGTCCGGATGTATTAATCGTAGTCAGTAGACATCTGACAAACGATGCATTTTGTAAATATATCAATAAGCATTATGATTTGTTTATCTTGGATGAATCACATACGTATAATCTGATGAACAATACAGCAGTTACAAGATTTTTAGCGTATTATCCTCCGATGATGTGTTATTTTTTAACTGCTACACCTAGACCAGCTAACCGAATTTATTGTAATAGTATTATTAATATTGCCAAGTTATCCGATCTAAAAAAAACTATCTATATAGTAGATAGTTTTTTTGAGCCATATTCCACAGACAATATTAGAAATATGGTAAAACGACTAGATGGACCATCTAATAAATATCATATATATACCGAGAAGTTATTATCTGTAGACGAGCCTAGAAACCAACTTATTCTTGATACCCTGGTAGAAGAATTCAAGTCAGGAACTATTAATAGAATTTTAGTTATTACTAAACTACGTGAACATATGGTATTCTTCTACAAACGATTATTAGATCTTTTCGGAGCAGAGGTTGTATTTATAGGAGACGCCCAAAATAGACGTACTCCAGATATGGTCAAATCGATTAAGGAACTAAATAGATTTATATTCGTATCCACCTTATTTTATTCCGGCACTGGTTTAGATATTCCGAGTTTGGATTCTTTGTTCATTTGCTCGGCAGTAATCAACAATATGCAAATAGAGCAATTACTAGGGAGGGTATGTCGAGAAACAGAACTATTAGATAGGACGGTATATGTATTTCCTAACACATCCATCAAAGAAATAAAGTACATGATAGGAAATTTCGTGCAACGAATTATTAGTCTGTCTGTAGATAAACTCGGATTTAAACAAGAAAGTTATCAGAAACATCAGGAATCTGAACCCGCTTCCGTACCAACATCCTCCAGAGAAGAACGTGTATTAAATAGAATATTTAACTCGCAAAATCGTTAAGAAGTTTAAGAGACGATCCACATGCTGAGCAGGCCAGTGTATTACCCCTCATAGTATTAATATAATCCAATGATACTTTTGTGATGTCGGAAATCTTAACCAATTTAGACTGACAGGCAGAACACGTCATACAATCATCATCGTCATCGATAACTGTAGTCTTGGGCTTCTTTTTGCGACTCTTCATTCCGGAACGCATATTGGTGCTATCCATTTAGGTAGTAAAAAATAAGTCAGAATATGCCCTATAACACGATCGTGCAAAACCTGGTATATCGTCTCTATCTTTATCACAATATAGTGTATCAACATCTTTATTATTGACCTCGTTTATCTTGGAACATGGAATGGGAACATTTTTGTTAACGGCCACCTTTGCCTTAATTCCAGATGTTGTAAAATTATAACTAAACAGTCTATCATCGACACAAATGAAATTCTTGTTTAGACGTTTGTAGTTTACGTATGCGGCTCGTTCTCGTCTCATTTTTTCAGATATTGCAGGTACTATAATATTAAAAATAAGAATGAAATAACATAGGATTAAAAATAAAGTTATCATGACTTCTAGTGCTGATTTAACTAACTTAAAAGAATTACTTAGTCTGTACAAAAGTTTGAGATTTTCAGATTCTGTGGCTATAGAGAAGTATAATTCTTTGGTAGAATGGGGAACATCTACTTACTGGAAAATAGGCGTACAAAAGGTAACTAATGTCGAGACGTCCATATCTGATTATTATGATGAGGTAAAAAATAAACCGTTTAATATTGATCCGGGGTATTATATTTTCTTACCAGTATATTTTGGAAGCGTCTTTATTTATTCAAAGGGTAAAAATATGGTAGAACTTGGATCTGGAAACTCTTTTCAAATACCGGATGAGATTCGAAGTGCGTGTAACAAAGTATTAGATAGTGATAACGGAATAGACTTTCTGAGATTTGTTTTGTTAAACAATAGATGGATAATGGAAGACGCTATATCAAAATACCAGTCTCCAGTTAATATATTTAAACTAGCTAGTGAGTACGGATTAAACATACCCAACTATTTAGAAATTGAAATAGAGGAAGACACATTATTTGACGATGAGTTATACTCTATTATGGAACGCTCTTTCGATGATACATTTCCAAAAATATCTATATCGTATATTAAGTTGGGAGAACTTAAGCGGCAAGTTGTAGACTTTTTCAAATTCTCATTCATGTATATTGAGTCAATCAAGGTAGATCGTATAGGAGATAATATTTTTATTCCTAGCGTTATAACAAAATCAGGAAAAAAGATATTAGTAAAAGATGTAGACCATTTAATACGATCCAAGGTTAGAGAACATACATTTGTAAAAGTAAAAAAGAAAAACACATTTTCCATTTTATACGACTATGATGGGAACGGAACAGAAACTAGAGGAGAAGTAATAAAACGAATTATAGACACTATAGGACGAGACTATTATGTTAATGGAAAGTATTTCTCTAAGGTTGGTATTGCAGGCTTAAAGCAATTGACTAATAAATTAGATATTAATGAGTGTGCAACTGTCGATGAGTTAGTTGATGAGATTAATAAATCCGGAACTGTAAAACGAAAAATAAAAAACCAATCAGTATTTGATTTAAGCAGAGAATGTTTGGGATATCCAGAAGCGGATTTTATAACGTTAGTTAATAACATGCGGTTCAAAATAGAAAATTGTAAGGTTGTAAATTTCAATATTGAAAATACTAATTGTTTAAATAACCCGAGTATTGAAACTATATATGGAAACTTCAACCAGTTCGTCTCAATCTTTAATACCGTTACCGATGTCAAAAAAAGATTATTCGAGTGAAATAATATGCGCCTTTGATATAGGTGCAAAAAATCCTGCCAGAACTGTTTTAGAAGTCAAGGATAACTCCGTTAGGGTATTGGATATATCAAAATTAGACTGGAGTTCTGATTGGGAAAGGCGCATAGCTCAAGATTTGTCACAATATGAATACACTACAGTTCTTCTAGAACGTCAGCCTAGAAGGTCACCGTACGTCAAATTTATCTATTTTATTAAAGGCTTTTTATATCATACATCTGCTGCCAAAGTTATTTGCGTCTCACCTGTCATGTCTGGTAATTCATATAGAGATCGAAAAAAGAGATCTGTTGAAGCATTTCTTGATTGGATGGACACATTCGGATTGCGAGACTCCGTTCCGGATAGACGCAAATTAGACGATGTAGCGGATAGTTTCAATTTGGCTATGAGATACGTATTAGATAAATGGAATACTAATTATACACCTTATAATAGGTGTAAATATAGAAATTACATAAAAAAAATGTAATAACGTTAGTAACGCCATTATGGATAATCTATTTACCTTTCTACATGAAATAGAAGATAGATATGCCAGAACTATTTTTAACTTTCATCTAATAAGTTGTGATGAAATAGGAGATATATATGGTCTTATGAAAGAACGCATTTCCTCAGAGGATATGTTTGACAATATAGTATATAATAAAGATATACATCCTGCCATTAAGAAACTAGTTTATTGCGACATCCAACTTACTAAACATATTATTAATCAGAATACGTATCCGGTATTTAACGATTCTTCACAAGTGAAATGTTGTCATTATTTCGATATAAACTCAAATAATAGCAATATTAGCTCTCGTACAGTAGAGATATTTGAGAGTGAAAAGTCATCTCTTGTATCATATATTAAAACTACCAATAAGAAGAGAAAGGTCAATTACGGCGAAATAAAGAAAACTGTACATGGAGGCACTAATGCAAATTACTTTTCCGGTAAAAAGTCTGATGAGTATCTGAGCACTACAGTCAGGTCCAACATTAATCAACCTTGGATCAAAACCATTTCTAAGAGAATGAGAGTAGATATCATTAATCACTCTATAGTAACGCGTGGAAAAAGCTCTATATTACAAACTATAGAAATTATTTTTACTAATAGAACATGTGTGAAAATATTCAAGGATTCTACTATGCACATTATTCTATCCAAGGACAAGGATGAAAAGGGATGTATAAACATGATTGATAAATTATTCTATGTATATTATAATTTATTTCTGTTGTTCGAGGATATCATCCAAAACGATTACTTTAAAGAAGTAGCTAATGTTGTAAACCATGTACTCATGGCTACGGCATTAGATGAGAAATTATTCCTAATTAAGAAAATGGCTGAACACGATGTTTATGGAGTTAGCAATTTCAAAATAGGGATGTTTAACCTGACATTTATTAAGTCGTTGGATCATACCGTTTTCCCCTCTCTGTTAGATGAGGATAGCAAAATAAAGTTTTTTAAGGGGAAAAAGCTCAATATTGTAGCATTACGATCTCTGGAGGATTGTACAAATTACGTGACTAAATCCGAGAATATGATAGAAATGATGAAGGAAAGATCGACTATTTTAAATAGCATAGATATAGAAACGGAATCGGTAGATCGTCTAAAAGAATTGCTTCTAAAATGAAAAAAAACACTGATTCAGAAATGGATCAACGACTCGGGTATAAGTTTTTGGTGCCTGATCCTAAAGCCGGAGTTTTTTATAGACCGTTACATTTCCAATATGTATCGTATTCTAATTTTATATTGCATCGATTGCATGAAATCTTGACCGTCAAGCGGCCACTCTTATCGTTTAAGAATAATACAGAACGAATTATGATAGAAATTAGCAATGTTAAAGTGACTCCTCCAGATTACTCACCTATAATTGCGAGTATTAAAGGTAAGAGTTATGACGCATTAGCCACGTTCACTGTAAATATCTTTAAAGAGGTAATGACCAAAGAGGGTATATCCATCACTAAAATAAGTAGTTATGAGGGAAAAGATTCTCATTTGATAAAAATTCCGCTACTAATAGGATATGGGAATAAAAATCCACTTGATACAGCCAAGTATCTTGTTCCTAATGTCATAGGTGGAGTCTTTATCAATAAACAATCTGTCGAAAAAGTAGGAATTAATCTAGTAGAAAAGATTACAACATGGCCAAAATTTAGGGTTGTTAAGCCAAACTCATTCACTTTCTCGTTTTCCTCCGTATCCCCTCCTAATGTATTACCGACAAGATATCGCCATTACAAGATATCTCTGGATATATCACAATTGGAAGCGTCGAATATATCATCGACAAAGACATTTATAACGGTCAATATTGTTTTGCTGTCTCAATATTTATCTAGAGTGAGTCTAGAATTCATTAGACGTAGTTTATCATACGATATGCCTCCAGAAGTTGTCTATCTAGTAAACGCGATAATAGATAGTGCTAAACGACTTACCGAATCTATTACTGACTTTAATATTGATACATACATTAATGACCTGGTGGAAGCTGAACACATTAAACAAAAATCTCAGTTAACGATTAACGAGTTTAAATATGAAATGCTGCATAACTTTTTACCTCATATGAACTATACACCCGATCAACTAAAGGGATTTTATATGATATCTTTACTAAGAAAGTTTCTCTACTGTATCTACCACACTTCTAGATATCCAGATAGAGATTCGATGGTTTGTCATCGCATCCTAACGTACGGCAAATATTTTGAGACGTTAGCACATGATGAATTAGAGAATTACATAGGTAACATCCGAAACGATATCATGAACAATCACAAGAACAGAGGCACTTACGCAGTAAACATTCATGTACTAACAACTCCTGGACTTAATCATGCATTTTCTAGTCTATTGAGTGGAAAGTTCAAAAAGTCAGACGGTAGTTATCGAACACATCCTCACTATTCATGGATGCAGAATATTTCTATTCCTAGAAGTGTTGGATTTTATCCGGATCAAGTAAAGATTTCAAAGATGTTTTCTGTCAGAAAATACCATCCAAGCCAATATCTTTACTTTTGTTCATCAGACGTTCCGGAAAGAGGTCCTCAGGTAGGTTTAGTATCTCAATTGTCTGTCTTGAGTTCCATTACAAATATACTAACGTCTGAGTATTTGGATTTGGAAAAGAAAATTTGTGAGTATATCAGATCATATTATAAAGATGATATAAGTTACTTTGAAACAGGATTTCCAATCACTATAGAAAATGCTCTAGTCGCATCTCTTAATCCAAATATGATATGTGATTTTGTAACTGACTTTAGACGTAGAAAACGGATGGGATTCTTCGGTAACTTGGAGGTAGGTATTACTTTAGTTAGGGATCACATGAATGAAATTCGCATTAATATTGGAGCAGGAAGATTAGTCAGACCATTCTTGGTTGTGGATAACGGAGAGCTCATGATGGATGTGTGTCCGGAGTTAGAAAGCAGATTAGACGACATGACATTCTCTGACATTCAGAAAGAGTTTCCACATGTCATCGAAATGGTAGATATAGAACAATTTACTTTTAGTAACGTATGTGAATCGGTTCAAAAATTTAGAATGATGTCAAAGGATGAAAGAAAGCAATACGATTTATGTGACTTTCCTGCCGAATTTAGAGATGGATATGTAGCATCTTCACTAGTGGGAATCAATCACAATTCTGGACCCAGAGCTATTCTTGGATGTGCTCAAGCTAAACAAGCTATCTCTTGTCTGAGTTCGGATATACGAAATAAAATAGACAATGGAATTCATTTGATGTATCCAGAGAGGCCAATTGTGATTAGTAAGGCTTTAGAAACTTCAAAGATTGCGGCTAATTGCTTCGGACAACATGTTACTATAGCATTAATGTCGTACAAAGGTATCAATCAAGAGGATGGAATTATCATCAAAAAACAATTTATTCAGAGAGGCGGTCTCGATATTGTTACAGCCAAGAAACATCAAGTAGAAATTCCATTGGAAAACTTTAATAACAAAGAAAGAGATAGGTCTAACGCCTATTCGAAATTAGAAAGTAATGGATTAGTTAGACTGAATGCTTTCTTGGAATCCGGAGACGCTATGGCAAGAAATATCTCATCAAGAACTCTTGAAGATGATTTTGCTAGAGATAATCAGATTAGCTTTGATGTTTCCGAGAAATATACAGATATGTACAAATCTCGCGTTGAACGAGTACAAGTAGAACTTACTGACAAAGTTAAGGTGCGAGTATTAACCATGAAAGAAAGAAGACCCATTCTAGGAGACAAATTTACTACTAGAACGAGTCAAAAGGGAACAGTCGCGTATATCGCAGATGAAACGGAACTTCCGTACGACGAAAATGGTATCACACCAGATGTCATTATTAATTCTACATCCATCTTCTCTAGAAAAACTATATCTATGTTGATAGAAGTTATTTTAACAGCCGCATATTCTACTAAGCCGTACAACAATAAGGGAGAAAACCGACCTGTCTGTTTTCCTAGTAGTAACGAAACATCTATCGATGCATATATGCAATTCGCTAAACAATGTTATGAGTATTCAAATCCGAAATTGTCCGAGGAAGAATTATCGGATAAAATCTTTTGTGAAAAGATTCTCTATGATCCTGAAACGGATAAGCCTTATGAATCCAAAGTATTTTTTGGACCAATTTATTACTTGCGTCTGAGACATTTAACTCAGGACAAGGCAACCGTTAGATGTAGAGGTAAAAAGACGAAGCTCATTAGACAAGCGAATGAGGGACGAAAACGTGGAGGAGGTATCAAGTTTGGAGAAATGGAGAGAGACTGTTTAATAGCACATGGTGCAGCCAATACTATTACAGAAGTTTTAAAAGACTCAGAAGAGGATTATCAAGATGTGTATATTTGTGAAAATTGTGGAGACATAGCAGCACAAATCAAAAGTATTAATACATGTCTTAGATGTTCAAAACTTAATCTCTCTCCTCTCTTAACAAAAATTGATACCACGCACGTATCTAAAGTATTTCTTACTCAAATGAACGCCAGAGGCGTAAAAGTTAAATTAGATTTCGAACGAAGGCCTCCTTCGTTTTATAAACCATTAGATAAAGTTGATCTTAAACCGTCTTTTCTGGTATAATATTGTTTAGTAGATACTCATCAAGATAAGCTAATTCACTAAACATATTATCGGATTCGGTATTGTTACTCGAGAATAGAGTTCGTTATGCTCCTGATATTCGGAAATCTGTGGAGTTTCAGGTTTTGGTGGAAGTGTAACTGCTACTTGGTGGGATACTGAAGGATATTTCAGAGAGTTGTGGATGTTCGGGTTCGACATCCACCGATGGTGTCACGCCACTAATCGGTTCGGTAACGTCTGTGGATGGAGGTGCTACTTCTACAGAACCTGTAGCCTCAGTTGTCAACGGAGATACATATTCAATGCGCGGAAATGTATAATTTGGTAATGGTTTCTCATGTGGATCTTAAGAAGAAGAGGTAAGATATCTACGAAAGATACCGATCACGTTTCTAGTTCTCTTTTGTAGAACTTTAACTTTTTCTTTCTCAGCATCTAGTTGATATTCCGACCTCTTCACGTTTCGCATGGGTTACCTCCGCAGTTTTTACAAGCGATTTCACGTTCCAGATCACGTTCAGCCTTCATACGTCTCTCCCTCTCTCTATCGAGTTTATCAGAGCAGTCTTTCTGAAGGCGATCGAACTCCATAAATTTCTCCAACGCTTTGATTGTTTCCATAGATTTCCGAAGTTTAGCTTCTAGGACGGCGATTCTTTTTTTTTTTTTTTTTTTTTTTTTCGAATTCACGGGGTACAACCGTTTCCATTACCACCATCTCTATGTTTCTTTTCTAGATCGGCAATCTTTCTCAATCTTTCTCAACATTTCATCCCCATACCTTTTCATTCCTCGAGTCTATTGTCGTCGAAATATCGTTCCAGCTCCTTTTCGACCTCAATAACTTTAGCACGTTGTTTCATCAAGCTCTCTCTTGTAGTACTATCATTTTTATCTGATTCCCTGACACGTTTAAGATCTTCATGTAATTGAGTCAGCTCTTGACGCAATCTCTTAACTAACTTCCTCTCTTGCTTCTTCGTCATAGTACTTACAATCACTATGGGATCCATTGTTACCACGTCTGTACTCGACGAGCTCACGTTTAAGAGATTCAATTTCCAGTTTGTATCGGTCCATGTCTCCATTGCTACACCACCATTAGATTTACAGGCTGCTAGTTGTCGTTCGAGATCAGAAATACGTGTTTTCTTGGAATGGATTTCGTCGATGTACTTGTCATGATTGGCATCGAAACACTTATTAAGTTCTTTTTTTCAATTCTACGATTTTATTTCTTTCGCGAGTCAATTCCCTCCTGTAGTAACTATCAGTTTTGTCAGATTCACGCTCTCTACGTAGACTTTCTTGTAAGTTACTAATTTGTTCCCTGGCATTACCGAGTTCAGTTTTATATGCCGAATAGAGTTCTGATTCATCCTTTGAGAAGATCTCTAGCGATCGTTCAAGATCCCTGATTCTAGTCTTTAGCCTATTTACCTCCTCAGAAGATGCTCCGTTACCGTTTTTACAATCGTTAAGATGTCTATCAAGATCCATGATTCTATCTCTTTTCCATATCAGCATTGATTTCATTATTACGTTCGCAGTCGTTCAACTGTATTTCAAGATCTGAGATTCTAGATTGTAATCTCTGTAGCATTTCCACGGCATTCACTCAGTTGTCTTTCAAGATCTGAGATTCTAGATTGGAGTCTGCTAATCTCTGTAAGATTTCCTCCTCCGCTCTCGATGCAGTCGGTCAACTTATTCTCTAGTTCTCTAATACGCGAACGCAGTGCATCAACTTCTTGTGTGTCTTCTTGATTGCGTGTGCATTCATCGAGTCTAGATTCGAGATCTCTAACGTGACGTCGTTCTTCCTCAAGTTCTCTGTGTACTACAGAAAGCGTGTCCCTATCTTGTTGATATTTAGCAATTTCTGATTCTAGAGTACTGATTCTACTCACGTATGTACTAATAGTTGTCTTAGCCTTATCAAGATCCTCCTTGTATTTGTCACATTCCTTGATATCCATACGAAGTCTGGACAGTTCCCATTCGACATTACGACGTTTATCGATTTCAGCTCGGAGATCGTCGTCGCGTTGTTTTAGCCACATACGACTAAGTTCAAGTTCTCGTTGACAAGATCCATCTACTTTTCCATCCCTAATAGTATCCAGTTCCTTTTCTAGTTCTGACCGCATTTCTCGTTCCATATCAAGAGATTCTCTCAATTCTCGTATAGTCTTCTTATCAATTTCTGATGAATCTGAACCATCATCTGTCCCATTTTGTTGCATATCCCTGAGTTCTTTGATCTCTGTTGTAAGTCTGTCGATTCTTTCGGTTTTATAAACAGAATCCCTTTCCAAAGTCCTAATCTTACTGAGTTTATCATTAAGTTCTTCATTCAATTCAGTGAGTTTTCTCTTGGCTTCTTCCAAGTCTGTTTTAAACTCTCCATCATTTCCGCATTCTTCCTCGCATTTATCTAACCATTCAATTAGTTTATTAATAACTAGTTGGTAATCAGCGATTCCTATAGCCGTTCTTGTATTTGTGGGAACATAATTAGGATCTTCTAATGGATTGTATGGCTTGATAGCATCATCTTTATCATTATTAGGTGGGGGATGGACAACCTTAATTGGTTGGTCCTCCTTATCTCCTCCAGTAGCATGTGGTTCTTCAATACCAGTATTAGTAATAGGCTTAGACAAATGCTTGTCGTACGCGGGCACTTCCTCATCCATCAAGTATTTATAATCGGGTTCTGTTTCAGAATATTCTTTTCTAAGAGACGCGACTTCAGGAGTTAGTAGAAGAACTCTGTTTCTGTATCTATCAACGCTGGAATCGATACTCAAGTTAAGGATAGCGAATACCTCATCGTCATCATCCGTATCTTCTGAAACGCCATCATATGACATTTCATGAAGTCTAACGTATTGATAAACAGAATCAGATTTAGTATTAAACAGATCCTTGACCTTTTTAGTAAATGCATATGTATATTTTAGATCTCCAGATTTCATAATATGATCGCATGCCTTAAATGTCAATGCTTCCATGATATAGTCTGGAACACTAATGGGTGACGAAAAAGATACAGCACCATATGCTACGTTGATAAATAGATCTGAACCACTAAGTAGATAATGATTAATGTTAAGGAAGAGGAAATATTCAGTATATAGATATGCCTTAGCATCATATCTTGTACTAAACACGCTAAACAGTTTATTGATGTGATCAATTTCCAACAGAACAATTAGAGCGGCAGGAATACCAACAAACATATTACCACATCCGTATTTTCTATGAATATCACATATCATATTAAAAAATCTTGATAGAAGAGCGAATATCTCGTCTGACTTAATGAGATGTAGTTCAGCAGCATAAGTCATAACTGTAAATAGAACATACTTTCCTGTAGTGTTGATTCTAGACTCCACATCAACACCATTATTAAAAATAGTTTTATATACATCTTTAATCTGCTCTCCGTTAATCGTCGAACGTTCTAGTATACGGAAACACTTTGATTTCTTATCTGTAGTTAATGACTTAGTGATATCACGAAGAATATTACGAATTACATTTCTTGTTTTTCTTGAGAGACCTGATTCAGAACTCAACTCATCGTTCCATAGTTTTTCTACCTCAGTGGCGAAATCTTTGGAGTGTTTGGTACATTTTTTAATAAGGTTCGTGACCTCCATTTATTATAAAAAATTTTTATTCAAAACTTAACTACAATCGGGTAATTATAAGATCGTAGATCTCCCATGTGGTGGAATACTACCATCTATCGCATGTTGATGGACAGTAGGTAATGGCCATGGGAACAGTAATGTTTGCATATTTATCTTTCTTGCTAGTATTACTGTATATTGTCCCAATGTTTCAATGTGATGTTCTAACCTATCAACTGCCACTGTATCACAACAATAATGTCCGATGGAATTAAGATTATGATCCAATGTGTTTAATATATGATTATCAAGTCTTATACGATCCGCGTCTTTTTTGACAGGATCAGGCTCTTCTACAGGAAGAAGTTTCGGCCTCTTATGATAGTCATGTCTGGGAAATGGTGGTCTAGGATGAGGATCAGGTATCGGAGTAGGTTTTGGATTATAATCATCATCATCATCATCATCATCATCATCATCATCATCATCTATGATATCATCATCTTCGATATTTATTTTGCTATCTTGATAATGTCCTATATCAGTTGCATTTTCAGCACTCGACTGAATATTAGTACATTCATTGTCTATTATTAACGTATTTCTAAACCCAAAATGTATATGTTGAACATCACTACTATAGTTGATGAGTCTTATAGCATGAATTCGCTTATCGTTATCGGGTTTATCTTCTGTCACCTTAACAATTCCTTTTTTATTAAACTCTGCATAATCATAACCATTTCTATTGTTTGTTCTAATATAAACGAGTATAGCATCATTGCTAAATTTTTCAATAGTATCAAAAACAGAATATCCTAAACCATATAATATATATTCAGGAACACTCAAACTAAATGTCCAGGATTCTCCTAAATACGTAAACTTTAATAGTGCTAAATCATTCAAAAATCTACCGCTTATAGATAGATAGTACATGAATGCGTATAGTAGTCTACCTATCTCTTTATTATGAAAACCGACATTACGATCATATATTTCGTGATATACATGTGACCCGTTTACGTTAAACCATAAATACATGGGTGATCCTATAAACATGAATTTATTTCTAATTCTCAGAGCCATAGTTAATTGACCGTGTAATATTTGTTTACATGCATACTTGATACGATCATTAATAAGATTTTTATCATTGCTCGTTATTTCAGAATCGTATATATAAGGAGTACCATCATGATTCTTACCAGATATTATACAAAATACTATATATAAAATATATTGACCCACGTTAGTAATCATGTAAATGTTTAATGTTTTAAATTTTGTATTTAATGATCCATCATCATATGCTAGCATGGTCTTGTGATATTCATTCTTTAAAATATAATATTGTGTTAGCCATTGCATTGGAGCTCCTAATGGAGATTTTCTATTCTCGTCCATTTTAGGATATGCTTTCATAAAGTCCCTAATAACTTCGTGAATAATGTTTCTATGTTTTCTACTGATGCATGTATTTGCTTCGATTTTTTTATCCCATGTTTCATCTATCATAGATTTAAACGCAGTAATGCTCGCAACATTAACATCTTGAACCATTGGTACAATTCCGTTCCATAAATTTATAATGTTCGCCATTTATATAACTCATTTTTTGAATATACTTTTAATTGAACAAAAGAGTTAAGTTACTCATATGGATGCCGTCCAGTCTGTACATCAATCTTTTTAGCCAGAGATATCATAGCCGCTCTTAGAGTTTCAGCGTGATTTTCCAACCTAAATAGAACTTCATCGTTGCGTTTACAACACTTTTCTATTTGTTCAAACTTTGTTGTTATATTAGTAATCTTTTTTTCCAAATTAGTTAGCCGTTGTTTGAGAGTTTCCTCATTATCGTCTCCATAGGCTTTAACAATTGCTTCGCGTTTAGTCTCTGGATTTTTAGCAGCCTTTGTAGAGAAAAATTCAGTTGCTGGAATTGCAAGATCGTCATCTCCGGGGAAAAGAGTTCCGTCCATTTAAAGTACAGATTTTAGAAACTGACACTCTGTGTTATTTATATTTGGCGCAATACATGGATTATAAATATCGATGTTAATAACATCAGAAAATGTAAAGTCTATACATTGTCGCATCGTGTTAAATTTTCTAATGGATCTAGTATTATTGGGTCCAACTTCTGCCTGAAATCCAAATATGGAAGCGGATACAAAACCGTTTCCTGGATAAACCACACATCTCCACTTTTGCTTTACATCAGAAATTGTGTCATTGACATCTTGAACTCTCCTATCTAATGCCGGTGTTCCACCTATAGATTTTGAATACTCGAATGCTGCATGAGTAGCATTGAATTCCTTAATATTGCCATAATTTTCATATATTGAGTAACTCTGGATAAAAAGTAAACACACCGCAGCCGTCGCTACTACAATAAAAAAAATTGATAGAGAGTTCATTTATAATCTATTAGAAGCTGATAAAATTTTTTTACACGCGTCAGACAATGCTTTAATAAATAGTTCAACATCTACTTTTGTCATATCGAACCGATGGTATGATTCTAACCTAGAATTACATCCGAAAAAGTTGACTATGTTCATAGTCATTAAGTCATTAACGAACAACATTCCAGACTCTGGATTATAAGACGATACTGTTTCGTCACAATCACCCACCTTAATCATGTGATTATGAATATTGGCTATTAGAGTACCTTCTAAGAAATCTATAATATCTTTGAAACACGATTTAAAATCAAACCACGAATATACTTCTACGAAGAAAGTTAGTTTACCCATAGGAGAGATAACTATAAATGGAGATCTAGATACAAAATCCGGATCTATGATAGTTTTAACATTATTATATTCTCTATTAAATACCTCCACATCTAAAAATGTTAATTTTGAAACTATGTCTTCGTTTATTACCGTACCTGAACTAAACGCTATAAGCTCTATTGTTTGAGAACTCTTTAAACGATATTCTTGAAATACATGTAACAAAGTTTCCTTTAACTCGGTCGGTTTATCTACCATAGTTACAGAATTTGTATCCTTATCTATAATATAATAATCAAAATCGTATAAAGTTATATAATTATCGTGTTCAGATTGTGATCTTTTCAAATAGACTAAAAACCCCATTTCTCTAGTAAGTATCTTATGTATATGTTTGTAAAATATCTTCATGGTGGGAATATGCTCTACAGCAGTTAGCCATTCCTCATTGACAGCTGTAGATGTATTATACAAAACTACTCCAATGTTTAACAAGGGCCATTTTACGAGATTATTAAATCCTTGTTTGATAAATGTAGCCAATGCGGGTTCGAGTTCAACGACGATTGAATTCTCTTCCCGTGGATGCTGCATGATGAACGACGGGATGTTGTTGTTCTATTGATTTGGAATTCTTTTTCGACTTTTTGTTTATATTAAATATTTTAAAATTTATGGCTGATAGTAATTCATGTACTACGGATAATGTAGACGTGTATTGCATATCGATATCTTTATTATTAGATAAATTTATCAATAAATGTGAGAAGTTTGCCTCGTTAAGGTCTTCCATTTAAATATTATATAAATATTTGTGTTTGTATTTTATTCGTCTTTTATGGGATAGTTTTTAACTAGTAAAGCTGTAATTACATACTTTGTCCGTAAAACATAAATATAAATACCCGCTTTTATCAAACGTTCCAAAAAGTCGGCAGCTGACATTTTTAACATGACATCTATTTTAAATACACTTAGGTTTTTAGAAAAAACATCATTTTATAATTGTAACGATTCAATAACTAAAGAAAAGATTAAGATTAAACATAAGGGAATGTTATTTGTATTTTATAAGCCAAAGCATTCTACCGTTGTTAAATACTTGTCTGGAGGAGGTATATATCATGATGATTTGGTTGTATTGGGGAAGGTAACAATTAATGATCTAAAGATGATGCTATTTTACATGGATTTATCATATCATGGAGTGACAAGTAGTGGAGCAATTTACAAATTGGGATCGTCTATCGATAGACTTTCTCTAAATAGGACTATTGTTACAAAAGTTAATAACAATTATAACAATTATAACAATTATAACAATTATAATTGTTATAATAATTATAATTGTTATAATTATGATGATACATTTTTTGACGATGATGATTGATCACTATTACACAATTTTGTTTTTGTACTTTCTAATATAGTGTTTAGGTTCTTTTTCATATGAGAATATTGACTTACTAAAATATCTATGTTTAACTTTTGTTCTATAACGTCCTTATCGGCGGTATCGGTACATATACGTAATTCACCTTCACAAAATACGGAGTCTTCGATAATAATAGCCAATCGATTATTGGATCTAGCTGTCTGTATCATATTCAACATGTTTAATATATCCTTTCGTTTCCCCTTTACAGGCATCGATCGTAGCATATTTTCCGCGTCTGAGATGGAAATGTTAAAACTGCAAAAATGCGTAATGTTAGCCCGTCCTAATATTGGTACGTGTCTATAAGTTTGGCATAGTAGAATAATAGACGTGTTTAAATGCCTTCCAAAGTTTAAGAATTCTATTAGAGTATTACATTTTGATAGTTTATCACCTACATCATCAAAAATAAGTAAAAAGTGTGCTGATTTTTTATGATTTTGTGCGACAGCAATACATTTTTCTATGTTACTTTTAGTTCGTATCAGATTATATTCTAGAGCTTCCTGACTACTAACGAAATTAATATGATTTGGCCAAATGTATCCATCATAATCTGGGTTATAAACGGGTGTAAACAAGAATATATGTTTATATTTTTTAACTAGTGTAGAAAACAGAGATAGTAAATAGATAGTTTTTCCAGATCCAGATCCTCCTGTTAAAACCATTCTAAACGGCATTTTTAATAAATTTTCTCTTGAAAATTGTTTTTCTTGAAAACAATTCATAATTATATTTACAGTTACTAAATTAATTTGATAATAAATCAAAATATGGAAAACTAAGGTCGTTAGTAGGGAGGAGAACAACGAAGGCATATCGTGATATAAATAACATTTATTATCATGATGACACCAGAAAACGACGAAGAGCAGACATCTGTGTTCTCCGCTACTGTTTACGGAGACAAAATTCAGGGAAAGAATAAACGCAAACGCGTGATTGGTCTATGTATTAGAATATCTATGGTTATTTCACTACTATCTATGATTACCATGTCCGCGTTTCTCATAGTGCGCCTAAATCAATGCATGTCTGCTAACAAGGCTGCTATTACTGACTCCGCTGTTGCCGTTGCTGCGGCATCATCTACTCATAGAAAGGTTGTGTCTAGCACTACACAATATGATCACAAAGAAAGCTGTAATGGTTTATATTACCAGGGTTCTTGTTATATATTACATTCAGACTATAAGTCATTCGAGGATGCTAAAGCAAACTGCGCTGCGGAATCATCAACACTACCCAATAAATCCGATGTCTTGACTACCTGGCTCATTGATTATGTTGAGGATACATGGGGATCTGATGGTAATCCAATTACAAAAACTACATCCGATTATCAAGATTCTGATGTATCACAAGAAGTTAGAAAGTATTTTTGTACATAAATAAATGAAATCGCTTAATAGACAAACTGTAAGTAGGTTTAGGAAGTTGTCGGTGCCGGCCGCTATAATGATGTTACTCTCAACCATTATTAGCGGCATAGGAACATTTCTGCATTACAGAGAAGAACTGATGCCTAGTGCTTGCGCCAATGGATGGATACAATACGATAAACATTGTTATCTGGATACCAACATTAAAATGTCTACGGATAATGCAGTTTATCAGTGTCGCAAATTACGAGCTAGATTGCCTAGACCTGATACTAGACATCTGAGAGTATTGTTTAGTATTTTTTATAAAGATTATTGGGTAAGTTTAAAAAAGACCAATGATAAATGGTTAGATATTAATAATGATAAAGATATAGATATTAGTAAATTAACAAATTTTAAGCAACTAAACAGCACGACGGATTCTGAGGCGTGTTATATATACAAGTCTGGAAAACTGGTTAAAACAGTATGTAAAAGTACTCAATCTGTACTATGCGTTAAAAGATTCTACAAGTGACAACAAAAAATGAATTAATAGTAAGTCGTTAACGTACGCCGCCATGGACGCCGCGTTTGTTATTACTCCAATGGGTGTGTTGACTATAACAGATACATTGTATGATGATCTCGATATCTCAATCATGGACTTTATAGGACCATACATTATAGGTAACATAAAAATTGTCCAAATAGATGTACGGGATATAAAATATTCCGACATGCAAAAATGCTACTTTAGCTATAAGGGTAAAATAGTTCCTCAGGATTCTAATGATTTGGCTAGATTCAACATTTATAGTATTTGTACAGCATACAGATCAAAAAATACCATCATCATAGCATGCGACTATGATATCATGTTAGATATAGAAGGTAAACATCAACCATTTTATCTATTCCCATCTATTGATGTTTTTAACGCTACAATCATAGAAGCGTATAATCTGTATACAGCTGGAGATTATCATCTGATCATCAATCCTTCAGATAATCTGAAAATGAAATTGTCGTTTAATTCTTCATTTTGTATATCAGACGGCAATGGATGGATTATAATTGATGGGAAATGTAATAGTAATTTTTTATCATAAAAGTTGTAAAGTAAATAATAAAACAATAAATATTGAACTAGTAGTATGTTGTATATTGAGCAATCAGAGATGATGCTGGTACCTCTTATCACGGTGACCGTAGTTGCGGGAACAATATTAGTATGTTATATATTATATATTTGTAGGAAAAAGATACGTACTGTCTATAATGACAATAAAATTATCATGACAAAATTAAAAAAGATAAAGAGTCCTAATTCCAGCAAATCTAGTAAATCAACTGATAGCGAATCAGACTGGGAGGATCACTGTAGTGCTATGGAACAAAACAATGACGTAGATAATATTTCTAGAAATGAGATATTGAACGATGATAGCTTCGCTGGTAGTTTAATATGGGATAACGAATCCAATATCATGGCGCCTAGCACAGAACACATTTACGATAGTGTTGCTGGAAGCACGCTGCTAATAAATAATGATCGTAATGAACAGACTATTTATCAGAATACTACAGTAGTAATTAATGATACAGAGACTGTTGAAATACTTAATGAAGATACCAAACAGATTCCTAGCTATTCTTCCAATCCTTTCGTAAATTATAATAAAACCAGTATTTGTAGCAAGTCAAATCCGTTCATTGCAGAACTCAACAATAAATTTAGTGATAATAATCCGTTTAGGAGAGCACATAGTGACGATTATCTTAATAAGCAACAAGATCATGAATACGATGATATAGAATCATCGGTTGTATCATTGGTCTGATTAGTTTCCTTTTTATAAAATTGAAGTAATATTTAGTATTAATTACCGCCGATGCATTATACAAATATGGAGATATTCCCTGTATTCGGCATTTCTAAAATTAGCAATTTTATTGCTAATAATGACTGTAGATATTATATAGATGTAGAGCATCAAAAAATTATATCTGATGAGATCAATAGACAGATGGATGAAACGGTACTTCTTACCAACATCTTAAGCGTAGAAGTTGTAAATGACAATGAGATGTACCATCTTATTCCCCATAGACTATCGACTATTATACTCTGTATTAGTTCTGTTGGAGGATGTGTTATCTCTATAGATAATGACGTCAATGACAAAAATATTCTAACATTTCCCATTGATCATGCTGTAATCATATCCCCACTGAGTAAATGTGTCGTAGTTAGCAAGGGCCCTACAACCATACTGGTTGTTAAAGCGGATATACCCAGCAAACGATTGGTAACATCGTTTACAAACGACATACTGTATGTAAACAATCTATCACTGATTAATTATTTACCGTCGTCTGTATTCATTATTAGACGAGTCACCGACTATTTGGATAGACACATATGTGATCAGATATTTGCTAATAATAAGTGGTATTCCATTATAACTATCGACGATAAGCAATATCCTATTCCATCAAATTGTATAGGTATGTCTTCTGCCAAGTACATAAATTCGAGCATCGAGCAAGATATTTTGATCCATGTTTGTAACCTCGAGCATCCATTCGACTCAGTCTACAAAAAAATGCAGTCGTACAATTCTCTACCTATCAAGGAACAAATATTGTATGGTAGAATTGATAATATAAATATGAGCATTAGTATTTCGGTGGATTAATAGATTTCTCTAGTATGGGATCATTAATCATCTCTAAATACATCATAAAAAAGCTATTATCAAATACTGTACTGAATGGATTCATTCTTTTCTCTTTTTATGAAACTCTGTTGTATATCTACGGATAAAACTAGAAGCAAAAAATCTGATAGGAAGAATAATGATTATATGGAGGAACACGATTATTATAAAATAACAATAGTTCCTGGTTCCTCTTCCACGTCTACTAGCTCGTGGTATTATACACATGCCTAGTAATAGTCTCTTTGCGTTGACGGAAAGCAGACTAGAAATAACAGGCCAAAATGTTCAGACACCATAATAGTTCCCAACCCAGATAATAACAGAGTTCCATCAACACATTCCTTTAAACTCAATCCCAAACCCAAAACCGTTAAAATGTATCCAGCCAATTGATAGTAGATAATGAGGTGTACAGCACATGATAATTTACACAGTAACCAAAATGAAAACACTTTAGTAATTATAAGAAATATAGACGGTAATGTCATCATCAACAATCCAATAATATGCCTGAGAGTAAACATTGACGGATAAAACAAAAATGCCCCGCATAACTCTATCATGGCAATAACGCAACCAAACACTTGTAAAATTCCTAAATTAGTAGAAAATACAACTGATATCGATGTATAAGCGATTTCGAGGAATAATAAGAACAAAGTAATTCCCGTAAAGATAAACATCAACATTGTTTGGTAATCATTAAACCAATTAGTATGACGTTGAATTAATTTCACAGTATATTTTATTCCAGTATTATCCCCGCATGTATACGTACCTGGTAAGATATCTTTATATTCCATAATCAATGAGACATCACTATCCGATAACGAATGAAGTCTAGCACTAGTATGCCATTTACTTAATATGGTCGTCTTGGAAGTTTTATTATAAGTTAAAATATCATGATTGTCCAATTTCCATCTAATATACTTTGTCGGATTATCTATAATACACGGAATAATGATGGTATCATTACATGCTGTATATTCTATAGTCTTTGTAGATGTTATAACCACAAAAGTACAGAGGTATATCAACAATATTCTAACTCTTAACATTTTTATTTATTTAAAATGATACCTTTGTTATTTATTTTATTCTTATTTTGCTAACGGTATCGAATGGCATAAGTTTGAAACGAGTGAAGAAATAATTTCTACTTACTTAATAGATTATGTGGTAACGGGTGTTATTAATGGGGATGTATATACATTTTCAAATAATGAACTAAACAAAACTGGGTTAACTAATAACAATAATTATATCACAACATCTATAAAAGTAGAGGATAAGGATACATTAGTAGTATGCGGAACCAATAACGGAAATCCCAAATGTTGGAAAATAGACGGTTCATACTACCCAAAACATATAGGTAGAGGATACGATCATCAAAATAGCAAAGTAACGATAATCAGTCACAATGAATGTGTACTATCCGACATAAACATATAAAAAGAAGGAATTAAACGATGGAGAAGATTTGACGGACCATGTGGTTATGATTAAACGAGTTAAGTTTTTTAAGAAGCCTTAGAAGAGAGGCTATTGGGTATGAGAATCCGAAATATTAAACCAGACAACCCCATATAATTTTATAGCTAAGAATGCCGCGAAGAATGGAACTAATAAAAACGGAAATATTTGTAGCACAACGAATAACTCCCAAACTGCATTCATGTTACACTATATAACACTACTTCGGTTAGATGTTTTAGAAAAAATAAATATCACCGTACCGTTTTGTTGTATAAAAATAACAATTAACAATTATCAATTTTTTTCTTTAATATTTTACGTGGTTGACCATTCTTGGTGGTAAAATAATCTCTTAGTGTTGGAATGGAATGCTGTTTAATGTTTCCACACTCATCGTATATTTTGACGTATGCAGTCACATCGTTTACGCAATAGTCAGACTGTAGTTCTATCATGCTTCCTACGTTAGAAGGAGGAACAGTTTTAAAGTCTCTTGGTTTTAATCTATTGTCATTAGTTTTCATGAAATCCTTTGTTTTATCCACTTCACATTTTAAATAAATGTCCACTATACATTCTTCTGTTAATTTTACTAGATCATCATGAGTCATAGAATTCATAGGTTCCGTAGTCCATGGATCCAAACTAGCAAACTTCGCGTATACGGTATCGCGATTAGTGTATACACCAACTGTATGAAAATTAAGAAAACAGTTTAATAAATCTACAGAAATATTTAATCCTCCGTTTGATACAGATGCGCCATATTTATGGATTTCGGATTCACACGTTGTTTGTCTAAGGGGTTCGTCTAGTGTTGCTTCTACATAGACTTCGATTCCCATATATTCTTTATTGCCAGAATCACATACCGATTTATCATACGCTGGTTCACTTGTTTGAAAACTAAATGGTAGTAGATACATCAAAATAATAAATAATAAGTACATTCTGCAATATTGTTATCGTAATTGGAAAATTGGTATTCAAGTGAGCTGGATTATGTGAGTATTGGATTGTATATTTTATTTTATATTTTATATTTTATATTTTATTTTATATTTTATATTTTATTTTATATTTTGTAGTAAGAATAGAATGCTAATGTCAAGTTTATTCGAATAGATGTCTTATTAAAAAACATATATAATAAATAACAATGGCTGAATGGCATAAAATTATCGAGGATATCTCAAAAAATAATAAGTTCGAGGATGCCGCCATCGTTGATTACAAGACTACAAAGAATGTTCTAGCGGCTATTCCTAACAGAACATTTGCAAAGATTAATCCGGGTGAAGTTATTCCCCTCATCACTAATCATAATATTCTAAAACCTCTTATTGGTCAGAAATTTTGTATTGTATATACTAACTCTCTAATGGATGAGAACACGTATGCTATGGAGTTGCTTACTGGGTACGCCCCTGTATCTCCGATCGTTATAGCGAGAACTCATACCGCACTTATATTTTTGATGGGTAAGCCAACAACATCCAGACGTGATGTGTATAGAACATGTAGAGATCACGCTACCCGTGTACGTGCAACTGGTAATTAAAATAAAAAGTAATATTCATATGTAGTGTCAATTTTAAATGATGATGAAATGGATAATATCCATATTGACGATGTCAATAATGCCGGTATTGACATACAGCTCATCGATTTTTAGATTTCATTCAGAGGATATTGAATTATGTTATGGGAATTTGTATTTTGATAGGATCTATAATAATGTAGTAAATATAAAATATATTCCTGAGCATATTCCATATAGATATAATTTTATTAATCGTACGTTCTCCGTAGATGAACTAGATGATAATGTCTTTTTTACACATGGTTATTTTTTAAAACACAAATATGGTTGTTCACTTAATCCTAGTTTGATTGTCTCATTATCAGGAAACTTAAAATATAATGATATACAATGCTCAGTAAATGTATCGTGTCTCATTAAAAATTTGGCAACGAGTACATCTACTATATTAACATCTAAACATAAGACTTATTCTCTATATCGGTCCATGTGTATTGCTATAATAGGATACGATTCTATTATATGGTATAAATATATAAATGACAGGTATAATGACATCTATGATTTTACTGCAATATGTATGCTAATAGCGTCTACATTGATAGTGATCATATACGTGTTTAAAAAAATAAAAATGAACTCTTAATTATGTTATACTATTAGAAATGGATAAAATCAAAATTACGATTGATTCAAAAATTGGTAATGTTGTTACCATATCGTATAACTTGGAAAAGATAACTATTGATGTCACGCCAAAAAAGAAAAAAGAAAAGGATGTATTATTAGCGCAATCAGTTGCTGTCGAAGAGGCAAAAGATGTCAAGGTGGAAGAAAAAAATATTATCGATATTGAAGATGACGATGATATGGATATAGAAAACACGTAATACGATCTATAAAAATAAGTATTAAATACTTTTTATTTACGGTACTCTTGTAGTGGTGATACCACTAATCGATTATTTTTTTTAAAAAAATACTTATTCTGATTCTTCTAGCCATTTCCGTGTTCGTTCGAATGCCACATCGACGTCAAAGATAGGGGAGTAGTTGAAATCTAGTTCTGCATTGTTGGTACGCACCTCAAATGTAGTGTTGGATATCTTCAACGTATAGTTGTTGAGTATTGATGGTTTTCTAAATAGAATTCTCTTTTCTTGCACGCGTACATTTTTAGCATCCATCTTGGAATCCTAGATCCTTGTTCTATTCCCAATGGTTTCATCAATAGAAGATTAAACATATCGTAAGAACACGATGGAGAGTAATCGTAGCAAAAGTAAGCATTTCCTTTAATCGCAGATCCCGGATACTGGATATATTTTGCAGCCAACACGTGCATCCATGCAACATTTCCTACATATACCCGGCTATGCACAGCGTCATCATCGACTGTACGATACATAATGTTACCGTGTTGCTTACATTGCTCGTAAAAGACTTTCGTCAATTTGTCTCCTTCTCCGTAAATTCCAGTGGGTCTTAGGCAACAAGTATACAATTTTGCGCCATTCATGATTACGGAATTATTGGCTTTCATAACCAGTTGCTCGGCCATACGTTTACTTTTTGCGTATACATGTCCTGGTGATATATCATAAAGGGTATGCTCATGACCGATGAATGGATTACCGTGTTTATTTGGTCCTATTGCTTCCATGCTACTAGTATAGATCAAATACTTGATTCCTAGGTCCACACAAGCTGCCAATATAGTCTGTGTTCCATAATAGTTTACTTTCATGATTTCATTATCAGTGTATTTTCCAAATACATCCACTAGAGCAGCCGTATGAATAATCAGATTTACCCCATCTAGCGCTTCTCTCACCTTATCAAAGTCGTTTATATCACATTGTATATAGTTTATAACCTTAACTTTCAAGGTTATTGGTTGTGGATCTTCTACAATATCTATGACTCTTATTTCTTGAACATCATCTGCGCTAATTAAAAGTTTTACTATATACCTGCCTAGAAATCCGGCACCGCCAGTAACCGCGTACACGGCCATTGCTGCCACTCATAATATCAGACTACTTATTCTATTTTACTAAATAATGGCTGTTTGTATAATAGACCACGATAATATCAGAGGAGTTATTTACGTTGAACAAGTCCATGGAAAAGATAAAGTTTTAGGATCAGTTATTGGATTAAAATCCGGAACGTATAGTTTGATAATTCATCGTTACGGAGATATTAGTCGAGGATGTGATTCCATAGGCAGTCCAGAAATATTTATCGGTAACATCTTTGTAAACAGATATGGTGTAGCATATGTTTATTTAGATACAGATGTAAATATATCTACAATTATTGGAAAGGCGTTATCTATTTCAAAAAATGATCAGAGATTAGCGTGTGGAGTTATTGGTATTTCGTACATAAATGAAAAGATAATACATTTTCTTACAATTAACGAGAATGGCGTTTGATATATCAGTTAATGCGTCTAAAACAATAAATGCATTAGTTTACTTTTCTACTTAGCAAGATAAATTAGTCATACGTAATGAAGTTAATGATATACACTACACTGTCGAATTTGATAGGGACAAAGTAGTTGATACGTTTATTTCATATAATAGACATAATGACTCCATAGAGATAAGAGGGGTGCTTCCAGAGGAAACTAATATTGGTCGCGTGGTTAATACGCCGGTTAGTATGACTTACTTGTATAATAAGTATAGTTTTAAACCGATTTTAGCAGAATATATAAGACACAGAAATACTATATCCGGCAACATTTATTCGGCATTGATGACGCTAGATGATTTGGTTATTAAACAGTATGGAGACATTGATCTATTATTTAATGAGAAACTTAAAGTAGACTCCGATTCGGGACTATTTGACTTTGTCAACTTTGTAAAGGATATGATATGTTGTGATTCTAGAATAGTAGTAGCTCTATCTAGTCTAGTATCTAAACATTGGGAATTGACAAATAAAAAGTATAGGTGTATGGCATTAGCCGAACATATAGCTGATAGTATTCCAATATCTGAGCTATCTAGACTACGATACAATCTATGTAAGTATCTACGCGGACACACTGATAGCATAGAGGATGAATTTGATCATTTTGAAGACGATGATTTGTCTACATGTTCTGCCGTAACCGATAGGGAAACGGATGTATAATTTTTTTTATAGTATGAAGGATATGATGGATATGATGATATGATGGATATGATGATATGATGGATATGATGGATATGATGGATATGATGGATATGATAAAAAAATATAATTGTTGTATCCATTCCCATTCAAATCACCTTATATGATTCTGTAACACAATGAAGGAGTCTCATAGATATATAGAGGTCAGATACTGGTTTGATAAACTTTTTATTCCACATGAGCATGTTTGACTTATGGTTAGACACACATACTTTAACAAATCACTGAAAATTGGAGTTAGGTATTCCTCTCAGAATCAGTTGCCGTTCTGGAACATTAAATGTATTTTTTATGATATACTCCAACGCATTTATGTGGGTATACAACAAGTCATTAATAATGAGTATTTCCAAGAGTTTTAGTTGTCTAGTATTTAACAAGAGAAGAGATTTCAACAGACTGTTTATGAACTCGAATACCGCCTCATTGTCGCTTATATTGATGACATGACATCGAATTCCCAATATCAATCTCATCAGTGATGAGTAGCTCAATCTTGTTATCGGGATCCAATTTCTAAAGATGTCATTAAACCCTCGATCGTGAATGGATTTATCATCATCGTTTTTATGTTGGACATGAGCTTAGTCCGTTTGTCCACATCTATATACGATGATTTCTGAATTATTTCATATATCTCTCGTTAACTCCAGGAACTTGTCAGGGATCTAACTTTAATATGTTCTCGTCTAAGAGATGAAAATCTTTGGATGGTTGCATGTGACTTTTCTCTAAAGGATGATGTTACCCGATCCTCTCTTAAATGACTCCATCTTATCCTTGGACAAGATGGACAGTCTATTTTCCTTAGATGGTTTAATATTTTTTACCCATGATCTATAAAGGTAGACAGACCTAATCGTCTCGGATGACCATATATTATTTTCAGTTTTATTATACGCATAAATTGTAAAAAATATGTTAGGTTTACGAAAATGTCTCGTGGGGCATTAATCGTTTTTGAAGGATTGGACAAATCTGGAAAAACAACACAATGTATGAACATCATGGAATCTATACCGGCAAACACGATAAAATATCTTAACTTTCCTCAGCGATCCACAGTCACTGGAAAGATGATAGATGACTATCTAACTCGTAAAAAAACCTATAATGATCATATAGTTAATCTATTATTTTGTGCAAATAGATGGGAGTTTGCATCTTTTATACAAGAACAACTAGAACAGGGAATTACTTTAATAGTTGACAGATACGCGTTCTCTGGAGTAGCGTATGCCACCGCTAAAGGCGCGTCAATGACTCTCAGTAAGAGTTATGAATCTGGATTGCCTAAACCCGACTTAGTTATATTCTTGGAATCTGGTAGCAAAGAAATTAATAGAAACATCGGCGAGGAAATTTATGAAGATGTTGAATTCCAACAAAAGGTATTACAAGAATATAAAAAAATGATTGAAGAAGGAGATATTCATTGGCAAATTATTTCTTCTGAATTCGAGGAAGATGTAAAGAAGGAGTTGATTAAGAATATAGTTATAGAGGCTATACACACGGTTACTGGACCAGTGGGGCAACTGTGGATGTAATAAAATGAAATTACATTTTTATAAATAGATGTTAGTACAGTGTTATAAATGGATGAAGCATATTACTCTGGCAACTTGGAATCAGTACTCGGGGATACGTGTCCGATATGCATACCGAACTCGCATCAATATCTCAATTAGTTATTGCCAAGATAGAAACTATAGATAATGATTATTAAACAAGGACATTGTAAATTTTATCATGTGTAGATCAAACTTGGATAATCCATTTATCTCTTTCCTAGATACTGCATATACTATCATAGATCAAGAGATCTATCAGAACGAGTTGATTAATTCATTAGACGATAATGAAATTATCGATTGTATAGTTAACAAGTTTATGAGCTTTTATAAGGATAACCTAGAAAATATGGTAGATGCTATCATTACTCTAAAATATTATAATTAATAATCCAGATTTTAAAACTACGTATGTGGAAGTACTCGGTTCCAGAATAGCTGATATAGATATTAAACAAGTGATACGTAAGAATATAATACAATTGTCTAATGATCCGCGAACGATATTTGTGAAAATATTAAAAAAAAATACTTTTTTTATTAAATGACGTCTCTTCGCGAATTTAGAAAATTATGCTGTGATATATATCACGCATCAGGATATAAAGAAAAATCTAAATTAATTAGAGACTTTATAACAGATAGAGATGATACCGATACATATTTGATCATTAAGCTATTGCTTCCCGGATTAGACGATAGAATGTATAACATGAACGATAAACAAATTATAAAATTATATAGTATAATATTTAAACAATCTCAGGAAGATATGCTACAAGATTTAGGATACGGATATATAGGAGACACTATTAGGACTTTCTTCAAAGAGAACACGGAAATCCGTCCACGAGATAAAAGCATTTTAACTTTAGAAGAAGTGGATAGTTTTTTAACTACGTTATCATCAGTAACTAAAGAATCACATCAAATAAAATTATTGACTGATATAGCATCTGTTTGTACATGTAATGATTTAAAATGTGTAGTCATGCTTATTGATAAAGATCTAAAAATTAAAGCGGGTCCTCGGTACGTGCTTAACGCTATTAGTCCTCATGCCTATGATGTTTTTAGAAAATCTAATAACTTGAAAGAGATAATAGAAAATGCAGCTAAACAAAATCTAGACTCTATATCTATTTCTGTTATGACTCCAATTAATCCCATGTTAGCGGAATCATGTGATTCTGTCAATAAGGCGTTTAAAAAATTTCCATCAGGAATGTTTGCGGAAGTCAAATACGATGGTGAAAGAGTACAAGTTCATAAAAAAAATAACGAGTTTGCATTCTTTAGTAGAAACATGAAACCAGTACTCTCTCATAAAGTGGATTATCTCAAAGAATACATACCGAAAGCATTTAAAAAAGCTACGTCTATCGTATTGGATTCTGAAATTGTTCTTGTAGACGAACATAATGTACCGCTACCGTTTGGAAGTTTAGGTATACACAAAAAGAAAGAATATAAAAACTCTAACATGTGTTTGTTCGTATTTGACTGTTTATACTTTGATGGATTCGATATGACAGACATTCCATTGTATGAACGAAGATCTTTTCTCAAAGATGTTATGGTCGAAATACCCAATAGAATAGTATTCTCAGAGTTGACGAATATTAGTAACGAGTCTCAGTTAACTGATGTATTAGATGATGCACTAACGAGAAAATTAGAAGGATTGGTCTTAAAAGATATTAATGGCGTATACGAACCGGGAAAGAGAAGATGGTTAAAAATAAAGCGAGACTATTTGAACGAGGGTTCCATGGCAGATTCTGCCGATTTAGTAGTACTAGGTGCCTACTATGGTAAAGGAGGAAAGGGTGGTATCATGGCAGTCTTTCTAATGGGTTGTTACGACAATGAATCCGGTAAATGGAAGACGGTAACTAAATGTTCCGGTCACGATGATAATACGTTAAGGGTTTTGCAAGACCAATTAACGATGGTTAAAATTAACAAGGATCCCAAAAAAATTCCAGAGTGGTTGGTAGTTAATAAAATCTATATTCCCGATTTTGTAGTAGATGATCCGAAACAATCTCAGATATGGGAAATTTCAGGAGCAGAGTTTACATCTTCCAAGTCACATACAGCGAATGGAATATCGATTAGATTTCCTAGATTTACTAGGATTAGAGAAGATAAAACGTGGAAAGAATCTACTCATCTAAACGATTTAGTAAACTTGACTAAATCTCTTAATAGTTACATATAAACTGAAAAATAAAATAACACTATTTTAGTTGGTAGTCGCCATGGATGGTGTTATCGTATACTGTCTAAATGCGTTAGTAAAACATGGCGAGGAAATAAATCATATAAAAAATGATTTCATGATTAAACCATGTTGTGAAAGAGTTTGTGAAAAAGTCAAGAACGTTCACATCGGCGGACAATCTAAAAACAATACAGTGATTGCAGATTTGCCATATCTGGATAATGCTGTATCAGATGTATGCAAATCAATATATAAAAAGAATGTATCAAGAATATCCAGATTTGCTAATTTGATAAAAATAGATGACGATGACAAGACTCCTACCGGCGTATATAATTATTTTAAACCTAAAGATGCTATTCCTGTTATTATATCCATAGGAAAGGATAAAGATGTCTGTGAACTATTAATCTCATCTGATAAAGCGTGTGCGTGTATAAAGTTAAATTTATATAAAGTAGCCATTCTTCCCATGGATGTTTCCTTTTTTACCAAAGGAAATGCATCATTGATTATTCTCCTGTTTGATTTCTCTATCGATGCGGCACCTCTCTTAAGAAGTGTAACCGATAATAATGTTATTATATCTAGACACCAGCGCCTACATGACGAGCTTCCGAGTTCCAATTGGTTCAAGTTTTACATAAGTATAAAGTCCGACTATTGTTCTATATTATATATGGTTGTTGATGGATCTATGATGTATGCGATAGCTGATAATAGAACTCACGCAATTATTAGCAAAAATATATTAGACAATACTACGATTAACGATGAGTGTAGATGCTGTTATTCTGAACCACAGATTAGGATTCTTGATAGAGATGAGATGCTCAATGGATCATCGTGTTATATGAACAGACATTGTATTATGATGAATTTACCTGATGTAGGCGAATTTGGATCTAGTATGTTGGGGAAATATGAACCTGACATGATTAAGATTGCTCTTTCGGTGGCTGGTAATTTAATAAGAAATCGAGACTACATTCCCGGGAGACGAGGCTATAGCTACTACGTTTACGGTATAGCCTCTAGATAATTTTTTTTAAGCACGAAATAAAAACATAATTTTAAACAATCTATTTCATACTATTTTGTGTGCTCACCATGAACATAAAGATAGATATATTAGTATTTCTGGTGATAAATTTACGGCGACTGCTAGGAGGGAAAATGAAGAAAGAAAAAATATCTACCTCTCCAAAAAGAAAAACTACTGATGTTATCAAACCTGATTATCTTGAGTACAATGACTTGTTAGATAGAGATGAGATGTCTACTATTCTAGAGGAATATTAGGCCTTAGAATAAAATATGGACGACTCTTAACGAAATTAGAAAATTCGATAATGATGTTGAAGAACAATTCGGTACTATAGAAGAACTCAAGCAGAAGCTTAGATTAAATTCTGAAGAGGGAGCAGATAATTTTATAGATTATATAAAGGTACAAAAACAGGATATCATCAAACTTACTGTATACGATTGCATATATCTATGATAGGATTGTGTGCGTGCGTGGTAGATGTTTGGAGAAATGAGAAACTGTTTTCTAGATGGAAATATTGGTTACAAGCGATTAAACTGTTTATTGATGATCACATGCTTGATAAGATAAAATCTATTGTAGAATAGACTAGTGTATGTGGAAATGTCATAGAAAGTTAAAAGTTAATGAGAGCAAAAATATATAAGGTTGTATTCCATATTTGTTATTTTTTTCTGTAATAGTTAGAAAATACATTCGATGGTCTATCTACCAGATTATTATGTGTTATAAGGTACTTTTCTCATAATAAACTAGAGTATGAGTAAGATAGTGTTTTTCAAAAACATATAAATCTAAAATTGATGGATGAGATATACAGCTATTAATTTCGAAAATATATTTTAATCTGATAACTTTAAACATGGATTTTTGATGGTGGTTTAAGTTTAAAAAAGATTTTGTTATTGTAGTATGATAATATCAAAAAGATGGATATAAAGAATTTACTGACTACATGTACTATTTTACATTACTACATTGGCTACGGCATATATACCTATTTCGTCACTTCCACACGCTCCGGTAAACGGGTGTCATGTGACGAGGGAGAATCTTGATAAGAGGCATAATCAATGTTGTAATCCGATGTCCACCTGGAGAATTTGCCAAGGTCAGATGTAGAGTTGGTAGTGATAACACAAAATGTGAACACTGCCCACCTCATACATATACCGCAATCCCCAATTATTCTAATAGATGTCATCAATGTAGAAAATGCCCAACAGGATCATTTGATAAGGTAAAGTGTACCGGAACACAGAACAAATGTTCGTGTCATCCTGGTTGGTATACGCTACTGATTCTTCACAGACTGAAGATTGTCGAGATTTGTGTACCAAAAAAGGAGATGTCCATGCGGATACTTTGGTGGAATAGATGAAGGAAATCCTATTTGTAAATCGTGTTGTGTTGGTGAATATTGCGACTACCTACGTAATTATAGACTTGATCCATTTCCTCCATGCAAACTATCTATCTAAATGTAATTAATTATGATTTTGATGATAATGTTACCATACATTATATCACTACTTGGTTAGTGTGTATTATTTAGTATGGAAGACCTATTAATAATTACTTATCTTTTGACGATCTTGTTATAATTATAATATAAAAATACTTATGACATAGTAACTCATAATTGCTGACGCGATAAATTCGTAATAATCTGTTTTGTTCAAATTTTTATAAGGAATCTACAGGCATAAAAATAAAAATATAATCTATAATATACTCTTACAACGCCATCATGAATAGCAGTGAATTAATTGCTGTTATTATGGATTTAGAAATAGTGGACGATTTTGTGATATTAATATAGTTATTAATGATGAAAGGATAAACGCGCATAGACTCATCCTATCTGGAGCCTCCGAATATTTTTTCCATTCTGTTTTCCAATAATTTTATCGATTCTAATGAATACGAAGTTAATCTAAGTCATTTAGATTATCAAAGTGTTAACTATTTGATCGATTACATTTATGGGATACCTTTGAGCCTAACTAACAATAACGTGAAATATATTCTTTCAACCGCTGATTTTTTTACAAATTGGATCTGTCATTACTGAGTGCGAAAAATACATACTTAAAAATCTTTGTTTTAGAAACTGTATCGATTTCTACATATACGCTGATAAATATAATAACAAGAAAATAGAATTAGCATCGTTTAACACAATATTACGAAATATTTTGAGACTCATCAACAATGAAAACTTTAAATACTTAACAGAGGAATCAATGATAAAAATTTTAAGCGATGATATGTTATATATAAAAAATGAGGATTTCACCCCACTGATTCTCATTAAATGGTTAGAGAGTACACCAACCATGTACCGTCGAGTTACTTAGATGCCTCAGAATATCATTTCTTTCCCCACAAGTTATAAAATCACTTTATAGTCATCGACTGGTTAGTTCAATCTACGAATGTATAACATTCTTAAACAATATAGCATTCTTGGATAAATCATTTCCTAGATACCATATCATCGAGTTGATATCTATCGGTATAAGTAATTCACATGATAAGATTTCCATAAACTGCTACAATCATAAAAAAATTCATGGGAAATGATATCTTCACGTAGATATAGGTGTAGTTTCGCAGTGACCGTCCTGGATAATATTATCTATATGATGGGTGGATATGATCAGTCCCTGTATAGAAGTTCAAAGGTTATAGCGTACAATACATGTACTAATTCTTGGATATATGATATACCAGAGCTAAAAATATCATCGTTCTAATTGCGGAGGAGTTGCCAATGACGAATACATTTATTGTATAGGCGGTATACGCGATCAGGAGTCATCGTTGATATCTAGTATCGATAGATGGAAGCCATCAAAACCATATTGGCAGAAGTATGCTAAAATGTGCGAACCAAAATGTGATATGGGGTTGCGATTTTAAACGGATTAATATATGTCATAGGTGGAGTCGTTAAAGGTGACACACATATACCAACGCACTAGAGAGTTTATCAGAAGATGGATGGATGAATCATCAACGTCTTCCAATAAAAATGTCCAATATGTCGACGATTGTTCATGCTGGAAAGATTTATATATCTAGAGGTTACAACAATAGTAGTGTAGTTAATGTAATATCGAATCTAGTCCTTAGCTATAATCCGATATATGATGAATGGACCAAATTATCATCATTAAATATTCCTAGAATTAATCCTGCTCTATGGTCAGTGTATAATAAATTATATGTAGGAGGAGTAATATCTGATGATGTTCAAACTAATACATCTGAAACATACGATAAAGAAAAAGATTGTTGGACATTGGATAATGGTCACTTGTTACCACATAATTATATAATGTATAAATGCGAACCGTTTAAACATAGATATCCATTGGAAAAAACACAGTACACGAATGATTTTCTAAAGTATTTGGAAAGTTTTATAGGTAGTTGATAGAACAAAATACATAATTTTGTAAAAATAAATCACTTTTTATACTAATATGACACAATTACCAATACTTTTGTTACTAATATCATTAGTATACGCTACACCTTCTCCTCAGACATCTAAAAAAATAGGTGATGATGCAACTATATCATGTAGTCGAAATAATACAAATTACTACGTTGTTATGAGTGCTTGGTATAAGGAGCCCAATTCCATTATTCTCTTAGCTGCCAAAAGCGACGTCTTGTATTTTGATAATTATACCAAGGATAAAATATCTTACGACTCTCCATACGATGATCTAGTTACAACTATCACAATTAAATCATTGACTGCTGGAGATGCCGGTACTTATATATGTGCATTCTTTATGACATCGACTACAAATGATACTGATAAAGTAGATTATGAAGAATACTCCATAGAGTTGATTGTAAATACAGATAGTGAATCGACTATAGACATAATACTATCTGGATCTACACCAGAAACTATTTCTGAGAAACCAGAGGATATAGATAATTCTAATTGCTCGTCTGTATTCGAAATCACGACTCCGGAACCAATTACTGATAATGTAGACGACCATACAGACACCGTCACATACACTAGTGATAGCATTAATACAGTAAATGCATCATCTGGAGAATCCACAACAGACGAGATTCCGGAACCAATTACTGATAAAGAAGAAGATCATACAGTAACAGACACTGTCTCATACACTACAGTAAGTACATCATCTGGAATTGTCACTACTAAATCAACCACCGATGATGCGGATCTTTATGATACATACAATGATAATGATACAGTACCGCCAACTACTGTAGGTGGTAGTACAACCTCTATTAGCAATTATAAAACCAAGGACTTCGTAGAAATATTTGGTATTACCACATTAATTATATTGTCAGCAGTGGCGATTTTCTGTATTACGTATTATATATGTAATAAACACCCACGTAAATACAAAACAGAGAACAAAGTCTAGATTTTTGACTTACATAAATATCTGGGATAATAAAATCTATCATATTGAGAGGACCATCTGGTTCAGGAAAGACAGCCATAACCAAAAGACTGTTAAAAGACTATGGGAATATATTTGGATTTGTGGTGTCCCATACCACTAGATTTCCTCGTCCTATGGAACGAGAAGGTGTTGATTACCTTACGTTAACAGAGAGGCCATCTGGAAGGGAATAGCCGCCGGAAACTTTCTAGAACATACTGAGTTTTTAGGAAATATTTACGGAACTTCTAAAACAGCTGTAAATACAGCGGTTATTAATAATCGTATTTGCGCGATGGATTTAAACATCAACGGTGTTAGAAGTCTTAAAAATACTTACCTAATGCATTACTTGGGTATATAAGACCTACCTCTCTTAAAATGGTTGAGACCAATCTTCGTCGTAGAAACACTGAAGCGGACGACGAATCTCATCGTCGCGTGATGTTGGCAAAAAACGGATATGGATGAGGTCAACGAAGCAGGTCTATTCGACACTATTATTATTGAAGATGATGTGAATTTAGCATATAGTAAGTGTTAATTCAGATACTACAGGACCGTATTAGAATGTATTTTAACACTAATTAGAGACTTAAGATTTGACTTAAAACTTGATAATTAATAATATAACTCGTTTTTATATGTGGCTATTTCAACGTCTAATGTATTAGTTAAATATTAAAACTTACCACGTAAAACTTAAAATTTAAAATGGTATTTCATTGACAGATCATACATTATGAAGTTTCAAGGACTTGTGTTAATTGACAATTGCAAAAATCAATGGGTCGTTGGACCATTAATAGGAAAAGGTGGATTCGGTAGTATTTATACTACTAATGACAATAATTATGTAGTAAAAATAGAGCCCAAAGCTAACGGATCATTATTTACCGAACAGGCATTTTATACTAGAGTACTTAAACCATCCGTTATCGAAGAATGGAAAAAATCTCACAATATAAAGCACGTAGGTCTTATCACATGCAAGGCATTTGGTTTATACAAATCCATTAATGTGGAATATCGATTCTTGGTAATAAATAGATTAGGTGCAGATCTAGATGCGGTGATCAGAGCCAATAATAATAGACTACCAGAAAGGTCGGTGATGTTGATCGGAATCGAAATCTTAAATACCATACAATTTATGCACGAGCAAGGATATTCTCACGGAGATATTAAAGCGAGTAATATAGTCTTGGATCAAATAGATAAGAATAAATTATATCTAGTGGATTACGGATTGGTTTCTAAATTCATGTCTAACGGCGAACATGTTCCATTTATAAGAAATCCAAATAAAATGGATAACGGTACTCTAGAATTTACACCTATAGATTCGCATAAAGGATACGTTGTATCTAGACGTGGTGATCTAGAAACACTTGGATATTGTATGATTAGATGGTTGGGAGGTATCTTGCCATGGACTAAGATATCTGAAACAAAGAATTCTGCATTAGTAAGTGCCGCAAAACAGAAATATGTTAACAATACTGCGACTTTGTTAATGACCAGTTTGCAATATGCACCTAGAGAATTGCTGCAATATATTACCATGGTAAACTCTTTGACATATTTTGAGGAACCCAATTACGACGAGTTTCGTCGAGTATTAATGAATGGAGTTATGAAAAATTTTTGTTGATAAAAAAATTAAAAAAATAACTTAGTTATTATCACTCTCGTGAGTACAATAGAAACATGGCGATGTTTTACGCACACGCTTTCGGTGGGTACGACGAGAACCTTCATGCATTTCCTGGAATATCATCGACGGTTGCCAATGATGTCAGGAAATATTCTGTTGTGTCAGTTTATAATAAAAAGTATAACATTGTAAAAAACAAATATATGTGGTGTAACAGTCAAGTGAACAAGAGATATATTGGAGCACTACTGCCTATGTTTGAATGCAATGAATATCTACAAATTGGAGATCCAATCCATGATCTAGAAGGAAATCAAATCTCTATTGTCACATATCGCCACAAAAACTACTATGCTCTAAGTGGAATTGGGTACGAGAGTCTAGACTTGTGTTTGGAAGGAGTAGGGATTCATCATTACGTACTTGAAACAGGAAACGCGGTATATGGAAAAGTTCAACATGAGTATTCTACTATCAAAGAGAAGGCCAAAGAAATGAATGCACTCAAACCAGGACCTATCATCGATTACCACGTCTGGATAGGAGATTGTGTCTGCCAAGTTACTACTGTAGACGTGCATGGAAAGGAAATTATGAGAATGAGATTCAAAAGGGGTGCGGTGCTTCCGATTCCAAATCTGGTAAAAGTTAAAGTTGGGGAGGAAAATGATACAATAAATCTTTCCACTTCCATATCAGCTCTCCTGAATTCCGGTGGCGGCACCATCGAGGTAACATCTAAGGAAGAACGTGTAGATTATGTACTCATGAAACGTTTGGAATCTATACATCATCTGTGGTCTGTAGTGTATGATCATCTTAATGTTGTGAATGGCGAAGAACGATGTTATGTACATATGCATTCATCTCATCAAAGTCCTATGCTGAGTACTGTAAAAACAAATTTGTACATGAAGACTATGGGAGCATGTCTTCAAATGGACTCCATGGAAGCTCTAGAGTATCTTAGTGAACTGAAGGAATCAGGTGGGCGGAGTCCCAGACCAGAATTGCAGAAATTTGAATATCCAGATGGAGTGAAAGACACTGAATCAATTGAGAGATTGGCAGAGGAGTTCTTCAATAGATCAGAACTTCAGGCCGGTGAATCAGTCAAATTTGGTAATTCTATTAATGTTAAACATACATCTGTTTCAGCTAAGCAACTAAGAACACGTATACGACAGCAGCTTCCTTCTATACTCTCATCTTTTGCCAACACAAAGGGTGGATATTTGTTCATTGGAGTTGATAATAATACACACAAAGTAATTGGATTCACGGTGGGTCATGACTACCTCAAACTGGTAGAGAGTGATATAGAAAAGTATATCCAAAAACTTCCTGTTGTGCATTTCTGCAAGAAAAAAGAGGACATCAAGTACGCATGTAGATTCATCAAGGTGTATAAACCTGGTGATGAGACTACCTCGACATATGTGTGCGCAATCAAAGTGGAAAGATGCTGCTGTGCTGTGTTTGCGGATTGGCCAGAATCATGGTACATGGATACTAGTGGTAGTATGAAGAAGTATTCTCCAGATGAATGGGTGTCACATATAAAATTTTAATTAGGGTAAGGTAAAACTATATATAATAACTAACAATTTGTGTATCATATAGACAATTAATTAGGTAACTGTTATCTCTTTTTAACTAACTAACTAACTAACTAACTCTTATATACTATTAATAATACATCTATTAATCATTGATTAGCTTATTGCTTTAATTGTTTTTGTAAACTAACACTGTTCATTGAAAAGGGATAACATGTTACAGAATATAAATTATATATGGATTTTTTTAAAAAGGAAATACTTGACTGGAGTATATATTTATTTCTTCATTACATAACACGTCTGTGTTCTAATTCTTCCAATTCTTCCACATCTCATATAATACAGGAATATAATCTTGTTCGAAAATATGAGAAAGTGGATAAAACAATAGTTGATTTTTTATCTAGGTGGCCAAATTTATTCCATATTTTAGAATATGGGGAAAATATTCTACATATTTATTTTATAGATGCTGCTAATACGAATATTATGATTTTTTTTCTAGATAGAGTATTAAATATTAATAAGAACCGTGGGTCATTTATACATAATCTCGGGTTATCATCCATTAATATAAAAGAATATGTATATCAATTAGTTAATAATGATCATCTAGATAATAGTATAAGACTAATGCTTGAAAATGGACGTAGAACAAGACATTTTTTGTCTTATATATTGGATACAGTTAATATCTATATAAGTATTTTAATAAATCATAGATTTTATATAGATGCCGAAGACAGTTACGGTTGTACATTATTACATAGATGTATATATAACTATAAGAAATCAGAATCAGAATCATATAATGAATTAATTAAGATATTGTTAAATAATGGATCAGATGTAGATAAAAAAGATACGTACGGAAACACACCGTTTATCCTATTATGTAAACACGATATCGACAACGCGGAATTGTTTGAGATATGTTTAGAGAATGCTAATATAGACTCTGTAGACTTTAATGGATATACACCTCTTCATTATGTCTCATGTCGTAATAAATATGATTTTGTAAAGTTATTAATTTCTAAAGGAGCAAATGTTAATGCACGTAATAGATTCGGAACTACTCCATTTTATTGTGGAATTATACACGGTATCTCGCTTATAAAACTATATTTGGAATCAGACACAGAGTTAGAAATAGATAATGAACATATAGTTCGTCATTTAATAATTTTTGATGCTGTTGAATCTTTAGATTATCTATTGTCCAGAGGAGTTATTGATATTAACTATCGTACTATATACAACGAAACATCTATTTACGACGCTGTCAGTTATAATGCGTATAATACGTTAGTCTATCTATTAAACAGAAATGGTGATTTTGAGACGATTACTACTAGTGGATGTACATGTATTTCGGAAGCAGTCGCGAACAACAACAAAATAATAATGGATATACTATTGTCTAAACGACCATCTTTGAAAATTATGATACCATCTATGATAGCAATTACTAAACATAAACAACATAATGCAGATTTATTGAAAATGTGTATAAAATATACTGCGTGTATGACCGATTATGATACTCTTATAGATGTACAATCGCTACATCAATATAAATGGTATATTTTAAAATGTTTTGATGAAATAGATATCATGAAGAGATGTTATATAAAAAATAAAACTGTATTCCAATTAGTTTTTTGTATCAAAGACATTAATACTTTAATGAGATACGGTAGACATCCTTCTTTCGTGAAATGTAATATTCTCGACGTATACGGAAGTTGTGTACGTAATATCATAGCATCTATTAGATATCGTCAGAGATTAATTAGTCTATTATCCAAGAAGCTGGATGCTGGAGATAAATGGTCGTGTTTTCCTAACGAAATAAAATATAAAATATTGGAAAACTTTAACGATAACGAACTGACCACATATCTGAAAATCTTATAAACACTATTAAAATATAAAATCTAAGTAGGATAAAATCACACTACATCATTGTTTCCTTTTAGTGCTCGACAGTGTATACTATTTTTAACACTCATAAATAAAAATGAAAACGATTTCCGTTGTTACGTTGTTATGCGTACTACCTGCTGTTGTTTATTCAACATGTACTGTACCCACTATGAATAACGCTAAATTAACGTCTACCGAAACATCGTTTAATGATAAACAGAAAGTTACGTTTACATGTGATTCAGGATATCATTCTTTGGATCCAAATGCTGTCTGTGAAACAGATAAATGGAAATACGAAAATCCATGCAAGAAAATGTGCACAGTTTCTGATTATGTCTCTGAACTATATGATAAGCCATTATACGAAGTGAATTCCACCATGACACTAAGTTGCAACGGTGAAACAAAATATTTTCGTTGTGAAGAAAAAAATGGAAATACTTCTTGGAATGATACTGTCACGTGTCCTAATGCGGAATGTCAACCTCTTCAATTAGAACACGGATCGTGTCAACCAGTTAAAGAAAAATACTCATTTGGGGAATATATGACTATCAACTGTGATGTTGGATATGAGGTTATTGGTGTTTCGTATATAAGTTGTACGGCTAATTCTTGGAATGTTATTCCATCATGTCAACAAAAATGTGATATACCGTCCCTATCTAATGGATTAATTTCCGGATCTACATTTTCTATCGGTGGCGTTATACATCTTAGTTGTAAAAGTGGTTTTACACTAACGGGGTCTTCATCATCCACATGTATCGACGGTAAATGGAATCCCATACTCCCAACATGTGTACGATCTAACGAAGAATTTGATCCAGTGGATGATGGTCCCGACGATGAGACAGATCTGAGCAAACTCTCGAAAGACGTTGTACAATATGAACAAGAAATAGAATCGTTAGAAGCAACTTATCATATAATCATAATGGCGTTGACAATTATGGGTGTCATATTTCTAATCTCCATTATAGTATTAGTTTGTTCCTGTGACAAAAATAATGACCAATATAAGTTCCATAAATTGCTACCGTGAATATAAATCCGTTAAAATAATTAATAATTAATAATTAATAACGAACAAGTATCAAAAGATTAAAGAATTAGCTAGAATCAATTAGATGTCTTCTTCAGTGGATGTTGATATCTACGATGCCGTTAGAGCATTTTTACTCAGGCACTATTATGACAAGAGATTTATTGTGTATGGAAGAAGTAACACCATATTACATAATATATACAGGCTATTTACAAGATGCACCGTTATACCGTTCGATGATATAGTACGTACTATGCCAAATGAATCACGTGTTAAACAATGGGTGATGGATACACTTAATGGTATAATGATGAATGAATTCGATACTGTATGTGTGGGTACCGGACTACGATTCATGGAAATGTTTTTCGATTACAATAAAAATAATCCCAAAAATAGCATCAACAATCAAATAATGTATGATATAATTAATAGCGTAGCCATAATTCTAGCTAATGAGAGATATAGAAGCGCGTTTAACGACGATAGAATATACATCCGTAGAACTATGATGGACAAATTGTACGAATACGCATCTCTAACTACTATTGGTACGATCACTGGAGGTGTTTGTTATTTTATCTGTTGATGCATCTAGTTAGTTTGTATAAATAATTATTTCGATATACTAGTTAAAATTTTAAGATTTTAAATGTATAAAAAACTAATAACGTTTTTATTTGTAATAGGTGCAGTTGCATCCTATTCGAATAATGAGTACACTCCGTTTAATAAACTGAGTGTAAAACTCTATATAGATGGAGTAGATAATATAGAAAATTCATATACTGATGATAATAATGAATTGGTGTTAAATTTTAAAGAGTACACAATTTCTATTATTACAGAGTCATGTGACGTCGGATTTGATTCCATAGATATAGATGTTATAAACGACTATAAAATTATTGATATGTATACCATTGACTCGTCTACTATTCAACGCAGAGGACATACGTGTAGAATATCTACCAAATTATCATGCCATTATGATAAGTACCCTTATATCCACAAATATGAGGGTGATGAACGACAATATTCTATTACCGCAGAGGGAAAATGCTATAAAGGAATAAAATATGAAATAAGTATGATGAACGATGATACTCTATTGAGAAAACATACTCTTAAAATTGGATTTACTTATATATTCGATCGTCATGGGCATAGTAATACATATTATTCAAAATATGATTTTTAAAAATTTAAAATATATTATCACTTCAGTGACAGTAGTCAAATAACAAACAACACCATGAGATATATTATAATTCTCGCAGTTTTGTTCATTAATAGTATACATGCTAAAATAACTAGTTATAAGTTTGAATCCGTCAATTTTGATTCCAAAATTGAATGGACTGGGGATGGTCTATACAATATATCCCTTAAAAATTATGGCATCAAGACGTGGCAAACAATGTATACAAATGTACCAGAAGGAACATACGACATATCCGGATTTCCAAAGAATGATTTCGTATCTTTCTGGGTTAAATTTGAACAAGGCGACTATAAAGTGGAAGAGTATTGTACGGGACTATGTGTCGAAGTAAAAATTGGACCACCAACTGTAATATTGACTGAATATGACGATCATATCAATTTGTTCATCGAGCATCCGTATGCTACTAGAGGTAGCAAGAAGATTCCTATTTACAAACGCGGTGACATGTGTGATATCTACTTGTTGTATACGGCTAACTTCACATTCGGAGATTCTGAAGAACCAGTAACATATGATATCGATGACTACGATTGCACGTCTACAGGTTGCAGTATAGACTTTGCCACAACAGAAAAAGTGTGTGTGACAGCACAGGGAGCCACAGAAGGGTTTCTCGAAAAAATTACTCCATGGAGTTCGGAAGTATGTCTGACACCTAAAAAGAATGTATATACGTGCGCAATTAGATCTAAAGAAGATGTTCCCAATTTCAAGGACAAAATAGCCAGAGTTATCACGAGAAAATTTAATAAACAGTCTCAATCTTATTTGACTAAATTTCTCGGTAGCACATCGAATGATGTTACAACTTTTCTTAGCATTCTTGACTAAATATTCATAACTAATTTTTATTAATGATACAAAAATGAAATAAACTGTATATTATACACTGGTTAACACCCTTGGCTCTAACCATTTTCAAGATGAGGTCCCTGATTATAGTCCTTCTGTTCCCCTCTATCATCTACTCAATGTCTATTAGACGATGCGAGAAGACTGAAGAGGAAACATGGGGATTAAAAATAGGGTTGTGTATAATTGCCAAAGATTTCTATCCCGAAAGAACTGATTGCAGTGTTCATCGCCCAACTGCAAGTGGAGGATTGATAACTGAAGGCAATGGATTCAGAGTAGTTATATATGATCAATGTACAGAACCCCATGACTTTATTATCACCGATACTCAACAAACACGTCTTGGATCATCTCATACATATATTAAATTCAGTAACATGAATACAGGTGTCCCATCTAGTATTCCAAAATGTTCCAGAACTCTCTGTATTTCTGTATATTGTGATCAAGAGGCGGGAGACATAAAATTTGAGGAGTATACTCAAGAATCAAGTGATATCAGTATTAGAGTTAAGTATGATTCATCATGTATTGATTATCTGGGTATTAATCAAAGTTTCATGAATGAATGTATTCGAAGAATTACAACATGGGATAGAGAATCATGCGTCAGAATTGATACACAGACTATAAATAAATATCTTAAGTCTTGCACCAACACAAAATTCGACCGTAATGTCTACAAAAGGTACATACTGAAGAGTAAAGCACTCCATGCTAAAACAGAGTTGTAATAGATATAAAATACTTTTTATAATAATTAGGCTAGAAAAATCTCACTCACATGTAATCTTAAAAAAATGATATGATAGTTCTTACAAGTAGCGATTGAGTTTTAAATGGATTCTATTAATTACCGGGGAACTTAACAATTCGTTCTGATCTACAGACATTGGTTAATAAATCATCTTATTTTGCCAATATATTAAAATGTGGAAACTCCACTAATAATATTACATTGTGCGACTTTCAAGATGATGTGATATATAGGGTTATACAGTTTTAACAATTATATAATAGAGATAGAAAGTACAAAAGATGTAGAATCAATGATATGGCACGCTAAACAGTTGGGTGTGGAATCATTGCTAAAAGAATGTCAAAATTATTTGCTTAGAATATTACGTATATAATTGTTTAGAAATTTATAGAATAACTAATATTAATACATTATCGTATATCTACAACGATATAAGAAACTTCATATTGGATAATATTACTATTAATATATAAGGATCCAGATTTTATATATTTGCCTAAATACATTATTATAGATTTACTAGGACAATCACCTAAATGTTTTTAACGAAGATAATGTGGTAAAGATTATATACACTTATATATCTTCCGATATCTACAAGGATATTCCATATCATCATTGTGTAAACTAAATAACGTTTTCTATGGCATTTAATAAGGACATTGGATATGTGGAAAAGTGATGTATGGAAGTTAGTACATTATCAACTTCTCCTTATTGATTGAAAATGAAAATATAAATAGTTTTTATGTATAGCGGTATCTACCCTATAGTTTTATTGCTTACTACTAACATGGATTCAGATACAGATACAGATACAGATACAGATACAGATACAGATACAGATACAGATGTAGAAGATATCATGAATGAAATAGATAGAGAGAAAGAAGAAATACTAAAAAATGTAGAAATTGAAAATAATAAAAACATTAACAAGAATCATCCCAGTGAATATATTAGAGAAGCACTTGTTATTAATACCAGTAGTAATAGTGATTCCATTGATAAAGAAGTTATAGAATATATCAGTCACGATGTAGGAATATAGATCATATCTACTAATTTTTATAATCGATACAAAACATAAAAACAACTCGTTATTACATAGCAGGTATGGAATCCTTCAAGTATTGTTTTGATAACGATGGTAAGAAATGGATTATCGGAAATACTTTATATTCTGGTAATTCAATACTCTATAAGGTCAGAAAAAATTTCACTAGTTCGTTCTACAATTACGTAATGAAGATAGATCATAAATCACACAAGCCATTGTTGTCCGAAATACGATTCTATATATCTGTATTGGATCCTTTGACTATCAACAACTGGACACGGGAACGTGGTATAAAGTATTTGGCTATTCCAGATCTGTATGGAATTGGAGAAACCGATGATTATATGTTCTTCATTATAAAGAATTTGGGAAGAGTATTCGCCCCAAAGGATAGTGAATCAGTTTTCGAAGCATGTGTCACTATGATAAACACGTTAGAGTTTATACACTCTCAAGGATTTACTCATGGAAAAATAGAACCGATGAATATACTGATTAGAAATAAACGTATTTCACTAATTGACTATTCTAGAACTAACAAACTATACAAAAGTGGAACACATATAGATTACAACGAGGACATGATAACTTCAGGAAATATCAATTATATGTGTGTAGACAATCATCTTGGAGCAACAGTTTCAAGACGAGGAGATTTAGAAATGTTGGGATATTGCATGATAGAATGGTTCGGTGGTAAACTTCCATGGAAAAACGAAAGTAGTATAAAAGTAATAAAACAAAAAAAAGAATATAAACAATTTATAGCTACTTTTTTTGAGGACTGTTTTCCTGAAGGAAATGAACCTCTGGAATTAGTTAGATATATAGAATTAGTATACATGTTAGATTATTCTCAAACTCCTAATTATGACAGACTACGTAGACTGTTTATACAAGATTGAAATTATATTCTTTTTTTTATAGAGTGTGGGGGTAGTGTTACGGATATCTAATATTAATATTAGACTATCTCTATCGCGCTACACGACCAATATCGATTACTATGGATATCTTCAGGGAAATCGCATCTTCTATGAAAGGAAAGAATGTATTCATTTCTCCAGCGTCAATCTCGTCAGTATTGACAATACTGTATTATGGAGCTAATGGATCCACTGCTGAACAGCTATCAAAATATGTAGAAAAGGAGGAGAACATGGATAAGGTTAGCGCTCAGAATATCTCATTCAAATCCATGAATAAAGTATATGGGCGATATTCTGCCGTGTTTAAAGATTCCTTTTTGGGAAAAATTGGCGATAAGTTTCAAACTGTTGACTTCACTGATTGTCGCACTATAGATGCAATCAATAAGTGTGTAGATATCTTTACTGAGGGAAAAATCAATCCACTATTGGATGAACCATTGTCTCCTGATACCTGTCTCCTAGCAATTAGTGCCGTATACTTTAAAGCAAAATGGTTGATGCCATTCGAAAAGGAATTTACCAGTGATTATCCCTTTTACGTATCTCCAACGGAAATGGTAGATGTAAGTATGATGTCTATTTACGGCGAGCCATTTAATCACGCATCTGTAAAAGAATCATTCGGTAACTTTTCAATCATAGAACTGCCATATGTTGGAGATACTAGTATGATGGTCATTCTTCCAAACAAGATTGATGGATTAGAATCCATAGAACAAAATCTAACAGATACAAATTTTAAGAAATGGTGTAACTCTCTGAAAGCTACGTTTATCGATGTGCACATTCCTAAGTTTAAGGTAATAGGTTCGTATAATCTTGTGGATACGCTAATAAAGTTGGGACTGACAGATGTGTTCTATTCAACTGGTGATTATATCAATATGTGTAATTCAGATGTGAGTGTTGACGCTATGATTCACAAAACGTATATAGATGTCAATGAAGAGTATACAGAAGCAGCTGCAGCAACTTCTGTACTAGTGGCAGACTGTGCATCAACAGTTACAAATGAGTTCTGTGCAGATCATCCGTTCATCTATGTGATTAGACATGTCGATGGTAAAATTCTTTTCGTTGGTAGATATTGCTCTCCAACAACTAATTAAGCACATTCTTAATATTAGAATATTATATAGTTAAGATTTTTACTAACAGGTTAACATTTTTTTTTAAAAATAGAAAAAACATGTGGTATTAGTGCAGGTCGTTATTCTTCCAATTGCAATTGGTAAGATGACGGCCAACTTTAGTACCCACGTCTTTTCACCACAACACTGTGGATGTGACAGACTGACCAGTATTGATGACGTCAGACAATGTTTGACTGAATATATTTATTGGTCGTCGTATGCATACCGCAACAGGCAATGCGCTGGACAACTGTATGACACACTCCTCTCTTTTAAAGATGATGCGGAATCAGTGTTCATCGACGTTCGTGAGCTGGTAAAAAATATGCCGTGGGATAATGTTAAGGATTGTACAGAGATCATCCGTTGTTATATACCGGATGAGCAAAAAACCATCAGAGAGATTTCGGCCATCATTGGACTTTGTGCATATGCTGCTACTTACTGGGGAGGTGAAGACCATCCCACTAGTAACAGTCTGAACGCATTGTTTGTGATGCTTGAGATGCTCAATTACATGGATTATACCATCATATTCTGGCGTATGAATTGATGAGTTACAGCTTGACATTTCTTCTTTCCTCCCTCTTCTTCTACCTTTCCCAGAAACAAACTTTTTTTACCCACTATAAAATAAAATGAGTATACTACCTGTTATATTTCTTCCTATATTTTTTTATTCTCCATTCGTTCAGACTTTTAACGTGCCTGAATGTATCGACAAAGGGCAATATTTTGCATCATTCATGGAGTTAGAAAACGAGCCAGTAATCTTACCATGTCCTCAAATAAATACGCTATCATCCGGATATAATATATTAGATATTTTATGGGAAAAACGAGGAGCGGATAATGATAGAATTATACAGATAGATAATGGTAGCAATATGCTAATTCTGAACCCGACACAATCAGACTCTGGTATTTATATATACATTACCACGAACGAAACCTACTGTGACATGATGTCGTTAAATTTGACAATCGTGTCTGTCTCAGAATCAAATATAGATCTTATCTCGTATCCACAAATAGTAAATGAGAGATCTACTGGTAAAATGGTATGTCCCAATATTAATGCATTTATTTCTAGTAACGTAAACACAGAATTATATGGAGCGGACATCGACGCCTTAGAAATAAGAGACTTAAACAACGGACACCTGGAATTATTACCATAGAAGATGTTAGAAAAAATGATGCTGGTTATTATACATGTGTTTTAGAATATATATATATATATATATATATATATATATATATATATATATGGGCAAAACATATAACGTAACCAGAATTATAAAATTAGAGGTACGGGATAGAATAATACCTCCTACTATGAAATTACCAGAAGGAGTAGTAACTTCAATAGGTAGTAATTTGACTATTGCATGTAGAGTATCGTTGAGACTTCCCACAACGGACACCGACGTCTTTTGGATAAGTAATGGTATATGTATTACGAAGAAGAAGACGAGGACGGAGACGGTAGAATAAGTGTAGCAAATAAAATCTATATGACCGATAAGAGACGTGTTATTACATCCTGGTTAAACATTAATCCTGTCAAGGAAGAAGATGCTACAACGTTTACGTGTATGGCGTTTACTATTCCTAGCATCAGCAAAACAGTTACTGTTAGTAAACGTGAATGTATGTTGTTACATTTCCATATCAATTGAGTTTATAAGAATTTTTTATACATTATCTTCCAACAAACAATTGACGAACGTATTGCTATGATTAACTCCCACAATACTATATATATTATTAATCATTAACTTGCAGACTATACCTAGTAGTGCTATTTTGACATACTCATGTTCTTGTGTAATCGCAGTATCTATATTATTAAAGTACGTAAATCTAGCTATAGTTTTATTATTTAATTTTAGATAATATACTGTCTCCGTATTTTTAAAAAATTACCACATCCTTTATTAAATCATGAATGGGAATTTCTGTGTCATCGTTAGTATATTGTGAACAACAAGAGCAGATATCTATAGGAAAGGGTGGAATGCGATACATTGATCTATGTAGTTTTAAAACATACGCGAACTTTGAAGAATTTATATAAATCATCTCACGAGATATTGCTCTCTGTCATATTCATACACCTGTATAAACTTTCTAGACATCTTACAATGTGTTATTTTATGATCATATTTACATATTTACTGGTATATCAAAGATGTTAGATTAGTTAATGGGAATCGTCTATAATAATGAATATTAAACAATTATAGGAGGAGTTTATACCTACAAAAACATCATAAAAATGAGTCATCGTCCGATTTATGTTTTAAATATACTAACATTACTACCTTCAGAAATTATATACGAAATATTATACATGCTGACAATTAACGATCTTTATAATATATAGTATCCACCTACCAAAGTATAATTGTATTTTTCTCATGTGATGTGTGTAAAAAACTGATATTATATAATTATCTTAGTACCTATGATGAAGATGAAGATGAAGATGAAGATGATGGTCCGTATATATTTTGTATCATTATCGTTATTGCTATTCCATAGTTACGCCATAGACATCGAAAATGAAATCACCGAATTCTTCAATAAAATGAGAGATACTCTACCAGCTAAAGACTCTAAATGGTTGAATCCAGTATGTATGTTTGGAGGCACAATGAATGATATGGCCGCTCTAGGAGAGCCATTCAGTGCAAAGTGTCCTCCTATTGAAGACAGTCTTTTATCGCATAGATATAAAGACTATGTGGTTAAATGGGAAAGGCTAGAAAAGAATAGACGGCGACAGGTTTCTAATAAACGTGTTAAACATGGTGATTTATGGATAGCCAACTATACATCTAAATTCAGTAACCGTAGGTATTTATGTACCGTAACCACAAAGAATGGTGACTGTGTTCAGGGTGTAGTTAGATCTCATGTGTGGAAACCTTCTTCATGCATTCCAAAAACATATGAACTAGGTACTTATGATAAGTATGGCATAGACTTATACTGTGGAATTCTTTATGCGAACCATTATAATAATATAACTTGGTATAAAGATAATAAGGAAATTAATATCGACGATTTTAAGTATTCACAAGCGGGAAAGGAATTAATTATTCATAATCCAGAGTTAGAAGATAGTGGAAGATACGACTGTTACGTTCATTACGACGACGTTAGAATCAAGAATGATATCGTAGTATCAAGATGTAAAATACTTACGGTTATACCGTCACAAGACCACAGGTTTAAACTAATACTAGATCCGAAAATCAACGTAACGATAGGAGAACCTGCCAATATAACATGCAGTGCTGTGTCAACGTCATTATTTGTCGACGATGTACTGATTGAATGGGAAAATCCATCCGGATGGATTATAGGATTAGATTTTGGTGTATACTCTATTTTAACTAGTAGAGGCGGTATCACCGAGGCGACTTTGTATTTTGAAAATGTTACTGAAGAATATATAGGCAATACATATACATGTCGTGGACACAACTATTATTTTGATAAAACTCTTACAACTACAGTAGTATTGGAGTAAATACACAATGCATTTTTATATACATTACTGAATTATTATTATTAATTATATCGTATTTGTGCTATAGAATGGATGAAGATACGCGACTATCTAGGTATTTGTATCTCACCGATAGAGAACATATAAATGTAGACTCTATTAAACAGTTGTGTAAAATATCAGATCCTAATGCATGTTATAGATGTGGATGTACGGCTTTACATGAGTACTTTTATAATTATAGATCAGTCAACGGAAAATACAAGTATAGATACAACGGTTACTATCAATATTATTCATCTAGCGATTATGAAAATTATAATGAATATTATTATGATGATTATGATAGAACTGGTATGAACAGTGAGAGTGATAATATATCAATCAAAACAGAATACGAGAATGAATATGAATTCTATGATGAAACACAAGATCAAAGTACACAACTAGTAGATTACGACATTAAACTCAAAACCAATGAGGATGATTTTGTTGATGAATTCTATGGTTATGATAGATCAGTGGGTGTCCATGATTATATAGATGTATCAATTAATAAAGTAGTATATGGAAGAGAGTCTCACGTAAGATGGTGGGATATATGGCAAGAACATAATGATGGCGTATACAGTATAGGAAAGGAGTGCATAGATAATATATACGAAGACAGACATACCGTAGACGAATTCTACAAGATAGACAGCGTATCAGATGTAGATGACGCAGAACATATATCTCAGATAACTAATGATGTATCTACACAAACATGGGAAAAGAAATCAGAGTTAGATAGATACATGGAAATGTATCCTCGTCATAGATATGGTAAGCATTCTGTCTTTAAGGGATTTTCTGACAAAGTTAGAAAAAATGATTTAGACATGAACGTGGTAAAAGAATTACTTTCTAACGGTGCATCTCTAACAATCAAGGATAGCAGTAATAAGGATCCAATTGCTGTTTATTTTAGAAGAACAATAATGAATTTAGAAATGATTGATATCATTAACAAACATACAACTATCTATGAACGCAGGTATATAGTACACTCCTATCTAAAAAATTATAGAAATTTCGATTATCCATTTTTCAGAAAGTTAGTTTTGACTAATAAACATTGTCTCAACAATTATTGTAATATAAGCGACGGCAAATATGGAACACCACTACATATATTAGCATCTAATAAAAAAATAATAACTCCTAATTACATGAAGTTATTAGTGTATAACGGAAATGATATAAACGCACGAGGTGAAGATACACAAATGCGAACTCCATTACACAAATATTTGTGTAAATTTGTATATCATAATATTGAATATGGTATCCGATACTATAATGAAAAGATTATAGACGCATTTATAGAGTTAGGAGCCGATCTAACTATTCCAAATGACGATGGAATGATACCAGTAGTTTACTGTATACACTCAAATGCCGAATATGGTTATAACAATATTACTAACATAAAGATAATACGTAAACTACTTAATCTTAGTAGACATGCGTCACATAATCTATTTAGAGATCGAGTCATGCACGATTATATAAGTAATACATATATTGATCTTGAGTGTTTAGATATCATTAGATCACTTGATGGGTACGATATTAATTGTTACTTTGAAGGACGTACACCACTTCATTGCGCTATACAATATAACTTCACTCAGATTGCTGAGTACTTATTAGATCGAGGAGCTGATATATCATTAAAGACAGACGATGGTAAAACTGTATTTGATTTATCGTTATGTAGTTACATTCCTCTTAAATGGACTAGCTTTTTGATTAGTCGTCTACCGCCTAAAAGTGTCATATGCTCACTGACTAACCATATAATAGATTATGTTCTTACGAACAATAGACGTATTATTTGGCAGAGTCAAATGATTAATAAGTACGTACTGTTACTGGACCCATCCTTTTATTATAGATTCAGAAATGTTATCGAAAACAAATTAGACCAATACAATAATCGTTATAATATGTTCGAACACGATAGGGACGTTAATGAAAAGTATGGCAAAGTCTTACATGACCTCGATACATATATCAAGGATGTACAAGTATTAAAATCTACTTCCATCACTAATAATATAACACTATACGACACTATTATAAATAATAAGTCAGAGTTTCCTATACGTTGTGTAAACGACAAACAATTAATTAATCTCATAAAATCCAATACATATCATAATCTTATCGAAAAAGTTATTAAAAATACATTAGAGAAATATACTTTAACTAATATAGTCCTCGAGTATATGATCTCATCTCGATCTCAATCATCTTATTTGAGTCGTATTCCTAATGAGATATTACTCGAAATATTATATAAACTCGACATGTACGATTTACGTAATCTATATACAAGATATATGAGAGAGAATGATATCACAGAGTATCATATAGAGAATACGAGGTCTGTTTCTACACAGACATGAATAATGAATACACATACAACGTTTTTTTTAATCTTAGATATAACACTAATTACATCAAGATTATATATTGAAATCGTAATTTGAGTTGTCTGATCATCATGGATATCGAAAATGATATACGTAACATTAGCAATCTTTTAGATGATTGATATATTATTATGCGATGTAATCATAACTATCGGAGATGTAGAAATTAAAGCGCATAAAACTATTTTGGTTGCCGGATCTACGTATTTTAAAACAATGTTCACAACATCTATGATAGCGAGAGATCTAGCAACTAGAGTAAATATACAGATGTTCGATAAAGATGCCGTCAAAAATATTGTACAGTACTTATACAATAGGTATATAAGTTCTATGAATGTGATAGACATATTAAAATGCACCGACTAAGAACGTAAAACGAACTATAGAATGTTATACAATGGGTGATGATAAGTAGAAGATGTTACCCGATATACCCATAGCATTATCTAGTTATGGCATGTGTGTATTAGATCAATACATATACATTATAAGCGGTCGTACCCAACACTGATTATACATCGGTACATACAGTAAATAGCATAGATATGGAGGAGGATACAAATATTTCAAATAAAGTTATGAGATACGCGCTGTCAATAATATATGGAAGACATTACCTAACTTCTGAACTGGAACTATAAATCCAGGCTCTCGCATAAAGATGAATATATATGTTGTATGCGACATCAAAGATGAAAAAATGTTAAGACTTATATATTTAGATATAACACGAATATGTATGACGGATGGGAATTGGTAACGATGACAGAAAGCAGATTGTCAGCTCTGCATACTATTCTTCATGACAATACCATAATGATGTTACATTGTTATGAAGCGTATATGTTACAAGATACATTTAATGTGCTTACGGAACATATATTTAGAAACATCTACTAACGATTTTTTATGCTTGTATTATTAATGGTATGTAATATGATTTAATTGATTGTGTACACGATACCAATTTGTCGAGTATGAATACGGAGTACAAACATAAACTGAAGTTTAACATTATTTATTTATGATATACATTATATACATTATATACATTATATACATTATATACATTATATACATTATATACATTATATACATTATATACATTATATACATTATATACATTATATACATTATATACATTATATACATTATATACATTATATACATTATATACATTATATATCGTTATTGTTTGGTCTATGCCATGGATATCTTTAAAGAACTAATCTTAAAACATACGGATGAAAATGTTTTGATTTCTCCAGTTTCCATTTTATCTACTTTATCTATTCTGAATCATGGAGCAGCTGGTTCTACAGCTGAACAACTATCAAAATATATAGAGAATATGAATGAGAATACACCCGATGATAAGAAGGATGACAATAATGACATGGACGTAGATATTCCGTATTGCGCGACACTAGCTACCGCAAATAAAATATACGGTAGTGATAGTATCGAGTTCCATGCCTCATTCCTACAAAAAATAAAAGACGATTTTCAAACTGTAAACTTTAATAATGCGAACCAAACAAAGGAACTAATCAACGAATGGGTTAAGACAATGACAAATGGTAAAATTAATTCCTTATTGACTAGTCCGCTATCCATTAATACTCGTATGATAGTTATTAGCGCCGTCCATTTTAAAGCAATGTGGAAATATCCATTTTCTAAACATCTTACATATACAGACAAGTTTTATATTTCTAAGAATATAGTTACCAGTGTTGATATGATGGTGGGTACCGAGAATGACTTGCAATATGTACATATTAATGAATTATTCGGAGGATTCTCTATTATCGATATTCCATACGAGGGAAACTCTAGTATGGTGATTATACTGCCGGACGACATAGAAGGTATATATAACATAGAAAAAAATATAACAGATGAAAAATTTAAAAAATGGTGTGGTATGTTATCTACTAAAAGTATAGACTTGTATATGCCAAAGTTTAAAGTGGAAATGACGGAACCGTATAATCTGGTACCGATTCTAGAAAATTTAGGACTTACTAATATATTTGGATATTATGCAGATTTTAGTAAGATGTGTAATGAAACTATCACTGTAGAAAAATTTCTACATACGACGTTTATAGATGTTAATGAGGAGTATACAGAAGTATCGGCCGTTACAGGAGTATTCATGACTAACTTTTCGATGGTATATCGTATGAAGGTCTACATAAACCATCCATTCATATACATGATTAAAGATAACACCGGACATACACTTTTTATAGGGAAATACTGCTATCCGCAATAAATATAAACAATAGACTTTTATCACGTTATCTCATGTATAAAATATTACAAATAGTATAGCATAAACTAAAGTCGATACATACATTAAAACTTAAATAATAATGTAATTTACAATTAATAGTATAAACTAAAAAAATTAAAAAATTAAAAACAATATCATTATTATAAGTAATATCAAAATGACGATATACGGATTAATAGCGTATCTTATATTCGTGACTTCATCCATCGCTAGTCCACTTTACATTCCCGTTATTCCGCCCATTTCGGAAGATAAATCGTTCAATAGTGTAGAGGTATTAGTTTCTTTGTTTCCCGATGACCAAAAAGACTATACAGTAACTTCTCAGTTCAATAACTACACTATCGGTACCAAAGACTGGACTATCAACGTACTATCCACACCTGATGGTCTGGACATACCATTGACTAATATAACTTATTGGTCACGGTTTACTATAGGTCGTGCATTGTTCAAATCAGAGTCTGAGGATATTTTCCAAAAGAAAATGAGTATTCTAGGTGTTTCTATAGAATGTAAGAAGCCGTCGACATTACTTACTTTTTTAACCGTGCGTAAAATGACTCGAGTATTTAATAGATTTCCAGATATGGCTTATTATCGAGGAGACTGTCTAGAAGCCGTTTATGTAACAATGACTTATAAAAATACTAAAACTGGAGAGACTGATTACACGTACCTCTCTAATGGGGGGTTGCCTGCATACTATCGTAATGGGGTCGATGGTTGATTATTGATTAGTATATTCCTTATATTCCTTATTCTTTTTATTCACACAAAAAGAACATTTTTATAAACATGAAACCACTGTCTAAATGTAATTATGATCTTGATTTATAGATGATGATCAGCCTTCAGAGGATTTTGACCAGTATGTTTAATATGAAAAAAAACATAACTATTAAGCGCTATTGCGCTATTGTGCTTAATTATTTTGCTCTATAAACTGAATATATAGCCACAATTATTGACGGGCTTGTTTGTGACCGACAATCATGAATTTTCAGAAATTATCTCTGGCTATATATCTTACGGTGACATGTTCGTGGTGTTATGAAACATGTATGAGAAAAACTGCGTTGTATCATGACATTCAATTGGAGCATGTAGAAGACAATAAAGATAGTGTAGCATCGCTACCGTACAAGTATCTACAAGTAGTCAAACAAAGAGAACGTAGTAGATTGTTGGCTACATTTAATTGGACGGATATAGCTGAGGGTGTTAGAAATGAGTTCATTAAAATATGTGATATCAACGGAACATATCTATATAATTATACTATTGCTGTTAGTATAATTATTGATTCCACGGAAGAACTACCAACAGTTACTCCAATTACAACATATGAACCTTCTATATATAATTATACTATCGATTATAGCACTGTTATTACTACTGAAGAACTACAAGTGACTCCAACATATGCACCTGTAACAACTCCTCTTCCAACATCAGCAGTTCCTTATGATCAACGATCGAATAACAATGTAAGTACTATATCTATTCAGGTACTGAGTAAAATATTGGGAGTCAATGAAACAGAATTAACTAATTATCTTATTATGCATAAAAATGACACTGTTGACAATAACACCATGGTTGATGATGAGACATCTGACAATAACACATTACATGGTAATATAGGATTTTTGGAAATAAATAATTGTTATAATGTTTCTGTGTCAGATGCTAGTTTTAGAATAACATTAGTAAACGATACTTCTGAAGAAATTTTGCTAATGCTAACAGGAACTAGTTCATCCGACACCTTCATATCTTCCACCAATATCACTGAATGTTTGAAAACATTAATCAATAATGTGTCGATTAATGATGTACTTATAACACAAAATATGAATGTAACATCTAATTGTGATAAATGCTCAATGAATTTGATGGCATCCGTTATTCCTGCAGTTAATGAATTTAACAATACGTTGATGAAAATTGGTGTAAAAGATGATGAAAACAATACGGTATATAAATATTATAATTGTAAACTAACTACAAATTCTACATGTGATGAGTTAATCAATTTAGATGAAGTCATTAACAACATAACTCTGACAAATATTATACACAATAGTGTTTCGACAACTAACAGCAGAAAAAGACGAGATCTGAATGATGAGTTTGAATTTTCCACTTCCAAGGAATTAGATTGTCTTTACGAATCATATGGTGTAAACGATGATATAAGTCATTGTTTTGCATCACCTAGACGTAGACGATCTGACGACAAAAAGGAGTACATGGACATGAAATTATTCGACCACGCGAAAAAAGATTTAGGAATAGACAGTGTTATTCCTAGAGGTACAACCCATTTCCAAGTAGGTGCATCTGGTGCAAGTGGTGGTGTTGTAGGAGATAGTTTCCCATTTCAAAATGTTAAATCGCGTGCCAGTCTATTGGCGGAAAAAATAATGCCTAGAGTACCTATTACTGCTACCGAAGCTGATCTATATGCAACTGTAAATAGACAACCCAAGTTACCAGCAGGTGTTAAAAGTACTCCGTTTACAGAGGCGCTTGTGTCTACGATAAACCAAAAGCTTTCTAATGTTAGAGAGGTAACTTATGCTTCGCTCAATCTGCCAGGATCAAGTGGCTATGTTCATAGACCATCTGATTCTGTTATTTATAGCAGTATAAGACGGTCACGTTTACCTAGTGATAGCGATAGTGATTATGAGGATATACAAACTGTTGTTAAGGAATATAATGAAAGATATGGTAGATCAGTCAGTAGAACACAGTCATCAAGTAGTGAAAGCGATTTTGAAGATATAGATACTGTTGTTAGGGAATATAGACAAAAATATGGCAATGCAATGGCAAAAGGACGTAGTAGTTCCCCTAAACCTGATCCATTATATAGTACTGTTAAGAAAACAACTAAAAGTCTATCTACTGGTGTAGACATAGTTACAAAACAATCAGACTATTCTCTATTACCTGACGTTAATACTGGCAGTTCTATTGTGTCACCTCTCACCAGAAAAGGAGCTACTAGACGACGACCTAGACGCCCTACAAATGATGGTCTACAGAGTCCAAATCCTCCTCTCCGTAATCCACTTCCTCAACATGATGATTATTCTCCTCCACAAGTACACAGACCTCCACCACTTCCTCCTAAACCAGTCCAAAATCCGCCACAACTTCCCCCTAGACCAGTAGGTCAATTACTACCTCCTCCTATAGATCAACCAGATAAAGGATTTAGTAAGTTTGTATCACCTAGACGGTGTAGAAGAGCAAGCTCTGGAGTCATATGTGGTATGATACAATCAAAACCAAACGATGATACCTATTCACTTCTTCAACGATCAAAAATTGAACCAGAATATGTGGAGGTTGGTAATGGTATACCCAAGAACAATGTTCCTGTAATAGGTAATAAACATAGTAAAAAATATACATCGACGATGTCAAAAATATCAACAAAATTTGATAAATCTACGGCATTTGGAGCAGCAATGTTACTAACTGGTCAGCAGGCCATTAGCCAACAGACTAGATCAACTACGTTGAGTAGAAAAGATCAGATGAGTAAGGAAGAAAAGATATTCGAAGCAGTTACAATGAGTCTATCAACTATAGGTTCAACGTTGACGTCTGCAGGTATGACGGGTGGTCCAAAACTAATGATTGCAGGAATGGCTATAACGGCTATAACTGGTATAATAGATACGATAAAAGATATATATTACATGTTTTCAGGACAGGAGAGGCCAGTAGATCCTGTTATTAAATTATTTAATAAGTACACTGGCTTAATGTCCGATAATAATAAAATGGGTGTAAGAAAATGTTTGACACCCGGTGACGACACACTTATTTATATCGCATACAGAAACGATACCAGTTTTAAACAGAATACGGATGCGATGGCTTTGTATTTCTTAGATGTTATCGATTCAGAGATCCTATATCTAAACACATCAAATTTAGTTCTAGAGTATCAACTAAAGGTGGCTTGCCCCATAGGAACATTAAGATCTGTAGATGTGGACATAACTGCGTATACAATATTATATGATACAGCGGATAATATTAAGAAATACAAGTTTATCAGAATGGCAACGCTACTATCCAAACATCCAGTTATTAGATTGACATGTGGTTTAGCAGCAACATTGGTGATTAAACCGTACGAGGTACCCATCAGTGATATGCAACTACTAAAAATGGCGACGCCTGGTGAACCAGAATCCACTAAATCTATACCATCCGATGTCTGTGATAGGTATCCTCTAAAGAAATTCTATCTTTTAGCTGGTGGTTGTCCCTATGATACATCTCAAACTTTTATTGTACATACTACTTGCAGTATTCTACTAAGAACAGCTACACGGGATCAGTTTAGAAACAGATGGGTGTTACAAAATCCATTTAGACAAGAAGGGACATATAAGCAACTGTTTACCTTTAGCAAATACGATTTTAACGACACCATAATCGATCCTAATGGTGTGGTGGGTCATGCTAGCTTTTGTACCAATAGAAGCAGCAACCAATGTTTCTGGTCCGAACCTATGATATTGGAAGATGTATCATCGTGTAGTTCTAGAACTAGAAAAATATACGTAAAACTGGGAATATTTAATGCTGAAGGTTTTAATAGTTTTGTACTAAATTGTCCAACTGGGTCTACACCTACATACATCAAACATAAAAATGCGGACAGTAACAATGTTATCATAGAGCTACCTGTAGGTGATTACGGCACAGCCAAATTGTATTCAGCAACAAAACCATCGAGGATAGCTGTGTTCTGCACACATAACTATGATAAACGATTCAAATCAGATATTATAGTTCTAATGTTTAATAAAAACAGCGGTATTCCATTTTGGAGCATGTACACAGGAAGTGTAACTAGTAAAAATAGAATGTTTGCCACATTGGCTAGAGGAATGCCGTTTAGATCAACGTATTGCGATAACAGACGACGATCAGGTTGTTATTATGCAGGAATACCATTTCATGAAGATAGTGTAGAAACAGATATACATTATGGACCAGAAATAATGTTAAAGGAAACATATGACATAAACAGTATTGACCCACGAGTTATAACAAAGTCAAAGACCCATTTTCCTGCTCCATTGAGTGTAAAATTCATGGTTGACAATTTAGGAAATGGATATGACAACCCTAATTCATTTTGGGAAGATGCTAAAACTAAGAAACGGACATATAGTGCAATGACGATAAAAGTCCTACCATGTACAGTGAGAAATAAAAATATAGACTTTGGATATAACTATGGAGATATTATTTCTAATATGGTTTATCTACAATCTACTAGTCAGGATTATGGAGATGGTACCAAATATACATTTAAATCCGTAACTAGATCAGATCATGAGTGTGAATCTAGCTTAGATCTAACGTCTAAGGAAGTAACTGTGACATGTCCTGCGTTTAGTATACCAAGAAATATATCAACATATGAAGGTCTATGCTTTAGTGTTACTACATCTAAAGATCATTGTGCTACAGGTATTGGTTGGTTAAAATCTAGTGGTTATGGGAAGGAAGATGCTGATAAACCACGTGCTTGTTTTCATCATTGGAATTATTACACACTGTCGTTGGATTATTACTGTTCATACGAAGATATTTGGAGAAGCACCTGGCCTGACTATGATCCATGTAAGTCATATATCCATATAGAGTATAGAGATACATGGATAGAATCTAATGTGTTACAGCAACCTCCTTACACATTCGAATTCATTCATGACAATTCTAACGAATATGTGGATAAAGAAATTAGTAACAAATTAAATGATCTGTACAATGAATACAAGAAGATTATGGAATATAGCGACGGATCATTGCCGGCGTCTATAAACAGATTAGCAAAGGCATTGACTTCAGAGGGTAGAGAAATAGCAAGTGTTAATATAGATGGTAATCTGTTAGATATCGCATATCAAGCAGATAAGGAAAAGATGGCCGACATACAGACAAGAATAAATGATATTATTAGAGATTTGTTTATACACACTCTATCAGACAAAGATATAAAAGACATTATAGAATCCGAAGAAGGTAAGAGATGTTGTATAATAGATGTTAAGAACAATCTTGTTAAAAAGTACTATTCTATTGATAATTATCTATGTGATACTTTAGATGATTATATATACACCTCTGTAGAATATAACAAATCCTATGTGTTAGTAAACGATACTTATATGAGCTATGACTATCTTGAATCATCAGGTGTAGTTGTTCTATCATGTTATGAAATGACTATAATCTCCTTGGATACAAAAGACGCCAAAGATGCTATAGAAGATGTGATAGTAGCAAGTGCGGTAGCCGAAGCATTGAATGACATGTTTAAGGAATTTGATAAAAACGTAAGTGCTATTATAATAAAAGAAGAAGATAATTATCTAAACAGTTCGCCCGATATCTACCATATAATATATATCATAGGTGGCACTATTCTGCTACTGTTAGTCATTATTTTAATATTGGCAATTTATATAGCGCGCAATAAATACAGAACCAGGAAATATGAAATAATGAAATATGACAATATGAGCATTAAATCTGAGCATCATGATAGTCTTGAAACAGTGTCTATGGAAATTATTGATAATCGGTACTAATAAAATAGTTTAACTCTTTTAGAACCAGTTTGGTACTGTAATTTCAGTTCATTACTCGTTGAGAATATTGATGATTTTTTTTAAATGAGTATCGGTAGTTACATATTACCATATCATCCATTATATAATCGATGATGCATGTATTAGAATACTTTCCGAATAAGTCTTCTAAATATTGTATTAATTATGAAAAACTATGCTATGTGAGTATGATTCAAAGATGTTTAATGATACGATACTAGATTTTATCTCTAGCGAGATTGTTTAGAATCATTTATCATAACTATGTTTAATAAATTCATCAACGAATATCGATAAAGACCTCTTGTAATTCGAGTATAGGAAGTAGTATTACCATATCAACTTCCGAGTTAACAATTACTCTAAAACATGAGGATTGTACTCCTGTCTTTATTGGAGATCACTATTCAGTCGTTGATAAACTAGTAACCTCAGGTTTCTTTACAAACGATAAAGTACAACATCAAGACCTCACAACACAGTGCAAGATTAATCTAGAAATCAAATGTAATTCTGGAGGAGAATCTAGACAACTAACACCCACGGCGAAGTATACTTTATGCCTCATTCAGAAACGGTAACTGTAGTAGGAGACTGTCTCTCTAATCTCGATGTATATATAATATATACCAATACGGACGCGATATATTCCGACATGAATGGCGTCGCTTATCATATGTTATATCCTAAATGTTGATCATATTCCACAAATGATTGTGAACGAGATTAAATCATCTAACAAATAATTAGTTTTTTATGACATTAACATATAATAAATAAATTAATCATTATTGACTTAACGATGACGAAAGTTATCATTATCTTAGGATTCTTGATTATTAATACAAATTCGTTGTGTCTATGAAATGTGAACAAGGTGTCTCATATTATAATGCACAAGAATTAAAGTGTTGTAAACTATCTAGCCAGGAACATATTCAGATTATCGATGTGATAAATACAGCGATACCATCTGTGGACATTGTCCAAGTGACACATTCACGTCAATATATAATCGTTCTCCTCGGTGTCATAGTTGTAGAGGTCACACCTTGTACACCTACCACAAATAGAATATGTCATTGTGACTCGAATAGTTATCGTCTCCTTAAAGCTTCTGATGGTAACTGTGTTACATGTGCTCCTAAAACAAAATATGGTCGTGTGTACGGAAAGAAAGGAGAAAATGATATGGAATACCATTTGTAAGAAATGTCGGAAGGGTACTTATTCAGATATTGTATCTGACTCTGATCAATGTAAACCTATGACAAGATAAGACTTACTCGCATCTACTGGATAGACATAAATATCCTCCTCGTAATAATGAAATATAATATACACTAATTATTAATATCAATCGAGTATTAACATATAAGTTATTTTTAAACCCCTTTTGGGTTCCGTCCTAAACGGCGTTTCGGTCTGTGTCGCCACCATGGTCACACCGAGCCTCTGCGTGCTCCTCCATCGAGGACGACTTCAACTATGACAGCTCGGTGGCGTCTGCCAGCGTGTACATACGAATGGCATTTCTAAGAAAAGTCTACGGTATCCTTTCTACAATTTCCTTTAACAACGGCAACAGCTGCAGTATTTTTATACTTTGAATGCATCGGACATTTATACAAGGGAGTCCTGTTCTAATATTGGCATCAATGTTCGGATCTATAGGCTTGATTTTCGCATTGACTTTACACAGACATGAACATCCCCTGAATCTGTACATACTTTGTGGATTTACACTGTTAGAATCTCTAACGCTGGCCTCTGTTGTTACTTTCTATGATGCACGTATCGTTATGCAAGCTTTCATGTTGACTACTGCAGTGTTTCTTGCTCTGACTACATATACTCTACAATCAAAGAGAGATTTCAGTAAACTTGTAACAGGATTGTTTGCTGCTTTCTGGATTTTAATTTTGTCAGGAGTCTTGAGGATAAAGTTTAAAATAGAATTAATAAAGAACATATAGGTCATTTTTTAAACATGGATAGAAACCAAGGTTGTTAGTTAATAATATACAAGATATTTTTTCTCACTCTGATCCATGTAAACCAAGGACGAGAGACACTCTCATTCCTCATTCACGACACCATTAAAAATGGAAATTAAAGCCCTCTATTAAGCACAGACGGCTACAGGTCTACCATCAGGTTACCTTCGTCTACCTTCACAATGGCCTCTCCTTGTGCCCAGTTCAGTCCCTGTCATTGCCACGCTACTAAGGACTCCCTGAATACCGTGACTGACGTCAGACATTGTCTGACTGAATACATCCTGTGGGTTTCTCATAGATGGACCCATAGAGAAAGCGCAGGGCCTCTCTACAGGCTTCTCATCTCTTTCAGAATTGATGCAATGGAGCTATTTGGTAGCGAGTTGAAGGAGTTCTCGAATTCACTTCCGTGGGACAATATCGACAATTGCGTGGAGATCATTAAATGTTTCATCAGAAATGACTCCATGAAAACCGCCAAAGAACTTTGTGCAATAATTGGACTTTGTACTCAATCAGCTATTGTCACTGGAAGAGTCTTCAATGATAAGTATATCGACATACTACTTATGCTGCGAAAGATTCTGAACGAGAACGACTATCTCACCCTCTTGGATCATATCCTCACTGCTAAATACTAAATCTCCTTCATGCTCTCTCACTAATACTCTTACTCACTACACTTTTTATCATCTTATGATGAATGATTGCCTTCATCATTTTTTCGTGGAATATAATATAGGAATAATTAGCACCAGAATAGCTATGGATATCTCGTTAAGAATATTCTCTATAAGAGACATAATGTAGACATAGTTATTATATCCTTCTTAGATAAGTGTTACGCTACTGGAAAGTTTCCATCGTTATTATTACATGAAGATGATATAATTAAACCAACATTGAGATTGGCTCTTATGTTAGCTGGATTGAATTACTGTAATAAATGCATCGAGTATAGAGGGATATAGCAATTCTCGATAATAGTCATGCAATATTTGAATGAGACTGATAATTTAGGTAATACAGTACTACACACATATCTTTCTAGATTATATATCGTTAAAAATCTGTAAGATGTATATTTCTCATAAGTATCCACTGTGTAATATTATTAATGGATATATAGATAACGCAATAGGGACTAATAGTATTGTAAAAGATATAATCGACTATTTACGTACATATCCAGATATCTATATTCCTACTAGTTTGCTGCGTAGTTGCATCATTGATATGCATGATTTATCAGGATTCAGAGATGAATTACTAAGTAAACTACAATCCCACAATAAGTAAGAATCAAATATCAAAAACTCACTTTTGATTTTTCTAGTCTTAAGTAATACATATATTTATTAATAGACCTATGAAATAAAAAAAGGTAACAATGGATTCGCGTATAGCTATTTACGTATTAGTATCGGCATCTCTTTTGTATCTTGTTAATTGTCACAAACTAGTACATTACTTCAATCTGAAAATAAATGGAAGTGATATAACTAATACAGCAGATATATTGCTGGACAATTATCCAATTATGACCTTTGATGGAAAGGATATTTATCCATCTATCTCGTTCATGGTCGGTAATAAACTTTTCCTAGATCTTTATAAAAATATCTTTGAAGAATTTTTCAGACTATTTCGAGTATCTGTAAGTAGTCAATACGAGGAATTAGAATATTATTATTCATGTGATTATACTAACAACCGTCCTACAATTAAACAACATTACTTTTATAACGGCGACGAATATACTGAAATTGATAGATCGAAAAAAGCCACTAATAAAAACAGTTGGTTAATTACTTCAGGCTTTAGACTACAAAAATGGTTCGATAGCGAAGATTGTATAATTTATCTCAGATCTTTAGTTAGAAGAATGGAAGACAGTAACAAAAACAGTAAAAAAACTTAGTACTTAGATATCGAAAAAATATATTTTTGTAGACTCTTGAGAATAGAAGGAAAACATGTACATAATTATAAAAAATGAAAATCAATGGCGAATAAGACAGTGCGATTCGCACCATGGAGTCGGTAGATTTCATGGCTGTCGATGAGCAGTTTCACGACGACCTCGATCTTTGGTCATTATCTTTGGTAGATGATTATAAAAAACATGGATTAGGTGTTGACTGTTATGTTCTAGAACCAGTTGTTGACAGGAAAATATTTGATAGATTTCTCCTTGAACCAATTTGTGATCCTGTAGATGTTCTGTATGATTATTTTAGGATTCATAGAGATAATATTGATCAGTATATAGTAGATAGACTGTTTGCATATATTACATATAAAGATATTATATCTGCATTAGTGTCAAAGAATTATATGGAAGATATTTTCTCTATAATTATTAAGAATTGTAATTCTGTGCAAGATCTCTTACTTTACTATCTATCTAATGCATATGTAGAAATAGACATTGTTGATCTTATGGTAGATCATGGGGCTGTAATATATAAAATAGAATGCTTGAATGCCTATTTTAGGGGAATATGTAAAAAGGAAAGTAGTGTTGTTGAGTTTATTTTGAATTGTGGTATCCCAGATGAAAATGATGTTAAATTAGATCTATATAAAATAATTCAGTATACTAGGGGATTCCTTGTAGATGAACCCACAGTATTAGAAATTTATAAGCTTTGTATCCCATATATTGAAGATATCAATCAACTAGATGCTGGTGGAAGGACCTTGCTTTATCGCGCTATCTATGCAGGTTATATAGATTTAGTATCATGGCTATTAGAAAATGGAGCAAATGTCAACGCAGTAATGAGTAATGGATATACATGTCTTGACGTGGCCGTGGATAGGGGATCTGTCATCGCCCGTAGGGAAGCACATCTTAAAATATTAGAAATATTGCTTAGAGAACCATTGTCTATTGACTGTATAAAATTAGCTATACTTAATAATACAATTGAAAACCATGATGTGATAAAGCTCTGTATCAAGTATTTTATGATGGTAGATTATTCACTTTGTAATGTGTATGCATCATCACTCTTTGATTATATAATTGATTGTAAACAAGAATTGGAGTACATTAGGCAGATGAAAATTCATAATACAACCATGTATGAGTTAATCTATAATAGAGACAAAAACAAGCATGCTTCCCATATTCTACATAGGTATTCTAAACATCCAGTTTTGACACAGTGTATCACTAAAGGATTCAAGATTTACACAGAAGTAACCGAGCAGGTCACTAAAGCTCTAAACAGACGTGCTCTAATAGATGAGATAATAAACAATGTATCAACTGATGACAATCTCCTATCAAAACTTCCATTAGAAATTAGGGATCTAATTGTTTCACAAGCTGTCATATAGAGTTCTATCCACCCACCTTTCTTGAAATGAGTTAATAGTCATAAGTTAGTTAAGTCATAAGTTAGTTAAGTCATAAGTTAGTTAAGTCATAAGTTAGTTAAGTCATAAGTTAGTTAAGTCATAAGTTAGTTAAGTCATAAGTTAGTTAAGTCATAAGTTAGTTTATAGTCTAACACTTCTAATTTTTATACCTTGATCTTTTTCTCTAATTATGAAAAAGTAAATCATTATGAAGATGGATGAAATGGACGAGATTGTGCGCATCGTTAACGATAGTATGTGGTACGTACCTAACGCATTTATGGACGACGGTGATAATGAAGGTCACATTTCTGTCAATAATGTCTGTCATATGTATCTCGCATTCTTTGATGTGGATATATCATCTCATCTGTTTAAATTAGTTATTAAACACTGCGATCTGAATAAACGACTAAAATGTGGTAACTCTCCATTACATTGCTATACGATGAATACACGATTTAATCCATCTGTATTAAAGATATTGTTACGCCACGGCATGCGTAACTTTGATAGCAAGGATAAAAAAGGACATATTCCTCTACACCACTATCTGATTCATTCACTATCAATCGATAACAAGATCTTTGATATACTAACGGACCCCATTGATGACTTTAGTAAATCATCCGATCTATTGCTGTGTTATCTTAGATATAAATTCAATGGGAGCTTAAACTATTACGTTCTGTACAAATTATTGACTAAAGGATCTGACCCTAATTGCGTCGATGAGGATGGACTCACTTCTCTTCATTACTACTGTAAACACATATCCGCGTTCCACGAAAGCAATTATTACAAGTCAAAGAGTCACACTAAGATGCGAGCTGAGAAGCGATTCATCTACGCGATAATAGATCATGGAGCAAACATTAACGCGGTTACGAAAATCGGAAATACGCCGTTACACACTTACCTTCAACAGTATACCAAACATAGTCCTCGTGTGGTGTATGCTCTTTTATCTCGAGGAGCCGATACGAGGATACGTAATAATCTTGATTGTACACCCATCATGGAATACATAAAGAACGATTGTGCAACAGGTCATATTCTCATAATGTTACTCAATTGGCACGAACAAAAATACGGGAAATTACAAAAGGAAGAAGGACAACATCTACTTTATCTATTCATAAAACATAATCAAGGATATGGAAGTCGCTCTCTCAATATACTACGGTATCTACTAGATAGATTCGACATTCAGAAAGACGAATACTATAATACAATGACTCCTCTTCATACCGCCTTCCAGAATTGCAATAACAATGTTGCCTCATACCTCGTATACATCGGATACGACATCAACCTTCCGACTAAAGACGATAAGACAGTATTCGACTTGGTGTTTGAAAACAGAAACATTATATACAAGGCGGATGTCGTTAATGACATTATCCACCACAGACTGAAAGTATCTCTACCTATGATTAAATCGTTGTTCTACAAGATGTCGGAGTTCTCTCCCTACGACGATCACTACGTAAAGAAGATAATAGCCTACTGCCTATTAAGGGACGAGTCATTTGCGGAACTACATACTAAATTCTGTTTAAACGAGGACTATAAAAGTGTATTTATGAAAAATATATCATTCGATAAGATAGATTCCATCATCGAAAAATGTAGTCGTGACATAAGTCTCCTCAAAGAGATTCGAATCTCAGACACCGACTTGTATACGGTATTGAGAACAGAAGACATCCGGTATCACACATATCTCGAAGCCATACATTCAGACAAACGCATTTCATTTCCCATGTACGACGATCTCATAGAACAGTGTCATCTATCGATGGAGCATAAAAGTAAACTCGTCGACAAAGCACTCAATAAATTAGAGTCTACCATCGATAGTCAATCTAGACTATCGTATTTGCCTCCGGAAATTATGCGCAATATCATAACCAAGCTAAGCGACTACCATCTAAACAGTATGTTGTACGGAAAGAACCATTACAAATATTATCCATGATAGAAAGAAAATATTTAAAAAATAATCTATATGATTGGAGAAGTAGGAAACAAACAGTAACAAGACGACGATTACTACATTATTAAATCATGAGGTCCGTATTATACTCGTATATATTGTTTCTCTCATGTATAATAATAAACGGAAGAGATATAGCACCACATGCACCATCCAATGGAAAGTGTAAAGACAACGAATACAGAAGCCGTAATCTATGTTGTCTATCGTGTCCTCCGGGAACTTACGCTTCCAGATTATGTGATAGCAAGACTAATACACAATGTACACCGTGTGGTTCGGATACCTTTACATCTCACAATAATCATTTACAGGCTTGTCTAAGTTGTAACGGAAGATGTGATAGTAATCAGGTAGAGACGCGATCGTGTAACACGACTCACAATAGAATCTGTGAATGCTCTCCAGGATATTATTGTCTTCTCAAAGGAGCATCAGGGTGTAGAACATGTATTTCTAAAACAAAGTGTGGAATAGGATACGGAGTATCCGGATACACGTCTACCGGAGACGTCATCTGTTCTCCGTGTGGTCCCGGAACATATTCTCACACCGTCTCTTCCACAGATAAATGCGAACCCGTCGTAACCAGCAATACATTTAACTATATCGATGTGGAAATTAACCTGTATCCAGTCAACGACACATCGTGTACTCGGACGACCACTACCGGTCTCAGCGAATCCATCTCAACGTCGGAACTAACTATTACCATGAATCATAAAGATTGTGATCCAGTCTTTCGTGCAGAATACTTCTCTGTCCTTAATAATGTAGCAACTTCAGGATTCTTTACAGGAGAAAATAGATATCAGAATACTTCAAAGATATGTACTCTGAATTTCGAGATTAAATGTAACAACAAAGATTCATCTTCCAAACAGTTAACGAAAACAAAGAATGATACTATCATGCCGCATTCAGAGACGGTAACTCTAGTGGGCGACTGTCTATCTAGCGTCGACATCTACATACTATATAGTAATACCAATACTCAAGACTACGAAAATGATACAATCTCTTATCATATGGGTAATGTTCTCGATGTCAATAGCCATATGCCCGCTAGTTGCGATATACATAAACTGATCACTAATTCCCAGAATCCCACCCACTTATAGTAAGTTTTTTTACCTATAAATAATAAATACAATAATTAATTTCTCGTAAAAGTAGAAAATATATTCTAATTTATTATATGGTAAGAAAGTAGAATCATCTAGAACAGTAATCAATCAATAGCAATCATGAAACAATATATTGTCCTGGCATGCATGTGCCTAGTGGCAGCTGCTATGCCTACTAGTCTTCAACAATCTTCATCCTCGTGTACTGAAGAAGAAAACAAACATCATATGGGAATCGATGTTATTATCAAAGTCACAAAGCAAGACCAAACACCGACCAATGATAAGATTTGTCAATCCGTAACGGAAGTTACAGAGACCGAAGATGATGAGGTATCCGAAGAAGTTGTAAAAGGAGATCCCACCACTTATTACACTATCGTCGGTGCGGGTCTTAACATGAACTTTGGATTCACCAAATGCCCAAAGATTTCATCCATCTCCGAATCCTCTGATGGAAACACTGTGAATACTAGATTGTCCAGCGTGTCACCGGGACAAGGTAAGGACTCTCCCGCGATCACGCGTGAAGAAGCTCTGGCTATGATCAAAGACTGTGAGATGTCTATCGACATCAGATGTAGCGAAGAAGAGAAAGACAGTGACATCAAGACCCATCCAGTACTTGGGTCTAACATCTCACATAAGAAAGTGAGTTACAAAGATATCATCGGTTCAACGATCGTTGATACAAAATGTGTCAAGAACCTAGAGTTTAGCGTACGTATCGGAGACATGTGTGAGGAATCATCTGAACTTGAAGTCAAGGATGGATTCAAGTATGTCGACGGATCGGCATCTGAAGGTGCAACCGATGATACTTCACTCATCGATTCAACAAAACTCAAAGCATGTGTCTGAATCGATAACTCTATTCATCTGAAAATGGATGAGTTGGGTTAATCGAACGATTCAGACACCGCACCACGAATTAAAAAAGACCGGGCACTATATTCCGGTTTGCAAAACAAAAATATTTAACTACATTCACAAAAAGTTACCTCTCGTTACTTCTTCTTTCTGTTTCAATATGTGATACGATATGATCACTATTCGTATTCTCTTGGTCTCATAAAAAAGTTTTACAAAAAAAAAAAAAAATATTTTTATTCTCTTTCTCTCTTCGATGGTCTCACAAAAATATTAAACCTCTTTCTGATGTCTCAACTATTTCGTAAACGATAACGTCCAACAATATATTCTCGTAGAGCTTATCAACATCCTTATACCAATCTAGGTTGTCAGACAATTGCATCATAAAATAATGTTTATAATTTACACGTTAACATCATATAATAAACGTATATAGTTAATATTTTTGGAATATAAATGATCTGTAAAATCCATGTAGGGGACACTGCTCACGTTTTTTCTCTAGTACATAATTTCACACAAGTTTTTATACAGACAAATTAATTCTCGTCCATATATTTTAAAACATTGACTTTTGTACTAAGAAAAATATCTTGACTAACCATCTCTTTCTCTCTTCGATGGGTCTCACAAAAATATTAAACCTCTTTCTGATGGAGTCGTAAAAAGTTTTTATCCTTTCTCTCTTCGATAGGTCTCACAAAAATATTAAACCTCTTTCTGATGGTCTCTATAAACGATTGATTTTTCTTACCCTCTAGAGTTTCCTACGGTCGTGGGTCACACATTTTTTTCTAGACACTAAATAAAATAGTAAAATTAAATTAATTATAAAATTATGTATATAATTTACTAAC
