# Supplementary material for: Exploring the key genomic variation in monkeypox virus during the 2022 outbreak
Source: BMC Genom Data. 2023 Nov 16;24:67. doi: 10.1186/s12863-023-01171-0 (PMC10652487; doi:10.1186/s12863-023-01171-0)
Supplement: Supplementary file 12 — Additional file 12. Full length sequence of RS9. [file 12863_2023_1171_MOESM12_ESM.docx]

**Additional file12. Full length sequence of RS9.**

GTTAGTAAATTATATACATAATTTTATAATTAATTTAATTTTACTATTTTATTTAGTGTCTAGAAAAAAATGTGTGACCCACGACCGTAGGAAACTCTAGAGGGTAAGAAAAATCAATCGTTTATAGAGACCATCAGAAAGAGGTTTAATATTTTTGTGAGACCTATCGAAGAGAGAAAGGATAAAAACTTTTTACGACTCCATCAGAAAGAGGTTTAATATTTTTGTGAGACCCATCGAAGAGAGAAAGAGATGGTTAGTCAAGATATTTTTCTTAGTACAAAAGTCAATGTTTTAAAATATATGGACGAGAATTAATTTGTCTGTATAAAAACTTGTGTGAAATTATGTACTAGAGAAAAAACGTGAGCAGTGTCCCCTACATGGATTTTACAGATCATTTATATTCCAAAAATATTAACTATATACGTTTATTATATGATGTTAACGTGTAAATTATAAACATTATTTTATGATGCAATTGTCTGACAACCTAGATTGGTATAAGGATGTTGATAAGCTCTACGAGAATATATTGTTGGACGTTATCGTTTACGAAATAGTTGAGACATCAGAAAGAGGTTTAATATTTTTGTGAGACCATCGAAGAGAGAAAGAGAATAAAAATATTTTTTTTTTTTTTGTAAAACTTTTTTATGAGACCAAGAGAATACGAATAGTGATCATATCGTATCACATATTGAAACAGAAAGAAGAAGTAACGAGAGGTA

ACTTTTTGTGAATGTAGTTAAATATTTTTGTTTTGCAAACCGGAATATAGTGCCCGGTCTTTTTTAATTC

GTGGTGCGGTGTCTGAATCGTTCGATTAACCCAACTCATCCATTTTCAGATGAATAGAGTTATCGATTCA

GACACATGCTTTGAGTTTTGTTGAATCGATGAGTGAAGTATCATCGGTTGCACCTTCAGATGCCGATCCG

TCGACATACTTGAATCCATCCTTGACTTCAAGTTCAGATGATTCCTCACACATGTCTCCGATACGTACGC

TAAACTCTAGGTTCTTGACACATTTTGTATCAACGATCGTTGAACCGATGATATCTTTGTAACTCACTTT

CTTATGTGAGATGTTAGACCCAAGTACTGGATGGGTCTTGATGTCACTGTCTTTCTCTTCTTCGCTACAT

CTGATGTCGATAGACATCTCACAGTCTTTGATCATAGCCAGAGCTTCTTCACGCGTGATCGCGGGAGAGT

CCTTACCTTGTCCCGGTGACACGCTGGACAATCTAGTATTCACAGTGTTTCCATCAGAGGATTCGGAGAT

GGATAAAATCTTTGGGCATTTGGTGAATCCAAAGTTCATGTTAAGACCCGCACCGACGATAGTGTAATAA

GTGGTGGGATCTCCTTTTACAACTTCTTCGGATACCTCATCATCTTCGGTCTCTGTAACTTCCGTTACGG

ATTGACAAATCTTATCATTGGTCGGTGTTTGGTCTTGCTTTGTGACTTTGATAATAACATCGATTCCCAT

ATGATGTTTGTTTTCTTCTTCAGTACACGAGGATGAAGATTGTTGAAGACTAGTAGGCATAGCAGCTGCC

ACTAGGCACATGCATGCCAGGACAATATATTGTTTCATGATTGCTATTGATTGATTACTGTTCTAGATGA

TTCTACTTTCTTACCATATAATAAATTAGAATATATTTTCTACTTTTACGAGAAATTAATTATTGTATTT

ATTATTTATAGGTAAAAAAACTTACTATAAGTGGGTGGGATTCTGGGAATTAGTGATCAGTTTATGTATA

TCGCAACTAGCGGGCATATGGCTATTGACATCGAGAACATTACCCATATGATAAGAGATTGTATCATTTT

CGTAGTCTTGAGTATTGGTATTACTATATAGTATGTAGATGTCGACGCTAGATAGACAGTCGCCCACTAG

AGTTACCGTCTCTGAATGCGGCATGATAGTATCATTCTTTGTTTTCGTTAACTGTTTGGAAGATGAATCT

TTGTTGTTACATTTAATCTCGAAATTCAGAGTACATATCTTTGAAGTATTCTGATATCTATTTTCTCCTG

TAAAGAATCCTGAAGTTGCTACATTATTAAGGACAGAGAAGTATTCTGCACGAAAGACTGGATCACAATC

TTTATGATTCATGGTAATAGTTAGTTCCGACGTTGAGATGGATTCGCTGAGACCGGTAGTGGTCGTCCGA

GTACACGATGTGTCGTTGACTGGATACAGGTTAATTTCCACATCGATATAGTTAAATGTATTGCTGGTTA

CGACGGGTTCGCATTTATCTGTGGAAGAGACGGTGTGAGAATATGTTCCGGGACCACACGGAGAACAGAT

GACGTCTCCGGTAGACGTGTATCCGGATACTCCGTATCCTATTCCACACTTTGTTTTAGAAATACATGTT

CTACACCCTGATGCTCCTTTGAGAAGACAATAATATCCTGGAGAGCATTCACAGATTCTATTGTGAGTCG

TGTTACACGATCGCGTCTCTACCTGATTACTATCACATCTTCCGTTACAACTTAGACAAGCCTGTAAATG

ATTATTGTGAGATGTAAAGGTATCCGAACCACACGGTGTACATTGTGTATTAGTCTTGCTATCACATAAT

CTGAAAGCGTAAGTTCCCGGAGGACACGATAGACAACATAGATTACGGCTTCTGTATTCGTTGTCTTTAC

ACTTTCCATTGGATGGTGCATGTGGTGCTATATCTCTTCCGTTTATTATTATACATGAGAGAAACAATAT

ATACGAGTATAATACGGACCTCATGATTTAATAATGTAGTAATCGTCGTCTTGTTACTGTTTGTTTCCTA

CTTCTCCAATCATATAGATTATTTTTTAAATATTTTCTTTCTATCATGGATAATATTTGTAATGGTTCTT

TCCGTACAACATACTGTTTAGATGGTAGTCGCTTAGCTTGGTTATGATATTGCGCATAATTTCCGGAGGC

AAATACGATAGTCTAGATTGACTATCGATGGTAGACTCTAATTTATTGAGTGCTTTGTCGACGAGTTTAC

TTTTATGCTCCATCGATAGATGACACTGTTCTATGAGATCGTCGTACATGGGAAATGAAATGCGTTTGTC

TGAATGTATGGCTTCGAGATATGTGTGATACCGAATGTCTTCTGTTCTCAATACCGTATACAAGTCGGTG

TCTGAGATTCGAATCTCTTTGAGGAGACTTATGTCACGACTACATTTTTCGATGATGGAATCTATCTTAT

CGAATGATATATTTTTCATAAATACACTTTTATAGTCCTCGTTTAAACAGAATTTAGTATGTAGTTCCGC

AAATGACTCGTCCCTTAATAGGCAGTAGGCTATTATCTTCTTTACGTAGTGATCGTCGTAGGGAGAGAAC

TCCGACATCTTGTAGAACAACGATTTAATCATAGGTAGAGATACTTTCAGTCTGTGGTGGATAATGTCAT

TAACGACATCCGCCTTGTATATAATGTTTCTGTTTTCAAACACCAAGTCGAATACTGTCTTATCGTCTTT

AGTCGGAAGGTTGATGTCGTATCCAATGTATACGAGGTATGAGGCAACATTGTTATTGCAATTCTGGAAG

GCGGTATGAAGAGGAGTCATTGTATTATAGTATTCGTCTTTCTGAATGTCGAATCTATCTAGTAGATACC

GTAGTATATTGAGAGAGCGACTTCCATATCCTTGATTATGTTTTATGAATAGATAAAGTAGATGTTGTCC

TTCTTCCTTTTGTAATTTCCCGTATTTTTGTTCGTGCCAATTGAGTAACATTATGAGAATATGACCTGTT

GCACAATCGTTCTTTATGTATTCCATGATGGGTGTACAATTAAGATTATTACGTATCCTCGTATCGGCTC

CTCGAGATAAAAGAGCATACACCACACGAGGACTATGTTTGGTATACTGTTGAAGGTAAGTGTGTAACGG

CGTATTTCCGATTTTCGTAACCGCGTTAATGTTTGCTCCATGATCTATTATCGCGTAGATGAATCGCTTC

TCAGCTCGCATCTTAGTGTGACTCTTTGACTTGTAATAATTGCTTTCGTGGAACGCGGATATGTGTTTAC

AGTAGTAATGAAGAGAAGTGAGTCCATCCTCATCGACGCAATTAGGGTCAGATCCTTTAGTCAATAATTT

GTACAGAACGTAATAGTTTAAGCTCCCATTGAATTTATATCTAAGATAACACAGCAATAGATCGGATGAT

TTACTAAAGTCATCAATGGGGTCCGTTAGTATATCAAAGATCTTGTTATCGATTGATAGTGAATGAATCA

GATAGTGGTGTAGAGGAATATGTCCTTTTTTATCCTTGCTATCAAAGTTACGCATGCCGTGGCGTAACAA

TATCTTTAATACAGATGGATTAAATCGTGTATTCATCGTATAGCAATGTAATGGAGAGTTACCACATTTT

AGTCGTTTATTCAGATCGCAGTGTTTAATAACTAATTTAAACAGATGAGATGATATATCCACATCAAAGA

ATGCGAGATACATATGACAGACATTATTGACAGAAATGTGACCTTCATTATCACCGTCGTCCATAAATGC

GTTAGGTACGTACCACATACTATCGTTAACGATGCGCACAATCTCGTCCATTTCATCCATCTTCATAATG

ATTTACTTTTTCATAATTAGAGAAAAAGATCAAGGTATAAAAATTAGAAGTGTTAGACTATAAACTAACT

TATGACTTAACTAACTTATGACTTAACTAACTTATGACTTAACTAACTTATGACTTAACTAACTTATGAC

TTAACTAACTTATGACTTAACTAACTTATGACTTAACTAACTTATGACTATTAACTCATTTCAAGAAAGG

TGGGTGGATAGAACTCTATATGACAGCTTGTGAAACAATTAGATCCCTAATTTCTAATGGAAGTTTTGAT

AGGAGATTGTCATCAGTTGATACATTGTTTATTATCTCATCTATTAGAGCACGTCTGTTTAGAGCTTTAG

TGACCTGCTCGGTTACTTCTGTGTAAATCTTGAATCCTTTAGTGATACACTGTGTCAAAACTGGATGTTT

AGAATACCTATGTAGAATATGGGAAGCATGCTTGTTTTTGTCTCTATTATAGATTAACTCATACATGGTT

GTATTATGAATTTTCATCTGCCTAATGTACTCCAATTCTTGTTTACAATCAATTATATAATCAAAGAGTG

ATGATGCATACACATTACAAAGTGAATAATCTACCATCATAAAATACTTGATACAGAGCTTTATCACATC

ATGGTTTTCAATTGTATTATTAAGTATAGCTAATTTTATACAGTCAATAGACAATGGTTCTCTAAGCAAT

ATTTCTAATATTTTAAGATGTGCTTCCCTACGGGCGATGACAGATCCCCTATCCACGGCCACGTCAAGAC

ATGTATATCCATTACTCATTACTGCGTTGACATTTGCTCCATTTTCTAATAGCCATGATACTAAATCTAT

ATAACCTGCATAGATAGCGCGATAAAGCAAGGTCCTTCCACCAGCATCTAGTTGATTGATATCTTCAATA

TATGGGATACAAAGCTTATAAATTTCTAATACTGTGGGTTCATCTACAAGGAATCCCCTAGTATACTGAA

TTATTTTATATAGATCTAATTTAACATCATTTTCATCTGGGATACCACAATTCAAAATAAACTCAACAAC

ACTACTTTCCTTTTTACATATTCCCCTAAAATAGGCATTCAAGCATTCTATTTTATATATTACAGCCCCA

TGATCTACCATAAGATCAACAATGTCTATTTCTACATATGCATTAGATAGATAGTAAAGTAAGAGATCTT

GCACAGAATTACAATTCTTAATAATTATAGAGAAAATATCTTCCATATAATTCTTTGACACTAATGCAGA

TATAATATCTTTATATGTAATATATGCAAACAGTCTATCTACTATATACTGATCAATATTATCTCTATGA

ATCCTAAAATAATCATACAGAACATCTACAGGATCACAAATTGGTTCAAGGAGAAATCTATCAAATATTT

TCCTGTCAACAACTGGTTCTAGAACATAACAGTCAACACCTAATCCATGTTTTTTATAATCATCTACCAA

AGATAATGACCAAAGATCGAGGTCGTCGTGAAACTGCTCATCGACAGCCATGAAATCTACCGACTCCATG

GTGCGAATCGCACTGTCTTATTCGCCATTGATTTTCATTTTTTATAATTATGTACATGTTTTCCTTCTAT

TCTCAAGAGTCTACAAAAATATATTTTTTCGATATCTAAGTACTAAGTTTTTTTACTGTTTTTGTTACTG

TCTTCCATTCTTCTAACTAAAGATCTGAGATAAATTATACAATCTTCGCTATCGAACCATTTTTGTAGTC

TAAAGCCTGAAGTAATTAACCAACTGTTTTTATTAGTGGCTTTTTTCGATCTATCCTCGTCCTCTCATCA

TCCTTATATTATTATCATTATTATCATAGTCTATTAAACACAAATCATCTACGTTTATAACAACATTCTC

ATTATTAATTAGTTCTGTAGAATATCTTTAATAATTTGGCTATACATCTGTTCAATACTATCTATTGATG

ATTTCTTTTTTAAGACTTAAACTAGTTATGGTAATGACGATGAAATCGAGTAGTAACTTCTAATAAAGAC

TTGATATCATTATCATATGTTTGATCGTCATAGTTAATAGTGTGGCTAAATGGTACTGTTAATAAGTTTA

TAGACAATATCATAGTATTTTCTTTCCAGAATTAGATTATTTTTTTAAATACTGATCCTCACAATTCCGT

GATGTAGCAGTAGTTGGTGCATGGTCTATATCGTTAAAATGTATCATATATAATAGTTTTCTGACGTGGA

GTACAGAATTTTCGATTAATGAGTTCATGGTAAGGAAGGGCAAATGCCTGTATATAATATACATAAGTTA

ATAGTTTTTTATCATATTTTCTAATACCATAATAAAAATTATCATTATTGCGTTTGGTAGTTCTGCCCTA

TCATCTATATCACTGTCACTCTCGCTCTCACTATATCTTCTAAAATTACAAACAACTGGATATTCGATAA

CAGCATTTGTGTAGTTTTTGTCTTTTACAGTATATACGTTATTGTCAAAATCTAAACAAATATTAGCATA

ATACATCTATCTATAAGATCAGGATCCATGTTCGAGCATACTAGCCATGTATATTTGTAACTTCGTCGTA

CAGCGTTAGATCAATAGAATAAACAATCGTGTGACGCAACTTTTTTACGATCTAGTTGTATGAGTTTATC

GTTTACATAAGCAATTAACGGCTTTAACAGATGATCTGAGTAATAATATACCTCTGTTATACGTTTAATG

TTCACGGTCTTAGTATTTTTAGATATCAATTGTGATTTACACCATATTCGACTCCCTTGTGTGTAACGTT

AGAAATTCTAAATCTATATTATCTATTACAGCGTAAAACACATTCAATATTGTATTGTTATTTTTATATT

ATTTACACAATTAACAATGTATTATTAGTTTATATTACTGAATTAATAATATAAAATTCCCAATCTTGTC

ATAAACACAAAATCCATTAAAAATGTCGATAAAATATCTGATGTTGTTGTTCGCTGCTATGATAATCAGA

TCATTAGCCGATAGTGGTAATGCTATCGAAACAACATCGCCAGAAATTACAAACGCTACAACAGATATTC

CAGCTATCAGATTATGTGGTCCAGAGGGAGATGGATATTGTTTACACGGTGACTGTATCCACGCTAGAGA

TATTGACGGTATGTATTGTAGATGCTCTCATGGTTATACAGGTATTAGATGTCAGCATGTAGTATTAGTA

GACTATCAACGTTCAGGAAAACCAGACACTACAACATCATATATCCCATCTCTAGGTATTGTGCTTGTAT

TAGTAGGCATTATTATTACGTGTTGTCTATTATCTGTTTATATGTTCACTCGAAGAACTAAACTACCTAT

ACAAGATATGGTTGTGTTATATTTTTTATAAAATTTTTTTATGAGTATTTTTACAAAAATGTATATGTAT

AAAAAAAATACTAAGTATACGATGTATCCTGTATTATTTGTATTTATCTAAACAATACTTCTGCCTCTAG

ATGGGATACAAAAATTTTTTATTTCAGCATATTAAAGTAAAATCTAGTTACCTTGAAAATGAATACAGTG

GGTGGTTCCGTATCACCAGTAAGAACATAATAGTCGAATACAGTATCCGATTGAGATTTTGCATACAATA

CTAGTCTAGAAAGAAATTTGTAATCATCCTCTGTGACAGGAGTCCATATATCTGTATCATCGTCTAGTTT

TTATCAGTGTCCTATGCTATATTCCTGTTATCATCATTAGTTAATGAAAATAACTCTCGTGCTTCAGAAA

AGTCAAATATTGTATCCATACATATATCTCCAAAACTATCACTTATACGTTTATCTTTAACGAACATATA

CCTAGATGGTTATTTACTAACAGACATTTTTTCAAGATCTATTGACAATAACTCCTATAGTTTCCACATC

AACCAAGTAATGATCATCTATTGTTATATAACAATAACATAACTCTTTTCCATTTTTATCAGTATCTATA

TCAACGTCGTTGTAGTGAATAGTAGTCATTGATCTATTATATGAAACGGATATGTCTAGTTAATATTTTC

TTTGATTTAAAGTCTATAGTCTTTACAAACATAATATCCTTATCCGACTTTATATTTCCTGTAGGGTGGC

ATAATTTTATTCTGCCTCCACAATCAGTGTTTCCAAATATATTACTAGACAATATTCCATATAGTTATTA

GTTAAGGGACCCAATTAGAACACGTACGCGCTTATTCATCATTTGGATCGTATTTCATAAAAGTTATTAT

GTTATAGATGTCAACACATTCTACATTTTTTTAATTGTCTATATAGTATTTTTCTGATATTTTCTATAAT

ATCAGAATTGTCTTCCATAGGAAGTTGTATACTATTCGGAATCAGTTACATGTTTAAATAATTCTCTGAT

GTCATTCCTTATACAATCAAATTCATTATTAAACAGTTTAATAGTCTGTAGACCTTTATCGTCGTACATA

TCCATTGTCTTATTAGTTACGCTTATTTTTATGGGTTTTACGTTGCTTTATTATATTTTATAATAATGAT

TGTTTGACAATGTCGTAGTATAGATATATTATTAGAGGAGGTATAATTATAAAAAGTTTCTGAGTACGAT

GTTATAAGAGGAGAGGACACATTAACATCATACATCAATTAACTACATTCTTATAACATTGTAATCAAAA

GAATTGCAATTTTGATGTATAACAACTGTCAATGGAATTGTATATTACAAATTACGGTATGTTGTAACAA

CAAATACCGATCGGTAATTGTCTCTGCCGCTGTAATAGAATTAATTATATATCTATTACACCGGCCTTGT

ATCATAATAAAGTTGTGGTAGTATGATCTCCATATTTATAATTTAGTACTTTGTATTTAGTTTTTTTGGA

ATCATAAAAAAAGTTTTACTAATTTAAAATTTAAAAAGTATTTACATTTTTCACTGTTTAGTCGCGGATA

TGGAATTCGATCCTGTCAAAATCAATACATCATCTATAGATCATGTAACAATATTACAATACATAGATGA

ACCAAATGATATAAGACTAACAGTATGCATTATCCGAAATGTTAATAACATTACATATTATATCAATATC

ACAAAAATAAATCCACATTTGGCTAATCGATTTCGGGCTTGGAAAAAACGTATCGCCGGAAGGGACTATA

TGACTAACTTATCTAGAGATACAGGAATACAACAATCAAAACTTACTGAAACTATACGTAACTGTCAAAA

AAATAAAAACATATATGGTCTATATATACACTACAATTTAGTTATTAATGTGGTTATTGATTGGATAACC

GATGTGATTGTTCAATCAATATTAAGAGGGTTGGTAAATTGGTACATAGCTAATAATACATATACTCCAA

ATACACCCAATAATACTACAACCATTTCTGAGTTGGATATCATCAAAATACTGGATAAATACGAGGACAT

GTATAGAGTAAGTAAAGAAAAAGAATGTGGAATTTGCTATGAAGTTGTTTACTCAAAACGATTAGAAAAC

GATAGATACTTTGGTTTATTGGATTCGTGTAATCATATATTTTGCATAACATGTATCAATATATGGCATA

GAACACGAAGAGAAACCGGTGCGTCAGATAATTGTCCTATATGCCGTACACGTTTTAAAAAAATAACAAT

GAGCAAGTTCTATAAGCTAGTTAACTAATAAATAAAAAGTTTAATTATCGACGATATATGTCGTTATTTT

TCTCTCATATGAAAGATTAATTTGATTCTAATATAATCTTCAGTATTGGATGAATCTCAATTCAAATTAA

TTCCATTAGATTAGATTAGATTAGATCATAAATAAAAATAGTAGCACGCACTACTTCAGCCAAATATTCT

TTTTTGAAACGCCATCTAGCGTAATGAGAACACAAGTGAACCTATAATGAGCAAATTTATTAGTATCGGT

TACATGAAGGACTTTACGTAGAGTGGTGATTCCTCCATCTGTGGTACGAACGGTTTCATCTTCTTTGATG

CCACCACCCAGATGTTCTATAAACTTGGTATCCTCGTCCGATTTCATATCATTTGCCAACCAATACATAT

AGCTAAACCCAGGCATACGTTCCACACATCCGGAACAATGAAATTCTCCAGAAGATGTTACAATGGCTAG

ATTTGGACATTTGGTTTCAACCGCGTTAACATATGAGTGAACACACCCATACATGAAAGCGATGAGAAAT

AGGATTCTCATCTTGCCAAAATATCACTATAAAAAATTTATTTATCAATTTTAAAGGTATAAAAAAATAC

TTATTGTTGCTCGAATATTTTGTATTTGATGGTATACGGAAGATTAGAAATGTAGGTATTATCATCAACT

GATTTTATGATGGTTTTATGAATTTTATTATGCTTCACTATTGCATCGGAAATAATATCATATGCTTCCA

CGTATATTTTATTTTGTTTTGACTCATAATACGCACGTAATTCTGGATTATTGGCATATCGATGAATAAT

TTTAGCTCCATGCTCAGTAAATATTAATGAGAACATAGTGTTGCCTCCTACCATTATTTTTTTCATCTCA

TTCAATTCTTGATTGCAGAGATCTATATAATCATTATAGCGTTGACTTATGGACTCTGGAATCTTAGACG

ATGTACAGTCATCTATAATCATGGTATATTTAATACATTGTTTTATAGCATAGGCATTATCTACGATATT

AGATACTTCACTCAATGAATCAATCACACAATCTAATGTAGGTTTATGACATAATAGCATTTTCAGCAGT

TCAATGTTTCTAGATTCGTTGATGGCAATGGTTATACATGTATATCCGTTATTTGATCTAATGTTGACAT

CTGAACCGGATTCTAGCAGTAAAGATACTAGAGATTGTTTATTATATCTAACAGCCTTGTGAAGAAGTGT

TTCTCCTCGTTTGTCAATCATGTTAATGTCTTTAAGATAAGGTAGACAAATGTTTATAGTACTAAGAATT

GGGCAAACATAAGACATGTCACAAAGACCCTTTTTGTATGTATAAGTGTAGAAATTATAACATCCATAGT

TGGATTCACATAGGTGTCCAATCGGGATCTCTCCATCATCGAGATGATTGACGGCATCTCCCCCTTCCTT

TTTTAGTAGATATTTCATCGTGTAAGAATCAATATTAATATTTCTAAAGTATCTGTGTATAGCCTCTTTA

TTTACCACAGCTCCATATTCCAACATGCATTCCACTAGAGGGATATCGATATCGCCGAATGTCATATACT

CAATTAGTATATGTTGGAGGACATCCGAGTTCATTGTTTTCAATATCAAAGAGATGGTTTCCTTATCATT

TCTCCATAGTGGTACAATACTACACATTATTCCGTACGGCTTTCCATTCTCCAAAAACAATTTTACCAAA

TCTAAATCTACATCTTTATTGTATCTATAATCACTATTTAGATAATCAGCCATAATTCCTCGAGTGCAAC

ATGTTAGATCGTCTATATATGAATAAGCCGTGTTATCTATTCCTTTCATTAACAATTTAACGATGTCTAT

ATCTATATGAGATGACTTAATATAATATTGAAGAGCTGTACAATAGTTTTTATCTATAGAAGACGGCTTG

ATTCCGTGATTAATTAGACATTTAACAACTTCCGGACGCACATATGCTCTCGTATCCGACTCTGAATACA

GATGAGCGATGATATACAGATGCAATACGGTACCACAATTTCGTGGTTGATAATCATCATACACGTATCC

GTACTCGTCATCCTCATAAAGAACACTGCATCCATTTTCTATGAACAAATCAATAATTTCAGGAACAGGA

TCATCTGTCATTACATAATTTTCTATAACTGAACGATGGTTTTCACATTTAACACTCAAGTCAAATCCAT

GTTCTACCAACACCTTTATCAAGTCAACGTCTACATTTTTTGATTTCATATAGCTGAATATATTAAAGTC

ATTTATGTTGCTATATCCAGTAGCTTCTAGTAGAGCCATCGCTATATCCTTATTGACTTTAACATGTCTA

CTATTTGTGTATTCTTCTATTGGGGTAAACTGTCTCCAATTTTTATGTAATGGATTAGTGCCACTATCTA

GTAGTAGTTTGACGACCTCAACATTATTACAATGCTCATTGAAAAGGTATGCGTGTAAAGCATTATTCTT

GAATTGGTTCCTGGTATCATTAGGATCTCTGTCTCTCAACATCTGTTTAAGTTCATCGAGAACCACCTCC

TCATTTTCCAGATAGTCAAACATTTTGACTGAATAGAAGTGAATGAGCTACTGTGAACTCTATACACCCG

CACAACTAATGTCATTAAATATCATTTTTGAATGTATTTATACCATGTCAAAAACTTGTACAATTATTAA

TAAAAATAATTAGTGTTTAAATTTTACCAGTTCCAGATTTTACACCTCCGTTAACACCTCCATTAACCCC

ACTTTTTACACCACTGGACGATCCTCCTCCCCACATTCCACTGCCACTAGATGTATAAGTTTTAGATCCT

TTATTACTACCATCATGTCCATGGATAAAGACACTCCACATGCCGCCACTACTACCCCCTTTAGACGACA

TATTAATAAGACAAGTTTAACAATAAAATTAATCACGAGTACCCTACTACCAACCACTATTATATGATTA

CAGTACCTTGACTAAAGTCTCTAGTCACAAGATCAATACTACCAACCTACGCTATTATATGATTATAGTT

TCTATTTTTATAGGAACGCGTACGAGAAAATCAAATGTCTAAGTTCTAACGGTAGTGTTGATAAACGATT

GTTATCCGCGGATACCTCATCTATCATGTTGTCTATTTTCTTACTTTGTTCTATTAACCTATTAGCATTA

TATATTATTTGATTATAAAACTTATATTGCTTATTAGCCCAATCTGTAAATATCGGATTATTAACATATC

GTTTCTTTGTAGGTTTATTTAACTTGTACATCACTGTAAGCATGTCCGTACCATTTATTTTAATTTGACA

CATATCAGCAATTTCTTTTTCGCAGTCGGTTATATATTCTATATAAGATGGATACGTATCACATATGTAC

TTATAGTCTACTAATATGAAGTACTTAATACATATTTTCAGTAACGATTTAGCCTTATTACCTATTAATA

AGTGCCTGTCGTTGGATAGGTAATCAACTGTTTTCTTAATACATTCGATGGTTGGTAATTTACTCAAAAT

AATTTCCAATATCTTAATATATATTTCTGCTATTTCTGGTATACATGCATGTGCCATTATAACACAAATA

CCAATACATGTAGACCCATATGTTGTTGTTATATTAATATCTGCGCCATTATCTATTAACCATTCTATTA

GGTCAACACTATGCGACTCGATACAATAATAAAGTATACTACGTCCATGTTTATCTATTTTGTTTATATC

ATCGATATACGGCTTACAAATTTCTAGTATCGATAACACTTCTGACTCGTGAATAAATAAGGTAGGGAAT

AACGGCATAATATTTATTATGTTATCATCATTAACAACTACGTTTCCATTTTTTAAAATATACTCTACAA

CTTTAGGATCCCTATTGTCAAATCTTTTAAAATATTTATTTATATGCTTAAATCTATATAATATAGCTCC

TTCCCTAATCATACATTTGATAACATTGATGTACACTGTATGATAAGATACATATTCTGACAATAGATCT

TGTATAGAATCTGTATATCTTTTAAGAATTGAGGATATTATGACATTATTACGTAAACTATTACACAATT

CTAAAATATAAAATGTATCATGGGCAGATAATAGTTTATCCACTATATAATTATCTATTTTATGATTTTT

CTTCCTATATTGTTTACGTAAATAGATAGATAGAATATGCATTAGTTCATTACCGCTATAGTTACTATCG

AATAACACGTCAAATATTTCCCGTTTAATATCGCATTTGTCAACATAATAATAGAGTATGGTACGTTCAC

GATAAGTATAATGACACATCTCGTTTTCGTGCGAAATTAAATAGTTTATCACGTCCAAAGATGTCACATA

ACCATCTTGTGACCTAGTAATAATATAATAATAGAGAACTGTTTTACCCATTCTATTATCATAATCAGTG

GTGTAGTCATAATCTAAATAATCAAACTCGTCATCCCAATTAAAATAAATATAATCAGTACATTGAATGG

GTATGATATTGTACCCATACTGTATGTTGCTACATGTAGGTATTCCTTTATCCAATAATAGTTTAAATAC

ATCTATATTAGGATTTGATGTTGTCGCGTATTTCTCTACAATATTAATACCATTTTTGATACTATTTATT

TCTATACCTTTCGAAATTAGTAATTTCAATAAGTCTATATCGATGTTATCAGAACATAGATATTCAAATA

TATCAAAATCATTGATATTTTTATAGTCGACTGACGACAATAACAAAATCACAACATCGTTTTTGATATT

ATTATTTTTTTTGGTAACGTATGCCTTTAATGGAGTTTCACCATCATACTCATATAATGGATTTGCACCA

CTTTCTATTAATGATTGTGCACTACTGGCATCGATGTTAAATGTTTTACAACTATCATAGAGTATCTTAT

CGTTAACCATGATTGGTTGTTGATGTTATCACATTTTTTGGTTTCTTTCATTTCAGTTATGTATGGATTT

AGCACGTTTGGGAAGCATGAGCTCATATGATTTCAGTACTGTAGTGTCAGTACTATTAGTTTCGATCAGA

TCAATGTCTAGATCTATAGAATCAAAACACAATAGGTCAGAAGATAATGAATATCTGTACGCTTCTTCTT

GTACTGTAACTTCTGGTTTTGTTAGATGGTTGCATCGTGCTTTAACGTCAATGGTACAAATTTTATCCTC

GCTTTGTGTATATCATATTCGTCTCTAGTATAAAATTCTATATTCAAATTATCATGCGATGTGTGTACGC

TAACGGTATCAATAAACGGAGCACAGCATTTAGTCAACAGTAATCCAAAATTTTTTAAAGTATATCTTAA

CGAAAGAAGTTGTCATCGTTAGAGTGTGGTAAATCATTGTCTACGGTACTAGATCCTCATAAGTGTATAT

ATCTAGAGTAATATTTAATTTATCAAATGGTTGATAATATGGATGTTGTGGCAATTTCCTAATACGGAAA

TAAGACATAAACACGCAATAAATCTAATTGCGGACATGTTACACTCCTTAAAAAATACGAATAAACACTT

TGGCTTTTAGTAAGTGTCATTTAACACTATACTCATATTAATCCATGGACTCATAATCTCTATACGGGAT

TAACGGATGTTCTATATACGGGGATGAGTAGTTCTCTTCTTTAACTTTATACTTTTTACTAATCATATTT

AGACTGATGTATGGGTAATAGTGTTTGAAGAGCTCGTTCTCATCATCAGAATAAATCAATATCTCTGTTT

TTTTGTTATACAGATGTATTACAGCCTCATATATTACGTAATAGAACGTGTAATCTACCTTATTAACTTT

CACCGCATAGTTGTTTGCAAATACGGTTAATCCTTTGACCTCGTCGATTTCCGACCAATCTGGGCGTATA

ATGAATCTTAACTTTAATTTCTTGTAATCATTCGAAATAATTTTTAGTTTGCATCCGTAGTTATCTCCTC

TATGTAACTGTAAATTTCTCAACGCGATATCTCCATTAATAATGATGTCGAATTCGTGTTGTATACCCAT

ACTGAATTGATGAACTAATACCGACGGTATTAATAGTAATTTACTTTTCATCTTTACATACTTGGTAATA

GTTTTACTATCATAAGTTTATAAATTCCACAAGCTACTATGGAATATACCAACCATCTTAGTATAGAACA

CATGTCTTAAAGTTATTAATTAATTACATGTTGTTTTATATATCGCTACGAATTTAAACAGAGGAATCAG

TTAGGAAGAAAAAATTATCTGTCATCATCATCATCTATTGGATAACATCTCTGTATTCTACGATAGAGTG

CTATTTTAAGATGTGACAGATCCGTGTCATCAAATATATACTCCATTAAAATGATTATTCCGGCAGCGAA

CTTGATATTGGATACATCACGACCTTTGTTAATATCCACGACAATAGACAGCAATCCCATTGTTCCATAA

ACAGTGAGTTTATCTTTCTTTGAAGTGATATTTTGTAGAGATCTTATAAAACTGTCGAATGACATCGTAT

TTATATCTTTAGCTAAATCATATATGTTACCATCATAATATCTAACAGCATCTATCTTAAACGTTTCCAT

CGCTGTAAAGACGTTTCCGATAGATGGTCTCGTTTCATCAGTCATACTGAGCCAACAAATGTAATCGTGT

ATAACATCTTTGATAGAATCAGACTCTAAAGAAAAGGAATCGGCTTTATTATACACATTCATGATAAACT

TAATGAAAAATGTTTTTCGTTGTTTAAGTTGGATGAATAGTATGTCTTAATAATTGTTATTATTTCACTA

ATTAATATTTAGTAACGAGTACACTCTATAAAAACGAGAATGACATAACTAATCATAACTAGTTATCAAA

GAATGTCTAGGACGCGTAATTTTTTATGGTATAGATCCTGTAAGCGTTGTCTGTATTCTGGAGCTATTTT

CTCTATCGCATTAGTGAGTTCAGAATATGTTATAAATTTAAATCGAATAACGAACATAACTTTAGTAAAG

TCGTCTATATTAACTCTTTTATTTTCTAGCCATCGTAATACCATGTTTAAGATAGTATATTCTCTAGTTA

CTACGATCTCATCGTTGTCTAGAATATCGCATACTGAATCTACATCCAATTTTAGAAATTGGTCTGTGCT

ACATATCTCTTCTATATTATTGTTGATGTATTGTCGTAGAAAACTATTACGTAGACCATTTTCTTTATAA

AACGAATATATAGTACTCCAATTATCTTTACCGATATATTTGCATACATAATCCATTCTCTCAATCACTA

CATCTTTAAGAGTTTGGTTGTTAAGATATTTGGCTAAACTATATAATTCTATTAGATCATCAACAGAATC

AGTATATATTTTTCTAGATCCAAAGATGAACTCTTTGGCATCCTCTATAATATTATCAGAAAAGATATTT

TCGTGTTTTAGTTTATCAAGATTTAACCTGTTCATATCCATGATTAACGACGTCATATAACCACATAAAA

TAAAAATCCATTTTCATTTTTAGCACAATACTATTCATAATTGATATTGATGTAATATTTTGTTACTTTG

AACGTAAAGACAGTACACGGGTCCGTATCTCCAACAAGCACGTAGTAATCAAATTTGGTGTTGTTAAACT

TCGCAATATTCATCAATTTAGATAGAAACATATACTCATCATCTGTTTTAGGAATCCATGTATTATTACC

ACTTTCCAACTTATCATTATCCCAGGCTATGTTTCGCCCATCATCGTTGCACAGAGTGAATAATTCTTTT

GTATTCGGTAGTTCAAATATATGATCCATGCATATATCGACAAAGCTATTGTAGATGTGATTTTTCCTAA

ATCTAATATAAAACTCGTTTACTAGCAAACATTTTCCTGATTTATCGACCAAGACACACATGGTTTCTAA

ATCTATCAAGTGGTGGGGATCCATAGTTATAACGCAGTAACATAAATTATTACCTTCTTGACTGTCGCTA

ATATCTATATACTTATTGTTATCGTATTGGATTCTACATATAGATGGCTTGTATATCAAAGATATAGAAC

ACATAACCAATTTATATTCTCGCTTTGTATTTTCGAATCTAAAGTTAAGAGATTTAGAAAACATTATATC

ATCGGATGATGATATCACTGTTTCCAGAGTAGGATATATTAAAGTCTTTAAAGATTTTGTCCGATTCAAA

TAAATCACTAAATAATATCCCATATTATCATCTGTTATAGTCGTGTCATTAAATCTATTATATTTTATGA

AAGATATATCACTGCTCACCTCTATATTTCGTACATTTTTAAACTGTTTGTATAATATCTCTCTAATACA

ATCAGATATATCTATTGTGTCGGTAGACGATACCGTTACATTTGAATTAATGGTGTTCCATTTTACAACT

TTTAACAAGTTGACCAATTCATTTCTAATAGTATCAAACTCTCCATGATTAAATATTTTAATAGTATCCA

TTTTATATCACTACGGACATAAACCATTGTATATTTTTTATGTTTATTAGTGTACACATTTTGGAAGTAA

GTTCCTGGATCGGATGTCACCGCAGTAATATTGTTGATTATTTCTGACATCGACGTATTATATAGTTTTT

TAATTCCATATCTTTTAGAAAAGTTAAACATCCTTATACAATTTGTGGAATTAATATTATGAATCATGGT

TTTTACACATAGATCTATTACAGGCGGAACATCAATTATTATGGCAGCAACTAGTATCATTTCTACATTG

TTTATGGTGATGTTTATCTTCTTCCAGCGCATATAGTCTAATATCGATTCAAACGCGTGATAGTTTATAC

CATTCAATATAATCGCTTCATCCTTTAGATGGTGATCCTGAATGTGTTTAAAAAATTATACGGAGACGCC

GTAATAATTTCCCCATTGATAGAAAATATCACGCTTTCCATTTTCTTGAAGTACTAAAAGTAATTATAAT

ATAATGTAAAGGTTTATATATTCAATATTTTTTATAAAAAAAATCATTTTGACATTAATTCCTTTTTAAA

TTTCAGTCTATCATCTATAGAAACATATTCTATGAATTTATAAAATGCTTTTACGTATCCTATCGTAGGT

GATAGAACCGCTAAAAAACCTATCGAATTTCTACAAAAGAATCTGTTATATGGTATAGGGAGAGTATAAA

ACATTAAATGTCCGTACTTATTAAAGTATTCAGTAGCCAATCCTAACTCTTTCGAATAATTATTAATGGC

TCTTATTCTGTACGAATCTATTTTTTTGAACAATGGACCTAGTGGTATATCTTGTTCTATGTATCTAAAA

TAATGTCTGACTAGATCCGTTAGTTTAATATCCGCAGTCATCTTGTCTAGAATGGCAAATCTAACTGCGG

GTTTAGGCGTAGGCGTTAGTTTAGTTTCTATATCTACATCTATGTCTTTATCTAACACCAAAAATATAAT

AGCTAATATTTTATTACAATCATCCGGATATTCTTCTACGATCTCACTAACTAATGTTTCTTTGGTTATA

CTAGTATAGTCACGATCAGACAAATAAAGAAAATCAGATGATCGATGAATAATACATTTAAATTCATCAT

CTGTAAGATTTTTGAGATGTCTCATTAAAATATTATTAGTGTCAGTTCTCATTATCATATATTGACAGCA

GCTATTACACTTATTTTATTTTTCTGTATTTTATTACTTTTCACCATATAGATCAGTCATTAGATCATCA

AAATACTTTTCAATCATCCTAAAGAGTATGGTGAACGAATCTTCCCATCTAATTTCTGAACGTCTACCAA

TGTCTCTAGCCACTTTGGCACTAATAGCGATCATTCGCTTAACATCTTCTACATTATTAACTGGTTGATT

CAATCTATCTAGCAATGGACCGTCGGATAGCGTCATTCTCATGTTCTTAATCAATGTACATACATCGTCA

TCATCTACCAATTCATCAAACAATATAAGCTTTTTAAAATCATCATTATAATAGGATGGATCGCCGTCAT

TTCTCCAAAGAATATATCTAATAAGTAGAGTCCTCATGCTTAGTAATTTAACTATTTTAGTTAACAACTA

TTTTTTATGTTAAATCAATTAGTAACACCGCTATGTTTAATACTTATTCATATTTTAGTTTTAGGATCGA

GAATCAATACAAAAATTAATACATCAATTTTGGAAATACTTAGTTTCCACGTAGTCAATGAAACATTTGA

GCTCATCGTAAAGGACGTTCTCGTACAAGACGTAACTATAAATTGGTTTATATTTGTTCAAGATAGATAC

AAATCCGATAACTTTTTTGACGAATTCTACGGGATTCACTTTAAAAGTGTCATACCGGGTTCTTTTTATT

CTTTTAAACAGATCGATTGTGTGATGTTGATTAGGTCTTTTACGAATTTGATACAGAATAGCGTTTACAT

ATCCACCATAGTAATCAATAGCCATTTGTTCGTATGTCATAAATTCTTTAATTATATGACACTGTGTATT

ATTTAGTTCGTCCTTGTTCATCATTAGGAATCTATCCAATATGGCAATTATATTAGAACTATAACTGCGT

TGTATGCGCATGTTGATGTGTCTGTTTATACAATCAATTATACTAGGATCCATACCACTACAATCGGGTA

AAATTGTAGCATCATATACCATTTCTAGTACTTTAGATTCATTGTTATCCATTGCAGAGGACGTCATGAT

CGAATCCAAAAAAATATATTATTTTTATGTTATTTTGTTAAAAATAATCATCAAATACTTCGTAAGATAC

TCCTTCATGAACATAATCAGTTACAAAACGTTTATATGAAGTAAAGTATCTACAATTTTTACAAAAGTCA

GGATGCATAAGTACAAAGTACGCGATAAACGGAATAATAATAGATTTATCTAGTTTATCTTTTTCTATCT

CTTTCATAGTTATATACATGGTCTCAGAAGTCGGATTATGTAACATCAGCTTCGATAAAATGACTGGGTT

ATTTAGTCTTACACATTCGCTCATACATGTATGACCGTTAACTATAGAGTCTACACTAAAATGATTGAAT

AATAGATAGTCTACCATTGTTTCGTATTCAGATAGTACAGCGTAGTACATGGCATCTTCACAAATTATAT

CATTATCTAATAGATATTTGACGCATCTTATGGATCCCACTTCAACAGCCATCTTAAAATCGGTAGAATC

ATATTGCTTTCCTTTATCGTTAATAATTTCTAGAACATCATCTCTATCATAAAAGATACAAATATTAACT

GTTTGATCAGTAATAACATTGCTAGTCGATATCAATTTGTTAATAAGATGCGCTGGGCTCAATGTCTTAA

TAAGAAGTGTAAGAGGACTATCTCCAAATTTGTTTTGTTTATTAACATCCGTTGATGGAAGTAAAAGATT

TATAATGTCTACATACTTGACTGTTTTAGAGCATACAATATGGAGAGGCGTATTTCCATCATGATCTGGT

TTTGAGGGACTAATTCCTAGTTTCATCATCCATGAGATTGTAGAAGCTTTTGGATTGTCTGACATAAGAT

GTCTATGAATATGATTTTTGCCAAATTTATCCACTATCCTGGCTTCGAATCCGATAGACATTATTTTTTT

AAACACTCTTTCTGAAGGATCTGTACACGCCAACAACGGACCACATCCTTCTTCATCAACCGAGTTGTTA

ATCTTGGCTCCATACTGTACCAATAAATTTATTCTCTCTATGACTTCATCATCTGTTCCCGAGAGATAAT

ATAGAGGTGTTTTATTATGTTTATCACATGCGTTTGGATCTGCGCCGTGCACCAGCAGCATCGCGACTAT

TCTATTATTATTAATTTTAGAAGCTATATGCAATGGATAATTTCCATCATCATCCGTCTCATTTGGAGAG

TATCCTCTATGAAGAAGTTCTTCTATAAATCGTTCATCTAGTCCTTTAATGCCACAATACGCATGTAGAA

TGTGATAATTTCCAGAGGGTTCGATAACTTGTAGCATATTCCTAAATACATCTAAATTTTTACTATTATA

TTTGGCATAAAGAGATAGATAATACTCGACCGACATAATGTTGTGTTGTCCATTATAGTATAAAAATTAA

TATTTCTATTTCTATATATTTGCAACAATTTACTCTCTATAACAAATATCATAACTTAGTTCTTTTATGT

CAAGAAGGCACTGGTTTAATTCATCTATAAATGTCACTCCATAACTACCACGCATACTATACTCAGAATT

ATGATAAAGATATTTATTCTTGGGGTGTAAGTAATGGGGATTAATCTTTGTTGGATCAGTCTCTAAGTTA

ACACATGTCACACATGATCCATTTATAGTTATATCACACGATGATGATTTATGAATTGATTCCGGAAGAT

CGCTATTGTATTTTGTAGTTCCACAATTCATTTCCATACATGTTATTGTCACACTAATATTATGATGAAC

TTTATCTAGCCGCTGAGTGGTAAACAACAGAACAGATAGTTTATTATCTTTACCAACACCCTCAGCCGCT

GCCACAAATCTCTGATCCGTATCCATGATGGTCATGTTTACTTTTAGTCCGTATCCAGTCAACACTATGT

TAGCATTTCTGTCGATATAGCTTTCACTCATATGACACTCACCAATAATTGTAGAATTAATGTCGTAATT

TACACCAATAGTGAGTTCGGCGACAAAGTACCAGTACCGGTAATCTTGTCGATGAGGACATATAGTATTC

TTGTATTCTACCGAATACCCGAGAGATGCGATACAAAAGAGTAAGACTAATTTGTAAACCATCTTACTCA

AAAATATGCGACAATAGGAAATCTATCTTATACACATAATTATTCTATCAATTTTACCAATTAGTTAGTG

TAATGTTAACAAAAATGTGGGATAATTTAATAGTTTTTCCTTACACAATTGACATACATGAGTCTGAGTT

CCTCGTTTTTGCTAATTATTTCGTCCAATTTATTATTCTTGACATCGTCAAGATCTTTTGTATAGGAGTC

AGACTTGTATTCAACATGTTTTTCTATAATCATCTTAGCTATTTCGGCATCATCCAATAGTACATTTTCC

AGATTAACAGAATAGATATTAATGTCGTATTTGAACAGAGCCTGTAACATCTCAATGTCTTTATTATCTA

TAGCCAATTTGATGTCCGGAATGAAGAGAAGGGAATTGGTGTTTGTTGACGTCATATAGTCGAGCAAGAG

AATCATCATATCCACGTGTCCATTTTTTATAGTGGTGTGAATACAACTAAGGAGAATAGCCAGATCAAAA

GGAGATGGTATCTCTGAAAGAAAGTAGGAAACAATACTTACATCATTAAGCATGACAGCATGATAAAATG

AAGTTTTCCATCCAGTTTTCCCATAGAACATCAGTCTCCAATTTTTCTTAACAAACAGTTTTACCGTTTG

CATGTTACCACTATCAACCGCATAATACAATGCGGTGTTTCCTTTGTCATCAAATTGTGAATCATCCATT

CCACTGAATAGCAAAATCTTTACTATTTTGGTATCTTCTAATGTGGCTGCCTGATGTAATGGAAATTCAT

TATCTAGAAGATTTTTCAATGCTCCAGCGTTCAACAACGTACATACTAGACGCACGTTATTATCAGCTAT

TGCATAATACAAGACACTATGACCATTGATATCCGCCTTAAATGCATCTTTGCTAGAGAGAAAGCTTTTC

AGTTGCTTAGACTTCCAAGTATTAATTCGTGACAGATCCATGTCTGAAACGAGACGCTAATTAGTGTATA

TTTTTTCATTTTTTATAATTTTGTCATATTGTACCAGAATTAATAATATCTCTAATAGATCTGATTAGTA

GATATATGGCTATCGCAAAACAACATATACACATTTAATAAAAATAATATTCATTAAGAAGATTCAGATT

CCACTGTACCCATCAATATAAAATAAAATAATTATTCCTTACATCGTACCCATAAACAATATATTAAGTA

TATTCCACCTTACCCATAAACAATATAAATCCAGTAATATCATGTCTAATGATGAACACAAATGGTGTAT

TAAATTCCAGTTCTTCAGGAGATGATCTCGCCGTAGCTACCATGATAGTAGATGCCTCCGCTACAGTTCC

TTGTTCGTCTACATCTATCTTTACATTCTGAAACATTTTATAAATATATAATGGGTCCCTAGTCATATGT

TTAAACGACGCCTTATCTGGATTAAACATACTAGGAGCCATCATTTCGGCTATCGACTTAATATCCCTCT

TGTTTTCGATAGAAAATCTAGGGAGTTTAAGATTGTACATTTTATTCCCTAATTGAGATGACCAATATTC

TAATTTTGCAGGCGTGATAGAATCTGTGAAATGGGTCATATTATCACCTATTGCCAGGTACATACTAATA

TTAGCATCCTTATACAGAAGGCGCACCATATCATATTCTTCGTCATCGATTGTGATTGTATTTCCTTGCA

ATTTAGTAACTACGTTCATCATGGGAACCGTTTTCGTACCGTACTTATTAGTAAAACTAGCATTGTGTGT

TTTAGTGATATCAAACGGATATTGCCACGTACCTTTAAAATATATAGTATTAATGATTGCCCATAGAGTA

TTATCGTCGAGCATAGTAGAATCAACTACATTAGACATACCAGATCTACGTTCTACTATAGAATTAATTT

TATTAACCGCATCTCGTCTAAAGTTTAATCTATATAGGCCGAATCTATGATATTGTTGATAATACGACGG

TTTAATACACACAGTACTATCGACGAAACTTTGATACGTTAGATCTGTGTACGTATATTTAGATGTTTTC

ATCTTAGCTAATCCGGATATTAATTCTGTAAATGCTGGACCCAGATCTCTTTTTCTCAAATTCATAGTAT

TCAATAATTCTACTCTAGTATTACCTGATGCAGACAATAGCGACATAAACATAGAAAACGAATACCCAAA

CGGTGAGAAGACAATATTATCATTATCATCCTCATCCCCATTTTGAATATTTTTATACGCTAATATACCA

GCATTGATAAATCCCTGCAGACGATATGCGGATACTGAACACGCTAATGATAGTATCAATAACGCAATCA

TGATTTTTATGGTATTAATAATTAACCTTATTTTTATGTTTGGTATAAAAATTATTGATGTCTACACATC

CTTTATAATCAACTCTAATCACTTTAACTTTTACAGTTTTCCCTACAAGTTTATCCCTATATTCAACATA

TCTATCCATATGCATCTCTTAACACTCTGCCAAGATAGCTTCAAAGTGAGGATAGTCAAAAAGATAAATA

TATAGAGTATAATCATTCTCGTATACTCTGCCCTTTATTACATCGCCCGCATTGGGCAACGAATAACAAA

ATGCAAGCATCGTGTTAACGGGCTCGTAAATTGGGATAAAATTATGTTTTTATTGTTTATCTATTTTATT

CAAGAGAATATTCAGGAAGTTCCTTTTCTGGTTGTATCTCGTCGCAGTATATATCATTTGTACATTGTTT

CATATTTTTTAATAGTCTACACCTTTTAGTAGGACTAGTATCGTACAATTCATAGCTGTATTTTGAATTC

CAATCACGTATAAAAATATCTTCCAATTGTTGACGAAGACCTAATCCATCATCCGGTGTAATATTAATAG

ATGCTCCACATATATCCGTAAAGTAATTTCCTGTCCAATTTGATGTACCTATATACGCCGTTTTATCGGT

TACCATATATTTTGCATGGTTTACCCTAGAATACGGAATGGGAGGATCAGCATCTGGTACAATAAATAGC

TTTACTTCTATATCTATGTTTTTAGATTTTAGCATAGCTATAGATCTTAAAAAGTTTCTCATGATAAACG

AAGATCGTTGCCAGCAACTAATCAATAGCTTAACGGATACTTGTCTGTCTATAGCGGATCTTCTTAATTC

ATCTTCTATATAAGGCCAAAACAAAATTTTACCCGCCTTTGAATAAATAATAGGAATAAAGTTCATAACA

GATACATAAACGAATTTACTCGCATTTCCGATACATGACAATAAAGCGGTTAAATCATTGGTTCTTTCCA

TAGTACATAATTGTTGTGGTGCAGAAGCAATAAATACAGAGTGTGGAACACCGCTTACGTTAATACTAAG

AGGATGATCTGTATTATAATACGACGGATAAAAGTTTTTCCAATTATATGGTAGATTGTTAACTCCAAGA

TACCAGTATACCTCAAAAATTTGAGTGAGATCCGCTGCCAAGTTCCTATTATTGAAGATCGCAATACCCA

ATTCCTTGACCTGAGTTAGTGATCTCCAATCCATGTTAGCGCTTCCTAAATAAATATGTGTATTATCAGA

TATCCAAAATTTTGTATGAAGAACTCCTCCTAGGATATTTGTAATATCTATGTATCGTACTTCAACTCCG

GCCATTTGTAGTCTTTCAACATCCTTTAATGGTTTGTTGGATTTATTGACGGCTACTCTAACTCTTACTC

CTCTTTTGGGTAATTGTACAATCTCGTTTAATATTACCGTGCCGAAATTCGTACCCACTTCATCCGATAA

ACTCCAATAAAAAGATGATATATCTAGTGTTTTTATGGTATTGGATAGAATTTCCCTCCACATGTTAAAT

GTAGTCAAATATACTTTATCAAATTGCATACCTATAGGAATAGTCTCTGTAATCACTGCGATTGTATTAT

CCGGATTCATTTTATTTGTTAAAAAAATAATCCTATATCACTTCACTCTATTAAAAATCCAAGTTTCTAT

TTCTTTCATGACTGATTTTTTAACTTCATCCGTTTCCTTATGAAGATGATGTTTGGCGCCTTCATAAATT

TTTATTTCCCTATTACAATTTGCATGTTGCATGAAATAATATGCACCTGAAACATCGCTAATCTCATTGT

TTGTTCCCTGGAGTATGAGAGTCGGGGTGTTAATCTTGGGAATTATTTTTCTAACCTTGTTGGTAGCCTT

CAAGACCTGACTAGCAAATCCAGCCTTAATTTTTTCATGATTGACTAATGGATCGTATTGGTATTTATAA

ACTTCATCCATATCTCTAGATACTGATTCTGGACATAGCTTTCCGACTGACGCATTTGGTGTAATGGTTC

CCATAAGTTTTGCAGCTAGCAGATTCAGTCTTGGAACAGCGTCTGCATTAACTAGAGGAGACATTAGAAT

CATTGCTGTAAACAAGTTTGGATTATCGCAAGCAGCTAGTATAGAAATTGTTGCTCCCATGGAATGACCC

AATAAGAAGACTGGAACTCCTGGATAAGTAGATTTAATAGTCACCACGTGCTGTACCACATCTCTAACAT

ACTTACCAAAGTCATCAATCATCATTTTTTCACCATTACTTCTTCCATGGCCAATATGATCATGTGAGAA

TACTAAAATTCCTAACGATGATATGTTTTCAGCTAGTTCGTCATAACGTCCAGAATGTTCACCAGCTCCA

TGACTTATGAATACTAATGCCTTAGGATATGTAATAGGTTTCCAATATTTACAATATATGTAATCATTGT

CCAGATTGAACATACAGTTTGTACTCATGATTCACTATATAACTATCAATATTAACAGTTCGTTTAATGA

TCATATTATTTTTATGTTTTATTGATAATTGTAAAAATATACAATTAAATCAATATAGAGGAAGGAGACG

GTACTGTATTTTGTGAGATAGTAATGGAGACTAAATCAGATTATGAGGATGCTGTTTTTTACTTTGTGGA

TGATGATGAAATATGTAGTCGCGACTCCATCATTGATCTAATAGATGAATATATCACGTGGAGAAATCAT

GTTATAGTGTTTAATAAAGATATTACCAGTTGTGGAAGACTGTACAAGGAATTGATAAAGTTCGATGATG

TCGCTATACGGTACTATGGTATTGATAAAATTAATGAGATTGTCGAGGCTATGAGTGAAGGAGACCACTA

CATCAATCTTACAGAAGTCCATGATCAGGAAAGTCTATTCGCTACCATAGGAATATGTGCTAAAATCACT

GAACATTGGGGATACAAAAAGATTTCAGAATCTAAATTCCAATCATTGGGAAACATTACAGATCTGATGA

CCGACGATAATATAAACATCTTGATACTTTTTCTAGAAAAAAAAATGAATTGATGATATAAGTGTCTTCA

TAACGCATTATTACGTTAGCATTCTATTATCCAGTGTTAAAAAAATTATCCTATCATGTATTTGAGAGTC

TTATATGTAGCAAACATGATAACTGCAATACCCATAATCTTTAGATATTCACGCGTGCTATGGATGGCAT

TATCCCGCGGTGCGGAAATGTACGTTATATAATCTACAAAATAATCATCGCATATAGTATGAGATAGTAG

AGTAAACATTTTTATCGTTTCTACTGGGTTCATACATCGTCTACCCAATTCGGTAATGAATGAAATTGTC

GCCAATCTTACACCCAAACCCTTGTTGTTCATTAGTATAGTATTAACTTCATTATTTATGTCATAAACTG

TAAATGATTCTGTAGATGCCATATCACACATGATATTCATGTCACTATTATAATCATTATTAACTTTATC

ACAATACGTGTTGATAATATCTACATATGATCTAGTTTTTGTGGGTAATTGTCTATACAAGTCGTCTAAA

CGTTGTTTACTCATATAGTATTGAACAGCCATCATTACATGGTCCCGTTCCGTTGATAGATAATCGAGTA

TGTTAGTAGACTTGTCAAATCTATATACCATATTTTCTGGAAGCGGATATACATAGTCGCGATCATCATT

ATCACTAGCCTCATCCTCTATATCATGTACATGTACATAATCTATGATATTATTATACATAAACATCGAC

AACATACTATTGTCTATTATCTAAGTCCTGTTGATCCAAACCCTTGATCTCCTCTATCTGTACTATCTAG

AGATTGTACTTCTTCAAGTTCTGGATAATATATACGTTGATAGATTAGCTGAGCTATTCTATCTCCAGTA

TTTACATTAAACGTACATTTTCCATTATTAATAAGAATGACTCCTATGCTTCCCCTATAATCTTCGTCTA

TTACACCGCCTCCTATATCAATGCCTTTTAGGGACAGACCAGACCTAGGAGCTATTCTACCATAGCAGAA

CTTAGGCATGGACATACTAATATCTGTCTTAATTAACTGTCGTTCTCCAGGAGGGATAGTATAATCGTAA

GCGCTATACAAATCATATCCGGCAGCACCCGGCGATTGCCTAGTAGGTGATTTAGCTCTGTTAGTTTCCT

TAACAAATCTAACTGGTGAGTTAATATTCATGTTGAACATAAAAAATATCATTTTATTTCAAAATTATTT

ACCATTCCATTCCATCCCATATATTCCATGAATAAGTGCGATTATTGTACACTTCTATAGTATCTATATA

CGATCCACGATAAAATCCTCCTATCAATAGCAGTTTATTATCCACTATGATCAATTCTGGATTATCCCTC

GGATAAATAGGATCATCTATCAGAGTCCATGTATTGCTGGATTCACAATAAAATTCCGCATTTCTACCAA

CCAAGAATAACCTTCTACCAAACACTAACGCACATGATTTATAATGAGGATAATAAGTGGATGGTCCAAA

CTGCCACTGATCATGATTGGGTAGCAAATATTCTGTAGTTGTATCAGTTTCAGAATGTCCTCCCATTACG

TATATAACATTGTTTATGGATGCCACTGCTGGATTACATCTAGGTTTCAGAAGACTCGGCATATTAACCC

AAGCAGCATCCCCGTGGAACCAACGCTCAACAGATGTGGGATTTGGTAGACCTCCTACTACGTATAATTT

ATTGTTAGCGGGTATCCCGCTAGCATACAGTCTGGGGCTATTCATCGGAGGAATTGGAATCCAATTGTTT

GATATATAATTTACCGCTATAGCATTGTTATGTATTTCATTGTTCATCCATCCACCGATAAGATATACTA

CTTCTCCAACATGAGTACTTGTACACATATGGAATATATCTATAATTTGATCCATGTTCATAGGATACTC

TATGAATGGATACTTGTATGATTTGCGTGGTTGTTTATCACAATGAAATATTATGTTACAGTCTAGTATC

CATTTTACATTATGTATACCTCTGGGAGAAAGATAATTTGACCTGATTACATTTTTGATAAGAAGTAGCA

GATTTCCTAATCTATTTCTTCGCCTCATATACCACTTAATGACAAAATCAACTACATAATCCTCATCTGG

AACATTTAGTTCGTCGCTTTCTAGAATAAGTTTCATAGATAGATAATCAAAATTGTCTATGATGTCATCT

TCCAGTTCCAAAAAGTGTTTGGTAATAAAGTCTTTAGTATGACATAAGAGATTGGATAGTCCGTATTCTA

TACCCATCATGTAACACTCGATACAATATTCCTTTCTAAAATCTCGTAGGATAAAGTTTATACAAGTGTA

GATGATAAATTCTACAGATGTTAATATAGAAGCACGTAATAAATTGACGACGTTATGACTATCTATATAT

ACCTTTCCAGTATATGAGTAAATAACTATAGAAGTTAGACTGTGAATGTCAAGGTCTAGACAAACCCTCG

TAACTGGATCTTTATTTTTTGTGTATTTTTGGCGTAAATGTGTGCAAAAGTATGGAGATAACTTTTTCAA

TATCGTAGAATTGACTATTATATTACCTCCTATAGCTTCAATAATTGTTTTGAATTTCTTAGTCGTGTAC

AATGCTAATATATTCTTACAGTACACAGTATTGACAAATATCGGCATTTATGTTTCTTTAAAAGTCAACA

TCTAAAGAAAAATGATTGTCTTCTTGAGACATAACTCCCATTTTTTGGTATTCACCCACACGTTTTTCGA

AAAAATTAGTTTTTCCTTCCAATGATATATTTTCCATGAAATCAAACGGATTGGTAACATTGTAAATTTT

TTTAAATCCCAATTCAGAAATCAATCTATCTGCGACGAATTCTATATATGTTTTCATCATTTCACAATTC

ATTCCTATGAGTTTAACTGGAAGAGCCACAGTAAGAAATTCTTGTTCAATGGATACCGCATTTGTTATAA

TAAATCTAACGGTTTCTTCACTCGGTGGATGCAATAAATGTTTAAACATCAAACATGCGAAATCGCAGTG

CAGACCCTCGTCTCTACTAATTAATTCGTTAGAAAACGTGAGTCCGGGCATTAGGCCACGCTTTTTAAGC

CAAAATATGGAAGCGAATGATCCGGAAAAGAAGATTCCTTCTACTGCAGCAAAGGCAATAAGTCTCTCTC

CATAACCGGCGCTGTCATGTATCCACTTTTGAGCCCAATCGGCCTTCTTTTTTACACAAGGCATCGTTTC

TATGGCATTAAAGAGGTAGTTTTTTTCATTACTATCTTTAACATAAGTATCGATCAAAAGACTATACATT

TCCGAATGAATGTTTTCAATGGCCATCTGAAATCCGTAGAAACATCTAGCCTCGGTAATCTGCACTTCTG

TACAAAATCGTTCTGCTAAATTTTCATTCACTATTCCGTCACTGGCTGCAAAAAACGCCAATACATGTTT

TATAAAATATTTTTCGTCTGGTGTTAGTTTATTCCAGTCATTGATATCTTTAGATATATCCACTTCTTCC

ACTGTCCAAAATGATGCCTCTGCCTTTTTATACATATTCCAGATGTCATGATATTGGATTGGGAAAATAA

CAAATCTATTTGGATTTGGTGCAAGGATAGGTTCCATAACTAAATTAACAATAGTAGTAATTTTTTTTCA

GTTATCTGTATGACGACTGTACTTGGATCTTTTGTATATCGCTATCGCCGCAATCACTACAATAATTACA

AGTATTATTGATAGCATTGTTATTACTACTATCATAATTAAATTATCGACATTCATGGGTGTTGAATAAT

CGTTATCATCATTTTGTAATTGTGACGTCATACTAGATAAATCATTTGTGAGATTGTTGTGGGAAGCGGG

CACGGAAGATGCATTATCATTATTATTTAACGCCTCCCATTCGGATTCACAAATGTTACGCACGTTCAAA

GTTTTATGGAAACTATAATTTTGTGAAAACAGATAACAAGAAAACTCGTCATCGTTCAAATTTTTAACGA

TAGTAAACCGATTAAACGTCGAGCTAATTTCTAACGCTAGCGACTCTGTTGGATATGGGTTTCCAGATAT

ATATCTTTTCAGTTCCCCTACGTATCTATAATCATCTGTAGGAAATGGAAGATATTTCCATTTATCTACT

GTTCCTAATATCATATGCGGTGGTGTAGAACCATTAAGCGCGAAAGATGTTATTTCGCATCGTATTTTAA

CTTCGCAATAATTTCTGGTTAGATAACGCACTCTACCAGTCAAGTCAATGATATTAGCCTTTACAGATAT

ATTCATAGTAGTCGTAACGATGACTCCATCTTTTAGATGTGATACTCCTTTGTATGTACCAGAATCTTCG

TACCTCAAACTCGATATATTTAAACAAGTTAATGATATATTAACGCGTTTTATGAATGATGATATATAAC

CAGAAGTTTTATCCTCTGTGGCTAGCGCTATAACCTTATCATTATAATACCAACTAGTGTAATTAATATG

TGACATGACAGTGTGGGTACAAATATGTACATTATCGTCTACGTCGTATTTGATACATCCGCATTCAGCC

AACAAATATAAAATTACAAAAACTCTAACGACGTTTGTACACATCTTGATGCGGTTTAATAAATGTTTTG

ATTTCAATTTATTGTAAAAAAAGATTCGGTTTTATACTGTTCGATATTCTCATTGCTTATATTCTCATCT

ATCATCTCCACACAGTCAAATCCATGGTTAACATGTACCTCATCAACCGGTAAAAGACTATCGGATTCTT

CTATCATCATAACTCGAGAATATTTAATTTGGTGGTCATTATTAATCAAGTCAATTATCTTATTTTTAAC

AAACGTAAGTATTTTACTCATTTTTTTATAAAAACTTTTAGAAATATACAGACTCTATCGTGTGTCTATA

TCTTCTTTTTATATCCAATGTATTTATGTCTGATTTTTCTTCATTTATCATATATAATGGTCCAAATTCT

ACACGTGCTTCGGATTCATCCAGATCATTAAGGTTCTTATAATCGCAACATCCTTCTCTTCCATCTTCTA

CATCTTCCTTCTTATTCTTAGCGTCACAGAATCTACCACAGCAGGATCCCATGACGAGAGTCACATTAAA

CTAATTCATTTTCAATTATAATATACTGATTAGTAATGACAATTAAAATAAAAATATTCTTCATAACCGG

TAAGAAAGTAAAAAGTTCACATTGAAACTATGTCAGTAGTTATACATCATGAGATGATATACTCTATTTT

GGTGGAGGATTATATGATATAATTCGTGGATAATCATTCTTAAGACACATTTCTTCATTCGTAAATCTTT

TCACATTAAATGAGTGTCCATATTTTGCAATTTCTTCATATGATGGCGGTGTACGTGGACGAGGCTGCTC

CTGTTCTTGTAGTCGTCGACTGTCGTGTTTGCGTTTAGATCCCTCCATTATCGCGATCGCGTAGTGAGTA

CTATTTATACCTTGTAATTAAATTTTTTTATTAATTAAACGTATAAAAACGTTCCGTATCTGTATTTAAG

AGCCAGATTTCGTCTAATAGAACAAATAGCTACAGTAAAAATAACTAGAATAATCGCTACACCCACTAGA

AACCACGGATCGTAATACGGCAATCGGTTTTCGATAATAGGTGGAACGTATATTTTATTTAAGGACTTAA

CAATTGTCTGTAAACCACAATTTGCTTCCGCCGATCCTGTATTAACTATCTGTAAAAGCATATGTTGACC

GGGAGGAGCCGAACATTCTCCGATATTCAATTTTTGTATATCTATAATGTTATTAACCTCCGCATACGCA

TTACAGTTCTTTTCTAGCTTGGATACTACACTAGGTACATCATCTAAATCTATTCCTATTTCCTCAGCGA

TAGCTCTTCTATCCTTTTCCGAAAGTAATGAAATCACTTCAATAAATGATTCAACCATGAGTGTGAAACT

AAGTCGAGAATTACTCATGCATTTGTTAGTTATTCGGAGCGCGCAATTTTTAAACTGTCCTATAACCTCT

CCTATATGAATAGCACAAGTGACATTAGTAGGGATAGAATGTTGAGCTAATTTTTGTAAATAACTATCTA

TAAAAAGATTATACAAAGTTTTAAACTCTTTAGTTTCTGCCATTTATCCAGTCTGAGAAAATGTCTCTCA

TAATAAATTTTTCCAAGAAACTAATTGGGTGAAGAATGGAAACCTTTAATCTATATTTATCACAGTCTGT

TTTGGTACACATGATGAATTCTTCTAATGCTGTACTAAATTCGATATCTTTTTCGATTTCTGGATATGTT

TTTAATAAAGTATGAACAAAGAAATGGAAATCGTAATACCAGTTATGTTCAACTTTGAAATTGTTTTTTA

TTTTCTTGTTAATGATTCCAGCCACTTGGGAAAAGTCAAAGTCGTTTAATGCCGATTTAATACGTTCATT

AAAAACAAACTTTTTATTCTTTAGATGAATTATTATTGGTTCATTGGAATCAAAAAGTAAGATATTATCG

GGTTTAAGATCTGCATGTAAAAAGTTGTCACAACAGGGTAGTTCGTAGATTTTAATGTATAACAGAGACA

TCTGTAAAAAGATAAACTTTATGTATTGTACCAAAGATTTAAATCCTAATTTGATAGCTAACTCGGTATC

TACTTTATCTGCCGAATACAGTGCTAGGGGAAAAATTATAATATTTCCTCTTTCGTATTCGTAATTAGTT

CTCTTTTCATGTTCGAAAAAGTGAAACATGCGGTTAAAATAGTTTATAACATTAATATTACTGTTAATAA

CTGCCGGATAAAAGTGGGATAGTAATTTCACGAATTTGATACTGTCCTTTCTCTCGTTAAACGCCTTTAG

AAAAACTTTAGAAGAATATCTCAATGAGAGTTCCTGACCATCCATAGTTTGTATCAATAATAGCAACATA

TGAAGAACCCGCTTATACAGAGTATGTAAAAATGTTAATTTATAGTTTAATCCCATGGCCCACGCACACA

CGATTAATTTTTTTTCATCTCCCTTTAGATTGTTGTATAGAAATTTGGGTACTGTAAACTCCGCCGTAGT

TTCCATGGGACTATATAATTTTGTGGCCTCGAATACAAATTTTACTACATAGTTATCTATCTTAAAGACT

ATACCATATCCTCCTGTAGATATGTGATAAAAATCGTCGTTTATAGGATAAAATCGTTTATCTTTTTGTT

GGAAAAAGGATGAATTAATGTAATCATTCTCTTCTATCTTTAGTAGTGTTTCCTTATTAAAATTCTTAAA

ATAATTTAACAATCTAACTGACGGAGCCCAATTTTGGTGTAAATCTAATTGGGACATTATGTTGTTAAAA

TATAAACAGTCTCCTAATATAACAGTATCTGATAATCTATGGGGAGACATCCATTGATATTCAGGGGATG

AATCATTGGCAACACCCATTTATTGTACAAAAAGCCCCAATTTACAAACGAAAGTCCAGGTTTGATAGAG

ATAAACTATTAACTATTTTGTCTCTGTTTTTAACACCTCCACAGTTTTTAATTTCTTTGGTAATGAAATT

ATTCACAATATCAGTATCTTCTTTATCTACCAGAGATTTTACTAACTTGATAACCTTGGCTGTCTCATTC

AATAGGGTAGTGATATTTGTATGTATGATATTGATATCTTTTTGAATTGTTTCTTTTAGAAGTGATTCTT

TGATGGTATCAGCATACGAATTACAATAATGCAGAAACTCAGTTAACATGCAGGAATTATAGTAAGCCAA

TTCCAATTGTTGCCTGTATTGTATTAGAGTATTAATATGCGCAATGATGTCCTTGCGTTTCTCTGATAGA

ATGCGAGCAGCGATTTTGGCGTTATCATTTGACGATATTTCTGGAATGACGAATCCTGTTTCTACTAACT

TCTTGGTAGGACAAAGTGAAACAATCAAGAAAATAGCTTCTCCTCCTATTTGTGGAAGAAATTGAACTCC

TCTAGATGATCTACTGACGATAGTATCTCCTTGACAGATATTGGACCGAACTACGGAAGTACCTGGAATG

TAAAGCCCTGAAACCCCCTCATTTTTTAAGCAGATTGTTGCCGTAAATCCTGCACTATGCCCAAGATAGA

GAGCTCCTTTGGTGAATCCATCACTATGTTTCAGTTTAACCAAGAAACAGTCAGCTGGTCTAAAATTTCC

ATCTCTATCTAATACAGAATCCAACTTGATGTCAGGGACTATGACCGGTTTAATGTTATATGTAACATTG

AGTAAATCCTTAAGTTCATAATCATCGTTGTCATCAGTTATGTACGATCCAAACAATGTTTCTACCGGCA

TGGTGGATACGAAGATGCTATCCATCAGAATGTTTCCCTGATTAGTATTTTCTATATAGCTATTCTTCTT

TAAACGATTTTCCGAATCAGTAACTATGTTCATTTTTTTAGGAGTAGGACGTCTAGCCAGTATGGAAGAG

GATTTTCTAGATACTCTCTTCAACATCTTTGATCTCAATGGAATGCAAAACCCCATGGTGTAACAACCAA

CGATAAAAATAATATTGTTTTTTCACTTTTTATAATTTTACCATCTGACTCATGGATTCATTAATATCTT

TACAAGAGCTACTAACGTATAATTCTTTATAACTAAACTGAGATATATACACCGGATCTATGGTTTCCAT

AATTGAGTAAATGAATGCTCGGCAATAACTAATGGCAAATGTATAGAACAACGAAATTATACTAGAGTTG

TTAAAGTTAATATTTTCTATGAGTTGTTCCAATAAATTATTTGTTGTGACTGCGTTCAAGTCATAAATTA

TCTTGATACTATCCAGTAAACAGTCTTTAAGTTCTGGAATATTATCATCCCATTGTAAAGCCCCTAGTTC

GACTATCGAATATCCTGCTCTGATAGCAGTTTCAATATCGACGGACGTCAATACTGTAATAAAGGTGGTA

GTATTGTCATCATCGTGATAAACTACGGGAATATGGTCGTTAGTAGGTACCGTGACTTTACACAACGCGA

TATATAACTTTCCTTTTGTACCATTTTTAACGTAGTTGGGACGTCCTGCAGGGTATTGTTTTGAAGAAAT

GATATCGAGAACAGATTTGATACGATATTTGTTGGATTCCTGATTATTCACTATAATATAATCTAGACAG

ATAGATGATTCGATAAATAGAGAAGGTATATCGTTGGTAGGATAATACATCCCCATTCCAGTATTCTCGG

ATACTCTATTGATGACACTAGTTAAGAACATGTCTTCTATTCTAGAAAACGAAAACATCCTACATGGACT

CATTAAAACTTCTAACGCTCCTGATTGTGTTTCGAATGCCTCGTACAAGGATTTCAAGGATGCCATAGAT

TCTTTGACCAACGATTTAGTATTGCGTTTAGCATCTGATTTTTTTATTAAATCAAATGGTCGGCTCTCTG

GTTTACTACCCCAATGATAACAATAGTCTTGTAAAGATAAACCGCAAGAAAATTTATACACATCCATCCA

AATAACCCTAGCACCGTCGGATGATATTAATGTATTATTATAGATTTTCCATCCACAGTTATTGGGCCAG

TATACTGTTAGCAACGGTATATCGAATAGATTACTCATGTAACCTACTAGAATGATAGTTCGTGTACTAG

TCATAATATCTTTAATCCAATCTAAGAAATCTAAAATTAGATCTTTTACACTATTAAAGTTAACAAAGGT

ATTACCCGGGTACGTGGATATCATATATGGTATTGGTCCATTATCAGTAATGGCTCCATAAACTGATACG

GCAATGGTTTTTATATGTGTTTGATCTAATGAGGACGAAATTCGCGCCCACAATTCATCTCTAGATATGC

ATTTAATATCGAACGGTAACACATCAATCTCGGGACGCGTATATGTTTCTAAATTCTTAATCCAAATATA

ATGATGACCTATATGCCCTATTATCATACTGTCAACTATAGTATACCTAGAGAACTTTCGATACATCTGC

TGTTTCCTGTAATCGTTAAATTTTACAAATCTATAACATGCTAAACCTTTTGACGACAGCCATTCATTAA

TTTCTGATATGGAATCTGTATTCTTAATACCGTATCGTTCTAAAGCCAGTGCTATATCTCCCTGTTCGTG

GGAACGCTTTCGTATAATATCGATCAATGGATAATATGAAGTTTTTGGAGAATAATATGATTCATGATCT

ATTTCGTCCATAAACAATCTAGACATAGGAATTGGAGGCGATGATCTTAATTTTGTGCAATGGGTCAATC

CTATAACTTCTAATATTGTAATATTCATCATCGACATAACACTATCTATGTTATCATCGTATATTAGTAT

ACCACGACCTTCTTCATTTCGTGCCAAAATGATATACAGTCTTAAATAATTACGCAATATCTCAATAGTT

TCATAATTGTTAGCTGTTTTCATCAAGGTTTGTATCCTGTTTAACATGATGGCGTTCTATAACGTCTCTA

TTTTCTATTTTTAATTTTTTTAAATTTTTAACGATTTACTGTGGCTAGATACCCAATCTCTTTCAAATAT

TTTTTTAGCCTTGCTTACAAGCTGTTTATCTATACTATTAAAACTGACGAATCCGTGATTTTGGTAATGG

GTTCCGTCGAAATTTGCCGAAGTGATATGAACATATTCGTCGTCGACTATTAACAATTTTGTATTATTCT

GAATAGTGAAAACCTTCACAGATAGATCATTTTGAACACACAACGCATCTAGACTTCTGGCGGTTGCCAT

AGAATATACGTCGTTCTTATCCCAATTACCAACTAGAAGTCTGATCTTAACTCCTCTATTAATGGCTGCT

TCTATAATGGAGTTGTAAATGTCAGGCCAATAGTAGCTATTACCGTCGACACGTGTAGTGGGAACTATGG

CCAAATGTTCAATATCTATACTAGTCTTAGCCGACTTGAGTTTATCAATAACTACATCAGTGTCTAGATC

TCTAGAATATCCCAATAGGTGTTCTGGAGAATCAGTAAAGAACACTCCACCTATAGGATTCTTAATATGA

TACGCAGTGCTAACTGGCAGACAACAAGCCGCAGAGCATAAATTCAACCATGAATTTTTTGCGCTATTAA

AGGCTTTAAAAGTATCAAATCTTCTACGAAGATCTGTGGCCAGCGGAGGATAATCAGAATATACGCCTAA

CGTTTTAATCGTATGTATAGATCCTCCAGTAAATGACGCGTTTCCTACATAACATCTTTCATCATCAGAC

ACCCAAAAACAACCGAGTAGTAGTCCCACATTATTTTTTTTATCTATATTAACGGTTATAAAATTTATAT

CCGGGGAGTGACTTTGTAGCTCTCCCAGATTTCTTTTCCCTCGTTCATCTAGCAAAACTATTATTTTAAT

CCCTTTTTCAGATACCTCTTTTAGTTTATCAAAAATAAGCGCTCCCCTAGTAGTACTCAGAGGATTACAA

CAAAAAGATGCTATGTATATATATTTCTTAGCTAGAGTGATAATTTCGTTAAAACATTCAAATGTTGTCA

AATGATCGGATCTAAAATCCATATTTTCTGGTAGTGTTTCTACCAGCCTACATTTTGCTCCCGCAGGTAC

CGATGCAAATGGCCACATTTAGTTAACATAAAAACTTATATATCCTGTTCTATCAACGATTCTAGAATAT

CATCGGCTATATCGCTAAAATTTTCATCAAAGTCGACATCACAACCTAACTCAGTCAATATATTAAGAAG

TTCCATGATGTCATCTTCGTCTATTTCTATATCCGTATCCATTGTAGATTGTTGACCGATTATCGAGTTT

AAATCATTACTAATACTCAATCCTTCAGAATACAATCTGTGTTTCATTGTAAATTTATAGGCGGTGTATT

TAAGTTGGTAGATTTTCAATTATGTATCAATATAGCAACAGTAGTTCTTGCTCCTCCTTGATTTTAGCAT

CCTCTTCATTATTTTCTTCTACGTACATAATCATGTCTAATACGTTAGACAACACACCGACGATGGTGGC

CGCCACAGACACGAATATGACTAGACCGATGACCATTTAAAAAATACTCTCTAGCTTTAACTTAAACTGT

ATCGATCATTCTTTTAGCACATGTATAATATAAAAACATTATTCTATTTCGAATTTAGGCTTCCAAAAAT

TTTTCATCCGTAAACCGATAATAATATATATAGACTTGTTAATAGTCGGAATAAATATATTAATGCTTAA

ACTATCATCATCTCCACGATTAGAGATACAATATTTACATTCTTTTTGCTGTTTCGAAACTTTATCAATA

CACGTTAATACAAACCCAGGAAGGAGATATTGAAACTGAGGCTGTTGAAAATGAAACGGCGAATACAATA

ATTCAGATAATGTAAAATCATGATTCCGTATTCTGATGATATTAGAACTGCTAATGGATGTCGATGGTAT

GTATCTAGGAGTATCTATTTTAACAAAGCATCGATTTGCTAATATACAATTATCCTTTTGATTAATTGTT

ATTTTATTCATATTCTTAAAAGGTTTCATATTTATCAATTCTTCTACATTAAAAATTTCCATTTTTAATT

TATCTAGCCCCGCAATACTCCTCATTACGTTTCATTTTTTGTCTAGAATACCCATTTTGTTCATCTTGGT

ACATAGATTATCCAATTGAGAAGCGCATTTAGTAGTTTTGTACATTTTAAGTTTATTAACGAATCGTCGA

AAACTAGTTATAGTTAACATTTTATTATTTGATACCCTGATATTAATACCCCTGCCGTTACTATTATTTA

TAACTGATGTAACCCACGTAACATTGGAATTAATTATCGATAGTAATGCATCGACACTTCCAAAATTGTC

TATTATAAACTCACCGATAATTTTTTTATTGCATGTTTTCATATTCATTAGGATTATCAAATCTTTAATC

TTATTACGATTGTATGCGTTGATATTACAAGACGTCATTCTAAAAGACGGAGGATTTCCATCAAATGCCA

GACAATCACGTACAAAGTACATGGAAATAGGTTTTGTTCTATTACGCATCATAGATTCATATAAAACACC

CGTAGAAATACTAATTTGTTTTACTCTATAAAATACTATTGCATCTATTTCATCGTTTTGTATAACGTCT

TTCCAAGTGTCAAATTCCAATTTTTTTTCATTGATAGTACCAAATTCTTCTATCTCTTTAACTACTTGCA

TAGATAGGTAATTACAGTGATGCCTACATGCCGTTTTTTGAAACTGAATAGATGCATCTAGAAGCGATGC

TACACTAGTCACGATCACCACTTTCATATTTAGAATATATGTATGTAAAAATATAGTAGAATTTCATTTT

GTTTTTTTCTATGCTATAAATGAATTCTCATTTTGTATCCGCACATACTCCGTTTTATATCAATACCAAA

GAAGGAAGATATCTGGTTCTAAAAGCCGTTAAAGTATGCGATGTTAGAACTGTAGAATGCGAAGGAAGTA

AAGCTTCCTGCGTACTCAAAGTAGATAAACCCTCATCACCCACGTGTGAGAGAAGACCTTCGTCCCCGTC

CAGATGCGAGAGAATGAATAACCCTGGAAAACAAGTCCCGTTTATGAGAACGGACATGTTACAAAATATG

TTTGCTGCTAATCGCGACAACGTAACGTCAAGACTTTTGAACTAAAATACAATTATATCTTTTTCGATAT

TAATAAATCCGTGTCTCCCGGGTTTTTTATCTCTTTCAGTATGTGAATAGATAGGTATTTTATCTCTATT

CATCATCGAATTTAAGAGATCCGATAAACATTGTTTGTATTCTCCAGATGTCAGCATCTGATACAACAAT

ATATGTGCACATAAACCTCTGGCACTTATTTCATGTACCTTCCCCTTATCACTAAGGAGAATAGTATTTG

AGAAATATGTATACATGATATTATCATGTATTAGATATACAGAATTTGTAACACTCTCGAAATCACACGA

TGTGTCGGCGTTAAGATCTAATATATCACTCGATAACACATTTTCATCTAGATACACTAGACATTTTTTA

AAGCTAAAATAGTCTTTAGTAGTAACAGTAACTATGCGATTATTTTCATCGATGATACATTTCATCGGCA

TATTATTACGCGTACCATCAAAGACTATACCATGTGTATATCTAACGTATTCTAGCATAGTTGCCATACG

TACATTAAACTTTTCAGGATCTTTGGATAGATCTTCCAATCTATCTATTTGAGAAAACATTTTTATCATG

TTCAATAGTTGAAACGTCGGATCCACTATATAGATATTATCTATAAAGATTTTAGGAACTATGTTCATGG

TATCCTGGCGAATATTAAAACTATCAATGATATGATTATCGTTTTCATCTTTTATCACCATATAGTTTCT

AAGATATGGGATTTTACTTAATATAATATTATTTCCCGTAATAAATTTTATTAGAAATGCCAAATCTATA

AGAAAAGTCCTAGAATTAGTCTGAAGAATATCTATATCACCGTACCGTATATTTGGATTAATTAGATATA

GAGAATATGATCCGTAACATATACAACTTTTATTATGACGTCTAAGATATTCTTCCATCAACTTATTAAC

ATTTTTGACTAGGGAAGATACATTATGACGTCCCATTACTTTTGCCTTGTCTATTACAGCGACGTTCATA

GAATTTAGCATATCTCTTGCCAATTCTTCCATTGATGTTACATTATAAGAAATTTTAGATGAAATTACAT

TTGGAGCTTTAATAGTAAGAACTCCTAATATATCCGTGTATGTGGTCACTAATACAGATTGTAGTTCTAT

AATCGTAAATAATTTACCTATATTATATGTTTGAGTTTGTTTAGAAAAGTAGCTAAGTATACGATCTTTT

ATTTCTGATGCCGATGTATCAACATCGAAAAAAAATCTTTTTTTATTCTTTTTTACTAACGATACGAATA

TGTCTTTGTTAAAAACAGTTATTTTCTGAATATTTCTAGCTTGTAATTTTAACATATGATATTCGTTCAC

ACTAGGTACTCTGCCTAAATAGGTTTCTATAATCTTTAATGTAATATTAGGAAGAGTATTCTGATCAGGA

TTCCTATTCATTTTGAGGATTTAAAACTCTGATTATTGTCTAATATGGTCTCAACACAAACTTTTTCACA

GAGTGATAGAGTTTTTGATAACTCGTTTTTCTTAAGAAATATAAAACTACTGTCTCCAGAGCTCGCTCTA

TCTTTTATTTTATCTAATTCGATACAAACTCCTGATACTGGTTCAGAAAGTAATTCATTAATTTTCAGTC

CTTTATAGAAGATATTTAATATAGATAATACAAAATCTTCAGTTCTTGATATCGATCTGATTGATCCTAG

AACTAGATATATTAATAACGTGCTCATTAGGCAGTTTATGGCAGCTTGATAATTAGATATAGTATATTCC

AGTTCATATTTATTAGATACCGCATTGCCCAGATTTTGATATTCTATGAATTCCTCTGAAAATAAATCCA

AAATAACTAGACATTCTATTTTTTGTGGATTAGTGTACTCTCTTCCCTCTATCATGTTCACTACTGGTGT

CCACAATGATAAATATCTAGAGGGAATATAATATAGTCCATATGATGCCAATCTAGCAATGTCGAATAAC

TGTAATTTTATTCTTCGCTCTTCATTATGAATTGAATCTTGAGGTATAAACCTAACACAAATTATATCAT

TAGACTTTTCGTATGTAATGTCTTTCATGTTATAAGTTTTTAATCCTGGAATAGAATCTATTTTAATGAG

GCTTTTAAACGCAGCGTTCTCCAACGAGTCAAAGCATAATACTCTGTTGGTTTTCTTATATTCAATATTA

CGATTTTCTTCTTTGAATGGAATAGGTTTTTGAATTAGTTTATAATTACAACATAATAGATAAGGAAGTG

TGTAAATAGTACGCGGAAAAAACATAATAGCTCCCCTGTTTTCATCCATGGTTTTAAGTAAATGATCACT

GGCTTCTTTAGTCAATGGATATTCGAACATTAACCGTTTCATCATCATTGGACAGAATCCATATTTCTTA

ATGTAAAGAGTGATCAAATCATTGTGTTTATTGTACCATCTTGTTGTAAATGTGTATTCGGTTATCGGAT

CTGCTCCTTTTTCTATTAAAGTATCGATATCGATCTCGTCTAAGAATTCAACTATATCGACATATTTCAT

TTGTATACACATAACCATTACTAACGTAGAATGTATAGGAAGAGATGTAACGGGAACAGGGTTTGTTGAT

TCGCAAACTATTCTAATACATAATTCTTCTGTTAATACGTCTTGCACGTAATCTATTATAGATGCCAAGA

TATCTATATAATTATTTTGTAAGATGATGTTAACAATGTGATCTATATAAGTAGTGTAATAATTCATGTA

TTTCGATATATGTTCCAACTCTGTCTTTGTGATGTCTAGTTTCGTAATATCTATAGCGTCCTCAAAAAAT

ATATTCGCATATATTCCCAAGTCTTCAGTTCTATCTTCTAAAAAATCTTCAACGTATGGAATATAATAAT

CTATTTTACCTCTTCTGATGTCATTAATGATATAGTTTTTGACACTATTTTCCGTCAATTGATTCTTATT

CACTATGTCTAAAAACCGGATAGCGTCCCTAGGACGAACTACTGCCATTAATATCTCTATTATAGCTTCT

GGACATAAATCATCTATTATACCAGAATTAATGGGAACTATTCCGTATCTATCTAACATAGTTTTAAGAA

AGTCAGAATCTAAGACCTGATGTTCATATATTGGTTCATACATGAAATGATCTCTATTGATGATAGTGAC

TATTTCATTCTCTGAAAATTGGTAACTCATTCTATACACGCTTTCCTTGTTGATAAAGGATAGTATATAC

TCAATGGAATTTGTACCAACAAACTGTTCTCTTATGAATCGTATATCATCATCTGAAATGATCATGTAAG

GCATACATTTAACAATAAGAGACTTGTCTCCTGTTATCAATATACTATTCTTGTGATAATTTATGTGTGC

GGCAAATTTGTCCACGTTCTTTAATTTTGTTATAGTAGATATCAAATCCAATGGAGATACAGTTCTTGGC

TTAAACAGATATAGTTTTTCTGGAACGAATTCTACAACATTATTATAAAGGACTTTGGGTATATAAGTGG

GATGAAATCCTATTTTAATTAATGCGATAGCCTTGTCCTCGTGCAGATATCCAAACGCTTTTGTGATAGT

ATGGCATTCATTGTCTAGAAACGCTCTACGAATATCTGTAACAGATATCATCTTTAGAGAATACTAGTCG

CGTTAATAGTACTAAAATTTGTATTTTTTAATCTATCTCAATAAAAAATTAATATGTATGATTCAATGTA

TAACTAAACTACTAACTGTTATTGATAACTAGAATCAGAATCTAATGATGACATAACTAAGAAGTTTATC

TACAGCCAATTTAGCTGCATTATTTTTAGCATCTCGTTTAGATTTTCCATCTGCCTTATCGAATACTCTT

CCGTCAATGTCTACACAGGCATAAAATGTAGGAGAGTTACTAGGCCCCACTGATTCAATACGAAAAGACC

AATCTCTCCTAGTTATTTGACAGTACTCATTAATAACGGTGACAGGGTTAACACCTTTCCAATAAATAAT

TTTTTTAACCGGAATAACATCATCAAAAGACTTATGATCCTCTCTCATTGATTTTTCGCGGGATACATCA

TCTATTATAGCATCAGCATCAGAATCTGTAGGCCGTGTATCAGCATCCATTGTCGTAGACCAACGAGGAG

GAGTATCGTTGGAGCTGTAAACCATAGCACTACGTTGAAGATCATACAGAGCTTTATTAACTTCTCGCTT

CTCCATATTAAGTTGTTTAGTTAGTTGTACAGCAGTAGCTCCTTAGTCCAATGTTTTTAATAACCGCACA

CAATCTCTGTGTCAGAACGCTCGTCAATATAGATCTTAGAAATTTTTTTAGAGAGAACTAACGCAACTAG

CAATAAAACTGATCTTATTTTATCATTTTTTTTATTCATCATCCTCTGGTGGTTCGTCGTTCCTATCGAA

TGTAGCTCTGATTAACCCGTCATCTATAGGTGATGCTGGTTCTGGAGATTCTGGAGGAGATGGATTATTA

TCTGGAAGAATCTCTGTTATTTCCTTGTTTTCATGTATCGATTGCGTTGTAACATTAAGATTGCGAAATG

CTCTAAATTTGGGAGGCTTAAAGTGTTGTTTACAATCTCTACACGCGTGTCTAACTAATGGAGGTTCATC

AGCGGCTCTAGTTTGAATCATCATCGGTGTAGTATTCCTACTTTTACAGTTAGGACACGGTGTATTGTAT

TTCTCGTCGAGAACGTTAAAATAATCGTTGTAACTCACATCCTTTATTTTATCTATATTGTATTCTACTC

CTTTCTTAATGCATTTTATACCGAACAAGAGATAGCGAAGGAATTCTTTTTCGGTACCGCTAGTACCCTT

AATCATATCACATAGTGTTTTATATTCTAAATGTGTGGCAATGGACGGTTTATTTCTATACGATAGTTTG

TTTTTGGAATCCTTTGAGTATTCTATACCAATATTATTCTTTGATTCGAATTTAGTTTCTTCGATATTAG

ATTTTGTATTACCTATATTCTTGATGTAGTACTTTGATGATTTTTCCATGGCCCATTCTATTAAGTTTTC

CAAGTTGGCATCATCCACATATTGTGATAGTAATTCTCGGATATCAGTAGTGACTACCGCCATTGATATT

TGTTCATTTGATGAGTAACTACTAATGTATACATTTTCCATTTATAACACTTATGTATTAACTTTGTTTA

TTTATATTTTTTCATTATTATGTTGATATTAATAATCGTATTGTGGTTATATGGCTACAATTTCATAATG

AGTTGAAGTCAGTGTCCTATGATCAATGACGATAGCTTTACTCTGAAAAGAAAGTATCAAATCGATAGTG

CAGAGTCAACAATGAAAATGGATAAGACGATGACAAAGTTTCAGAATAGAGTCAAAATGGTAAAAGAAAT

AAATCAGACGATAAGAGCAGCACAAACTCATTACGAGACATTGAAACTAGGATATATAAAATTTAAGGGA

ATGATTAGGACTACTACTCTAGAAGATATAGCACCATCTATTCCAAATAATCAGAAAACTTATAAACTAT

TCTCGGACATTTCAGTCATTGGCAAAGCATCACAGAATCCGAGTAAGATGATATATGCTCGCTGCTTTAC

ATGTTTCCCAATTTGTTTGGAGATGACCATAGATTCATTTGTTATAGAATGCATCCAACATTGTTCATGA

TATAGTTGAATCATGTATGCCTGTTCGTATGCCTGTGGCTAAGATACTGTGTAAAGAAATGGTAAATAAA

TACTTTGAGAATCTTTAAGAGTGCATTGACTTTGTTAGTGAATAGGCATTCCATCTTTCTCCAATACTAA

TTCAAATTGTTAAATTAATAATGGAATAGTATAAATAGTTATTAGTGATAGGATAGTAAACATAATTATT

AGAATAGTAGTGTAGTATCATAGATAACTCTCTTCTATAAAAAATGGATTTTATTCGTAGAAAGTATCTT

ATATACACAGTAGAAAATAATATAGATTTTTTAAAGGATGATACATTAAGTAAAGTAAACAATTTTACCC

TCAATCATGTACTAGCTCTCAAGTATCTAGTTAGCAATTTTCCTCAACATGTTATTACTAAGGATGTATT

AGCTAATACCAATTTTTTTGTTTTCATACATATGGTACGATGCTGTAAAGTATACGAAGCGGTTTTACGA

CACGCATTTGATGCACCCACGTTGTACGTTAAAGCATTGACTAAGAATTATTTATCGTTTAGTAACACAA

TACAGTCGTACAAGGAAACAGTGCATAAACTAACACAAGATGAAAAATTTTTAGAGGTTGCCGAATACAT

GGACGAATTAGGAGAACTTATAGGCGTAAATTATGACTTAGTTCTTAATCCATTATTTCACGGAGGGGAA

CCCATCAAAGATATGGAAATCATTTTTTTAAAACTGTTTAAGAAAACAGACTTCAAAGTTGTTAAAAAAT

TAAGTGTTATAAGATTACTTATTTGGGCATACCTAAGCAAGAAAGATACAGGCATAGAGTTTGCGGATAA

TGATAGACAAGATATATATACTCTATTTCAACAAACTGGTAGAATAGTCCATAGCAATCTAACAGAAACG

TTTAGGGATTATATCTTTCCCGGAGATAAGACTAGCTATTGGGTGTGGTTAAACGAAAGTATAGCTAATG

ATGCGGATATCGTTATTAATAGACCCGCCATTACCATGTATGATAAAATTCTTAGTTATATATACTCTGA

GATAAAACAGGGACGCGTTAATAAAAACATGCTTAAGTTAGTTTATATCTTTGAGCCTGAAAAAGATATC

AGAGAACTTCTGCTAGAAATCATATATGATATTCCTGGAGATATCCTATCTATTATTGATGCAAAAAACG

ACGATTGGAAAAAATATTTTATTAGTTTTTACAAAGCTAATTTTATTAACGGTAATACATTTATTAGTGA

TAGAACGTTTAACGATGACTTATTCAGAGTTGTTGTTCAAATAGATCCCGAATATTTCGATAATGAACGA

ATTATGTCTTTATTCTATACGAGTGCTGCGGACATTAAACGATTTGATGAGTTAGATATTAATAACAGTT

ATATATCTAATATAATTTATGAGGTGAACGATATCACATTAGATACAATGGATGATATGAAGAAGTGTCA

AATCTTTAACGAGGATACGTTGTATTATGTTAAGGAATACAATACATACCTGTTTTTGCACGAGTCGGAT

CCCATGGTCATAGAGAACGGAATACTAAAGAAACTGTCATCTATAAAATCCAAGAGTAGACGGCTGAACT

TGTTTAGCAAAAACATTTTAAAATATTATTTAGACGGACAATTGGCTCGTCTAGGTCTTGTGTTAGATGA

TTATAAAGGAGACTTATTAGTTAAAATGATAAACCATCTCAAATCTGTGGAGGATGTATCCGCATTCGTT

AGATTTTCTACAGATAAAAACCCTAGTATTCTTCCATCGCTAATCAAAACTATTTTAGCTAGTTATAATA

TTTCCATCATCGTCTTATTTCAAAGGTTTTTAAGAGATAATCTATATCATGTAGAAGAATTCTTGGATAA

AAGCATCCATCTAACCAAGACGGATAAGAAATATATACTTCAATTGATAAGGCACGGTAGATCATAGAAC

AAACCAAATATATTATTAATAATTTGTATATACATAGATATAATTATCACATATTAAAAAATAACACATT

TTTGATAAATGGAAACCGTTGCAACAATTCAGACTCCCACCAAATTAATGAATAAAGAAAATGCAGAAAT

GATTTTGGAAAAAATTGTTAATCATATAGCTATGTATATTAGTGACGAATCAATATATTCAGAAAATAAT

CCTGAATATATTGATTTTCGTAACAGATACGGAGACTATAGATCTCTCATTATAAAAAGTGATCACGAGT

TTGTAAAGCTATGTAAAGATCATGCAGAGAAAAGTTCTCCAGAAACGCAACAAATGATTATCAAACACAT

ATACGAACAATATCTTATTCCAGTATCTGAAGTACTATTAAAACCTATAATGTCCATGGGTGACATATTT

ACATATAACGGATGTAAAGACAATGAATGGATGCTAGAACAACTCTCTACCCTAAACTTTAACAATCTCT

ACACATGGAACTCATGTAGCATAGGCAATGTAACGCGTCTGTTTTATACATTTTTTAGTTATCTGATGAA

AGATAAACTAAATATATAAGTATAATCCCATTCTAATACTTTAACCTGATGTATTATTACCTGCATCTTA

TTAGAATATTAACCTAACTAAAAGACATAAAAAGCGGTAGGATATAAATATTATGGCCGCAACCGTTCCG

CGTTTTGACGATGTGTACAAAAATGCACAAAGAAGAATTCTAGATCAAGAAACATTTTTTAGTAGAGGTC

TAAGTAGACCGTTAATGAAAAACACATATCTATTTGATAATTACGCGTATGGATGGATACCAGAAACTGC

AATTTGGAGTAGTAGATACGCAAACCTAGATGCTAGTGACTATTATCCCATTTCGTTGGGATTACTTAAA

AAGTTTGAATTTCTCATGTCTCTATATAAAGGTCCTATTCCCGTATATGAAGAAAAAGTAAATACTGAAT

TCATTGCTAATGGATCTTTCTCCGGTAGATACGTATCATATCTTAGAAAGTTTTCTGCCCTTCCAACAAA

CGAGTTTATTAGTTTTTTATTATTGACCTCCATCCCTATCTATAATATCTTATTCTGGTTTAAAAACACA

CAGTTTGATATTACTAAACACACATTATTCAGATACGTCTATACAGATAATACCAAACACCTTGCGTTGG

CTAGGTATATACATCAAACAGGAGACTATAAGCCTTTGTTTAGTCGTCTCAAAGAGAATTATATATTTAC

TGGTCCCGTTCCAATAGGTATCAAAGATATAGATCACCCTAATCTTAGTAGAGCAAGAAGTCCATCCGAT

TATGAGACATTAGCTAATATTAGTACTATATTGTACTTTACCAAGTATGATCCAGTATTAATGTTTTTAT

TGTTTTACGTACCTGGGTATTCAATTACTACAAAAATTACTCCAGCCGTAGAATATCTAATGGATAAACT

GAATCTAACAAAGAGCGACGTACAACTGTTGTAAATTATTTTATGCTTCGTAAAATGTAGGTCTTGAACC

AAACATTCTTTGAAAAAATGAGATGCATAAAACTTTATTATCCAATAGATTAACTATTTCAGACGTCAAT

CGTTTAAAGTAAACTTCGTAAAATATTCTTTGATTGCTGCCGAGTTTAAAACTTCTATCGATAATTGTTT

CATATGTTTTAATATTTACAAGTTTTTTGGTCCATGGTACATTAGCTGGACAGATATATGCAAAATAATA

TCGTTCTCCAAGTTCTATAGTCTCTGGATTGTTTTTATTATATTCAGTAACCAAATACATATTAGGGTTA

TCTGCGGATTTATAATTTGAGTGATGCATTCGACTCAACATAAATAATTCTAGAGGAGACGATCTACTAT

CAAATTCGGATCGTAAATCTGTTTCTAAAGAACGGAGAATATCTATACATACCTGATTAGAATTCATCCG

TCCTTCAGACAACATCTCAGACAGTCTGGTCTTGTATGTCTTAATCATATTCTTATGAAACTTGGAAACA

TCTCTTCTAGTTTCACTAGTACCTTTATTAATTCTCTCAGGTACAGATTTTGAATTCGACGATGCCGAGT

ATTTCATCGTTGTATATTTCTTCTTCGATTGCATAATCAAATTCTTATATACCGCCTCAAACTCTATTTT

AAAATTATTAAACAATACTCTACTATTAATCAGTCGTTCTAACTCCTTTGCTATTTCTATGGACTTATCT

ACATCTTGACTGTCTATCTCTGTAAACACGGAGTCGGTATCTCCATACACGCTACGAAAACGAAATCTAT

AATCTATAGGCAACGATGTTTTCACAATCGGATTAATATCTCTATCGTCCATATAAAATGGATTACTTAA

TGTATTGGCAAACCGTAACATACCGTTGGATAACTCTGCTCCATTTAGTACCGATTCTAGATACAATATC

ATTCTACGTCCTATGGATGTGCAACTCTTAGCCGAAGCGTATGAGTATAGAGCACTATTTCTAAATCCCA

TCAGACCATATACTGAGTTGGCTACTATCTTGTACGTATATTGCATGGAATCATAGATGGCCTTTTCAGT

TGAACTGGTAGCCTGTTTTAACATCTTTTTATATCTGGCTCTCTCTGCCAAAAATGTTCTTAATAGTCTA

GGAATGGTTCCTTCTATTGATCTATCGAAAATTGCTATTTCAGAGATGAGGTTCGGTAGTCTAGGTTCAC

AATGAACCGTAATATATCTAGGAGGTGGATATTTCTGAAGCAAGAGTTGATTATTTATTTCTTCTTCCAA

TCTATTGGTACTAACAACGACACCGACTAATGTTTCCGGAGATAGATTTCCAAAGATACACACATTAGGA

TACAGACTGTTATAATCAAAGATTAATACATTATTACTAAACATTTTTTGTTTTGGAGCAAATACCTTAC

CGCCTTCATAAGGAAACTTTTGTTTTGTTTCTGATCTAACTAAGATAGTTTTAGTTTCCAACAATAGCTT

TAACAGTGGACCCTTGATGATTGTACTCGCTCTATATTCGAATACCATGGATTGAGGAAGCACATATGTT

GCCGCACCAGCGTCTGTTTTTGTTTCTACTCCATAATACTCCCACAAATACTGACACAAACAAGCATCAT

GAATACAGTATCTAGCCATATCTAAAGCTATGTTTAGATTATAATCCTTATACATCTGAGCTAAATCAAT

GTCATCCTTTCCGAAAGATAATTTATATATATCATTAGGTAAAGTAGGACATGATAGTACGACTTTAAAT

CCATTTTCCAAAATATCTTTACGAATTACTTTACATATAATATCCTCATCAACAGTCACGTAATTACCTG

TGGTTAAAACCTTTGCAAATGTATCGGCTTTGCCTTTCGCGTCCGTAGTATCGTCACCGATGAACGTCAT

TTCTCTAACTCCTCTATTTAATACTTTACCCATGCAACTGAACGCGTTCTTGGATATAGAATCCAATTTG

TACGAATCCAATTTTTCAGATTTTTGAATGAATGAATATAGATCGAAAAATATAGTTCCATTATTGTTAT

TAACGTGAAACGTAGTATTGGCCATGCCGCATACTCCCTTATGACTAGACTGATTTCTCTCATAAATACA

GAGATGTACAGCTTCCTTTTTGTCTGGAGATCTAAAGATAATCTTCTCTCCTGTTAATAACTCTAGACGA

TTAGTAATATATCTCAGATCAAAGTTATGTCCGTTAAAGGTAACGACGTAGTCGAACGTTAGTTCCAACA

ATTGTTTAGCTATTCGTAACAAAACTATTTCAGAACATAGAACTAGTTCTCGTTCGTAATCCATTTCCAT

TAGCGACTGTATCCTCAAACATCCTCTATCGACGGCTTCTTGTATTTCCTGTTCCGTTAACATCTCTTCA

TTAATGAGCGTAAACAGTAATCGTTTACCACTTAAATCGATATAACAGTAACTTGTATGCGAGATTGGGT

TAATAAATACAGAAGGAAACTTCTTATCGAAGTGACACTCTATATCTAGAAATAAGTACGATCTTGGGAT

ATCGAATCTAGGTATTTCTTTAGCGAAACAGTTACGTGGATCGTCACAATGATAACATCCATTGTTAATC

TTTGTCAAATATTGCTCGTCCAACGAGTAACATCCGTCTGGAGATATCCCGTTAGAAATATAAAACCAAC

TAATATTGAAAAATTCATCCATGGTGGCATTTTGTATGCTGCGTTTCTTTGGCTCTTCTATCAACCACAT

ATCTGCGACGGAGCATTTTCTATCTTTAATATCTAGATTATAACTTATTGTCTCGTCAATGTCTATAGTT

CTCATCTTTCCCATCGGCCTCGCATTAAATGGAGGAGGAGATAATGACTGATATATTTCGTCCGTCACTA

CGTAATAAAAGTAATGAGGAAATCGTATAAATACGGTCTCGCCATTTCGACATCTGGATTTCAGATATAA

AAATCTGTTTTCACCGTGACTTTCAAACCAATTAATACACCTAACATCCATTTCTAGAATTTAGAAATAT

ATTTTCATTTAAATGAATCCCAAACATTGGGGAAGAGCCGTATGGACCATTATTTTTATAGTACTTTCGC

AAGCGGGTTTAGACGGCAACATAGAAGCGTGTAAACGAAAACTATATACTATAGTCAGCACTCTTCCATG

TCCTGCATGTAGACGACACGCGACTATCGCTATAGAGAACAATAATGTCATGTCTAGCGATGATCTGAAT

TATATTTATTATTTTTTCATCAGATTATTTAACAATTTGGCATTTGATCCCAAATACGCAATCGATGTGT

CAAAGGTTAAACCTTTATAAACTTAACCCATTATAAAACTTATGATTAGTCACGACTGAAATAACCGCGT

GATTATTTTTTGGTATAATTCTACACGGCATGGTTTCTGTGACTATGAATTCAACACCTGTTATCTTAGT

GAAATCTTTAACAAACAGCAAGGGTTCGTCAAAGACATAAAACTCATTGTTTACGATCGAAATAGACCCC

CTATCACACTTAAAATAAAAAATATCCTTATCCTTTACCACCAAATAAAATTCTGATTGGTCAATGTGAA

TGTATTCACTTAACAGTTCCACAAATTTATTTATTAACTCCGAGGCACATACATCGTCGGTATTTTTTAT

GACAAACTTTACTCTTCCAGCATCCGTTTCTAAAAAAATATTAACGAGTTCCATTTATATCATCCAATAT

TATTGAAATGACGTTGATGGACAGATGATATAAATAAGAAGGTACAGTACCTTTGTCCACCATCTCCTCC

AATTCATACTCTATTTTGTCATTAACTTTAATGTGTGAAAACAGTACGCCACATGCTTCCATGACAGTGT

GTAACACTTTGGATACAAAATGTTTGACATTAGTATAATTGTCCAAGACTGTCAATCTATAATAGATAGT

AGCTATAATATATTCTATGATGGTATTGAAGAAGATGACAACCTTGGCATATTGATCATTTAACACAGAC

ATGGTATCAACAAATAGCTTAAATGAAAGAGAATCAGTAATTGGAATAAGCGTCTTCTCGATGTAGTGTC

CGTATACCAACATGTCTGATATTTTGATGTATTCCATTAAATTATTTAGTTTTTTCTTTTTATTCTCGTT

AAACAGAATTTCTGTCAATGGACCCCAACATCGTTGACCTATTAAGTTTTGATTGATTTTTCCGTGTAAG

GCGTATCTAGTCAGATCGTATAGCCTATCCAATAATCCATCGTCTGTGCGTAGATCACATCGTACACTTT

TTAATTTTCTATAGAAGAGTGACAGACATCTGGAGCAATTACAGACAGCAATTTCTTTATTCTCTACAGA

TGTAAGATACTTGAAGATATTCCTATGATGATGCAGAATTTTGGATAACACGGTATTGATGGTATCTGTT

ACCATAATTCCTTTGACTGATAGTGTCAAAGTACAAGATTTCCAATCTTTTGCAATTTTCAGTACCATTA

TCTTTGTTTTGATATCTATATCAGACAGCATGGTACGTCTGACAACACAGGGATTAAGACGGAAAGATGA

AATGATTCTCTCAACATCTTCAATAGATACCTTGCTATTTTTTTTGGCATTATCTATATGTGAGAGAATA

TCCTCTAGAGAATCAGTATCCTTTTTGATGATAGTGGATCTCAATGACATGGGACGTCTAAACCTTCTTA

TTCTATCACCAGATTGCATGGTGATTTGTCTTCTTTCTTTTATCATGATGTAATCTCTAAATTCATCGGC

AAATTGTCTATATCTAAAATCATAATATGAGATGTTTACCTCTACAAATATCTGTTCGTCCAATGTTAGA

GTATCTATATCAGTTTTGTATTCCAAATTAAACATGGCAACGGATTTAATTTTATATTCCTCTATTAAGT

CCTCGTCGATAATAACAGAATGTAGATAATCATTTAATCCATCGTACATGGTTGGAAGATGCTCGTTGAC

AAAATCTTTAATTGTCTTGATGAAGGTGGGACTATATCTAACATCTTGATTAATAAAATTTATAACATTG

TCCATAGGATACTTTGTAACTAGTTTTATACACATCTCTTCATTGGTAAGTTTAGACAGAATATCGTGAA

CAGGTGGTATATTATATTCATCAGATATACGAAGAATAATGTCCAAATCTATATTGTTTAATATATTATA

TAGATGTAGTGTAGCTCCTACAGGAATATCTTTAACTAAGTCAATGATTTCATCAACAGTTAGATCTATT

TTAAAGTTAATCATATAGGCATTGATTTTTAAAAGGTATGTAGCCTTGACTACATTCTCATTAATTAACC

ATTCCAAGTCACTGTGTGTAAGAAGATTATATTCTATCATAAGCTTGACTACATTTGGTCCCGATACCAT

TAAAGAATTCTTATGATATAAGGAAACAGCTTTTAGGTACTCATCTACTCTACAAGAATTTTGGAGAGCC

TTAACGATATCAGTGACGTTTATTATTTCAGGAGGAAAGAACCTAACATTGAGAATATCTGAATTAATAG

CTTCCAGATACAGTGATTTTGGCAATAGTCCGTGTAATCCATAATCCAGTAACACGAGCTGGTGCTTGCT

AGACACCTTTTCAATGTTTAATTTTTTTGAAATAAGCTTTGATAAAGCCTTCCTCGCAAATTCCGGATAC

ATGAACATGTCGCCAACATGATTAAGTATTGTTTTTCATTATTTTTATATTTTCTCAACAAGTTCTCAAT

ACCCCAATAGATAATAGAATATCACCCAATGCGTCCATGTTGTCTATTTCCAACAGGTCGCTATATCCAC

CAATAGAAGTTTTCCCAAAAAAGATTCTAGGAACAGTTCTACCACCAGTAATTTGTTCAAAATAGTCACG

CAATTCATTTTCGGGTTTAAATTCTTTAATATCTACAATTTCATACGCTCCTCTTTTGAAACTAAACTTA

TTTAGAATATCCAGTGCGTTTCTACAAAAAGGACATGTAAACTTGACAAAAATTGTCACTTTGTTATTGG

CCAACCTTTGTTGTACAAATTCCTCGGCCATTTTTAATATTTAAGTGATACAAAACTATCTCGACTTATT

TAACTCTTTAGTCGAGATATATGGACACAGATAGCTATATGATAACCAACTACAGAAGACAAACGCTATA

AAAAACATAATTACGACGAGCATATTTATAAATATTTTTATTCAGTATTACTTGATATAGTAATATTAGG

CACAGTCAAACATTCAACCACTCTAGATACATTAACTCTCTCATTTTCTTTAACAAATTCTGCAATATCT

TCGTAAAAAGATTCTTGAAACTTTTTAGAATATCTATCGACTCTAGATGAAATAGCGTTCGTCAACATAC

TATGTTTTGTATACATAAAGGCGCCCATTTTAACAGTTTCTAGTGACAAAATGCTAGCGATCCTAGGATC

CTTTAGAATCACATAGATTGACGATTCGTCTCTCTTAGTAACTCTAGTAAAATAATCATACAATCTAGTA

CGCGAAATAATATTATCCTTGACTTGAGGAGATCTAAACAATCTAGTTTTGAGAACATCGATAAGTTCAT

CGGGAATTACATACATACTATCTTTAATAGAACTCTTTTCATCCAGTTGAATGGATTCGTCCTTAACCAA

CTGATTAATGAGATCTTCTATTTTATCATTTTCTAGATGATATGTATGTCCATTAAAGTTAAATTGTGTA

GCGCTTCTTTTTAGCCTAGCAGCCAATACTTTAACATCACTAATATCGATATACAAAGGAGATGATTTAT

CGATGGTATTAAGAATTCGTTTTTCGACATCCGTCAAAACCAATTCCTTTTTGCCTGTATCATCCAGTTT

GCCATTCTTTGTAAAGAAATTATTTTCTACTAGACTATTAATAAGACTGATAAGGATTCCTCCATAATTG

CACAATCCAAACTTTTTCACAAAACTAGACTTTACGAGATCTACAGGAATGCGTACTTCAGGTTTCTTAG

CTTGTGATTTTTTCTTTTGTGGACATTTTCTAGTGACCAACTCATCTACCATTTCATTGATTTTAGCAGT

GAAATAAGCTTTCAATGCACGGGCACTGATACTATTGAAAACGAGTTGATCTTCAAATTCCGCCATTTAA

GTTCACCAAACAACTTTTAAATACAAATATATCAATAGTAGTAGAATAAGAACTATAAAAAAAATAATAA

TTAACCAATACCAACCCCAACAACCTGTATTATTAGTTGATGTGACAGTTTTCTCATCACTTAGAACAGA

TTTAACAATTTCTATAAAGTCTGTCAAATCATCTTCCTGAGAACCCATAAATACACCAAATATAGCAGCG

TACAACTTATCCATTTATACATTGAATATTGGCTTTTCTTTATCGCTATCTTCATCATATTCATCATCAA

TATCAACAAGTCCCAGATTACGAACCAGATCTTCTTCTACATTTTCAGTCATTGATACGCGTTCACTATC

TCCAGAGAGTCCGATAACGTTAGCCACTACTTCTCTATCAATGATTAGTTTCTTGAGCGCGAATGTAATT

TTTGTTTCCGTTCCGGATCTATAGAAAACTACAGGTGTAATAATTGCCTTGGCTAATTGTCTTTCTCTTT

TACTGAGTGATTCTAGTTCACCTTCTATAGATCTGAGAATGGATGATTCTCCAGTCGAAACATATTCTAC

CATGGCTCCGTTTAATTTGTTGATGAAGATGGATTCATCCTTAAATGTTTTCTCTGTAATAGTTTCCGCC

GAAAGACTATGCAAAGAATTTGGAATGCGTTCCTTGTGTGTAATGTTTCCATAGACAGCTTCTAGAAGTT

GATACAACATAGGACTAGCCGCGGTAACTTTTATTTTTAGAAAGTATCCATCGCTTCTATCTTGTTTAGA

TTTATTTTTATAAAGTTTAGTCTCTCCTTCCAACATAATAAAAGTGGAAGTCATCTGACTAGATAAACTA

TCAGTAAGTTTTATAGAGATAGATGAACAATTAGCGTATTGAGAAGCATTTAGTGTAACGCATTCGATAC

ATTTTGCATTAGATTTACTAATCGATTTTGCATACTCTATAACACCCGCACAAGTCTGTAGAGAATCGCT

AGATGCTGTAGGTCTTGGTGAAGTTTCAACTCTCTTCTTGATTACCTTACTCATGATTAAACCTAAATAA

TTGTACTTTGTAATATAATGATATATATTTTCACTTTATCTCATTTGAGAATAAAAATGTTTTTGTTAAC

CACTGCATGATGTACAGATTTCGGAATCGCAAACCACTTGTGGTTTTATTTTATCCTTGTCCAATGTGAA

TTGAATGGGAGCGGATGCGGGTTTCGTACGTAGATAGTACATTCCCGTTTTTAGACCGAGACTCCATCCG

TAAAAATGCATACTCGTTAGTTTGGAATAACTCGGATCTGCTATATGGATATTCATAGATTGACTTTGAT

CGATGAAGGCTCCCCTGTCTGCAGCCATTTTTATGATCGTCTTTTGTGGAATTTCCCAAATAGTTTTATA

AACTCGCTTAATATCTTCTGGAAGGTTTGTATTCTGAATGGATCCACCATCTACCATAATCCTATTCTTG

ATCTCATCATTCCATAATTTTCTCTCGGTTAAAACTCTAAGGAGATGCGGGTTAACTACTTGGAATTCTC

CAGACAATACTCTCCGAGTGTAAATATTACTGGTATACGGTTCCACCGACTCATTATTTCCCAAAATTTG

AGCAGTTGATGCAGTCGGCATAGGTGCCACCAATAAACTATTTCTAAGACCGTATGTTCTGATTTTATCT

TTTAGAGGTTCCCAATTCCAAAGATCCGACGGTACAACATTCCAAAGATCATATTGTAGAATACCGTTAC

TGGCGTACGATCCTACATATGTATCATATGGTCCTTCCTTCTCAGCTAGTTTACAACTCGCCTCTAATGC

ACCGTAATAAATGGTTTCAAAGATCTTCTTATTTAGATCTTGTGCTTCCAGGCTATCAAATGGATAATTT

AAGAGAATAAACGCGTCCGCTAATCCTTGAACACCAATACCGATAGGTCTATGTCTCTTATTAGAGATTT

CAGCTTCTGGAATAGGATAATAATTAATATCTATAATTTTATTGAGATTTCTGACAATTACTTTGACCAC

ATCCTTCAGTTTGAGAAAATCAAATCGCCCATCTATTACAAACATGTTCAATGCAACAGATGCCAGATTA

CACACGGCTACCTCATTAGCATCCGCATATTGTATTATCTCAGTGCAAAGATTACTACACTTGATGGTTC

CTAAATTTTGTTGATTACTCTTTTTGTTACACGCATCCTTATAAAGAATGAATGGAGTACCAGTTTCAAT

CTGAGATTCTATAATCGCTTTCCAGACGACTCGAGCCTTTATTATACATTTGTATCTCCTTTCTCTTTCG

TATAGTGTATACAATCGTTCGAACTCGTCTCCCCAAACATTGTCCAATCCAGGACATTCATCCGGACACA

TCAACGACCACTCTCCGTCATCCTTCACTCGTTTCATAAAGAGATCAGGAATCCAAAGAGCTATAAATAG

ATCTCTTGTTCTATGTTCATCGTTTCCTGTATTCTTTTTAAGATCGAGGAACGCCATAATATCAGAATGC

CACGGTTCCAAGTATATGGCCATAACTCCAGGCCGTTTGTTTCCTCCCTGATCTATGTATCTAGCGGTGT

TATTATAAACTCTCAACATTGGAATAATACCGTTTGATATACCATTGGTACCGGAGATATAGCTTCCACT

GGCACGAATATTACTAATTGATAGACCTATTCCCCCTGCCATTTTAGAGATTAATGCGCATCGTTTTAAC

GTGTCATAGATGCCTTCTATGCTATCATCGATCATGTTAAGTAGAAAACAGCTAGACATTTGGTGACGAG

TAGTTCCCGCATTAAATAAGGTAGGAGAAGCGTGCGTAAACCATTTTTCAGAAAGTAGATTGTACGTCTC

AATAGCTGAGTCTATATCCCATTGATGAATTCCTACTGCGACACGCATTAACATGTGCTGAGGTCTTTCA

ACAATTTTGTTGTTTATTTTCAACAAGTAGGATTTTTCCAAAGTTTTAAAACCAAAATAGTTGTATGAAA

AGTCTCGTTCGTAAATAATAACCGAATTGAGCTTATCCTTATATTTGTTAACTATATCCATGGTAATACT

TGAAATAATCGGAGAATGTTTCCCATTTTTAGGATTAACATAGTTGAATAAATCCTCCATCACTTCACTA

AATAGTTTTTTTGTTTCCTTGTGTAGATTTGATATGGCTATTCTGGCGGCTAGAATGGCATAATCCGGAT

GTTGTGTAGTACAAGTGGCTGCTATTTCGGCTGCCAGAGTGTCCAATTCTACCGTTGTTACTCCATTATA

TATTCCTTGAATAACCTTCATAGCTATTTTAATAGGATCTATATGATCAGTGTTTAAGCCATAGCACAAT

TTTCTAATACGAGACGTGATTTTATCAAACATGACATTTTCCTTGTATCCATTTCGTTTAATGACAAACA

TTTTTGTTGGTGTAATAAAAAAAATTATTTAATTTTTCATTAATAGGGATTTGACGTATGTAGCGTACAA

AATTATCGTTCCTGGTATATAGATAAAGAGTCCTATATATTTGAAAATCGTTACGGTTCGATTAAACTTT

AATGATTGCATTGTGAATATATCATTAGGATTTAACTCCTTGACTATCATGGCGGTGCCAGAAATTACCA

TCAAAAGCATTAATACAGTTATGCAGATCGCAGTTAGAACGGTTATAGCATCCACCATTTATATCTAAAA

ATTAGATCAAAGAATATGTGACAACGTCCTAGTTGTATACTGAGAATTGACGAAACAATGTTTCTTACAT

ATTTTTTTCTTATTAGTAACCGACTTAATAGTAGGAACTGGAAAACTAGACTTGATTATTCTATAAGTAT

AGATACCCTTCCAAATAATGTTCTCTTTGATAAAAGTTCCAGAAAATGTAGAATTTTTTAAAAAGTTATC

TTTTGCTATTACTAATATCGTGGTTAGACGCTTATTATTAATATGAGTGATGAAATCCACACCGCTTCTA

GATATCGCTTTTATTTCCACATTAGATGGTAAATCCAATAGTGAAACTATCTTTTTAGGAATGTATGGAC

TCGCGTTTAGAGGAGTGAACGTCTTCGGAGTAGTAAAGGATGATTCGTCAAATGAATAAACAATTTCACA

AATGGATGTTAATGTATTAGTAGGAAATTTTTTGACGCTAGTGGAATTGAAGATTCTAATGGATGATGTT

CTACCTATTTCATCCGATAACATGTTAATTTCCAATACCAACGGTTTTAATATTTCGATGATATACGGTA

GTCTCTCTTTCGGACTTATATAGCTTATTCCACAATACGAGTCATTATATACTCCAAAAAACAAAATAAC

TAGTATAAAATCTGTATCGAATGGGAAAAACGAAATTATCGATATAGGTATAGAATCCGGAACATTGAAC

GTATTAATACTTAATTCTTTTTCAGTGGTAAGAACCGATAGGTTATTGACATTGTATGGTTTTAAATATT

CTATAACTTGAGACTTGATAGATATTAATGACGAATTGAAAATTATTTTTATCACCACGTGTGTTTCAGG

ATCATCGTCGACGCCAGTTAACCAACCGAATGGAGTAAAATAAATATCATTAATATATGCTCTAGATATT

AGTATTTTTATTAATCCTTTGATTATCATCTTCTCGTACGCGAATGATTCCATGATCAAGAGTGATTTGA

GAACATCCTCCGGAGTATTAATGGGTTTAGTAAACAGTCCATCGTTGCAATAATAAAAGTTGTCCAAGTT

AAAGGATATTATGCATTCGTTTAAAGATATCACCTCATCTAACGGAGACAATTTTTTGGTAGGTTTTAGA

GACTTTGAAGCTACTTGTTTAACAAAGTTATTCATCGTCGTCTACTATTCTATTTAATTTTGTAGTTAAT

TTATCACATATCACATTAATTGACTTTTTGGTCCACTTTTCCATACGTTTATATTCTTTTAATCCTGCGT

TATCCGTTTCCGTTATATACAGGGATAGATCTTGCAAGTTAAATAGAATGCTCTTAAATAATGTCATTTT

TTTATCCGCTAAAAATTTAAAGAATGTATAAACTTTTTTCAAAGATTTAAAACTTTTAGGTGGAGTTCTG

GTACACAATATCATAAACAAACTAATAAACATCCCACATTCAGATTCCAACAATTGATTAACTTCCACAT

TAATACAGCCTATTTTCGCTCCAAATGTACATTCGAAAAATCTGAATAAAACATCAATATCGCAATTTGT

ATTATCCAATACAGAATGTCTGTGATTCGTGTTAAAACCATCGGAAAAAGAATAGAAATAAAAATTATTA

TAATGGTGGAATTCAGTTGGAATATTGCCTCCGGAGTCATAAAAGGATACTAAACATTGTTTTTTATCGT

AAATTACACATTTCCAATGAGACAAATAACAAAATCCAAACATTACAAATCTAGAGGTAGAACTTTTAAT

TTTGTCTTTAAGTATATACGATAAGATATGTTTATTCATAAACGCGTCAAATTTTTCATGAATAGCTAAG

GAGTTTAAGAATCTCATGTCAAATTGTCCTATATAATCCACTTCGGATCCATAAGCAAACTGAGAGACTA

AGTTCTTAATACTTCGATTGCTCATCCAGGCTCCTCTCTCAGGCTCTATTTTCATCTTGACGACCTTTGG

ATTTTCACCAGTATGTATTCCTTTACGTGATAAATCATCAATTTTCAAATCCATTTGTGAGAAGTCTATC

GCCTTAGATACTTTTTCCCGTAGTTGAGGTTTAAAGAAATACGCTAACGGTATACTAGTAGGTAACTCAA

AGACATCATATATAGAATGGTAACGCGTCGTTAACTCGTCGGTTAACTCTTTCTTTTGATCGAGTTCATC

GCTACTATTGGGTCTGCTCAGGTGCCCCGACTCTACTAGTTCCAACATCATACCGATAGGAATACAAGAC

ACTTTGCCAGCGGTTGTAGATTTATCATATTTCTCCACCACATATCCGTTACAATTTGTTAAGAATTTAG

ATACATCTATATTGCTACATAATCCAGCTAGTGAATATATATGACATAATAAATTGGTAAATCCTAGTTC

TGGTATTTTACTAATTACTAAATCTGTATATCTTTCCATTTATCATGGAAAAGAATTTACCAGATATCTT

CTTTTTTCCAAACTGCGTTAATGTATTCTCTTACAAATATTCACAAGATGAATTCAGTAATATGAGTAAA

ACGGAACGTGATAATTTCTCATTGGCTGTGTTTCCAGTGATAAAACATAGATGGCATAACGCACACGTTG

TAAAACATAAAGGAATATACAAAGTTAGTACAGAAGCACGTGGAAAAAAAGTATCTCCTCCATCACTAGG

AAAACCCGCACATATAAACCTAATGTCGAAGCAATATATATATAGTGAGTATGCAATAAGCTTTGAATGT

TATAGTTTTCTAAAATGTATAACAAATACAGAAATCAATTCGTTCGATGAGTATATATTAAGAGGACTAT

TAGAAGCTGGTAATAGTTTACAGATATTTTCCAATTCCGTAGGTAAACGAATAGATACTATAGGTGTACT

AGGGAATAAGTATCCATTTAGCAAAATTCCATTGGCCTCATTAACTCCTAAAGCACAACGAGAGATATTT

TTAGCGTGGATTTCTCATAGACCTGTAGTTTTAACTGGAGGAACCGGAGTGGGTAAGACGTCACAGGTAC

CCAAGTTATTGCTTTGGTTTAATTATTTATTTGGTGGATTCTCTTCTCTAGATAAAATCACTGACTTTCA

CGAAAGACCAGTCATTCTATCTCTTCCTAGGATAGCTTTAGTTAGATTGCATAGCAATACCATTTTAAAA

TCATTGGGATTTAAGGTACTAGATGGATCTCCTATCTCTTTACGGTACGGATCTATACCGGAAGAATTAA

TAAACAAACAACCAAAAAAATATGGAATTGTATTTTCTACCCATAAGTTATCTCTAACAAAACTATTTAG

TTATGGCACTATTATTATAGACGAAGTTCATGAGCATGATCAAATAGGAGATATTATTATAGCAGTAGCG

AGAAAACATCATACGAAAATAGATTCTATGTTTTTAATGACTGCCACGTTAGAGGATGACAGGGAACGTC

TAAAAATATTTTTACCTAATCCCGCATTTATACATATTCCTGGAGATACACTGTTTAAAATTAGCGAGGT

ATTTATTCATAATAAGATAAATCCATCTTCCAGAATGGCATATATAGAAGAAGAAAAGAGAAATTTAGTT

ACTGCTATACAGATGTATACTCCTCCTGATGGATCATCCGGTATAGTCTTTGTGGCATCCGTTGCACAGT

GTCACGAATATAAATCATATTTAGAAAAAAGATTACCGTATGATATGTATATTATTCATGGTAAGGTCTT

AGATATAGACGAAATATTAGAAAAAGTGTATTCATCACCTAATGTATCGATAATTATTTCTACTCCTTAT

TTGGAATCCAGCGTTACTATACGCAATGTTACACACATTTATGATATGGGTAGAGTTTTTGTCCCCGCTC

CTTTTGGAGGATCACAACAATTTATTTCTAAATCTATGAGAGATCAACGAAAAGGAAGAGTAGGAAGAGT

TAATCCTGGAACATACGTATATTTCTATGATCTGTCTTATATGAAATCTATACAGCGAATAGATTCAGAA

TTTCTACATAATTATATATTGTACGCTAATAAGTTTAATCTAACACTCCCCGAAGATTTGTTTATAATCC

CTACAAATTTGGATATTCTATGGCGTACAAAGGAATATATAGACTCGTTCGATATTAGTACAGAAACATG

GAATAAATTATTATCCAATTATTATATGAAGATGATAGAGTATGCTAAACTTTATGTACTAAGTCCTATT

CTCGCTGAGGAGTTGGATAACTTTGAGAGGACGGGAGAATTAACTAGTATTGTACAAGAAGCCATTTTAT

CTCTAAATTTACAAATTAAGATTTTAAAATTTAAACATAAAGATGATGATACGTATATACACTTTTGTAG

AATATTATTCGGTGTCTATAACGGAACAAACGCTACTATATATTATCATAGACCTCTAACGGGATATATG

AATATGATTTCAGATACTATATTTGTTCCTGTAGATAATAACTAAAAATCAAAATCTAATGACCACATCT

TTTTTTAGAGATGAAAAATTTTCCACATCTCCTTTTGTAGACACGACTAAACATTTTGCAGAAAAAAGTT

TATTATTATTTAGATAATCGTATACTTCATCAGTGTAGATAGTAAATGTGAACAGATAAAAGGTATTCTT

GCTCAATAGATTGGTAAATTCCATAGAATATATTAATCCTTTCTTCTTGAGATCCCACATCATTTCAACC

AAAGACGTTTTATCCAATGATTTACCTCGTACTATACCACATACAAAACTAGATTTTGCAGTGATGTCGT

ACCTGGTATTCCTACCAAACAAAATTTTACTTTTAGTTCTTTTAGAAAATTCTAAGGTAGAATCTCTATT

TGTCAATATGTCATCTATGGAATTACCACTAGCAAAAAATGATAGAAATATATATTGATACATCGCAGCT

GGTTTTGATCTACTATACTTTAAAAACGAATCAGATTCCATAATTGCTTGTATATCATCAGCTGAAAAAC

TATGTTTTACACGTATTCCTTCGGCATTTCTTTTTAATGATATATCTTGTTTAGACAATGATAAAGTTAT

CATGTCCATGAGAGACGCGTCTCCGTATCGTATAAATATTTCATTAGATGTTAGACGCTTCATTAGGGGT

ATACTTCTATAAGGTTTCTTAATTAGTCCATCATTGGTTGCGTCAAGAACTACTATCTGATGTTGTTGGG

TATCTCTAGTGTTACACATGGCCTTACTAAAGTTTGGGTAAATAACTATGATATCTCTATTAATTATAGA

TGTATATATTTCATTCGTCAAGGATATTAATATCGACTTACTATCGTCATTAATACGTGTAATGTAATCA

TATAAATCATGCGATAGCCAAGGAAAATTCAAATAGATGTTCATCATATAATCGTCGCTATAATTCATAT

TAATACTTTGACATTGACTAATTTGTAATATAGCCTCGCCACGAAGAAAGCTCTCGTATTCAGTTTCATC

GATAAAGGATACCGTTAAATATAACTGGTTGCCGATAGTCTCATAGTCTATTAAGTGGTAAGTTTCGTAT

AAATACAGAATCCCTAAAATATTATCTAATGTGGGATTAATCCTTACCATAACTGTATAAAATGGAGCCG

GAGTCATAACTATTTTACCGTTTGTACTTACTGGAATAGATGAAGGAATAATCTCCGGACATGATGGTAA

AGACCCAAATGTCTGTTTGAAGAAATCCAATGTTCCAGGTCCTAATCTCTTGACAAAAATTACGATATTC

GATCCCGATATCCTTTGCATTCTATTTACCAGCATATCACGAACTATATTAAGATTATCTATCATGTCTA

TTCTCCCACCGTTATATAAATCGCCTCCGCTAAGAAACGTTAGTATATCCATACAATGGAATACTTCATT

TCTAAAATAGTATTCGTTTTCTAATTCTTTAATGTGAAATCGTATACTAGAAAGGGAAAAATTATCTTTG

AGTTTTCCATTAGAAAAGAACCACGAAACTAATGTTCTGATTGCGTCTGACTCCGTCGCTGAATTAATAG

ATTTACACCAAAAACTCATATAACTTCTAGATGTAGAAGCATTCGCTAAAAAATTAGTAGAATCAAAGGA

TATAAGTAGATGTTCCAACAAGTGAGCAATTCCCAAGATTTCATCTATATCATTCTCGAATCCGAAATTA

GAAATTCCCAAGTAGATATCCTTTTTCATCCGATCATTGATGAAAATACGAACTTTATTCGGTAAGACGA

TCATTTACTAAGGAGTAAAATAGGAAGTAACGTTCGTATATCGTTATCGTCGTATAAATTAAAGGTGTGT

TTTTTGCCATTAAGAGACATTATAATTTTACCAATATTGGAATTATAATATAGGTGTATTTGAGCACTAG

AAACGGTCGATGCATCGGTAAATATAGCTGTATCTAATGTTCTAGTCGGTATTTCTTCATTTCGCTGTCT

AATGATAGCGTTTTCTCTATCTGTTTCCATTACAGCTGCCTGAAGTTTATTGGTCGGATAATATGTAAAA

TAATAAGAAATACATACGAATAACAAAAATAAAATAAGATATAATAAAGATGCCATTTAGAGATCTAATT

TTGTTCAACTTGTCCAAATTCCTACTTACAGAAGATGAGGAATCGTTGGAGATAGTATCTTCCTTATGTA

GAGGATTTGAAATATCTTACGATGACTTAATATCGTACTTTCCAGATAGGAAATACCATAAATATATTTC

TAAGGTATTTGAACATGTAGATTTATCGGAGGAATTAAGTATGGAATTCCATGATACAACTCTGAGAGAT

TTAGTATATCTTAGATTGTACAAGTATTCCAAGTATATACGGCCGTGTTATAAATTAGGAGATAATCTAA

AAGGTATAGTTGTTATAAAGGACAGAAATATATATATTAGAGAAGCAAATGATGACTTGATAGAATATCT

CCTCAAGGAATACACTCCTCAGATTTATACATATTCTAATGAGCGAGTTCCCATAGCTGGTTCAAAATTA

ATTCTTTGTGGATTTTCTCAAGTTACATTTATGGCGTATACAACGTCGCATATAACAACAAATAAAAAGG

TAGATGTTCTCGTTTCCAAAAAATGTATAGATGAACTAGTCGATCCAATAAATTATCAAATACTTCAAAA

TTTATTTGATAAAGGAAGCGGAACAATAAACAAAATACTCAGGAAGATATTTTATTCGGTAACAGGTGGC

CAAACTCCATAGGTAGCTTTTTCTATTTCGGATTTTAGAATTTCCAAATTCACCAGCGATTTATCGGTTT

TGGTGAAATCCAAGGATTTATTAATGTCCACAAATGCCATTTGTTTTGTCTGTGGATTGTATTTGAAAAT

GGAAACGATGTAGTTAGATAGATGCGCGGCGAAGTTTCCTATTAGGGTTCCGCGCTTCACGTCACCCAAC

ATACTTGAATCACCATCCTTTAAAAAAAATGATAAGATATCAACATGGAGTATATCATACTCGGATTTTA

ATTCTTCTACTGCCTCACTGACATTTTCACAAATACTACAATACGGTTTACCGAAAATAATCAGTACGTT

CTTCATTTATGGGTATCAAAAACTTAAAATCGTTACTGCTGGAAAATAAATCACTGACGATATTAGATGA

TAATTTATACAAAGTATACAATGGAATATTTGTGGATACAATGAGTATTTATATAGCCGTCGCCAATTGT

GTCAGAAACTTAGAAGAGTTAACTACGGTATTCATAAAATACGTAAACGGATGGGTAAAAAAGGGAGGAC

ATGTAACCCTTTTTATCGATAGAGGAAGTATAAAAATTAAACAAGACGTTAGAGACAAGAGACGTAAATA

TTCTAAATTAACCAAGGACAGAAAAATGTTAGAATTAGAAAAGTGTACATCCGAAATACAAAATGTTACC

GGATTTATGGAAGAAGAAATAAAGGCAGAAATGCAATTAAAAATCGATAAACTCACATTTCAAATATATT

TATCTGATTATGATAACATAAAAATATCATTGAATGAGATACTAACACATTTCAACAATAATGAGAATGT

TACATTATTTTATTGTGATGAACGAGACGCAGAATTCGTTATGTGTCTAGAGGCTAAAACACAGTTCTCT

ACCACAGGAGAATGGCCGTTAATAATAAGTACCGATCAGGATACTATGCTATTCGCGTCTGCTGATAATC

ATCCTAAGATGATAAAAAACTTAACTCAACTGTTTAAATTTGTTCCCTCGGCAGAGGATAACTATTTAGC

AAAATTAACTGCATTAGTGAATGGATGTGATTTCTTTCCTGGACTCTATGGGGCATCTATAACACCCAAC

AACTTAAACAAAATACAATTGTTTAGTGATTTTACAATCGATAATATAGTCACTAGTTTGGCAATTAAAA

ATTATTATAGAAAGACTAACTCTACCGTAGACGTGCGTAATATTGTTACGTTTATAAACGATTACGCTAA

TTTAGACGATGTCTACTCGTATATTCCTCCTTGTCAATGCACTGTTCAAGAATTTATATTCTCCGCATTA

GATGAAAAATGGAATGAATTTAAATCATCTTATTTAGAGAGCGTGCCGTTACCCTGCCAATTAATGTACG

CATTAGAACCACGTAAGGAGATTGATGTTTCAGAAGTTAAAACTTTATCATCTTATATAGATTTCGAAAA

TACTAAATCAGATATCGATGTTATAAAATCTATATCCTCGATTTTTGGATATTCTAACGAAAACTGTAAC

ACCATAGTGTTCGGCATCTATAAGGATAATTTACTACTGAGTATAAATAATTCATTTTACTTTAACGATA

GTCTGTTAATAACCAATACTAAAAGTGATAATATAATAAATATAGGTTACTAGATTAAAAAATGGTGTTC

CAGCTCGTGTGTTCTACATGCGGCAAAGATATTTCTCACGAACGATATAAATTGATTATACGAAAAAAAT

CATTAAAGGATGTACTAGTCAGTGTAAAGAACGAATGTTGTAGGTTAAAATTATCTACACAAATAGAACC

TCAACGTAACTTAACAGTGCAACCTCTATTGGATATAAACTAATGGATCCGGTTAATTTTATCAAGACAT

ATGCGCCTAGAGGTTCTATTATTTTTATTAATTATGCCATGTCATTAACTAGTCATTTGAATCCATCGAT

AGAAAAACATGTGGGTATTTATTATGGTACGTTATTATCGGAACACTTGGTAGTTGAATCTACCTATAGA

AAAGGAGTTAGAATAGTCCCATTGGATAGATTTTTTGAAGGATATCTTAGTGCAAAAGTATACATGTTAG

AGAATATTCAAGTTATGAAAATAGCAGCTGATATGTCGTTAACTTTACTAGGTATTCCATATGGATTTGG

TCATGATAGAATGTATTGTTTTAAATTGGTAGCTGAATGTTATAAAAATGCCGGTATTGATACATCGTCT

AAACGAATATTAGGTAAAGATATTTTTCTGAGCCAAAACTTTACAGATGATAATAGATGGATAAAGATAT

ATGATTCTAATAATTTAACATTTTGGCAAATTGATTACCTTAAAGGGTGAGTTAATATGCATAACTACTC

CTCCGTTGTTTTTTCCCTCGTTCTTTTTCTTAACGTTGTTTGCCATCACTCTCATAATGTAAAGATATTC

TAAAATGGTAAACTTTTGCATATCGGATGCAGAAATTGGTATAAATGTTGTAATTGTATTATTTCCCGTC

AATGGACTAGTCACAGCTCCATCAGTTTTATATCCTTTAGAGTATTTCTCACTCGTGTCTAGCATTCTAG

AGCATTCCATGATCTGTTTATCGTTGATATTGGCCGGAAAGATAGATTTTTTATTTTTTATTATATTACT

ATTGGCAATTGTAGATATAACTTCTGGTAAATATTTTTCTACCTTTTCAATCTCTTCTATTTTCAAGCCG

GCTATATATTCTGCTATATTGTTACTAGTATCAATACCTTTTCTGGCTAAGAAGTCATATGTGGTATTCA

CTATATCAGTTTTAACTGGTAGTTCCATTAGCCTTTCCACTTCTGCAGAATAATTAGAAATTGGTTCTTT

ACCAGAAAATCCAGCTACTATAATAGGCTCACCGATGATCATTGGCAAAATCCTATATTGTACCAGATTA

ATGAGAGCATATTTCATTTCCAATAATTCTGCTAGTTCTTGAGACATTGATTTATTTGATGAATCTATTT

GGTTCTCTAGATACTCTACCATTTCTGCCGCATACAATAACTTGTTAGATAAAATCAGGGTTATCAAAGT

GTTTAGTGTGGCTAGAATAGTGGGCTTGCACGTATTAAAGAATGCTGTAGTATGAGTAAACCGTTTTAAC

GAATTATATAGTCTCCAGAAATCTGTGGCGTTGCATACATGAACTGAATGACATCGAAGATTGTCCAATA

TTTTTAATAGCTGCTCTTTGTCCATTATTTCTATATTTGACTCGCAACAATTGTAGATACCATTAATCAC

TGATTCCTTTTTCGATGCCGGACAATAGCACAATTGTTTAGCTTTGGACTCTATGTATTCAGAATTAATA

GATATATCTCTCAATACAGATTGCACTATACATTTTGAAACTATGTCAAAAATTGTAGAACGACGCTGTT

CTGTAGCCATTTAACTTTAAATAATTTACAAAAATTTAAAATGAGCATCCGTATAAAAATCGATAAATTG

CGCCAAATTGTGGCATATTTTTCAGAGTTCAGCGAAGAAGTGTCTATAAATGTAGACTTGACGGATGAAT

TAATGTATATTTTTGCCGCCTTGGGCGGATCTGTAAACATTTGGGCCATTATACCTCTCAGTGCATCAGT

GTTCTACCGCGGAGCCGAAAATATTGTGTTTAACCTTCCAGTGTCCAAGGTAAAATCGTGTTTGTGTAGT

TTTCACAATGATGCTATCATAAATATAGAACCTGATCTGGAAAATAATCTAGTAAAACTTTCTAGTTATC

ATGTAGTAAGTGTCGATTGTAACAAGGAACTGATGCCTATTAGGACAGATACTACTATTTGTCTAAGTAT

AGATCAAAAGAAATCTTACGTATTTAATTTTCACAAGTATGAAGAAAAATGTTGTGGTAGAACCGTCATT

CATCTAGAATGGTTGTTGGGCTTTATCAAGTGTATTAGTCAGCATCAGCATTTGGCTATTATGTTTAAAG

ATGACAATATTATTATGAAGACTCCTGGTAATACTGATGCGTTTTCCAGGGAATATTCTATGACTGAATG

TTCTCAAGAACTACAAAAGTTTTCTTTCAAAATAGCTATCTCGTCTCTCAACAAACTACGAGGATTCAAA

AAGAGAGTCAATGTTTTTGAAACTAGAATCGTAATGGATAATGACGATAACATTCTAGGAATGTTGTTTT

CGGATAGAGTTCAATCCTTTAAGATTAACATCTTTATGGCGTTTTTAGACTAATACTTTCAATGAGATAA

ATATGGGTGGCGGAGTAAGTGTTGAGCTCCCTAAACGGGATCCACCTCCGGGAGTACCCACTGATGAGAT

GTTATTAAACGTGGATAAAATGCATGACGTGATAGCTCCCGCTAAGCTTTTAGAATATGTGCATATAGGA

CCACTAACAAAAGATAAAGAGGATAAAGTAAAGAAAAGATATCCAGAGTTTAGATTAGTCAACACAGGAC

CCGGTGGTCTTTCGGCATTATTAAGACAATCATATAATGGAACCGCACCCAATTGCTGTCGCACTTTTAA

TCGTACTCATTATTGGAAGAAGGATGGAAAGATATCAGATAAGTATGAAGAGGGTGCAGTATTAGAATCG

TGTTGGCCCGACGTCCACGACACTGGAAAATGCGATGTTGATTTATTCGACTGGTGTCAGGGGGATACGT

TCGATATAAACATATGCCATCAGTGGATCGGTTCAGCCTTTAATAGGAGTGATAGAACTGTAGAGGGTCG

ACAATCGTTAATAAATCTGTATAATAAGATGCAAAGATTATGTAGTAAAGATGCTAGTGTACCAATATGT

GAATTATTTTTGCATCATTTACGCGCACACAATACAGAAGATAGTAAAGAGATGATCGATTATATTCTAA

GACAACAGTCGGCGGACTTTAAACAGAAATATATGAGATGTAGTTATCCCACTAGAGATAAGTTAGAAGA

GTCATTAAAATATGCGGAACCTCGAGAATGTTGGGATCCAGAGTGTTCGAATGCCAATGTTAATTTCTTA

CTAACACGTAATTATAATAATTTAGGACTTTGCAATATTGTACGATGTAATACGAGCGTGAATAACTTAC

AGATGGATAAAACTTCCTCATTAAGATTATCATGTGGATTAAGCAATAGTGATAGATTTTCTACTGTTCC

CGTCAATAGAGCAAAAGTAGTTCAACATAATATTAAACATTCGTTCGACCTAAAATTGCATTTGATCAGT

TTATTATCTCTCTTGGTAATATGGATACTAATTGTAGCTATTTAAATGGGTGCCGCAGCAAGCATACAGA

CGACTGTGAATACACTCAGTGAACGTATCTCGTCTAAATTAGAACAAGAAGCGAACGCTAGTGCTCAAAC

AAAATGTGATATAGAAATCGGAAATTTTTATATCCGACAAAACCATGGATGTAACATCACTGTTAAAAAT

ATGTGCTCTGCGGACGCGGATGCTCAGTTGGATGCTGTGTTATCAGCCGCTACAGAAACATATAGTGGAT

TAACACCGGAACAAAAAGCATACGTACCAGCTATGTTTACTGCTGCGTTAAACATTCAGACGAGTGTAAA

CACTGTTGTTAGAGATTTTGAAAATTATGTGAAACAGACTTGTAATTCTAGCGCTGTTGTCGATAACAAA

TTAAAGATACAAAACGTAATTATAGATGAATGTTACGGAGCCCCAGGATCTCCAACAAATTTGGAATTTA

TTAATACAGGATCTAGCAAAGGAAATTGTGCCATTAAGGCGTTGATGCAATTGACTACTAAGGCCACTAC

TCAAATAGCACCTAGACAAGTTGCTGGTACAGGAGTTCAGTTTTATATGATTGTTATCGGTGTTATAATA

TTGGCAGCGTTGTTTATGTACTATGCCAAGCGTATGCTGTTCACATCCACCAATGATAAAATCAAACTTA

TTTTAGCCAATAAGGAAAACGTCCATTGGACTACTTACATGGACACATTCTTTAGAACTTCTCCGATGAT

TATTGCTACCACGGATATACAAAACTGAAAATATATTGATAATATTTTAATAGATTAACATGGAAGTTAT

CGCTGATCGTCTAGACGATATAGTGAAACAAAATATAGCGGATGAAAAATTTGTAGATTTTGTTATACAC

GGTCTAGAGCATCAATGTCCTGCTATACTTCGACCATTAATTAGGTTGTTTATTGATATACTATTATTTG

TTATAGTAATTTATATTTTTACGGTACGTCTAGTAAGTAGAAATTATCAAATATTGTTGGTGTTGGTGGC

GCTAGTCATCACATTAACTATTTTTTTATTACTTTATACTATAATAGTACTAGACTGACTTCTAACAAAC

ATCTCACCTGCCATAAATAAATGCTTGATATTAAAGTCTTCTATTTCTAACACTATTCCATCTGTGGAAA

ATAATACTCTGACATTATCGCTAATTGATACATCGGTAAGTGATATGCCTATAAAGTAATAATCTTCTTT

GGGCACATATACCAGTGTACCAGGTTCTAACAACCTATTTACTGGTGCTCCTGTAGCATACTTTTTTTTT

ACCTTGAGAATATCCATTGTTTGCTTGGTCAATAGTGATATGTGATTTTTTATCAACCACTCAAAAAAGT

AATTGGAGTGTTCATATCCTCTACGGGCTATTGTCTCATGACCGTGTATGAAATTTAAGTAACACGACTG

TGGTAGATTTGTTCTATAGAGCCGGTTGCCGCAAATAGATAGAACTACCAATATGTCTGTACAAATGTTA

AACATTAATTGATTAACAGAAAAAACAATGTTCGTTCTGGGAATAGAAACCAGATTAAAACAAAATTCAT

TAGAATATATGCCACGTTTATACATGGAATATAAAATAACTACAGTTTGAAAAATAACAGTATCATTTAA

ACATTTAACTTGCGGGGTTAATCTCACAACTTTACTGTTTTTGAACTGTTCAAAATATAGCATAGATCCA

TGAGAAATACGTTTAGCCGCCTTTAATAGAGGAAATCCAACCGCCTTTCTGGATCTCACCAACGACGATA

GTTCTGACCAGCAACTCATTTCTTCATCATCCACCTGTTTTAACATATAATAGGCAGGAGATAGATATCC

ATCATTGCAATATTCCTTCTCGTAGGCACACAATCTAATATTGATAAAATCTCCATTCTCTTCTCTGTAT

TTATTATCTTGTCTCGGTGGCTGATTAGGCTGTGGTCTATCGTTGTTGAATCTATTTTGGTCATTAAATC

TTTCATTTCTTCCTGGTATATTTCTATCACCTCGTTTGGTTGGATTTTTGTCTATATTATCGTTTGTAAC

ATCGGTACGGGTATTCATTTATCACAAAAAAAACTTCTCTAAATGAGTCTACTACTAGAAAACCTCATCG

AAGAAGATACCATATTTTTTGCAGGAAGTATATCTGAGTATGATGATTTACAAATGGTTATTGCTGGTGC

AAAATCCAAATTTCCAAGATCTATGCTTTCTATTTTTAATATAGTACCTAGAACGATGTCAAAATATGAG

TTGGAGTTGATTCATAACGAGAATATCACAGGGGCAATGTTTACCACAATGTATAATATAAGAAACAATT

TGGGTCTAGGCGATGATAAACTAACTATTGAAGCCATTGAAAACTATTTCTTGGATCCTAACAATGAGGT

TATGCCTCTTATCATTAATAATACGGATATGACTACCGTCATTCCTAAAAAAAGTGGTAGGAGAAAGAAT

AAGAACATGGTTATCTTCCGTCAAGGATCATCACCTATCTTGTGTATTTTCGAAACTCGTAAAAAGATTA

ATATTTATAAAGAAAATATGGAATCCGTATCGACTAAGTATACACCTATCGGAGACAACAAGGCTTTGAT

ATCTAAATATGCGGGAATTAATATCCTGAATGTGTATTCTCCTTCCACGTCCATGAGATTGAATGCCATT

TACGGATTCACCAATAAAAATAAACTAGAGAAACTTAGTACTAATAAGGAACTAGAATCGTATAGTTCTA

GCCCTCTTCAAGAACCCATTAGGTTAAATGATTTTCTGGGACTATTGGAATGTGTTAAAAAGAATATTCC

TCTAACAGATATTCCGACAAAGGATTGATTACTATAAATGGAGAATGTTCCTAATGTATACTTTAATCCT

GTGTTTATAGAGCCCACGTTTAAACATTCTTTATTAAGTGTTTATAAACACAGATTAATAGTTTTATTTG

AAGTATTCGTTGTATTCATTCTAATATATGTATTTTTTAGATCTGAATTAAATATGTTCTTCATGCCTAA

ACGAAAAATACCCGATCCTATTGATAGATTACGACGTGCTAATCTAGCGTGTGAAGACGATAAATTAATG

ATCTATGGATTACCATGGATAACAACTCAAACATCTGCGTTATCAATAAATAGTAAACCGATAGTGTATA

AAGATTGTGCAAAGCTTTTGCGATCAATAAATGGATCACAACCAGTATCTCTTAACGATGTTCTTCGCAG

ATGATGATTCATTTTTTAAGTATTTTGCTAGTCAAGATGATGAATCTTCATTATCTGATATATTGCAAAT

CACTCAATATCTAGACTTTCTGTTATTATTATTGATCCAATCAAAAAATAAATTAGAAGCTGTGGGTCAT

TGTTATGAATCTCTTTCAGAGGAATACAGACAATTGACAAAATTCACAGACTCTCAAGATTTTAAAAAAC

TGTTTAACAAGGTCCCTATTGTTACAGATGGAAGGGTCAAACTTAATAAAGGATATTTGTTCGACTTTGT

GATTAGTTTGATGCGATTCAAAAAAGAATCAGCTCTAGCTACCACCGCAATAGATCCTGTTAGATACATA

GATCCTCGTCGTGATATCGCATTTTCTAACGTGATGGATATATTAAAGTCGAATAAAGTTGAAAAATAAT

TAATTCTTTATTGTTATCATGAACGGCGGACATATTCAGTTGATAATCGGCCCCATGTTTTCAGGTAAAA

GTACAGAATTAATTAGACGAGTTAGACGTTATCAAATAGCTCAATATAAATGTGTGACTATAAAATATTC

TAACGATAATAGATACGGAACGGGACTATGGACACATGATAAGAATAATTTTGCAGCATTGGAAGTAACT

AAACTATGTGATGTCTTGGAAGCAATTACAGATTTCTCCGTGATAGGTATAGATGAAGGACAGTTCTTTC

CAGACATTGTTGAATTCTGTGAGCGTATGGCAAACGAAGGAAAAATAGTTATAGTAGCCGCGCTCGATGG

GACATTTCAACGTAGACCGTTTAATAATATTTTGAATCTTATTCCATTATCTGAAATGGTGGTAAAACTA

ACTGCAGTGTGTATGAAATGCTTTAAGGAGGCTTCCTTTTCTAAACGATTAGGTACAGAAACCGAGATAG

AAATAATAGGAGGTAATGATATGTATCAATCTGTGTGTAGAAAGTGTTACATCGACTCATAATATTATAT

TTTTTATCTAAAAAACTAAAAATAAACATTGATTAAATTTTAATATAATACTTAAAAATGGATGTTGTGT

CGTTAGATAAACCGTTTATGTATTTTGAGGAAATTGATAATGAGTTAGATTACGAACCAGAAAGTGCAAA

TGAGGTCGCAAAAAAACTGCCGTATCAAGGACAGTTAAAACTATTACTAGGAGAATTATTTTTTCTTAGT

AAGTTACAGCGACACGGTATATTAGATGGCGCCACCGTAGTGTATATAGGATCTGCTCCAGGTACACATA

TACGTTATTTGAGAGATCATTTCTATAATTTAGGAGTGATCATCAAATGGATGCTAATTGACGGCCGCCA

TCATGATCCTATTCTAAATGGATTGCGTGATGTGACTCTAGTGACTCGGTTTGTTGATGAGGAATATCTA

CGATCCATCAAAAAACAACTACATCCTTCTAAGATTATTTTAATTTCTGATGTGCGATCCAAACGAGGAG

GAAATGAACCTAGTACTGCGGATTTACTAAGTAATTATGCTCTACAAAATGTCATGATTAGTATTTTAAA

CCCCGTGGCGTCTAGTCTTAAATGGAGATGCCCGTTTCCAGATCAATGGATCAAGGACTTTTATATCCCA

CACGGTAATAAAATGTTACAACCTTTTGCTCCTTCATATTCAGCTGAAATGAGATTATTAAGTATTTATA

CCGGTGAGAATATGAGACTGACTCGAGTTACCAAATCAGACGCTGTAAATTATGAAAAAAAGATGTATTA

CCTTAATAAGATAGTCCGCAACAAAGTAGTTATTAACTTTGATTATCCTAATCAGGAATATGACTATTTT

CACATGTACTTTATGTTGAGGACCGTATACTGCAATAAAACATTTCCTACTACTAAAGCAAAGATACTAT

TTCTACAACAATCTATATTTCGTTTCTTAAATATTCCAACGACATCAACTGAAAAAGTTAGTCATGAACC

AATACAACGTAAAATATCTAGCAAAGATTCTATGTCTAAAAACAGAAATAGCAAGAGATCCGTACGCGGT

AATAAATAGAAACGTACTACTGAGATATACTACCGATATAGAGTATAATGATTTAGTTACTTTAATAACC

GTTAGACATAAAATTGATTCTATGAAAACTGTGTTTCAGGTATTTAACGAATCATCCATAAATTATACTC

CGGTTGATGATGATTATGGAGAACCAATCATTATAACATCGTATCTTCAAAAAGGTCATAACAAGTTTCC

TGTAAATTTTCTATACATAGATGTGGTAATATCTGACTTATTTCCTAGCTTTGTTAGACTAGATACTACA

GAAACTAATATAGTTAATAGTGTACTACAAACAGGCGATGGTAAAAAGACTCTTCGTCTTCCTAAAATGT

TAGAGACGGAAATAGTTGTCAAGATTCTCTATCGTCCTAATATACCATTAAAAATTGTTAGATTTTTCCG

CAATAACATGGTAACTGGAGTAGAGATAGCCGATAGATCTGTTATTTCAGTCGCTGATTAATCAATTAGT

AGAGATGAGATAAGAACATTATAATAATCAATAATATATCTTATATCTGTTTAGAAAAATGCTAATATTA

AAATAGCTAACGCTAGTAATCCAATCGGAAGCCATTTGATATCTATAATAGGGTATCTAATTTCCTGATT

CAGATAGCGTACGGCTATATTCTCGGTAGCTACTCGTTTGGAATCACAGACATTATTTACATCTAATTTA

CTATCTGTAATGGAAACGTTTCCCAATGAAATGGTACAATCAGATACATTACATCTTGATATATTTTTTT

TTAAAGAGGCTGGTAACAACGCATCGCTTCGTTTACATGGCTCGTACCAACAATAATAGGGTAATCTTGT

ATCTATTCCTATCCGTACTATACTTTTATCAGGATAAATACATTTACATCGTATATCGTCTTTGTTAGTA

TCACAGAATGCATAAATTTGTTCGTCCGTCATGATAAAAATTTAAAGTGTAAATATAACTATTATTTTTA

TAGTTATAATAAAAAGGGAAATTTGATTGTATACCTTCGGTTCTTTAAAAGAAACTGACTTGATAAAAAT

GGCTGTAATCTCTAAGGTTACGTATAGTCTATACGATCAAAAAGAGATTAATGCCACAGATATTATCATT

AGTCATATTAAAAATGACGACGATATCGGTACCGTTAAAGATGGTAGACTAGGTGCTATGGATGGGGCAT

TATGTAAAACTTGTGGGAAAACGGAATTGGAATGTTTCGGTCACTGGGGTAAAGTAAGTATTTATAAAAC

TCATATAGTTAAGCCTGAATTTATTTCAGAAATTATTCGTTTACTGAATCATATATGTATTCATTGCGGA

TTATTGCGTTCACGAGAACCGTATTCCGACGATATTAACCTAAAAGAGTTATCGGTACACGCTCTTAGGA

GATTAAAGGATAAAATATTATCCAAGAAAAAGTCATGTTGGAACAGCGAATGTATGCAACCGTATCAAAA

AATTACTTTTTCAAAGAAAAAGGTTTGTTTCGTCAACAAGTTGGATGATATTAACGTTCCTAATTCTCTC

ATCTATCAAAAGTTAATTTCTATTCATGAAAAGTTTTGGCCATTATTAGAAATTCATCAATATCCAGCTA

ACTTATTTTATACAGACTACTTTCCCATCCCTCCGTTGATTATTAGACCGGCTATTAGTTTTTGGATAGA

TAGTATACCCAAAGAGACAAATGAATTAACTTACTTATTAGGTATGATCGTTAAGAATTGTAACTTGAAT

GCTGATGAACAGGTTATCCAGAAGGCGGTAATAGAATACGATGATATTAAAATTATTTCTAATAACACTA

CCAGTATCAATTTATCATATATCACATCCGGCAAAAATAATATGATTAGAAGTTATATCGTCGCTCGGCG

AAAAGATCAGACCGCTAGATCCGTAATTGGTCCCAGTACATCTATCACCGTTAATGAGGTAGGAATGCCC

ACATATATTAGAAATACACTTACAGAAAAGATATTTGTTAATGCCTTTACAGTGGATAAAGTTAAACAAC

TATTAGCATCAAACCAAGTTAAATTTTACTTTAATAAACGATTAAACCAATTAACAAGAATACGTCAAGG

AAAGTTTATCAAAAATAAAATACATTTATTGCCTGGTGATTGGGTAGAAGTAGCTGTTCAAGAATATACA

AGTATTATTTTTGGAAGACAACCGTCTCTACATAGATACAACGTCATCGCTTCATCTATCAGAGCTACCG

AAGGAGATACTATCAAAATATCTCCCGGAATTGCCAACTCTCAAAATGCTGATTTTGACGGAGATGAAGA

ATGGATGATATTGGAGCAAAATCCTAAAGCCGTAGTTGAACAAAGTATTCTTATGTATCCGACAACGTTA

CTCAAACACGATATTCATGGAGCCCCCGTTTATGGATCTATTCAAGATGAAATCGTAGCAGCGTATTCAT

TGTTTAGGATACAAGATCTTTGTTTAGATGAAGTATTGAACATCTTGGGGAAATATGGAAGAGAGTTCGA

TCCTAAAGGTAAATGTAAATTCAGCGGTAAAGATATCTATACTTACTTGATAGGTGAAAAGATTAATTAT

CCGGGTCTCTTAAAGGATGGTGAAATTATTGCAAACGACGTAGATAGTAATTTTGTTGTAGCTATGAGGC

ATCTGTCATTGGCTGGACTCTTATCCGATCATAAATCGAACGTGGAAGGTATCAACTTTATTATCAAGTC

ATCTTATGTTTTTAAGAGATATCTATCTATATACGGTTTTGGGGTGACATTCAAAGATCTGAGACCAAAT

TCGACGTTCACTAATAAATTGGAGGCTATCAACGTAGAAAAAATAGAACTTATCAAAGAAGCATACGCCA

AATATCTCAAAGATGTAAGAGACGGGAAAATAGTTCCATTATCTAAAGCTTTAGAGGCGGACTACTTGGA

ATCCATGTTATCCAACTTGACAAATCTTAATATCAGAGAGATAGAAGAACATATGAGACAAACGCTGATA

GATGATCCAGATAATAACCTCCTGAAAATGGCCAAAGCGGGTTATAAAGTAAATCCCACAGAACTAATGT

ATATTCTAGGTACTTATGGACAACAGAGGATAGATGGCGAACCAGCAGAGACTCGAGTATTGGGTAGAGT

CTTACCTTACTATCTTCCAGACTCTAAGGATCCAGAAGGAAGAGGTTATATTCTTAATTCTTTAACAAAA

GGATTAACGGGTTCTCAATATTACTTTTTGATGCTGGTTGCAAGATCTCAATCTACTGATATTGTCTGTG

AAACATCACGTACCGGAACACTGGCTAGAAAAATCATTAAAAAGATGGAGGATATGGTGGTCGACGGATA

CGGACAAGTAGTTATAGGTAATACGCTCATCAAGTACGCAGCCAATTATACCAAAATTCTAGGCTCAGTA

TGTAAACCTGTAGATCTTATCTATCCAGATGAGTCCATGACTTGGTATTTGGAAATTAGTGCTTTGTGGA

ATAAAATAAAACAGGGATTCGTTTACTCTCAGAAACAGAAACTTGCAAAGAAGACATTGGCGCCGTTTAA

TTTCCTAGTATTCGTCAAACCCACCACTGAGGATAATGCTATTAAGGTTAAGGATCTGTACGATATGATT

CATAACGTCATTGATGATGTGAGAGAGAAATACTTCTTTACGGTATCTAATATAGATTTTATGGAGTATA

TATTCTTGACGCATCTTAATCCTTCTAGAATTAGAATTACAAAAGAAACGGCTATTACTATCTTTGAAAA

GTTCTATGAAAAACTCAATTATACTCTAGGTGGTGGAACTCCTATTGGAATTATTTCTGCACAGGTATTG

TCTGAGAAGTTTACACAACAAGCCCTGTCCAGTTTTCACACTACTGAAAAGAGTGGTGCTGTAAAACAAA

AACTTGGTTTCAACGAGTTTAATAACTTGACTAATTTGAGTAAGAATAAGACCGAAATTATCACTCTGGT

ATCCGATGATATCTCTAAACTTCAATCTGTTAAGATTAATTTCGAATTTGTATGTTTGGGAGAATTAAAT

CCAGACATCACTCTTCGAAAAGAAACAGATAGATATGTAGTAGACATAATAGTCAATAGATTATACATCA

AGAGAGCAGAAATAACCGAATTAGTCGTCGAATATATGATTGAACGATTTATCTCCTTTAGCGTCATTGT

AAAGGAATGGGGTATGGAGACATTCATTGAGGACGAGGATAATATTAGATTTACTATCTACCTAAATTTC

GTTGAACCGGAGGAATTGAATCTTAGTAAGTTTATGATGGTTCTTCCAGGTGCCGCCAACAAGGGCAAGA

TTAGTAAATTCAAGATTCCTATCTCTGACTATACGGGATATAACGACTTCAATCAAACAAAAAAGCTCAA

TAAGATGACTGTAGAACTCATGAATCTAAAAGAATTGGGTTCTTTCGATTTGGAGAACGTCAACGTGTAT

CCTGGAGTATGGAATACATACGATATCTTTGGTATTGAGGCCGCTCGTGGATACTTGTGCGAAGCCATGT

TAAACACCTATGGAGAAGGTTTCGATTATCTGTACCAGCCTTGTGATCTTCTCGCTAGTTTACTATGTGC

TAGTTACGAACCAGAATCAGTTAATAAATTCAAGTTCGGTGCAGCTAGTACTCTTAAGAGAGCTACGTTC

GGAGATAATAAAGCATTGTTAAACGCGGCTCTTCATAAAAAGTCAGAACCTATTAACGATAATAGTAGCT

GCCACTTTTTTAGCAAGGTCCCTAATATAGGAACTGGATATTACAAATACTTTATCGACTTGGGTCTTCT

CATGAGAATGGAAAGGAAACTATCTGATAAGATATCTTCTCAAAAGATCAAGGAGATAGAAGAAACAGAA

GACTTTTAATTCTTATCAATAACATATTTTTCTATGATCTGTCTTTTAAACGATGGATTTTCCACAAATG

CGCCTCTCAAGTCCCTCATAGAATGATACACGTATAAAAAATATAGCATAGGTGATGACTCCTTATTTTT

AGACATTAGATATGCCAAAATCATAGCCCCGCTTCTATTTACTCCTGCAACACAATGAACCAACACGGGC

TCGTTTCGTTGATCACATTTAGATAAGAAGGCGGTCACGTCGTCAAAATATTTACTAATATCAGTAGTTG

TATCATCTACCAACGGTATATGAATAATATTAATATTAGAGTTAGGTAATGTATATTTATCCATCGTCAA

ATTTAAAACATATTTGAACTTAACTTCAGATGATGGTGCATCCATAGCATTTTTATAATTTCCCAAATAC

ACATTATTTGTTACTCTTGTCATTATAGTGGGAGATTTGGCTCTGTGCATATCTCCAGTTGAACGTAGTA

GTAAGTATTTATACAAACTTTTCTTATCCATTTATAACGTACAAATGGATAAAACTACTTTATCAGTAAA

CGCATGCAATTTAGAATACGTTAGAGAAAAGGCTATAGTAGGCGTACAAGCAGCCAAGACATCAACACTT

ATATTTTTTGTTATTATATTGGCAATTAGTGCGCTATTACTCTGGTTTCAGACGTCTGATAATCCAGTCT

TTAATGAATTAACGAGATATATGCGAATTAAAAATACGGTTAACGATTGGAAATCATTAACGGATAGCAA

AACAAAATTAGAAAGCGATAGAGGTAGACTTCTAGCCGCTGGTAAGGATGATATATTCGAATTCAAATGT

GTGGATTTCGGCGCCTATTTTATAGCTATGCGATTGGATAAGAAAACATATCTGCCGCAAGCTATTAGGC

GAGGTACTGGAGACGCGTGGATGGTTAAAAAGGCGGCAAAAGTCGATCCATCTGCTCAACAATTTTGTCA

GTATTTGATAAAACACAAGTCTAATAATGTTATTACTTGTGGTAATGAGATGTTAAATGAATTAGGTTAT

AGCGGTTATTTTATGTCACCGCATTGGTGTTCCGATCTTAGTAATATGGAATAAGTGTTAGATAAATGCG

GTAACAAATGTTCCTGTAAGGAACCATAACAGTTTAGATTTAACATTAAAGATGAGCATAAACATAATAA

ACAAAATTACAATCAAACCTATAACATTAATATCAAACAATCCAAAAAATGAAATCAATGGAGTAGTAAA

CGTGTACATAACTCCTGGATAACGTTTAGCAGCTACCGTTCCTATTCTAGACCAAAAATTTGGTTTCATG

GTTTCGAAGCGGTGTTCTGCAACAAGACGAGGATCGTGTTCTACATATTTGGCAGAGTTATCCATTATTT

GCCTGTTAATCTTCATTTCGTTTTCGATTCTGGCTATTTCAAAATAAAATCCCGATGATAGACCTCCAGA

CTTTATAATTTCATCTACGATGTTCAGCGCCGTAGTAACTCTAATAATATAGGCGGATAAGCTAACATCA

TACCCTCCTGTATATGTAAATATGGCATGATCTTTGTCTATTACAAGCTCGGTTTTAACTTTATTTCCTG

TAATAATTTCTCTCATCTGTAGGATATCTATTTTCTTGTCATGTATTGCCTTCAAGACGGGACGAAGAAA

CGTAATATCCTCAATAACGTTATCGTTTTCTATAATAACTACATATTCTACATTTTTATTTTCTAGCTCG

GTAAAAAATTTAGAATCCCATAGGGCTAAATGTCTAGCGATATTTCTTTTCGTTTCCTCTGTACACATAG

TGTTACAAAACCCTGAAAAGAAGTGAGTATACTTGTCATCATCTCTAATATTTCCTCCAGTCCATTGTAT

AAACACATAATCCTTGTAATGATCTGGATCATCATTGACTATCACAACATCTCTTTTTTCTTGCATAACT

TCATTGTCCTTCACATCATCGAACTTCTGATCATTAATATGCTCATGAACATTAGGAAATGTTTCTGATG

GAGGTCTATCAATAACTGGCACAACAATAACAGGAGTTTTCACCGCCGCCATTTAGTTATTGAAATTAAT

CATATACAACTCTCTAATACGAGTTATATTTTCGTCTATCCATTGTTTCACATTGACATATTTCGACAAA

AAGATATAAAATGCGTATTCCAATGCTTCTCTGTTTAATGAATTACTAAAATATACAAACACGTCACTGT

CTGGTAATAAATAATATCTTAGAATATTGTAACAATTTATTTTGTATTGCACATGTTCGTGATCTATGAG

TTCTTCTTCAAATGGCATAGGATCTCCGAATCTGAAAACGTATAAATAGGAGTTAGAATAATAATATTTG

AGAGTATTGGTAATGTATAAACTCTTTAGCGGTATAATTAGTTTTTTTCTCTCGATTTCTATTTTTAGAT

GTGATGGAAAAATGACTAATTTTGTAGCATTAGTATCATGAACTCTAATCAAAATCTTAATATCTTCGTC

ACATGTTAGCTCTTTGAAGTTTTTAAGAGATGCATCAGTTGGTTTTACAGATGGAGTAGGTGCAACAATT

TTTTGTTTAATGCATGCATGTATTGGAGCCATTGTCTTAACTATAATGGTGCTTGTATCGAAAAACTTTA

ATGCGGATAACGGAAGCTCTTCGCCGCGACTTTCTACGTCGTAATTGGGTTCTAATGCCGATCTCTGAAT

GGATACTAGTTTTCTAAGTTCTAATGTAATTCTCTGAAAATGTAAATCCAATTCCTCCGGCATTATAGAT

GTGTATACATCGGTAAATAAAACTATAGTATCCAACGATCCCTTCTCGCAAATTCTAGTCTTAACCAAGA

AATCGTATATAACTACGGAGATGGCGTATTTAAGAGTGGATTCTTCTACCGTTTTGTTCTTGGATTTCAT

ATAAGAAACTATAAAGTCCGCACTACTGTTAAGAATGATCACTAACGCAACTATATAGTTCAAATTAAGC

ATCTTGGAAACATAAAATAACTCTGTAGATGATACTTGACTTTCGAATAAGTTTGCAGACAAACGAAGAA

AGAACAGACCTCTCTTAATTTCAGAAGAAAACTTTTTTTCGTATTCCTGACGTCTAGAGTTTATATCAAT

AAGAAAGTTAAGAATTAGTCGGTTAATGTTGTATTTCATTACCCAAGTTTGAGATTTCATAATATTGTCA

AAAGACATGATAATATTAAAGATAAAGCGCTGACTATGAACGAAATAGCTATATGGTTCGCTCAAGAATA

TAGTCTTGTTAAACGTGGAAACGATAACTGTATTTTTAATCACGTCAGCGGCATCTAAATTAAATATAGG

TATATTTATTCCACACACTCTACAATATGCCACACCATCTTCATAATAAATAAATTCGTTAGCAAAATTA

TTAATTTTAGTGAAATAGTTAGCGTCAACTTTCATAGCTTCCTTCAATCTAATTTGATGCTCACATGGCG

CGAATTCTACTCTAACATCCCTTTTCCATGCCTCAGGTTCATCGATCTCTATAATATCTAGTTTCTTGCG

TTTCACAAACACAGGCTCGTCTCTCGCGATGAGATCTGTATAGTAACTATGTAAATGATAACTAGATAGA

AAGATGTAGCTATATAGATGACGATCCTTTAAGAGAGGTATAATAACTTTACCCCAATCAGATAGACTGT

TGTTATGGTCTTCGGAAAAAGAATTTTTATAAATTTTTCCAGTATTTTCTAAATATACGTACTTGATATC

TAAGAAATCCTTAATAATAATAGGAATGGATAATCCGTCTATTTTATAAAGAAATACATATCGCATATTA

TACTTTTTTTTGGAAATTGGAATACCGATGTGTCTACATAAATACGCAAAGTCTAAATATTTTTTAGAGA

ATCTTAGTTGGTCCAAATTCTTTTCCAAGTACGGTAATAGATTTTTCATATTGAACGGTATCTTCTTGAT

CTCTGGTTCTAATTCCGCATTAAATGATGAAACTAAGTCACTATTTTTATAACTAACGATTACATCACCT

CTAACATCATCATTTACCAGGATACTGATCTTCTTTTGTCGTAAATACATGTCTAATGTGTTAAAAAAAA

GATCATACAAGTTATACGTCATTTCATCTGTAGTATTCTTGTCATTGAAGGATAAACTCGTACTAATCTC

TTCTTTAACAGTCTGTTCAAATTTATATCCTATATATGAAAAAATAGCAACCAGTGTTTGATCATCCGCG

TCAATATTCTGTTCTATCGTAGTGTATAACAATCTTATATCTTCTTCTGTGATAGTCGATACGTTATAAA

GGTTGATAACGAAAATATTTTTATTTCGTGAAATAAAGTCATTGTAGGATTTTGGACTTATATTCGTGTC

TAGTAGATATGATTTTATTTTTGGAATGATCTCAATTAAAATAGTCTCTTTAGAGTCCATTTAAAGTTAC

AAACAACTAGGAAATTGGTTTATGATGTATAATTTTTTTAGTTTTTATAGATTCTTTATTCTATACTTAA

AAAATGAAAATAAATACAAAGGTTCTTGAGGGTTGTGTTAATTGAAAGCGATAAATAATCATAAATTATT

TCATTATCGCGATATCCGTTAAGTTTGTATCGTAATGGCGTGGTCAATTACGAATAAAGCGGATACTAGT

AGTTTCACAAAGATGGCTGAAATCAGAGCTCATCTAAGAAATAGCGCTGAAAATAAAGATAAAAACGAGG

ATATTTTCCCGGAAGATGTAATAATTCCATCTACTAAGCCCAAAACCAAACGAACCACTACTCCTCGTAA

ACCAGCGGCTACTAAAAGATCAACCAAAAAGGATAAAGAAAAGGAGGAAGTGGAAGAAGTAGTTATAGAG

GAATATCATCAAACAACTGAAGAAAATTCTCCACCTCCGTCATCATCTCCTGGAGTCGGCGACATTGTAG

AAAGCGTGGCCGCTGTAGAGCTCGATGATAGCGACGGGGATGATGAACCTATGGTACAAGTTGAAGCTGG

TAAAGTAAATCATAGTGCTAGAAGCGATCTCTCTGACCTAAAGGTGGCTACCGACAATATCGTTAAAGAT

CTTAAGAAAATTATTACTAGAATCTCTGCAGTATCGACTGTTCTAGAGGATGTTCAAGCAGCTGGTATCT

CTAGACAATTTACTTCTATGACTAAAGCTATTACAACACTATCTGATCTAGTCACCGAGGGAAAATCTAA

AGTTGTTCGTAAAAAAGTTAAAACTTGTAAGAAGTAAATGCGTGCACTTTTTTATAAAGATGGTAAACTG

TTTACCGATAATAATTTTTTAAATCCTGTATCAGACGATAATCCAGCGTATGAGGTTTTGCAACATGTTA

AAATTCCTACTCATTTAACAGATGTAGTAGTATATGAACAAACGTGGGAAGAGGCATTAACTAGATTAAT

TTTTGTGGGAAGTGATTCAAAAGGACGTAGACAATACTTTTACGGAAAAATGCATATACAGAATCGCAAT

GCTAAAAGAGATCGTATTTTTGTTAGAGTATATAACGTTATGAAACGAATTAATTGTTTTATAAACAAAA

ATATAAAGAAATCGTCCACAGATTCCAATTATCAGTTGGCGGTTTTTATGTTAATGGAAACTATGTTTTT

TATTAGATTTGGTAAAATGAAATATCTTAAGGAGAATGAAACAGTAGGGTTATTAACACTAAAAAATAAA

CACATAGAAATAAGTCCCGATGAAATAGTTATCAAGTTTGTAGGAAAGGACAAAGTTTCACATGAATTTG

TTGTTCATAAGTCTAATAGACTATATAAACCGCTATTGAAACTGACTGATGATTCTAGTCCCGAAGAATT

TCTGTTCAACAAACTAAGTGAACGAAAGGTATATGAATGTATCAAACAGTTTGGTATTAGAATCAAGGAT

CTCCGAACGTATGGAGTCAATTATACGTTTTTATATAATTTTTGGACAAATGTAAAGTCCGTATCTCCTC

TTCCATCACCAAAAAAGTTGATAGCATTAACTATCAAACAAACTGCTGAAGTGGTAGGTCATACTCCATC

AATTTCAAAAAGAGCTTATATGGCAACGACTATTTTAGAAATGGTAAAGGATAAAAATTTTTTAGACGTA

GTATCTAAAACTACGTTCGATGAATTCCTATCTATAGTCGTAGATCACGTTAAATCATCTACGGATGGAT

GATAATAGATCTTTACACAAATAATTACAAGACCGATAAATGGAAATGGATAAACGGATGAAATCTCTCG

CTATGACAGCTTTCTTCGGAGAGCTAAACACGTTAGATATTATGGCATTGATAATGTCTATATTTAAACA

CCATCCAAACAATACCATTTTTTCAGTGGATAAGGATGGTCAATTTATGATTGATTTCGAATACGATAAT

TATAAGGCTTCTCAATATTTGGATCTGACCCTCACTCCGATATCTGGAAATGAATGCAAGACTCACGCAT

CTAGTATAGCCGAACAATTGGCGTGTGTGGATATTATTAAAGAGGATATTAGCGAATATATCAAAACTAC

TCCCCGTCTTAAACGATTTATAAAAAAATACCGCAATAGATCATATACTCGTATCAGTCGAGATACAGAA

AAGCTTAAAATAGCTCTAGCTAAAGGCATAGATTACGAATATATAAAAGACGCTTGTTAATAAGTAAATG

AAAAAAAACTAGTCGTTTATAATAAAACACAATATGGATGCCAACATAGTATCATCTTCTACTATTGCGA

CGTATATAGACGCTTTAGCGAAGAATGCTTCAGAATTAGAACAGAGGTCTACCGCATACGAAATAAATAA

TGAATTGGAACTAGTATTTATTAAACCGCCATTGATTACGTTGACAAATGTAGTAAATATCTCCACGATT

CAGGAATCGTTTATTCGATTTACCGTTACTAATAAGGAAGGTATCAAAATTAGAACTAAGATTCCATTAT

CTAAGGTACATGGTCTAGATGTAAAAAATGTGCAGTTGGTAGATGCTATAGATAACATAGTTTGGGAAAA

GAAATCATTAGTGACGGAAAATCGTCTTCACAAAGAATGCTTGTTGAGACTATCAACAGAGGAACGTCAT

ATATTTTTGGATTACAAGAAATATGGATCCTCTATCCGACTAGAATTAGTCAATCTTATTCAAGCAAAAA

CAAAAAACTTTACGATAGACTTTAAGCTAAAATATTTTCTAGGATCTGGCGCTCAATCTAAAAGTTCTTT

ATTGCACGCTATTAATCATCCAAAGTCAAGGCCTAATACATCTCTGGAAATAGAATTTACACCTAGAGAC

AATGAAACAGTTCCATATGATGAACTAATAAAGGAATTGACGACTCTCTCGCGTCATATATTTATGGCTT

CTCCAGAGAATGTAATTCTTTCTCCACCTATTAACGCACCTATAAAGACTTTTATGTTGCCTAAACAAGA

TATAGTAGGTCTGGATCTGGAAAATCTATATGCCGTAACTAAGACTGACGGCATTCCTATAACTATCAGA

GTTACATCAAAAGGGTTGTATTGTTATTTTACACATCTTGGTTATATTATTAGATATCCAGTTAAGAGAA

CAATAGATTCCGAAGTAGTAGTCTTTGGTGAGGCAGTTAAGGATAAGAACTGGACCGTATATCTCATTAA

GCTAATAGAGCCCGTAAATGCAATCAGTGATAGACTAGAAGAAAGTAAGTATGTTGAATCTAAACTAGTG

GATATTTGTGATCGGATAGTATTCAAGTCAAAGAAATACGAAGGTCCGTTTACTACAACTAGTGAAGTCG

TCGATATGTTATCTACATATTTACCAAAGCAACCAGAAGGTGTTATTCTGTTCTATTCAAAGGGACCTAA

ATCTAACATTGATTTTAAAATCAAAAAGGAGAATACTATAGACCAAACTGCAAATGTAGTATTTAGGTAC

ATGTCCAGTGAACCAATTATCTTTGGAGAGTCGTCTATCTTTATAGAGTATAAGAAATTTACCAACGATA

AAGGCTTTCCTAAAGAATATGGTTCTGGTAAGATTGTGTTATATAACGGCGTTAATTATCTAAATAATAT

CTATTGTTTGGAATATATTAATACACATAATGAAGTGGGTATTAAGTCCGTTGTTGTACCTATTAAGTTT

ATAGCAGAATTCTTAGTCAATGGAGAAATACTTAAACCTAGAATCGATAAAACCATGAAATATATTAACT

CAGAAGACTATTATGGAAATCAACATAATATCATAGTCGAACATTTAAGAGATCAAAGCATCAAAATAGG

AGATGTCTTTAACGAGGATAAACTATCGGATGTTGGACATCAATACGCTGCCAACAACGATAAATTTAGA

TTAAATCCAGAAGTTAGTTATTTTACTAATAAACGAACTAGAGGGCCGTTGGGAATTTTATCAAACTACG

TCAAGACTCTTCTTATTTCTATGTATTGTTCCAAAACATTTTTAGACGATTCCAACAAACGAAAGGTATT

AGCGATTGATTTTGGAAACGGTGCTGACCTGGAAAAATACTTTTATGGAGAGATTGCGTTATTGGTAGCG

ACGGATCCGGATGCTGATGCTATAGCTAGAGGAAATGAAAGATACAACAAATTAAATTCTGGAATTAAAA

CCAAGTACTACAAATTTGACTACATTCAGGAAACTATTCGATCCGATACATTTGTCTCTAGTGTCAGAGA

AGTATTCTATTTTGGAAAGTTTAATATCATTGACTGGCAGTTCGCTATTCATTATTCTTTTCATCCAAGA

CATTATGCTACAGTCATGAATAACTTATCCGAACTAACTGCTTCTGGAGGCAAGGTATTAATTACTACCA

TGGATGGAGACAAATTATCAAAATTAACCGATAAAAAGACTTTTATAATTCATAAGAATCTACCTAGTAG

CGAAAACTATATGTCTGTAGAAAAAATAGCTGATGATAGAATAGTGGTATATAATCCATCAACAATGTCT

ACTCCAATGACTGAATACATTATCAAAAAGAACGATATAGTCAGAGTGTTTAACGAATACGGATTTGTTC

TTGTAGATAATGTTGATTTCGCTACAATTATAGAACGAAGTAAAAAGTTTATTAATGGCGCATCTACAAT

GGAAGATAGACCGTCTACAAGAAACTTTTTCGAACTAAATAGAGGAGCCATTAAATGTGAAGGTTTAGAT

GTCGAAGACTTACTTAGTTACTATGTTGTTTATGTCTTTTCTAAGCGGTAAATAATAATATGGTATGGGT

TCTGATATCCCCGTTCTAAATGCATTAAATAATTCCAATAGAGCGATTTTTGTTCCTATAGGACCTTCCA

ACTGTGGATACTCTGTATTATTAATAGATATATTAATACTTTTGTAGGGTAACAGAGGTTCTACGTCTTC

TAAAAATAAAAGTTTTATAACATCTGGCCTGTTCATAAATAAAAACTTGGCGATTCTATATATACTCTTA

TTATCAAATCTAGCCATTGTCTTATAGATGTGAGCTACTGTAGGTGTACCATTTGATTTTCTTTCTAATA

CTATATATTTCTCTCGAAGAAGTTCTTGCAGATCATCTGGGAATAAAATACTACTGTTGAGTAAATCAGT

TATTTTTTTTATATCGATATTGATGGACATTTTTATAGTTAAGGATAATAAGTATCCCAAAGTAGATAAC

GACGATAACGAAGTATTTATACTTTTAGGAAATCACAATGACTTTATCAGATCAAAATTAACAAAATTAA

AGGAGCATGTATTTTTTTCTGAATATATTGTGACTCCAGATACATATGGATCTTTATGCGTCGAATTAAA

TGGGTCTAGTTTTCAGCACGGTGGTAGATATATAGAGGTGGAGGAATTTATAGATGCTGGAAGACAAGTT

AGATGGTGTTCTACATCCAATCATATATCTGAAGATATACACACTGATAAATTTGTCATTTATGATATTT

ATACGTTTGATTCGTTCAAGAATAAACGATTGGTATTTGTACAGGTACCTCCATCATTAGGAGATGATAG

CTATTTAACTAATCCGTTATTGTCTCCGTATTATCGTAATTCAGTAGCCAGACAAATGGTCAATGATATG

ATTTTTAATCAAGATTCATTTTTAAAATATTTATTAGAACATCTGATTAGAAGCCACTATAGAGTTTCTA

AACATATAACAATAGTTAGATACAAGGATACCGAAGAATTAAATCTAACAAGAATATGTTATAATAGAGA

TAAGTTTAAGGCGTTTGTATTCGCTTGGTTTAACGGCGTTTCGGAAAATGAAAAGGTACTAGATACGTAT

AAAAAGGTATCTGATTTGATATAATGAATTCAGTGACTATATCACACGCACCATATACTATTACTTATCA

CGATGATTGGGAACCAGTAATGAGTCAATTGGTAGAGTTTTATAACGAAGTAGCCAGTTGGTTGCTACGC

GACGAGACGTCGCCTATTCCTGATAAGTTCTTTATACAATTGAAACAGCCGCTTAGAAATAAACGAGTAT

GTGTGTGTGGTATAGATCCGTATCCAAAAGATGGAACTGGTGTACCGTTCGAATCACCAAATTTTACAAA

AAAATCAATTAAGGAGATAGCTTCATCTATATCTAGATTAACCGGAGTAATTGATTATAAAGGTTATAAC

CTTAATATAATAGACGGGGTTATACCCTGGAATTATTACTTAAGTTGTAAATTAGGAGAAACAAAAAGTC

ACGCGATTTACTGGGATAAGATTTCCAAGTTACTGCTACAGCATATAACTAAACACGTTAGTGTTCTTTA

TTGTTTGGGTAAAACAGATTTCTCGAATATACGGGCAAAGTTAGAATCCCCGGTAACTACCATAGTGGGA

TATCATCCAGCGGCCAGAGACCACCAATTCGAGAAAGATCGATCATTTGAAATTATCAACGTTTTACTGG

AATTAGACAACAAGACACCTATAAATTGGGCTCAAGGGTTTATTTATTAATGCTTTAGTGAAATTTTAAC

TTGTGTTCTAAATGGATGCGGCTATTAGAGGTAATGATGTTATCTTTGTTCTTAAGACTATAGGTGTCCC

GTCAGCATGCAGACAAAATGAAGATCCAAGATTCGTAGAAGCATTTAAATGCGACGAGTTAGAAAGATAT

ATTGATAATAATCCAGAATGTACACTATTCGAAAGTCTTAGGGATGAGGAAGCATACTCTATAGTCAGAA

TTTTCATGGATGTAGATTTAGACGCGTGTCTAGACGAAATAGATTATTTAACGGCTATTCAAGATTTTAT

TATCGAGGTGTCAAACTGTGTAGCTAGATTCGCATTTACAGAATGCGGTGCCATTCATGAAAATGTAATA

AAATCCATGAGATCTAATTTTTCATTGACTAAGTCTACAAATAGAGATAAAACAAGTTTTCATATTATCT

TTTTAGACACGTATACCACTATGGATACATTGATAGCTATGAAACGAACACTATTAGAATTAAGTAGATC

ATCTGAAAATCCACTAACAAGATCGATAGACACTGCCGTATATAGGAGAAAAACAACTCTTCGGGTTGTA

GGTACTAGGAAAAATCCAAATTGCGACACTATTCATGTAATGCAACCACCTCACGATAATATAGAAGATT

ACCTATTCACTTACGTGGATATGAACAACAATAGTTATTACTTTTCTCTACAACGACGATTGGAGGATTT

AGTTCCTGATAAGTTATGGGAACCAGGGTTTATTTCGTTCGAAGACGCTATAAAAAGAGTTTCAAAAATA

TTCATTAATTCTATAATAAACTTTAATGATCTCGATGAAAATAATTTTACAACGGTACCACTGGTCATAG

ATTATGTAACACCTTGTGCATTATGTAAAAAACGATCGCATAAACATCCGCATCAACTATCGTTGGAAAA

TGGTGCTATTAGAATTTACAAAACTGGTAATCCACATAGTTGTAAAGTTAAAATTGTTCCGTTGGATGGT

AATAAACTGTTTAATATTGCACAAAGAATTTTAGACACTAACTCTGTTTTATTAACCGAACGAGGAGACC

ATATAGTTTGGATTAATAATTCATGGAAATTTAACAGCGAAGAACCCTTGATAACAAAACTAATTCTATC

AATAAGACATCAACTACCTAAGGAATATTCAAGCGAATTACTCTGTCCGAGGAAACGAAAGACTGTAGAA

GCTAACATACGAGACATGTTAGTAGATTCAGTAGAGACCGATACCTATCCGGATAAACTTCCGTTTAAAA

ATGGTGTATTGGACCTGGTAGACGGAATGTTTTACTCTGGAGATGATGCTAAAAAATATACGTGTACTGT

ATCGACCGGATTTAAATTTGACGATACAAAATTCGTCGAAGACAGTCCAGAAATGGAAGAGTTAATGAAT

ATCATTAACGATATCCAACCATTAACGGATGAAAATAAGAAAAATAGAGAGCTGTATGAAAAAACATTAT

CTAGTTGTTTATGTGGTGCTACCAAAGGATGTTTAACATTCTTTTTTGGAGAAACCGCAACTGGGAAGTC

GACAACCAAACGTTTGTTAAAGTCTGCTATCGGTGACCTGTTTGTCGAGACGGGTCAAACAATTTTAACA

GATGTATTGGATAAAGGACCTAATCCATTTATCGCTAATATGCATTTAAAAAGATCTGTATTCTGTAGCG

AACTACCTGATTTTGCATGTAGTGGATCAAAGAAAATTAGATCTGATAATATTAAAAAGTTGACAGAACC

TTGTGTCATTGGAAGACCGTGTTTCTCCAATAAAATTAATAATAGAAACCATGCGACAATCATTATCGAT

ACTAATTACAAACCTGTCTTTGATAGGATAGATAACGCATTAATGAGAAGAATTGCCGTCGTGCGATTCA

GAACACACTTTTCTCAACCTTCTGGTAGAGAGGCTGCTGAAAATAATGACGCGTACGATAAAGTCAAACT

ATTAGACGAGGGATTAGATGGTAAAATACAGAATAATAGATATAGATTCGCATTTCTATACTTGTTGGTT

AAATGGTACAAAAAATATCATATTCCTATTATGAAACTATATCCTACACCGGAAGAGATTCCGGACTTTG

CATTCTATCTCAAAATAGGTACTCTGTTGGTATCTAGCTCTGTAAAGCATATTCCATTAATGACGGACCT

CTCCAAAAAGGGATATATATTGTACGATAATGTGGTTACTCTTCCGTTGACTACTTTCCAACAGAAAATA

TCCAAGTATTTTAATTCTAGACTATTTGGACACGATATAGAGAGCTTCATCAATAGACATAAGAAATTTG

CCAATGTTAGTGATGAATATCTGCAATATATATTCATAGAGGATATTTCATCTCCGTAAATATATGCCAT

ATATTTATAGAATATATCACATATCTAAATGAATACCGGAATCATAGATTTATTTGATAATCATGTTGAT

AGTATACCAACTATATTACCTCATCAGTTAGCTACTTTAGATTATCTAGTTAGAACTATCATAGATGAGA

ACAGAAGCGTGTTATTGTTCCATATTATGGGATCGGGTAAAACAATAATCGCTTTGTTGTTCGCCTTGGT

AGCTTCCAGATTTAAAAAGGTTTACATTTTAGTACCGAACATCAACATCTTAAAAATTTTCAATTATAAT

ATGGGTGTAGCTATGAACTTGTTTAATGACGAATTCATAGCTGAGAATATCTTTATTCATTCCACAACAA

GTTTTTATTCTCTTAATTATAACGATAACGTCATTAATTATAACGGATTAAGTCGCTACAATAACTCTAT

TTTTATCGTTGATGAGGCACATAATATTTTTGGGAATAATACTGGAGAACTTATGACCGTGATAAAAAAT

AAAAACAAGATTCCTTTTCTACTATTGTCTGGATCTCCCATTACTAACACACCTAATACGCTGGGTCATA

TTATAGATTTAATGTCCGAAGAGACGATAGATTTTGGTGAGATTATTAGTCGTGGTAAGAAAGTAATTCA

GACACTTCTTAACGAACGCGGAGTGAATGTACTCAAGGATTTGCTTAAAGGAAGAATATCATATTACGAA

ATGCCGGACAAAGATCTACCAACAATAAGATATCACGGACGTAAATTTCTAGATACTCGAGTAGTATATT

GTCACATGTCTAAACTTCAAGAGAAAGATTATATGATTACTAGACGGCAGCTATGTTATCATGAAATGTT

TGATAAAAATATGTATAACGTGTCAATGGCAGTATTGGGACAACTTAATCTGATGAATAATTTAGATACG

TTATTTCAGGAACAGGATAAGGAATTGTACCCAAATCTGAAAATAAATAATGGAGTGTTATACGGTGAAG

AATTGGTAACGTTAAACATTAGTTCCAAATTTAAGTACTTTATCAATCGGATACAGACACTCAAGGGAAA

ACACTTTATATACTTCTCTAATTCTACATATGGTGGATTGGTAATTAAATATATCATGCTCAGTAATGGA

TATTCTGAATATAATGGTTCTCAGGGAACTAATCCACATATGATAAACGGCAAACCAAAAACATTTGCTA

TCGTTACTAGTAAAATGAAATCGTCTTTAGAGGATCTATTAGATGTGTATAATTCTCCTGAAAACGATGA

TGGCAATCAATTGATGTTTTTGTTTTCGTCAAACATTATGTCTGAATCCTATACTCTGAAAGAGGTAAGG

CATATTTGGTTTATGACTATCCCGGATACTTTTTCTCAATACAACCAAATTCTTGGACGATCTATTAGAA

AATTCTCTTACGTCGATATTTCTGAACCCGTTAATGTATATCTTTTAGCAGCCGTATATTCAGATTTCAA

TGACGAAGTGACGTCATTAAACGATTATACACAGGATGAATTGATTAATGTTTTACCCTTTGACATCAAA

AAGCTGTTGTATCTAAAATTTAAGACTAAAGAAACGAATAGAATATACTCTATTCTTCAAGAGATGTCTG

AAACGTATTCTCTTCCACCACATCCATCAATTGTAAAAGTTTTATTGGGAGAATTGGTCAGACAATTTTT

TTATAATAATTCTCGTATTAAGTATAACGACTCCAAGTTACTTAAAATGGTTACATCAGTTATAAAAAAT

AAAGAAGACGCTAGGAATTACATAGATGATATTGTAAACGGTCACTTCTTTGTATCGAATAAAGTATTTG

ATAAATCTCTTTTATACAAATACGAAAACGATATTATTACAGTACCGTTTAGACTTTCCTACGAACCATT

TGTTTGGGGAGTTAACTTTCGTAAAGAATATAATGTGGTATCTTCTCCATAAAACTGATGAGATATATAA

AGAAATAAATGTCGAGCTTTGTTACCAATGGATATCTTCCAGTTACATTGGAACCACATGAGTTGACGTT

AGACATAAAAACTAATATTAGGAATGCCGTATATAAGGCGTATCTCCATAGAGAAATTAGTGGTAAAATG

GCCAAGAAAATAGAAATTCGTGAAGACGTGGAATTACCTCTCGGTGAAATAGTTAATAATTCTGTAGTTA

TAAACGTTCCGTGTGTAATAACCTACGCATATTATCACGTTGGGGATATAGTCAGAGGAACATTAAACAT

CGAAGATGAATCAAATGTAACTATTCAATGTGGAGATTTAATCTGTAAACTAAGTAGAGATTCGGGTACT

GTATCATTTAGCGATTCAAAGTACTGCTTTTTTCGAAATGGTAATGCGTATGATAACGGCATCGAAGTCT

CCGCCGTTCTAATGGAGGCTCAACAAGGTACCGAATCTAGTTTTGTTTTTCTCGCGAATATCGTTGACTC

ATAAGAAAGAGAATAGCGGTGAGTATAAATACGAATACTATGGCAATAATTGCGAATGTTTTATTCCCTT

CGATATATTTTTGATAATATGAAAAACATGCCTCTCTCAAATCAGACAACCATTTCATAAAATAGTTCTC

TCGCACTGGTGAGGTGGTTGCAGCTCGTATAATCTCCCCAGAATAATATACTTGCGTGTCGTCGTTCAAT

TTATACGGATTTCTATAATTCTCTGTTATATAATGAGGTTTACCCTCATGATTAGACGACGACAATAGTG

TTCTGAATTTAGATAGTTGATCAGAATGAATGTTTATTGGTGTTGGAAAAATTATCCATGCTGCGTCTGC

AGAGTGGTTGATAGTTGTTCCTAGATATGTAAAATAATCCAACGTACTAGGTAGCAAATTGTCTAGATAA

AATACTGAATCAAATGGCGCAGACATATTAGCGGATCTAATGGAATCCAATTGATTGACTATCTTTTGAA

AATATACATTTTTATGATCTGATACTTGTAAGAATATAGCAATAATGATAATTCCATCATCGTGTTTTTT

TGCCTCTTCATAAGAACTATATTTTTTCTTATTCCAATGAACCAGATTAATCTCTCCAGAGTATTTGTAT

ACATCTATCAAGTGATTGGATCCATAATCGTCTTCCTTTCCCCAATATATATGTATTGTTGATAACACAT

ATTCATTGGGGAGAAACCCTCCACTTATATATCCTCCTTTAAAATTAATCCTTACTAGTTTTCCAGTATT

CTGGATAGTGGTTGGTTTCGACTCATTATAATGTATGTCTAACGTCTTCAATCGCGCGTCAGAAATTGCT

TTTTTAGTTTCTATATTAATAGGAGATAGTTGTTGAGGCATAGTAAAAATGAAATGATAACTGTCTAGAA

ATAGCTCTTAGTATGGGATTTACAATGGATGAGGAAGTGATATTTGAAACTCCTAGAGAATTAATATCTA

TTAAACGAATAAAAGATATTCCAAGATCAAAAGACACGCACGTGTTTGCTGCGTGTATAACAAGTGACGG

ATATCCGTTAATAGGAGCTAGAAGAACTTCATTCGCATTCCAGGCGATATTATCTCAACAAAATTCAGAT

TCTATCTTTAGAGTATCCACTAAACTATTACGGTTTATGTACTACAATGAACTAAGAGAAATCTTTAGAC

GGTTGAGAAAAGGTTCTATCAACAATATCGATCCTCACTTCGAAGAGTTAATATTATTGGGTGGTAAACT

AGATAAAAAGGAATCTATTAAAGATTGTTTAAGAAGAGAATTAAAAGAGGAAAGTGATGAACATATAACA

GTAAAAGAATTCGGAAATGTAATTCTAAAACTTACAACGAGTGATAAATTATTTAATAAAGTATATATAG

GTTATTGCATGGCATGTTTTATTAATCAATCGTTGGAGGATTTATCACATACTAGTATTTACAATGTAGA

AATTAGAAAGATTAAATCGTTAAATGATTGTATTAACGACGATAAATACGAATATCTGTCTTATATTTAT

AATATACTAATTAATAGTAAATGAGCTTTTACAGATCTAGTATAATTAGTCAGATTATTAAGTATAATAG

ACGACTAGCTAAGTCTATTATTTGCGAGGATGACTCTCAAATTATTACACTCACGGCATTCGTTAACCAA

TGCCTATGGTGTCATAAACGAGTATCCGTGTCCGCTATTTTATTAACTACTGATAACAAAATATTAGTAT

GTAACAGACGAGATAGTTTTCTCTATTCTGAAATAATTAGAACTAGAAACATGTATAGAAAGAAACGATT

ATTTCTGAATTATTCCAATTATTTGAACAAACAGGAAAGAAGTATACTATCGTCATTTTTTTCTCTAGAT

CCAGCTACTGCTGATAATGATAGAATAAACGCTATTTATCCGGGTGGTATACCCAAAAGGGGTGAGAACG

TTCCAGAGTGTTTATCCAGGGAAATCAAAGAAGAAGTTAATATAGACAATTCTTTTGTATTCATAGACAC

TCGTTTTTTTATTCATGGTATCATAGAAGATACCATTATTAACAAATTTTTTGAGGTAATTTTCTTTGTT

GGACGAATATCTCTAACGAGTGATCAAATTATTGATACCTTTAAAAGTAATCATGAAATAAAGGATCTAA

TATTTTTAGATCCAAATTCAGGTAATGGACTCCAATACGAAATTGCAAAATATGCTCTAGATACTGCAAA

ACTTAAATGTTACGGTCATAGAGGATGTTATTATGAATCATTAAAAAAATTAACTGAGGATGATTGATTA

GAAAATATAAATTAATTTACCATCGTGTATTTTTATAACGGGATTGTCTGGCATATCATGTAGATAGTTA

CCGTCTACATCGTATACTCTACCATCTACGCCTTTAAATCCTCTATTTATTGATATTAATCTATTAGAAT

TGGAATACCAAATATTAGTACCCTCAATTAGTTTATTGGTAATATTTTTTTTAGACGATAGATCGATGGC

TCTTGAAACCAAGGTTTTCCAACCGGACTCATTGTCTATCGGTGAGAAGTCTTTTTCATTAGCATGAATC

CATTCTAATGATGTATGTTTAAACACTCTAAACAATTGTACAAATTCTTTTGATTTGTTTTGAATGATTT

CAAATAGGTCTTCGTCTACAGTAGGCATACCATTAGATAATCTAGCCATTATAAAGTGCACGTTTACATA

TCTACGTTCTGGAGGAGTAAGAACGTGACTATTGAGACGAATGGCTCTTCCTACTATCTGACGAAGAGAC

GCCTCGTTCCATGTCATATCTAAAATGAAGATATCATTGATTGAGAAGAAACTAATACCCTCGCCTCCGC

TAGAAGAGAATACGCATGTTTTAATGCATTCTCCGTTAGTGTTTGATTCTTGGTTAAACTCAGCCACCGC

CTTGATTCTAGTATCTTTTGTTCTAGATGAGAACTCTATATTAGAGATACCAAAGACTTTGAAATATAGT

AATAAGATTTCTATTCCTGACTGATTAACAAATGGTTCAAAGACTAGACATTTACCATGGGATGCTAATA

TTCCCAAACATACATCTATAAATTTGACGCTTTTCTCTTTTAATTCAGTAAATAGAGAGATATCAGCCGC

AATAGCATCCCCTCCCAATAGTTCTCCCTTTTTAAAGGTGTCTAATGCGGATTTAGAAAATTCTCTATCT

CTTAATGAATTTTTAAAATCATTATATAGGGTTGCTATCTCTTGTGCGTATTCTCCCGGATCACGATTTT

GTCTTTCAGGAAAGCTATCGAATGTAAACGTAGTAGCCATACGTCTCAGAATTCTAAATGATGATATACC

AGTTTTTATTTCTGCGAGTTTAGCCTTTTGATAAATCTCTTCTTGCTTTTTTGACATATTAACGTATCGC

ATTAATACTGTTTTCTTAGCGAATGATGCAGACCCTTCCACATCATCAAAAATAGAAAACTCGTTATTAA

CTATGTACGAACATAGGCCTCCTAGTTTGGAGACTAATTCTTTTTCATCGACTAGACGTTTATTCTCAAA

TAGCGATTGGTGTTGTAAGGATCCTGGTCGCAGTAAGTTAACCAACATGGTGAATTCTTGCACACTATTA

ACGATAGGTGTAGCCGATAAACAAATCATCTTATGGTTTTTTAACGCAATGGTCTTAGATAAAAAATTAT

ATACTGACCGAGTAGGACGGATCTTACCATCTTCTTTGATTAATGATTTAGAAATGAAGTTATGACATTC

ATCAATGATGACGCATATTCTACTCTTGGAATTAATAGTTTTGATATTAGTAAAAAATTTATTTCTAAAA

TTTTGATCATCGTAATTAATAAAAATACAATCCTTCGTTATCTCTGGAGCGTATCTGAGTATAGTGTTTA

TCCAAGGATCTTCTATCAAAGCCTTTTTTACCAATAAGATAATTGCCCAATTCGTATAAATATCCTTAAG

ATGTTTGAGAATATATACAGTAGTCATTGTTTTACCGACACCTGTTTCATGGAACAATAAAAGAGAATGC

ATACTGTCTAATCCTAAGAAAACTCTTGCTACAAAATGTTGATAATCCTTGAGGCGTACTACGTCTGACC

CCATCATTTCAACGGGCATATTAGTAGTTCTGCGTAAGGCATAATCGATATAGGCCGCGTGTGATTTACT

CATTTATGAGTGATAAGTAATAACTATGTTTTAAAAATCACAGCAGTAGTTTAACTAGCCTTCTCTGATG

TTTGTTTTCGATACTTTTTGAATCAGAAGTCATACTAGAATAAAGCAGCGAGTGAACGTAATAGAGAGCT

TCGTATACTCTATTCGAAAACTCTAAGAACTTATTAATGAATTCCGTATCCACTGGATCGTTTAAAATAC

TAAATTGAACAGTGTTCACATCCTTCCAAGACGAAGACTTAGTGACGGACTTAACATGAGACATAAATAA

ATCCAAATTTTTTTTATAAACATCACTAGCCACCATAATGGCGCTATCTTTCAACCAACTATCGCTTACG

CATTTTAACAGTCTAACATTTTTAAAGAGACTACAATATATTCTCATAGTATCGATTACACCTCTACCGA

ATAGAGTGGGAAGTTTAATAATACAATATTTTTCGTTTACAAAATCAAATAATGGTCGAAACACGTCGAA

GGTTAACATCTTATAATCGCTAATGTATAGATTGTTTTCAGTGAGATGATTATTAGATTTAATAGCATCT

CGTTCACGTTTGAACAGTTTATTGCGTGCGCTGAGGTCGGCAACTACGGCATCCGCTCTAGTACTCCTCC

CATAATACTTTACGCTATTAATCTTTAAAATTTCATAGACTTTATCTAGATCGCTTTCTGGTAACATGAT

ATCATGTGTAAAAAGTTTTAACATGTCGGTCGGCATTCTATTTAGATCATTAACTCTAGAAATCTGAAGA

AAGTAATTAGCTCCATATTCCAGACTAGGTAATGGGCTTTTACCTAAAGACAAGTTAAGTTCTGGCAATG

TTTCATAAAATGGAAGAAGGACATGTGTCCCCTCCCGGATATTTTTTACAATTTCATCCATTTACAACTC

TATAGTTTGTTTTCATTATTATTAGTTATTATCTCCCATAATCTTGGTAATACTTACACCTTGATCATAA

GATACCTTATACAGGTCATTACATACAACTACCAATTGTTTTTGTACATAATAGATTGGATGATTGATAT

CCATGGTGGAATAAACTACTCGAACAGATAGTTTATCTTTCCCCCTAGATACATTGGCCGTAATAGTTGT

CGGCCTAAAGAATATCTTTGGTGTAAAGTTAAAAGTTAGGGTTCTTGTTCCATTATTGCTTTTTGTCAGT

AGTTCGTTATAAATTCTCGAGATGGGCCCGTTCTCTGAATATAGAACATCATTTCCAAATCTAACTTCTA

GTCTAGAAATAATATCGGTCTTATTTTTAAAATCTATTCCCTTGATGAATGGATCGTTAATAAACAAATC

CTTGGCCTTTGATTCGGCTGATCTATTATCTCCGTTATAGACGTTACGTTGACTAGTCCAAAGACTTACA

GGAATAGATGTATCGATGATGTTGATAGTATGTGATATGTGAGCAAAGACTGTTCTCTTGGTGGCGTCGC

TATATGTTCCAGTAATGGCGGAAAACTTTTTAGAAATGTTATATATAAAAGAATTTTTTCGGGTTCCAAA

CATTAACAGATTAGTATGAAGATAAACACTCATATTATCAGGAACATTATCAATTTTTACATAAACATCG

GCATCTTGAATAGAAACAACACCATCTTCTGGAACCTCTACGATCTCGGCAGATTCCGGATAACCAGTCG

GTGGACCATCACTAACAATAACTAGATCATCCAACAATCTACTCACATATGCGTCTATATAATCTTTTTC

ATCTTGTGAGTACCCTGGATACGAAATAAATTTGTTATCAGTATTTCCATAATAAGGTTTAGTATAAACA

GAGAGAGATGTTGCTGCATGAACTTCGGTTACTGTCGCCGTTGGTTGGTTTATTTGACCTATTACTCTCC

TAGGTTTCTCTATAAATGATGGTTTAATTTGTACATTCTTAACCATATATCCAATAAAGCTCAATTCAGG

AACATAAACAAATTCTTTGTTGAACGTTTCAAAGTCGAACGAAGAGTCACGAATAACGATATCGGATACT

GGATTGAAGGTCACCGTTACGGTAATTTTTGAATCGGATAGTTTAAGACTACTGAATGTATCTTCCACAT

CAAACGGAGTTTTAATATAAACGTATACTGTAGATGGTTCTTTAATAGTGTCATTAGGAGTTAGGCCAAT

AGAAATATCATTAAGTTCACTAGAATATCCAGAATGTTTCAAAGCAATTGTATTATTGATACAATTATTA

TATAATTCTTCGCCCTCAATTTCCCAAATAACACCGTTACACGAAGAGACAGATACATGATTAATACATT

TATATCCAACATATGGCACGTAACCGAATCTTCCCATACCTTTAACTTCTGGAAGTTCCAAACTCAGAAC

CAAATGATTAAGCGCAGTAATATACTGATCCCTAATTTCGAAGCTAGCGATAGCCTGATTGTCTGGCCCA

TCGTTTGTCATAACTCCGGATAGAGAAATATATTGCGGCATATATAAAGTTGGAATTTGACTATCAACTG

CGAAGACATTAGACCGTTTAATAAAGTCATCCCCACCGATCAAAGAATTAATGATAGTATTATTCATTTT

CTATTTAAAATGGAAAAAGCTTACAATAAACTCCGTAGAGAAATATCTATAATTTGTGAGTTTTCCTTAA

AGTAACAGCTTCCGTAAACACCGTCTTTATCTCTTAGTAAGTTTATTGTATTTATGACCTTTTCCTTATC

TTCATAGAATACTAAAGGCAATAAAGAAATTTTTGGTTCTTCTCTAAGAGCTACGTGAGACTTAACCATA

GACGCCAACGAATCCCTACATATTTTAGAACAGAAATACCCAACTTCACCACCCTTGAATGTCTCAATAC

TAATAGGTCTAAAAACCAAATCTTGATTACAAAACCAACACTTATCAATTACACTATTTGTCTTAATAGA

CATATCTGCCATAGATTTATAATACTTTGGTAGTATACAAGCGAGTGCTTCTTCTTTAGCGGGCTTAAAG

ACTGCTTTAGGTGCTGAAATAACCACATCTGGAAGACTTACTCGCTTAGCCATTTAATTACGGAACTATT

TTTTTATACTTCTAATGAACAAGTAGAAAACCTCTCATCTACAAAAACATACTCGTGTCCATAATCCTCT

ACCATAGTAACACGTTTTTTAGATCTCATATGTGCTAAAAAGTTTTCCCATACTAATTGGTTACTATTAT

TTTTCGTATAATTTTTAACAGTTTGAGGTTTTAGATTTTTAGTTACAGAAGTGATATCGAATATTTTATC

CAAAAAGAATGAGTAATTAATTGTCTTAGAAGGAGTGTTTTCTTGGCAAAAGAATACCAAGTGCTTAAAT

ATTTCTACTACTTCATTAATCTTTTCTGTACTCAGATTCAGTTTCTCATCTTTTACTTGATTGATTATTT

CAAAGACTAACTTATAATCCTTTTTATTTATTCTCTCGTTAGCCTTAAGAAAACTAGATACAAAATTTGC

ATCTACATCATCCGTGGATATTTGATTTTTTTCCATGATATCCAATAGTTCCGAGATAATTTCTCCAGAA

CATTGATGAGACAATAATCTCCGCAATACATTTCTCAAATGAATAAGTTTATTAGACACGTGGAAGTTTG

ACTTTTTTTGTACCTTTGTACATTTTTGAAATACAGACTCGCAAAAAATACAATATTCATATCCTTGTTC

AGATACTATACCGTTATGTCTACAACAGCTACATAATCGTAGATTCATGTTAACACTCTACGTATCTCGT

CGTCCAATATTTTATATAAAAACATTTTATTTCTAGACGTTGTCAGAAAATCCTGTAATATTTTTAGTTT

TTTTGGTTGTGAATAAAGTATCGCCCTAATAATATTGGTACCGTCTTCCGACAATATAGTAGTTAAATTA

TCCGAGCATGTAGAAGAACACCGCTTAGGCGGATTCAGTACAATGTTATATTTTTCGTACCAACTCATTT

AAATATCATAATCTAAAATAGTTCTGTAATATGTCTAGCGCTAATATATTGATCATAATCCTGTGCATAA

ATTAAGATACAACAATGTCTTGAAATCATCGACATGGCTTCTTCCATAGTTAGAAGATCATCGTCAAAGT

TAGCAACGTGATTCATCAACATTTGCTGTTTTGAGGCAGCAAATACTGAACCATCACCATTCAACCATTC

ATAAAAACCATCGTCTGAATCCATTGATAATTTCTTGTACTGGTTTTTGAGAGCTCGCATCAATCTAGCA

TTTCTAGCTCCCGGATTGAAAACAGAAAGAGGATCGTACATCCAAGGTCCATTTTCTGTAAATAGAATCG

TATAATGTCCCTTCAAGAAGATATCAGACGATCCACAATCAAAGAATTGGTCTCCGAGTTTGTAACAGAC

TGCGGACTTTAACCTATACATGATACCGTTTAGCATGATTTCTGGTGATACGTCAATCGGAGTATCATCT

ATTAGAGATCTAAAGCCGGTGTAACATTCTCCGCCAAACATATTCTTATTCTGACGTCGTTCTACATAAA

ACATCATTGCTCCATTAACGATAACAGGTGAATGAACAGCACTACCCATCACATTAGTTCCCAATGGATC

AATGTGTGTAACTCCAGAACATCTTCCATAGCCTATGTTAGGAGGAGCGAACACCACTCTTCCACTATTG

CCATCGAATGCCATAGAATAAATATCCTTGGAATTGATAGAAATCGGACTGTCGGATGTTGTTATCATCT

TCATAGGATTAACAACGATGTATGGTGCAGCCTGAAGTTTCATATCGTAACTGATGCCGTTCATAGGTCT

AGCCACAGAAACCAACGTAGGTCTAAATCCAACTATAGACAAAATAGAAGCTAATATCTGTTCCTCATCT

GTCATAACTTGAGAGCATCCAGTATGAATAATCTTCATTAGATGGGGATCTACCGCATCATCATCGTTAC

AATAAAAAATTCCCATTCTAATGTTCATAATTGCTTTTCTAATCATGGTATGAATGTTTGCTCTCTGAAT

CTCTGTGGAAATTAGATCTGATACACCTGTAATCACTATCGGATTATCCTCCGTAAGACGATTAACCAAC

AACATATAATTATAAGACTTTACTCTTCTAAATTCATAAAGTTGCTGGATTAGACTATATGTGTCTCCAT

GTACATACGCGTTCTCGAGCGCAGGAAGTTTAATACCGAATAGTGCCATCAGAATAGGATGAATGTAGTA

ATTAGTTTCTGGTTTTCTATAAATAAAAGACAAATCTTGTGAACTAGACATATCGGTAAAATGCATGGAT

TGGAATCGTGTAGTCGACAGAAGAATATGATGATTAGATGGAGAGTATATTTTATCTAACTCTTTGAGTT

GGTCACCGATTCTAGGACTAGCTCGAGAATGAATAAGTACTAAGGGATGAGTACATTTCACAGAAACACT

GGCGTTGTTCAACGTACTCTTTACATGGGAAAGGAGTTGAAATAGCTCGTTTCTATTTGTCCTGACAATA

TTTAGTTTATTCATAATATTAAGCATATCCTGAATAGTAAAGTTAGATGTGTCATACTTGTTAGTAGTTA

GATATTTAGCAATTGCATTCCCATCATTTCTCAATCTCGTACTCCAATCATGTGTGGATGCTACTTCGTC

GATGGAAACCATACAATCCTTTTTGATAGGCTGTTGAGATTGATCATTTCCTGTACGTTTAGGTTTGGTA

CGTTGATTTCTAGCCCCTGCTGATATAAAGTCATCGTCTACAATTTGGGATAATGAATTACATACACTAC

AAGACAAAGATTTATCAGAAGTGTGAATATGATCTTCATCTACCAAAGAAAGAGTTTGATTAGTATAACT

AGATTTTAGTCCCGCGTTAGATGTTAAAAAAACATCGCTATTGACCACGGCTTCCATTATTTATATTCGT

AGTTTTTACTCGAAAGCGTGATTTTAATATCCAATCTTATTACTTTTGGAATCGTTCAAAACCTTTGACT

AGTTGTAGAATTTGATCTATTGCCCTACGCGTATACTCCCTTGCATCATATACGTTCGTCACCAGATCGT

TTGTTTCGGCCTGAAGTTGACGCATATCTTTTTCAACACTCGACATGAGATCCTTAAGGGTCATATCGTC

TAGATTTTGTTGAGATGCTGCTCCTGGATTTGGATTTTGTTGTGCTGTTGTACATACTGTACCACCAGTA

GGTGTAGGAGTACATACAGTGGCCACAATAGGAGGTTGAAGAGGTGTAACCGTTGGAGTAGTACAAGAAA

TACTTCCATCCGATTGTTGTGTACATGTGGTTGTTGGTAACGTCTGAGAAGGTTGGGTAGATGGCGGTGT

CGTCATCTTTTGATCTTTATTAAATTTAGAGATAATATCCTGAACAGTATTGCTCGGCGTCAACGCTGGA

AGGAGTGTACTCGCCGGCGCATCAGTATCTGTAGACAACCAATCAAAAAGATTAGACATATCAGATGATG

TATTAGTTTGTTGACGTGGTTTTAGTACAGGAGCAGTACTACTAGGTAGAAGAATAGGAGCCGGTGTAGG

TGTCGGAACCGGCTGTGGAGTTATATGAATAGTTGGTTGTAGCGGTTGGGTAGGCTGTCTGCTGGCGGTC

ATCATATTATCTCTAGCTAGTTGTTCTCGCAACTGTCTTTGATAATACGACTCTTGAGACTTTAGTCCTA

TTTCAATCGCTTCATCCTTTTTCGTATCCGGATCCTTTTCTTCAGAATAATAGATTGACGACTTTGGTGT

AGAGGATTCTGCCAGCCCCTGTGAGAACTTGTTAAAGAAGTCCATTTAAGGCTTTAAAATTGAATTGCGA

TTATAAGATTAAATGGCAGACACGGACGATATTATCGACTATGAATCCGATGATCTCACCGAATACGAGG

ATGATGAAGAAGATGGAGAGTCACTAGAAACTAGTGATATAGATCCCAAATCTTCTTATAAGATTGTAGA

ATCAACATCCACTCATATAGAAGATGCGCATTCCAATCTTAAACATATAGGGAATCATATATCTGCTCTT

AAACGACGCTATACTAGACGTATAAGTCTATTTGAAATAGCGGGTATAATAGCAGAAAGCTATAACTTGC

TTCAACGAGGAAGATTACCTCTAGTTTCAGAATTTTCTGACGAAACGATGAAGCAAAATATGCTACATGT

AATTATACAAGAGATAGAGGAGGGTTCTTGTCCTATAGTCATCGAAAAGAACGGAGAATTGTTGTCGGTA

AACGATTTTGATAAAGATGGTCTAAAATTCCATCTAGACTATATTATCAAAATTTGGAAACTTCAAAAAC

GATATTAGAATTTATACGAATATCGTTCTCTAAATGTCACAATCAAGTCTCTCATATTCAGCAGTTTATT

GTCGTACTTTATATCGTGTTCATTAACGATATTTTGCAAAATAGTAATGATTCTATCTTCCTTCGATAGA

TATTCTTCAGAGATTATTGTCTTATATTCTTTCTTGTTATCCGATATGAATTTGATAAGACTTTGAACAT

TATTAATACCCGTCTGTTTAATTTTTTCTATAGATATTTTAGTTTTGGTAGATTCTATGGTGTCTGTTAA

TAGGCATCCAACATCGACATTCGACGTCAATTGTCTATAAATCAGAGTATAAATTTTAGAAATAACATTA

GCAAATTGTTGTGCGTTGATGTCGTTATTCTGAAACAGTATGATTTTAGGTAGCATTTTCTTAACAAAGA

GAACGTATTTATTGTTACTCAGTTGAACAGATGATATATCCAGATTACTAACGCATCTGATTCCATATAC

CAAACTTTCAGAAGAAATGGTGTACAATTGTTTGTATTCATTCAATGTCTCCTTTTCAGAAATTAGTTTA

GAGTCGAATACTGCAATAATTTTCAAGAGATAGTTTTCATCAGATAAGATTTTATTTAGTGTAGATATGA

TAAAACTATTGTTTTGTTGGAGAACTTGATACGCCGCATTCTCTGTAGTCGACGCTCTCAAATGGGAAAC

AATCTCTATTATTTTTTTGGAATCGGATACTATATCTTCGGTATCTTGACGCAGTCTAGTATACATAGAG

TTAAGAGAAATTAGAGTTTGTACATTAAGCAACATGTCTCTAAATGTGGCTACAAACTTTTCTTTTTCCA

CATCATCTAGTTTATTATATACCGATTTCACAACGGCACCAGATTTAAGGAACCAGAATGAAAAACTCTG

ATAACTACAATATTTCATCATAGTTACGATTTTATCATCTTCTATAGTTGGTGTGATAACACATACCTTT

TTCTCCAAGACTGGAACCAACGTCATAAAAATGTTTAAATCAAAATCCATATCAACATCTGATGCGCTAA

GACCAGTCTCGCGTTCAAGATTATCTTTACTAATGGTGACGAACTCATCGTATAGAACTCTAAGTTTGTC

CATTATTTATTTACAGATTTAGTTGTTTAATTTATTTGTGCTCTTCCAGAGTTGGGATAGTATTTTTCTA

ACGTCGGTATTATATTATTAGGATCTACGTTCATATGTATCATAATATTAATCATCCACGTTTTGATAAA

TCTATCTTTAGCTTCTGAAATAACGTATTTAAACAAAGGAGAAAAATATTTAGTTACGGCATCAGACGCG

ATAACATTTTTTGTAAATGTAACGTATTTAGACGACAGATCTTCGTTAAAAAGTTTTCCATCTATGTAGA

ATCCATCGGTTGTTAACACCATTCCCGCGTCAGAGTGAATAGGAGTTTGAATAGTTTGTTTTGGAAATAG

ATCCTTCAATAACTTATAGTTGGGTGGGAAAAAATCGATTTTATCACTAGACTCTTTCTTTTTTACTATC

ATTACCTCATGAACTATTTCTTGAATGAGTATATGTATTTTCTTTCCTATATCGGTCGCGTTCATTGGAA

AATATATCATGTCGTTAACTATAAGAATATTTTTATCCTCGTTTACAAACTGAATAATATCAGATATAGT

TCGTAAACGAACTATATCATCACCAGCACAACATCTAACTATATGATATCCACTAGTTTCCTTTAGCCGT

TTATTATCTTGTTCCATATTAGCAGTCATTCCATCATTTAAGAAGGCGTCAAAGATAATAGGGAGAAATG

ACATTTTGGATTCTGTTACGACTTTACCAAAATTAAGGATATACGGACTTACTATCTTTTTCTCAACGTC

GATTTGATGAACACACGATGAAAATGTACTTCGATGAGATTGATCATGTAGAAAACAACAAGGGATACAA

TATTTCCGCATATCATGAAATATATTAAGAAATCCCACTTTATTATATTTCCCCAAAGGATCAATGCATG

TAAACATTATACCGTTATCATTAATAAAGACTTCTTTCTCATCGGATCTGTAAAAGTTGTTACTGATTTT

TTTCATTCCAGGATCTAGATAATTAATAATAATGGGTTTTCTATTCTTATTCTTTGTATTTTGACATATC

CTAGACCAGTAAACAGTTTCCACTTTGGTAAAATCAGAAGACTTTTGAACGCTATTAAACATGGCATTAA

TGGCAATAACTAAAAATGTAAAATATTTTTCTATGTTAGGAATATGGTTTTTCACTTTAATAGATATATG

GTTTTTTGCCAAAATGATAGATATTTTTTTATCCGATGATAGTAAAATATTATTAGTCGCCGTCTCTATA

AAAATGAAGCTAGTCTCGATATCCAATTTTATTCTAGAATTGATAGGAGTCGCCAAATGTACCTTATACG

TTATATCTCCCTTGATGCGTTCCATTTGTGTATCTATATCGGACACAAGATCTGTAAATAGTTTTACGTT

ATTAATCATCACGGTATCGCCATCGCTAGATAATGCTAATGTACTATCCAAGTCCCAAATGGAGAGATTT

AACTGTTCATCGTTTAGAATAAAATGATTACCTGTCATATTAATAAAGTGTTCATCGTATCTAGATAACA

ACGACTTATAATTAATGTCCAAGTCTTGAACTCGCTGAATGATCTTTTTTAACCCAGTTAGTTTTAGATT

GGTACGAAATATATTGTTAAACTTTGATTCTACAGTAATGTCCAAATCTAGTTGTGGAAATACTTCCATC

AACATTGTTTCAAACTTGATAATATTATTATCTACATCTTCGTACGATCCAAATTCCGGAATAGATGTAT

CGCACGCTCTGGCCACCCAGATAACCAAAAAGTCACACGCTCCAGAATATACATTGTATAAAAAGCTATC

GTTTTTTAGTAGTGTTTTTTTCTGAGTATATACGAAAGGATTAAAAATAGTATTATCAACGTAACTATAT

TCCAAATTATTCTTATGAGAATAGATAATAATATCGTCCTTAATATCTAACAAATTTCCTAAATATCCCT

TTAATTGAGTCATTCGAAGCGTTAATAAAATATGTCTCTTAACTATTTCCGGCCGTTGTATATTTAAATG

ACTTCGTAAGAAATAATATATAGGCGACTTCTCATCTATGTAATCATATGGAGTGAGATATAGGGCTCGT

TCTACCTCCTGCCCCTTACCCACCTGTAATACCAATTGCGGACTCACTATATATCGCATATTTATATCGT

GGGGTAAAGTGAAAATCTACTACCGATGATGTAAGTCTTACAATGTTCGAACCAGTACCAGATCTTAATT

TGGAGGCCTCCGTAGAACTAGGGGAGGTAAATATAGATCAAACAACACCTATGATAAAGGAGAATAGCGG

TTTTATATCCCGTAGTAGACGTCTATTCGCCCATAGATCTAAGGATGATGAGAGAAAACTAGCACTACGA

TTCTTTTTACAAAGACTTTATTTTTTAGATCATAGAGAGATTCATTATTTGTTCAGATGCGTCGACGCTG

TAAAAGACGTCACTATTACCAAAAAAAATAACATTATCGTGGCGCCTTATATAGCACTTTTAACTATCGC

ATCAAAAGGATGCAAACTTACAGAAACAATGATTGAAGCATTCTTTCCAGAACTATATAATGAACATAGT

AAGAAATTCAAATTCAACTCTCAAGTATCCATCATCCAAGAAAAACTCGGATACCAGTCTGGAAACTATC

ACGTTTATGATTTTGAACCGTATTACTCTACAGTAGCTCTGGCTATTCGAGATGAACATTCATCTGGCAT

TTTTAATATCCGTCAAGAGAGTTATCTTGTAAGTTCATTATCTGAAATAACATATAGATTTTATCTAATT

AATCTAAAATCTGATCTTGTTCAATGGAGTGCTAGTACGGGCGCTGTAATTAATCAAATGGTAAATACTG

TATTGATTACAGTGTATGAAAAATTACAACTGGCCATAGAAAATGATTCACAATTTACATGTTCATTGGC

TGTGGAATCAGAACTTCCAATAAAATTACTTAAAGATAGAAATGAATTATTTACAAAATTCATTAACGAG

TTAAAAAAGACCAGTTCATTCAAGATAAGCAAACGTGATAAGGATACGCTATTAAAACATTTTACTTATG

ACTGGAGTTAGAATTTATAGACGACACATTTCGTTTATCATTGTTACTATTACTATCATTATTAGTATTC

TTCTTGTCATCTTGTTCAGAAATATACAGCAATGCTATACCTAATACTAAATACATTATCATGCTTGCAA

TGGCTCTAACAACAACGAACCAAAATGAATTTGGTCGTAGCTTTTGTTCACAAAAATACATAAAGAAATG

TCTACATAAATCTATGGCGCCATTGGCTACTTGAAATAGCGCCAGTCCTCCTACAGATTTTAATATAGCT

GTATAACATGACATTTATTCATCATCAAAAGAGACAGAGTCACCATCTGTCATATTTAGATTTTTTTTCA

TGTGTTCAAAGTATCCTCTACTCATTTCATTATAATAGTTTATCATGCTTAGAATTTTAGGACGGATCAA

TGAGTAAGACTTGACTAGATCGTCAGTAGTAATTTGTGCATCATCTATTCTGCATCCGCTTCGTCGAATA

ATGTATAGCATCGCTTTGAGATTCTCCATAGCTATCAAGTCTTTATATAATGACATGGAAATATCTGTGA

ATGCTTTATACTTCTCCAACATCGATGCCTTAACATCATCACATACTTTAGCATTGAAAATACGTTCTAT

TGTGTAGATGGATGTAGCAAGATTTTTAAACAACAATGCCATCTTACATGATGATTGTCTCAAGTCTCCA

ATCGTTTGTTTAGAACGATTAGCTACAGAGTCCAATGCTTGGCTAACTAGCATATTATTATCTTTAGAAA

TTGTATTCTTCAATGAGGCGTTTATCATATCTGTGATTTCGTTAGTCATATTACAGTCTGACTGGGTTGT

AATGTTATCCAACATATCACCTATGGATACGGTACACGTACCAGCATTTGTAATAATCCTATCTAAGATG

TTGTATGGCATTGCGCAGAAAATATCTTCTCCTGTAATATCTCCACTCTCGATAAATCTACTCAGATTAT

TCTTAAATGCCTTATTCTCTGGAGAAAAGATATCAGTGTCCATCATTTCATTAATAGTATACGCAGAAAA

GATACCACGAGTATCAATTCTATCCAAGATACTTATCGGTTCCGAGTCACAGATAATTGTTTCCTCTCCT

TCGGGAGATCCTGCATAGAAATATCTAGGACAATAGTTTCTATACTGTCTGTAACTCTGATAATCTCTAA

AGTCACTAACTGATACCATGAAATTGAGAAGATCAAACGCTGAAGTAATCAATTTTTCTGCCTCGTTTTT

ACTACAACTAGTTTTCATCAATGTAGTGACGATGTATTGTTTAGTTACTCTTGGTCTAATACTGATGATA

GAGATATTATTGCTTCCCATAATGGATCTTCTAGTAGTCACCTTAAAGCCCATTGATGCGAATAGCAGAT

AGATAAAGTCTTGGTATGACTCCTTTCTAATATAGTACGGACTACCTTTGTCACCCAACTTTATACCCAC

ATAAGCCATAACAACCTCTTTAATAGCCGTTTCATGAGGTTTATCAGCCATGAGCCTGAGTAGTTGAAAG

AATCGCATGAATCCCGTCTCAGAAAGTCCTATATGCATGATAGATTTATCTTTCCTGGGAAACTCTCGTA

TAGTTATAGATGAAATACTCTTCAAAGTTTCTGAAATAAGATTAGTAACAGTCTTACCTCCGACTACTCT

GGGTAACAAACATACTCTAATAGGTGTTTTCTCTGCGGAGATAATATCAGAAAGGATAGAGCAATAAGTA

GTATTATTGTGATTATAAAGACCGAATACATAACAGGTAGAATTTATAAACATCATGTCCTGAAGGGTTT

TAGACTTGTATTCCTCGTAATCTATACCGTCCCAAAACATGGATTTGGTAACTTTGATAGCCGTAGATCT

TTGTTCCTTCGCTAACAGGTTAAAGAAATTAATAAAGAATTTGTTGTTTCTATTTATGTCCACAAATTGC

ACGTTTGGAAGCGCCACGGTTACATTCACTGCAGCATTTTGAGGATCGCGAGTATGAAGTACGATGTTAT

TGTTTACTGGTATATCTGGAAAGAAATCTACCAGTCTAGGAATAAGAGATTGATATCGCATAGAAATAGT

AAAGTTTATAATCTCATCATTGAAGATTACTCTGTTACCATTGTAATAAATTGGTACTCTATCATAATCA

TCGACAAAGTACTGTTCATACATGATGAGATGTTTATATGTTGGCATAGTAGTGAGATCGACGTTTGGTA

ATGGCAATGTATTAAGATTAACTCCATAATGTCTAGCAGCATCTGCGATGTTATAAGTGATGTCAAAGCG

GGGTTGATCTTGTGCTGTTATATATTGTCTAACACCTATAAGATTATCAAAATCTTGTCTGCTTAATACA

CCGTTAACAATTTTTGCCTTGAATTCTTTTATTGGTGCATTAATAACATCCTTATAGAGGATGTTAAACA

AATAAGTATTATCAAAGTTAAGATCTGGGTATTTCTTTTCTGCTAGAACATCCATTGAGTCGGAGCCATC

TGGTTTAATATAACCACCGATAAATCTAGCTCTGTATTCTGTATCCGTCAATCTAATATTAAGAAGGTGT

TGAGTGAAAGGTGGAAGATCGTAAAAGCTGTGAGTATTAATAATAGGGTTAGTTTCCGAACTAATGTTAA

TTGGATGATTAATAATATTTATATTTCCAGCGTTAAGTGTAACATTAAACAGTTTTAATTCACGTGACGT

GGTATCAATTAAATAATTAATGCCCAATTTGGATATAGTAGCCTGAAGCTCATCTTGTTTAGTTACGGAT

CCTAATGAGTTATTAAGAAATACATCGAACGGATGAACGAAGGTTGTTTTAAGTTGGTCACATACTTTGT

AATCTAGACATAGATGTGGAAGAACGGTAGAAACTATACGAAATAGATATTCAGAGTCCTCTAATTGATC

AAGAGTAACTATTGACTTAATAGGCATCATTTATTTAGTATTAAATGACGACCGTACCAGTGACAGATAT

ACAAAACGACTTAATTACAGAGTTTTCAGAAGATAATTATCCATCTAACAAAAATTATGAAATAACTCTT

CGCCAAATGTCTATTCTAACTCACGTTAACAACGTGGTAGATAGAGAACATAATGCCGCCGTAGTGTCAT

CTCCAGAGGAAATATCATCACAACTTAATGAAGATCTATTTCCAGATGATGATTCACCGGCCACTATTAT

CGAACGAGTACAACCTCATACTACTATTATTGACGATACGCCACCTCCTACTTTTCGTAGAGAGTTATTG

ATATCGGAACAACGTCAACAACGAGAAAAAAGATTTAATATTACAGTATCAAAAAATTCTGAAGCAATAA

TGGAATCTAGATCTATGATAACTTCTATGCCAACACAAACACCATCCTTGGGAGTAGTTTATGATAAAGA

TAAAAGAATTCAGATGCTAGAGGATGAAGTGGTTAATCTTAGAAATCAACGATCTAATACAAAATCATCT

GATAATTTAGATAATTTTACCAGAATACTATTTGGTAAGACTCCGTATAAATCAACCGAAGTTAATAAGC

GTATAGCCATCGTTAATTATGCAAATTTGAACGGGTCCCCCTTATCAGTCGAGGACTTGGATGTCTGTTC

GGAGGATGAAATAGATAGAATCTATAAAACGATTAAACAATATCACGAAAGTAGAAAACGAAAAATTATC

GTCACTAACGTGATTATTATTGTCATAAACATTATTGAGCAGGCATTGCTAAAACTCGGATTTGAAGAAA

TCAAAGGACTGAGTACCGATATCACTTCAGAAATTATCGATGTGGAGATCGGAGATGACTGCGATGCTGT

AGCATCAAAACTAGGAATCGGTAACAGTCCGGTTCTTAATATTGTATTGTTTATACTCAAGATATTCGTT

AAACGAATTAAAATTATTTAATTTAATACATTCCCATATCCAGACAACAATCGTCTGGATTAATCTGTTC

CTGTCGTCTCATACCGGACGACATATTAATCTTTTTATTAGTGGGCATCTTTTTAGATGGTTTCTTTTTC

CCAGCATTAACTGATTCGATACCTAGAAGATCGTGATTGATTTCTCCGACCATTCCACGAACTTCTAATT

GGCCGTCTCTAACGGTACCATAAACTATTTTACCAGCATTAGTAACAGCTTGGACAATCTGACCATCCAT

TGCGTTGAATGATGTAGTTGCTGTTGTTCTACGTCTAGGAGCACCAGAGGTATTTTTAGAGCTCTTGGAT

GTTGATGTAGAAGACGAGGATTTTGATTTTGGTTTACATGTAATACATTTTGAACTCTTTGATTTTGTAT

CACATGCACCGGCAGTCACATCTGTTTGAGAATTAAGATTATTGTTGCCTCCTTTGACGGCTGCATCTCC

ACCGATCTGCGCTAGTAGATTTTTAAGCTGTGGTGTAATCTTATTAACTGTTTCAATATAATCATCGTAA

CTACTTCTAACGGCTAAATTTTTTTTATCCGCCATTTAGAAGCTAAAAATATTTTTATTTATGCAGAAGA

TTTAACTAGATTATACAATGAACTAATATGATCCTTTTCTAGATTATTTACGAACTTGGTATTTCTTGTT

TCTGGAGGAGGAGAATTTAAATTCGGACTTGGATTCGGATTTTGTGGGTTCTTGATCTTATTATACAGCG

TGTATAGGATGGTGACGGTAACTGCTACACAAATACCGATCAACAGAAGAATACCAATCATTTATTGACA

ATAACTTCACTATGATCAAGTATGTAATAATCATCTTTTCACTAAGTAAGTAGTAATAATGATTCAACAA

TGACACGATATATGGACGATAATAATTTAGTTCATGGAAATATCGCTATGATTGGTGTGAATGACTCCGC

TAACTCTGTGGGGTGCACAGTGCTTTCCCCACATAGAATAAATTAGCATTCCGACTGTGATAATAATACC

AAGTATAAACGCCATAATACTCAATACTTTCCATGTACGAGTGGGACTGGTAGACTTACTAAAGTCAATA

AAGGCGAAGATACACGAAAGAATCAAAAGAATGATTCCAGCGATTAGCACGCCAGAAAAATAATTTCCAA

TCATAAGCATCATGTCCATTTAACTAATAAAAATTTTAAATCGCCGAATAAACAAAGTGGAATATAAACC

ATATAAAAACAATAGTTTGTACTGCAAAAATAATATCTATTTTTGTTTTCGAAGATATGGTAAAATTAAA

TAGTAGTACACAGCATGTTATAACTAACAGCAGCAACGGCTCGTAATTACTTATCATTTACTAGACGAAA

AGGTGGTGGGATATTTTCTTGCTCAAATAATACGAATATATCACCCATCCATTTTATACGATGTTTATAT

ACTCTAATCTTTAATAGATCTATAGATGACGGGTTTACCAATAATATAGATTTTATCGATTCATCTAATT

TAAACCCTTCCTTAAACGTGAATGATCTATTATCTGGCATAATGATGACCCTACCTGATGAATCTGACAA

TGTACTGGGCCATGTAGAATAAATTATCAACGAATTATCGTCTACGAACATTTATATCATTTGTTTTAAT

TTTAGGACGTGAATAAATAGATATAAAATAGAAAATAACAGATATTACAACCAGTGTTATGGACGCACCC

AACCATGTAGGCAGTTTTATTTTATCGTTTACTACAGGTTCTCCTGGATGTACGTCACCAACTGCAGACG

TAGTTCTAGTACAATTAGACGTAAGTTCCGCTTGGGAATTTTTTAACGCTAAAGAGTTAACGTTGATCGT

ACACCCAACGTATTTACATCTAGTTCTTTGAACATCTTGATTATAATATAACCATTTTCTATCTCTAGAT

TCGTCAGTGCACTCATGTAACCAACATACCCTAGGTCCTAAATATTTATCTCCGGAATTAGATTTTGGAT

AATTCGCGCACCAACAATTTCTATTTCCTTTATGGTCGTTACAAAAGACGTATAATGCCGTATCCCCAAA

AGTAAAATAATCAGGACGAATAATTCTAATAAACTCAGAACAATATCTCGCATCCATATGTTTGGAGCAA

ATATCGGAATAAGTAGACATAGCCGGTTTCCGTTTTACACGTAACCATTCTAAACAATTGGGGTTTCCAG

GATCGTTTCTACAAAAACCAGTCATGAAATCGTCACAATGTTCTGTCTTGTAATTATTATTAAATATTTT

TGGACAGTGTTTGGTATTTGTCTTAGAACAACATTTTGCCACGCTATCACTATCACCCAGGAGATAATCC

TTTTTTATAAAATGACATCGTTGCCCGGATGCTATATAATCAGTAGCATATTTTAAATCCTTAATATATT

CAGGAGTTACCTCGTTCTGATAATAGATTAATGATCCAGGACGAAATTTGAAAGAACTACATGGTTCTCC

ATGAATTAATACATATTGTTTAGCAAATTCAGGAACTATAAAACTACTACAATGATCTATCGACATACCA

TCTATCAAACAAAATTTGGGTTTAATTTCTCCTGGAGACGTTTCATAATAATACATATAACTTTCTTCGG

CAAACCTAACAGCTCTATTATATTCAGGATAATTAAAATCTAATACCATATATTTGTCTCGTATATCTGC

TATTCCTGTCTCTATTTTGATTCTATTAAGAGTAACAGCTGCCCCCATTCTTAATAATCATCAGTATTTA

AACTGTTAAATGTTGGTATATCAACATCTATCTTATTTCCCGCAGTATAAGGTTTGTTGCAGGTATACTG

TTCAGGAATGGGTACATTTATACTTCTTTTATAGTCCTGTCTTTCGATGTTCATCACAAATGCAAAGAAC

AGAATAAACAAAATAATGTAAGAAATAATATTAAATATCTGTGAATTCGTAAATACATTGATTGCCATAA

TAATTACAGCAGCTACAATACACACAATAGACATTCCCACAGTGTTGCCATTACCTCCACGATACATTTG

AGTTACTAAGCAATAGGTAATAACTAAGCTAGTAAGAGGCAATAGAAAAGATGAGATAAATATCATCAAT

ATAGAGATTAGAGGAGGGCTATATAGAGCCAAGACGAACAAAATCAAACCGAGTAACGTTCTAACATCAT

TATTTTTGAAGATTCCCAAATAATCATTCATTATTCCTCCATAATCGTTTTGCATCATACCCCCATCTTT

AGGCATAAACGATTGCTGCTGTTCCTCTGTAAATAAATCTTTATCAAGCACTCCAGCACCCGCAGAGAAG

TCATCAAGCATATTGTAATATCTTAAATAACTCATTTATATATTAAAAAATGTCACTATTAAAGATGGAG

TATAATCTTTATGCCGAACTAAAAAAAATGACTTGTGGTCAGACCATAAGTCTTTTTAATGAAGACGGCG

ATTTCGTAGAAGTTGAACCAGGATCATCCTTTAAGTTTCTAATACCTAAGGGATTTTACTCCTCTCCTTG

TGTAAAGACGAGTCTAGTATTCAAGACATTAACAACGACCGATAATAAAATTACTAGTATCAATCCAACA

AATGCGCCAAAGTTATATCCTCTTCAACGCAAAGTCGTATCTGAAGTAGTTTCTAATATGAGGAAAATGA

TCGAATTAAAACGTCCTCTATACATCACTCTTCACTTGGCATGTGGATTTGGTAAGACTATTACCACGTG

TTATCTTATGACCACACACGGCAGAAAAACCATCATTTGCGTACCCAATAAAATGTTAATACATCAATGG

AAGACACAGGTAGAGGCAGTCGGATTGGAACATAAGATATCTATAGATGGAGTTAGTAGTCTATTAAAGG

AACTAAAGACTCAAAGTCCGGATGTATTAATCGTAGTCAGTAGACATCTGACAAACGATGCATTTTGTAA

ATATATCAATAAGCATTATGATTTGTTTATCTTGGATGAATCACATACGTATAATCTGATGAACAATACA

GCAGTTACAAGATTTTTAGCGTATTATCCTCCGATGATGTGTTATTTTTTAACTGCTACACCTAGACCAG

CTAACCAAATTTATTGTAATAGTATTATTAATATTGCCAAGTTATCCGATCTAAAAAAAACTATCTATAT

AGTAGATAGTTTTTTTGAGCCATATTCCACAGACAATATTAGAAATATGGTAAAACGACTAGATGGACCA

TCTAATAAATATCATATATATACCGAGAAGTTATTATCTGTAGACGAGCCTAGAAACCAACTTATTCTTG

ATACCCTGGTAGAAGAATTCAAGTCAGGAACTATTAATAGAATTTTAGTTATTACTAAACTACGTGAACA

TATGGTATTCTTCTACAAACGATTATTAGATCTTTTCGGAGCAGAGGTTGTATTTATAGGAGACGCCCAA

AATAGACGTACTCCAGATATGGTCAAATCGATTAAGGAACTAAATAGATTTATATTCGTATCCACCTTAT

TTTATTCCGGCACTGGTTTAGATATTCCGAGTTTGGATTCTTTGTTCATTTGCTCGGCAGTAATCAACAA

TATGCAAATAGAGCAATTACTAGGGAGGGTATGTCGAGAAACAGAACTATTAGATAGGACGGTATATGTA

TTTCCTAACACATCCATCAAAAAAATAAAGTACATGATAGGAAATTTCGTGCAACGAATTATTAGTCTGT

CTGTAGATAAACTCGGATTTAAACAAGAAAGTTATCAGAAACATCAGGAATCTGAACCCGCTTCCGTACC

AACATCCTCCAGAGAAGAACGTGTATTAAATAGAATATTTAACTCGCAAAATCGTTAAGAAGTTTAAGAG

ACGATCCACATGCTGAGCAGGCCAGTGTATTACCCCTCATAGTATTAATATAATCCAATGATACTTTTGT

GATGTCGGAAATCTTAACCAATTTAGACTGACAGGCAGAACACGTCATACAATCATCATCGTCATCGATA

ACTGTAGTCTTGGGCTTCTTTTTGCGACTCTTCATTCCGGAACGCATATTGGTGCTATCCATTTAGGTAG

TAAAAAATAAGTCAGAATATGCCCTATAACACGATCGTGCAAAACCTGGTATATCGTCTCTATCTTTATC

ACAATATAGTGTATCAACATCTTTATTATTGACCTCGTTTATCTTGGAACATGGAATGGGAACATTTTTG

TTAACGGCCACCTTTGCCTTAATTCCAGATGTTGTAAAATTATAACTAAACAGTCTATCATCGACACAAA

TGAAATTCTTGTTTAGACGTTTGTAGTTTACGTATGCGGCTCGTTCTCGTCTCATTTTTTCAGATATTGC

AGGTACTATAATATTAAAAATAAGAATGAAATAACATAGGATTAAAAATAAAGTTATCATGACTTCTAGT

GCTGATTTAACTAACTTAAAAGAATTACTTAGTCTGTACAAAAGTTTGAGATTTTCAGATTCTGTGGCTA

TAGAGAAGTATAATTCTTTGGTAGAATGGGGAACATCTACTTACTGGAAAATAGGCGTACAAAAGGTAAC

TAATGTCGAGACGTCCATATCTGATTATTATGATGAGGTAAAAAATAAACCGTTTAATATTGATCCGGGG

TATTATATTTTCTTACCAGTATATTTTGGAAGCGTCTTTATTTATTCAAAGGGTAAAAATATGGTAGAAC

TTGGATCTGGAAACTCTTTTCAAATACCGGATGAGATTCGAAGTGCGTGTAACAAAGTATTAGATAGTGA

TAACGGAATAGACTTTCTGAGATTTGTTTTGTTAAACAATAGATGGATAATGGAAGACGCTATATCAAAA

TACCAGTCTCCAGTTAATATATTTAAACTAGCTAGTGAGTACGGATTAAACATACCCAACTATTTAGAAA

TTGAAATAGAGGAAGACACATTATTTGACGATGAGTTATACTCTATTATGGAACGCTCTTTCGATGATAC

ATTTCCAAAAATATCTATATCGTATATTAAGTTGGGAGAACTTAAGCGGCAAGTTGTAGACTTTTTCAAA

TTCTCATTCATGTATATTGAGTCAATCAAGGTAGATCGTATAGGAGATAATATTTTTATTCCTAGCGTTA

TAACAAAATCAGGAAAAAAGATATTAGTAAAAGATGTAGACCATTTAATACGATCCAAGGTTAGAGAACA

TACATTTGTAAAAGTAAAAAAGAAAAACACATTTTCCATTTTATACGACTATGATGGGAACGGAACAGAA

ACTAGAGGAGAAGTAATAAAACGAATTATAGACACTATAGGACGAGACTATTATGTTAATGGAAAGTATT

TCTCTAAGGTTGGTATTGCAGGCTTAAAGCAATTGACTAATAAATTAGATATTAATGAGTGTGCAACTGT

CGATGAGTTAGTTGATGAGATTAATAAATCCGGAACTGTAAAACGAAAAATAAAAAACCAATCAGTATTT

GATTTAAGCAGAGAATGTTTGGGATATCCAGAAGCGGATTTTATAACGTTAGTTAATAACATGCGGTTCA

AAATAGAAAATTGTAAGGTTGTAAATTTCAATATTGAAAATACTAATTGTTTAAATAACCCGAGTATTGA

AACTATATATGGAAACTTCAACCAGTTCGTCTCAATCTTTAATACCGTTACCGATGTCAAAAAAAGATTA

TTCGAGTGAAATAATATGCGCCTTTGATATAGGTGCAAAAAATCCTGCCAGAACTGTTTTAGAAGTCAAG

GATAACTCCGTTAGGGTATTGGATATATCAAAATTAGACTGGAGTTCTGATTGGGAAAGGCGCATAGCTC

AAGATTTGTCACAATATGAATACACTACAGTTCTTCTAGAACGTCAGCCTAGAAGGTCACCGTACGTCAA

ATTTATCTATTTTATTAAAGGCTTTTTATATCATACATCTGCTGCCAAAGTTATTTGCGTCTCACCTGTC

ATGTCTGGTAATTCATATAGAGATCGAAAAAAGAGATCTGTTGAAGCATTTCTTGATTGGATGGACACAT

TCGGATTGCGAGACTCCGTTCCGGATAGACGCAAATTAGACGATGTAGCGGATAGTTTCAATTTGGCTAT

GAGATACGTATTAGATAAATGGAATACTAATTATACACCTTATAATAGGTGTAAATATAGAAATTACATA

AAAAAAATGTAATAACGTTAGTAACGCCATTATGGATAATCTATTTACCTTTCTACATGAAATAGAAGAT

AGATATGCCAGAACTATTTTTAACTTTCATCTAATAAGTTGTGATGAAATAGGAGATATATATGGTCTTA

TGAAAGAACGCATTTCCTCAGAGGATATGTTTGACAATATAGTATATAATAAAGATATACATCCTGCCAT

TAAGAAACTAGTTTATTGCGACATCCAACTTACTAAACATATTATTAATCAGAATACGTATCCGGTATTT

AACGATTCTTCACAAGTGAAATGTTGTCATTATTTCGATATAAACTCAAATAATAGCAATATTAGCTCTC

GTACAGTAGAGATATTTGAGAGTGAAAAGTCATCTCTTGTATCATATATTAAAACTACCAATAAGAAGAG

AAAGGTCAATTACGGCGAAATAAAGAAAACTGTACATGGAGGCACTAATGCAAATTACTTTTCCGGTAAA

AAGTCTGATGAGTATCTGAGCACTACAGTCAGGTCCAACATTAATCAACCTTGGATCAAAACCATTTCTA

AGAGAATGAGAGTAGATATCATTAATCACTCTATAGTAACGCGTGGAAAAAGCTCTATATTACAAACTAT

AGAAATTATTTTTACTAATAGAACATGTGTGAAAATATTCAAGGATTCTACTATGCACATTATTCTATCC

AAGGACAAGGATGAAAAGGGATGTATAAACATGATTGATAAATTATTCTATGTATATTATAATTTATTTC

TGTTGTTCGAGGATATCATCCAAAACGATTACTTTAAAGAAGTAGCTAATGTTGTAAACCATGTACTCAT

GGCTACGGCATTAGATGAGAAATTATTCCTAATTAAGAAAATGGCTGAACACGATGTTTATGGAGTTAGC

AATTTCAAAATAGGGATGTTTAACCTGACATTTATTAAGTTGTTGGATCATACCGTTTTCCCCTCTCTGT

TAGATGAGGATAGCAAAATAAAGTTTTTTAAGGGGAAAAAGCTCAATATTGTAGCATTACGATCTCTGGA

GGATTGTACAAATTACGTGACTAAATCCGAGAATATGATAGAAATGATGAAGGAAAGATCGACTATTTTA

AATAGCATAGATATAGAAACGGAATCGGTAGATCGTCTAAAAGAATTGCTTCTAAAATGAAAAAAAACAC

TGATTCAGAAATGGATCAACGACTCGGGTATAAGTTTTTGGTGCCTGATCCTAAAGCCGGAGTTTTTTAT

AGACCGTTACATTTCCAATATGTATCGTATTCTAATTTTATATTGCATCGATTGCATGAAATCTTGACCG

TCAAGCGGCCACTCTTATCGTTTAAGAATAATACAGAACGAATTATGATAGAAATTAGCAATGTTAAAGT

GACTCCTCCAGATTACTCACCTATAATTGCGAGTATTAAAGGTAAGAGTTATGACGCATTAGCCACGTTC

ACTGTAAATATCTTTAAAGAGGTAATGACCAAAGAGGGTATATCCATCACTAAAATAAGTAGTTATGAGG

GAAAAGATTCTCATTTGATAAAAATTCCGCTACTAATAGGATATGGGAATAAAAATCCACTTGATACAGC

CAAGTATCTTGTTCCTAATGTCATAGGTGGAGTCTTTATCAATAAACAATCTGTCGAAAAAGTAGGAATT

AATCTAGTAGAAAAGATTACAACATGGCCAAAATTTAGGGTTGTTAAGCCAAACTCATTCACTTTCTCGT

TTTCCTCCGTATCCCCTCCTAATGTATTACCGACAAGATATCGCCATTACAAGATATCTCTGGATATATC

ACAATTGGAAGCGTCGAATATATCATCGACAAAGACATTTATAACGGTCAATATTGTTTTGCTGTCTCAA

TATTTATCTAGAGTGAGTCTAGAATTCATTAGACGTAGTTTATCATACGATATGCCTCCAGAAGTTGTCT

ATCTAGTAAACGCGATAATAGATAGTGCTAAACGACTTACCGAATCTATTACTGACTTTAATATTGATAC

ATACATTAATGACCTGGTGGAAGCTGAACACATTAAACAAAAATCTCAGTTAACGATTAACGAGTTTAAA

TATGAAATGCTGCATAACTTTTTACCTCATATGAACTATACACCCGATCAACTAAAGGGATTTTATATGA

TATCTTTACTAAGAAAGTTTCTCTACTGTATCTACCACACTTCTAGATATCCAGATAGAGATTCGATGGT

TTGTCATCGCATCCTAACGTACGGCAAATATTTTGAGACGTTAGCACATGATGAATTAGAGAATTACATA

GGTAACATCCGAAACGATATCATGAACAATCACAAGAACAGAGGCACTTACGCAGTAAACATTCATGTAC

TAACAACTCCTGGACTTAATCATGCATTTTCTAGTCTATTGAGTGGAAAGTTCAAAAAGTCAGACGGTAG

TTATCGAACACATCCTCACTATTCATGGATGCAGAATATTTCTATTCCTAGAAGTGTTGGATTTTATCCG

GATCAAGTAAAGATTTCAAAGATGTTTTCTGTCAGAAAATACCATCCAAGCCAATATCTTTACTTTTGTT

CATCAGACGTTCCGGAAAGAGGTCCTCAGGTAGGTTTAGTATCTCAATTGTCTGTCTTGAGTTCCATTAC

AAATATACTAACGTCTGAGTATTTGGATTTGGAAAAGAAAATTTGTGAGTATATCAGATCATATTATAAA

GATGATATAAGTTACTTTGAAACAGGATTTCCAATCACTATAGAAAATGCTCTAGTCGCATCTCTTAATC

CAAATATGATATGTGATTTTGTAACTGACTTTAGACGTAGAAAACGGATGGGATTCTTCGGTAACTTGGA

GGTAGGTATTACTTTAGTTAGGGATCACATGAATGAAATTCGCATTAATATTGGAGCAGGAAGATTAGTC

AGACCATTCTTGGTTGTGGATAACGGAGAGCTCATGATGGATGTGTGTCCGGAGTTAGAAAGCAGATTAG

ACGACATGACATTCTCTGACATTCAGAAAGAGTTTCCACATGTCATCGAAATGGTAGATATAGAACAATT

TACTTTTAGTAACGTATGTGAATCGGTTCAAAAATTTAGAATGATGTCAAAGGATGAAAGAAAGCAATAC

GATTTATGTGACTTTCCTGCCGAATTTAGAGATGGATATGTAGCATCTTCACTAGTGGGAATCAATCACA

ATTCTGGACCCAGAGCTATTCTTGGATGTGCTCAAGCTAAACAAGCTATCTCTTGTCTGAGTTCGGATAT

ACGAAATAAAATAGACAATGGAATTCATTTGATGTATCCAGAGAGGCCAATTGTGATTAGTAAGGCTTTA

GAAACTTCAAAGATTGCGGCTAATTGCTTCGGACAACATGTTACTATAGCATTAATGTCGTACAAAGGTA

TCAATCAAGAGGATGGAATTATCATCAAAAAACAATTTATTCAGAGAGGCGGTCTCGATATTGTTACAGC

CAAGAAACATCAAGTAGAAATTCCATTGGAAAACTTTAATAACAAAGAAAGAGATAGGTCTAACGCCTAT

TCGAAATTAGAAAGTAATGGATTAGTTAGACTGAATGCTTTCTTGGAATCCGGAGACGCTATGGCAAGAA

ATATCTCATCAAGAACTCTTGAAGATGATTTTGCTAGAGATAATCAGATTAGCTTTGATGTTTCCGAGAA

ATATACAGATATGTACAAATCTCGCGTTGAACGAGTACAAGTAGAACTTACTGACAAAGTTAAGGTGCGA

GTATTAACCATGAAAGAAAGAAGACCCATTCTAGGAGACAAATTTACTACTAGAACGAGTCAAAAGGGAA

CAGTCGCGTATATCGCAGATGAAACGGAACTTCCGTACGACGAAAATGGTATCACACCAGATGTCATTAT

TAATTCTACATCCATCTTCTCTAGAAAAACTATATCTATGTTGATAGAAGTTATTTTAACAGCCGCATAT

TCTACTAAGCCGTACAACAATAAGGGAGAAAACCGACCTGTCTGTTTTCCTAGTAGTAACGAAACATCTA

TCGATGCATATATGCAATTCGCTAAACAATGTTATGAGTATTCAAATCCGAAATTGTCCGAGGAAGAATT

ATCGGATAAAATCTTTTGTGAAAAGATTCTCTATGATCCTGAAACGGATAAGCCTTATGAATCCAAAGTA

TTTTTTGGACCAATTTATTACTTGCGTCTGAGACATTTAACTCAGGACAAGGCAACCGTTAGATGTAGAG

GTAAAAAGACGAAGCTCATTAGACAAGCGAATGAGGGACGAAAACGTGGAGGAGGTATCAAGTTTGGAGA

AATGGAGAGAGACTGTTTAATAGCACATGGTGCAGCCAATACTATTACAGAAGTTTTAAAAGACTCAGAA

GAGGATTATCAAGATGTGTATATTTGTGAAAATTGTGGAGACATAGCAGCACAAATCAAAAGTATTAATA

CATGTCTTAGATGTTCAAAACTTAATCTCTCTCCTCTCTTAACAAAAATTGATACCACGCACGTATCTAA

AGTATTTCTTACTCAAATGAACGCCAGAGGCGTAAAAGTTAAATTAGATTTCGAACGAAGGCCTCCTTCG

TTTTATAAACCATTAGATAAAGTTGATCTTAAACCGTCTTTTCTGGTATAATATTGTTTAGTAGATACTC

ATCAAGATAAGCTAATTCACTAAACATATTATCGGATTCGGTATTGTTACTCGAGAATAGAGTTCGTTAT

GCTCCTGATATTCGGAAATCTGTGGAGTTTCAGGTTTTGGTGGAAGTGTAACTGCTACTTGGTGGGATAC

TGAAGGATATTTCAGAGAGTTGTGGATGTTCGGGTTCGACATCCACCGATGGTGTCACGCCACTAATCGG

TTCGGTAACGTCTGTGGATGGAGGTGCTACTTCTACAGAACCTGTAGCCTCAGTTGTCAACGGAGATACA

TATTCAATGCGCGGAAATGTATAATTTGGTAATGGTTTCTCATGTGGATCTTAAGAAGAAGAGGTAAGAT

ATCTACGAAAGATACCGATCACGTTTCTAGTTCTCTTTTGTAGAACTTTAACTTTTTCTTTCTCAGCATC

TAGTTGATATTCCGACCTCTTCACGTTTCGCATGGGTTACCTCCGCAGTTTTTACAAGCGATTTCACGTT

CCAGATCACGTTCAGCCTTCATACGTCTCTCCCTCTCTCTATCGAGTTTATCAGAGCAGTCTTTCTGAAG

GCGATCGAACTCCATAAATTTCTCCAACGCTTTGATTGTTTCCATAGATTTCCGAAGTTTAGCTTCTAGG

ACGGCGATTCTTTTTTTTTTTTTTTTTTTTTTTTTTTCGAATTCACGGGGTACAACCGTTTCCATTACCACCATCTCT

ATGTTTCTTTTCTAGATCGGCAATCTTTCTCAACATTTCATCCCCATACCTTTTCATTCCTCGAGTCTAT

TGTCGTCGAAATATCGTTCCAGCTCCTTTTCGACCTCAATAACTTTAGCACGTTGTTTCATCAAGCTCTC

TCTTGTAGTACTATCATTTTTATCTGATTCCCTGACACGTTTAAGATCTTCATGTAATTGAGTCAGCTCT

TGACGCAATCTCTTAACTAACTTCCTCTCTTGCTTCTTCGTCATAGTACTTACAATCACTATGGGATCCA

TTGTTACCACGTCTGTACTCGACGAGCTCACGTTTAAGAGATTCAATTTCCAGTTTGTATCGGTCCATGT

CTCCATTGCTACACCACCATTAGATTTACAGGCTGCTAGTTGTCGTTCGAGATCAGAAATACGTGTTTTC

TTGGAATGGATTTCGTCGATGTACTTGTCATGATTGGCATCGAAACACTTATTAAGTTCTTTTTTTCAAT

TCTACGATTTTATTTCTTTCGCGAGTCAATTCCCTCCTGTAGTAACTATCAGTTTTGTCAGATTCACGCT

CTCTACGTAGACTTTCTTGTAAGTTACTAATTTGTTCCCTGGCATTACCGAGTTCAGTTTTATATGCCGA

ATAGAGTTCTGATTCATCCTTTGAGAAGATCTCTAGCGATCGTTCAAGATCCCTGATTCTAGTCTTTAGC

CTATTTACCTCCTCAGAAGATGCTCCGTTACCGTTTTTACAATCGTTAAGATGTCTATCAAGATCCATGA

TTCTATCTCTTTTCCATATCAGCATTGATTTCATTATTACGTTCGCAGTCGTTCAACTGTATTTCAAGAT

CTGAGATTCTAGATTGTAATCTCTGTAGCATTTCCACGGCATTCACTCAGTTGTCTTTCAAGATCTGAGA

TTCTAGATTGGAGTCTGCTAATCTCTGTAAGATTTCCTCCTCCGCTCTCGATGCAGTCGGTCAACTTATT

CTCTAGTTCTCTAATACGCGAACGCAGTGCATCAACTTCTTGTGTGTCTTCTTGATTGCGTGTGCATTCA

TCGAGTCTAGATTCGAGATCTCTAACGTGACGTCGTTCTTCCTCAAGTTCTCTGTGTACTACAGAAAGCG

TGTCCCTATCTTGTTGATATTTAGCAATTTCTGATTCTAGAGTACTGATTCTACTCACGTATGTACTAAT

AGTTGTCTTAGCCTTATCAAGATCCTCCTTGTATTTGTCACATTCCTTGATATCCATACGAAGTCTGGAC

AGTTCCCATTCGACATTACGACGTTTATCGATTTCAGCTCGGAGATCGTCGTCGCGTTGTTTTAGCCACA

TACGACTAAGTTCAAGTTCTCGTTGACAAGATCCATCTACTTTTCCATCCCTAATAGTATCCAGTTCCTT

TTCTAGTTCTGACCGCATTTCTCGTTCCATATCAAGAGATTCTCTCAATTCTCGTATAGTCTTCTTATCA

ATTTCTGATGAATCTGAACCATCATCTGTCCCATTTTGTTGCATATCCCTGAGTTCTTTGATCTCTGTTG

TAAGTCTGTCGATTCTTTCGGTTTTATAAACAGAATCCCTTTCCAAAGTCCTAATCTTACTGAGTTTATC

ATTAAGTTCTTCATTCAATTCAGTGAGTTTTCTCTTGGCTTCTTCCAAGTCTGTTTTAAACTCTCCATCA

TTTCCGCATTCTTCCTCGCATTTATCTAACCATTCAATTAGTTTATTAATAACTAGTTGGTAATCAGCGA

TTCCTATAGCCGTTCTTGTATTTGTGGGAACATAATTAGGATCTTCTAATGGATTGTATGGCTTGATAGC

ATCATCTTTATCATTATTAGGTGGGGGATGGACAACCTTAATTGGTTGGTCCTCCTTATCTCCTCCAGTA

GCATGTGGTTCTTCAATACCAGTATTAGTAATAGGCTTAGACAAATGCTTGTCGTACGCGGGCACTTCCT

CATCCATCAAGTATTTATAATCGGGTTCTGTTTCAGAATATTCTTTTCTAAGAGACGCGACTTCAGGAGT

TAGTAGAAGAACTCTGTTTCTGTATCTATCAACGCTGGAATCGATACTCAAGTTAAGGATAGCGAATACC

TCATCGTCATCATCCGTATCTTCTGAAACGCCATCATATGACATTTCATGAAGTCTAACGTATTGATAAA

CAGAATCAGATTTAGTATTAAACAGATCCTTGACCTTTTTAGTAAATGCATATGTATATTTTAGATCTCC

AGATTTCATAATATGATCGCATGCCTTAAATGTCAATGCTTCCATGATATAGTCTGGAACACTAATGGGT

GACGAAAAAGATACAGCACCATATGCTACGTTGATAAATAGATCTGAACCACTAAGTAGATAATGATTAA

TGTTAAGGAAGAGGAAATATTCAGTATATAGATATGCCTTAGCATCATATCTTGTACTAAACACGCTAAA

CAGTTTATTGATGTGATCAATTTCCAACAGAACAATTAGAGCGGCAGGAATACCAACAAACATATTACCA

CATCCGTATTTTCTATGAATATCACATATCATATTAAAAAATCTTGATAGAAGAGCGAATATCTCGTCTG

ACTTAATGAGATGTAGTTCAGCAGCATAAGTCATAACTGTAAATAGAACATACTTTCCTGTAGTGTTGAT

TCTAGACTCCACATCAACACCATTATTAAAAATAGTTTTATATACATCTTTAATCTGCTCTCCGTTAATC

GTCGAACGTTCTAGTATACGGAAACACTTTGATTTCTTATCTGTAGTTAATGACTTAGTGATATCACGAA

GAATATTACGAATTACATTTCTTGTTTTTCTTGAGAGACCTGATTCAGAACTCAACTCATCGTTCCATAG

TTTTTCTACCTCAGTGGCGAAATCTTTGGAGTGTTTGGTACATTTTTTAATAAGGTTCGTGACCTCCATT

TATTATAAAAAATTTTTATTCAAAACTTAACTACAATCGGGTAATTATAAGATCGTAGATCTCCCATGTG

GTGGAATACTACCATCTATCGCATGTTGATGGACAGTAGGTAATGGCCATGGGAACAGTAATGTTTGCAT

ATTTATCTTTCTTGCTAGTATTACTGTATATTGTCCCAATGTTTCAATGTGATGTTCTAACCTATCAACT

GCCACTGTATCACAACAATAATGTCCGATGGAATTAAGATTATGATCCAATGTGTTTAATATATGATTAT

CAAGTCTTATACGATCCGCGTCTTTTTTGACAGGATCAGGCTCTTCTACAGGAAGAAGTTTCGGCCTCTT

ATGATAGTCATGTCTGGGAAATGGTGGTCTAGGATGAGGATCAGGTATCGGAGTAGGTTTTGGATTATAATCA

TCATCATCATCATCATCATCATCATCATCATCATCATCTATGATATCATCATCTTCGATATTTATTTTGC

TATCTTGATAATGTCCTATATCAGTTGCATTTTCAGCACTCGACTGAATATTAGTACATTCATTGTCTAT

TATTAACGTATTTCTAAACCCAAAATGTATATGTTGAACATCACTACTATAGTTGATGAGTCTTATAGCA

TGAATTCGCTTATCGTTATCGGGTTTATCTTCTGTCACCTTAACAATTCCTTTTTTATTAAACTCTGCAT

AATCATAACCATTTCTATTGTTTGTTCTAATATAAACGAGTATAGCATCATTGCTAAATTTTTCAATAGT

ATCAAAAACAGAATATCCTAAACCATATAATATATATTCAGGAACACTCAAACTAAATGTCCAGGATTCT

CCTAAATACGTAAACTTTAATAGTGCTAAATCATTCAAAAATCTACCGCTTATAGATAGATAGTACATGA

ATGCGTATAGTAGTCTACCTATCTCTTTATTATGAAAACCGACATTACGATCATATATTTCGTGATATAC

ATGTGACCCGTTTACGTTAAACCATAAATACATGGGTGATCCTATAAACATGAATTTATTTCTAATTCTC

AGAGCCATAGTTAATTGACCGTGTAATATTTGTTTACATGCATACTTGATACGATCATTAATAAGATTTT

TATCATTGCTCGTTATTTCAGAATCGTATATATAAGGAGTACCATCATGATTCTTACCAGATATTATACA

AAATACTATATATAAAATATATTGACCCACGTTAGTAATCATGTAAATGTTTAATGTTTTAAATTTTGTA

TTTAATGATCCATCATCATATGCTAGCATGGTCTTGTGATATTCATTCTTTAAAATATAATATTGTGTTA

GCCATTGCATTGGAGCTCCTAATGGAGATTTTCTATTCTCGTCCATTTTAGGATATGCTTTCATAAAGTC

CCTAATAACTTCGTGAATAATGTTTCTATGTTTTCTACTGATGCATGTATTTGCTTCGATTTTTTTATCC

CATGTTTCATCTATCATAGATTTAAACGCAGTAATGCTCGCAACATTAACATCTTGAACCATTGGTACAA

TTCCGTTCCATAAATTTATAATGTTCGCCATTTATATAACTCATTTTTTGAATATACTTTTAATTGAACA

AAAGAGTTAAGTTACTCATATGGATGCCGTCCAGTCTGTACATCAATCTTTTTAGCCAGAGATATCATAG

CCGCTCTTAGAGTTTCAGCGTGATTTTCCAACCTAAATAGAACTTCATCGTTGCGTTTACAACACTTTTC

TATTTGTTCAAACTTTGTTGTTATATTAGTAATCTTTTTTTCCAAATTAGTTAGCCGTTGTTTGAGAGTT

TCCTCATTATCGTCTCCATAGGCTTTAACAATTGCTTCGCGTTTAGTCTCTGGATTTTTAGCAGCCTTTG

TAGAGAAAAATTCAGTTGCTGGAATTGCAAGATCGTCATCTCCGGGGAAAAGAGTTCCGTCCATTTAAAG

TACAGATTTTAGAAACTGACACTCTGTGTTATTTATATTTGGCGCAATACATGGATTATAAATATCGATG

TTAATAACATCAGAAAATGTAAAGTCTATACATTGTCGCATCGTGTTAAATTTTCTAATGGATCTAGTAT

TATTGGGTCCAACTTCTGCCTGAAATCCAAATATGGAAGCGGATACAAAACCGTTTCCTGGATAAACCAC

ACATCTCCACTTTTGCTTTACATCAGAAATTGTGTCATTGACATCTTGAACTCTCCTATCTAATGCCGGT

GTTCCACCTATAGATTTTGAATACTCGAATGCTGCATGAGTAGCATTGAATTCCTTAATATTGCCATAAT

TTTCATATATTGAGTAACTCTGGATAAAAAGTAAACACACCGCAGCCGTCGCTACTACAATAAAAAAAAT

TGATAGAGAGTTCATTTATAATCTATTAGAAGCTGATAAAATTTTTTTACACGCGTCAGACAATGCTTTA

ATAAATAGTTCAACATCTACTTTTGTCATATCGAACCGATGGTATGATTCTAACCTAGAATTACATCCGA

AAAAGTTGACTATGTTCATAGTCATTAAGTCATTAACGAACAACATTCCAGACTCTGGATTATAAGACGA

TACTGTTTCGTCACAATCACCCACCTTAATCATGTGATTATGAATATTGGCTATTAGAGTACCTTCTAAG

AAATCTATAATATCTTTGAAACACGATTTAAAATCAAACCACGAATATACTTCTACGAAGAAAGTTAGTT

TACCCATAGGAGAGATAACTATAAATGGAGATCTAGATACAAAATCCGGATCTATGATAGTTTTAACATT

ATTATATTCTCTATTAAATACCTCCACATCTAAAAATGTTAATTTTGAAACTATGTCTTCGTTTATTACC

GTACCTGAACTAAACGCTATAAGCTCTATTGTTTGAGAACTCTTTAAACGATATTCTTGAAATACATGTA

ACAAAGTTTCCTTTAACTCGGTCGGTTTATCTACCATAGTTACAGAATTTGTATCCTTATCTATAATATA

ATAATCAAAATCGTATAAAGTTATATAATTATCGTGTTCAGATTGTGATCTTTTCAAATAGACTAAAAAC

CCCATTTCTCTAGTAAGTATCTTATGTATATGTTTGTAAAATATCTTCATGGTGGGAATATGCTCTACAG

CAGTTAGCCATTCCTCATTGACAGCTGTAGATGTATTATACAAAACTACTCCAATGTTTAACAAGGGCCA

TTTTACGAGATTATTAAATCCTTGTTTGATAAATGTAGCCAATGCGGGTTCGAGTTCAACGACGATTGAA

TTCTCTTCCCGTGGATGCTGCATGATGAACGACGGGATGTTGTTGTTCTATTGATTTGGAATTCTTTTTC

GACTTTTTGTTTATATTAAATATTTTAAAATTTATGGCTGATAGTAATTCATGTACTACGGATAATGTAG

ACGTGTATTGCATATCGATATCTTTATTATTAGATAAATTTATCAATAAATGTGAGAAGTTTGCCTCGTT

AAGGTCTTCCATTTAAATATTATATAAATATTTGTGTTTGTATTTTATTCGTCTTTTATGGGATAGTTTT

TAACTAGTAAAGCTGTAATTACATACTTTGTCCGTAAAACATAAATATAAATACCCGCTTTTATCAAACG

TTCCAAAAAGTCGGCAGCTGACATTTTTAACATGACATCTATTTTAAATACACTTAGGTTTTTAGAAAAA

ACATCATTTTATAATTGTAACGATTCAATAACTAAAGAAAAGATTAAGATTAAACATAAGGGAATGTTAT

TTGTATTTTATAAGCCAAAGCATTCTACCGTTGTTAAATACTTGTCTGGAGGAGGTATATATCATGATGA

TTTGGTTGTATTGGGGAAGGTAACAATTAATGATCTAAAGATGATGCTATTTTACATGGATTTATCATAT

CATGGAGTGACAAGTAGTGGAGCAATTTACAAATTGGGATCGTCTATCGATAGACTTTCTCTAAATAGGA

CTATTGTTACAAAAGTTAATAACAATTATAACAATTATAACAATTATAACAATTATAATTGTTATAATAA

TTATAATTGTTATAATTATGATGATACATTTTTTGACGATGATGATTGATCACTATTACACAATTTTGTT

TTTGTACTTTCTAATATAGTGTTTAGGTTCTTTTTCATATGAGAATATTGACTTACTAAAATATCTATGT

TTAACTTTTGTTCTATAACGTCCTTATCGGCGGTATCGGTACATATACGTAATTCACCTTCACAAAATAC

GGAGTCTTCGATAATAATAGCCAATCGATTATTGGATCTAGCTGTCTGTATCATATTCAACATGTTTAAT

ATATCCTTTCGTTTCCCCTTTACAGGCATCGATCGTAGCATATTTTCCGCGTCTGAGATGGAAATGTTAA

AACTGCAAAAATGCGTAATGTTAGCCCGTCCTAATATTGGTACGTGTCTATAAGTTTGGCATAGTAGAAT

AATAGACGTGTTTAAATGCCTTCCAAAGTTTAAGAATTCTATTAGAGTATTACATTTTGATAGTTTATCA

CCTACATCATCAAAAATAAGTAAAAAGTGTGCTGATTTTTTATGATTTTGTGCGACAGCAATACATTTTT

CTATGTTACTTTTAGTTCGTATCAGATTATATTCTAGAGCTTCCTGACTACTAACGAAATTAATATGATT

TGGCCAAATGTATCCATCATAATCTGGGTTATAAACGGGTGTAAACAAGAATATATGTTTATATTTTTTA

ACTAGTGTAGAAAACAGAGATAGTAAATAGATAGTTTTTCCAGATCCAGATCCTCCTGTTAAAACCATTC

TAAACGGCATTTTTAATAAATTTTCTCTTGAAAATTGTTTTTCTTGAAAACAATTCATAATTATATTTAC

AGTTACTAAATTAATTTGATAATAAATCAAAATATGGAAAACTAAGGTCGTTAGTAGGGAGGAGAACAAC

GAAGGCATATCGTGATATAAATAACATTTATTATCATGATGACACCAGAAAACGACGAAGAGCAGACATC

TGTGTTCTCCGCTACTGTTTACGGAGACAAAATTCAGGGAAAGAATAAACGCAAACGCGTGATTGGTCTA

TGTATTAGAATATCTATGGTTATTTCACTACTATCTATGATTACCATGTCCGCGTTTCTCATAGTGCGCC

TAAATCAATGCATGTCTGCTAACAAGGCTGCTATTACTGACTCCGCTGTTGCCGTTGCTGCGGCATCATC

TACTCATAGAAAGGTTGTGTCTAGCACTACACAATATGATCACAAAGAAAGCTGTAATGGTTTATATTAC

CAGGGTTCTTGTTATATATTACATTCAGACTATAAGTCATTCGAGGATGCTAAAGCAAACTGCGCTGCGG

AATCATCAACACTACCCAATAAATCCGATGTCTTGACTACCTGGCTCATTGATTATGTTGAGGATACATG

GGGATCTGATGGTAATCCAATTACAAAAACTACATCCGATTATCAAGATTCTGATGTATCACAAGAAGTT

AGAAAGTATTTTTGTACATAAATAAATGAAATCGCTTAATAGACAAACTGTAAGTAGGTTTAGGAAGTTG

TCGGTGCCGGCCGCTATAATGATGTTACTCTCAACCATTATTAGCGGCATAGGAACATTTCTGCATTACA

GAGAAGAACTGATGCCTAGTGCTTGCGCCAATGGATGGATACAATACGATAAACATTGTTATCTGGATAC

CAACATTAAAATGTCTACGGATAATGCAGTTTATCAGTGTCGCAAATTACGAGCTAGATTGCCTAGACCT

GATACTAGACATCTGAGAGTATTGTTTAGTATTTTTTATAAAGATTATTGGGTAAGTTTAAAAAAGACCA

ATGATAAATGGTTAGATATTAATAATGATAAAGATATAGATATTAGTAAATTAACAAATTTTAAGCAACT

AAACAGCACGACGGATTCTGAGGCGTGTTATATATACAAGTCTGGAAAACTGGTTAAAACAGTATGTAAA

AGTACTCAATCTGTACTATGCGTTAAAAGATTCTACAAGTGACAACAAAAAATGAATTAATAGTAAGTCG

TTAACGTACGCCGCCATGGACGCCGCGTTTGTTATTACTCCAATGGGTGTGTTGACTATAACAGATACAT

TGTATGATGATCTCGATATCTCAATCATGGACTTTATAGGACCATACATTATAGGTAACATAAAAATTGT

CCAAATAGATGTACGGGATATAAAATATTCCGACATGCAAAAATGCTACTTTAGCTATAAGGGTAAAATA

GTTCCTCAGGATTCTAATGATTTGGCTAGATTCAACATTTATAGTATTTGTACAGCATACAGATCAAAAA

ATACCATCATCATAGCATGCGACTATGATATCATGTTAGATATAGAAGGTAAACATCAACCATTTTATCT

ATTCCCATCTATTGATGTTTTTAACGCTACAATCATAGAAGCGTATAATCTGTATACAGCTGGAGATTAT

CATCTGATCATCAATCCTTCAGATAATCTGAAAATGAAATTGTCGTTTAATTCTTCATTTTGTATATCAG

ACGGCAATGGATGGATTATAATTGATGGGAAATGTAATAGTAATTTTTTATCATAAAAGTTGTAAAGTAA

ATAATAAAACAATAAATATTGAACTAGTAGTATGTTGTATATTGAGCAATCAGAGATGATGCTGGTACCT

CTTATCACGGTGACCGTAGTTGCGGGAACAATATTAGTATGTTATATATTATATATTTGTAGGAAAAAGA

TACGTACTGTCTATAATGACAATAAAATTATCATGACAAAATTAAAAAAGATAAAGAGTCCTAATTCCAG

CAAATCTAGTAAATCAACTGATAGCGAATCAGACTGGGAGGATCACTGTAGTGCTATGGAACAAAACAAT

GACGTAGATAATATTTCTAGAAATGAGATATTGAACGATGATAGCTTCGCTGGTAGTTTAATATGGGATA

ACGAATCCAATATCATGGCGCCTAGCACAGAACACATTTACGATAGTGTTGCTGGAAGCACGCTGCTAAT

AAATAATGATCGTAATGAACAGACTATTTATCAGAATACTACAGTAGTAATTAATGATACAGAGACTGTT

GAAATACTTAATGAAGATACCAAACAGATTCCTAGCTATTCTTCCAATCCTTTCGTAAATTATAATAAAA

CCAGTATTTGTAGCAAGTCAAATCCGTTCATTGCAGAACTCAACAATAAATTTAGTGATAATAATCCGTT

TAGGAGAGCACATAGTGACGATTATCTTAATAAGCAACAAGATCATGAATACGATGATATAGAATCATCG

GTTGTATCATTGGTCTGATTAGTTTCCTTTTTATAAAATTGAAGTAATATTTAGTATTAATTACCGCCGA

TGCATTATACAAATATGGAGATATTCCCTGTATTCGGCATTTCTAAAATTAGCAATTTTATTGCTAATAA

TGACTGTAGATATTATATAGATGTAGAGCATCAAAAAATTATATCTGATGAGATCAATAGACAGATGGAT

GAAACGGTACTTCTTACCAACATCTTAAGCGTAGAAGTTGTAAATGACAATGAGATGTACCATCTTATTC

CCCATAGACTATCGACTATTATACTCTGTATTAGTTCTGTTGGAGGATGTGTTATCTCTATAGATAATGA

CGTCAATGACAAAAATATTCTAACATTTCCCATTGATCATGCTGTAATCATATCCCCACTGAGTAAATGT

GTCGTAGTTAGCAAGGGCCCTACAACCATACTGGTTGTTAAAGCGGATATACCCAGCAAACGATTGGTAA

CATCGTTTACAAACGACATACTGTATGTAAACAATCTATCACTGATTAATTATTTACCGTCGTCTGTATT

CATTATTAGACGAGTCACCGACTATTTGGATAGACACATATGTGATCAGATATTTGCTAATAATAAGTGG

TATTCCATTATAACTATCGACGATAAGCAATATCCTATTCCATCAAATTGTATAGGTATGTCTTCTGCCA

AGTACATAAATTCGAGCATCGAGCAAGATATTTTGATCCATGTTTGTAACCTCGAGCATCCATTCGACTC

AGTCTACAAAAAAATGCAGTCGTACAATTCTCTACCTATCAAGGAACAAATATTGTATGGTAGAATTGAT

AATATAAATATGAGCATTAGTATTTCGGTGGATTAATAGATTTCTCTAGTATGGGATCATTAATCATCTC

TAAATACATCATAAAAAAGCTATTATCAAATACTGTACTGAATGGATTCATTCTTTTCTCTTTTTATGAA

ACTCTGTTGTATATCTACGGATAAAACTAGAAGCAAAAAATCTGATAGGAAGAATAATGATTATATGGAG

GAACACGATTATTATAAAATAACAATAGTTCCTGGTTCCTCTTCCACGTCTACTAGCTCGTGGTATTATA

CACATGCCTAGTAATAGTCTCTTTGCGTTGACGGAAAGCAGACTAGAAATAACAGGCCAAAATGTTCAGA

CACCATAATAGTTCCCAACCCAGATAATAACAGAGTTCCATCAACACATTCCTTTAAACTCAATCCCAAA

CCCAAAACCGTTAAAATGTATCCAGCCAATTGATAGTAGATAATGAGGTGTACAGCACATGATAATTTAC

ACAGTAACCAAAATGAAAACACTTTAGTAATTATAAGAAATATAGACGGTAATGTCATCATCAACAATCC

AATAATATGCCTGAGAGTAAACATTGACGGATAAAACAAAAATGCCCCGCATAACTCTATCATGGCAATA

ACGCAACCAAACACTTGTAAAATTCCTAAATTAGTAGAAAATACAACTGATATCGATGTATAAGCGATTT

CGAGGAATAATAAGAACAAAGTAATTCCCGTAAAGATAAACATCAACATTGTTTGGTAATCATTAAACCA

ATTAGTATGACGTTGAATTAATTTCACAGTATATTTTATTCCAGTATTATCCCCGCATGTATACGTACCT

GGTAAGATATCTTTATATTCCATAATCAATGAGACATCACTATCCGATAACGAATGAAGTCTAGCACTAG

TATGCCATTTACTTAATATGGTCGTCTTGGAAGTTTTATTATAAGTTAAAATATCATGATTGTCCAATTT

CCATCTAATATACTTTGTCGGATTATCTATAATACACGGAATAATGATGGTATCATTACATGCTGTATAT

TCTATAGTCTTTGTAGATGTTATAACCACAAAAGTACAGAGGTATATCAACAATATTCTAACTCTTAACA

TTTTTATTTATTTAAAATGATACCTTTGTTATTTATTTTATTCTTATTTTGCTAACGGTATCGAATGGCA

TAAGTTTGAAACGAGTGAAGAAATAATTTCTACTTACTTAATAGATTATGTGGTAACGGGTGTTATTAAT

GGGGATGTATATACATTTTCAAATAATGAACTAAACAAAACTGGGTTAACTAATAACAATAATTATATCA

CAACATCTATAAAAGTAGAGGATAAGGATACATTAGTAGTATGCGGAACCAATAACGGAAATCCCAAATG

TTGGAAAATAGACGGTTCATACTACCCAAAACATATAGGTAGAGGATACGATCATCAAAATAGCAAAGTA

ACGATAATCAGTCACAATGAATGTGTACTATCCGACATAAACATATAAAAAGAAGGAATTAAACGATGGA

GAAGATTTGACGGACCATGTGGTTATGATTAAACGAGTTAAGTTTTTTAAGAAGCCTTAGAAGAGAGGCT

ATTGGGTATGAGAATCCGAAATATTAAACCAGACAACCCCATATAATTTTATAGCTAAGAATGCCGCGAA

GAATGGAACTAATAAAAACGGAAATATTTGTAGCACAACGAATAACTCCCAAACTGCATTCATGTTACAC

TATATAACACTACTTCGGTTAGATGTTTTAGAAAAAATAAATATCACCGTACCGTTTTGTTGTATAAAAA

TAACAATTAACAATTATCAATTTTTTTCTTTAATATTTTACGTGGTTGACCATTCTTGGTGGTAAAATAA

TCTCTTAGTGTTGGAATGGAATGCTGTTTAATGTTTCCACACTCATCGTATATTTTGACGTATGCAGTCA

CATCGTTTACGCAATAGTCAGACTGTAGTTCTATCATGCTTCCTACGTTAGAAGGAGGAACAGTTTTAAA

GTCTCTTGGTTTTAATCTATTGTCATTAGTTTTCATGAAATCCTTTGTTTTATCCACTTCACATTTTAAA

TAAATGTCCACTATACATTCTTCTGTTAATTTTACTAGATCATCATGAGTCATAGAATTCATAGGTTCCG

TAGTCCATGGATCCAAACTAGCAAACTTCGCGTATACGGTATCGCGATTAGTGTATACACCAACTGTATG

AAAATTAAGAAAACAGTTTAATAAATCTACAGAAATATTTAATCCTCCGTTTGATACAGATGCGCCATAT

TTATGGATTTCGGATTCACACGTTGTTTGTCTAAGGGGTTCGTCTAGTGTTGCTTCTACATAGACTTCGA

TTCCCATATATTCTTTATTGCCAGAATCACATACCGATTTATCATACGCTGGTTCACTTGTTTGAAAACT

AAATGGTAGTAGATACATCAAAATAATAAATAATAAGTACATTCTGCAATATTGTTATCGTAATTGGAAA

ATTGGTATTCAAGTGAGCTGGATTATGTGAGTATTGGATTGTATATTTTATTTTATATTTTATATTTTAT

ATTTTATTTTATATTTTATATTTTATTTTATATTTTGTAGTAAGAATAGAATGCTAATGTCAAGTTTATT

CGAATAGATGTCTTATTAAAAAACATATATAATAAATAACAATGGCTGAATGGCATAAAATTATCGAGGA

TATCTCAAAAAATAATAAGTTCGAGGATGCCGCCATCGTTGATTACAAGACTACAAAGAATGTTCTAGCG

GCTATTCCTAACAGAACATTTGCAAAGATTAATCCGGGTGAAGTTATTCCCCTCATCACTAATCATAATA

TTCTAAAACCTCTTATTGGTCAGAAATTTTGTATTGTATATACTAACTCTCTAATGGATGAGAACACGTA

TGCTATGGAGTTGCTTACTGGGTACGCCCCTGTATCTCCGATCGTTATAGCGAGAACTCATACCGCACTT

ATATTTTTGATGGGTAAGCCAACAACATCCAGACGTGATGTGTATAGAACATGTAGAGATCACGCTACCC

GTGTACGTGCAACTGGTAATTAAAATAAAAAGTAATATTCATATGTAGTGTCAATTTTAAATGATGATGA

AATGGATAATATCCATATTGACGATGTCAATAATGCCGGTATTGACATACAGCTCATCGATTTTTAGATT

TCATTCAGAGGATATTGAATTATGTTATGGGAATTTGTATTTTGATAGGATCTATAATAATGTAGTAAAT

ATAAAATATATTCCTGAGCATATTCCATATAGATATAATTTTATTAATCGTACGTTCTCCGTAGATGAAC

TAGATGATAATGTCTTTTTTACACATGGTTATTTTTTAAAACACAAATATGGTTGTTCACTTAATCCTAG

TTTGATTGTCTCATTATCAGGAAACTTAAAATATAATGATATACAATGCTCAGTAAATGTATCGTGTCTC

ATTAAAAATTTGGCAACGAGTACATCTACTATATTAACATCTAAACATAAGACTTATTCTCTATATCGGT

CCATGTGTATTGCTATAATAGGATACGATTCTATTATATGGTATAAATATATAAATGACAGGTATAATGA

CATCTATGATTTTACTGCAATATGTATGCTAATAGCGTCTACATTGATAGTGATCATATACGTGTTTAAA

AAAATAAAAATGAACTCTTAATTATGTTATACTATTAGAAATGGATAAAATCAAAATTACGATTGATTCA

AAAATTGGTAATGTTGTTACCATATCGTATAACTTGGAAAAGATAACTATTGATGTCACGCCAAAAAAGA

AAAAAGAAAAGGATGTATTATTAGCGCAATCAGTTGCTGTCGAAGAGGCAAAAGATGTCAAGGTGGAAGA

AAAAAATATTATCGATATTGAAGATGACGATGATATGGATATAGAAAACACGTAATACGATCTATAAAAA

TAAGTATTAAATACTTTTTATTTACGGTACTCTTGTAGTGGTGATACCACTAATCGATTATTTTTTTTAA

AAAAATACTTATTCTGATTCTTCTAGCCATTTCCGTGTTCGTTCGAATGCCACATCGACGTCAAAAATAG

GGGAGTAGTTGAAATCTAGTTCTGCATTGTTGGTACGCACCTCAAATGTAGTGTTGGATATCTTCAACGT

ATAGTTGTTGAGTATTGATGGTTTTCTAAATAGAATTCTCTTCATATCATTCTTGCACGCGTACATTTTT

AGCATCCATCTTGGAATCCTAGATCCTTGTTCTATTCCCAATGGTTTCATCAATAGAAGATTAAACATAT

CGTAAGAACACGATGGAGAGTAATCGTAGCAAAAGTAAGCATTTCCTTTAATCGCAGATCCCGGATACTG

GATATATTTTGCAGCCAACACGTGCATCCATGCAACATTTCCTACATATACCCGGCTATGCACAGCGTCA

TCATCGACTGTACGATACATAATGTTACCGTGTTGCTTACATTGCTCGTAAAAGACTTTCGTCAATTTGT

CTCCTTCTCCGTAAATTCCAGTGGGTCTTAGGCAACAAGTATACAATTTTGCGCCATTCATGATTACGGA

ATTATTGGCTTTCATAACCAGTTGCTCGGCCATACGTTTACTTTTTGCGTATACATGTCCTGGTGATATA

TCATAAAGGGTATGCTCATGACCGATGAATGGATTACCGTGTTTATTTGGTCCTATTGCTTCCATGCTAC

TAGTATAGATCAAATACTTGATTCCTAGGTCCACACAAGCTGCCAATATAGTCTGTGTTCCATAATAGTT

TACTTTCATGATTTCATTATCAGTGTATTTTCCAAATACATCCACTAGAGCAGCCGTATGAATAATCAGA

TTTACCCCATCTAGCGCTTCTCTCACCTTATCAAAGTCGTTTATATCACATTGTATATAGTTTATAACCT

TAACTTTCGAGGTTATTGGTTGTGGATCTTCTACAATATCTATGACTCTTATTTCTTGAACATCATCTGC

GCTAATTAAAAGTTTTACTATATACCTGCCTAGAAATCCGGCACCGCCAGTAACCGCGTACACGGCCATT

GCTGCCACTCATAATATCAGACTACTTATTCTATTTTACTAAATAATGGCTGTTTGTATAATAGACCACG

ATAATATCAGAGGAGTTATTTACGTTGAACAAGTCCATGGAAAAGATAAAGTTTTAGGATCAGTTATTGG

ATTAAAATCCGGAACGTATAGTTTGATAATTCATCGTTACGGAGATATTAGTCGAGGATGTGATTCCATA

GGCAGTCCAGAAATATTTATCGGTAACATCTTTGTAAACAGATATGGTGTAGCATATGTTTATTTAGATA

CAGATGTAAATATATCTACAATTATTGGAAAGGCGTTATCTATTTCAAAAAATGATCAGAGATTAGCGTG

TGGAGTTATTGGTATTTCGTACATAAATGAAAAGATAATACATTTTCTTACAATTAACGAGAATGGCGTT

TGATATATCAGTTAATGCGTCTAAAACAATAAATGCATTAGTTTACTTTTCTACTCAGCAAGATAAATTA

GTCATACGTAATGAAGTTAATGATATACACTACACTGTCGAATTTGATAGGGACAAAGTAGTTGATACGT

TTATTTCATATAATAGACATAATGACTCCATAGAGATAAGAGGGGTGCTTCCAGAGGAAACTAATATTGG

TCGCGTGGTTAATACGCCGGTTAGTATGACTTACTTGTATAATAAGTATAGTTTTAAACCGATTTTAGCA

GAATATATAAGACACAGAAATACTATATCCGGCAACATTTATTCGGCATTGATGACGCTAGATGATTTGG

TTATTAAACAGTATGGAGACATTGATCTATTATTTAATGAGAAACTTAAAGTAGACTCCGATTCGGGACT

ATTTGACTTTGTCAACTTTGTAAAGGATATGATATGTTGTGATTCTAGAATAGTAGTAGCTCTATCTAGT

CTAGTATCTAAACATTGGGAATTGACAAATAAAAAGTATAGGTGTATGGCATTAGCCGAACATATAGCTG

ATAGTATTCCAATATCTGAGCTATCTAGACTACGATACAATCTATGTAAGTATCTACGCGGACACACTGA

TAGCATAGAGGATGAATTTGATTATTTTGAAGACGATGATTTGTCTACATGTTCTGCCGTAACCGATAGG

GAAACGGATGTATAATTTTTTTTATAGTATGAAGGATATGATGGATATGATGATATGATGGATATGATGG

ATATGATGGATATGATGGATATGATAAAAAAATATAATTGTTGTATCCATTCCCATTCAAATCACCTTAT

ATGATTCTGTAACACAATGAAGGAGTCTCATAGATATATAGAGGTCAGATACTGGTTTGATAAACTTTTT

ATTCCACATGAGCATGTTTGACTTATGGTTAGACACACATACTTTAACAAATCACTGAAAATTGGAGTTA

GGTATTCCTCTCAGAATCAGTTGCCGTTCTGGAACATTAAATGTATTTTTTATGATATACTCCAACGCAT

TTATGTGGGTATACAACAAGTCATTAATAATGAGTATTTCCAAGAGTTTTAGTTGTCTAGTATTTAACAA

GAGAAGAGATTTCAACAGACTGTTTATGAACTCGAATACCGCCTCATTGTCGCTTATATTGATGACATGA

CATCGAATTCCCAATATCAATCTCATCAGTGATGAGTAGCTCAATCTTGTTATCGGGATCCAATTTCTAA

AGATGTCATTAAACCCTCGATCGTGAATGGATTTATCATCATCGTTTTTATGTTGGACATGAGCTTAGTC

CGTTTGTCCACATCTATATACGATGATTTCTGAATTATTTCATATATCTCTCGTTAACTCCAGGAACTTG

TCAGGGATCTAACTTTAATATGTTCTCGTCTAAGAGATGAAAATCTTTGGATGGTTGCATGTGACTTTTC

TCTAAAGGATGATGTTACCCGATCCTCTCTTAAATGACTCCATCTTATCCTTGGACAAGATGGACAGTCT

ATTTTCCTTAGATGGTTTAATATTTTTTACCCATGATCTATAAAGGTAGACAGACCTAATCGTCTCGGAT

GACCATATATTATTTTCCGTTTTATTATACGCATAAATTGTAAAAAATATGTTAGGTTTACGAAAATGTC

TCGTGGGGCATTAATCGTTTTTGAAGGATTGGACAAATCTGGAAAAACAACACAATGTATGAACATCATG

GAATCTATACCGGCAAACACGATAAAATATCTTAACTTTCCTCAGCGATCCACAGTCACTGGAAAGATGA

TAGATGACTATCTAACTCGTAAAAAAACCTATAATGATCATATAGTTAATCTATTATTTTGTGCAAATAG

ATGGGAGTTTGCATCTTTTATACAAGAACAACTAGAACAGGGAATTACTTTAATAGTTGACAGATACGCG

TTCTCTGGAGTAGCGTATGCCACCGCTAAAGGCGCGTCAATGACTCTCAGTAAGAGTTATGAATCTGGAT

TGCCTAAACCCGACTTAGTTATATTCTTGGAATCTGGTAGCAAAGAAATTAATAGAAACATCGGCGAGGA

AATTTATGAAGATGTTGAATTCCAACAAAAGGTATTACAAGAATATAAAAAAATGATTGAAGAAGGAGAT

ATTCATTGGCAAATTATTTCTTCTGAATTCGAGGAAGATGTAAAGAAGGAGTTGATTAAGAATATAGTTA

TAGAGGCTATACACACGGTTACTGGACCAGTGGGGCAACTGTGGATGTAATAAAATGAAATTACATTTTT

ATAAATAGATGTTAGTACAGTGTTATAAATGGATGAAGCATATTACTCTGGCAACTTGGAATCAGTACTC

GGGGATACGTGTCCGATATGCATACCGAACTCGCATCAATATCTCAATTAGTTATTGCCAAGATAGAAAC

TATAGATAATGATTATTAAACAAGGACATTGTAAATTTTATCATGTGTAGATCAAACTTGGATAATCCAT

TTATCTCTTTCCTAGATACTGCATATACTATCATAGATCAAGAGATCTATCAGAACGAGTTGATTAATTC

ATTAGACGATAATGAAATTATCGATTGTATAGTTAACAAGTTTATGAGCTTTTATAAGGATAACCTAGAA

AATATGGTAGATGCTATCATTACTCTAAAATATTATAATTAATAATCCAGATTTTAAAACTACGTATGTG

GAAGTACTCGGTTCCAGAATAGCTGATATAGATATTAAACAAGTGATACGTAAGAATATAATACAATTGT

CTAATGATCCGCGAACGATATTTGTGAAAATATTAAAAAAAAATACTTTTTTTATTAAATGACGTCTCTT

CGCGAATTTAGAAAATTATGCTGTGATATATATCACGCATCAGGATATAAAGAAAAATCTAAATTAATTA

GAGACTTTATAACAGATAGAGATGATACCGATACATATTTGATCATTAAGCTATTGCTTCCCGGATTAGA

CGATAGAATGTATAACATGAACGATAAACAAATTATAAAATTATATAGTATAATATTTAAACAATCTCAG

GAAGATATGCTACAAGATTTAGGATACGGATATATAGGAGACACTATTAGGACTTTCTTCAAAGAGAACA

CGGAAATCCGTCCACGAGATAAAAGCATTTTAACTTTAGAAGAAGTGGATAGTTTTTTAACTACGTTATC

ATCAGTAACTAAAGAATCACATCAAATAAAATTATTGACTGATATAGCATCTGTTTGTACATGTAATGAT

TTAAAATGTGTAGTCATGCTTATTGATAAAGATCTAAAAATTAAAGCGGGTCCTCGGTACGTGCTTAACG

CTATTAGTCCTCATGCCTATGATGTTTTTAGAAAATCTAATAACTTGAAAGAGATAATAGAAAATGCAGC

TAAACAAAATCTAGACTCTATATCTATTTCTGTTATGACTCCAATTAATCCCATGTTAGCGGAATCATGT

GATTCTGTCAATAAGGCGTTTAAAAAATTTCCATCAGGAATGTTTGCGGAAGTCAAATACGATGGTGAAA

GAGTACAAGTTCATAAAAAAAATAACGAGTTTGCATTCTTTAGTAGAAACATGAAACCAGTACTCTCTCA

TAAAGTGGATTATCTCAAAGAATACATACCGAAAGCATTTAAAAAAGCTACGTCTATCGTATTGGATTCT

GAAATTGTTCTTGTAGACGAACATAATGTACCGCTACCGTTTGGAAGTTTAGGTATACACAAAAAGAAAG

AATATAAAAACTCTAACATGTGTTTGTTCGTATTTGACTGTTTATACTTTGATGGATTCGATATGACAGA

CATTCCATTGTATGAACGAAGATCTTTTCTCAAAGATGTTATGGTCGAAATACCCAATAGAATAGTATTC

TCAGAGTTGACGAATATTAGTAACGAGTCTCAGTTAACTGATGTATTAGATGATGCACTAACGAGAAAAT

TAGAAGGATTGGTCTTAAAAGATATTAATGGCGTATACGAACCGGGAAAGAGAAGATGGTTAAAAATAAA

GCGAGACTATTTGAACGAGGGTTCCATGGCAGATTCTGCCGATTTAGTAGTACTAGGTGCCTACTATGGT

AAAGGAGGAAAGGGTGGTATCATGGCAGTCTTTCTAATGGGTTGTTACGACGATGAATCCGGTAAATGGA

AGACGGTAACTAAATGTTCCGGTCACGATGATAATACGTTAAGGGTTTTGCAAGACCAATTAACGATGGT

TAAAATTAACAAGGATCCCAAAAAAATTCCAGAGTGGTTGGTAGTTAATAAAATCTATATTCCCGATTTT

GTAGTAGATGATCCGAAACAATCTCAGATATGGGAAATTTCAGGAGCAGAGTTTACATCTTCCAAGTCAC

ATACAGCGAATGGAATATCGATTAGATTTCCTAGATTTACTAGGATTAGAGAAGATAAAACGTGGAAAGA

ATCTACTCATCTAAACGATTTAGTAAACTTGACTAAATCTCTTAATAGTTACATATAAACTGAAAAATAA

AATAACACTATTTTAGTTGGTAGTCGCCATGGATGGTGTTATCGTATACTGTCTAAATGCGTTAGTAAAA

CATGGCGAGGAAATAAATCATATAAAAAATGATTTCATGATTAAACCATGTTGTGAAAGAGTTTGTGAAA

AAGTCAAGAACGTTCACATCGGCGGACAATCTAAAAACAATACAGTGATTGCAGATTTGCCATATCTGGA

TAATGCTGTATCAGATGTATGCAAATCAATATATAAAAAGAATGTATCAAGAATATCCAGATTTGCTAAT

TTGATAAAAATAGATGACGATGACAAGACTCCTACCGGCGTATATAATTATTTTAAACCTAAAGATGCTA

TTCCTGTTATTATATCCATAGGAAAGGATAAAGATGTCTGTGAACTATTAATCTCATCTGATAAAGCGTG

TGCGTGTATAAAGTTAAATTTATATAAAGTAGCCATTCTTCCCATGGATGTTTCCTTTTTTACCAAAGGA

AATGCATCATTGATTATTCTCCTGTTTGATTTCTCTATCGATGCGGCACCTCTCTTAAGAAGTGTAACCG

ATAATAATGTTATTATATCTAGACACCAGCGCCTACATGACGAGCTTCCGAGTTCCAATTGGTTCAAGTT

TTACATAAGTATAAAGTCCGACTATTGTTCTATATTATATATGGTTGTTGATGGATCTATGATGTATGCG

ATAGCTGATAATAGAACTCACGCAATTATTAGCAAAAATATATTAGACAATACTACGATTAACGATGAGT

GTAGATGCTGTTATTCTGAACCACAGATTAGGATTCTTGATAGAGATGAGATGCTCAATGGATCATCGTG

TTATATGAACAGACATTGTATTATGATGAATTTACCTGATGTAGGCGAATTTGGATCTAGTATGTTGGGG

AAATATGAACCTGACATGATTAAGATTGCTCTTTCGGTGGCTGGTAATTTAATAAGAAATCGAGACTACA

TTCCCGGGAGACGAGGCTATAGCTACTACGTTTACGGTATAGCCTCTAGATAATTTTTTTTAAGCACGAA

ATAAAAACATAATTTTAAACAATCTATTTCATACTATTTTGTGTGCTCACCATGAACATAAAGATAGATA

TATTAGTATTTCTGGTGATAAATTTACGGCGACTGCTAGGAGGGAAAATGAAGAAAGAAAAAATATCTAC

CTCTCCAAAAAGAAAAACTACTGATGTTATCAAACCTGATTATCTTGAGTACAATGACTTGTTAGATAGA

GATGAGATGTCTACTATTCTAGAGGAATATTAGGCCTTAGAATAAAATATGGACGACTCTTAACGAAATT

AGAAAATTCGATAATGATGTTGAAGAACAATTCGGTACTATAGAAGAACTCAAGCAGAAGCTTAGATTAA

ATTCTGAAGAGAGAGCAGATAATTTTATAGATTATATAAAGGTACAAAAACAGGATATCATCAAACTTAC

TGTATACGATTGCATATATCTATGATAGGATTGTGTGCGTGCGTGGTAGATGTTTGGAGAAATGAGAAAC

TGTTTTCTAGATGGAAATATTGGTTACAAGCGATTAAACTGTTTATTGATGATCACATGCTTGATAAGAT

AAAATCTATTGTAGAATAGACTAGTGTATGTGGAAATGTCATAGAAAGTTAAAAGTTAATGAGAGCAAAA

ATATATAAGGTTGTATTCCATATTTGTTATTTTTTTCTGTAATAGTTAGAAAATACATTCGATGGTCTAT

CTACCAGATTATTATGTGTTATAAGGTACTTTTCTCATAATAAACTAGAGTATGAGTAAGATAGTGTTTT

TCAAAAACATATAAATCTAAAATTGATGGATGAGATATACAGCTATTAATTTCGAAAATATATTTTAATC

TGATAACTTTAAACATGGATTTTTGATGGTGGTTTAAGTTTAAAAAAGATTTTGTTATTGTAGTATGATA

ATATCAAAAAGATGGATATAAAGAATTTACTGACTACATGTACTATTTTACATTACTACATTGGCTACGG

CATATATACCTATTTCGTCACTTCCACACGCTCCGGTAAACGGGTGTCATGTGACGAGGGAGAATCTTGA

TAAGAGGCATAATCAATGTTGTAATCCGATGTCCACCTGGAGAATTTGCCAAGGTCAGATGTAGAGTTGG

TAGTGATAACACAAAATGTGAACACTGCCCACCTCATACATATACCGCAATCCCCAATTATTCTAATAGA

TGTCATCAATGTAGAAAATGCCCAACAGGATCATTTGATAAGGTAAAGTGTACCGGAACACAGAACAAAT

GTTCGTGTCATCCTGGTTGGTATACGCTACTGATTCTTCACAGACTGAAGATTGTCGAGATTTGTGTACC

AAAAAAGGAGATGTCCATGCGGATACTTTGGTGGAATAGATGAAGGAAATCCTATTTGTAAATCGTGTTG

TGTTGGTGAATATTGCGACTACCTACGTAATTATAGACTTGATCCATTTCCTCCATGCAAACTATCTATC

TAAATGTAATTAATTATGATTTTGATGATAATGTTACCATACATTATATCACTACTTGGTTAGTGTGTAT

TATTTAGTATGGAAGACCTATTAATAATTACTTATCTTTTGACGATCTTGTTATAATTATAATATAAAAA

TACTTATGACATAGTAACTCATAATTGCTGACGCGATAAATTCGTAATAATCTGTTTTGTTCAAATTTTT

ATAAGGAATCTACAGGCATAAAAATAAAAATATAATCTATAATATACTCTTACAACGCCATCATGAATAG

CAGTGAATTAATTGCTGTTATTATGGATTTAGAAATAGTGGACGATTTTGTGATATTAATATAGTTATTA

ATGATGAAAGGATAAACGCGCATAGACTCATCCTATCTGGAGCCTCCGAATATTTTTTCCATTCTGTTTT

CCAATAATTTTATCGATTCTAATGAATACGAAGTTAATCTAAGTCATTTAGATTATCAAAGTGTTAACTA

TTTGATCGATTACATTTATGGGATACCTTTGAGCCTAACTAACAATAACGTGAAATATATTCTTTCAACC

GCTGATTTTTTTACAAATTGGATCTGTCATTACTGAGTGCGAAAAATACATACTTAAAAATCTTTGTTTT

AGAAACTGTATCGATTTCTACATATACGCTGATAAATATAATAACAAGAAAATAGAATTAGCATCGTTTA

ACACAATATTACGAAATATTTTGAGACTCATCAACAATGAAAACTTTAAATACTTAACAGAGGAATCAAT

GATAAAAATTTTAAGCGATGATATGTTATATATAAAAAATGAGGATTTCACCCCACTGATTCTCATTAAA

TGGTTAGAGAGTACACCAACCATGTACCGTCGAGTTACTTAGATGCCTCAGAATATCATTTCTTTCCCCA

CAAGTTATAAAATCACTTTATAGTCATCGACTGGTTAGTTCAATCTACGAATGTATAACATTCTTAAACA

ATATAGCATTCTTGGATAAATCATTTCCTAGATACCATATCATCGAGTTGATATCTATCGGTATAAGTAA

TTCACATGATAAGATTTCCATAAACTGCTACAATCATAAAAAAATTCATGGGAAATGATATCTTCACGTA

GATATAGGTGTAGTTTCGCAGTGACCGTCCTGGATAATATTATCTATATGATGGGTGGATATGATCAGTC

CCTGTATAGAAGTTCAAAGGTTATAGCGTACAATACATGTACTAATTCTTGGATATATGATATACCAGAG

CTAAAAATATCATCGTTCTAATTGCGGAGGAGTTGCCAATGACGAATACATTTATTGTATAGGCGGTATA

CGCGATCAGGAGTCATCGTTGATATCTAGTATCGATAGATGGAAGCCATCAAAACCATATTGGCAGAAGT

ATGCTAAAATGTGCGAACCAAAATGTGATATGGGGTTGCGATTTTAAACGGATTAATATATGTCATAGGT

GGAGTCGTTAAAGGTGACACACATATACCAACGCACTAGAGAGTTTATCAGAAGATGGATGGATGAATCA

TCAACGTCTTCCAATAAAAATGTCCAATATGTCGACGATTGTTCATGCTGGAAAGATTTATATATCTAGA

GGTTACAACAATAGTAGTGTAGTTAATGTAATATCGAATCTAGTCCTTAGCTATAATCCGATATATGATG

AATGGACCAAATTATCATCATTAAATATTCCTAGAATTAATCCTGCTCTATGGTCAGTGTATAATAAATT

ATATGTAGGAGGAGTAATATCTGATGATGTTCAAACTAATACATCTGAAACATACGATAAAGAAAAAGAT

TGTTGGACATTGGATAATGGTCACTTGTTACCACATAATTATATAATGTATAAATGCGAACCGTTTAAAC

ATAGATATCCATTGGAAAAAACACAGTACACGAATGATTTTCTAAAGTATTTGGAAAGTTTTATAGGTAG

TTGATAGAACAAAATACATAATTTTGTAAAAATAAATCACTTTTTATACTAATATGACACAATTACCAAT

ACTTTTGTTACTAATATCATTAGTATACGCTACACCTTCTCCTCAGACATCTAAAAAAATAGGTGATGAT

GCAACTATATCATGTAGTCGAAATAATACAAATTACTACGTTGTTATGAGTGCTTGGTATAAGGAGCCCA

ATTCCATTATTCTCTTAGCTGCCAAAAGCGACGTCTTGTATTTTGATAATTATACCAAGGATAAAATATC

TTACGACTCTCCATACGATGATCTAGTTACAACTATCACAATTAAATCATTGACTGCTGGAGATGCCGGT

ACTTATATATGTGCATTCTTTATGACATCGACTACAAATGATACTGATAAAGTAGATTATGAAGAATACT

CCATAGAGTTGATTGTAAATACAGATAGTGAATCGACTATAGACATAATACTATCTGGATCTACACCAGA

AACTATTTCTGAGAAACCAGAGGATATAGATAATTCTAATTGCTCGTCTGTATTCGAAATCACGACTCCG

GAACCAATTACTGATAATGTAGACGACCATACAGACACCGTCACATACACTAGTGATAGCATTAATACAG

TAAATGCATCATCTGGAGAATCCACAACAGACGAGATTCCGGAACCAATTACTGATAAAGAAGAAGATCA

TACAGTAACAGACACTGTCTCATACACTACAGTAAGTACATCATCTGGAATTGTCACTACTAAATCAACC

ACCGATGATGCGGATCTTTATGATACATACAATGATAATGATACAGTACCGCCAACTACTGTAGGTGGTA

GTACAACCTCTATTAGCAATTATAAAACCAAGGACTTCGTAGAAATATTTGGTATTACCACATTAATTAT

ATTGTCAGCAGTGGCGATTTTCTGTATTACGTATTATATATGTAATAAACACCCACGTAAATACAAAACA

GAGAACAAAGTCTAGATTTTTGACTTACATAAATATCTGGGATAATAAAATCTATCATATTGAGAGGACC

ATCTGGTTCAGGAAAGACAGCCATAACCAAAAGACTGTTAAAAGACTATGGGAATATATTTGGATTTGTG

GTGTCCCATACCACTAGATTTCCTCGTCCTATGGAACGAGAAGGTGTTGATTACCTTACGTTAACAGAGA

GGCCATCTGGAAGGGAATAGCCGCCGGAAACTTTCTAGAACATACTGAGTTTTTAGGAAATATTTACGGA

ACTTCTAAAACAGCTGTAAATACAGCGGTTATTAATAATCGTATTTGCGCGATGGATTTAAACATCAACG

GTGTTAGAAGTCTTAAAAATACTTACCTAATGCATTACTTGGGTATATAAGACCTACCTCTCTTAAAATG

GTTGAGACCAATCTTCGTCGTAGAAACACTGAAGCGGACGACGAATCTCATCGTCGCGTGATGTTGGCAA

AAAACGGATATGGATGAGGTCAACGAAGCAGGTCTATTCGACACTATTATTATTGAAGATGATGTGAATT

TAGCATATAGTAAGTGTTAATTCAGATACTACAGGACCGTATTAGAATGTATTTTAACACTAATTAGAGA

CTTAAGATTTGACTTAAAACTTGATAATTAATAATATAACTCGTTTTTATATGTGGCTATTTCAACGTCT

AATGTATTAGTTAAATATTAAAACTTACCACGTAAAACTTAAAATTTAAAATGGTATTTCATTGACAGAT

CATACATTATGAAGTTTCAAGGACTTGTGTTAATTGACAATTGCAAAAATCAATGGGTCGTTGGACCATT

AATAGGAAAAGGTGGATTCGGTAGTATTTATACTACTAATGACAATAATTATGTAGTAAAAATAGAGCCC

AAAGCTAACGGATCATTATTTACCGAACAGGCATTTTATACTAGAGTACTTAAACCATCCGTTATCGAAG

AATGGAAAAAATCTCACAATATAAAGCACGTAGGTCTTATCACATGCAAGGCATTTGGTTTATACAAATC

CATTAATGTGGAATATCGATTCTTGGTAATAAATAGATTAGGTGCAGATCTAGATGCGGTGATCAGAGCC

AATAATAATAGACTACCAGAAAGGTCGGTGATGTTGATCGGAATCGAAATCTTAAATACCATACAATTTA

TGCACGAGCAAGGATATTCTCACGGAGATATTAAAGCGAGTAATATAGTCTTGGATCAAATAGATAAGAA

TAAATTATATCTAGTGGATTACGGATTGGTTTCTAAATTCATGTCTAACGGCGAACATGTTCCATTTATA

AGAAATCCAAATAAAATGGATAACGGTACTCTAGAATTTACACCTATAGATTCGCATAAAGGATACGTTG

TATCTAGACGTGGTGATCTAGAAACACTTGGATATTGTATGATTAGATGGTTGGGAGGTATCTTGCCATG

GACTAAGATATCTGAAACAAAGAATTCTGCATTAGTAAGTGCCGCAAAACAGAAATATGTTAACAATACT

GCGACTTTGTTAATGACCAGTTTGCAATATGCACCTAGAGAATTGCTGCAATATATTACCATGGTAAACT

CTTTGACATATTTTGAGGAACCCAATTACGACGAGTTTCGTCGAGTATTAATGAATGGAGTTATGAAAAA

TTTTTGTTGATAAAAAAATTAAAAAAATAACTTAGTTATTATCACTCTCGTGAGTACAATAGAAACATGG

CGATGTTTTACGCACACGCTTTCGGTGGGTACGACGAGAACCTTCATGCATTTCCTGGAATATCATCGAC

GGTTGCCAATGATGTCAGGAAATATTCTGTTGTGTCAGTTTATAATAAAAAGTATAACATTGTAAAAAAC

AAATATATGTGGTGTAACAGTCAAGTGAACAAGAGATATATTGGAGCACTACTGCCTATGTTTGAATGCA

ATGAATATCTACAAATTGGAGATCCAATCCATGATCTAGAAGGAAATCAAATCTCTATTGTCACATATCG

CCACAAAAACTACTATGCTCTAAGTGGAATTGGGTACGAGAGTCTAGACTTGTGTTTGGAAGGAGTAGGG

ATTCATCATCACGTACTTGAAACAGGAAACGCGGTATATGGAAAAGTTCAACATGAGTATTCTACTATCA

AAGAGAAGGCCAAAGAAATGAATGCACTCAAACCAGGACCTATCATCGATTACCACGTCTGGATAGGAGA

TTGTGTCTGCCAAGTTACTACTGTAGACGTGCATGGAAAGGAAATTATGAGAATGAGATTCAAAAGGGGT

GCGGTGCTTCCGATTCCAAATCTGGTAAAAGTTAAAGTTGGGGAGGAAAATGATACAATAAATCTTTCCA

CTTCCATATCAGCTCTCCTAAATTCCGGTGGCGGCACCATCGAGGTAACATCTAAGGAAGAACGTGTAGA

TTATGTACTCATGAAACGTTTGGAATCTATACATCATTTGTGGTCTGTAGTGTATGATCATCTTAATGTT

GTGAATGGCGAAGAACGATGTTATGTACATATGCATTCATCTCATCAAAGTCCTATGCTGAGTACTGTAA

AAACAAATTTGTACATGAAGACTATGGGAGCATGTCTTCAAATGGACTCCATGGAAGCTCTAGAGTATCT

TAGTGAACTGAAGGAATCAGGTGGGCGGAGTCCCAGACCAGAATTGCAGAAATTTGAATATCCAGATGGA

GTGAAAGACACTGAATCAATTGAGAGATTGGCAGAGGAGTTCTTCAATAGATCAGAACTTCAGGCCGGTG

AATCAGTCAAATTTGGTAATTCTATTAATGTTAAACATACATCTGTTTCAGCTAAGCAACTAAGAACACG

TATACGACAGCAGCTTCCTTCTATACTCTCATCTTTTGCCAACACAAAGGGTGGATATTTGTTCATTGGA

GTTGATAATAATACACACAAAGTAATTGGATTCACGGTGGGTCATGACTACCTCAAACTGGTAGAGAGTG

ATATAGAAAAGTATATCCAAAAACTTCCTGTTGTGCATTTCTGCAAGAAAAAAGAGGACATCAAGTACGC

ATGTAGATTCATCAAGGTGTATAAACCTGGTGATGAGACTACCTCGACATATGTGTGCGCAATCAAAGTG

GAAAGATGCTGCTGTGCTGTGTTTGCGGATTGGCCAGAATCATGGTACATGGATACTAGTGGTAGTATGA

AGAAGTATTCTCCAGATGAATGGGTGTCACATATAAAATTTTAATTAGGGTAAGGTAAAACTATATATAA

TAACTAACAATTTGTGTATCATATAGACAATTAATTAGGTAACTGTTATCTCTTTTTAACTAACTAACTA

ACTAACTAACTCTTATATACTATTAATAATACATCTATTAATCATTGATTAGCTTATTGCTTTAATTGTT

TTTGTAAACTAACACTGTTCATTGAAAAGGGATAACATGTTACAGAATATAAATTATATATGGATTTTTT

TAAAAAGGAAATACTTGACTGGAGTATATATTTATTTCTTCATTACATAACACGTCTGTGTTCTAATTCT

TCCAATTCTTCCACATCTCATATAATACAGGAATATAATCTTGTTCGAAAATATGAGAAAGTGGATAAAA

CAATAGTTGATTTTTTATCTAGGTGGCCAAATTTATTCCATATTTTAGAATATGGGGAAAATATTCTACA

TATTTATTTTATAGATGCTGCTAATACGAATATTATGATTTTTTTTCTAGATAGAGTATTAAATATTAAT

AAGAACCGTGGGTCATTTATACATAATCTCGGGTTATCATCCATTAATATAAAAGAATATGTATATCAAT

TAGTTAATAATGATCATCTAGATAATAGTATAAGACTAATGCTTGAAAATGGACGTAGAACAAGACATTT

TTTGTCTTATATATTGGATACAGTTAATATCTATATAAGTATTTTAATAAATCATAGATTTTATATAGAT

GCCGAAGACAGTTACGGTTGTACATTATTACATAGATGTATATATAACTATAAGAAATCAGAATCAGAAT

CATATAATGAATTAATTAAGATATTGTTAAATAATGGATCAGATGTAGATAAAAAAGATACGTACGGAAA

CACACCGTTTATCCTATTATGTAAACACGATATCGACAACGCGGAATTGTTTGAGATATGTTTAGAGAAT

GCTAATATAGACTCTGTAGACTTTAATGGATATACACCTCTTCATTATGTCTCATGTCGTAATAAATATG

ATTTTGTAAAGTTATTAATTTCTAAAGGAGCAAATGTTAATGCACGTAATAGATTCGGAACTACTCCATT

TTATTGTGGAATTATACACGGTATCTCGCTTATAAAACTATATTTGGAATCAGACACAGAGTTAGAAATA

GATAATGAACATATAGTTCGTCATTTAATAATTTTTGATGCTGTTGAATCTTTAGATTATCTATTGTCCA

GAGGAGTTATTGATATTAACTATCGTACTATATACAACGAAACATCTATTTACGACGCTGTCAGTTATAA

TGCGTATAATACGTTAGTCTATCTATTAAACAGAAATGGTGATTTTGAGACGATTACTACTAGTGGATGT

ACATGTATTTCGGAAGCAGTCGCGAACAACAACAAAATAATAATGGATATACTATTGTCTAAACGACCAT

CTTTGAAAATTATGATACCATCTATGATAGCAATTACTAAACATAAACAACATAATGCAGATTTATTGAA

AATGTGTATAAAATATACTGCGTGTATGACCGATTATGATACTCTTATAGATGTACAATCGCTACATCAA

TATAAATGGTATATTTTAAAATGTTTTGATGAAATAGATATCATGAAGAGATGTTATATAAAAAATAAAA

CTGTATTCCAATTAGTTTTTTGTATCAAAGACATTAATACTTTAATGAGATACGGTAGACATCCTTCTTT

CGTGAAATGTAATATTCTTGACGTATACGGAAGTTGTGTACGTAATATCATAGCATCTATTAGATATCGT

CAGAGATTAATTAGTCTATTATCCAAGAAGCTGGATGCTGGAGATAAATGGTCGTGTTTTCCTAACGAAA

TAAAATATAAAATATTGGAAAACTTTAACGATAACGAACTGACCACATATCTGAAAATCTTATAAACACT

ATTAAAATATAAAATCTAAGTAGGATAAAATCACACTACATCATTGTTTCCTTTTAGTGCTCGACAGTGT

ATACTATTTTTAACACTCATAAATAAAAATGAAAACGATTTCCGTTGTTACGTTGTTATGCGTACTACCT

GCTGTTGTTTATTCAACATGTACTGTACCCACTATGAATAACGCTAAATTAACGTCTACCGAAACATCGT

TTAATGATAAACAGAAAGTTACGTTTACATGTGATTCAGGATATCATTCTTTGGATCCAAATGCTGTCTG

TGAAACAGATAAATGGAAATACGAAAATCCATGCAAGAAAATGTGCACAGTTTCTGATTATGTCTCTGAA

CTATATGATAAGCCATTATACGAAGTGAATTCCACCATGACACTAAGTTGCAACGGTGAAACAAAATATT

TTCGTTGTGAAGAAAAAAATGGAAATACTTCTTGGAATGATACTGTCACGTGTCCTAATGCGGAATGTCA

ACCTCTTCAATTAGAACACGGATCGTGTCAACCAGTTAAAGAAAAATACTCATTTGGGGAATATATGACT

ATCAACTGTGATGTTGGATATGAGGTTATTGGTGTTTCGTATATAAGTTGTACGGCTAATTCTTGGAATG

TTATTCCATCATGTCAACAAAAATGTGATATACCGTCCCTATCTAATGGATTAATTTCCGGATCTACATT

TTCTATCGGTGGCGTTATACATCTTAGTTGTAAAAGTGGTTTTACACTAACGGGGTCTCCATCATCCACA

TGTATCGACGGTAAATGGAATCCCATACTCCCAACATGTGTACGATCTAACGAAGAATTTGATCCAGTGG

ATGATGGTCCCGACGATGAGACAGATCTGAGCAAACTCTCGAAAGACGTTGTACAATATGAACAAGAAAT

AGAATCGTTAGAAGCAACTTATCATATAATCATAATGGCGTTGACAATTATGGGTGTCATATTTCTAATC

TCCATTATAGTATTAGTTTGTTCCTGTGACAAAAATAATGACCAATATAAGTTCCATAAATTGCTACCGT

GAATATAAATCCGTTAAAATAATTAATAATTAATAATTAATAACGAACAAGTATCAAAAGATTAAAGAAT

TAGCTAGAATCAATTAGATGTCTTCTTCAGTGGATGTTGATATCTACGATGCCGTTAGAGCATTTTTACT

CAGGCACTATTATGACAAGAGATTTATTGTGTATGGAAGAAGTAACACCATATTACATAATATATACAGG

CTATTTACAAGATGCACCGTTATACCGTTCGATGATATAGTACGTACTATGCCAAATGAATCACGTGTTA

AACAATGGGTGATGGATACACTTAATGGTATAATGATGAATGAATTCGATACTGTATGTGTGGGTACCGG

ACTACGATTCATGGAAATGTTTTTCGATTACAATAAAAATAATCCCAAAAATAGCATCAACAATCAAATA

ATGTATGATATAATTAATAGCGTAGCCATAATTCTAGCTAATGAGAGATATAGAAGCGCGTTTAACGACG

ATAGAATATACATCCGTAGAACTATGATGGACAAATTGTACGAATACGCATCTCTAACTACTATTGGTAC

GATCACTGGAGGTGTTTGTTATTTTATCTGTTGATGCATCTAGTTAGTTTGTATAAATAATTATTTCGAT

ATACTAGTTAAAATTTTAAGATTTTAAATGTATAAAAAACTAATAACGTTTTTATTTGTAATAGGTGCAG

TTGCATCCTATTCGAATAATGAGTACACTCCGTTTAATAAACTGAGTGTAAAACTCTATATAGATGGAGT

AGATAATATAGAAAATTCATATACTGATGATAATAATGAATTGGTGTTAAATTTTAAAGAGTACACAATT

TCTATTATTACAGAGTCATGTGACGTCGGATTTGATTCCATAGATATAGATGTTATAAACGACTATAAAA

TTATTGATATGTATACCATTGACTCGTCTACTATTCAACGCAGAGGACATACGTGTAGAATATCTACCAA

ATTATCATGCCATTATGATAAGTACCCTTATATCCACAAATATGAGGGTGATGAACGACAATATTCTATT

ACCGCAGAGGGAAAATGCTATAAAGGAATAAAATATGAAATAAGTATGATGAACGATGATACTCTATTGA

GAAAACATACTCTTAAAATTGGATTTACTTATATATTCGATCGTCATGGGCATAGTAATACATATTATTC

AAAATATGATTTTTAAAAATTTAAAATATATTATCACTTCAGTGACAGTAGTCAAATAACAAACAACACC

ATGAGATATATTATAATTCTCGCAGTTTTGTTCATTAATAGTATACATGCTAAAATAACTAGTTATAAGT

TTGAATCCGTCAATTTTGATTCCAAAATTGAATGGACTGGGGATGGTCTATACAATATATCCCTTAAAAA

TTATGGCATCAAGACGTGGCAAACAATGTATACAAATGTACCAGAAGGAACATACGACATATCCGGATTT

CCAAAGAATGATTTCGTATCTTTCTGGGTTAAATTTGAACAAGGCGACTATAAAGTGGAAGAGTATTGTA

CGGGACTATGTGTCGAAGTAAAAATTGGACCACCAACTGTAATATTGACTGAATATGACGATCATATCAA

TTTGTTCATCGAGCATCCGTATGCTACTAGAGGTAGCAAGAAGATTCCTATTTACAAACGCGGTGACATG

TGTGATATCTACTTGTTGTATACGGCTAACTTCACATTCGGAGATTCTGAAGAACCAGTAACATATGATA

TCGATGACTACGATTGCACGTCTACAGGTTGCAGTATAGACTTTGCCACAACAGAAAAAGTGTGTGTGAC

AGCACAGGGAGCCACAGAAGGGTTTCTCGAAAAAATTACTCCATGGAGTTCGGAAGTATGTCTGACACCT

AAAAAGAATGTATATACGTGCGCAATTAGATCTAAAGAAGATGTTCCCAATTTCAAGGACAAAATAGCCA

GAGTTATCACGAGAAAATTTAATAAACAGTCTCAATCTTATTTGACTAAATTTCTCGGTAGCACATCGAA

TGATGTTACAACTTTTTTTAGCATTCTTGACTAAATATTCATAACTAATTTTTATTAATGATACAAAAAT

GAAATAAACTGTATATTATACACTGGTTAACGCCCTTGGCTCTAACCATTTTCAAGATGAGGTCCCTGAT

TATAGTCCTTCTGTTCCCCTCTATCATCTACTCAATGTCTATTAGACGATGCGAGAAGACTGAAGAGGAA

ACATGGGGATTAAAAATAGGGTTGTGTATAATTGCCAAAGATTTCTATCCCGAAAGAACTGATTGCAGTG

TTCATCGCCCAACTGCAAGTGGAGGATTGATAACTGAAGGCAATGGATTCAGAGTAGTTATATATGATCA

ATGTACAGAACCCCATGACTTTATTATCACCGATACTCAACAAACACGTCTTGGATCATCTCATACATAT

ATTAAATTCAGTAACATGAATACAGGTGTCCCATCTAGTATTCCAAAATGTTCCAGAACTCTCTGTATTT

CTGTATATTGTGATCAAGAGGCGGGAGACATAAAATTTGAGGAGTATACTCAAGAATCAAGTGATATCAG

TATTAGAGTTAAGTATGATTCATCATGTATTGATTATCTGGGTATTAATCAAAGTTTCATGAATGAATGT

ATTCGAAGAATTACAACATGGGATAGAGAATCATGCGTCAGAATTGATACACAGACTATAAATAAATATC

TTAAGTCTTGCACCAACACAAAATTCGACCGTAATGTCTACAAAAGGTACATACTGAAGAGTAAAGCACT

CCATGCTAAAACAGAGTTGTAATAGATATAAAATACTTTTTATAATAATTAGGCTAGAAAAATCTCACTC

ACATGTAATCTTAAAAAAATGATATGATAGTTCTTACAAGTAGCGATTGAGTTTTAAATGGATTCTATTA

ATTACCGGGGAACTTAACAATTCGTTCTGATCTACAGACATTGGTTAATAAATCATCTTATTTTGCCAAT

ATATTAAAATGTGGAAACTCCACTAATAATATTACATTGTGCGACTTTCAAGATGATGTGATATATAGGG

TTATACAGTTTTAACAATTATATAATAGAGATAGAAAGTACAAAAGATGTAGAATCAATGATATGGCACG

CTAAACAGTTGGGTGTGGAATCATTGCTAAAAGAATGTCAAAATTATTTGCTTAGAATATTACGTATATA

ATTGTTTAGAAATTTATAGAATAACTAATATTAATACATTATCGTATATCTACAACGATATAAGAAACTT

CATATTGGATAATATTACTATTAATATATAAGGATCCAGATTTTATATATTTGCCTAAATACATTATTAT

AGATTTACTAGGACAATCACCTAAATGTTTTTAACGAAGATAATGTGGTAAAGATTATATACACTTATAT

ATCTTCCGATATCTACAAGGATATTCCATATCATCATTGTGTAAACTAAATAACGTTTTCTATGGCATTT

AATAAGGACATTGGATATGTGGAAAAGTGATGTATGGAAGTTAGTACATTATCAACTTCTCCTTATTGAT

TGAAAATGAAAATATAAATAGTTTTTATGTATAGCGGTATCTACCCTATAGTTTTATTGCTTACTACTAA

CATGGATTCAGATACAGATACAGATACAGATACAGATACAGATACAGATACAGATACAGATGTAGAAGAT

ATCATGAATGAAATAGATAGAGAGAAAGAAGAAATACTAAAAAATGTAGAAATTGAAAATAATAAAAACA

TTAACAAGAATCATCCCAGTGAATATATTAGAGAAGCACTTGTTATTAATACCAGTAGTAATAGTGATTC

CATTGATAAAGAAGTTATAGAATATATCAGTCACGATGTAGGAATATAGATCATATCTACTAATTTTTAT

AATCGATACAAAACATAAAAACAACTCGTTATTACATAGCAGGTATGGAATCCTTCAAGTATTGTTTTGA

TAACGATGGTAAGAAATGGATTATCGGAAATACTTTATATTCTGGTAATTCAATACTCTATAAGGTCAGA

AAAAATTTCACTAGTTCGTTCTACAATTACGTAATGAAGATAGATCATAAATCACACAAGCCATTGTTGT

CCGAAATACGATTCTATATATCTGTATTGGATCCTTTGACTATCAACAACTGGACACGAGAACGTGGTAT

AAAGTATTTGGCTATTCCAGATCTGTATGGAATTGGAGAAACCGATGATTATATGTTCTTCATTATAAAG

AATTTGGGAAGAGTATTCGCCCCAAAGGATAGTGAATCAGTTTTCGAAGCATGTGTCACTATGATAAACA

CGTTAGAGTTTATACACTCTCAAGGATTTACTCATGGAAAAATAGAACCGATGAATATACTGATTAGAAA

TAAACGTATTTCACTAATTGACTATTCTAGAACTAACAAACTATACAAAAGTGGAACACATATAGATTAC

AACGAGGACATGATAACTTCAGGAAATATCAATTATATGTGTGTAGACAATCATCTTGGAGCAACAGTTT

CAAGACGAGGAGATTTAGAAATGTTGGGATATTGCATGATAGAATGGTTCGGTGGTAAACTTCCATGGAA

AAACGAAAGTAGTATAAAAGTAATAAAACAAAAAAAAGAATATAAACAATTTATAGCTACTTTTTTTGAG

GACTGTTTTCCTGAAGGAAATGAACCTCTGGAATTAGTTAGATATATAGAATTAGTATACATGTTAGATT

ATTCTCAAACTCCTAATTATGACAGACTACGTAGACTGTTTATACAAGATTGAAATTATATTCTTTTTTT

TATAGAGTGTGGGGGTAGTGTTACGGATATCTAATATTAATATTAGACTATCTCTATCGCGCTACACGAC

CAATATCGATTACTATGGATATCTTCAGGGAAATCGCATCTTCTATGAAAGGAAAGAATGTATTCATTTC

TCCAGCGTCAATCTCGTCAGTATTGACAATACTGTATTATGGAGCTAATGGATCCACTGCTGAACAGCTA

TCAAAATATGTAGAAAAGGAGGAGAACATGGATAAGGTTAGCGCTCAGAATATCTCATTCAAATCCATGA

ATAAAGTATATGGGCGATATTCTGCCGTGTTTAAAGATTCCTTTTTGGGAAAAATTGGCGATAAGTTTCA

AACTGTTGACTTCACTGATTGTCGCACTATAGATGCAATCAATAAGTGTGTAGATATCTTTACTGAGGGA

AAAATCAATCCACTATTGGATGAACCATTGTCTCCTGATACCTGTCTCCTAGCAATTAGTGCCGTATACT

TTAAAGCAAAATGGTTGATGCCATTCGAAAAGGAATTTACCAGTGATTATCCCTTTTACGTATCTCCAAC

GGAAATGGTAGATGTAAGTATGATGTCTATTTACGGCGAGCCATTTAATCACGCATCTGTAAAAGAATCA

TTCGGTAACTTTTCAATCATAGAACTGCCATATGTTGGAGATACTAGTATGATGGTCATTCTTCCAAACA

AGATTGATGGATTAGAATCCATAGAACAAAATCTAACAGATACAAATTTTAAGAAATGGTGTAACTCTCT

GAAAGCTACGTTTATCGATGTGCACATTCCTAAGTTTAAGGTAATAGGTTCGTATAATCTTGTGGATACG

CTAATAAAGTTGGGACTGACAGATGTGTTCTATTCAACTGGTGATTATATCAATATGTGTAATTCAGATG

TGAGTGTTGACGCTATGATTCACAAAACGTATATAGATGTCAATGAAGAGTATACAGAAGCAGCTGCAGC

AACTTCTGTACTAGTGGCAGACTGTGCATCAACAGTTACAAATGAGTTCTGTGCAGATCATCCGTTCATC

TATGTGATTAGACATGTCGATGGTAAAATTCTTTTCGTTGGTAGATATTGCTCTCCAACAACTAATTAAG

CACATTCTTAATATTAGAATATTATATAGTTAAGATTTTTACTAACAGGTTAACATTTTTTTTTAAAAAT

AGAAAAAACATGTGGTATTAGTGCAGGTCGTTATTCTTCCAATTGCAATTGGTAAGATGACGGCCAACTT

TAGTACCCACGTCTTTTCACCACAACACTGTGGATGTGACAGACTGACCAGTATTGATGACGTCAGACAA

TGTTTGACTGAATATATTTATTGGTCGTCGTATGCATACCGCAACAGGCAATGCGCTGGACAACTGTATG

ACACACTCCTCTCTTTTAAAGATGATGCGGAATCAGTGTTCATCGACGTTCGTGAGCTGGTAAAAAATAT

GCCGTGGGATAATGTTAAGGATTGTACAGAGATCATCCGTTGTTATATACCGGATGAGCAAAAAACCATC

AGAGAGATTTCGGCCATCATTGGACTTTGTGCATATGCTGCTACTTACTGGGGAGGTGAAGACCATCCCA

CTAGTAACAGTCTGAACGCATTGTTTGTGATGCTTGAGATGCTCAATTACATGGATTATACCATCATATT

CTGGCGTATGAATTGATGAGTTACAGCTTGACATTTCTTCTTTCCTCCCTCTTCTTCTACCTTTCCCAGA

AACAAACTTTTTTTACCCACTATAAAATAAAATGAGTATACTACCTGTTATATTTCTTCCTATATTTTTT

TATTCTCCATTCGTTCAGACTTTTAACGTGCCTGAATGTATCGACAAAGGGCAATATTTTGCATCATTCA

TGGAGTTAGAAAACGAGCCAGTAATCTTACCATGTCCTCAAATAAATACGCTATCATCCGGATATAATAT

ATTAGATATTTTATGGGAAAAACGAGGAGCGGATAATGATAGAATTATACAGATAGATAATGGTAGCAAT

ATGCTAATTCTGAACCCGACACAATCAGACTCTGGTATTTATATATACATTACCACGAACGAAACCTACT

GTGACATGATGTCGTTAAATTTGACAATCGTGTCTGTCTCAGAATCAAATATAGATCTTATCTCGTATCC

ACAAATAGTAAATGAGAGATCTACTGGTAAAATGGTATGTCCCAATATTAATGCATTTATTTCTAGTAAC

GTAAACACAGAATTATATGGAGCGGACATCGACGCCTTAGAAATAAGAGACTTAAACAACGGACACCTGG

AATTATTACCATAGAAGATGTTAGAAAAAATGATGCTGGTTATTATACATGTGTTTTAGAATATATAT

ATATATATATATATATATATATATATATATATATATATATGGGCAAAACATATAACGTAACCAGAATTAT

AAAATTAGAGGTACGGGATAGAATAATACCTCCTACTATGAAATTACCAGAAGGAGTAGTAACTTCAATA

GGTAGTAATTTGACTATTGCATGTAGAGTATCGTTGAGACTTCCCACAACGGACACCGACGTCTTTTGGA

TAAGTAATGGTATATGTATTACGAAGAAGAAGACGAGGACGGAGACGGTAGAATAAGTGTAGCAAATAAA

ATCTATATGACCGATAAGAGACGTGTTATTACATCCTGGTTAAACATTAATCCTGTCAAGGAAGAAGATG

CTACAACGTTTACGTGTATGGCGTTTACTATTCCTAGCATCAGCAAAACAGTTACTGTTAGTAAACGTGA

ATGTATGTTGTTACATTTCCATATCAATTGAGTTTATAAGAATTTTTTATACATTATCTTCCAACAAACA

ATTGACGAACGTATTGCTATGATTAACTCCCACAATACTATATATATTATTAATCATTAACTTGCAGACT

ATACCTAGTAGTGCTATTTTGACATACTCATGTTCTTGTGTAATCGCAGTATCTATATTATTAAAGTACG

TAAATCTAGCTATAGTTTTATTATTTAATTTTAGATAATATACTGTCTCCGTATTTTTAAAAAATTACCA

CATCCTTTATTAAATCATGAATGGGAATTTCTGTGTCATCGTTAGTATATTGTGAACAACAAGAGCAGAT

ATCTATAGGAAAGGGTGGAATGCGATACATTGATCTATGTAGTTTTAAAACATACGCGAACTTTGAAGAA

TTTATATAAATCATCTCACGAGATATTGCTCTCTGTCATATTCATACACCTGTATAAACTTTCTAGACAT

CTTACAATGTGTTATTTTATGATCATATTTACATATTTACTGGTATATCAAAGATGTTAGATTAGTTAAT

GGGAATCGTCTATAATAATGAATATTAAACAATTATAGGAGGAGTTTATACCTACAAAAACATCATAAAA

ATGAGTCATCGTCCGATTTATGTTTTAAATATACTAACATTACTACCTTCAGAAATTATATACGAAATAT

TATACATGCTGACAATTAACGATCTTTATAATATATAGTATCCACCTACCAAAGTATAATTGTATTTTTC

TCATGTGATGTGTGTAAAAAACTGATATTATATAATTATCTTAGTACCTATGATGAAGATGAAGATGAAG

ATGAAGATGATGGTCCGTATATATTTTGTATCATTATCGTTATTGCTATTCCATAGTTACGCCATAGACA

TCGAAAATGAAATCACCGAATTCTTCAATAAAATGAGAGATACTCTACCAGCTAAAGACTCTAAATGGTT

GAATCCAGTATGTATGTTTGGAGGCACAATGAATGATATGGCCGCTCTAGGAGAGCCATTCAGTGCAAAG

TGTCCTCCTATTGAAGACAGTCTTTTATCGCATAGATATAAAGACTATGTGGTTAAATGGGAAAGGCTAG

AAAAGAATAGACGGCGACAGGTTTCTAATAAACGTGTTAAACATGGTGATTTATGGATAGCCAACTATAC

ATCTAAATTCAGTAACCGTAGGTATTTATGTACCGTAACCACAAAGAATGGTGACTGTGTTCAGGGTGTA

GTTAGATCTCATGTGTGGAAACCTTCTTCATGCATTCCAAAAACATATGAACTAGGTACTTATGATAAGT

ATGGCATAGACTTATACTGTGGAATTCTTTATGCGAACCATTATAATAATATAACTTGGTATAAAGATAA

TAAGGAAATTAATATCGACGATTTTAAGTATTCACAAGCGGGAAAGGAATTAATTATTCATAATCCAGAG

TTAGAAGATAGTGGAAGATACGACTGTTACGTTCATTACGACGACGTTAGAATCAAGAATGATATCGTAG

TATCAAGATGTAAAATACTTACGGTTATACCGTCACAAGACCACAGGTTTAAACTAATACTAGATCCGAA

AATCAACGTAACGATAGGAGAACCTGCCAATATAACATGCAGTGCTGTGTCAACGTCATTATTTGTCGAC

GATGTACTGATTGAATGGGAAAATCCATCCGGATGGATTATAGGATTAGATTTTGGTGTATACTCTATTT

TAACTAGTAGAGGCGGTATCACCGAGGCGACTTTGTATTTTGAAAATGTTACTGAAGAATATATAGGCAA

TACATATACATGTCGTGGACACAACTATTATTTTGATAAAACTCTTACAACTACAGTAGTATTGGAGTAA

ATACACAATGCATTTTTATATACATTACTGAATTATTATTATTAATTATATCGTATTTGTGCTATAGAAT

GGATGAAGATACGCGACTATCTAGGTATTTGTATCTCACCGATAGAGAACATATAAATGTAGACTCTATT

AAACAGTTGTGTAAAATATCAGATCCTAATGCATGTTATAGATGTGGATGTACGGCTTTACATGAGTACT

TTTATAATTATAGATCAGTCAACGGAAAATACAAGTATAGATACAACGGTTACTATCAATATTATTCATC

TAGCGATTATGAAAATTATAATGAATATTATTATGATGATTATGATAGAACTGGTATGAACAGTGAGAGT

GATAATATATCAATCAAAACAGAATACGAGAATGAATATGAATTCTATGATGAAACACAAGATCAAAGTA

CACAACTAGTAGATTACGACATTAAACTCAAAACCAATGAGGATGATTTTGTTGATGAATTCTATGGTTA

TGATAGATCAGTGGGTGTCCATGATTATATAGATGTATCAATTAATAAAGTAGTATATGGAAGAGAGTCT

CACGTAAGATGGTGGGATATATGGCAAGAACATAATGATGGCGTATACAGTATAGGAAAGGAGTGCATAG

ATAATATATACGAAGACAGACATACCGTAGACGAATTCTACAAGATAGACAGCGTATCAGATGTAGATGA

CGCAGAACATATATCTCAGATAACTAATGATGTATCTACACAAACATGGGAAAAGAAATCAGAGTTAGAT

AGATACATGGAAATGTATCCTCGTCATAGATATGGTAAGCATTCTGTCTTTAAGGGATTTTCTGACAAAG

TTAGAAAAAATGATTTAGACATGAACGTGGTAAAAGAATTACTTTCTAACGGTGCATCTCTAACAATCAA

GGATAGCAGTAATAAGGATCCAATTGCTGTTTATTTTAGAAGAACAATAATGAATTTAGAAATGATTGAT

ATCATTAACAAACATACAACTATCTATGAACGCAGGTATATAGTACACTCCTATCTAAAAAATTATAGAA

ATTTCGATTATCCATTTTTCAGAAAGTTAGTTTTGACTAATAAACATTGTCTCAACAATTATTGTAATAT

AAGCGACGGCAAATATGGAACACCACTACATATATTAGCATCTAATAAAAAAATAATAACTCCTAATTAC

ATGAAGTTATTAGTGTATAACGGAAATGATATAAACGCACGAGGTGAAGATACACAAATGCGAACTCCAT

TACACAAATATTTGTGTAAATTTGTATATCATAATATTGAATATGGTATCCGATACTATAATGAAAAGAT

TATAGACGCATTTATAGAGTTAGGAGCCGATCTAACTATTCCAAATGACGATGGAATGATACCAGTAGTT

TACTGTATACACTCAAATGCCGAATATGGTTATAACAATATTACTAACATAAAGATAATACGTAAACTAC

TTAATCTTAGTAGACATGCGTCACATAATCTATTTAGAGATCGAGTCATGCACGATTATATAAGTAATAC

ATATATTGATCTTGAGTGTTTAGATATCATTAGATCACTTGATGGGTACGATATTAATTGTTACTTTGAA

GGACGTACACCACTTCATTGCGCTATACAATATAACTTCACTCAGATTGCTGAGTACTTATTAGATCGAG

GAGCTGATATATCATTAAAGACAGACGATGGTAAAACTGTATTTGATTTATCGTTATGTAGTTACATTCC

TCTTAAATGGACTAGCTTTTTGATTAGTCGTCTACCGCCTAAAAGTGTCATATGCTCACTGACTAACCAT

ATAATAGATTATGTTCTTACGAACAATAGACGTATTATTTGGCAGAGTCAAATGATTAATAAGTACGTAC

TGTTACTGGACCCATCCTTTTATTATAGATTCAGAAATGTTATCGAAAACAAATTAGACCAATACAATAA

TCGTTATAATATGTTCGAACACGATAGGGACGTTAATGAAAAGTATGGCAAAGTCTTACATGACCTCGAT

ACATATATCAAGGATGTACAAGTATTAAAATCTACTTCCATCACTAATAATATAACACTATACGACACTA

TTATAAATAATAAGTCAGAGTTTCCTATACGTCGTGTAAACGACAAACAATTAATTAATCTCATAAAATC

CAATACATATCATAATCTTATCGAAAAAGTTATTAAAAATACATTAGAGAAATATACTTTAACTAATATA

GTCCTCGAGTATATGATCTCATCTCGATCTCAATCATCTTATTTGAGTCGTATTCCTAATGAGATATTAC

TCGAAATATTATATAAACTCGACATGTACGATTTACGTAATCTATATACAAGATATATGAGAGAGAATGA

TATCACAGAGTATCATATAGAGAATACGAGGTCTGTTTCTACACAGACATGAATAATGAATACACATACA

ACGTTTTTTTTAATCTTAGATATAACACTAATTACATCAAGATTATATATTGAAATCGTAATTTGAGTTG

TCTGATCATCATGGATATCGAAAATAATATACGTAACATTAGCAATCTTTTAGATGATTGATATATTATT

ATGCGATGTAATCATAACTATCGGAGATGTAGAAATTAAAGCGCATAAAACTATTTTGGTTGCCGGATCT

ACGTATTTTAAAACAATGTTCACAACATCTATGATAGCGAGAGATCTAGCAACTAGAGTAAATATACAGA

TGTTCGATAAAGATGCCGTCAAAAATATTGTACAGTACTTATACAATAGGTATATAAGTTCTATGAATGT

GATAGACATATTAAAATGCACCGACTAAGAACGTAAAACGAACTATAGAATGTTATACAATGGGTGATGA

TAAGTAGAAGATGTTACCCGATATACCCATAGCATTATCTAGTTATGGCATGTGTGTATTAGATCAATAC

ATATACATTATAAGCGGTCGTACCCAACACTGATTATACATCGGTACATACAGTAAATAGCATAGATATG

GAGGAGGATACAAATATTTCAAATAAAGTTATGAGATACGCGCTGTCAATAATATATGGAAGACATTACC

TAACTTCTGAACTGGAACTATAAATCCAGGCTCTCGCATAAAGATGAATATATATGTTGTATGCGACATC

AAAGATGAAAAAATGTTAAGACTTATATATTTAGATATAACACGAATATGTATGACGGATGGGAATTGGT

AACGATGACAGAAAGCAGATTGTCAGCTCTGCATACTATTCTTCATGACAATACCATAATGATGTTACAT

TGTTATGAAGCGTATATGTTACAAGATACATTTAATGTGCTTACGGAACATATATTTAGAAACATCTACT

AACGATTTTTTATGCTTGTATTATTAATGGTATGTAATATGATTTAATTGATTGTGTACACGATACCAAT

TTGTCGAGTATGAATACGGAGTACAAACATAAACTGAAGTTTAACATTATTTATTTATGATATACATTATATACATTATATACATTATATACATTATATACATTATATACATTATATACATTATATACATTATATACATTATATACATTATATACATTATATACATTATATACATTATATACATTATATACATTATATACATTATATATCGTTATTGTTTGGTCTATGCCATGGATATCTTTAAAGAACTAATCTTAAAACATACGGATGAAAATGTTTTGATTTCTCCAGTTTCCATTTTATCTACTTTATCTATTCTGAATCATGGAGCAGCTGGTTCTACAGCTGAACAACTATCAAAATATATAGAGAATATGAATGAGAAT

ACACCCGATGATAAGAAGGATGACAATAATGACATGGACGTAGATATTCCGTATTGCGCGACACTAGCTA

CCGCAAATAAAATATACGGTAGTGATAGTATCGAGTTCCATGCCTCATTCCTACAAAAAATAAAAGACGA

TTTTCAAACTGTAAACTTTAATAATGCGAACCAAACAAAGGAACTAATCAACGAATGGGTTAAGACAATG

ACAAATGGTAAAATTAATTCCTTATTGACTAGTCCGCTATCCATTAATACTCGTATGATAGTTATTAGCG

CCGTCCATTTTAAAGCAATGTGGAAATATCCATTTTCTAAACATCTTACATATACAGACAAGTTTTATAT

TTCTAAGAATATAGTTACCAGTGTTGATATGATGGTGGGTACCGAGAATGACTTGCAATATGTACATATT

AATGAATTATTCGGAGGATTCTCTATTATCGATATTCCATACGAGGGAAACTCTAGTATGGTGATTATAC

TGCCGGACGACATAGAAGGTATATATAACATAGAAAAAAATATAACAGATGAAAAATTTAAAAAATGGTG

TGGTATGTTATCTACTAAAAGTATAGACTTGTATATGCCAAAGTTTAAAGTGGAAATGACGGAACCGTAT

AATCTGGTACCGATTCTAGAAAATTTAGGACTTACTAATATATTTGGATATTATGCAGATTTTAGTAAGA

TGTGTAATGAAACTATCACTGTAGAAAAATTTCTACATACGACGTTTATAGATGTTAATGAGGAGTATAC

AGAAGTATCGGCCGTTACAGGAGTATTCATGACTAACTTTTCGATGGTATATCGTATGAAGGTCTACATA

AACCATCCATTCATATACATGATTAAAGATAACACCGGACATACACTTTTTATAGGGAAATACTGCTATC

CGCAATAAATATAAACAATAGACTTTTATCACGTTATCTCATGTATAAAATATTACAAATAGTATAGCAT

AAACTAAAGTCGATACATACATTAAAACTTAAATAATAATGTAATTTACAATTAATAGTATAAACTAAAA

AAATTAAAAAATTAAAAACAATATCATTATTATAAGTAATATCAAAATGACGATATACGGATTAATAGCG

TATCTTATATTCGTGACTTCATCCATCGCTAGTCCACTTTACATTCCCGTTATTCCGCCCATTTCGGAAG

ATAAATCGTTCAATAGTGTAGAGGTATTAGTTTCTTTGTTTCCCGATGACCAAAAAGACTATACAGTAAC

TTCTCAGTTCAATAACTACACTATCGGTACCAAAGACTGGACTATCAACGTACTATCCACACCTGATGGT

CTGGACATACCATTGACTAATATAACTTATTGGTCACGGTTTACTATAGGTCGTGCATTGTTCAAATCAG

AGTCTGAGGATATTTTCCAAAAGAAAATGAGTATTCTAGGTGTTTCTATAGAATGTAAGAAGCCGTCGAC

ATTACTTACTTTTTTAACCGTGCGTAAAATGACTCGAGTATTTAATAGATTTCCAGATATGGCTTATTAT

CGAGGAGACTGTCTAGAAGCCGTTTATGTAACAATGACTTATAAAAATACTAAAACTGGAGAGACTGATT

ACACGTACCTCTCTAATGGGGGGTTGCCTGCATACTATCGTAATGGGGTCGATGGTTGATTATTGATTAG

TATATTCCTTATATTCCTTATTCTTTTTATTCACACAAAAAGAACATTTTTATAAACATGAAACCACTGT

CTAAATGTAATTATGATCTTGATTTATAGATGATGATCAGCCTTCAGAGGATTTTGACCAGTATGTTTAA

TATGAAAAAAAACATAACTATTAAGCGCTATTGCGCTATTGTGCTTAATTATTTTGCTCTATAAACTGAA

TATATAGCCACAATTATTGACGGGCTTGTTTGTGACCGACAATCATGAATTTTCAGAAATTATCTCTGGC

TATATATCTTACGGTGACATGTTCGTGGTGTTATGAAACATGTATGAGAAAAACTGCGTTGTATCATGAC

ATTCAATTGGAGCATGTAGAAGACAATAAAGATAGTGTAGCATCGCTACCGTACAAGTATCTACAAGTAG

TCAAACAAAGAGAACGTAGTAGATTGTTGGCTACATTTAATTGGACGGATATAGCTGAGGGTGTTAGAAA

TGAGTTCATTAAAATATGTGATATCAACGGAACATATCTATATAATTATACTATTGCTGTTAGTATAATT

ATTGATTCCACGGAAGAACTACCAACAGTTACTCCAATTACAACATATGAACCTTCTATATATAATTATA

CTATCGATTATAGCACTGTTATTACTACTGAAGAACTACAAGTGACTCCAACATATGCACCTGTAACAAC

TCCTCTTCCAACATCAGCAGTTCCTTATGATCAACGATCGAATAACAATGTAAGTACTATATCTATTCAG

GTACTGAGTAAAATATTGGGAGTCAATGAAACAGAATTAACTAATTATCTTATTATGCATAAAAATGACA

CTGTTGACAATAACACCATGGTTGATGATGAGACATCTAACAATAACACATTACATGGTAATATAGGATT

TTTGGAAATAAATAATTGTTATAATGTTTCTGTGTCAGATGCTAGTTTTAGAATAACATTAGTAAACGAT

ACTTCTGAAGAAATTTTGCTAATGCTAACAGGAACTAGTTCATCCGACACCTTCATATCTTCCACCAATA

TCACTGAATGTTTGAAAACATTAATCAATAATGTGTCGATTAATGATGTACTTATAACACAAAATATGAA

TGTAACATCTAATTGTGATAAATGCTCAATGAATTTGATGGCATCCGTTATTCCTGCAGTTAATGAATTT

AACAATACGTTGATGAAAATTGGTGTAAAAGATGATGAAAACAATACGGTATATAAATATTATAATTGTA

AACTAACTACAAATTCTACATGTGATGAGTTAATCAATTTAGATGAAGTCATTAACAACATAACTCTGAC

AAATATTATACACAATAGTGTTTCGACAACTAACAGCAGAAAAAGACGAGATCTGAATGATGAGTTTGAA

TTTTCCACTTCCAAGGAATTAGATTGTCTTTACGAATCATATGGTGTAAACGATGATATAAGTCATTGTT

TTGCATCACCTAGACGTAGACGATCTGACGACAAAAAGGAGTACATGGACATGAAATTATTCGACCACGC

GAAAAAAGATTTAGGAATAGACAGTGTTATTCCTAGAGGTACAACCCATTTCCAAGTAGGTGCATCTGGT

GCAAGTGGTGGTGTTGTAGGAGATAGTTTCCCATTTCAAAATGTTAAATCGCGTGCCAGTCTATTGGCGG

AAAAAATAATGCCTAGAGTACCTATTACTGCTACCGAAGCTGATCTATATGCAACTGTAAATAGACAACC

CAAGTTACCAGCAGGTGTTAAAAGTACTCCGTTTACAGAGGCGCTTGTGTCTACGATAAACCAAAAGCTT

TCTAATGTTAGAGAGGTAACTTATGCTTCGCTCAATCTGCCAGGATCAAGTGGCTATGTTCATAGACCAT

CTGATTCTGTTATTTATAGCAGTATAAGACGGTCACGTTTACCTAGTGATAGCGATAGTGATTATGAGGA

TATACAAACTGTTGTTAAGGAATATAATGAAAGATATGGTAGATCAGTCAGTAGAACACAGTCATCAAGT

AGTGAAAGCGATTTTGAAGATATAGATACTGTTGTTAGGGAATATAGACAAAAATATGGCAATGCAATGG

CAAAAGGACGTAGTAGTTCCCCTAAACCTGATCCATTATATAGTACTGTTAAGAAAACAACTAAAAGTCT

ATCTACTGGTGTAGACATAGTTACAAAACAATCAGACTATTCTCTATTACCTGACGTTAATACTGGCAGT

TCTATTGTGTCACCTCTCACCAGAAAAGGAGCTACTAGACGACGACCTAGACGCCCTACAAATGATGGTC

TACAGAGTCCAAATCCTCCTCTCCGTAATCCACTTCCTCAACATGATGATTATTCTCCTCCACAAGTACA

CAGACCTCCACCACTTCCTCCTAAACCAGTCCAAAATTCGCCACAACTTCCCCCTAGACCAGTAGGTCAA

TTACTACCTCCTCCTATAGATCAACCAGATAAAGGATTTAGTAAGTTTGTATCACCTAGACGGTGTAGAA

GAGCAAGCTCTGGAGTCATATGTGGTATGATACAATCAAAACCAAACGATGATACCTATTCACTTCTTCA

ACGATCAAAAATTGAACCAGAATATGTGGAGGTTGGTAATGGTATACCCAAGAACAATGTTCCTGTAATA

GGTAATAAACATAGTAAAAAATATACATCGACGATGTCAAAAATATCAACAAAATTTGATAAATCTACGG

CATTTGGAGCAGCAATGTTACTAACTGGTCAGCAGGCCATTAGCCAACAGACTAGATCAACTACGTTGAG

TAGAAAAGATCAGATGAGTAAGGAAGAAAAGATATTCGAAGCAGTTACAATGAGTCTATCAACTATAGGT

TCAACGTTGACGTCTGCAGGTATGACGGGTGGTCCAAAACTAATGATTGCAGGAATGGCTATAACGGCTA

TAACTGGTATAATAGATACGATAAAAGATATATATTACATGTTTTCAGGACAGGAGAGGCCAGTAGATCC

TGTTATTAAATTATTTAATAAGTACACTGGCTTAATGTCCGATAATAATAAAATGGGTGTAAGAAAATGT

TTGACACCCGGTGACGACACACTTATTTATATCGCATACAGAAACGATACCAGTTTTAAACAGAATACGG

ATGCGATGGCTTTGTATTTCTTAGATGTTATCGATTCAGAGATCCTATATCTAAACACATCAAATTTAGT

TCTAGAGTATCAACTAAAGGTGGCTTGCCCCATAGGAACATTAAGATCTGTAGATGTGGACATAACTGCG

TATACAATATTATATGATACAGCGGATAATATTAAGAAATACAAGTTTATCAGAATGGCAACGCTACTAT

CCAAACATCCAGTTATTAGATTGACATGTGGTTTAGCAGCAACATTGGTGATTAAACCGTACGAGGTACC

CATCAGTGATATGCAACTACTAAAAATGGCGACGCCTGGTGAACCAGAATCCACTAAATCTATACCATCC

GATGTCTGTGATAGGTATCCTCTAAAGAAATTCTATCTTTTAGCTGGTGGTTGTCCCTATGATACATCTC

AAACTTTTATTGTACATACTACTTGCAGTATTCTACTAAGAACAGCTACACGGGATCAGTTTAGAAACAG

ATGGGTGTTACAAAATCCATTTAGACAAGAAGGGACATATAAGCAACTGTTTACCTTTAGCAAATACGAT

TTTAACGACACCATAATCGATCCTAATGGTGTGGTGGGTCATGCTAGCTTTTGTACCAATAGAAGCAGCA

ACCAATGTTTCTGGTCCGAACCTATGATATTGGAAGATGTATCATCGTGTAGTTCTAGAACTAGAAAAAT

ATACGTAAAACTGGGAATATTTAATGCTGAAGGTTTTAATAGTTTTGTACTAAATTGTCCAACTGGGTCT

ACACCTACATACATCAAACATAAAAATGCGGACAGTAACAATGTTATCATAGAGCTACCTGTAGGTGATT

ACGGCACAGCCAAATTGTATTCAGCAACAAAACCATCGAGGATAGCTGTGTTCTGCACACATAACTATGA

TAAACGATTCAAATCAGATATTATAGTTCTAATGTTTAATAAAAACAGCGGTATTCCATTTTGGAGCATG

TACACAGGAAGTGTAACTAGTAAAAATAGAATGTTTGCCACATTGGCTAGAGGAATGCCGTTTAGATCAA

CGTATTGCGATAACAGACGACGATCAGGTTGTTATTATGCAGGAATACCATTTCATGAAGATAGTGTAGA

AACAGATATACATTATGGACCAGAAATAATGTTAAAGGAAACATATGACATAAACAGTATTGACCCACGA

GTTATAACAAAGTCAAAGACCCATTTTCCTGCTCCATTGAGTGTAAAATTCATGGTTGACAATTTAGGAA

ATGGATATGACAACCCTAATTCATTTTGGGAAGATGCTAAAACTAAGAAACGGACATATAGTGCAATGAC

GATAAAAGTCCTACCATGTACAGTGAGAAATAAAAATATAGACTTTGGATATAACTATGGAGATATTATT

TCTAATATGGTTTATCTACAATCTACTAGTCAGGATTATGGAGATGGTACCAAATATACATTTAAATCCG

TAACTAGATCAGATCATGAGTGTGAATCTAGCTTAGATCTAACGTCTAAGGAAGTAACTGTGACATGTCC

TGCGTTTAGTATACCAAGAAATATATCAACATATGAAGGTCTATGCTTTAGTGTTACTACATCTAAAGAT

CATTGTGCTACAGGTATTGGTTGGTTAAAATCTAGTGGTTATGGGAAGGAAGATGCTGATAAACCACGTG

CTTGTTTTCATCATTGGAATTATTACACACTGTCGTTGGATTATTACTGTTCATACGAAGATATTTGGAG

AAGCACCTGGCCTGACTATGATCCATGTAAGTCATATATCCATATAGAGTATAGAGATACATGGATAGAA

TCTAATGTGTTACAGCAACCTCCTTACACATTCGAATTCATTCATGACAATTCTAACGAATATGTGGATA

AAGAAATTAGTAACAAATTAAATGATCTGTACAATGAATACAAGAAGATTATGGAATATAGCGACGGATC

ATTGCCGGCGTCTATAAACAGATTAGCAAAGGCATTGACTTCAGAGGGTAGAGAAATAGCAAGTGTTAAT

ATAGATGGTAATCTGTTAGATATCGCATATCAAGCAGATAAGGAAAAGATGGCCGACATACAGACAAGAA

TAAATGATATTATTAGAGATTTGTTTATACACACTCTATCAGACAAAGATATAAAAGACATTATAGAATC

CGAAGAAGGTAAGAGATGTTGTATAATAGATGTTAAGAACAATCTTGTTAAAAAGTACTATTCTATTGAT

AATTATCTATGTGATACTTTAGATGATTATATATACACCTCTGTAGAATATAACAAATCCTATGTGTTAG

TAAACGATACTTATATAAGCTATGACTATCTTGAATCATCAGGTGTAGTTGTTCTATCATGTTATGAAAT

GACTATAATCTCCTTGGATACAAAAGACGCCAAAGATGCTATAGAAGATGTGATAGTAGCAAGTGCGGTA

GCCGAAGCATTGAATGACATGTTTAAGGAATTTGATAAAAACGTAAGTGCTATTATAATAAAAGAAGAAG

ATAATTATCTAAACAGTTCGCCCGATATCTACCATATAATATATATCATAGGTGGCACTATTCTGCTACT

GTTAGTCATTATTTTAATATTGGCAATTTATATAGCGCGCAATAAATACAGAACCAGGAAATATGAAATA

ATGAAATATGACAATATGAGCATTAAATCTGAGCATCATGATAGTCTTGAAACAGTGTCTATGGAAATTA

TTGATAATCGGTACTAATAAAATAGTTTAACTCTTTTAGAACCAGTTTGGTACTGTAATTTCAGTTCATT

ACTCGTTGAGAATATTGATGATTTTTTTTAAATGAGTATCGGTAGTTACATATTACCATATCATCCATTA

TATAATCGATGATGCATGTATTAGAATACTTTCCGAATAAGTCTTCTAAATATTGTATTAATTATGAAAA

ACTATGCTATGTGAGTATGATTCAAAGATGTTTAATGATACGATACTAGATTTTATCTCTAGCGAGATTG

TTTAGAATCATTTATCATAACTATGTTTAATAAATTCATCAACGAATATCGATAAAGACCTCTTGTAATT

CGAGTATAGGAAGTAGTATTACCATATCAACTTCCGAGTTAACAATTACTCTAAAACATGAGGATTGTAC

TCCTGTCTTTATTGGAGATCACTATTTAGTCGTTGATAAACTAGTAACCTCAGGTTTCTTTACAAACGAT

AAAGTACAACATCAAGACCTCACAACACAGTGCAAGATTAATCTAGAAATCAAATGTAATTCTGGAGGAG

AATCTAGACAACTAACACCCACGGCGAAGTATACTTTATGCCTCATTCAGAAACGGTAACTGTAGTAGGA

GACTGTCTCTCTAATCTCGATGTATATATAATATATACCAATACGGACGCGATATATTCCGACATGAATG

GCGTCGCTTATCATATGTTATATCCTAAATGTTGATCATATTCCACAAATGATTGTGAACGAGATTAAAT

CATCTAACAAATAATTAGTTTTTTATGACATTAACATATAATAAATAAATTAATCATTATTGACTTAACG

ATGACGAAAGTTATCATTATCTTAGGATTCTTGATTATTAATACAAATTCGTTGTGTCTATGAAATGTGA

ACAAGGTGTCTCATATTATAATGCACAAGAATTAAAGTGTTGTAAACTATCTAGCCAGGAACATATTCAG

ATTATCGATGTGATAAATACAGCGATACCATCTGTGGACATTGTCCAAGTGACACATTCACGTCAATATA

TAATCGTTCTCCTCGGTGTCATAGTTGTAGAGGTCACACCTTGTACACCTACCACAAATAGAATATGTCA

TTGTGACTCGAATAGTTATCGTCTCCTTAAAGCTTCTGATGGTAACTGTGTTACATGTGCTCCTAAAACA

AAATATGGTCGTGTGTACGGAAAGAAAGGAGAAAATGATATGGAATACCATTTGTAAGAAATGTCGGAAG

GGTACTTATTCAGATATTGTATCTGACTCTGATCAATGTAAACCTATGACAAGATAAGACTTACTCGCAT

CTACTGGATAGACATAAATATCCTCCTCGTAATAATGAAATATAATATACACTAATTATTAATATCAATC

GAGTATTAACATATAAGTTATTTTTAAACCCCTTTTGGGTTCCGTCCTAAACGGCGTTTCGGTCTGTGTC

GCCACCATGGTCACACCGAGCCTCTGCGTGCTCCTCCATCGAGGACGACTTCAACTATGACAGCTCGGTG

GCGTCTGCCAGCGTGTACATACGAATGGCATTTCTAAGAAAAGTCTACGGTATCCTTTCTACAATTTCCT

TTAACAACGGCAACAGCTGCAGTATTTTTATACTTTGAATGCATCGGACATTTATACAAGGGAGTCCTGT

TCTAATATTGGCATCAATGTTCGGATCTATAGGCTTGATTTTCGCATTGACTTTACACAGACATGAACAT

CCCCTGAATCTGTACATACTTTGTGGATTTACACTGTTAGAATCTCTAACGCTGGCCTCTGTTGTTACTT

TCTATGATGCACGTATCGTTATGCAAGCTTTCATGTTGACTACTGCAGTGTTTCTTGCTCTGACTACATA

TACTCTACAATCAAAGAGAGATTTCAGTAAACTTGTAACAGGATTGTTTGCTGCTTTCTGGATTTTAATT

TTGTCAGGAGTCTTGAGGATAAAGTTTAAAATAGAATTAATAAAGAACATATAGGTCATTTTTTAAACAT

GGATAGAAACCAAGGTTGTTAGTTAATAATATACAAGATATTTTTTCTCACTCTGATCCATGTAAACCAA

GGACGAGAGACACTCTCATTCCTCATTCACGACACCATTAAAAATGGAAATTAAAGCCCTCTATTAAGCA

CAGACGGCTACAGGTCTACCATCAGGTTACCTTCGTCTACCTTCACAATGGCCTCTCCTTGTGCCCAGTT

CAGTCCCTGTCATTGCCACGCTACTAAGGACTCCCTGAATACCGTGACTGACGTCAGACATTGTCTGACT

GAATACATCCTGTGGGTTTCTCATAGATGGACCCATAGAGAAAGCGCAGGGCCTCTCTACAGGCTTCTCA

TCTCTTTCAGAATTGATGCAATGGAGCTATTTGGTAGCGAGTTGAAGGAGTTCTCGAATTCACTTCCGTG

GGACAATATCGACAATTGCGTGGAGATCATTAAATGTTTCATCAGAAATGACTCCATGAAAACCGCCAAA

GAACTTTGTGCAATAATTGGACTTTGTACTCAATCAGCTATTGTCACTGGAAGAGTCTTCAATGATAAGT

ATATCGACATACTACTTATGCTGCGAAAGATTCTGAACGAGAACGACTATCTCACCCTCTTGGATCATAT

CCTCACTGCTAAATACTAAATCTCCTTCATGCTCTCTCACTAATACTCTTACTCACTACACTTTTTATCA

TCTTATGATGAATGATTGCCTTCATCATTTTTTCGTGGAATATAATATAGGAATAATTAGCACCAGAATA

GCTATGGATATCTCGTTAAGAATATTCTCTATAAGAGACATAATGTAGACATAGTTATTATATCCTTCTT

AGATAAGTGTTACGCTACTGGAAAGTTTCCATCGTTATTATTACATGAAGATGATATAATTAAACCAACA

TTGAGATTGGCTCTTATGTTAGCTGGATTGAATTACTGTAATAAATGCATCGAGTATAGAGGGATATAGC

AATTCTCGATAATAGTCATGCAATATTTGAATGAGACTGATAATTTAGGTAATACAGTACTACACACATA

TCTTTCTAGATTATATATCGTTAAAAATCTGTAAGATGTATATTTCTCATAAGTATCCACTGTGTAATAT

TATTAATGGATATATAGATAACGCAATAGGGACTAATAGTATTGTAAAAGATATAATCGACTATTTACGT

ACATATCCAGATATCTATATTCCTACTAGTTTGCTGCGTAGTTGCATCATTGATATGCATGATTTATCAG

GATTCAGAGATGAATTACTAAGTAAACTACAATCCCACAATAAGTAAGAATCAAATATCAAAAACTCACT

TTTGATTTTTCTAGTCTTAAGTAATACATATATTTATTAATAGACCTATGAAATAAAAAAAGGTAACAAT

GGATTCGCGTATAGCTATTTACGTATTAGTATCGGCATCTCTTTTGTATCTTGTTAATTGTCACAAACTA

GTACATTACTTCAATCTGAAAATAAATGGAAGTGATATAACTAATACAGCAGATATATTGCTGGACAATT

ATCCAATTATGACCTTTGATGGAAAGGATATTTATCCATCTATCTCGTTCATGGTCGGTAATAAACTTTT

CCTAGATCTTTATAAAAATATCTTTGAAGAATTTTTCAGACTATTTCGAGTATCTGTAAGTAGTCAATAC

GAGGAATTAGAATATTATTATTCATGTGATTATACTAACAACCGTCCTACAATTAAACAACATTACTTTT

ATAACGGCGACGAATATACTGAAATTGATAGATCGAAAAAAGCCACTAATAAAAACAGTTGGTTAATTAC

TTCAGGCTTTAGACTACAAAAATGGTTCGATAGCGAAGATTGTATAATTTATCTCAGATCTTTAGTTAGA

AGAATGGAAGACAGTAACAAAAACAGTAAAAAAACTTAGTACTTAGATATCGAAAAAATATATTTTTGTA

GACTCTTGAGAATAGAAGGAAAACATGTACATAATTATAAAAAATGAAAATCAATGGCGAATAAGACAGT

GCGATTCGCACCATGGAGTCGGTAGATTTCATGGCTGTCGATGAGCAGTTTCACGACGACCTCGATCTTT

GGTCATTATCTTTGGTAGATGATTATAAAAAACATGGATTAGGTGTTGACTGTTATGTTCTAGAACCAGT

TGTTGACAGGAAAATATTTGATAGATTTCTCCTTGAACCAATTTGTGATCCTGTAGATGTTCTGTATGAT

TATTTTAGGATTCATAGAGATAATATTGATCAGTATATAGTAGATAGACTGTTTGCATATATTACATATA

AAGATATTATATCTGCATTAGTGTCAAAGAATTATATGGAAGATATTTTCTCTATAATTATTAAGAATTG

TAATTCTGTGCAAGATCTCTTACTTTACTATCTATCTAATGCATATGTAGAAATAGACATTGTTGATCTT

ATGGTAGATCATGGGGCTGTAATATATAAAATAGAATGCTTGAATGCCTATTTTAGGGGAATATGTAAAA

AGGAAAGTAGTGTTGTTGAGTTTATTTTGAATTGTGGTATCCCAGATGAAAATGATGTTAAATTAGATCT

ATATAAAATAATTCAGTATACTAGGGGATTCCTTGTAGATGAACCCACAGTATTAGAAATTTATAAGCTT

TGTATCCCATATATTGAAGATATCAATCAACTAGATGCTGGTGGAAGGACCTTGCTTTATCGCGCTATCT

ATGCAGGTTATATAGATTTAGTATCATGGCTATTAGAAAATGGAGCAAATGTCAACGCAGTAATGAGTAA

TGGATATACATGTCTTGACGTGGCCGTGGATAGGGGATCTGTCATCGCCCGTAGGGAAGCACATCTTAAA

ATATTAGAAATATTGCTTAGAGAACCATTGTCTATTGACTGTATAAAATTAGCTATACTTAATAATACAA

TTGAAAACCATGATGTGATAAAGCTCTGTATCAAGTATTTTATGATGGTAGATTATTCACTTTGTAATGT

GTATGCATCATCACTCTTTGATTATATAATTGATTGTAAACAAGAATTGGAGTACATTAGGCAGATGAAA

ATTCATAATACAACCATGTATGAGTTAATCTATAATAGAGACAAAAACAAGCATGCTTCCCATATTCTAC

ATAGGTATTCTAAACATCCAGTTTTGACACAGTGTATCACTAAAGGATTCAAGATTTACACAGAAGTAAC

CGAGCAGGTCACTAAAGCTCTAAACAGACGTGCTCTAATAGATGAGATAATAAACAATGTATCAACTGAT

GACAATCTCCTATCAAAACTTCCATTAGAAATTAGGGATCTAATTGTTTCACAAGCTGTCATATAGAGTT

CTATCCACCCACCTTTCTTGAAATGAGTTAATAGTCATAAGTTAGTTAAGTCATAAGTTAGTTAAGTCAT

AAGTTAGTTAAGTCATAAGTTAGTTAAGTCATAAGTTAGTTAAGTCATAAGTTAGTTAAGTCATAAGTTA

GTTAAGTCATAAGTTAGTTTATAGTCTAACACTTCTAATTTTTATACCTTGATCTTTTTCTCTAATTATG

AAAAAGTAAATCATTATGAAGATGGATGAAATGGACGAGATTGTGCGCATCGTTAACGATAGTATGTGGT

ACGTACCTAACGCATTTATGGACGACGGTGATAATGAAGGTCACATTTCTGTCAATAATGTCTGTCATAT

GTATCTCGCATTCTTTGATGTGGATATATCATCTCATCTGTTTAAATTAGTTATTAAACACTGCGATCTG

AATAAACGACTAAAATGTGGTAACTCTCCATTACATTGCTATACGATGAATACACGATTTAATCCATCTG

TATTAAAGATATTGTTACGCCACGGCATGCGTAACTTTGATAGCAAGGATAAAAAAGGACATATTCCTCT

ACACCACTATCTGATTCATTCACTATCAATCGATAACAAGATCTTTGATATACTAACGGACCCCATTGAT

GACTTTAGTAAATCATCCGATCTATTGCTGTGTTATCTTAGATATAAATTCAATGGGAGCTTAAACTATT

ACGTTCTGTACAAATTATTGACTAAAGGATCTGACCCTAATTGCGTCGATGAGGATGGACTCACTTCTCT

TCATTACTACTGTAAACACATATCCGCGTTCCACGAAAGCAATTATTACAAGTCAAAGAGTCACACTAAG

ATGCGAGCTGAGAAGCGATTCATCTACGCGATAATAGATCATGGAGCAAACATTAACGCGGTTACGAAAA

TCGGAAATACGCCGTTACACACTTACCTTCAACAGTATACCAAACATAGTCCTCGTGTGGTGTATGCTCT

TTTATCTCGAGGAGCCGATACGAGGATACGTAATAATCTTAATTGTACACCCATCATGGAATACATAAAG

AACGATTGTGCAACAGGTCATATTCTCATAATGTTACTCAATTGGCACGAACAAAAATACGGGAAATTAC

AAAAGGAAGAAGGACAACATCTACTTTATCTATTCATAAAACATAATCAAGGATATGGAAGTCGCTCTCT

CAATATACTACGGTATCTACTAGATAGATTCGACATTCAGAAAGACGAATACTATAATACAATGACTCCT

CTTCATACCGCCTTCCAGAATTGCAATAACAATGTTGCCTCATACCTCGTATACATTGGATACGACATCA

ACCTTCCGACTAAAGACGATAAGACAGTATTCGACTTGGTGTTTGAAAACAGAAACATTATATACAAGGC

GGATGTCGTTAATGACATTATCCACCACAGACTGAAAGTATCTCTACCTATGATTAAATCGTTGTTCTAC

AAGATGTCGGAGTTCTCTCCCTACGACGATCACTACGTAAAGAAGATAATAGCCTACTGCCTATTAAGGG

ACGAGTCATTTGCGGAACTACATACTAAATTCTGTTTAAACGAGGACTATAAAAGTGTATTTATGAAAAA

TATATCATTCGATAAGATAGATTCCATCATCGAAAAATGTAGTCGTGACATAAGTCTCCTCAAAGAGATT

CGAATCTCAGACACCGACTTGTATACGGTATTGAGAACAGAAGACATTCGGTATCACACATATCTCGAAG

CCATACATTCAGACAAACGCATTTCATTTCCCATGTACGACGATCTCATAGAACAGTGTCATCTATCGAT

GGAGCATAAAAGTAAACTCGTCGACAAAGCACTCAATAAATTAGAGTCTACCATCGATAGTCAATCTAGA

CTATCGTATTTGCCTCCGGAAATTATGCGCAATATCATAACCAAGCTAAGCGACTACCATCTAAACAGTA

TGTTGTACGGAAAGAACCATTACAAATATTATCCATGATAGAAAGAAAATATTTAAAAAATAATCTATAT

GATTGGAGAAGTAGGAAACAAACAGTAACAAGACGACGATTACTACATTATTAAATCATGAGGTCCGTAT

TATACTCGTATATATTGTTTCTCTCATGTATAATAATAAACGGAAGAGATATAGCACCACATGCACCATC

CAATGGAAAGTGTAAAGACAACGAATACAGAAGCCGTAATCTATGTTGTCTATCGTGTCCTCCGGGAACT

TACGCTTTCAGATTATGTGATAGCAAGACTAATACACAATGTACACCGTGTGGTTCGGATACCTTTACAT

CTCACAATAATCATTTACAGGCTTGTCTAAGTTGTAACGGAAGATGTGATAGTAATCAGGTAGAGACGCG

ATCGTGTAACACGACTCACAATAGAATCTGTGAATGCTCTCCAGGATATTATTGTCTTCTCAAAGGAGCA

TCAGGGTGTAGAACATGTATTTCTAAAACAAAGTGTGGAATAGGATACGGAGTATCCGGATACACGTCTA

CCGGAGACGTCATCTGTTCTCCGTGTGGTCCCGGAACATATTCTCACACCGTCTCTTCCACAGATAAATG

CGAACCCGTCGTAACCAGCAATACATTTAACTATATCGATGTGGAAATTAACCTGTATCCAGTCAACGAC

ACATCGTGTACTCGGACGACCACTACCGGTCTCAGCGAATCCATCTCAACGTCGGAACTAACTATTACCA

TGAATCATAAAGATTGTGATCCAGTCTTTCGTGCAGAATACTTCTCTGTCCTTAATAATGTAGCAACTTC

AGGATTCTTTACAGGAGAAAATAGATATCAGAATACTTCAAAGATATGTACTCTGAATTTCGAGATTAAA

TGTAACAACAAAGATTCATCTTCCAAACAGTTAACGAAAACAAAGAATGATACTATCATGCCGCATTCAG

AGACGGTAACTCTAGTGGGCGACTGTCTATCTAGCGTCGACATCTACATACTATATAGTAATACCAATAC

TCAAGACTACGAAAATGATACAATCTCTTATCATATGGGTAATGTTCTCGATGTCAATAGCCATATGCCC

GCTAGTTGCGATATACATAAACTGATCACTAATTCCCAGAATCCCACCCACTTATAGTAAGTTTTTTTAC

CTATAAATAATAAATACAATAATTAATTTCTCGTAAAAGTAGAAAATATATTCTAATTTATTATATGGTA

AGAAAGTAGAATCATCTAGAACAGTAATCAATCAATAGCAATCATGAAACAATATATTGTCCTGGCATGC

ATGTGCCTAGTGGCAGCTGCTATGCCTACTAGTCTTCAACAATCTTCATCCTCGTGTACTGAAGAAGAAA

ACAAACATCATATGGGAATCGATGTTATTATCAAAGTCACAAAGCAAGACCAAACACCGACCAATGATAA

GATTTGTCAATCCGTAACGGAAGTTACAGAGACCGAAGATGATGAGGTATCCGAAGAAGTTGTAAAAGGA

GATCCCACCACTTATTACACTATCGTCGGTGCGGGTCTTAACATGAACTTTGGATTCACCAAATGCCCAA

AGATTTTATCCATCTCCGAATCCTCTGATGGAAACACTGTGAATACTAGATTGTCCAGCGTGTCACCGGG

ACAAGGTAAGGACTCTCCCGCGATCACGCGTGAAGAAGCTCTGGCTATGATCAAAGACTGTGAGATGTCT

ATCGACATCAGATGTAGCGAAGAAGAGAAAGACAGTGACATCAAGACCCATCCAGTACTTGGGTCTAACA

TCTCACATAAGAAAGTGAGTTACAAAGATATCATCGGTTCAACGATCGTTGATACAAAATGTGTCAAGAA

CCTAGAGTTTAGCGTACGTATCGGAGACATGTGTGAGGAATCATCTGAACTTGAAGTCAAGGATGGATTC

AAGTATGTCGACGGATCGGCATCTGAAGGTGCAACCGATGATACTTCACTCATCGATTCAACAAAACTCA

AAGCATGTGTCTGAATCGATAACTCTATTCATCTGAAAATGGATGAGTTGGGTTAATCGAACGATTCAGA

CACCGCACCACGAATTAAAAAAGACCGGGCACTATATTCCGGTTTGCAAAACAAAAATATTTAACTACAT

TCACAAAAAGTTACCTCTCGTTACTTCTTCTTTCTGTTTCAATATGTGATACGATATGATCACTATTCGT

ATTCTCTTGGTCTCATAAAAAAGTTTTACAAAAAAAAAAAAAATATTTTTATTCTCTTTCTCTCTTCGAT

GGTCTCACAAAAATATTAAACCTCTTTCTGATGTCTCAACTATTTCGTAAACGATAACGTCCAACAATAT

ATTCTCGTAGAGCTTATCAACATCCTTATACCAATCTAGGTTGTCAGACAATTGCATCATAAAATAATGT

TTATAATTTACACGTTAACATCATATAATAAACGTATATAGTTAATATTTTTGGAATATAAATGATCTGT

AAAATCCATGTAGGGGACACTGCTCACGTTTTTTCTCTAGTACATAATTTCACACAAGTTTTTATACAGA

CAAATTAATTCTCGTCCATATATTTTAAAACATTGACTTTTGTACTAAGAAAAATATCTTGACTAACCAT

CTCTTTCTCTCTTCGATGGGTCTCACAAAAATATTAAACCTCTTTCTGATGGAGTCGTAAAAAGTTTTTA

TCCTTTCTCTCTTCGATAGGTCTCACAAAAATATTAAACCTCTTTCTGATGGTCTCTATAAACGATTGAT

TTTTCTTACCCTCTAGAGTTTCCTACGGTCGTGGGTCACACATTTTTTTCTAGACACTAAATAAAATAGT

AAAAT

TAAATTAATTATAAAATTATGTATATAATTTACTAAC
