# Supplementary material for: Exploring the key genomic variation in monkeypox virus during the 2022 outbreak
Source: BMC Genom Data. 2023 Nov 16;24:67. doi: 10.1186/s12863-023-01171-0 (PMC10652487; doi:10.1186/s12863-023-01171-0)
Supplement: Supplementary file 13 — Additional file 13. Full length sequence of RS10. [file 12863_2023_1171_MOESM13_ESM.docx]

**Additional file13. Full length sequence of RS10.**

gttagtaaattatatacataattttaTAATTAATTTAATTTTACTATTTTATTTAGTGTCTAGAAAAAAATGTGTGACCCACGACCGTAGGAAACTCTAGAGGGTAAGAAAAATCAATCGTTTATAGAGACCATCAGAAAGAGGTTTAATATTTTTGTGAGACCTATCGAAGAGAGAAAGGATAAAAACTTTTTACGACTCCATCAGAAAGAGGTTTAATATTTTTGTGAGACCCATCGAAGAGAGAAAGAGATGGTTAGTCAAGATATTTTTCTTAGTACAAAAGTCAATGTTTTAAAATATATGGACGAGAATTAATTTGTCTGTATAAAAACTTGTGTGAAATTATGTACTAGAGAAAAAACGTGAGCAGTGTCCCCTACATGGATTTTACAGATCATTTATATTCCAAAAATATTAACTATATACGTTTATTATATGATGTTAACGTGTAAATTATAAACATTATTTTATGATGCAATTGTCTGACAACCTAGATTGGTATAAGGATGTTGATAAGCTCTACGAGAATATATTGTTGGACGTTATCGTTTACGAAATAGTTGAGACATCAGAAAGAGGTTTAATATTTTTGTGAGACCATCGAAGAGAGAAAGAGAATAAAAATATTTTTTTTTTTTTTGTAAAACTTTTTT

ATGAGACCAAGAGAATACGAATAGTGATCATATCGTATCACATATTGAAACAGAAAGAAGAAGTAACGAG

AGGTAACTTTTTGTGAATGTAGTTAAATATTTTTGTTTTGCAAACCGGAATATAGTGCCCGGTCTTTTTT

AATTCGTGGTGCGGTGTCTGAATCGTTCGATTAACCCAACTCATCCATTTTCAGATGAATAGAGTTATCG

ATTCAGACACATGCTTTGAGTTTTGTTGAATCGATGAGTGAAGTATCATCGGTTGCACCTTCAGATGCCG

ATCCGTCGACATACTTGAATCCATCCTTGACTTCAAGTTCAGATGATTCCTCACACATGTCTCCGATACG

TACGCTAAACTCTAGGTTCTTGACACATTTTGTATCAACGATCGTTGAACCGATGATATCTTTGTAACTC

ACTTTCTTATGTGAGATGTTAGACCCAAGTACTGGATGGGTCTTGATGTCACTGTCTTTCTCTTCTTCGC

TACATCTGATGTCGATAGACATCTCACAGTCTTTGATCATAGCCAGAGCTTCTTCACGCGTGATCGCGGG

AGAGTCCTTACCTTGTCCCGGTGACACGCTGGACAATCTAGTATTCACAGTGTTTCCATCAGAGGATTCG

GAGATGGATAAAATCTTTGGGCATTTGGTGAATCCAAAGTTCATGTTAAGACCCGCACCGACGATAGTGT

AATAAGTGGTGGGATCTCCTTTTACAACTTCTTCGGATACCTCATCATCTTCGGTCTCTGTAACTTCCGT

TACGGATTGACAAATCTTATCATTGGTCGGTGTTTGGTCTTGCTTTGTGACTTTGATAATAACATCGATT

CCCATATGATGTTTGTTTTCTTCTTCAGTACACGAGGATGAAGATTGTTGAAGACTAGTAGGCATAGCAG

CTGCCACTAGGCACATGCATGCCAGGACAATATATTGTTTCATGATTGCTATTGATTGATTACTGTTCTA

GATGATTCTACTTTCTTACCATATAATAAATTAGAATATATTTTCTACTTTTACGAGAAATTAATTATTG

TATTTATTATTTATAGGTAAAAAAACTTACTATAAGTGGGTGGGATTCTGGGAATTAGTGATCAGTTTAT

GTATATCGCAACTAGCGGGCATATGGCTATTGACATCGAGAACATTACCCATATGATAAGAGATTGTATC

ATTTTCGTAGTCTTGAGTATTGGTATTACTATATAGTATGTAGATGTCGACGCTAGATAGACAGTCGCCC

ACTAGAGTTACCGTCTCTGAATGCGGCATGATAGTATCATTCTTTGTTTTCGTTAACTGTTTGGAAGATG

AATCTTTGTTGTTACATTTAATCTCGAAATTCAGAGTACATATCTTTGAAGTATTCTGATATCTATTTTC

TCCTGTAAAGAATCCTGAAGTTGCTACATTATTAAGGACAGAGAAGTATTCTGCACGAAAGACTGGATCA

CAATCTTTATGATTCATGGTAATAGTTAGTTCCGACGTTGAGATGGATTCGCTGAGACCGGTAGTGGTCG

TCCGAGTACACGATGTGTCGTTGACTGGATACAGGTTAATTTCCACATCGATATAGTTAAATGTATTGCT

GGTTACGACGGGTTCGCATTTATCTGTGGAAGAGACGGTGTGAGAATATGTTCCGGGACCACACGGAGAA

CAGATGACGTCTCCGGTAGACGTGTATCCGGATACTCCGTATCCTATTCCACACTTTGTTTTAGAAATAC

ATGTTCTACACCCTGATGCTCCTTTGAGAAGACAATAATATCCTGGAGAGCATTCACAGATTCTATTGTG

AGTCGTGTTACACGATCGCGTCTCTACCTGATTACTATCACATCTTCCGTTACAACTTAGACAAGCCTGT

AAATGATTATTGTGAGATGTAAAGGTATCCGAACCACACGGTGTACATTGTGTATTAGTCTTGCTATCAC

ATAATCTGAAAGCGTAAGTTCCCGGAGGACACGATAGACAACATAGATTACGGCTTCTGTATTCGTTGTC

TTTACACTTTCCATTGGATGGTGCATGTGGTGCTATATCTCTTCCGTTTATTATTATACATGAGAGAAAC

AATATATACGAGTATAATACGGACCTCATGATTTAATAATGTAGTAATCGTCGTCTTGTTACTGTTTGTT

TCCTACTTCTCCAATCATATAGATTATTTTTTAAATATTTTCTTTCTATCATGGATAATATTTGTAATGG

TTCTTTCCGTACAACATACTGTTTAGATGGTAGTCGCTTAGCTTGGTTATGATATTGCGCATAATTTCCG

GAGGCAAATACGATAGTCTAGATTGACTATCGATGGTAGACTCTAATTTATTGAGTGCTTTGTCGACGAG

TTTACTTTTATGCTCCATCGATAGATGACACTGTTCTATGAGATCGTCGTACATGGGAAATGAAATGCGT

TTGTCTGAATGTATGGCTTCGAGATATGTGTGATACCGAATGTCTTCTGTTCTCAATACCGTATACAAGT

CGGTGTCTGAGATTCGAATCTCTTTGAGGAGACTTATGTCACGACTACATTTTTCGATGATGGAATCTAT

CTTATCGAATGATATATTTTTCATAAATACACTTTTATAGTCCTCGTTTAAACAGAATTTAGTATGTAGT

TCCGCAAATGACTCGTCCCTTAATAGGCAGTAGGCTATTATCTTCTTTACGTAGTGATCGTCGTAGGGAG

AGAACTCCGACATCTTGTAGAACAACGATTTAATCATAGGTAGAGATACTTTCAGTCTGTGGTGGATAAT

GTCATTAACGACATCCGCCTTGTATATAATGTTTCTGTTTTCAAACACCAAGTCGAATACTGTCTTATCG

TCTTTAGTCGGAAGGTTGATGTCGTATCCAATGTATACGAGGTATGAGGCAACATTGTTATTGCAATTCT

GGAAGGCGGTATGAAGAGGAGTCATTGTATTATAGTATTCGTCTTTCTGAATGTCGAATCTATCTAGTAG

ATACCGTAGTATATTGAGAGAGCGACTTCCATATCCTTGATTATGTTTTATGAATAGATAAAGTAGATGT

TGTCCTTCTTCCTTTTGTAATTTCCCGTATTTTTGTTCGTGCCAATTGAGTAACATTATGAGAATATGAC

CTGTTGCACAATCGTTCTTTATGTATTCCATGATGGGTGTACAATTAAGATTATTACGTATCCTCGTATC

GGCTCCTCGAGATAAAAGAGCATACACCACACGAGGACTATGTTTGGTATACTGTTGAAGGTAAGTGTGT

AACGGCGTATTTCCGATTTTCGTAACCGCGTTAATGTTTGCTCCATGATCTATTATCGCGTAGATGAATC

GCTTCTCAGCTCGCATCTTAGTGTGACTCTTTGACTTGTAATAATTGCTTTCGTGGAACGCGGATATGTG

TTTACAGTAGTAATGAAGAGAAGTGAGTCCATCCTCATCGACGCAATTAGGGTCAGATCCTTTAGTCAAT

AATTTGTACAGAACGTAATAGTTTAAGCTCCCATTGAATTTATATCTAAGATAACACAGCAATAGATCGG

ATGATTTACTAAAGTCATCAATGGGGTCCGTTAGTATATCAAAGATCTTGTTATCGATTGATAGTGAATG

AATCAGATAGTGGTGTAGAGGAATATGTCCTTTTTTATCCTTGCTATCAAAGTTACGCATGCCGTGGCGT

AACAATATCTTTAATACAGATGGATTAAATCGTGTATTCATCGTATAGCAATGTAATGGAGAGTTACCAC

ATTTTAGTCGTTTATTCAGATCGCAGTGTTTAATAACTAATTTAAACAGATGAGATGATATATCCACATC

AAAGAATGCGAGATACATATGACAGACATTATTGACAGAAATGTGACCTTCATTATCACCGTCGTCCATA

AATGCGTTAGGTACGTACCACATACTATCGTTAACGATGCGCACAATCTCGTCCATTTCATCCATCTTCA

TAATGATTTACTTTTTCATAATTAGAGAAAAAGATCAAGGTATAAAAATTAGAAGTGTTAGACTATAAAC

TAACTTATGACTTAACTAACTTATGACTTAACTAACTTATGACTTAACTAACTTATGACTTAACTAACTT

ATGACTTAACTAACTTATGACTTAACTAACTTATGACTTAACTAACTTATGACTATTAACTCATTTCAAG

AAAGGTGGGTGGATAGAACTCTATATGACAGCTTGTGAAACAATTAGATCCCTAATTTCTAATGGAAGTT

TTGATAGGAGATTGTCATCAGTTGATACATTGTTTATTATCTCATCTATTAGAGCACGTCTGTTTAGAGC

TTTAGTGACCTGCTCGGTTACTTCTGTGTAAATCTTGAATCCTTTAGTGATACACTGTGTCAAAACTGGA

TGTTTAGAATACCTATGTAGAATATGGGAAGCATGCTTGTTTTTGTCTCTATTATAGATTAACTCATACA

TGGTTGTATTATGAATTTTCATCTGCCTAATGTACTCCAATTCTTGTTTACAATCAATTATATAATCAAA

GAGTGATGATGCATACACATTACAAAGTGAATAATCTACCATCATAAAATACTTGATACAGAGCTTTATC

ACATCATGGTTTTCAATTGTATTATTAAGTATAGCTAATTTTATACAGTCAATAGACAATGGTTCTCTAA

GCAATATTTCTAATATTTTAAGATGTGCTTCCCTACGGGCGATGACAGATCCCCTATCCACGGCCACGTC

AAGACATGTATATCCATTACTCATTACTGCGTTGACATTTGCTCCATTTTCTAATAGCCATGATACTAAA

TCTATATAACCTGCATAGATAGCGCGATAAAGCAAGGTCCTTCCACCAGCATCTAGTTGATTGATATCTT

CAATATATGGGATACAAAGCTTATAAATTTCTAATACTGTGGGTTCATCTACAAGGAATCCCCTAGTATA

CTGAATTATTTTATATAGATCTAATTTAACATCATTTTCATCTGGGATACCACAATTCAAAATAAACTCA

ACAACACTACTTTCCTTTTTACATATTCCCCTAAAATAGGCATTCAAGCATTCTATTTTATATATTACAG

CCCCATGATCTACCATAAGATCAACAATGTCTATTTCTACATATGCATTAGATAGATAGTAAAGTAAGAG

ATCTTGCACAGAATTACAATTCTTAATAATTATAGAGAAAATATCTTCCATATAATTCTTTGACACTAAT

GCAGATATAATATCTTTATATGTAATATATGCAAACAGTCTATCTACTATATACTGATCAATATTATCTC

TATGAATCCTAAAATAATCATACAGAACATCTACAGGATCACAAATTGGTTCAAGGAGAAATCTATCAAA

TATTTTCCTGTCAACAACTGGTTCTAGAACATAACAGTCAACACCTAATCCATGTTTTTTATAATCATCT

ACCAAAGATAATGACCAAAGATCGAGGTCGTCGTGAAACTGCTCATCGACAGCCATGAAATCTACCGACT

CCATGGTGCGAATCGCACTGTCTTATTCGCCATTGATTTTCATTTTTTATAATTATGTACATGTTTTCCT

TCTATTCTCAAGAGTCTACAAAAATATATTTTTTCGATATCTAAGTACTAAGTTTTTTTACTGTTTTTGT

TACTGTCTTCCATTCTTCTAACTAAAGATCTGAGATAAATTATACAATCTTCGCTATCGAACCATTTTTG

TAGTCTAAAGCCTGAAGTAATTAACCAACTGTTTTTATTAGTGGCTTTTTTCGATCTATCCTCGTCCTCT

CATCATCCTTATATTATTATCATTATTATCATAGTCTATTAAACACAAATCATCTACGTTTATAACAACA

TTCTCATTATTAATTAGTTCTGTAGAATATCTTTAATAATTTGGCTATACATCTGTTCAATACTATCTAT

TGATGATTTCTTTTTTAAGACTTAAACTAGTTATGGTAATGACGATGAAATCGAGTAGTAACTTCTAATA

AAGACTTGATATCATTATCATATGTTTGATCGTCATAGTTAATAGTGTGGCTAAATGGTACTGTTAATAA

GTTTATAGACAATATCATAGTATTTTCTTTCCAGAATTAGATTATTTTTTTAAATACTGATCCTCACAAT

TCCGTGATGTAGCAGTAGTTGGTGCATGGTCTATATCGTTAAAATGTATCATATATAATAGTTTTCTGAC

GTGGAGTACAGAATTTTCGATTAATGAGTTCATGGTAAGGAAGGGCAAATGCCTGTATATAATATACATA

AGTTAATAGTTTTTTATCATATTTTCTAATACCATAATAAAAATTATCATTATTGCGTTTGGTAGTTCTG

CCCTATCATCTATATCACTGTCACTCTCGCTCTCACTATATCTTCTAAAATTACAAACAACTGGATATTC

GATAACAGCATTTGTGTAGTTTTTGTCTTTTACAGTATATACGTTATTGTCAAAATCTAAACAAATATTA

GCATAATACATCTATCTATAAGATCAGGATCCATGTTCGAGCATACTAGCCATGTATATTTGTAACTTCG

TCGTACAGCGTTAGATCAATAGAATAAACAATCGTGTGACGCAACTTTTTTACGATCTAGTTGTATGAGT

TTATCGTTTACATAAGCAATTAACGGCTTTAACAGATGATCTGAGTAATAATATACCTCTGTTATACGTT

TAATGTTCACGGTCTTAGTATTTTTAGATATCAATTGTGATTTACACCATATTCGACTCCCTTGTGTGTA

ACGTTAGAAATTCTAAATCTATATTATCTATTACAGCGTAAAACACATTCAATATTGTATTGTTATTTTT

ATATTATTTACACAATTAACAATGTATTATTAGTTTATATTACTGAATTAATAATATAAAATTCCCAATC

TTGTCATAAACACAAAATCCATTAAAAATGTCGATAAAATATCTGATGTTGTTGTTCGCTGCTATGATAA

TCAGATCATTAGCCGATAGTGGTAATGCTATCGAAACAACATCGCCAGAAATTACAAACGCTACAACAGA

TATTCCAGCTATCAGATTATGTGGTCCAGAGGGAGATGGATATTGTTTACACGGTGACTGTATCCACGCT

AGAGATATTGACGGTATGTATTGTAGATGCTCTCATGGTTATACAGGTATTAGATGTCAGCATGTAGTAT

TAGTAGACTATCAACGTTCAGGAAAACCAGACACTACAACATCATATATCCCATCTCTAGGTATTGTGCT

TGTATTAGTAGGCATTATTATTACGTGTTGTCTATTATCTGTTTATATGTTCACTCGAAGAACTAAACTA

CCTATACAAGATATGGTTGTGTTATATTTTTTATAAAATTTTTTTATGAGTATTTTTACAAAAATGTATA

TGTATAAAAAAAATACTAAGTATACGATGTATCCTGTATTATTTGTATTTATCTAAACAATACTTCTGCC

TCTAGATGGGATACAAAAATTTTTTATTTCAGCATATTAAAGTAAAATCTAGTTACCTTGAAAATGAATA

CAGTGGGTGGTTCCGTATCACCAGTAAGAACATAATAGTCGAATACAGTATCCGATTGAGATTTTGCATA

CAATACTAGTCTAGAAAGAAATTTGTAATCATCCTCTGTGACAGGAGTCCATATATCTGTATCATCGTCT

AGTTTTTATCAGTGTCCTATGCTATATTCCTGTTATCATCATTAGTTAATGAAAATAACTCTCGTGCTTC

AGAAAAGTCAAATATTGTATCCATACATATATCTCCAAAACTATCACTTATACGTTTATCTTTAACGAAC

ATATACCTAGATGGTTATTTACTAACAGACATTTTTTCAAGATCTATTGACAATAACTCCTATAGTTTCC

ACATCAACCAAGTAATGATCATCTATTGTTATATAACAATAACATAACTCTTTTCCATTTTTATCAGTAT

CTATATCAACGTCGTTGTAGTGAATAGTAGTCATTGATCTATTATATGAAACGGATATGTCTAGTTAATA

TTTTCTTTGATTTAAAGTCTATAGTCTTTACAAACATAATATCCTTATCCGACTTTATATTTCCTGTAGG

GTGGCATAATTTTATTCTGCCTCCACAATCAGTGTTTCCAAATATATTACTAGACAATATTCCATATAGT

TATTAGTTAAGGGACCCAATTAGAACACGTACGCGCTTATTCATCATTTGGATCGTATTTCATAAAAGTT

ATTATGTTATAGATGTCAACACATTCTACATTTTTTTAATTGTCTATATAGTATTTTTCTGATATTTTCT

ATAATATCAGAATTGTCTTCCATAGGAAGTTGTATACTATTCGGAATCAGTTACATGTTTAAATAATTCT

CTGATGTCATTCCTTATACAATCAAATTCATTATTAAACAGTTTAATAGTCTGTAGACCTTTATCGTCGT

ACATATCCATTGTCTTATTAGTTACGCTTATTTTTATGGGTTTTACGTTGCTTTATTATATTTTATAATA

ATGATTGTTTGACAATGTCGTAGTATAGATATATTATTAGAGGAGGTATAATTATAAAAAGTTTCTGAGT

ACGATGTTATAAGAGGAGAGGACACATTAACATCATACATCAATTAACTACATTCTTATAACATTGTAAT

CAAAAGAATTGCAATTTTGATGTATAACAACTGTCAATGGAATTGTATATTACAAATTACGGTATGTTGT

AACAACAAATACCGATCGGTAATTGTCTCTGCCGCTGTAATAGAATTAATTATATATCTATTACACCGGC

CTTGTATCATAATAAAGTTGTGGTAGTATGATCTCCATATTTATAATTTAGTACTTTGTATTTAGTTTTT

TTGGAATCATAAAAAAAGTTTTACTAATTTAAAATTTAAAAAGTATTTACATTTTTCACTGTTTAGTCGC

GGATATGGAATTCGATCCTGTCAAAATCAATACATCATCTATAGATCATGTAACAATATTACAATACATA

GATGAACCAAATGATATAAGACTAACAGTATGCATTATCCGAAATGTTAATAACATTACATATTATATCA

ATATCACAAAAATAAATCCACATTTGGCTAATCGATTTCGGGCTTGGAAAAAACGTATCGCCGGAAGGGA

CTATATGACTAACTTATCTAGAGATACAGGAATACAACAATCAAAACTTACTGAAACTATACGTAACTGT

CAAAAAAATAAAAACATATATGGTCTATATATACACTACAATTTAGTTATTAATGTGGTTATTGATTGGA

TAACCGATGTGATTGTTCAATCAATATTAAGAGGGTTGGTAAATTGGTACATAGCTAATAATACATATAC

TCCAAATACACCCAATAATACTACAACCATTTCTGAGTTGGATATCATCAAAATACTGGATAAATACGAG

GACATGTATAGAGTAAGTAAAGAAAAAGAATGTGGAATTTGCTATGAAGTTGTTTACTCAAAACGATTAG

AAAACGATAGATACTTTGGTTTATTGGATTCGTGTAATCATATATTTTGCATAACATGTATCAATATATG

GCATAGAACACGAAGAGAAACCGGTGCGTCAGATAATTGTCCTATATGCCGTACACGTTTTAAAAAAATA

ACAATGAGCAAGTTCTATAAGCTAGTTAACTAATAAATAAAAAGTTTAATTATCGACGATATATGTCGTT

ATTTTTCTCTCATATGAAAGATTAATTTGATTCTAATATAATCTTCAGTATTGGATGAATCTCAATTCAA

ATTAATTCCATTAGATTAGATTAGATTAGATCATAAATAAAAATAGTAGCACGCACTACTTCAGCCAAAT

ATTCTTTTTTGAAACGCCATCTAGCGTAATGAGAACACAAGTGAACCTATAATGAGCAAATTTATTAGTA

TCGGTTACATGAAGGACTTTACGTAGAGTGGTGATTCCTCCATCTGTGGTACGAACGGTTTCATCTTCTT

TGATGCCACCACCCAGATGTTCTATAAACTTGGTATCCTCGTCCGATTTCATATCATTTGCCAACCAATA

CATATAGCTAAACCCAGGCATACGTTCCACACATCCGGAACAATGAAATTCTCCAGAAGATGTTACAATG

GCTAGATTTGGACATTTGGTTTCAACCGCGTTAACATATGAGTGAACACACCCATACATGAAAGCGATGA

GAAATAGGATTCTCATCTTGCCAAAATATCACTATAAAAAATTTATTTATCAATTTTAAAGGTATAAAAA

AATACTTATTGTTGCTCGAATATTTTGTATTTGATGGTATACGGAAGATTAGAAATGTAGGTATTATCAT

CAACTGATTTTATGATGGTTTTATGAATTTTATTATGCTTCACTATTGCATCGGAAATAATATCATATGC

TTCCACGTATATTTTATTTTGTTTTGACTCATAATACGCACGTAATTCTGGATTATTGGCATATCGATGA

ATAATTTTAGCTCCATGCTCAGTAAATATTAATGAGAACATAGTGTTGCCTCCTACCATTATTTTTTTCA

TCTCATTCAATTCTTGATTGCAGAGATCTATATAATCATTATAGCGTTGACTTATGGACTCTGGAATCTT

AGACGATGTACAGTCATCTATAATCATGGTATATTTAATACATTGTTTTATAGCATAGGCATTATCTACG

ATATTAGATACTTCACTCAATGAATCAATCACACAATCTAATGTAGGTTTATGACATAATAGCATTTTCA

GCAGTTCAATGTTTCTAGATTCGTTGATGGCAATGGTTATACATGTATATCCGTTATTTGATCTAATGTT

GACATCTGAACCGGATTCTAGCAGTAAAGATACTAGAGATTGTTTATTATATCTAACAGCCTTGTGAAGA

AGTGTTTCTCCTCGTTTGTCAATCATGTTAATGTCTTTAAGATAAGGTAGACAAATGTTTATAGTACTAA

GAATTGGGCAAACATAAGACATGTCACAAAGACCCTTTTTGTATGTATAAGTGTAGAAATTATAACATCC

ATAGTTGGATTCACATAGGTGTCCAATCGGGATCTCTCCATCATCGAGATGATTGACGGCATCTCCCCCT

TCCTTTTTTAGTAGATATTTCATCGTGTAAGAATCAATATTAATATTTCTAAAGTATCTGTGTATAGCCT

CTTTATTTACCACAGCTCCATATTCCAACATGCATTCCACTAGAGGGATATCGATATCGCCGAATGTCAT

ATACTCAATTAGTATATGTTGGAGGACATCCGAGTTCATTGTTTTCAATATCAAAGAGATGGTTTCCTTA

TCATTTCTCCATAGTGGTACAATACTACACATTATTCCGTACGGCTTTCCATTCTCCAAAAACAATTTTA

CCAAATCTAAATCTACATCTTTATTGTATCTATAATCACTATTTAGATAATCAGCCATAATTCCTCGAGT

GCAACATGTTAGATCGTCTATATATGAATAAGCCGTGTTATCTATTCCTTTCATTAACAATTTAACGATG

TCTATATCTATATGAGATGACTTAATATAATATTGAAGAGCTGTACAATAGTTTTTATCTATAGAAGACG

GCTTGATTCCGTGATTAATTAGACATTTAACAACTTCCGGACGCACATATGCTCTCGTATCCGACTCTGA

ATACAGATGAGCGATGATATACAGATGCAATACGGTACCACAATTTCGTGGTTGATAATCATCATACACG

TATCCGTACTCGTCATCCTCATAAAGAACACTGCATCCATTTTCTATGAACAAATCAATAATTTCAGGAA

CAGGATCATCTGTCATTACATAATTTTCTATAACTGAACGATGGTTTTCACATTTAACACTCAAGTCAAA

TCCATGTTCTACCAACACCTTTATCAAGTCAACGTCTACATTTTTTGATTTCATATAGCTGAATATATTA

AAGTCATTTATGTTGCTATATCCAGTAGCTTCTAGTAGAGCCATCGCTATATCCTTATTGACTTTAACAT

GTCTACTATTTGTGTATTCTTCTATTGGGGTAAACTGTCTCCAATTTTTATGTAATGGATTAGTGCCACT

ATCTAGTAGTAGTTTGACGACCTCAACATTATTACAATGCTCATTGAAAAGGTATGCGTGTAAAGCATTA

TTCTTGAATTGGTTCCTGGTATCATTAGGATCTCTGTCTCTCAACATCTGTTTAAGTTCATCGAGAACCA

CCTCCTCATTTTCCAGATAGTCAAACATTTTGACTGAATAGAAGTGAATGAGCTACTGTGAACTCTATAC

ACCCGCACAACTAATGTCATTAAATATCATTTTTGAATGTATTTATACCATGTCAAAAACTTGTACAATT

ATTAATAAAAATAATTAGTGTTTAAATTTTACCAGTTCCAGATTTTACACCTCCGTTAACACCTCCATTA

ACCCCACTTTTTACACCACTGGACGATCCTCCTCCCCACATTCCACTGCCACTAGATGTATAAGTTTTAG

ATCCTTTATTACTACCATCATGTCCATGGATAAAGACACTCCACATGCCGCCACTACTACCCCCTTTAGA

CGACATATTAATAAGACAAGTTTAACAATAAAATTAATCACGAGTACCCTACTACCAACCACTATTATAT

GATTACAGTACCTTGACTAAAGTCTCTAGTCACAAGATCAATACTACCAACCTACGCTATTATATGATTA

TAGTTTCTATTTTTATAGGAACGCGTACGAGAAAATCAAATGTCTAAGTTCTAACGGTAGTGTTGATAAA

CGATTGTTATCCGCGGATACCTCATCTATCATGTTGTCTATTTTCTTACTTTGTTCTATTAACCTATTAG

CATTATATATTATTTGATTATAAAACTTATATTGCTTATTAGCCCAATCTGTAAATATCGGATTATTAAC

ATATCGTTTCTTTGTAGGTTTATTTAACTTGTACATCACTGTAAGCATGTCCGTACCATTTATTTTAATT

TGACACATATCAGCAATTTCTTTTTCGCAGTCGGTTATATATTCTATATAAGATGGATACGTATCACATA

TGTACTTATAGTCTACTAATATGAAGTACTTAATACATATTTTCAGTAACGATTTAGCCTTATTACCTAT

TAATAAGTGCCTGTCGTTGGATAGGTAATCAACTGTTTTCTTAATACATTCGATGGTTGGTAATTTACTC

AAAATAATTTCCAATATCTTAATATATATTTCTGCTATTTCTGGTATACATGCATGTGCCATTATAACAC

AAATACCAATACATGTAGACCCATATGTTGTTGTTATATTAATATCTGCGCCATTATCTATTAACCATTC

TATTAGGTCAACACTATGCGACTCGATACAATAATAAAGTATACTACGTCCATGTTTATCTATTTTGTTT

ATATCATCGATATACGGCTTACAAATTTCTAGTATCGATAACACTTCTGACTCGTGAATAAATAAGGTAG

GGAATAACGGCATAATATTTATTATGTTATCATCATTAACAACTACGTTTCCATTTTTTAAAATATACTC

TACAACTTTAGGATCCCTATTGTCAAATCTTTTAAAATATTTATTTATATGCTTAAATCTATATAATATA

GCTCCTTCCCTAATCATACATTTGATAACATTGATGTACACTGTATGATAAGATACATATTCTGACAATA

GATCTTGTATAGAATCTGTATATCTTTTAAGAATTGAGGATATTATGACATTATTACGTAAACTATTACA

CAATTCTAAAATATAAAATGTATCATGGGCAGATAATAGTTTATCCACTATATAATTATCTATTTTATGA

TTTTTCTTCCTATATTGTTTACGTAAATAGATAGATAGAATATGCATTAGTTCATTACCGCTATAGTTAC

TATCGAATAACACGTCAAATATTTCCCGTTTAATATCGCATTTGTCAACATAATAATAGAGTATGGTACG

TTCACGATAAGTATAATGACACATCTCGTTTTCGTGCGAAATTAAATAGTTTATCACGTCCAAAGATGTC

ACATAACCATCTTGTGACCTAGTAATAATATAATAATAGAGAACTGTTTTACCCATTCTATTATCATAAT

CAGTGGTGTAGTCATAATCTAAATAATCAAACTCGTCATCCCAATTAAAATAAATATAATCAGTACATTG

AATGGGTATGATATTGTACCCATACTGTATGTTGCTACATGTAGGTATTCCTTTATCCAATAATAGTTTA

AATACATCTATATTAGGATTTGATGTTGTCGCGTATTTCTCTACAATATTAATACCATTTTTGATACTAT

TTATTTCTATACCTTTCGAAATTAGTAATTTCAATAAGTCTATATCGATGTTATCAGAACATAGATATTC

AAATATATCAAAATCATTGATATTTTTATAGTCGACTGACGACAATAACAAAATCACAACATCGTTTTTG

ATATTATTATTTTTTTTGGTAACGTATGCCTTTAATGGAGTTTCACCATCATACTCATATAATGGATTTG

CACCACTTTCTATTAATGATTGTGCACTACTGGCATCGATGTTAAATGTTTTACAACTATCATAGAGTAT

CTTATCGTTAACCATGATTGGTTGTTGATGTTATCACATTTTTTGGTTTCTTTCATTTCAGTTATGTATG

GATTTAGCACGTTTGGGAAGCATGAGCTCATATGATTTCAGTACTGTAGTGTCAGTACTATTAGTTTCGA

TCAGATCAATGTCTAGATCTATAGAATCAAAACACAATAGGTCAGAAGATAATGAATATCTGTACGCTTC

TTCTTGTACTGTAACTTCTGGTTTTGTTAGATGGTTGCATCGTGCTTTAACGTCAATGGTACAAATTTTA

TCCTCGCTTTGTGTATATCATATTCGTCTCTAGTATAAAATTCTATATTCAAATTATCATGCGATGTGTG

TACGCTAACGGTATCAATAAACGGAGCACAGCATTTAGTCAACAGTAATCCAAAATTTTTTAAAGTATAT

CTTAACGAAAGAAGTTGTCATCGTTAGAGTGTGGTAAATCATTGTCTACGGTACTAGATCCTCATAAGTG

TATATATCTAGAGTAATATTTAATTTATCAAATGGTTGATAATATGGATGTTGTGGCAATTTCCTAATAC

GGAAATAAGACATAAACACGCAATAAATCTAATTGCGGACATGTTACACTCCTTAAAAAATACGAATAAA

CACTTTGGCTTTTAGTAAGTGTCATTTAACACTATACTCATATTAATCCATGGACTCATAATCTCTATAC

GGGATTAACGGATGTTCTATATACGGGGATGAGTAGTTCTCTTCTTTAACTTTATACTTTTTACTAATCA

TATTTAGACTGATGTATGGGTAATAGTGTTTGAAGAGCTCGTTCTCATCATCAGAATAAATCAATATCTC

TGTTTTTTTGTTATACAGATGTATTACAGCCTCATATATTACGTAATAGAACGTGTAATCTACCTTATTA

ACTTTCACCGCATAGTTGTTTGCAAATACGGTTAATCCTTTGACCTCGTCGATTTCCGACCAATCTGGGC

GTATAATGAATCTTAACTTTAATTTCTTGTAATCATTCGAAATAATTTTTAGTTTGCATCCGTAGTTATC

TCCTCTATGTAACTGTAAATTTCTCAACGCGATATCTCCATTAATAATGATGTCGAATTCGTGTTGTATA

CCCATACTGAATTGATGAACTAATACCGACGGTATTAATAGTAATTTACTTTTCATCTTTACATACTTGG

TAATAGTTTTACTATCATAAGTTTATAAATTCCACAAGCTACTATGGAATATACCAACCATCTTAGTATA

GAACACATGTCTTAAAGTTATTAATTAATTACATGTTGTTTTATATATCGCTACGAATTTAAACAGAGGA

ATCAGTTAGGAAGAAAAAATTATCTGTCATCATCATCATCTATTGGATAACATCTCTGTATTCTACGATA

GAGTGCTATTTTAAGATGTGACAGATCCGTGTCATCAAATATATACTCCATTAAAATGATTATTCCGGCA

GCGAACTTGATATTGGATACATCACGACCTTTGTTAATATCCACGACAATAGACAGCAATCCCATTGTTC

CATAAACAGTGAGTTTATCTTTCTTTGAAGTGATATTTTGTAGAGATCTTATAAAACTGTCGAATGACAT

CGTATTTATATCTTTAGCTAAATCATATATGTTACCATCATAATATCTAACAGCATCTATCTTAAACGTT

TCCATCGCTGTAAAGACGTTTCCGATAGATGGTCTCGTTTCATCAGTCATACTGAGCCAACAAATGTAAT

CGTGTATAACATCTTTGATAGAATCAGACTCTAAAGAAAAGGAATCGGCTTTATTATACACATTCATGAT

AAACTTAATGAAAAATGTTTTTCGTTGTTTAAGTTGGATGAATAGTATGTCTTAATAATTGTTATTATTT

CACTAATTAATATTTAGTAACGAGTACACTCTATAAAAACGAGAATGACATAACTAATCATAACTAGTTA

TCAAAGAATGTCTAGGACGCGTAATTTTTTATGGTATAGATCCTGTAAGCGTTGTCTGTATTCTGGAGCT

ATTTTCTCTATCGCATTAGTGAGTTCAGAATATGTTATAAATTTAAATCGAATAACGAACATAACTTTAG

TAAAGTCGTCTATATTAACTCTTTTATTTTCTAGCCATCGTAATACCATGTTTAAGATAGTATATTCTCT

AGTTACTACGATCTCATCGTTGTCTAGAATATCGCATACTGAATCTACATCCAATTTTAGAAATTGGTCT

GTGCTACATATCTCTTCTATATTATTGTTGATGTATTGTCGTAGAAAACTATTACGTAGACCATTTTCTT

TATAAAACGAATATATAGTACTCCAATTATCTTTACCGATATATTTGCATACATAATCCATTCTCTCAAT

CACTACATCTTTAAGAGTTTGGTTGTTAAGATATTTGGCTAAACTATATAATTCTATTAGATCATCAACA

GAATCAGTATATATTTTTCTAGATCCAAAGATGAACTCTTTGGCATCCTCTATAATATTATCAGAAAAGA

TATTTTCGTGTTTTAGTTTATCAAGATTTAACCTGTTCATATCCATGATTAACGACGTCATATAACCACA

TAAAATAAAAATCCATTTTCATTTTTAGCACAATACTATTCATAATTGATATTGATGTAATATTTTGTTA

CTTTGAACGTAAAGACAGTACACGGGTCCGTATCTCCAACAAGCACGTAGTAATCAAATTTGGTGTTGTT

AAACTTCGCAATATTCATCAATTTAGATAGAAACATATACTCATCATCTGTTTTAGGAATCCATGTATTA

TTACCACTTTCCAACTTATCATTATCCCAGGCTATGTTTCGCCCATCATCGTTGCACAGAGTGAATAATT

CTTTTGTATTCGGTAGTTCAAATATATGATCCATGCATATATCGACAAAGCTATTGTAGATGTGATTTTT

CCTAAATCTAATATAAAACTCGTTTACTAGCAAACATTTTCCTGATTTATCGACCAAGACACACATGGTT

TCTAAATCTATCAAGTGGTGGGGATCCATAGTTATAACGCAGTAACATAAATTATTACCTTCTTGACTGT

CGCTAATATCTATATACTTATTGTTATCGTATTGGATTCTACATATAGATGGCTTGTATATCAAAGATAT

AGAACACATAACCAATTTATATTCTCGCTTTGTATTTTCGAATCTAAAGTTAAGAGATTTAGAAAACATT

ATATCATCGGATGATGATATCACTGTTTCCAGAGTAGGATATATTAAAGTCTTTAAAGATTTTGTCCGAT

TCAAATAAATCACTAAATAATATCCCATATTATCATCTGTTATAGTCGTGTCATTAAATCTATTATATTT

TATGAAAGATATATCACTGCTCACCTCTATATTTCGTACATTTTTAAACTGTTTGTATAATATCTCTCTA

ATACAATCAGATATATCTATTGTGTCGGTAGACGATACCGTTACATTTGAATTAATGGTGTTCCATTTTA

CAACTTTTAACAAGTTGACCAATTCATTTCTAATAGTATCAAACTCTCCATGATTAAATATTTTAATAGT

ATCCATTTTATATCACTACGGACATAAACCATTGTATATTTTTTATGTTTATTAGTGTACACATTTTGGA

AGTAAGTTCCTGGATCGGATGTCACCGCAGTAATATTGTTGATTATTTCTGACATCGACGTATTATATAG

TTTTTTAATTCCATATCTTTTAGAAAAGTTAAACATCCTTATACAATTTGTGGAATTAATATTATGAATC

ATGGTTTTTACACATAGATCTATTACAGGCGGAACATCAATTATTATGGCAGCAACTAGTATCATTTCTA

CATTGTTTATGGTGATGTTTATCTTCTTCCAGCGCATATAGTCTAATATCGATTCAAACGCGTGATAGTT

TATACCATTCAATATAATCGCTTCATCCTTTAGATGGTGATCCTGAATGTGTTTAAAAAATTATACGGAG

ACGCCGTAATAATTTCCCCATTGATAGAAAATATCACGCTTTCCATTTTCTTGAAGTACTAAAAGTAATT

ATAATATAATGTAAAGGTTTATATATTCAATATTTTTTATAAAAAAAATCATTTTGACATTAATTCCTTT

TTAAATTTCAGTCTATCATCTATAGAAACATATTCTATGAATTTATAAAATGCTTTTACGTATCCTATCG

TAGGTGATAGAACCGCTAAAAAACCTATCGAATTTCTACAAAAGAATCTGTTATATGGTATAGGGAGAGT

ATAAAACATTAAATGTCCGTACTTATTAAAGTATTCAGTAGCCAATCCTAACTCTTTCGAATAATTATTA

ATGGCTCTTATTCTGTACGAATCTATTTTTTTGAACAATGGACCTAGTGGTATATCTTGTTCTATGTATC

TAAAATAATGTCTGACTAGATCCGTTAGTTTAATATCCGCAGTCATCTTGTCTAGAATGGCAAATCTAAC

TGCGGGTTTAGGCGTAGGCGTTAGTTTAGTTTCTATATCTACATCTATGTCTTTATCTAACACCAAAAAT

ATAATAGCTAATATTTTATTACAATCATCCGGATATTCTTCTACGATCTCACTAACTAATGTTTCTTTGG

TTATACTAGTATAGTCACGATCAGACAAATAAAGAAAATCAGATGATCGATGAATAATACATTTAAATTC

ATCATCTGTAAGATTTTTGAGATGTCTCATTAAAATATTATTAGTGTCAGTTCTCATTATCATATATTGA

CAGCAGCTATTACACTTATTTTATTTTTCTGTATTTTATTACTTTTCACCATATAGATCAGTCATTAGAT

CATCAAAATACTTTTCAATCATCCTAAAGAGTATGGTGAACGAATCTTCCCATCTAATTTCTGAACGTCT

ACCAATGTCTCTAGCCACTTTGGCACTAATAGCGATCATTCGCTTAACATCTTCTACATTATTAACTGGT

TGATTCAATCTATCTAGCAATGGACCGTCGGATAGCGTCATTCTCATGTTCTTAATCAATGTACATACAT

CGTCATCATCTACCAATTCATCAAACAATATAAGCTTTTTAAAATCATCATTATAATAGGATGGATCGCC

GTCATTTCTCCAAAGAATATATCTAATAAGTAGAGTCCTCATGCTTAGTAATTTAACTATTTTAGTTAAC

AACTATTTTTTATGTTAAATCAATTAGTAACACCGCTATGTTTAATACTTATTCATATTTTAGTTTTAGG

ATCGAGAATCAATACAAAAATTAATACATCAATTTTGGAAATACTTAGTTTCCACGTAGTCAATGAAACA

TTTGAGCTCATCGTAAAGGACGTTCTCGTACAAGACGTAACTATAAATTGGTTTATATTTGTTCAAGATA

GATACAAATCCGATAACTTTTTTGACGAATTCTACGGGATTCACTTTAAAAGTGTCATACCGGGTTCTTT

TTATTCTTTTAAACAGATCGATTGTGTGATGTTGATTAGGTCTTTTACGAATTTGATACAGAATAGCGTT

TACATATCCACCATAGTAATCAATAGCCATTTGTTCGTATGTCATAAATTCTTTAATTATATGACACTGT

GTATTATTTAGTTCGTCCTTGTTCATCATTAGGAATCTATCCAATATGGCAATTATATTAGAACTATAAC

TGCGTTGTATGCGCATGTTGATGTGTCTGTTTATACAATCAATTATACTAGGATCCATACCACTACAATC

GGGTAAAATTGTAGCATCATATACCATTTCTAGTACTTTAGATTCATTGTTATCCATTGCAGAGGACGTC

ATGATCGAATCCAAAAAAATATATTATTTTTATGTTATTTTGTTAAAAATAATCATCAAATACTTCGTAA

GATACTCCTTCATGAACATAATCAGTTACAAAACGTTTATATGAAGTAAAGTATCTACAATTTTTACAAA

AGTCAGGATGCATAAGTACAAAGTACGCGATAAACGGAATAATAATAGATTTATCTAGTTTATCTTTTTC

TATCTCTTTCATAGTTATATACATGGTCTCAGAAGTCGGATTATGTAACATCAGCTTCGATAAAATGACT

GGGTTATTTAGTCTTACACATTCGCTCATACATGTATGACCGTTAACTATAGAGTCTACACTAAAATGAT

TGAATAATAGATAGTCTACCATTGTTTCGTATTCAGATAGTACAGCGTAGTACATGGCATCTTCACAAAT

TATATCATTATCTAATAGATATTTGACGCATCTTATGGATCCCACTTCAACAGCCATCTTAAAATCGGTA

GAATCATATTGCTTTCCTTTATCGTTAATAATTTCTAGAACATCATCTCTATCATAAAAGATACAAATAT

TAACTGTTTGATCAGTAATAACATTGCTAGTCGATATCAATTTGTTAATAAGATGCGCTGGGCTCAATGT

CTTAATAAGAAGTGTAAGAGGACTATCTCCAAATTTGTTTTGTTTATTAACATCCGTTGATGGAAGTAAA

AGATTTATAATGTCTACATACTTGACTGTTTTAGAGCATACAATATGGAGAGGCGTATTTCCATCATGAT

CTGGTTTTGAGGGACTAATTCCTAGTTTCATCATCCATGAGATTGTAGAAGCTTTTGGATTGTCTGACAT

AAGATGTCTATGAATATGATTTTTGCCAAATTTATCCACTATCCTGGCTTCGAATCCGATAGACATTATT

TTTTTAAACACTCTTTCTGAAGGATCTGTACACGCCAACAACGGACCACATCCTTCTTCATCAACCGAGT

TGTTAATCTTGGCTCCATACTGTACCAATAAATTTATTCTCTCTATGACTTCATCATCTGTTCCCGAGAG

ATAATATAGAGGTGTTTTATTATGTTTATCACATGCGTTTGGATCTGCGCCGTGCACCAGCAGCATCGCG

ACTATTCTATTATTATTAATTTTAGAAGCTATATGCAATGGATAATTTCCATCATCATCCGTCTCATTTG

GAGAGTATCCTCTATGAAGAAGTTCTTCTATAAATCGTTCATCTAGTCCTTTAATGCCACAATACGCATG

TAGAATGTGATAATTTCCAGAGGGTTCGATAACTTGTAGCATATTCCTAAATACATCTAAATTTTTACTA

TTATATTTGGCATAAAGAGATAGATAATACTCGACCGACATAATGTTGTGTTGTCCATTATAGTATAAAA

ATTAATATTTCTATTTCTATATATTTGCAACAATTTACTCTCTATAACAAATATCATAACTTAGTTCTTT

TATGTCAAGAAGGCACTGGTTTAATTCATCTATAAATGTCACTCCATAACTACCACGCATACTATACTCA

GAATTATGATAAAGATATTTATTCTTGGGGTGTAAGTAATGGGGATTAATCTTTGTTGGATCAGTCTCTA

AGTTAACACATGTCACACATGATCCATTTATAGTTATATCACACGATGATGATTTATGAATTGATTCCGG

AAGATCGCTATTGTATTTTGTAGTTCCACAATTCATTTCCATACATGTTATTGTCACACTAATATTATGA

TGAACTTTATCTAGCCGCTGAGTGGTAAACAACAGAACAGATAGTTTATTATCTTTACCAACACCCTCAG

CCGCTGCCACAAATCTCTGATCCGTATCCATGATGGTCATGTTTACTTTTAGTCCGTATCCAGTCAACAC

TATGTTAGCATTTCTGTCGATATAGCTTTCACTCATATGACACTCACCAATAATTGTAGAATTAATGTCG

TAATTTACACCAATAGTGAGTTCGGCGACAAAGTACCAGTACCGGTAATCTTGTCGATGAGGACATATAG

TATTCTTGTATTCTACCGAATACCCGAGAGATGCGATACAAAAGAGTAAGACTAATTTGTAAACCATCTT

ACTCAAAAATATGCGACAATAGGAAATCTATCTTATACACATAATTATTCTATCAATTTTACCAATTAGT

TAGTGTAATGTTAACAAAAATGTGGGATAATTTAATAGTTTTTCCTTACACAATTGACATACATGAGTCT

GAGTTCCTCGTTTTTGCTAATTATTTCGTCCAATTTATTATTCTTGACATCGTCAAGATCTTTTGTATAG

GAGTCAGACTTGTATTCAACATGTTTTTCTATAATCATCTTAGCTATTTCGGCATCATCCAATAGTACAT

TTTCCAGATTAACAGAATAGATATTAATGTCGTATTTGAACAGAGCCTGTAACATCTCAATGTCTTTATT

ATCTATAGCCAATTTGATGTCCGGAATGAAGAGAAGGGAATTGGTGTTTGTTGACGTCATATAGTCGAGC

AAGAGAATCATCATATCCACGTGTCCATTTTTTATAGTGGTGTGAATACAACTAAGGAGAATAGCCAGAT

CAAAAGGAGATGGTATCTCTGAAAGAAAGTAGGAAACAATACTTACATCATTAAGCATGACAGCATGATA

AAATGAAGTTTTCCATCCAGTTTTCCCATAGAACATCAGTCTCCAATTTTTCTTAACAAACAGTTTTACC

GTTTGCATGTTACCACTATCAACCGCATAATACAATGCGGTGTTTCCTTTGTCATCAAATTGTGAATCAT

CCATTCCACTGAATAGCAAAATCTTTACTATTTTGGTATCTTCTAATGTGGCTGCCTGATGTAATGGAAA

TTCATTATCTAGAAGATTTTTCAATGCTCCAGCGTTCAACAACGTACATACTAGACGCACGTTATTATCA

GCTATTGCATAATACAAGACACTATGACCATTGATATCCGCCTTAAATGCATCTTTGCTAGAGAGAAAGC

TTTTCAGTTGCTTAGACTTCCAAGTATTAATTCGTGACAGATCCATGTCTGAAACGAGACGCTAATTAGT

GTATATTTTTTCATTTTTTATAATTTTGTCATATTGTACCAGAATTAATAATATCTCTAATAGATCTGAT

TAGTAGATATATGGCTATCGCAAAACAACATATACACATTTAATAAAAATAATATTCATTAAGAAGATTC

AGATTCCACTGTACCCATCAATATAAAATAAAATAATTATTCCTTACATCGTACCCATAAACAATATATT

AAGTATATTCCACCTTACCCATAAACAATATAAATCCAGTAATATCATGTCTAATGATGAACACAAATGG

TGTATTAAATTCCAGTTCTTCAGGAGATGATCTCGCCGTAGCTACCATGATAGTAGATGCCTCCGCTACA

GTTCCTTGTTCGTCTACATCTATCTTTACATTCTGAAACATTTTATAAATATATAATGGGTCCCTAGTCA

TATGTTTAAACGACGCCTTATCTGGATTAAACATACTAGGAGCCATCATTTCGGCTATCGACTTAATATC

CCTCTTGTTTTCGATAGAAAATCTAGGGAGTTTAAGATTGTACATTTTATTCCCTAATTGAGATGACCAA

TATTCTAATTTTGCAGGCGTGATAGAATCTGTGAAATGGGTCATATTATCACCTATTGCCAGGTACATAC

TAATATTAGCATCCTTATACAGAAGGCGCACCATATCATATTCTTCGTCATCGATTGTGATTGTATTTCC

TTGCAATTTAGTAACTACGTTCATCATGGGAACCGTTTTCGTACCGTACTTATTAGTAAAACTAGCATTG

TGTGTTTTAGTGATATCAAACGGATATTGCCACGTACCTTTAAAATATATAGTATTAATGATTGCCCATA

GAGTATTATCGTCGAGCATAGTAGAATCAACTACATTAGACATACCAGATCTACGTTCTACTATAGAATT

AATTTTATTAACCGCATCTCGTCTAAAGTTTAATCTATATAGGCCGAATCTATGATATTGTTGATAATAC

GACGGTTTAATACACACAGTACTATCGACGAAACTTTGATACGTTAGATCTGTGTACGTATATTTAGATG

TTTTCATCTTAGCTAATCCGGATATTAATTCTGTAAATGCTGGACCCAGATCTCTTTTTCTCAAATTCAT

AGTATTCAATAATTCTACTCTAGTATTACCTGATGCAGACAATAGCGACATAAACATAGAAAACGAATAC

CCAAACGGTGAGAAGACAATATTATCATTATCATCCTCATCCCCATTTTGAATATTTTTATACGCTAATA

TACCAGCATTGATAAATCCCTGCAGACGATATGCGGATACTGAACACGCTAATGATAGTATCAATAACGC

AATCATGATTTTTATGGTATTAATAATTAACCTTATTTTTATGTTTGGTATAAAAATTATTGATGTCTAC

ACATCCTTTATAATCAACTCTAATCACTTTAACTTTTACAGTTTTCCCTACAAGTTTATCCCTATATTCA

ACATATCTATCCATATGCATCTCTTAACACTCTGCCAAGATAGCTTCAAAGTGAGGATAGTCAAAAAGAT

AAATATATAGAGTATAATCATTCTCGTATACTCTGCCCTTTATTACATCGCCCGCATTGGGCAACGAATA

ACAAAATGCAAGCATCGTGTTAACGGGCTCGTAAATTGGGATAAAATTATGTTTTTATTGTTTATCTATT

TTATTCAAGAGAATATTCAGGAAGTTCCTTTTCTGGTTGTATCTCGTCGCAGTATATATCATTTGTACAT

TGTTTCATATTTTTTAATAGTCTACACCTTTTAGTAGGACTAGTATCGTACAATTCATAGCTGTATTTTG

AATTCCAATCACGTATAAAAATATCTTCCAATTGTTGACGAAGACCTAATCCATCATCCGGTGTAATATT

AATAGATGCTCCACATATATCCGTAAAGTAATTTCCTGTCCAATTTGATGTACCTATATACGCCGTTTTA

TCGGTTACCATATATTTTGCATGGTTTACCCTAGAATACGGAATGGGAGGATCAGCATCTGGTACAATAA

ATAGCTTTACTTCTATATCTATGTTTTTAGATTTTAGCATAGCTATAGATCTTAAAAAGTTTCTCATGAT

AAACGAAGATCGTTGCCAGCAACTAATCAATAGCTTAACGGATACTTGTCTGTCTATAGCGGATCTTCTT

AATTCATCTTCTATATAAGGCCAAAACAAAATTTTACCCGCCTTTGAATAAATAATAGGAATAAAGTTCA

TAACAGATACATAAACGAATTTACTCGCATTTCCGATACATGACAATAAAGCGGTTAAATCATTGGTTCT

TTCCATAGTACATAATTGTTGTGGTGCAGAAGCAATAAATACAGAGTGTGGAACACCGCTTACGTTAATA

CTAAGAGGATGATCTGTATTATAATACGACGGATAAAAGTTTTTCCAATTATATGGTAGATTGTTAACTC

CAAGATACCAGTATACCTCAAAAATTTGAGTGAGATCCGCTGCCAAGTTCCTATTATTGAAGATCGCAAT

ACCCAATTCCTTGACCTGAGTTAGTGATCTCCAATCCATGTTAGCGCTTCCTAAATAAATATGTGTATTA

TCAGATATCCAAAATTTTGTATGAAGAACTCCTCCTAGGATATTTGTAATATCTATGTATCGTACTTCAA

CTCCGGCCATTTGTAGTCTTTCAACATCCTTTAATGGTTTGTTGGATTTATTGACGGCTACTCTAACTCT

TACTCCTCTTTTGGGTAATTGTACAATCTCGTTTAATATTACCGTGCCGAAATTCGTACCCACTTCATCC

GATAAACTCCAATAAAAAGATGATATATCTAGTGTTTTTATGGTATTGGATAGAATTTCCCTCCACATGT

TAAATGTAGTCAAATATACTTTATCAAATTGCATACCTATAGGAATAGTCTCTGTAATCACTGCGATTGT

ATTATCCGGATTCATTTTATTTGTTAAAAAAATAATCCTATATCACTTCACTCTATTAAAAATCCAAGTT

TCTATTTCTTTCATGACTGATTTTTTAACTTCATCCGTTTCCTTATGAAGATGATGTTTGGCGCCTTCAT

AAATTTTTATTTCCCTATTACAATTTGCATGTTGCATGAAATAATATGCACCTGAAACATCGCTAATCTC

ATTGTTTGTTCCCTGGAGTATGAGAGTCGGGGTGTTAATCTTGGGAATTATTTTTCTAACCTTGTTGGTA

GCCTTCAAGACCTGACTAGCAAATCCAGCCTTAATTTTTTCATGATTGACTAATGGATCGTATTGGTATT

TATAAACTTCATCCATATCTCTAGATACTGATTCTGGACATAGCTTTCCGACTGACGCATTTGGTGTAAT

GGTTCCCATAAGTTTTGCAGCTAGCAGATTCAGTCTTGGAACAGCGTCTGCATTAACTAGAGGAGACATT

AGAATCATTGCTGTAAACAAGTTTGGATTATCGCAAGCAGCTAGTATAGAAATTGTTGCTCCCATGGAAT

GACCCAATAAGAAGACTGGAACTCCTGGATAAGTAGATTTAATAGTCACCACGTGCTGTACCACATCTCT

AACATACTTACCAAAGTCATCAATCATCATTTTTTCACCATTACTTCTTCCATGGCCAATATGATCATGT

GAGAATACTAAAATTCCTAACGATGATATGTTTTCAGCTAGTTCGTCATAACGTCCAGAATGTTCACCAG

CTCCATGACTTATGAATACTAATGCCTTAGGATATGTAATAGGTTTCCAATATTTACAATATATGTAATC

ATTGTCCAGATTGAACATACAGTTTGTACTCATGATTCACTATATAACTATCAATATTAACAGTTCGTTT

AATGATCATATTATTTTTATGTTTTATTGATAATTGTAAAAATATACAATTAAATCAATATAGAGGAAGG

AGACGGTACTGTATTTTGTGAGATAGTAATGGAGACTAAATCAGATTATGAGGATGCTGTTTTTTACTTT

GTGGATGATGATGAAATATGTAGTCGCGACTCCATCATTGATCTAATAGATGAATATATCACGTGGAGAA

ATCATGTTATAGTGTTTAATAAAGATATTACCAGTTGTGGAAGACTGTACAAGGAATTGATAAAGTTCGA

TGATGTCGCTATACGGTACTATGGTATTGATAAAATTAATGAGATTGTCGAGGCTATGAGTGAAGGAGAC

CACTACATCAATCTTACAGAAGTCCATGATCAGGAAAGTCTATTCGCTACCATAGGAATATGTGCTAAAA

TCACTGAACATTGGGGATACAAAAAGATTTCAGAATCTAAATTCCAATCATTGGGAAACATTACAGATCT

GATGACCGACGATAATATAAACATCTTGATACTTTTTCTAGAAAAAAAAATGAATTGATGATATAAGTGT

CTTCATAACGCATTATTACGTTAGCATTCTATTATCCAGTGTTAAAAAAATTATCCTATCATGTATTTGA

GAGTCTTATATGTAGCAAACATGATAACTGCAATACCCATAATCTTTAGATATTCACGCGTGCTATGGAT

GGCATTATCCCGCGGTGCGGAAATGTACGTTATATAATCTACAAAATAATCATCGCATATAGTATGAGAT

AGTAGAGTAAACATTTTTATCGTTTCTACTGGGTTCATACATCGTCTACCCAATTCGGTAATGAATGAAA

TTGTCGCCAATCTTACACCCAAACCCTTGTTGTTCATTAGTATAGTATTAACTTCATTATTTATGTCATA

AACTGTAAATGATTCTGTAGATGCCATATCACACATGATATTCATGTCACTATTATAATCATTATTAACT

TTATCACAATACGTGTTGATAATATCTACATATGATCTAGTTTTTGTGGGTAATTGTCTATACAAGTCGT

CTAAACGTTGTTTACTCATATAGTATTGAACAGCCATCATTACATGGTCCCGTTCCGTTGATAGATAATC

GAGTATGTTAGTAGACTTGTCAAATCTATATACCATATTTTCTGGAAGCGGATATACATAGTCGCGATCA

TCATTATCACTAGCCTCATCCTCTATATCATGTACATGTACATAATCTATGATATTATTATACATAAACA

TCGACAACATACTATTGTCTATTATCTAAGTCCTGTTGATCCAAACCCTTGATCTCCTCTATCTGTACTA

TCTAGAGATTGTACTTCTTCAAGTTCTGGATAATATATACGTTGATAGATTAGCTGAGCTATTCTATCTC

CAGTATTTACATTAAACGTACATTTTCCATTATTAATAAGAATGACTCCTATGCTTCCCCTATAATCTTC

GTCTATTACACCGCCTCCTATATCAATGCCTTTTAGGGACAGACCAGACCTAGGAGCTATTCTACCATAG

CAGAACTTAGGCATGGACATACTAATATCTGTCTTAATTAACTGTCGTTCTCCAGGAGGGATAGTATAAT

CGTAAGCGCTATACAAATCATATCCGGCAGCACCCGGCGATTGCCTAGTAGGTGATTTAGCTCTGTTAGT

TTCCTTAACAAATCTAACTGGTGAGTTAATATTCATGTTGAACATAAAAAATATCATTTTATTTCAAAAT

TATTTACCATTCCATTCCATCCCATATATTCCATGAATAAGTGCGATTATTGTACACTTCTATAGTATCT

ATATACGATCCACGATAAAATCCTCCTATCAATAGCAGTTTATTATCCACTATGATCAATTCTGGATTAT

CCCTCGGATAAATAGGATCATCTATCAGAGTCCATGTATTGCTGGATTCACAATAAAATTCCGCATTTCT

ACCAACCAAGAATAACCTTCTACCAAACACTAACGCACATGATTTATAATGAGGATAATAAGTGGATGGT

CCAAACTGCCACTGATCATGATTGGGTAGCAAATATTCTGTAGTTGTATCAGTTTCAGAATGTCCTCCCA

TTACGTATATAACATTGTTTATGGATGCCACTGCTGGATTACATCTAGGTTTCAGAAGACTCGGCATATT

AACCCAAGCAGCATCCCCGTGGAACCAACGCTCAACAGATGTGGGATTTGGTAGACCTCCTACTACGTAT

AATTTATTGTTAGCGGGTATCCCGCTAGCATACAGTCTGGGGCTATTCATCGGAGGAATTGGAATCCAAT

TGTTTGATATATAATTTACCGCTATAGCATTGTTATGTATTTCATTGTTCATCCATCCACCGATAAGATA

TACTACTTCTCCAACATGAGTACTTGTACACATATGGAATATATCTATAATTTGATCCATGTTCATAGGA

TACTCTATGAATGGATACTTGTATGATTTGCGTGGTTGTTTATCACAATGAAATATTATGTTACAGTCTA

GTATCCATTTTACATTATGTATACCTCTGGGAGAAAGATAATTTGACCTGATTACATTTTTGATAAGAAG

TAGCAGATTTCCTAATCTATTTCTTCGCCTCATATACCACTTAATGACAAAATCAACTACATAATCCTCA

TCTGGAACATTTAGTTCGTCGCTTTCTAGAATAAGTTTCATAGATAGATAATCAAAATTGTCTATGATGT

CATCTTCCAGTTCCAAAAAGTGTTTGGTAATAAAGTCTTTAGTATGACATAAGAGATTGGATAGTCCGTA

TTCTATACCCATCATGTAACACTCGATACAATATTCCTTTCTAAAATCTCGTAGGATAAAGTTTATACAA

GTGTAGATGATAAATTCTACAGATGTTAATATAGAAGCACGTAATAAATTGACGACGTTATGACTATCTA

TATATACCTTTCCAGTATATGAGTAAATAACTATAGAAGTTAGACTGTGAATGTCAAGGTCTAGACAAAC

CCTCGTAACTGGATCTTTATTTTTTGTGTATTTTTGGCGTAAATGTGTGCAAAAGTATGGAGATAACTTT

TTCAATATCGTAGAATTGACTATTATATTACCTCCTATAGCTTCAATAATTGTTTTGAATTTCTTAGTCG

TGTACAATGCTAATATATTCTTACAGTACACAGTATTGACAAATATCGGCATTTATGTTTCTTTAAAAGT

CAACATCTAAAGAAAAATGATTGTCTTCTTGAGACATAACTCCCATTTTTTGGTATTCACCCACACGTTT

TTCGAAAAAATTAGTTTTTCCTTCCAATGATATATTTTCCATGAAATCAAACGGATTGGTAACATTGTAA

ATTTTTTTAAATCCCAATTCAGAAATCAATCTATCTGCGACGAATTCTATATATGTTTTCATCATTTCAC

AATTCATTCCTATGAGTTTAACTGGAAGAGCCACAGTAAGAAATTCTTGTTCAATGGATACCGCATTTGT

TATAATAAATCTAACGGTTTCTTCACTCGGTGGATGCAATAAATGTTTAAACATCAAACATGCGAAATCG

CAGTGCAGACCCTCGTCTCTACTAATTAATTCGTTAGAAAACGTGAGTCCGGGCATTAGGCCACGCTTTT

TAAGCCAAAATATGGAAGCGAATGATCCGGAAAAGAAGATTCCTTCTACTGCAGCAAAGGCAATAAGTCT

CTCTCCATAACCGGCGCTGTCATGTATCCACTTTTGAGCCCAATCGGCCTTCTTTTTTACACAAGGCATC

GTTTCTATGGCATTAAAGAGGTAGTTTTTTTCATTACTATCTTTAACATAAGTATCGATCAAAAGACTAT

ACATTTCCGAATGAATGTTTTCAATGGCCATCTGAAATCCGTAGAAACATCTAGCCTCGGTAATCTGCAC

TTCTGTACAAAATCGTTCTGCTAAATTTTCATTCACTATTCCGTCACTGGCTGCAAAAAACGCCAATACA

TGTTTTATAAAATATTTTTCGTCTGGTGTTAGTTTATTCCAGTCATTGATATCTTTAGATATATCCACTT

CTTCCACTGTCCAAAATGATGCCTCTGCCTTTTTATACATATTCCAGATGTCATGATATTGGATTGGGAA

AATAACAAATCTATTTGGATTTGGTGCAAGGATAGGTTCCATAACTAAATTAACAATAGTAGTAATTTTT

TTTCAGTTATCTGTATGACGACTGTACTTGGATCTTTTGTATATCGCTATCGCCGCAATCACTACAATAA

TTACAAGTATTATTGATAGCATTGTTATTACTACTATCATAATTAAATTATCGACATTCATGGGTGTTGA

ATAATCGTTATCATCATTTTGTAATTGTGACGTCATACTAGATAAATCATTTGTGAGATTGTTGTGGGAA

GCGGGCACGGAAGATGCATTATCATTATTATTTAACGCCTCCCATTCGGATTCACAAATGTTACGCACGT

TCAAAGTTTTATGGAAACTATAATTTTGTGAAAACAGATAACAAGAAAACTCGTCATCGTTCAAATTTTT

AACGATAGTAAACCGATTAAACGTCGAGCTAATTTCTAACGCTAGCGACTCTGTTGGATATGGGTTTCCA

GATATATATCTTTTCAGTTCCCCTACGTATCTATAATCATCTGTAGGAAATGGAAGATATTTCCATTTAT

CTACTGTTCCTAATATCATATGCGGTGGTGTAGAACCATTAAGCGCGAAAGATGTTATTTCGCATCGTAT

TTTAACTTCGCAATAATTTCTGGTTAGATAACGCACTCTACCAGTCAAGTCAATGATATTAGCCTTTACA

GATATATTCATAGTAGTCGTAACGATGACTCCATCTTTTAGATGTGATACTCCTTTGTATGTACCAGAAT

CTTCGTACCTCAAACTCGATATATTTAAACAAGTTAATGATATATTAACGCGTTTTATGAATGATGATAT

ATAACCAGAAGTTTTATCCTCTGTGGCTAGCGCTATAACCTTATCATTATAATACCAACTAGTGTAATTA

ATATGTGACATGACAGTGTGGGTACAAATATGTACATTATCGTCTACGTCGTATTTGATACATCCGCATT

CAGCCAACAAATATAAAATTACAAAAACTCTAACGACGTTTGTACACATCTTGATGCGGTTTAATAAATG

TTTTGATTTCAATTTATTGTAAAAAAAGATTCGGTTTTATACTGTTCGATATTCTCATTGCTTATATTCT

CATCTATCATCTCCACACAGTCAAATCCATGGTTAACATGTACCTCATCAACCGGTAAAAGACTATCGGA

TTCTTCTATCATCATAACTCGAGAATATTTAATTTGGTGGTCATTATTAATCAAGTCAATTATCTTATTT

TTAACAAACGTAAGTATTTTACTCATTTTTTTATAAAAACTTTTAGAAATATACAGACTCTATCGTGTGT

CTATATCTTCTTTTTATATCCAATGTATTTATGTCTGATTTTTCTTCATTTATCATATATAATGGTCCAA

ATTCTACACGTGCTTCGGATTCATCCAGATCATTAAGGTTCTTATAATCGCAACATCCTTCTCTTCCATC

TTCTACATCTTCCTTCTTATTCTTAGCGTCACAGAATCTACCACAGCAGGATCCCATGACGAGAGTCACA

TTAAACTAATTCATTTTCAATTATAATATACTGATTAGTAATGACAATTAAAATAAAAATATTCTTCATA

ACCGGTAAGAAAGTAAAAAGTTCACATTGAAACTATGTCAGTAGTTATACATCATGAGATGATATACTCT

ATTTTGGTGGAGGATTATATGATATAATTCGTGGATAATCATTCTTAAGACACATTTCTTCATTCGTAAA

TCTTTTCACATTAAATGAGTGTCCATATTTTGCAATTTCTTCATATGATGGCGGTGTACGTGGACGAGGC

TGCTCCTGTTCTTGTAGTCGTCGACTGTCGTGTTTGCGTTTAGATCCCTCCATTATCGCGATCGCGTAGT

GAGTACTATTTATACCTTGTAATTAAATTTTTTTATTAATTAAACGTATAAAAACGTTCCGTATCTGTAT

TTAAGAGCCAGATTTCGTCTAATAGAACAAATAGCTACAGTAAAAATAACTAGAATAATCGCTACACCCA

CTAGAAACCACGGATCGTAATACGGCAATCGGTTTTCGATAATAGGTGGAACGTATATTTTATTTAAGGA

CTTAACAATTGTCTGTAAACCACAATTTGCTTCCGCCGATCCTGTATTAACTATCTGTAAAAGCATATGT

TGACCGGGAGGAGCCGAACATTCTCCGATATTCAATTTTTGTATATCTATAATGTTATTAACCTCCGCAT

ACGCATTACAGTTCTTTTCTAGCTTGGATACTACACTAGGTACATCATCTAAATCTATTCCTATTTCCTC

AGCGATAGCTCTTCTATCCTTTTCCGAAAGTAATGAAATCACTTCAATAAATGATTCAACCATGAGTGTG

AAACTAAGTCGAGAATTACTCATGCATTTGTTAGTTATTCGGAGCGCGCAATTTTTAAACTGTCCTATAA

CCTCTCCTATATGAATAGCACAAGTGACATTAGTAGGGATAGAATGTTGAGCTAATTTTTGTAAATAACT

ATCTATAAAAAGATTATACAAAGTTTTAAACTCTTTAGTTTCTGCCATTTATCCAGTCTGAGAAAATGTC

TCTCATAATAAATTTTTCCAAGAAACTAATTGGGTGAAGAATGGAAACCTTTAATCTATATTTATCACAG

TCTGTTTTGGTACACATGATGAATTCTTCTAATGCTGTACTAAATTCGATATCTTTTTCGATTTCTGGAT

ATGTTTTTAATAAAGTATGAACAAAGAAATGGAAATCGTAATACCAGTTATGTTCAACTTTGAAATTGTT

TTTTATTTTCTTGTTAATGATTCCAGCCACTTGGGAAAAGTCAAAGTCGTTTAATGCCGATTTAATACGT

TCATTAAAAACAAACTTTTTATTCTTTAGATGAATTATTATTGGTTCATTGGAATCAAAAAGTAAGATAT

TATCGGGTTTAAGATCTGCATGTAAAAAGTTGTCACAACAGGGTAGTTCGTAGATTTTAATGTATAACAG

AGACATCTGTAAAAAGATAAACTTTATGTATTGTACCAAAGATTTAAATCCTAATTTGATAGCTAACTCG

GTATCTACTTTATCTGCCGAATACAGTGCTAGGGGAAAAATTATAATATTTCCTCTTTCGTATTCGTAAT

TAGTTCTCTTTTCATGTTCGAAAAAGTGAAACATGCGGTTAAAATAGTTTATAACATTAATATTACTGTT

AATAACTGCCGGATAAAAGTGGGATAGTAATTTCACGAATTTGATACTGTCCTTTCTCTCGTTAAACGCC

TTTAGAAAAACTTTAGAAGAATATCTCAATGAGAGTTCCTGACCATCCATAGTTTGTATCAATAATAGCA

ACATATGAAGAACCCGCTTATACAGAGTATGTAAAAATGTTAATTTATAGTTTAATCCCATGGCCCACGC

ACACACGATTAATTTTTTTTCATCTCCCTTTAGATTGTTGTATAGAAATTTGGGTACTGTAAACTCCGCC

GTAGTTTCCATGGGACTATATAATTTTGTGGCCTCGAATACAAATTTTACTACATAGTTATCTATCTTAA

AGACTATACCATATCCTCCTGTAGATATGTGATAAAAATCGTCGTTTATAGGATAAAATCGTTTATCTTT

TTGTTGGAAAAAGGATGAATTAATGTAATCATTCTCTTCTATCTTTAGTAGTGTTTCCTTATTAAAATTC

TTAAAATAATTTAACAATCTAACTGACGGAGCCCAATTTTGGTGTAAATCTAATTGGGACATTATGTTGT

TAAAATATAAACAGTCTCCTAATATAACAGTATCTGATAATCTATGGGGAGACATCCATTGATATTCAGG

GGATGAATCATTGGCAACACCCATTTATTGTACAAAAAGCCCCAATTTACAAACGAAAGTCCAGGTTTGA

TAGAGATAAACTATTAACTATTTTGTCTCTGTTTTTAACACCTCCACAGTTTTTAATTTCTTTGGTAATG

AAATTATTCACAATATCAGTATCTTCTTTATCTACCAGAGATTTTACTAACTTGATAACCTTGGCTGTCT

CATTCAATAGGGTAGTGATATTTGTATGTATGATATTGATATCTTTTTGAATTGTTTCTTTTAGAAGTGA

TTCTTTGATGGTATCAGCATACGAATTACAATAATGCAGAAACTCAGTTAACATGCAGGAATTATAGTAA

GCCAATTCCAATTGTTGCCTGTATTGTATTAGAGTATTAATATGCGCAATGATGTCCTTGCGTTTCTCTG

ATAGAATGCGAGCAGCGATTTTGGCGTTATCATTTGACGATATTTCTGGAATGACGAATCCTGTTTCTAC

TAACTTCTTGGTAGGACAAAGTGAAACAATCAAGAAAATAGCTTCTCCTCCTATTTGTGGAAGAAATTGA

ACTCCTCTAGATGATCTACTGACGATAGTATCTCCTTGACAGATATTGGACCGAACTACGGAAGTACCTG

GAATGTAAAGCCCTGAAACCCCCTCATTTTTTAAGCAGATTGTTGCCGTAAATCCTGCACTATGCCCAAG

ATAGAGAGCTCCTTTGGTGAATCCATCACTATGTTTCAGTTTAACCAAGAAACAGTCAGCTGGTCTAAAA

TTTCCATCTCTATCTAATACAGAATCCAACTTGATGTCAGGGACTATGACCGGTTTAATGTTATATGTAA

CATTGAGTAAATCCTTAAGTTCATAATCATCGTTGTCATCAGTTATGTACGATCCAAACAATGTTTCTAC

CGGCATGGTGGATACGAAGATGCTATCCATCAGAATGTTTCCCTGATTAGTATTTTCTATATAGCTATTC

TTCTTTAAACGATTTTCCGAATCAGTAACTATGTTCATTTTTTTAGGAGTAGGACGTCTAGCCAGTATGG

AAGAGGATTTTCTAGATACTCTCTTCAACATCTTTGATCTCAATGGAATGCAAAACCCCATGGTGTAACA

ACCAACGATAAAAATAATATTGTTTTTTCACTTTTTATAATTTTACCATCTGACTCATGGATTCATTAAT

ATCTTTACAAGAGCTACTAACGTATAATTCTTTATAACTAAACTGAGATATATACACCGGATCTATGGTT

TCCATAATTGAGTAAATGAATGCTCGGCAATAACTAATGGCAAATGTATAGAACAACGAAATTATACTAG

AGTTGTTAAAGTTAATATTTTCTATGAGTTGTTCCAATAAATTATTTGTTGTGACTGCGTTCAAGTCATA

AATTATCTTGATACTATCCAGTAAACAGTCTTTAAGTTCTGGAATATTATCATCCCATTGTAAAGCCCCT

AGTTCGACTATCGAATATCCTGCTCTGATAGCAGTTTCAATATCGACGGACGTCAATACTGTAATAAAGG

TGGTAGTATTGTCATCATCGTGATAAACTACGGGAATATGGTCGTTAGTAGGTACCGTGACTTTACACAA

CGCGATATATAACTTTCCTTTTGTACCATTTTTAACGTAGTTGGGACGTCCTGCAGGGTATTGTTTTGAA

GAAATGATATCGAGAACAGATTTGATACGATATTTGTTGGATTCCTGATTATTCACTATAATATAATCTA

GACAGATAGATGATTCGATAAATAGAGAAGGTATATCGTTGGTAGGATAATACATCCCCATTCCAGTATT

CTCGGATACTCTATTGATGACACTAGTTAAGAACATGTCTTCTATTCTAGAAAACGAAAACATCCTACAT

GGACTCATTAAAACTTCTAACGCTCCTGATTGTGTTTCGAATGCCTCGTACAAGGATTTCAAGGATGCCA

TAGATTCTTTGACCAACGATTTAGTATTGCGTTTAGCATCTGATTTTTTTATTAAATCAAATGGTCGGCT

CTCTGGTTTACTACCCCAATGATAACAATAGTCTTGTAAAGATAAACCGCAAGAAAATTTATACACATCC

ATCCAAATAACCCTAGCACCGTCGGATGATATTAATGTATTATTATAGATTTTCCATCCACAGTTATTGG

GCCAGTATACTGTTAGCAACGGTATATCGAATAGATTACTCATGTAACCTACTAGAATGATAGTTCGTGT

ACTAGTCATAATATCTTTAATCCAATCTAAGAAATCTAAAATTAGATCTTTTACACTATTAAAGTTAACA

AAGGTATTACCCGGGTACGTGGATATCATATATGGTATTGGTCCATTATCAGTAATGGCTCCATAAACTG

ATACGGCAATGGTTTTTATATGTGTTTGATCTAATGAGGACGAAATTCGCGCCCACAATTCATCTCTAGA

TATGCATTTAATATCGAACGGTAACACATCAATCTCGGGACGCGTATATGTTTCTAAATTCTTAATCCAA

ATATAATGATGACCTATATGCCCTATTATCATACTGTCAACTATAGTATACCTAGAGAACTTTCGATACA

TCTGCTGTTTCCTGTAATCGTTAAATTTTACAAATCTATAACATGCTAAACCTTTTGACGACAGCCATTC

ATTAATTTCTGATATGGAATCTGTATTCTTAATACCGTATCGTTCTAAAGCCAGTGCTATATCTCCCTGT

TCGTGGGAACGCTTTCGTATAATATCGATCAATGGATAATATGAAGTTTTTGGAGAATAATATGATTCAT

GATCTATTTCGTCCATAAACAATCTAGACATAGGAATTGGAGGCGATGATCTTAATTTTGTGCAATGGGT

CAATCCTATAACTTCTAATATTGTAATATTCATCATCGACATAACACTATCTATGTTATCATCGTATATT

AGTATACCACGACCTTCTTCATTTCGTGCCAAAATGATATACAGTCTTAAATAATTACGCAATATCTCAA

TAGTTTCATAATTGTTAGCTGTTTTCATCAAGGTTTGTATCCTGTTTAACATGATGGCGTTCTATAACGT

CTCTATTTTCTATTTTTAATTTTTTTAAATTTTTAACGATTTACTGTGGCTAGATACCCAATCTCTTTCA

AATATTTTTTTAGCCTTGCTTACAAGCTGTTTATCTATACTATTAAAACTGACGAATCCGTGATTTTGGT

AATGGGTTCCGTCGAAATTTGCCGAAGTGATATGAACATATTCGTCGTCGACTATTAACAATTTTGTATT

ATTCTGAATAGTGAAAACCTTCACAGATAGATCATTTTGAACACACAACGCATCTAGACTTCTGGCGGTT

GCCATAGAATATACGTCGTTCTTATCCCAATTACCAACTAGAAGTCTGATCTTAACTCCTCTATTAATGG

CTGCTTCTATAATGGAGTTGTAAATGTCAGGCCAATAGTAGCTATTACCGTCGACACGTGTAGTGGGAAC

TATGGCCAAATGTTCAATATCTATACTAGTCTTAGCCGACTTGAGTTTATCAATAACTACATCAGTGTCT

AGATCTCTAGAATATCCCAATAGGTGTTCTGGAGAATCAGTAAAGAACACTCCACCTATAGGATTCTTAA

TATGATACGCAGTGCTAACTGGCAGACAACAAGCCGCAGAGCATAAATTCAACCATGAATTTTTTGCGCT

ATTAAAGGCTTTAAAAGTATCAAATCTTCTACGAAGATCTGTGGCCAGCGGAGGATAATCAGAATATACG

CCTAACGTTTTAATCGTATGTATAGATCCTCCAGTAAATGACGCGTTTCCTACATAACATCTTTCATCAT

CAGACACCCAAAAACAACCGAGTAGTAGTCCCACATTATTTTTTTTATCTATATTAACGGTTATAAAATT

TATATCCGGGGAGTGACTTTGTAGCTCTCCCAGATTTCTTTTCCCTCGTTCATCTAGCAAAACTATTATT

TTAATCCCTTTTTCAGATACCTCTTTTAGTTTATCAAAAATAAGCGCTCCCCTAGTAGTACTCAGAGGAT

TACAACAAAAAGATGCTATGTATATATATTTCTTAGCTAGAGTGATAATTTCGTTAAAACATTCAAATGT

TGTCAAATGATCGGATCTAAAATCCATATTTTCTGGTAGTGTTTCTACCAGCCTACATTTTGCTCCCGCA

GGTACCGATGCAAATGGCCACATTTAGTTAACATAAAAACTTATATATCCTGTTCTATCAACGATTCTAG

AATATCATCGGCTATATCGCTAAAATTTTCATCAAAGTCGACATCACAACCTAACTCAGTCAATATATTA

AGAAGTTCCATGATGTCATCTTCGTCTATTTCTATATCCGTATCCATTGTAGATTGTTGACCGATTATCG

AGTTTAAATCATTACTAATACTCAATCCTTCAGAATACAATCTGTGTTTCATTGTAAATTTATAGGCGGT

GTATTTAAGTTGGTAGATTTTCAATTATGTATCAATATAGCAACAGTAGTTCTTGCTCCTCCTTGATTTT

AGCATCCTCTTCATTATTTTCTTCTACGTACATAATCATGTCTAATACGTTAGACAACACACCGACGATG

GTGGCCGCCACAGACACGAATATGACTAGACCGATGACCATTTAAAAAATACTCTCTAGCTTTAACTTAA

ACTGTATCGATCATTCTTTTAGCACATGTATAATATAAAAACATTATTCTATTTCGAATTTAGGCTTCCA

AAAATTTTTCATCCGTAAACCGATAATAATATATATAGACTTGTTAATAGTCGGAATAAATATATTAATG

CTTAAACTATCATCATCTCCACGATTAGAGATACAATATTTACATTCTTTTTGCTGTTTCGAAACTTTAT

CAATACACGTTAATACAAACCCAGGAAGGAGATATTGAAACTGAGGCTGTTGAAAATGAAACGGCGAATA

CAATAATTCAGATAATGTAAAATCATGATTCCGTATTCTGATGATATTAGAACTGCTAATGGATGTCGAT

GGTATGTATCTAGGAGTATCTATTTTAACAAAGCATCGATTTGCTAATATACAATTATCCTTTTGATTAA

TTGTTATTTTATTCATATTCTTAAAAGGTTTCATATTTATCAATTCTTCTACATTAAAAATTTCCATTTT

TAATTTATCTAGCCCCGCAATACTCCTCATTACGTTTCATTTTTTGTCTAGAATACCCATTTTGTTCATC

TTGGTACATAGATTATCCAATTGAGAAGCGCATTTAGTAGTTTTGTACATTTTAAGTTTATTAACGAATC

GTCGAAAACTAGTTATAGTTAACATTTTATTATTTGATACCCTGATATTAATACCCCTGCCGTTACTATT

ATTTATAACTGATGTAACCCACGTAACATTGGAATTAATTATCGATAGTAATGCATCGACACTTCCAAAA

TTGTCTATTATAAACTCACCGATAATTTTTTTATTGCATGTTTTCATATTCATTAGGATTATCAAATCTT

TAATCTTATTACGATTGTATGCGTTGATATTACAAGACGTCATTCTAAAAGACGGAGGATTTCCATCAAA

TGCCAGACAATCACGTACAAAGTACATGGAAATAGGTTTTGTTCTATTACGCATCATAGATTCATATAAA

ACACCCGTAGAAATACTAATTTGTTTTACTCTATAAAATACTATTGCATCTATTTCATCGTTTTGTATAA

CGTCTTTCCAAGTGTCAAATTCCAATTTTTTTTCATTGATAGTACCAAATTCTTCTATCTCTTTAACTAC

TTGCATAGATAGGTAATTACAGTGATGCCTACATGCCGTTTTTTGAAACTGAATAGATGCATCTAGAAGC

GATGCTACACTAGTCACGATCACCACTTTCATATTTAGAATATATGTATGTAAAAATATAGTAGAATTTC

ATTTTGTTTTTTTCTATGCTATAAATGAATTCTCATTTTGTATCCGCACATACTCCGTTTTATATCAATA

CCAAAGAAGGAAGATATCTGGTTCTAAAAGCCGTTAAAGTATGCGATGTTAGAACTGTAGAATGCGAAGG

AAGTAAAGCTTCCTGCGTACTCAAAGTAGATAAACCCTCATCACCCACGTGTGAGAGAAGACCTTCGTCC

CCGTCCAGATGCGAGAGAATGAATAACCCTGGAAAACAAGTCCCGTTTATGAGAACGGACATGTTACAAA

ATATGTTTGCTGCTAATCGCGACAACGTAACGTCAAGACTTTTGAACTAAAATACAATTATATCTTTTTC

GATATTAATAAATCCGTGTCTCCCGGGTTTTTTATCTCTTTCAGTATGTGAATAGATAGGTATTTTATCT

CTATTCATCATCGAATTTAAGAGATCCGATAAACATTGTTTGTATTCTCCAGATGTCAGCATCTGATACA

ACAATATATGTGCACATAAACCTCTGGCACTTATTTCATGTACCTTCCCCTTATCACTAAGGAGAATAGT

ATTTGAGAAATATGTATACATGATATTATCATGTATTAGATATACAGAATTTGTAACACTCTCGAAATCA

CACGATGTGTCGGCGTTAAGATCTAATATATCACTCGATAACACATTTTCATCTAGATACACTAGACATT

TTTTAAAGCTAAAATAGTCTTTAGTAGTAACAGTAACTATGCGATTATTTTCATCGATGATACATTTCAT

CGGCATATTATTACGCGTACCATCAAAGACTATACCATGTGTATATCTAACGTATTCTAGCATAGTTGCC

ATACGTACATTAAACTTTTCAGGATCTTTGGATAGATCTTCCAATCTATCTATTTGAGAAAACATTTTTA

TCATGTTCAATAGTTGAAACGTCGGATCCACTATATAGATATTATCTATAAAGATTTTAGGAACTATGTT

CATGGTATCCTGGCGAATATTAAAACTATCAATGATATGATTATCGTTTTCATCTTTTATCACCATATAG

TTTCTAAGATATGGGATTTTACTTAATATAATATTATTTCCCGTAATAAATTTTATTAGAAATGCCAAAT

CTATAAGAAAAGTCCTAGAATTAGTCTGAAGAATATCTATATCACCGTACCGTATATTTGGATTAATTAG

ATATAGAGAATATGATCCGTAACATATACAACTTTTATTATGACGTCTAAGATATTCTTCCATCAACTTA

TTAACATTTTTGACTAGGGAAGATACATTATGACGTCCCATTACTTTTGCCTTGTCTATTACAGCGACGT

TCATAGAATTTAGCATATCTCTTGCCAATTCTTCCATTGATGTTACATTATAAGAAATTTTAGATGAAAT

TACATTTGGAGCTTTAATAGTAAGAACTCCTAATATATCCGTGTATGTGGTCACTAATACAGATTGTAGT

TCTATAATCGTAAATAATTTACCTATATTATATGTTTGAGTTTGTTTAGAAAAGTAGCTAAGTATACGAT

CTTTTATTTCTGATGCCGATGTATCAACATCGAAAAAAAATCTTTTTTTATTCTTTTTTACTAACGATAC

GAATATGTCTTTGTTAAAAACAGTTATTTTCTGAATATTTCTAGCTTGTAATTTTAACATATGATATTCG

TTCACACTAGGTACTCTGCCTAAATAGGTTTCTATAATCTTTAATGTAATATTAGGAAGAGTATTCTGAT

CAGGATTCCTATTCATTTTGAGGATTTAAAACTCTGATTATTGTCTAATATGGTCTCAACACAAACTTTT

TCACAGAGTGATAGAGTTTTTGATAACTCGTTTTTCTTAAGAAATATAAAACTACTGTCTCCAGAGCTCG

CTCTATCTTTTATTTTATCTAATTCGATACAAACTCCTGATACTGGTTCAGAAAGTAATTCATTAATTTT

CAGTCCTTTATAGAAGATATTTAATATAGATAATACAAAATCTTCAGTTCTTGATATCGATCTGATTGAT

CCTAGAACTAGATATATTAATAACGTGCTCATTAGGCAGTTTATGGCAGCTTGATAATTAGATATAGTAT

ATTCCAGTTCATATTTATTAGATACCGCATTGCCCAGATTTTGATATTCTATGAATTCCTCTGAAAATAA

ATCCAAAATAACTAGACATTCTATTTTTTGTGGATTAGTGTACTCTCTTCCCTCTATCATGTTCACTACT

GGTGTCCACAATGATAAATATCTAGAGGGAATATAATATAGTCCATATGATGCCAATCTAGCAATGTCGA

ATAACTGTAATTTTATTCTTCGCTCTTCATTATGAATTGAATCTTGAGGTATAAACCTAACACAAATTAT

ATCATTAGACTTTTCGTATGTAATGTCTTTCATGTTATAAGTTTTTAATCCTGGAATAGAATCTATTTTA

ATGAGGCTTTTAAACGCAGCGTTCTCCAACGAGTCAAAGCATAATACTCTGTTGGTTTTCTTATATTCAA

TATTACGATTTTCTTCTTTGAATGGAATAGGTTTTTGAATTAGTTTATAATTACAACATAATAGATAAGG

AAGTGTGTAAATAGTACGCGGAAAAAACATAATAGCTCCCCTGTTTTCATCCATGGTTTTAAGTAAATGA

TCACTGGCTTCTTTAGTCAATGGATATTCGAACATTAACCGTTTCATCATCATTGGACAGAATCCATATT

TCTTAATGTAAAGAGTGATCAAATCATTGTGTTTATTGTACCATCTTGTTGTAAATGTGTATTCGGTTAT

CGGATCTGCTCCTTTTTCTATTAAAGTATCGATATCGATCTCGTCTAAGAATTCAACTATATCGACATAT

TTCATTTGTATACACATAACCATTACTAACGTAGAATGTATAGGAAGAGATGTAACGGGAACAGGGTTTG

TTGATTCGCAAACTATTCTAATACATAATTCTTCTGTTAATACGTCTTGCACGTAATCTATTATAGATGC

CAAGATATCTATATAATTATTTTGTAAGATGATGTTAACAATGTGATCTATATAAGTAGTGTAATAATTC

ATGTATTTCGATATATGTTCCAACTCTGTCTTTGTGATGTCTAGTTTCGTAATATCTATAGCGTCCTCAA

AAAATATATTCGCATATATTCCCAAGTCTTCAGTTCTATCTTCTAAAAAATCTTCAACGTATGGAATATA

ATAATCTATTTTACCTCTTCTGATGTCATTAATGATATAGTTTTTGACACTATTTTCCGTCAATTGATTC

TTATTCACTATGTCTAAAAACCGGATAGCGTCCCTAGGACGAACTACTGCCATTAATATCTCTATTATAG

CTTCTGGACATAAATCATCTATTATACCAGAATTAATGGGAACTATTCCGTATCTATCTAACATAGTTTT

AAGAAAGTCAGAATCTAAGACCTGATGTTCATATATTGGTTCATACATGAAATGATCTCTATTGATGATA

GTGACTATTTCATTCTCTGAAAATTGGTAACTCATTCTATACACGCTTTCCTTGTTGATAAAGGATAGTA

TATACTCAATGGAATTTGTACCAACAAACTGTTCTCTTATGAATCGTATATCATCATCTGAAATGATCAT

GTAAGGCATACATTTAACAATAAGAGACTTGTCTCCTGTTATCAATATACTATTCTTGTGATAATTTATG

TGTGCGGCAAATTTGTCCACGTTCTTTAATTTTGTTATAGTAGATATCAAATCCAATGGAGATACAGTTC

TTGGCTTAAACAGATATAGTTTTTCTGGAACGAATTCTACAACATTATTATAAAGGACTTTGGGTATATA

AGTGGGATGAAATCCTATTTTAATTAATGCGATAGCCTTGTCCTCGTGCAGATATCCAAACGCTTTTGTG

ATAGTATGGCATTCATTGTCTAGAAACGCTCTACGAATATCTGTAACAGATATCATCTTTAGAGAATACT

AGTCGCGTTAATAGTACTAAAATTTGTATTTTTTAATCTATCTCAATAAAAAATTAATATGTATGATTCA

ATGTATAACTAAACTACTAACTGTTATTGATAACTAGAATCAGAATCTAATGATGACATAACTAAGAAGT

TTATCTACAGCCAATTTAGCTGCATTATTTTTAGCATCTCGTTTAGATTTTCCATCTGCCTTATCGAATA

CTCTTCCGTCAATGTCTACACAGGCATAAAATGTAGGAGAGTTACTAGGCCCCACTGATTCAATACGAAA

AGACCAATCTCTCCTAGTTATTTGACAGTACTCATTAATAACGGTGACAGGGTTAACACCTTTCCAATAA

ATAATTTTTTTAACCGGAATAACATCATCAAAAGACTTATGATCCTCTCTCATTGATTTTTCGCGGGATA

CATCATCTATTATAGCATCAGCATCAGAATCTGTAGGCCGTGTATCAGCATCCATTGTCGTAGACCAACG

AGGAGGAGTATCGTTGGAGCTGTAAACCATAGCACTACGTTGAAGATCATACAGAGCTTTATTAACTTCT

CGCTTCTCCATATTAAGTTGTTTAGTTAGTTGTACAGCAGTAGCTCCTTAGTCCAATGTTTTTAATAACC

GCACACAATCTCTGTGTCAGAACGCTCGTCAATATAGATCTTAGAAATTTTTTTAGAGAGAACTAACGCA

ACTAGCAATAAAACTGATCTTATTTTATCATTTTTTTTATTCATCATCCTCTGGTGGTTCGTCGTTCCTA

TCGAATGTAGCTCTGATTAACCCGTCATCTATAGGTGATGCTGGTTCTGGAGATTCTGGAGGAGATGGAT

TATTATCTGGAAGAATCTCTGTTATTTCCTTGTTTTCATGTATCGATTGCGTTGTAACATTAAGATTGCG

AAATGCTCTAAATTTGGGAGGCTTAAAGTGTTGTTTACAATCTCTACACGCGTGTCTAACTAATGGAGGT

TCATCAGCGGCTCTAGTTTGAATCATCATCGGTGTAGTATTCCTACTTTTACAGTTAGGACACGGTGTAT

TGTATTTCTCGTCGAGAACGTTAAAATAATCGTTGTAACTCACATCCTTTATTTTATCTATATTGTATTC

TACTCCTTTCTTAATGCATTTTATACCGAACAAGAGATAGCGAAGGAATTCTTTTTCGGTACCGCTAGTA

CCCTTAATCATATCACATAGTGTTTTATATTCTAAATGTGTGGCAATGGACGGTTTATTTCTATACGATA

GTTTGTTTTTGGAATCCTTTGAGTATTCTATACCAATATTATTCTTTGATTCGAATTTAGTTTCTTCGAT

ATTAGATTTTGTATTACCTATATTCTTGATGTAGTACTTTGATGATTTTTCCATGGCCCATTCTATTAAG

TTTTCCAAGTTGGCATCATCCACATATTGTGATAGTAATTCTCGGATATCAGTAGTGACTACCGCCATTG

ATATTTGTTCATTTGATGAGTAACTACTAATGTATACATTTTCCATTTATAACACTTATGTATTAACTTT

GTTTATTTATATTTTTTCATTATTATGTTGATATTAATAATCGTATTGTGGTTATATGGCTACAATTTCA

TAATGAGTTGAAGTCAGTGTCCTATGATCAATGACGATAGCTTTACTCTGAAAAGAAAGTATCAAATCGA

TAGTGCAGAGTCAACAATGAAAATGGATAAGACGATGACAAAGTTTCAGAATAGAGTCAAAATGGTAAAA

GAAATAAATCAGACGATAAGAGCAGCACAAACTCATTACGAGACATTGAAACTAGGATATATAAAATTTA

AGGGAATGATTAGGACTACTACTCTAGAAGATATAGCACCATCTATTCCAAATAATCAGAAAACTTATAA

ACTATTCTCGGACATTTCAGTCATTGGCAAAGCATCACAGAATCCGAGTAAGATGATATATGCTCGCTGC

TTTACATGTTTCCCAATTTGTTTGGAGATGACCATAGATTCATTTGTTATAGAATGCATCCAACATTGTT

CATGATATAGTTGAATCATGTATGCCTGTTCGTATGCCTGTGGCTAAGATACTGTGTAAAGAAATGGTAA

ATAAATACTTTGAGAATCTTTAAGAGTGCATTGACTTTGTTAGTGAATAGGCATTCCATCTTTCTCCAAT

ACTAATTCAAATTGTTAAATTAATAATGGAATAGTATAAATAGTTATTAGTGATAGGATAGTAAACATAA

TTATTAGAATAGTAGTGTAGTATCATAGATAACTCTCTTCTATAAAAAATGGATTTTATTCGTAGAAAGT

ATCTTATATACACAGTAGAAAATAATATAGATTTTTTAAAGGATGATACATTAAGTAAAGTAAACAATTT

TACCCTCAATCATGTACTAGCTCTCAAGTATCTAGTTAGCAATTTTCCTCAACATGTTATTACTAAGGAT

GTATTAGCTAATACCAATTTTTTTGTTTTCATACATATGGTACGATGCTGTAAAGTATACGAAGCGGTTT

TACGACACGCATTTGATGCACCCACGTTGTACGTTAAAGCATTGACTAAGAATTATTTATCGTTTAGTAA

CACAATACAGTCGTACAAGGAAACAGTGCATAAACTAACACAAGATGAAAAATTTTTAGAGGTTGCCGAA

TACATGGACGAATTAGGAGAACTTATAGGCGTAAATTATGACTTAGTTCTTAATCCATTATTTCACGGAG

GGGAACCCATCAAAGATATGGAAATCATTTTTTTAAAACTGTTTAAGAAAACAGACTTCAAAGTTGTTAA

AAAATTAAGTGTTATAAGATTACTTATTTGGGCATACCTAAGCAAGAAAGATACAGGCATAGAGTTTGCG

GATAATGATAGACAAGATATATATACTCTATTTCAACAAACTGGTAGAATAGTCCATAGCAATCTAACAG

AAACGTTTAGGGATTATATCTTTCCCGGAGATAAGACTAGCTATTGGGTGTGGTTAAACGAAAGTATAGC

TAATGATGCGGATATCGTTATTAATAGACCCGCCATTACCATGTATGATAAAATTCTTAGTTATATATAC

TCTGAGATAAAACAGGGACGCGTTAATAAAAACATGCTTAAGTTAGTTTATATCTTTGAGCCTGAAAAAG

ATATCAGAGAACTTCTGCTAGAAATCATATATGATATTCCTGGAGATATCCTATCTATTATTGATGCAAA

AAACGACGATTGGAAAAAATATTTTATTAGTTTTTACAAAGCTAATTTTATTAACGGTAATACATTTATT

AGTGATAGAACGTTTAACGATGACTTATTCAGAGTTGTTGTTCAAATAGATCCCGAATATTTCGATAATG

AACGAATTATGTCTTTATTCTATACGAGTGCTGCGGACATTAAACGATTTGATGAGTTAGATATTAATAA

CAGTTATATATCTAATATAATTTATGAGGTGAACGATATCACATTAGATACAATGGATGATATGAAGAAG

TGTCAAATCTTTAACGAGGATACGTTGTATTATGTTAAGGAATACAATACATACCTGTTTTTGCACGAGT

CGGATCCCATGGTCATAGAGAACGGAATACTAAAGAAACTGTCATCTATAAAATCCAAGAGTAGACGGCT

GAACTTGTTTAGCAAAAACATTTTAAAATATTATTTAGACGGACAATTGGCTCGTCTAGGTCTTGTGTTA

GATGATTATAAAGGAGACTTATTAGTTAAAATGATAAACCATCTCAAATCTGTGGAGGATGTATCCGCAT

TCGTTAGATTTTCTACAGATAAAAACCCTAGTATTCTTCCATCGCTAATCAAAACTATTTTAGCTAGTTA

TAATATTTCCATCATCGTCTTATTTCAAAGGTTTTTAAGAGATAATCTATATCATGTAGAAGAATTCTTG

GATAAAAGCATCCATCTAACCAAGACGGATAAGAAATATATACTTCAATTGATAAGGCACGGTAGATCAT

AGAACAAACCAAATATATTATTAATAATTTGTATATACATAGATATAATTATCACATATTAAAAAATAAC

ACATTTTTGATAAATGGAAACCGTTGCAACAATTCAGACTCCCACCAAATTAATGAATAAAGAAAATGCA

GAAATGATTTTGGAAAAAATTGTTAATCATATAGCTATGTATATTAGTGACGAATCAATATATTCAGAAA

ATAATCCTGAATATATTGATTTTCGTAACAGATACGGAGACTATAGATCTCTCATTATAAAAAGTGATCA

CGAGTTTGTAAAGCTATGTAAAGATCATGCAGAGAAAAGTTCTCCAGAAACGCAACAAATGATTATCAAA

CACATATACGAACAATATCTTATTCCAGTATCTGAAGTACTATTAAAACCTATAATGTCCATGGGTGACA

TATTTACATATAACGGATGTAAAGACAATGAATGGATGCTAGAACAACTCTCTACCCTAAACTTTAACAA

TCTCTACACATGGAACTCATGTAGCATAGGCAATGTAACGCGTCTGTTTTATACATTTTTTAGTTATCTG

ATGAAAGATAAACTAAATATATAAGTATAATCCCATTCTAATACTTTAACCTGATGTATTATTACCTGCA

TCTTATTAGAATATTAACCTAACTAAAAGACATAAAAAGCGGTAGGATATAAATATTATGGCCGCAACCG

TTCCGCGTTTTGACGATGTGTACAAAAATGCACAAAGAAGAATTCTAGATCAAGAAACATTTTTTAGTAG

AGGTCTAAGTAGACCGTTAATGAAAAACACATATCTATTTGATAATTACGCGTATGGATGGATACCAGAA

ACTGCAATTTGGAGTAGTAGATACGCAAACCTAGATGCTAGTGACTATTATCCCATTTCGTTGGGATTAC

TTAAAAAGTTTGAATTTCTCATGTCTCTATATAAAGGTCCTATTCCCGTATATGAAGAAAAAGTAAATAC

TGAATTCATTGCTAATGGATCTTTCTCCGGTAGATACGTATCATATCTTAGAAAGTTTTCTGCCCTTCCA

ACAAACGAGTTTATTAGTTTTTTATTATTGACCTCCATCCCTATCTATAATATCTTATTCTGGTTTAAAA

ACACACAGTTTGATATTACTAAACACACATTATTCAGATACGTCTATACAGATAATACCAAACACCTTGC

GTTGGCTAGGTATATACATCAAACAGGAGACTATAAGCCTTTGTTTAGTCGTCTCAAAGAGAATTATATA

TTTACTGGTCCCGTTCCAATAGGTATCAAAGATATAGATCACCCTAATCTTAGTAGAGCAAGAAGTCCAT

CCGATTATGAGACATTAGCTAATATTAGTACTATATTGTACTTTACCAAGTATGATCCAGTATTAATGTT

TTTATTGTTTTACGTACCTGGGTATTCAATTACTACAAAAATTACTCCAGCCGTAGAATATCTAATGGAT

AAACTGAATCTAACAAAGAGCGACGTACAACTGTTGTAAATTATTTTATGCTTCGTAAAATGTAGGTCTT

GAACCAAACATTCTTTGAAAAAATGAGATGCATAAAACTTTATTATCCAATAGATTAACTATTTCAGACG

TCAATCGTTTAAAGTAAACTTCGTAAAATATTCTTTGATTGCTGCCGAGTTTAAAACTTCTATCGATAAT

TGTTTCATATGTTTTAATATTTACAAGTTTTTTGGTCCATGGTACATTAGCTGGACAGATATATGCAAAA

TAATATCGTTCTCCAAGTTCTATAGTCTCTGGATTGTTTTTATTATATTCAGTAACCAAATACATATTAG

GGTTATCTGCGGATTTATAATTTGAGTGATGCATTCGACTCAACATAAATAATTCTAGAGGAGACGATCT

ACTATCAAATTCGGATCGTAAATCTGTTTCTAAAGAACGGAGAATATCTATACATACCTGATTAGAATTC

ATCCGTCCTTCAGACAACATCTCAGACAGTCTGGTCTTGTATGTCTTAATCATATTCTTATGAAACTTGG

AAACATCTCTTCTAGTTTCACTAGTACCTTTATTAATTCTCTCAGGTACAGATTTTGAATTCGACGATGC

CGAGTATTTCATCGTTGTATATTTCTTCTTCGATTGCATAATCAAATTCTTATATACCGCCTCAAACTCT

ATTTTAAAATTATTAAACAATACTCTACTATTAATCAGTCGTTCTAACTCCTTTGCTATTTCTATGGACT

TATCTACATCTTGACTGTCTATCTCTGTAAACACGGAGTCGGTATCTCCATACACGCTACGAAAACGAAA

TCTATAATCTATAGGCAACGATGTTTTCACAATCGGATTAATATCTCTATCGTCCATATAAAATGGATTA

CTTAATGTATTGGCAAACCGTAACATACCGTTGGATAACTCTGCTCCATTTAGTACCGATTCTAGATACA

ATATCATTCTACGTCCTATGGATGTGCAACTCTTAGCCGAAGCGTATGAGTATAGAGCACTATTTCTAAA

TCCCATCAGACCATATACTGAGTTGGCTACTATCTTGTACGTATATTGCATGGAATCATAGATGGCCTTT

TCAGTTGAACTGGTAGCCTGTTTTAACATCTTTTTATATCTGGCTCTCTCTGCCAAAAATGTTCTTAATA

GTCTAGGAATGGTTCCTTCTATTGATCTATCGAAAATTGCTATTTCAGAGATGAGGTTCGGTAGTCTAGG

TTCACAATGAACCGTAATATATCTAGGAGGTGGATATTTCTGAAGCAAGAGTTGATTATTTATTTCTTCT

TCCAATCTATTGGTACTAACAACGACACCGACTAATGTTTCCGGAGATAGATTTCCAAAGATACACACAT

TAGGATACAGACTGTTATAATCAAAGATTAATACATTATTACTAAACATTTTTTGTTTTGGAGCAAATAC

CTTACCGCCTTCATAAGGAAACTTTTGTTTTGTTTCTGATCTAACTAAGATAGTTTTAGTTTCCAACAAT

AGCTTTAACAGTGGACCCTTGATGATTGTACTCGCTCTATATTCGAATACCATGGATTGAGGAAGCACAT

ATGTTGCCGCACCAGCGTCTGTTTTTGTTTCTACTCCATAATACTCCCACAAATACTGACACAAACAAGC

ATCATGAATACAGTATCTAGCCATATCTAAAGCTATGTTTAGATTATAATCCTTATACATCTGAGCTAAA

TCAATGTCATCCTTTCCGAAAGATAATTTATATATATCATTAGGTAAAGTAGGACATGATAGTACGACTT

TAAATCCATTTTCCAAAATATCTTTACGAATTACTTTACATATAATATCCTCATCAACAGTCACGTAATT

ACCTGTGGTTAAAACCTTTGCAAATGTATCGGCTTTGCCTTTCGCGTCCGTAGTATCGTCACCGATGAAC

GTCATTTCTCTAACTCCTCTATTTAATACTTTACCCATGCAACTGAACGCGTTCTTGGATATAGAATCCA

ATTTGTACGAATCCAATTTTTCAGATTTTTGAATGAATGAATATAGATCGAAAAATATAGTTCCATTATT

GTTATTAACGTGAAACGTAGTATTGGCCATGCCGCATACTCCCTTATGACTAGACTGATTTCTCTCATAA

ATACAGAGATGTACAGCTTCCTTTTTGTCTGGAGATCTAAAGATAATCTTCTCTCCTGTTAATAACTCTA

GACGATTAGTAATATATCTCAGATCAAAGTTATGTCCGTTAAAGGTAACGACGTAGTCGAACGTTAGTTC

CAACAATTGTTTAGCTATTCGTAACAAAACTATTTCAGAACATAGAACTAGTTCTCGTTCGTAATCCATT

TCCATTAGCGACTGTATCCTCAAACATCCTCTATCGACGGCTTCTTGTATTTCCTGTTCCGTTAACATCT

CTTCATTAATGAGCGTAAACAGTAATCGTTTACCACTTAAATCGATATAACAGTAACTTGTATGCGAGAT

TGGGTTAATAAATACAGAAGGAAACTTCTTATCGAAGTGACACTCTATATCTAGAAATAAGTACGATCTT

GGGATATCGAATCTAGGTATTTCTTTAGCGAAACAGTTACGTGGATCGTCACAATGATAACATCCATTGT

TAATCTTTGTCAAATATTGCTCGTCCAACGAGTAACATCCGTCTGGAGATATCCCGTTAGAAATATAAAA

CCAACTAATATTGAAAAATTCATCCATGGTGGCATTTTGTATGCTGCGTTTCTTTGGCTCTTCTATCAAC

CACATATCTGCGACGGAGCATTTTCTATCTTTAATATCTAGATTATAACTTATTGTCTCGTCAATGTCTA

TAGTTCTCATCTTTCCCATCGGCCTCGCATTAAATGGAGGAGGAGATAATGACTGATATATTTCGTCCGT

CACTACGTAATAAAAGTAATGAGGAAATCGTATAAATACGGTCTCGCCATTTCGACATCTGGATTTCAGA

TATAAAAATCTGTTTTCACCGTGACTTTCAAACCAATTAATACACCTAACATCCATTTCTAGAATTTAGA

AATATATTTTCATTTAAATGAATCCCAAACATTGGGGAAGAGCCGTATGGACCATTATTTTTATAGTACT

TTCGCAAGCGGGTTTAGACGGCAACATAGAAGCGTGTAAACGAAAACTATATACTATAGTCAGCACTCTT

CCATGTCCTGCATGTAGACGACACGCGACTATCGCTATAGAGAACAATAATGTCATGTCTAGCGATGATC

TGAATTATATTTATTATTTTTTCATCAGATTATTTAACAATTTGGCATTTGATCCCAAATACGCAATCGA

TGTGTCAAAGGTTAAACCTTTATAAACTTAACCCATTATAAAACTTATGATTAGTCACGACTGAAATAAC

CGCGTGATTATTTTTTGGTATAATTCTACACGGCATGGTTTCTGTGACTATGAATTCAACACCTGTTATC

TTAGTGAAATCTTTAACAAACAGCAAGGGTTCGTCAAAGACATAAAACTCATTGTTTACGATCGAAATAG

ACCCCCTATCACACTTAAAATAAAAAATATCCTTATCCTTTACCACCAAATAAAATTCTGATTGGTCAAT

GTGAATGTATTCACTTAACAGTTCCACAAATTTATTTATTAACTCCGAGGCACATACATCGTCGGTATTT

TTTATGACAAACTTTACTCTTCCAGCATCCGTTTCTAAAAAAATATTAACGAGTTCCATTTATATCATCC

AATATTATTGAAATGACGTTGATGGACAGATGATATAAATAAGAAGGTACAGTACCTTTGTCCACCATCT

CCTCCAATTCATACTCTATTTTGTCATTAACTTTAATGTGTGAAAACAGTACGCCACATGCTTCCATGAC

AGTGTGTAACACTTTGGATACAAAATGTTTGACATTAGTATAATTGTCCAAGACTGTCAATCTATAATAG

ATAGTAGCTATAATATATTCTATGATGGTATTGAAGAAGATGACAACCTTGGCATATTGATCATTTAACA

CAGACATGGTATCAACAAATAGCTTAAATGAAAGAGAATCAGTAATTGGAATAAGCGTCTTCTCGATGTA

GTGTCCGTATACCAACATGTCTGATATTTTGATGTATTCCATTAAATTATTTAGTTTTTTCTTTTTATTC

TCGTTAAACAGAATTTCTGTCAATGGACCCCAACATCGTTGACCTATTAAGTTTTGATTGATTTTTCCGT

GTAAGGCGTATCTAGTCAGATCGTATAGCCTATCCAATAATCCATCGTCTGTGCGTAGATCACATCGTAC

ACTTTTTAATTTTCTATAGAAGAGTGACAGACATCTGGAGCAATTACAGACAGCAATTTCTTTATTCTCT

ACAGATGTAAGATACTTGAAGATATTCCTATGATGATGCAGAATTTTGGATAACACGGTATTGATGGTAT

CTGTTACCATAATTCCTTTGACTGATAGTGTCAAAGTACAAGATTTCCAATCTTTTGCAATTTTCAGTAC

CATTATCTTTGTTTTGATATCTATATCAGACAGCATGGTACGTCTGACAACACAGGGATTAAGACGGAAA

GATGAAATGATTCTCTCAACATCTTCAATAGATACCTTGCTATTTTTTTTGGCATTATCTATATGTGAGA

GAATATCCTCTAGAGAATCAGTATCCTTTTTGATGATAGTGGATCTCAATGACATGGGACGTCTAAACCT

TCTTATTCTATCACCAGATTGCATGGTGATTTGTCTTCTTTCTTTTATCATGATGTAATCTCTAAATTCA

TCGGCAAATTGTCTATATCTAAAATCATAATATGAGATGTTTACCTCTACAAATATCTGTTCGTCCAATG

TTAGAGTATCTATATCAGTTTTGTATTCCAAATTAAACATGGCAACGGATTTAATTTTATATTCCTCTAT

TAAGTCCTCGTCGATAATAACAGAATGTAGATAATCATTTAATCCATCGTACATGGTTGGAAGATGCTCG

TTGACAAAATCTTTAATTGTCTTGATGAAGGTGGGACTATATCTAACATCTTGATTAATAAAATTTATAA

CATTGTCCATAGGATACTTTGTAACTAGTTTTATACACATCTCTTCATTGGTAAGTTTAGACAGAATATC

GTGAACAGGTGGTATATTATATTCATCAGATATACGAAGAATAATGTCCAAATCTATATTGTTTAATATA

TTATATAGATGTAGTGTAGCTCCTACAGGAATATCTTTAACTAAGTCAATGATTTCATCAACAGTTAGAT

CTATTTTAAAGTTAATCATATAGGCATTGATTTTTAAAAGGTATGTAGCCTTGACTACATTCTCATTAAT

TAACCATTCCAAGTCACTGTGTGTAAGAAGATTATATTCTATCATAAGCTTGACTACATTTGGTCCCGAT

ACCATTAAAGAATTCTTATGATATAAGGAAACAGCTTTTAGGTACTCATCTACTCTACAAGAATTTTGGA

GAGCCTTAACGATATCAGTGACGTTTATTATTTCAGGAGGAAAGAACCTAACATTGAGAATATCTGAATT

AATAGCTTCCAGATACAGTGATTTTGGCAATAGTCCGTGTAATCCATAATCCAGTAACACGAGCTGGTGC

TTGCTAGACACCTTTTCAATGTTTAATTTTTTTGAAATAAGCTTTGATAAAGCCTTCCTCGCAAATTCCG

GATACATGAACATGTCGCCAACATGATTAAGTATTGTTTTTCATTATTTTTATATTTTCTCAACAAGTTC

TCAATACCCCAATAGATAATAGAATATCACCCAATGCGTCCATGTTGTCTATTTCCAACAGGTCGCTATA

TCCACCAATAGAAGTTTTCCCAAAAAAGATTCTAGGAACAGTTCTACCACCAGTAATTTGTTCAAAATAG

TCACGCAATTCATTTTCGGGTTTAAATTCTTTAATATCTACAATTTCATACGCTCCTCTTTTGAAACTAA

ACTTATTTAGAATATCCAGTGCGTTTCTACAAAAAGGACATGTAAACTTGACAAAAATTGTCACTTTGTT

ATTGGCCAACCTTTGTTGTACAAATTCCTCGGCCATTTTTAATATTTAAGTGATACAAAACTATCTCGAC

TTATTTAACTCTTTAGTCGAGATATATGGACACAGATAGCTATATGATAACCAACTACAGAAGACAAACG

CTATAAAAAACATAATTACGACGAGCATATTTATAAATATTTTTATTCAGTATTACTTGATATAGTAATA

TTAGGCACAGTCAAACATTCAACCACTCTAGATACATTAACTCTCTCATTTTCTTTAACAAATTCTGCAA

TATCTTCGTAAAAAGATTCTTGAAACTTTTTAGAATATCTATCGACTCTAGATGAAATAGCGTTCGTCAA

CATACTATGTTTTGTATACATAAAGGCGCCCATTTTAACAGTTTCTAGTGACAAAATGCTAGCGATCCTA

GGATCCTTTAGAATCACATAGATTGACGATTCGTCTCTCTTAGTAACTCTAGTAAAATAATCATACAATC

TAGTACGCGAAATAATATTATCCTTGACTTGAGGAGATCTAAACAATCTAGTTTTGAGAACATCGATAAG

TTCATCGGGAATTACATACATACTATCTTTAATAGAACTCTTTTCATCCAGTTGAATGGATTCGTCCTTA

ACCAACTGATTAATGAGATCTTCTATTTTATCATTTTCTAGATGATATGTATGTCCATTAAAGTTAAATT

GTGTAGCGCTTCTTTTTAGCCTAGCAGCCAATACTTTAACATCACTAATATCGATATACAAAGGAGATGA

TTTATCGATGGTATTAAGAATTCGTTTTTCGACATCCGTCAAAACCAATTCCTTTTTGCCTGTATCATCC

AGTTTGCCATTCTTTGTAAAGAAATTATTTTCTACTAGACTATTAATAAGACTGATAAGGATTCCTCCAT

AATTGCACAATCCAAACTTTTTCACAAAACTAGACTTTACGAGATCTACAGGAATGCGTACTTCAGGTTT

CTTAGCTTGTGATTTTTTCTTTTGTGGACATTTTCTAGTGACCAACTCATCTACCATTTCATTGATTTTA

GCAGTGAAATAAGCTTTCAATGCACGGGCACTGATACTATTGAAAACGAGTTGATCTTCAAATTCCGCCA

TTTAAGTTCACCAAACAACTTTTAAATACAAATATATCAATAGTAGTAGAATAAGAACTATAAAAAAAAT

AATAATTAACCAATACCAACCCCAACAACCTGTATTATTAGTTGATGTGACAGTTTTCTCATCACTTAGA

ACAGATTTAACAATTTCTATAAAGTCTGTCAAATCATCTTCCTGAGAACCCATAAATACACCAAATATAG

CAGCGTACAACTTATCCATTTATACATTGAATATTGGCTTTTCTTTATCGCTATCTTCATCATATTCATC

ATCAATATCAACAAGTCCCAGATTACGAACCAGATCTTCTTCTACATTTTCAGTCATTGATACGCGTTCA

CTATCTCCAGAGAGTCCGATAACGTTAGCCACTACTTCTCTATCAATGATTAGTTTCTTGAGCGCGAATG

TAATTTTTGTTTCCGTTCCGGATCTATAGAAAACTACAGGTGTAATAATTGCCTTGGCTAATTGTCTTTC

TCTTTTACTGAGTGATTCTAGTTCACCTTCTATAGATCTGAGAATGGATGATTCTCCAGTCGAAACATAT

TCTACCATGGCTCCGTTTAATTTGTTGATGAAGATGGATTCATCCTTAAATGTTTTCTCTGTAATAGTTT

CCGCCGAAAGACTATGCAAAGAATTTGGAATGCGTTCCTTGTGTGTAATGTTTCCATAGACAGCTTCTAG

AAGTTGATACAACATAGGACTAGCCGCGGTAACTTTTATTTTTAGAAAGTATCCATCGCTTCTATCTTGT

TTAGATTTATTTTTATAAAGTTTAGTCTCTCCTTCCAACATAATAAAAGTGGAAGTCATCTGACTAGATA

AACTATCAGTAAGTTTTATAGAGATAGATGAACAATTAGCGTATTGAGAAGCATTTAGTGTAACGCATTC

GATACATTTTGCATTAGATTTACTAATCGATTTTGCATACTCTATAACACCCGCACAAGTCTGTAGAGAA

TCGCTAGATGCTGTAGGTCTTGGTGAAGTTTCAACTCTCTTCTTGATTACCTTACTCATGATTAAACCTA

AATAATTGTACTTTGTAATATAATGATATATATTTTCACTTTATCTCATTTGAGAATAAAAATGTTTTTG

TTAACCACTGCATGATGTACAGATTTCGGAATCGCAAACCACTTGTGGTTTTATTTTATCCTTGTCCAAT

GTGAATTGAATGGGAGCGGATGCGGGTTTCGTACGTAGATAGTACATTCCCGTTTTTAGACCGAGACTCC

ATCCGTAAAAATGCATACTCGTTAGTTTGGAATAACTCGGATCTGCTATATGGATATTCATAGATTGACT

TTGATCGATGAAGGCTCCCCTGTCTGCAGCCATTTTTATGATCGTCTTTTGTGGAATTTCCCAAATAGTT

TTATAAACTCGCTTAATATCTTCTGGAAGGTTTGTATTCTGAATGGATCCACCATCTACCATAATCCTAT

TCTTGATCTCATCATTCCATAATTTTCTCTCGGTTAAAACTCTAAGGAGATGCGGGTTAACTACTTGGAA

TTCTCCAGACAATACTCTCCGAGTGTAAATATTACTGGTATACGGTTCCACCGACTCATTATTTCCCAAA

ATTTGAGCAGTTGATGCAGTCGGCATAGGTGCCACCAATAAACTATTTCTAAGACCGTATGTTCTGATTT

TATCTTTTAGAGGTTCCCAATTCCAAAGATCCGACGGTACAACATTCCAAAGATCATATTGTAGAATACC

GTTACTGGCGTACGATCCTACATATGTATCATATGGTCCTTCCTTCTCAGCTAGTTTACAACTCGCCTCT

AATGCACCGTAATAAATGGTTTCAAAGATCTTCTTATTTAGATCTTGTGCTTCCAGGCTATCAAATGGAT

AATTTAAGAGAATAAACGCGTCCGCTAATCCTTGAACACCAATACCGATAGGTCTATGTCTCTTATTAGA

GATTTCAGCTTCTGGAATAGGATAATAATTAATATCTATAATTTTATTGAGATTTCTGACAATTACTTTG

ACCACATCCTTCAGTTTGAGAAAATCAAATCGCCCATCTATTACAAACATGTTCAATGCAACAGATGCCA

GATTACACACGGCTACCTCATTAGCATCCGCATATTGTATTATCTCAGTGCAAAGATTACTACACTTGAT

GGTTCCTAAATTTTGTTGATTACTCTTTTTGTTACACGCATCCTTATAAAGAATGAATGGAGTACCAGTT

TCAATCTGAGATTCTATAATCGCTTTCCAGACGACTCGAGCCTTTATTATACATTTGTATCTCCTTTCTC

TTTCGTATAGTGTATACAATCGTTCGAACTCGTCTCCCCAAACATTGTCCAATCCAGGACATTCATCCGG

ACACATCAACGACCACTCTCCGTCATCCTTCACTCGTTTCATAAAGAGATCAGGAATCCAAAGAGCTATA

AATAGATCTCTTGTTCTATGTTCATCGTTTCCTGTATTCTTTTTAAGATCGAGGAACGCCATAATATCAG

AATGCCACGGTTCCAAGTATATGGCCATAACTCCAGGCCGTTTGTTTCCTCCCTGATCTATGTATCTAGC

GGTGTTATTATAAACTCTCAACATTGGAATAATACCGTTTGATATACCATTGGTACCGGAGATATAGCTT

CCACTGGCACGAATATTACTAATTGATAGACCTATTCCCCCTGCCATTTTAGAGATTAATGCGCATCGTT

TTAACGTGTCATAGATGCCTTCTATGCTATCATCGATCATGTTAAGTAGAAAACAGCTAGACATTTGGTG

ACGAGTAGTTCCCGCATTAAATAAGGTAGGAGAAGCGTGCGTAAACCATTTTTCAGAAAGTAGATTGTAC

GTCTCAATAGCTGAGTCTATATCCCATTGATGAATTCCTACTGCGACACGCATTAACATGTGCTGAGGTC

TTTCAACAATTTTGTTGTTTATTTTCAACAAGTAGGATTTTTCCAAAGTTTTAAAACCAAAATAGTTGTA

TGAAAAGTCTCGTTCGTAAATAATAACCGAATTGAGCTTATCCTTATATTTGTTAACTATATCCATGGTA

ATACTTGAAATAATCGGAGAATGTTTCCCATTTTTAGGATTAACATAGTTGAATAAATCCTCCATCACTT

CACTAAATAGTTTTTTTGTTTCCTTGTGTAGATTTGATATGGCTATTCTGGCGGCTAGAATGGCATAATC

CGGATGTTGTGTAGTACAAGTGGCTGCTATTTCGGCTGCCAGAGTGTCCAATTCTACCGTTGTTACTCCA

TTATATATTCCTTGAATAACCTTCATAGCTATTTTAATAGGATCTATATGATCAGTGTTTAAGCCATAGC

ACAATTTTCTAATACGAGACGTGATTTTATCAAACATGACATTTTCCTTGTATCCATTTCGTTTAATGAC

AAACATTTTTGTTGGTGTAATAAAAAAAATTATTTAATTTTTCATTAATAGGGATTTGACGTATGTAGCG

TACAAAATTATCGTTCCTGGTATATAGATAAAGAGTCCTATATATTTGAAAATCGTTACGGTTCGATTAA

ACTTTAATGATTGCATTGTGAATATATCATTAGGATTTAACTCCTTGACTATCATGGCGGTGCCAGAAAT

TACCATCAAAAGCATTAATACAGTTATGCAGATCGCAGTTAGAACGGTTATAGCATCCACCATTTATATC

TAAAAATTAGATCAAAGAATATGTGACAACGTCCTAGTTGTATACTGAGAATTGACGAAACAATGTTTCT

TACATATTTTTTTCTTATTAGTAACCGACTTAATAGTAGGAACTGGAAAACTAGACTTGATTATTCTATA

AGTATAGATACCCTTCCAAATAATGTTCTCTTTGATAAAAGTTCCAGAAAATGTAGAATTTTTTAAAAAG

TTATCTTTTGCTATTACTAATATCGTGGTTAGACGCTTATTATTAATATGAGTGATGAAATCCACACCGC

TTCTAGATATCGCTTTTATTTCCACATTAGATGGTAAATCCAATAGTGAAACTATCTTTTTAGGAATGTA

TGGACTCGCGTTTAGAGGAGTGAACGTCTTCGGAGTAGTAAAGGATGATTCGTCAAATGAATAAACAATT

TCACAAATGGATGTTAATGTATTAGTAGGAAATTTTTTGACGCTAGTGGAATTGAAGATTCTAATGGATG

ATGTTCTACCTATTTCATCCGATAACATGTTAATTTCCAATACCAACGGTTTTAATATTTCGATGATATA

CGGTAGTCTCTCTTTCGGACTTATATAGCTTATTCCACAATACGAGTCATTATATACTCCAAAAAACAAA

ATAACTAGTATAAAATCTGTATCGAATGGGAAAAACGAAATTATCGATATAGGTATAGAATCCGGAACAT

TGAACGTATTAATACTTAATTCTTTTTCAGTGGTAAGAACCGATAGGTTATTGACATTGTATGGTTTTAA

ATATTCTATAACTTGAGACTTGATAGATATTAATGACGAATTGAAAATTATTTTTATCACCACGTGTGTT

TCAGGATCATCGTCGACGCCAGTTAACCAACCGAATGGAGTAAAATAAATATCATTAATATATGCTCTAG

ATATTAGTATTTTTATTAATCCTTTGATTATCATCTTCTCGTACGCGAATGATTCCATGATCAAGAGTGA

TTTGAGAACATCCTCCGGAGTATTAATGGGTTTAGTAAACAGTCCATCGTTGCAATAATAAAAGTTGTCC

AAGTTAAAGGATATTATGCATTCGTTTAAAGATATCACCTCATCTAACGGAGACAATTTTTTGGTAGGTT

TTAGAGACTTTGAAGCTACTTGTTTAACAAAGTTATTCATCGTCGTCTACTATTCTATTTAATTTTGTAG

TTAATTTATCACATATCACATTAATTGACTTTTTGGTCCACTTTTCCATACGTTTATATTCTTTTAATCC

TGCGTTATCCGTTTCCGTTATATACAGGGATAGATCTTGCAAGTTAAATAGAATGCTCTTAAATAATGTC

ATTTTTTTATCCGCTAAAAATTTAAAGAATGTATAAACTTTTTTCAAAGATTTAAAACTTTTAGGTGGAG

TTCTGGTACACAATATCATAAACAAACTAATAAACATCCCACATTCAGATTCCAACAATTGATTAACTTC

CACATTAATACAGCCTATTTTCGCTCCAAATGTACATTCGAAAAATCTGAATAAAACATCAATATCGCAA

TTTGTATTATCCAATACAGAATGTCTGTGATTCGTGTTAAAACCATCGGAAAAAGAATAGAAATAAAAAT

TATTATAATGGTGGAATTCAGTTGGAATATTGCCTCCGGAGTCATAAAAGGATACTAAACATTGTTTTTT

ATCGTAAATTACACATTTCCAATGAGACAAATAACAAAATCCAAACATTACAAATCTAGAGGTAGAACTT

TTAATTTTGTCTTTAAGTATATACGATAAGATATGTTTATTCATAAACGCGTCAAATTTTTCATGAATAG

CTAAGGAGTTTAAGAATCTCATGTCAAATTGTCCTATATAATCCACTTCGGATCCATAAGCAAACTGAGA

GACTAAGTTCTTAATACTTCGATTGCTCATCCAGGCTCCTCTCTCAGGCTCTATTTTCATCTTGACGACC

TTTGGATTTTCACCAGTATGTATTCCTTTACGTGATAAATCATCAATTTTCAAATCCATTTGTGAGAAGT

CTATCGCCTTAGATACTTTTTCCCGTAGTTGAGGTTTAAAGAAATACGCTAACGGTATACTAGTAGGTAA

CTCAAAGACATCATATATAGAATGGTAACGCGTCGTTAACTCGTCGGTTAACTCTTTCTTTTGATCGAGT

TCATCGCTACTATTGGGTCTGCTCAGGTGCCCCGACTCTACTAGTTCCAACATCATACCGATAGGAATAC

AAGACACTTTGCCAGCGGTTGTAGATTTATCATATTTCTCCACCACATATCCGTTACAATTTGTTAAGAA

TTTAGATACATCTATATTGCTACATAATCCAGCTAGTGAATATATATGACATAATAAATTGGTAAATCCT

AGTTCTGGTATTTTACTAATTACTAAATCTGTATATCTTTCCATTTATCATGGAAAAGAATTTACCAGAT

ATCTTCTTTTTTCCAAACTGCGTTAATGTATTCTCTTACAAATATTCACAAGATGAATTCAGTAATATGA

GTAAAACGGAACGTGATAATTTCTCATTGGCTGTGTTTCCAGTGATAAAACATAGATGGCATAACGCACA

CGTTGTAAAACATAAAGGAATATACAAAGTTAGTACAGAAGCACGTGGAAAAAAAGTATCTCCTCCATCA

CTAGGAAAACCCGCACATATAAACCTAATGTCGAAGCAATATATATATAGTGAGTATGCAATAAGCTTTG

AATGTTATAGTTTTCTAAAATGTATAACAAATACAGAAATCAATTCGTTCGATGAGTATATATTAAGAGG

ACTATTAGAAGCTGGTAATAGTTTACAGATATTTTCCAATTCCGTAGGTAAACGAATAGATACTATAGGT

GTACTAGGGAATAAGTATCCATTTAGCAAAATTCCATTGGCCTCATTAACTCCTAAAGCACAACGAGAGA

TATTTTTAGCGTGGATTTCTCATAGACCTGTAGTTTTAACTGGAGGAACCGGAGTGGGTAAGACGTCACA

GGTACCCAAGTTATTGCTTTGGTTTAATTATTTATTTGGTGGATTCTCTTCTCTAGATAAAATCACTGAC

TTTCACGAAAGACCAGTCATTCTATCTCTTCCTAGGATAGCTTTAGTTAGATTGCATAGCAATACCATTT

TAAAATCATTGGGATTTAAGGTACTAGATGGATCTCCTATCTCTTTACGGTACGGATCTATACCGGAAGA

ATTAATAAACAAACAACCAAAAAAATATGGAATTGTATTTTCTACCCATAAGTTATCTCTAACAAAACTA

TTTAGTTATGGCACTATTATTATAGACGAAGTTCATGAGCATGATCAAATAGGAGATATTATTATAGCAG

TAGCGAGAAAACATCATACGAAAATAGATTCTATGTTTTTAATGACTGCCACGTTAGAGGATGACAGGGA

ACGTCTAAAAATATTTTTACCTAATCCCGCATTTATACATATTCCTGGAGATACACTGTTTAAAATTAGC

GAGGTATTTATTCATAATAAGATAAATCCATCTTCCAGAATGGCATATATAGAAGAAGAAAAGAGAAATT

TAGTTACTGCTATACAGATGTATACTCCTCCTGATGGATCATCCGGTATAGTCTTTGTGGCATCCGTTGC

ACAGTGTCACGAATATAAATCATATTTAGAAAAAAGATTACCGTATGATATGTATATTATTCATGGTAAG

GTCTTAGATATAGACGAAATATTAGAAAAAGTGTATTCATCACCTAATGTATCGATAATTATTTCTACTC

CTTATTTGGAATCCAGCGTTACTATACGCAATGTTACACACATTTATGATATGGGTAGAGTTTTTGTCCC

CGCTCCTTTTGGAGGATCACAACAATTTATTTCTAAATCTATGAGAGATCAACGAAAAGGAAGAGTAGGA

AGAGTTAATCCTGGAACATACGTATATTTCTATGATCTGTCTTATATGAAATCTATACAGCGAATAGATT

CAGAATTTCTACATAATTATATATTGTACGCTAATAAGTTTAATCTAACACTCCCCGAAGATTTGTTTAT

AATCCCTACAAATTTGGATATTCTATGGCGTACAAAGGAATATATAGACTCGTTCGATATTAGTACAGAA

ACATGGAATAAATTATTATCCAATTATTATATGAAGATGATAGAGTATGCTAAACTTTATGTACTAAGTC

CTATTCTCGCTGAGGAGTTGGATAACTTTGAGAGGACGGGAGAATTAACTAGTATTGTACAAGAAGCCAT

TTTATCTCTAAATTTACAAATTAAGATTTTAAAATTTAAACATAAAGATGATGATACGTATATACACTTT

TGTAGAATATTATTCGGTGTCTATAACGGAACAAACGCTACTATATATTATCATAGACCTCTAACGGGAT

ATATGAATATGATTTCAGATACTATATTTGTTCCTGTAGATAATAACTAAAAATCAAAATCTAATGACCA

CATCTTTTTTTAGAGATGAAAAATTTTCCACATCTCCTTTTGTAGACACGACTAAACATTTTGCAGAAAA

AAGTTTATTATTATTTAGATAATCGTATACTTCATCAGTGTAGATAGTAAATGTGAACAGATAAAAGGTA

TTCTTGCTCAATAGATTGGTAAATTCCATAGAATATATTAATCCTTTCTTCTTGAGATCCCACATCATTT

CAACCAAAGACGTTTTATCCAATGATTTACCTCGTACTATACCACATACAAAACTAGATTTTGCAGTGAT

GTCGTACCTGGTATTCCTACCAAACAAAATTTTACTTTTAGTTCTTTTAGAAAATTCTAAGGTAGAATCT

CTATTTGTCAATATGTCATCTATGGAATTACCACTAGCAAAAAATGATAGAAATATATATTGATACATCG

CAGCTGGTTTTGATCTACTATACTTTAAAAACGAATCAGATTCCATAATTGCTTGTATATCATCAGCTGA

AAAACTATGTTTTACACGTATTCCTTCGGCATTTCTTTTTAATGATATATCTTGTTTAGACAATGATAAA

GTTATCATGTCCATGAGAGACGCGTCTCCGTATCGTATAAATATTTCATTAGATGTTAGACGCTTCATTA

GGGGTATACTTCTATAAGGTTTCTTAATTAGTCCATCATTGGTTGCGTCAAGAACTACTATCTGATGTTG

TTGGGTATCTCTAGTGTTACACATGGCCTTACTAAAGTTTGGGTAAATAACTATGATATCTCTATTAATT

ATAGATGTATATATTTCATTCGTCAAGGATATTAATATCGACTTACTATCGTCATTAATACGTGTAATGT

AATCATATAAATCATGCGATAGCCAAGGAAAATTCAAATAGATGTTCATCATATAATCGTCGCTATAATT

CATATTAATACTTTGACATTGACTAATTTGTAATATAGCCTCGCCACGAAGAAAGCTCTCGTATTCAGTT

TCATCGATAAAGGATACCGTTAAATATAACTGGTTGCCGATAGTCTCATAGTCTATTAAGTGGTAAGTTT

CGTATAAATACAGAATCCCTAAAATATTATCTAATGTGGGATTAATCCTTACCATAACTGTATAAAATGG

AGCCGGAGTCATAACTATTTTACCGTTTGTACTTACTGGAATAGATGAAGGAATAATCTCCGGACATGAT

GGTAAAGACCCAAATGTCTGTTTGAAGAAATCCAATGTTCCAGGTCCTAATCTCTTGACAAAAATTACGA

TATTCGATCCCGATATCCTTTGCATTCTATTTACCAGCATATCACGAACTATATTAAGATTATCTATCAT

GTCTATTCTCCCACCGTTATATAAATCGCCTCCGCTAAGAAACGTTAGTATATCCATACAATGGAATACT

TCATTTCTAAAATAGTATTCGTTTTCTAATTCTTTAATGTGAAATCGTATACTAGAAAGGGAAAAATTAT

CTTTGAGTTTTCCATTAGAAAAGAACCACGAAACTAATGTTCTGATTGCGTCTGACTCCGTCGCTGAATT

AATAGATTTACACCAAAAACTCATATAACTTCTAGATGTAGAAGCATTCGCTAAAAAATTAGTAGAATCA

AAGGATATAAGTAGATGTTCCAACAAGTGAGCAATTCCCAAGATTTCATCTATATCATTCTCGAATCCGA

AATTAGAAATTCCCAAGTAGATATCCTTTTTCATCCGATCATTGATGAAAATACGAACTTTATTCGGTAA

GACGATCATTTACTAAGGAGTAAAATAGGAAGTAACGTTCGTATATCGTTATCGTCGTATAAATTAAAGG

TGTGTTTTTTGCCATTAAGAGACATTATAATTTTACCAATATTGGAATTATAATATAGGTGTATTTGAGC

ACTAGAAACGGTCGATGCATCGGTAAATATAGCTGTATCTAATGTTCTAGTCGGTATTTCTTCATTTCGC

TGTCTAATGATAGCGTTTTCTCTATCTGTTTCCATTACAGCTGCCTGAAGTTTATTGGTCGGATAATATG

TAAAATAATAAGAAATACATACGAATAACAAAAATAAAATAAGATATAATAAAGATGCCATTTAGAGATC

TAATTTTGTTCAACTTGTCCAAATTCCTACTTACAGAAGATGAGGAATCGTTGGAGATAGTATCTTCCTT

ATGTAGAGGATTTGAAATATCTTACGATGACTTAATATCGTACTTTCCAGATAGGAAATACCATAAATAT

ATTTCTAAGGTATTTGAACATGTAGATTTATCGGAGGAATTAAGTATGGAATTCCATGATACAACTCTGA

GAGATTTAGTATATCTTAGATTGTACAAGTATTCCAAGTATATACGGCCGTGTTATAAATTAGGAGATAA

TCTAAAAGGTATAGTTGTTATAAAGGACAGAAATATATATATTAGAGAAGCAAATGATGACTTGATAGAA

TATCTCCTCAAGGAATACACTCCTCAGATTTATACATATTCTAATGAGCGAGTTCCCATAGCTGGTTCAA

AATTAATTCTTTGTGGATTTTCTCAAGTTACATTTATGGCGTATACAACGTCGCATATAACAACAAATAA

AAAGGTAGATGTTCTCGTTTCCAAAAAATGTATAGATGAACTAGTCGATCCAATAAATTATCAAATACTT

CAAAATTTATTTGATAAAGGAAGCGGAACAATAAACAAAATACTCAGGAAGATATTTTATTCGGTAACAG

GTGGCCAAACTCCATAGGTAGCTTTTTCTATTTCGGATTTTAGAATTTCCAAATTCACCAGCGATTTATC

GGTTTTGGTGAAATCCAAGGATTTATTAATGTCCACAAATGCCATTTGTTTTGTCTGTGGATTGTATTTG

AAAATGGAAACGATGTAGTTAGATAGATGCGCGGCGAAGTTTCCTATTAGGGTTCCGCGCTTCACGTCAC

CCAACATACTTGAATCACCATCCTTTAAAAAAAATGATAAGATATCAACATGGAGTATATCATACTCGGA

TTTTAATTCTTCTACTGCCTCACTGACATTTTCACAAATACTACAATACGGTTTACCGAAAATAATCAGT

ACGTTCTTCATTTATGGGTATCAAAAACTTAAAATCGTTACTGCTGGAAAATAAATCACTGACGATATTA

GATGATAATTTATACAAAGTATACAATGGAATATTTGTGGATACAATGAGTATTTATATAGCCGTCGCCA

ATTGTGTCAGAAACTTAGAAGAGTTAACTACGGTATTCATAAAATACGTAAACGGATGGGTAAAAAAGGG

AGGACATGTAACCCTTTTTATCGATAGAGGAAGTATAAAAATTAAACAAGACGTTAGAGACAAGAGACGT

AAATATTCTAAATTAACCAAGGACAGAAAAATGTTAGAATTAGAAAAGTGTACATCCGAAATACAAAATG

TTACCGGATTTATGGAAGAAGAAATAAAGGCAGAAATGCAATTAAAAATCGATAAACTCACATTTCAAAT

ATATTTATCTGATTATGATAACATAAAAATATCATTGAATGAGATACTAACACATTTCAACAATAATGAG

AATGTTACATTATTTTATTGTGATGAACGAGACGCAGAATTCGTTATGTGTCTAGAGGCTAAAACACAGT

TCTCTACCACAGGAGAATGGCCGTTAATAATAAGTACCGATCAGGATACTATGCTATTCGCGTCTGCTGA

TAATCATCCTAAGATGATAAAAAACTTAACTCAACTGTTTAAATTTGTTCCCTCGGCAGAGGATAACTAT

TTAGCAAAATTAACTGCATTAGTGAATGGATGTGATTTCTTTCCTGGACTCTATGGGGCATCTATAACAC

CCAACAACTTAAACAAAATACAATTGTTTAGTGATTTTACAATCGATAATATAGTCACTAGTTTGGCAAT

TAAAAATTATTATAGAAAGACTAACTCTACCGTAGACGTGCGTAATATTGTTACGTTTATAAACGATTAC

GCTAATTTAGACGATGTCTACTCGTATATTCCTCCTTGTCAATGCACTGTTCAAGAATTTATATTCTCCG

CATTAGATGAAAAATGGAATGAATTTAAATCATCTTATTTAGAGAGCGTGCCGTTACCCTGCCAATTAAT

GTACGCATTAGAACCACGTAAGGAGATTGATGTTTCAGAAGTTAAAACTTTATCATCTTATATAGATTTC

GAAAATACTAAATCAGATATCGATGTTATAAAATCTATATCCTCGATTTTTGGATATTCTAACGAAAACT

GTAACACCATAGTGTTCGGCATCTATAAGGATAATTTACTACTGAGTATAAATAATTCATTTTACTTTAA

CGATAGTCTGTTAATAACCAATACTAAAAGTGATAATATAATAAATATAGGTTACTAGATTAAAAAATGG

TGTTCCAGCTCGTGTGTTCTACATGCGGCAAAGATATTTCTCACGAACGATATAAATTGATTATACGAAA

AAAATCATTAAAGGATGTACTAGTCAGTGTAAAGAACGAATGTTGTAGGTTAAAATTATCTACACAAATA

GAACCTCAACGTAACTTAACAGTGCAACCTCTATTGGATATAAACTAATGGATCCGGTTAATTTTATCAA

GACATATGCGCCTAGAGGTTCTATTATTTTTATTAATTATGCCATGTCATTAACTAGTCATTTGAATCCA

TCGATAGAAAAACATGTGGGTATTTATTATGGTACGTTATTATCGGAACACTTGGTAGTTGAATCTACCT

ATAGAAAAGGAGTTAGAATAGTCCCATTGGATAGATTTTTTGAAGGATATCTTAGTGCAAAAGTATACAT

GTTAGAGAATATTCAAGTTATGAAAATAGCAGCTGATATGTCGTTAACTTTACTAGGTATTCCATATGGA

TTTGGTCATGATAGAATGTATTGTTTTAAATTGGTAGCTGAATGTTATAAAAATGCCGGTATTGATACAT

CGTCTAAACGAATATTAGGTAAAGATATTTTTCTGAGCCAAAACTTTACAGATGATAATAGATGGATAAA

GATATATGATTCTAATAATTTAACATTTTGGCAAATTGATTACCTTAAAGGGTGAGTTAATATGCATAAC

TACTCCTCCGTTGTTTTTTCCCTCGTTCTTTTTCTTAACGTTGTTTGCCATCACTCTCATAATGTAAAGA

TATTCTAAAATGGTAAACTTTTGCATATCGGATGCAGAAATTGGTATAAATGTTGTAATTGTATTATTTC

CCGTCAATGGACTAGTCACAGCTCCATCAGTTTTATATCCTTTAGAGTATTTCTCACTCGTGTCTAGCAT

TCTAGAGCATTCCATGATCTGTTTATCGTTGATATTGGCCGGAAAGATAGATTTTTTATTTTTTATTATA

TTACTATTGGCAATTGTAGATATAACTTCTGGTAAATATTTTTCTACCTTTTCAATCTCTTCTATTTTCA

AGCCGGCTATATATTCTGCTATATTGTTACTAGTATCAATACCTTTTCTGGCTAAGAAGTCATATGTGGT

ATTCACTATATCAGTTTTAACTGGTAGTTCCATTAGCCTTTCCACTTCTGCAGAATAATTAGAAATTGGT

TCTTTACCAGAAAATCCAGCTACTATAATAGGCTCACCGATGATCATTGGCAAAATCCTATATTGTACCA

GATTAATGAGAGCATATTTCATTTCCAATAATTCTGCTAGTTCTTGAGACATTGATTTATTTGATGAATC

TATTTGGTTCTCTAGATACTCTACCATTTCTGCCGCATACAATAACTTGTTAGATAAAATCAGGGTTATC

AAAGTGTTTAGTGTGGCTAGAATAGTGGGCTTGCACGTATTAAAGAATGCTGTAGTATGAGTAAACCGTT

TTAACGAATTATATAGTCTCCAGAAATCTGTGGCGTTGCATACATGAACTGAATGACATCGAAGATTGTC

CAATATTTTTAATAGCTGCTCTTTGTCCATTATTTCTATATTTGACTCGCAACAATTGTAGATACCATTA

ATCACTGATTCCTTTTTCGATGCCGGACAATAGCACAATTGTTTAGCTTTGGACTCTATGTATTCAGAAT

TAATAGATATATCTCTCAATACAGATTGCACTATACATTTTGAAACTATGTCAAAAATTGTAGAACGACG

CTGTTCTGTAGCCATTTAACTTTAAATAATTTACAAAAATTTAAAATGAGCATCCGTATAAAAATCGATA

AATTGCGCCAAATTGTGGCATATTTTTCAGAGTTCAGCGAAGAAGTGTCTATAAATGTAGACTTGACGGA

TGAATTAATGTATATTTTTGCCGCCTTGGGCGGATCTGTAAACATTTGGGCCATTATACCTCTCAGTGCA

TCAGTGTTCTACCGCGGAGCCGAAAATATTGTGTTTAACCTTCCAGTGTCCAAGGTAAAATCGTGTTTGT

GTAGTTTTCACAATGATGCTATCATAAATATAGAACCTGATCTGGAAAATAATCTAGTAAAACTTTCTAG

TTATCATGTAGTAAGTGTCGATTGTAACAAGGAACTGATGCCTATTAGGACAGATACTACTATTTGTCTA

AGTATAGATCAAAAGAAATCTTACGTATTTAATTTTCACAAGTATGAAGAAAAATGTTGTGGTAGAACCG

TCATTCATCTAGAATGGTTGTTGGGCTTTATCAAGTGTATTAGTCAGCATCAGCATTTGGCTATTATGTT

TAAAGATGACAATATTATTATGAAGACTCCTGGTAATACTGATGCGTTTTCCAGGGAATATTCTATGACT

GAATGTTCTCAAGAACTACAAAAGTTTTCTTTCAAAATAGCTATCTCGTCTCTCAACAAACTACGAGGAT

TCAAAAAGAGAGTCAATGTTTTTGAAACTAGAATCGTAATGGATAATGACGATAACATTCTAGGAATGTT

GTTTTCGGATAGAGTTCAATCCTTTAAGATTAACATCTTTATGGCGTTTTTAGACTAATACTTTCAATGA

GATAAATATGGGTGGCGGAGTAAGTGTTGAGCTCCCTAAACGGGATCCACCTCCGGGAGTACCCACTGAT

GAGATGTTATTAAACGTGGATAAAATGCATGACGTGATAGCTCCCGCTAAGCTTTTAGAATATGTGCATA

TAGGACCACTAACAAAAGATAAAGAGGATAAAGTAAAGAAAAGATATCCAGAGTTTAGATTAGTCAACAC

AGGACCCGGTGGTCTTTCGGCATTATTAAGACAATCATATAATGGAACCGCACCCAATTGCTGTCGCACT

TTTAATCGTACTCATTATTGGAAGAAGGATGGAAAGATATCAGATAAGTATGAAGAGGGTGCAGTATTAG

AATCGTGTTGGCCCGACGTCCACGACACTGGAAAATGCGATGTTGATTTATTCGACTGGTGTCAGGGGGA

TACGTTCGATATAAACATATGCCATCAGTGGATCGGTTCAGCCTTTAATAGGAGTGATAGAACTGTAGAG

GGTCGACAATCGTTAATAAATCTGTATAATAAGATGCAAAGATTATGTAGTAAAGATGCTAGTGTACCAA

TATGTGAATTATTTTTGCATCATTTACGCGCACACAATACAGAAGATAGTAAAGAGATGATCGATTATAT

TCTAAGACAACAGTCGGCGGACTTTAAACAGAAATATATGAGATGTAGTTATCCCACTAGAGATAAGTTA

GAAGAGTCATTAAAATATGCGGAACCTCGAGAATGTTGGGATCCAGAGTGTTCGAATGCCAATGTTAATT

TCTTACTAACACGTAATTATAATAATTTAGGACTTTGCAATATTGTACGATGTAATACGAGCGTGAATAA

CTTACAGATGGATAAAACTTCCTCATTAAGATTATCATGTGGATTAAGCAATAGTGATAGATTTTCTACT

GTTCCCGTCAATAGAGCAAAAGTAGTTCAACATAATATTAAACATTCGTTCGACCTAAAATTGCATTTGA

TCAGTTTATTATCTCTCTTGGTAATATGGATACTAATTGTAGCTATTTAAATGGGTGCCGCAGCAAGCAT

ACAGACGACTGTGAATACACTCAGTGAACGTATCTCGTCTAAATTAGAACAAGAAGCGAACGCTAGTGCT

CAAACAAAATGTGATATAGAAATCGGAAATTTTTATATCCGACAAAACCATGGATGTAACATCACTGTTA

AAAATATGTGCTCTGCGGACGCGGATGCTCAGTTGGATGCTGTGTTATCAGCCGCTACAGAAACATATAG

TGGATTAACACCGGAACAAAAAGCATACGTACCAGCTATGTTTACTGCTGCGTTAAACATTCAGACGAGT

GTAAACACTGTTGTTAGAGATTTTGAAAATTATGTGAAACAGACTTGTAATTCTAGCGCTGTTGTCGATA

ACAAATTAAAGATACAAAACGTAATTATAGATGAATGTTACGGAGCCCCAGGATCTCCAACAAATTTGGA

ATTTATTAATACAGGATCTAGCAAAGGAAATTGTGCCATTAAGGCGTTGATGCAATTGACTACTAAGGCC

ACTACTCAAATAGCACCTAGACAAGTTGCTGGTACAGGAGTTCAGTTTTATATGATTGTTATCGGTGTTA

TAATATTGGCAGCGTTGTTTATGTACTATGCCAAGCGTATGCTGTTCACATCCACCAATGATAAAATCAA

ACTTATTTTAGCCAATAAGGAAAACGTCCATTGGACTACTTACATGGACACATTCTTTAGAACTTCTCCG

ATGATTATTGCTACCACGGATATACAAAACTGAAAATATATTGATAATATTTTAATAGATTAACATGGAA

GTTATCGCTGATCGTCTAGACGATATAGTGAAACAAAATATAGCGGATGAAAAATTTGTAGATTTTGTTA

TACACGGTCTAGAGCATCAATGTCCTGCTATACTTCGACCATTAATTAGGTTGTTTATTGATATACTATT

ATTTGTTATAGTAATTTATATTTTTACGGTACGTCTAGTAAGTAGAAATTATCAAATATTGTTGGTGTTG

GTGGCGCTAGTCATCACATTAACTATTTTTTTATTACTTTATACTATAATAGTACTAGACTGACTTCTAA

CAAACATCTCACCTGCCATAAATAAATGCTTGATATTAAAGTCTTCTATTTCTAACACTATTCCATCTGT

GGAAAATAATACTCTGACATTATCGCTAATTGATACATCGGTAAGTGATATGCCTATAAAGTAATAATCT

TCTTTGGGCACATATACCAGTGTACCAGGTTCTAACAACCTATTTACTGGTGCTCCTGTAGCATACTTTT

TTTTTACCTTGAGAATATCCATTGTTTGCTTGGTCAATAGTGATATGTGATTTTTTATCAACCACTCAAA

AAAGTAATTGGAGTGTTCATATCCTCTACGGGCTATTGTCTCATGACCGTGTATGAAATTTAAGTAACAC

GACTGTGGTAGATTTGTTCTATAGAGCCGGTTGCCGCAAATAGATAGAACTACCAATATGTCTGTACAAA

TGTTAAACATTAATTGATTAACAGAAAAAACAATGTTCGTTCTGGGAATAGAAACCAGATTAAAACAAAA

TTCATTAGAATATATGCCACGTTTATACATGGAATATAAAATAACTACAGTTTGAAAAATAACAGTATCA

TTTAAACATTTAACTTGCGGGGTTAATCTCACAACTTTACTGTTTTTGAACTGTTCAAAATATAGCATAG

ATCCATGAGAAATACGTTTAGCCGCCTTTAATAGAGGAAATCCAACCGCCTTTCTGGATCTCACCAACGA

CGATAGTTCTGACCAGCAACTCATTTCTTCATCATCCACCTGTTTTAACATATAATAGGCAGGAGATAGA

TATCCATCATTGCAATATTCCTTCTCGTAGGCACACAATCTAATATTGATAAAATCTCCATTCTCTTCTC

TGTATTTATTATCTTGTCTCGGTGGCTGATTAGGCTGTGGTCTATCGTTGTTGAATCTATTTTGGTCATT

AAATCTTTCATTTCTTCCTGGTATATTTCTATCACCTCGTTTGGTTGGATTTTTGTCTATATTATCGTTT

GTAACATCGGTACGGGTATTCATTTATCACAAAAAAAACTTCTCTAAATGAGTCTACTACTAGAAAACCT

CATCGAAGAAGATACCATATTTTTTGCAGGAAGTATATCTGAGTATGATGATTTACAAATGGTTATTGCT

GGTGCAAAATCCAAATTTCCAAGATCTATGCTTTCTATTTTTAATATAGTACCTAGAACGATGTCAAAAT

ATGAGTTGGAGTTGATTCATAACGAGAATATCACAGGGGCAATGTTTACCACAATGTATAATATAAGAAA

CAATTTGGGTCTAGGCGATGATAAACTAACTATTGAAGCCATTGAAAACTATTTCTTGGATCCTAACAAT

GAGGTTATGCCTCTTATCATTAATAATACGGATATGACTACCGTCATTCCTAAAAAAAGTGGTAGGAGAA

AGAATAAGAACATGGTTATCTTCCGTCAAGGATCATCACCTATCTTGTGTATTTTCGAAACTCGTAAAAA

GATTAATATTTATAAAGAAAATATGGAATCCGTATCGACTAAGTATACACCTATCGGAGACAACAAGGCT

TTGATATCTAAATATGCGGGAATTAATATCCTGAATGTGTATTCTCCTTCCACGTCCATGAGATTGAATG

CCATTTACGGATTCACCAATAAAAATAAACTAGAGAAACTTAGTACTAATAAGGAACTAGAATCGTATAG

TTCTAGCCCTCTTCAAGAACCCATTAGGTTAAATGATTTTCTGGGACTATTGGAATGTGTTAAAAAGAAT

ATTCCTCTAACAGATATTCCGACAAAGGATTGATTACTATAAATGGAGAATGTTCCTAATGTATACTTTA

ATCCTGTGTTTATAGAGCCCACGTTTAAACATTCTTTATTAAGTGTTTATAAACACAGATTAATAGTTTT

ATTTGAAGTATTCGTTGTATTCATTCTAATATATGTATTTTTTAGATCTGAATTAAATATGTTCTTCATG

CCTAAACGAAAAATACCCGATCCTATTGATAGATTACGACGTGCTAATCTAGCGTGTGAAGACGATAAAT

TAATGATCTATGGATTACCATGGATAACAACTCAAACATCTGCGTTATCAATAAATAGTAAACCGATAGT

GTATAAAGATTGTGCAAAGCTTTTGCGATCAATAAATGGATCACAACCAGTATCTCTTAACGATGTTCTT

CGCAGATGATGATTCATTTTTTAAGTATTTTGCTAGTCAAGATGATGAATCTTCATTATCTGATATATTG

CAAATCACTCAATATCTAGACTTTCTGTTATTATTATTGATCCAATCAAAAAATAAATTAGAAGCTGTGG

GTCATTGTTATGAATCTCTTTCAGAGGAATACAGACAATTGACAAAATTCACAGACTCTCAAGATTTTAA

AAAACTGTTTAACAAGGTCCCTATTGTTACAGATGGAAGGGTCAAACTTAATAAAGGATATTTGTTCGAC

TTTGTGATTAGTTTGATGCGATTCAAAAAAGAATCAGCTCTAGCTACCACCGCAATAGATCCTGTTAGAT

ACATAGATCCTCGTCGTGATATCGCATTTTCTAACGTGATGGATATATTAAAGTCGAATAAAGTTGAAAA

ATAATTAATTCTTTATTGTTATCATGAACGGCGGACATATTCAGTTGATAATCGGCCCCATGTTTTCAGG

TAAAAGTACAGAATTAATTAGACGAGTTAGACGTTATCAAATAGCTCAATATAAATGTGTGACTATAAAA

TATTCTAACGATAATAGATACGGAACGGGACTATGGACACATGATAAGAATAATTTTGCAGCATTGGAAG

TAACTAAACTATGTGATGTCTTGGAAGCAATTACAGATTTCTCCGTGATAGGTATAGATGAAGGACAGTT

CTTTCCAGACATTGTTGAATTCTGTGAGCGTATGGCAAACGAAGGAAAAATAGTTATAGTAGCCGCGCTC

GATGGGACATTTCAACGTAGACCGTTTAATAATATTTTGAATCTTATTCCATTATCTGAAATGGTGGTAA

AACTAACTGCAGTGTGTATGAAATGCTTTAAGGAGGCTTCCTTTTCTAAACGATTAGGTACAGAAACCGA

GATAGAAATAATAGGAGGTAATGATATGTATCAATCTGTGTGTAGAAAGTGTTACATCGACTCATAATAT

TATATTTTTTATCTAAAAAACTAAAAATAAACATTGATTAAATTTTAATATAATACTTAAAAATGGATGT

TGTGTCGTTAGATAAACCGTTTATGTATTTTGAGGAAATTGATAATGAGTTAGATTACGAACCAGAAAGT

GCAAATGAGGTCGCAAAAAAACTGCCGTATCAAGGACAGTTAAAACTATTACTAGGAGAATTATTTTTTC

TTAGTAAGTTACAGCGACACGGTATATTAGATGGCGCCACCGTAGTGTATATAGGATCTGCTCCAGGTAC

ACATATACGTTATTTGAGAGATCATTTCTATAATTTAGGAGTGATCATCAAATGGATGCTAATTGACGGC

CGCCATCATGATCCTATTCTAAATGGATTGCGTGATGTGACTCTAGTGACTCGGTTTGTTGATGAGGAAT

ATCTACGATCCATCAAAAAACAACTACATCCTTCTAAGATTATTTTAATTTCTGATGTGCGATCCAAACG

AGGAGGAAATGAACCTAGTACTGCGGATTTACTAAGTAATTATGCTCTACAAAATGTCATGATTAGTATT

TTAAACCCCGTGGCGTCTAGTCTTAAATGGAGATGCCCGTTTCCAGATCAATGGATCAAGGACTTTTATA

TCCCACACGGTAATAAAATGTTACAACCTTTTGCTCCTTCATATTCAGCTGAAATGAGATTATTAAGTAT

TTATACCGGTGAGAATATGAGACTGACTCGAGTTACCAAATCAGACGCTGTAAATTATGAAAAAAAGATG

TATTACCTTAATAAGATAGTCCGCAACAAAGTAGTTATTAACTTTGATTATCCTAATCAGGAATATGACT

ATTTTCACATGTACTTTATGTTGAGGACCGTATACTGCAATAAAACATTTCCTACTACTAAAGCAAAGAT

ACTATTTCTACAACAATCTATATTTCGTTTCTTAAATATTCCAACGACATCAACTGAAAAAGTTAGTCAT

GAACCAATACAACGTAAAATATCTAGCAAAGATTCTATGTCTAAAAACAGAAATAGCAAGAGATCCGTAC

GCGGTAATAAATAGAAACGTACTACTGAGATATACTACCGATATAGAGTATAATGATTTAGTTACTTTAA

TAACCGTTAGACATAAAATTGATTCTATGAAAACTGTGTTTCAGGTATTTAACGAATCATCCATAAATTA

TACTCCGGTTGATGATGATTATGGAGAACCAATCATTATAACATCGTATCTTCAAAAAGGTCATAACAAG

TTTCCTGTAAATTTTCTATACATAGATGTGGTAATATCTGACTTATTTCCTAGCTTTGTTAGACTAGATA

CTACAGAAACTAATATAGTTAATAGTGTACTACAAACAGGCGATGGTAAAAAGACTCTTCGTCTTCCTAA

AATGTTAGAGACGGAAATAGTTGTCAAGATTCTCTATCGTCCTAATATACCATTAAAAATTGTTAGATTT

TTCCGCAATAACATGGTAACTGGAGTAGAGATAGCCGATAGATCTGTTATTTCAGTCGCTGATTAATCAA

TTAGTAGAGATGAGATAAGAACATTATAATAATCAATAATATATCTTATATCTGTTTAGAAAAATGCTAA

TATTAAAATAGCTAACGCTAGTAATCCAATCGGAAGCCATTTGATATCTATAATAGGGTATCTAATTTCC

TGATTCAGATAGCGTACGGCTATATTCTCGGTAGCTACTCGTTTGGAATCACAGACATTATTTACATCTA

ATTTACTATCTGTAATGGAAACGTTTCCCAATGAAATGGTACAATCAGATACATTACATCTTGATATATT

TTTTTTTAAAGAGGCTGGTAACAACGCATCGCTTCGTTTACATGGCTCGTACCAACAATAATAGGGTAAT

CTTGTATCTATTCCTATCCGTACTATACTTTTATCAGGATAAATACATTTACATCGTATATCGTCTTTGT

TAGTATCACAGAATGCATAAATTTGTTCGTCCGTCATGATAAAAATTTAAAGTGTAAATATAACTATTAT

TTTTATAGTTATAATAAAAAGGGAAATTTGATTGTATACCTTCGGTTCTTTAAAAGAAACTGACTTGATA

AAAATGGCTGTAATCTCTAAGGTTACGTATAGTCTATACGATCAAAAAGAGATTAATGCCACAGATATTA

TCATTAGTCATATTAAAAATGACGACGATATCGGTACCGTTAAAGATGGTAGACTAGGTGCTATGGATGG

GGCATTATGTAAAACTTGTGGGAAAACGGAATTGGAATGTTTCGGTCACTGGGGTAAAGTAAGTATTTAT

AAAACTCATATAGTTAAGCCTGAATTTATTTCAGAAATTATTCGTTTACTGAATCATATATGTATTCATT

GCGGATTATTGCGTTCACGAGAACCGTATTCCGACGATATTAACCTAAAAGAGTTATCGGTACACGCTCT

TAGGAGATTAAAGGATAAAATATTATCCAAGAAAAAGTCATGTTGGAACAGCGAATGTATGCAACCGTAT

CAAAAAATTACTTTTTCAAAGAAAAAGGTTTGTTTCGTCAACAAGTTGGATGATATTAACGTTCCTAATT

CTCTCATCTATCAAAAGTTAATTTCTATTCATGAAAAGTTTTGGCCATTATTAGAAATTCATCAATATCC

AGCTAACTTATTTTATACAGACTACTTTCCCATCCCTCCGTTGATTATTAGACCGGCTATTAGTTTTTGG

ATAGATAGTATACCCAAAGAGACAAATGAATTAACTTACTTATTAGGTATGATCGTTAAGAATTGTAACT

TGAATGCTGATGAACAGGTTATCCAGAAGGCGGTAATAGAATACGATGATATTAAAATTATTTCTAATAA

CACTACCAGTATCAATTTATCATATATCACATCCGGCAAAAATAATATGATTAGAAGTTATATCGTCGCT

CGGCGAAAAGATCAGACCGCTAGATCCGTAATTGGTCCCAGTACATCTATCACCGTTAATGAGGTAGGAA

TGCCCACATATATTAGAAATACACTTACAGAAAAGATATTTGTTAATGCCTTTACAGTGGATAAAGTTAA

ACAACTATTAGCATCAAACCAAGTTAAATTTTACTTTAATAAACGATTAAACCAATTAACAAGAATACGT

CAAGGAAAGTTTATCAAAAATAAAATACATTTATTGCCTGGTGATTGGGTAGAAGTAGCTGTTCAAGAAT

ATACAAGTATTATTTTTGGAAGACAACCGTCTCTACATAGATACAACGTCATCGCTTCATCTATCAGAGC

TACCGAAGGAGATACTATCAAAATATCTCCCGGAATTGCCAACTCTCAAAATGCTGATTTTGACGGAGAT

GAAGAATGGATGATATTGGAGCAAAATCCTAAAGCCGTAGTTGAACAAAGTATTCTTATGTATCCGACAA

CGTTACTCAAACACGATATTCATGGAGCCCCCGTTTATGGATCTATTCAAGATGAAATCGTAGCAGCGTA

TTCATTGTTTAGGATACAAGATCTTTGTTTAGATGAAGTATTGAACATCTTGGGGAAATATGGAAGAGAG

TTCGATCCTAAAGGTAAATGTAAATTCAGCGGTAAAGATATCTATACTTACTTGATAGGTGAAAAGATTA

ATTATCCGGGTCTCTTAAAGGATGGTGAAATTATTGCAAACGACGTAGATAGTAATTTTGTTGTAGCTAT

GAGGCATCTGTCATTGGCTGGACTCTTATCCGATCATAAATCGAACGTGGAAGGTATCAACTTTATTATC

AAGTCATCTTATGTTTTTAAGAGATATCTATCTATATACGGTTTTGGGGTGACATTCAAAGATCTGAGAC

CAAATTCGACGTTCACTAATAAATTGGAGGCTATCAACGTAGAAAAAATAGAACTTATCAAAGAAGCATA

CGCCAAATATCTCAAAGATGTAAGAGACGGGAAAATAGTTCCATTATCTAAAGCTTTAGAGGCGGACTAC

TTGGAATCCATGTTATCCAACTTGACAAATCTTAATATCAGAGAGATAGAAGAACATATGAGACAAACGC

TGATAGATGATCCAGATAATAACCTCCTGAAAATGGCCAAAGCGGGTTATAAAGTAAATCCCACAGAACT

AATGTATATTCTAGGTACTTATGGACAACAGAGGATAGATGGCGAACCAGCAGAGACTCGAGTATTGGGT

AGAGTCTTACCTTACTATCTTCCAGACTCTAAGGATCCAGAAGGAAGAGGTTATATTCTTAATTCTTTAA

CAAAAGGATTAACGGGTTCTCAATATTACTTTTTGATGCTGGTTGCAAGATCTCAATCTACTGATATTGT

CTGTGAAACATCACGTACCGGAACACTGGCTAGAAAAATCATTAAAAAGATGGAGGATATGGTGGTCGAC

GGATACGGACAAGTAGTTATAGGTAATACGCTCATCAAGTACGCAGCCAATTATACCAAAATTCTAGGCT

CAGTATGTAAACCTGTAGATCTTATCTATCCAGATGAGTCCATGACTTGGTATTTGGAAATTAGTGCTTT

GTGGAATAAAATAAAACAGGGATTCGTTTACTCTCAGAAACAGAAACTTGCAAAGAAGACATTGGCGCCG

TTTAATTTCCTAGTATTCGTCAAACCCACCACTGAGGATAATGCTATTAAGGTTAAGGATCTGTACGATA

TGATTCATAACGTCATTGATGATGTGAGAGAGAAATACTTCTTTACGGTATCTAATATAGATTTTATGGA

GTATATATTCTTGACGCATCTTAATCCTTCTAGAATTAGAATTACAAAAGAAACGGCTATTACTATCTTT

GAAAAGTTCTATGAAAAACTCAATTATACTCTAGGTGGTGGAACTCCTATTGGAATTATTTCTGCACAGG

TATTGTCTGAGAAGTTTACACAACAAGCCCTGTCCAGTTTTCACACTACTGAAAAGAGTGGTGCTGTAAA

ACAAAAACTTGGTTTCAACGAGTTTAATAACTTGACTAATTTGAGTAAGAATAAGACCGAAATTATCACT

CTGGTATCCGATGATATCTCTAAACTTCAATCTGTTAAGATTAATTTCGAATTTGTATGTTTGGGAGAAT

TAAATCCAGACATCACTCTTCGAAAAGAAACAGATAGATATGTAGTAGACATAATAGTCAATAGATTATA

CATCAAGAGAGCAGAAATAACCGAATTAGTCGTCGAATATATGATTGAACGATTTATCTCCTTTAGCGTC

ATTGTAAAGGAATGGGGTATGGAGACATTCATTGAGGACGAGGATAATATTAGATTTACTATCTACCTAA

ATTTCGTTGAACCGGAGGAATTGAATCTTAGTAAGTTTATGATGGTTCTTCCAGGTGCCGCCAACAAGGG

CAAGATTAGTAAATTCAAGATTCCTATCTCTGACTATACGGGATATAACGACTTCAATCAAACAAAAAAG

CTCAATAAGATGACTGTAGAACTCATGAATCTAAAAGAATTGGGTTCTTTCGATTTGGAGAACGTCAACG

TGTATCCTGGAGTATGGAATACATACGATATCTTTGGTATTGAGGCCGCTCGTGGATACTTGTGCGAAGC

CATGTTAAACACCTATGGAGAAGGTTTCGATTATCTGTACCAGCCTTGTGATCTTCTCGCTAGTTTACTA

TGTGCTAGTTACGAACCAGAATCAGTTAATAAATTCAAGTTCGGTGCAGCTAGTACTCTTAAGAGAGCTA

CGTTCGGAGATAATAAAGCATTGTTAAACGCGGCTCTTCATAAAAAGTCAGAACCTATTAACGATAATAG

TAGCTGCCACTTTTTTAGCAAGGTCCCTAATATAGGAACTGGATATTACAAATACTTTATCGACTTGGGT

CTTCTCATGAGAATGGAAAGGAAACTATCTGATAAGATATCTTCTCAAAAGATCAAGGAGATAGAAGAAA

CAGAAGACTTTTAATTCTTATCAATAACATATTTTTCTATGATCTGTCTTTTAAACGATGGATTTTCCAC

AAATGCGCCTCTCAAGTCCCTCATAGAATGATACACGTATAAAAAATATAGCATAGGTGATGACTCCTTA

TTTTTAGACATTAGATATGCCAAAATCATAGCCCCGCTTCTATTTACTCCTGCAACACAATGAACCAACA

CGGGCTCGTTTCGTTGATCACATTTAGATAAGAAGGCGGTCACGTCGTCAAAATATTTACTAATATCAGT

AGTTGTATCATCTACCAACGGTATATGAATAATATTAATATTAGAGTTAGGTAATGTATATTTATCCATC

GTCAAATTTAAAACATATTTGAACTTAACTTCAGATGATGGTGCATCCATAGCATTTTTATAATTTCCCA

AATACACATTATTTGTTACTCTTGTCATTATAGTGGGAGATTTGGCTCTGTGCATATCTCCAGTTGAACG

TAGTAGTAAGTATTTATACAAACTTTTCTTATCCATTTATAACGTACAAATGGATAAAACTACTTTATCA

GTAAACGCATGCAATTTAGAATACGTTAGAGAAAAGGCTATAGTAGGCGTACAAGCAGCCAAGACATCAA

CACTTATATTTTTTGTTATTATATTGGCAATTAGTGCGCTATTACTCTGGTTTCAGACGTCTGATAATCC

AGTCTTTAATGAATTAACGAGATATATGCGAATTAAAAATACGGTTAACGATTGGAAATCATTAACGGAT

AGCAAAACAAAATTAGAAAGCGATAGAGGTAGACTTCTAGCCGCTGGTAAGGATGATATATTCGAATTCA

AATGTGTGGATTTCGGCGCCTATTTTATAGCTATGCGATTGGATAAGAAAACATATCTGCCGCAAGCTAT

TAGGCGAGGTACTGGAGACGCGTGGATGGTTAAAAAGGCGGCAAAAGTCGATCCATCTGCTCAACAATTT

TGTCAGTATTTGATAAAACACAAGTCTAATAATGTTATTACTTGTGGTAATGAGATGTTAAATGAATTAG

GTTATAGCGGTTATTTTATGTCACCGCATTGGTGTTCCGATCTTAGTAATATGGAATAAGTGTTAGATAA

ATGCGGTAACAAATGTTCCTGTAAGGAACCATAACAGTTTAGATTTAACATTAAAGATGAGCATAAACAT

AATAAACAAAATTACAATCAAACCTATAACATTAATATCAAACAATCCAAAAAATGAAATCAATGGAGTA

GTAAACGTGTACATAACTCCTGGATAACGTTTAGCAGCTACCGTTCCTATTCTAGACCAAAAATTTGGTT

TCATGGTTTCGAAGCGGTGTTCTGCAACAAGACGAGGATCGTGTTCTACATATTTGGCAGAGTTATCCAT

TATTTGCCTGTTAATCTTCATTTCGTTTTCGATTCTGGCTATTTCAAAATAAAATCCCGATGATAGACCT

CCAGACTTTATAATTTCATCTACGATGTTCAGCGCCGTAGTAACTCTAATAATATAGGCGGATAAGCTAA

CATCATACCCTCCTGTATATGTAAATATGGCATGATCTTTGTCTATTACAAGCTCGGTTTTAACTTTATT

TCCTGTAATAATTTCTCTCATCTGTAGGATATCTATTTTCTTGTCATGTATTGCCTTCAAGACGGGACGA

AGAAACGTAATATCCTCAATAACGTTATCGTTTTCTATAATAACTACATATTCTACATTTTTATTTTCTA

GCTCGGTAAAAAATTTAGAATCCCATAGGGCTAAATGTCTAGCGATATTTCTTTTCGTTTCCTCTGTACA

CATAGTGTTACAAAACCCTGAAAAGAAGTGAGTATACTTGTCATCATCTCTAATATTTCCTCCAGTCCAT

TGTATAAACACATAATCCTTGTAATGATCTGGATCATCATTGACTATCACAACATCTCTTTTTTCTTGCA

TAACTTCATTGTCCTTCACATCATCGAACTTCTGATCATTAATATGCTCATGAACATTAGGAAATGTTTC

TGATGGAGGTCTATCAATAACTGGCACAACAATAACAGGAGTTTTCACCGCCGCCATTTAGTTATTGAAA

TTAATCATATACAACTCTCTAATACGAGTTATATTTTCGTCTATCCATTGTTTCACATTGACATATTTCG

ACAAAAAGATATAAAATGCGTATTCCAATGCTTCTCTGTTTAATGAATTACTAAAATATACAAACACGTC

ACTGTCTGGTAATAAATAATATCTTAGAATATTGTAACAATTTATTTTGTATTGCACATGTTCGTGATCT

ATGAGTTCTTCTTCAAATGGCATAGGATCTCCGAATCTGAAAACGTATAAATAGGAGTTAGAATAATAAT

ATTTGAGAGTATTGGTAATGTATAAACTCTTTAGCGGTATAATTAGTTTTTTTCTCTCGATTTCTATTTT

TAGATGTGATGGAAAAATGACTAATTTTGTAGCATTAGTATCATGAACTCTAATCAAAATCTTAATATCT

TCGTCACATGTTAGCTCTTTGAAGTTTTTAAGAGATGCATCAGTTGGTTTTACAGATGGAGTAGGTGCAA

CAATTTTTTGTTTAATGCATGCATGTATTGGAGCCATTGTCTTAACTATAATGGTGCTTGTATCGAAAAA

CTTTAATGCGGATAACGGAAGCTCTTCGCCGCGACTTTCTACGTCGTAATTGGGTTCTAATGCCGATCTC

TGAATGGATACTAGTTTTCTAAGTTCTAATGTAATTCTCTGAAAATGTAAATCCAATTCCTCCGGCATTA

TAGATGTGTATACATCGGTAAATAAAACTATAGTATCCAACGATCCCTTCTCGCAAATTCTAGTCTTAAC

CAAGAAATCGTATATAACTACGGAGATGGCGTATTTAAGAGTGGATTCTTCTACCGTTTTGTTCTTGGAT

TTCATATAAGAAACTATAAAGTCCGCACTACTGTTAAGAATGATCACTAACGCAACTATATAGTTCAAAT

TAAGCATCTTGGAAACATAAAATAACTCTGTAGATGATACTTGACTTTCGAATAAGTTTGCAGACAAACG

AAGAAAGAACAGACCTCTCTTAATTTCAGAAGAAAACTTTTTTTCGTATTCCTGACGTCTAGAGTTTATA

TCAATAAGAAAGTTAAGAATTAGTCGGTTAATGTTGTATTTCATTACCCAAGTTTGAGATTTCATAATAT

TGTCAAAAGACATGATAATATTAAAGATAAAGCGCTGACTATGAACGAAATAGCTATATGGTTCGCTCAA

GAATATAGTCTTGTTAAACGTGGAAACGATAACTGTATTTTTAATCACGTCAGCGGCATCTAAATTAAAT

ATAGGTATATTTATTCCACACACTCTACAATATGCCACACCATCTTCATAATAAATAAATTCGTTAGCAA

AATTATTAATTTTAGTGAAATAGTTAGCGTCAACTTTCATAGCTTCCTTCAATCTAATTTGATGCTCACA

TGGCGCGAATTCTACTCTAACATCCCTTTTCCATGCCTCAGGTTCATCGATCTCTATAATATCTAGTTTC

TTGCGTTTCACAAACACAGGCTCGTCTCTCGCGATGAGATCTGTATAGTAACTATGTAAATGATAACTAG

ATAGAAAGATGTAGCTATATAGATGACGATCCTTTAAGAGAGGTATAATAACTTTACCCCAATCAGATAG

ACTGTTGTTATGGTCTTCGGAAAAAGAATTTTTATAAATTTTTCCAGTATTTTCTAAATATACGTACTTG

ATATCTAAGAAATCCTTAATAATAATAGGAATGGATAATCCGTCTATTTTATAAAGAAATACATATCGCA

TATTATACTTTTTTTTGGAAATTGGAATACCGATGTGTCTACATAAATACGCAAAGTCTAAATATTTTTT

AGAGAATCTTAGTTGGTCCAAATTCTTTTCCAAGTACGGTAATAGATTTTTCATATTGAACGGTATCTTC

TTGATCTCTGGTTCTAATTCCGCATTAAATGATGAAACTAAGTCACTATTTTTATAACTAACGATTACAT

CACCTCTAACATCATCATTTACCAGGATACTGATCTTCTTTTGTCGTAAATACATGTCTAATGTGTTAAA

AAAAAGATCATACAAGTTATACGTCATTTCATCTGTAGTATTCTTGTCATTGAAGGATAAACTCGTACTA

ATCTCTTCTTTAACAGTCTGTTCAAATTTATATCCTATATATGAAAAAATAGCAACCAGTGTTTGATCAT

CCGCGTCAATATTCTGTTCTATCGTAGTGTATAACAATCTTATATCTTCTTCTGTGATAGTCGATACGTT

ATAAAGGTTGATAACGAAAATATTTTTATTTCGTGAAATAAAGTCATTGTAGGATTTTGGACTTATATTC

GTGTCTAGTAGATATGATTTTATTTTTGGAATGATCTCAATTAAAATAGTCTCTTTAGAGTCCATTTAAA

GTTACAAACAACTAGGAAATTGGTTTATGATGTATAATTTTTTTAGTTTTTATAGATTCTTTATTCTATA

CTTAAAAAATGAAAATAAATACAAAGGTTCTTGAGGGTTGTGTTAATTGAAAGCGATAAATAATCATAAA

TTATTTCATTATCGCGATATCCGTTAAGTTTGTATCGTAATGGCGTGGTCAATTACGAATAAAGCGGATA

CTAGTAGTTTCACAAAGATGGCTGAAATCAGAGCTCATCTAAGAAATAGCGCTGAAAATAAAGATAAAAA

CGAGGATATTTTCCCGGAAGATGTAATAATTCCATCTACTAAGCCCAAAACCAAACGAACCACTACTCCT

CGTAAACCAGCGGCTACTAAAAGATCAACCAAAAAGGATAAAGAAAAGGAGGAAGTGGAAGAAGTAGTTA

TAGAGGAATATCATCAAACAACTGAAGAAAATTCTCCACCTCCGTCATCATCTCCTGGAGTCGGCGACAT

TGTAGAAAGCGTGGCCGCTGTAGAGCTCGATGATAGCGACGGGGATGATGAACCTATGGTACAAGTTGAA

GCTGGTAAAGTAAATCATAGTGCTAGAAGCGATCTCTCTGACCTAAAGGTGGCTACCGACAATATCGTTA

AAGATCTTAAGAAAATTATTACTAGAATCTCTGCAGTATCGACTGTTCTAGAGGATGTTCAAGCAGCTGG

TATCTCTAGACAATTTACTTCTATGACTAAAGCTATTACAACACTATCTGATCTAGTCACCGAGGGAAAA

TCTAAAGTTGTTCGTAAAAAAGTTAAAACTTGTAAGAAGTAAATGCGTGCACTTTTTTATAAAGATGGTA

AACTGTTTACCGATAATAATTTTTTAAATCCTGTATCAGACGATAATCCAGCGTATGAGGTTTTGCAACA

TGTTAAAATTCCTACTCATTTAACAGATGTAGTAGTATATGAACAAACGTGGGAAGAGGCATTAACTAGA

TTAATTTTTGTGGGAAGTGATTCAAAAGGACGTAGACAATACTTTTACGGAAAAATGCATATACAGAATC

GCAATGCTAAAAGAGATCGTATTTTTGTTAGAGTATATAACGTTATGAAACGAATTAATTGTTTTATAAA

CAAAAATATAAAGAAATCGTCCACAGATTCCAATTATCAGTTGGCGGTTTTTATGTTAATGGAAACTATG

TTTTTTATTAGATTTGGTAAAATGAAATATCTTAAGGAGAATGAAACAGTAGGGTTATTAACACTAAAAA

ATAAACACATAGAAATAAGTCCCGATGAAATAGTTATCAAGTTTGTAGGAAAGGACAAAGTTTCACATGA

ATTTGTTGTTCATAAGTCTAATAGACTATATAAACCGCTATTGAAACTGACTGATGATTCTAGTCCCGAA

GAATTTCTGTTCAACAAACTAAGTGAACGAAAGGTATATGAATGTATCAAACAGTTTGGTATTAGAATCA

AGGATCTCCGAACGTATGGAGTCAATTATACGTTTTTATATAATTTTTGGACAAATGTAAAGTCCGTATC

TCCTCTTCCATCACCAAAAAAGTTGATAGCATTAACTATCAAACAAACTGCTGAAGTGGTAGGTCATACT

CCATCAATTTCAAAAAGAGCTTATATGGCAACGACTATTTTAGAAATGGTAAAGGATAAAAATTTTTTAG

ACGTAGTATCTAAAACTACGTTCGATGAATTCCTATCTATAGTCGTAGATCACGTTAAATCATCTACGGA

TGGATGATAATAGATCTTTACACAAATAATTACAAGACCGATAAATGGAAATGGATAAACGGATGAAATC

TCTCGCTATGACAGCTTTCTTCGGAGAGCTAAACACGTTAGATATTATGGCATTGATAATGTCTATATTT

AAACACCATCCAAACAATACCATTTTTTCAGTGGATAAGGATGGTCAATTTATGATTGATTTCGAATACG

ATAATTATAAGGCTTCTCAATATTTGGATCTGACCCTCACTCCGATATCTGGAAATGAATGCAAGACTCA

CGCATCTAGTATAGCCGAACAATTGGCGTGTGTGGATATTATTAAAGAGGATATTAGCGAATATATCAAA

ACTACTCCCCGTCTTAAACGATTTATAAAAAAATACCGCAATAGATCATATACTCGTATCAGTCGAGATA

CAGAAAAGCTTAAAATAGCTCTAGCTAAAGGCATAGATTACGAATATATAAAAGACGCTTGTTAATAAGT

AAATGAAAAAAAACTAGTCGTTTATAATAAAACACAATATGGATGCCAACATAGTATCATCTTCTACTAT

TGCGACGTATATAGACGCTTTAGCGAAGAATGCTTCAGAATTAGAACAGAGGTCTACCGCATACGAAATA

AATAATGAATTGGAACTAGTATTTATTAAACCGCCATTGATTACGTTGACAAATGTAGTAAATATCTCCA

CGATTCAGGAATCGTTTATTCGATTTACCGTTACTAATAAGGAAGGTATCAAAATTAGAACTAAGATTCC

ATTATCTAAGGTACATGGTCTAGATGTAAAAAATGTGCAGTTGGTAGATGCTATAGATAACATAGTTTGG

GAAAAGAAATCATTAGTGACGGAAAATCGTCTTCACAAAGAATGCTTGTTGAGACTATCAACAGAGGAAC

GTCATATATTTTTGGATTACAAGAAATATGGATCCTCTATCCGACTAGAATTAGTCAATCTTATTCAAGC

AAAAACAAAAAACTTTACGATAGACTTTAAGCTAAAATATTTTCTAGGATCTGGCGCTCAATCTAAAAGT

TCTTTATTGCACGCTATTAATCATCCAAAGTCAAGGCCTAATACATCTCTGGAAATAGAATTTACACCTA

GAGACAATGAAACAGTTCCATATGATGAACTAATAAAGGAATTGACGACTCTCTCGCGTCATATATTTAT

GGCTTCTCCAGAGAATGTAATTCTTTCTCCACCTATTAACGCACCTATAAAGACTTTTATGTTGCCTAAA

CAAGATATAGTAGGTCTGGATCTGGAAAATCTATATGCCGTAACTAAGACTGACGGCATTCCTATAACTA

TCAGAGTTACATCAAAAGGGTTGTATTGTTATTTTACACATCTTGGTTATATTATTAGATATCCAGTTAA

GAGAACAATAGATTCCGAAGTAGTAGTCTTTGGTGAGGCAGTTAAGGATAAGAACTGGACCGTATATCTC

ATTAAGCTAATAGAGCCCGTAAATGCAATCAGTGATAGACTAGAAGAAAGTAAGTATGTTGAATCTAAAC

TAGTGGATATTTGTGATCGGATAGTATTCAAGTCAAAGAAATACGAAGGTCCGTTTACTACAACTAGTGA

AGTCGTCGATATGTTATCTACATATTTACCAAAGCAACCAGAAGGTGTTATTCTGTTCTATTCAAAGGGA

CCTAAATCTAACATTGATTTTAAAATCAAAAAGGAGAATACTATAGACCAAACTGCAAATGTAGTATTTA

GGTACATGTCCAGTGAACCAATTATCTTTGGAGAGTCGTCTATCTTTATAGAGTATAAGAAATTTACCAA

CGATAAAGGCTTTCCTAAAGAATATGGTTCTGGTAAGATTGTGTTATATAACGGCGTTAATTATCTAAAT

AATATCTATTGTTTGGAATATATTAATACACATAATGAAGTGGGTATTAAGTCCGTTGTTGTACCTATTA

AGTTTATAGCAGAATTCTTAGTCAATGGAGAAATACTTAAACCTAGAATCGATAAAACCATGAAATATAT

TAACTCAGAAGACTATTATGGAAATCAACATAATATCATAGTCGAACATTTAAGAGATCAAAGCATCAAA

ATAGGAGATGTCTTTAACGAGGATAAACTATCGGATGTTGGACATCAATACGCTGCCAACAACGATAAAT

TTAGATTAAATCCAGAAGTTAGTTATTTTACTAATAAACGAACTAGAGGGCCGTTGGGAATTTTATCAAA

CTACGTCAAGACTCTTCTTATTTCTATGTATTGTTCCAAAACATTTTTAGACGATTCCAACAAACGAAAG

GTATTAGCGATTGATTTTGGAAACGGTGCTGACCTGGAAAAATACTTTTATGGAGAGATTGCGTTATTGG

TAGCGACGGATCCGGATGCTGATGCTATAGCTAGAGGAAATGAAAGATACAACAAATTAAATTCTGGAAT

TAAAACCAAGTACTACAAATTTGACTACATTCAGGAAACTATTCGATCCGATACATTTGTCTCTAGTGTC

AGAGAAGTATTCTATTTTGGAAAGTTTAATATCATTGACTGGCAGTTCGCTATTCATTATTCTTTTCATC

CAAGACATTATGCTACAGTCATGAATAACTTATCCGAACTAACTGCTTCTGGAGGCAAGGTATTAATTAC

TACCATGGATGGAGACAAATTATCAAAATTAACCGATAAAAAGACTTTTATAATTCATAAGAATCTACCT

AGTAGCGAAAACTATATGTCTGTAGAAAAAATAGCTGATGATAGAATAGTGGTATATAATCCATCAACAA

TGTCTACTCCAATGACTGAATACATTATCAAAAAGAACGATATAGTCAGAGTGTTTAACGAATACGGATT

TGTTCTTGTAGATAATGTTGATTTCGCTACAATTATAGAACGAAGTAAAAAGTTTATTAATGGCGCATCT

ACAATGGAAGATAGACCGTCTACAAGAAACTTTTTCGAACTAAATAGAGGAGCCATTAAATGTGAAGGTT

TAGATGTCGAAGACTTACTTAGTTACTATGTTGTTTATGTCTTTTCTAAGCGGTAAATAATAATATGGTA

TGGGTTCTGATATCCCCGTTCTAAATGCATTAAATAATTCCAATAGAGCGATTTTTGTTCCTATAGGACC

TTCCAACTGTGGATACTCTGTATTATTAATAGATATATTAATACTTTTGTAGGGTAACAGAGGTTCTACG

TCTTCTAAAAATAAAAGTTTTATAACATCTGGCCTGTTCATAAATAAAAACTTGGCGATTCTATATATAC

TCTTATTATCAAATCTAGCCATTGTCTTATAGATGTGAGCTACTGTAGGTGTACCATTTGATTTTCTTTC

TAATACTATATATTTCTCTCGAAGAAGTTCTTGCAGATCATCTGGGAATAAAATACTACTGTTGAGTAAA

TCAGTTATTTTTTTTATATCGATATTGATGGACATTTTTATAGTTAAGGATAATAAGTATCCCAAAGTAG

ATAACGACGATAACGAAGTATTTATACTTTTAGGAAATCACAATGACTTTATCAGATCAAAATTAACAAA

ATTAAAGGAGCATGTATTTTTTTCTGAATATATTGTGACTCCAGATACATATGGATCTTTATGCGTCGAA

TTAAATGGGTCTAGTTTTCAGCACGGTGGTAGATATATAGAGGTGGAGGAATTTATAGATGCTGGAAGAC

AAGTTAGATGGTGTTCTACATCCAATCATATATCTGAAGATATACACACTGATAAATTTGTCATTTATGA

TATTTATACGTTTGATTCGTTCAAGAATAAACGATTGGTATTTGTACAGGTACCTCCATCATTAGGAGAT

GATAGCTATTTAACTAATCCGTTATTGTCTCCGTATTATCGTAATTCAGTAGCCAGACAAATGGTCAATG

ATATGATTTTTAATCAAGATTCATTTTTAAAATATTTATTAGAACATCTGATTAGAAGCCACTATAGAGT

TTCTAAACATATAACAATAGTTAGATACAAGGATACCGAAGAATTAAATCTAACAAGAATATGTTATAAT

AGAGATAAGTTTAAGGCGTTTGTATTCGCTTGGTTTAACGGCGTTTCGGAAAATGAAAAGGTACTAGATA

CGTATAAAAAGGTATCTGATTTGATATAATGAATTCAGTGACTATATCACACGCACCATATACTATTACT

TATCACGATGATTGGGAACCAGTAATGAGTCAATTGGTAGAGTTTTATAACGAAGTAGCCAGTTGGTTGC

TACGCGACGAGACGTCGCCTATTCCTGATAAGTTCTTTATACAATTGAAACAGCCGCTTAGAAATAAACG

AGTATGTGTGTGTGGTATAGATCCGTATCCAAAAGATGGAACTGGTGTACCGTTCGAATCACCAAATTTT

ACAAAAAAATCAATTAAGGAGATAGCTTCATCTATATCTAGATTAACCGGAGTAATTGATTATAAAGGTT

ATAACCTTAATATAATAGACGGGGTTATACCCTGGAATTATTACTTAAGTTGTAAATTAGGAGAAACAAA

AAGTCACGCGATTTACTGGGATAAGATTTCCAAGTTACTGCTACAGCATATAACTAAACACGTTAGTGTT

CTTTATTGTTTGGGTAAAACAGATTTCTCGAATATACGGGCAAAGTTAGAATCCCCGGTAACTACCATAG

TGGGATATCATCCAGCGGCCAGAGACCACCAATTCGAGAAAGATCGATCATTTGAAATTATCAACGTTTT

ACTGGAATTAGACAACAAGACACCTATAAATTGGGCTCAAGGGTTTATTTATTAATGCTTTAGTGAAATT

TTAACTTGTGTTCTAAATGGATGCGGCTATTAGAGGTAATGATGTTATCTTTGTTCTTAAGACTATAGGT

GTCCCGTCAGCATGCAGACAAAATGAAGATCCAAGATTCGTAGAAGCATTTAAATGCGACGAGTTAGAAA

GATATATTGATAATAATCCAGAATGTACACTATTCGAAAGTCTTAGGGATGAGGAAGCATACTCTATAGT

CAGAATTTTCATGGATGTAGATTTAGACGCGTGTCTAGACGAAATAGATTATTTAACGGCTATTCAAGAT

TTTATTATCGAGGTGTCAAACTGTGTAGCTAGATTCGCATTTACAGAATGCGGTGCCATTCATGAAAATG

TAATAAAATCCATGAGATCTAATTTTTCATTGACTAAGTCTACAAATAGAGATAAAACAAGTTTTCATAT

TATCTTTTTAGACACGTATACCACTATGGATACATTGATAGCTATGAAACGAACACTATTAGAATTAAGT

AGATCATCTGAAAATCCACTAACAAGATCGATAGACACTGCCGTATATAGGAGAAAAACAACTCTTCGGG

TTGTAGGTACTAGGAAAAATCCAAATTGCGACACTATTCATGTAATGCAACCACCTCACGATAATATAGA

AGATTACCTATTCACTTACGTGGATATGAACAACAATAGTTATTACTTTTCTCTACAACGACGATTGGAG

GATTTAGTTCCTGATAAGTTATGGGAACCAGGGTTTATTTCGTTCGAAGACGCTATAAAAAGAGTTTCAA

AAATATTCATTAATTCTATAATAAACTTTAATGATCTCGATGAAAATAATTTTACAACGGTACCACTGGT

CATAGATTATGTAACACCTTGTGCATTATGTAAAAAACGATCGCATAAACATCCGCATCAACTATCGTTG

GAAAATGGTGCTATTAGAATTTACAAAACTGGTAATCCACATAGTTGTAAAGTTAAAATTGTTCCGTTGG

ATGGTAATAAACTGTTTAATATTGCACAAAGAATTTTAGACACTAACTCTGTTTTATTAACCGAACGAGG

AGACCATATAGTTTGGATTAATAATTCATGGAAATTTAACAGCGAAGAACCCTTGATAACAAAACTAATT

CTATCAATAAGACATCAACTACCTAAGGAATATTCAAGCGAATTACTCTGTCCGAGGAAACGAAAGACTG

TAGAAGCTAACATACGAGACATGTTAGTAGATTCAGTAGAGACCGATACCTATCCGGATAAACTTCCGTT

TAAAAATGGTGTATTGGACCTGGTAGACGGAATGTTTTACTCTGGAGATGATGCTAAAAAATATACGTGT

ACTGTATCGACCGGATTTAAATTTGACGATACAAAATTCGTCGAAGACAGTCCAGAAATGGAAGAGTTAA

TGAATATCATTAACGATATCCAACCATTAACGGATGAAAATAAGAAAAATAGAGAGCTGTATGAAAAAAC

ATTATCTAGTTGTTTATGTGGTGCTACCAAAGGATGTTTAACATTCTTTTTTGGAGAAACCGCAACTGGG

AAGTCGACAACCAAACGTTTGTTAAAGTCTGCTATCGGTGACCTGTTTGTCGAGACGGGTCAAACAATTT

TAACAGATGTATTGGATAAAGGACCTAATCCATTTATCGCTAATATGCATTTAAAAAGATCTGTATTCTG

TAGCGAACTACCTGATTTTGCATGTAGTGGATCAAAGAAAATTAGATCTGATAATATTAAAAAGTTGACA

GAACCTTGTGTCATTGGAAGACCGTGTTTCTCCAATAAAATTAATAATAGAAACCATGCGACAATCATTA

TCGATACTAATTACAAACCTGTCTTTGATAGGATAGATAACGCATTAATGAGAAGAATTGCCGTCGTGCG

ATTCAGAACACACTTTTCTCAACCTTCTGGTAGAGAGGCTGCTGAAAATAATGACGCGTACGATAAAGTC

AAACTATTAGACGAGGGATTAGATGGTAAAATACAGAATAATAGATATAGATTCGCATTTCTATACTTGT

TGGTTAAATGGTACAAAAAATATCATATTCCTATTATGAAACTATATCCTACACCGGAAGAGATTCCGGA

CTTTGCATTCTATCTCAAAATAGGTACTCTGTTGGTATCTAGCTCTGTAAAGCATATTCCATTAATGACG

GACCTCTCCAAAAAGGGATATATATTGTACGATAATGTGGTTACTCTTCCGTTGACTACTTTCCAACAGA

AAATATCCAAGTATTTTAATTCTAGACTATTTGGACACGATATAGAGAGCTTCATCAATAGACATAAGAA

ATTTGCCAATGTTAGTGATGAATATCTGCAATATATATTCATAGAGGATATTTCATCTCCGTAAATATAT

GCCATATATTTATAGAATATATCACATATCTAAATGAATACCGGAATCATAGATTTATTTGATAATCATG

TTGATAGTATACCAACTATATTACCTCATCAGTTAGCTACTTTAGATTATCTAGTTAGAACTATCATAGA

TGAGAACAGAAGCGTGTTATTGTTCCATATTATGGGATCGGGTAAAACAATAATCGCTTTGTTGTTCGCC

TTGGTAGCTTCCAGATTTAAAAAGGTTTACATTTTAGTACCGAACATCAACATCTTAAAAATTTTCAATT

ATAATATGGGTGTAGCTATGAACTTGTTTAATGACGAATTCATAGCTGAGAATATCTTTATTCATTCCAC

AACAAGTTTTTATTCTCTTAATTATAACGATAACGTCATTAATTATAACGGATTAAGTCGCTACAATAAC

TCTATTTTTATCGTTGATGAGGCACATAATATTTTTGGGAATAATACTGGAGAACTTATGACCGTGATAA

AAAATAAAAACAAGATTCCTTTTCTACTATTGTCTGGATCTCCCATTACTAACACACCTAATACGCTGGG

TCATATTATAGATTTAATGTCCGAAGAGACGATAGATTTTGGTGAGATTATTAGTCGTGGTAAGAAAGTA

ATTCAGACACTTCTTAACGAACGCGGAGTGAATGTACTCAAGGATTTGCTTAAAGGAAGAATATCATATT

ACGAAATGCCGGACAAAGATCTACCAACAATAAGATATCACGGACGTAAATTTCTAGATACTCGAGTAGT

ATATTGTCACATGTCTAAACTTCAAGAGAAAGATTATATGATTACTAGACGGCAGCTATGTTATCATGAA

ATGTTTGATAAAAATATGTATAACGTGTCAATGGCAGTATTGGGACAACTTAATCTGATGAATAATTTAG

ATACGTTATTTCAGGAACAGGATAAGGAATTGTACCCAAATCTGAAAATAAATAATGGAGTGTTATACGG

TGAAGAATTGGTAACGTTAAACATTAGTTCCAAATTTAAGTACTTTATCAATCGGATACAGACACTCAAG

GGAAAACACTTTATATACTTCTCTAATTCTACATATGGTGGATTGGTAATTAAATATATCATGCTCAGTA

ATGGATATTCTGAATATAATGGTTCTCAGGGAACTAATCCACATATGATAAACGGCAAACCAAAAACATT

TGCTATCGTTACTAGTAAAATGAAATCGTCTTTAGAGGATCTATTAGATGTGTATAATTCTCCTGAAAAC

GATGATGGCAATCAATTGATGTTTTTGTTTTCGTCAAACATTATGTCTGAATCCTATACTCTGAAAGAGG

TAAGGCATATTTGGTTTATGACTATCCCGGATACTTTTTCTCAATACAACCAAATTCTTGGACGATCTAT

TAGAAAATTCTCTTACGTCGATATTTCTGAACCCGTTAATGTATATCTTTTAGCAGCCGTATATTCAGAT

TTCAATGACGAAGTGACGTCATTAAACGATTATACACAGGATGAATTGATTAATGTTTTACCCTTTGACA

TCAAAAAGCTGTTGTATCTAAAATTTAAGACTAAAGAAACGAATAGAATATACTCTATTCTTCAAGAGAT

GTCTGAAACGTATTCTCTTCCACCACATCCATCAATTGTAAAAGTTTTATTGGGAGAATTGGTCAGACAA

TTTTTTTATAATAATTCTCGTATTAAGTATAACGACTCCAAGTTACTTAAAATGGTTACATCAGTTATAA

AAAATAAAGAAGACGCTAGGAATTACATAGATGATATTGTAAACGGTCACTTCTTTGTATCGAATAAAGT

ATTTGATAAATCTCTTTTATACAAATACGAAAACGATATTATTACAGTACCGTTTAGACTTTCCTACGAA

CCATTTGTTTGGGGAGTTAACTTTCGTAAAGAATATAATGTGGTATCTTCTCCATAAAACTGATGAGATA

TATAAAGAAATAAATGTCGAGCTTTGTTACCAATGGATATCTTCCAGTTACATTGGAACCACATGAGTTG

ACGTTAGACATAAAAACTAATATTAGGAATGCCGTATATAAGGCGTATCTCCATAGAGAAATTAGTGGTA

AAATGGCCAAGAAAATAGAAATTCGTGAAGACGTGGAATTACCTCTCGGTGAAATAGTTAATAATTCTGT

AGTTATAAACGTTCCGTGTGTAATAACCTACGCATATTATCACGTTGGGGATATAGTCAGAGGAACATTA

AACATCGAAGATGAATCAAATGTAACTATTCAATGTGGAGATTTAATCTGTAAACTAAGTAGAGATTCGG

GTACTGTATCATTTAGCGATTCAAAGTACTGCTTTTTTCGAAATGGTAATGCGTATGATAACGGCATCGA

AGTCTCCGCCGTTCTAATGGAGGCTCAACAAGGTACCGAATCTAGTTTTGTTTTTCTCGCGAATATCGTT

GACTCATAAGAAAGAGAATAGCGGTGAGTATAAATACGAATACTATGGCAATAATTGCGAATGTTTTATT

CCCTTCGATATATTTTTGATAATATGAAAAACATGCCTCTCTCAAATCAGACAACCATTTCATAAAATAG

TTCTCTCGCACTGGTGAGGTGGTTGCAGCTCGTATAATCTCCCCAGAATAATATACTTGCGTGTCGTCGT

TCAATTTATACGGATTTCTATAATTCTCTGTTATATAATGAGGTTTACCCTCATGATTAGACGACGACAA

TAGTGTTCTGAATTTAGATAGTTGATCAGAATGAATGTTTATTGGTGTTGGAAAAATTATCCATGCTGCG

TCTGCAGAGTGGTTGATAGTTGTTCCTAGATATGTAAAATAATCCAACGTACTAGGTAGCAAATTGTCTA

GATAAAATACTGAATCAAATGGCGCAGACATATTAGCGGATCTAATGGAATCCAATTGATTGACTATCTT

TTGAAAATATACATTTTTATGATCTGATACTTGTAAGAATATAGCAATAATGATAATTCCATCATCGTGT

TTTTTTGCCTCTTCATAAGAACTATATTTTTTCTTATTCCAATGAACCAGATTAATCTCTCCAGAGTATT

TGTATACATCTATCAAGTGATTGGATCCATAATCGTCTTCCTTTCCCCAATATATATGTATTGTTGATAA

CACATATTCATTGGGGAGAAACCCTCCACTTATATATCCTCCTTTAAAATTAATCCTTACTAGTTTTCCA

GTATTCTGGATAGTGGTTGGTTTCGACTCATTATAATGTATGTCTAACGTCTTCAATCGCGCGTCAGAAA

TTGCTTTTTTAGTTTCTATATTAATAGGAGATAGTTGTTGAGGCATAGTAAAAATGAAATGATAACTGTC

TAGAAATAGCTCTTAGTATGGGATTTACAATGGATGAGGAAGTGATATTTGAAACTCCTAGAGAATTAAT

ATCTATTAAACGAATAAAAGATATTCCAAGATCAAAAGACACGCACGTGTTTGCTGCGTGTATAACAAGT

GACGGATATCCGTTAATAGGAGCTAGAAGAACTTCATTCGCATTCCAGGCGATATTATCTCAACAAAATT

CAGATTCTATCTTTAGAGTATCCACTAAACTATTACGGTTTATGTACTACAATGAACTAAGAGAAATCTT

TAGACGGTTGAGAAAAGGTTCTATCAACAATATCGATCCTCACTTCGAAGAGTTAATATTATTGGGTGGT

AAACTAGATAAAAAGGAATCTATTAAAGATTGTTTAAGAAGAGAATTAAAAGAGGAAAGTGATGAACATA

TAACAGTAAAAGAATTCGGAAATGTAATTCTAAAACTTACAACGAGTGATAAATTATTTAATAAAGTATA

TATAGGTTATTGCATGGCATGTTTTATTAATCAATCGTTGGAGGATTTATCACATACTAGTATTTACAAT

GTAGAAATTAGAAAGATTAAATCGTTAAATGATTGTATTAACGACGATAAATACGAATATCTGTCTTATA

TTTATAATATACTAATTAATAGTAAATGAGCTTTTACAGATCTAGTATAATTAGTCAGATTATTAAGTAT

AATAGACGACTAGCTAAGTCTATTATTTGCGAGGATGACTCTCAAATTATTACACTCACGGCATTCGTTA

ACCAATGCCTATGGTGTCATAAACGAGTATCCGTGTCCGCTATTTTATTAACTACTGATAACAAAATATT

AGTATGTAACAGACGAGATAGTTTTCTCTATTCTGAAATAATTAGAACTAGAAACATGTATAGAAAGAAA

CGATTATTTCTGAATTATTCCAATTATTTGAACAAACAGGAAAGAAGTATACTATCGTCATTTTTTTCTC

TAGATCCAGCTACTGCTGATAATGATAGAATAAACGCTATTTATCCGGGTGGTATACCCAAAAGGGGTGA

GAACGTTCCAGAGTGTTTATCCAGGGAAATCAAAGAAGAAGTTAATATAGACAATTCTTTTGTATTCATA

GACACTCGTTTTTTTATTCATGGTATCATAGAAGATACCATTATTAACAAATTTTTTGAGGTAATTTTCT

TTGTTGGACGAATATCTCTAACGAGTGATCAAATTATTGATACCTTTAAAAGTAATCATGAAATAAAGGA

TCTAATATTTTTAGATCCAAATTCAGGTAATGGACTCCAATACGAAATTGCAAAATATGCTCTAGATACT

GCAAAACTTAAATGTTACGGTCATAGAGGATGTTATTATGAATCATTAAAAAAATTAACTGAGGATGATT

GATTAGAAAATATAAATTAATTTACCATCGTGTATTTTTATAACGGGATTGTCTGGCATATCATGTAGAT

AGTTACCGTCTACATCGTATACTCTACCATCTACGCCTTTAAATCCTCTATTTATTGATATTAATCTATT

AGAATTGGAATACCAAATATTAGTACCCTCAATTAGTTTATTGGTAATATTTTTTTTAGACGATAGATCG

ATGGCTCTTGAAACCAAGGTTTTCCAACCGGACTCATTGTCTATCGGTGAGAAGTCTTTTTCATTAGCAT

GAATCCATTCTAATGATGTATGTTTAAACACTCTAAACAATTGTACAAATTCTTTTGATTTGTTTTGAAT

GATTTCAAATAGGTCTTCGTCTACAGTAGGCATACCATTAGATAATCTAGCCATTATAAAGTGCACGTTT

ACATATCTACGTTCTGGAGGAGTAAGAACGTGACTATTGAGACGAATGGCTCTTCCTACTATCTGACGAA

GAGACGCCTCGTTCCATGTCATATCTAAAATGAAGATATCATTGATTGAGAAGAAACTAATACCCTCGCC

TCCGCTAGAAGAGAATACGCATGTTTTAATGCATTCTCCGTTAGTGTTTGATTCTTGGTTAAACTCAGCC

ACCGCCTTGATTCTAGTATCTTTTGTTCTAGATGAGAACTCTATATTAGAGATACCAAAGACTTTGAAAT

ATAGTAATAAGATTTCTATTCCTGACTGATTAACAAATGGTTCAAAGACTAGACATTTACCATGGGATGC

TAATATTCCCAAACATACATCTATAAATTTGACGCTTTTCTCTTTTAATTCAGTAAATAGAGAGATATCA

GCCGCAATAGCATCCCCTCCCAATAGTTCTCCCTTTTTAAAGGTGTCTAATGCGGATTTAGAAAATTCTC

TATCTCTTAATGAATTTTTAAAATCATTATATAGGGTTGCTATCTCTTGTGCGTATTCTCCCGGATCACG

ATTTTGTCTTTCAGGAAAGCTATCGAATGTAAACGTAGTAGCCATACGTCTCAGAATTCTAAATGATGAT

ATACCAGTTTTTATTTCTGCGAGTTTAGCCTTTTGATAAATCTCTTCTTGCTTTTTTGACATATTAACGT

ATCGCATTAATACTGTTTTCTTAGCGAATGATGCAGACCCTTCCACATCATCAAAAATAGAAAACTCGTT

ATTAACTATGTACGAACATAGGCCTCCTAGTTTGGAGACTAATTCTTTTTCATCGACTAGACGTTTATTC

TCAAATAGCGATTGGTGTTGTAAGGATCCTGGTCGCAGTAAGTTAACCAACATGGTGAATTCTTGCACAC

TATTAACGATAGGTGTAGCCGATAAACAAATCATCTTATGGTTTTTTAACGCAATGGTCTTAGATAAAAA

ATTATATACTGACCGAGTAGGACGGATCTTACCATCTTCTTTGATTAATGATTTAGAAATGAAGTTATGA

CATTCATCAATGATGACGCATATTCTACTCTTGGAATTAATAGTTTTGATATTAGTAAAAAATTTATTTC

TAAAATTTTGATCATCGTAATTAATAAAAATACAATCCTTCGTTATCTCTGGAGCGTATCTGAGTATAGT

GTTTATCCAAGGATCTTCTATCAAAGCCTTTTTTACCAATAAGATAATTGCCCAATTCGTATAAATATCC

TTAAGATGTTTGAGAATATATACAGTAGTCATTGTTTTACCGACACCTGTTTCATGGAACAATAAAAGAG

AATGCATACTGTCTAATCCTAAGAAAACTCTTGCTACAAAATGTTGATAATCCTTGAGGCGTACTACGTC

TGACCCCATCATTTCAACGGGCATATTAGTAGTTCTGCGTAAGGCATAATCGATATAGGCCGCGTGTGAT

TTACTCATTTATGAGTGATAAGTAATAACTATGTTTTAAAAATCACAGCAGTAGTTTAACTAGCCTTCTC

TGATGTTTGTTTTCGATACTTTTTGAATCAGAAGTCATACTAGAATAAAGCAGCGAGTGAACGTAATAGA

GAGCTTCGTATACTCTATTCGAAAACTCTAAGAACTTATTAATGAATTCCGTATCCACTGGATCGTTTAA

AATACTAAATTGAACAGTGTTCACATCCTTCCAAGACGAAGACTTAGTGACGGACTTAACATGAGACATA

AATAAATCCAAATTTTTTTTATAAACATCACTAGCCACCATAATGGCGCTATCTTTCAACCAACTATCGC

TTACGCATTTTAACAGTCTAACATTTTTAAAGAGACTACAATATATTCTCATAGTATCGATTACACCTCT

ACCGAATAGAGTGGGAAGTTTAATAATACAATATTTTTCGTTTACAAAATCAAATAATGGTCGAAACACG

TCGAAGGTTAACATCTTATAATCGCTAATGTATAGATTGTTTTCAGTGAGATGATTATTAGATTTAATAG

CATCTCGTTCACGTTTGAACAGTTTATTGCGTGCGCTGAGGTCGGCAACTACGGCATCCGCTCTAGTACT

CCTCCCATAATACTTTACGCTATTAATCTTTAAAATTTCATAGACTTTATCTAGATCGCTTTCTGGTAAC

ATGATATCATGTGTAAAAAGTTTTAACATGTCGGTCGGCATTCTATTTAGATCATTAACTCTAGAAATCT

GAAGAAAGTAATTAGCTCCATATTCCAGACTAGGTAATGGGCTTTTACCTAAAGACAAGTTAAGTTCTGG

CAATGTTTCATAAAATGGAAGAAGGACATGTGTCCCCTCCCGGATATTTTTTACAATTTCATCCATTTAC

AACTCTATAGTTTGTTTTCATTATTATTAGTTATTATCTCCCATAATCTTGGTAATACTTACACCTTGAT

CATAAGATACCTTATACAGGTCATTACATACAACTACCAATTGTTTTTGTACATAATAGATTGGATGATT

GATATCCATGGTGGAATAAACTACTCGAACAGATAGTTTATCTTTCCCCCTAGATACATTGGCCGTAATA

GTTGTCGGCCTAAAGAATATCTTTGGTGTAAAGTTAAAAGTTAGGGTTCTTGTTCCATTATTGCTTTTTG

TCAGTAGTTCGTTATAAATTCTCGAGATGGGCCCGTTCTCTGAATATAGAACATCATTTCCAAATCTAAC

TTCTAGTCTAGAAATAATATCGGTCTTATTTTTAAAATCTATTCCCTTGATGAATGGATCGTTAATAAAC

AAATCCTTGGCCTTTGATTCGGCTGATCTATTATCTCCGTTATAGACGTTACGTTGACTAGTCCAAAGAC

TTACAGGAATAGATGTATCGATGATGTTGATAGTATGTGATATGTGAGCAAAGACTGTTCTCTTGGTGGC

GTCGCTATATGTTCCAGTAATGGCGGAAAACTTTTTAGAAATGTTATATATAAAAGAATTTTTTCGGGTT

CCAAACATTAACAGATTAGTATGAAGATAAACACTCATATTATCAGGAACATTATCAATTTTTACATAAA

CATCGGCATCTTGAATAGAAACAACACCATCTTCTGGAACCTCTACGATCTCGGCAGATTCCGGATAACC

AGTCGGTGGACCATCACTAACAATAACTAGATCATCCAACAATCTACTCACATATGCGTCTATATAATCT

TTTTCATCTTGTGAGTACCCTGGATACGAAATAAATTTGTTATCAGTATTTCCATAATAAGGTTTAGTAT

AAACAGAGAGAGATGTTGCTGCATGAACTTCGGTTACTGTCGCCGTTGGTTGGTTTATTTGACCTATTAC

TCTCCTAGGTTTCTCTATAAATGATGGTTTAATTTGTACATTCTTAACCATATATCCAATAAAGCTCAAT

TCAGGAACATAAACAAATTCTTTGTTGAACGTTTCAAAGTCGAACGAAGAGTCACGAATAACGATATCGG

ATACTGGATTGAAGGTCACCGTTACGGTAATTTTTGAATCGGATAGTTTAAGACTACTGAATGTATCTTC

CACATCAAACGGAGTTTTAATATAAACGTATACTGTAGATGGTTCTTTAATAGTGTCATTAGGAGTTAGG

CCAATAGAAATATCATTAAGTTCACTAGAATATCCAGAATGTTTCAAAGCAATTGTATTATTGATACAAT

TATTATATAATTCTTCGCCCTCAATTTCCCAAATAACACCGTTACACGAAGAGACAGATACATGATTAAT

ACATTTATATCCAACATATGGCACGTAACCGAATCTTCCCATACCTTTAACTTCTGGAAGTTCCAAACTC

AGAACCAAATGATTAAGCGCAGTAATATACTGATCCCTAATTTCGAAGCTAGCGATAGCCTGATTGTCTG

GCCCATCGTTTGTCATAACTCCGGATAGAGAAATATATTGCGGCATATATAAAGTTGGAATTTGACTATC

AACTGCGAAGACATTAGACCGTTTAATAAAGTCATCCCCACCGATCAAAGAATTAATGATAGTATTATTC

ATTTTCTATTTAAAATGGAAAAAGCTTACAATAAACTCCGTAGAGAAATATCTATAATTTGTGAGTTTTC

CTTAAAGTAACAGCTTCCGTAAACACCGTCTTTATCTCTTAGTAAGTTTATTGTATTTATGACCTTTTCC

TTATCTTCATAGAATACTAAAGGCAATAAAGAAATTTTTGGTTCTTCTCTAAGAGCTACGTGAGACTTAA

CCATAGACGCCAACGAATCCCTACATATTTTAGAACAGAAATACCCAACTTCACCACCCTTGAATGTCTC

AATACTAATAGGTCTAAAAACCAAATCTTGATTACAAAACCAACACTTATCAATTACACTATTTGTCTTA

ATAGACATATCTGCCATAGATTTATAATACTTTGGTAGTATACAAGCGAGTGCTTCTTCTTTAGCGGGCT

TAAAGACTGCTTTAGGTGCTGAAATAACCACATCTGGAAGACTTACTCGCTTAGCCATTTAATTACGGAA

CTATTTTTTTATACTTCTAATGAACAAGTAGAAAACCTCTCATCTACAAAAACATACTCGTGTCCATAAT

CCTCTACCATAGTAACACGTTTTTTAGATCTCATATGTGCTAAAAAGTTTTCCCATACTAATTGGTTACT

ATTATTTTTCGTATAATTTTTAACAGTTTGAGGTTTTAGATTTTTAGTTACAGAAGTGATATCGAATATT

TTATCCAAAAAGAATGAGTAATTAATTGTCTTAGAAGGAGTGTTTTCTTGGCAAAAGAATACCAAGTGCT

TAAATATTTCTACTACTTCATTAATCTTTTCTGTACTCAGATTCAGTTTCTCATCTTTTACTTGATTGAT

TATTTCAAAGACTAACTTATAATCCTTTTTATTTATTCTCTCGTTAGCCTTAAGAAAACTAGATACAAAA

TTTGCATCTACATCATCCGTGGATATTTGATTTTTTTCCATGATATCCAATAGTTCCGAGATAATTTCTC

CAGAACATTGATGAGACAATAATCTCCGCAATACATTTCTCAAATGAATAAGTTTATTAGACACGTGGAA

GTTTGACTTTTTTTGTACCTTTGTACATTTTTGAAATACAGACTCGCAAAAAATACAATATTCATATCCT

TGTTCAGATACTATACCGTTATGTCTACAACAGCTACATAATCGTAGATTCATGTTAACACTCTACGTAT

CTCGTCGTCCAATATTTTATATAAAAACATTTTATTTCTAGACGTTGTCAGAAAATCCTGTAATATTTTT

AGTTTTTTTGGTTGTGAATAAAGTATCGCCCTAATAATATTGGTACCGTCTTCCGACAATATAGTAGTTA

AATTATCCGAGCATGTAGAAGAACACCGCTTAGGCGGATTCAGTACAATGTTATATTTTTCGTACCAACT

CATTTAAATATCATAATCTAAAATAGTTCTGTAATATGTCTAGCGCTAATATATTGATCATAATCCTGTG

CATAAATTAAGATACAACAATGTCTTGAAATCATCGACATGGCTTCTTCCATAGTTAGAAGATCATCGTC

AAAGTTAGCAACGTGATTCATCAACATTTGCTGTTTTGAGGCAGCAAATACTGAACCATCACCATTCAAC

CATTCATAAAAACCATCGTCTGAATCCATTGATAATTTCTTGTACTGGTTTTTGAGAGCTCGCATCAATC

TAGCATTTCTAGCTCCCGGATTGAAAACAGAAAGAGGATCGTACATCCAAGGTCCATTTTCTGTAAATAG

AATCGTATAATGTCCCTTCAAGAAGATATCAGACGATCCACAATCAAAGAATTGGTCTCCGAGTTTGTAA

CAGACTGCGGACTTTAACCTATACATGATACCGTTTAGCATGATTTCTGGTGATACGTCAATCGGAGTAT

CATCTATTAGAGATCTAAAGCCGGTGTAACATTCTCCGCCAAACATATTCTTATTCTGACGTCGTTCTAC

ATAAAACATCATTGCTCCATTAACGATAACAGGTGAATGAACAGCACTACCCATCACATTAGTTCCCAAT

GGATCAATGTGTGTAACTCCAGAACATCTTCCATAGCCTATGTTAGGAGGAGCGAACACCACTCTTCCAC

TATTGCCATCGAATGCCATAGAATAAATATCCTTGGAATTGATAGAAATCGGACTGTCGGATGTTGTTAT

CATCTTCATAGGATTAACAACGATGTATGGTGCAGCCTGAAGTTTCATATCGTAACTGATGCCGTTCATA

GGTCTAGCCACAGAAACCAACGTAGGTCTAAATCCAACTATAGACAAAATAGAAGCTAATATCTGTTCCT

CATCTGTCATAACTTGAGAGCATCCAGTATGAATAATCTTCATTAGATGGGGATCTACCGCATCATCATC

GTTACAATAAAAAATTCCCATTCTAATGTTCATAATTGCTTTTCTAATCATGGTATGAATGTTTGCTCTC

TGAATCTCTGTGGAAATTAGATCTGATACACCTGTAATCACTATCGGATTATCCTCCGTAAGACGATTAA

CCAACAACATATAATTATAAGACTTTACTCTTCTAAATTCATAAAGTTGCTGGATTAGACTATATGTGTC

TCCATGTACATACGCGTTCTCGAGCGCAGGAAGTTTAATACCGAATAGTGCCATCAGAATAGGATGAATG

TAGTAATTAGTTTCTGGTTTTCTATAAATAAAAGACAAATCTTGTGAACTAGACATATCGGTAAAATGCA

TGGATTGGAATCGTGTAGTCGACAGAAGAATATGATGATTAGATGGAGAGTATATTTTATCTAACTCTTT

GAGTTGGTCACCGATTCTAGGACTAGCTCGAGAATGAATAAGTACTAAGGGATGAGTACATTTCACAGAA

ACACTGGCGTTGTTCAACGTACTCTTTACATGGGAAAGGAGTTGAAATAGCTCGTTTCTATTTGTCCTGA

CAATATTTAGTTTATTCATAATATTAAGCATATCCTGAATAGTAAAGTTAGATGTGTCATACTTGTTAGT

AGTTAGATATTTAGCAATTGCATTCCCATCATTTCTCAATCTCGTACTCCAATCATGTGTGGATGCTACT

TCGTCGATGGAAACCATACAATCCTTTTTGATAGGCTGTTGAGATTGATCATTTCCTGTACGTTTAGGTT

TGGTACGTTGATTTCTAGCCCCTGCTGATATAAAGTCATCGTCTACAATTTGGGATAATGAATTACATAC

ACTACAAGACAAAGATTTATCAGAAGTGTGAATATGATCTTCATCTACCAAAGAAAGAGTTTGATTAGTA

TAACTAGATTTTAGTCCCGCGTTAGATGTTAAAAAAACATCGCTATTGACCACGGCTTCCATTATTTATA

TTCGTAGTTTTTACTCGAAAGCGTGATTTTAATATCCAATCTTATTACTTTTGGAATCGTTCAAAACCTT

TGACTAGTTGTAGAATTTGATCTATTGCCCTACGCGTATACTCCCTTGCATCATATACGTTCGTCACCAG

ATCGTTTGTTTCGGCCTGAAGTTGACGCATATCTTTTTCAACACTCGACATGAGATCCTTAAGGGTCATA

TCGTCTAGATTTTGTTGAGATGCTGCTCCTGGATTTGGATTTTGTTGTGCTGTTGTACATACTGTACCAC

CAGTAGGTGTAGGAGTACATACAGTGGCCACAATAGGAGGTTGAAGAGGTGTAACCGTTGGAGTAGTACA

AGAAATACTTCCATCCGATTGTTGTGTACATGTGGTTGTTGGTAACGTCTGAGAAGGTTGGGTAGATGGC

GGTGTCGTCATCTTTTGATCTTTATTAAATTTAGAGATAATATCCTGAACAGTATTGCTCGGCGTCAACG

CTGGAAGGAGTGTACTCGCCGGCGCATCAGTATCTGTAGACAACCAATCAAAAAGATTAGACATATCAGA

TGATGTATTAGTTTGTTGACGTGGTTTTAGTACAGGAGCAGTACTACTAGGTAGAAGAATAGGAGCCGGT

GTAGGTGTCGGAACCGGCTGTGGAGTTATATGAATAGTTGGTTGTAGCGGTTGGGTAGGCTGTCTGCTGG

CGGTCATCATATTATCTCTAGCTAGTTGTTCTCGCAACTGTCTTTGATAATACGACTCTTGAGACTTTAG

TCCTATTTCAATCGCTTCATCCTTTTTCGTATCCGGATCCTTTTCTTCAGAATAATAGATTGACGACTTT

GGTGTAGAGGATTCTGCCAGCCCCTGTGAGAACTTGTTAAAGAAGTCCATTTAAGGCTTTAAAATTGAAT

TGCGATTATAAGATTAAATGGCAGACACGGACGATATTATCGACTATGAATCCGATGATCTCACCGAATA

CGAGGATGATGAAGAAGATGGAGAGTCACTAGAAACTAGTGATATAGATCCCAAATCTTCTTATAAGATT

GTAGAATCAACATCCACTCATATAGAAGATGCGCATTCCAATCTTAAACATATAGGGAATCATATATCTG

CTCTTAAACGACGCTATACTAGACGTATAAGTCTATTTGAAATAGCGGGTATAATAGCAGAAAGCTATAA

CTTGCTTCAACGAGGAAGATTACCTCTAGTTTCAGAATTTTCTGACGAAACGATGAAGCAAAATATGCTA

CATGTAATTATACAAGAGATAGAGGAGGGTTCTTGTCCTATAGTCATCGAAAAGAACGGAGAATTGTTGT

CGGTAAACGATTTTGATAAAGATGGTCTAAAATTCCATCTAGACTATATTATCAAAATTTGGAAACTTCA

AAAACGATATTAGAATTTATACGAATATCGTTCTCTAAATGTCACAATCAAGTCTCTCATATTCAGCAGT

TTATTGTCGTACTTTATATCGTGTTCATTAACGATATTTTGCAAAATAGTAATGATTCTATCTTCCTTCG

ATAGATATTCTTCAGAGATTATTGTCTTATATTCTTTCTTGTTATCCGATATGAATTTGATAAGACTTTG

AACATTATTAATACCCGTCTGTTTAATTTTTTCTATAGATATTTTAGTTTTGGTAGATTCTATGGTGTCT

GTTAATAGGCATCCAACATCGACATTCGACGTCAATTGTCTATAAATCAGAGTATAAATTTTAGAAATAA

CATTAGCAAATTGTTGTGCGTTGATGTCGTTATTCTGAAACAGTATGATTTTAGGTAGCATTTTCTTAAC

AAAGAGAACGTATTTATTGTTACTCAGTTGAACAGATGATATATCCAGATTACTAACGCATCTGATTCCA

TATACCAAACTTTCAGAAGAAATGGTGTACAATTGTTTGTATTCATTCAATGTCTCCTTTTCAGAAATTA

GTTTAGAGTCGAATACTGCAATAATTTTCAAGAGATAGTTTTCATCAGATAAGATTTTATTTAGTGTAGA

TATGATAAAACTATTGTTTTGTTGGAGAACTTGATACGCCGCATTCTCTGTAGTCGACGCTCTCAAATGG

GAAACAATCTCTATTATTTTTTTGGAATCGGATACTATATCTTCGGTATCTTGACGCAGTCTAGTATACA

TAGAGTTAAGAGAAATTAGAGTTTGTACATTAAGCAACATGTCTCTAAATGTGGCTACAAACTTTTCTTT

TTCCACATCATCTAGTTTATTATATACCGATTTCACAACGGCACCAGATTTAAGGAACCAGAATGAAAAA

CTCTGATAACTACAATATTTCATCATAGTTACGATTTTATCATCTTCTATAGTTGGTGTGATAACACATA

CCTTTTTCTCCAAGACTGGAACCAACGTCATAAAAATGTTTAAATCAAAATCCATATCAACATCTGATGC

GCTAAGACCAGTCTCGCGTTCAAGATTATCTTTACTAATGGTGACGAACTCATCGTATAGAACTCTAAGT

TTGTCCATTATTTATTTACAGATTTAGTTGTTTAATTTATTTGTGCTCTTCCAGAGTTGGGATAGTATTT

TTCTAACGTCGGTATTATATTATTAGGATCTACGTTCATATGTATCATAATATTAATCATCCACGTTTTG

ATAAATCTATCTTTAGCTTCTGAAATAACGTATTTAAACAAAGGAGAAAAATATTTAGTTACGGCATCAG

ACGCGATAACATTTTTTGTAAATGTAACGTATTTAGACGACAGATCTTCGTTAAAAAGTTTTCCATCTAT

GTAGAATCCATCGGTTGTTAACACCATTCCCGCGTCAGAGTGAATAGGAGTTTGAATAGTTTGTTTTGGA

AATAGATCCTTCAATAACTTATAGTTGGGTGGGAAAAAATCGATTTTATCACTAGACTCTTTCTTTTTTA

CTATCATTACCTCATGAACTATTTCTTGAATGAGTATATGTATTTTCTTTCCTATATCGGTCGCGTTCAT

TGGAAAATATATCATGTCGTTAACTATAAGAATATTTTTATCCTCGTTTACAAACTGAATAATATCAGAT

ATAGTTCGTAAACGAACTATATCATCACCAGCACAACATCTAACTATATGATATCCACTAGTTTCCTTTA

GCCGTTTATTATCTTGTTCCATATTAGCAGTCATTCCATCATTTAAGAAGGCGTCAAAGATAATAGGGAG

AAATGACATTTTGGATTCTGTTACGACTTTACCAAAATTAAGGATATACGGACTTACTATCTTTTTCTCA

ACGTCGATTTGATGAACACACGATGAAAATGTACTTCGATGAGATTGATCATGTAGAAAACAACAAGGGA

TACAATATTTCCGCATATCATGAAATATATTAAGAAATCCCACTTTATTATATTTCCCCAAAGGATCAAT

GCATGTAAACATTATACCGTTATCATTAATAAAGACTTCTTTCTCATCGGATCTGTAAAAGTTGTTACTG

ATTTTTTTCATTCCAGGATCTAGATAATTAATAATAATGGGTTTTCTATTCTTATTCTTTGTATTTTGAC

ATATCCTAGACCAGTAAACAGTTTCCACTTTGGTAAAATCAGAAGACTTTTGAACGCTATTAAACATGGC

ATTAATGGCAATAACTAAAAATGTAAAATATTTTTCTATGTTAGGAATATGGTTTTTCACTTTAATAGAT

ATATGGTTTTTTGCCAAAATGATAGATATTTTTTTATCCGATGATAGTAAAATATTATTAGTCGCCGTCT

CTATAAAAATGAAGCTAGTCTCGATATCCAATTTTATTCTAGAATTGATAGGAGTCGCCAAATGTACCTT

ATACGTTATATCTCCCTTGATGCGTTCCATTTGTGTATCTATATCGGACACAAGATCTGTAAATAGTTTT

ACGTTATTAATCATCACGGTATCGCCATCGCTAGATAATGCTAATGTACTATCCAAGTCCCAAATGGAGA

GATTTAACTGTTCATCGTTTAGAATAAAATGATTACCTGTCATATTAATAAAGTGTTCATCGTATCTAGA

TAACAACGACTTATAATTAATGTCCAAGTCTTGAACTCGCTGAATGATCTTTTTTAACCCAGTTAGTTTT

AGATTGGTACGAAATATATTGTTAAACTTTGATTCTACAGTAATGTCCAAATCTAGTTGTGGAAATACTT

CCATCAACATTGTTTCAAACTTGATAATATTATTATCTACATCTTCGTACGATCCAAATTCCGGAATAGA

TGTATCGCACGCTCTGGCCACCCAGATAACCAAAAAGTCACACGCTCCAGAATATACATTGTATAAAAAG

CTATCGTTTTTTAGTAGTGTTTTTTTCTGAGTATATACGAAAGGATTAAAAATAGTATTATCAACGTAAC

TATATTCCAAATTATTCTTATGAGAATAGATAATAATATCGTCCTTAATATCTAACAAATTTCCTAAATA

TCCCTTTAATTGAGTCATTCGAAGCGTTAATAAAATATGTCTCTTAACTATTTCCGGCCGTTGTATATTT

AAATGACTTCGTAAGAAATAATATATAGGCGACTTCTCATCTATGTAATCATATGGAGTGAGATATAGGG

CTCGTTCTACCTCCTGCCCCTTACCCACCTGTAATACCAATTGCGGACTCACTATATATCGCATATTTAT

ATCGTGGGGTAAAGTGAAAATCTACTACCGATGATGTAAGTCTTACAATGTTCGAACCAGTACCAGATCT

TAATTTGGAGGCCTCCGTAGAACTAGGGGAGGTAAATATAGATCAAACAACACCTATGATAAAGGAGAAT

AGCGGTTTTATATCCCGTAGTAGACGTCTATTCGCCCATAGATCTAAGGATGATGAGAGAAAACTAGCAC

TACGATTCTTTTTACAAAGACTTTATTTTTTAGATCATAGAGAGATTCATTATTTGTTCAGATGCGTCGA

CGCTGTAAAAGACGTCACTATTACCAAAAAAAATAACATTATCGTGGCGCCTTATATAGCACTTTTAACT

ATCGCATCAAAAGGATGCAAACTTACAGAAACAATGATTGAAGCATTCTTTCCAGAACTATATAATGAAC

ATAGTAAGAAATTCAAATTCAACTCTCAAGTATCCATCATCCAAGAAAAACTCGGATACCAGTCTGGAAA

CTATCACGTTTATGATTTTGAACCGTATTACTCTACAGTAGCTCTGGCTATTCGAGATGAACATTCATCT

GGCATTTTTAATATCCGTCAAGAGAGTTATCTTGTAAGTTCATTATCTGAAATAACATATAGATTTTATC

TAATTAATCTAAAATCTGATCTTGTTCAATGGAGTGCTAGTACGGGCGCTGTAATTAATCAAATGGTAAA

TACTGTATTGATTACAGTGTATGAAAAATTACAACTGGCCATAGAAAATGATTCACAATTTACATGTTCA

TTGGCTGTGGAATCAGAACTTCCAATAAAATTACTTAAAGATAGAAATGAATTATTTACAAAATTCATTA

ACGAGTTAAAAAAGACCAGTTCATTCAAGATAAGCAAACGTGATAAGGATACGCTATTAAAACATTTTAC

TTATGACTGGAGTTAGAATTTATAGACGACACATTTCGTTTATCATTGTTACTATTACTATCATTATTAG

TATTCTTCTTGTCATCTTGTTCAGAAATATACAGCAATGCTATACCTAATACTAAATACATTATCATGCT

TGCAATGGCTCTAACAACAACGAACCAAAATGAATTTGGTCGTAGCTTTTGTTCACAAAAATACATAAAG

AAATGTCTACATAAATCTATGGCGCCATTGGCTACTTGAAATAGCGCCAGTCCTCCTACAGATTTTAATA

TAGCTGTATAACATGACATTTATTCATCATCAAAAGAGACAGAGTCACCATCTGTCATATTTAGATTTTT

TTTCATGTGTTCAAAGTATCCTCTACTCATTTCATTATAATAGTTTATCATGCTTAGAATTTTAGGACGG

ATCAATGAGTAAGACTTGACTAGATCGTCAGTAGTAATTTGTGCATCATCTATTCTGCATCCGCTTCGTC

GAATAATGTATAGCATCGCTTTGAGATTCTCCATAGCTATCAAGTCTTTATATAATGACATGGAAATATC

TGTGAATGCTTTATACTTCTCCAACATCGATGCCTTAACATCATCACATACTTTAGCATTGAAAATACGT

TCTATTGTGTAGATGGATGTAGCAAGATTTTTAAACAACAATGCCATCTTACATGATGATTGTCTCAAGT

CTCCAATCGTTTGTTTAGAACGATTAGCTACAGAGTCCAATGCTTGGCTAACTAGCATATTATTATCTTT

AGAAATTGTATTCTTCAATGAGGCGTTTATCATATCTGTGATTTCGTTAGTCATATTACAGTCTGACTGG

GTTGTAATGTTATCCAACATATCACCTATGGATACGGTACACGTACCAGCATTTGTAATAATCCTATCTA

AGATGTTGTATGGCATTGCGCAGAAAATATCTTCTCCTGTAATATCTCCACTCTCGATAAATCTACTCAG

ATTATTCTTAAATGCCTTATTCTCTGGAGAAAAGATATCAGTGTCCATCATTTCATTAATAGTATACGCA

GAAAAGATACCACGAGTATCAATTCTATCCAAGATACTTATCGGTTCCGAGTCACAGATAATTGTTTCCT

CTCCTTCGGGAGATCCTGCATAGAAATATCTAGGACAATAGTTTCTATACTGTCTGTAACTCTGATAATC

TCTAAAGTCACTAACTGATACCATGAAATTGAGAAGATCAAACGCTGAAGTAATCAATTTTTCTGCCTCG

TTTTTACTACAACTAGTTTTCATCAATGTAGTGACGATGTATTGTTTAGTTACTCTTGGTCTAATACTGA

TGATAGAGATATTATTGCTTCCCATAATGGATCTTCTAGTAGTCACCTTAAAGCCCATTGATGCGAATAG

CAGATAGATAAAGTCTTGGTATGACTCCTTTCTAATATAGTACGGACTACCTTTGTCACCCAACTTTATA

CCCACATAAGCCATAACAACCTCTTTAATAGCCGTTTCATGAGGTTTATCAGCCATGAGCCTGAGTAGTT

GAAAGAATCGCATGAATCCCGTCTCAGAAAGTCCTATATGCATGATAGATTTATCTTTCCTGGGAAACTC

TCGTATAGTTATAGATGAAATACTCTTCAAAGTTTCTGAAATAAGATTAGTAACAGTCTTACCTCCGACT

ACTCTGGGTAACAAACATACTCTAATAGGTGTTTTCTCTGCGGAGATAATATCAGAAAGGATAGAGCAAT

AAGTAGTATTATTGTGATTATAAAGACCGAATACATAACAGGTAGAATTTATAAACATCATGTCCTGAAG

GGTTTTAGACTTGTATTCCTCGTAATCTATACCGTCCCAAAACATGGATTTGGTAACTTTGATAGCCGTA

GATCTTTGTTCCTTCGCTAACAGGTTAAAGAAATTAATAAAGAATTTGTTGTTTCTATTTATGTCCACAA

ATTGCACGTTTGGAAGCGCCACGGTTACATTCACTGCAGCATTTTGAGGATCGCGAGTATGAAGTACGAT

GTTATTGTTTACTGGTATATCTGGAAAGAAATCTACCAGTCTAGGAATAAGAGATTGATATCGCATAGAA

ATAGTAAAGTTTATAATCTCATCATTGAAGATTACTCTGTTACCATTGTAATAAATTGGTACTCTATCAT

AATCATCGACAAAGTACTGTTCATACATGATGAGATGTTTATATGTTGGCATAGTAGTGAGATCGACGTT

TGGTAATGGCAATGTATTAAGATTAACTCCATAATGTCTAGCAGCATCTGCGATGTTATAAGTGATGTCA

AAGCGGGGTTGATCTTGTGCTGTTATATATTGTCTAACACCTATAAGATTATCAAAATCTTGTCTGCTTA

ATACACCGTTAACAATTTTTGCCTTGAATTCTTTTATTGGTGCATTAATAACATCCTTATAGAGGATGTT

AAACAAATAAGTATTATCAAAGTTAAGATCTGGGTATTTCTTTTCTGCTAGAACATCCATTGAGTCGGAG

CCATCTGGTTTAATATAACCACCGATAAATCTAGCTCTGTATTCTGTATCCGTCAATCTAATATTAAGAA

GGTGTTGAGTGAAAGGTGGAAGATCGTAAAAGCTGTGAGTATTAATAATAGGGTTAGTTTCCGAACTAAT

GTTAATTGGATGATTAATAATATTTATATTTCCAGCGTTAAGTGTAACATTAAACAGTTTTAATTCACGT

GACGTGGTATCAATTAAATAATTAATGCCCAATTTGGATATAGTAGCCTGAAGCTCATCTTGTTTAGTTA

CGGATCCTAATGAGTTATTAAGAAATACATCGAACGGATGAACGAAGGTTGTTTTAAGTTGGTCACATAC

TTTGTAATCTAGACATAGATGTGGAAGAACGGTAGAAACTATACGAAATAGATATTCAGAGTCCTCTAAT

TGATCAAGAGTAACTATTGACTTAATAGGCATCATTTATTTAGTATTAAATGACGACCGTACCAGTGACA

GATATACAAAACGACTTAATTACAGAGTTTTCAGAAGATAATTATCCATCTAACAAAAATTATGAAATAA

CTCTTCGCCAAATGTCTATTCTAACTCACGTTAACAACGTGGTAGATAGAGAACATAATGCCGCCGTAGT

GTCATCTCCAGAGGAAATATCATCACAACTTAATGAAGATCTATTTCCAGATGATGATTCACCGGCCACT

ATTATCGAACGAGTACAACCTCATACTACTATTATTGACGATACGCCACCTCCTACTTTTCGTAGAGAGT

TATTGATATCGGAACAACGTCAACAACGAGAAAAAAGATTTAATATTACAGTATCAAAAAATTCTGAAGC

AATAATGGAATCTAGATCTATGATAACTTCTATGCCAACACAAACACCATCCTTGGGAGTAGTTTATGAT

AAAGATAAAAGAATTCAGATGCTAGAGGATGAAGTGGTTAATCTTAGAAATCAACGATCTAATACAAAAT

CATCTGATAATTTAGATAATTTTACCAGAATACTATTTGGTAAGACTCCGTATAAATCAACCGAAGTTAA

TAAGCGTATAGCCATCGTTAATTATGCAAATTTGAACGGGTCCCCCTTATCAGTCGAGGACTTGGATGTC

TGTTCGGAGGATGAAATAGATAGAATCTATAAAACGATTAAACAATATCACGAAAGTAGAAAACGAAAAA

TTATCGTCACTAACGTGATTATTATTGTCATAAACATTATTGAGCAGGCATTGCTAAAACTCGGATTTGA

AGAAATCAAAGGACTGAGTACCGATATCACTTCAGAAATTATCGATGTGGAGATCGGAGATGACTGCGAT

GCTGTAGCATCAAAACTAGGAATCGGTAACAGTCCGGTTCTTAATATTGTATTGTTTATACTCAAGATAT

TCGTTAAACGAATTAAAATTATTTAATTTAATACATTCCCATATCCAGACAACAATCGTCTGGATTAATC

TGTTCCTGTCGTCTCATACCGGACGACATATTAATCTTTTTATTAGTGGGCATCTTTTTAGATGGTTTCT

TTTTCCCAGCATTAACTGATTCGATACCTAGAAGATCGTGATTGATTTCTCCGACCATTCCACGAACTTC

TAATTGGCCGTCTCTAACGGTACCATAAACTATTTTACCAGCATTAGTAACAGCTTGGACAATCTGACCA

TCCATTGCGTTGAATGATGTAGTTGCTGTTGTTCTACGTCTAGGAGCACCAGAGGTATTTTTAGAGCTCT

TGGATGTTGATGTAGAAGACGAGGATTTTGATTTTGGTTTACATGTAATACATTTTGAACTCTTTGATTT

TGTATCACATGCACCGGCAGTCACATCTGTTTGAGAATTAAGATTATTGTTGCCTCCTTTGACGGCTGCA

TCTCCACCGATCTGCGCTAGTAGATTTTTAAGCTGTGGTGTAATCTTATTAACTGTTTCAATATAATCAT

CGTAACTACTTCTAACGGCTAAATTTTTTTTATCCGCCATTTAGAAGCTAAAAATATTTTTATTTATGCA

GAAGATTTAACTAGATTATACAATGAACTAATATGATCCTTTTCTAGATTATTTACGAACTTGGTATTTC

TTGTTTCTGGAGGAGGAGAATTTAAATTCGGACTTGGATTCGGATTTTGTGGGTTCTTGATCTTATTATA

CAGCGTGTATAGGATGGTGACGGTAACTGCTACACAAATACCGATCAACAGAAGAATACCAATCATTTAT

TGACAATAACTTCACTATGATCAAGTATGTAATAATCATCTTTTCACTAAGTAAGTAGTAATAATGATTC

AACAATGACACGATATATGGACGATAATAATTTAGTTCATGGAAATATCGCTATGATTGGTGTGAATGAC

TCCGCTAACTCTGTGGGGTGCACAGTGCTTTCCCCACATAGAATAAATTAGCATTCCGACTGTGATAATA

ATACCAAGTATAAACGCCATAATACTCAATACTTTCCATGTACGAGTGGGACTGGTAGACTTACTAAAGT

CAATAAAGGCGAAGATACACGAAAGAATCAAAAGAATGATTCCAGCGATTAGCACGCCAGAAAAATAATT

TCCAATCATAAGCATCATGTCCATTTAACTAATAAAAATTTTAAATCGCCGAATAAACAAAGTGGAATAT

AAACCATATAAAAACAATAGTTTGTACTGCAAAAATAATATCTATTTTTGTTTTCGAAGATATGGTAAAA

TTAAATAGTAGTACACAGCATGTTATAACTAACAGCAGCAACGGCTCGTAATTACTTATCATTTACTAGA

CGAAAAGGTGGTGGGATATTTTCTTGCTCAAATAATACGAATATATCACCCATCCATTTTATACGATGTT

TATATACTCTAATCTTTAATAGATCTATAGATGACGGGTTTACCAATAATATAGATTTTATCGATTCATC

TAATTTAAACCCTTCCTTAAACGTGAATGATCTATTATCTGGCATAATGATGACCCTACCTGATGAATCT

GACAATGTACTGGGCCATGTAGAATAAATTATCAACGAATTATCGTCTACGAACATTTATATCATTTGTT

TTAATTTTAGGACGTGAATAAATAGATATAAAATAGAAAATAACAGATATTACAACCAGTGTTATGGACG

CACCCAACCATGTAGGCAGTTTTATTTTATCGTTTACTACAGGTTCTCCTGGATGTACGTCACCAACTGC

AGACGTAGTTCTAGTACAATTAGACGTAAGTTCCGCTTGGGAATTTTTTAACGCTAAAGAGTTAACGTTG

ATCGTACACCCAACGTATTTACATCTAGTTCTTTGAACATCTTGATTATAATATAACCATTTTCTATCTC

TAGATTCGTCAGTGCACTCATGTAACCAACATACCCTAGGTCCTAAATATTTATCTCCGGAATTAGATTT

TGGATAATTCGCGCACCAACAATTTCTATTTCCTTTATGGTCGTTACAAAAGACGTATAATGCCGTATCC

CCAAAAGTAAAATAATCAGGACGAATAATTCTAATAAACTCAGAACAATATCTCGCATCCATATGTTTGG

AGCAAATATCGGAATAAGTAGACATAGCCGGTTTCCGTTTTACACGTAACCATTCTAAACAATTGGGGTT

TCCAGGATCGTTTCTACAAAAACCAGTCATGAAATCGTCACAATGTTCTGTCTTGTAATTATTATTAAAT

ATTTTTGGACAGTGTTTGGTATTTGTCTTAGAACAACATTTTGCCACGCTATCACTATCACCCAGGAGAT

AATCCTTTTTTATAAAATGACATCGTTGCCCGGATGCTATATAATCAGTAGCATATTTTAAATCCTTAAT

ATATTCAGGAGTTACCTCGTTCTGATAATAGATTAATGATCCAGGACGAAATTTGAAAGAACTACATGGT

TCTCCATGAATTAATACATATTGTTTAGCAAATTCAGGAACTATAAAACTACTACAATGATCTATCGACA

TACCATCTATCAAACAAAATTTGGGTTTAATTTCTCCTGGAGACGTTTCATAATAATACATATAACTTTC

TTCGGCAAACCTAACAGCTCTATTATATTCAGGATAATTAAAATCTAATACCATATATTTGTCTCGTATA

TCTGCTATTCCTGTCTCTATTTTGATTCTATTAAGAGTAACAGCTGCCCCCATTCTTAATAATCATCAGT

ATTTAAACTGTTAAATGTTGGTATATCAACATCTATCTTATTTCCCGCAGTATAAGGTTTGTTGCAGGTA

TACTGTTCAGGAATGGGTACATTTATACTTCTTTTATAGTCCTGTCTTTCGATGTTCATCACAAATGCAA

AGAACAGAATAAACAAAATAATGTAAGAAATAATATTAAATATCTGTGAATTCGTAAATACATTGATTGC

CATAATAATTACAGCAGCTACAATACACACAATAGACATTCCCACAGTGTTGCCATTACCTCCACGATAC

ATTTGAGTTACTAAGCAATAGGTAATAACTAAGCTAGTAAGAGGCAATAGAAAAGATGAGATAAATATCA

TCAATATAGAGATTAGAGGAGGGCTATATAGAGCCAAGACGAACAAAATCAAACCGAGTAACGTTCTAAC

ATCATTATTTTTGAAGATTCCCAAATAATCATTCATTATTCCTCCATAATCGTTTTGCATCATACCCCCA

TCTTTAGGCATAAACGATTGCTGCTGTTCCTCTGTAAATAAATCTTTATCAAGCACTCCAGCACCCGCAG

AGAAGTCATCAAGCATATTGTAATATCTTAAATAACTCATTTATATATTAAAAAATGTCACTATTAAAGA

TGGAGTATAATCTTTATGCCGAACTAAAAAAAATGACTTGTGGTCAGACCATAAGTCTTTTTAATGAAGA

CGGCGATTTCGTAGAAGTTGAACCAGGATCATCCTTTAAGTTTCTAATACCTAAGGGATTTTACTCCTCT

CCTTGTGTAAAGACGAGTCTAGTATTCAAGACATTAACAACGACCGATAATAAAATTACTAGTATCAATC

CAACAAATGCGCCAAAGTTATATCCTCTTCAACGCAAAGTCGTATCTGAAGTAGTTTCTAATATGAGGAA

AATGATCGAATTAAAACGTCCTCTATACATCACTCTTCACTTGGCATGTGGATTTGGTAAGACTATTACC

ACGTGTTATCTTATGACCACACACGGCAGAAAAACCATCATTTGCGTACCCAATAAAATGTTAATACATC

AATGGAAGACACAGGTAGAGGCAGTCGGATTGGAACATAAGATATCTATAGATGGAGTTAGTAGTCTATT

AAAGGAACTAAAGACTCAAAGTCCGGATGTATTAATCGTAGTCAGTAGACATCTGACAAACGATGCATTT

TGTAAATATATCAATAAGCATTATGATTTGTTTATCTTGGATGAATCACATACGTATAATCTGATGAACA

ATACAGCAGTTACAAGATTTTTAGCGTATTATCCTCCGATGATGTGTTATTTTTTAACTGCTACACCTAG

ACCAGCTAACCAAATTTATTGTAATAGTATTATTAATATTGCCAAGTTATCCGATCTAAAAAAAACTATC

TATATAGTAGATAGTTTTTTTGAGCCATATTCCACAGACAATATTAGAAATATGGTAAAACGACTAGATG

GACCATCTAATAAATATCATATATATACCGAGAAGTTATTATCTGTAGACGAGCCTAGAAACCAACTTAT

TCTTGATACCCTGGTAGAAGAATTCAAGTCAGGAACTATTAATAGAATTTTAGTTATTACTAAACTACGT

GAACATATGGTATTCTTCTACAAACGATTATTAGATCTTTTCGGAGCAGAGGTTGTATTTATAGGAGACG

CCCAAAATAGACGTACTCCAGATATGGTCAAATCGATTAAGGAACTAAATAGATTTATATTCGTATCCAC

CTTATTTTATTCCGGCACTGGTTTAGATATTCCGAGTTTGGATTCTTTGTTCATTTGCTCGGCAGTAATC

AACAATATGCAAATAGAGCAATTACTAGGGAGGGTATGTCGAGAAACAGAACTATTAGATAGGACGGTAT

ATGTATTTCCTAACACATCCATCAAAAAAATAAAGTACATGATAGGAAATTTCGTGCAACGAATTATTAG

TCTGTCTGTAGATAAACTCGGATTTAAACAAGAAAGTTATCAGAAACATCAGGAATCTGAACCCGCTTCC

GTACCAACATCCTCCAGAGAAGAACGTGTATTAAATAGAATATTTAACTCGCAAAATCGTTAAGAAGTTT

AAGAGACGATCCACATGCTGAGCAGGCCAGTGTATTACCCCTCATAGTATTAATATAATCCAATGATACT

TTTGTGATGTCGGAAATCTTAACCAATTTAGACTGACAGGCAGAACACGTCATACAATCATCATCGTCAT

CGATAACTGTAGTCTTGGGCTTCTTTTTGCGACTCTTCATTCCGGAACGCATATTGGTGCTATCCATTTA

GGTAGTAAAAAATAAGTCAGAATATGCCCTATAACACGATCGTGCAAAACCTGGTATATCGTCTCTATCT

TTATCACAATATAGTGTATCAACATCTTTATTATTGACCTCGTTTATCTTGGAACATGGAATGGGAACAT

TTTTGTTAACGGCCACCTTTGCCTTAATTCCAGATGTTGTAAAATTATAACTAAACAGTCTATCATCGAC

ACAAATGAAATTCTTGTTTAGACGTTTGTAGTTTACGTATGCGGCTCGTTCTCGTCTCATTTTTTCAGAT

ATTGCAGGTACTATAATATTAAAAATAAGAATGAAATAACATAGGATTAAAAATAAAGTTATCATGACTT

CTAGTGCTGATTTAACTAACTTAAAAGAATTACTTAGTCTGTACAAAAGTTTGAGATTTTCAGATTCTGT

GGCTATAGAGAAGTATAATTCTTTGGTAGAATGGGGAACATCTACTTACTGGAAAATAGGCGTACAAAAG

GTAACTAATGTCGAGACGTCCATATCTGATTATTATGATGAGGTAAAAAATAAACCGTTTAATATTGATC

CGGGGTATTATATTTTCTTACCAGTATATTTTGGAAGCGTCTTTATTTATTCAAAGGGTAAAAATATGGT

AGAACTTGGATCTGGAAACTCTTTTCAAATACCGGATGAGATTCGAAGTGCGTGTAACAAAGTATTAGAT

AGTGATAACGGAATAGACTTTCTGAGATTTGTTTTGTTAAACAATAGATGGATAATGGAAGACGCTATAT

CAAAATACCAGTCTCCAGTTAATATATTTAAACTAGCTAGTGAGTACGGATTAAACATACCCAACTATTT

AGAAATTGAAATAGAGGAAGACACATTATTTGACGATGAGTTATACTCTATTATGGAACGCTCTTTCGAT

GATACATTTCCAAAAATATCTATATCGTATATTAAGTTGGGAGAACTTAAGCGGCAAGTTGTAGACTTTT

TCAAATTCTCATTCATGTATATTGAGTCAATCAAGGTAGATCGTATAGGAGATAATATTTTTATTCCTAG

CGTTATAACAAAATCAGGAAAAAAGATATTAGTAAAAGATGTAGACCATTTAATACGATCCAAGGTTAGA

GAACATACATTTGTAAAAGTAAAAAAGAAAAACACATTTTCCATTTTATACGACTATGATGGGAACGGAA

CAGAAACTAGAGGAGAAGTAATAAAACGAATTATAGACACTATAGGACGAGACTATTATGTTAATGGAAA

GTATTTCTCTAAGGTTGGTATTGCAGGCTTAAAGCAATTGACTAATAAATTAGATATTAATGAGTGTGCA

ACTGTCGATGAGTTAGTTGATGAGATTAATAAATCCGGAACTGTAAAACGAAAAATAAAAAACCAATCAG

TATTTGATTTAAGCAGAGAATGTTTGGGATATCCAGAAGCGGATTTTATAACGTTAGTTAATAACATGCG

GTTCAAAATAGAAAATTGTAAGGTTGTAAATTTCAATATTGAAAATACTAATTGTTTAAATAACCCGAGT

ATTGAAACTATATATGGAAACTTCAACCAGTTCGTCTCAATCTTTAATACCGTTACCGATGTCAAAAAAA

GATTATTCGAGTGAAATAATATGCGCCTTTGATATAGGTGCAAAAAATCCTGCCAGAACTGTTTTAGAAG

TCAAGGATAACTCCGTTAGGGTATTGGATATATCAAAATTAGACTGGAGTTCTGATTGGGAAAGGCGCAT

AGCTCAAGATTTGTCACAATATGAATACACTACAGTTCTTCTAGAACGTCAGCCTAGAAGGTCACCGTAC

GTCAAATTTATCTATTTTATTAAAGGCTTTTTATATCATACATCTGCTGCCAAAGTTATTTGCGTCTCAC

CTGTCATGTCTGGTAATTCATATAGAGATCGAAAAAAGAGATCTGTTGAAGCATTTCTTGATTGGATGGA

CACATTCGGATTGCGAGACTCCGTTCCGGATAGACGCAAATTAGACGATGTAGCGGATAGTTTCAATTTG

GCTATGAGATACGTATTAGATAAATGGAATACTAATTATACACCTTATAATAGGTGTAAATATAGAAATT

ACATAAAAAAAATGTAATAACGTTAGTAACGCCATTATGGATAATCTATTTACCTTTCTACATGAAATAG

AAGATAGATATGCCAGAACTATTTTTAACTTTCATCTAATAAGTTGTGATGAAATAGGAGATATATATGG

TCTTATGAAAGAACGCATTTCCTCAGAGGATATGTTTGACAATATAGTATATAATAAAGATATACATCCT

GCCATTAAGAAACTAGTTTATTGCGACATCCAACTTACTAAACATATTATTAATCAGAATACGTATCCGG

TATTTAACGATTCTTCACAAGTGAAATGTTGTCATTATTTCGATATAAACTCAAATAATAGCAATATTAG

CTCTCGTACAGTAGAGATATTTGAGAGTGAAAAGTCATCTCTTGTATCATATATTAAAACTACCAATAAG

AAGAGAAAGGTCAATTACGGCGAAATAAAGAAAACTGTACATGGAGGCACTAATGCAAATTACTTTTCCG

GTAAAAAGTCTGATGAGTATCTGAGCACTACAGTCAGGTCCAACATTAATCAACCTTGGATCAAAACCAT

TTCTAAGAGAATGAGAGTAGATATCATTAATCACTCTATAGTAACGCGTGGAAAAAGCTCTATATTACAA

ACTATAGAAATTATTTTTACTAATAGAACATGTGTGAAAATATTCAAGGATTCTACTATGCACATTATTC

TATCCAAGGACAAGGATGAAAAGGGATGTATAAACATGATTGATAAATTATTCTATGTATATTATAATTT

ATTTCTGTTGTTCGAGGATATCATCCAAAACGATTACTTTAAAGAAGTAGCTAATGTTGTAAACCATGTA

CTCATGGCTACGGCATTAGATGAGAAATTATTCCTAATTAAGAAAATGGCTGAACACGATGTTTATGGAG

TTAGCAATTTCAAAATAGGGATGTTTAACCTGACATTTATTAAGTTGTTGGATCATACCGTTTTCCCCTC

TCTGTTAGATGAGGATAGCAAAATAAAGTTTTTTAAGGGGAAAAAGCTCAATATTGTAGCATTACGATCT

CTGGAGGATTGTACAAATTACGTGACTAAATCCGAGAATATGATAGAAATGATGAAGGAAAGATCGACTA

TTTTAAATAGCATAGATATAGAAACGGAATCGGTAGATCGTCTAAAAGAATTGCTTCTAAAATGAAAAAA

AACACTGATTCAGAAATGGATCAACGACTCGGGTATAAGTTTTTGGTGCCTGATCCTAAAGCCGGAGTTT

TTTATAGACCGTTACATTTCCAATATGTATCGTATTCTAATTTTATATTGCATCGATTGCATGAAATCTT

GACCGTCAAGCGGCCACTCTTATCGTTTAAGAATAATACAGAACGAATTATGATAGAAATTAGCAATGTT

AAAGTGACTCCTCCAGATTACTCACCTATAATTGCGAGTATTAAAGGTAAGAGTTATGACGCATTAGCCA

CGTTCACTGTAAATATCTTTAAAGAGGTAATGACCAAAGAGGGTATATCCATCACTAAAATAAGTAGTTA

TGAGGGAAAAGATTCTCATTTGATAAAAATTCCGCTACTAATAGGATATGGGAATAAAAATCCACTTGAT

ACAGCCAAGTATCTTGTTCCTAATGTCATAGGTGGAGTCTTTATCAATAAACAATCTGTCGAAAAAGTAG

GAATTAATCTAGTAGAAAAGATTACAACATGGCCAAAATTTAGGGTTGTTAAGCCAAACTCATTCACTTT

CTCGTTTTCCTCCGTATCCCCTCCTAATGTATTACCGACAAGATATCGCCATTACAAGATATCTCTGGAT

ATATCACAATTGGAAGCGTCGAATATATCATCGACAAAGACATTTATAACGGTCAATATTGTTTTGCTGT

CTCAATATTTATCTAGAGTGAGTCTAGAATTCATTAGACGTAGTTTATCATACGATATGCCTCCAGAAGT

TGTCTATCTAGTAAACGCGATAATAGATAGTGCTAAACGACTTACCGAATCTATTACTGACTTTAATATT

GATACATACATTAATGACCTGGTGGAAGCTGAACACATTAAACAAAAATCTCAGTTAACGATTAACGAGT

TTAAATATGAAATGCTGCATAACTTTTTACCTCATATGAACTATACACCCGATCAACTAAAGGGATTTTA

TATGATATCTTTACTAAGAAAGTTTCTCTACTGTATCTACCACACTTCTAGATATCCAGATAGAGATTCG

ATGGTTTGTCATCGCATCCTAACGTACGGCAAATATTTTGAGACGTTAGCACATGATGAATTAGAGAATT

ACATAGGTAACATCCGAAACGATATCATGAACAATCACAAGAACAGAGGCACTTACGCAGTAAACATTCA

TGTACTAACAACTCCTGGACTTAATCATGCATTTTCTAGTCTATTGAGTGGAAAGTTCAAAAAGTCAGAC

GGTAGTTATCGAACACATCCTCACTATTCATGGATGCAGAATATTTCTATTCCTAGAAGTGTTGGATTTT

ATCCGGATCAAGTAAAGATTTCAAAGATGTTTTCTGTCAGAAAATACCATCCAAGCCAATATCTTTACTT

TTGTTCATCAGACGTTCCGGAAAGAGGTCCTCAGGTAGGTTTAGTATCTCAATTGTCTGTCTTGAGTTCC

ATTACAAATATACTAACGTCTGAGTATTTGGATTTGGAAAAGAAAATTTGTGAGTATATCAGATCATATT

ATAAAGATGATATAAGTTACTTTGAAACAGGATTTCCAATCACTATAGAAAATGCTCTAGTCGCATCTCT

TAATCCAAATATGATATGTGATTTTGTAACTGACTTTAGACGTAGAAAACGGATGGGATTCTTCGGTAAC

TTGGAGGTAGGTATTACTTTAGTTAGGGATCACATGAATGAAATTCGCATTAATATTGGAGCAGGAAGAT

TAGTCAGACCATTCTTGGTTGTGGATAACGGAGAGCTCATGATGGATGTGTGTCCGGAGTTAGAAAGCAG

ATTAGACGACATGACATTCTCTGACATTCAGAAAGAGTTTCCACATGTCATCGAAATGGTAGATATAGAA

CAATTTACTTTTAGTAACGTATGTGAATCGGTTCAAAAATTTAGAATGATGTCAAAGGATGAAAGAAAGC

AATACGATTTATGTGACTTTCCTGCCGAATTTAGAGATGGATATGTAGCATCTTCACTAGTGGGAATCAA

TCACAATTCTGGACCCAGAGCTATTCTTGGATGTGCTCAAGCTAAACAAGCTATCTCTTGTCTGAGTTCG

GATATACGAAATAAAATAGACAATGGAATTCATTTGATGTATCCAGAGAGGCCAATTGTGATTAGTAAGG

CTTTAGAAACTTCAAAGATTGCGGCTAATTGCTTCGGACAACATGTTACTATAGCATTAATGTCGTACAA

AGGTATCAATCAAGAGGATGGAATTATCATCAAAAAACAATTTATTCAGAGAGGCGGTCTCGATATTGTT

ACAGCCAAGAAACATCAAGTAGAAATTCCATTGGAAAACTTTAATAACAAAGAAAGAGATAGGTCTAACG

CCTATTCGAAATTAGAAAGTAATGGATTAGTTAGACTGAATGCTTTCTTGGAATCCGGAGACGCTATGGC

AAGAAATATCTCATCAAGAACTCTTGAAGATGATTTTGCTAGAGATAATCAGATTAGCTTTGATGTTTCC

GAGAAATATACAGATATGTACAAATCTCGCGTTGAACGAGTACAAGTAGAACTTACTGACAAAGTTAAGG

TGCGAGTATTAACCATGAAAGAAAGAAGACCCATTCTAGGAGACAAATTTACTACTAGAACGAGTCAAAA

GGGAACAGTCGCGTATATCGCAGATGAAACGGAACTTCCGTACGACGAAAATGGTATCACACCAGATGTC

ATTATTAATTCTACATCCATCTTCTCTAGAAAAACTATATCTATGTTGATAGAAGTTATTTTAACAGCCG

CATATTCTACTAAGCCGTACAACAATAAGGGAGAAAACCGACCTGTCTGTTTTCCTAGTAGTAACGAAAC

ATCTATCGATGCATATATGCAATTCGCTAAACAATGTTATGAGTATTCAAATCCGAAATTGTCCGAGGAA

GAATTATCGGATAAAATCTTTTGTGAAAAGATTCTCTATGATCCTGAAACGGATAAGCCTTATGAATCCA

AAGTATTTTTTGGACCAATTTATTACTTGCGTCTGAGACATTTAACTCAGGACAAGGCAACCGTTAGATG

TAGAGGTAAAAAGACGAAGCTCATTAGACAAGCGAATGAGGGACGAAAACGTGGAGGAGGTATCAAGTTT

GGAGAAATGGAGAGAGACTGTTTAATAGCACATGGTGCAGCCAATACTATTACAGAAGTTTTAAAAGACT

CAGAAGAGGATTATCAAGATGTGTATATTTGTGAAAATTGTGGAGACATAGCAGCACAAATCAAAAGTAT

TAATACATGTCTTAGATGTTCAAAACTTAATCTCTCTCCTCTCTTAACAAAAATTGATACCACGCACGTA

TCTAAAGTATTTCTTACTCAAATGAACGCCAGAGGCGTAAAAGTTAAATTAGATTTCGAACGAAGGCCTC

CTTCGTTTTATAAACCATTAGATAAAGTTGATCTTAAACCGTCTTTTCTGGTATAATATTGTTTAGTAGA

TACTCATCAAGATAAGCTAATTCACTAAACATATTATCGGATTCGGTATTGTTACTCGAGAATAGAGTTC

GTTATGCTCCTGATATTCGGAAATCTGTGGAGTTTCAGGTTTTGGTGGAAGTGTAACTGCTACTTGGTGG

GATACTGAAGGATATTTCAGAGAGTTGTGGATGTTCGGGTTCGACATCCACCGATGGTGTCACGCCACTA

ATCGGTTCGGTAACGTCTGTGGATGGAGGTGCTACTTCTACAGAACCTGTAGCCTCAGTTGTCAACGGAG

ATACATATTCAATGCGCGGAAATGTATAATTTGGTAATGGTTTCTCATGTGGATCTTAAGAAGAAGAGGT

AAGATATCTACGAAAGATACCGATCACGTTTCTAGTTCTCTTTTGTAGAACTTTAACTTTTTCTTTCTCA

GCATCTAGTTGATATTCCGACCTCTTCACGTTTCGCATGGGTTACCTCCGCAGTTTTTACAAGCGATTTC

ACGTTCCAGATCACGTTCAGCCTTCATACGTCTCTCCCTCTCTCTATCGAGTTTATCAGAGCAGTCTTTC

TGAAGGCGATCGAACTCCATAAATTTCTCCAACGCTTTGATTGTTTCCATAGATTTCCGAAGTTTAGCTT

CTAGGACGGCGATTCTTTTTTTTTTTTTTTTTTTTTTTTTTTCGAATTCACGGGGTACAACCGTTTCCAT

TACCACCATCTCTATGTTTCTTTTCTAGATCGGCAATCTTTCTCAACATTTCATCCCCATACCTTTTCAT

TCCTCGAGTCTATTGTCGTCGAAATATCGTTCCAGCTCCTTTTCGACCTCAATAACTTTAGCACGTTGTT

TCATCAAGCTCTCTCTTGTAGTACTATCATTTTTATCTGATTCCCTGACACGTTTAAGATCTTCATGTAA

TTGAGTCAGCTCTTGACGCAATCTCTTAACTAACTTCCTCTCTTGCTTCTTCGTCATAGTACTTACAATC

ACTATGGGATCCATTGTTACCACGTCTGTACTCGACGAGCTCACGTTTAAGAGATTCAATTTCCAGTTTG

TATCGGTCCATGTCTCCATTGCTACACCACCATTAGATTTACAGGCTGCTAGTTGTCGTTCGAGATCAGA

AATACGTGTTTTCTTGGAATGGATTTCGTCGATGTACTTGTCATGATTGGCATCGAAACACTTATTAAGT

TCTTTTTTTCAATTCTACGATTTTATTTCTTTCGCGAGTCAATTCCCTCCTGTAGTAACTATCAGTTTTG

TCAGATTCACGCTCTCTACGTAGACTTTCTTGTAAGTTACTAATTTGTTCCCTGGCATTACCGAGTTCAG

TTTTATATGCCGAATAGAGTTCTGATTCATCCTTTGAGAAGATCTCTAGCGATCGTTCAAGATCCCTGAT

TCTAGTCTTTAGCCTATTTACCTCCTCAGAAGATGCTCCGTTACCGTTTTTACAATCGTTAAGATGTCTA

TCAAGATCCATGATTCTATCTCTTTTCCATATCAGCATTGATTTCATTATTACGTTCGCAGTCGTTCAAC

TGTATTTCAAGATCTGAGATTCTAGATTGTAATCTCTGTAGCATTTCCACGGCATTCACTCAGTTGTCTT

TCAAGATCTGAGATTCTAGATTGGAGTCTGCTAATCTCTGTAAGATTTCCTCCTCCGCTCTCGATGCAGT

CGGTCAACTTATTCTCTAGTTCTCTAATACGCGAACGCAGTGCATCAACTTCTTGTGTGTCTTCTTGATT

GCGTGTGCATTCATCGAGTCTAGATTCGAGATCTCTAACGTGACGTCGTTCTTCCTCAAGTTCTCTGTGT

ACTACAGAAAGCGTGTCCCTATCTTGTTGATATTTAGCAATTTCTGATTCTAGAGTACTGATTCTACTCA

CGTATGTACTAATAGTTGTCTTAGCCTTATCAAGATCCTCCTTGTATTTGTCACATTCCTTGATATCCAT

ACGAAGTCTGGACAGTTCCCATTCGACATTACGACGTTTATCGATTTCAGCTCGGAGATCGTCGTCGCGT

TGTTTTAGCCACATACGACTAAGTTCAAGTTCTCGTTGACAAGATCCATCTACTTTTCCATCCCTAATAG

TATCCAGTTCCTTTTCTAGTTCTGACCGCATTTCTCGTTCCATATCAAGAGATTCTCTCAATTCTCGTAT

AGTCTTCTTATCAATTTCTGATGAATCTGAACCATCATCTGTCCCATTTTGTTGCATATCCCTGAGTTCT

TTGATCTCTGTTGTAAGTCTGTCGATTCTTTCGGTTTTATAAACAGAATCCCTTTCCAAAGTCCTAATCT

TACTGAGTTTATCATTAAGTTCTTCATTCAATTCAGTGAGTTTTCTCTTGGCTTCTTCCAAGTCTGTTTT

AAACTCTCCATCATTTCCGCATTCTTCCTCGCATTTATCTAACCATTCAATTAGTTTATTAATAACTAGT

TGGTAATCAGCGATTCCTATAGCCGTTCTTGTATTTGTGGGAACATAATTAGGATCTTCTAATGGATTGT

ATGGCTTGATAGCATCATCTTTATCATTATTAGGTGGGGGATGGACAACCTTAATTGGTTGGTCCTCCTT

ATCTCCTCCAGTAGCATGTGGTTCTTCAATACCAGTATTAGTAATAGGCTTAGACAAATGCTTGTCGTAC

GCGGGCACTTCCTCATCCATCAAGTATTTATAATCGGGTTCTGTTTCAGAATATTCTTTTCTAAGAGACG

CGACTTCAGGAGTTAGTAGAAGAACTCTGTTTCTGTATCTATCAACGCTGGAATCGATACTCAAGTTAAG

GATAGCGAATACCTCATCGTCATCATCCGTATCTTCTGAAACGCCATCATATGACATTTCATGAAGTCTA

ACGTATTGATAAACAGAATCAGATTTAGTATTAAACAGATCCTTGACCTTTTTAGTAAATGCATATGTAT

ATTTTAGATCTCCAGATTTCATAATATGATCGCATGCCTTAAATGTCAATGCTTCCATGATATAGTCTGG

AACACTAATGGGTGACGAAAAAGATACAGCACCATATGCTACGTTGATAAATAGATCTGAACCACTAAGT

AGATAATGATTAATGTTAAGGAAGAGGAAATATTCAGTATATAGATATGCCTTAGCATCATATCTTGTAC

TAAACACGCTAAACAGTTTATTGATGTGATCAATTTCCAACAGAACAATTAGAGCGGCAGGAATACCAAC

AAACATATTACCACATCCGTATTTTCTATGAATATCACATATCATATTAAAAAATCTTGATAGAAGAGCG

AATATCTCGTCTGACTTAATGAGATGTAGTTCAGCAGCATAAGTCATAACTGTAAATAGAACATACTTTC

CTGTAGTGTTGATTCTAGACTCCACATCAACACCATTATTAAAAATAGTTTTATATACATCTTTAATCTG

CTCTCCGTTAATCGTCGAACGTTCTAGTATACGGAAACACTTTGATTTCTTATCTGTAGTTAATGACTTA

GTGATATCACGAAGAATATTACGAATTACATTTCTTGTTTTTCTTGAGAGACCTGATTCAGAACTCAACT

CATCGTTCCATAGTTTTTCTACCTCAGTGGCGAAATCTTTGGAGTGTTTGGTACATTTTTTAATAAGGTT

CGTGACCTCCATTTATTATAAAAAATTTTTATTCAAAACTTAACTACAATCGGGTAATTATAAGATCGTA

GATCTCCCATGTGGTGGAATACTACCATCTATCGCATGTTGATGGACAGTAGGTAATGGCCATGGGAACA

GTAATGTTTGCATATTTATCTTTCTTGCTAGTATTACTGTATATTGTCCCAATGTTTCAATGTGATGTTC

TAACCTATCAACTGCCACTGTATCACAACAATAATGTCCGATGGAATTAAGATTATGATCCAATGTGTTT

AATATATGATTATCAAGTCTTATACGATCCGCGTCTTTTTTGACAGGATCAGGCTCTTCTACAGGAAGAA

GTTTCGGCCTCTTATGATAGTCATGTCTGGGAAATGGTGGTCTAGGATGAGGATCAGGTATCGGAGTAGG

TTTTGGATTATAATCATCATCATCATCATCATCATCATCATCATCATCATCATCTATGATATCATCATCT

TCGATATTTATTTTGCTATCTTGATAATGTCCTATATCAGTTGCATTTTCAGCACTCGACTGAATATTAG

TACATTCATTGTCTATTATTAACGTATTTCTAAACCCAAAATGTATATGTTGAACATCACTACTATAGTT

GATGAGTCTTATAGCATGAATTCGCTTATCGTTATCGGGTTTATCTTCTGTCACCTTAACAATTCCTTTT

TTATTAAACTCTGCATAATCATAACCATTTCTATTGTTTGTTCTAATATAAACGAGTATAGCATCATTGC

TAAATTTTTCAATAGTATCAAAAACAGAATATCCTAAACCATATAATATATATTCAGGAACACTCAAACT

AAATGTCCAGGATTCTCCTAAATACGTAAACTTTAATAGTGCTAAATCATTCAAAAATCTACCGCTTATA

GATAGATAGTACATGAATGCGTATAGTAGTCTACCTATCTCTTTATTATGAAAACCGACATTACGATCAT

ATATTTCGTGATATACATGTGACCCGTTTACGTTAAACCATAAATACATGGGTGATCCTATAAACATGAA

TTTATTTCTAATTCTCAGAGCCATAGTTAATTGACCGTGTAATATTTGTTTACATGCATACTTGATACGA

TCATTAATAAGATTTTTATCATTGCTCGTTATTTCAGAATCGTATATATAAGGAGTACCATCATGATTCT

TACCAGATATTATACAAAATACTATATATAAAATATATTGACCCACGTTAGTAATCATGTAAATGTTTAA

TGTTTTAAATTTTGTATTTAATGATCCATCATCATATGCTAGCATGGTCTTGTGATATTCATTCTTTAAA

ATATAATATTGTGTTAGCCATTGCATTGGAGCTCCTAATGGAGATTTTCTATTCTCGTCCATTTTAGGAT

ATGCTTTCATAAAGTCCCTAATAACTTCGTGAATAATGTTTCTATGTTTTCTACTGATGCATGTATTTGC

TTCGATTTTTTTATCCCATGTTTCATCTATCATAGATTTAAACGCAGTAATGCTCGCAACATTAACATCT

TGAACCATTGGTACAATTCCGTTCCATAAATTTATAATGTTCGCCATTTATATAACTCATTTTTTGAATA

TACTTTTAATTGAACAAAAGAGTTAAGTTACTCATATGGATGCCGTCCAGTCTGTACATCAATCTTTTTA

GCCAGAGATATCATAGCCGCTCTTAGAGTTTCAGCGTGATTTTCCAACCTAAATAGAACTTCATCGTTGC

GTTTACAACACTTTTCTATTTGTTCAAACTTTGTTGTTATATTAGTAATCTTTTTTTCCAAATTAGTTAG

CCGTTGTTTGAGAGTTTCCTCATTATCGTCTCCATAGGCTTTAACAATTGCTTCGCGTTTAGTCTCTGGA

TTTTTAGCAGCCTTTGTAGAGAAAAATTCAGTTGCTGGAATTGCAAGATCGTCATCTCCGGGGAAAAGAG

TTCCGTCCATTTAAAGTACAGATTTTAGAAACTGACACTCTGTGTTATTTATATTTGGCGCAATACATGG

ATTATAAATATCGATGTTAATAACATCAGAAAATGTAAAGTCTATACATTGTCGCATCGTGTTAAATTTT

CTAATGGATCTAGTATTATTGGGTCCAACTTCTGCCTGAAATCCAAATATGGAAGCGGATACAAAACCGT

TTCCTGGATAAACCACACATCTCCACTTTTGCTTTACATCAGAAATTGTGTCATTGACATCTTGAACTCT

CCTATCTAATGCCGGTGTTCCACCTATAGATTTTGAATACTCGAATGCTGCATGAGTAGCATTGAATTCC

TTAATATTGCCATAATTTTCATATATTGAGTAACTCTGGATAAAAAGTAAACACACCGCAGCCGTCGCTA

CTACAATAAAAAAAATTGATAGAGAGTTCATTTATAATCTATTAGAAGCTGATAAAATTTTTTTACACGC

GTCAGACAATGCTTTAATAAATAGTTCAACATCTACTTTTGTCATATCGAACCGATGGTATGATTCTAAC

CTAGAATTACATCCGAAAAAGTTGACTATGTTCATAGTCATTAAGTCATTAACGAACAACATTCCAGACT

CTGGATTATAAGACGATACTGTTTCGTCACAATCACCCACCTTAATCATGTGATTATGAATATTGGCTAT

TAGAGTACCTTCTAAGAAATCTATAATATCTTTGAAACACGATTTAAAATCAAACCACGAATATACTTCT

ACGAAGAAAGTTAGTTTACCCATAGGAGAGATAACTATAAATGGAGATCTAGATACAAAATCCGGATCTA

TGATAGTTTTAACATTATTATATTCTCTATTAAATACCTCCACATCTAAAAATGTTAATTTTGAAACTAT

GTCTTCGTTTATTACCGTACCTGAACTAAACGCTATAAGCTCTATTGTTTGAGAACTCTTTAAACGATAT

TCTTGAAATACATGTAACAAAGTTTCCTTTAACTCGGTCGGTTTATCTACCATAGTTACAGAATTTGTAT

CCTTATCTATAATATAATAATCAAAATCGTATAAAGTTATATAATTATCGTGTTCAGATTGTGATCTTTT

CAAATAGACTAAAAACCCCATTTCTCTAGTAAGTATCTTATGTATATGTTTGTAAAATATCTTCATGGTG

GGAATATGCTCTACAGCAGTTAGCCATTCCTCATTGACAGCTGTAGATGTATTATACAAAACTACTCCAA

TGTTTAACAAGGGCCATTTTACGAGATTATTAAATCCTTGTTTGATAAATGTAGCCAATGCGGGTTCGAG

TTCAACGACGATTGAATTCTCTTCCCGTGGATGCTGCATGATGAACGACGGGATGTTGTTGTTCTATTGA

TTTGGAATTCTTTTTCGACTTTTTGTTTATATTAAATATTTTAAAATTTATGGCTGATAGTAATTCATGT

ACTACGGATAATGTAGACGTGTATTGCATATCGATATCTTTATTATTAGATAAATTTATCAATAAATGTG

AGAAGTTTGCCTCGTTAAGGTCTTCCATTTAAATATTATATAAATATTTGTGTTTGTATTTTATTCGTCT

TTTATGGGATAGTTTTTAACTAGTAAAGCTGTAATTACATACTTTGTCCGTAAAACATAAATATAAATAC

CCGCTTTTATCAAACGTTCCAAAAAGTCGGCAGCTGACATTTTTAACATGACATCTATTTTAAATACACT

TAGGTTTTTAGAAAAAACATCATTTTATAATTGTAACGATTCAATAACTAAAGAAAAGATTAAGATTAAA

CATAAGGGAATGTTATTTGTATTTTATAAGCCAAAGCATTCTACCGTTGTTAAATACTTGTCTGGAGGAG

GTATATATCATGATGATTTGGTTGTATTGGGGAAGGTAACAATTAATGATCTAAAGATGATGCTATTTTA

CATGGATTTATCATATCATGGAGTGACAAGTAGTGGAGCAATTTACAAATTGGGATCGTCTATCGATAGA

CTTTCTCTAAATAGGACTATTGTTACAAAAGTTAATAACAATTATAACAATTATAACAATTATAACAATT

ATAATTGTTATAATAATTATAATTGTTATAATTATGATGATACATTTTTTGACGATGATGATTGATCACT

ATTACACAATTTTGTTTTTGTACTTTCTAATATAGTGTTTAGGTTCTTTTTCATATGAGAATATTGACTT

ACTAAAATATCTATGTTTAACTTTTGTTCTATAACGTCCTTATCGGCGGTATCGGTACATATACGTAATT

CACCTTCACAAAATACGGAGTCTTCGATAATAATAGCCAATCGATTATTGGATCTAGCTGTCTGTATCAT

ATTCAACATGTTTAATATATCCTTTCGTTTCCCCTTTACAGGCATCGATCGTAGCATATTTTCCGCGTCT

GAGATGGAAATGTTAAAACTGCAAAAATGCGTAATGTTAGCCCGTCCTAATATTGGTACGTGTCTATAAG

TTTGGCATAGTAGAATAATAGACGTGTTTAAATGCCTTCCAAAGTTTAAGAATTCTATTAGAGTATTACA

TTTTGATAGTTTATCACCTACATCATCAAAAATAAGTAAAAAGTGTGCTGATTTTTTATGATTTTGTGCG

ACAGCAATACATTTTTCTATGTTACTTTTAGTTCGTATCAGATTATATTCTAGAGCTTCCTGACTACTAA

CGAAATTAATATGATTTGGCCAAATGTATCCATCATAATCTGGGTTATAAACGGGTGTAAACAAGAATAT

ATGTTTATATTTTTTAACTAGTGTAGAAAACAGAGATAGTAAATAGATAGTTTTTCCAGATCCAGATCCT

CCTGTTAAAACCATTCTAAACGGCATTTTTAATAAATTTTCTCTTGAAAATTGTTTTTCTTGAAAACAAT

TCATAATTATATTTACAGTTACTAAATTAATTTGATAATAAATCAAAATATGGAAAACTAAGGTCGTTAG

TAGGGAGGAGAACAACGAAGGCATATCGTGATATAAATAACATTTATTATCATGATGACACCAGAAAACG

ACGAAGAGCAGACATCTGTGTTCTCCGCTACTGTTTACGGAGACAAAATTCAGGGAAAGAATAAACGCAA

ACGCGTGATTGGTCTATGTATTAGAATATCTATGGTTATTTCACTACTATCTATGATTACCATGTCCGCG

TTTCTCATAGTGCGCCTAAATCAATGCATGTCTGCTAACAAGGCTGCTATTACTGACTCCGCTGTTGCCG

TTGCTGCGGCATCATCTACTCATAGAAAGGTTGTGTCTAGCACTACACAATATGATCACAAAGAAAGCTG

TAATGGTTTATATTACCAGGGTTCTTGTTATATATTACATTCAGACTATAAGTCATTCGAGGATGCTAAA

GCAAACTGCGCTGCGGAATCATCAACACTACCCAATAAATCCGATGTCTTGACTACCTGGCTCATTGATT

ATGTTGAGGATACATGGGGATCTGATGGTAATCCAATTACAAAAACTACATCCGATTATCAAGATTCTGA

TGTATCACAAGAAGTTAGAAAGTATTTTTGTACATAAATAAATGAAATCGCTTAATAGACAAACTGTAAG

TAGGTTTAGGAAGTTGTCGGTGCCGGCCGCTATAATGATGTTACTCTCAACCATTATTAGCGGCATAGGA

ACATTTCTGCATTACAGAGAAGAACTGATGCCTAGTGCTTGCGCCAATGGATGGATACAATACGATAAAC

ATTGTTATCTGGATACCAACATTAAAATGTCTACGGATAATGCAGTTTATCAGTGTCGCAAATTACGAGC

TAGATTGCCTAGACCTGATACTAGACATCTGAGAGTATTGTTTAGTATTTTTTATAAAGATTATTGGGTA

AGTTTAAAAAAGACCAATGATAAATGGTTAGATATTAATAATGATAAAGATATAGATATTAGTAAATTAA

CAAATTTTAAGCAACTAAACAGCACGACGGATTCTGAGGCGTGTTATATATACAAGTCTGGAAAACTGGT

TAAAACAGTATGTAAAAGTACTCAATCTGTACTATGCGTTAAAAGATTCTACAAGTGACAACAAAAAATG

AATTAATAGTAAGTCGTTAACGTACGCCGCCATGGACGCCGCGTTTGTTATTACTCCAATGGGTGTGTTG

ACTATAACAGATACATTGTATGATGATCTCGATATCTCAATCATGGACTTTATAGGACCATACATTATAG

GTAACATAAAAATTGTCCAAATAGATGTACGGGATATAAAATATTCCGACATGCAAAAATGCTACTTTAG

CTATAAGGGTAAAATAGTTCCTCAGGATTCTAATGATTTGGCTAGATTCAACATTTATAGTATTTGTACA

GCATACAGATCAAAAAATACCATCATCATAGCATGCGACTATGATATCATGTTAGATATAGAAGGTAAAC

ATCAACCATTTTATCTATTCCCATCTATTGATGTTTTTAACGCTACAATCATAGAAGCGTATAATCTGTA

TACAGCTGGAGATTATCATCTGATCATCAATCCTTCAGATAATCTGAAAATGAAATTGTCGTTTAATTCT

TCATTTTGTATATCAGACGGCAATGGATGGATTATAATTGATGGGAAATGTAATAGTAATTTTTTATCAT

AAAAGTTGTAAAGTAAATAATAAAACAATAAATATTGAACTAGTAGTATGTTGTATATTGAGCAATCAGA

GATGATGCTGGTACCTCTTATCACGGTGACCGTAGTTGCGGGAACAATATTAGTATGTTATATATTATAT

ATTTGTAGGAAAAAGATACGTACTGTCTATAATGACAATAAAATTATCATGACAAAATTAAAAAAGATAA

AGAGTCCTAATTCCAGCAAATCTAGTAAATCAACTGATAGCGAATCAGACTGGGAGGATCACTGTAGTGC

TATGGAACAAAACAATGACGTAGATAATATTTCTAGAAATGAGATATTGAACGATGATAGCTTCGCTGGT

AGTTTAATATGGGATAACGAATCCAATATCATGGCGCCTAGCACAGAACACATTTACGATAGTGTTGCTG

GAAGCACGCTGCTAATAAATAATGATCGTAATGAACAGACTATTTATCAGAATACTACAGTAGTAATTAA

TGATACAGAGACTGTTGAAATACTTAATGAAGATACCAAACAGATTCCTAGCTATTCTTCCAATCCTTTC

GTAAATTATAATAAAACCAGTATTTGTAGCAAGTCAAATCCGTTCATTGCAGAACTCAACAATAAATTTA

GTGATAATAATCCGTTTAGGAGAGCACATAGTGACGATTATCTTAATAAGCAACAAGATCATGAATACGA

TGATATAGAATCATCGGTTGTATCATTGGTCTGATTAGTTTCCTTTTTATAAAATTGAAGTAATATTTAG

TATTAATTACCGCCGATGCATTATACAAATATGGAGATATTCCCTGTATTCGGCATTTCTAAAATTAGCA

ATTTTATTGCTAATAATGACTGTAGATATTATATAGATGTAGAGCATCAAAAAATTATATCTGATGAGAT

CAATAGACAGATGGATGAAACGGTACTTCTTACCAACATCTTAAGCGTAGAAGTTGTAAATGACAATGAG

ATGTACCATCTTATTCCCCATAGACTATCGACTATTATACTCTGTATTAGTTCTGTTGGAGGATGTGTTA

TCTCTATAGATAATGACGTCAATGACAAAAATATTCTAACATTTCCCATTGATCATGCTGTAATCATATC

CCCACTGAGTAAATGTGTCGTAGTTAGCAAGGGCCCTACAACCATACTGGTTGTTAAAGCGGATATACCC

AGCAAACGATTGGTAACATCGTTTACAAACGACATACTGTATGTAAACAATCTATCACTGATTAATTATT

TACCGTCGTCTGTATTCATTATTAGACGAGTCACCGACTATTTGGATAGACACATATGTGATCAGATATT

TGCTAATAATAAGTGGTATTCCATTATAACTATCGACGATAAGCAATATCCTATTCCATCAAATTGTATA

GGTATGTCTTCTGCCAAGTACATAAATTCGAGCATCGAGCAAGATATTTTGATCCATGTTTGTAACCTCG

AGCATCCATTCGACTCAGTCTACAAAAAAATGCAGTCGTACAATTCTCTACCTATCAAGGAACAAATATT

GTATGGTAGAATTGATAATATAAATATGAGCATTAGTATTTCGGTGGATTAATAGATTTCTCTAGTATGG

GATCATTAATCATCTCTAAATACATCATAAAAAAGCTATTATCAAATACTGTACTGAATGGATTCATTCT

TTTCTCTTTTTATGAAACTCTGTTGTATATCTACGGATAAAACTAGAAGCAAAAAATCTGATAGGAAGAA

TAATGATTATATGGAGGAACACGATTATTATAAAATAACAATAGTTCCTGGTTCCTCTTCCACGTCTACT

AGCTCGTGGTATTATACACATGCCTAGTAATAGTCTCTTTGCGTTGACGGAAAGCAGACTAGAAATAACA

GGCCAAAATGTTCAGACACCATAATAGTTCCCAACCCAGATAATAACAGAGTTCCATCAACACATTCCTT

TAAACTCAATCCCAAACCCAAAACCGTTAAAATGTATCCAGCCAATTGATAGTAGATAATGAGGTGTACA

GCACATGATAATTTACACAGTAACCAAAATGAAAACACTTTAGTAATTATAAGAAATATAGACGGTAATG

TCATCATCAACAATCCAATAATATGCCTGAGAGTAAACATTGACGGATAAAACAAAAATGCCCCGCATAA

CTCTATCATGGCAATAACGCAACCAAACACTTGTAAAATTCCTAAATTAGTAGAAAATACAACTGATATC

GATGTATAAGCGATTTCGAGGAATAATAAGAACAAAGTAATTCCCGTAAAGATAAACATCAACATTGTTT

GGTAATCATTAAACCAATTAGTATGACGTTGAATTAATTTCACAGTATATTTTATTCCAGTATTATCCCC

GCATGTATACGTACCTGGTAAGATATCTTTATATTCCATAATCAATGAGACATCACTATCCGATAACGAA

TGAAGTCTAGCACTAGTATGCCATTTACTTAATATGGTCGTCTTGGAAGTTTTATTATAAGTTAAAATAT

CATGATTGTCCAATTTCCATCTAATATACTTTGTCGGATTATCTATAATACACGGAATAATGATGGTATC

ATTACATGCTGTATATTCTATAGTCTTTGTAGATGTTATAACCACAAAAGTACAGAGGTATATCAACAAT

ATTCTAACTCTTAACATTTTTATTTATTTAAAATGATACCTTTGTTATTTATTTTATTCTTATTTTGCTA

ACGGTATCGAATGGCATAAGTTTGAAACGAGTGAAGAAATAATTTCTACTTACTTAATAGATTATGTGGT

AACGGGTGTTATTAATGGGGATGTATATACATTTTCAAATAATGAACTAAACAAAACTGGGTTAACTAAT

AACAATAATTATATCACAACATCTATAAAAGTAGAGGATAAGGATACATTAGTAGTATGCGGAACCAATA

ACGGAAATCCCAAATGTTGGAAAATAGACGGTTCATACTACCCAAAACATATAGGTAGAGGATACGATCA

TCAAAATAGCAAAGTAACGATAATCAGTCACAATGAATGTGTACTATCCGACATAAACATATAAAAAGAA

GGAATTAAACGATGGAGAAGATTTGACGGACCATGTGGTTATGATTAAACGAGTTAAGTTTTTTAAGAAG

CCTTAGAAGAGAGGCTATTGGGTATGAGAATCCGAAATATTAAACCAGACAACCCCATATAATTTTATAG

CTAAGAATGCCGCGAAGAATGGAACTAATAAAAACGGAAATATTTGTAGCACAACGAATAACTCCCAAAC

TGCATTCATGTTACACTATATAACACTACTTCGGTTAGATGTTTTAGAAAAAATAAATATCACCGTACCG

TTTTGTTGTATAAAAATAACAATTAACAATTATCAATTTTTTTCTTTAATATTTTACGTGGTTGACCATT

CTTGGTGGTAAAATAATCTCTTAGTGTTGGAATGGAATGCTGTTTAATGTTTCCACACTCATCGTATATT

TTGACGTATGCAGTCACATCGTTTACGCAATAGTCAGACTGTAGTTCTATCATGCTTCCTACGTTAGAAG

GAGGAACAGTTTTAAAGTCTCTTGGTTTTAATCTATTGTCATTAGTTTTCATGAAATCCTTTGTTTTATC

CACTTCACATTTTAAATAAATGTCCACTATACATTCTTCTGTTAATTTTACTAGATCATCATGAGTCATA

GAATTCATAGGTTCCGTAGTCCATGGATCCAAACTAGCAAACTTCGCGTATACGGTATCGCGATTAGTGT

ATACACCAACTGTATGAAAATTAAGAAAACAGTTTAATAAATCTACAGAAATATTTAATCCTCCGTTTGA

TACAGATGCGCCATATTTATGGATTTCGGATTCACACGTTGTTTGTCTAAGGGGTTCGTCTAGTGTTGCT

TCTACATAGACTTCGATTCCCATATATTCTTTATTGCCAGAATCACATACCGATTTATCATACGCTGGTT

CACTTGTTTGAAAACTAAATGGTAGTAGATACATCAAAATAATAAATAATAAGTACATTCTGCAATATTG

TTATCGTAATTGGAAAATTGGTATTCAAGTGAGCTGGATTATGTGAGTATTGGATTGTATATTTTATTTT

ATATTTTATATTTTATATTTTATTTTATATTTTATATTTTATTTTATATTTTGTAGTAAGAATAGAATGC

TAATGTCAAGTTTATTCGAATAGATGTCTTATTAAAAAACATATATAATAAATAACAATGGCTGAATGGC

ATAAAATTATCGAGGATATCTCAAAAAATAATAAGTTCGAGGATGCCGCCATCGTTGATTACAAGACTAC

AAAGAATGTTCTAGCGGCTATTCCTAACAGAACATTTGCAAAGATTAATCCGGGTGAAGTTATTCCCCTC

ATCACTAATCATAATATTCTAAAACCTCTTATTGGTCAGAAATTTTGTATTGTATATACTAACTCTCTAA

TGGATGAGAACACGTATGCTATGGAGTTGCTTACTGGGTACGCCCCTGTATCTCCGATCGTTATAGCGAG

AACTCATACCGCACTTATATTTTTGATGGGTAAGCCAACAACATCCAGACGTGATGTGTATAGAACATGT

AGAGATCACGCTACCCGTGTACGTGCAACTGGTAATTAAAATAAAAAGTAATATTCATATGTAGTGTCAA

TTTTAAATGATGATGAAATGGATAATATCCATATTGACGATGTCAATAATGCCGGTATTGACATACAGCT

CATCGATTTTTAGATTTCATTCAGAGGATATTGAATTATGTTATGGGAATTTGTATTTTGATAGGATCTA

TAATAATGTAGTAAATATAAAATATATTCCTGAGCATATTCCATATAGATATAATTTTATTAATCGTACG

TTCTCCGTAGATGAACTAGATGATAATGTCTTTTTTACACATGGTTATTTTTTAAAACACAAATATGGTT

GTTCACTTAATCCTAGTTTGATTGTCTCATTATCAGGAAACTTAAAATATAATGATATACAATGCTCAGT

AAATGTATCGTGTCTCATTAAAAATTTGGCAACGAGTACATCTACTATATTAACATCTAAACATAAGACT

TATTCTCTATATCGGTCCATGTGTATTGCTATAATAGGATACGATTCTATTATATGGTATAAATATATAA

ATGACAGGTATAATGACATCTATGATTTTACTGCAATATGTATGCTAATAGCGTCTACATTGATAGTGAT

CATATACGTGTTTAAAAAAATAAAAATGAACTCTTAATTATGTTATACTATTAGAAATGGATAAAATCAA

AATTACGATTGATTCAAAAATTGGTAATGTTGTTACCATATCGTATAACTTGGAAAAGATAACTATTGAT

GTCACGCCAAAAAAGAAAAAAGAAAAGGATGTATTATTAGCGCAATCAGTTGCTGTCGAAGAGGCAAAAG

ATGTCAAGGTGGAAGAAAAAAATATTATCGATATTGAAGATGACGATGATATGGATATAGAAAACACGTA

ATACGATCTATAAAAATAAGTATTAAATACTTTTTATTTACGGTACTCTTGTAGTGGTGATACCACTAAT

CGATTATTTTTTTTAAAAAAATACTTATTCTGATTCTTCTAGCCATTTCCGTGTTCGTTCGAATGCCACA

TCGACGTCAAAAATAGGGGAGTAGTTGAAATCTAGTTCTGCATTGTTGGTACGCACCTCAAATGTAGTGT

TGGATATCTTCAACGTATAGTTGTTGAGTATTGATGGTTTTCTAAATAGAATTCTCTTCATATCATTCTT

GCACGCGTACATTTTTAGCATCCATCTTGGAATCCTAGATCCTTGTTCTATTCCCAATGGTTTCATCAAT

AGAAGATTAAACATATCGTAAGAACACGATGGAGAGTAATCGTAGCAAAAGTAAGCATTTCCTTTAATCG

CAGATCCCGGATACTGGATATATTTTGCAGCCAACACGTGCATCCATGCAACATTTCCTACATATACCCG

GCTATGCACAGCGTCATCATCGACTGTACGATACATAATGTTACCGTGTTGCTTACATTGCTCGTAAAAG

ACTTTCGTCAATTTGTCTCCTTCTCCGTAAATTCCAGTGGGTCTTAGGCAACAAGTATACAATTTTGCGC

CATTCATGATTACGGAATTATTGGCTTTCATAACCAGTTGCTCGGCCATACGTTTACTTTTTGCGTATAC

ATGTCCTGGTGATATATCATAAAGGGTATGCTCATGACCGATGAATGGATTACCGTGTTTATTTGGTCCT

ATTGCTTCCATGCTACTAGTATAGATCAAATACTTGATTCCTAGGTCCACACAAGCTGCCAATATAGTCT

GTGTTCCATAATAGTTTACTTTCATGATTTCATTATCAGTGTATTTTCCAAATACATCCACTAGAGCAGC

CGTATGAATAATCAGATTTACCCCATCTAGCGCTTCTCTCACCTTATCAAAGTCGTTTATATCACATTGT

ATATAGTTTATAACCTTAACTTTCGAGGTTATTGGTTGTGGATCTTCTACAATATCTATGACTCTTATTT

CTTGAACATCATCTGCGCTAATTAAAAGTTTTACTATATACCTGCCTAGAAATCCGGCACCGCCAGTAAC

CGCGTACACGGCCATTGCTGCCACTCATAATATCAGACTACTTATTCTATTTTACTAAATAATGGCTGTT

TGTATAATAGACCACGATAATATCAGAGGAGTTATTTACGTTGAACAAGTCCATGGAAAAGATAAAGTTT

TAGGATCAGTTATTGGATTAAAATCCGGAACGTATAGTTTGATAATTCATCGTTACGGAGATATTAGTCG

AGGATGTGATTCCATAGGCAGTCCAGAAATATTTATCGGTAACATCTTTGTAAACAGATATGGTGTAGCA

TATGTTTATTTAGATACAGATGTAAATATATCTACAATTATTGGAAAGGCGTTATCTATTTCAAAAAATG

ATCAGAGATTAGCGTGTGGAGTTATTGGTATTTCGTACATAAATGAAAAGATAATACATTTTCTTACAAT

TAACGAGAATGGCGTTTGATATATCAGTTAATGCGTCTAAAACAATAAATGCATTAGTTTACTTTTCTAC

TCAGCAAGATAAATTAGTCATACGTAATGAAGTTAATGATATACACTACACTGTCGAATTTGATAGGGAC

AAAGTAGTTGATACGTTTATTTCATATAATAGACATAATGACTCCATAGAGATAAGAGGGGTGCTTCCAG

AGGAAACTAATATTGGTCGCGTGGTTAATACGCCGGTTAGTATGACTTACTTGTATAATAAGTATAGTTT

TAAACCGATTTTAGCAGAATATATAAGACACAGAAATACTATATCCGGCAACATTTATTCGGCATTGATG

ACGCTAGATGATTTGGTTATTAAACAGTATGGAGACATTGATCTATTATTTAATGAGAAACTTAAAGTAG

ACTCCGATTCGGGACTATTTGACTTTGTCAACTTTGTAAAGGATATGATATGTTGTGATTCTAGAATAGT

AGTAGCTCTATCTAGTCTAGTATCTAAACATTGGGAATTGACAAATAAAAAGTATAGGTGTATGGCATTA

GCCGAACATATAGCTGATAGTATTCCAATATCTGAGCTATCTAGACTACGATACAATCTATGTAAGTATC

TACGCGGACACACTGATAGCATAGAGGATGAATTTGATTATTTTGAAGACGATGATTTGTCTACATGTTC

TGCCGTAACCGATAGGGAAACGGATGTATAATTTTTTTTATAGTATGAAGGATATGATGGATATGATGAT

ATGATGGATATGATGGATATGATGGATATGATGGATATGATAAAAAAATATAA

TTGTTGTATCCATTCCCATTCAAATCACCTTATATGATTCTGTAACACAATGAAGGAGTCTCATAGATAT

ATAGAGGTCAGATACTGGTTTGATAAACTTTTTATTCCACATGAGCATGTTTGACTTATGGTTAGACACA

CATACTTTAACAAATCACTGAAAATTGGAGTTAGGTATTCCTCTCAGAATCAGTTGCCGTTCTGGAACAT

TAAATGTATTTTTTATGATATACTCCAACGCATTTATGTGGGTATACAACAAGTCATTAATAATGAGTAT

TTCCAAGAGTTTTAGTTGTCTAGTATTTAACAAGAGAAGAGATTTCAACAGACTGTTTATGAACTCGAAT

ACCGCCTCATTGTCGCTTATATTGATGACATGACATCGAATTCCCAATATCAATCTCATCAGTGATGAGT

AGCTCAATCTTGTTATCGGGATCCAATTTCTAAAGATGTCATTAAACCCTCGATCGTGAATGGATTTATC

ATCATCGTTTTTATGTTGGACATGAGCTTAGTCCGTTTGTCCACATCTATATACGATGATTTCTGAATTA

TTTCATATATCTCTCGTTAACTCCAGGAACTTGTCAGGGATCTAACTTTAATATGTTCTCGTCTAAGAGA

TGAAAATCTTTGGATGGTTGCATGTGACTTTTCTCTAAAGGATGATGTTACCCGATCCTCTCTTAAATGA

CTCCATCTTATCCTTGGACAAGATGGACAGTCTATTTTCCTTAGATGGTTTAATATTTTTTACCCATGAT

CTATAAAGGTAGACAGACCTAATCGTCTCGGATGACCATATATTATTTTCCGTTTTATTATACGCATAAA

TTGTAAAAAATATGTTAGGTTTACGAAAATGTCTCGTGGGGCATTAATCGTTTTTGAAGGATTGGACAAA

TCTGGAAAAACAACACAATGTATGAACATCATGGAATCTATACCGGCAAACACGATAAAATATCTTAACT

TTCCTCAGCGATCCACAGTCACTGGAAAGATGATAGATGACTATCTAACTCGTAAAAAAACCTATAATGA

TCATATAGTTAATCTATTATTTTGTGCAAATAGATGGGAGTTTGCATCTTTTATACAAGAACAACTAGAA

CAGGGAATTACTTTAATAGTTGACAGATACGCGTTCTCTGGAGTAGCGTATGCCACCGCTAAAGGCGCGT

CAATGACTCTCAGTAAGAGTTATGAATCTGGATTGCCTAAACCCGACTTAGTTATATTCTTGGAATCTGG

TAGCAAAGAAATTAATAGAAACATCGGCGAGGAAATTTATGAAGATGTTGAATTCCAACAAAAGGTATTA

CAAGAATATAAAAAAATGATTGAAGAAGGAGATATTCATTGGCAAATTATTTCTTCTGAATTCGAGGAAG

ATGTAAAGAAGGAGTTGATTAAGAATATAGTTATAGAGGCTATACACACGGTTACTGGACCAGTGGGGCA

ACTGTGGATGTAATAAAATGAAATTACATTTTTATAAATAGATGTTAGTACAGTGTTATAAATGGATGAA

GCATATTACTCTGGCAACTTGGAATCAGTACTCGGGGATACGTGTCCGATATGCATACCGAACTCGCATC

AATATCTCAATTAGTTATTGCCAAGATAGAAACTATAGATAATGATTATTAAACAAGGACATTGTAAATT

TTATCATGTGTAGATCAAACTTGGATAATCCATTTATCTCTTTCCTAGATACTGCATATACTATCATAGA

TCAAGAGATCTATCAGAACGAGTTGATTAATTCATTAGACGATAATGAAATTATCGATTGTATAGTTAAC

AAGTTTATGAGCTTTTATAAGGATAACCTAGAAAATATGGTAGATGCTATCATTACTCTAAAATATTATA

ATTAATAATCCAGATTTTAAAACTACGTATGTGGAAGTACTCGGTTCCAGAATAGCTGATATAGATATTA

AACAAGTGATACGTAAGAATATAATACAATTGTCTAATGATCCGCGAACGATATTTGTGAAAATATTAAA

AAAAAATACTTTTTTTATTAAATGACGTCTCTTCGCGAATTTAGAAAATTATGCTGTGATATATATCACG

CATCAGGATATAAAGAAAAATCTAAATTAATTAGAGACTTTATAACAGATAGAGATGATACCGATACATA

TTTGATCATTAAGCTATTGCTTCCCGGATTAGACGATAGAATGTATAACATGAACGATAAACAAATTATA

AAATTATATAGTATAATATTTAAACAATCTCAGGAAGATATGCTACAAGATTTAGGATACGGATATATAG

GAGACACTATTAGGACTTTCTTCAAAGAGAACACGGAAATCCGTCCACGAGATAAAAGCATTTTAACTTT

AGAAGAAGTGGATAGTTTTTTAACTACGTTATCATCAGTAACTAAAGAATCACATCAAATAAAATTATTG

ACTGATATAGCATCTGTTTGTACATGTAATGATTTAAAATGTGTAGTCATGCTTATTGATAAAGATCTAA

AAATTAAAGCGGGTCCTCGGTACGTGCTTAACGCTATTAGTCCTCATGCCTATGATGTTTTTAGAAAATC

TAATAACTTGAAAGAGATAATAGAAAATGCAGCTAAACAAAATCTAGACTCTATATCTATTTCTGTTATG

ACTCCAATTAATCCCATGTTAGCGGAATCATGTGATTCTGTCAATAAGGCGTTTAAAAAATTTCCATCAG

GAATGTTTGCGGAAGTCAAATACGATGGTGAAAGAGTACAAGTTCATAAAAAAAATAACGAGTTTGCATT

CTTTAGTAGAAACATGAAACCAGTACTCTCTCATAAAGTGGATTATCTCAAAGAATACATACCGAAAGCA

TTTAAAAAAGCTACGTCTATCGTATTGGATTCTGAAATTGTTCTTGTAGACGAACATAATGTACCGCTAC

CGTTTGGAAGTTTAGGTATACACAAAAAGAAAGAATATAAAAACTCTAACATGTGTTTGTTCGTATTTGA

CTGTTTATACTTTGATGGATTCGATATGACAGACATTCCATTGTATGAACGAAGATCTTTTCTCAAAGAT

GTTATGGTCGAAATACCCAATAGAATAGTATTCTCAGAGTTGACGAATATTAGTAACGAGTCTCAGTTAA

CTGATGTATTAGATGATGCACTAACGAGAAAATTAGAAGGATTGGTCTTAAAAGATATTAATGGCGTATA

CGAACCGGGAAAGAGAAGATGGTTAAAAATAAAGCGAGACTATTTGAACGAGGGTTCCATGGCAGATTCT

GCCGATTTAGTAGTACTAGGTGCCTACTATGGTAAAGGAGGAAAGGGTGGTATCATGGCAGTCTTTCTAA

TGGGTTGTTACGACGATGAATCCGGTAAATGGAAGACGGTAACTAAATGTTCCGGTCACGATGATAATAC

GTTAAGGGTTTTGCAAGACCAATTAACGATGGTTAAAATTAACAAGGATCCCAAAAAAATTCCAGAGTGG

TTGGTAGTTAATAAAATCTATATTCCCGATTTTGTAGTAGATGATCCGAAACAATCTCAGATATGGGAAA

TTTCAGGAGCAGAGTTTACATCTTCCAAGTCACATACAGCGAATGGAATATCGATTAGATTTCCTAGATT

TACTAGGATTAGAGAAGATAAAACGTGGAAAGAATCTACTCATCTAAACGATTTAGTAAACTTGACTAAA

TCTCTTAATAGTTACATATAAACTGAAAAATAAAATAACACTATTTTAGTTGGTAGTCGCCATGGATGGT

GTTATCGTATACTGTCTAAATGCGTTAGTAAAACATGGCGAGGAAATAAATCATATAAAAAATGATTTCA

TGATTAAACCATGTTGTGAAAGAGTTTGTGAAAAAGTCAAGAACGTTCACATCGGCGGACAATCTAAAAA

CAATACAGTGATTGCAGATTTGCCATATCTGGATAATGCTGTATCAGATGTATGCAAATCAATATATAAA

AAGAATGTATCAAGAATATCCAGATTTGCTAATTTGATAAAAATAGATGACGATGACAAGACTCCTACCG

GCGTATATAATTATTTTAAACCTAAAGATGCTATTCCTGTTATTATATCCATAGGAAAGGATAAAGATGT

CTGTGAACTATTAATCTCATCTGATAAAGCGTGTGCGTGTATAAAGTTAAATTTATATAAAGTAGCCATT

CTTCCCATGGATGTTTCCTTTTTTACCAAAGGAAATGCATCATTGATTATTCTCCTGTTTGATTTCTCTA

TCGATGCGGCACCTCTCTTAAGAAGTGTAACCGATAATAATGTTATTATATCTAGACACCAGCGCCTACA

TGACGAGCTTCCGAGTTCCAATTGGTTCAAGTTTTACATAAGTATAAAGTCCGACTATTGTTCTATATTA

TATATGGTTGTTGATGGATCTATGATGTATGCGATAGCTGATAATAGAACTCACGCAATTATTAGCAAAA

ATATATTAGACAATACTACGATTAACGATGAGTGTAGATGCTGTTATTCTGAACCACAGATTAGGATTCT

TGATAGAGATGAGATGCTCAATGGATCATCGTGTTATATGAACAGACATTGTATTATGATGAATTTACCT

GATGTAGGCGAATTTGGATCTAGTATGTTGGGGAAATATGAACCTGACATGATTAAGATTGCTCTTTCGG

TGGCTGGTAATTTAATAAGAAATCGAGACTACATTCCCGGGAGACGAGGCTATAGCTACTACGTTTACGG

TATAGCCTCTAGATAATTTTTTTTAAGCACGAAATAAAAACATAATTTTAAACAATCTATTTCATACTAT

TTTGTGTGCTCACCATGAACATAAAGATAGATATATTAGTATTTCTGGTGATAAATTTACGGCGACTGCT

AGGAGGGAAAATGAAGAAAGAAAAAATATCTACCTCTCCAAAAAGAAAAACTACTGATGTTATCAAACCT

GATTATCTTGAGTACAATGACTTGTTAGATAGAGATGAGATGTCTACTATTCTAGAGGAATATTAGGCCT

TAGAATAAAATATGGACGACTCTTAACGAAATTAGAAAATTCGATAATGATGTTGAAGAACAATTCGGTA

CTATAGAAGAACTCAAGCAGAAGCTTAGATTAAATTCTGAAGAGAGAGCAGATAATTTTATAGATTATAT

AAAGGTACAAAAACAGGATATCATCAAACTTACTGTATACGATTGCATATATCTATGATAGGATTGTGTG

CGTGCGTGGTAGATGTTTGGAGAAATGAGAAACTGTTTTCTAGATGGAAATATTGGTTACAAGCGATTAA

ACTGTTTATTGATGATCACATGCTTGATAAGATAAAATCTATTGTAGAATAGACTAGTGTATGTGGAAAT

GTCATAGAAAGTTAAAAGTTAATGAGAGCAAAAATATATAAGGTTGTATTCCATATTTGTTATTTTTTTC

TGTAATAGTTAGAAAATACATTCGATGGTCTATCTACCAGATTATTATGTGTTATAAGGTACTTTTCTCA

TAATAAACTAGAGTATGAGTAAGATAGTGTTTTTCAAAAACATATAAATCTAAAATTGATGGATGAGATA

TACAGCTATTAATTTCGAAAATATATTTTAATCTGATAACTTTAAACATGGATTTTTGATGGTGGTTTAA

GTTTAAAAAAGATTTTGTTATTGTAGTATGATAATATCAAAAAGATGGATATAAAGAATTTACTGACTAC

ATGTACTATTTTACATTACTACATTGGCTACGGCATATATACCTATTTCGTCACTTCCACACGCTCCGGT

AAACGGGTGTCATGTGACGAGGGAGAATCTTGATAAGAGGCATAATCAATGTTGTAATCCGATGTCCACC

TGGAGAATTTGCCAAGGTCAGATGTAGAGTTGGTAGTGATAACACAAAATGTGAACACTGCCCACCTCAT

ACATATACCGCAATCCCCAATTATTCTAATAGATGTCATCAATGTAGAAAATGCCCAACAGGATCATTTG

ATAAGGTAAAGTGTACCGGAACACAGAACAAATGTTCGTGTCATCCTGGTTGGTATACGCTACTGATTCT

TCACAGACTGAAGATTGTCGAGATTTGTGTACCAAAAAAGGAGATGTCCATGCGGATACTTTGGTGGAAT

AGATGAAGGAAATCCTATTTGTAAATCGTGTTGTGTTGGTGAATATTGCGACTACCTACGTAATTATAGA

CTTGATCCATTTCCTCCATGCAAACTATCTATCTAAATGTAATTAATTATGATTTTGATGATAATGTTAC

CATACATTATATCACTACTTGGTTAGTGTGTATTATTTAGTATGGAAGACCTATTAATAATTACTTATCT

TTTGACGATCTTGTTATAATTATAATATAAAAATACTTATGACATAGTAACTCATAATTGCTGACGCGAT

AAATTCGTAATAATCTGTTTTGTTCAAATTTTTATAAGGAATCTACAGGCATAAAAATAAAAATATAATC

TATAATATACTCTTACAACGCCATCATGAATAGCAGTGAATTAATTGCTGTTATTATGGATTTAGAAATA

GTGGACGATTTTGTGATATTAATATAGTTATTAATGATGAAAGGATAAACGCGCATAGACTCATCCTATC

TGGAGCCTCCGAATATTTTTTCCATTCTGTTTTCCAATAATTTTATCGATTCTAATGAATACGAAGTTAA

TCTAAGTCATTTAGATTATCAAAGTGTTAACTATTTGATCGATTACATTTATGGGATACCTTTGAGCCTA

ACTAACAATAACGTGAAATATATTCTTTCAACCGCTGATTTTTTTACAAATTGGATCTGTCATTACTGAG

TGCGAAAAATACATACTTAAAAATCTTTGTTTTAGAAACTGTATCGATTTCTACATATACGCTGATAAAT

ATAATAACAAGAAAATAGAATTAGCATCGTTTAACACAATATTACGAAATATTTTGAGACTCATCAACAA

TGAAAACTTTAAATACTTAACAGAGGAATCAATGATAAAAATTTTAAGCGATGATATGTTATATATAAAA

AATGAGGATTTCACCCCACTGATTCTCATTAAATGGTTAGAGAGTACACCAACCATGTACCGTCGAGTTA

CTTAGATGCCTCAGAATATCATTTCTTTCCCCACAAGTTATAAAATCACTTTATAGTCATCGACTGGTTA

GTTCAATCTACGAATGTATAACATTCTTAAACAATATAGCATTCTTGGATAAATCATTTCCTAGATACCA

TATCATCGAGTTGATATCTATCGGTATAAGTAATTCACATGATAAGATTTCCATAAACTGCTACAATCAT

AAAAAAATTCATGGGAAATGATATCTTCACGTAGATATAGGTGTAGTTTCGCAGTGACCGTCCTGGATAA

TATTATCTATATGATGGGTGGATATGATCAGTCCCTGTATAGAAGTTCAAAGGTTATAGCGTACAATACA

TGTACTAATTCTTGGATATATGATATACCAGAGCTAAAAATATCATCGTTCTAATTGCGGAGGAGTTGCC

AATGACGAATACATTTATTGTATAGGCGGTATACGCGATCAGGAGTCATCGTTGATATCTAGTATCGATA

GATGGAAGCCATCAAAACCATATTGGCAGAAGTATGCTAAAATGTGCGAACCAAAATGTGATATGGGGTT

GCGATTTTAAACGGATTAATATATGTCATAGGTGGAGTCGTTAAAGGTGACACACATATACCAACGCACT

AGAGAGTTTATCAGAAGATGGATGGATGAATCATCAACGTCTTCCAATAAAAATGTCCAATATGTCGACG

ATTGTTCATGCTGGAAAGATTTATATATCTAGAGGTTACAACAATAGTAGTGTAGTTAATGTAATATCGA

ATCTAGTCCTTAGCTATAATCCGATATATGATGAATGGACCAAATTATCATCATTAAATATTCCTAGAAT

TAATCCTGCTCTATGGTCAGTGTATAATAAATTATATGTAGGAGGAGTAATATCTGATGATGTTCAAACT

AATACATCTGAAACATACGATAAAGAAAAAGATTGTTGGACATTGGATAATGGTCACTTGTTACCACATA

ATTATATAATGTATAAATGCGAACCGTTTAAACATAGATATCCATTGGAAAAAACACAGTACACGAATGA

TTTTCTAAAGTATTTGGAAAGTTTTATAGGTAGTTGATAGAACAAAATACATAATTTTGTAAAAATAAAT

CACTTTTTATACTAATATGACACAATTACCAATACTTTTGTTACTAATATCATTAGTATACGCTACACCT

TCTCCTCAGACATCTAAAAAAATAGGTGATGATGCAACTATATCATGTAGTCGAAATAATACAAATTACT

ACGTTGTTATGAGTGCTTGGTATAAGGAGCCCAATTCCATTATTCTCTTAGCTGCCAAAAGCGACGTCTT

GTATTTTGATAATTATACCAAGGATAAAATATCTTACGACTCTCCATACGATGATCTAGTTACAACTATC

ACAATTAAATCATTGACTGCTGGAGATGCCGGTACTTATATATGTGCATTCTTTATGACATCGACTACAA

ATGATACTGATAAAGTAGATTATGAAGAATACTCCATAGAGTTGATTGTAAATACAGATAGTGAATCGAC

TATAGACATAATACTATCTGGATCTACACCAGAAACTATTTCTGAGAAACCAGAGGATATAGATAATTCT

AATTGCTCGTCTGTATTCGAAATCACGACTCCGGAACCAATTACTGATAATGTAGACGACCATACAGACA

CCGTCACATACACTAGTGATAGCATTAATACAGTAAATGCATCATCTGGAGAATCCACAACAGACGAGAT

TCCGGAACCAATTACTGATAAAGAAGAAGATCATACAGTAACAGACACTGTCTCATACACTACAGTAAGT

ACATCATCTGGAATTGTCACTACTAAATCAACCACCGATGATGCGGATCTTTATGATACATACAATGATA

ATGATACAGTACCGCCAACTACTGTAGGTGGTAGTACAACCTCTATTAGCAATTATAAAACCAAGGACTT

CGTAGAAATATTTGGTATTACCACATTAATTATATTGTCAGCAGTGGCGATTTTCTGTATTACGTATTAT

ATATGTAATAAACACCCACGTAAATACAAAACAGAGAACAAAGTCTAGATTTTTGACTTACATAAATATC

TGGGATAATAAAATCTATCATATTGAGAGGACCATCTGGTTCAGGAAAGACAGCCATAACCAAAAGACTG

TTAAAAGACTATGGGAATATATTTGGATTTGTGGTGTCCCATACCACTAGATTTCCTCGTCCTATGGAAC

GAGAAGGTGTTGATTACCTTACGTTAACAGAGAGGCCATCTGGAAGGGAATAGCCGCCGGAAACTTTCTA

GAACATACTGAGTTTTTAGGAAATATTTACGGAACTTCTAAAACAGCTGTAAATACAGCGGTTATTAATA

ATCGTATTTGCGCGATGGATTTAAACATCAACGGTGTTAGAAGTCTTAAAAATACTTACCTAATGCATTA

CTTGGGTATATAAGACCTACCTCTCTTAAAATGGTTGAGACCAATCTTCGTCGTAGAAACACTGAAGCGG

ACGACGAATCTCATCGTCGCGTGATGTTGGCAAAAAACGGATATGGATGAGGTCAACGAAGCAGGTCTAT

TCGACACTATTATTATTGAAGATGATGTGAATTTAGCATATAGTAAGTGTTAATTCAGATACTACAGGAC

CGTATTAGAATGTATTTTAACACTAATTAGAGACTTAAGATTTGACTTAAAACTTGATAATTAATAATAT

AACTCGTTTTTATATGTGGCTATTTCAACGTCTAATGTATTAGTTAAATATTAAAACTTACCACGTAAAA

CTTAAAATTTAAAATGGTATTTCATTGACAGATCATACATTATGAAGTTTCAAGGACTTGTGTTAATTGA

CAATTGCAAAAATCAATGGGTCGTTGGACCATTAATAGGAAAAGGTGGATTCGGTAGTATTTATACTACT

AATGACAATAATTATGTAGTAAAAATAGAGCCCAAAGCTAACGGATCATTATTTACCGAACAGGCATTTT

ATACTAGAGTACTTAAACCATCCGTTATCGAAGAATGGAAAAAATCTCACAATATAAAGCACGTAGGTCT

TATCACATGCAAGGCATTTGGTTTATACAAATCCATTAATGTGGAATATCGATTCTTGGTAATAAATAGA

TTAGGTGCAGATCTAGATGCGGTGATCAGAGCCAATAATAATAGACTACCAGAAAGGTCGGTGATGTTGA

TCGGAATCGAAATCTTAAATACCATACAATTTATGCACGAGCAAGGATATTCTCACGGAGATATTAAAGC

GAGTAATATAGTCTTGGATCAAATAGATAAGAATAAATTATATCTAGTGGATTACGGATTGGTTTCTAAA

TTCATGTCTAACGGCGAACATGTTCCATTTATAAGAAATCCAAATAAAATGGATAACGGTACTCTAGAAT

TTACACCTATAGATTCGCATAAAGGATACGTTGTATCTAGACGTGGTGATCTAGAAACACTTGGATATTG

TATGATTAGATGGTTGGGAGGTATCTTGCCATGGACTAAGATATCTGAAACAAAGAATTCTGCATTAGTA

AGTGCCGCAAAACAGAAATATGTTAACAATACTGCGACTTTGTTAATGACCAGTTTGCAATATGCACCTA

GAGAATTGCTGCAATATATTACCATGGTAAACTCTTTGACATATTTTGAGGAACCCAATTACGACGAGTT

TCGTCGAGTATTAATGAATGGAGTTATGAAAAATTTTTGTTGATAAAAAAATTAAAAAAATAACTTAGTT

ATTATCACTCTCGTGAGTACAATAGAAACATGGCGATGTTTTACGCACACGCTTTCGGTGGGTACGACGA

GAACCTTCATGCATTTCCTGGAATATCATCGACGGTTGCCAATGATGTCAGGAAATATTCTGTTGTGTCA

GTTTATAATAAAAAGTATAACATTGTAAAAAACAAATATATGTGGTGTAACAGTCAAGTGAACAAGAGAT

ATATTGGAGCACTACTGCCTATGTTTGAATGCAATGAATATCTACAAATTGGAGATCCAATCCATGATCT

AGAAGGAAATCAAATCTCTATTGTCACATATCGCCACAAAAACTACTATGCTCTAAGTGGAATTGGGTAC

GAGAGTCTAGACTTGTGTTTGGAAGGAGTAGGGATTCATCATCACGTACTTGAAACAGGAAACGCGGTAT

ATGGAAAAGTTCAACATGAGTATTCTACTATCAAAGAGAAGGCCAAAGAAATGAATGCACTCAAACCAGG

ACCTATCATCGATTACCACGTCTGGATAGGAGATTGTGTCTGCCAAGTTACTACTGTAGACGTGCATGGA

AAGGAAATTATGAGAATGAGATTCAAAAGGGGTGCGGTGCTTCCGATTCCAAATCTGGTAAAAGTTAAAG

TTGGGGAGGAAAATGATACAATAAATCTTTCCACTTCCATATCAGCTCTCCTAAATTCCGGTGGCGGCAC

CATCGAGGTAACATCTAAGGAAGAACGTGTAGATTATGTACTCATGAAACGTTTGGAATCTATACATCAT

TTGTGGTCTGTAGTGTATGATCATCTTAATGTTGTGAATGGCGAAGAACGATGTTATGTACATATGCATT

CATCTCATCAAAGTCCTATGCTGAGTACTGTAAAAACAAATTTGTACATGAAGACTATGGGAGCATGTCT

TCAAATGGACTCCATGGAAGCTCTAGAGTATCTTAGTGAACTGAAGGAATCAGGTGGGCGGAGTCCCAGA

CCAGAATTGCAGAAATTTGAATATCCAGATGGAGTGAAAGACACTGAATCAATTGAGAGATTGGCAGAGG

AGTTCTTCAATAGATCAGAACTTCAGGCCGGTGAATCAGTCAAATTTGGTAATTCTATTAATGTTAAACA

TACATCTGTTTCAGCTAAGCAACTAAGAACACGTATACGACAGCAGCTTCCTTCTATACTCTCATCTTTT

GCCAACACAAAGGGTGGATATTTGTTCATTGGAGTTGATAATAATACACACAAAGTAATTGGATTCACGG

TGGGTCATGACTACCTCAAACTGGTAGAGAGTGATATAGAAAAGTATATCCAAAAACTTCCTGTTGTGCA

TTTCTGCAAGAAAAAAGAGGACATCAAGTACGCATGTAGATTCATCAAGGTGTATAAACCTGGTGATGAG

ACTACCTCGACATATGTGTGCGCAATCAAAGTGGAAAGATGCTGCTGTGCTGTGTTTGCGGATTGGCCAG

AATCATGGTACATGGATACTAGTGGTAGTATGAAGAAGTATTCTCCAGATGAATGGGTGTCACATATAAA

ATTTTAATTAGGGTAAGGTAAAACTATATATAATAACTAACAATTTGTGTATCATATAGACAATTAATTA

GGTAACTGTTATCTCTTTTTAACTAACTAACTAACTAACTAACTCTTATATACTATTAATAATACATCTA

TTAATCATTGATTAGCTTATTGCTTTAATTGTTTTTGTAAACTAACACTGTTCATTGAAAAGGGATAACA

TGTTACAGAATATAAATTATATATGGATTTTTTTAAAAAGGAAATACTTGACTGGAGTATATATTTATTT

CTTCATTACATAACACGTCTGTGTTCTAATTCTTCCAATTCTTCCACATCTCATATAATACAGGAATATA

ATCTTGTTCGAAAATATGAGAAAGTGGATAAAACAATAGTTGATTTTTTATCTAGGTGGCCAAATTTATT

CCATATTTTAGAATATGGGGAAAATATTCTACATATTTATTTTATAGATGCTGCTAATACGAATATTATG

ATTTTTTTTCTAGATAGAGTATTAAATATTAATAAGAACCGTGGGTCATTTATACATAATCTCGGGTTAT

CATCCATTAATATAAAAGAATATGTATATCAATTAGTTAATAATGATCATCTAGATAATAGTATAAGACT

AATGCTTGAAAATGGACGTAGAACAAGACATTTTTTGTCTTATATATTGGATACAGTTAATATCTATATA

AGTATTTTAATAAATCATAGATTTTATATAGATGCCGAAGACAGTTACGGTTGTACATTATTACATAGAT

GTATATATAACTATAAGAAATCAGAATCAGAATCATATAATGAATTAATTAAGATATTGTTAAATAATGG

ATCAGATGTAGATAAAAAAGATACGTACGGAAACACACCGTTTATCCTATTATGTAAACACGATATCGAC

AACGCGGAATTGTTTGAGATATGTTTAGAGAATGCTAATATAGACTCTGTAGACTTTAATGGATATACAC

CTCTTCATTATGTCTCATGTCGTAATAAATATGATTTTGTAAAGTTATTAATTTCTAAAGGAGCAAATGT

TAATGCACGTAATAGATTCGGAACTACTCCATTTTATTGTGGAATTATACACGGTATCTCGCTTATAAAA

CTATATTTGGAATCAGACACAGAGTTAGAAATAGATAATGAACATATAGTTCGTCATTTAATAATTTTTG

ATGCTGTTGAATCTTTAGATTATCTATTGTCCAGAGGAGTTATTGATATTAACTATCGTACTATATACAA

CGAAACATCTATTTACGACGCTGTCAGTTATAATGCGTATAATACGTTAGTCTATCTATTAAACAGAAAT

GGTGATTTTGAGACGATTACTACTAGTGGATGTACATGTATTTCGGAAGCAGTCGCGAACAACAACAAAA

TAATAATGGATATACTATTGTCTAAACGACCATCTTTGAAAATTATGATACCATCTATGATAGCAATTAC

TAAACATAAACAACATAATGCAGATTTATTGAAAATGTGTATAAAATATACTGCGTGTATGACCGATTAT

GATACTCTTATAGATGTACAATCGCTACATCAATATAAATGGTATATTTTAAAATGTTTTGATGAAATAG

ATATCATGAAGAGATGTTATATAAAAAATAAAACTGTATTCCAATTAGTTTTTTGTATCAAAGACATTAA

TACTTTAATGAGATACGGTAGACATCCTTCTTTCGTGAAATGTAATATTCTTGACGTATACGGAAGTTGT

GTACGTAATATCATAGCATCTATTAGATATCGTCAGAGATTAATTAGTCTATTATCCAAGAAGCTGGATG

CTGGAGATAAATGGTCGTGTTTTCCTAACGAAATAAAATATAAAATATTGGAAAACTTTAACGATAACGA

ACTGACCACATATCTGAAAATCTTATAAACACTATTAAAATATAAAATCTAAGTAGGATAAAATCACACT

ACATCATTGTTTCCTTTTAGTGCTCGACAGTGTATACTATTTTTAACACTCATAAATAAAAATGAAAACG

ATTTCCGTTGTTACGTTGTTATGCGTACTACCTGCTGTTGTTTATTCAACATGTACTGTACCCACTATGA

ATAACGCTAAATTAACGTCTACCGAAACATCGTTTAATGATAAACAGAAAGTTACGTTTACATGTGATTC

AGGATATCATTCTTTGGATCCAAATGCTGTCTGTGAAACAGATAAATGGAAATACGAAAATCCATGCAAG

AAAATGTGCACAGTTTCTGATTATGTCTCTGAACTATATGATAAGCCATTATACGAAGTGAATTCCACCA

TGACACTAAGTTGCAACGGTGAAACAAAATATTTTCGTTGTGAAGAAAAAAATGGAAATACTTCTTGGAA

TGATACTGTCACGTGTCCTAATGCGGAATGTCAACCTCTTCAATTAGAACACGGATCGTGTCAACCAGTT

AAAGAAAAATACTCATTTGGGGAATATATGACTATCAACTGTGATGTTGGATATGAGGTTATTGGTGTTT

CGTATATAAGTTGTACGGCTAATTCTTGGAATGTTATTCCATCATGTCAACAAAAATGTGATATACCGTC

CCTATCTAATGGATTAATTTCCGGATCTACATTTTCTATCGGTGGCGTTATACATCTTAGTTGTAAAAGT

GGTTTTACACTAACGGGGTCTCCATCATCCACATGTATCGACGGTAAATGGAATCCCATACTCCCAACAT

GTGTACGATCTAACGAAGAATTTGATCCAGTGGATGATGGTCCCGACGATGAGACAGATCTGAGCAAACT

CTCGAAAGACGTTGTACAATATGAACAAGAAATAGAATCGTTAGAAGCAACTTATCATATAATCATAATG

GCGTTGACAATTATGGGTGTCATATTTCTAATCTCCATTATAGTATTAGTTTGTTCCTGTGACAAAAATA

ATGACCAATATAAGTTCCATAAATTGCTACCGTGAATATAAATCCGTTAAAATAATTAATAATTAATAAT

TAATAACGAACAAGTATCAAAAGATTAAAGAATTAGCTAGAATCAATTAGATGTCTTCTTCAGTGGATGT

TGATATCTACGATGCCGTTAGAGCATTTTTACTCAGGCACTATTATGACAAGAGATTTATTGTGTATGGA

AGAAGTAACACCATATTACATAATATATACAGGCTATTTACAAGATGCACCGTTATACCGTTCGATGATA

TAGTACGTACTATGCCAAATGAATCACGTGTTAAACAATGGGTGATGGATACACTTAATGGTATAATGAT

GAATGAATTCGATACTGTATGTGTGGGTACCGGACTACGATTCATGGAAATGTTTTTCGATTACAATAAA

AATAATCCCAAAAATAGCATCAACAATCAAATAATGTATGATATAATTAATAGCGTAGCCATAATTCTAG

CTAATGAGAGATATAGAAGCGCGTTTAACGACGATAGAATATACATCCGTAGAACTATGATGGACAAATT

GTACGAATACGCATCTCTAACTACTATTGGTACGATCACTGGAGGTGTTTGTTATTTTATCTGTTGATGC

ATCTAGTTAGTTTGTATAAATAATTATTTCGATATACTAGTTAAAATTTTAAGATTTTAAATGTATAAAA

AACTAATAACGTTTTTATTTGTAATAGGTGCAGTTGCATCCTATTCGAATAATGAGTACACTCCGTTTAA

TAAACTGAGTGTAAAACTCTATATAGATGGAGTAGATAATATAGAAAATTCATATACTGATGATAATAAT

GAATTGGTGTTAAATTTTAAAGAGTACACAATTTCTATTATTACAGAGTCATGTGACGTCGGATTTGATT

CCATAGATATAGATGTTATAAACGACTATAAAATTATTGATATGTATACCATTGACTCGTCTACTATTCA

ACGCAGAGGACATACGTGTAGAATATCTACCAAATTATCATGCCATTATGATAAGTACCCTTATATCCAC

AAATATGAGGGTGATGAACGACAATATTCTATTACCGCAGAGGGAAAATGCTATAAAGGAATAAAATATG

AAATAAGTATGATGAACGATGATACTCTATTGAGAAAACATACTCTTAAAATTGGATTTACTTATATATT

CGATCGTCATGGGCATAGTAATACATATTATTCAAAATATGATTTTTAAAAATTTAAAATATATTATCAC

TTCAGTGACAGTAGTCAAATAACAAACAACACCATGAGATATATTATAATTCTCGCAGTTTTGTTCATTA

ATAGTATACATGCTAAAATAACTAGTTATAAGTTTGAATCCGTCAATTTTGATTCCAAAATTGAATGGAC

TGGGGATGGTCTATACAATATATCCCTTAAAAATTATGGCATCAAGACGTGGCAAACAATGTATACAAAT

GTACCAGAAGGAACATACGACATATCCGGATTTCCAAAGAATGATTTCGTATCTTTCTGGGTTAAATTTG

AACAAGGCGACTATAAAGTGGAAGAGTATTGTACGGGACTATGTGTCGAAGTAAAAATTGGACCACCAAC

TGTAATATTGACTGAATATGACGATCATATCAATTTGTTCATCGAGCATCCGTATGCTACTAGAGGTAGC

AAGAAGATTCCTATTTACAAACGCGGTGACATGTGTGATATCTACTTGTTGTATACGGCTAACTTCACAT

TCGGAGATTCTGAAGAACCAGTAACATATGATATCGATGACTACGATTGCACGTCTACAGGTTGCAGTAT

AGACTTTGCCACAACAGAAAAAGTGTGTGTGACAGCACAGGGAGCCACAGAAGGGTTTCTCGAAAAAATT

ACTCCATGGAGTTCGGAAGTATGTCTGACACCTAAAAAGAATGTATATACGTGCGCAATTAGATCTAAAG

AAGATGTTCCCAATTTCAAGGACAAAATAGCCAGAGTTATCACGAGAAAATTTAATAAACAGTCTCAATC

TTATTTGACTAAATTTCTCGGTAGCACATCGAATGATGTTACAACTTTTTTTAGCATTCTTGACTAAATA

TTCATAACTAATTTTTATTAATGATACAAAAATGAAATAAACTGTATATTATACACTGGTTAACGCCCTT

GGCTCTAACCATTTTCAAGATGAGGTCCCTGATTATAGTCCTTCTGTTCCCCTCTATCATCTACTCAATG

TCTATTAGACGATGCGAGAAGACTGAAGAGGAAACATGGGGATTAAAAATAGGGTTGTGTATAATTGCCA

AAGATTTCTATCCCGAAAGAACTGATTGCAGTGTTCATCGCCCAACTGCAAGTGGAGGATTGATAACTGA

AGGCAATGGATTCAGAGTAGTTATATATGATCAATGTACAGAACCCCATGACTTTATTATCACCGATACT

CAACAAACACGTCTTGGATCATCTCATACATATATTAAATTCAGTAACATGAATACAGGTGTCCCATCTA

GTATTCCAAAATGTTCCAGAACTCTCTGTATTTCTGTATATTGTGATCAAGAGGCGGGAGACATAAAATT

TGAGGAGTATACTCAAGAATCAAGTGATATCAGTATTAGAGTTAAGTATGATTCATCATGTATTGATTAT

CTGGGTATTAATCAAAGTTTCATGAATGAATGTATTCGAAGAATTACAACATGGGATAGAGAATCATGCG

TCAGAATTGATACACAGACTATAAATAAATATCTTAAGTCTTGCACCAACACAAAATTCGACCGTAATGT

CTACAAAAGGTACATACTGAAGAGTAAAGCACTCCATGCTAAAACAGAGTTGTAATAGATATAAAATACT

TTTTATAATAATTAGGCTAGAAAAATCTCACTCACATGTAATCTTAAAAAAATGATATGATAGTTCTTAC

AAGTAGCGATTGAGTTTTAAATGGATTCTATTAATTACCGGGGAACTTAACAATTCGTTCTGATCTACAG

ACATTGGTTAATAAATCATCTTATTTTGCCAATATATTAAAATGTGGAAACTCCACTAATAATATTACAT

TGTGCGACTTTCAAGATGATGTGATATATAGGGTTATACAGTTTTAACAATTATATAATAGAGATAGAAA

GTACAAAAGATGTAGAATCAATGATATGGCACGCTAAACAGTTGGGTGTGGAATCATTGCTAAAAGAATG

TCAAAATTATTTGCTTAGAATATTACGTATATAATTGTTTAGAAATTTATAGAATAACTAATATTAATAC

ATTATCGTATATCTACAACGATATAAGAAACTTCATATTGGATAATATTACTATTAATATATAAGGATCC

AGATTTTATATATTTGCCTAAATACATTATTATAGATTTACTAGGACAATCACCTAAATGTTTTTAACGA

AGATAATGTGGTAAAGATTATATACACTTATATATCTTCCGATATCTACAAGGATATTCCATATCATCAT

TGTGTAAACTAAATAACGTTTTCTATGGCATTTAATAAGGACATTGGATATGTGGAAAAGTGATGTATGG

AAGTTAGTACATTATCAACTTCTCCTTATTGATTGAAAATGAAAATATAAATAGTTTTTATGTATAGCGG

TATCTACCCTATAGTTTTATTGCTTACTACTAACATGGATTCAGATACAGATACAGATACAGATACAGAT

ACAGATACAGATACAGATACAGATGTAGAAGATATCATGAATGAAATAGATAGAGAGAAAGAAGAAATAC

TAAAAAATGTAGAAATTGAAAATAATAAAAACATTAACAAGAATCATCCCAGTGAATATATTAGAGAAGC

ACTTGTTATTAATACCAGTAGTAATAGTGATTCCATTGATAAAGAAGTTATAGAATATATCAGTCACGAT

GTAGGAATATAGATCATATCTACTAATTTTTATAATCGATACAAAACATAAAAACAACTCGTTATTACAT

AGCAGGTATGGAATCCTTCAAGTATTGTTTTGATAACGATGGTAAGAAATGGATTATCGGAAATACTTTA

TATTCTGGTAATTCAATACTCTATAAGGTCAGAAAAAATTTCACTAGTTCGTTCTACAATTACGTAATGA

AGATAGATCATAAATCACACAAGCCATTGTTGTCCGAAATACGATTCTATATATCTGTATTGGATCCTTT

GACTATCAACAACTGGACACGAGAACGTGGTATAAAGTATTTGGCTATTCCAGATCTGTATGGAATTGGA

GAAACCGATGATTATATGTTCTTCATTATAAAGAATTTGGGAAGAGTATTCGCCCCAAAGGATAGTGAAT

CAGTTTTCGAAGCATGTGTCACTATGATAAACACGTTAGAGTTTATACACTCTCAAGGATTTACTCATGG

AAAAATAGAACCGATGAATATACTGATTAGAAATAAACGTATTTCACTAATTGACTATTCTAGAACTAAC

AAACTATACAAAAGTGGAACACATATAGATTACAACGAGGACATGATAACTTCAGGAAATATCAATTATA

TGTGTGTAGACAATCATCTTGGAGCAACAGTTTCAAGACGAGGAGATTTAGAAATGTTGGGATATTGCAT

GATAGAATGGTTCGGTGGTAAACTTCCATGGAAAAACGAAAGTAGTATAAAAGTAATAAAACAAAAAAAA

GAATATAAACAATTTATAGCTACTTTTTTTGAGGACTGTTTTCCTGAAGGAAATGAACCTCTGGAATTAG

TTAGATATATAGAATTAGTATACATGTTAGATTATTCTCAAACTCCTAATTATGACAGACTACGTAGACT

GTTTATACAAGATTGAAATTATATTCTTTTTTTTATAGAGTGTGGGGGTAGTGTTACGGATATCTAATAT

TAATATTAGACTATCTCTATCGCGCTACACGACCAATATCGATTACTATGGATATCTTCAGGGAAATCGC

ATCTTCTATGAAAGGAAAGAATGTATTCATTTCTCCAGCGTCAATCTCGTCAGTATTGACAATACTGTAT

TATGGAGCTAATGGATCCACTGCTGAACAGCTATCAAAATATGTAGAAAAGGAGGAGAACATGGATAAGG

TTAGCGCTCAGAATATCTCATTCAAATCCATGAATAAAGTATATGGGCGATATTCTGCCGTGTTTAAAGA

TTCCTTTTTGGGAAAAATTGGCGATAAGTTTCAAACTGTTGACTTCACTGATTGTCGCACTATAGATGCA

ATCAATAAGTGTGTAGATATCTTTACTGAGGGAAAAATCAATCCACTATTGGATGAACCATTGTCTCCTG

ATACCTGTCTCCTAGCAATTAGTGCCGTATACTTTAAAGCAAAATGGTTGATGCCATTCGAAAAGGAATT

TACCAGTGATTATCCCTTTTACGTATCTCCAACGGAAATGGTAGATGTAAGTATGATGTCTATTTACGGC

GAGCCATTTAATCACGCATCTGTAAAAGAATCATTCGGTAACTTTTCAATCATAGAACTGCCATATGTTG

GAGATACTAGTATGATGGTCATTCTTCCAAACAAGATTGATGGATTAGAATCCATAGAACAAAATCTAAC

AGATACAAATTTTAAGAAATGGTGTAACTCTCTGAAAGCTACGTTTATCGATGTGCACATTCCTAAGTTT

AAGGTAATAGGTTCGTATAATCTTGTGGATACGCTAATAAAGTTGGGACTGACAGATGTGTTCTATTCAA

CTGGTGATTATATCAATATGTGTAATTCAGATGTGAGTGTTGACGCTATGATTCACAAAACGTATATAGA

TGTCAATGAAGAGTATACAGAAGCAGCTGCAGCAACTTCTGTACTAGTGGCAGACTGTGCATCAACAGTT

ACAAATGAGTTCTGTGCAGATCATCCGTTCATCTATGTGATTAGACATGTCGATGGTAAAATTCTTTTCG

TTGGTAGATATTGCTCTCCAACAACTAATTAAGCACATTCTTAATATTAGAATATTATATAGTTAAGATT

TTTACTAACAGGTTAACATTTTTTTTTAAAAATAGAAAAAACATGTGGTATTAGTGCAGGTCGTTATTCT

TCCAATTGCAATTGGTAAGATGACGGCCAACTTTAGTACCCACGTCTTTTCACCACAACACTGTGGATGT

GACAGACTGACCAGTATTGATGACGTCAGACAATGTTTGACTGAATATATTTATTGGTCGTCGTATGCAT

ACCGCAACAGGCAATGCGCTGGACAACTGTATGACACACTCCTCTCTTTTAAAGATGATGCGGAATCAGT

GTTCATCGACGTTCGTGAGCTGGTAAAAAATATGCCGTGGGATAATGTTAAGGATTGTACAGAGATCATC

CGTTGTTATATACCGGATGAGCAAAAAACCATCAGAGAGATTTCGGCCATCATTGGACTTTGTGCATATG

CTGCTACTTACTGGGGAGGTGAAGACCATCCCACTAGTAACAGTCTGAACGCATTGTTTGTGATGCTTGA

GATGCTCAATTACATGGATTATACCATCATATTCTGGCGTATGAATTGATGAGTTACAGCTTGACATTTC

TTCTTTCCTCCCTCTTCTTCTACCTTTCCCAGAAACAAACTTTTTTTACCCACTATAAAATAAAATGAGT

ATACTACCTGTTATATTTCTTCCTATATTTTTTTATTCTCCATTCGTTCAGACTTTTAACGTGCCTGAAT

GTATCGACAAAGGGCAATATTTTGCATCATTCATGGAGTTAGAAAACGAGCCAGTAATCTTACCATGTCC

TCAAATAAATACGCTATCATCCGGATATAATATATTAGATATTTTATGGGAAAAACGAGGAGCGGATAAT

GATAGAATTATACAGATAGATAATGGTAGCAATATGCTAATTCTGAACCCGACACAATCAGACTCTGGTA

TTTATATATACATTACCACGAACGAAACCTACTGTGACATGATGTCGTTAAATTTGACAATCGTGTCTGT

CTCAGAATCAAATATAGATCTTATCTCGTATCCACAAATAGTAAATGAGAGATCTACTGGTAAAATGGTA

TGTCCCAATATTAATGCATTTATTTCTAGTAACGTAAACACAGAATTATATGGAGCGGACATCGACGCCT

TAGAAATAAGAGACTTAAACAACGGACACCTGGAATTATTACCATAGAAGATGTTAGAAAAAATGATGCT

GGTTATTATACATGTGTTTTAGAATATATATATATATATATATATATATATATATATATATATA

TATGGGCAAAACATATAACGTAACCAGAATTATAAAATTAGAGGTACGGGATAGAATAATACCTCCTACT

ATGAAATTACCAGAAGGAGTAGTAACTTCAATAGGTAGTAATTTGACTATTGCATGTAGAGTATCGTTGA

GACTTCCCACAACGGACACCGACGTCTTTTGGATAAGTAATGGTATATGTATTACGAAGAAGAAGACGAG

GACGGAGACGGTAGAATAAGTGTAGCAAATAAAATCTATATGACCGATAAGAGACGTGTTATTACATCCT

GGTTAAACATTAATCCTGTCAAGGAAGAAGATGCTACAACGTTTACGTGTATGGCGTTTACTATTCCTAG

CATCAGCAAAACAGTTACTGTTAGTAAACGTGAATGTATGTTGTTACATTTCCATATCAATTGAGTTTAT

AAGAATTTTTTATACATTATCTTCCAACAAACAATTGACGAACGTATTGCTATGATTAACTCCCACAATA

CTATATATATTATTAATCATTAACTTGCAGACTATACCTAGTAGTGCTATTTTGACATACTCATGTTCTT

GTGTAATCGCAGTATCTATATTATTAAAGTACGTAAATCTAGCTATAGTTTTATTATTTAATTTTAGATA

ATATACTGTCTCCGTATTTTTAAAAAATTACCACATCCTTTATTAAATCATGAATGGGAATTTCTGTGTC

ATCGTTAGTATATTGTGAACAACAAGAGCAGATATCTATAGGAAAGGGTGGAATGCGATACATTGATCTA

TGTAGTTTTAAAACATACGCGAACTTTGAAGAATTTATATAAATCATCTCACGAGATATTGCTCTCTGTC

ATATTCATACACCTGTATAAACTTTCTAGACATCTTACAATGTGTTATTTTATGATCATATTTACATATT

TACTGGTATATCAAAGATGTTAGATTAGTTAATGGGAATCGTCTATAATAATGAATATTAAACAATTATA

GGAGGAGTTTATACCTACAAAAACATCATAAAAATGAGTCATCGTCCGATTTATGTTTTAAATATACTAA

CATTACTACCTTCAGAAATTATATACGAAATATTATACATGCTGACAATTAACGATCTTTATAATATATA

GTATCCACCTACCAAAGTATAATTGTATTTTTCTCATGTGATGTGTGTAAAAAACTGATATTATATAATT

ATCTTAGTACCTATGATGAAGATGAAGATGAAGATGAAGATGATGGTCCGTATATATTTTGTATCATTAT

CGTTATTGCTATTCCATAGTTACGCCATAGACATCGAAAATGAAATCACCGAATTCTTCAATAAAATGAG

AGATACTCTACCAGCTAAAGACTCTAAATGGTTGAATCCAGTATGTATGTTTGGAGGCACAATGAATGAT

ATGGCCGCTCTAGGAGAGCCATTCAGTGCAAAGTGTCCTCCTATTGAAGACAGTCTTTTATCGCATAGAT

ATAAAGACTATGTGGTTAAATGGGAAAGGCTAGAAAAGAATAGACGGCGACAGGTTTCTAATAAACGTGT

TAAACATGGTGATTTATGGATAGCCAACTATACATCTAAATTCAGTAACCGTAGGTATTTATGTACCGTA

ACCACAAAGAATGGTGACTGTGTTCAGGGTGTAGTTAGATCTCATGTGTGGAAACCTTCTTCATGCATTC

CAAAAACATATGAACTAGGTACTTATGATAAGTATGGCATAGACTTATACTGTGGAATTCTTTATGCGAA

CCATTATAATAATATAACTTGGTATAAAGATAATAAGGAAATTAATATCGACGATTTTAAGTATTCACAA

GCGGGAAAGGAATTAATTATTCATAATCCAGAGTTAGAAGATAGTGGAAGATACGACTGTTACGTTCATT

ACGACGACGTTAGAATCAAGAATGATATCGTAGTATCAAGATGTAAAATACTTACGGTTATACCGTCACA

AGACCACAGGTTTAAACTAATACTAGATCCGAAAATCAACGTAACGATAGGAGAACCTGCCAATATAACA

TGCAGTGCTGTGTCAACGTCATTATTTGTCGACGATGTACTGATTGAATGGGAAAATCCATCCGGATGGA

TTATAGGATTAGATTTTGGTGTATACTCTATTTTAACTAGTAGAGGCGGTATCACCGAGGCGACTTTGTA

TTTTGAAAATGTTACTGAAGAATATATAGGCAATACATATACATGTCGTGGACACAACTATTATTTTGAT

AAAACTCTTACAACTACAGTAGTATTGGAGTAAATACACAATGCATTTTTATATACATTACTGAATTATT

ATTATTAATTATATCGTATTTGTGCTATAGAATGGATGAAGATACGCGACTATCTAGGTATTTGTATCTC

ACCGATAGAGAACATATAAATGTAGACTCTATTAAACAGTTGTGTAAAATATCAGATCCTAATGCATGTT

ATAGATGTGGATGTACGGCTTTACATGAGTACTTTTATAATTATAGATCAGTCAACGGAAAATACAAGTA

TAGATACAACGGTTACTATCAATATTATTCATCTAGCGATTATGAAAATTATAATGAATATTATTATGAT

GATTATGATAGAACTGGTATGAACAGTGAGAGTGATAATATATCAATCAAAACAGAATACGAGAATGAAT

ATGAATTCTATGATGAAACACAAGATCAAAGTACACAACTAGTAGATTACGACATTAAACTCAAAACCAA

TGAGGATGATTTTGTTGATGAATTCTATGGTTATGATAGATCAGTGGGTGTCCATGATTATATAGATGTA

TCAATTAATAAAGTAGTATATGGAAGAGAGTCTCACGTAAGATGGTGGGATATATGGCAAGAACATAATG

ATGGCGTATACAGTATAGGAAAGGAGTGCATAGATAATATATACGAAGACAGACATACCGTAGACGAATT

CTACAAGATAGACAGCGTATCAGATGTAGATGACGCAGAACATATATCTCAGATAACTAATGATGTATCT

ACACAAACATGGGAAAAGAAATCAGAGTTAGATAGATACATGGAAATGTATCCTCGTCATAGATATGGTA

AGCATTCTGTCTTTAAGGGATTTTCTGACAAAGTTAGAAAAAATGATTTAGACATGAACGTGGTAAAAGA

ATTACTTTCTAACGGTGCATCTCTAACAATCAAGGATAGCAGTAATAAGGATCCAATTGCTGTTTATTTT

AGAAGAACAATAATGAATTTAGAAATGATTGATATCATTAACAAACATACAACTATCTATGAACGCAGGT

ATATAGTACACTCCTATCTAAAAAATTATAGAAATTTCGATTATCCATTTTTCAGAAAGTTAGTTTTGAC

TAATAAACATTGTCTCAACAATTATTGTAATATAAGCGACGGCAAATATGGAACACCACTACATATATTA

GCATCTAATAAAAAAATAATAACTCCTAATTACATGAAGTTATTAGTGTATAACGGAAATGATATAAACG

CACGAGGTGAAGATACACAAATGCGAACTCCATTACACAAATATTTGTGTAAATTTGTATATCATAATAT

TGAATATGGTATCCGATACTATAATGAAAAGATTATAGACGCATTTATAGAGTTAGGAGCCGATCTAACT

ATTCCAAATGACGATGGAATGATACCAGTAGTTTACTGTATACACTCAAATGCCGAATATGGTTATAACA

ATATTACTAACATAAAGATAATACGTAAACTACTTAATCTTAGTAGACATGCGTCACATAATCTATTTAG

AGATCGAGTCATGCACGATTATATAAGTAATACATATATTGATCTTGAGTGTTTAGATATCATTAGATCA

CTTGATGGGTACGATATTAATTGTTACTTTGAAGGACGTACACCACTTCATTGCGCTATACAATATAACT

TCACTCAGATTGCTGAGTACTTATTAGATCGAGGAGCTGATATATCATTAAAGACAGACGATGGTAAAAC

TGTATTTGATTTATCGTTATGTAGTTACATTCCTCTTAAATGGACTAGCTTTTTGATTAGTCGTCTACCG

CCTAAAAGTGTCATATGCTCACTGACTAACCATATAATAGATTATGTTCTTACGAACAATAGACGTATTA

TTTGGCAGAGTCAAATGATTAATAAGTACGTACTGTTACTGGACCCATCCTTTTATTATAGATTCAGAAA

TGTTATCGAAAACAAATTAGACCAATACAATAATCGTTATAATATGTTCGAACACGATAGGGACGTTAAT

GAAAAGTATGGCAAAGTCTTACATGACCTCGATACATATATCAAGGATGTACAAGTATTAAAATCTACTT

CCATCACTAATAATATAACACTATACGACACTATTATAAATAATAAGTCAGAGTTTCCTATACGTCGTGT

AAACGACAAACAATTAATTAATCTCATAAAATCCAATACATATCATAATCTTATCGAAAAAGTTATTAAA

AATACATTAGAGAAATATACTTTAACTAATATAGTCCTCGAGTATATGATCTCATCTCGATCTCAATCAT

CTTATTTGAGTCGTATTCCTAATGAGATATTACTCGAAATATTATATAAACTCGACATGTACGATTTACG

TAATCTATATACAAGATATATGAGAGAGAATGATATCACAGAGTATCATATAGAGAATACGAGGTCTGTT

TCTACACAGACATGAATAATGAATACACATACAACGTTTTTTTTAATCTTAGATATAACACTAATTACAT

CAAGATTATATATTGAAATCGTAATTTGAGTTGTCTGATCATCATGGATATCGAAAATAATATACGTAAC

ATTAGCAATCTTTTAGATGATTGATATATTATTATGCGATGTAATCATAACTATCGGAGATGTAGAAATT

AAAGCGCATAAAACTATTTTGGTTGCCGGATCTACGTATTTTAAAACAATGTTCACAACATCTATGATAG

CGAGAGATCTAGCAACTAGAGTAAATATACAGATGTTCGATAAAGATGCCGTCAAAAATATTGTACAGTA

CTTATACAATAGGTATATAAGTTCTATGAATGTGATAGACATATTAAAATGCACCGACTAAGAACGTAAA

ACGAACTATAGAATGTTATACAATGGGTGATGATAAGTAGAAGATGTTACCCGATATACCCATAGCATTA

TCTAGTTATGGCATGTGTGTATTAGATCAATACATATACATTATAAGCGGTCGTACCCAACACTGATTAT

ACATCGGTACATACAGTAAATAGCATAGATATGGAGGAGGATACAAATATTTCAAATAAAGTTATGAGAT

ACGCGCTGTCAATAATATATGGAAGACATTACCTAACTTCTGAACTGGAACTATAAATCCAGGCTCTCGC

ATAAAGATGAATATATATGTTGTATGCGACATCAAAGATGAAAAAATGTTAAGACTTATATATTTAGATA

TAACACGAATATGTATGACGGATGGGAATTGGTAACGATGACAGAAAGCAGATTGTCAGCTCTGCATACT

ATTCTTCATGACAATACCATAATGATGTTACATTGTTATGAAGCGTATATGTTACAAGATACATTTAATG

TGCTTACGGAACATATATTTAGAAACATCTACTAACGATTTTTTATGCTTGTATTATTAATGGTATGTAA

TATGATTTAATTGATTGTGTACACGATACCAATTTGTCGAGTATGAATACGGAGTACAAACATAAACTGA

AGTTTAACATTATTTATTTATGATATACATTATATACATTATATACATTATATACATTATATACATTATA

TACATTATATACATTATATACATTATATACATTATATACATTATATATCGTTATTGTTTGGTCTATGCCA

TGGATATCTTTAAAGAACTAATCTTAAAACATACGGATGAAAATGTTTTGATTTCTCCAGTTTCCATTTT

ATCTACTTTATCTATTCTGAATCATGGAGCAGCTGGTTCTACAGCTGAACAACTATCAAAATATATAGAG

AATATGAATGAGAATACACCCGATGATAAGAAGGATGACAATAATGACATGGACGTAGATATTCCGTATT

GCGCGACACTAGCTACCGCAAATAAAATATACGGTAGTGATAGTATCGAGTTCCATGCCTCATTCCTACA

AAAAATAAAAGACGATTTTCAAACTGTAAACTTTAATAATGCGAACCAAACAAAGGAACTAATCAACGAA

TGGGTTAAGACAATGACAAATGGTAAAATTAATTCCTTATTGACTAGTCCGCTATCCATTAATACTCGTA

TGATAGTTATTAGCGCCGTCCATTTTAAAGCAATGTGGAAATATCCATTTTCTAAACATCTTACATATAC

AGACAAGTTTTATATTTCTAAGAATATAGTTACCAGTGTTGATATGATGGTGGGTACCGAGAATGACTTG

CAATATGTACATATTAATGAATTATTCGGAGGATTCTCTATTATCGATATTCCATACGAGGGAAACTCTA

GTATGGTGATTATACTGCCGGACGACATAGAAGGTATATATAACATAGAAAAAAATATAACAGATGAAAA

ATTTAAAAAATGGTGTGGTATGTTATCTACTAAAAGTATAGACTTGTATATGCCAAAGTTTAAAGTGGAA

ATGACGGAACCGTATAATCTGGTACCGATTCTAGAAAATTTAGGACTTACTAATATATTTGGATATTATG

CAGATTTTAGTAAGATGTGTAATGAAACTATCACTGTAGAAAAATTTCTACATACGACGTTTATAGATGT

TAATGAGGAGTATACAGAAGTATCGGCCGTTACAGGAGTATTCATGACTAACTTTTCGATGGTATATCGT

ATGAAGGTCTACATAAACCATCCATTCATATACATGATTAAAGATAACACCGGACATACACTTTTTATAG

GGAAATACTGCTATCCGCAATAAATATAAACAATAGACTTTTATCACGTTATCTCATGTATAAAATATTA

CAAATAGTATAGCATAAACTAAAGTCGATACATACATTAAAACTTAAATAATAATGTAATTTACAATTAA

TAGTATAAACTAAAAAAATTAAAAAATTAAAAACAATATCATTATTATAAGTAATATCAAAATGACGATA

TACGGATTAATAGCGTATCTTATATTCGTGACTTCATCCATCGCTAGTCCACTTTACATTCCCGTTATTC

CGCCCATTTCGGAAGATAAATCGTTCAATAGTGTAGAGGTATTAGTTTCTTTGTTTCCCGATGACCAAAA

AGACTATACAGTAACTTCTCAGTTCAATAACTACACTATCGGTACCAAAGACTGGACTATCAACGTACTA

TCCACACCTGATGGTCTGGACATACCATTGACTAATATAACTTATTGGTCACGGTTTACTATAGGTCGTG

CATTGTTCAAATCAGAGTCTGAGGATATTTTCCAAAAGAAAATGAGTATTCTAGGTGTTTCTATAGAATG

TAAGAAGCCGTCGACATTACTTACTTTTTTAACCGTGCGTAAAATGACTCGAGTATTTAATAGATTTCCA

GATATGGCTTATTATCGAGGAGACTGTCTAGAAGCCGTTTATGTAACAATGACTTATAAAAATACTAAAA

CTGGAGAGACTGATTACACGTACCTCTCTAATGGGGGGTTGCCTGCATACTATCGTAATGGGGTCGATGG

TTGATTATTGATTAGTATATTCCTTATATTCCTTATTCTTTTTATTCACACAAAAAGAACATTTTTATAA

ACATGAAACCACTGTCTAAATGTAATTATGATCTTGATTTATAGATGATGATCAGCCTTCAGAGGATTTT

GACCAGTATGTTTAATATGAAAAAAAACATAACTATTAAGCGCTATTGCGCTATTGTGCTTAATTATTTT

GCTCTATAAACTGAATATATAGCCACAATTATTGACGGGCTTGTTTGTGACCGACAATCATGAATTTTCA

GAAATTATCTCTGGCTATATATCTTACGGTGACATGTTCGTGGTGTTATGAAACATGTATGAGAAAAACT

GCGTTGTATCATGACATTCAATTGGAGCATGTAGAAGACAATAAAGATAGTGTAGCATCGCTACCGTACA

AGTATCTACAAGTAGTCAAACAAAGAGAACGTAGTAGATTGTTGGCTACATTTAATTGGACGGATATAGC

TGAGGGTGTTAGAAATGAGTTCATTAAAATATGTGATATCAACGGAACATATCTATATAATTATACTATT

GCTGTTAGTATAATTATTGATTCCACGGAAGAACTACCAACAGTTACTCCAATTACAACATATGAACCTT

CTATATATAATTATACTATCGATTATAGCACTGTTATTACTACTGAAGAACTACAAGTGACTCCAACATA

TGCACCTGTAACAACTCCTCTTCCAACATCAGCAGTTCCTTATGATCAACGATCGAATAACAATGTAAGT

ACTATATCTATTCAGGTACTGAGTAAAATATTGGGAGTCAATGAAACAGAATTAACTAATTATCTTATTA

TGCATAAAAATGACACTGTTGACAATAACACCATGGTTGATGATGAGACATCTAACAATAACACATTACA

TGGTAATATAGGATTTTTGGAAATAAATAATTGTTATAATGTTTCTGTGTCAGATGCTAGTTTTAGAATA

ACATTAGTAAACGATACTTCTGAAGAAATTTTGCTAATGCTAACAGGAACTAGTTCATCCGACACCTTCA

TATCTTCCACCAATATCACTGAATGTTTGAAAACATTAATCAATAATGTGTCGATTAATGATGTACTTAT

AACACAAAATATGAATGTAACATCTAATTGTGATAAATGCTCAATGAATTTGATGGCATCCGTTATTCCT

GCAGTTAATGAATTTAACAATACGTTGATGAAAATTGGTGTAAAAGATGATGAAAACAATACGGTATATA

AATATTATAATTGTAAACTAACTACAAATTCTACATGTGATGAGTTAATCAATTTAGATGAAGTCATTAA

CAACATAACTCTGACAAATATTATACACAATAGTGTTTCGACAACTAACAGCAGAAAAAGACGAGATCTG

AATGATGAGTTTGAATTTTCCACTTCCAAGGAATTAGATTGTCTTTACGAATCATATGGTGTAAACGATG

ATATAAGTCATTGTTTTGCATCACCTAGACGTAGACGATCTGACGACAAAAAGGAGTACATGGACATGAA

ATTATTCGACCACGCGAAAAAAGATTTAGGAATAGACAGTGTTATTCCTAGAGGTACAACCCATTTCCAA

GTAGGTGCATCTGGTGCAAGTGGTGGTGTTGTAGGAGATAGTTTCCCATTTCAAAATGTTAAATCGCGTG

CCAGTCTATTGGCGGAAAAAATAATGCCTAGAGTACCTATTACTGCTACCGAAGCTGATCTATATGCAAC

TGTAAATAGACAACCCAAGTTACCAGCAGGTGTTAAAAGTACTCCGTTTACAGAGGCGCTTGTGTCTACG

ATAAACCAAAAGCTTTCTAATGTTAGAGAGGTAACTTATGCTTCGCTCAATCTGCCAGGATCAAGTGGCT

ATGTTCATAGACCATCTGATTCTGTTATTTATAGCAGTATAAGACGGTCACGTTTACCTAGTGATAGCGA

TAGTGATTATGAGGATATACAAACTGTTGTTAAGGAATATAATGAAAGATATGGTAGATCAGTCAGTAGA

ACACAGTCATCAAGTAGTGAAAGCGATTTTGAAGATATAGATACTGTTGTTAGGGAATATAGACAAAAAT

ATGGCAATGCAATGGCAAAAGGACGTAGTAGTTCCCCTAAACCTGATCCATTATATAGTACTGTTAAGAA

AACAACTAAAAGTCTATCTACTGGTGTAGACATAGTTACAAAACAATCAGACTATTCTCTATTACCTGAC

GTTAATACTGGCAGTTCTATTGTGTCACCTCTCACCAGAAAAGGAGCTACTAGACGACGACCTAGACGCC

CTACAAATGATGGTCTACAGAGTCCAAATCCTCCTCTCCGTAATCCACTTCCTCAACATGATGATTATTC

TCCTCCACAAGTACACAGACCTCCACCACTTCCTCCTAAACCAGTCCAAAATTCGCCACAACTTCCCCCT

AGACCAGTAGGTCAATTACTACCTCCTCCTATAGATCAACCAGATAAAGGATTTAGTAAGTTTGTATCAC

CTAGACGGTGTAGAAGAGCAAGCTCTGGAGTCATATGTGGTATGATACAATCAAAACCAAACGATGATAC

CTATTCACTTCTTCAACGATCAAAAATTGAACCAGAATATGTGGAGGTTGGTAATGGTATACCCAAGAAC

AATGTTCCTGTAATAGGTAATAAACATAGTAAAAAATATACATCGACGATGTCAAAAATATCAACAAAAT

TTGATAAATCTACGGCATTTGGAGCAGCAATGTTACTAACTGGTCAGCAGGCCATTAGCCAACAGACTAG

ATCAACTACGTTGAGTAGAAAAGATCAGATGAGTAAGGAAGAAAAGATATTCGAAGCAGTTACAATGAGT

CTATCAACTATAGGTTCAACGTTGACGTCTGCAGGTATGACGGGTGGTCCAAAACTAATGATTGCAGGAA

TGGCTATAACGGCTATAACTGGTATAATAGATACGATAAAAGATATATATTACATGTTTTCAGGACAGGA

GAGGCCAGTAGATCCTGTTATTAAATTATTTAATAAGTACACTGGCTTAATGTCCGATAATAATAAAATG

GGTGTAAGAAAATGTTTGACACCCGGTGACGACACACTTATTTATATCGCATACAGAAACGATACCAGTT

TTAAACAGAATACGGATGCGATGGCTTTGTATTTCTTAGATGTTATCGATTCAGAGATCCTATATCTAAA

CACATCAAATTTAGTTCTAGAGTATCAACTAAAGGTGGCTTGCCCCATAGGAACATTAAGATCTGTAGAT

GTGGACATAACTGCGTATACAATATTATATGATACAGCGGATAATATTAAGAAATACAAGTTTATCAGAA

TGGCAACGCTACTATCCAAACATCCAGTTATTAGATTGACATGTGGTTTAGCAGCAACATTGGTGATTAA

ACCGTACGAGGTACCCATCAGTGATATGCAACTACTAAAAATGGCGACGCCTGGTGAACCAGAATCCACT

AAATCTATACCATCCGATGTCTGTGATAGGTATCCTCTAAAGAAATTCTATCTTTTAGCTGGTGGTTGTC

CCTATGATACATCTCAAACTTTTATTGTACATACTACTTGCAGTATTCTACTAAGAACAGCTACACGGGA

TCAGTTTAGAAACAGATGGGTGTTACAAAATCCATTTAGACAAGAAGGGACATATAAGCAACTGTTTACC

TTTAGCAAATACGATTTTAACGACACCATAATCGATCCTAATGGTGTGGTGGGTCATGCTAGCTTTTGTA

CCAATAGAAGCAGCAACCAATGTTTCTGGTCCGAACCTATGATATTGGAAGATGTATCATCGTGTAGTTC

TAGAACTAGAAAAATATACGTAAAACTGGGAATATTTAATGCTGAAGGTTTTAATAGTTTTGTACTAAAT

TGTCCAACTGGGTCTACACCTACATACATCAAACATAAAAATGCGGACAGTAACAATGTTATCATAGAGC

TACCTGTAGGTGATTACGGCACAGCCAAATTGTATTCAGCAACAAAACCATCGAGGATAGCTGTGTTCTG

CACACATAACTATGATAAACGATTCAAATCAGATATTATAGTTCTAATGTTTAATAAAAACAGCGGTATT

CCATTTTGGAGCATGTACACAGGAAGTGTAACTAGTAAAAATAGAATGTTTGCCACATTGGCTAGAGGAA

TGCCGTTTAGATCAACGTATTGCGATAACAGACGACGATCAGGTTGTTATTATGCAGGAATACCATTTCA

TGAAGATAGTGTAGAAACAGATATACATTATGGACCAGAAATAATGTTAAAGGAAACATATGACATAAAC

AGTATTGACCCACGAGTTATAACAAAGTCAAAGACCCATTTTCCTGCTCCATTGAGTGTAAAATTCATGG

TTGACAATTTAGGAAATGGATATGACAACCCTAATTCATTTTGGGAAGATGCTAAAACTAAGAAACGGAC

ATATAGTGCAATGACGATAAAAGTCCTACCATGTACAGTGAGAAATAAAAATATAGACTTTGGATATAAC

TATGGAGATATTATTTCTAATATGGTTTATCTACAATCTACTAGTCAGGATTATGGAGATGGTACCAAAT

ATACATTTAAATCCGTAACTAGATCAGATCATGAGTGTGAATCTAGCTTAGATCTAACGTCTAAGGAAGT

AACTGTGACATGTCCTGCGTTTAGTATACCAAGAAATATATCAACATATGAAGGTCTATGCTTTAGTGTT

ACTACATCTAAAGATCATTGTGCTACAGGTATTGGTTGGTTAAAATCTAGTGGTTATGGGAAGGAAGATG

CTGATAAACCACGTGCTTGTTTTCATCATTGGAATTATTACACACTGTCGTTGGATTATTACTGTTCATA

CGAAGATATTTGGAGAAGCACCTGGCCTGACTATGATCCATGTAAGTCATATATCCATATAGAGTATAGA

GATACATGGATAGAATCTAATGTGTTACAGCAACCTCCTTACACATTCGAATTCATTCATGACAATTCTA

ACGAATATGTGGATAAAGAAATTAGTAACAAATTAAATGATCTGTACAATGAATACAAGAAGATTATGGA

ATATAGCGACGGATCATTGCCGGCGTCTATAAACAGATTAGCAAAGGCATTGACTTCAGAGGGTAGAGAA

ATAGCAAGTGTTAATATAGATGGTAATCTGTTAGATATCGCATATCAAGCAGATAAGGAAAAGATGGCCG

ACATACAGACAAGAATAAATGATATTATTAGAGATTTGTTTATACACACTCTATCAGACAAAGATATAAA

AGACATTATAGAATCCGAAGAAGGTAAGAGATGTTGTATAATAGATGTTAAGAACAATCTTGTTAAAAAG

TACTATTCTATTGATAATTATCTATGTGATACTTTAGATGATTATATATACACCTCTGTAGAATATAACA

AATCCTATGTGTTAGTAAACGATACTTATATAAGCTATGACTATCTTGAATCATCAGGTGTAGTTGTTCT

ATCATGTTATGAAATGACTATAATCTCCTTGGATACAAAAGACGCCAAAGATGCTATAGAAGATGTGATA

GTAGCAAGTGCGGTAGCCGAAGCATTGAATGACATGTTTAAGGAATTTGATAAAAACGTAAGTGCTATTA

TAATAAAAGAAGAAGATAATTATCTAAACAGTTCGCCCGATATCTACCATATAATATATATCATAGGTGG

CACTATTCTGCTACTGTTAGTCATTATTTTAATATTGGCAATTTATATAGCGCGCAATAAATACAGAACC

AGGAAATATGAAATAATGAAATATGACAATATGAGCATTAAATCTGAGCATCATGATAGTCTTGAAACAG

TGTCTATGGAAATTATTGATAATCGGTACTAATAAAATAGTTTAACTCTTTTAGAACCAGTTTGGTACTG

TAATTTCAGTTCATTACTCGTTGAGAATATTGATGATTTTTTTTAAATGAGTATCGGTAGTTACATATTA

CCATATCATCCATTATATAATCGATGATGCATGTATTAGAATACTTTCCGAATAAGTCTTCTAAATATTG

TATTAATTATGAAAAACTATGCTATGTGAGTATGATTCAAAGATGTTTAATGATACGATACTAGATTTTA

TCTCTAGCGAGATTGTTTAGAATCATTTATCATAACTATGTTTAATAAATTCATCAACGAATATCGATAA

AGACCTCTTGTAATTCGAGTATAGGAAGTAGTATTACCATATCAACTTCCGAGTTAACAATTACTCTAAA

ACATGAGGATTGTACTCCTGTCTTTATTGGAGATCACTATTTAGTCGTTGATAAACTAGTAACCTCAGGT

TTCTTTACAAACGATAAAGTACAACATCAAGACCTCACAACACAGTGCAAGATTAATCTAGAAATCAAAT

GTAATTCTGGAGGAGAATCTAGACAACTAACACCCACGGCGAAGTATACTTTATGCCTCATTCAGAAACG

GTAACTGTAGTAGGAGACTGTCTCTCTAATCTCGATGTATATATAATATATACCAATACGGACGCGATAT

ATTCCGACATGAATGGCGTCGCTTATCATATGTTATATCCTAAATGTTGATCATATTCCACAAATGATTG

TGAACGAGATTAAATCATCTAACAAATAATTAGTTTTTTATGACATTAACATATAATAAATAAATTAATC

ATTATTGACTTAACGATGACGAAAGTTATCATTATCTTAGGATTCTTGATTATTAATACAAATTCGTTGT

GTCTATGAAATGTGAACAAGGTGTCTCATATTATAATGCACAAGAATTAAAGTGTTGTAAACTATCTAGC

CAGGAACATATTCAGATTATCGATGTGATAAATACAGCGATACCATCTGTGGACATTGTCCAAGTGACAC

ATTCACGTCAATATATAATCGTTCTCCTCGGTGTCATAGTTGTAGAGGTCACACCTTGTACACCTACCAC

AAATAGAATATGTCATTGTGACTCGAATAGTTATCGTCTCCTTAAAGCTTCTGATGGTAACTGTGTTACA

TGTGCTCCTAAAACAAAATATGGTCGTGTGTACGGAAAGAAAGGAGAAAATGATATGGAATACCATTTGT

AAGAAATGTCGGAAGGGTACTTATTCAGATATTGTATCTGACTCTGATCAATGTAAACCTATGACAAGAT

AAGACTTACTCGCATCTACTGGATAGACATAAATATCCTCCTCGTAATAATGAAATATAATATACACTAAT

TATTAATATCAATCGAGTATTAACATATAAGTTATTTTTAAACCCCTTTTGGGTTCCGTCCTAAACGGCG

TTTCGGTCTGTGTCGCCACCATGGTCACACCGAGCCTCTGCGTGCTCCTCCATCGAGGACGACTTCAACT

ATGACAGCTCGGTGGCGTCTGCCAGCGTGTACATACGAATGGCATTTCTAAGAAAAGTCTACGGTATCCT

TTCTACAATTTCCTTTAACAACGGCAACAGCTGCAGTATTTTTATACTTTGAATGCATCGGACATTTATA

CAAGGGAGTCCTGTTCTAATATTGGCATCAATGTTCGGATCTATAGGCTTGATTTTCGCATTGACTTTAC

ACAGACATGAACATCCCCTGAATCTGTACATACTTTGTGGATTTACACTGTTAGAATCTCTAACGCTGGC

CTCTGTTGTTACTTTCTATGATGCACGTATCGTTATGCAAGCTTTCATGTTGACTACTGCAGTGTTTCTT

GCTCTGACTACATATACTCTACAATCAAAGAGAGATTTCAGTAAACTTGTAACAGGATTGTTTGCTGCTT

TCTGGATTTTAATTTTGTCAGGAGTCTTGAGGATAAAGTTTAAAATAGAATTAATAAAGAACATATAGGT

CATTTTTTAAACATGGATAGAAACCAAGGTTGTTAGTTAATAATATACAAGATATTTTTTCTCACTCTGA

TCCATGTAAACCAAGGACGAGAGACACTCTCATTCCTCATTCACGACACCATTAAAAATGGAAATTAAAG

CCCTCTATTAAGCACAGACGGCTACAGGTCTACCATCAGGTTACCTTCGTCTACCTTCACAATGGCCTCT

CCTTGTGCCCAGTTCAGTCCCTGTCATTGCCACGCTACTAAGGACTCCCTGAATACCGTGACTGACGTCA

GACATTGTCTGACTGAATACATCCTGTGGGTTTCTCATAGATGGACCCATAGAGAAAGCGCAGGGCCTCT

CTACAGGCTTCTCATCTCTTTCAGAATTGATGCAATGGAGCTATTTGGTAGCGAGTTGAAGGAGTTCTCG

AATTCACTTCCGTGGGACAATATCGACAATTGCGTGGAGATCATTAAATGTTTCATCAGAAATGACTCCA

TGAAAACCGCCAAAGAACTTTGTGCAATAATTGGACTTTGTACTCAATCAGCTATTGTCACTGGAAGAGT

CTTCAATGATAAGTATATCGACATACTACTTATGCTGCGAAAGATTCTGAACGAGAACGACTATCTCACC

CTCTTGGATCATATCCTCACTGCTAAATACTAAATCTCCTTCATGCTCTCTCACTAATACTCTTACTCAC

TACACTTTTTATCATCTTATGATGAATGATTGCCTTCATCATTTTTTCGTGGAATATAATATAGGAATAA

TTAGCACCAGAATAGCTATGGATATCTCGTTAAGAATATTCTCTATAAGAGACATAATGTAGACATAGTT

ATTATATCCTTCTTAGATAAGTGTTACGCTACTGGAAAGTTTCCATCGTTATTATTACATGAAGATGATA

TAATTAAACCAACATTGAGATTGGCTCTTATGTTAGCTGGATTGAATTACTGTAATAAATGCATCGAGTA

TAGAGGGATATAGCAATTCTCGATAATAGTCATGCAATATTTGAATGAGACTGATAATTTAGGTAATACA

GTACTACACACATATCTTTCTAGATTATATATCGTTAAAAATCTGTAAGATGTATATTTCTCATAAGTAT

CCACTGTGTAATATTATTAATGGATATATAGATAACGCAATAGGGACTAATAGTATTGTAAAAGATATAA

TCGACTATTTACGTACATATCCAGATATCTATATTCCTACTAGTTTGCTGCGTAGTTGCATCATTGATAT

GCATGATTTATCAGGATTCAGAGATGAATTACTAAGTAAACTACAATCCCACAATAAGTAAGAATCAAAT

ATCAAAAACTCACTTTTGATTTTTCTAGTCTTAAGTAATACATATATTTATTAATAGACCTATGAAATAA

AAAAAGGTAACAATGGATTCGCGTATAGCTATTTACGTATTAGTATCGGCATCTCTTTTGTATCTTGTTA

ATTGTCACAAACTAGTACATTACTTCAATCTGAAAATAAATGGAAGTGATATAACTAATACAGCAGATAT

ATTGCTGGACAATTATCCAATTATGACCTTTGATGGAAAGGATATTTATCCATCTATCTCGTTCATGGTC

GGTAATAAACTTTTCCTAGATCTTTATAAAAATATCTTTGAAGAATTTTTCAGACTATTTCGAGTATCTG

TAAGTAGTCAATACGAGGAATTAGAATATTATTATTCATGTGATTATACTAACAACCGTCCTACAATTAA

ACAACATTACTTTTATAACGGCGACGAATATACTGAAATTGATAGATCGAAAAAAGCCACTAATAAAAAC

AGTTGGTTAATTACTTCAGGCTTTAGACTACAAAAATGGTTCGATAGCGAAGATTGTATAATTTATCTCA

GATCTTTAGTTAGAAGAATGGAAGACAGTAACAAAAACAGTAAAAAAACTTAGTACTTAGATATCGAAAA

AATATATTTTTGTAGACTCTTGAGAATAGAAGGAAAACATGTACATAATTATAAAAAATGAAAATCAATG

GCGAATAAGACAGTGCGATTCGCACCATGGAGTCGGTAGATTTCATGGCTGTCGATGAGCAGTTTCACGA

CGACCTCGATCTTTGGTCATTATCTTTGGTAGATGATTATAAAAAACATGGATTAGGTGTTGACTGTTAT

GTTCTAGAACCAGTTGTTGACAGGAAAATATTTGATAGATTTCTCCTTGAACCAATTTGTGATCCTGTAG

ATGTTCTGTATGATTATTTTAGGATTCATAGAGATAATATTGATCAGTATATAGTAGATAGACTGTTTGC

ATATATTACATATAAAGATATTATATCTGCATTAGTGTCAAAGAATTATATGGAAGATATTTTCTCTATA

ATTATTAAGAATTGTAATTCTGTGCAAGATCTCTTACTTTACTATCTATCTAATGCATATGTAGAAATAG

ACATTGTTGATCTTATGGTAGATCATGGGGCTGTAATATATAAAATAGAATGCTTGAATGCCTATTTTAG

GGGAATATGTAAAAAGGAAAGTAGTGTTGTTGAGTTTATTTTGAATTGTGGTATCCCAGATGAAAATGAT

GTTAAATTAGATCTATATAAAATAATTCAGTATACTAGGGGATTCCTTGTAGATGAACCCACAGTATTAG

AAATTTATAAGCTTTGTATCCCATATATTGAAGATATCAATCAACTAGATGCTGGTGGAAGGACCTTGCT

TTATCGCGCTATCTATGCAGGTTATATAGATTTAGTATCATGGCTATTAGAAAATGGAGCAAATGTCAAC

GCAGTAATGAGTAATGGATATACATGTCTTGACGTGGCCGTGGATAGGGGATCTGTCATCGCCCGTAGGG

AAGCACATCTTAAAATATTAGAAATATTGCTTAGAGAACCATTGTCTATTGACTGTATAAAATTAGCTAT

ACTTAATAATACAATTGAAAACCATGATGTGATAAAGCTCTGTATCAAGTATTTTATGATGGTAGATTAT

TCACTTTGTAATGTGTATGCATCATCACTCTTTGATTATATAATTGATTGTAAACAAGAATTGGAGTACA

TTAGGCAGATGAAAATTCATAATACAACCATGTATGAGTTAATCTATAATAGAGACAAAAACAAGCATGC

TTCCCATATTCTACATAGGTATTCTAAACATCCAGTTTTGACACAGTGTATCACTAAAGGATTCAAGATT

TACACAGAAGTAACCGAGCAGGTCACTAAAGCTCTAAACAGACGTGCTCTAATAGATGAGATAATAAACA

ATGTATCAACTGATGACAATCTCCTATCAAAACTTCCATTAGAAATTAGGGATCTAATTGTTTCACAAGC

TGTCATATAGAGTTCTATCCACCCACCTTTCTTGAAATGAGTTAATAGTCATAAGTTAGTTAAGTCATAA

GTTAGTTAAGTCATAAGTTAGTTAAGTCATAAGTTAGTTAAGTCATAAGTTAGTTAAGTCATAAGTTAGT

TAAGTCATAAGTTAGTTAAGTCATAAGTTAGTTTATAGTCTAACACTTCTAATTTTTATACCTTGATCTT

TTTCTCTAATTATGAAAAAGTAAATCATTATGAAGATGGATGAAATGGACGAGATTGTGCGCATCGTTAA

CGATAGTATGTGGTACGTACCTAACGCATTTATGGACGACGGTGATAATGAAGGTCACATTTCTGTCAAT

AATGTCTGTCATATGTATCTCGCATTCTTTGATGTGGATATATCATCTCATCTGTTTAAATTAGTTATTA

AACACTGCGATCTGAATAAACGACTAAAATGTGGTAACTCTCCATTACATTGCTATACGATGAATACACG

ATTTAATCCATCTGTATTAAAGATATTGTTACGCCACGGCATGCGTAACTTTGATAGCAAGGATAAAAAA

GGACATATTCCTCTACACCACTATCTGATTCATTCACTATCAATCGATAACAAGATCTTTGATATACTAA

CGGACCCCATTGATGACTTTAGTAAATCATCCGATCTATTGCTGTGTTATCTTAGATATAAATTCAATGG

GAGCTTAAACTATTACGTTCTGTACAAATTATTGACTAAAGGATCTGACCCTAATTGCGTCGATGAGGAT

GGACTCACTTCTCTTCATTACTACTGTAAACACATATCCGCGTTCCACGAAAGCAATTATTACAAGTCAA

AGAGTCACACTAAGATGCGAGCTGAGAAGCGATTCATCTACGCGATAATAGATCATGGAGCAAACATTAA

CGCGGTTACGAAAATCGGAAATACGCCGTTACACACTTACCTTCAACAGTATACCAAACATAGTCCTCGT

GTGGTGTATGCTCTTTTATCTCGAGGAGCCGATACGAGGATACGTAATAATCTTAATTGTACACCCATCA

TGGAATACATAAAGAACGATTGTGCAACAGGTCATATTCTCATAATGTTACTCAATTGGCACGAACAAAA

ATACGGGAAATTACAAAAGGAAGAAGGACAACATCTACTTTATCTATTCATAAAACATAATCAAGGATAT

GGAAGTCGCTCTCTCAATATACTACGGTATCTACTAGATAGATTCGACATTCAGAAAGACGAATACTATA

ATACAATGACTCCTCTTCATACCGCCTTCCAGAATTGCAATAACAATGTTGCCTCATACCTCGTATACAT

TGGATACGACATCAACCTTCCGACTAAAGACGATAAGACAGTATTCGACTTGGTGTTTGAAAACAGAAAC

ATTATATACAAGGCGGATGTCGTTAATGACATTATCCACCACAGACTGAAAGTATCTCTACCTATGATTA

AATCGTTGTTCTACAAGATGTCGGAGTTCTCTCCCTACGACGATCACTACGTAAAGAAGATAATAGCCTA

CTGCCTATTAAGGGACGAGTCATTTGCGGAACTACATACTAAATTCTGTTTAAACGAGGACTATAAAAGT

GTATTTATGAAAAATATATCATTCGATAAGATAGATTCCATCATCGAAAAATGTAGTCGTGACATAAGTC

TCCTCAAAGAGATTCGAATCTCAGACACCGACTTGTATACGGTATTGAGAACAGAAGACATTCGGTATCA

CACATATCTCGAAGCCATACATTCAGACAAACGCATTTCATTTCCCATGTACGACGATCTCATAGAACAG

TGTCATCTATCGATGGAGCATAAAAGTAAACTCGTCGACAAAGCACTCAATAAATTAGAGTCTACCATCG

ATAGTCAATCTAGACTATCGTATTTGCCTCCGGAAATTATGCGCAATATCATAACCAAGCTAAGCGACTA

CCATCTAAACAGTATGTTGTACGGAAAGAACCATTACAAATATTATCCATGATAGAAAGAAAATATTTAA

AAAATAATCTATATGATTGGAGAAGTAGGAAACAAACAGTAACAAGACGACGATTACTACATTATTAAAT

CATGAGGTCCGTATTATACTCGTATATATTGTTTCTCTCATGTATAATAATAAACGGAAGAGATATAGCA

CCACATGCACCATCCAATGGAAAGTGTAAAGACAACGAATACAGAAGCCGTAATCTATGTTGTCTATCGT

GTCCTCCGGGAACTTACGCTTTCAGATTATGTGATAGCAAGACTAATACACAATGTACACCGTGTGGTTC

GGATACCTTTACATCTCACAATAATCATTTACAGGCTTGTCTAAGTTGTAACGGAAGATGTGATAGTAAT

CAGGTAGAGACGCGATCGTGTAACACGACTCACAATAGAATCTGTGAATGCTCTCCAGGATATTATTGTC

TTCTCAAAGGAGCATCAGGGTGTAGAACATGTATTTCTAAAACAAAGTGTGGAATAGGATACGGAGTATC

CGGATACACGTCTACCGGAGACGTCATCTGTTCTCCGTGTGGTCCCGGAACATATTCTCACACCGTCTCT

TCCACAGATAAATGCGAACCCGTCGTAACCAGCAATACATTTAACTATATCGATGTGGAAATTAACCTGT

ATCCAGTCAACGACACATCGTGTACTCGGACGACCACTACCGGTCTCAGCGAATCCATCTCAACGTCGGA

ACTAACTATTACCATGAATCATAAAGATTGTGATCCAGTCTTTCGTGCAGAATACTTCTCTGTCCTTAAT

AATGTAGCAACTTCAGGATTCTTTACAGGAGAAAATAGATATCAGAATACTTCAAAGATATGTACTCTGA

ATTTCGAGATTAAATGTAACAACAAAGATTCATCTTCCAAACAGTTAACGAAAACAAAGAATGATACTAT

CATGCCGCATTCAGAGACGGTAACTCTAGTGGGCGACTGTCTATCTAGCGTCGACATCTACATACTATAT

AGTAATACCAATACTCAAGACTACGAAAATGATACAATCTCTTATCATATGGGTAATGTTCTCGATGTCA

ATAGCCATATGCCCGCTAGTTGCGATATACATAAACTGATCACTAATTCCCAGAATCCCACCCACTTATA

GTAAGTTTTTTTACCTATAAATAATAAATACAATAATTAATTTCTCGTAAAAGTAGAAAATATATTCTAA

TTTATTATATGGTAAGAAAGTAGAATCATCTAGAACAGTAATCAATCAATAGCAATCATGAAACAATATA

TTGTCCTGGCATGCATGTGCCTAGTGGCAGCTGCTATGCCTACTAGTCTTCAACAATCTTCATCCTCGTG

TACTGAAGAAGAAAACAAACATCATATGGGAATCGATGTTATTATCAAAGTCACAAAGCAAGACCAAACA

CCGACCAATGATAAGATTTGTCAATCCGTAACGGAAGTTACAGAGACCGAAGATGATGAGGTATCCGAAG

AAGTTGTAAAAGGAGATCCCACCACTTATTACACTATCGTCGGTGCGGGTCTTAACATGAACTTTGGATT

CACCAAATGCCCAAAGATTTTATCCATCTCCGAATCCTCTGATGGAAACACTGTGAATACTAGATTGTCC

AGCGTGTCACCGGGACAAGGTAAGGACTCTCCCGCGATCACGCGTGAAGAAGCTCTGGCTATGATCAAAG

ACTGTGAGATGTCTATCGACATCAGATGTAGCGAAGAAGAGAAAGACAGTGACATCAAGACCCATCCAGT

ACTTGGGTCTAACATCTCACATAAGAAAGTGAGTTACAAAGATATCATCGGTTCAACGATCGTTGATACA

AAATGTGTCAAGAACCTAGAGTTTAGCGTACGTATCGGAGACATGTGTGAGGAATCATCTGAACTTGAAG

TCAAGGATGGATTCAAGTATGTCGACGGATCGGCATCTGAAGGTGCAACCGATGATACTTCACTCATCGA

TTCAACAAAACTCAAAGCATGTGTCTGAATCGATAACTCTATTCATCTGAAAATGGATGAGTTGGGTTAA

TCGAACGATTCAGACACCGCACCACGAATTAAAAAAGACCGGGCACTATATTCCGGTTTGCAAAACAAAA

ATATTTAACTACATTCACAAAAAGTTACCTCTCGTTACTTCTTCTTTCTGTTTCAATATGTGATACGATA

TGATCACTATTCGTATTCTCTTGGTCTCATAAAAAAGTTTTACAAAAAAAAAAAAAATATTTTTATTCTC

TTTCTCTCTTCGATGGTCTCACAAAAATATTAAACCTCTTTCTGATGTCTCAACTATTTCGTAAACGATA

ACGTCCAACAATATATTCTCGTAGAGCTTATCAACATCCTTATACCAATCTAGGTTGTCAGACAATTGCA

TCATAAAATAATGTTTATAATTTACACGTTAACATCATATAATAAACGTATATAGTTAATATTTTTGGAA

TATAAATGATCTGTAAAATCCATGTAGGGGACACTGCTCACGTTTTTTCTCTAGTACATAATTTCACACA

AGTTTTTATACAGACAAATTAATTCTCGTCCATATATTTTAAAACATTGACTTTTGTACTAAGAAAAATA

TCTTGACTAACCATCTCTTTCTCTCTTCGATGGGTCTCACAAAAATATTAAACCTCTTTCTGATGGAGTC

GTAAAAAGTTTTTATCCTTTCTCTCTTCGATAGGTCTCACAAAAATATTAAACCTCTTTCTGATGGTCTC

TATAAACGATTGATTTTTCTTACCCTCTAGAGTTTCCTACGGTCGTGGGTCACACATTTTTTTCTAGACA

CTAAATAAAATAGTAAAATTAAATTAATTA

TAAAATTATGTATATAATTTACTAAC
