# Supplementary material for: Exploring the key genomic variation in monkeypox virus during the 2022 outbreak
Source: BMC Genom Data. 2023 Nov 16;24:67. doi: 10.1186/s12863-023-01171-0 (PMC10652487; doi:10.1186/s12863-023-01171-0)
Supplement: Supplementary file 18 — Additional file 18. [file 12863_2023_1171_MOESM18_ESM.docx]

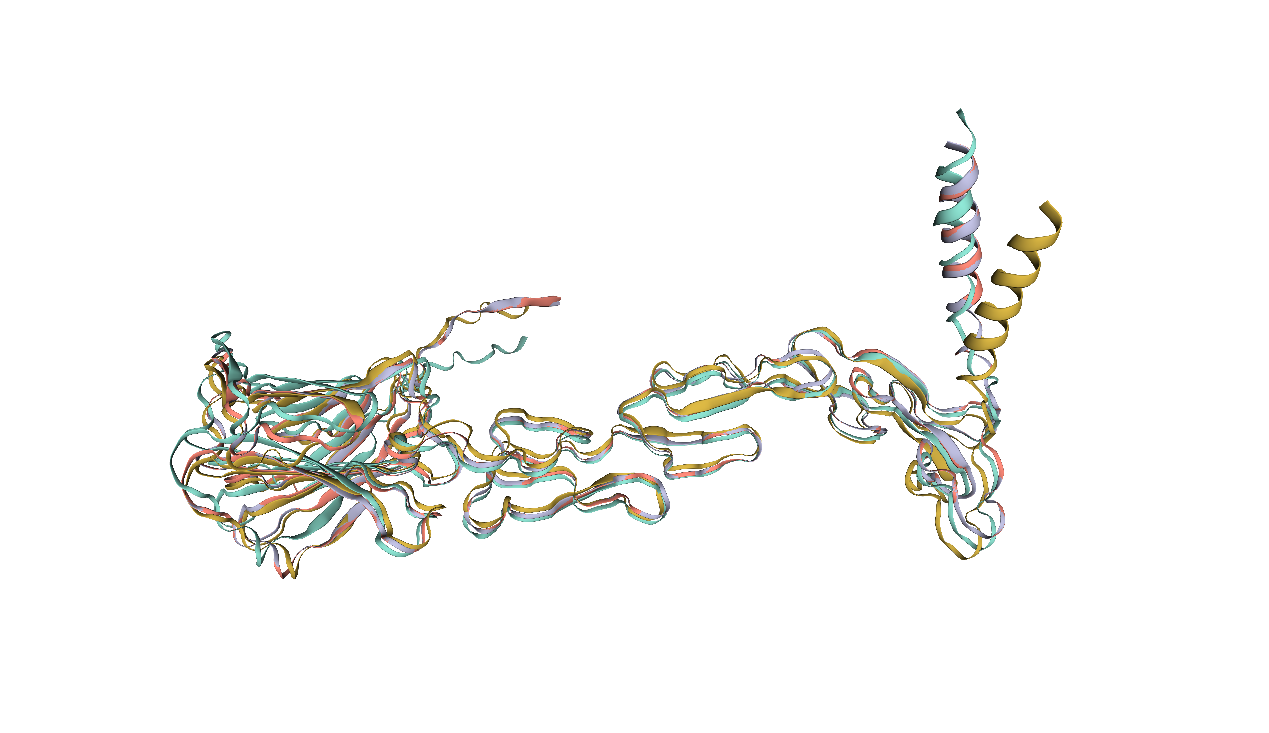


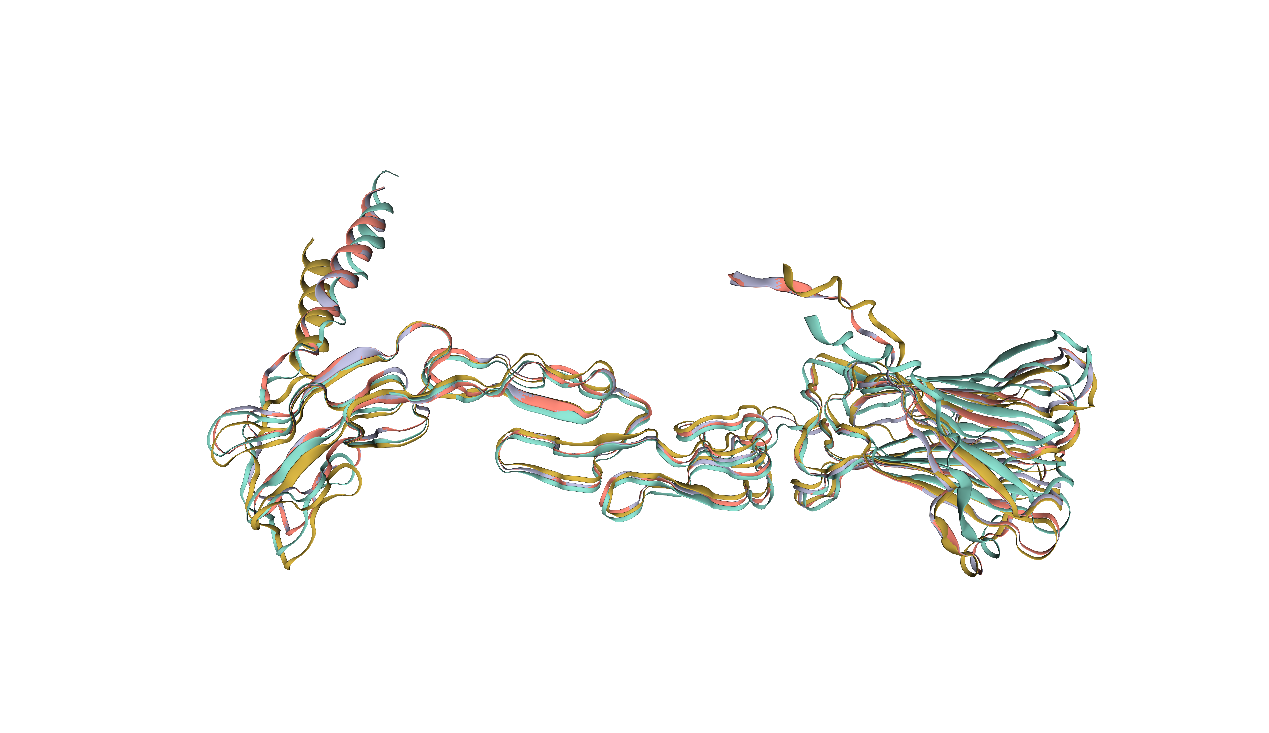


**The full picture of J2L and the rotation after 180°.**

**
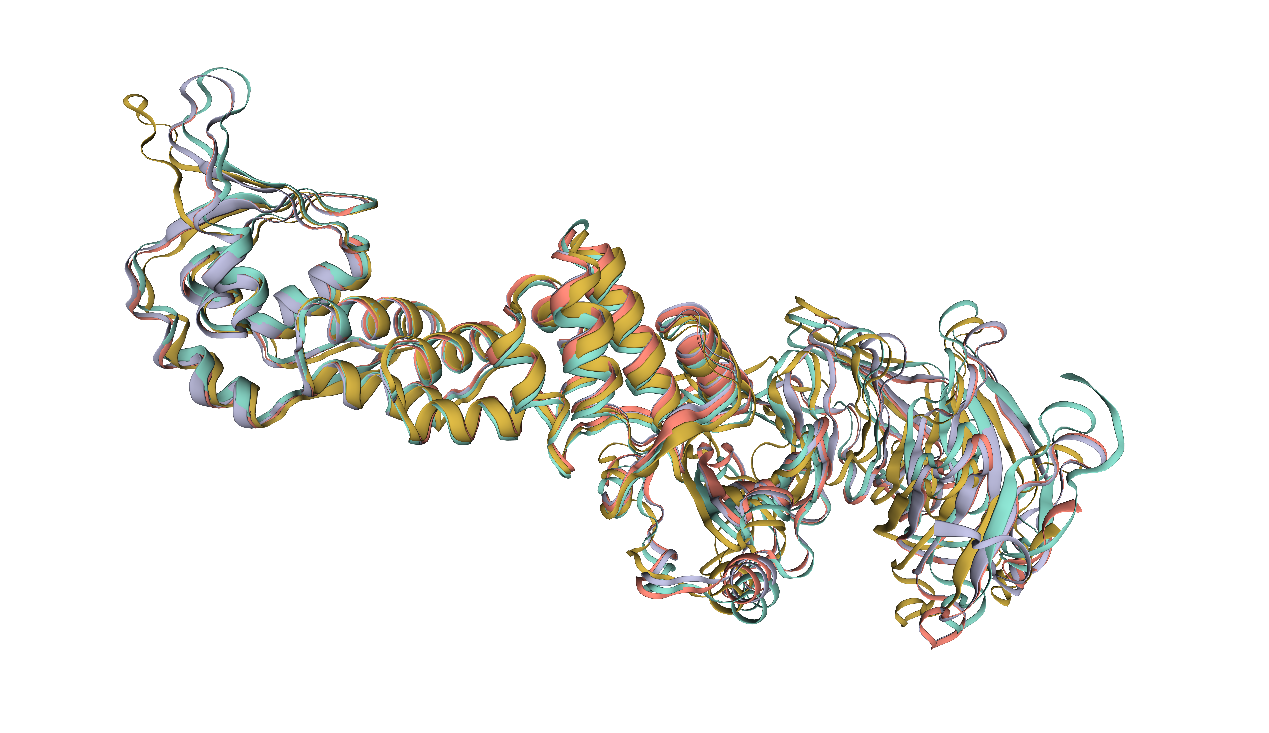
**

**
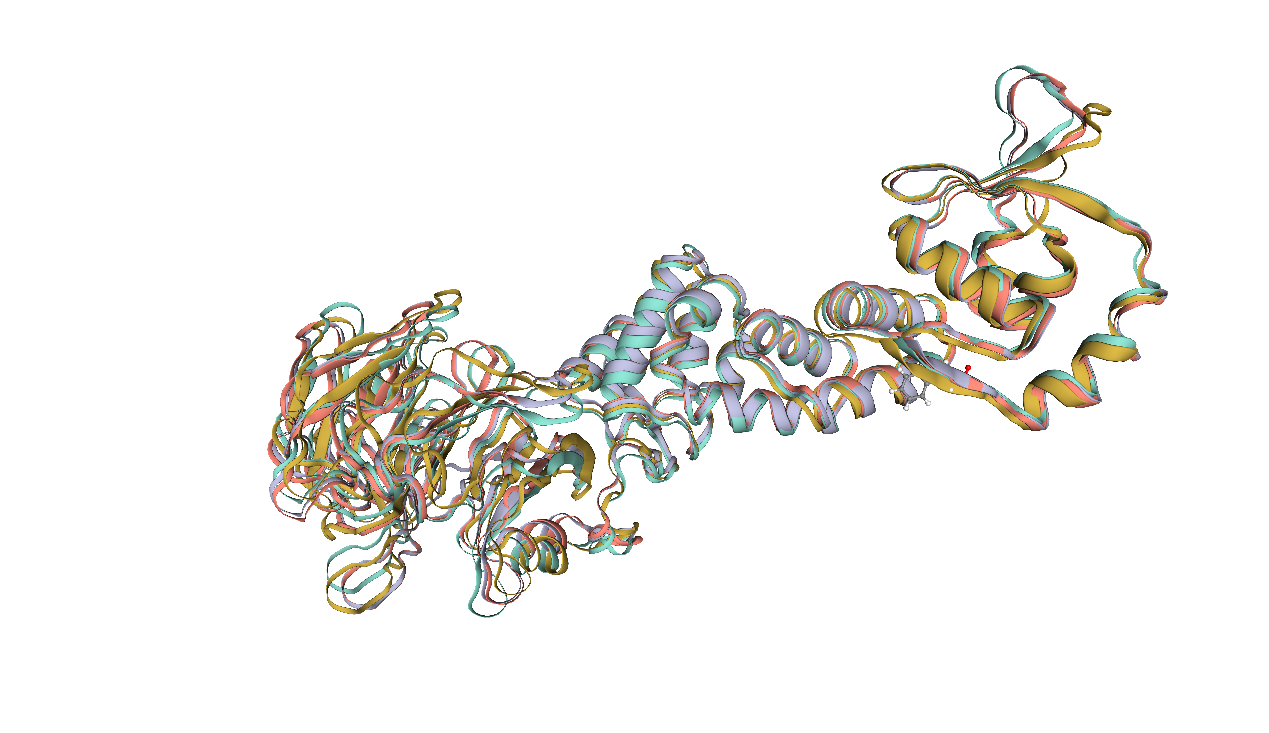
**

**The full picture of C9L and the rotation after 180°.**

**
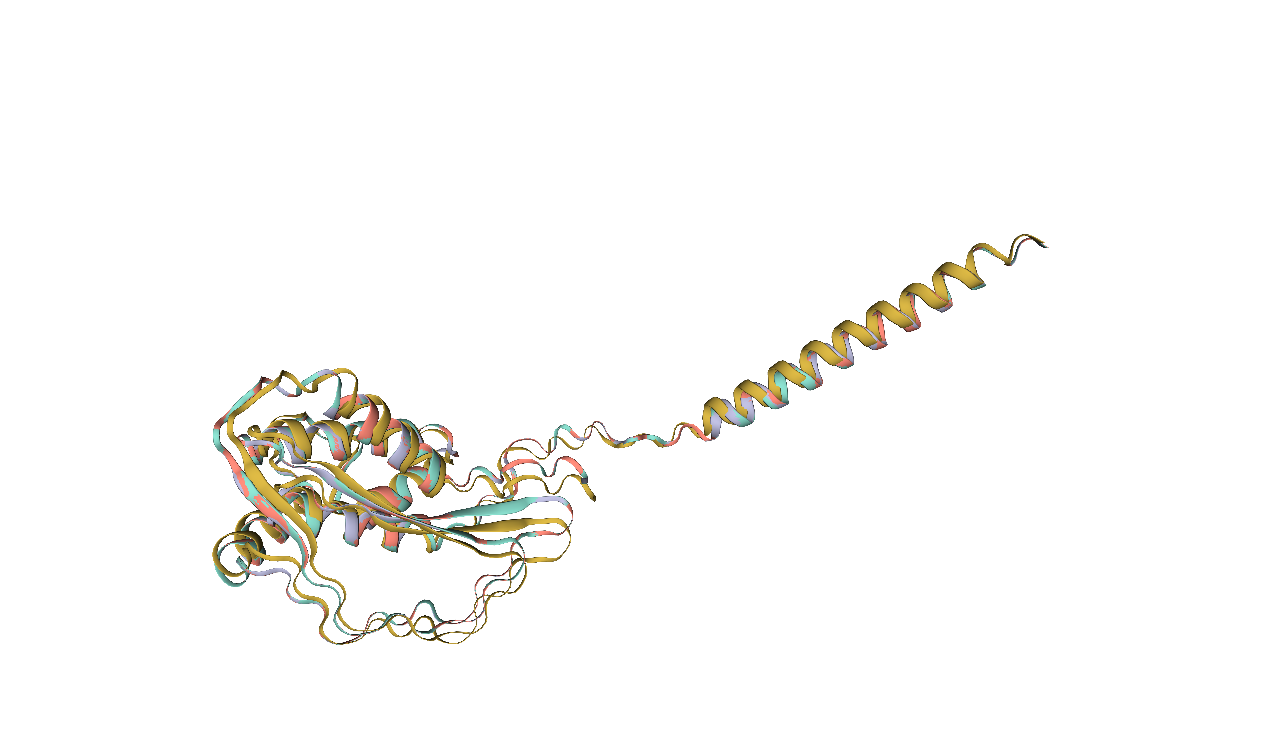
**

**
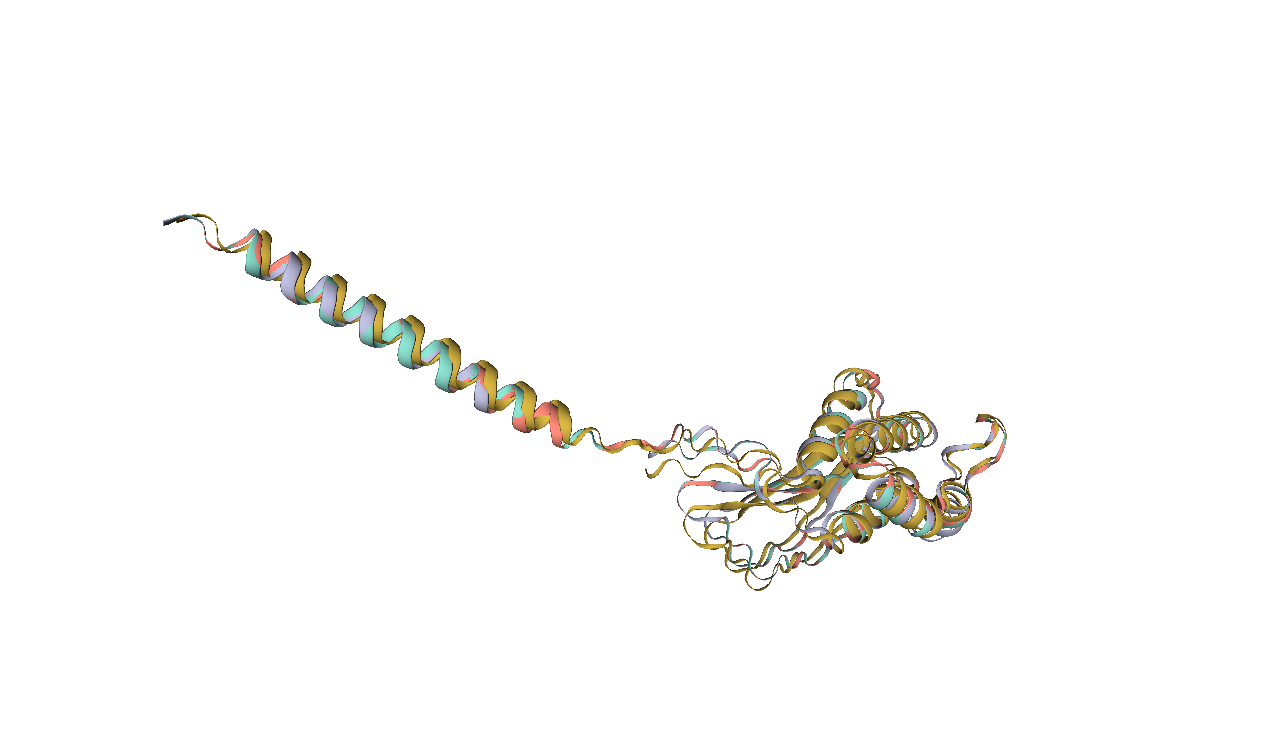
**

**The full picture of C15L and the rotation after 180****°.**

**
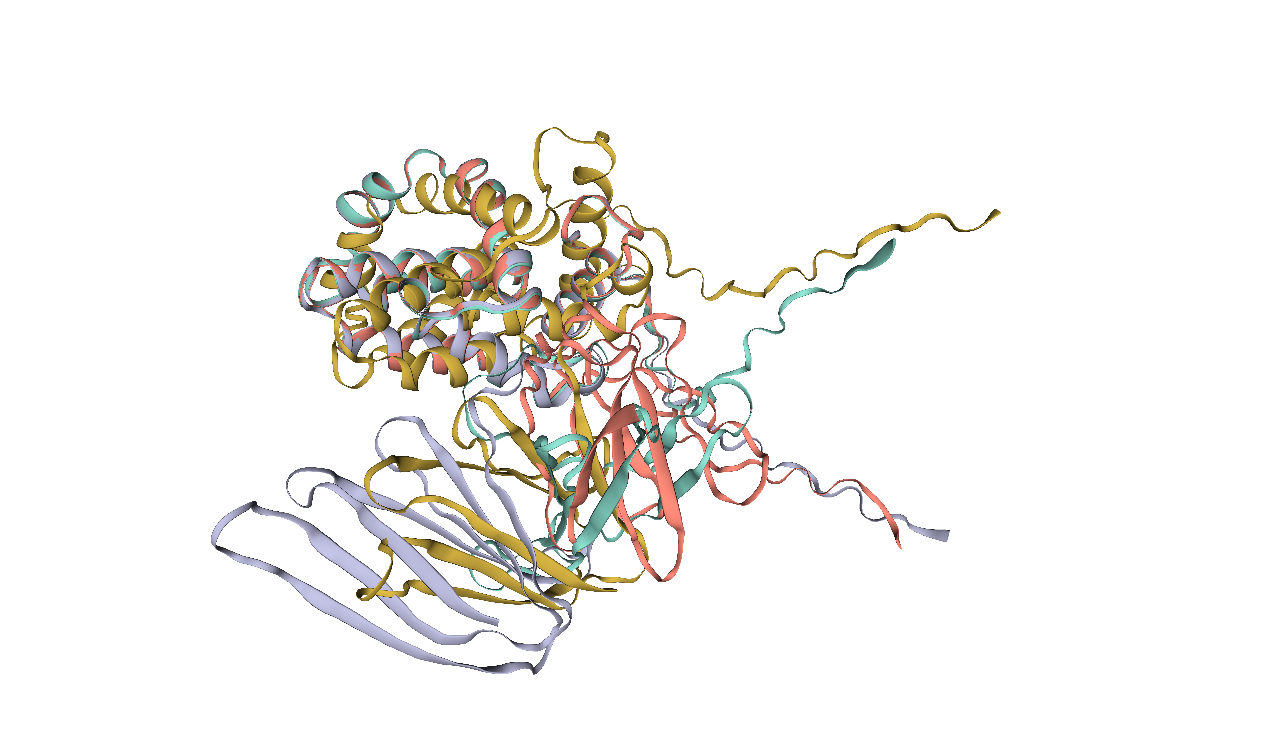
**

**
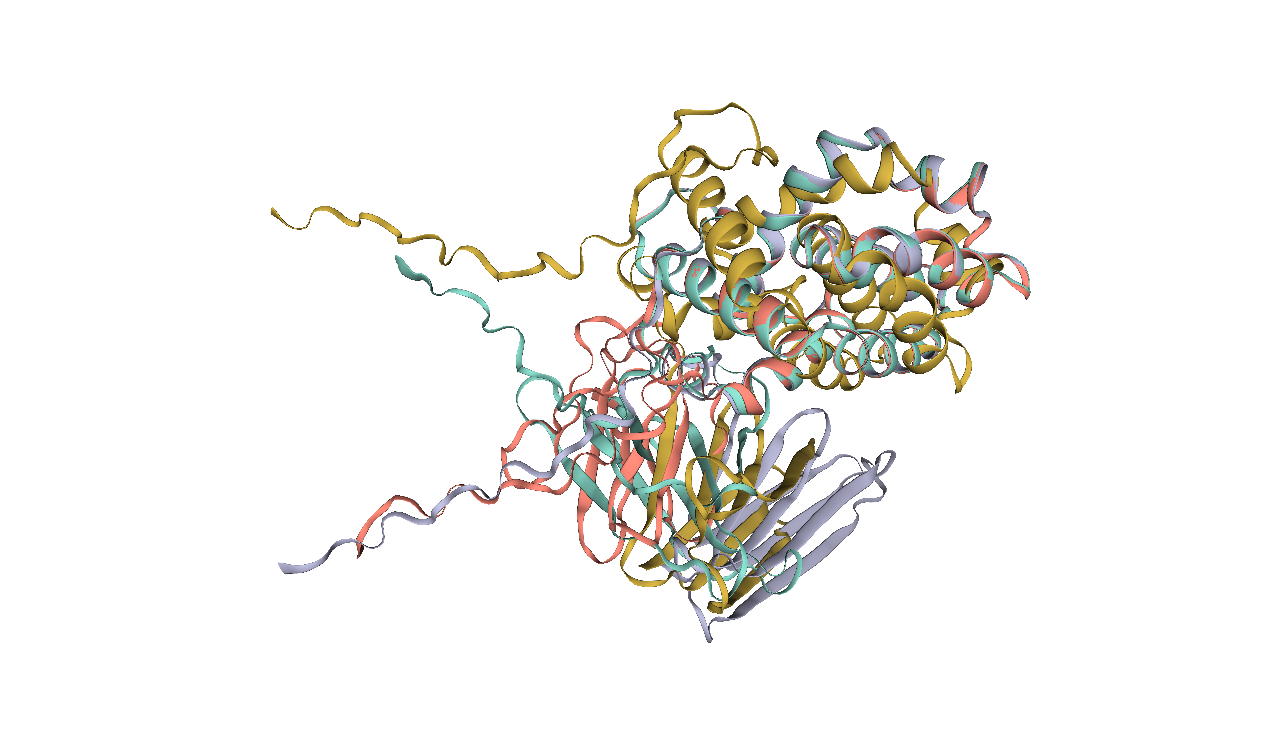
**

**The full picture of A47R and the rotation after 180°.**
